# A Study into the Collision-induced Dissociation (CID) Behavior of Cross-Linked Peptides*[Fn FN2]

**DOI:** 10.1074/mcp.M115.049296

**Published:** 2015-12-30

**Authors:** Sven H. Giese, Lutz Fischer, Juri Rappsilber

**Affiliations:** From the ‡Department of Bioanalytics, Institute of Biotechnology, Technische Universität Berlin, 13355 Berlin, Germany;; §Wellcome Trust Centre for Cell Biology, School of Biological Sciences, University of Edinburgh, Edinburgh EH9 3BF, United Kingdom

## Abstract

Cross-linking/mass spectrometry resolves protein–protein interactions or protein folds by help of distance constraints. Cross-linkers with specific properties such as isotope-labeled or collision-induced dissociation (CID)-cleavable cross-linkers are in frequent use to simplify the identification of cross-linked peptides. Here, we analyzed the mass spectrometric behavior of 910 unique cross-linked peptides in high-resolution MS1 and MS2 from published data and validate the observation by a ninefold larger set from currently unpublished data to explore if detailed understanding of their fragmentation behavior would allow computational delivery of information that otherwise would be obtained via isotope labels or CID cleavage of cross-linkers. Isotope-labeled cross-linkers reveal cross-linked and linear fragments in fragmentation spectra. We show that fragment mass and charge alone provide this information, alleviating the need for isotope-labeling for this purpose. Isotope-labeled cross-linkers also indicate cross-linker-containing, albeit not specifically cross-linked, peptides in MS1. We observed that acquisition can be guided to better than twofold enrich cross-linked peptides with minimal losses based on peptide mass and charge alone. By help of CID-cleavable cross-linkers, individual spectra with only linear fragments can be recorded for each peptide in a cross-link. We show that cross-linked fragments of ordinary cross-linked peptides can be linearized computationally and that a simplified subspectrum can be extracted that is enriched in information on one of the two linked peptides. This allows identifying candidates for this peptide in a simplified database search as we propose in a search strategy here. We conclude that the specific behavior of cross-linked peptides in mass spectrometers can be exploited to relax the requirements on cross-linkers.

Cross-linking/mass spectrometry extends the use of mass-spectrometry-based proteomics from identification (1, 2), quantification (3), and characterization of protein complexes (4) into resolving protein structures and protein–protein interactions (5–8). Chemical reagents (cross-linkers) covalently connect amino acid pairs that are within a cross-linker-specific distance range in the native three-dimensional structure of a protein or protein complex. A cross-linking/mass spectrometry experiment is typically conducted in four steps: (1) cross-linking of the target protein or complex, (2) protein digestion (usually with trypsin), (3) LC-MS analysis, and (4) database search. The digested peptide mixture consists of linear and cross-linked peptides, and the latter can be enriched by strong cation exchange (9) or size exclusion chromatography (10). Cross-linked peptides are of high value as they provide direct information on the structure and interactions of proteins.

Cross-linked peptides fragment under collision-induced dissociation (CID) conditions primarily into b- and y-ions, as do their linear counterparts. An important difference regarding database searches between linear and cross-linked peptides stems from not knowing which peptides might be cross-linked. Therefore, one has to consider each single peptide and all pairwise combinations of peptides in the database. Having *n* peptides leads to (*n*2 + *n*)/2 possible pairwise combinations. This leads to two major challenges: With increasing size of the database, search time and the risk of identifying false positives increases. One way of circumventing these problems is to use MS2-cleavable cross-linkers (11, 12), at the cost of limited experimental design and choice of cross-linker.

In a first database search approach (13), all pairwise combinations of peptides in a database were considered in a concatenated and linearized form. Thereby, all possible single bond fragments are considered in one of the two database entries per peptide pair, and the cross-link can be identified by a normal protein identification algorithm. Already, the second search approach split the peptides for the purpose of their identification (14). Linear fragments were used to retrieve candidate peptides from the database that are then matched based on the known mass of the cross-linked pair and scored as a pair against the spectrum. Isotope-labeled cross-linkers were used to sort the linear and cross-linked fragments apart. Many other search tools and approaches have been developed since (10, 15–19); see (20) for a more detailed list, at least some of which follow the general idea of an open modification search (21–24).

As a general concept for open modification search of cross-linked peptides, cross-linked peptides represent two peptides, each with an unknown modification given by the mass of the other peptide and the cross-linker. One identifies both peptides individually and then matches them based on knowing the mass of cross-linked pair (14, 22, 24). Alternatively, one peptide is identified first and, using that peptide and the cross-linker as a modification mass, the second peptide is identified from the database (21, 23). An important element of the open modification search approach is that it essentially converts the quadratic search space of the cross-linked peptides into a linear search space of modified peptides. Still, many peptides and many modification positions have to be considered, especially when working with large databases or when using highly reactive cross-linkers with limited amino acid selectivity (25).

We hypothesize that detailed knowledge of the fragmentation behavior of cross-linked peptides might reveal ways to improve the identification of cross-linked peptides. Detailed analyses of the fragmentation behavior of linear peptides exist (26–28), and the analysis of the fragmentation behavior of cross-linked peptides has guided the design of scores (24, 29). Further, cross-link-specific ions have been observed from higher energy collision dissociation (HCD) data (30). Isotope-labeled cross-linkers are used to distinguish cross-linked from linear fragments, generally in low-resolution MS2 of cross-linked peptides (14).

We compared the mass spectrometric behavior of cross-linked peptides to that of linear peptides, using 910 high-resolution fragment spectra matched to unique cross-linked peptides from multiple different public datasets at 5% peptide-spectrum match (PSM)^1^ false discovery rate (FDR). In addition, we repeated all experiments with a larger sample set that contains 8,301 spectra—also including data from ongoing studies from our lab (Supplemental material S9-S12). This paper presents the mass spectrometric signature of cross-linked peptides that we identified in our analysis and the resulting heuristics that are incorporated into an integrated strategy for the analysis and identification of cross-linked peptides. We present computational strategies that indicate the possibility of alleviating the need for mass-spectrometrically restricted cross-linker choice.

## EXPERIMENTAL PROCEDURES

### 

#### 

##### Spectra Collection and Filtering

We collected database search results from experiments that were acquired and described in previous publications (31–33) (Pride: PXD002142, PXD001835, PXD001454) and accumulated cross-linked and linear peptide spectrum matches (PSMs). All data were acquired in CID mode on hybrid linear iontrap-Orbitrap mass spectrometers (LTQ Orbitrap Velos, Thermo Scientific, Bremen, Germany). The cross-linker in all searches was bis(sulfosuccinimidyl)suberate or its isotopic variant bis(sulfosuccinimidyl)suberate-d4. A typical search was performed using Xi (ERI Edinburgh, UK) and the following parameters: MS accuracy, 6 ppm; MS/MS accuracy, 20 ppm; enzyme, trypsin; maximum missed cleavages, 4; maximum number of modifications, 3; fixed modification, carbamidomethylation on cysteine; variable modifications, oxidation on methionine; and modification by the hydrolyzed or the ammonia reacted cross-linker on lysine, serine, threonine, tyrosine, and the protein N terminus. Cross-linking was allowed to involve lysine, serine, threonine, tyrosine, and the protein N terminus. To ensure the analysis of high-quality data, we extracted 910 PSMs to unique cross-linked peptides at a 5% FDR cutoff using XiFDR (v. 1.0.4.13, (31)). Along with the 910 cross-linked PSMs, we extracted 4,161 linear PSMs from the cross-linking acquisitions as a reference data set for linear peptides. Detailed information about each PSM is available in the Supplemental Table S1 along with the annotation of the cross-linked peptides (Supplementary File S2). In addition, we repeated all experiments with a larger sample set that contains 8,301 spectra—also including data from ongoing studies from our lab (Supplemental material S9-S12). To provide a comparison on the specific mass-spectrometric properties, we included search results from a linear peptide identification experiment using MaxQuant from a cyclin-dependent kinase (CDK)-regulated chicken chromatin dataset (34) on our machine with 1% FDR.

##### Data Extraction

Software written in Python (2.7, www.python.org) was used to extract relevant fragmentation information from the local PostgreSQL database containing details about search settings and spectra annotations. For each PSM involving a cross-linked peptide, the match score, peptide sequence (alpha and beta), precursor charge, experimental mass, and cross-link position (alpha and beta peptide) were extracted. In addition, the identified fragments were stored with each PSM. For each fragment, the *m/z*, charge, fragment type, intensity, and associated isotope cluster information were stored. When isotope clusters were identified, the summed intensity over all isotope peaks was used instead of the intensity of the monoisotopic peak. Similarly, linear PSMs were extracted. After extracting all fragments, the intensity for each fragment was normalized by division by the most intense fragment from the respective PSM. In addition, the respective intensity rank for each matched fragment was stored. A high rank refers to a high intensity and a rank of one to the lowest intensity in that PSM. For example, a spectrum containing three matched peaks with fictive intensities (10, 3, 1) was first normalized by the base peak to arrive at (1, 0.3, 0.1). Then, the ranks were derived such that the intensities are converted to (3, 2, 1). To compare the ranked intensity among peptides of different length (as done in Figs. 3*A* and 3*B*), the rank was further normalized by the number of matched peaks per spectrum. Thereby, the highest intense peak received a normalized rank value of 1. For the fictive example, this led to peak intensities of (1, 0.66, 0.33). We then compared b- or y-ion intensities for fragments in relation to the linker position or the peptide length, disregarding the specific ion index information (*e.g*. y7).

##### Similarity Computation of Linear and Cross-Linked Spectra

The similarity comparison of two spectra was realized via an adapted ranked dot product scoring scheme. The ranked dot product is usually used in spectral library searches where acquired spectra are compared *versus* annotated spectra from previous database identifications (35). Here, we define the ranked dot product as follows:

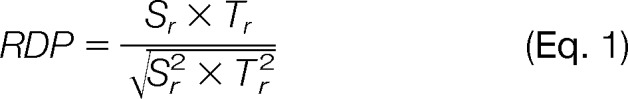
 where *S_r_* × *T_r_* is the scalar product of the two vectors *S_r_* and *T_r_* that represent the identified ions of the source and target peptide, respectively. Usually, the vectors *S_r_* and *T_r_* contain binned intensity values from the observed spectrum and the target spectrum from the spectral library. Here, we adapted the scoring scheme such that only nonlossy, b- and y-ion intensities were compared. For example, for a peptide of length five, the vectors *S_r_* and *T_r_* have length eight. Moreover, instead of using actual intensity values, we replaced intensity values by intensity ranks (35). If a specific ion type was present in the source but not in the target peptide, the intensity for that particular ion in the target peptide was set to zero and *vice versa*. Otherwise, the intensity for each ion was derived via its rank. To evaluate the scoring behavior, a reference similarity distribution of random pairings of cross-linked peptides was computed. The reference distribution was derived by computing the similarity of 1,000 random peptide combinations. We made sure that no comparison of peptides with the same sequence is included. The resulting random score distribution was used to evaluate all other score distributions.

##### Evaluation of the Predictive Power to Distinguish Linear and Cross-Linked Fragments

Based on the ground truth of 910 PSMs, we evaluated the predictive power of the relative fragment mass and the charge state as indicators whether or not a fragment is cross-linker containing. The applied constraints were the fragment mass divided by the precursor mass, the charge state of the fragment, and the combination of both. Only fragments with isotope clusters were used for this analysis. The performance of the classification was evaluated via the sensitivity, defined as *sn* = $\frac{TP}{TP+FN\prime }$, and the specificity, defined as *sp* = $\frac{TP}{TP+FP\prime }$, where a true positive (*TP*) is a fragment that was annotated as cross-linker containing and is also predicted as such, a false positive (*FP*) is a fragment that was annotated as linear but was predicted as cross-linked, and a true negative (*TN*) is a fragment that was annotated as linear and was also predicted linear. Lastly, a false negative (*FN*) is a fragment that was annotated as cross-linked but was not recognized as such.

## RESULTS AND DISCUSSION

### 

#### 

##### Mass and Charge of Cross-Linked Peptides Can Be Used to Direct Data-Dependent Acquisition

Digestion of cross-linked proteins yields both linear and cross-linked peptides. We wondered if the signals of cross-linked peptides either in MS1 or MS2 differed systematically from those of linear peptides. Note that we are focusing here on the most frequent form of cross-linked peptides: tryptic peptides that are cross-linked via lysine residues or serine, threonine, and tyrosine.

**Fig. 1.**
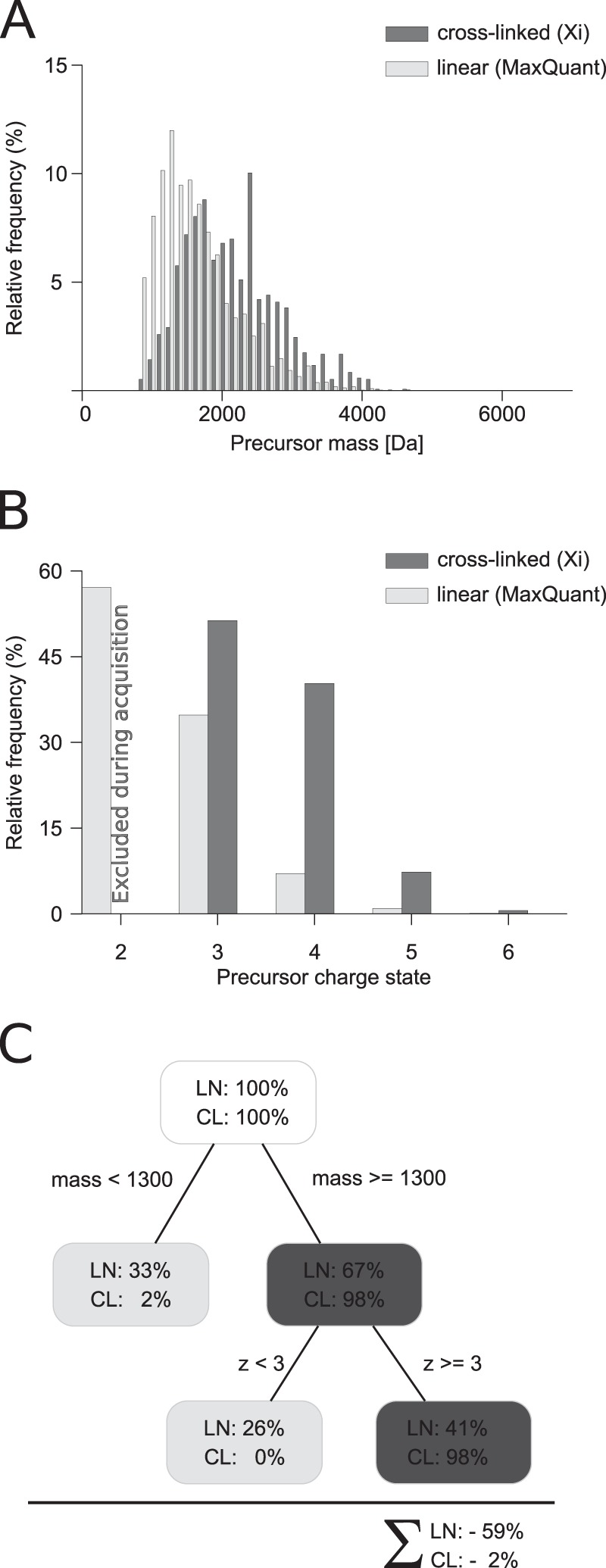
**Precursor properties of linear and cross-linked peptides.** (*A*) Comparison of precursor masses from linear and cross-linked identifications. (*B*) Comparison of the charge state from cross-linking acquisitions (charge state 1 and 2 were excluded during acquisition) and noncross-linked acquisitions (charge state 1 was excluded). (*C*) Decision tree to enrich for cross-linked peptides. The cross-linking results are derived from 1,255 PSMs identified with a 5% false discovery rate and a minimum peptide length of 4. The linear identifications contain 14,361 PSMs with a 1% false discovery rate.

The precursor masses of (tryptic, Lys/Ser/Thr/Tyr-linked) cross-linked peptides and (tryptic) linear peptides have a large overlap in their mass distribution (Fig. 1*A*). However, in the margin area, *i.e.* considering all masses up to 1,300 Da, linear peptides are more frequently observed than cross-linked peptides. Given a mass cutoff of *e.g.* 1,300 Da, it is possible to reduce the complexity of the sample dramatically, *i.e.* 33.3% of the linear spectra can be disregarded. This benefit comes with a loss of 2% in unique cross-linked peptides. Often, these hits are disputable because both or one of the peptides in the cross-linked product is rather short. In these cases, reliable identification is usually not possible. Thus, restricting acquisition to precursors above 1,300 Da appears a viable strategy to enrich for cross-linked peptides. Cross-linked peptides having a larger size than linear peptides can be rationalized by them being a pair of peptides. In addition, a protease-cleavage site is frequently blocked when using lysine-reactive cross-linkers and trypsin. Cross-linked peptides would then be expected to be a pair of peptides each having a missed cleavage site and thus in total four-times the mass of a linear peptide on average. This was already utilized in sample preparation by enriching for cross-linked peptides in size exclusion chromatography (10).

Cross-linked peptides are often higher charged than linear peptides (Fig. 1*B*), as was noted based on smaller sample sizes previously (9, 14). We investigated this here in detail based on our set of 910 PSMs. All our data in cross-link analyses were acquired excluding charge states 1 and 2, based on our initial observations (9). Therefore, nothing more can be said here on the occurrence of cross-linked peptides in these charge states. Looking at linear peptides from noncross-linked samples, more than half (57%) are doubly charged. This supports the current strategy of at least excluding doubly charged precursors during data acquisition (9, 14). Adding triply charged precursors to the exclusion (14) further improves on this by removing an additional 35% of linear peptides. However, excluding triply charged precursors from fragmentation analysis also reduces the number of identified cross-linked peptides by almost half (48%). Given this considerable loss of cross-linked peptides, it appears advisable to exclude only doubly and not also triply charged precursors from the analysis, at least when working with ionization conditions similar to ours (9).

In summary, an enrichment of 2.3-fold could be achieved for cross-linked over linear peptides. This is based solely on MS1 peak characteristics and comes at no additional experimental costs. It should be noted that this is comparable and possibly complementary to the fold enrichment achieved by the currently widely used chromatographic enrichment strategies, strong cation exchange (9) or size exclusion chromatography (10) for cross-linking experiments. In chromatographic methods, about 50% of the linear peptides never reach the mass spectrometer. In the acquisition-based approach, they do but are not selected for MS2.

##### Mass and Charge Reveal the Cross-Link Status of Fragments without Using Isotopes

**Fig. 2.**
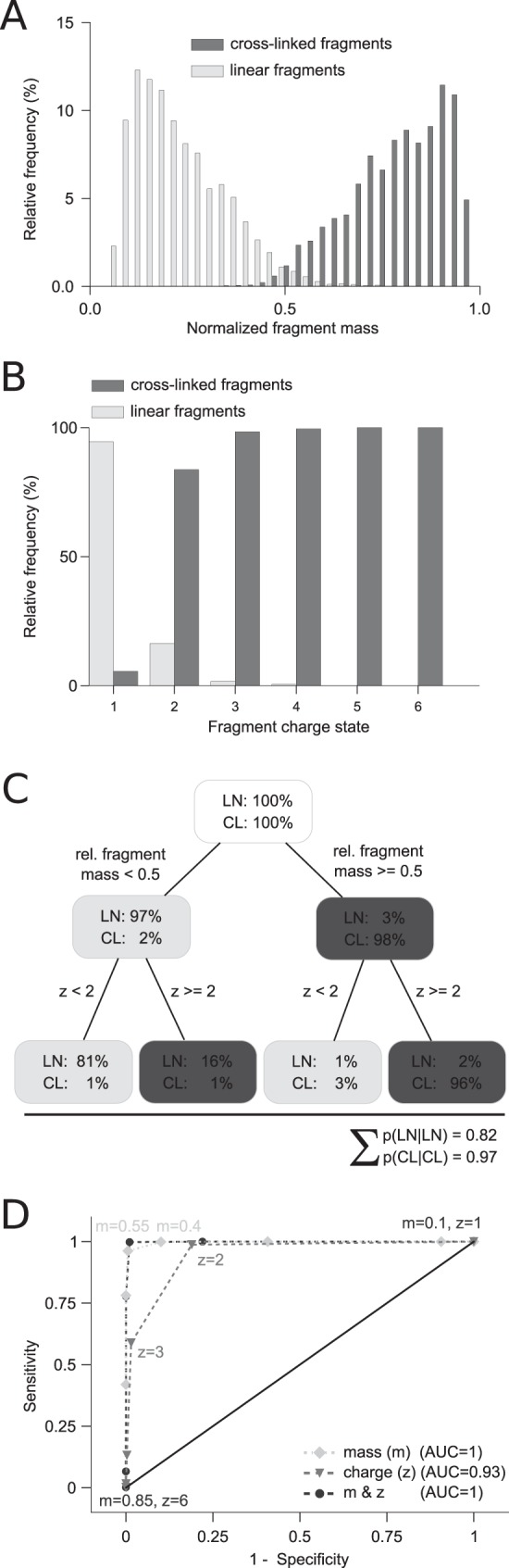
**Fragment properties of cross-linked peptides.** (*A*) Comparison of cross-linked and linear fragment masses of cross-linked peptides normalized by their precursor mass. (*B*) Distribution of assigned charge states from isotope clusters distinguished in cross-linked and linear fragments of cross-linked peptides. (*C*) Decision tree visualizing the process to decide if a fragment is cross-linked or linear based on charge and mass. (*D*) Receiver operating characteristic curve showing the sensitivity (*TP*/(*TP* + *FN*)) and specificity (*TN*/(*FP* + *TN*)) for assigning a cross-linked fragment as cross-linked and linear fragments as noncross-linked. Thresholds are annotated and based on charge and/or mass. The data were derived from 910 high-confidence identifications with a 5% false discovery rate (FDR) and a minimum peptide length of 6. Abbreviations: TP, true positives; FN, false negatives; TN, true negatives; FP, false positives.

Extending the mass and charge analysis to fragments (Fig. 2) leads to the observation that linear fragments can be distinguished from cross-linked fragments with high confidence. We define the normalized fragment mass as the fragment mass divided by the precursor mass. Looking at the normalized fragment mass reveals that the distributions for cross-linked and linear fragments are very well separated. Linear fragments tend to have a smaller mass than 50% of the precursor mass. In contrast, cross-linked fragments tend to have a larger mass than 50% of the precursor mass. Very few linear fragments (2.5%) and cross-linked fragments (1.6%) are not following this rule. Consequently, the mass-based prediction is highly successful. Setting the decision boundary to 50% precursor mass yields a sensitivity of 0.99 and a specificity of 0.97. The corresponding receiver operating characteristic curve yields an area under the curve of 0.996 (Fig. 2*D*) by relative fragment mass alone.

In addition to the fragment mass, the charge state distribution differs for linear and cross-linked fragments (Fig. 2*D*). Essentially all fragments with charge state one are linear. Similarly, the vast majority of fragments that are triply or higher charged are cross-linked. If the fragment is doubly charged, the probability that the fragment is cross-linked is four-times higher than being linear. Hence, cross-linked and linear fragments can be very well separated by evaluating the charge state of the fragment, reaching a sensitivity of 0.98 and a specificity of 0.81, respectively. The charged-based prediction yields an overall area under the curve of 0.93. A combined approach of normalized fragment mass and charge state to detect cross-linked fragment species provides additional resolving power and increases the area under the curve to ∼1 (Fig. 2*D*).

One of the first search algorithms for the identification of cross-links by database searching builds on the idea of knowing which fragments are linear and which are cross-linked (14). The cross-link status of the fragments was assessed through isotope labeling. Using isotope-labeled cross-linker, cross-linked fragments experience a mass shift in the fragmentation spectra of light and heavy cross-linked peptides. In contrast, linear fragments are observed with identical mass in both fragmentation spectra. While being intriguing, this approach for determining the cross-link status of fragments has a number of inherent setbacks: (1) Both peaks of a labeled cross-linked peptide have to be selected for fragmentation; selecting only one does not yield the required information. (2) The MS1 signal of the cross-linked peptide is split into two, whereas other peptides are seen with their original intensity. (3) The choice of cross-linker is limited. (4) Any use of isotope labeling increases the complexity of the sample. (5) Use of isotopes for this purpose complicates their use for quantitation. We here present an alternative to isotope labeling for high-resolution fragmentation spectra. If the fragment charge can be determined and thus also the fragment mass, isotopes are not needed to determine the cross-link status of fragments. By using high-resolution data, the search algorithm can benefit for free from the confident distinction of linear and cross-linked fragments. This leaves isotopes for quantification of cross-links (31).

##### Cross-Linked Peptides Fragment Similar to the Corresponding Linear Peptides

**Fig. 3.**
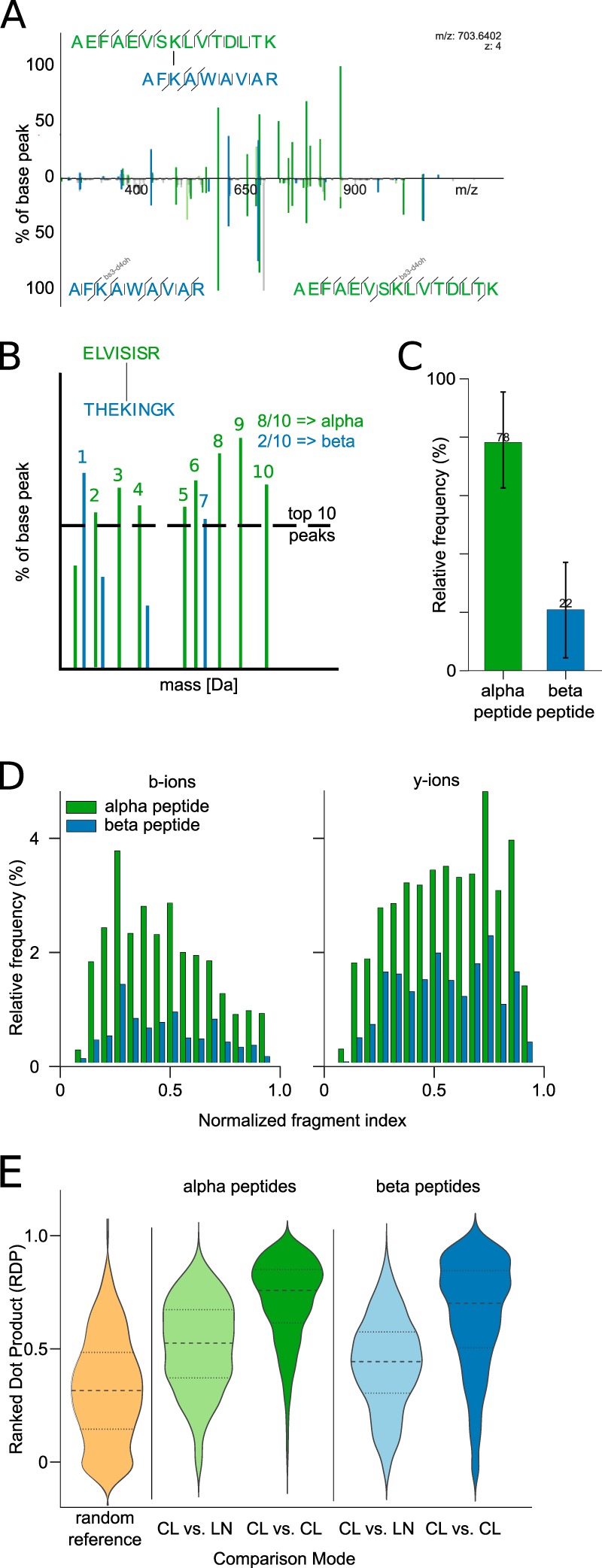
**Fragmentation patterns of cross-linked peptides.** (*A*) Spectral comparison of a cross-linked peptide (*upper part*) and an overlay of the individual linear peptide spectra (*lower part*). Equivalent fragments from the cross-linked peptide and the respective linear peptides have been aligned to facilitate direct comparison (Supplemental Fig. S6 shows the individual spectra with annotations). (*B*) Visualization of an idealized (hypothetical) cross-linked peptide spectrum that is divided into alpha and beta peptides. The alpha peptide is defined as the peptide that has more ions among the ten most intense ions. (*C*) Distribution of annotated fragment peaks among the ten most intense ions of identified ions. The height refers to the mean with the standard deviation as error bars. (*D*) Quantitative analysis of b- and y-ion fragment peak intensities of alpha and beta peptides, respectively. (*E*) Quantitative comparison of the spectral similarity between linear (LN) and cross-linked (CL) peptides. A reference distribution is derived by randomly comparing spectra of cross-linked peptides. The data for (*B–E*) was derived from 910 high-confidence identifications with a 5% false discovery rate (FDR). Abbreviations: CL, cross-linked; LN, linear.

Cross-linked peptides are expected to fragment like linear peptides to generate b- and y-ions under CID conditions. However, the extent to which this fragmentation is affected by the cross-link or the presence of two peptides in close proximity in the gas phase is not immediately clear. As a first step, we compared the fragmentation spectrum of the cross-linked peptide pair AEFAEVSKLVTDLTK–AFKAWAVAR with those obtained for both peptides individually (Fig. 3*A*, see Supplemental Fig. S6 for annotation of the individual spectra). For ease of comparison, the b- and y-ion signals of the noncross-linked peptides were moved to the same *m/z* value of the corresponding b- or y-ion in the cross-linked peptide. The two fragmentation spectra of the linear peptides together show a marked resemblance to the fragmentation spectrum of the cross-linked peptide pair, albeit some fragment yields are affected by the linkage. Furthermore, there was no dominant presence of double fragmentation observed. This means that despite a cross-linked peptide being more complex and having more parameters, its fragmentation follows essentially the same rules as apply to linear peptides. In essence, the cross-linked peptide fragmented like two linear peptides, each bearing the respective other peptide as a modification. This opens the prospect of at least initially dealing with both peptides individually during the identification process. Even if the final evaluation of matches should be done as a cross-linked pair, first candidates could be extracted from a linear instead of a quadratic search space.

Cross-linked peptide CID spectra contain fragments from two peptides but at unequal contribution. Usually, one of the two partners of a cross-linked peptide shows superior fragmentation, measured in the number of fragments and their intensities. Asymmetric sequence coverage of the two peptides in a cross-link has been observed previously, under HCD fragmentation conditions (30). We call the more dominant fragmented peptide the alpha peptide and the submissive peptide the beta peptide. Formally, the alpha peptide was defined as the peptide with more identified ions among the ten most intense peaks (Fig. 3*B*). On average, 78% of the fragments within the ten highest intense matched fragments are attributed to the alpha peptide (Fig. 3*C*). Alpha peptides show consistently higher intensities for b- and y-ions, whereas y-ions for both peptides are more intense than b-ions (Fig. 3*D*). As the two peptides differ in the intensity of their fragments, one could envision to use intensity as a means to separate the otherwise superimposed fragmentation spectra of both peptides of the cross-link. This suggests the possibility of separating the fragmentation spectra of alpha and beta peptides computationally, similarly to the use of MS2-cleavable cross-linkers experimentally (11, 12). MS2-cleavable cross-linkers, in addition, provide a route to the mass of the alpha and beta peptides but restrict the choice of the cross-linker. Also, normal cross-linkers cleave to some extent under HCD fragmentation at the bond between the cross-linker and the peptide (30). At least under our experimental conditions, this is a rare event (10% of cross-linked peptides) (Supplemental Table S8).

Investigating more systematically the fragmentation similarity of peptides in cross-links and their linear counterparts reinforced the above conclusions. A quantitative view at the spectral similarity was achieved through exhaustive comparisons of cross-linked and linear peptides through the ranked dot product. For the systematic assessment of the spectral similarity, we used the linear peptide identifications from cross-link database searches and compared the spectra to all cross-linked peptides with the same sequence (Fig. 3*E*). Peptides in cross-links display large spectral similarity to their linear counterparts, regardless if alpha or beta peptides are considered. However, subspectra for a peptide in a cross-link look more alike, independent of the partner peptide or link position, than to the spectra of the corresponding linear peptide. Beta peptides generally perform less well in these comparisons. They tend to have less intense ions and also fewer ions. This reduces the overlap of beta peptide fragment ions between spectra. With a higher overlap in fragment ions, the spectral similarity increases and *vice versa*. Factors that potentially influence the fragmentation are the charge state, cross-linked residue, or the partner peptide. Of these, the highest influence on the fragmentation behavior comes from the charge state with minor, but present, effects from the other factors (see Supplemental Fig. S4).

##### Uncross-Linking Peptides by Data Analysis Resolves the n^2^ Problem of Their Identification

In order to identify a pair of peptides that are cross-linked, one needs to consider the pairwise combination of all peptides in a database. As databases become bigger, this space grows quadratically. An exhaustive database construction could be avoided if a few candidates for at least one of the two peptides could be identified in a simplified first search. Ideally, one was to isolate the fragment peaks of one peptide. An adapted linear search can then retrieve candidates for this peptide without having to actually select a single one as the final match. Once having candidates for this peptide, one would know the mass of the corresponding second peptide, extract all mass matches from the original database of linear peptides, and construct a concentrated “bonsai” database of peptide pairs that would largely enrich for the cross-linked peptide. As observed above, intensity enriches fragment ions of the alpha peptide over those of the beta peptide. So, a stepwise extraction of candidates appears possible.

**Fig. 4.**
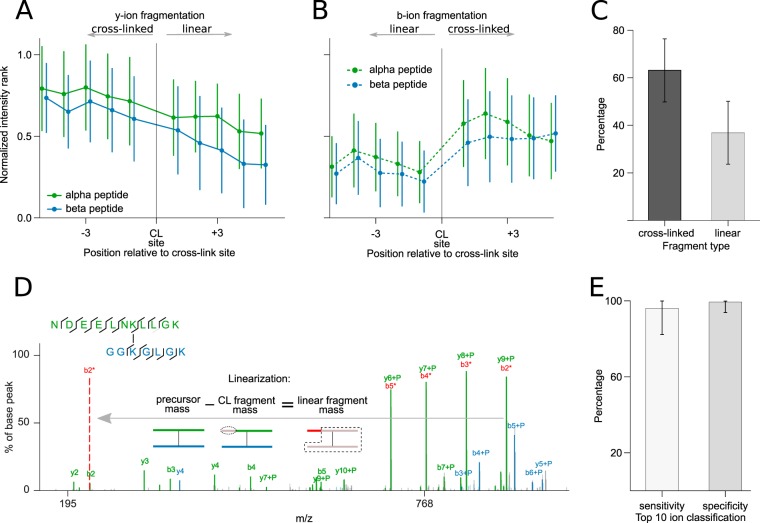
**Cross-linked peptide fragmentation patterns.** Influence of the cross-link site (CL site) on y-ion (*A*) and b-ion yield (*B*), respectively. Longer y-ions are located to the left of the cross-link site; longer b-ions are located to the right of the cross-link site. Fragment intensities were transformed to ranks, with high intensities having a higher rank, and then normalized by the number of fragments in a spectrum. Error bars correspond to the standard deviation of all measured intensities at a relative position. (*C*) Distribution of cross-linker containing and linear fragments in cross-linked peptide spectra, respectively. (*D*) Example spectrum reflecting preferred cleavage of cross-linked fragments and an exemplary linearization of the cross-linked y7-ion of the alpha peptide. As shown in the pictogram of the linearization process, the y9-ion is transformed to the b2 ion (which was also observed as low intense peak) by subtracting the fragment mass from the precursor mass. Similarly, the y8 ion can be transformed to the b3 ion, which is indicated by the annotation with a '*' in the spectrum. (*E*) Sensitivity and specificity of correctly assigned cross-linked and linear fragments by their charge and mass from the top ten identified ions. The underlying data were extracted from 910 PSMs at a 5% FDR. For bar plots, the height and error bars refer to the mean and the standard deviation of all evaluated PSMs. The linear alpha peptides are also shown in Supplemental Fig. S7.

Extracting candidates for the alpha peptide as a linear peptide without knowing its peptide mass is complicated by the intense presence of cross-linked fragments and by the presence of fragments of the beta peptide. The dominance of the alpha peptide suggests the possibility of extracting a subspectrum that enriches for fragmentation information of this peptide. This could be achieved by simply taking the ten most intense fragments. However, this means that one looks primarily at cross-linked fragments (Fig. 4). For y-ions, longer fragments were seen with higher intensities (Fig. 4*A*). This favors cross-linked fragments that tend to be larger. For b-ions, there is no continuous effect. Instead, the cross-link site appears to exert a direct effect, leading primarily to cross-linked b-ions (Fig. 4*B*). The apparent influence of the link site on b-ions can be mechanistically explained through the presence of the second peptide. Charge in fragments is primarily carried by basic residues. y-ions of tryptic peptides have one by default at their C terminus. b-ions lack this terminal basic residue. However, cross-linked b-ions are modified by the second peptide. In this way, like y-ions they carry a C-terminal basic residue. The general dominance of cross-linked fragments (Fig. 4*C*) complicates the identification of the alpha peptide as they can only be used if the modification mass is known. However, this mass is inaccessible. The only solution would be to uncross-link the fragments.

Importantly, cross-linked fragments can be converted during data processing into their linear counterparts. Above, we established a reliable method to distinguish signals of cross-linked and not cross-linked fragments. The challenge is in converting cross-linked fragments into not cross-linked ones. Interestingly, any fragment also carries with its mass the information of the matching counterpart that is missing to make the whole peptide. Looking at this relation as a formula and resolving this formula to the missing fragment defines the mass of the fragment as the mass of the peptide less the mass of the observed fragment. If the peptide is cross-linked and the fragment is as well, then the missing fragment is linear. In this way, we can convert a cross-linked fragment into a linear fragment. Since cross-linked fragments are generally observed more frequently (Fig. 4*C*), the linearization step provides a valuable information gain. As all fragment ions are linearized, the matching of fragments can be done entirely free of having to consider cross-links. In consequence, the processed MS2 spectrum contains the fragments of two linear peptides and thus provides much of the value of CID-cleavable cross-linkers.

The linearization is straightforward and highly reliable. For instance, the alpha peptide fragment ions y6+P, y7+P, y8+P, and y9+P (+P refers to the cross-linked partner peptide) (Fig. 4*D*) fulfill the 50% precursor rule, *i.e.* their fragment mass is larger than 50% of the precursor mass (note that they are the base peaks). To remove the dependence of P—and perform a simple linear matching—the cross-linked ions need to be linearized: The y6+P-ion is converted into its complementary b5-ion. y7+P becomes b4, y8+P becomes b3, and y9+P becomes b2. Note that the b-ions were also observed on their own in our example spectrum. However, this is not always the case, and they would not have made it under the ten most intense ions on their own. After the linearization, we only have linearized fragments in the spectrum that can be matched by standard database search approaches. We established above that 60–100% of the top ten matched peaks in the fragmentation spectrum of cross-linked peptides derived from alpha peptide fragmentation. Among these, we detected the cross-linked fragments with high success (∼98% average per spectrum) by their charge or mass alone (Fig. 4*E*). In consequence, we can extract a subspectrum that is largely enriched in linear fragments of the alpha peptide, thus substituting further aspects of CID-cleavable cross-linkers.

Open modification search also resolves the *n*^2^ problem, but it is still necessary to look for large modification masses. Knowing which fragments are cross-linked (and knowing how to linearize them) allows us to simplify the open modification search paradigm: Instead of considering wide gap mass ranges or all possible modification sites, a standard linear search is sufficient to identify candidates for at least one of the two peptides in the cross-linked peptide. Considering open modifications is computationally expensive. Possibly as a consequence, the prevalence of secondary cross-link reactions, *i.e.* serine, threonine, or tyrosine cross-links with bis(sulfosuccinimidyl)suberate, are generally neglected (30). Our results show that these reactions make up ∼14% of all cross-links and thus contribute largely to the outcome of an analysis (Supplemental Fig. S3). Identifying peptides with multiple cross-link sites becomes even more challenging if photoactivatable cross-linkers, such as sulfosuccinimidyl 4,4′-azipentanoate (sulfo-SDA) are used (36). Sulfo-SDA links some nucleophilic amino acids (lysine, serine, threonine, tyrosine, and the protein N terminus) with any other amino acid by having a standard N-hydroxysuccinimide (NHS)-activated ester on one side and a highly reactive diazirine group on the other. Therefore, open modification search paradigms would need to consider almost as many linkable residues as there are amino acids in the peptide to generate the right theoretical spectrum for each cross-linkable site. Existing search engines could utilize the highly reliable linearization process to avoid the probing of all possible cross-link sites.

##### An Integrated Search Strategy for Cross-Linked Peptides

With the above observations and concepts in hand, an integrated search strategy becomes possible. The quadratic search problem of cross-linked peptides can be simplified if the database size is decreased. Instead of combining exhaustively all peptides of the database, we first identify a set of candidates for one peptide. In a second step, all peptides can be extracted from the database that complete these candidates to obtain the mass of the observed cross-linked peptide. Combining these two sets of candidate linear peptides gives a focused database of candidate cross-linked peptides. The final identification is done against this largely reduced database. The stepwise candidate extraction is facilitated by the asymmetric fragmentation yield of cross-linked peptides. One peptide tends to give more intense fragment signals. An intensity cutoff can enrich, therefore, for information of one peptide in a simplified subspectrum comprising the *n* most intense peaks, *e.g. n* = 10. Unfortunately, cross-linked fragments contribute the majority to this subset of signals. However, using charge and relative mass as indicators, these can confidently be revealed and then converted into linear fragments. This removes any dependence of fragments on knowing the other peptide. Candidates for the first peptide can now be identified based on linear fragment data alone. Having a small set of candidates of the first peptide allows calculating the mass of the respective partner peptide candidates by simple algebra from the mass of the cross-link. Consequently, candidates for the second peptide can be extracted from the database by mass look-up. In this way, an initial set of peptide pair candidates is generated guided by data rather than following exhaustive combination of all peptides in the database. Exhaustive database search in this hugely reduced peptide pair database then allows identifying the final match.

**Fig. 5.**
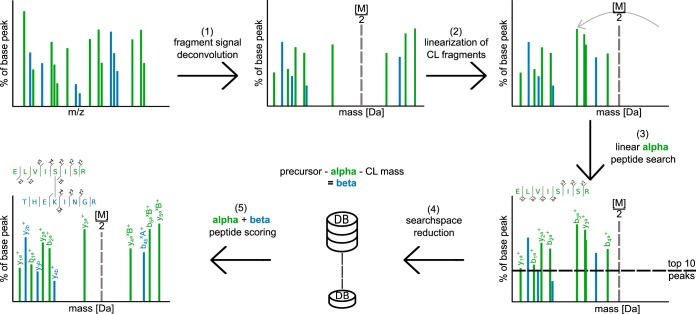
**A search strategy for the identification of cross-linked peptides based on their CID behavior.** (*1*) A mass spectrum is processed by peak picking, deisotoping, resolving losses, and decharging. (*2*) Putative cross-linked fragment peaks are converted to linear fragment peaks. (*3*) The top ten peaks are extracted and matched against a linear database version. (*4*) *n* candidates for the alpha peptide are extracted. For each alpha peptide candidate, *m* beta peptide candidates are extracted such that each alpha/beta pair adds up to the precursor mass. (*5*) The combined identifications of alpha and beta peptides are then scored together.

We have implemented this search strategy in Xi and used it successfully in several studies (33, 37–44). In concrete terms (Fig. 5), we start with the full spectrum of all peaks from a MS2 scan. After charge state assignment and removal of isotopic peaks (1) the linearization of alpha peptide candidate ions is performed (2). The decision whether or not a fragment is going to be linearized depends on the relative precursor mass and the charge. If either the relative precursor mass is > = 0.5 or the charge state > = 2, the given fragment will be linearized. After the linearization, the ten highest ion signals are selected for a dedicated linear database search for alpha peptide candidates (3). The first search is a means to extract a moderate number of candidates for the alpha peptide without knowing the mass or location of the cross-link. A small number of peaks is usually sufficient to extract the true alpha peptide as one of the candidates from the database. However, the actual identification is done on the full spectrum together with the beta peptide. The list of alpha peptides is used to generate corresponding beta peptides by a precursor mass filter (4). Corresponding beta peptides are extracted by subtracting the alpha peptide mass and the cross-linker mass from the measured precursor, as also explained above. Finally, the matching peptide pairs (alpha + beta peptide candidates) are reevaluated on the initial, untreated spectrum to localize the cross-link site and perform a scoring with all fragment ions present. Only in this step, the final alpha and beta peptide pairing/scoring is done. The final match for any given spectrum is the one with the highest scoring pair. After all spectra have been processed, separate FDR estimation needs to be performed. Elements of our stepwise identification have been described previously (21).

##### Influence of the Sample Size on Our Analysis

We analyzed the precursor and fragment information of 910 PSMs that were identified in a collection of published experiments conducted in our lab. In addition, we challenged the presented analysis with all ongoing studies of our lab—yielding a total of 8,301 PSMs—to question if our set of 910 PSMs was large enough to arrive at general conclusions (see Supplemental material S9-S12). For example, the enrichment possibilities during acquisition for cross-linked peptides have been investigated in Fig. 1—coming to the conclusion that a charge and mass based selection filter greatly enriches cross-linked peptides (Supplemental Fig. S9). Based on 910 PSMs our analysis arrives at excluding 59% of linear peptides at the expense of losing 7% cross-linked peptides. Based on all our data, we conclude 59% of linear peptides can be excluded at the expense of losing 4% cross-linked peptides. As a second example, to distinguish cross-linked from linear fragments, we introduced a mass cutoff of 50% precursor mass: In 910 PSMs, 2.5% of linear fragments and 1.6% of cross-linked fragments were not following this 50% rule. For the larger collection (8,301 PSMs), 2.25% of linear fragments and 1.8% of cross-linked fragments did not follow this rule. Finally, 77% of the top ten fragment peaks derive from the alpha peptide in 910 PSMs. This contrasts to 80% in 8,301 PSMs. Fragment peak intensity is hence a reliable filter to assign a subset of fragments to one of the two linked peptides. In summary, the 8,301 PSMs confirm the observations made on the basis of 910 PSMs, suggesting that our analysis was not limited by sample size.

## CONCLUSION

In this paper, we developed computational solutions to three experimental problems, building upon in-depth data mining of MS1 and MS2 properties of cross-linked peptides (1). The enrichment of cross-linked peptides is crucial to the success of cross-linking experiments. We show that focused acquisition can reach similar enrichment success for cross-linked peptides as chromatographic methods (2). Fragmentation spectra of cross-linked peptides contain fragments of two peptides. We find that fragments of the alpha peptide can be enriched through selection of the most intense peaks. Computationally, this parallels at least in part the use of MS2-cleavable cross-linkers. A benefit of doing this computationally is not relying on cross-linker properties and thus potentially being universally applicable (3). Finally, fragmentation spectra of cross-linked peptides contain linear and cross-linked fragments. We show that cross-linked fragments have a distinguishable signal in CID (mass and charge). Thus, there is no need for labeling strategies to recognize cross-linked fragments. Our resulting search strategy sees the linearization of cross-linked fragments to collect enough evidence to extract candidates for one of the cross-linked peptides before the other, an approach that avoids the large search space of cross-linked peptides. In conclusion, computational approaches prove highly valuable in complementing experimental strategies in the endeavor of simplifying the identification of cross-linked peptides.

## Supplementary Material

Supplemental Data

## Footnotes

^1^ The abbreviations used are:

PSM: peptide spectrum match.
