# Supplementary material for: A Study into the Collision-induced Dissociation (CID) Behavior of Cross-Linked Peptides
Source: Mol Cell Proteomics. 2015 Dec 30;15(3):1094–104. doi: 10.1074/mcp.M115.049296 (PMC4813691; doi:10.1074/mcp.M115.049296)
Supplement: Supplemental Data [file supp_15_3_1094__index.html]

A study into the CID behavior of cross-linked peptides — A Study into the Collision-induced Dissociation (CID) Behavior of Cross-Linked Peptides — CID Behavior of Cross-Linked Peptides — Supplemental Data 

# A Study into the Collision-induced Dissociation (CID) Behavior of Cross-Linked Peptides

## Supplemental Data

- Supplementary Material (.pdf, 2.2 MB) - Supplementary Material
- Supplementary Table S1 (.xlsx, 124 KB) - Supplementary Table 1 containing XiFDR results.
- Supplementary File S2 (.pdf, 4.1 MB) - Supplementary File S2 containg the annotated spectra.
