# Supplementary material for: A Study into the Collision-induced Dissociation (CID) Behavior of Cross-Linked Peptides
Source: Mol Cell Proteomics. 2015 Dec 30;15(3):1094–104. doi: 10.1074/mcp.M115.049296 (PMC4813691; doi:10.1074/mcp.M115.049296)
Supplement: Supplemental Data [file 10.1074_M115.049296_mcp.M115.049296-3.pdf]

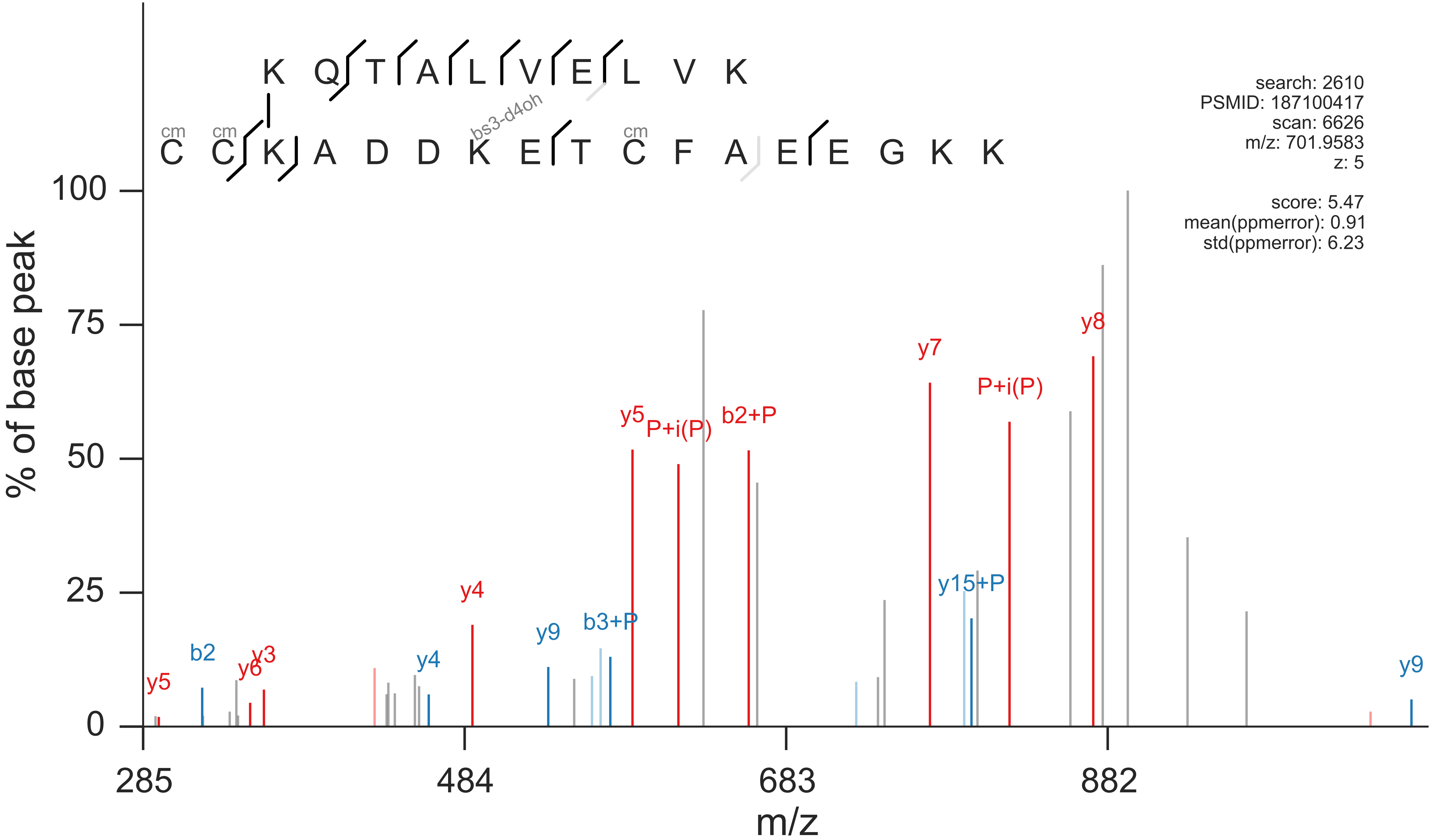
K Q T A L V E L V K
cm cm
C C K A D D K E T C F A E E G K K
cm
bs3-d4oh
search: 2610
PSMID: 187100417
scan: 6626
m/z: 701.9583
z: 5
score: 5.47
mean(ppmerror): 0.91
std(ppmerror): 6.23
% of base peak
m/z
0
25
50
75
100
285
484
683
882
y5
b2
y6
y3
y4
y4
y9
b3+P
y5
P+i(P)
b2+P
y7
y15+P
P+i(P)
y8
y9

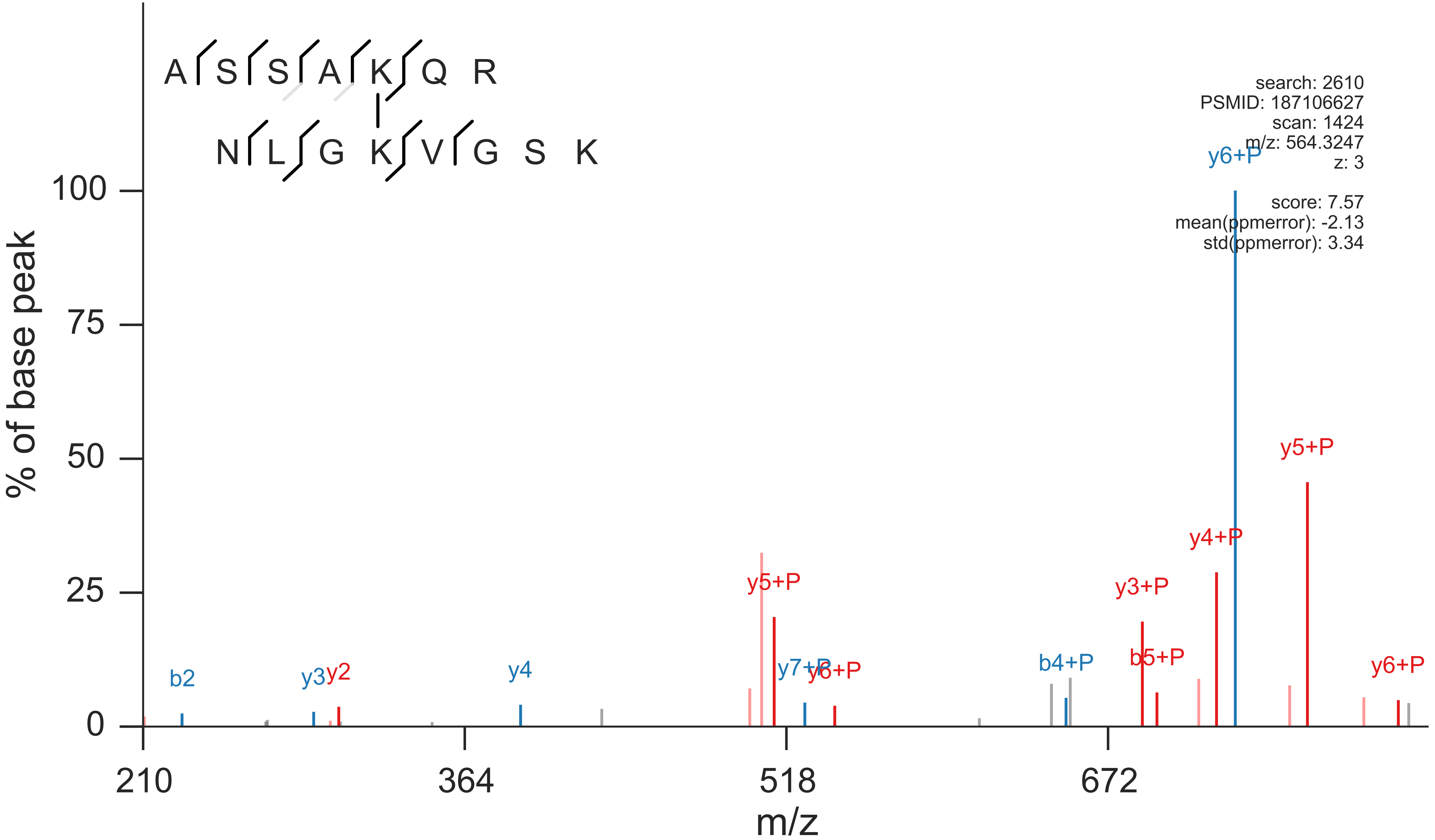
A S S A K Q R
N L G K V G S K
search: 2610
PSMID: 187106627
scan: 1424
m/z: 564.3247
z: 3
score: 7.57
mean(ppmerror): -2.13
std(ppmerror): 3.34
% of base peak
0
25
50
75
100
m/z
210
364
518
672
b2
y3
y2
y4
y5+P
y7+P
y6+P
b4+P
y3+P
b5+P
y4+P
y6+P
y5+P
y6+P

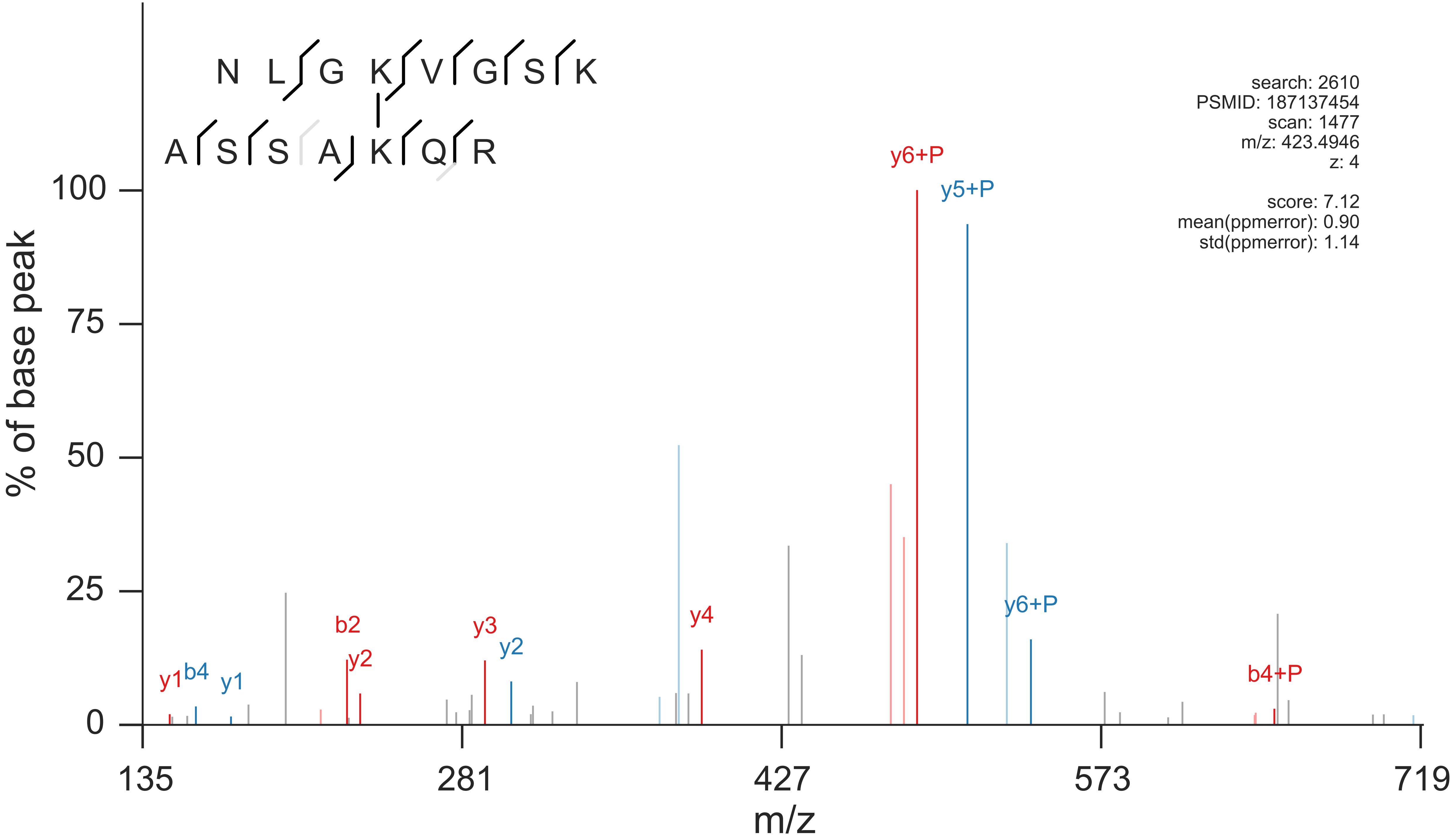
N L G K V G S K
A S S A K Q R
search: 2610
PSMID: 187137454
scan: 1477
m/z: 423.4946
z: 4
score: 7.12
mean(ppmerror): 0.90
std(ppmerror): 1.14
% of base peak
m/z
100
75
50
25
0
135
281
427
573
719
y6+P
y5+P
y6+P
b4+P
y4
y3
y2
b2
y2
y1
b4
y1

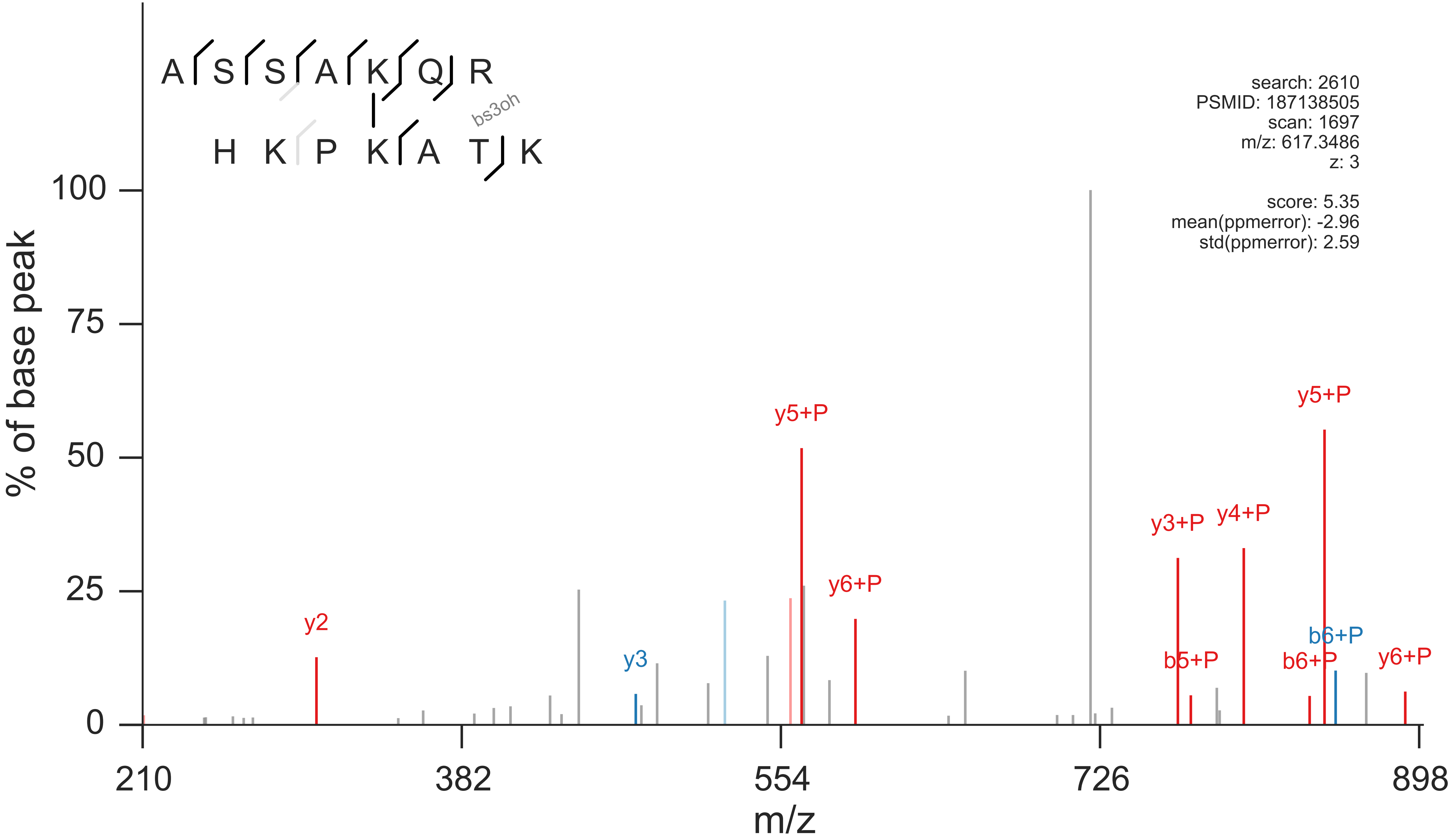
A S S A K Q R
H K P K A T K
bs3oh
search: 2610
PSMID: 187138505
scan: 1697
m/z: 617.3486
z: 3
score: 5.35
mean(ppmerror): -2.96
std(ppmerror): 2.59
% of base peak
100
75
50
25
0
210
382
554
726
898
m/z
y2
y3
y5+P
y6+P
y3+P
y4+P
b5+P
b6+P
y5+P
b6+P
y6+P

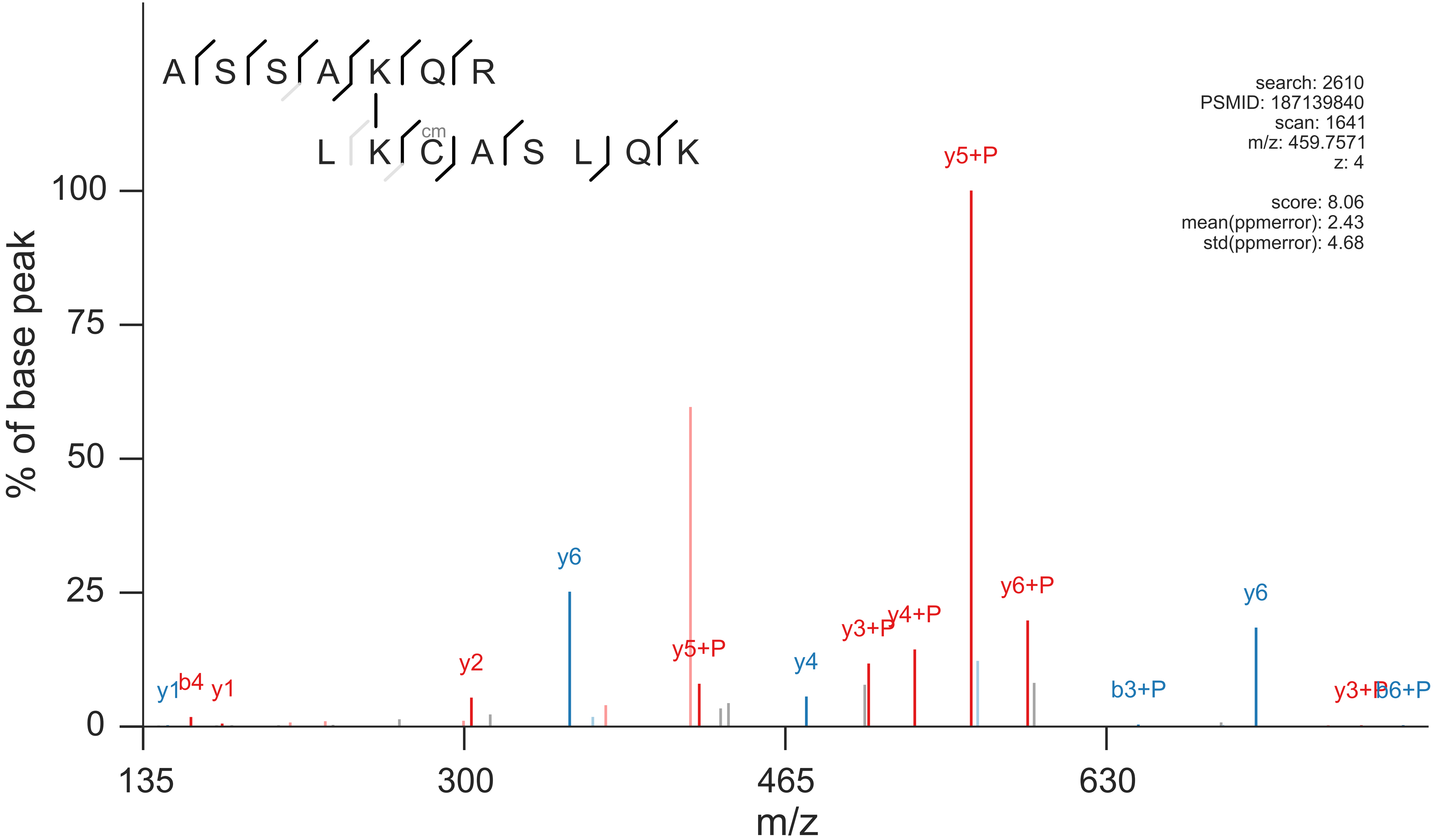
A S S A K Q R
L K C A S L Q K
cm
search: 2610
PSMID: 187139840
scan: 1641
m/z: 459.7571
z: 4
score: 8.06
mean(ppmerror): 2.43
std(ppmerror): 4.68
% of base peak
m/z
0
25
50
75
100
135
300
465
630
y1
b4
y1
y2
y6
y5+P
y4
y3+P
y4+P
y5+P
y6+P
b3+P
y6
y3+P
b6+P

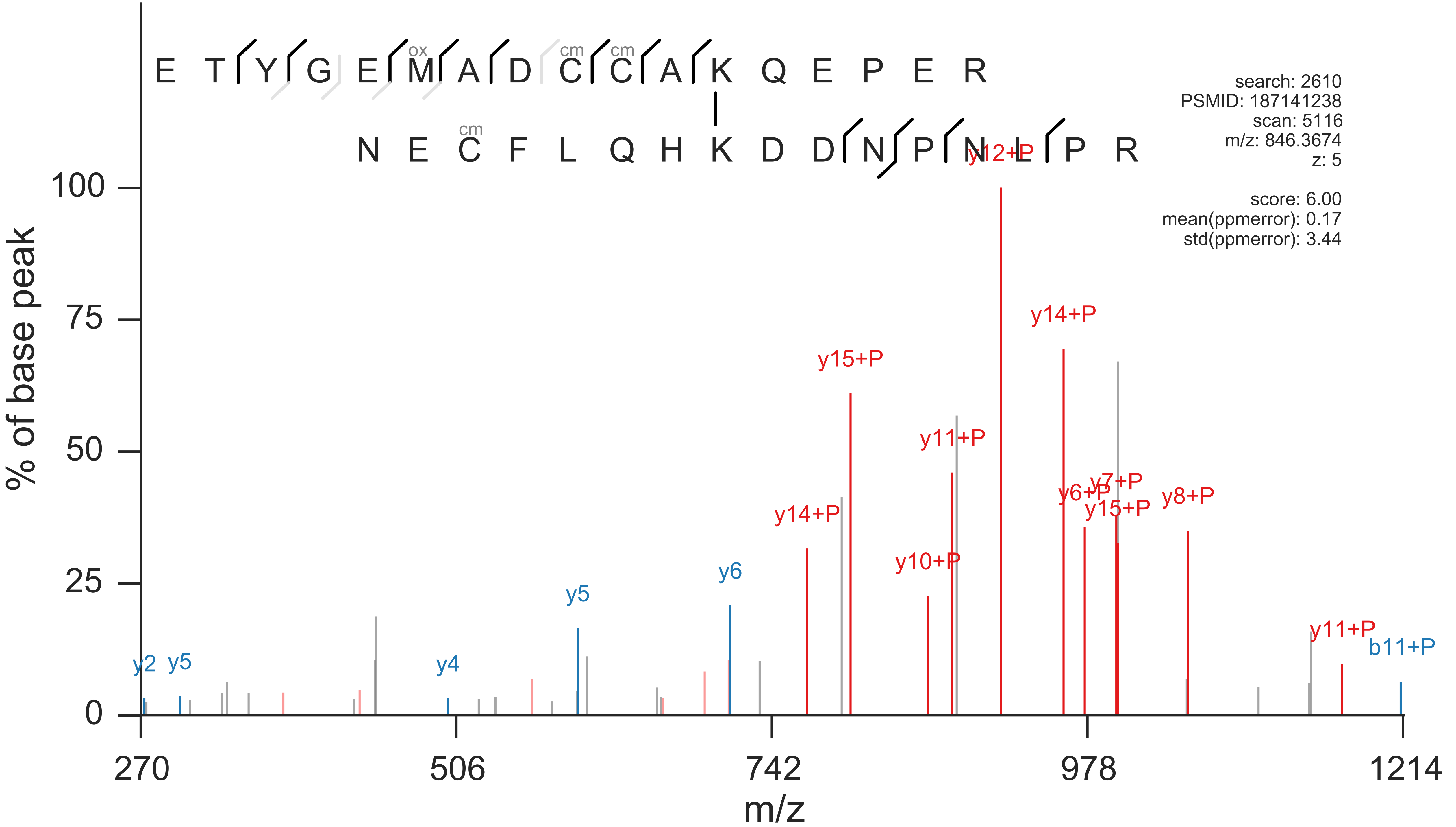

E T Y G E M A D C C A K Q E P E R
ox
cm
cm
N E C F L Q H K D D N P N L P R
cm
search: 2610
PSMID: 187141238
scan: 5116
m/z: 846.3674
z: 5
score: 6.00
mean(ppmerror): 0.17
std(ppmerror): 3.44
% of base peak
m/z
100
75
50
25
0
270
506
742
978
1214
y2
y5
y4
y5
y6
y14+P
y15+P
y10+P
y11+P
y12+P
y14+P
y6+P
y7+P
y15+P
y8+P
y11+P
b11+P

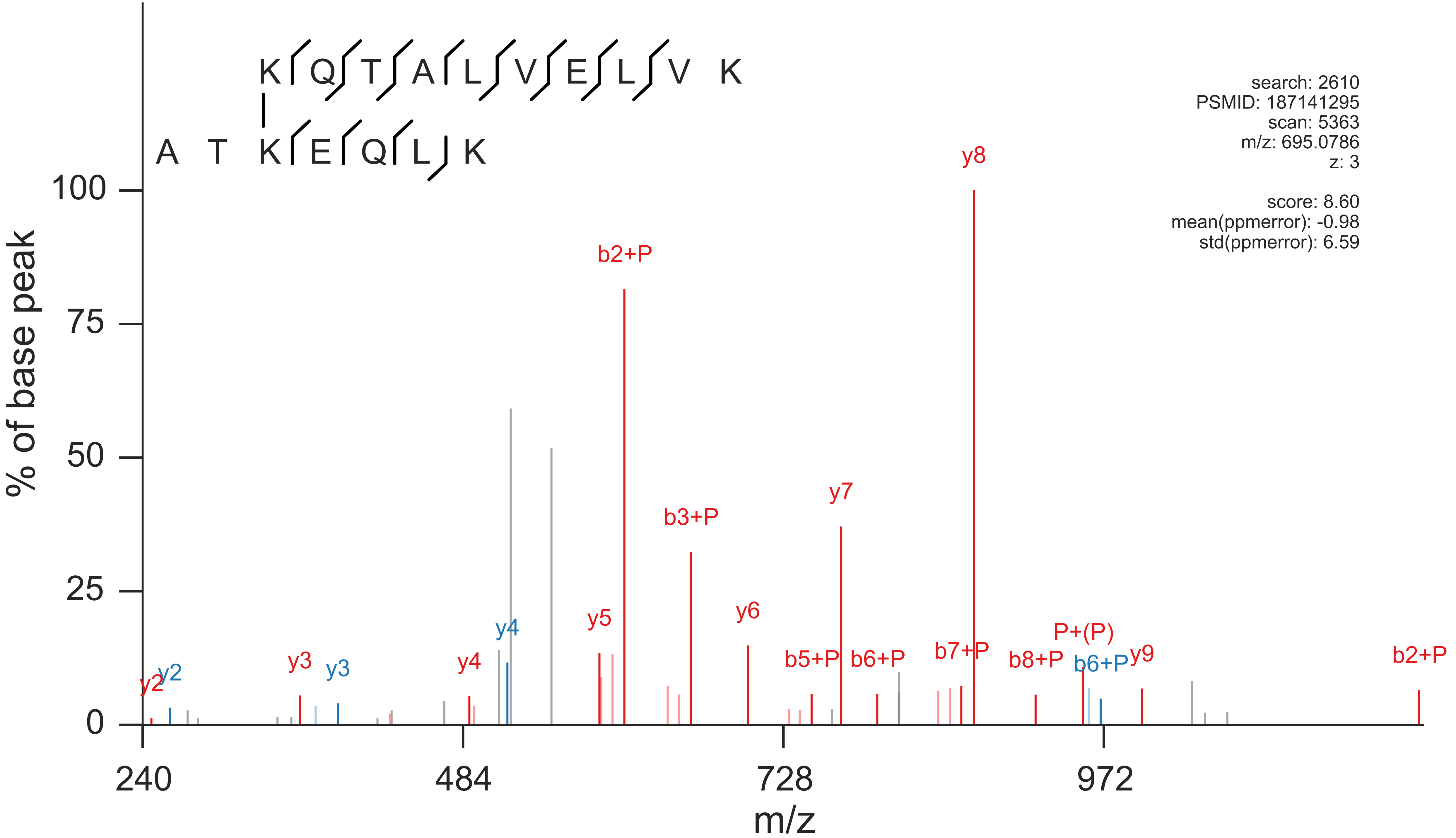

K Q T A L V E L V K
A T K E Q L K
search: 2610
PSMID: 187141295
scan: 5363
m/z: 695.0786
z: 3
score: 8.60
mean(ppmerror): -0.98
std(ppmerror): 6.59
% of base peak
m/z
y8
b2+P
y7
b3+P
y6
y5
y4
y3
y2
b5+P
b6+P
b7+P
b8+P
P+(P)
b6+P
y9

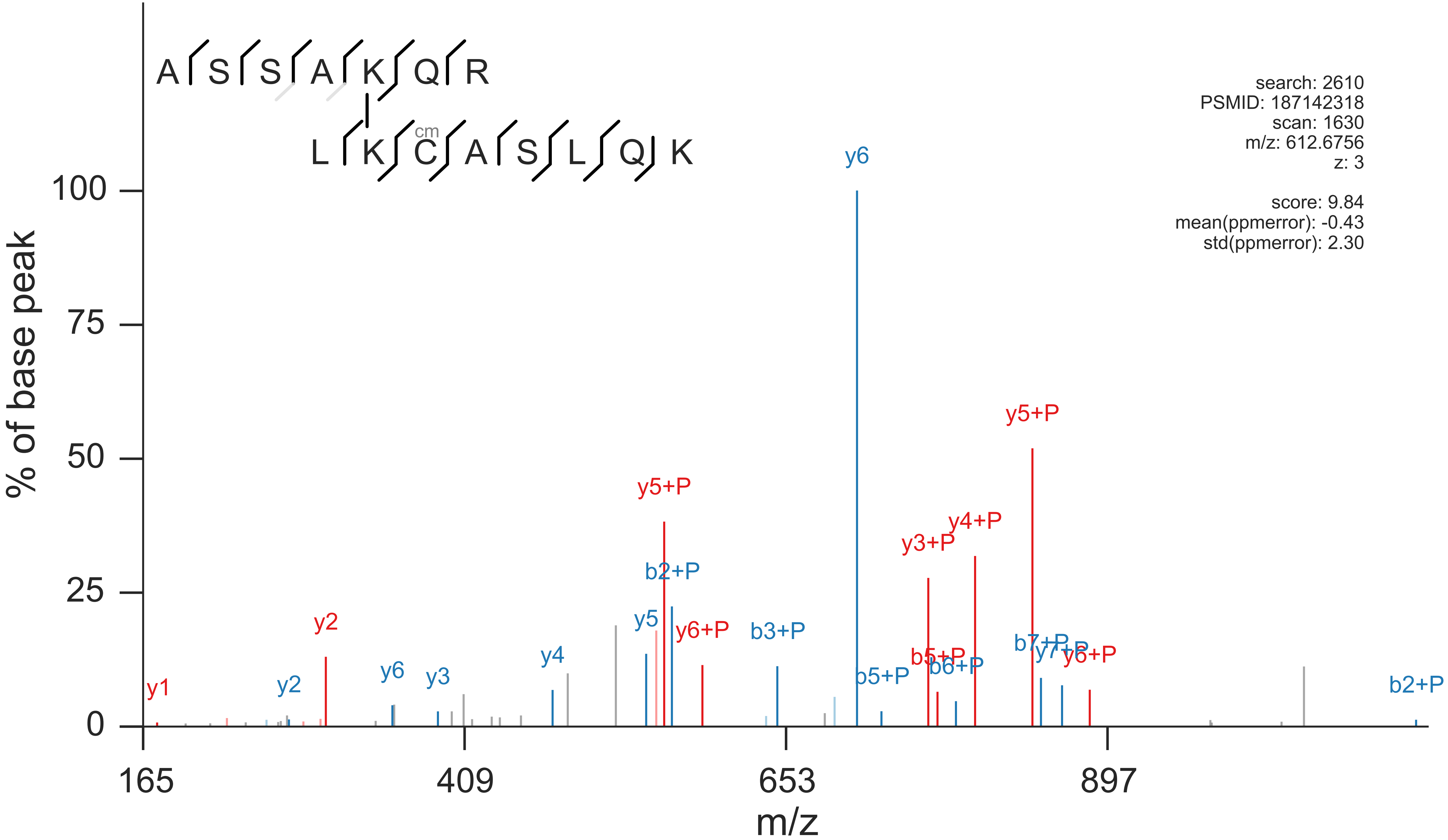

A S S A K Q R
L K C A S L Q K
cm
search: 2610
PSMID: 187142318
scan: 1630
m/z: 612.6756
z: 3
score: 9.84
mean(ppmerror): -0.43
std(ppmerror): 2.30
% of base peak
m/z
100
75
50
25
0
165
409
653
897
y1
y2
y6
y3
y4
y5
y5+P
b2+P
y6+P
b3+P
b5+P
y3+P
y4+P
b6+P
y5+P
b7+P
y7+P
y6+P
b2+P

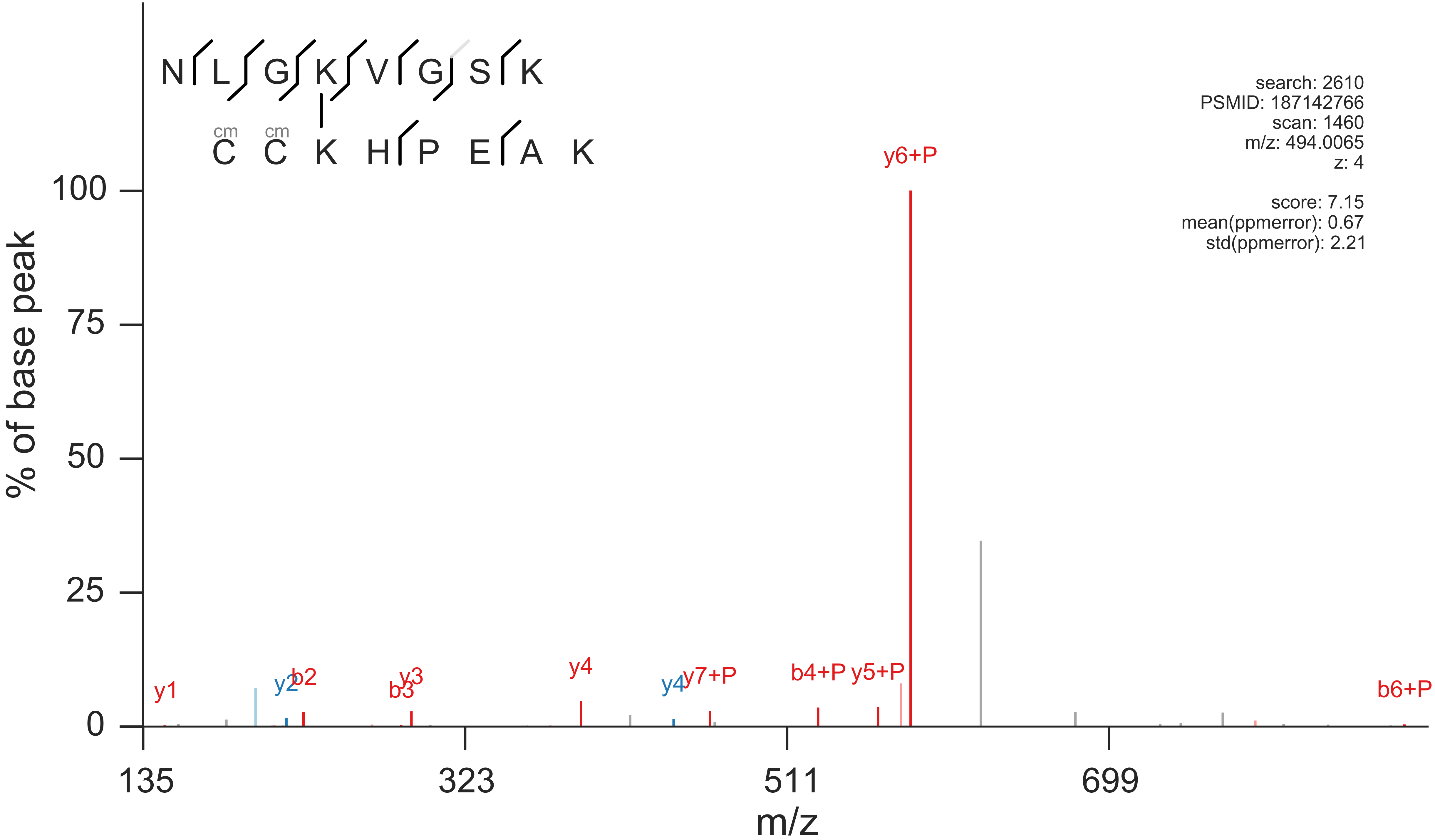

N L G K V G S K
C C K H P E A K
cm cm
search: 2610
PSMID: 187142766
scan: 1460
m/z: 494.0065
z: 4
score: 7.15
mean(ppmerror): 0.67
std(ppmerror): 2.21
y6+P
y1
y2
b2
b3
y3
y4
y4
y7+P
b4+P
y5+P
b6+P
% of base peak
100
75
50
25
0
135
323
511
699
m/z

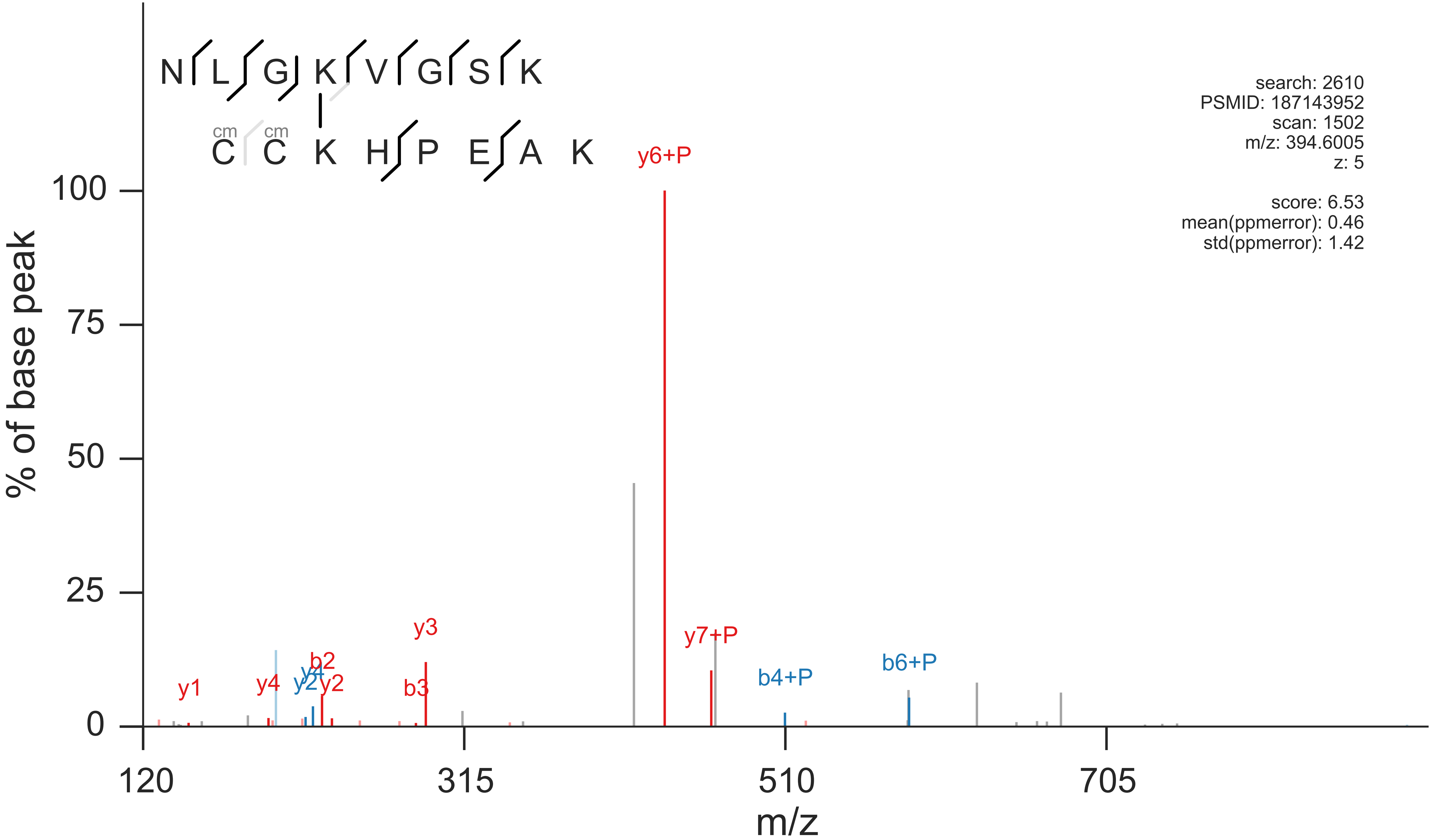

N L G K V G S K
cm cm
C C K H P E A K
search: 2610
PSMID: 187143952
scan: 1502
m/z: 394.6005
z: 5
score: 6.53
mean(ppmerror): 0.46
std(ppmerror): 1.42
y6+P
y7+P
b4+P
b6+P
y3
b3
b2
y4
y2
y2
y4
y1
% of base peak
100
75
50
25
0
120
315
510
705
m/z

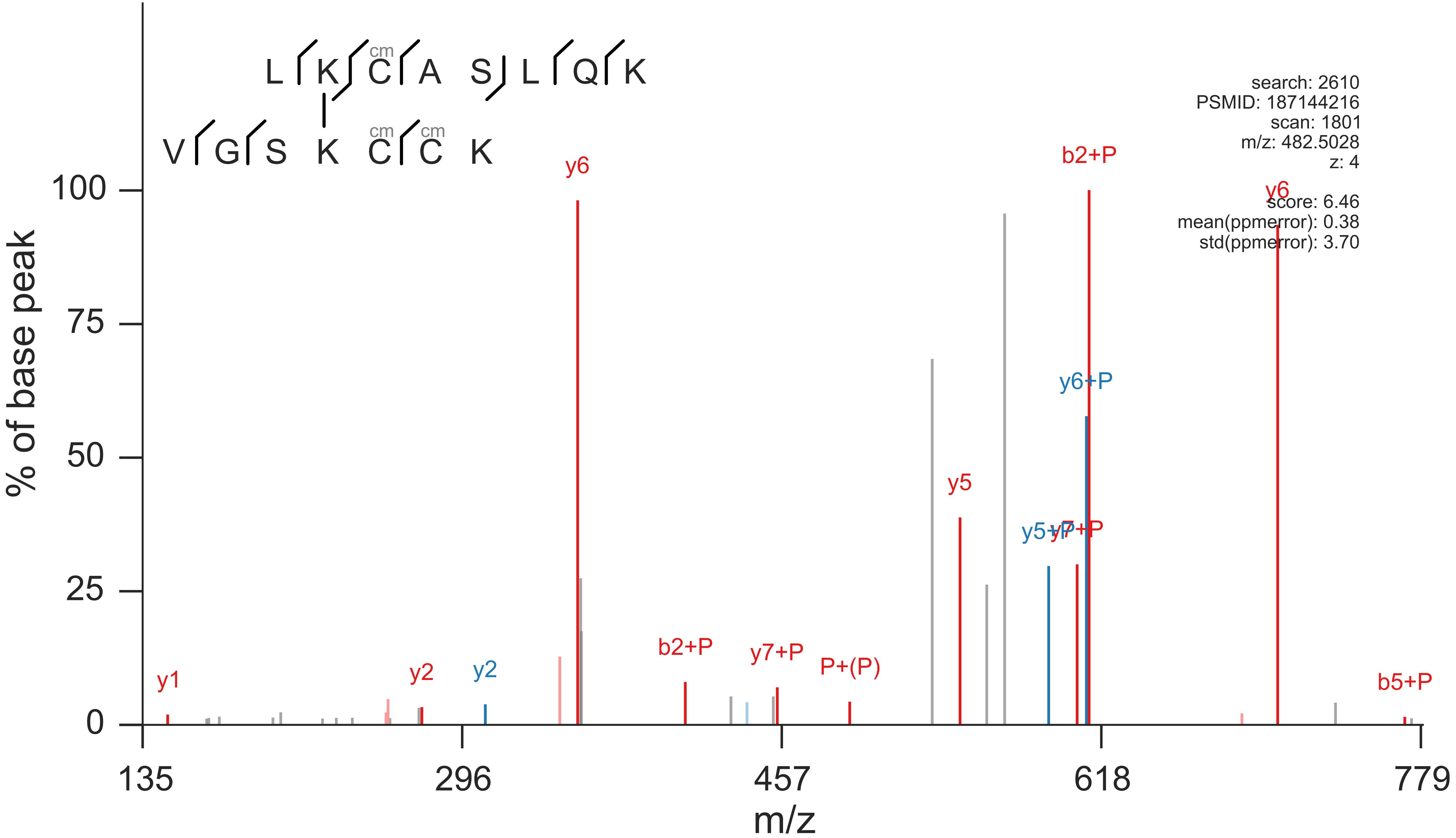
L K C A S L Q K
cm
V G S K C C K
cm cm
search: 2610
PSMID: 187144216
scan: 1801
m/z: 482.5028
z: 4
score: 6.46
mean(ppmerror): 0.38
std(ppmerror): 3.70
% of base peak
0
25
50
75
100
135
296
457
618
779
m/z
y1
y2
y2
y6
b2+P
y7+P
P+(P)
y5
y5+P
y7+P
y6+P
b2+P
y6
b5+P

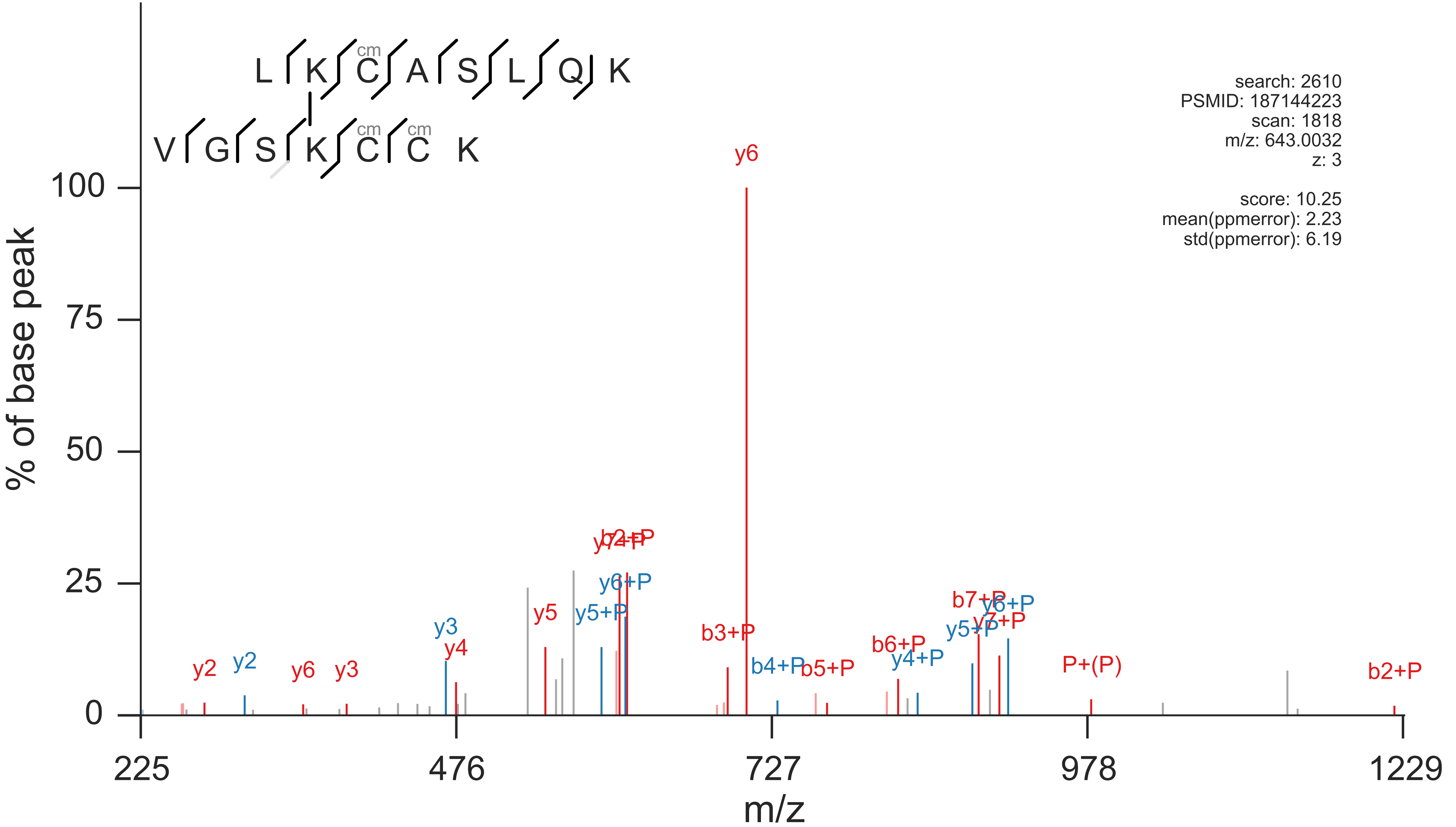
L K C A S L Q K
V G S K C C K
cm
search: 2610
PSMID: 187144223
scan: 1818
m/z: 643.0032
z: 3
score: 10.25
mean(ppmerror): 2.23
std(ppmerror): 6.19
% of base peak
m/z
0
25
50
75
100
225
476
727
978
1229
y6
y2
y3
y4
y5
y5+P
y6+P
b3+P
b4+P
b5+P
b6+P
y4+P
b7+P
y7+P
P+(P)
b2+P

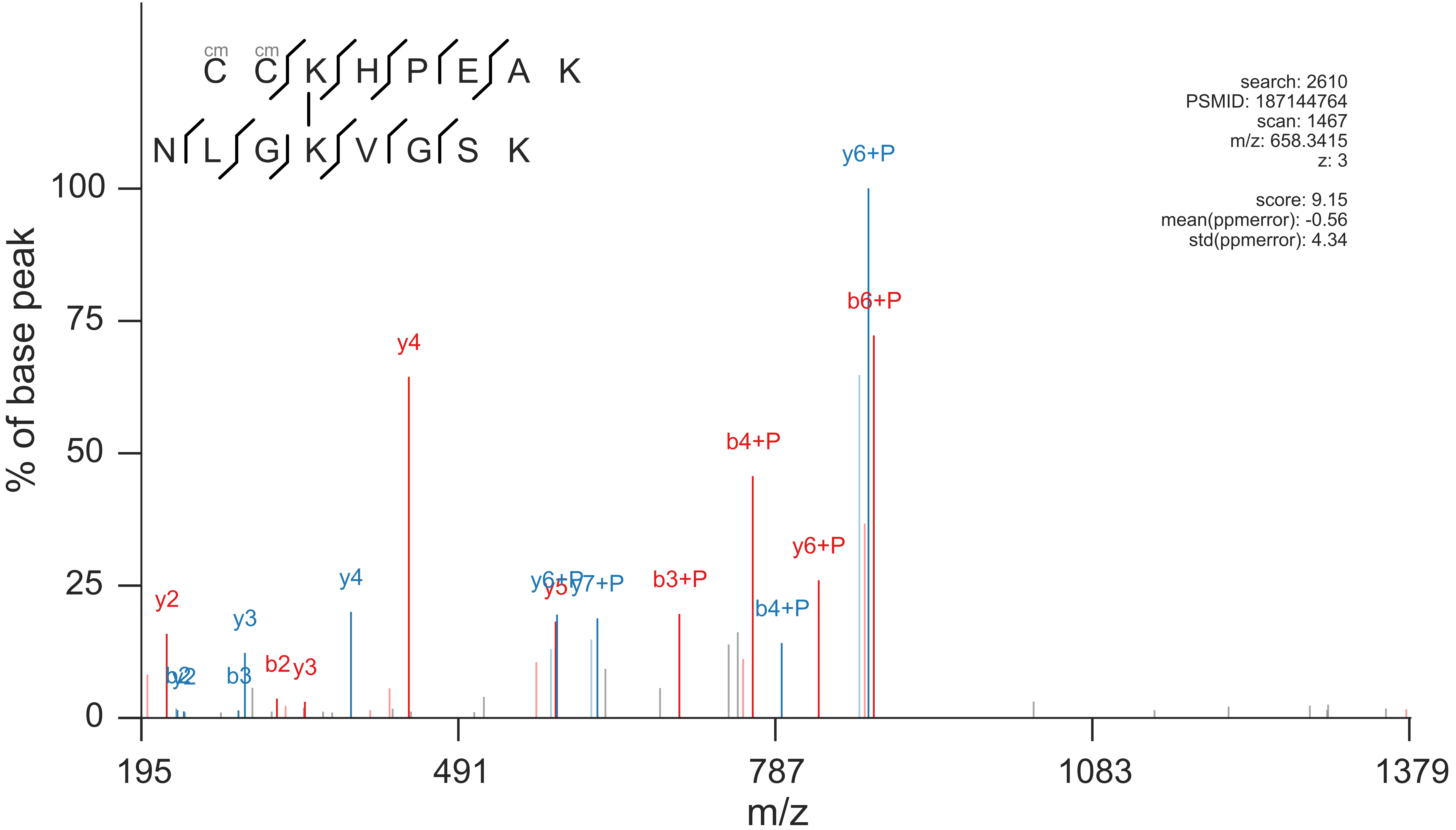

cm cm
C C K H P E A K
N L G K V G S K
search: 2610
PSMID: 187144764
scan: 1467
m/z: 658.3415
z: 3
score: 9.15
mean(ppmerror): -0.56
std(ppmerror): 4.34
% of base peak
100
75
50
25
0
195
491
787
1083
1379
m/z
y6+P
b6+P
y4
b4+P
y6+P
y2
y3
y4
y6+P
y5
y7+P
b3+P
b4+P
b2
y3
b3

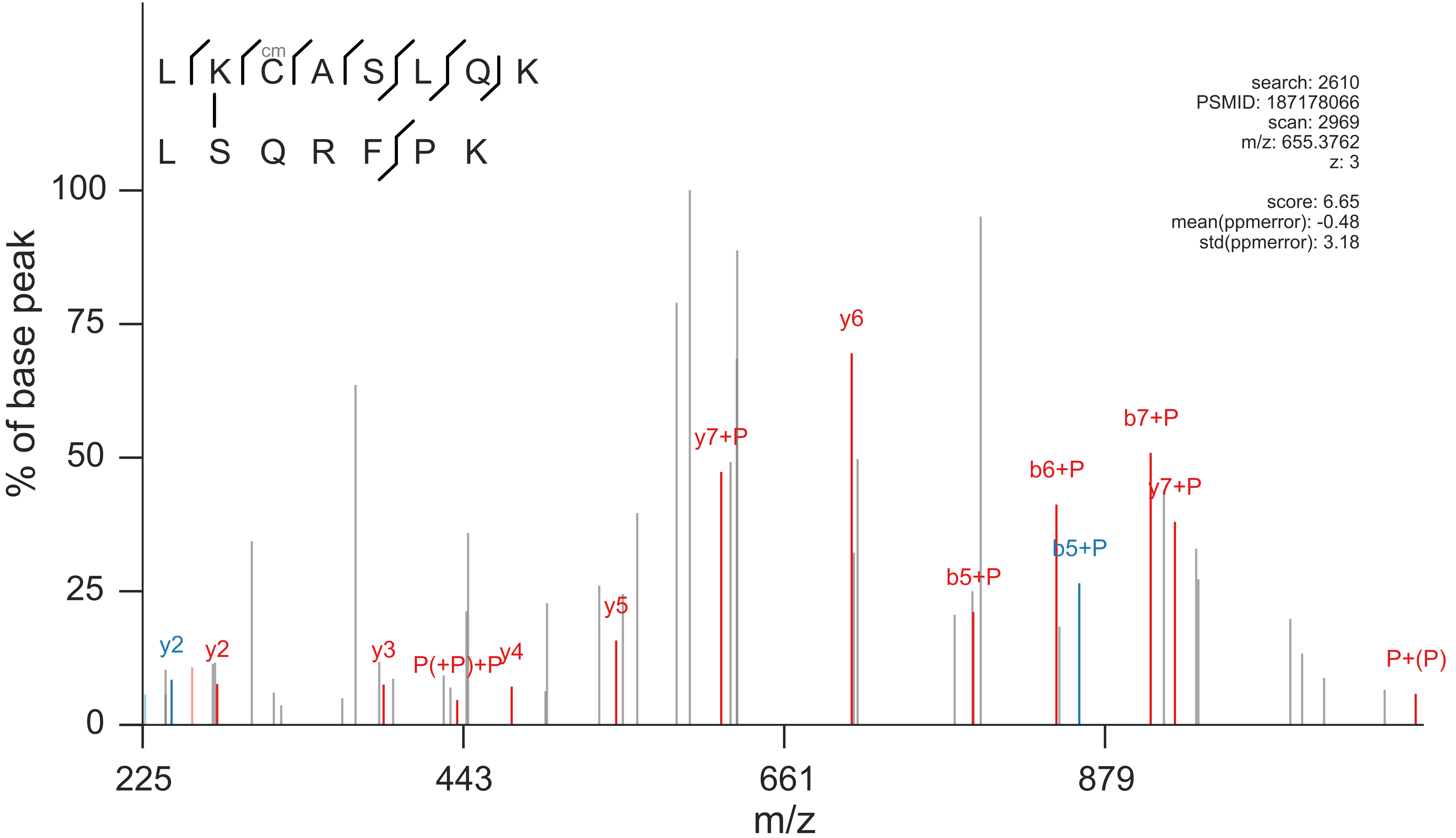

L K C A S L Q K
cm
L S Q R F P K
search: 2610
PSMID: 187178066
scan: 2969
m/z: 655.3762
z: 3
score: 6.65
mean(ppmerror): -0.48
std(ppmerror): 3.18
% of base peak
100
75
50
25
0
y2
y2
y3
P(+P)+P
y4
y5
y7+P
y6
b5+P
b6+P
b5+P
b7+P
y7+P
P+(P)
225
443
661
879
m/z

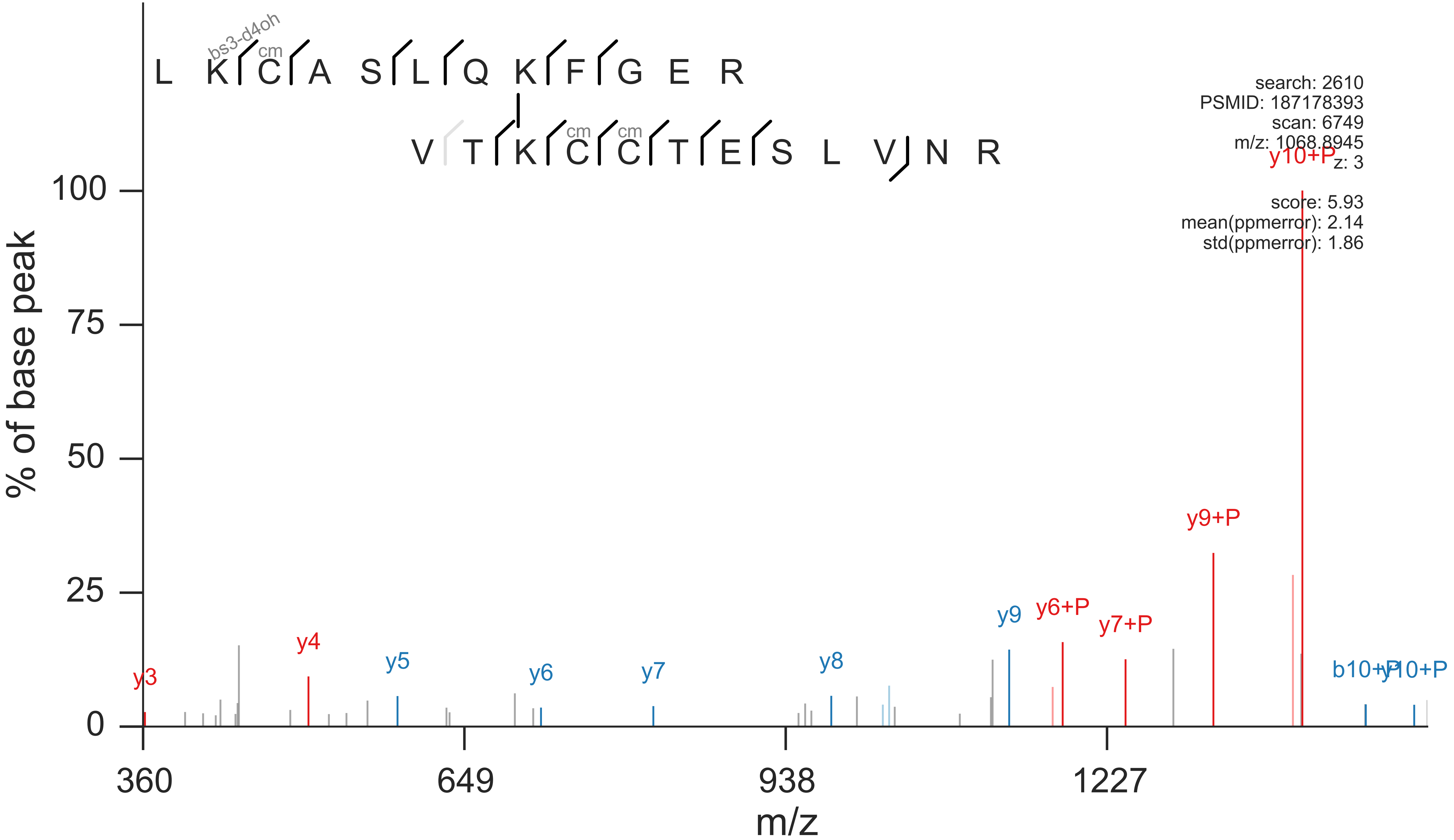

bs3-d4oh
cm
L K C A S L Q K F G E R
cm cm
V T K C C T E S L V N R
search: 2610
PSMID: 187178393
scan: 6749
m/z: 1068.8945
z: 3
score: 5.93
mean(ppmerror): 2.14
std(ppmerror): 1.86
% of base peak
100
75
50
25
0
y10+P
y9+P
y9
y6+P
y7+P
y3
y4
y5
y6
y7
y8
b10+P
y10+P
360
649
938
1227
m/z

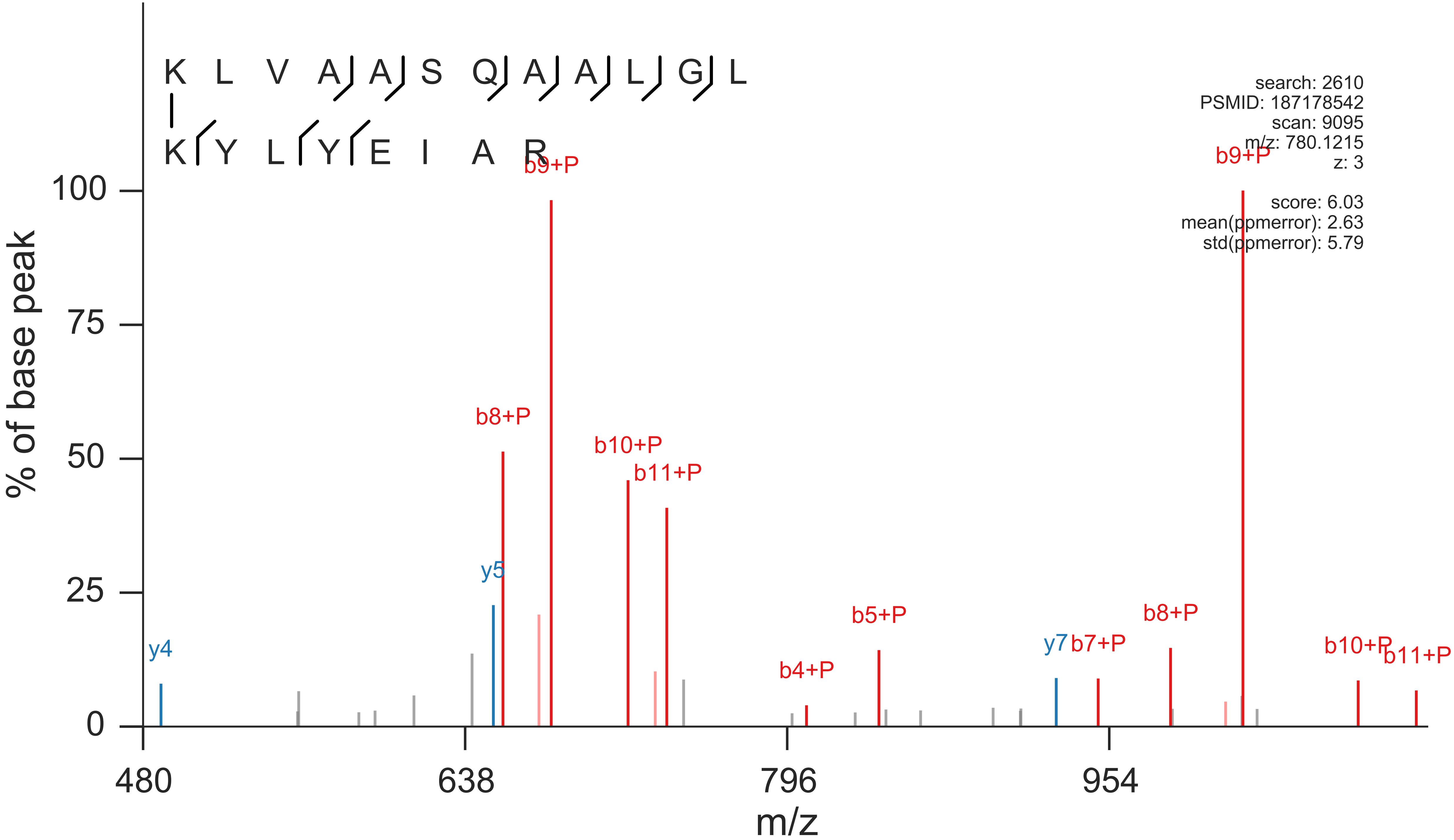

K L V A A S Q A A L G L
K Y L Y E I A R
search: 2610
PSMID: 187178542
scan: 9095
m/z: 780.1215
z: 3
score: 6.03
mean(ppmerror): 2.63
std(ppmerror): 5.79
% of base peak
m/z
0
25
50
75
100
480
638
796
954
y4
y5
y7
b4+P
b5+P
b7+P
b8+P
b9+P
b10+P
b11+P
b8+P
b9+P
b10+P
b11+P

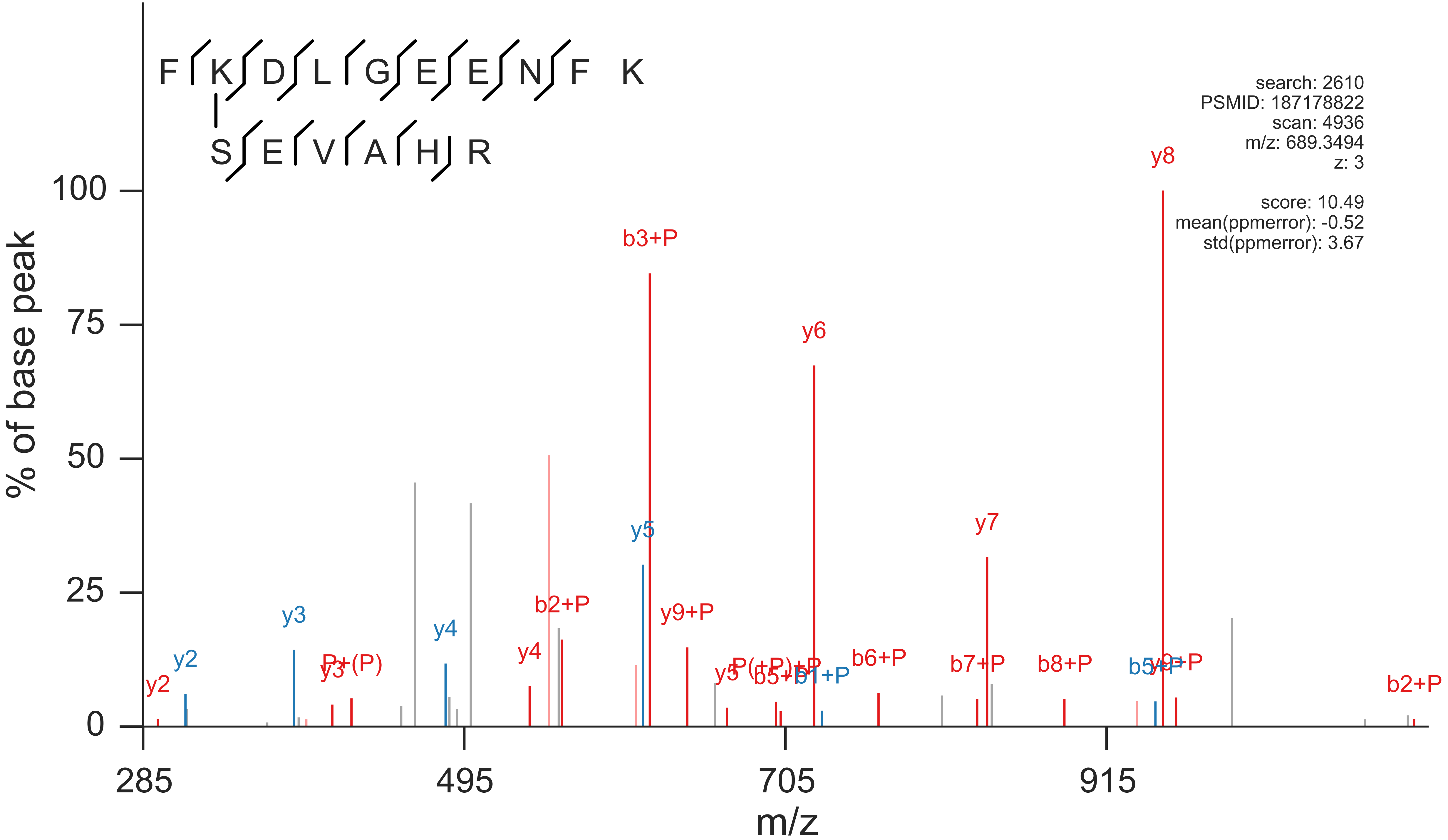

F K D L G E E N F K
S E V A H R
search: 2610
PSMID: 187178822
scan: 4936
m/z: 689.3494
z: 3
score: 10.49
mean(ppmerror): -0.52
std(ppmerror): 3.67
% of base peak
m/z
0
25
50
75
100
285
495
705
915
y8
b3+P
y6
y7
y5
y2
y3
y4
b2+P
y9+P
b6+P
b7+P
b8+P

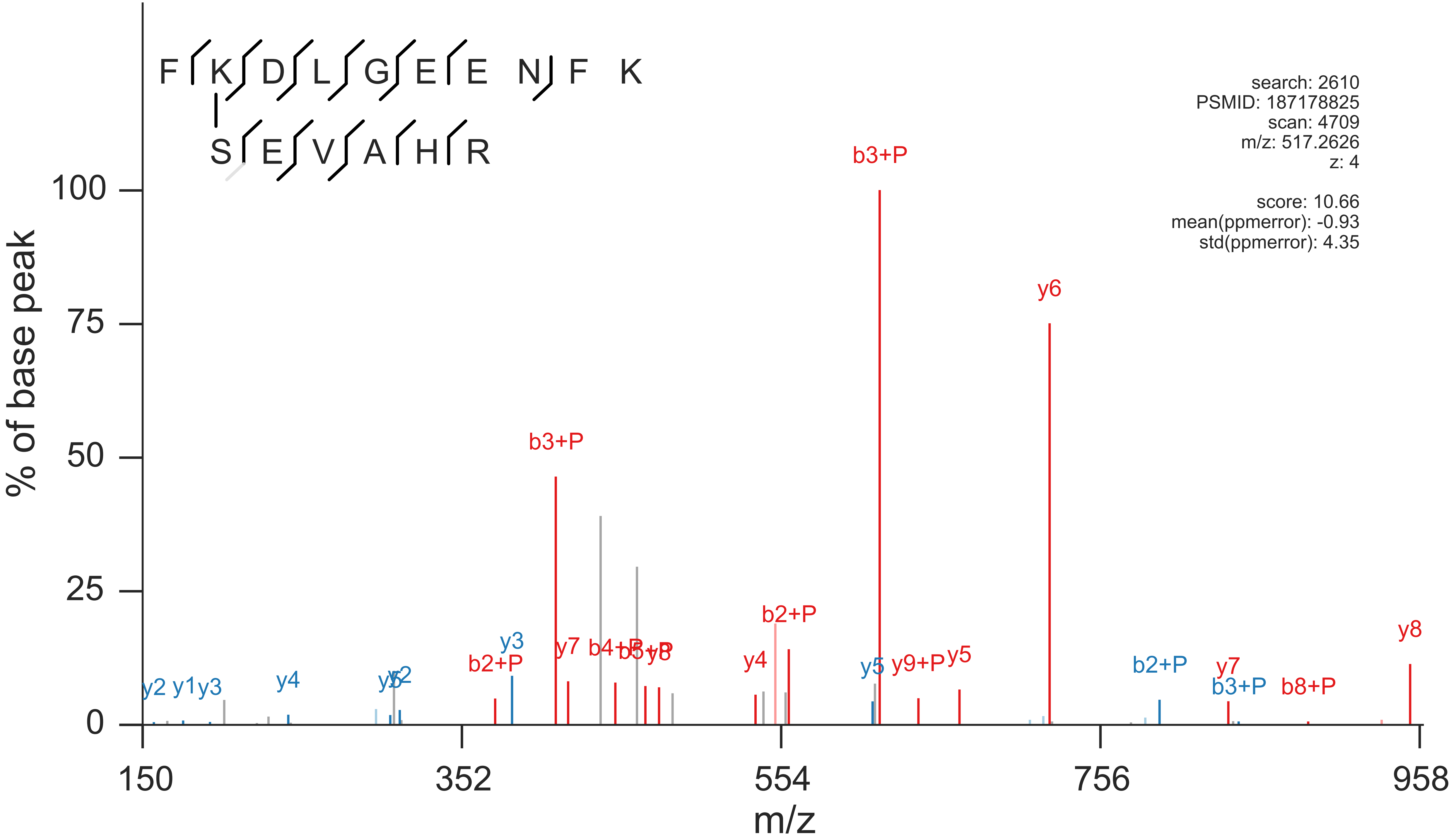

F K D L G E E N F K
S E V A H R
search: 2610
PSMID: 187178825
scan: 4709
m/z: 517.2626
z: 4
score: 10.66
mean(ppmerror): -0.93
std(ppmerror): 4.35
% of base peak
m/z
100
75
50
25
0
150
352
554
756
958
b3+P
y6
b3+P
b2+P
y3
y7
b4+P
y8
y4
y5
y9+P
y5
b2+P
y7
b3+P
b8+P
y8
y2
y1
y3
y4
y2

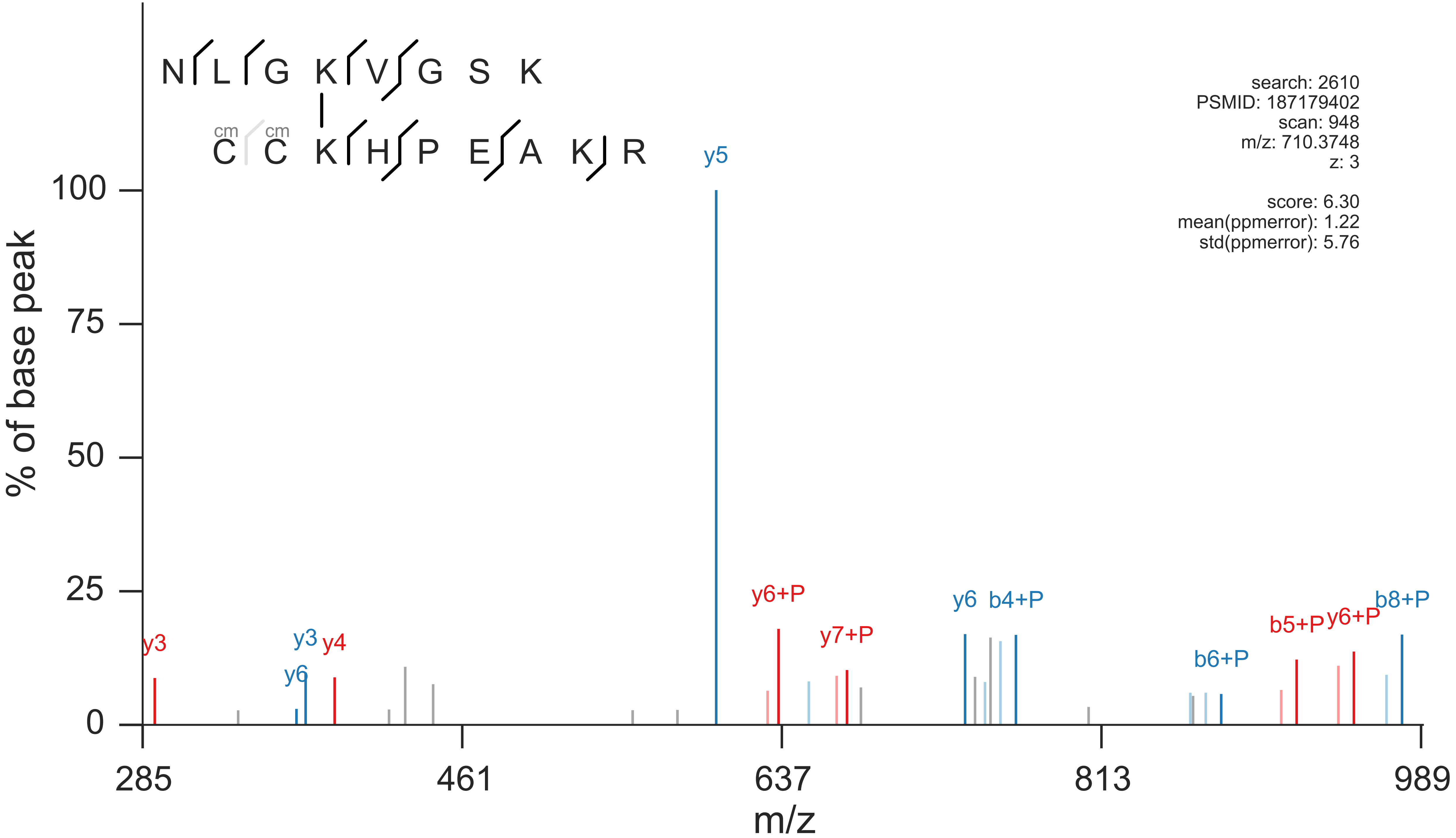
N L G K V G S K
cm cm
C C K H P E A K R
search: 2610
PSMID: 187179402
scan: 948
m/z: 710.3748
z: 3
score: 6.30
mean(ppmerror): 1.22
std(ppmerror): 5.76
% of base peak
100
75
50
25
0
y5
y3
y3
y6
y4
y6+P
y7+P
y6
b4+P
b6+P
b5+P
y6+P
b8+P
285
461
637
813
989
m/z

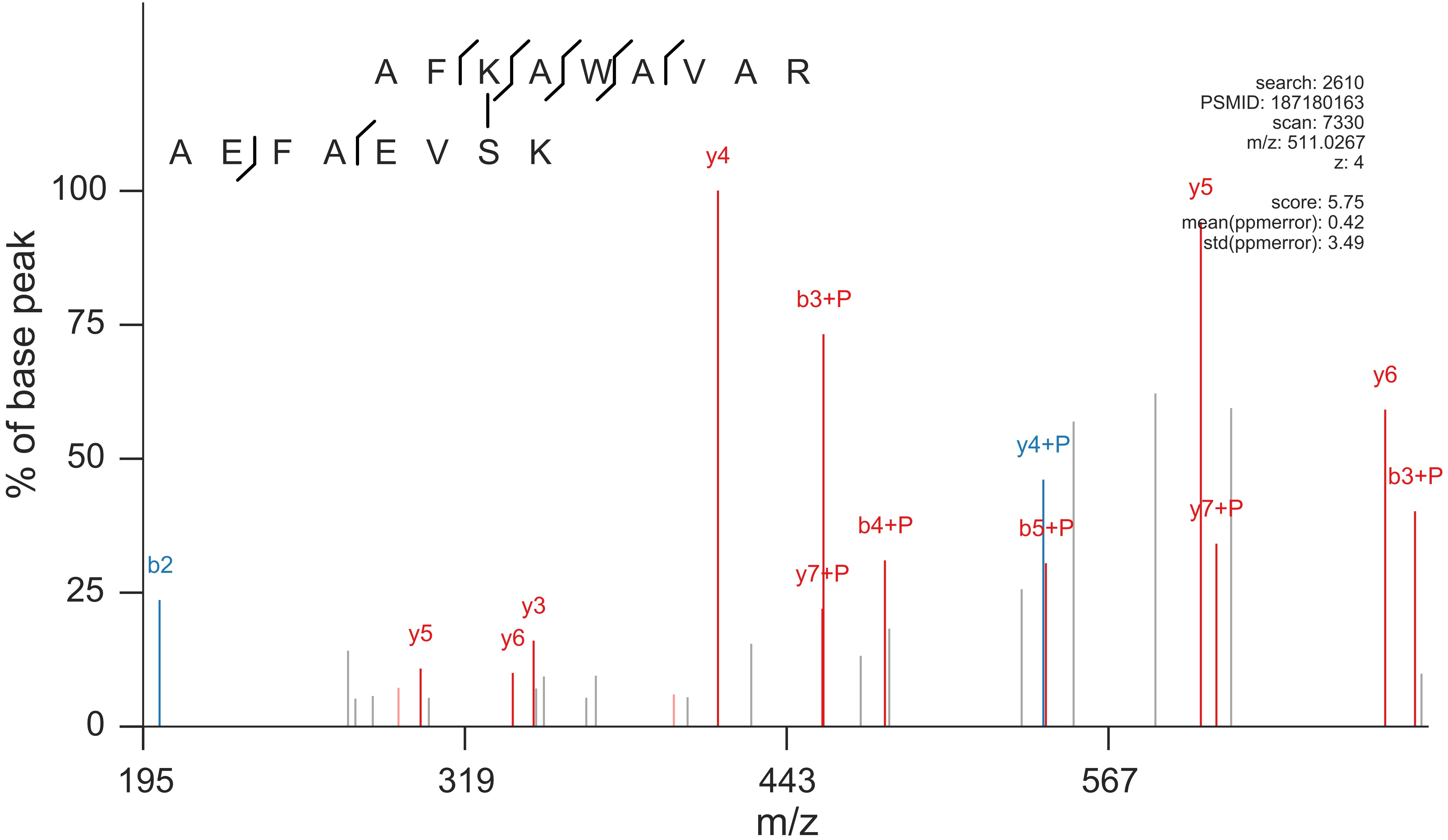

A F K A W A V A R
A E F A E V S K
search: 2610
PSMID: 187180163
scan: 7330
m/z: 511.0267
z: 4
score: 5.75
mean(ppmerror): 0.42
std(ppmerror): 3.49
% of base peak
m/z
100
75
50
25
0
195
319
443
567
b2
y5
y6
y3
y4
b3+P
y7+P
b4+P
y4+P
b5+P
y5
y7+P
y6
b3+P

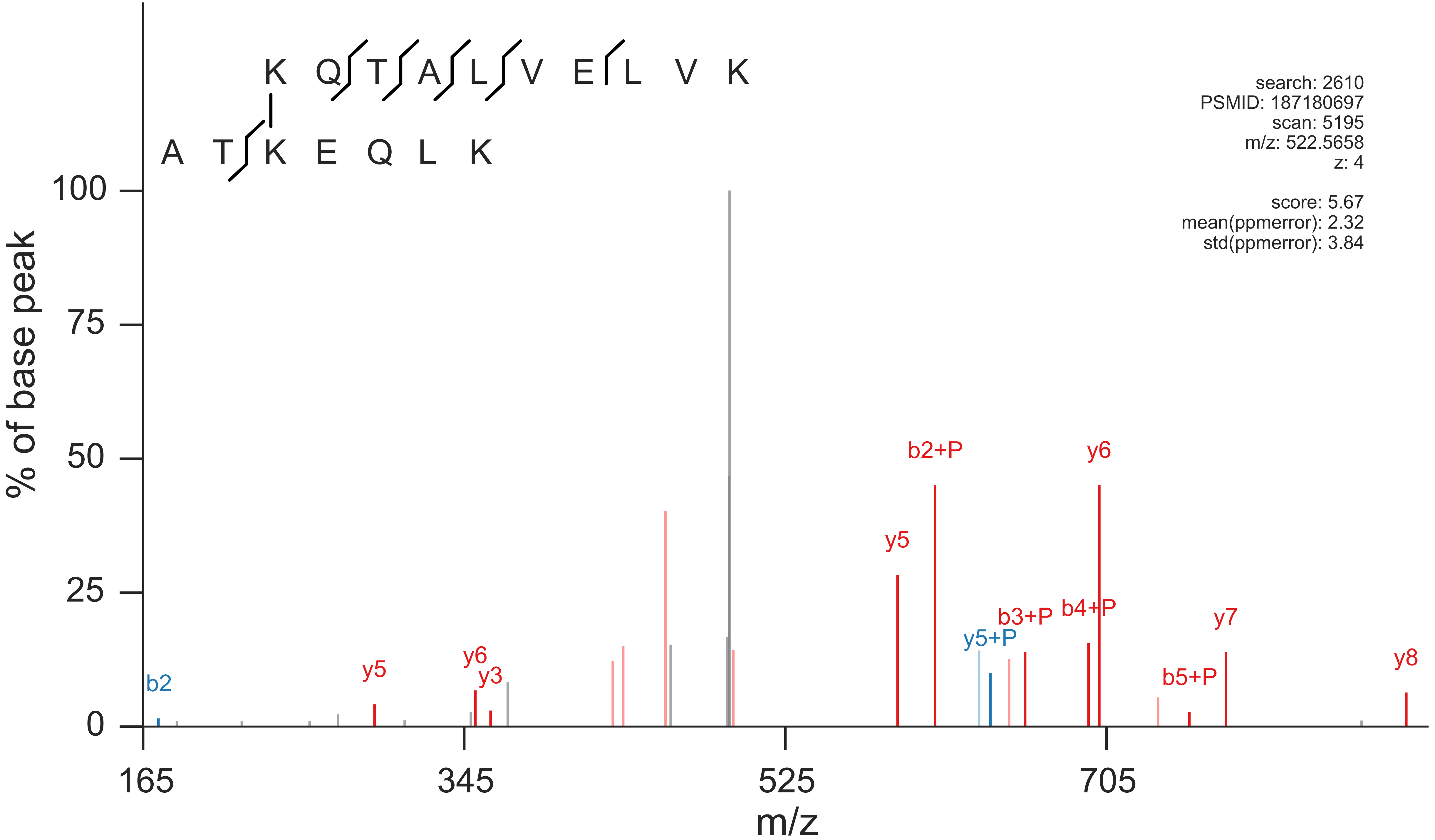

K Q T A L V E L V K
A T K E Q L K
search: 2610
PSMID: 187180697
scan: 5195
m/z: 522.5658
z: 4
score: 5.67
mean(ppmerror): 2.32
std(ppmerror): 3.84
% of base peak
100
75
50
25
0
b2
y5
y6
y3
y5
b2+P
y5+P
b3+P
b4+P
y6
b5+P
y7
y8
165
345
525
705
m/z

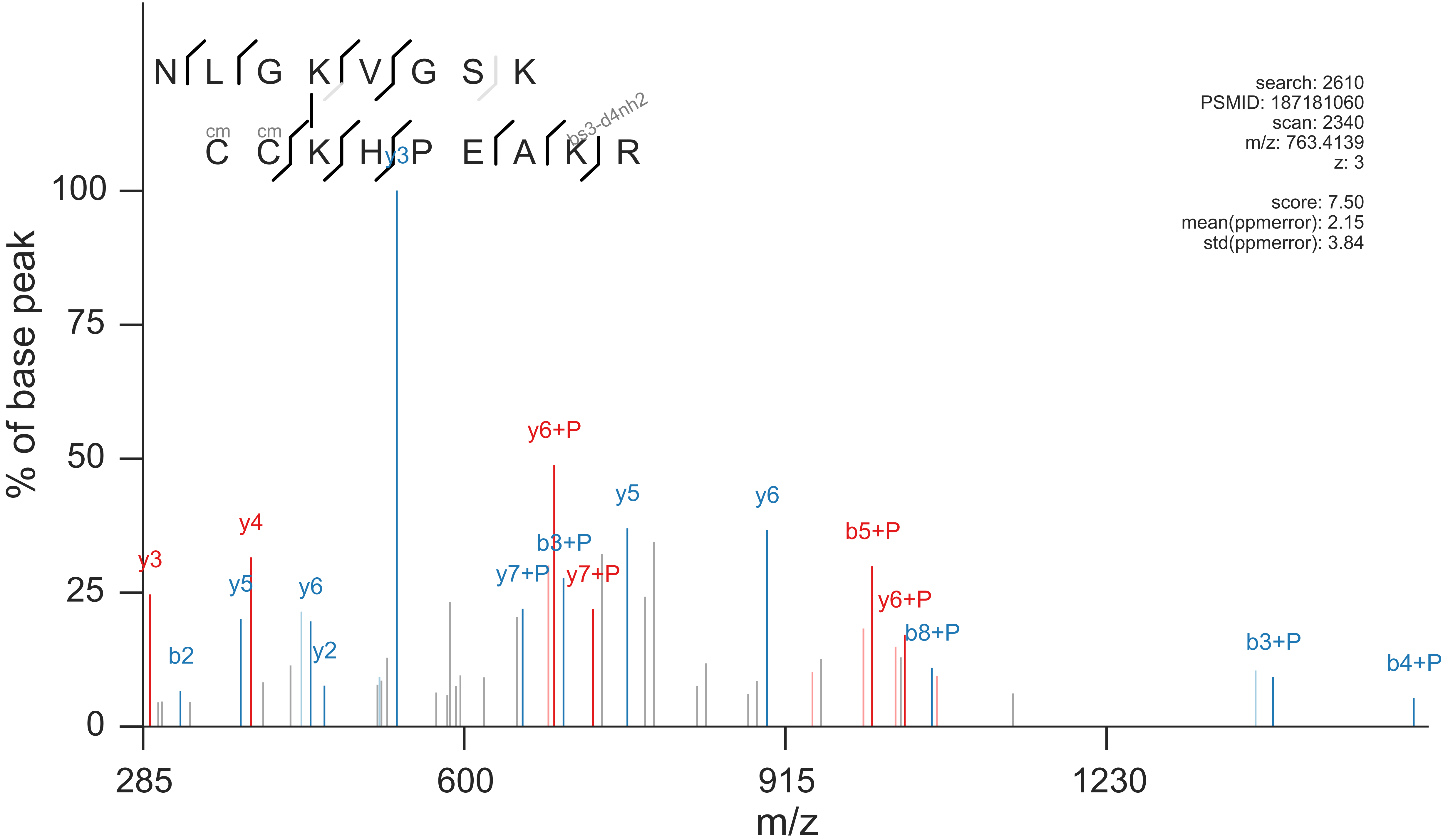
N L G K V G S K
cm cm
C C K H P E A K R
bs3-d4nh2
search: 2610
PSMID: 187181060
scan: 2340
m/z: 763.4139
z: 3
score: 7.50
mean(ppmerror): 2.15
std(ppmerror): 3.84
% of base peak
m/z
0
25
50
75
100
285
600
915
1230
y3
b2
y5
y4
y6
y2
y3
y7+P
y6+P
b3+P
y7+P
y5
y6
b5+P
y6+P
b8+P
b3+P
b4+P

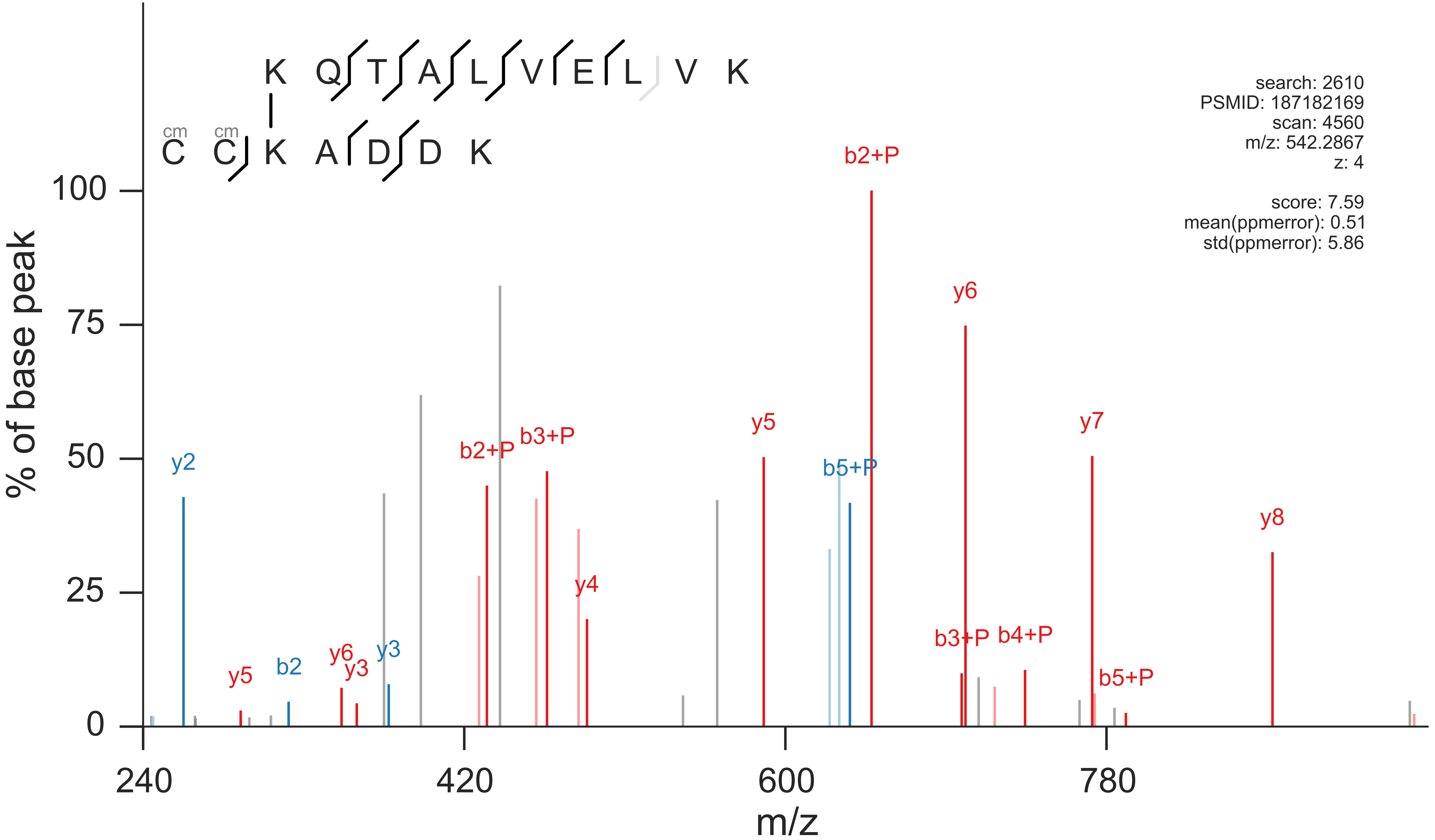
K Q T A L V E L V K
cm C cm C K A D D K
search: 2610
PSMID: 187182169
scan: 4560
m/z: 542.2867
z: 4
score: 7.59
mean(ppmerror): 0.51
std(ppmerror): 5.86
% of base peak
m/z
100
75
50
25
0
240
420
600
780
y2
y5
b2
y6
y3
y3
b2+P
b3+P
y4
y5
b5+P
b2+P
y6
b3+P
b4+P
y7
b5+P
y8

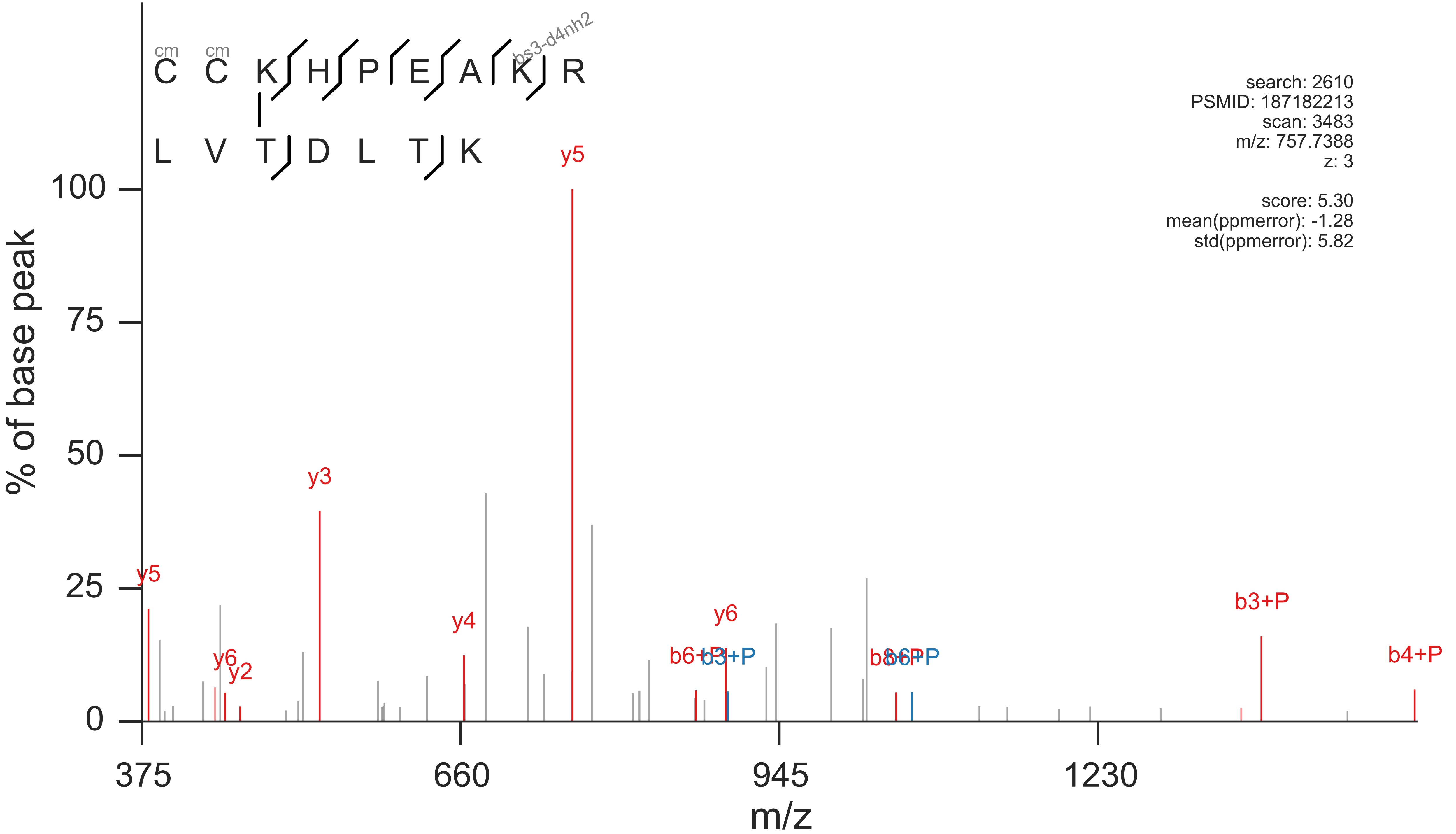

cm cm
C C K H P E A K R
bs3-d4nh2
L V T D L T K
search: 2610
PSMID: 187182213
scan: 3483
m/z: 757.7388
z: 3
score: 5.30
mean(ppmerror): -1.28
std(ppmerror): 5.82
% of base peak
m/z
y5
y3
y4
y6
y2
b6+P
b3+P
b3+P
b4+P

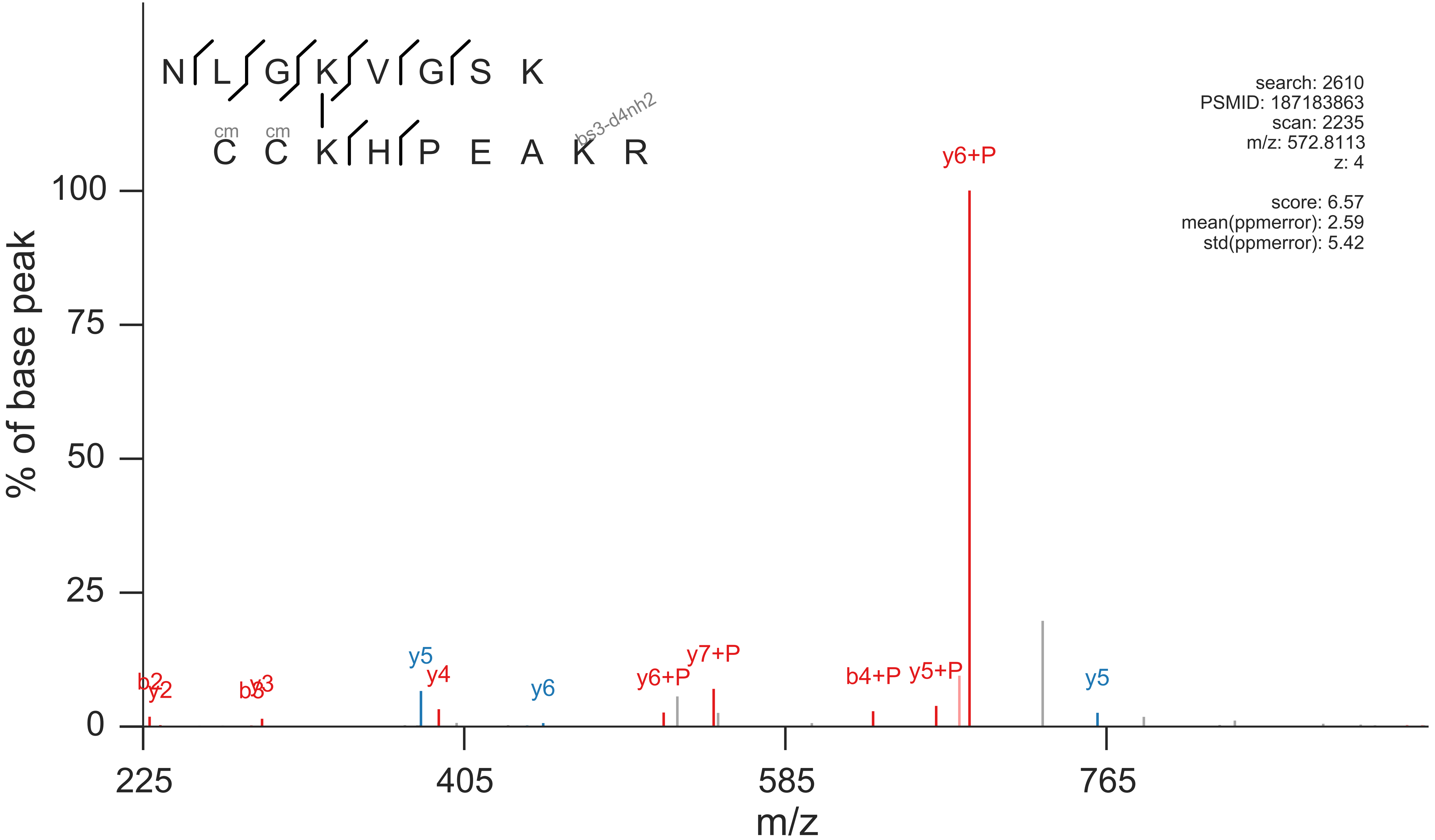

N L G K V G S K
cm cm
C C K H P E A K R
bs3-d4nh2
search: 2610
PSMID: 187183863
scan: 2235
m/z: 572.8113
z: 4
score: 6.57
mean(ppmerror): 2.59
std(ppmerror): 5.42
% of base peak
100
75
50
25
0
225
405
585
765
m/z
y6+P
b2
y2
b3
y3
y5
y4
y6
y6+P
y7+P
b4+P
y5+P
y5

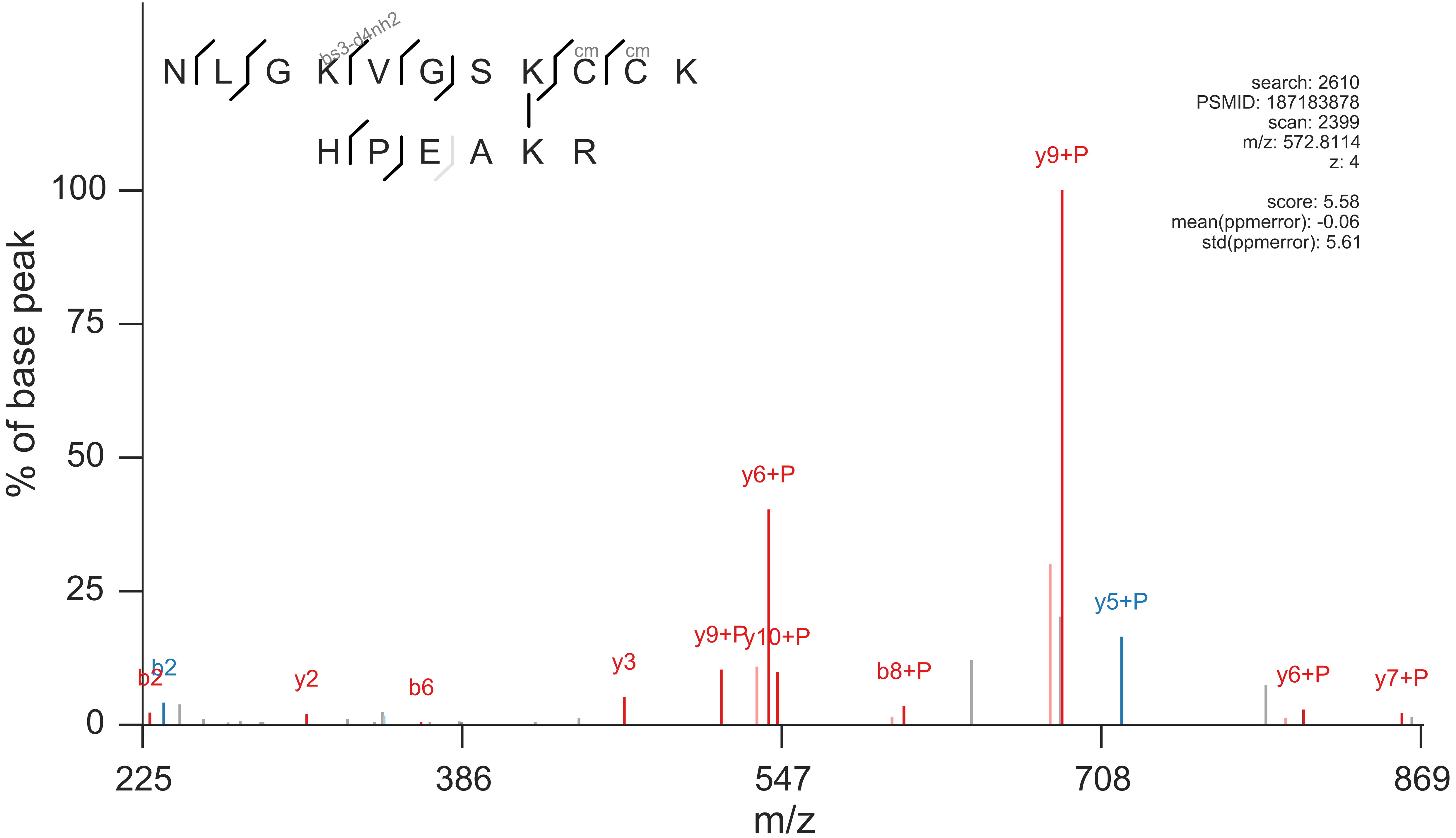

NLGKVGSKCCK
bs3-d4nh2
cm
cm
HPEAKR
search: 2610
PSMID: 187183878
scan: 2399
m/z: 572.8114
z: 4
score: 5.58
mean(ppmerror): -0.06
std(ppmerror): 5.61
% of base peak
m/z
y9+P
y6+P
y5+P
y9+P
y10+P
y3
b8+P
y6+P
y7+P
b2
b2
y2
b6
0
25
50
75
100
225
386
547
708
869

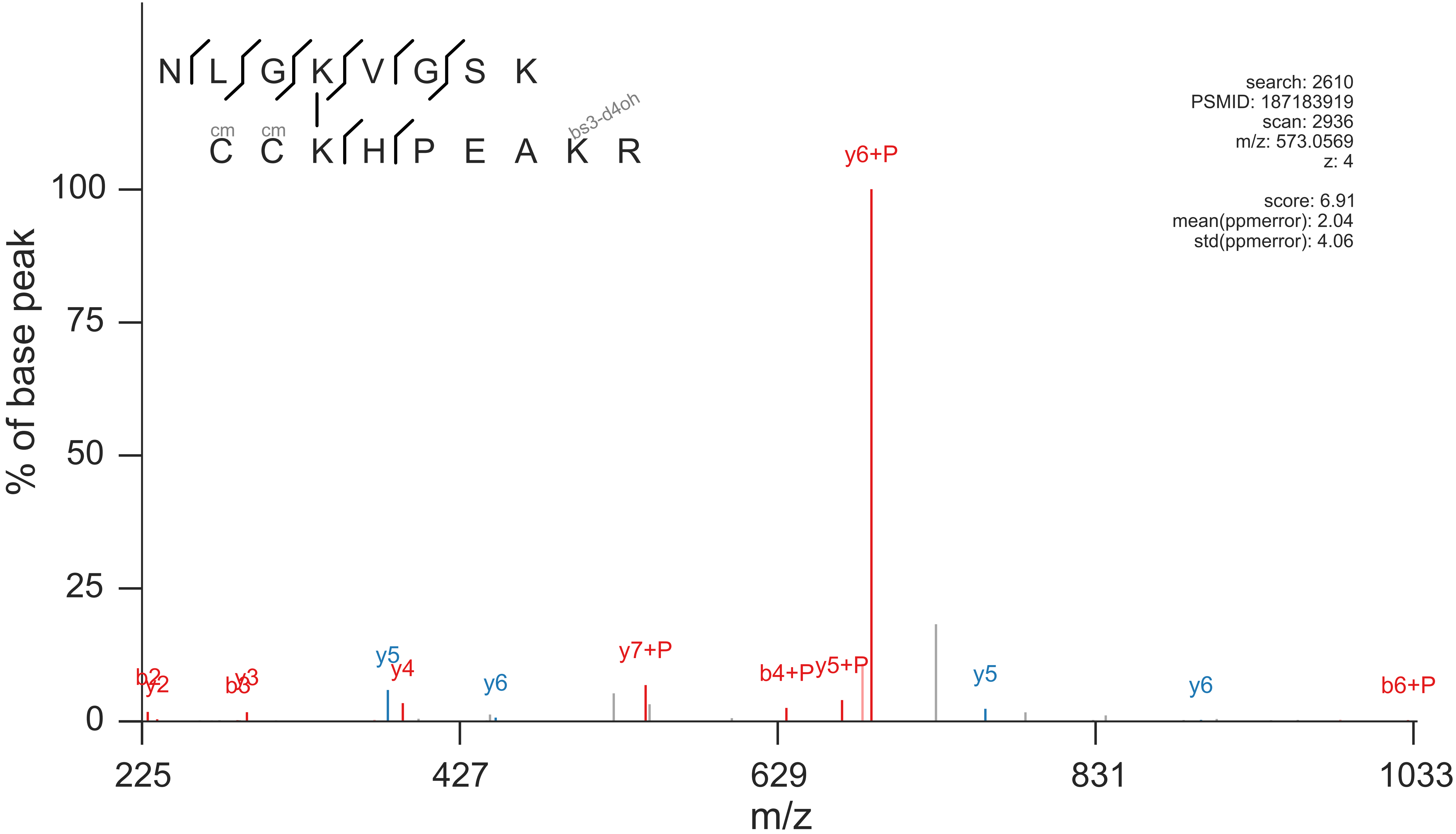

N L G K V G S K
cm cm
C C K H P E A K R
bs3-d4oh
search: 2610
PSMID: 187183919
scan: 2936
m/z: 573.0569
z: 4
score: 6.91
mean(ppmerror): 2.04
std(ppmerror): 4.06
% of base peak
m/z
0
25
50
75
100
225
427
629
831
1033
y6+P
b2
y2
b3
y3
y5
y4
y6
y7+P
b4+P
y5+P
y5
y6
b6+P

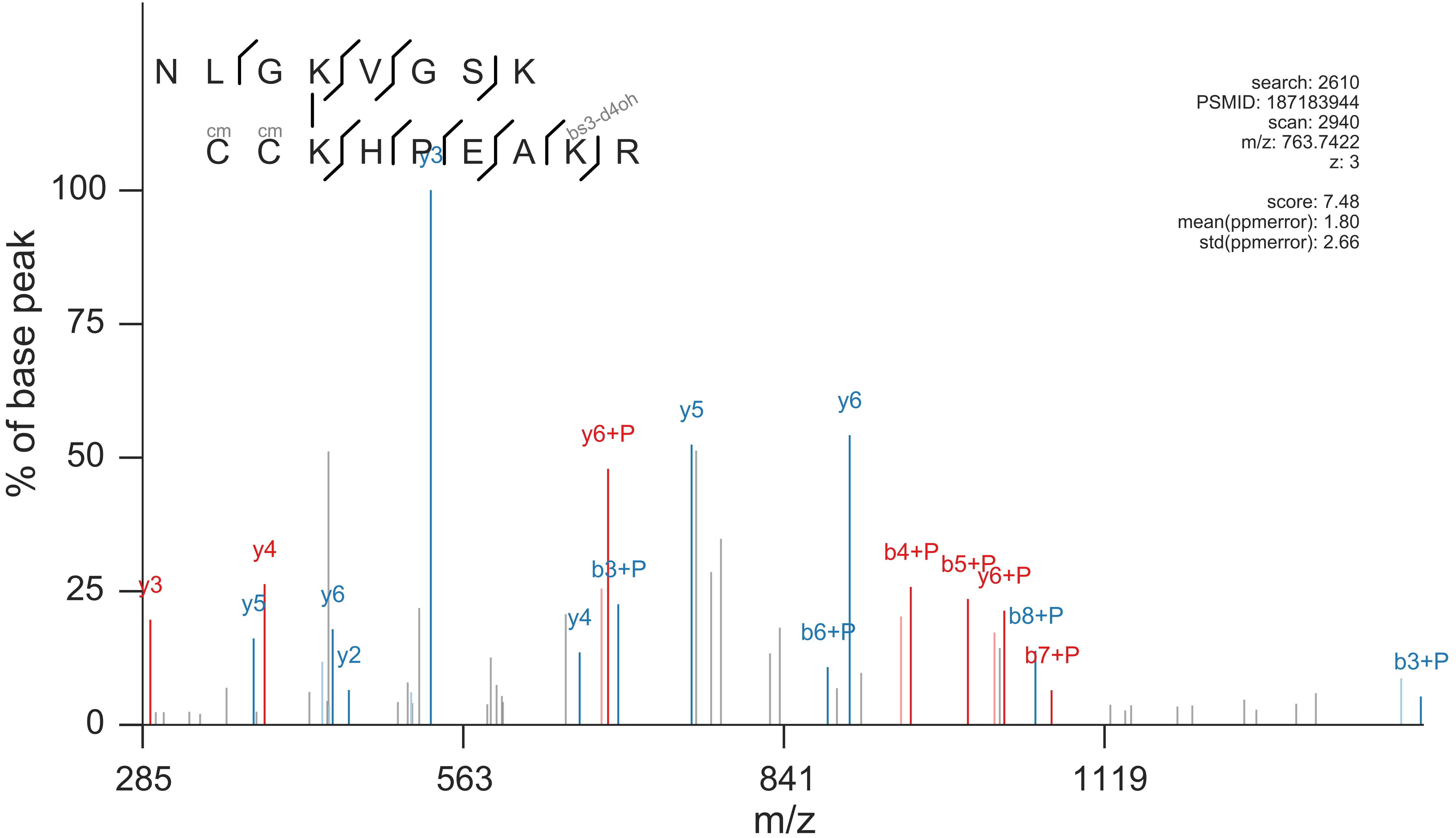

N L G K V G S K
C C K H P E A K R
cm cm
bs3-d4oh
search: 2610
PSMID: 187183944
scan: 2940
m/z: 763.7422
z: 3
score: 7.48
mean(ppmerror): 1.80
std(ppmerror): 2.66
% of base peak
m/z
0
25
50
75
100
285
563
841
1119
y3
y4
y5
y6
y2
y3
y4
y6+P
b3+P
y5
y6
b6+P
b4+P
b5+P
y6+P
b8+P
b7+P
b3+P

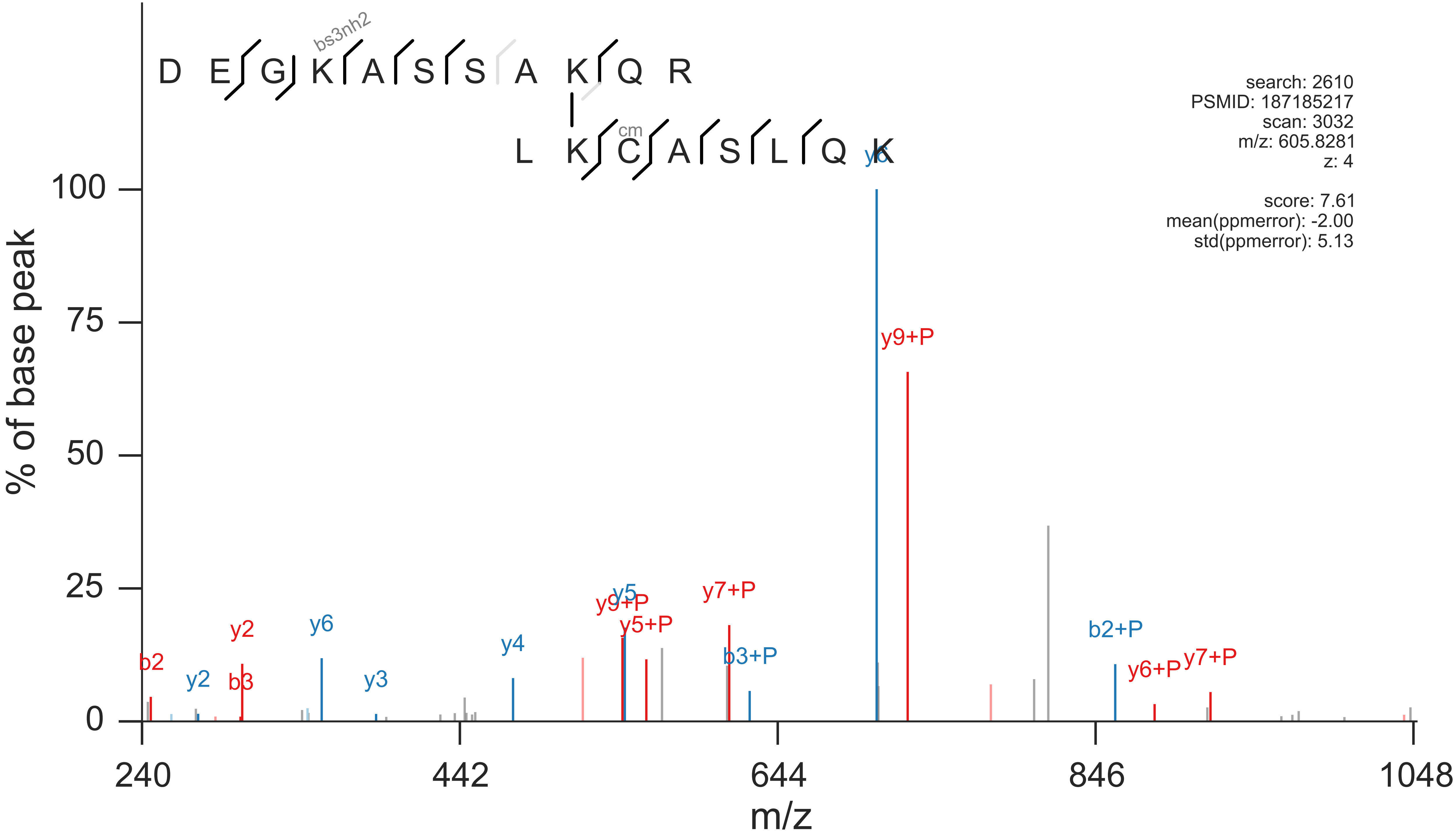

bs3nh2
D E G K A S S A K Q R
cm
L K C A S L Q K
search: 2610
PSMID: 187185217
scan: 3032
m/z: 605.8281
z: 4
score: 7.61
mean(ppmerror): -2.00
std(ppmerror): 5.13
% of base peak
0
25
50
75
100
m/z
240
442
644
846
1048
b2
y2
b3
y2
y6
y3
y4
y9+P
y5
y5+P
y7+P
b3+P
y6
y9+P
b2+P
y6+P
y7+P

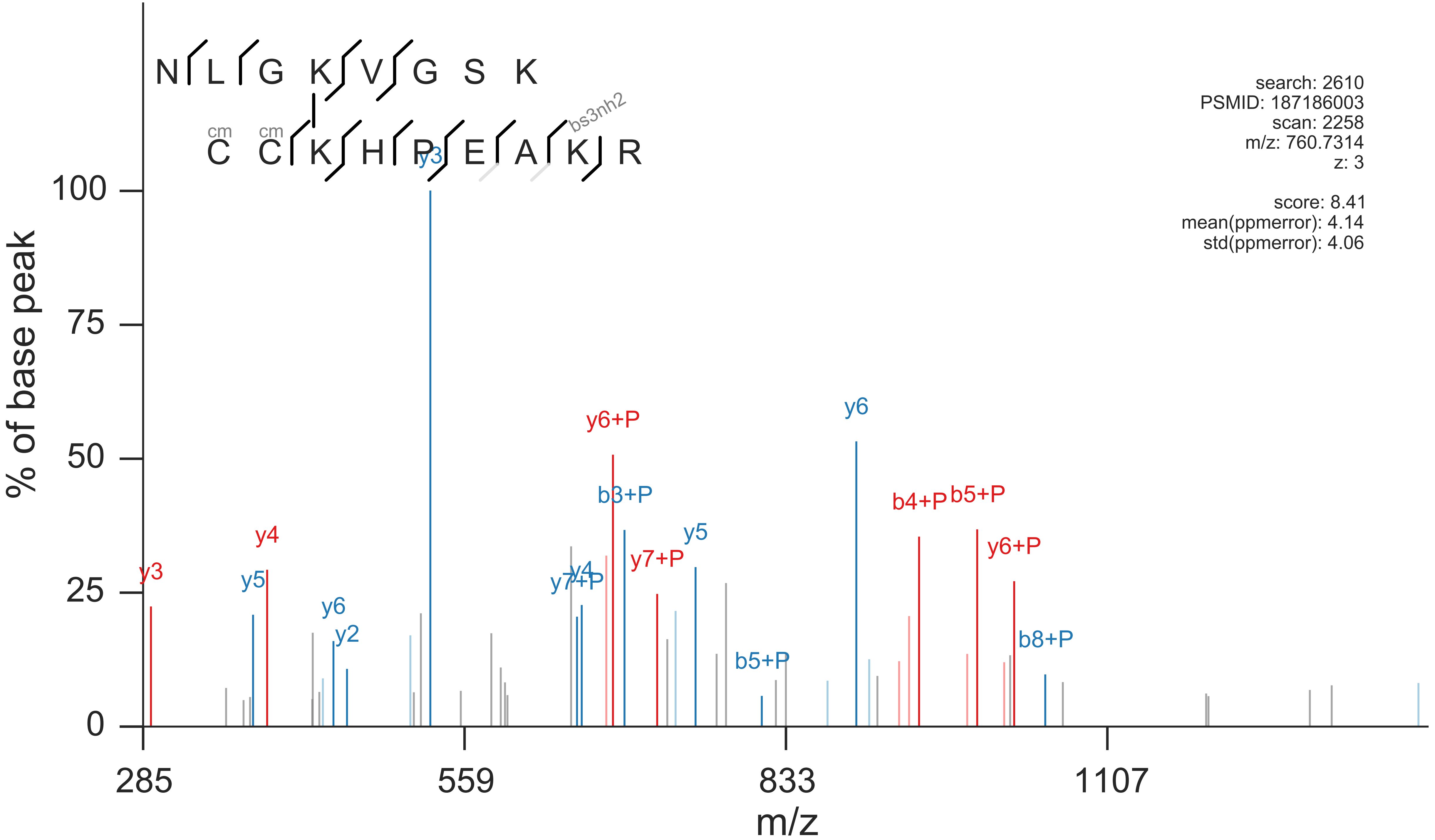

N L G K V G S K
cm cm
C C K H P E A K R
bs3nh2
search: 2610
PSMID: 187186003
scan: 2258
m/z: 760.7314
z: 3
score: 8.41
mean(ppmerror): 4.14
std(ppmerror): 4.06
% of base peak
100
75
50
25
0
285
559
833
1107
m/z
y3
y6+P
y6
b3+P
b4+P
b5+P
y4
y5
y7+P
y6+P
y3
y5
y6
y2
y7+P
y4
b5+P
b8+P

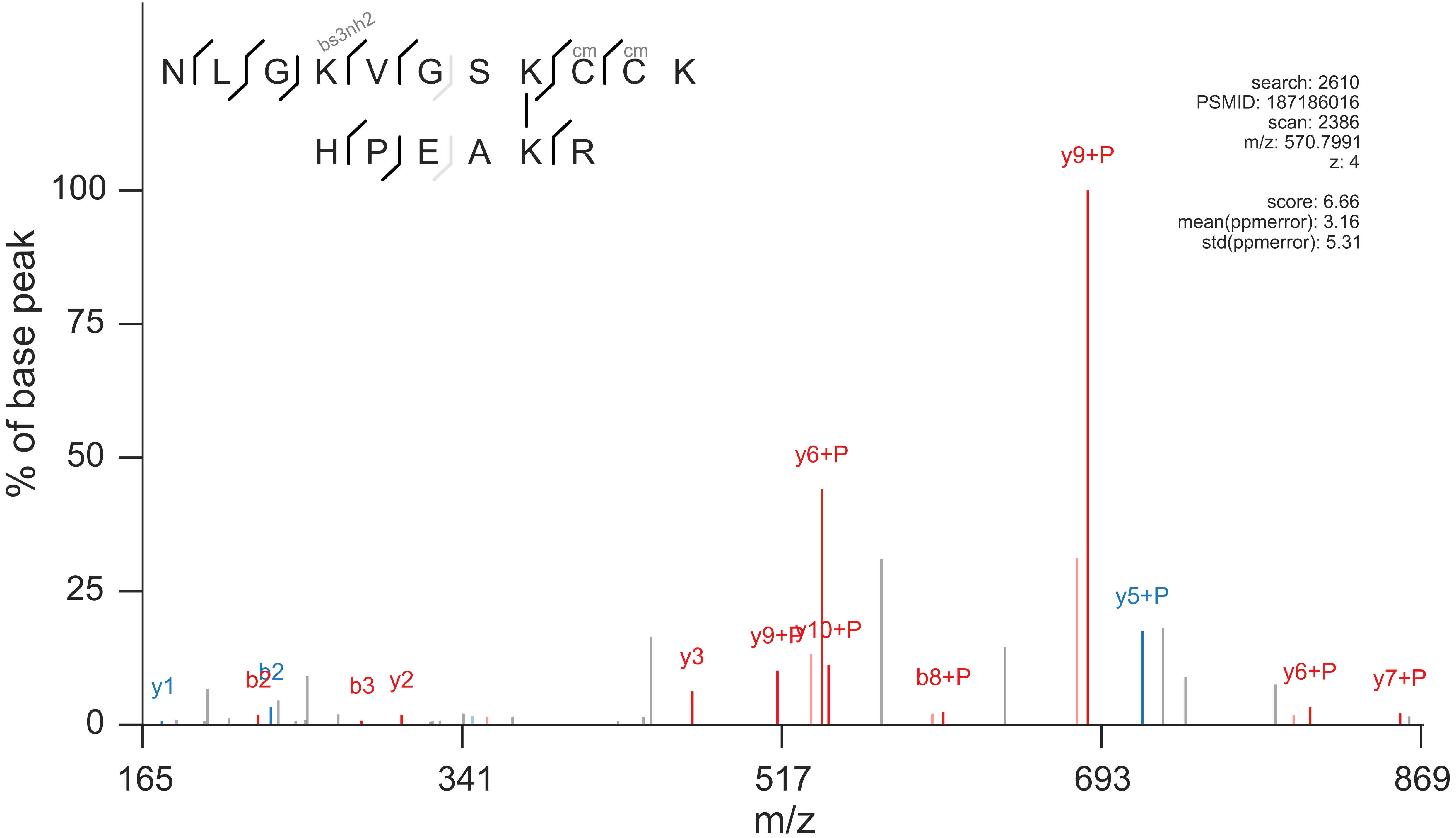

bs3nh2
cm
cm
N L G K V G S K C C K
H P E A K R
search: 2610
PSMID: 187186016
scan: 2386
m/z: 570.7991
z: 4
score: 6.66
mean(ppmerror): 3.16
std(ppmerror): 5.31
% of base peak
100
75
50
25
0
y9+P
y6+P
y5+P
y9+P
y10+P
y3
y1
b2
b2
b3
y2
b8+P
y6+P
y7+P
165
341
517
693
869
m/z

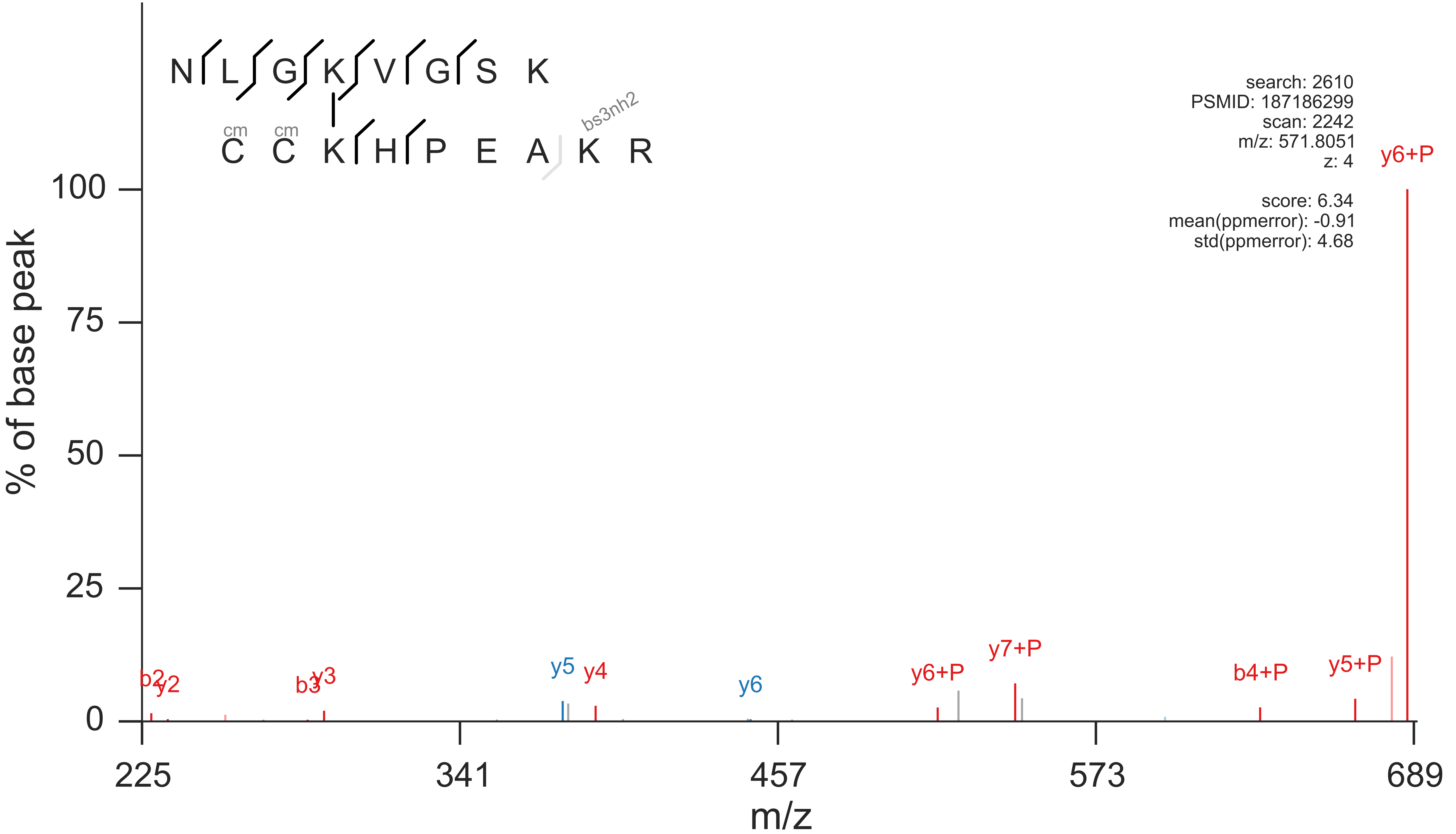

N L G K V G S K
C C K H P E A K R
cm
cm
bs3nh2
search: 2610
PSMID: 187186299
scan: 2242
m/z: 571.8051
z: 4
score: 6.34
mean(ppmerror): -0.91
std(ppmerror): 4.68
% of base peak
m/z
100
75
50
25
0
225
341
457
573
689
b2
y2
b3
y3
y5
y4
y6
y6+P
y7+P
b4+P
y5+P
y6+P

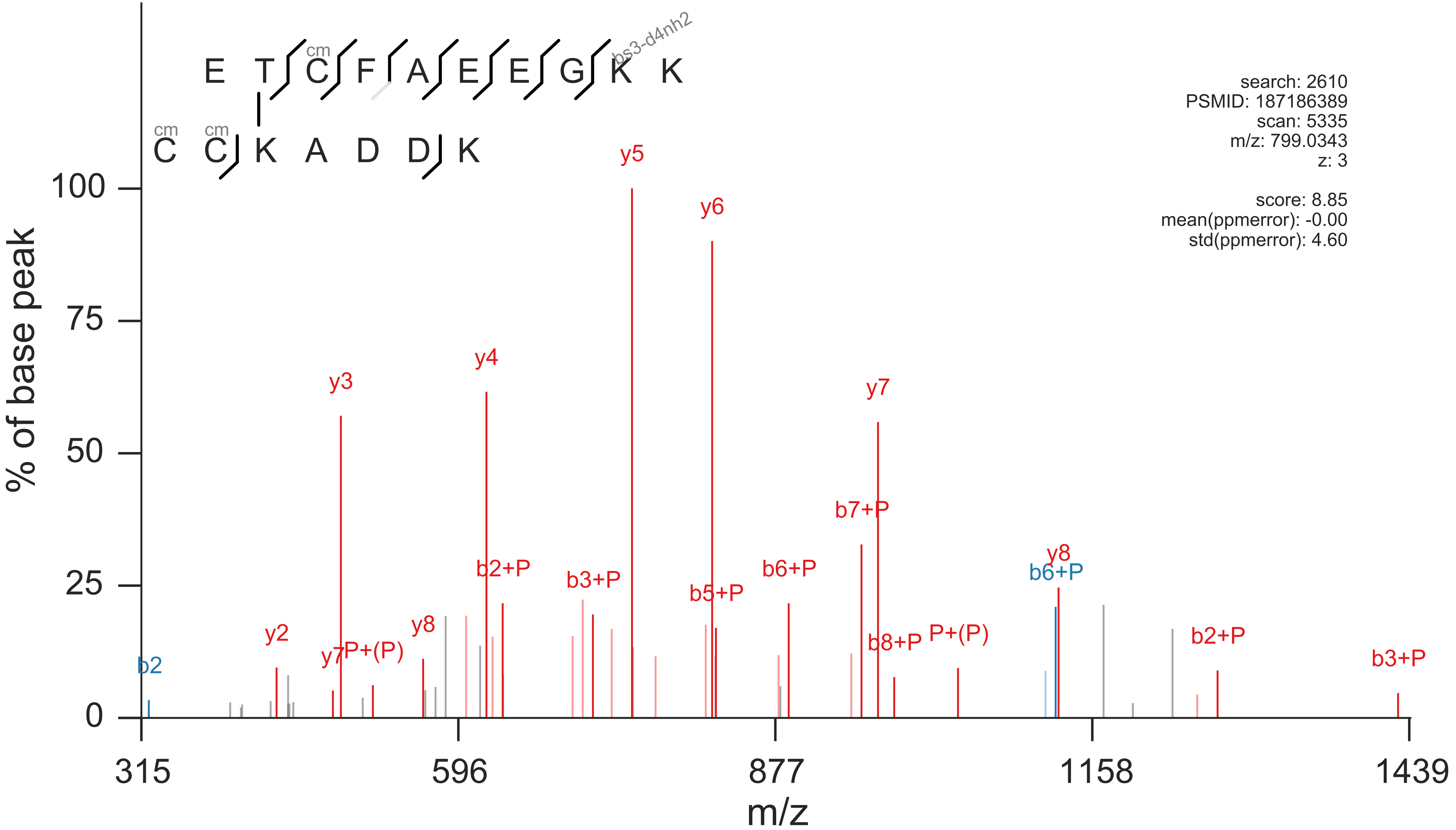

E T C F A E E G K K
cm
bs3-d4nh2
C C K A D D K
cm cm
search: 2610
PSMID: 187186389
scan: 5335
m/z: 799.0343
z: 3
score: 8.85
mean(ppmerror): -0.00
std(ppmerror): 4.60
% of base peak
100
75
50
25
0
m/z
315
596
877
1158
1439
b2
y2
y3
y7P+(P)
y8
y4
b2+P
b3+P
y5
b5+P
y6
b6+P
b7+P
y7
b8+P
P+(P)
y8
b6+P
b2+P
b3+P

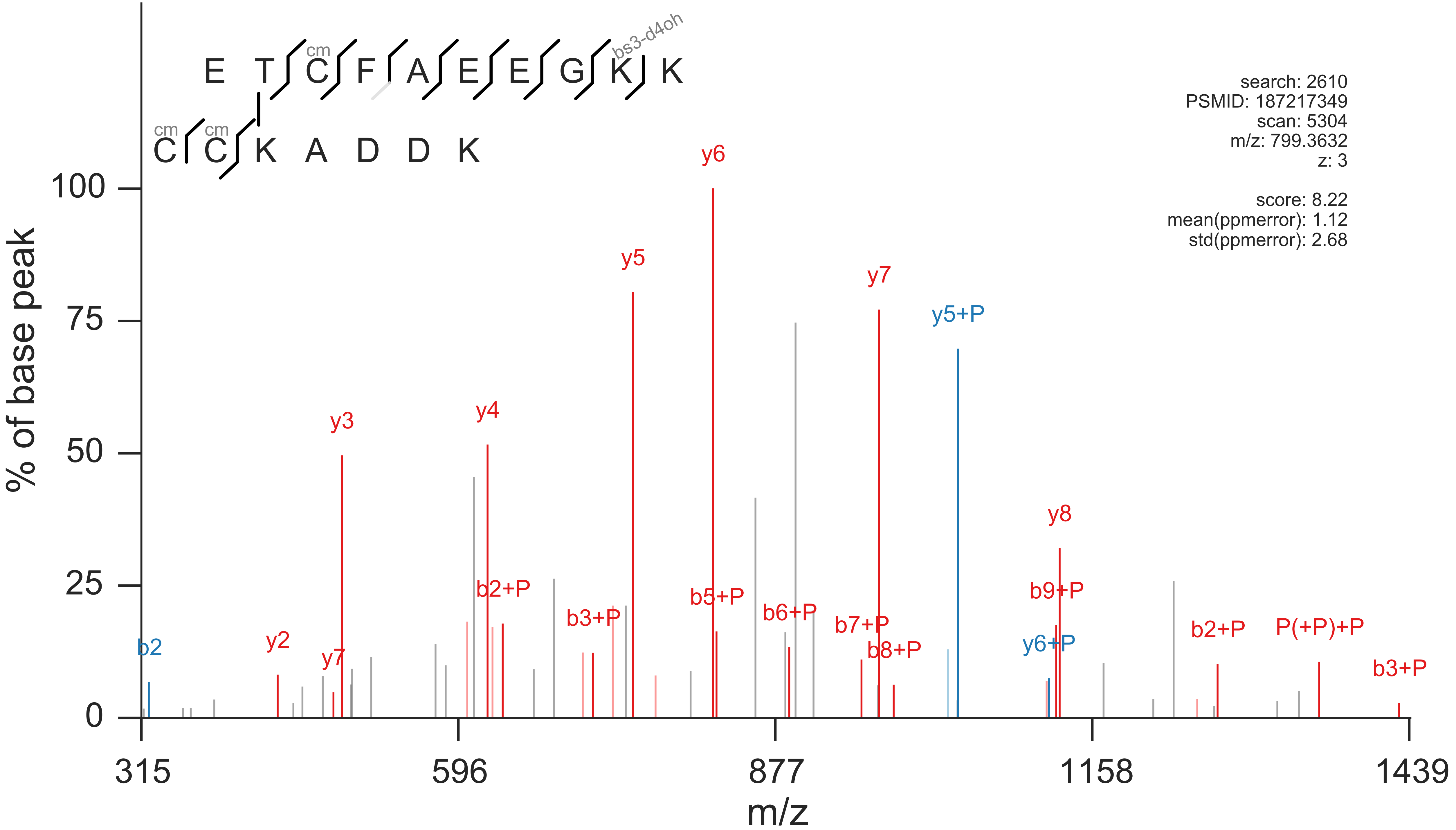

E T C F A E E G K K
cm
bs3-d4oh
C C K A D D K
cm cm
search: 2610
PSMID: 187217349
scan: 5304
m/z: 799.3632
z: 3
score: 8.22
mean(ppmerror): 1.12
std(ppmerror): 2.68
% of base peak
m/z
315
596
877
1158
1439
0
25
50
75
100
y6
y5
y7
y5+P
y3
y4
y8
b2+P
b3+P
b5+P
b6+P
b7+P
b8+P
b9+P
y6+P
b2+P
P(+P)+P
b3+P
b2
y2
y7

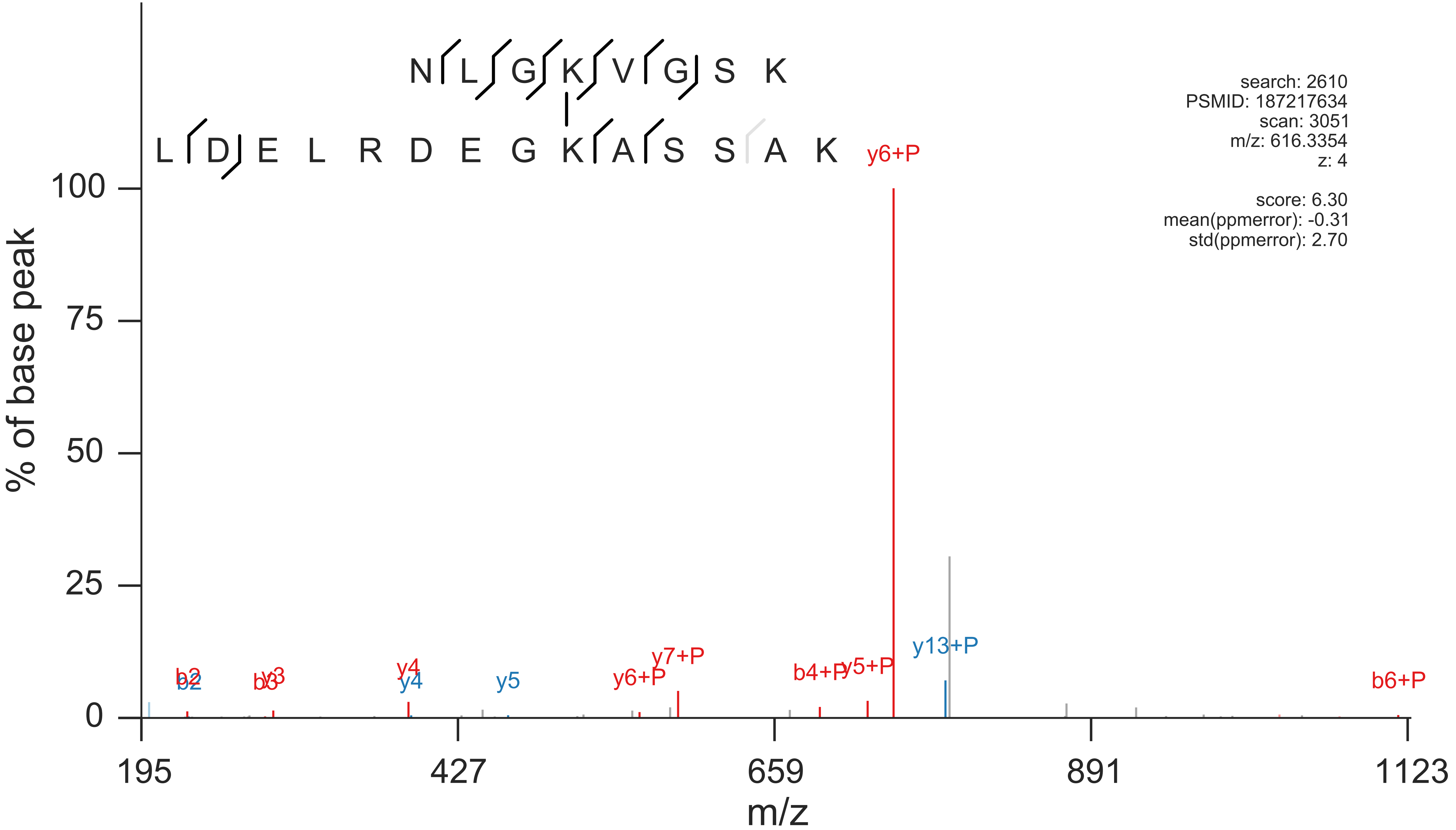

N L G K V G S K
L D E L R D E G K A S S A K
search: 2610
PSMID: 187217634
scan: 3051
m/z: 616.3354
z: 4
score: 6.30
mean(ppmerror): -0.31
std(ppmerror): 2.70
% of base peak
m/z
100
75
50
25
0
195
427
659
891
1123
y6+P
b2
b2
b3
y3
y4
y4
y5
y6+P
y7+P
b4+P
y5+P
y13+P
b6+P

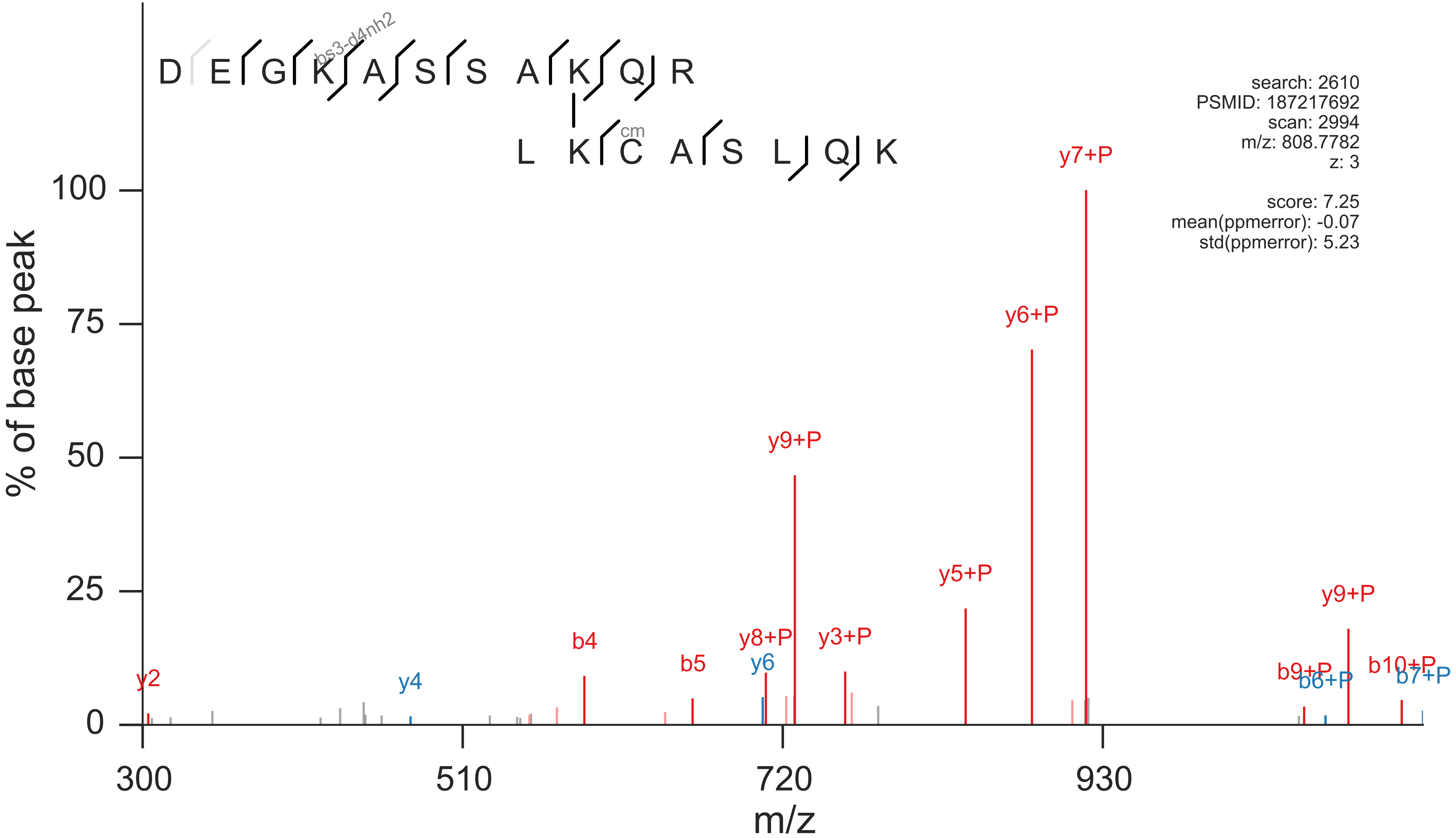

D E G K A S S A K Q R
bs3-d4nh2
L K C A S L Q K
cm
search: 2610
PSMID: 187217692
scan: 2994
m/z: 808.7782
z: 3
score: 7.25
mean(ppmerror): -0.07
std(ppmerror): 5.23
% of base peak
0
25
50
75
100
300
510
720
930
m/z
y7+P
y6+P
y9+P
y5+P
y9+P
y8+P
y3+P
b4
b5
y6
y4
y2
b9+P
b6+P
b10+P
b7+P

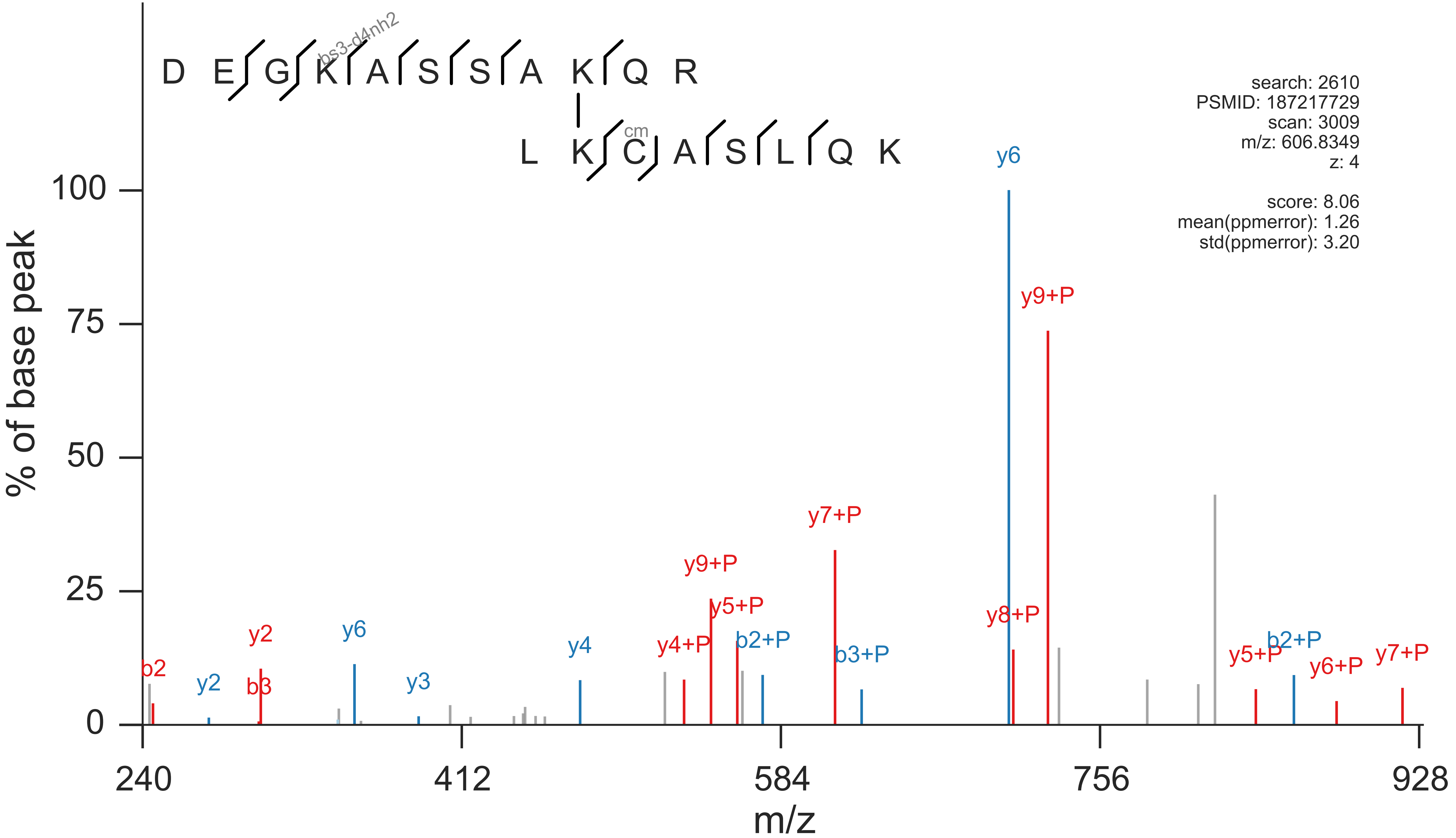

D E G K A S S A K Q R
bs3-d4nh2
L K C A S L Q K
cm
search: 2610
PSMID: 187217729
scan: 3009
m/z: 606.8349
z: 4
score: 8.06
mean(ppmerror): 1.26
std(ppmerror): 3.20
% of base peak
m/z
100
75
50
25
0
240
412
584
756
928
y6
y9+P
y7+P
y9+P
y5+P
y8+P
y4+P
b2+P
b3+P
y5+P
b2+P
y6+P
y7+P
y2
y6
y4
b2
y2
b3
y3

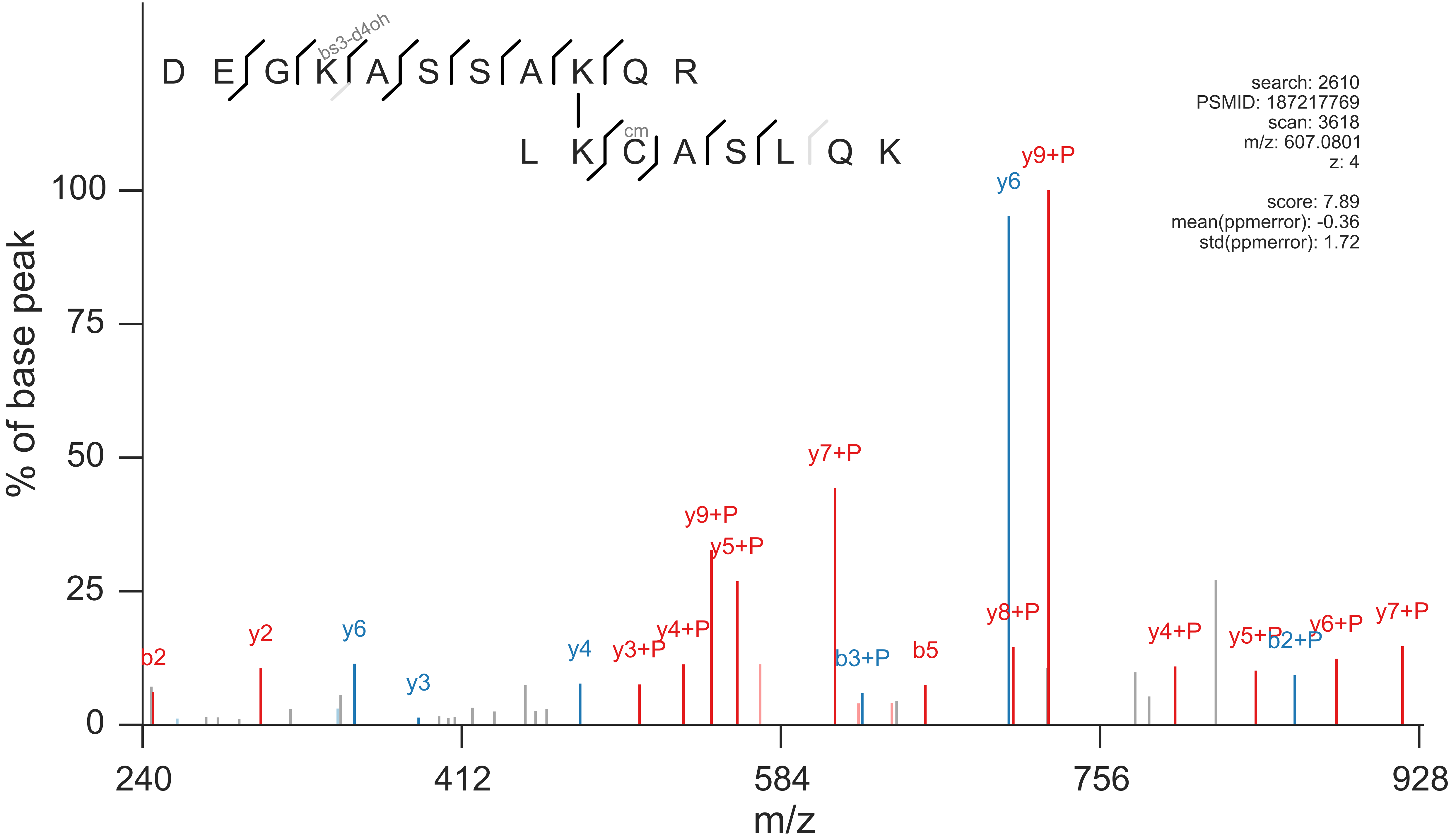

D E G K A S S A K Q R
bs3-d4oh
L K C A S L Q K
cm
search: 2610
PSMID: 187217769
scan: 3618
m/z: 607.0801
z: 4
score: 7.89
mean(ppmerror): -0.36
std(ppmerror): 1.72
% of base peak
m/z
100
75
50
25
0
240
412
584
756
928
b2
y2
y6
y3
y4
y3+P
y4+P
y9+P
y5+P
y7+P
b3+P
b5
y6
y9+P
y8+P
y4+P
y5+P
b2+P
y6+P
y7+P

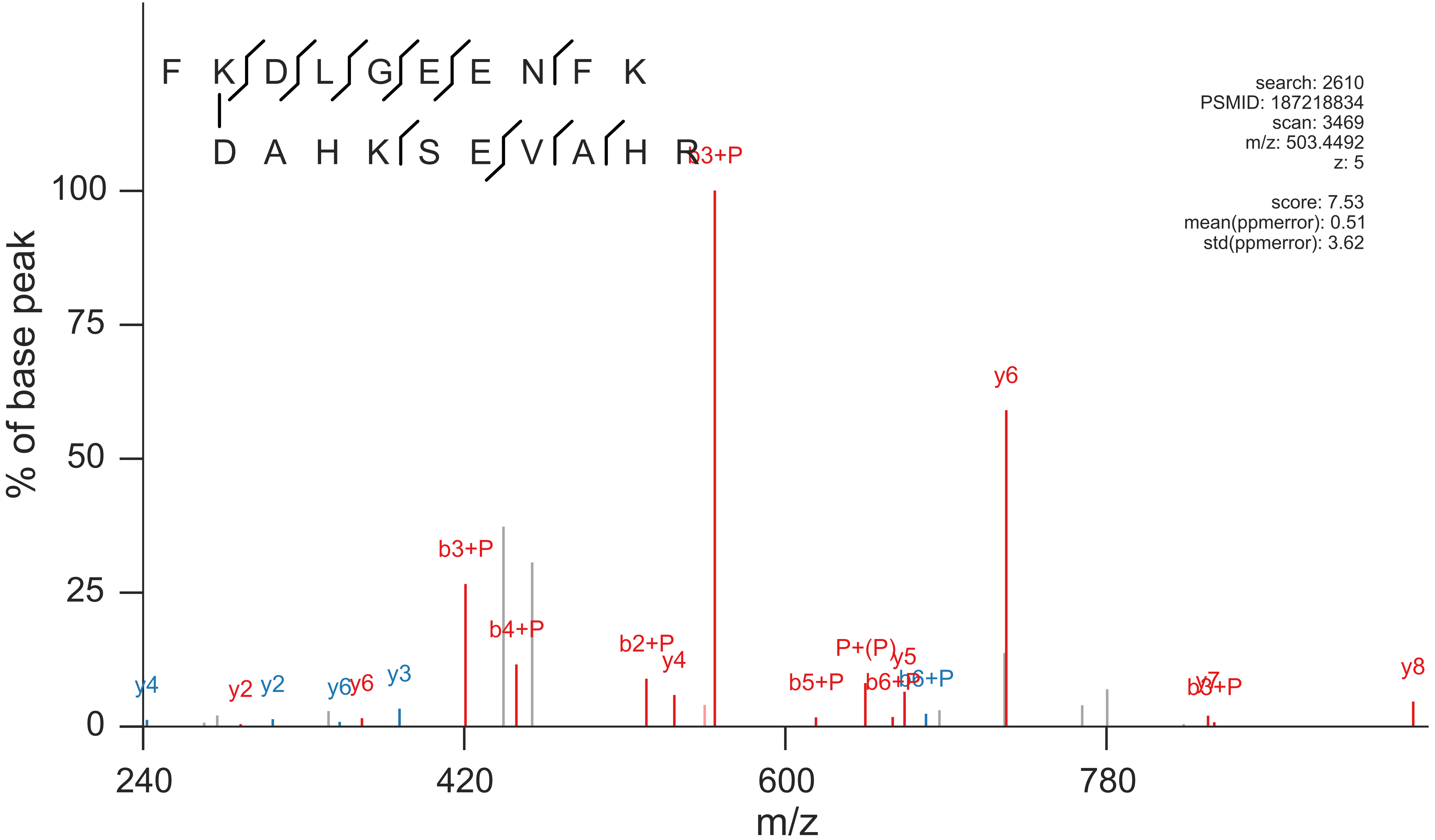

F K D L G E E N F K
D A H K S E V A H R
search: 2610
PSMID: 187218834
scan: 3469
m/z: 503.4492
z: 5
score: 7.53
mean(ppmerror): 0.51
std(ppmerror): 3.62
% of base peak
m/z
0
25
50
75
100
240
420
600
780
b3+P
y6
b3+P
b4+P
b2+P
y4
P+(P)
y5
b5+P
b6+P
b6+P
y8
b3+P
y7
y4
y2
y2
y6
y6
y3

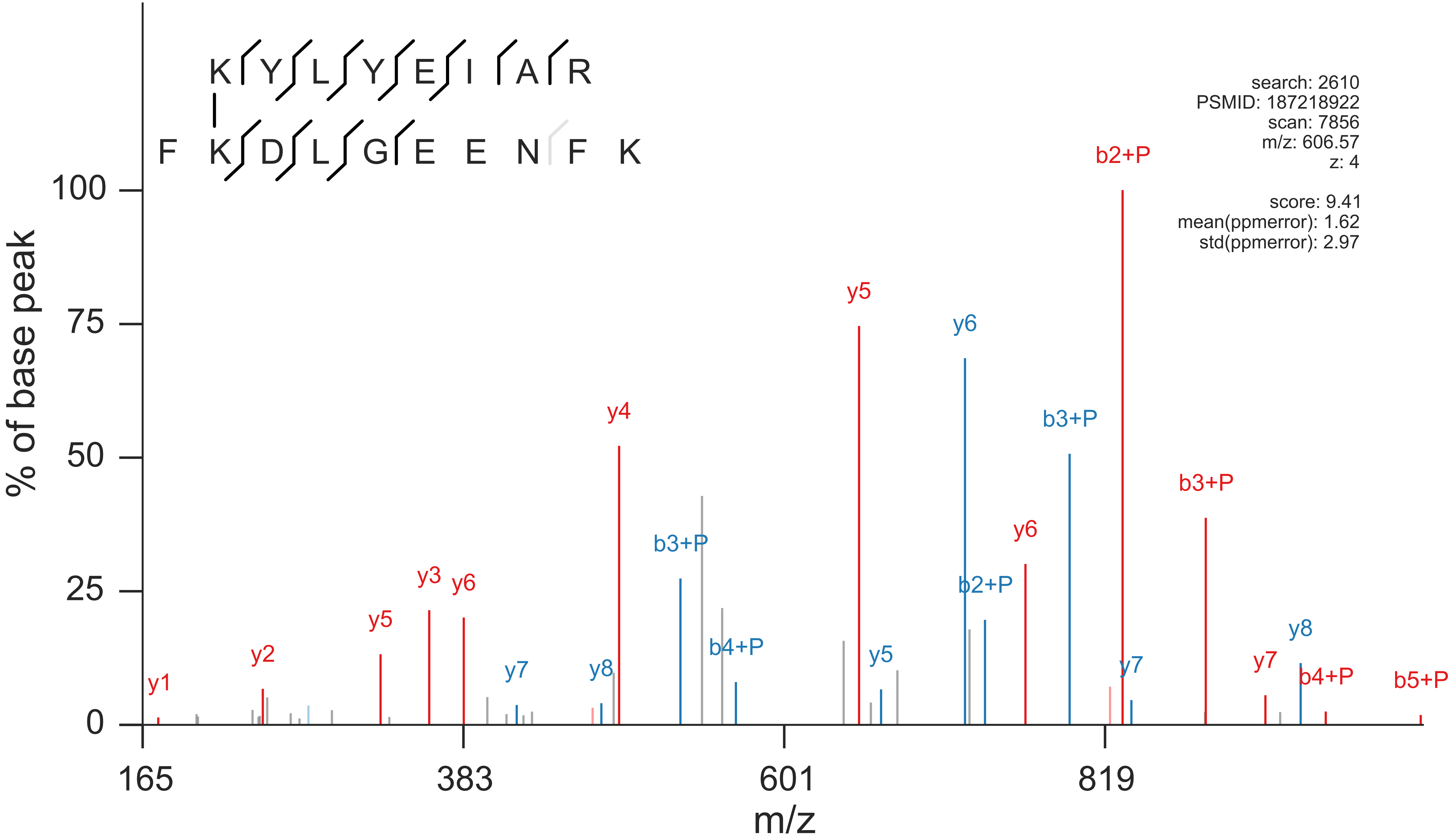
K Y L Y E I A R
F K D L G E E N F K
search: 2610
PSMID: 187218922
scan: 7856
m/z: 606.57
z: 4
score: 9.41
mean(ppmerror): 1.62
std(ppmerror): 2.97
% of base peak
m/z
165
383
601
819
0
25
50
75
100
y1
y2
y5
y3
y6
y7
y8
y4
b3+P
b4+P
y5
y5
y6
b2+P
b3+P
y6
b2+P
y7
b3+P
y7
y8
b4+P
b5+P

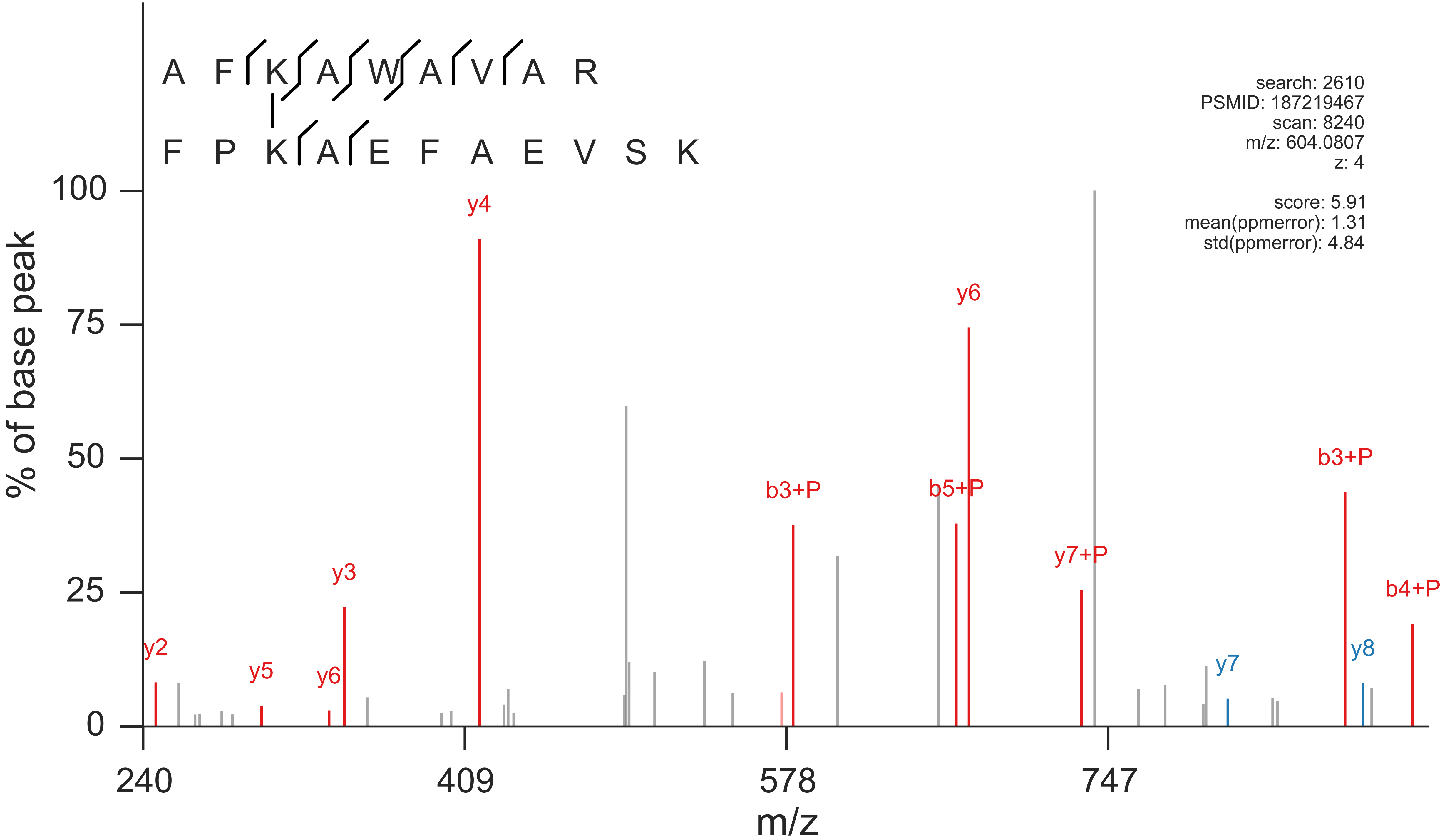

A F K A W A V A R
F P K A E F A E V S K
search: 2610
PSMID: 187219467
scan: 8240
m/z: 604.0807
z: 4
score: 5.91
mean(ppmerror): 1.31
std(ppmerror): 4.84
% of base peak
100
75
50
25
0
y4
y6
y3
y2
y5
y6
b3+P
b5+P
y7+P
b3+P
b4+P
y7
y8
240
409
578
747
m/z

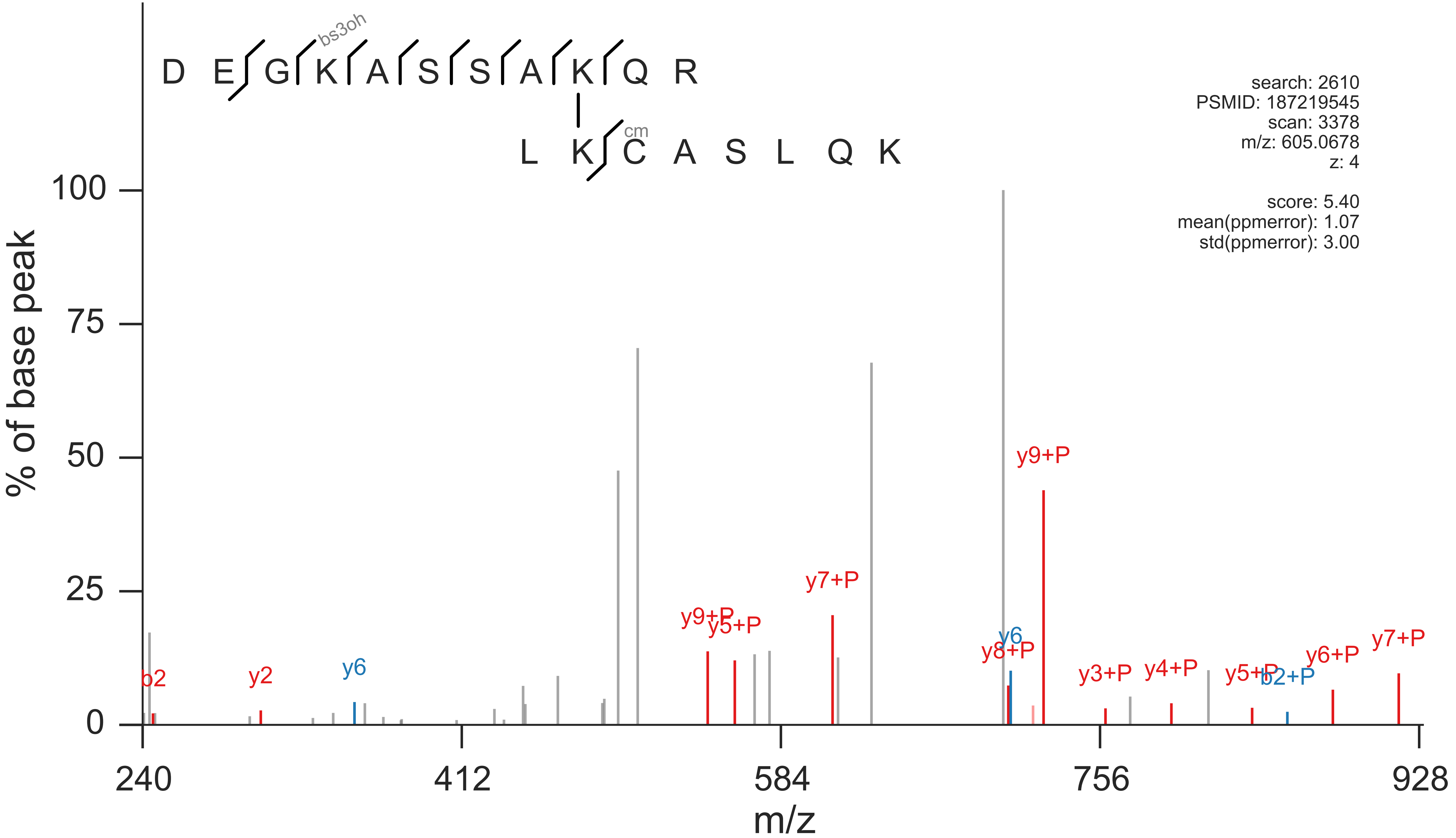

D E G K A S S A K Q R
bs3oh
L K C A S L Q K
cm
search: 2610
PSMID: 187219545
scan: 3378
m/z: 605.0678
z: 4
score: 5.40
mean(ppmerror): 1.07
std(ppmerror): 3.00
% of base peak
m/z
0
25
50
75
100
240
412
584
756
928
b2
y2
y6
y9+P
y5+P
y7+P
y8+P
y6
y9+P
y3+P
y4+P
y5+P
b2+P
y6+P
y7+P

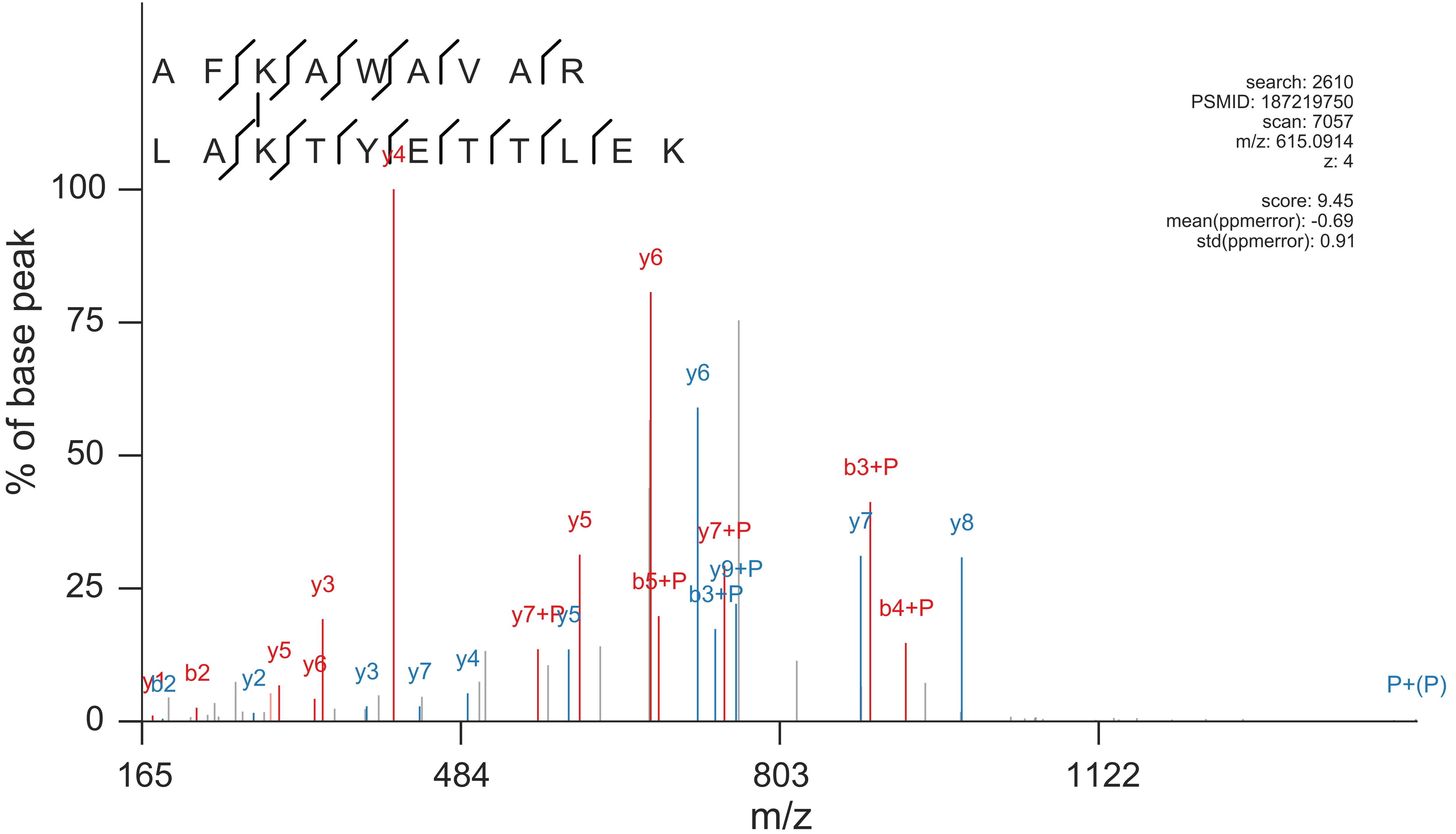
A F K A W A V A R
L A K T Y E T T L E K
search: 2610
PSMID: 187219750
scan: 7057
m/z: 615.0914
z: 4
score: 9.45
mean(ppmerror): -0.69
std(ppmerror): 0.91
% of base peak
m/z
0
25
50
75
100
165
484
803
1122
y1
b2
b2
y2
y5
y6
y3
y3
y7
y4
y4
y7+P
y5
y5
y6
b5+P
y6
y7+P
y9+P
b3+P
b3+P
y7
b4+P
y8
P+(P)

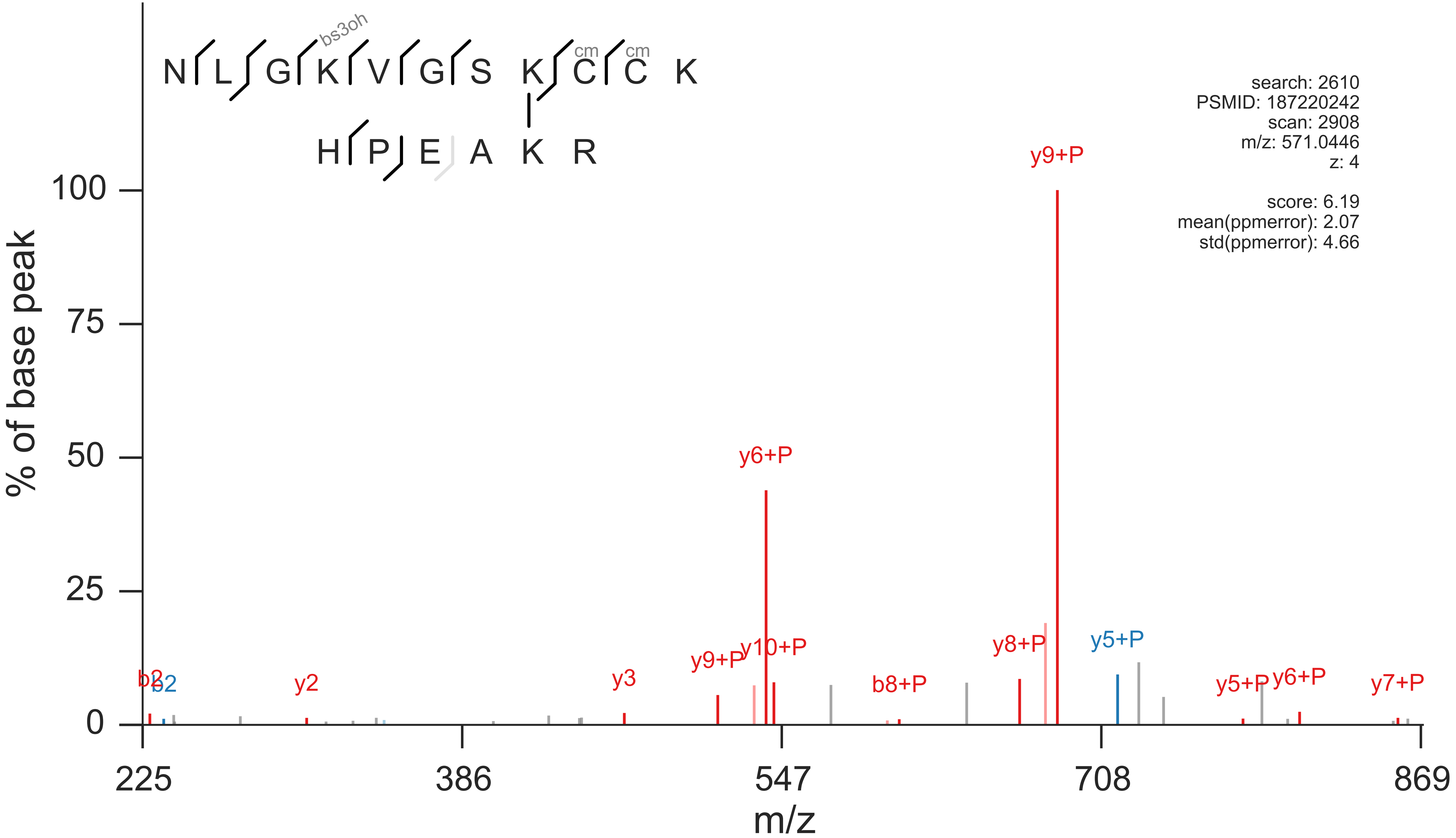
N L G K V G S K C C K
bs3oh
cm
cm
H P E A K R
search: 2610
PSMID: 187220242
scan: 2908
m/z: 571.0446
z: 4
score: 6.19
mean(ppmerror): 2.07
std(ppmerror): 4.66
% of base peak
100
75
50
25
0
y9+P
y6+P
y10+P
y9+P
y8+P
y5+P
b2
b2
y2
y3
b8+P
y5+P
y6+P
y7+P
225
386
547
708
869
m/z

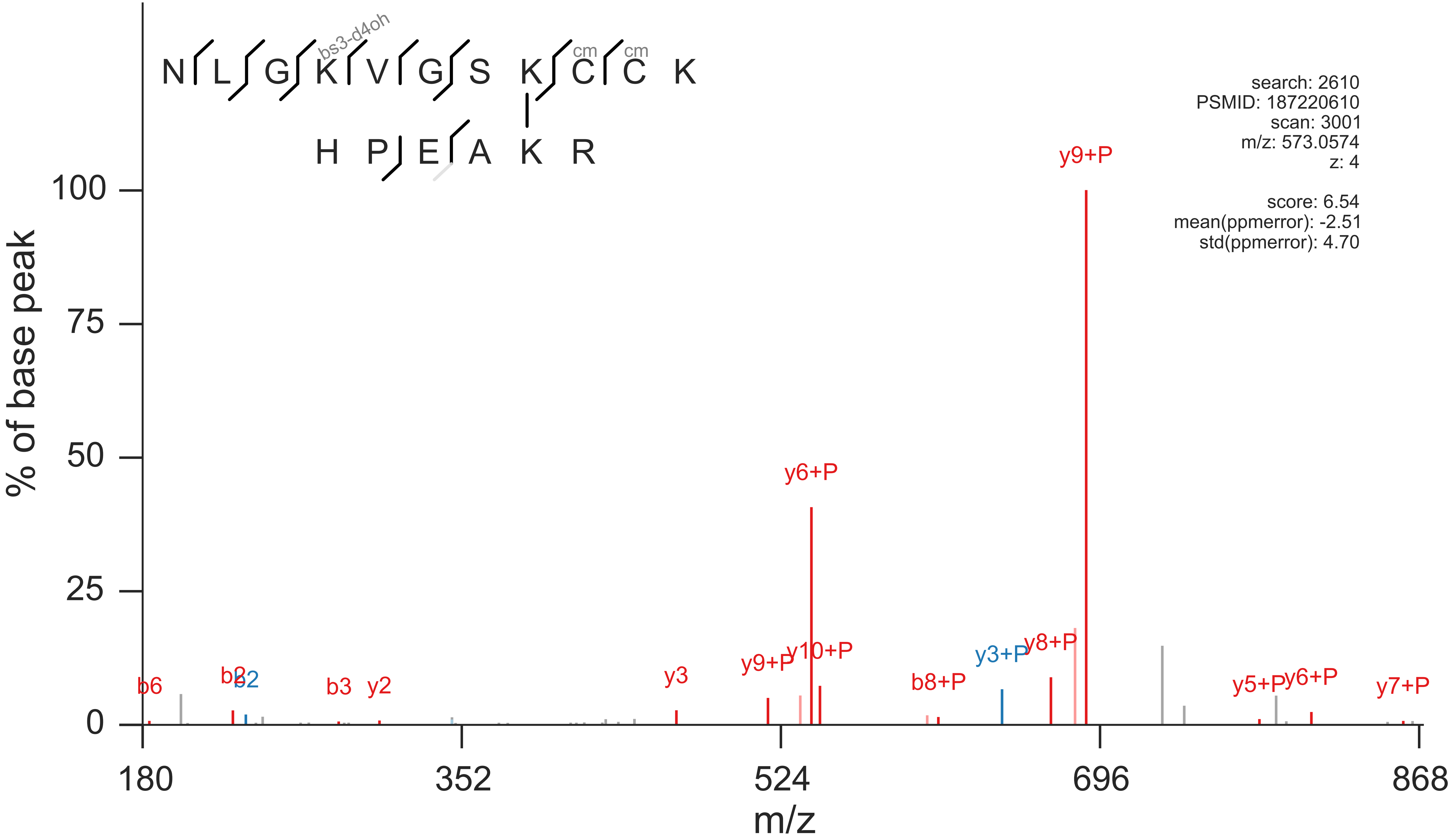

N L G K V G S K C C K
bs3-d4oh
cm
cm
H P E A K R
search: 2610
PSMID: 187220610
scan: 3001
m/z: 573.0574
z: 4
score: 6.54
mean(ppmerror): -2.51
std(ppmerror): 4.70
% of base peak
m/z
y9+P
y6+P
y10+P
y9+P
y8+P
y3+P
b8+P
y3
b6
b2
b2
b3
y2
y5+P
y6+P
y7+P
0
25
50
75
100
180
352
524
696
868

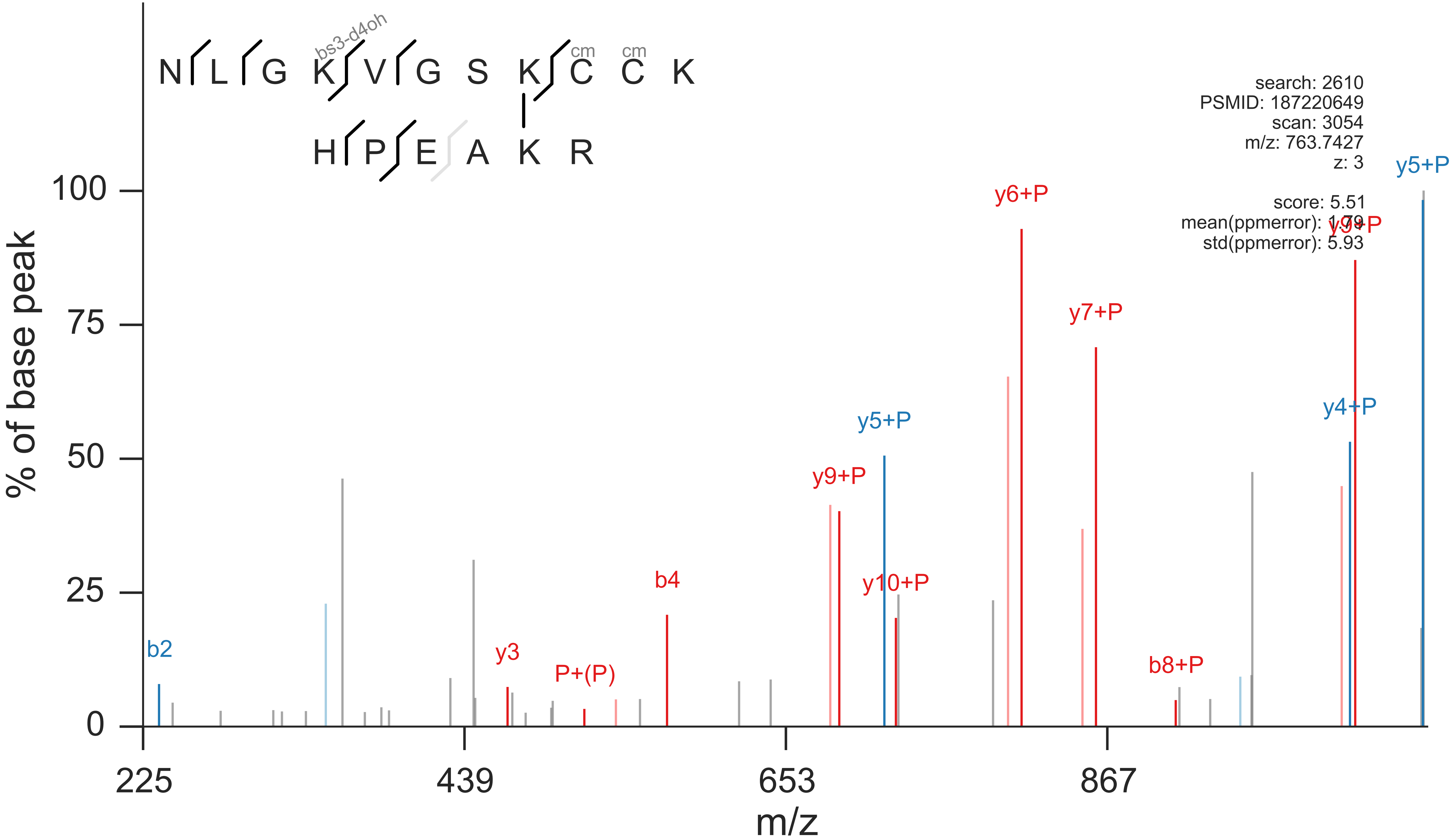

N L G K V G S K C C K
bs3-d4oh
cm
cm
H P E A K R
search: 2610
PSMID: 187220649
scan: 3054
m/z: 763.7427
z: 3
score: 5.51
mean(ppmerror): 1.79
std(ppmerror): 5.93
% of base peak
m/z
100
75
50
25
0
225
439
653
867
b2
y3
P+(P)
b4
y9+P
y5+P
y10+P
y6+P
y7+P
b8+P
y4+P
y9+P
y5+P

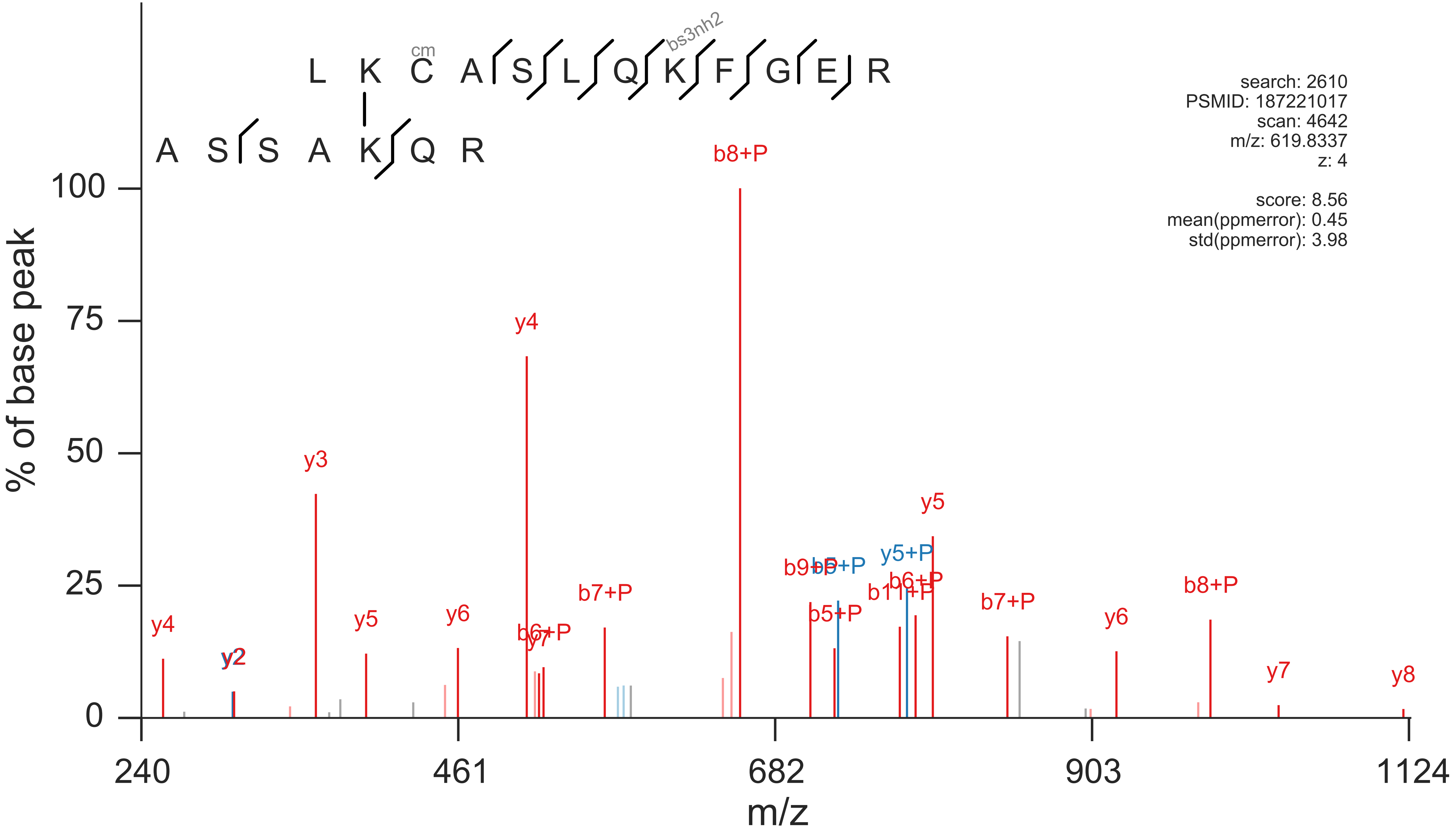
L K C A S L Q K F G E R
cm
bs3nh2
A S S A K Q R
search: 2610
PSMID: 187221017
scan: 4642
m/z: 619.8337
z: 4
score: 8.56
mean(ppmerror): 0.45
std(ppmerror): 3.98
% of base peak
m/z
0
25
50
75
100
240
461
682
903
1124
y4
y2
y3
y5
y6
y4
b6+P
y7
b7+P
b8+P
b9+P
b5+P
y5+P
b11+P
b6+P
y5
b7+P
y6
b8+P
y7
y8

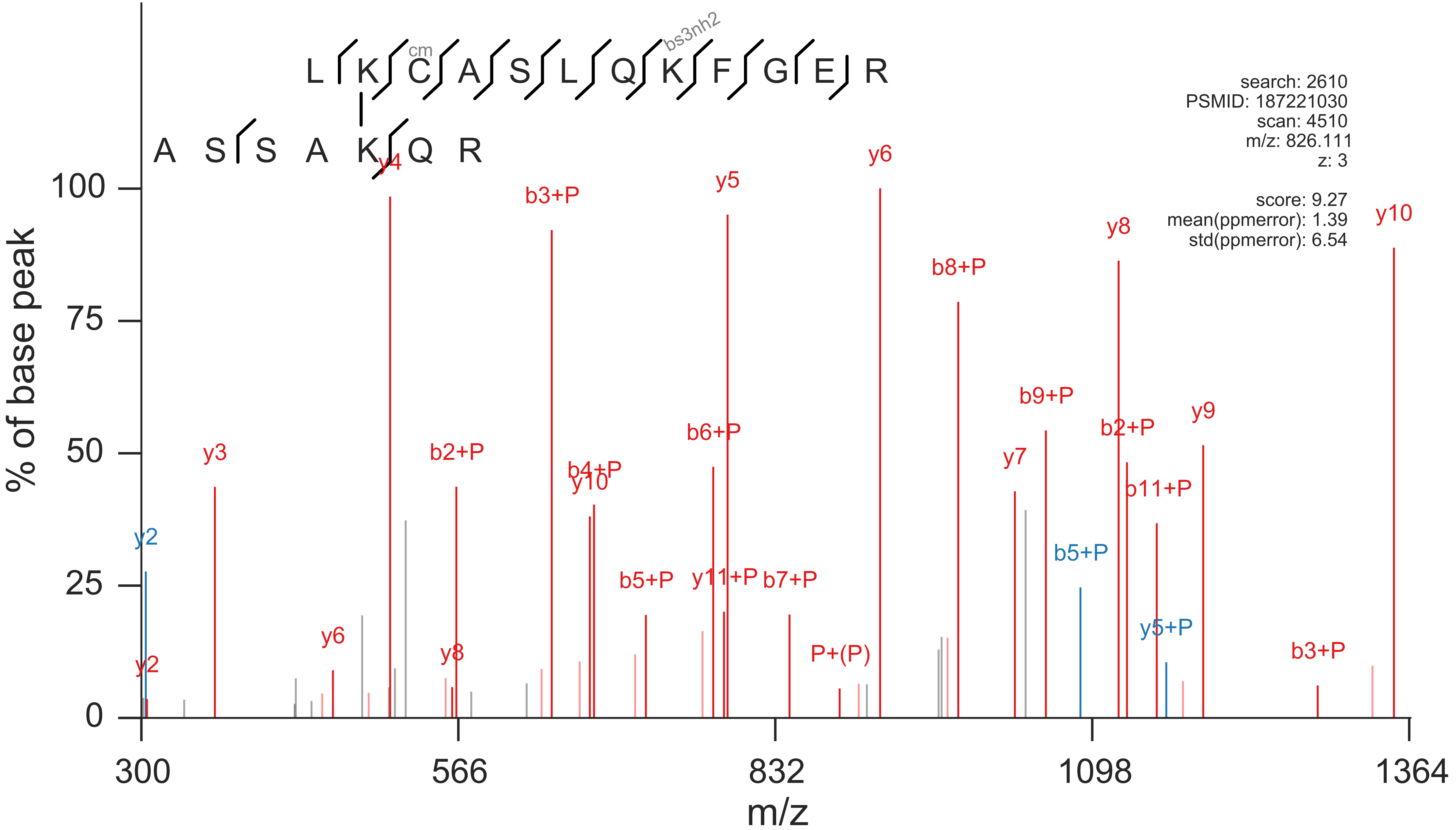

L K C A S L Q K F G E R
cm
bs3nh2
A S S A K Q R
search: 2610
PSMID: 187221030
scan: 4510
m/z: 826.111
z: 3
score: 9.27
mean(ppmerror): 1.39
std(ppmerror): 6.54
% of base peak
m/z
0
25
50
75
100
300
566
832
1098
1364
y2
y2
y3
y6
y4
b2+P
y8
b3+P
b4+P
y10
b5+P
b6+P
y11+P
y5
b7+P
P+(P)
y6
b8+P
y7
b9+P
b5+P
y8
b2+P
b11+P
y5+P
y9
b3+P
y10

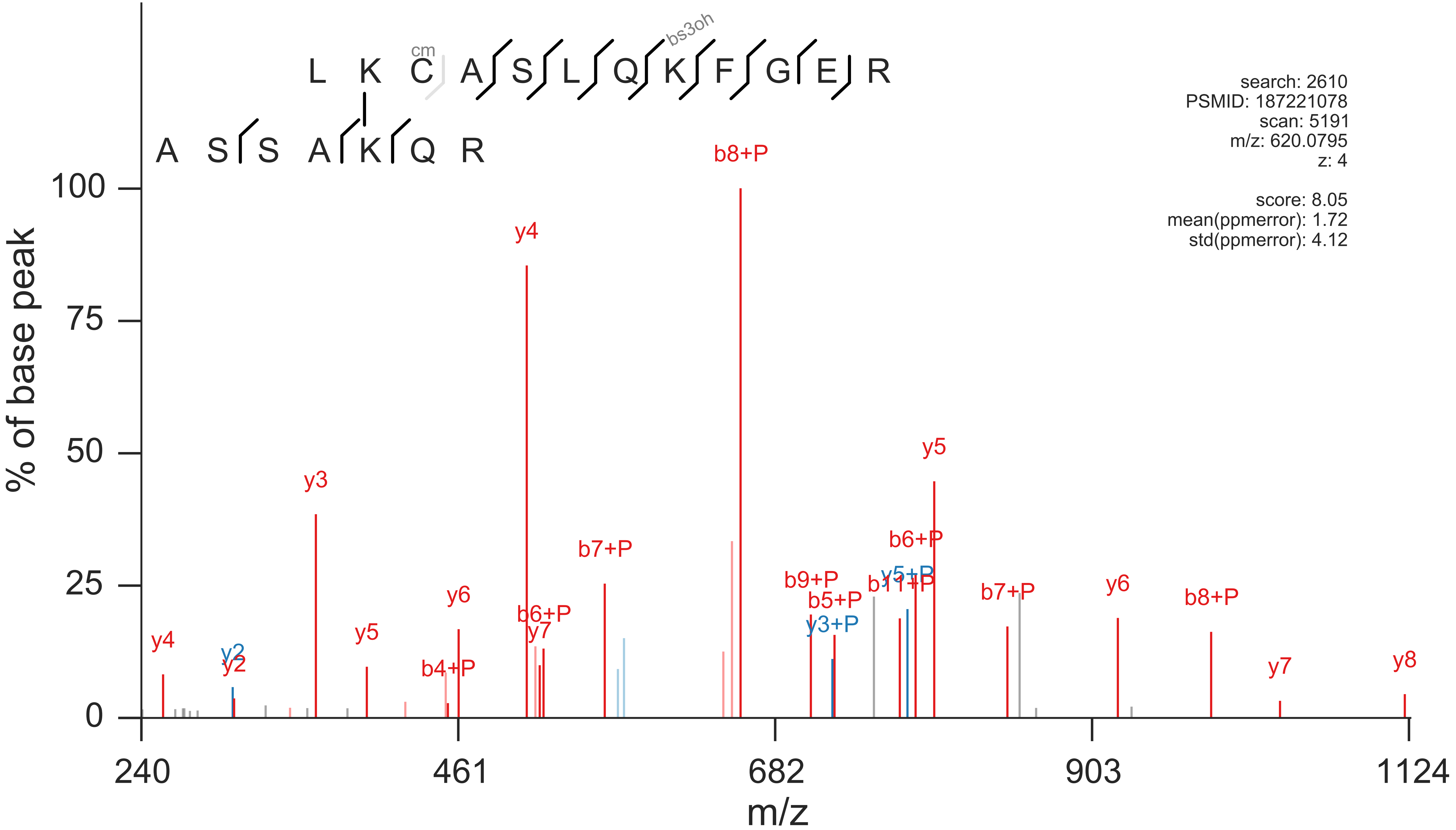

L K C A S L Q K F G E R
cm
bs3oh
A S S A K Q R
search: 2610
PSMID: 187221078
scan: 5191
m/z: 620.0795
z: 4
score: 8.05
mean(ppmerror): 1.72
std(ppmerror): 4.12
% of base peak
m/z
0
25
50
75
100
240
461
682
903
1124
b8+P
y4
y5
y3
b6+P
b7+P
y5+P
b11+P
b9+P
b5+P
y3+P
b7+P
y6
b8+P
y6
b6+P
y7
y5
y4
y2
y2
b4+P
y7
y8

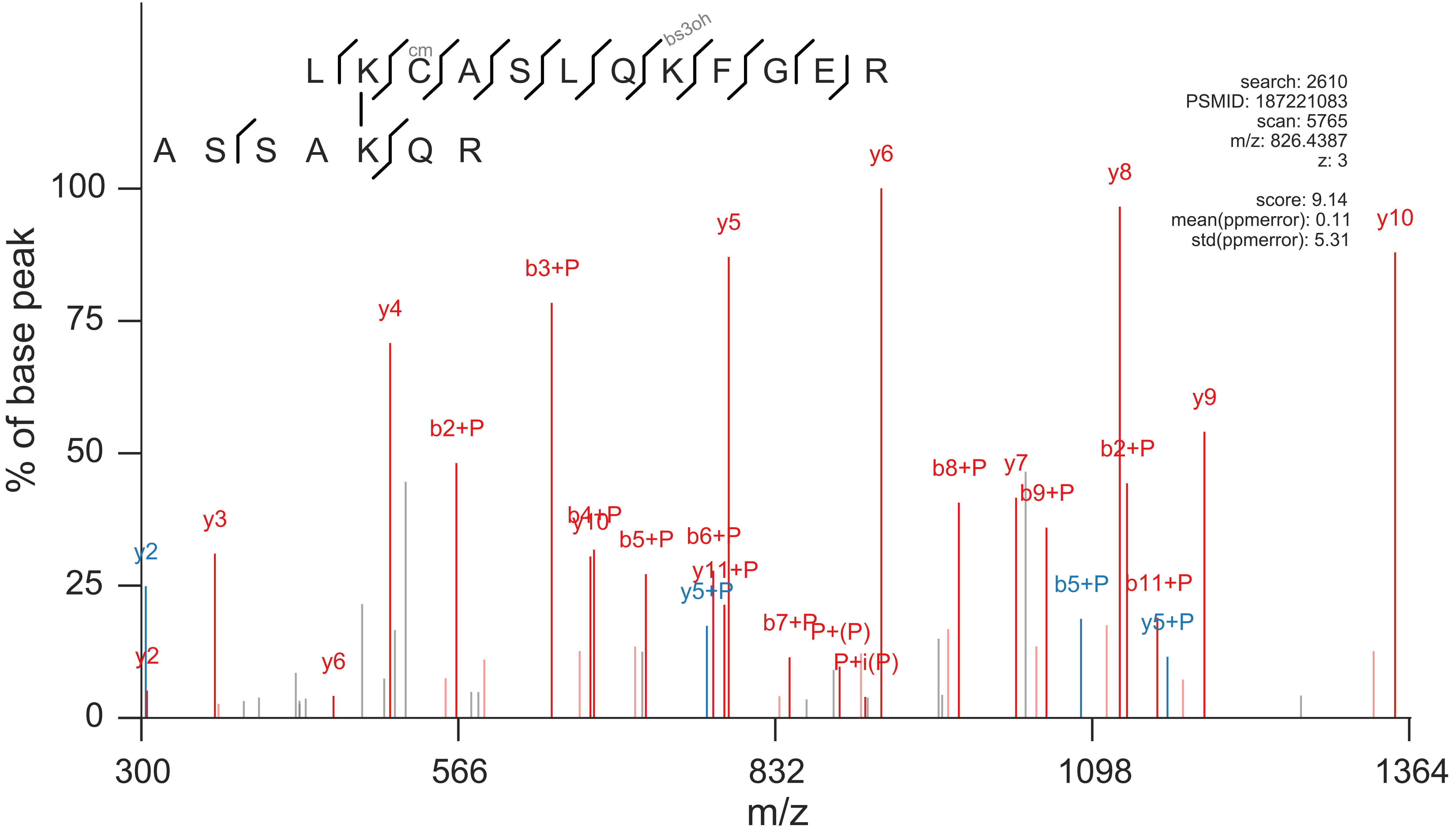
L K C A S L Q K F G E R
cm
bs3oh
A S S A K Q R
search: 2610
PSMID: 187221083
scan: 5765
m/z: 826.4387
z: 3
score: 9.14
mean(ppmerror): 0.11
std(ppmerror): 5.31
% of base peak
m/z
0
25
50
75
100
300
566
832
1098
1364
y2
y2
y3
y6
y4
b2+P
b3+P
b4+P
y10
b5+P
b6+P
y5
y11+P
y5+P
b7+P
P+(P)
P+i(P)
y6
b8+P
y7
b9+P
b5+P
y8
b2+P
b11+P
y5+P
y9
y10

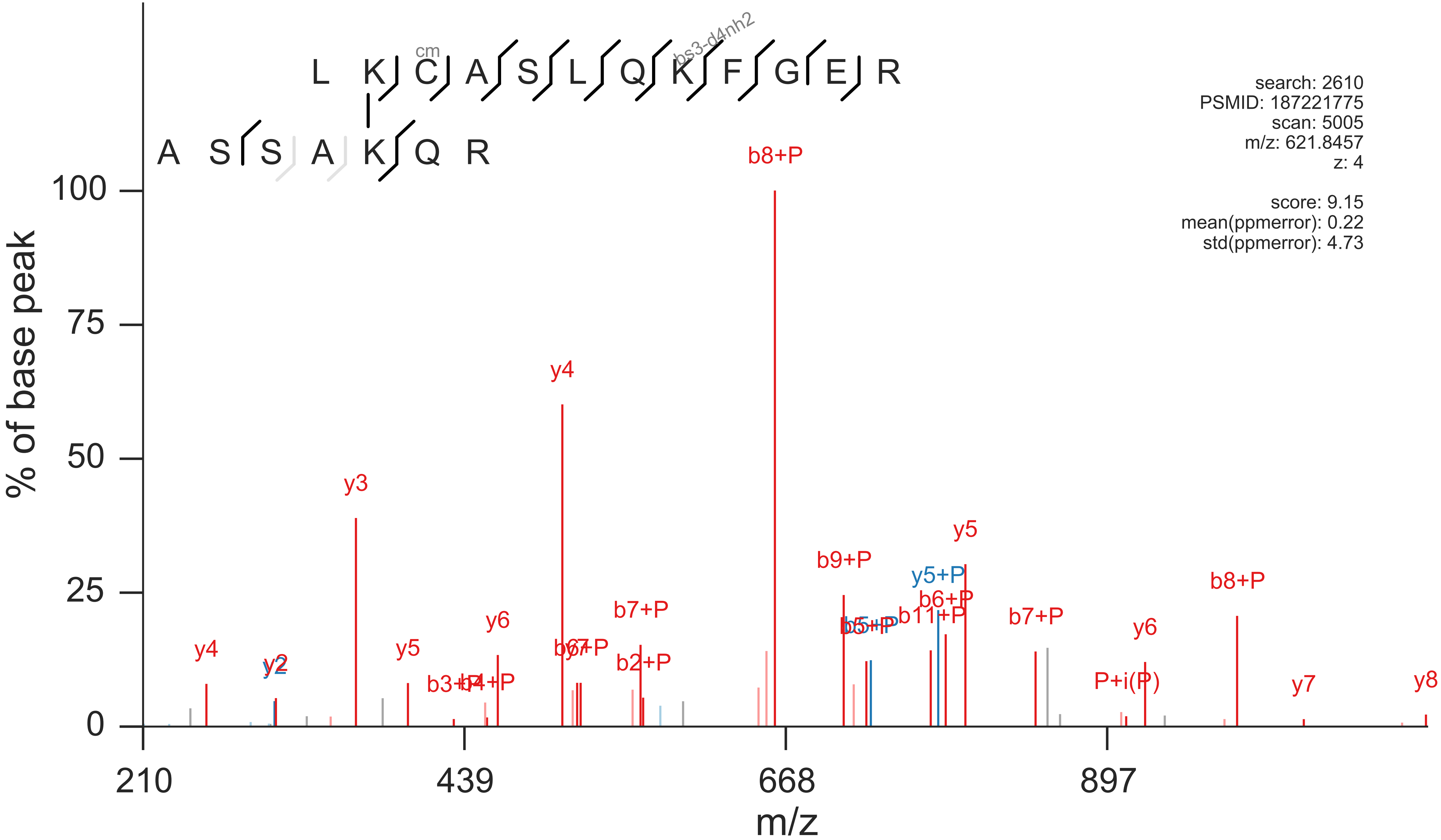

L K C A S L Q K F G E R
cm
bs3-d4nh2
A S S A K Q R
search: 2610
PSMID: 187221775
scan: 5005
m/z: 621.8457
z: 4
score: 9.15
mean(ppmerror): 0.22
std(ppmerror): 4.73
% of base peak
m/z
0
25
50
75
100
210
439
668
897
b8+P
y4
y3
y5
b9+P
y5+P
b6+P
b11+P
b7+P
b8+P
y6
y4
y2
y5
y6
b7+P
b2+P
P+i(P)
y7
y8

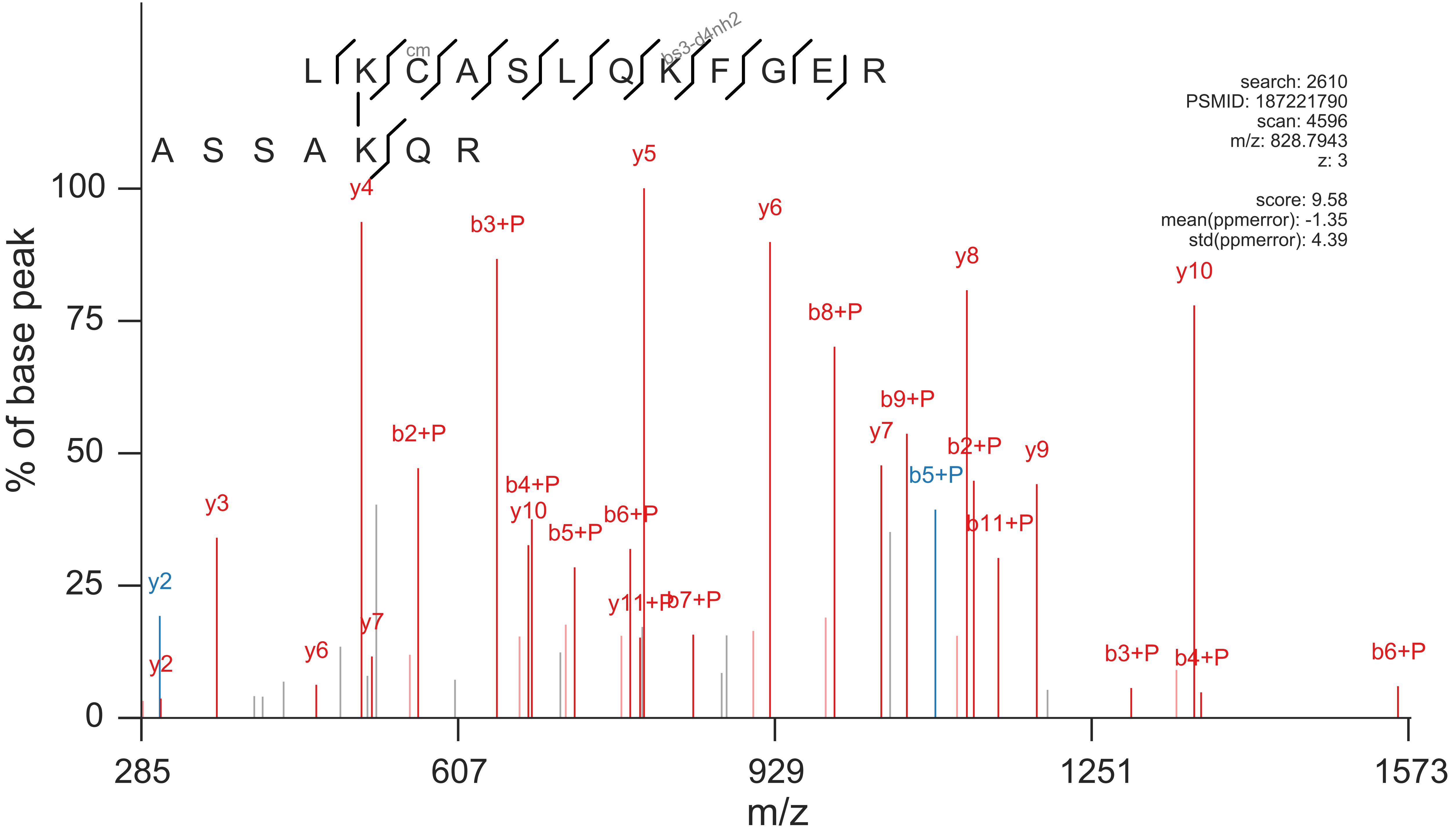

L K C A S L Q K F G E R
cm
bs3-d4nh2
A S S A K Q R
search: 2610
PSMID: 187221790
scan: 4596
m/z: 828.7943
z: 3
score: 9.58
mean(ppmerror): -1.35
std(ppmerror): 4.39
% of base peak
m/z
100
75
50
25
0
285
607
929
1251
1573
y2
y2
y3
y6
y4
y7
b2+P
b3+P
b4+P
y10
b5+P
b6+P
y11+P
b7+P
y5
y6
b8+P
y7
b9+P
b5+P
y8
b2+P
b11+P
y9
y10
b3+P
b4+P
b6+P

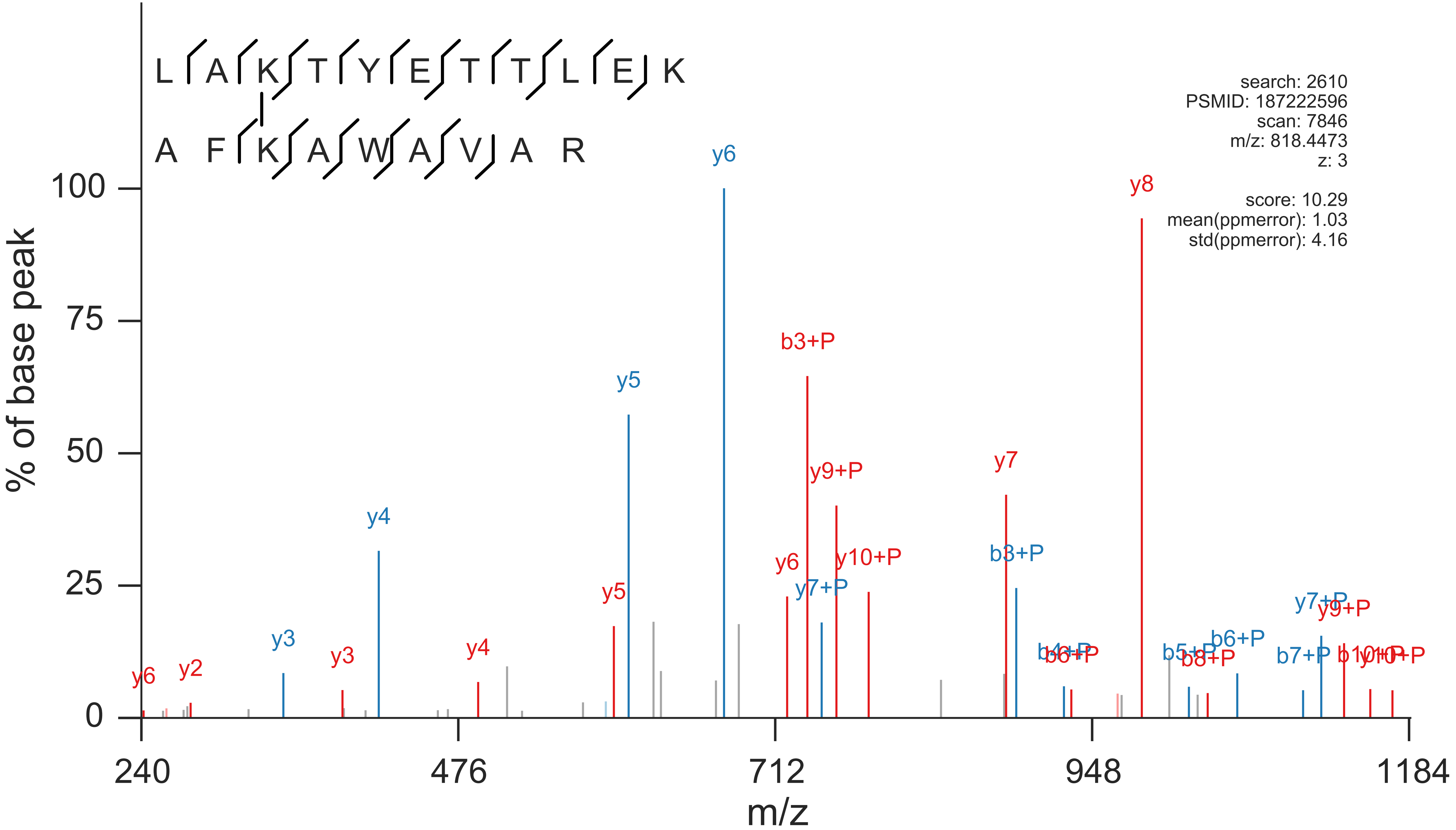
L A K T Y E T T L E K
A F K A W A V A R
search: 2610
PSMID: 187222596
scan: 7846
m/z: 818.4473
z: 3
score: 10.29
mean(ppmerror): 1.03
std(ppmerror): 4.16
% of base peak
0
25
50
75
100
m/z
240
476
712
948
1184
y6
y2
y3
y3
y4
y4
y5
y5
y6
y6
b3+P
y7+P
y9+P
y10+P
y7
b3+P
y8
b5+P
b8+P
b6+P
b7+P
y7+P
y9+P

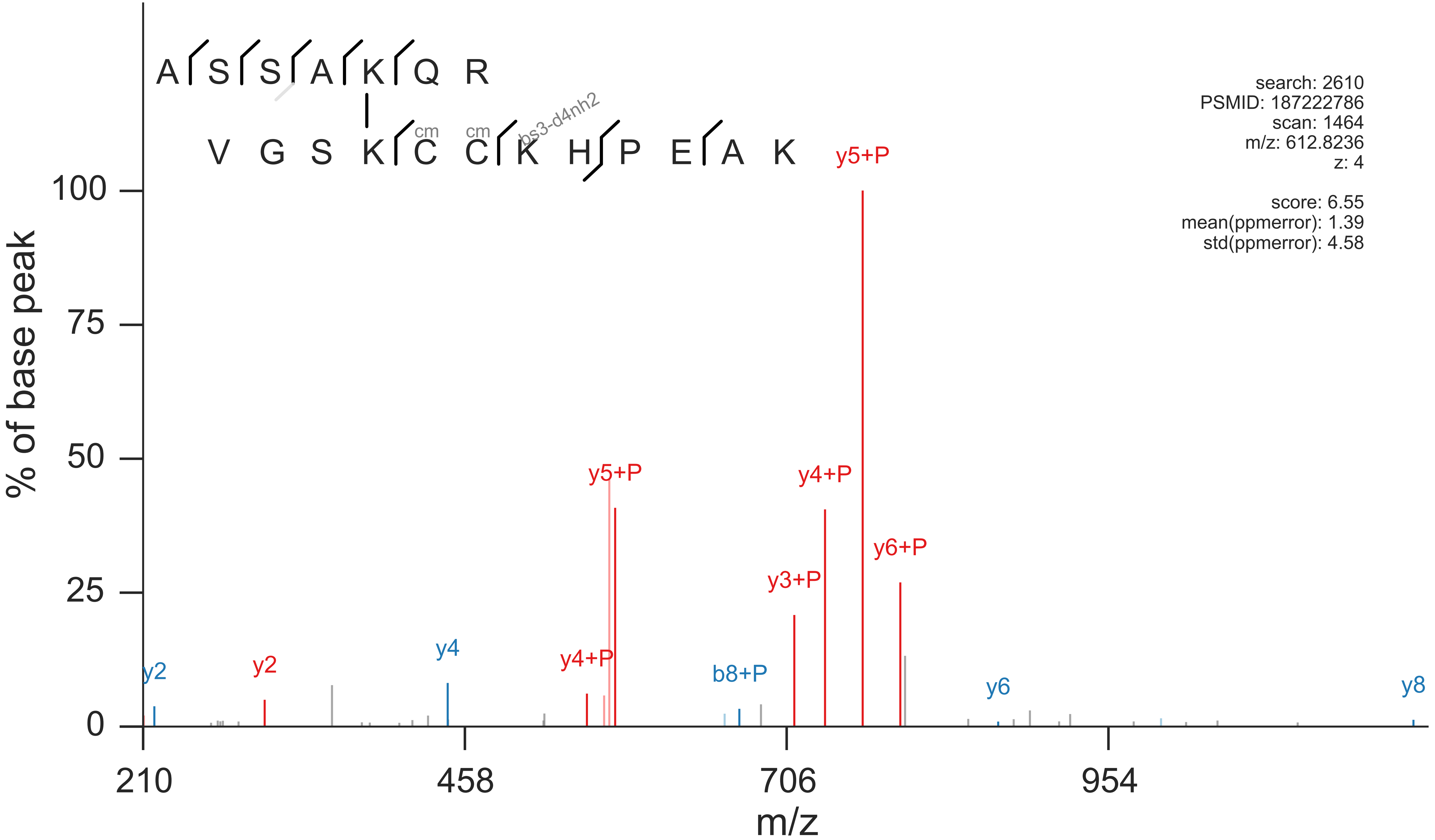

A S S A K Q R
V G S K C C K H P E A K
cm
cm
bs3-d4nh2
search: 2610
PSMID: 187222786
scan: 1464
m/z: 612.8236
z: 4
score: 6.55
mean(ppmerror): 1.39
std(ppmerror): 4.58
% of base peak
m/z
100
75
50
25
0
210
458
706
954
y5+P
y5+P
y4+P
y6+P
y3+P
y2
y2
y4
y4+P
b8+P
y6
y8

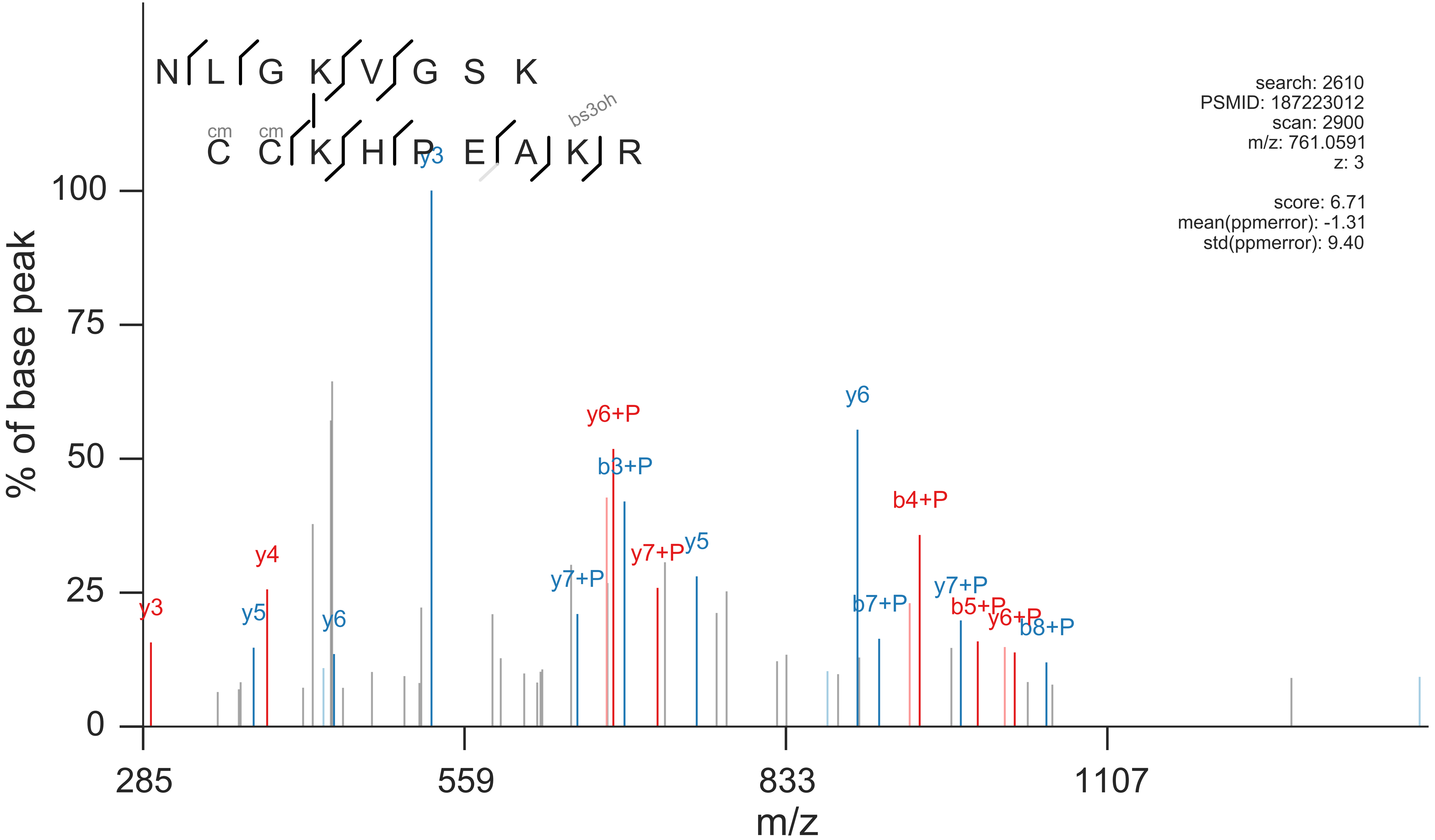

N L G K V G S K
cm cm bs3oh
C C K H P E A K R
search: 2610
PSMID: 187223012
scan: 2900
m/z: 761.0591
z: 3
score: 6.71
mean(ppmerror): -1.31
std(ppmerror): 9.40
% of base peak
m/z
0
25
50
75
100
285
559
833
1107
y3
y4
y5
y6
y3
y7+P
y6+P
b3+P
y7+P
y5
y6
b7+P
b4+P
y7+P
b5+P
y6+P
b8+P

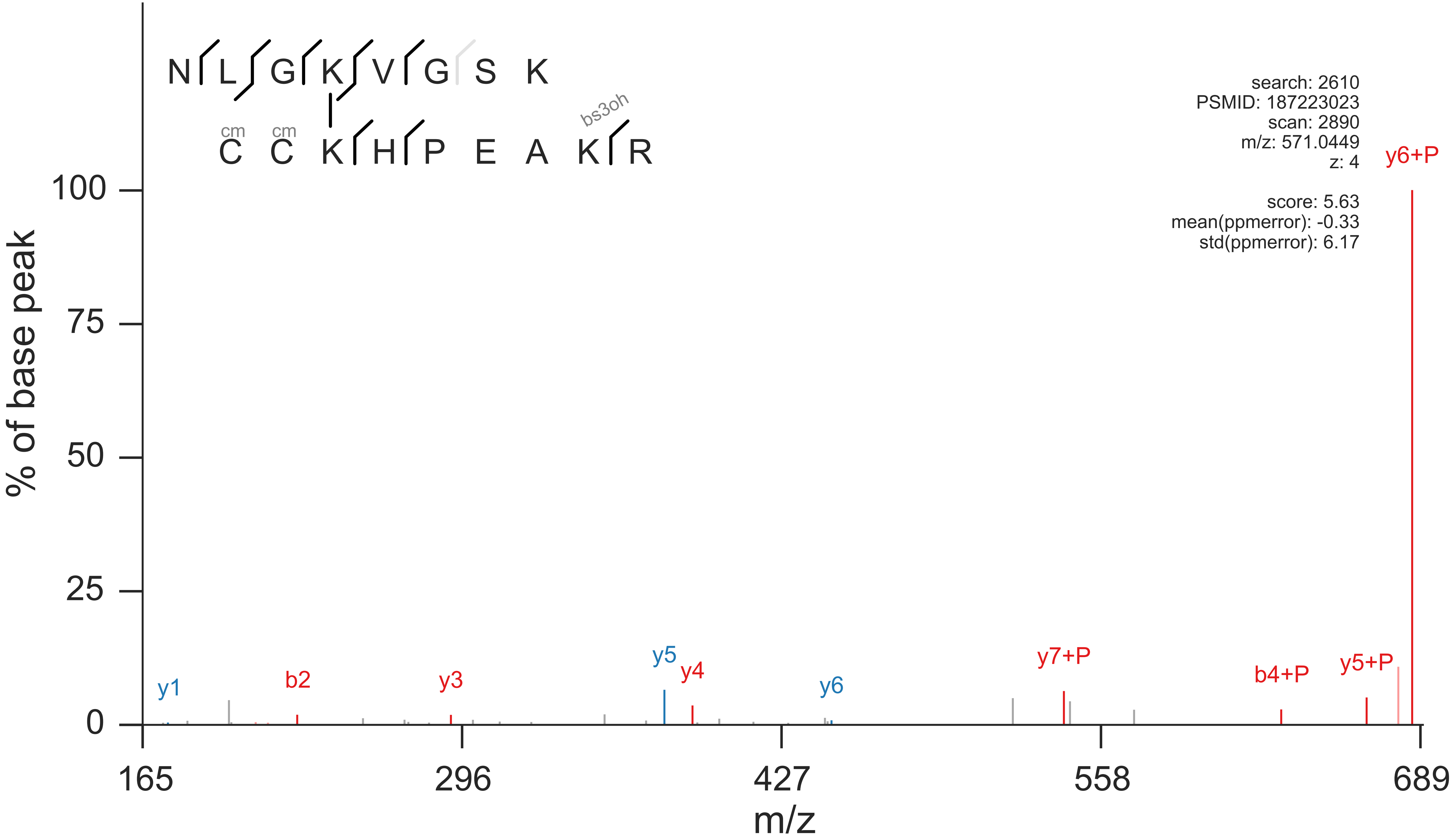

N L G K V G S K
cm cm bs3oh
C C K H P E A K R
search: 2610
PSMID: 187223023
scan: 2890
m/z: 571.0449
z: 4
score: 5.63
mean(ppmerror): -0.33
std(ppmerror): 6.17
% of base peak
100
75
50
25
0
m/z
165
296
427
558
689
y1
b2
y3
y5
y4
y6
y7+P
b4+P
y5+P
y6+P

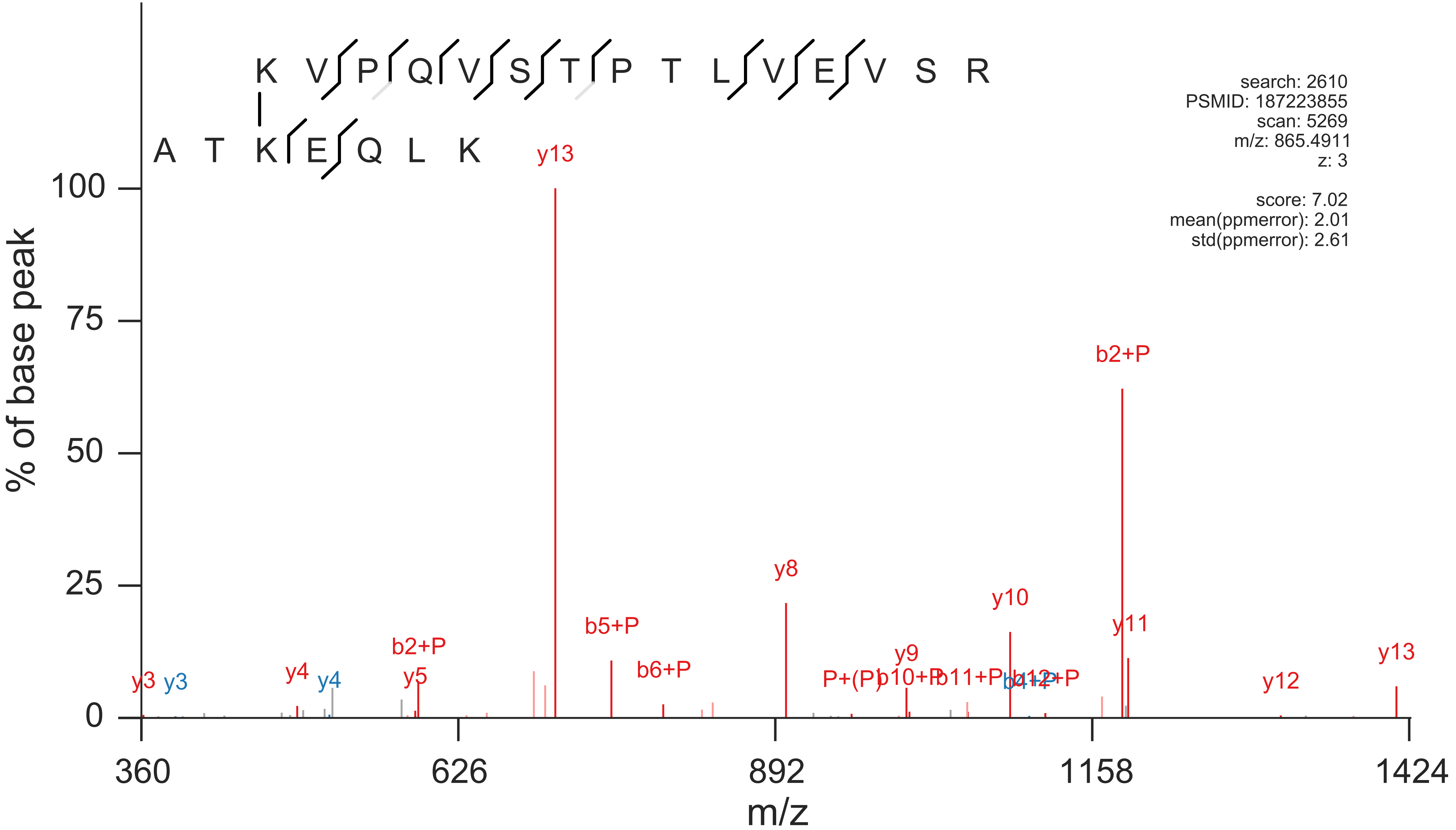
K V P Q V S T P T L V E V S R
A T K E Q L K
search: 2610
PSMID: 187223855
scan: 5269
m/z: 865.4911
z: 3
score: 7.02
mean(ppmerror): 2.01
std(ppmerror): 2.61
% of base peak
m/z
y13
b2+P
y8
y10
y11
b5+P
b6+P
y9
b2+P
y5
y4
y4
y3
y3
y12
y13

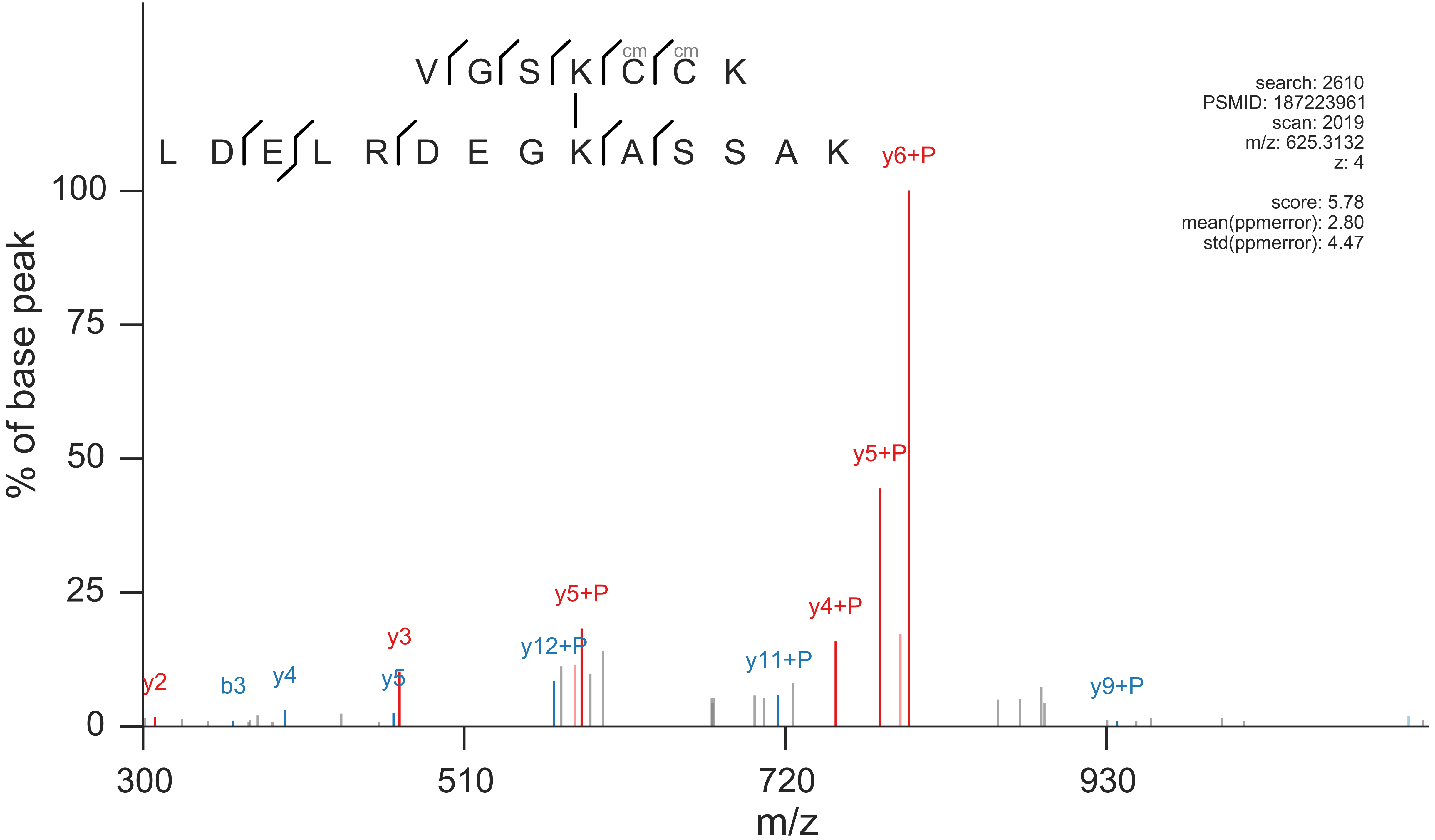
V G S K C C K
cm
cm
L D E L R D E G K A S S A K
search: 2610
PSMID: 187223961
scan: 2019
m/z: 625.3132
z: 4
score: 5.78
mean(ppmerror): 2.80
std(ppmerror): 4.47
% of base peak
m/z
100
75
50
25
0
300
510
720
930
y6+P
y5+P
y5+P
y4+P
y3
y12+P
y11+P
y9+P
y2
b3
y4
y5

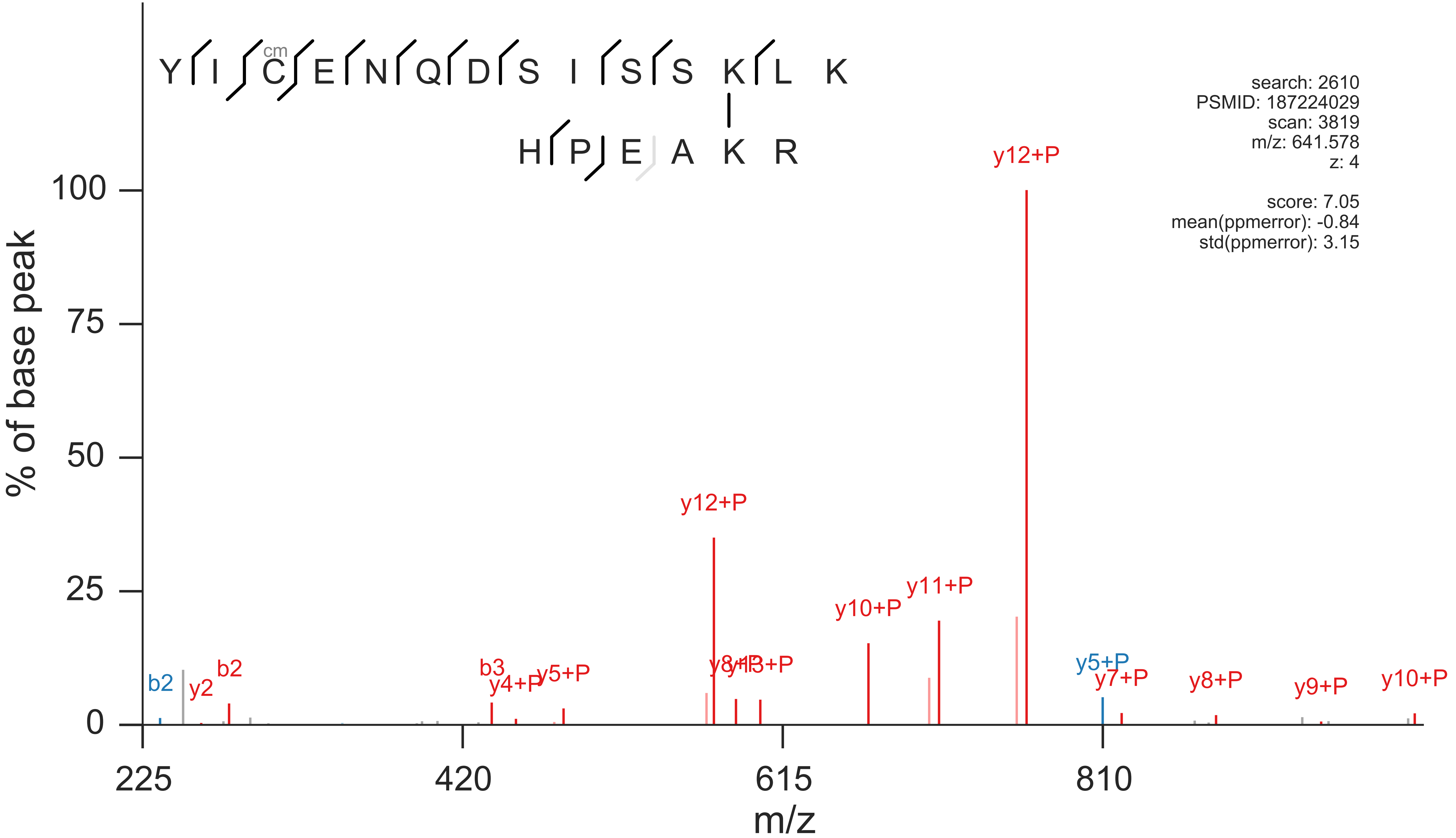

Y I C E N Q D S I S S K L K
cm
H P E A K R
search: 2610
PSMID: 187224029
scan: 3819
m/z: 641.578
z: 4
score: 7.05
mean(ppmerror): -0.84
std(ppmerror): 3.15
% of base peak
m/z
y12+P
y11+P
y10+P
b2
y2
b3
y4+P
y5+P
y7+P
y8+P
y9+P

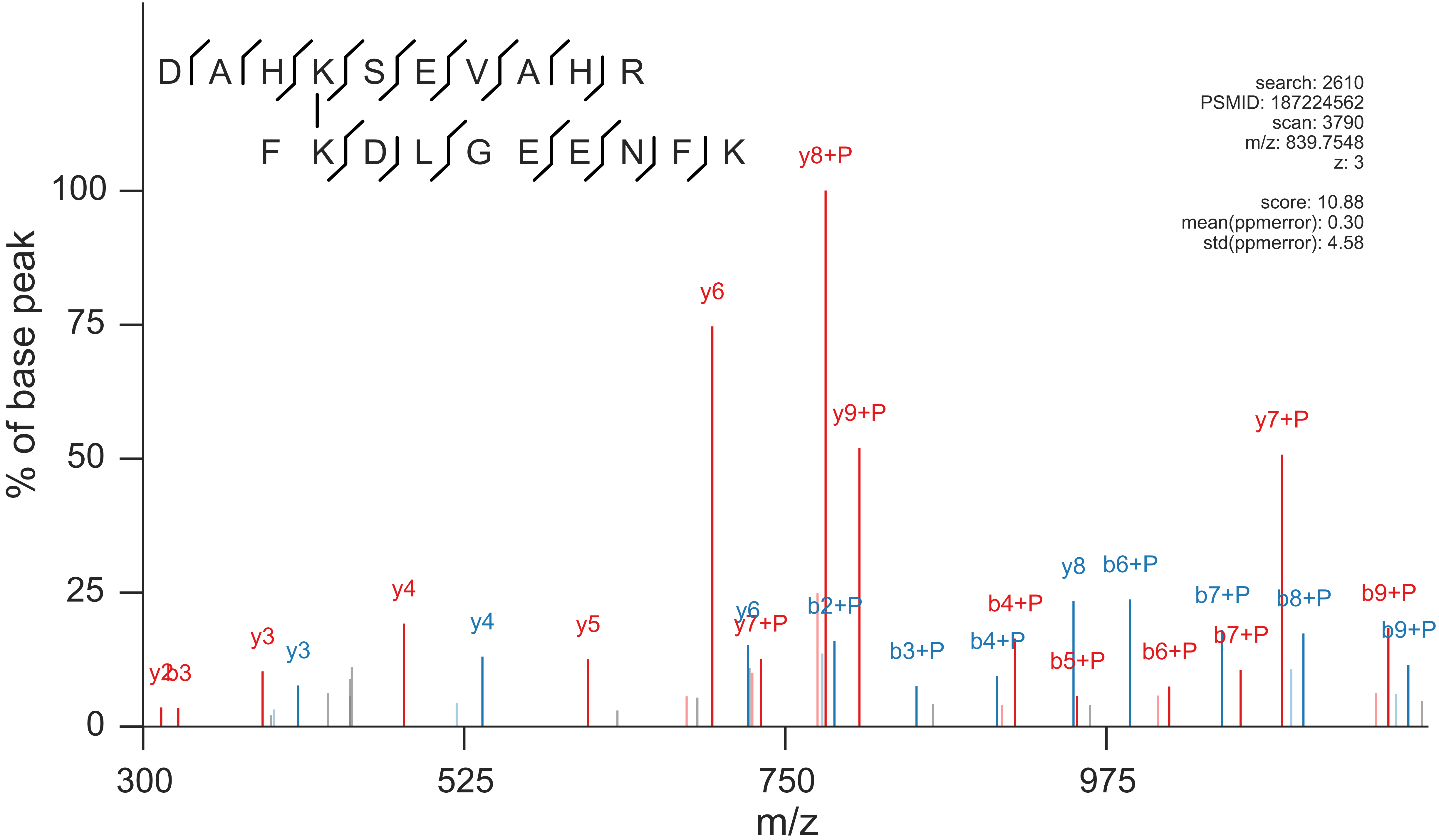

D A H K S E V A H R
F K D L G E E N F K
search: 2610
PSMID: 187224562
scan: 3790
m/z: 839.7548
z: 3
score: 10.88
mean(ppmerror): 0.30
std(ppmerror): 4.58
% of base peak
m/z
100
75
50
25
0
300
525
750
975
y8+P
y6
y9+P
y7+P
y4
y3
y5
y6
y7+P
b2+P
b3+P
b4+P
b4+P
y8
b6+P
b5+P
b6+P
b7+P
b7+P
b8+P
b9+P
b9+P
y4
y3

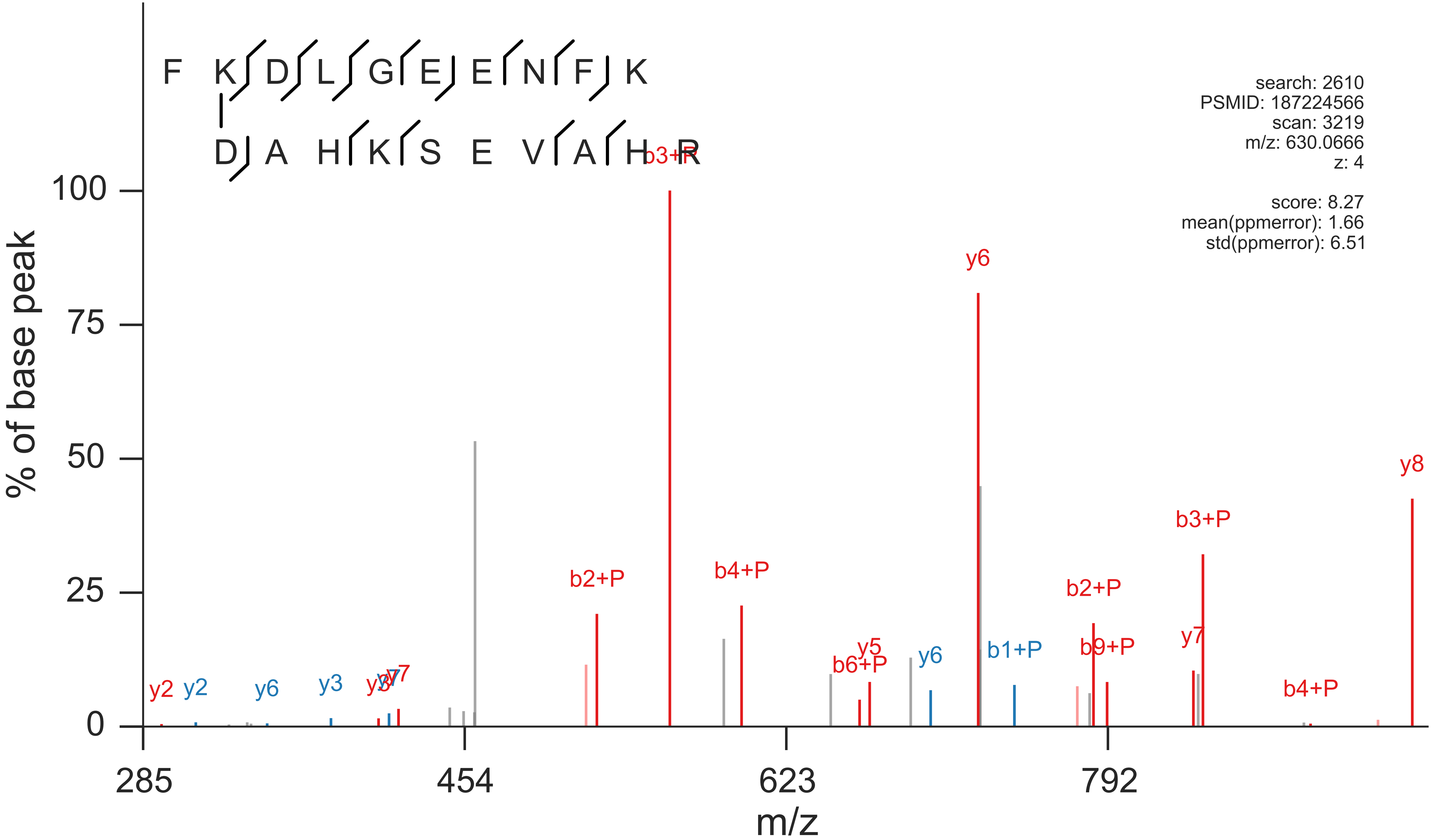

F K D L G E E N F K
D A H K S E V A H R
search: 2610
PSMID: 187224566
scan: 3219
m/z: 630.0666
z: 4
score: 8.27
mean(ppmerror): 1.66
std(ppmerror): 6.51
% of base peak
m/z
0
25
50
75
100
285
454
623
792
b3+P
y6
y8
b3+P
b2+P
b4+P
b2+P
b9+P
y7
b4+P
y5
b6+P
y6
b1+P
y2
y2
y6
y3
y3
y7

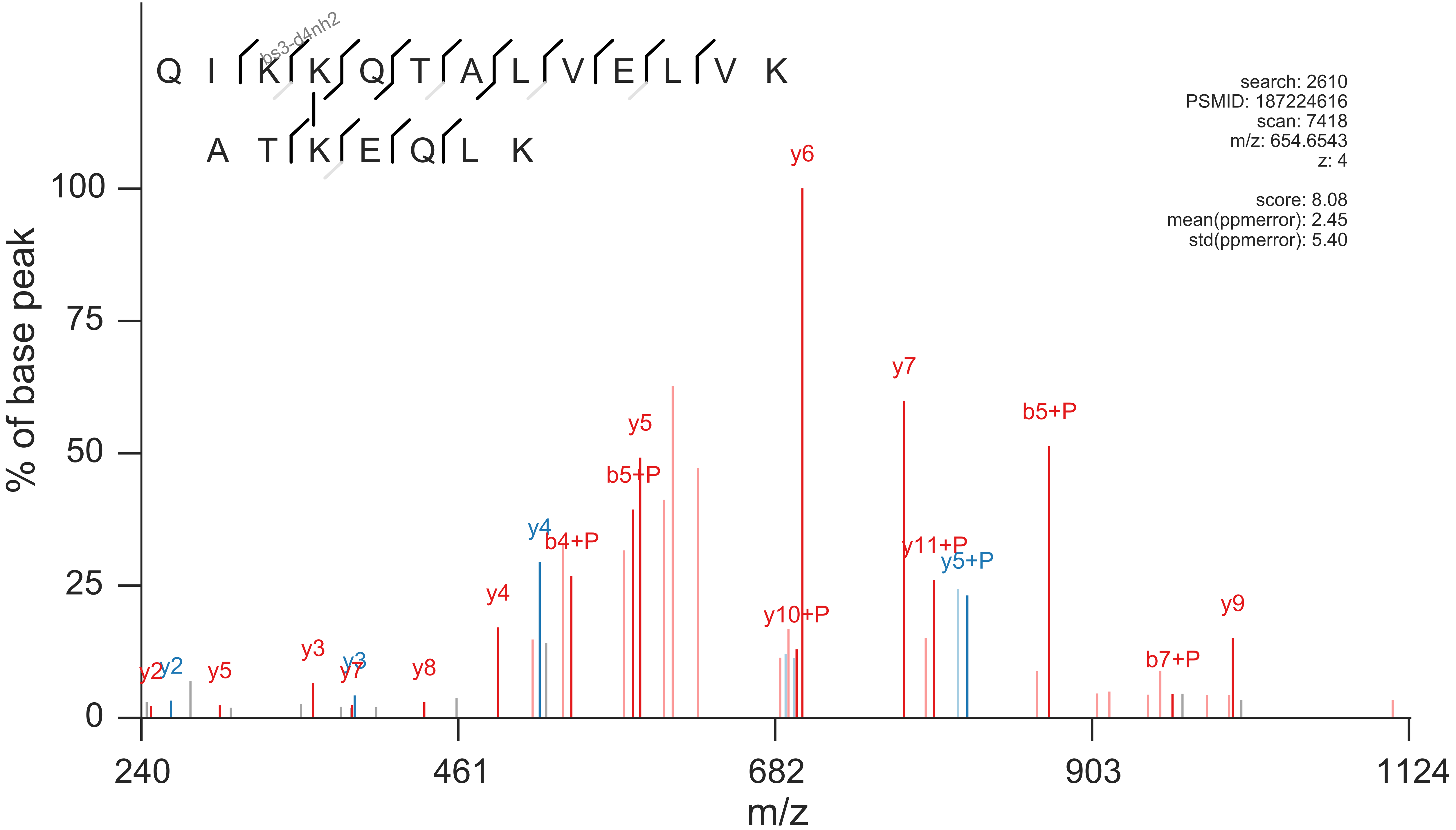

bs3-d4nh2
Q I K K Q T A L V E L V K
A T K E Q L K
search: 2610
PSMID: 187224616
scan: 7418
m/z: 654.6543
z: 4
score: 8.08
mean(ppmerror): 2.45
std(ppmerror): 5.40
% of base peak
m/z
0
25
50
75
100
240
461
682
903
1124
y6
y7
b5+P
y5
b5+P
y4
b4+P
y11+P
y5+P
y4
y10+P
y9
b7+P
y3
y3
y7
y8
y2
y2
y5

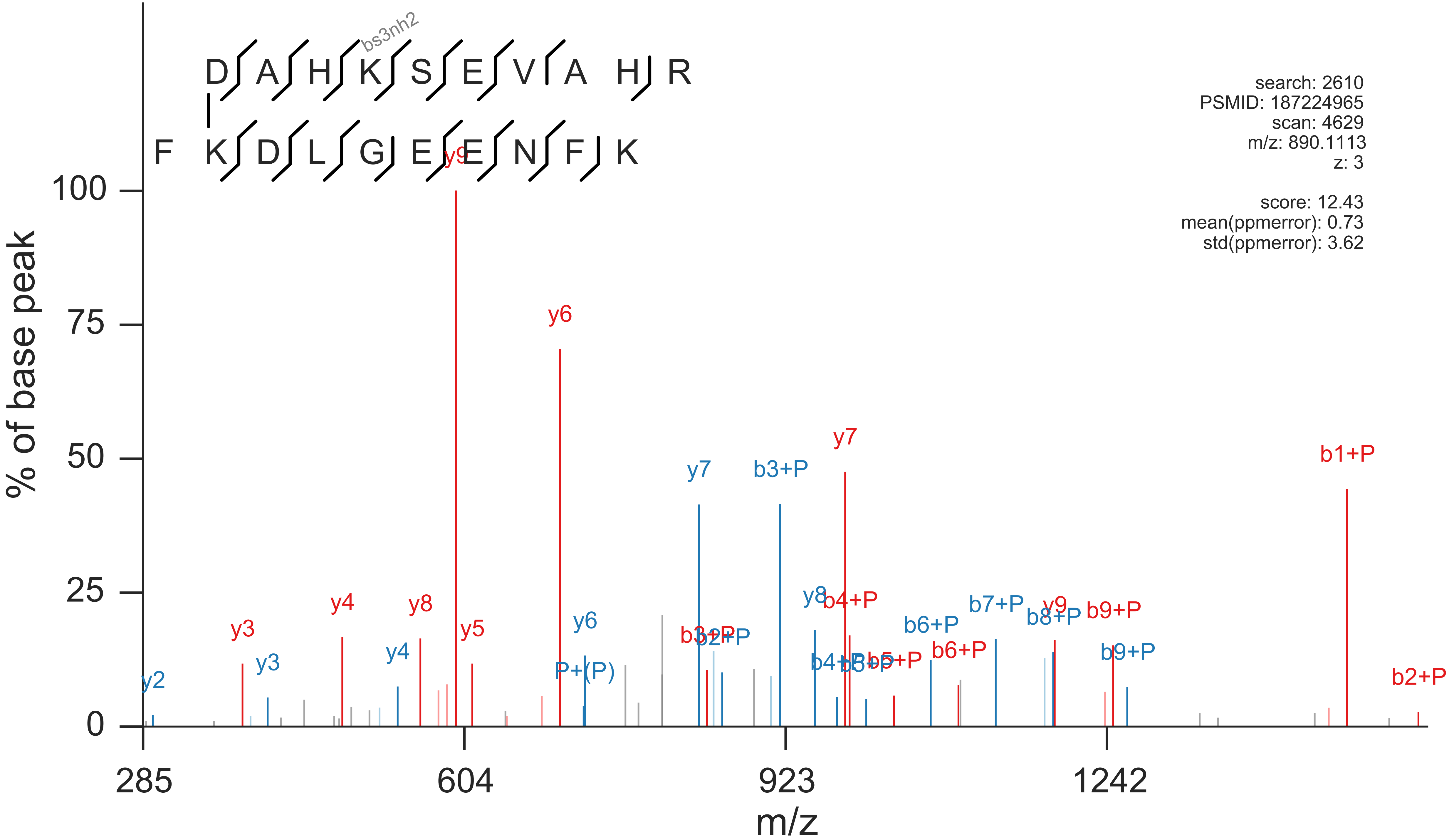

D A H K S E V A H R
F K D L G E E N F K
bs3nh2
search: 2610
PSMID: 187224965
scan: 4629
m/z: 890.1113
z: 3
score: 12.43
mean(ppmerror): 0.73
std(ppmerror): 3.62
% of base peak
m/z
0
25
50
75
100
285
604
923
1242
y9
y6
y7
b3+P
y7
b1+P
y2
y3
y4
y8
y5
y6
P+(P)
y8
b4+P
b6+P
b7+P
b8+P
b9+P
b9+P
b6+P
b2+P

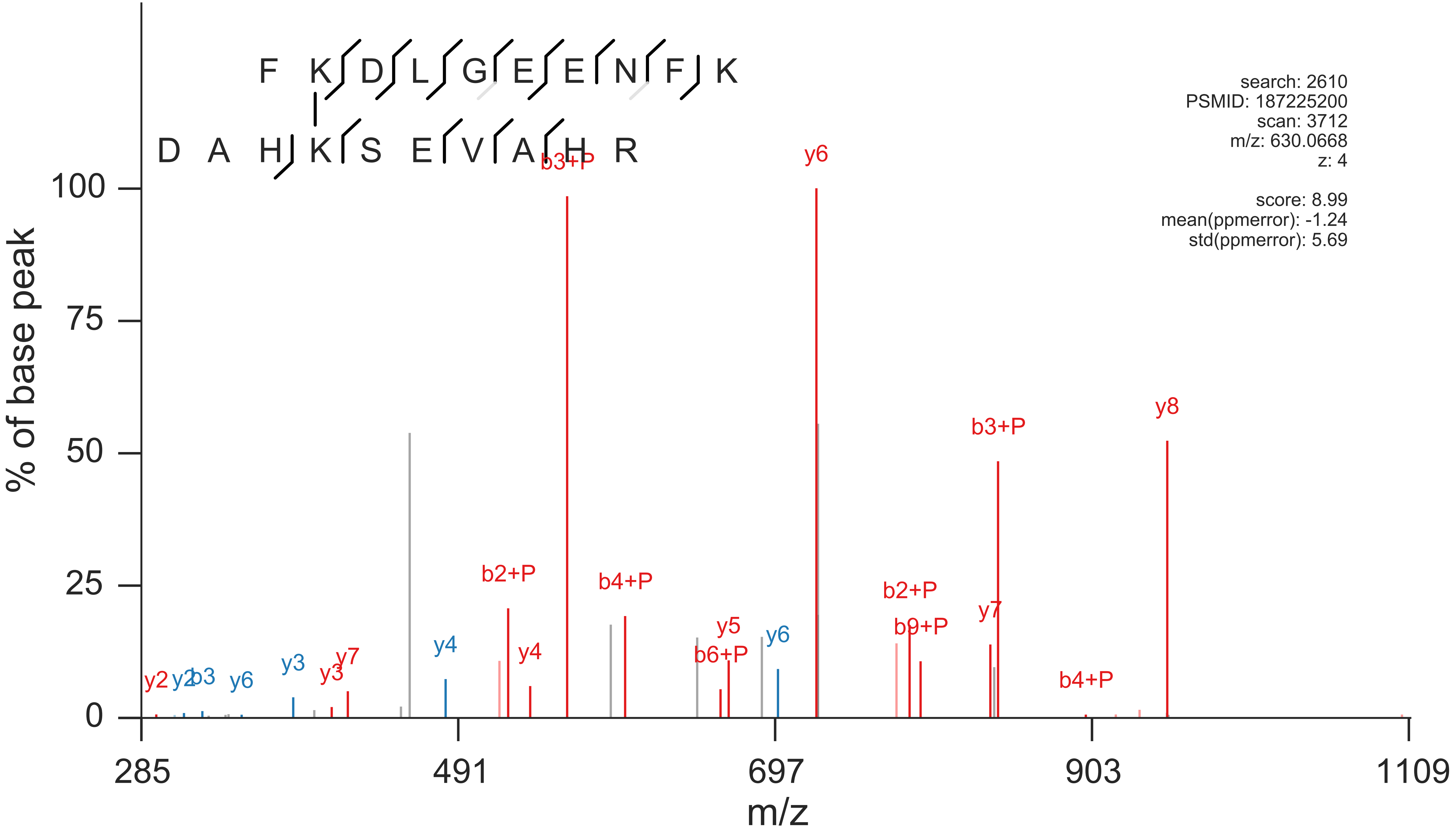

F K D L G E E N F K
D A H K S E V A H R
search: 2610
PSMID: 187225200
scan: 3712
m/z: 630.0668
z: 4
score: 8.99
mean(ppmerror): -1.24
std(ppmerror): 5.69
% of base peak
m/z
0
25
50
75
100
285
491
697
903
1109
y2
y2
b3
y6
y3
y3
y7
y4
b2+P
y4
b3+P
b4+P
y5
b6+P
y6
y6
b2+P
b9+P
y7
b3+P
b4+P
y8

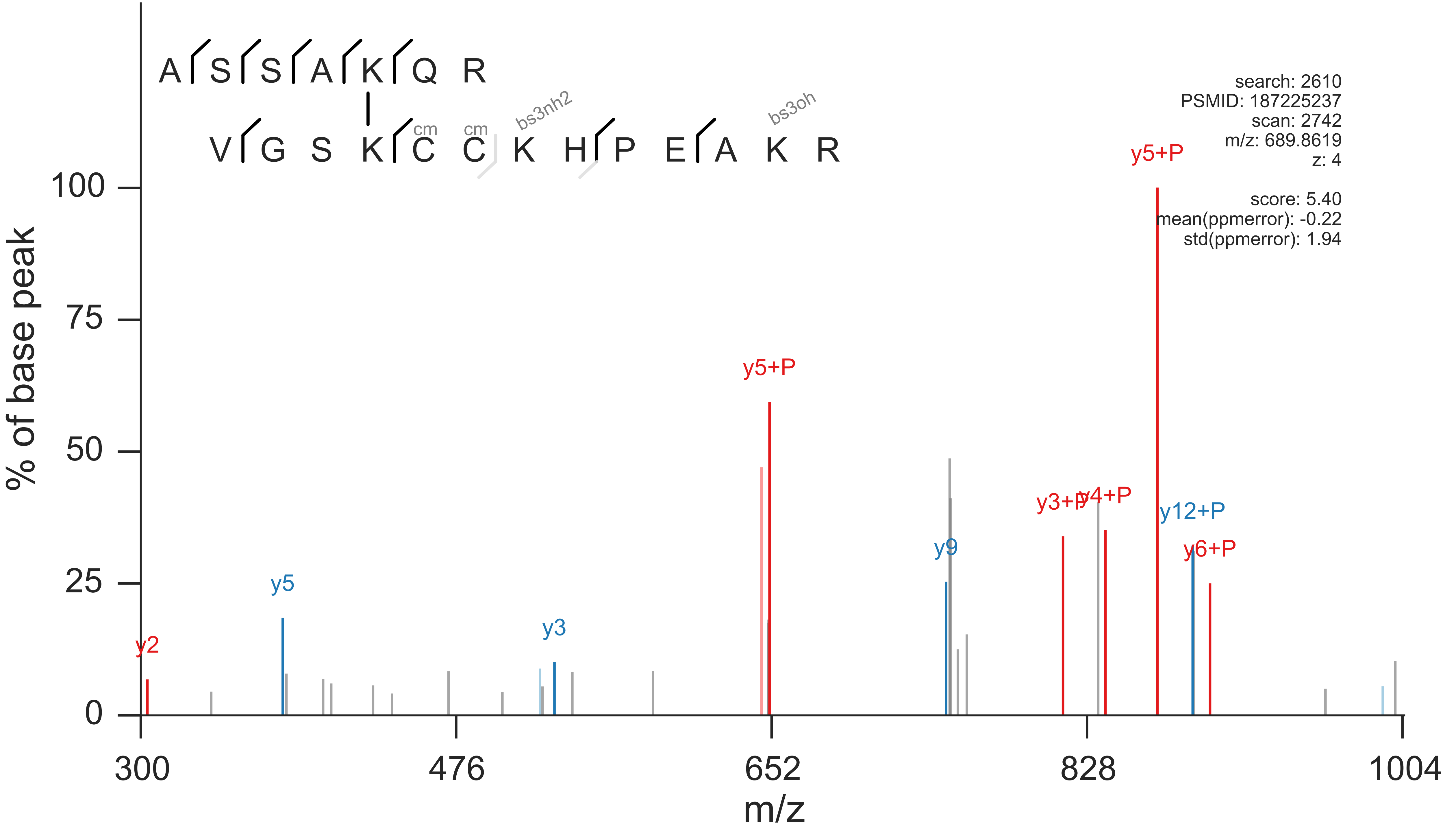
A S S A K Q R
V G S K C C K H P E A K R
cm
cm
bs3nh2
bs3oh
search: 2610
PSMID: 187225237
scan: 2742
m/z: 689.8619
z: 4
score: 5.40
mean(ppmerror): -0.22
std(ppmerror): 1.94
% of base peak
0
25
50
75
100
m/z
300
476
652
828
1004
y2
y5
y3
y5+P
y9
y3+P
y4+P
y5+P
y12+P
y6+P

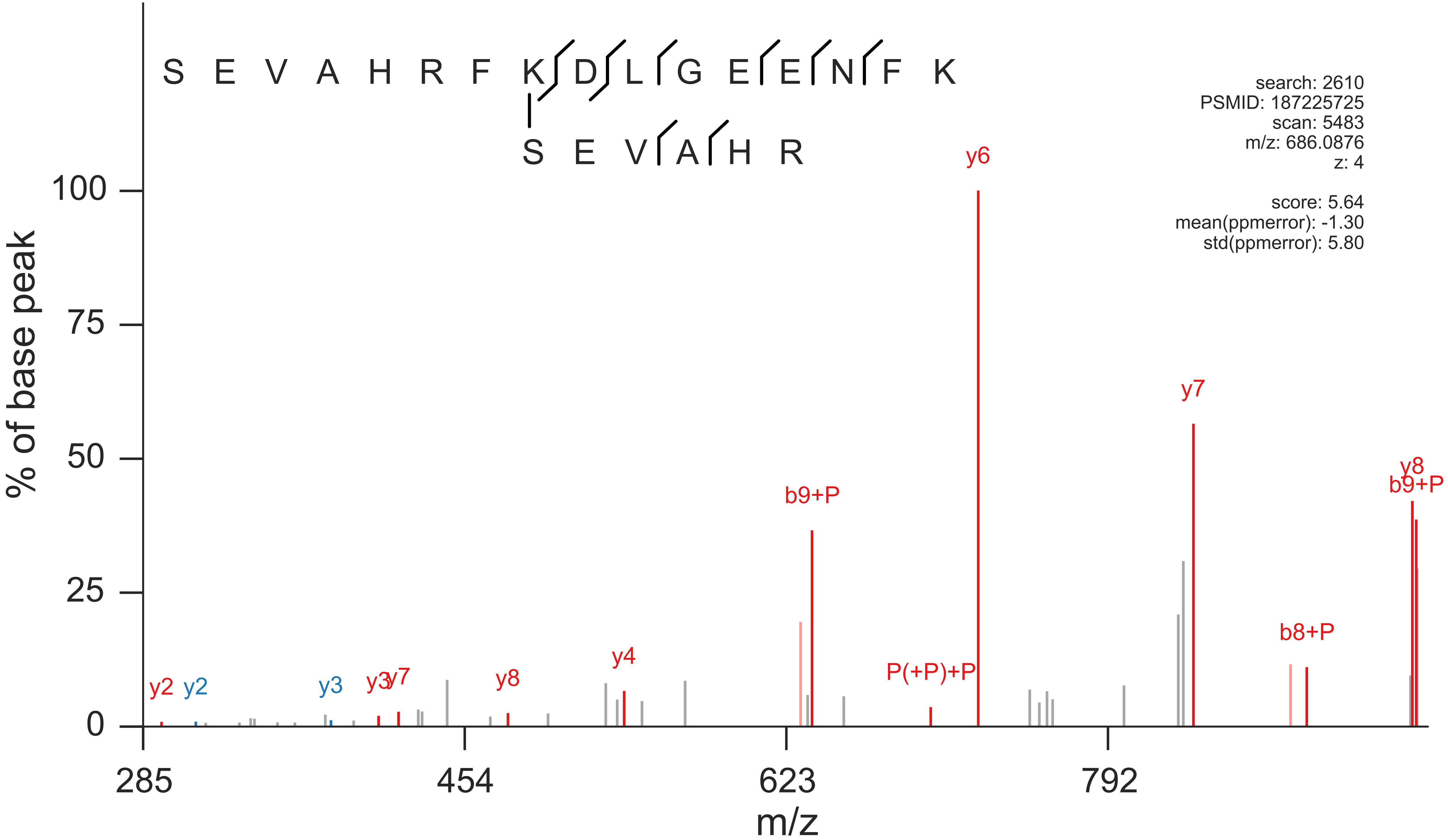

S E V A H R F K D L G E E N F K
S E V A H R
search: 2610
PSMID: 187225725
scan: 5483
m/z: 686.0876
z: 4
score: 5.64
mean(ppmerror): -1.30
std(ppmerror): 5.80
% of base peak
100
75
50
25
0
285
454
623
792
m/z
y6
y7
y8
b9+P
b9+P
b8+P
P(+P)+P
y4
y8
y3
y7
y3
y2
y2

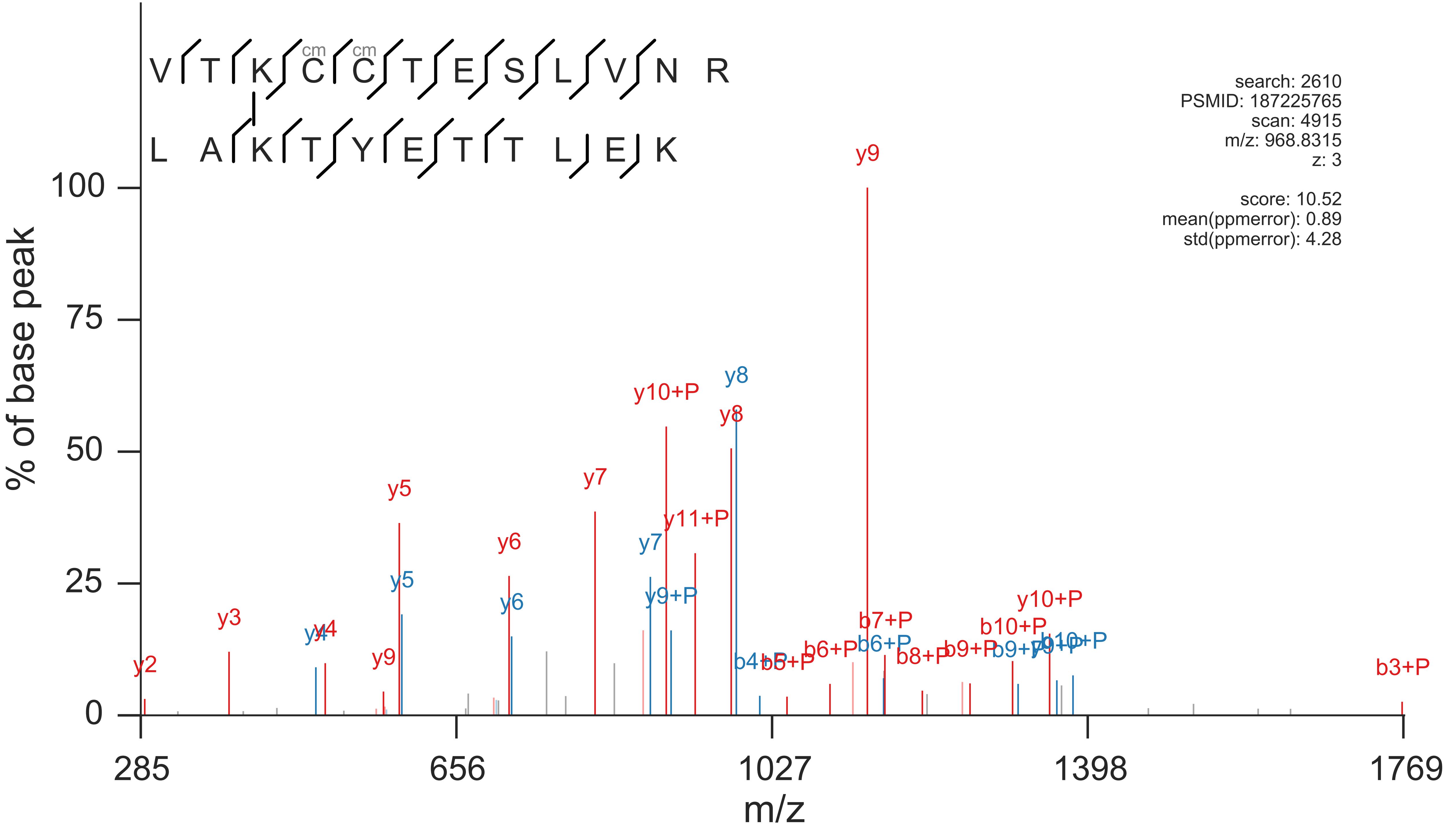
V T K C C T E S L V N R
L A K T Y E T T L E K
search: 2610
PSMID: 187225765
scan: 4915
m/z: 968.8315
z: 3
score: 10.52
mean(ppmerror): 0.89
std(ppmerror): 4.28
% of base peak
m/z
285
656
1027
1398
1769
0
25
50
75
100

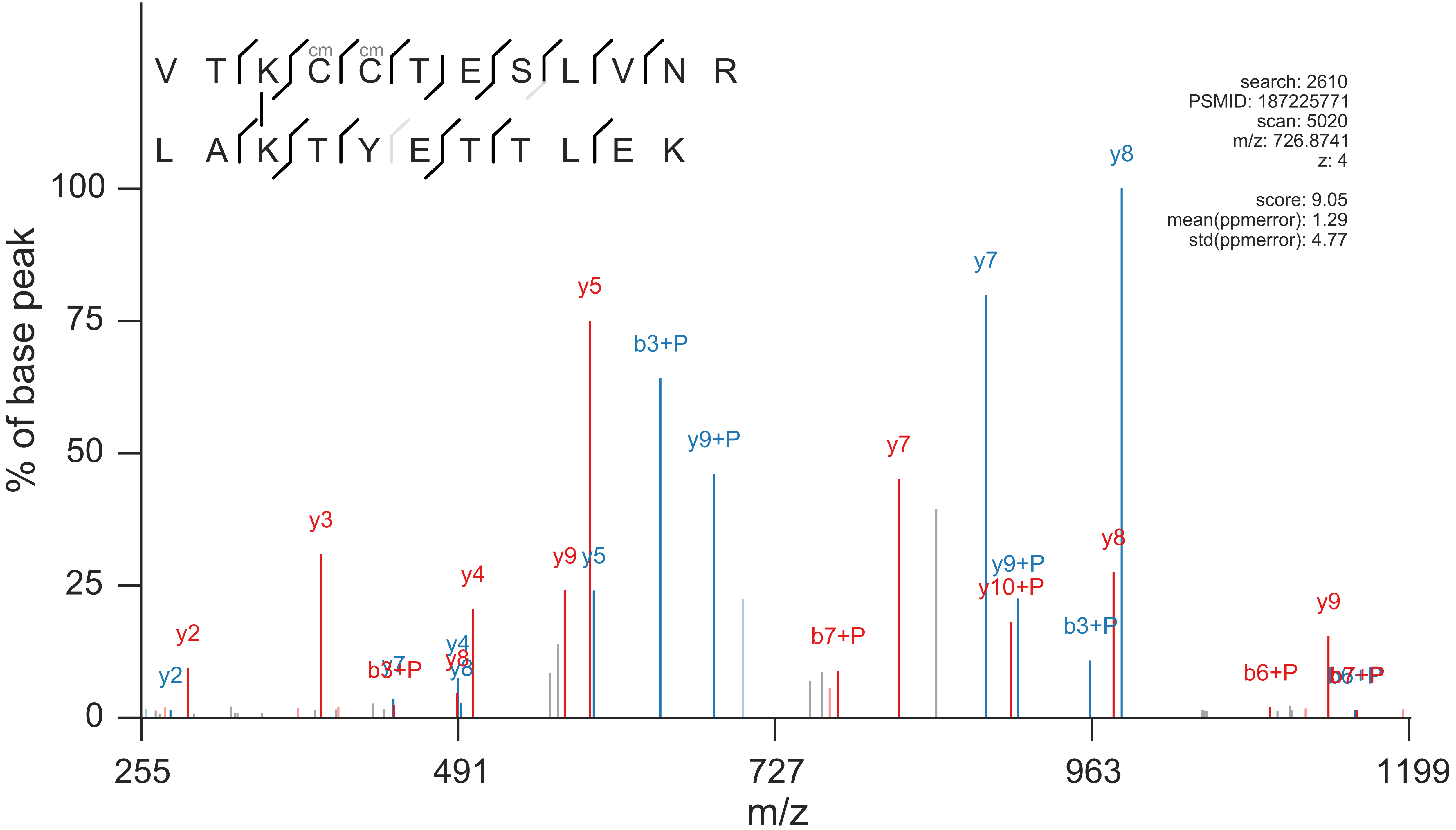

V T K C C T E S L V N R
cm cm
L A K T Y E T T L E K
search: 2610
PSMID: 187225771
scan: 5020
m/z: 726.8741
z: 4
score: 9.05
mean(ppmerror): 1.29
std(ppmerror): 4.77
% of base peak
m/z
0
25
50
75
100
255
491
727
963
1199
y2
y2
y3
b3+P
y7
y4
y8
y8
y4
y9
y5
y5
b3+P
y9+P
b7+P
y7
y7
y9+P
y10+P
y8
b3+P
y8
b6+P
y9

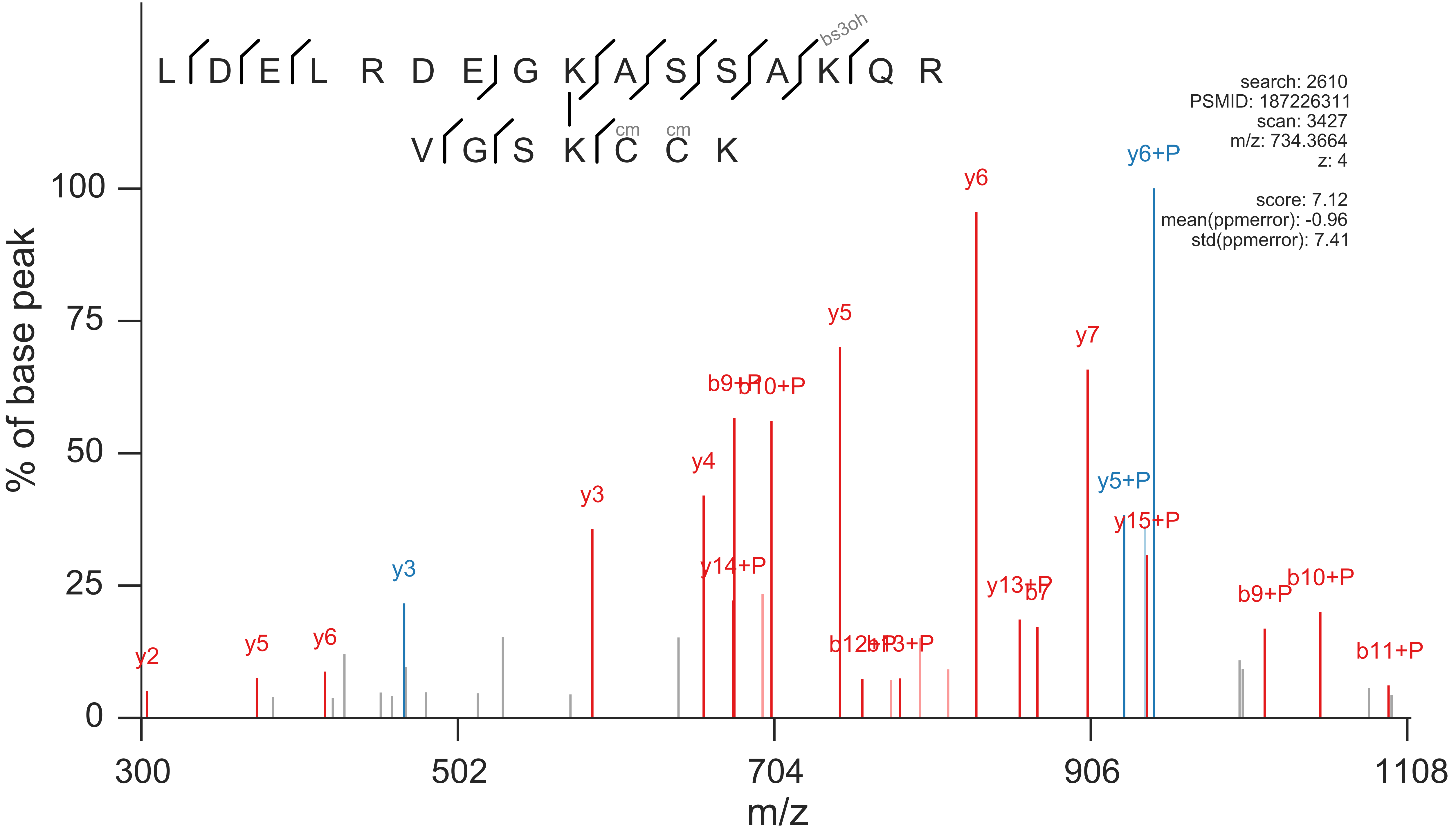

L D E L R D E G K A S S A K Q R
bs3oh
V G S K C C K
cm
cm
search: 2610
PSMID: 187226311
scan: 3427
m/z: 734.3664
z: 4
score: 7.12
mean(ppmerror): -0.96
std(ppmerror): 7.41
% of base peak
m/z
100
75
50
25
0
300
502
704
906
1108
y2
y5
y6
y3
y3
y4
b9+P
b10+P
y14+P
y5
b12+P
b13+P
y6
y13+P
b7
y7
y5+P
y15+P
y6+P
b9+P
b10+P
b11+P

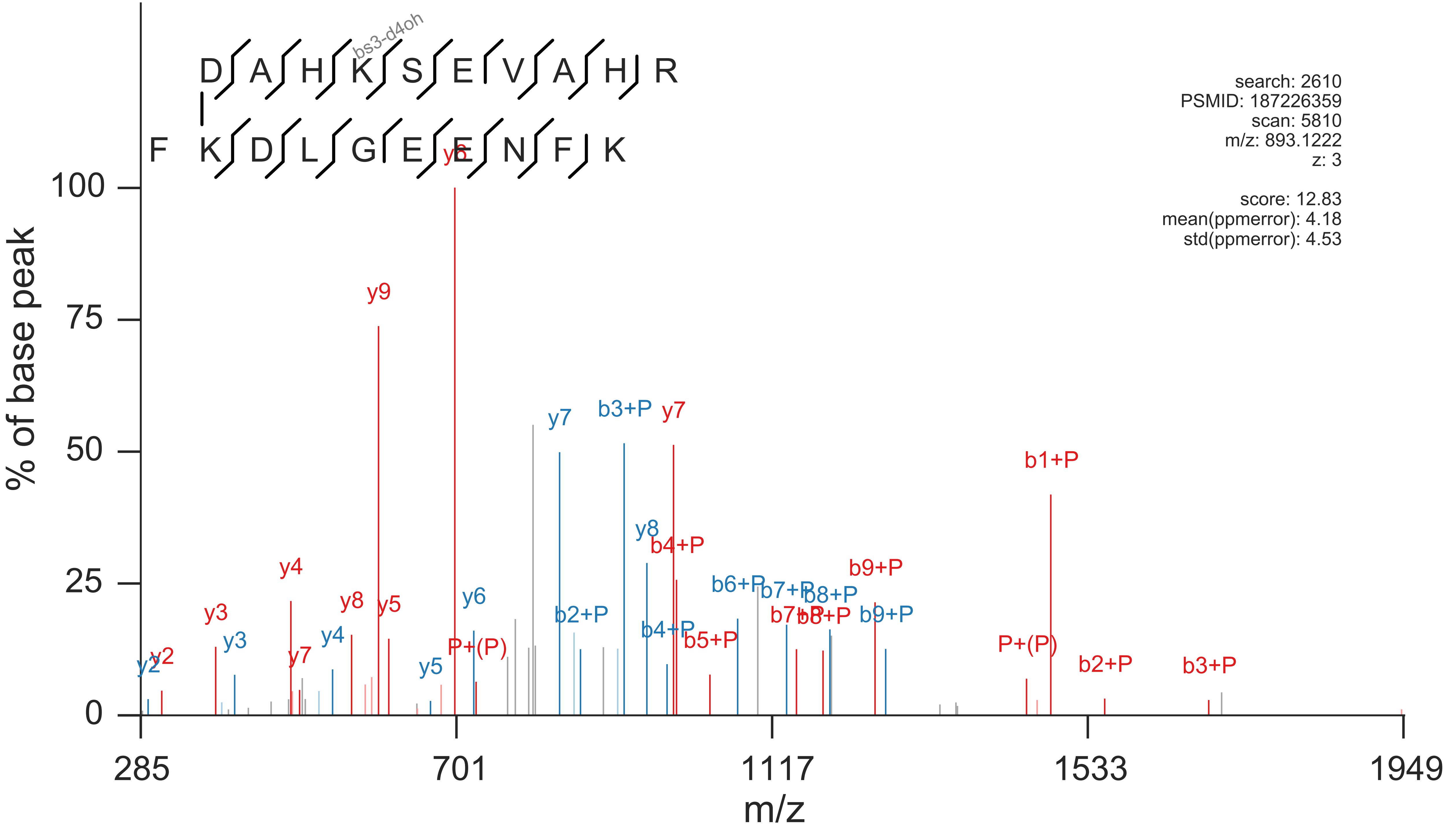

D A H K S E V A H R
F K D L G E E N F K
bs3-d4oh
search: 2610
PSMID: 187226359
scan: 5810
m/z: 893.1222
z: 3
score: 12.83
mean(ppmerror): 4.18
std(ppmerror): 4.53
% of base peak
m/z
0
25
50
75
100
285
701
1117
1533
1949
y2
y3
y4
y5
y6
y7
y8
y9
b1+P
b2+P
b3+P
b4+P
b5+P
b6+P
b7+P
b8+P
b9+P
P+(P)

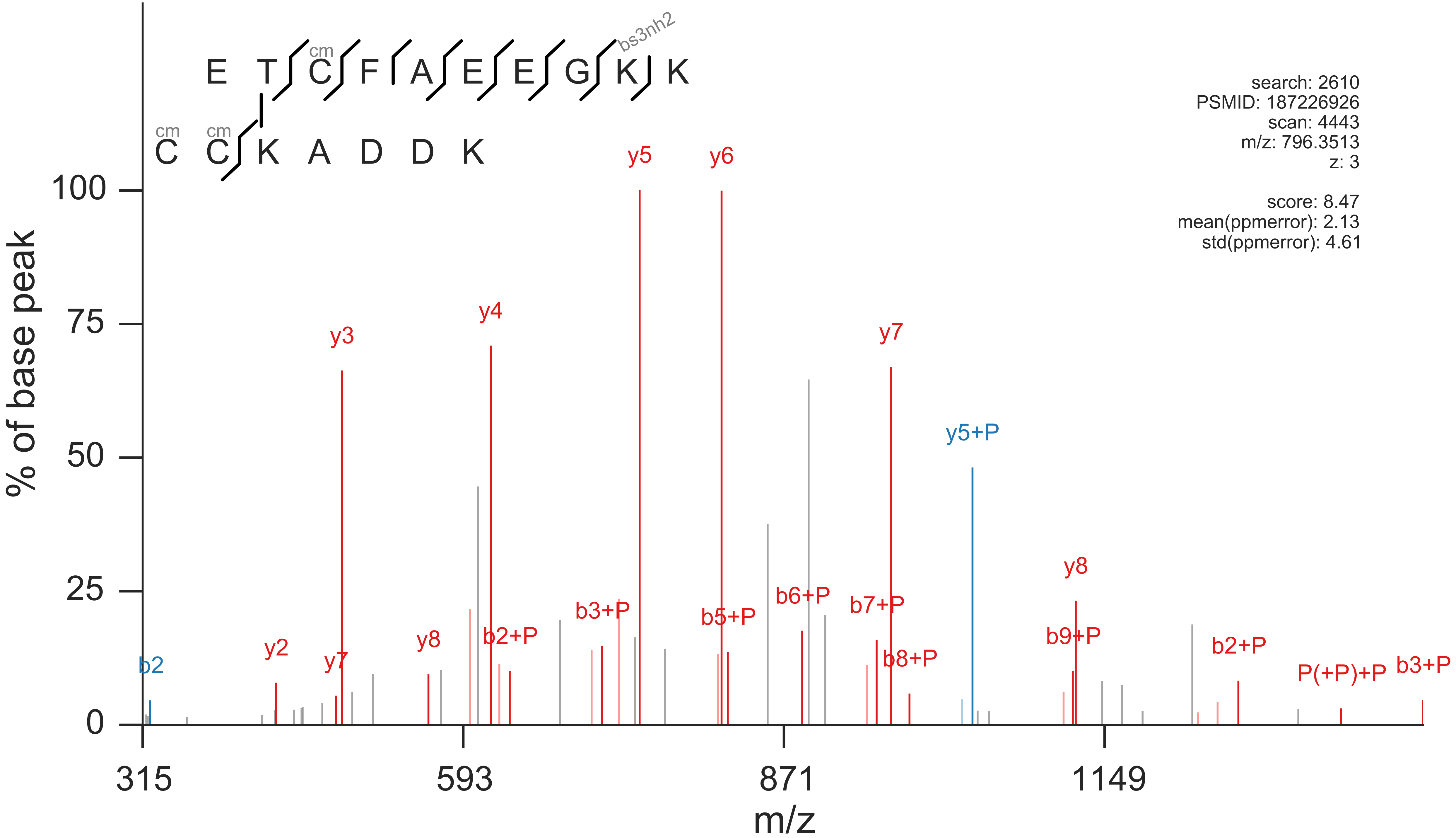

E T C F A E E G K K
cm
bs3nh2
C C K A D D K
cm cm
search: 2610
PSMID: 187226926
scan: 4443
m/z: 796.3513
z: 3
score: 8.47
mean(ppmerror): 2.13
std(ppmerror): 4.61
% of base peak
100
75
50
25
0
315
593
871
1149
m/z
b2
y2
y7
y3
y8
y4
b2+P
b3+P
y5
b5+P
y6
b6+P
b7+P
y7
b8+P
y5+P
y8
b9+P
b2+P
P(+P)+P
b3+P

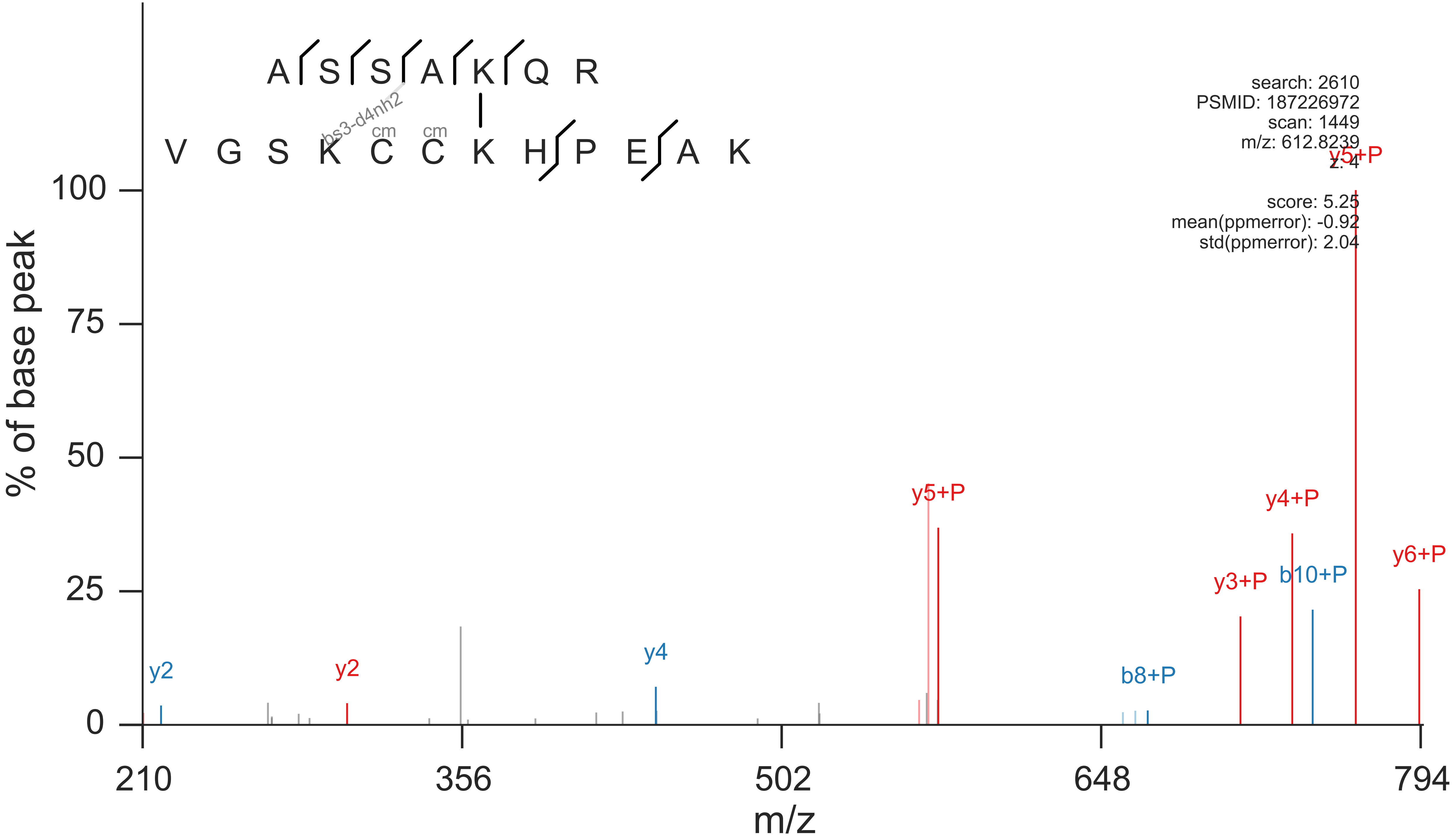
A S S A K Q R
V G S K C C K H P E A K
bs3-d4nh2
cm
cm
search: 2610
PSMID: 187226972
scan: 1449
m/z: 612.8239
z: 4
score: 5.25
mean(ppmerror): -0.92
std(ppmerror): 2.04
% of base peak
100
75
50
25
0
y2
y2
y4
y5+P
y4+P
y3+P
b10+P
y5+P
y6+P
b8+P
210
356
502
648
794
m/z

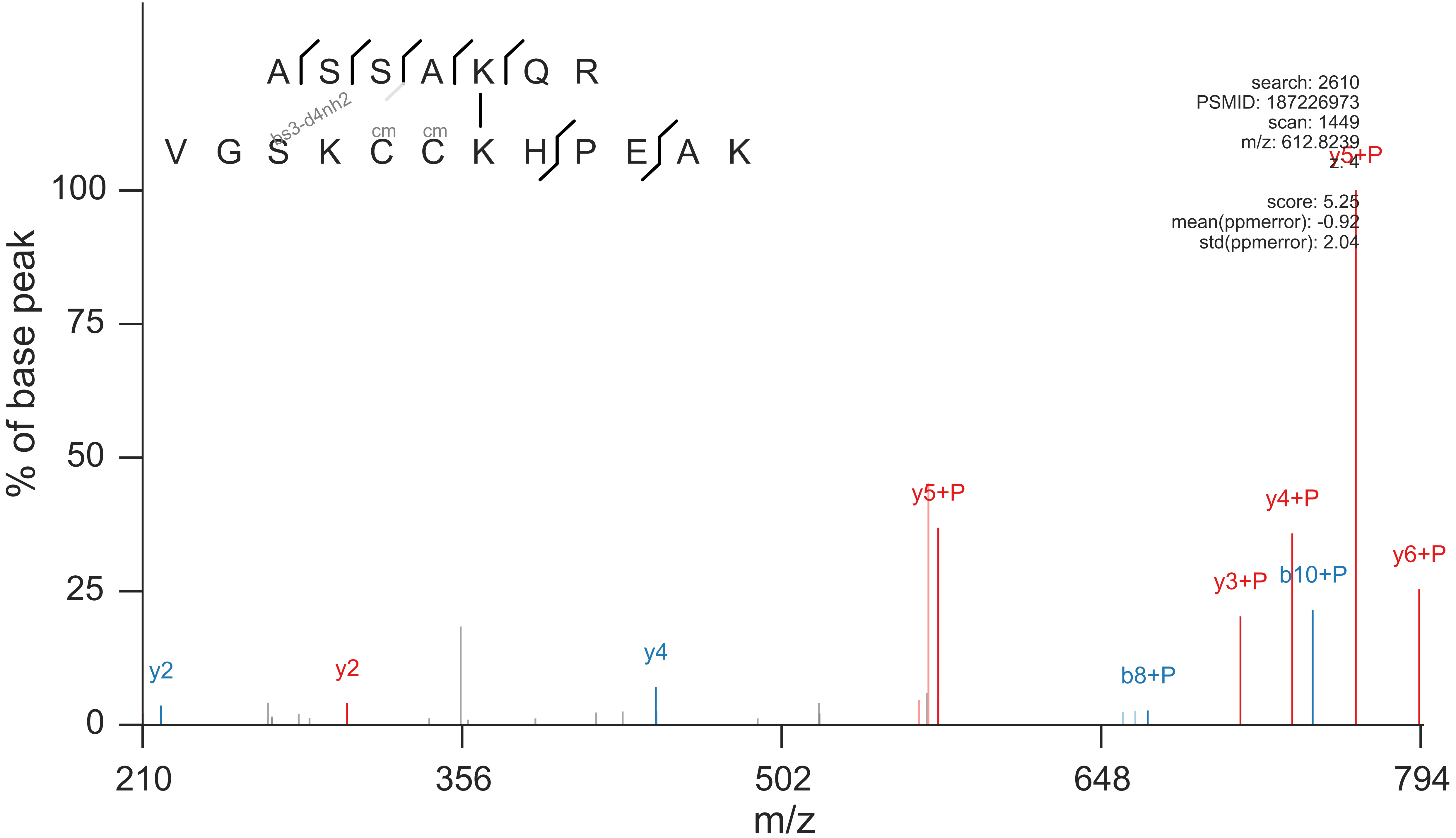

A S S A K Q R
V G S K C C K H P E A K
bs3-d4nh2
cm
cm
search: 2610
PSMID: 187226973
scan: 1449
m/z: 612.8239
z: 4
score: 5.25
mean(ppmerror): -0.92
std(ppmerror): 2.04
% of base peak
m/z
y2
y2
y4
y5+P
y5+P
y4+P
y3+P
b10+P
y6+P
b8+P

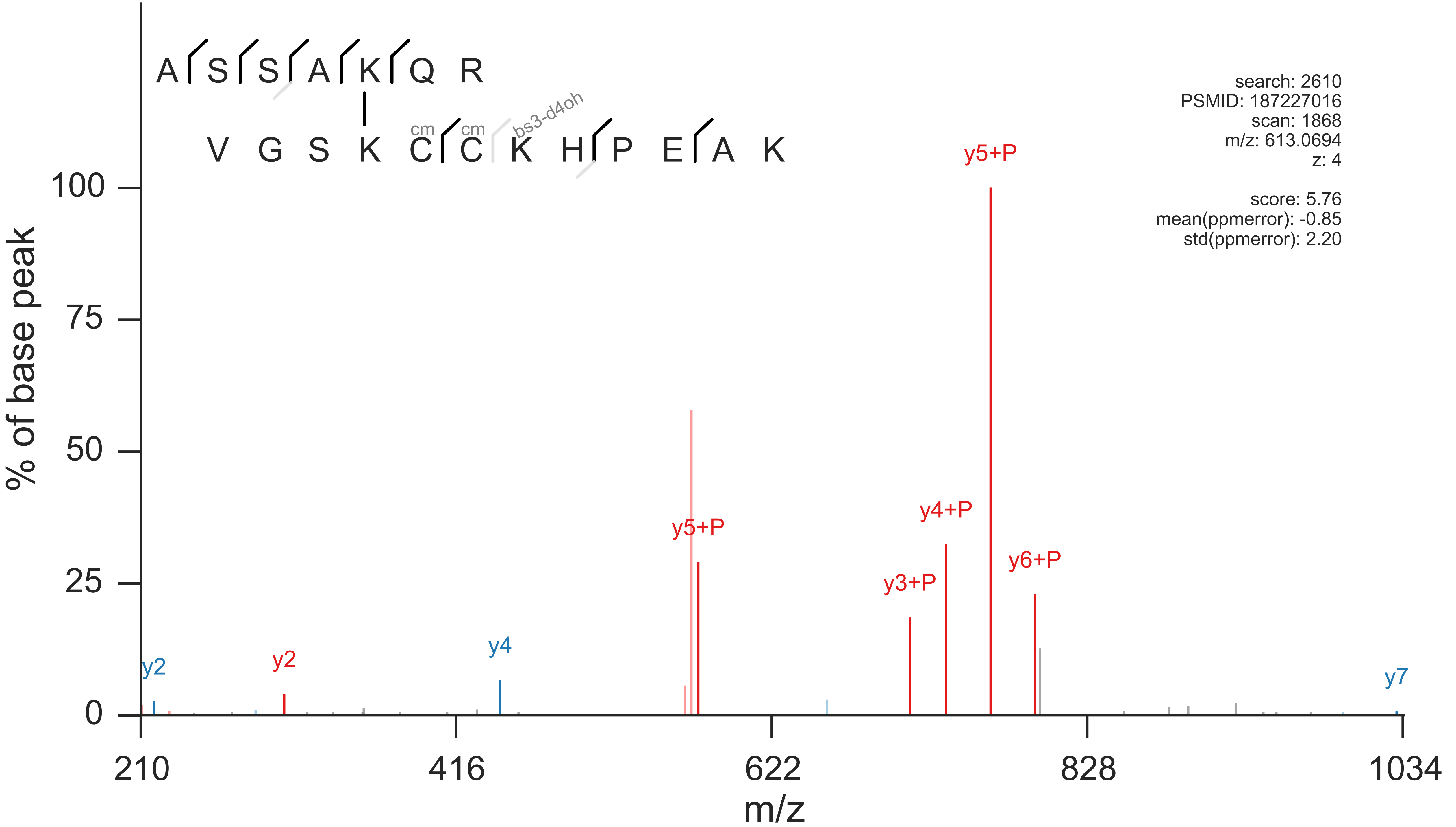

A S S A K Q R
V G S K C C K H P E A K
cm
cm
bs3-d4oh
search: 2610
PSMID: 187227016
scan: 1868
m/z: 613.0694
z: 4
score: 5.76
mean(ppmerror): -0.85
std(ppmerror): 2.20
% of base peak
100
75
50
25
0
y5+P
y5+P
y4+P
y3+P
y6+P
y2
y2
y4
y7
210
416
622
828
1034
m/z

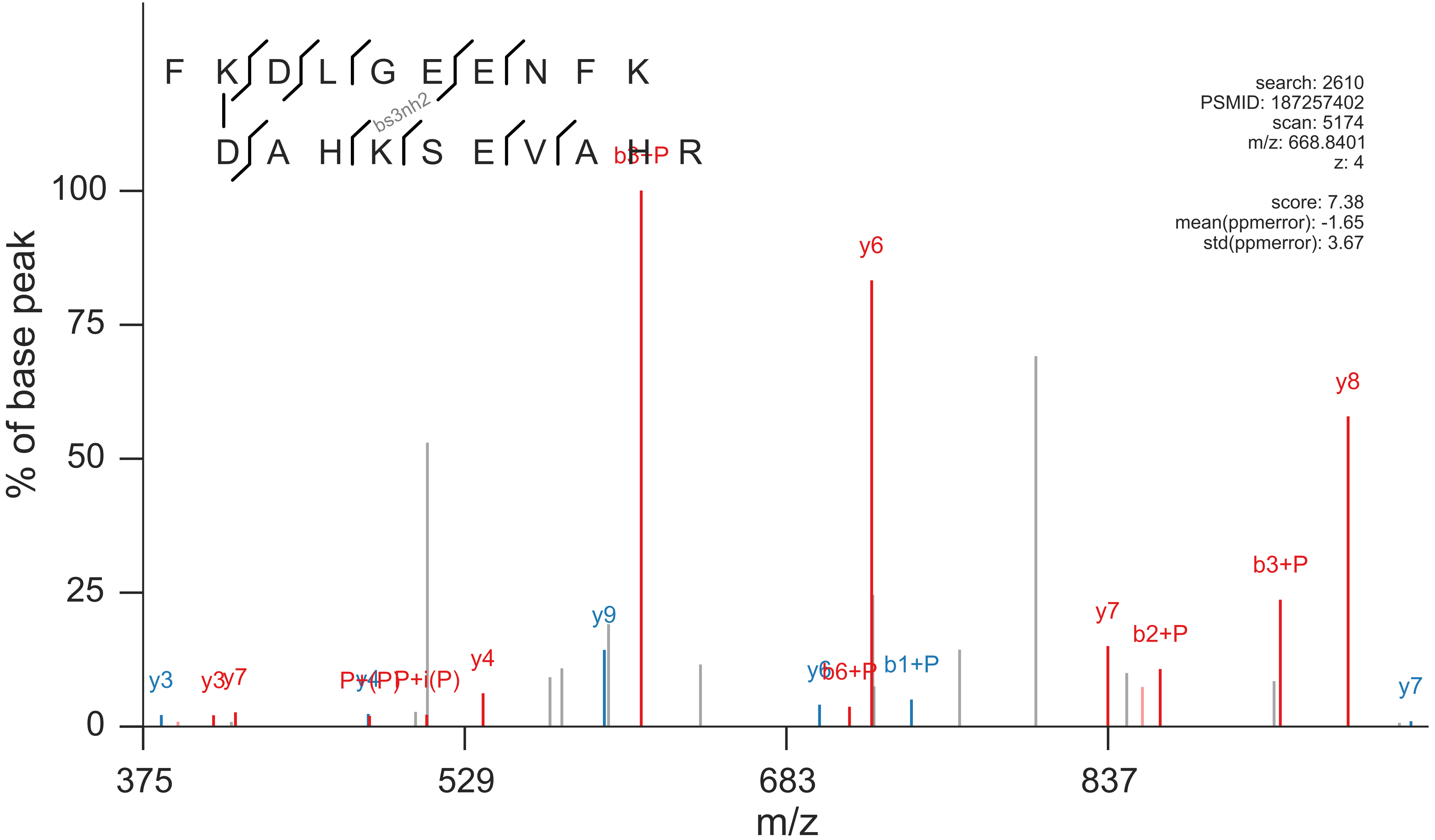

F K D L G E E N F K
D A H K S E V A H R
bs3nh2
search: 2610
PSMID: 187257402
scan: 5174
m/z: 668.8401
z: 4
score: 7.38
mean(ppmerror): -1.65
std(ppmerror): 3.67
% of base peak
100
75
50
25
0
375
529
683
837
m/z
b3+P
y6
y8
y9
y7
b2+P
b3+P
y3
y3
y7
y4
y4
b1+P
y7

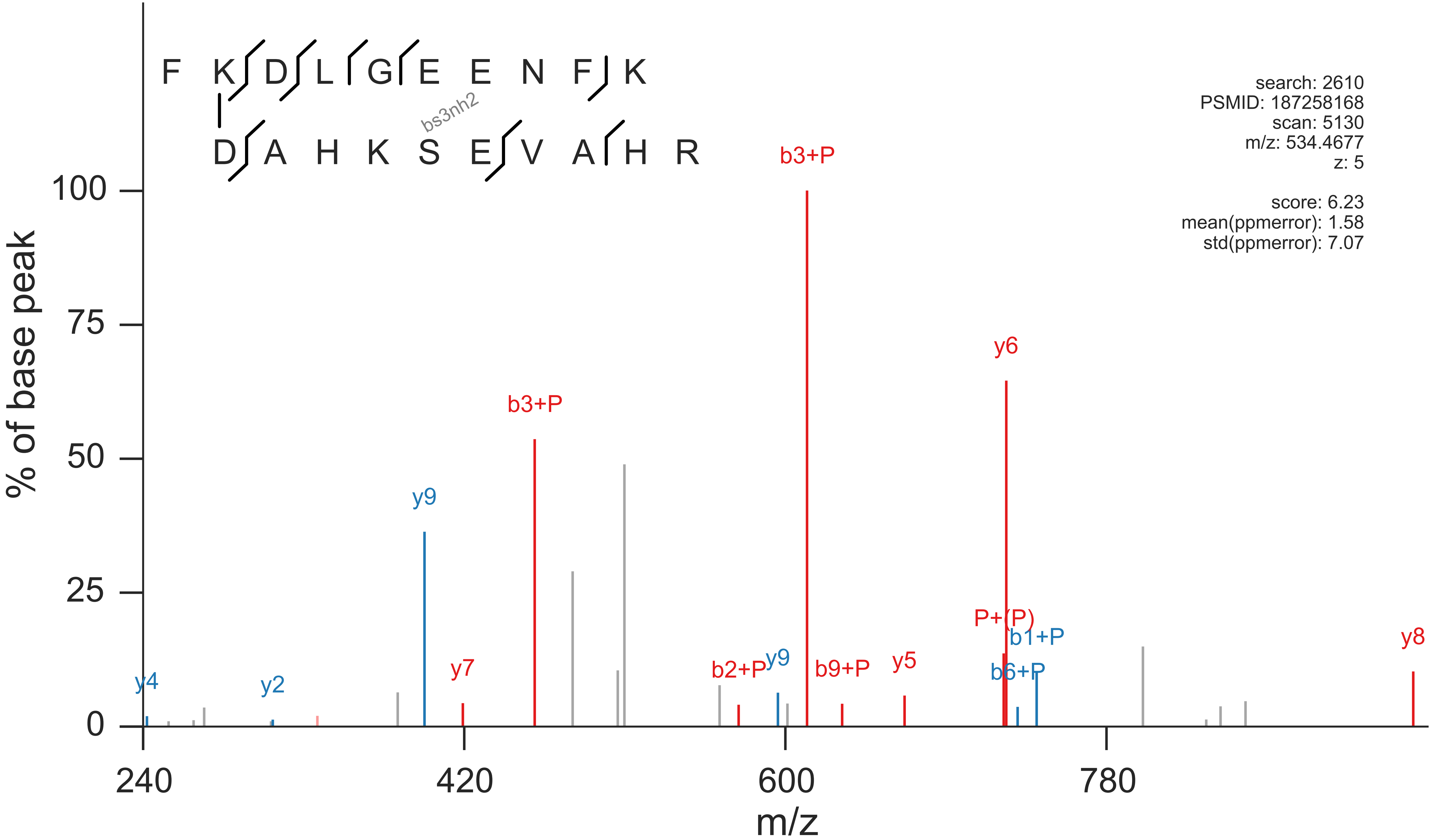

F K D L G E E N F K
D A H K S E V A H R
bs3nh2
search: 2610
PSMID: 187258168
scan: 5130
m/z: 534.4677
z: 5
score: 6.23
mean(ppmerror): 1.58
std(ppmerror): 7.07
% of base peak
m/z
b3+P
y6
b3+P
y9
y7
y4
y2
b2+P
y9
b9+P
y5
P+(P)
b1+P
b6+P
y8
0
25
50
75
100
240
420
600
780

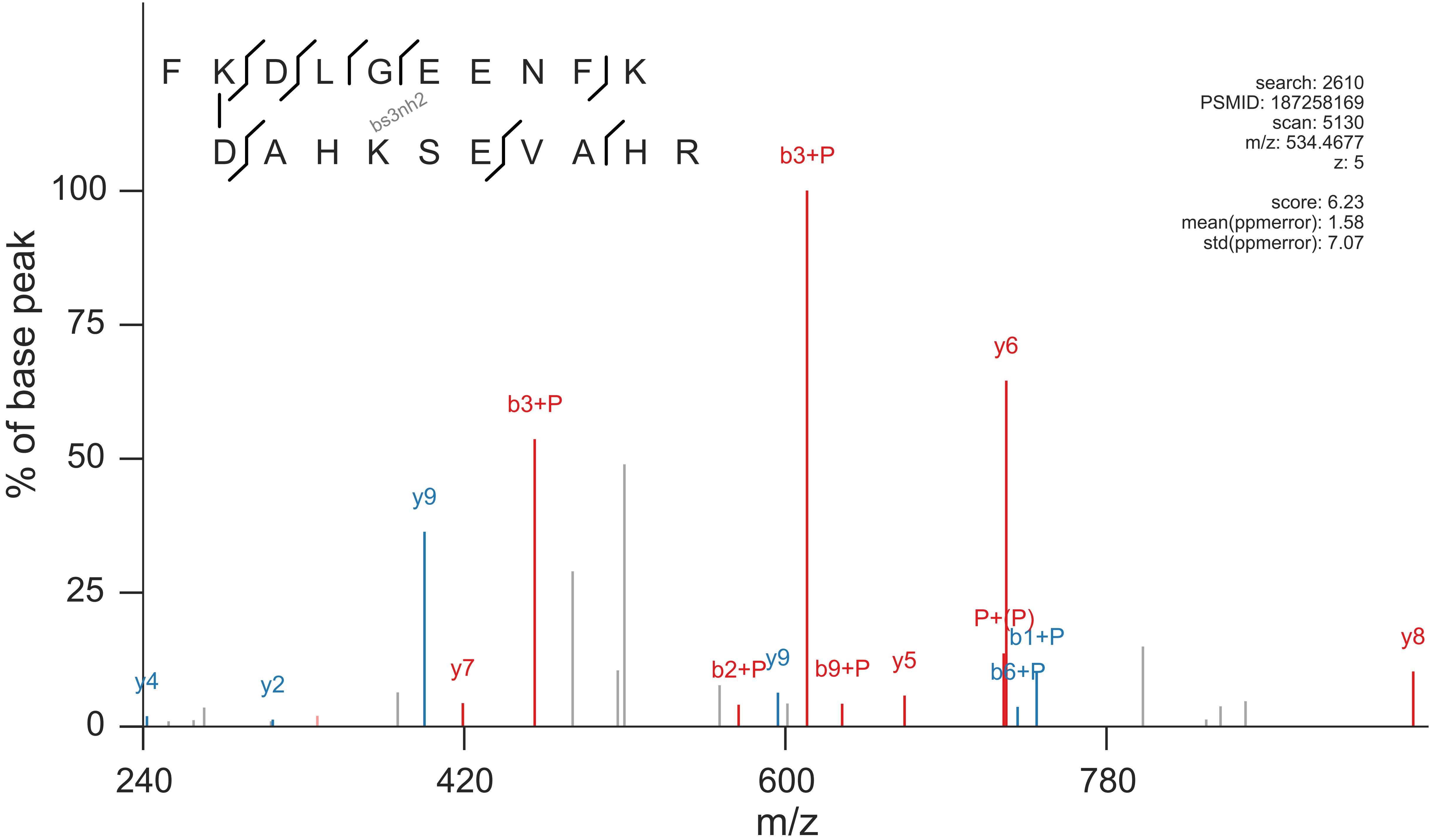

F K D L G E E N F K
D A H K S E V A H R
bs3nh2
search: 2610
PSMID: 187258169
scan: 5130
m/z: 534.4677
z: 5
score: 6.23
mean(ppmerror): 1.58
std(ppmerror): 7.07
% of base peak
m/z
100
75
50
25
0
240
420
600
780
b3+P
y6
b3+P
y9
y7
y4
y2
b2+P
y9
b9+P
y5
P+(P)
b1+P
b6+P
y8

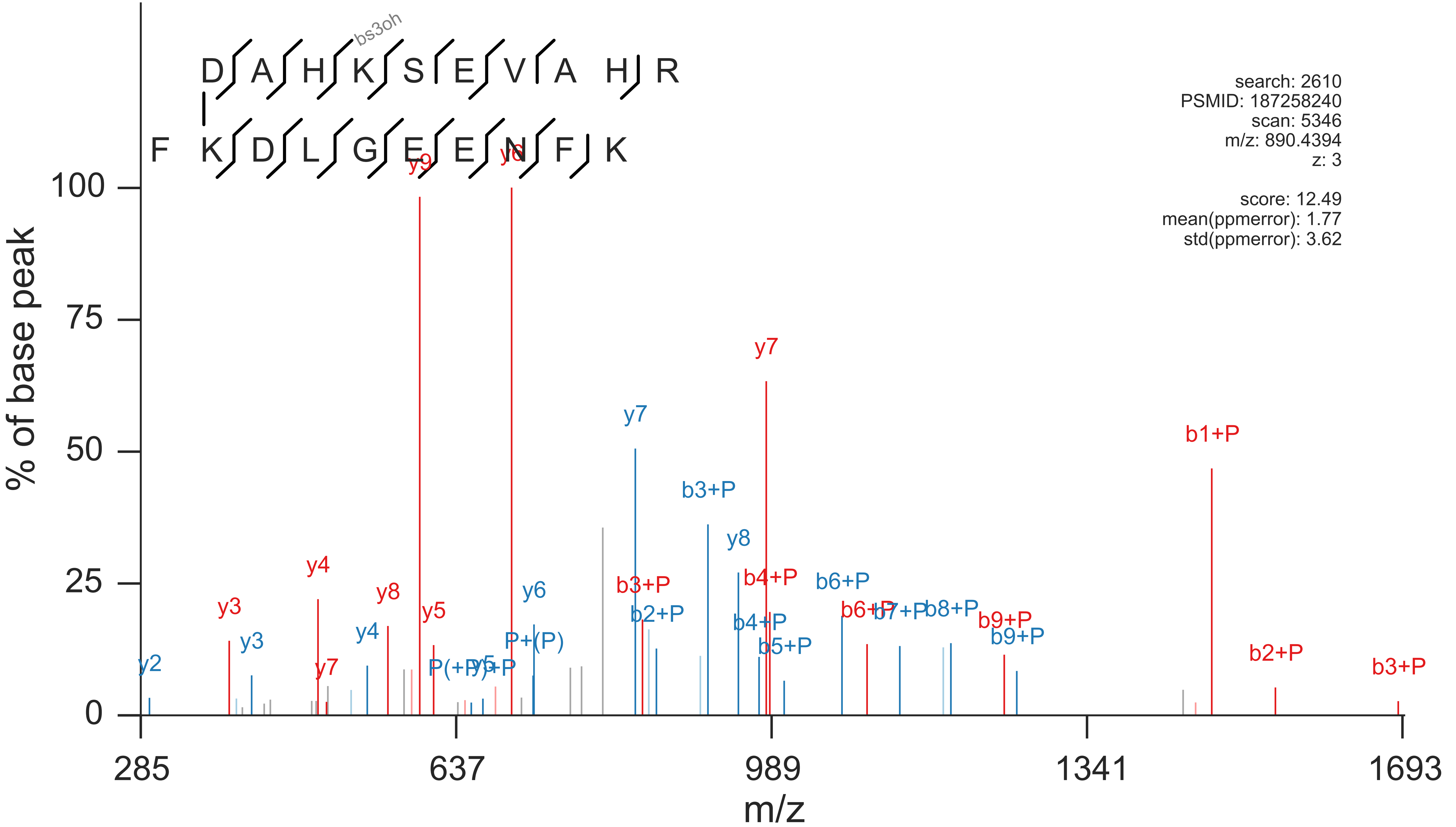

bs3oh
D A H K S E V A H R
F K D L G E E N F K
search: 2610
PSMID: 187258240
scan: 5346
m/z: 890.4394
z: 3
score: 12.49
mean(ppmerror): 1.77
std(ppmerror): 3.62
% of base peak
m/z
0
25
50
75
100
285
637
989
1341
1693
y9
y6
y7
y7
b1+P
b3+P
y8
b4+P
b3+P
b6+P
y4
y3
y8
y5
y6
b2+P
b4+P
b6+P
b7+P
b8+P
b9+P
b9+P
y3
y4
b5+P
y2
y7
P+(P)
b2+P
b3+P

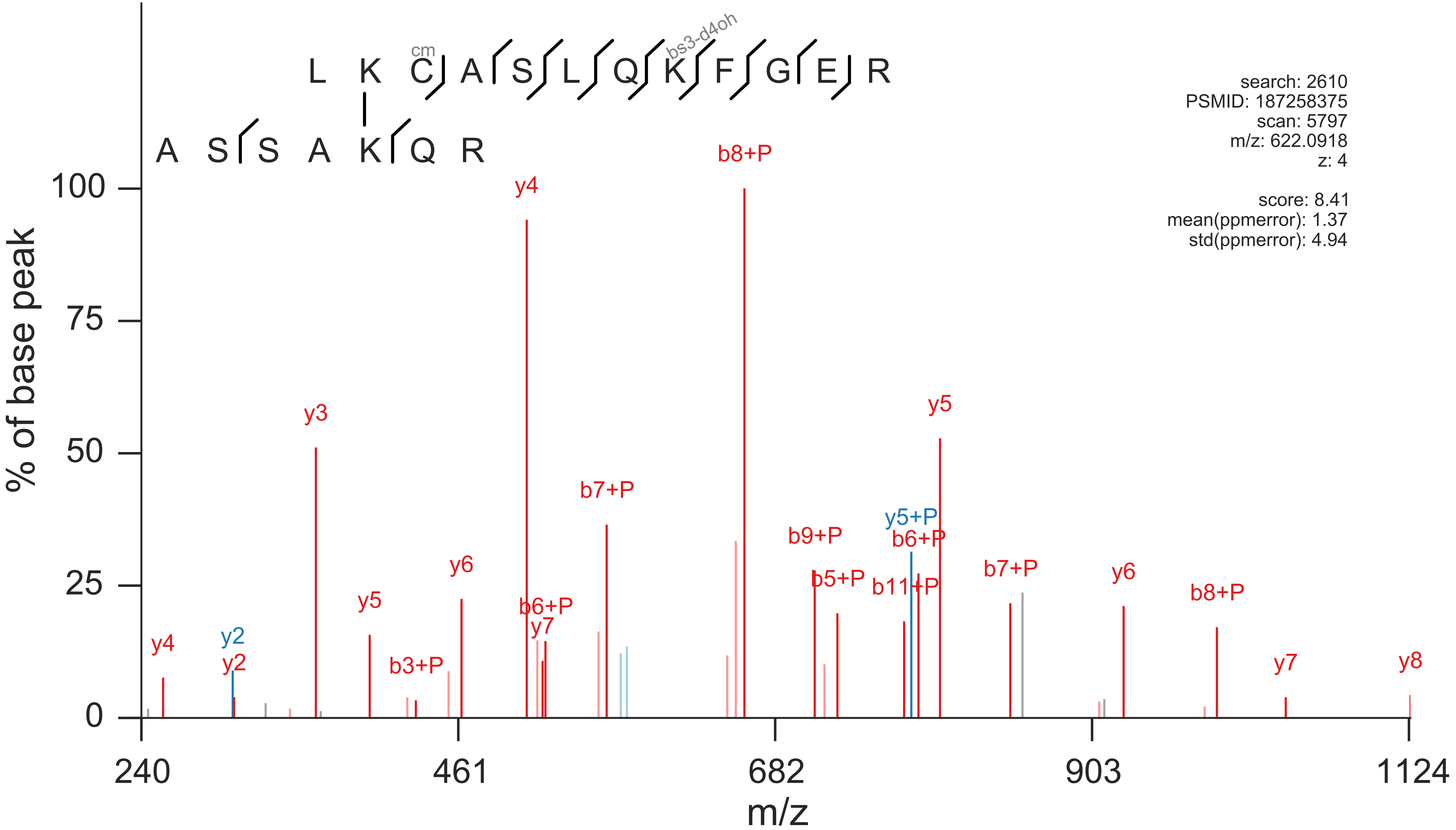

L K C A S L Q K F G E R
cm
bs3-d4oh
A S S A K Q R
search: 2610
PSMID: 187258375
scan: 5797
m/z: 622.0918
z: 4
score: 8.41
mean(ppmerror): 1.37
std(ppmerror): 4.94
% of base peak
m/z
0
25
50
75
100
240
461
682
903
1124
y4
y2
y2
y3
y5
b3+P
y6
y4
b6+P
y7
b7+P
b8+P
b9+P
b5+P
y5+P
b6+P
b11+P
y5
b7+P
y6
b8+P
y7
y8

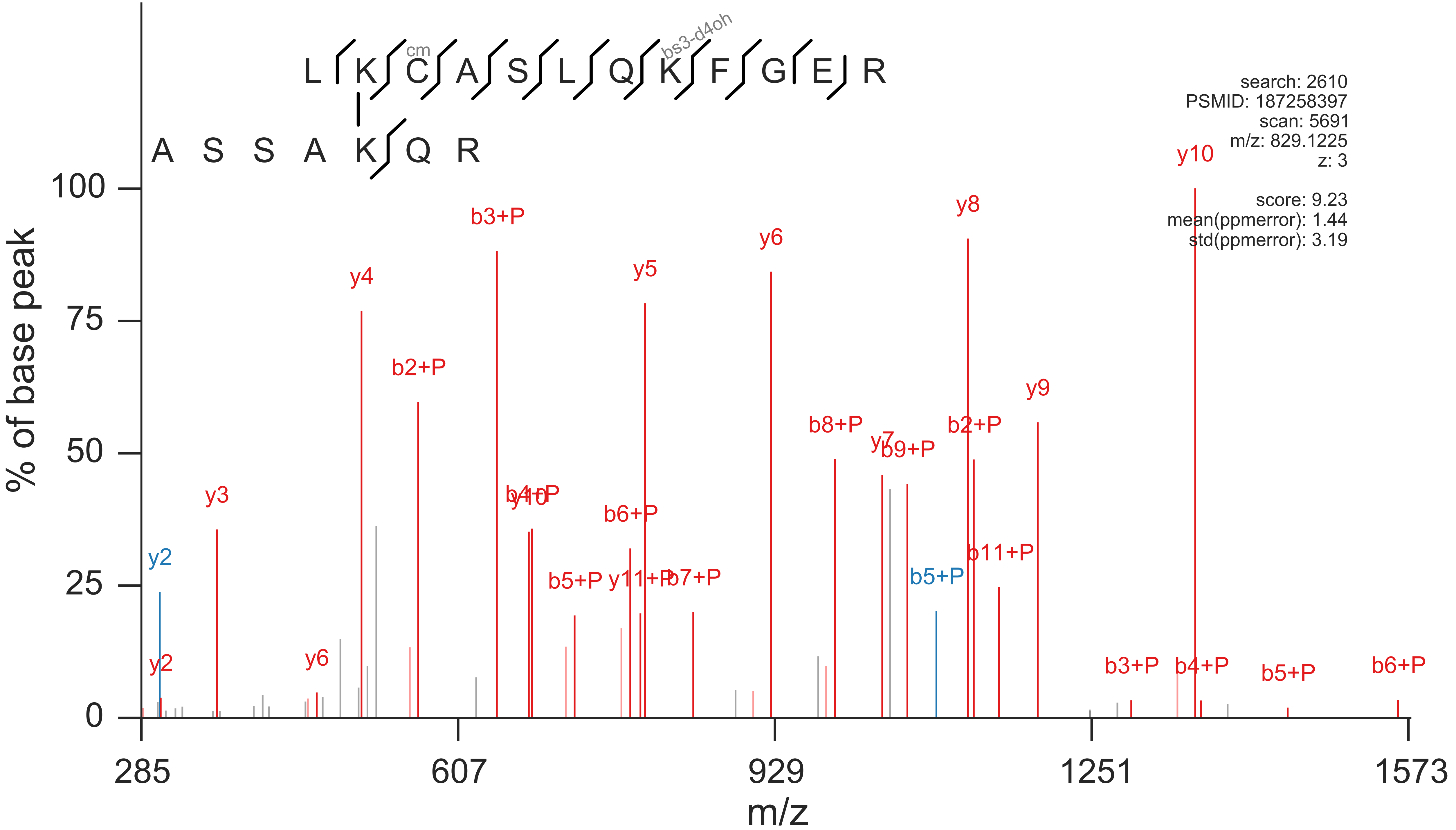

L K C A S L Q K F G E R
cm
bs3-d4oh
A S S A K Q R
search: 2610
PSMID: 187258397
scan: 5691
m/z: 829.1225
z: 3
score: 9.23
mean(ppmerror): 1.44
std(ppmerror): 3.19
% of base peak
m/z
0
25
50
75
100
285
607
929
1251
1573
y10
y8
y6
y5
y4
b3+P
b2+P
y9
b8+P
y7
b9+P
b2+P
b4+P
y10
y3
b6+P
b11+P
y2
b5+P
y11+P
b7+P
b5+P
y2
y6
b3+P
b4+P
b5+P
b6+P

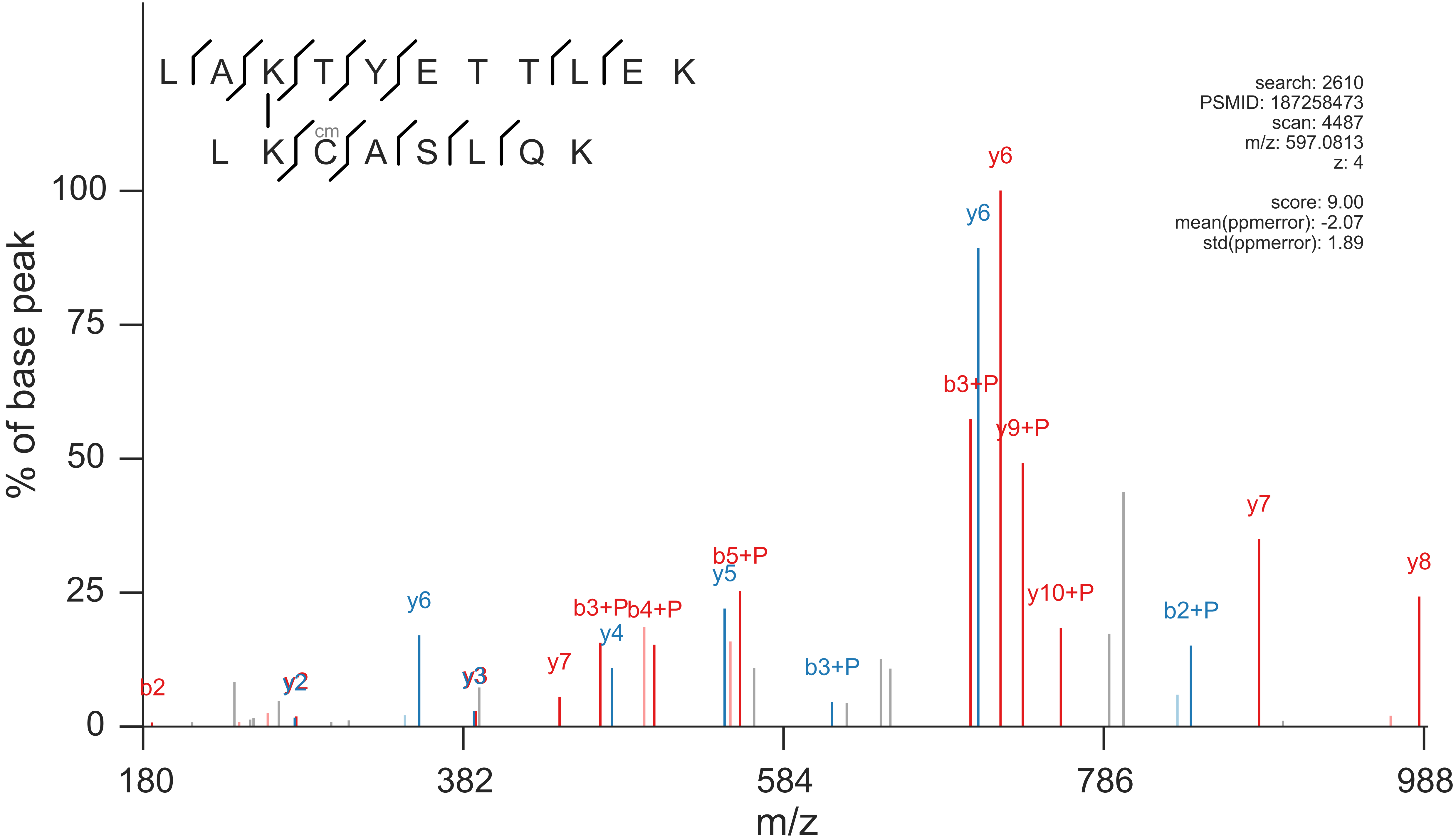

L A K T Y E T T L E K
L K C A S L Q K
cm
search: 2610
PSMID: 187258473
scan: 4487
m/z: 597.0813
z: 4
score: 9.00
mean(ppmerror): -2.07
std(ppmerror): 1.89
% of base peak
m/z
100
75
50
25
0
180
382
584
786
988
y6
y6
b3+P
y9+P
y7
y8
y10+P
b2+P
b5+P
y5
b3+P
b3+P
b4+P
y4
y7
y6
y3
y2
b2

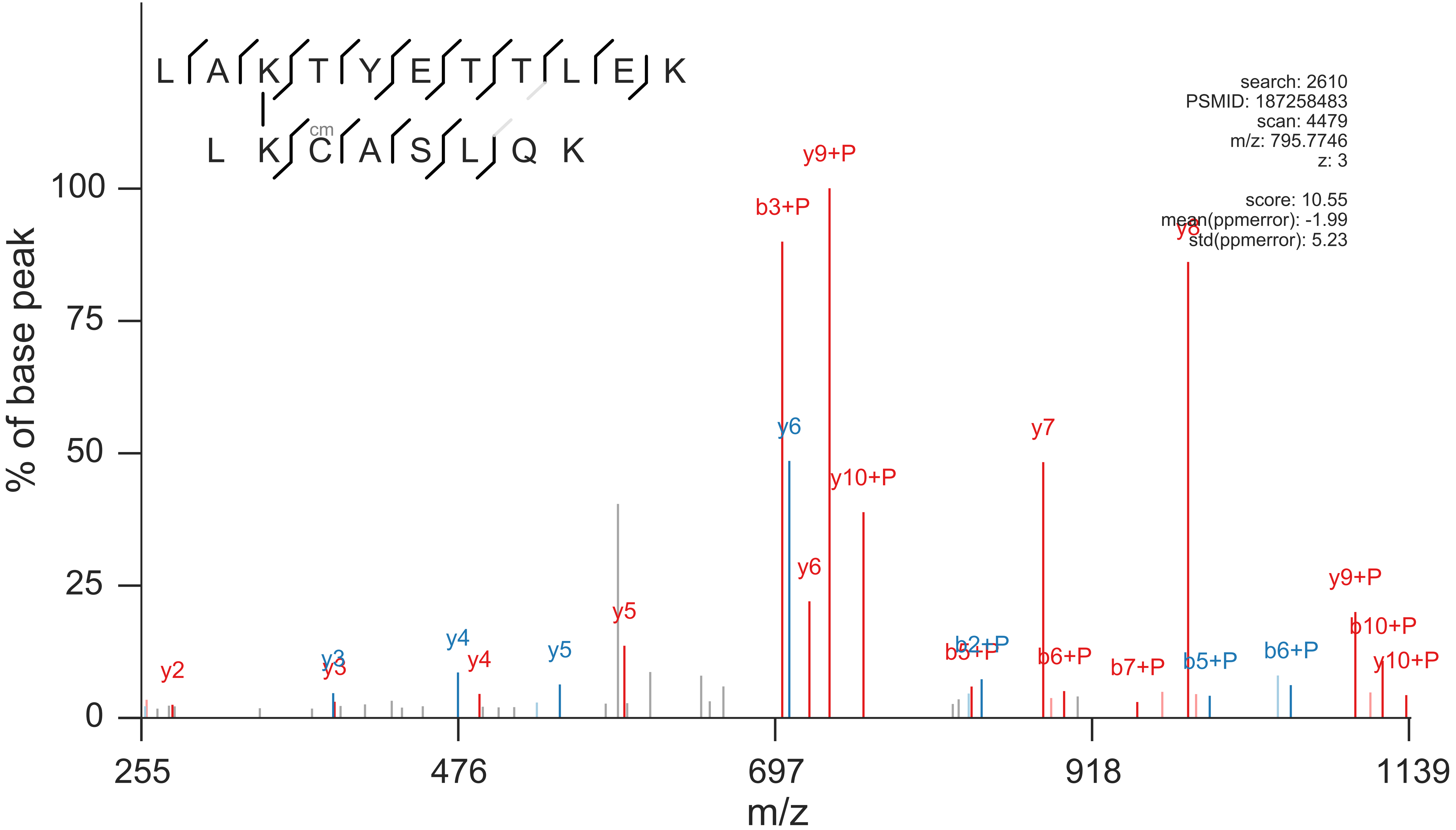

L A K T Y E T T L E K
L K C A S L Q K
cm
search: 2610
PSMID: 187258483
scan: 4479
m/z: 795.7746
z: 3
score: 10.55
mean(ppmerror): -1.99
std(ppmerror): 5.23
% of base peak
m/z
0
25
50
75
100
255
476
697
918
1139
y2
y3
y3
y4
y4
y5
y5
b3+P
y9+P
y6
y6
y10+P
y7
y8
b2+P
b5+P
b6+P
b7+P
b5+P
b6+P
y9+P
b10+P
y10+P

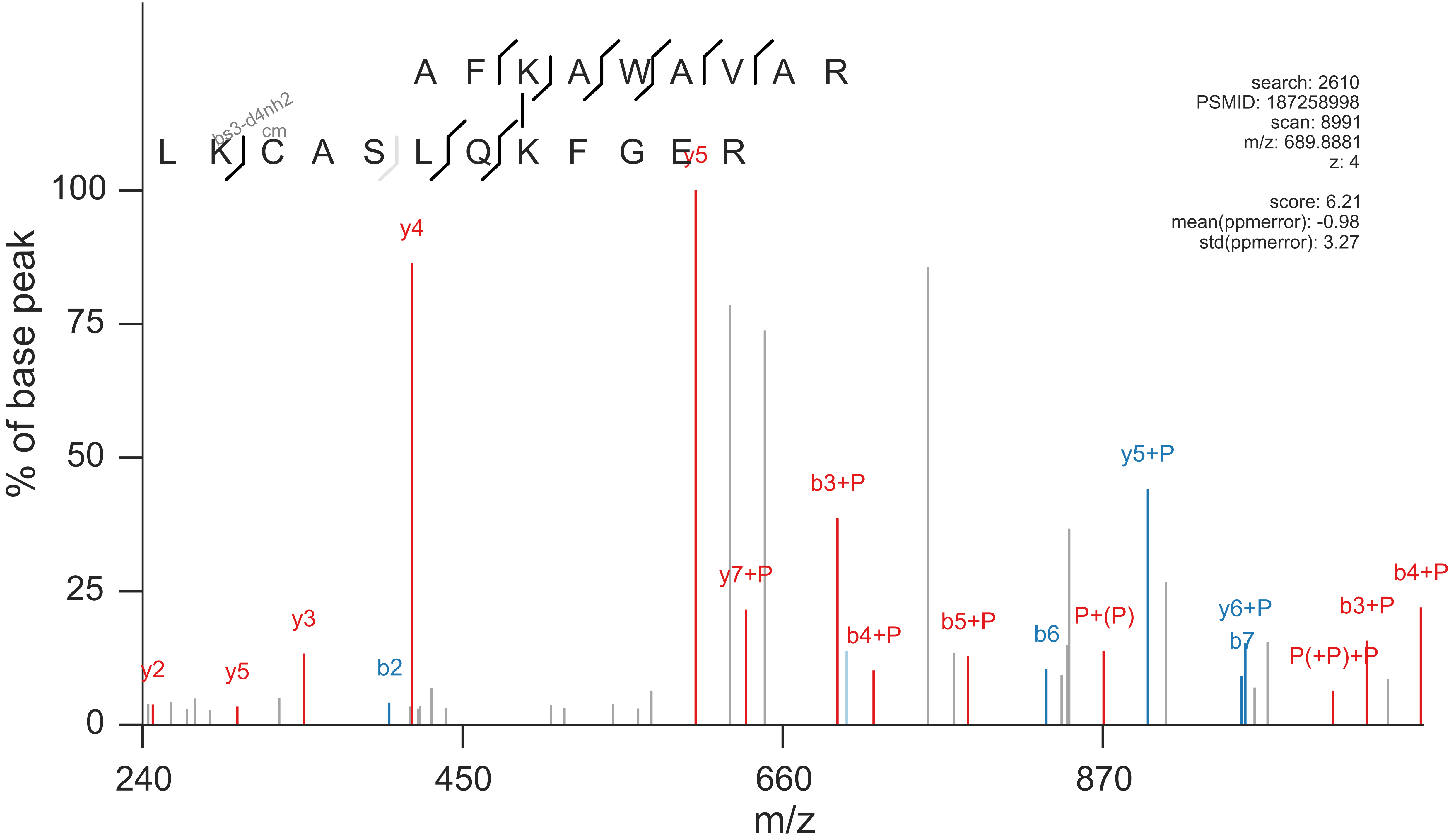

A F K A W A V A R
L K C A S L Q K F G E R
bs3-d4nh2
cm
search: 2610
PSMID: 187258998
scan: 8991
m/z: 689.8881
z: 4
score: 6.21
mean(ppmerror): -0.98
std(ppmerror): 3.27
% of base peak
m/z
100
75
50
25
0
240
450
660
870
y2
y5
y3
b2
y4
y5
y7+P
b3+P
b4+P
b5+P
b6
P+(P)
y5+P
y6+P
b7
P(+P)+P
b3+P
b4+P

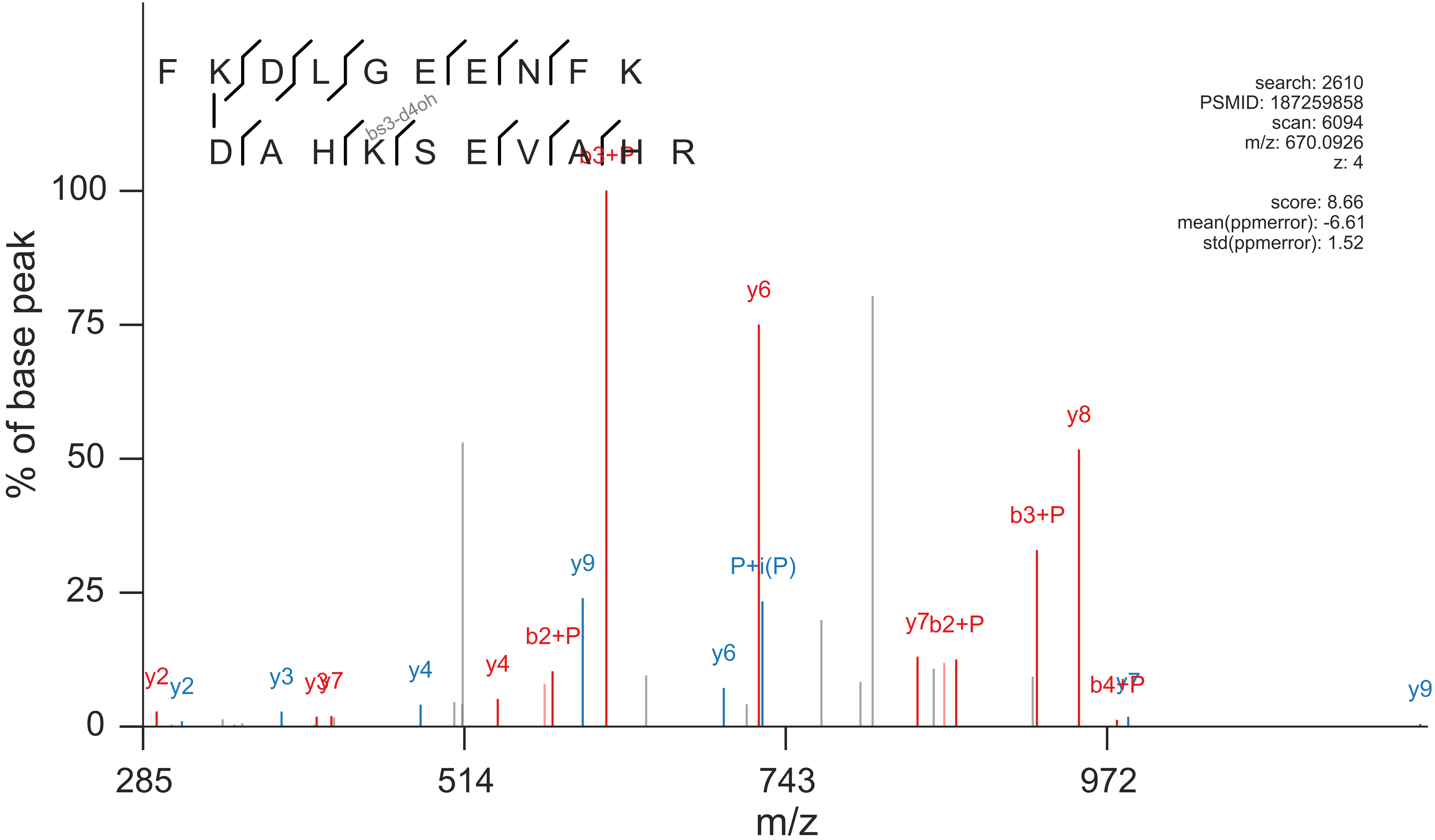

F K D L G E E N F K
D A H K S E V A H R
bs3-d4oh
search: 2610
PSMID: 187259858
scan: 6094
m/z: 670.0926
z: 4
score: 8.66
mean(ppmerror): -6.61
std(ppmerror): 1.52
% of base peak
m/z
b3+P
y6
y8
b3+P
y9
P+i(P)
y7
b2+P
b2+P
y4
y6
y4
y3
y3
y7
y2
y2
b4+P
y7
y9
0
25
50
75
100
285
514
743
972

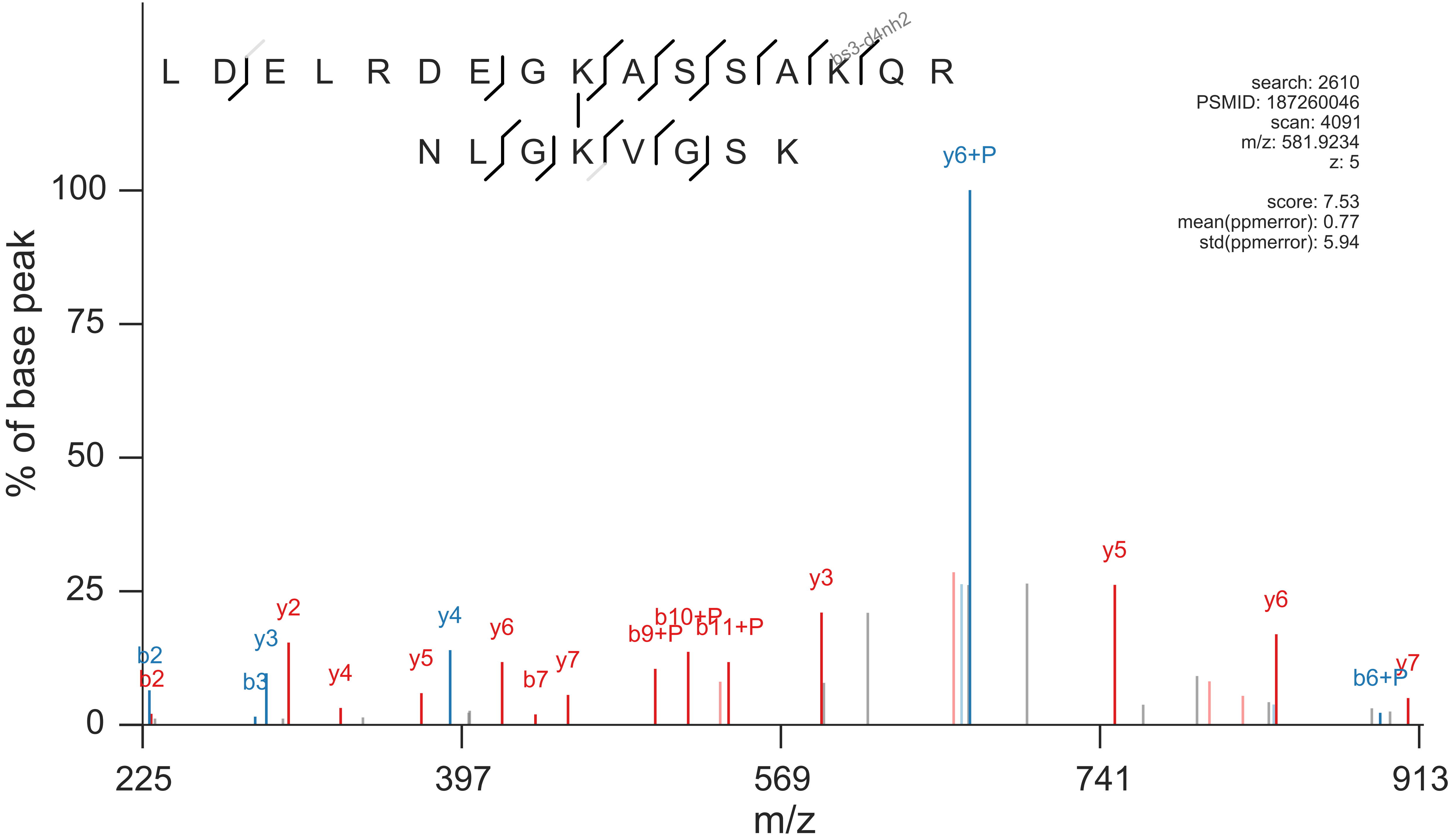

L D E L R D E G K A S S A K Q R
bs3-d4nh2
N L G K V G S K
search: 2610
PSMID: 187260046
scan: 4091
m/z: 581.9234
z: 5
score: 7.53
mean(ppmerror): 0.77
std(ppmerror): 5.94
% of base peak
100
75
50
25
0
225
397
569
741
913
m/z
y6+P
y5
y6
y3
b10+P
b9+P
b11+P
y2
y4
y6
y3
b2
b2
b3
y4
y5
b7
y7
b6+P
y7

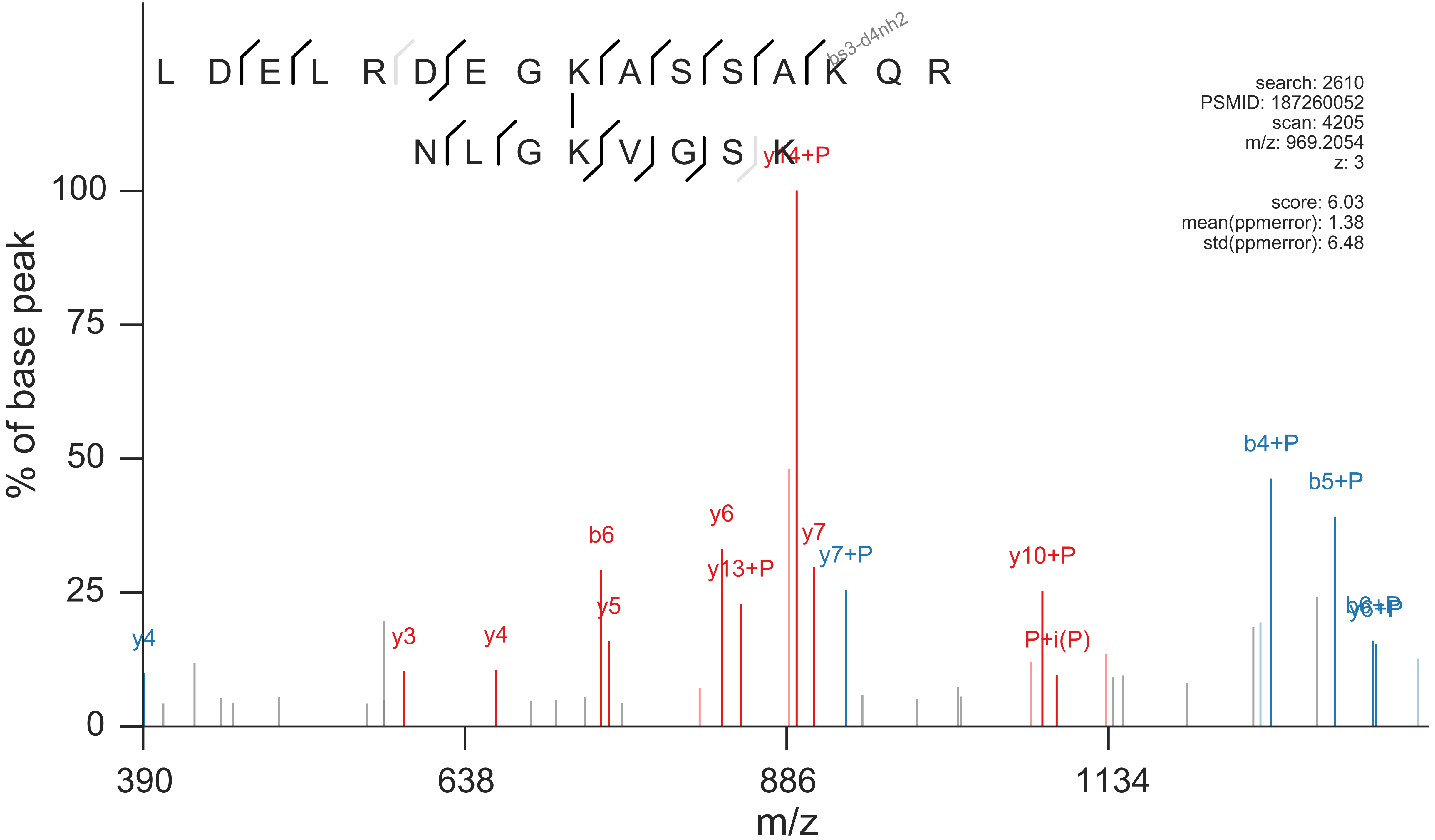
L D E L R D E G K A S S A K Q R
bs3-d4nh2
N L G K V G S K
search: 2610
PSMID: 187260052
scan: 4205
m/z: 969.2054
z: 3
score: 6.03
mean(ppmerror): 1.38
std(ppmerror): 6.48
% of base peak
100
75
50
25
0
390
638
886
1134
m/z
y14+P
b4+P
b5+P
b6+P
y6+P
y6
b6
y7
y7+P
y10+P
y13+P
y5
y4
y3
y4
P+i(P)

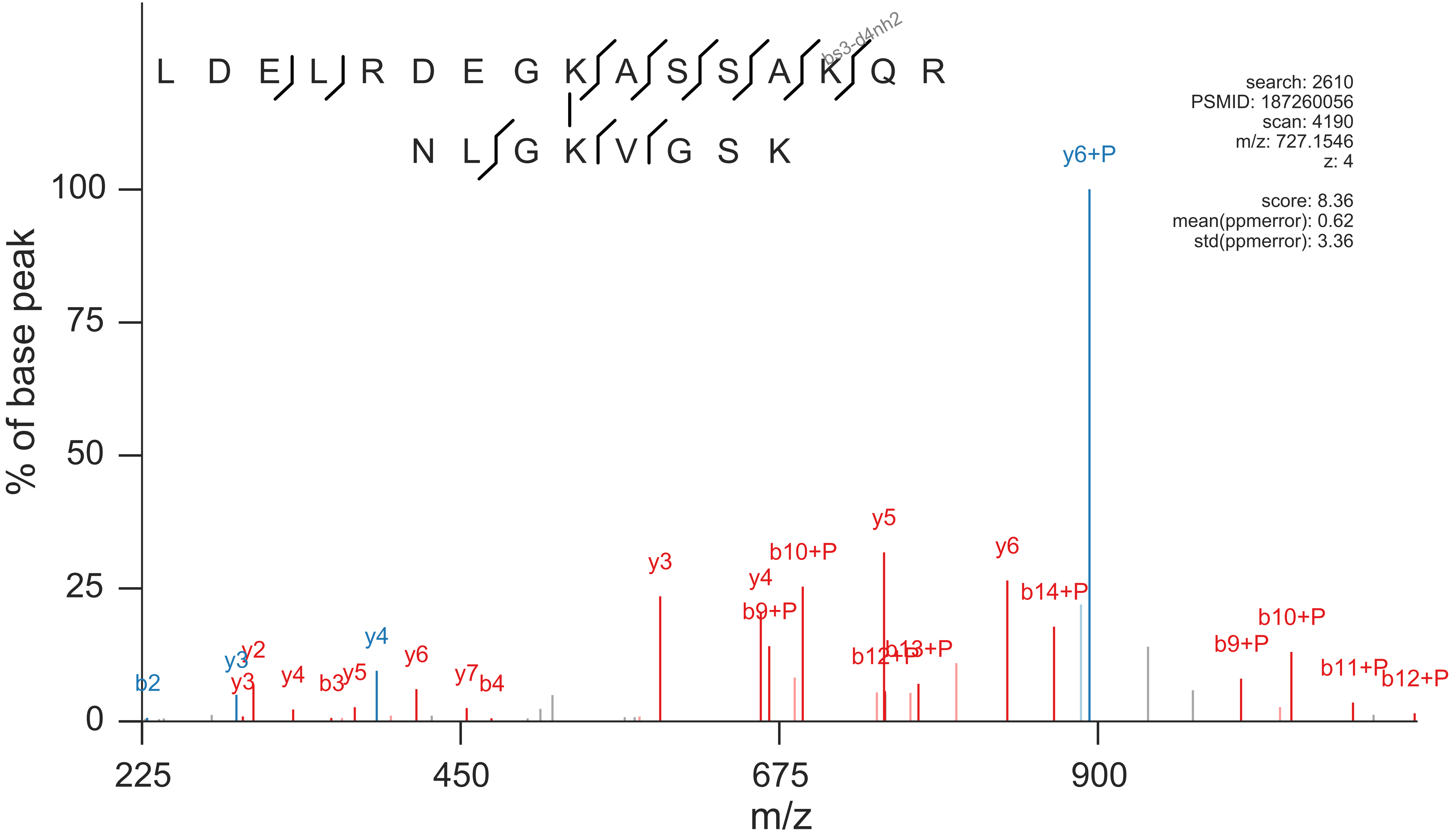

L D E L R D E G K A S S A K Q R
N L G K V G S K
bs3-d4nh2
search: 2610
PSMID: 187260056
scan: 4190
m/z: 727.1546
z: 4
score: 8.36
mean(ppmerror): 0.62
std(ppmerror): 3.36
% of base peak
m/z
0
25
50
75
100
225
450
675
900
y6+P
y5
b10+P
y6
y3
y4
b14+P
b9+P
b10+P
b13+P
b12+P
b9+P
b11+P
b12+P
y4
y3
y2
y3
b2
y4
y6
b3
y5
y7
b4

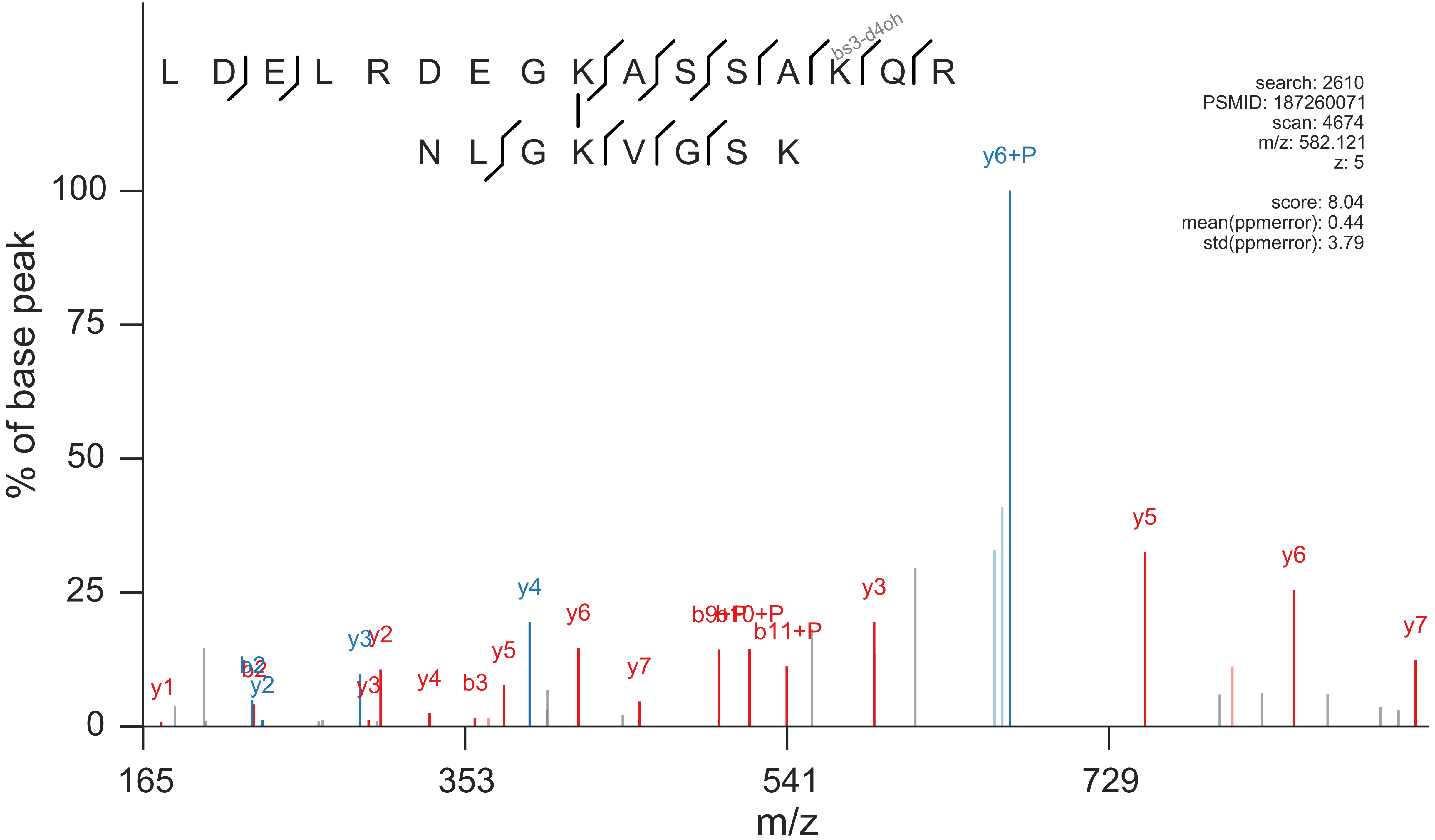

L D E L R D E G K A S S A K Q R
bs3-d4oh
N L G K V G S K
search: 2610
PSMID: 187260071
scan: 4674
m/z: 582.121
z: 5
score: 8.04
mean(ppmerror): 0.44
std(ppmerror): 3.79
% of base peak
100
75
50
25
0
165
353
541
729
m/z
y6+P
y5
y6
y7
y4
y6
y3
b9+P
b10+P
b11+P
y3
y2
y7
b2
b2
y2
y1
y4
b3
y5
y3

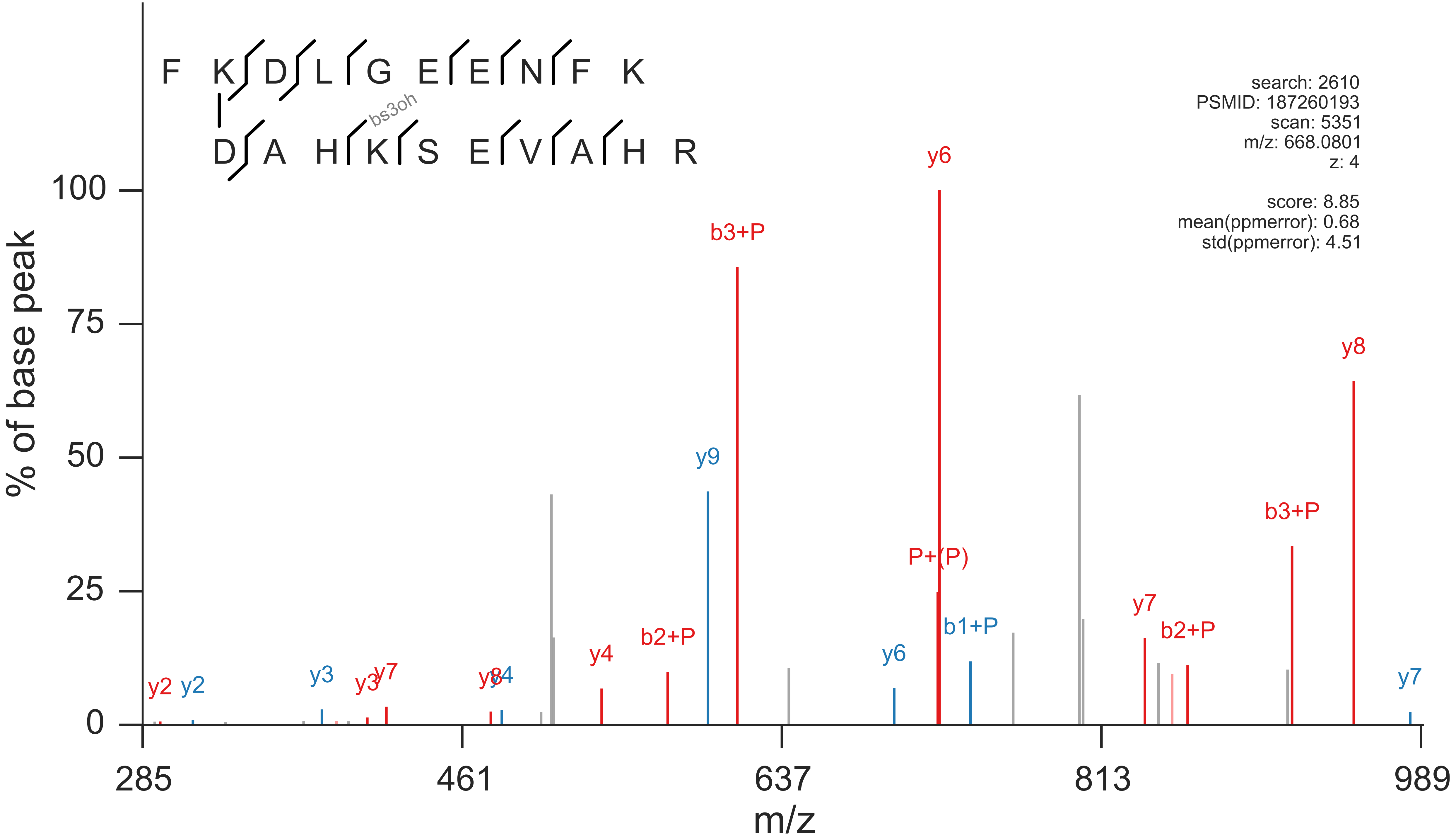

F K D L G E E N F K
D A H K S E V A H R
bs3oh
search: 2610
PSMID: 187260193
scan: 5351
m/z: 668.0801
z: 4
score: 8.85
mean(ppmerror): 0.68
std(ppmerror): 4.51
% of base peak
m/z
y6
b3+P
y8
y9
b3+P
P+(P)
y7
b2+P
b1+P
y6
y4
b2+P
y2
y2
y3
y3
y7
y8
y4
y7

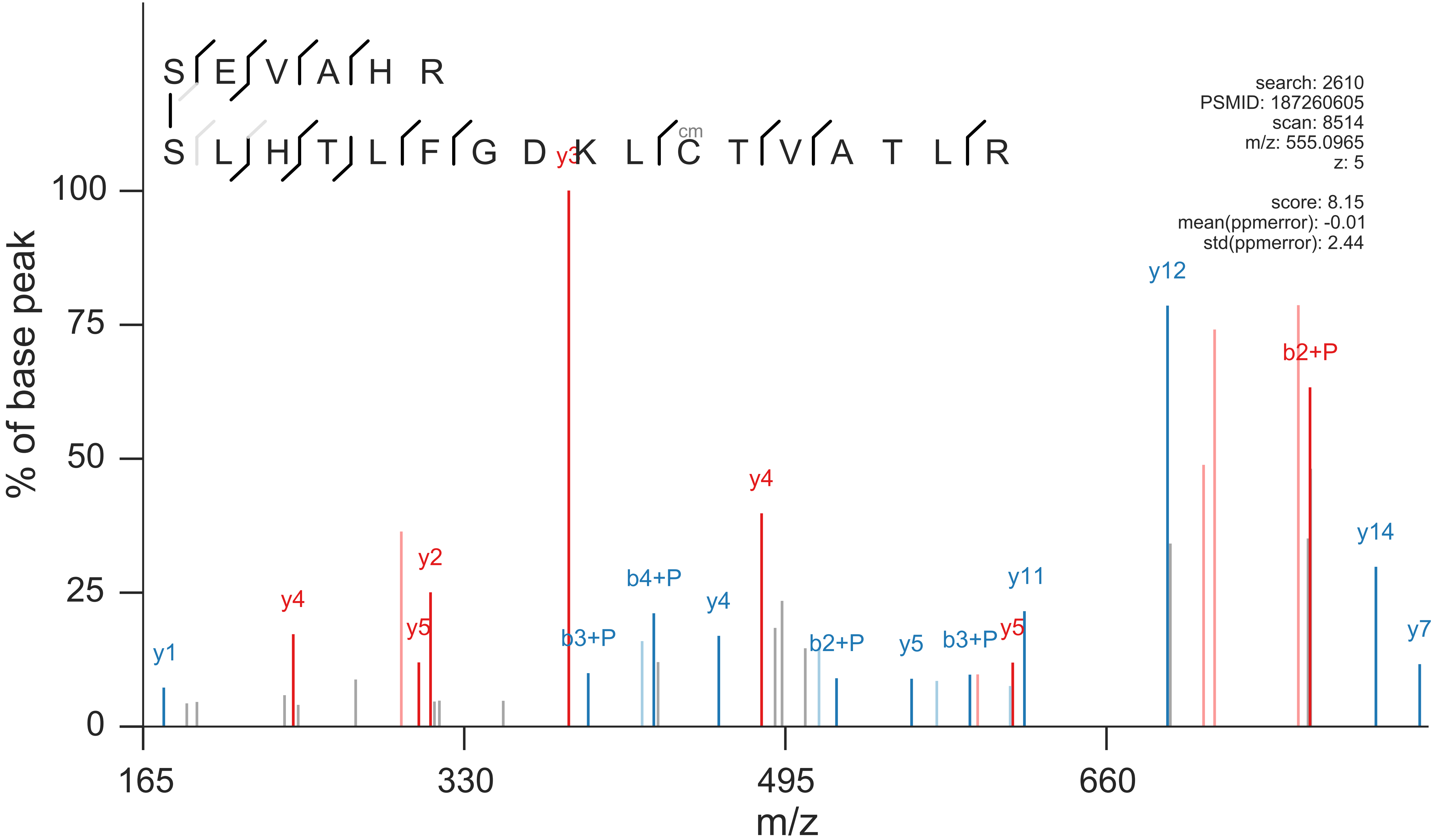
S E V A H R
S L H T L F G D K L C T V A T L R
cm
search: 2610
PSMID: 187260605
scan: 8514
m/z: 555.0965
z: 5
score: 8.15
mean(ppmerror): -0.01
std(ppmerror): 2.44
% of base peak
m/z
0
25
50
75
100
165
330
495
660
y1
y4
y5
y2
y3
b3+P
b4+P
y4
y4
b2+P
y5
b3+P
y5
y11
y12
b2+P
y14
y7

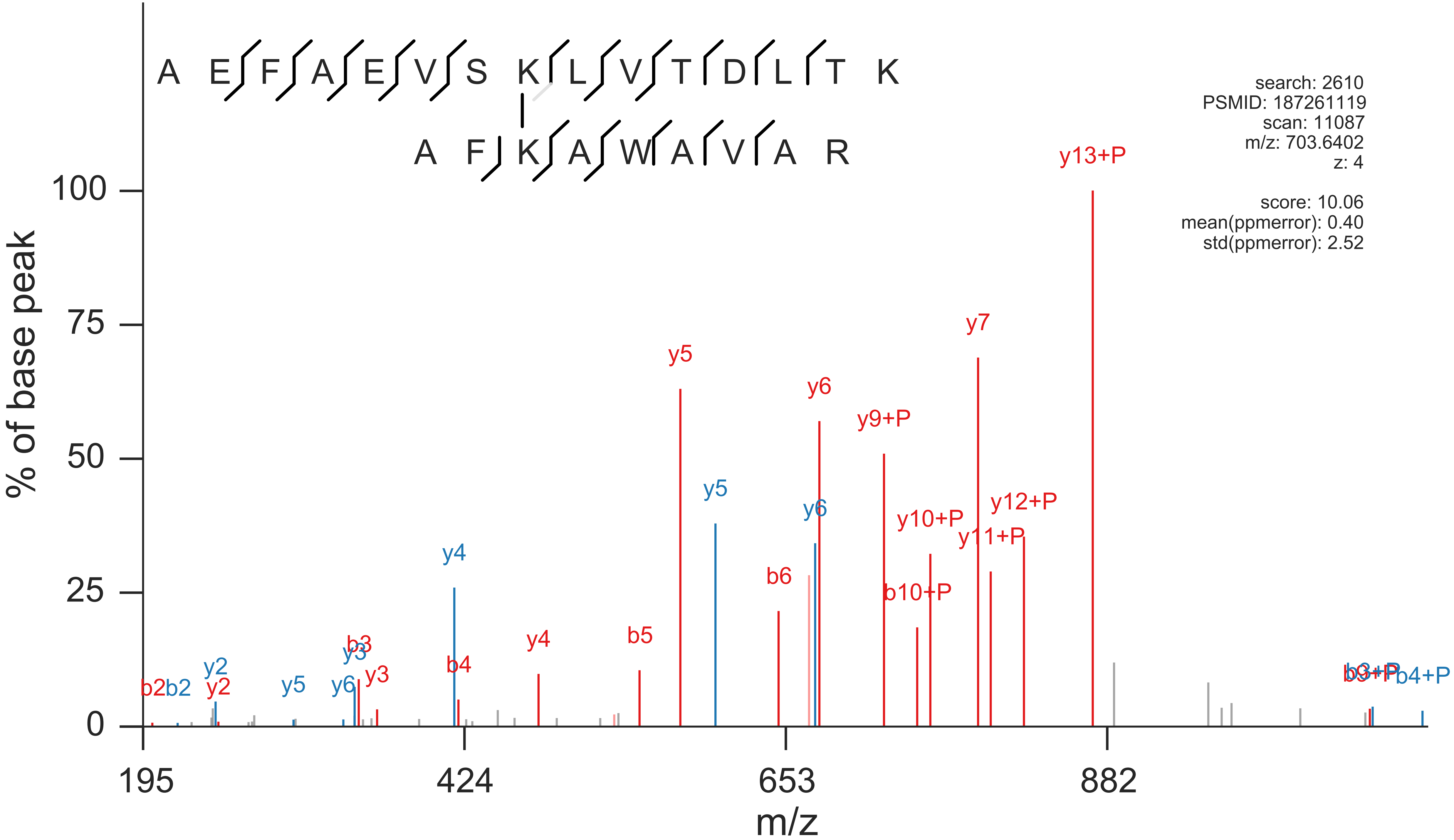

A E F A E V S K L V T D L T K
A F K A W A V A R
search: 2610
PSMID: 187261119
scan: 11087
m/z: 703.6402
z: 4
score: 10.06
mean(ppmerror): 0.40
std(ppmerror): 2.52
% of base peak
m/z
0
25
50
75
100
195
424
653
882
y13+P
y7
y5
y6
y9+P
y5
y6
y12+P
y10+P
y11+P
y4
b6
b10+P
b5
y4
b4
b3
y3
y3
y2
y2
y6
y5
b2
b2
b3+P
b4+P

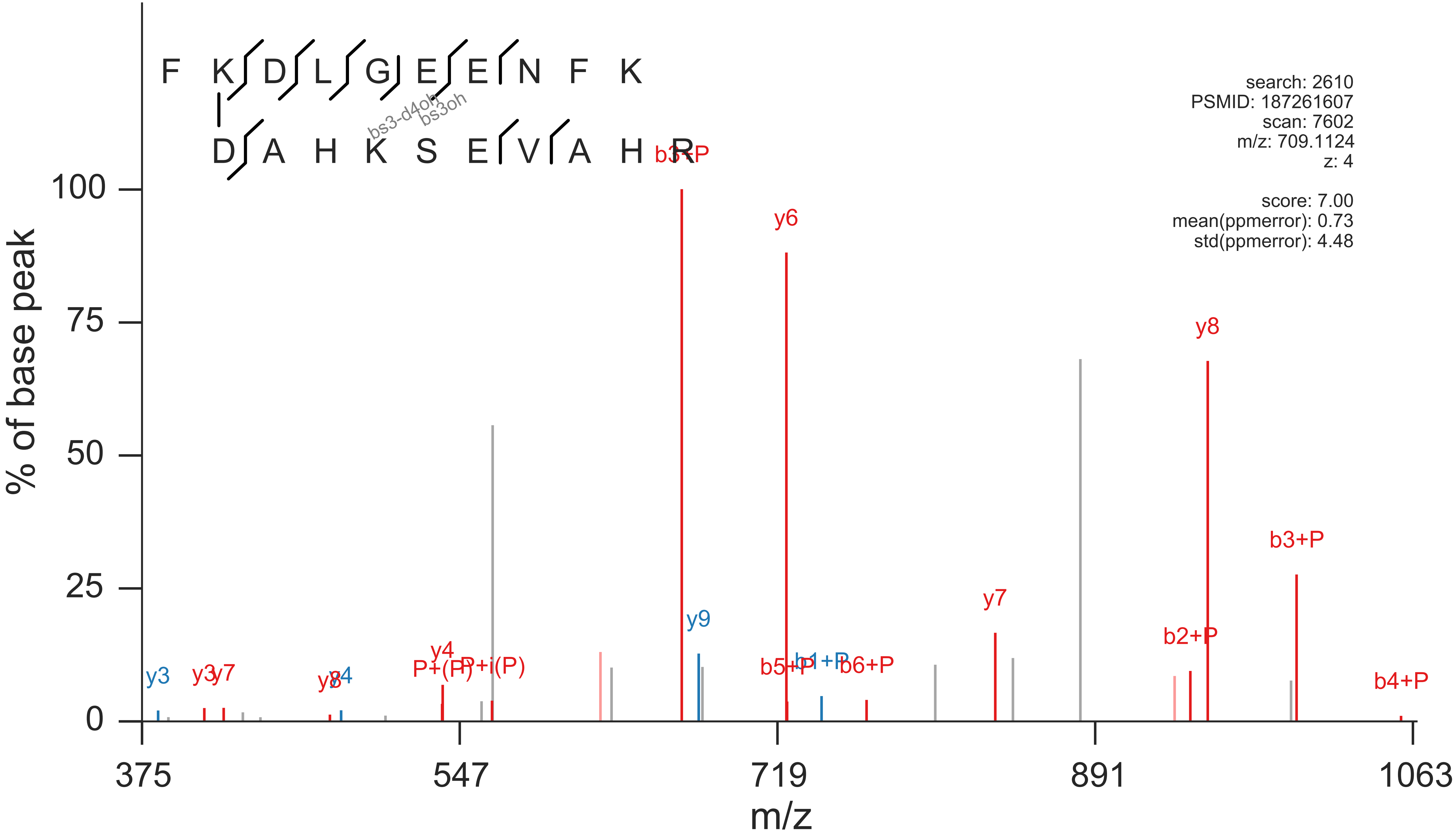

F K D L G E E N F K
D A H K S E V A H R
bs3-d4oh
bs3oh
search: 2610
PSMID: 187261607
scan: 7602
m/z: 709.1124
z: 4
score: 7.00
mean(ppmerror): 0.73
std(ppmerror): 4.48
% of base peak
m/z
100
75
50
25
0
375
547
719
891
1063
b3+P
y6
y8
b3+P
y7
y9
b2+P
y4
b4+P
y3
y3
y7
y8
y4
P+(P)
P+i(P)
b5+P
b1+P
b6+P

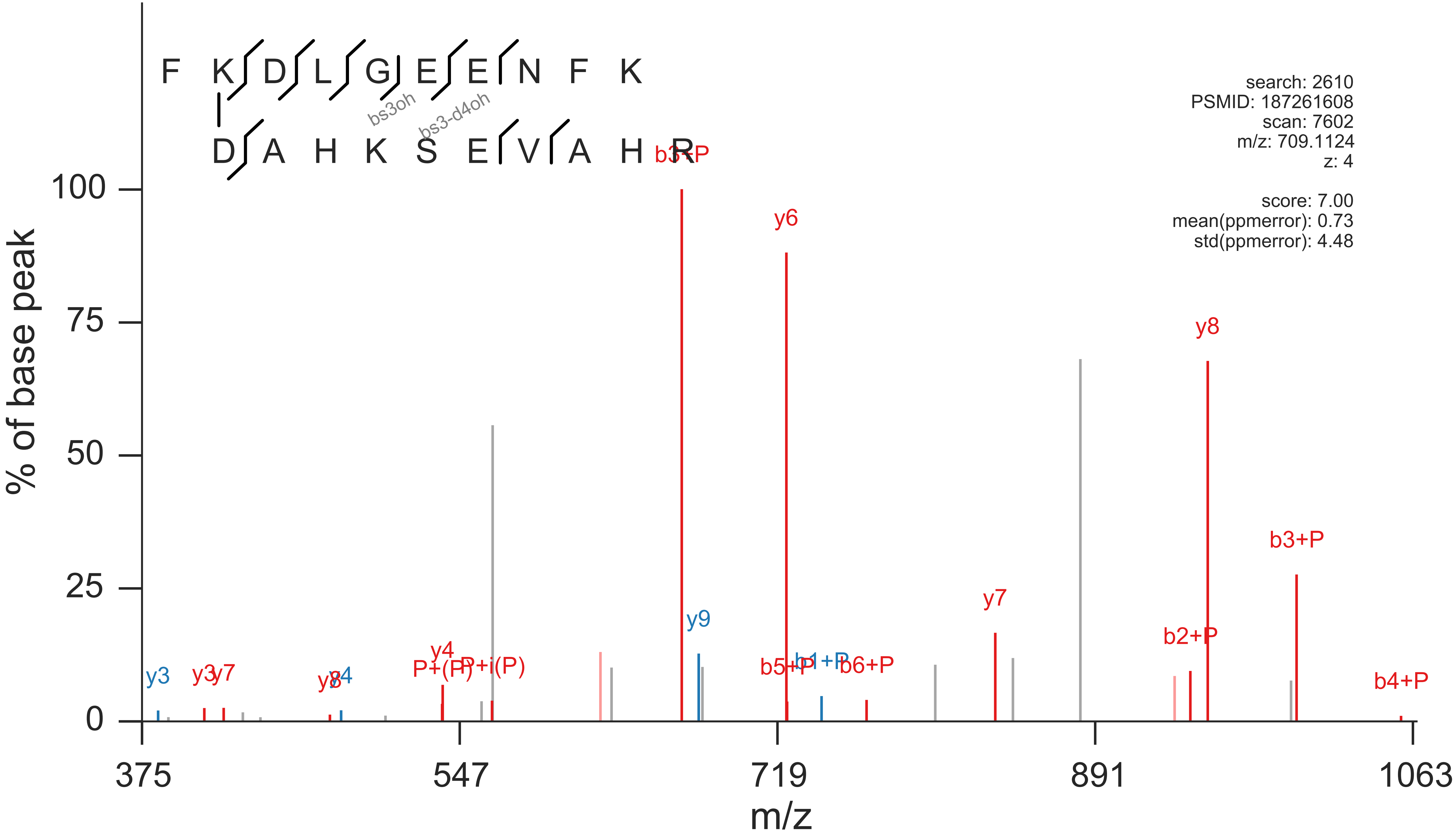

F K D L G E E N F K
D A H K S E V A H R
bs3oh
bs3-d4oh
search: 2610
PSMID: 187261608
scan: 7602
m/z: 709.1124
z: 4
score: 7.00
mean(ppmerror): 0.73
std(ppmerror): 4.48
% of base peak
m/z
100
75
50
25
0
375
547
719
891
1063
b3+P
y6
y8
b3+P
y7
b2+P
b4+P
y9
b6+P
b5+P
b1+P
y4
P+i(P)
y3
y3
y7
y8
y4

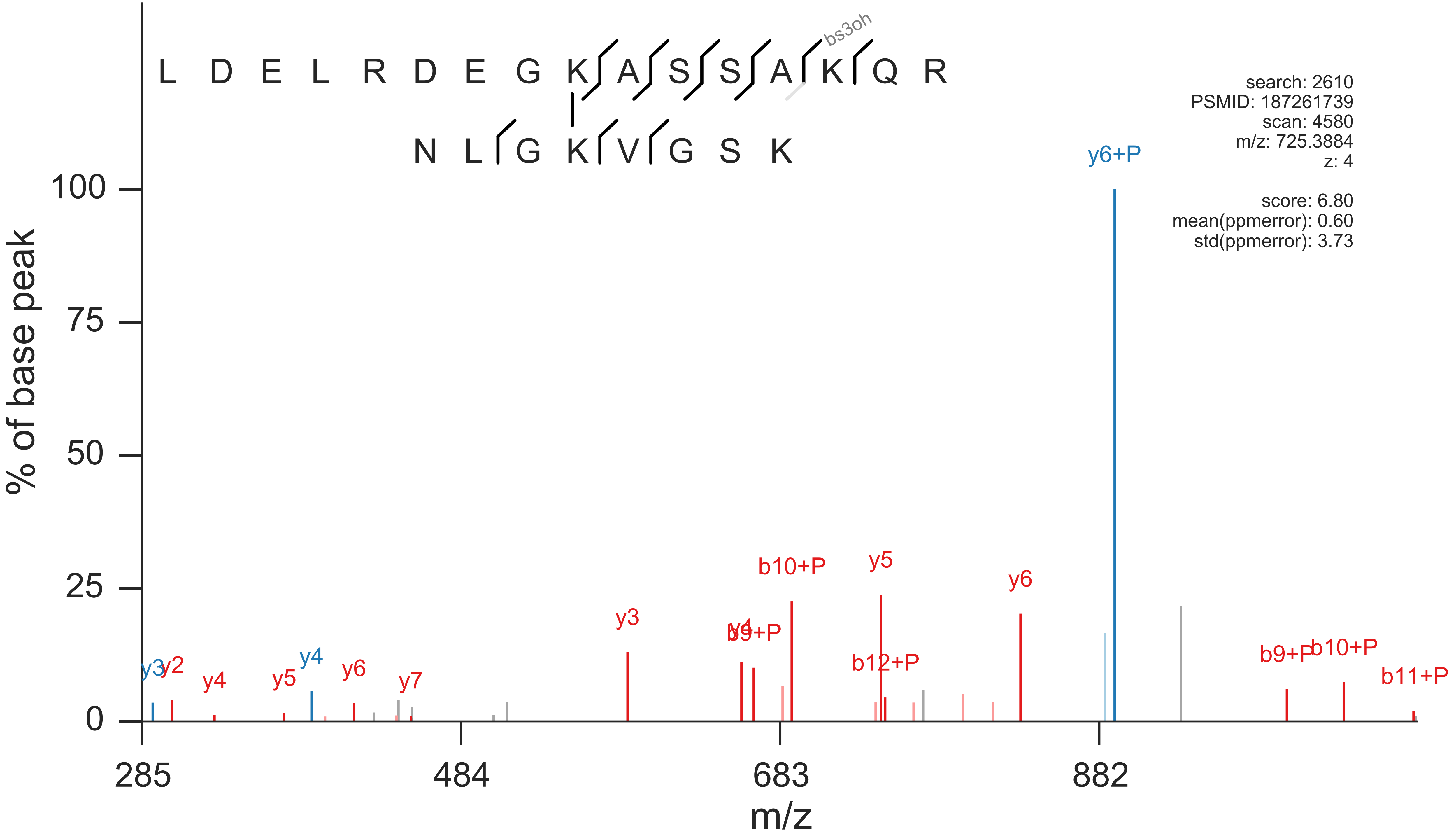

L D E L R D E G K A S S A K Q R
N L G K V G S K
bs3oh
search: 2610
PSMID: 187261739
scan: 4580
m/z: 725.3884
z: 4
score: 6.80
mean(ppmerror): 0.60
std(ppmerror): 3.73
% of base peak
m/z
y6+P
b10+P
y5
y6
y3
b9+P
b12+P
b9+P
b10+P
b11+P
y3
y2
y4
y5
y4
y6
y7

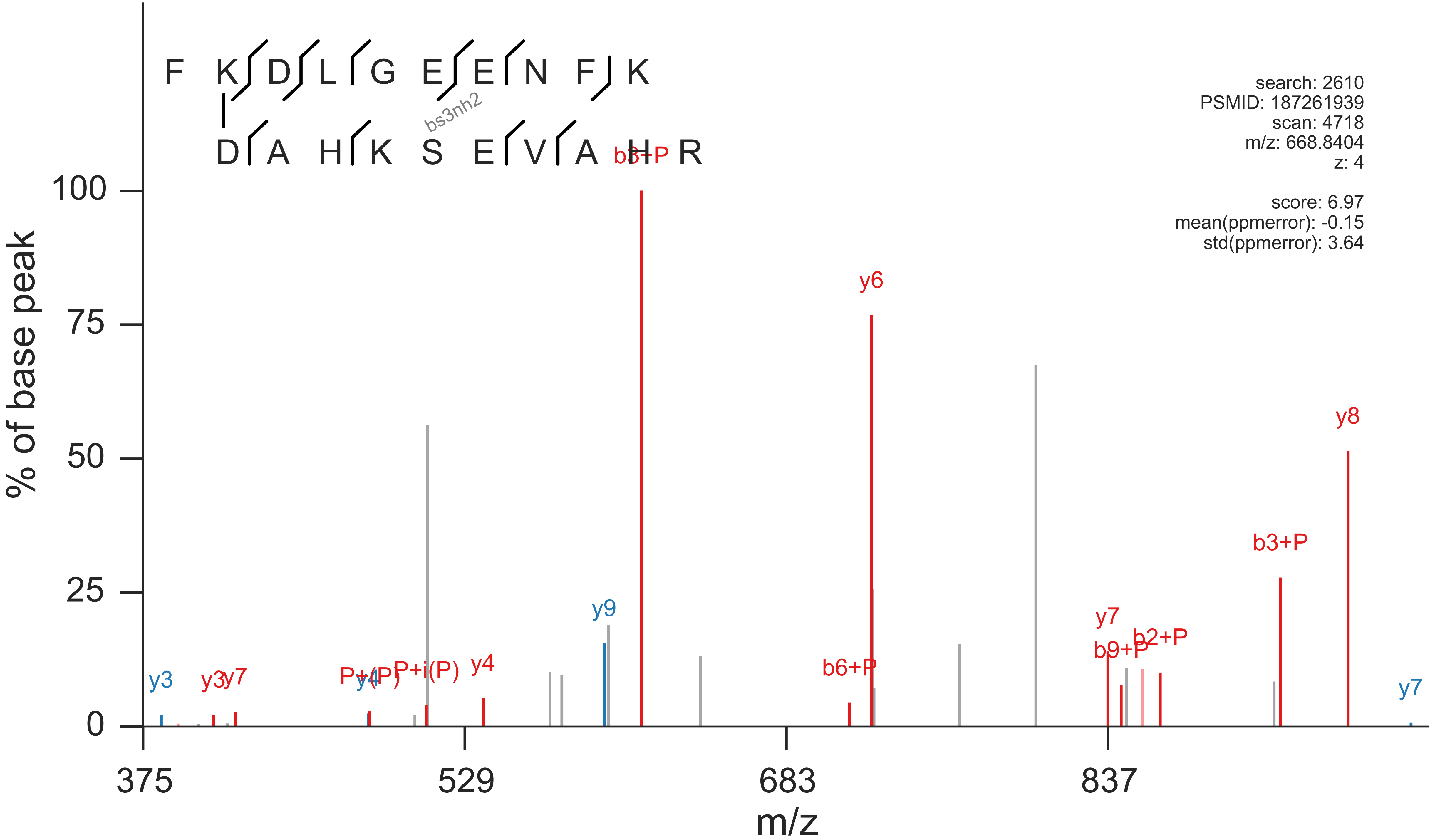

F K D L G E E N F K
D A H K S E V A H R
bs3nh2
search: 2610
PSMID: 187261939
scan: 4718
m/z: 668.8404
z: 4
score: 6.97
mean(ppmerror): -0.15
std(ppmerror): 3.64
% of base peak
m/z
0
25
50
75
100
375
529
683
837
b3+P
y6
y8
b3+P
y9
y7
b9+P
b2+P
b6+P
y4
P+i(P)
y3
y7
y3
y7

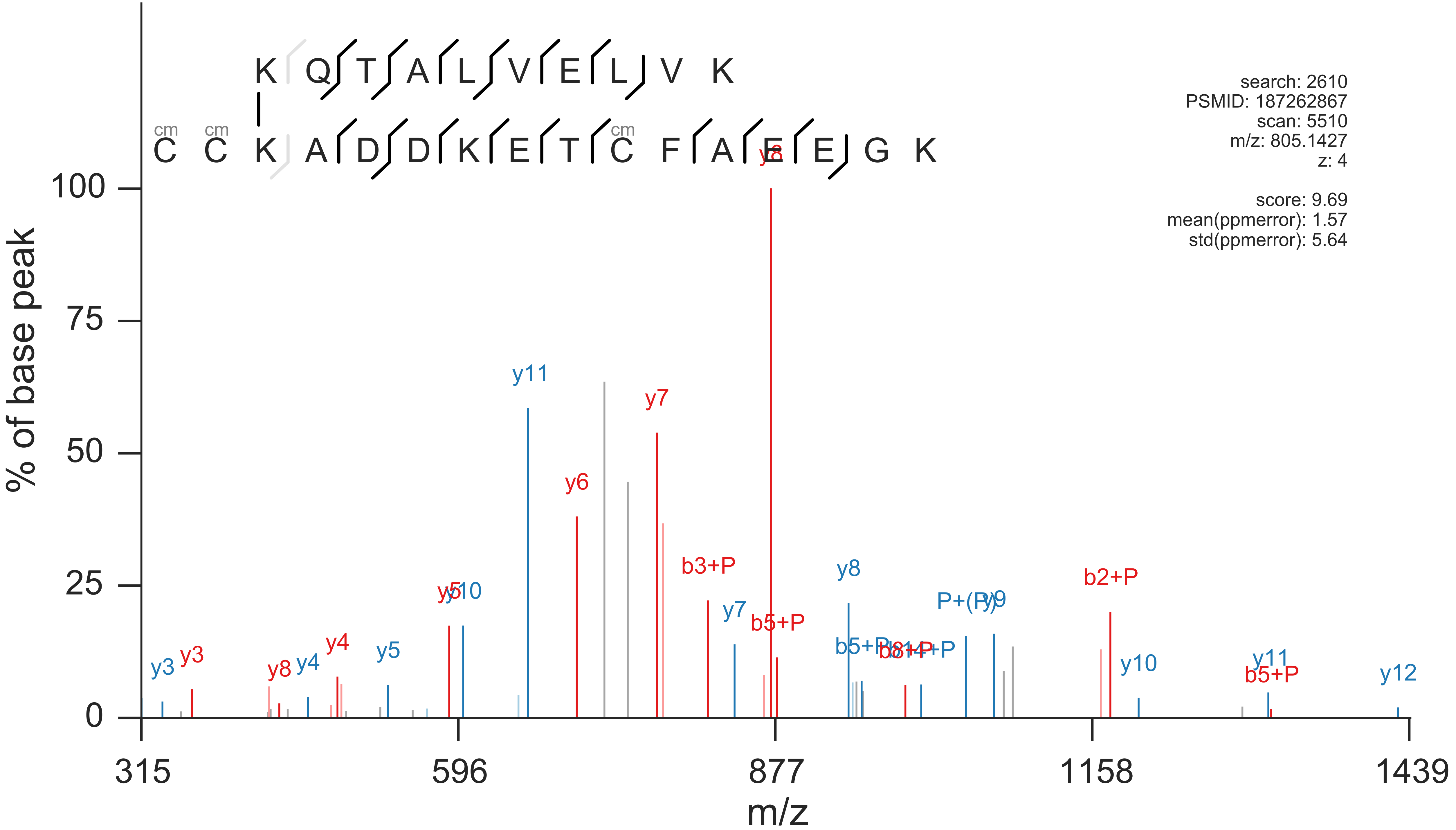

K Q T A L V E L V K
cm cm cm
C C K A D D K E T C F A E E G K
search: 2610
PSMID: 187262867
scan: 5510
m/z: 805.1427
z: 4
score: 9.69
mean(ppmerror): 1.57
std(ppmerror): 5.64
% of base peak
100
75
50
25
0
m/z
315
596
877
1158
1439
y3 y3 y8 y4 y4 y5 y5 y10 y11 y6 y7 b3+P y7 b5+P y8 y8 b5+P P+(P) y9 b2+P y10 y11 b5+P y12

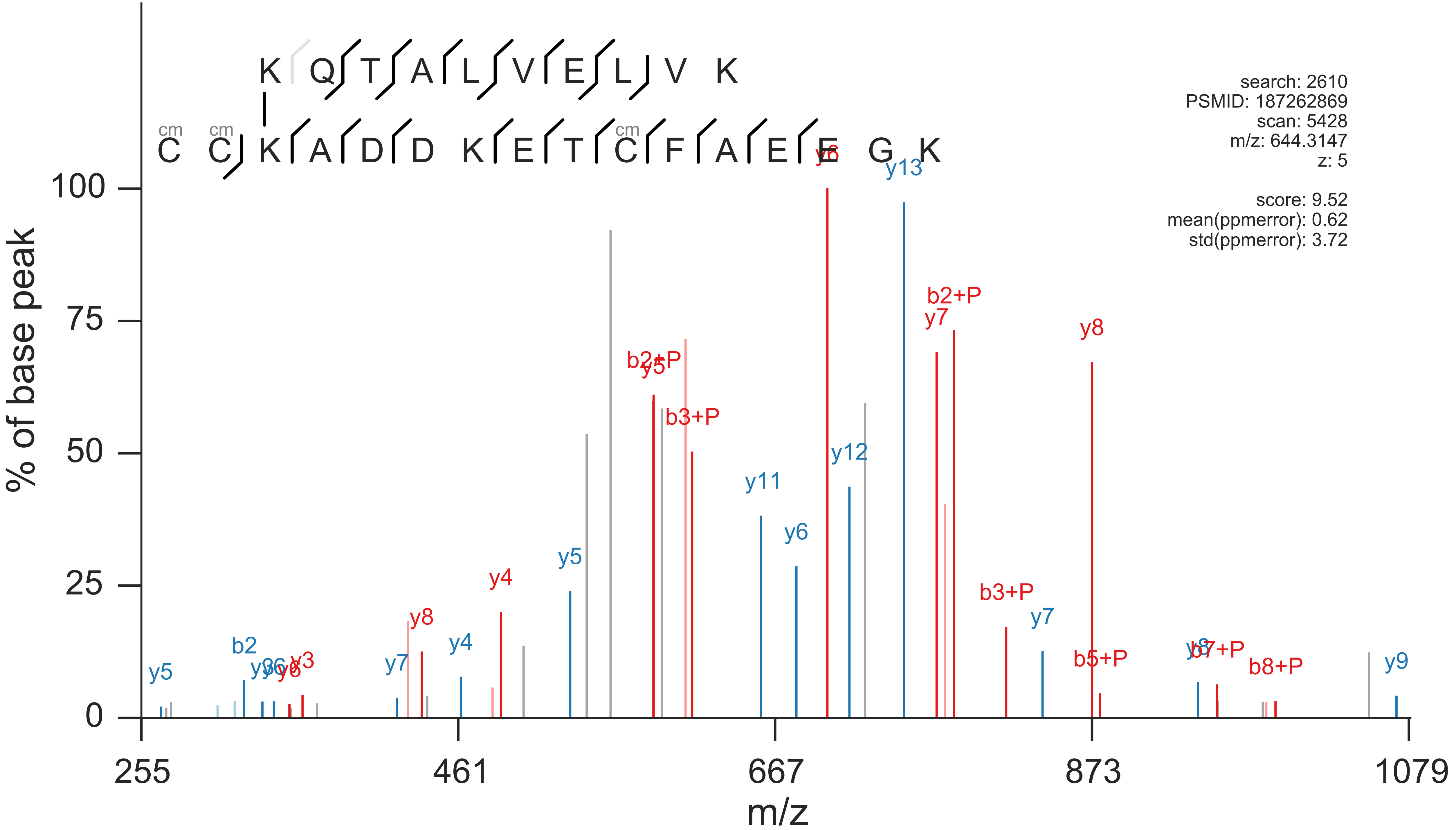
K Q T A L V E L V K
cm cm cm
C C K A D D K E T C F A E E G K
search: 2610
PSMID: 187262869
scan: 5428
m/z: 644.3147
z: 5
score: 9.52
mean(ppmerror): 0.62
std(ppmerror): 3.72
% of base peak
m/z
0
25
50
75
100
255
461
667
873
1079

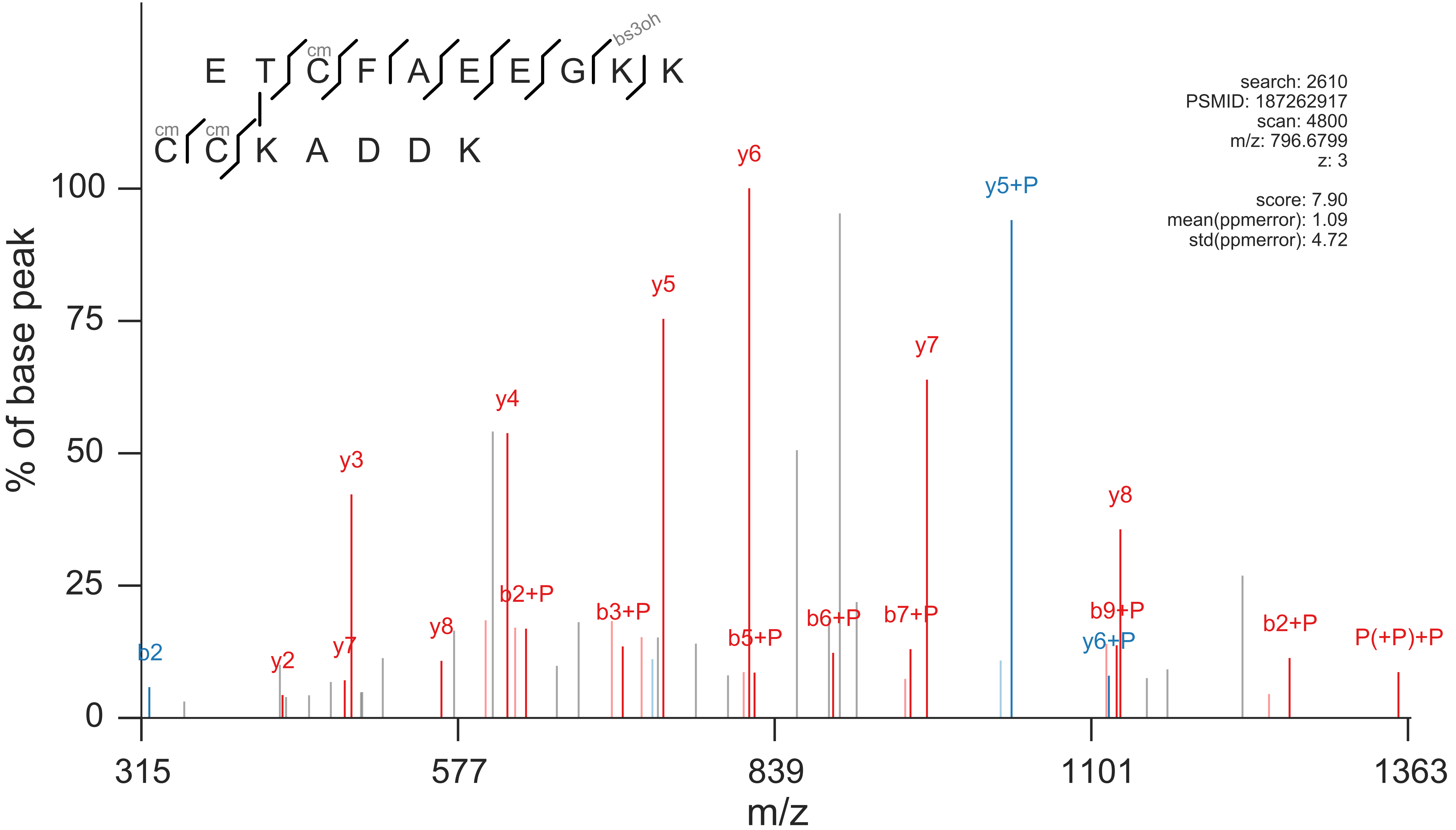

E T C F A E E G K K
cm
bs3oh
C C K A D D K
cm cm
search: 2610
PSMID: 187262917
scan: 4800
m/z: 796.6799
z: 3
score: 7.90
mean(ppmerror): 1.09
std(ppmerror): 4.72
% of base peak
m/z
0
25
50
75
100
315
577
839
1101
1363
b2
y2
y7
y3
y8
y4
b2+P
b3+P
y5
b5+P
y6
b6+P
b7+P
y7
y5+P
y6+P
b9+P
y8
b2+P
P(+P)+P

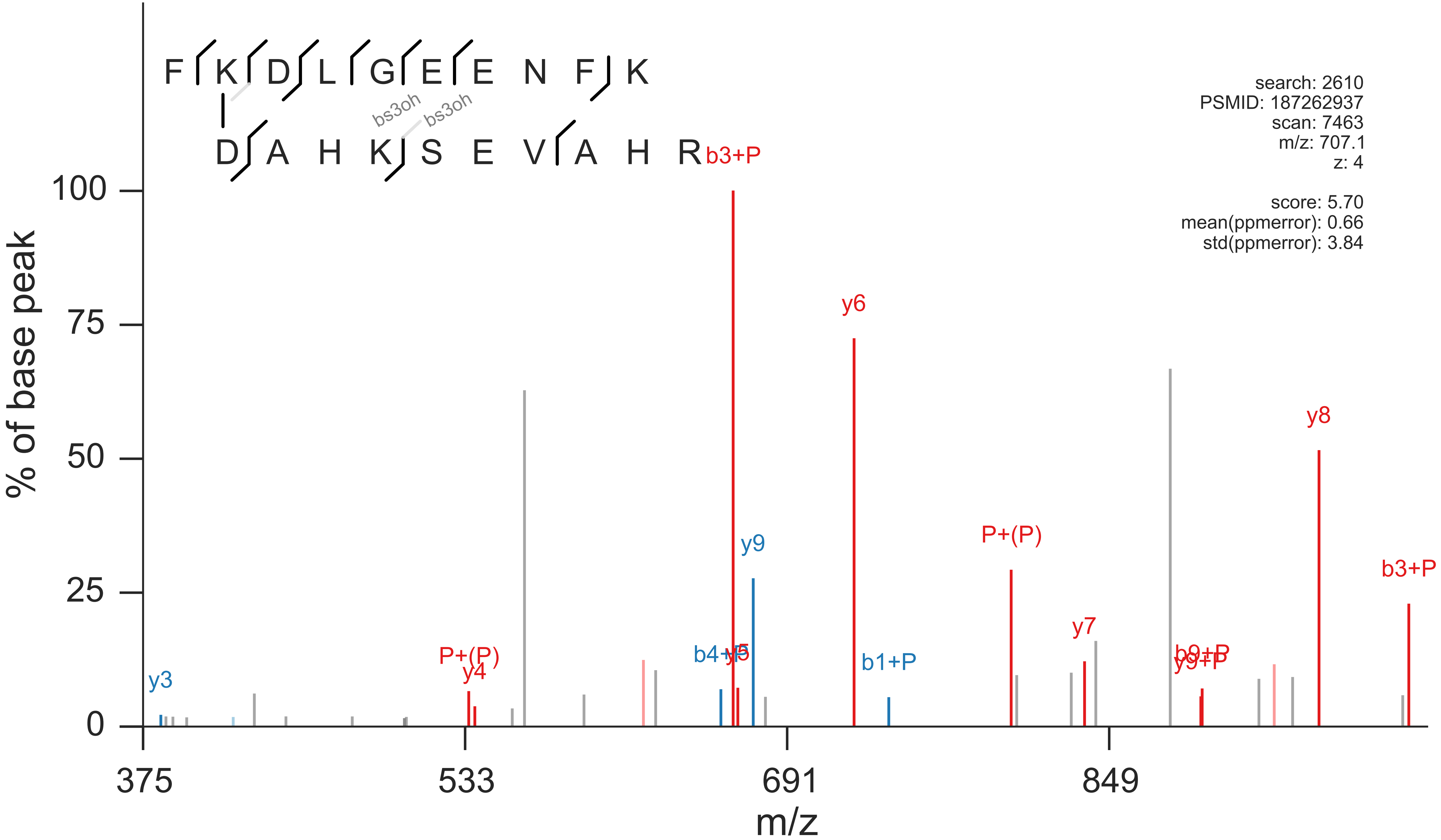

F K D L G E E N F K
bs3oh
bs3oh
D A H K S E V A H R
search: 2610
PSMID: 187262937
scan: 7463
m/z: 707.1
z: 4
score: 5.70
mean(ppmerror): 0.66
std(ppmerror): 3.84
% of base peak
m/z
b3+P
y6
y8
y9
P+(P)
b3+P
y7
b9+P
y9+P
b4+y5
b1+P
P+(P)
y4
y3
0
25
50
75
100
375
533
691
849

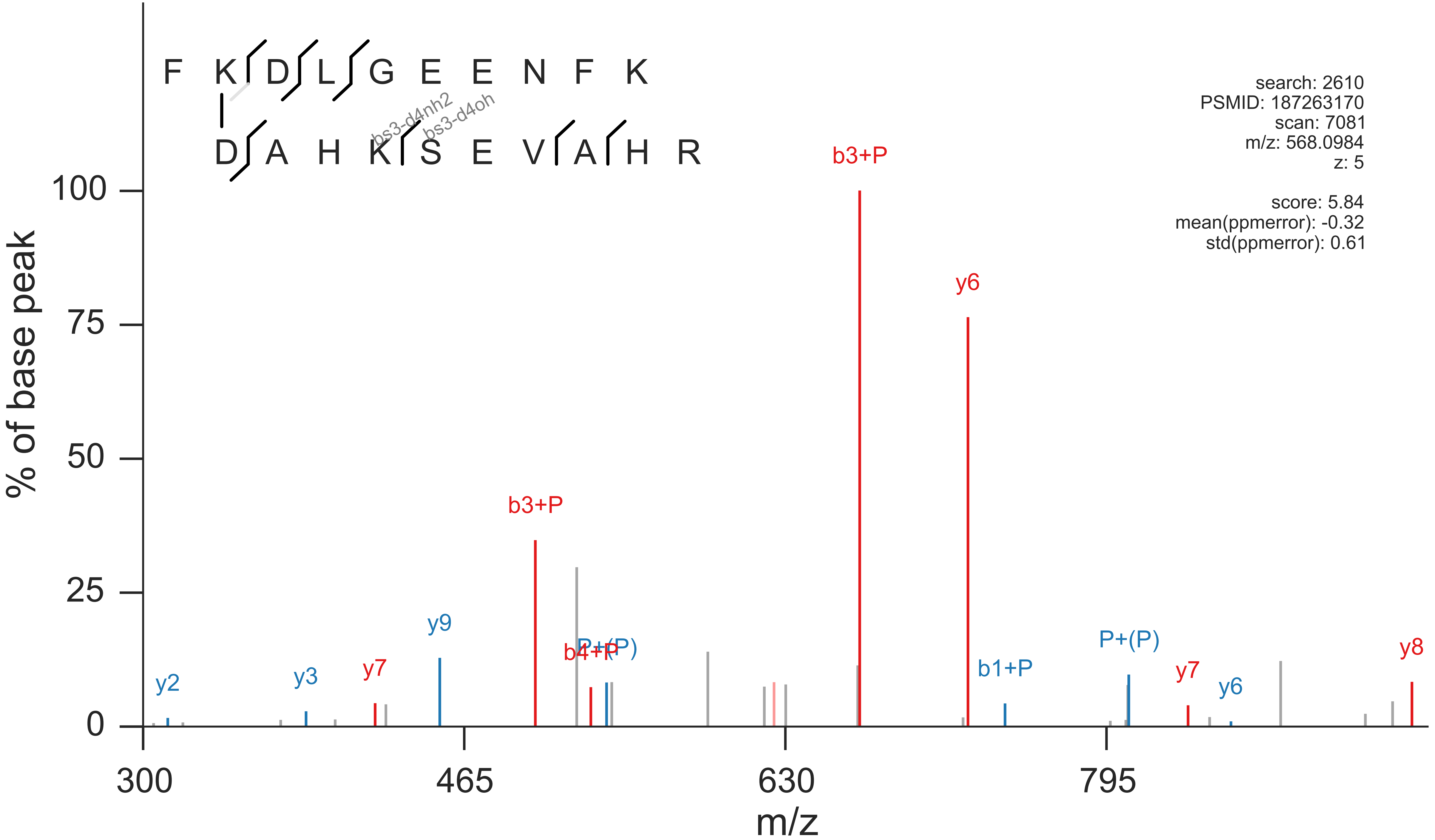

F K D L G E E N F K
D A H K S E V A H R
bs3-d4nh2
bs3-d4oh
search: 2610
PSMID: 187263170
scan: 7081
m/z: 568.0984
z: 5
score: 5.84
mean(ppmerror): -0.32
std(ppmerror): 0.61
% of base peak
m/z
0
25
50
75
100
300
465
630
795
b3+P
y6
b3+P
y9
b4+P
P+(P)
y2
y3
y7
b1+P
P+(P)
y7
y6
y8

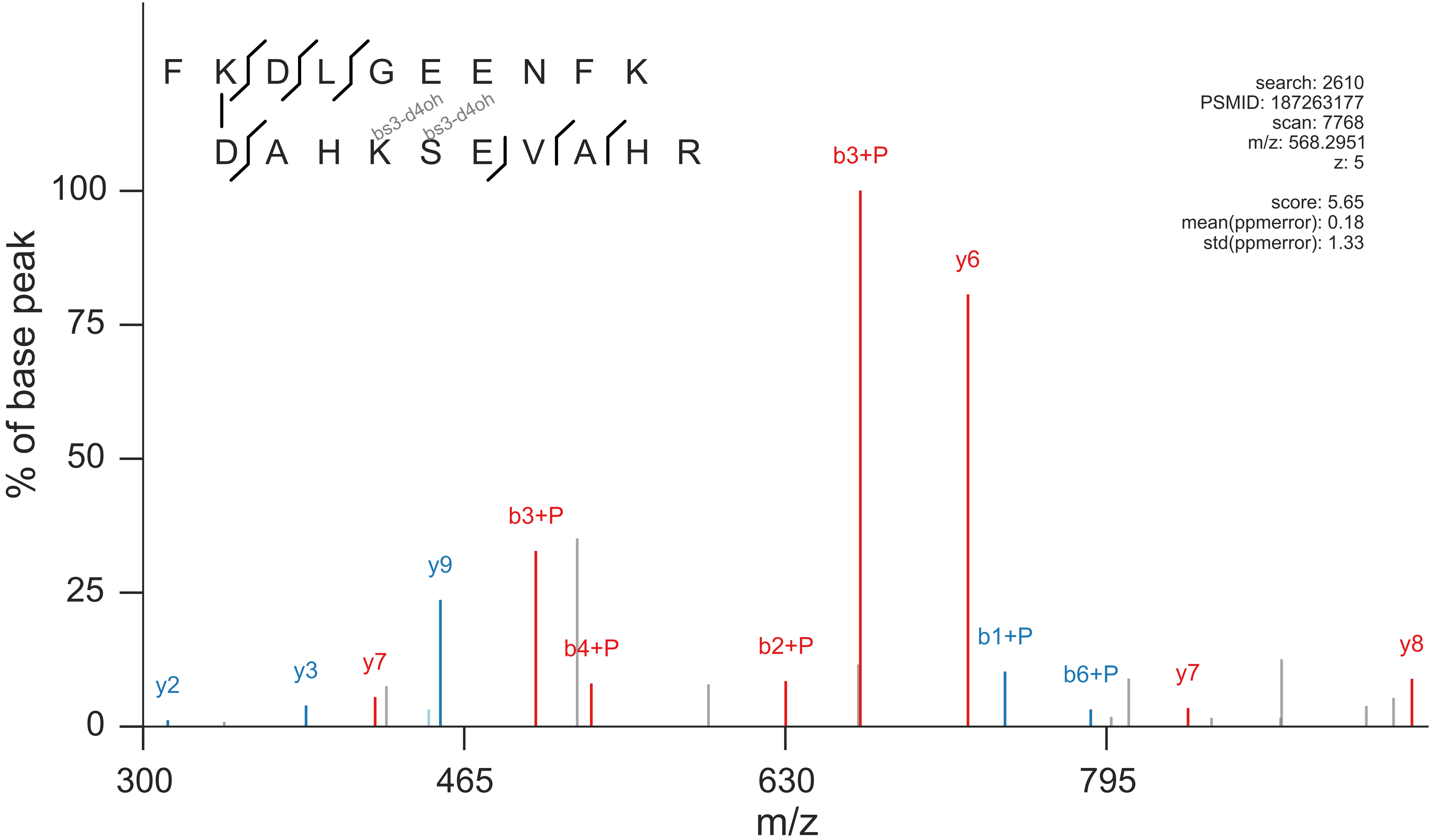

F K D L G E E N F K
D A H K S E V A H R
bs3-d4oh
bs3-d4oh
search: 2610
PSMID: 187263177
scan: 7768
m/z: 568.2951
z: 5
score: 5.65
mean(ppmerror): 0.18
std(ppmerror): 1.33
% of base peak
m/z
100
75
50
25
0
300
465
630
795
y2
y3
y7
y9
b3+P
b4+P
b2+P
b3+P
y6
b1+P
b6+P
y7
y8

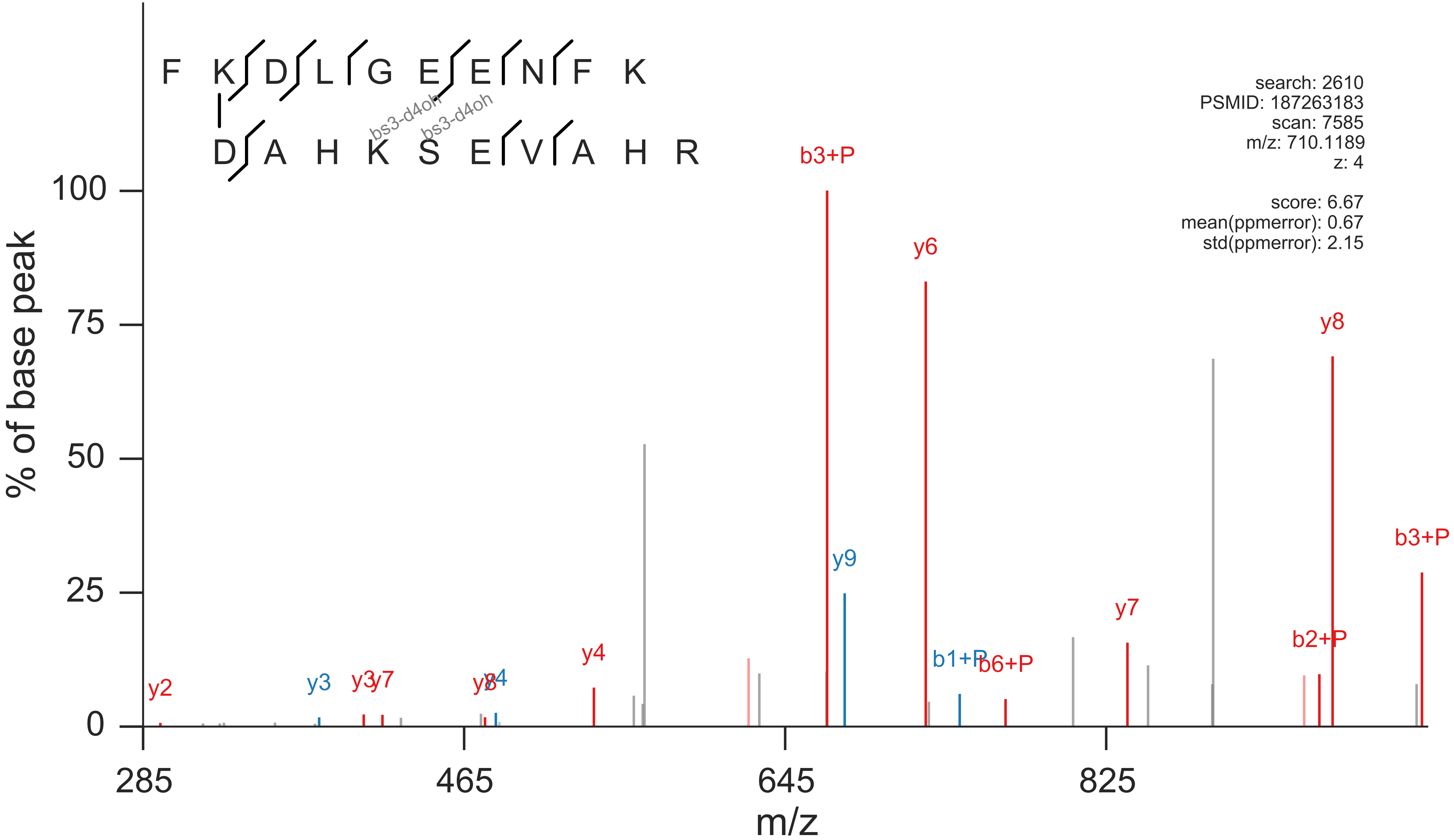
F K D L G E E N F K
D A H K S E V A H R
bs3-d4oh
bs3-d4oh
search: 2610
PSMID: 187263183
scan: 7585
m/z: 710.1189
z: 4
score: 6.67
mean(ppmerror): 0.67
std(ppmerror): 2.15
% of base peak
m/z
0
25
50
75
100
285
465
645
825
b3+P
y6
y8
b3+P
y9
y7
b2+P
b1+P
b6+P
y4
y2
y3
y3
y7
y8
y4

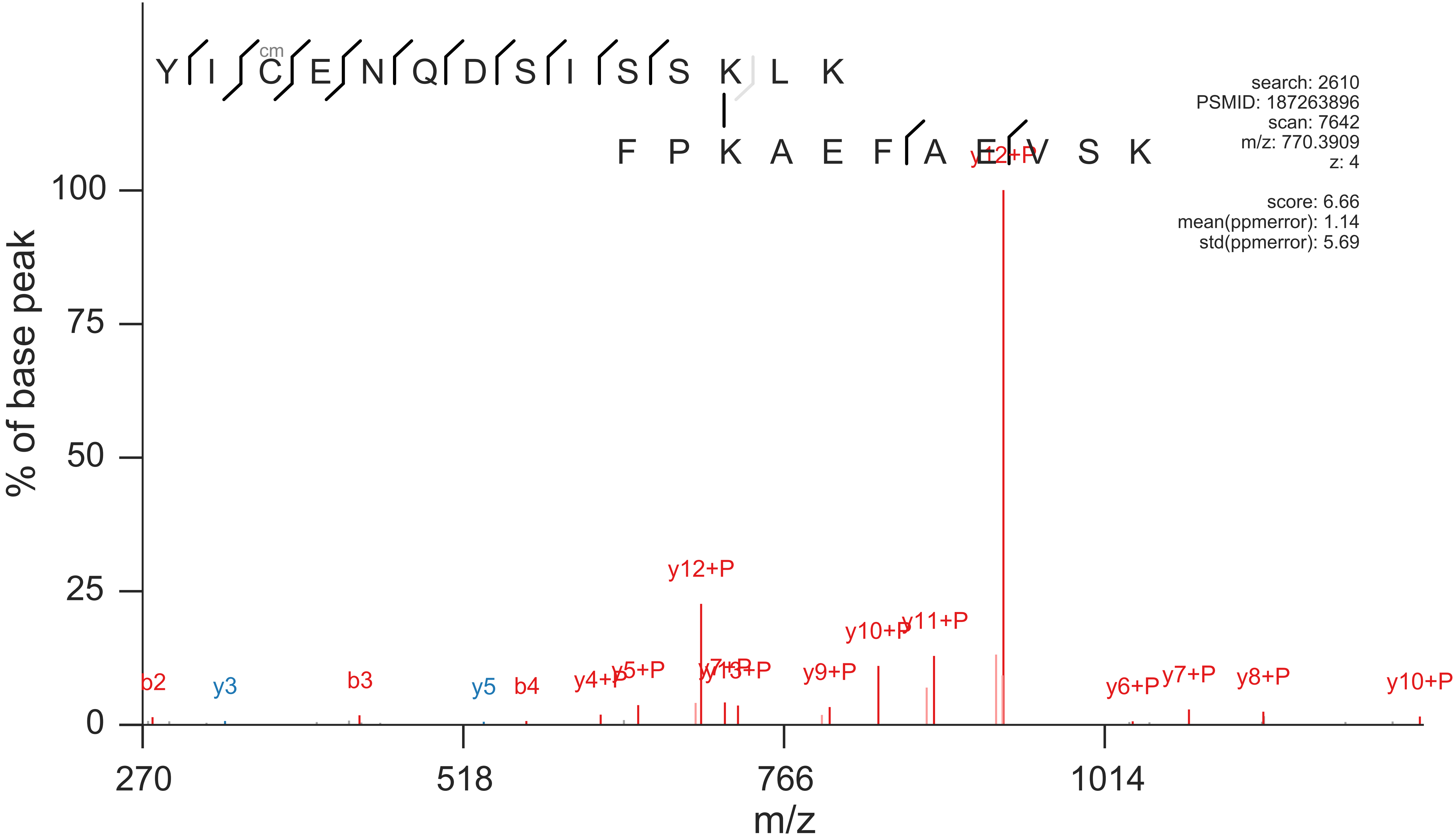

Y I C E N Q D S I S S K L K
cm
F P K A E F A E V S K
search: 2610
PSMID: 187263896
scan: 7642
m/z: 770.3909
z: 4
score: 6.66
mean(ppmerror): 1.14
std(ppmerror): 5.69
% of base peak
m/z
100
75
50
25
0
270
518
766
1014
b2
y3
b3
y5
b4
y4+P
y5+P
y12+P
y7+P
y13+P
y9+P
y10+P
y11+P
y12+P
y6+P
y7+P
y8+P
y10+P

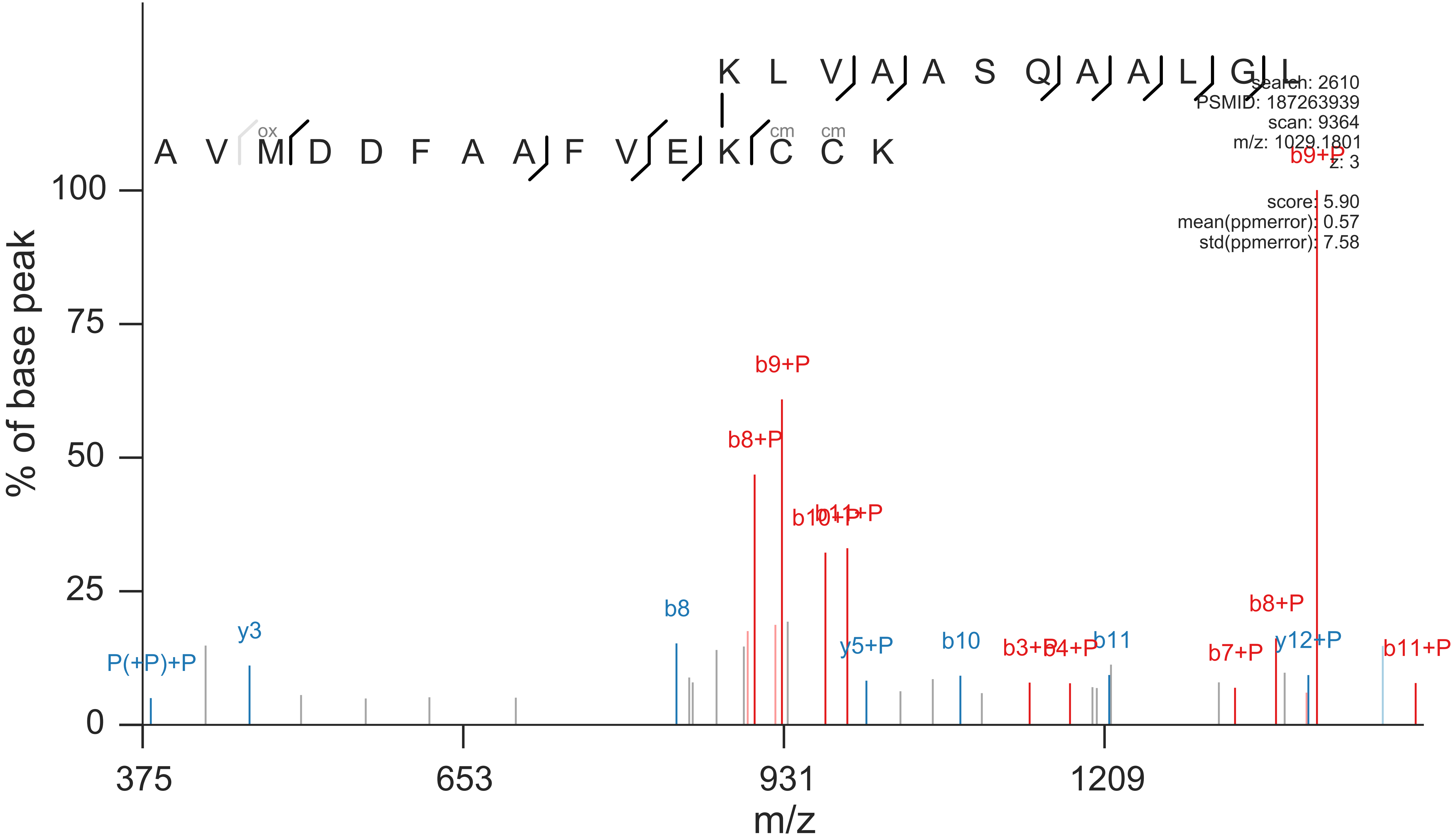

K L V A A S Q A A L G L
A V M D D F A A F V E K C C K
ox
cm
cm
search: 2610
PSMID: 187263939
scan: 9364
m/z: 1029.1801
z: 3
score: 5.90
mean(ppmerror): 0.57
std(ppmerror): 7.58
% of base peak
m/z
b9+P
b8+P
b10+P
b11+P
b8
y3
P(+P)+P
y5+P
b10
b3+P
b4+P
b11
b7+P
y12+P

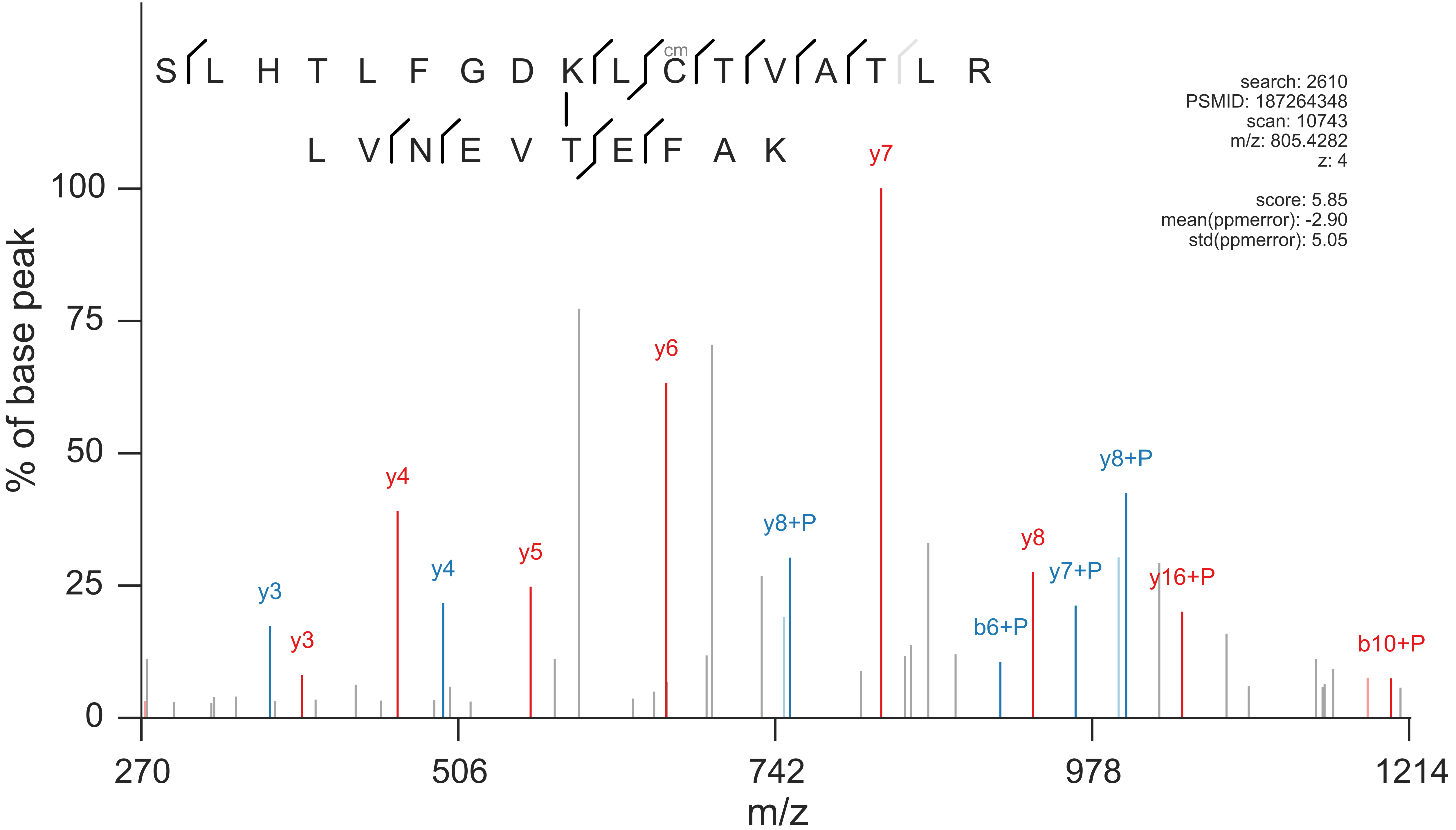
S L H T L F G D K L C T V A T L R
cm
L V N E V T E F A K
search: 2610
PSMID: 187264348
scan: 10743
m/z: 805.4282
z: 4
score: 5.85
mean(ppmerror): -2.90
std(ppmerror): 5.05
% of base peak
100
75
50
25
0
m/z
270
506
742
978
1214
y3
y3
y4
y4
y5
y6
y8+P
y7
b6+P
y8
y7+P
y8+P
y16+P
b10+P

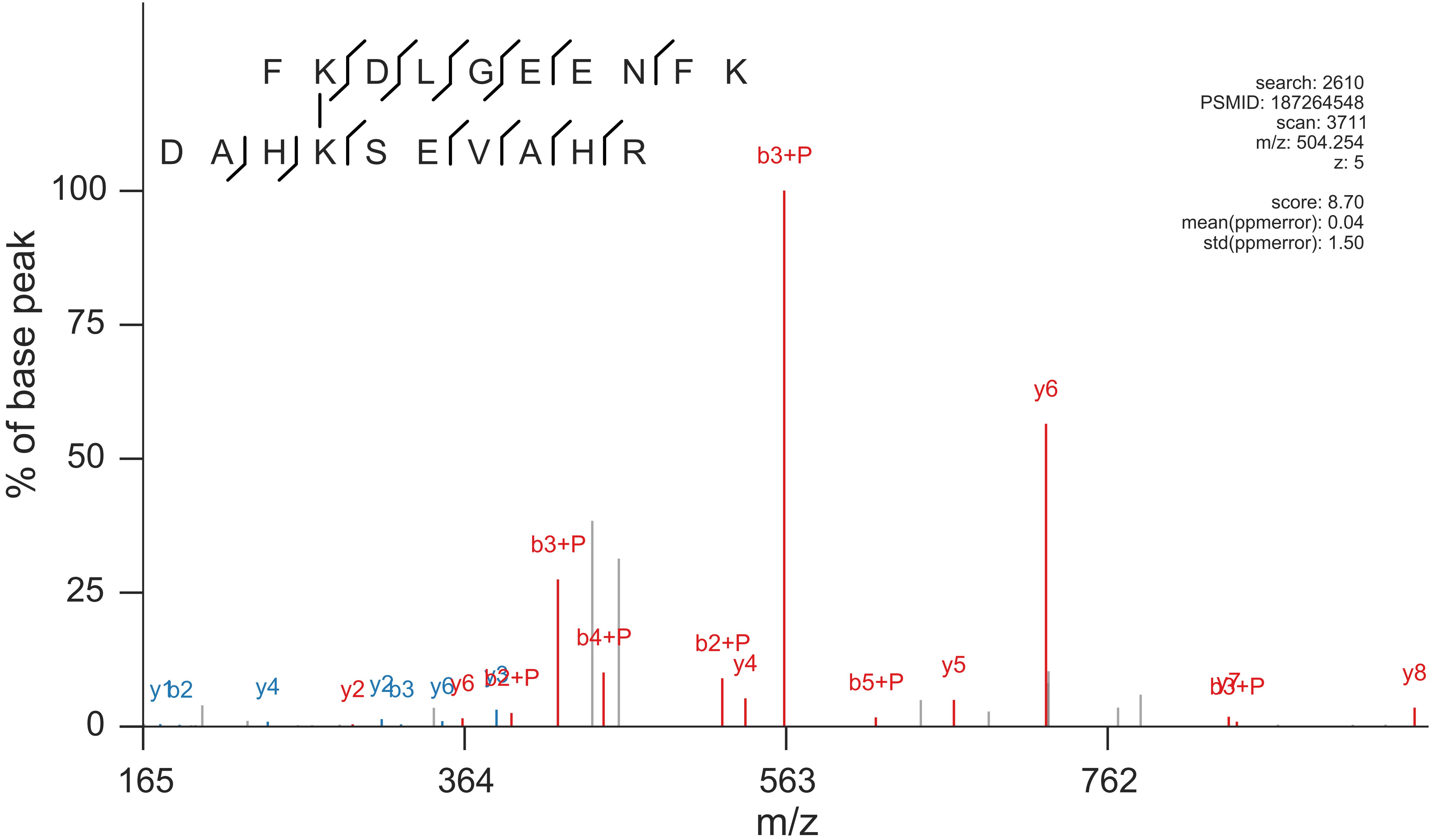
F K D L G E E N F K
D A H K S E V A H R
search: 2610
PSMID: 187264548
scan: 3711
m/z: 504.254
z: 5
score: 8.70
mean(ppmerror): 0.04
std(ppmerror): 1.50
% of base peak
m/z
0
25
50
75
100
165
364
563
762
b3+P
y6
b3+P
b4+P
b2+P
y4
b5+P
y5
y8
y2
y6
b2+P
y1
b2
y4
y2
b3
y6
y3
y7
b3+P

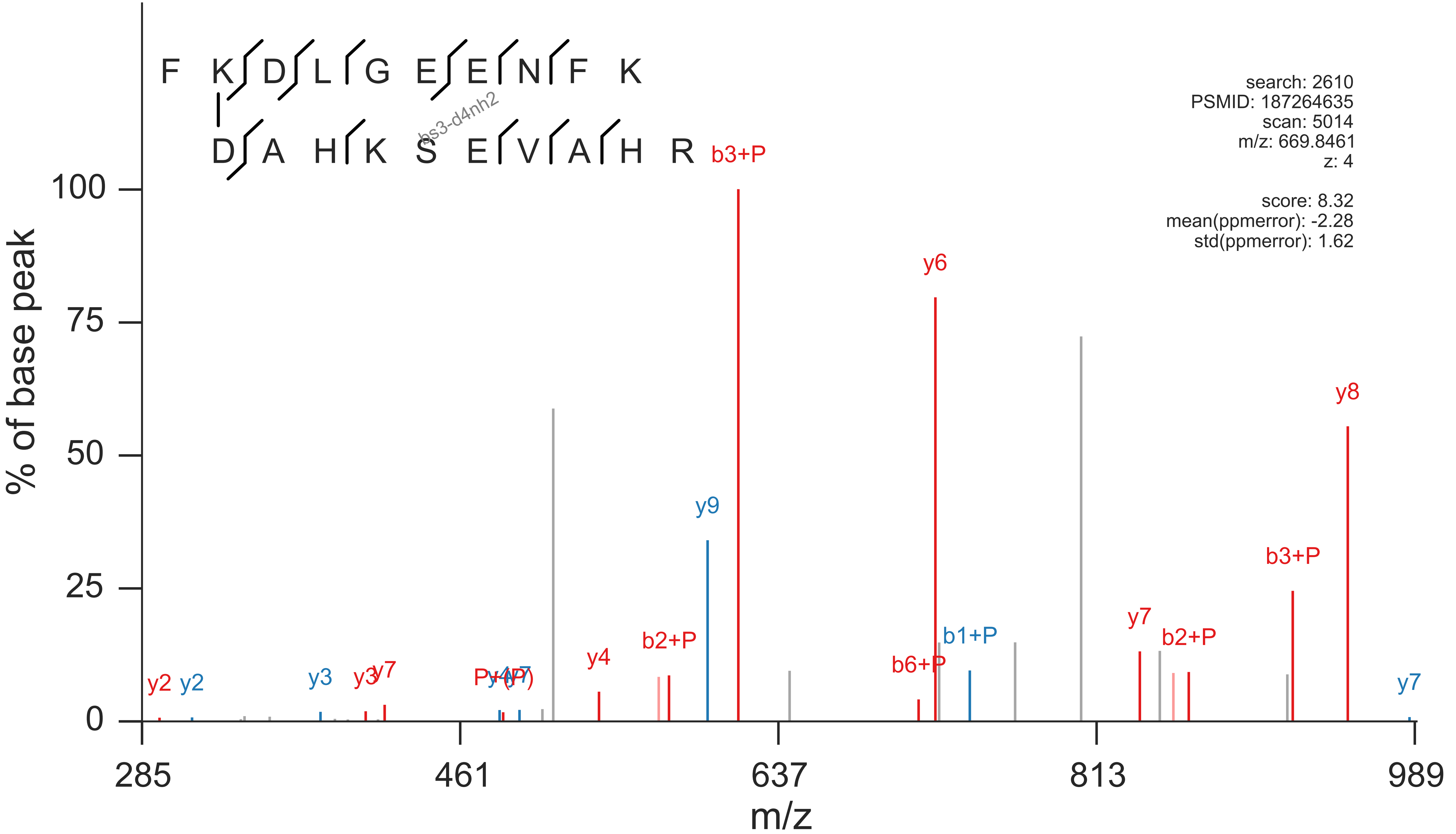

F K D L G E E N F K
D A H K S E V A H R
bs3-d4nh2
search: 2610
PSMID: 187264635
scan: 5014
m/z: 669.8461
z: 4
score: 8.32
mean(ppmerror): -2.28
std(ppmerror): 1.62
% of base peak
m/z
0
25
50
75
100
285
461
637
813
989
b3+P
y6
y8
y9
b3+P
y7
b2+P
b1+P
b6+P
b2+P
y4
y2
y2
y3
y3
y7
y7

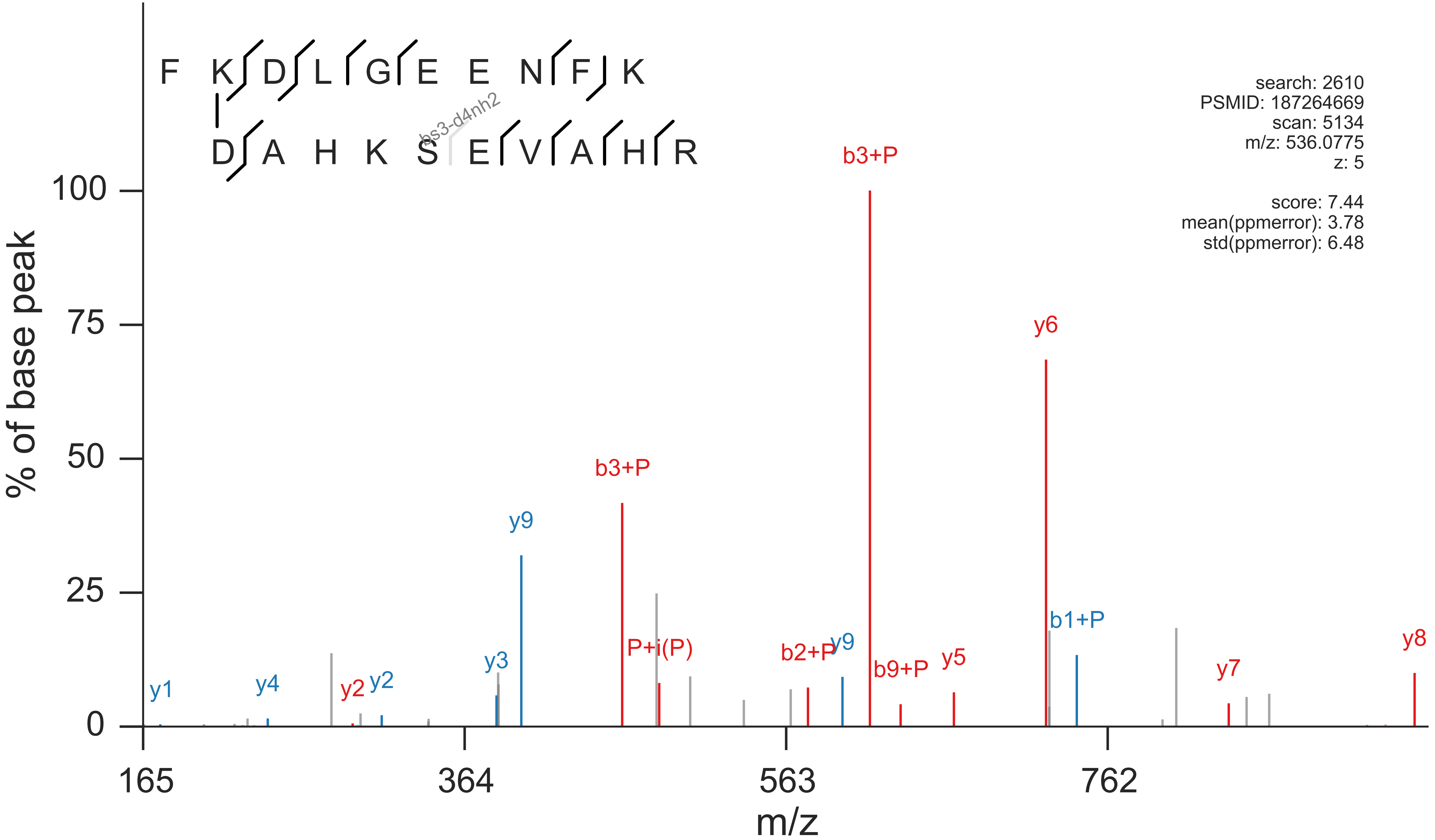

F K D L G E E N F K
D A H K S E V A H R
bs3-d4nh2
search: 2610
PSMID: 187264669
scan: 5134
m/z: 536.0775
z: 5
score: 7.44
mean(ppmerror): 3.78
std(ppmerror): 6.48
% of base peak
m/z
0
25
50
75
100
165
364
563
762
b3+P
y6
b3+P
y9
b1+P
P+i(P)
b2+P
y9
y8
y3
b9+P
y5
y7
y1
y4
y2
y2

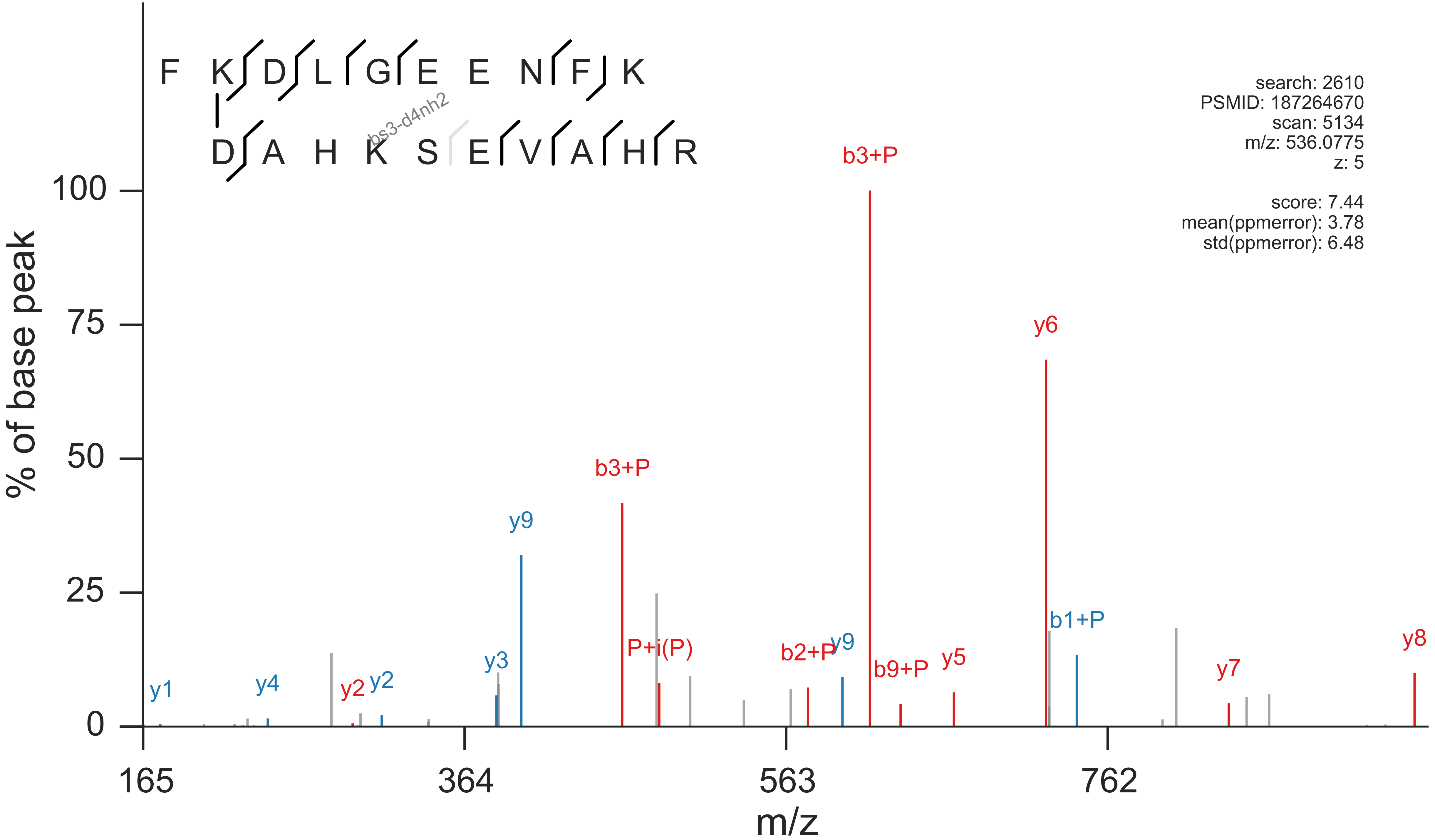

F K D L G E E N F K
D A H K S E V A H R
bs3-d4nh2
search: 2610
PSMID: 187264670
scan: 5134
m/z: 536.0775
z: 5
score: 7.44
mean(ppmerror): 3.78
std(ppmerror): 6.48
% of base peak
m/z
100
75
50
25
0
165
364
563
762
b3+P
y6
b3+P
y9
b1+P
P+i(P)
b2+P
y9
y8
y3
b9+P
y5
y7
y1
y4
y2
y2

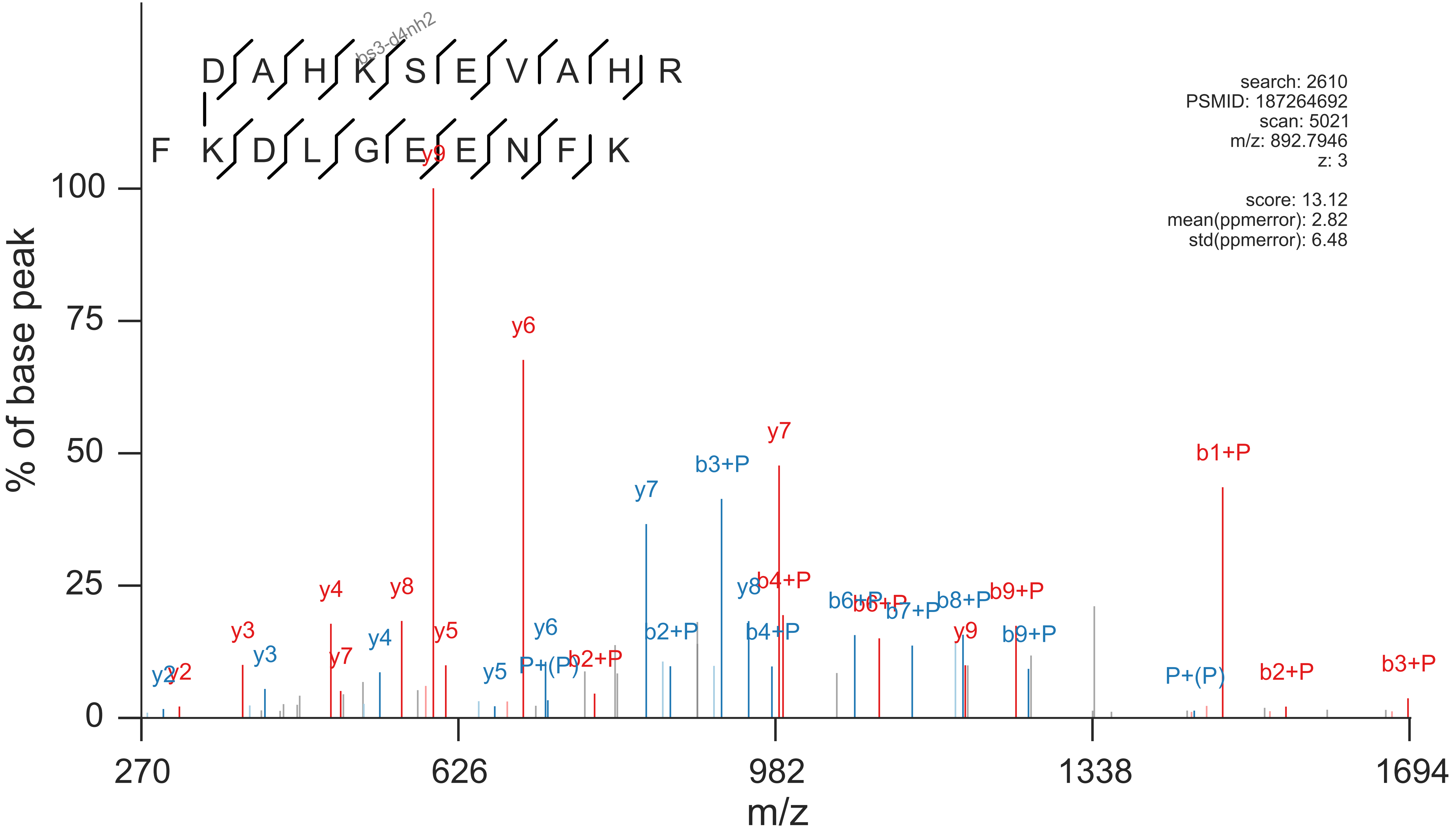

D A H K S E V A H R
F K D L G E E N F K
bs3-d4nh2
search: 2610
PSMID: 187264692
scan: 5021
m/z: 892.7946
z: 3
score: 13.12
mean(ppmerror): 2.82
std(ppmerror): 6.48
% of base peak
m/z

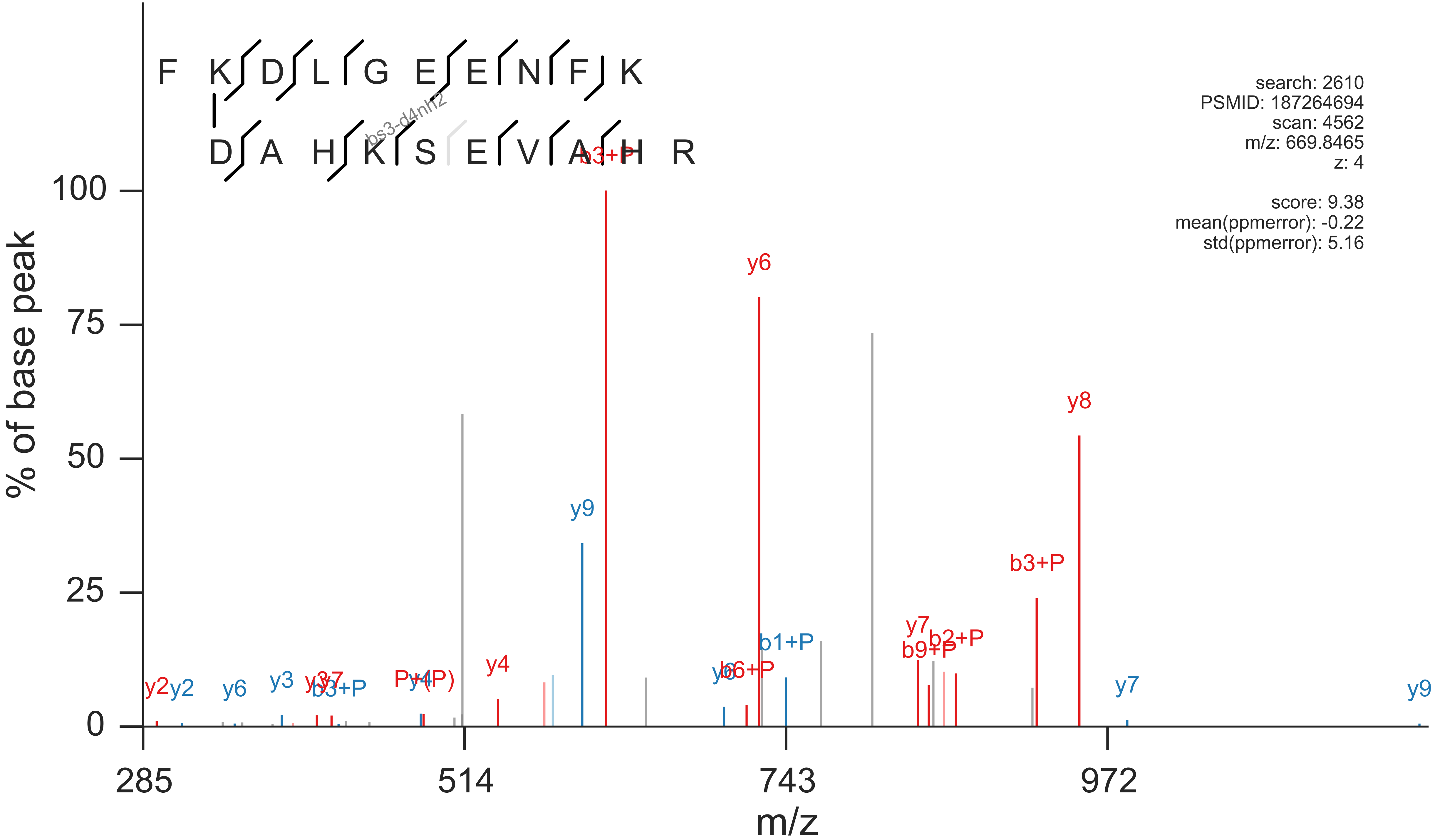

F K D L G E E N F K
D A H K S E V A H R
bs3-d4nh2
search: 2610
PSMID: 187264694
scan: 4562
m/z: 669.8465
z: 4
score: 9.38
mean(ppmerror): -0.22
std(ppmerror): 5.16
% of base peak
m/z
0
25
50
75
100
285
514
743
972
b3+P
y6
y8
y9
b3+P
y7
b9+P
b2+P
b1+P
b6+P
y4
P+(P)
y2
y6
y3
y7
y9

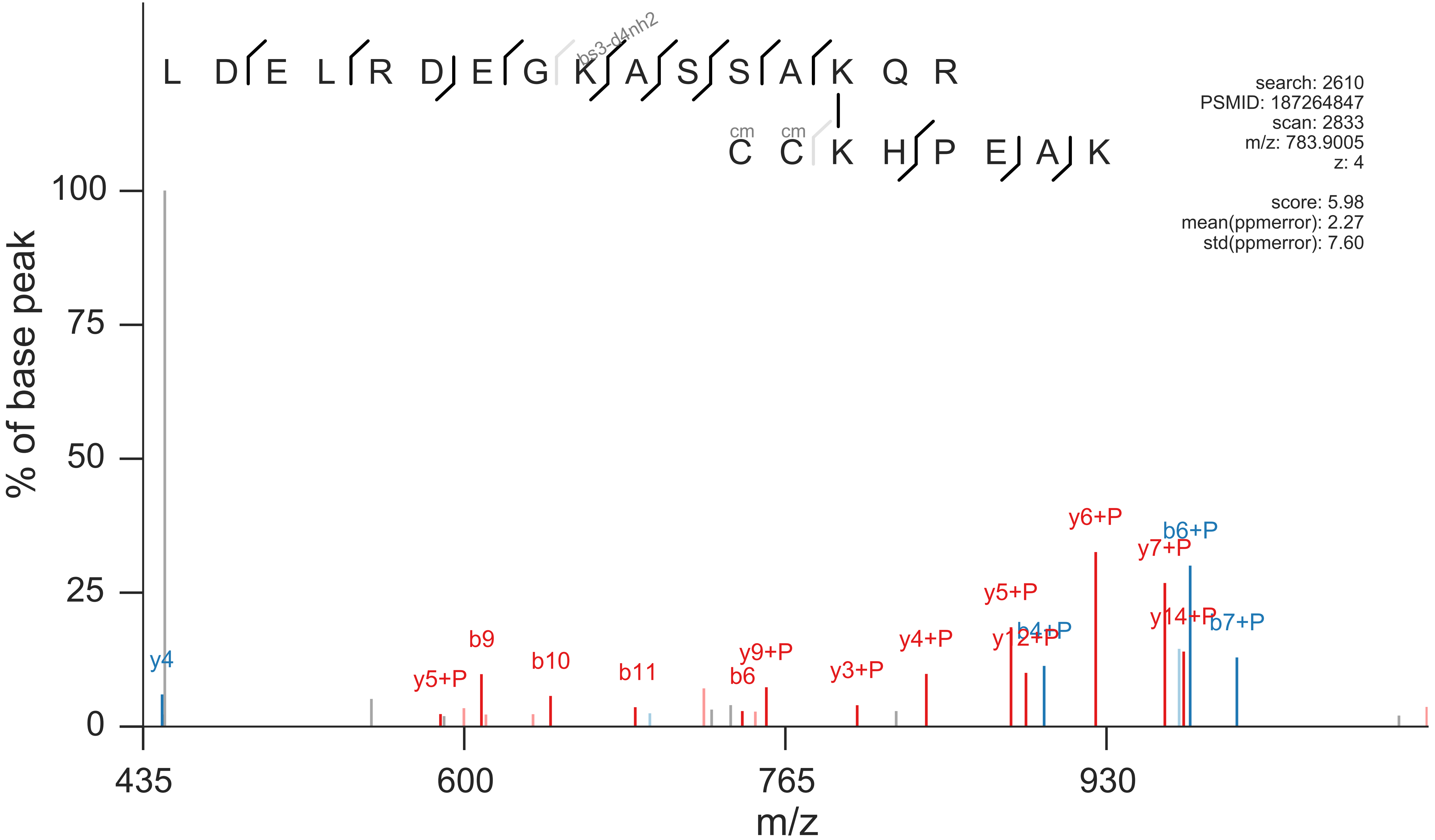

L D E L R D E G K A S S A K Q R
bs3-d4nh2
cm cm
C C K H P E A K
search: 2610
PSMID: 187264847
scan: 2833
m/z: 783.9005
z: 4
score: 5.98
mean(ppmerror): 2.27
std(ppmerror): 7.60
% of base peak
100
75
50
25
0
435
600
765
930
m/z
y4
y5+P
b9
b10
b11
b6
y9+P
y3+P
y4+P
y5+P
y12+P
b4+P
y6+P
y7+P
b6+P
y14+P
b7+P

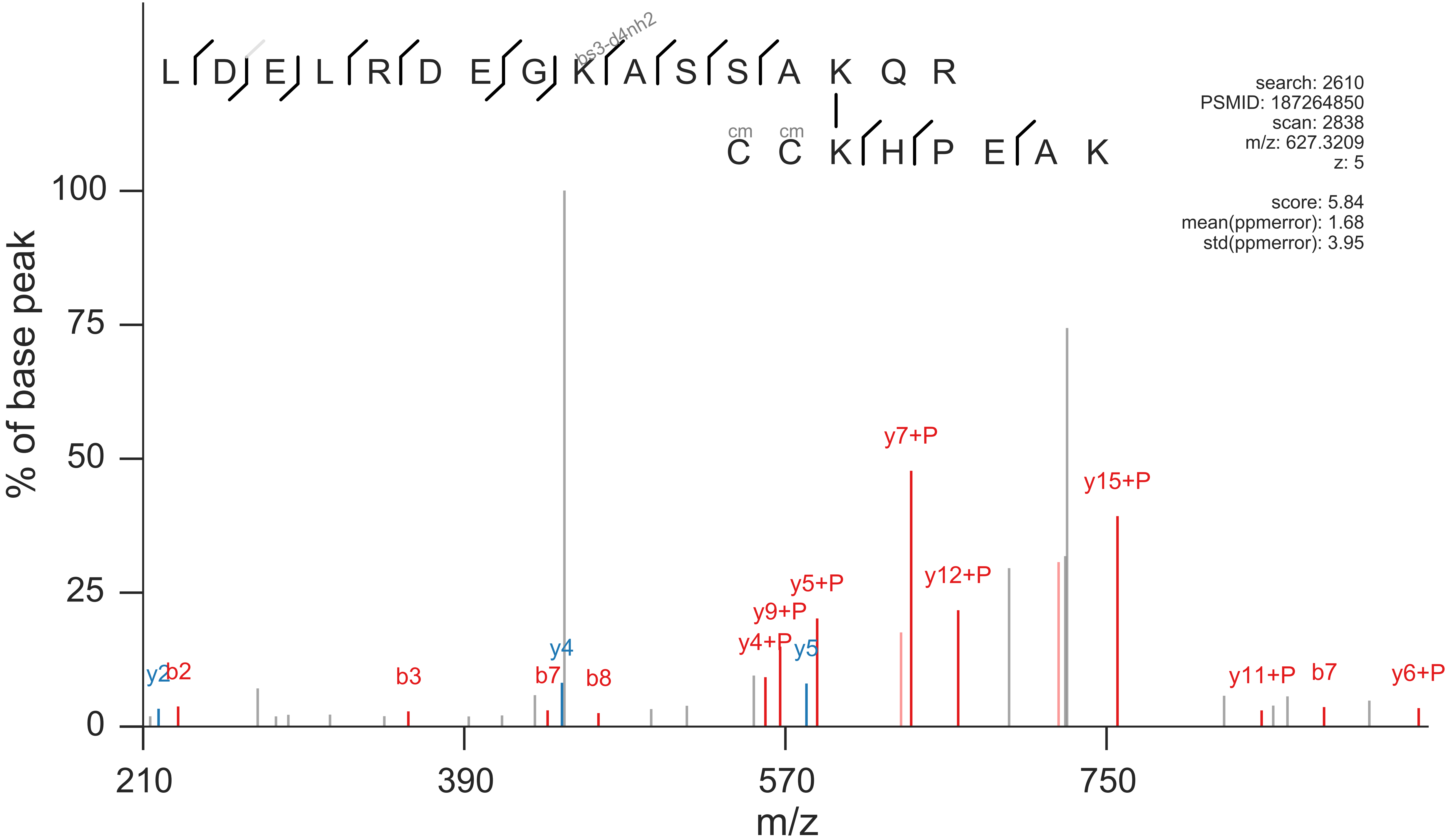

L D E L R D E G K A S S A K Q R
bs3-d4nh2
cm cm
C C K H P E A K
search: 2610
PSMID: 187264850
scan: 2838
m/z: 627.3209
z: 5
score: 5.84
mean(ppmerror): 1.68
std(ppmerror): 3.95
% of base peak
m/z
100
75
50
25
0
210
390
570
750
y2
b2
b3
b7
y4
b8
y4+P
y9+P
y5
y5+P
y7+P
y12+P
y15+P
y11+P
b7
y6+P

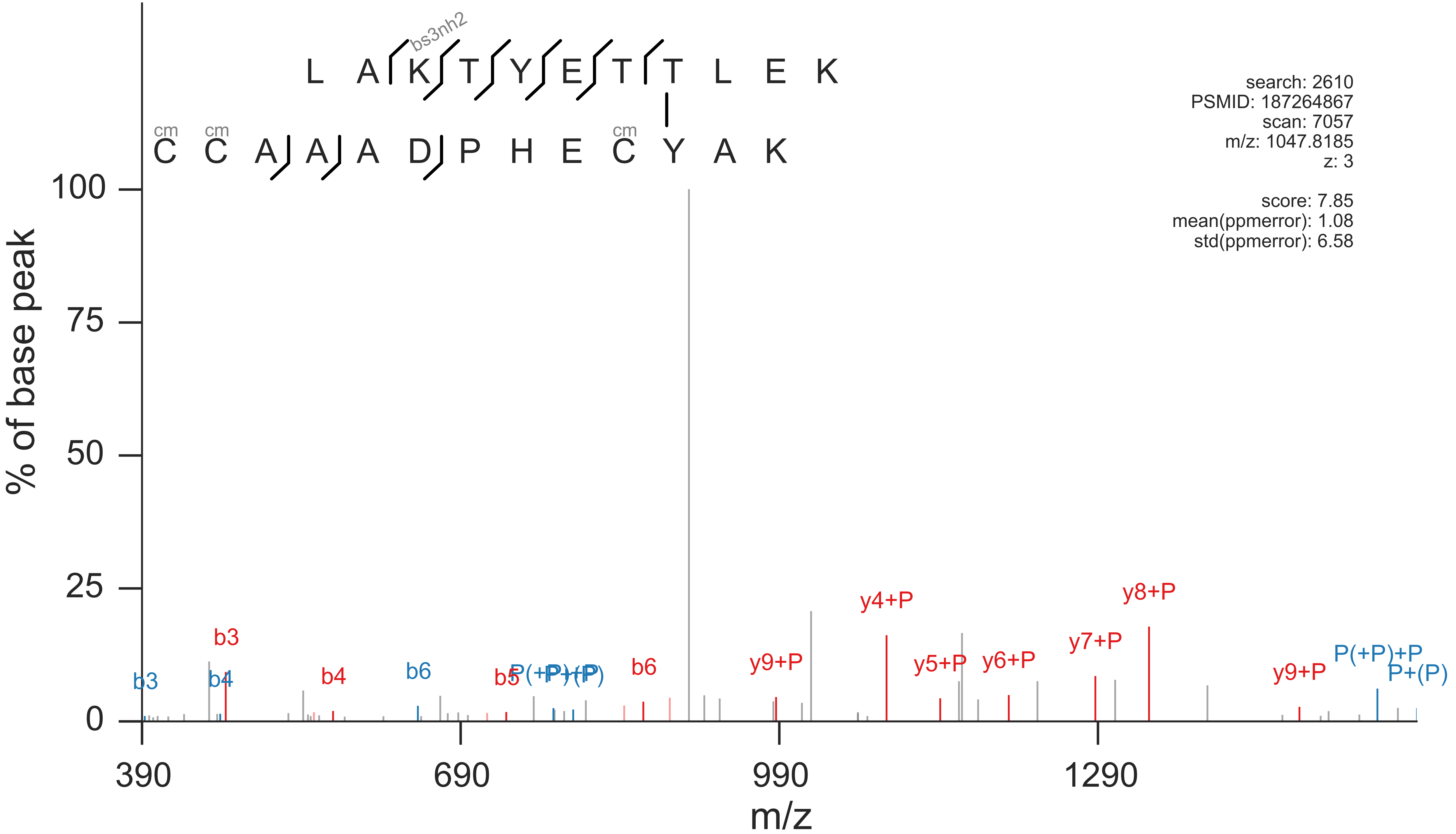

bs3nh2
L A K T Y E T T L E K
cm cm cm
C C A A A D P H E C Y A K
search: 2610
PSMID: 187264867
scan: 7057
m/z: 1047.8185
z: 3
score: 7.85
mean(ppmerror): 1.08
std(ppmerror): 6.58
% of base peak
100
75
50
25
0
390
690
990
1290
m/z
b3
b4
b6
b5
b6
y9+P
y4+P
y5+P
y6+P
y7+P
y8+P
y9+P
P(+P)+P
P+(P)

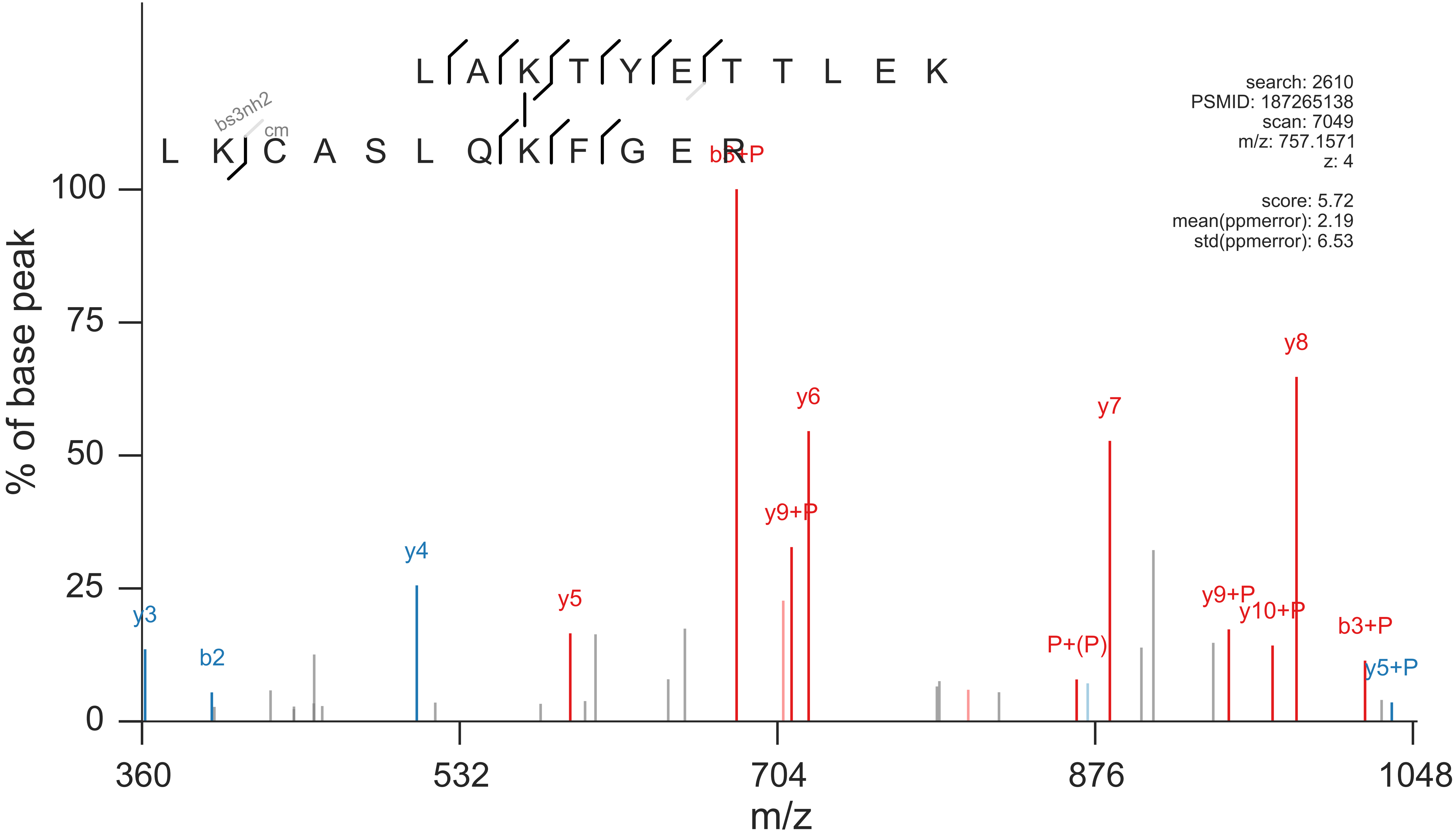

L A K T Y E T T L E K
L K C A S L Q K F G E R
bs3nh2
cm
search: 2610
PSMID: 187265138
scan: 7049
m/z: 757.1571
z: 4
score: 5.72
mean(ppmerror): 2.19
std(ppmerror): 6.53
% of base peak
m/z
b3+P
y8
y6
y7
y9+P
y4
y5
y3
b2
P+(P)
y9+P
y10+P
b3+P
y5+P

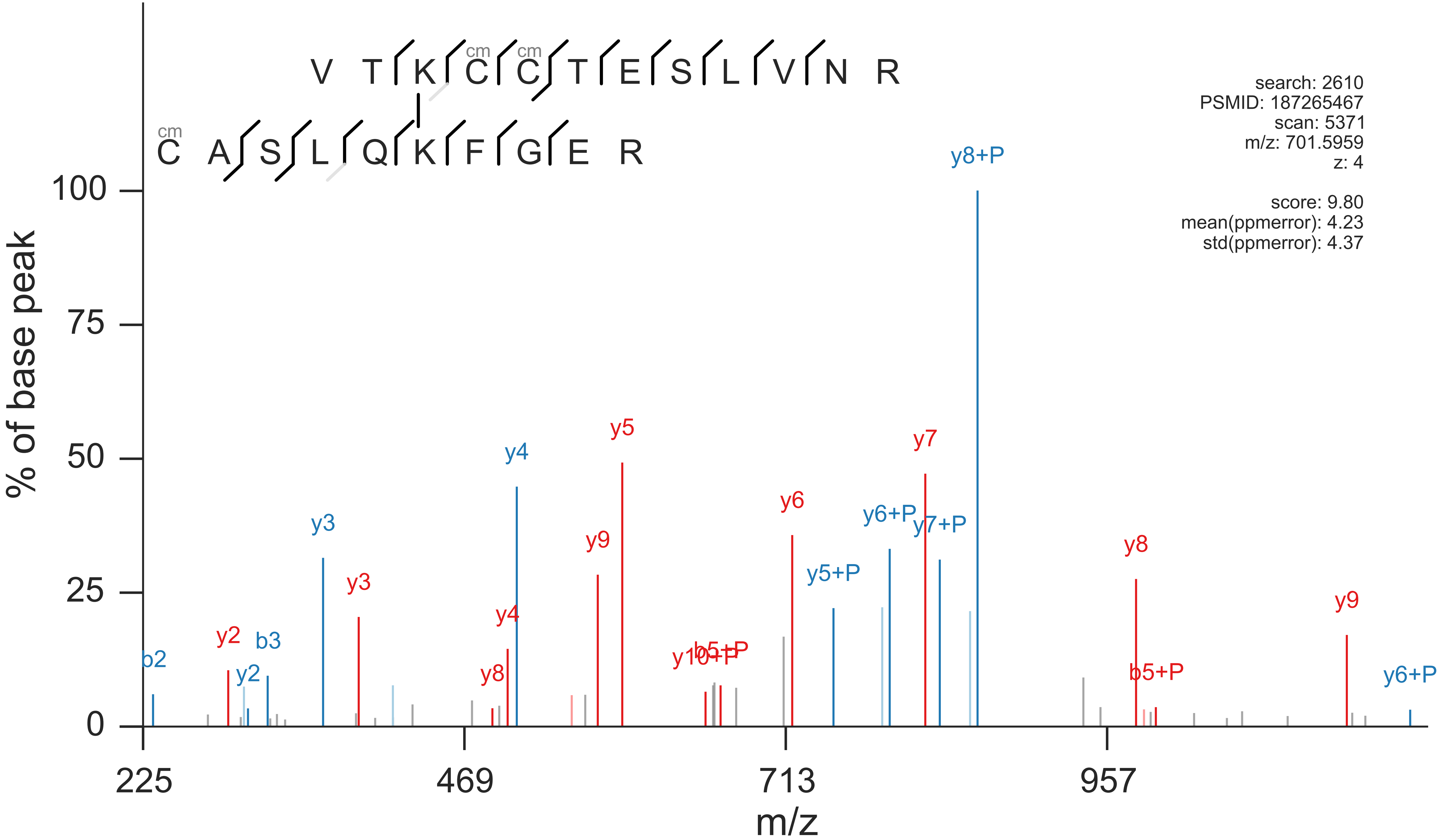

V T K C C T E S L V N R
C A S L Q K F G E R
cm
search: 2610
PSMID: 187265467
scan: 5371
m/z: 701.5959
z: 4
score: 9.80
mean(ppmerror): 4.23
std(ppmerror): 4.37
% of base peak
m/z
y8+P
y5
y4
y7
y6
y6+P
y7+P
y3
y9
y8
y5+P
y3
y9
b2
y2
b3
y2
y4
y8
y10+P
b5+P
b5+P
y6+P

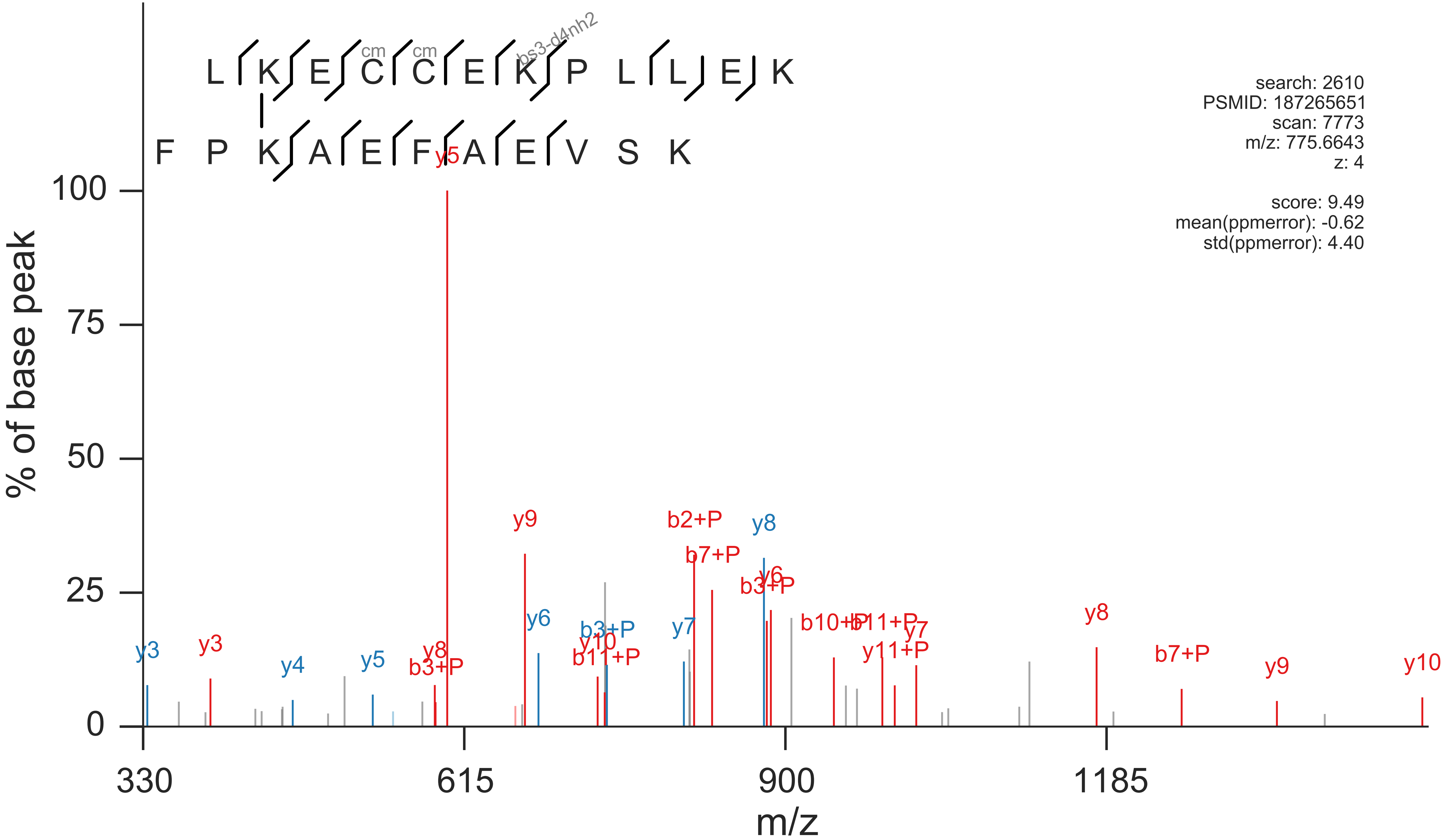

L K E C C E K P L L E K
cm
cm
bs3-d4nh2
F P K A E F A E V S K
search: 2610
PSMID: 187265651
scan: 7773
m/z: 775.6643
z: 4
score: 9.49
mean(ppmerror): -0.62
std(ppmerror): 4.40
% of base peak
m/z
100
75
50
25
0
330
615
900
1185
y3
y3
y4
y5
y8
b3+P
y5
y9
y6
b3+P
y10
b11+P
y7
b2+P
b7+P
y8
y6
b3+P
b10+P
b11+P
y7
y11+P
y8
b7+P
y9
y10

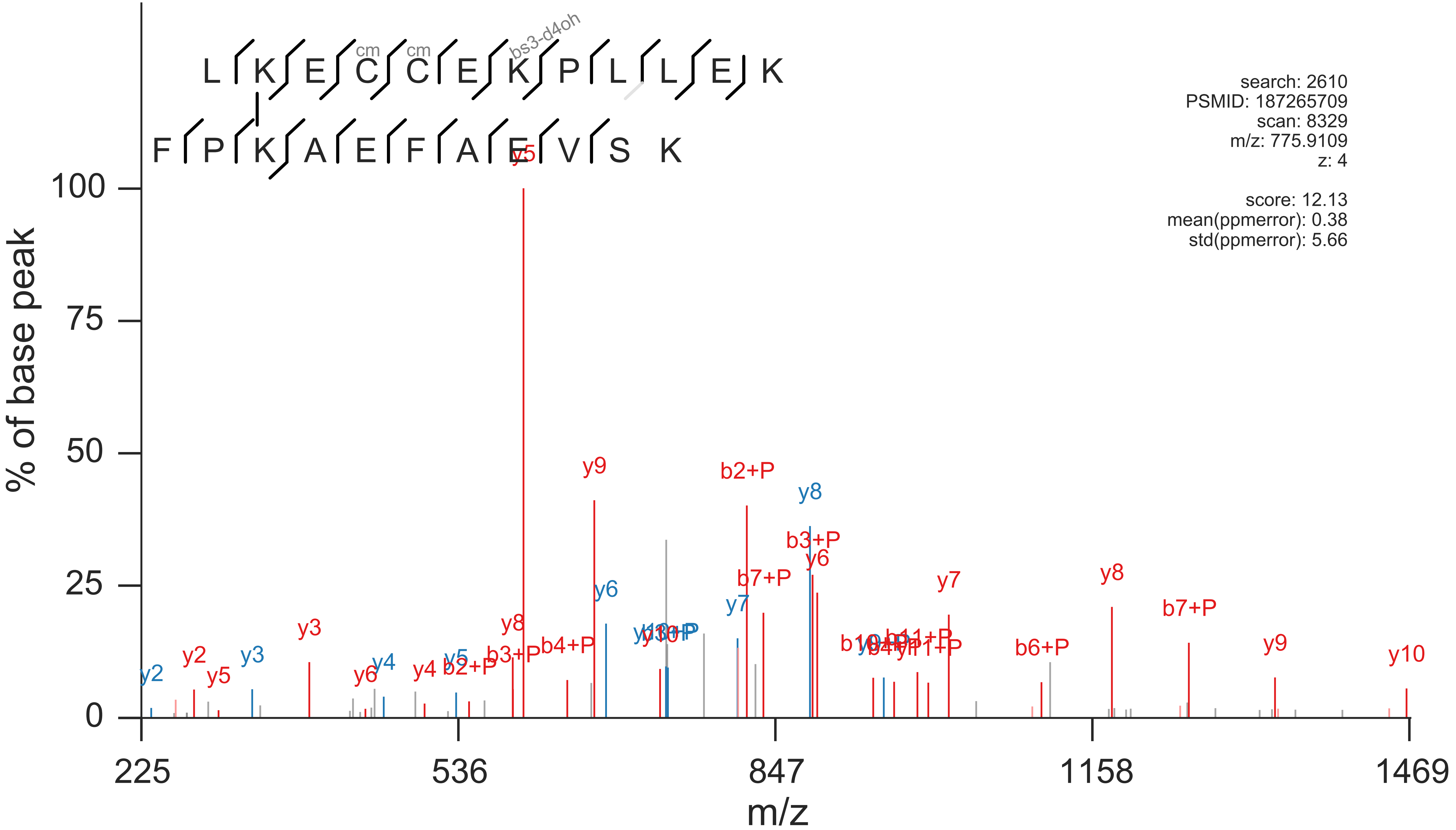

L K E C C E K P L L E K
cm
cm
bs3-d4oh
F P K A E F A E V S K
search: 2610
PSMID: 187265709
scan: 8329
m/z: 775.9109
z: 4
score: 12.13
mean(ppmerror): 0.38
std(ppmerror): 5.66
% of base peak
m/z

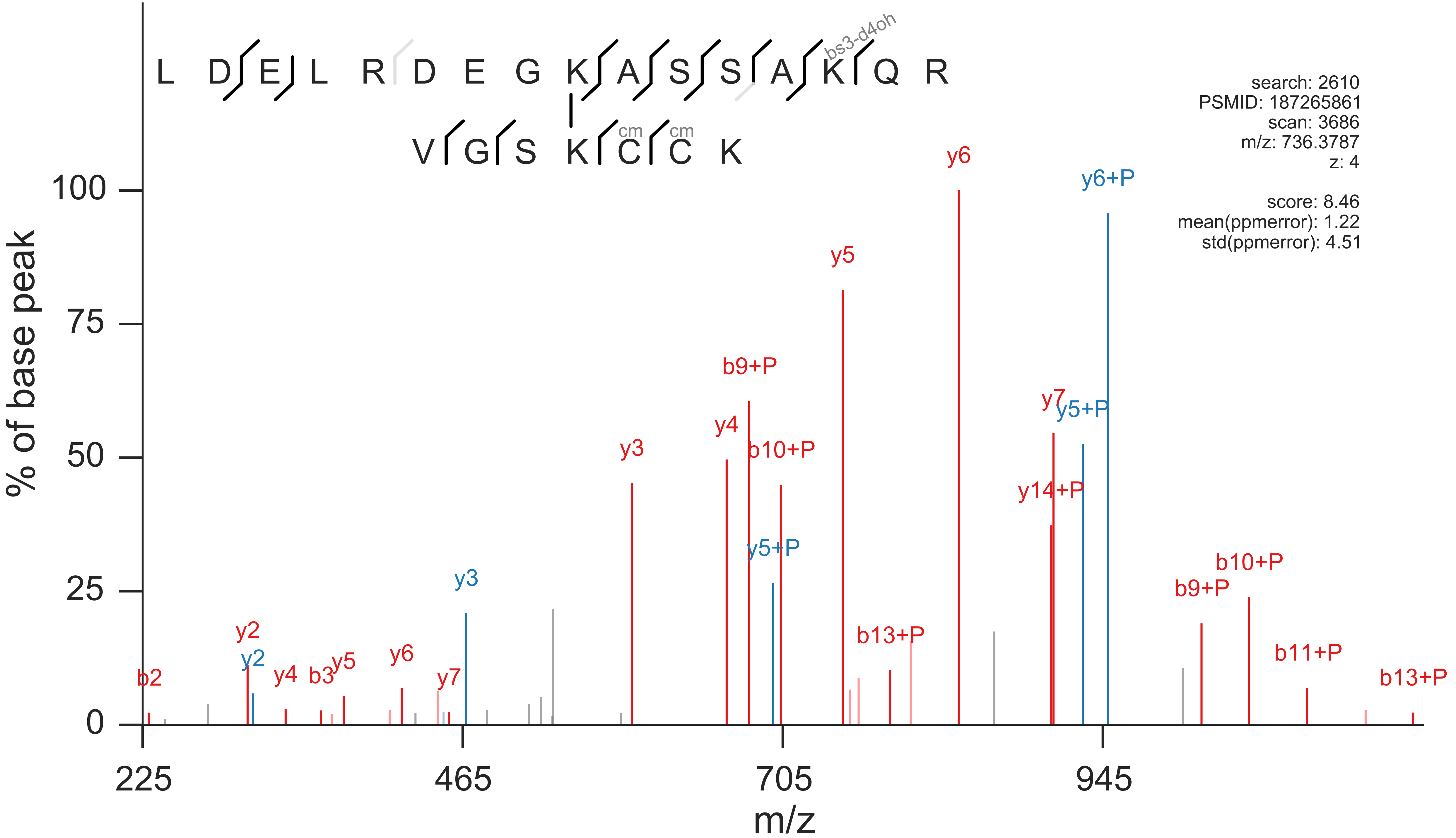
L D E L R D E G K A S S A K Q R
bs3-d4oh
V G S K C C K
cm
cm
search: 2610
PSMID: 187265861
scan: 3686
m/z: 736.3787
z: 4
score: 8.46
mean(ppmerror): 1.22
std(ppmerror): 4.51
% of base peak
m/z
0
25
50
75
100
225
465
705
945
b2
y2
y2
y4
b3
y5
y6
y7
y3
y3
y4
b9+P
b10+P
y5+P
y5
b13+P
y6
y7
y5+P
y14+P
y6+P
b9+P
b10+P
b11+P
b13+P

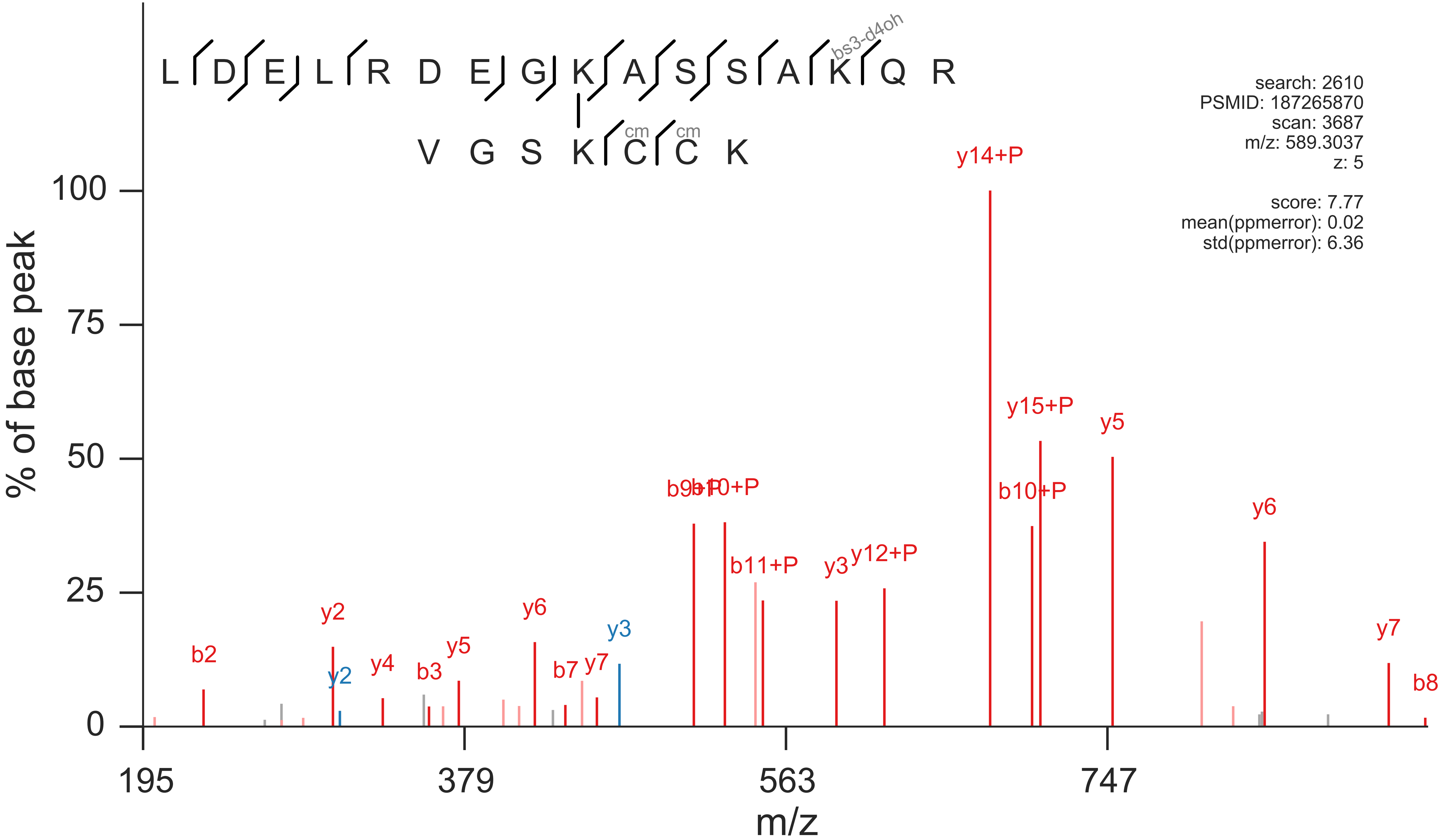

L D E L R D E G K A S S A K Q R
bs3-d4oh
V G S K C C K
cm
cm
search: 2610
PSMID: 187265870
scan: 3687
m/z: 589.3037
z: 5
score: 7.77
mean(ppmerror): 0.02
std(ppmerror): 6.36
% of base peak
m/z
0
25
50
75
100
195
379
563
747
y14+P
y15+P
y5
b10+P
y6
b11+P
y3
y12+P
y7
b8
b2
y2
y4
b3
y5
y6
b7
y7
y3

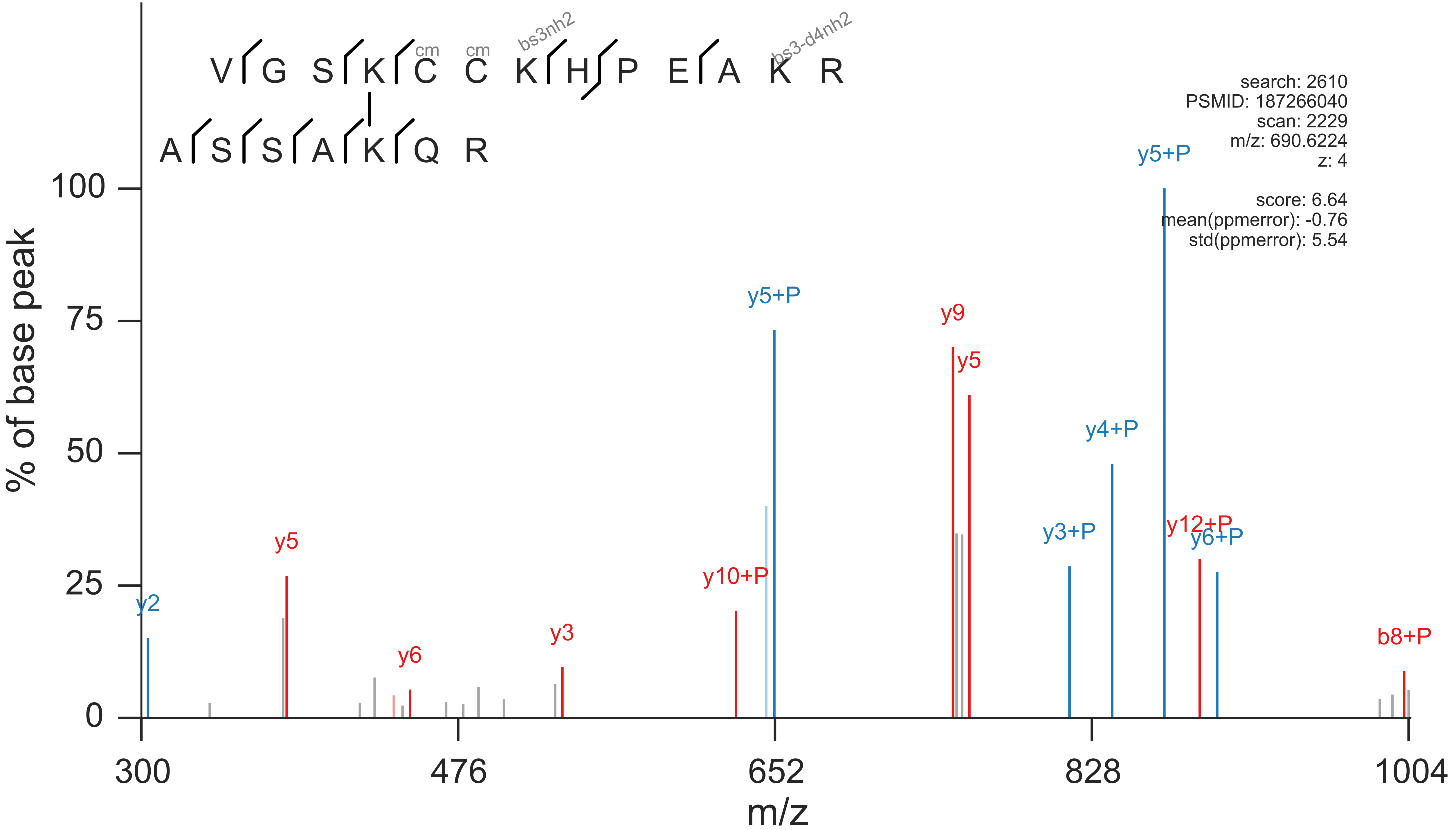

V G S K C C K H P E A K R
cm cm bs3nh2 bs3-d4nh2
A S S A K Q R
search: 2610
PSMID: 187266040
scan: 2229
m/z: 690.6224
z: 4
score: 6.64
mean(ppmerror): -0.76
std(ppmerror): 5.54
% of base peak
m/z
y2
y5
y6
y3
y10+P
y5+P
y9
y5
y3+P
y4+P
y5+P
y12+P
y6+P
b8+P
100
75
50
25
0
300
476
652
828
1004

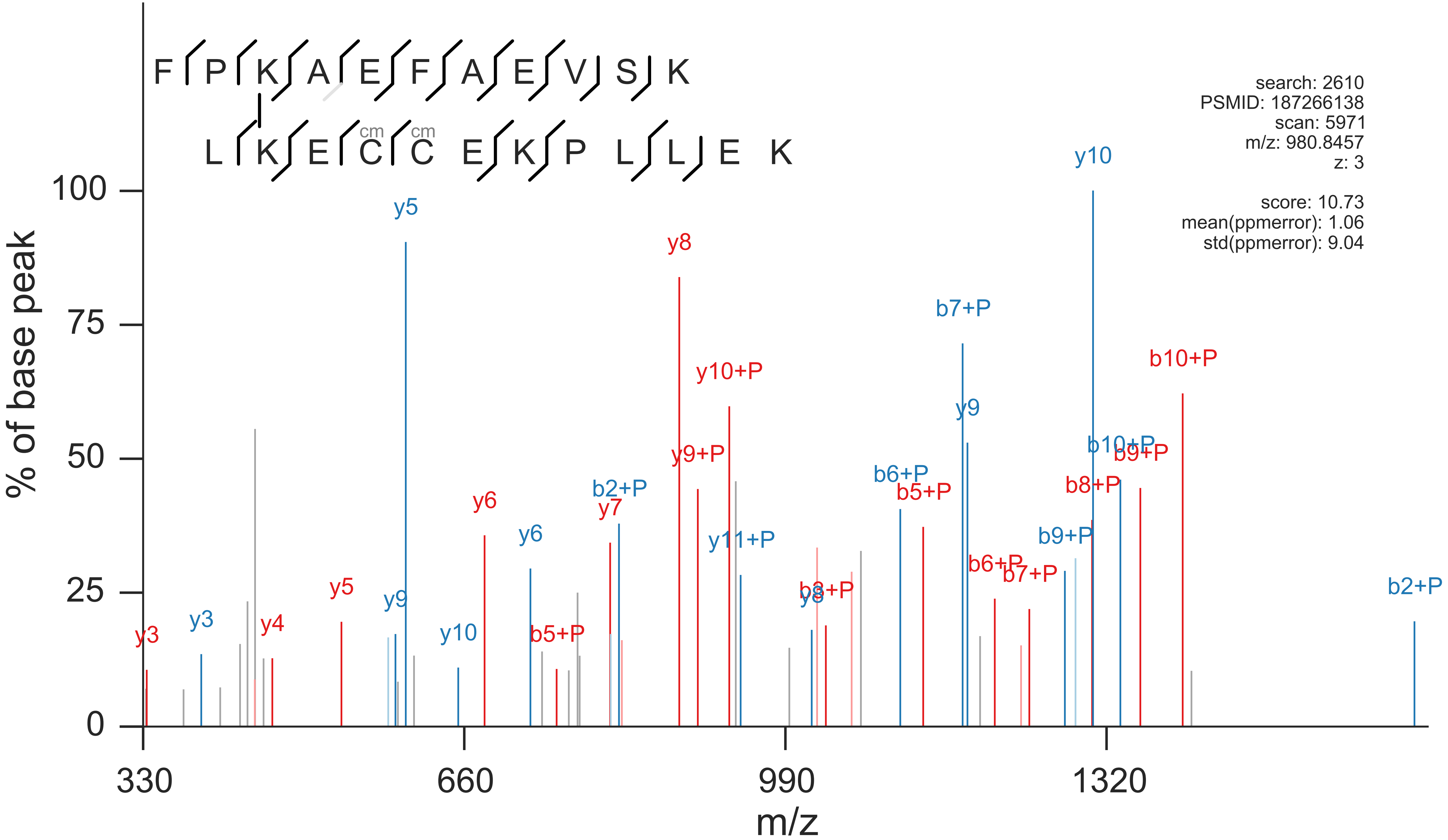

F P K A E F A E V S K
L K E C C E K P L L E K
cm
cm
search: 2610
PSMID: 187266138
scan: 5971
m/z: 980.8457
z: 3
score: 10.73
mean(ppmerror): 1.06
std(ppmerror): 9.04
% of base peak
m/z
0
25
50
75
100
330
660
990
1320
y3
y3
y4
y5
y9
y5
y10
y6
y6
b5+P
y7
b2+P
y8
y9+P
y10+P
y11+P
b3+P
y8
b6+P
b5+P
b7+P
y9
b6+P
b7+P
b9+P
b8+P
y10
b10+P
b9+P
b10+P
b2+P

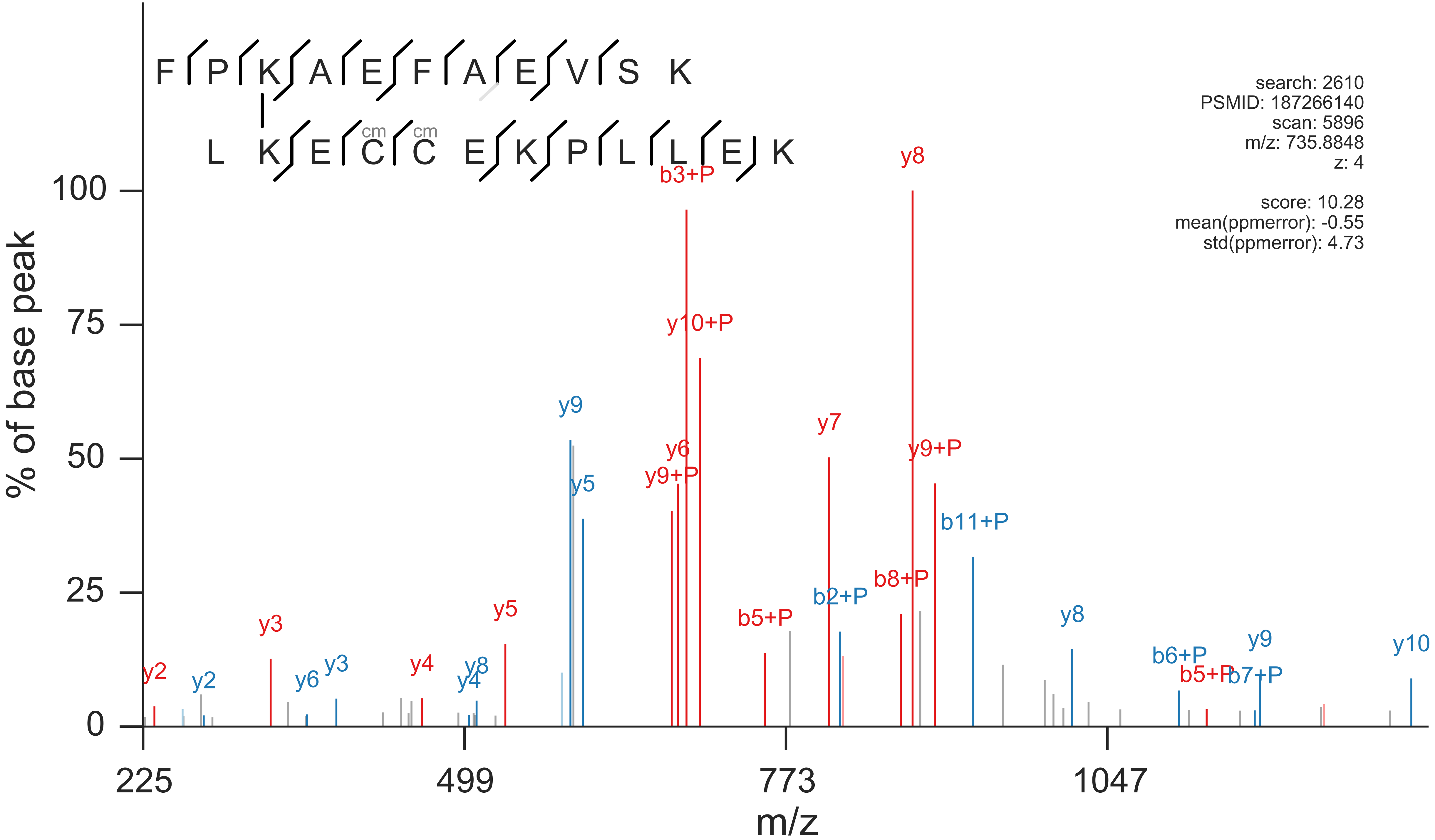

F P K A E F A E V S K
L K E C C E K P L L E K
cm cm
search: 2610
PSMID: 187266140
scan: 5896
m/z: 735.8848
z: 4
score: 10.28
mean(ppmerror): -0.55
std(ppmerror): 4.73
% of base peak
m/z
0
25
50
75
100
225
499
773
1047
y2
y2
y3
y6
y3
y4
y8
y4
y5
y9
y5
y6
y9+P
b3+P
y10+P
b5+P
y7
b2+P
b8+P
y8
y9+P
b11+P
y8
b6+P
b5+P
b7+P
y9
y10

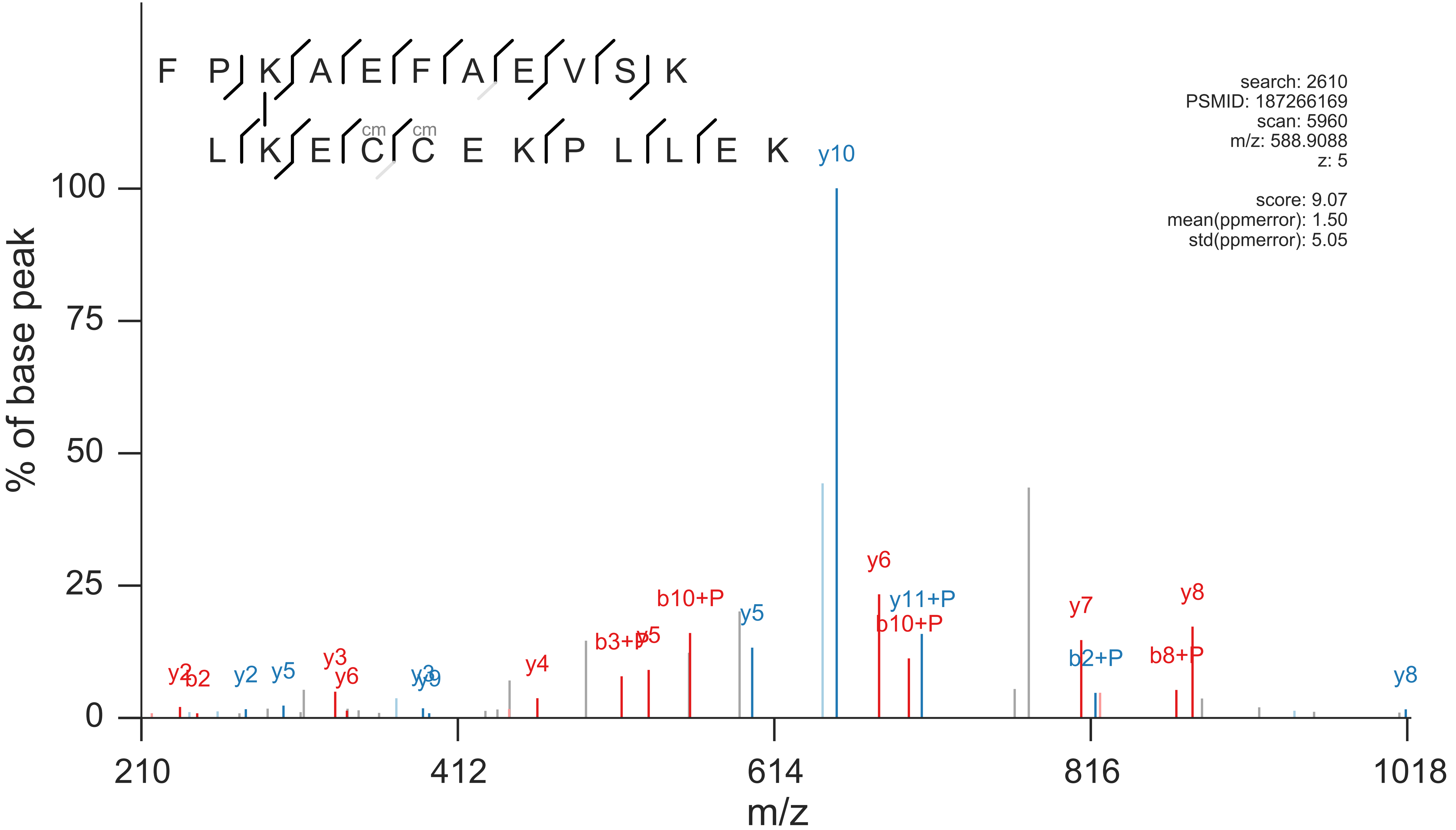

F P K A E F A E V S K
L K E C C E K P L L E K
cm
cm
search: 2610
PSMID: 187266169
scan: 5960
m/z: 588.9088
z: 5
score: 9.07
mean(ppmerror): 1.50
std(ppmerror): 5.05
% of base peak
m/z
0
25
50
75
100
210
412
614
816
1018
y10
y6
y11+P
b10+P
b10+P
y5
b3+P
y5
y7
y8
b2+P
b8+P
y8
y4
y3
y6
y2
b2
y2
y5
y3
y9

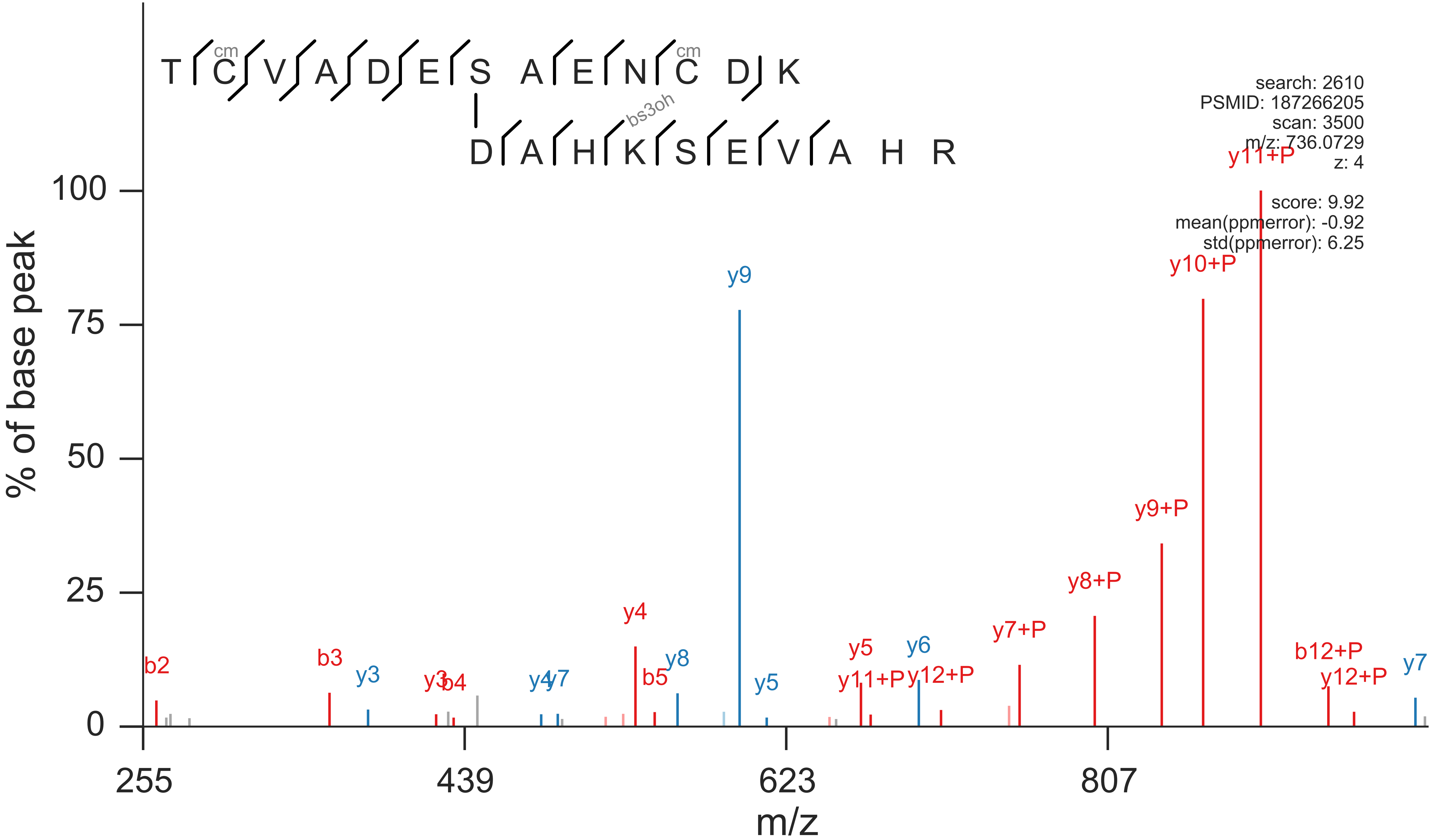

T C V A D E S A E N C D K
cm
cm
D A H K S E V A H R
bs3oh
search: 2610
PSMID: 187266205
scan: 3500
m/z: 736.0729
z: 4
score: 9.92
mean(ppmerror): -0.92
std(ppmerror): 6.25
% of base peak
m/z
0
25
50
75
100
255
439
623
807
b2
b3
y3
y3
b4
y4
y7
y4
b5
y8
y9
y5
y5
y11+P
y6
y12+P
y7+P
y8+P
y9+P
y10+P
y11+P
b12+P
y12+P
y7

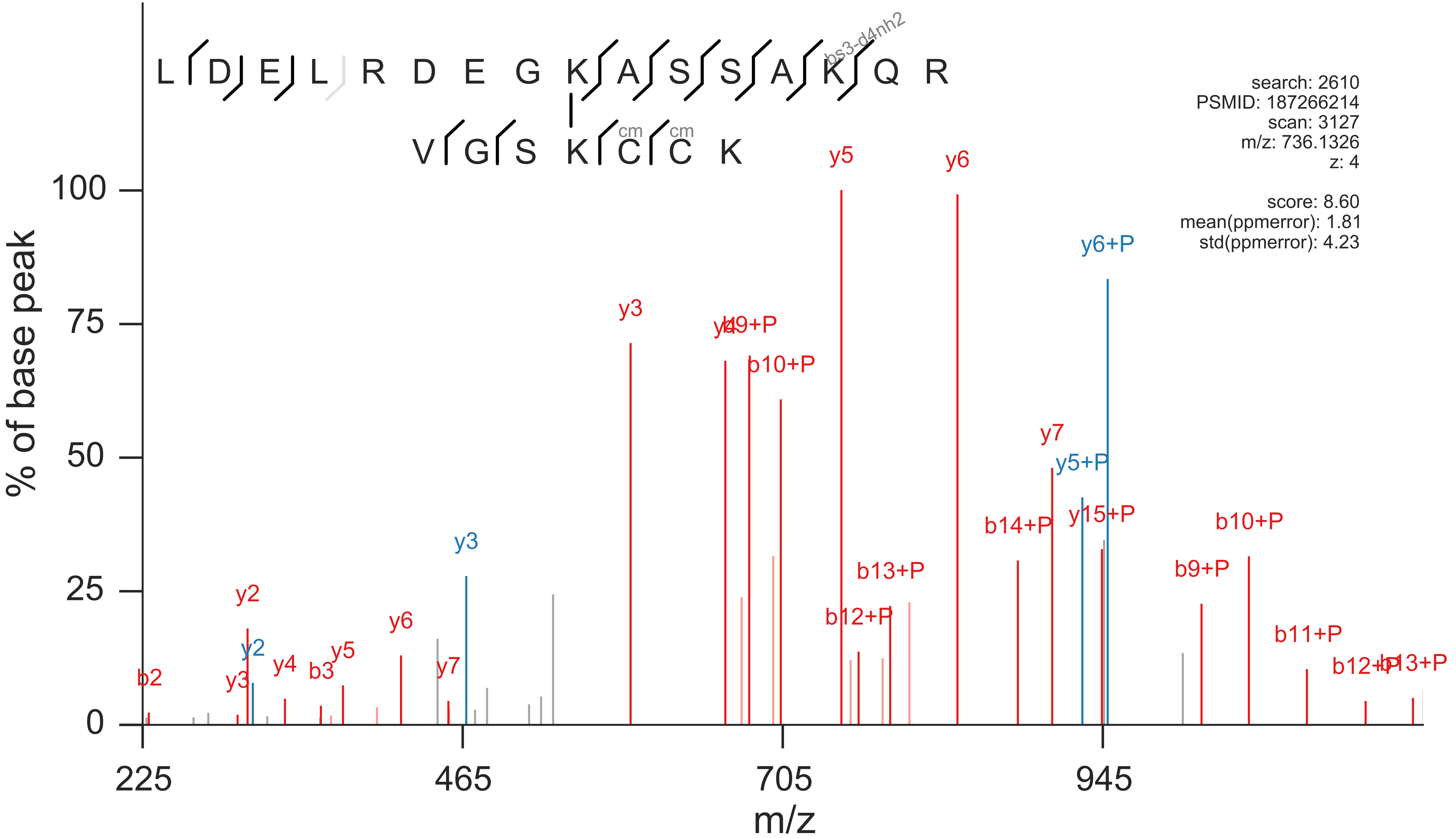

L D E L R D E G K A S S A K Q R
bs3-d4nh2
V G S K C C K
cm
cm
search: 2610
PSMID: 187266214
scan: 3127
m/z: 736.1326
z: 4
score: 8.60
mean(ppmerror): 1.81
std(ppmerror): 4.23
% of base peak
m/z
0
25
50
75
100
225
465
705
945
y5
y6
y6+P
y3
y4
b9+P
b10+P
y7
y5+P
y15+P
b14+P
b13+P
b12+P
b10+P
b9+P
b11+P
b12+P
b13+P
y2
y2
y3
b2
y3
y4
b3
y5
y6
y7

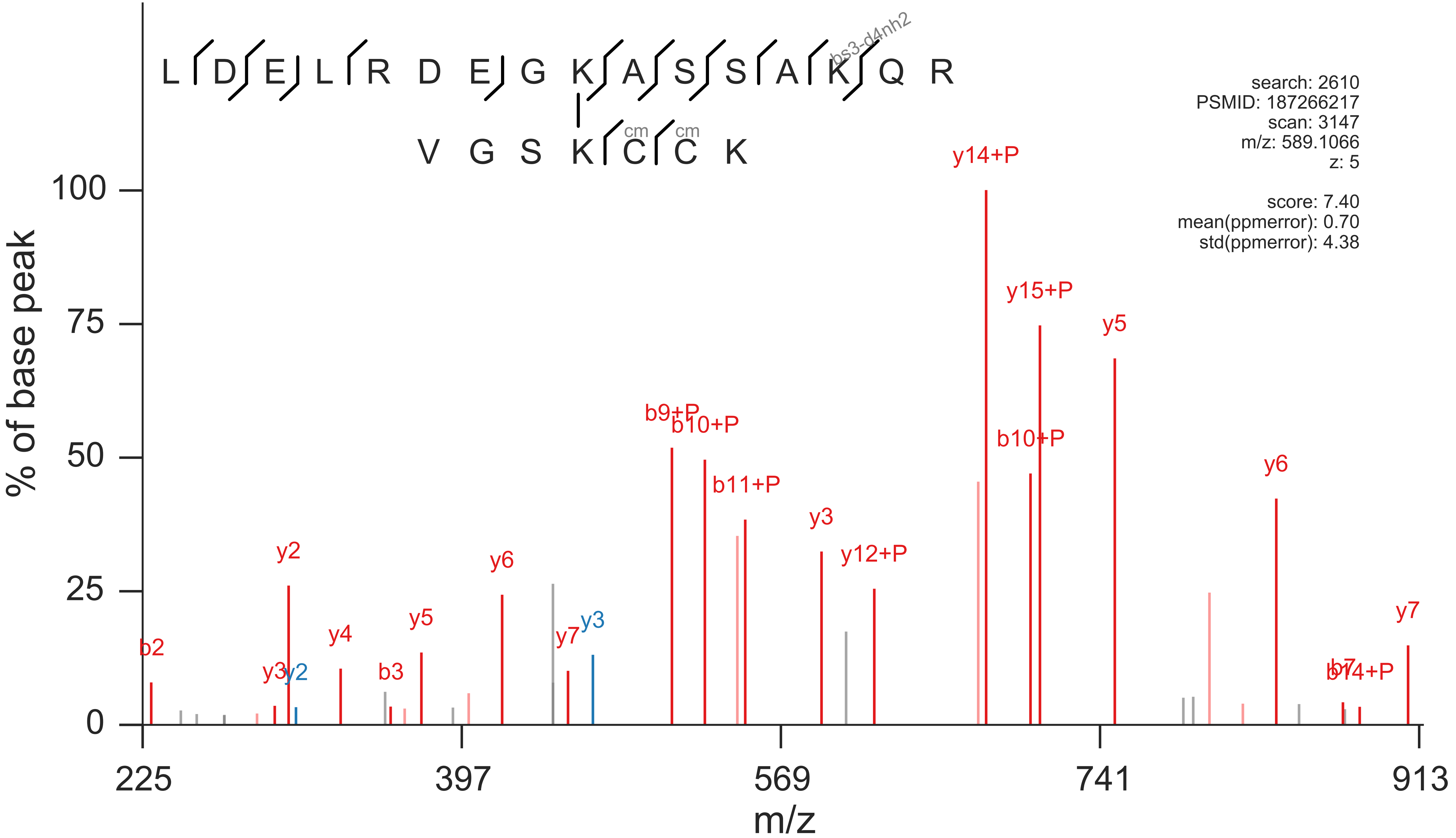

L D E L R D E G K A S S A K Q R
bs3-d4nh2
V G S K C C K
cm
cm
search: 2610
PSMID: 187266217
scan: 3147
m/z: 589.1066
z: 5
score: 7.40
mean(ppmerror): 0.70
std(ppmerror): 4.38
% of base peak
m/z
100
75
50
25
0
225
397
569
741
913
b2
y3
y2
y2
y4
b3
y5
y6
y7
y3
b9+P
b10+P
b11+P
y3
y12+P
y14+P
b10+P
y15+P
y5
y6
b7
b14+P
y7

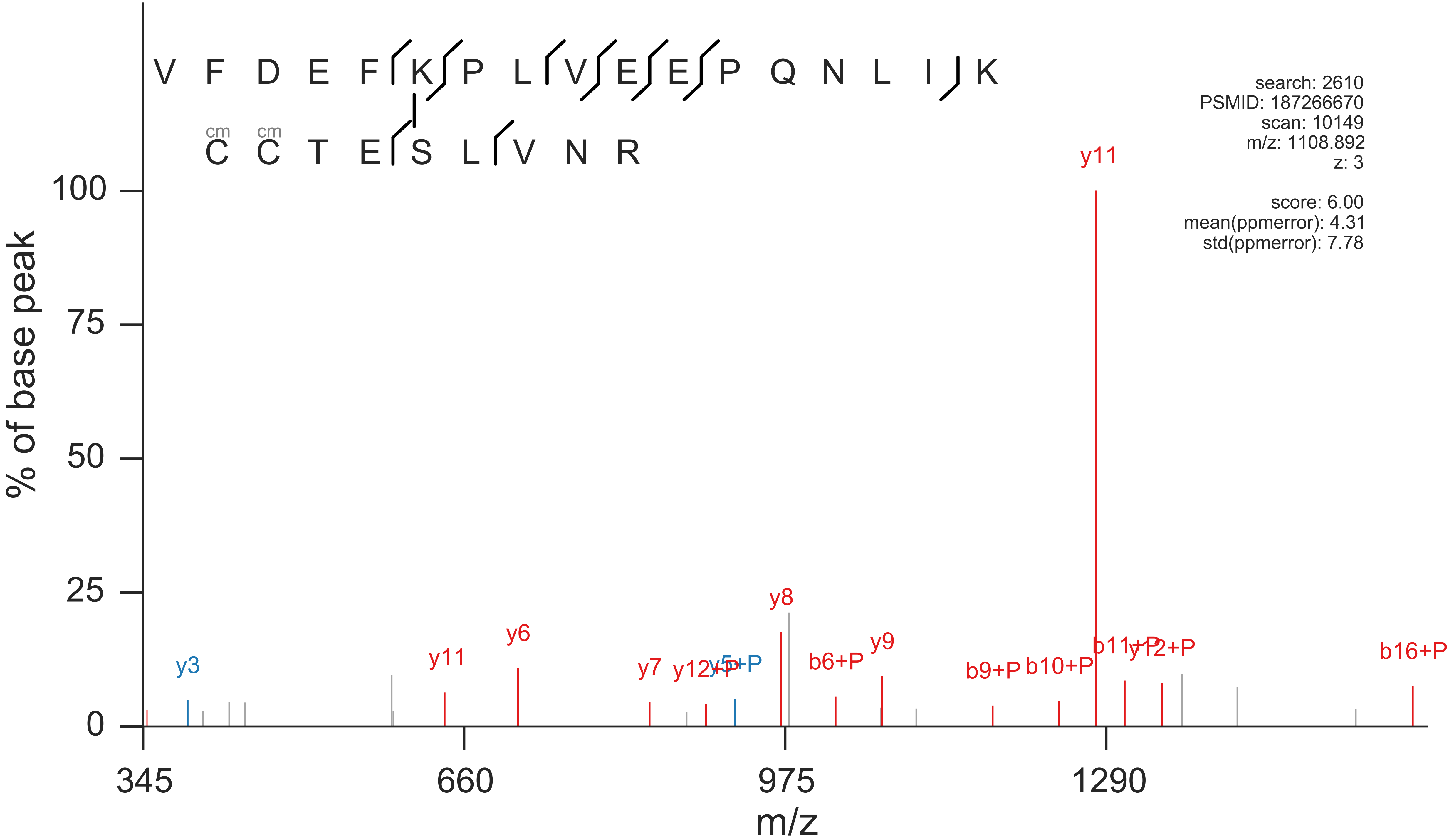

V F D E F K P L V E E P Q N L I K
cm cm
C C T E S L V N R
search: 2610
PSMID: 187266670
scan: 10149
m/z: 1108.892
z: 3
score: 6.00
mean(ppmerror): 4.31
std(ppmerror): 7.78
% of base peak
100
75
50
25
0
345
660
975
1290
m/z
y3
y11
y6
y7
y12+P
y5+P
y8
b6+P
y9
b9+P
b10+P
y11
b11+P
y12+P
b16+P

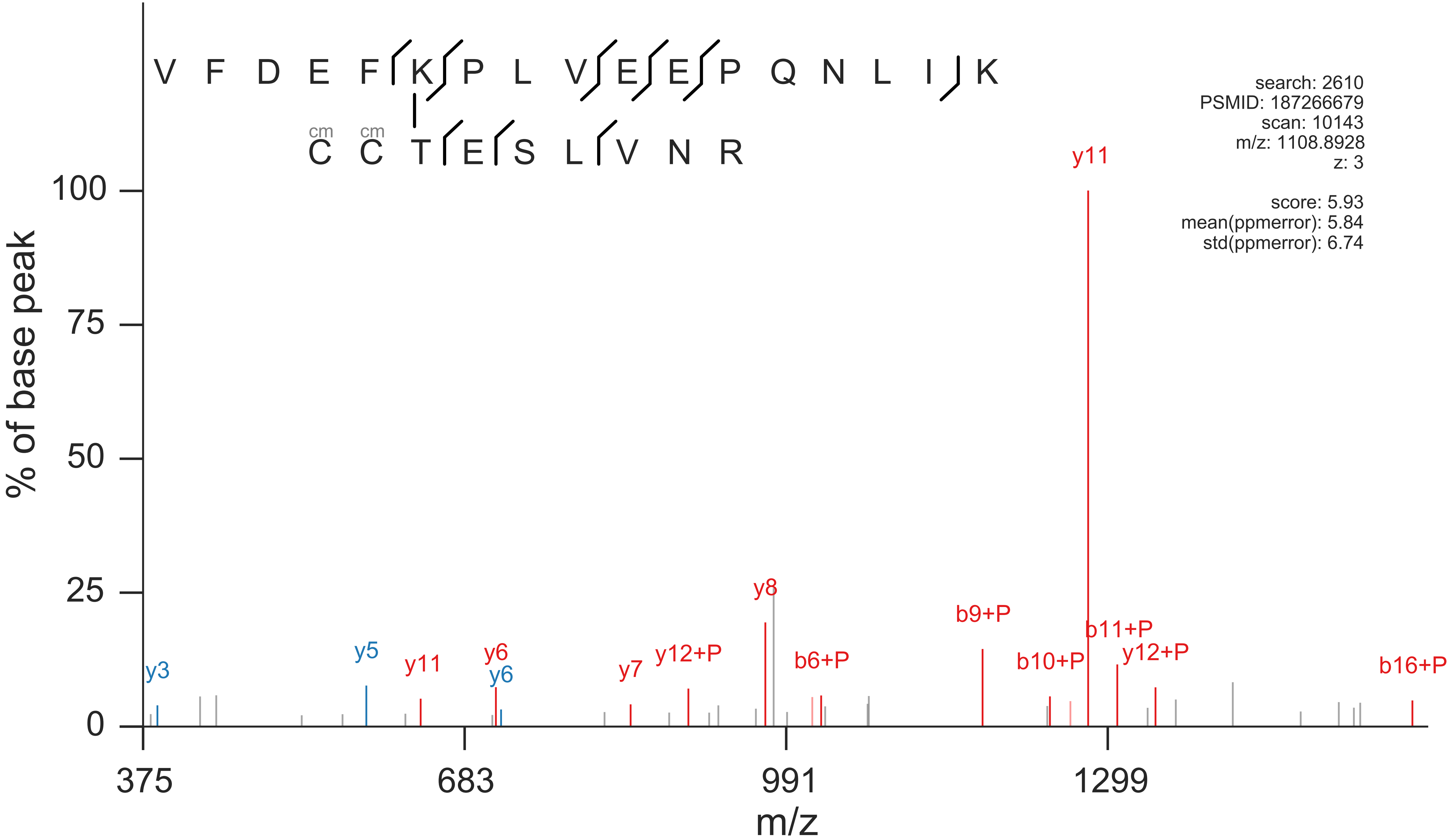

V F D E F K P L V E E P Q N L I K
cm C cm C T E S L V N R
search: 2610
PSMID: 187266679
scan: 10143
m/z: 1108.8928
z: 3
score: 5.93
mean(ppmerror): 5.84
std(ppmerror): 6.74
% of base peak
100
75
50
25
0
375
683
991
1299
m/z
y3
y5
y11
y6
y6
y7
y12+P
y8
b6+P
b9+P
b10+P
y11
b11+P
y12+P
b16+P

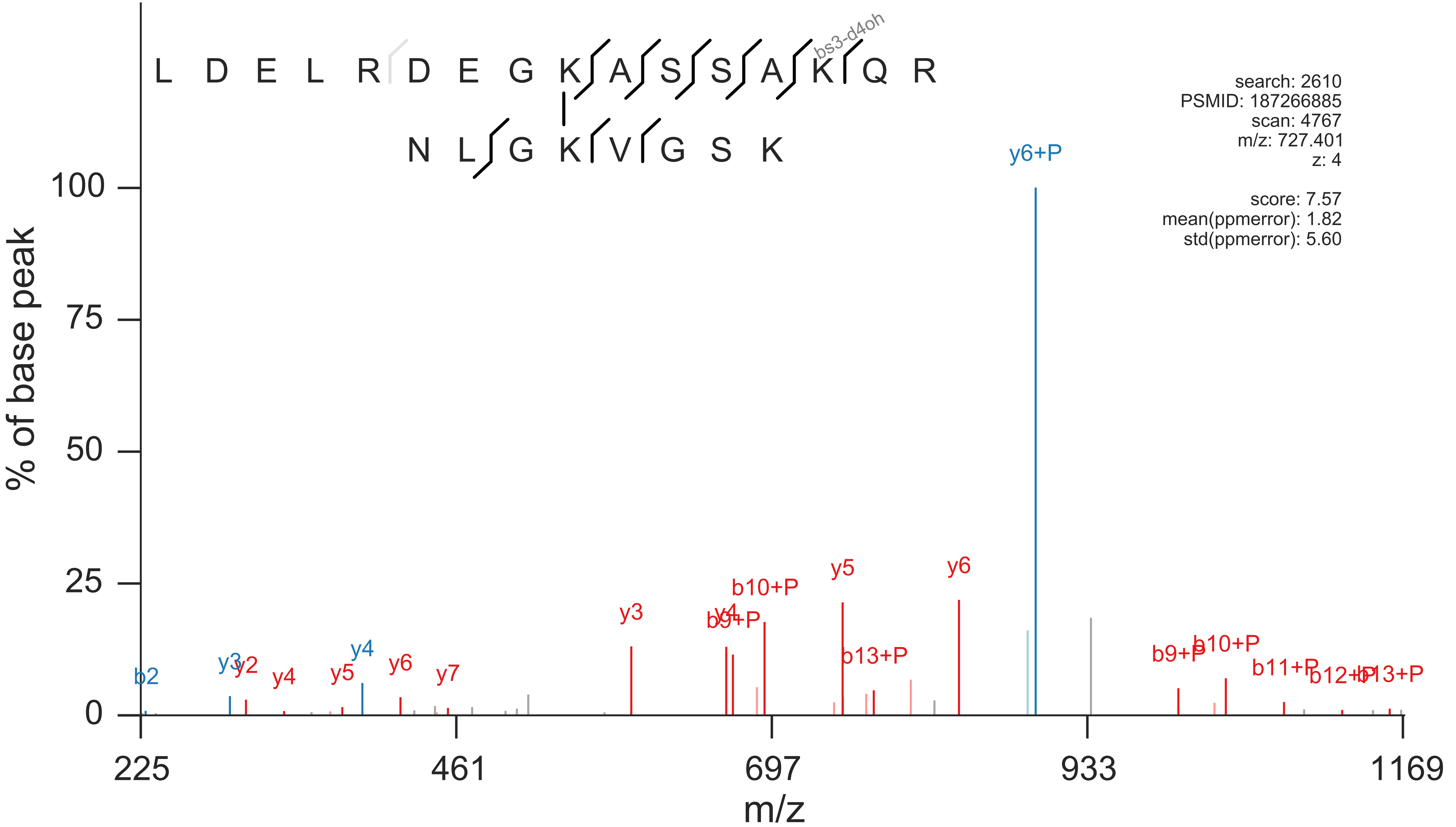

L D E L R D E G K A S S A K Q R
N L G K V G S K
bs3-d4oh
search: 2610
PSMID: 187266885
scan: 4767
m/z: 727.401
z: 4
score: 7.57
mean(ppmerror): 1.82
std(ppmerror): 5.60
y6+P
% of base peak
100
75
50
25
0
m/z
225
461
697
933
1169
b2
y3
y2
y4
y5
y4
y6
y7
y3
b9+P
b10+P
y5
b13+P
y6
b9+P
b10+P
b11+P
b12+P
b13+P

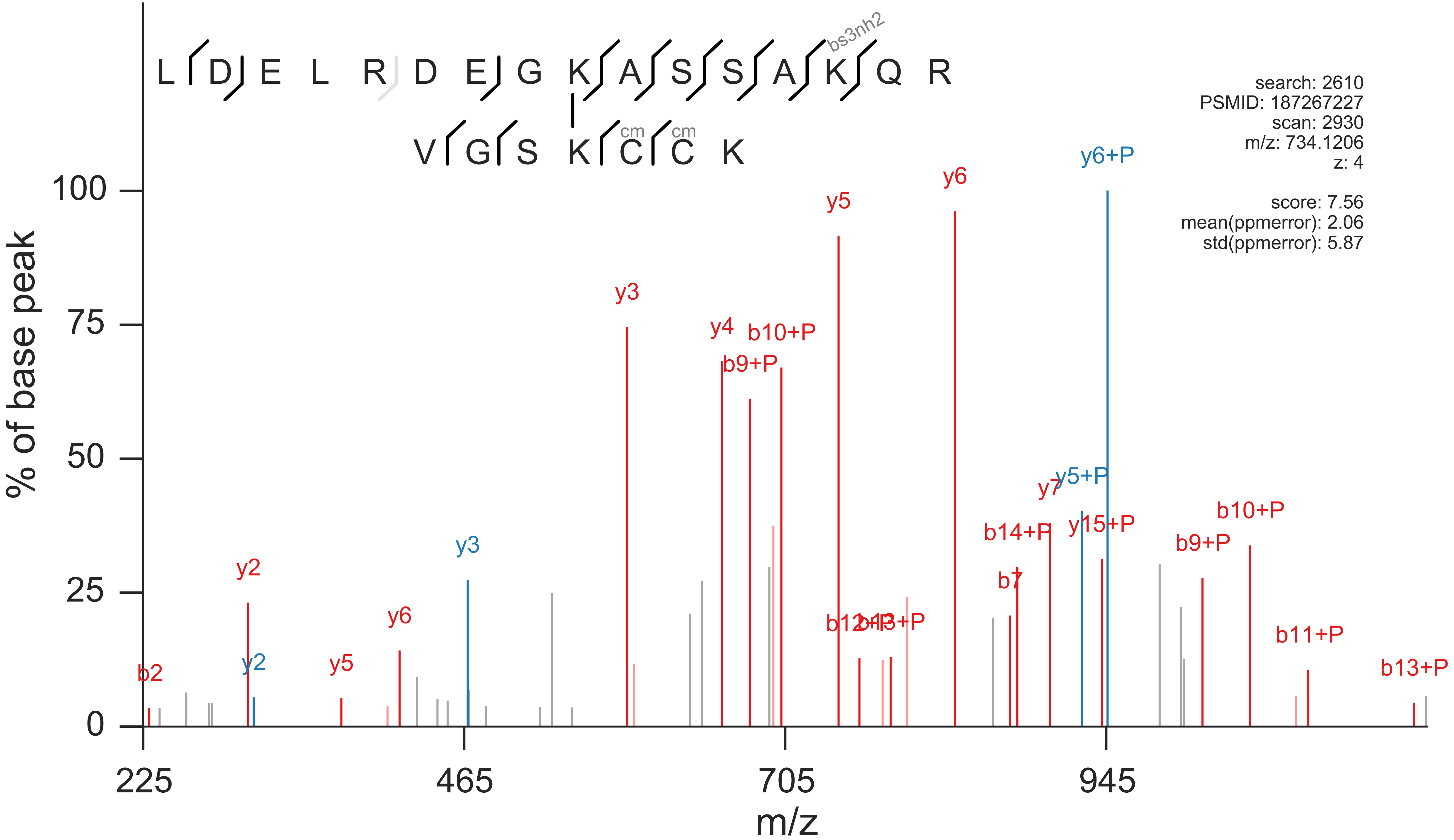

L D E L R D E G K A S S A K Q R
bs3nh2
V G S K C C K
cm
cm
search: 2610
PSMID: 187267227
scan: 2930
m/z: 734.1206
z: 4
score: 7.56
mean(ppmerror): 2.06
std(ppmerror): 5.87
% of base peak
m/z
0
25
50
75
100
225
465
705
945
y6+P
y6
y5
y3
y4
b10+P
b9+P
y7
y5+P
b14+P
y15+P
b10+P
b9+P
b7
b11+P
b13+P
y2
y3
y6
y2
y5
b2

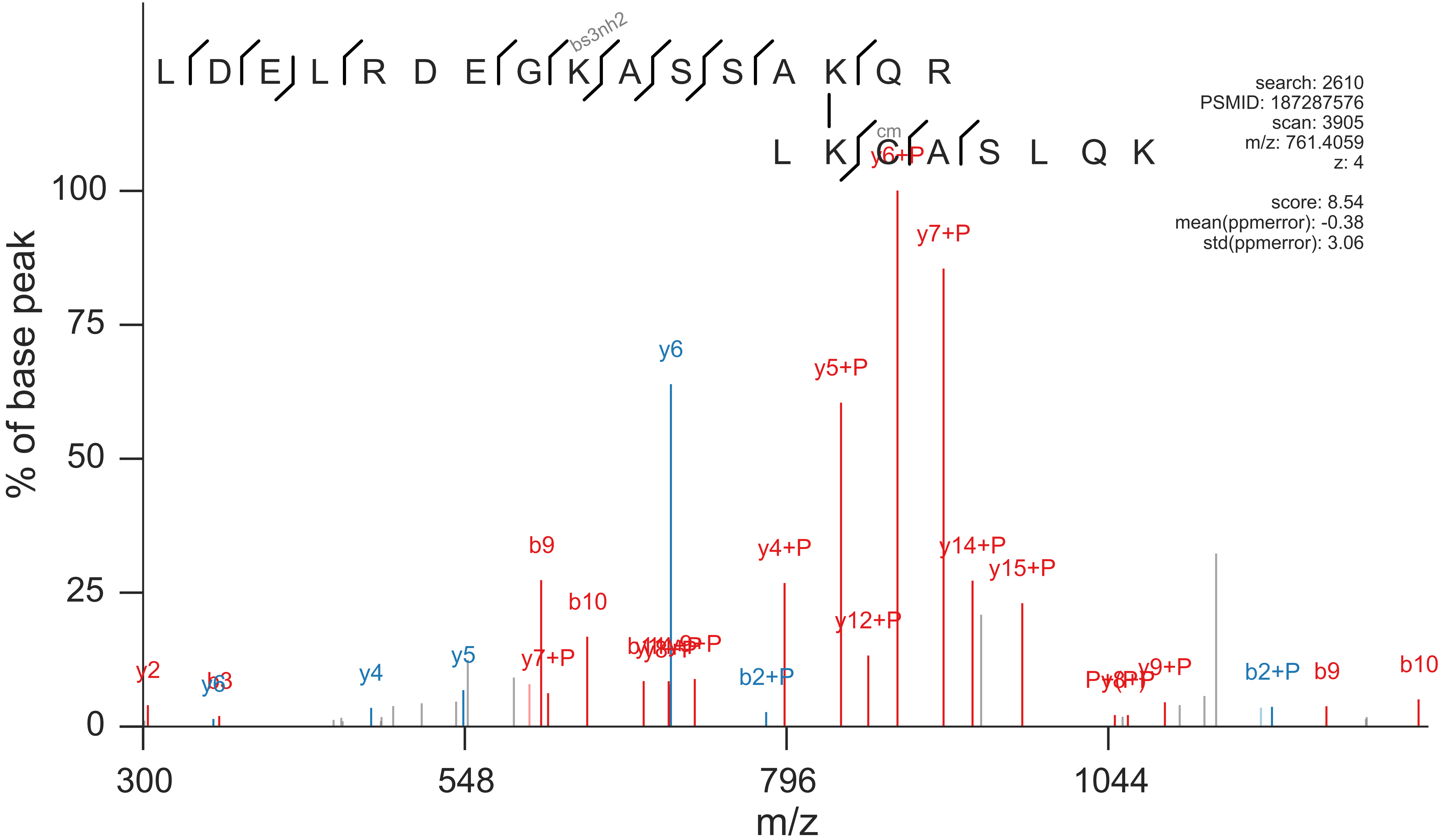

L D E L R D E G K A S S A K Q R
bs3nh2
L K C A S L Q K
cm
search: 2610
PSMID: 187287576
scan: 3905
m/z: 761.4059
z: 4
score: 8.54
mean(ppmerror): -0.38
std(ppmerror): 3.06
% of base peak
m/z
0
25
50
75
100
300
548
796
1044
y6
y5+P
y6+P
y7+P
b9
b10
y4+P
y14+P
y15+P
y12+P
y2
b3
y4
y5
y7+P
b2+P
y9+P
b2+P
b9
b10

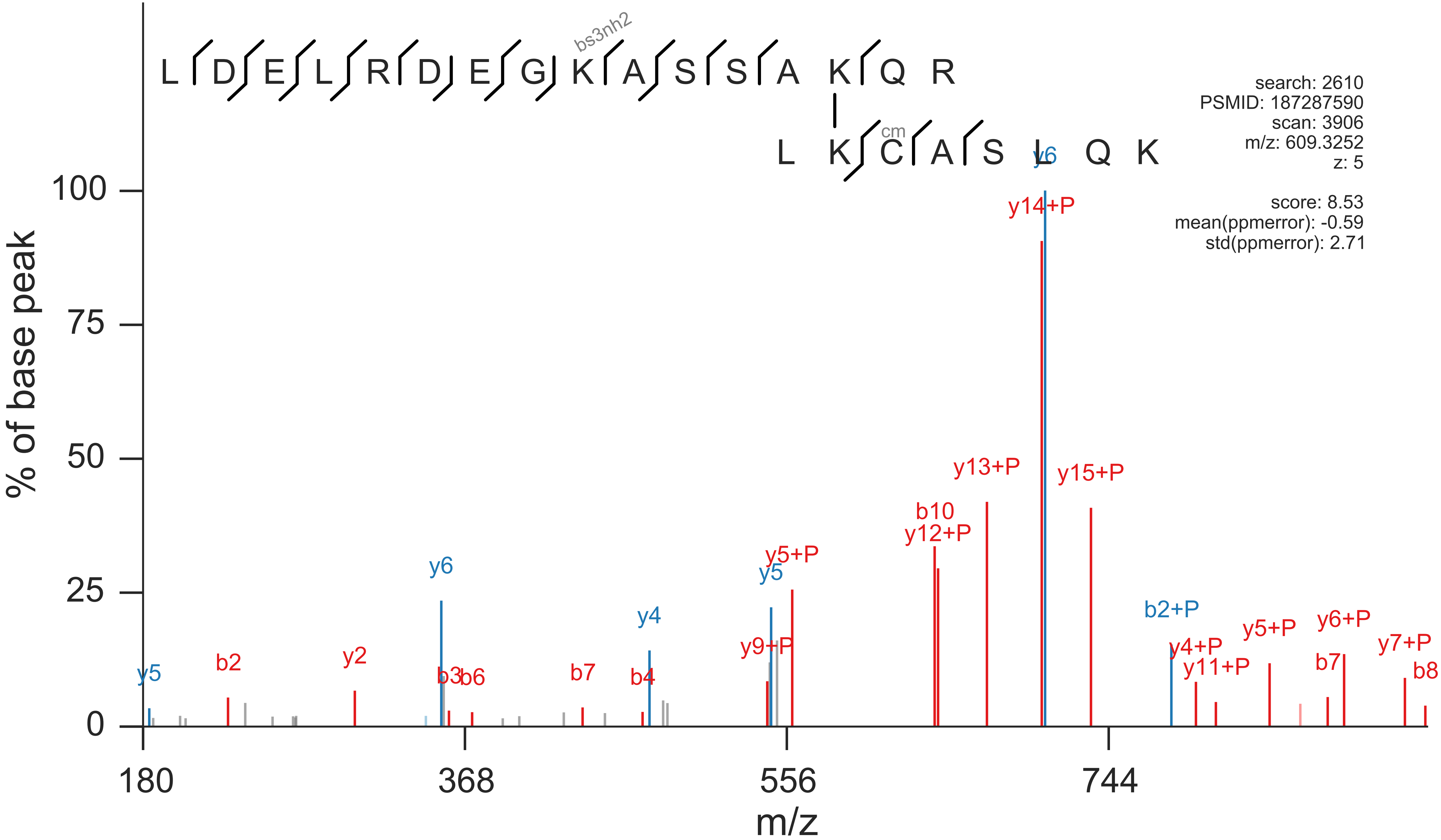

bs3nh2
L D E L R D E G K A S S A K Q R
cm
L K C A S L Q K
search: 2610
PSMID: 187287590
scan: 3906
m/z: 609.3252
z: 5
score: 8.53
mean(ppmerror): -0.59
std(ppmerror): 2.71
% of base peak
m/z
y6
y14+P
y13+P
y15+P
b10
y12+P
y5+P
y5
y6
y4
y9+P
b2+P
y4+P
y11+P
y5+P
y6+P
y7+P
b7
b8
y5
b2
y2
b3
b6
b7
b4

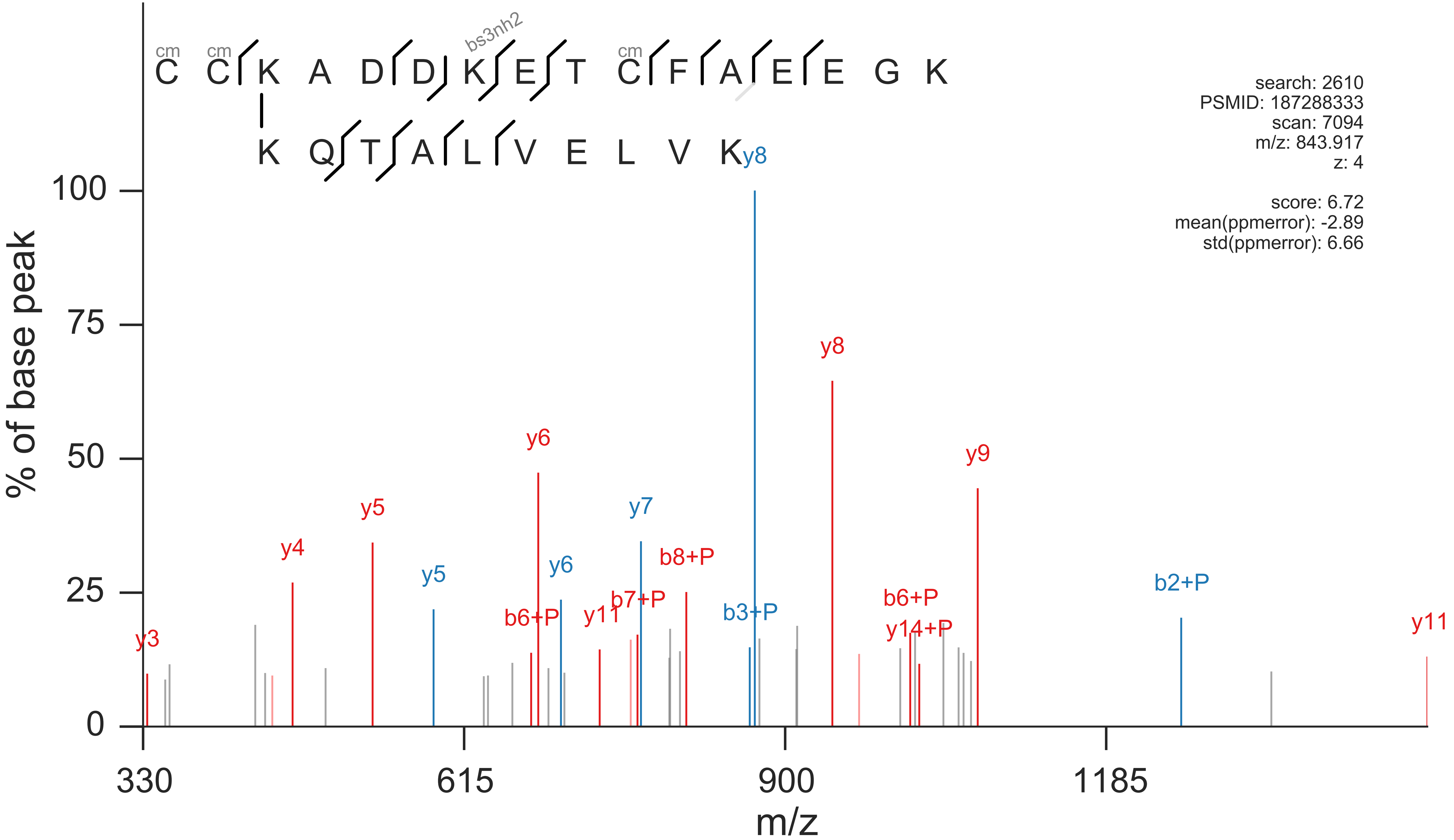

cm cm bs3nh2 cm
C C K A D D K E T C F A E E G K
K Q T A L V E L V K
search: 2610
PSMID: 187288333
scan: 7094
m/z: 843.917
z: 4
score: 6.72
mean(ppmerror): -2.89
std(ppmerror): 6.66
% of base peak
m/z
0
25
50
75
100
330
615
900
1185
y8
y8
y6
y9
y5
y4
y7
y5
y6
b8+P
b7+P
b6+P
y11
b3+P
b6+P
y14+P
b2+P
y11
y3

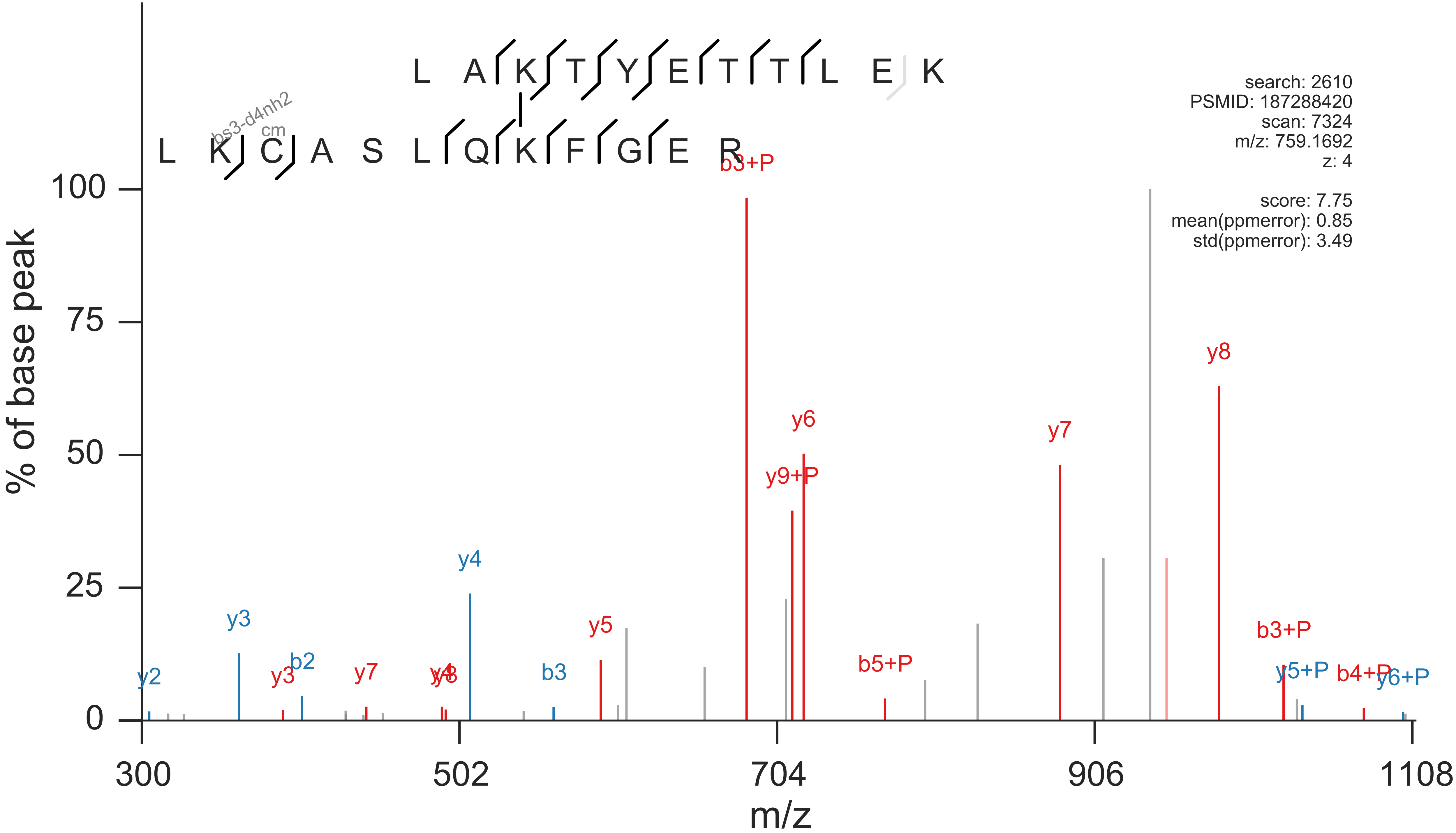
L A K T Y E T T L E K
L K C A S L Q K F G E R
bs3-d4nh2
cm
search: 2610
PSMID: 187288420
scan: 7324
m/z: 759.1692
z: 4
score: 7.75
mean(ppmerror): 0.85
std(ppmerror): 3.49
% of base peak
m/z
100
75
50
25
0
300
502
704
906
1108
b3+P
y8
y6
y7
y9+P
y4
y3
y5
b5+P
b3+P
y5+P
b4+P
y6+P
y2
y3
b2
y7
y4
y8
b3

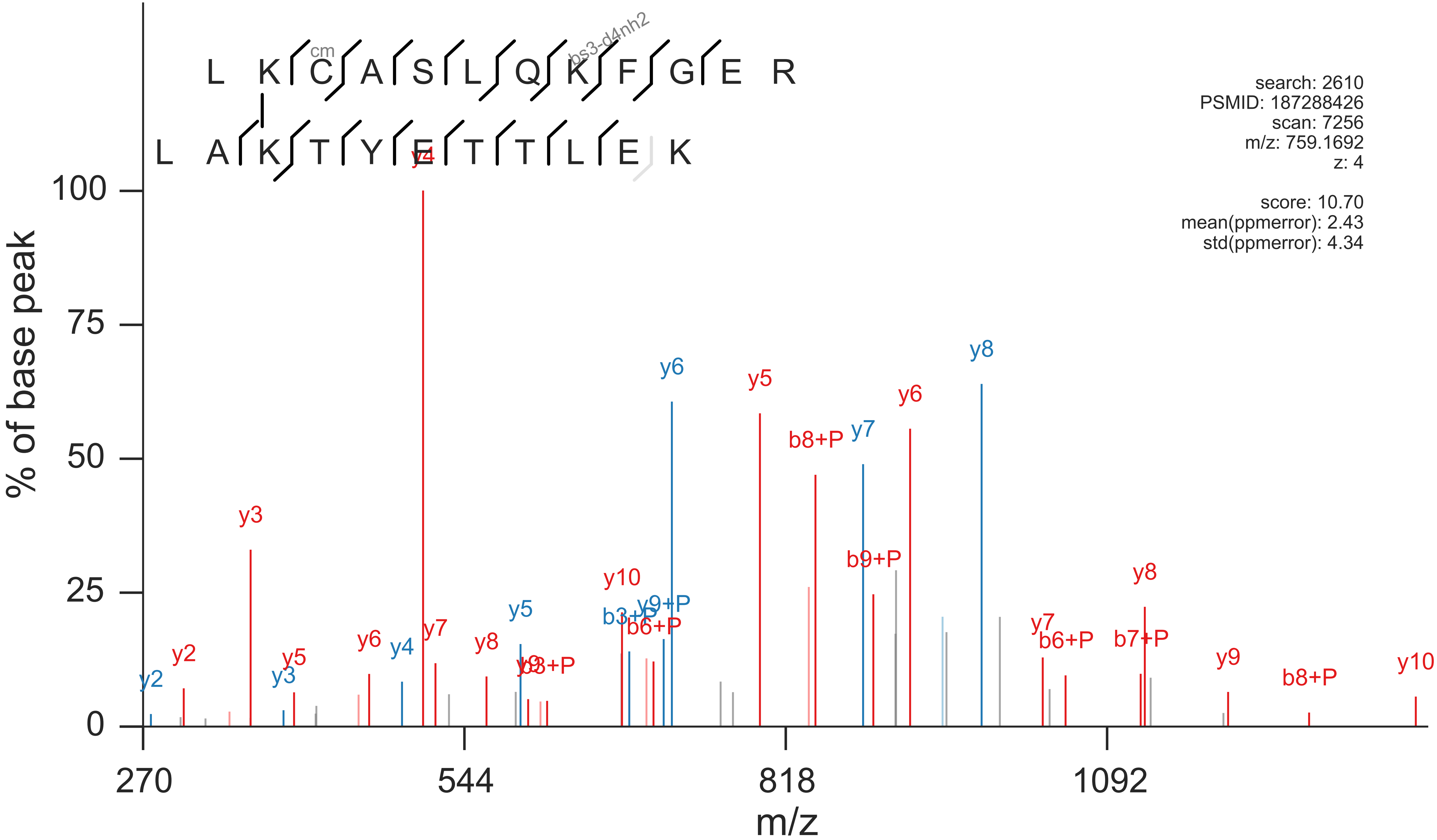
L K C A S L Q K F G E R
cm
bs3-d4nh2
L A K T Y E T T L E K
search: 2610
PSMID: 187288426
scan: 7256
m/z: 759.1692
z: 4
score: 10.70
mean(ppmerror): 2.43
std(ppmerror): 4.34
% of base peak
m/z
100
75
50
25
0
270
544
818
1092

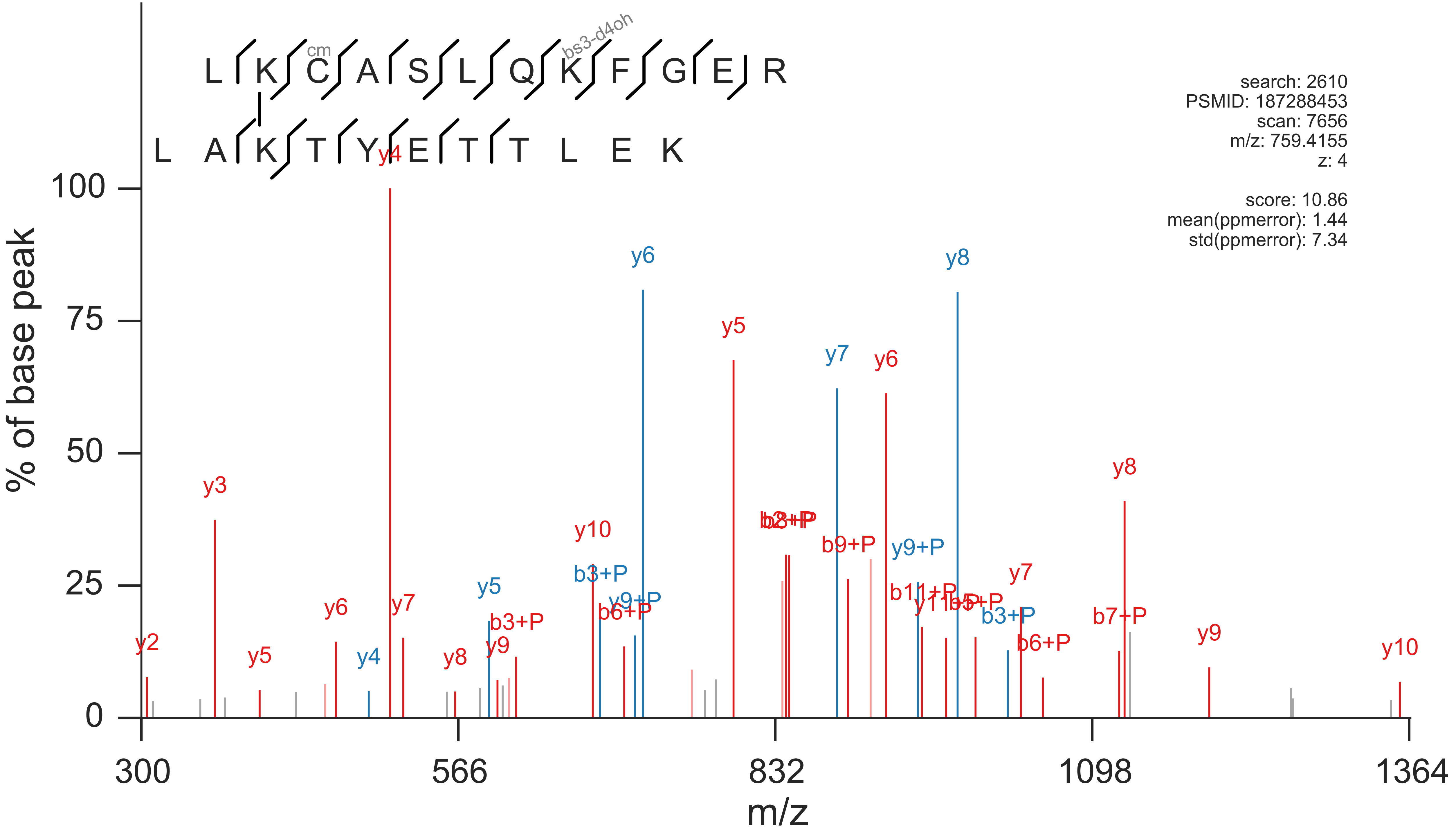

L K C A S L Q K F G E R
cm
bs3-d4oh
L A K T Y E T T L E K
search: 2610
PSMID: 187288453
scan: 7656
m/z: 759.4155
z: 4
score: 10.86
mean(ppmerror): 1.44
std(ppmerror): 7.34
% of base peak
m/z
0
25
50
75
100
300
566
832
1098
1364

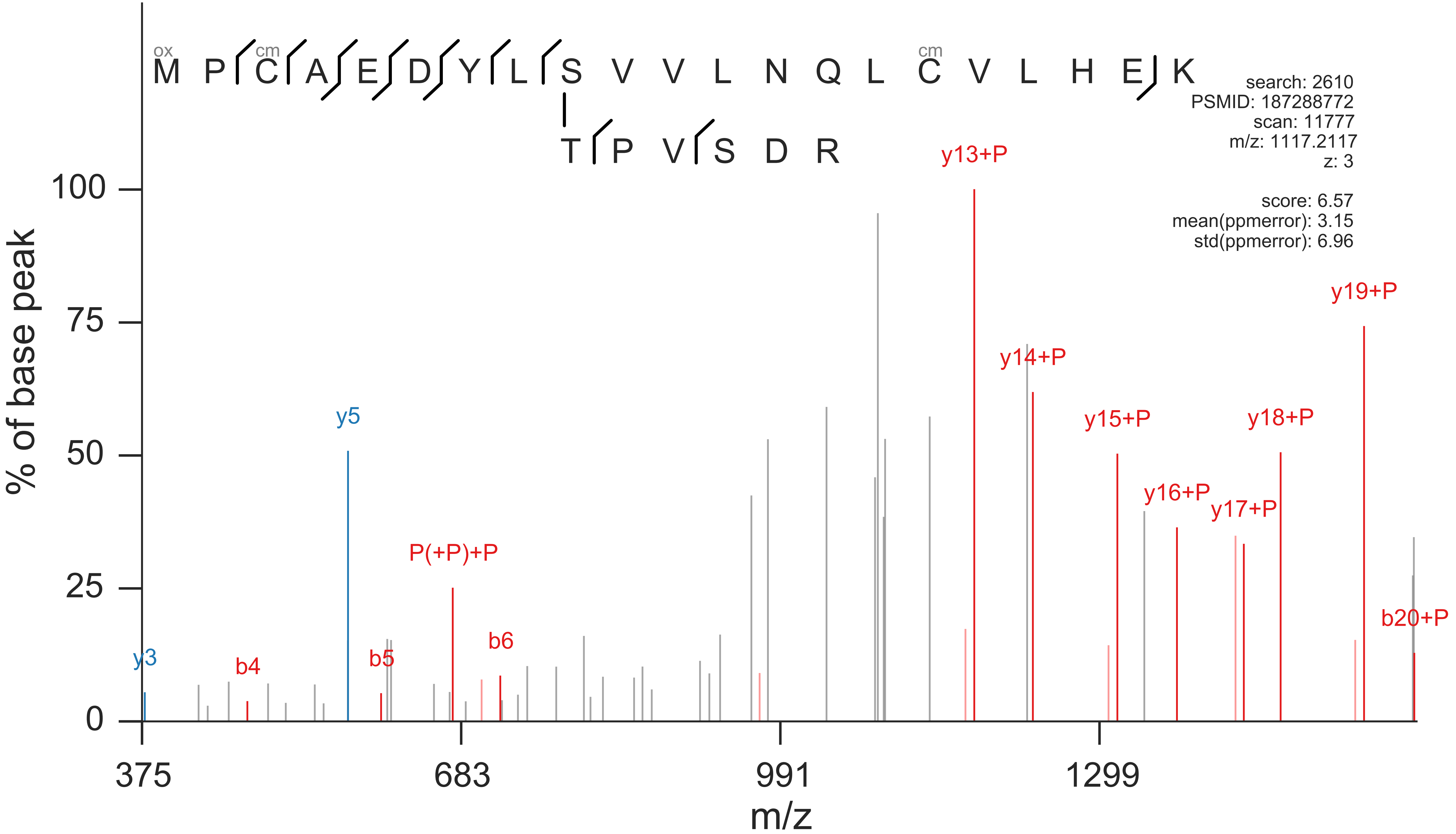

M P C A E D Y L S V V L N Q L C V L H E K
T P V S D R
ox
cm
cm
search: 2610
PSMID: 187288772
scan: 11777
m/z: 1117.2117
z: 3
score: 6.57
mean(ppmerror): 3.15
std(ppmerror): 6.96
% of base peak
m/z
y3
b4
y5
b5
P(+P)+P
b6
y13+P
y14+P
y15+P
y16+P
y17+P
y18+P
y19+P
b20+P
0
25
50
75
100
375
683
991
1299

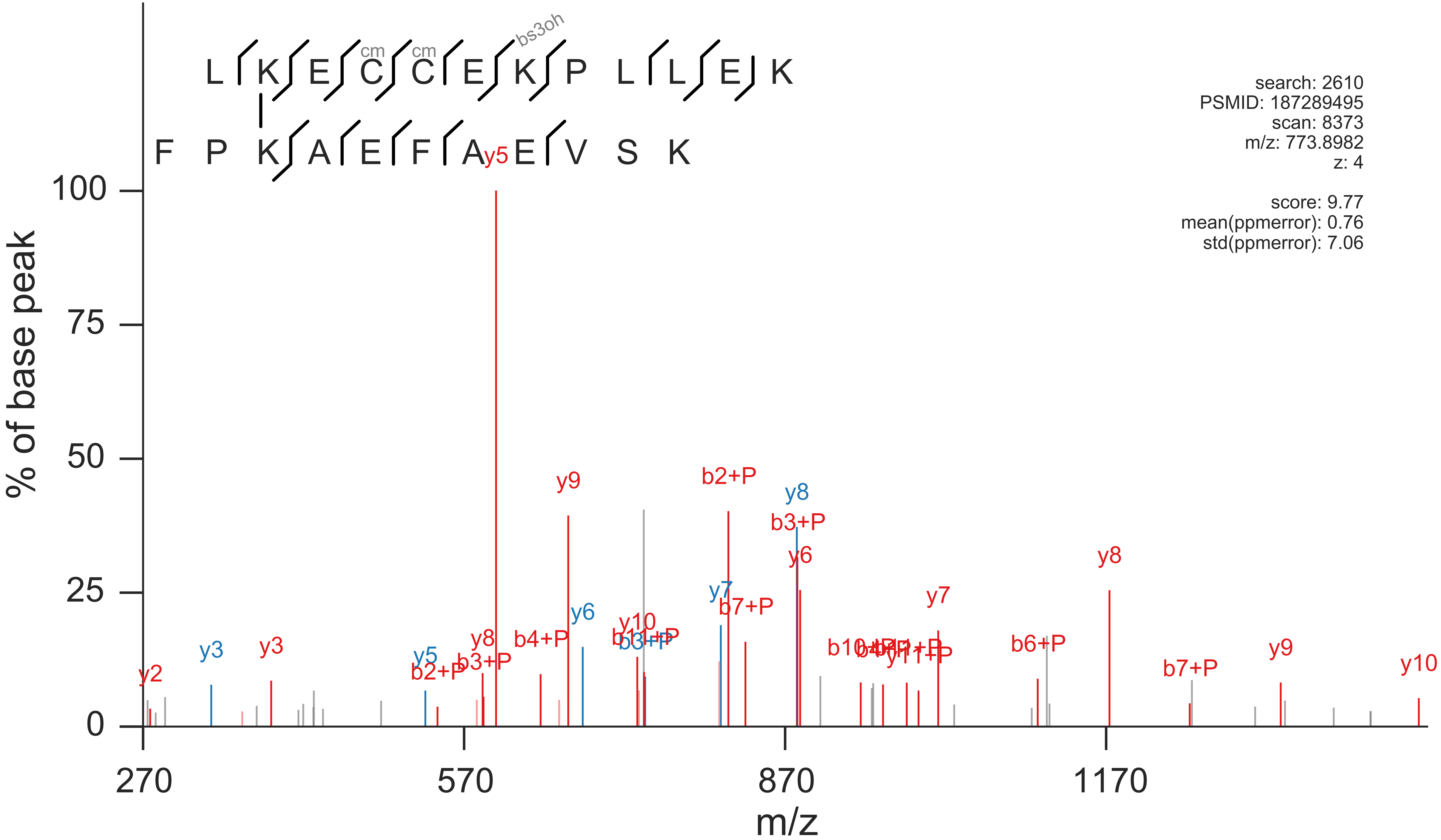

L K E C C E K P L L E K
cm
cm
bs3oh
F P K A E F A E V S K
search: 2610
PSMID: 187289495
scan: 8373
m/z: 773.8982
z: 4
score: 9.77
mean(ppmerror): 0.76
std(ppmerror): 7.06
% of base peak
m/z
0
25
50
75
100
270
570
870
1170
y2
y3
y3
y5
b2+P
y8
b3+P
y5
b4+P
y9
y6
y10
b3+P
y7
b2+P
b7+P
y8
b3+P
y6
y7
b6+P
y8
b7+P
y9
y10

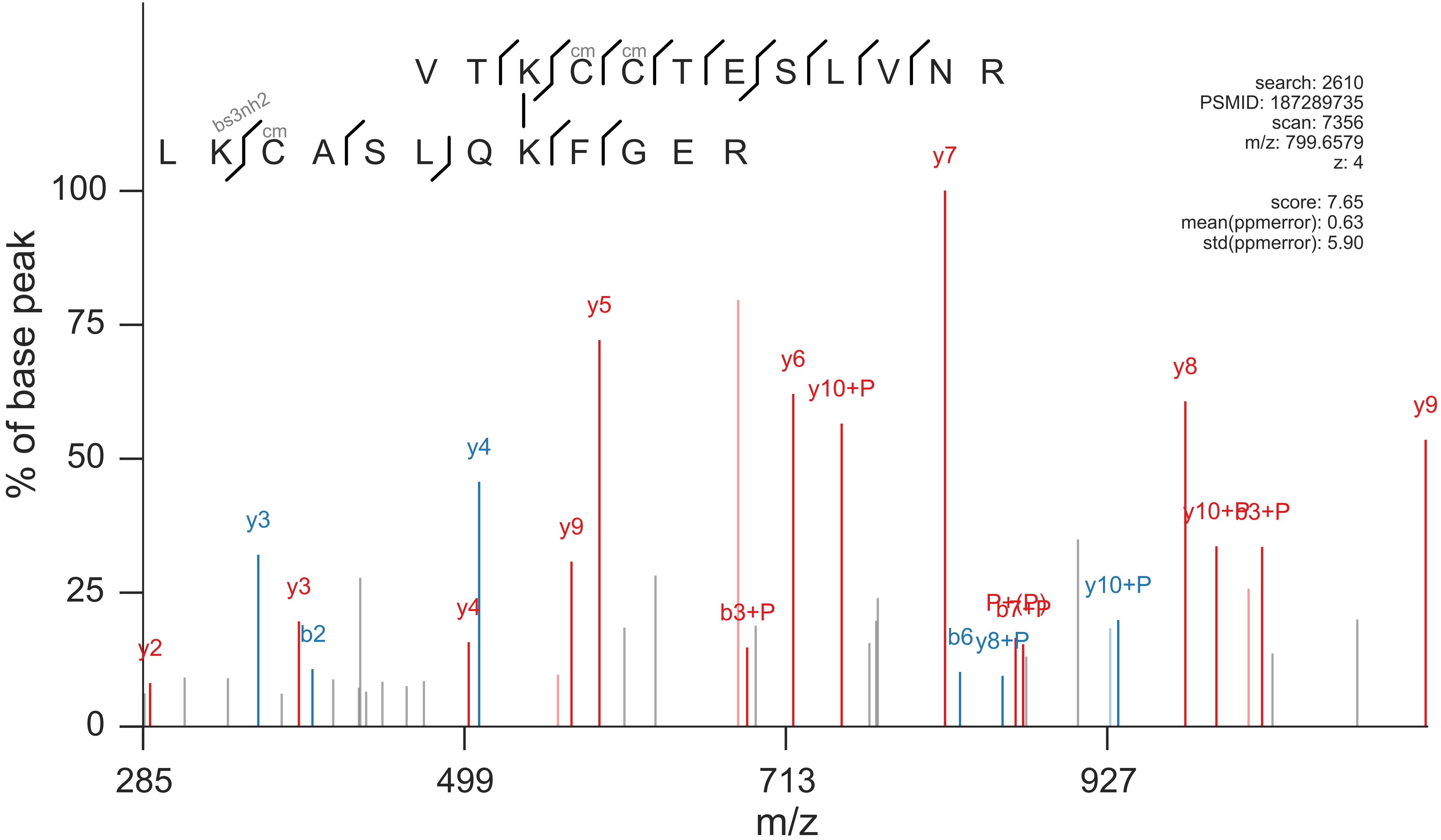
V T K C C T E S L V N R
L K C A S L Q K F G E R
bs3nh2
cm
search: 2610
PSMID: 187289735
scan: 7356
m/z: 799.6579
z: 4
score: 7.65
mean(ppmerror): 0.63
std(ppmerror): 5.90
% of base peak
m/z
0
25
50
75
100
285
499
713
927
y2
y3
y3
b2
y4
y4
y9
y5
b3+P
y6
y10+P
y7
b6
y8+P
b7+P
y10+P
y8
y10+P
b3+P
y9

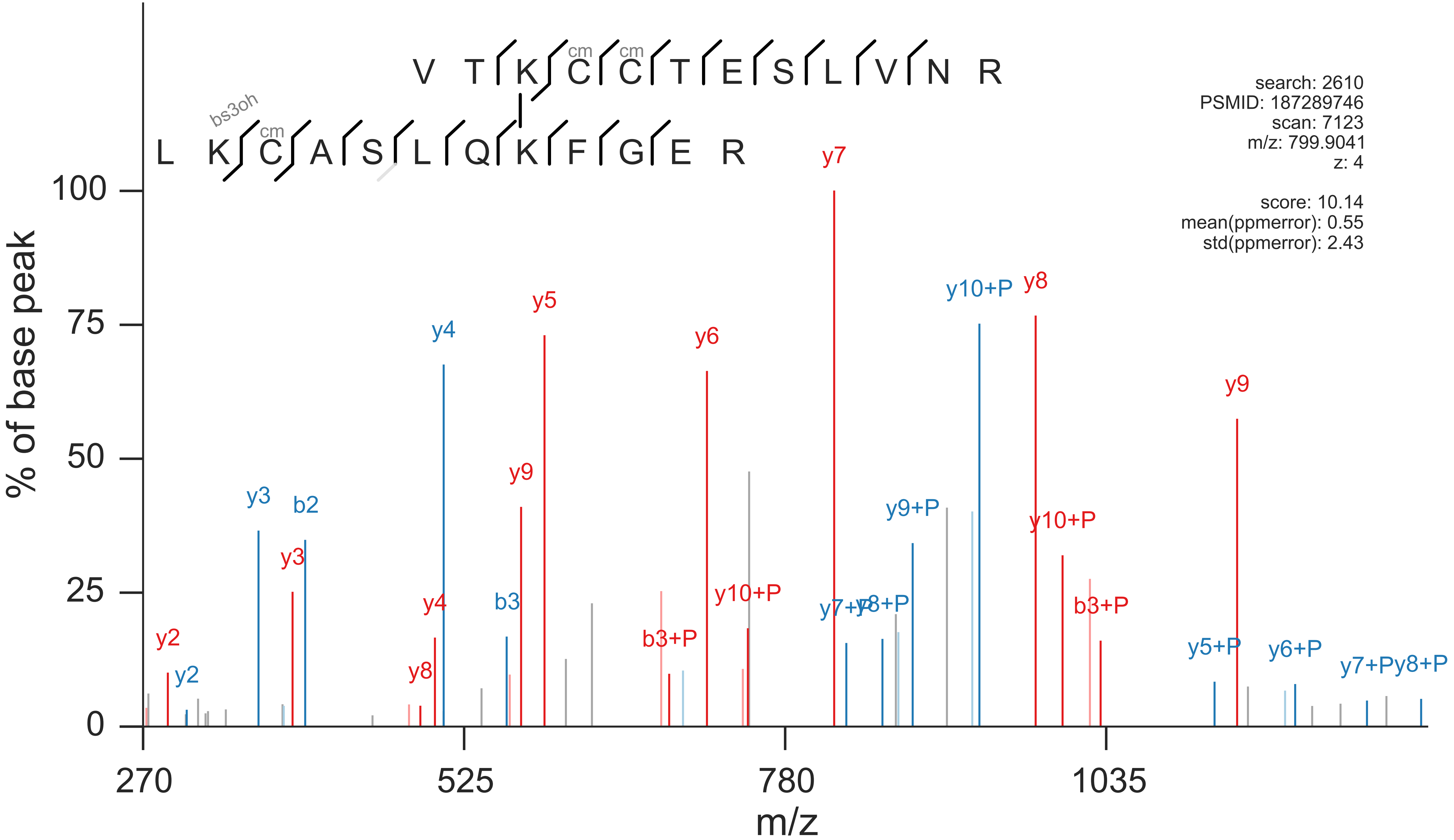

V T K C C T E S L V N R
L K C A S L Q K F G E R
cm
cm
cm
bs3oh
search: 2610
PSMID: 187289746
scan: 7123
m/z: 799.9041
z: 4
score: 10.14
mean(ppmerror): 0.55
std(ppmerror): 2.43
% of base peak
m/z
0
25
50
75
100
270
525
780
1035
y2
y2
y3
b2
y3
y4
y8
y4
b3
y9
y5
b3+P
y6
y10+P
y7
y7+P
y8+P
y9+P
y10+P
y8
y10+P
b3+P
y9
y5+P
y6+P
y7+P
y8+P

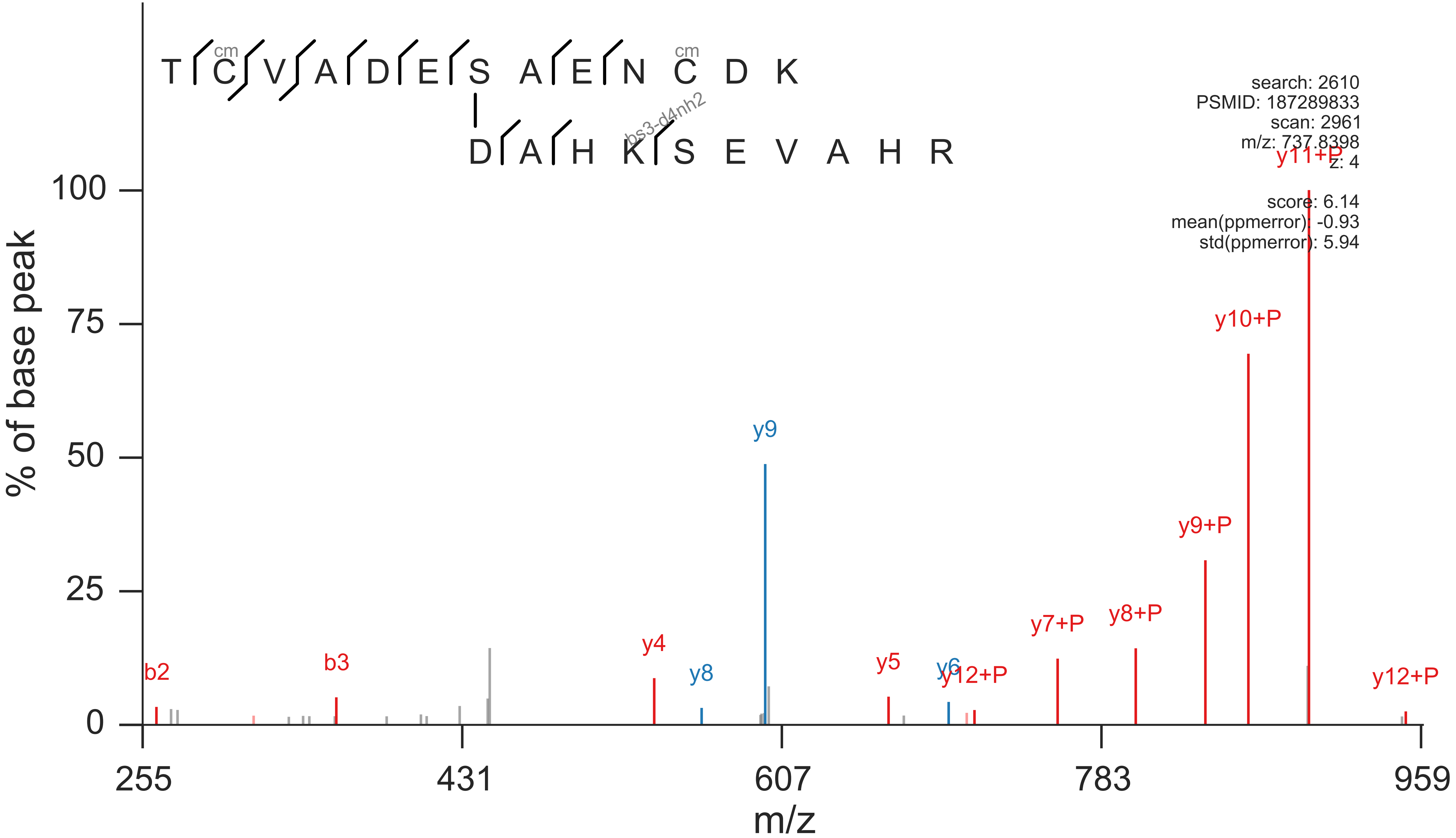

T C V A D E S A E N C D K
cm
cm
D A H K S E V A H R
bs3-d4nh2
search: 2610
PSMID: 187289833
scan: 2961
m/z: 737.8398
z: 4
score: 6.14
mean(ppmerror): -0.93
std(ppmerror): 5.94
% of base peak
100
75
50
25
0
255
431
607
783
959
m/z
b2
b3
y4
y8
y9
y5
y6
y12+P
y7+P
y8+P
y9+P
y10+P
y11+P
y12+P

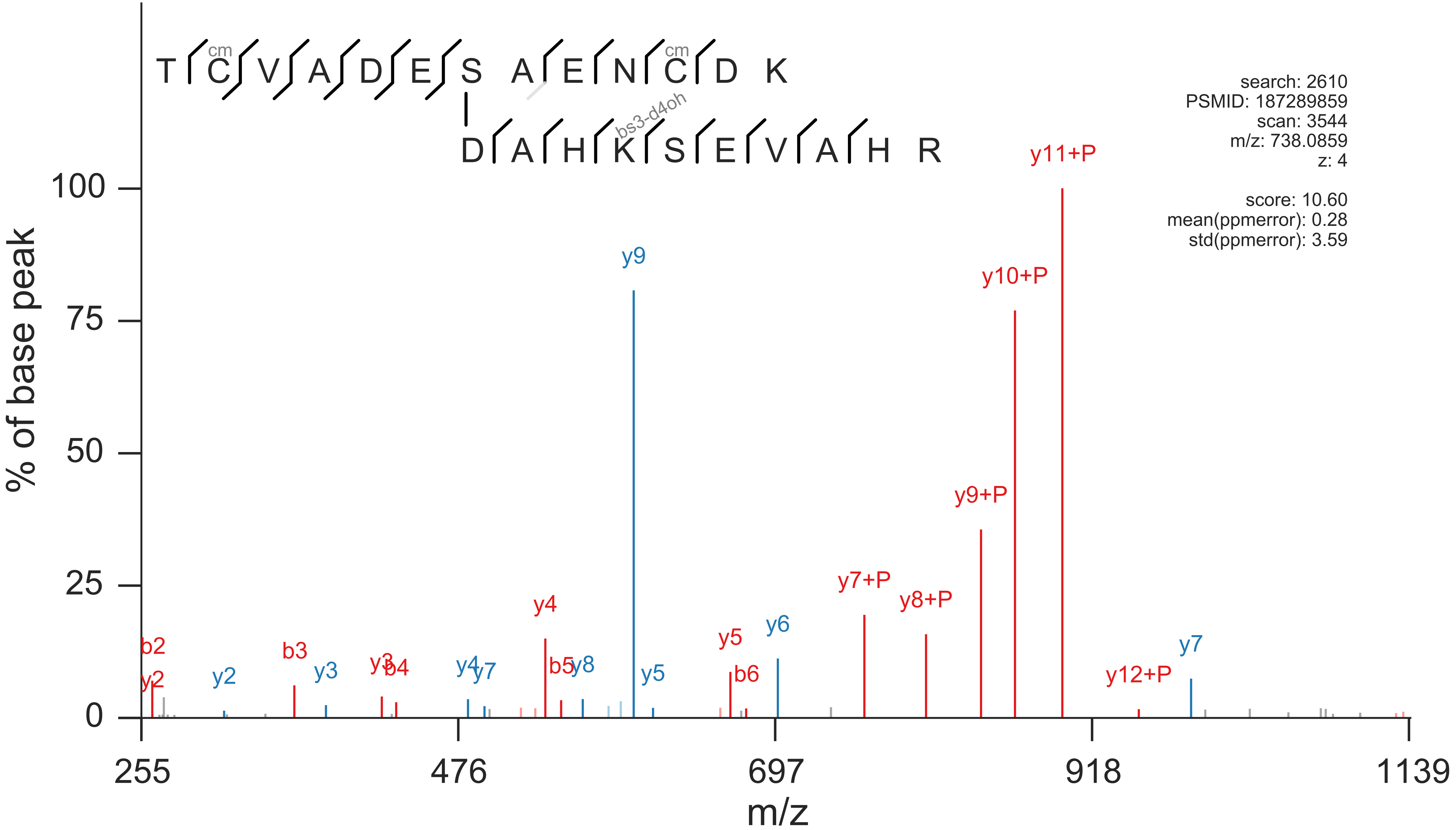

T C V A D E S A E N C D K
cm
cm
bs3-d4oh
D A H K S E V A H R
search: 2610
PSMID: 187289859
scan: 3544
m/z: 738.0859
z: 4
score: 10.60
mean(ppmerror): 0.28
std(ppmerror): 3.59
% of base peak
m/z
y11+P
y10+P
y9
y9+P
y7+P
y8+P
y4
y5
y6
y7
y12+P
b2
y2
b3
y3
b4
y4
y7
b5
y8
b6

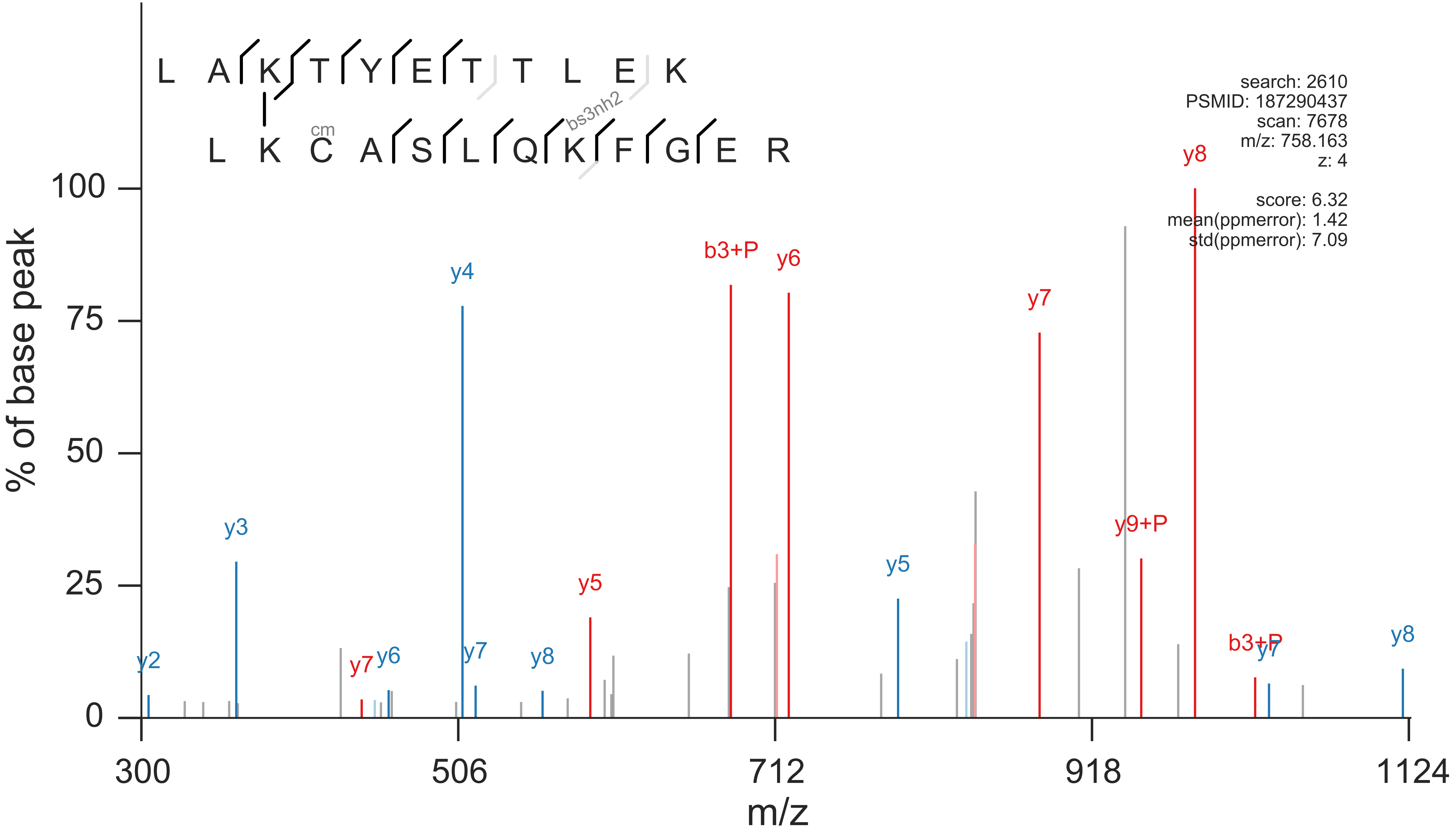

L A K T Y E T T L E K
L K C A S L Q K F G E R
cm
bs3nh2
search: 2610
PSMID: 187290437
scan: 7678
m/z: 758.163
z: 4
score: 6.32
mean(ppmerror): 1.42
std(ppmerror): 7.09
% of base peak
m/z
0
25
50
75
100
300
506
712
918
1124
y2
y3
y7
y6
y4
y7
y8
y5
b3+P
y6
y5
y7
y9+P
y8
b3+P
y7
y8

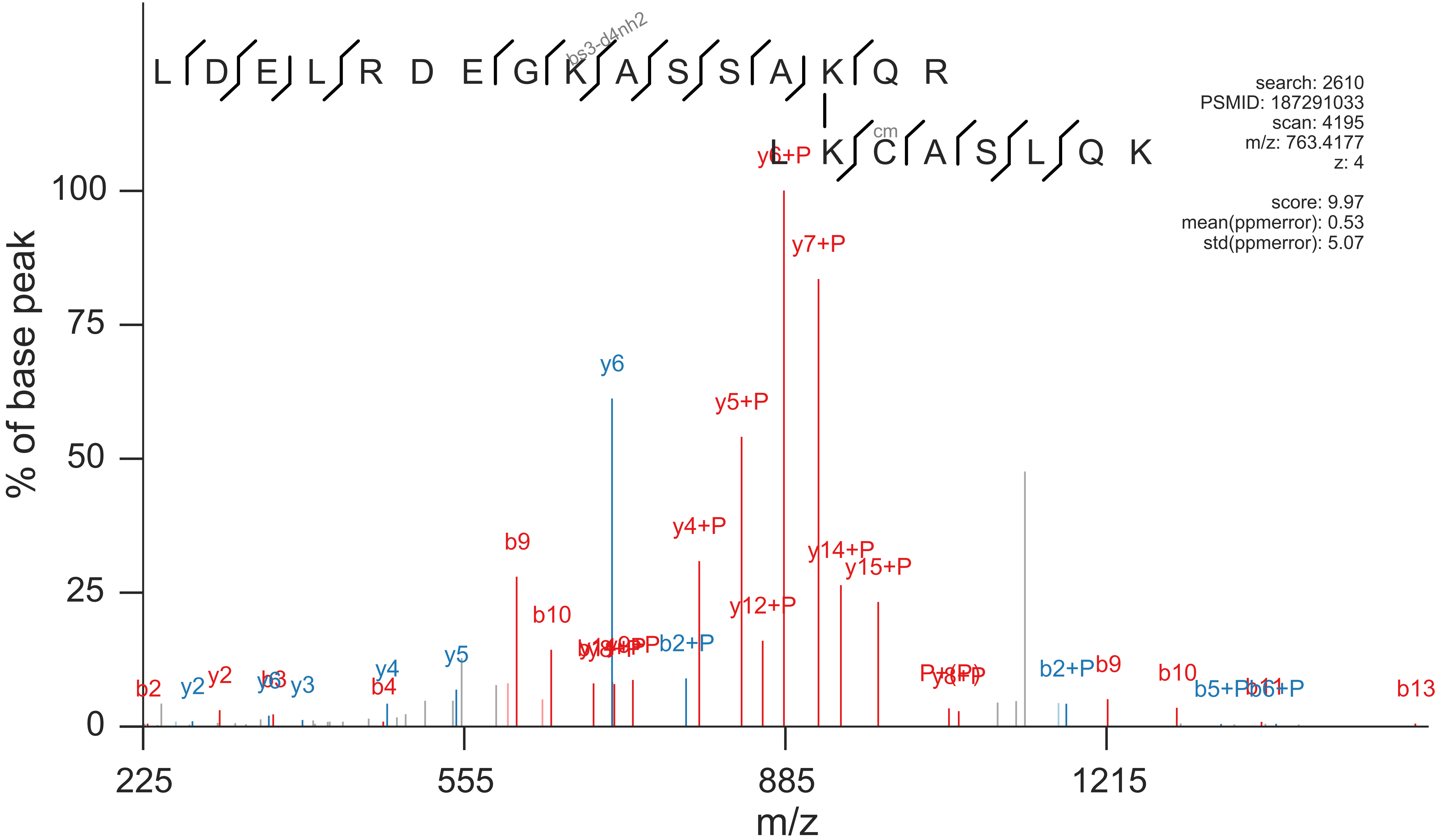

L D E L R D E G K A S S A K Q R
bs3-d4nh2
L K C A S L Q K
cm
search: 2610
PSMID: 187291033
scan: 4195
m/z: 763.4177
z: 4
score: 9.97
mean(ppmerror): 0.53
std(ppmerror): 5.07
% of base peak
m/z
100
75
50
25
0
225
555
885
1215
y6+P
y7+P
y6
y5+P
y4+P
b9
y14+P
y15+P
y12+P
b10
b2+P
y5
y4
b4
b2
y2
y2
y3
b2+P
b9
b10
b13

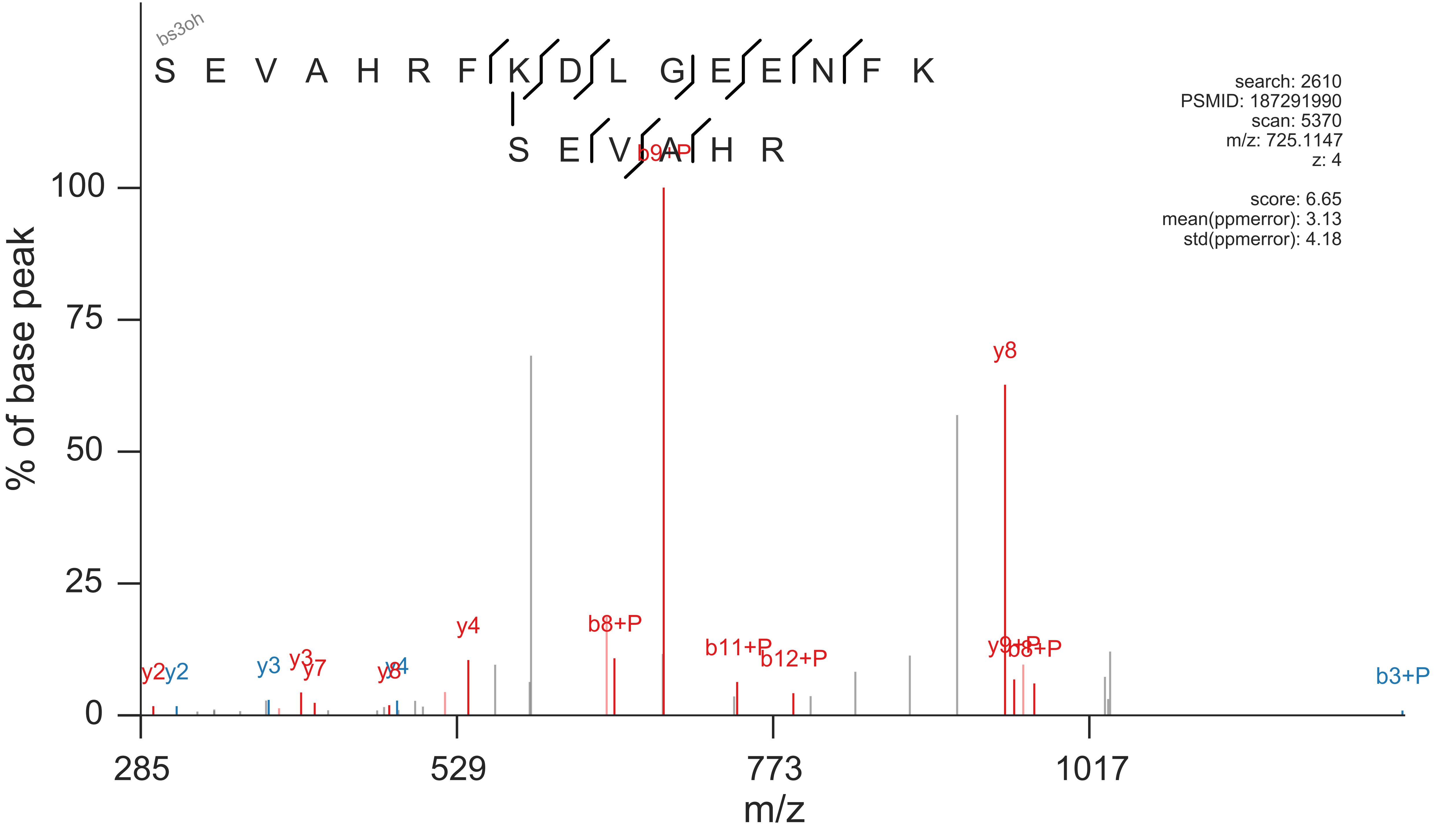

bs3oh
S E V A H R F K D L G E E N F K
S E V A H R
search: 2610
PSMID: 187291990
scan: 5370
m/z: 725.1147
z: 4
score: 6.65
mean(ppmerror): 3.13
std(ppmerror): 4.18
% of base peak
m/z
b9+P
y8
y4
b8+P
b11+P
b12+P
y9+P
b8+P
y2
y2
y3
y3
y7
y8
y4
b3+P
0
25
50
75
100
285
529
773
1017

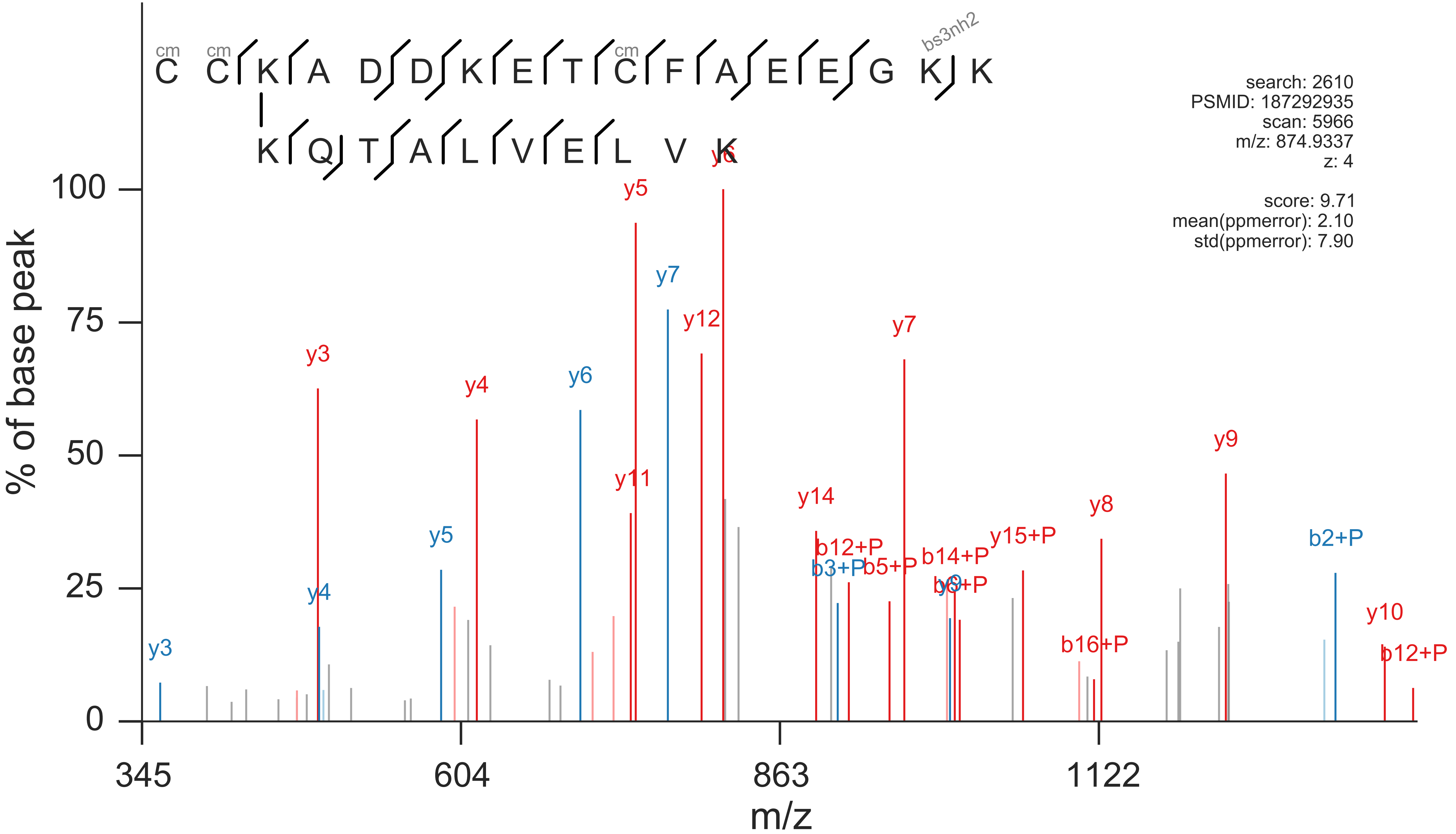
C C K A D D K E T C F A E E G K K
cm cm cm bs3nh2
K Q T A L V E L V K
search: 2610
PSMID: 187292935
scan: 5966
m/z: 874.9337
z: 4
score: 9.71
mean(ppmerror): 2.10
std(ppmerror): 7.90
% of base peak
m/z
0
25
50
75
100
345
604
863
1122
y3
y4
y5
y6
y7
y8
y9
y10
y11
y12
y14
y15+P
b2+P
b3+P
b5+P
b12+P
b14+P
b16+P

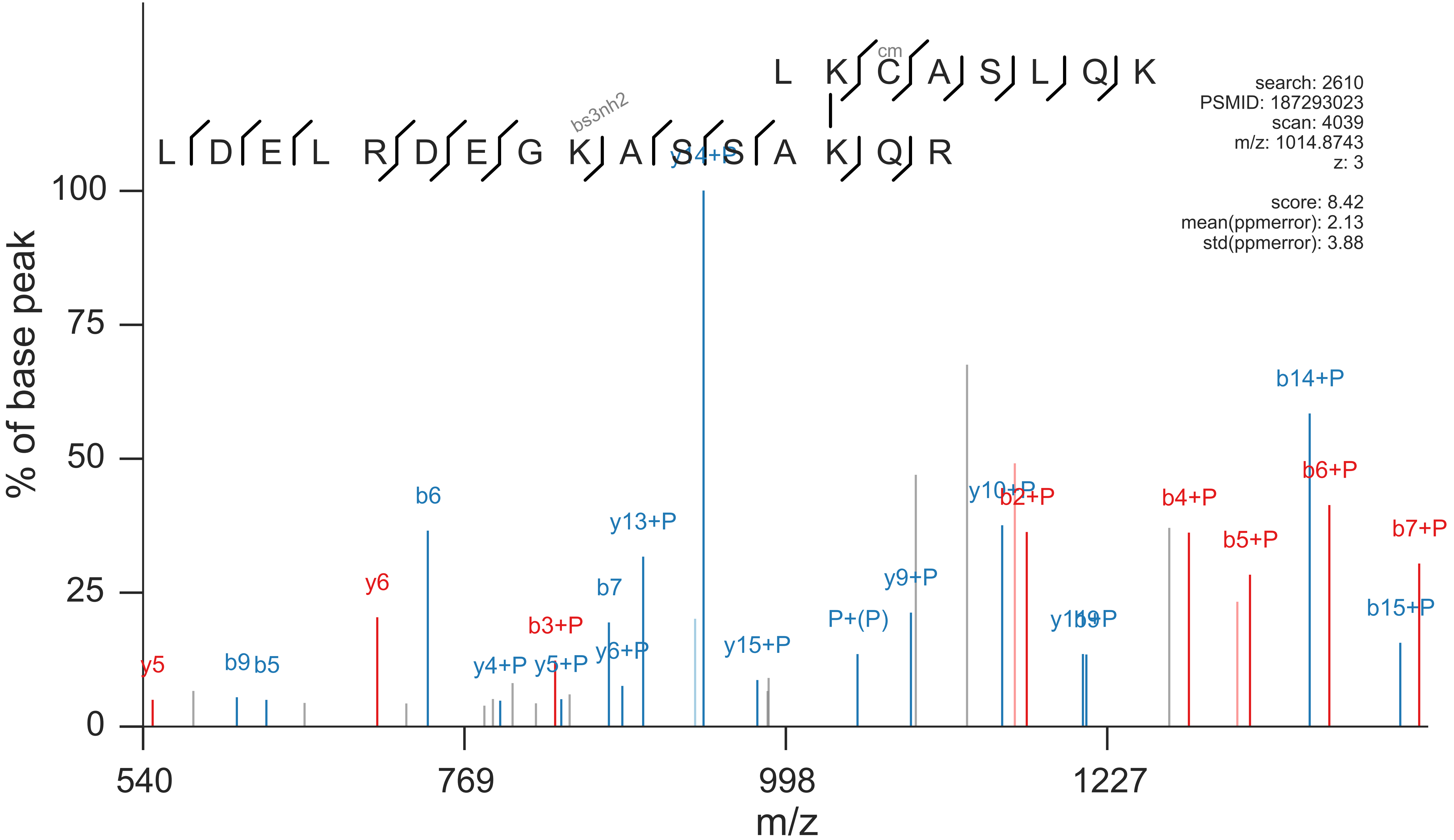

L K C A S L Q K
cm
L D E L R D E G K A S S A K Q R
bs3nh2
search: 2610
PSMID: 187293023
scan: 4039
m/z: 1014.8743
z: 3
score: 8.42
mean(ppmerror): 2.13
std(ppmerror): 3.88
% of base peak
100
75
50
25
0
540
769
998
1227
m/z
y5
b9
b5
y6
b6
y4+P
b3+P
y5+P
b7
y6+P
y13+P
y14+P
y15+P
P+(P)
y9+P
y10+P
b2+P
b4+P
b5+P
b14+P
b6+P
b7+P
b15+P

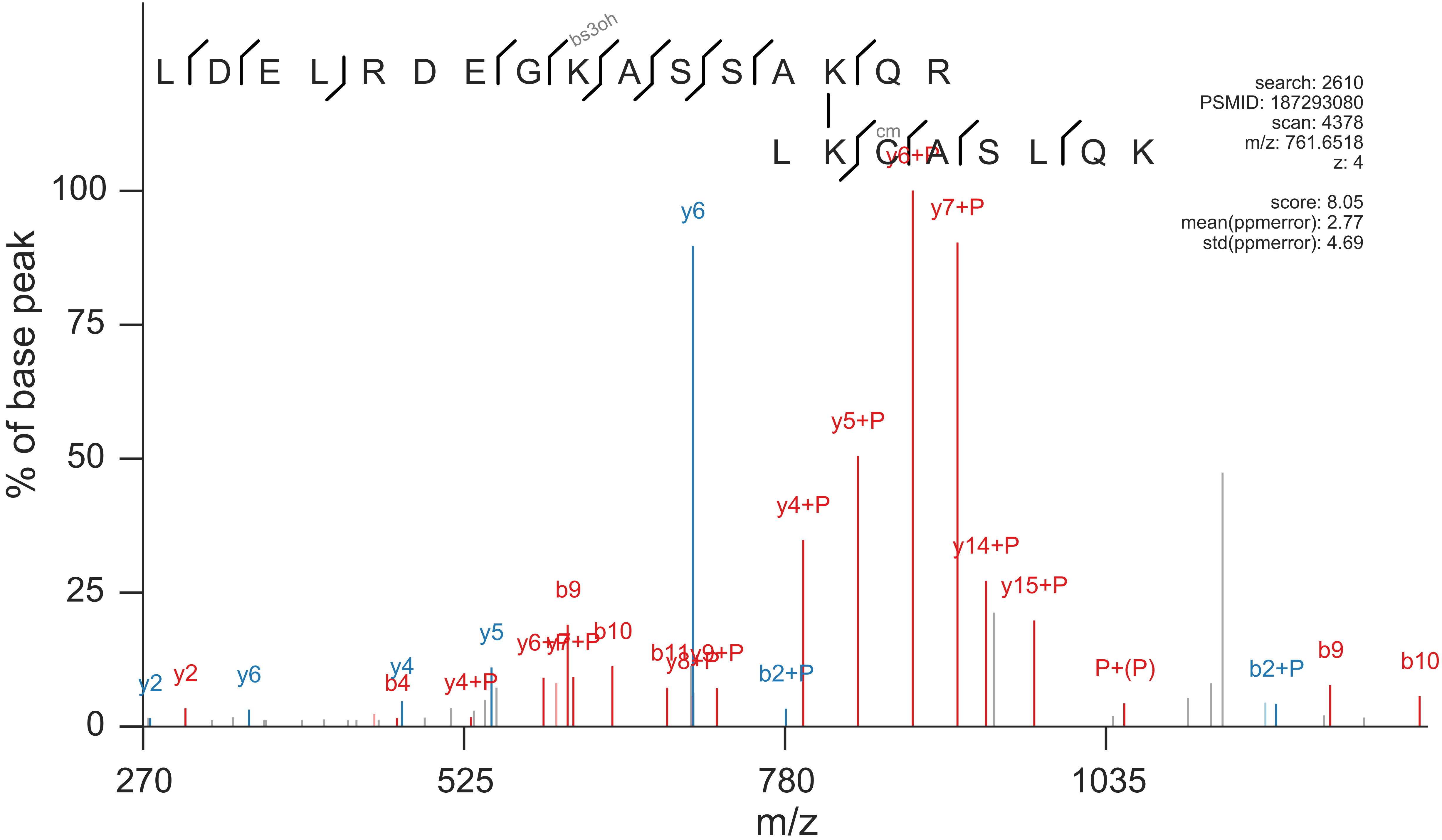

L D E L R D E G K A S S A K Q R
bs3oh
L K C A S L Q K
cm
search: 2610
PSMID: 187293080
scan: 4378
m/z: 761.6518
z: 4
score: 8.05
mean(ppmerror): 2.77
std(ppmerror): 4.69
% of base peak
m/z
0
25
50
75
100
270
525
780
1035
y6
y7+P
y5+P
y4+P
y14+P
y15+P
b9
b10
y5
y4
b4
y4+P
y2
y2
y6
b2+P
P+(P)
b2+P
b9
b10

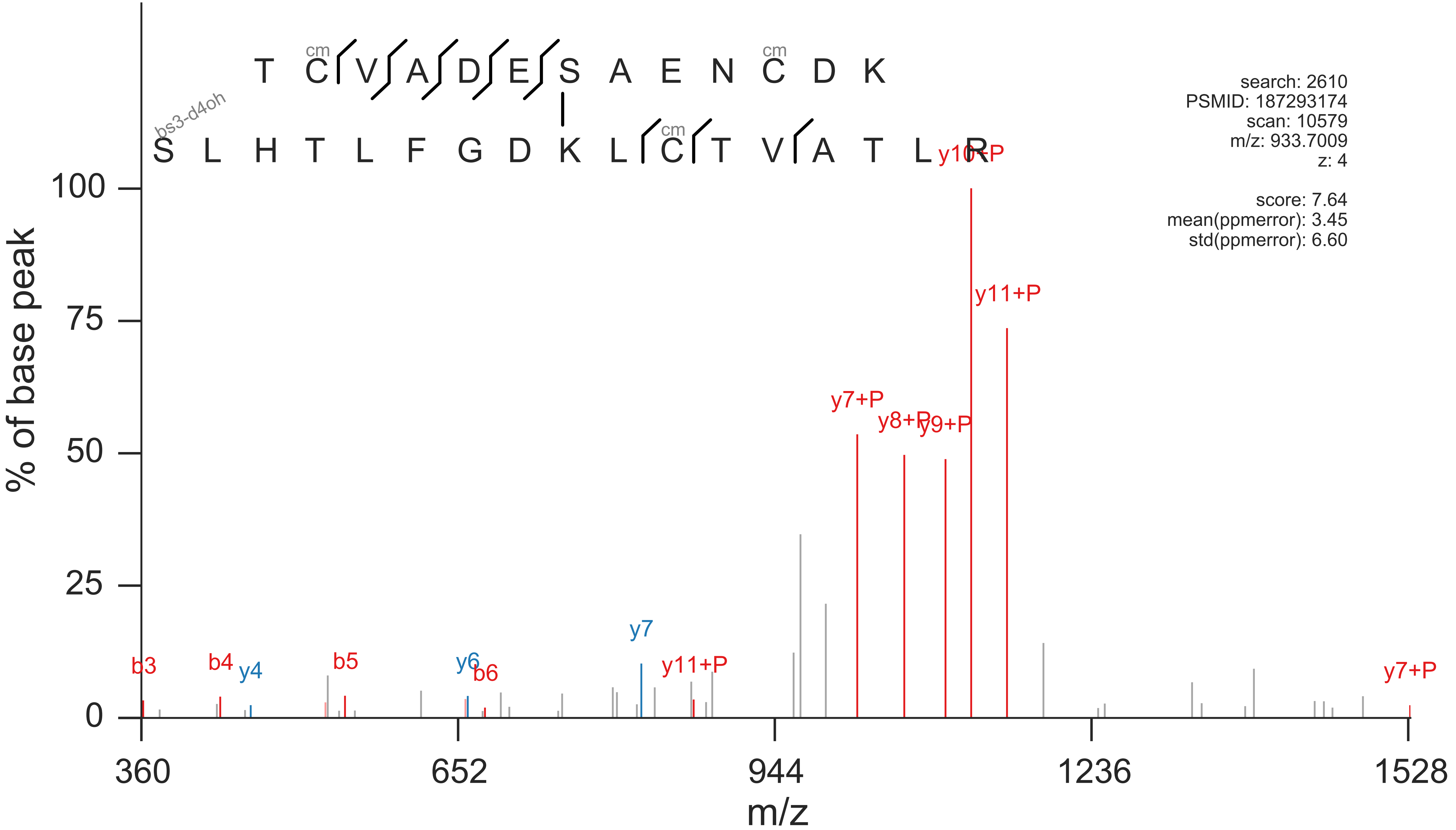

T C V A D E S A E N C D K
cm
bs3-d4oh
S L H T L F G D K L C T V A T L R
search: 2610
PSMID: 187293174
scan: 10579
m/z: 933.7009
z: 4
score: 7.64
mean(ppmerror): 3.45
std(ppmerror): 6.60
% of base peak
m/z
0
25
50
75
100
360
652
944
1236
1528
b3
b4
y4
b5
y6
b6
y7
y11+P
y7+P
y8+P
y9+P
y10+P
y11+P
y7+P

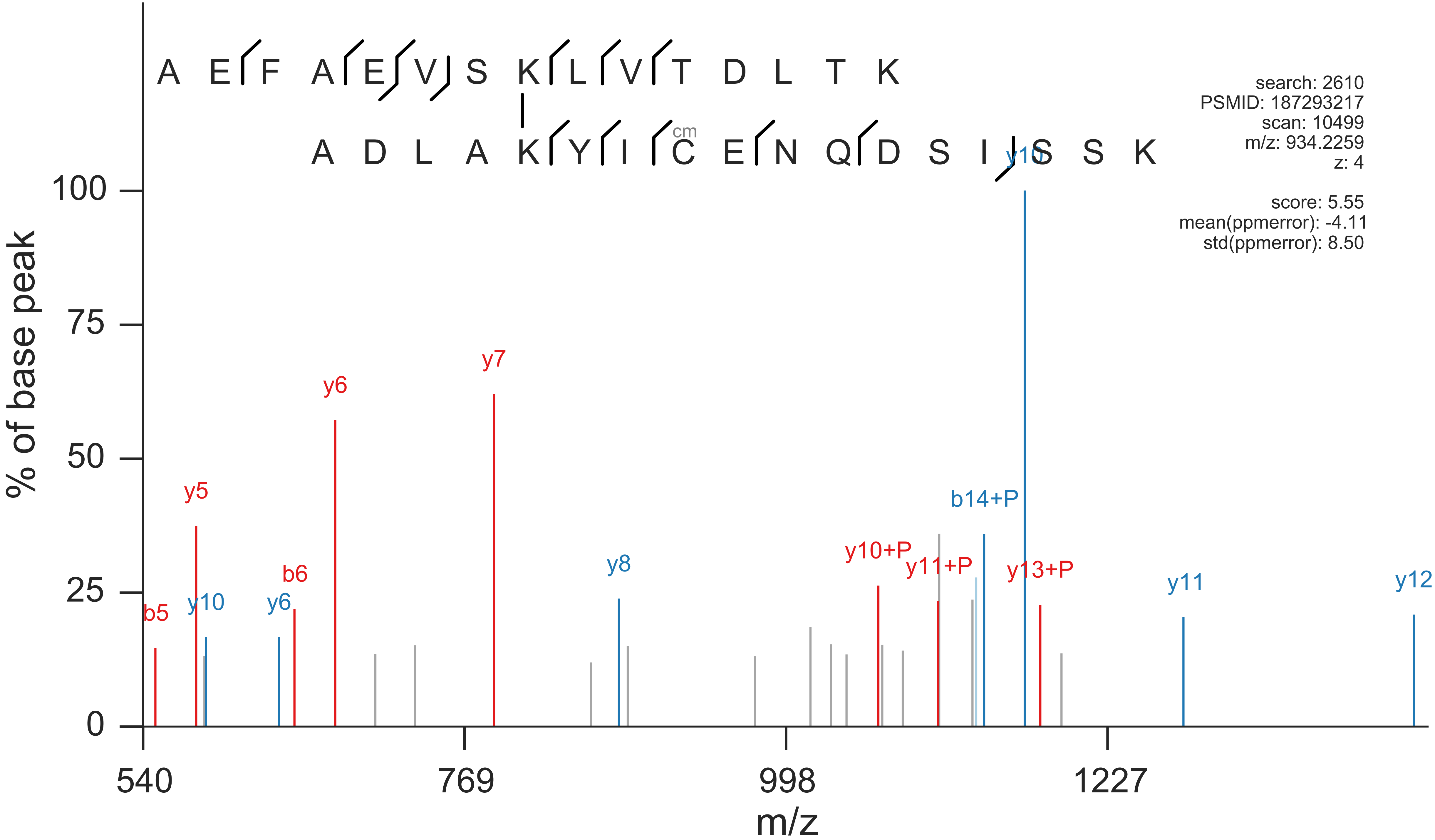

A E F A E V S K L V T D L T K
A D L A K Y I C E N Q D S I S S K
cm
search: 2610
PSMID: 187293217
scan: 10499
m/z: 934.2259
z: 4
score: 5.55
mean(ppmerror): -4.11
std(ppmerror): 8.50
% of base peak
m/z
0
25
50
75
100
540
769
998
1227
b5
y5
y10
y6
b6
y6
y7
y8
y10+P
y11+P
b14+P
y10
y13+P
y11
y12

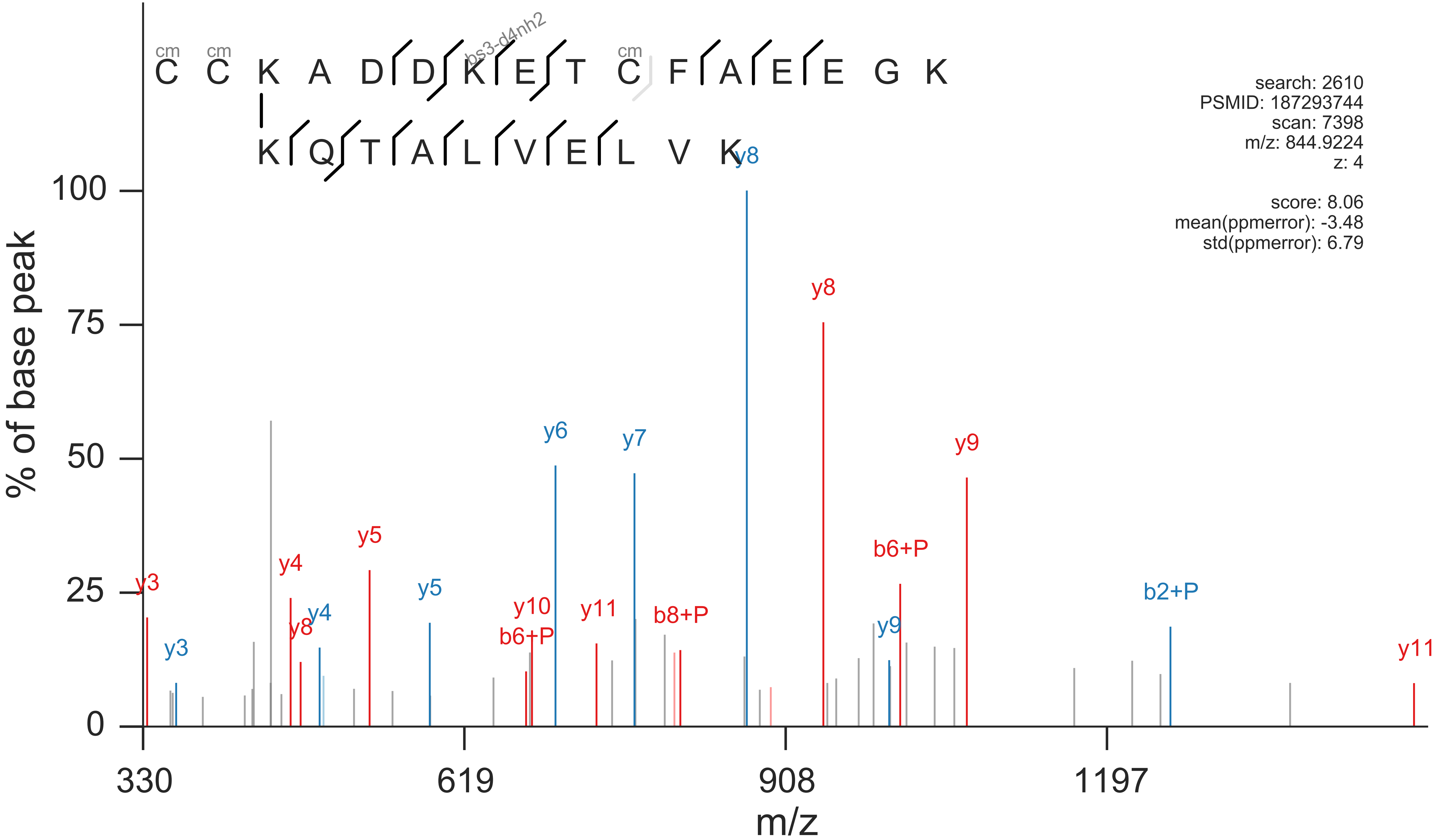

cm cm cm
C C K A D D K E T C F A E E G K
bs3-d4nh2
K Q T A L V E L V K
search: 2610
PSMID: 187293744
scan: 7398
m/z: 844.9224
z: 4
score: 8.06
mean(ppmerror): -3.48
std(ppmerror): 6.79
% of base peak
m/z
0
25
50
75
100
330
619
908
1197
y3
y3
y4
y8
y4
y5
y5
y10
b6+P
y6
y11
y7
b8+P
y8
y8
y9
b6+P
y9
b2+P
y11

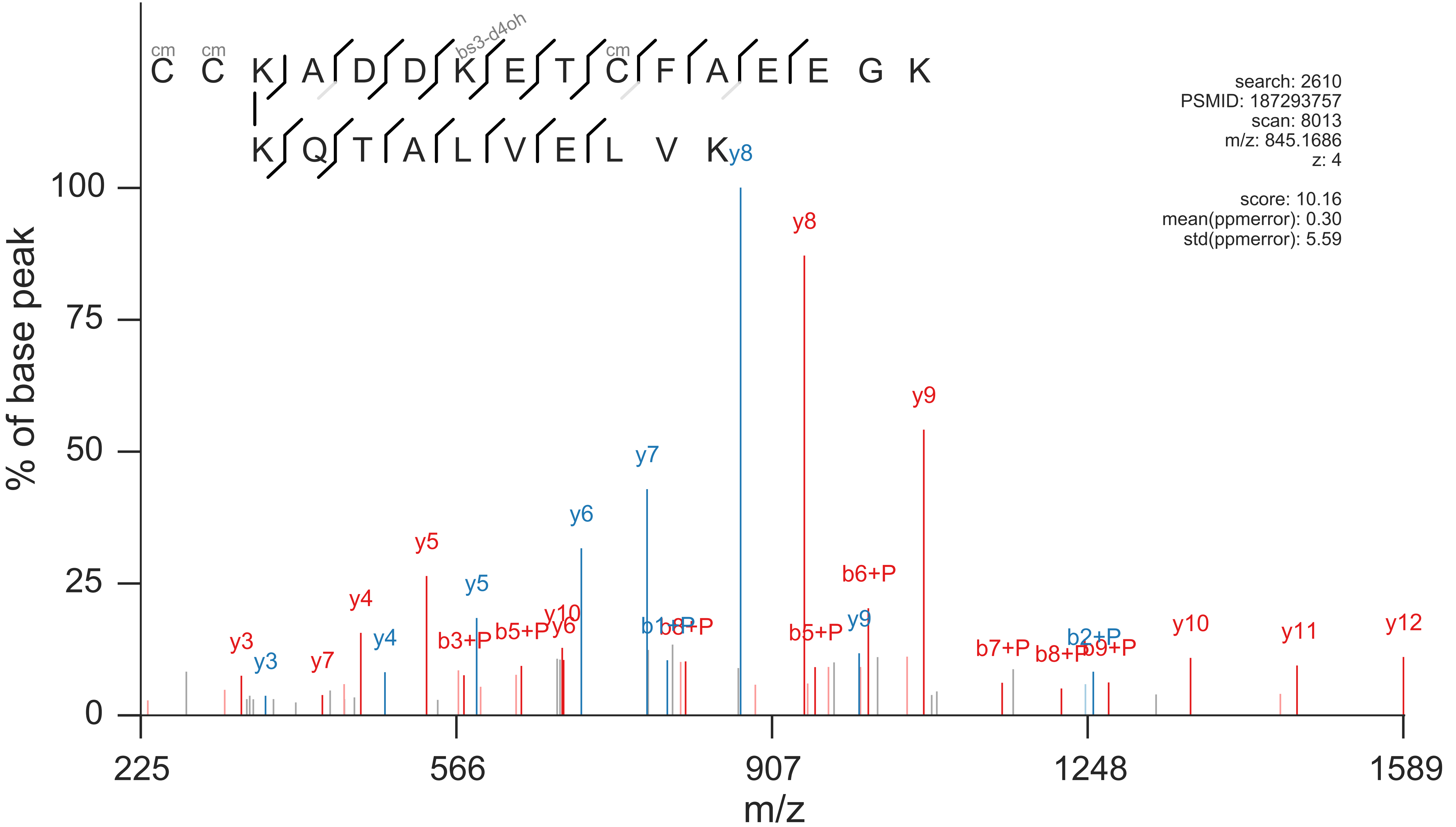

C C K A D D K E T C F A E E G K
K Q T A L V E L V K
cm cm cm
bs3-d4oh
search: 2610
PSMID: 187293757
scan: 8013
m/z: 845.1686
z: 4
score: 10.16
mean(ppmerror): 0.30
std(ppmerror): 5.59
% of base peak
m/z
100
75
50
25
0
225
566
907
1248
1589
y8
y9
y7
y6
y5
y4
y3
y10
y11
y12
b3+P
b5+P
b6+P
b7+P
b8+P
b9+P
b2+P

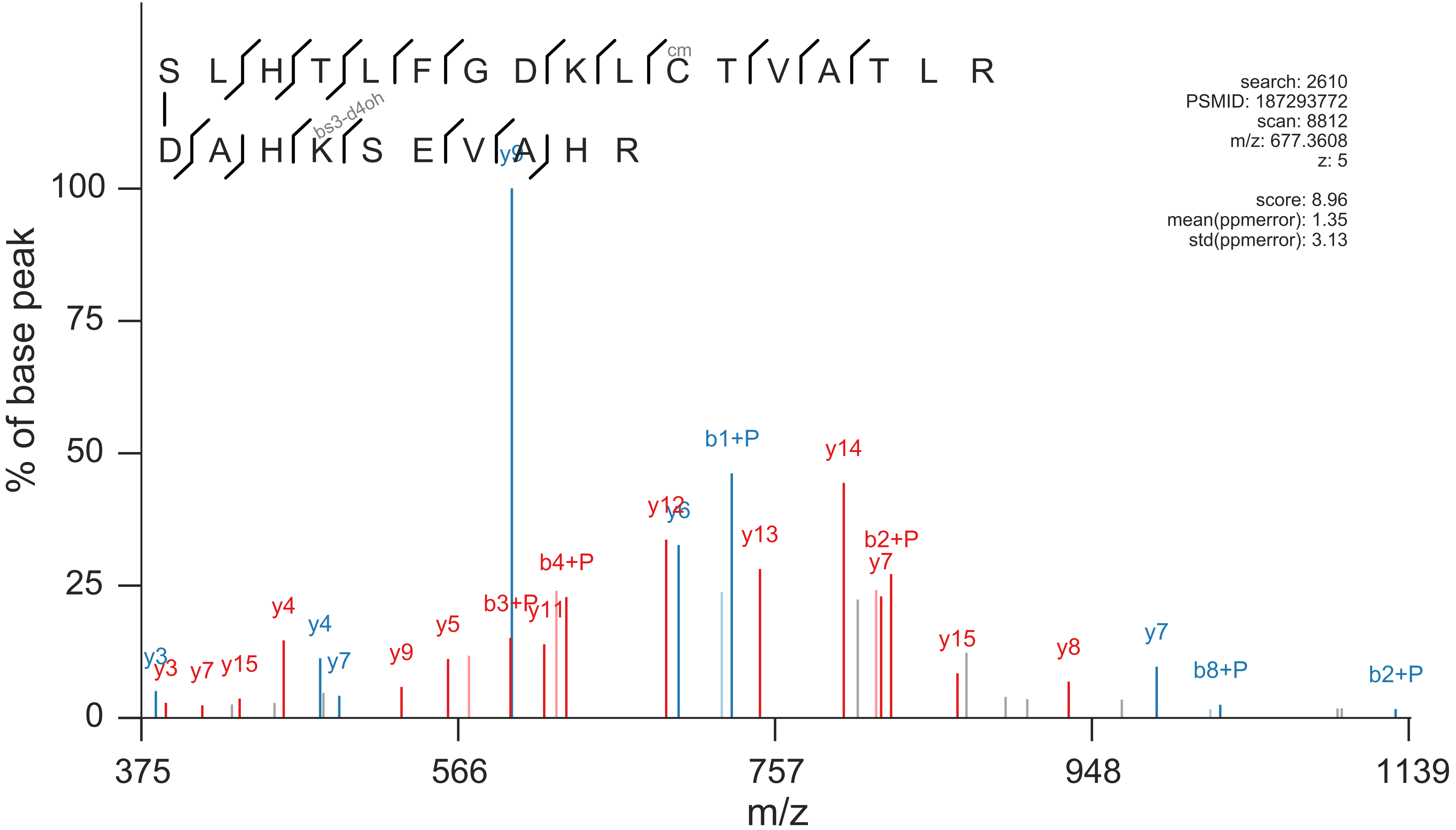

S L H T L F G D K L C T V A T L R
cm
D A H K S E V A H R
bs3-d4oh
search: 2610
PSMID: 187293772
scan: 8812
m/z: 677.3608
z: 5
score: 8.96
mean(ppmerror): 1.35
std(ppmerror): 3.13
% of base peak
m/z
0
25
50
75
100
375
566
757
948
1139
y9
b1+P
y14
y12
y6
y13
b2+P
y7
b4+P
b3+P
y11
y4
y4
y5
y15
y8
y7
b8+P
b2+P
y3
y3
y7
y15
y7
y9

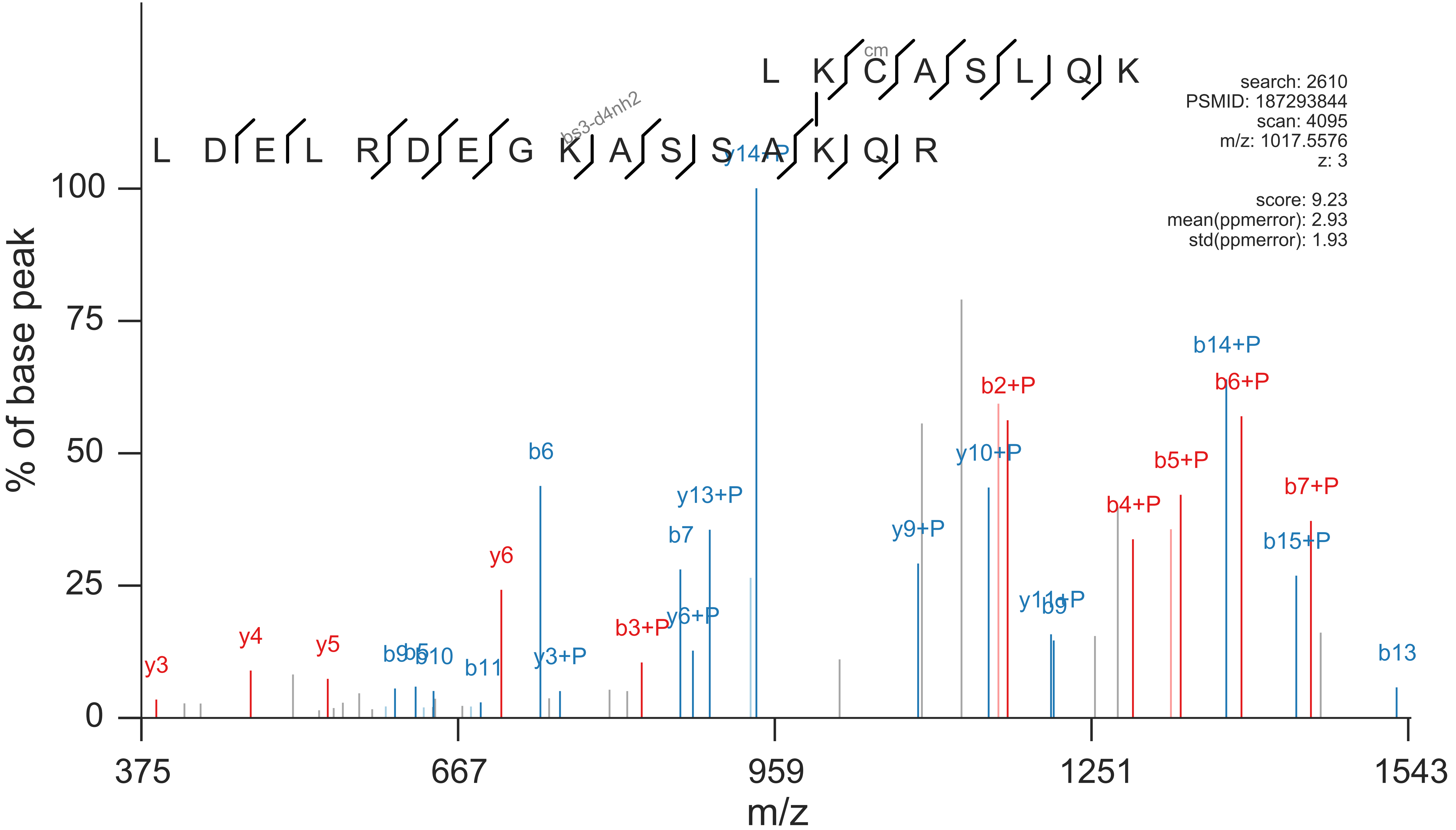
L K C A S L Q K
cm
L D E L R D E G K A S S A K Q R
bs3-d4nh2
search: 2610
PSMID: 187293844
scan: 4095
m/z: 1017.5576
z: 3
score: 9.23
mean(ppmerror): 2.93
std(ppmerror): 1.93
% of base peak
m/z
y14+P
b14+P
b2+P
b6+P
b6
y10+P
b5+P
y13+P
b4+P
b7+P
b7
y9+P
b15+P
y6
y11+P
b9
y6+P
b3+P
y4
y5
b13
b9
b5
b10
y3+P
y3
b11

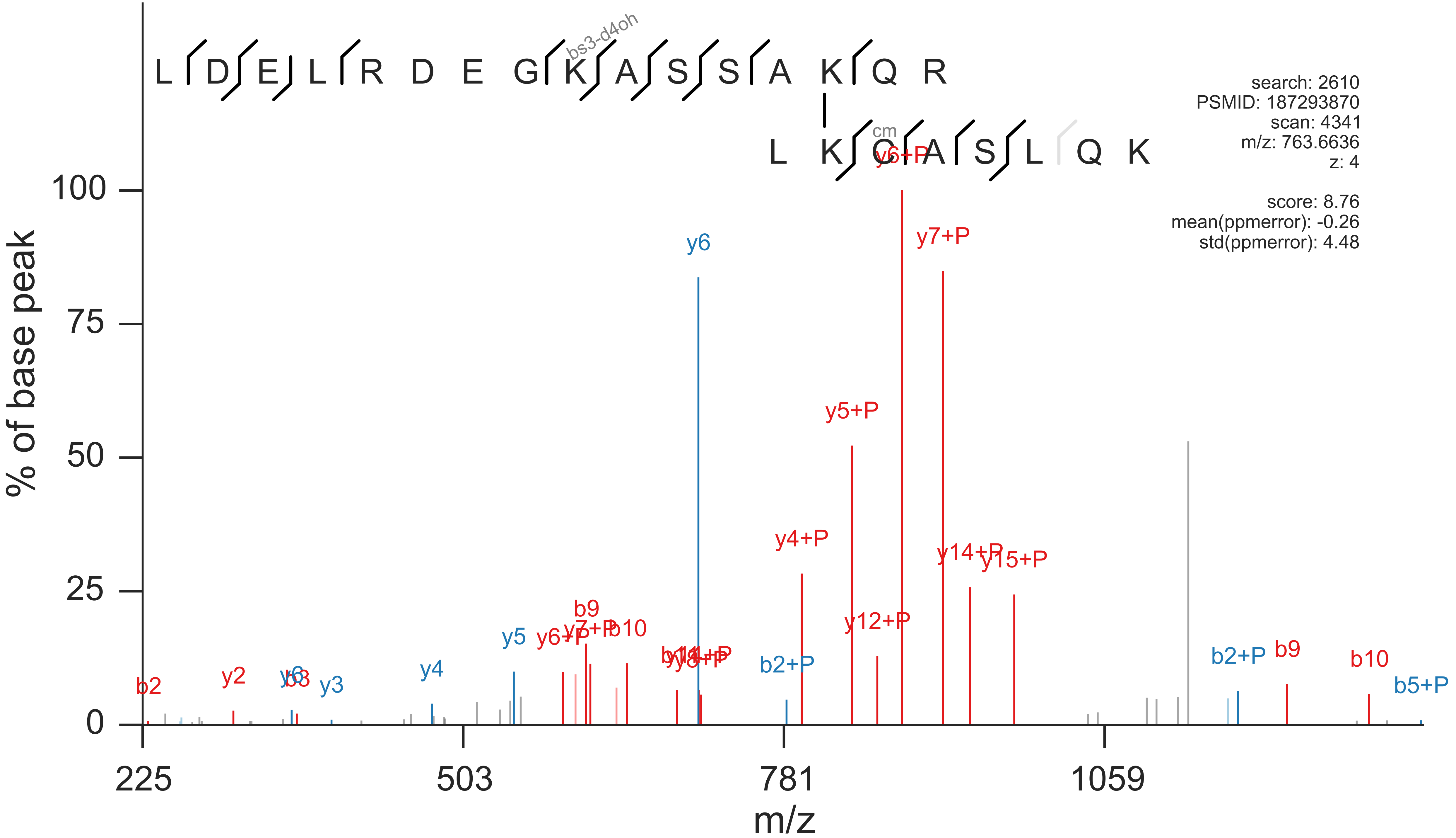

search: 2610
PSMID: 187293870
scan: 4341
m/z: 763.6636
z: 4
score: 8.76
mean(ppmerror): -0.26
std(ppmerror): 4.48
bs3-d4oh
cm
% of base peak
m/z

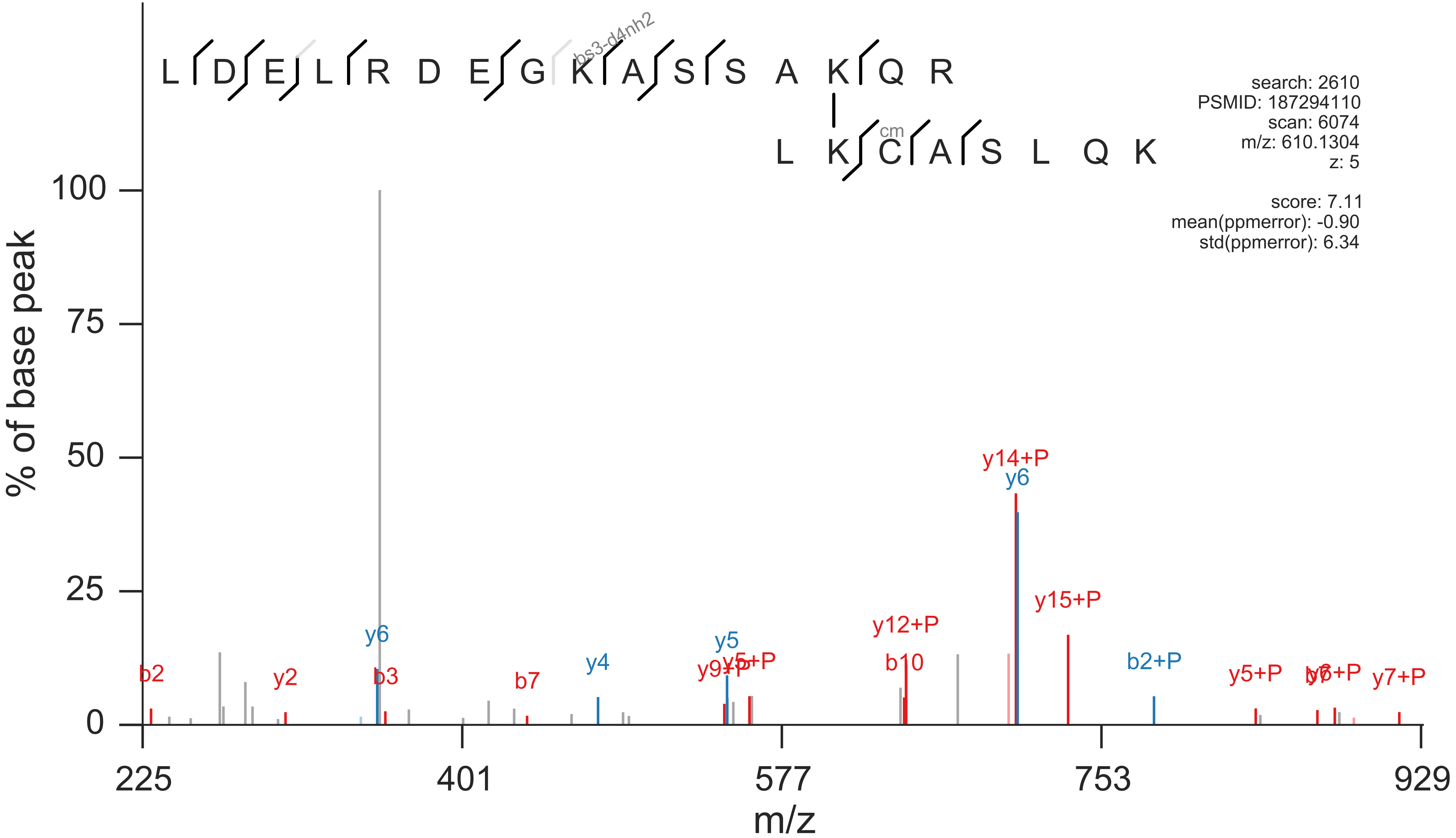

L D E L R D E G K A S S A K Q R
bs3-d4nh2
L K C A S L Q K
cm
search: 2610
PSMID: 187294110
scan: 6074
m/z: 610.1304
z: 5
score: 7.11
mean(ppmerror): -0.90
std(ppmerror): 6.34
% of base peak
m/z
y14+P
y6
y15+P
y12+P
b10
b2+P
y5+P
y7+P
y6
y5
y4
y2
b2
b3
b7

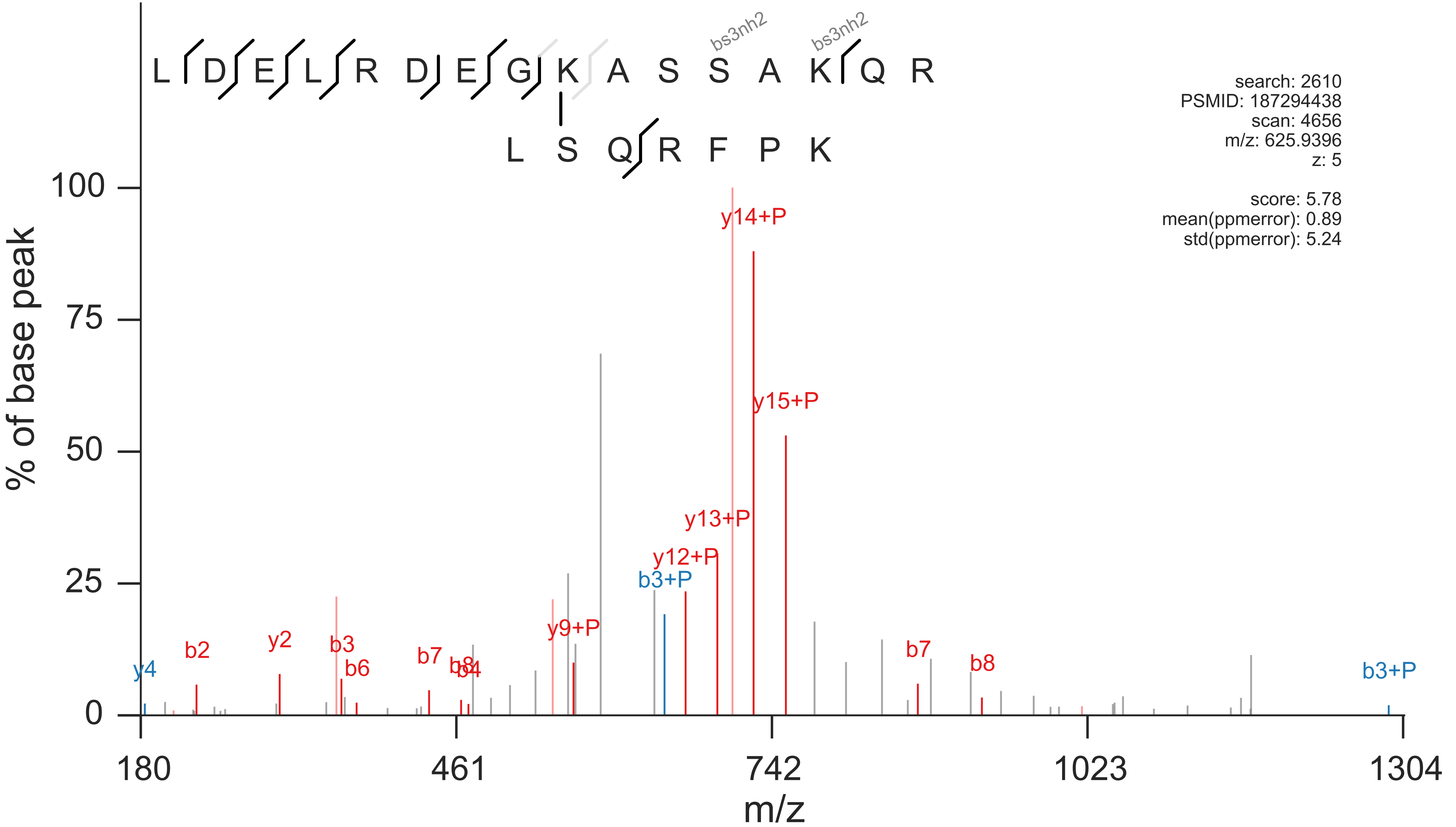

L D E L R D E G K A S S A K Q R
bs3nh2
bs3nh2
L S Q R F P K
search: 2610
PSMID: 187294438
scan: 4656
m/z: 625.9396
z: 5
score: 5.78
mean(ppmerror): 0.89
std(ppmerror): 5.24
% of base peak
0
25
50
75
100
m/z
180
461
742
1023
1304
y14+P
y15+P
y13+P
y12+P
b3+P
y9+P
y4
b2
y2
b3
b6
b7
b8
b4
b7
b8
b3+P

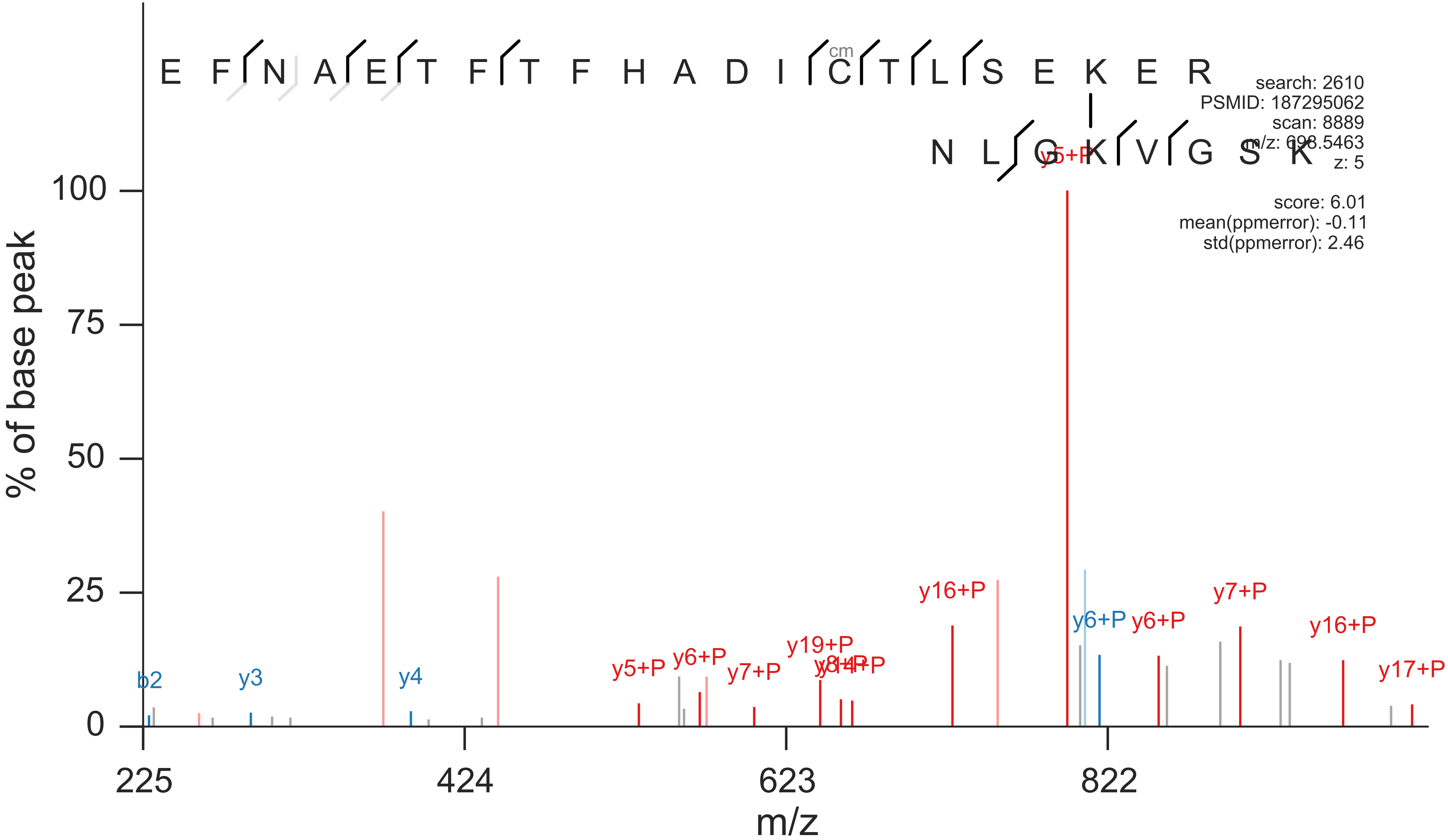

E F N A E T F T F H A D I C T L S E K E R
cm
N L G K V G S K
search: 2610
PSMID: 187295062
scan: 8889
m/z: 698.5463
z: 5
score: 6.01
mean(ppmerror): -0.11
std(ppmerror): 2.46
% of base peak
m/z
225
424
623
822
0
25
50
75
100
b2
y3
y4
y5+P
y6+P
y7+P
y19+P
y8+P
y16+P
y5+P
y6+P
y6+P
y7+P
y16+P
y17+P

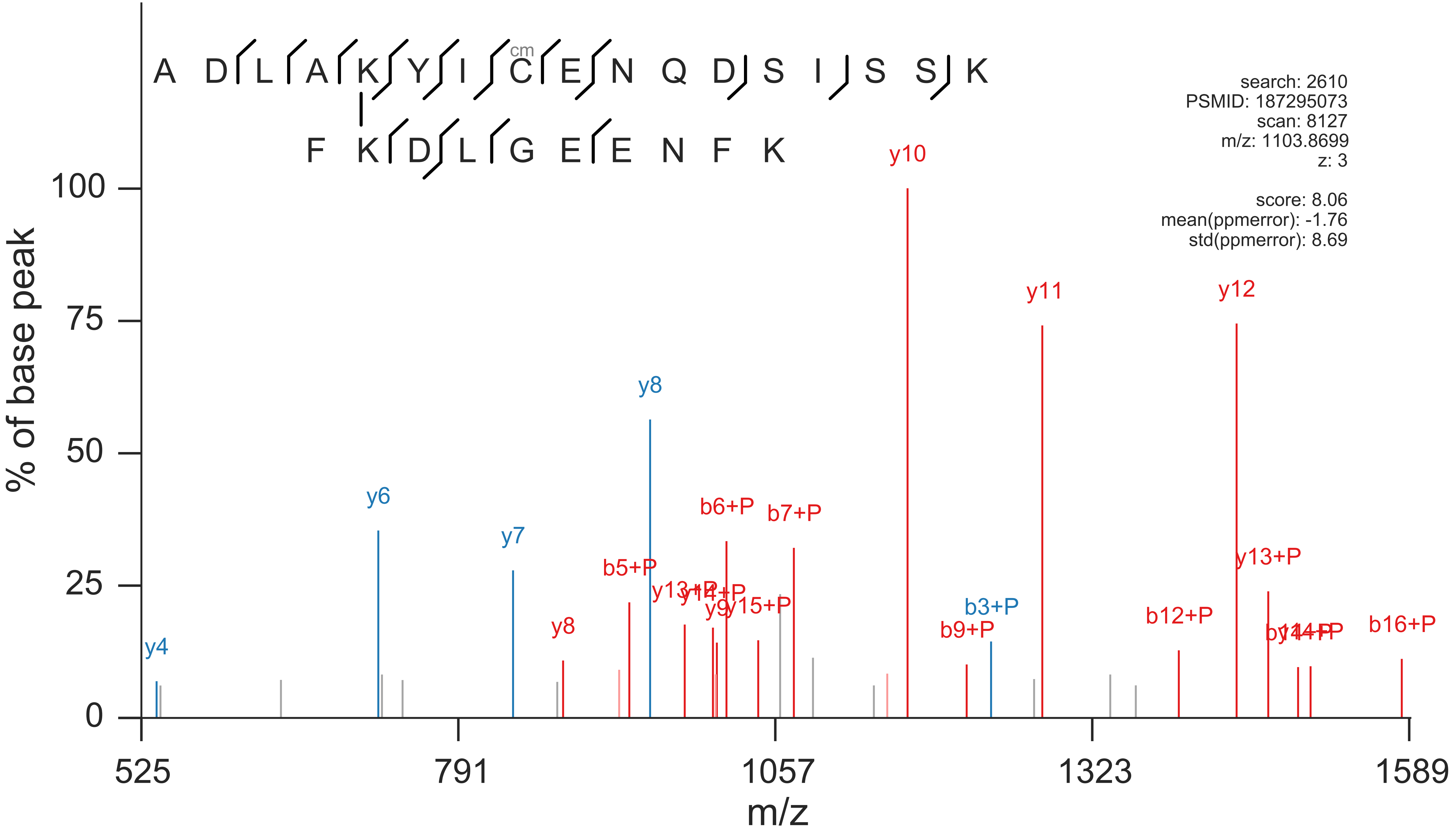

A D L A K Y I C E N Q D S I S S K
cm
F K D L G E E N F K
search: 2610
PSMID: 187295073
scan: 8127
m/z: 1103.8699
z: 3
score: 8.06
mean(ppmerror): -1.76
std(ppmerror): 8.69
% of base peak
m/z
0
25
50
75
100
525
791
1057
1323
1589
y4
y6
y7
y8
y8
b5+P
b6+P
b7+P
y10
y11
y12
b3+P
b9+P
b12+P
y13+P
b16+P

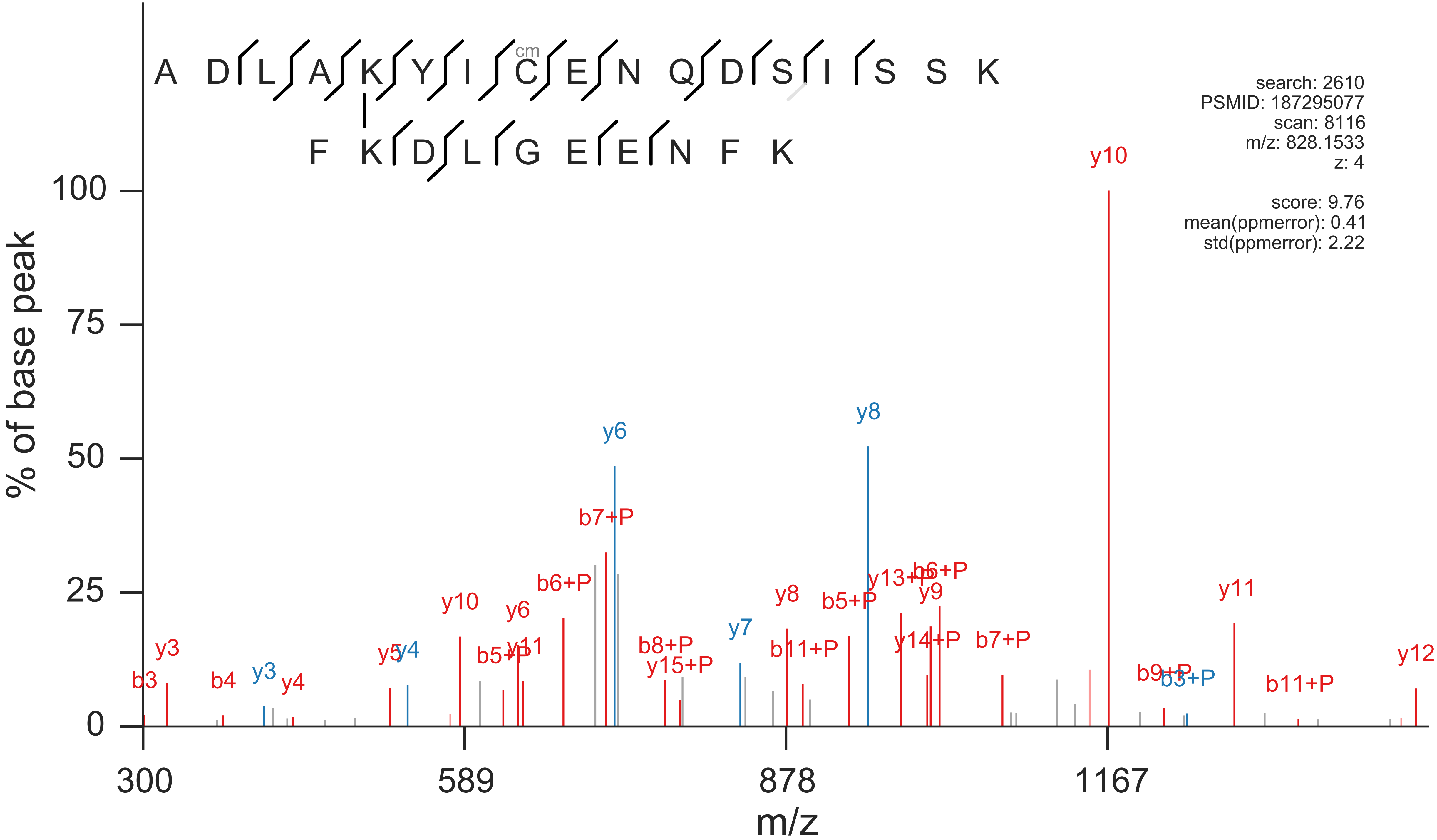

A D L A K Y I C E N Q D S I S S K
cm
F K D L G E E N F K
search: 2610
PSMID: 187295077
scan: 8116
m/z: 828.1533
z: 4
score: 9.76
mean(ppmerror): 0.41
std(ppmerror): 2.22
% of base peak
m/z

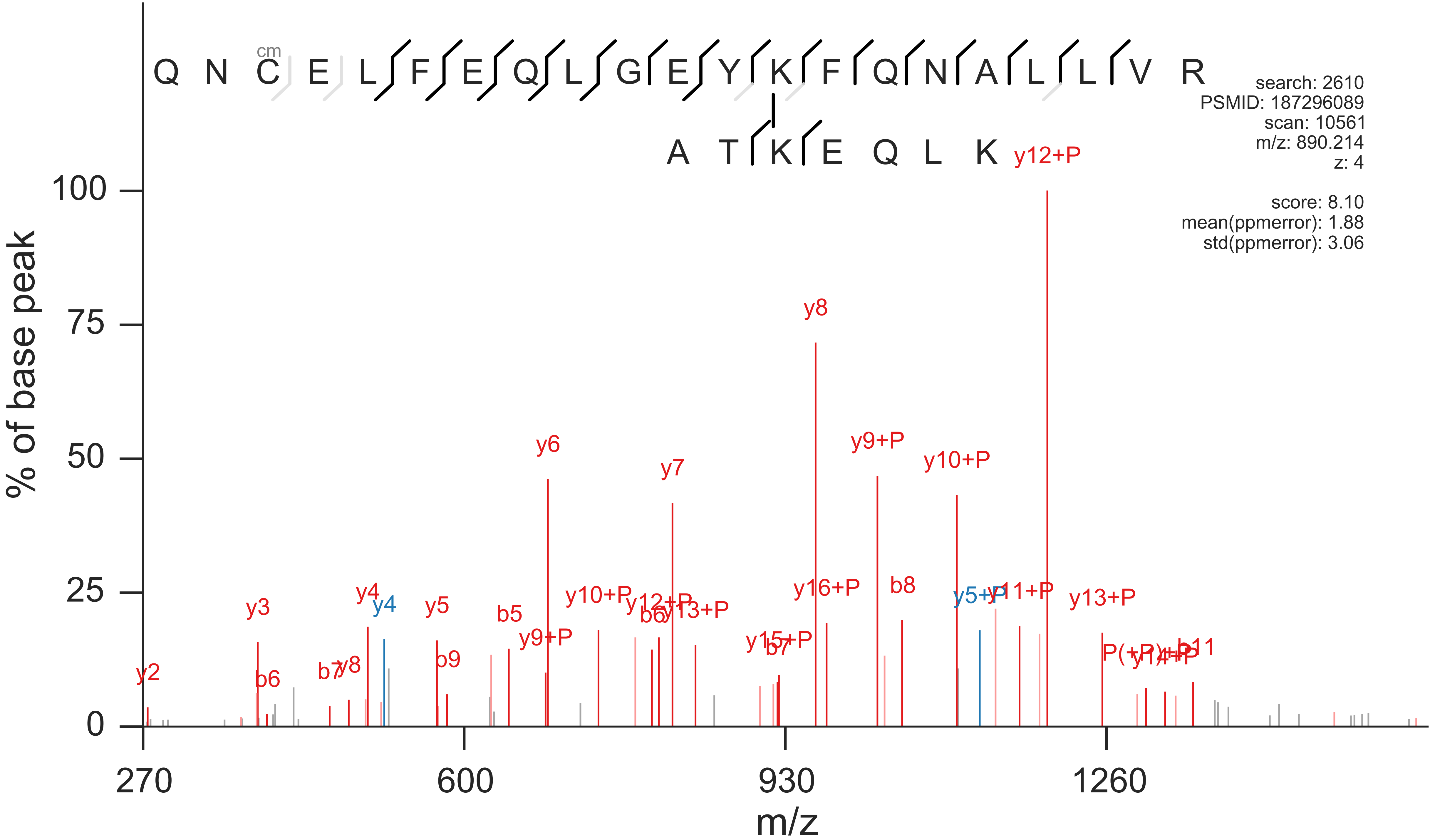

Q N C E L F E Q L G E Y K F Q N A L L V R
cm
A T K E Q L K
search: 2610
PSMID: 187296089
scan: 10561
m/z: 890.214
z: 4
score: 8.10
mean(ppmerror): 1.88
std(ppmerror): 3.06
% of base peak
m/z
y12+P
y8
y6
y7
y9+P
y10+P
y13+P
y16+P
b8
y5+P
y11+P
y4
y3
y5
b5
y2
b6
b7
y8
b9
y9+P
y10+P
y12+P
b6
y13+P
y15+P
b7
P(+P)
y14+P
b11
0
25
50
75
100
270
600
930
1260

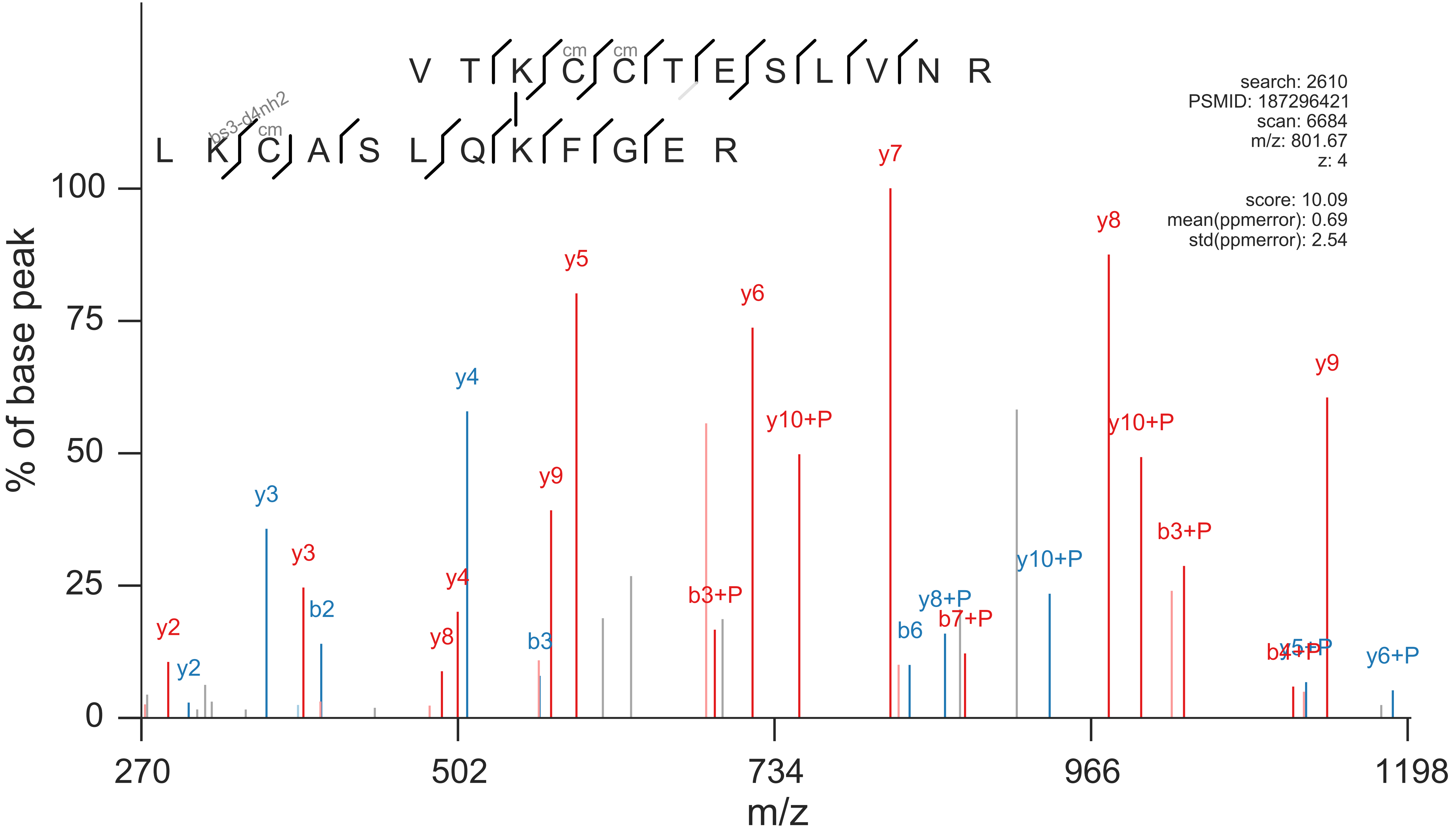
V T K C C T E S L V N R
cm cm
L K C A S L Q K F G E R
bs3-d4nh2
cm
search: 2610
PSMID: 187296421
scan: 6684
m/z: 801.67
z: 4
score: 10.09
mean(ppmerror): 0.69
std(ppmerror): 2.54
% of base peak
m/z
0
25
50
75
100
270
502
734
966
1198
y2
y2
y3
y3
b2
y8
y4
y4
b3
y9
y5
b3+P
y6
y10+P
y7
b6
y8+P
b7+P
y10+P
y8
y10+P
b3+P
y9
b4+P
y5+P
y6+P

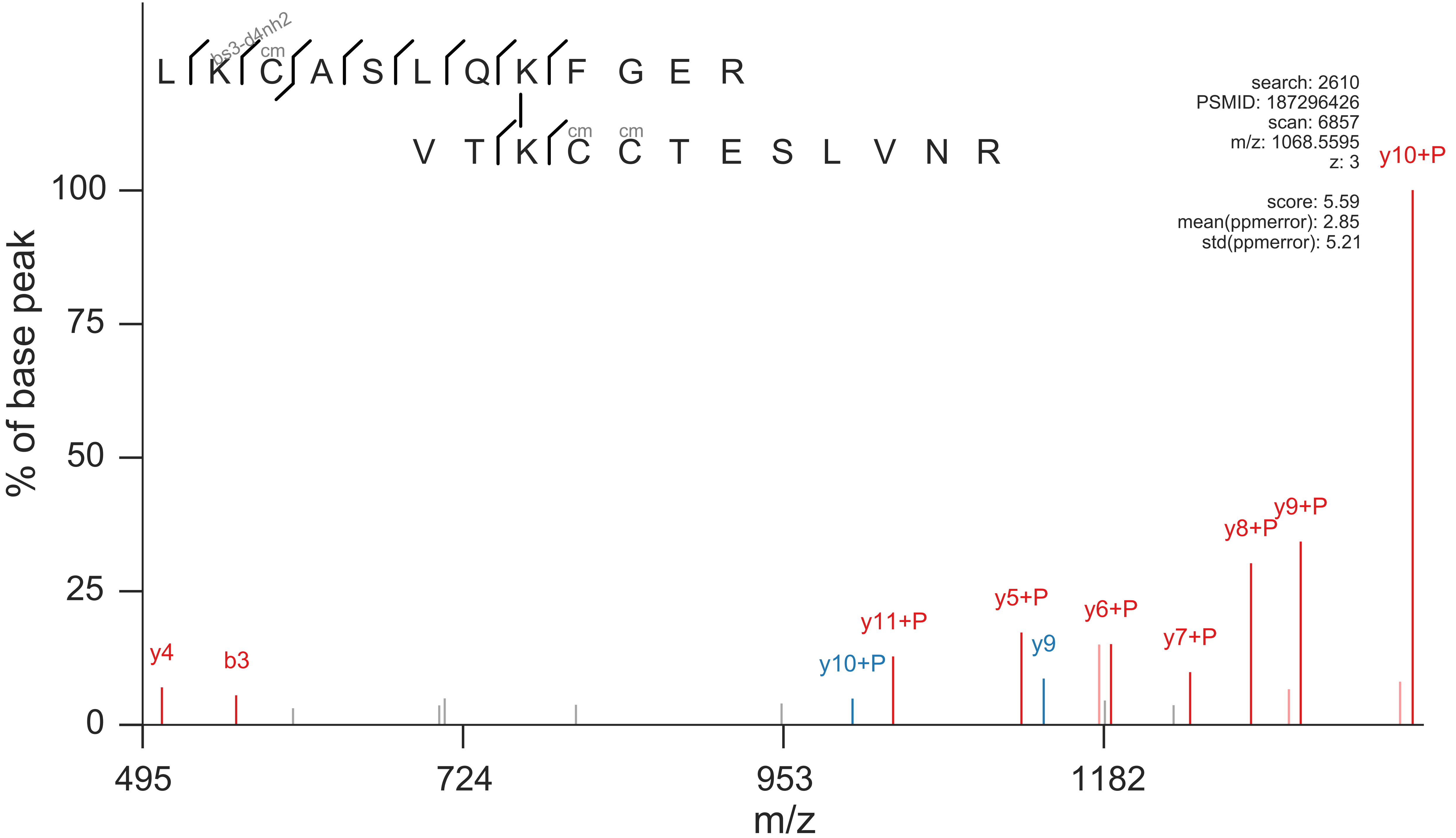

bs3-d4nh2
cm
L K C A S L Q K F G E R
cm cm
V T K C C T E S L V N R
search: 2610
PSMID: 187296426
scan: 6857
m/z: 1068.5595
z: 3
score: 5.59
mean(ppmerror): 2.85
std(ppmerror): 5.21
% of base peak
100
75
50
25
0
495
724
953
1182
m/z
y4
b3
y10+P
y11+P
y5+P
y9
y6+P
y7+P
y8+P
y9+P
y10+P

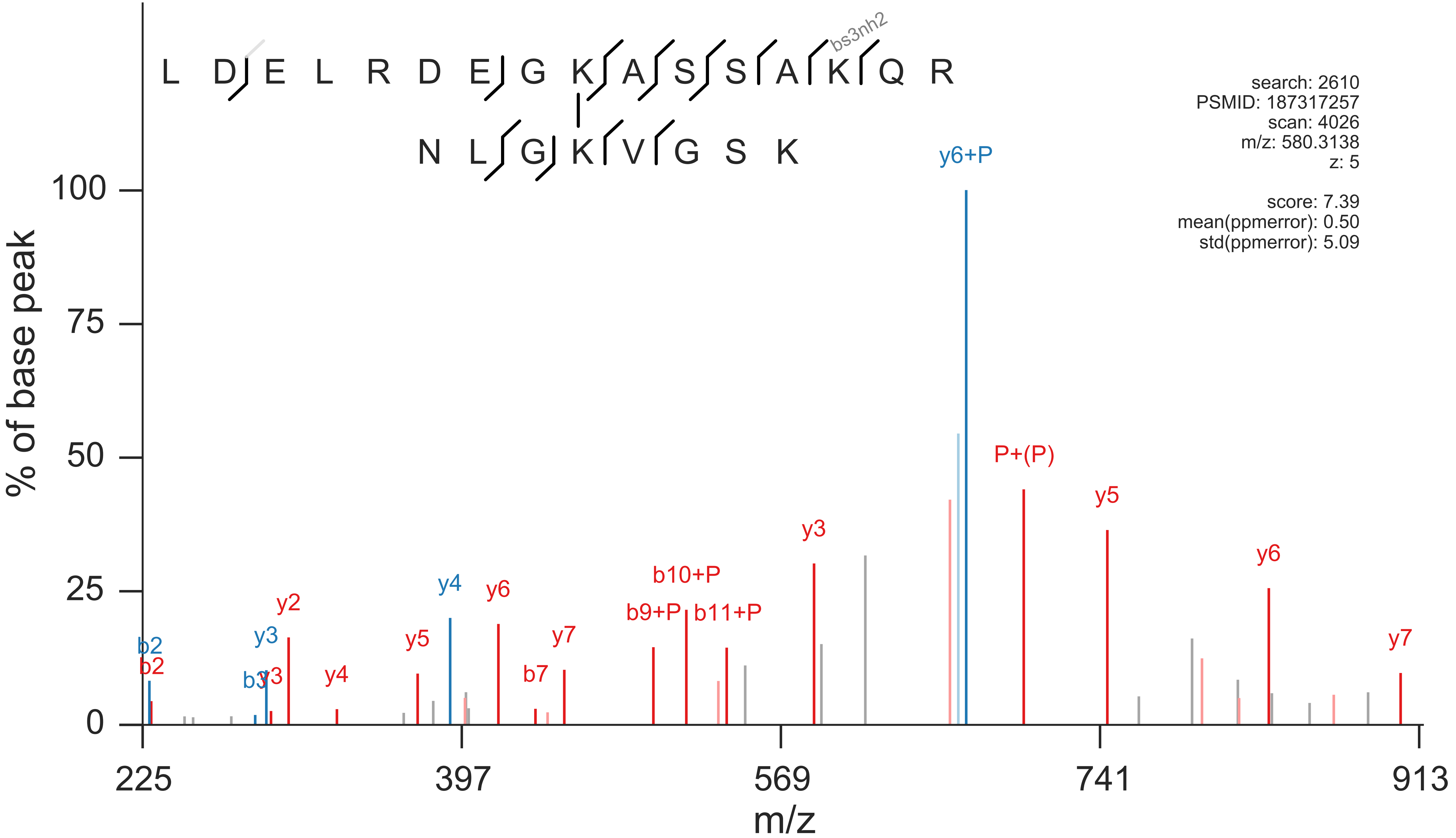

L D E L R D E G K A S S A K Q R
N L G K V G S K
bs3nh2
search: 2610
PSMID: 187317257
scan: 4026
m/z: 580.3138
z: 5
score: 7.39
mean(ppmerror): 0.50
std(ppmerror): 5.09
% of base peak
100
75
50
25
0
225
397
569
741
913
m/z
y6+P
P+(P)
y5
y3
y6
b10+P
b9+P
b11+P
y4
y6
y7
y2
y3
b2
b2
b3
y3
y4
y5
b7
y7

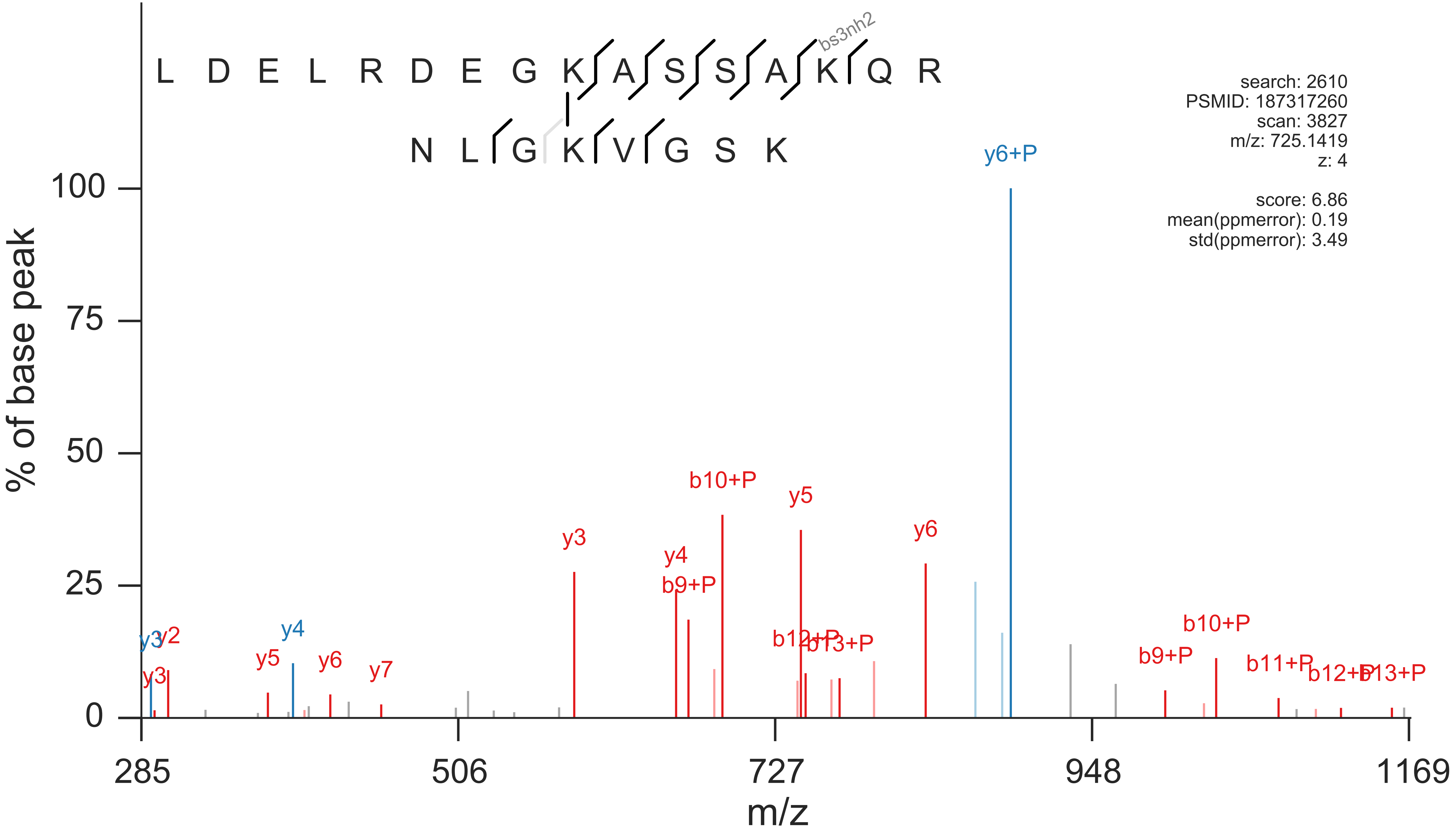

L D E L R D E G K A S S A K Q R
bs3nh2
N L G K V G S K
search: 2610
PSMID: 187317260
scan: 3827
m/z: 725.1419
z: 4
score: 6.86
mean(ppmerror): 0.19
std(ppmerror): 3.49
% of base peak
m/z
0
25
50
75
100
285
506
727
948
1169
y6+P
b10+P
y5
y6
y3
y4
b9+P
b12+P
b13+P
y3
y2
y4
y5
y6
y7
b10+P
b9+P
b11+P
b12+P
b13+P

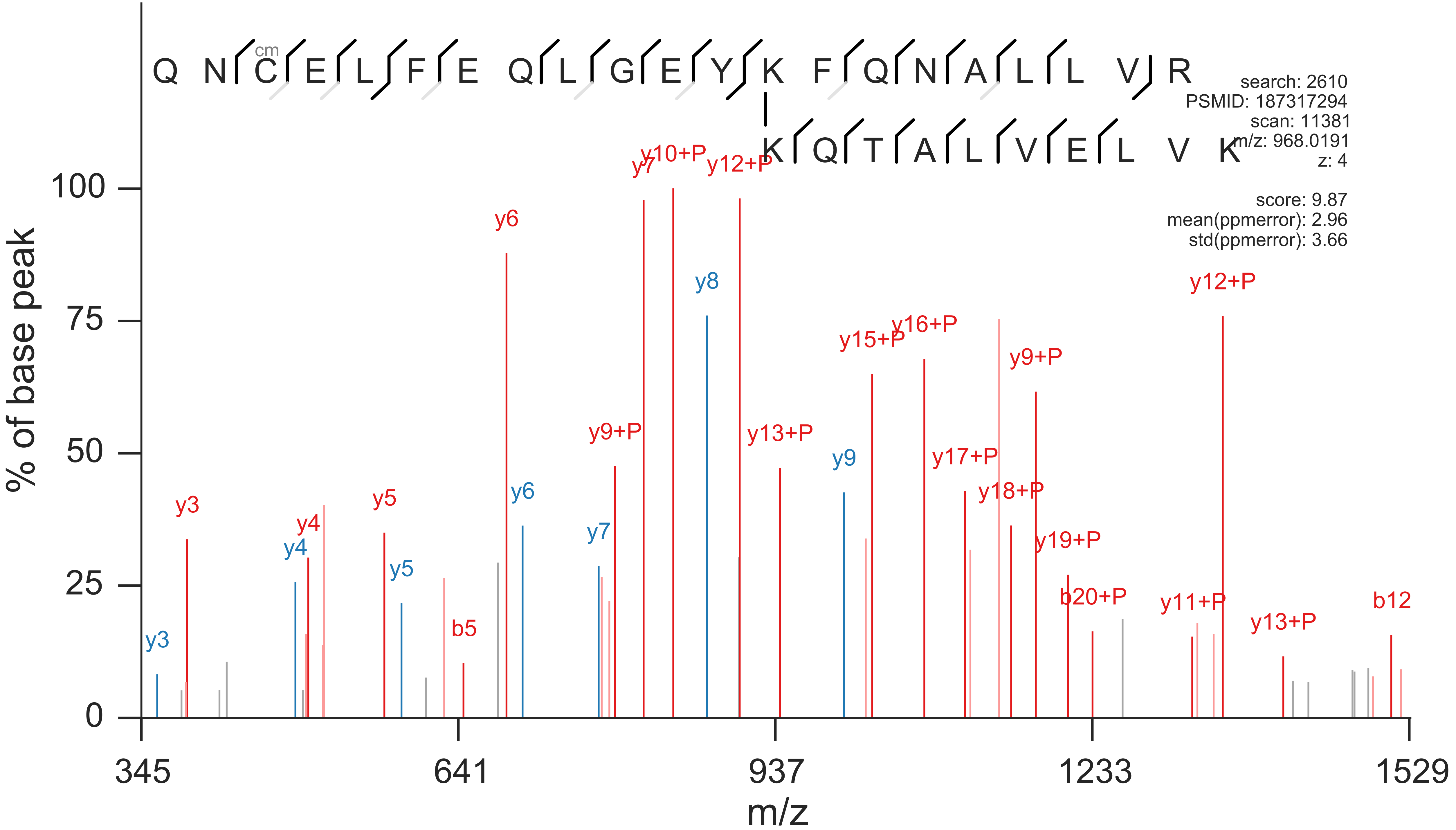

Q N C E L F E Q L G E Y K F Q N A L L V R
cm
K Q T A L V E L V K
search: 2610
PSMID: 187317294
scan: 11381
m/z: 968.0191
z: 4
score: 9.87
mean(ppmerror): 2.96
std(ppmerror): 3.66
% of base peak
m/z
0
25
50
75
100
345
641
937
1233
1529
y3
y3
y4
y4
y5
y5
b5
y6
y6
y7
y7
y9+P
y10+P
y12+P
y8
y13+P
y9
y15+P
y16+P
y17+P
y18+P
y9+P
y19+P
b20+P
y11+P
y12+P
y13+P
b12

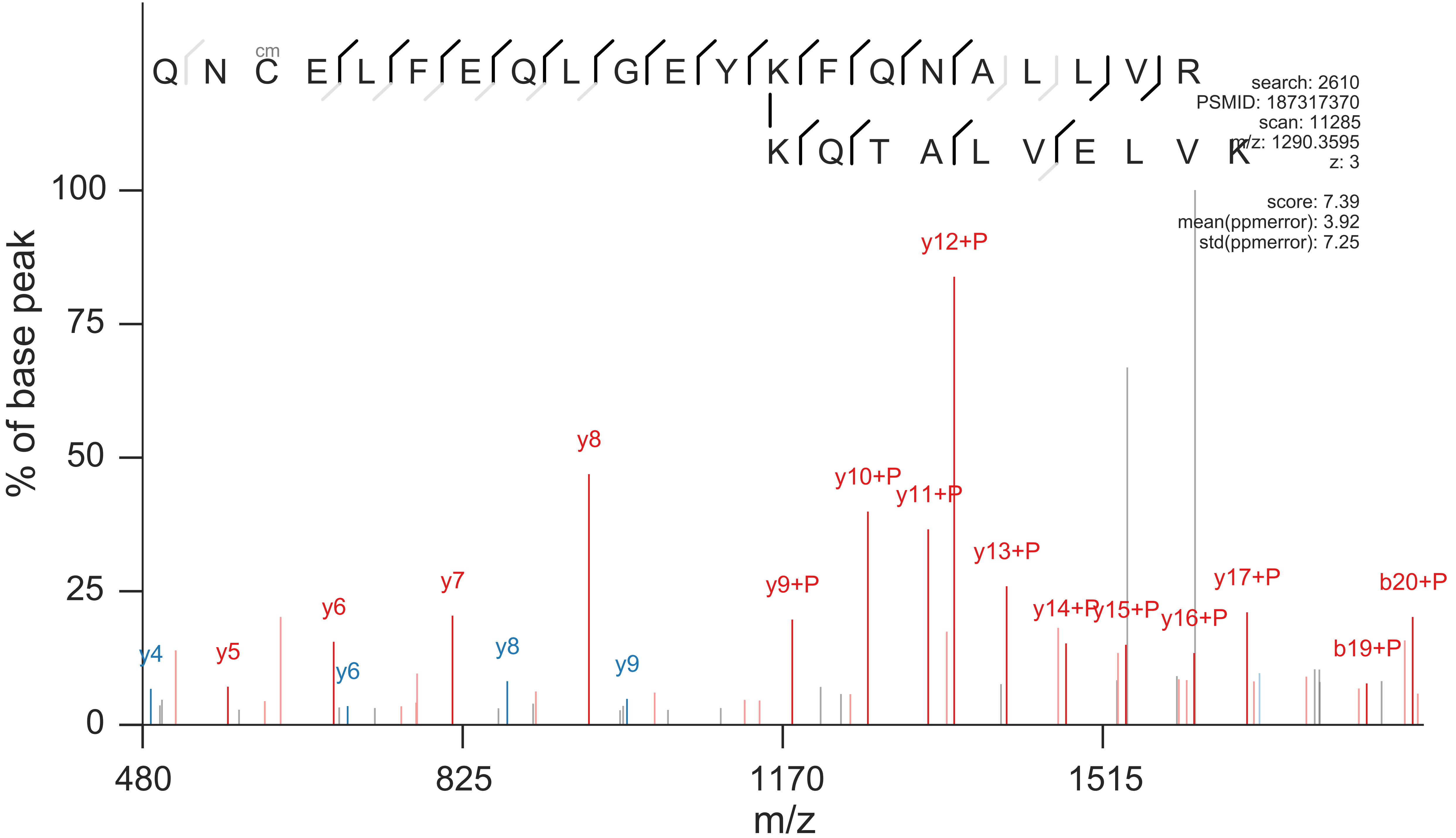

Q N C E L F E Q L G E Y K F Q N A L L V R
cm
K Q T A L V E L V K
search: 2610
PSMID: 187317370
scan: 11285
m/z: 1290.3595
z: 3
score: 7.39
mean(ppmerror): 3.92
std(ppmerror): 7.25
% of base peak
m/z
0
25
50
75
100
480
825
1170
1515
y4
y5
y6
y6
y7
y8
y8
y9
y9+P
y10+P
y11+P
y12+P
y13+P
y14+P
y15+P
y16+P
y17+P
b19+P
b20+P

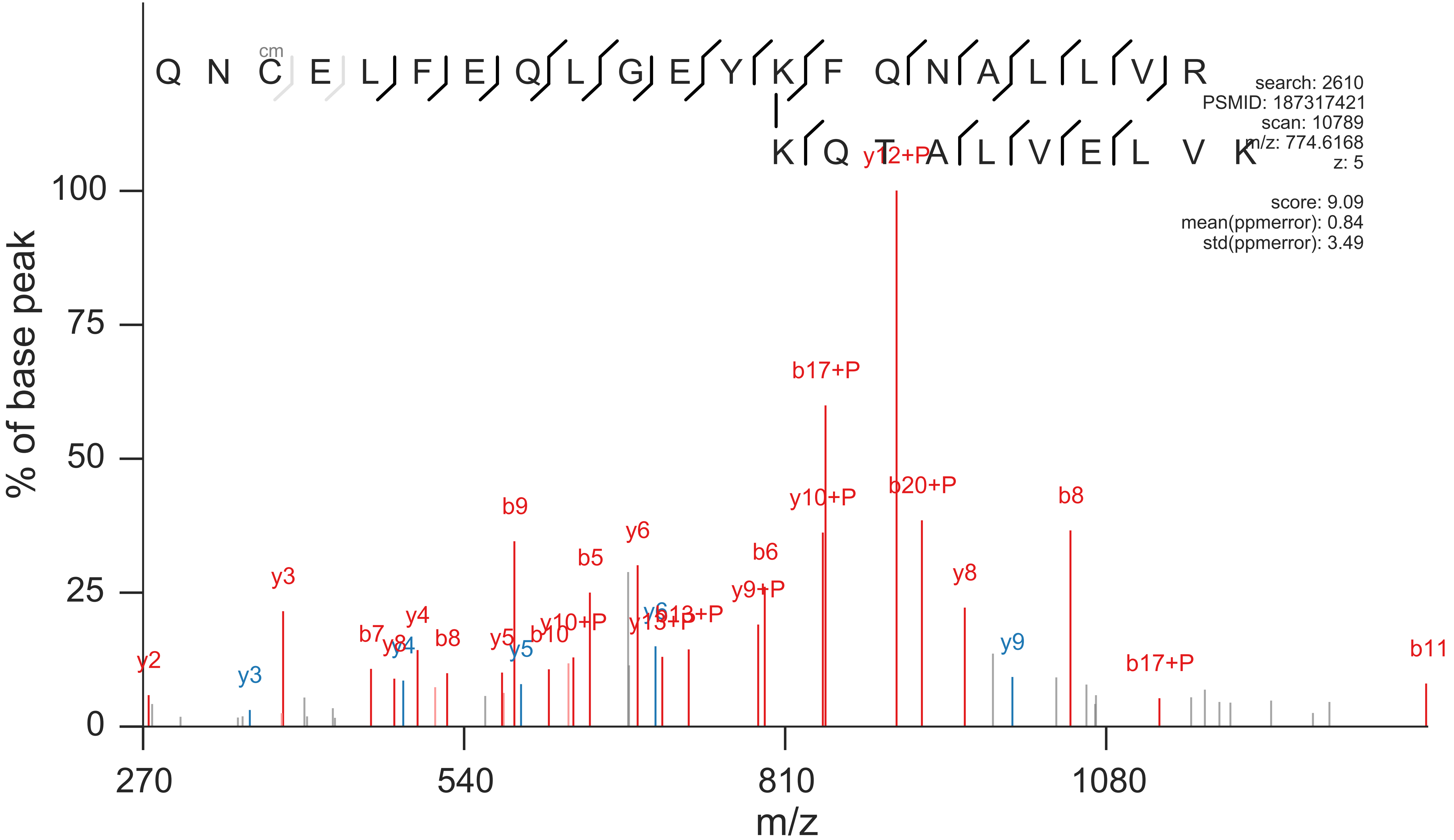

Q N C E L F E Q L G E Y K F Q N A L L V R
cm
K Q T A L V E L V K
search: 2610
PSMID: 187317421
scan: 10789
m/z: 774.6168
z: 5
score: 9.09
mean(ppmerror): 0.84
std(ppmerror): 3.49
% of base peak
m/z
0
25
50
75
100
270
540
810
1080
y12+P
b17+P
b20+P
y10+P
b8
b9
y6
b5
b6
y3
y8
y9+P
y4
b7
y8
y4
b8
y5
y5
b10
y10+P
y6
b13+P
y13+P
y9
y2
y3
b17+P
b11

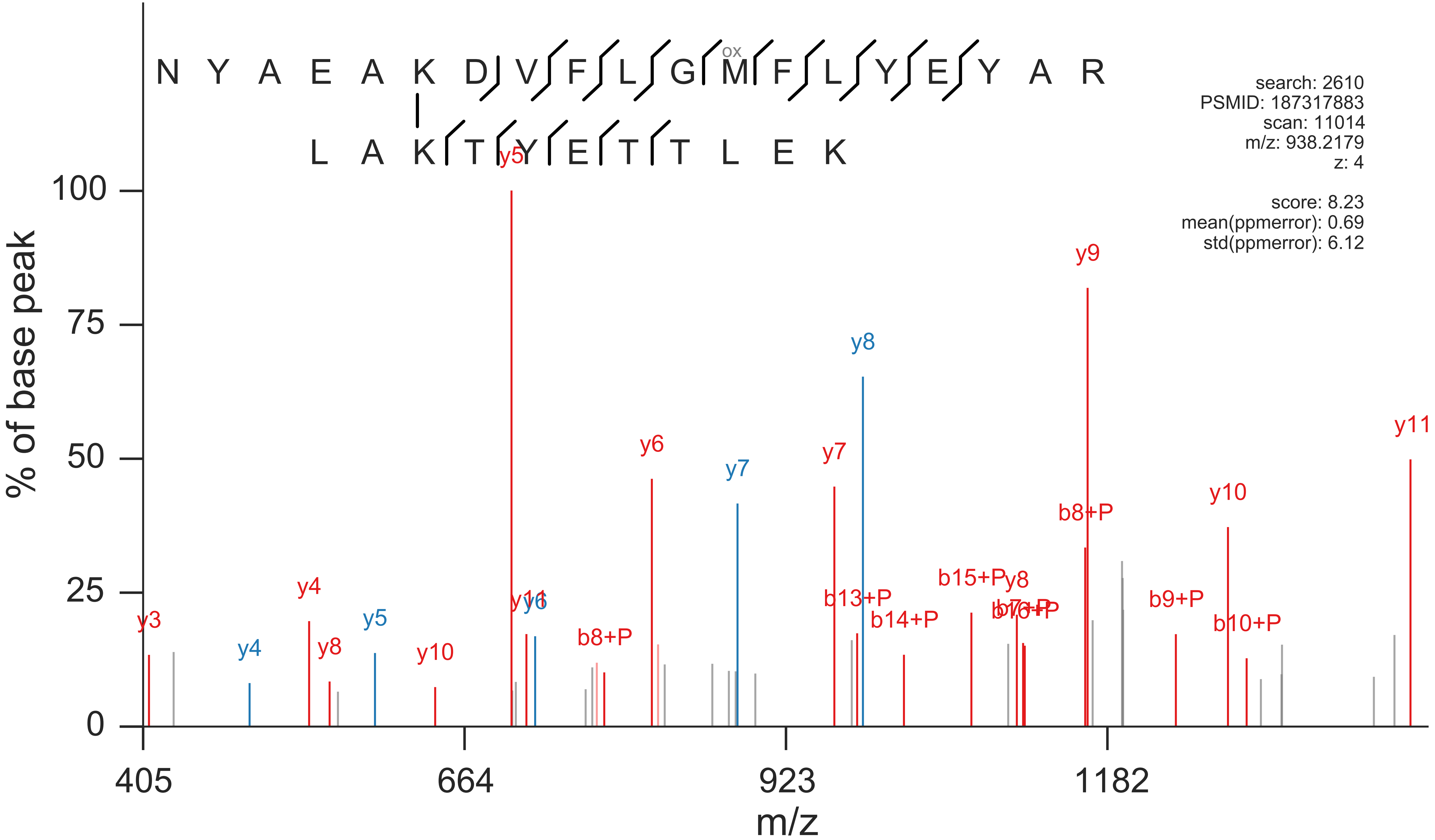

N Y A E A K D V F L G M F L Y E Y A R
ox
L A K T Y E T T T L E K
search: 2610
PSMID: 187317883
scan: 11014
m/z: 938.2179
z: 4
score: 8.23
mean(ppmerror): 0.69
std(ppmerror): 6.12
% of base peak
m/z
0
25
50
75
100
405
664
923
1182
y3
y4
y4
y8
y5
y10
y5
y11
y6
b8+P
y6
y7
y7
y8
b13+P
b14+P
b15+P
y8
b7+P
b16+P
y9
b8+P
b9+P
y10
b10+P
y11

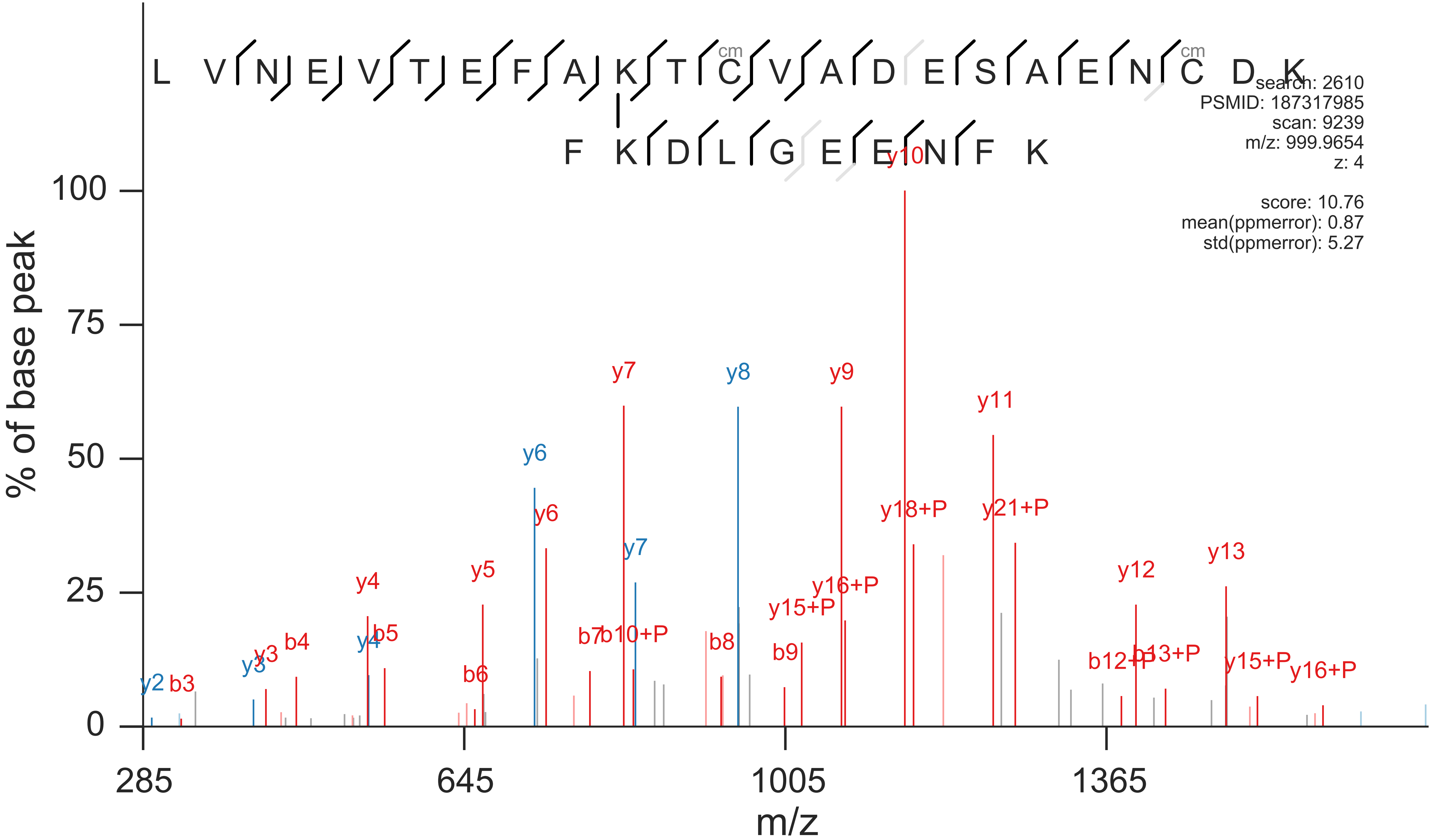

L V N E V T E F A K T C V A D E S A E N C D K
cm
cm
F K D L G E E N F K
search: 2610
PSMID: 187317985
scan: 9239
m/z: 999.9654
z: 4
score: 10.76
mean(ppmerror): 0.87
std(ppmerror): 5.27
% of base peak
100
75
50
25
0
285
645
1005
1365
m/z
y2
b3
y3
y3
b4
y4
y4
b5
y5
b6
y6
y6
y7
y7
b7
b10+P
y8
b8
b9
y9
y15+P
y16+P
y10
y18+P
y11
y21+P
y12
b12+P
b13+P
y13
y15+P
y16+P

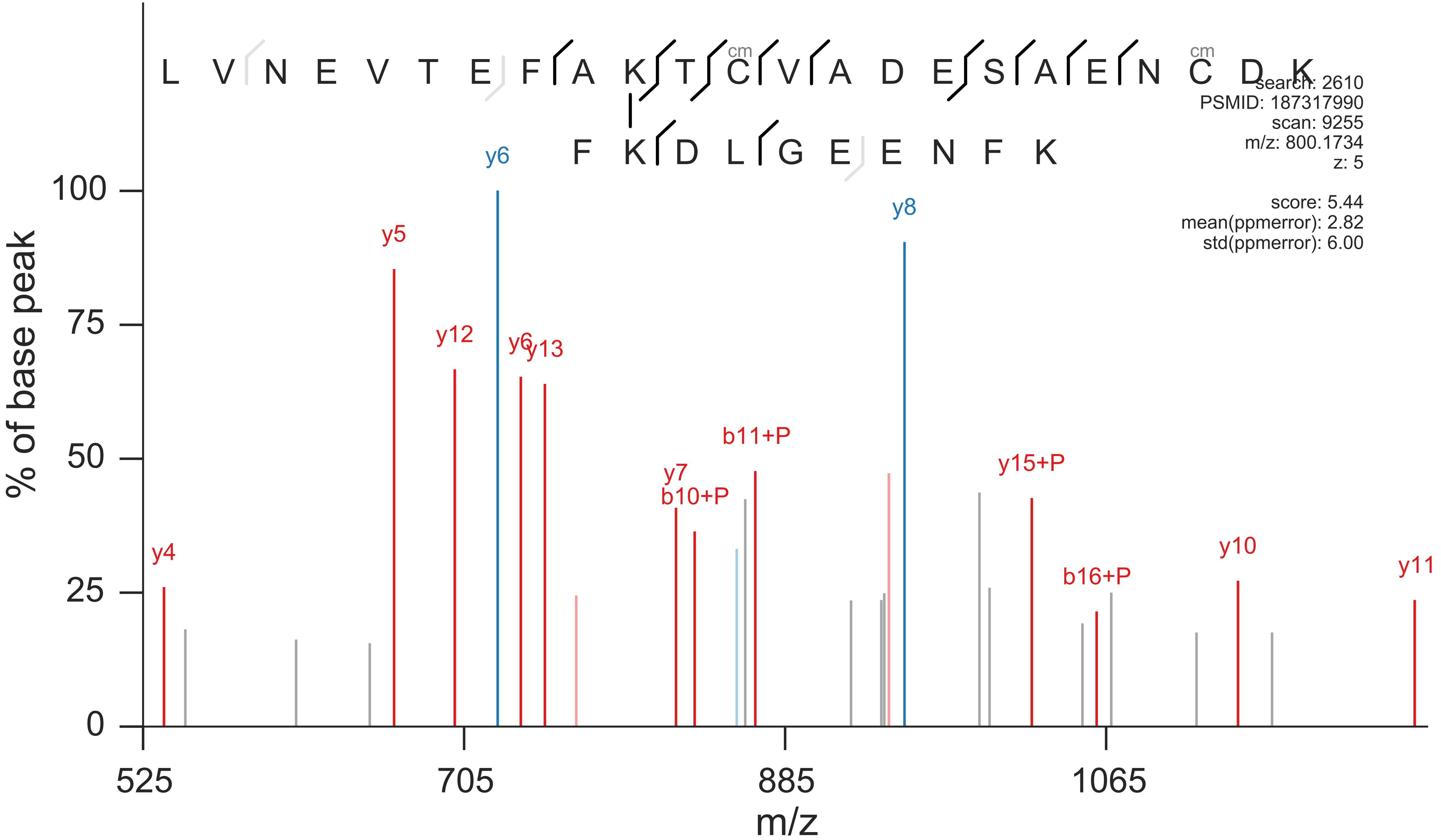

L V N E V T E F A K T C V A D E S A E N C D K
cm
cm
F K D L G E E N F K
search: 2610
PSMID: 187317990
scan: 9255
m/z: 800.1734
z: 5
score: 5.44
mean(ppmerror): 2.82
std(ppmerror): 6.00
% of base peak
m/z
0
25
50
75
100
525
705
885
1065
y4
y5
y12
y6
y6
y13
y7
b10+P
b11+P
y8
y15+P
b16+P
y10
y11

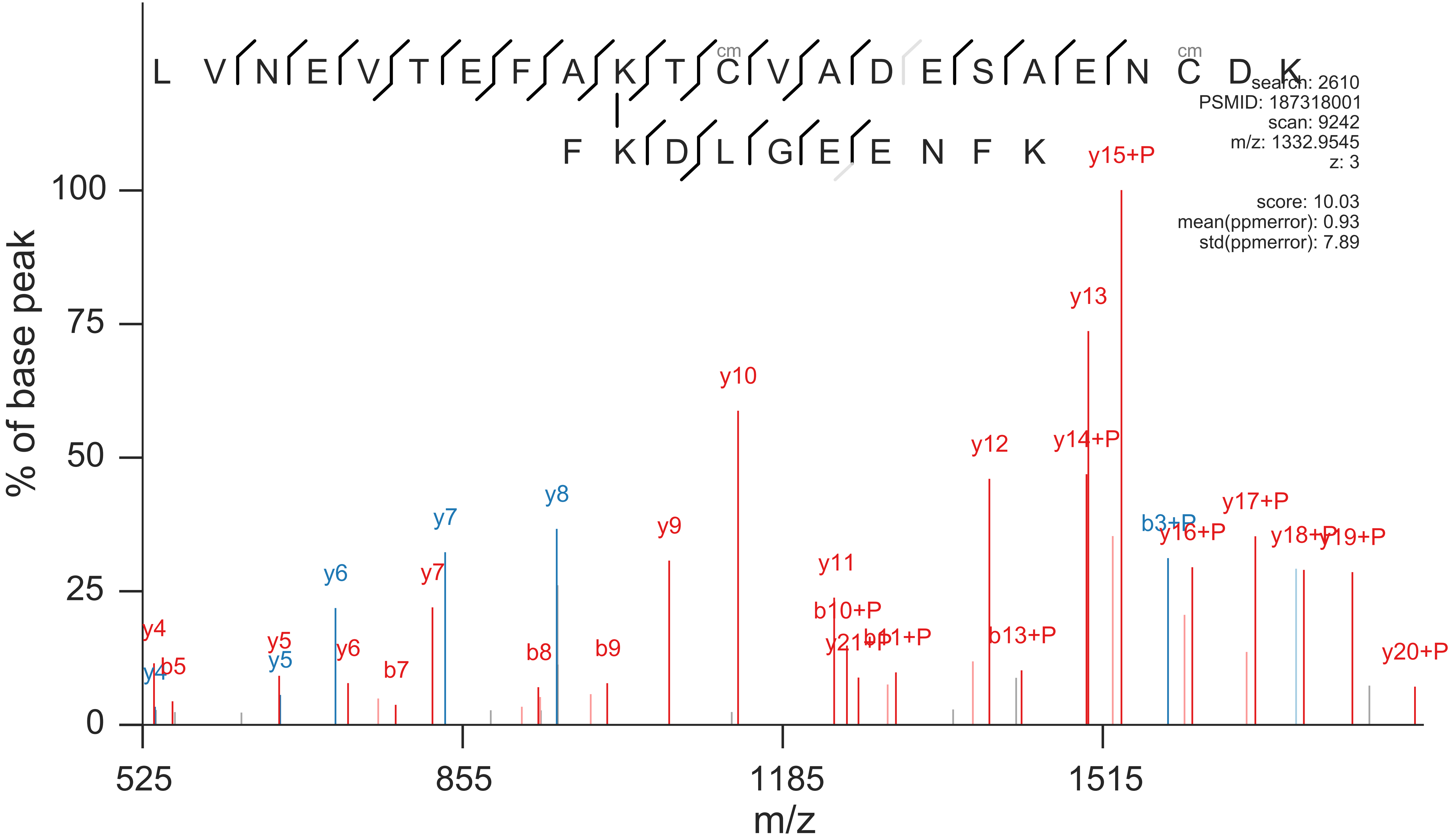

L V N E V T E F A K T C V A D E S A E N C D K
cm
cm
F K D L G E E N F K
search: 2610
PSMID: 187318001
scan: 9242
m/z: 1332.9545
z: 3
score: 10.03
mean(ppmerror): 0.93
std(ppmerror): 7.89
% of base peak
m/z
0
25
50
75
100
525
855
1185
1515
y15+P
y13
y10
y12
y14+P
y8
y7
y9
y17+P
b3+P
y16+P
y18+P
y19+P
y6
y7
y11
b10+P
y4
y5
y6
b8
b9
y21+P
b11+P
b13+P
y20+P
y5
b5
y4
b7

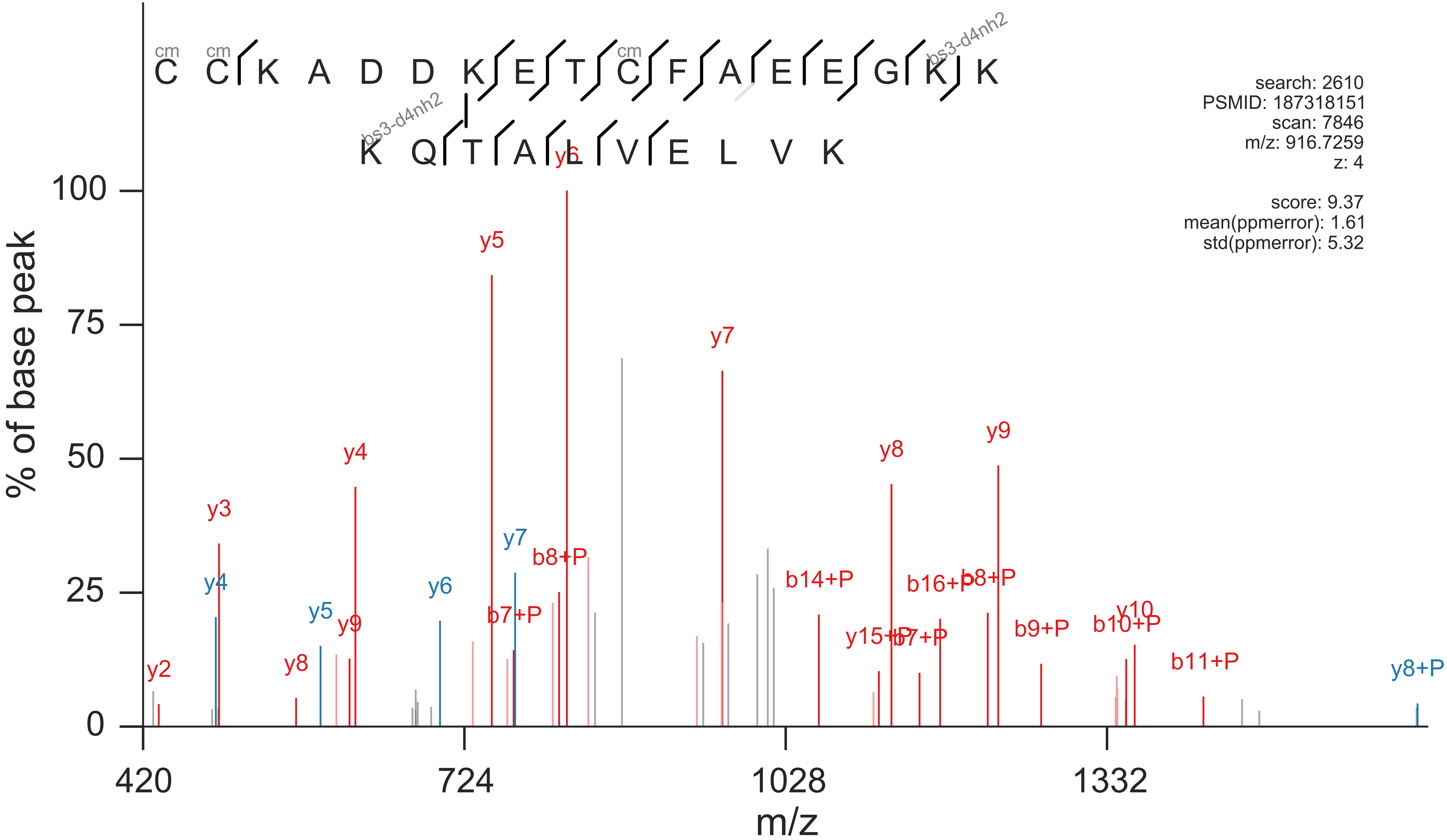

C C K A D D K E T C F A E E G K K
K Q T A L V E L V K
bs3-d4nh2
bs3-d4nh2
search: 2610
PSMID: 187318151
scan: 7846
m/z: 916.7259
z: 4
score: 9.37
mean(ppmerror): 1.61
std(ppmerror): 5.32
% of base peak
m/z

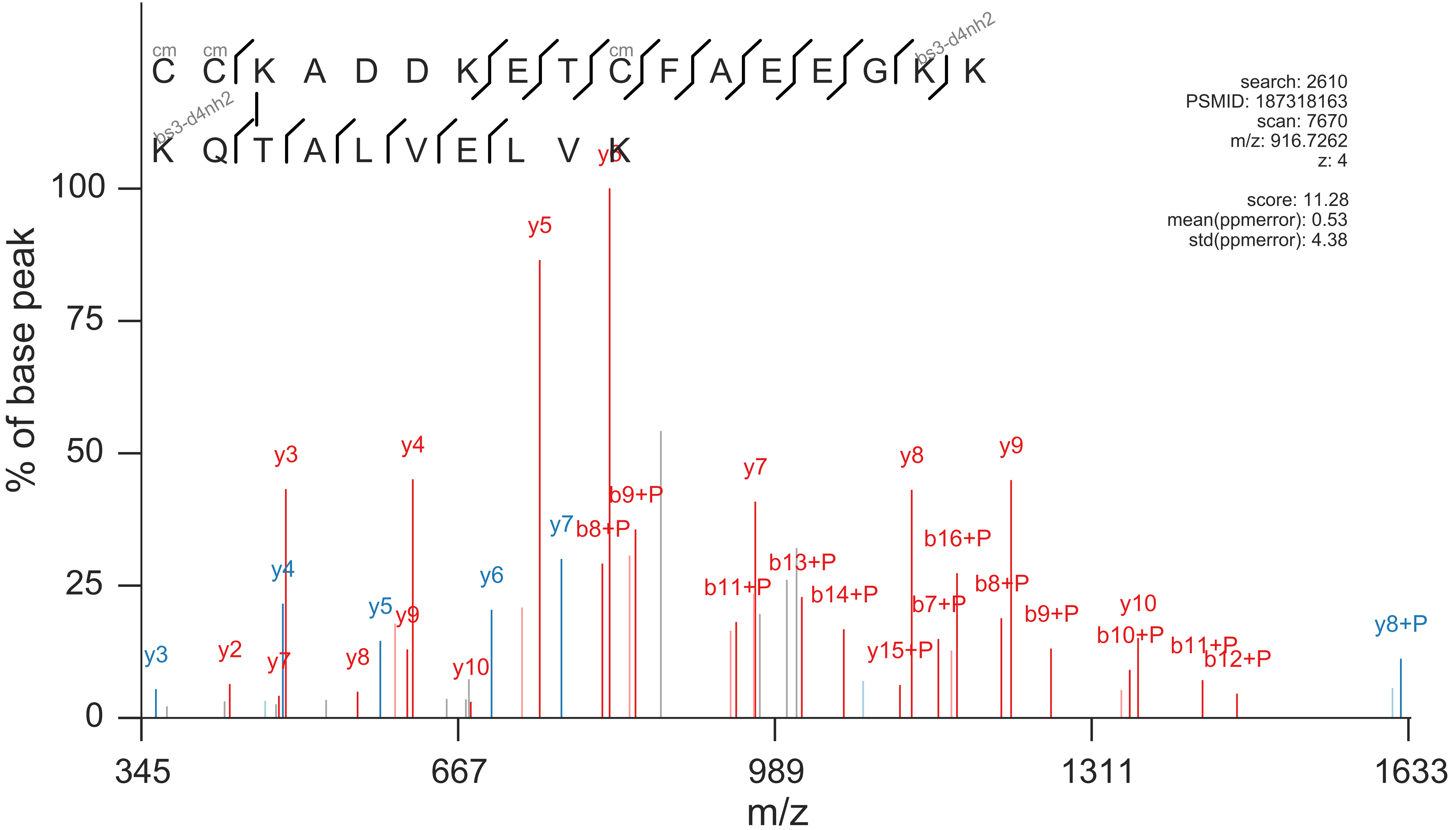

C C K A D D K E T C F A E E G K K
K Q T A L V E L V K
cm
bs3-d4nh2
search: 2610
PSMID: 187318163
scan: 7670
m/z: 916.7262
z: 4
score: 11.28
mean(ppmerror): 0.53
std(ppmerror): 4.38
% of base peak
m/z
0
25
50
75
100
345
667
989
1311
1633
y2
y3
y4
y5
y6
y7
y8
y9
y10
b8+P
b9+P
b10+P
b11+P
b12+P
b13+P
b14+P
b16+P
b7+P
y15+P
y8+P

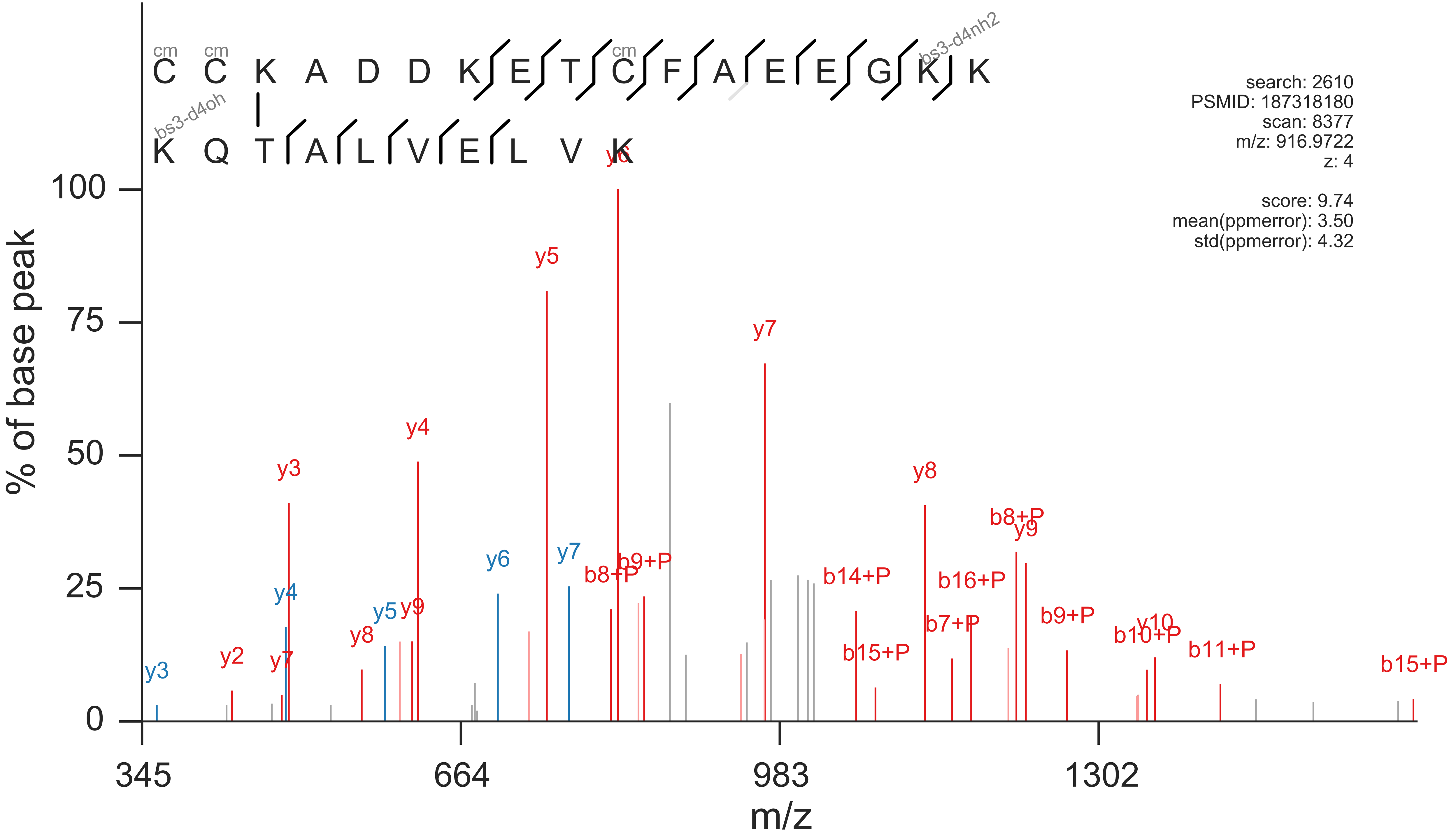
C C K A D D K E T C F A E E G K K
cm cm cm
bs3-d4nh2
bs3-d4oh
K Q T A L V E L V K
search: 2610
PSMID: 187318180
scan: 8377
m/z: 916.9722
z: 4
score: 9.74
mean(ppmerror): 3.50
std(ppmerror): 4.32
% of base peak
m/z
0
25
50
75
100
345
664
983
1302
y3
y2
y7
y4
y3
y8
y5
y9
y4
y6
y5
y7
b8+P
b9+P
y6
y7
b14+P
b15+P
y8
b7+P
b16+P
b8+P
y9
b9+P
b10+P
y10
b11+P
b15+P

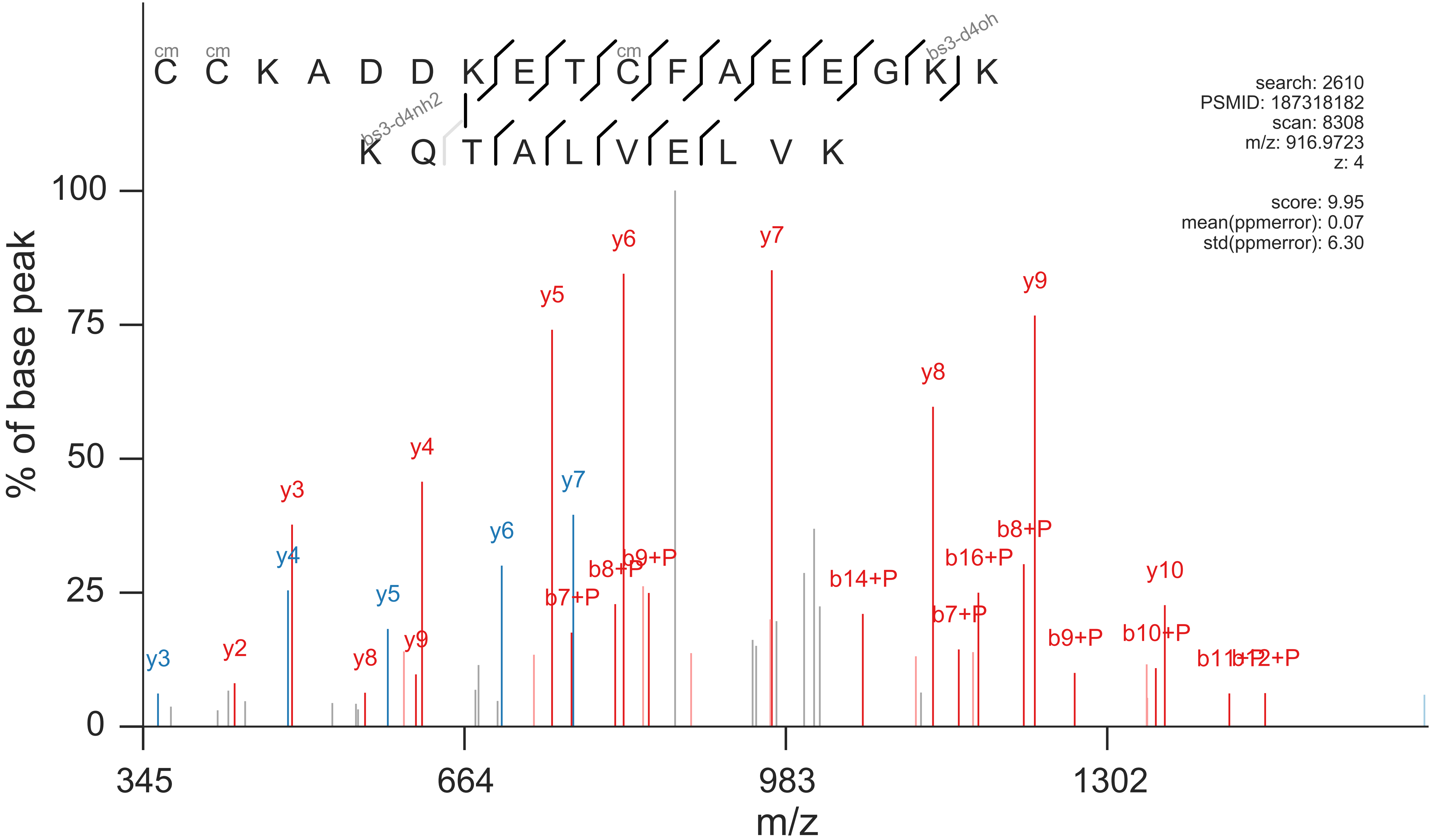

cm cm cm
C C K A D D K E T C F A E E G K K
bs3-d4oh
bs3-d4nh2
K Q T A L V E L V K
search: 2610
PSMID: 187318182
scan: 8308
m/z: 916.9723
z: 4
score: 9.95
mean(ppmerror): 0.07
std(ppmerror): 6.30
% of base peak
m/z
345
664
983
1302
0
25
50
75
100
y2 y3 y4 y5 y6 y7 y8 y9 y10
b7+P b8+P b9+P b10+P b11+P b12+P b14+P b16+P

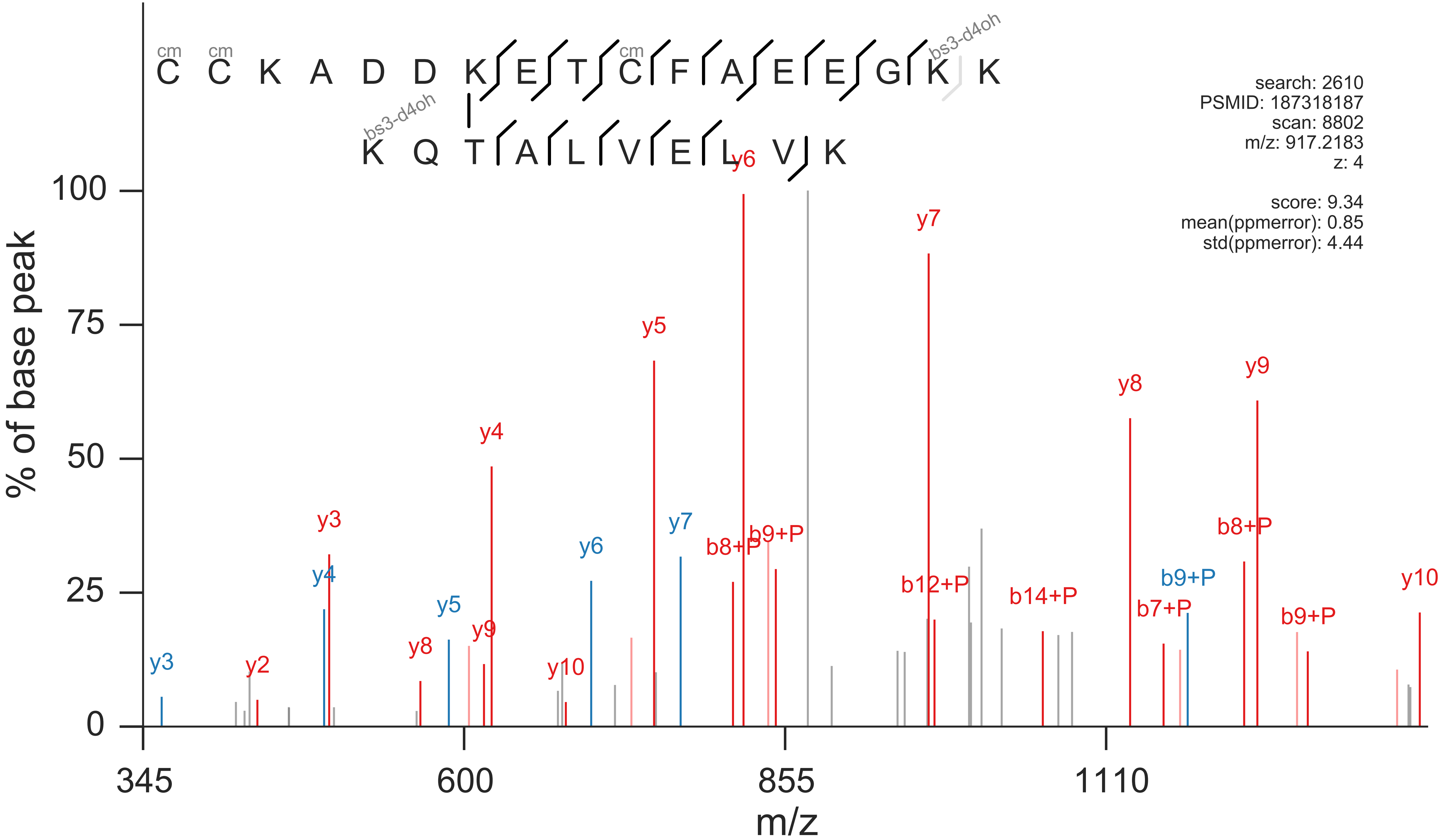

cm cm cm
C C K A D D K E T C F A E E G K K
bs3-d4oh
K Q T A L V E L V K
search: 2610
PSMID: 187318187
scan: 8802
m/z: 917.2183
z: 4
score: 9.34
mean(ppmerror): 0.85
std(ppmerror): 4.44
% of base peak
m/z

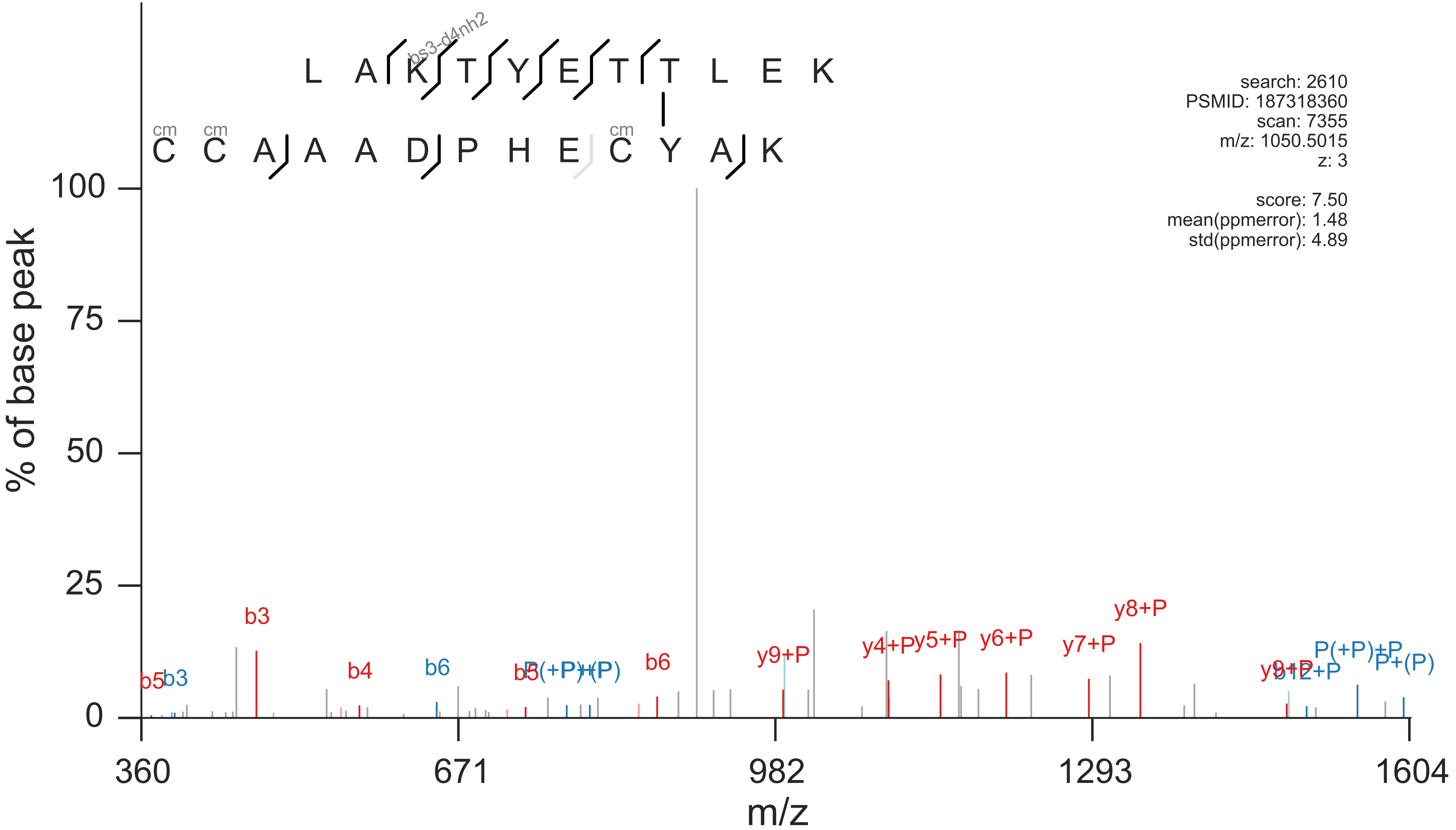

L A K T Y E T T L E K
bs3-d4nh2
C C A A A D P H E C Y A K
cm cm cm
search: 2610
PSMID: 187318360
scan: 7355
m/z: 1050.5015
z: 3
score: 7.50
mean(ppmerror): 1.48
std(ppmerror): 4.89
% of base peak
100
75
50
25
0
m/z
360
671
982
1293
1604
b5
b3
b3
b4
b6
b5
P(+P)+(P)
b6
y9+P
y4+P
y5+P
y6+P
y7+P
y8+P
y9+P
b12+P
P(+P)+P
P+(P)

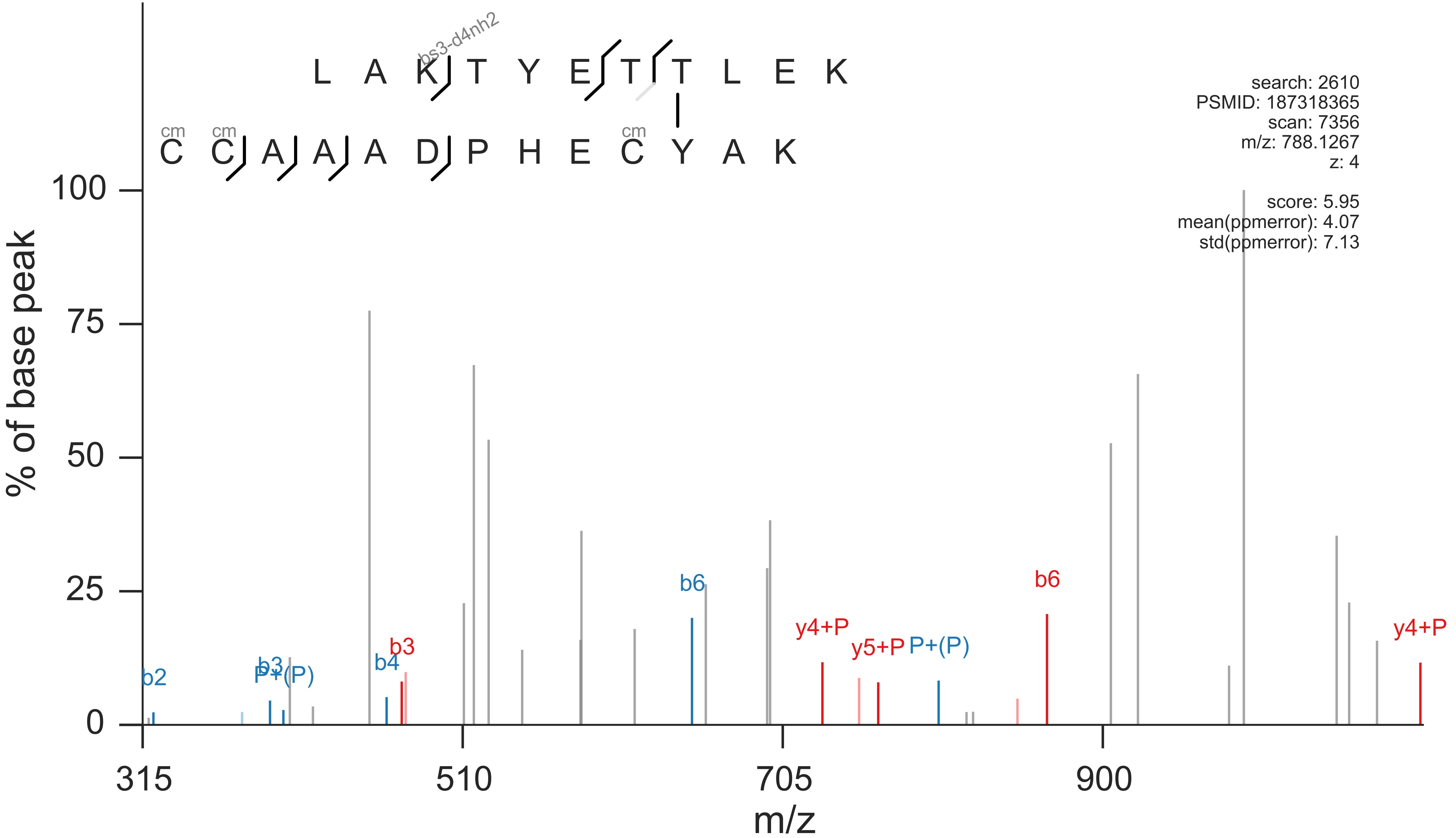

L A K T Y E T T L E K
bs3-d4nh2
C C A A A D P H E C Y A K
cm
cm
cm
search: 2610
PSMID: 187318365
scan: 7356
m/z: 788.1267
z: 4
score: 5.95
mean(ppmerror): 4.07
std(ppmerror): 7.13
% of base peak
m/z
b2
b3
P+(P)
b4
b3
b6
y4+P
y5+P
P+(P)
b6
y4+P

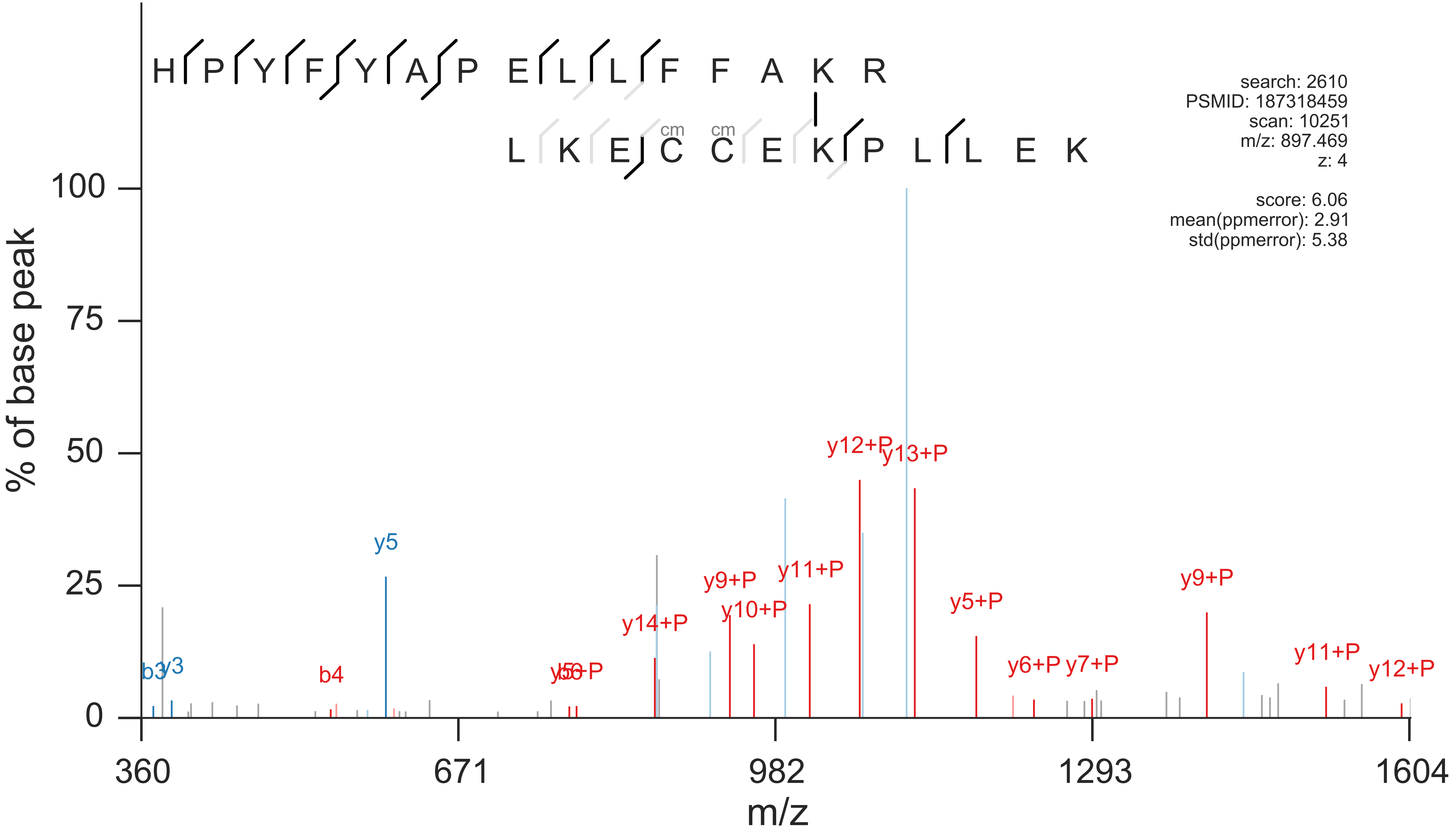

H P Y F Y A P E L L F F A K R
L K E C C E K P L L E K
cm
cm
search: 2610
PSMID: 187318459
scan: 10251
m/z: 897.469
z: 4
score: 6.06
mean(ppmerror): 2.91
std(ppmerror): 5.38
% of base peak
m/z
y5
y12+P
y13+P
y11+P
y9+P
y10+P
y14+P
y5+P
y9+P
y6+P
y7+P
y11+P
y12+P
b4
b3
y3

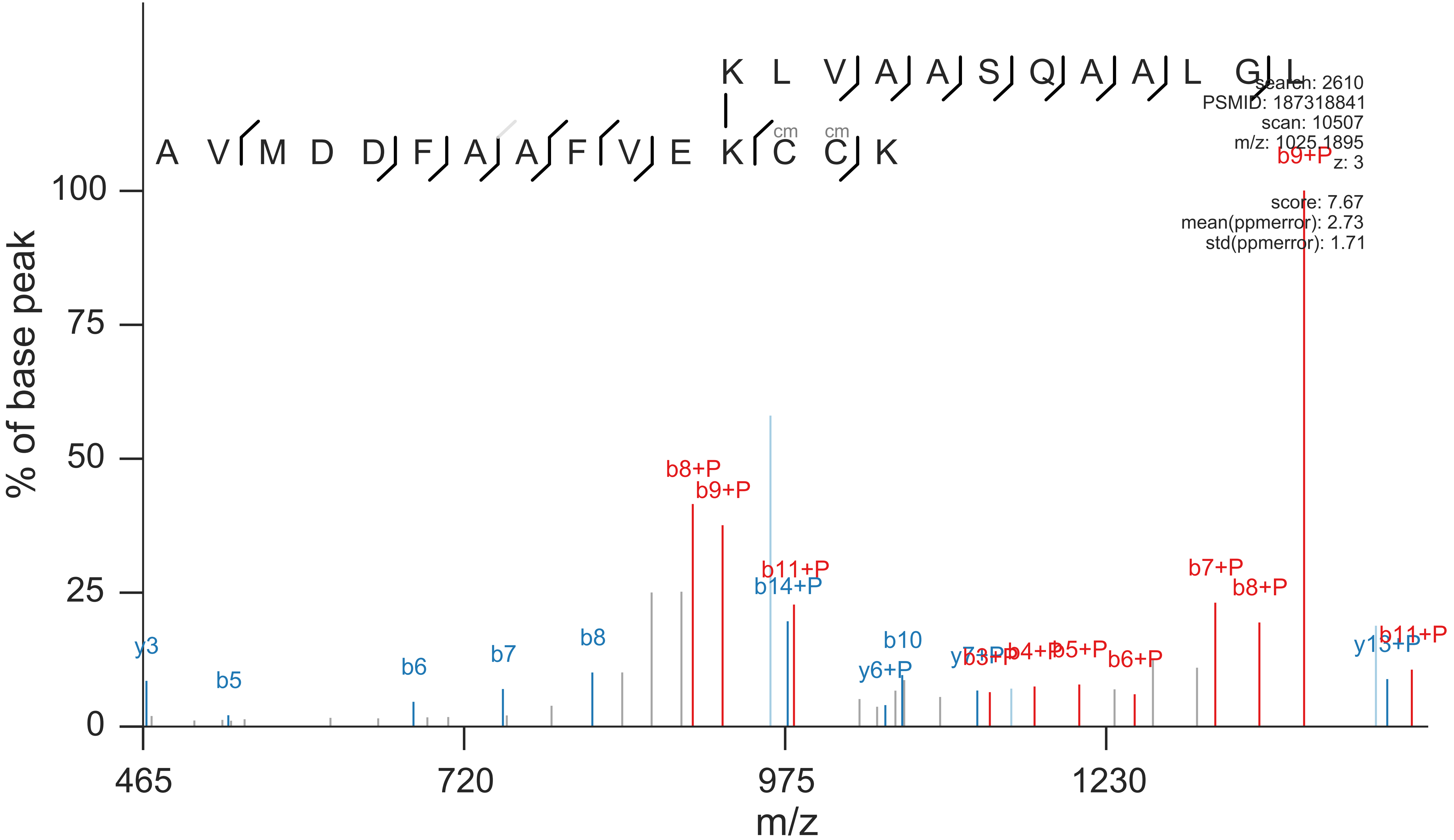
K L V A A S Q A A L G L
A V M D D F A A F V E K C C K
cm
cm
search: 2610
PSMID: 187318841
scan: 10507
m/z: 1025.1895
z: 3
score: 7.67
mean(ppmerror): 2.73
std(ppmerror): 1.71
% of base peak
m/z
100
75
50
25
0
465
720
975
1230
y3
b5
b6
b7
b8
b8+P
b9+P
b11+P
b14+P
y6+P
b10
b3+P
b4+P
b5+P
b6+P
b7+P
b8+P
b9+P
b11+P

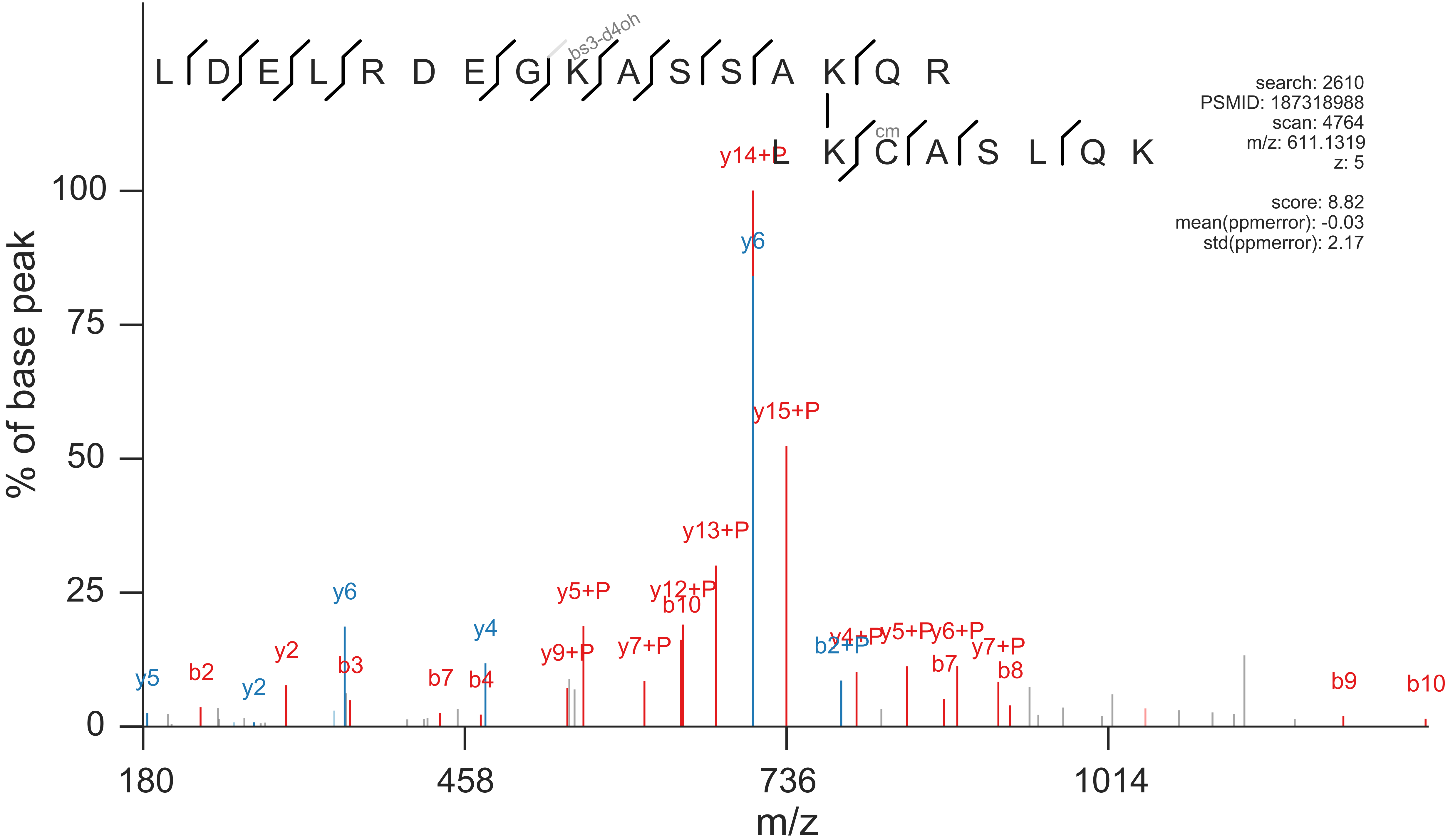
L D E L R D E G K A S S A K Q R
bs3-d4oh
L K C A S L Q K
cm
search: 2610
PSMID: 187318988
scan: 4764
m/z: 611.1319
z: 5
score: 8.82
mean(ppmerror): -0.03
std(ppmerror): 2.17
% of base peak
m/z
0
25
50
75
100
180
458
736
1014
y14+P
y6
y15+P
y13+P
y12+P
b10
y5+P
y9+P
y7+P
y6
y4
y2
y5
b2
y2
b3
b7
b4
b2+P
y4+P
y5+P
y6+P
y7+P
b7
b8
b9
b10

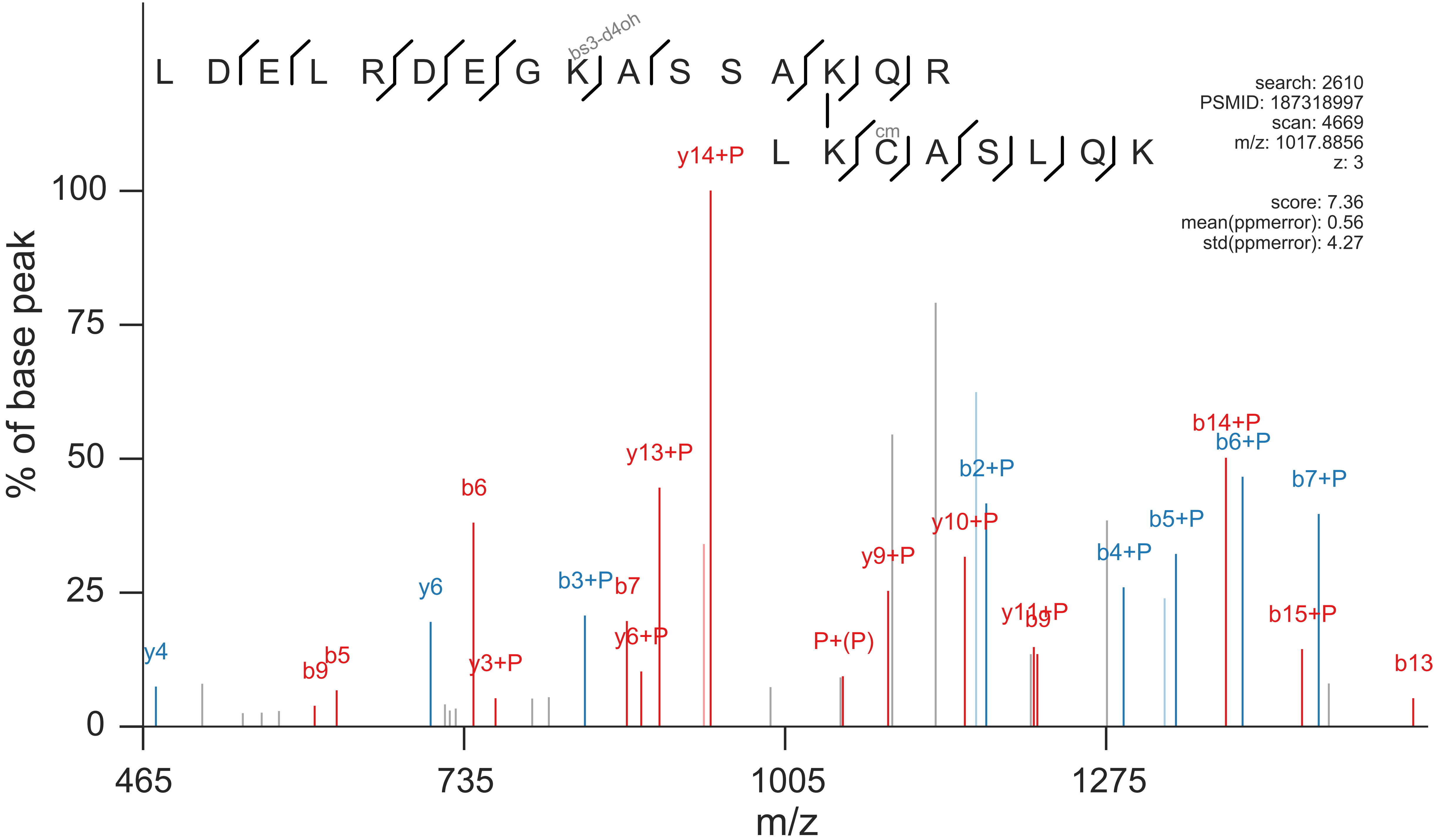

bs3-d4oh
L D E L R D E G K A S S A K Q R
cm
L K C A S L Q K
search: 2610
PSMID: 187318997
scan: 4669
m/z: 1017.8856
z: 3
score: 7.36
mean(ppmerror): 0.56
std(ppmerror): 4.27
% of base peak
100
75
50
25
0
465
735
1005
1275
m/z
y14+P
y13+P
b6
y6
b3+P
b7
y6+P
y3+P
y4
b9
b5
P+(P)
y9+P
y10+P
b2+P
y11+P
b9
b4+P
b5+P
b14+P
b6+P
b7+P
b15+P
b13

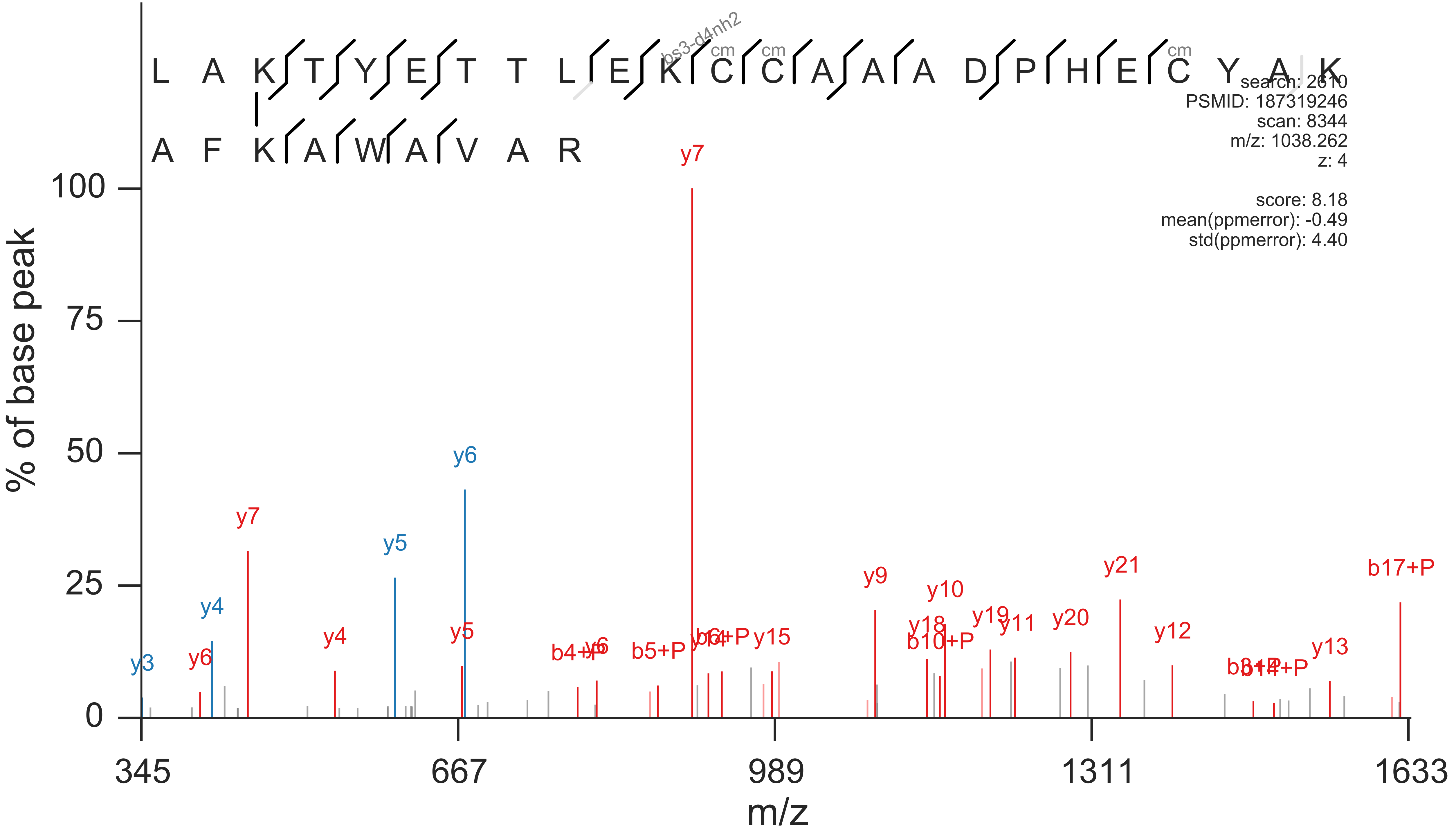

L A K T Y E T T L E K C C A A A D P H E C Y A K
A F K A W A V A R
bs3-d4nh2
cm
search: 2610
PSMID: 187319246
scan: 8344
m/z: 1038.262
z: 4
score: 8.18
mean(ppmerror): -0.49
std(ppmerror): 4.40
% of base peak
m/z
100
75
50
25
0
345
667
989
1311
1633
y3
y4
y5
y6
y6
y7
y4
y5
y7
b4+P
b5+P
y15
y9
y10
y18
b10+P
y19
y11
y20
y21
y12
y13
b17+P

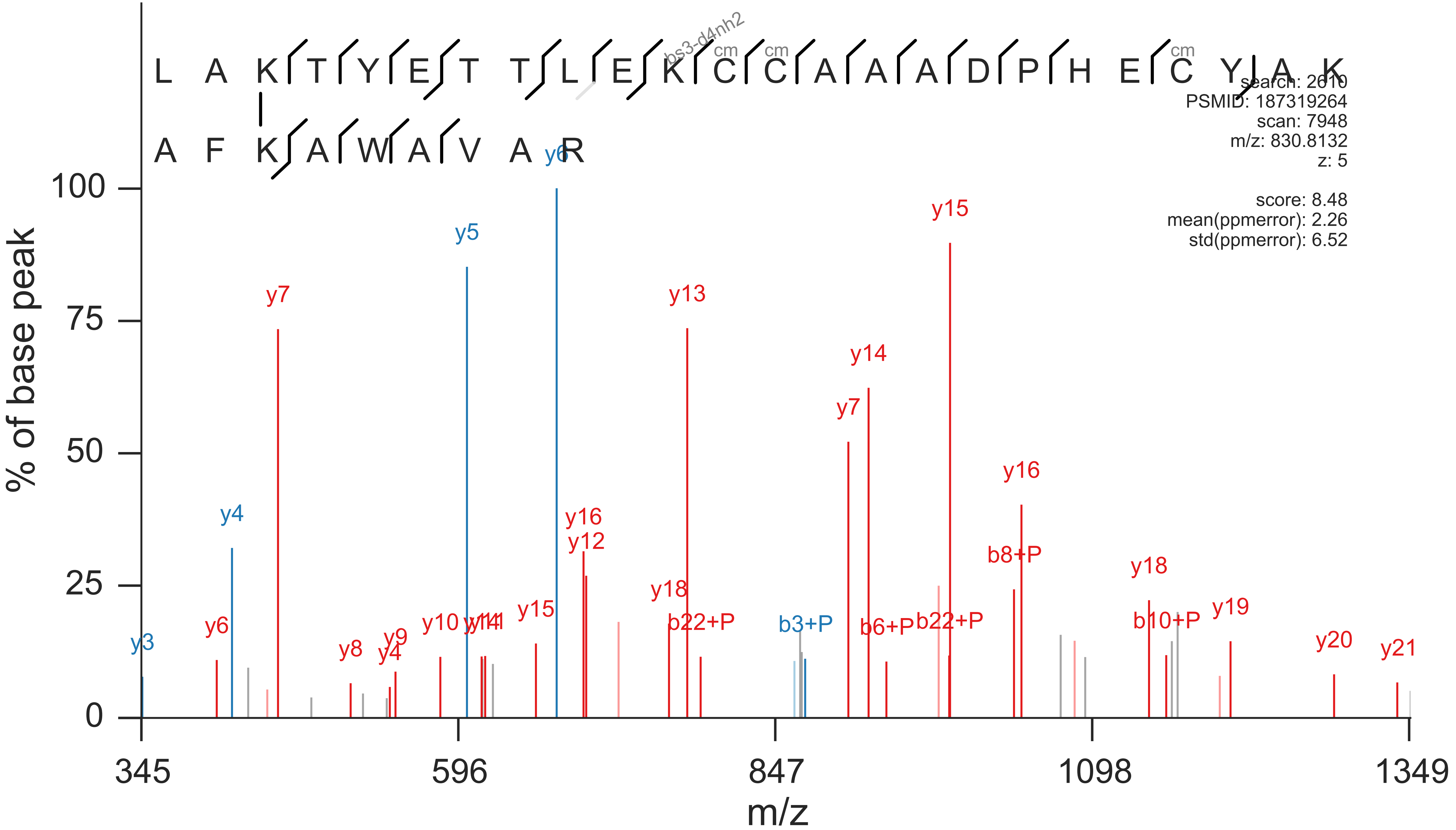

L A K T Y E T T L E K C C A A A D P H E C Y A K
A F K A W A V A R
bs3-d4nh2
cm
search: 2610
PSMID: 187319264
scan: 7948
m/z: 830.8132
z: 5
score: 8.48
mean(ppmerror): 2.26
std(ppmerror): 6.52
% of base peak
m/z
y3 y6 y4 y7 y8 y4 y9 y10 y11 y14 y5 y15 y6 y16 y12 y18 y13 b22+P b3+P y7 y14 b6+P b22+P y15 b8+P y16 y18 b10+P y19 y20 y21
0
25
50
75
100
345
596
847
1098
1349

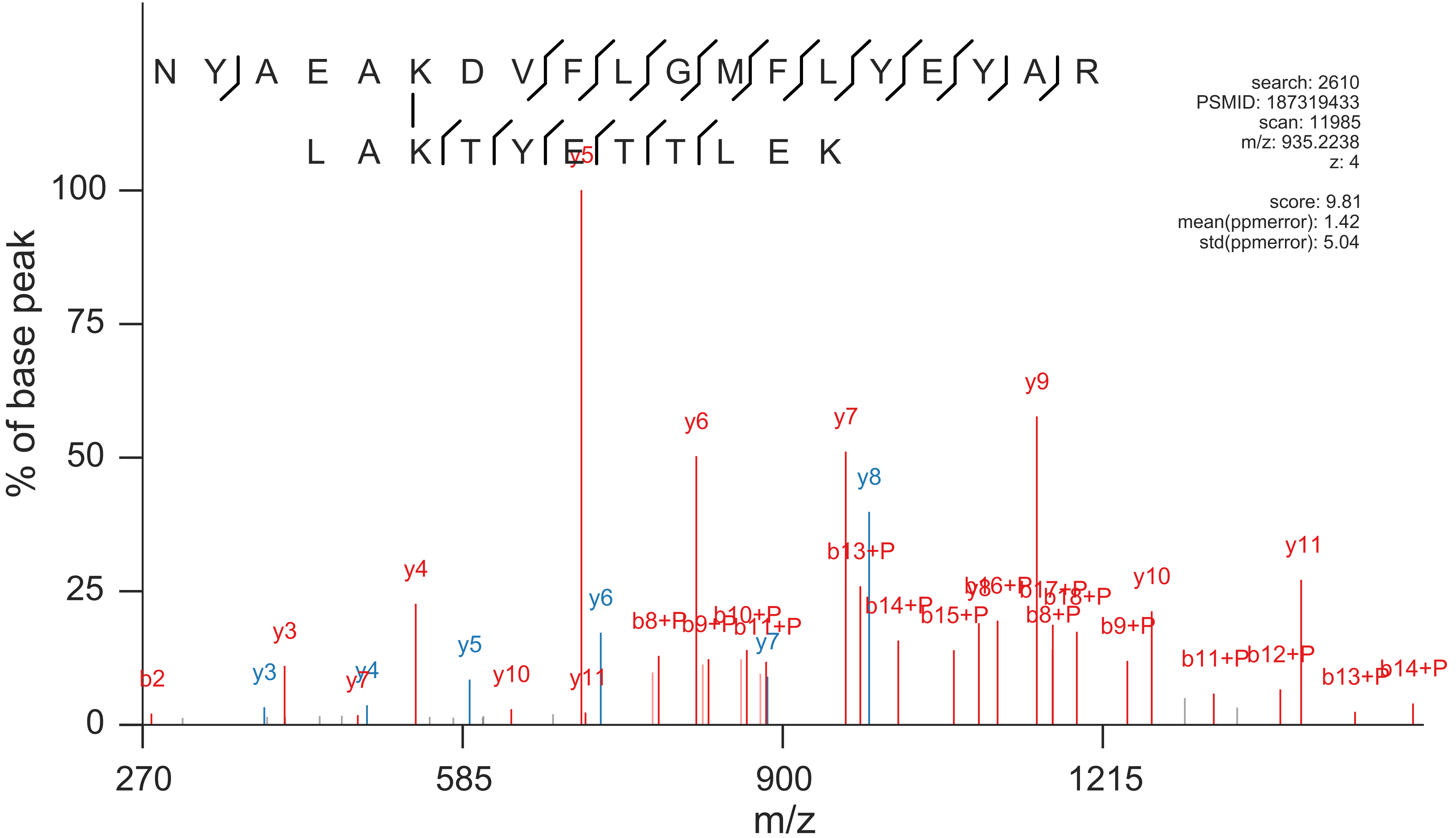

N Y A E A K D V F L G M F L Y E Y A R
L A K T Y E T T L E K
search: 2610
PSMID: 187319433
scan: 11985
m/z: 935.2238
z: 4
score: 9.81
mean(ppmerror): 1.42
std(ppmerror): 5.04
% of base peak
m/z
0
25
50
75
100
270
585
900
1215
y5
y6
y7
y9
y8
b13+P
y4
y3
b2
y10
y11
b8+P
b9+P
b10+P
b11+P
b14+P
b15+P
b16+P
b17+P
b18+P
b8+P
b9+P
y10
y11
b11+P
b12+P
b13+P
b14+P

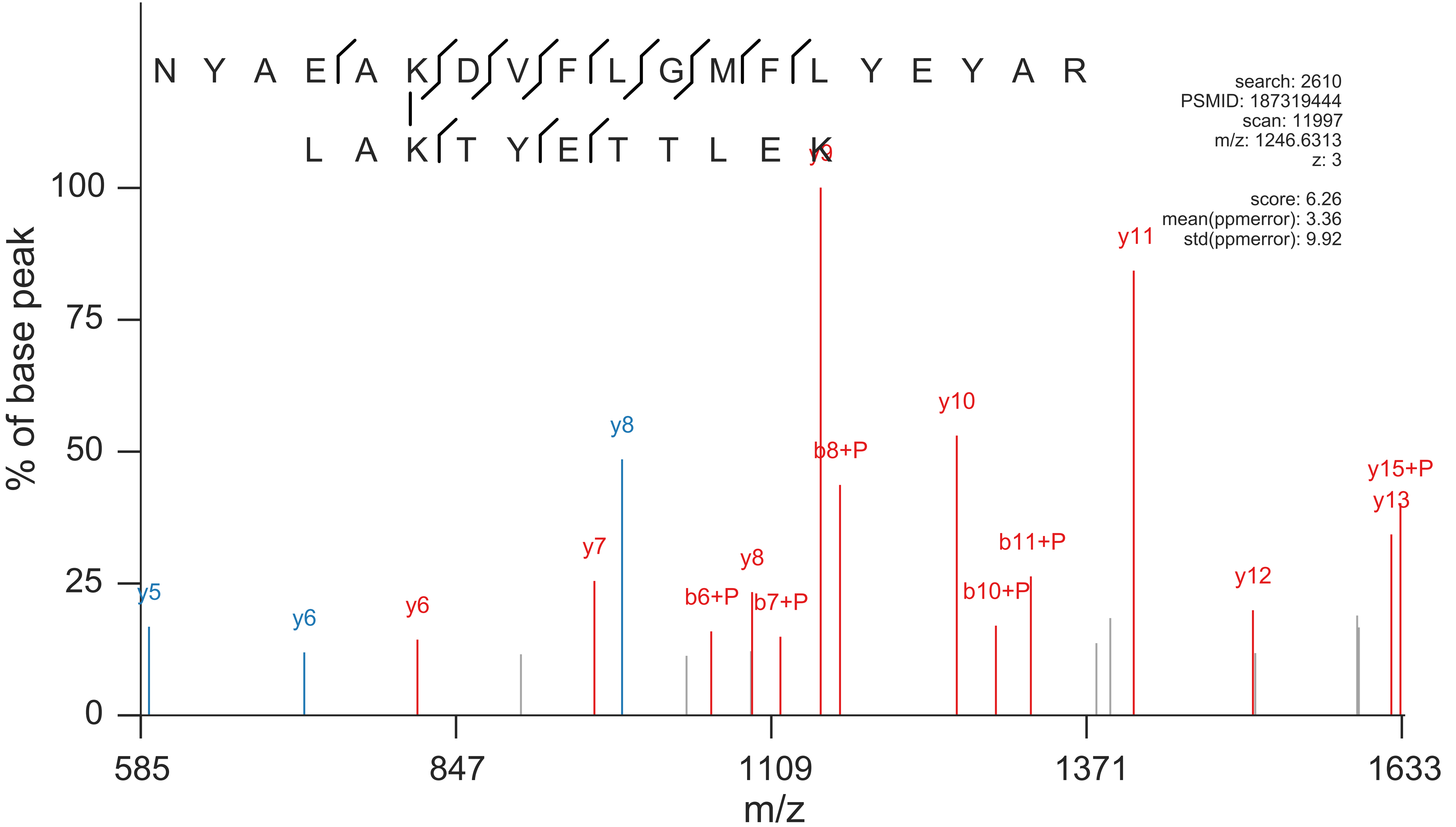

N Y A E A K D V F L G M F L Y E Y A R
L A K T Y E T T L E K
search: 2610
PSMID: 187319444
scan: 11997
m/z: 1246.6313
z: 3
score: 6.26
mean(ppmerror): 3.36
std(ppmerror): 9.92
% of base peak
m/z
0
25
50
75
100
585
847
1109
1371
1633
y5
y6
y6
y7
y8
b6+P
y8
b7+P
y9
b8+P
y10
b10+P
b11+P
y11
y12
y13
y15+P

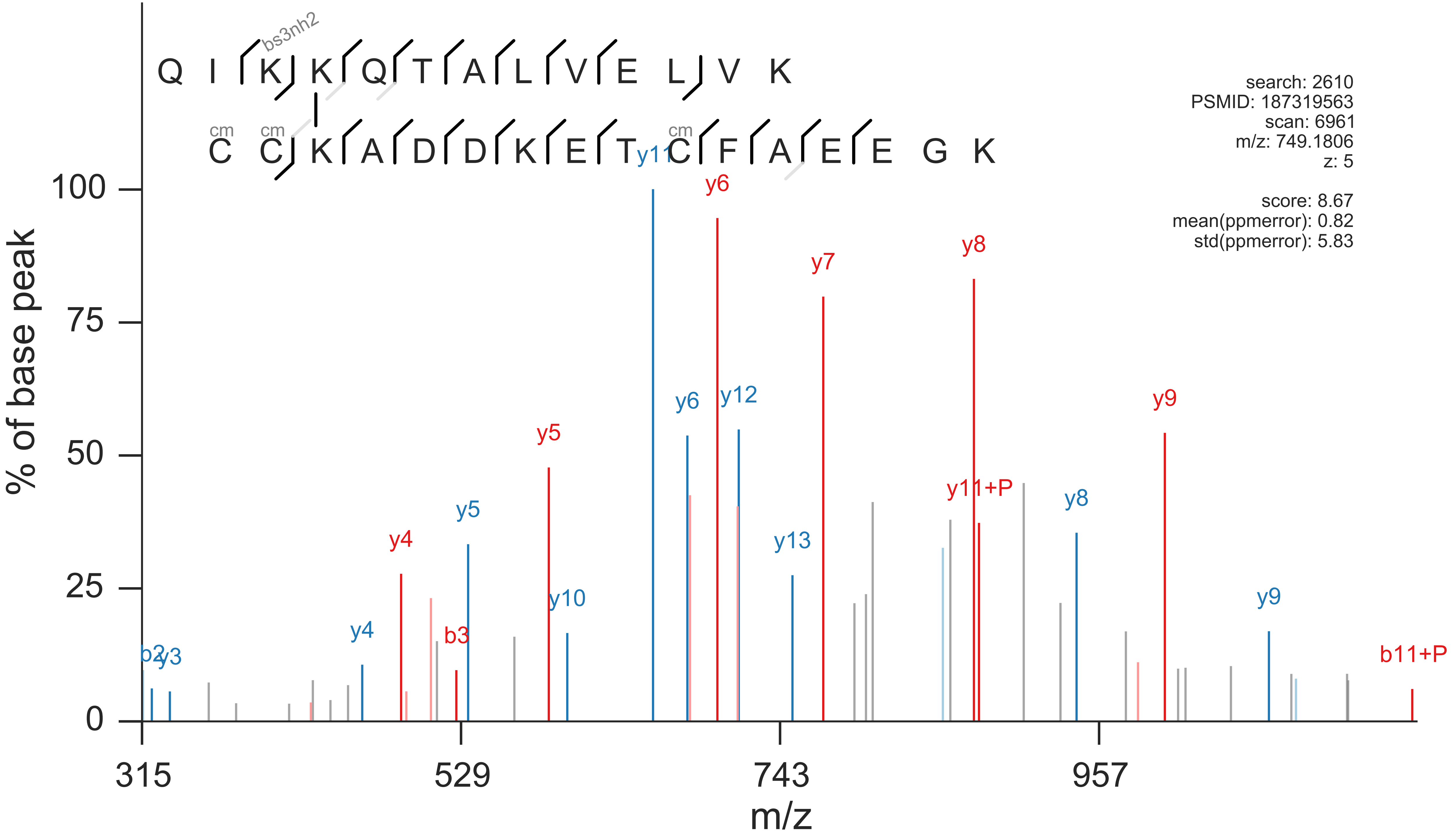

Q I K K Q T A L V E L V K
bs3nh2
cm cm cm
C C K A D D K E T C F A E E G K
search: 2610
PSMID: 187319563
scan: 6961
m/z: 749.1806
z: 5
score: 8.67
mean(ppmerror): 0.82
std(ppmerror): 5.83
% of base peak
m/z
100
75
50
25
0
315
529
743
957
y11
y6
y7
y8
y9
y5
y4
b3
y11+P
b11+P
y6
y12
y13
y8
y9
y10
y5
y4
b2
y3

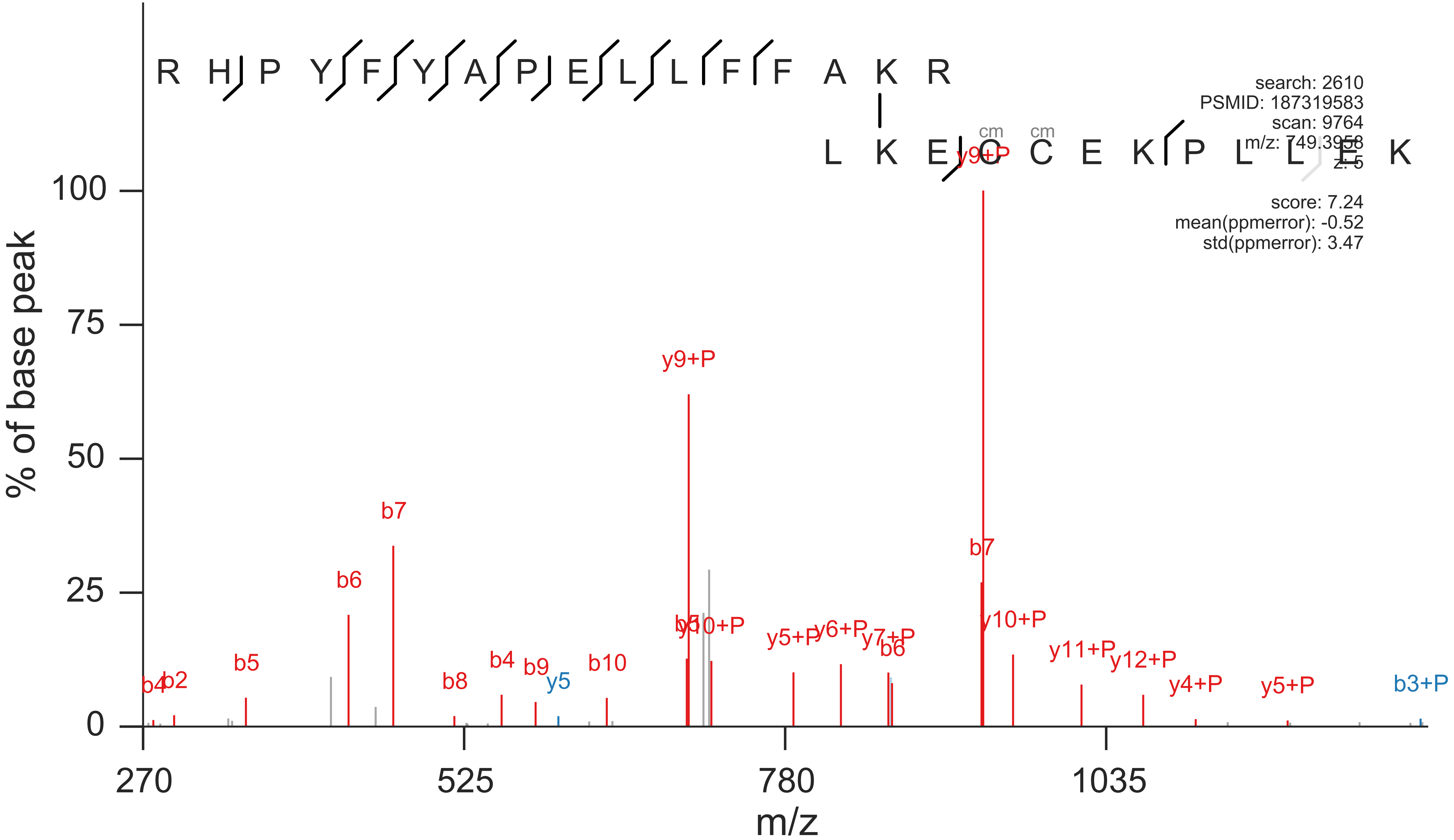

R H P Y F Y A P E L L F F A K R
L K E C E K P L L E K
cm cm
search: 2610
PSMID: 187319583
scan: 9764
m/z: 749.3958
z: 5
score: 7.24
mean(ppmerror): -0.52
std(ppmerror): 3.47
% of base peak
100
75
50
25
0
270
525
780
1035
m/z
y9+P
y9+P
b7
b7
b6
b6
b5
b4
b2
b8
b4
b9
y5
b10
y10+P
y5+P
y6+P
y7+P
y10+P
y11+P
y12+P
y4+P
y5+P
b3+P

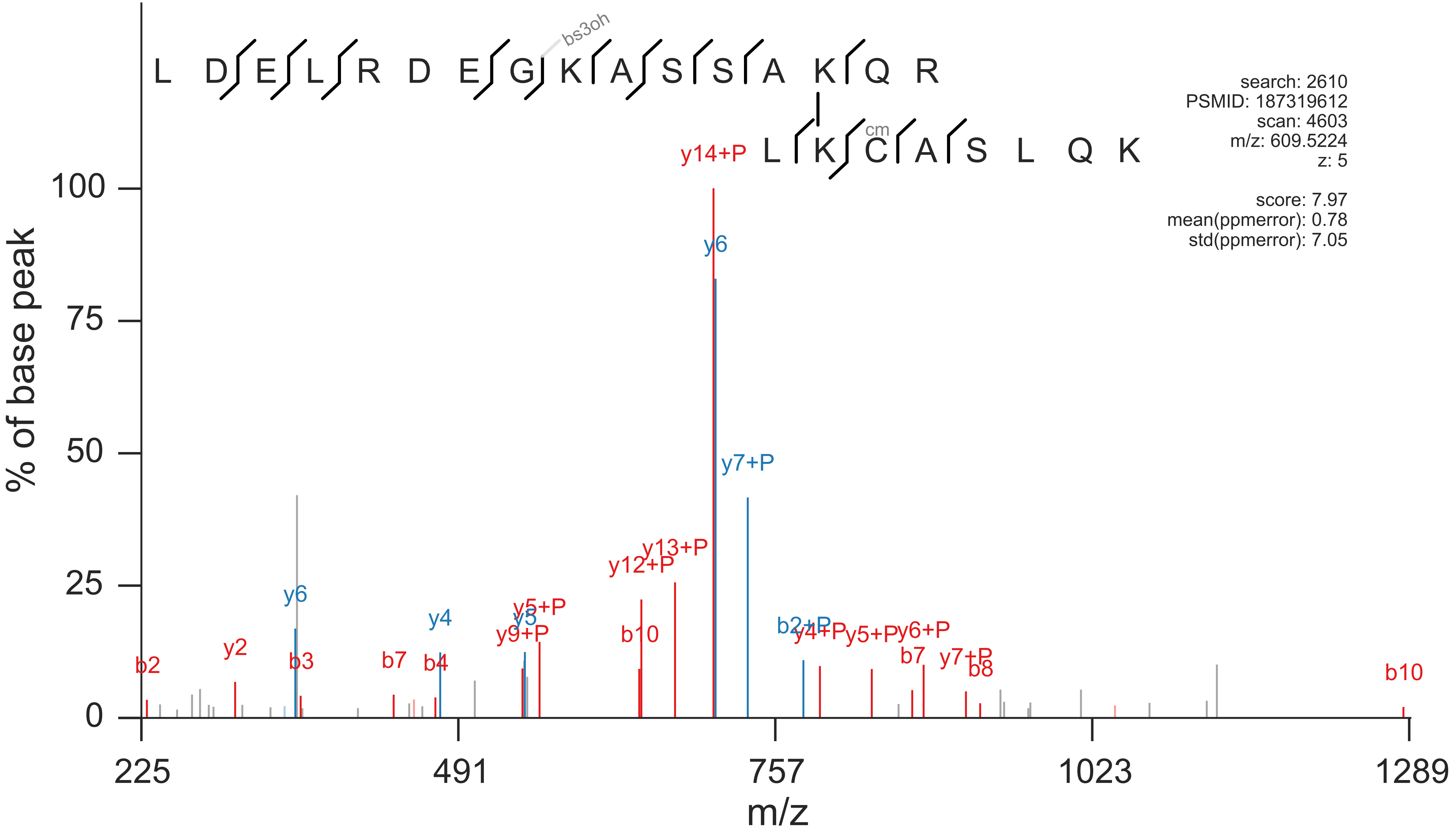

L D E L R D E G K A S S A K Q R
bs3oh
L K C A S L Q K
cm
search: 2610
PSMID: 187319612
scan: 4603
m/z: 609.5224
z: 5
score: 7.97
mean(ppmerror): 0.78
std(ppmerror): 7.05
% of base peak
m/z
100
75
50
25
0
225
491
757
1023
1289
y14+P
y6
y7+P
y13+P
y12+P
b10
y6
y4
y5+P
y5
y9+P
y2
b2
b3
b7
b4
b2+P
y4+P
y5+P
y6+P
b7
y7+P
b8
b10

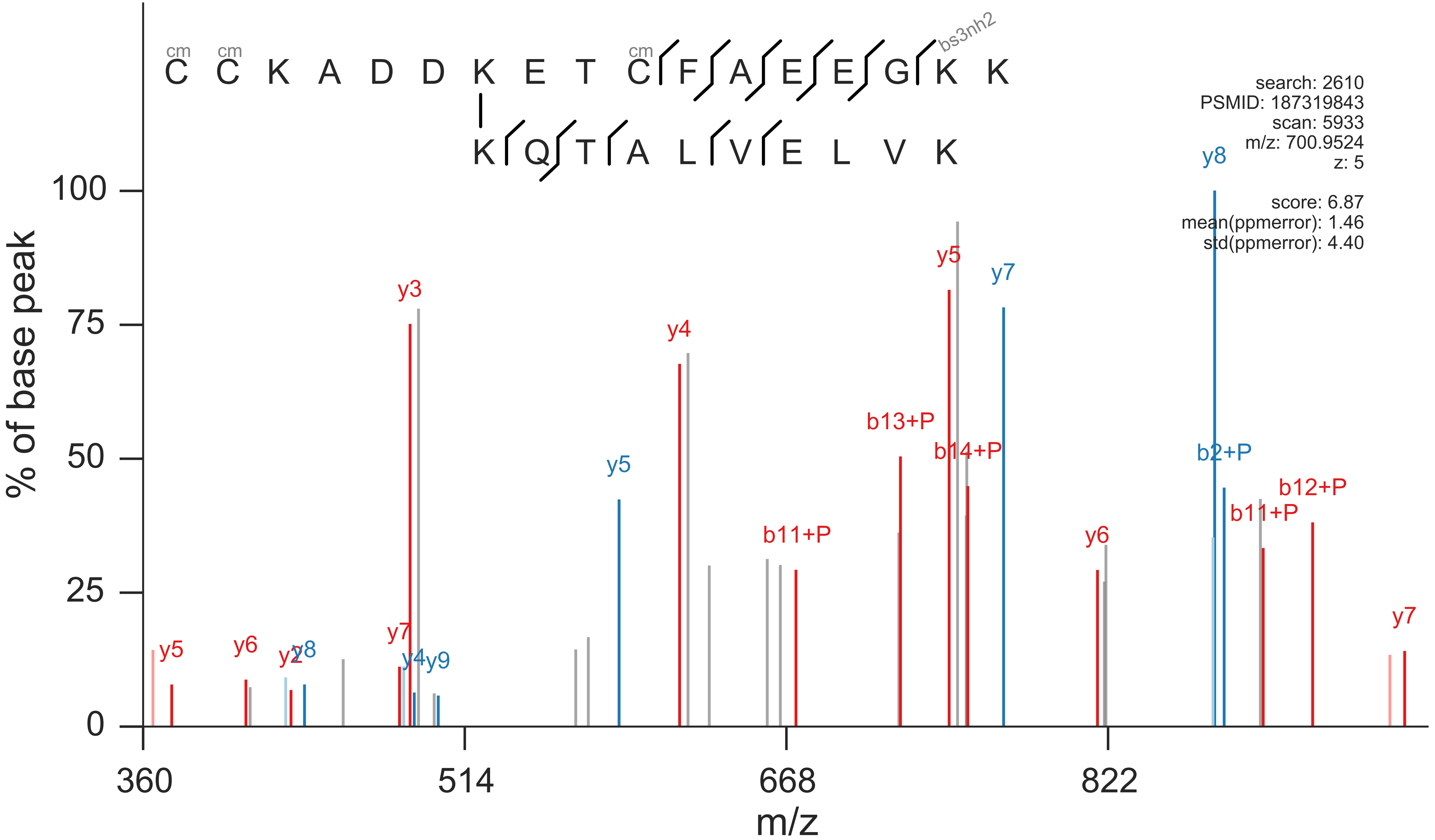

cm cm cm
C C K A D D K E T C F A E E G K K
bs3nh2
K Q T A L V E L V K
search: 2610
PSMID: 187319843
scan: 5933
m/z: 700.9524
z: 5
score: 6.87
mean(ppmerror): 1.46
std(ppmerror): 4.40
% of base peak
m/z
y5 y6 y2 y8 y7 y4 y9 y3 y5 y4 b11+P b13+P y5 b14+P y7 y6 y8 b2+P b11+P b12+P y7
0 25 50 75 100
360 514 668 822

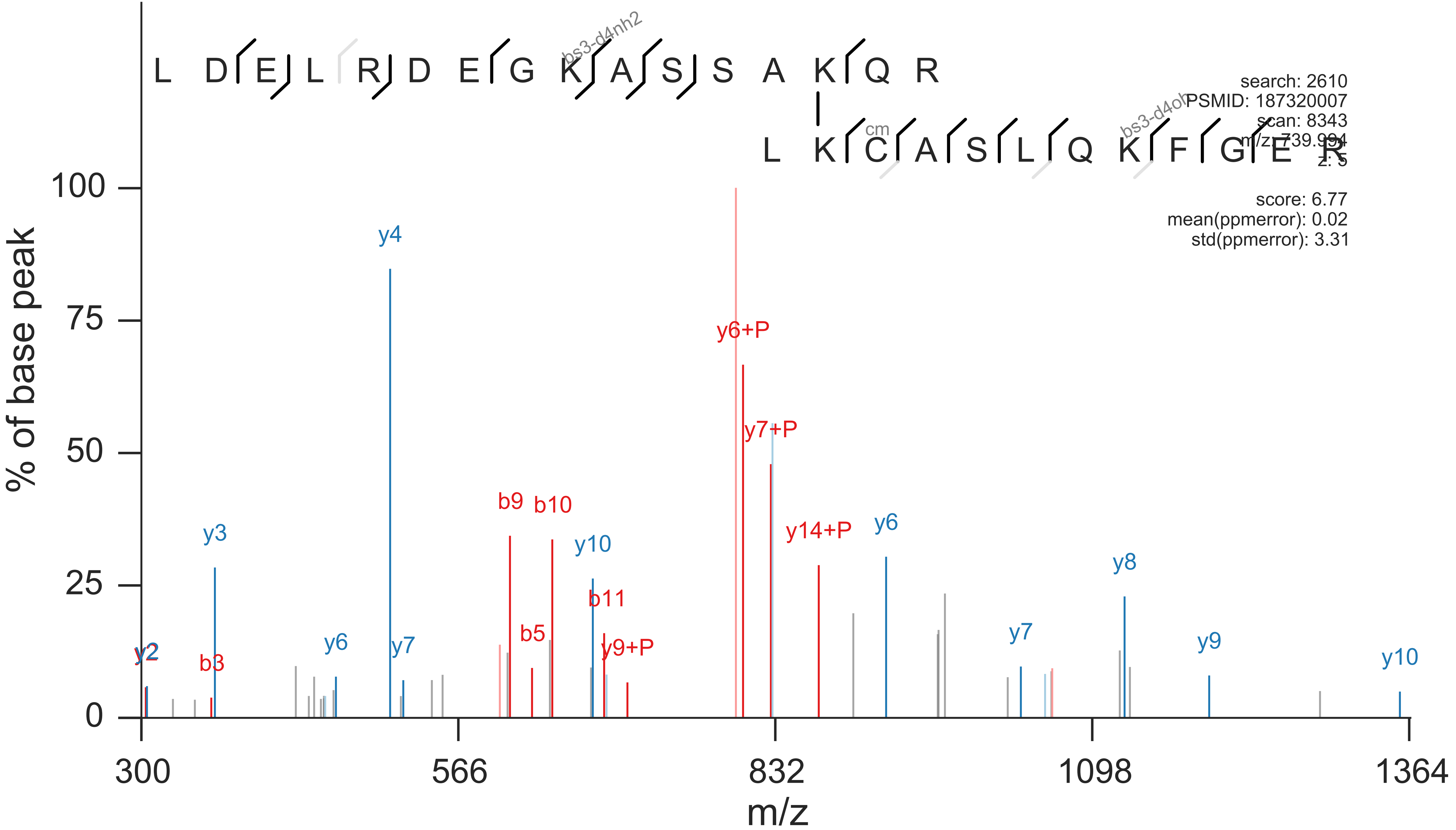

L D E L R D E G K A S S A K Q R
bs3-d4nh2
L K C A S L Q K F G E R
cm
bs3-d4oh
search: 2610
PSMID: 187320007
scan: 8343
m/z: 739.994
z: 5
score: 6.77
mean(ppmerror): 0.02
std(ppmerror): 3.31
% of base peak
m/z
y4
y6+P
y7+P
b9
b10
y10
y14+P
y6
y3
y8
b11
b5
y9+P
y6
y7
y7
y9
y10
y2
b3

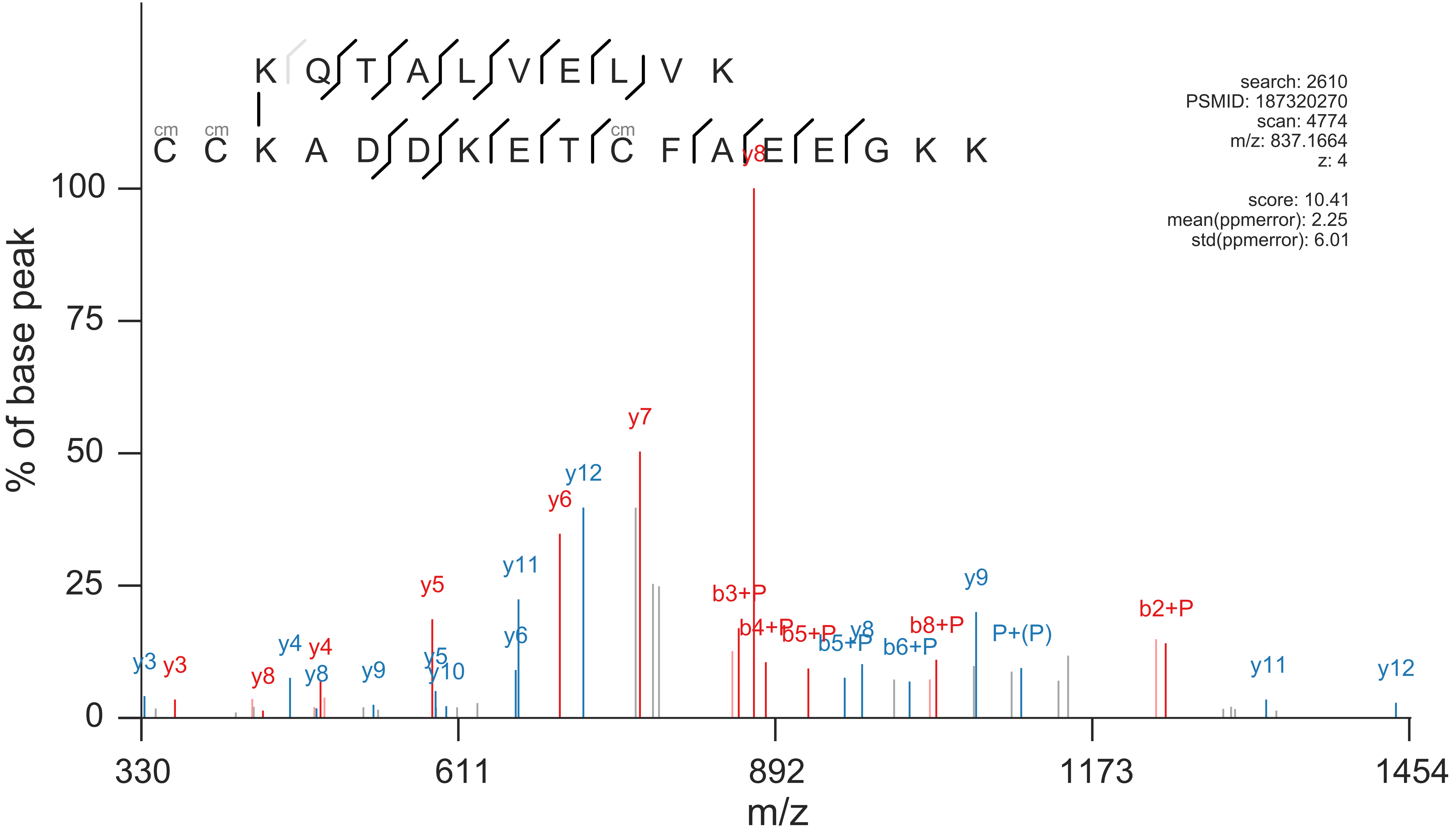

K Q T A L V E L V K
cm cm cm
C C K A D D K E T C F A E E G K K
search: 2610
PSMID: 187320270
scan: 4774
m/z: 837.1664
z: 4
score: 10.41
mean(ppmerror): 2.25
std(ppmerror): 6.01
% of base peak
100
75
50
25
0
y3 y3 y8 y4 y4 y8 y9 y5 y5 y10 y11 y6 y6 y12 y7 b3+P y8 b4+P b5+P b5+P y8 b6+P b8+P y9 P+(P) b2+P y11 y12
330
611
892
1173
1454
m/z

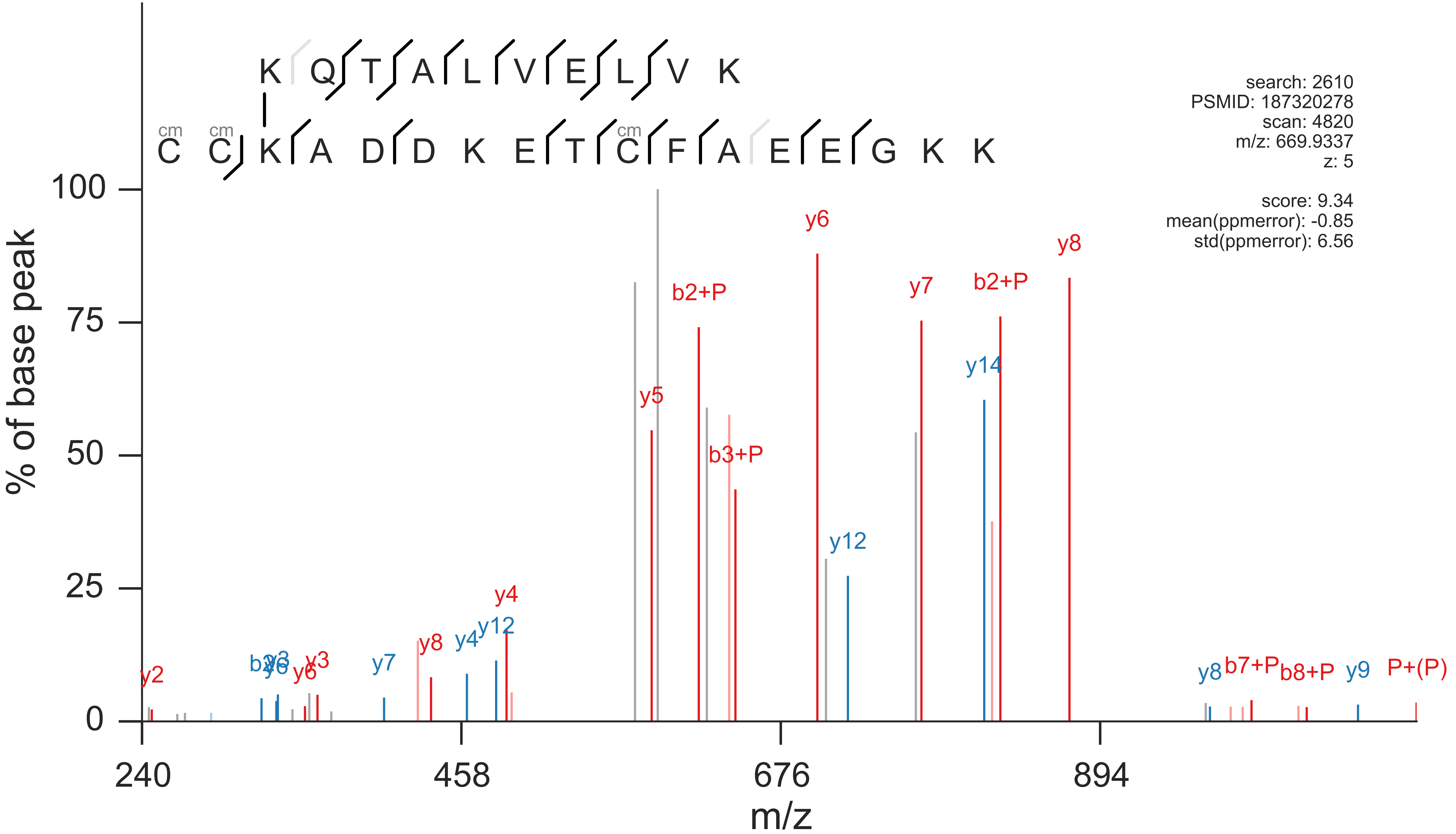

K Q T A L V E L V K
cm cm cm
C C K A D D K E T C F A E E G K K
search: 2610
PSMID: 187320278
scan: 4820
m/z: 669.9337
z: 5
score: 9.34
mean(ppmerror): -0.85
std(ppmerror): 6.56
% of base peak
m/z
0
25
50
75
100
240
458
676
894
y2
y7
y8
y4
y12
y4
y5
b2+P
b3+P
y6
y12
y7
b2+P
y14
y8
y8
b7+P
b8+P
y9
P+(P)

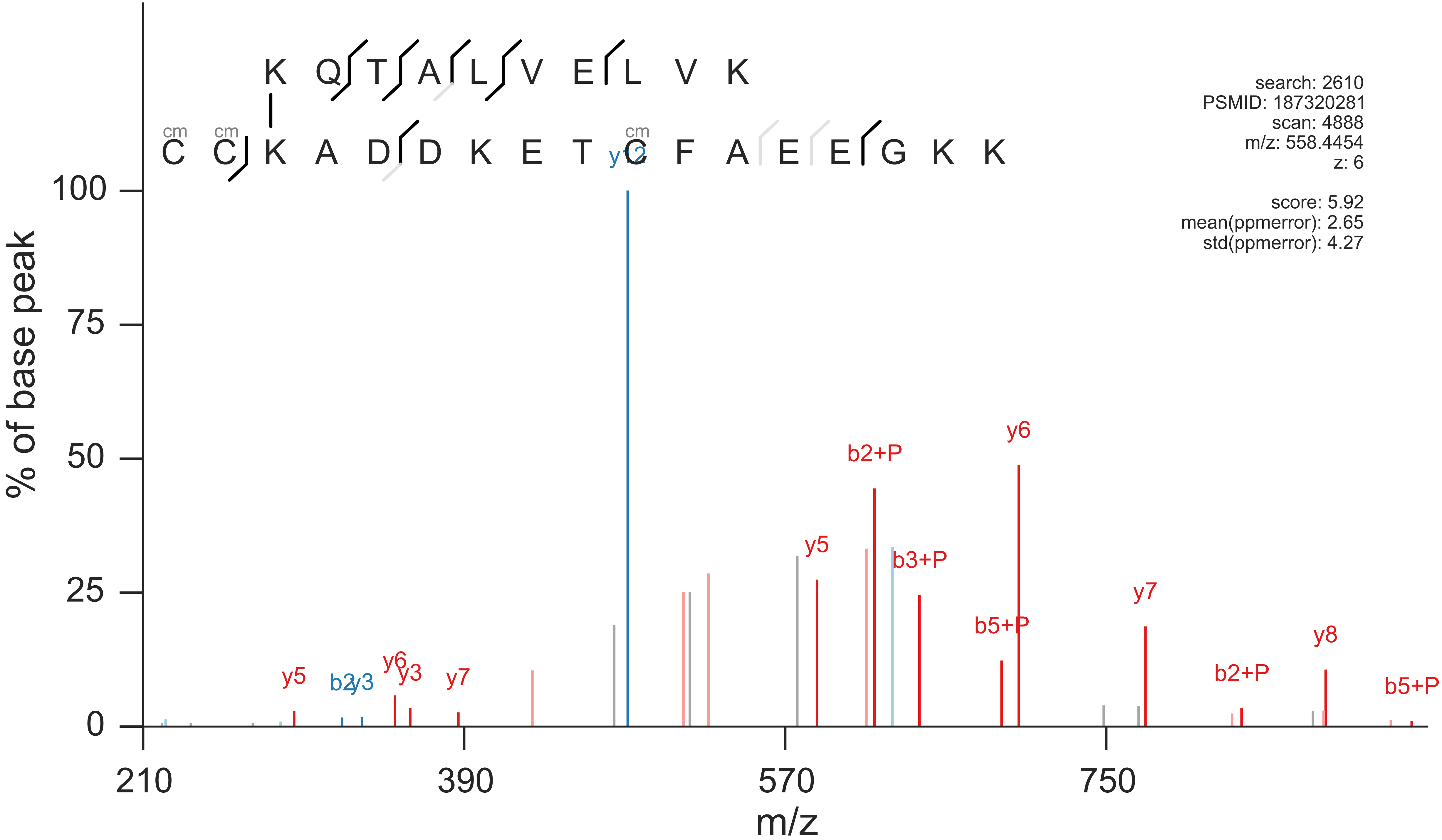

K Q T A L V E L V K
cm cm cm
C C K A D D K E T C F A E E G K K
search: 2610
PSMID: 187320281
scan: 4888
m/z: 558.4454
z: 6
score: 5.92
mean(ppmerror): 2.65
std(ppmerror): 4.27
% of base peak
m/z
100
75
50
25
0
210
390
570
750
y12
y5
b2
y3
y6
y3
y7
y5
b2+P
b3+P
y6
b5+P
y7
b2+P
y8
b5+P

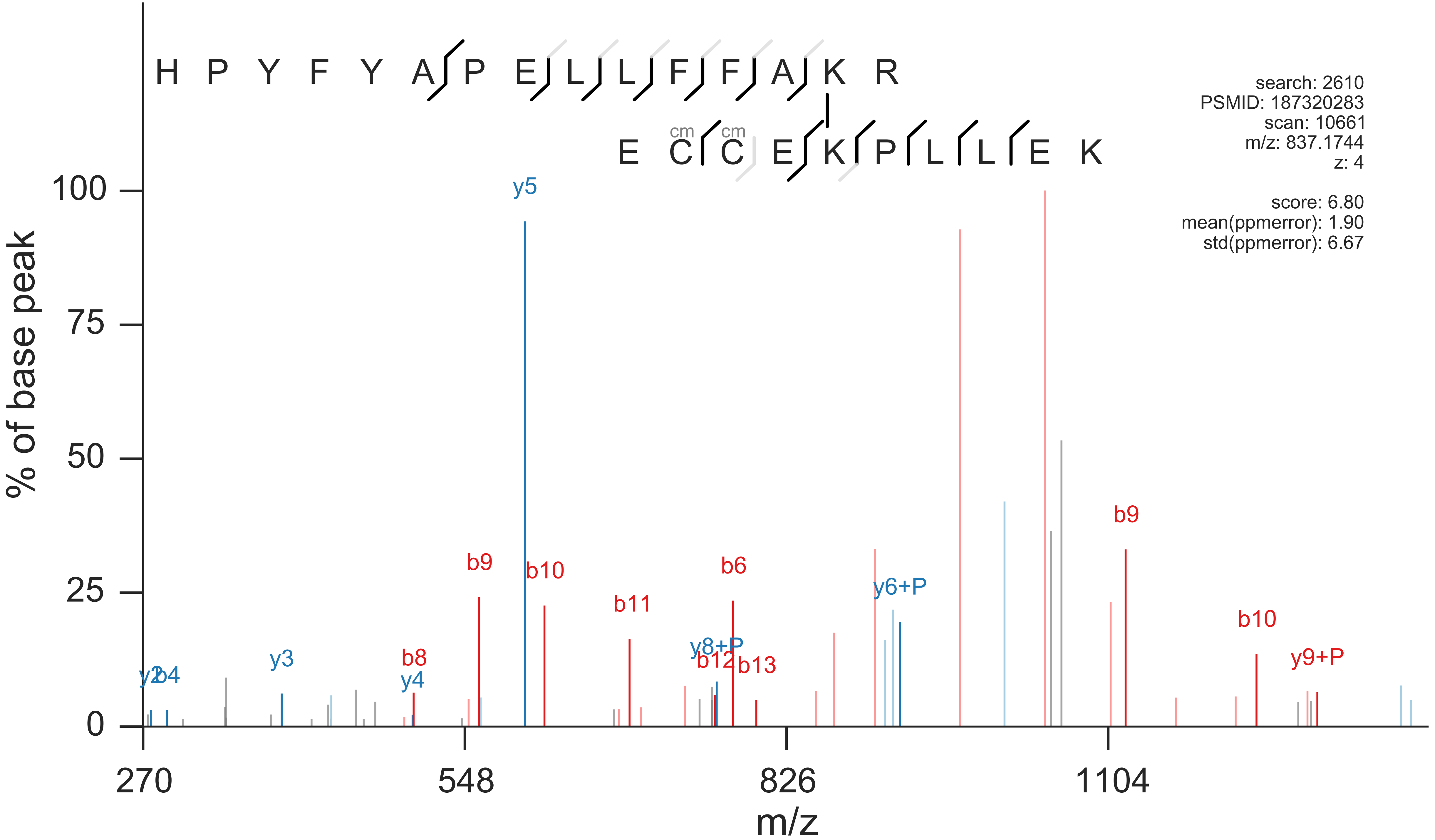

H P Y F Y A P E L L F F A K R
E C C E K P L L E K
cm
cm
search: 2610
PSMID: 187320283
scan: 10661
m/z: 837.1744
z: 4
score: 6.80
mean(ppmerror): 1.90
std(ppmerror): 6.67
% of base peak
m/z
0
25
50
75
100
270
548
826
1104
y5
b9
b10
b11
b6
y6+P
b9
b10
y9+P
y8+P
b12
b13
b8
y4
y3
y2
b4

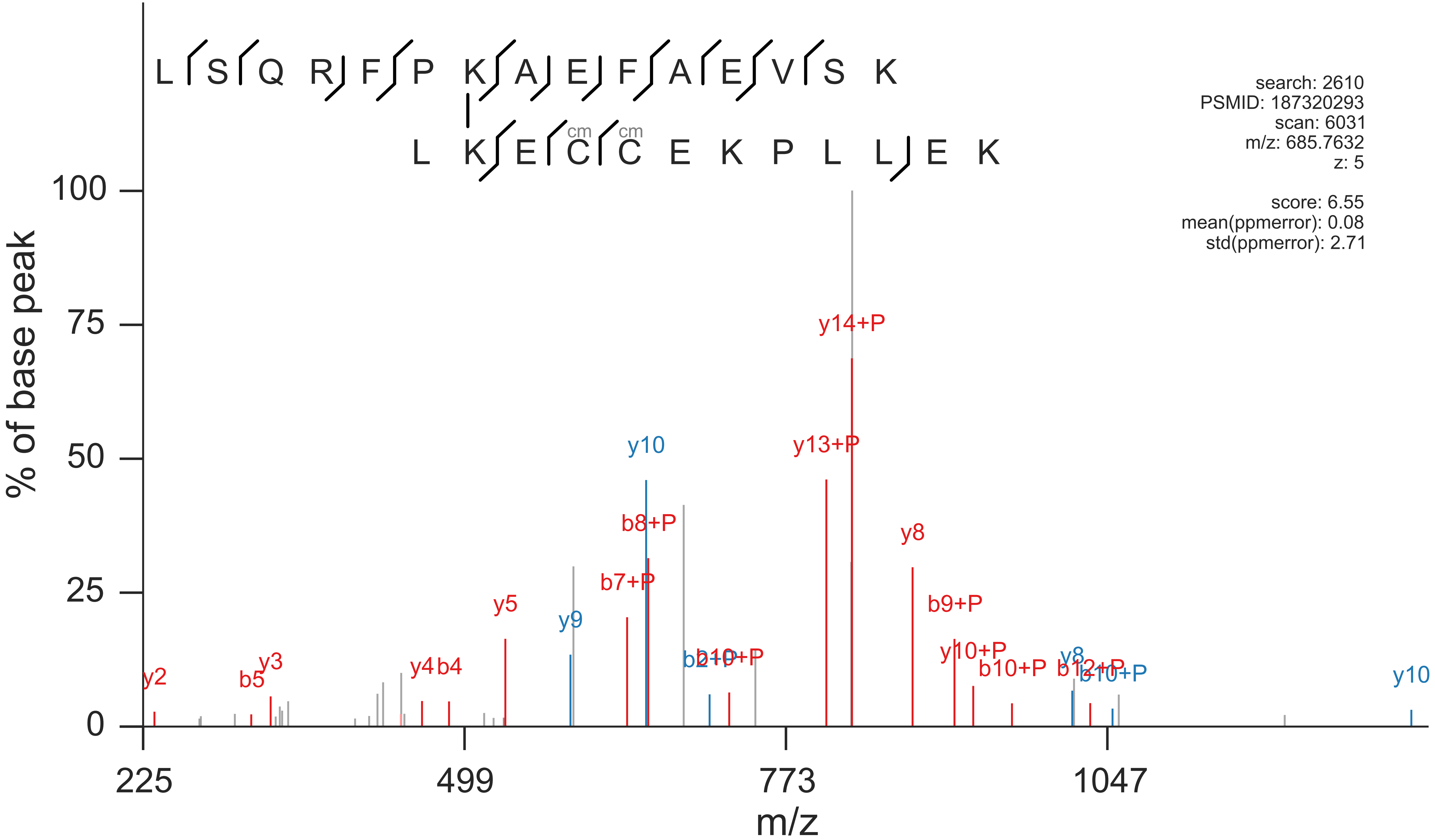

L S Q R F P K A E F A E V S K
L K E C C E K P L L E K
cm
cm
search: 2610
PSMID: 187320293
scan: 6031
m/z: 685.7632
z: 5
score: 6.55
mean(ppmerror): 0.08
std(ppmerror): 2.71
% of base peak
m/z
100
75
50
25
0
225
499
773
1047
y14+P
y13+P
y10
b8+P
y8
b7+P
b9+P
y5
y9
y10+P
b10+P
y2
b5
y3
y4
b4
y8
y10

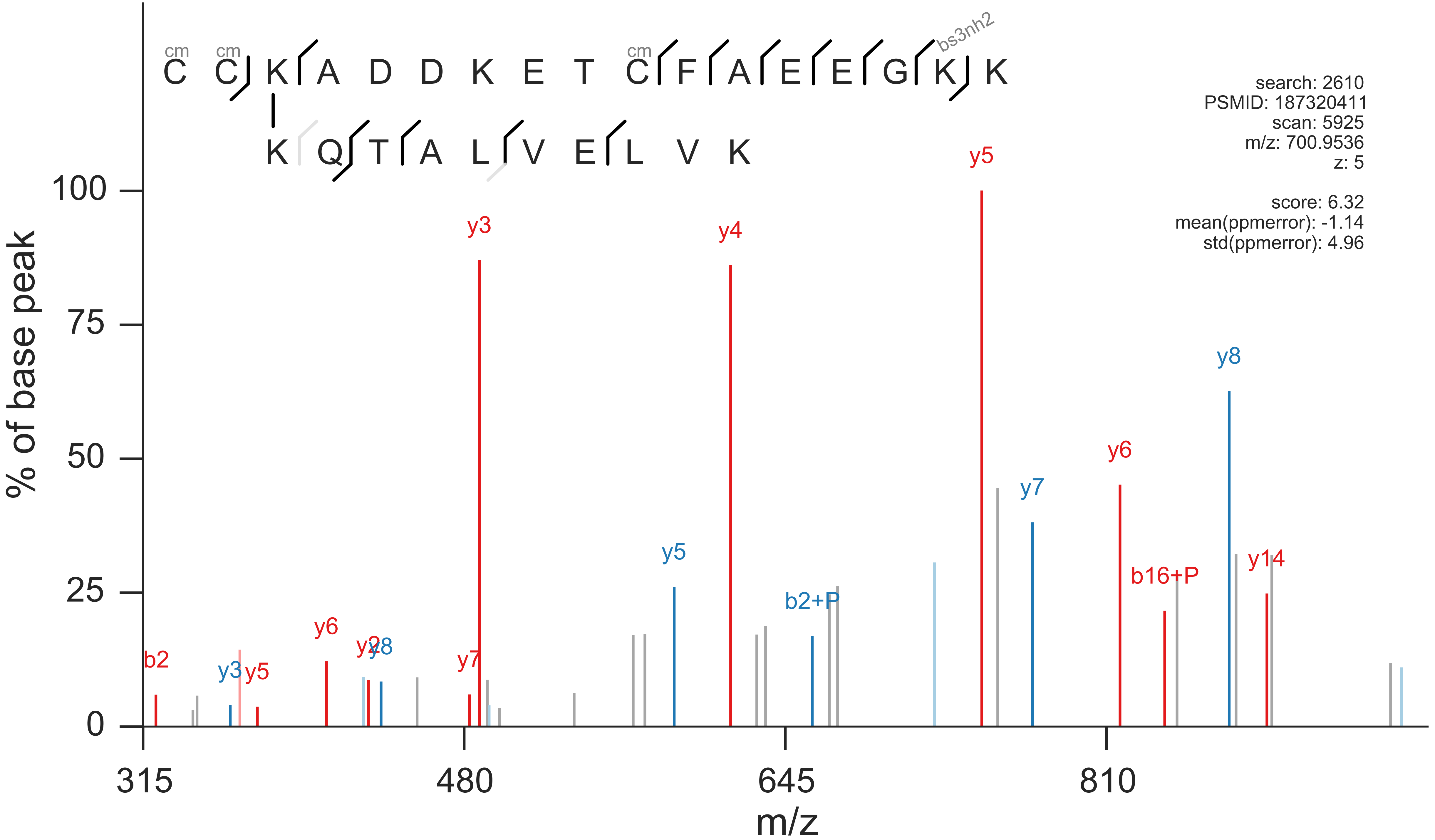

cm cm cm
C C K A D D K E T C F A E E G K K
bs3nh2
K Q T A L V E L V K
search: 2610
PSMID: 187320411
scan: 5925
m/z: 700.9536
z: 5
score: 6.32
mean(ppmerror): -1.14
std(ppmerror): 4.96
% of base peak
m/z
0
25
50
75
100
315
480
645
810
b2
y3
y5
y6
y2
y8
y7
y3
y5
y4
b2+P
y5
y7
y6
b16+P
y8
y14

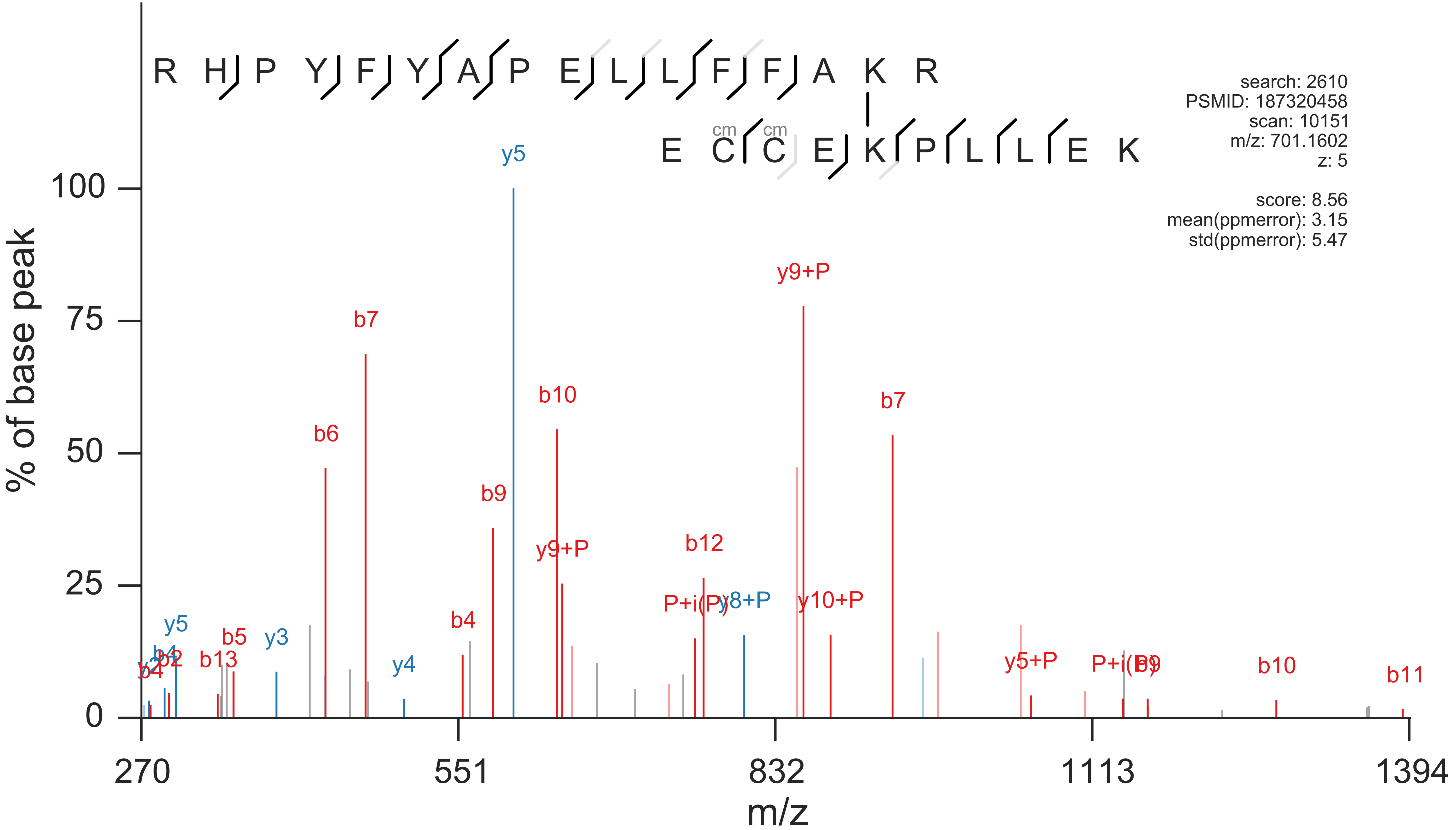

R H P Y F Y A P E L L F F A K R
E C C E K P L L E K
cm cm
search: 2610
PSMID: 187320458
scan: 10151
m/z: 701.1602
z: 5
score: 8.56
mean(ppmerror): 3.15
std(ppmerror): 5.47
% of base peak
m/z
0
25
50
75
100
270
551
832
1113
1394
y5
y9+P
b7
b10
b7
b6
b9
y9+P
b12
P+i(P)
y8+P
y10+P
b4
y5
b5
y3
b13
y4
y5+P
b10
b11

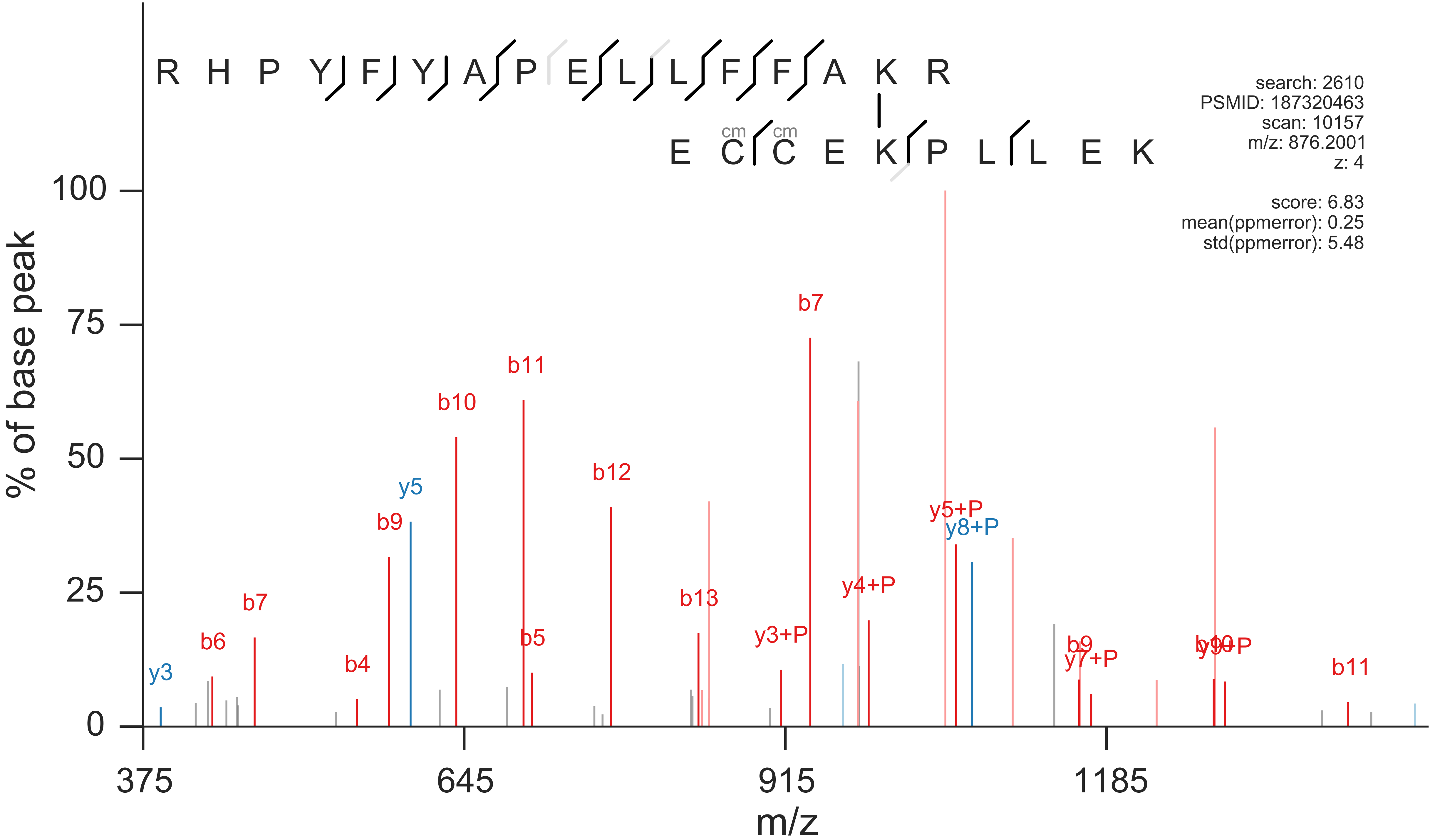

R H P Y F Y A P E L L F F A K R
E C C E K P L L E K
cm cm
search: 2610
PSMID: 187320463
scan: 10157
m/z: 876.2001
z: 4
score: 6.83
mean(ppmerror): 0.25
std(ppmerror): 5.48
% of base peak
m/z
0
25
50
75
100
375
645
915
1185
y3
b6
b7
b4
b9
y5
b10
b11
b5
b12
b13
y3+P
b7
y4+P
y5+P
y8+P
b9
y7+P
b10
y9+P
b11

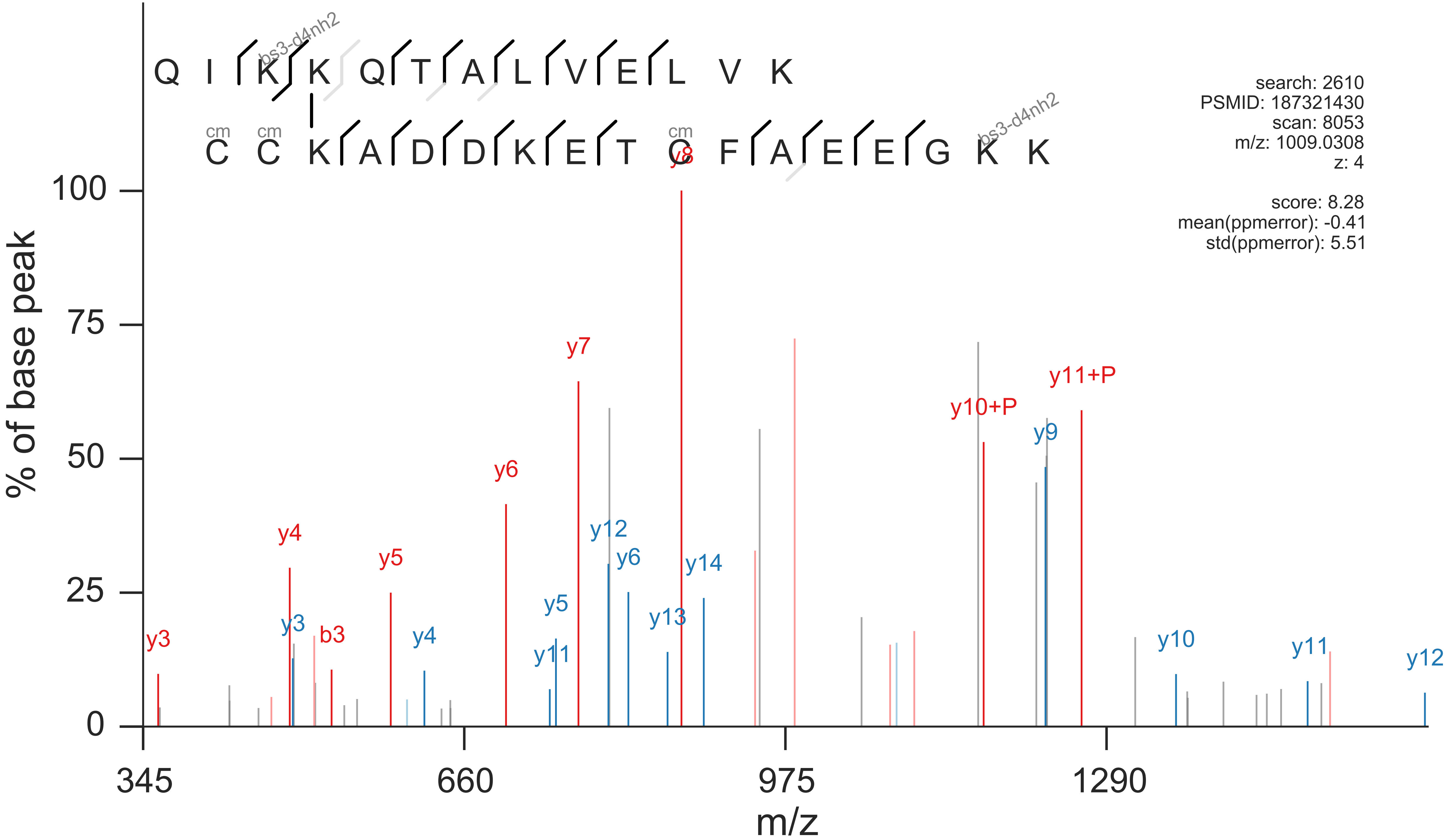

Q I K K Q T A L V E L V K
bs3-d4nh2
cm cm cm
C C K A D D K E T C F A E E G K K
bs3-d4nh2
search: 2610
PSMID: 187321430
scan: 8053
m/z: 1009.0308
z: 4
score: 8.28
mean(ppmerror): -0.41
std(ppmerror): 5.51
% of base peak
m/z
y3
y4
y5
y6
y7
y8
b3
y10+P
y11+P
y9
y10
y11
y12
y13
y14

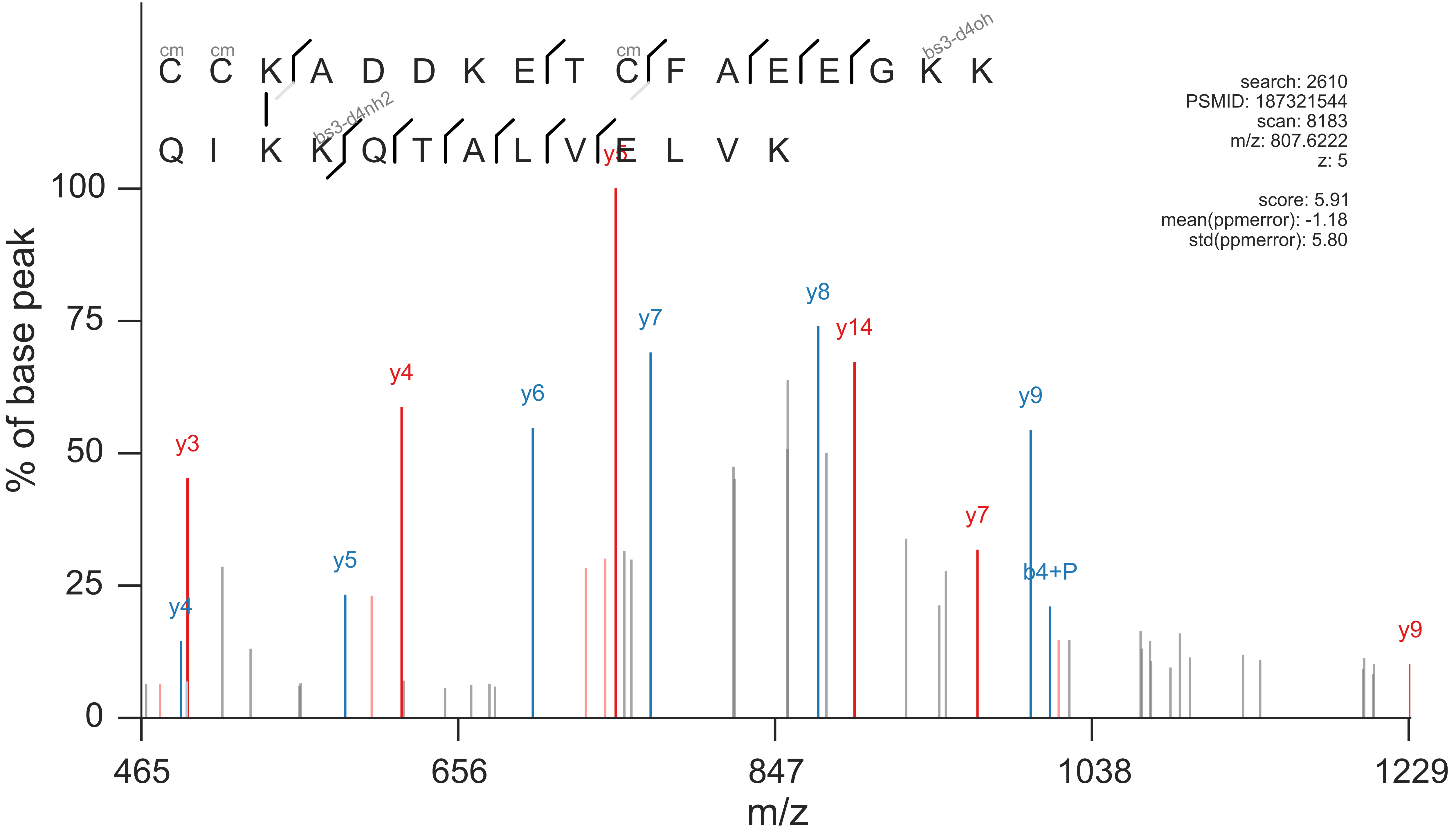

C C K A D D K E T C F A E E G K K
cm cm cm
bs3-d4oh
Q I K K Q T A L V E L V K
bs3-d4nh2
search: 2610
PSMID: 187321544
scan: 8183
m/z: 807.6222
z: 5
score: 5.91
mean(ppmerror): -1.18
std(ppmerror): 5.80
% of base peak
m/z
y3 y4 y5 y4 y6 y5 y7 y8 y14 y7 y9 b4+P y9

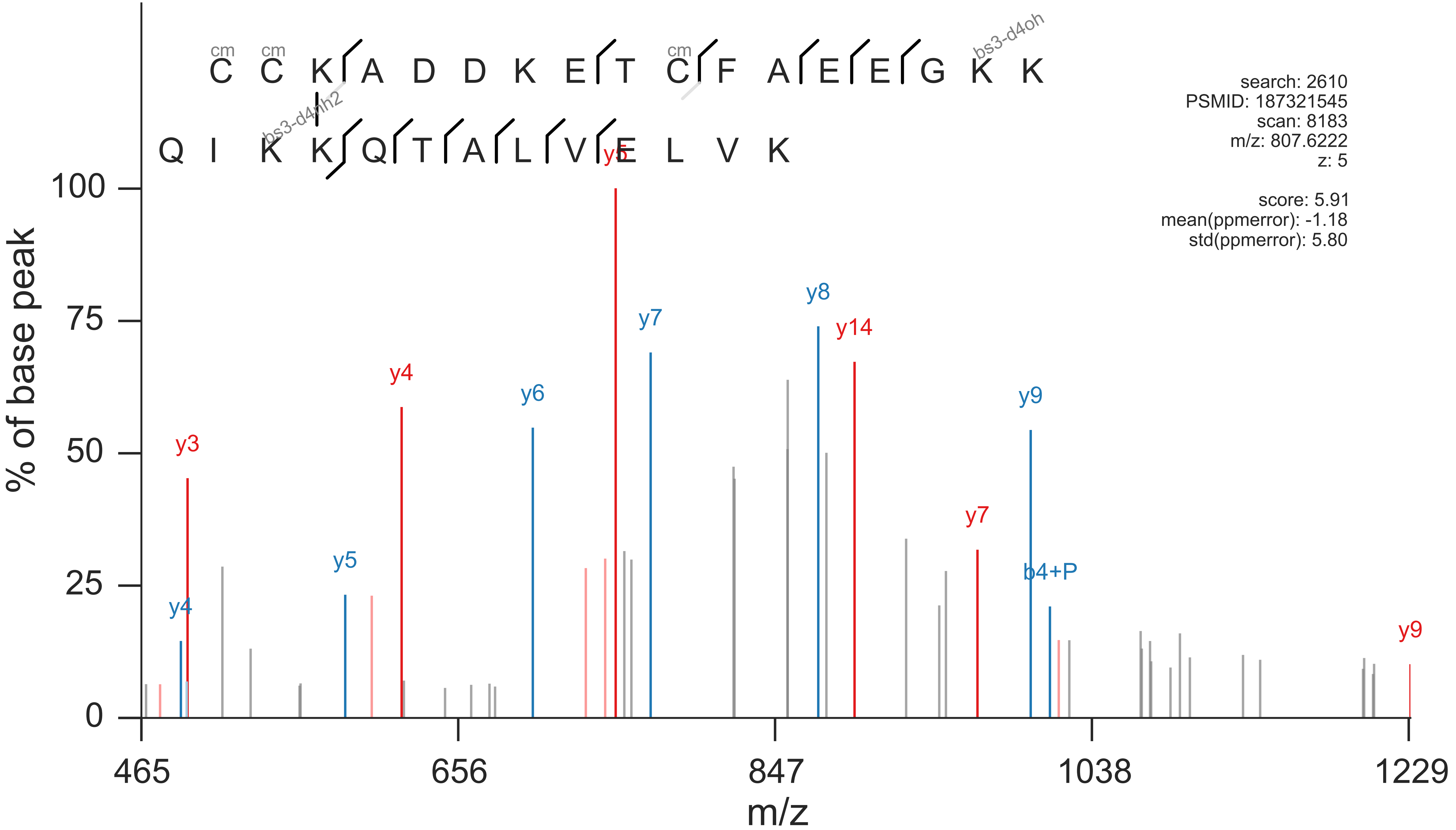

cm cm cm
C C K A D D K E T C F A E E G K K
bs3-d4oh
bs3-d4nh2
Q I K K Q T A L V E L V K
search: 2610
PSMID: 187321545
scan: 8183
m/z: 807.6222
z: 5
score: 5.91
mean(ppmerror): -1.18
std(ppmerror): 5.80
% of base peak
100
75
50
25
0
y3
y4
y5
y4
y6
y5
y7
y8
y14
y7
y9
b4+P
y9
465
656
847
1038
1229
m/z

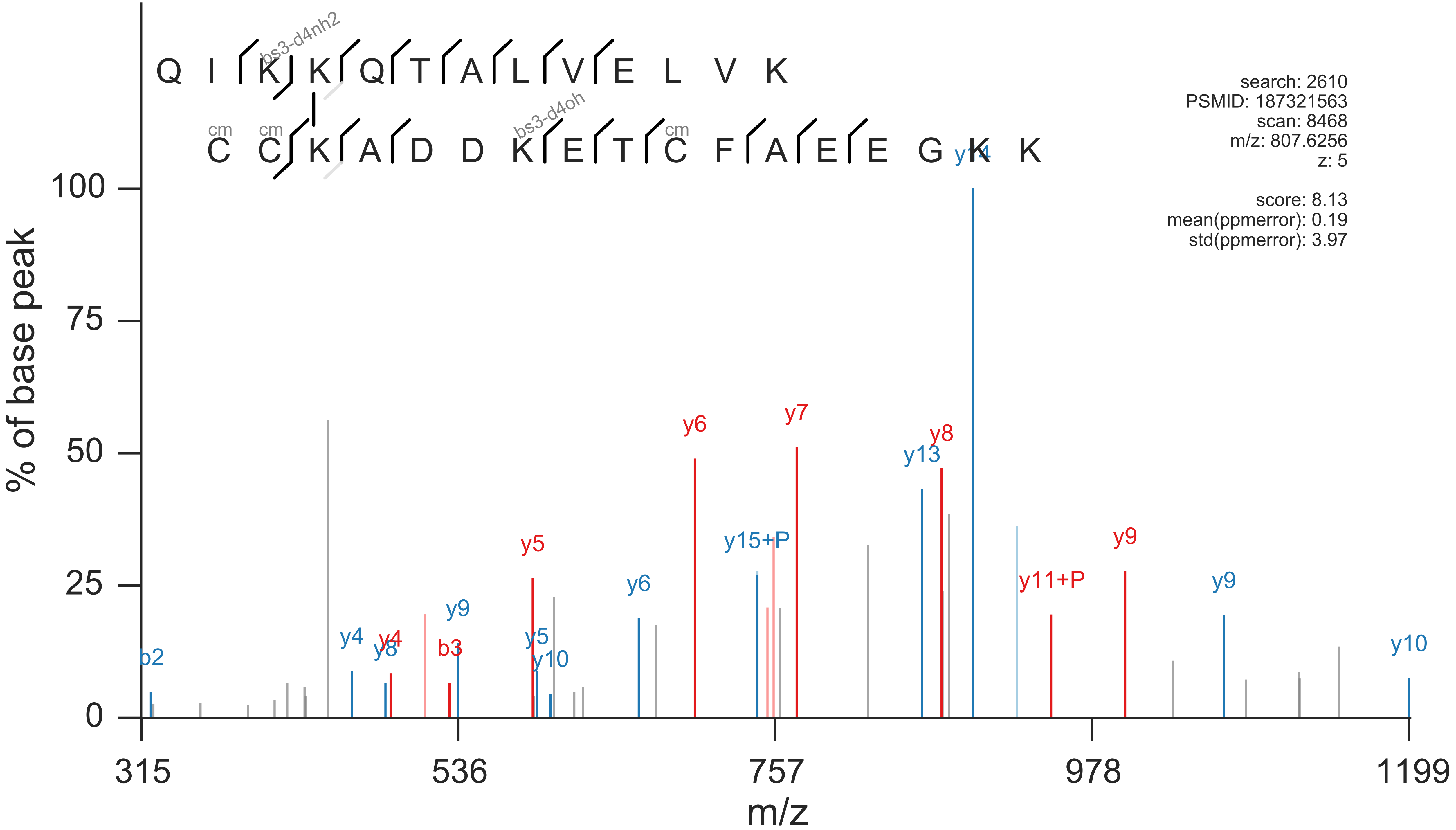

Q I K K Q T A L V E L V K
bs3-d4nh2
cm cm cm
C C K A D D K E T C F A E E G K K
bs3-d4oh
search: 2610
PSMID: 187321563
scan: 8468
m/z: 807.6256
z: 5
score: 8.13
mean(ppmerror): 0.19
std(ppmerror): 3.97
% of base peak
m/z
0
25
50
75
100
315
536
757
978
1199
b2
y4
y8
y4
y9
b3
y5
y5
y10
y6
y6
y15+P
y7
y13
y8
y14
y11+P
y9
y9
y10

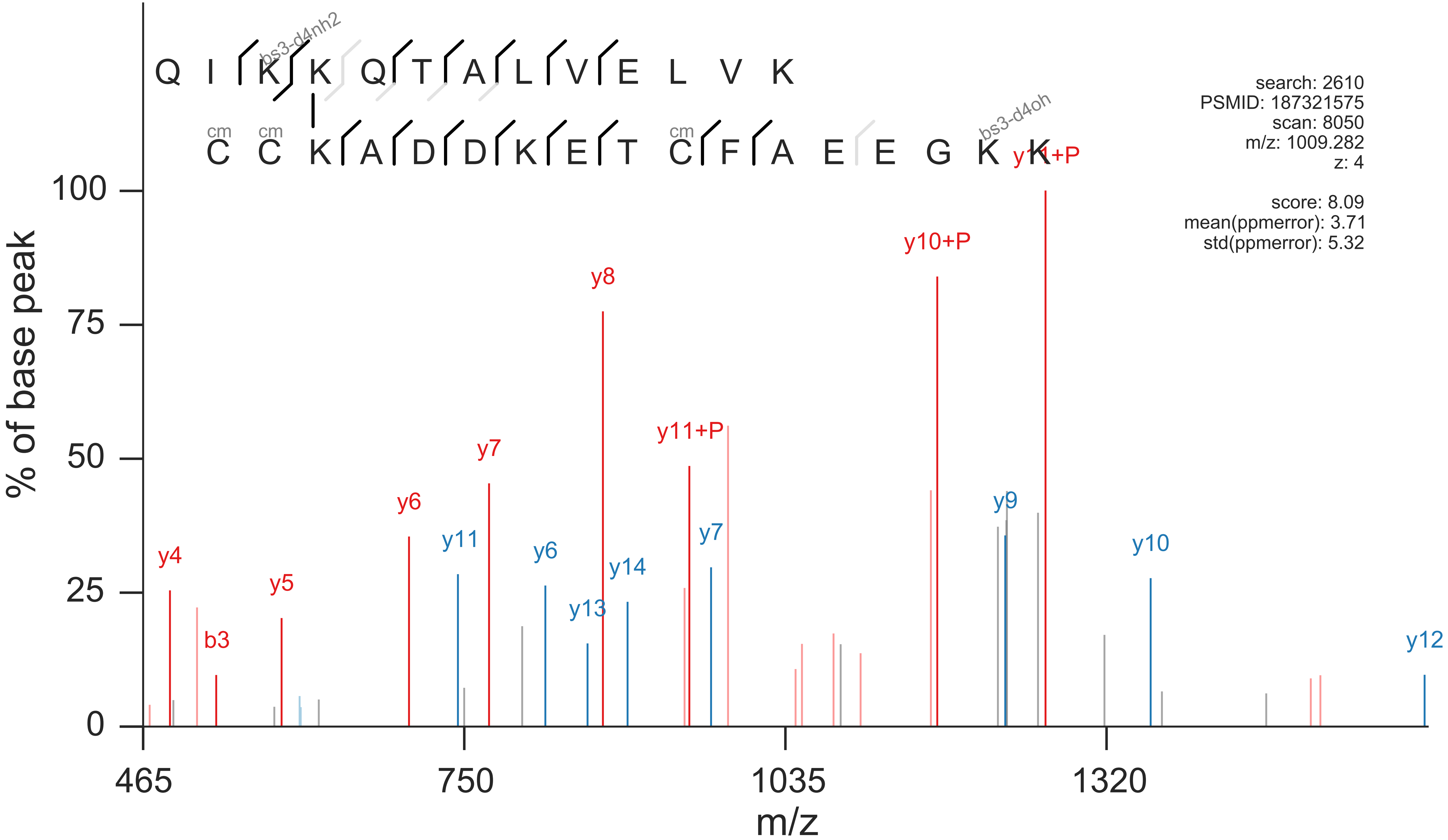

Q I K K Q T A L V E L V K
bs3-d4nh2
cm C cm C K A D D K E T cm C F A E E G K K
bs3-d4oh
search: 2610
PSMID: 187321575
scan: 8050
m/z: 1009.282
z: 4
score: 8.09
mean(ppmerror): 3.71
std(ppmerror): 5.32
% of base peak
m/z
0
25
50
75
100
465
750
1035
1320
y4
b3
y5
y6
y11
y7
y6
y13
y8
y14
y11+P
y7
y10+P
y9
y11+P
y10
y12

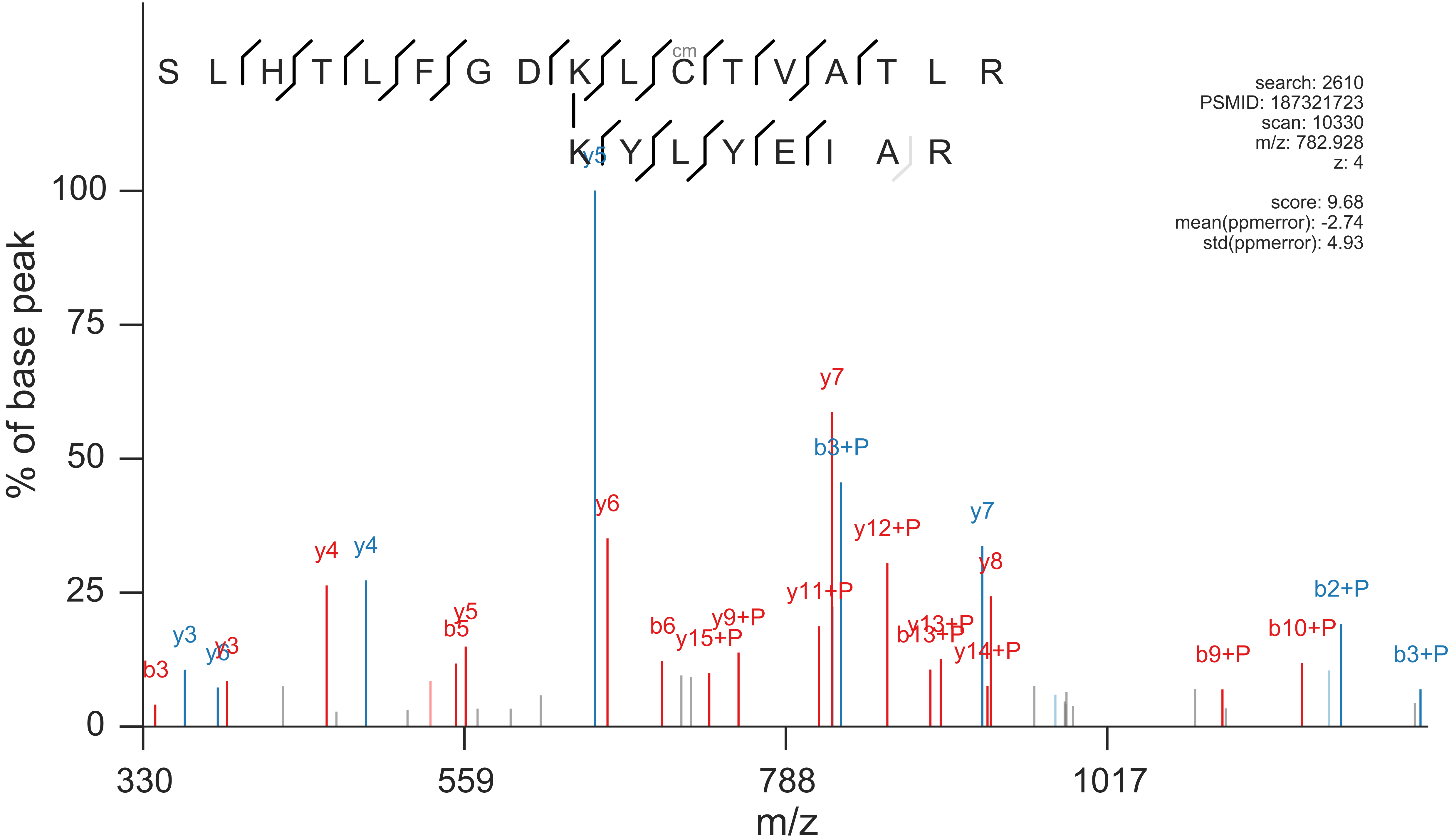

S L H T L F G D K L C T V A T L R
cm
K Y L Y E I A R
search: 2610
PSMID: 187321723
scan: 10330
m/z: 782.928
z: 4
score: 9.68
mean(ppmerror): -2.74
std(ppmerror): 4.93
% of base peak
m/z
100
75
50
25
0
330
559
788
1017
b3
y3
y3
y4
y4
b5
y5
y5
y6
b6
y15+P
y9+P
y11+P
y7
b3+P
y12+P
b13+P
y13+P
y7
y8
y14+P
b9+P
b10+P
b2+P
b3+P

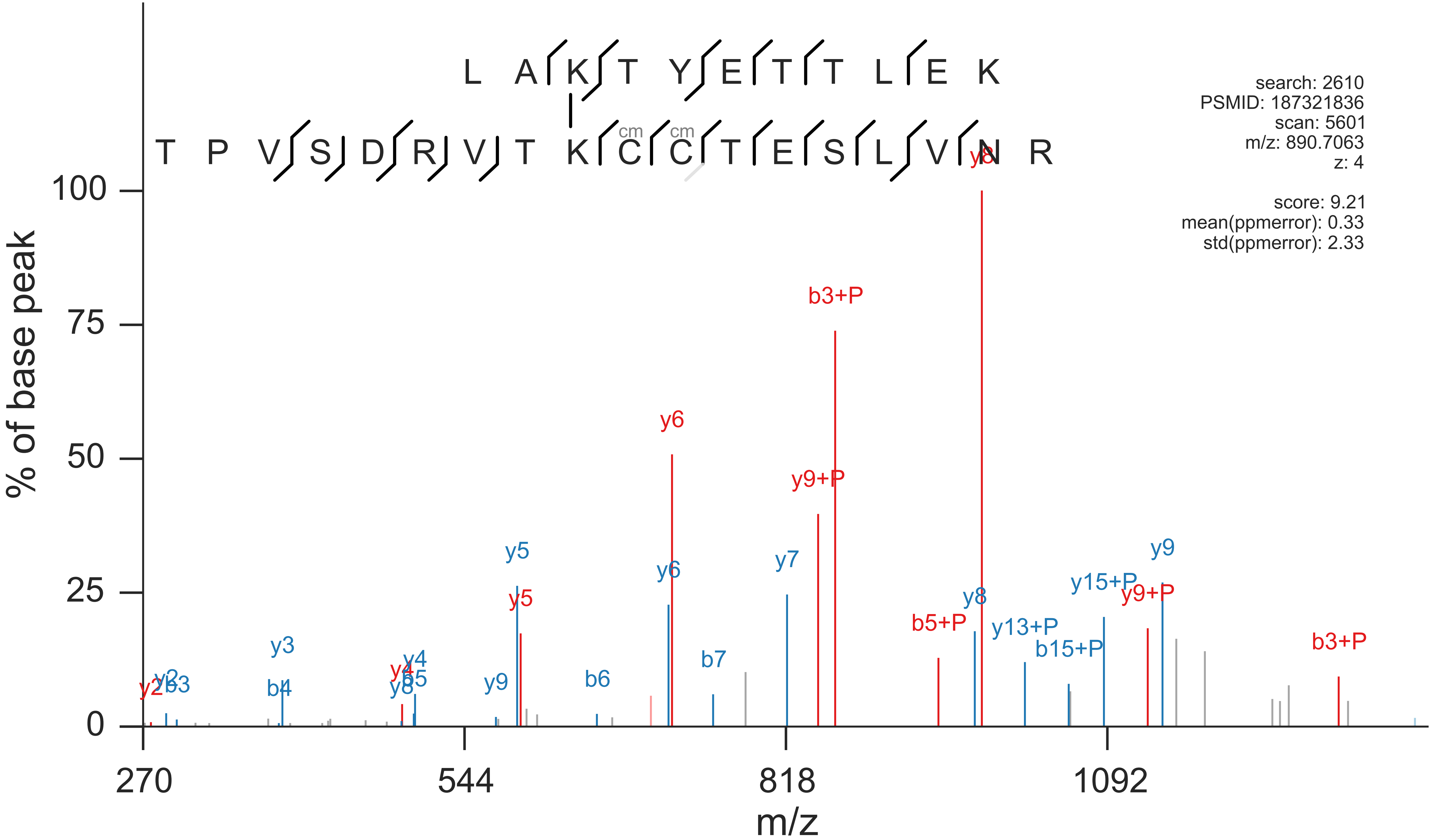
L A K T Y E T T L E K
T P V S D R V T K C C T E S L V N R
cm
cm
search: 2610
PSMID: 187321836
scan: 5601
m/z: 890.7063
z: 4
score: 9.21
mean(ppmerror): 0.33
std(ppmerror): 2.33
% of base peak
m/z
100
75
50
25
0
270
544
818
1092
y8
b3+P
y6
y9+P
y5
y5
y6
y7
y9
y15+P
y9+P
b5+P
y8
y13+P
b15+P
b3+P
y3
b4
y4
y4
b5
y8
y9
b6
b7
y2
y2
b3

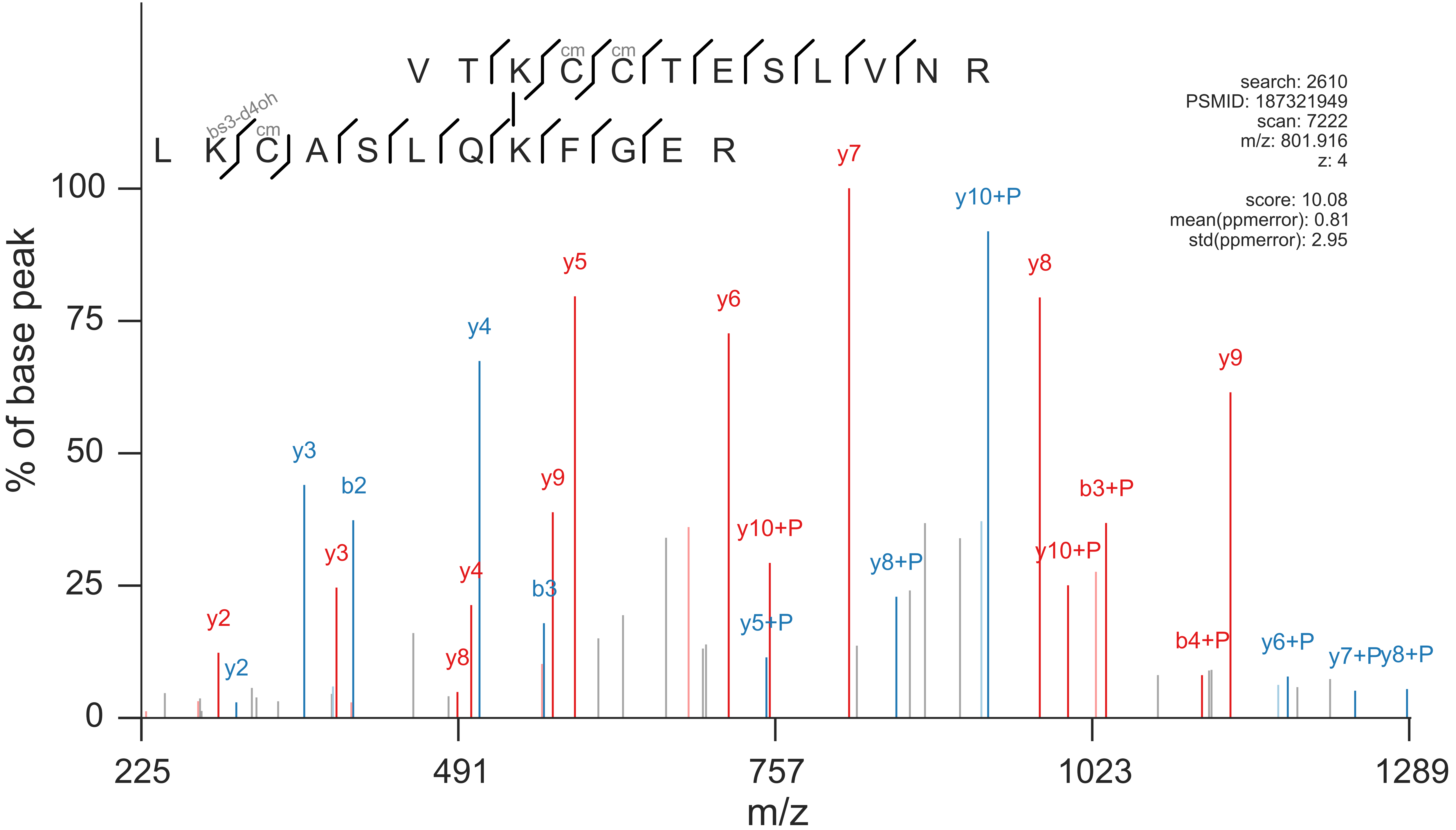
V T K C C T E S L V N R
cm
cm
L K C A S L Q K F G E R
bs3-d4oh
cm
search: 2610
PSMID: 187321949
scan: 7222
m/z: 801.916
z: 4
score: 10.08
mean(ppmerror): 0.81
std(ppmerror): 2.95
% of base peak
100
75
50
25
0
225
491
757
1023
1289
m/z
y2
y2
y3
y3
b2
y8
y4
y4
b3
y9
y5
y6
y10+P
y5+P
y7
y8+P
y10+P
y8
y10+P
b3+P
b4+P
y9
y6+P
y7+P
y8+P

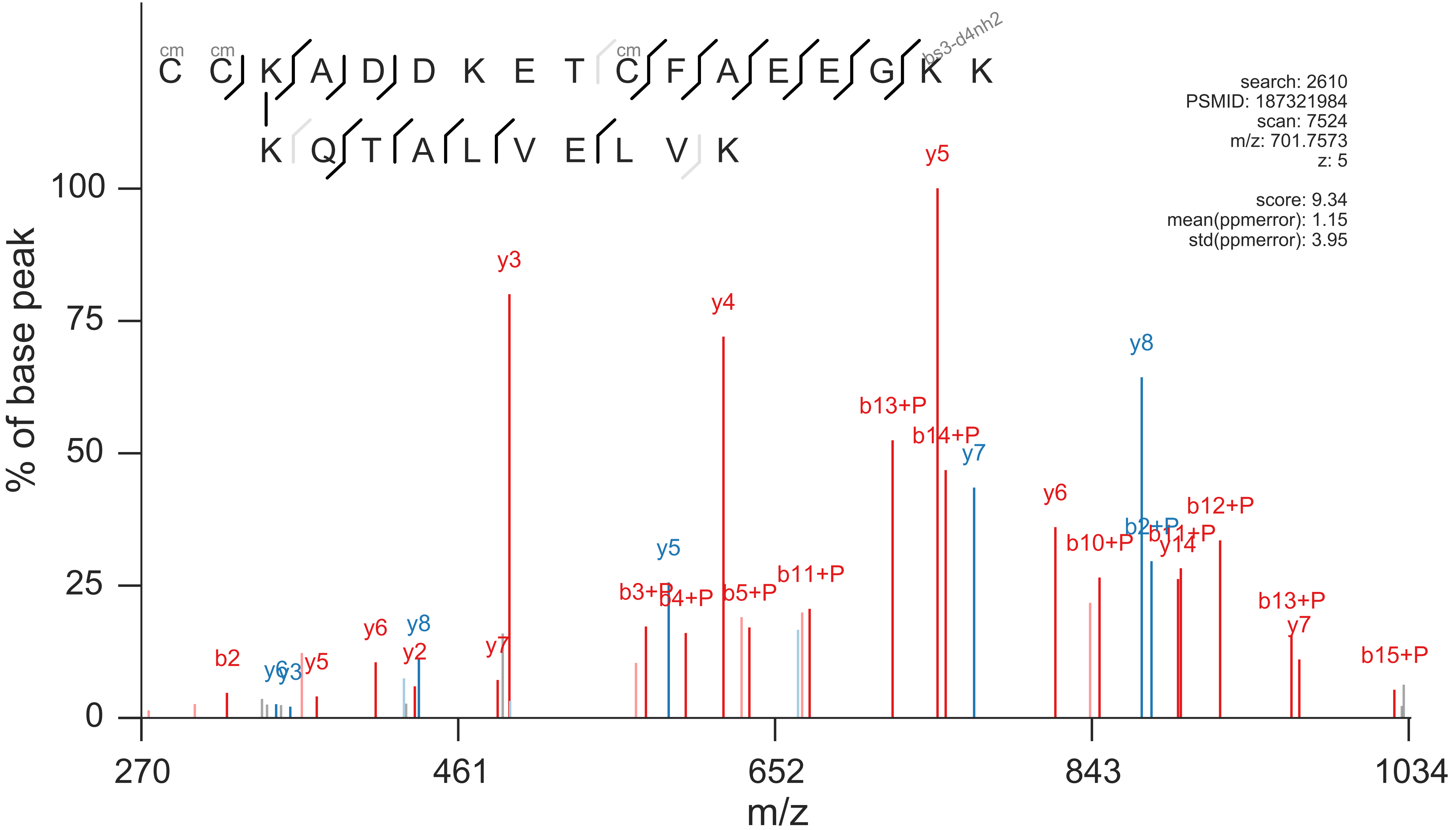

cm cm cm
C C K A D D K E T C F A E E G K K
bs3-d4nh2
K Q T A L V E L V K
search: 2610
PSMID: 187321984
scan: 7524
m/z: 701.7573
z: 5
score: 9.34
mean(ppmerror): 1.15
std(ppmerror): 3.95
% of base peak
m/z
0
25
50
75
100
270
461
652
843
1034
y3
y4
y5
b13+P
b14+P
y7
y8
y6
b12+P
b2+P
b11+P
y14
b10+P
y5
b11+P
b3+P
b4+P
b5+P
b13+P
y7
b15+P
y6
y8
y2
y7
b2
y6
y3
y5

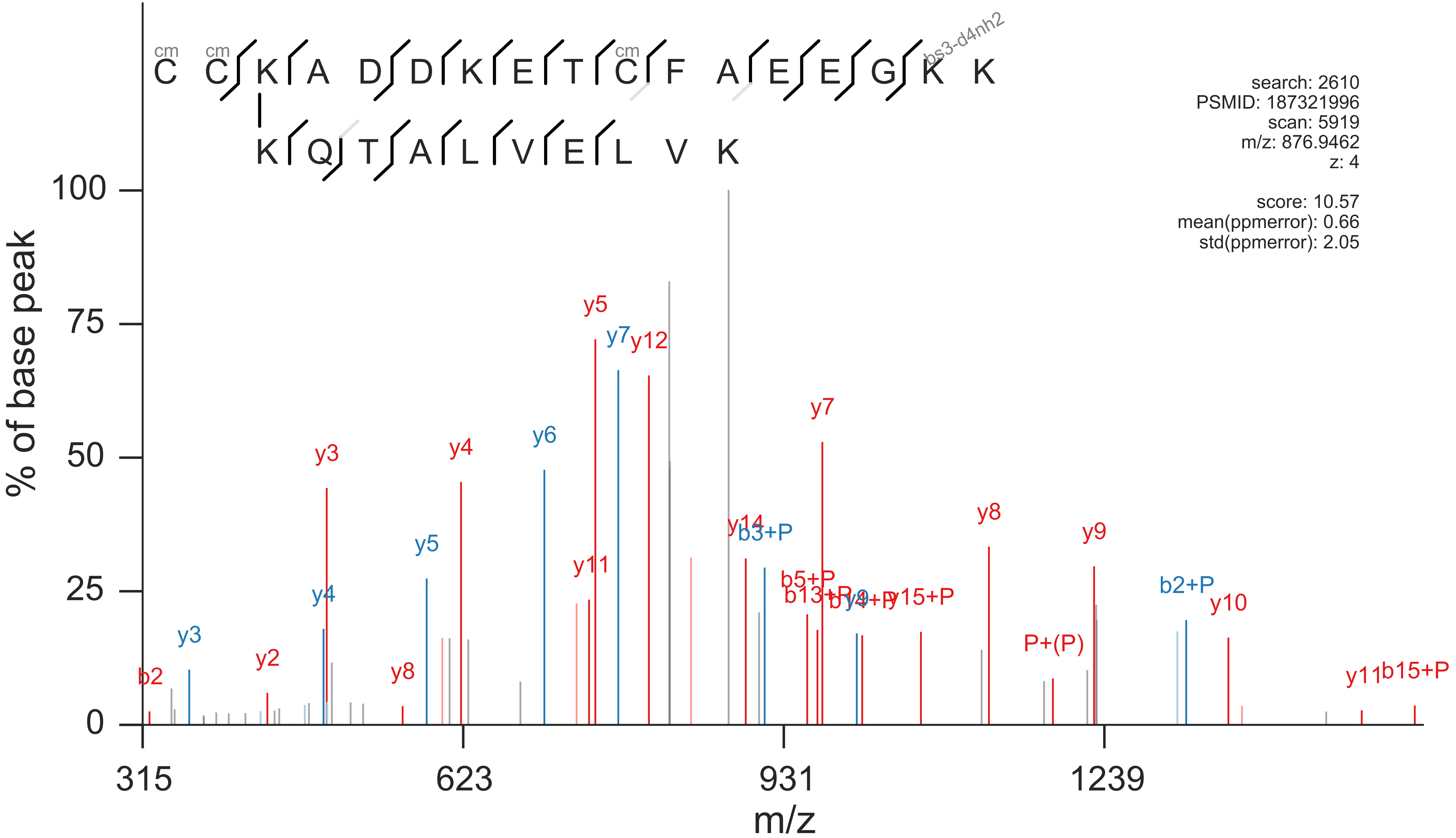

cm cm cm
C C K A D D K E T C F A E E G K K
bs3-d4nh2
K Q T A L V E L V K
search: 2610
PSMID: 187321996
scan: 5919
m/z: 876.9462
z: 4
score: 10.57
mean(ppmerror): 0.66
std(ppmerror): 2.05
% of base peak
100
75
50
25
0
315
623
931
1239
m/z
b2
y3
y2
y4
y3
y8
y5
y4
y6
y5
y7
y12
y11
y7
y14
b3+P
b5+P
b13+P
b14+P
y15+P
y8
y9
P+(P)
b2+P
y10
y11
b15+P

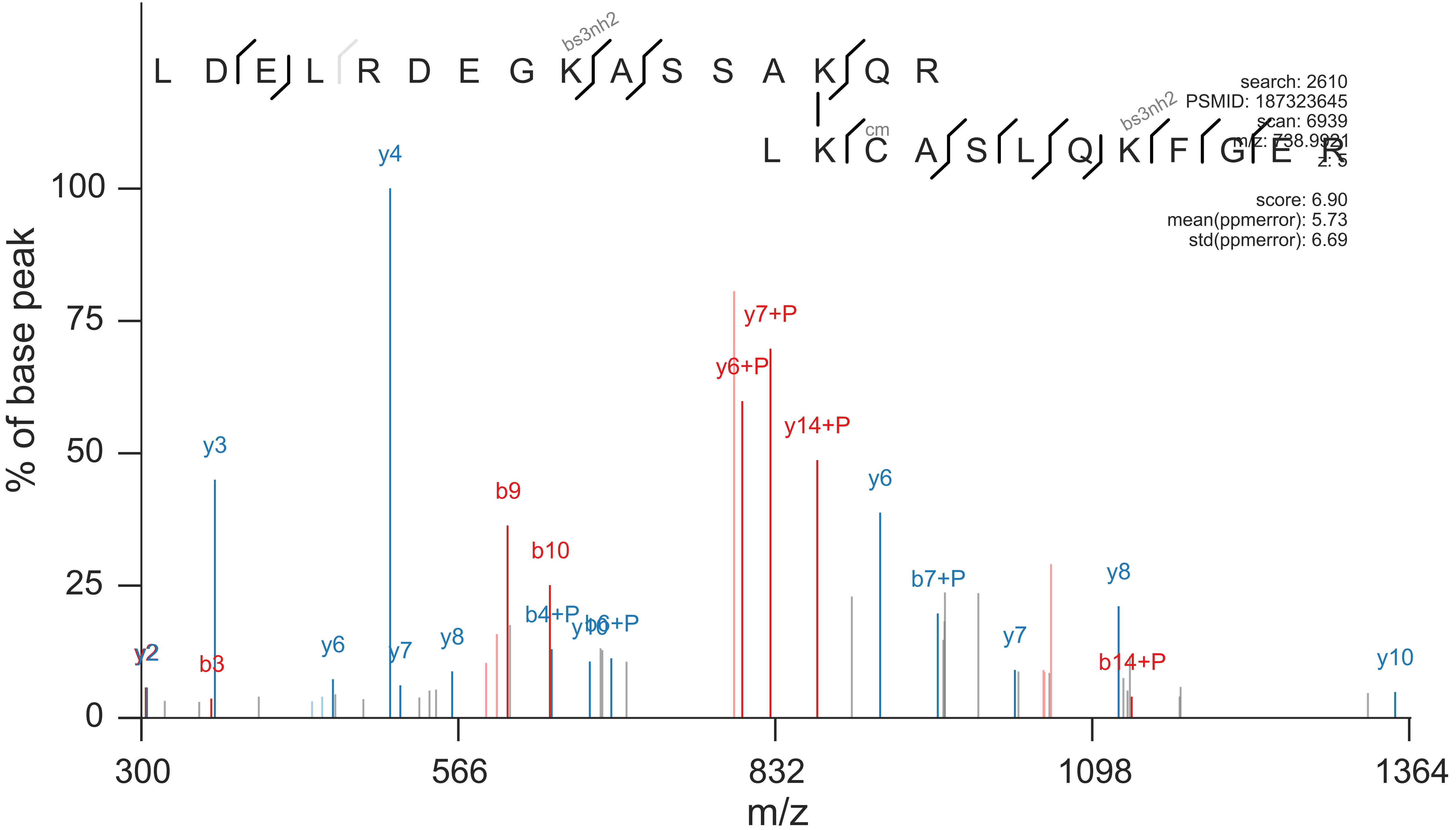

L D E L R D E G K A S S A K Q R
bs3nh2
L K C A S L Q K F G E R
cm
bs3nh2
search: 2610
PSMID: 187323645
scan: 6939
z: 5
score: 6.90
mean(ppmerror): 5.73
std(ppmerror): 6.69
% of base peak
m/z
y4
y3
y2
b3
y6
y7
y8
b9
b10
b4+P
b6+P
y7+P
y6+P
y14+P
y6
b7+P
y7
y8
b14+P
y10

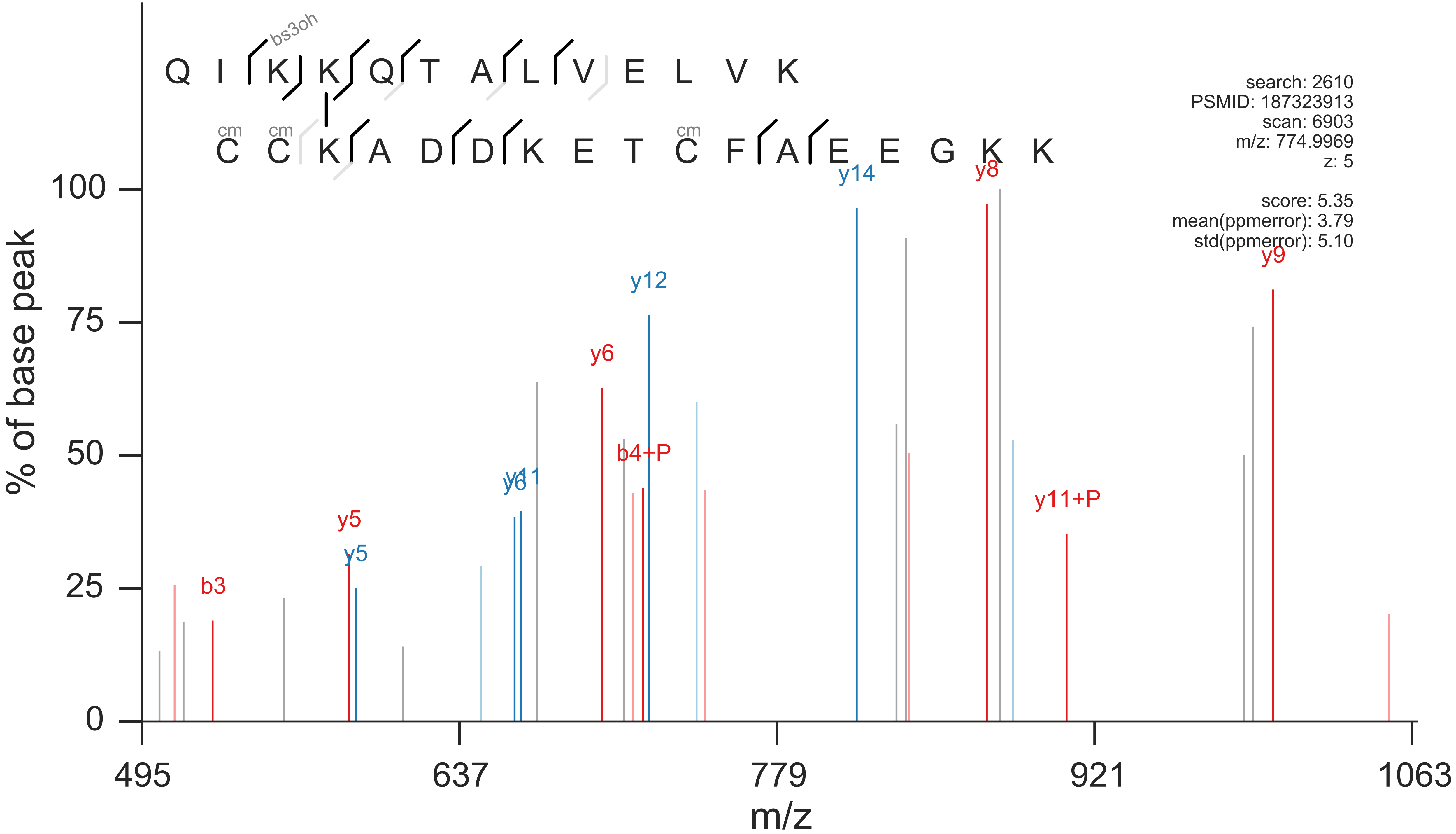

Q I K K Q T A L V E L V K
bs3oh
C C K A D D K E T C F A E E G K K
cm cm cm
search: 2610
PSMID: 187323913
scan: 6903
m/z: 774.9969
z: 5
score: 5.35
mean(ppmerror): 3.79
std(ppmerror): 5.10
% of base peak
m/z
0
25
50
75
100
495
637
779
921
1063
b3
y5
y5
y6
y11
y6
b4+P
y12
y14
y8
y11+P
y9

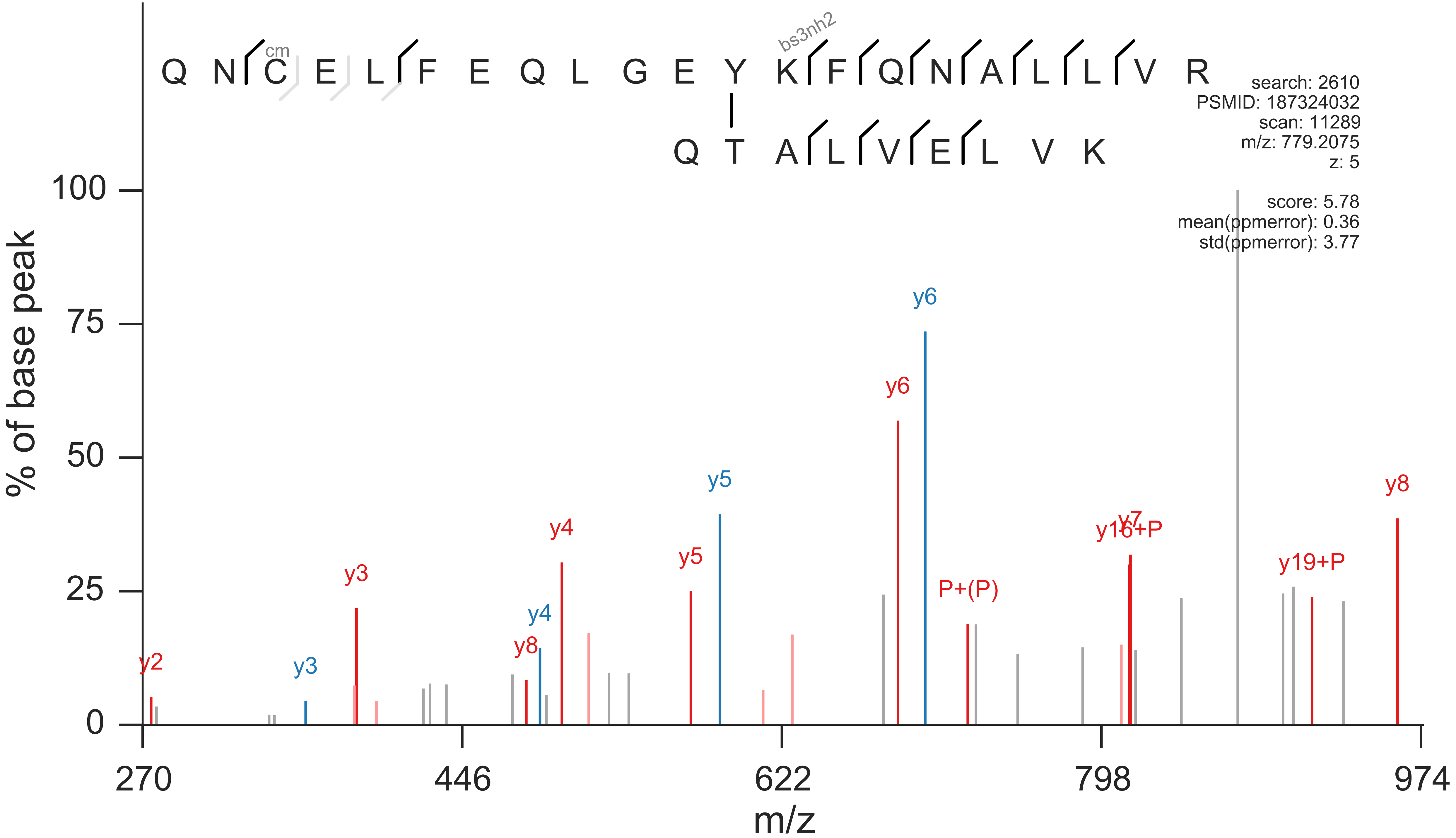

Q N C E L F E Q L G E Y K F Q N A L L V R
cm
bs3nh2
Q T A L V E L V K
search: 2610
PSMID: 187324032
scan: 11289
m/z: 779.2075
z: 5
score: 5.78
mean(ppmerror): 0.36
std(ppmerror): 3.77
% of base peak
100
75
50
25
0
270
446
622
798
974
m/z
y2
y3
y3
y8
y4
y4
y5
y5
y6
y6
P+(P)
y7
y16+P
y19+P
y8

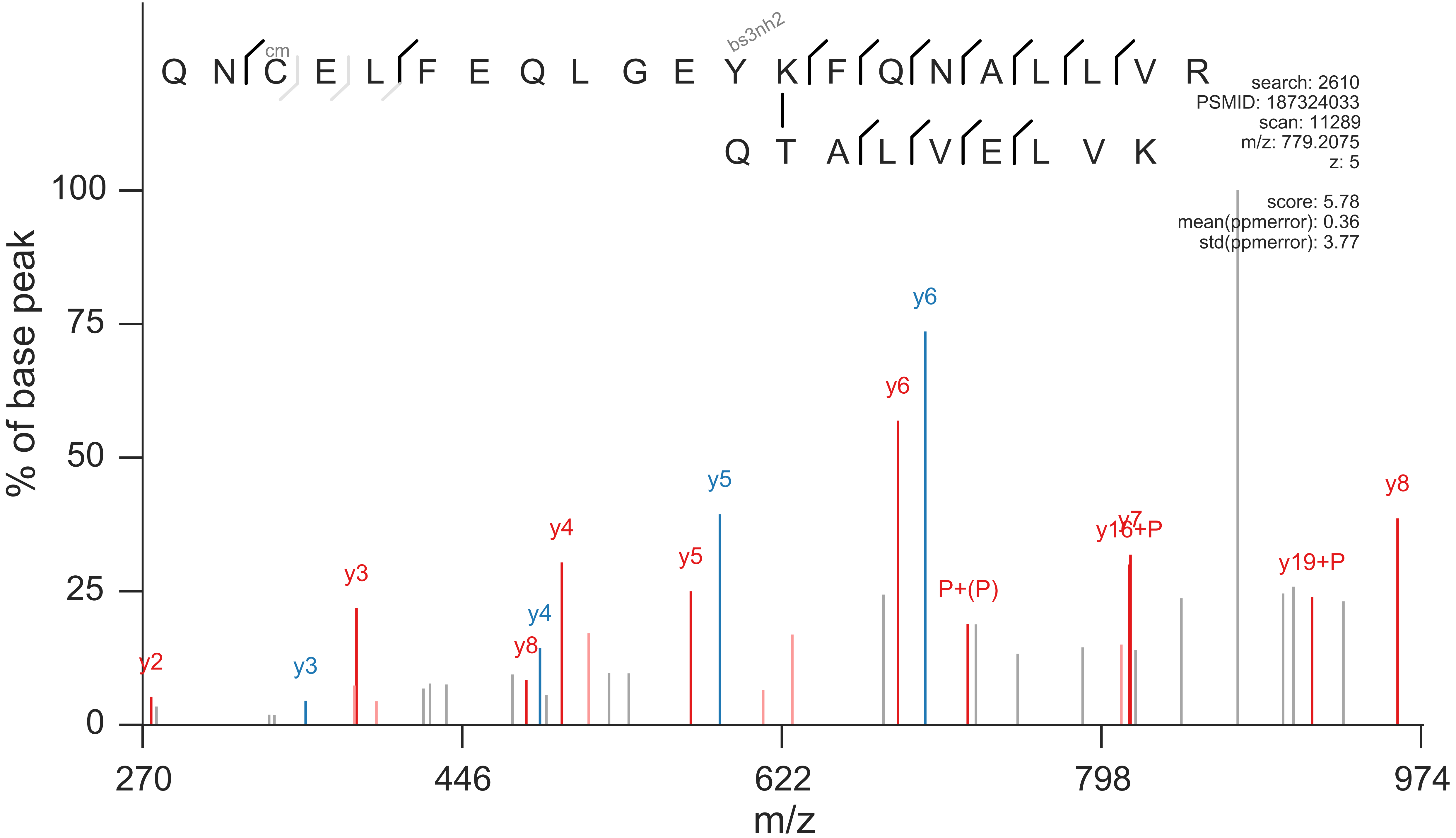

Q N C E L F E Q L G E Y K F Q N A L L V R
cm
bs3nh2
Q T A L V E L V K
search: 2610
PSMID: 187324033
scan: 11289
m/z: 779.2075
z: 5
score: 5.78
mean(ppmerror): 0.36
std(ppmerror): 3.77
% of base peak
100
75
50
25
0
y2
y3
y3
y4
y8
y4
y5
y5
y6
y6
P+(P)
y7
y16+P
y19+P
y8
270
446
622
798
974
m/z

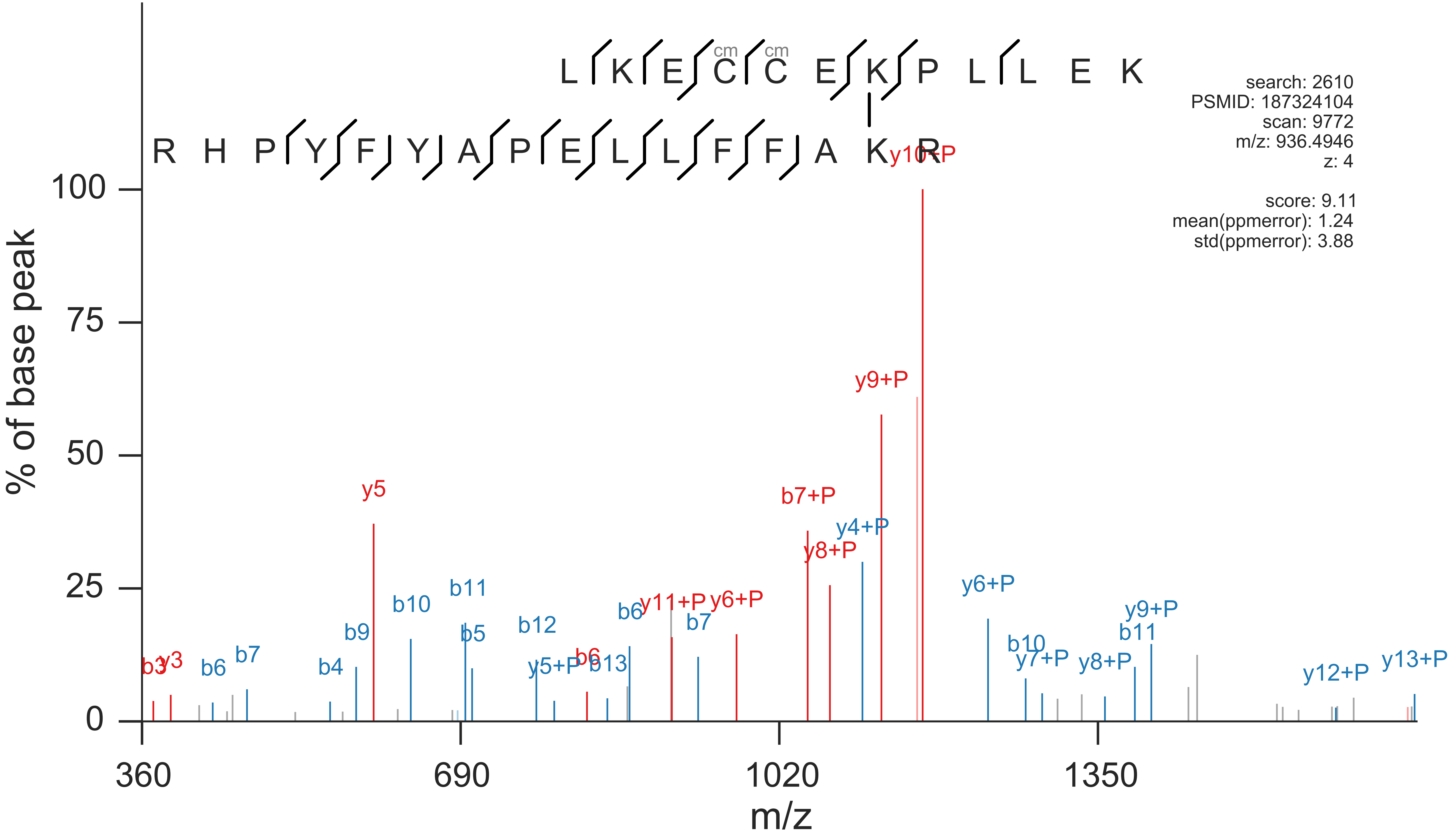

L K E C C E K P L L E K
cm cm
R H P Y F Y A P E L L F F A K R
search: 2610
PSMID: 187324104
scan: 9772
m/z: 936.4946
z: 4
score: 9.11
mean(ppmerror): 1.24
std(ppmerror): 3.88
% of base peak
m/z
0
25
50
75
100
360
690
1020
1350
y10+P
y9+P
y5
b7+P
y4+P
y8+P
b11
b10
b9
b5
b12
b6
y11+P
y6+P
b7
y6+P
b3
y3
b6
b7
b4
y5+P
b6
b13
b10
y7+P
y8+P
b11
y9+P
y12+P
y13+P

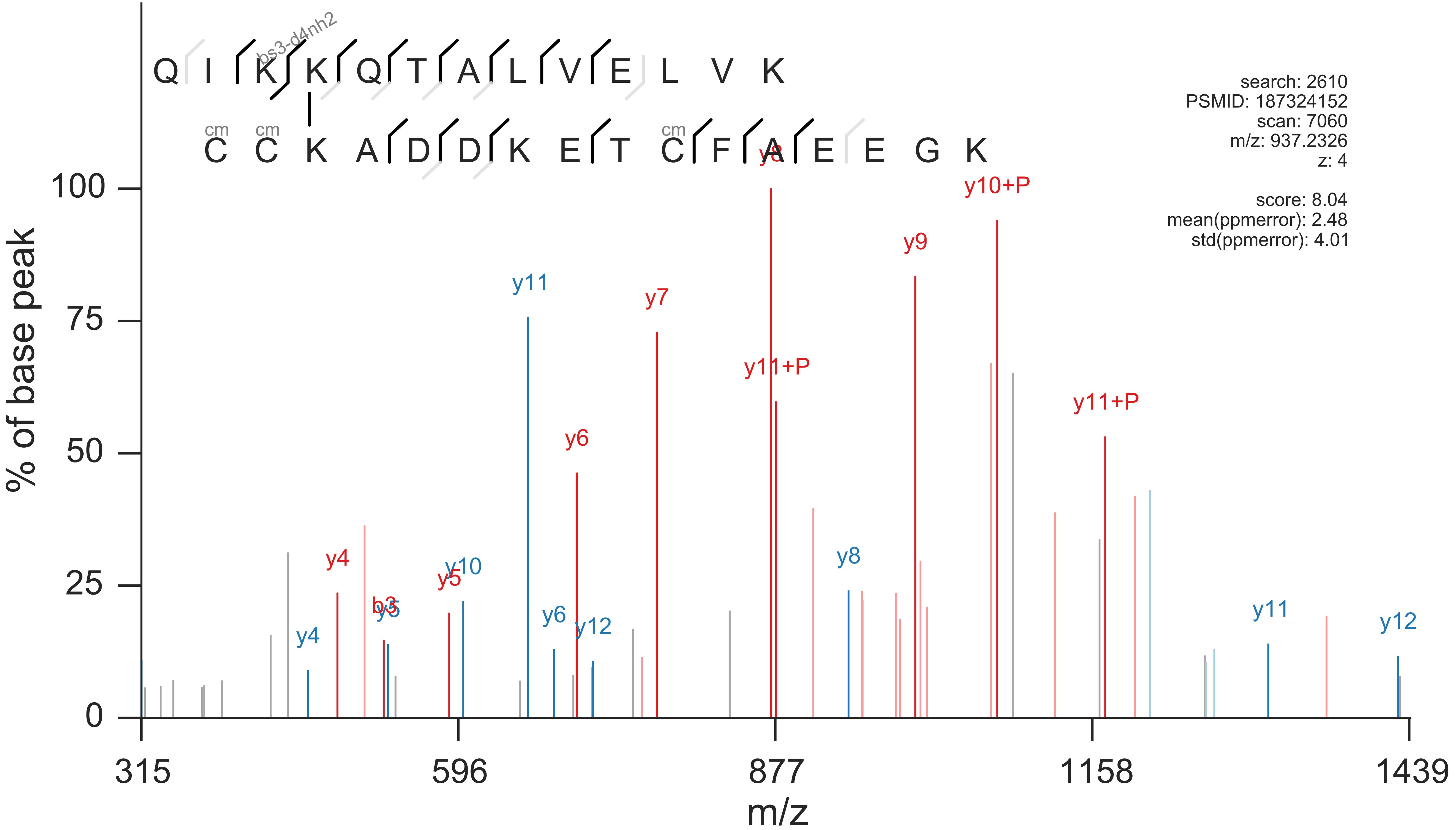

bs3-d4nh2
Q I K K Q T A L V E L V K
cm cm cm
C C K A D D K E T C F A E E G K
search: 2610
PSMID: 187324152
scan: 7060
m/z: 937.2326
z: 4
score: 8.04
mean(ppmerror): 2.48
std(ppmerror): 4.01
% of base peak
100
75
50
25
0
y8
y10+P
y9
y11
y7
y11+P
y6
y11+P
y4
y10
y5
b3
y5
y4
y6
y12
y8
y11
y12
315
596
877
1158
1439
m/z

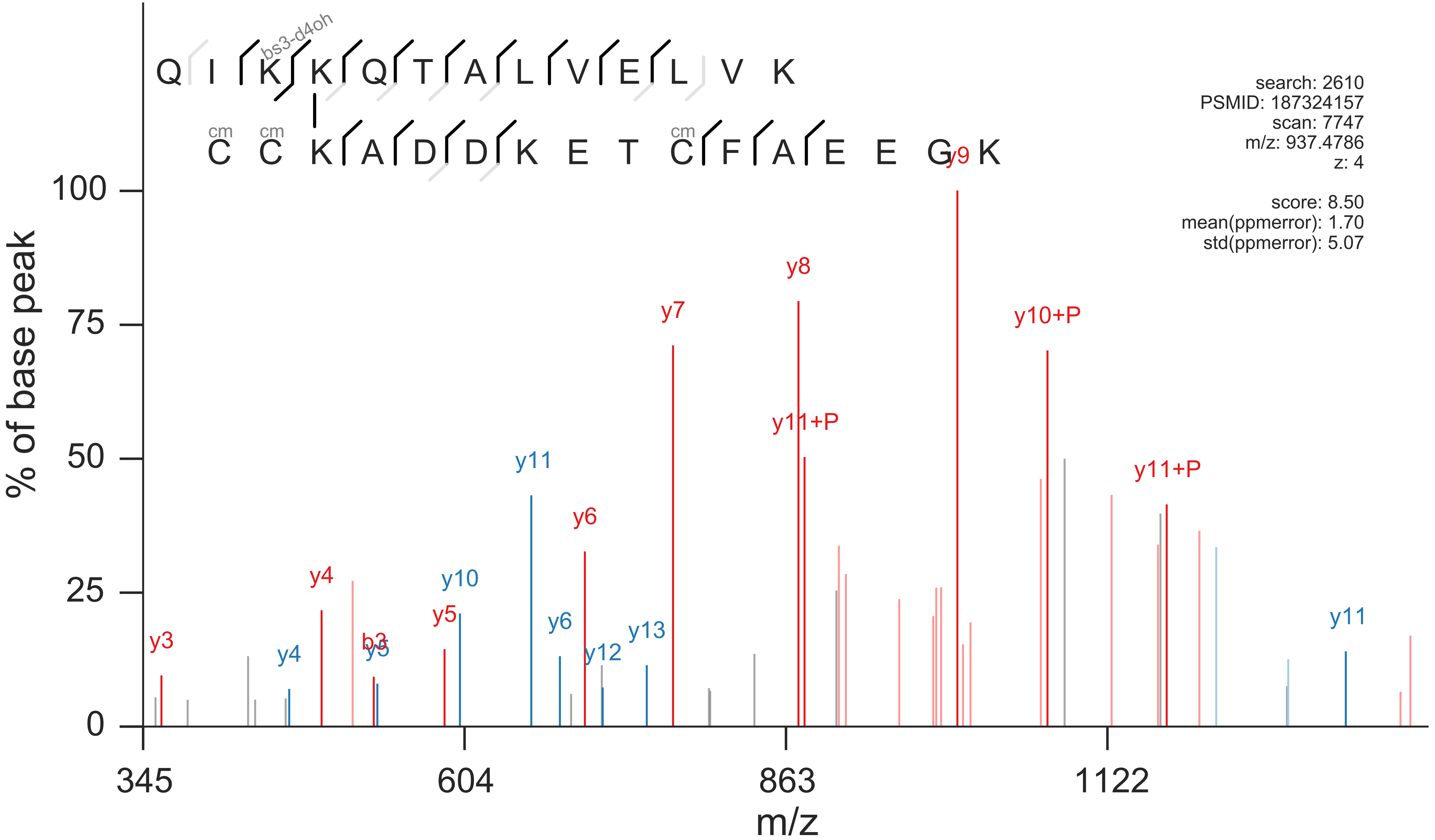

search: 2610
PSMID: 187324157
scan: 7747
m/z: 937.4786
z: 4
score: 8.50
mean(ppmerror): 1.70
std(ppmerror): 5.07
Q I K K Q T A L V E L V K
bs3-d4oh
C C K A D D K E T C F A E E G K
cm cm cm
% of base peak
m/z
0
25
50
75
100
345
604
863
1122
y3 y4 b3 y5 y6 y7 y8 y9 y10+P y11+P y11+P
y4 y5 y10 y11 y6 y12 y13 y11

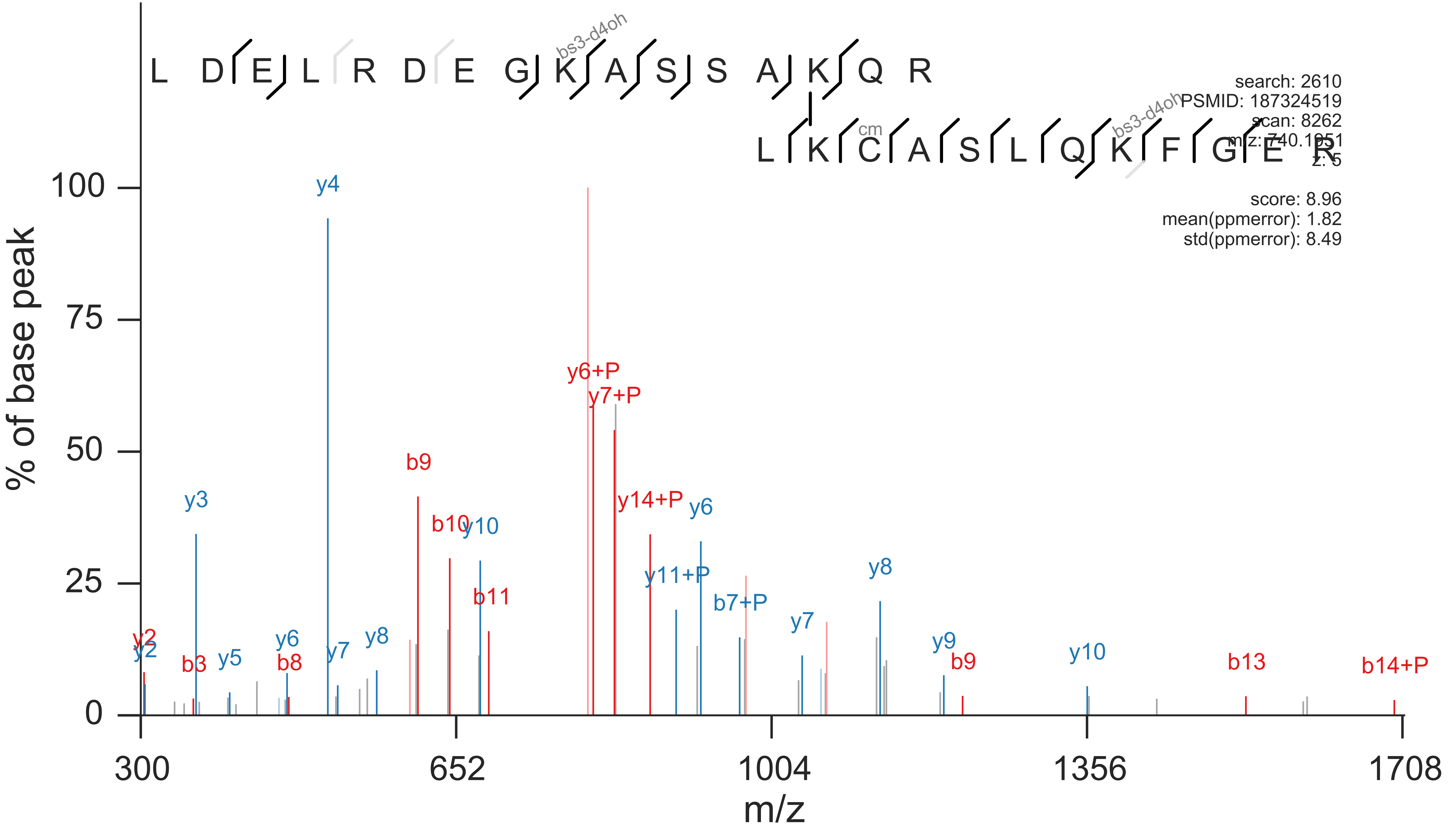

L D E L R D E G K A S S A K Q R
L K C A S L Q K F G E R
bs3-d4oh
cm
bs3-d4oh
search: 2610
PSMID: 187324519
scan: 8262
m/z: 740.1951
z: 5
score: 8.96
mean(ppmerror): 1.82
std(ppmerror): 8.49
% of base peak
m/z
y4
y6+P
y7+P
b9
y14+P
y6
y3
b10
y10
y11+P
b11
b7+P
y8
y7
y2
y2
b3
y5
y6
b8
y7
y8
y9
b9
y10
b13
b14+P
0
25
50
75
100
300
652
1004
1356
1708

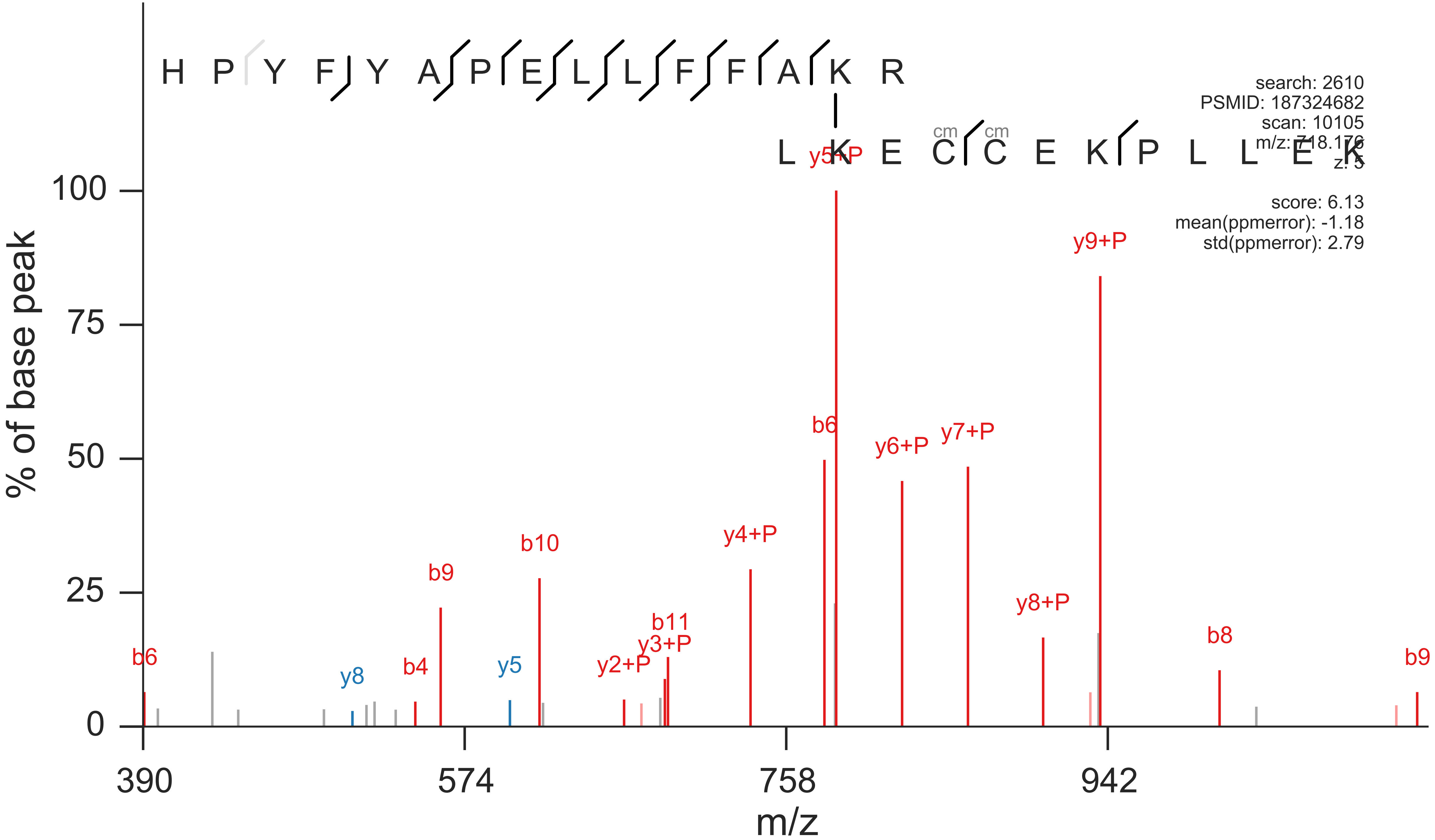

H P Y F Y A P E L L F F A K R
L K E C C E K P L L E K
cm cm
search: 2610
PSMID: 187324682
scan: 10105
m/z: 718.176
z: 5
score: 6.13
mean(ppmerror): -1.18
std(ppmerror): 2.79
% of base peak
m/z
0
25
50
75
100
390
574
758
942
b6
y8
b4
b9
y5
b10
y2+P
y3+P
b11
y4+P
b6
y5+P
y6+P
y7+P
y8+P
y9+P
b8
b9

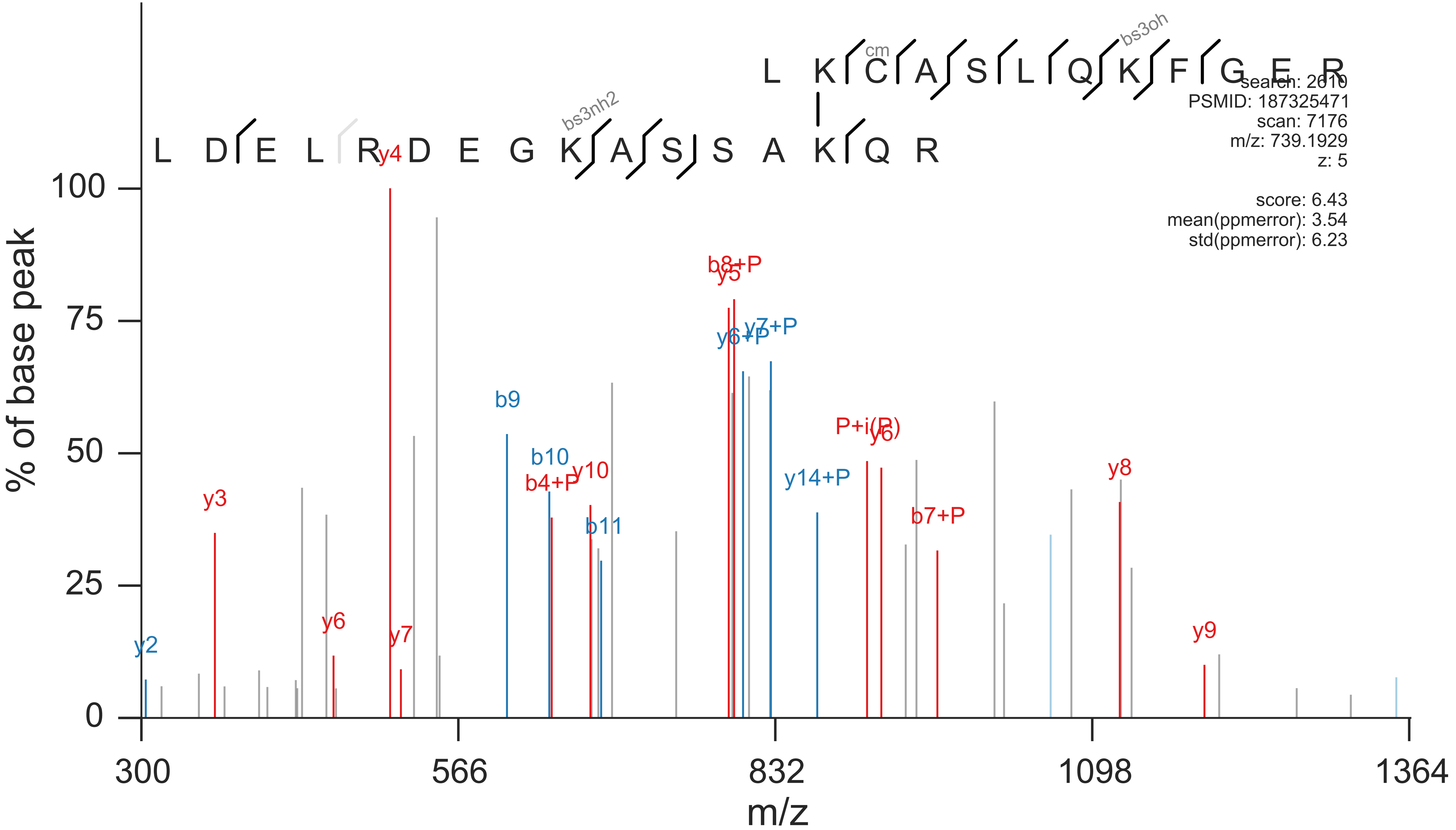

L K C A S L Q K F G E R
cm
bs3oh
L D E L R D E G K A S S A K Q R
bs3nh2
search: 2610
PSMID: 187325471
scan: 7176
m/z: 739.1929
z: 5
score: 6.43
mean(ppmerror): 3.54
std(ppmerror): 6.23
% of base peak
m/z
y2
y3
y6
y4
y7
b9
b10
b4+P
y10
b11
b8+P
y5
y6+P
y7+P
y14+P
P+i(P)
y6
b7+P
y8
y9

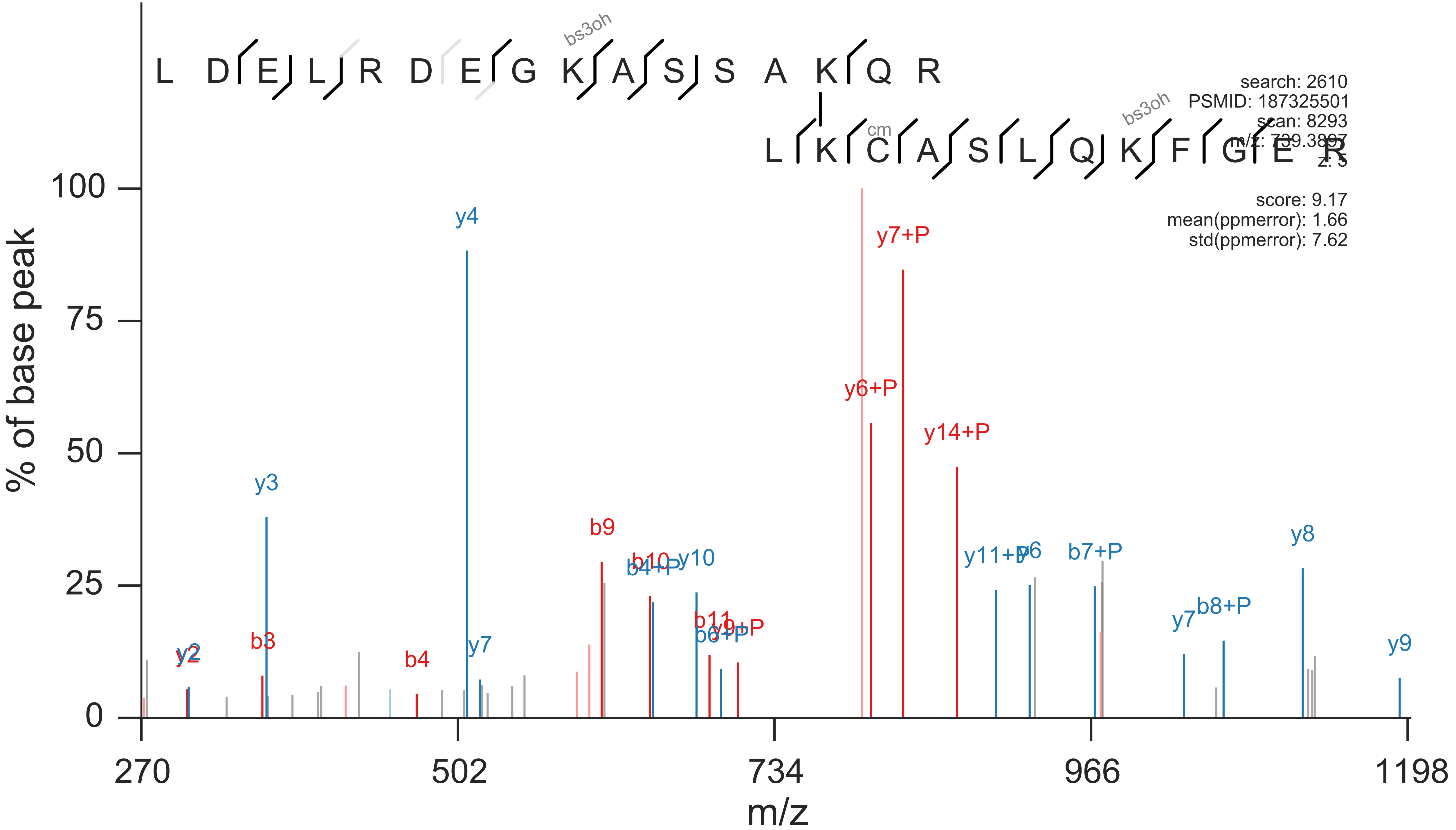

L D E L R D E G K A S S A K Q R
L K C A S L Q K F G E R
bs3oh
cm
search: 2610
PSMID: 187325501
scan: 8293
z: 3
score: 9.17
mean(ppmerror): 1.66
std(ppmerror): 7.62
% of base peak
m/z
y4
y7+P
y6+P
y14+P
y3
b9
b10
b4+P
y10
y11+P
y6
b7+P
y8
b8+P
y7
y9
b11
b6+P
y9+P
y2
b3
b4
y7
100
75
50
25
0
270
502
734
966
1198

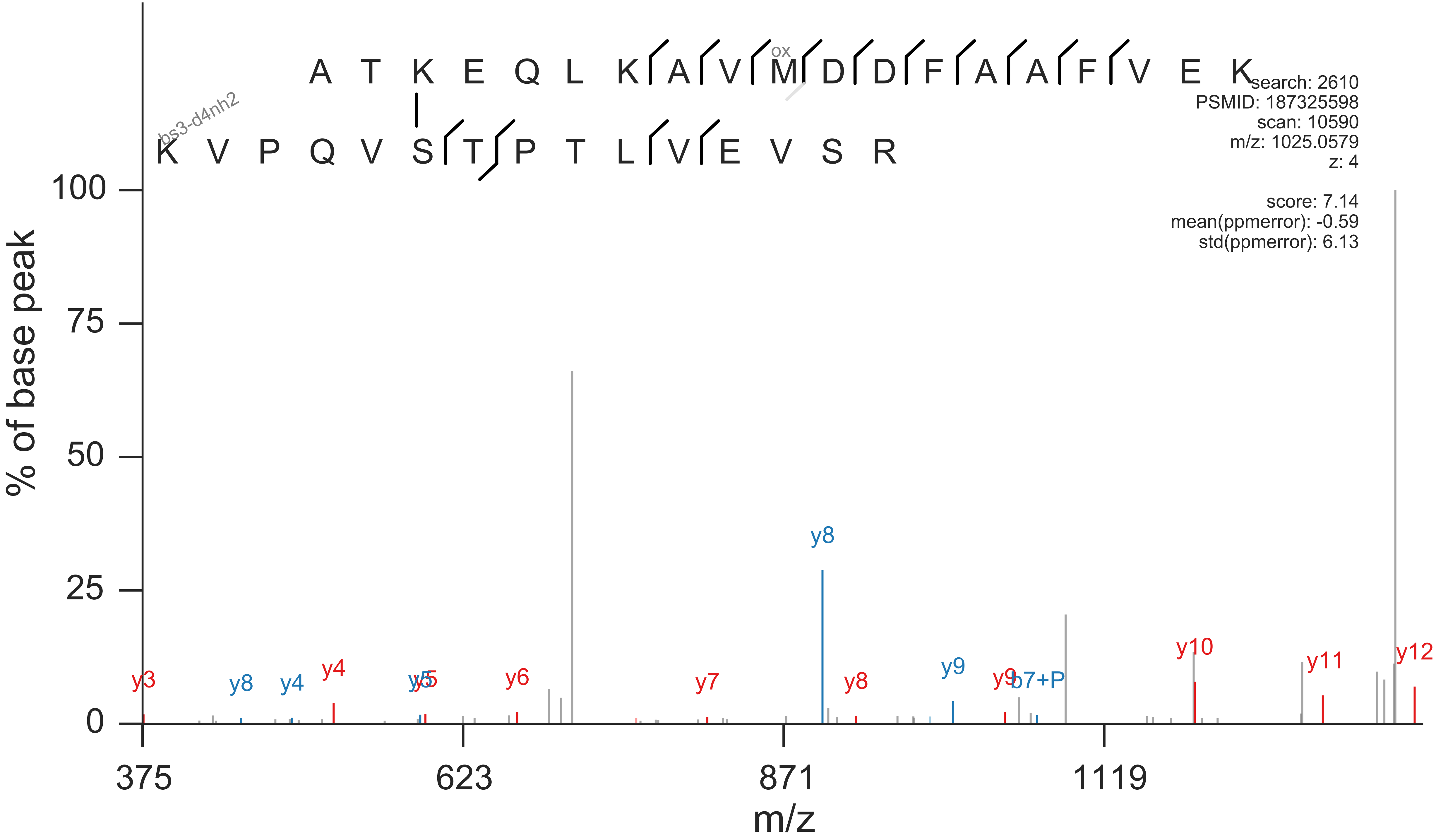
A T K E Q L K A V M D D F A A F V E K
ox
bs3-d4nh2
K V P Q V S T P T L V E V S R
search: 2610
PSMID: 187325598
scan: 10590
m/z: 1025.0579
z: 4
score: 7.14
mean(ppmerror): -0.59
std(ppmerror): 6.13
% of base peak
100
75
50
25
0
y3
y8
y4
y4
y5
y5
y6
y7
y8
y8
y9
y9
b7+P
y10
y11
y12
375
623
871
1119
m/z

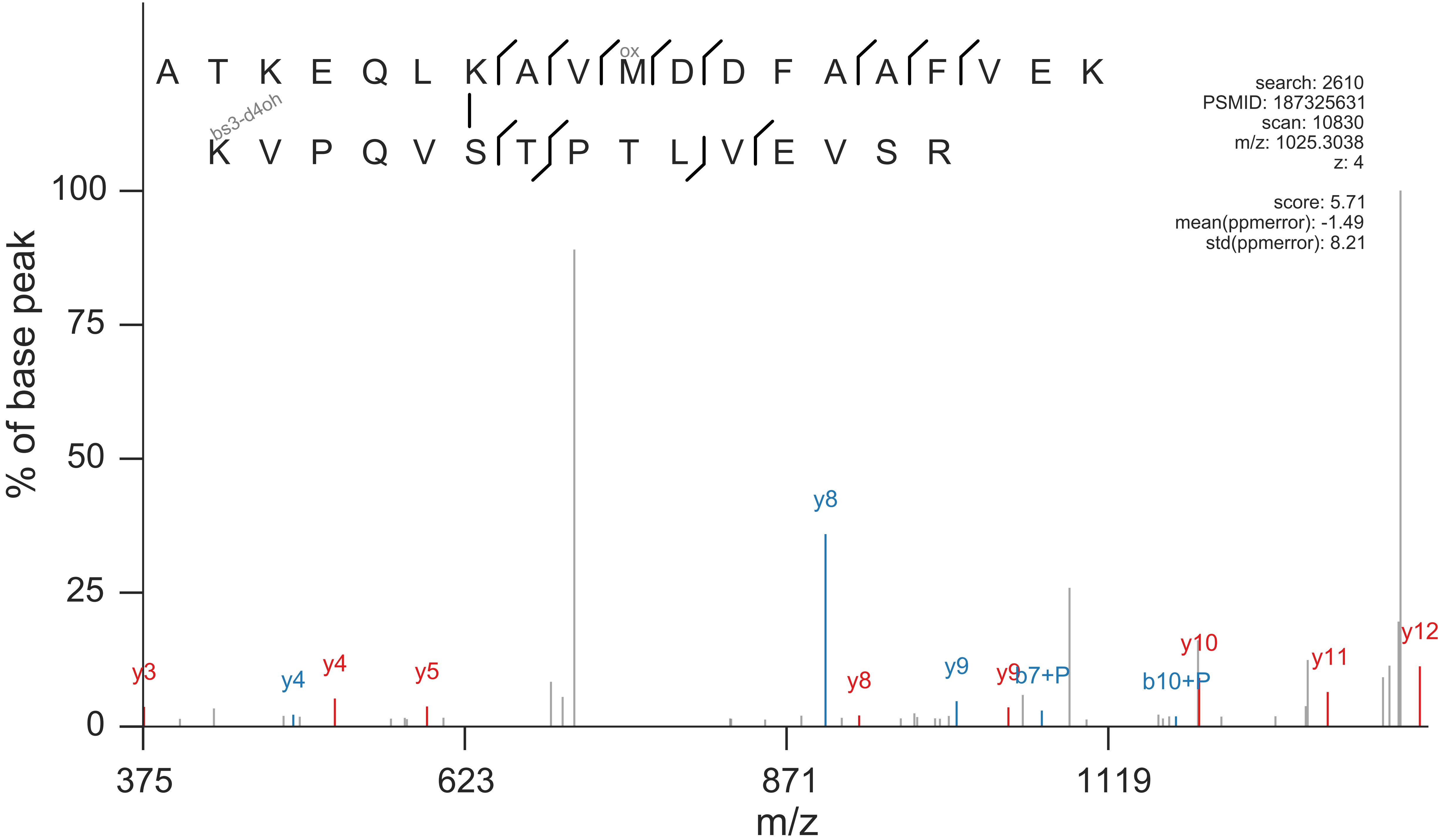

A T K E Q L K A V M D D F A A F V E K
ox
bs3-d4oh
K V P Q V S T P T L V E V S R
search: 2610
PSMID: 187325631
scan: 10830
m/z: 1025.3038
z: 4
score: 5.71
mean(ppmerror): -1.49
std(ppmerror): 8.21
% of base peak
100
75
50
25
0
y8
y3
y4
y4
y5
y8
y9
y9
b7+P
b10+P
y10
y11
y12
375
623
871
1119
m/z

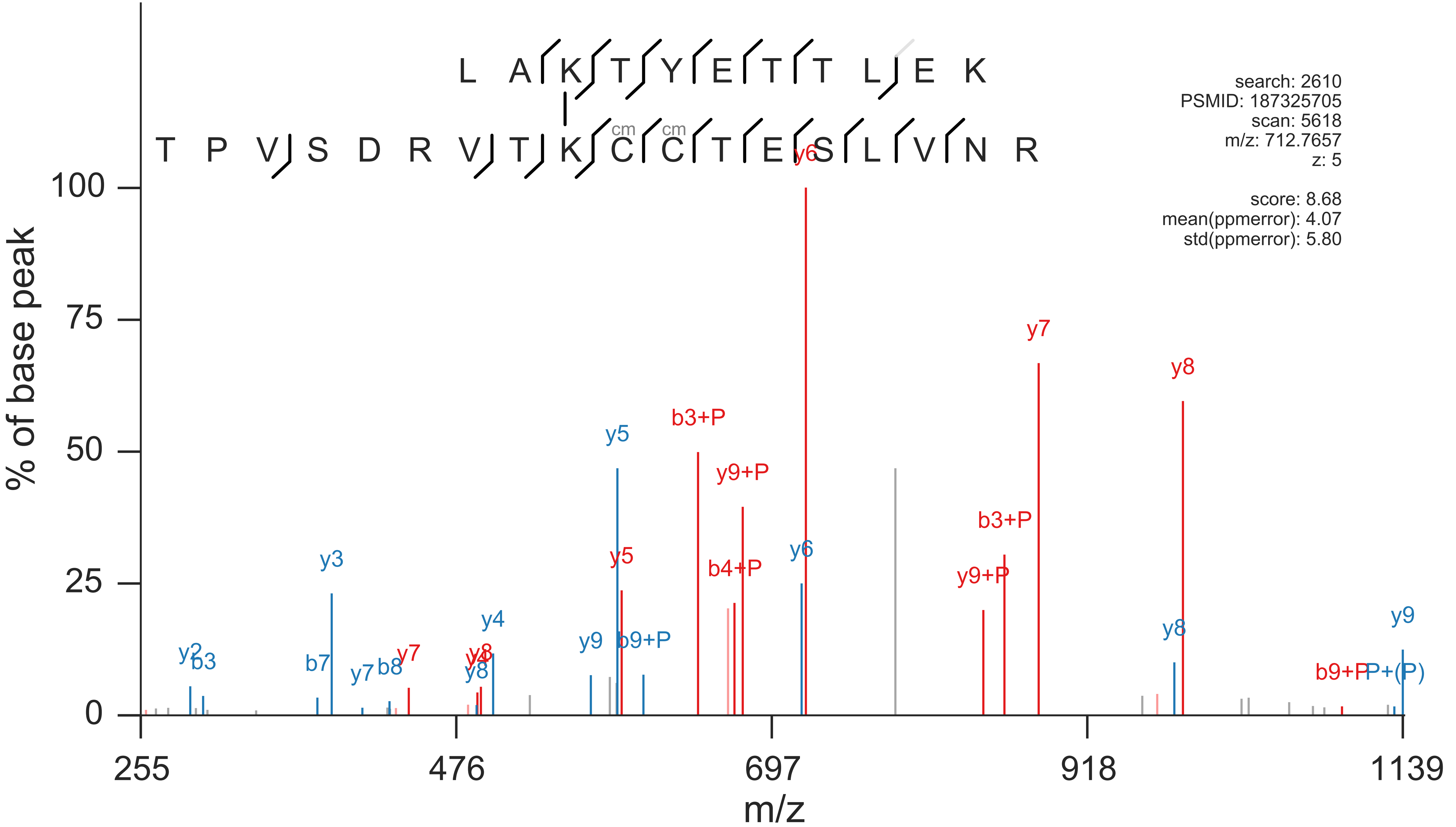

L A K T Y E T T L E K
T P V S D R V T K C C T E S L V N R
cm
cm
search: 2610
PSMID: 187325705
scan: 5618
m/z: 712.7657
z: 5
score: 8.68
mean(ppmerror): 4.07
std(ppmerror): 5.80
% of base peak
m/z
0
25
50
75
100
255
476
697
918
1139
y2
b3
y3
b7
y7
b8
y7
y4
y8
y8
y4
y9
y5
b9+P
y5
b3+P
y9+P
b4+P
y6
y6
y9+P
b3+P
y7
y8
y8
y9
b9+P
P+(P)

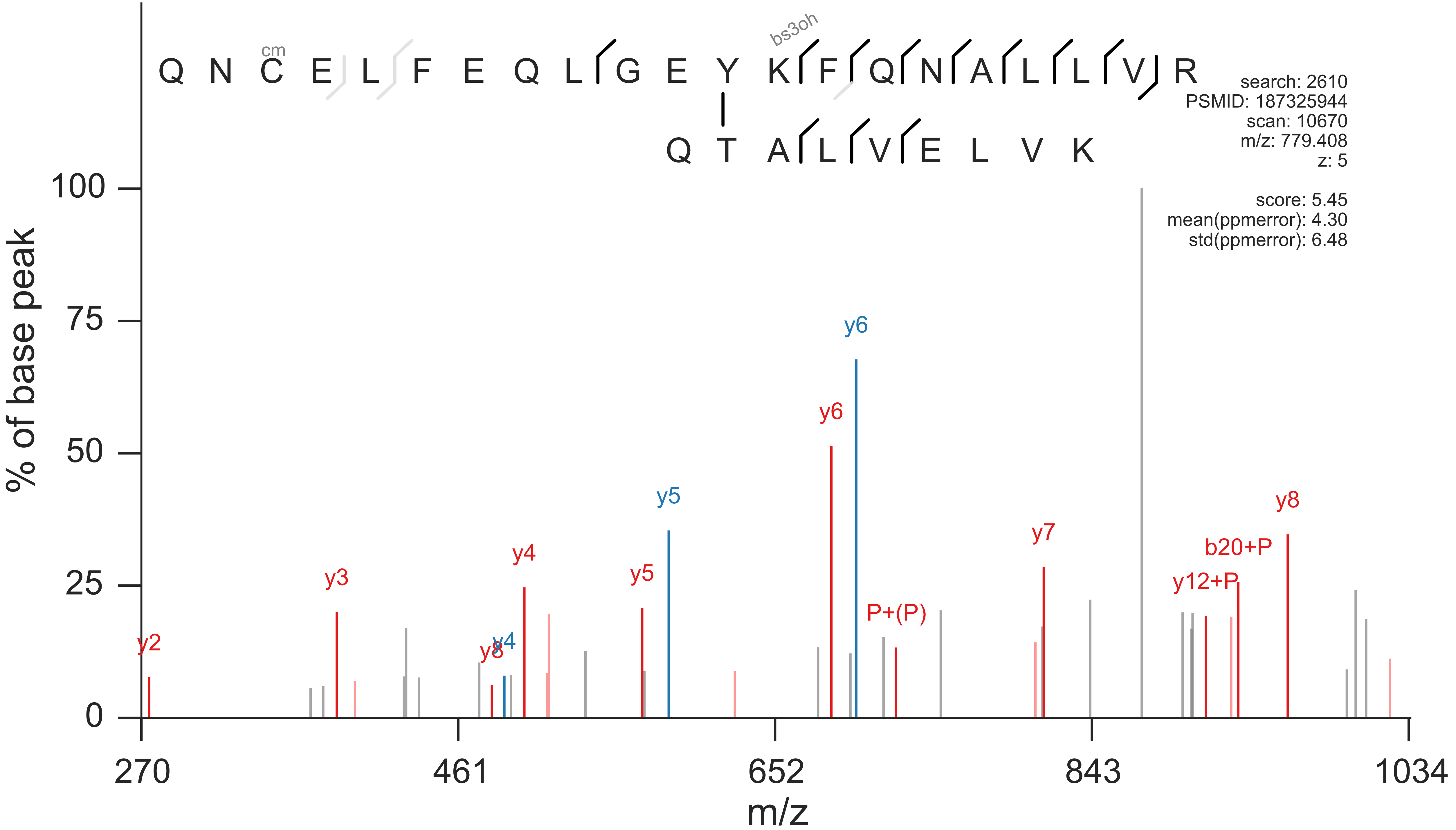

Q N C E L F E Q L G E Y K F Q N A L L V R
cm
bs3oh
Q T A L V E L V K
search: 2610
PSMID: 187325944
scan: 10670
m/z: 779.408
z: 5
score: 5.45
mean(ppmerror): 4.30
std(ppmerror): 6.48
% of base peak
100
75
50
25
0
270
461
652
843
1034
m/z
y2
y3
y8
y4
y4
y5
y5
y6
y6
P+(P)
y7
y12+P
b20+P
y8

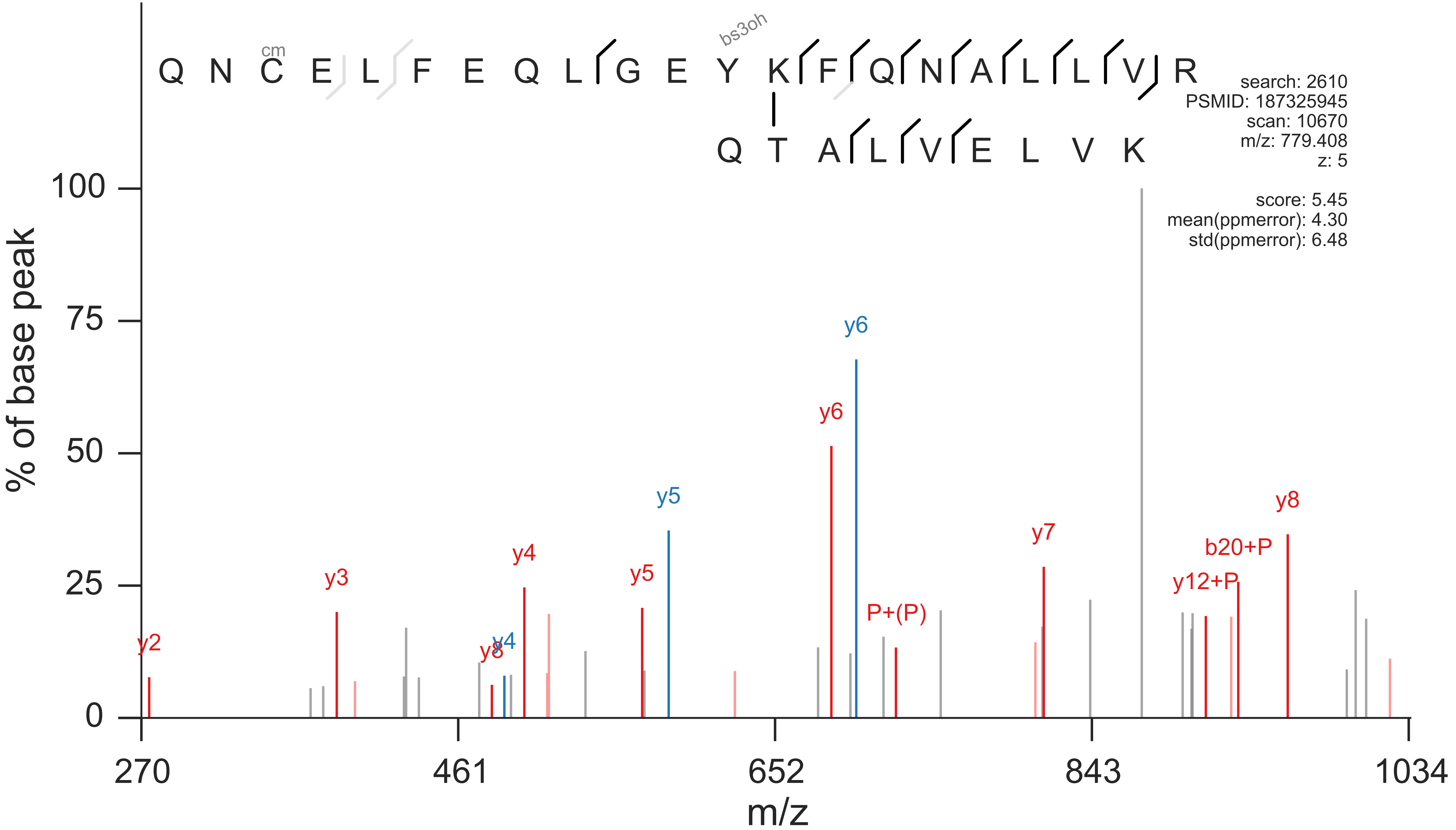

Q N C E L F E Q L G E Y K F Q N A L L V R
cm
bs3oh
Q T A L V E L V K
search: 2610
PSMID: 187325945
scan: 10670
m/z: 779.408
z: 5
score: 5.45
mean(ppmerror): 4.30
std(ppmerror): 6.48
% of base peak
m/z
0
25
50
75
100
270
461
652
843
1034
y2
y3
y8
y4
y4
y5
y5
y6
y6
P+(P)
y7
y12+P
b20+P
y8

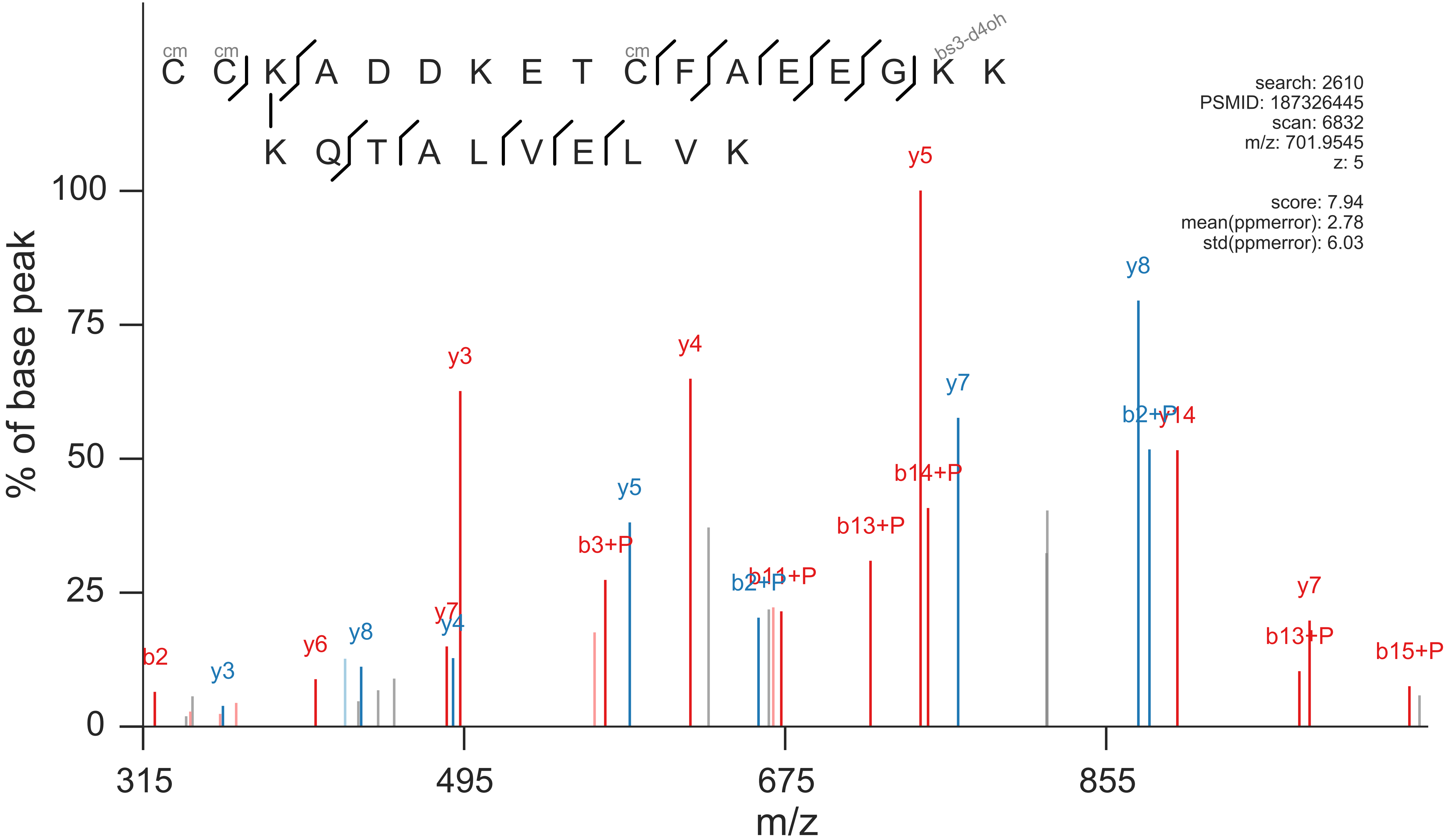
C C K A D D K E T C F A E E G K K
cm cm cm
bs3-d4oh
K Q T A L V E L V K
search: 2610
PSMID: 187326445
scan: 6832
m/z: 701.9545
z: 5
score: 7.94
mean(ppmerror): 2.78
std(ppmerror): 6.03
% of base peak
m/z
0
25
50
75
100
315
495
675
855
b2
y3
y6
y8
y7
y4
y3
b3+P
y5
y4
b2+P
b11+P
b13+P
y5
b14+P
y7
y8
b2+P
y14
y7
b13+P
b15+P

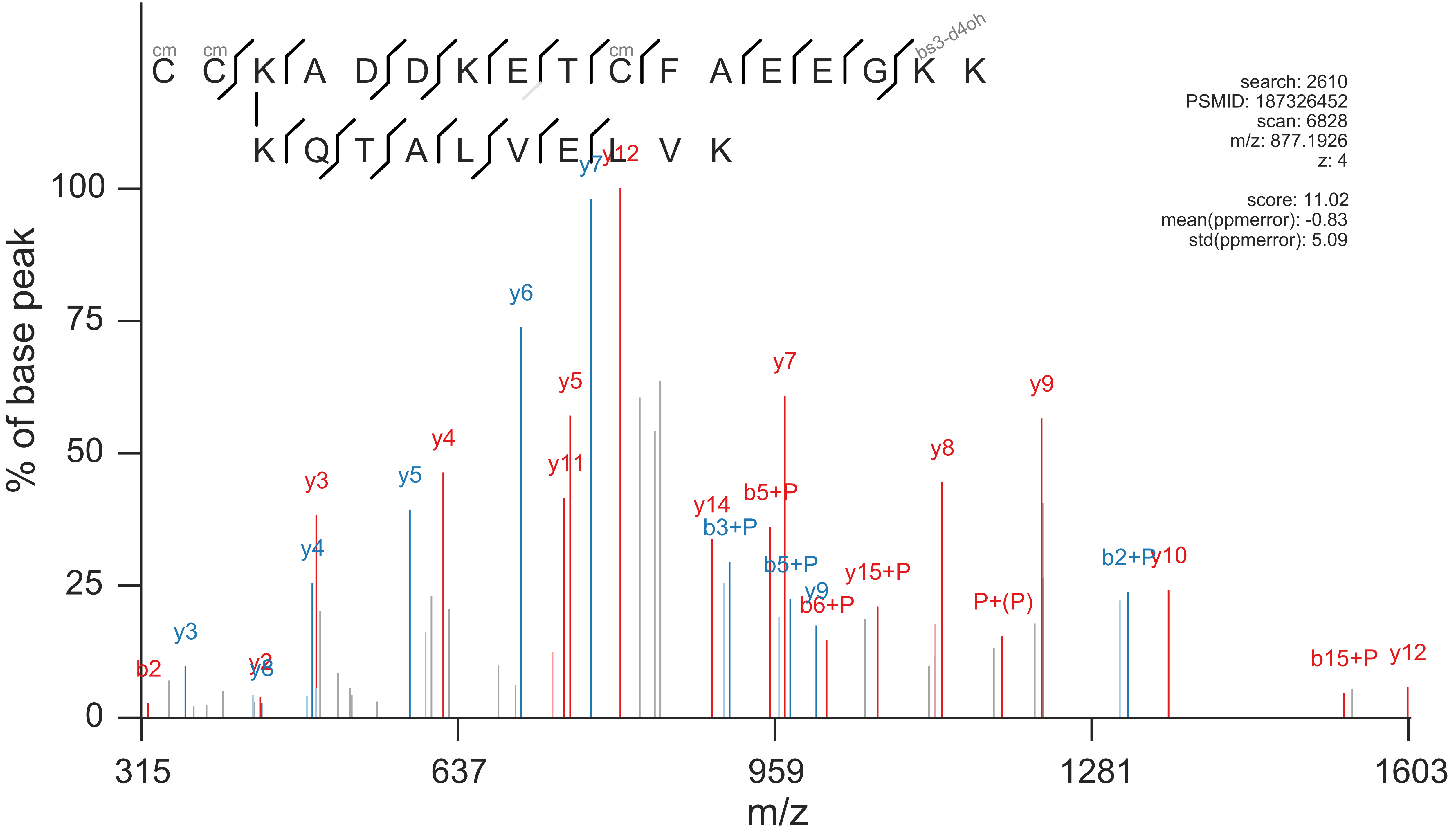

CCKADDKETCFAEEGKK
KQTALVELVK
bs3-d4oh
search: 2610
PSMID: 187326452
scan: 6828
m/z: 877.1926
z: 4
score: 11.02
mean(ppmerror): -0.83
std(ppmerror): 5.09
% of base peak
m/z

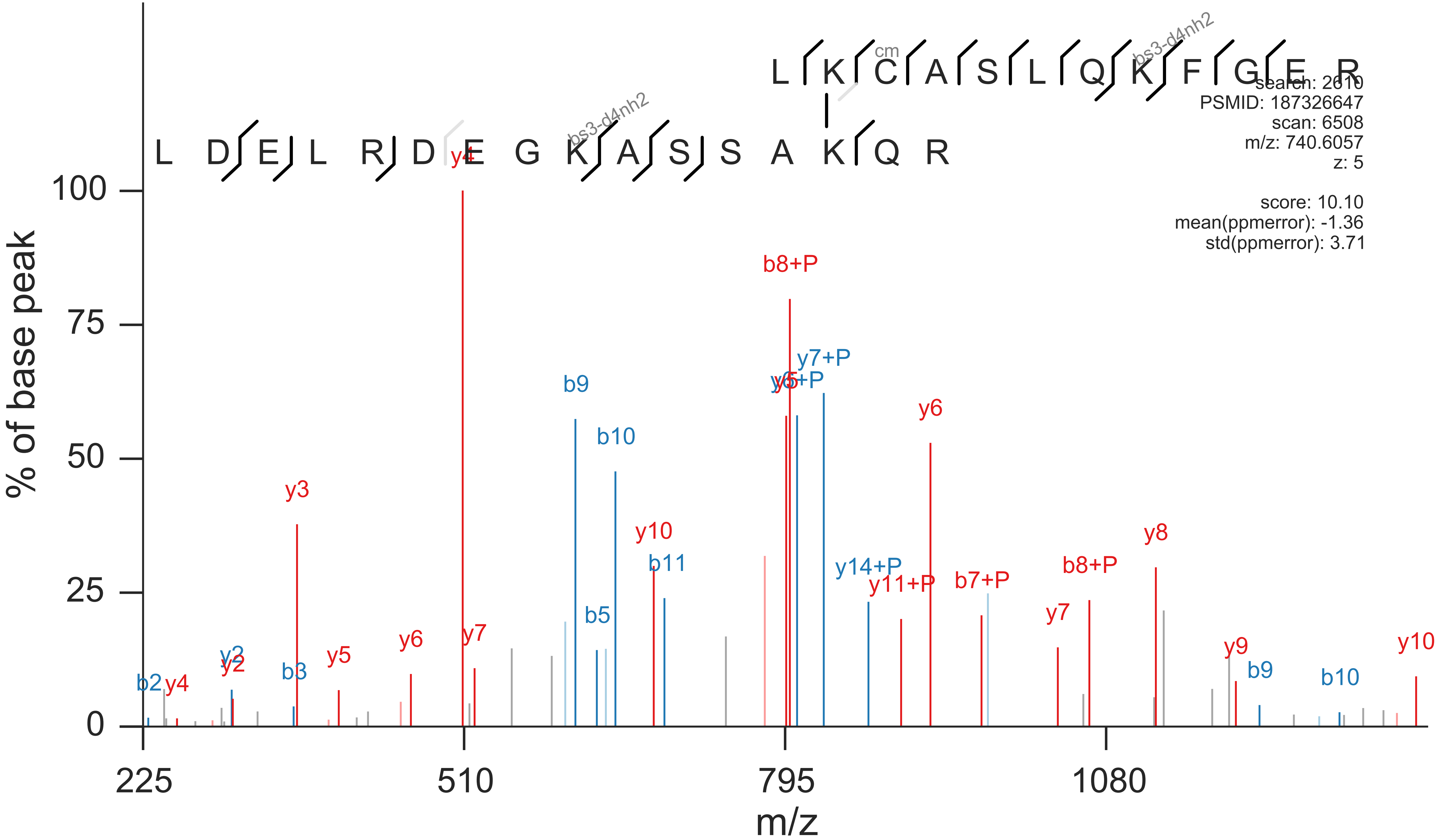

L K C A S L Q K F G E R
cm
bs3-d4nh2
L D E L R D E G K A S S A K Q R
bs3-d4nh2
search: 2610
PSMID: 187326647
scan: 6508
m/z: 740.6057
z: 5
score: 10.10
mean(ppmerror): -1.36
std(ppmerror): 3.71
% of base peak
m/z

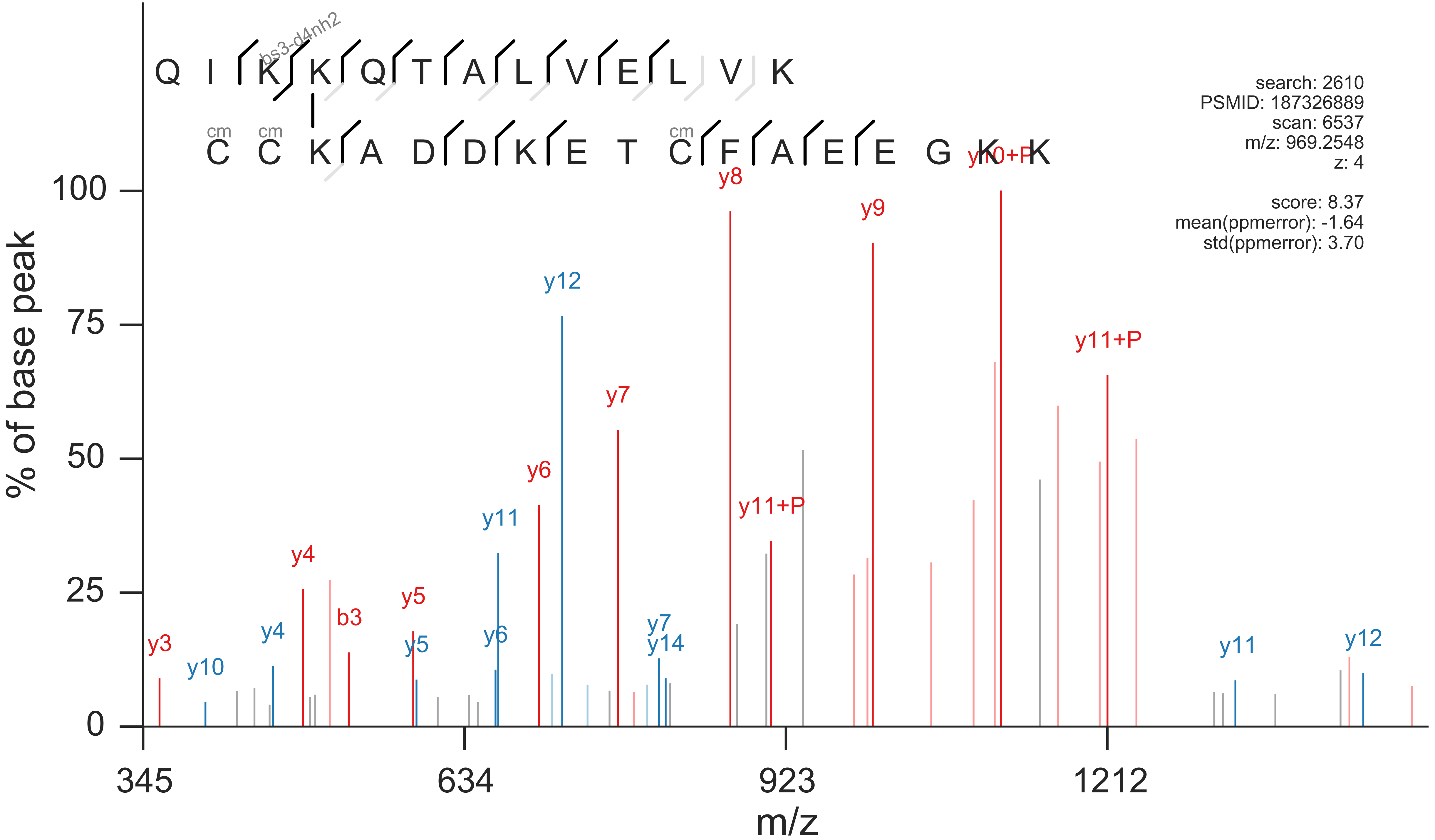

search: 2610
PSMID: 187326889
scan: 6537
m/z: 969.2548
z: 4
score: 8.37
mean(ppmerror): -1.64
std(ppmerror): 3.70
Q I K K Q T A L V E L V K
bs3-d4nh2
C C K A D D K E T C F A E E G K K
cm cm cm
% of base peak
m/z
0 25 50 75 100
345 634 923 1212
y3 y10 y4 y4 b3 y5 y5 y11 y6 y6 y12 y7 y7 y14 y8 y11+P y9 y10+P y11+P y11 y12

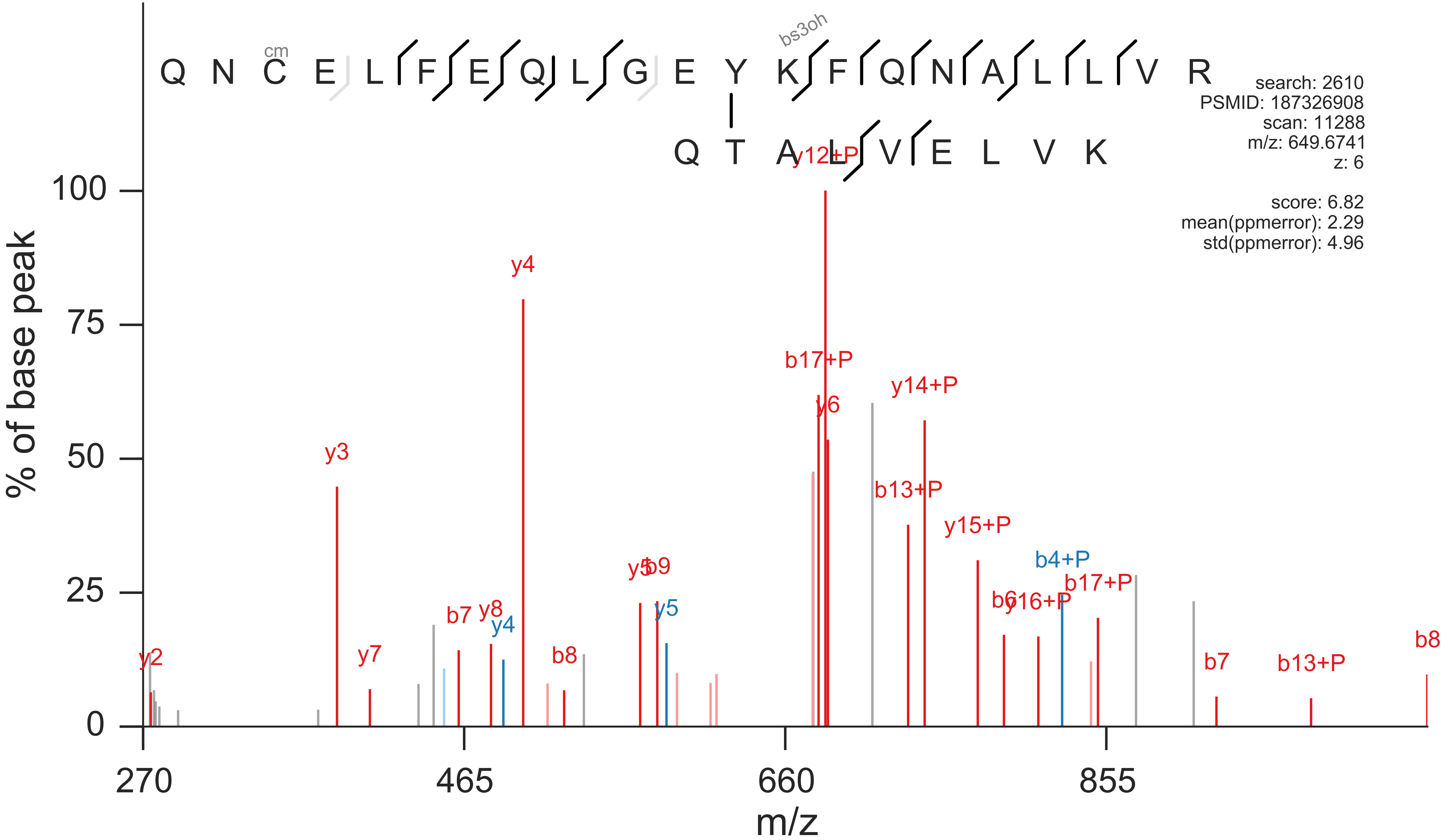

Q N C E L F E Q L G E Y K F Q N A L L V R
cm
bs3oh
Q T A L V E L V K
search: 2610
PSMID: 187326908
scan: 11288
m/z: 649.6741
z: 6
score: 6.82
mean(ppmerror): 2.29
std(ppmerror): 4.96
% of base peak
m/z
0
25
50
75
100
270
465
660
855
y2
y3
y7
b7
y8
y4
y4
b8
y5
b9
y5
y12+P
b17+P
y6
y14+P
b13+P
y15+P
b4+P
b6
y16+P
b17+P
b7
b13+P
b8

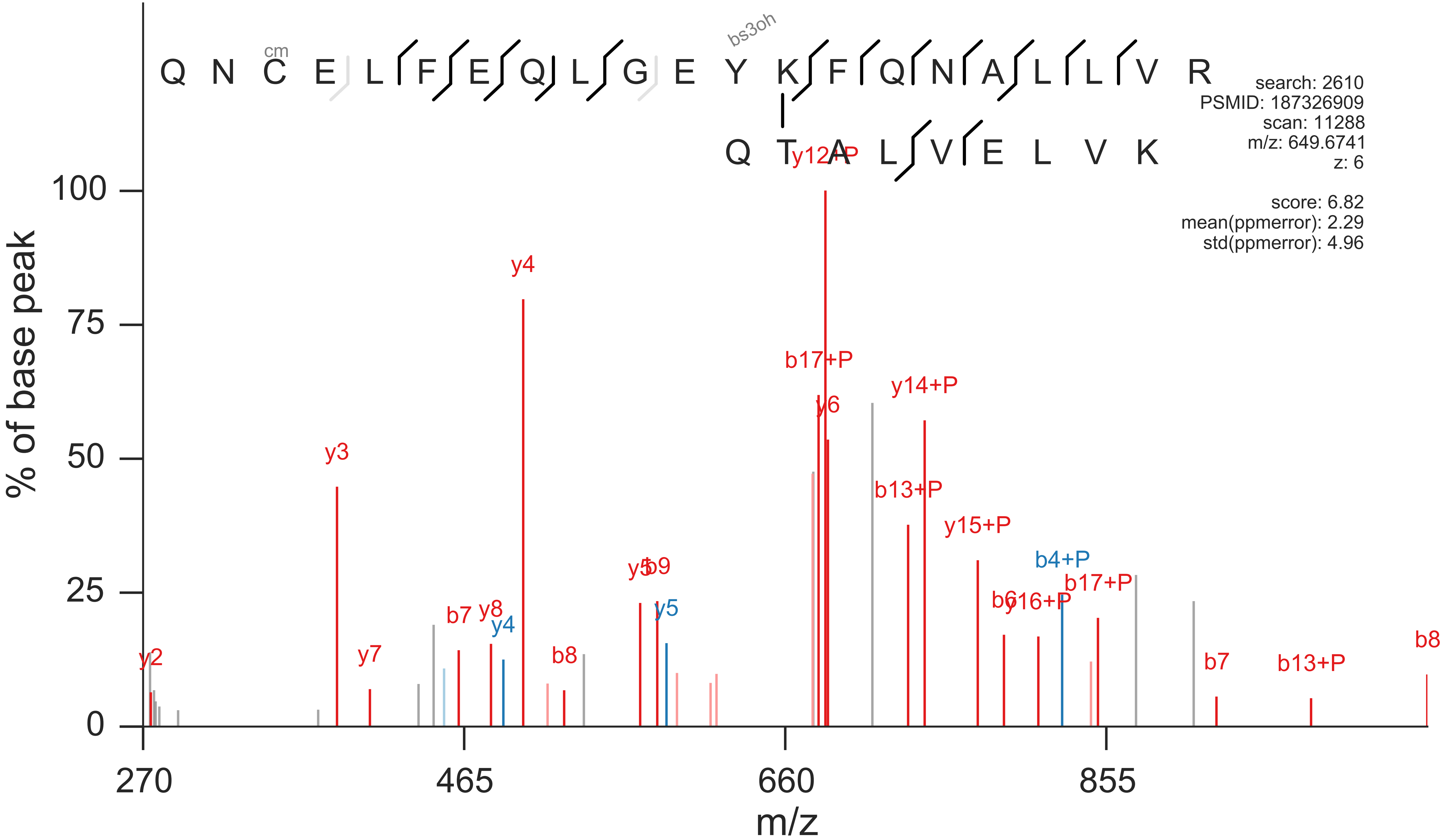

Q N C E L F E Q L G E Y K F Q N A L L V R
cm
bs3oh
Q T A L V E L V K
search: 2610
PSMID: 187326909
scan: 11288
m/z: 649.6741
z: 6
score: 6.82
mean(ppmerror): 2.29
std(ppmerror): 4.96
% of base peak
m/z
0
25
50
75
100
270
465
660
855
y2
y3
y7
b7
y8
y4
y4
b8
y5
b9
y5
y12+P
b17+P
y6
y14+P
b13+P
y15+P
b4+P
b6
y16+P
b17+P
b7
b13+P
b8

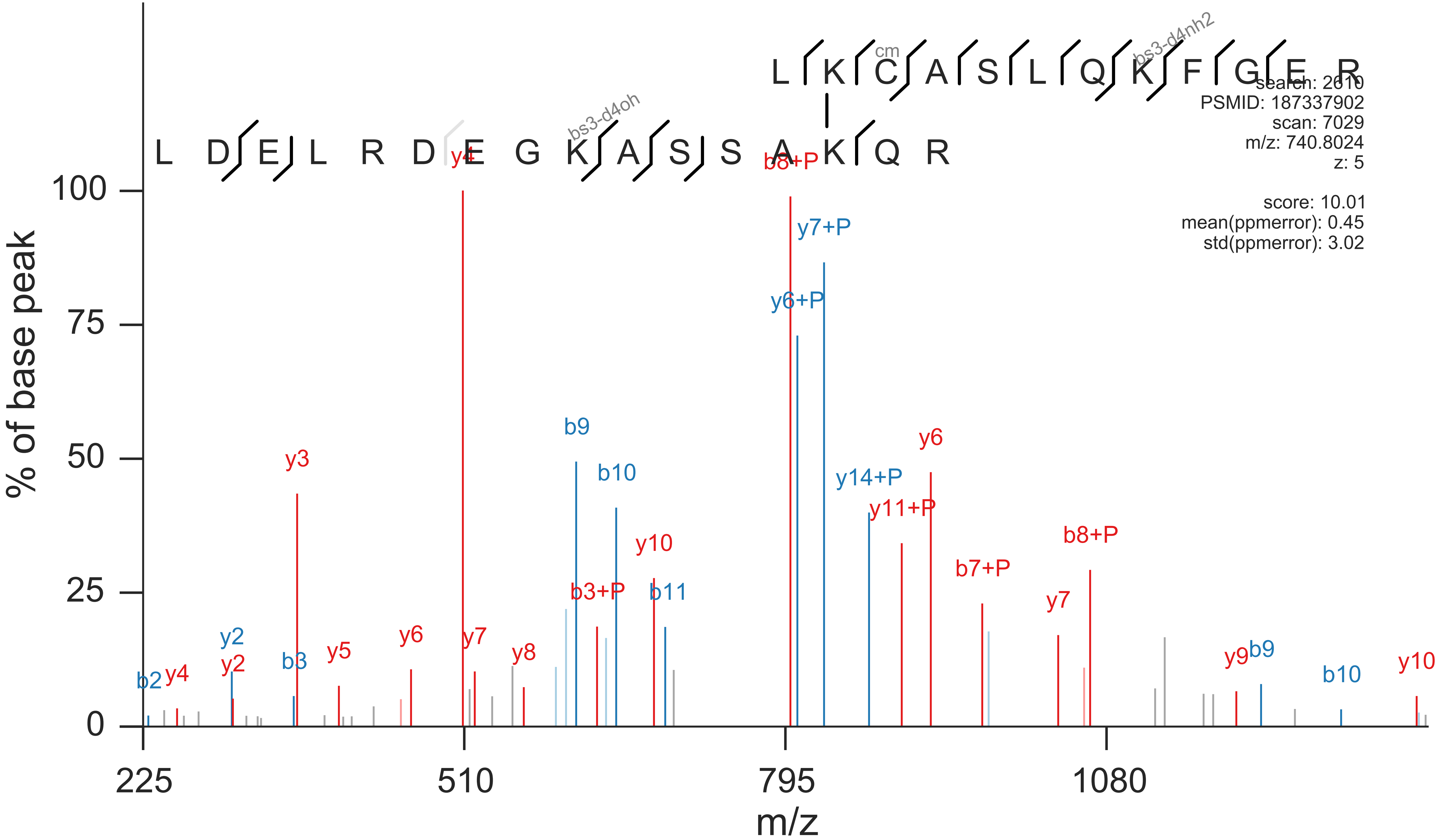

L K C A S L Q K F G E R
cm
bs3-d4nh2
L D E L R D E G K A S S A K Q R
bs3-d4oh
search: 2610
PSMID: 187337902
scan: 7029
m/z: 740.8024
z: 5
score: 10.01
mean(ppmerror): 0.45
std(ppmerror): 3.02
% of base peak
m/z
0
25
50
75
100
225
510
795
1080
b2
y4
y2
y2
b3
y3
y5
y6
y4
y7
y8
b9
b10
b3+P
y10
b11
b8+P
y7+P
y6+P
y14+P
y11+P
y6
b7+P
b8+P
y7
y9
b9
b10
y10

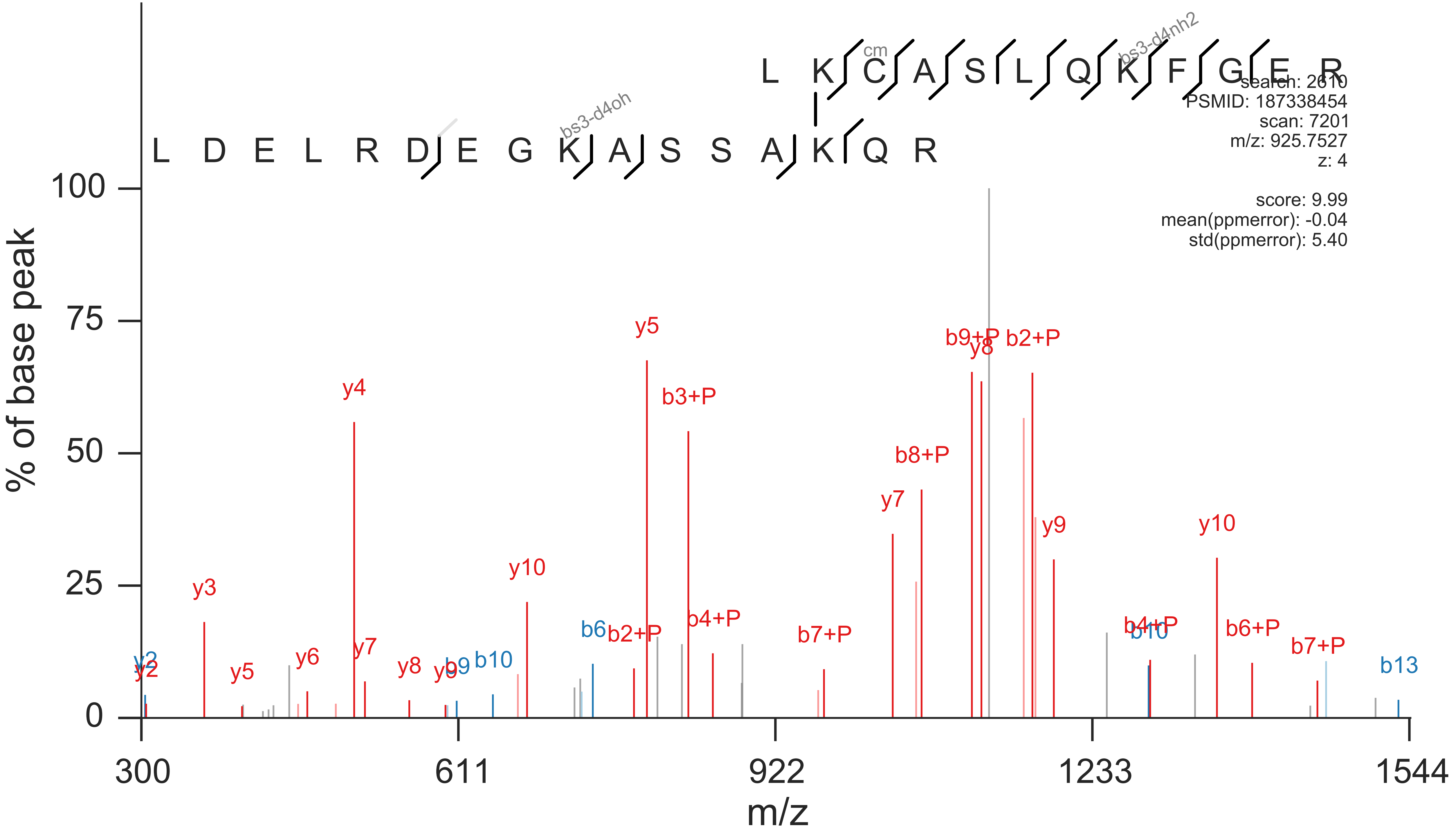

L K C A S L Q K F G E R
cm
bs3-d4nh2
L D E L R D E G K A S S A K Q R
bs3-d4oh
search: 2610
PSMID: 187338454
scan: 7201
m/z: 925.7527
z: 4
score: 9.99
mean(ppmerror): -0.04
std(ppmerror): 5.40
% of base peak
m/z

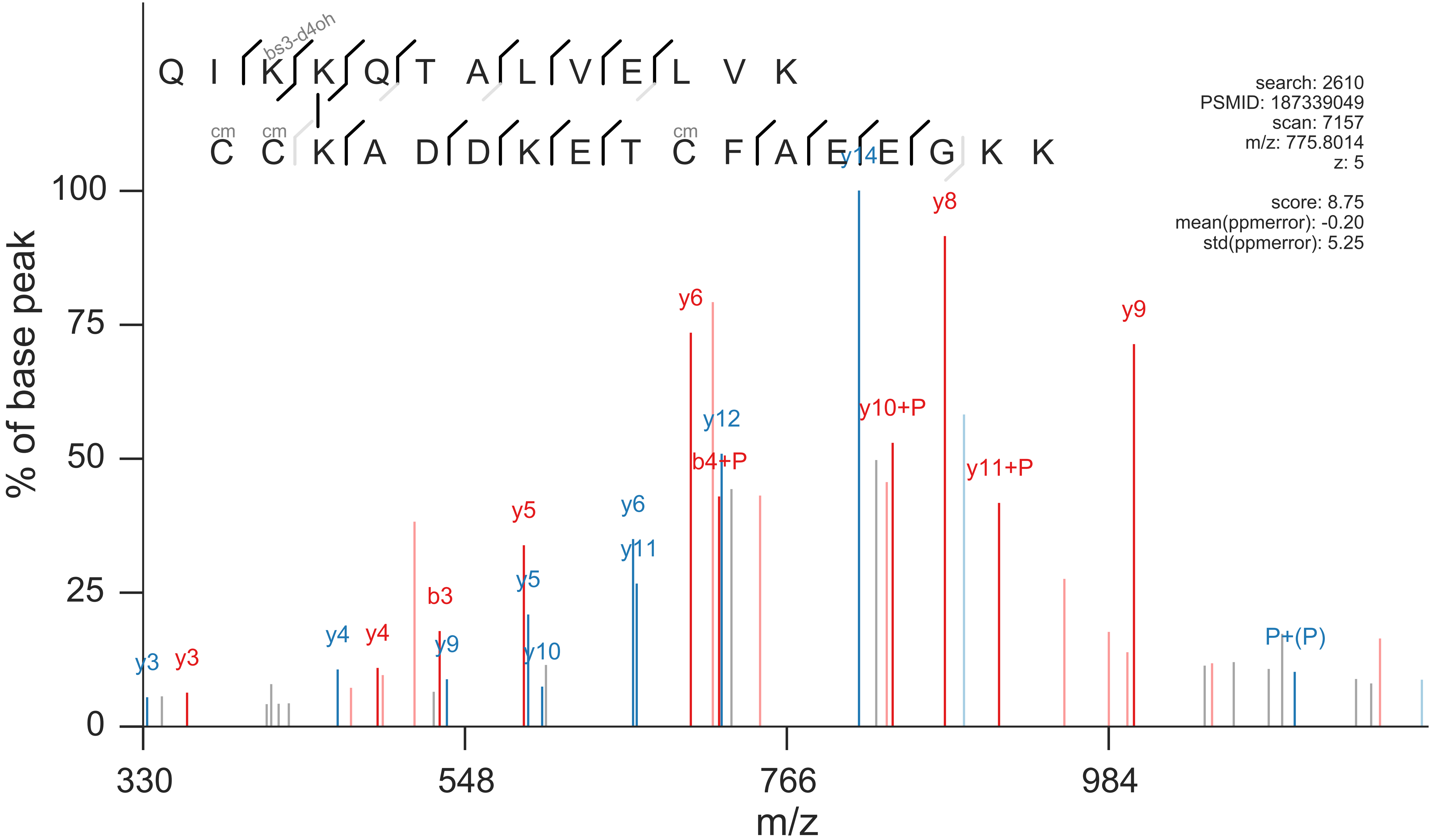

bs3-d4oh
Q I K K Q T A L V E L V K
cm cm cm
C C K A D D K E T C F A E E G K K
search: 2610
PSMID: 187339049
scan: 7157
m/z: 775.8014
z: 5
score: 8.75
mean(ppmerror): -0.20
std(ppmerror): 5.25
% of base peak
m/z
0
25
50
75
100
330
548
766
984
y3
y3
y4
y4
b3
y9
y5
y5
y10
y6
y11
y6
y12
b4+P
y14
y10+P
y8
y11+P
y9
P+(P)

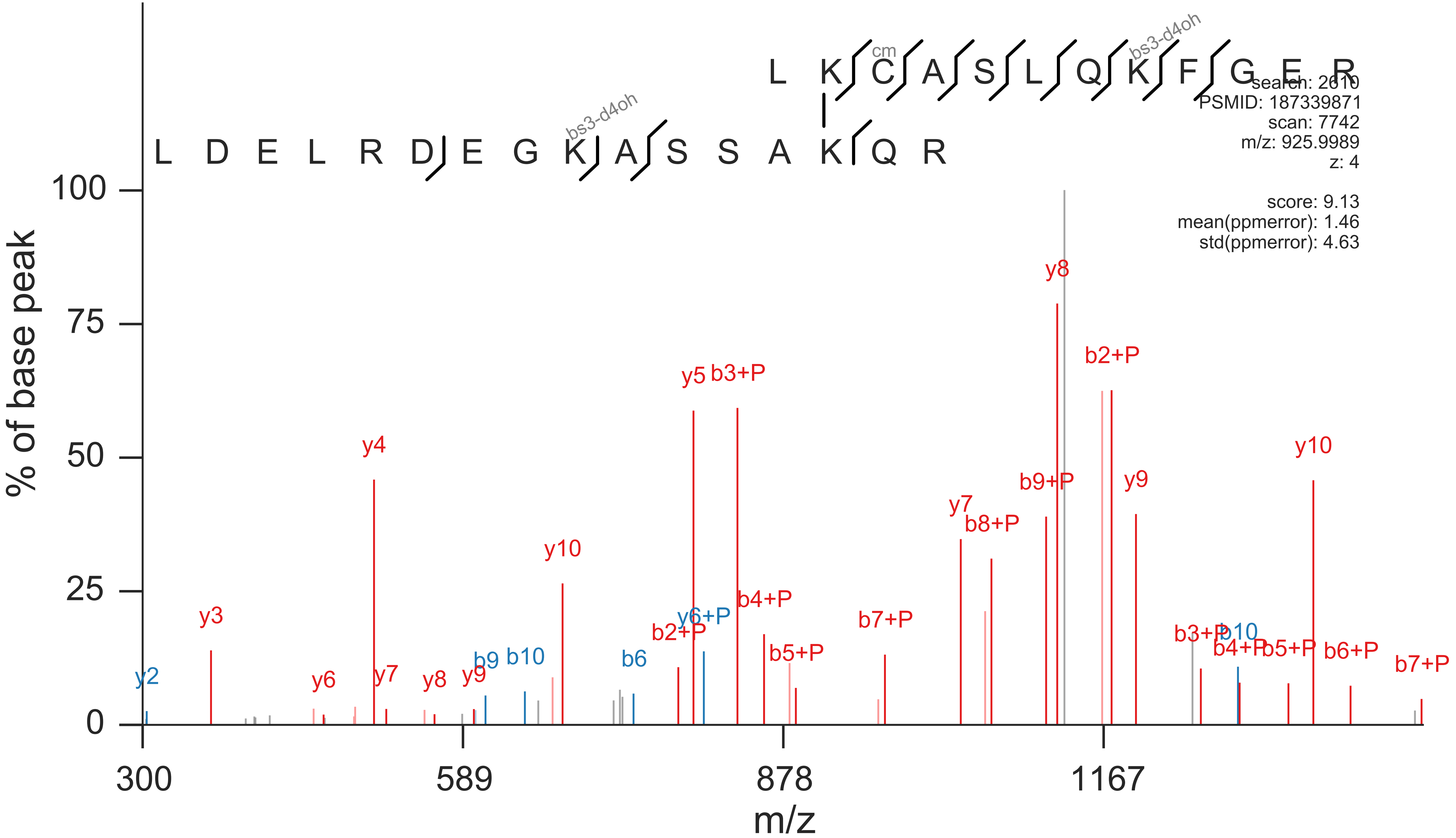

L K C A S L Q K F G E R
cm
bs3-d4oh
L D E L R D E G K A S S A K Q R
bs3-d4oh
search: 2610
PSMID: 187339871
scan: 7742
m/z: 925.9989
z: 4
score: 9.13
mean(ppmerror): 1.46
std(ppmerror): 4.63
% of base peak
m/z
0
25
50
75
100
300
589
878
1167
y2
y3
y6
y4
y7
y8
y9
b9
b10
y10
b6
b2+P
y5
y6+P
b3+P
b4+P
b5+P
b7+P
y7
b8+P
b9+P
y8
b2+P
y9
b3+P
b10
b4+P
b5+P
y10
b6+P
b7+P

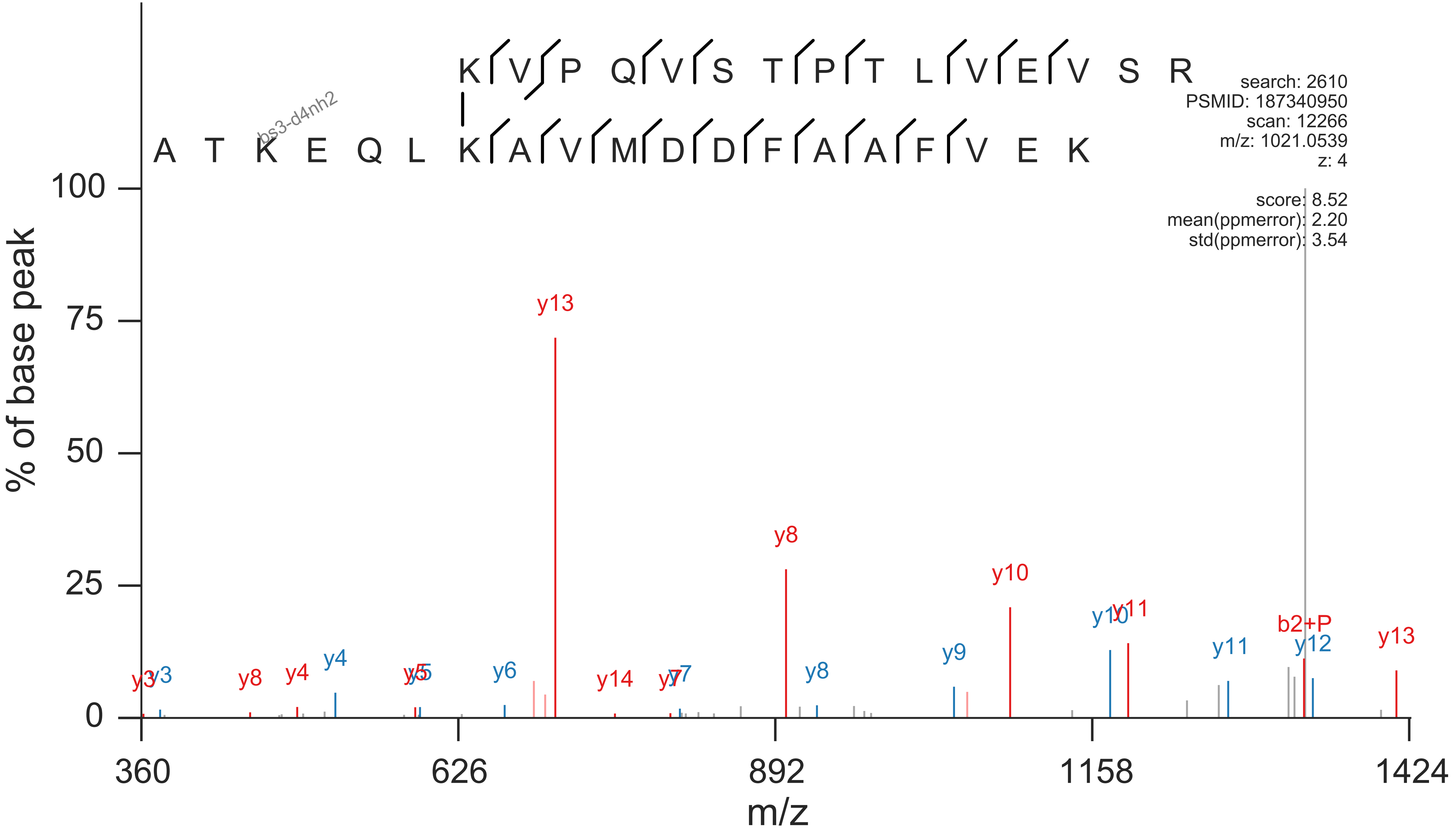

K V P Q V S T P T L V E V S R
A T K E Q L K A V M D D F A A F V E K
bs3-d4nh2
search: 2610
PSMID: 187340950
scan: 12266
m/z: 1021.0539
z: 4
score: 8.52
mean(ppmerror): 2.20
std(ppmerror): 3.54
% of base peak
m/z
100
75
50
25
0
360
626
892
1158
1424
y13
y8
y10
y11
y10
b2+P
y12
y13
y11
y9
y8
y7
y7
y14
y6
y5
y5
y4
y4
y8
y3
y3

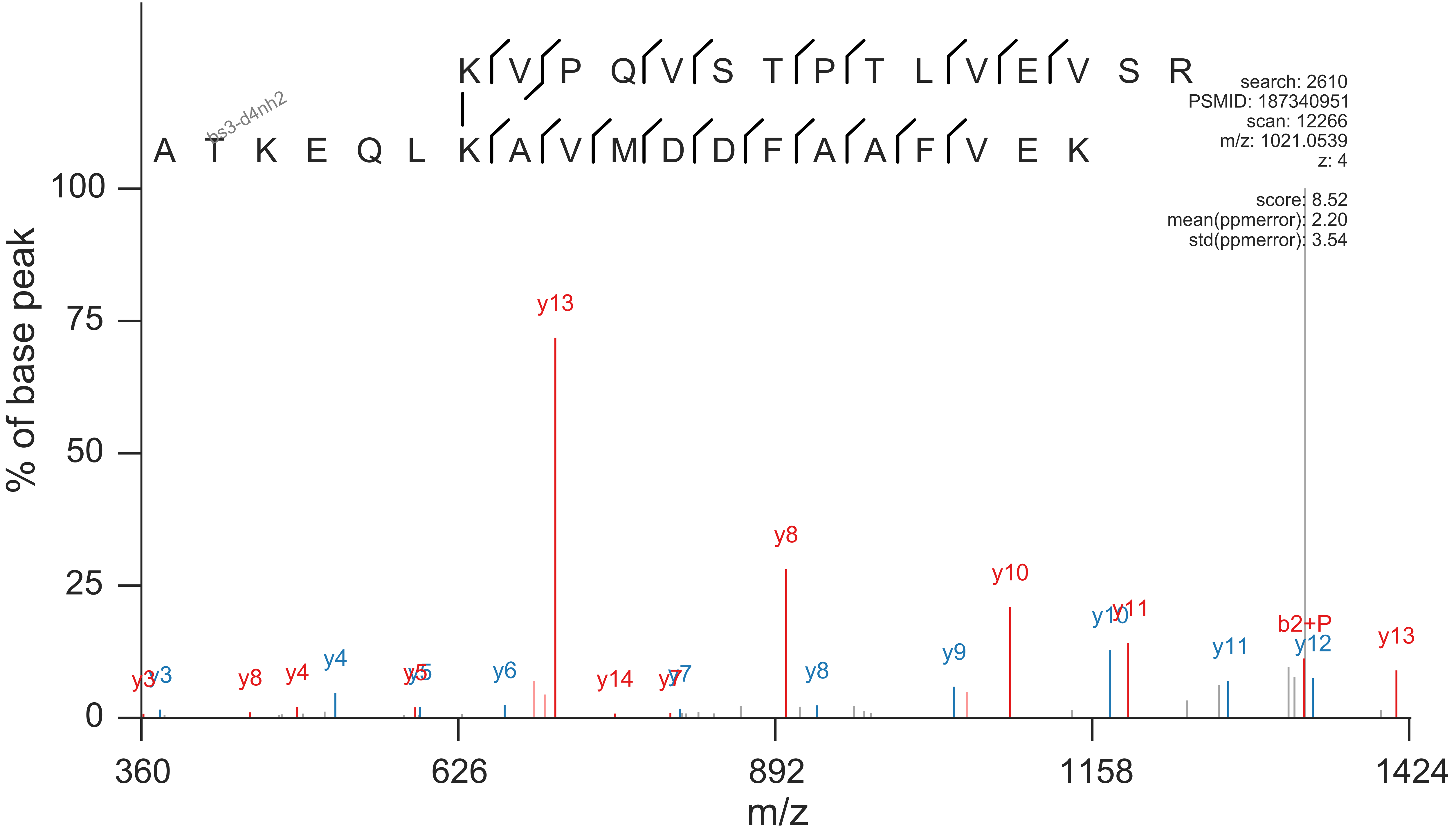

K V P Q V S T P T L V E V S R
A T K E Q L K A V M D D F A A F V E K
bs3-d4nh2
search: 2610
PSMID: 187340951
scan: 12266
m/z: 1021.0539
z: 4
score: 8.52
mean(ppmerror): 2.20
std(ppmerror): 3.54
% of base peak
m/z
0
25
50
75
100
360
626
892
1158
1424
y13
y8
y10
y11
y10
y11
b2+P
y12
y13
y9
y8
y7
y14
y6
y5
y4
y3

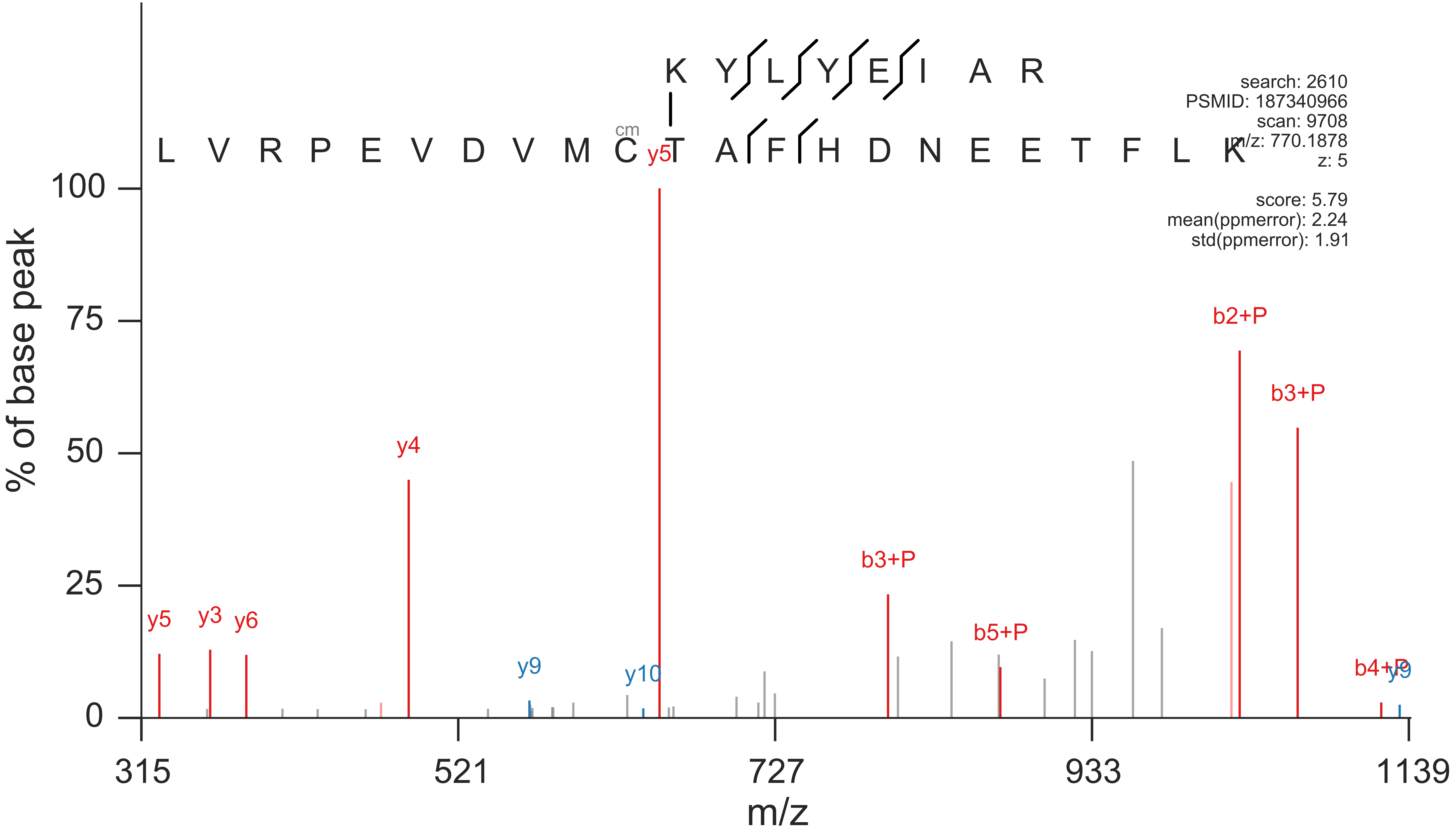

K Y L Y E I A R
L V R P E V D V M C T A F H D N E E T F L K
cm
search: 2610
PSMID: 187340966
scan: 9708
m/z: 770.1878
z: 5
score: 5.79
mean(ppmerror): 2.24
std(ppmerror): 1.91
% of base peak
m/z
100
75
50
25
0
315
521
727
933
1139
y5
y3
y6
y4
y9
y10
y5
b3+P
b5+P
b2+P
b3+P
b4+P
y9

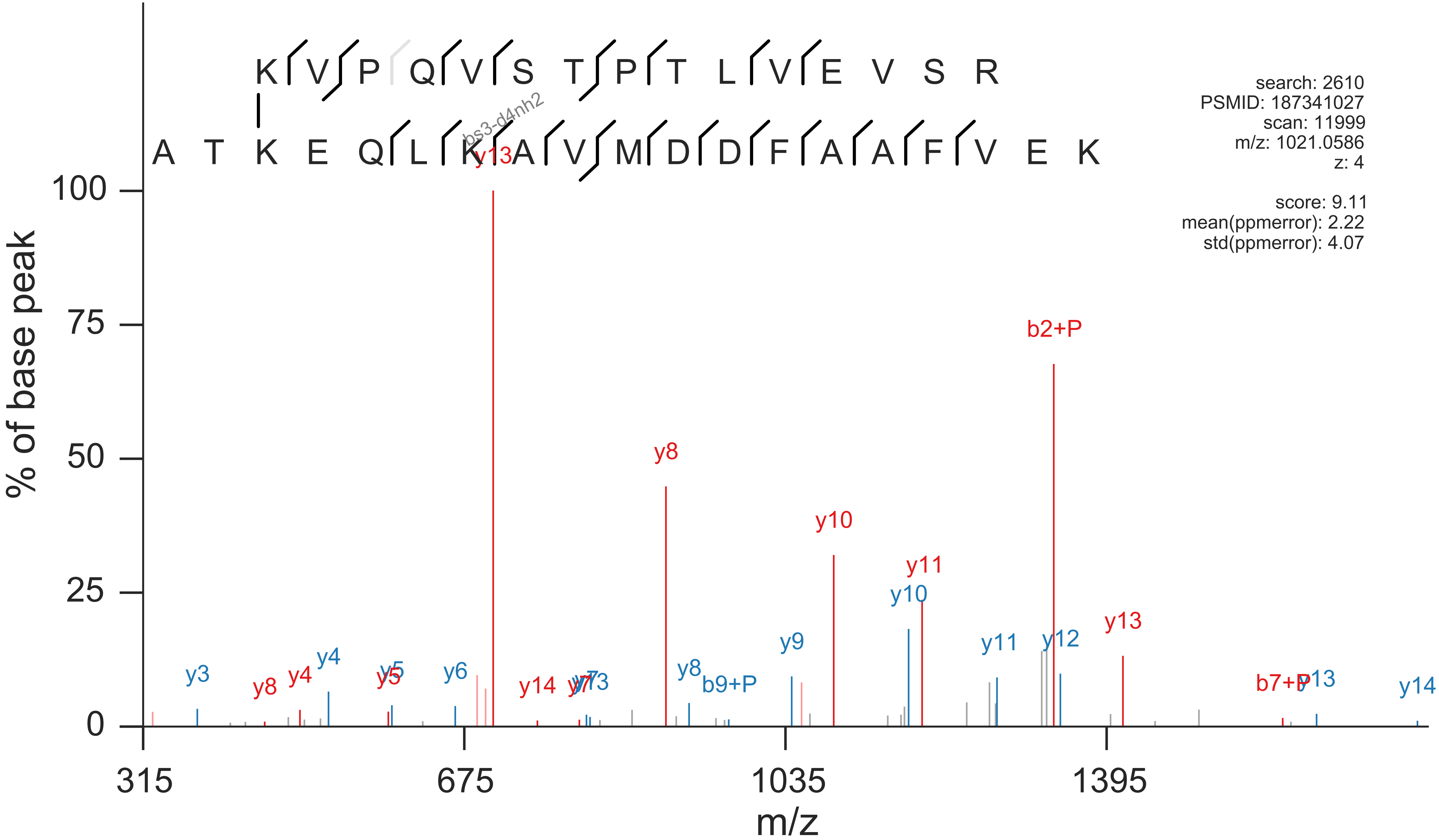

K V P Q V S T P T L V E V S R
A T K E Q L K A V M D D F A A F V E K
bs3-d4nh2
search: 2610
PSMID: 187341027
scan: 11999
m/z: 1021.0586
z: 4
score: 9.11
mean(ppmerror): 2.22
std(ppmerror): 4.07
% of base peak
m/z
0
25
50
75
100
315
675
1035
1395
y13
y8
y10
y11
y10
b2+P
y13
y3
y8
y4
y4
y5
y5
y6
y14
y7
y13
y8
b9+P
y9
y11
y12
b7+P
y13
y14

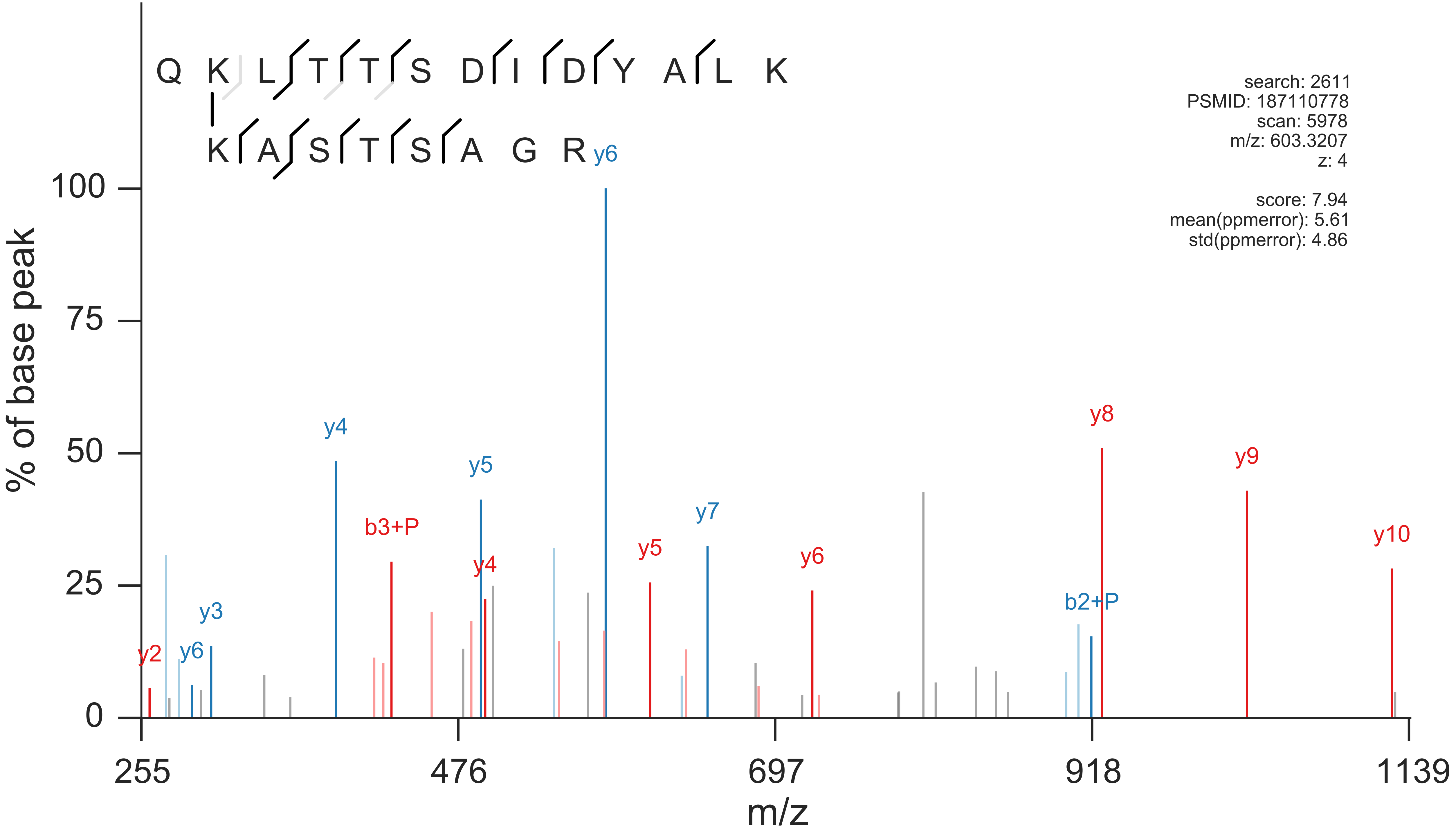
Q K L T T S D I D Y A L K
K A S T S A G R
search: 2611
PSMID: 187110778
scan: 5978
m/z: 603.3207
z: 4
score: 7.94
mean(ppmerror): 5.61
std(ppmerror): 4.86
% of base peak
m/z
100
75
50
25
0
255
476
697
918
1139
y2
y6
y3
y4
b3+P
y5
y4
y6
y5
y7
y6
b2+P
y8
y9
y10

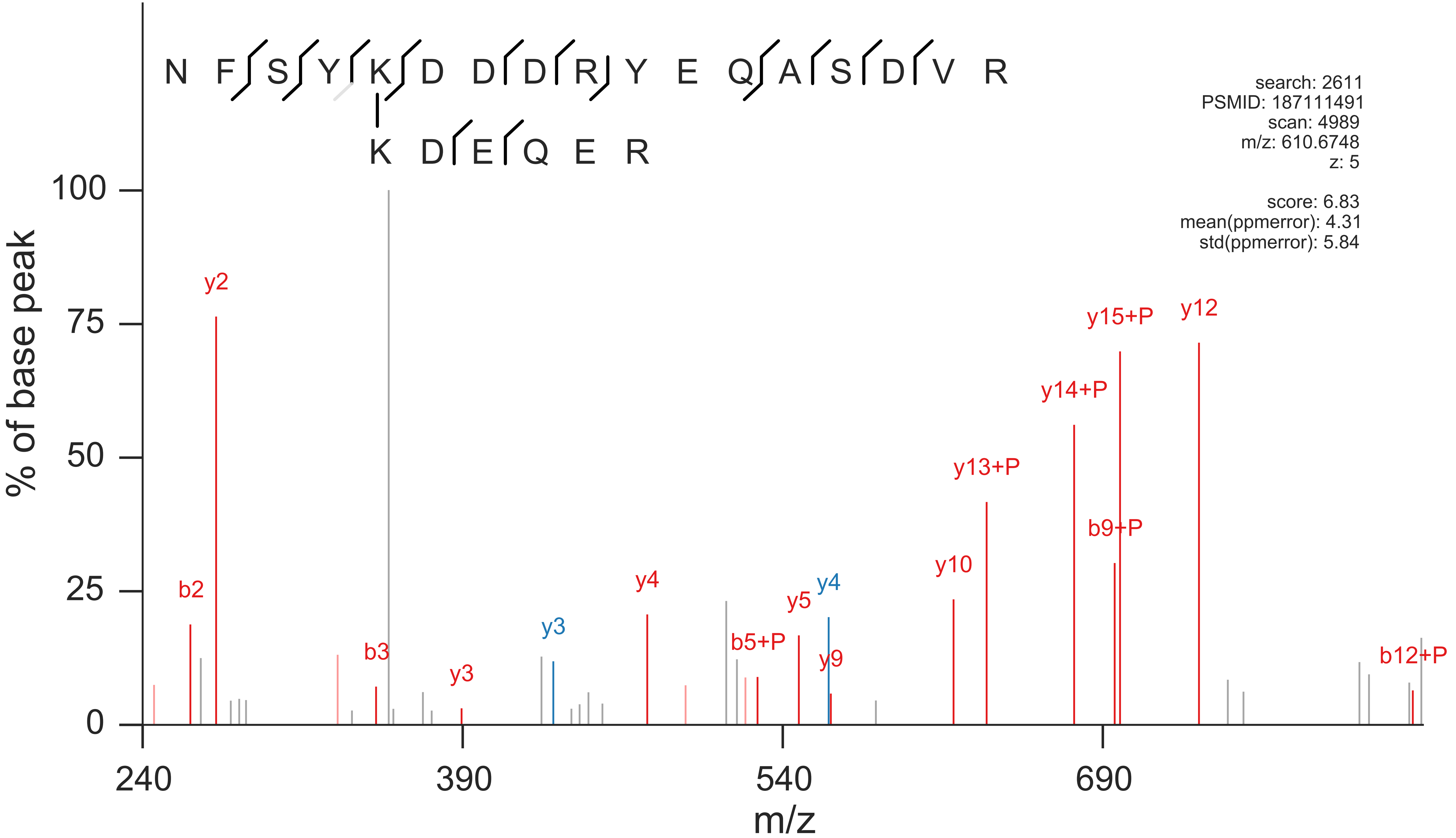

N F S Y K D D D R Y E Q A S D V R
K D E Q E R
search: 2611
PSMID: 187111491
scan: 4989
m/z: 610.6748
z: 5
score: 6.83
mean(ppmerror): 4.31
std(ppmerror): 5.84
% of base peak
m/z
y2
b2
b3
y3
y3
y4
b5+P
y5
y4
y9
y10
y13+P
y14+P
y15+P
b9+P
y12
b12+P

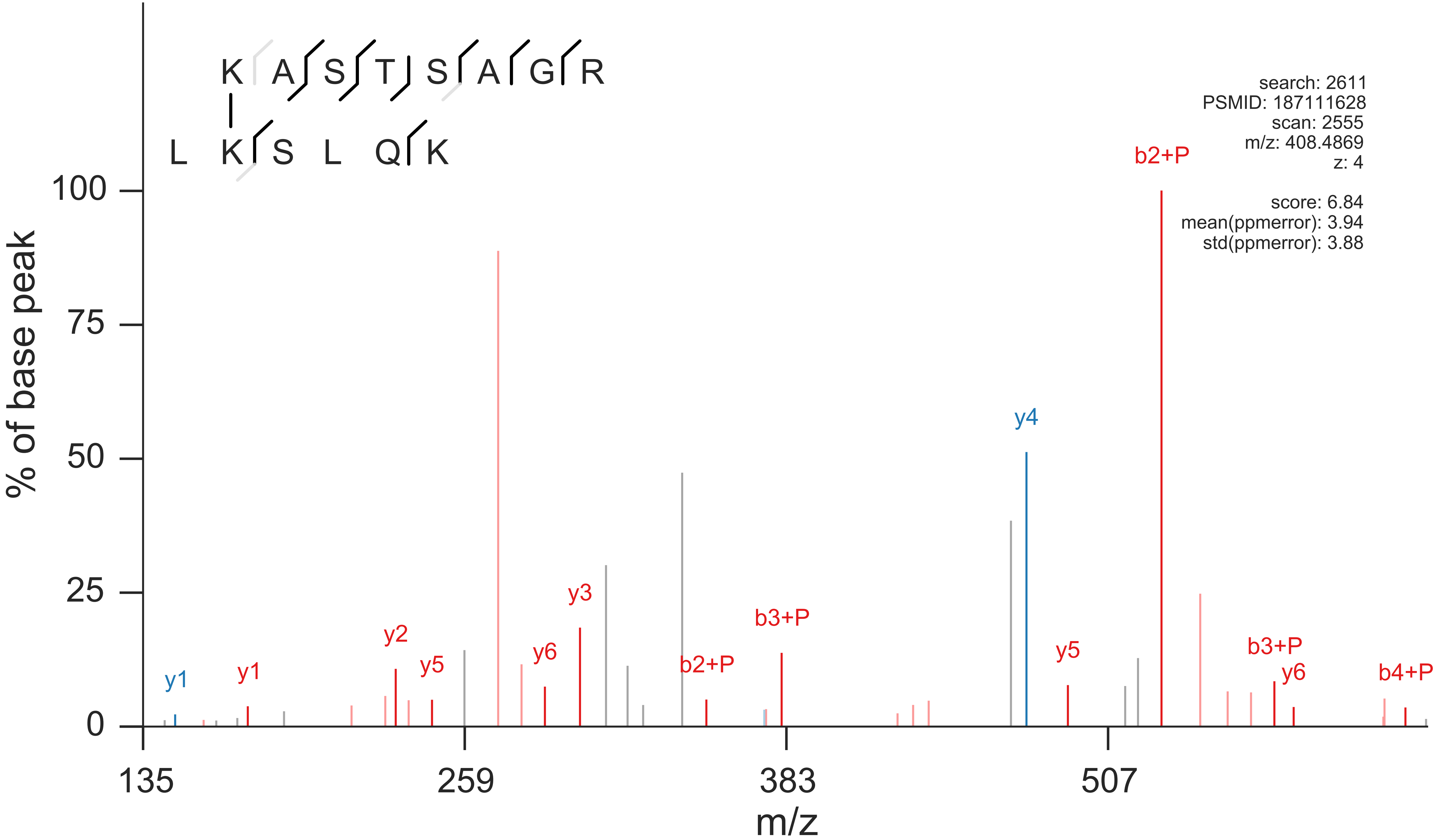

K A S T S A G R
L K S L Q K
search: 2611
PSMID: 187111628
scan: 2555
m/z: 408.4869
z: 4
score: 6.84
mean(ppmerror): 3.94
std(ppmerror): 3.88
% of base peak
100
75
50
25
0
135
259
383
507
m/z
y1
y1
y2
y5
y6
y3
b2+P
b3+P
y4
y5
b2+P
b3+P
y6
b4+P

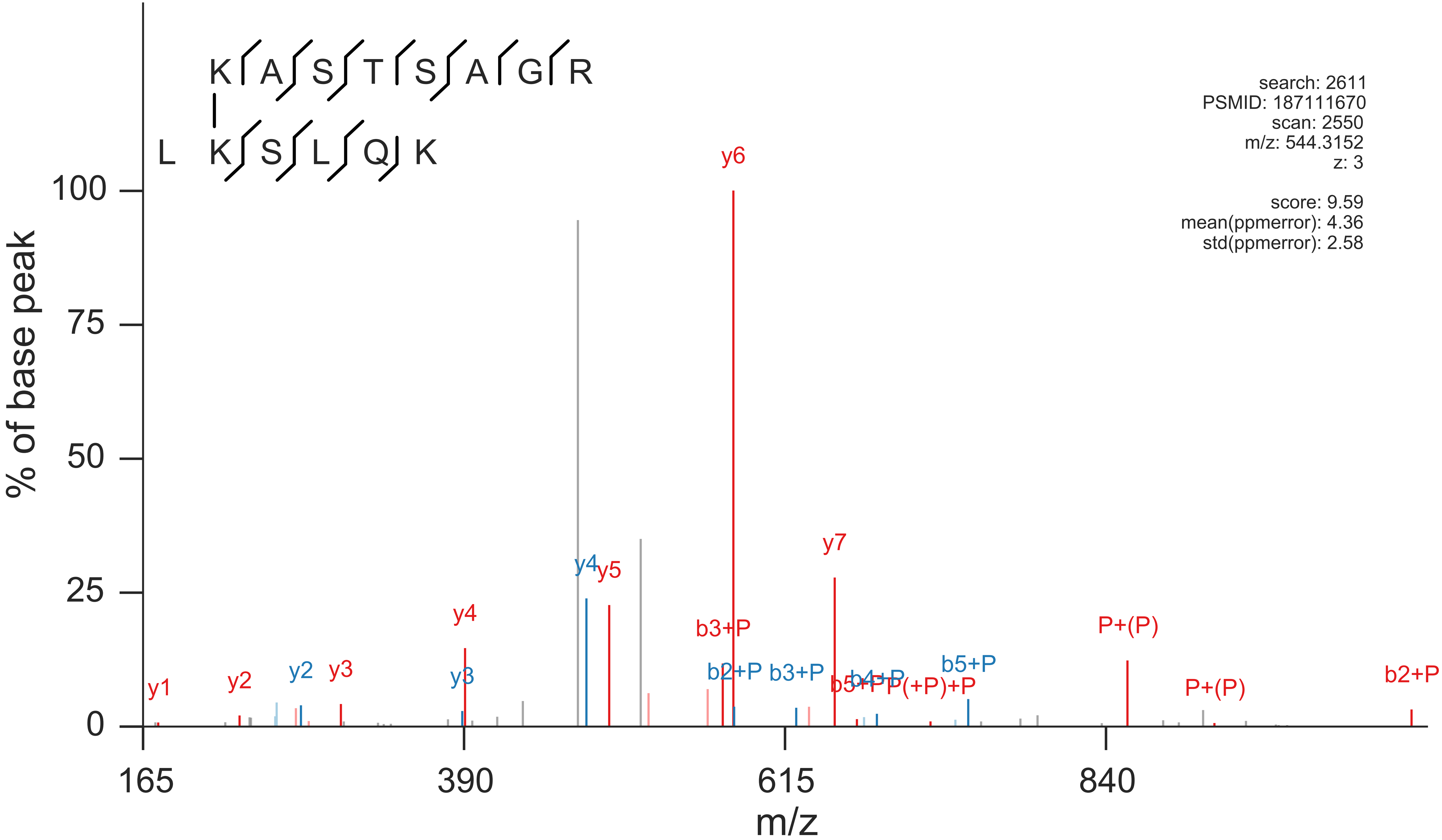

K A S T S A G R
L K S L Q K
search: 2611
PSMID: 187111670
scan: 2550
m/z: 544.3152
z: 3
score: 9.59
mean(ppmerror): 4.36
std(ppmerror): 2.58
% of base peak
100
75
50
25
0
165
390
615
840
m/z
y1
y2
y2
y3
y3
y4
y4
y5
y6
y7
b3+P
b2+P
b3+P
b4+P
b5+P
b5+P
P(+P)+P
P+(P)
P+(P)
b2+P

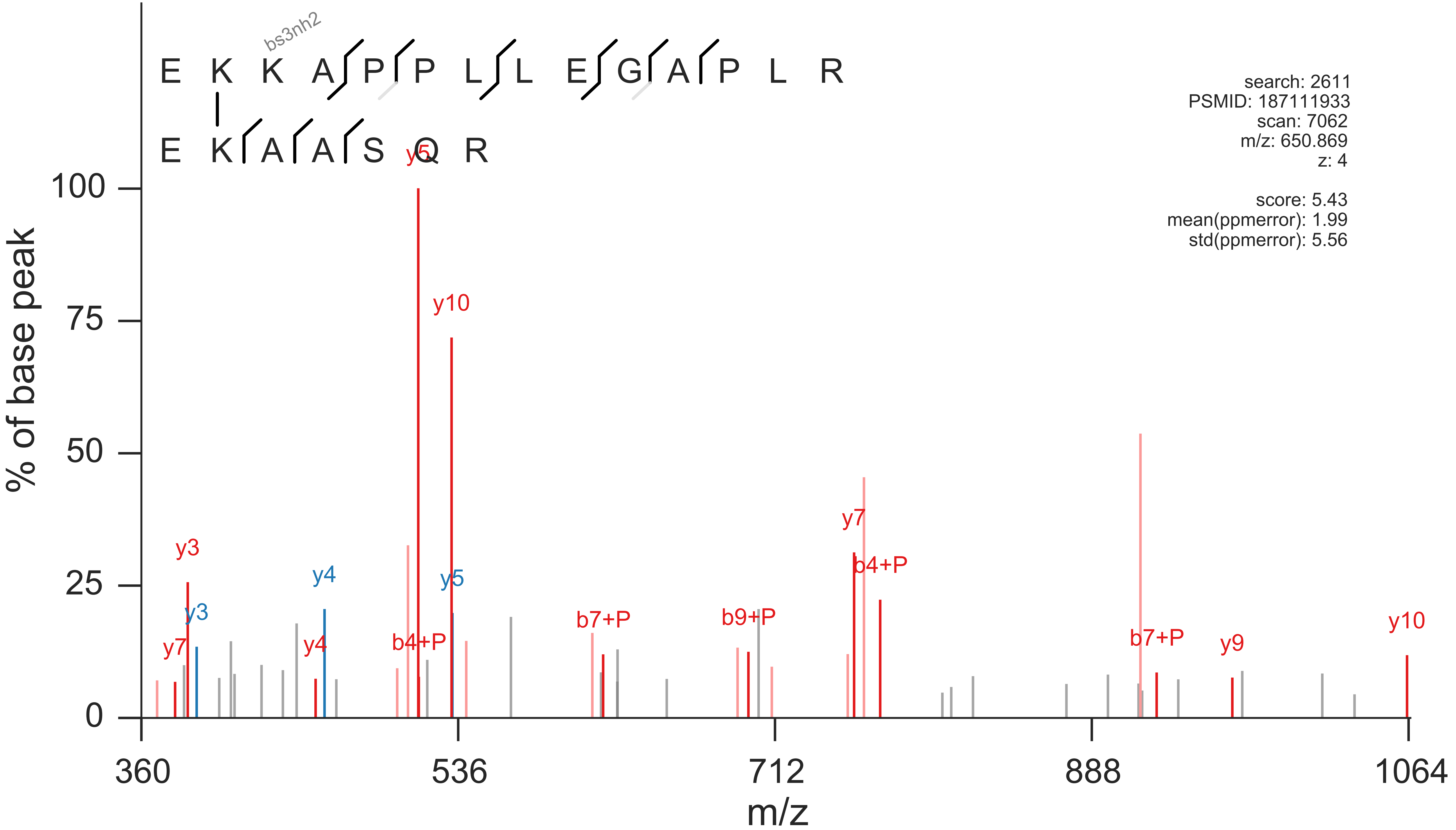

bs3nh2
E K K A P P L L E G A P L R
E K A A S Q R
search: 2611
PSMID: 187111933
scan: 7062
m/z: 650.869
z: 4
score: 5.43
mean(ppmerror): 1.99
std(ppmerror): 5.56
% of base peak
m/z
100
75
50
25
0
360
536
712
888
1064
y5
y10
y3
y7
y4
b4+P
b7+P
b9+P
b4+P
b7+P
y9
y10

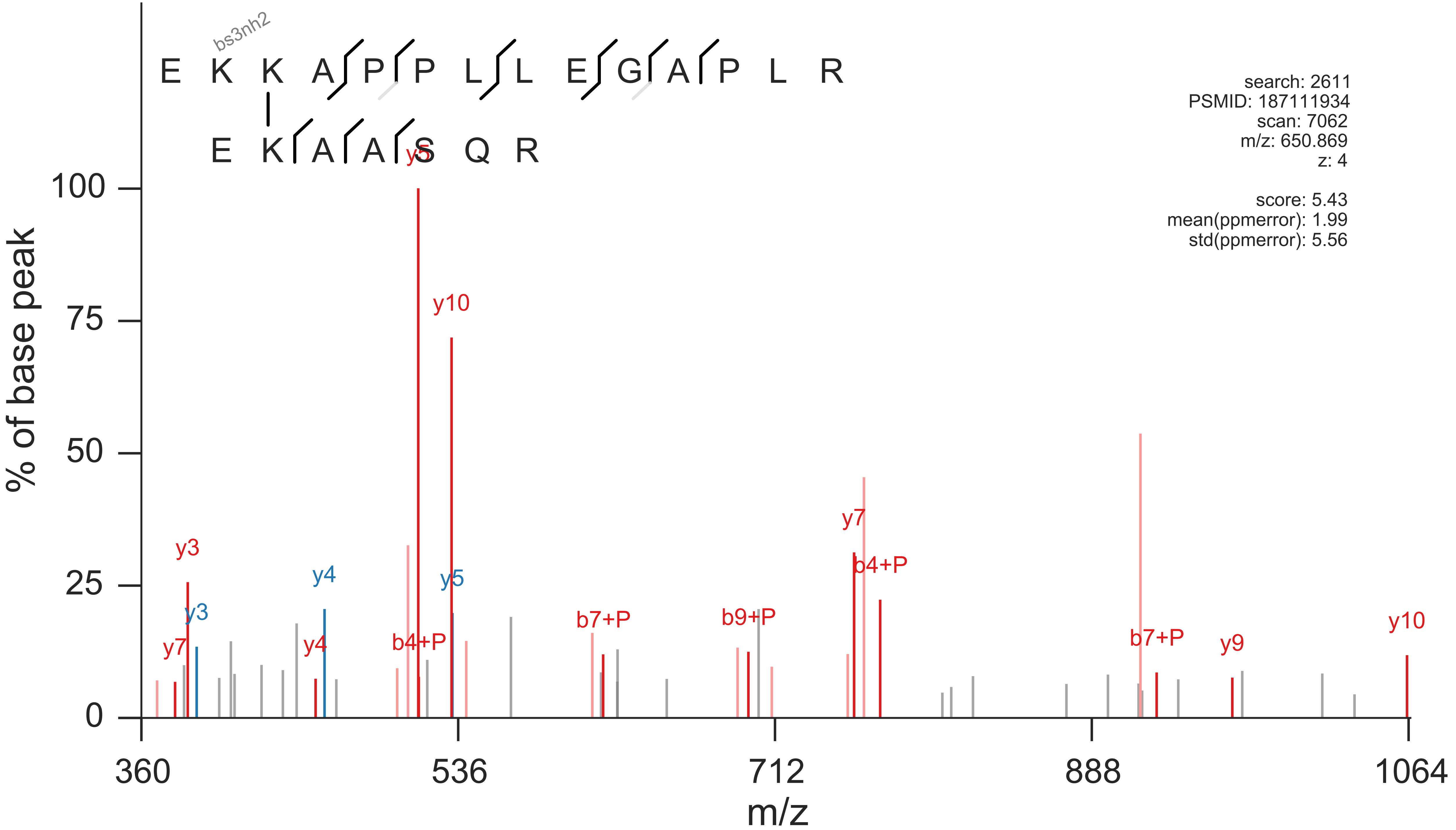

bs3nh2
E K K A P P L L E G A P L R
E K A A S Q R
search: 2611
PSMID: 187111934
scan: 7062
m/z: 650.869
z: 4
score: 5.43
mean(ppmerror): 1.99
std(ppmerror): 5.56
% of base peak
m/z
y3
y7
y3
y4
y4
y5
b4+P
y10
y5
b7+P
b9+P
y7
b4+P
b7+P
y9
y10

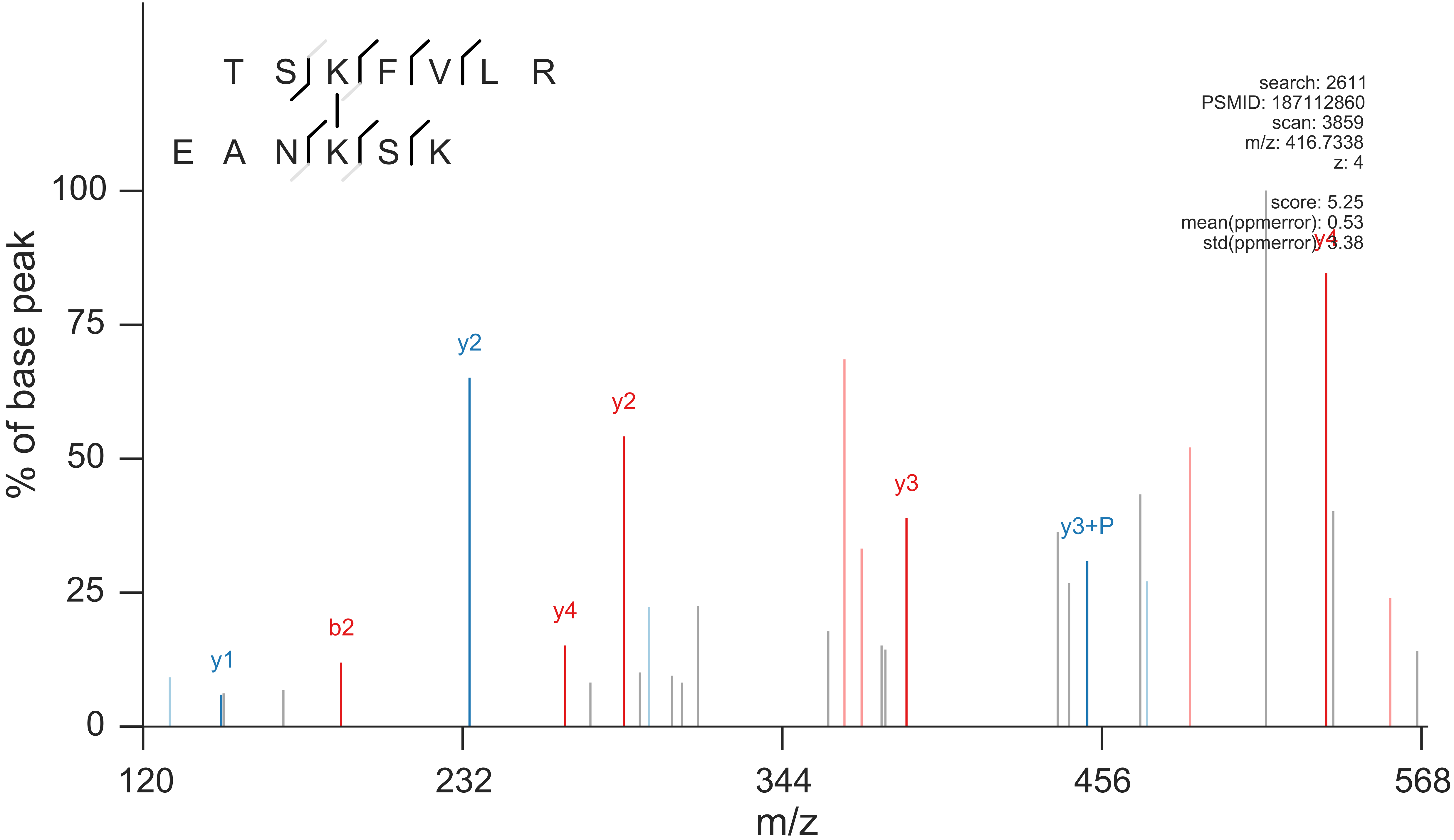

T S K F V L R
E A N K S K
search: 2611
PSMID: 187112860
scan: 3859
m/z: 416.7338
z: 4
score: 5.25
mean(ppmerror): 0.53
std(ppmerror): 3.38
% of base peak
m/z
0
25
50
75
100
120
232
344
456
568
y1
b2
y2
y4
y2
y3
y3+P
y4

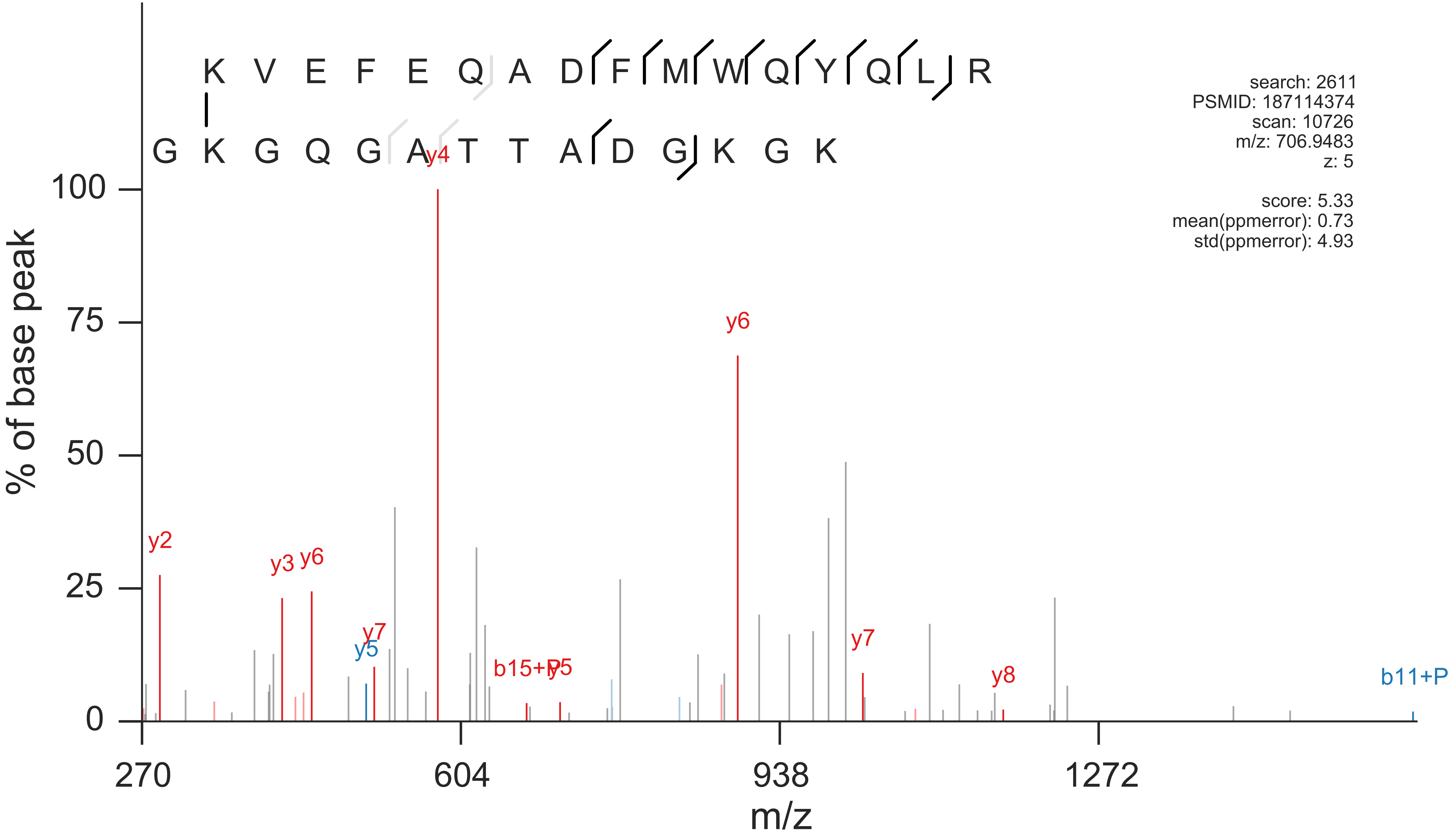
K V E F E Q A D F M W Q Y Q L R
G K G Q G A T T A D G K G K
search: 2611
PSMID: 187114374
scan: 10726
m/z: 706.9483
z: 5
score: 5.33
mean(ppmerror): 0.73
std(ppmerror): 4.93
% of base peak
100
75
50
25
0
y4
y6
y2
y3
y6
y7
y5
b15+P
y5
y7
y8
b11+P
270
604
938
1272
m/z

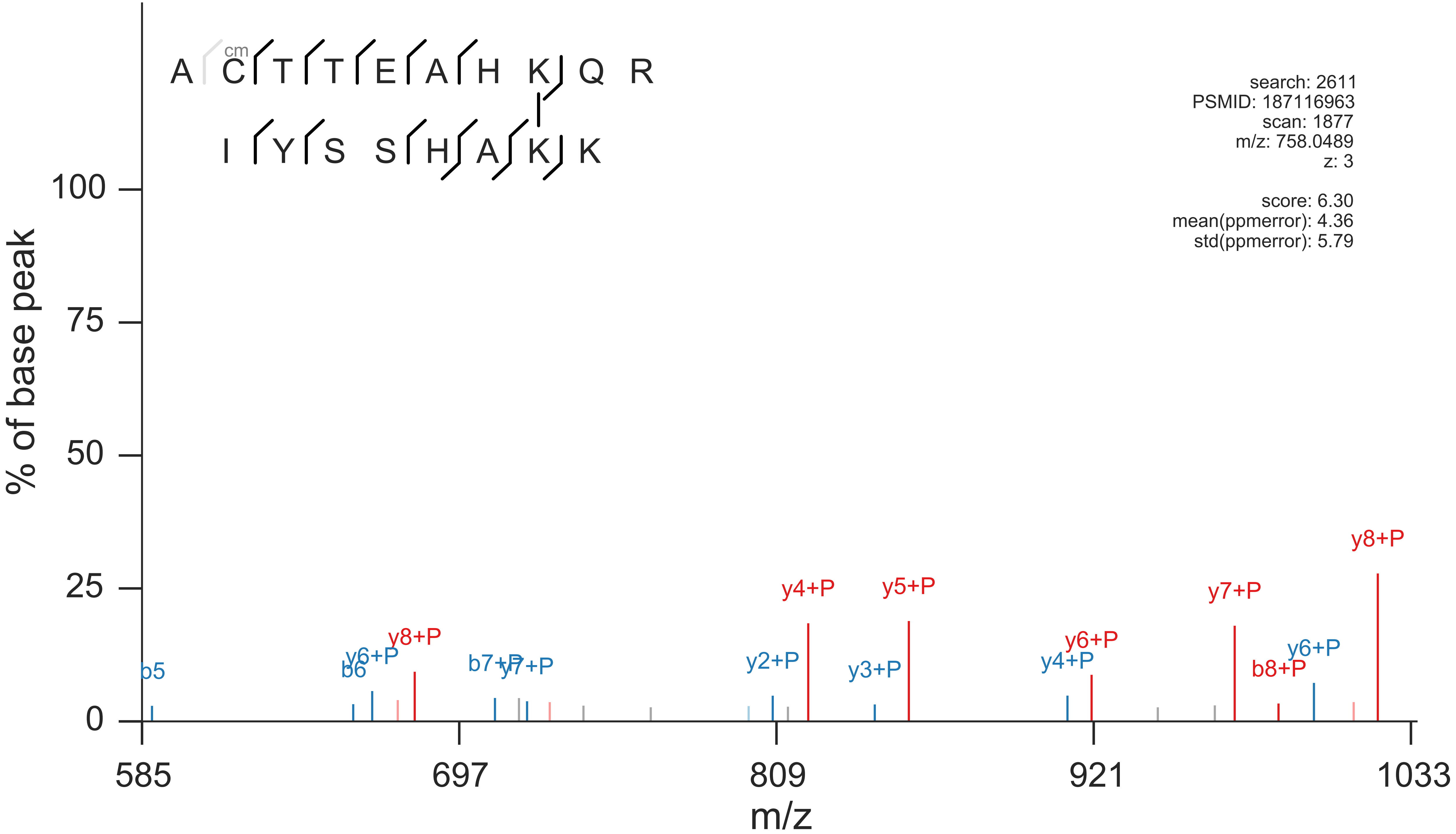

A C T T E A H K Q R
cm
I Y S S H A K K
search: 2611
PSMID: 187116963
scan: 1877
m/z: 758.0489
z: 3
score: 6.30
mean(ppmerror): 4.36
std(ppmerror): 5.79
% of base peak
100
75
50
25
0
b5
b6
y6+P
y8+P
b7+P
y7+P
y2+P
y4+P
y3+P
y5+P
y4+P
y6+P
y7+P
b8+P
y6+P
y8+P
585
697
809
921
1033
m/z

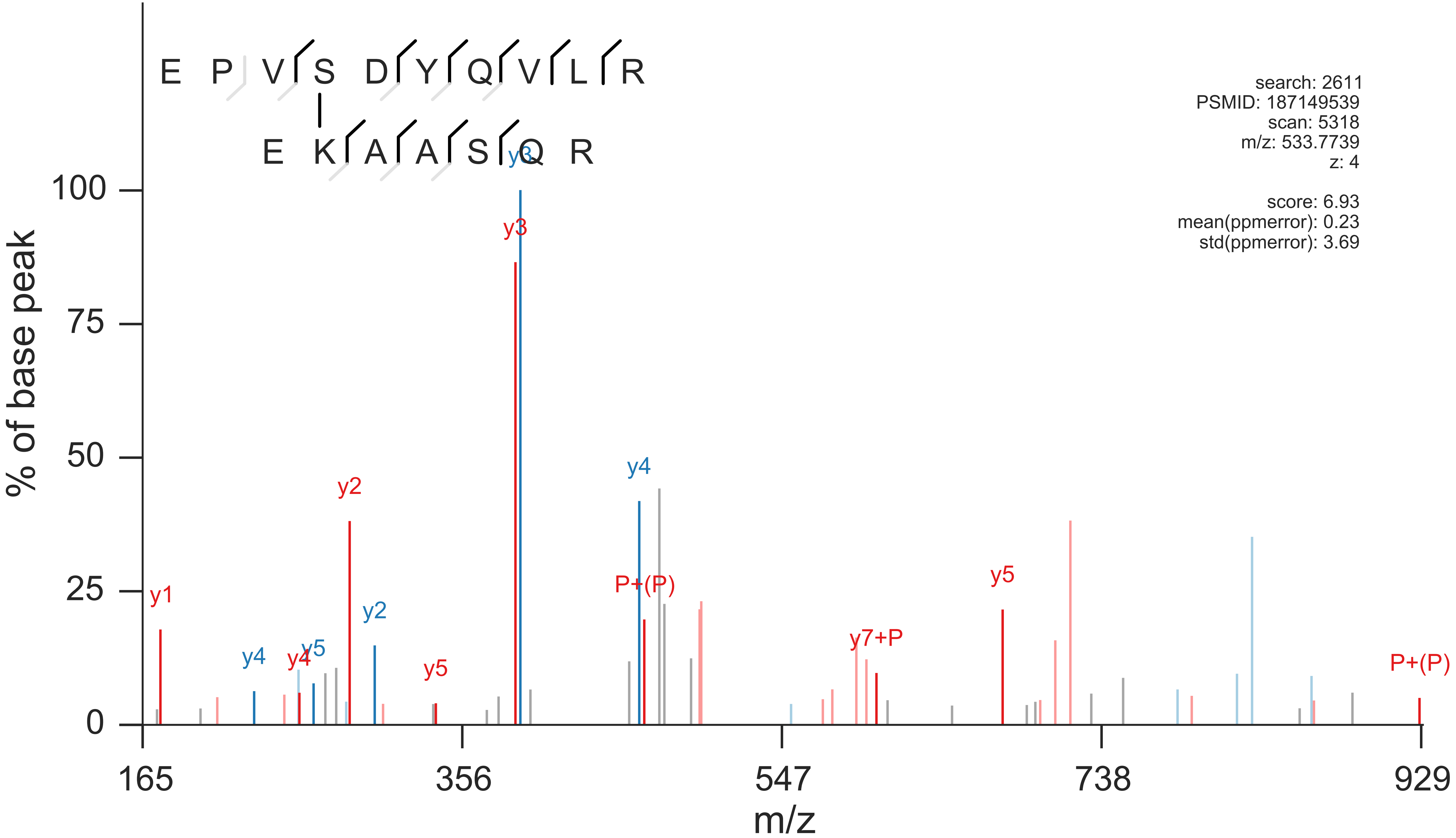

E P V S D Y Q V L R
E K A A S Q R
search: 2611
PSMID: 187149539
scan: 5318
m/z: 533.7739
z: 4
score: 6.93
mean(ppmerror): 0.23
std(ppmerror): 3.69
% of base peak
100
75
50
25
0
y1
y4
y4
y5
y2
y2
y5
y3
y3
y4
P+(P)
y7+P
y5
P+(P)
165
356
547
738
929
m/z

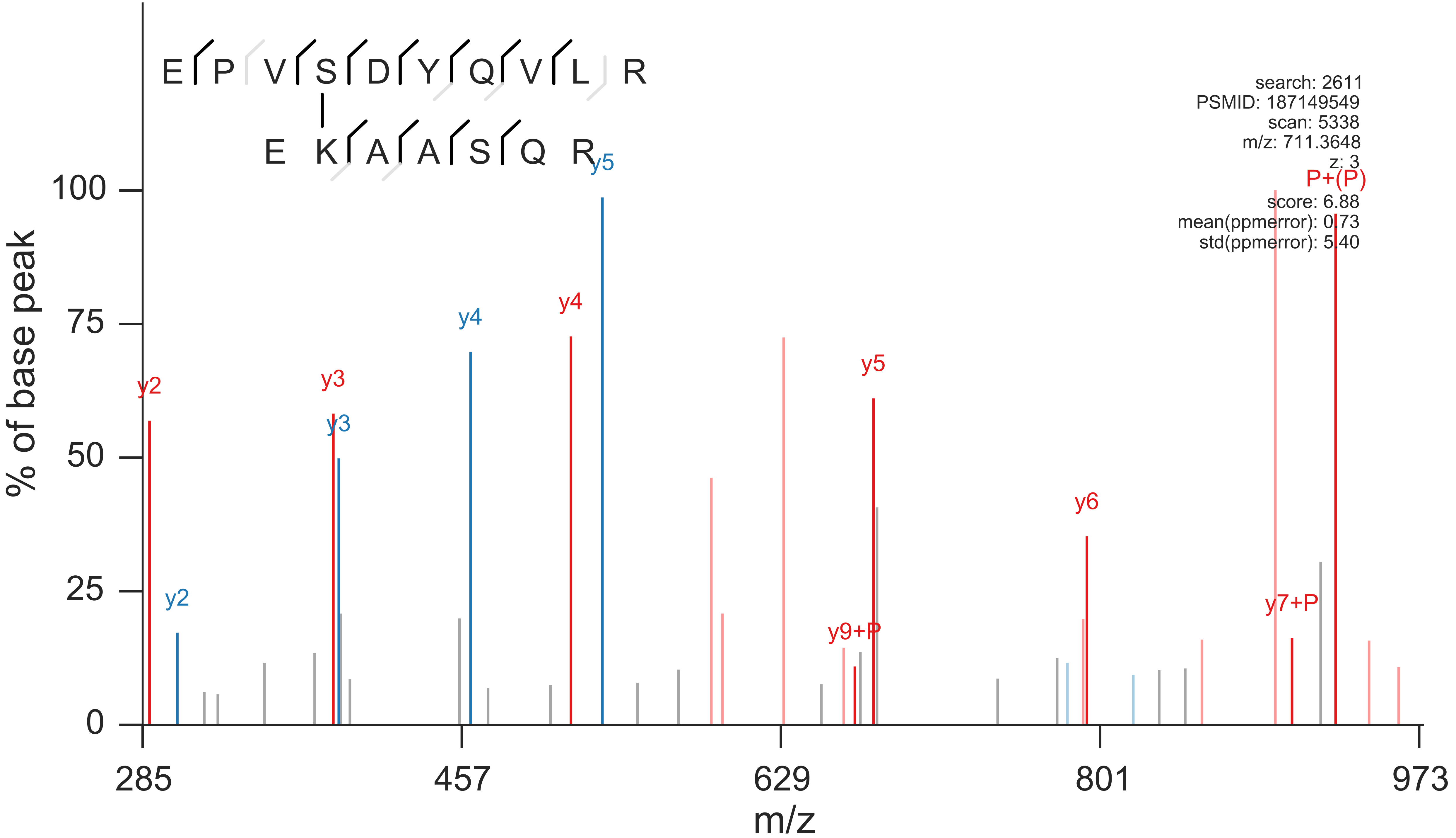

E P V S D Y Q V L R
E K A A S Q R
search: 2611
PSMID: 187149549
scan: 5338
m/z: 711.3648
z: 3
score: 6.88
mean(ppmerror): 0.73
std(ppmerror): 5.40
P+(P)
y2
y3
y4
y5
y6
y9+P
y7+P
% of base peak
m/z
100
75
50
25
0
285
457
629
801
973

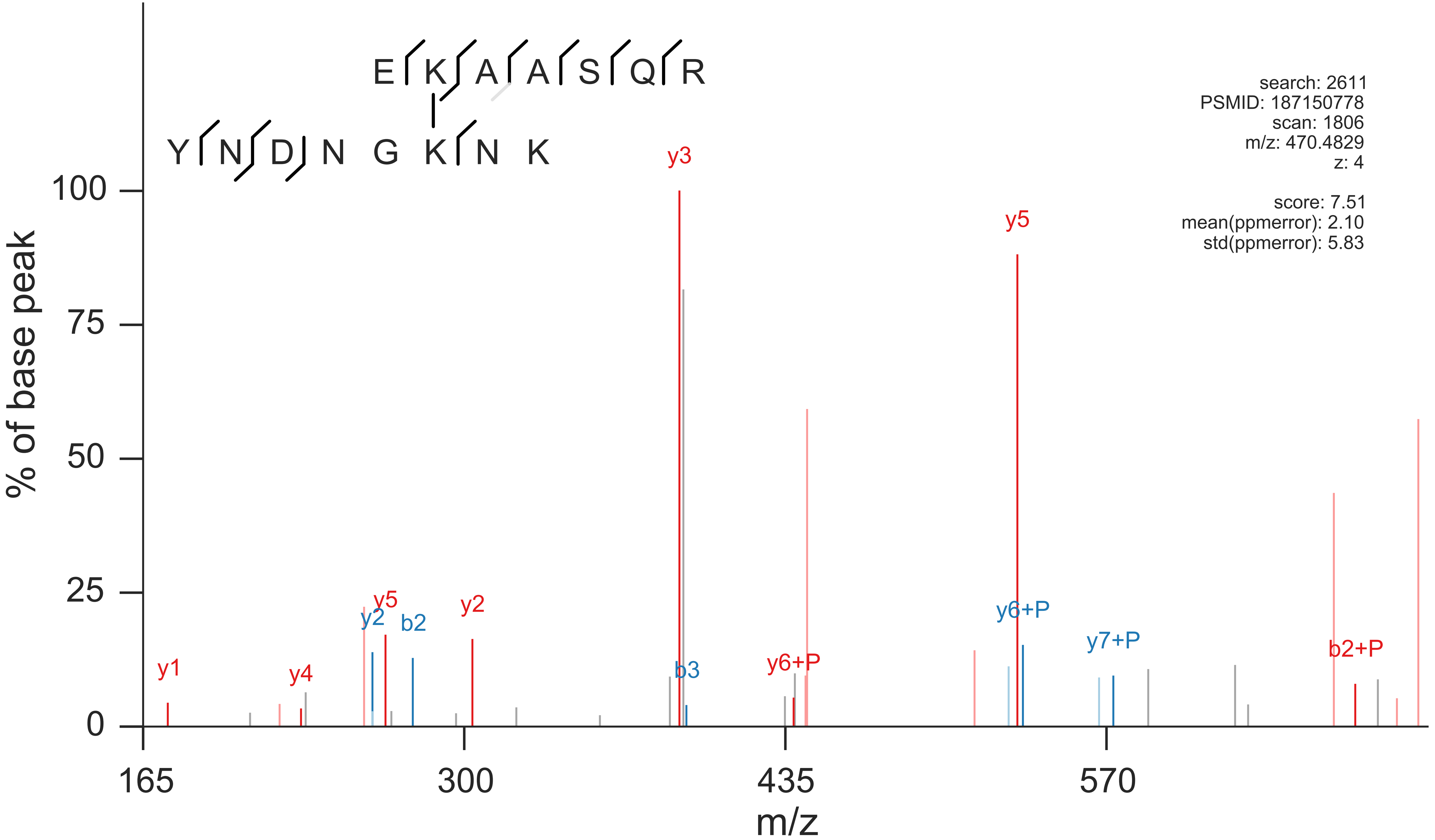

E K A A S Q R
Y N D N G K N K
search: 2611
PSMID: 187150778
scan: 1806
m/z: 470.4829
z: 4
score: 7.51
mean(ppmerror): 2.10
std(ppmerror): 5.83
% of base peak
100
75
50
25
0
y3
y5
y1
y4
y2
y5
b2
y2
b3
y6+P
y6+P
y7+P
b2+P
165
300
435
570
m/z

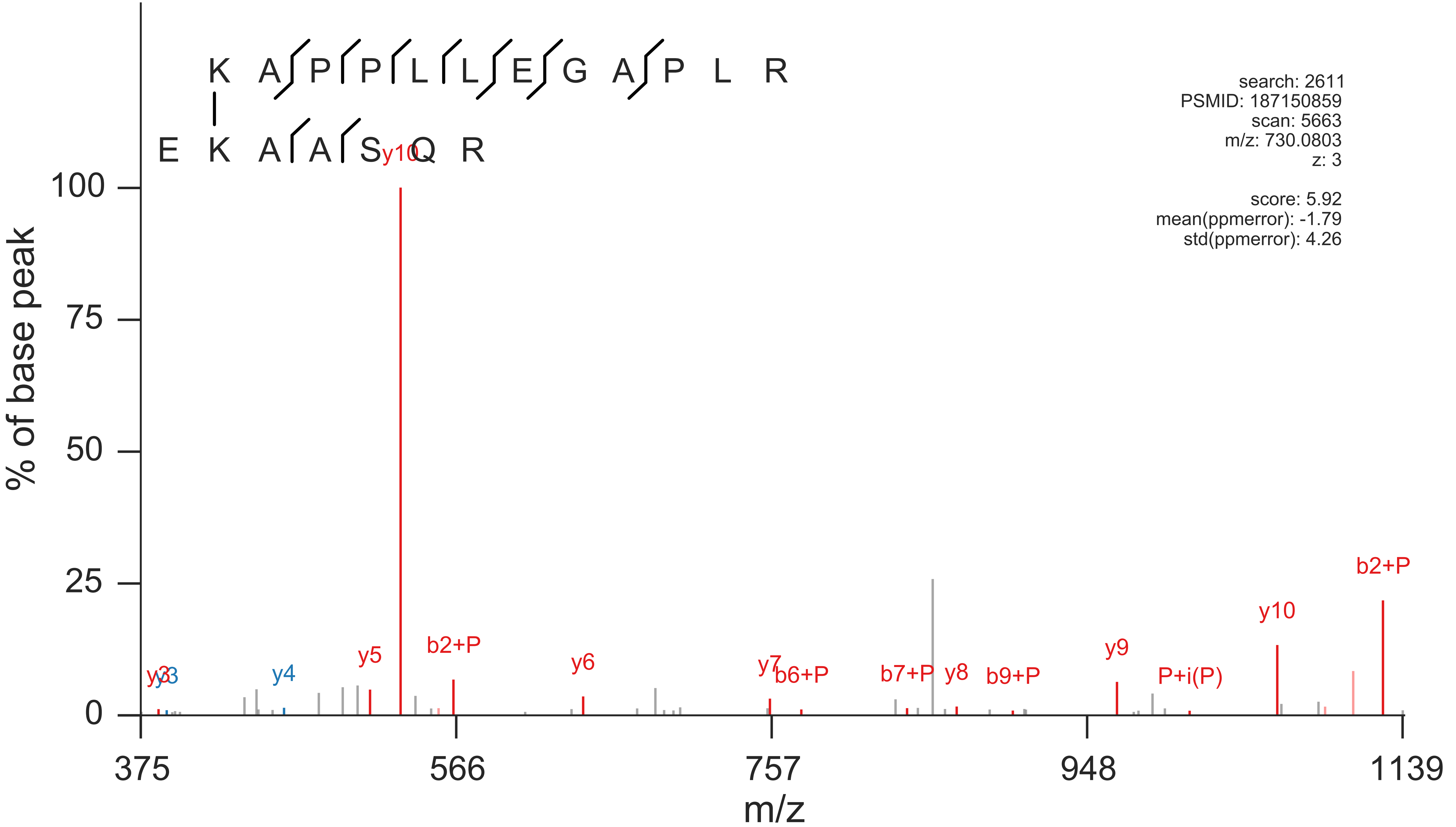

K A P P L L E G A P L R
E K A A S Q R
search: 2611
PSMID: 187150859
scan: 5663
m/z: 730.0803
z: 3
score: 5.92
mean(ppmerror): -1.79
std(ppmerror): 4.26
% of base peak
100
75
50
25
0
y10
y3
y3
y4
y5
b2+P
y6
y7
b6+P
b7+P
y8
b9+P
y9
P+i(P)
y10
b2+P
375
566
757
948
1139
m/z

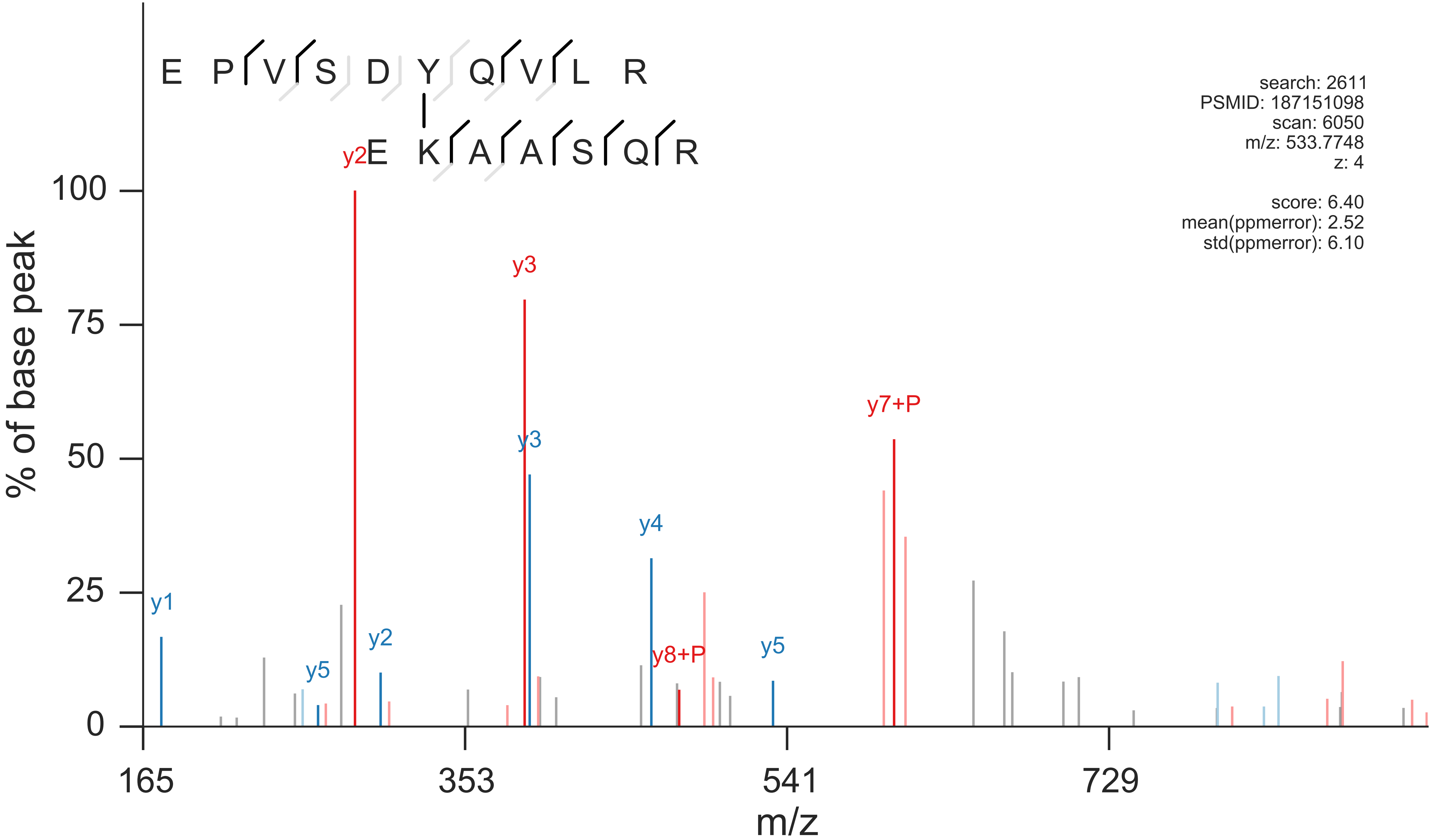

E P V S D Y Q V L R
E K A A S Q R
search: 2611
PSMID: 187151098
scan: 6050
m/z: 533.7748
z: 4
score: 6.40
mean(ppmerror): 2.52
std(ppmerror): 6.10
% of base peak
100
75
50
25
0
y1
y5
y2
y2
y3
y3
y4
y8+P
y5
y7+P
165
353
541
729
m/z

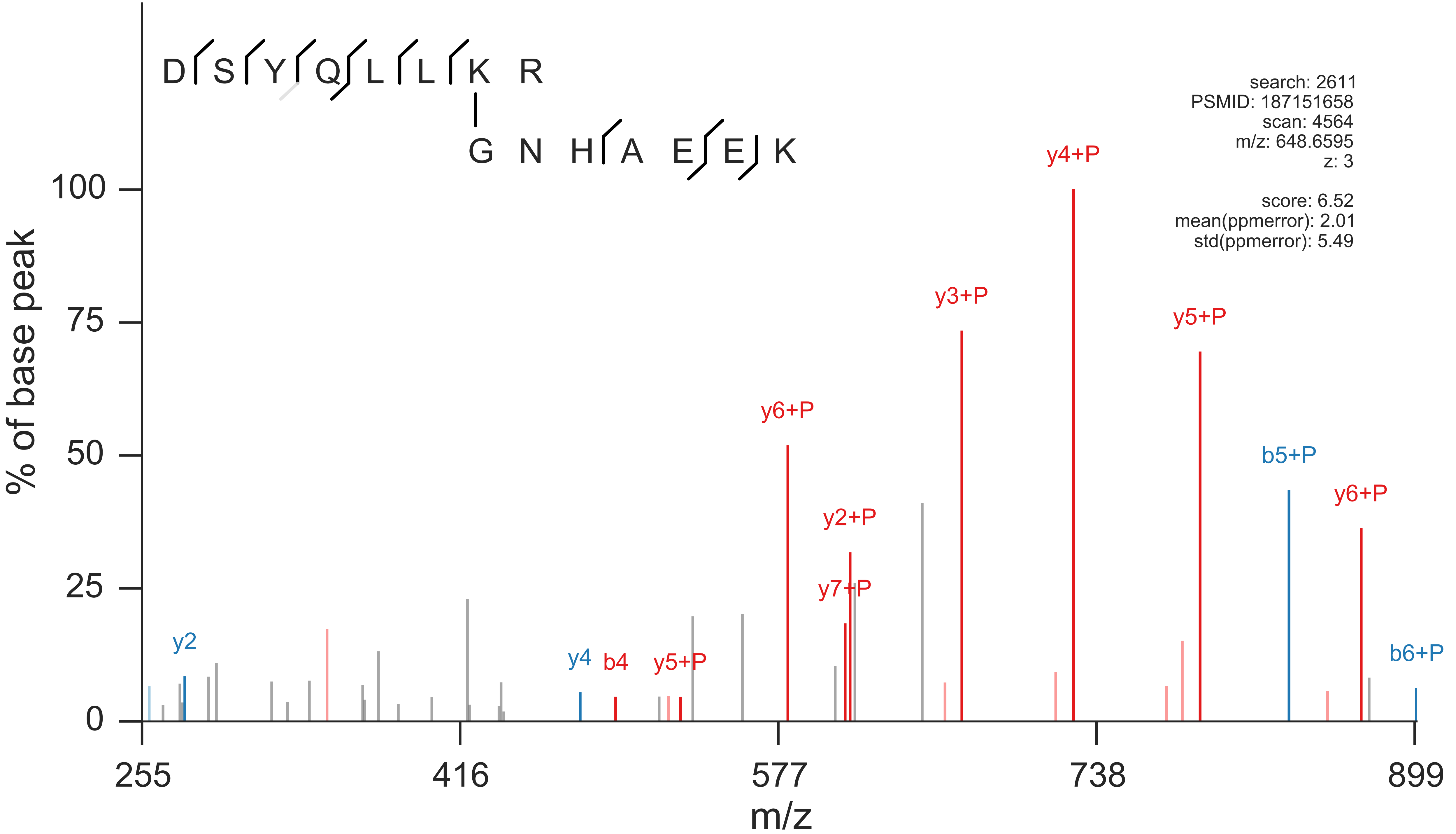

D S Y Q L L K R
G N H A E E K
search: 2611
PSMID: 187151658
scan: 4564
m/z: 648.6595
z: 3
score: 6.52
mean(ppmerror): 2.01
std(ppmerror): 5.49
% of base peak
m/z
y2
y4
b4
y5+P
y6+P
y7+P
y2+P
y3+P
y4+P
y5+P
b5+P
y6+P
b6+P
0
25
50
75
100
255
416
577
738
899

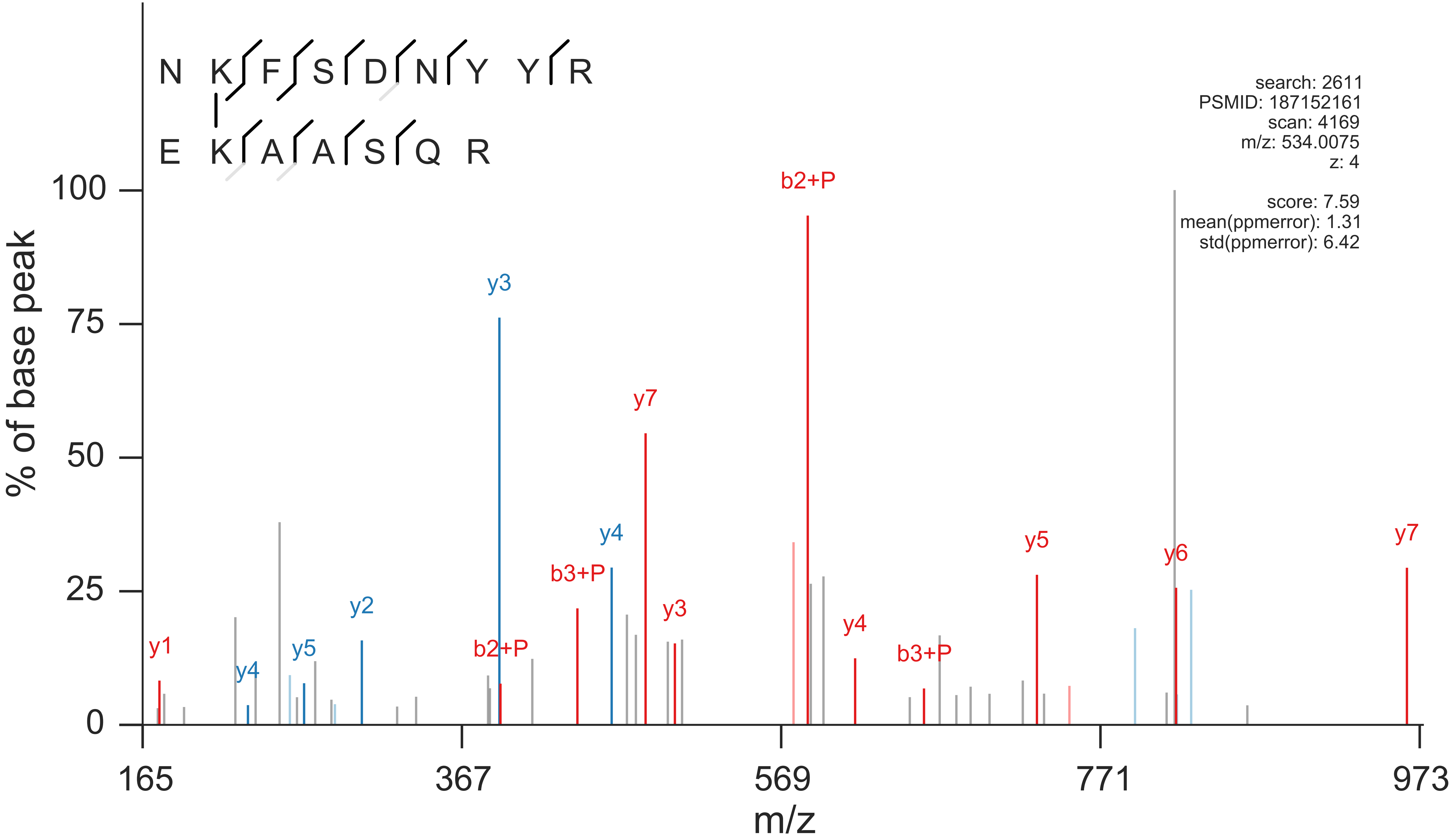

N K F S D N Y Y R
E K A A S Q R
search: 2611
PSMID: 187152161
scan: 4169
m/z: 534.0075
z: 4
score: 7.59
mean(ppmerror): 1.31
std(ppmerror): 6.42
% of base peak
m/z
0
25
50
75
100
165
367
569
771
973
y1
y4
y5
y2
b2+P
y3
b3+P
y4
y7
y3
b2+P
y4
b3+P
y5
y6
y7

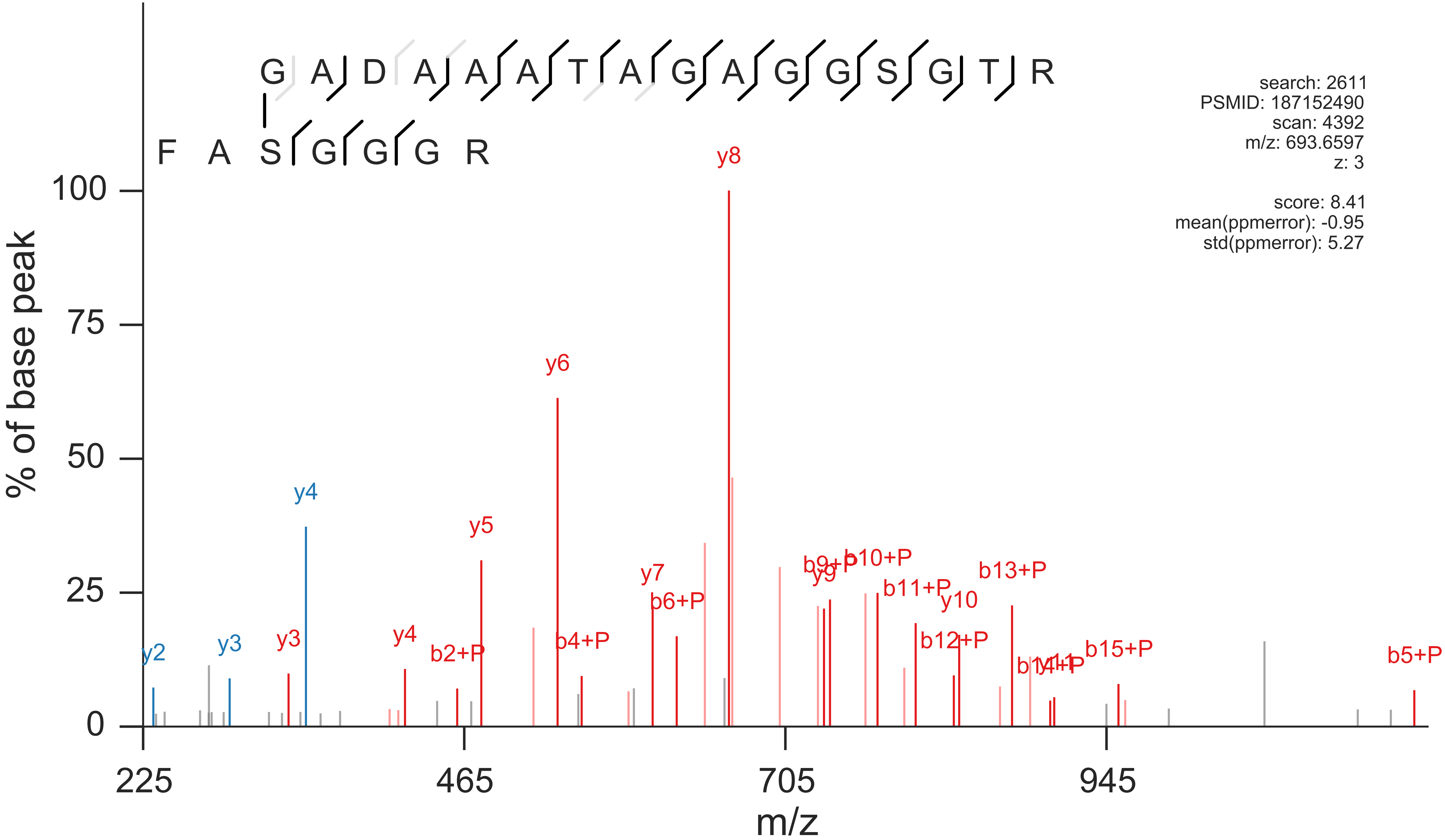
G A D A A A T A G A G G S G T R
F A S G G G R
search: 2611
PSMID: 187152490
scan: 4392
m/z: 693.6597
z: 3
score: 8.41
mean(ppmerror): -0.95
std(ppmerror): 5.27
% of base peak
m/z
y2
y3
y4
y3
y4
b2+P
y5
y6
b4+P
y7
b6+P
y8
b9+P
y9
b10+P
b11+P
y10
b12+P
b13+P
b15+P
b5+P
0
25
50
75
100
225
465
705
945

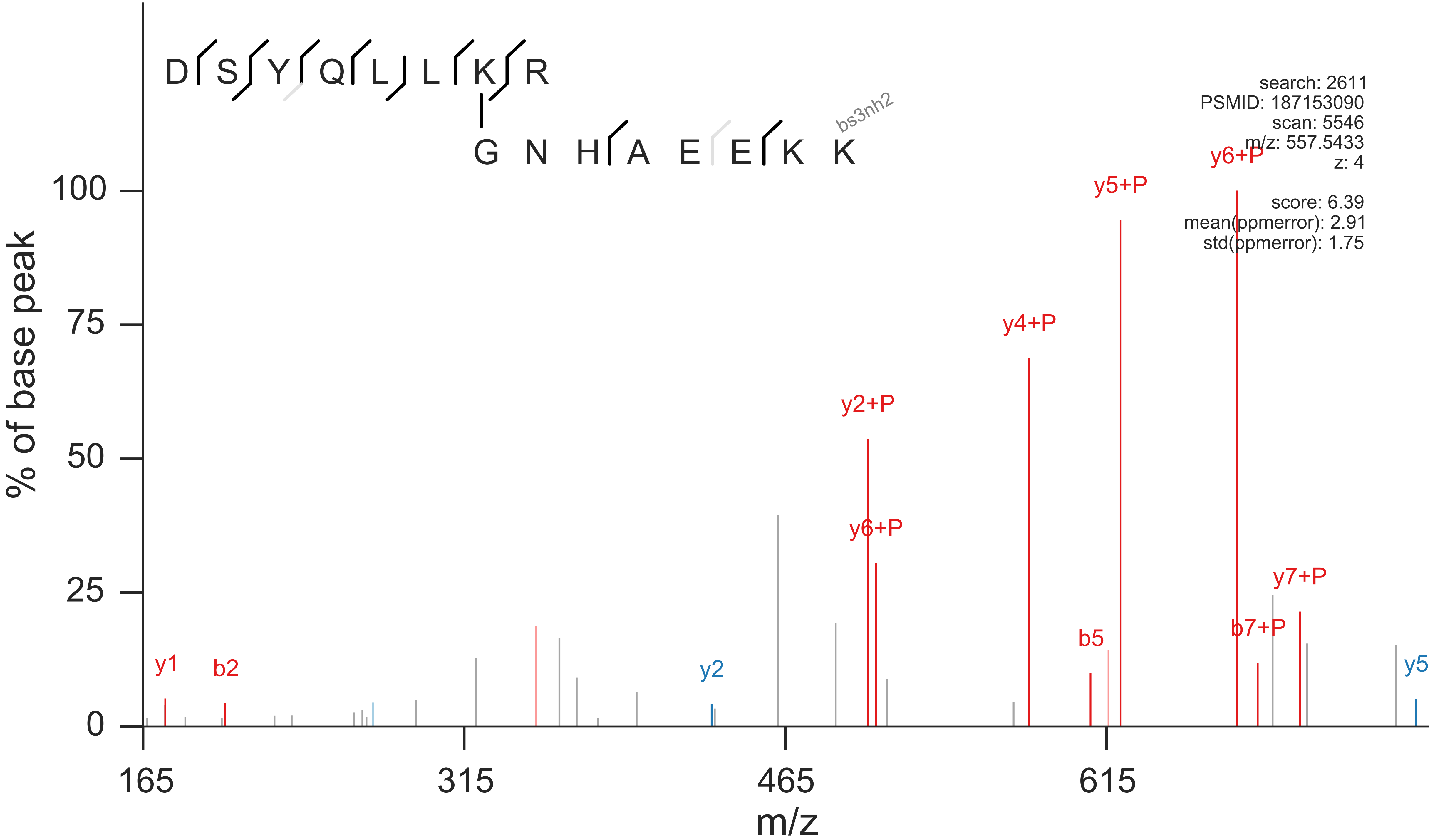

D S Y Q L L K R
G N H A E E K K
bs3nh2
search: 2611
PSMID: 187153090
scan: 5546
m/z: 557.5433
z: 4
score: 6.39
mean(ppmerror): 2.91
std(ppmerror): 1.75
% of base peak
m/z
y1
b2
y2
y2+P
y6+P
y4+P
y5+P
b5
b7+P
y7+P
y5
0
25
50
75
100
165
315
465
615

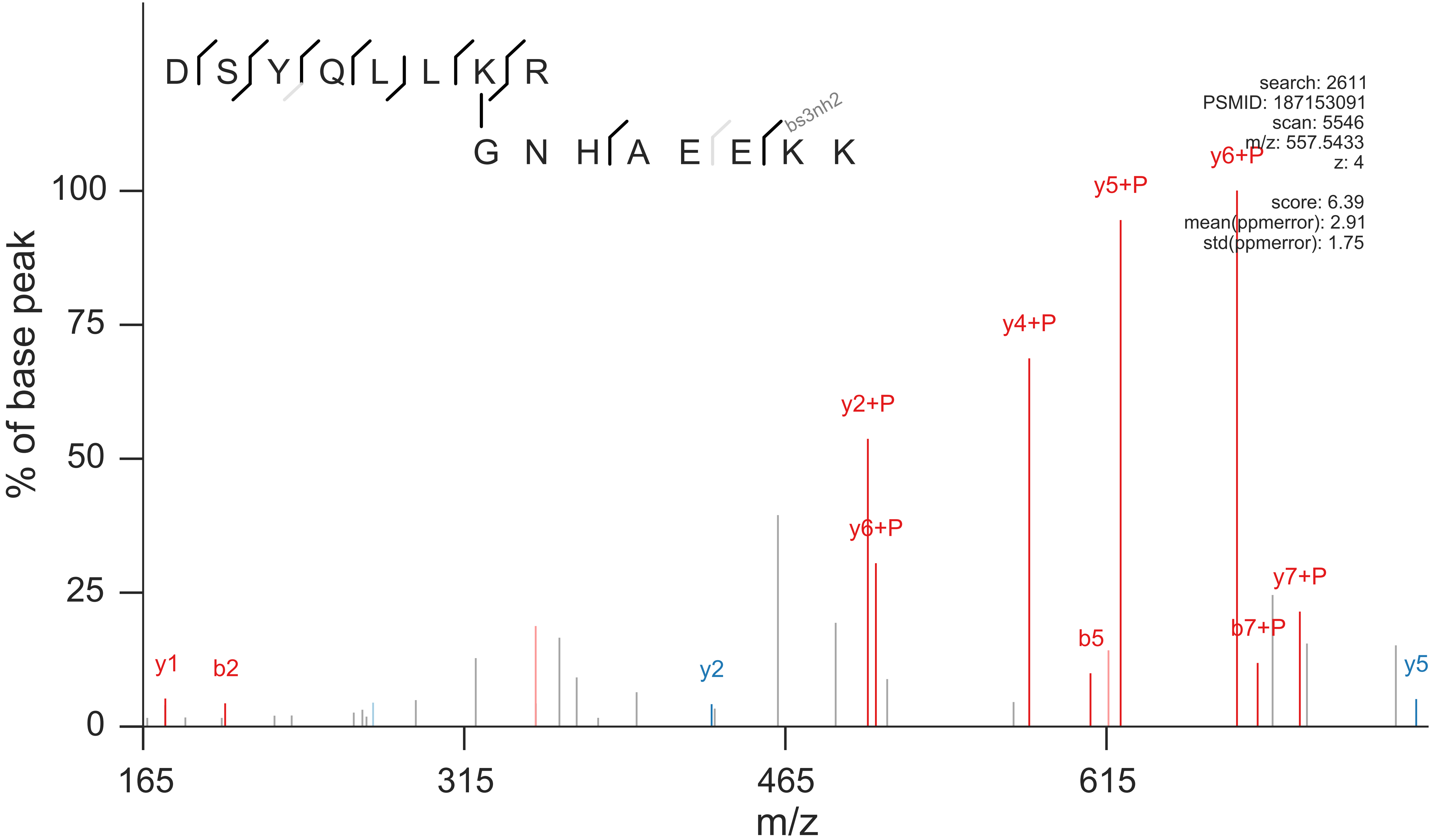

D S Y Q L L K R
G N H A E E K K
bs3nh2
search: 2611
PSMID: 187153091
scan: 5546
m/z: 557.5433
z: 4
score: 6.39
mean(ppmerror): 2.91
std(ppmerror): 1.75
% of base peak
100
75
50
25
0
165
315
465
615
m/z
y1
b2
y2
y2+P
y6+P
y4+P
y5+P
y6+P
b5
b7+P
y7+P
y5

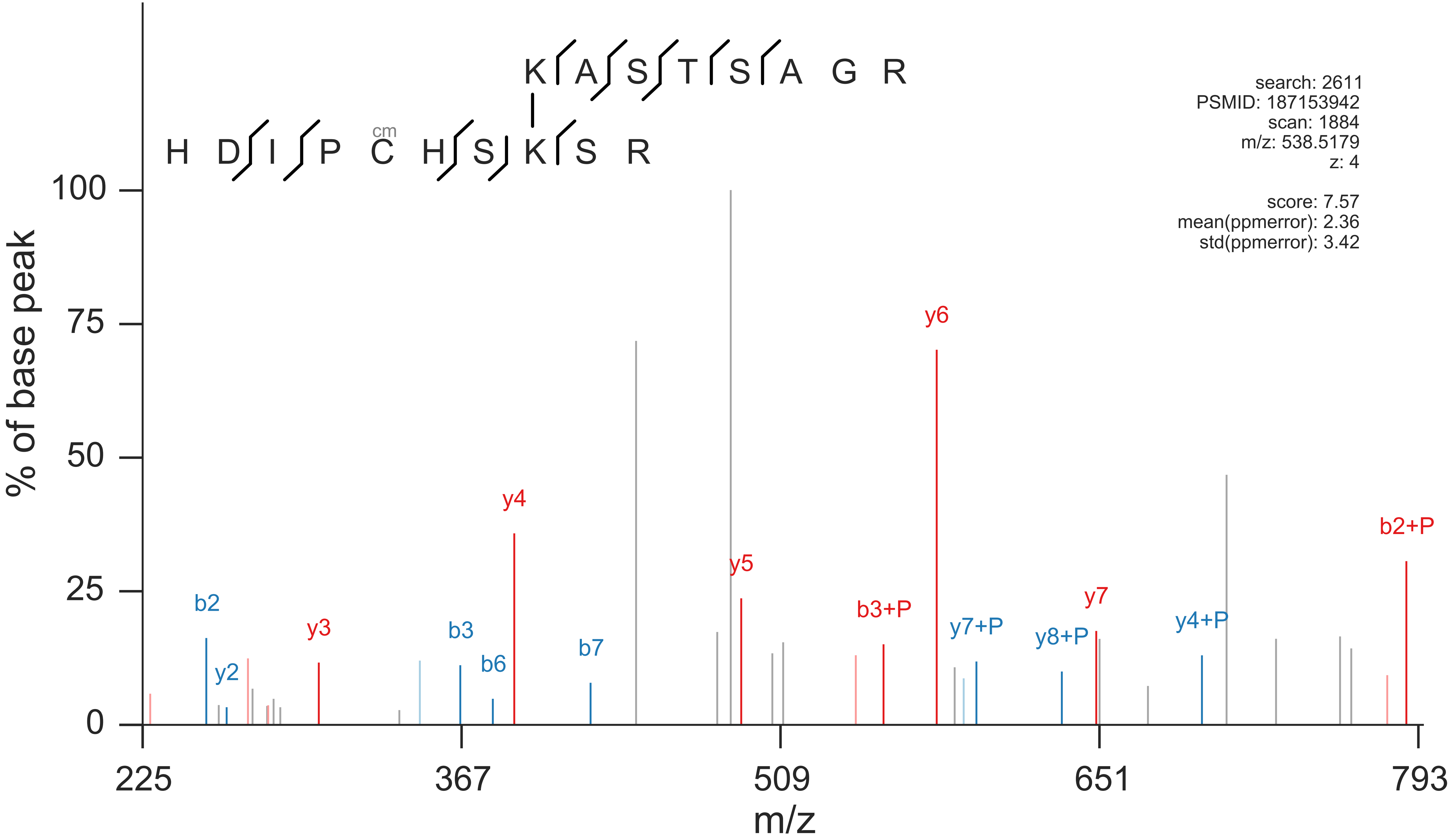

K A S T S A G R
H D I P C H S K S R
cm
search: 2611
PSMID: 187153942
scan: 1884
m/z: 538.5179
z: 4
score: 7.57
mean(ppmerror): 2.36
std(ppmerror): 3.42
% of base peak
100
75
50
25
0
b2
y2
y3
b3
b6
y4
b7
y5
b3+P
y6
y7+P
y8+P
y7
y4+P
b2+P
225
367
509
651
793
m/z

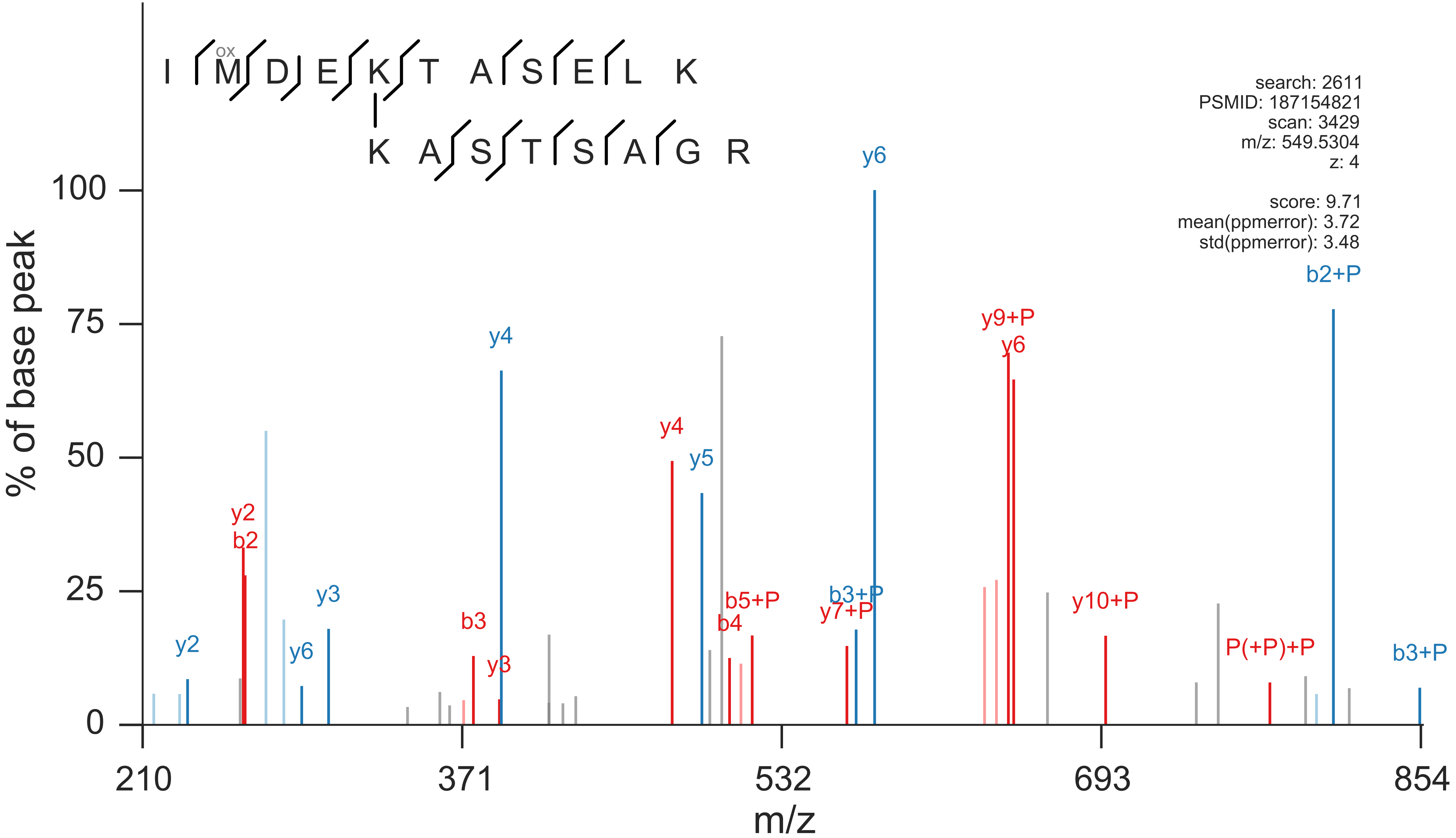

I M(ox) D E K T A S E L K
K A S T S A G R
search: 2611
PSMID: 187154821
scan: 3429
m/z: 549.5304
z: 4
score: 9.71
mean(ppmerror): 3.72
std(ppmerror): 3.48
% of base peak
m/z
0
25
50
75
100
210
371
532
693
854
y2
b2
y3
y6
b3
y4
y5
b4
b5+P
b3+P
y7+P
y9+P
y10+P
P(+P)+P
b2+P

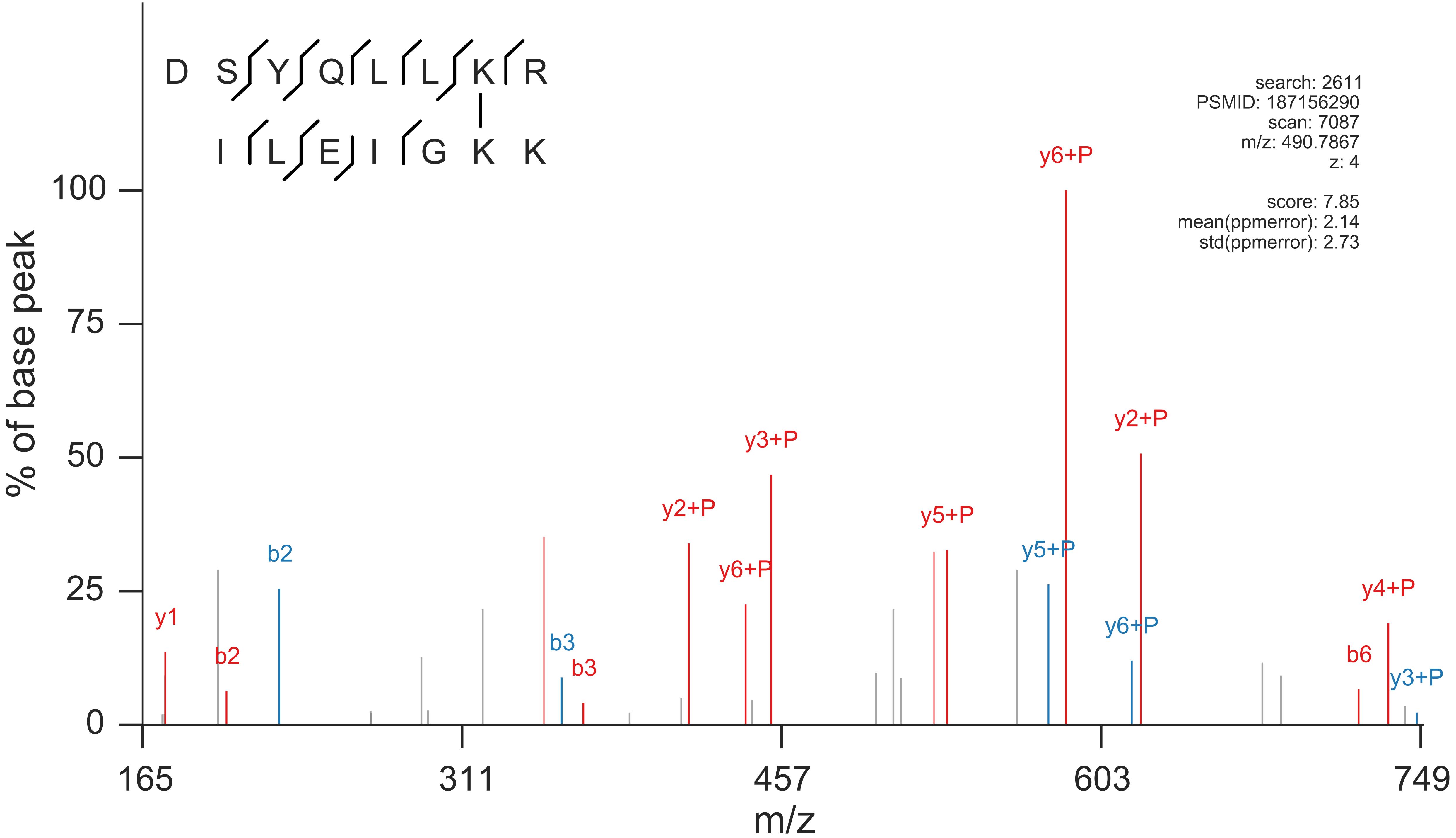

D S Y Q L L K R
I L E I G K K
search: 2611
PSMID: 187156290
scan: 7087
m/z: 490.7867
z: 4
score: 7.85
mean(ppmerror): 2.14
std(ppmerror): 2.73
% of base peak
m/z
0
25
50
75
100
165
311
457
603
749
y1
b2
b2
b3
b3
y2+P
y6+P
y3+P
y5+P
y5+P
y6+P
y2+P
y6+P
b6
y4+P
y3+P

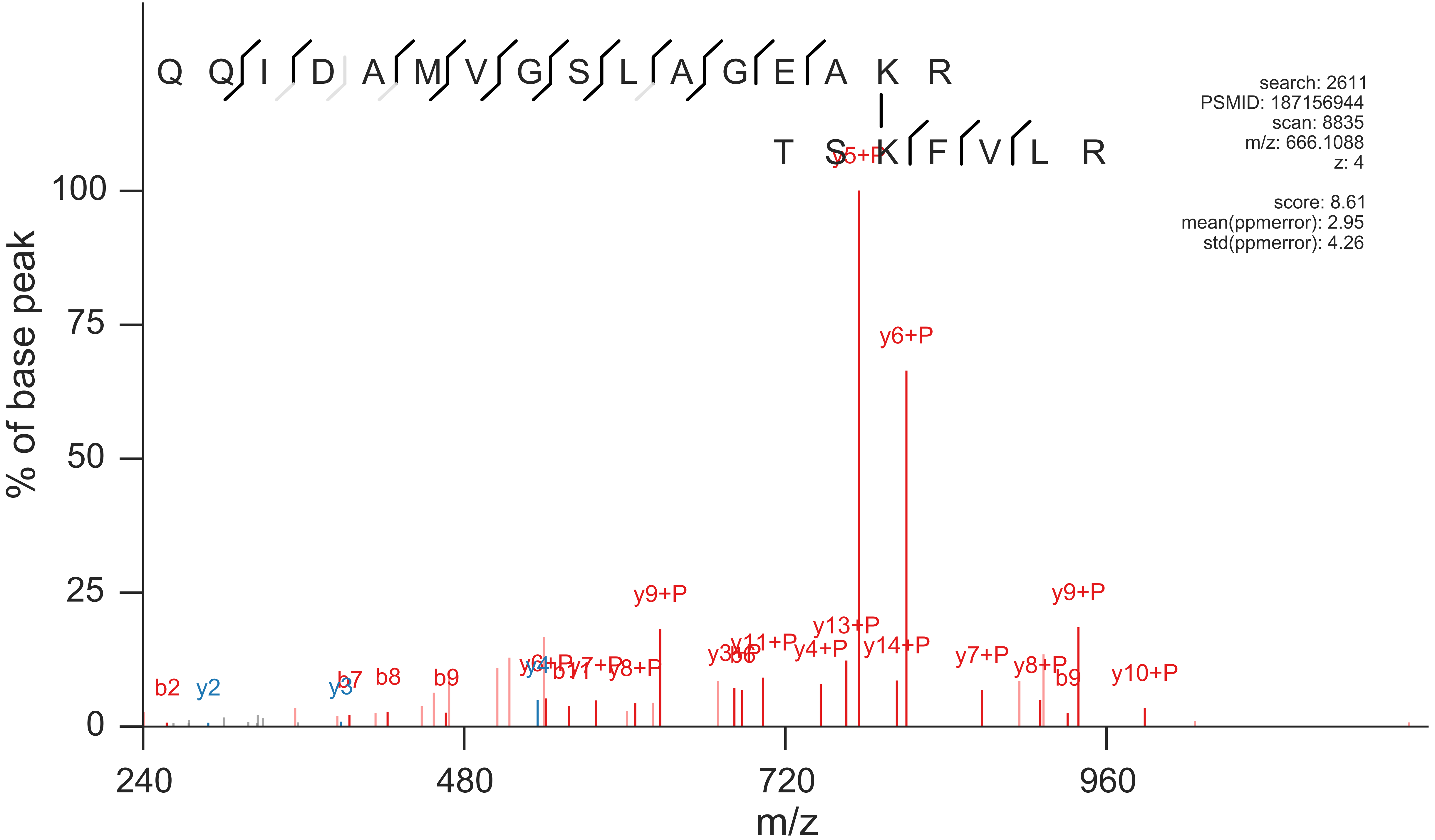

Q Q I D A M V G S L A G E A K R
T S K F V L R
search: 2611
PSMID: 187156944
scan: 8835
m/z: 666.1088
z: 4
score: 8.61
mean(ppmerror): 2.95
std(ppmerror): 4.26
% of base peak
m/z
y5+P
y6+P
y9+P
y13+P
y14+P
y11+P
y4+P
y7+P
y8+P
y10+P
b2
y2
b7
y3
b8
b9
b11
b6

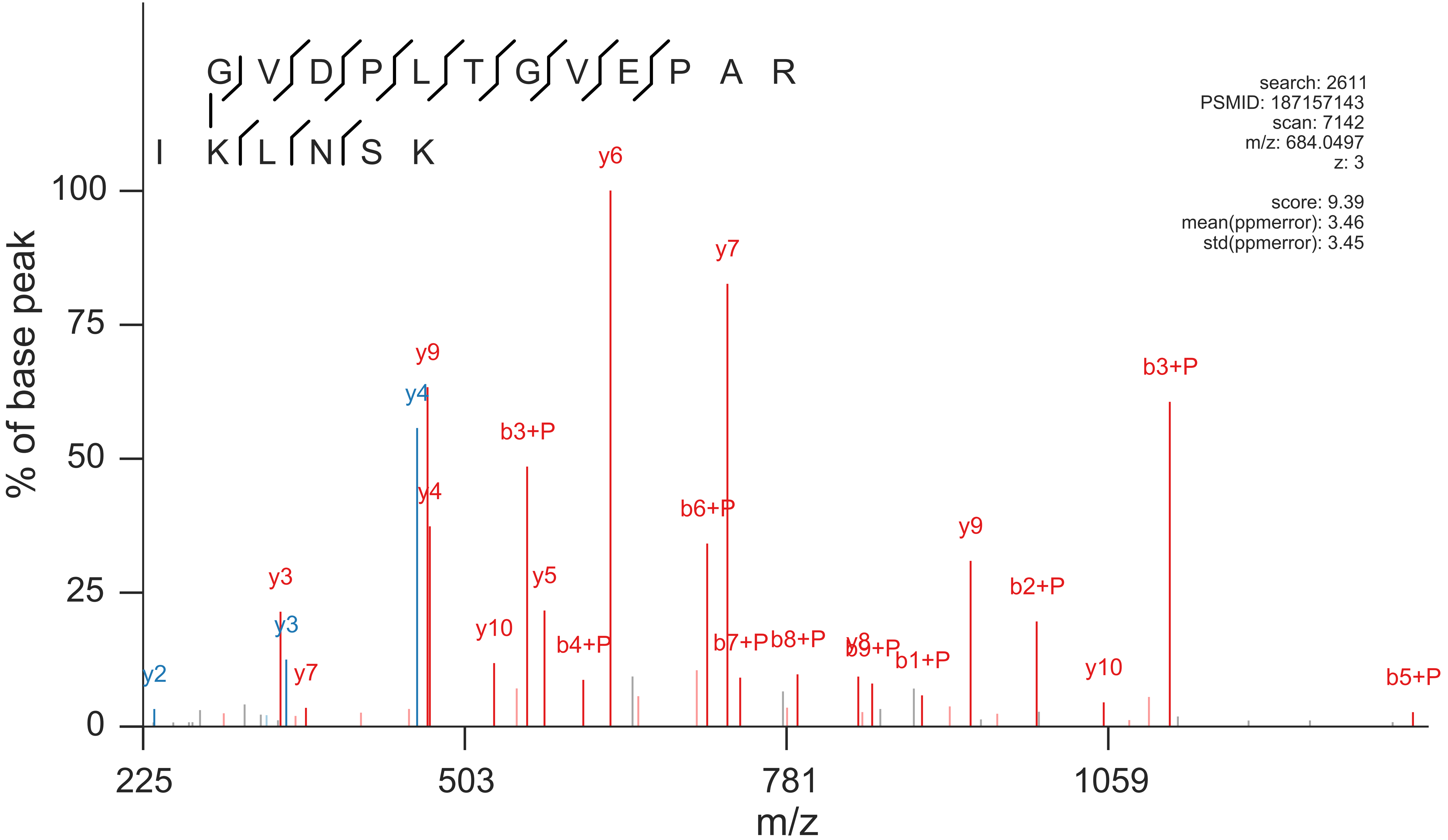

G V D P L T G V E P A R
I K L N S K
search: 2611
PSMID: 187157143
scan: 7142
m/z: 684.0497
z: 3
score: 9.39
mean(ppmerror): 3.46
std(ppmerror): 3.45
% of base peak
m/z
y6
y7
y9
y4
b3+P
y4
b6+P
y9
b3+P
b2+P
y3
y5
y3
y10
b4+P
b7+P
b8+P
y8
b9+P
b1+P
y10
b5+P
y2
y7
100
75
50
25
0
225
503
781
1059

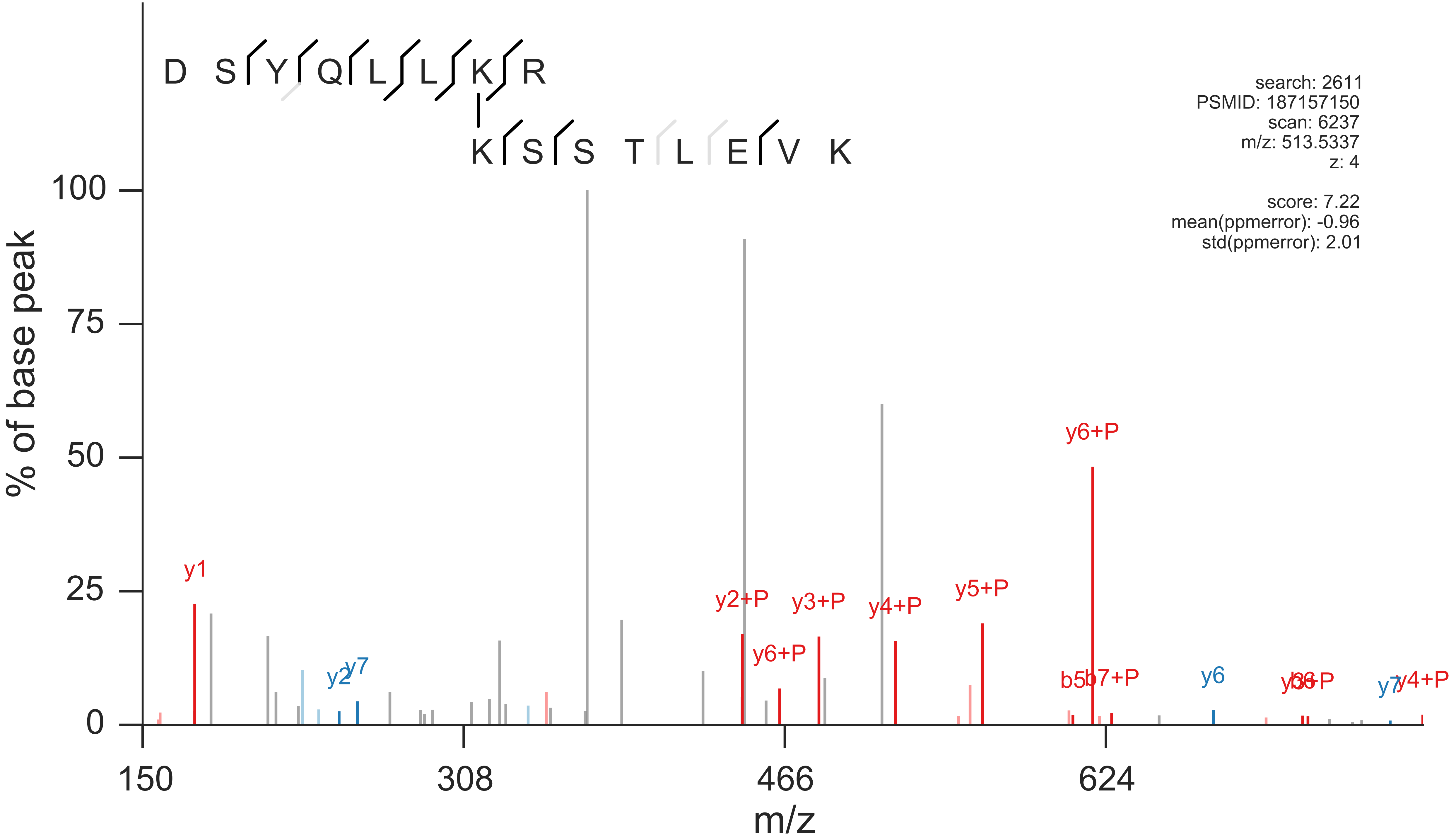

D S Y Q L L K R
K S S T L E V K
search: 2611
PSMID: 187157150
scan: 6237
m/z: 513.5337
z: 4
score: 7.22
mean(ppmerror): -0.96
std(ppmerror): 2.01
% of base peak
100
75
50
25
0
150
308
466
624
m/z
y1
y2
y7
y2+P
y6+P
y3+P
y4+P
y5+P
y6+P
b5
b7+P
y6
y7
y4+P

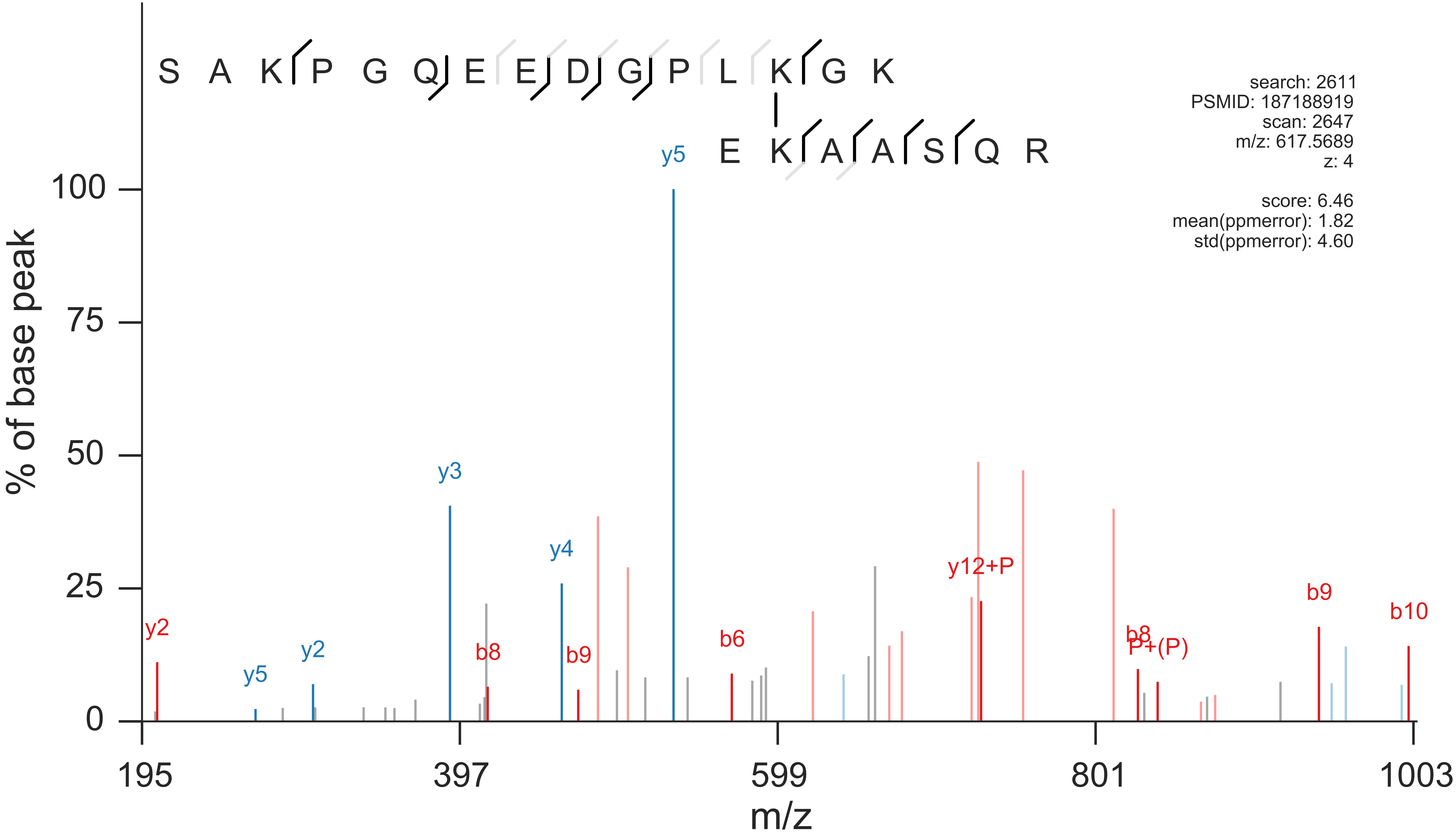

S A K P G Q E E D G P L K G K
E K A A S Q R
search: 2611
PSMID: 187188919
scan: 2647
m/z: 617.5689
z: 4
score: 6.46
mean(ppmerror): 1.82
std(ppmerror): 4.60
% of base peak
m/z
0
25
50
75
100
195
397
599
801
1003
y2
y5
y2
y3
b8
y4
b9
y5
b6
y12+P
b8
P+(P)
b9
b10

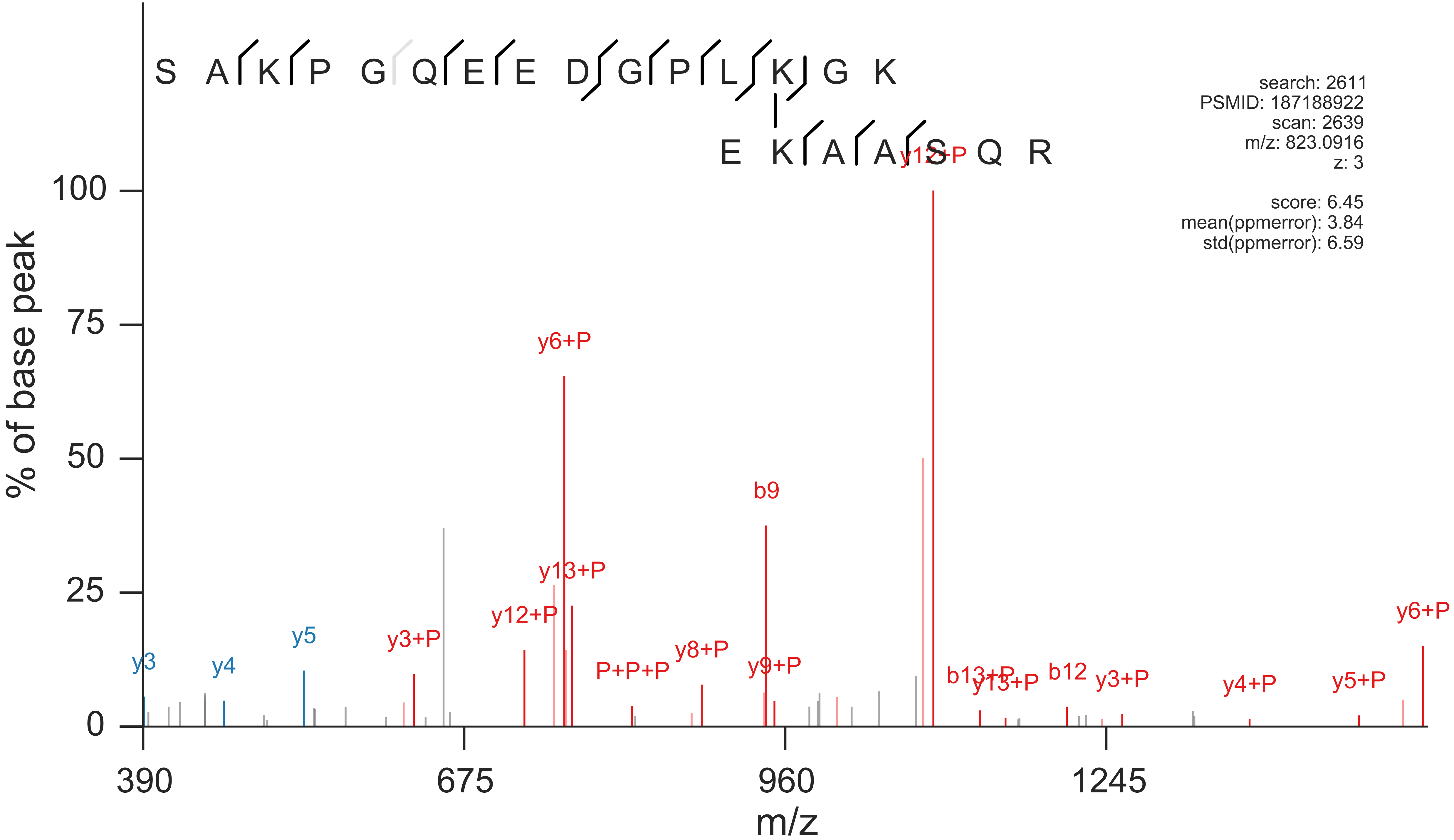

S A K P G Q E E D G P L K G K
E K A A S Q R
search: 2611
PSMID: 187188922
scan: 2639
m/z: 823.0916
z: 3
score: 6.45
mean(ppmerror): 3.84
std(ppmerror): 6.59
% of base peak
m/z
y3
y4
y5
y3+P
y12+P
y13+P
y6+P
P+P+P
y8+P
b9
y9+P
y12+P
b13+P
y13+P
b12
y3+P
y4+P
y5+P
y6+P

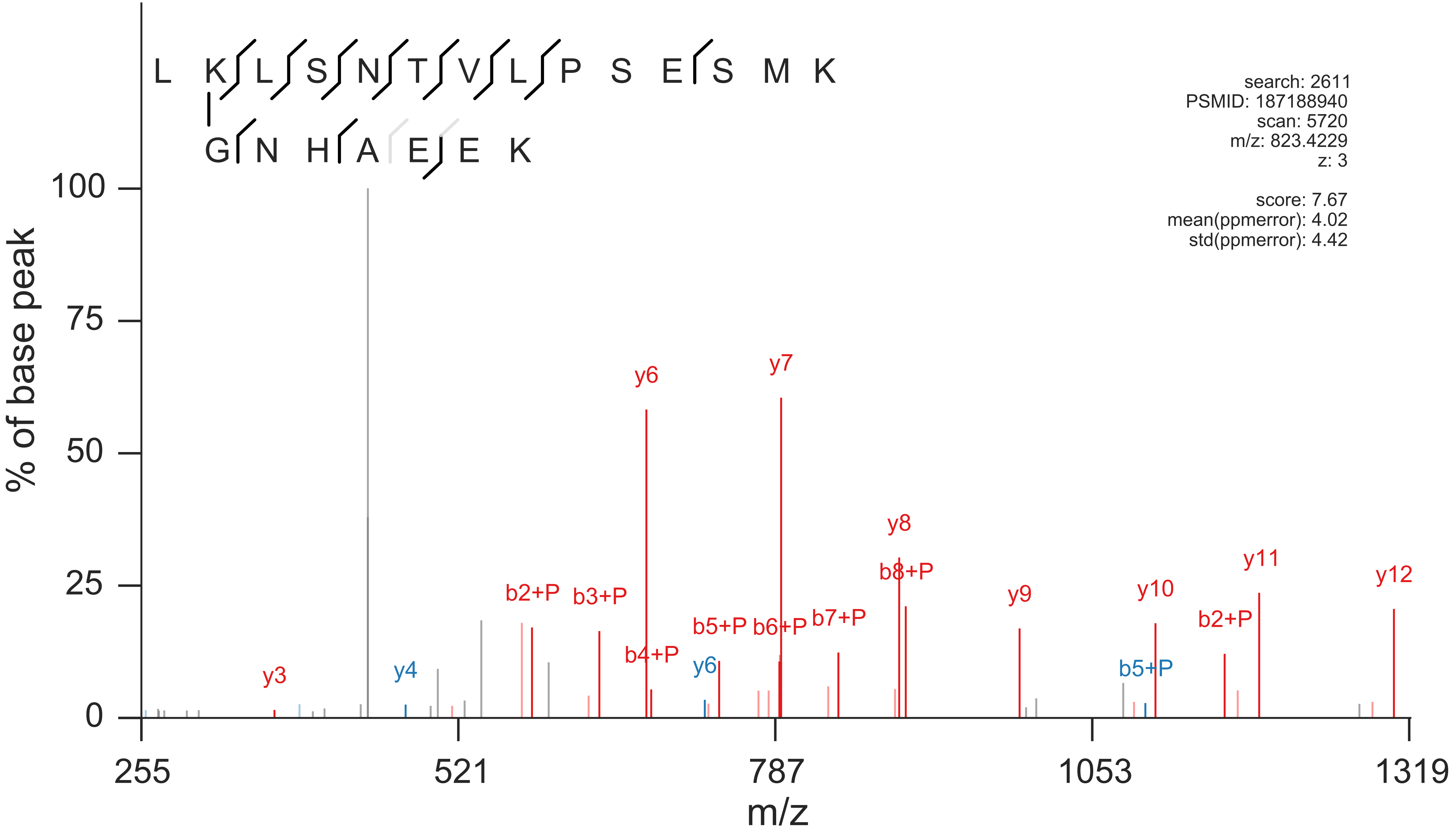

L K L S N T V L P S E S M K
G N H A E E K
search: 2611
PSMID: 187188940
scan: 5720
m/z: 823.4229
z: 3
score: 7.67
mean(ppmerror): 4.02
std(ppmerror): 4.42
% of base peak
m/z
y3
y4
b2+P
b3+P
y6
b4+P
b5+P
y6
b6+P
y7
b7+P
y8
b8+P
y9
b5+P
y10
b2+P
y11
y12

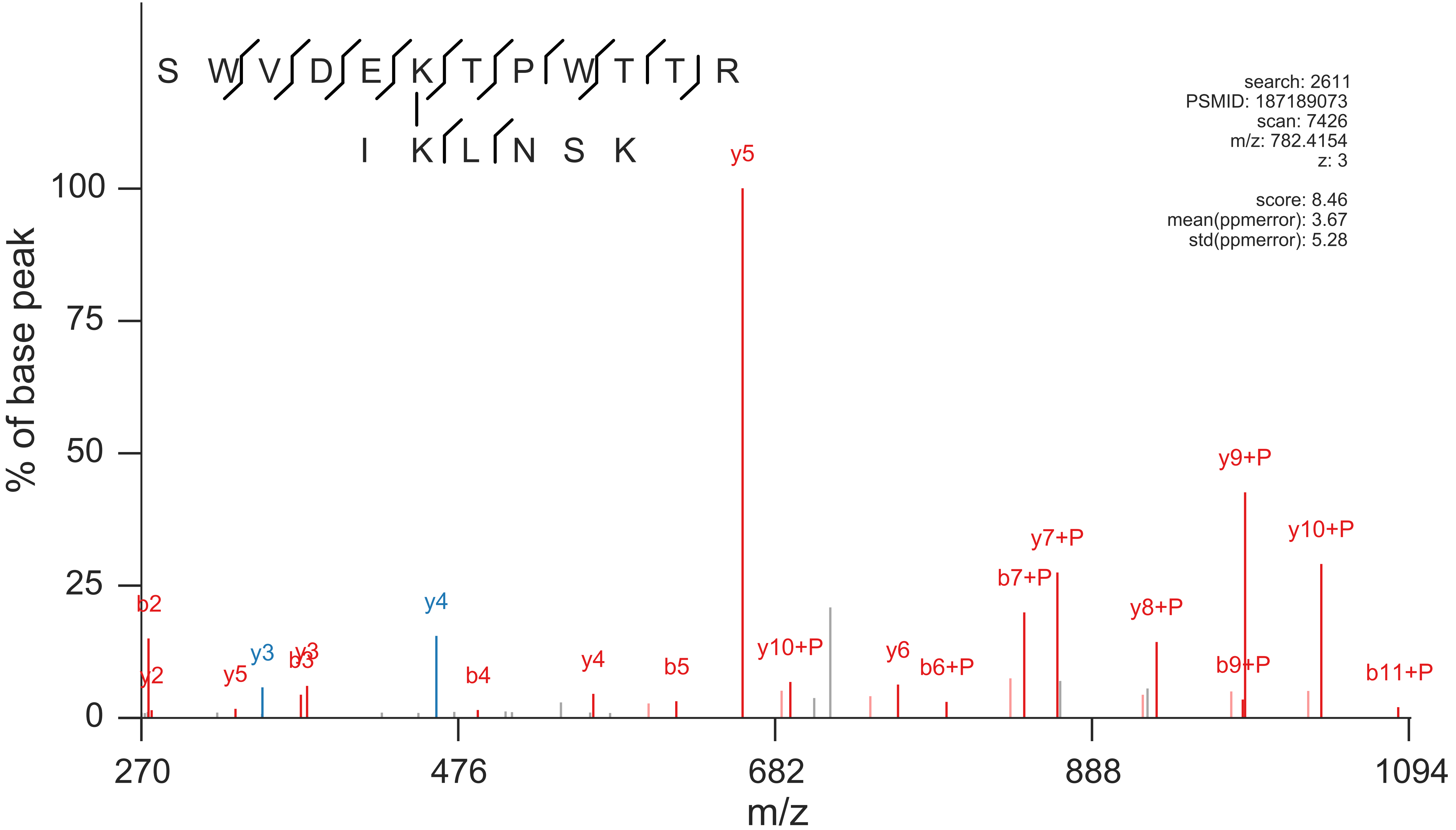

S W V D E K T P W T T R
I K L N S K
search: 2611
PSMID: 187189073
scan: 7426
m/z: 782.4154
z: 3
score: 8.46
mean(ppmerror): 3.67
std(ppmerror): 5.28
% of base peak
m/z
0
25
50
75
100
270
476
682
888
1094
b2
y2
y5
y3
b3
y3
y4
b4
y4
b5
y5
y10+P
y6
b6+P
b7+P
y7+P
y8+P
b9+P
y9+P
y10+P
b11+P

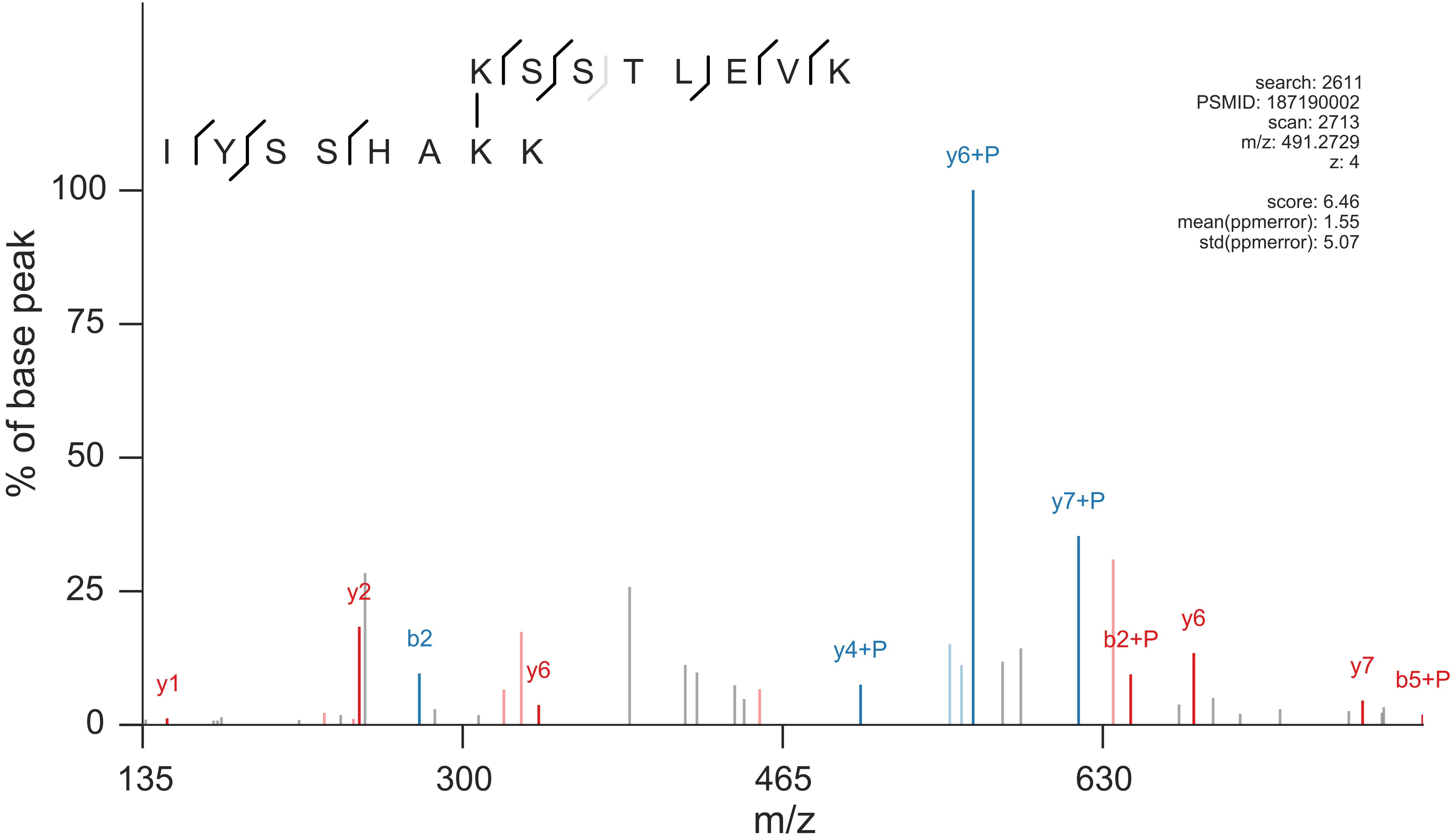

K S S T L E V K
I Y S S H A K K
search: 2611
PSMID: 187190002
scan: 2713
m/z: 491.2729
z: 4
score: 6.46
mean(ppmerror): 1.55
std(ppmerror): 5.07
% of base peak
100
75
50
25
0
y1
y2
b2
y6
y4+P
y6+P
y7+P
b2+P
y6
y7
b5+P
135
300
465
630
m/z

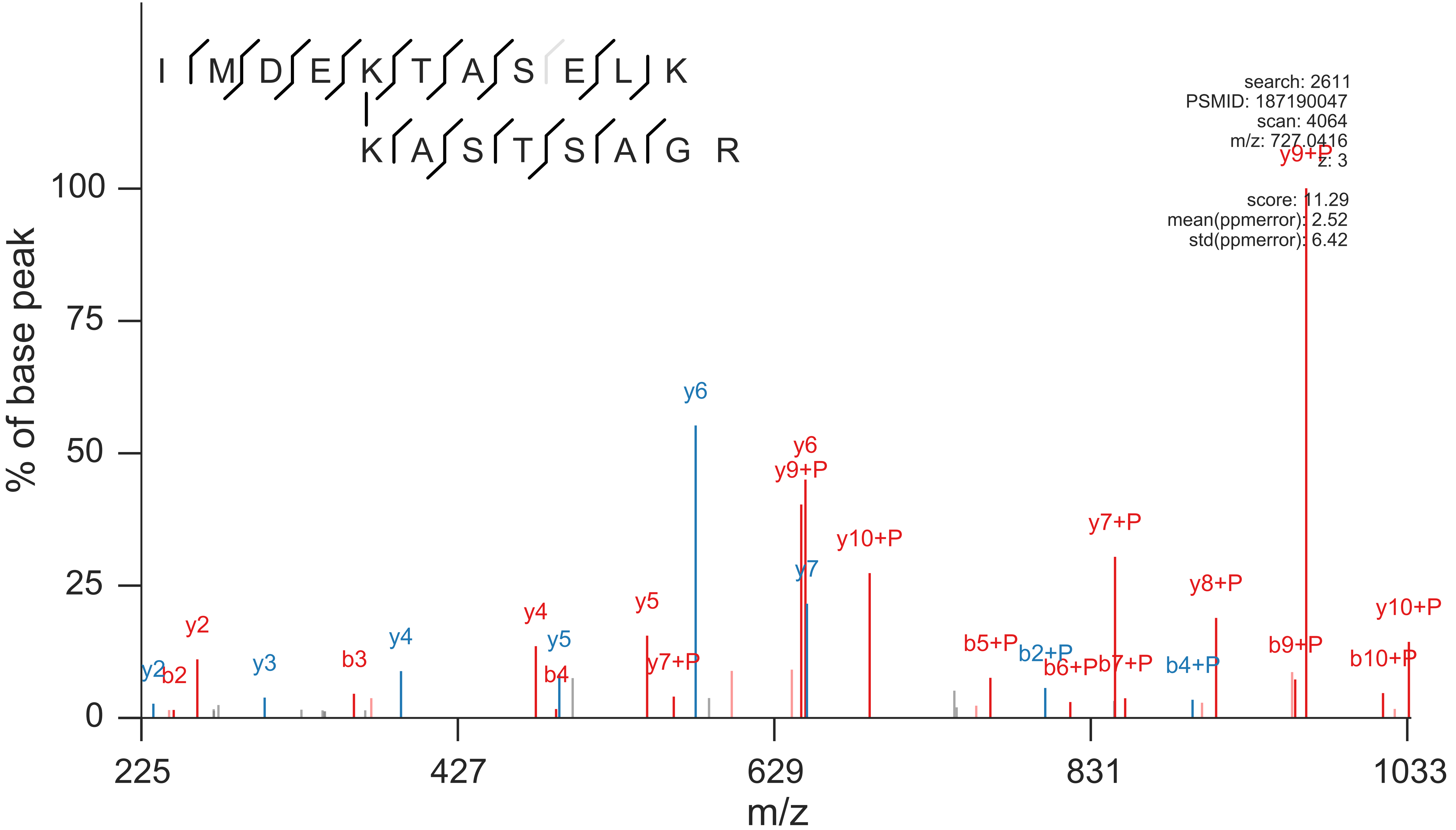

I M D E K T A S E L K
K A S T S A G R
search: 2611
PSMID: 187190047
scan: 4064
m/z: 727.0416
z: 3
score: 11.29
mean(ppmerror): 2.52
std(ppmerror): 6.42
% of base peak
m/z
0
25
50
75
100
225
427
629
831
1033
y2
b2
y2
y3
b3
y4
y4
y5
b4
y5
y7+P
y6
y6
y9+P
y7
y10+P
b5+P
b2+P
b6+P
b7+P
y7+P
b4+P
y8+P
b9+P
y9+P
b10+P
y10+P

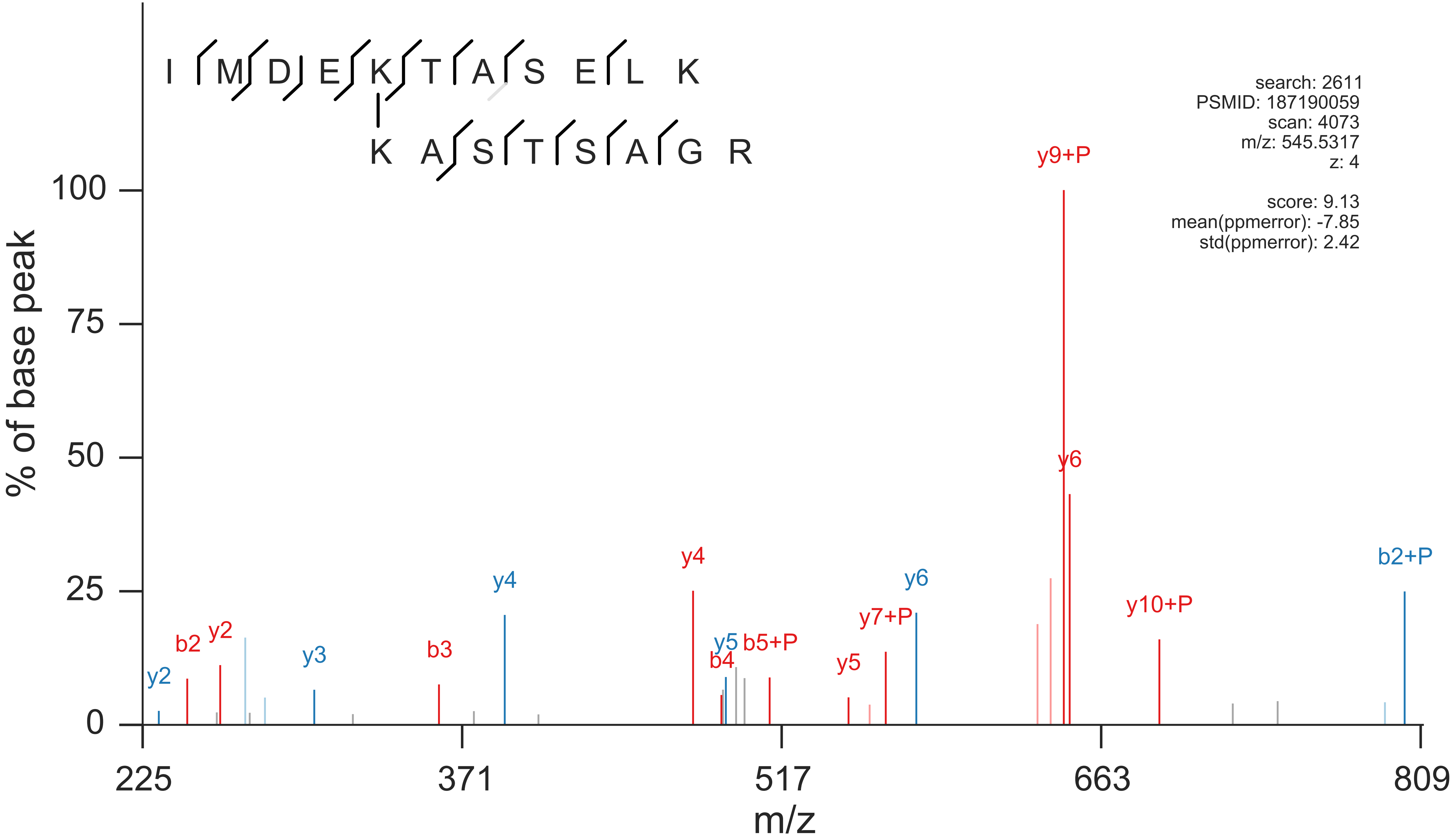

I M D E K T A S E L K
K A S T S A G R
search: 2611
PSMID: 187190059
scan: 4073
m/z: 545.5317
z: 4
score: 9.13
mean(ppmerror): -7.85
std(ppmerror): 2.42
% of base peak
m/z
100
75
50
25
0
225
371
517
663
809
y9+P
y6
y4
b2+P
y6
y10+P
y7+P
y4
y5
b5+P
b4
y5
b3
y3
b2
y2
y2

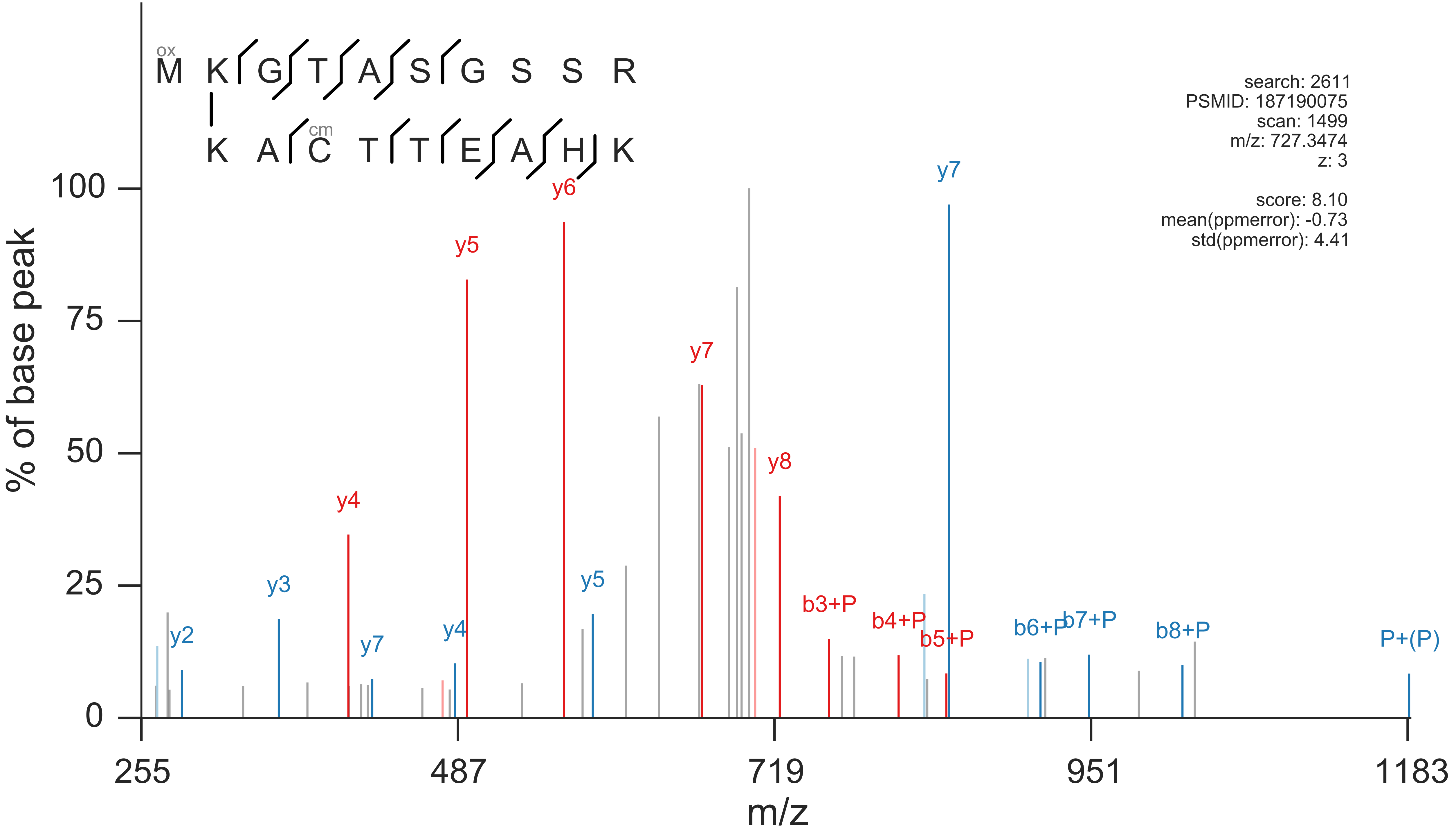

M K G T A S G S S R
K A C T T E A H K
ox
cm
search: 2611
PSMID: 187190075
scan: 1499
m/z: 727.3474
z: 3
score: 8.10
mean(ppmerror): -0.73
std(ppmerror): 4.41
% of base peak
m/z
0
25
50
75
100
255
487
719
951
1183
y2
y3
y4
y7
y4
y5
y6
y5
y7
y8
y7
b3+P
b4+P
b5+P
b6+P
b7+P
b8+P
P+(P)

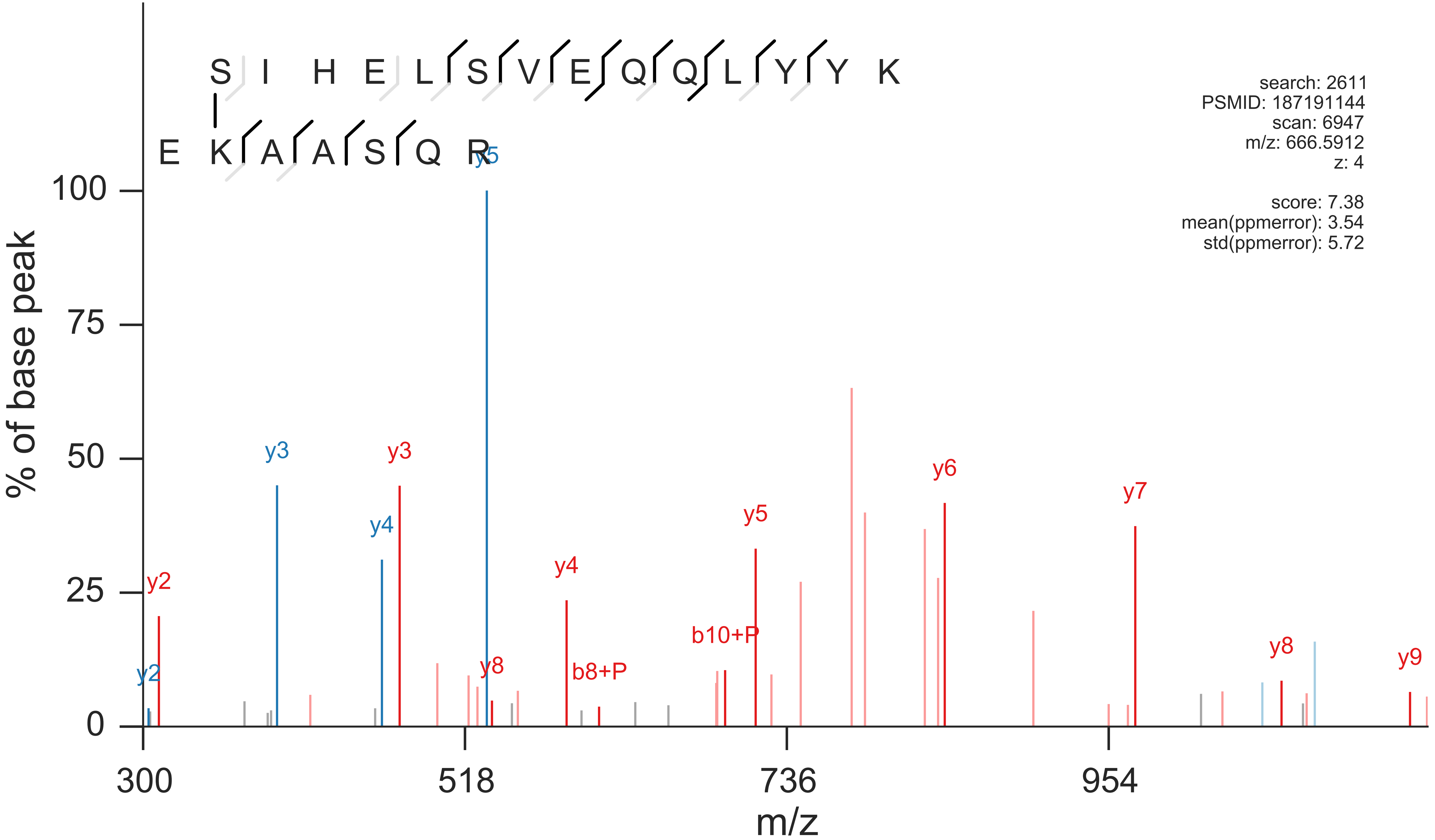

S I H E L S V E Q Q L Y Y K
E K A A S Q R
search: 2611
PSMID: 187191144
scan: 6947
m/z: 666.5912
z: 4
score: 7.38
mean(ppmerror): 3.54
std(ppmerror): 5.72
% of base peak
100
75
50
25
0
y5
y3
y3
y6
y7
y4
y5
y4
y2
b10+P
y8
y8
y9
y2
b8+P
300
518
736
954
m/z

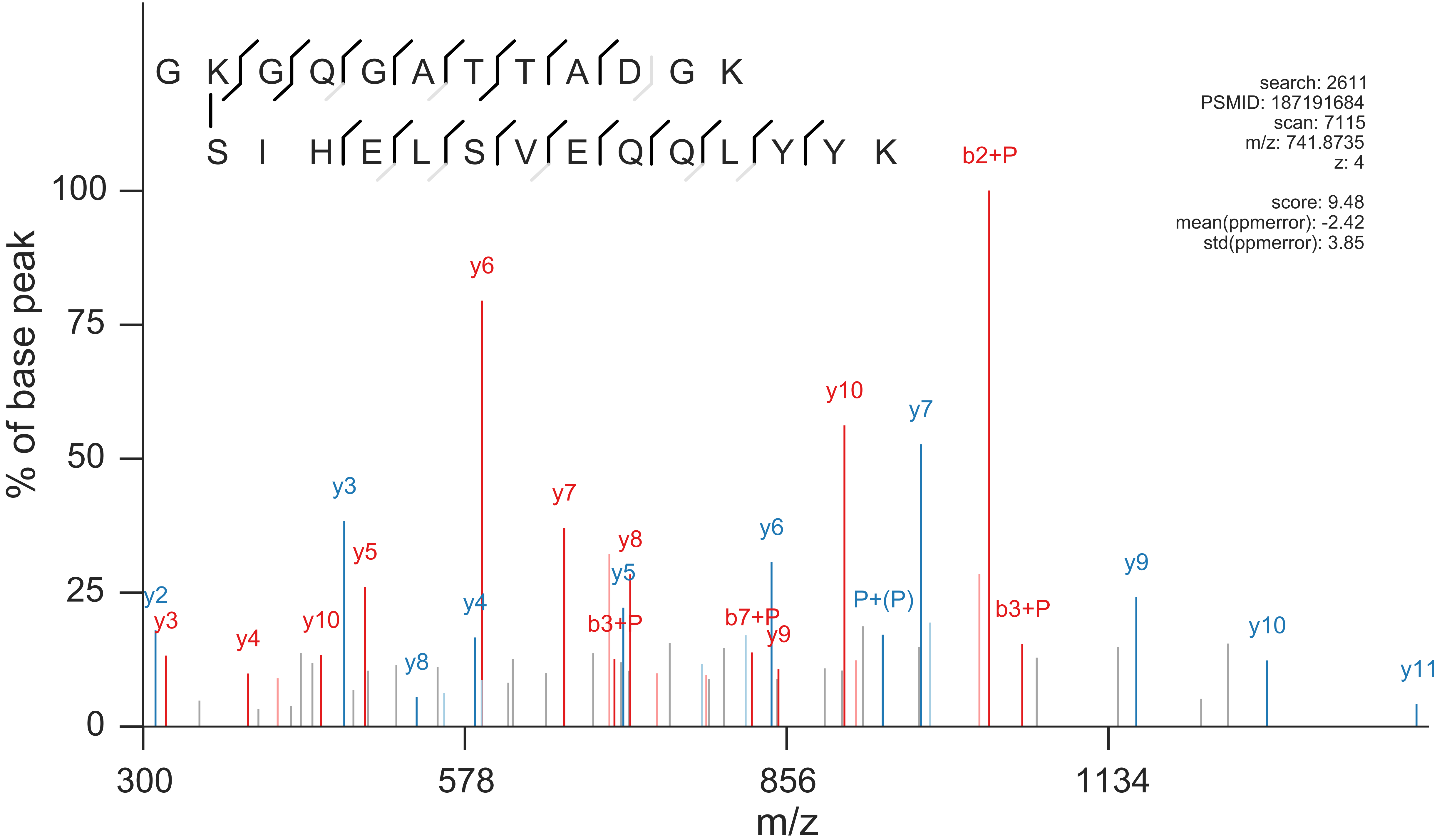
G K G Q G A T T A D G K
S I H E L S V E Q Q L Y Y K
search: 2611
PSMID: 187191684
scan: 7115
m/z: 741.8735
z: 4
score: 9.48
mean(ppmerror): -2.42
std(ppmerror): 3.85
% of base peak
m/z
100
75
50
25
0
300
578
856
1134
b2+P
y6
y10
y7
y3
y7
y8
y6
y5
y5
y9
y2
y3
y4
y10
y4
y8
b3+P
b7+P
y9
P+(P)
b3+P
y10
y11

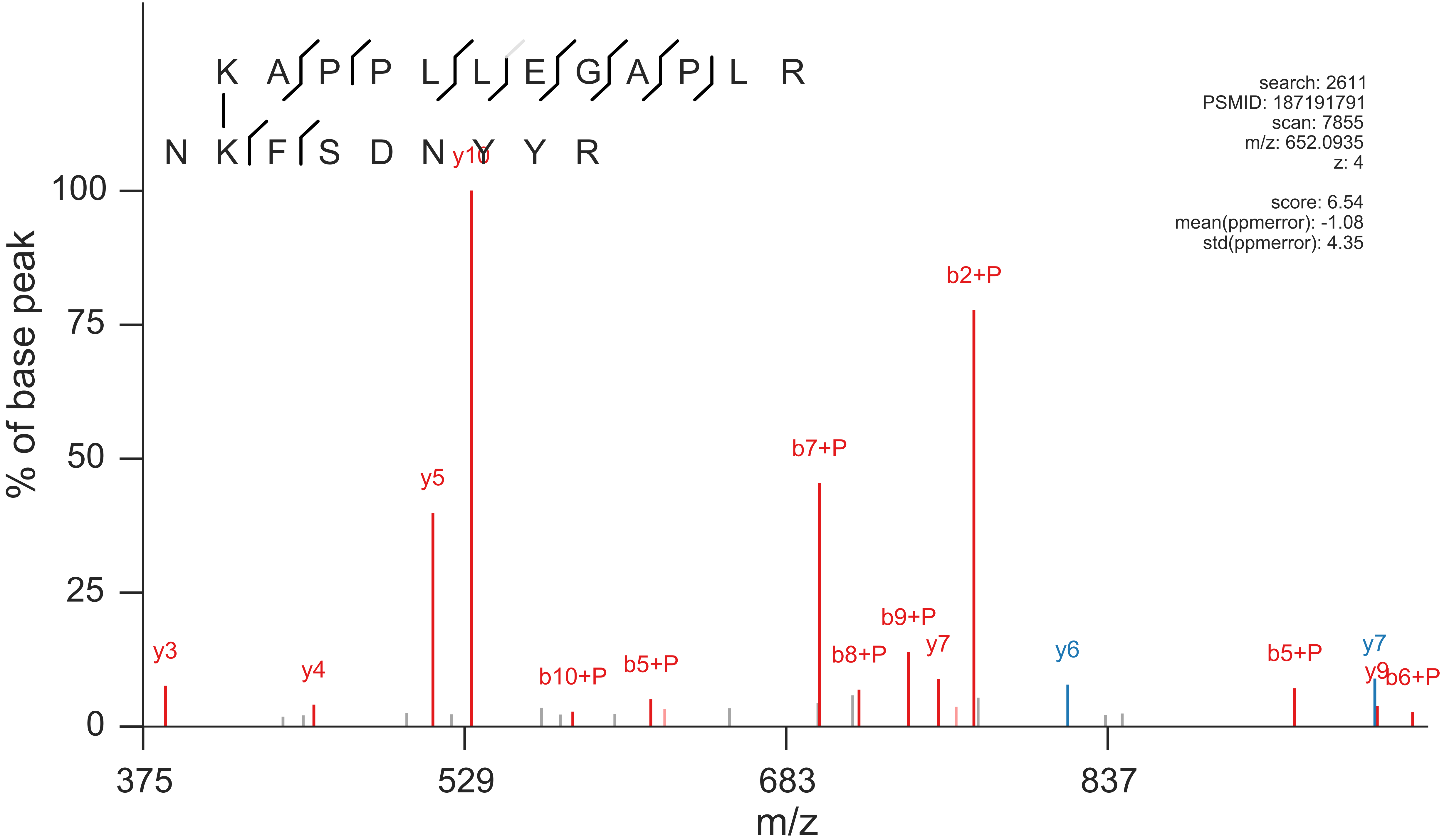

K A P P L L E G A P L R
N K F S D N Y Y R
search: 2611
PSMID: 187191791
scan: 7855
m/z: 652.0935
z: 4
score: 6.54
mean(ppmerror): -1.08
std(ppmerror): 4.35
% of base peak
100
75
50
25
0
y3
y4
y5
y10
b10+P
b5+P
b7+P
b8+P
b9+P
y7
b2+P
y6
b5+P
y7
y9
b6+P
375
529
683
837
m/z

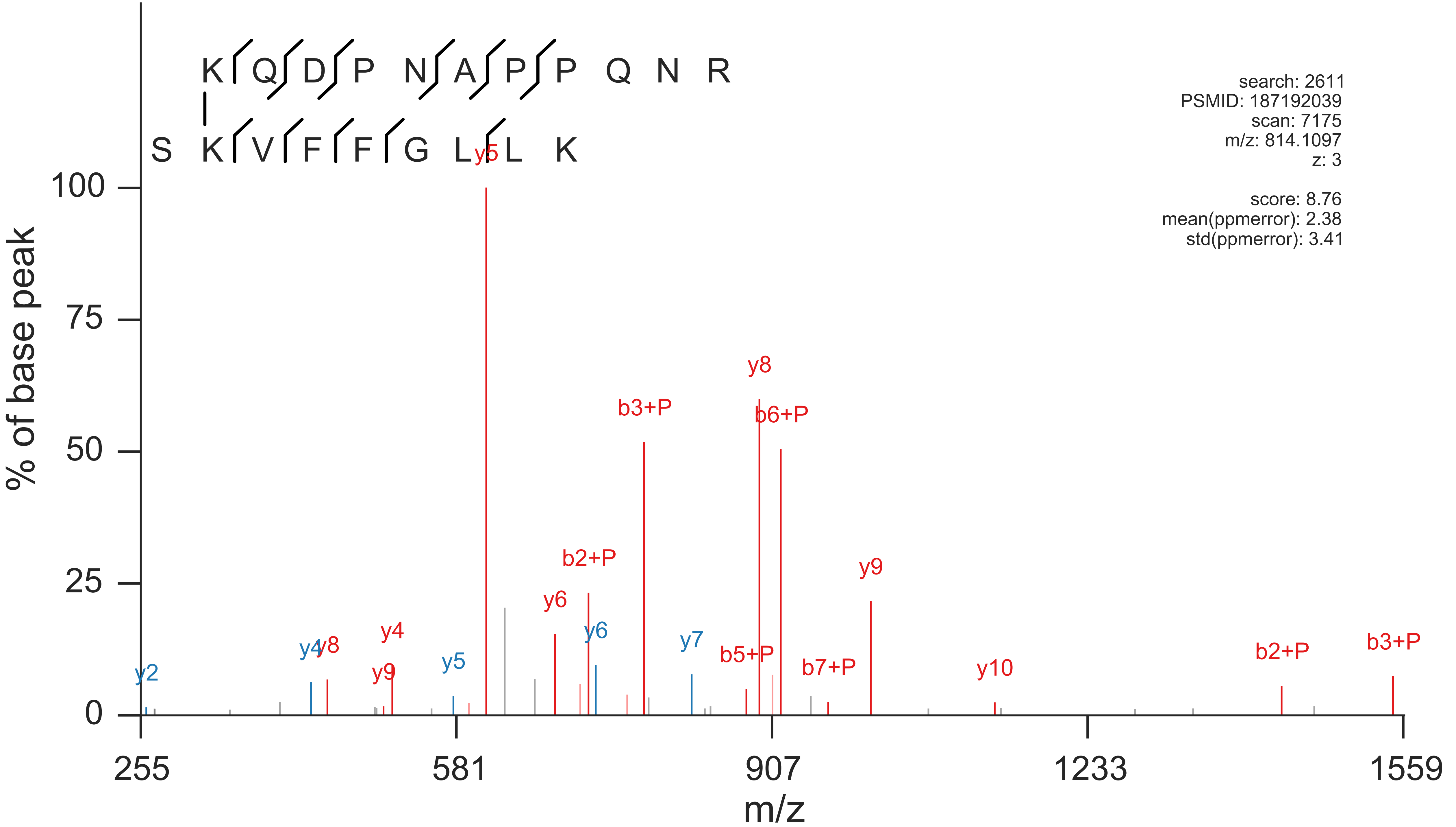

K Q D P N A P P Q N R
S K V F F G L L K
search: 2611
PSMID: 187192039
scan: 7175
m/z: 814.1097
z: 3
score: 8.76
mean(ppmerror): 2.38
std(ppmerror): 3.41
% of base peak
100
75
50
25
0
255
581
907
1233
1559
m/z
y2
y4
y8
y4
y9
y5
y5
y6
b2+P
y6
b3+P
y7
b5+P
y8
b6+P
b7+P
y9
y10
b2+P
b3+P

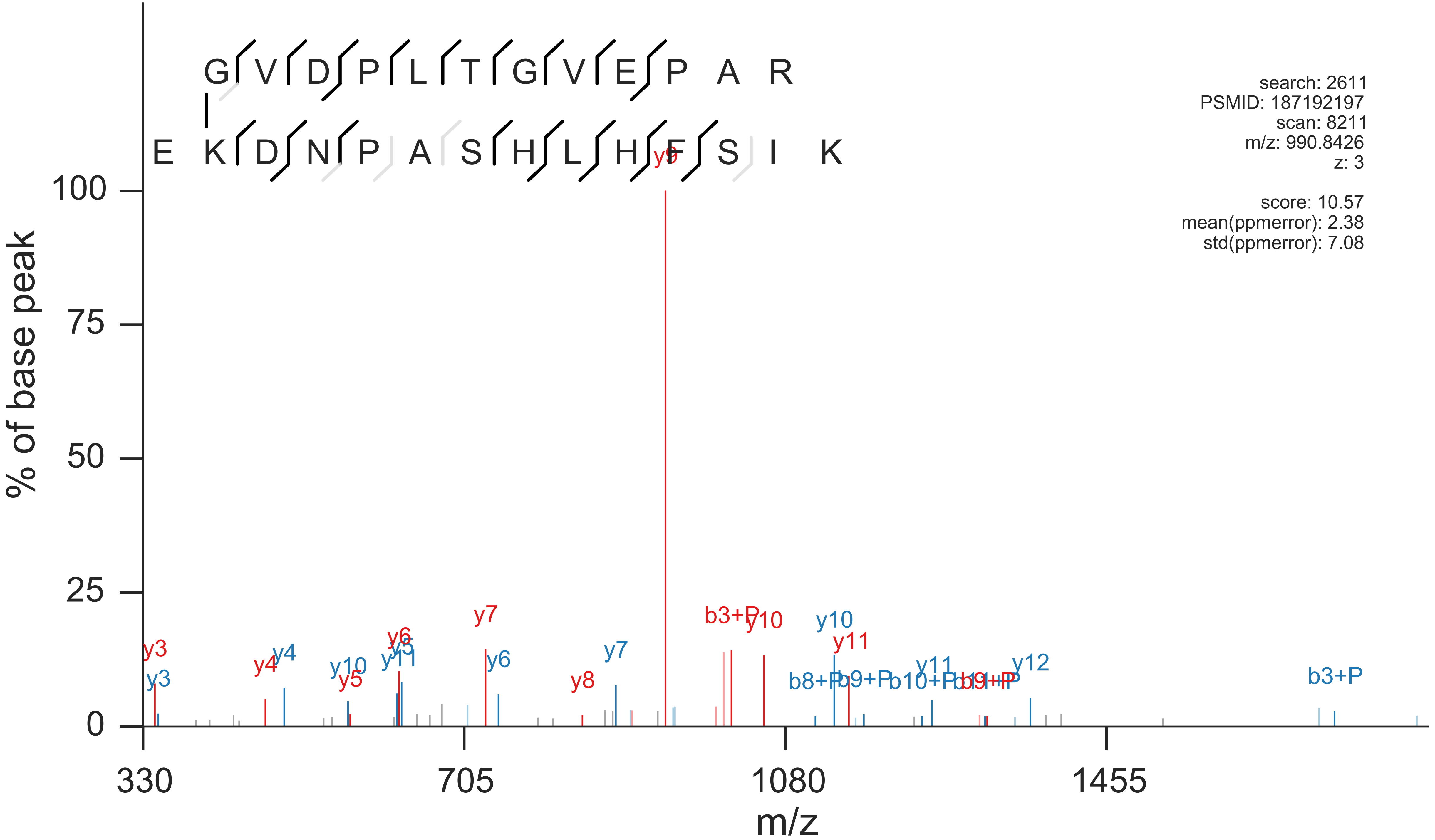
G V D P L T G V E P A R
E K D N P A S H L H F S I K
search: 2611
PSMID: 187192197
scan: 8211
m/z: 990.8426
z: 3
score: 10.57
mean(ppmerror): 2.38
std(ppmerror): 7.08
% of base peak
m/z
0
25
50
75
100
330
705
1080
1455
y3
y4
y5
y6
y7
y8
y9
y10
y11
b3+P
y3
y4
y10
y5
y11
y6
y7
y10
b8+P
b9+P
b10+P
y11
y12
b3+P

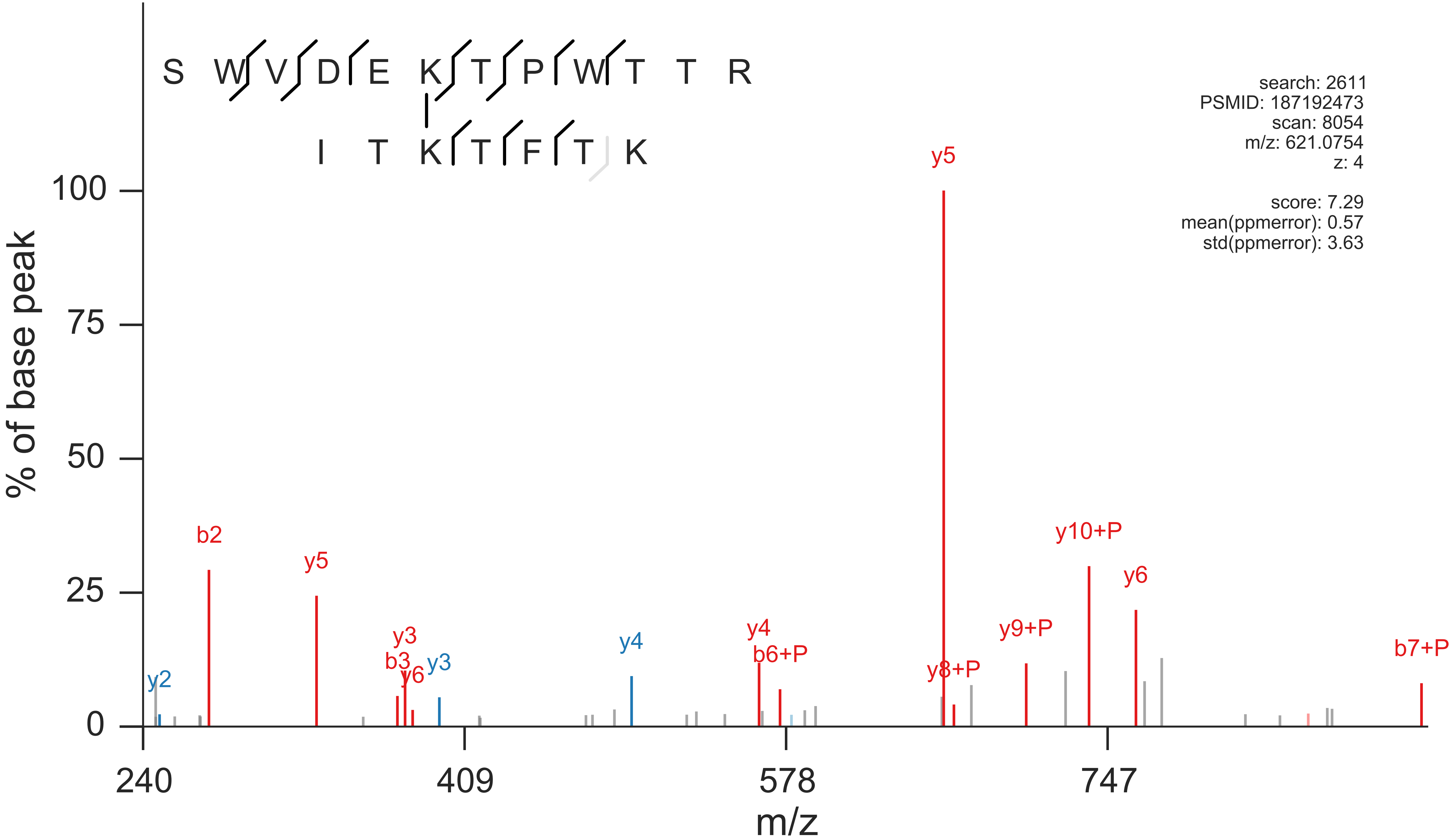
S W V D E K T P W T T R
I T K T F T K
search: 2611
PSMID: 187192473
scan: 8054
m/z: 621.0754
z: 4
score: 7.29
mean(ppmerror): 0.57
std(ppmerror): 3.63
% of base peak
100
75
50
25
0
240
409
578
747
m/z
y5
b2
y5
y3
b3
y6
y3
y2
y4
y4
b6+P
y8+P
y9+P
y10+P
y6
b7+P

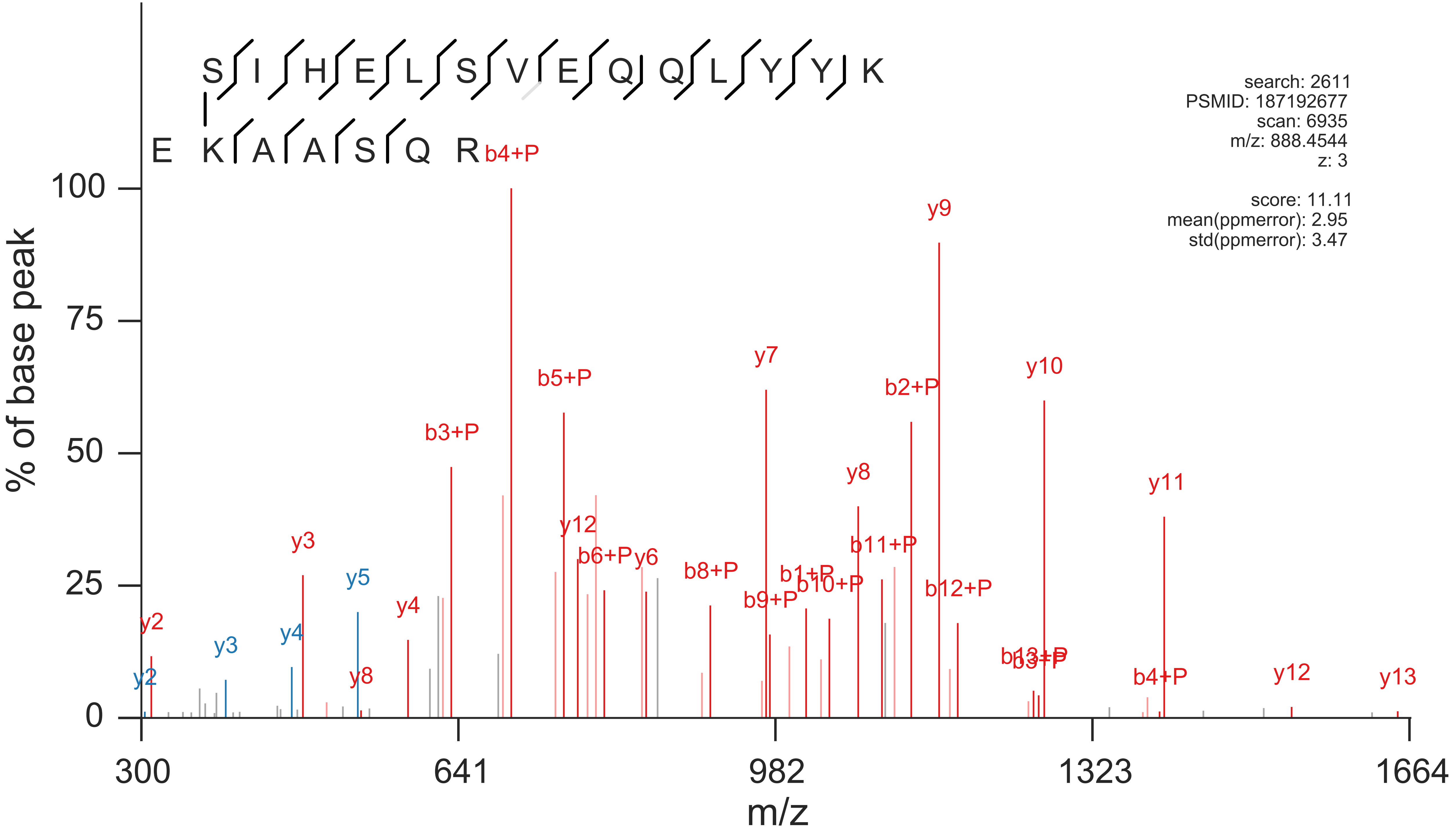

S I H E L S V E Q Q L Y Y K
E K A A S Q R
search: 2611
PSMID: 187192677
scan: 6935
m/z: 888.4544
z: 3
score: 11.11
mean(ppmerror): 2.95
std(ppmerror): 3.47
% of base peak
m/z
100
75
50
25
0
300
641
982
1323
1664
b4+P
y9
y7
y10
b5+P
b2+P
b3+P
y8
y11
y12
b6+P
y6
b8+P
b11+P
b1+P
b10+P
b9+P
b12+P
y3
y5
y4
y2
y8
y12
y13
b4+P

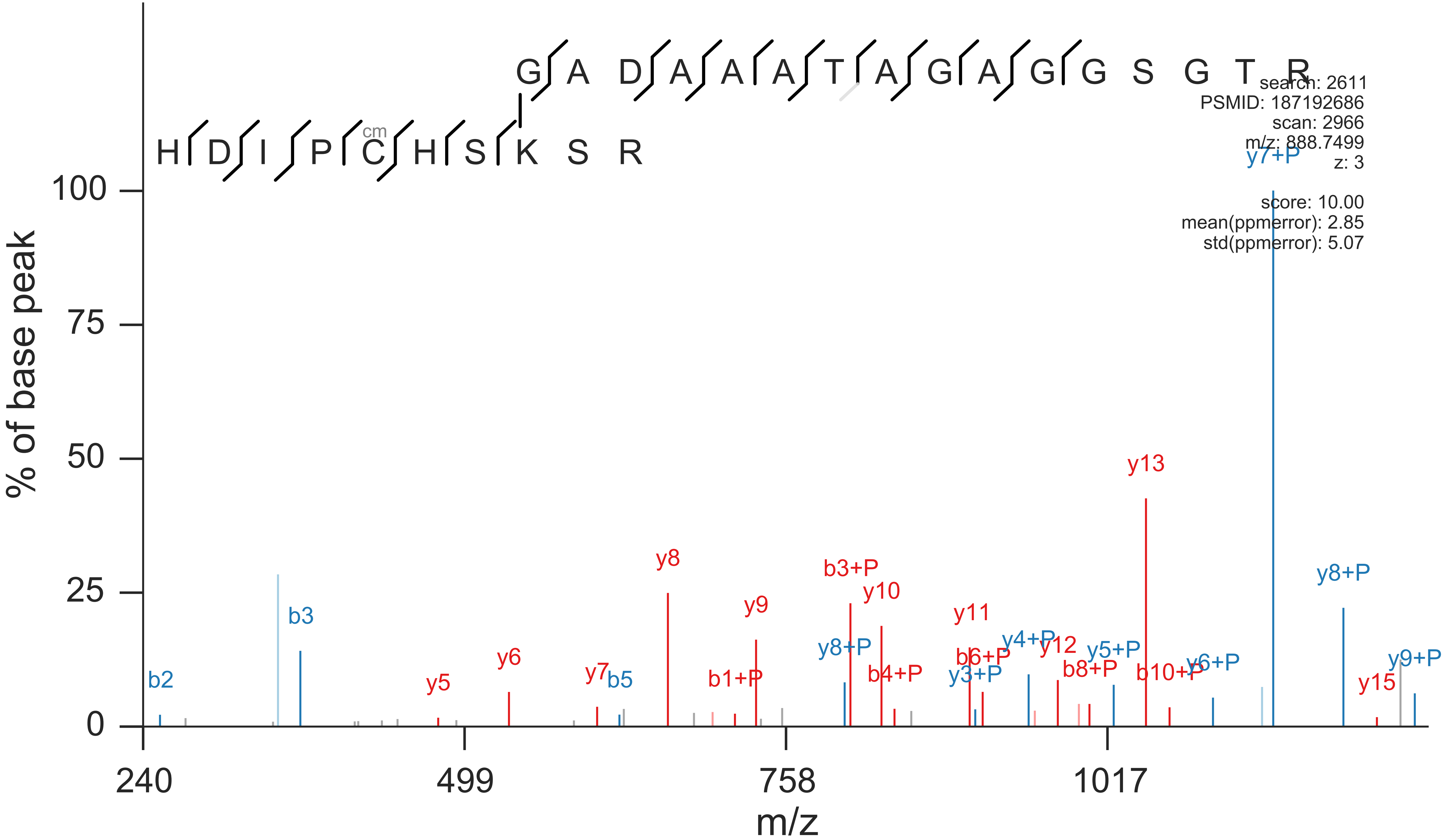
G A D A A A T A G A G G S G T R
H D I P C H S K S R
cm
search: 2611
PSMID: 187192686
scan: 2966
m/z: 888.7499
z: 3
score: 10.00
mean(ppmerror): 2.85
std(ppmerror): 5.07
% of base peak
m/z
100
75
50
25
0
240
499
758
1017
b2
b3
y5
y6
y7
b5
y8
b1+P
y9
y8+P
b3+P
y10
b4+P
y11
y3+P
b6+P
y4+P
y12
b8+P
y5+P
y13
b10+P
y6+P
y7+P
y8+P
y15
y9+P

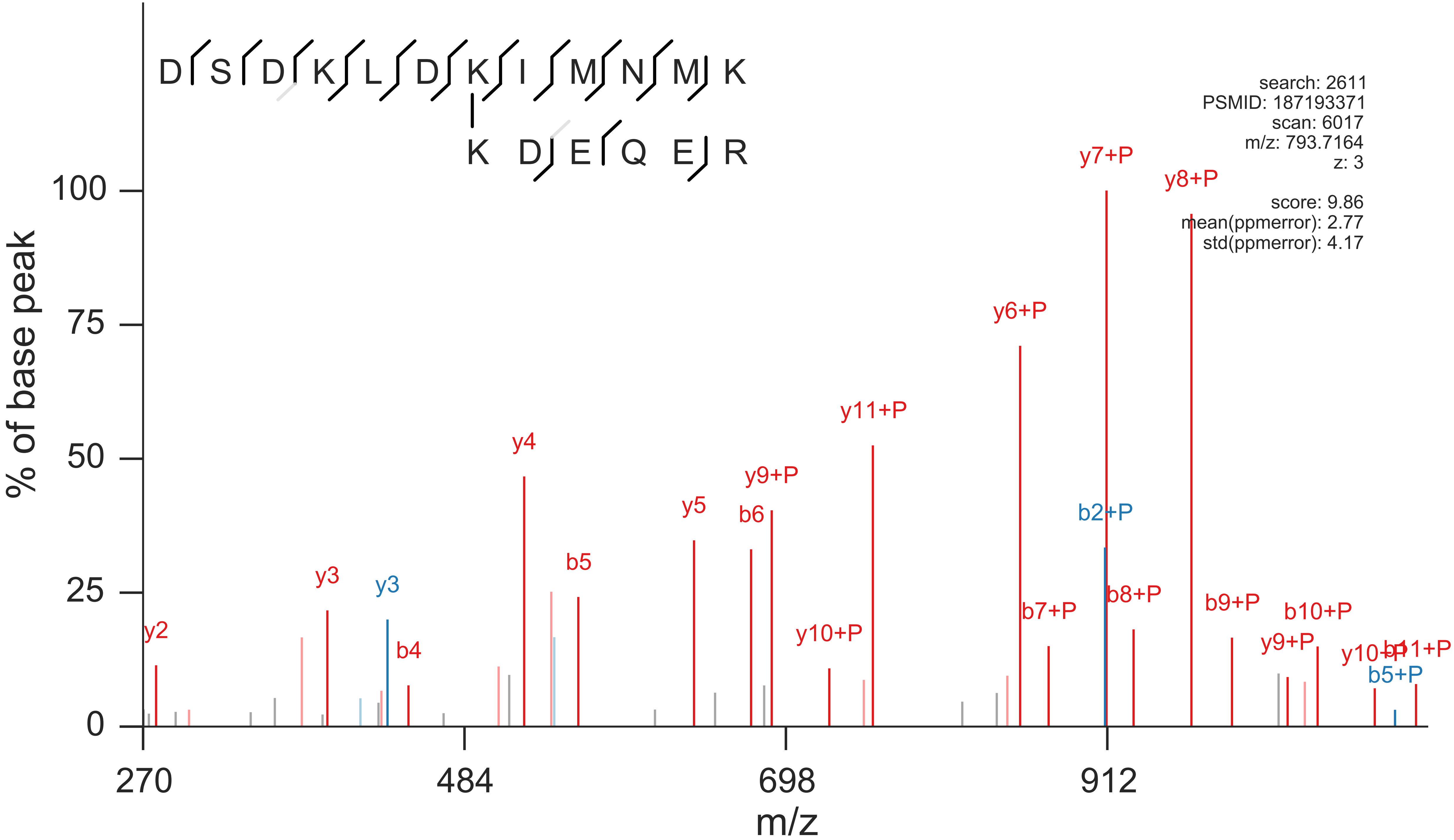

D S D K L D K I M N M K
K D E Q E R
search: 2611
PSMID: 187193371
scan: 6017
m/z: 793.7164
z: 3
score: 9.86
mean(ppmerror): 2.77
std(ppmerror): 4.17
% of base peak
m/z
0
25
50
75
100
270
484
698
912
y2
y3
y3
b4
y4
b5
y5
b6
y9+P
y10+P
y11+P
y6+P
b7+P
y7+P
b2+P
b8+P
y8+P
b9+P
y9+P
b10+P
y10+P
b11+P
b5+P

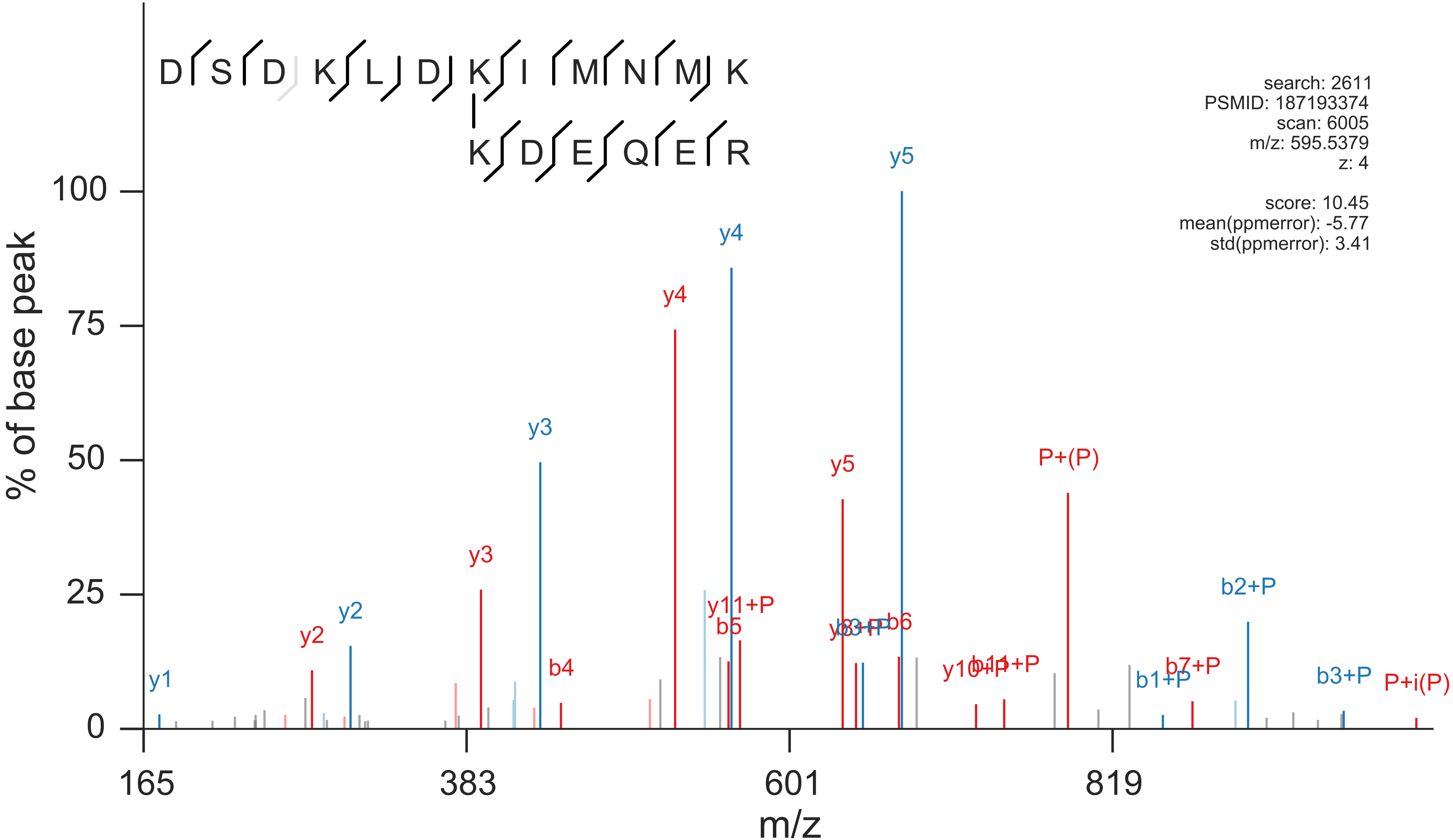

D S D K L D K I M N M K
K D E Q E R
search: 2611
PSMID: 187193374
scan: 6005
m/z: 595.5379
z: 4
score: 10.45
mean(ppmerror): -5.77
std(ppmerror): 3.41
% of base peak
0
25
50
75
100
m/z
165
383
601
819
y1
y2
y2
y3
y3
b4
y4
y4
y11+P
b5
y5
y5
b6
P+(P)
b2+P
b1+P
b7+P
b3+P
P+i(P)

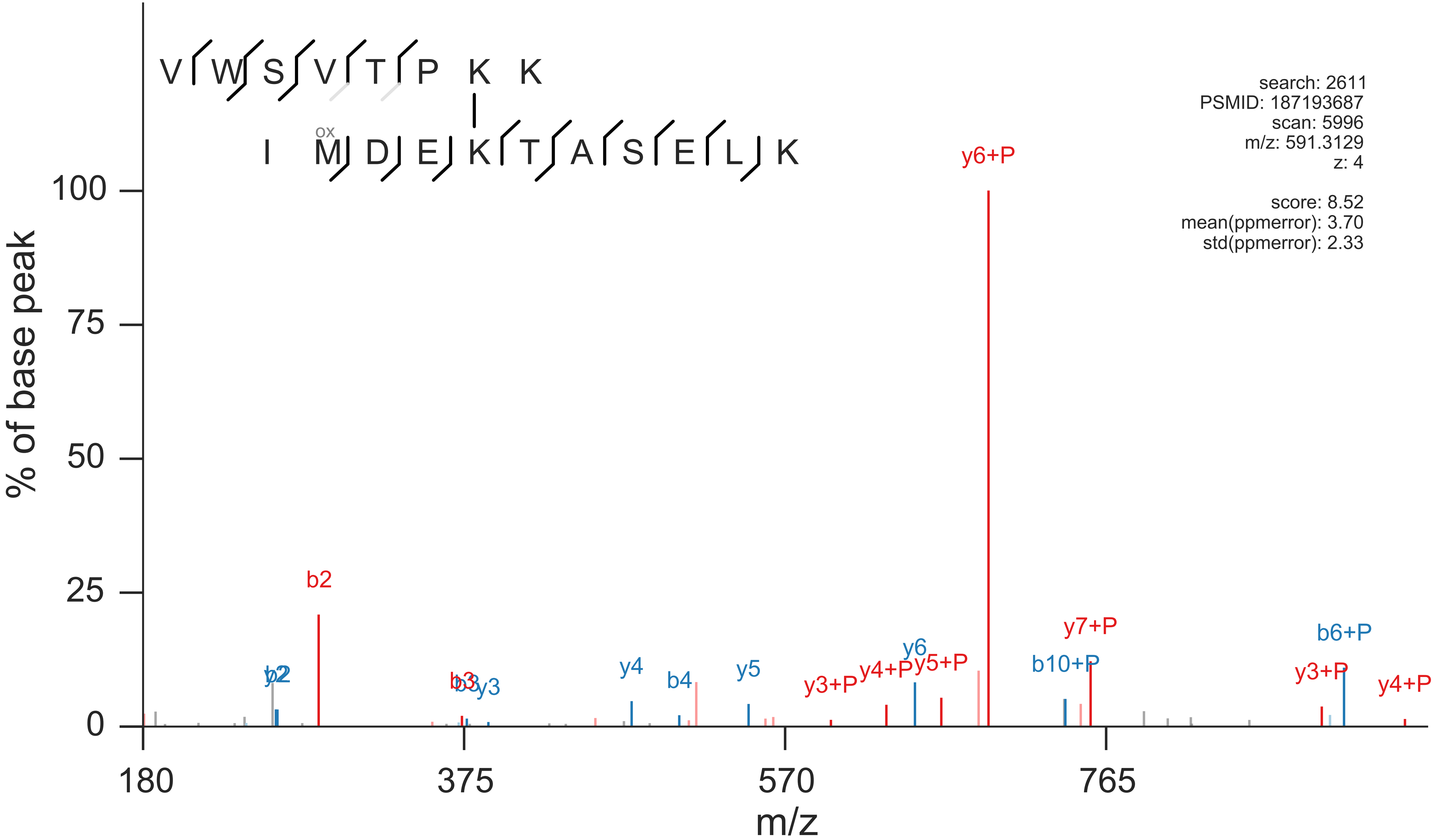

V W S V T P K K
I M D E K T A S E L K
ox
search: 2611
PSMID: 187193687
scan: 5996
m/z: 591.3129
z: 4
score: 8.52
mean(ppmerror): 3.70
std(ppmerror): 2.33
% of base peak
m/z
0
25
50
75
100
180
375
570
765
y6+P
b2
y7+P
b6+P
y6
b10+P
y4
b4
y5
y3+P
y4+P
y5+P
b3
y3
y2

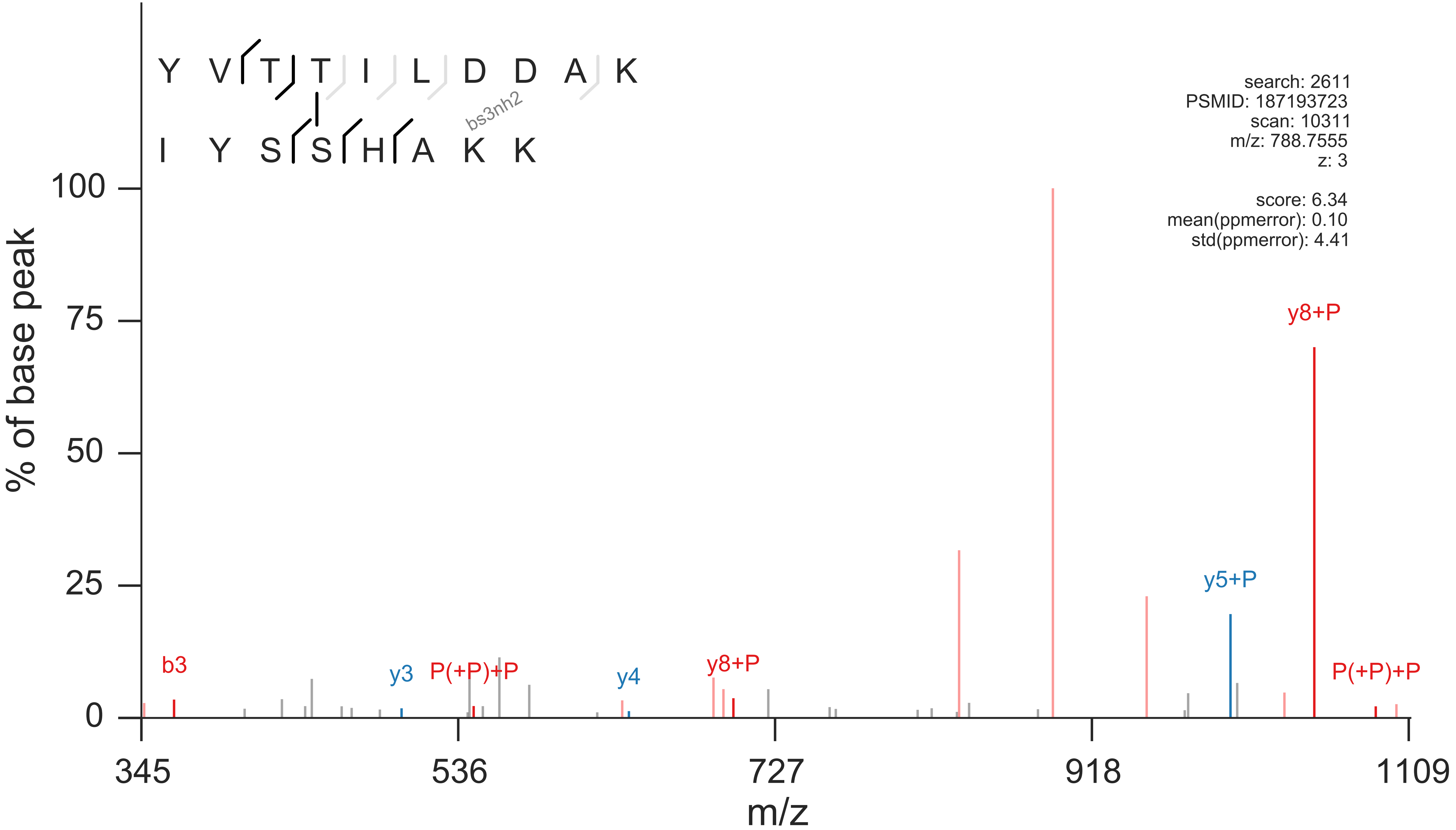

Y V T T I L D D A K
I Y S S H A K K
bs3nh2
search: 2611
PSMID: 187193723
scan: 10311
m/z: 788.7555
z: 3
score: 6.34
mean(ppmerror): 0.10
std(ppmerror): 4.41
% of base peak
100
75
50
25
0
b3
y3
P(+P)+P
y4
y8+P
y5+P
y8+P
P(+P)+P
345
536
727
918
1109
m/z

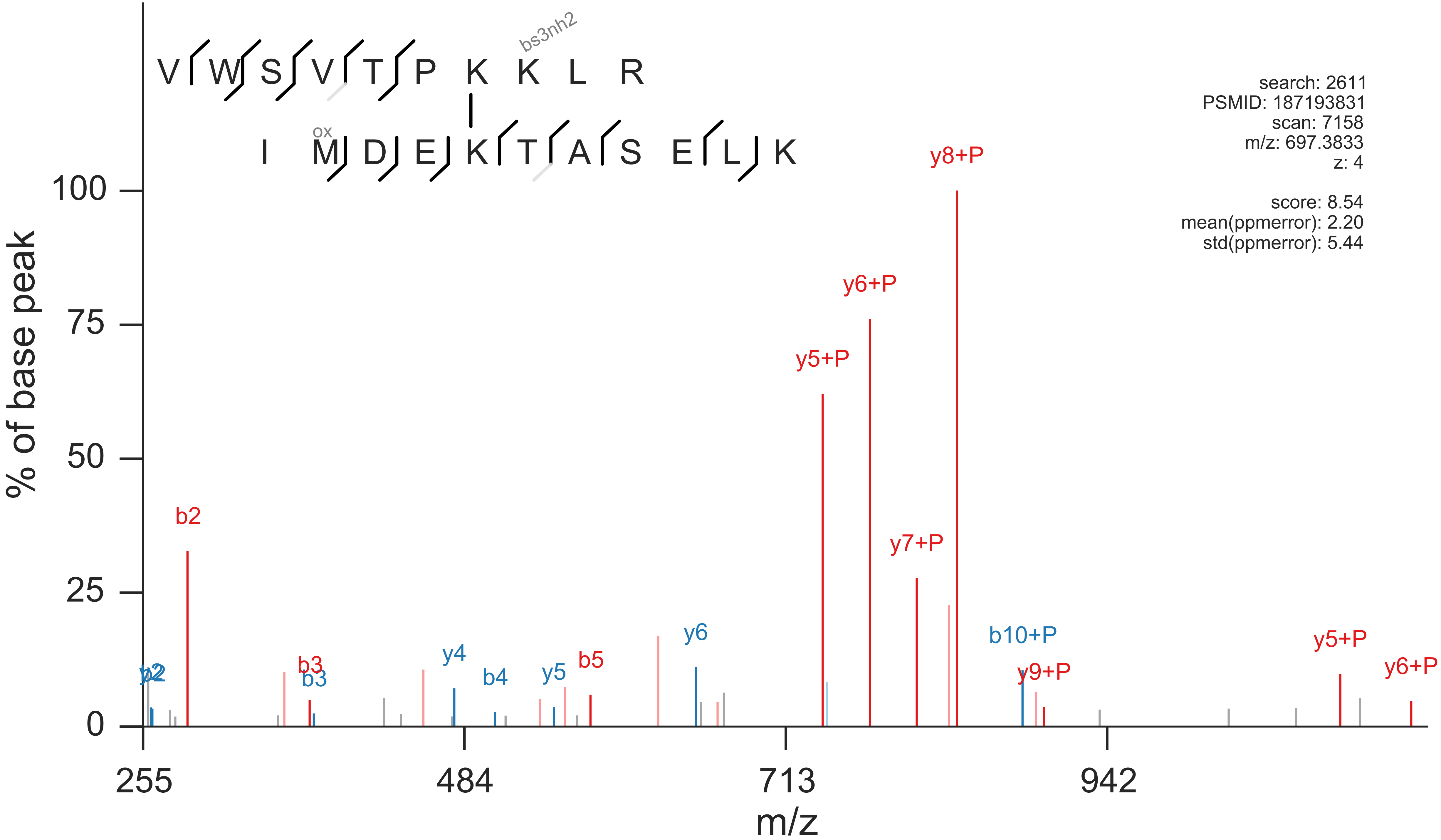
V W S V T P K K L R
bs3nh2
I M D E K T A S E L K
ox
search: 2611
PSMID: 187193831
scan: 7158
m/z: 697.3833
z: 4
score: 8.54
mean(ppmerror): 2.20
std(ppmerror): 5.44
% of base peak
m/z
0
25
50
75
100
255
484
713
942
y8+P
y6+P
y5+P
y7+P
b2
y2
b3
y4
b4
y5
b5
y6
b10+P
y9+P
y5+P
y6+P

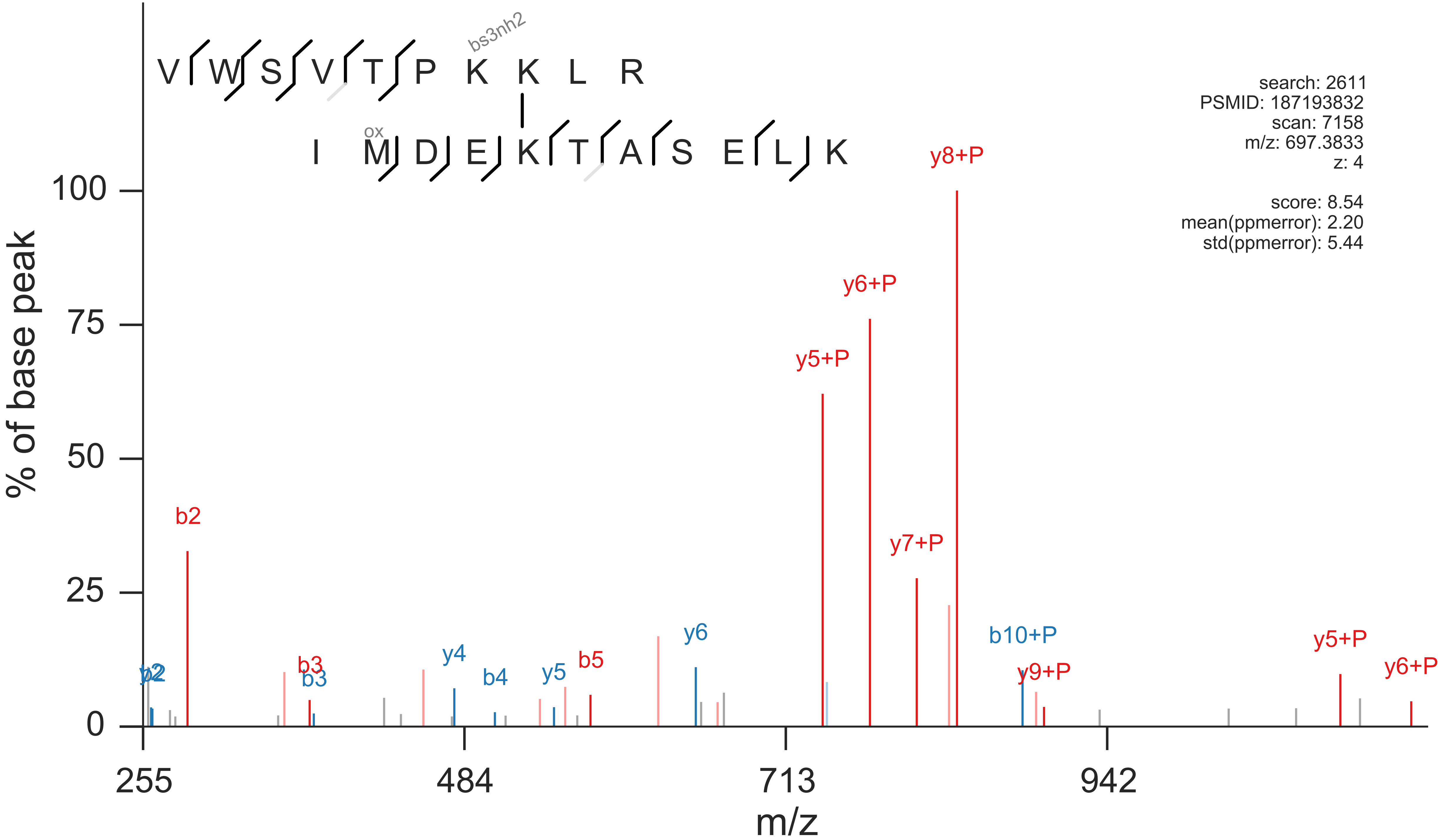

V W S V T P K K L R
bs3nh2
I M D E K T A S E L K
ox
search: 2611
PSMID: 187193832
scan: 7158
m/z: 697.3833
z: 4
score: 8.54
mean(ppmerror): 2.20
std(ppmerror): 5.44
% of base peak
m/z
100
75
50
25
0
255
484
713
942
b2
b3
b5
y5+P
y6+P
y7+P
y8+P
y9+P
y2
b2
b3
y4
b4
y5
y6
b10+P

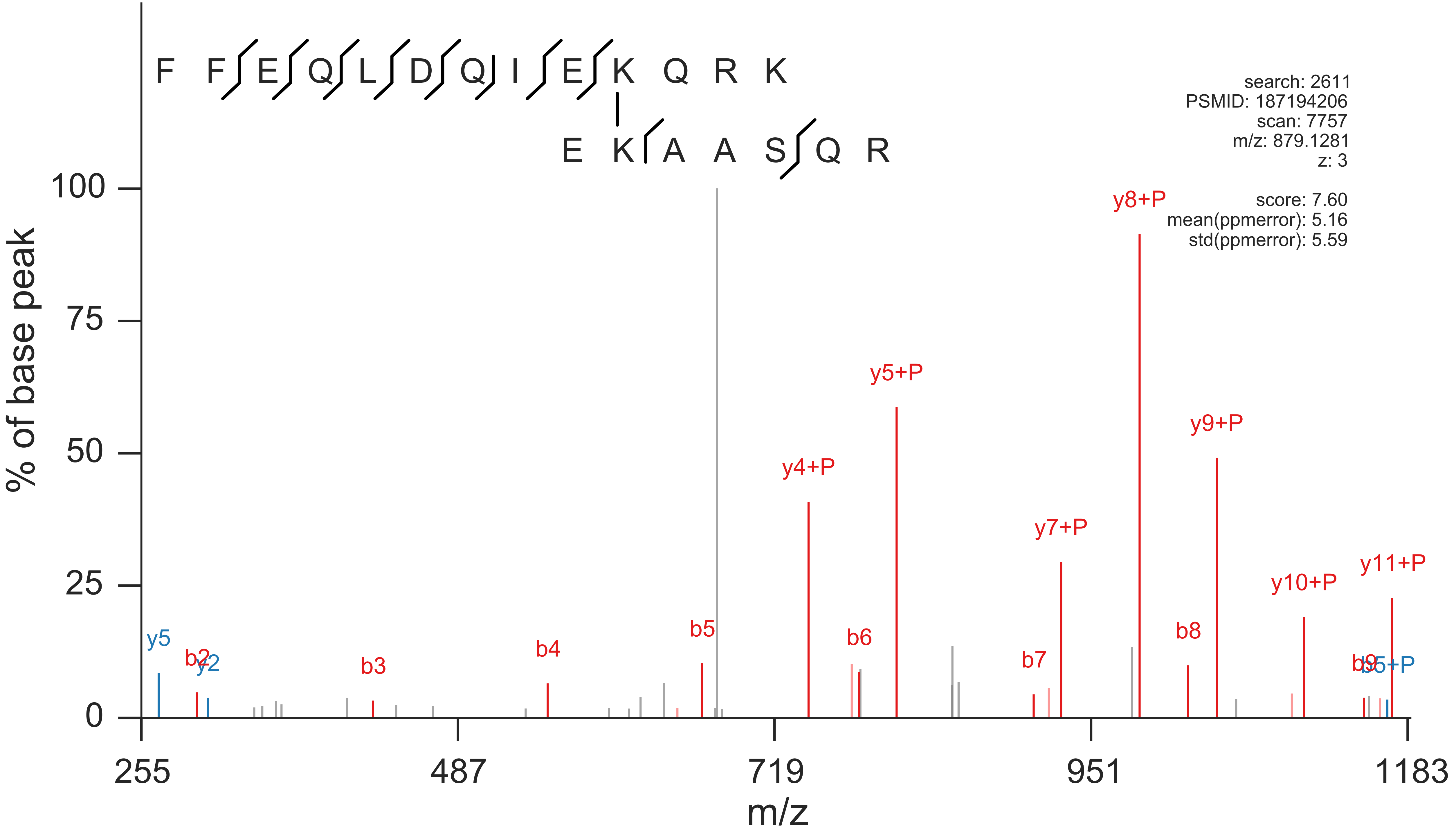

F F E Q L D Q I E K Q R K
E K A A S Q R
search: 2611
PSMID: 187194206
scan: 7757
m/z: 879.1281
z: 3
score: 7.60
mean(ppmerror): 5.16
std(ppmerror): 5.59
% of base peak
100
75
50
25
0
y5
b2
y2
b3
b4
b5
y4+P
b6
y5+P
b7
y7+P
y8+P
b8
y9+P
y10+P
b9
b5+P
y11+P
255
487
719
951
1183
m/z

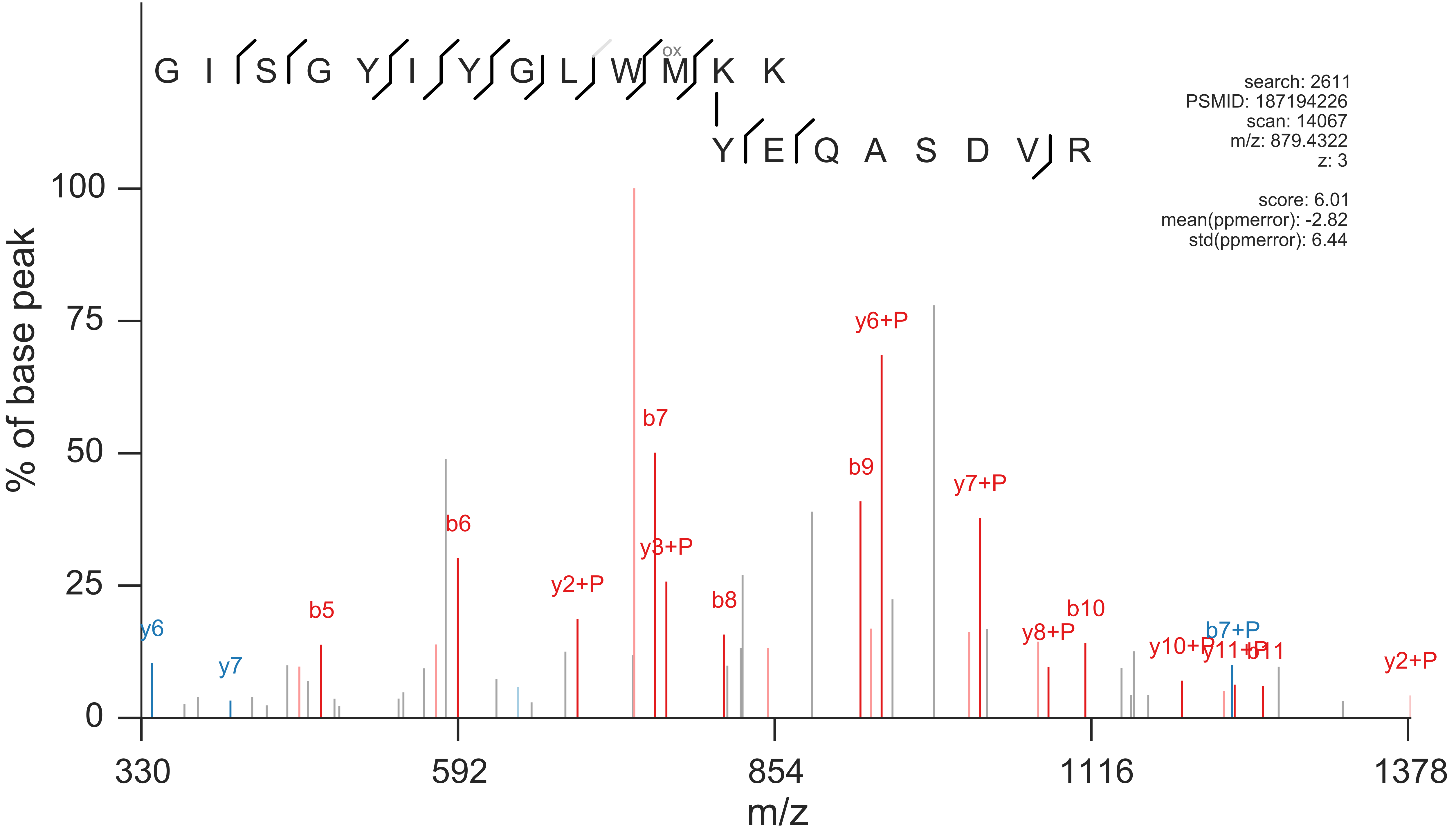
G I S G Y I Y G L W M K K
ox
Y E Q A S D V R
search: 2611
PSMID: 187194226
scan: 14067
m/z: 879.4322
z: 3
score: 6.01
mean(ppmerror): -2.82
std(ppmerror): 6.44
% of base peak
m/z
y6
y7
b5
b6
y2+P
b7
y3+P
b8
b9
y6+P
y7+P
y8+P
b10
y10+P
b7+P
y11+P
b11
y2+P

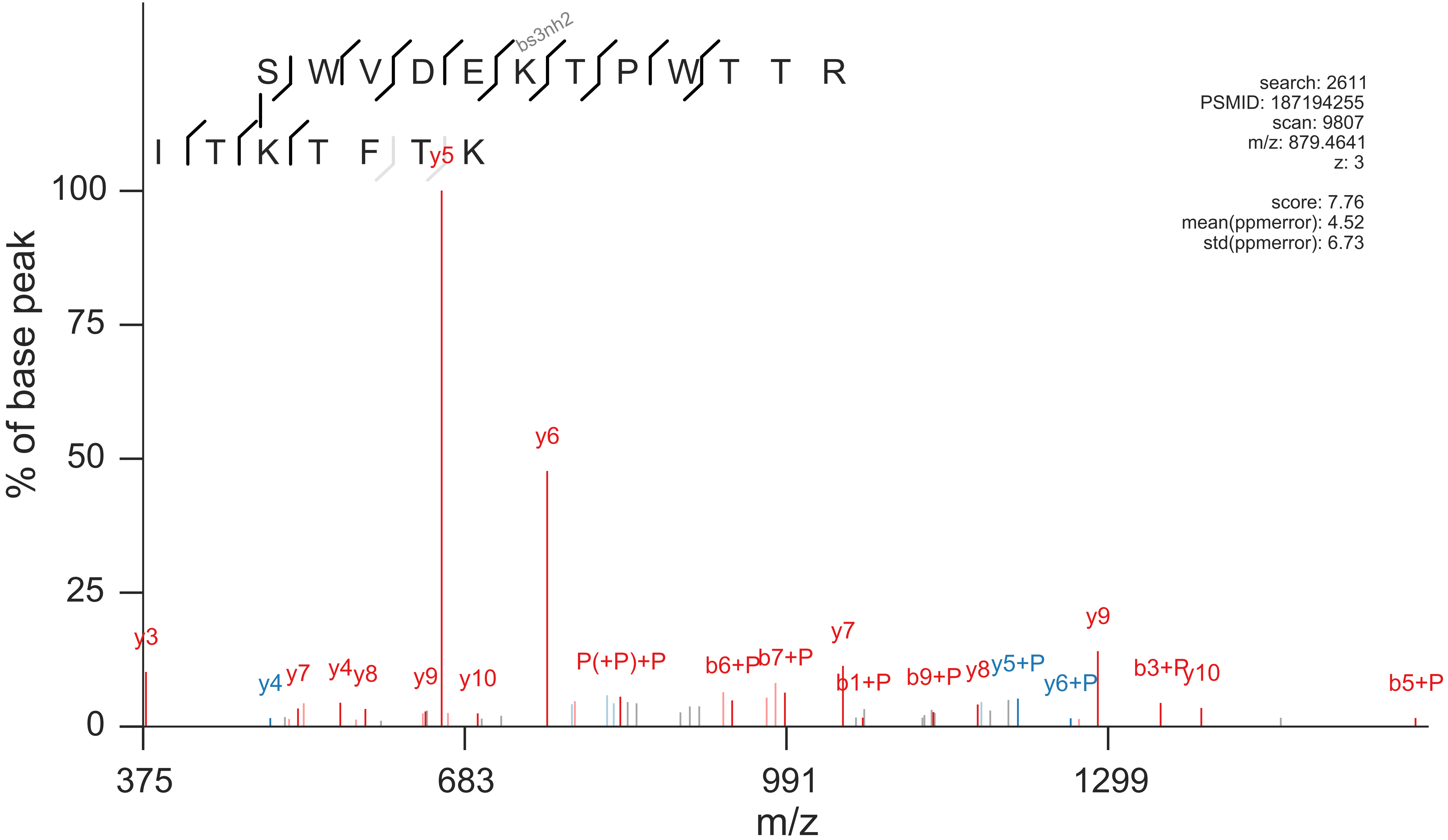

S W V D E K T P W T T R
bs3nh2
I T K T F T K
search: 2611
PSMID: 187194255
scan: 9807
m/z: 879.4641
z: 3
score: 7.76
mean(ppmerror): 4.52
std(ppmerror): 6.73
% of base peak
m/z

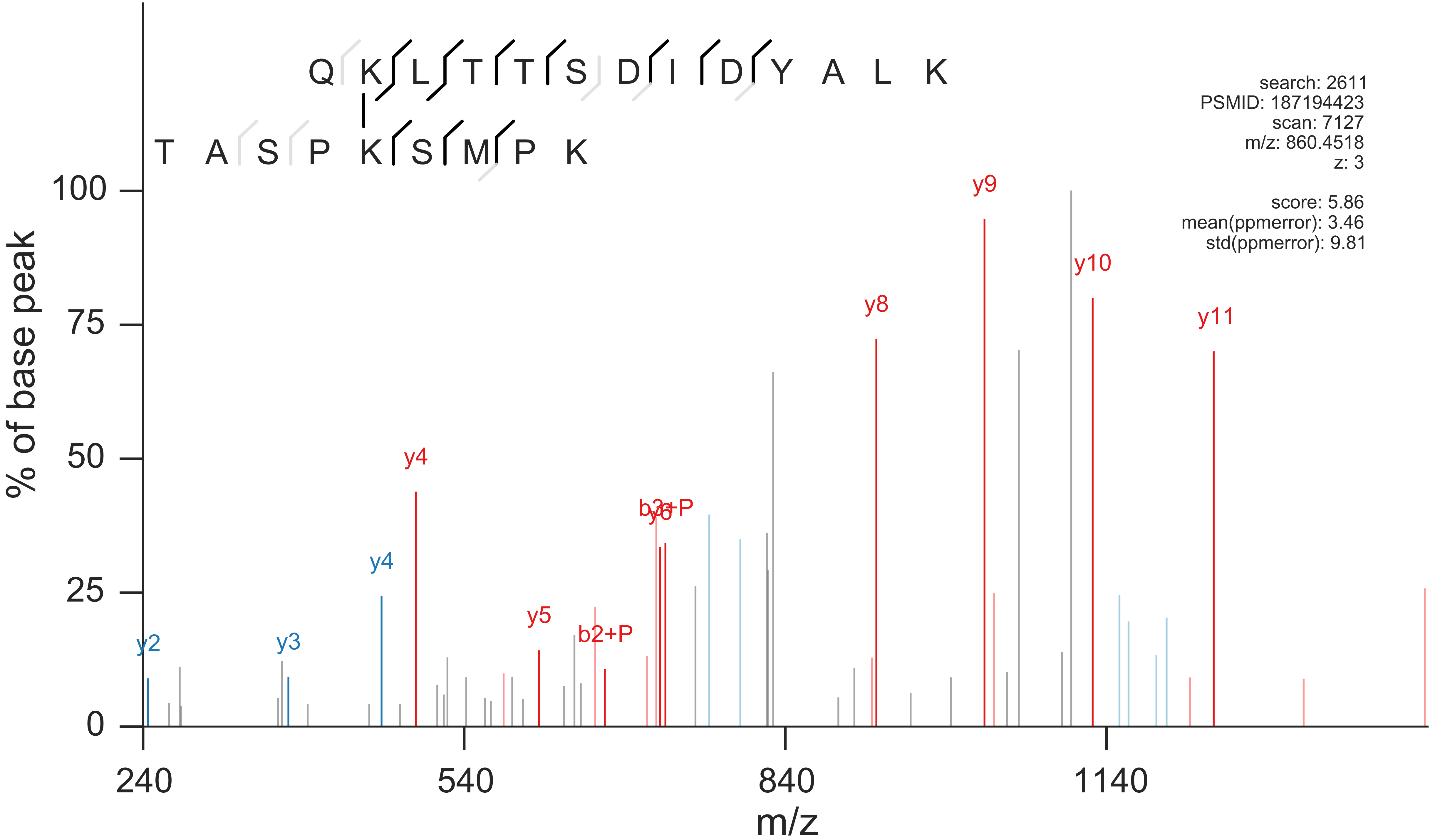

Q K L T T S D I D Y A L K
T A S P K S M P K
search: 2611
PSMID: 187194423
scan: 7127
m/z: 860.4518
z: 3
score: 5.86
mean(ppmerror): 3.46
std(ppmerror): 9.81
% of base peak
m/z
0
25
50
75
100
240
540
840
1140
y2
y3
y4
y4
y5
b2+P
b3+P
y6
y8
y9
y10
y11

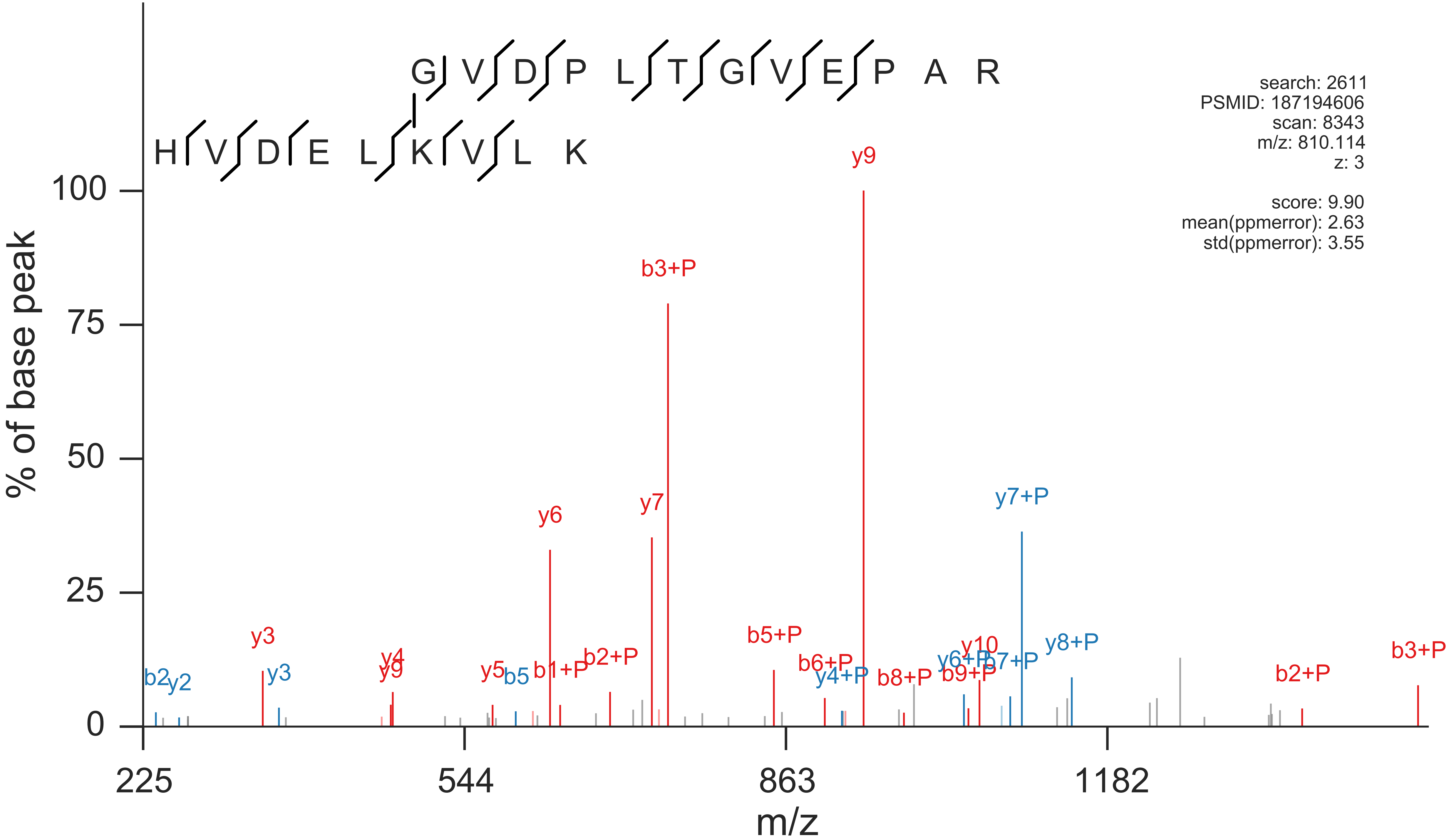

G V D P L T G V E P A R
H V D E L K V L K
search: 2611
PSMID: 187194606
scan: 8343
m/z: 810.114
z: 3
score: 9.90
mean(ppmerror): 2.63
std(ppmerror): 3.55
% of base peak
m/z
y9
b3+P
y6
y7
y7+P
y3
b5+P
y10
y8+P
b3+P
b2+P
b6+P
y6+P
b7+P
b2+P
y4
y9
y5
b5
b1+P
b2
y2
y3
y4+P
b8+P
b9+P

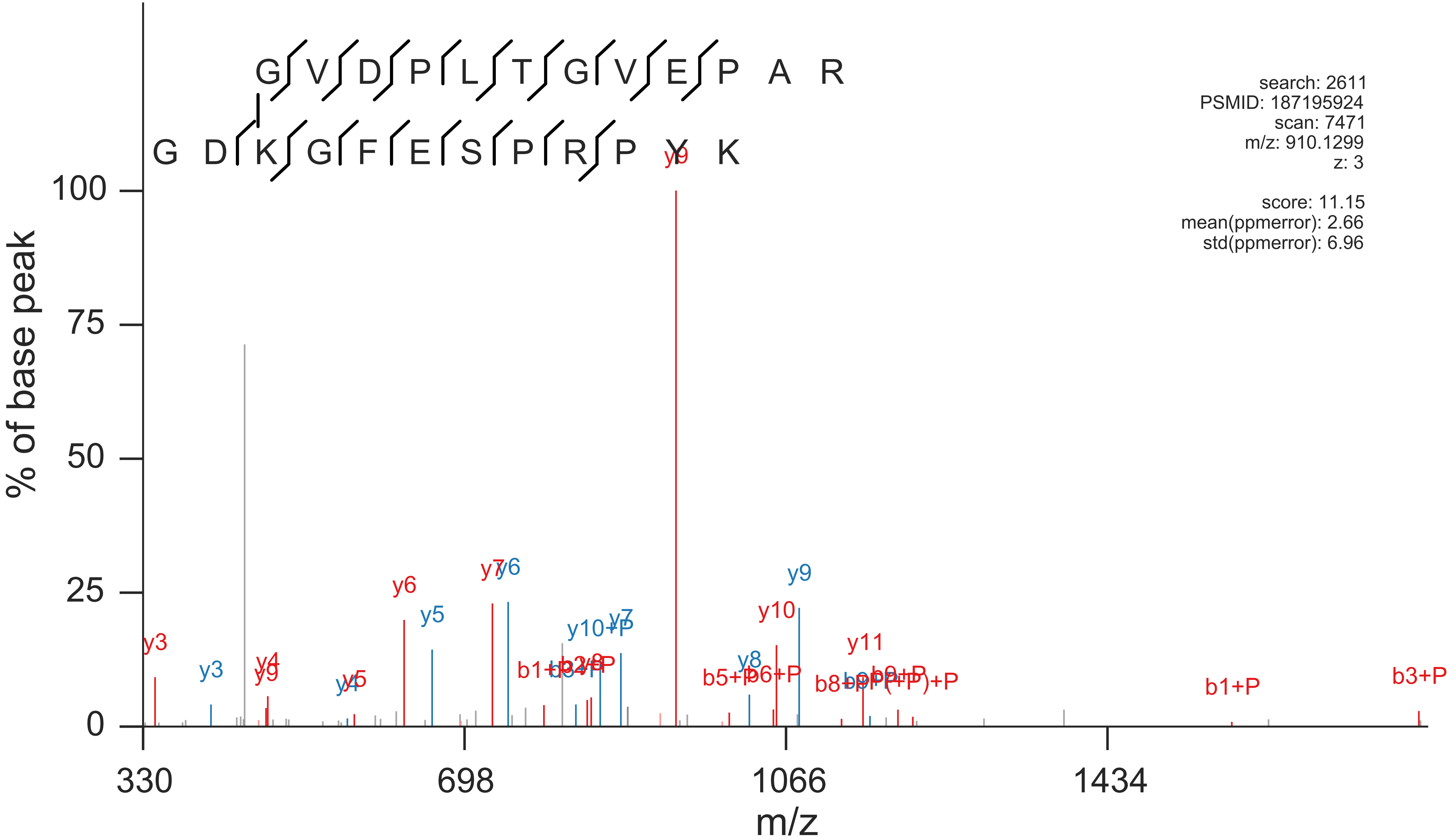

G V D P L T G V E P A R
G D K G F E S P R P Y K
search: 2611
PSMID: 187195924
scan: 7471
m/z: 910.1299
z: 3
score: 11.15
mean(ppmerror): 2.66
std(ppmerror): 6.96
% of base peak
m/z
0
25
50
75
100
330
698
1066
1434
y3
y4
y5
y6
y7
y8
y9
y10
y11
y10+P
b1+P
b3+P
b5+P
b6+P

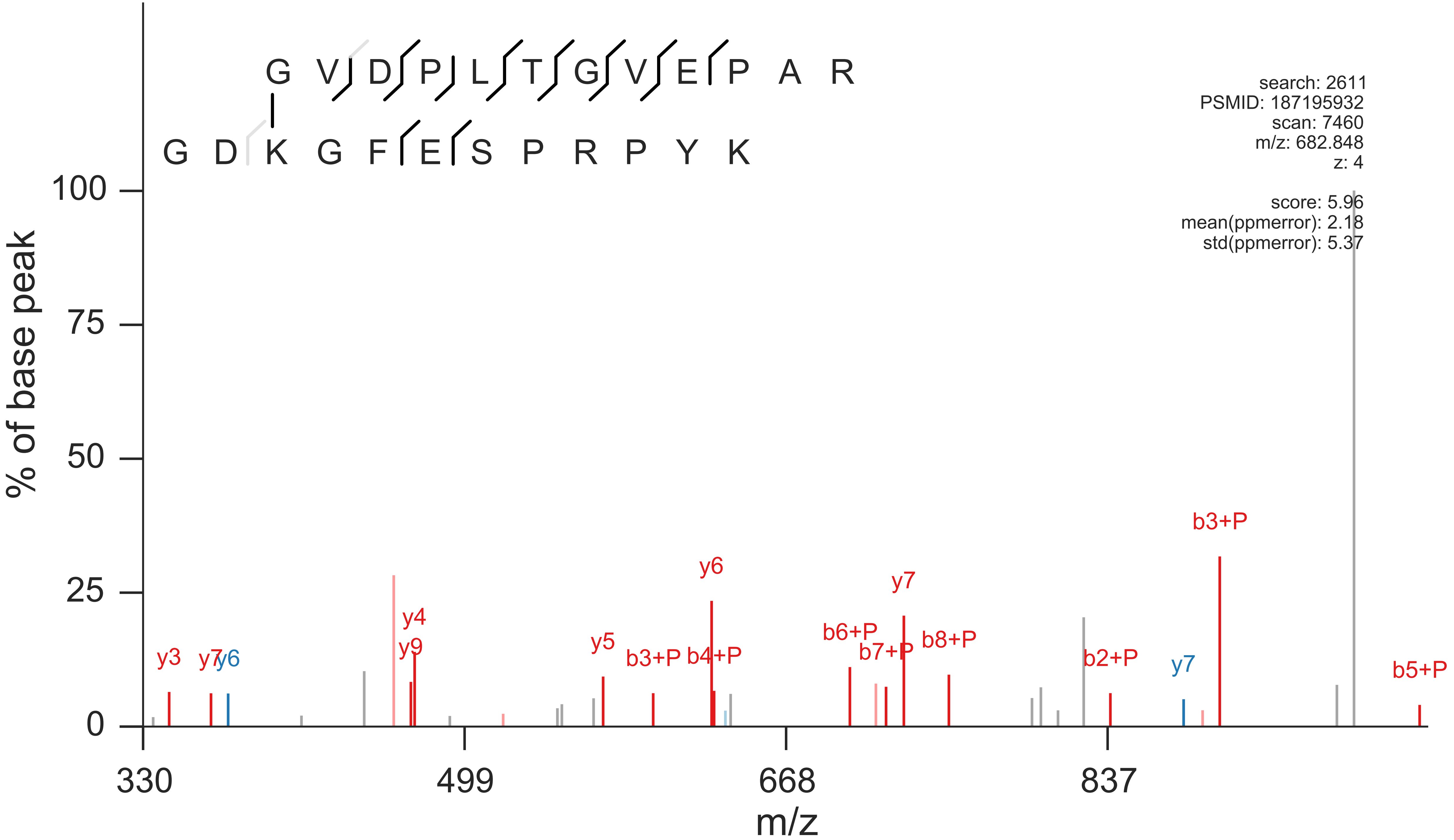

G V D P L T G V E P A R
G D K G F E S P R P Y K
search: 2611
PSMID: 187195932
scan: 7460
m/z: 682.848
z: 4
score: 5.96
mean(ppmerror): 2.18
std(ppmerror): 5.37
% of base peak
100
75
50
25
0
330
499
668
837
m/z
y3
y7
y6
y4
y9
y5
b3+P
b4+P
y6
b6+P
b7+P
y7
b8+P
b2+P
y7
b3+P
b5+P

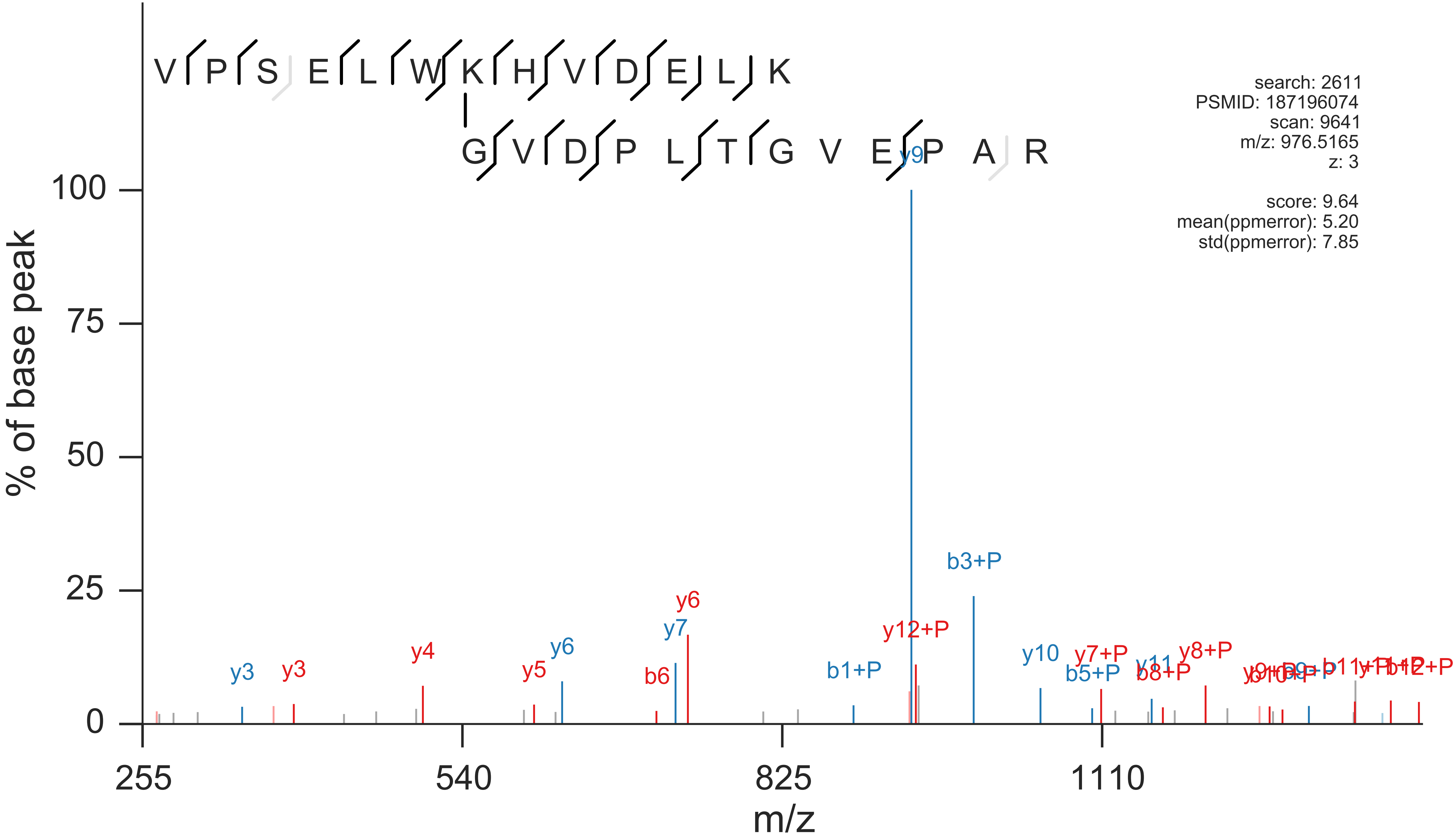

V P S E L W K H V D E L K
G V D P L T G V E P A R
search: 2611
PSMID: 187196074
scan: 9641
m/z: 976.5165
z: 3
score: 9.64
mean(ppmerror): 5.20
std(ppmerror): 7.85
% of base peak
m/z

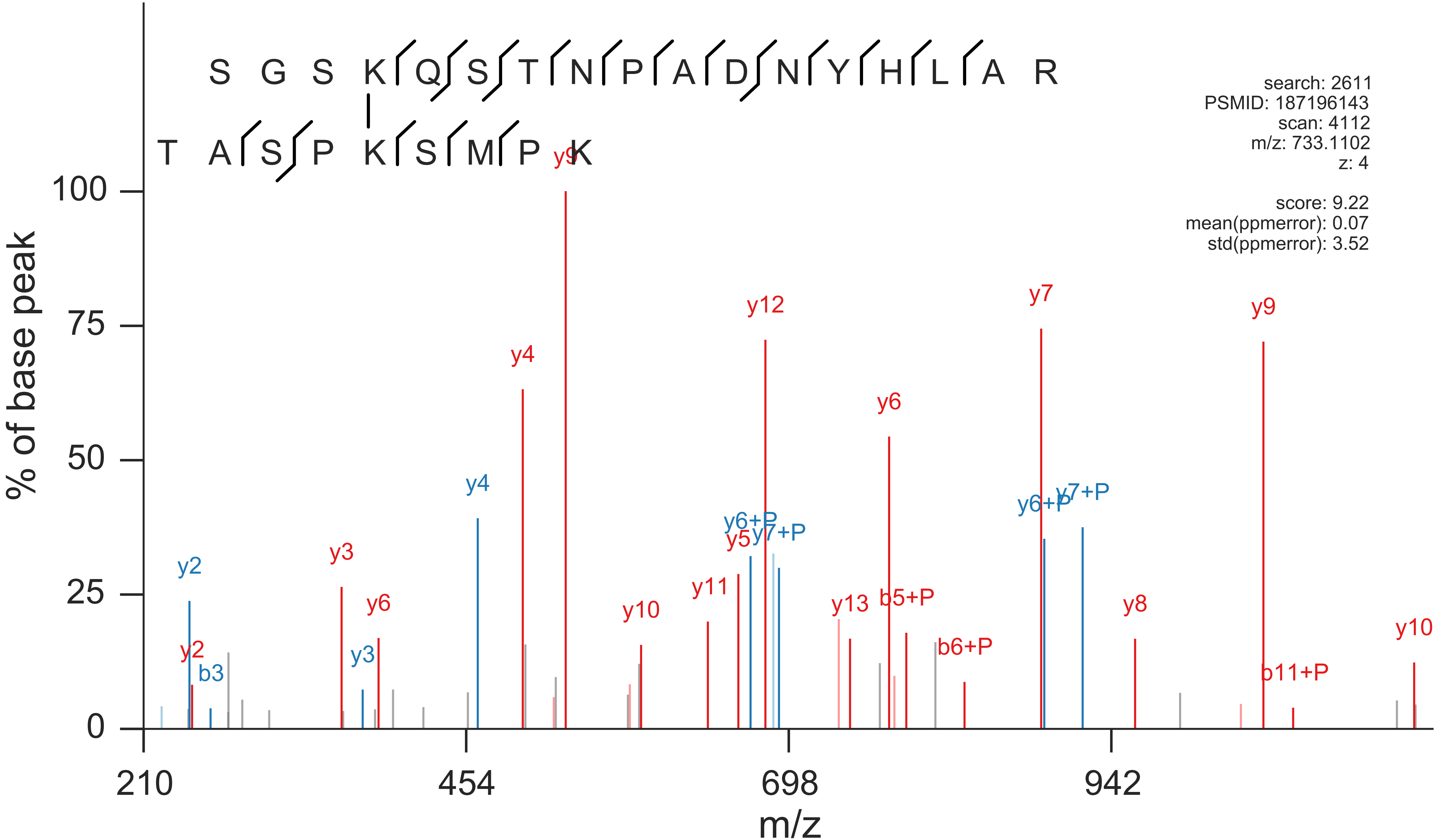

S G S K Q S T N P A D N Y H L A R
T A S P K S M P K
search: 2611
PSMID: 187196143
scan: 4112
m/z: 733.1102
z: 4
score: 9.22
mean(ppmerror): 0.07
std(ppmerror): 3.52
% of base peak
100
75
50
25
0
210
454
698
942
m/z
y2
b3
y3
y6
y4
y9
y10
y11
y5
y12
y6+P
y7+P
y13
b5+P
b6+P
y7
y8
y10
b11+P

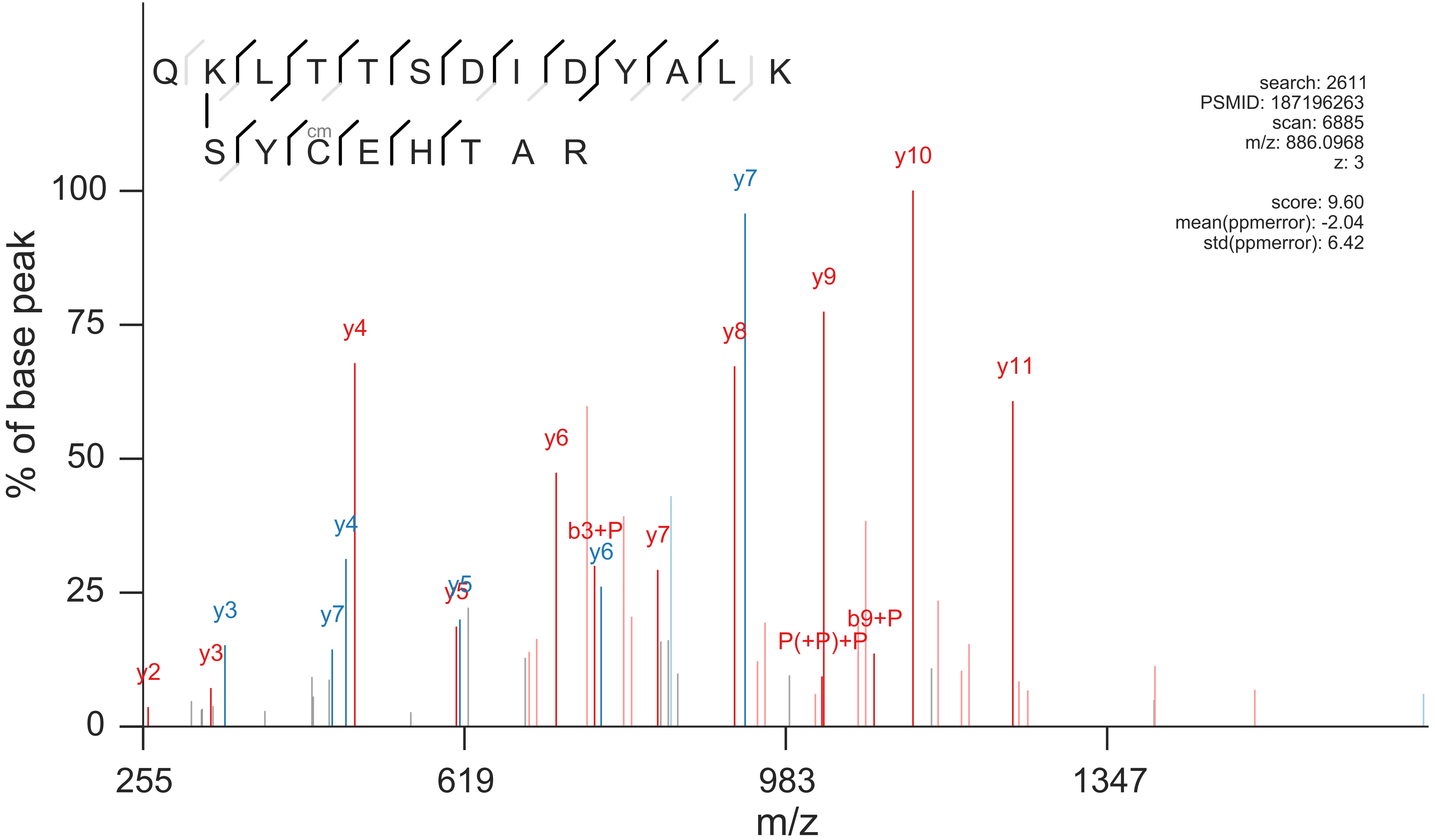

Q K L T T S D I D Y A L K
S Y C E H T A R
cm
search: 2611
PSMID: 187196263
scan: 6885
m/z: 886.0968
z: 3
score: 9.60
mean(ppmerror): -2.04
std(ppmerror): 6.42
% of base peak
m/z
100
75
50
25
0
255
619
983
1347
y2
y3
y3
y7
y4
y4
y5
y5
y6
b3+P
y6
y7
y8
y7
y9
y10
y11
P(+P)+P
b9+P

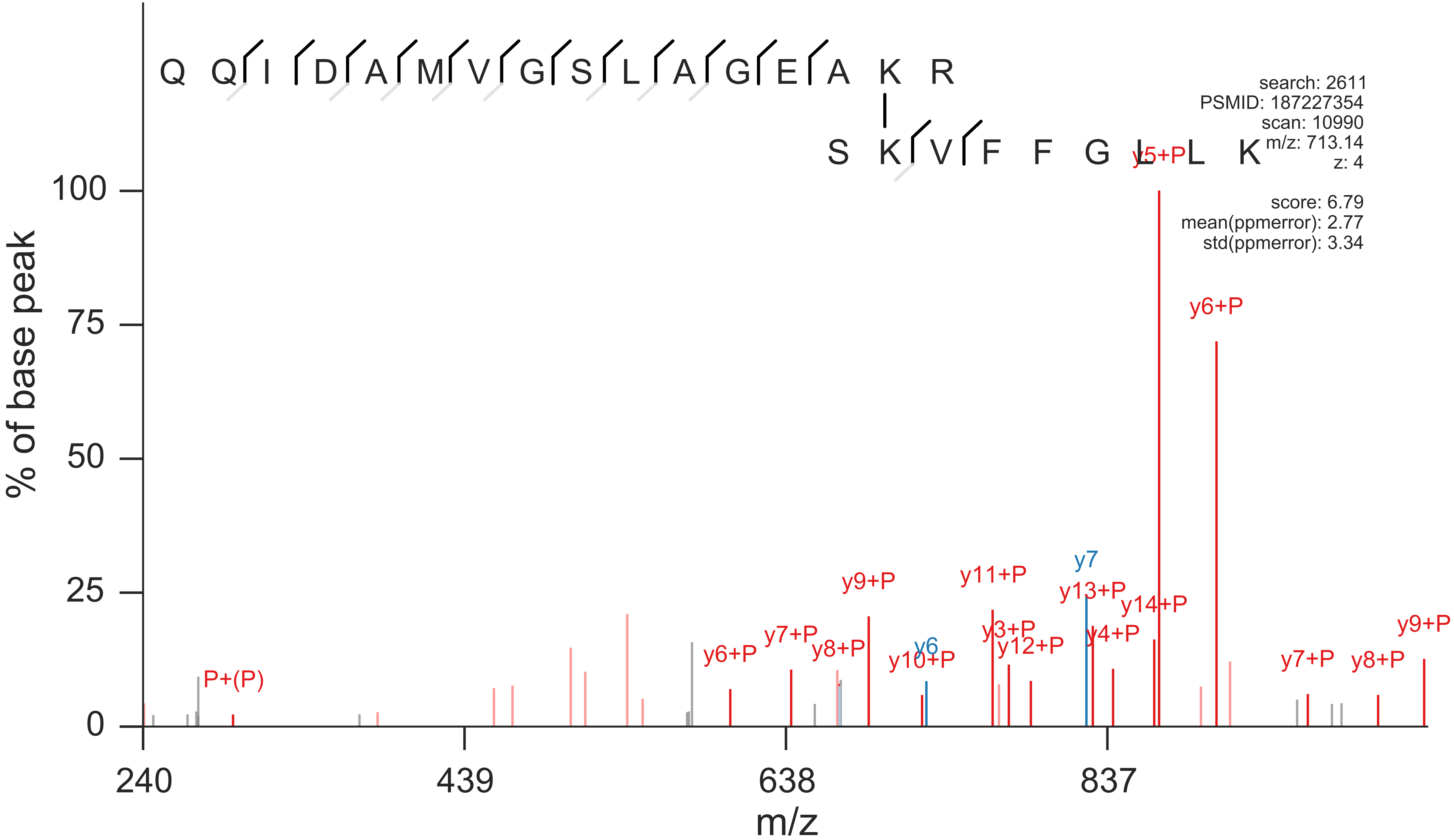

Q Q I D A M V G S L A G E A K R
S K V F F G L L K
search: 2611
PSMID: 187227354
scan: 10990
m/z: 713.14
z: 4
score: 6.79
mean(ppmerror): 2.77
std(ppmerror): 3.34
% of base peak
m/z
0
25
50
75
100
240
439
638
837
P+(P)
y6+P
y7+P
y8+P
y9+P
y6
y10+P
y11+P
y3+P
y12+P
y7
y13+P
y4+P
y14+P
y5+P
y6+P
y7+P
y8+P
y9+P

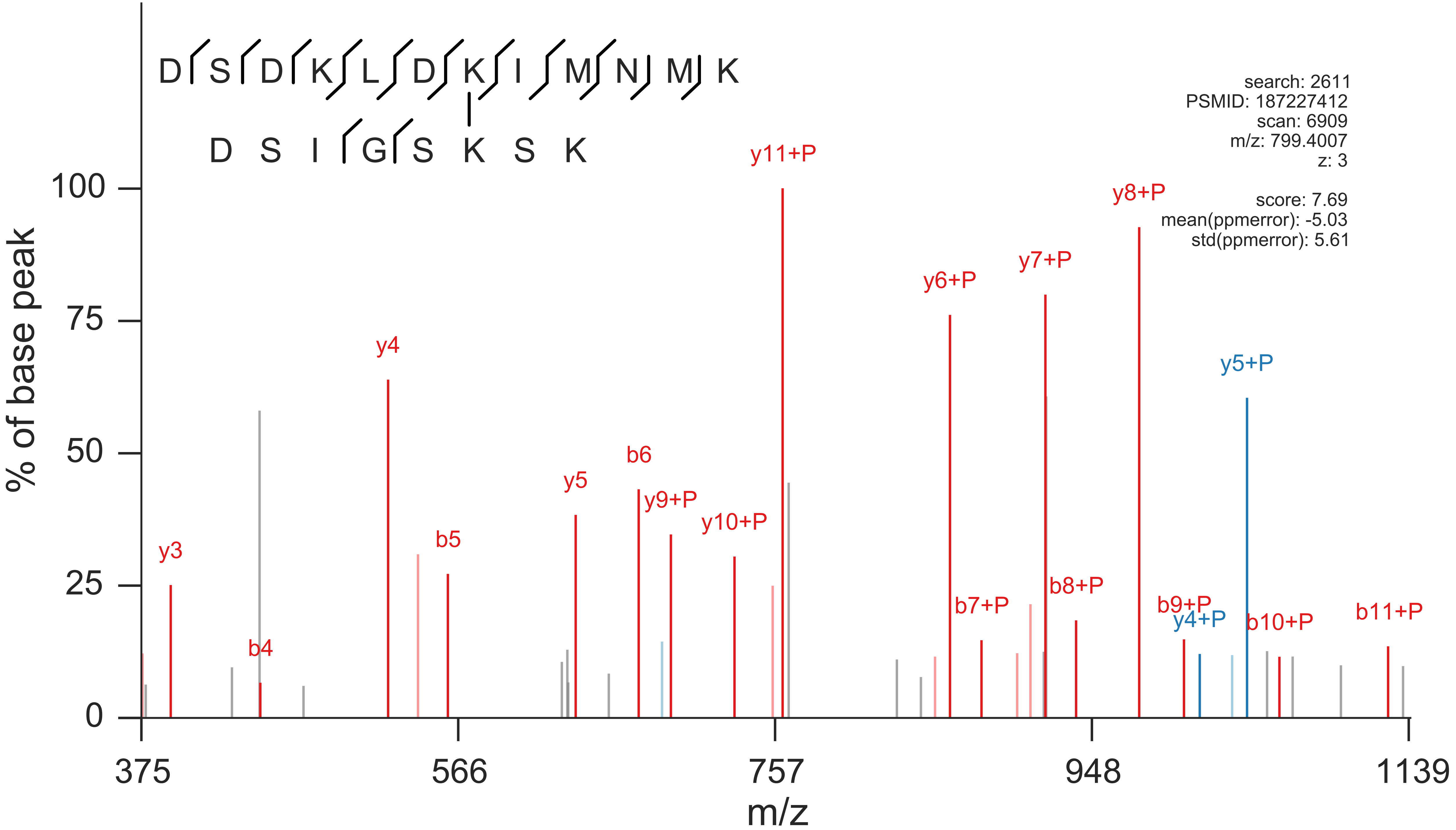

D S D K L D K I M N M K
D S I G S K S K
search: 2611
PSMID: 187227412
scan: 6909
m/z: 799.4007
z: 3
score: 7.69
mean(ppmerror): -5.03
std(ppmerror): 5.61
% of base peak
m/z
0
25
50
75
100
375
566
757
948
1139
y3
b4
y4
b5
y5
b6
y9+P
y10+P
y11+P
y6+P
b7+P
y7+P
b8+P
y8+P
b9+P
y4+P
y5+P
b10+P
b11+P

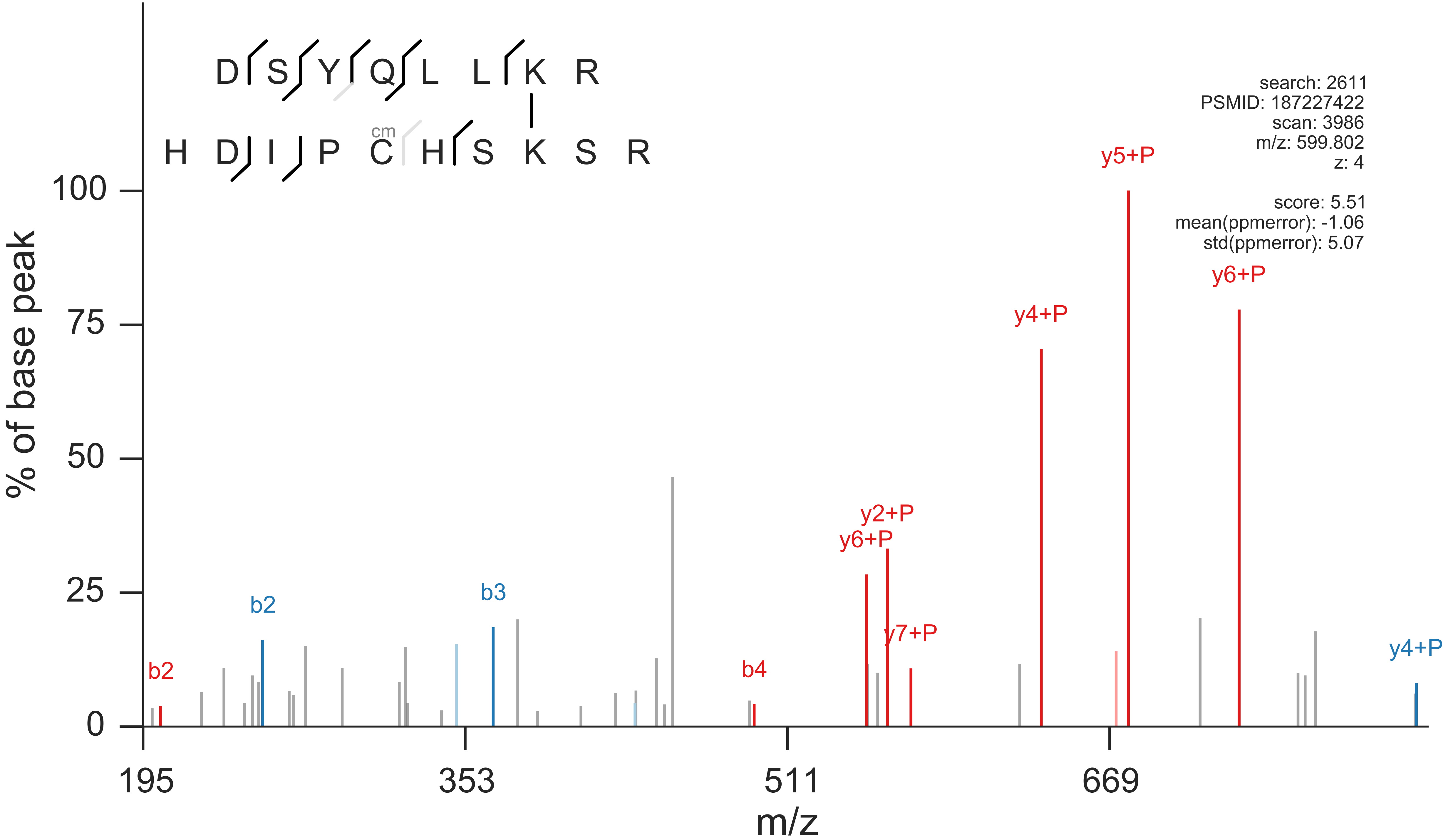

D S Y Q L L K R
H D I P C H S K S R
cm
search: 2611
PSMID: 187227422
scan: 3986
m/z: 599.802
z: 4
score: 5.51
mean(ppmerror): -1.06
std(ppmerror): 5.07
% of base peak
100
75
50
25
0
195
353
511
669
m/z
b2
b2
b3
b4
y6+P
y2+P
y7+P
y4+P
y5+P
y6+P
y4+P

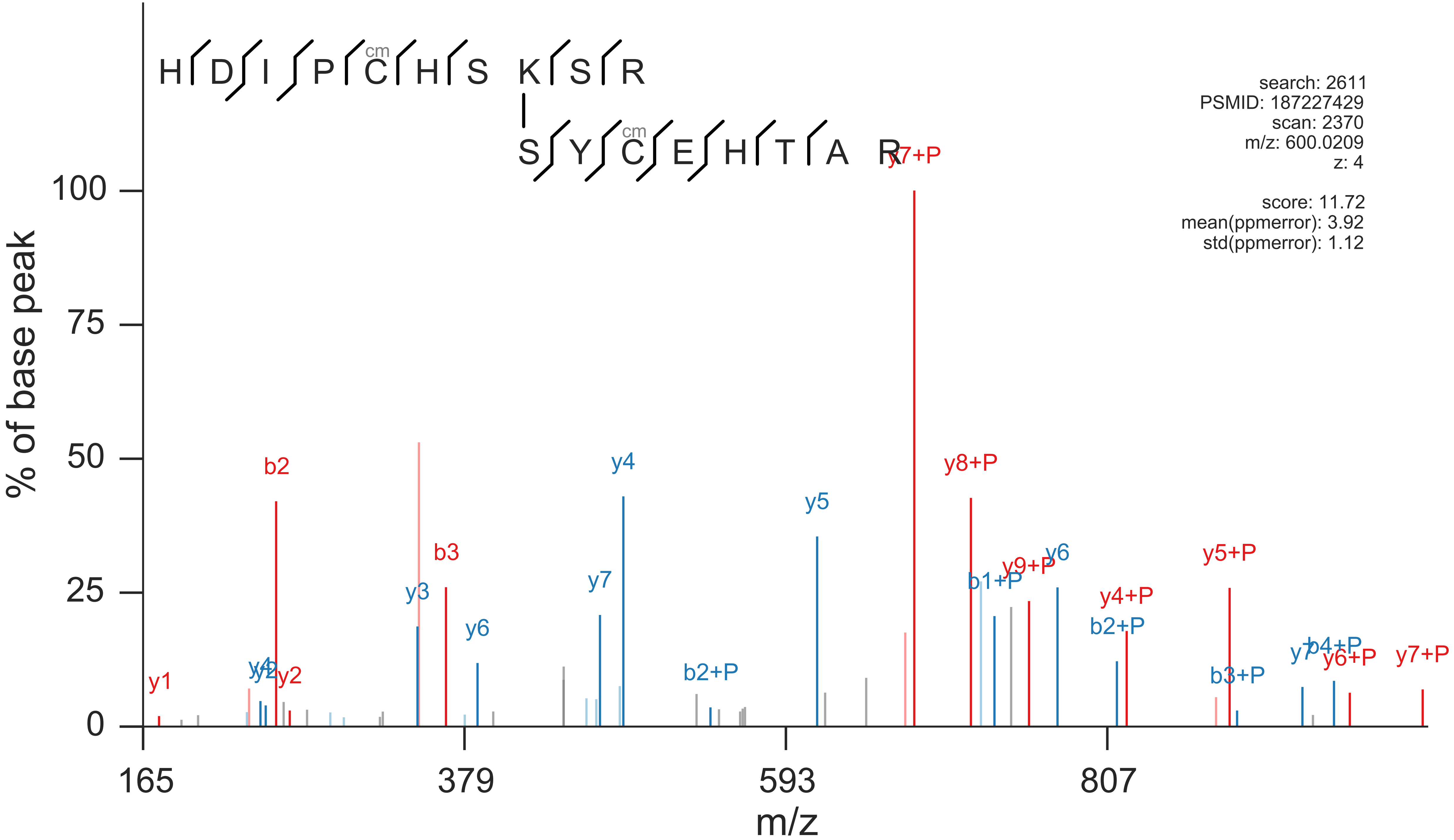
H D I P C H S K S R
cm
S Y C E H T A R
cm
search: 2611
PSMID: 187227429
scan: 2370
m/z: 600.0209
z: 4
score: 11.72
mean(ppmerror): 3.92
std(ppmerror): 1.12
% of base peak
100
75
50
25
0
165
379
593
807
m/z
y7+P
b2
y4
y5
y8+P
b3
y3
y7
y6
b1+P
y9+P
y6
y5+P
y4+P
b2+P
y6
y1
y2
b2+P
b3+P
y6+P
y7+P

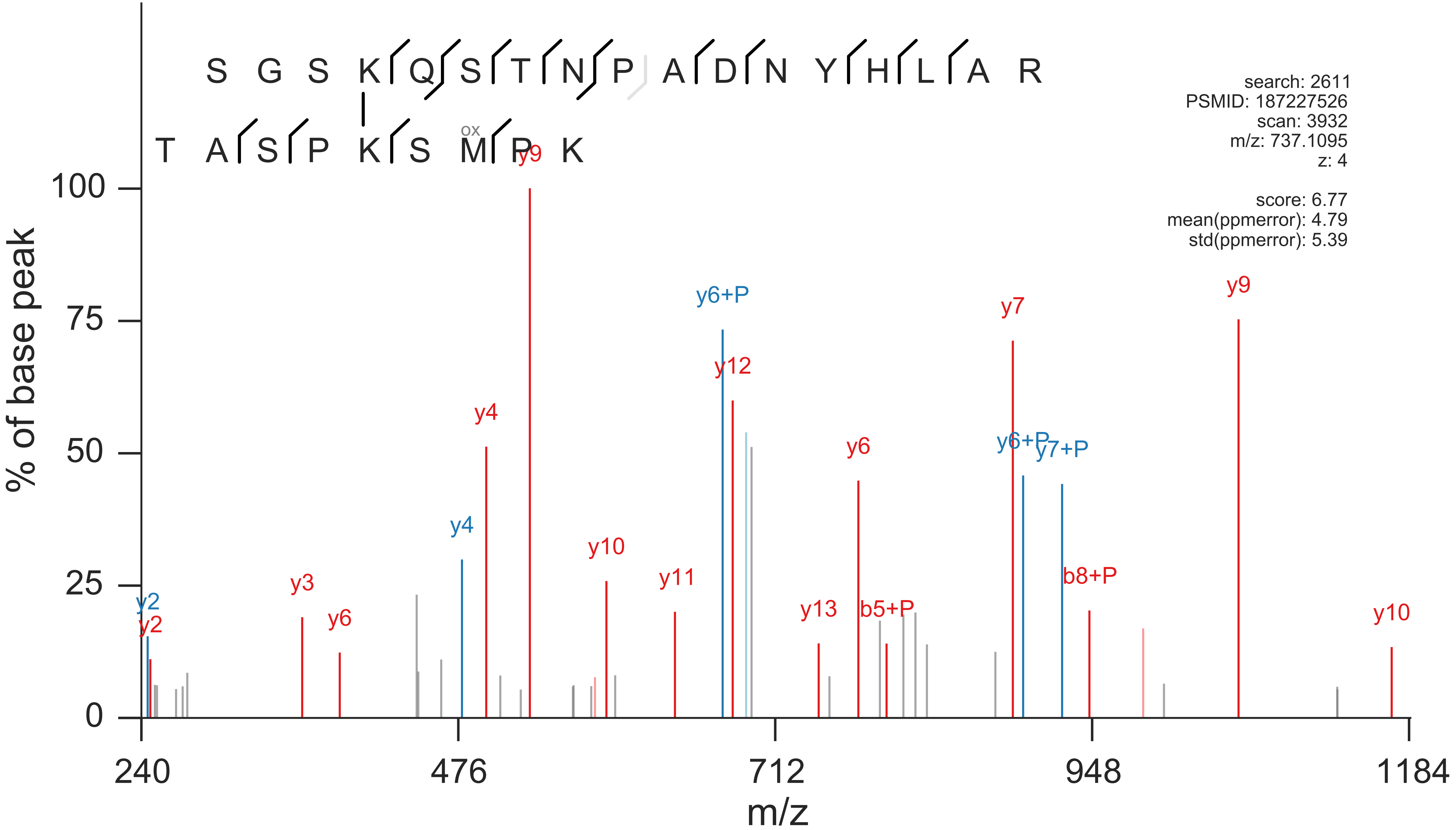

S G S K Q S T N P A D N Y H L A R
T A S P K S M P K
ox
search: 2611
PSMID: 187227526
scan: 3932
m/z: 737.1095
z: 4
score: 6.77
mean(ppmerror): 4.79
std(ppmerror): 5.39
% of base peak
m/z
0
25
50
75
100
240
476
712
948
1184
y2
y2
y3
y6
y4
y4
y9
y10
y11
y6+P
y12
y13
y6
b5+P
y7
y6+P
y7+P
b8+P
y9
y10

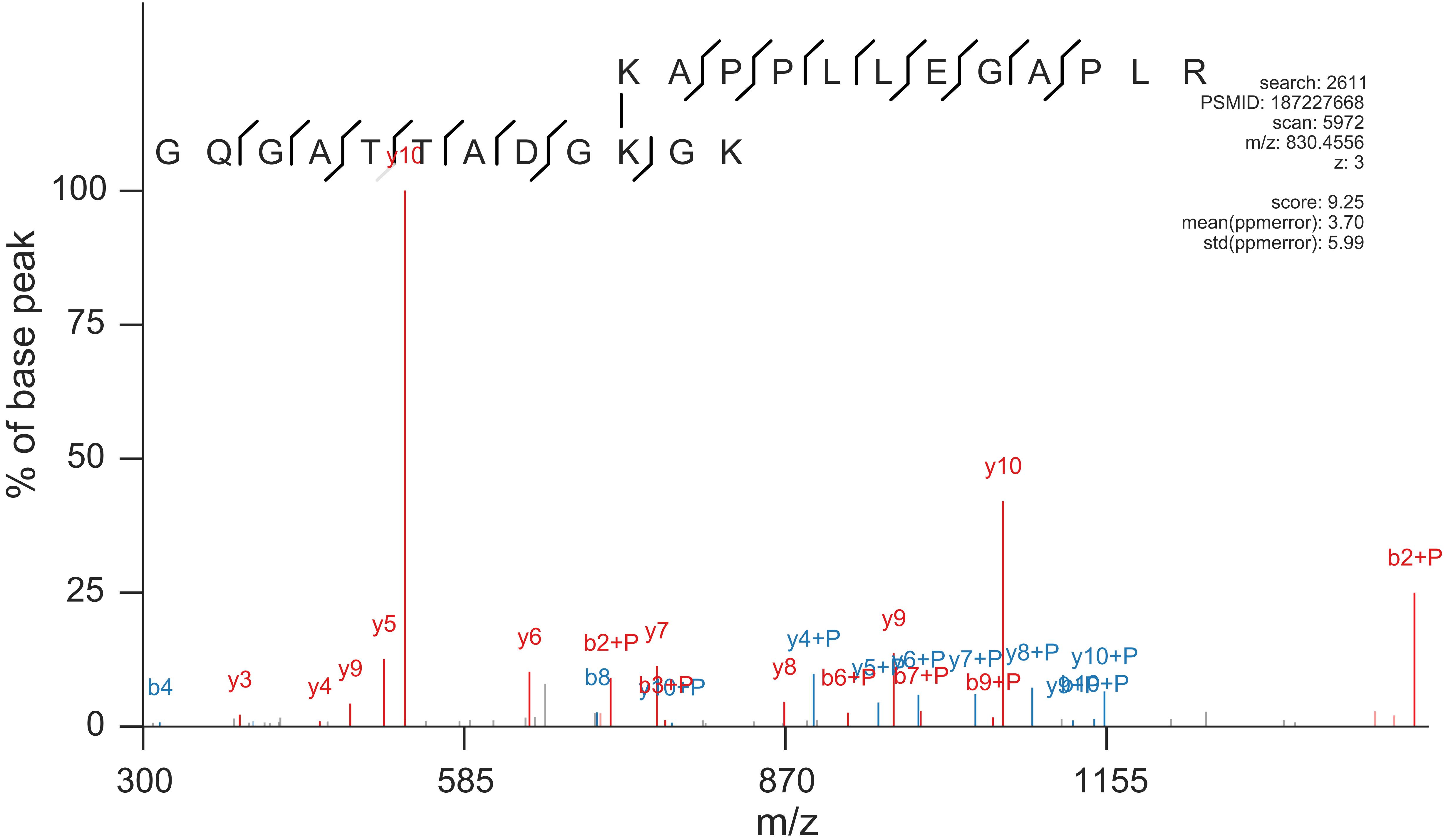
K A P P L L E G A P L R
G Q G A T T A D G K G K
search: 2611
PSMID: 187227668
scan: 5972
m/z: 830.4556
z: 3
score: 9.25
mean(ppmerror): 3.70
std(ppmerror): 5.99
% of base peak
m/z
100
75
50
25
0
300
585
870
1155
b4
y3
y4
y9
y5
y10
y6
b2+P
b8
y7
y8
y4+P
b6+P
y9
y6+P
b7+P
y7+P
y8+P
b9+P
y10+P
b2+P

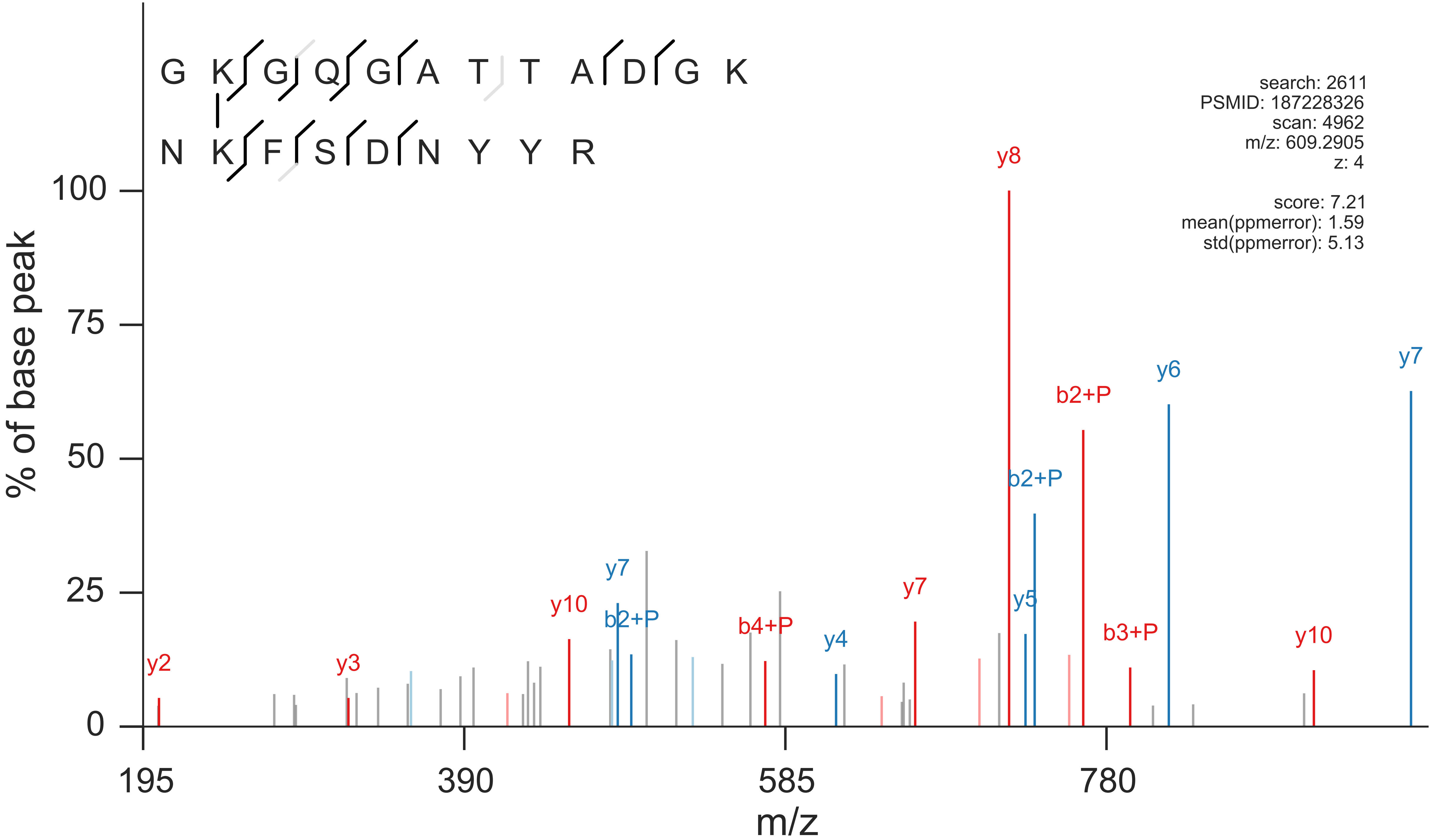

G K G Q G A T T A D G K
N K F S D N Y Y R
search: 2611
PSMID: 187228326
scan: 4962
m/z: 609.2905
z: 4
score: 7.21
mean(ppmerror): 1.59
std(ppmerror): 5.13
% of base peak
m/z
y2
y3
y10
y7
b2+P
b4+P
y4
y7
y8
y5
b2+P
b2+P
b3+P
y6
y10
y7

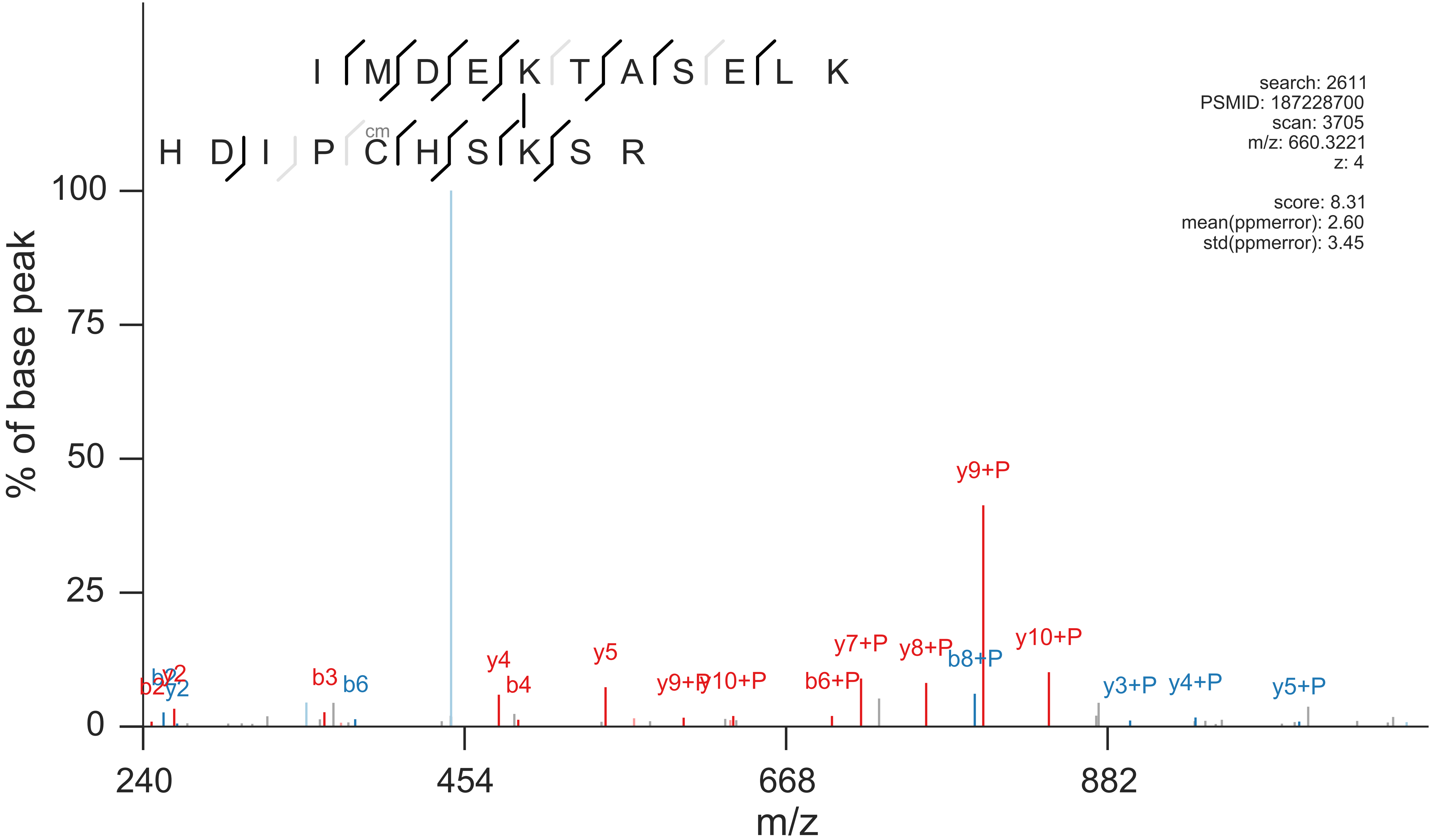

I M D E K T A S E L K
H D I P C H S K S R
cm
search: 2611
PSMID: 187228700
scan: 3705
m/z: 660.3221
z: 4
score: 8.31
mean(ppmerror): 2.60
std(ppmerror): 3.45
% of base peak
100
75
50
25
0
240
454
668
882
m/z
y9+P
y10+P
y7+P
y8+P
b8+P
y4
y5
b3
b6
b4
y9+P
y10+P
b6+P
y3+P
y4+P
y5+P
b2
y2
y2

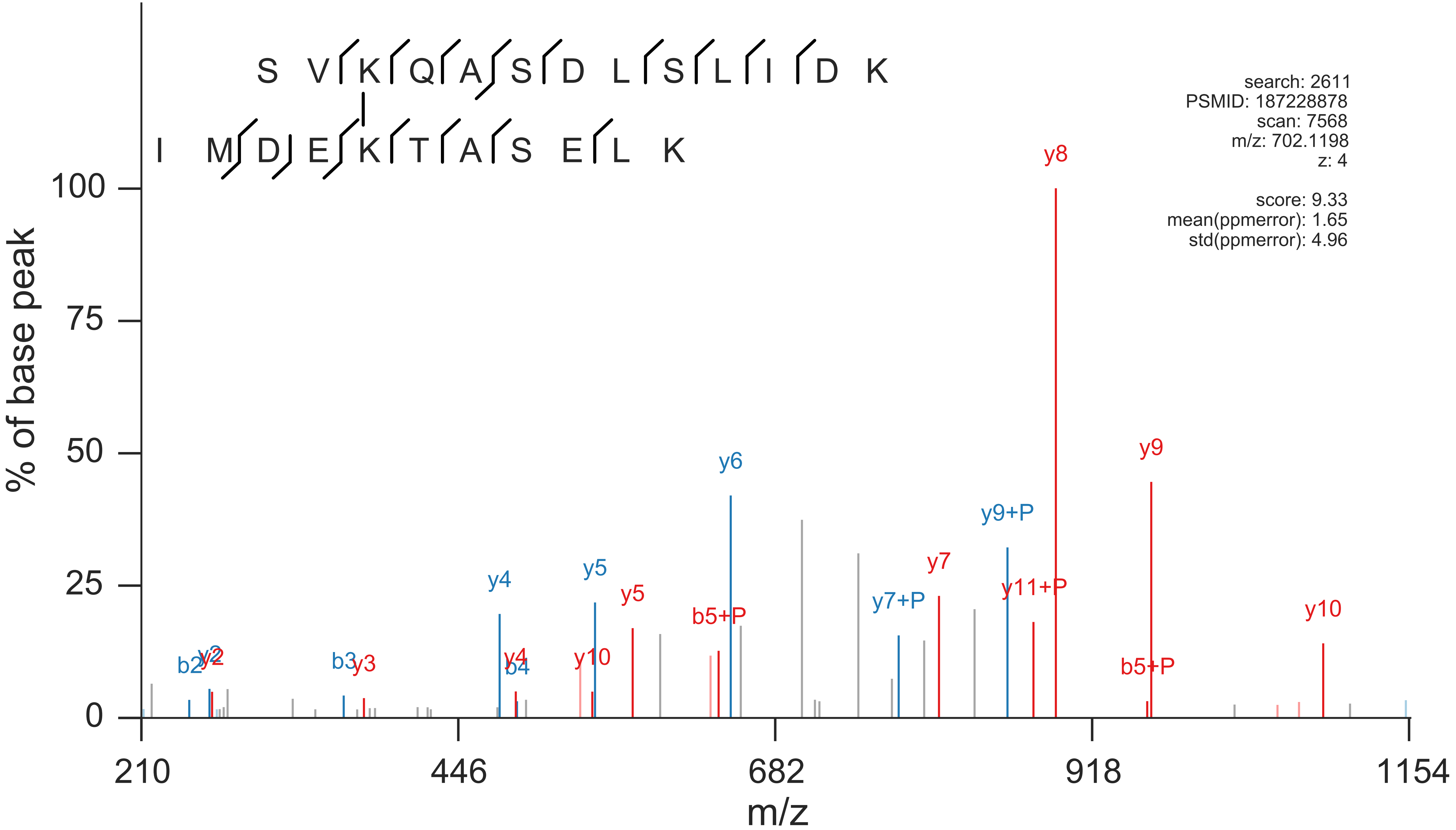

S V K Q A S D L S L I D K
I M D E K T A S E L K
search: 2611
PSMID: 187228878
scan: 7568
m/z: 702.1198
z: 4
score: 9.33
mean(ppmerror): 1.65
std(ppmerror): 4.96
% of base peak
m/z
0
25
50
75
100
210
446
682
918
1154
b2
y2
y2
b3
y3
y4
y4
b4
y5
y10
y5
b5+P
y6
y7+P
y7
y9+P
y11+P
y8
b5+P
y9
y10

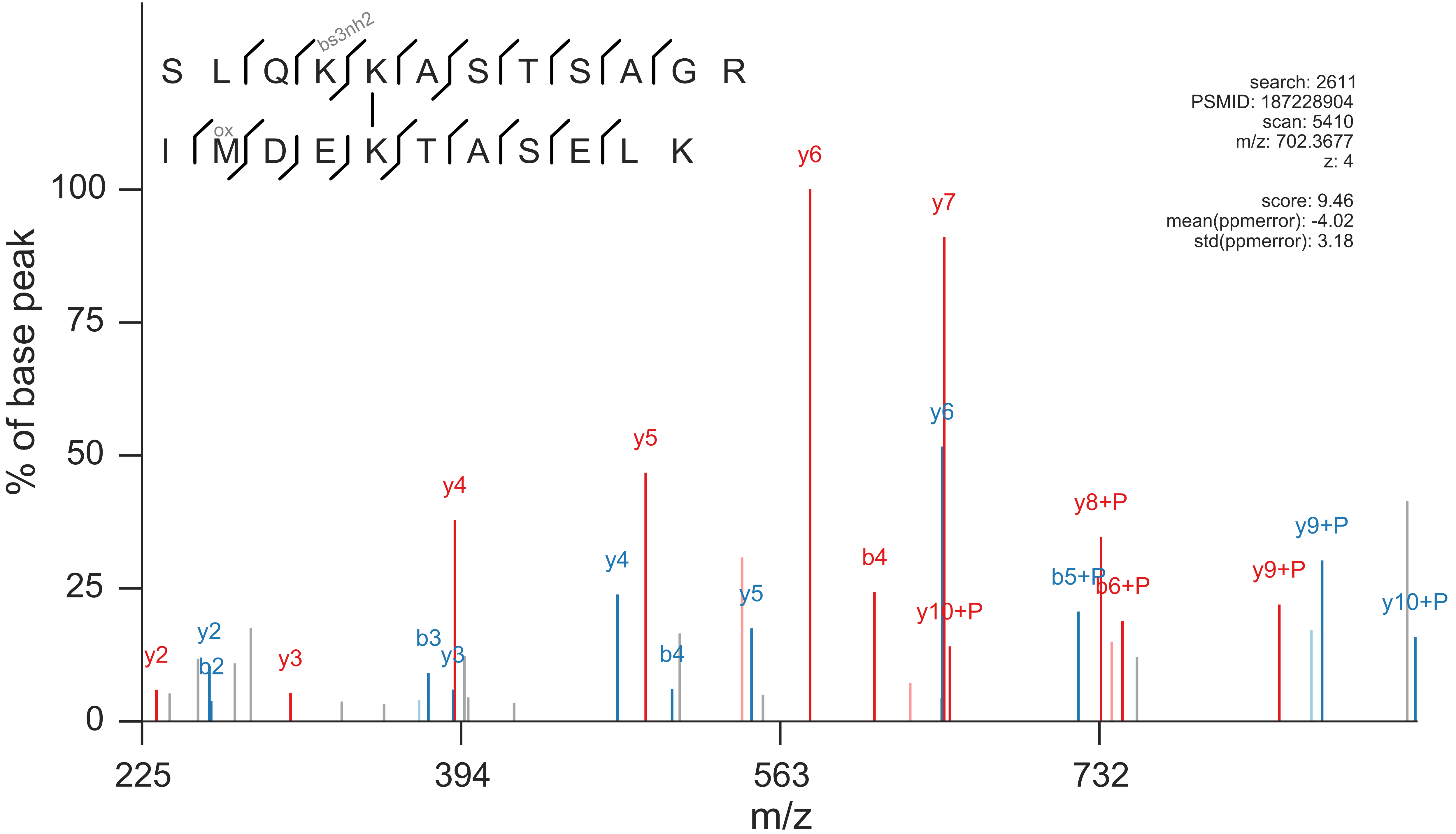

S L Q K K A S T S A G R
bs3nh2
I M D E K T A S E L K
ox
search: 2611
PSMID: 187228904
scan: 5410
m/z: 702.3677
z: 4
score: 9.46
mean(ppmerror): -4.02
std(ppmerror): 3.18
% of base peak
m/z
100
75
50
25
0
225
394
563
732
y2
y2
b2
y3
b3
y3
y4
y4
y5
b4
y5
y6
b4
y7
y6
y10+P
b5+P
y8+P
b6+P
y9+P
y9+P
y10+P

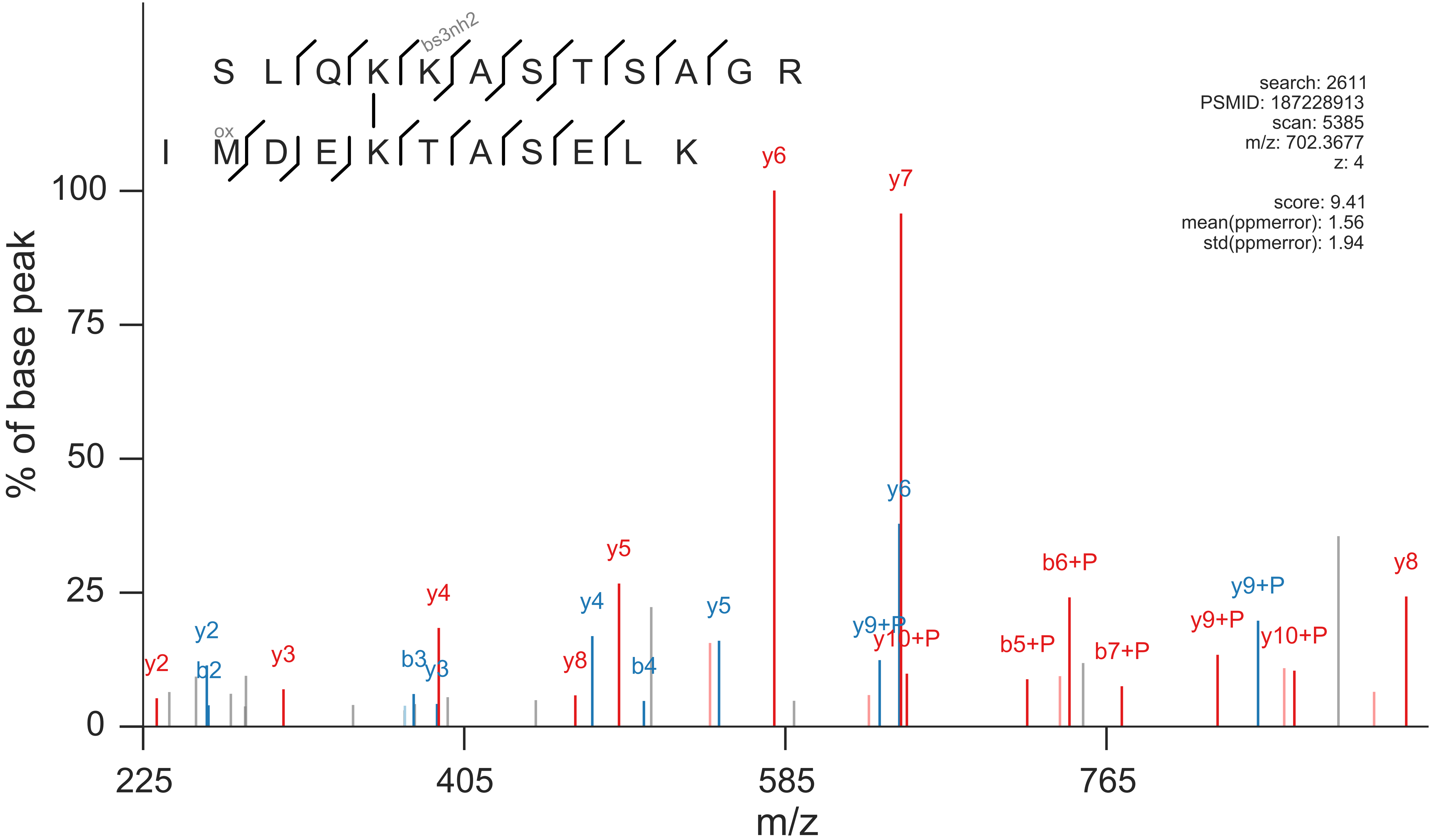

bs3nh2
S L Q K K A S T S A G R
ox
I M D E K T A S E L K
search: 2611
PSMID: 187228913
scan: 5385
m/z: 702.3677
z: 4
score: 9.41
mean(ppmerror): 1.56
std(ppmerror): 1.94
% of base peak
m/z
y2
y2
b2
y3
b3
y3
y4
y8
y4
y5
b4
y5
y6
y9+P
y6
y7
y10+P
b5+P
b6+P
b7+P
y9+P
y9+P
y10+P
y8

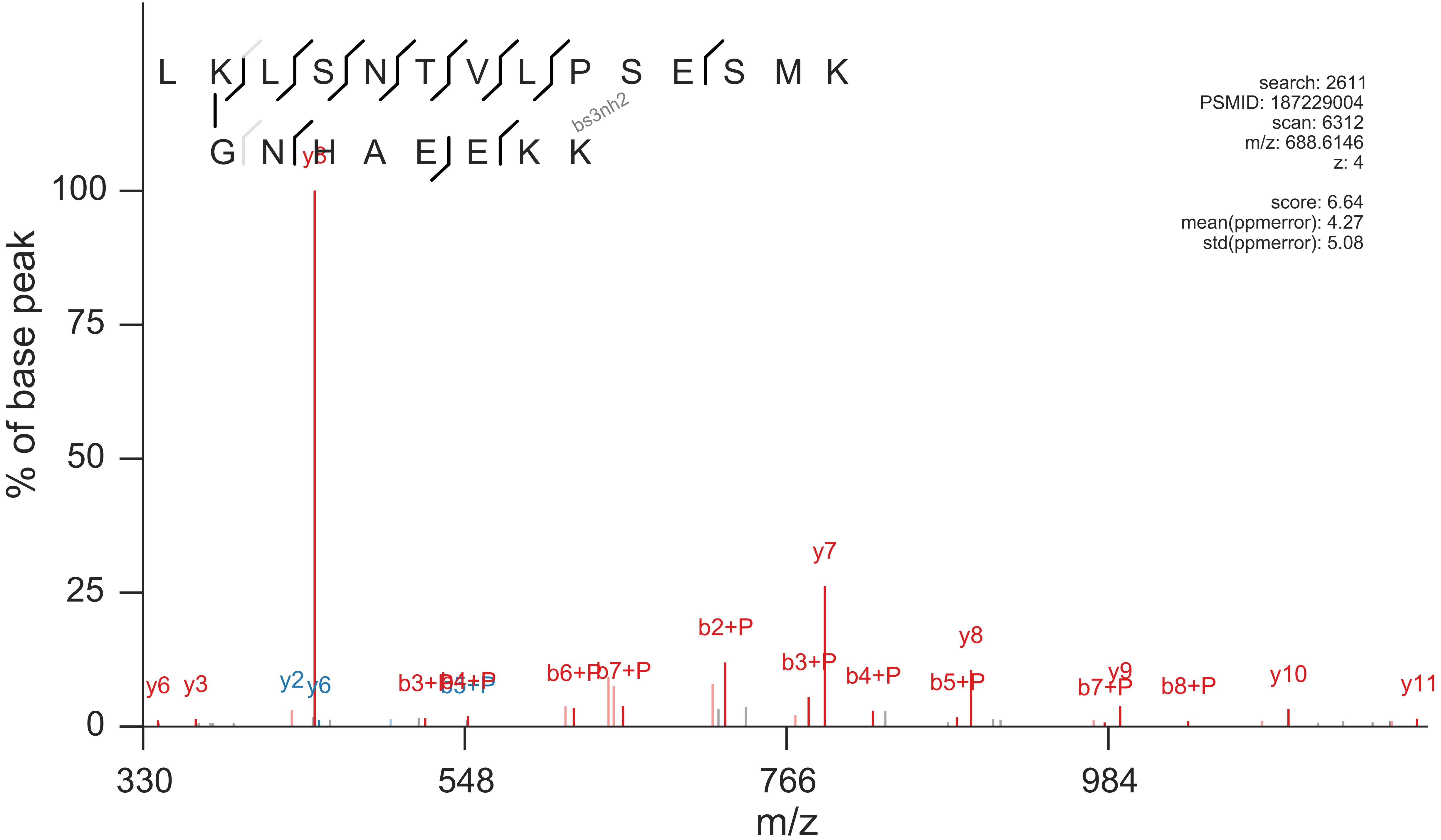

L K L S N T V L P S E S M K
G N H A E E K K
bs3nh2
search: 2611
PSMID: 187229004
scan: 6312
m/z: 688.6146
z: 4
score: 6.64
mean(ppmerror): 4.27
std(ppmerror): 5.08
% of base peak
100
75
50
25
0
330
548
766
984
m/z
y3
y7
b2+P
y8
b3+P
b4+P
b5+P
b6+P
b7+P
y9
b8+P
y10
y11
y6
y2
b3+P
b4+P
b5+P

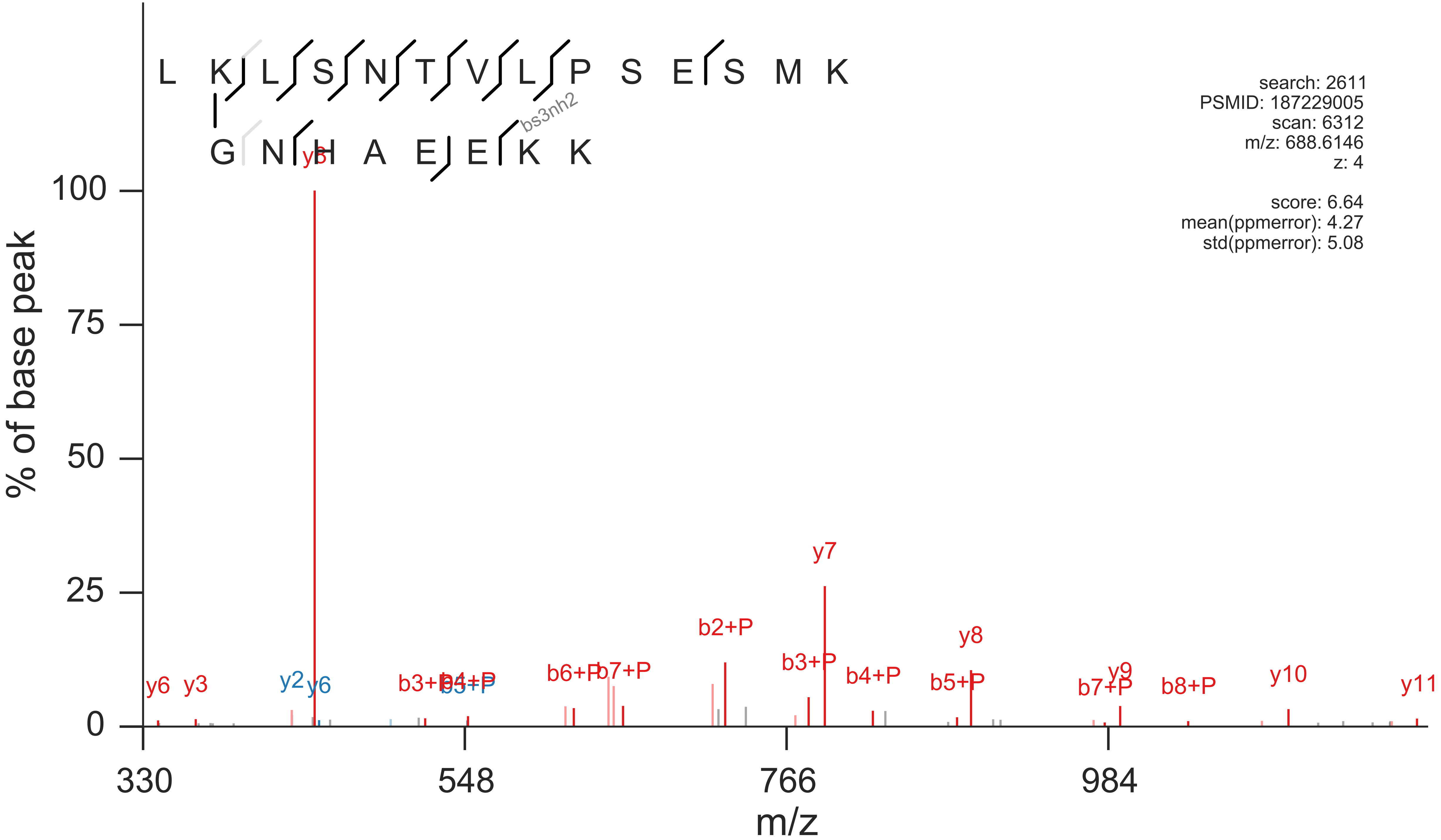
L K L S N T V L P S E S M K
G N H A E E K K
bs3nh2
search: 2611
PSMID: 187229005
scan: 6312
m/z: 688.6146
z: 4
score: 6.64
mean(ppmerror): 4.27
std(ppmerror): 5.08
% of base peak
m/z
0
25
50
75
100
330
548
766
984
y3
y7
b2+P
b3+P
y8
b4+P
b5+P
y9
b7+P
b8+P
y10
y11
y6
y3
y2
y6
b6+P
b7+P

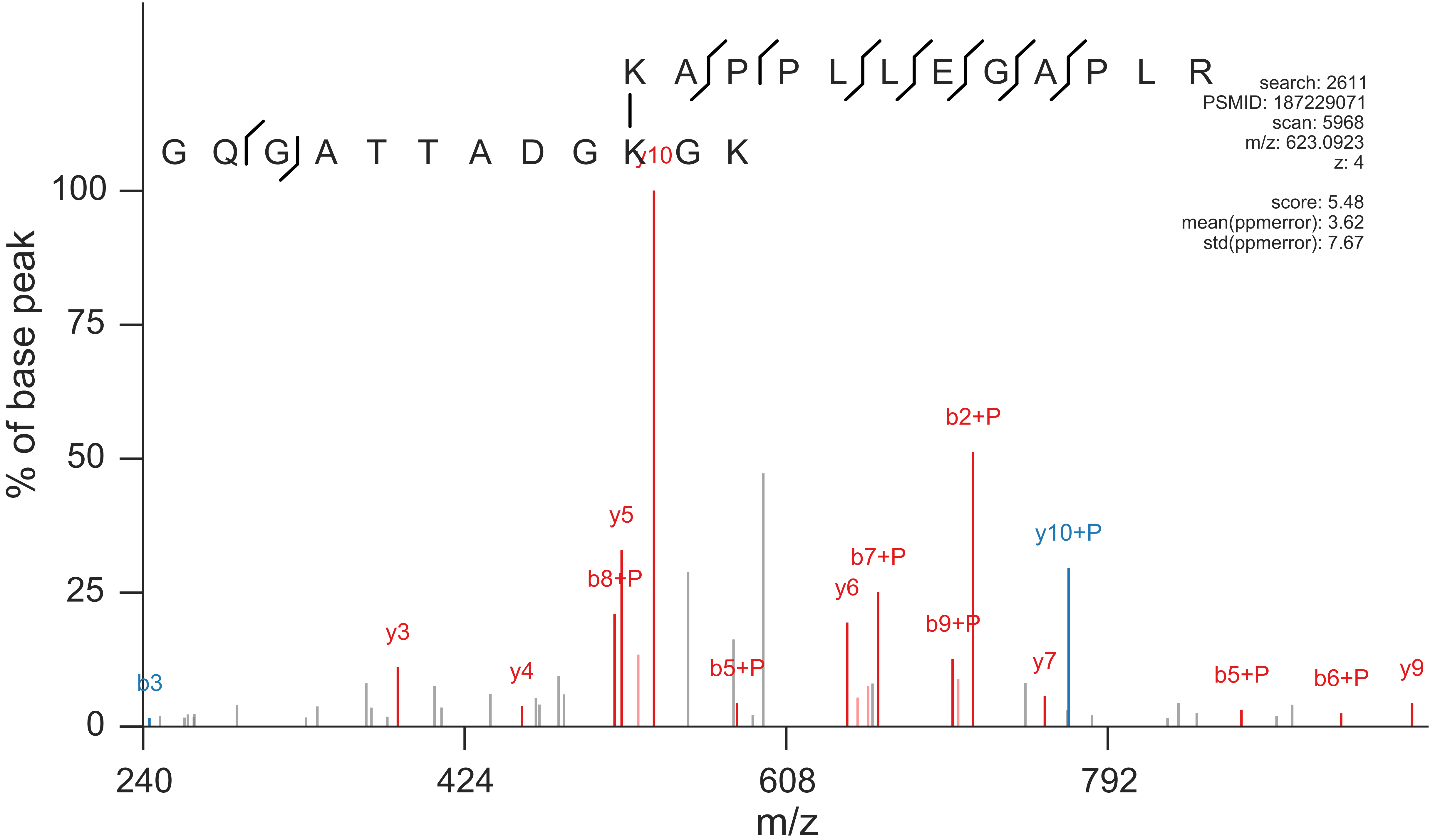

K A P P L L E G A P L R
G Q G A T T A D G K G K
search: 2611
PSMID: 187229071
scan: 5968
m/z: 623.0923
z: 4
score: 5.48
mean(ppmerror): 3.62
std(ppmerror): 7.67
% of base peak
m/z
0
25
50
75
100
240
424
608
792
b3
y3
y4
b8+P
y5
y10
b5+P
y6
b7+P
b9+P
b2+P
y7
y10+P
b5+P
b6+P
y9

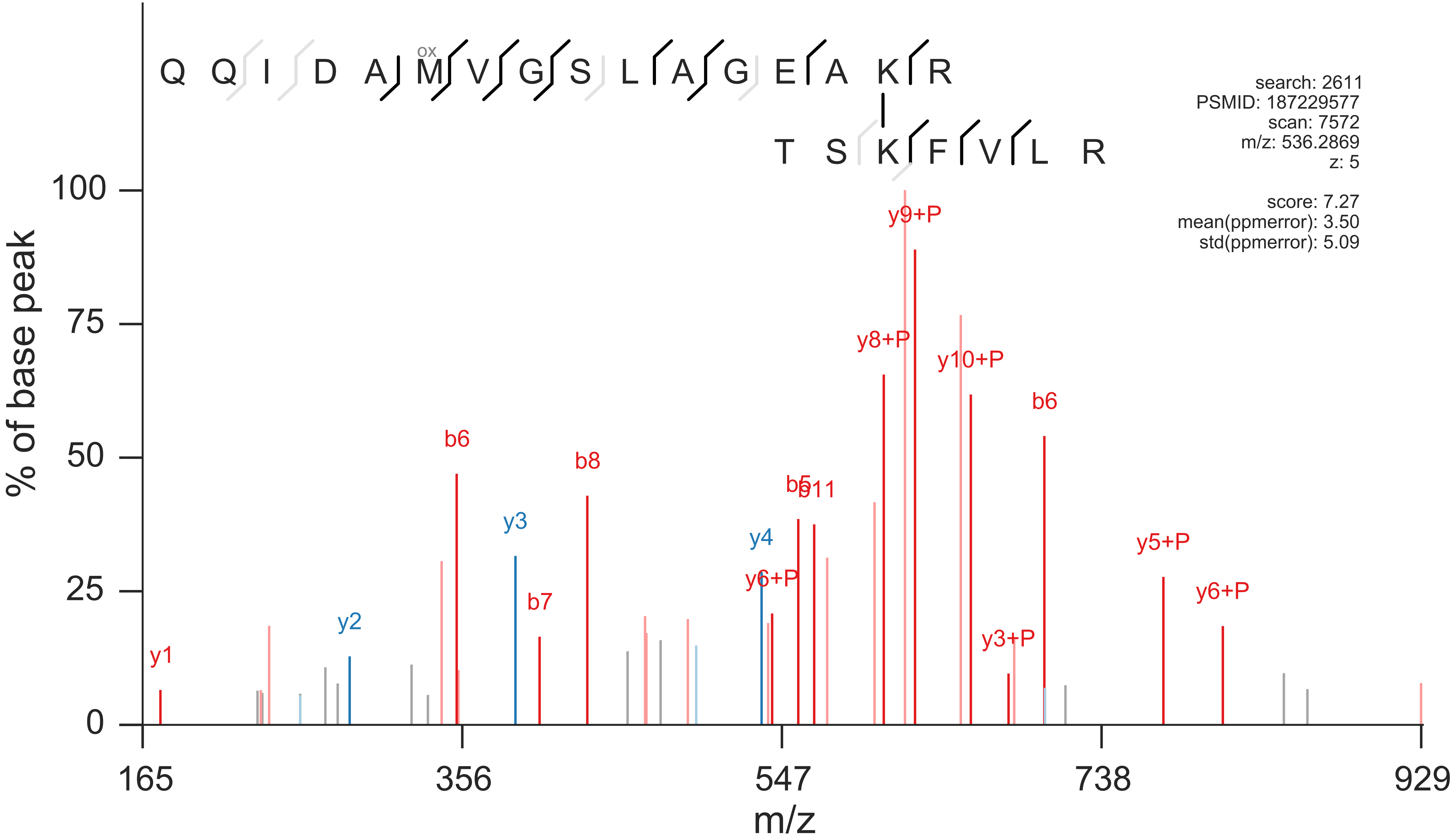

Q Q I D A M V G S L A G E A K R
ox
T S K F V L R
search: 2611
PSMID: 187229577
scan: 7572
m/z: 536.2869
z: 5
score: 7.27
mean(ppmerror): 3.50
std(ppmerror): 5.09
% of base peak
100
75
50
25
0
165
356
547
738
929
m/z
y1
y2
b6
y3
b7
b8
y4
y6+P
b5
b11
y8+P
y9+P
y10+P
y3+P
b6
y5+P
y6+P

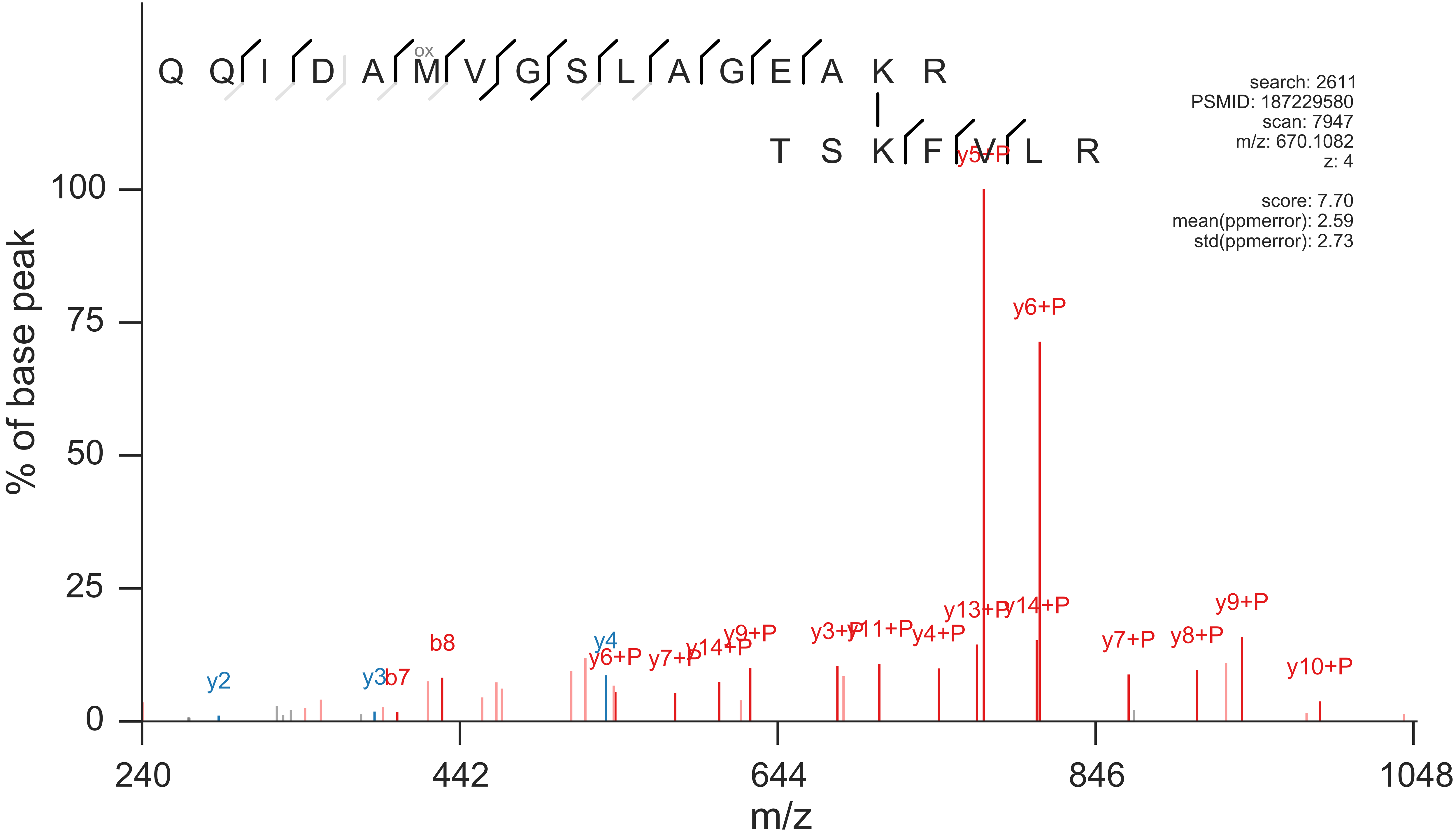

Q Q I D A M V G S L A G E A K R
ox
T S K F V L R
search: 2611
PSMID: 187229580
scan: 7947
m/z: 670.1082
z: 4
score: 7.70
mean(ppmerror): 2.59
std(ppmerror): 2.73
% of base peak
m/z
y5+P
y6+P
y13+P
y14+P
y9+P
y3+P
y11+P
y4+P
y7+P
y8+P
y10+P
b8
b7
y2
y3
y4

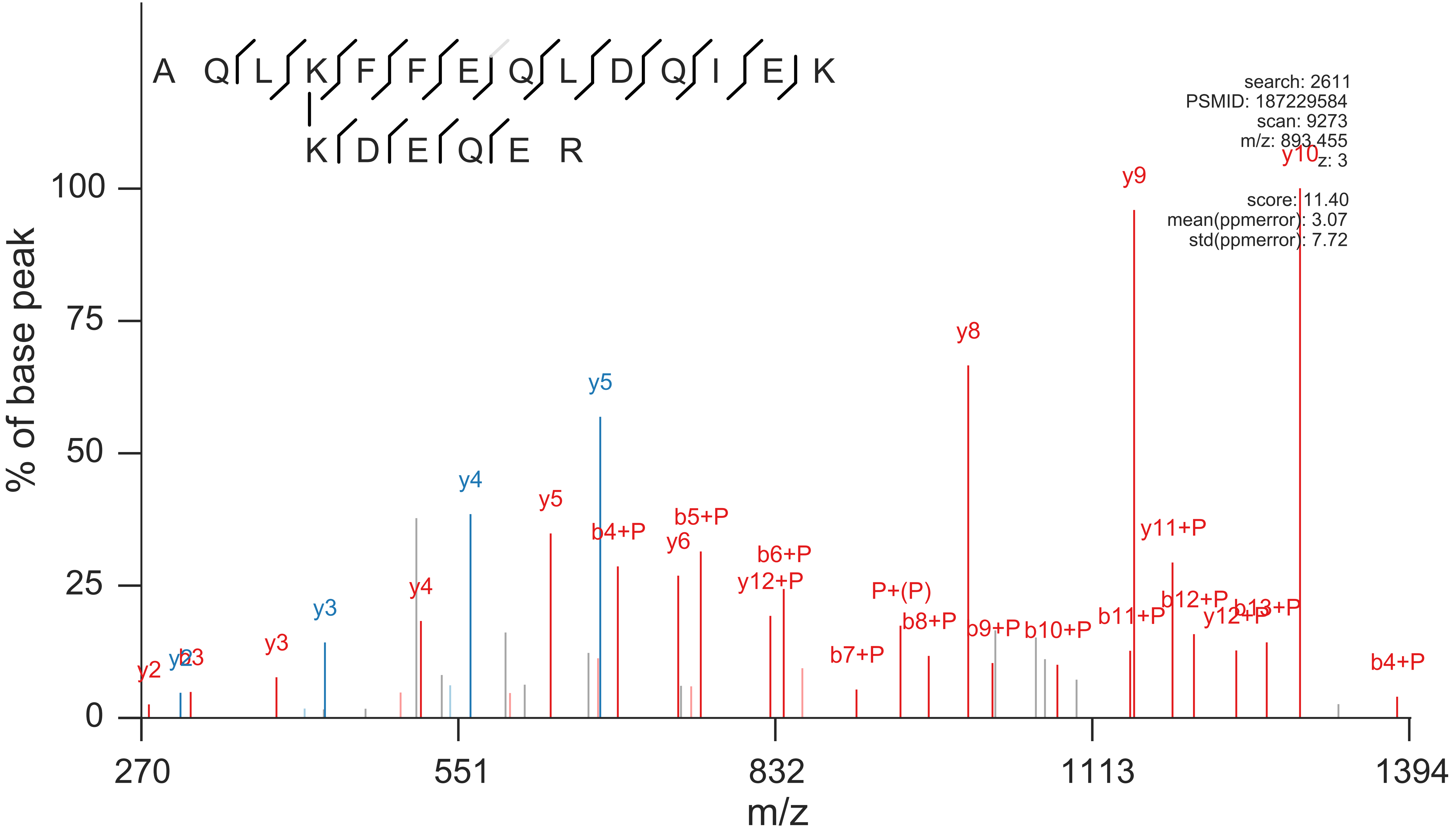

A Q L K F F E Q L D Q I E K
K D E Q E R
search: 2611
PSMID: 187229584
scan: 9273
m/z: 893.455
z: 3
score: 11.40
mean(ppmerror): 3.07
std(ppmerror): 7.72
% of base peak
m/z
0
25
50
75
100
270
551
832
1113
1394
y2
y2
b3
y3
y3
y4
y4
y5
y5
b4+P
y6
b5+P
b6+P
y12+P
b7+P
P+(P)
b8+P
y8
b9+P
b10+P
b11+P
y9
y11+P
b12+P
y12+P
b13+P
y10
b4+P

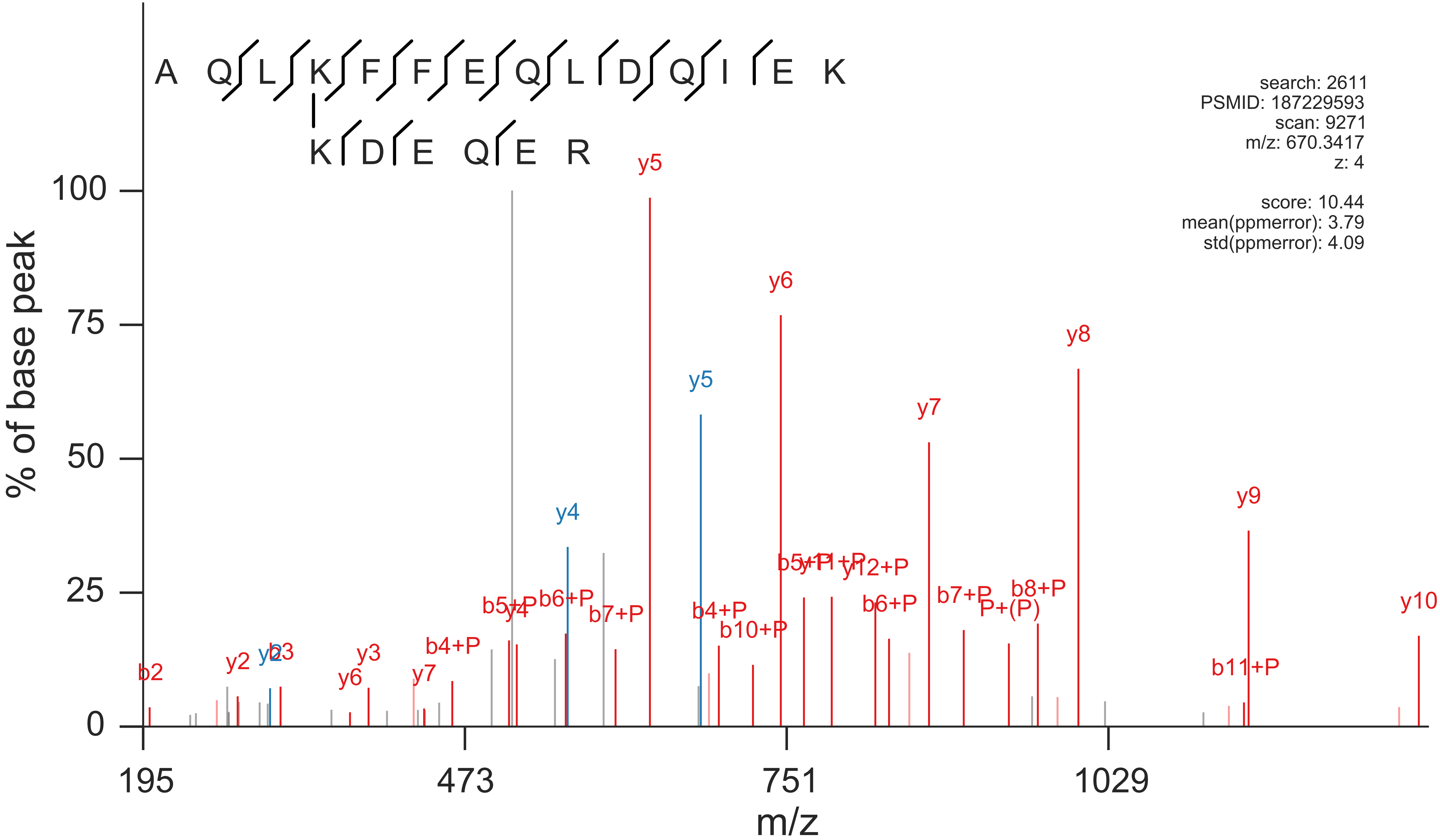

A Q L K F F E Q L D Q I E K
K D E Q E R
search: 2611
PSMID: 187229593
scan: 9271
m/z: 670.3417
z: 4
score: 10.44
mean(ppmerror): 3.79
std(ppmerror): 4.09
% of base peak
m/z
0
25
50
75
100
195
473
751
1029
y5
y6
y8
y7
y9
y10
y4
y5
b2
y2
y3
y6
y7
b4+P
b6+P
b7+P
b4+P
b10+P
b6+P
b7+P
b8+P
P+(P)
b11+P
y12+P

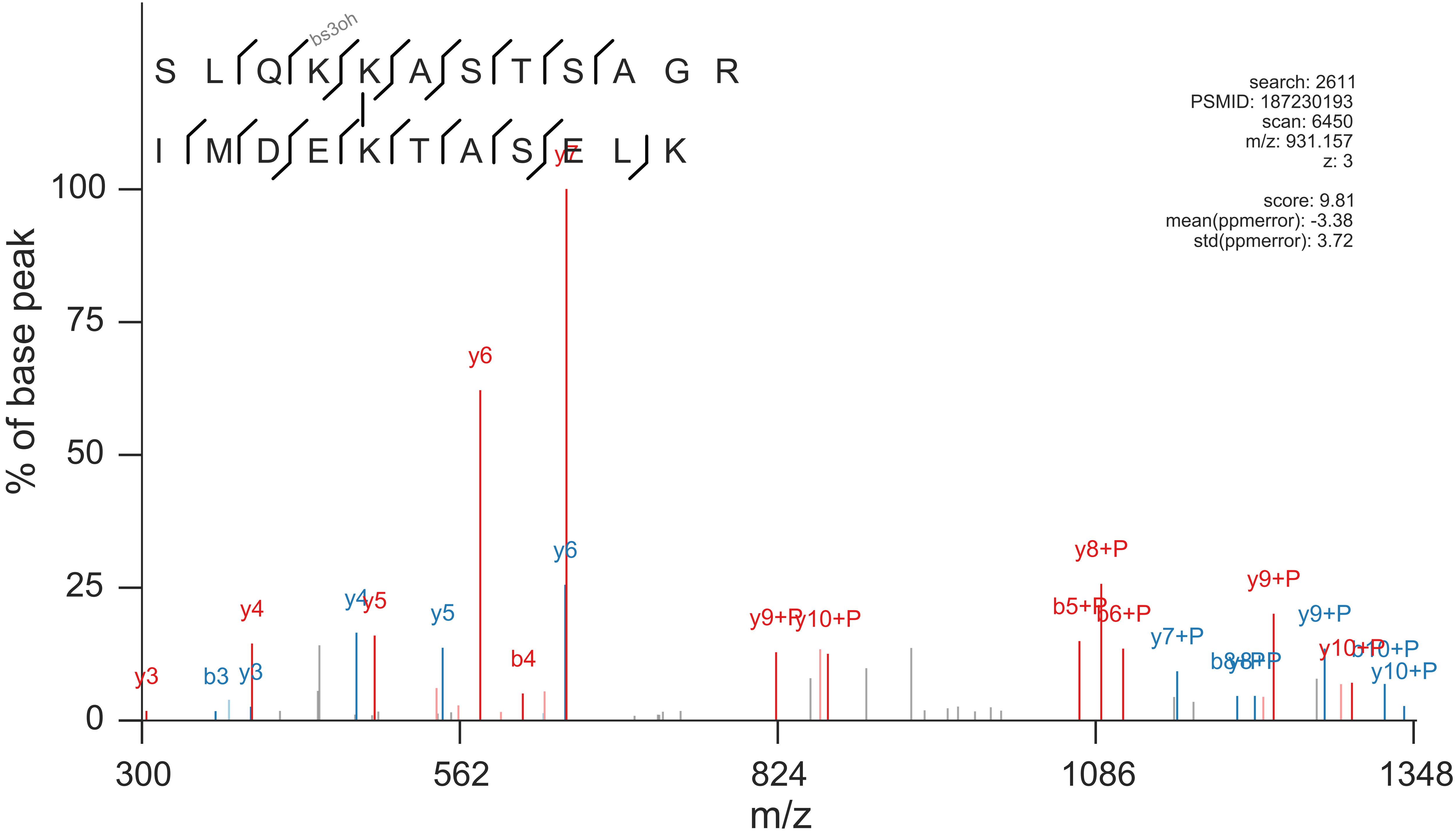

bs3oh
S L Q K K A S T S A G R
I M D E K T A S E L K
search: 2611
PSMID: 187230193
scan: 6450
m/z: 931.157
z: 3
score: 9.81
mean(ppmerror): -3.38
std(ppmerror): 3.72
% of base peak
m/z
0
25
50
75
100
300
562
824
1086
1348
y3
b3
y3
y4
y4
y5
y5
y6
b4
y7
y6
y9+P
y10+P
b5+P
y8+P
b6+P
y7+P
y9+P
y9+P
y10+P
b10+P
y10+P

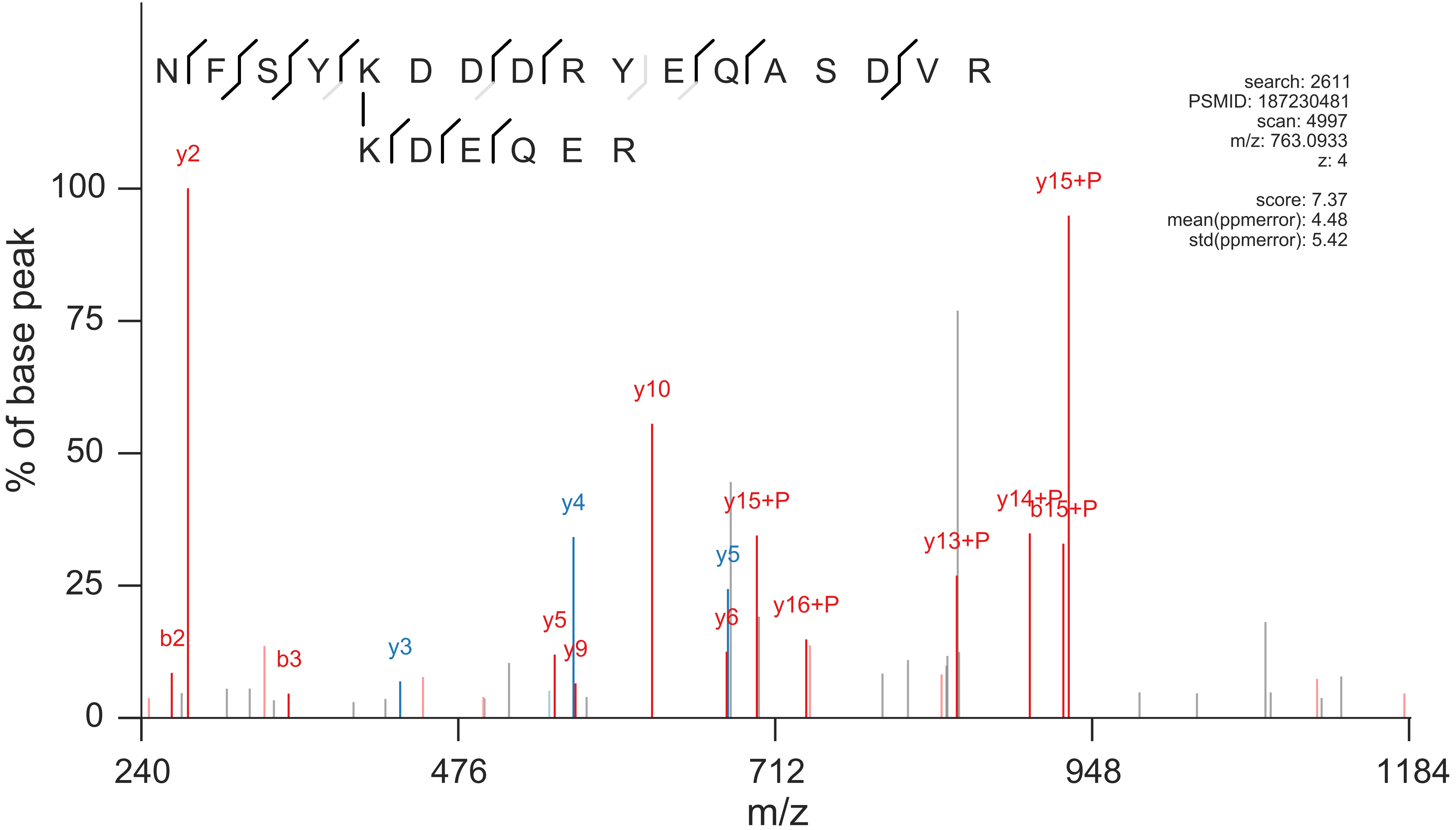

N F S Y K D D D R Y E Q A S D V R
K D E Q E R
search: 2611
PSMID: 187230481
scan: 4997
m/z: 763.0933
z: 4
score: 7.37
mean(ppmerror): 4.48
std(ppmerror): 5.42
% of base peak
100
75
50
25
0
y2
b2
b3
y3
y4
y5
y9
y10
y15+P
y5
y6
y16+P
y13+P
y14+P
b15+P
y15+P
240
476
712
948
1184
m/z

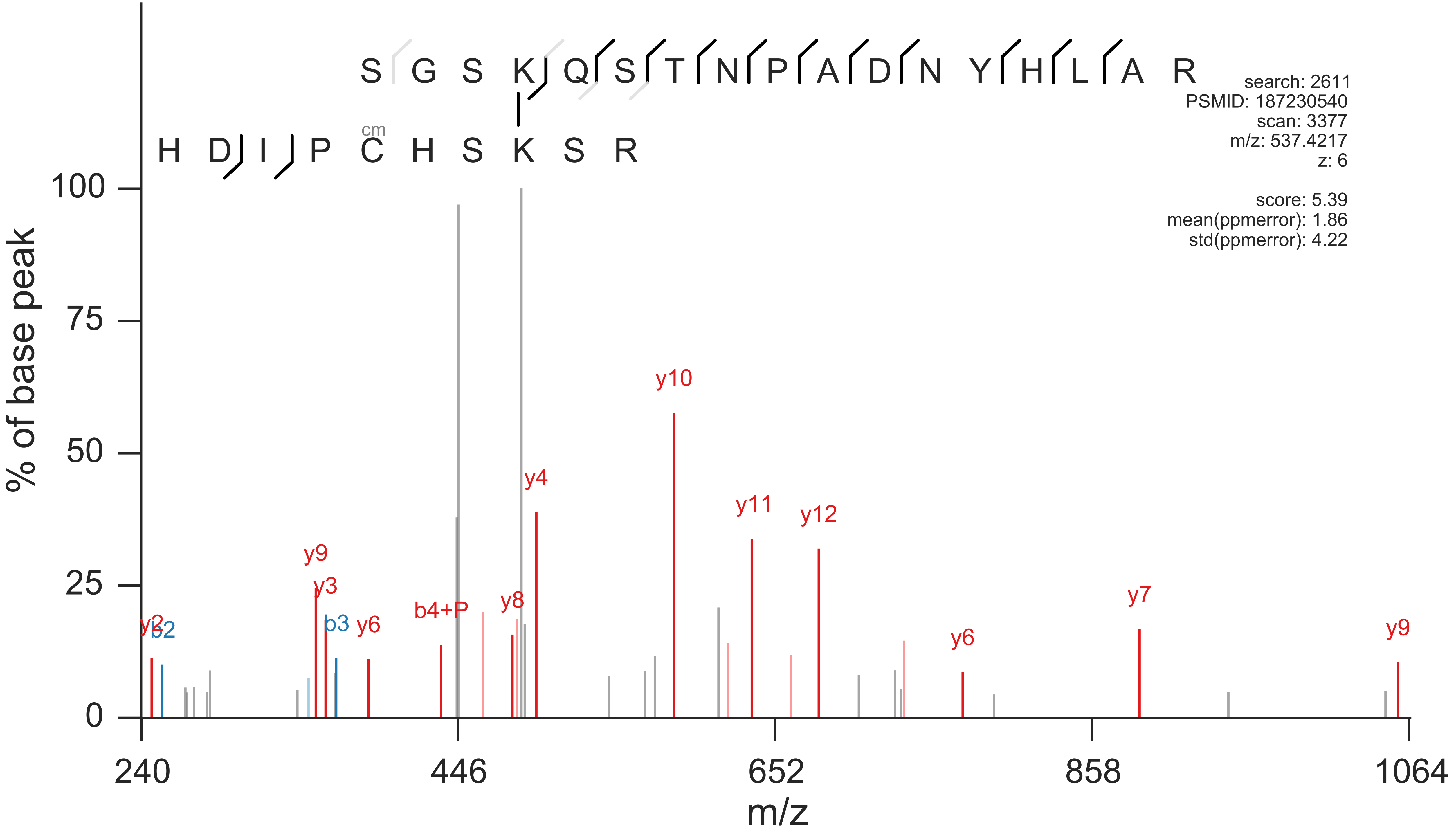
S G S K Q S T N P A D N Y H L A R
H D I P C H S K S R
cm
search: 2611
PSMID: 187230540
scan: 3377
m/z: 537.4217
z: 6
score: 5.39
mean(ppmerror): 1.86
std(ppmerror): 4.22
% of base peak
m/z
100
75
50
25
0
240
446
652
858
1064
y2
b2
y9
y3
b3
y6
b4+P
y8
y4
y10
y11
y12
y6
y7
y9

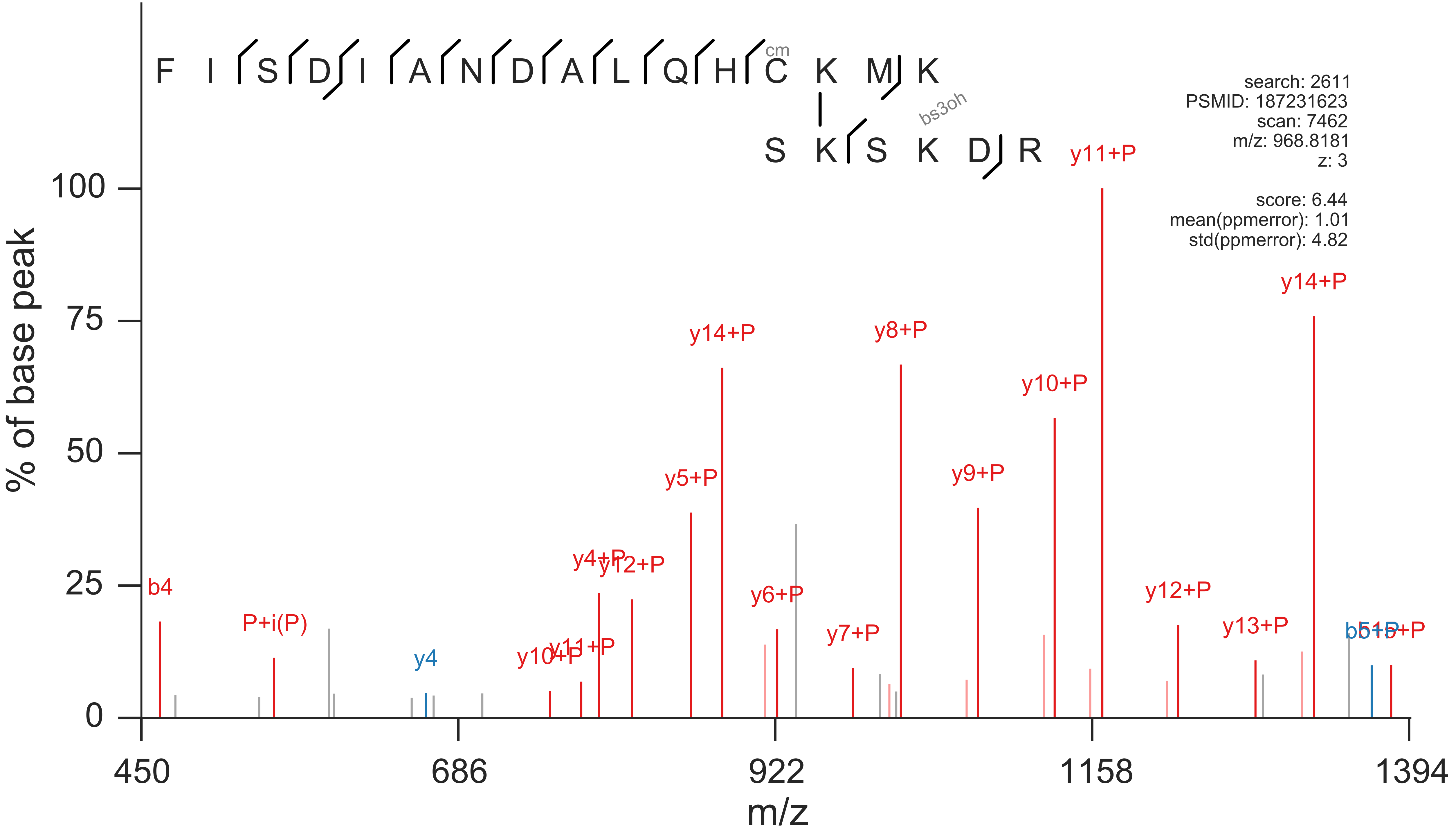

F I S D I A N D A L Q H C K M K
cm
S K S K D R
bs3oh
search: 2611
PSMID: 187231623
scan: 7462
m/z: 968.8181
z: 3
score: 6.44
mean(ppmerror): 1.01
std(ppmerror): 4.82
% of base peak
m/z
100
75
50
25
0
450
686
922
1158
1394
b4
P+i(P)
y4
y10+P
y11+P
y4+P
y12+P
y5+P
y14+P
y6+P
y7+P
y8+P
y9+P
y10+P
y11+P
y12+P
y13+P
y14+P

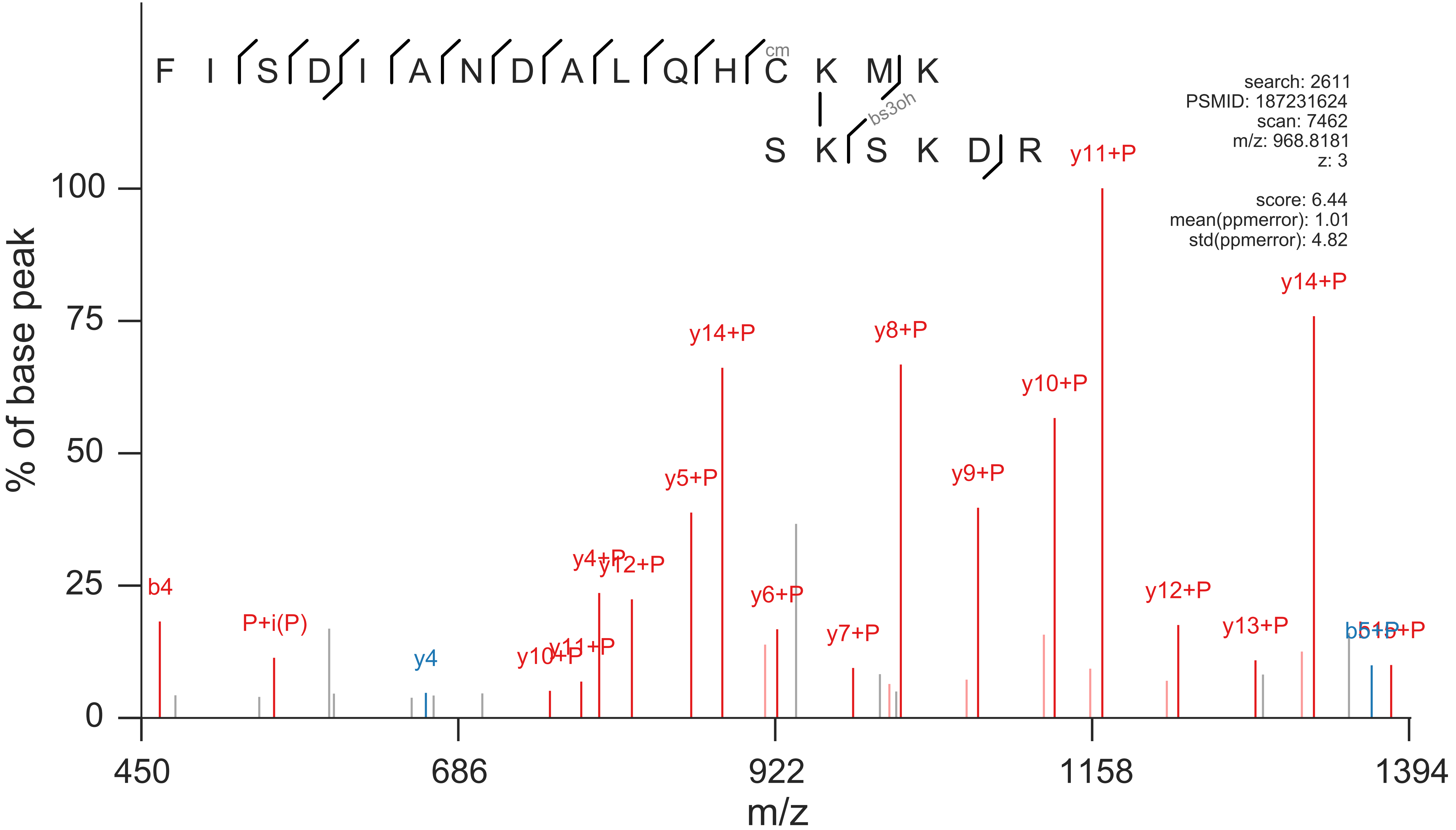

F I S D I A N D A L Q H C K M K
cm
bs3oh
S K S K D R
search: 2611
PSMID: 187231624
scan: 7462
m/z: 968.8181
z: 3
score: 6.44
mean(ppmerror): 1.01
std(ppmerror): 4.82
% of base peak
m/z
100
75
50
25
0
450
686
922
1158
1394
b4
P+i(P)
y4
y10+P
y11+P
y4+P
y12+P
y5+P
y14+P
y6+P
y7+P
y8+P
y9+P
y10+P
y11+P
y12+P
y13+P
y14+P

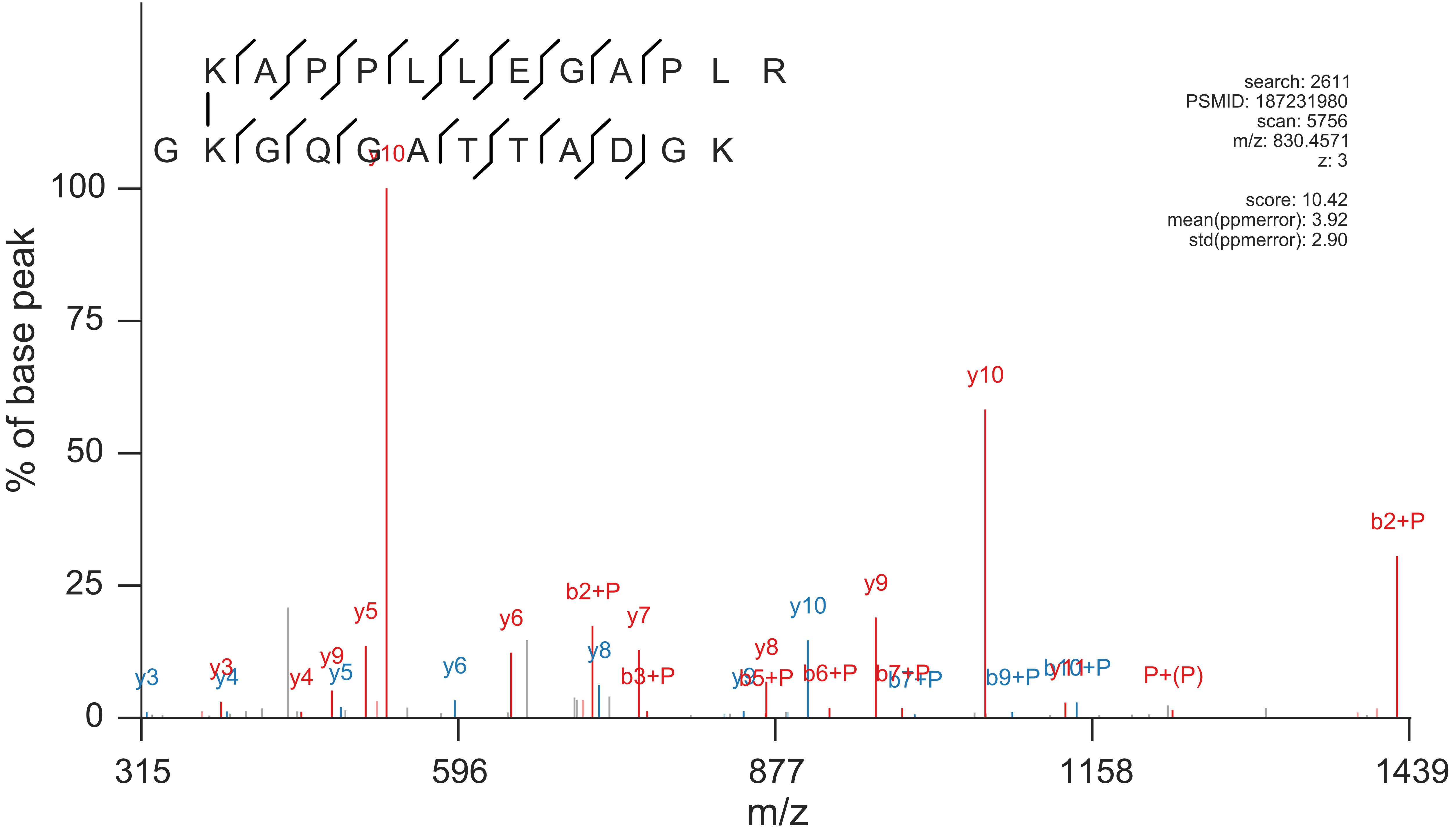
K A P P L L E G A P L R
G K G Q G A T T A D G K
search: 2611
PSMID: 187231980
scan: 5756
m/z: 830.4571
z: 3
score: 10.42
mean(ppmerror): 3.92
std(ppmerror): 2.90
% of base peak
m/z
y10
y5
y6
b2+P
y7
y9
y10
b2+P
y3
y4
y9
y5
y6
y8
y8
b3+P
b6+P
b7+P
b9+P
P+(P)

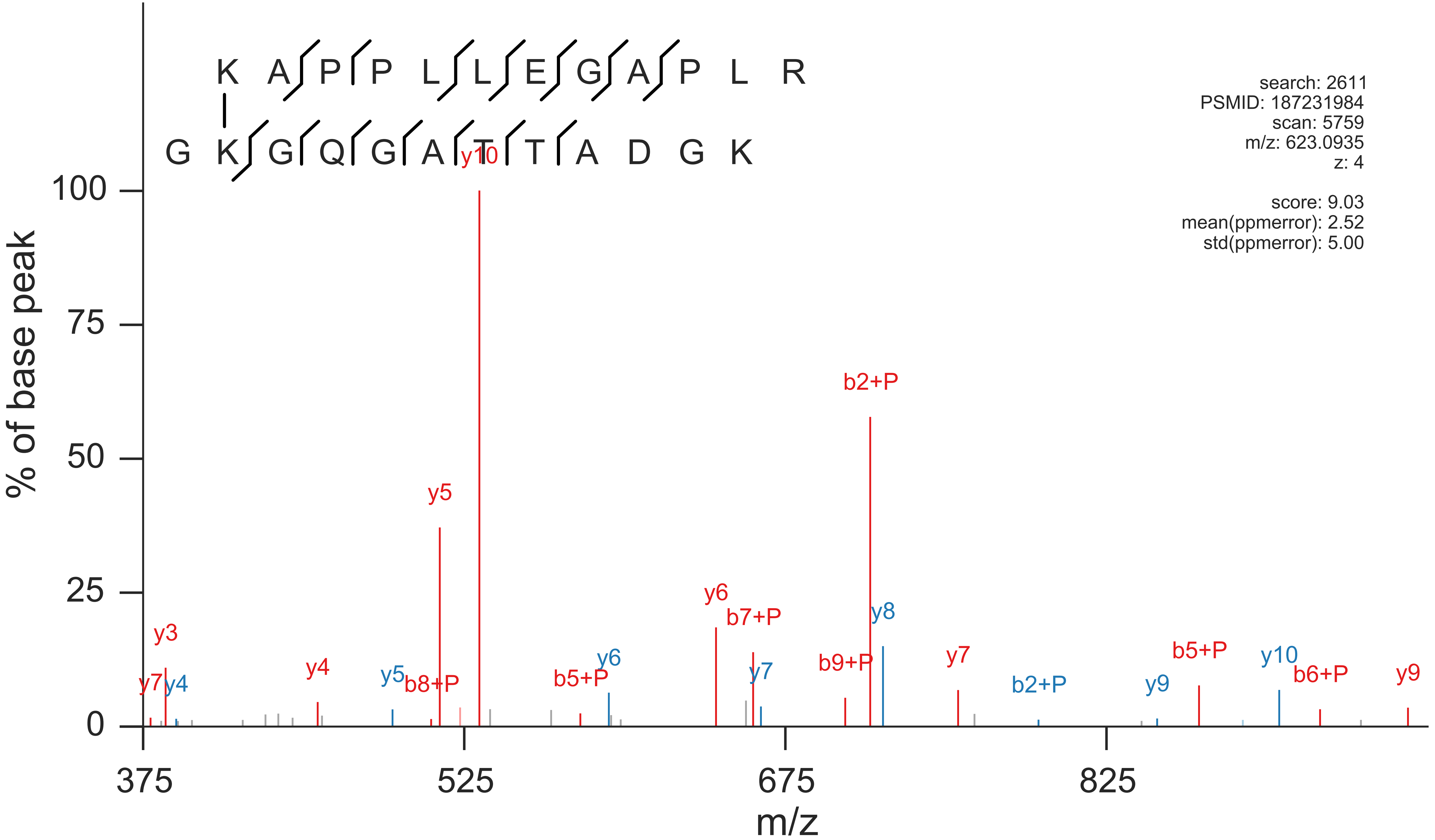
K A P P L L E G A P L R
G K G Q G A T T A D G K
search: 2611
PSMID: 187231984
scan: 5759
m/z: 623.0935
z: 4
score: 9.03
mean(ppmerror): 2.52
std(ppmerror): 5.00
% of base peak
m/z
0
25
50
75
100
375
525
675
825
y10
b2+P
y5
y6
b7+P
y8
y3
y7
y4
y4
y5
b8+P
b5+P
y6
y7
b9+P
y7
b2+P
y9
b5+P
y10
b6+P
y9

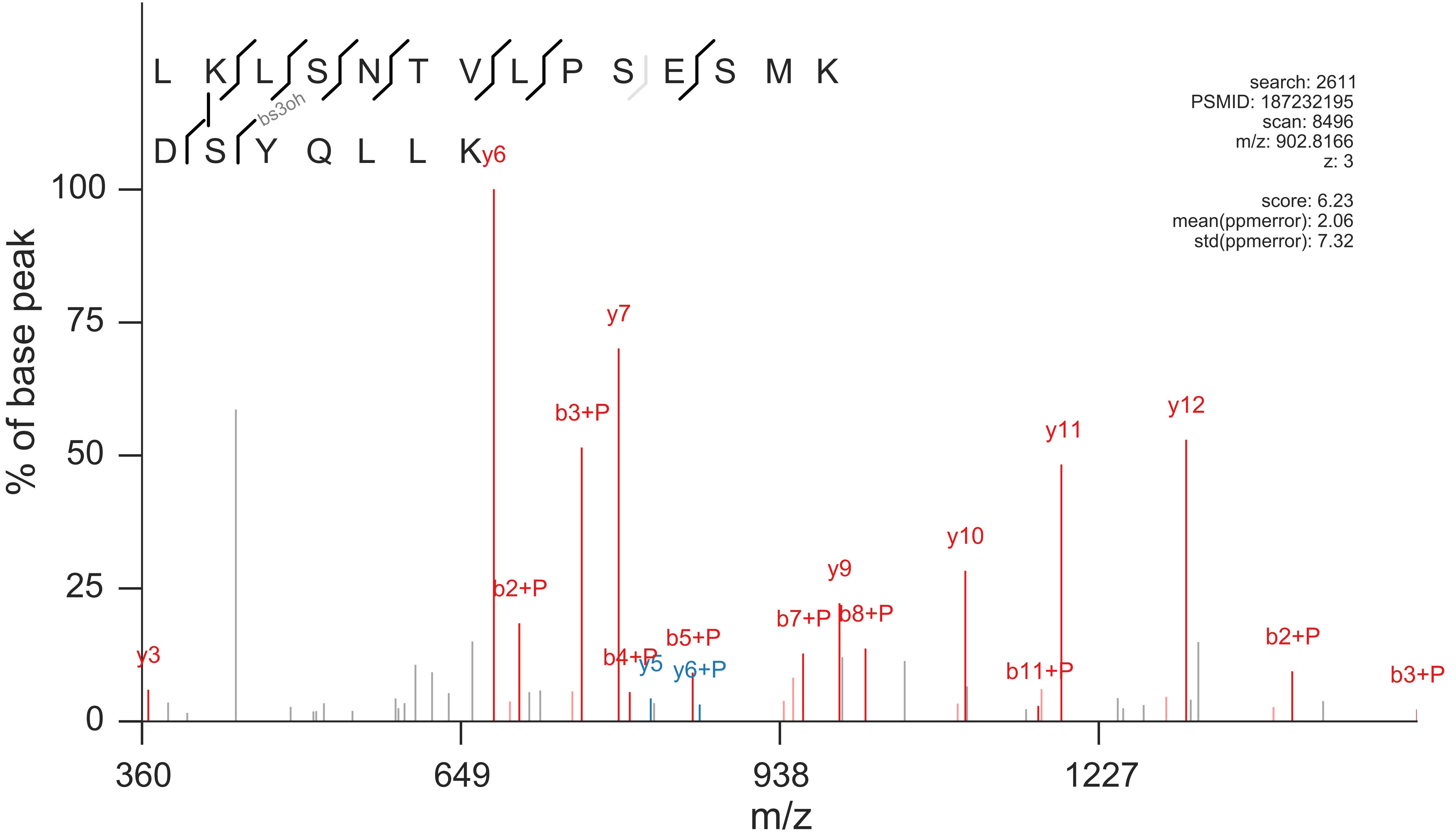

L K L S N T V L P S E S M K
D S Y Q L L K
bs3oh
search: 2611
PSMID: 187232195
scan: 8496
m/z: 902.8166
z: 3
score: 6.23
mean(ppmerror): 2.06
std(ppmerror): 7.32
% of base peak
100
75
50
25
0
m/z
360
649
938
1227
y6
y7
b3+P
y12
y11
y10
y9
b2+P
b8+P
b7+P
b5+P
b4+P
y5
y6+P
y3
b11+P
b2+P
b3+P

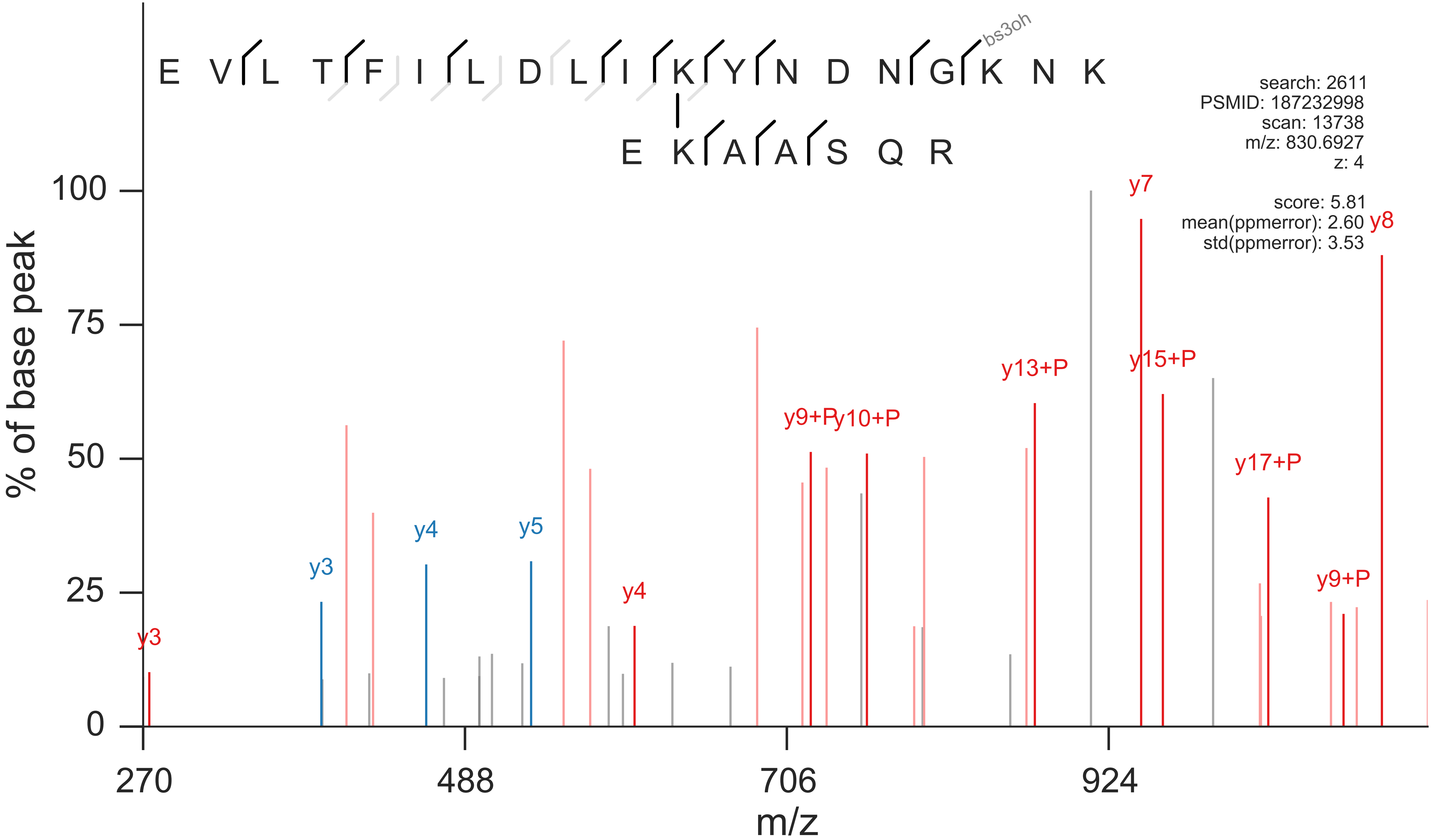

E V L T F I L D L I K Y N D N G K N K
bs3oh
E K A A S Q R
search: 2611
PSMID: 187232998
scan: 13738
m/z: 830.6927
z: 4
score: 5.81
mean(ppmerror): 2.60
std(ppmerror): 3.53
% of base peak
m/z
0
25
50
75
100
270
488
706
924
y3
y3
y4
y5
y4
y9+P
y10+P
y13+P
y7
y15+P
y17+P
y9+P
y8

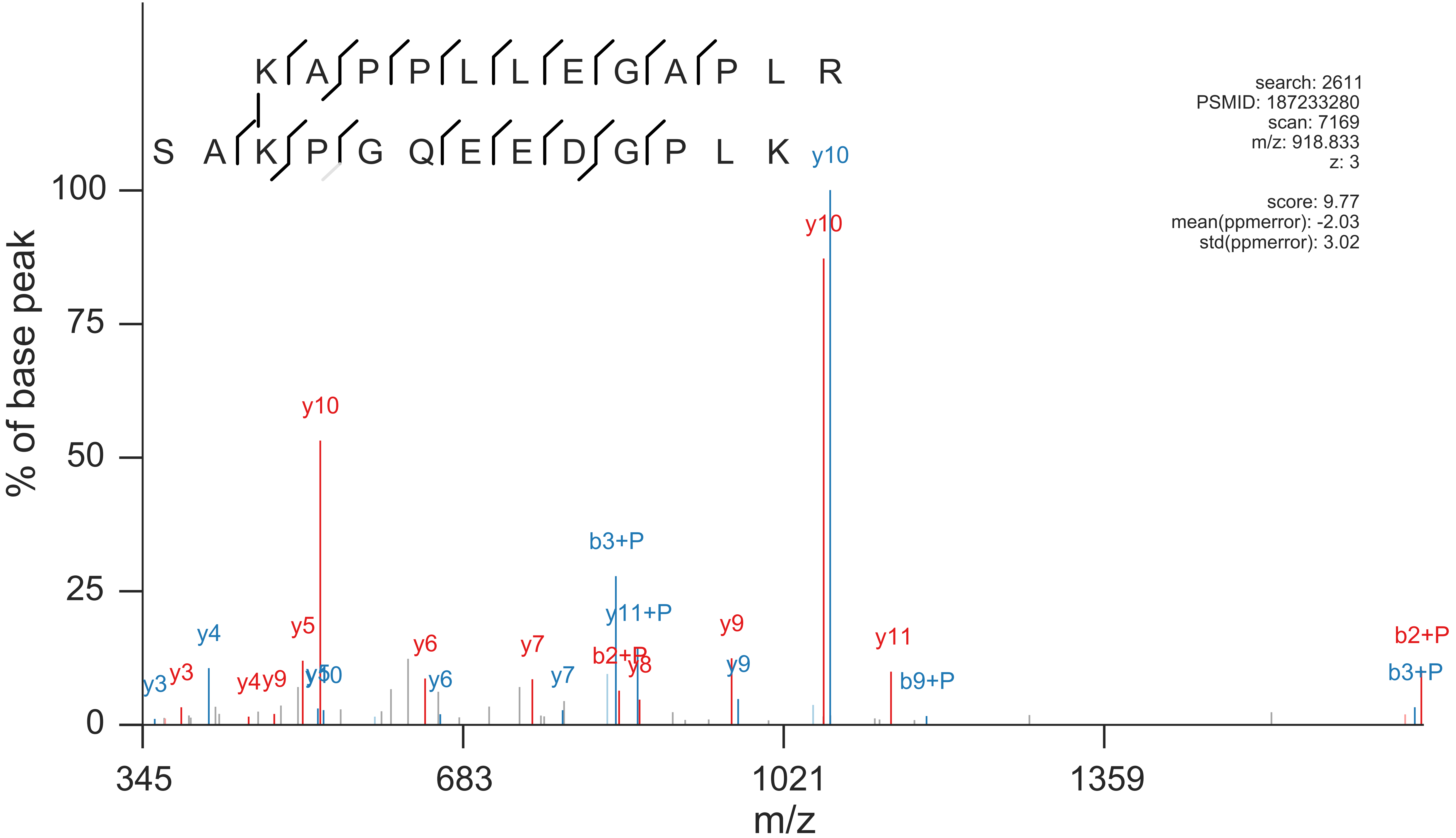

K A P P L L E G A P L R
S A K P G Q E E D G P L K
search: 2611
PSMID: 187233280
scan: 7169
m/z: 918.833
z: 3
score: 9.77
mean(ppmerror): -2.03
std(ppmerror): 3.02
% of base peak
m/z

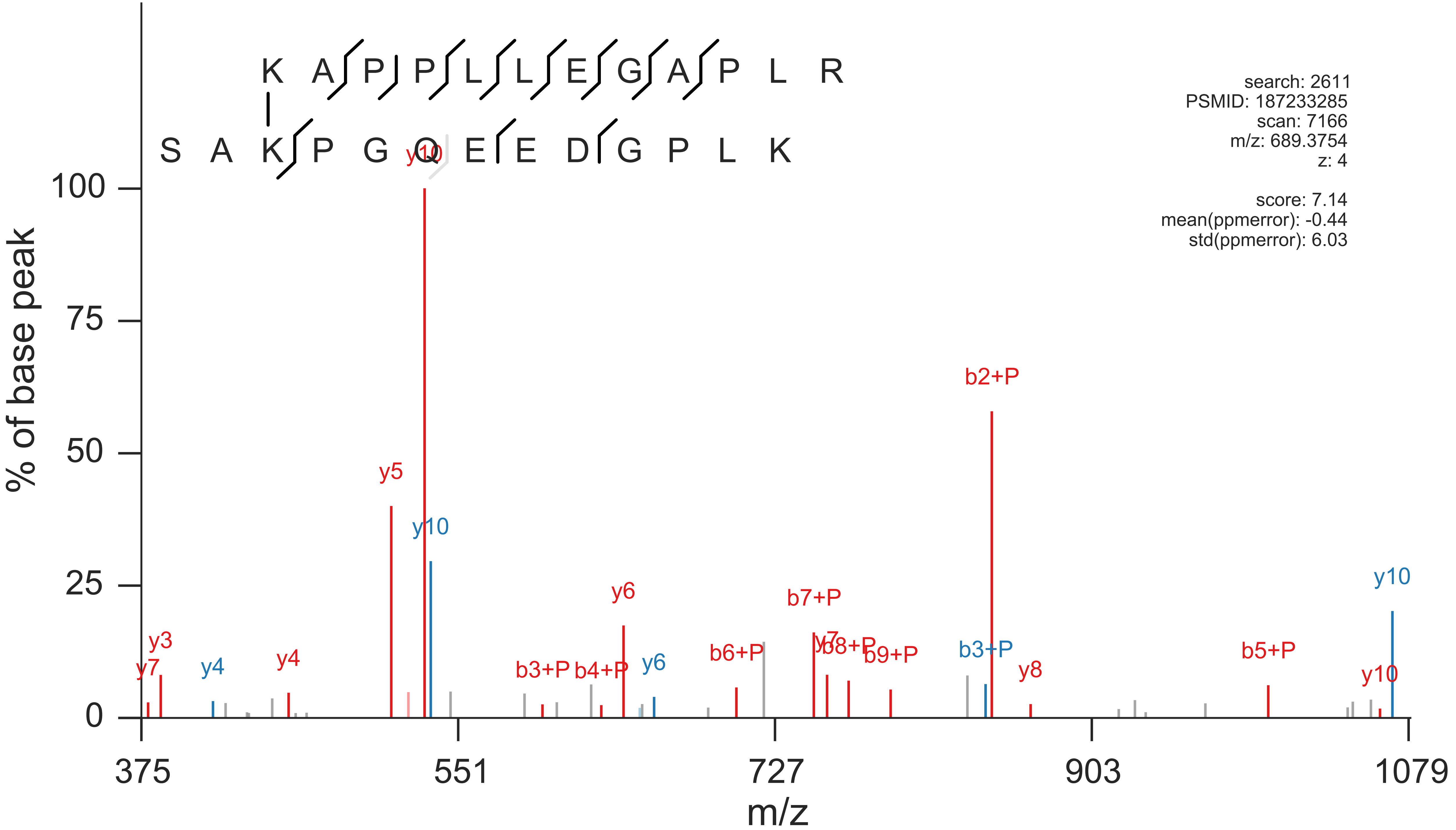
K A P P L L E G A P L R
S A K P G Q E E D G P L K
search: 2611
PSMID: 187233285
scan: 7166
m/z: 689.3754
z: 4
score: 7.14
mean(ppmerror): -0.44
std(ppmerror): 6.03
% of base peak
m/z
y7
y3
y4
y4
y5
y10
y10
b3+P
b4+P
y6
y6
b6+P
b7+P
y7
b8+P
b9+P
b3+P
b2+P
y8
b5+P
y10
y10
0
25
50
75
100
375
551
727
903
1079

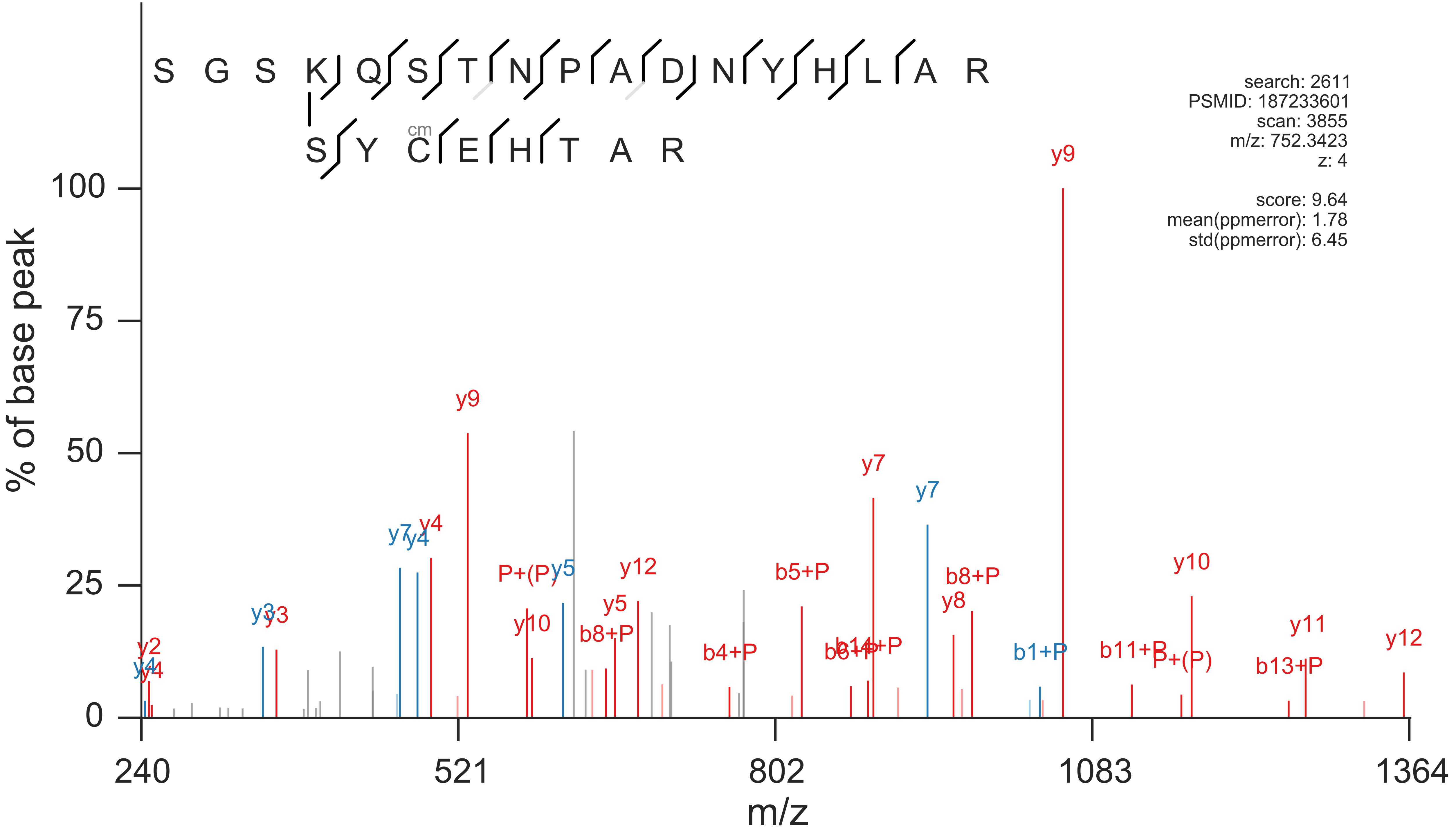
S G S K Q S T N P A D N Y H L A R
S Y C E H T A R
cm
search: 2611
PSMID: 187233601
scan: 3855
m/z: 752.3423
z: 4
score: 9.64
mean(ppmerror): 1.78
std(ppmerror): 6.45
% of base peak
m/z
0
25
50
75
100
240
521
802
1083
1364
y9
y7
y4
y5
y12
y10
y3
y2
b8+P
b4+P
b5+P
b8+P
y8
b1+P
b11+P
P+(P)
b13+P
y11

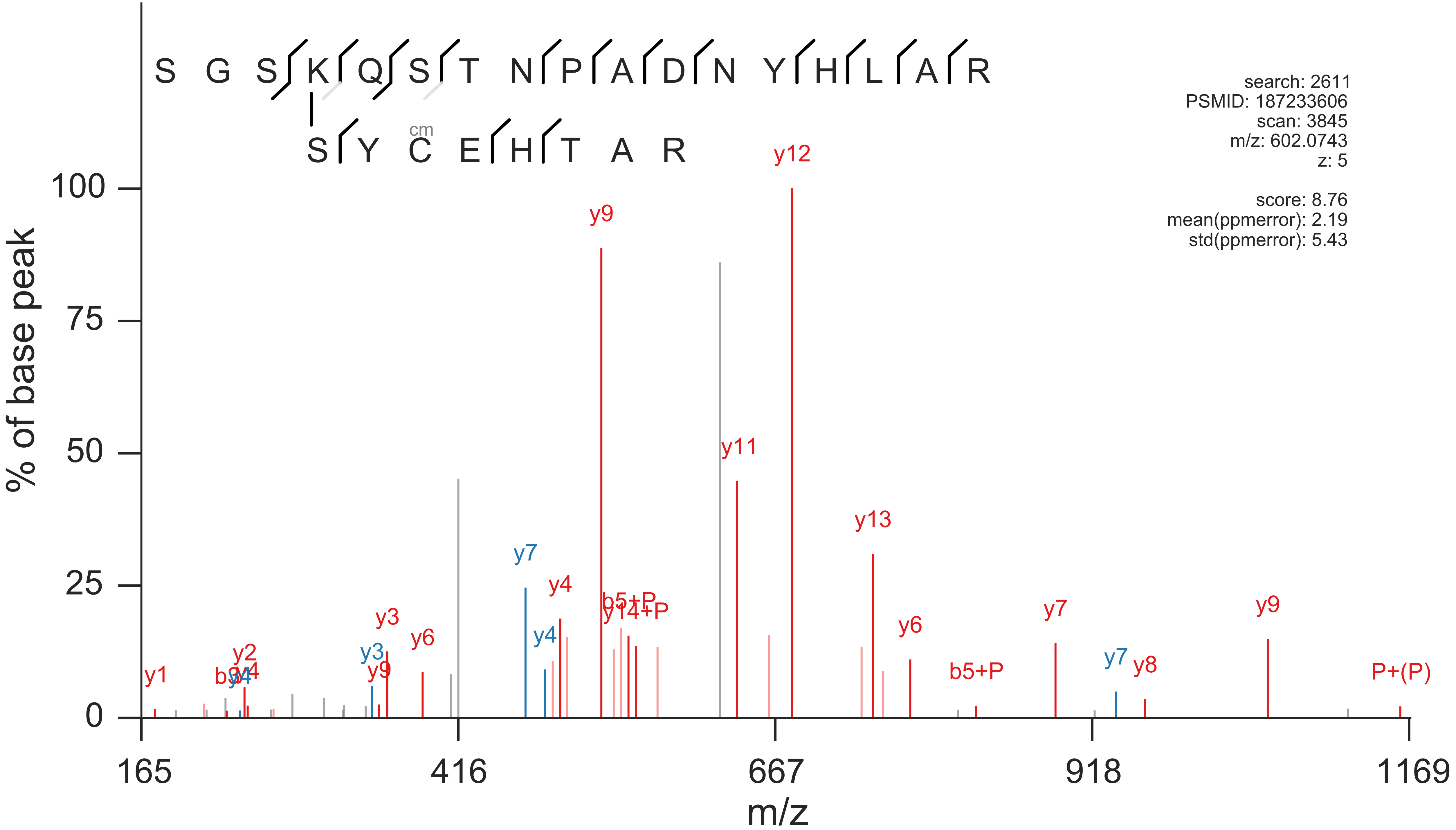

S G S K Q S T N P A D N Y H L A R
S Y C E H T A R
cm
search: 2611
PSMID: 187233606
scan: 3845
m/z: 602.0743
z: 5
score: 8.76
mean(ppmerror): 2.19
std(ppmerror): 5.43
% of base peak
m/z
y12
y9
y11
y13
y7
y4
b5+P
y14+P
y3
y6
y2
y1
b4
y4
y3
y9
y4
y6
y7
b5+P
y7
y8
y9
P+(P)

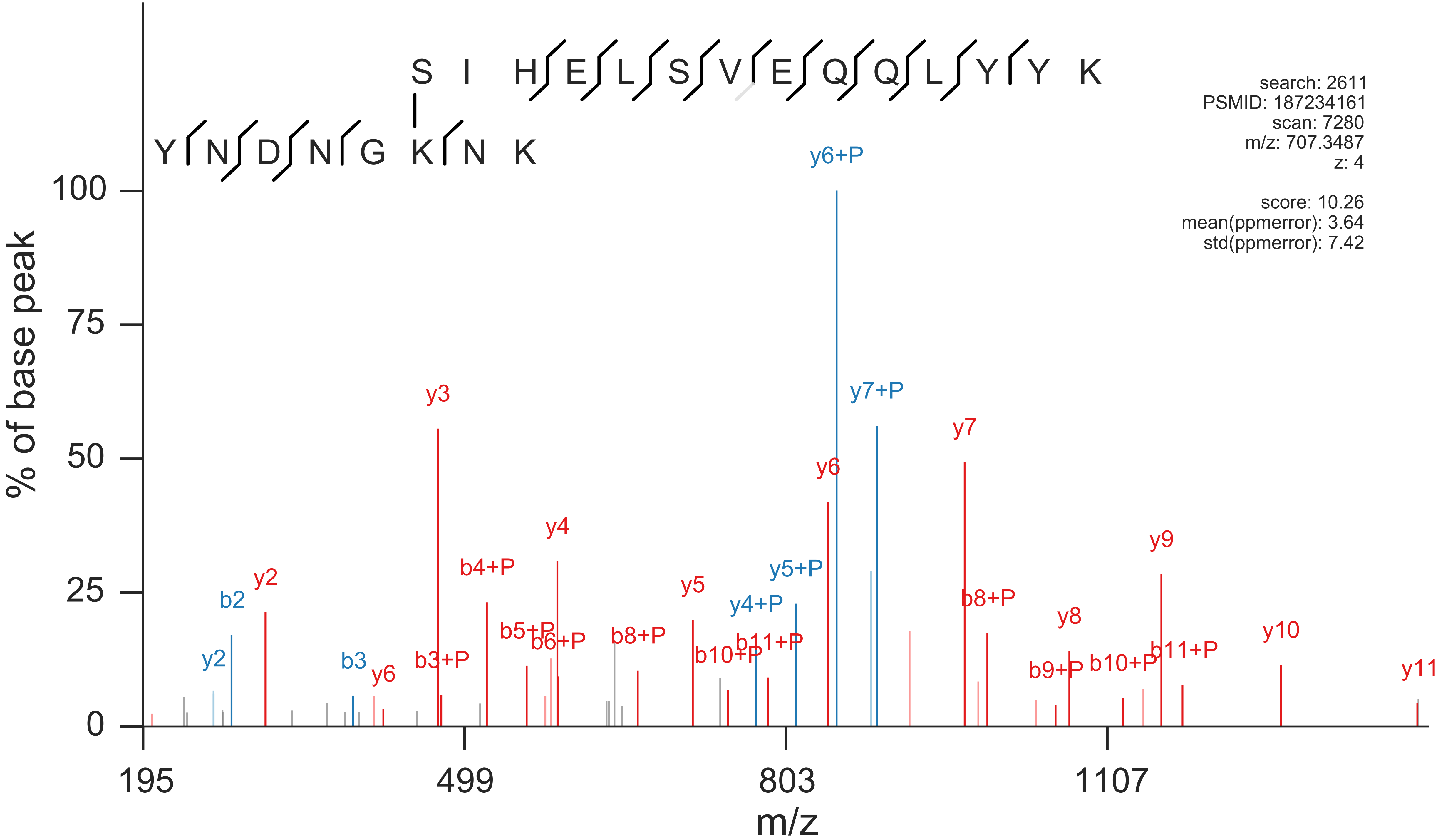

S I H E L S V E Q Q L Y Y K
Y N D N G K N K
search: 2611
PSMID: 187234161
scan: 7280
m/z: 707.3487
z: 4
score: 10.26
mean(ppmerror): 3.64
std(ppmerror): 7.42
% of base peak
m/z
100
75
50
25
0
195
499
803
1107
y6+P
y7+P
y3
y7
y6
y4
y9
b4+P
y2
y5+P
b2
y5
y4+P
b8+P
y8
b5+P
b6+P
b8+P
b11+P
y10
b10+P
b11+P
y2
b3
b3+P
b9+P
b10+P
y11
y6

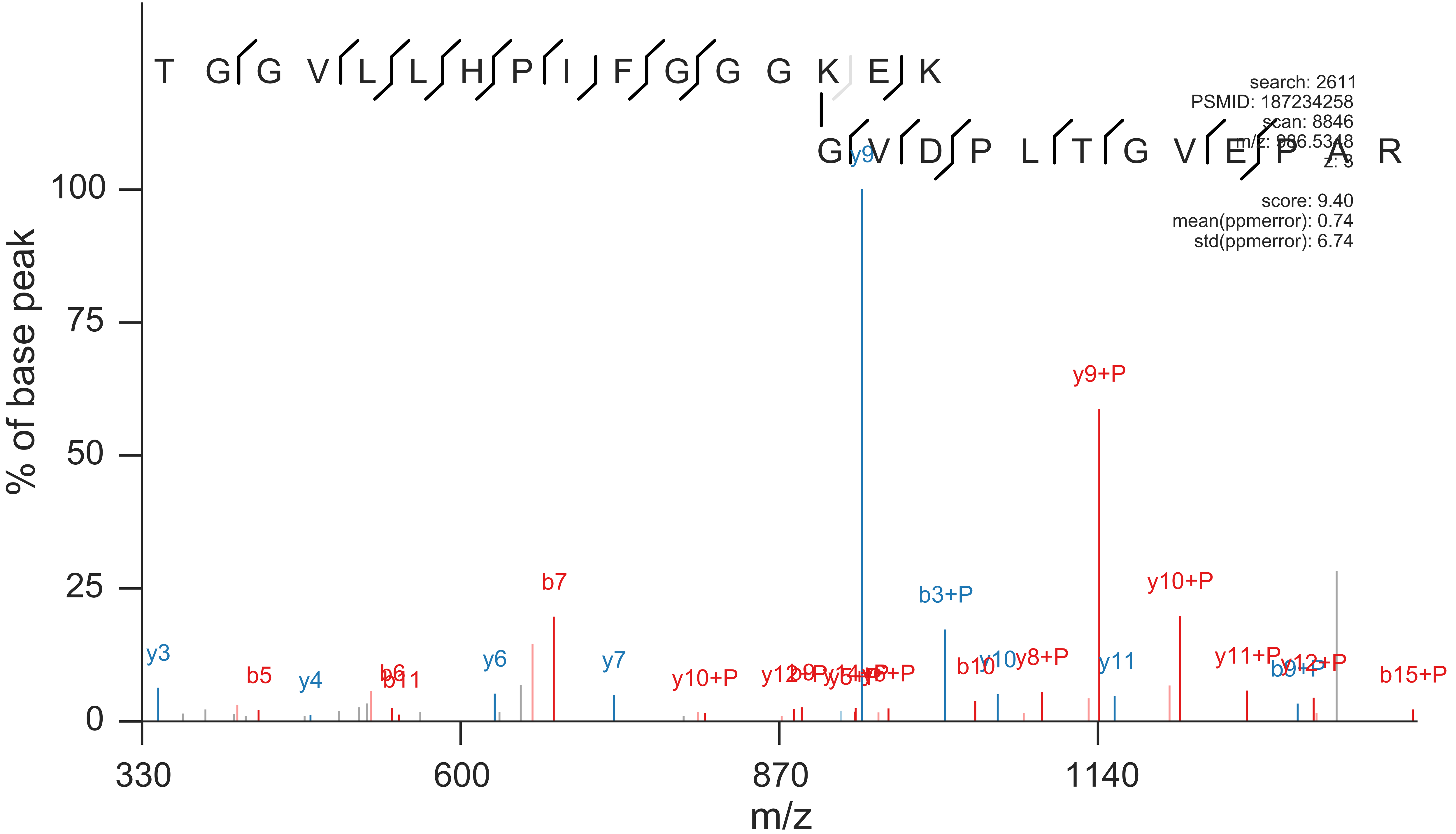
T G G V L L H P I F G G G K E K
G V D P L T G V E P A R
search: 2611
PSMID: 187234258
scan: 8846
score: 9.40
mean(ppmerror): 0.74
std(ppmerror): 6.74
% of base peak
m/z
y9
y9+P
b7
b3+P
y10+P
y3
b5
y4
b6
b11
y6
y7
b10
y10
y8+P
y11
y11+P
y12+P
b15+P

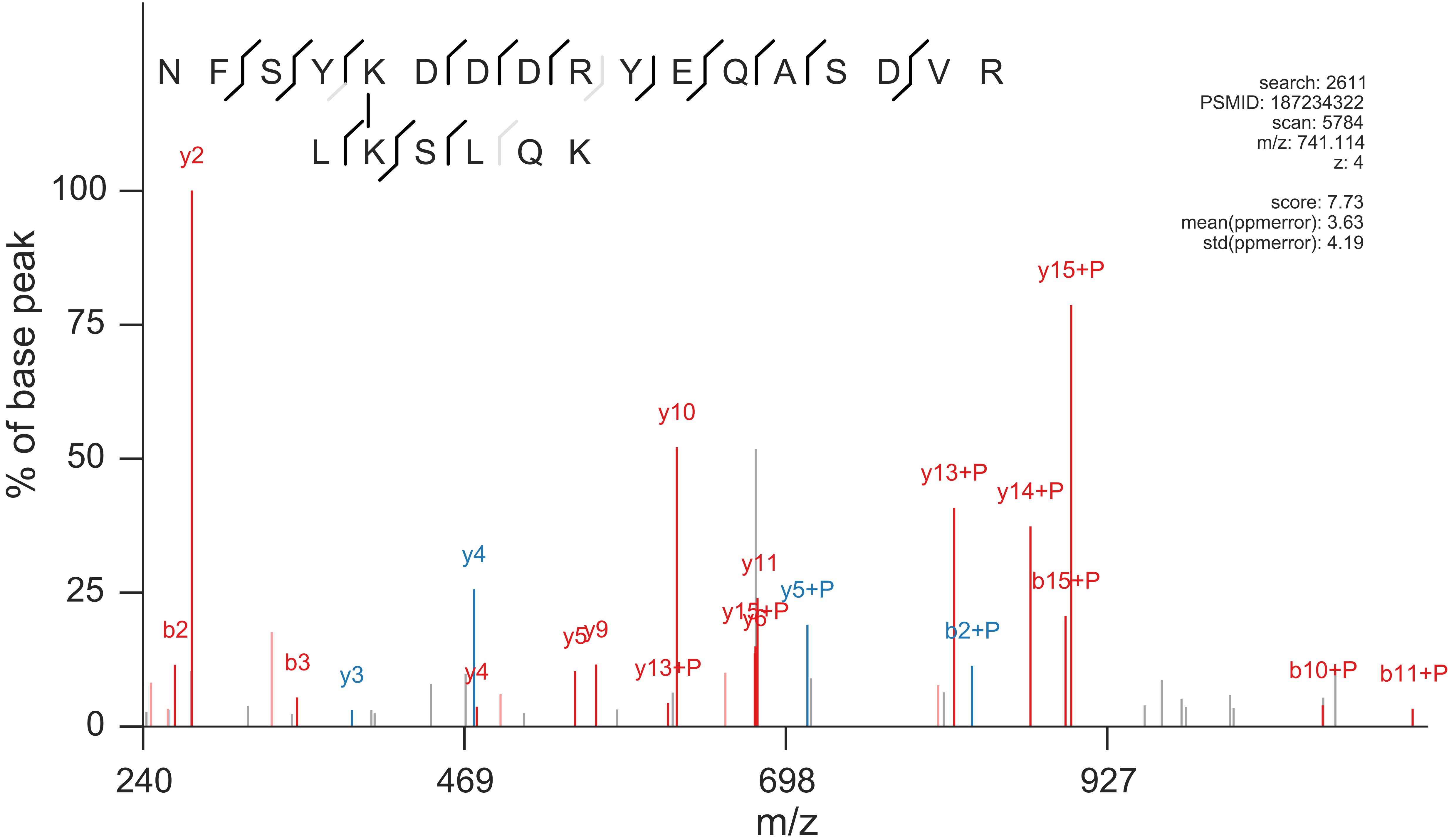

N F S Y K D D D R Y E Q A S D V R
L K S L Q K
search: 2611
PSMID: 187234322
scan: 5784
m/z: 741.114
z: 4
score: 7.73
mean(ppmerror): 3.63
std(ppmerror): 4.19
% of base peak
100
75
50
25
0
240
469
698
927
m/z
y2
b2
b3
y3
y4
y4
y5
y9
y10
y13+P
y11
y6
y5+P
b2+P
y13+P
y14+P
b15+P
y15+P
b10+P
b11+P

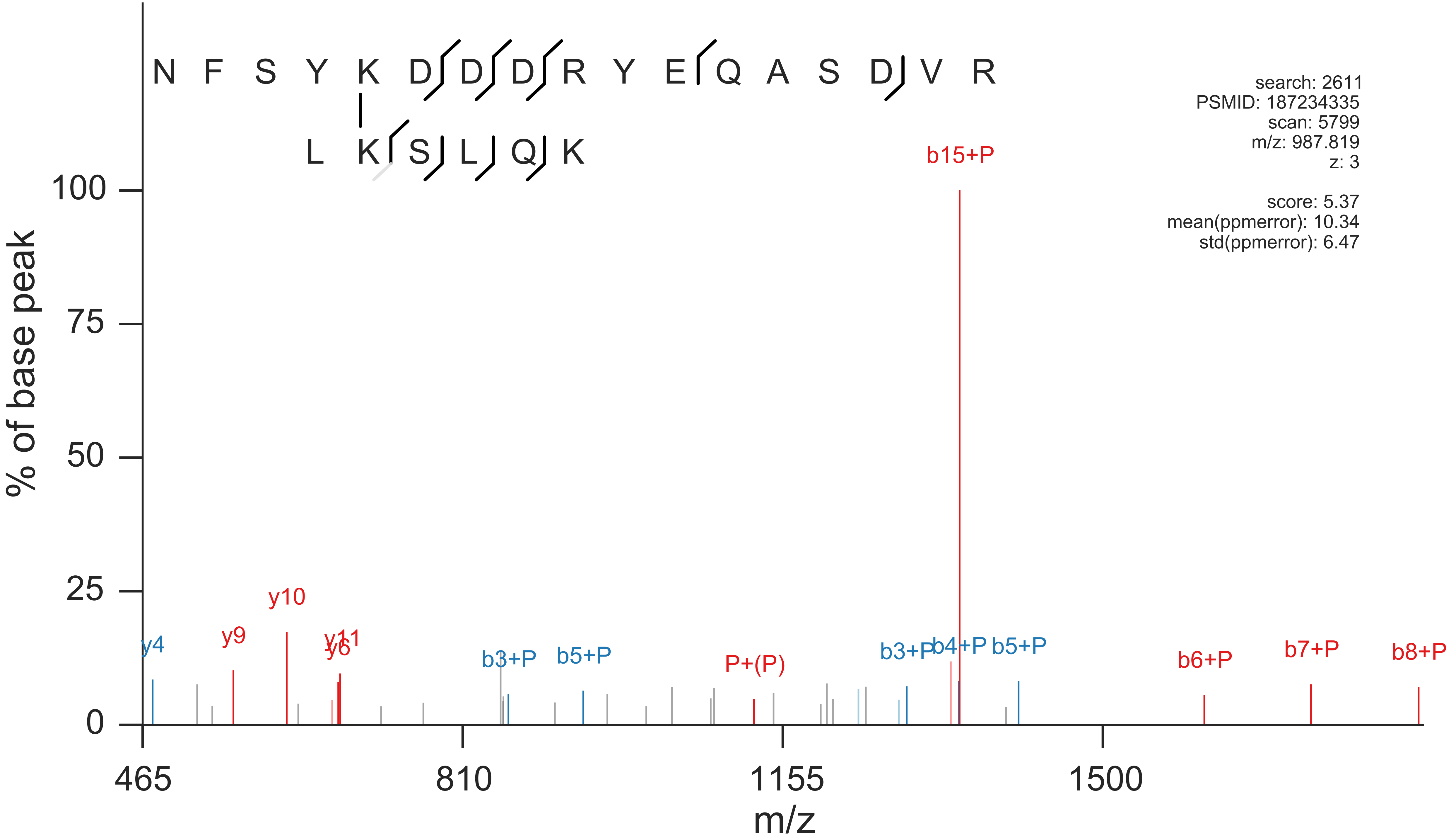
N F S Y K D D D R Y E Q A S D V R
L K S L Q K
search: 2611
PSMID: 187234335
scan: 5799
m/z: 987.819
z: 3
score: 5.37
mean(ppmerror): 10.34
std(ppmerror): 6.47
b15+P
y4
y9
y10
y11
y6
b3+P
b5+P
P+(P)
b3+P
b4+P
b5+P
b6+P
b7+P
b8+P
% of base peak
0
25
50
75
100
465
810
1155
1500
m/z

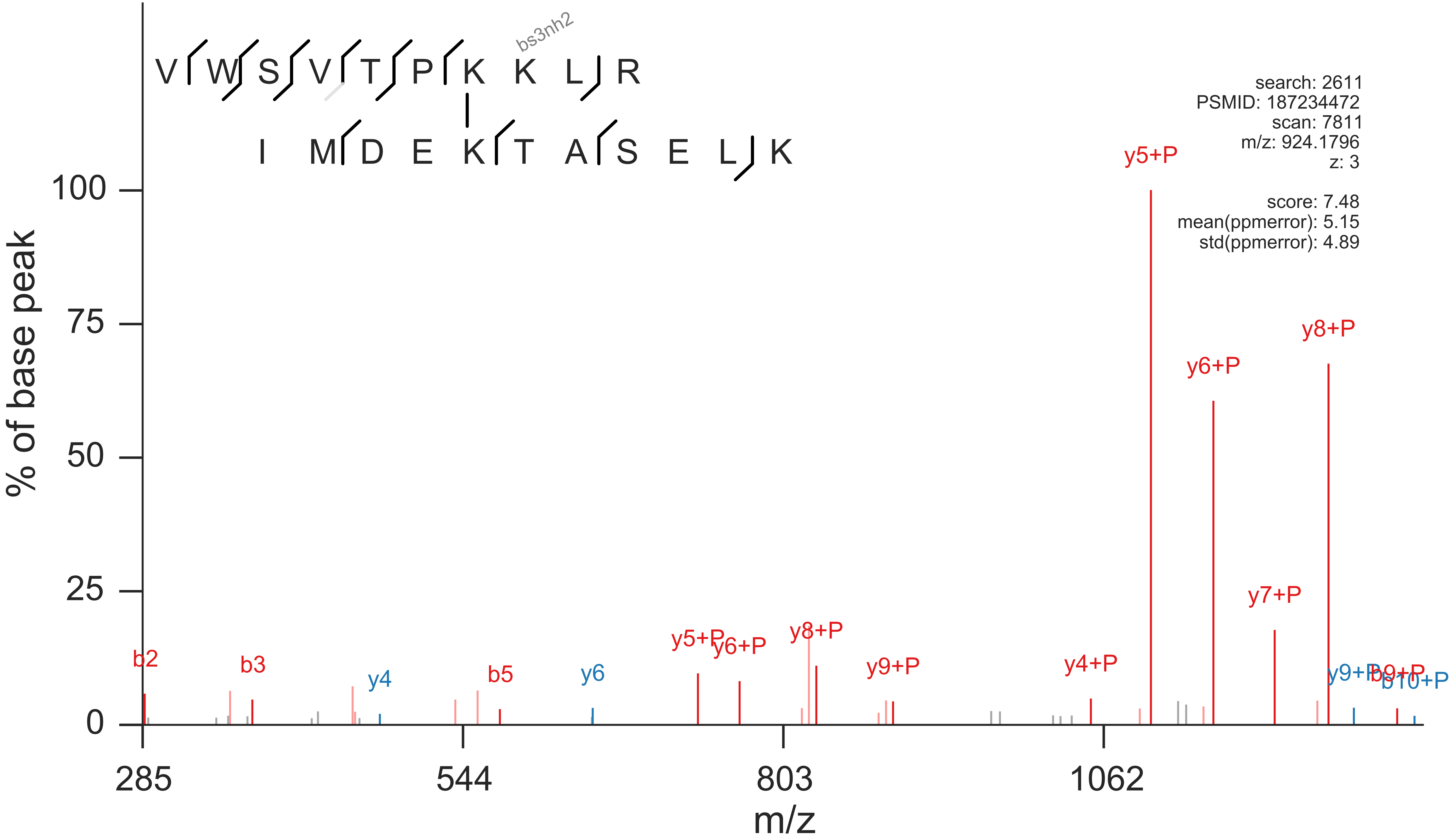

V W S V T P K K L R
bs3nh2
I M D E K T A S E L K
search: 2611
PSMID: 187234472
scan: 7811
m/z: 924.1796
z: 3
score: 7.48
mean(ppmerror): 5.15
std(ppmerror): 4.89
% of base peak
m/z
0
25
50
75
100
285
544
803
1062
b2
b3
y4
b5
y6
y5+P
y6+P
y8+P
y9+P
y4+P
y5+P
y6+P
y7+P
y8+P
y9+P
b9+P
b10+P

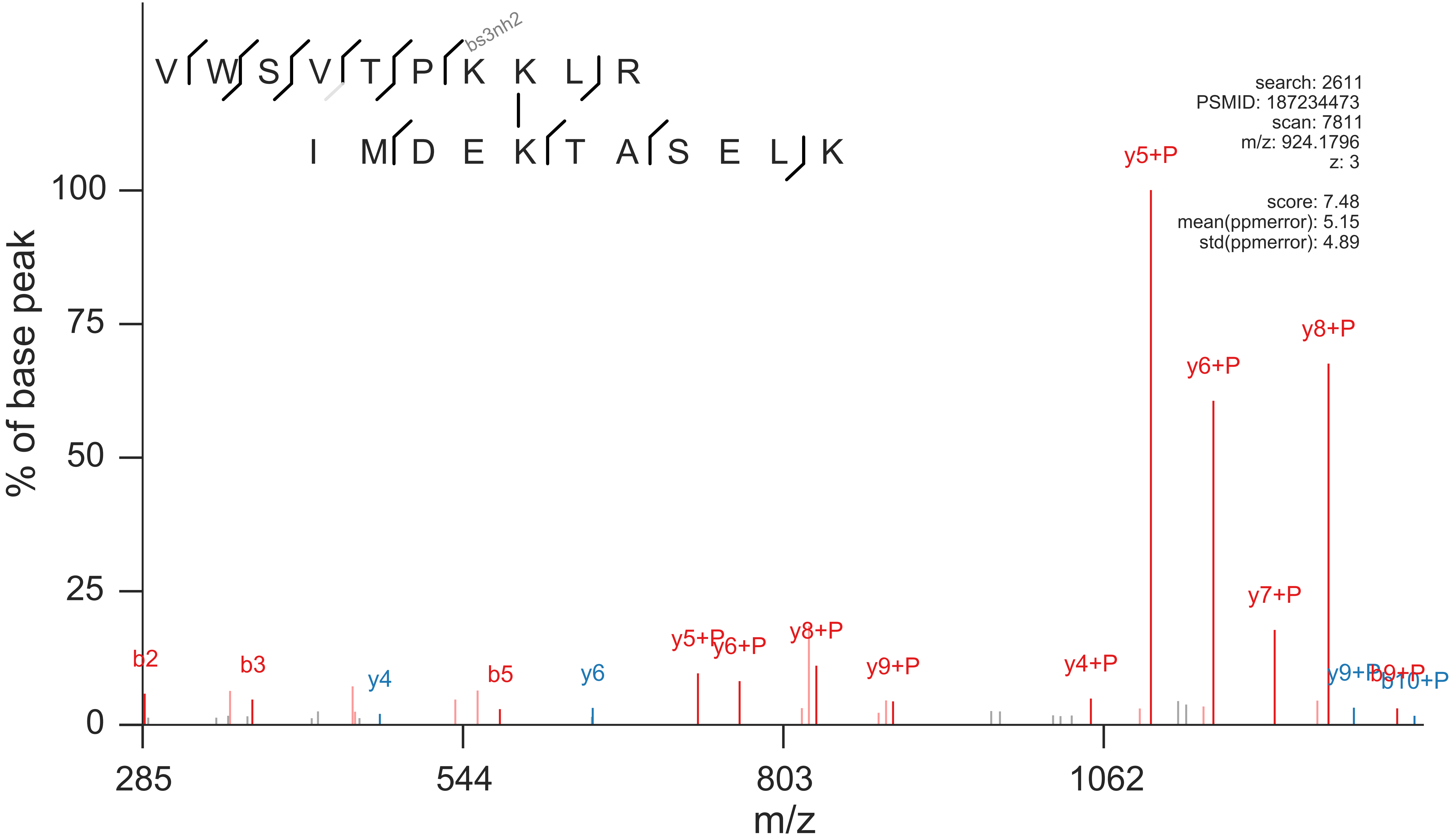

V W S V T P K K L R
bs3nh2
I M D E K T A S E L K
search: 2611
PSMID: 187234473
scan: 7811
m/z: 924.1796
z: 3
score: 7.48
mean(ppmerror): 5.15
std(ppmerror): 4.89
% of base peak
100
75
50
25
0
y5+P
y6+P
y8+P
y7+P
b2
b3
y4
b5
y6
y5+P
y6+P
y8+P
y9+P
y4+P
y9+P
b9+P
b10+P
285
544
803
1062
m/z

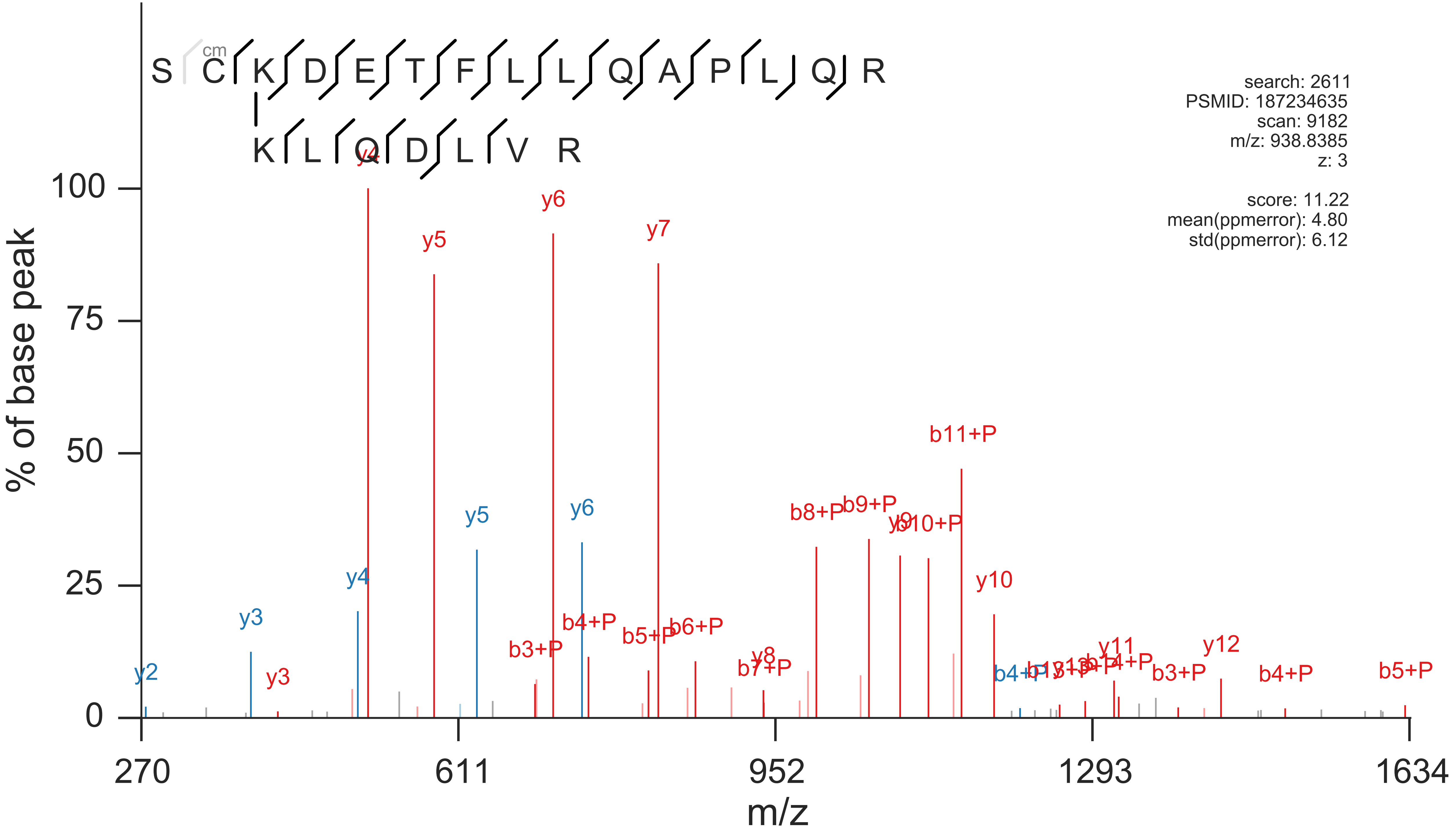

S C K D E T F L L Q A P L Q R
cm
K L Q D L V R
search: 2611
PSMID: 187234635
scan: 9182
m/z: 938.8385
z: 3
score: 11.22
mean(ppmerror): 4.80
std(ppmerror): 6.12
% of base peak
m/z
0
25
50
75
100
270
611
952
1293
1634
y2
y3
y4
y5
y6
y7
y8
y9
y10
y11
y12
b3+P
b4+P
b5+P
b6+P
b7+P
b8+P
b9+P
b10+P
b11+P

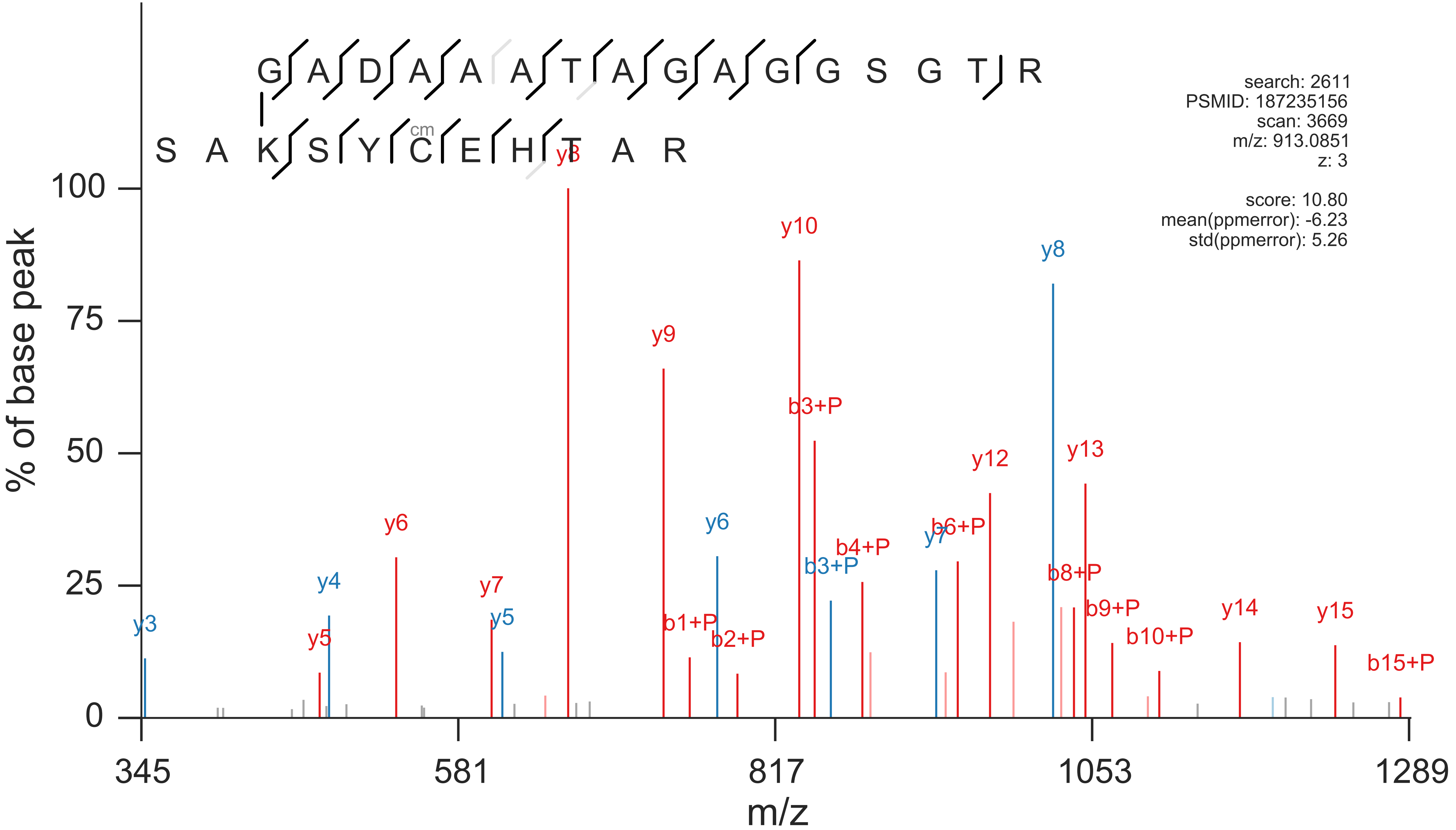

G A D A A A T A G A G G S G T R
S A K S Y C E H T A R
cm
search: 2611
PSMID: 187235156
scan: 3669
m/z: 913.0851
z: 3
score: 10.80
mean(ppmerror): -6.23
std(ppmerror): 5.26
% of base peak
m/z
y3 y4 y5 y6 y7 y8 y9 y10 y12 y13 y14 y15
b1+P b2+P b3+P b4+P b6+P b8+P b9+P b10+P b15+P

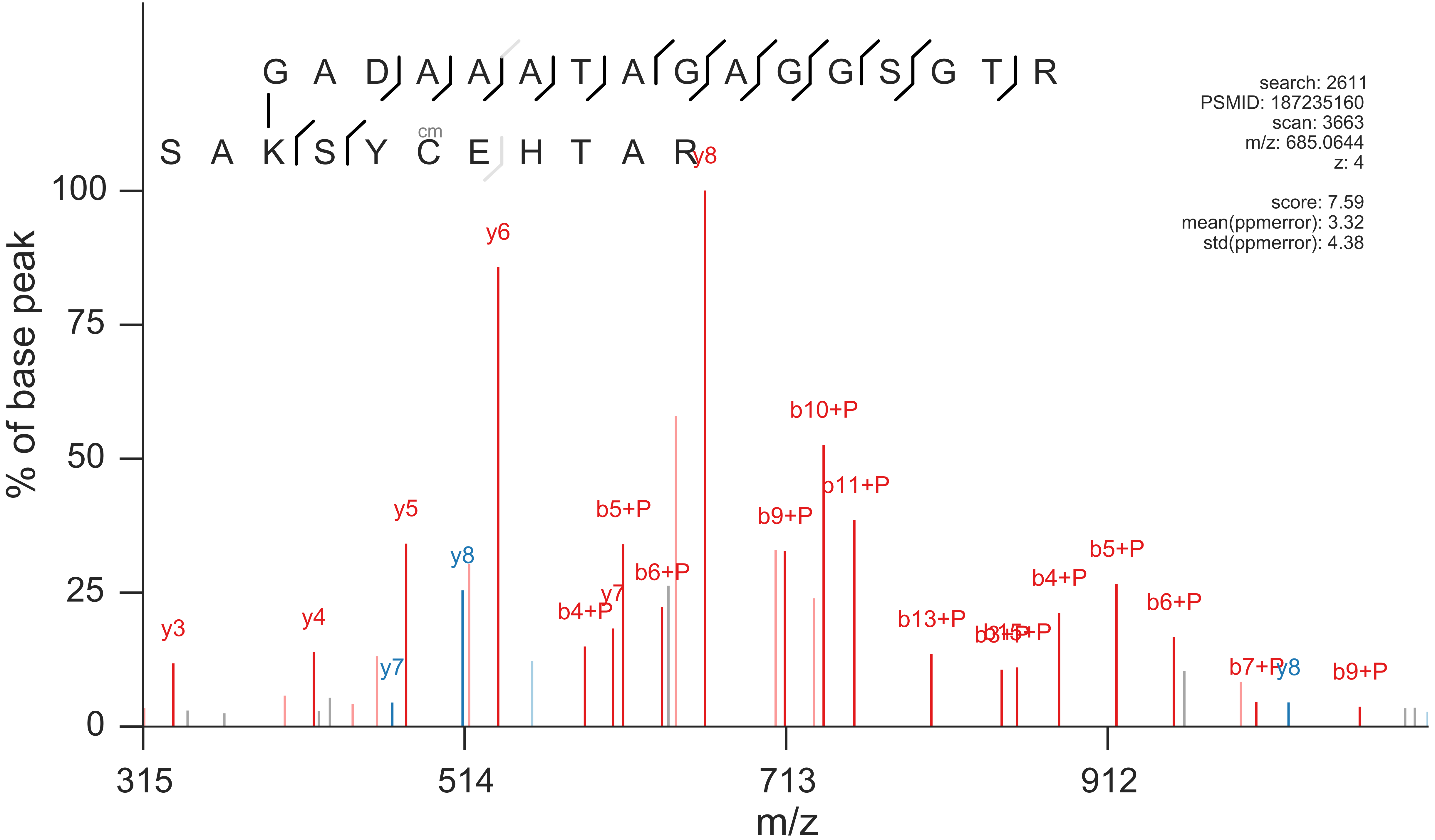

G A D A A A T A G A G G S G T R
S A K S Y C E H T A R
cm
search: 2611
PSMID: 187235160
scan: 3663
m/z: 685.0644
z: 4
score: 7.59
mean(ppmerror): 3.32
std(ppmerror): 4.38
% of base peak
m/z
0
25
50
75
100
315
514
713
912
y3
y4
y7
y5
y8
y6
b4+P
y7
b5+P
b6+P
y8
b9+P
b10+P
b11+P
b13+P
b4+P
b5+P
b6+P
b7+P
y8
b9+P

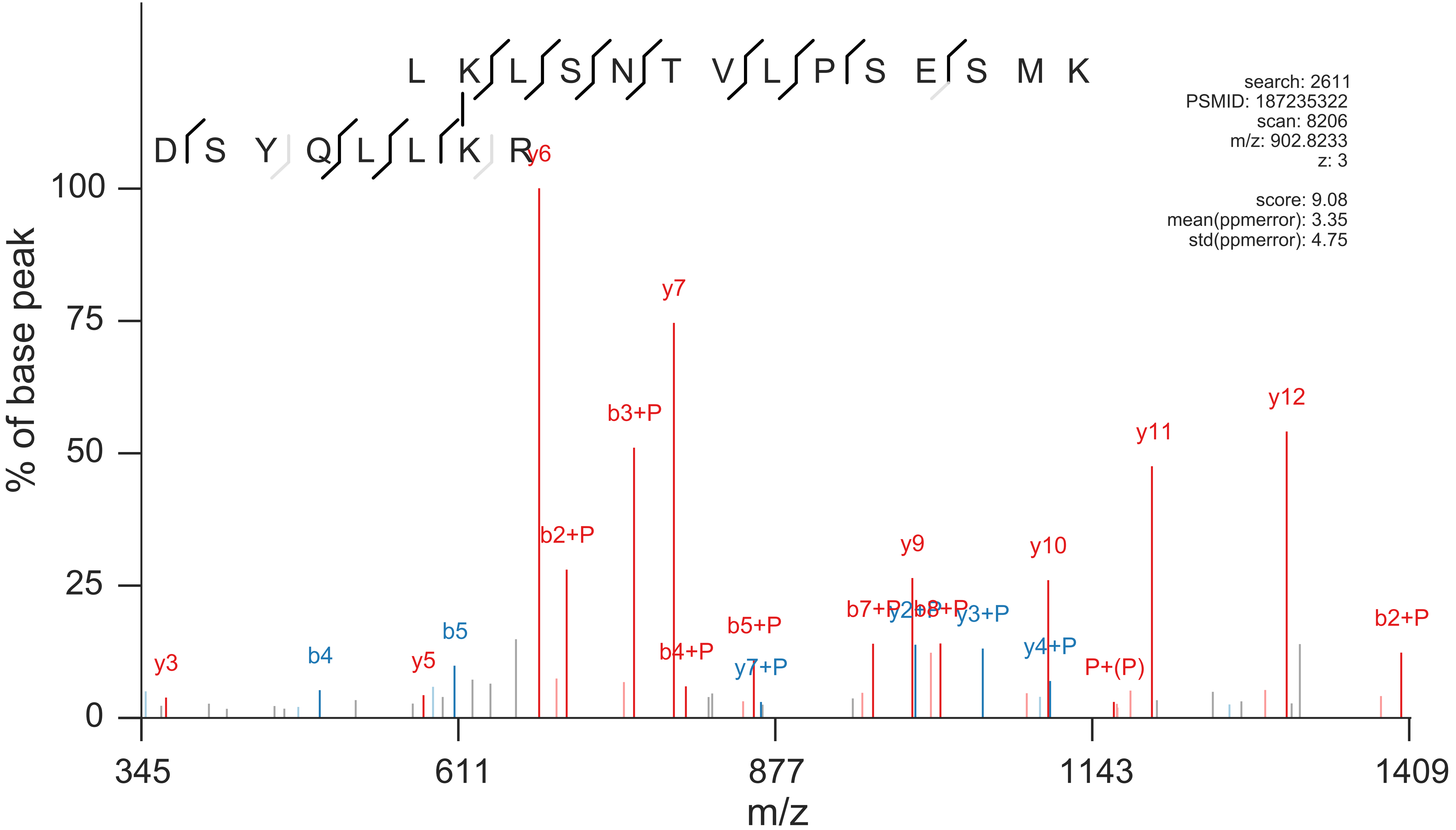

L K L S N T V L P S E S M K
D S Y Q L L K R
search: 2611
PSMID: 187235322
scan: 8206
m/z: 902.8233
z: 3
score: 9.08
mean(ppmerror): 3.35
std(ppmerror): 4.75
% of base peak
m/z
y6
y7
b3+P
y12
y11
b2+P
y9
y10
y3
b4
y5
b5
b4+P
y7+P
b5+P
b7+P
y2
b8+P
y3+P
y4+P
P+(P)
b2+P

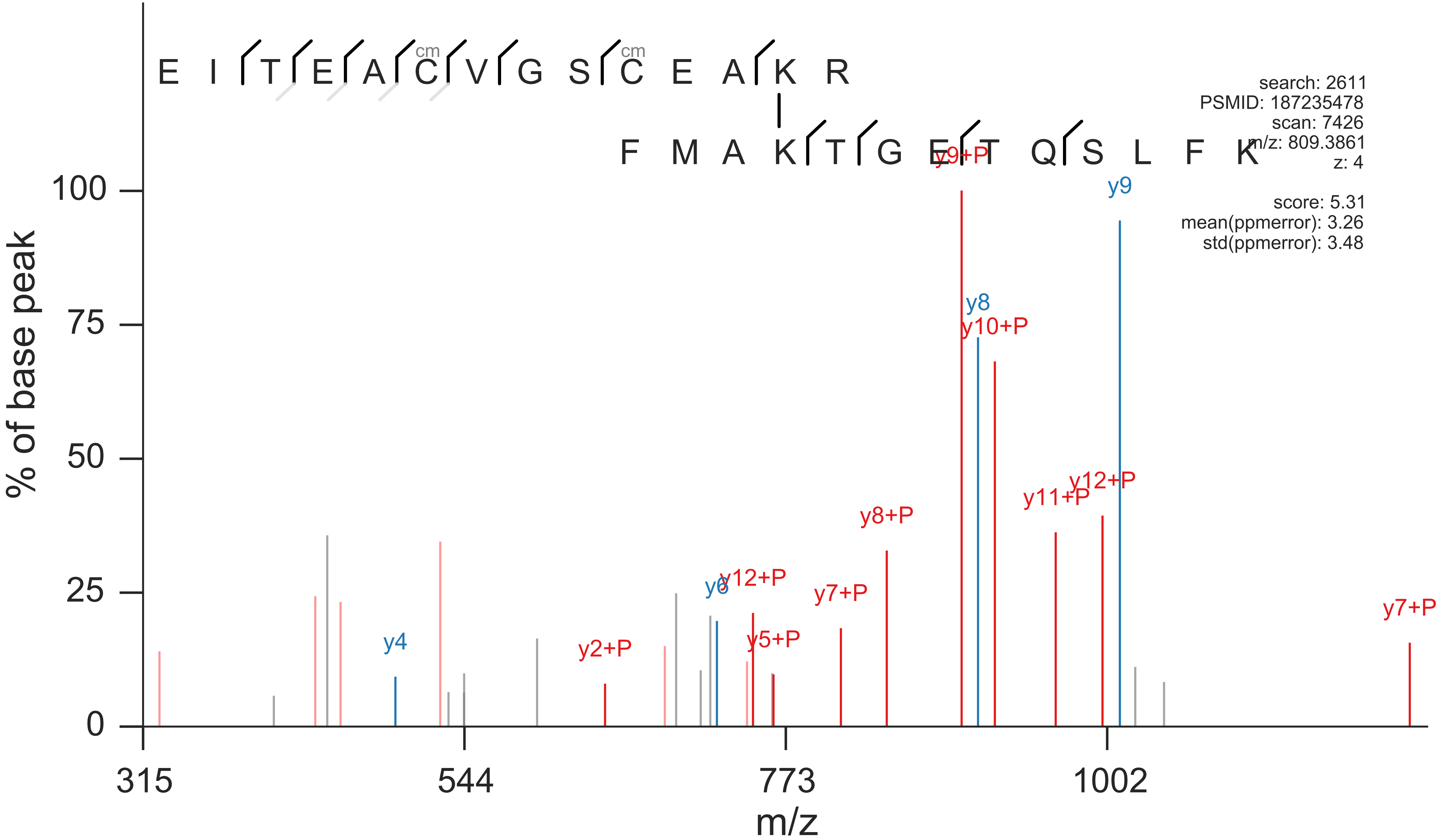

E I T E A C V G S C E A K R
cm
cm
F M A K T G E T Q S L F K
search: 2611
PSMID: 187235478
scan: 7426
m/z: 809.3861
z: 4
score: 5.31
mean(ppmerror): 3.26
std(ppmerror): 3.48
% of base peak
m/z
100
75
50
25
0
315
544
773
1002
y9+P
y9
y8
y10+P
y12+P
y11+P
y8+P
y6
y12+P
y7+P
y5+P
y4
y2+P
y7+P

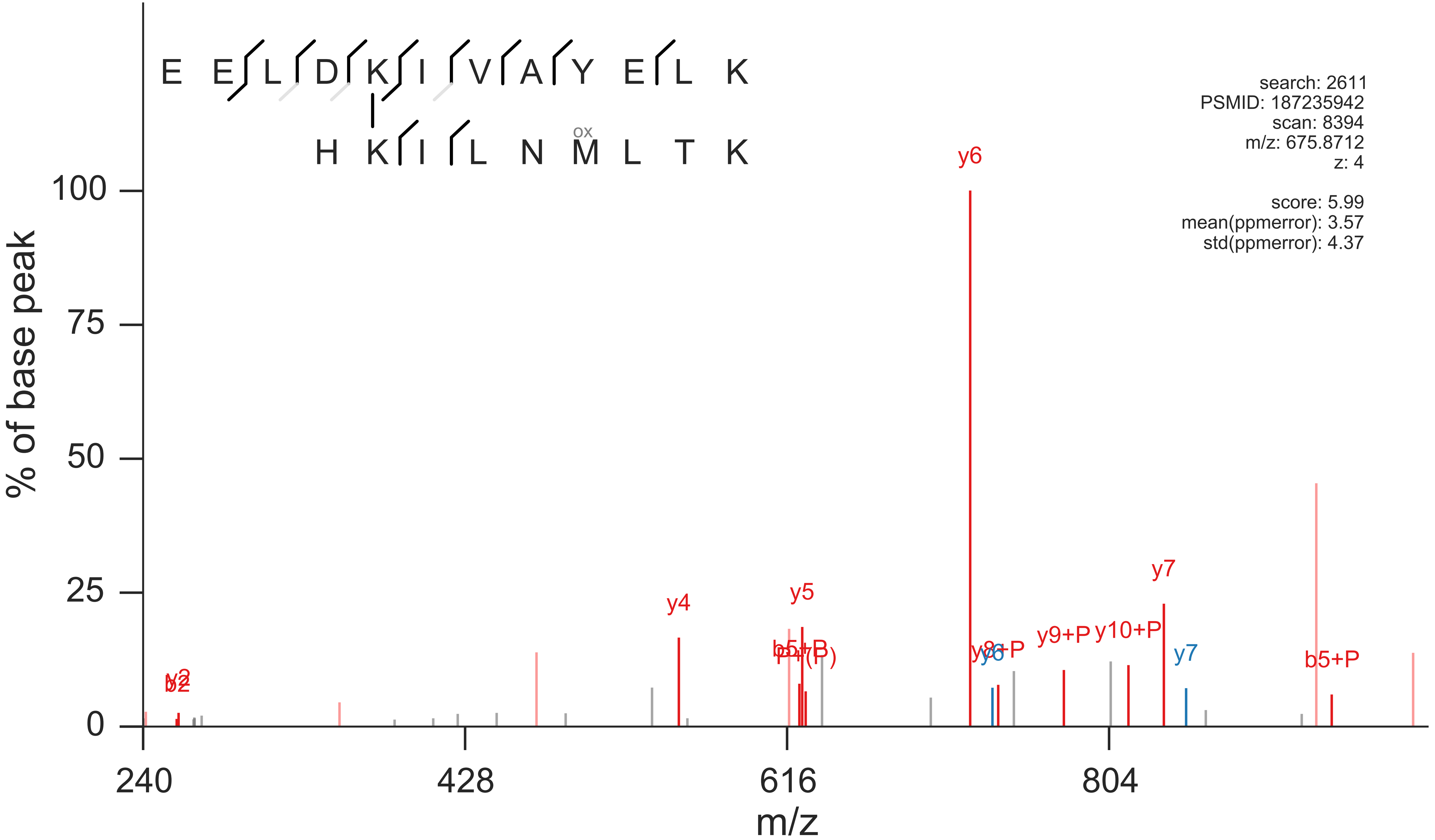

E E L D K I V A Y E L K
H K I L N M L T K
ox
search: 2611
PSMID: 187235942
scan: 8394
m/z: 675.8712
z: 4
score: 5.99
mean(ppmerror): 3.57
std(ppmerror): 4.37
% of base peak
m/z
y6
y7
y5
y4
y9+P
y10+P
b5+P
y2
b2

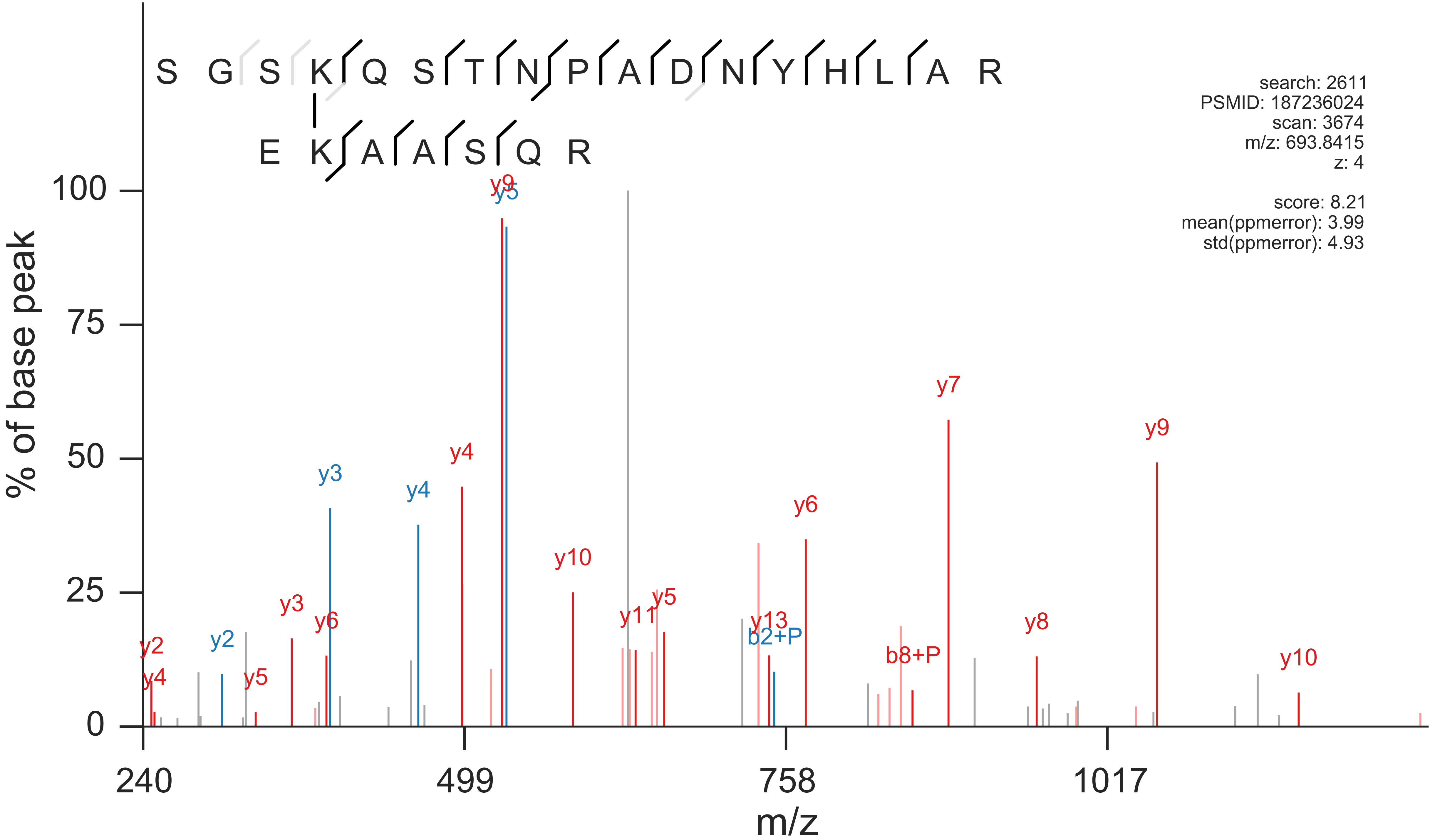
S G S K Q S T N P A D N Y H L A R
E K A A S Q R
search: 2611
PSMID: 187236024
scan: 3674
m/z: 693.8415
z: 4
score: 8.21
mean(ppmerror): 3.99
std(ppmerror): 4.93
% of base peak
m/z
0
25
50
75
100
240
499
758
1017
y2
y4
y2
y5
y3
y6
y3
y4
y4
y9
y5
y10
y11
y5
y13
b2+P
y6
b8+P
y7
y8
y9
y10

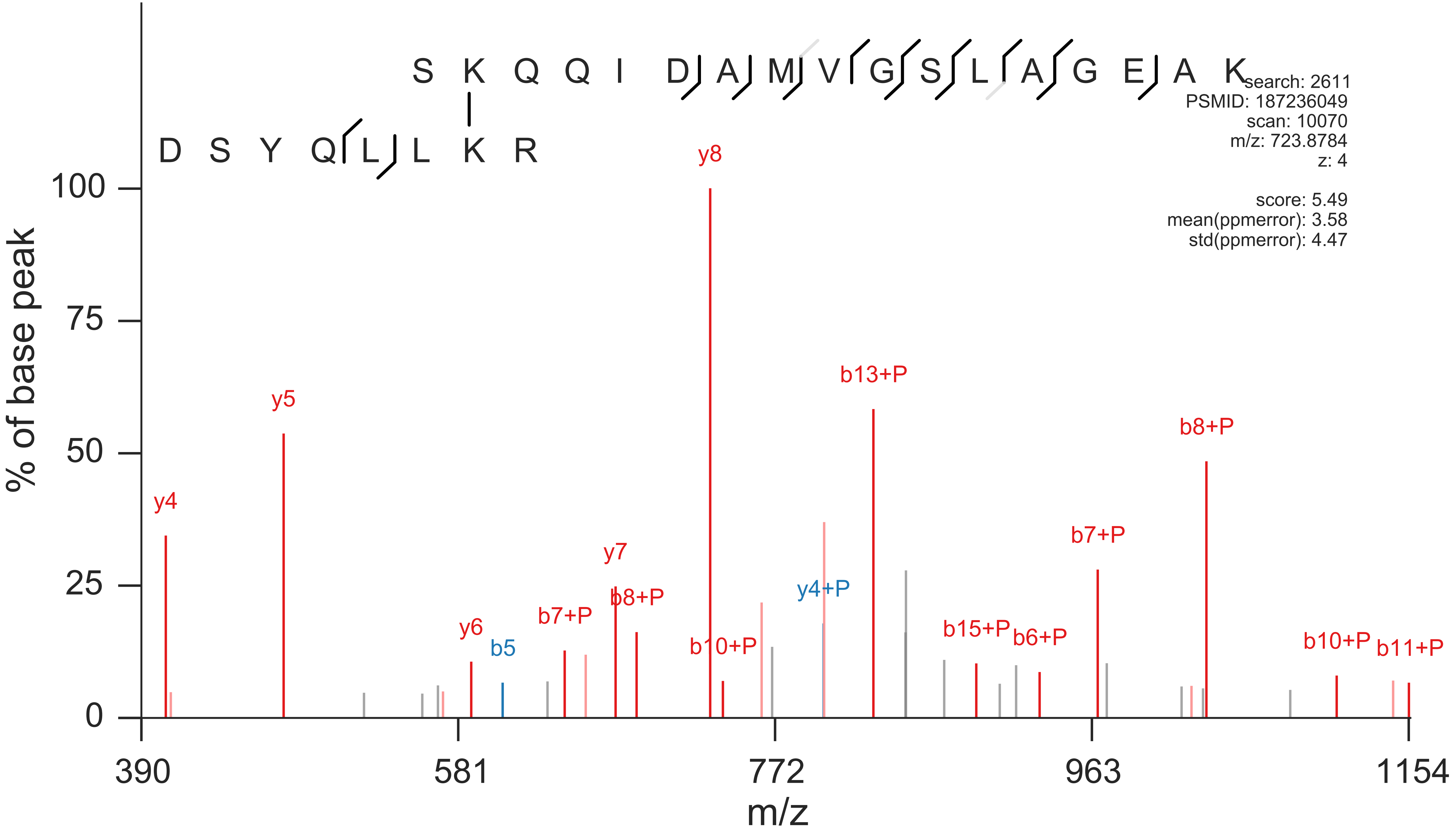

S K Q Q I D A M V G S L A G E A K
D S Y Q L L K R
search: 2611
PSMID: 187236049
scan: 10070
m/z: 723.8784
z: 4
score: 5.49
mean(ppmerror): 3.58
std(ppmerror): 4.47
% of base peak
m/z
y4
y5
y6
b5
b7+P
y7
b8+P
y8
b10+P
y4+P
b13+P
b15+P
b6+P
b7+P
b8+P
b10+P
b11+P

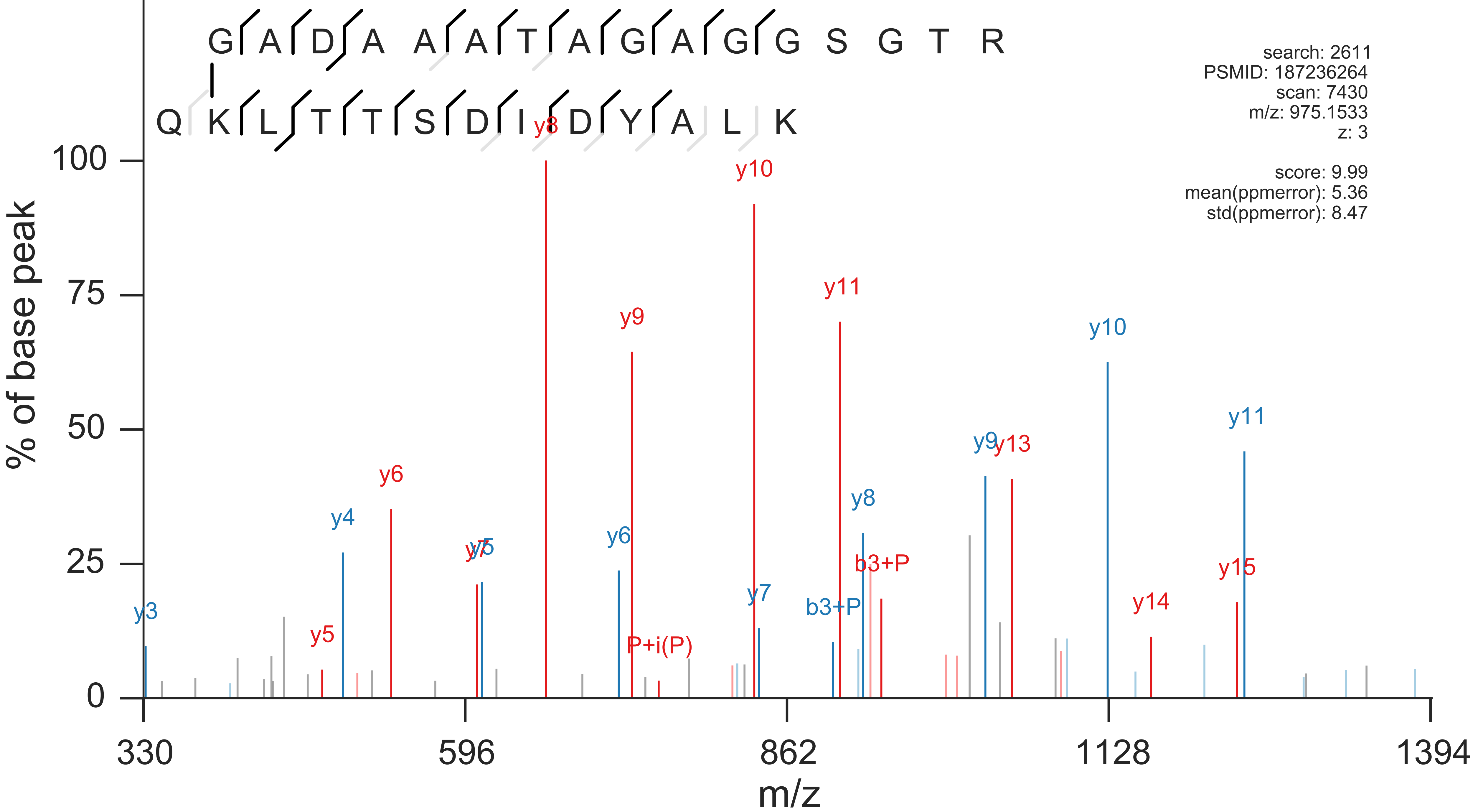

G A D A A A T A G A G G S G T R
Q K L T T S D I D Y A L K
search: 2611
PSMID: 187236264
scan: 7430
m/z: 975.1533
z: 3
score: 9.99
mean(ppmerror): 5.36
std(ppmerror): 8.47
% of base peak
m/z
0
25
50
75
100
330
596
862
1128
1394
y3
y4
y5
y6
y7
y8
y9
y10
y11
y13
y14
y15
b3+P
P+i(P)

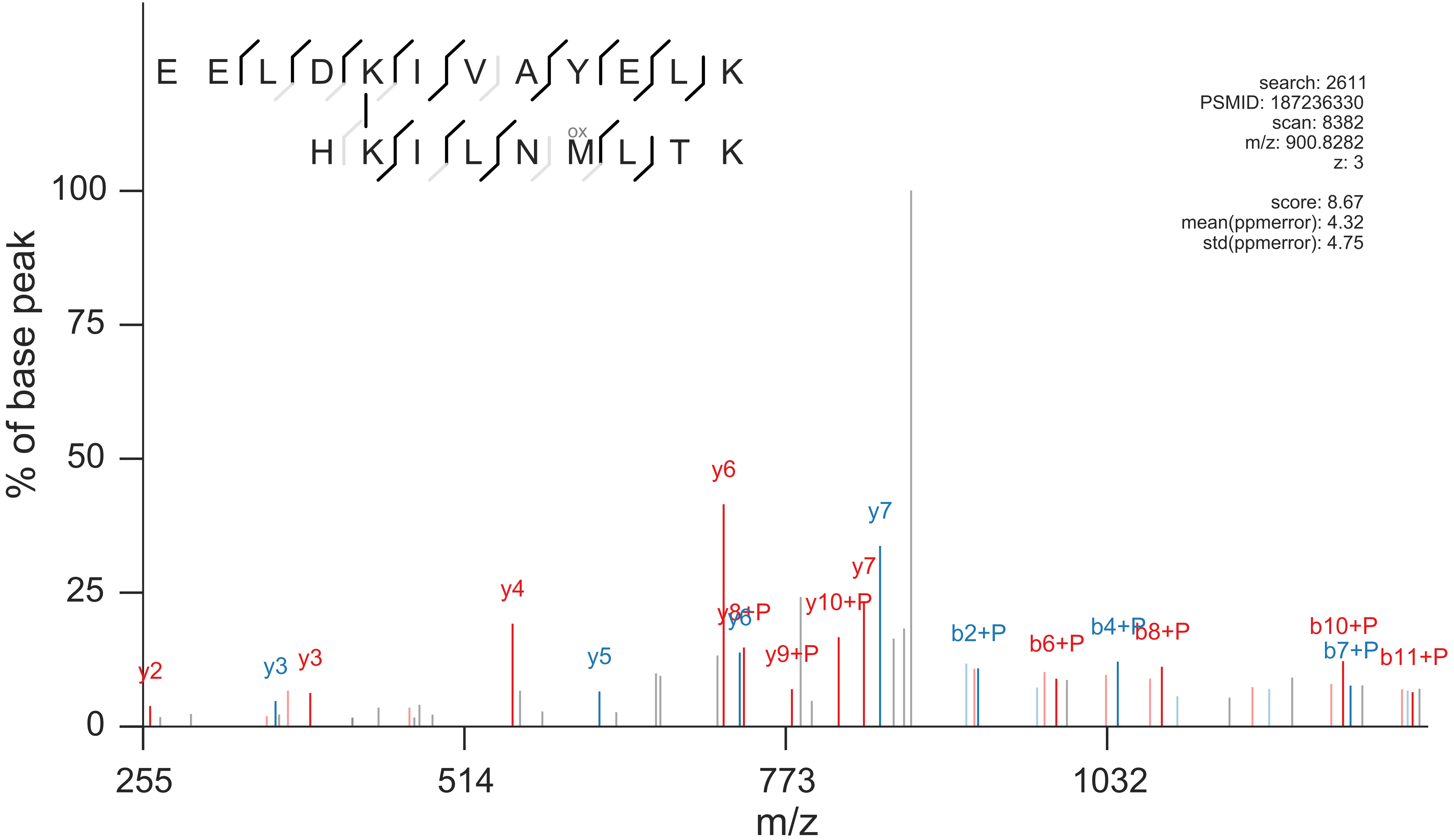
E E L D K I V A Y E L K
H K I L N M L T K
ox
search: 2611
PSMID: 187236330
scan: 8382
m/z: 900.8282
z: 3
score: 8.67
mean(ppmerror): 4.32
std(ppmerror): 4.75
% of base peak
100
75
50
25
0
255
514
773
1032
m/z
y2
y3
y3
y4
y5
y6
y8+P
y6
y9+P
y10+P
y7
y7
b2+P
b6+P
b4+P
b8+P
b10+P
b7+P
b11+P

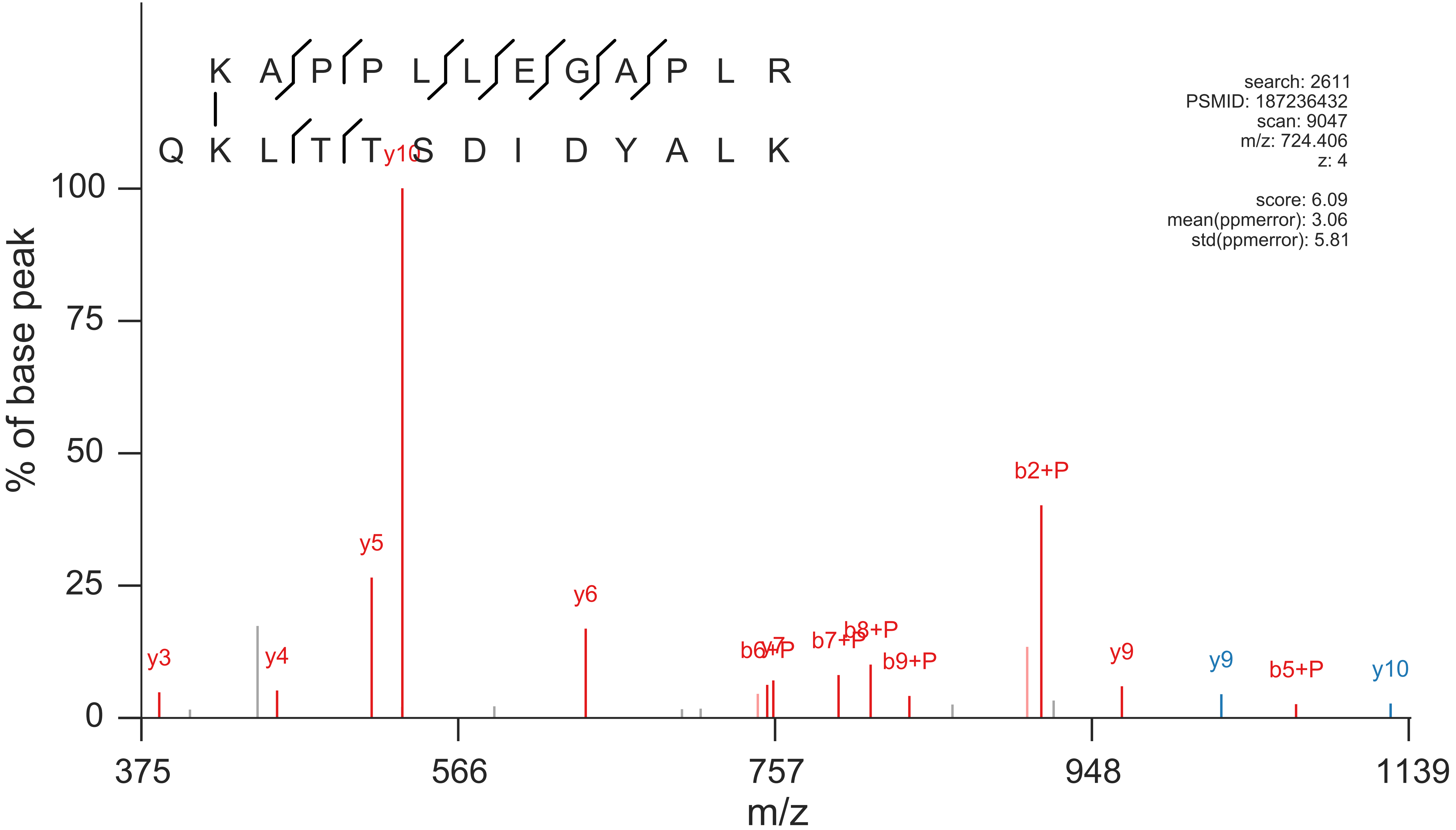
K A P P L L E G A P L R
Q K L T T S D I D Y A L K
search: 2611
PSMID: 187236432
scan: 9047
m/z: 724.406
z: 4
score: 6.09
mean(ppmerror): 3.06
std(ppmerror): 5.81
% of base peak
m/z
100
75
50
25
0
375
566
757
948
1139
y10
y3
y4
y5
y6
b6+P
y7
b7+P
b8+P
b9+P
b2+P
y9
y9
b5+P
y10

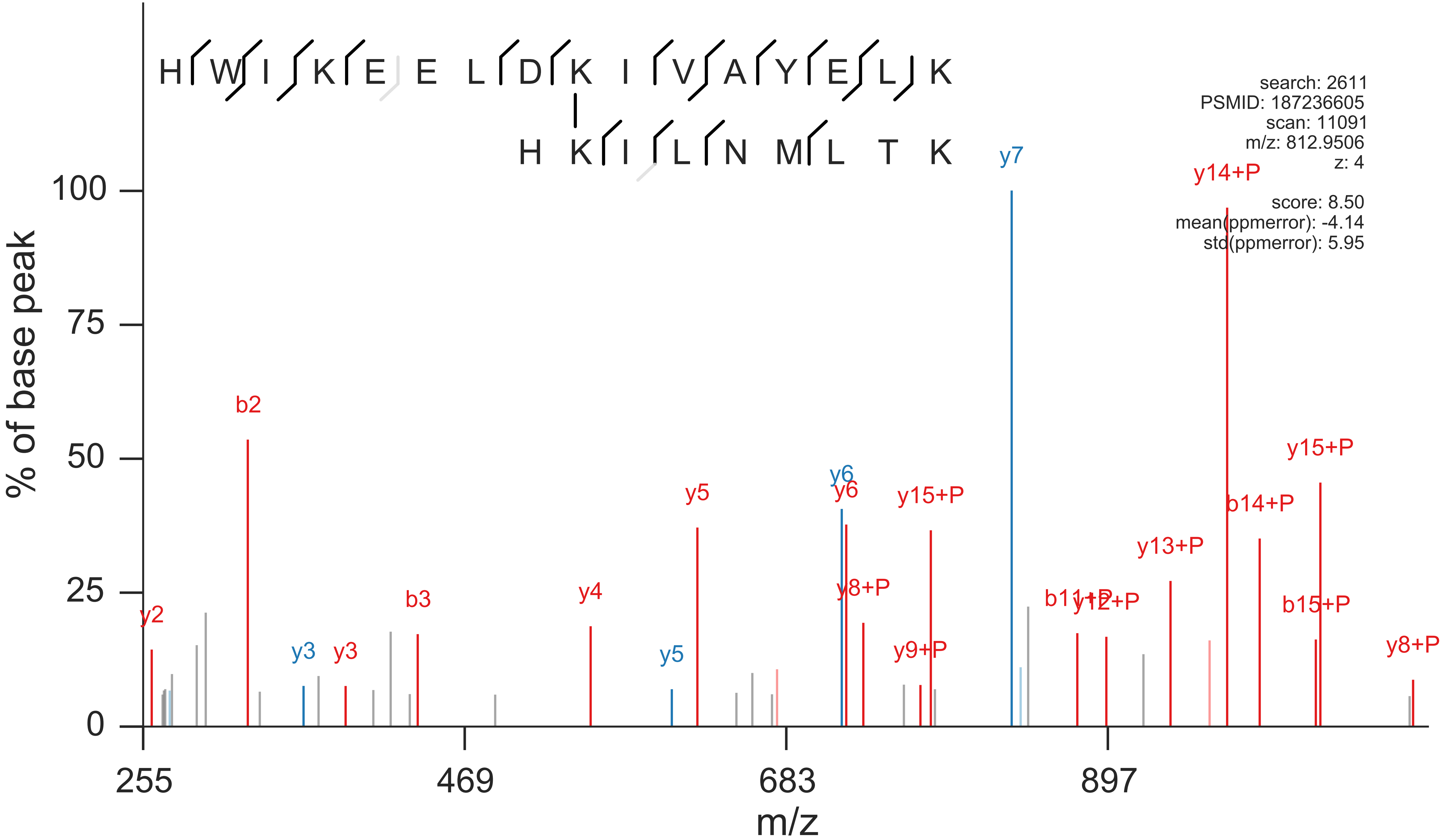

H W I K E E L D K I V A Y E L K
H K I L N M L T K
search: 2611
PSMID: 187236605
scan: 11091
m/z: 812.9506
z: 4
score: 8.50
mean(ppmerror): -4.14
std(ppmerror): 5.95
% of base peak
m/z

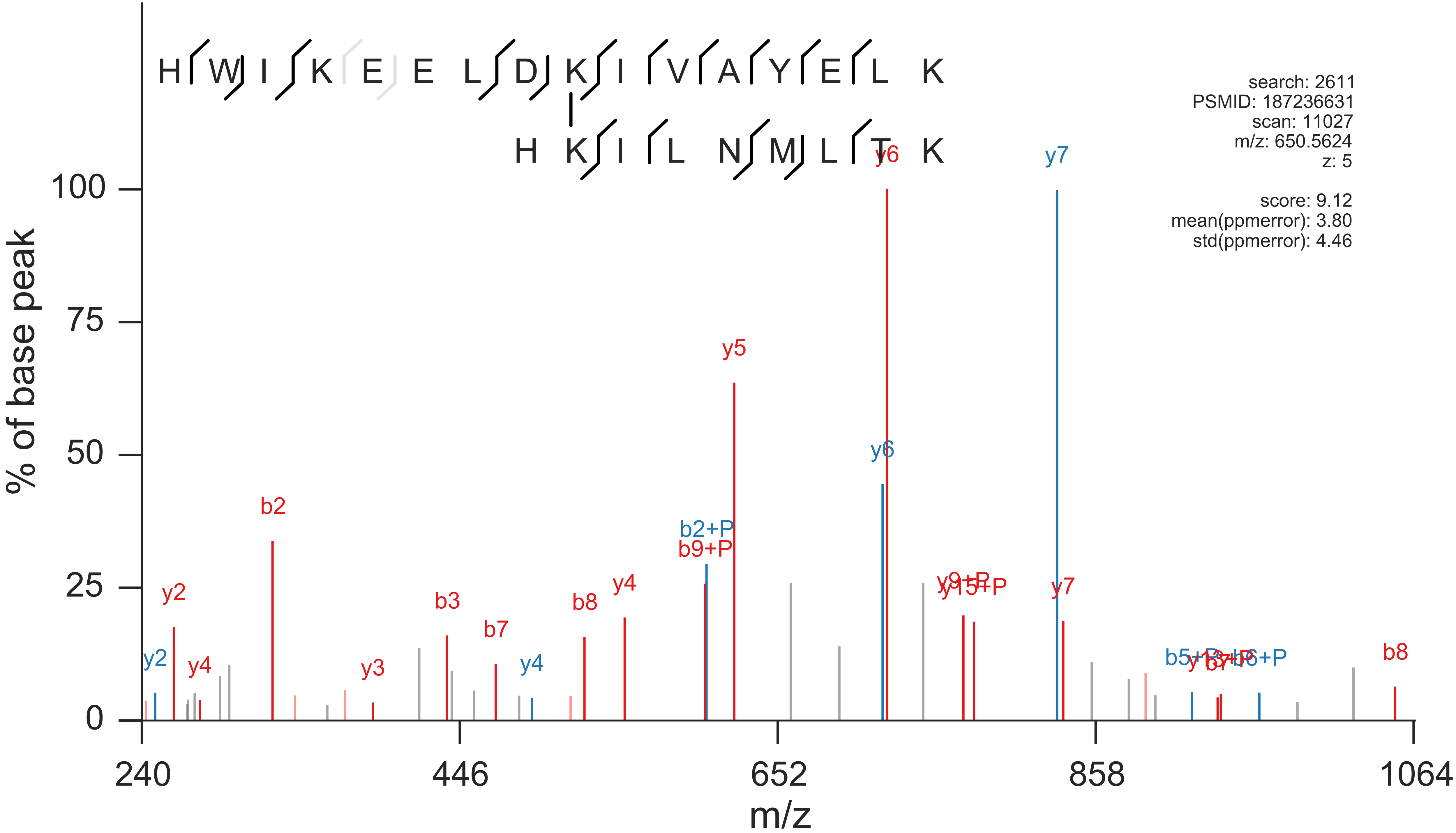

H W I K E E L D K I V A Y E L K
H K I L N M L T K
search: 2611
PSMID: 187236631
scan: 11027
m/z: 650.5624
z: 5
score: 9.12
mean(ppmerror): 3.80
std(ppmerror): 4.46
% of base peak
m/z
0
25
50
75
100
240
446
652
858
1064
y2
y2
y4
b2
y3
b3
b7
y4
b8
y4
b2+P
b9+P
y5
y6
y6
y7
y7
y15+P
b8

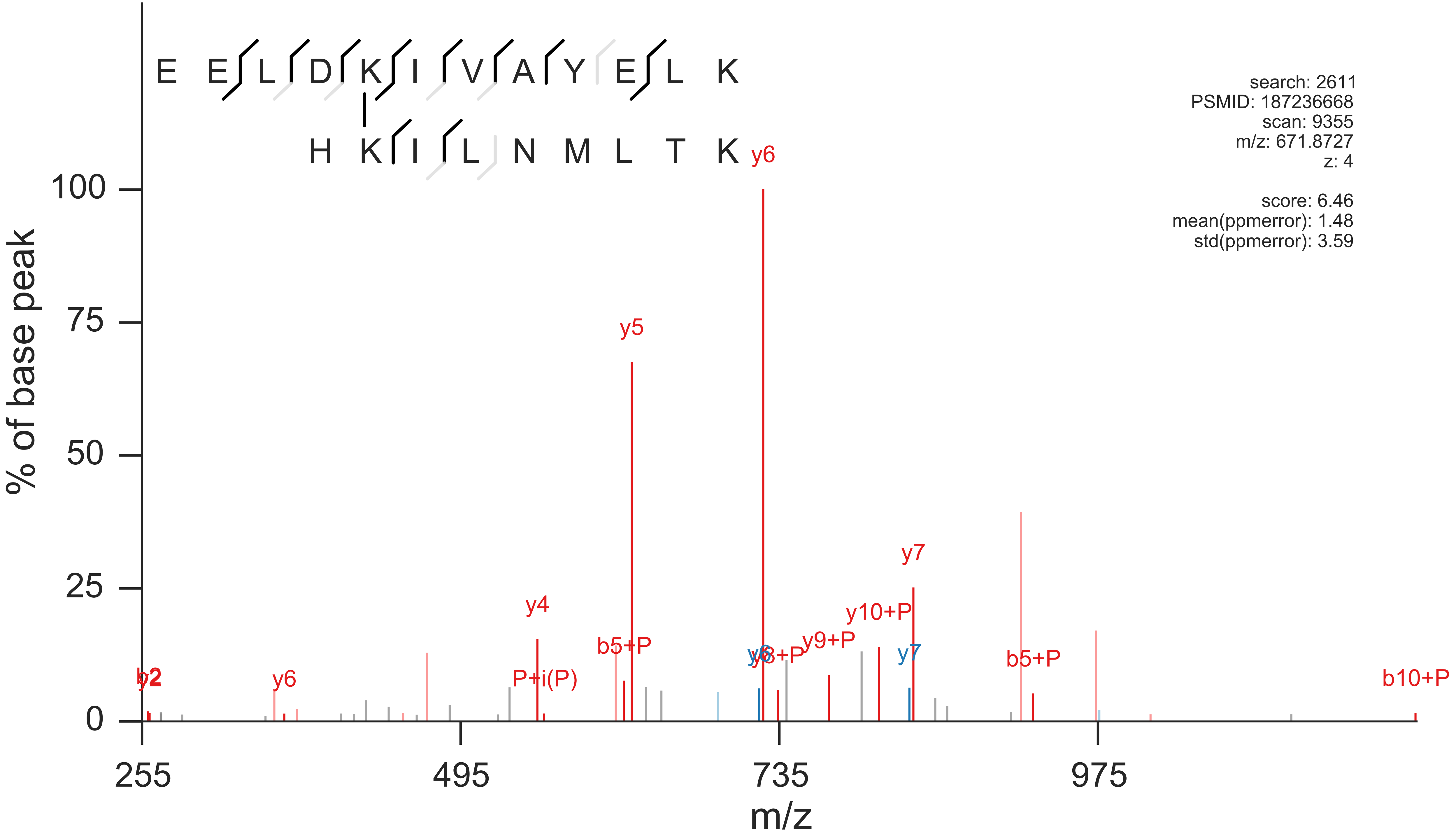

E E L D K I V A Y E L K
H K I L N M L T K
search: 2611
PSMID: 187236668
scan: 9355
m/z: 671.8727
z: 4
score: 6.46
mean(ppmerror): 1.48
std(ppmerror): 3.59
% of base peak
m/z
0
25
50
75
100
255
495
735
975
y6
y5
y7
y4
y10+P
y9+P
b5+P
y8+P
y6
y7
b5+P
P+i(P)
b2
y6
b10+P

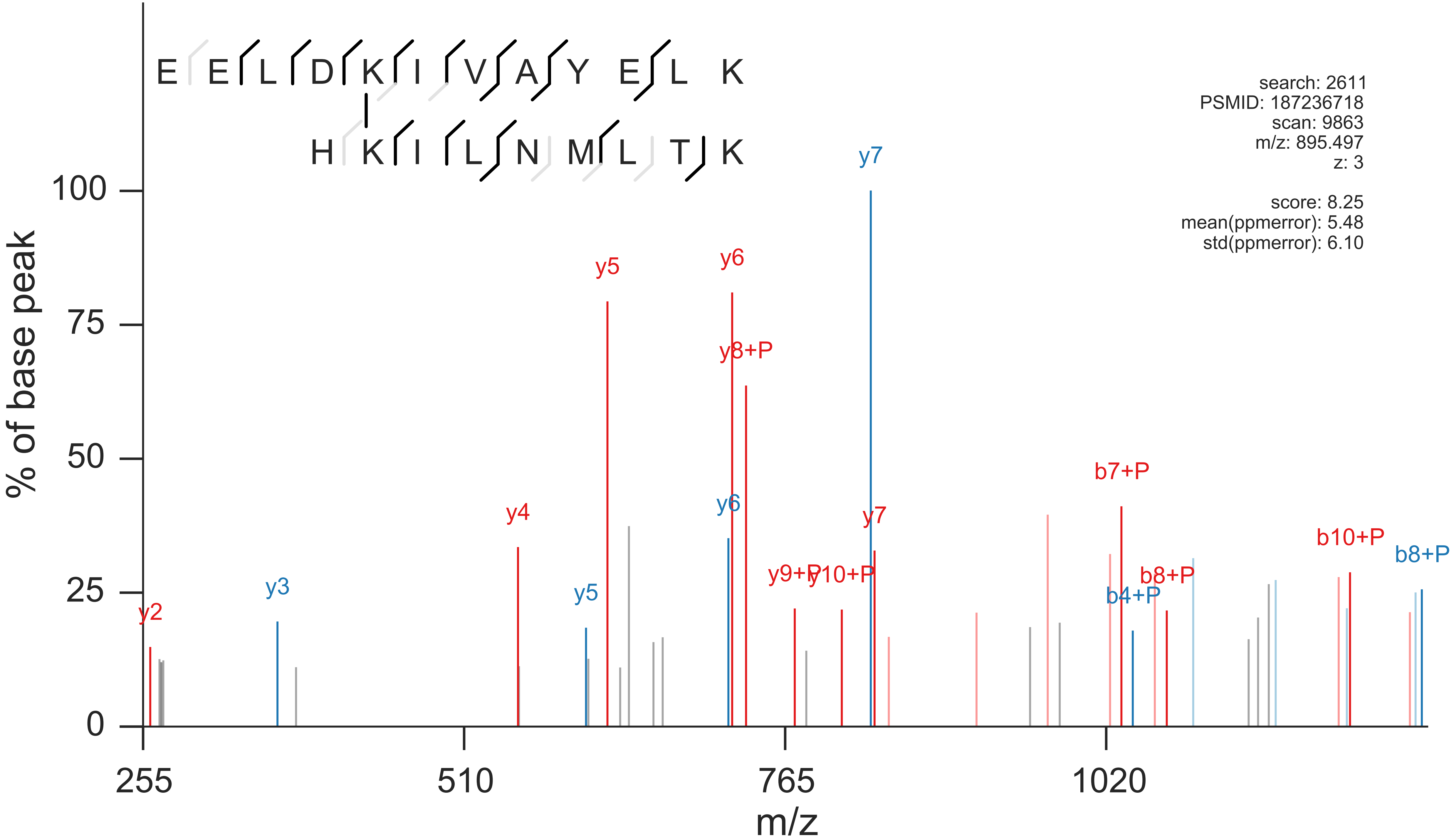

E E L D K I V A Y E L K
H K I L N M L T K
search: 2611
PSMID: 187236718
scan: 9863
m/z: 895.497
z: 3
score: 8.25
mean(ppmerror): 5.48
std(ppmerror): 6.10
% of base peak
m/z
0
25
50
75
100
255
510
765
1020
y2
y3
y4
y5
y5
y6
y6
y8+P
y7
y7
y9+P
y10+P
b7+P
b8+P
b4+P
b10+P
b8+P

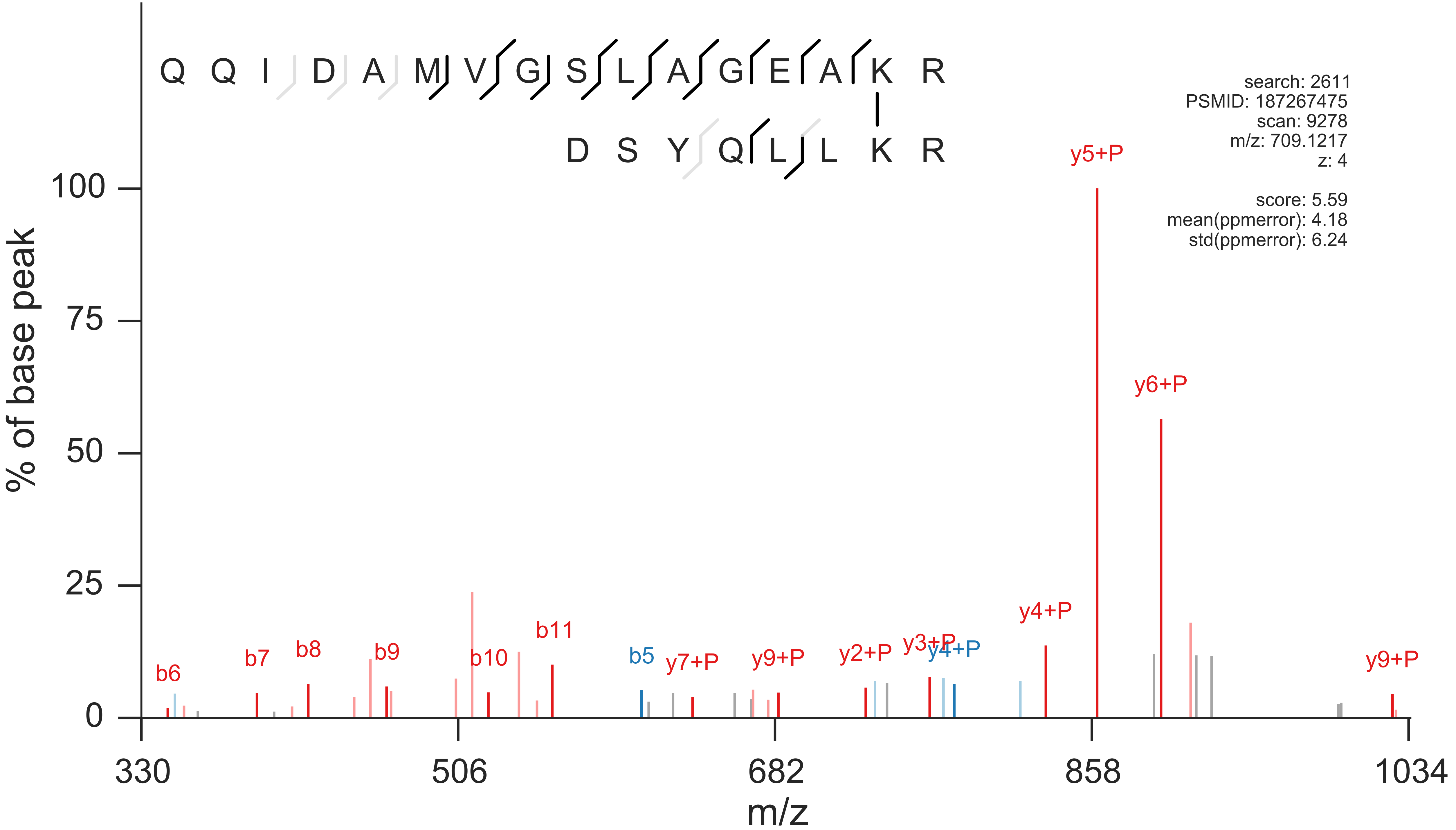

Q Q I D A M V G S L A G E A K R
D S Y Q L L K R
search: 2611
PSMID: 187267475
scan: 9278
m/z: 709.1217
z: 4
score: 5.59
mean(ppmerror): 4.18
std(ppmerror): 6.24
% of base peak
0
25
50
75
100
m/z
330
506
682
858
1034
y5+P
y6+P
y4+P
b6
b7
b8
b9
b10
b11
b5
y7+P
y9+P
y2+P
y3+P
y4+P
y9+P

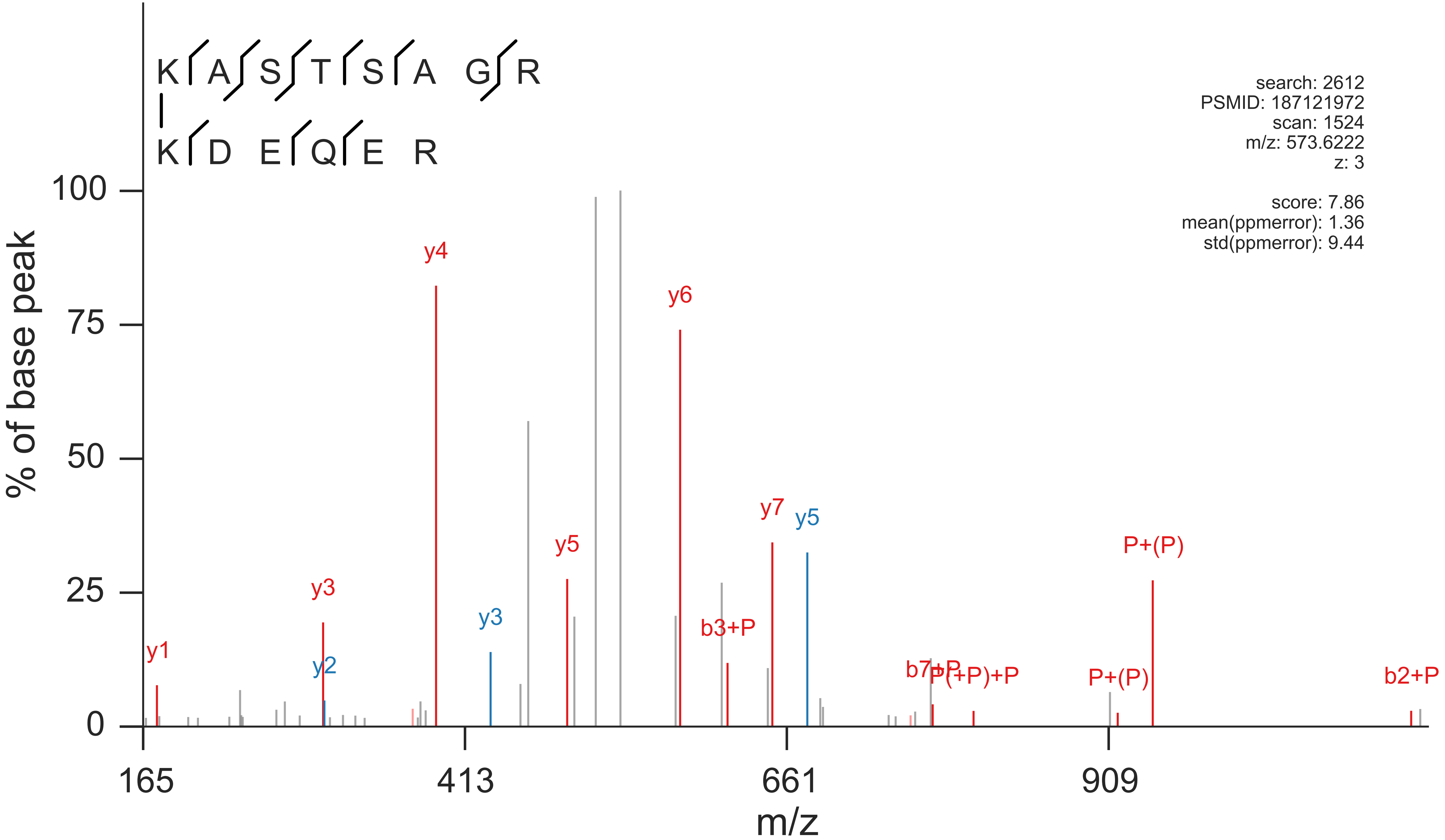

K A S T S A G R
K D E Q E R
search: 2612
PSMID: 187121972
scan: 1524
m/z: 573.6222
z: 3
score: 7.86
mean(ppmerror): 1.36
std(ppmerror): 9.44
% of base peak
100
75
50
25
0
y1
y3
y2
y4
y3
y5
y6
b3+P
y7
y5
b7+P
P(+P)+P
P+(P)
P+(P)
b2+P
165
413
661
909
m/z

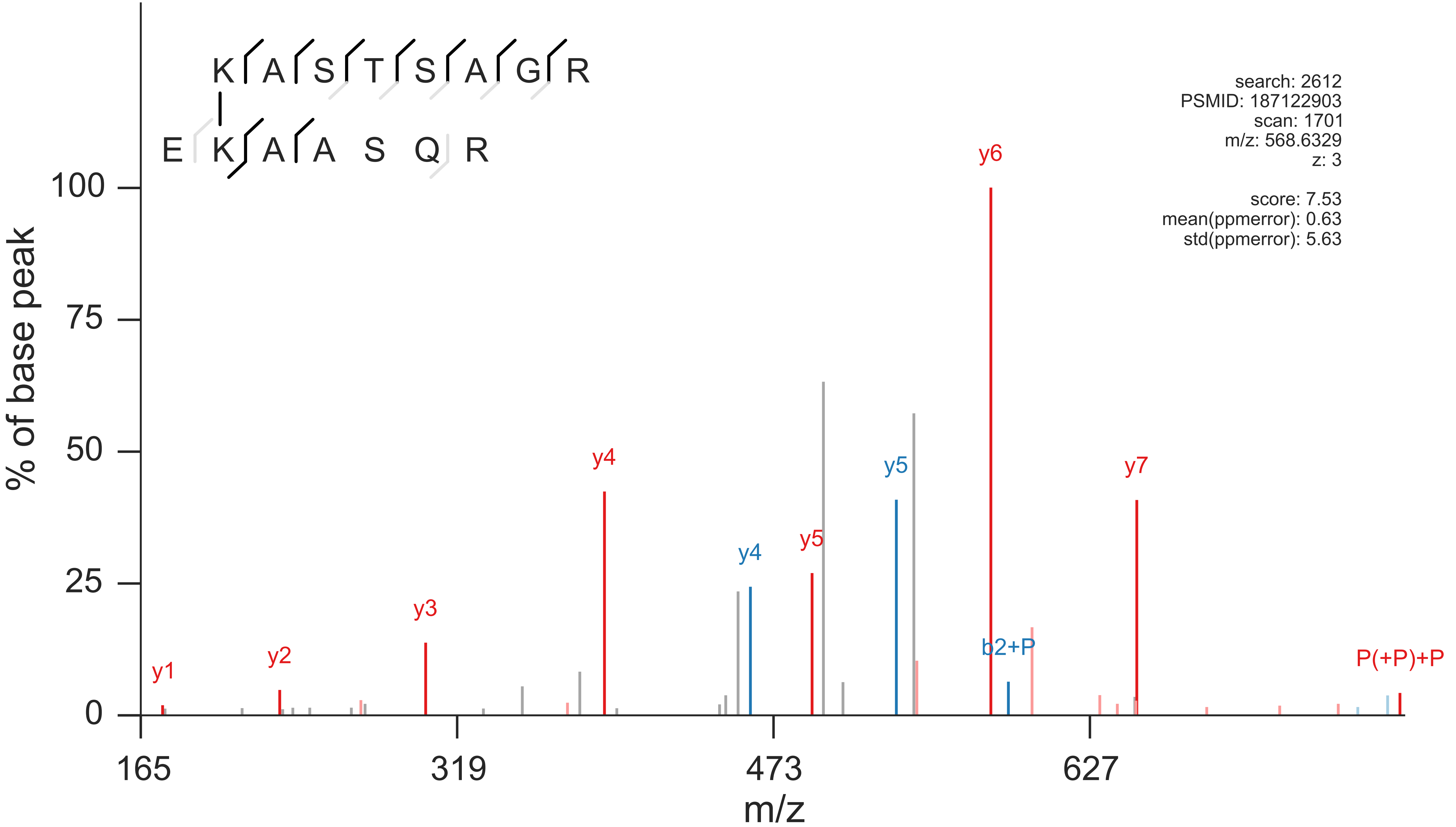
K A S T S A G R
E K A A S Q R
search: 2612
PSMID: 187122903
scan: 1701
m/z: 568.6329
z: 3
score: 7.53
mean(ppmerror): 0.63
std(ppmerror): 5.63
% of base peak
m/z
100
75
50
25
0
165
319
473
627
y1
y2
y3
y4
y4
y5
y5
y6
b2+P
y7
P(+P)+P

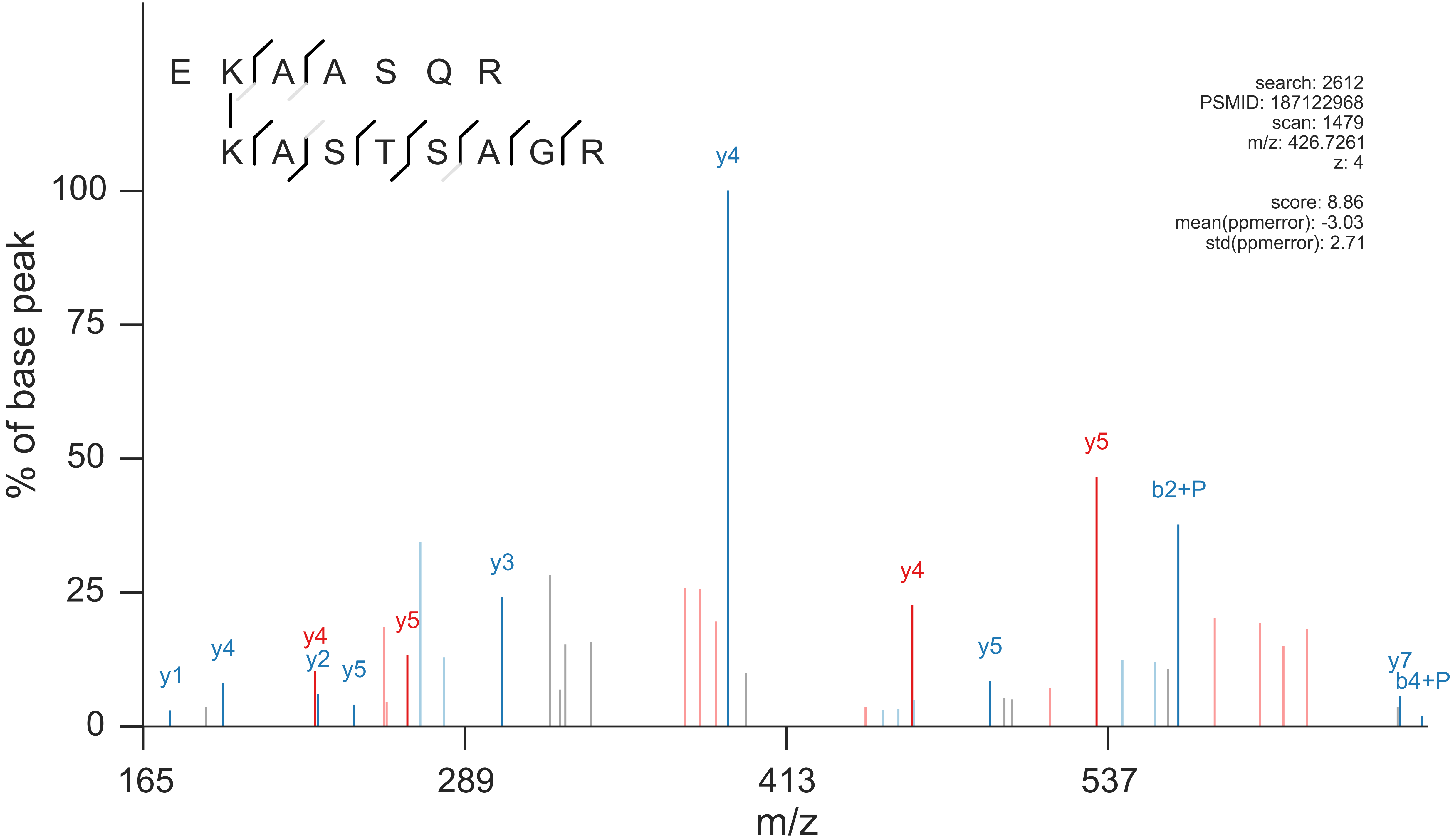

E K A A S Q R
K A S T S A G R
search: 2612
PSMID: 187122968
scan: 1479
m/z: 426.7261
z: 4
score: 8.86
mean(ppmerror): -3.03
std(ppmerror): 2.71
% of base peak
100
75
50
25
0
165
289
413
537
m/z
y1
y4
y4
y2
y5
y5
y3
y4
y4
y5
y5
b2+P
y7
b4+P

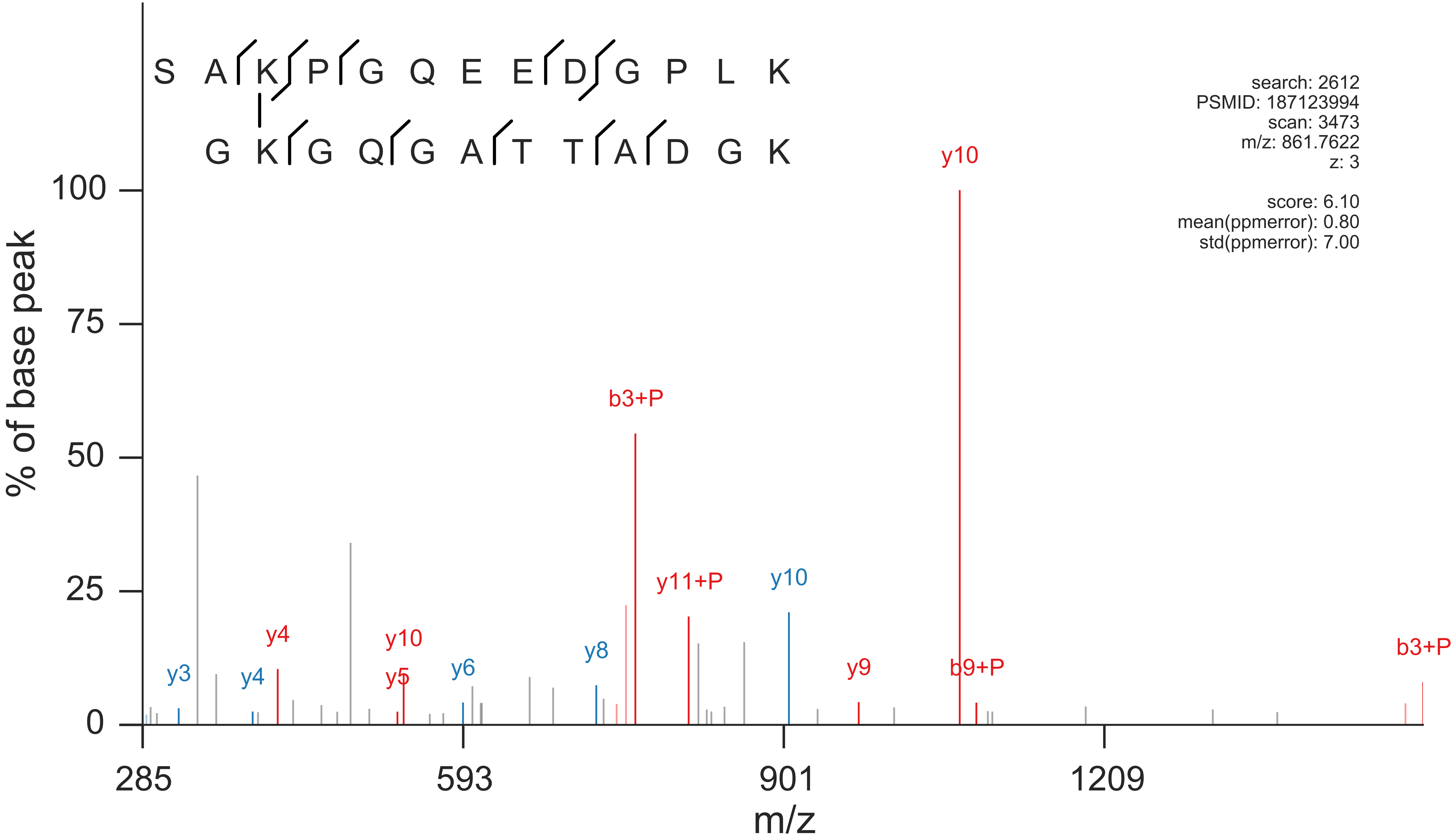

S A K P G Q E E D G P L K
G K G Q G A T T A D G K
search: 2612
PSMID: 187123994
scan: 3473
m/z: 861.7622
z: 3
score: 6.10
mean(ppmerror): 0.80
std(ppmerror): 7.00
% of base peak
100
75
50
25
0
285
593
901
1209
m/z
y10
b3+P
y11+P
y10
y4
y10
y5
y3
y4
y6
y8
y9
b9+P
b3+P

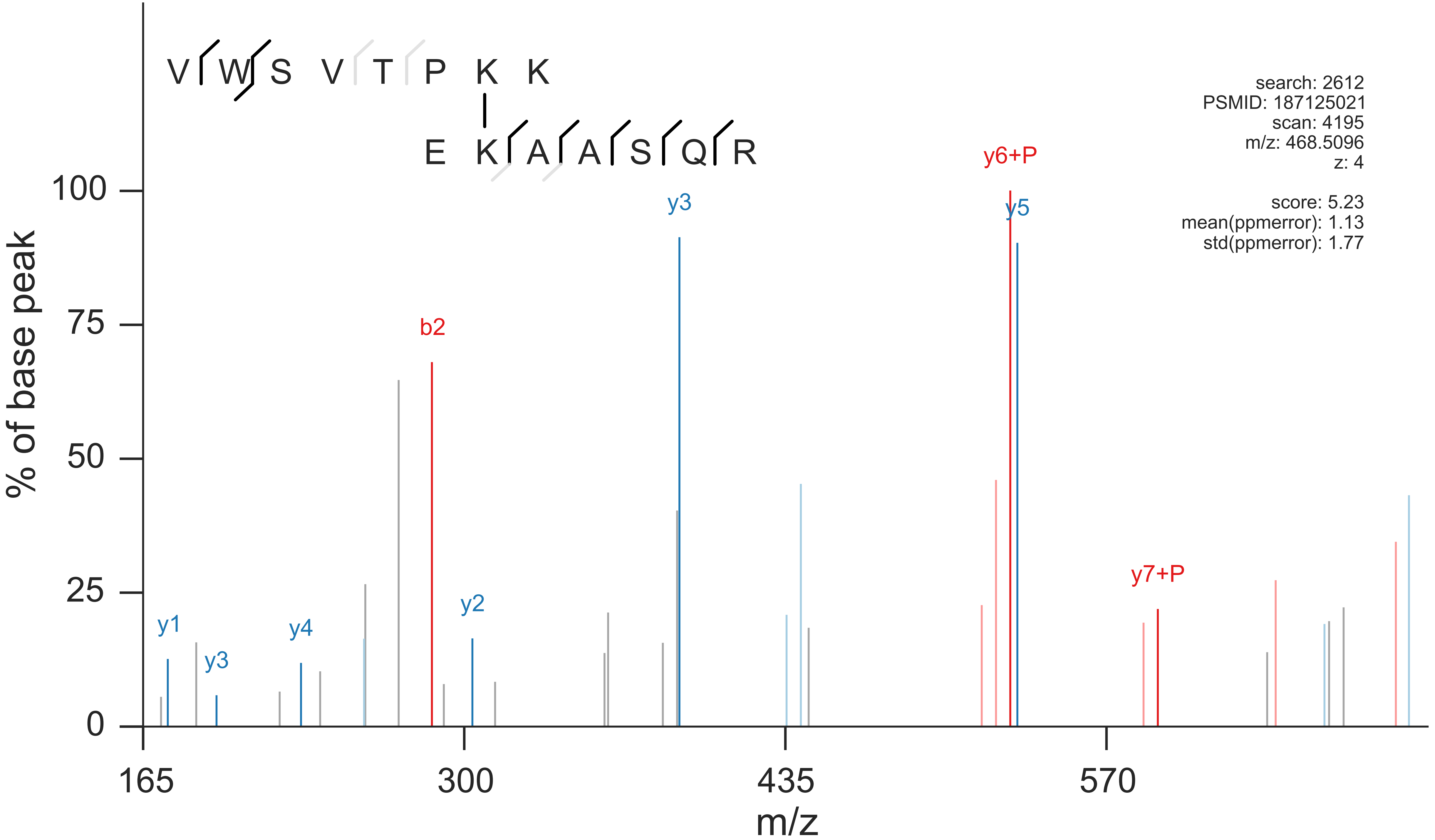

V W S V T P K K
E K A A S Q R
search: 2612
PSMID: 187125021
scan: 4195
m/z: 468.5096
z: 4
score: 5.23
mean(ppmerror): 1.13
std(ppmerror): 1.77
% of base peak
m/z
y1
y3
y4
b2
y2
y3
y6+P
y5
y7+P
0
25
50
75
100
165
300
435
570

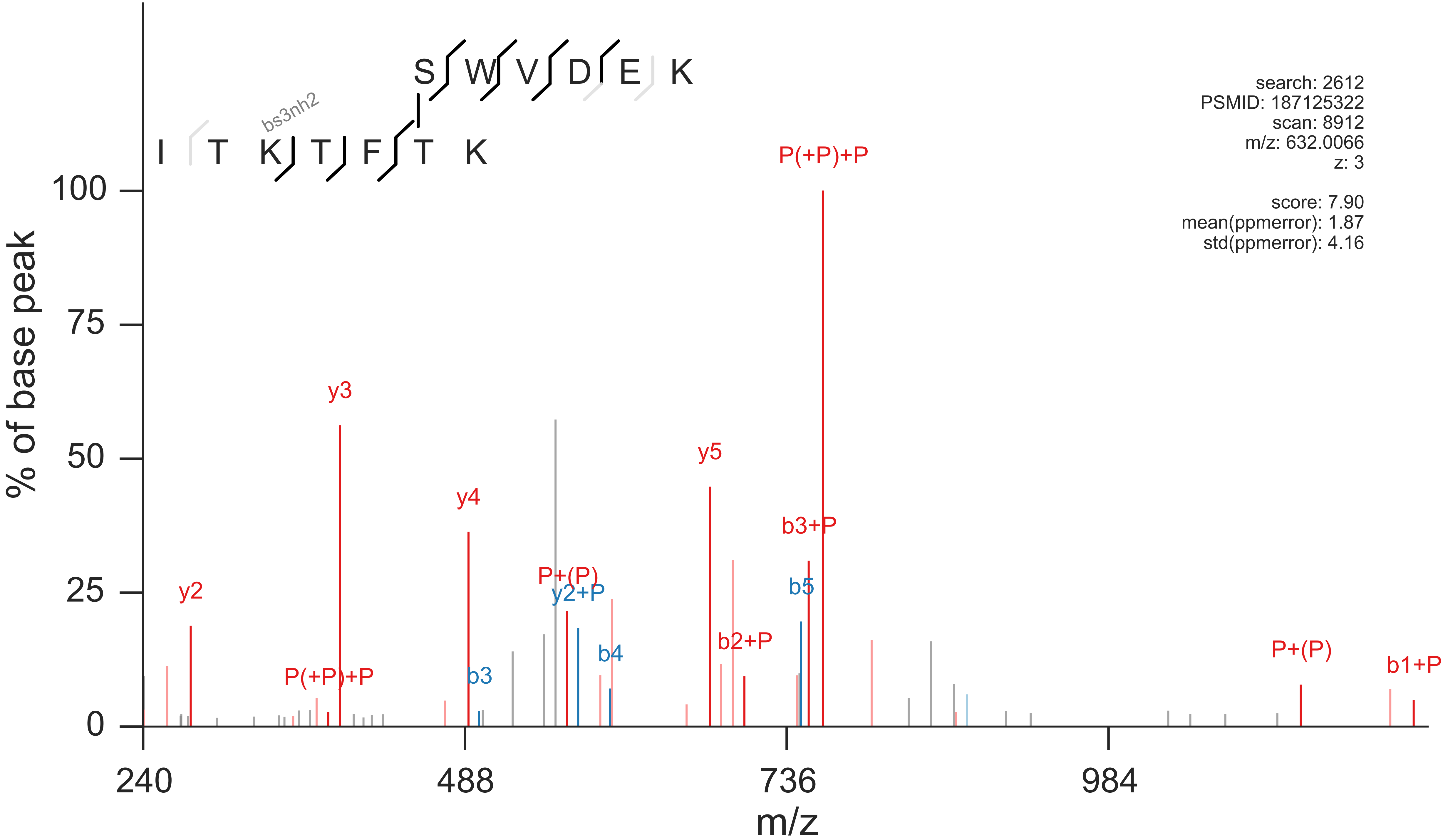
S W V D E K
I T K T F T K
bs3nh2
search: 2612
PSMID: 187125322
scan: 8912
m/z: 632.0066
z: 3
score: 7.90
mean(ppmerror): 1.87
std(ppmerror): 4.16
% of base peak
100
75
50
25
0
240
488
736
984
m/z
P(+P)+P
y3
y5
y4
b3+P
P+(P)
y2+P
b5
y2
b2+P
b4
P(+P)+P
b3
P+(P)
b1+P

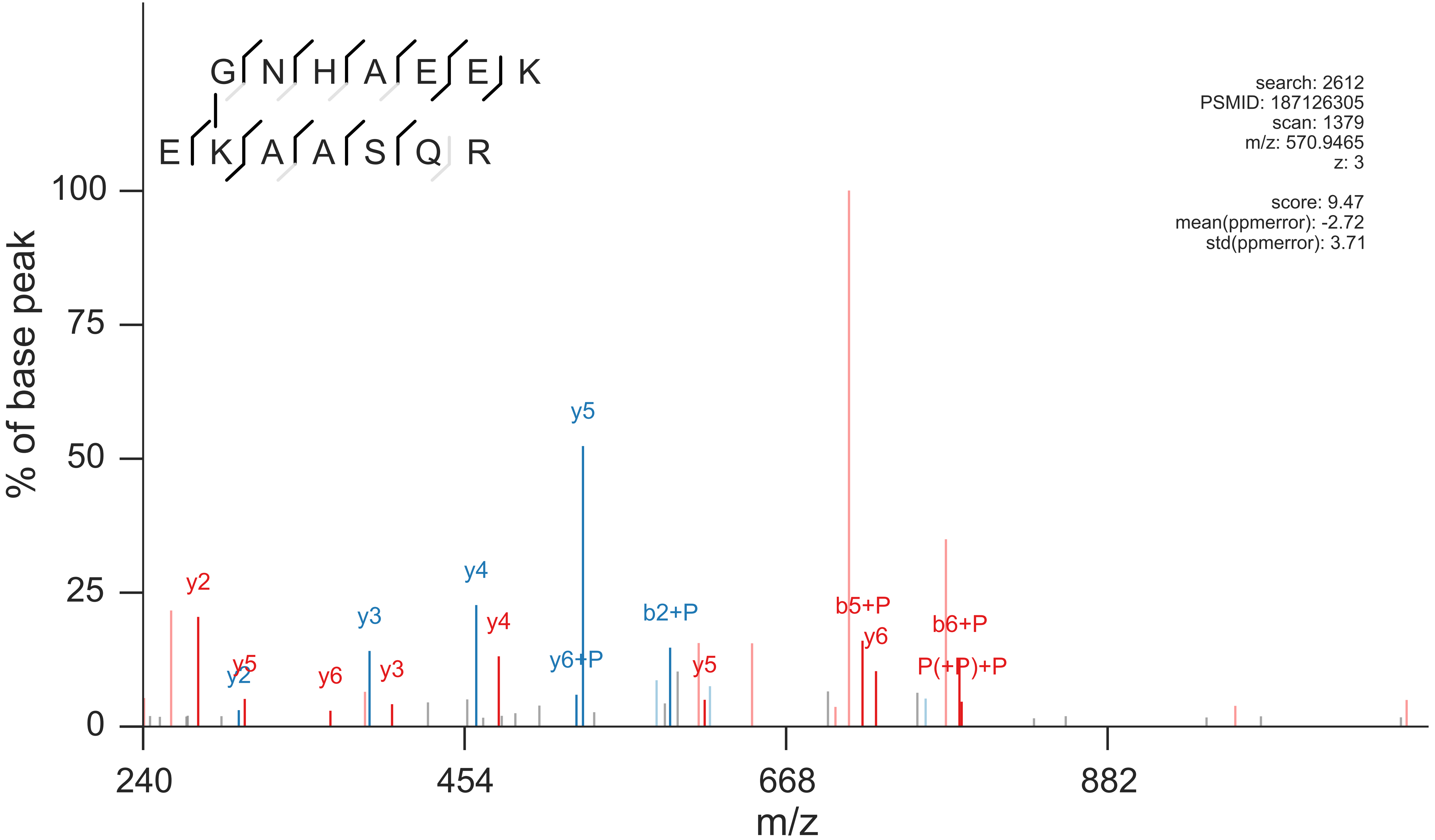
G N H A E E K
E K A A S Q R
search: 2612
PSMID: 187126305
scan: 1379
m/z: 570.9465
z: 3
score: 9.47
mean(ppmerror): -2.72
std(ppmerror): 3.71
% of base peak
100
75
50
25
0
240
454
668
882
m/z
y2
y5
y2
y6
y3
y3
y4
y4
y6+P
y5
b2+P
y5
b5+P
y6
b6+P
P(+P)+P

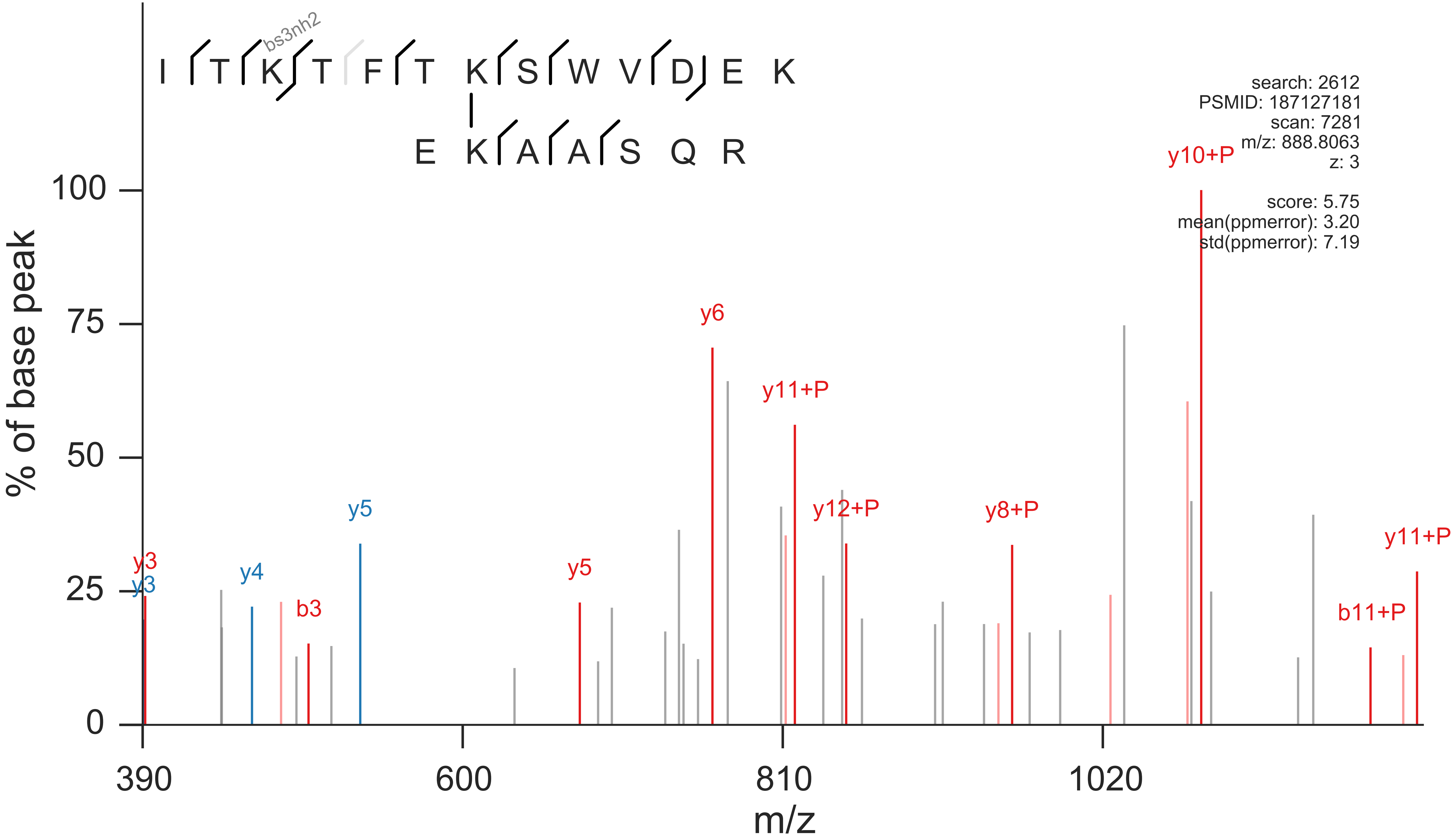

I T K T F T K S W V D E K
bs3nh2
E K A A S Q R
search: 2612
PSMID: 187127181
scan: 7281
m/z: 888.8063
z: 3
score: 5.75
mean(ppmerror): 3.20
std(ppmerror): 7.19
% of base peak
m/z
y3
y3
y4
b3
y5
y5
y6
y11+P
y12+P
y8+P
y10+P
b11+P
y11+P
0
25
50
75
100
390
600
810
1020

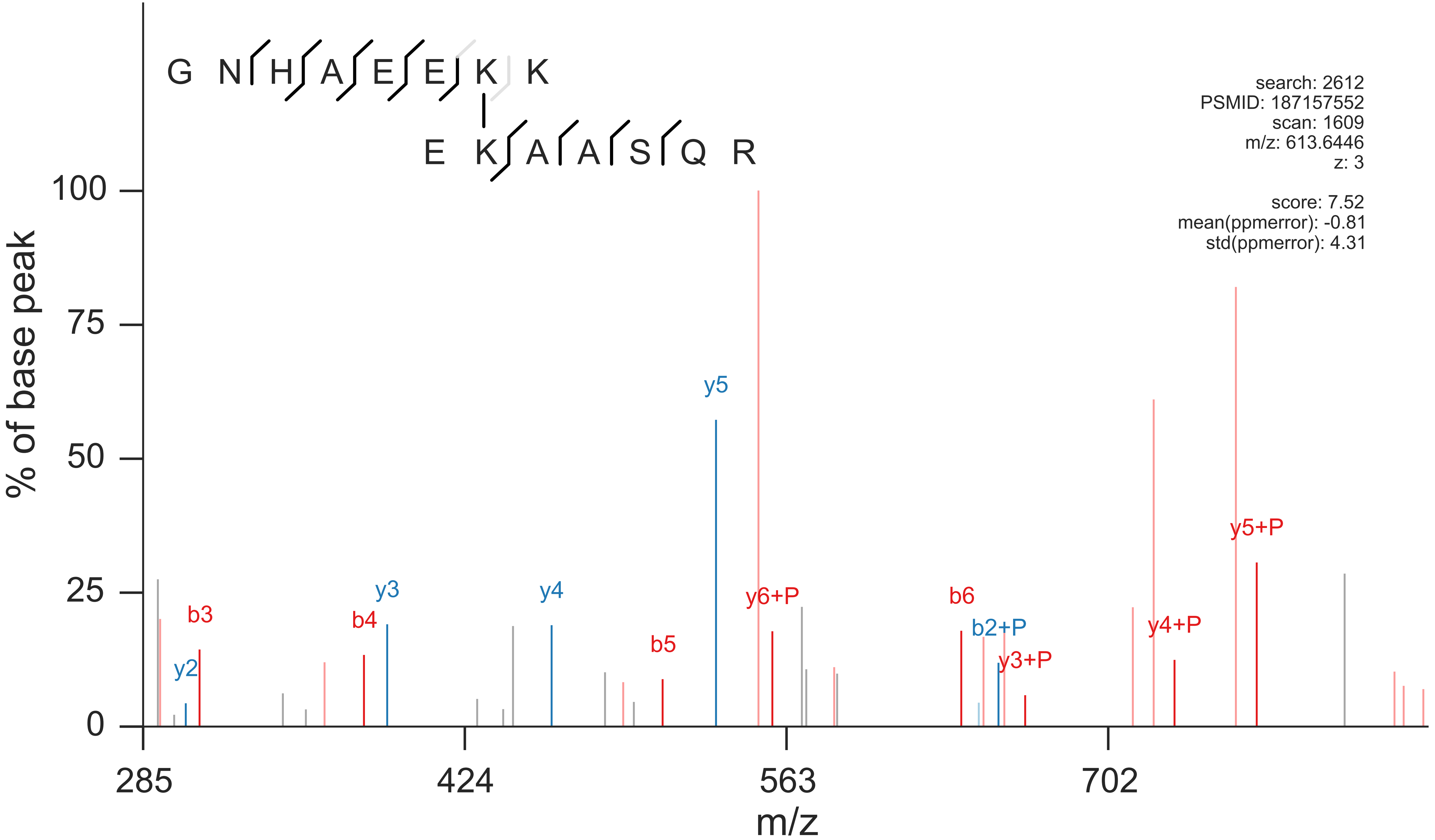
G N H A E E K K
E K A A S Q R
search: 2612
PSMID: 187157552
scan: 1609
m/z: 613.6446
z: 3
score: 7.52
mean(ppmerror): -0.81
std(ppmerror): 4.31
% of base peak
100
75
50
25
0
y5
y5+P
y3
y4
y6+P
b6
b3
b4
b2+P
y4+P
b5
y3+P
y2
285
424
563
702
m/z

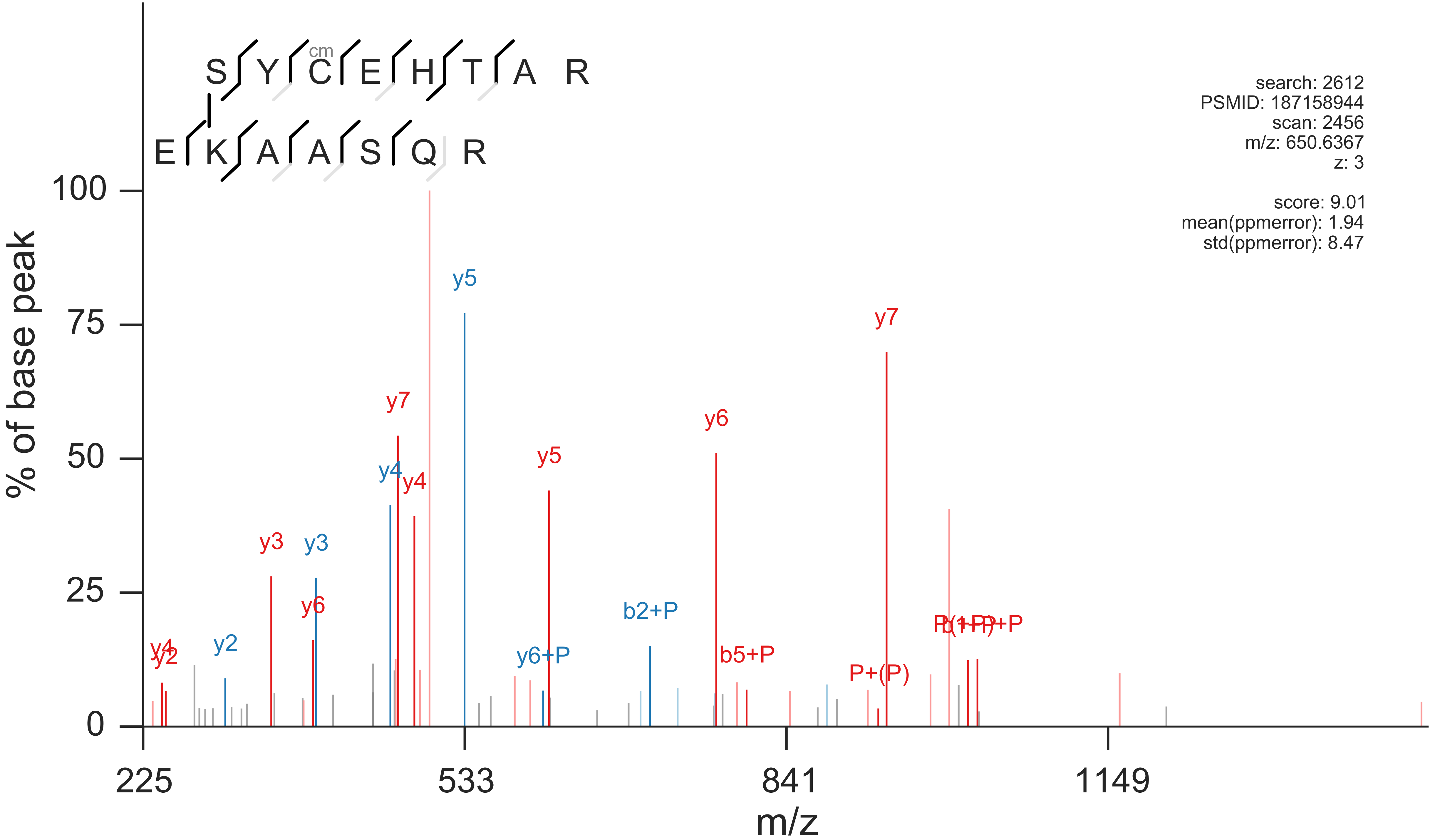

S Y C E H T A R
cm
E K A A S Q R
search: 2612
PSMID: 187158944
scan: 2456
m/z: 650.6367
z: 3
score: 9.01
mean(ppmerror): 1.94
std(ppmerror): 8.47
% of base peak
100
75
50
25
0
y5
y7
y7
y6
y5
y4
y4
y3
y3
y6
y2
y4
y2
b2+P
y6+P
b5+P
P+(P)
225
533
841
1149
m/z

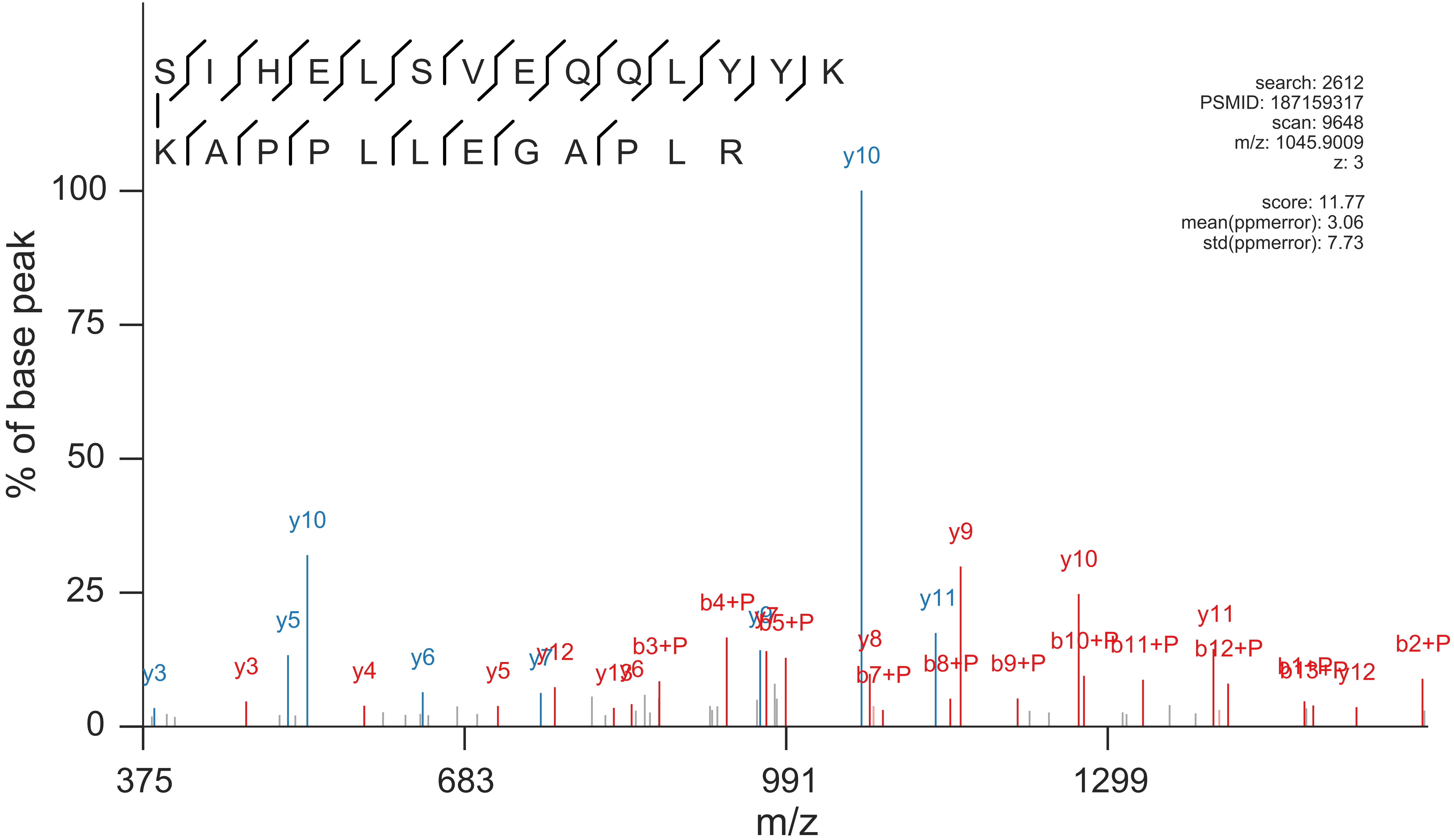

S I H E L S V E Q Q L Y Y K
K A P P L L E G A P L R
search: 2612
PSMID: 187159317
scan: 9648
m/z: 1045.9009
z: 3
score: 11.77
mean(ppmerror): 3.06
std(ppmerror): 7.73
% of base peak
m/z

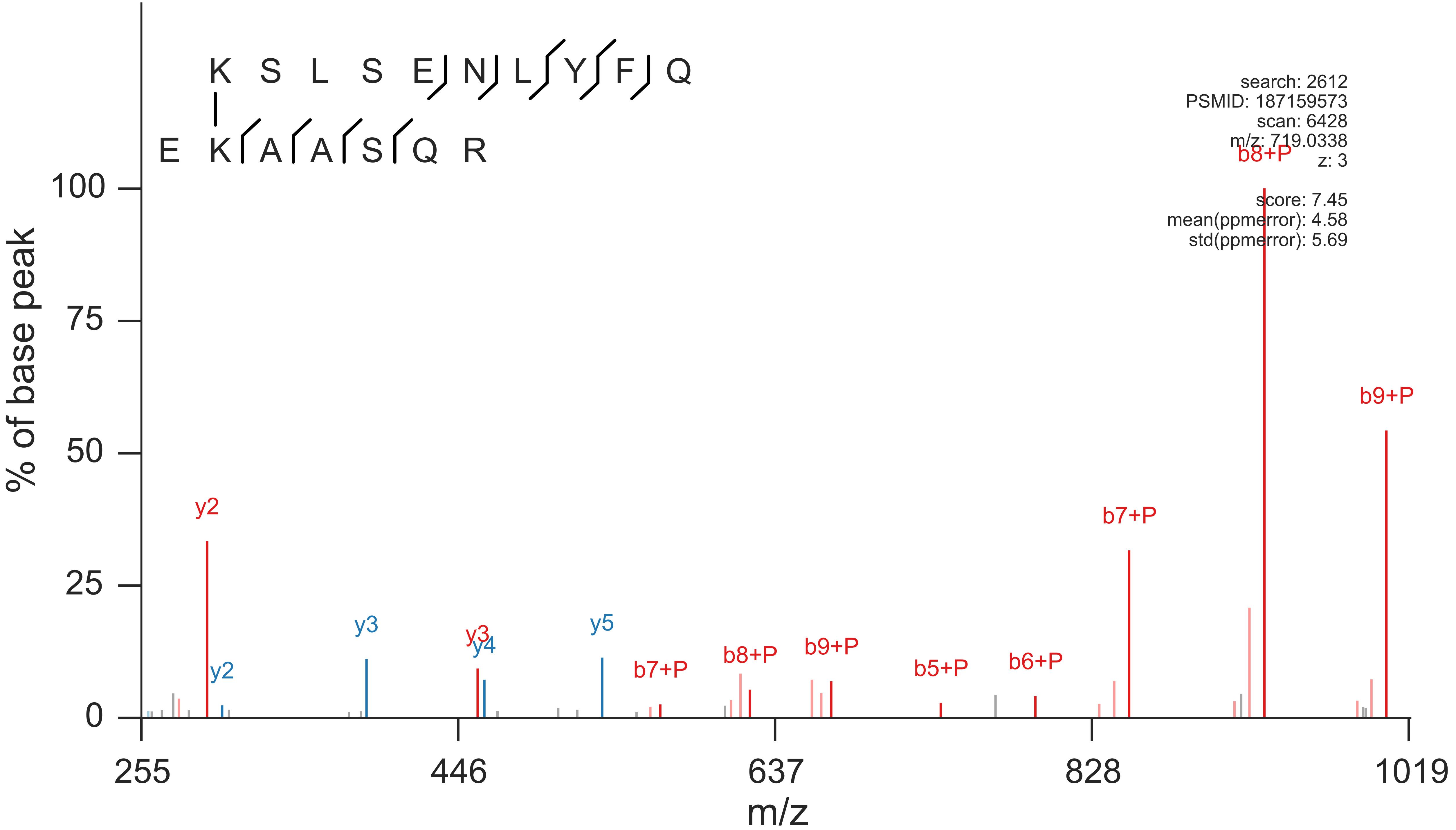

K S L S E N L Y F Q
E K A A S Q R
search: 2612
PSMID: 187159573
scan: 6428
m/z: 719.0338
z: 3
score: 7.45
mean(ppmerror): 4.58
std(ppmerror): 5.69
% of base peak
100
75
50
25
0
y2
y2
y3
y3
y4
y5
b7+P
b8+P
b9+P
b5+P
b6+P
b7+P
b8+P
b9+P
255
446
637
828
1019
m/z

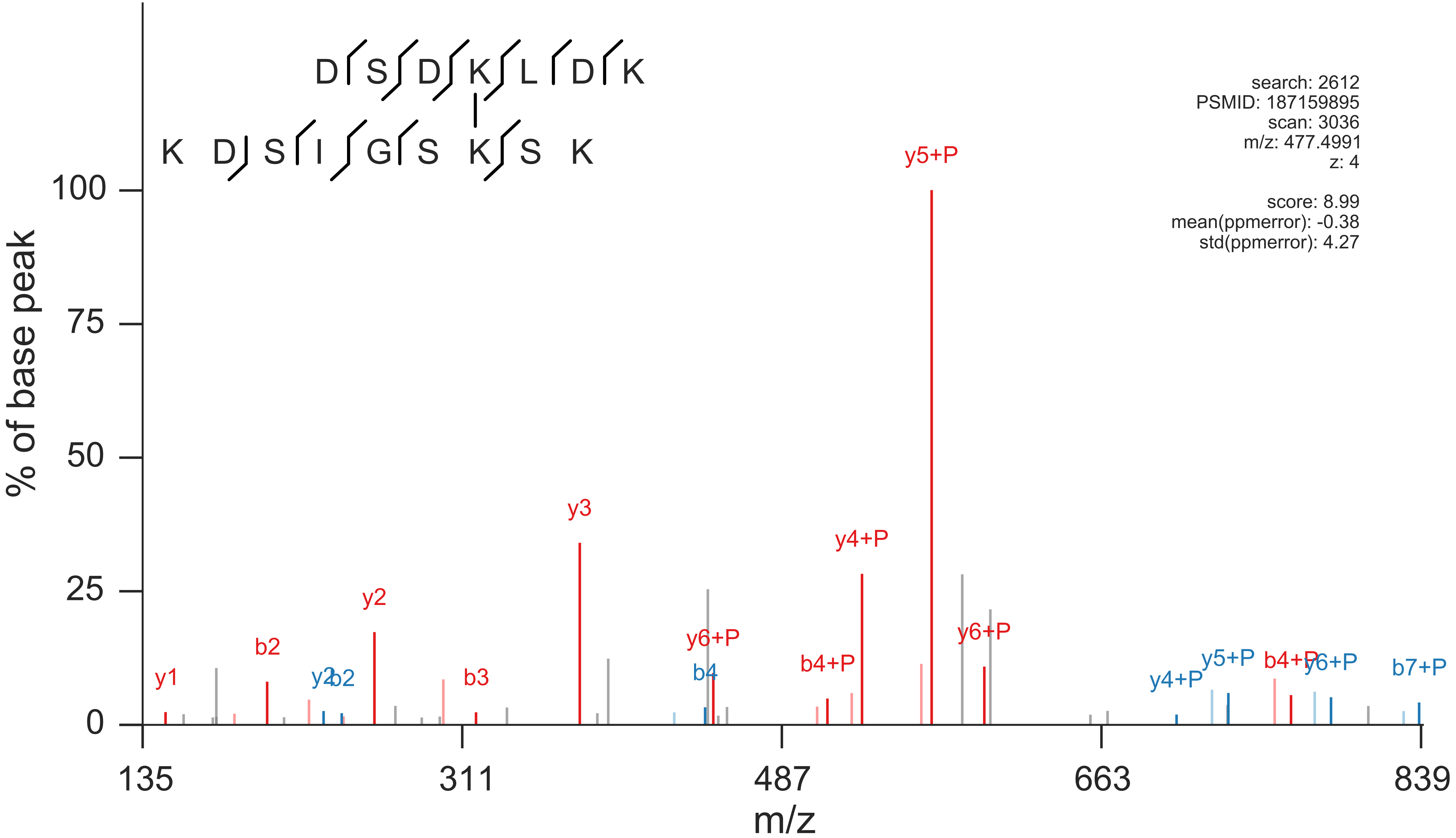

D S D K L D K
K D S I G S K S K
search: 2612
PSMID: 187159895
scan: 3036
m/z: 477.4991
z: 4
score: 8.99
mean(ppmerror): -0.38
std(ppmerror): 4.27
% of base peak
m/z
100
75
50
25
0
135
311
487
663
839
y1
b2
y2
b2
y2
b3
y3
b4
y6+P
b4+P
y4+P
y5+P
y6+P
y4+P
y5+P
b4+P
y6+P
b7+P

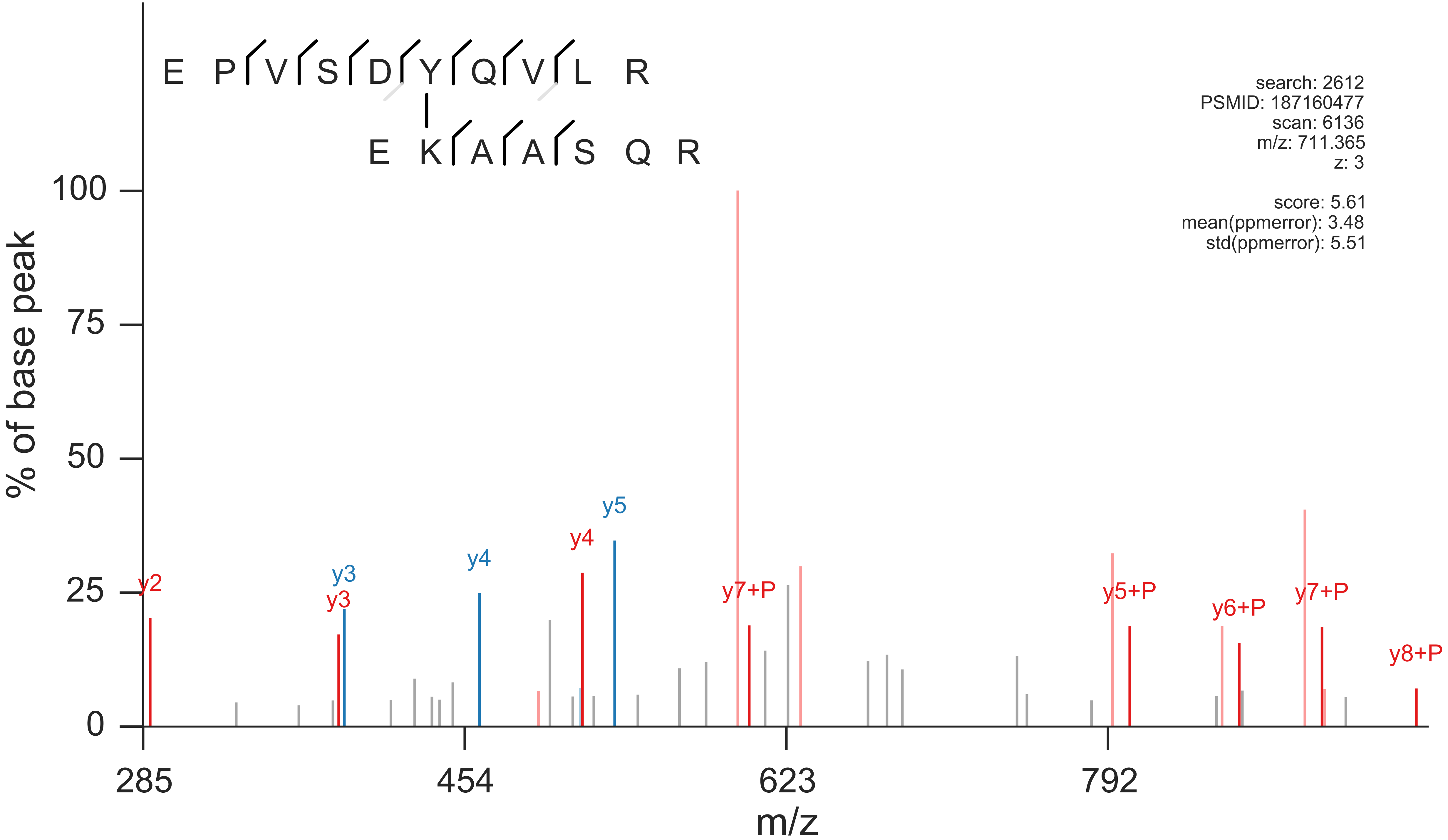

E P V S D Y Q V L R
E K A A S Q R
search: 2612
PSMID: 187160477
scan: 6136
m/z: 711.365
z: 3
score: 5.61
mean(ppmerror): 3.48
std(ppmerror): 5.51
% of base peak
100
75
50
25
0
y2
y3
y3
y4
y4
y5
y7+P
y5+P
y6+P
y7+P
y8+P
285
454
623
792
m/z

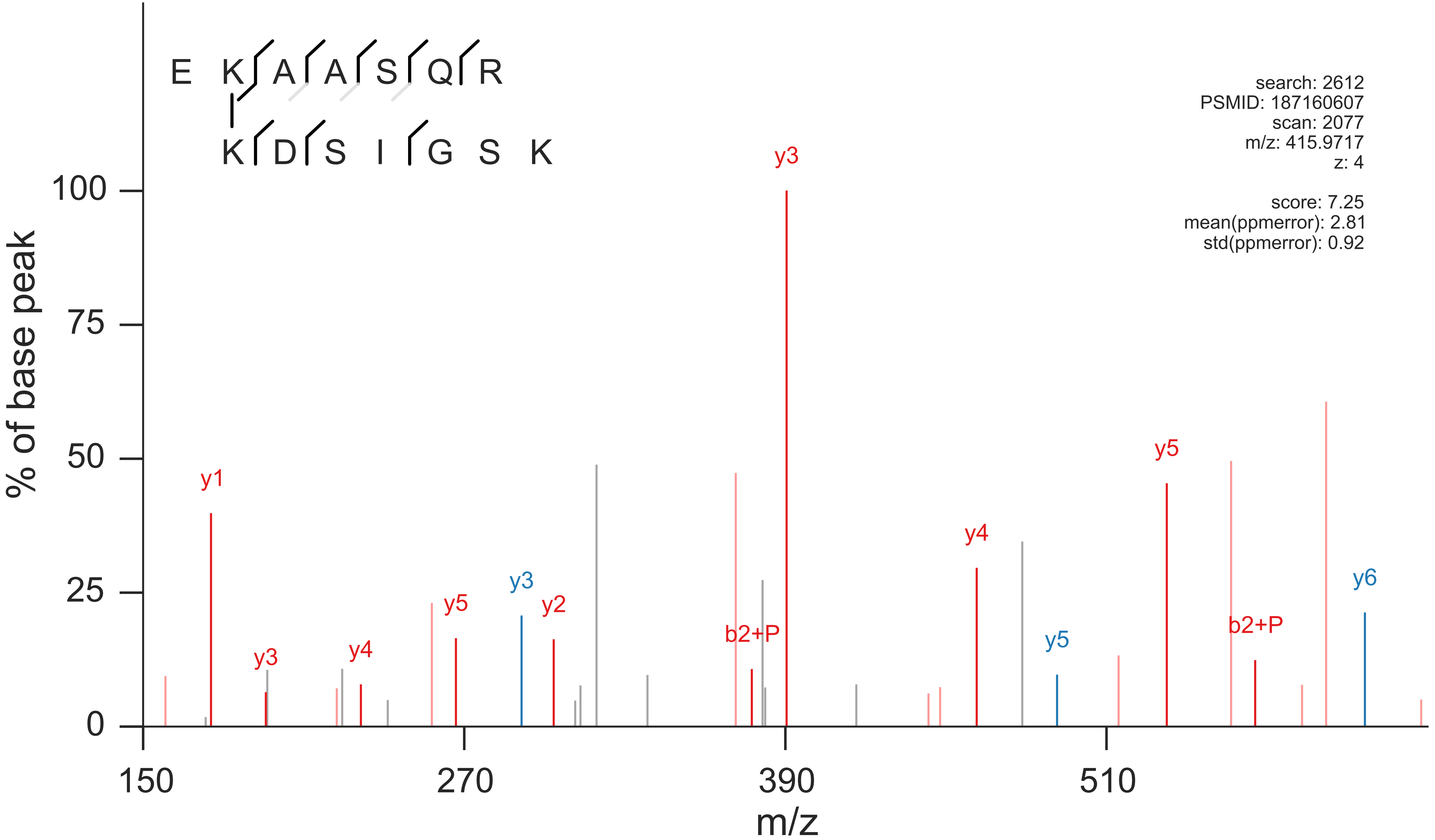

E K A A S Q R
K D S I G S K
search: 2612
PSMID: 187160607
scan: 2077
m/z: 415.9717
z: 4
score: 7.25
mean(ppmerror): 2.81
std(ppmerror): 0.92
% of base peak
m/z
0
25
50
75
100
150
270
390
510
y1
y3
y4
y5
y3
y2
b2+P
y3
y4
y5
y5
b2+P
y6

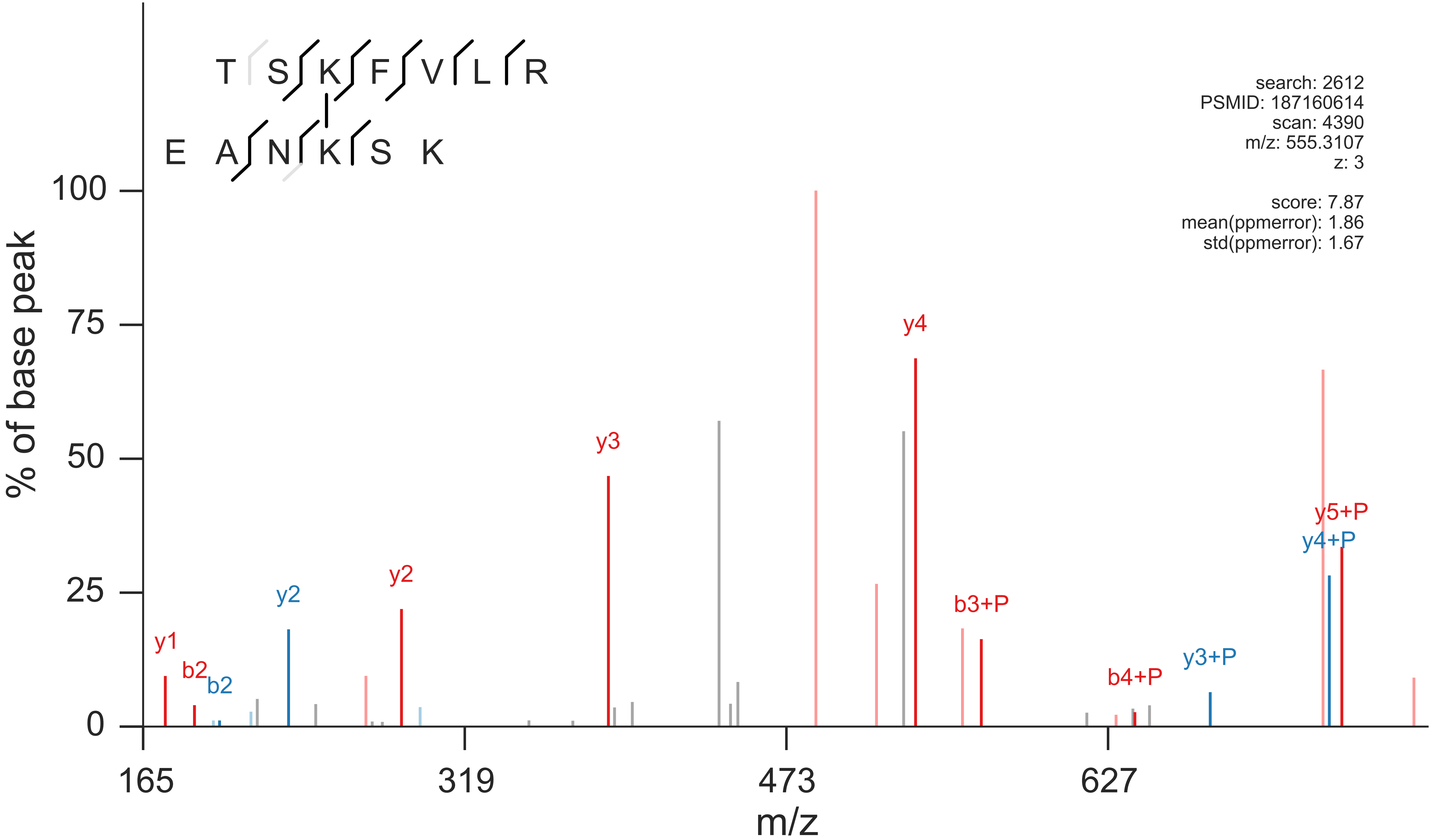
T S K F V L R
E A N K S K
search: 2612
PSMID: 187160614
scan: 4390
m/z: 555.3107
z: 3
score: 7.87
mean(ppmerror): 1.86
std(ppmerror): 1.67
% of base peak
100
75
50
25
0
y1
b2
b2
y2
y2
y3
y4
b3+P
b4+P
y3+P
y4+P
y5+P
165
319
473
627
m/z

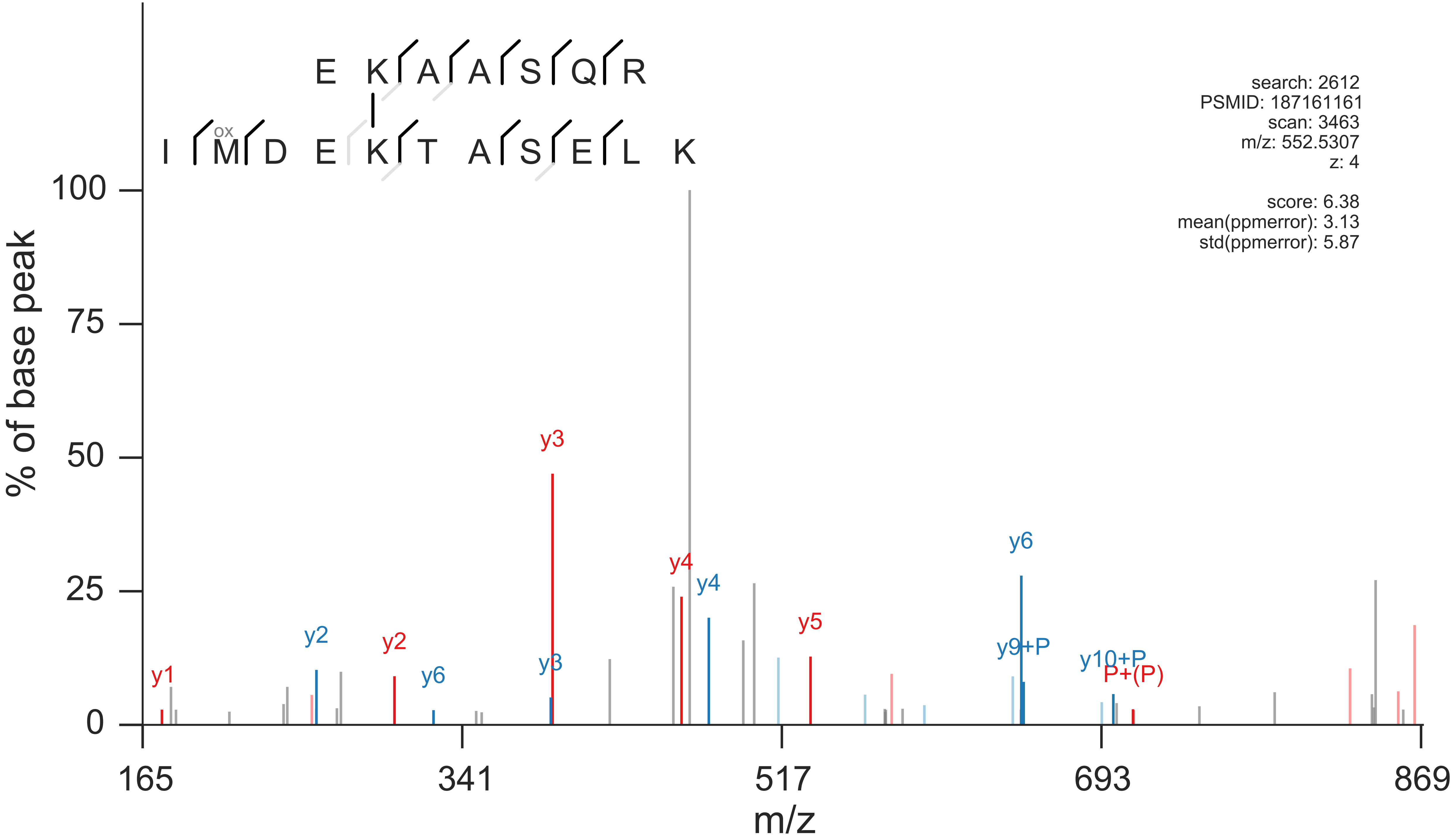

E K A A S Q R
I M D E K T A S E L K
ox
search: 2612
PSMID: 187161161
scan: 3463
m/z: 552.5307
z: 4
score: 6.38
mean(ppmerror): 3.13
std(ppmerror): 5.87
% of base peak
m/z
100
75
50
25
0
165
341
517
693
869
y1
y2
y2
y6
y3
y3
y4
y4
y5
y6
y9+P
y10+P
P+(P)

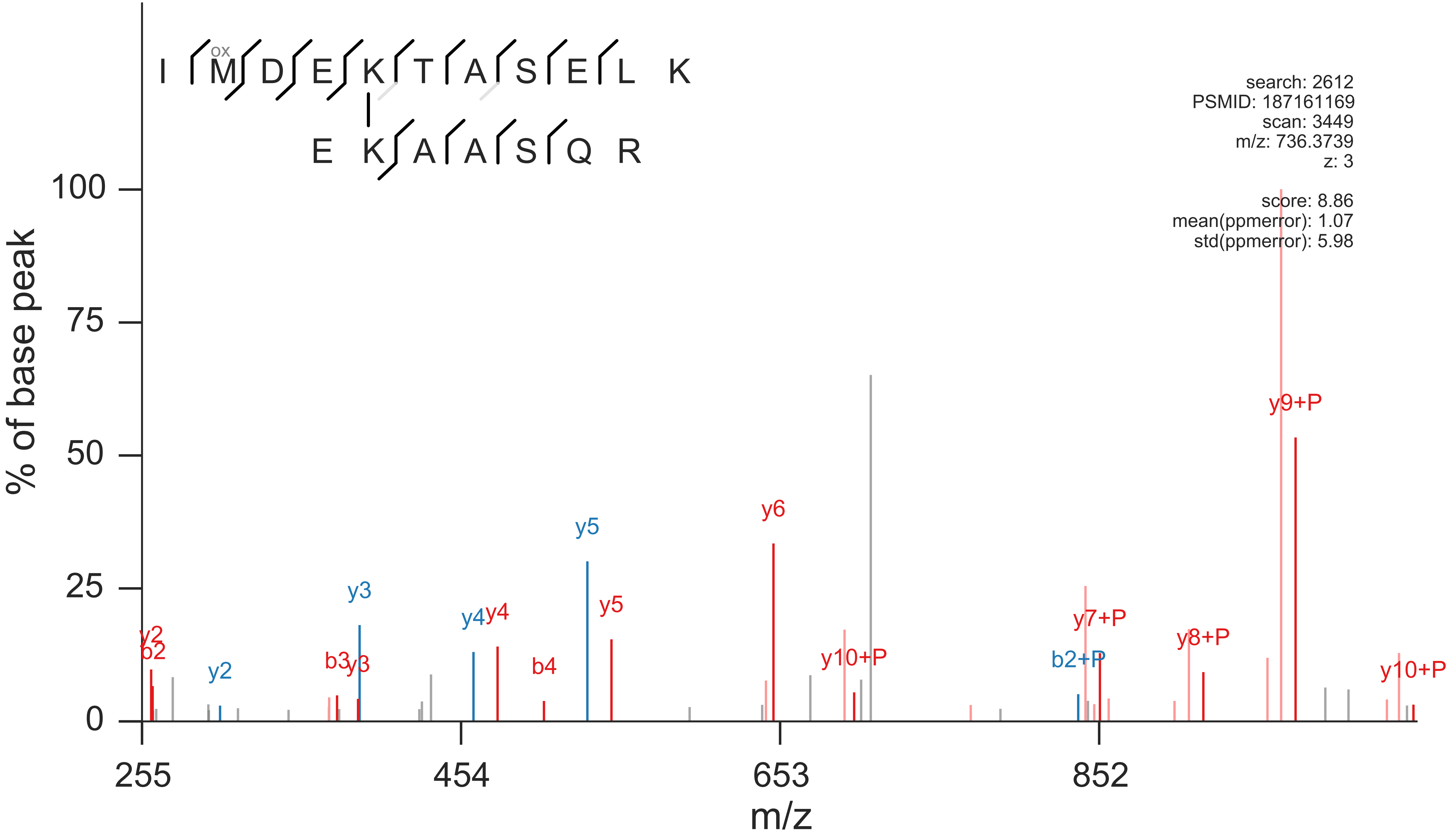

I M D E K T A S E L K
ox
E K A A S Q R
search: 2612
PSMID: 187161169
scan: 3449
m/z: 736.3739
z: 3
score: 8.86
mean(ppmerror): 1.07
std(ppmerror): 5.98
% of base peak
m/z
0
25
50
75
100
255
454
653
852
y2
b2
y2
b3
y3
y3
y4
y4
b4
y5
y5
y6
y10+P
b2+P
y7+P
y8+P
y9+P
y10+P

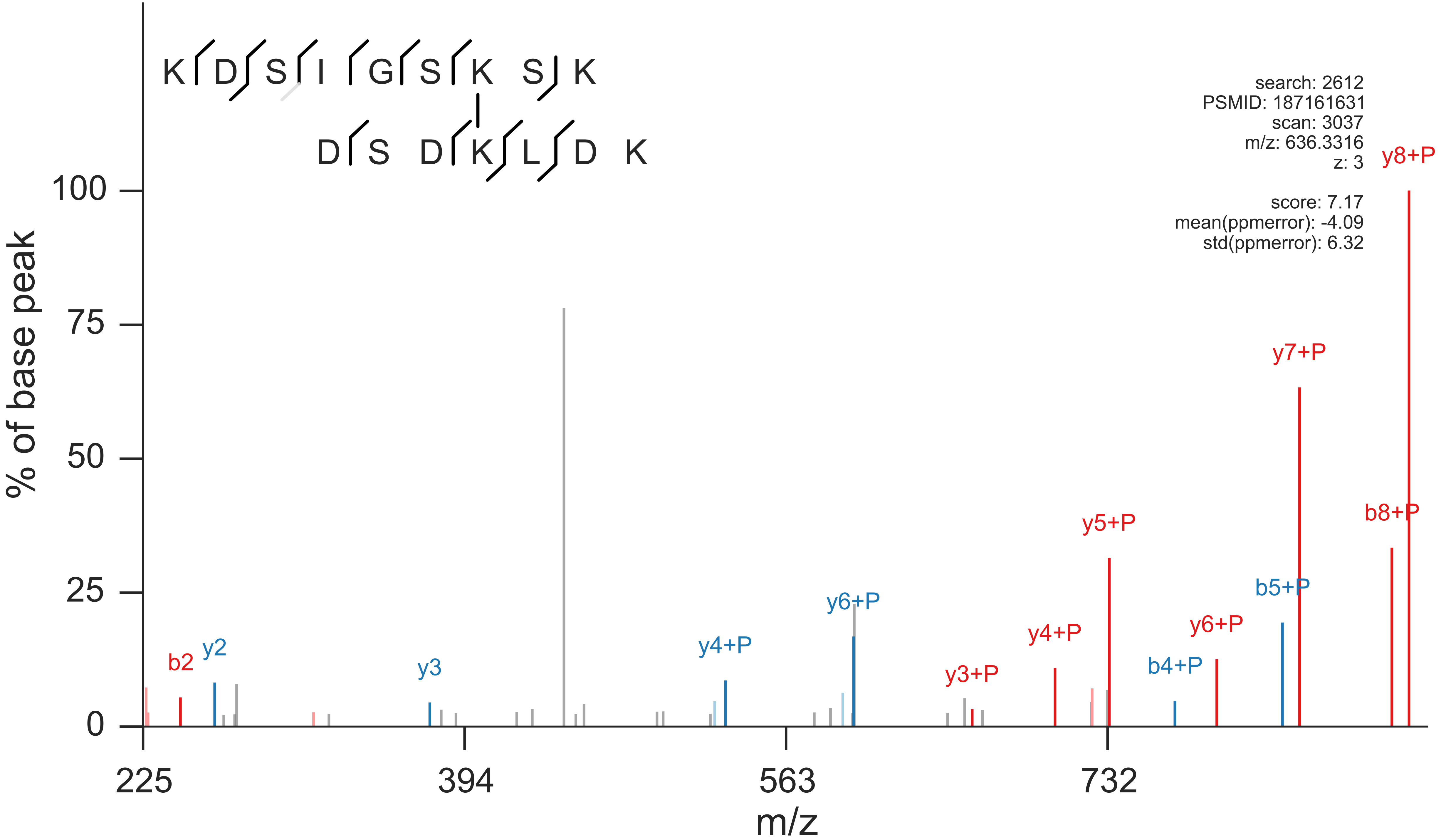

K D S I G S K S K
D S D K L D K
search: 2612
PSMID: 187161631
scan: 3037
m/z: 636.3316
z: 3
score: 7.17
mean(ppmerror): -4.09
std(ppmerror): 6.32
% of base peak
100
75
50
25
0
225
394
563
732
m/z
b2
y2
y3
y4+P
y6+P
y3+P
y4+P
y5+P
b4+P
y6+P
b5+P
y7+P
b8+P
y8+P

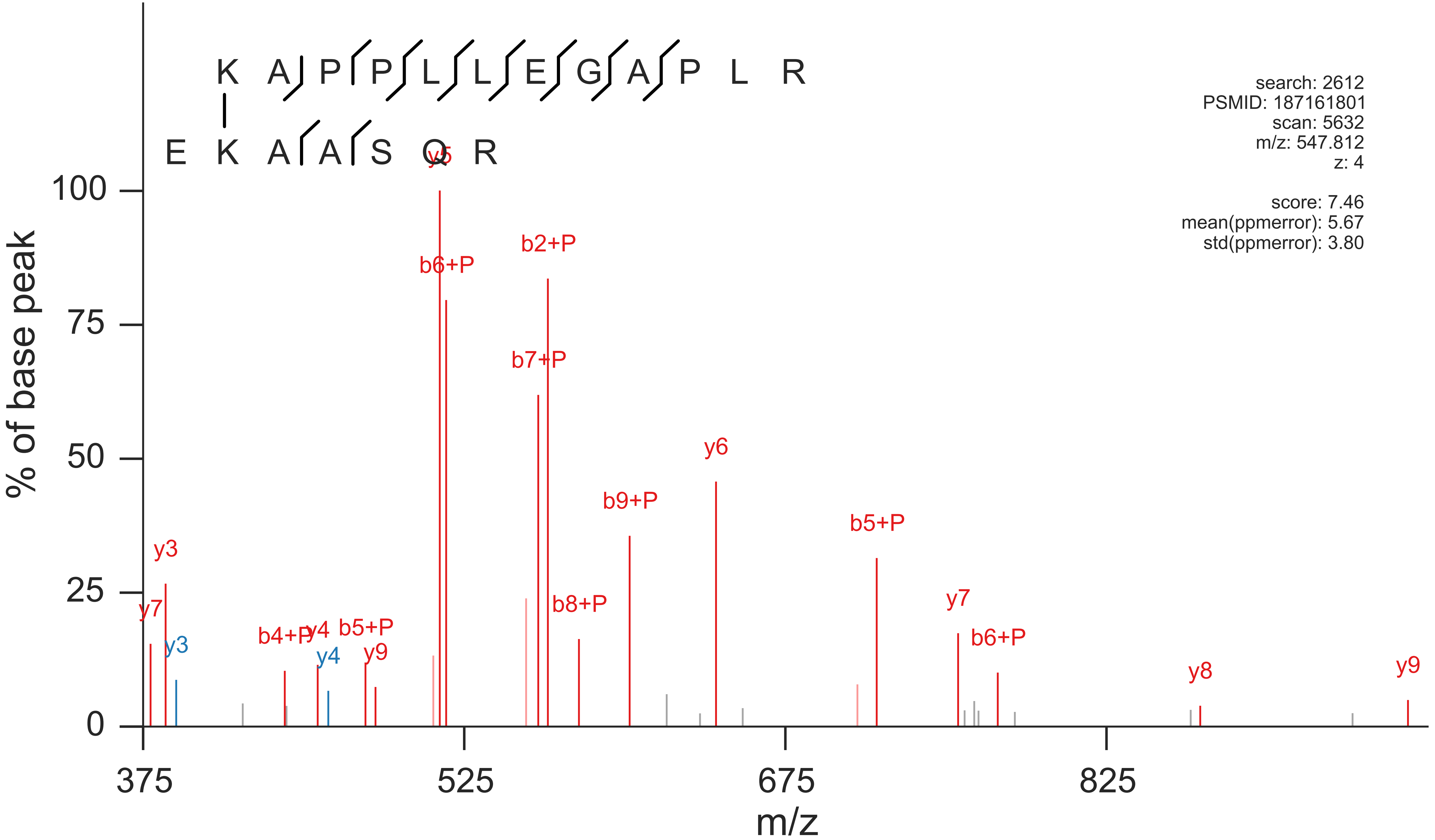

K A P P L L E G A P L R
E K A A S Q R
search: 2612
PSMID: 187161801
scan: 5632
m/z: 547.812
z: 4
score: 7.46
mean(ppmerror): 5.67
std(ppmerror): 3.80
% of base peak
m/z
100
75
50
25
0
375
525
675
825
y5
b6+P
b2+P
b7+P
y6
b9+P
b5+P
y3
y7
b8+P
y3
b4+P
y4
b5+P
y4
y9
y7
b6+P
y8
y9

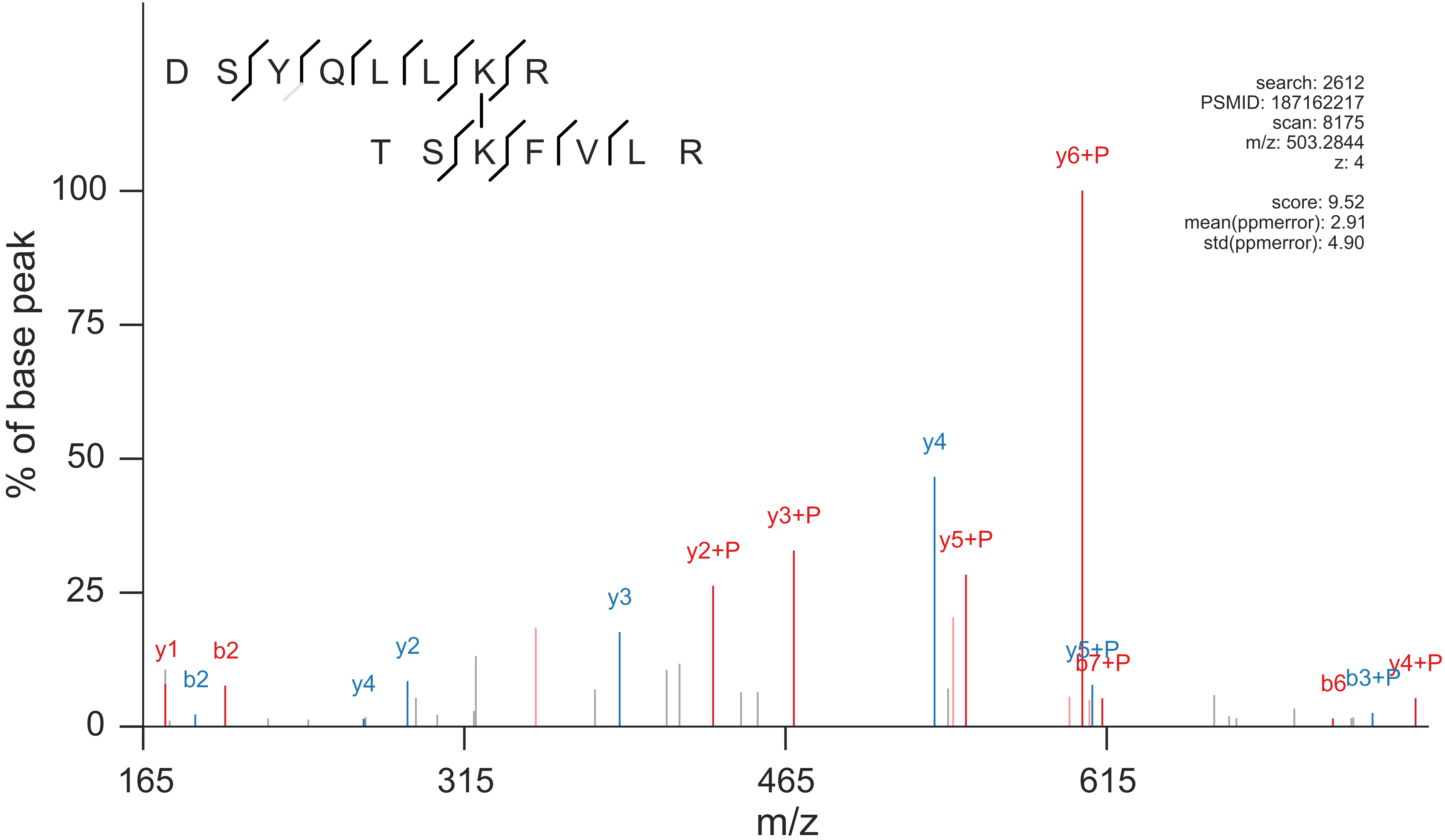

D S Y Q L L K R
T S K F V L R
search: 2612
PSMID: 187162217
scan: 8175
m/z: 503.2844
z: 4
score: 9.52
mean(ppmerror): 2.91
std(ppmerror): 4.90
y6+P
y4
y3+P
y2+P
y5+P
y3
y1
b2
b2
y2
y4
y5+P
b7+P
b6
b3+P
y4+P
% of base peak
0
25
50
75
100
165
315
465
615
m/z

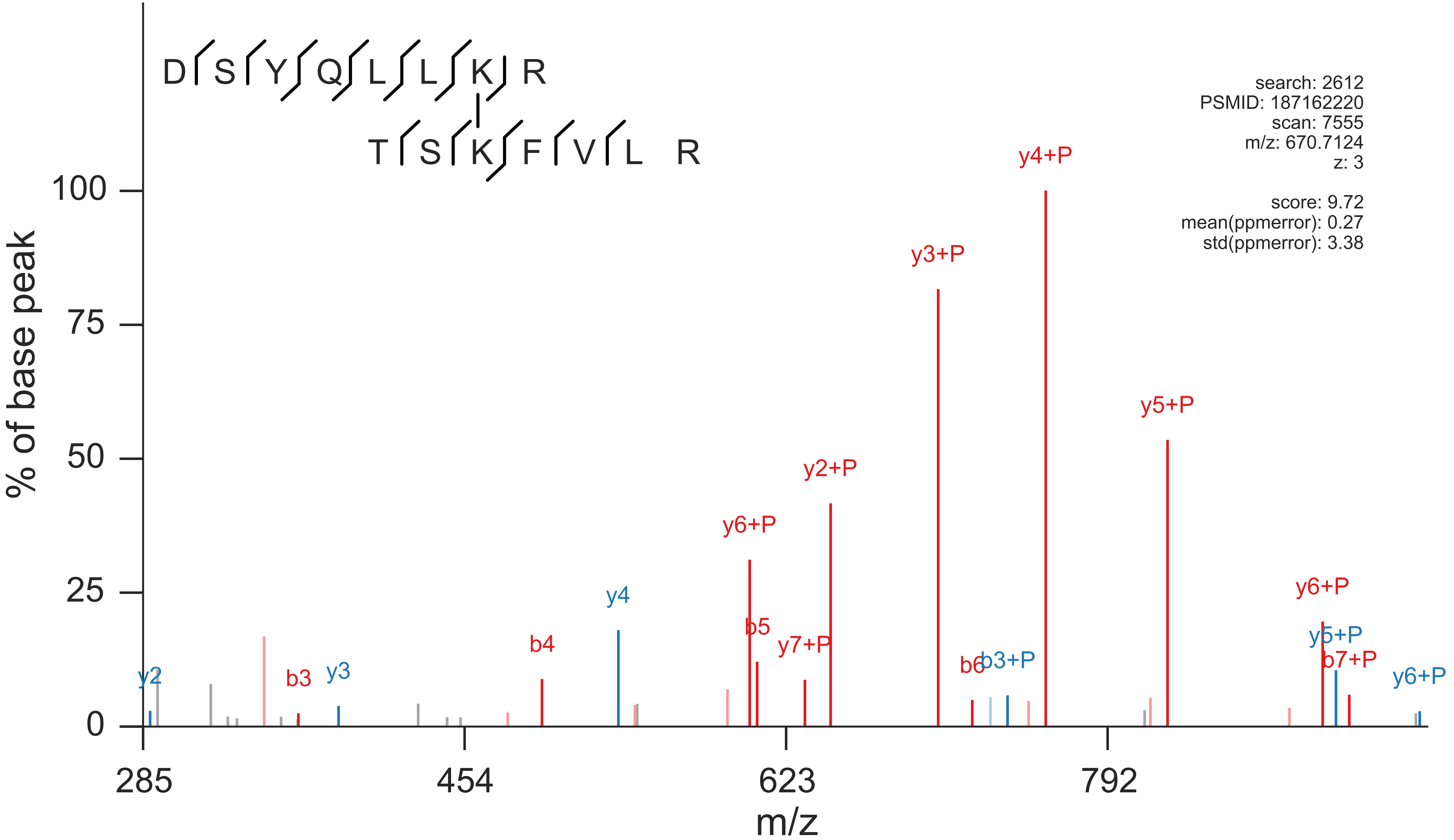

D S Y Q L L K R
T S K F V L R
search: 2612
PSMID: 187162220
scan: 7555
m/z: 670.7124
z: 3
score: 9.72
mean(ppmerror): 0.27
std(ppmerror): 3.38
% of base peak
100
75
50
25
0
285
454
623
792
m/z
y4+P
y3+P
y5+P
y2+P
y6+P
y6+P
y4
b5
y7+P
b4
y5+P
b7+P
b6
b3+P
y6+P
y2
b3
y3

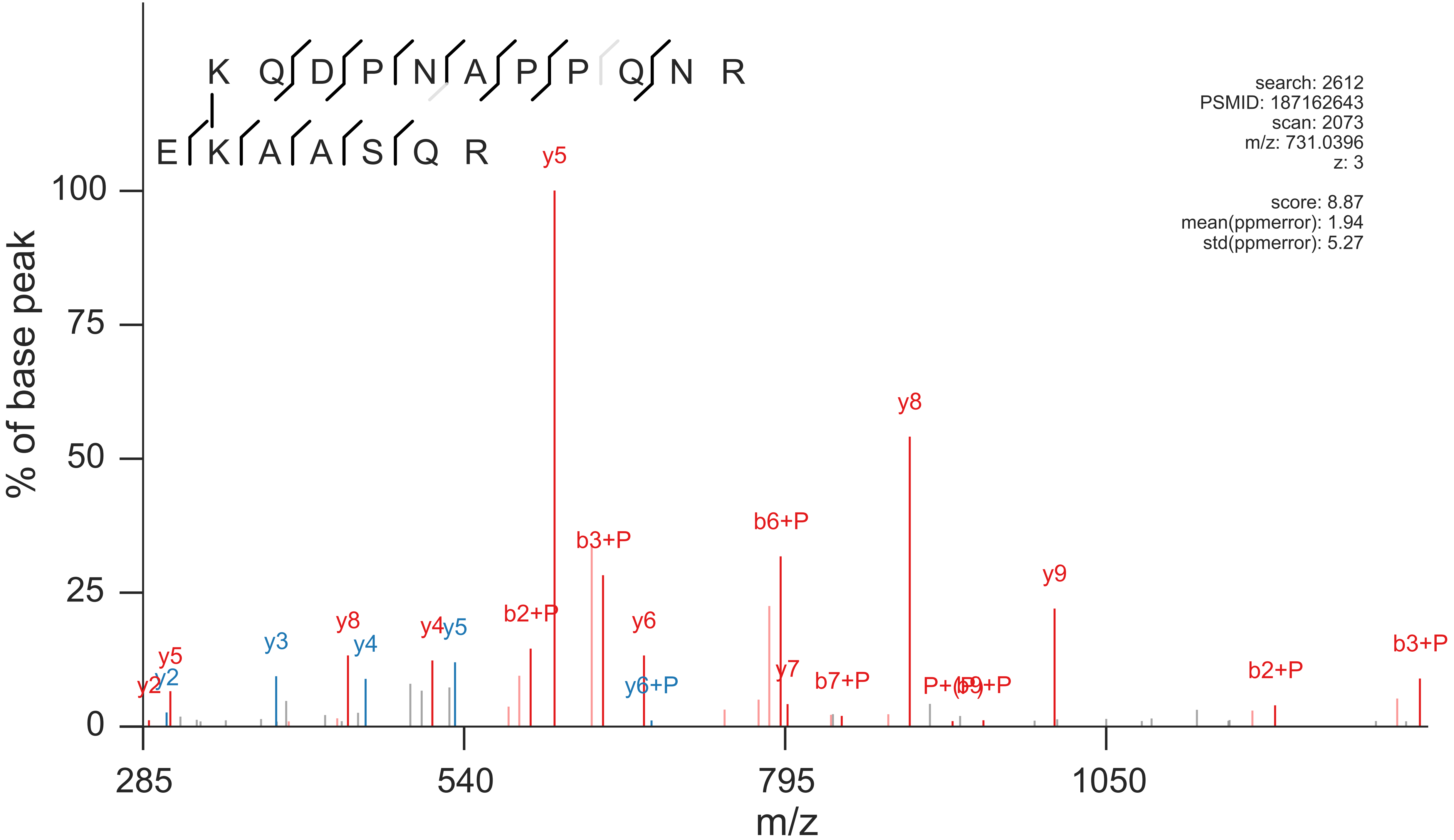

K Q D P N A P P Q N R
E K A A S Q R
search: 2612
PSMID: 187162643
scan: 2073
m/z: 731.0396
z: 3
score: 8.87
mean(ppmerror): 1.94
std(ppmerror): 5.27
% of base peak
m/z
100
75
50
25
0
285
540
795
1050
y5
y8
b6+P
b3+P
y9
y2
y2
y5
y3
y8
y4
y4
y5
b2+P
y6
y6+P
y7
b7+P
b2+P
b3+P

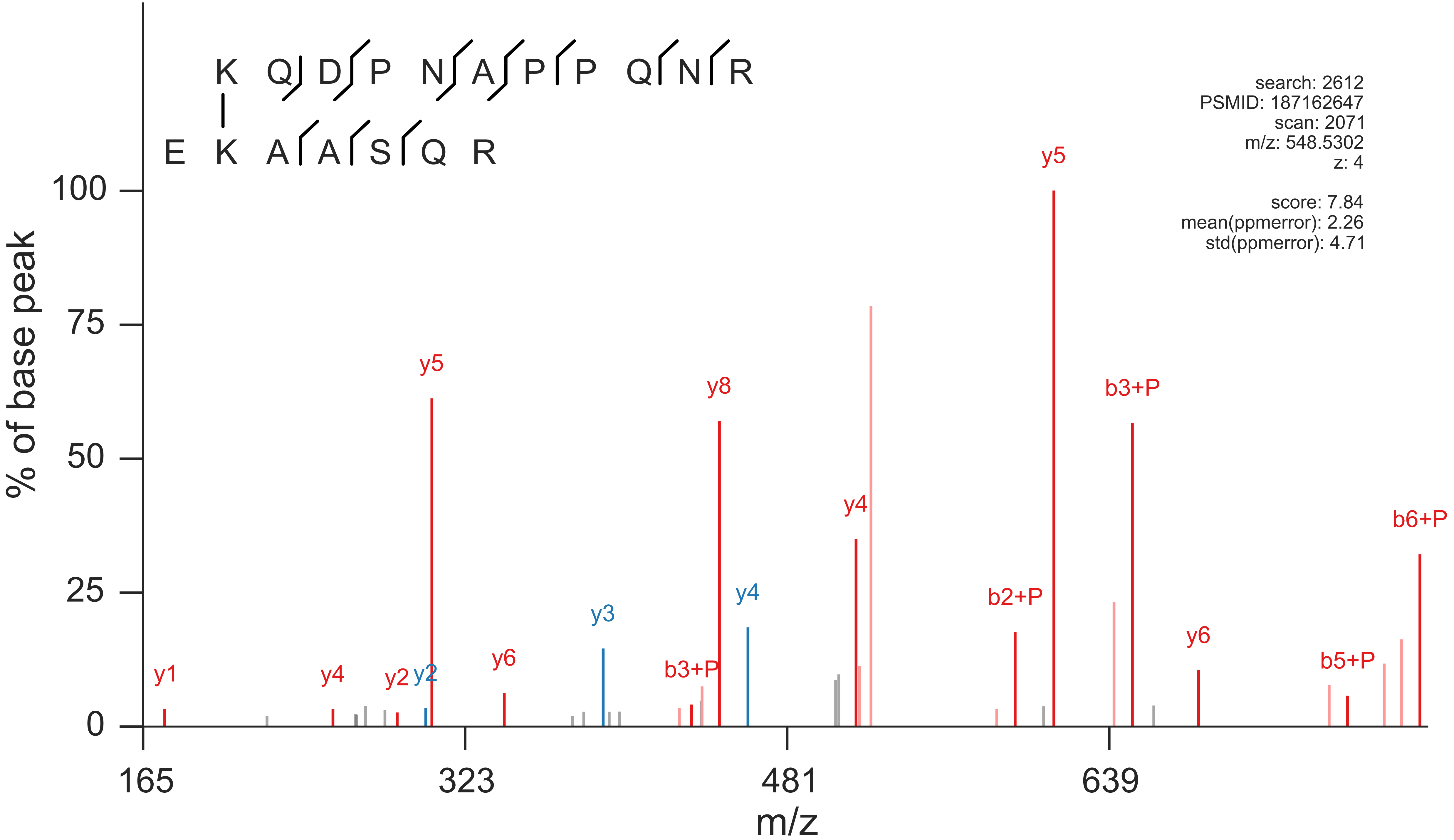

K Q D P N A P P Q N R
E K A A S Q R
search: 2612
PSMID: 187162647
scan: 2071
m/z: 548.5302
z: 4
score: 7.84
mean(ppmerror): 2.26
std(ppmerror): 4.71
% of base peak
m/z
0
25
50
75
100
165
323
481
639
y1
y4
y2
y2
y5
y6
y3
b3+P
y8
y4
y4
b2+P
y5
b3+P
y6
b5+P
b6+P

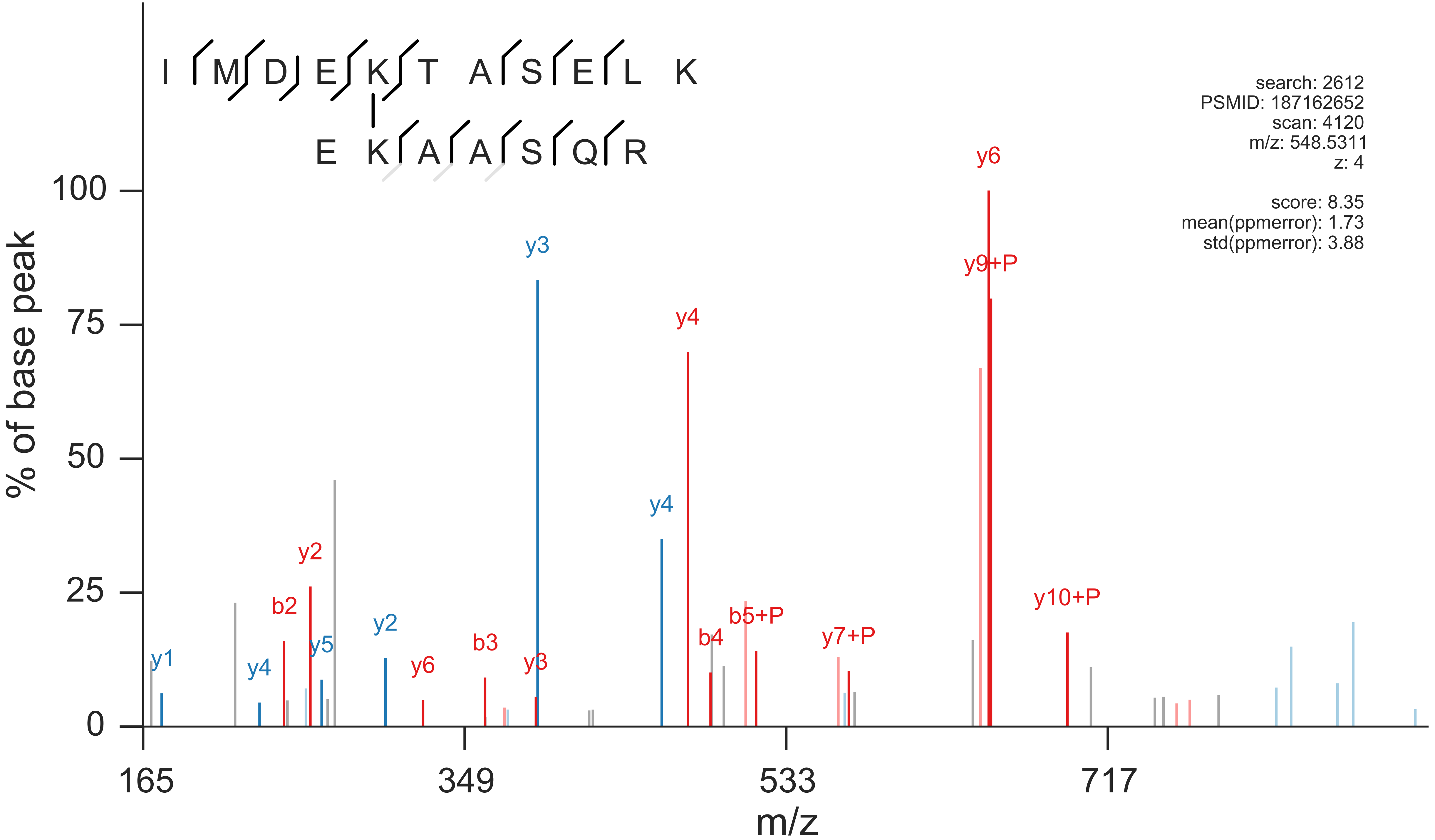

I M D E K T A S E L K
E K A A S Q R
search: 2612
PSMID: 187162652
scan: 4120
m/z: 548.5311
z: 4
score: 8.35
mean(ppmerror): 1.73
std(ppmerror): 3.88
% of base peak
m/z
0
25
50
75
100
165
349
533
717
y6
y9+P
y3
y4
y4
y2
b2
y5
y1
y4
y2
y6
b3
y3
b4
b5+P
y7+P
y10+P

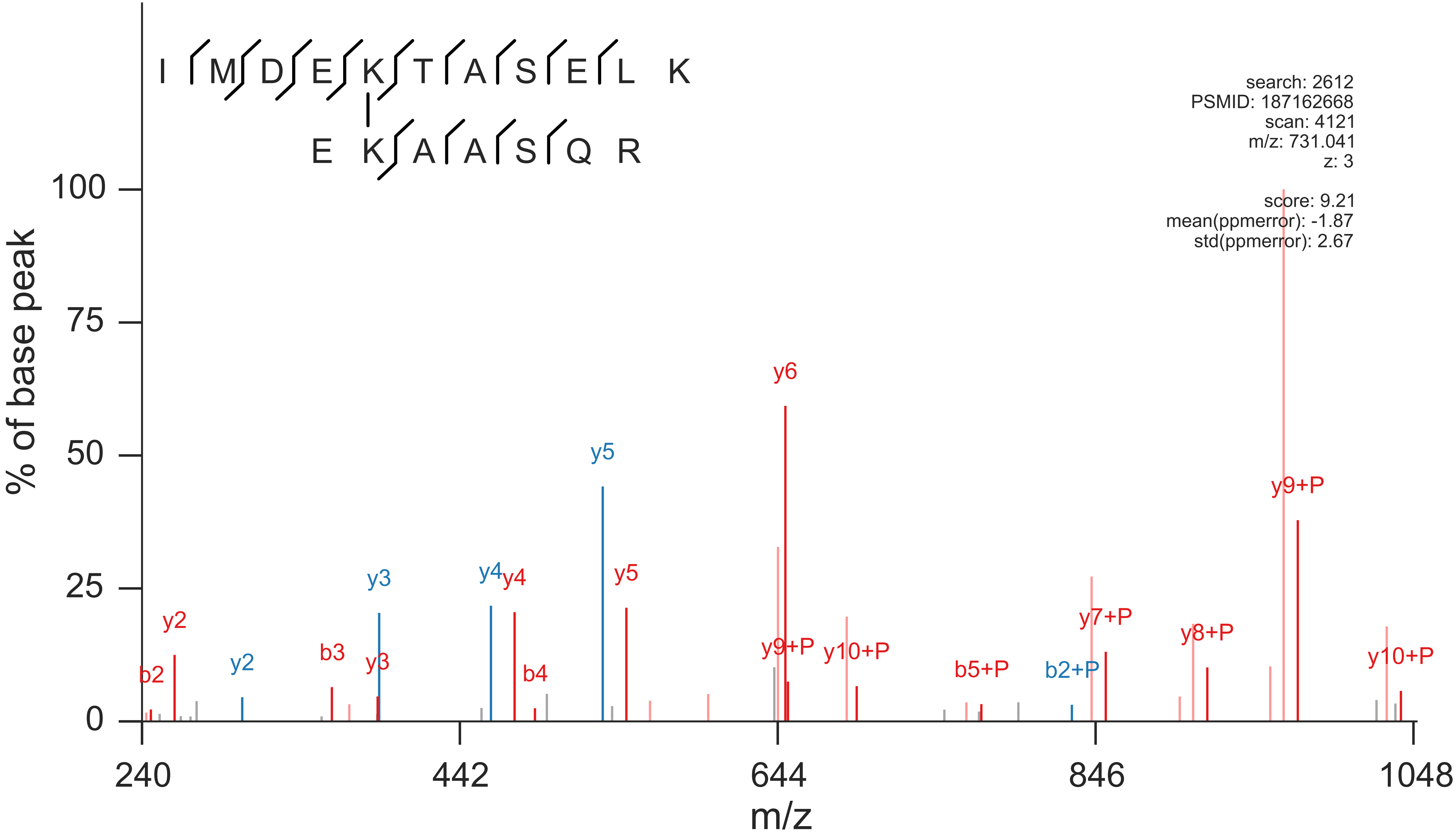

I M D E K T A S E L K
E K A A S Q R
search: 2612
PSMID: 187162668
scan: 4121
m/z: 731.041
z: 3
score: 9.21
mean(ppmerror): -1.87
std(ppmerror): 2.67
% of base peak
100
75
50
25
0
240
442
644
846
1048
m/z
y6
y5
y9+P
y4
y3
y4
y5
y2
b3
y2
y3
b2
b4
y9+P
y10+P
y7+P
y8+P
b5+P
b2+P
y10+P

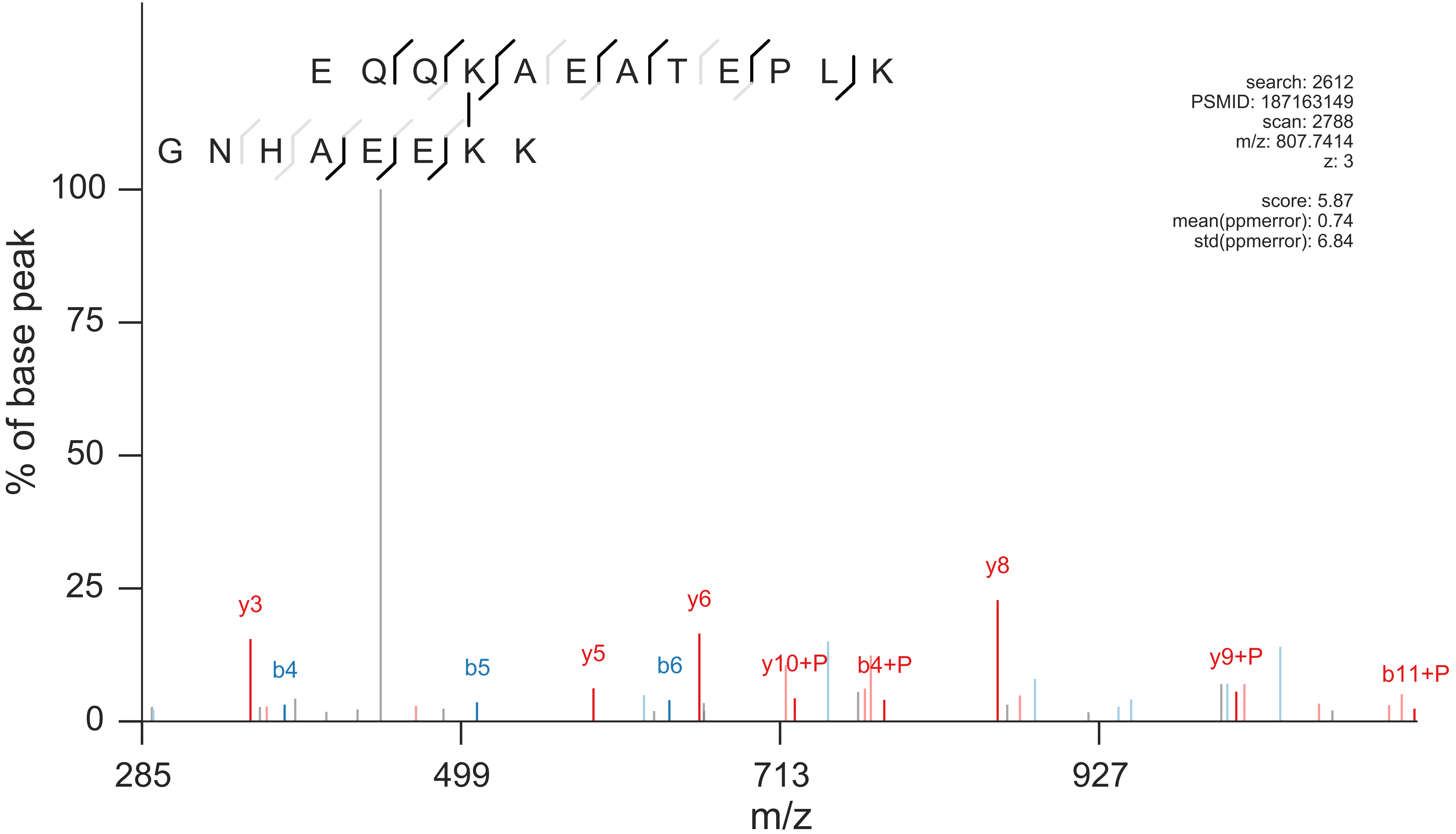
E Q Q K A E A T E P L K
G N H A E E K K
search: 2612
PSMID: 187163149
scan: 2788
m/z: 807.7414
z: 3
score: 5.87
mean(ppmerror): 0.74
std(ppmerror): 6.84
% of base peak
100
75
50
25
0
y3
b4
b5
y5
b6
y6
y10+P
b4+P
y8
y9+P
b11+P
285
499
713
927
m/z

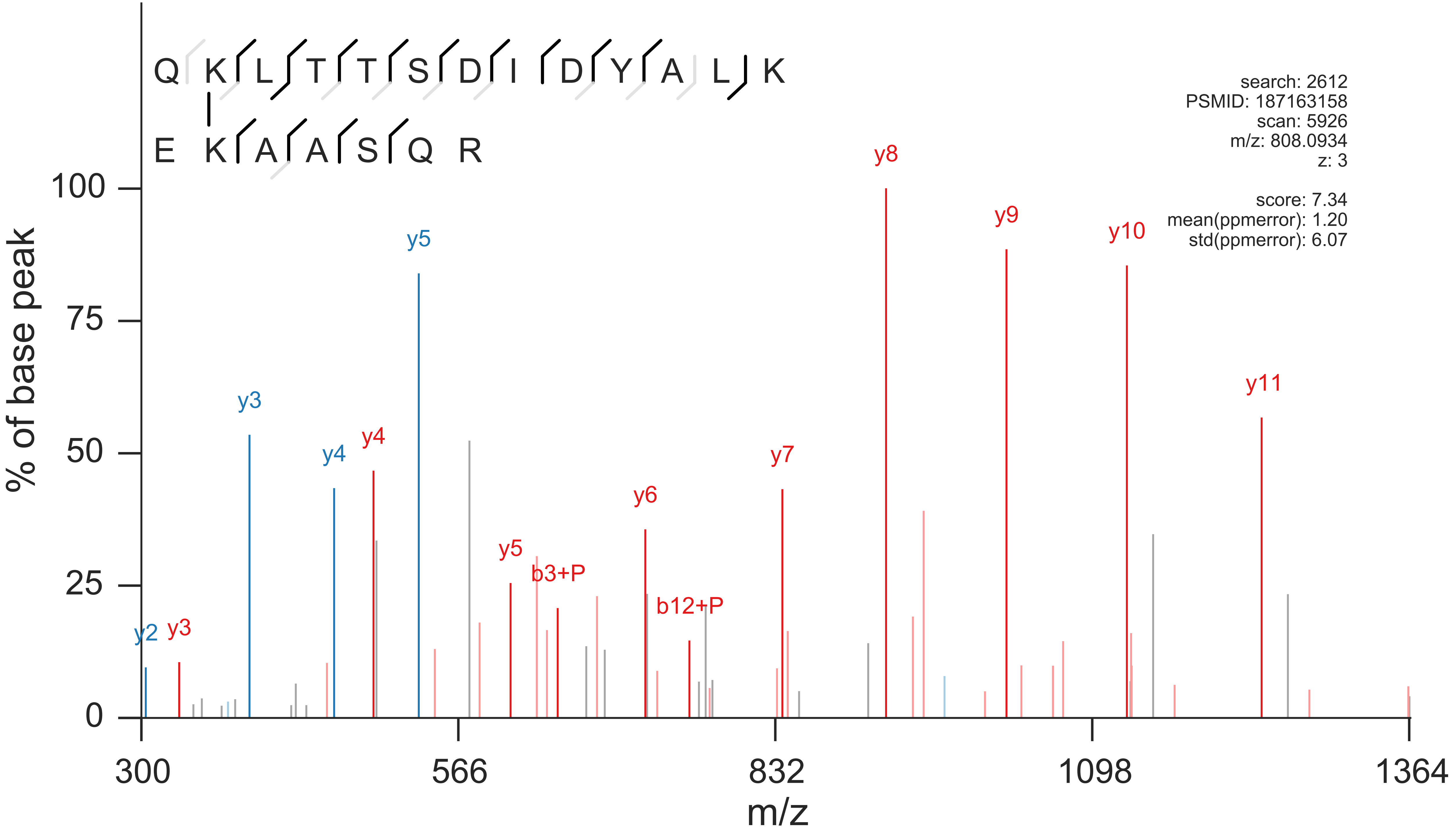

Q K L T T S D I D Y A L K
E K A A S Q R
search: 2612
PSMID: 187163158
scan: 5926
m/z: 808.0934
z: 3
score: 7.34
mean(ppmerror): 1.20
std(ppmerror): 6.07
% of base peak
m/z
y2
y3
y3
y4
y4
y5
y5
b3+P
y6
b12+P
y7
y8
y9
y10
y11
0
25
50
75
100
300
566
832
1098
1364

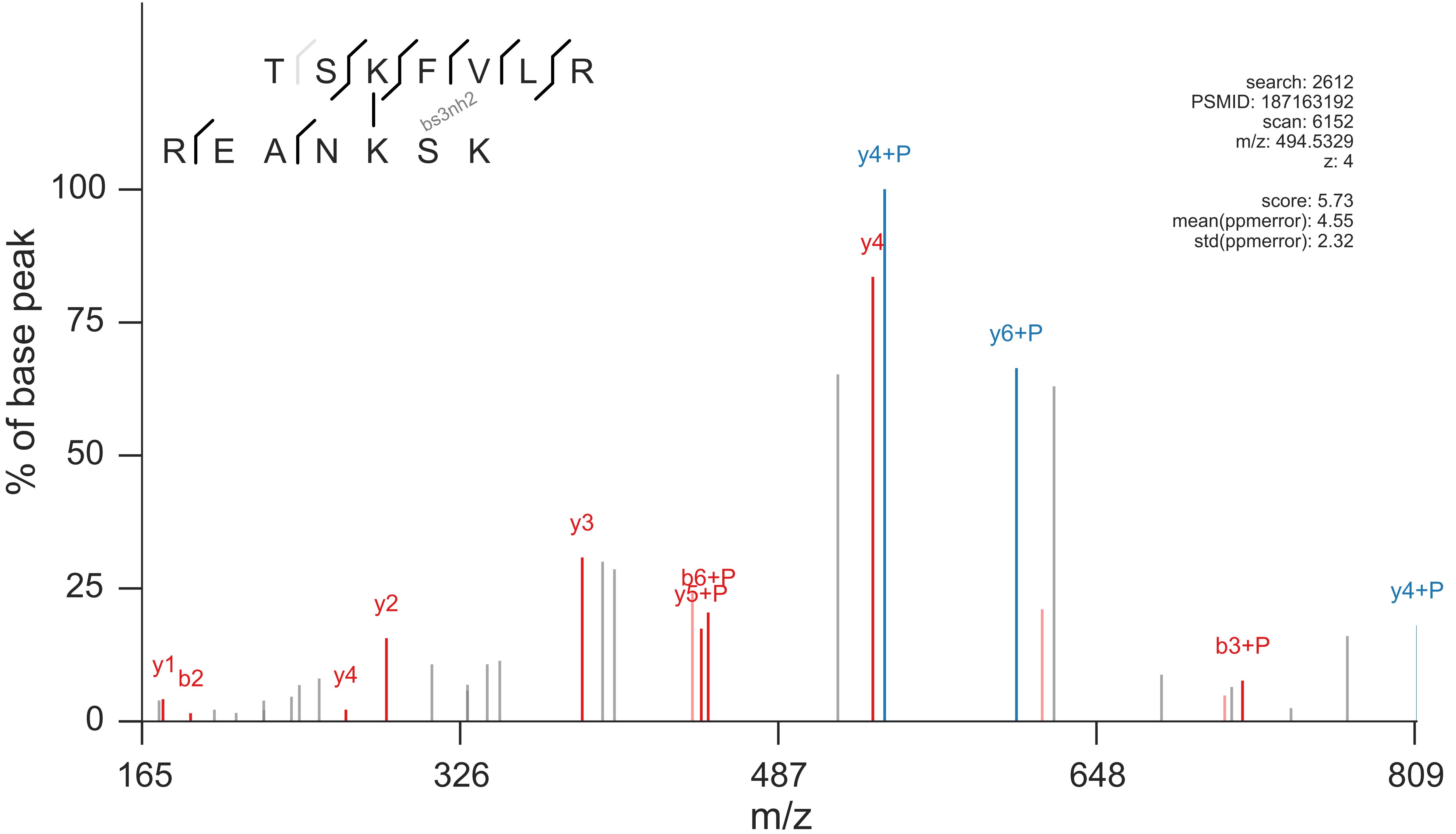

T S K F V L R
R E A N K S K
bs3nh2
search: 2612
PSMID: 187163192
scan: 6152
m/z: 494.5329
z: 4
score: 5.73
mean(ppmerror): 4.55
std(ppmerror): 2.32
% of base peak
m/z
0
25
50
75
100
165
326
487
648
809
y1
b2
y4
y2
y3
b6+P
y5+P
y4
y4+P
y6+P
b3+P
y4+P

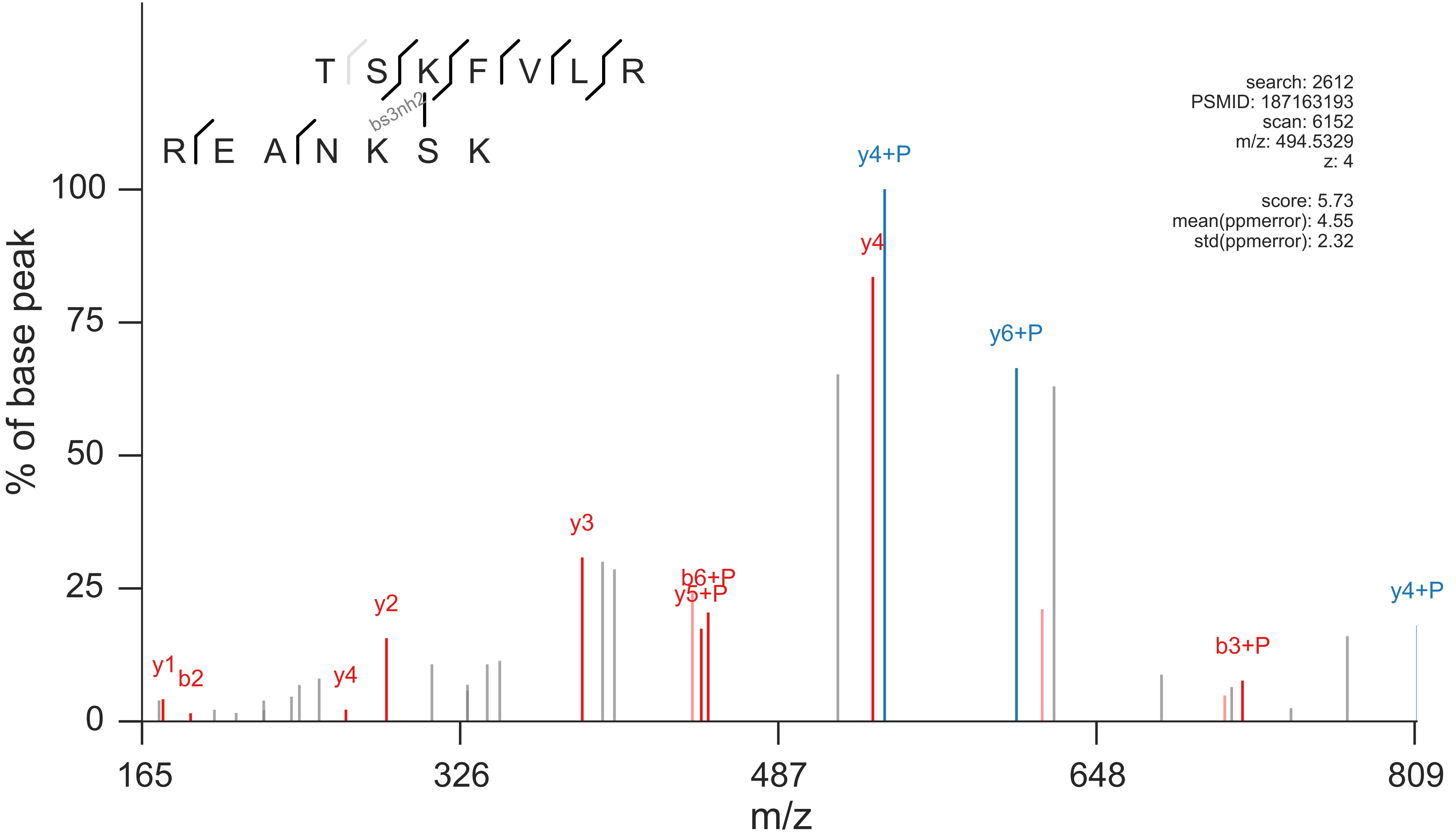

T S K F V L R
R E A N K S K
bs3nh2
search: 2612
PSMID: 187163193
scan: 6152
m/z: 494.5329
z: 4
score: 5.73
mean(ppmerror): 4.55
std(ppmerror): 2.32
% of base peak
m/z
0
25
50
75
100
165
326
487
648
809
y1
b2
y4
y2
y3
b6+P
y5+P
y4
y4+P
y6+P
b3+P
y4+P

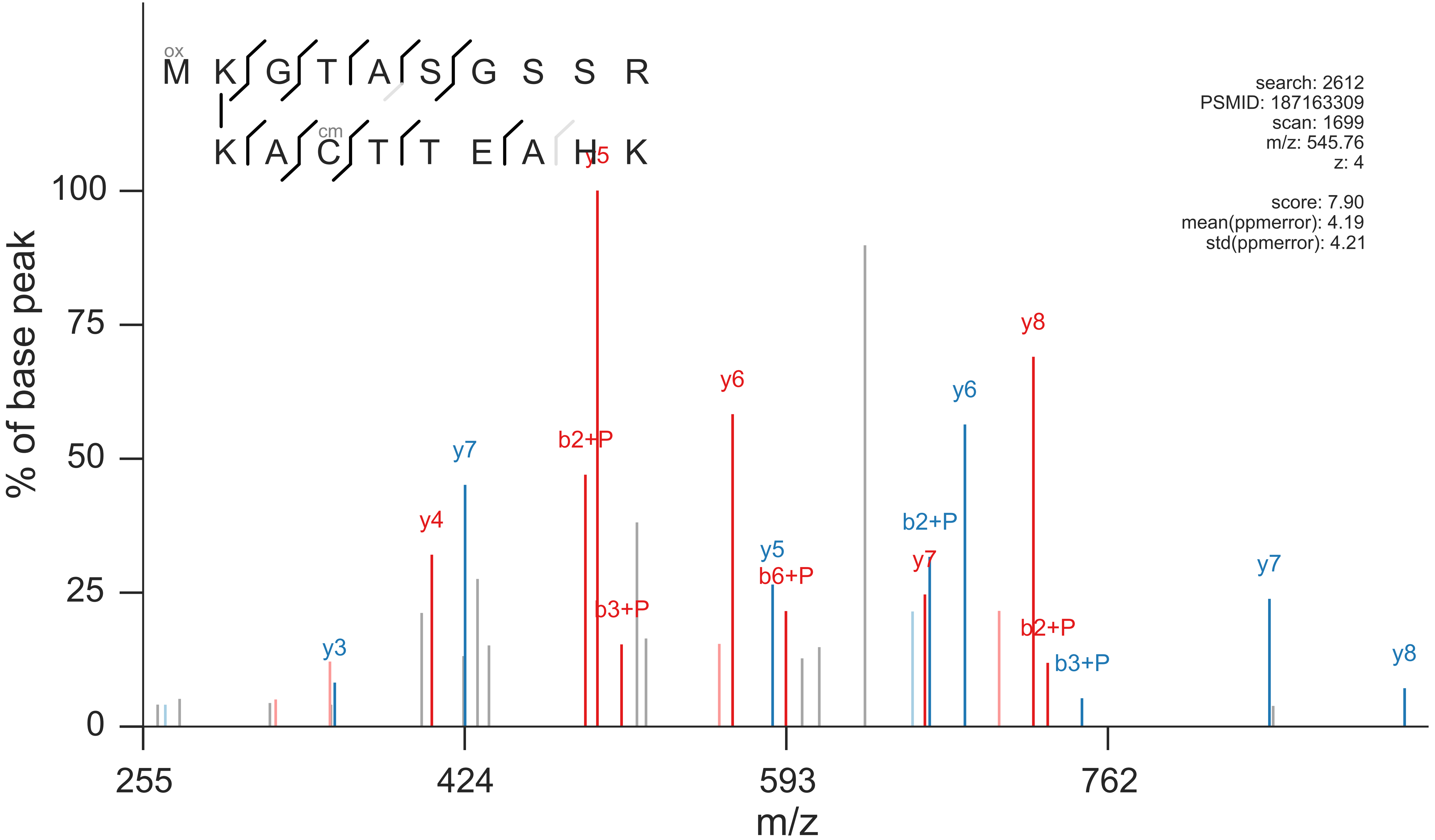

ox
M K G T A S G S S R
K A cm C T T E A H K
search: 2612
PSMID: 187163309
scan: 1699
m/z: 545.76
z: 4
score: 7.90
mean(ppmerror): 4.19
std(ppmerror): 4.21
% of base peak
100
75
50
25
0
255
424
593
762
m/z
y3
y4
y7
b2+P
y5
b3+P
y6
y5
b6+P
y7
b2+P
y6
y8
b2+P
b3+P
y7
y8

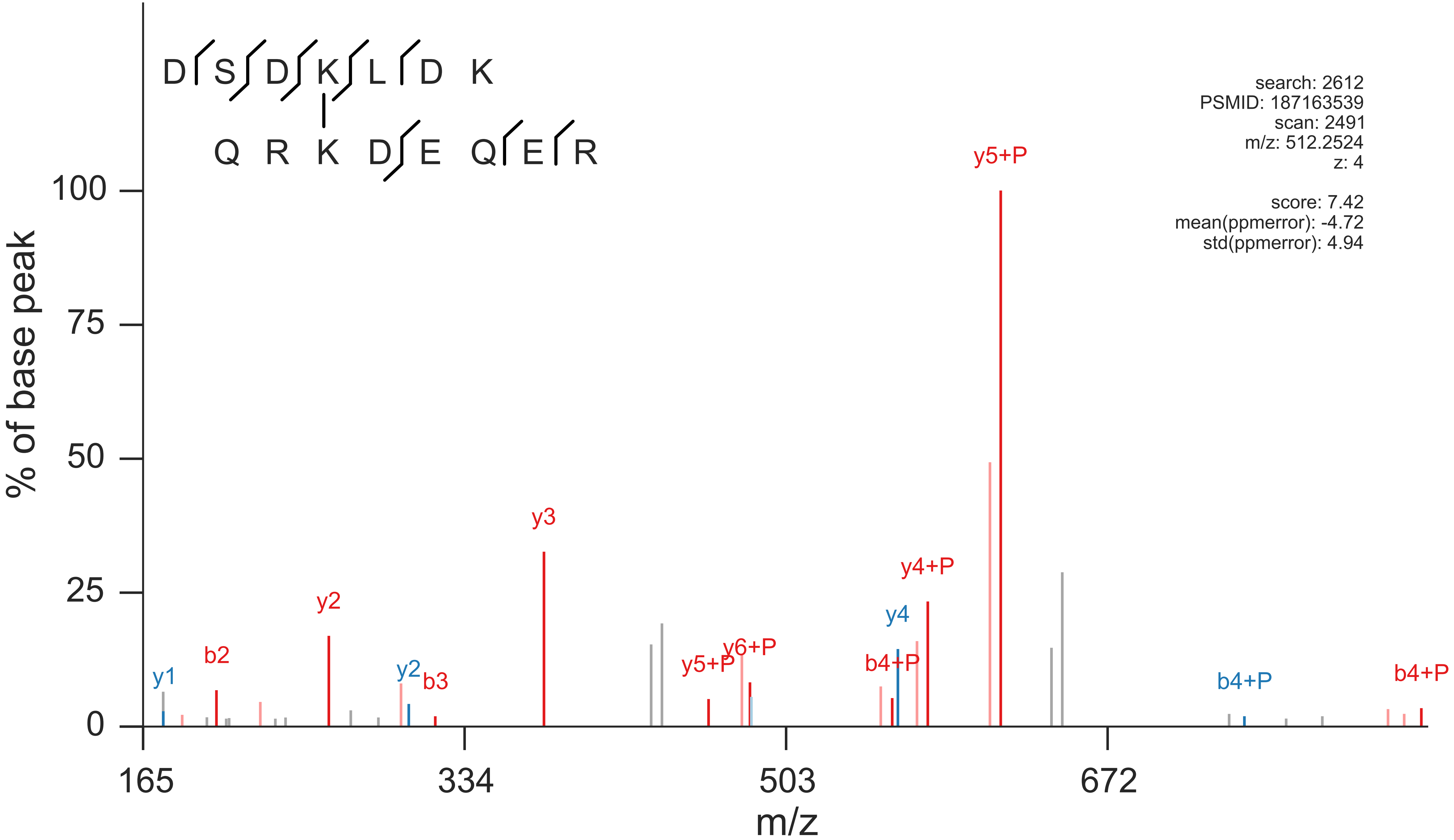

D S D K L D K
Q R K D E Q E R
search: 2612
PSMID: 187163539
scan: 2491
m/z: 512.2524
z: 4
score: 7.42
mean(ppmerror): -4.72
std(ppmerror): 4.94
% of base peak
100
75
50
25
0
165
334
503
672
m/z
y5+P
y3
y4+P
y2
y4
y6+P
y5+P
b4+P
b2
y1
y2
b3
b4+P
b4+P

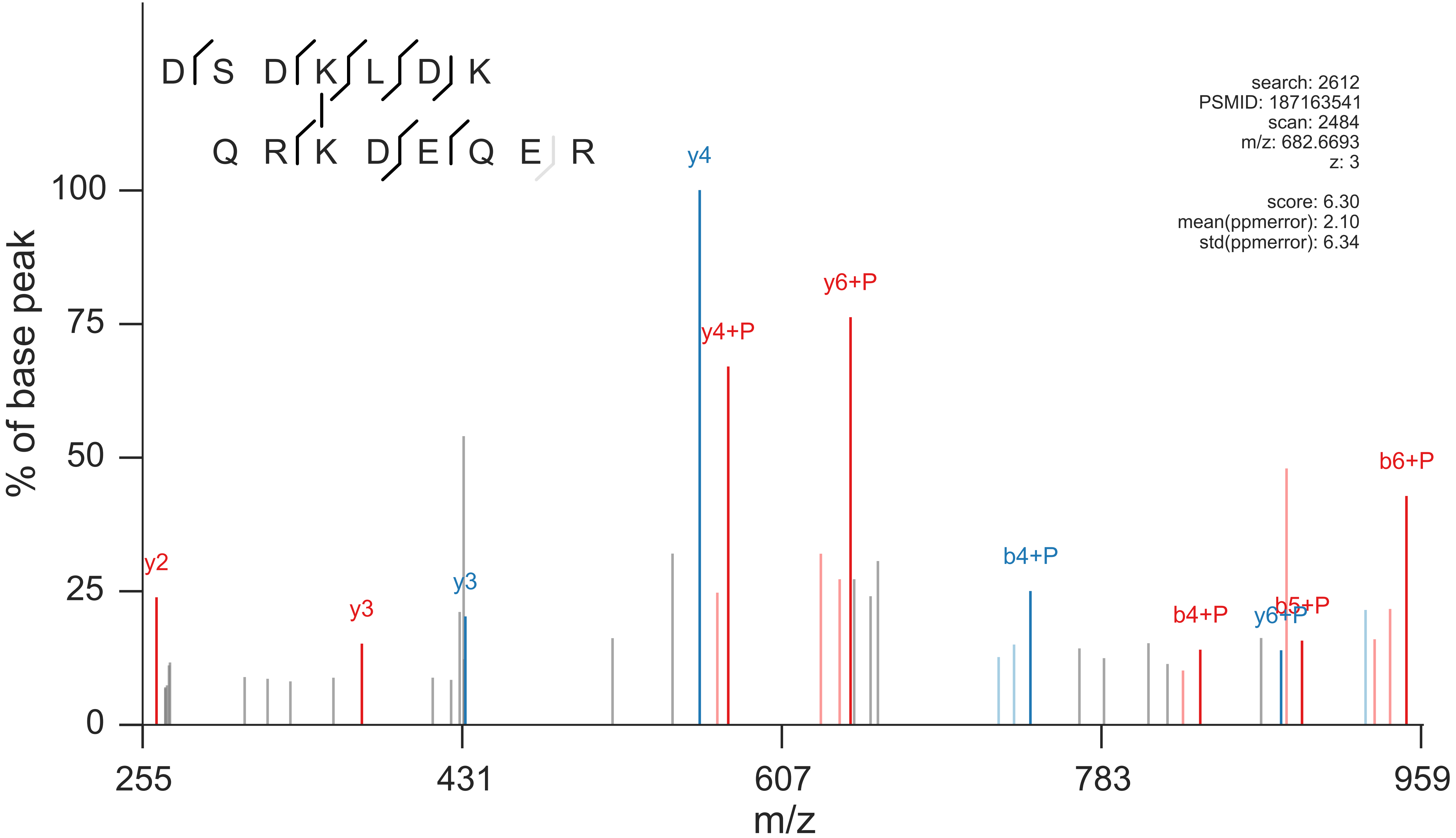

D S D K L D K
Q R K D E Q E R
search: 2612
PSMID: 187163541
scan: 2484
m/z: 682.6693
z: 3
score: 6.30
mean(ppmerror): 2.10
std(ppmerror): 6.34
% of base peak
m/z
y2
y3
y3
y4
y4+P
y6+P
b4+P
b4+P
y6+P
b5+P
b6+P
0
25
50
75
100
255
431
607
783
959

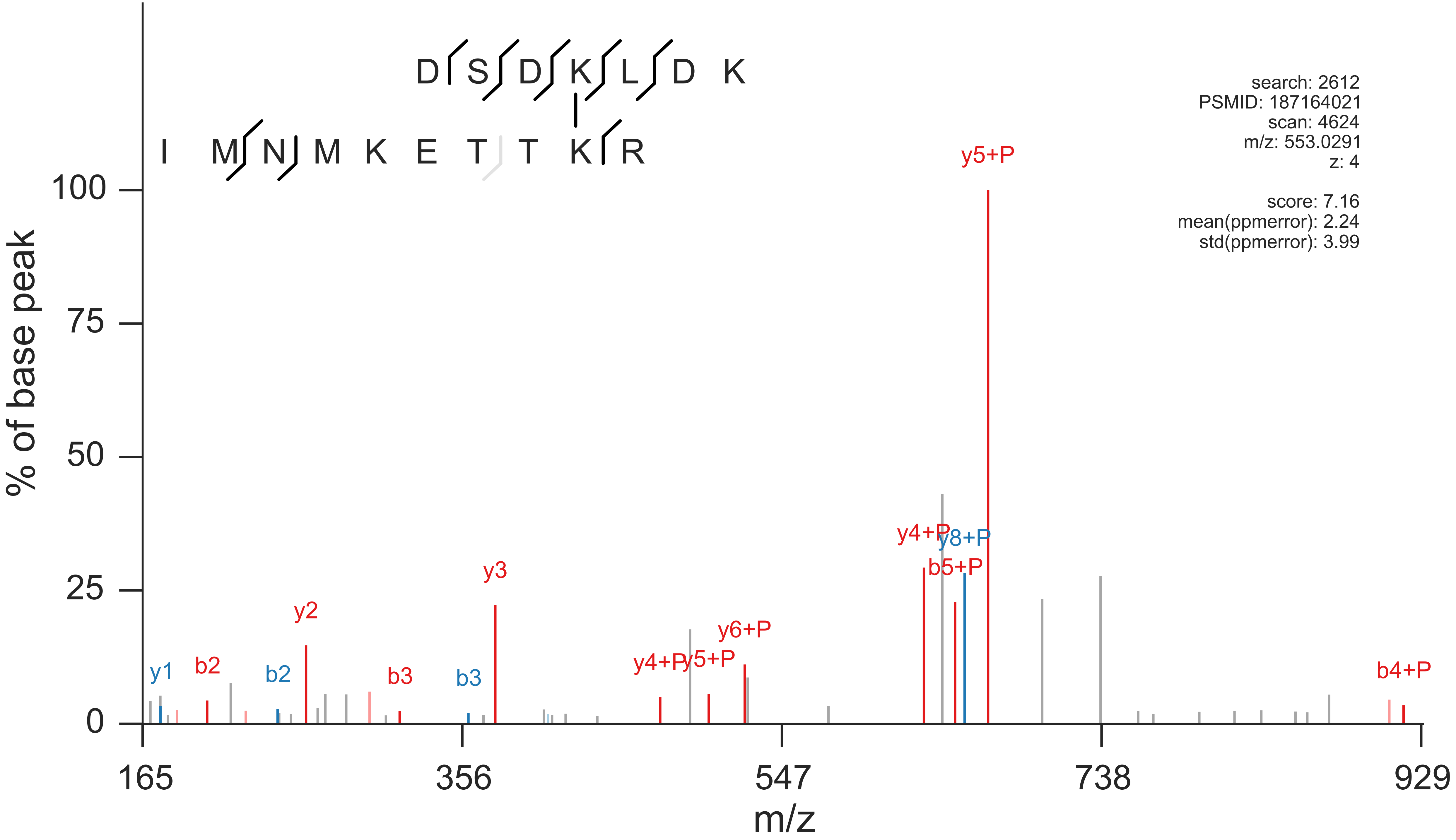

D S D K L D K
I M N M K E T T K R
search: 2612
PSMID: 187164021
scan: 4624
m/z: 553.0291
z: 4
score: 7.16
mean(ppmerror): 2.24
std(ppmerror): 3.99
% of base peak
m/z
y1
b2
b2
y2
b3
b3
y3
y4+P
y5+P
y6+P
y4+P
y8+P
b5+P
y5+P
b4+P

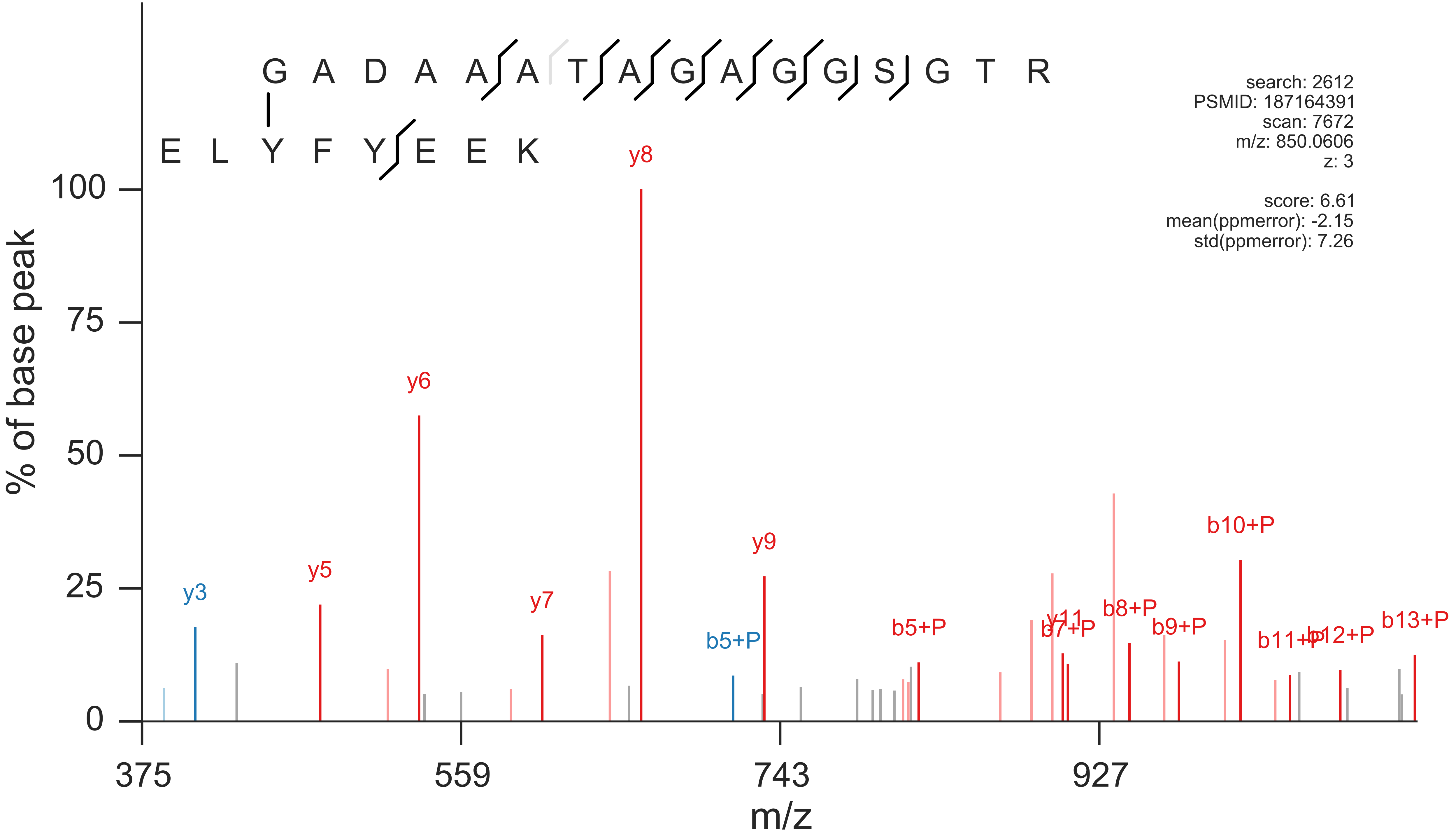
G A D A A A T A G A G G S G T R
E L Y F Y E E K
search: 2612
PSMID: 187164391
scan: 7672
m/z: 850.0606
z: 3
score: 6.61
mean(ppmerror): -2.15
std(ppmerror): 7.26
% of base peak
0
25
50
75
100
375
559
743
927
m/z
y3
y5
y6
y7
y8
y9
b5+P
b5+P
y11
b7+P
b8+P
b9+P
b10+P
b11+P
b12+P
b13+P

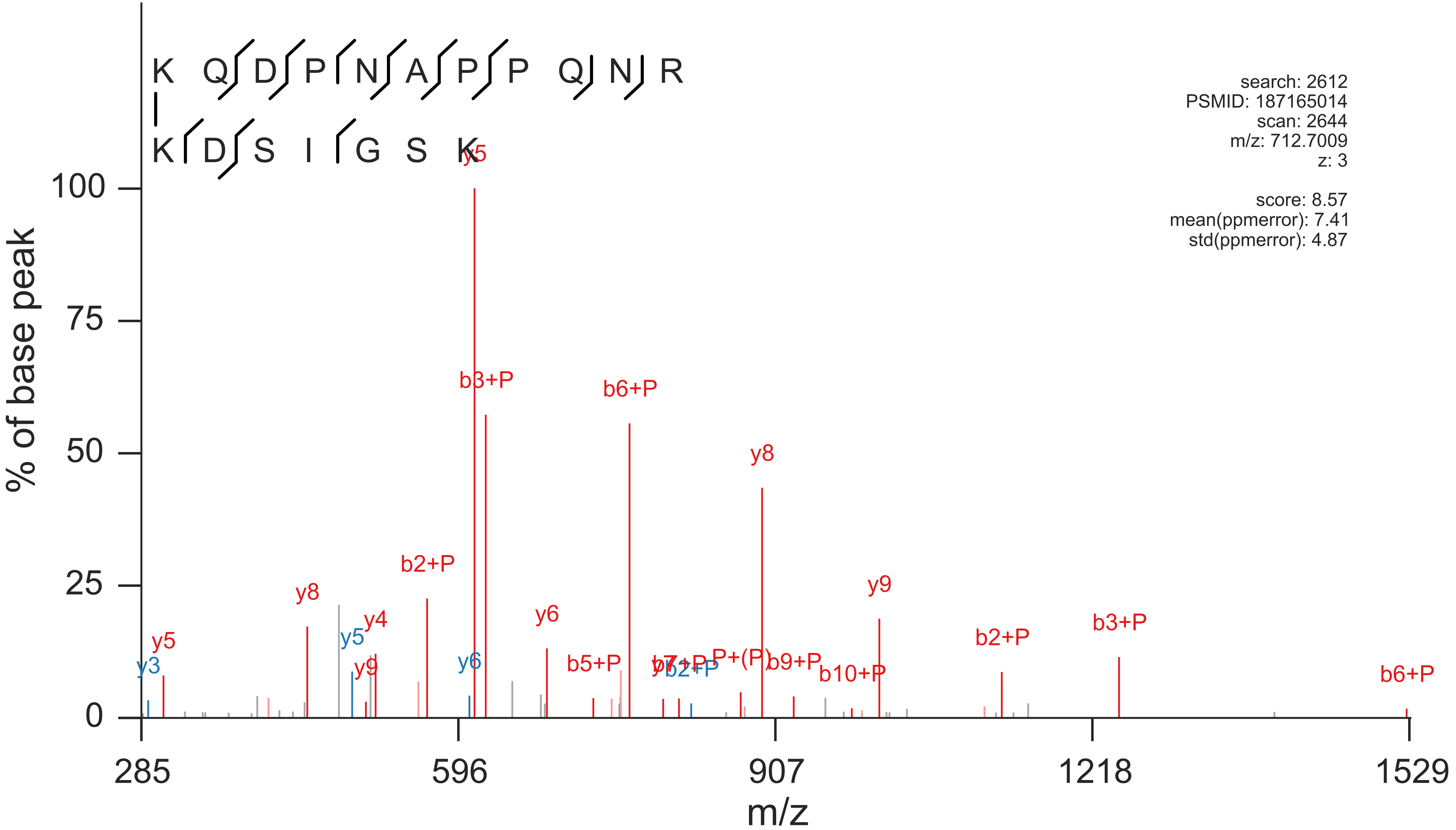

K Q D P N A P P Q N R
K D S I G S K
search: 2612
PSMID: 187165014
scan: 2644
m/z: 712.7009
z: 3
score: 8.57
mean(ppmerror): 7.41
std(ppmerror): 4.87
% of base peak
m/z
0
25
50
75
100
285
596
907
1218
1529
y5
b3+P
b6+P
y8
b2+P
y9
b3+P
b2+P
y8
y4
y5
y6
y3
y5
y9
y6
b5+P
b9+P
b10+P
b6+P

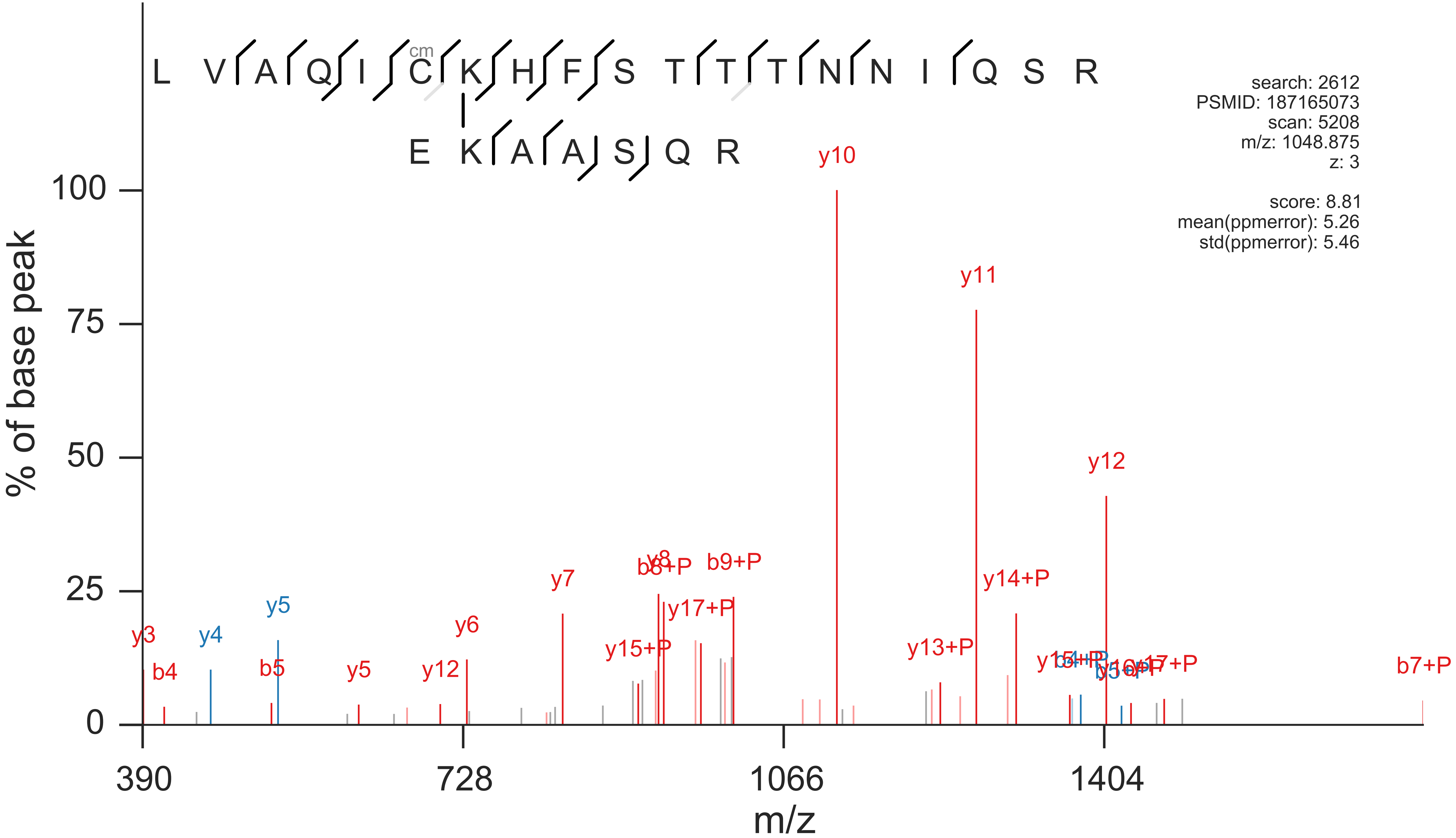

L V A Q I C K H F S T T T N N I Q S R
cm
E K A A S Q R
search: 2612
PSMID: 187165073
scan: 5208
m/z: 1048.875
z: 3
score: 8.81
mean(ppmerror): 5.26
std(ppmerror): 5.46
% of base peak
m/z
0
25
50
75
100
390
728
1066
1404
y10
y11
y12
y14+P
y7
b9+P
y17+P
y15+P
y13+P
y6
y5
y4
y3
b4
b5
y5
y12
b7+P

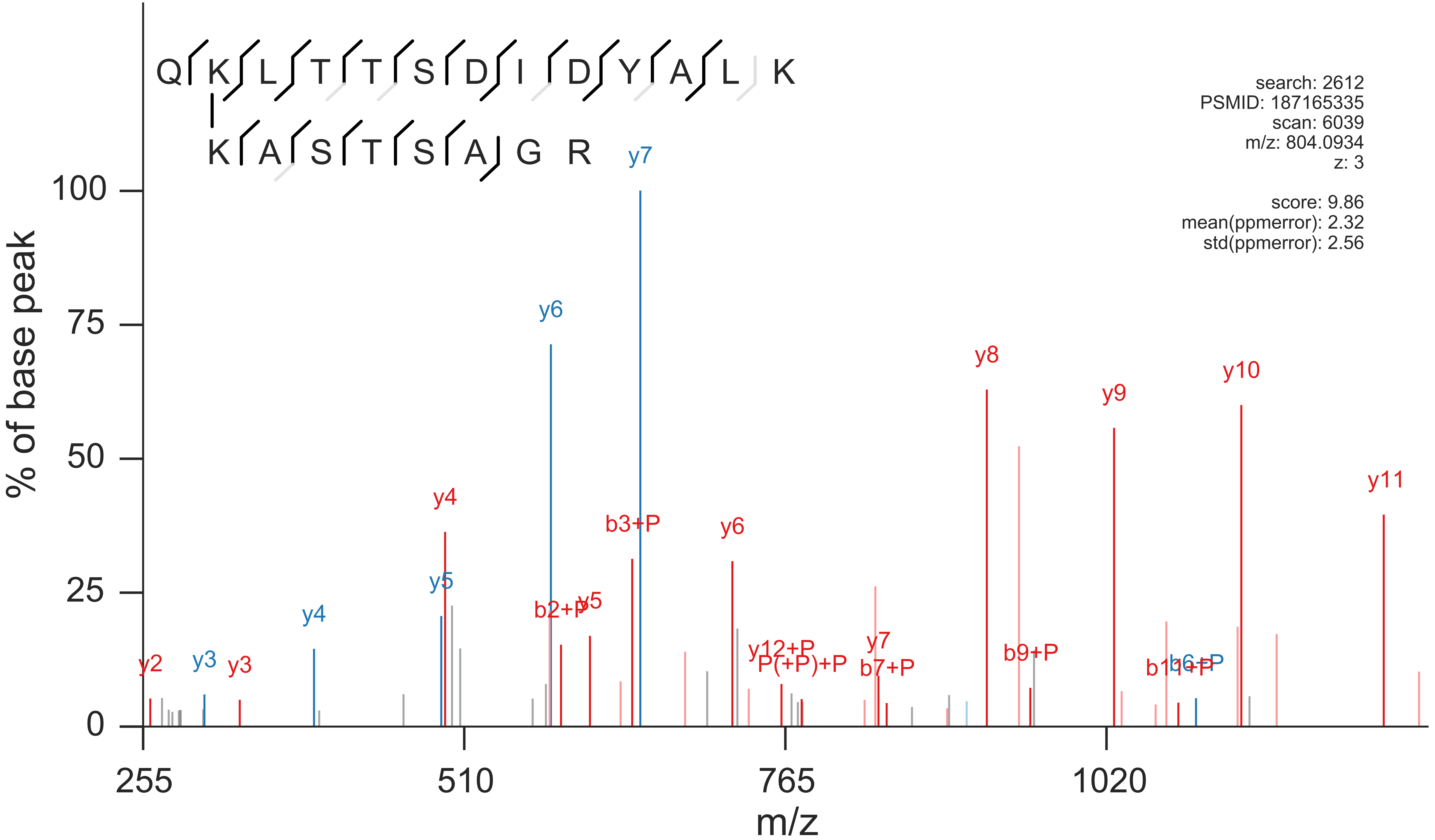

Q K L T T S D I D Y A L K
K A S T S A G R
search: 2612
PSMID: 187165335
scan: 6039
m/z: 804.0934
z: 3
score: 9.86
mean(ppmerror): 2.32
std(ppmerror): 2.56
% of base peak
m/z
0
25
50
75
100
255
510
765
1020
y2
y3
y3
y4
y4
y5
y6
y7
b2+P
y5
b3+P
y6
y12+P
P(+P)+P
y7
b7+P
y8
b9+P
y9
y10
y11

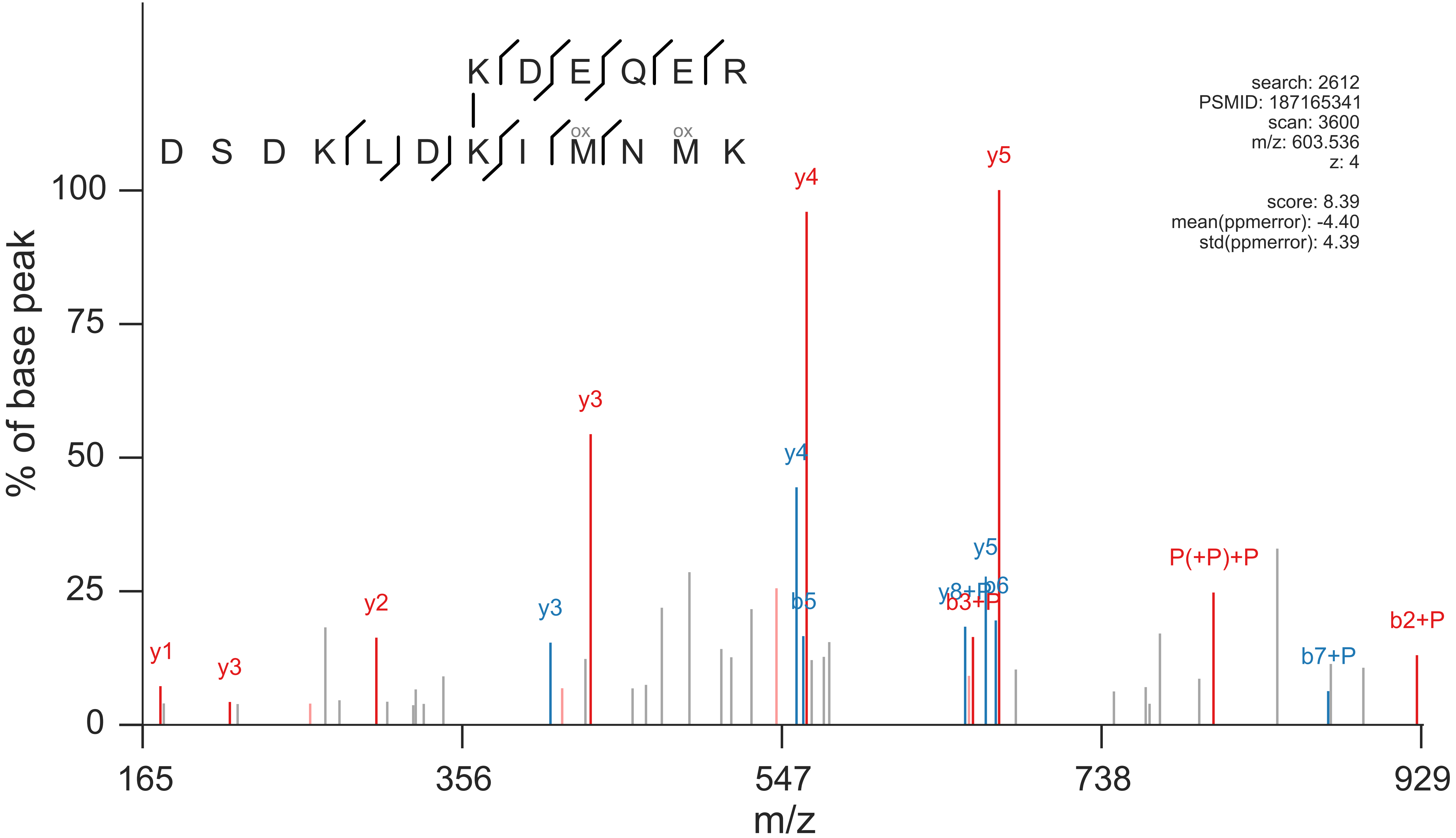

K D E Q E R
D S D K L D K I M N M K
ox
ox
search: 2612
PSMID: 187165341
scan: 3600
m/z: 603.536
z: 4
score: 8.39
mean(ppmerror): -4.40
std(ppmerror): 4.39
% of base peak
100
75
50
25
0
165
356
547
738
929
m/z
y1
y3
y2
y3
y3
y4
y4
b5
y5
y5
y8+P
b6
b3+P
P(+P)+P
b7+P
b2+P

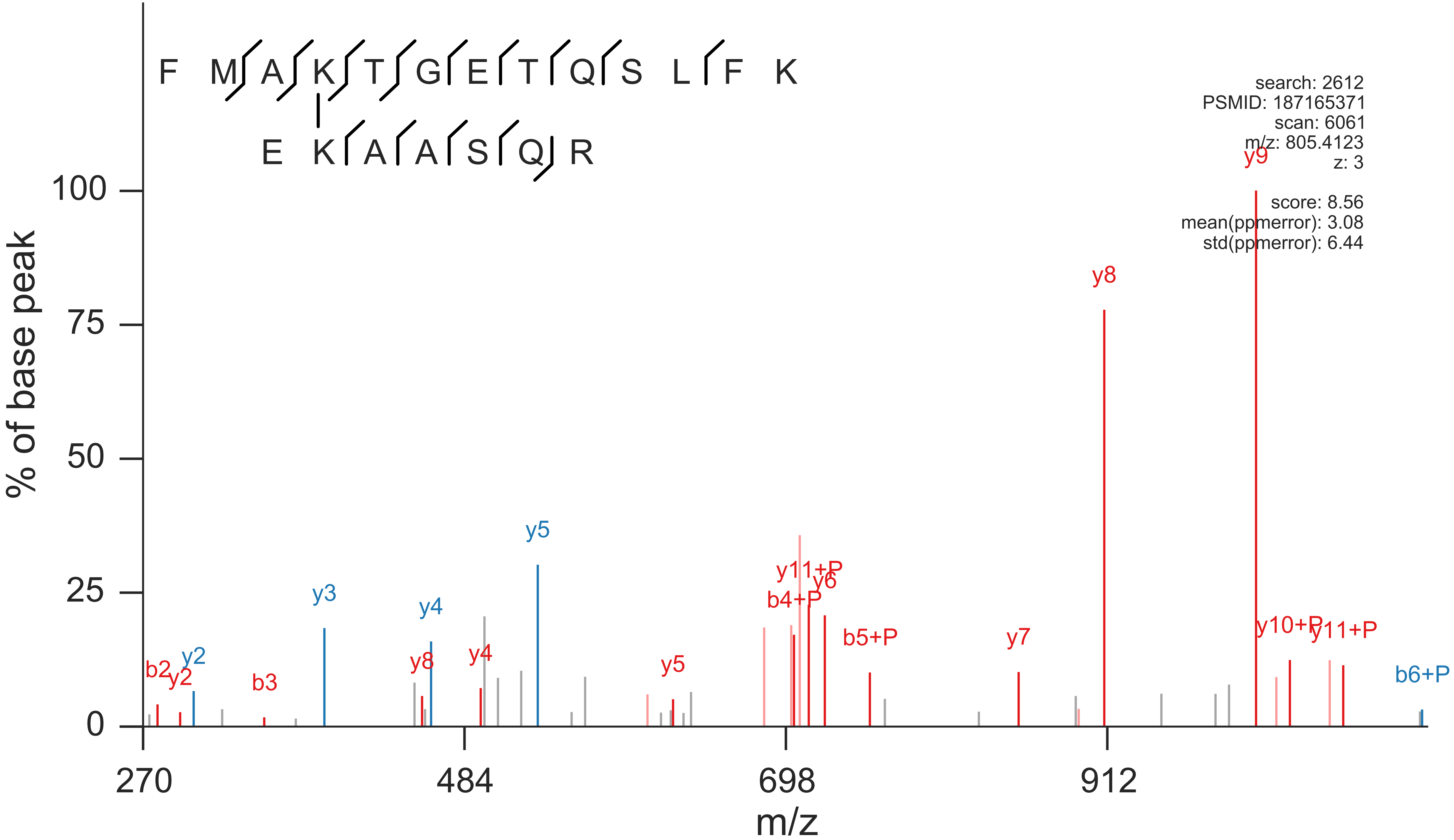
F M A K T G E T Q S L F K
E K A A S Q R
search: 2612
PSMID: 187165371
scan: 6061
m/z: 805.4123
z: 3
score: 8.56
mean(ppmerror): 3.08
std(ppmerror): 6.44
% of base peak
m/z
y9
y8
y5
y3
y4
y2
b2
y2
b3
y8
y4
y5
y11+P
y6
b4+P
b5+P
y7
y10+P
y11+P
b6+P
0
25
50
75
100
270
484
698
912

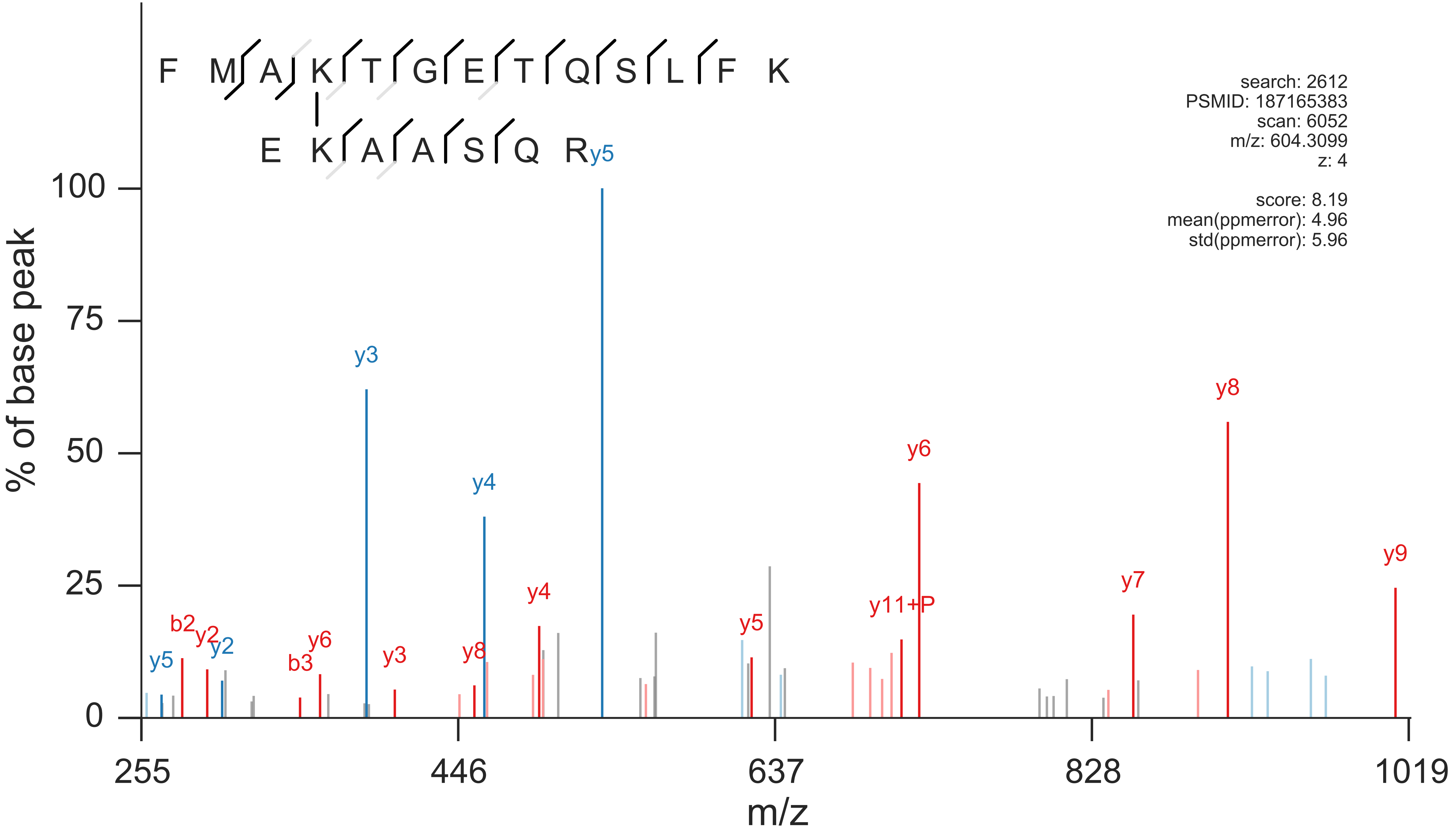

F M A K T G E T Q S L F K
E K A A S Q R
search: 2612
PSMID: 187165383
scan: 6052
m/z: 604.3099
z: 4
score: 8.19
mean(ppmerror): 4.96
std(ppmerror): 5.96
% of base peak
m/z
0
25
50
75
100
255
446
637
828
1019
y5
y3
y4
y2
b2
y2
b3
y6
y3
y8
y4
y5
y11+P
y6
y7
y8
y9

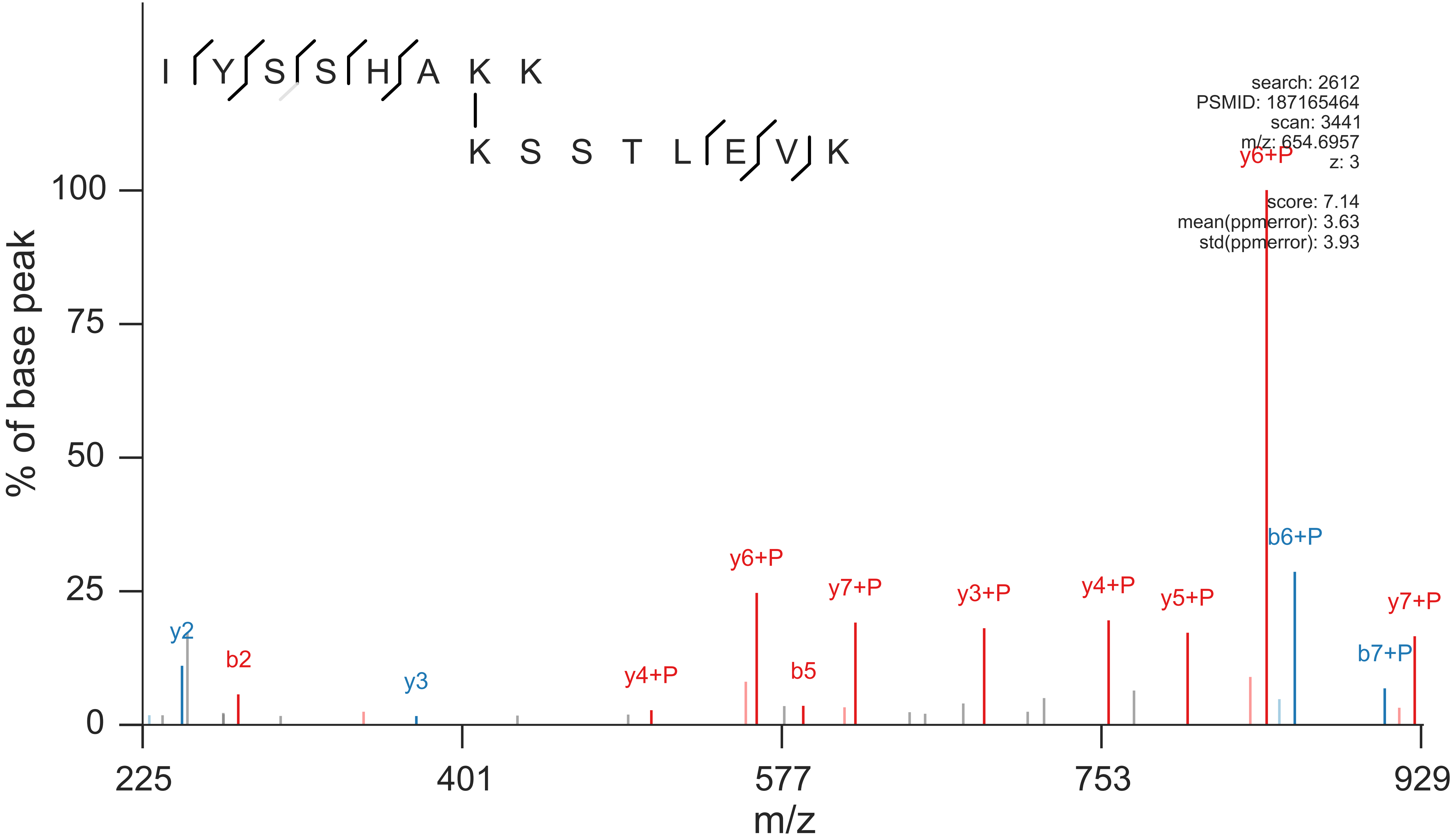

I Y S S H A K K
K S S T L E V K
search: 2612
PSMID: 187165464
scan: 3441
m/z: 654.6957
z: 3
score: 7.14
mean(ppmerror): 3.63
std(ppmerror): 3.93
% of base peak
100
75
50
25
0
m/z
225
401
577
753
929
y2
b2
y3
y4+P
y6+P
b5
y7+P
y3+P
y4+P
y5+P
y6+P
b6+P
b7+P
y7+P

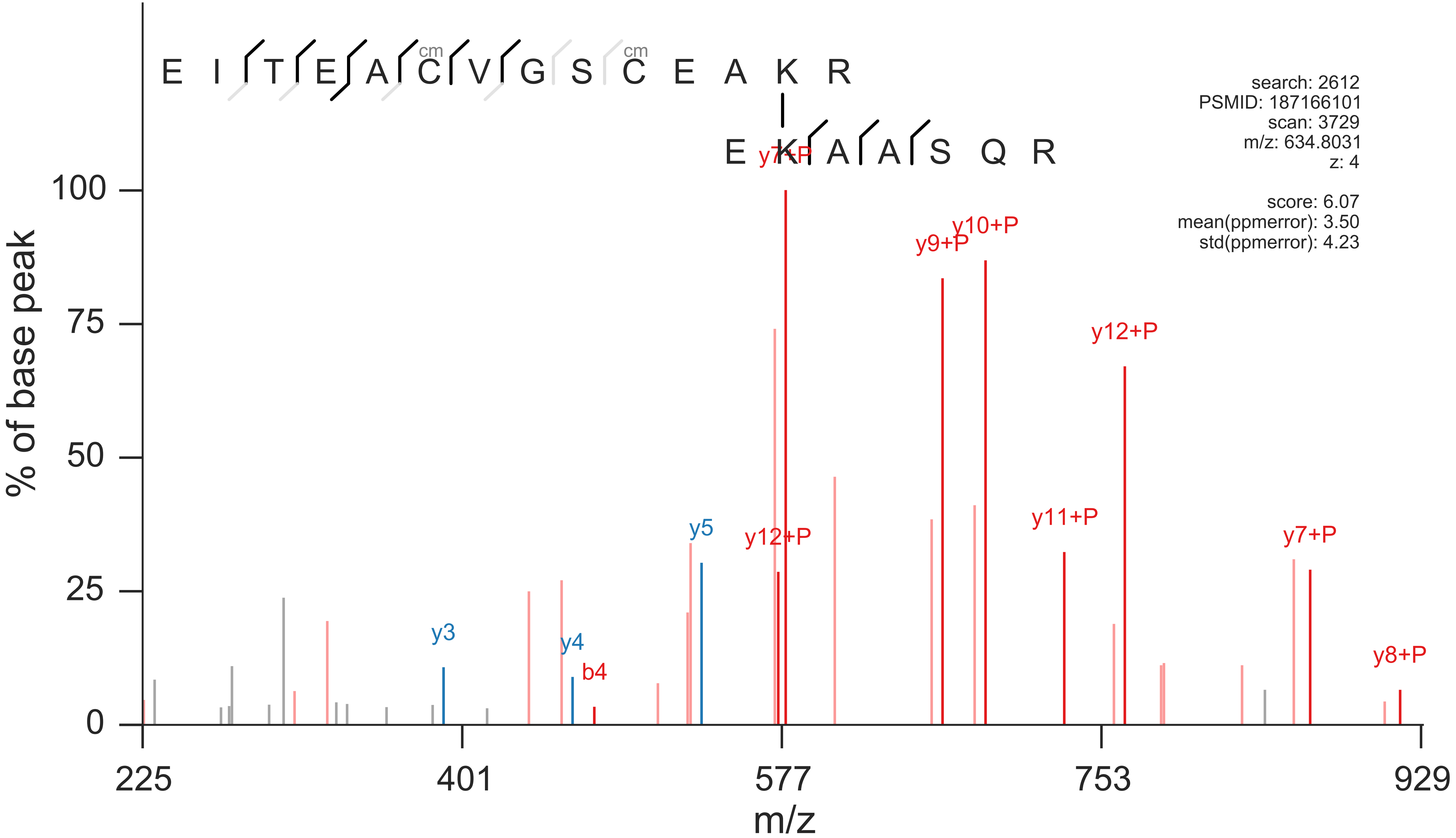

E I T E A C V G S C E A K R
cm
cm
E K A A S Q R
search: 2612
PSMID: 187166101
scan: 3729
m/z: 634.8031
z: 4
score: 6.07
mean(ppmerror): 3.50
std(ppmerror): 4.23
% of base peak
m/z
y3
y4
b4
y5
y12+P
y7+P
y9+P
y10+P
y11+P
y12+P
y7+P
y8+P
0
25
50
75
100
225
401
577
753
929

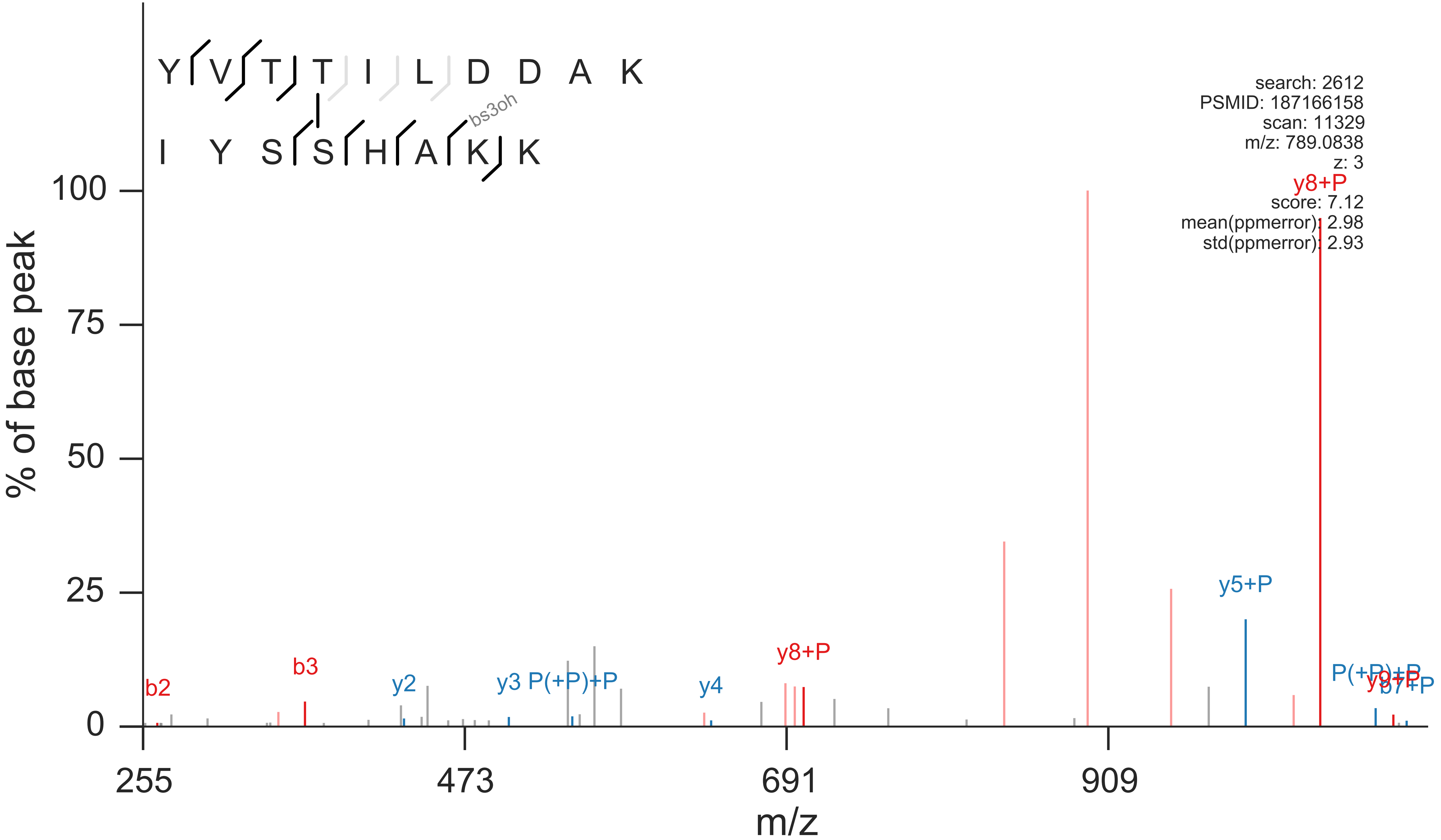

Y V T T I L D D A K
I Y S S H A K K
bs3oh
search: 2612
PSMID: 187166158
scan: 11329
m/z: 789.0838
z: 3
score: 7.12
mean(ppmerror): 2.98
std(ppmerror): 2.93
% of base peak
m/z
b2
b3
y2
y3 P(+P)+P
y4
y8+P
y5+P
y8+P
P(+P)+P
y9+P
b7+P

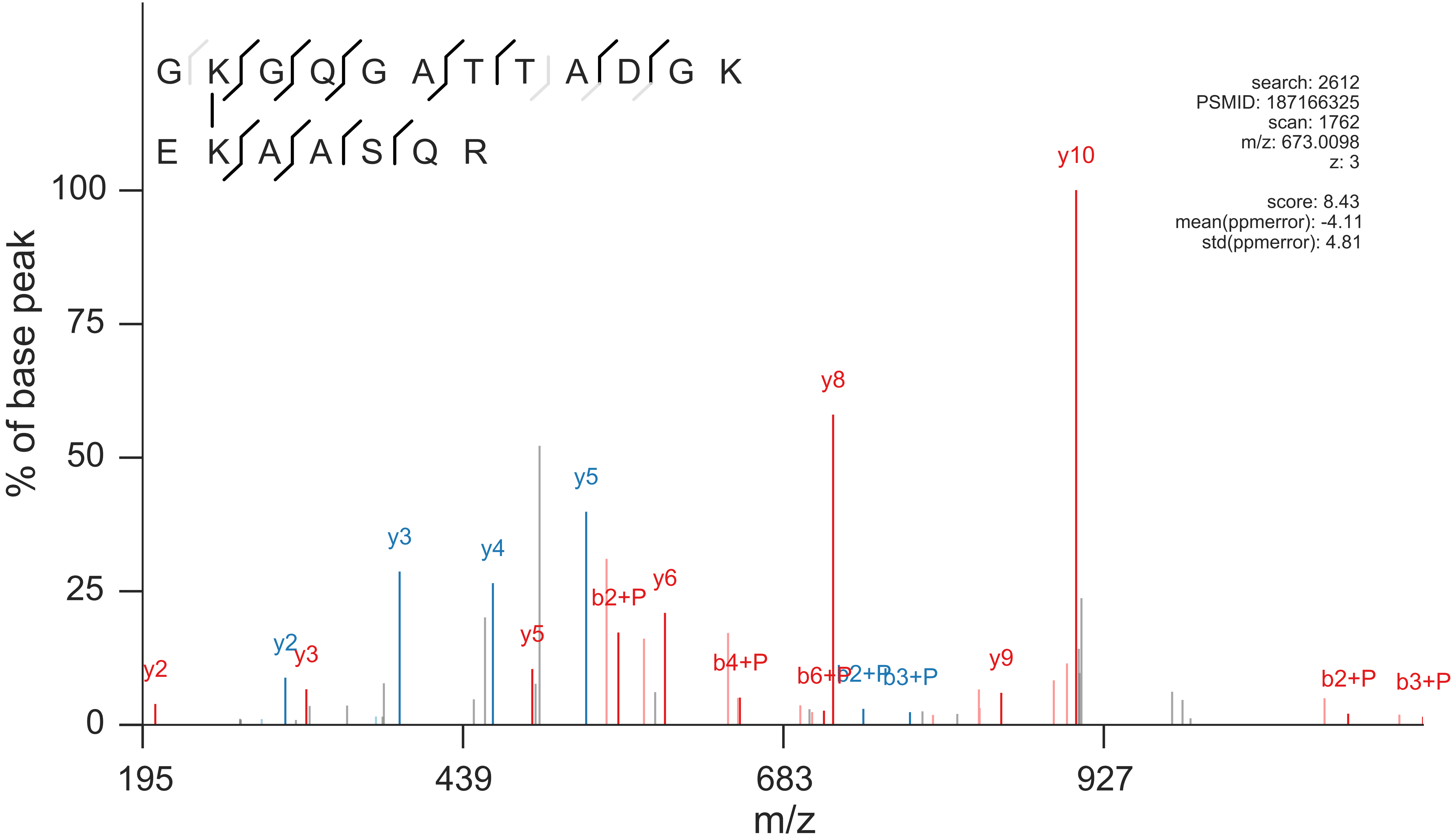
G K G Q G A T T A D G K
E K A A S Q R
search: 2612
PSMID: 187166325
scan: 1762
m/z: 673.0098
z: 3
score: 8.43
mean(ppmerror): -4.11
std(ppmerror): 4.81
% of base peak
m/z
y2
y2
y3
y3
y4
y5
y5
b2+P
y6
b4+P
b6+P
b2+P
b3+P
y8
y9
y10
b2+P
b3+P
0
25
50
75
100
195
439
683
927

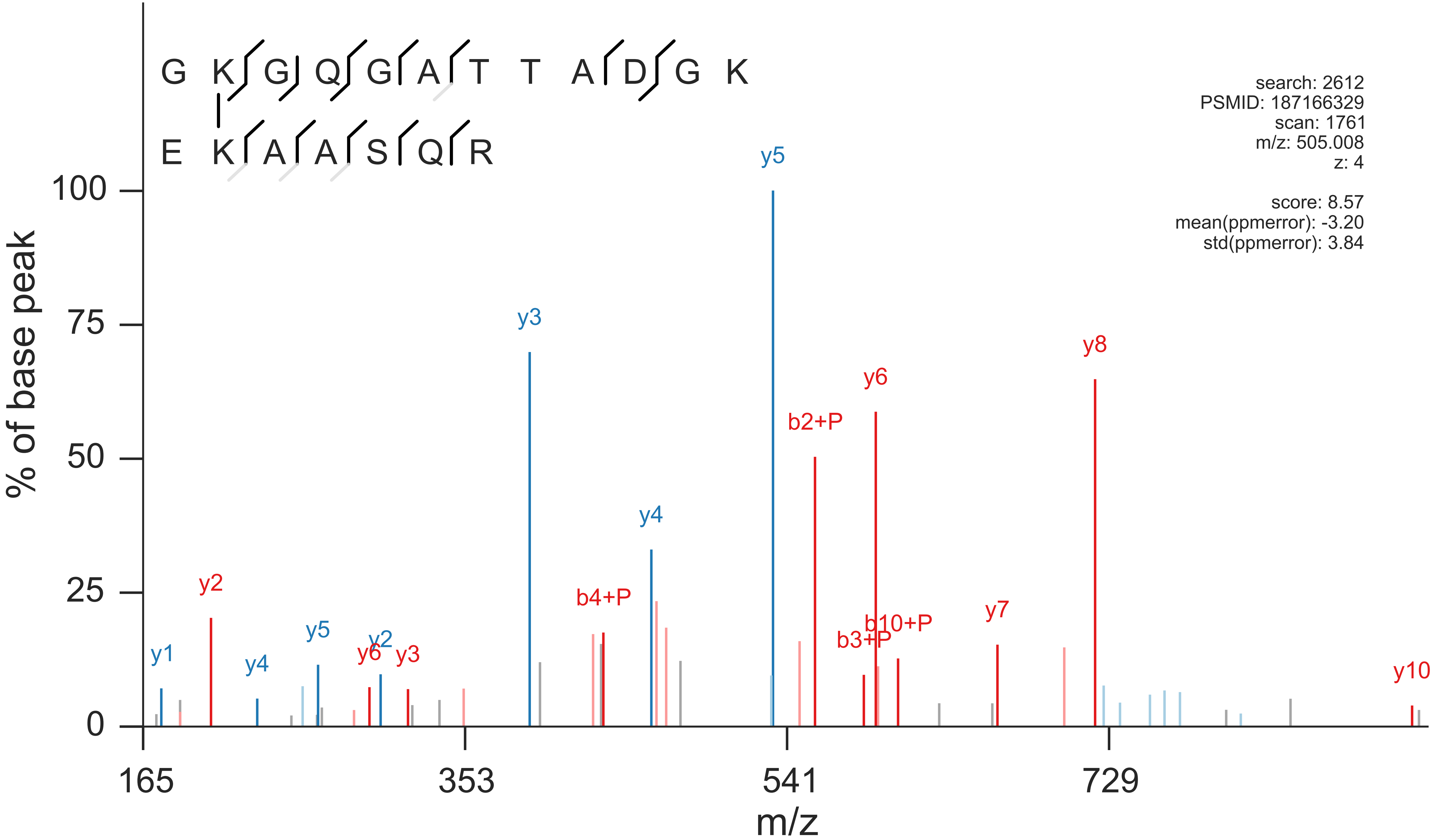

G K G Q G A T T A D G K
E K A A S Q R
search: 2612
PSMID: 187166329
scan: 1761
m/z: 505.008
z: 4
score: 8.57
mean(ppmerror): -3.20
std(ppmerror): 3.84
% of base peak
m/z
0
25
50
75
100
165
353
541
729
y1
y2
y4
y5
y6
y2
y3
y3
b4+P
y4
y5
b2+P
y6
b3+P
b10+P
y7
y8
y10

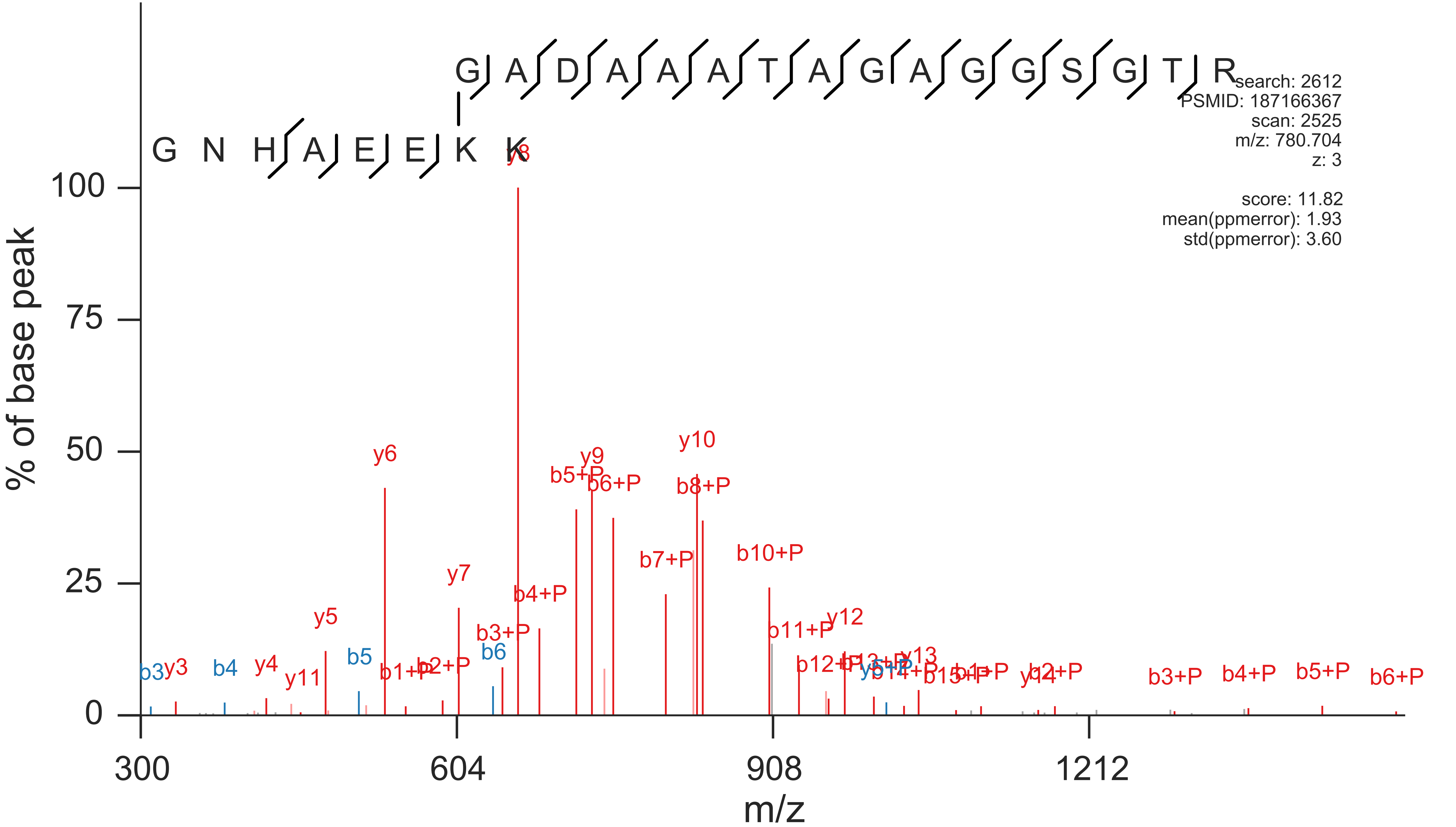

G A D A A A T A G A G G S G T R
G N H A E E K K
search: 2612
PSMID: 187166367
scan: 2525
m/z: 780.704
z: 3
score: 11.82
mean(ppmerror): 1.93
std(ppmerror): 3.60
% of base peak
m/z
0
25
50
75
100
300
604
908
1212
y3
y4
y5
y6
y7
y8
y9
y10
y11
y12
y13
y14
b3
b4
b5
b6
b1+P
b2+P
b3+P
b4+P
b5+P
b6+P
b7+P
b8+P
b10+P
b11+P
b12+P

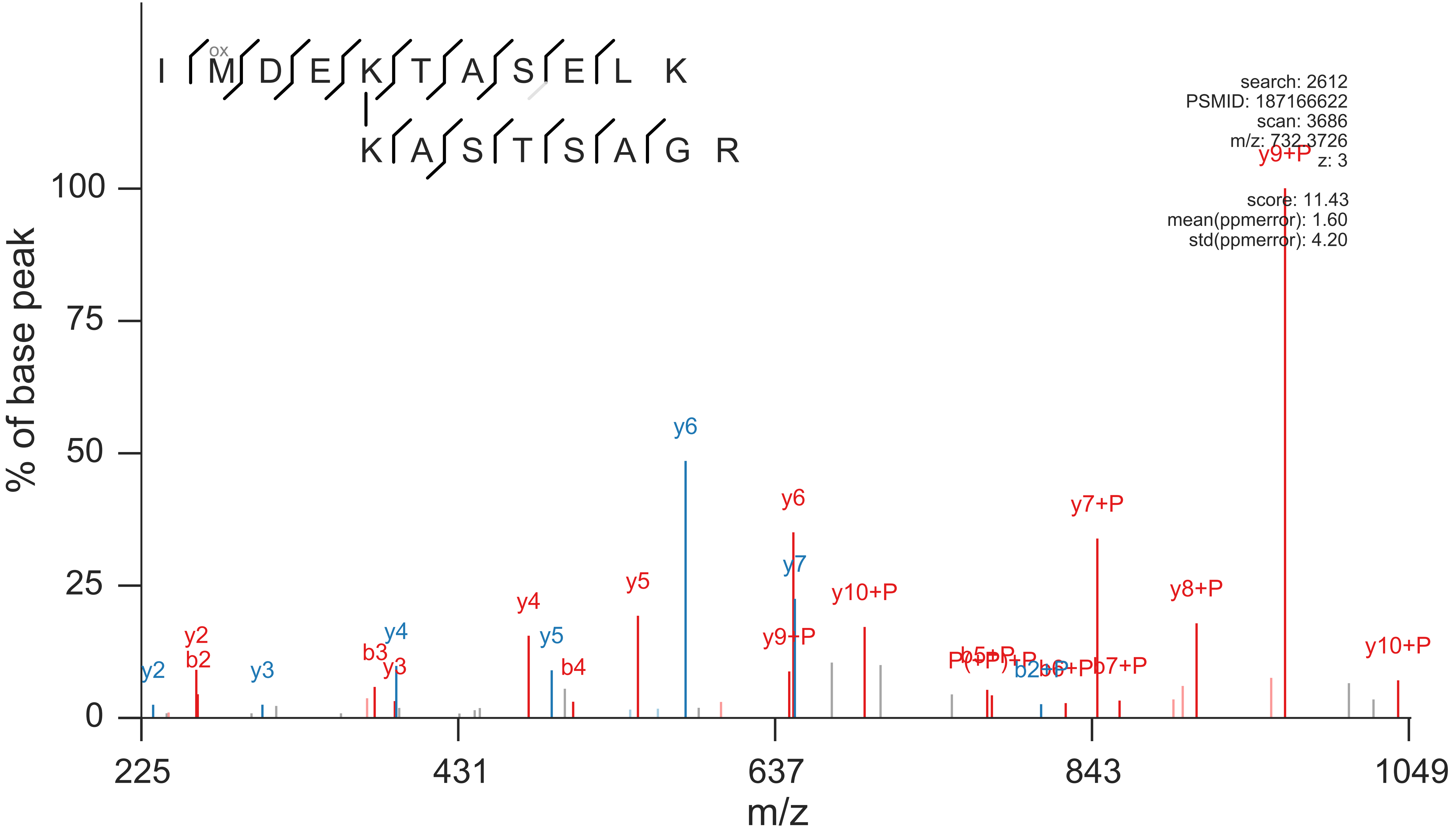

I M D E K T A S E L K
ox
K A S T S A G R
search: 2612
PSMID: 187166622
scan: 3686
m/z: 732.3726
z: 3
score: 11.43
mean(ppmerror): 1.60
std(ppmerror): 4.20
% of base peak
m/z
0
25
50
75
100
225
431
637
843
1049
y2
y2
b2
y3
b3
y4
y3
y4
y5
b4
y5
y6
y6
y7
y9+P
y10+P
y7+P
y8+P
y9+P
y10+P
b7+P

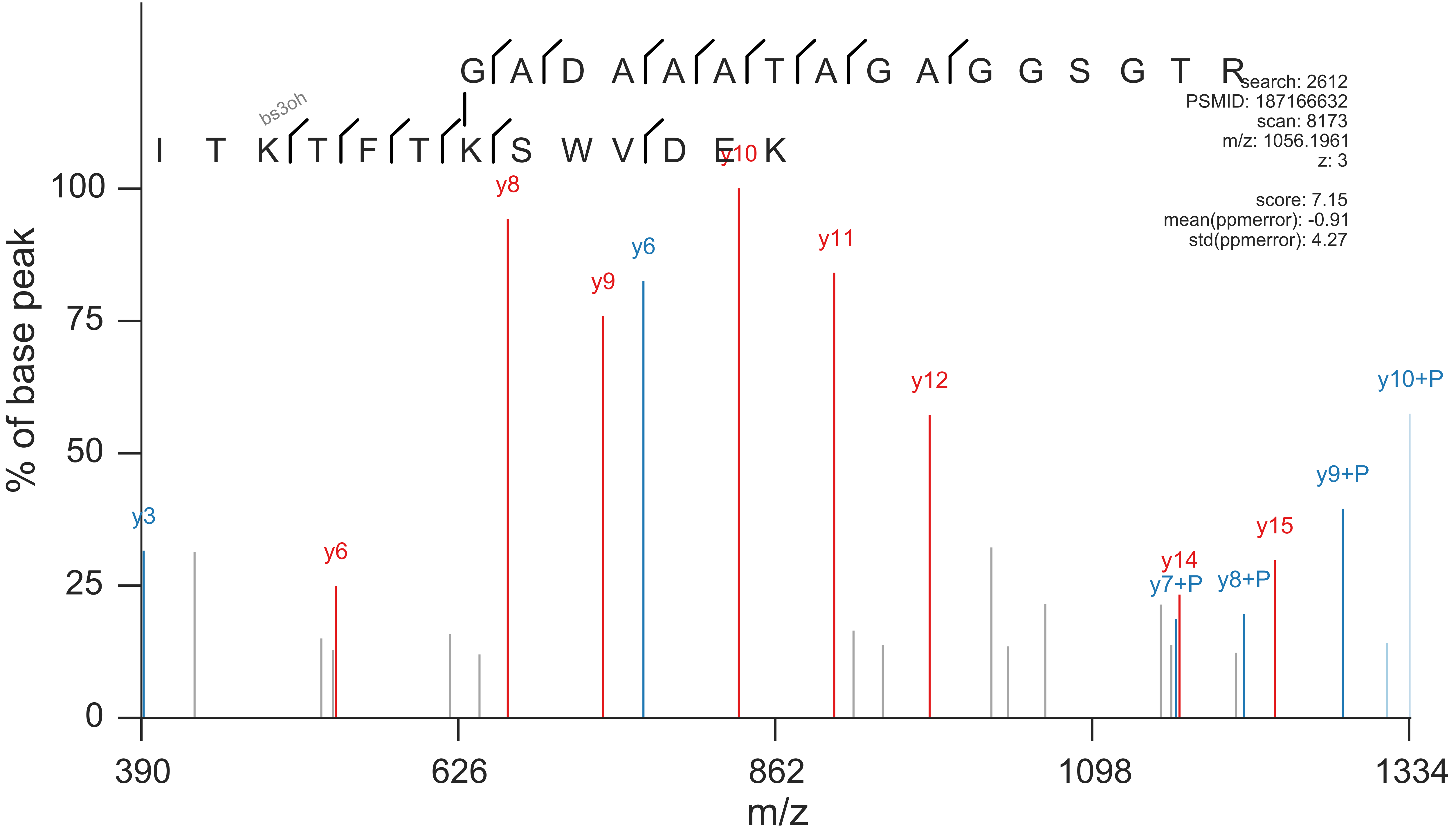

G A D A A A T A G A G G S G T R
I T K T F T K S W V D E K
bs3oh
search: 2612
PSMID: 187166632
scan: 8173
m/z: 1056.1961
z: 3
score: 7.15
mean(ppmerror): -0.91
std(ppmerror): 4.27
% of base peak
100
75
50
25
0
y3
y6
y8
y9
y6
y10
y11
y12
y14
y7+P
y8+P
y15
y9+P
y10+P
390
626
862
1098
1334
m/z

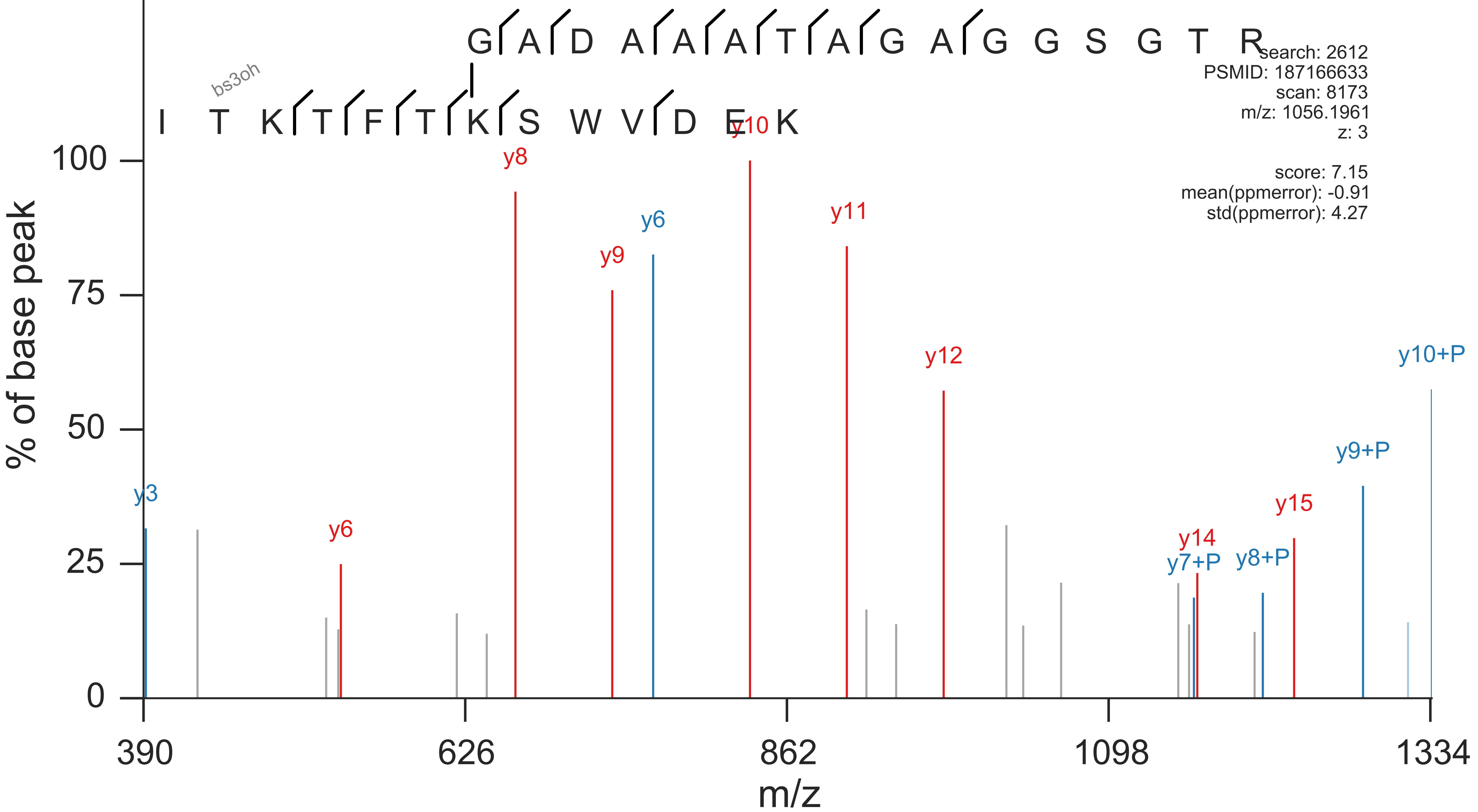

G A D A A A T A G A G G S G T R
I T K T F T K S W V D E K
bs3oh
search: 2612
PSMID: 187166633
scan: 8173
m/z: 1056.1961
z: 3
score: 7.15
mean(ppmerror): -0.91
std(ppmerror): 4.27
% of base peak
m/z
y3
y6
y8
y9
y6
y10
y11
y12
y14
y7+P
y8+P
y15
y9+P
y10+P

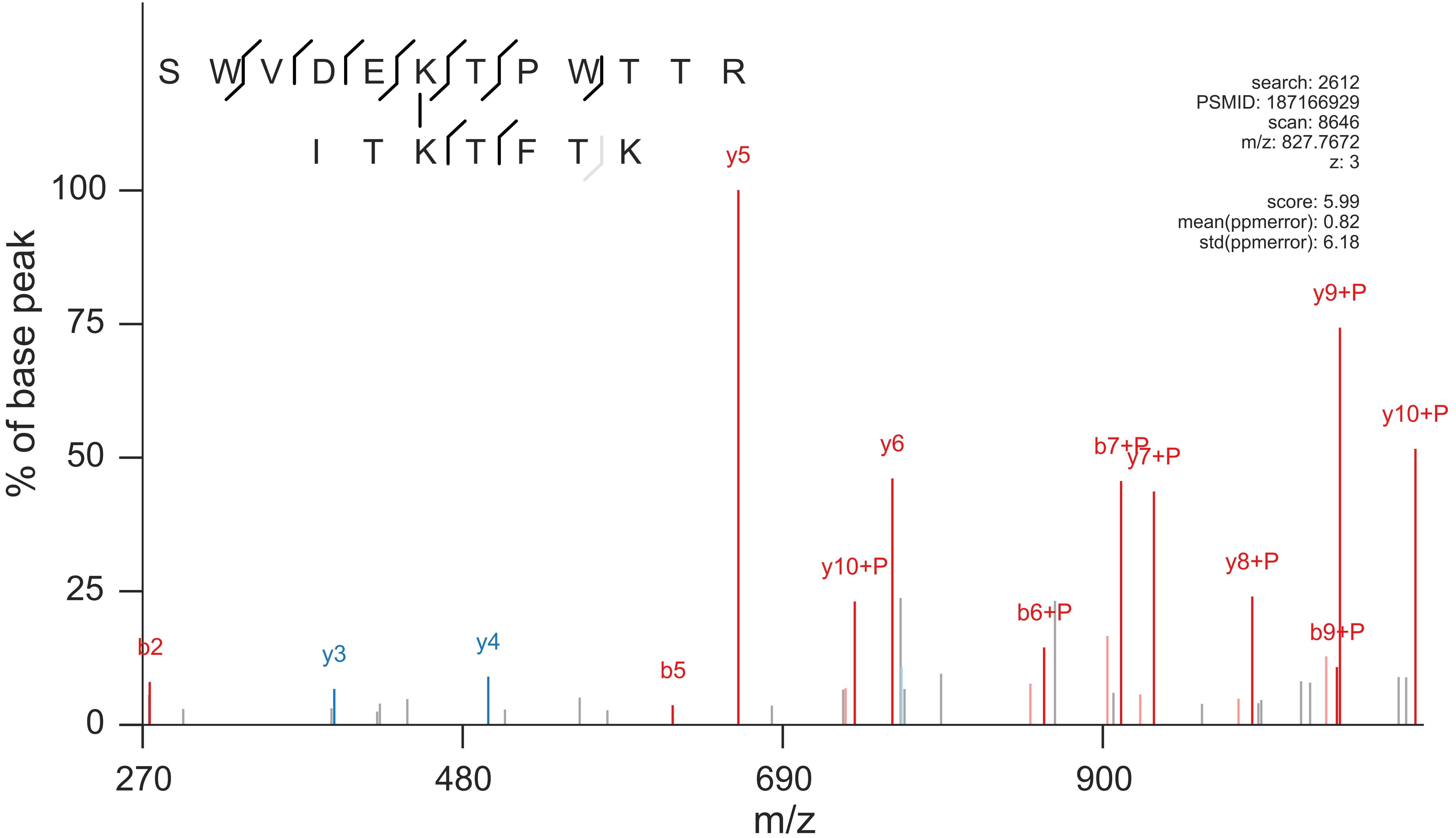

S W V D E K T P W T T R
I T K T F T K
search: 2612
PSMID: 187166929
scan: 8646
m/z: 827.7672
z: 3
score: 5.99
mean(ppmerror): 0.82
std(ppmerror): 6.18
% of base peak
m/z
y5
y6
y10+P
b7+P
y7+P
y9+P
y10+P
y8+P
b6+P
b9+P
b2
y3
y4
b5
100
75
50
25
0
270
480
690
900

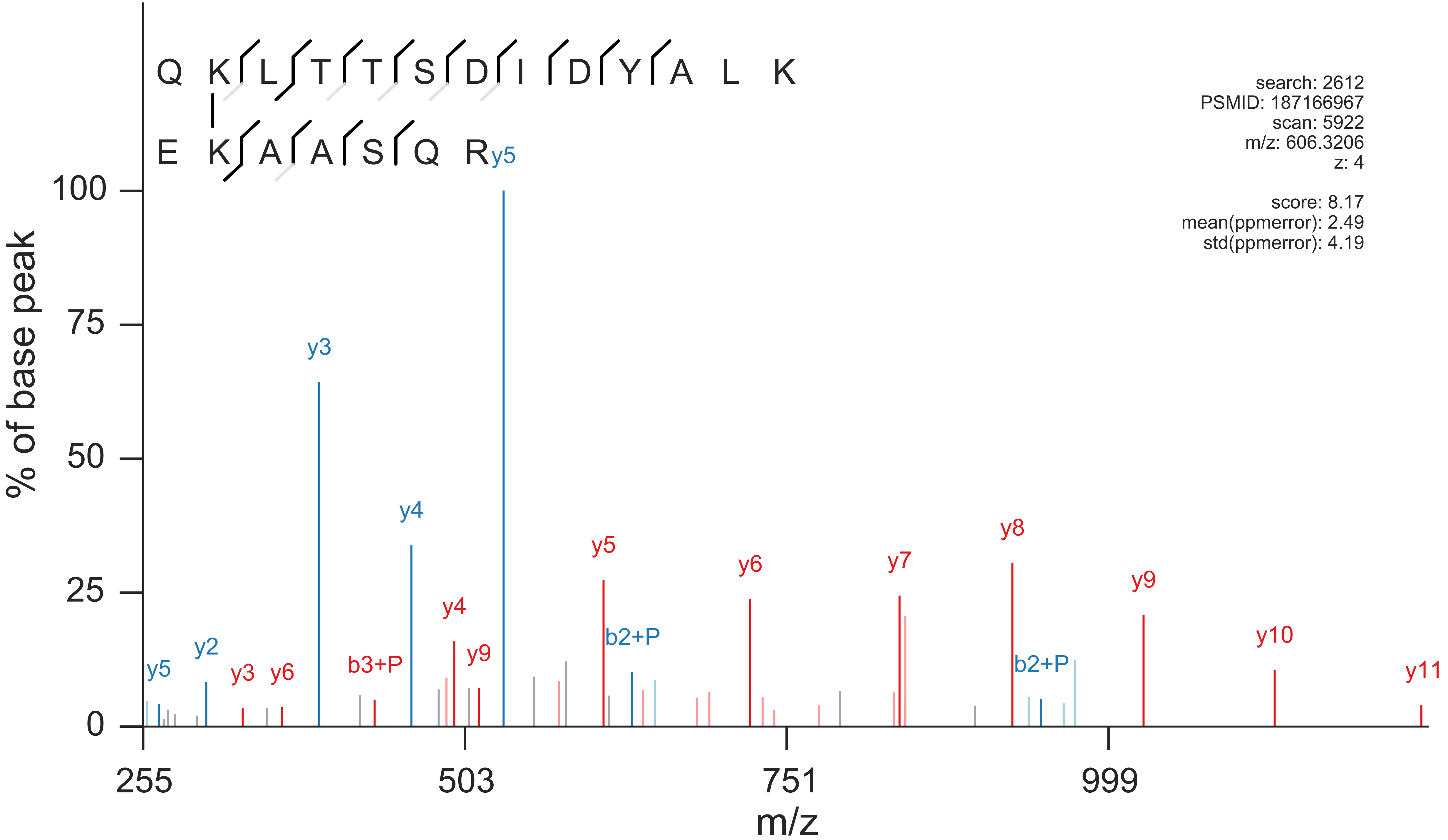

Q K L T T S D I D Y A L K
E K A A S Q R
search: 2612
PSMID: 187166967
scan: 5922
m/z: 606.3206
z: 4
score: 8.17
mean(ppmerror): 2.49
std(ppmerror): 4.19
% of base peak
m/z
0
25
50
75
100
255
503
751
999
y5
y2
y3
y6
y3
b3+P
y4
y4
y9
y5
y5
b2+P
y6
y7
y8
b2+P
y9
y10
y11

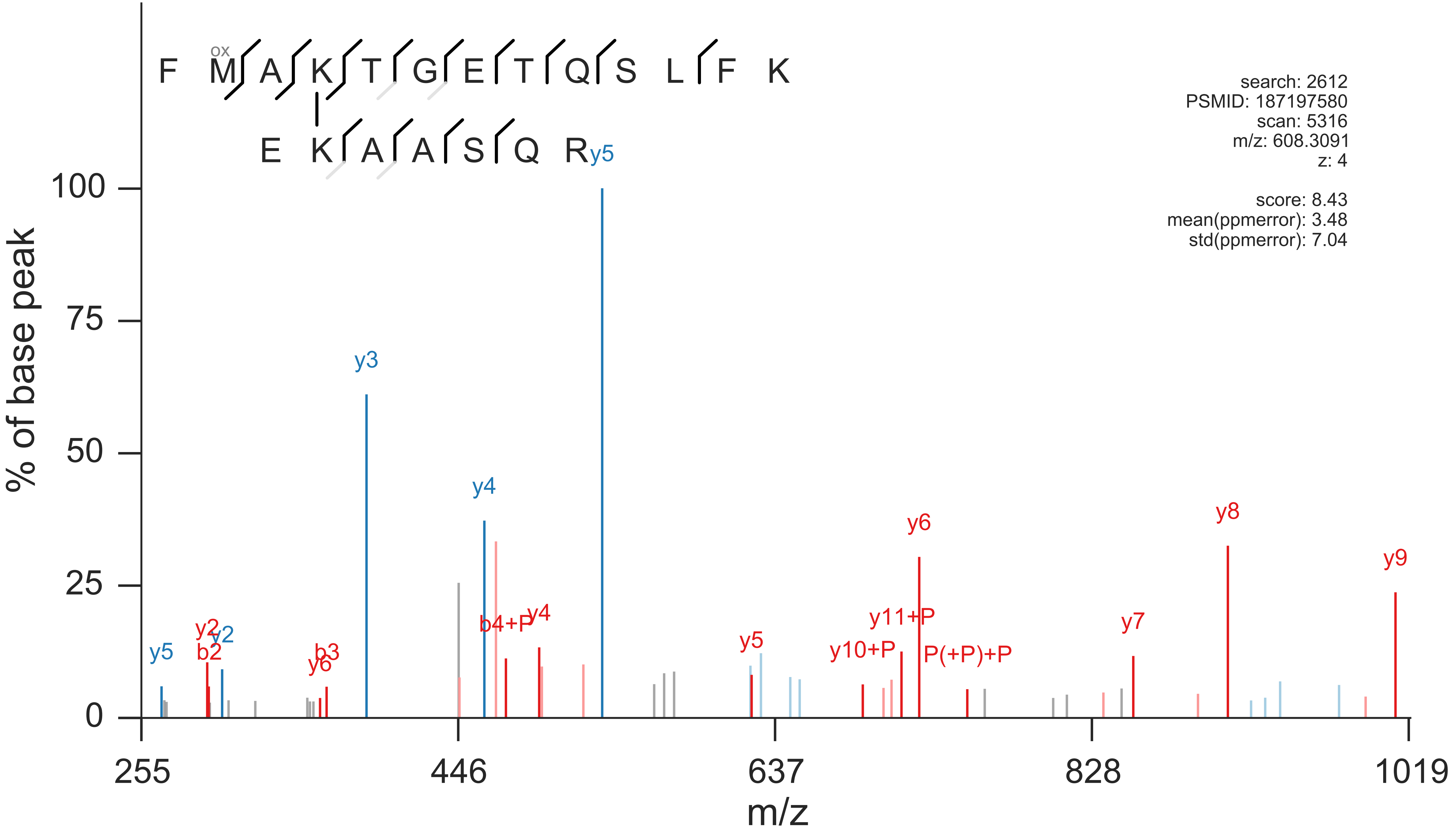

F M A K T G E T Q S L F K
ox
E K A A S Q R
search: 2612
PSMID: 187197580
scan: 5316
m/z: 608.3091
z: 4
score: 8.43
mean(ppmerror): 3.48
std(ppmerror): 7.04
% of base peak
m/z
100
75
50
25
0
255
446
637
828
1019
y5
y3
y4
y2
b2
y2
b3
y6
b4+P
y4
y5
y10+P
y11+P
y6
P(+P)+P
y7
y8
y9

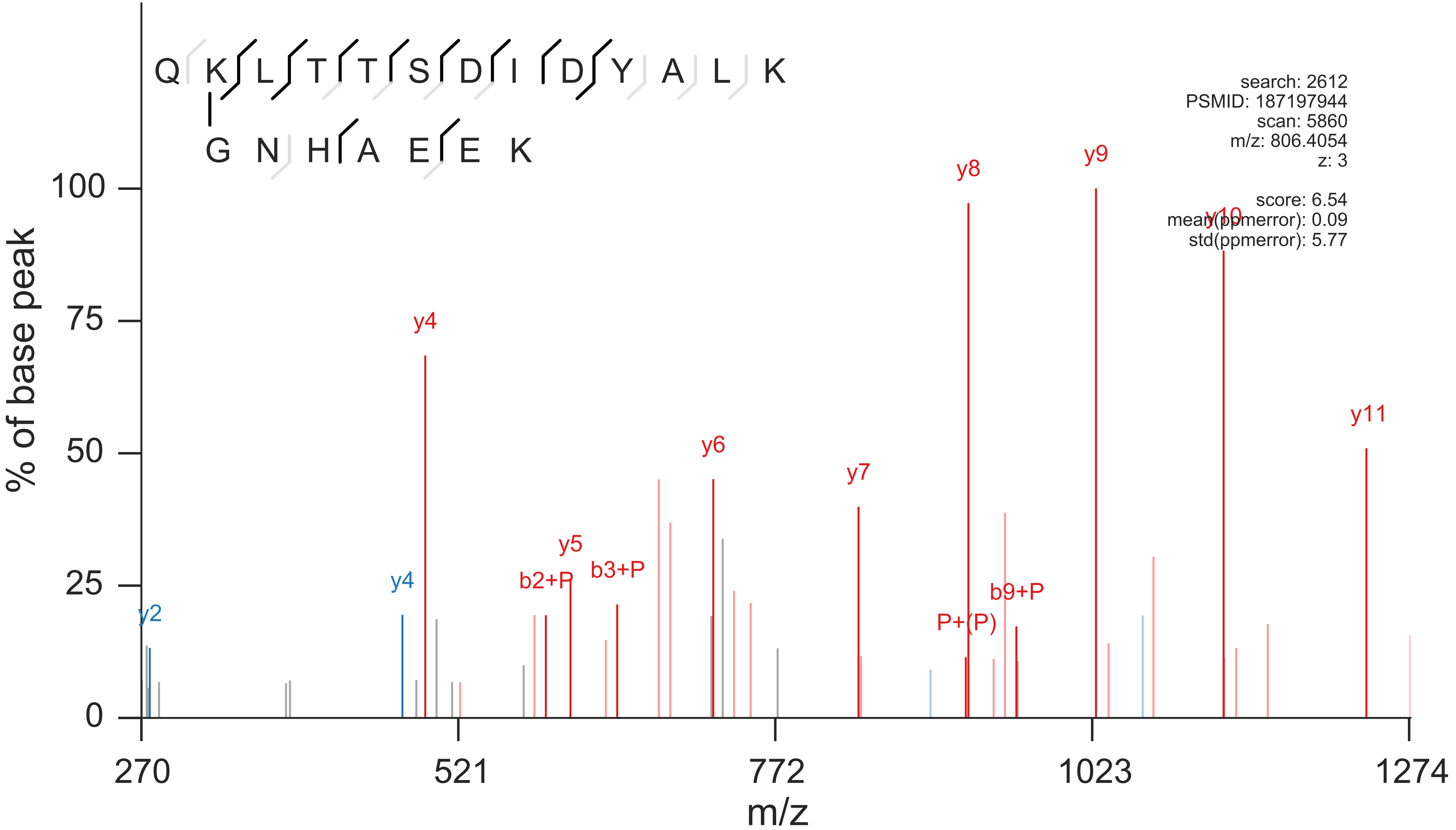

Q K L T T S D I D Y A L K
G N H A E E K
search: 2612
PSMID: 187197944
scan: 5860
m/z: 806.4054
z: 3
score: 6.54
mean(ppmerror): 0.09
std(ppmerror): 5.77
% of base peak
m/z
0
25
50
75
100
270
521
772
1023
1274
y2
y4
y4
b2+P
y5
b3+P
y6
y7
P+(P)
y8
b9+P
y9
y10
y11

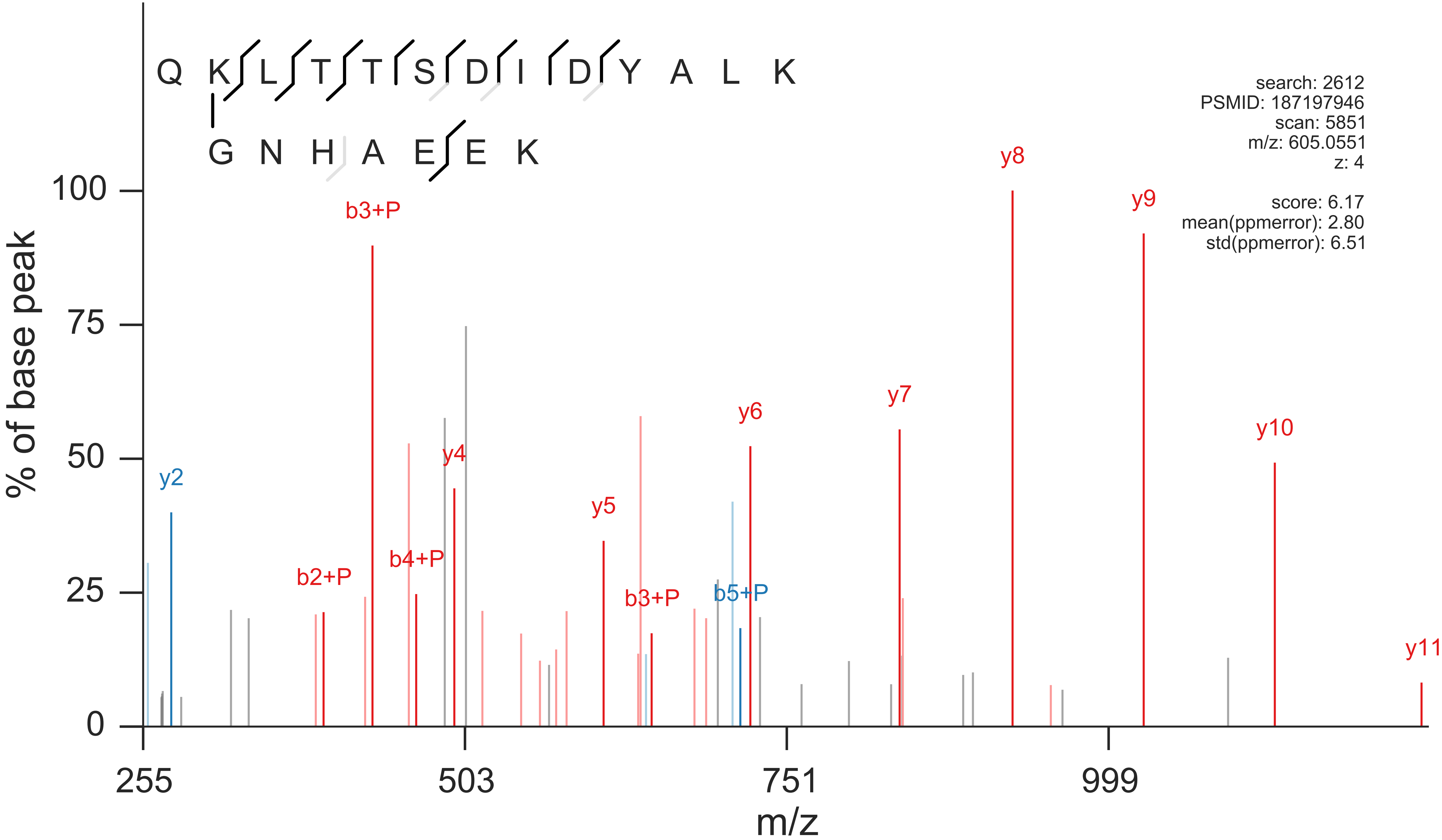
Q K L T T S D I D Y A L K
G N H A E E K
search: 2612
PSMID: 187197946
scan: 5851
m/z: 605.0551
z: 4
score: 6.17
mean(ppmerror): 2.80
std(ppmerror): 6.51
% of base peak
m/z
y2
b2+P
b3+P
b4+P
y4
y5
b3+P
b5+P
y6
y7
y8
y9
y10
y11
0
25
50
75
100
255
503
751
999

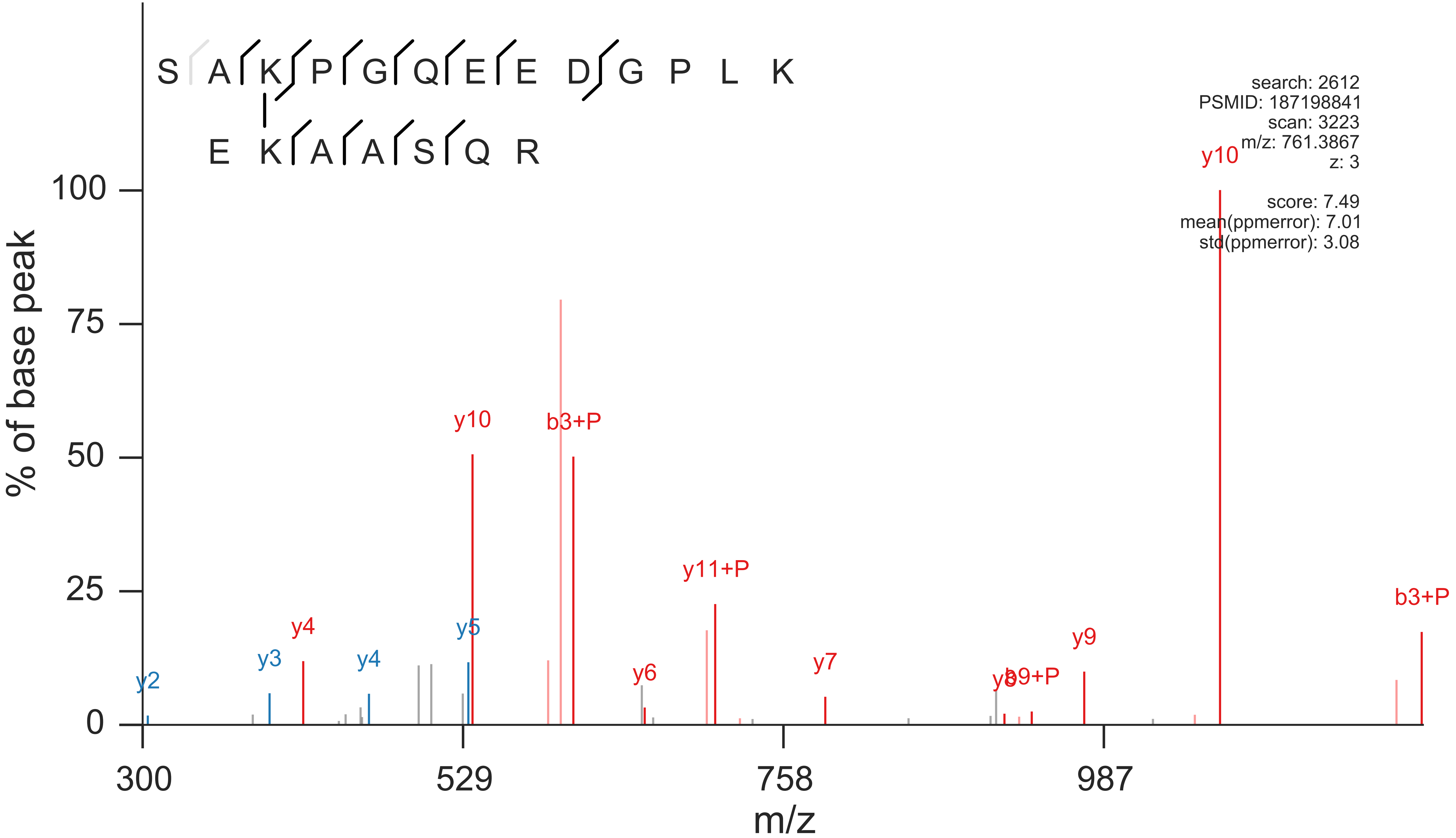

S A K P G Q E E D G P L K
E K A A S Q R
search: 2612
PSMID: 187198841
scan: 3223
m/z: 761.3867
z: 3
score: 7.49
mean(ppmerror): 7.01
std(ppmerror): 3.08
% of base peak
m/z
100
75
50
25
0
300
529
758
987
y2
y3
y4
y4
y5
y10
b3+P
y6
y11+P
y7
y8
b9+P
y9
y10
b3+P

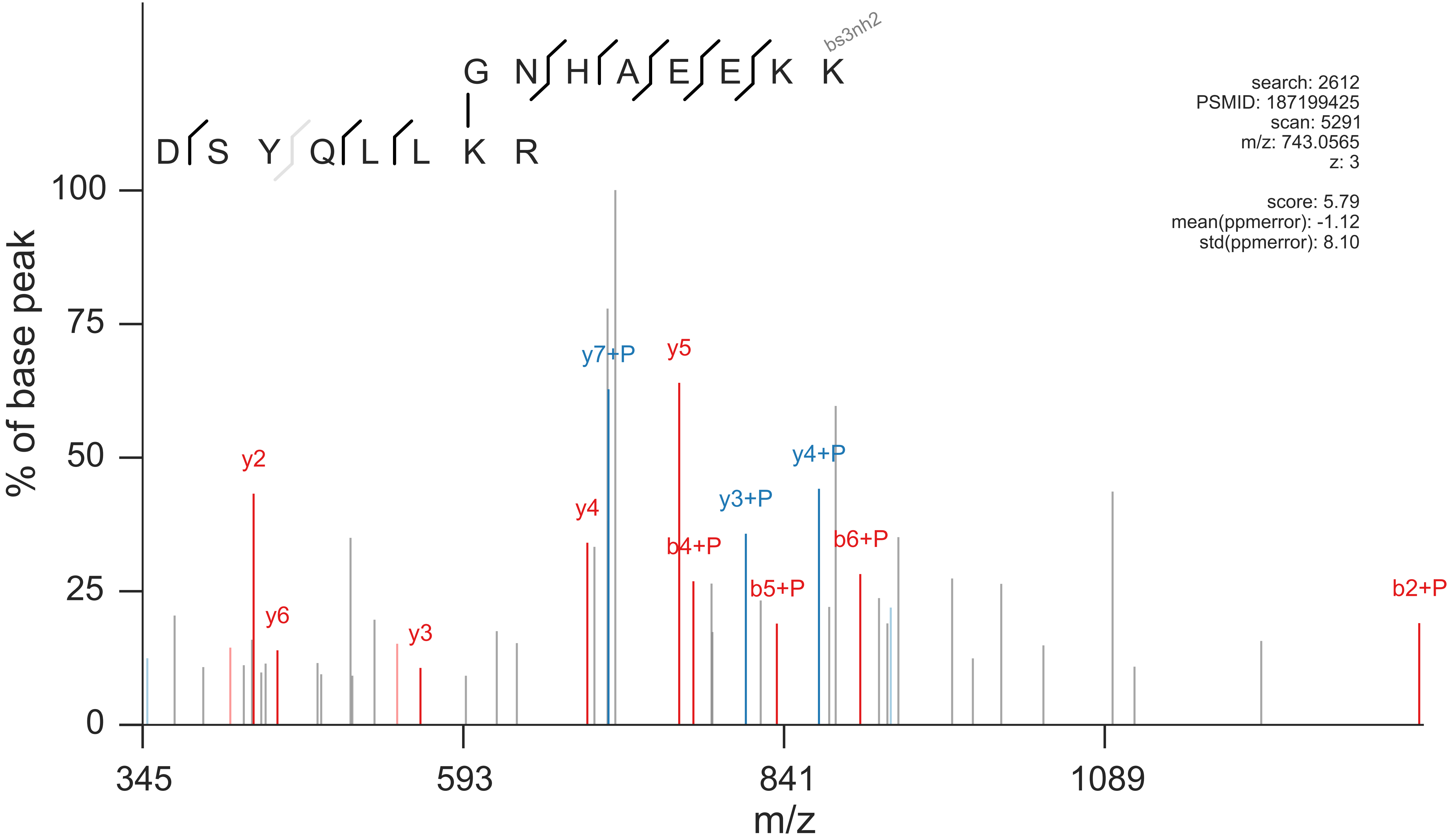

G N H A E E K K
bs3nh2
D S Y Q L L K R
search: 2612
PSMID: 187199425
scan: 5291
m/z: 743.0565
z: 3
score: 5.79
mean(ppmerror): -1.12
std(ppmerror): 8.10
% of base peak
100
75
50
25
0
y2
y6
y3
y4
y7+P
y5
b4+P
y3+P
b5+P
y4+P
b6+P
b2+P
345
593
841
1089
m/z

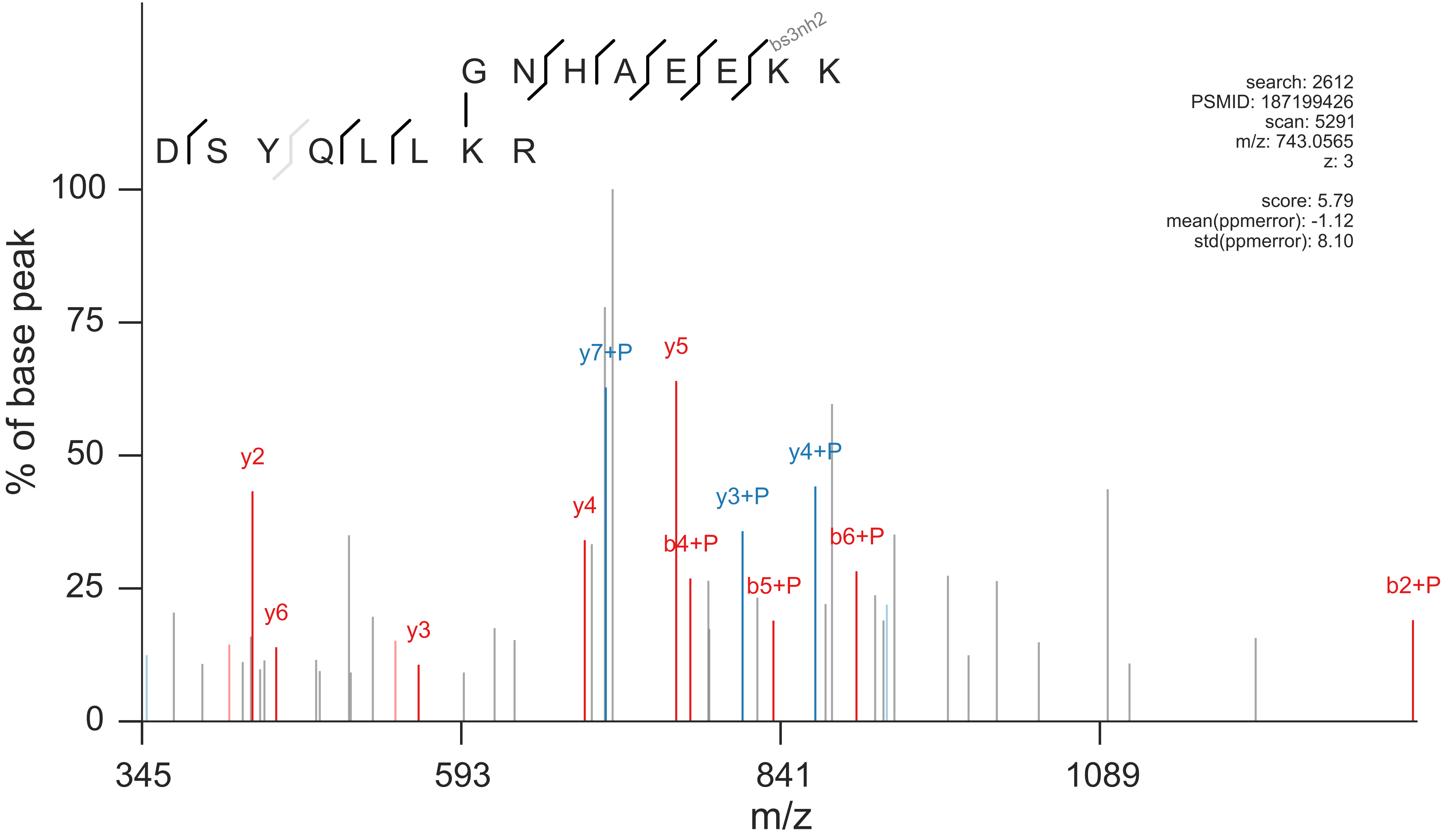
G N H A E E K K
D S Y Q L L K R
bs3nh2
search: 2612
PSMID: 187199426
scan: 5291
m/z: 743.0565
z: 3
score: 5.79
mean(ppmerror): -1.12
std(ppmerror): 8.10
% of base peak
100
75
50
25
0
y2
y6
y3
y4
y7+P
y5
b4+P
y3+P
b5+P
y4+P
b6+P
b2+P
345
593
841
1089
m/z

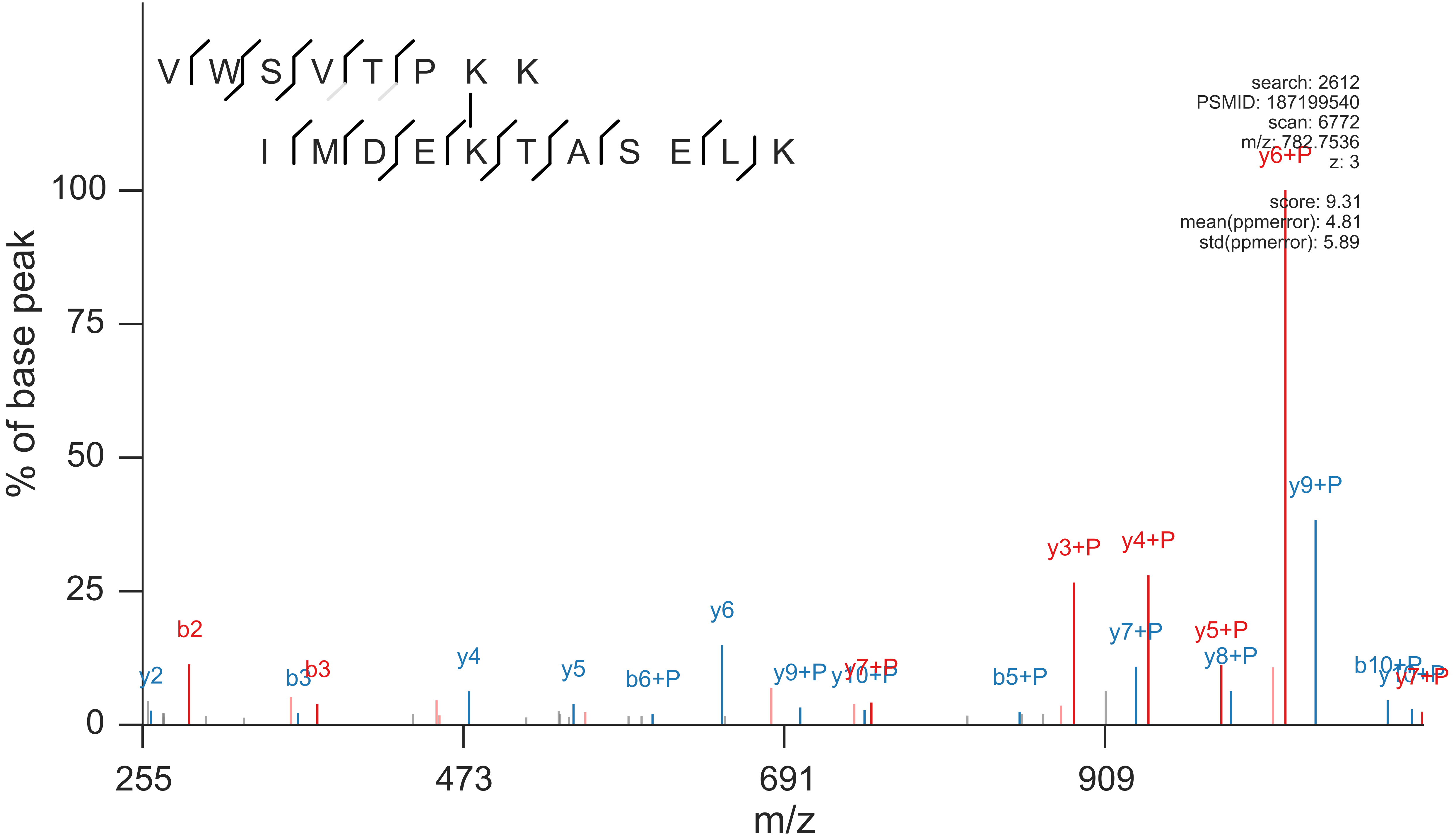

V W S V T P K K
I M D E K T A S E L K
search: 2612
PSMID: 187199540
scan: 6772
m/z: 782.7536
z: 3
score: 9.31
mean(ppmerror): 4.81
std(ppmerror): 5.89
% of base peak
100
75
50
25
0
255
473
691
909
m/z
y6+P
y9+P
y3+P
y4+P
y7+P
y5+P
y8+P
b10+P
y7+P
y6
b2
y4
y5
b6+P
y9+P
y10+P
y7+P
b5+P
y2
b3
b3

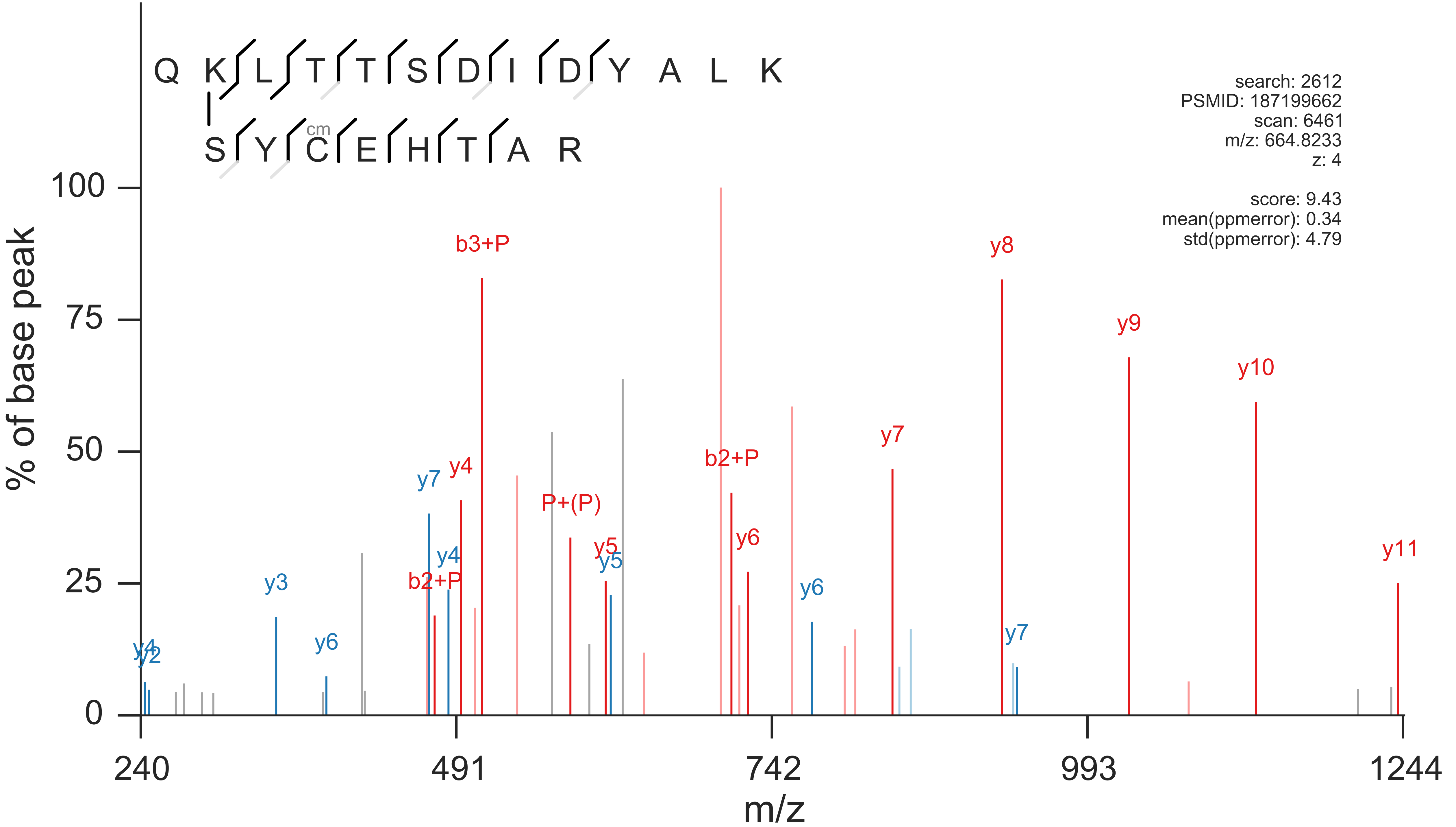

Q K L T T S D I D Y A L K
S Y C E H T A R
cm
search: 2612
PSMID: 187199662
scan: 6461
m/z: 664.8233
z: 4
score: 9.43
mean(ppmerror): 0.34
std(ppmerror): 4.79
% of base peak
m/z
100
75
50
25
0
240
491
742
993
1244
b3+P
y8
y9
y10
y7
b2+P
y4
y7
P+(P)
y5
y5
y6
y4
b2+P
y3
y6
y6
y11
y7
y4
y2

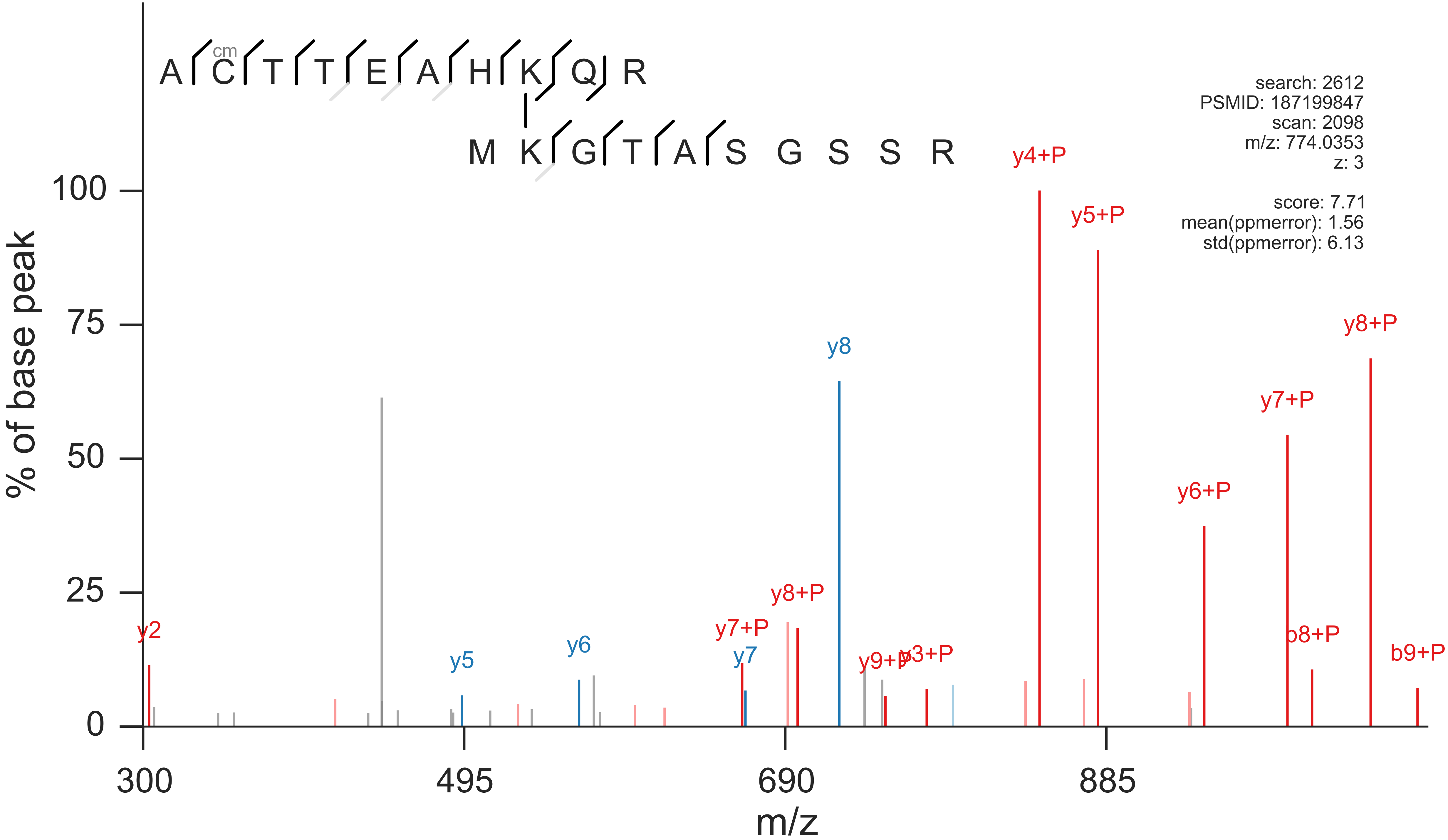

A C T T E A H K Q R
cm
M K G T A S G S S R
search: 2612
PSMID: 187199847
scan: 2098
m/z: 774.0353
z: 3
score: 7.71
mean(ppmerror): 1.56
std(ppmerror): 6.13
% of base peak
100
75
50
25
0
300
495
690
885
m/z
y2
y5
y6
y7
y7+P
y8+P
y8
y9+P
y3+P
y4+P
y5+P
y6+P
y7+P
b8+P
y8+P
b9+P

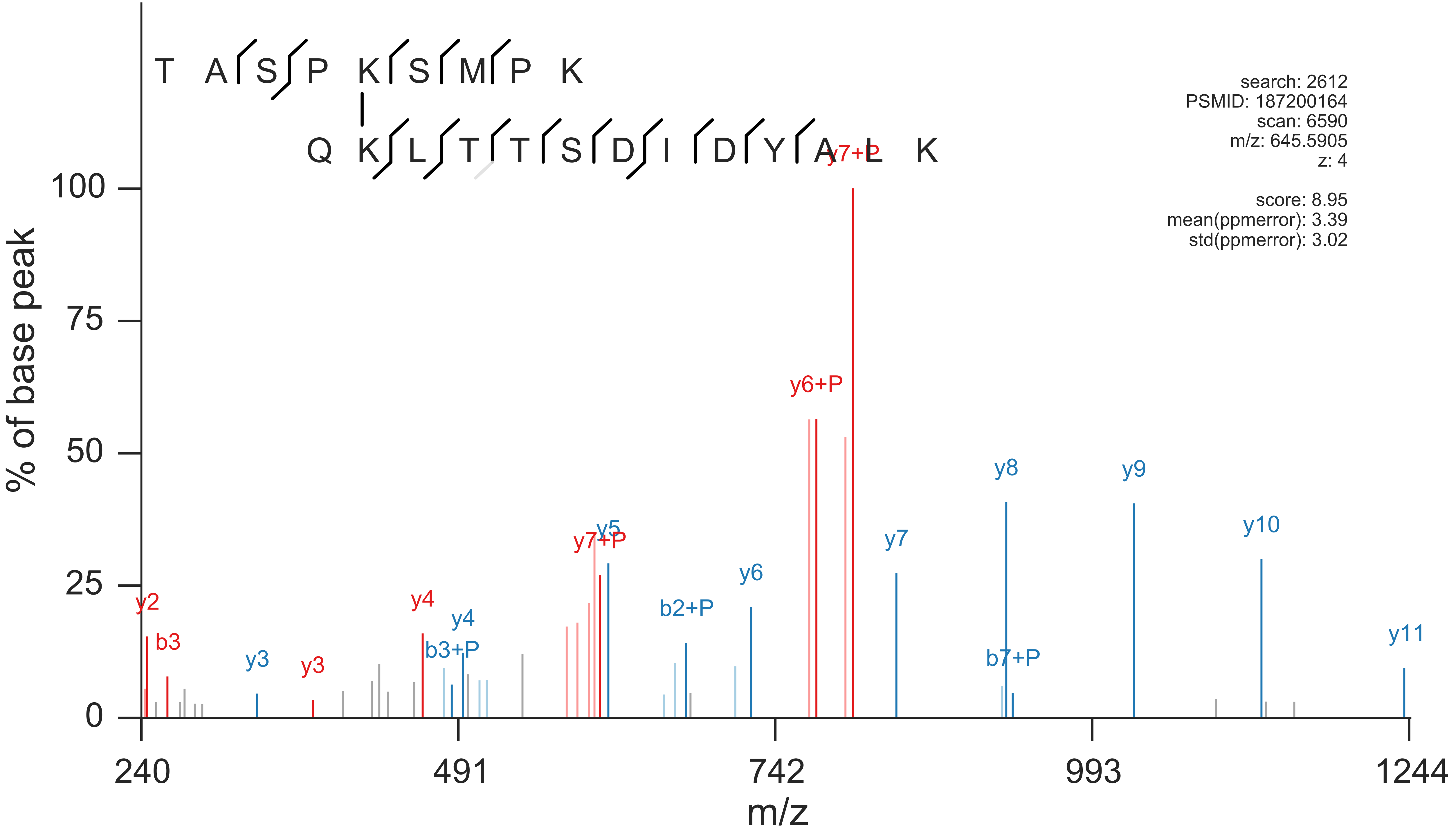

T A S P K S M P K
Q K L T T S D I D Y A L K
search: 2612
PSMID: 187200164
scan: 6590
m/z: 645.5905
z: 4
score: 8.95
mean(ppmerror): 3.39
std(ppmerror): 3.02
% of base peak
100
75
50
25
0
240
491
742
993
1244
m/z
y2
b3
y3
y3
y4
y4
b3+P
y7+P
y5
b2+P
y6
y6+P
y7+P
y7
y8
b7+P
y9
y10
y11

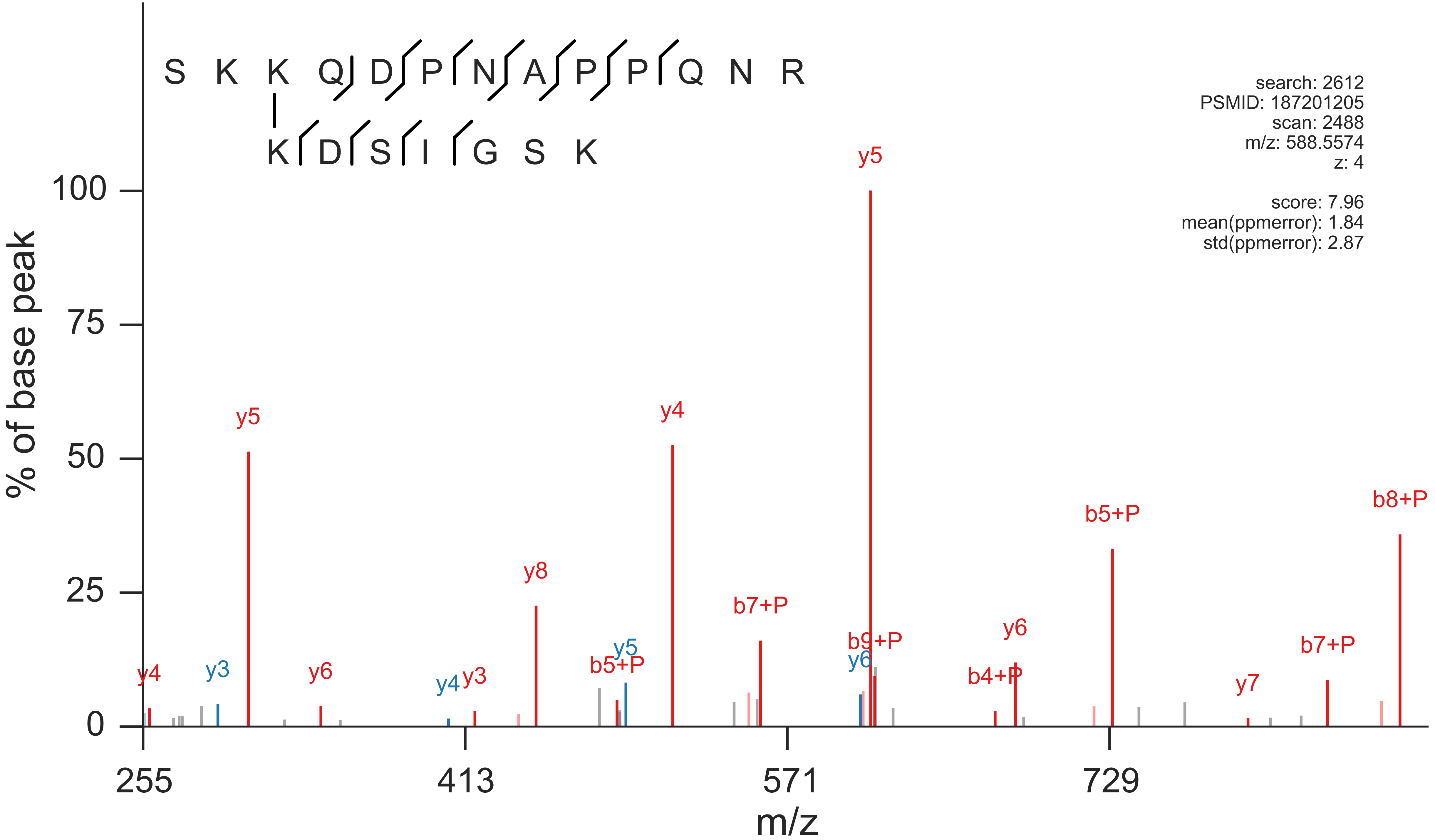
S K K Q D P N A P P Q N R
K D S I G S K
search: 2612
PSMID: 187201205
scan: 2488
m/z: 588.5574
z: 4
score: 7.96
mean(ppmerror): 1.84
std(ppmerror): 2.87
% of base peak
m/z
0
25
50
75
100
255
413
571
729
y4
y3
y5
y6
y4
y3
y8
b5+P
y5
y4
b7+P
b9+P
y6
y5
b4+P
y6
b5+P
y7
b7+P
b8+P

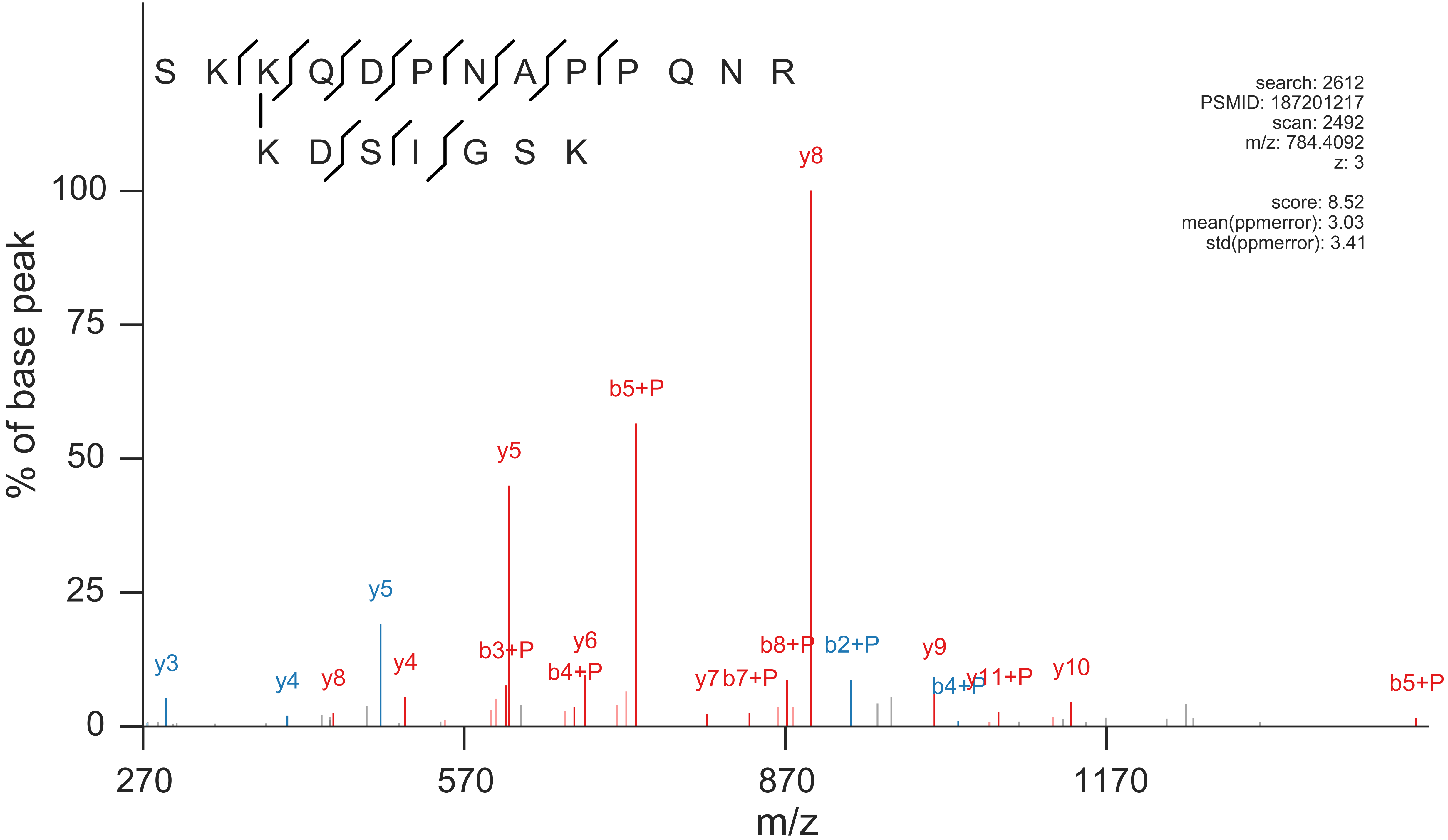

S K K Q D P N A P P Q N R
K D S I G S K
search: 2612
PSMID: 187201217
scan: 2492
m/z: 784.4092
z: 3
score: 8.52
mean(ppmerror): 3.03
std(ppmerror): 3.41
% of base peak
m/z
0
25
50
75
100
270
570
870
1170
y8
b5+P
y5
y5
y3
y4
y8
y4
b3+P
y6
b4+P
y7
b7+P
b8+P
b2+P
y9
b4+P
y11+P
y10
b5+P

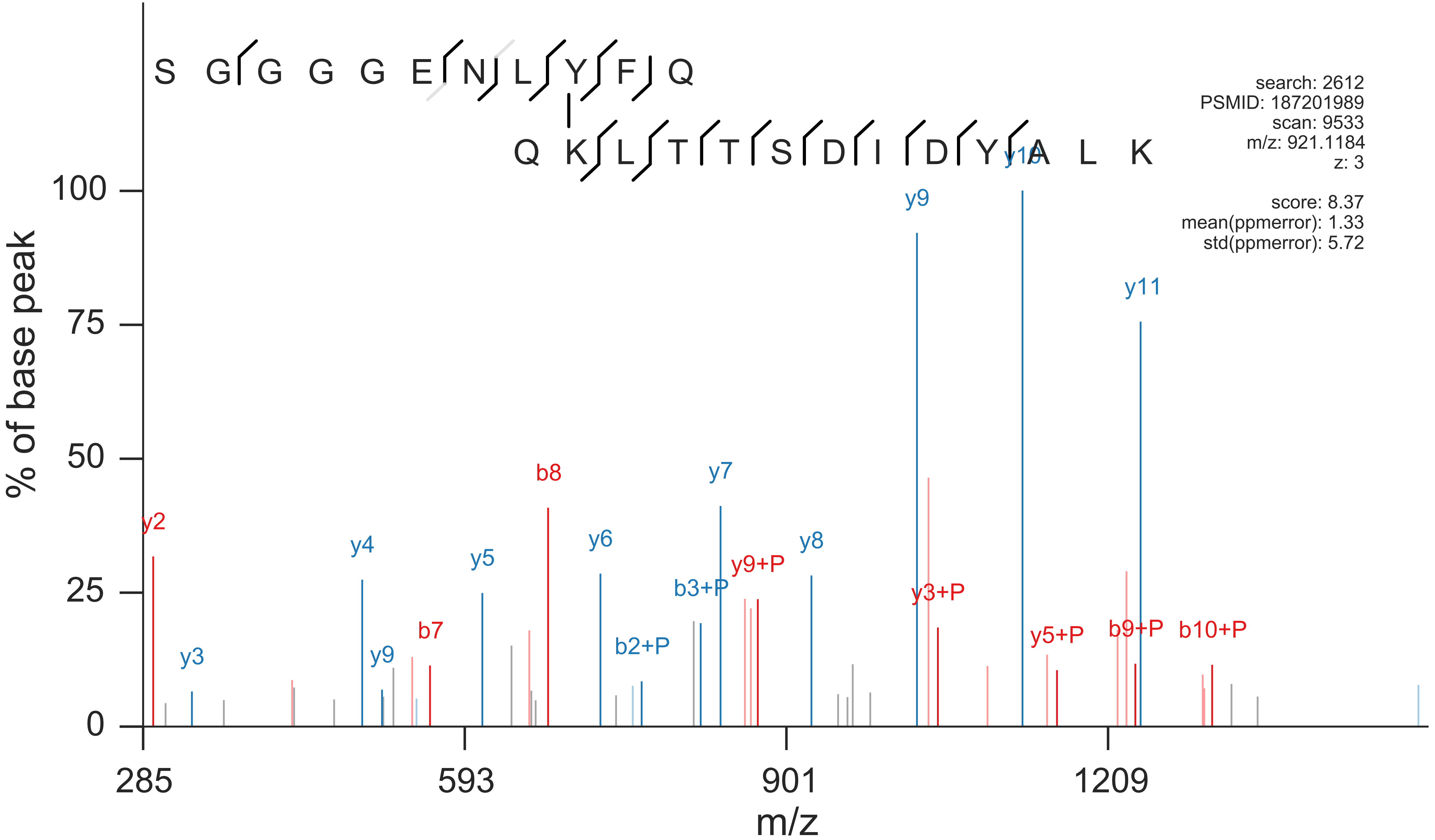

S G G G G E N L Y F Q
Q K L T T S D I D Y A L K
search: 2612
PSMID: 187201989
scan: 9533
m/z: 921.1184
z: 3
score: 8.37
mean(ppmerror): 1.33
std(ppmerror): 5.72
% of base peak
m/z
y2
y3
y4
y9
b7
y5
b8
y6
b2+P
b3+P
y7
y9+P
y8
y9
y3+P
y10
y5+P
y11
b9+P
b10+P

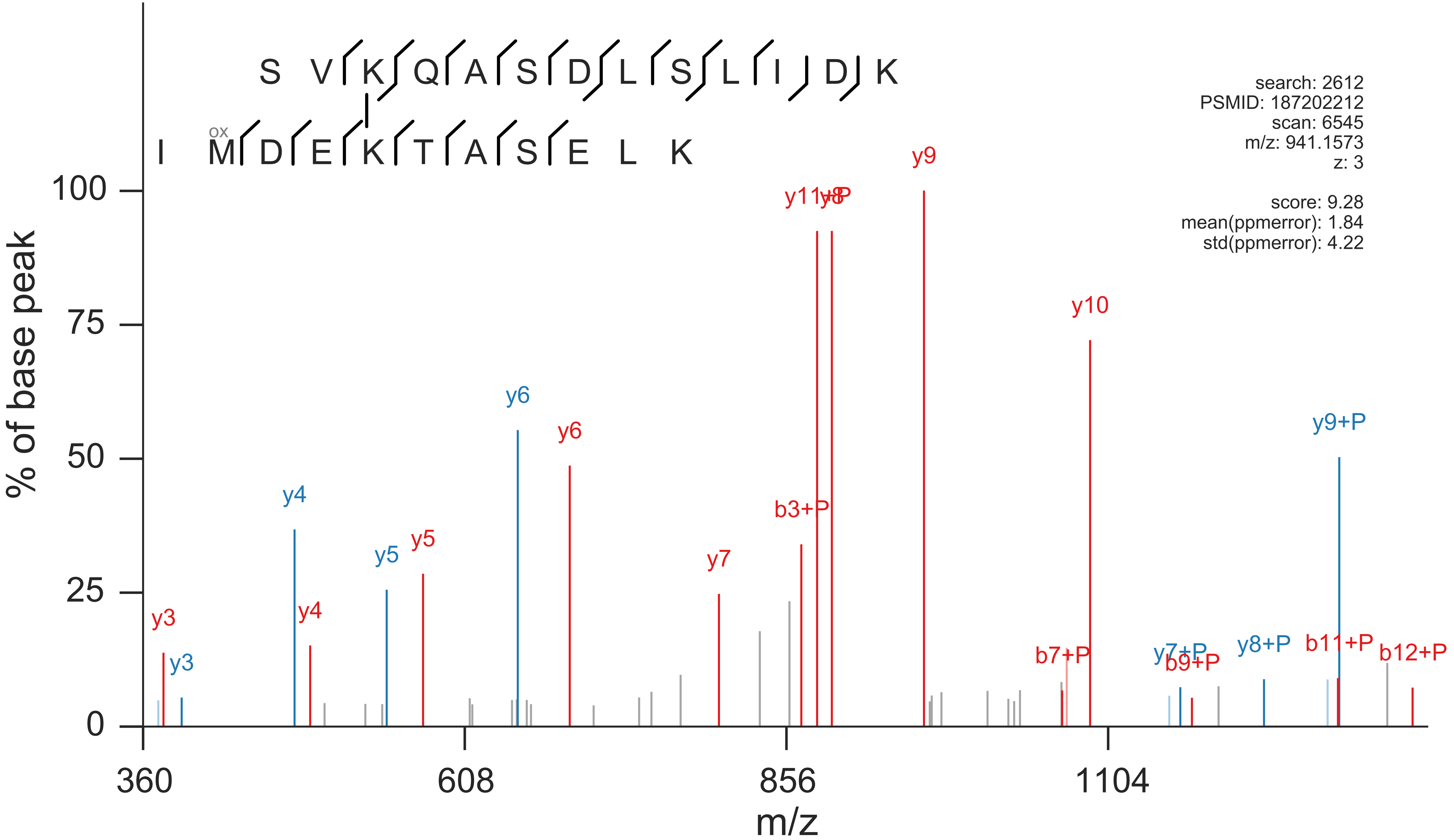

S V K Q A S D L S L I D K
I M D E K T A S E L K
ox
search: 2612
PSMID: 187202212
scan: 6545
m/z: 941.1573
z: 3
score: 9.28
mean(ppmerror): 1.84
std(ppmerror): 4.22
% of base peak
m/z

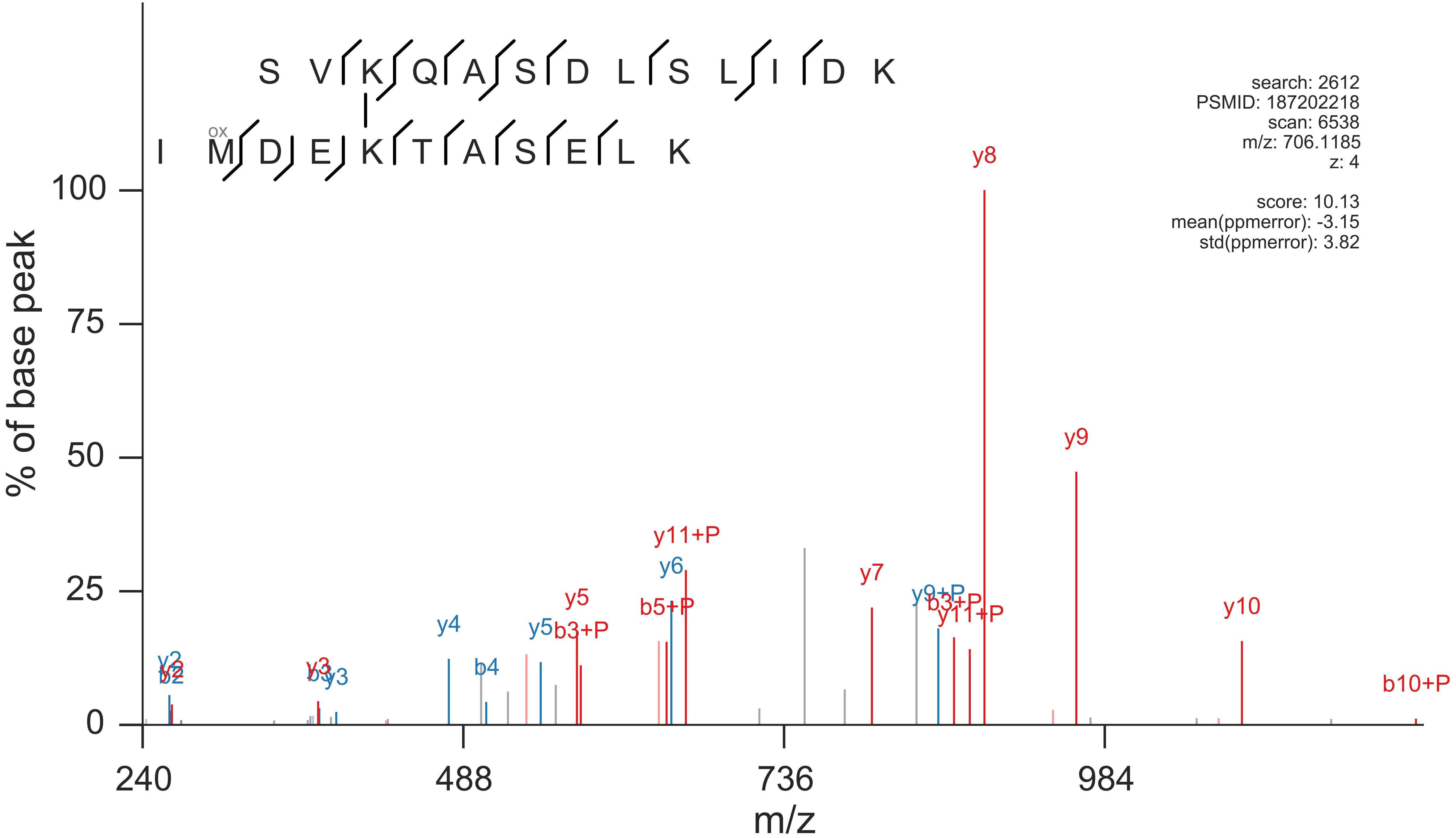

S V K Q A S D L S L I D K
I M D E K T A S E L K
ox
search: 2612
PSMID: 187202218
scan: 6538
m/z: 706.1185
z: 4
score: 10.13
mean(ppmerror): -3.15
std(ppmerror): 3.82
% of base peak
m/z
y8
y9
y11+P
y6
y7
y9+P
b3+P
y11+P
y5
b5+P
y4
y5
b3+P
y10
b10+P
y2
b2
y3
b3
y3
b4

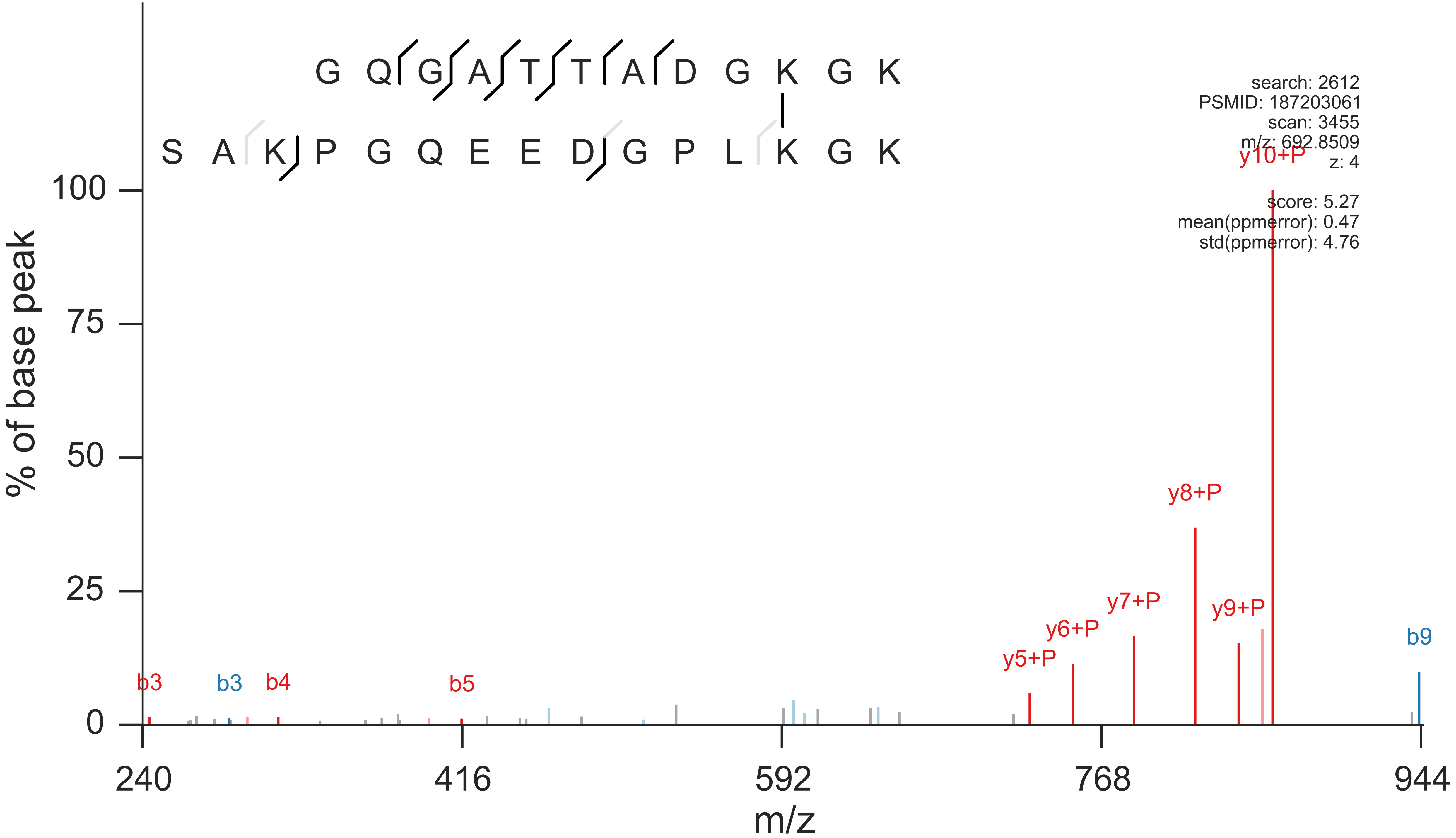
G Q G A T T A D G K G K
S A K P G Q E E D G P L K G K
search: 2612
PSMID: 187203061
scan: 3455
m/z: 692.8509
z: 4
score: 5.27
mean(ppmerror): 0.47
std(ppmerror): 4.76
% of base peak
100
75
50
25
0
240
416
592
768
944
m/z
b3
b3
b4
b5
y5+P
y6+P
y7+P
y8+P
y9+P
y10+P
b9

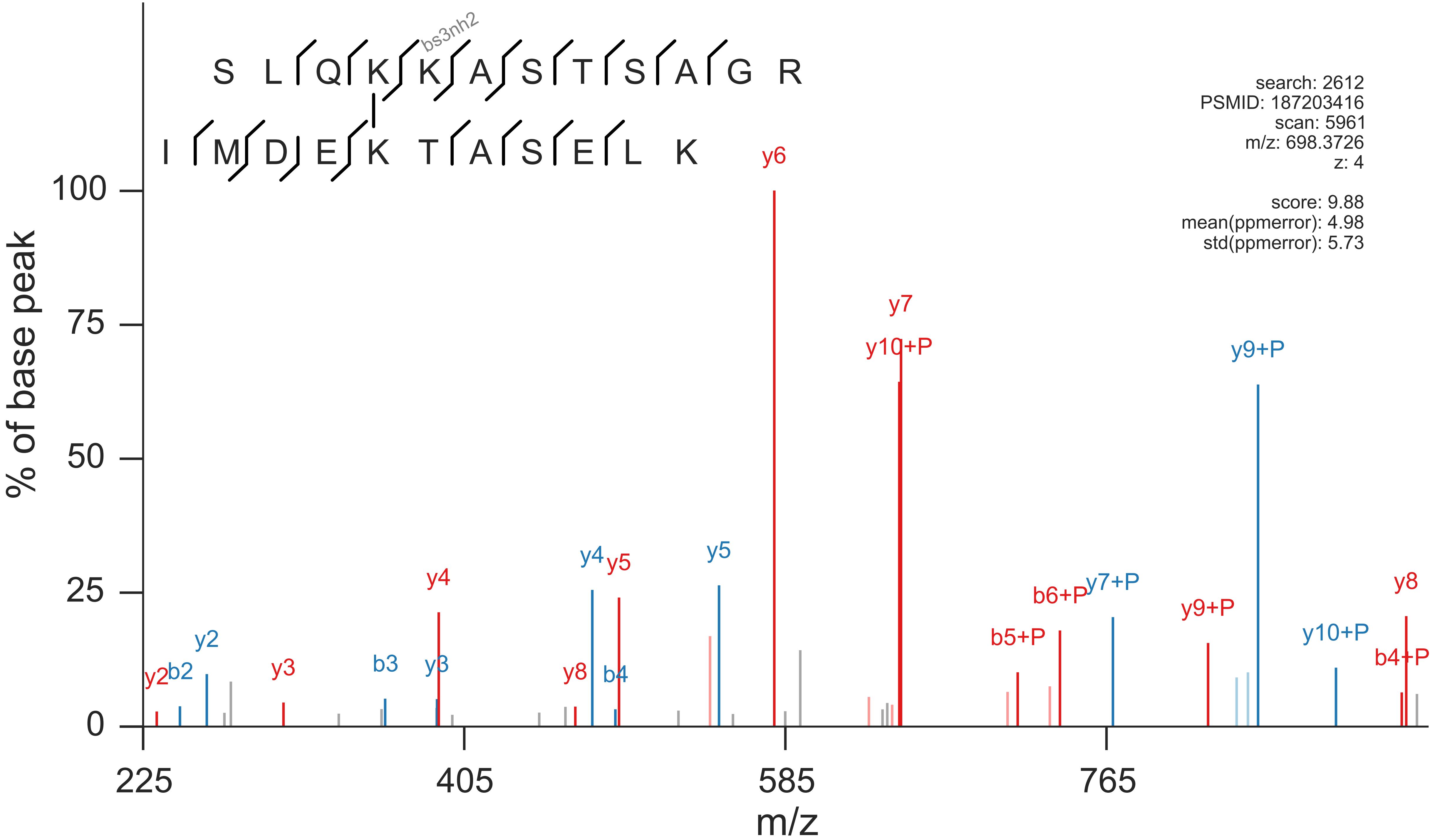

S L Q K K A S T S A G R
bs3nh2
I M D E K T A S E L K
search: 2612
PSMID: 187203416
scan: 5961
m/z: 698.3726
z: 4
score: 9.88
mean(ppmerror): 4.98
std(ppmerror): 5.73
% of base peak
m/z

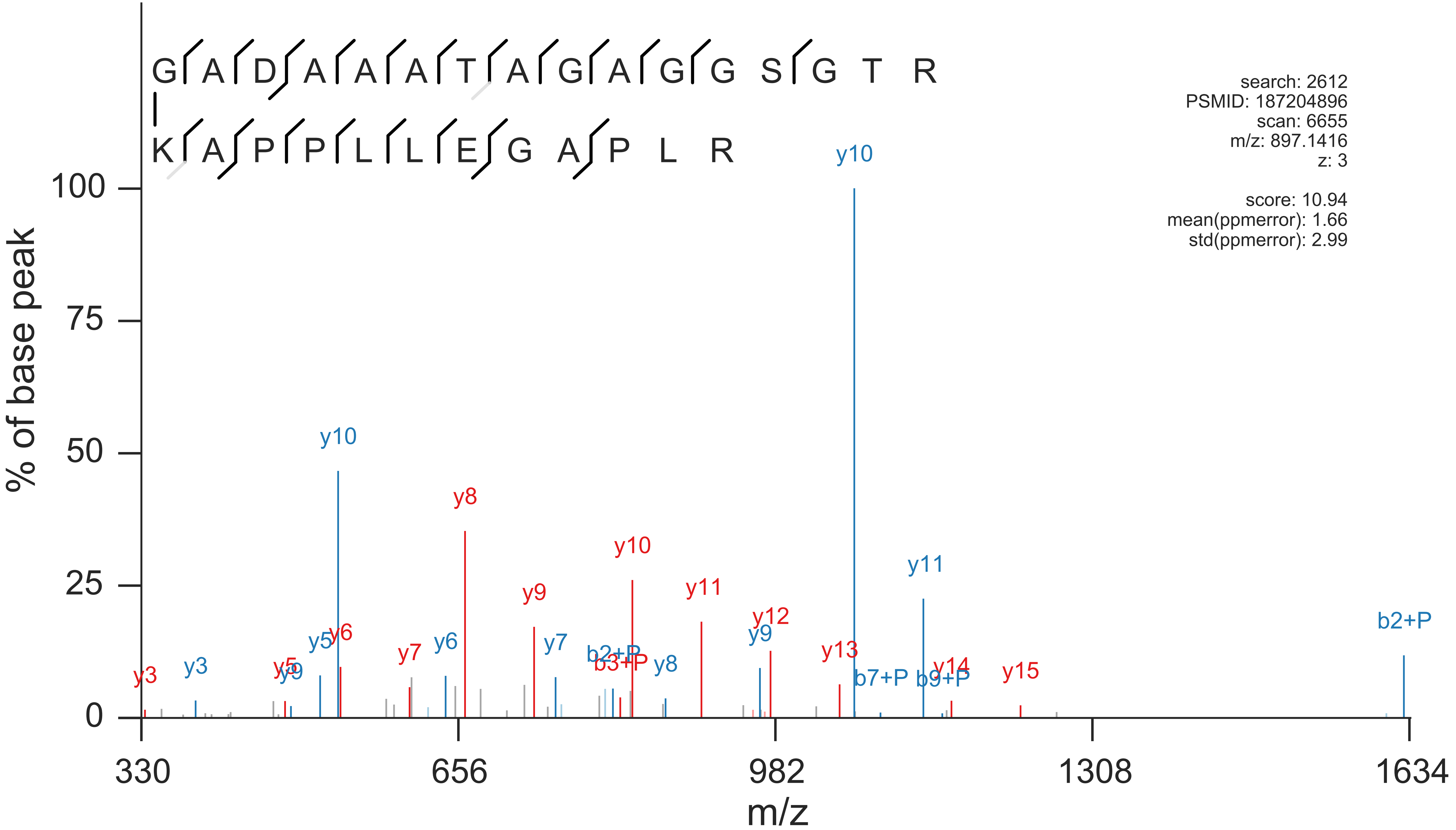

G A D A A A T A G A G G S G T R
K A P P L L E G A P L R
search: 2612
PSMID: 187204896
scan: 6655
m/z: 897.1416
z: 3
score: 10.94
mean(ppmerror): 1.66
std(ppmerror): 2.99
% of base peak
m/z
0
25
50
75
100
330
656
982
1308
1634
y3
y3
y5
y9
y5
y6
y10
y7
y6
y8
y9
y7
b2+P
b3+P
y10
y8
y11
y9
y12
y13
y10
b7+P
y11
b9+P
y14
y15
b2+P

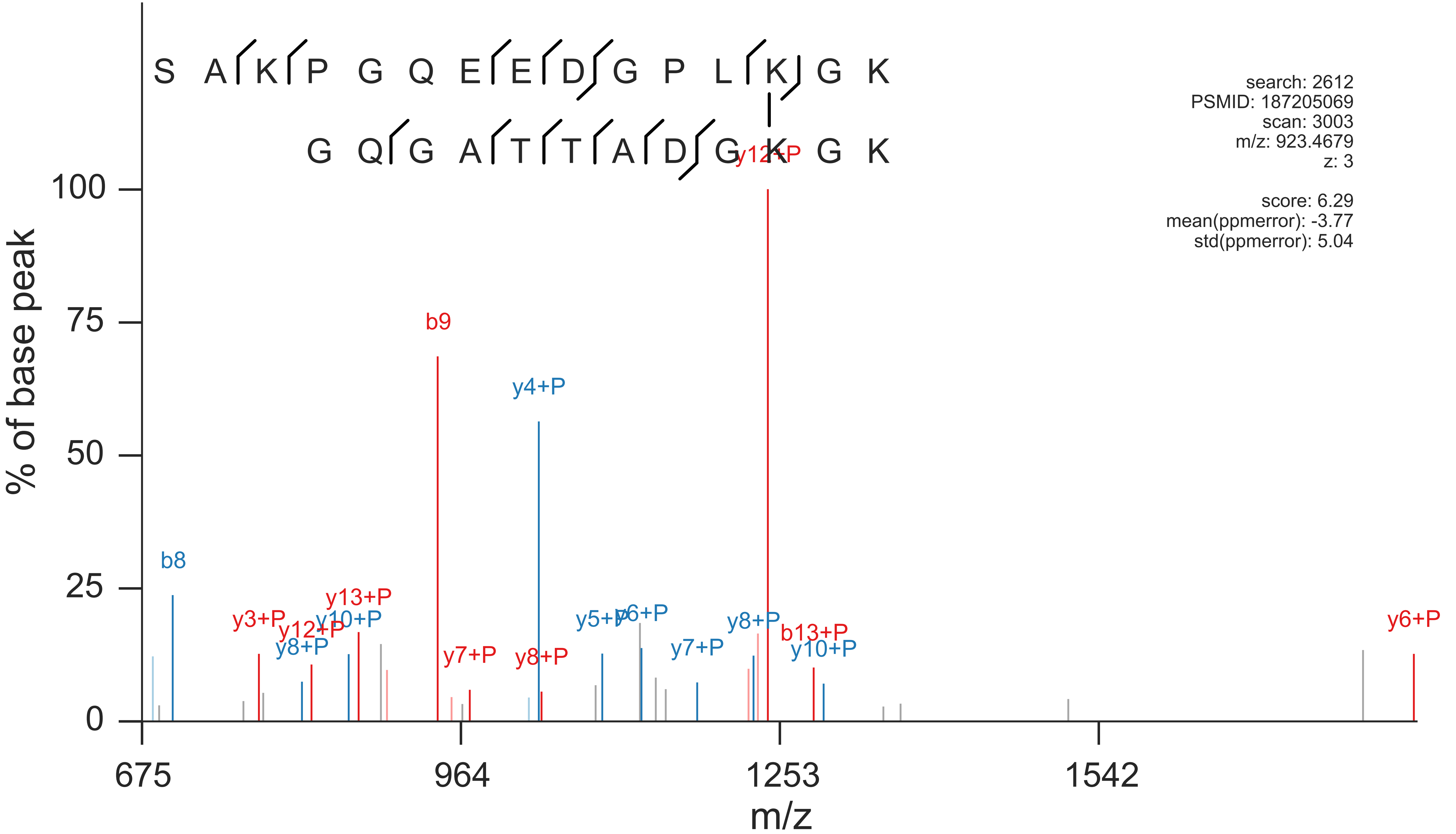

S A K P G Q E E D G P L K G K
G Q G A T T A D G K G K
search: 2612
PSMID: 187205069
scan: 3003
m/z: 923.4679
z: 3
score: 6.29
mean(ppmerror): -3.77
std(ppmerror): 5.04
% of base peak
m/z
b8
b9
y4+P
y12+P
y3+P
y12+P
y8+P
y10+P
y13+P
y7+P
y8+P
y5+P
y6+P
y7+P
y8+P
b13+P
y10+P
y6+P

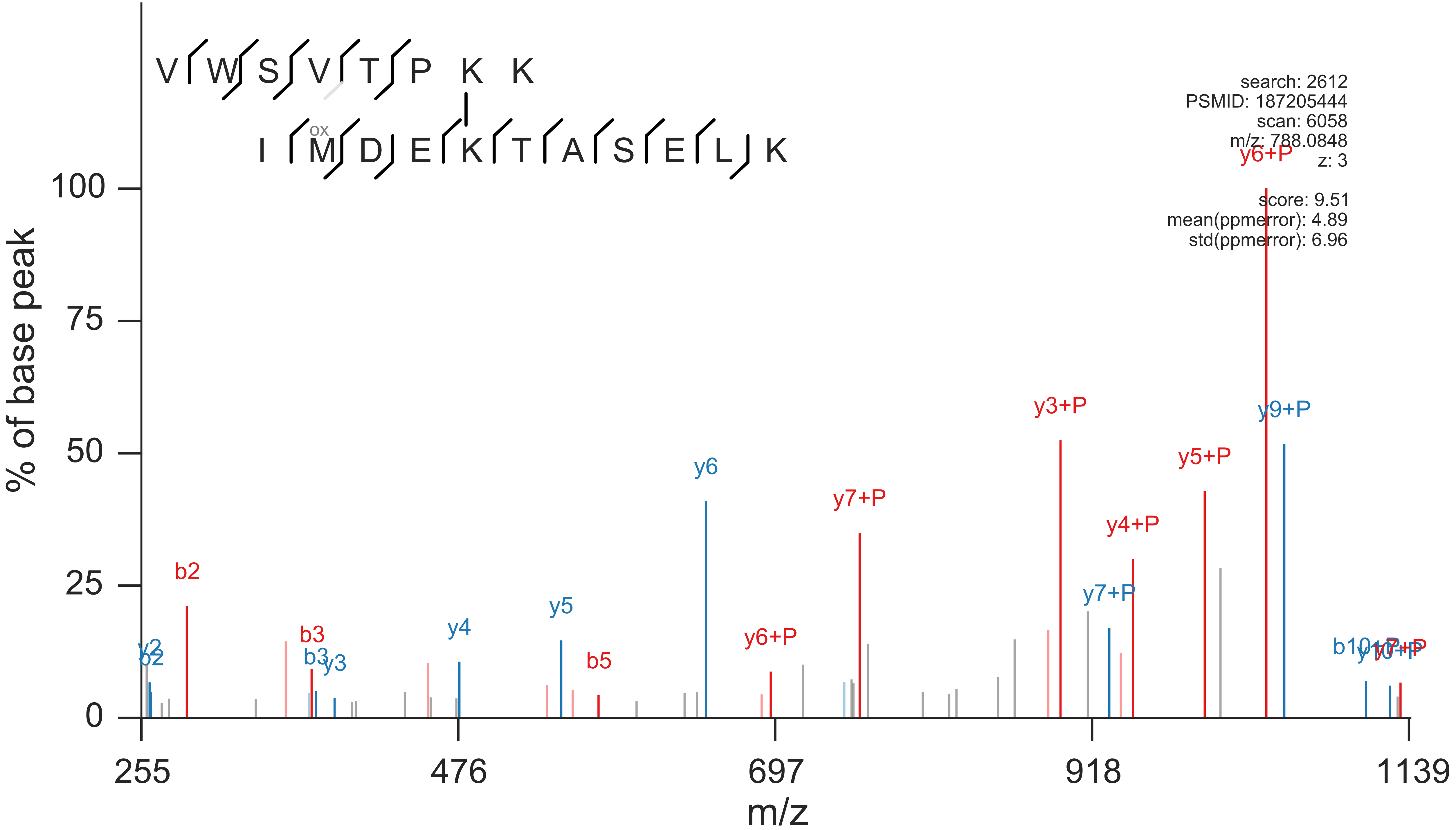

V W S V T P K K
I M D E K T A S E L K
ox
search: 2612
PSMID: 187205444
scan: 6058
m/z: 788.0848
z: 3
score: 9.51
mean(ppmerror): 4.89
std(ppmerror): 6.96
% of base peak
m/z
0
25
50
75
100
255
476
697
918
1139
b2
b3
b5
y2
b2
b3
y3
y4
y5
y6
y6+P
y7+P
y3+P
y7+P
y4+P
y5+P
y6+P
y9+P

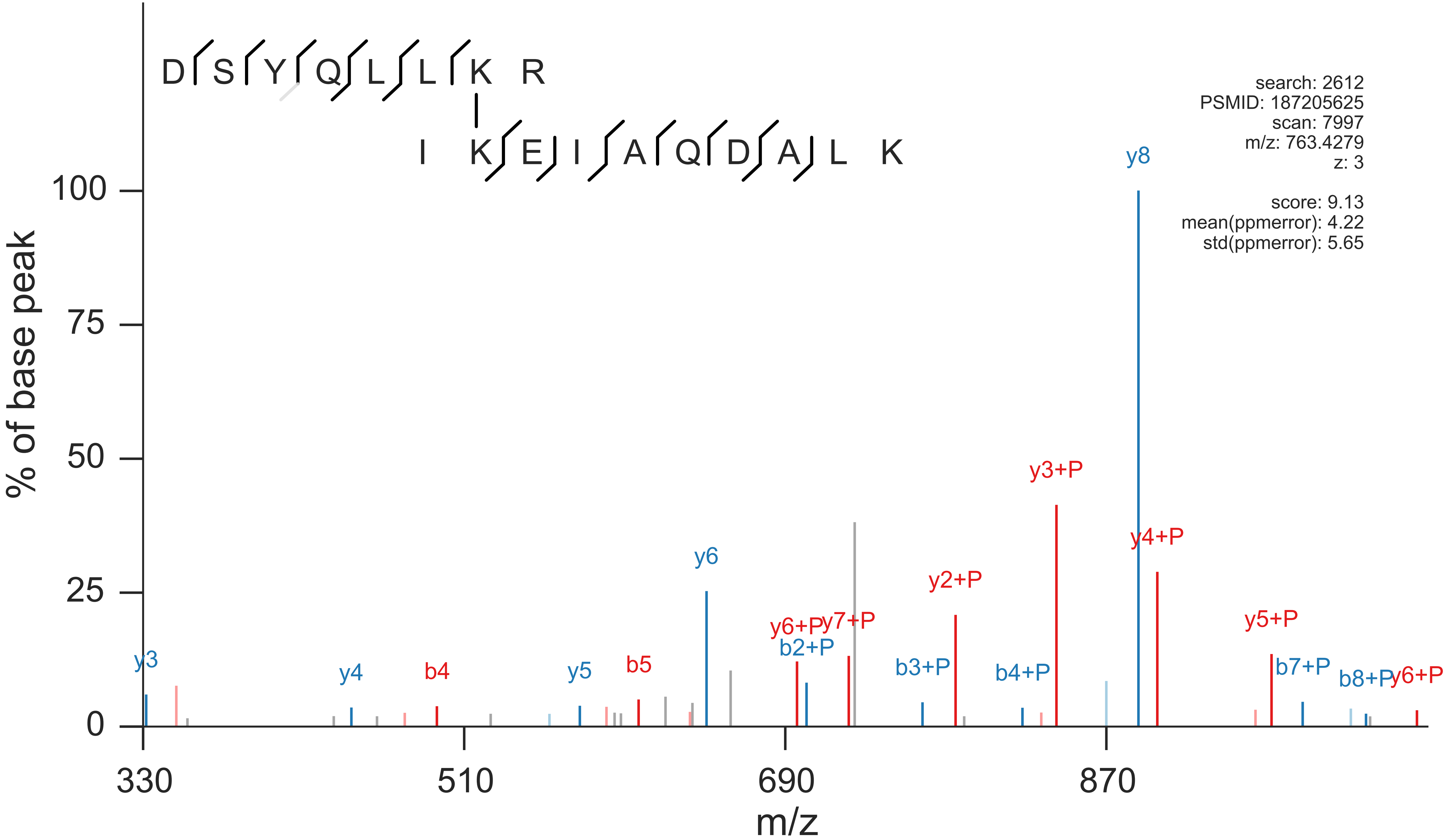

D S Y Q L L K R
I K E I A Q D A L K
search: 2612
PSMID: 187205625
scan: 7997
m/z: 763.4279
z: 3
score: 9.13
mean(ppmerror): 4.22
std(ppmerror): 5.65
% of base peak
100
75
50
25
0
y8
y3+P
y4+P
y6
y2+P
y6+P
y7+P
y5+P
b2+P
y3
y4
b4
y5
b5
b3+P
b4+P
b7+P
b8+P
y6+P
330
510
690
870
m/z

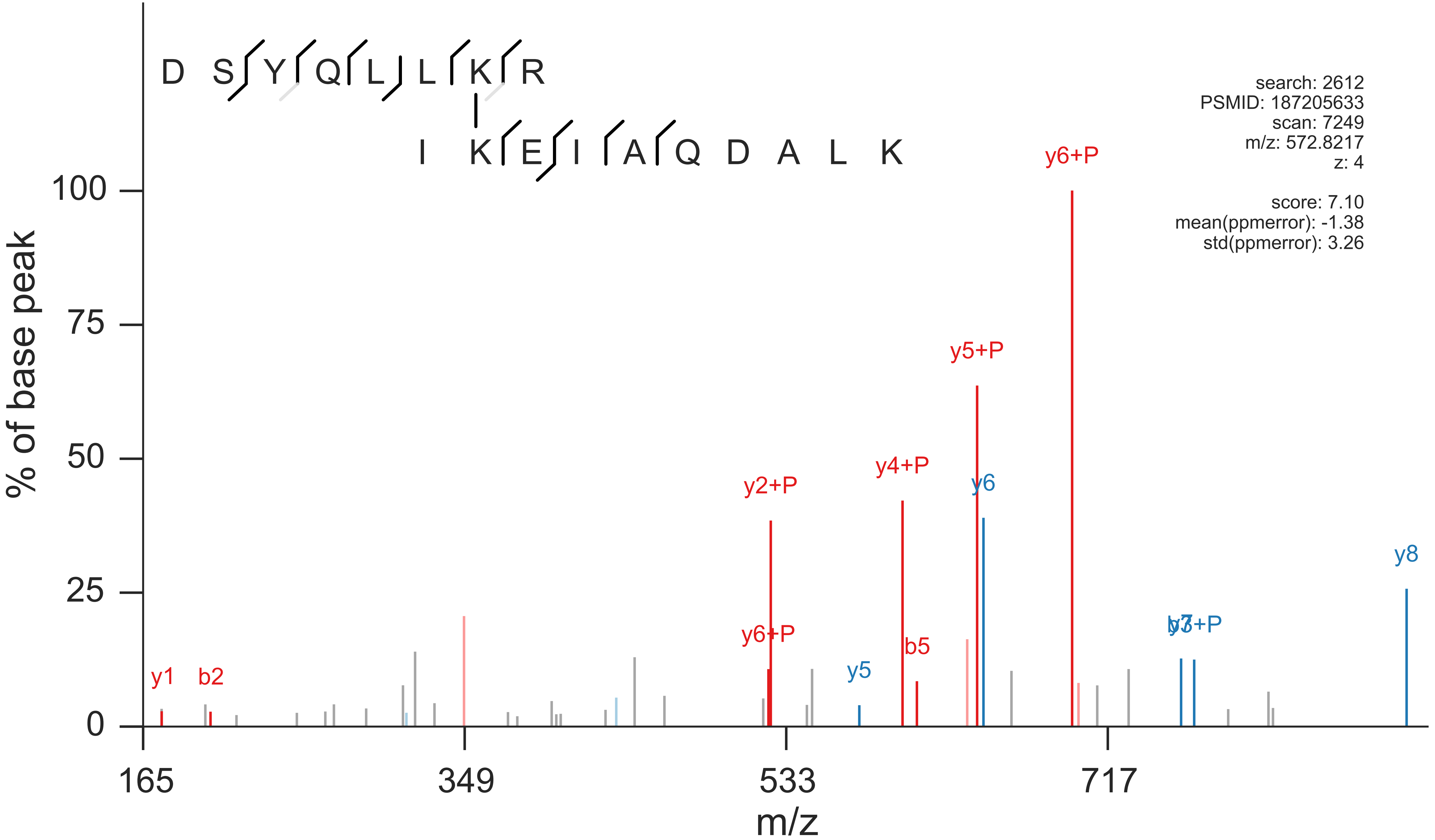

D S Y Q L L K R
I K E I A Q D A L K
search: 2612
PSMID: 187205633
scan: 7249
m/z: 572.8217
z: 4
score: 7.10
mean(ppmerror): -1.38
std(ppmerror): 3.26
% of base peak
m/z
100
75
50
25
0
165
349
533
717
y1
b2
y2+P
y6+P
y5
y4+P
b5
y5+P
y6
y6+P
y7+P
y8

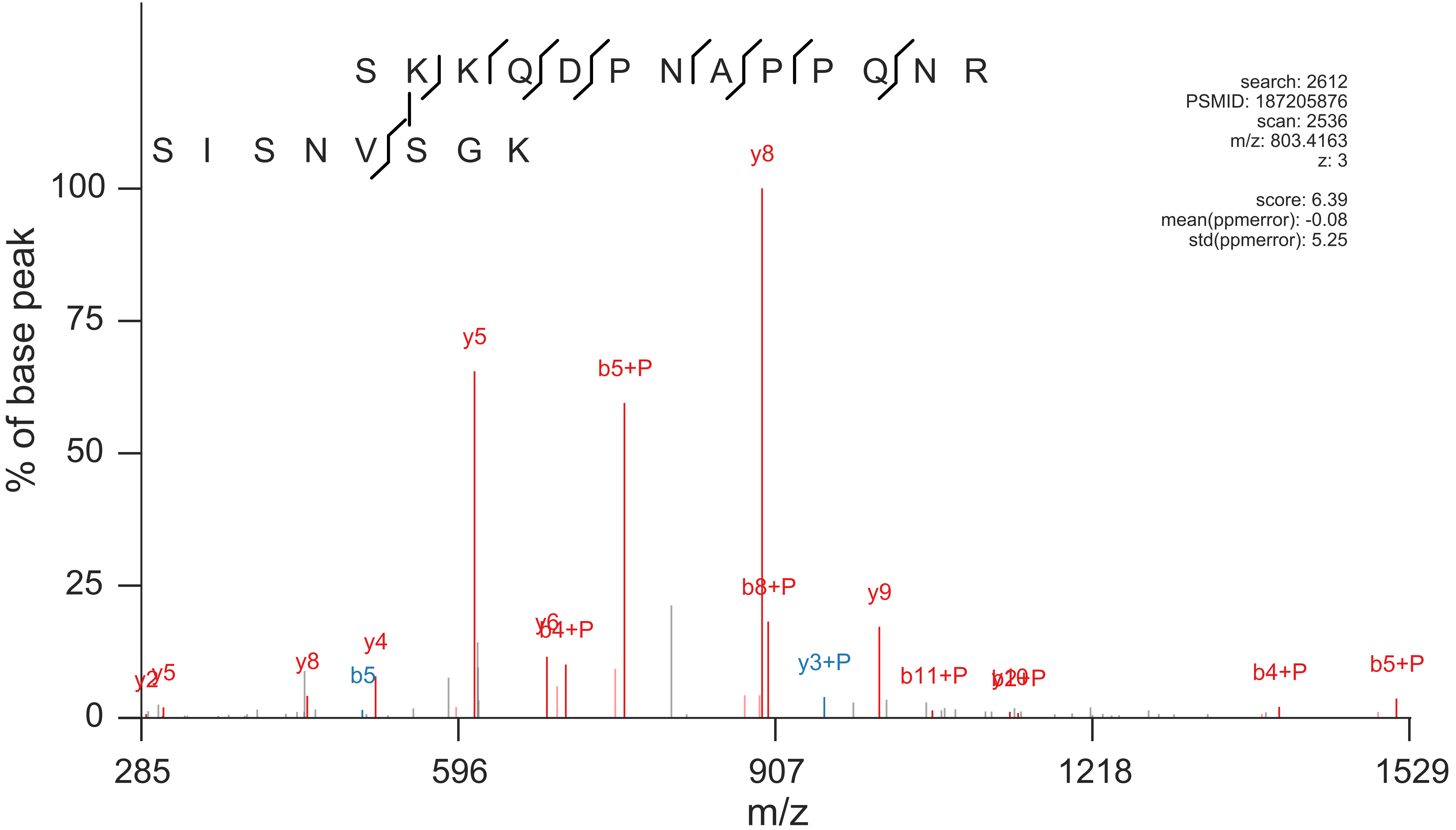

S K K Q D P N A P P Q N R
S I S N V S G K
search: 2612
PSMID: 187205876
scan: 2536
m/z: 803.4163
z: 3
score: 6.39
mean(ppmerror): -0.08
std(ppmerror): 5.25
% of base peak
m/z
y8
y5
b5+P
b8+P
y9
y6
b4+P
y4
b5
y3+P
b11+P
y2
b4+P
b5+P

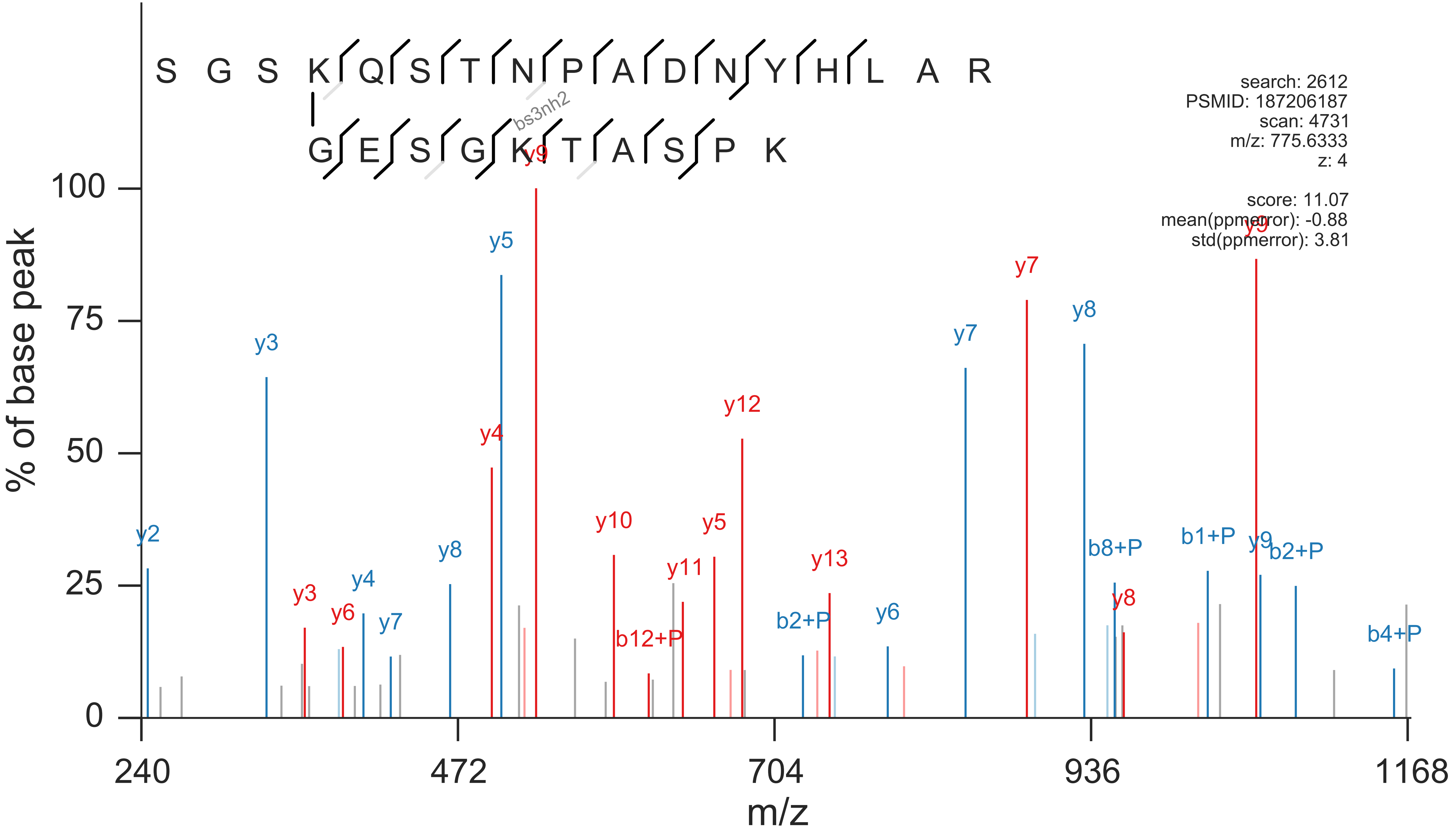
S G S K Q S T N P A D N Y H L A R
G E S G K T A S P K
bs3nh2
search: 2612
PSMID: 187206187
scan: 4731
m/z: 775.6333
z: 4
score: 11.07
mean(ppmerror): -0.88
std(ppmerror): 3.81
% of base peak
m/z
0
25
50
75
100
240
472
704
936
1168
y2
y3
y3
y6
y4
y7
y8
y4
y5
y9
y10
b12+P
y11
y5
y12
b2+P
y13
y6
y7
y7
y8
b8+P
y8
b1+P
y9
y9
b2+P
b4+P

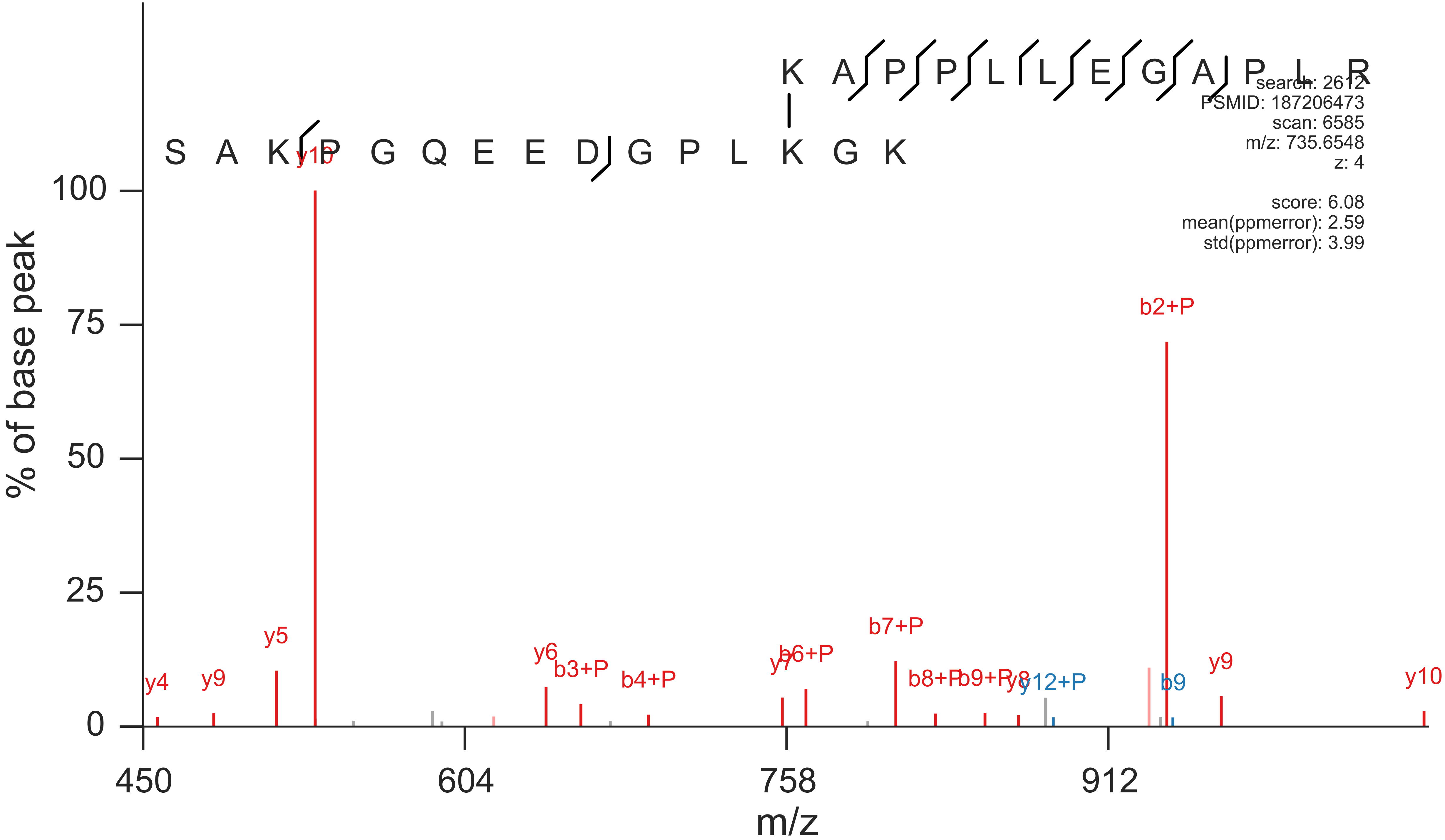

K A P P L L E G A P L R
S A K P G Q E E D G P L K G K
search: 2612
PSMID: 187206473
scan: 6585
m/z: 735.6548
z: 4
score: 6.08
mean(ppmerror): 2.59
std(ppmerror): 3.99
% of base peak
m/z
100
75
50
25
0
450
604
758
912
y10
b2+P
y4
y9
y5
y6
b3+P
b4+P
y7
b6+P
b7+P
b8+P
b9+P
y8
y12+P
b9
y9
y10

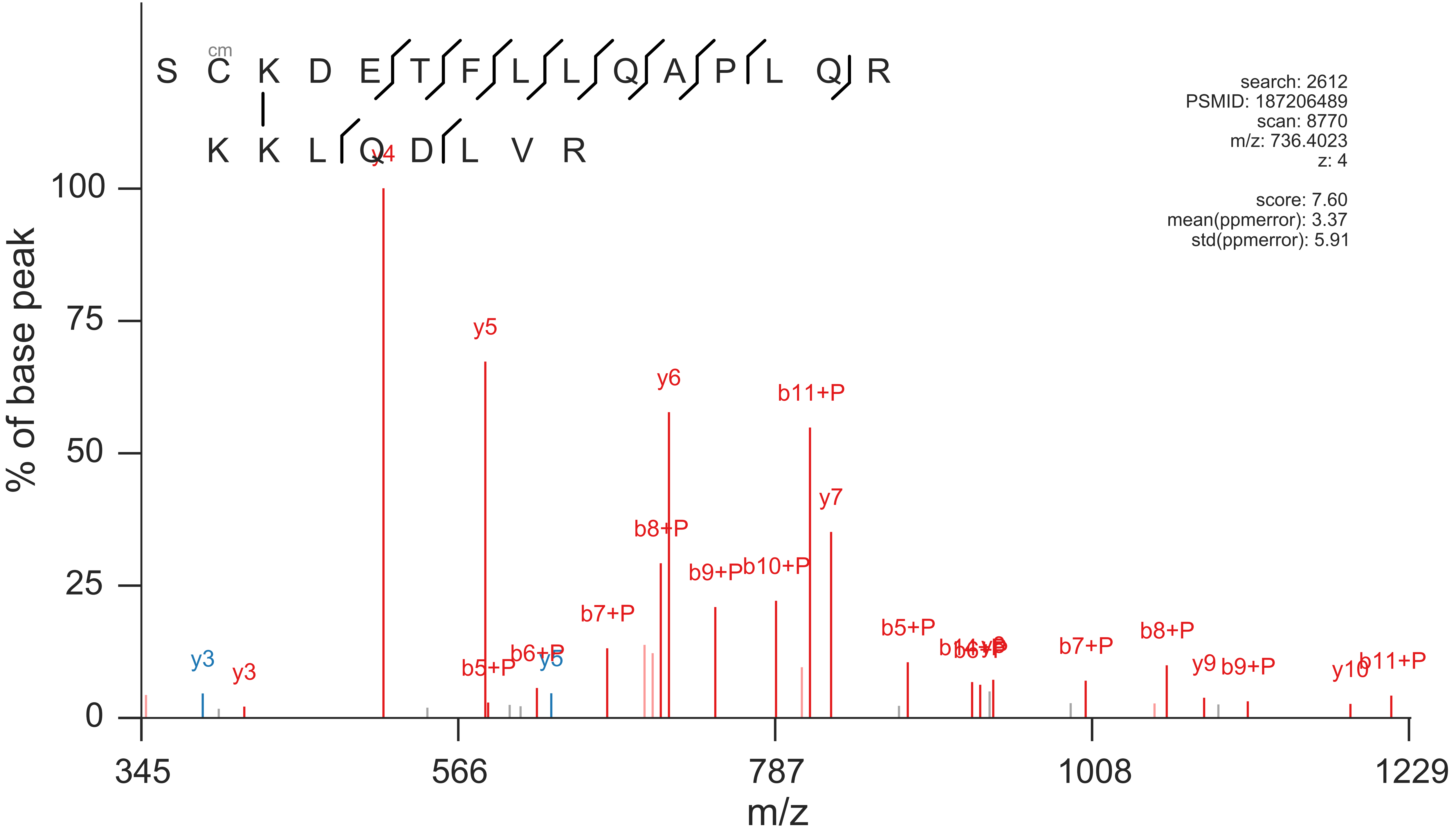

S C K D E T F L L Q A P L Q R
cm
K K L Q D L V R
search: 2612
PSMID: 187206489
scan: 8770
m/z: 736.4023
z: 4
score: 7.60
mean(ppmerror): 3.37
std(ppmerror): 5.91
% of base peak
m/z
y4
y5
y6
b11+P
y7
b8+P
b9+P
b10+P
b7+P
b5+P
b6+P
y3
y3
y5
b5+P
b7+P
b8+P
y9
b9+P
y10
b11+P

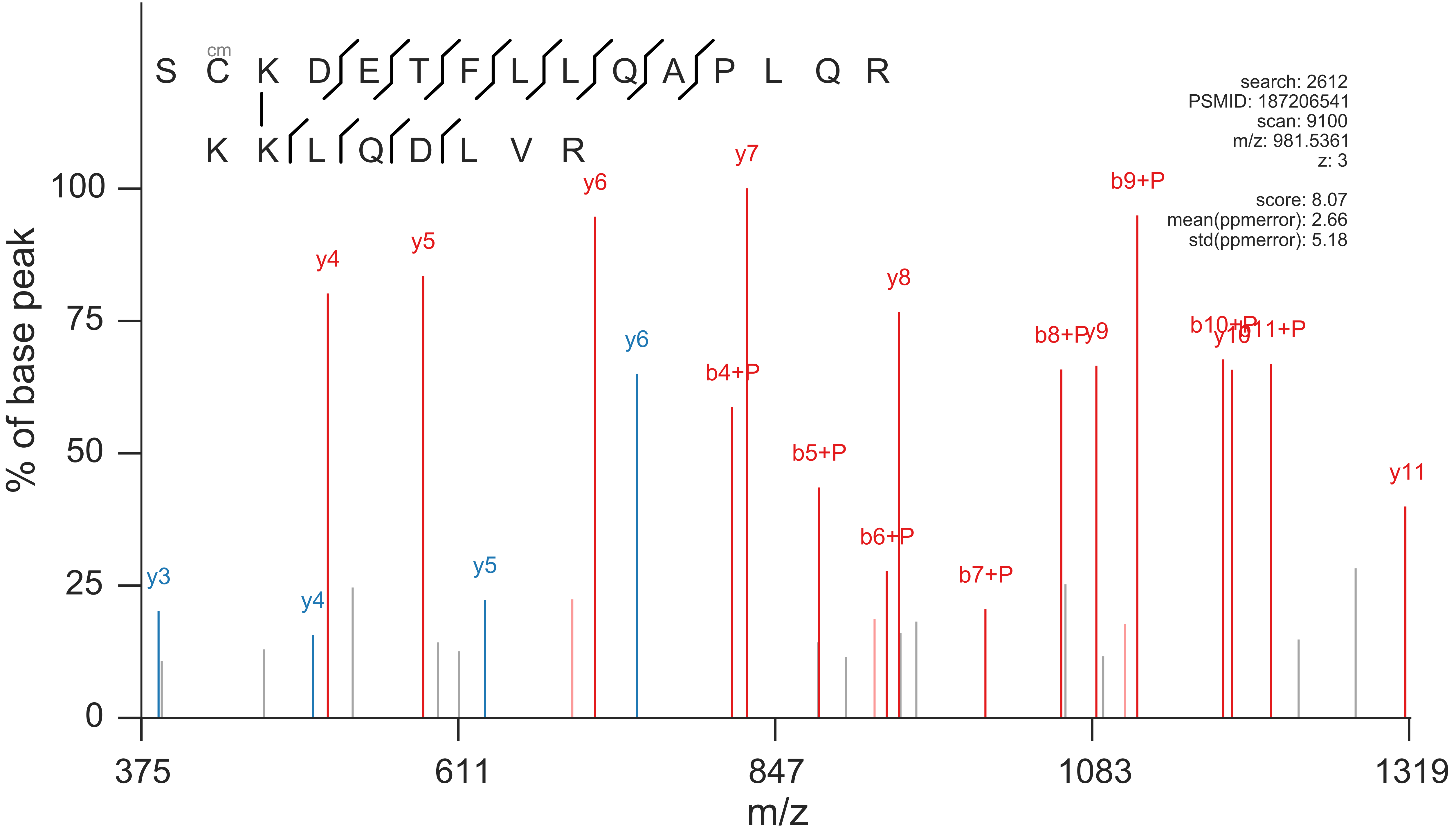

S C K D E T F L L Q A P L Q R
cm
K K L Q D L V R
search: 2612
PSMID: 187206541
scan: 9100
m/z: 981.5361
z: 3
score: 8.07
mean(ppmerror): 2.66
std(ppmerror): 5.18
% of base peak
m/z
0
25
50
75
100
375
611
847
1083
1319
y3
y4
y4
y5
y5
y6
y6
y7
b4+P
b5+P
b6+P
y8
b7+P
b8+P
y9
b9+P
b10+P
y10
b11+P
y11

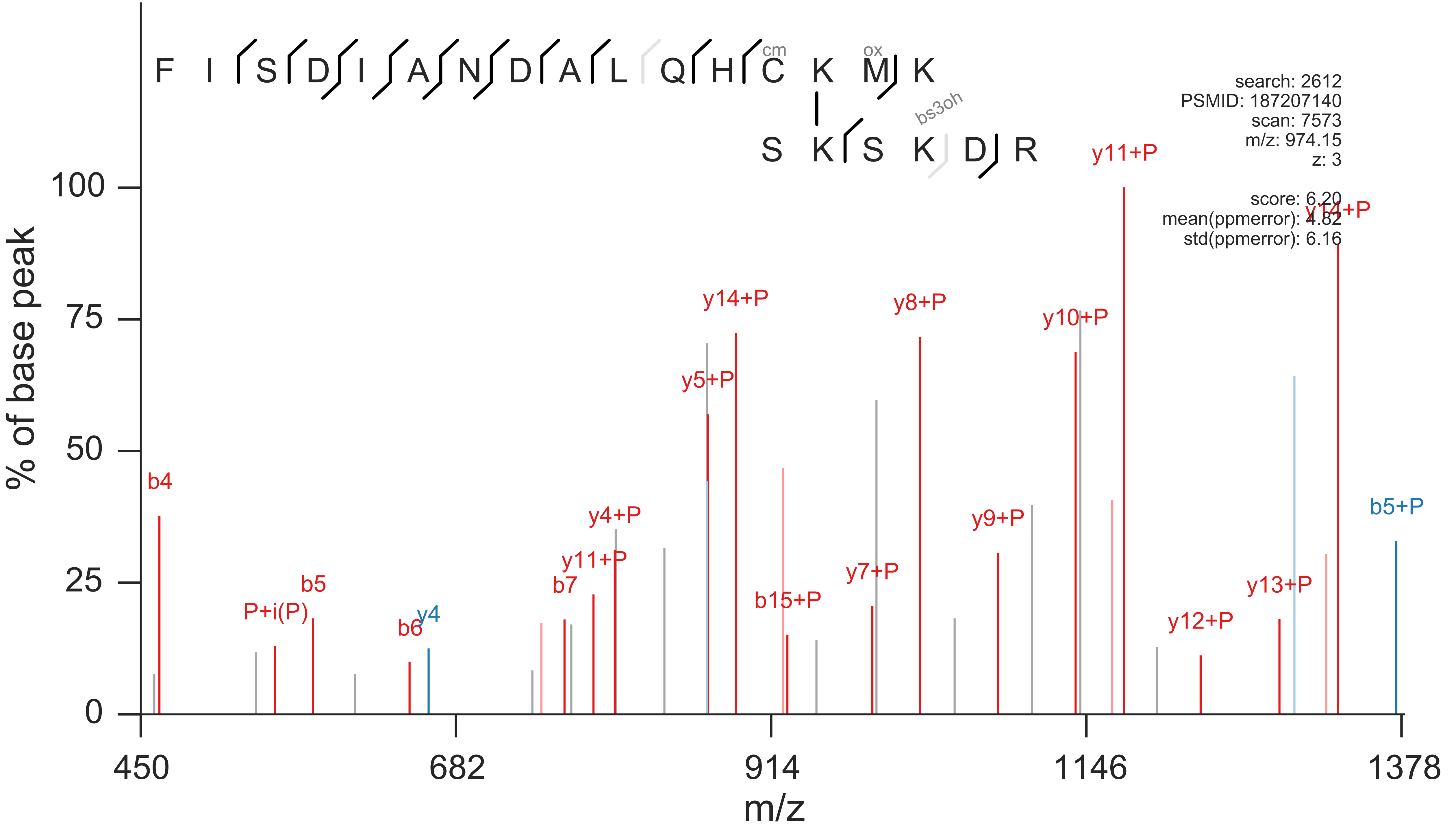

F I S D I A N D A L Q H C K M K
cm
ox
S K S K D R
bs3oh
search: 2612
PSMID: 187207140
scan: 7573
m/z: 974.15
z: 3
score: 6.20
mean(ppmerror): 4.82
std(ppmerror): 6.16
% of base peak
100
75
50
25
0
m/z
450
682
914
1146
1378
b4
P+i(P)
b5
b6
y4
b7
y11+P
y4+P
y5+P
y14+P
b15+P
y7+P
y8+P
y9+P
y10+P
y11+P
y12+P
y13+P
y14+P
b5+P

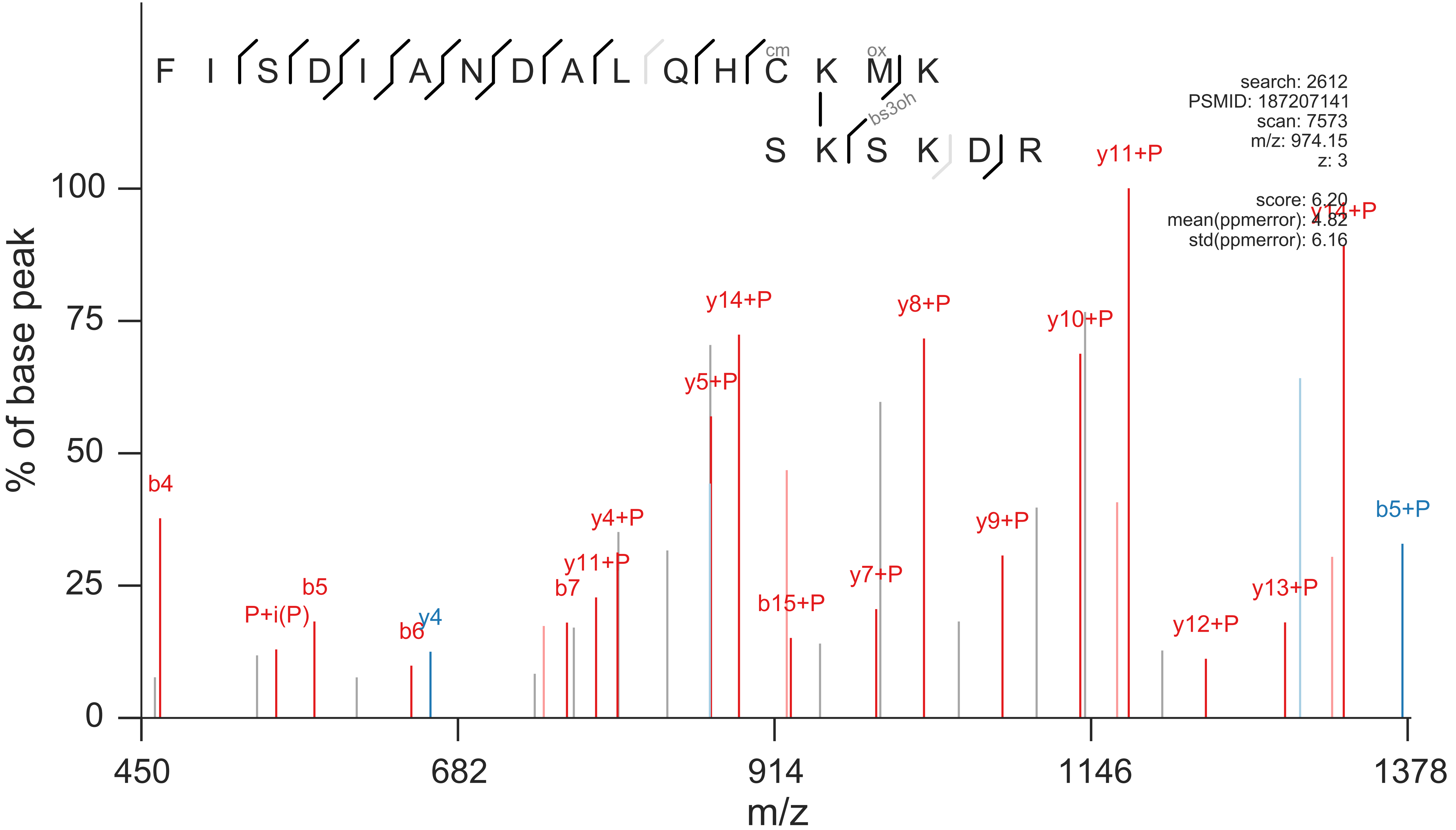

F I S D I A N D A L Q H C K M K
cm
ox
S K S K D R
bs3oh
search: 2612
PSMID: 187207141
scan: 7573
m/z: 974.15
z: 3
score: 6.20
mean(ppmerror): 4.82
std(ppmerror): 6.16
% of base peak
m/z
0
25
50
75
100
450
682
914
1146
1378
b4
P+i(P)
b5
b6
y4
b7
y11+P
y4+P
y5+P
y14+P
b15+P
y7+P
y8+P
y9+P
y10+P
y11+P
y12+P
y13+P
y14+P
b5+P

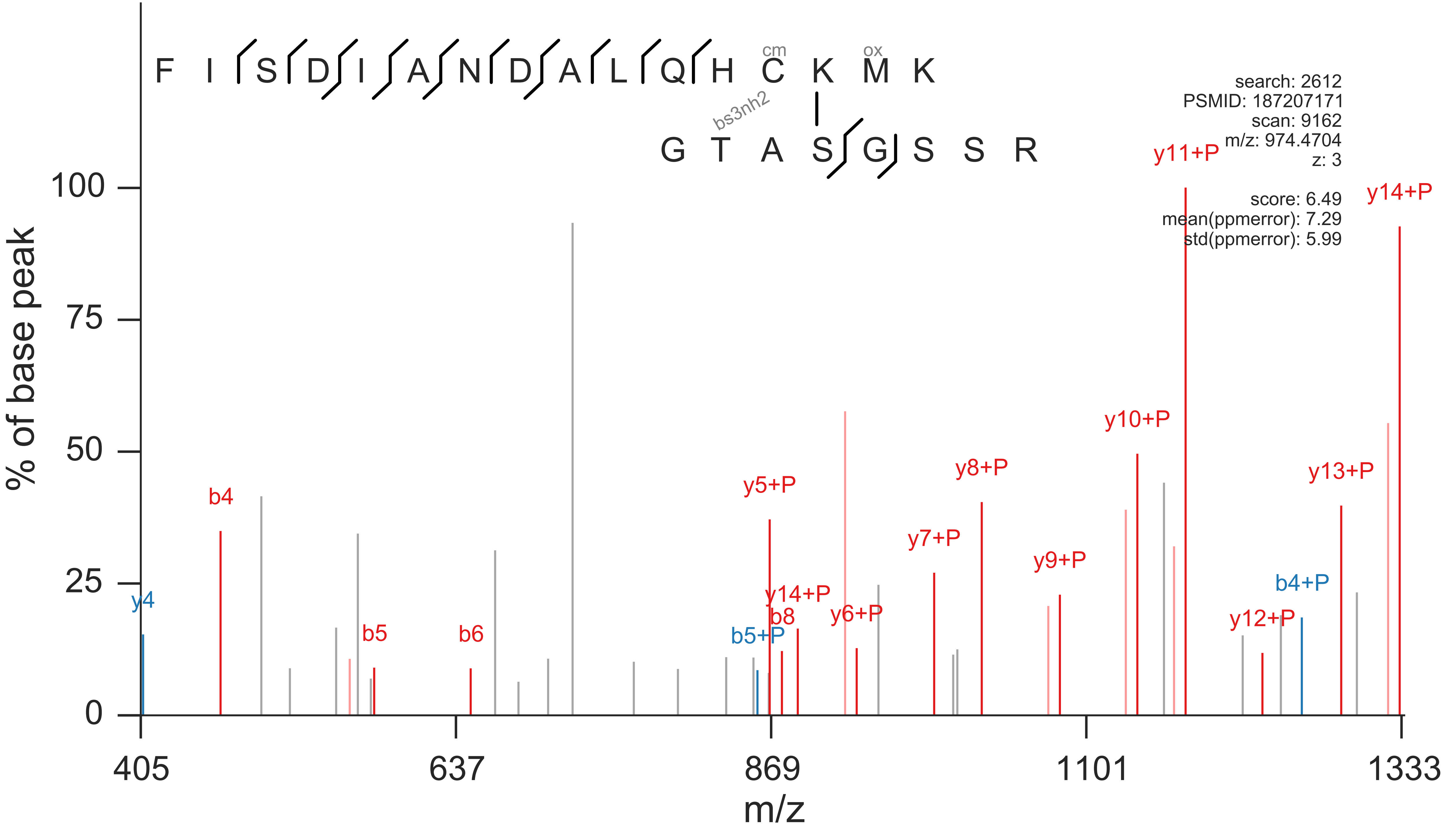
F I S D I A N D A L Q H C K M K
cm
ox
bs3nh2
G T A S G S S R
search: 2612
PSMID: 187207171
scan: 9162
m/z: 974.4704
z: 3
score: 6.49
mean(ppmerror): 7.29
std(ppmerror): 5.99
% of base peak
m/z
y4
b4
b5
b6
b5+P
y5+P
y14+P
b8
y6+P
y7+P
y8+P
y9+P
y10+P
y11+P
y12+P
b4+P
y13+P
y14+P
0
25
50
75
100
405
637
869
1101
1333

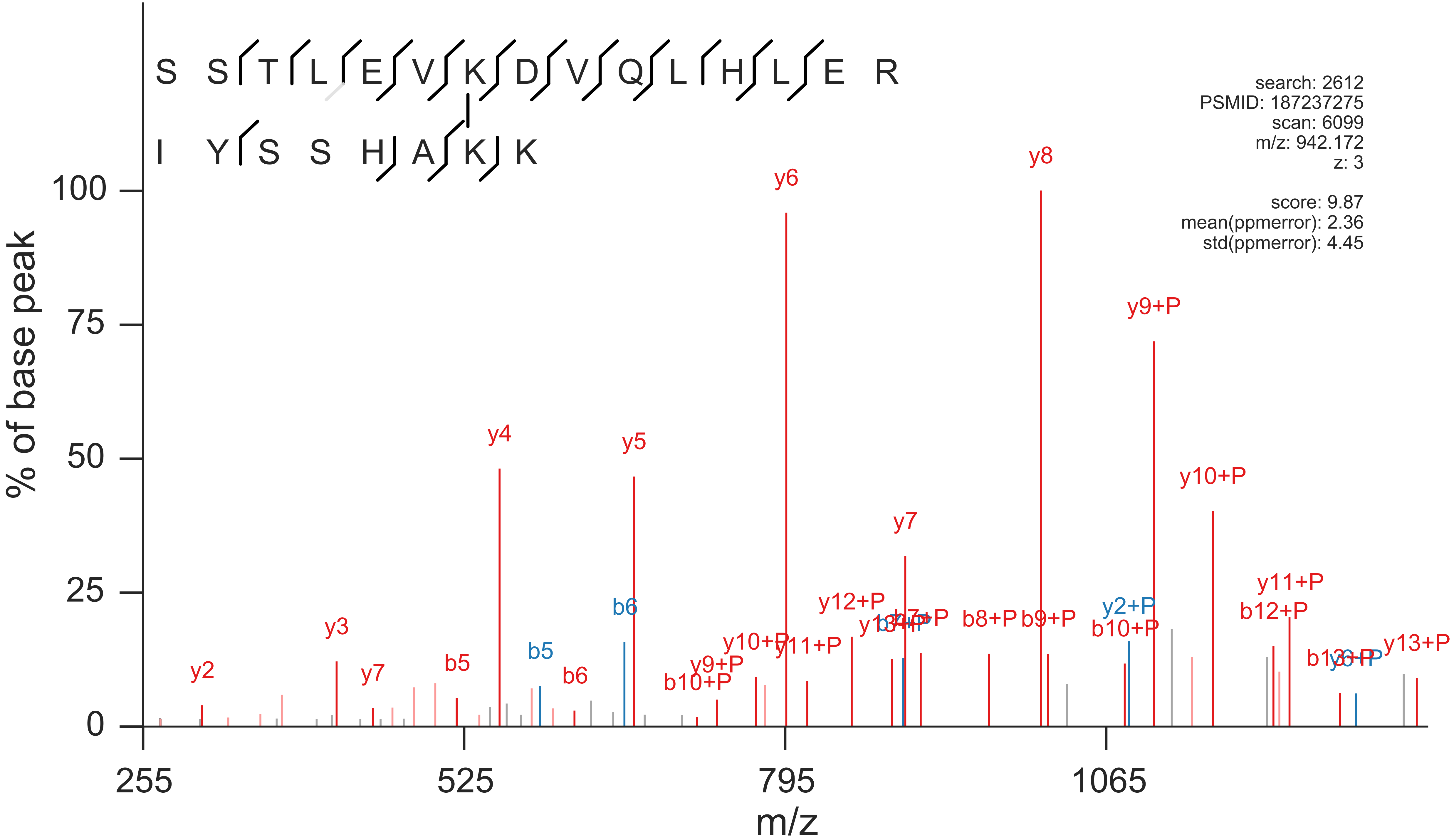
S S T L E V K D V Q L H L E R
I Y S S H A K K
search: 2612
PSMID: 187237275
scan: 6099
m/z: 942.172
z: 3
score: 9.87
mean(ppmerror): 2.36
std(ppmerror): 4.45
% of base peak
m/z
0
25
50
75
100
255
525
795
1065
y2
y3
y7
b5
y4
b5
b6
b6
y5
y6
b10+P
y9+P
y10+P
y11+P
y12+P
y7
b8+P
b9+P
y8
y2+P
b10+P
y9+P
y10+P
y11+P
b12+P
y13+P

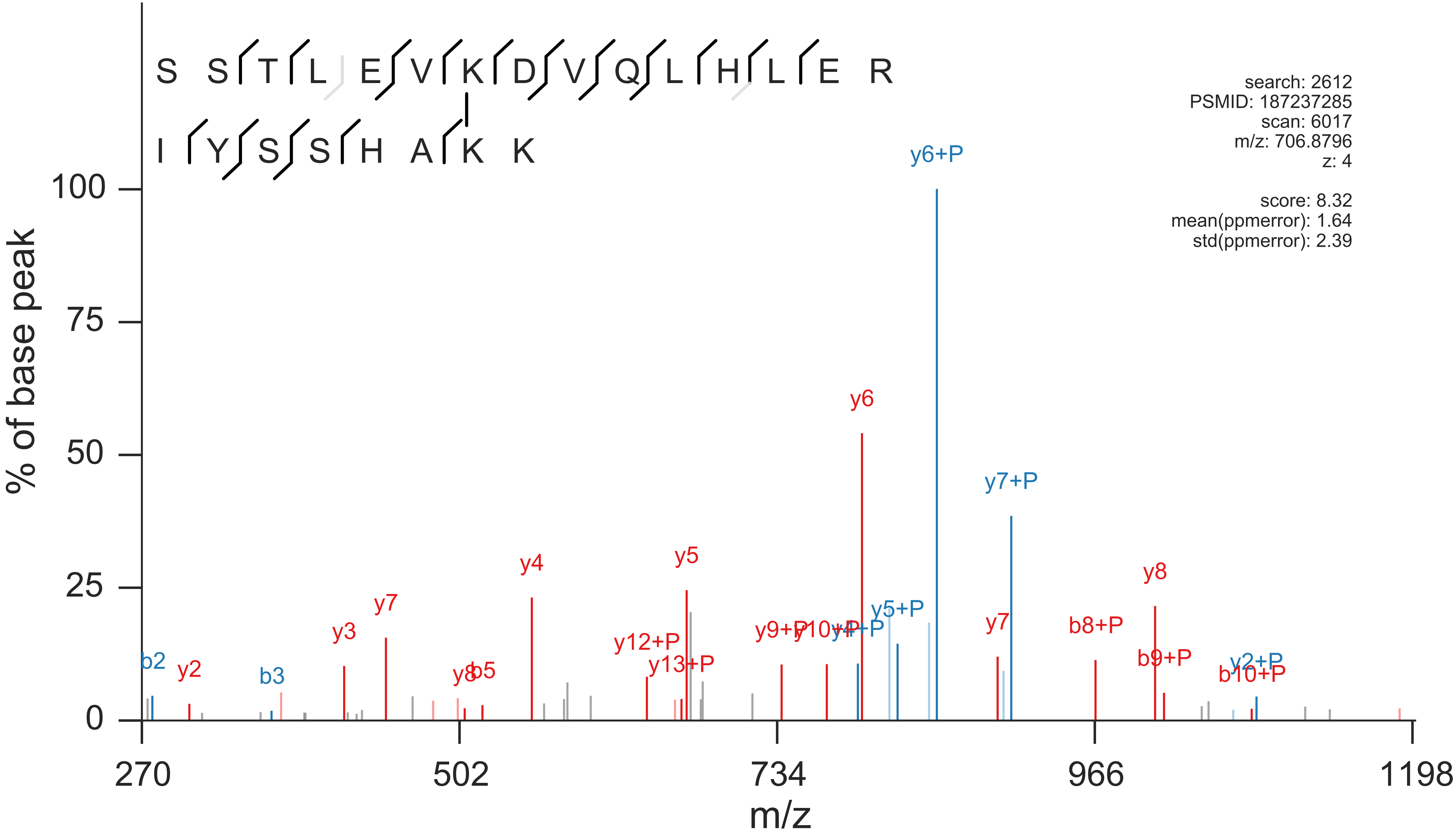

S S T L E V K D V Q L H L E R
I Y S S H A K K
search: 2612
PSMID: 187237285
scan: 6017
m/z: 706.8796
z: 4
score: 8.32
mean(ppmerror): 1.64
std(ppmerror): 2.39
% of base peak
m/z
y6+P
y6
y7+P
y8
y5
y4
y7
y3
y2
b2
b3
y8
b5
y12+P
y13+P
y9+P
y10+P
y4+P
y5+P
y7
b8+P
b9+P
y2+P
b10+P
0
25
50
75
100
270
502
734
966
1198

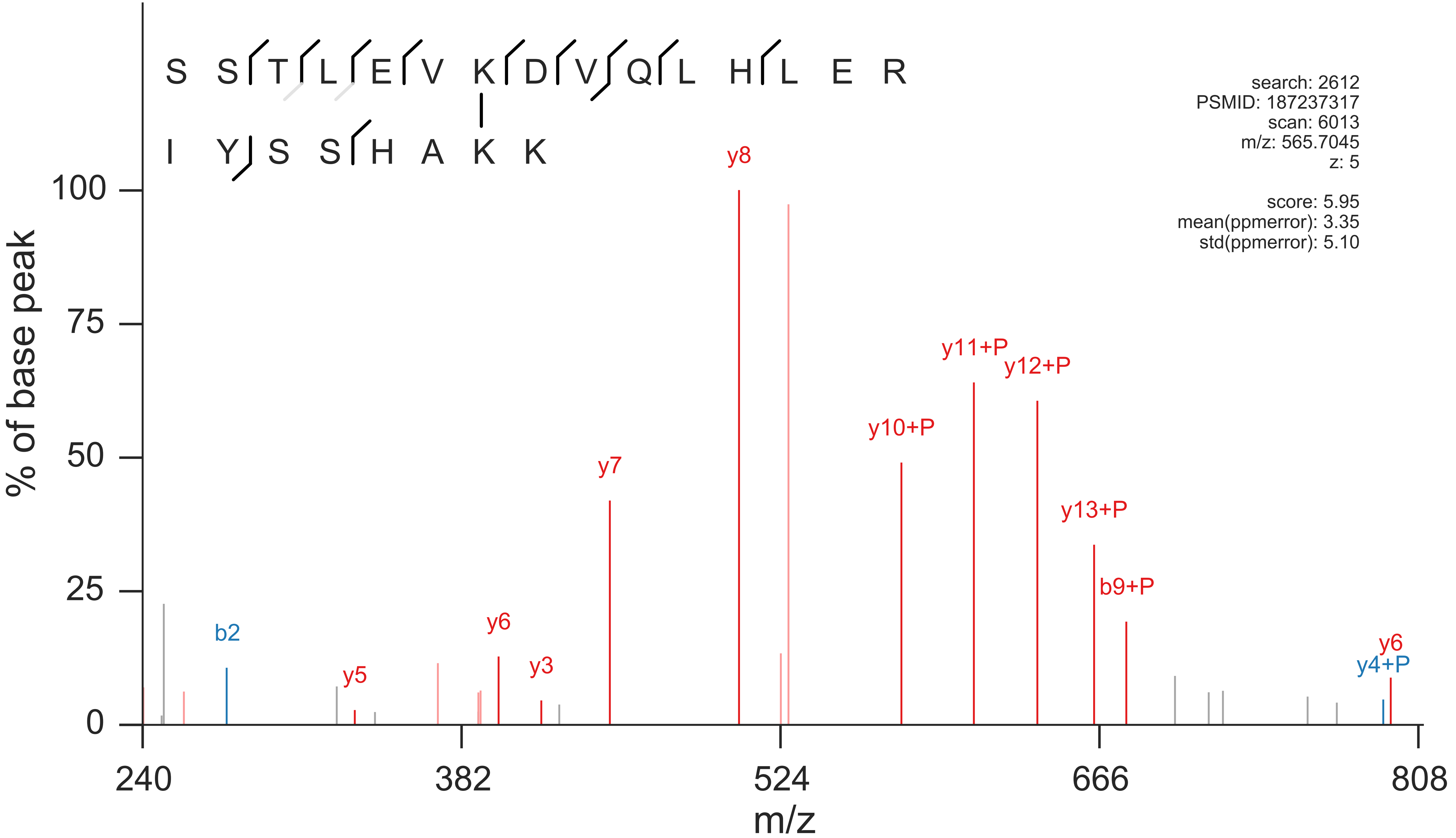

S S T L E V K D V Q L H L E R
I Y S S H A K K
search: 2612
PSMID: 187237317
scan: 6013
m/z: 565.7045
z: 5
score: 5.95
mean(ppmerror): 3.35
std(ppmerror): 5.10
% of base peak
100
75
50
25
0
b2
y5
y6
y3
y7
y8
y10+P
y11+P
y12+P
y13+P
b9+P
y6
y4+P
240
382
524
666
808
m/z

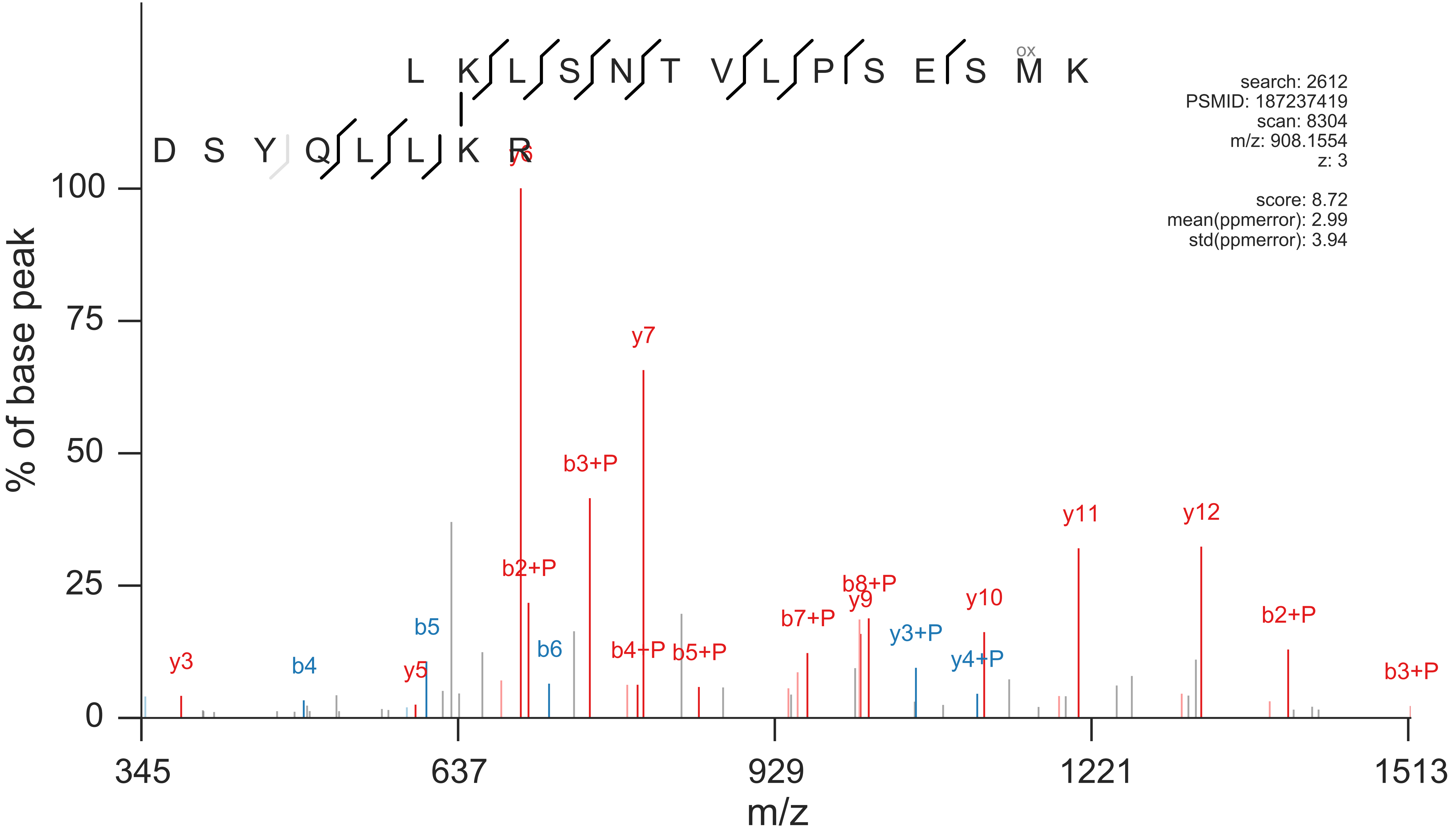

L K L S N T V L P S E S M K
ox
D S Y Q L L K R
search: 2612
PSMID: 187237419
scan: 8304
m/z: 908.1554
z: 3
score: 8.72
mean(ppmerror): 2.99
std(ppmerror): 3.94
% of base peak
100
75
50
25
0
y6
y7
b3+P
y11
y12
b2+P
b8+P
y9
y10
b7+P
b2+P
y3+P
b5
b6
b4+P
b5+P
y4+P
y3
b4
y5
b3+P
345
637
929
1221
1513
m/z

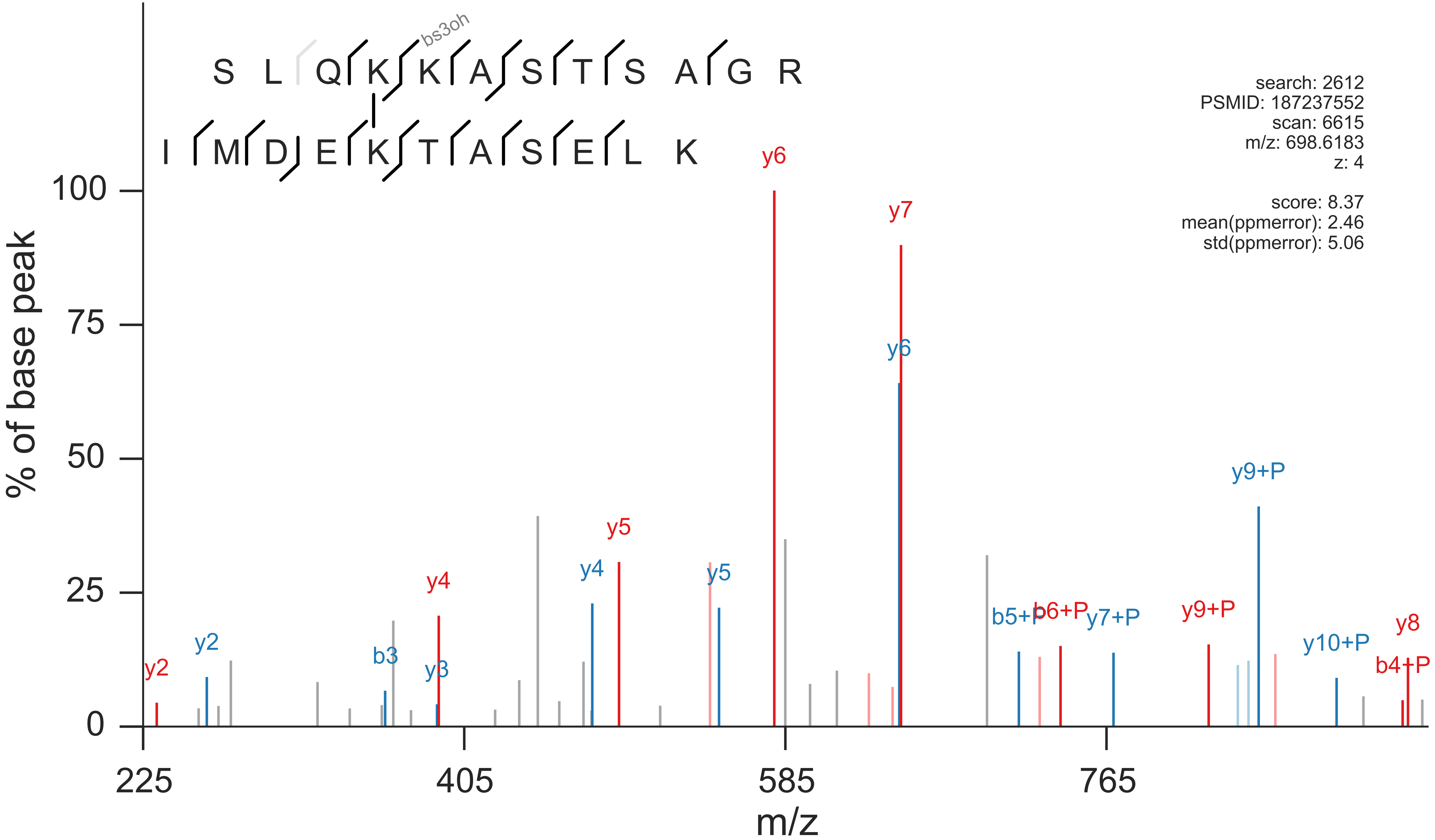

S L Q K K A S T S A G R
bs3oh
I M D E K T A S E L K
search: 2612
PSMID: 187237552
scan: 6615
m/z: 698.6183
z: 4
score: 8.37
mean(ppmerror): 2.46
std(ppmerror): 5.06
% of base peak
m/z
y2
y2
b3
y3
y4
y4
y5
y5
y6
y6
y7
b5+P
b6+P
y7+P
y9+P
y9+P
y10+P
y8
b4+P

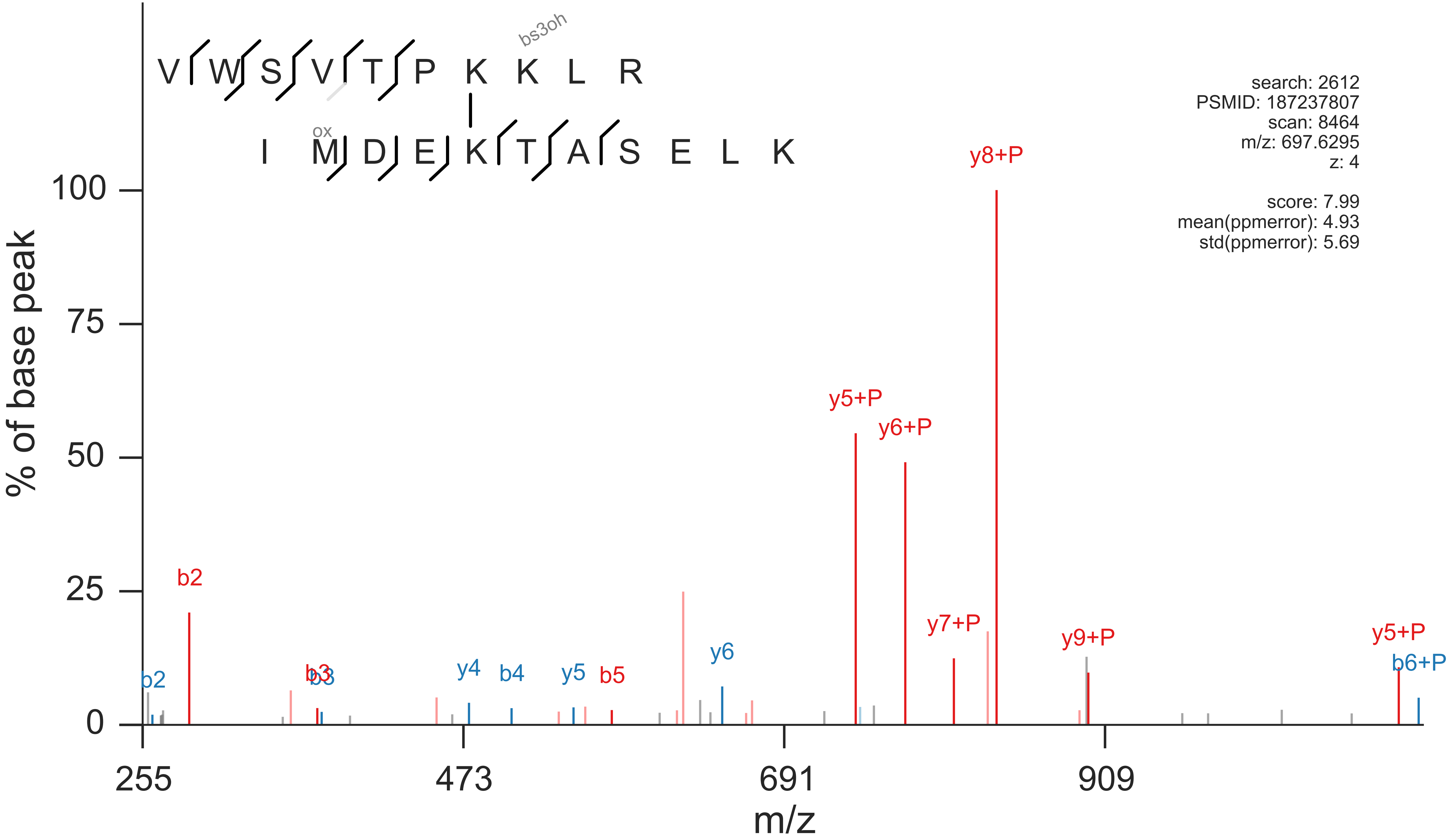

V W S V T P K K L R
bs3oh
I M D E K T A S E L K
ox
search: 2612
PSMID: 187237807
scan: 8464
m/z: 697.6295
z: 4
score: 7.99
mean(ppmerror): 4.93
std(ppmerror): 5.69
% of base peak
m/z
100
75
50
25
0
255
473
691
909
b2
b2
b3
b3
y4
b4
y5
b5
y6
y5+P
y6+P
y7+P
y8+P
y9+P
y5+P
b6+P

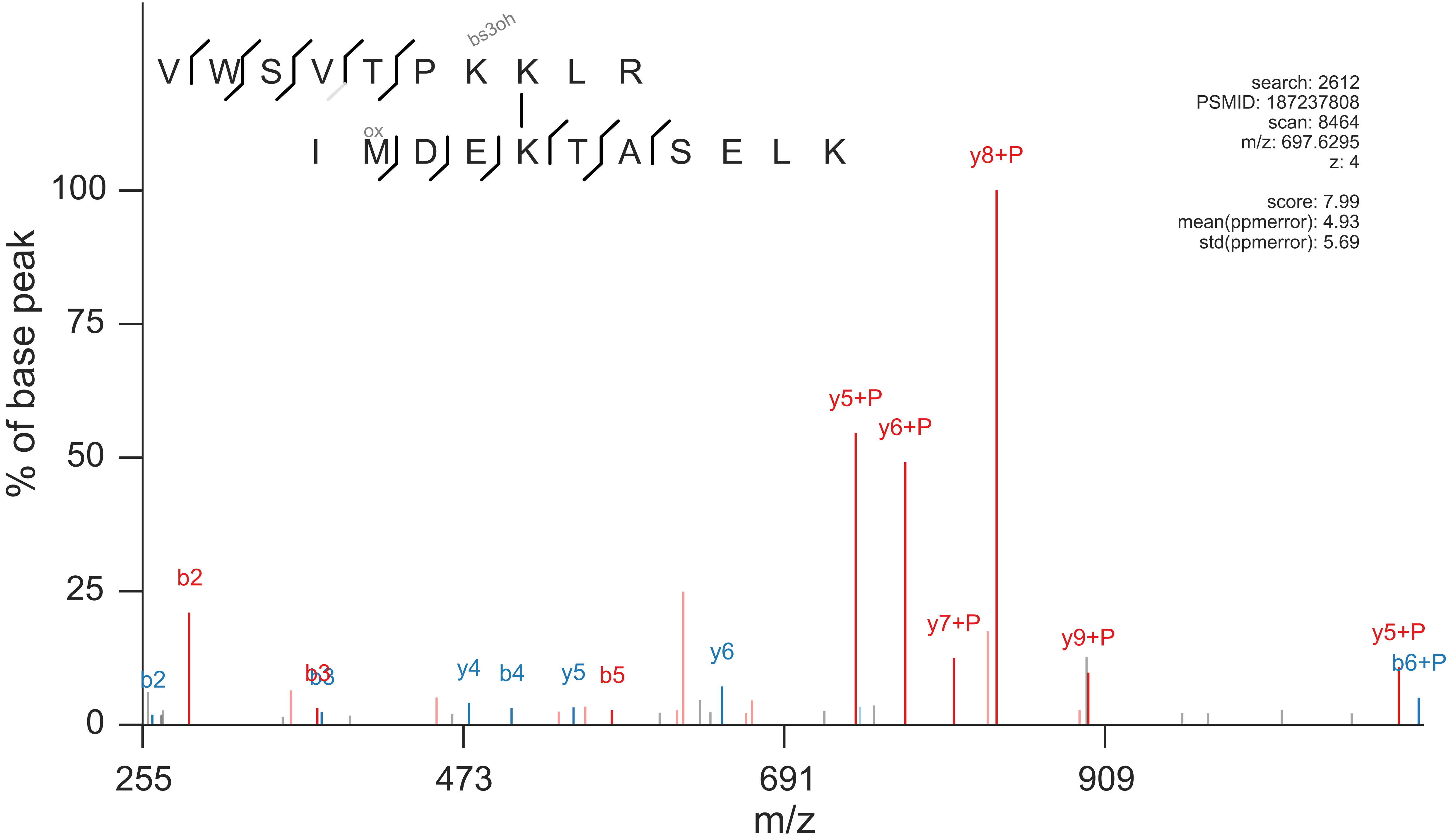

V W S V T P K K L R
bs3oh
I M D E K T A S E L K
ox
search: 2612
PSMID: 187237808
scan: 8464
m/z: 697.6295
z: 4
score: 7.99
mean(ppmerror): 4.93
std(ppmerror): 5.69
% of base peak
m/z
100
75
50
25
0
255
473
691
909
b2
b3
y4
b4
y5
b5
y6
y5+P
y6+P
y7+P
y8+P
y9+P
b6+P

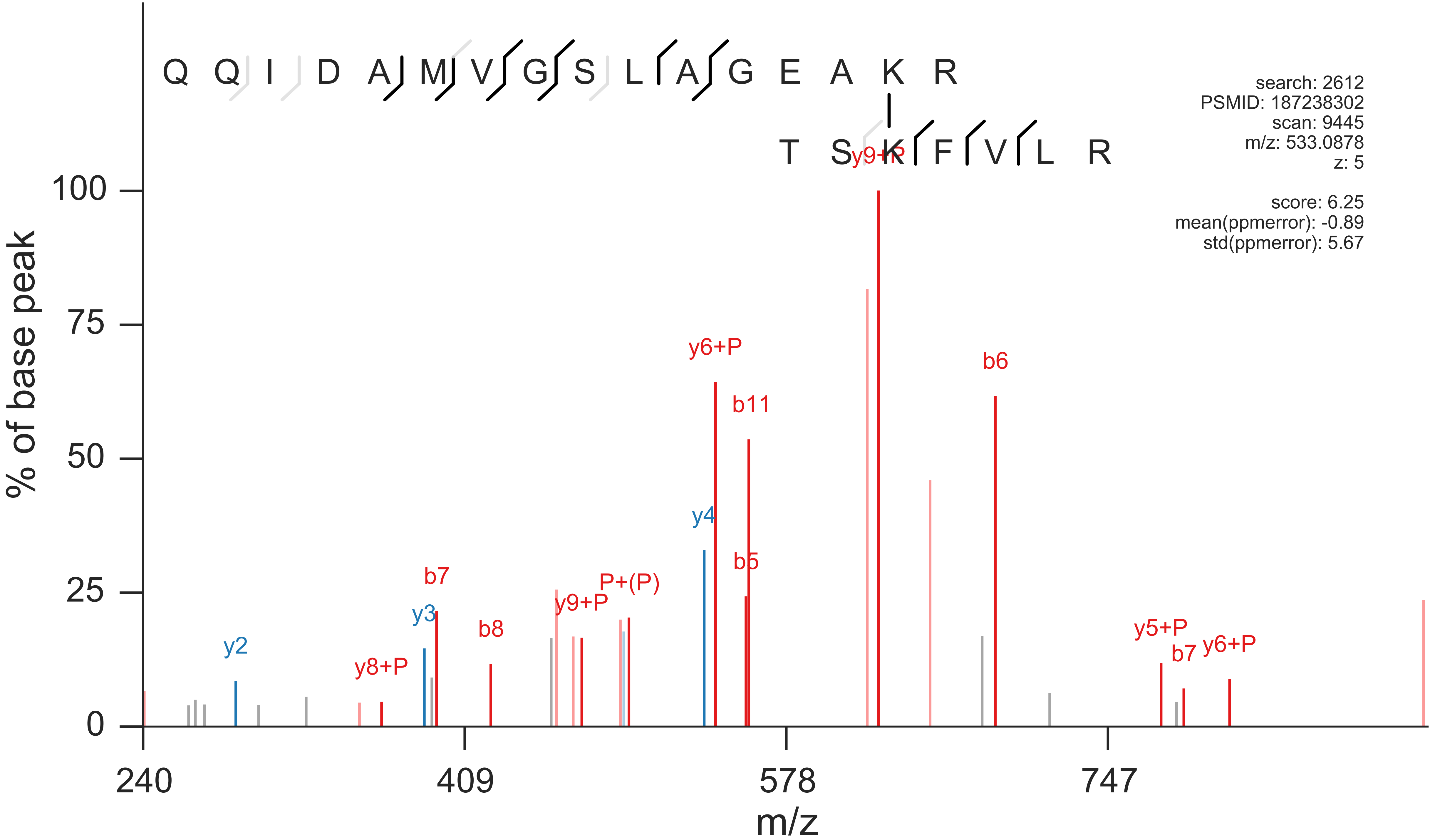
Q Q I D A M V G S L A G E A K R
T S K F V L R
search: 2612
PSMID: 187238302
scan: 9445
m/z: 533.0878
z: 5
score: 6.25
mean(ppmerror): -0.89
std(ppmerror): 5.67
% of base peak
m/z
0
25
50
75
100
240
409
578
747
y2
y3
y4
y8+P
b7
b8
y9+P
P+(P)
y6+P
b11
b5
y9+P
b6
y5+P
b7
y6+P

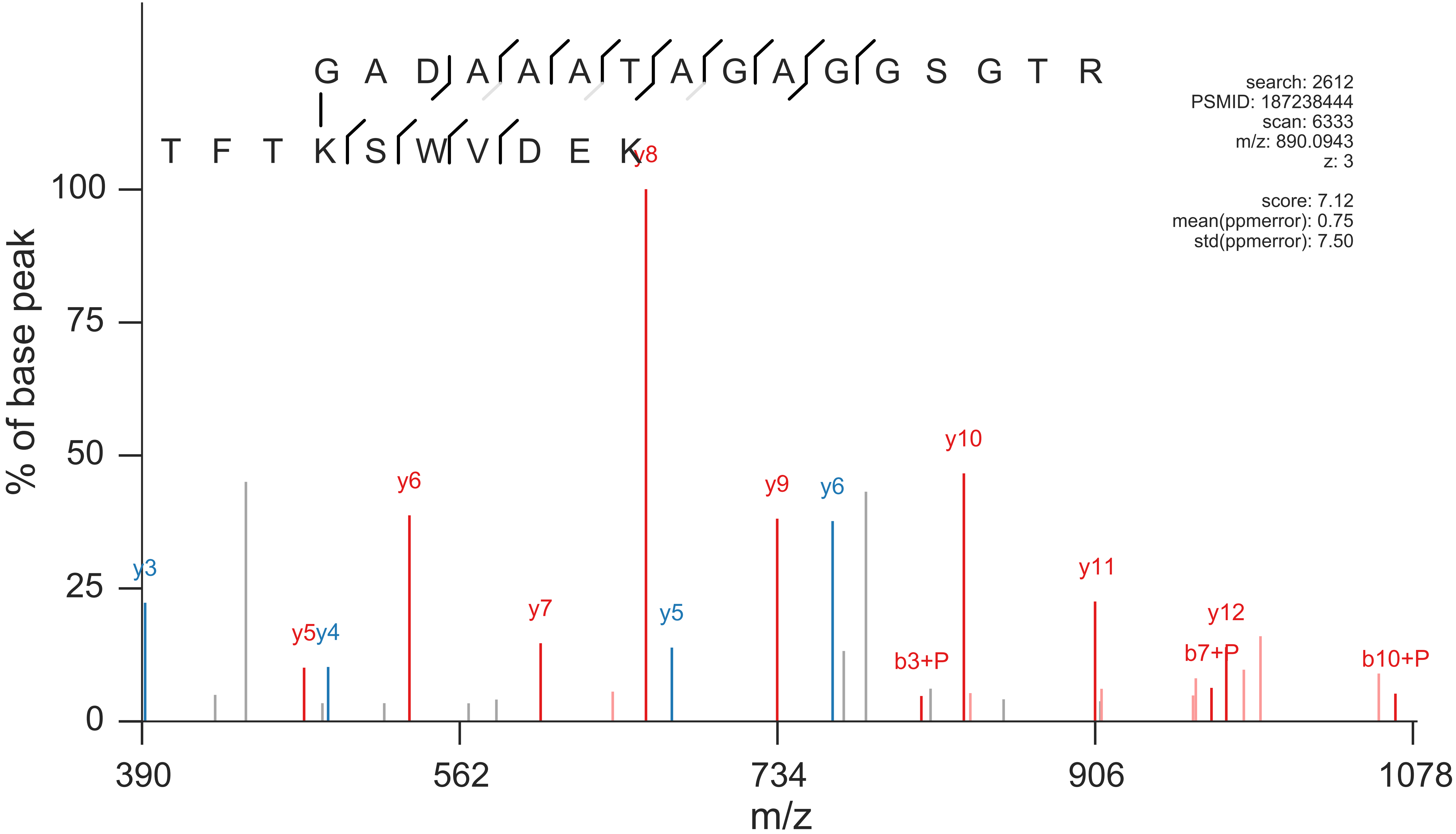
G A D A A A T A G A G G S G T R
T F T K S W V D E K
search: 2612
PSMID: 187238444
scan: 6333
m/z: 890.0943
z: 3
score: 7.12
mean(ppmerror): 0.75
std(ppmerror): 7.50
% of base peak
m/z
100
75
50
25
0
390
562
734
906
1078
y3
y5
y4
y6
y7
y8
y5
y9
y6
b3+P
y10
y11
y12
b7+P
b10+P

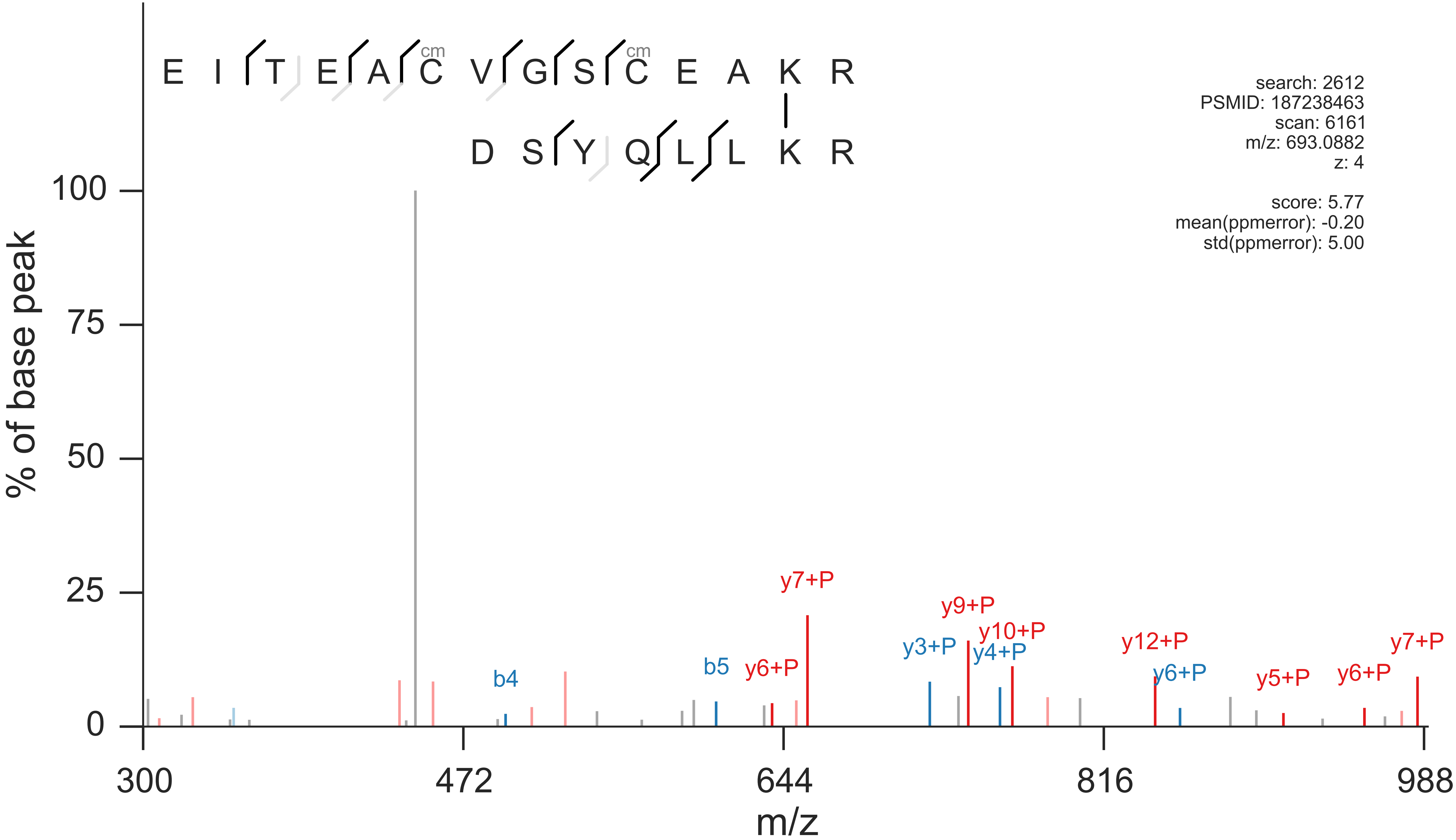

E I T E A C V G S C E A K R
cm
cm
D S Y Q L L K R
search: 2612
PSMID: 187238463
scan: 6161
m/z: 693.0882
z: 4
score: 5.77
mean(ppmerror): -0.20
std(ppmerror): 5.00
% of base peak
100
75
50
25
0
300
472
644
816
988
m/z
b4
b5
y6+P
y7+P
y3+P
y9+P
y10+P
y4+P
y12+P
y6+P
y5+P
y6+P
y7+P

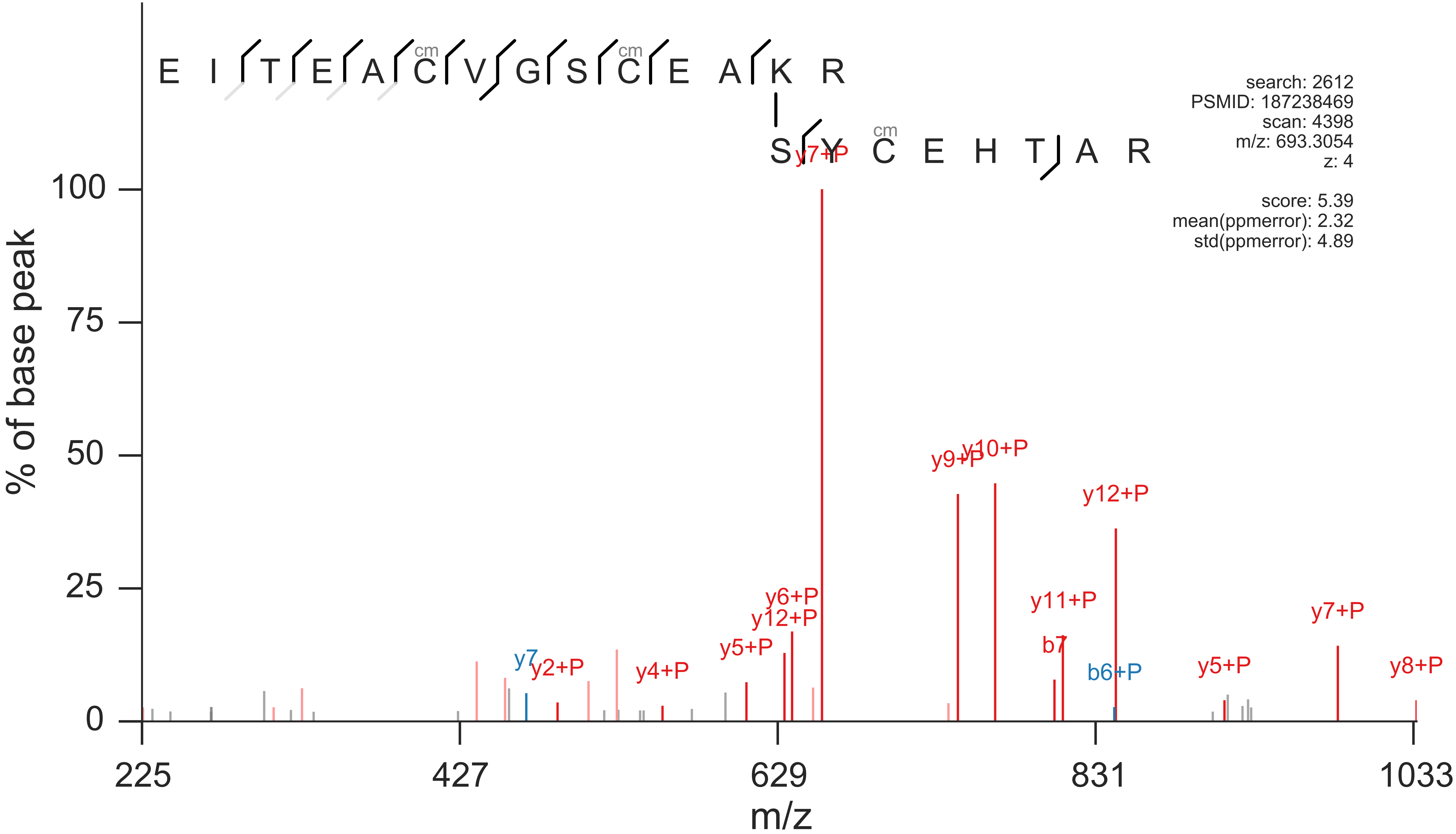

E I T E A C V G S C E A K R
S Y C E H T A R
cm
search: 2612
PSMID: 187238469
scan: 4398
m/z: 693.3054
z: 4
score: 5.39
mean(ppmerror): 2.32
std(ppmerror): 4.89
% of base peak
m/z
y7+P
y9+P
y10+P
y12+P
y11+P
y6+P
y12+P
y5+P
y4+P
y2+P
y7
b7
b6+P
y5+P
y7+P
y8+P

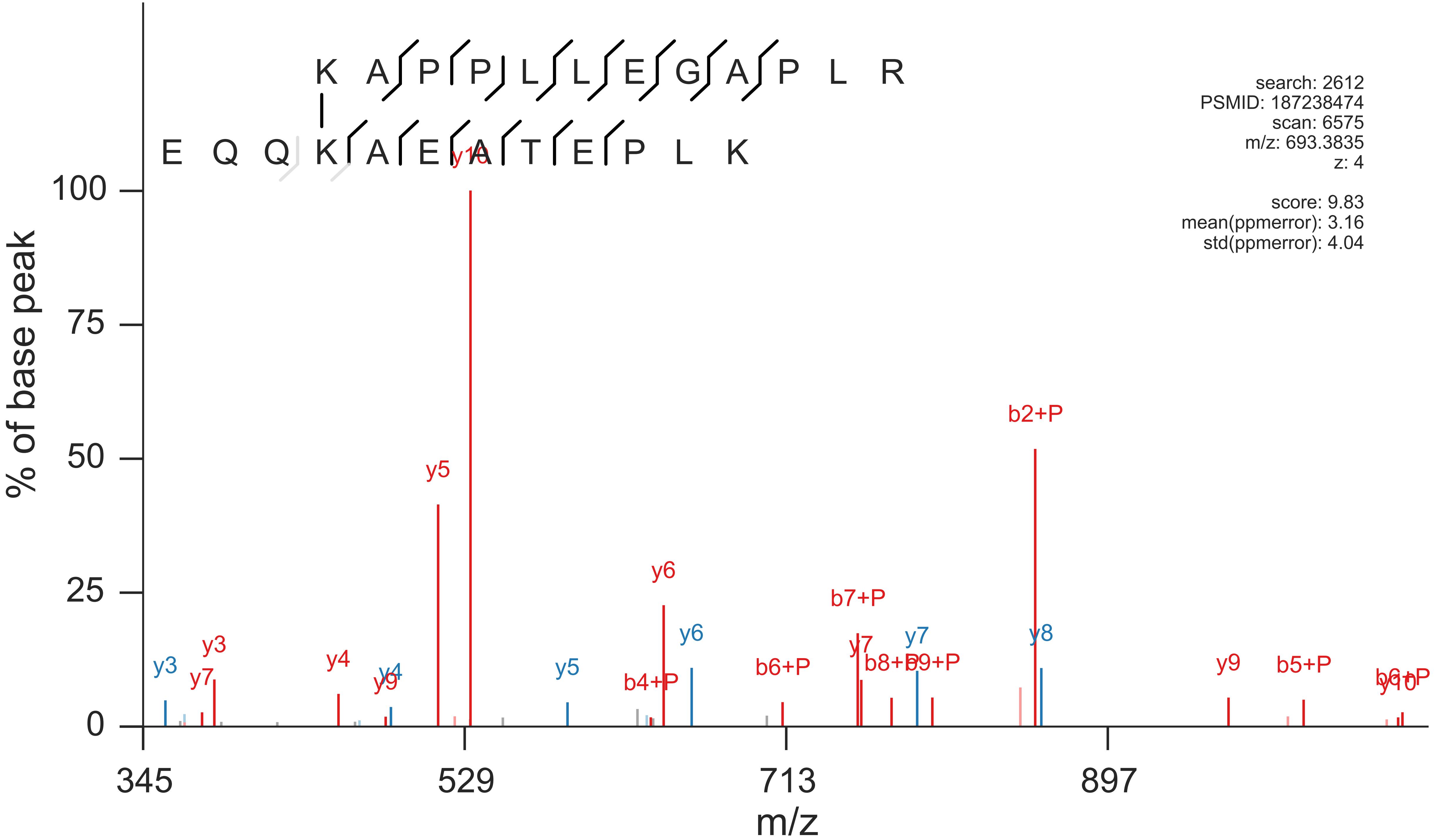

K A P P L L E G A P L R
E Q Q K A E A T E P L K
search: 2612
PSMID: 187238474
scan: 6575
m/z: 693.3835
z: 4
score: 9.83
mean(ppmerror): 3.16
std(ppmerror): 4.04
% of base peak
m/z
0
25
50
75
100
345
529
713
897
y3
y7
y3
y4
y9
y4
y5
y10
y5
b4+P
y6
y6
b6+P
y7
b7+P
b8+P
b9+P
y7
b2+P
y8
y9
b5+P
y10
b6+P

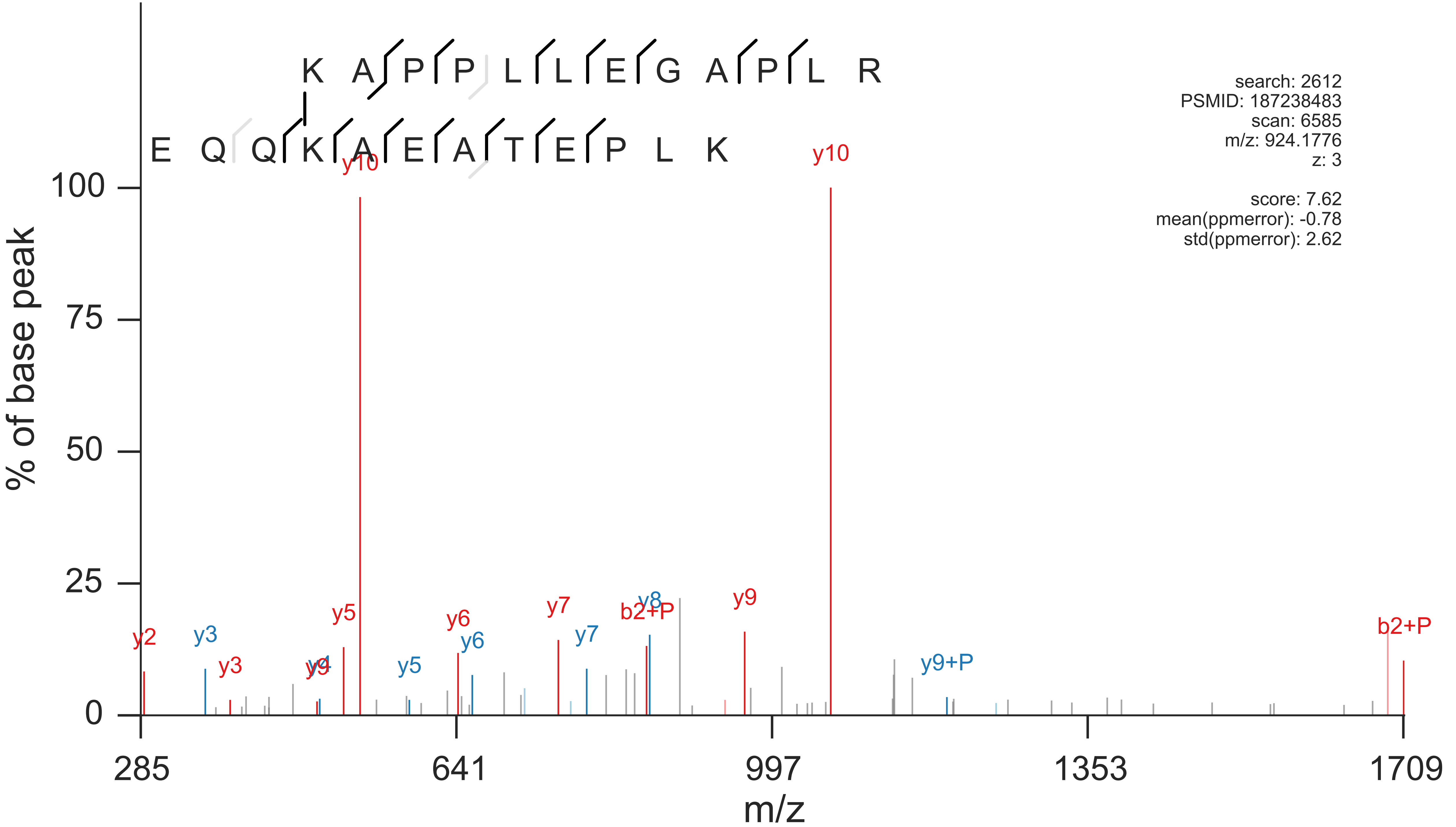

K A P P L L E G A P L R
E Q Q K A E A T E P L K
search: 2612
PSMID: 187238483
scan: 6585
m/z: 924.1776
z: 3
score: 7.62
mean(ppmerror): -0.78
std(ppmerror): 2.62
% of base peak
m/z
y2
y3
y4
y5
y6
y7
y8
y9
y10
b2+P
y9+P
100
75
50
25
0
285
641
997
1353
1709

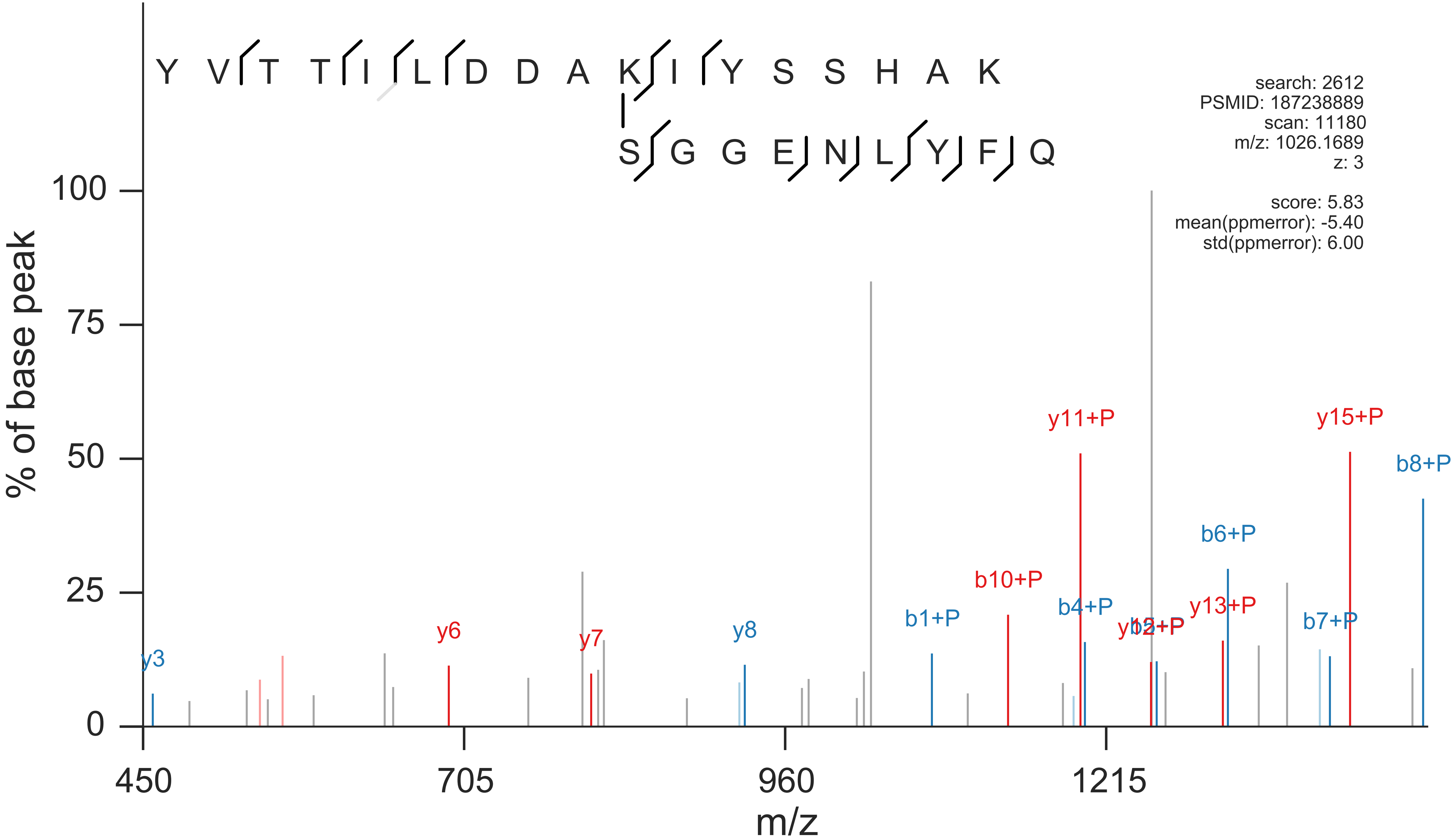
Y V T T I L D D A K I Y S S H A K
S G G E N L Y F Q
search: 2612
PSMID: 187238889
scan: 11180
m/z: 1026.1689
z: 3
score: 5.83
mean(ppmerror): -5.40
std(ppmerror): 6.00
% of base peak
m/z
100
75
50
25
0
450
705
960
1215
y3
y6
y7
y8
b1+P
b10+P
y11+P
b4+P
y12+P
b5+P
y13+P
b6+P
b7+P
y15+P
b8+P

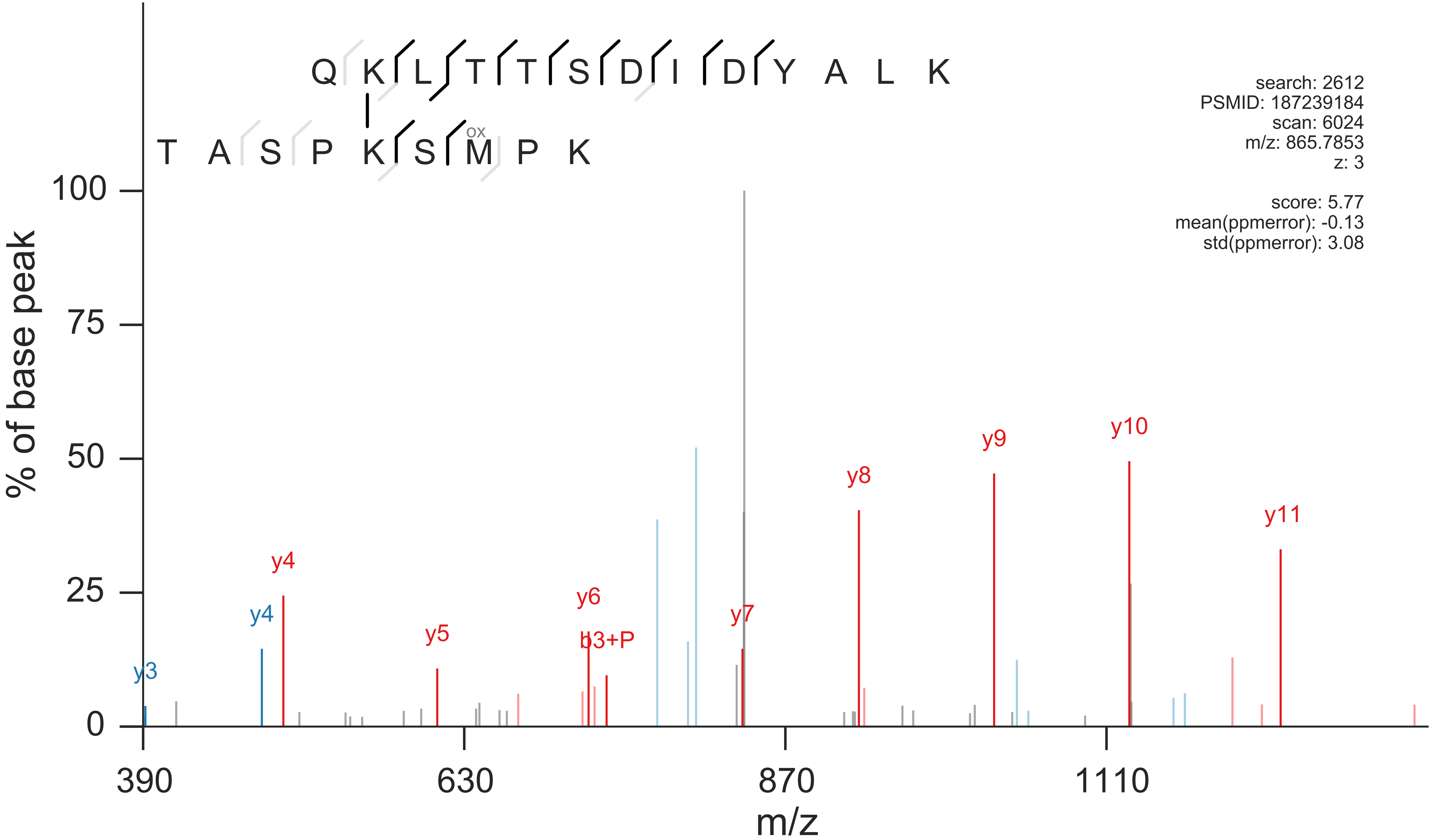

Q K L T T S D I D Y A L K
T A S P K S M P K
ox
search: 2612
PSMID: 187239184
scan: 6024
m/z: 865.7853
z: 3
score: 5.77
mean(ppmerror): -0.13
std(ppmerror): 3.08
% of base peak
m/z
100
75
50
25
0
390
630
870
1110
y3
y4
y4
y5
y6
b3+P
y7
y8
y9
y10
y11

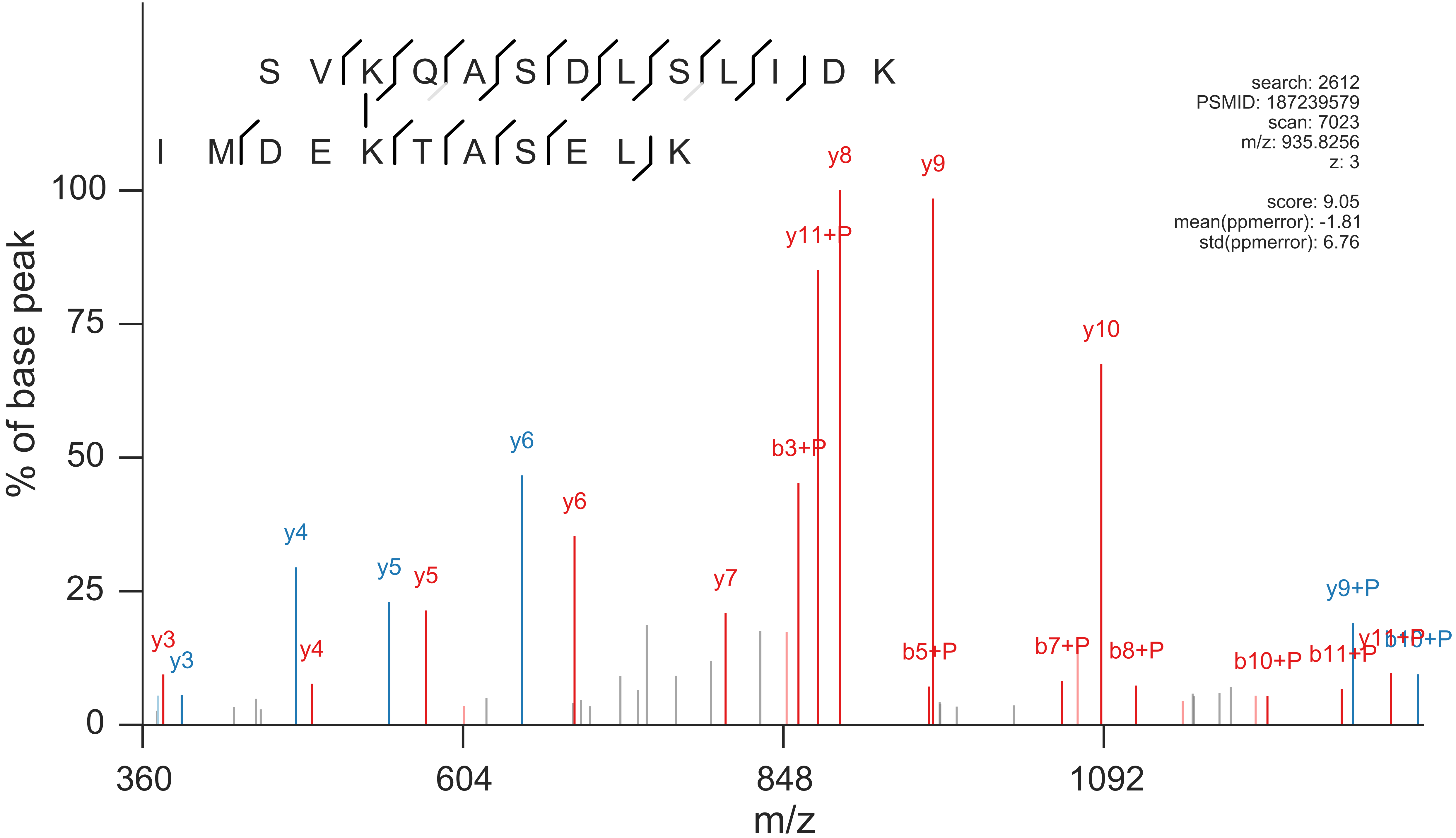

S V K Q A S D L S L I D K
I M D E K T A S E L K
search: 2612
PSMID: 187239579
scan: 7023
m/z: 935.8256
z: 3
score: 9.05
mean(ppmerror): -1.81
std(ppmerror): 6.76
% of base peak
m/z
360
604
848
1092
0
25
50
75
100
y3
y3
y4
y4
y5
y5
y6
y6
y7
b3+P
y11+P
y8
y9
b5+P
y10
b7+P
b8+P
b10+P
b11+P
y9+P

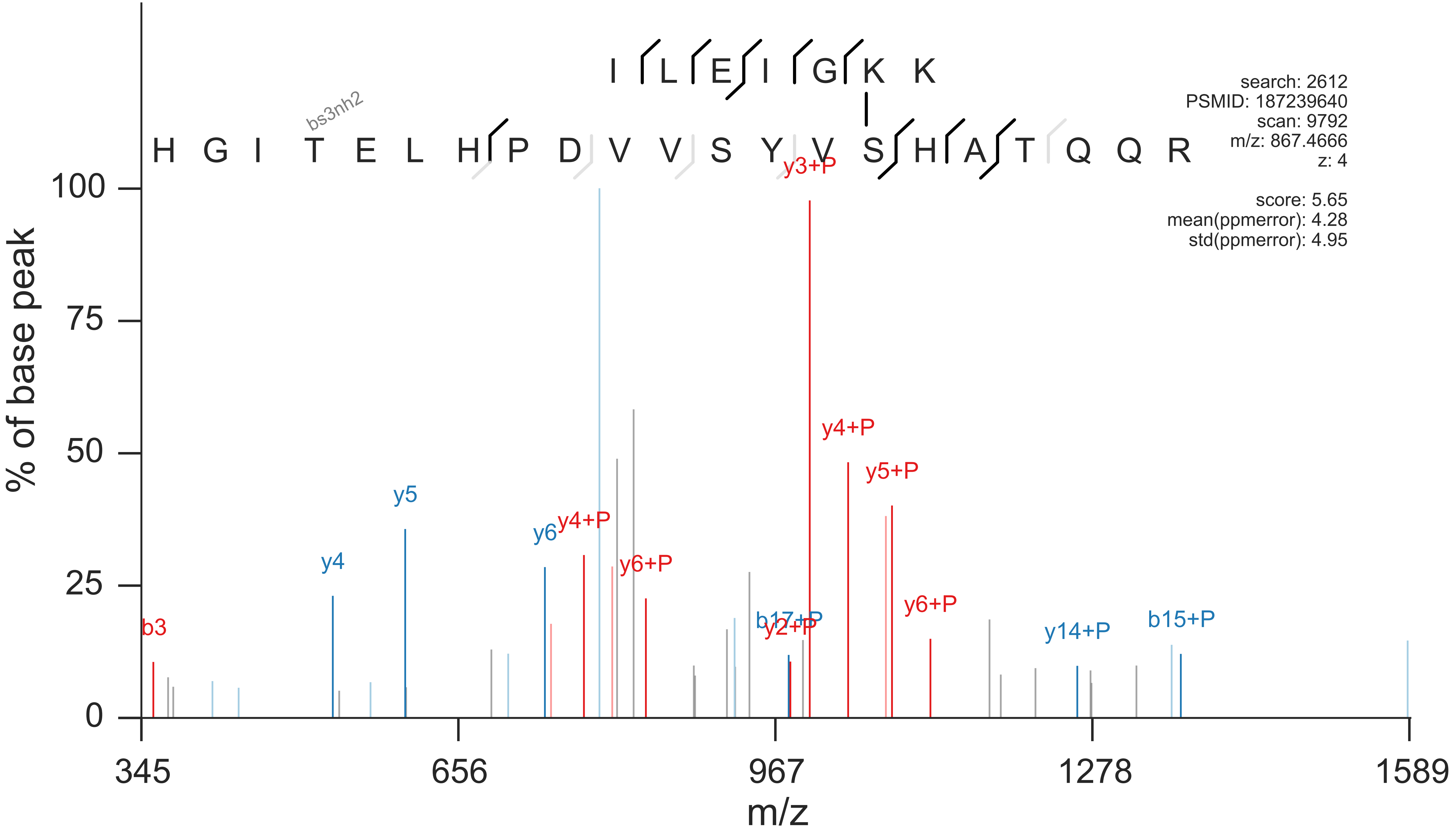

I L E I G K K
H G I T E L H P D V V S Y V S H A T Q Q R
bs3nh2
search: 2612
PSMID: 187239640
scan: 9792
m/z: 867.4666
z: 4
score: 5.65
mean(ppmerror): 4.28
std(ppmerror): 4.95
% of base peak
m/z
0
25
50
75
100
345
656
967
1278
1589
b3
y4
y5
y6
y4+P
y6+P
y3+P
y4+P
y5+P
y6+P
b17+P
y2+P
y14+P
b15+P

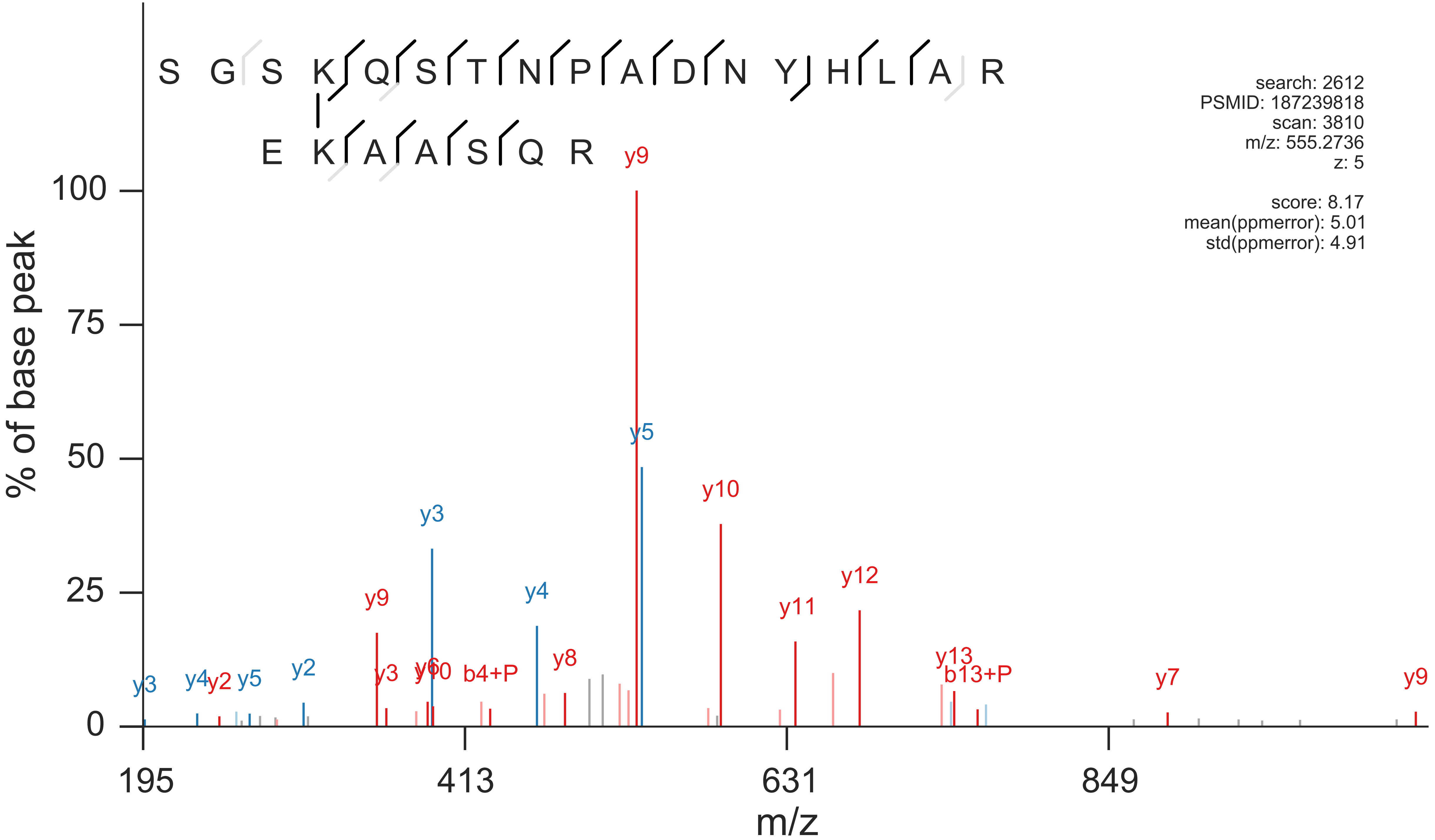

S G S K Q S T N P A D N Y H L A R
E K A A S Q R
search: 2612
PSMID: 187239818
scan: 3810
m/z: 555.2736
z: 5
score: 8.17
mean(ppmerror): 5.01
std(ppmerror): 4.91
% of base peak
100
75
50
25
0
195
413
631
849
m/z
y9
y5
y10
y3
y12
y4
y11
y9
y8
y13
b13+P
y7
y9
y3
y4
y2
y5
y2
y3
y6
y10
b4+P

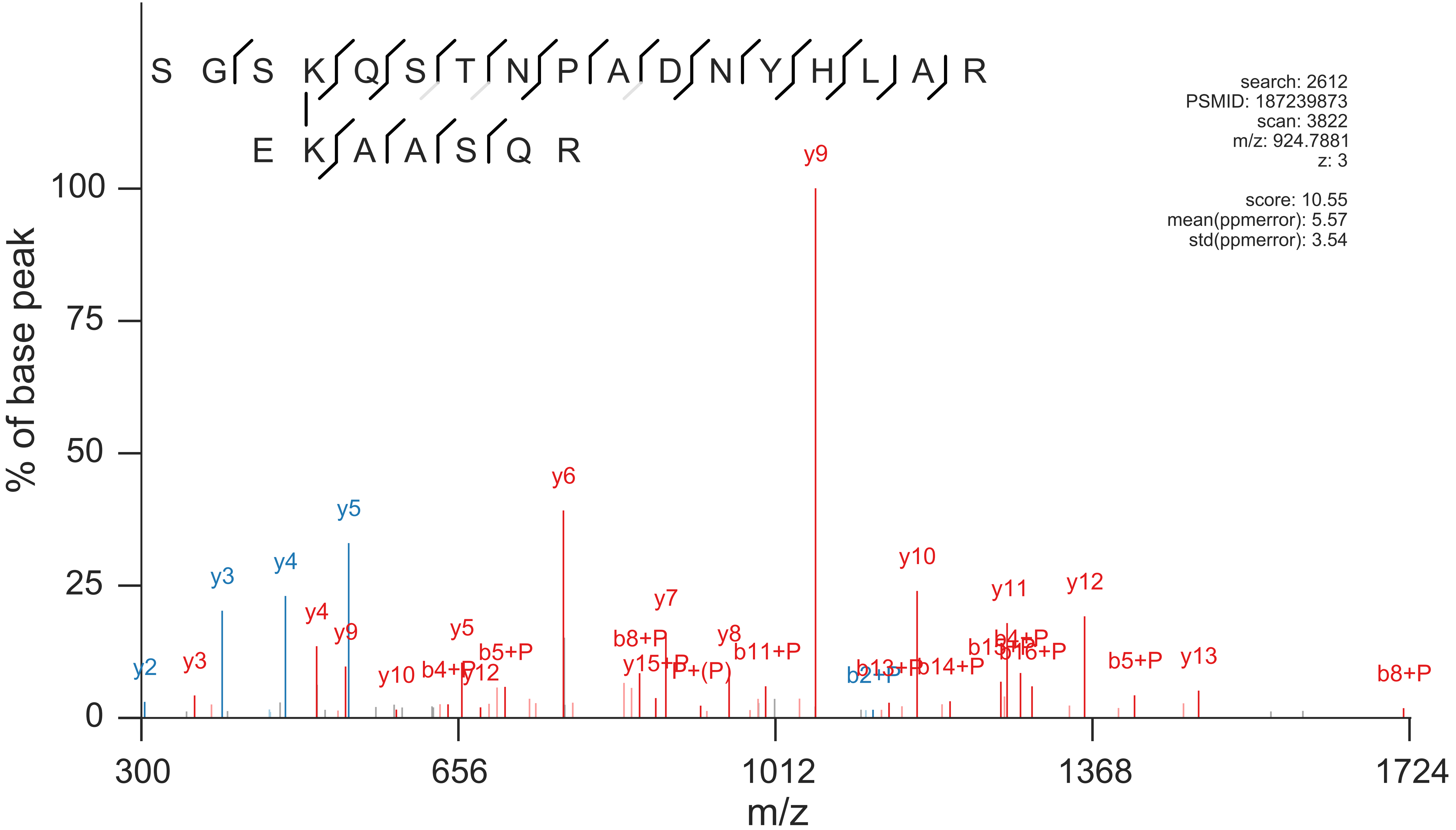

S G S K Q S T N P A D N Y H L A R
E K A A S Q R
search: 2612
PSMID: 187239873
scan: 3822
m/z: 924.7881
z: 3
score: 10.55
mean(ppmerror): 5.57
std(ppmerror): 3.54
% of base peak
m/z

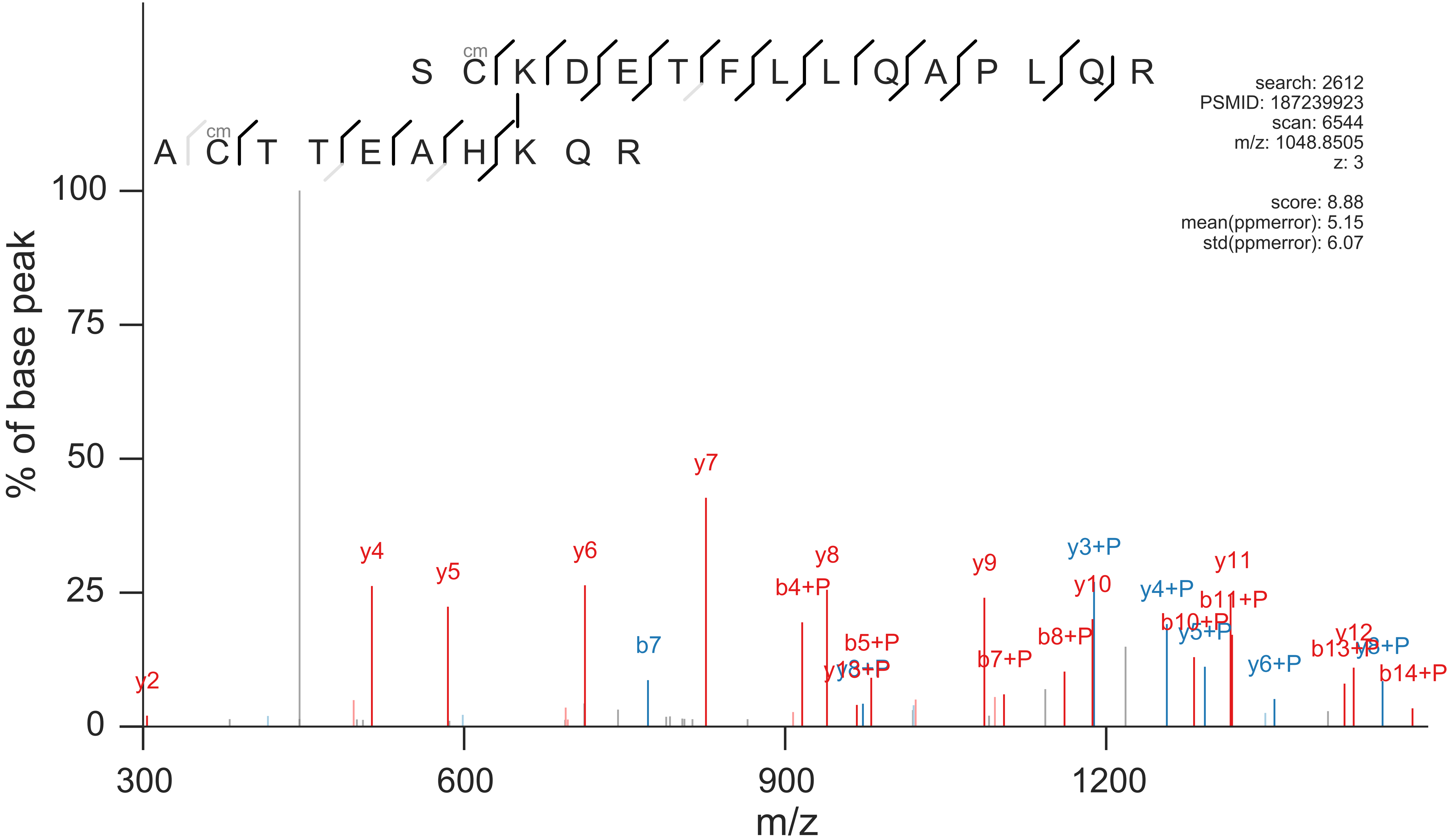

S C K D E T F L L Q A P L Q R
A C T T E A H K Q R
cm
search: 2612
PSMID: 187239923
scan: 6544
m/z: 1048.8505
z: 3
score: 8.88
mean(ppmerror): 5.15
std(ppmerror): 6.07
% of base peak
m/z
100
75
50
25
0
300
600
900
1200
y2
y4
y5
y6
b7
y7
y8
b4+P
b5+P
y9
b7+P
b8+P
y10
y3+P
y4+P
y11
b11+P
b10+P
y5+P
y6+P
y12
b13+P
b14+P

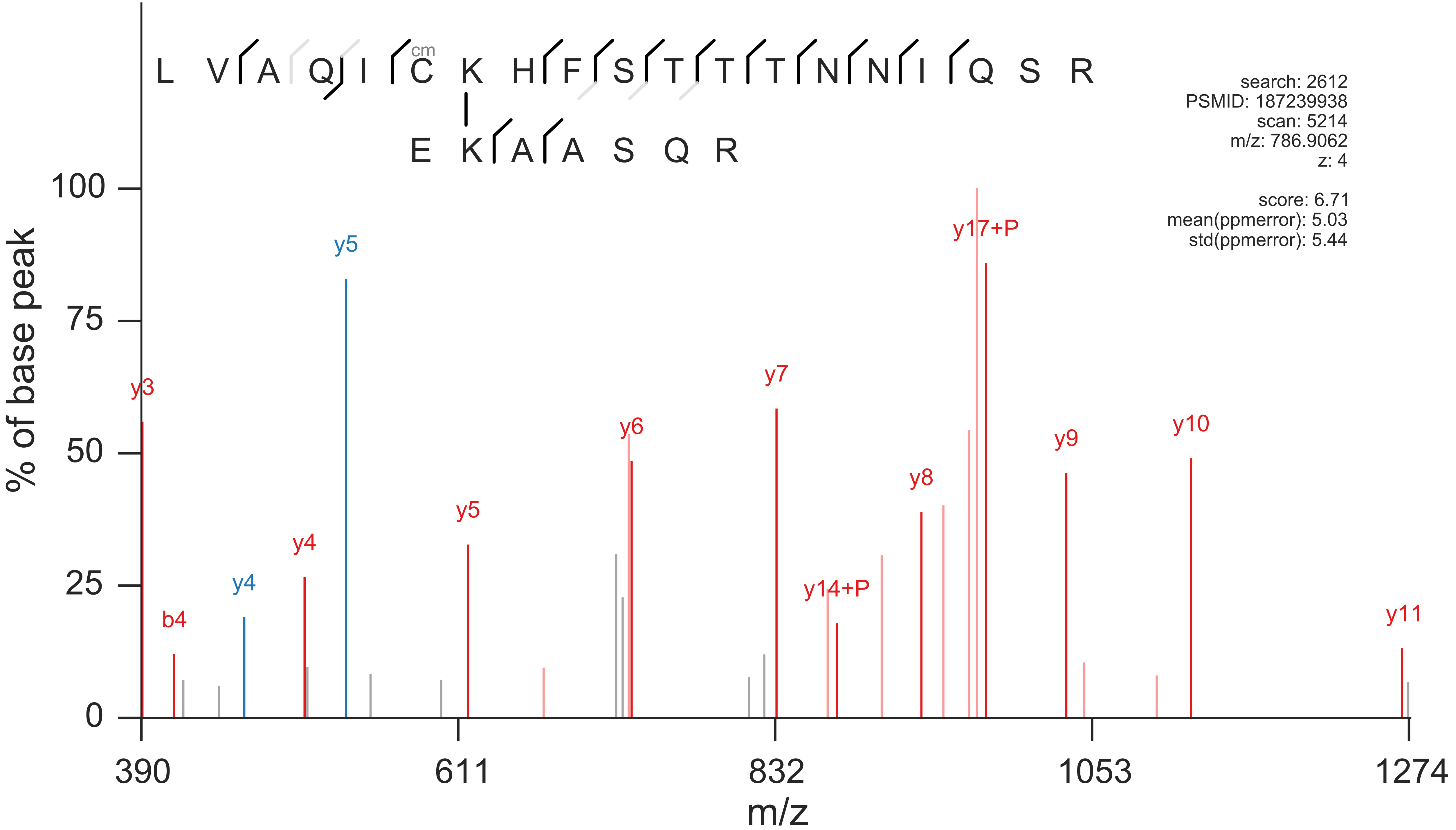

L V A Q I C K H F S T T T N N I Q S R
cm
E K A A S Q R
search: 2612
PSMID: 187239938
scan: 5214
m/z: 786.9062
z: 4
score: 6.71
mean(ppmerror): 5.03
std(ppmerror): 5.44
% of base peak
m/z
y3
b4
y4
y4
y5
y5
y6
y7
y14+P
y8
y17+P
y9
y10
y11
0
25
50
75
100
390
611
832
1053
1274

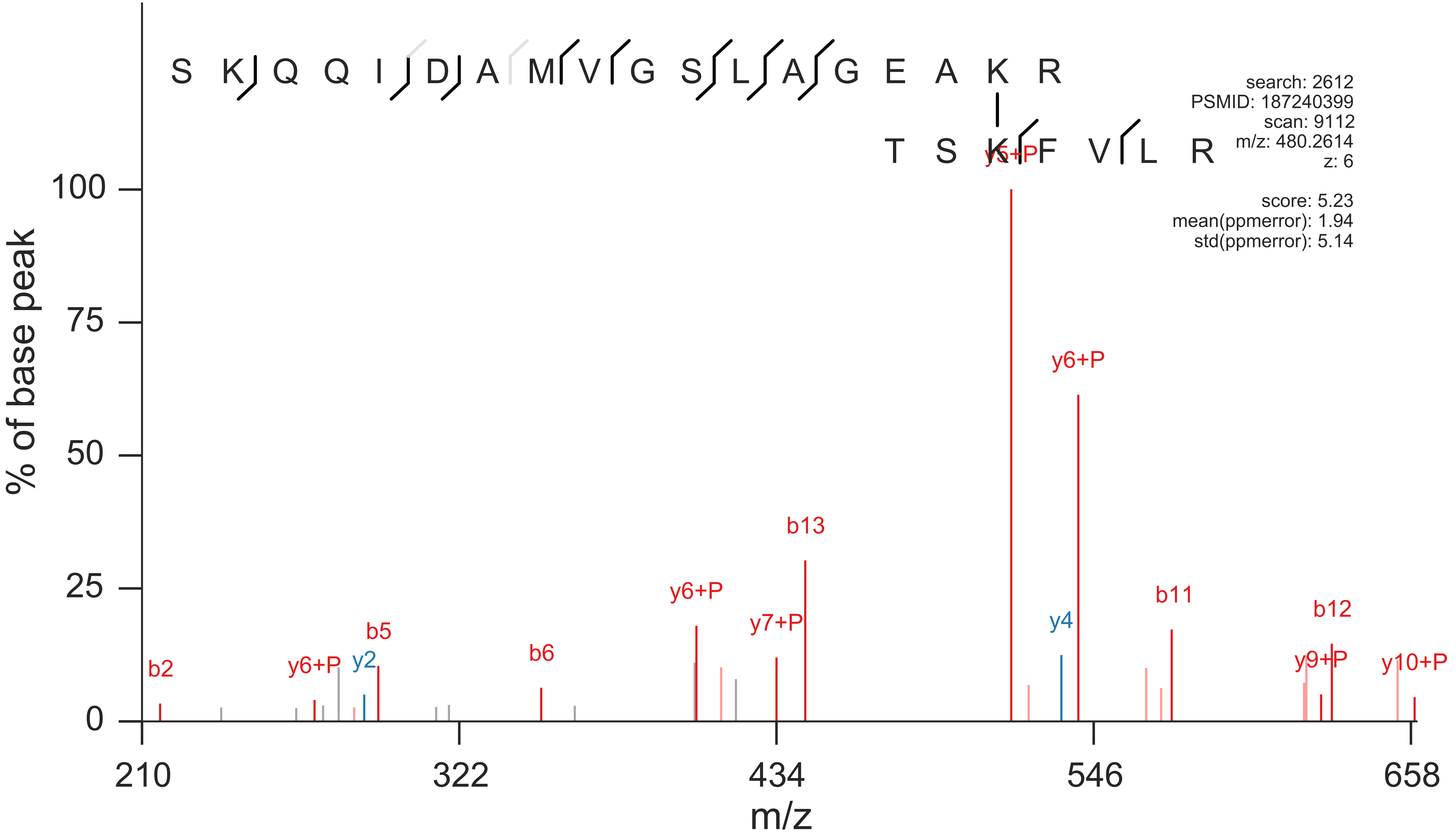

S K Q Q I D A M V G S L A G E A K R
T S K F V L R
search: 2612
PSMID: 187240399
scan: 9112
m/z: 480.2614
z: 6
score: 5.23
mean(ppmerror): 1.94
std(ppmerror): 5.14
% of base peak
m/z
y5+P
y6+P
b13
b11
b12
y4
y6+P
y7+P
b6
b5
y2
y6+P
b2
y9+P
y10+P

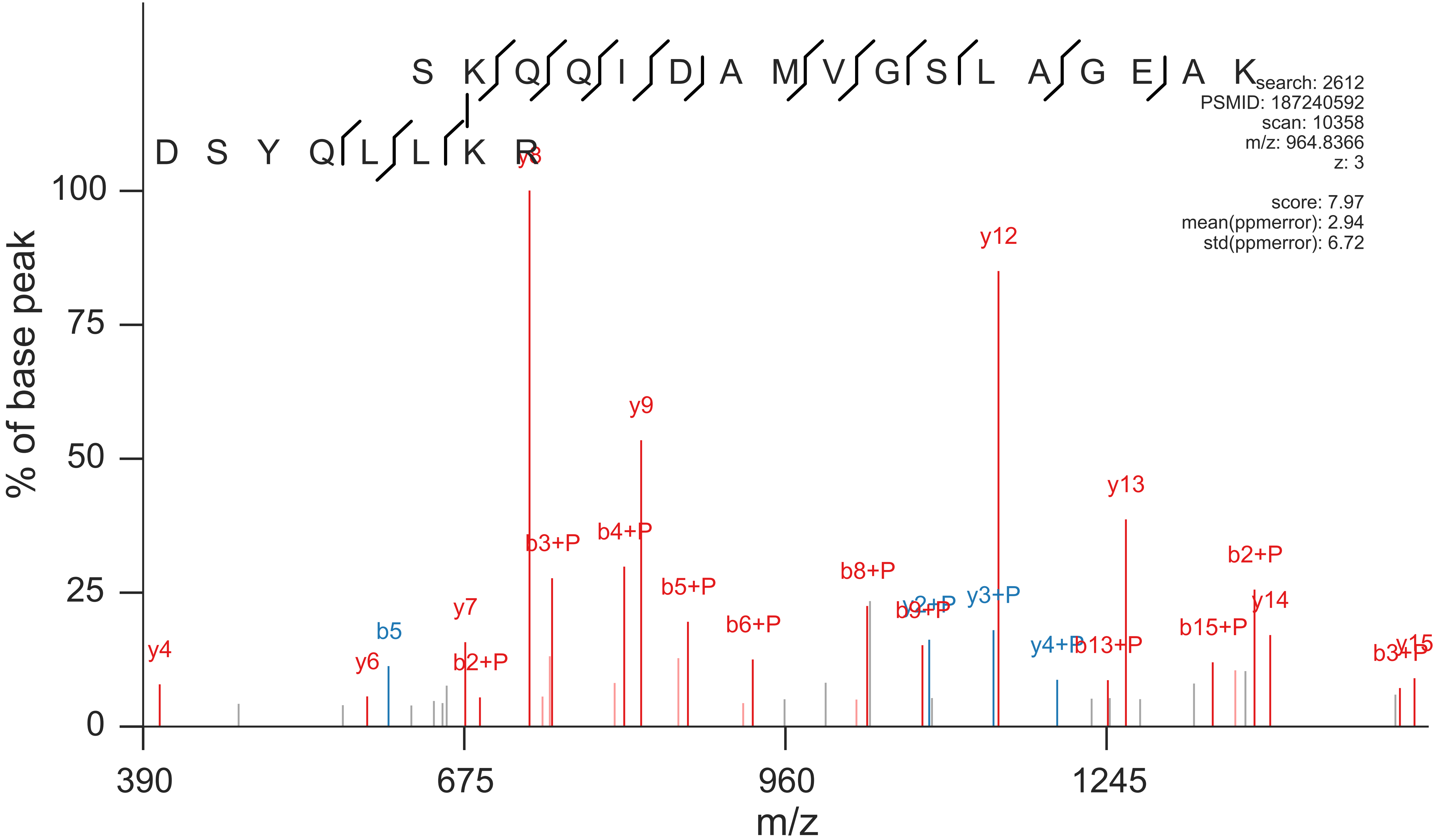

S K Q Q I D A M V G S L A G E A K
D S Y Q L L K R
search: 2612
PSMID: 187240592
scan: 10358
m/z: 964.8366
z: 3
score: 7.97
mean(ppmerror): 2.94
std(ppmerror): 6.72
% of base peak
m/z
y4
y6
b5
y7
b2+P
y8
b3+P
b4+P
y9
b5+P
b6+P
b8+P
b9+P
y2+P
y3+P
y12
y4+P
b13+P
y13
b15+P
b2+P
y14
b3+P
y15
0
25
50
75
100
390
675
960
1245

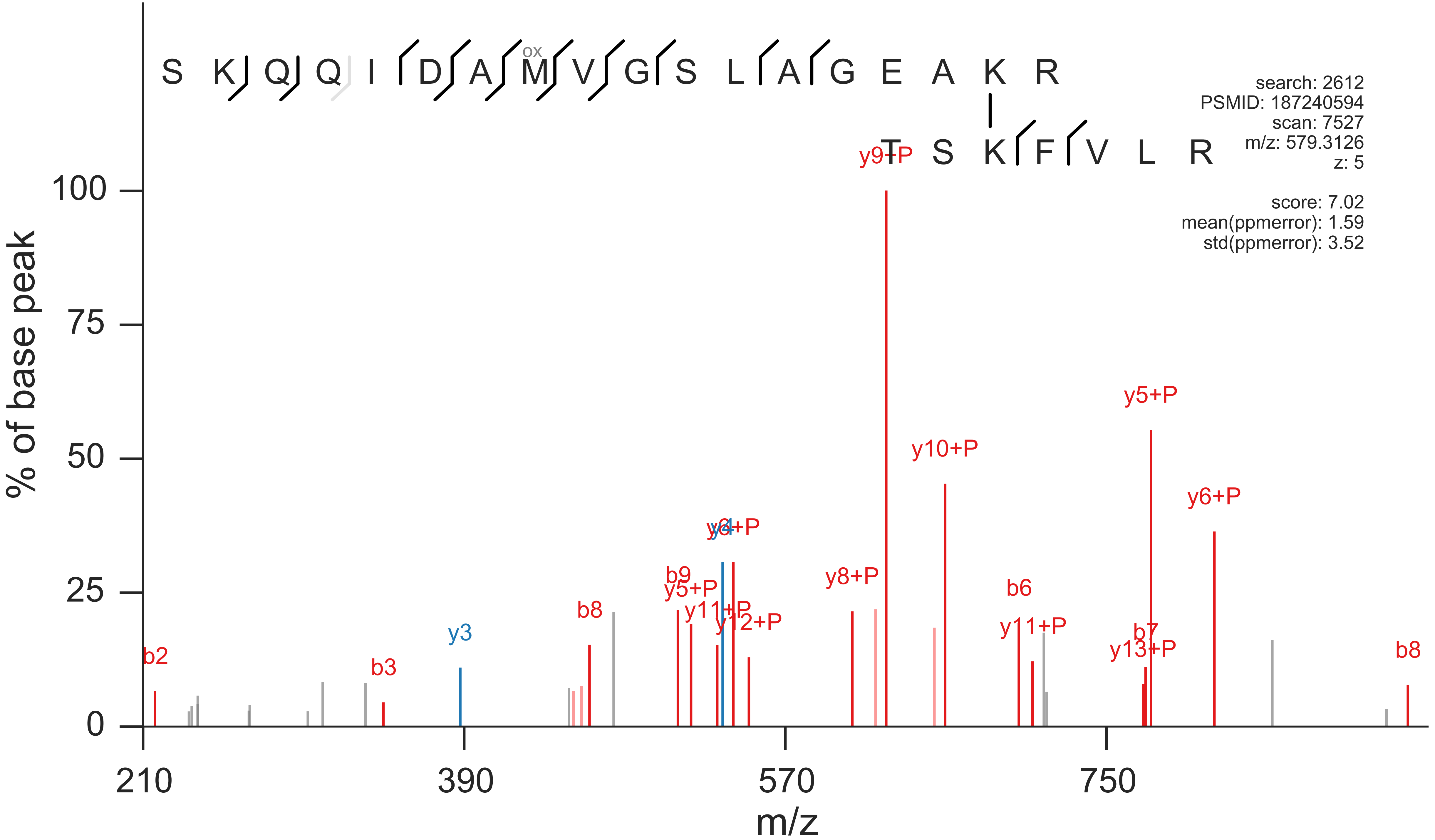

S K Q Q I D A M V G S L A G E A K R
ox
T S K F V L R
search: 2612
PSMID: 187240594
scan: 7527
m/z: 579.3126
z: 5
score: 7.02
mean(ppmerror): 1.59
std(ppmerror): 3.52
% of base peak
m/z
0
25
50
75
100
210
390
570
750
b2
b3
y3
b8
b9
y5+P
y11+P
y12+P
y4
y6+P
y8+P
y9+P
y10+P
b6
y11+P
b7
y13+P
y5+P
y6+P
b8

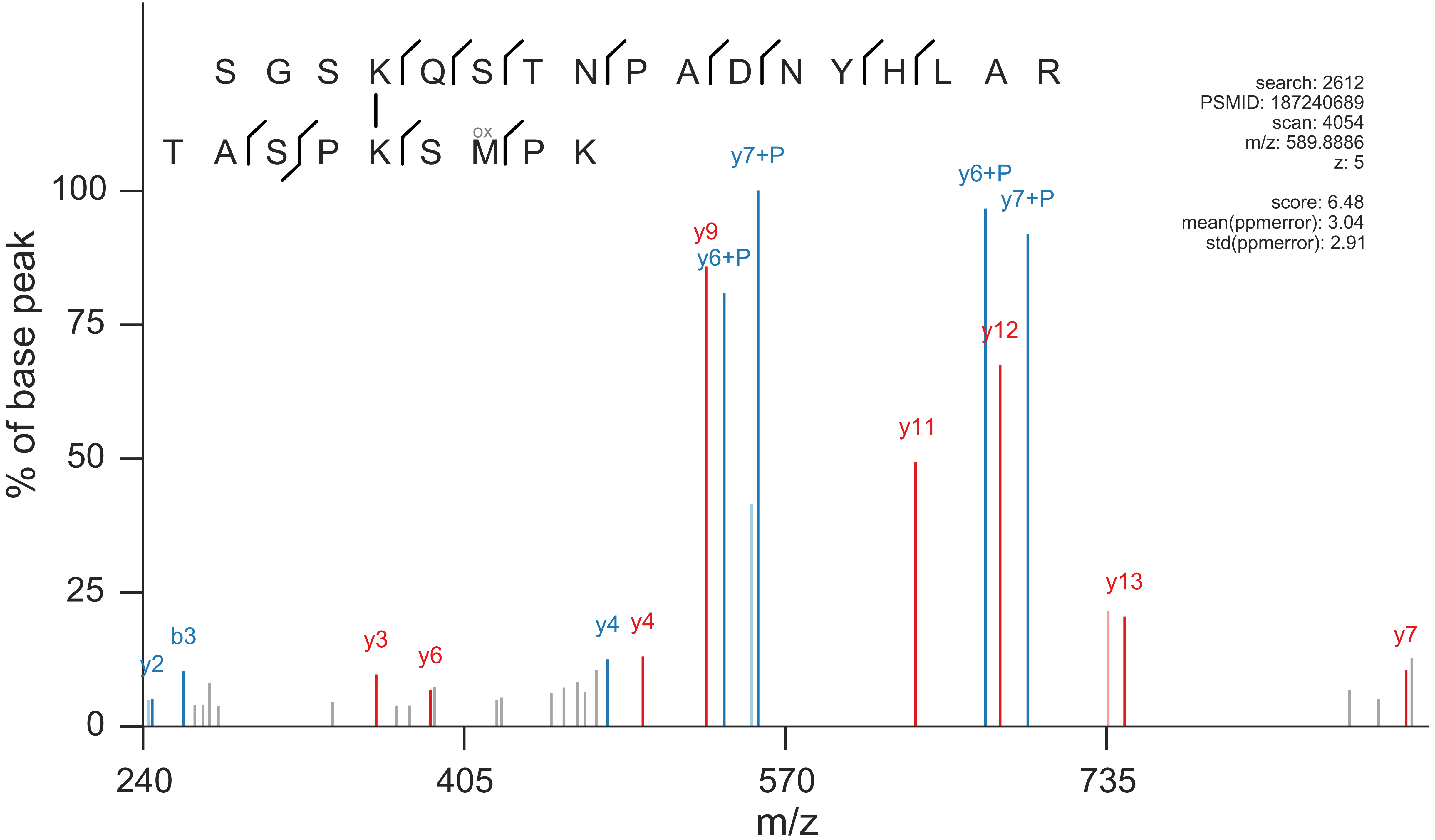

S G S K Q S T N P A D N Y H L A R
T A S P K S M P K
ox
search: 2612
PSMID: 187240689
scan: 4054
m/z: 589.8886
z: 5
score: 6.48
mean(ppmerror): 3.04
std(ppmerror): 2.91
% of base peak
m/z
y2
b3
y3
y6
y4
y4
y9
y6+P
y7+P
y11
y6+P
y12
y7+P
y13
y7
0
25
50
75
100
240
405
570
735

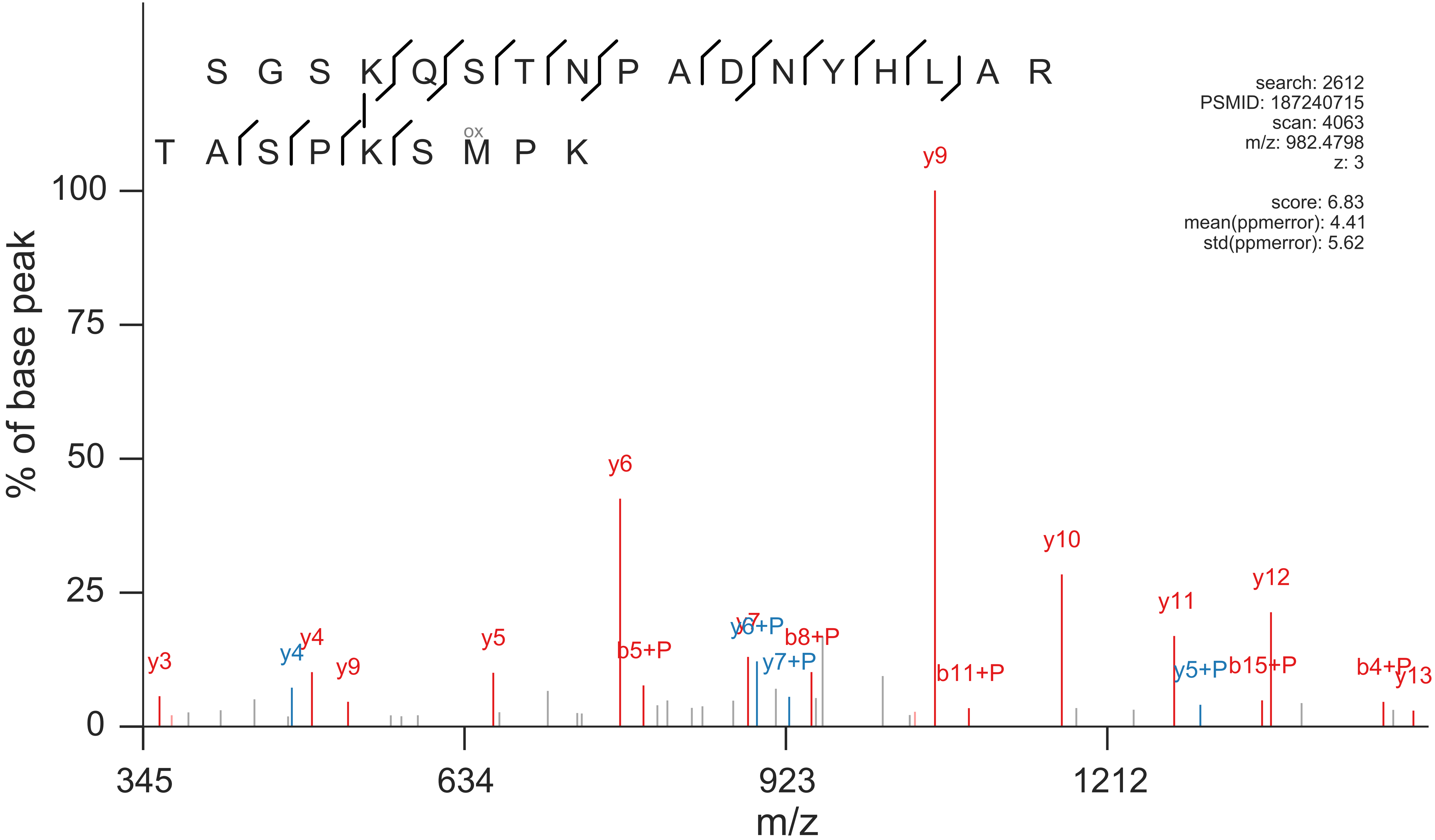

S G S K Q S T N P A D N Y H L A R
T A S P K S M P K
ox
search: 2612
PSMID: 187240715
scan: 4063
m/z: 982.4798
z: 3
score: 6.83
mean(ppmerror): 4.41
std(ppmerror): 5.62
% of base peak
100
75
50
25
0
y9
y6
y10
y12
y11
y3
y4
y4
y9
y5
b5+P
y7
y6+P
b8+P
y7+P
b11+P
y5+P
b15+P
b4+P
y13
345
634
923
1212
m/z

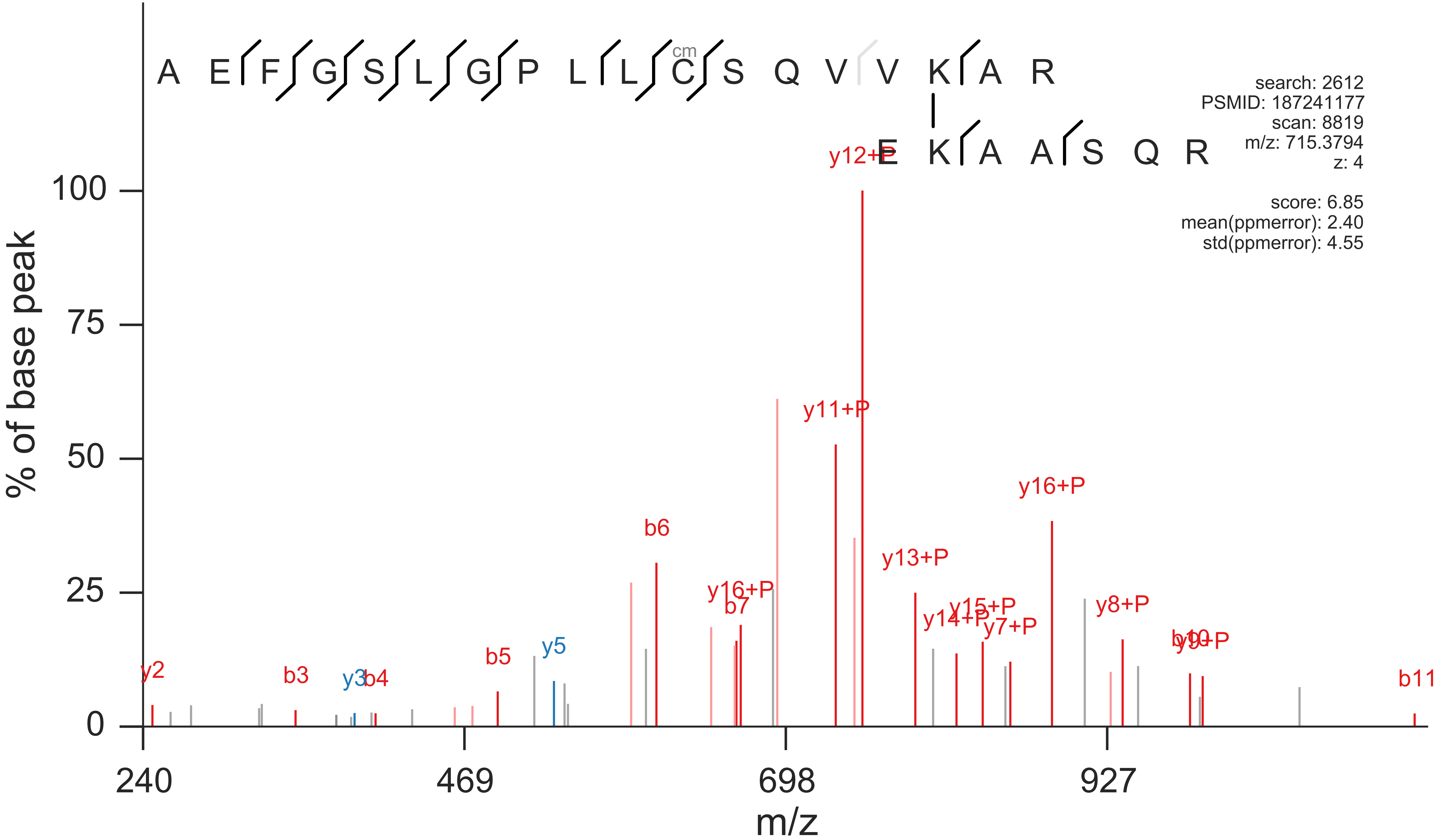

A E F G S L G P L L C S Q V V K A R
cm
E K A A S Q R
search: 2612
PSMID: 187241177
scan: 8819
m/z: 715.3794
z: 4
score: 6.85
mean(ppmerror): 2.40
std(ppmerror): 4.55
% of base peak
0
25
50
75
100
m/z
240
469
698
927
y2
b3
y3
b4
b5
y5
b6
y16+P
b7
y11+P
y12+P
y13+P
y14+P
y15+P
y7+P
y16+P
y8+P
b10
y9+P
b11

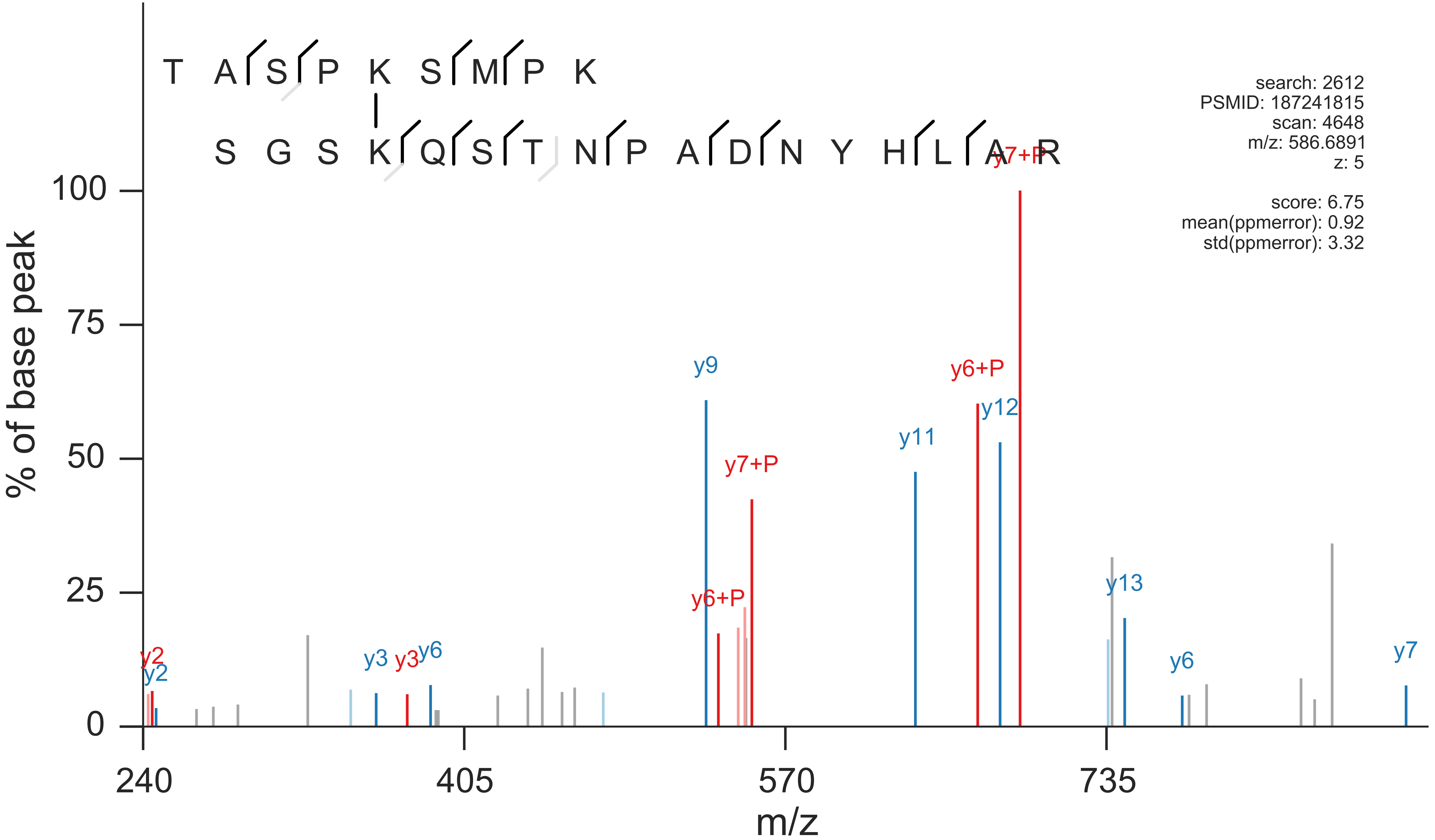

T A S P K S M P K
S G S K Q S T N P A D N Y H L A R
search: 2612
PSMID: 187241815
scan: 4648
m/z: 586.6891
z: 5
score: 6.75
mean(ppmerror): 0.92
std(ppmerror): 3.32
% of base peak
m/z
y9
y6+P
y12
y11
y7+P
y13
y2
y3
y6
y7

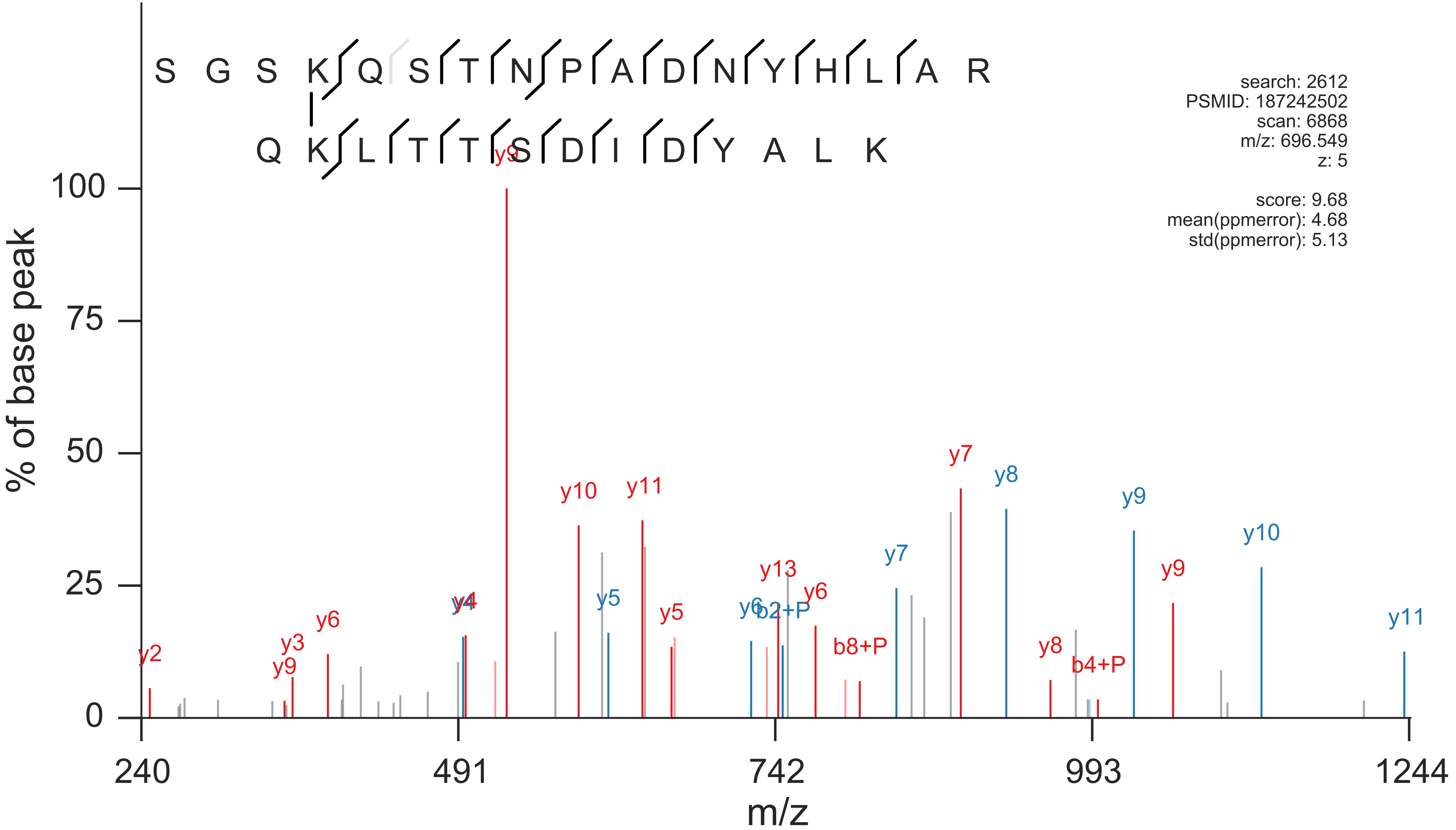

S G S K Q S T N P A D N Y H L A R
Q K L T T S D I D Y A L K
search: 2612
PSMID: 187242502
scan: 6868
m/z: 696.549
z: 5
score: 9.68
mean(ppmerror): 4.68
std(ppmerror): 5.13
% of base peak
m/z

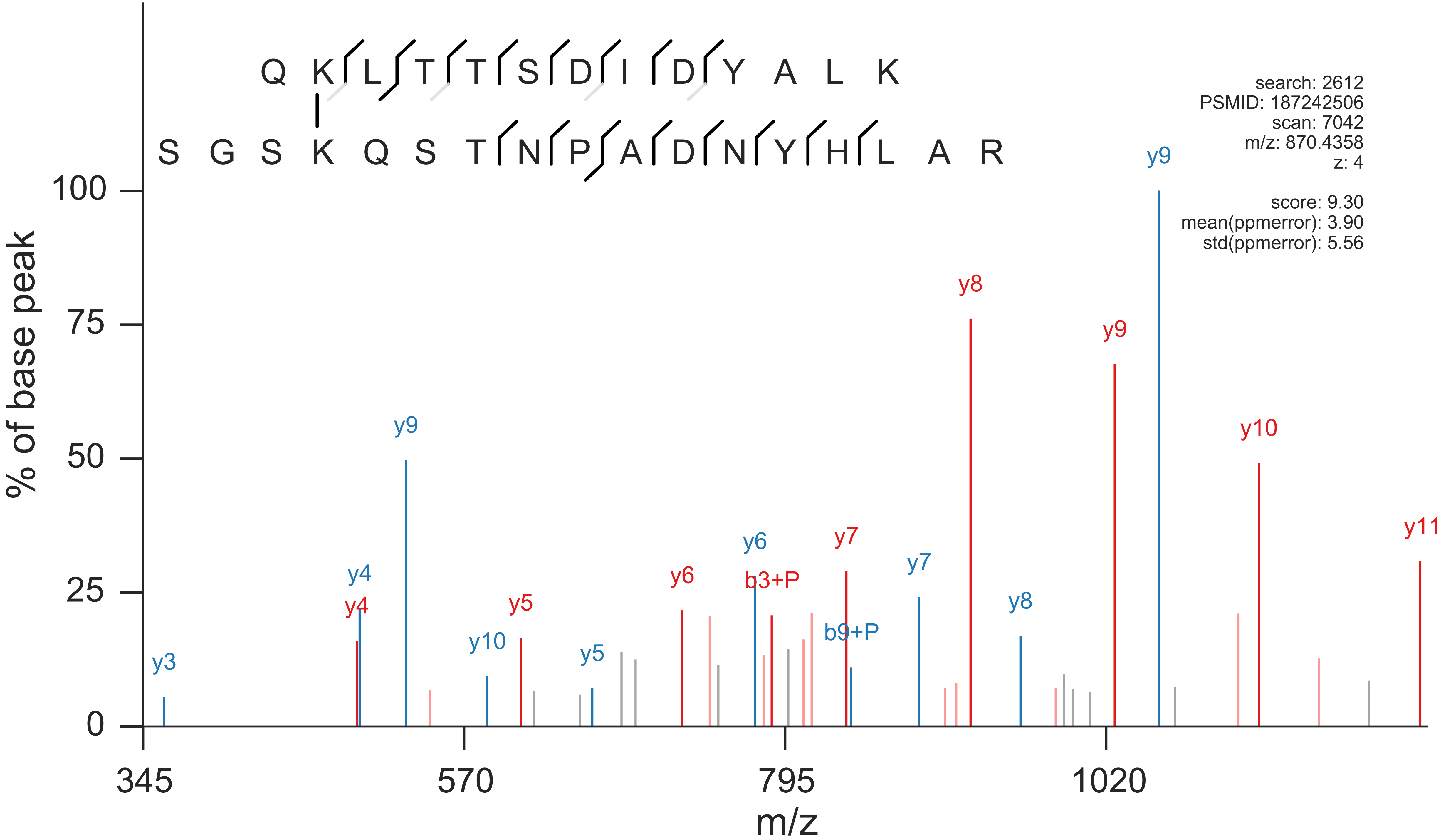

Q K L T T S D I D Y A L K
S G S K Q S T N P A D N Y H L A R
search: 2612
PSMID: 187242506
scan: 7042
m/z: 870.4358
z: 4
score: 9.30
mean(ppmerror): 3.90
std(ppmerror): 5.56
% of base peak
m/z
100
75
50
25
0
345
570
795
1020
y3
y4
y4
y9
y10
y5
y5
y6
y6
b3+P
y7
b9+P
y7
y8
y8
y9
y9
y10
y11

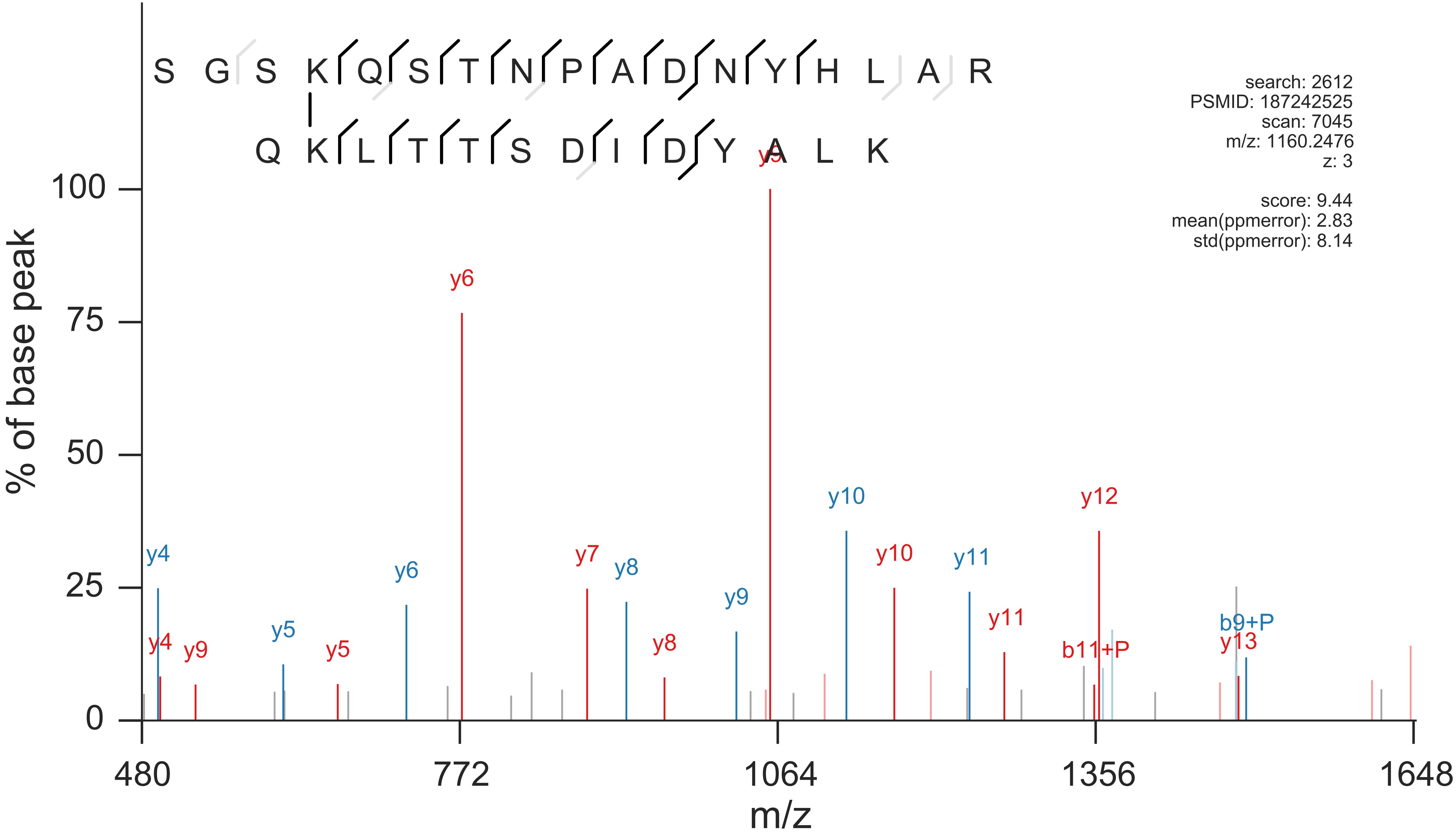

S G S K Q S T N P A D N Y H L A R
Q K L T T S D I D Y A L K
search: 2612
PSMID: 187242525
scan: 7045
m/z: 1160.2476
z: 3
score: 9.44
mean(ppmerror): 2.83
std(ppmerror): 8.14
% of base peak
m/z
0
25
50
75
100
480
772
1064
1356
1648
y6
y10
y12
y4
y6
y7
y8
y10
y11
y9
y11
y4
y9
y5
y5
y8
b11+P
b9+P
y13

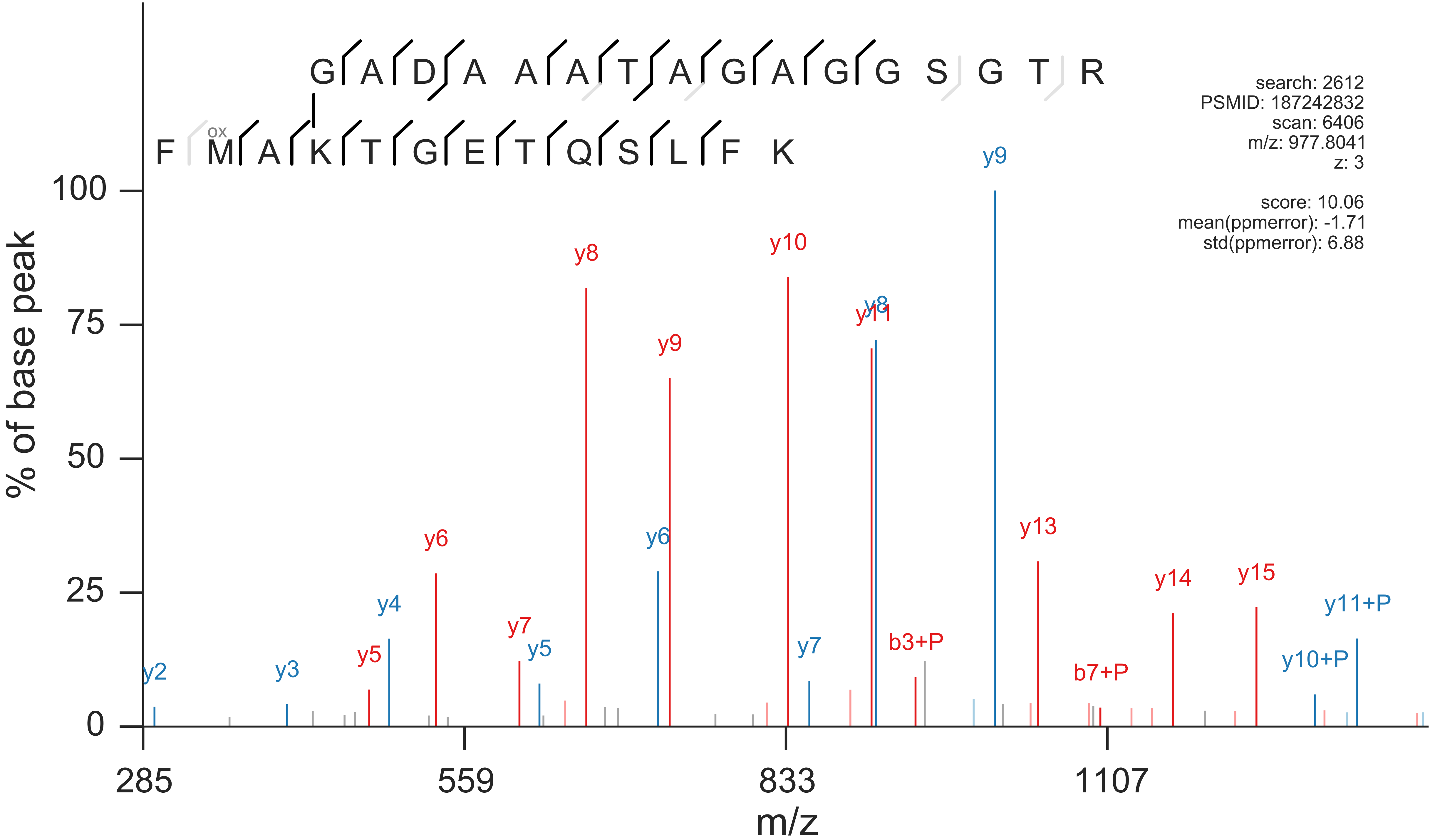

G A D A A A T A G A G G S G T R
F oxM A K T G E T Q S L F K
search: 2612
PSMID: 187242832
scan: 6406
m/z: 977.8041
z: 3
score: 10.06
mean(ppmerror): -1.71
std(ppmerror): 6.88
% of base peak
m/z
0
25
50
75
100
285
559
833
1107
y2
y3
y5
y4
y6
y7
y5
y8
y6
y9
y10
y7
y11
y8
b3+P
y9
y13
b7+P
y14
y15
y10+P
y11+P

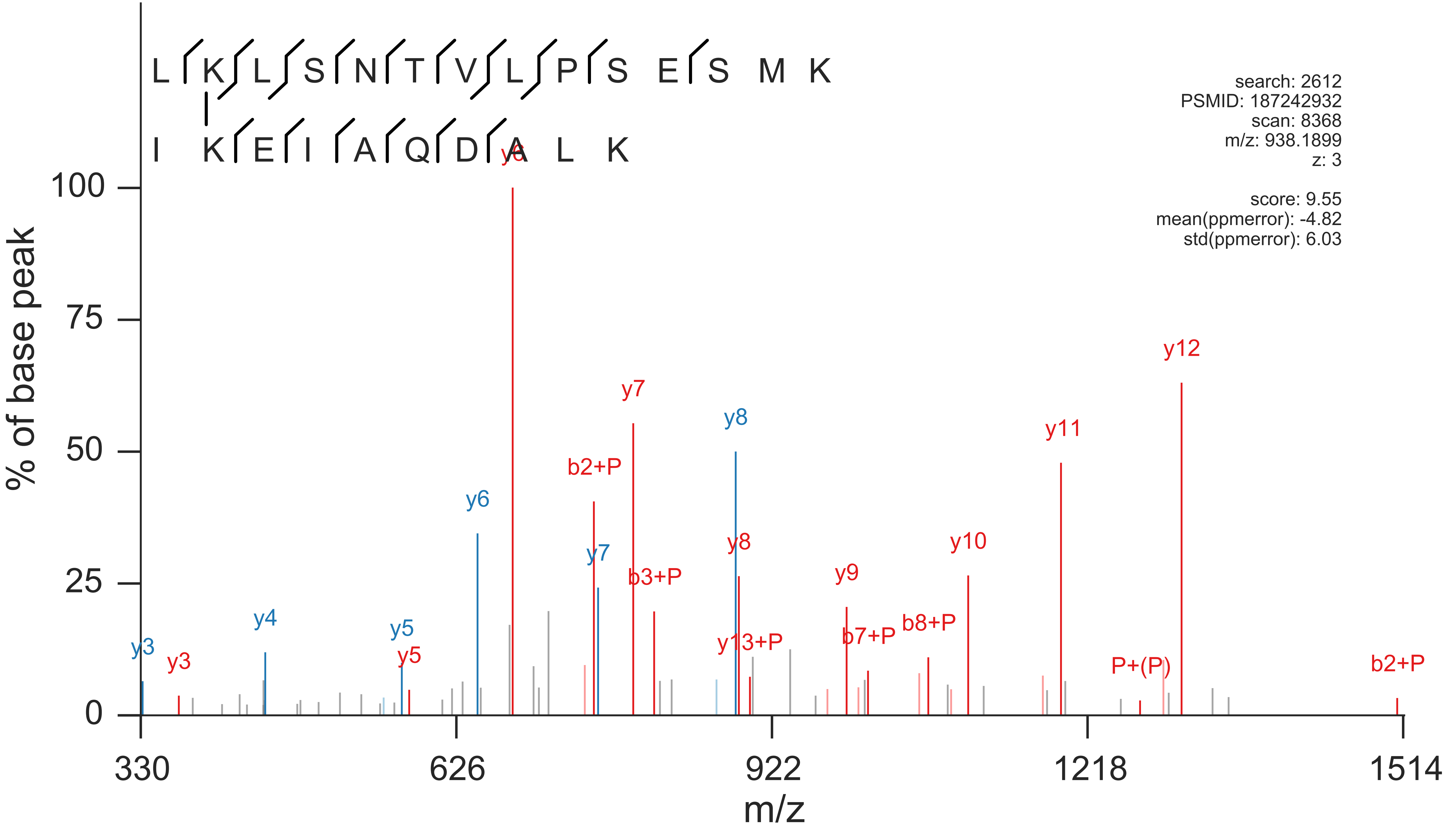

L K L S N T V L P S E S M K
I K E I A Q D A L K
search: 2612
PSMID: 187242932
scan: 8368
m/z: 938.1899
z: 3
score: 9.55
mean(ppmerror): -4.82
std(ppmerror): 6.03
% of base peak
m/z
100
75
50
25
0
330
626
922
1218
1514
y3
y3
y4
y5
y5
y6
y6
b2+P
y7
y7
b3+P
y8
y8
y13+P
y9
b7+P
b8+P
y10
y11
P+(P)
y12
b2+P

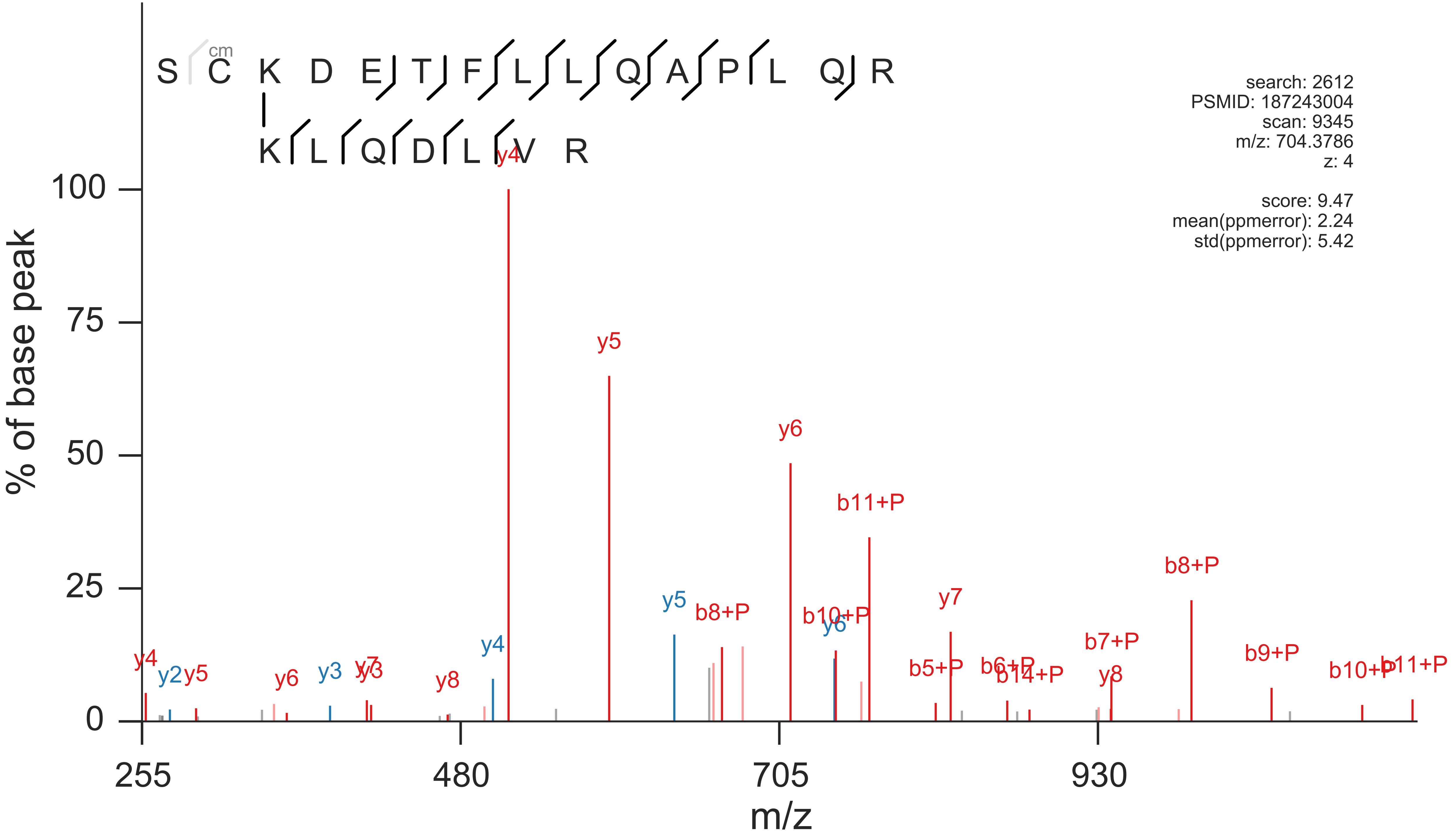

S C K D E T F L L Q A P L Q R
cm
K L Q D L V R
search: 2612
PSMID: 187243004
scan: 9345
m/z: 704.3786
z: 4
score: 9.47
mean(ppmerror): 2.24
std(ppmerror): 5.42
% of base peak
m/z
0
25
50
75
100
255
480
705
930
y4
y5
y6
b11+P
y7
b8+P
y5
b8+P
b10+P
y6
y4
y2
y3
y7
y3
y8
b5+P
b6+P
b14+P
b7+P
y8
b9+P
b10+P
b11+P

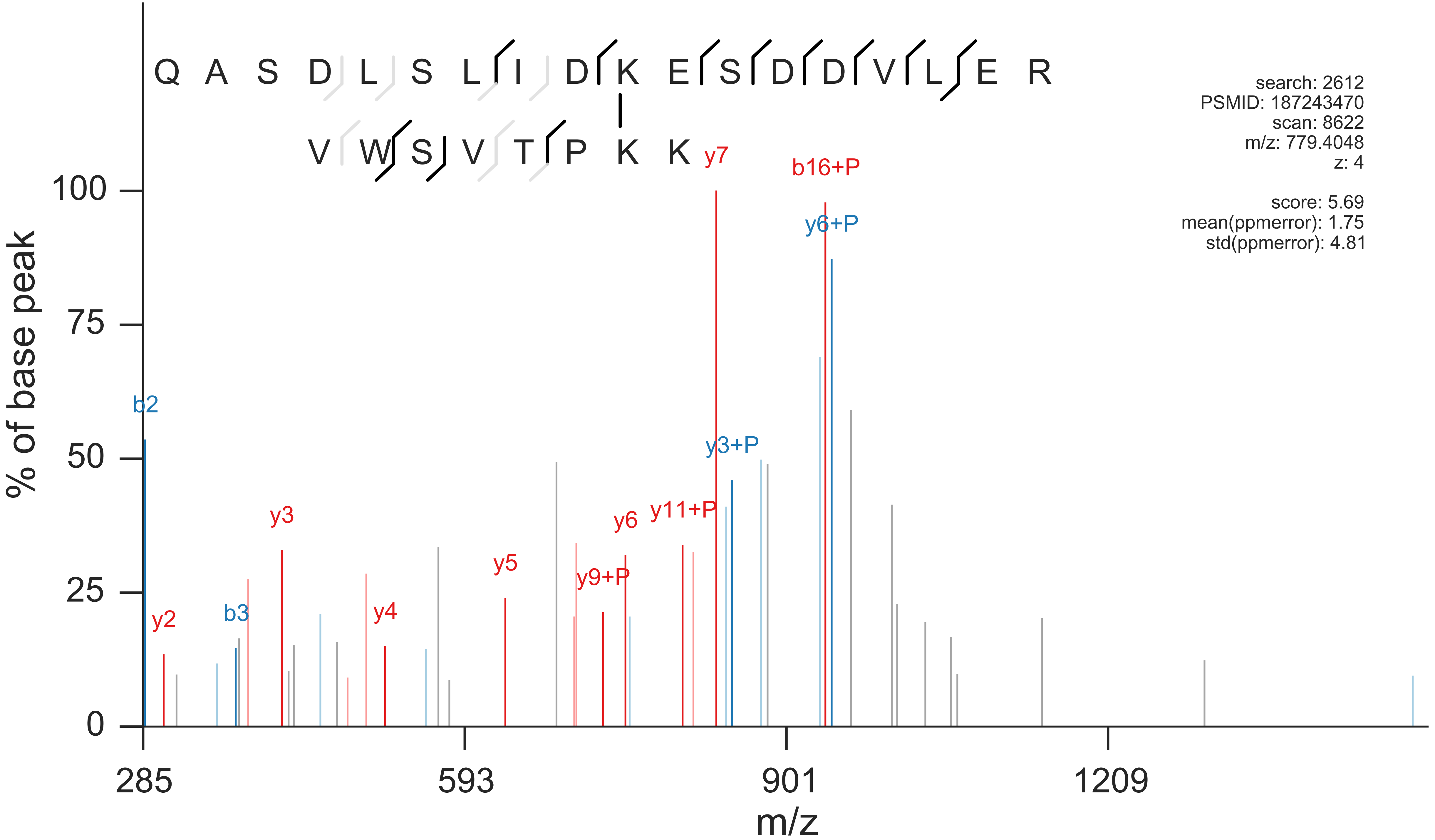
Q A S D L S L I D K E S D D V L E R
V W S V T P K K
search: 2612
PSMID: 187243470
scan: 8622
m/z: 779.4048
z: 4
score: 5.69
mean(ppmerror): 1.75
std(ppmerror): 4.81
% of base peak
m/z
0
25
50
75
100
285
593
901
1209
b2
y2
b3
y3
y4
y5
y9+P
y6
y11+P
y7
y3+P
b16+P
y6+P

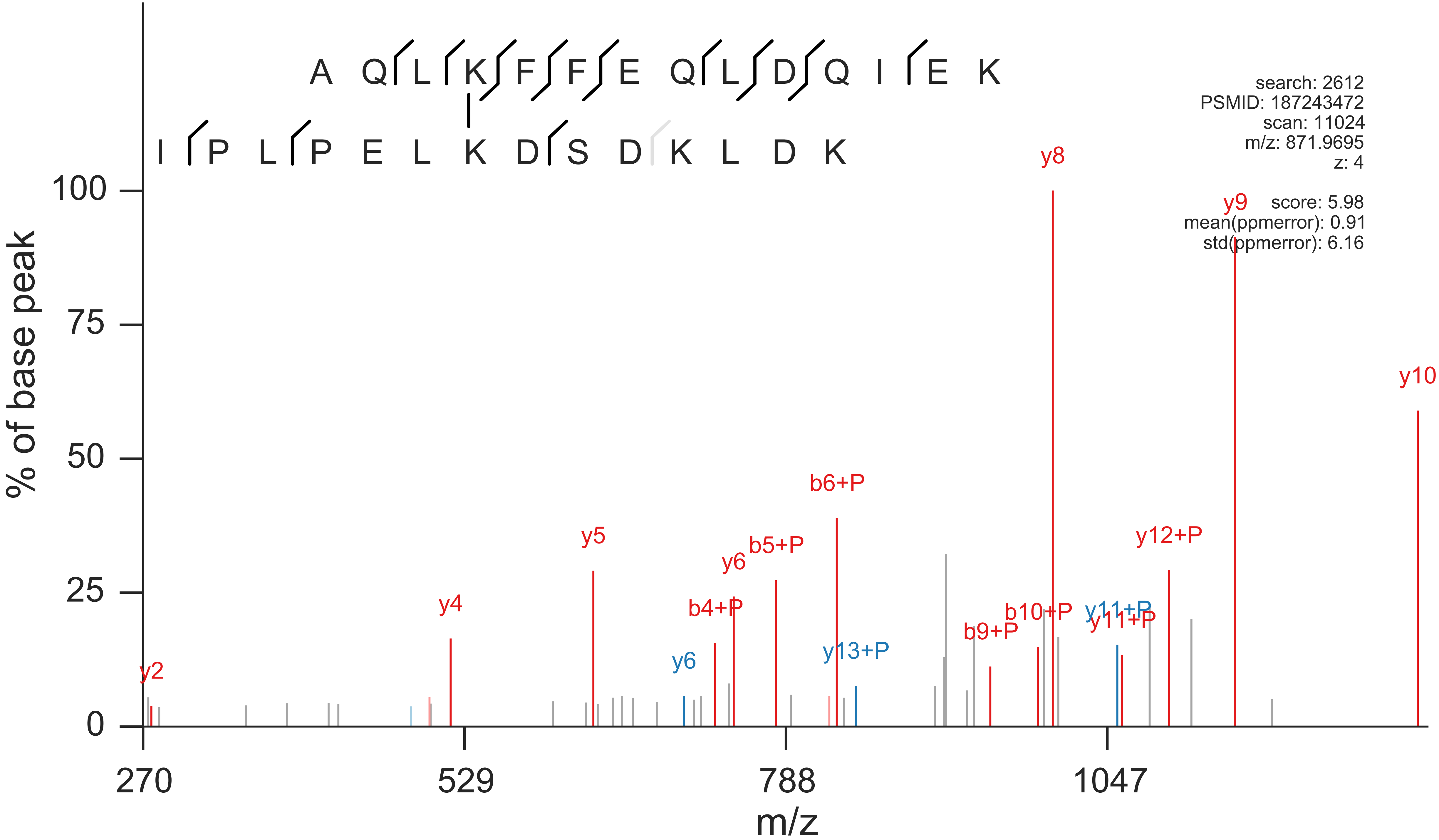

A Q L K F F E Q L D Q I E K
I P L P E L K D S D K L D K
search: 2612
PSMID: 187243472
scan: 11024
m/z: 871.9695
z: 4
score: 5.98
mean(ppmerror): 0.91
std(ppmerror): 6.16
% of base peak
m/z
y2
y4
y5
y6
b4+P
y6
b5+P
b6+P
y13+P
b9+P
b10+P
y8
y11+P
y11+P
y12+P
y9
y10
0
25
50
75
100
270
529
788
1047

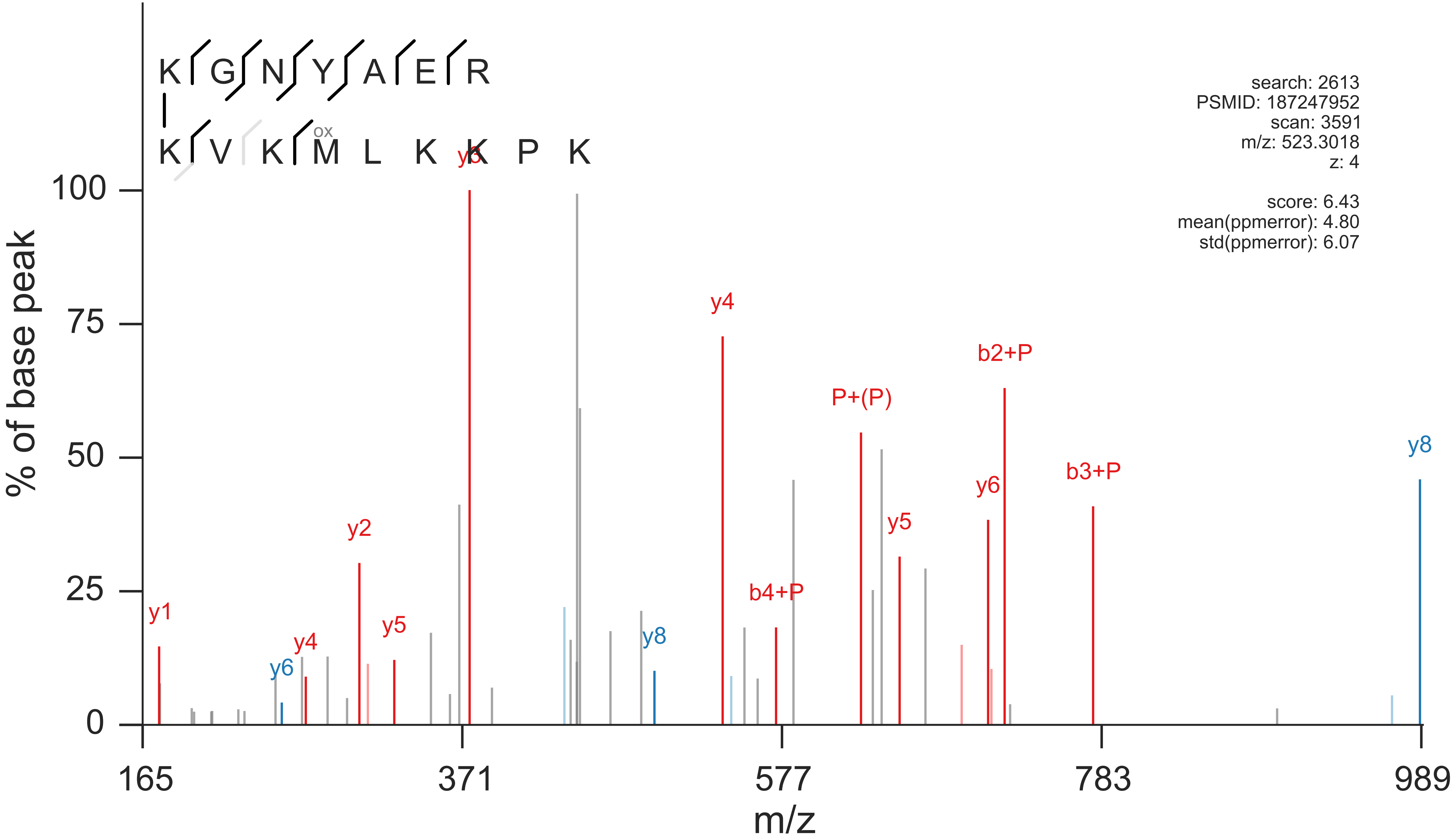

K G N Y A E R
K V K M L K K P K
ox
search: 2613
PSMID: 187247952
scan: 3591
m/z: 523.3018
z: 4
score: 6.43
mean(ppmerror): 4.80
std(ppmerror): 6.07
% of base peak
m/z
100
75
50
25
0
165
371
577
783
989
y1
y6
y4
y2
y5
y3
y8
y4
b4+P
P+(P)
y5
y6
b2+P
b3+P
y8

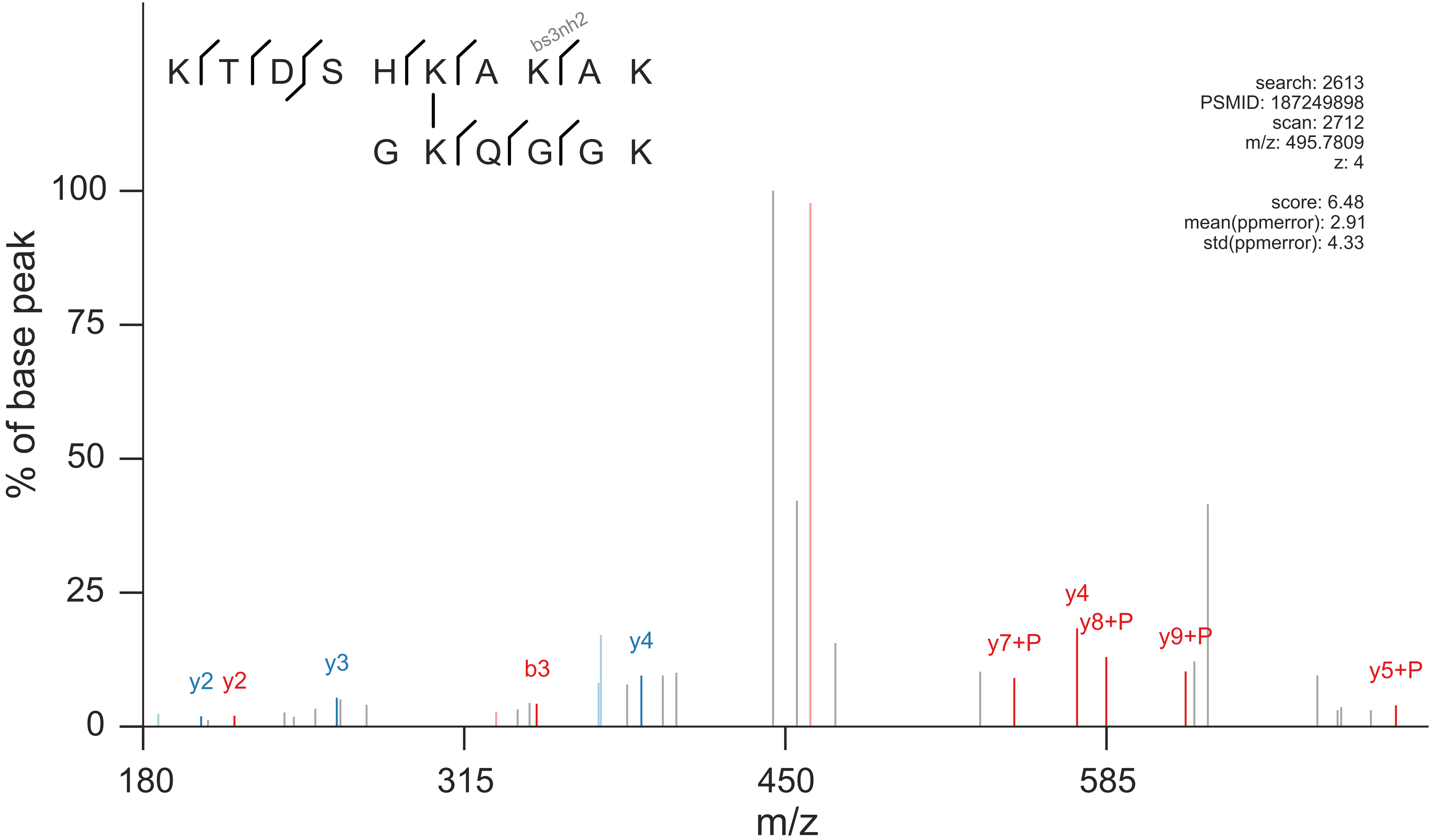

K T D S H K A K A K
bs3nh2
G K Q G G K
search: 2613
PSMID: 187249898
scan: 2712
m/z: 495.7809
z: 4
score: 6.48
mean(ppmerror): 2.91
std(ppmerror): 4.33
% of base peak
100
75
50
25
0
y2 y2
y3
b3
y4
y7+P
y4
y8+P
y9+P
y5+P
180
315
450
585
m/z

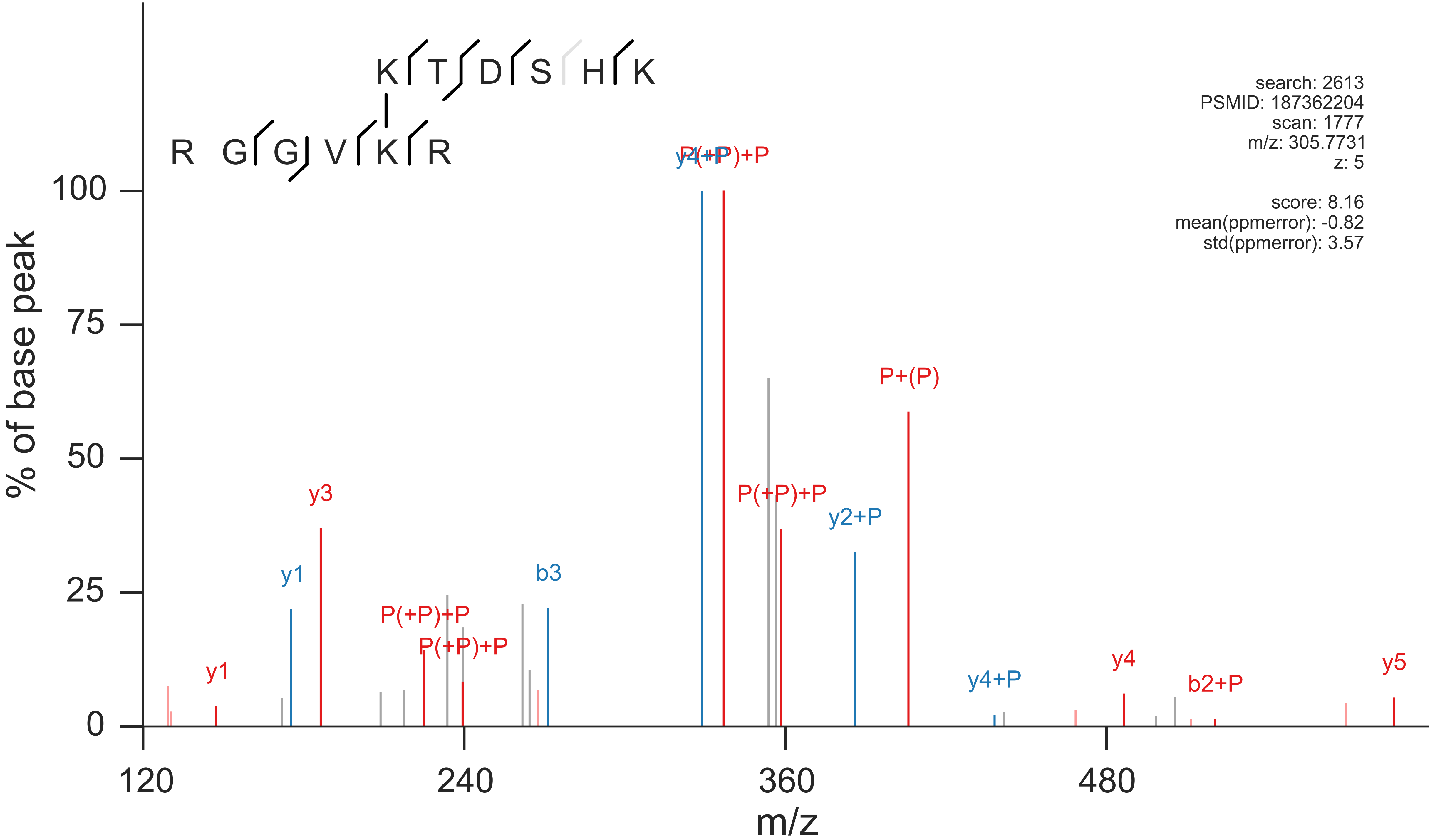

K T D S H K
R G G V K R
search: 2613
PSMID: 187362204
scan: 1777
m/z: 305.7731
z: 5
score: 8.16
mean(ppmerror): -0.82
std(ppmerror): 3.57
% of base peak
m/z
0
25
50
75
100
120
240
360
480
y1
y1
y3
P(+P)+P
P(+P)+P
b3
y4+P
P(+P)+P
P(+P)+P
y2+P
P+(P)
y4+P
y4
b2+P
y5

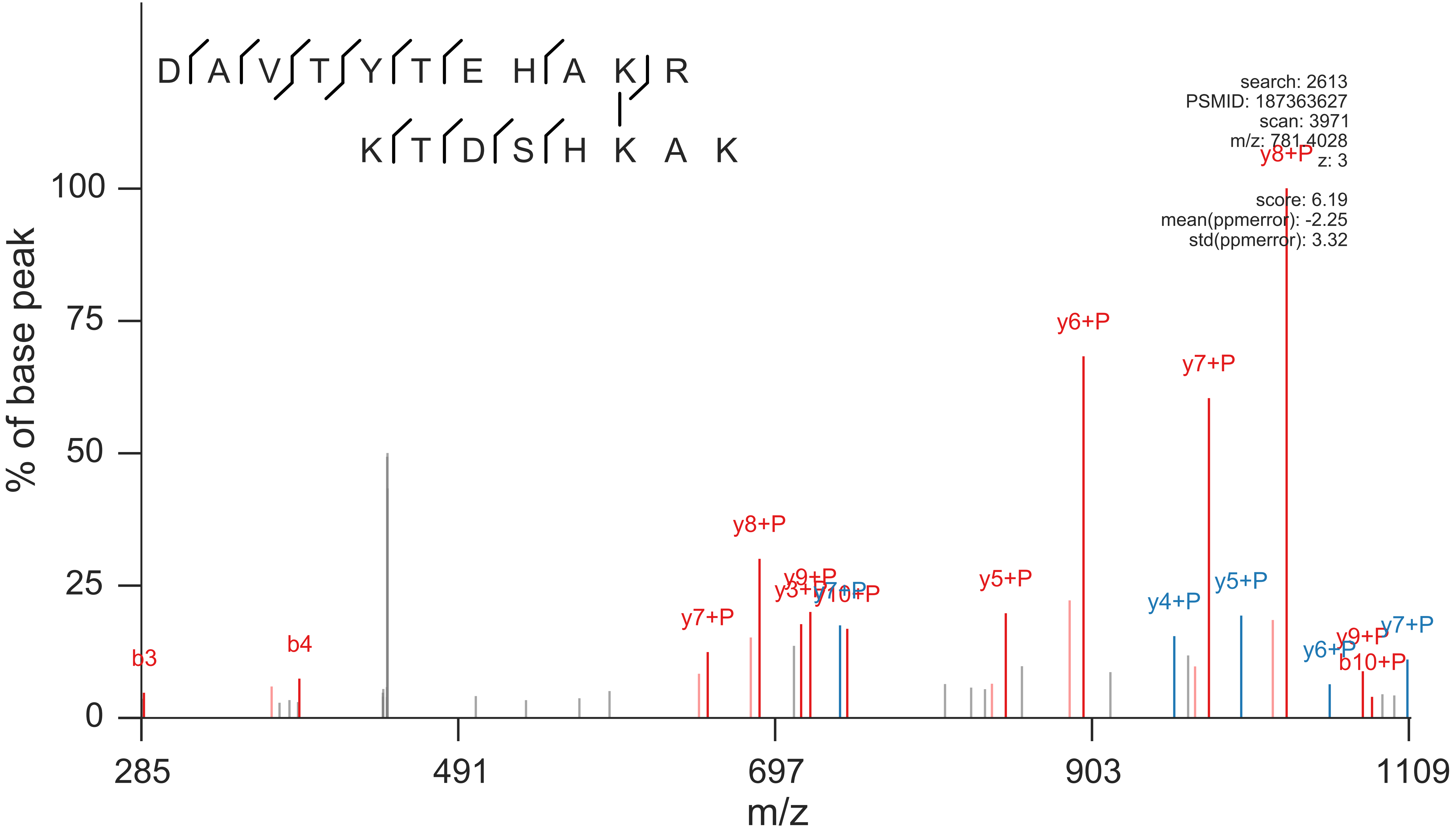

D A V T Y T E H A K R
K T D S H K A K
search: 2613
PSMID: 187363627
scan: 3971
m/z: 781.4028
z: 3
score: 6.19
mean(ppmerror): -2.25
std(ppmerror): 3.32
% of base peak
100
75
50
25
0
285
491
697
903
1109
m/z
b3
b4
y7+P
y8+P
y3+P
y9+P
y7+P
y10+P
y5+P
y6+P
y7+P
y4+P
y5+P
y8+P
y6+P
y9+P
b10+P
y7+P

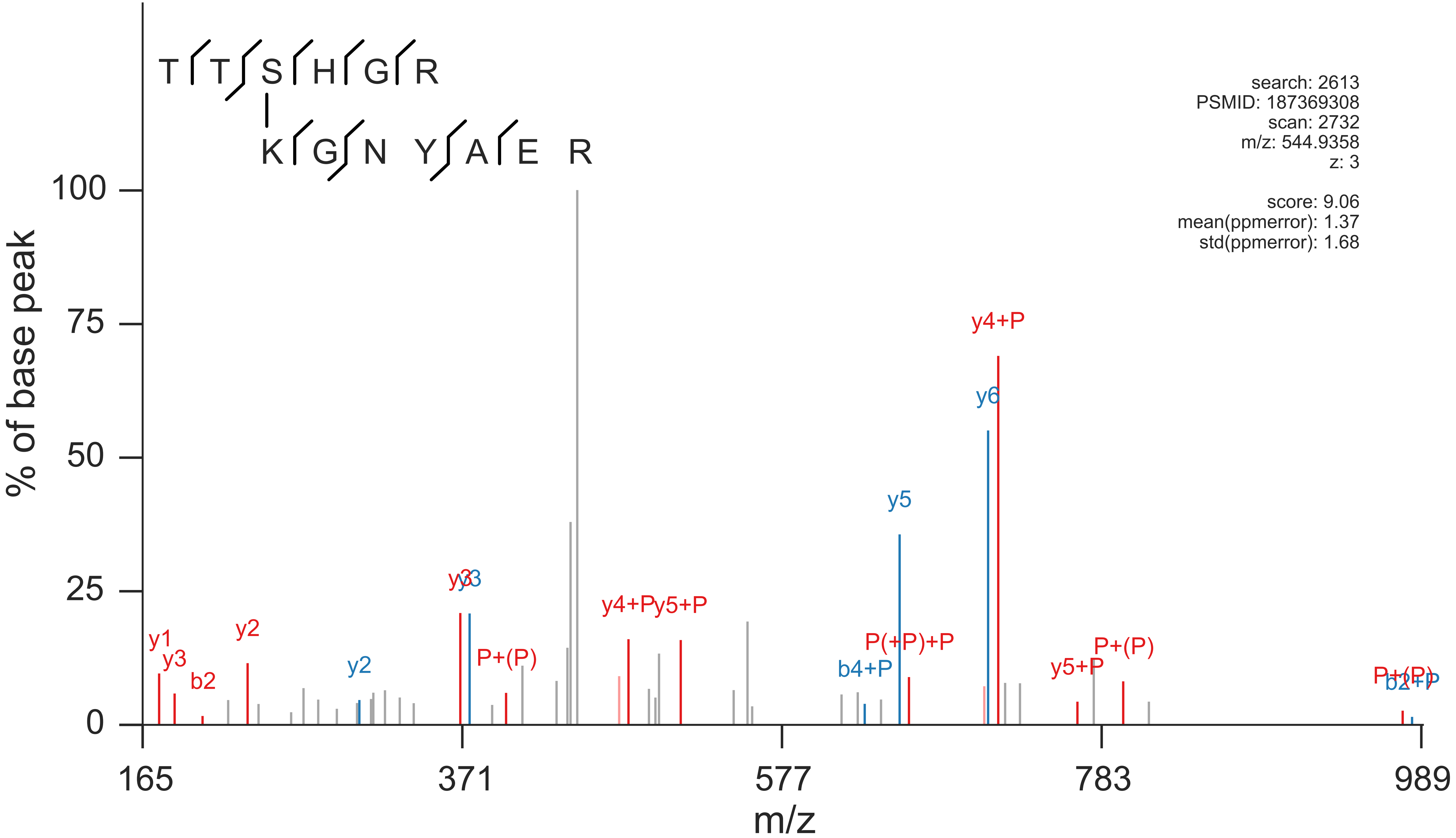

T T S H G R
K G N Y A E R
search: 2613
PSMID: 187369308
scan: 2732
m/z: 544.9358
z: 3
score: 9.06
mean(ppmerror): 1.37
std(ppmerror): 1.68
% of base peak
100
75
50
25
0
165
371
577
783
989
m/z
y1
y3
b2
y2
y2
y3
y3
P+(P)
y4+P
y5+P
b4+P
P(+P)+P
y5
y6
y4+P
y5+P
P+(P)
P+(P)
b2+P

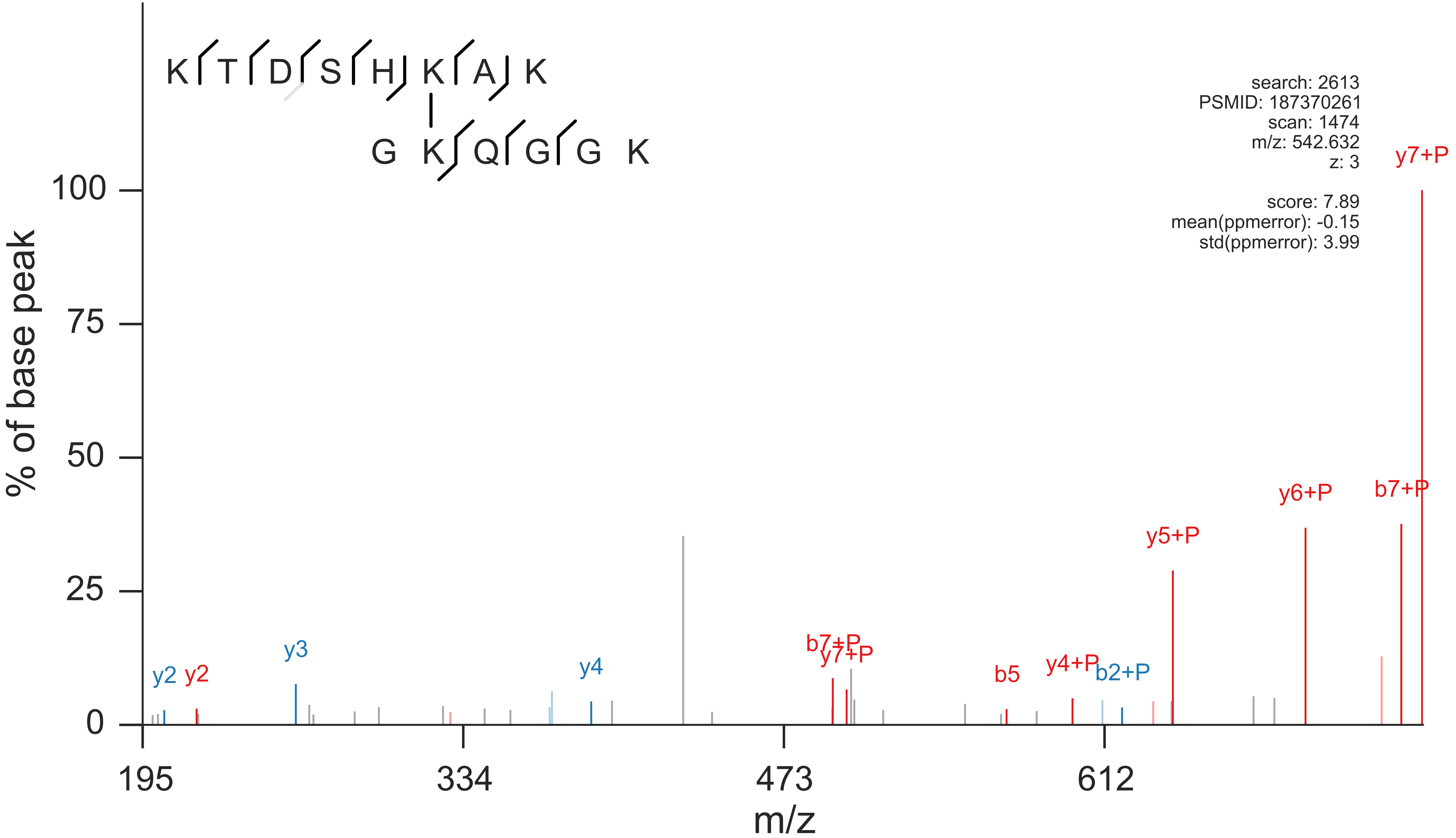
K T D S H K A K
G K Q G G K
search: 2613
PSMID: 187370261
scan: 1474
m/z: 542.632
z: 3
score: 7.89
mean(ppmerror): -0.15
std(ppmerror): 3.99
% of base peak
0
25
50
75
100
195
334
473
612
m/z
y2
y2
y3
y4
b7+P
y7+P
b5
y4+P
b2+P
y5+P
y6+P
b7+P
y7+P

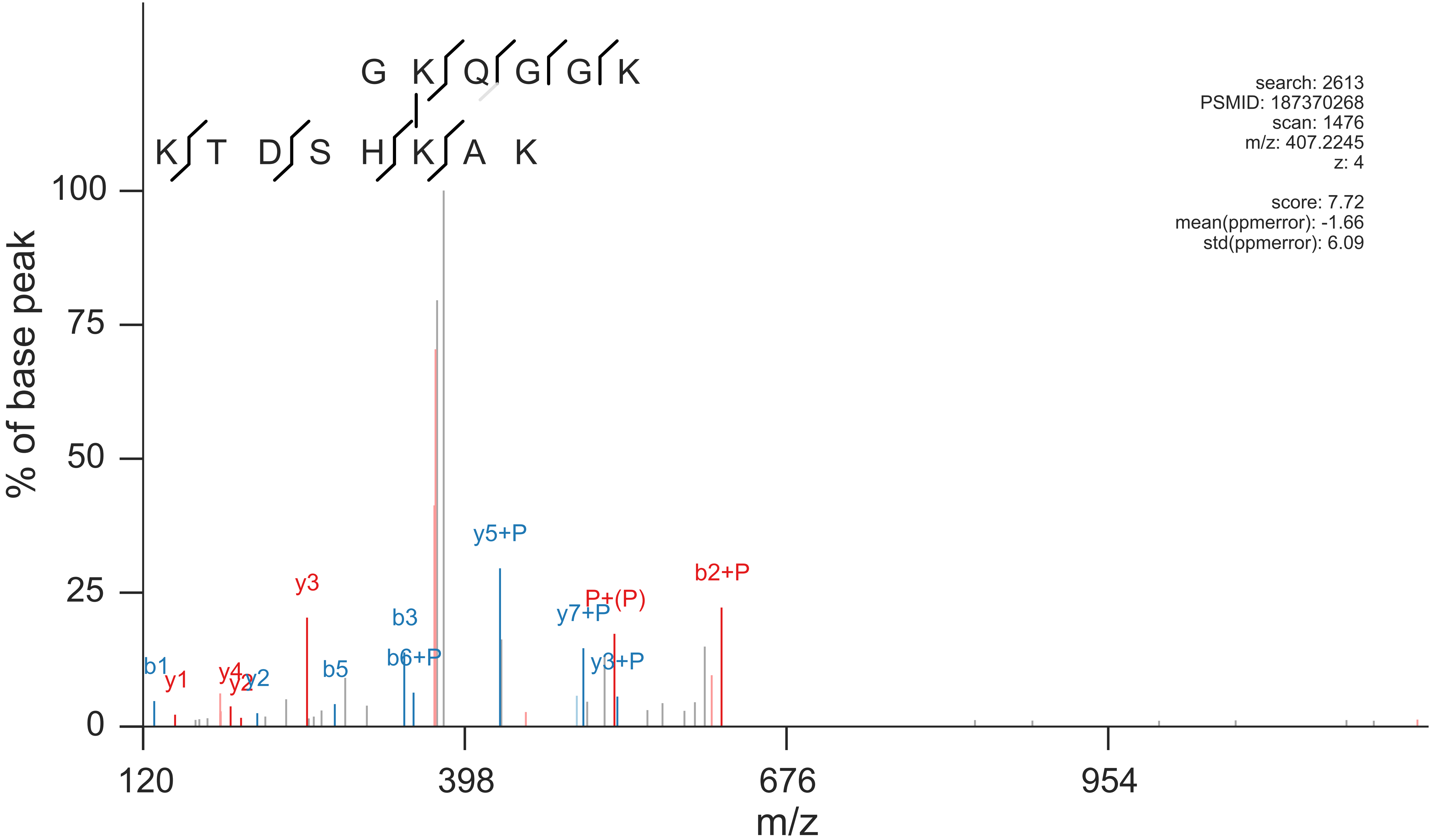

G K Q G G K
K T D S H K A K
search: 2613
PSMID: 187370268
scan: 1476
m/z: 407.2245
z: 4
score: 7.72
mean(ppmerror): -1.66
std(ppmerror): 6.09
% of base peak
100
75
50
25
0
y5+P
b2+P
P+(P)
y7+P
y3
b3
b6+P
y3+P
b1
y1
y4
y2
y2
b5
120
398
676
954
m/z

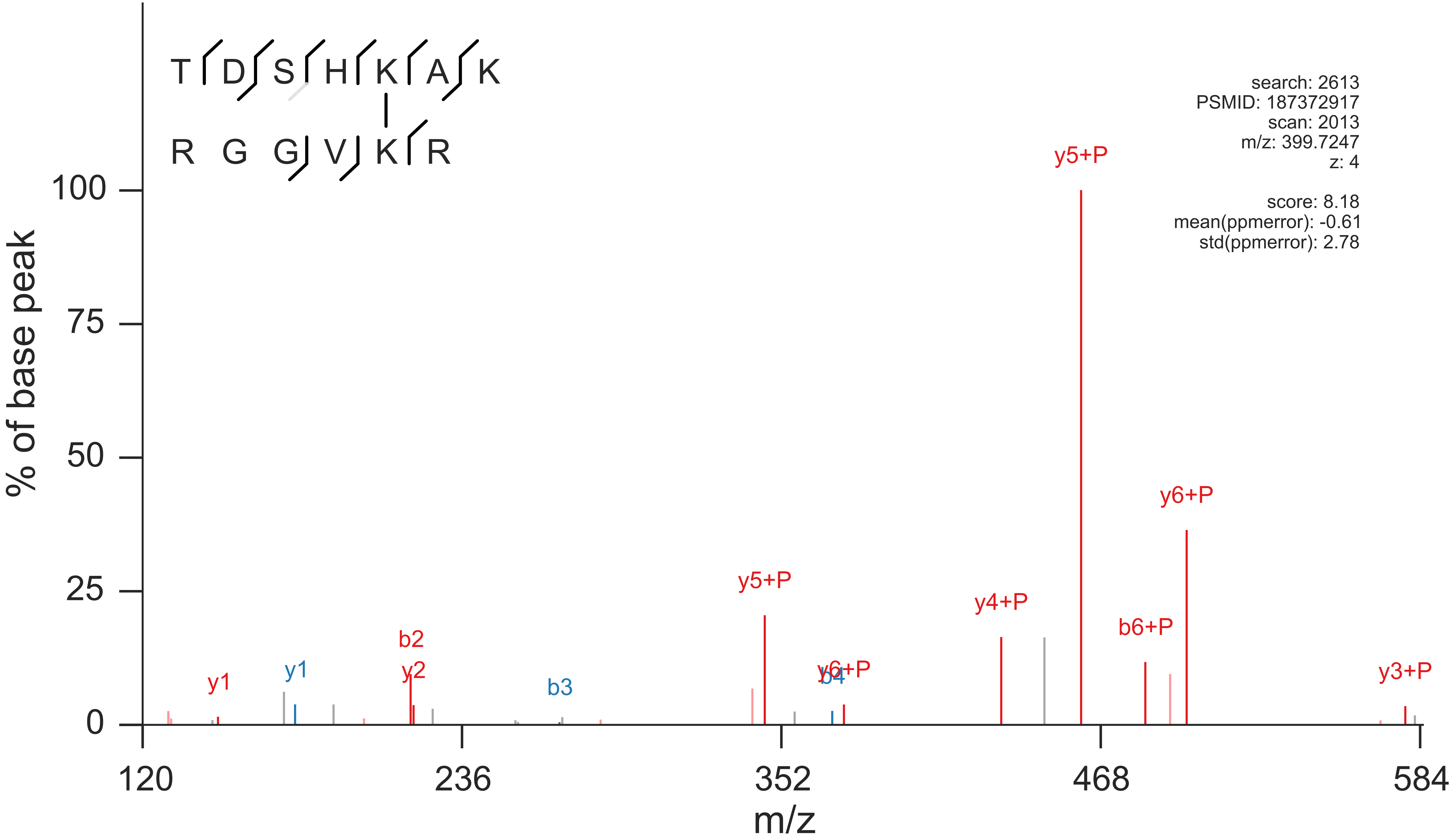
T D S H K A K
R G G V K R
search: 2613
PSMID: 187372917
scan: 2013
m/z: 399.7247
z: 4
score: 8.18
mean(ppmerror): -0.61
std(ppmerror): 2.78
% of base peak
100
75
50
25
0
y5+P
y6+P
y5+P
y4+P
b6+P
b2
y1
y1
y2
b3
y6+P
b4
y3+P
120
236
352
468
584
m/z

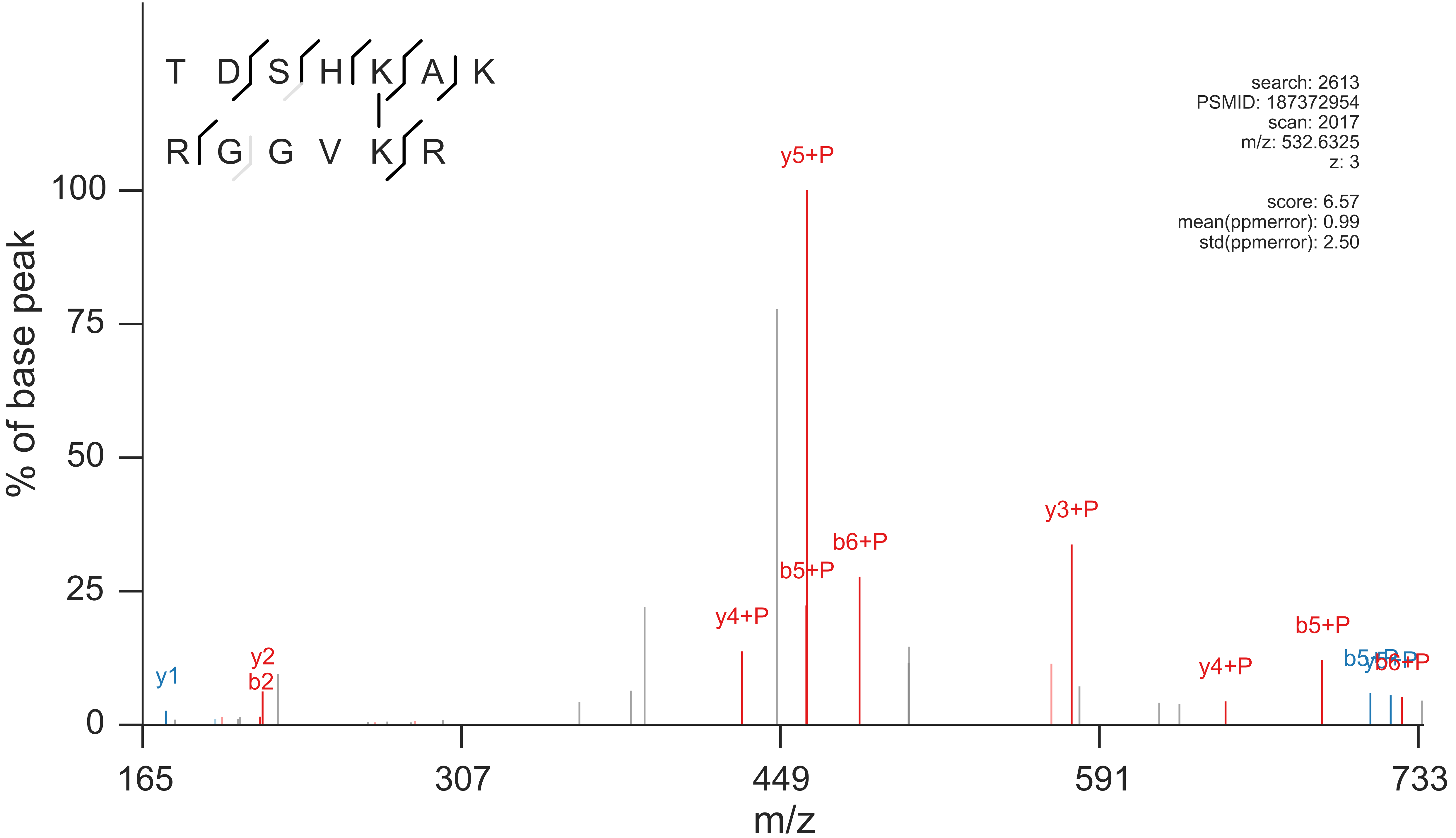

T D S H K A K
R G G V K R
search: 2613
PSMID: 187372954
scan: 2017
m/z: 532.6325
z: 3
score: 6.57
mean(ppmerror): 0.99
std(ppmerror): 2.50
% of base peak
m/z
y1
y2
b2
y4+P
y5+P
b5+P
b6+P
y3+P
y4+P
b5+P
0
25
50
75
100
165
307
449
591
733

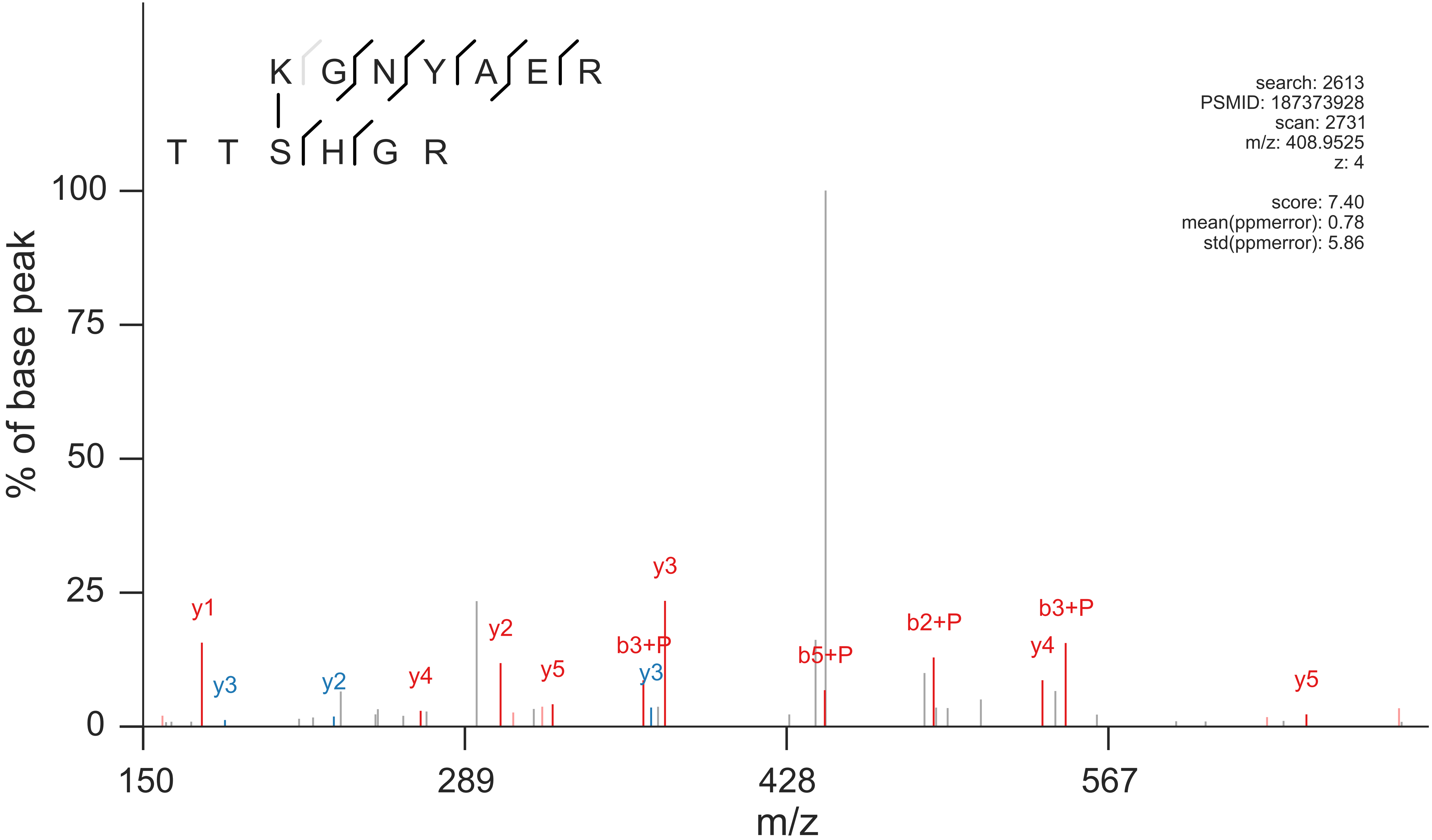

K G N Y A E R
T T S H G R
search: 2613
PSMID: 187373928
scan: 2731
m/z: 408.9525
z: 4
score: 7.40
mean(ppmerror): 0.78
std(ppmerror): 5.86
% of base peak
100
75
50
25
0
150
289
428
567
m/z
y1
y3
y2
y4
y2
y5
b3+P
y3
y3
b5+P
b2+P
y4
b3+P
y5

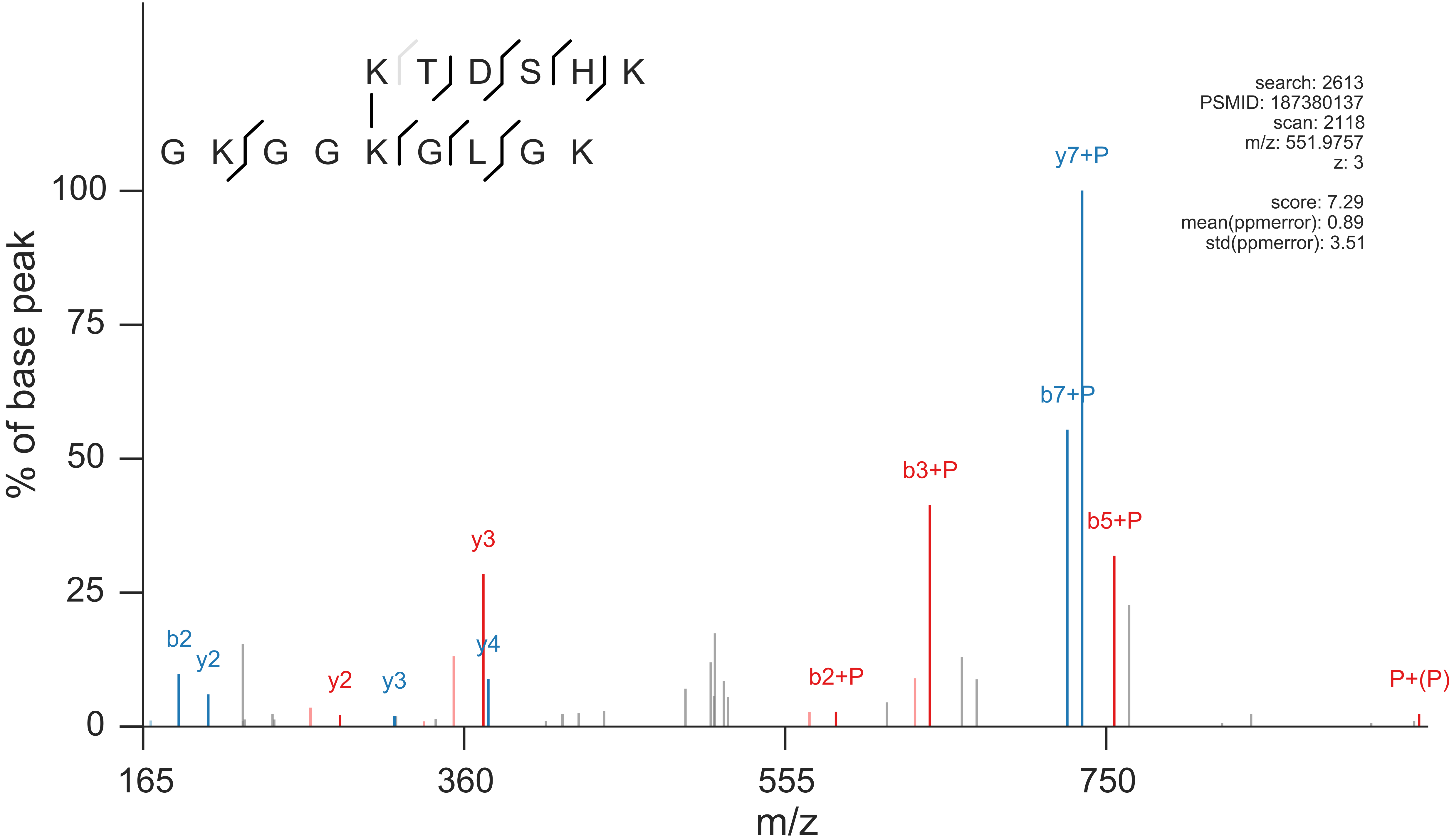
K T D S H K
G K G G K G L G K
search: 2613
PSMID: 187380137
scan: 2118
m/z: 551.9757
z: 3
score: 7.29
mean(ppmerror): 0.89
std(ppmerror): 3.51
% of base peak
m/z
0
25
50
75
100
165
360
555
750
y7+P
b7+P
b3+P
b5+P
y3
b2
y2
y2
y3
y4
b2+P
P+(P)

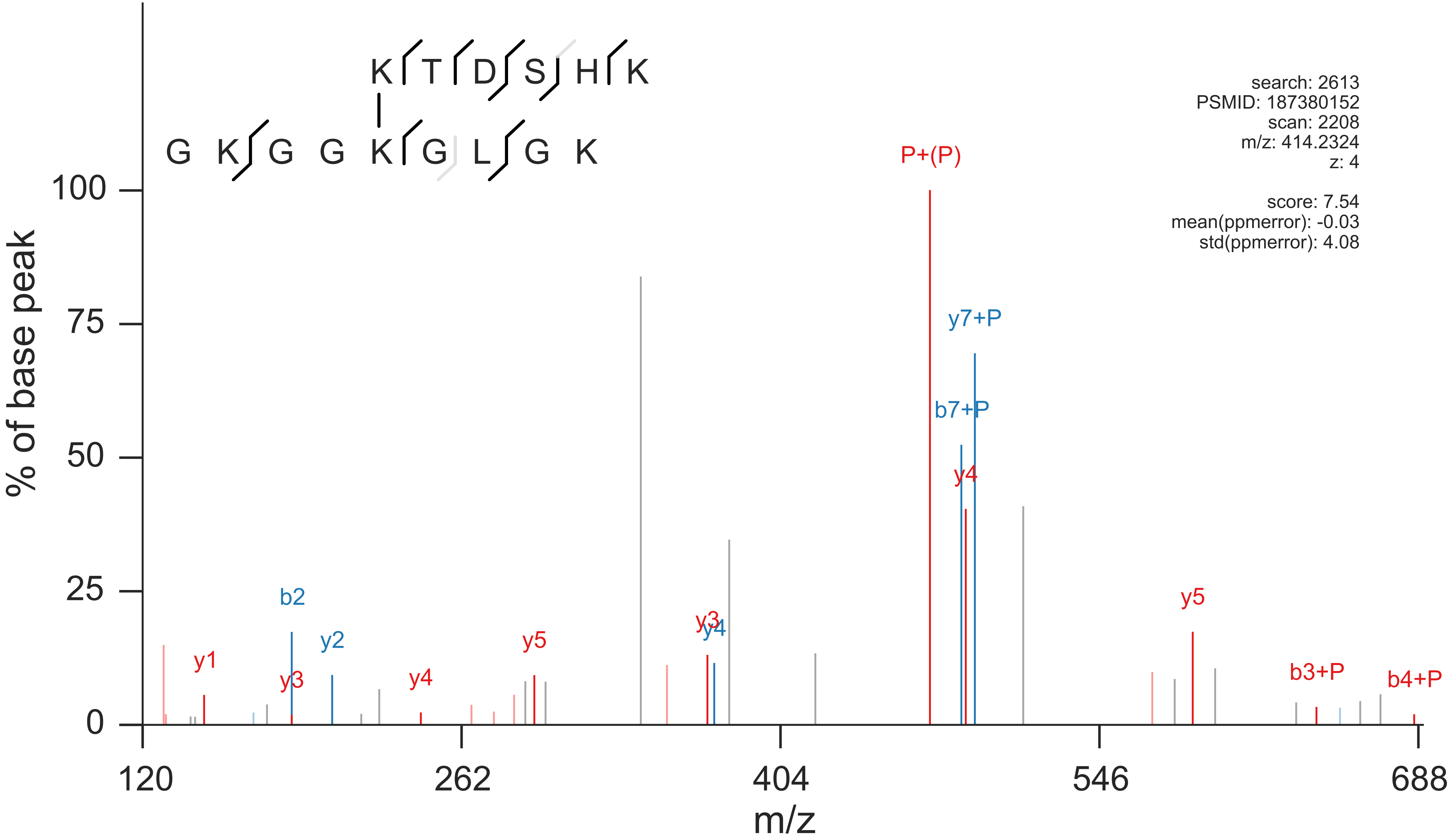

K T D S H K
G K G G K G L G K
search: 2613
PSMID: 187380152
scan: 2208
m/z: 414.2324
z: 4
score: 7.54
mean(ppmerror): -0.03
std(ppmerror): 4.08
P+(P)
y7+P
b7+P
y4
y5
y3
y4
b3+P
b4+P
b2
y2
y1
y3
y4
y5
% of base peak
100
75
50
25
0
120
262
404
546
688
m/z

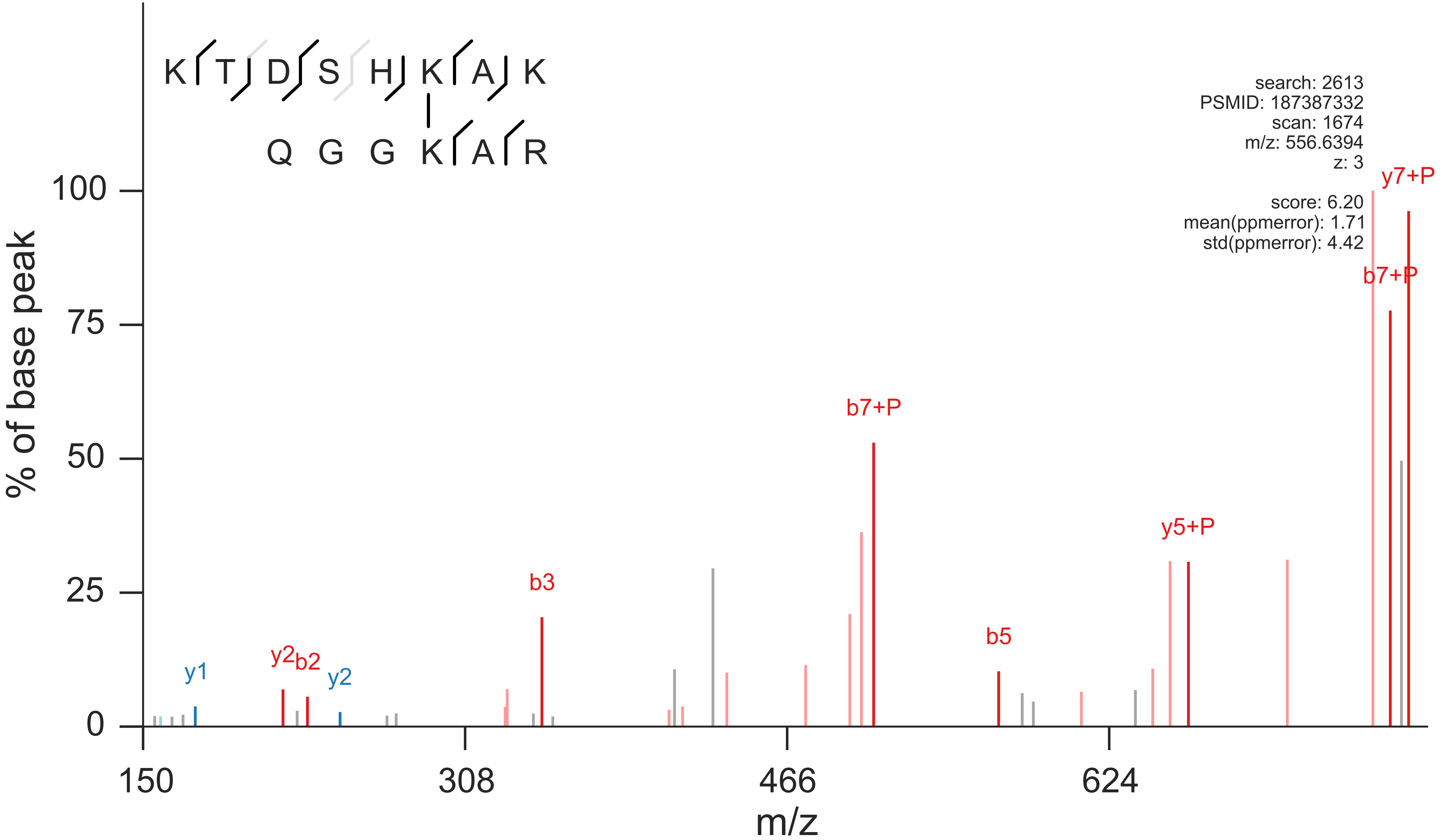
K T D S H K A K
Q G G K A R
search: 2613
PSMID: 187387332
scan: 1674
m/z: 556.6394
z: 3
score: 6.20
mean(ppmerror): 1.71
std(ppmerror): 4.42
% of base peak
m/z
100
75
50
25
0
150
308
466
624
y1
y2
b2
y2
b3
b7+P
b5
y5+P
b7+P
y7+P

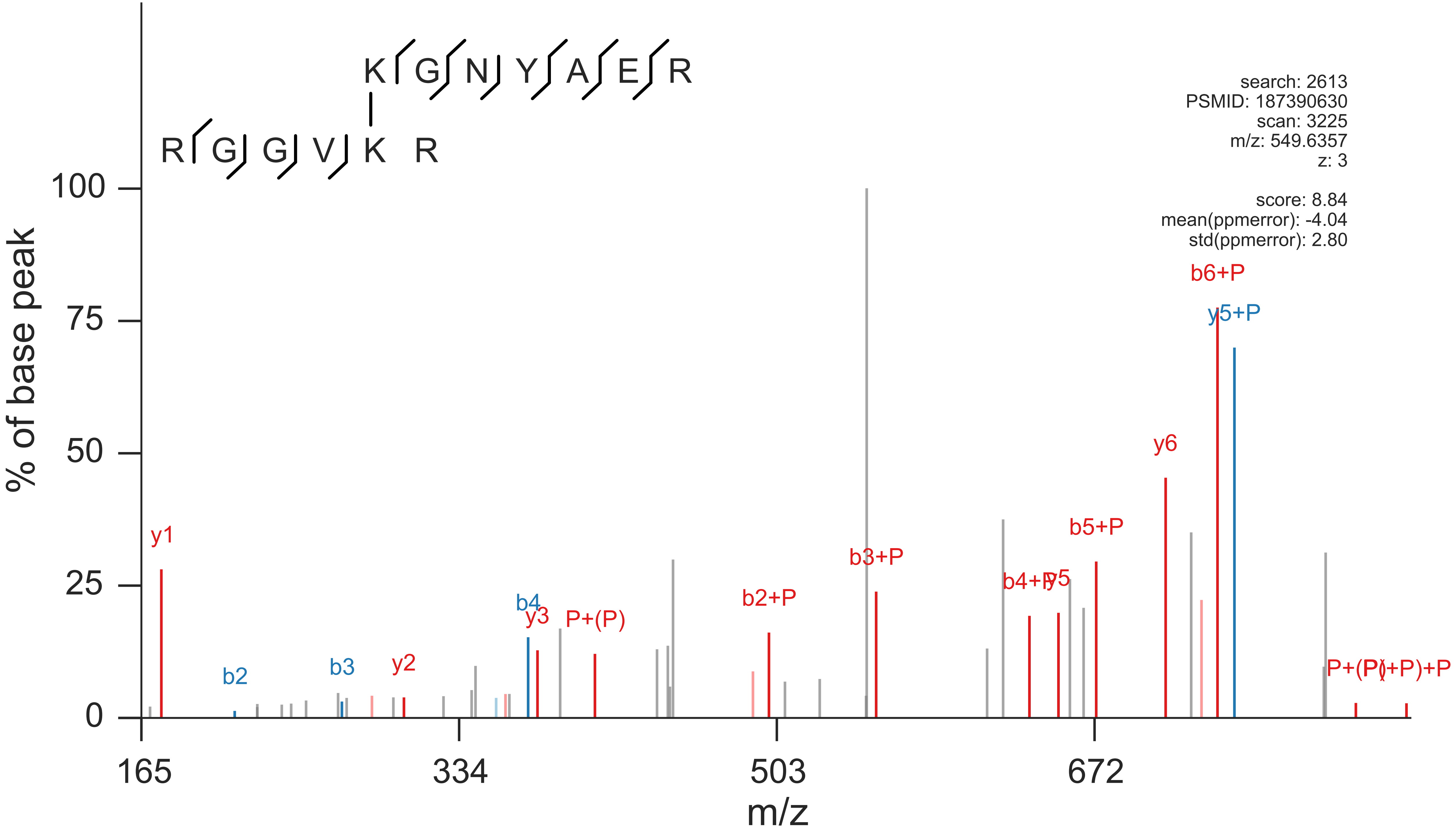

K G N Y A E R
R G G V K R
search: 2613
PSMID: 187390630
scan: 3225
m/z: 549.6357
z: 3
score: 8.84
mean(ppmerror): -4.04
std(ppmerror): 2.80
% of base peak
m/z
100
75
50
25
0
165
334
503
672
y1
b2
b3
y2
b4
y3
P+(P)
b2+P
b3+P
b4+P
y5
b5+P
y6
b6+P
y5+P
P+(P)+P)+P

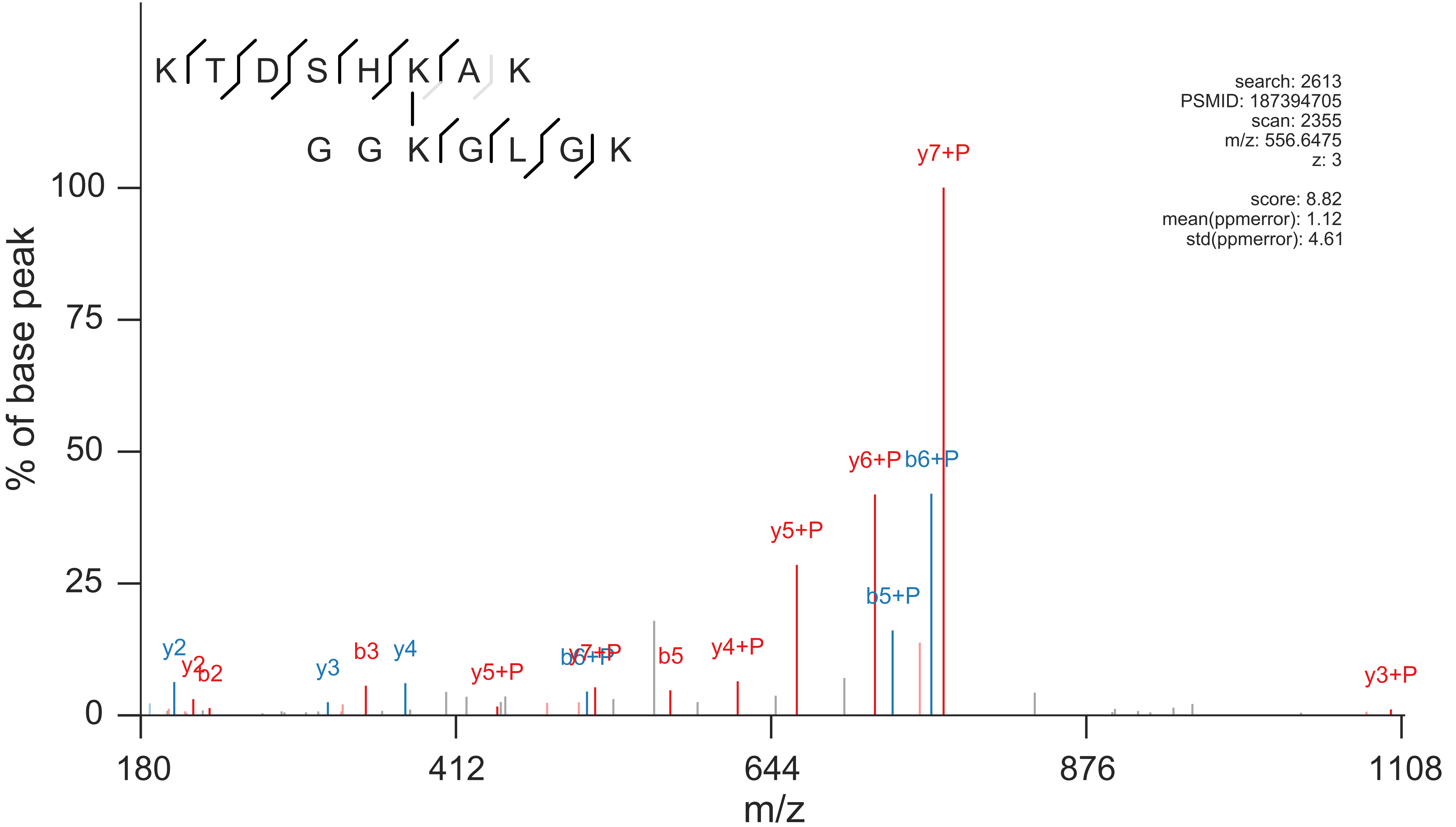

K T D S H K A K
G G K G L G K
search: 2613
PSMID: 187394705
scan: 2355
m/z: 556.6475
z: 3
score: 8.82
mean(ppmerror): 1.12
std(ppmerror): 4.61
y7+P
y6+P
b6+P
y5+P
b5+P
y2
y2
b2
y3
b3
y4
y5+P
b6+P
y7+P
b5
y4+P
y3+P
% of base peak
100
75
50
25
0
180
412
644
876
1108
m/z

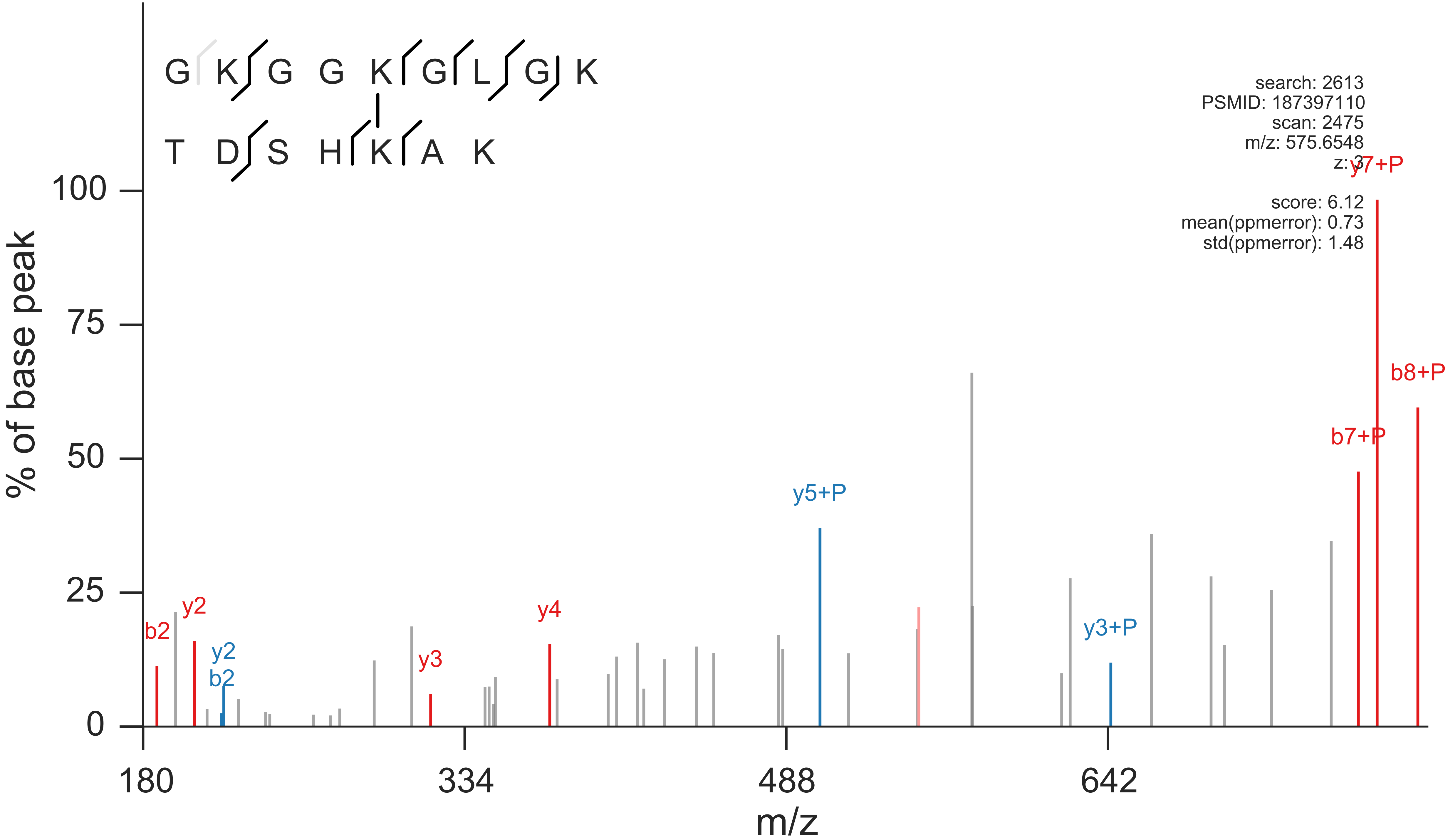
G K G G K G L G K
T D S H K A K
search: 2613
PSMID: 187397110
scan: 2475
m/z: 575.6548
z: 3
score: 6.12
mean(ppmerror): 0.73
std(ppmerror): 1.48
y7+P
b8+P
b7+P
y5+P
y3+P
y4
y3
y2
b2
y2
b2
% of base peak
100
75
50
25
0
180
334
488
642
m/z

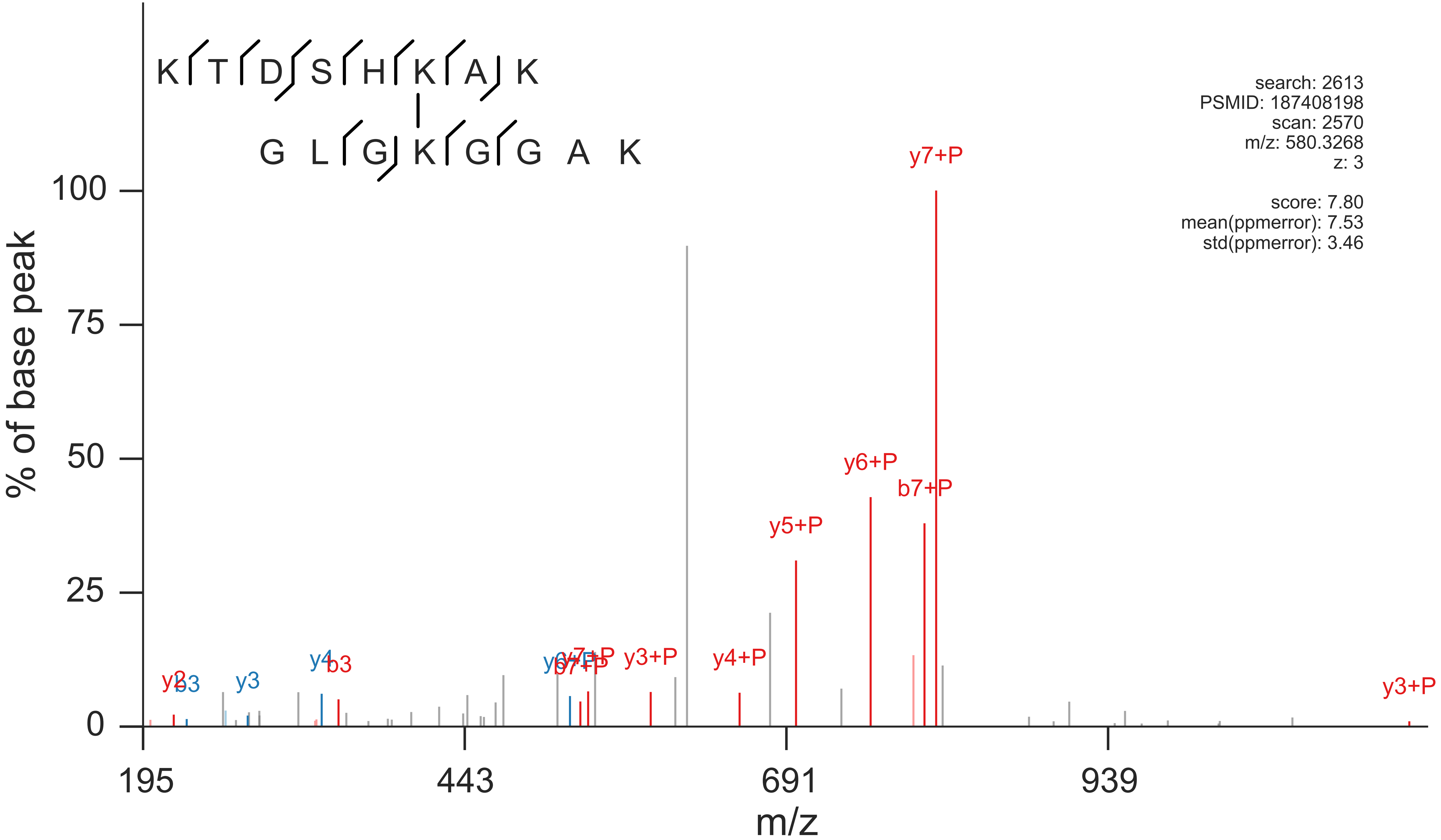
K T D S H K A K
G L G K G G A K
search: 2613
PSMID: 187408198
scan: 2570
m/z: 580.3268
z: 3
score: 7.80
mean(ppmerror): 7.53
std(ppmerror): 3.46
y7+P
y6+P
b7+P
y5+P
y3+P
y4+P
y4
b3
y2
b3
y3
% of base peak
100
75
50
25
0
195
443
691
939
m/z

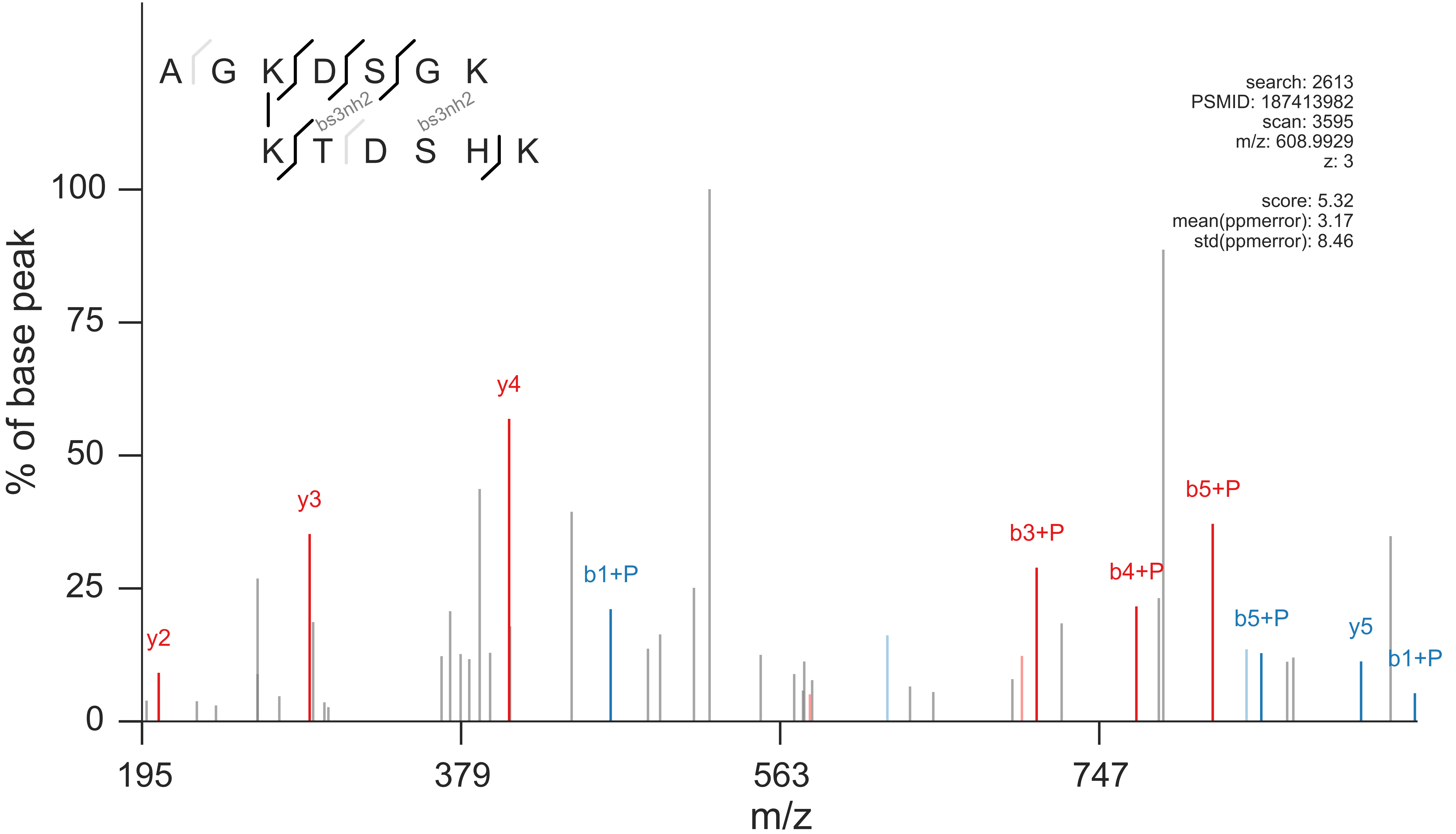

A G K D S G K
K T D S H K
bs3nh2
bs3nh2
search: 2613
PSMID: 187413982
scan: 3595
m/z: 608.9929
z: 3
score: 5.32
mean(ppmerror): 3.17
std(ppmerror): 8.46
% of base peak
100
75
50
25
0
y2
y3
y4
b1+P
b3+P
b4+P
b5+P
b5+P
y5
b1+P
195
379
563
747
m/z

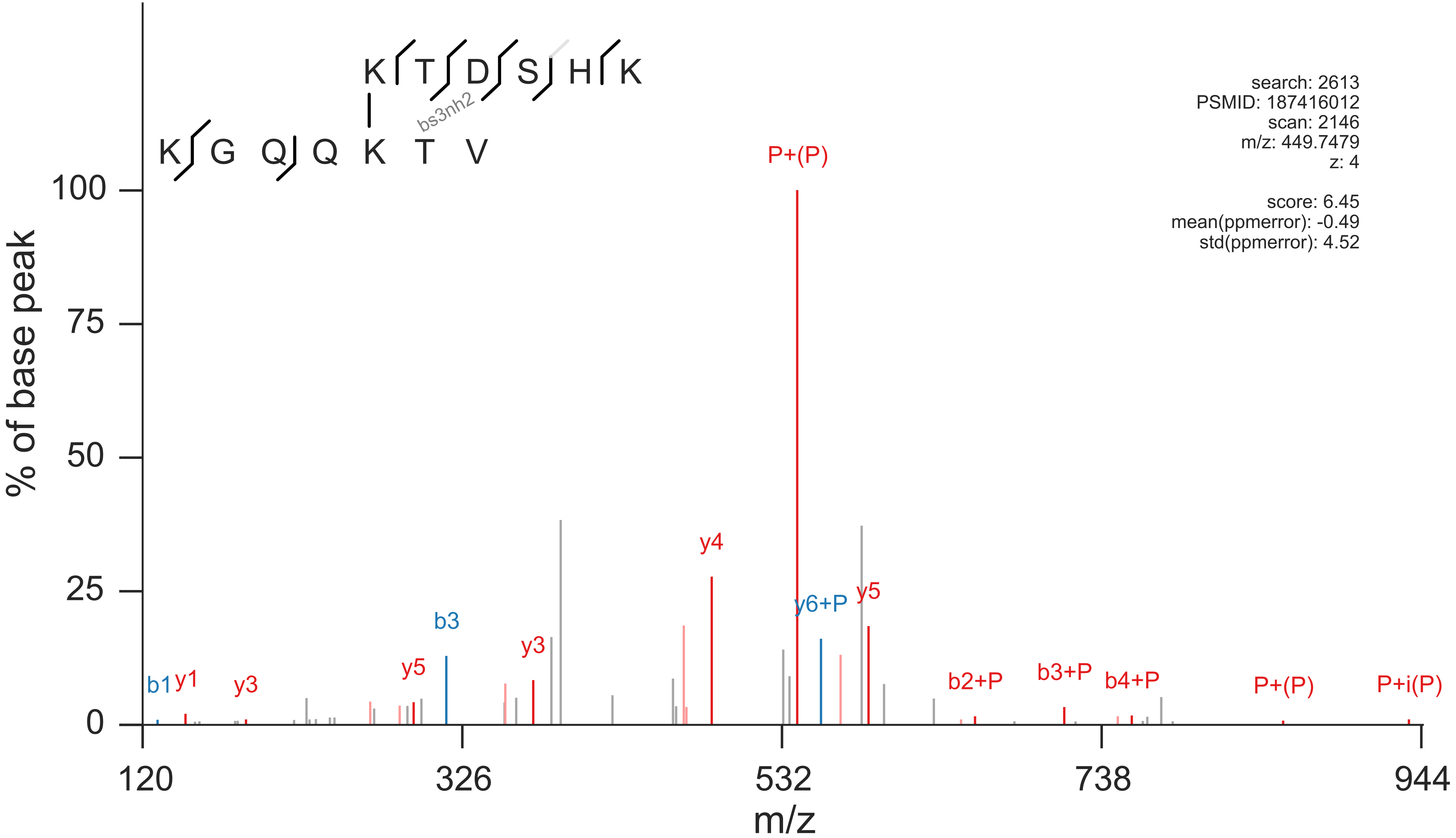

K T D S H K
K G Q Q K T V
bs3nh2
search: 2613
PSMID: 187416012
scan: 2146
m/z: 449.7479
z: 4
score: 6.45
mean(ppmerror): -0.49
std(ppmerror): 4.52
P+(P)
y4
y6+P
y5
b3
y3
y5
b1
y1
y3
b2+P
b3+P
b4+P
P+(P)
P+i(P)
% of base peak
100
75
50
25
0
120
326
532
738
944
m/z

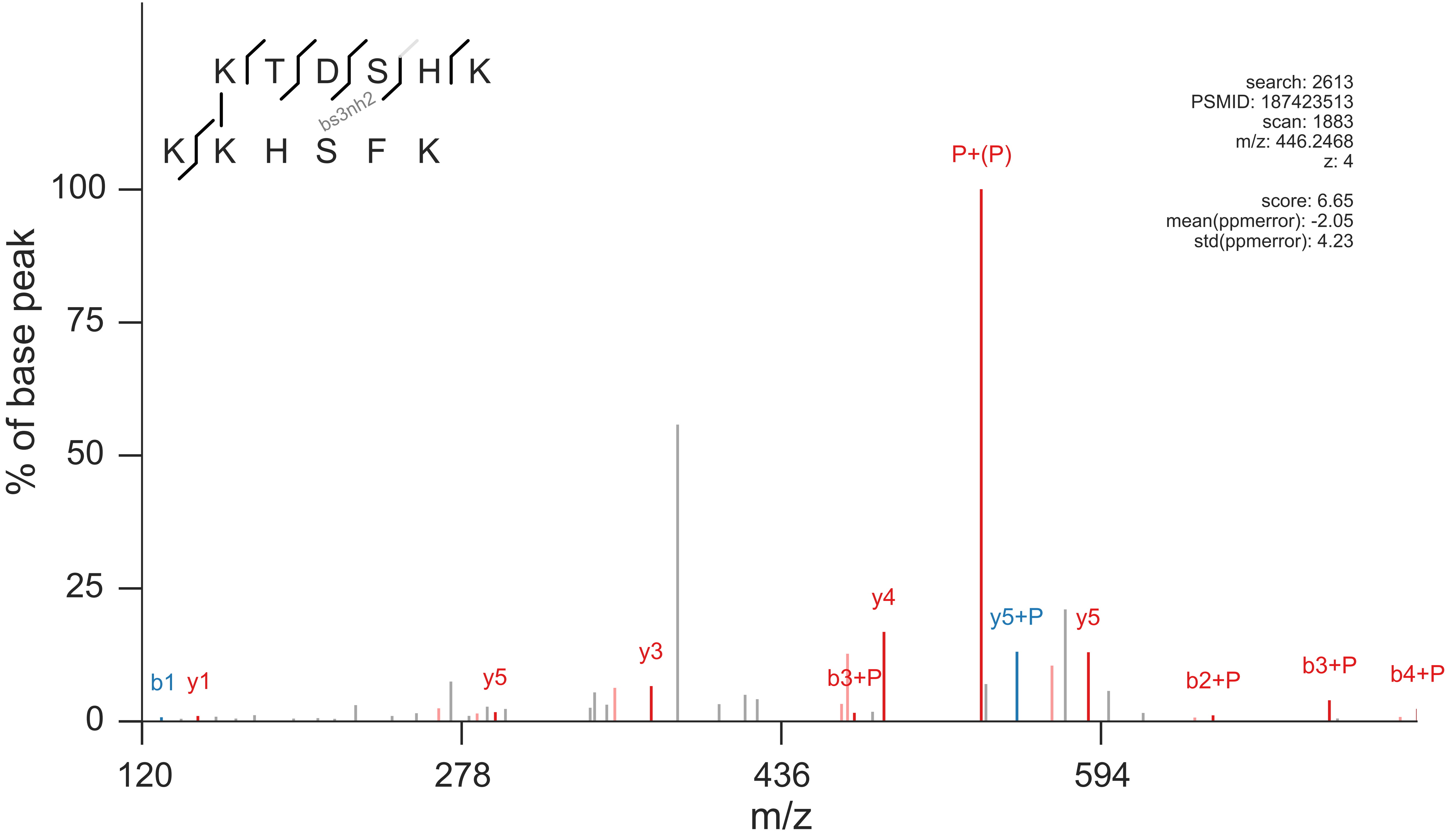

K T D S H K
bs3nh2
K K H S F K
search: 2613
PSMID: 187423513
scan: 1883
m/z: 446.2468
z: 4
score: 6.65
mean(ppmerror): -2.05
std(ppmerror): 4.23
P+(P)
% of base peak
100
75
50
25
0
b1
y1
y5
y3
b3+P
y4
y5+P
y5
b2+P
b3+P
b4+P
120
278
436
594
m/z

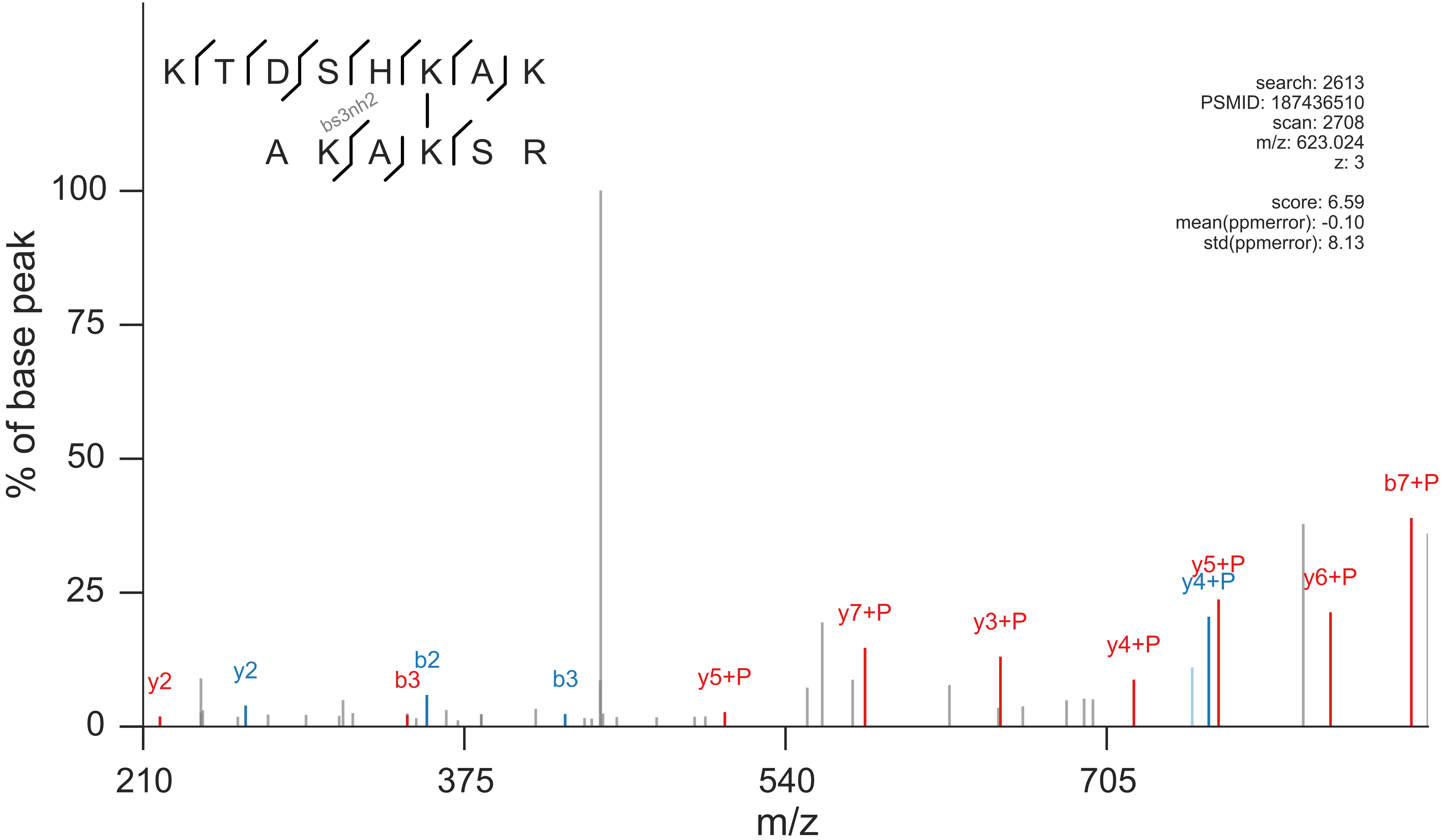
K T D S H K A K
bs3nh2
A K A K S R
search: 2613
PSMID: 187436510
scan: 2708
m/z: 623.024
z: 3
score: 6.59
mean(ppmerror): -0.10
std(ppmerror): 8.13
% of base peak
100
75
50
25
0
210
375
540
705
m/z
y2
y2
b3
b2
b3
y5+P
y7+P
y3+P
y4+P
y4+P
y5+P
y6+P
b7+P

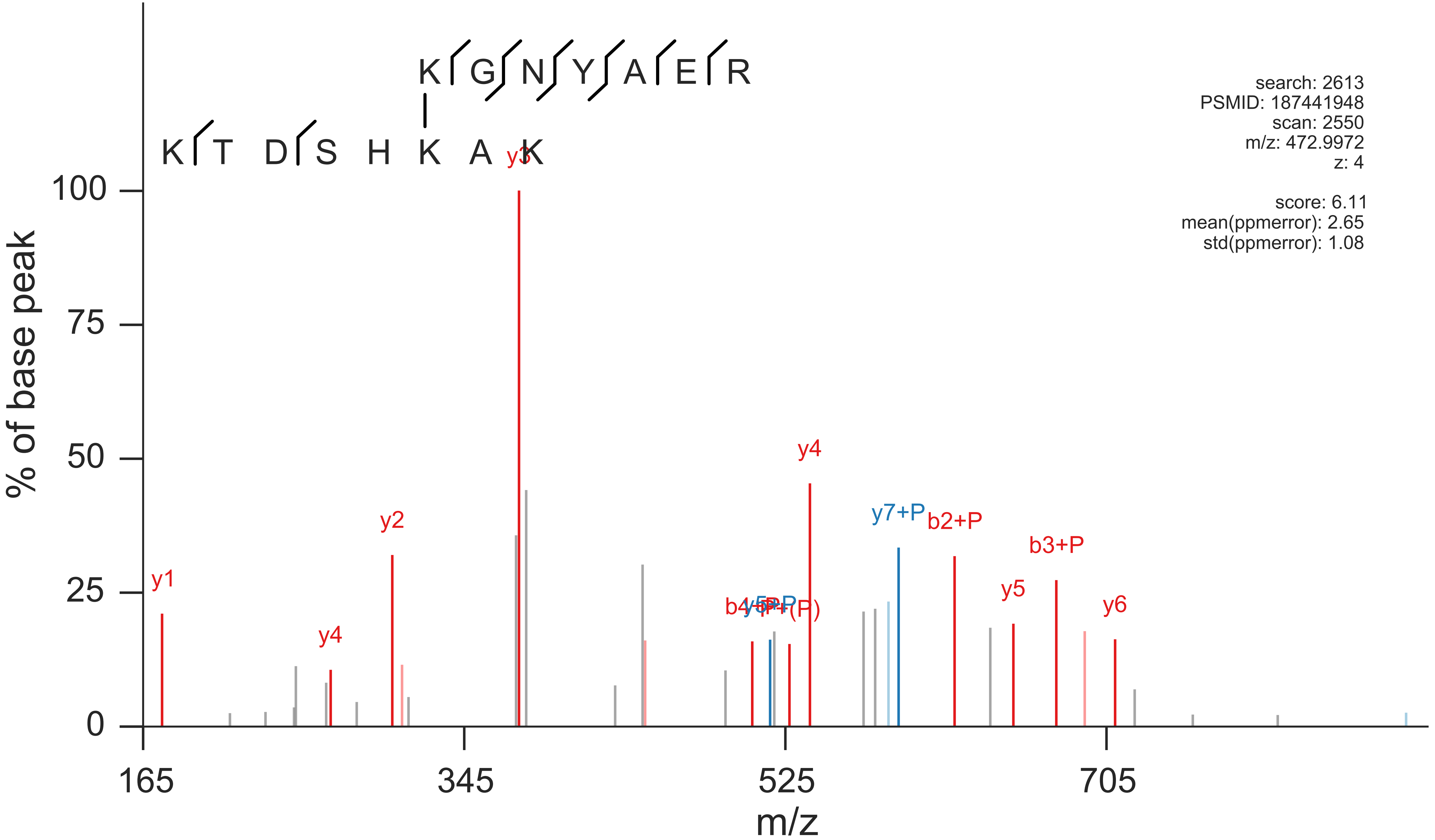

K G N Y A E R
K T D S H K A K
search: 2613
PSMID: 187441948
scan: 2550
m/z: 472.9972
z: 4
score: 6.11
mean(ppmerror): 2.65
std(ppmerror): 1.08
% of base peak
m/z
y1
y4
y2
y3
y4
y7+P
b2+P
b3+P
y5
y6

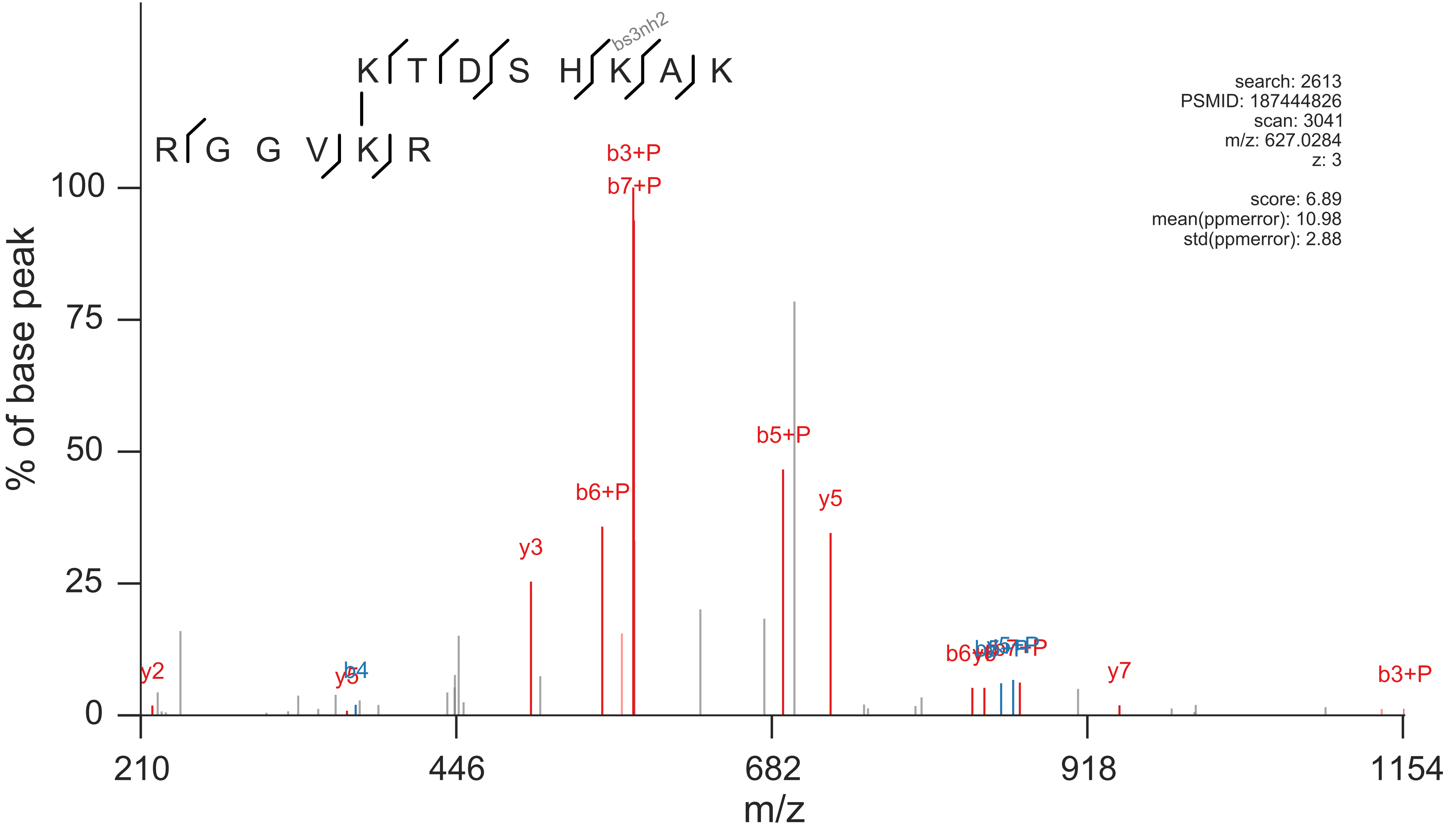

K T D S H K A K
bs3nh2
R G G V K R
search: 2613
PSMID: 187444826
scan: 3041
m/z: 627.0284
z: 3
score: 6.89
mean(ppmerror): 10.98
std(ppmerror): 2.88
b3+P
b7+P
b5+P
y5
b6+P
y3
y2
b4
y7
% of base peak
100
75
50
25
0
210
446
682
918
1154
m/z

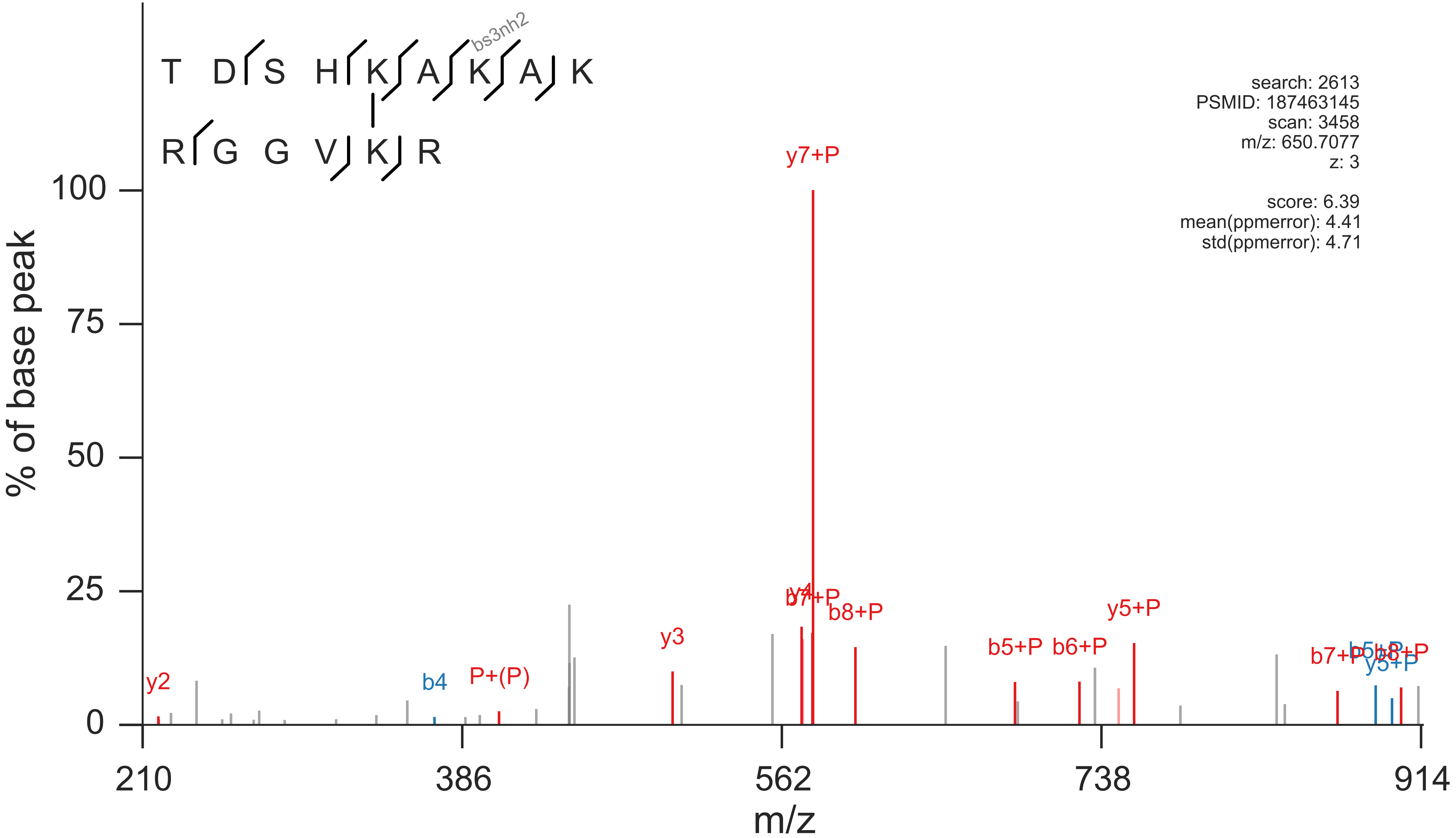

T D S H K A K A K
R G G V K R
bs3nh2
search: 2613
PSMID: 187463145
scan: 3458
m/z: 650.7077
z: 3
score: 6.39
mean(ppmerror): 4.41
std(ppmerror): 4.71
y7+P
y2
b4
P+(P)
y3
y4
b7+P
b8+P
b5+P
b6+P
y5+P
% of base peak
100
75
50
25
0
210
386
562
738
914
m/z

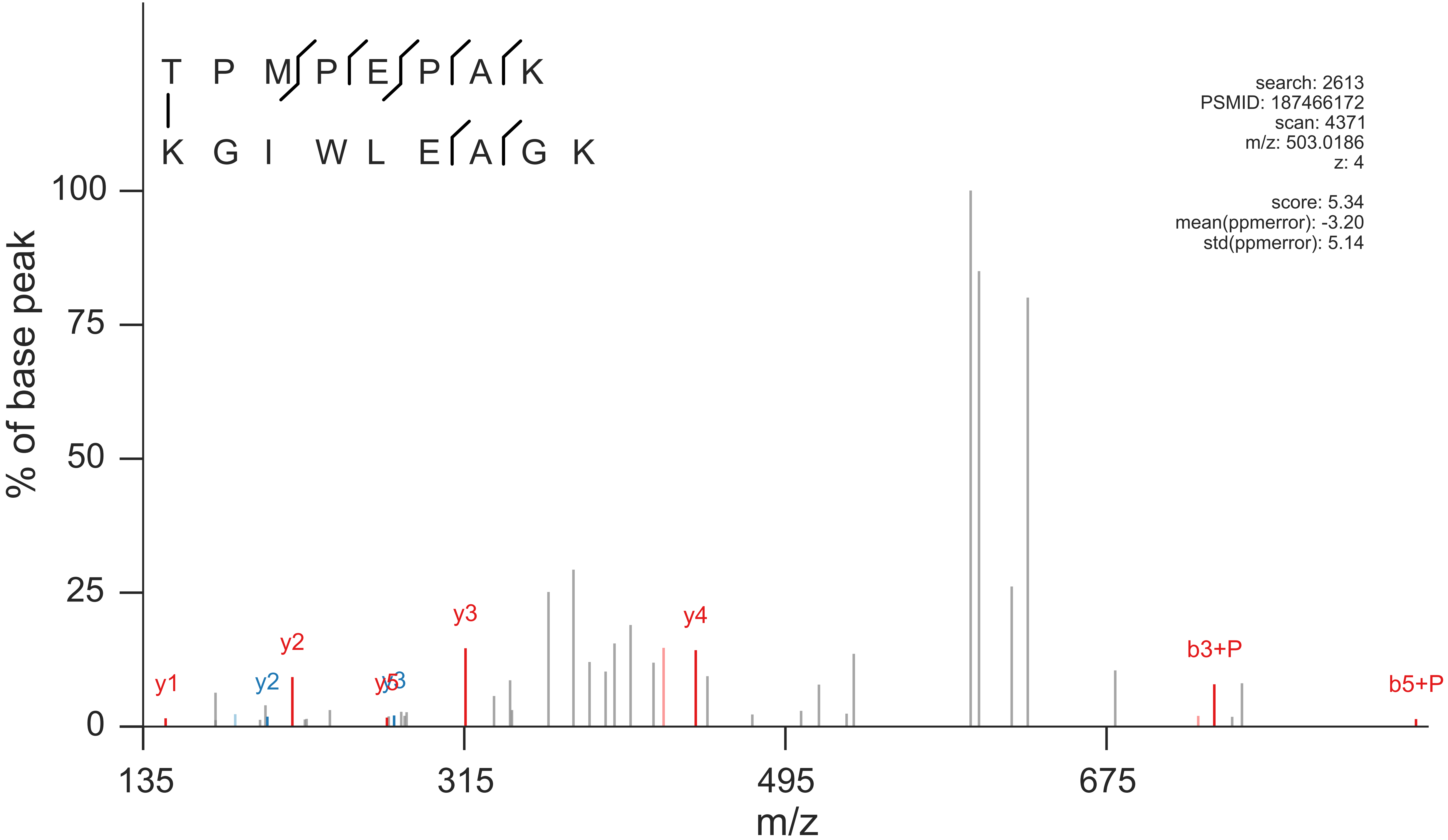

T P M P E P A K
K G I W L E A G K
search: 2613
PSMID: 187466172
scan: 4371
m/z: 503.0186
z: 4
score: 5.34
mean(ppmerror): -3.20
std(ppmerror): 5.14
% of base peak
100
75
50
25
0
y1
y2
y2
y5
y3
y3
y4
b3+P
b5+P
135
315
495
675
m/z

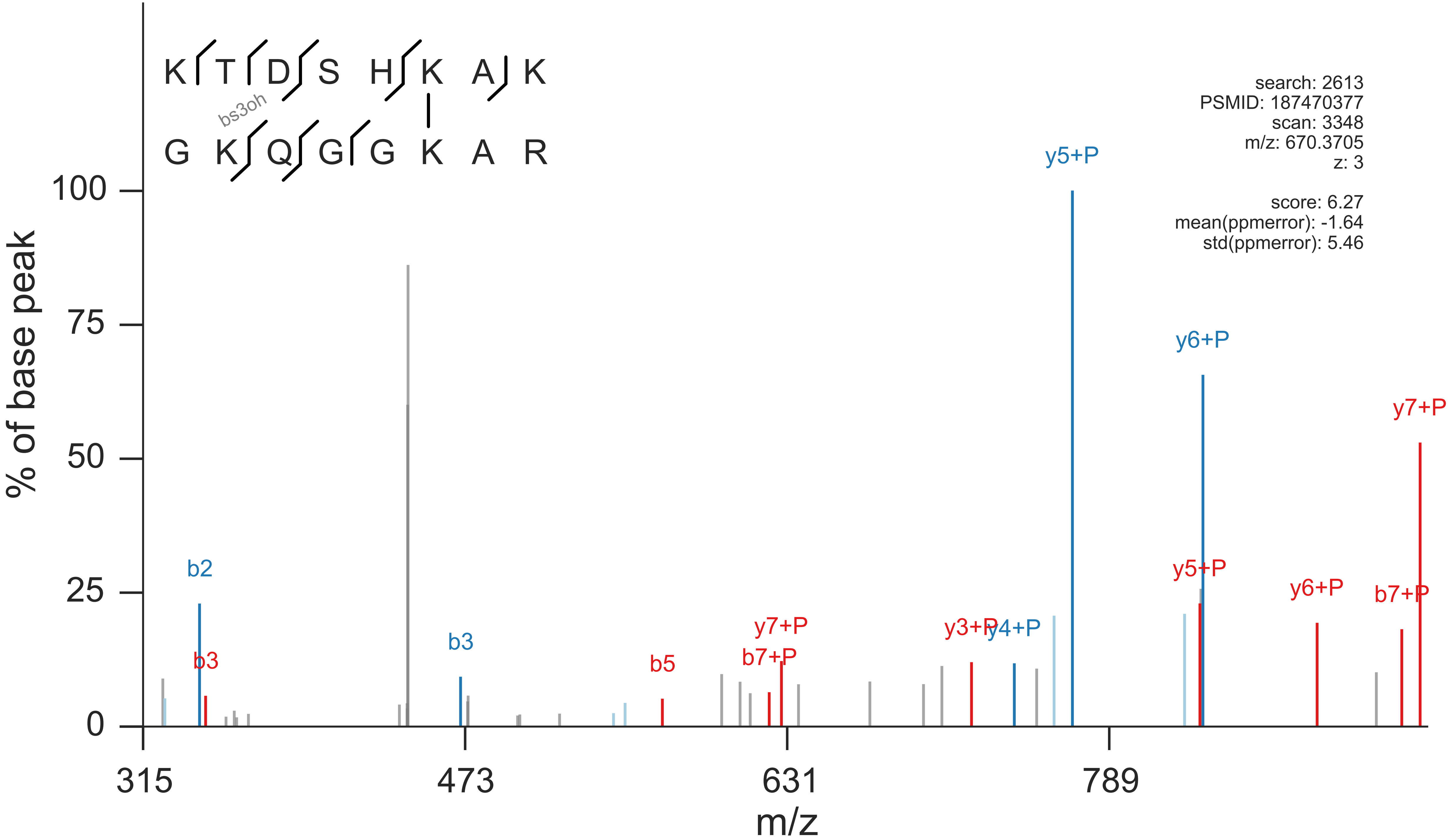
K T D S H K A K
G K Q G G K A R
bs3oh
search: 2613
PSMID: 187470377
scan: 3348
m/z: 670.3705
z: 3
score: 6.27
mean(ppmerror): -1.64
std(ppmerror): 5.46
% of base peak
m/z
y5+P
y6+P
y7+P
b2
b3
y5+P
y6+P
b7+P
y7+P
b7+P
y3+P
y4+P
b5
0
25
50
75
100
315
473
631
789

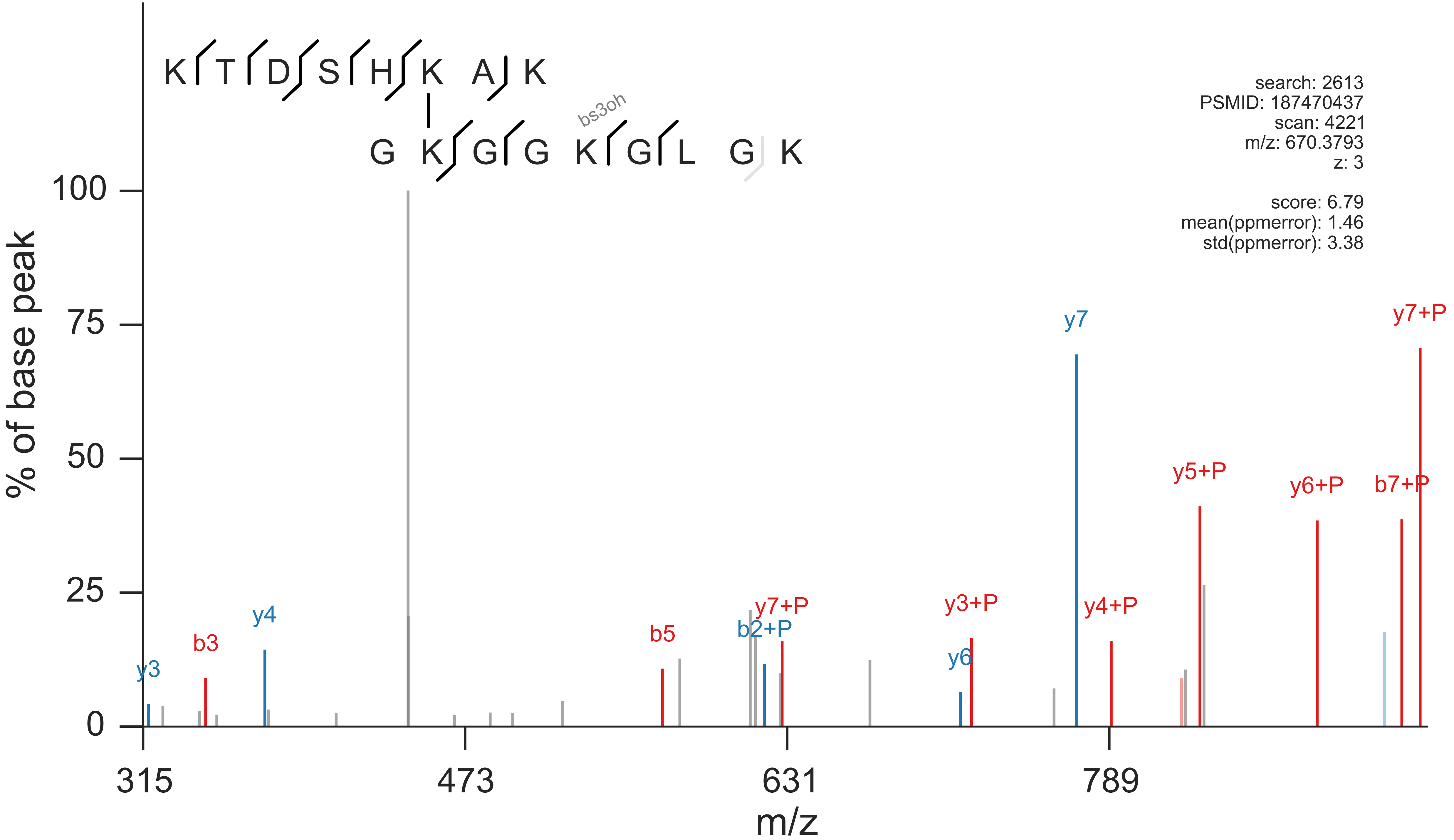

K T D S H K A K
G K G G K G L G K
bs3oh
search: 2613
PSMID: 187470437
scan: 4221
m/z: 670.3793
z: 3
score: 6.79
mean(ppmerror): 1.46
std(ppmerror): 3.38
% of base peak
m/z
100
75
50
25
0
315
473
631
789
y3
b3
y4
b5
b2+P
y7+P
y6
y3+P
y7
y4+P
y5+P
y6+P
b7+P
y7+P

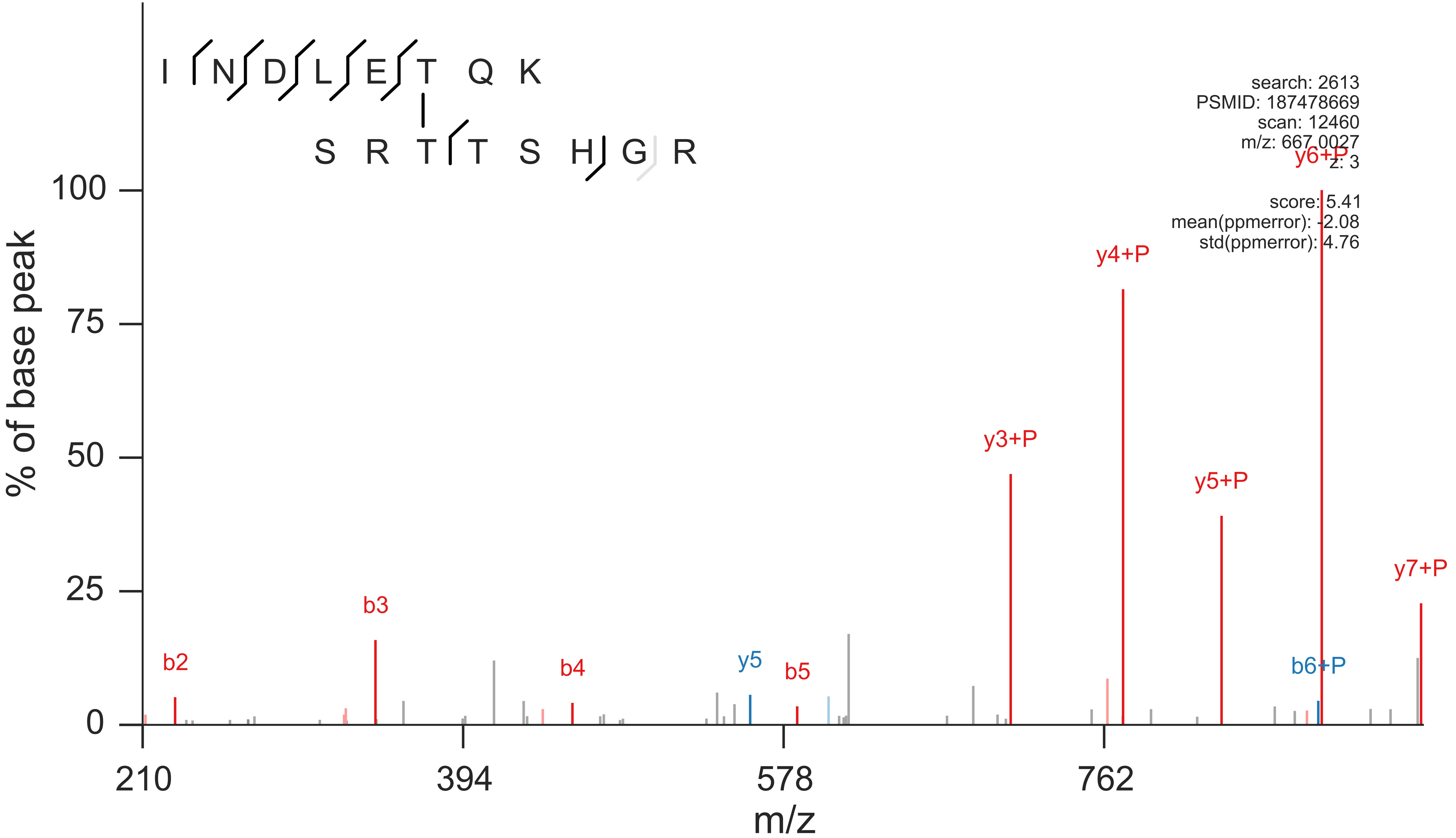

I N D L E T Q K
S R T T S H G R
search: 2613
PSMID: 187478669
scan: 12460
m/z: 667.0027
z: 3
score: 5.41
mean(ppmerror): -2.08
std(ppmerror): 4.76
% of base peak
m/z
100
75
50
25
0
210
394
578
762
b2
b3
b4
y5
b5
y3+P
y4+P
y5+P
y6+P
b6+P
y7+P

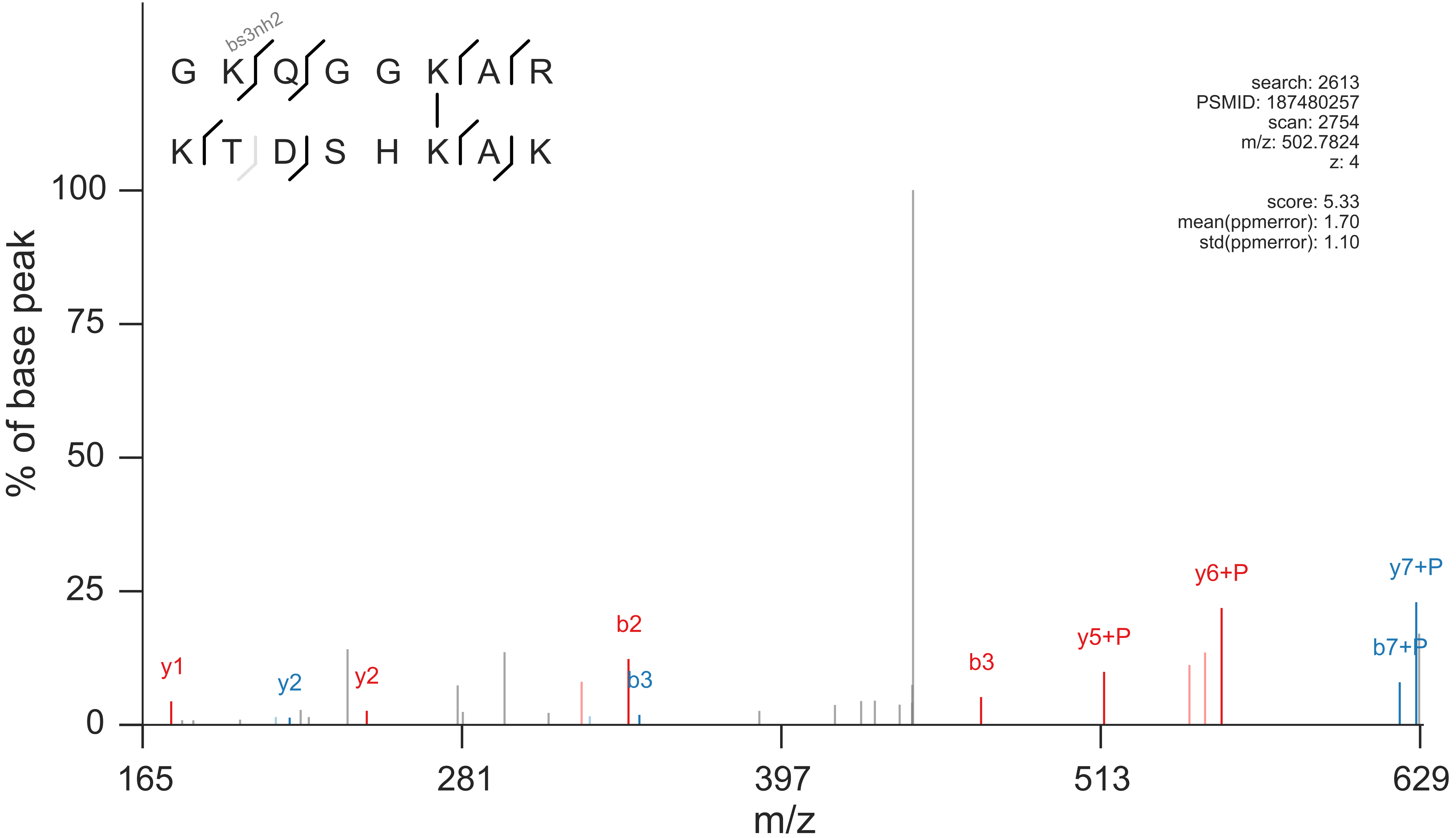

G K Q G G K A R
K T D S H K A K
bs3nh2
search: 2613
PSMID: 187480257
scan: 2754
m/z: 502.7824
z: 4
score: 5.33
mean(ppmerror): 1.70
std(ppmerror): 1.10
% of base peak
100
75
50
25
0
y1
y2
y2
b2
b3
b3
y5+P
y6+P
b7+P
y7+P
165
281
397
513
629
m/z

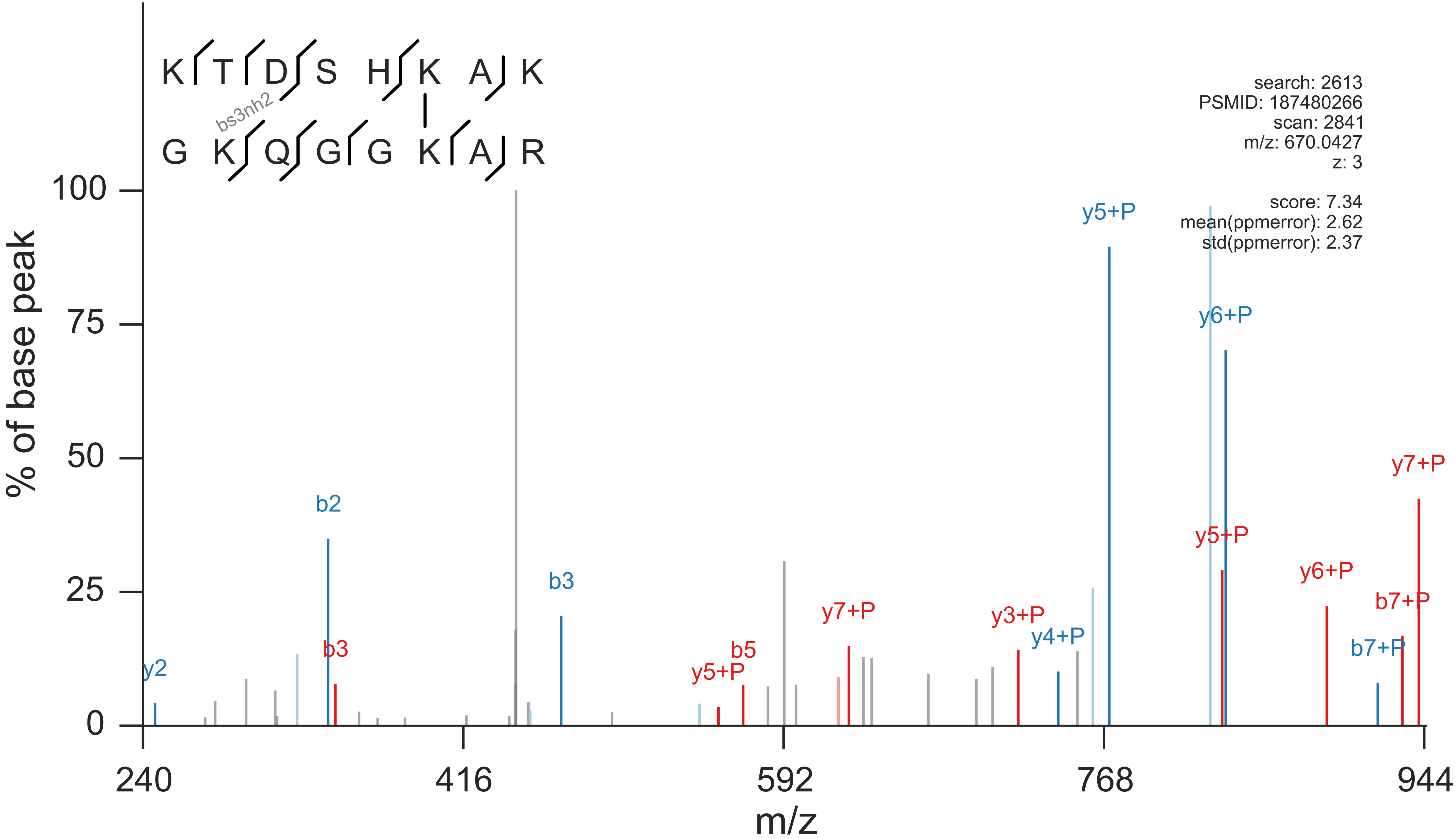

K T D S H K A K
G K Q G G K A R
bs3nh2
search: 2613
PSMID: 187480266
scan: 2841
m/z: 670.0427
z: 3
score: 7.34
mean(ppmerror): 2.62
std(ppmerror): 2.37
% of base peak
m/z
0
25
50
75
100
240
416
592
768
944
y2
b2
b3
b3
y5+P
b5
y7+P
y3+P
y4+P
y5+P
y6+P
y5+P
y6+P
b7+P
b7+P
y7+P

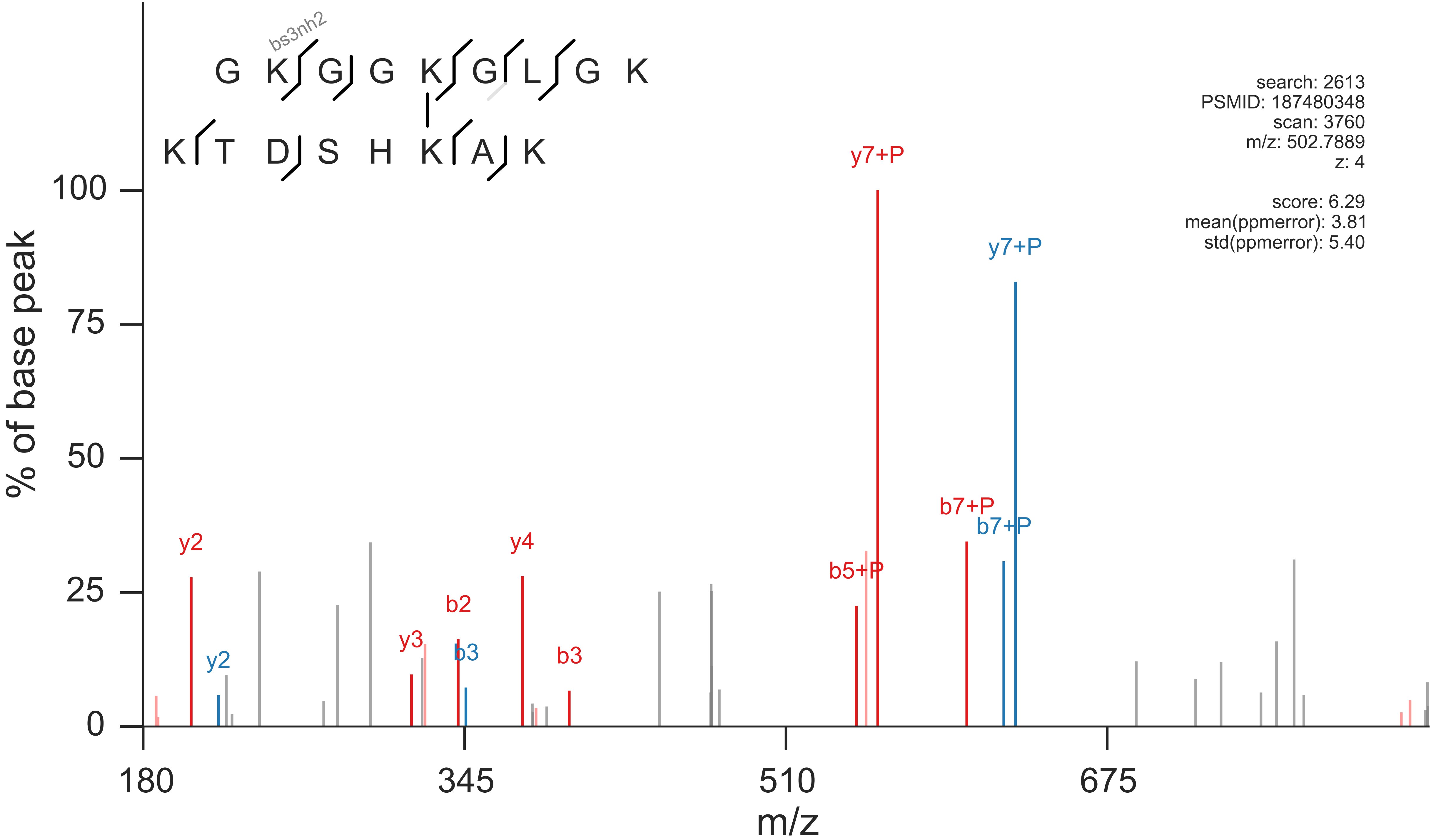

bs3nh2
G K G G K G L G K
K T D S H K A K
search: 2613
PSMID: 187480348
scan: 3760
m/z: 502.7889
z: 4
score: 6.29
mean(ppmerror): 3.81
std(ppmerror): 5.40
y7+P
y7+P
b7+P
b7+P
b5+P
y2
y4
b2
y3
b3
y2
b3
% of base peak
100
75
50
25
0
180
345
510
675
m/z

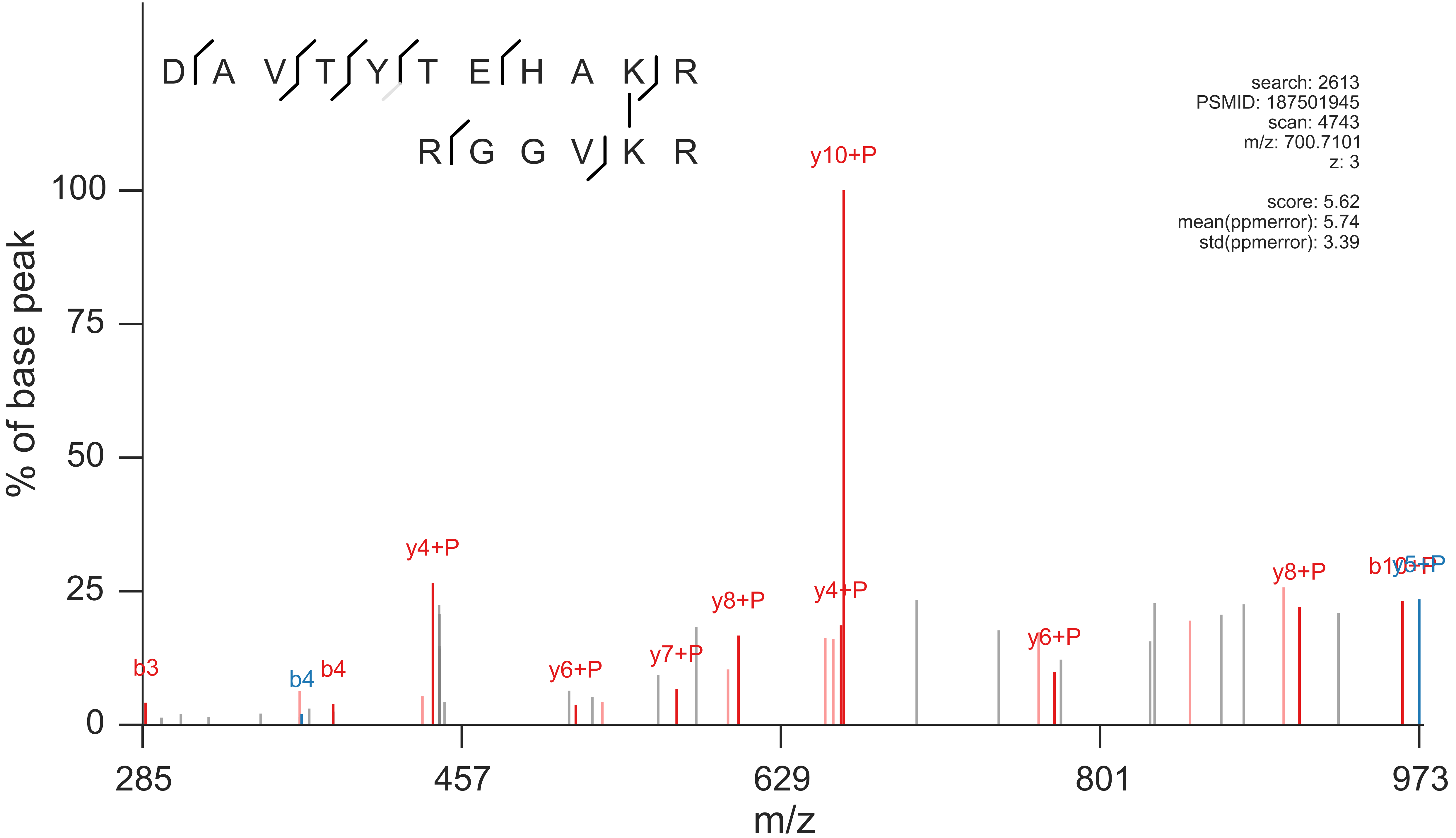

D A V T Y T E H A K R
R G G V K R
search: 2613
PSMID: 187501945
scan: 4743
m/z: 700.7101
z: 3
score: 5.62
mean(ppmerror): 5.74
std(ppmerror): 3.39
y10+P
y4+P
y8+P
y4+P
y7+P
y6+P
y6+P
y8+P
b3
b4
b4
% of base peak
100
75
50
25
0
285
457
629
801
973
m/z

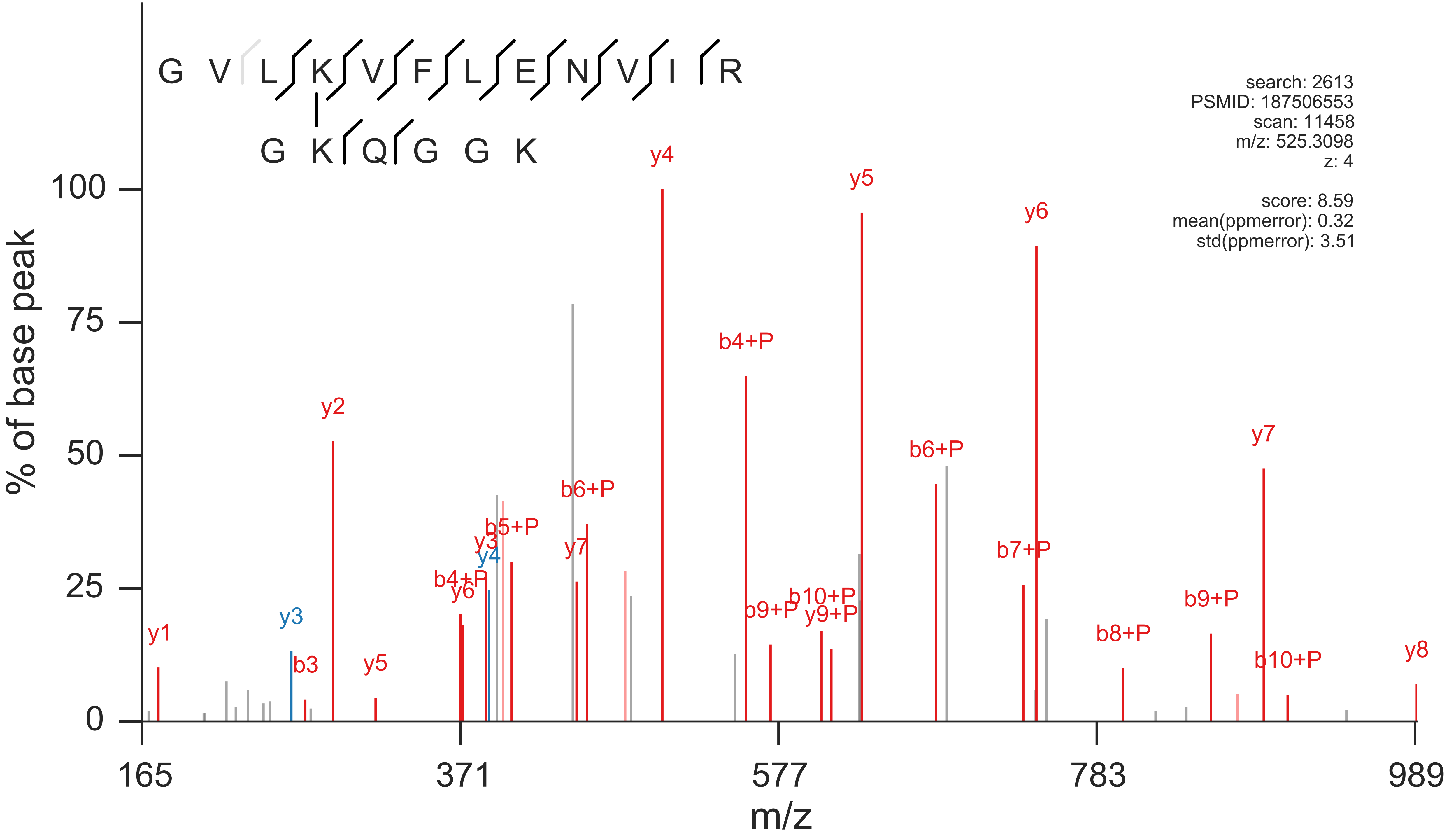
G V L K V F L E N V I R
G K Q G G K
search: 2613
PSMID: 187506553
scan: 11458
m/z: 525.3098
z: 4
score: 8.59
mean(ppmerror): 0.32
std(ppmerror): 3.51
% of base peak
m/z
165
371
577
783
989
0
25
50
75
100
y1
y3
b3
y2
y5
b4+P
y6
y3
y4
b5+P
b6+P
y7
y4
b4+P
b9+P
b10+P
y9+P
y5
b6+P
y6
b7+P
b8+P
b9+P
y7
b10+P
y8

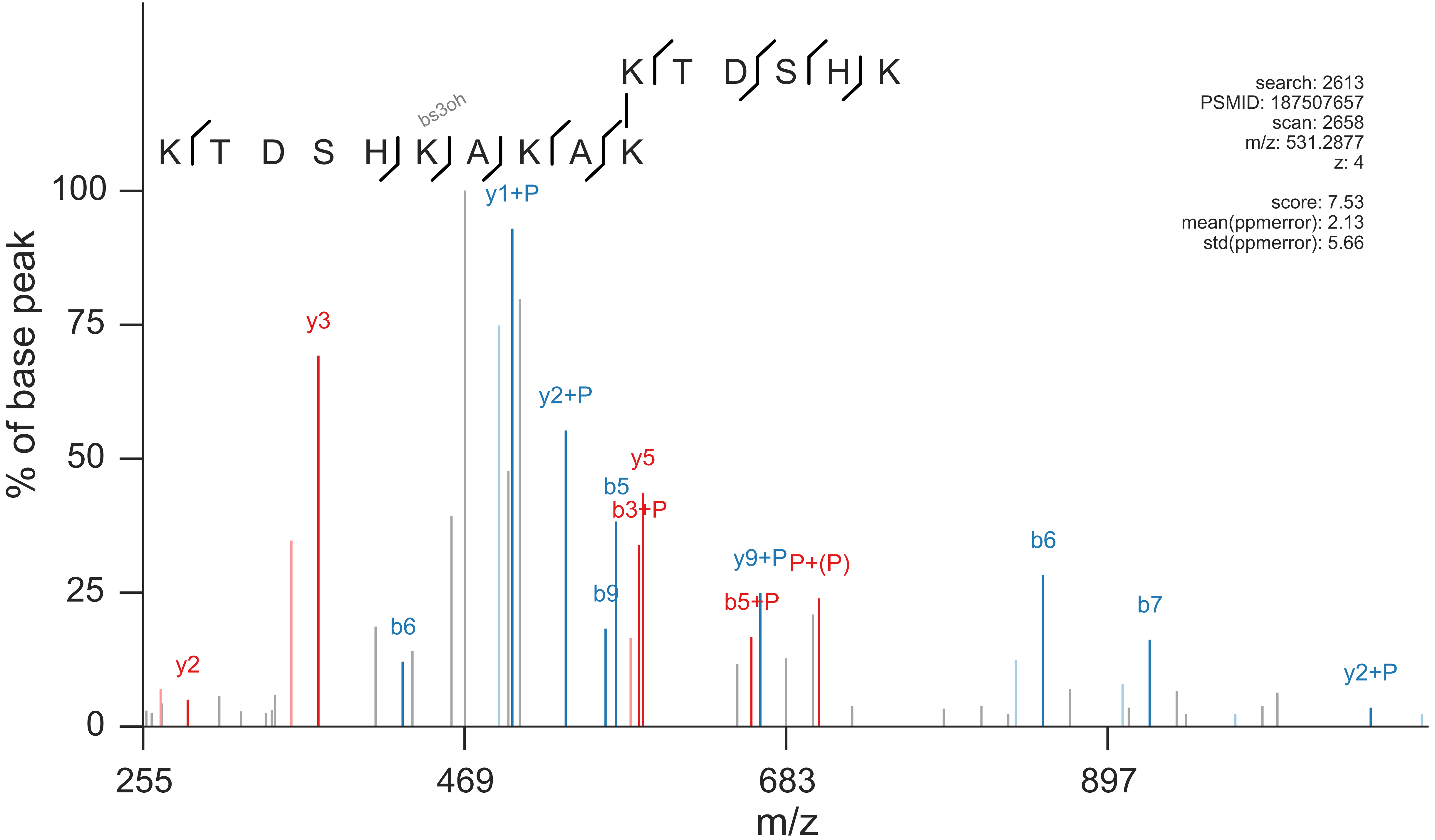

K T D S H K
K T D S H K A K A K
bs3oh
search: 2613
PSMID: 187507657
scan: 2658
m/z: 531.2877
z: 4
score: 7.53
mean(ppmerror): 2.13
std(ppmerror): 5.66
% of base peak
100
75
50
25
0
y1+P
y3
y2+P
y5
b5
b3+P
b6
y9+P
P+(P)
b9
b5+P
b6
b7
y2
y2+P
255
469
683
897
m/z

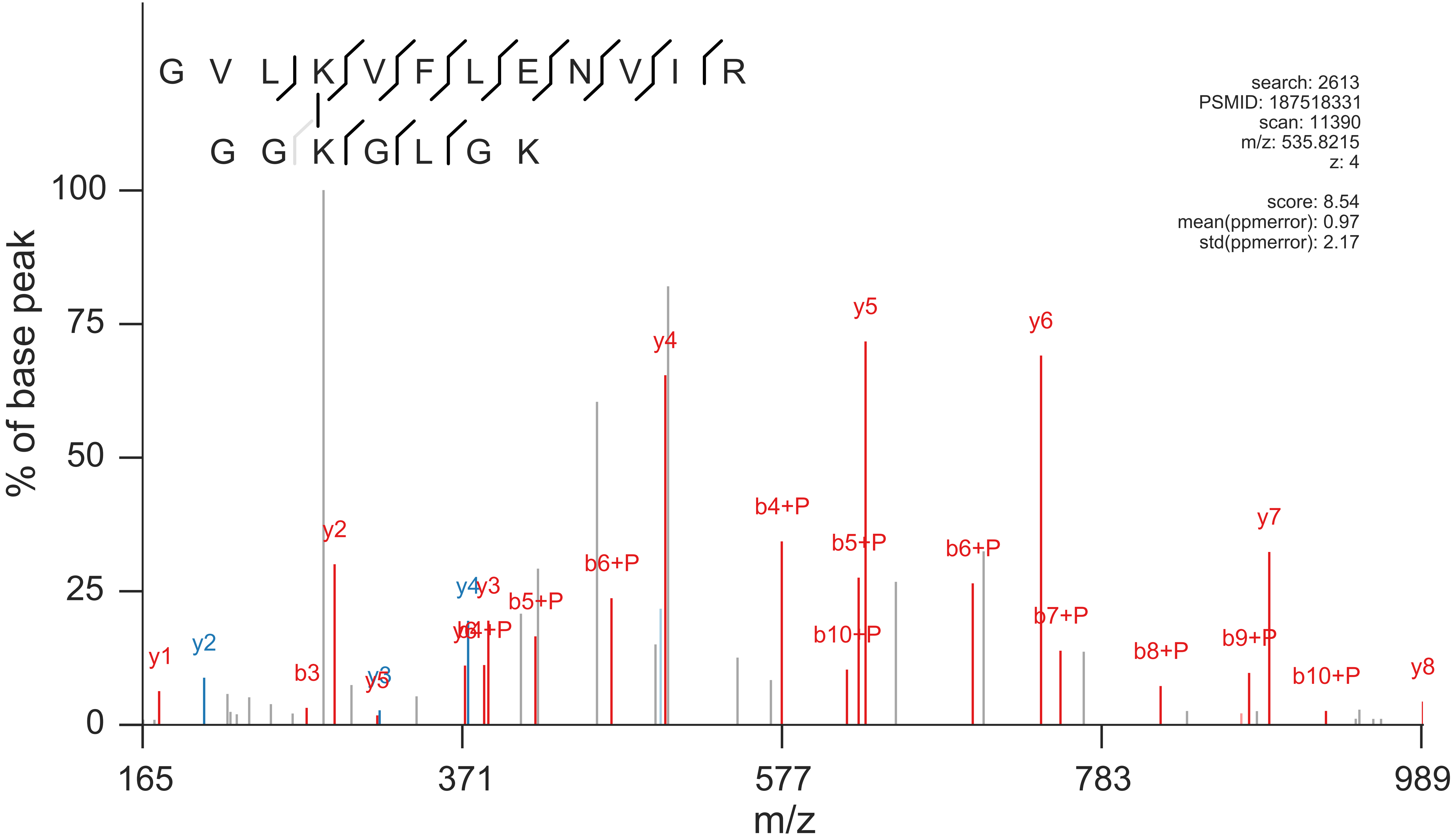
G V L K V F L E N V I R
G G K G L G K
search: 2613
PSMID: 187518331
scan: 11390
m/z: 535.8215
z: 4
score: 8.54
mean(ppmerror): 0.97
std(ppmerror): 2.17
% of base peak
m/z
0
25
50
75
100
165
371
577
783
989
y1
y2
b3
y2
y3
y4
y3
b5+P
b6+P
y4
b4+P
b5+P
b10+P
y5
b6+P
y6
b7+P
b8+P
b9+P
y7
b10+P
y8

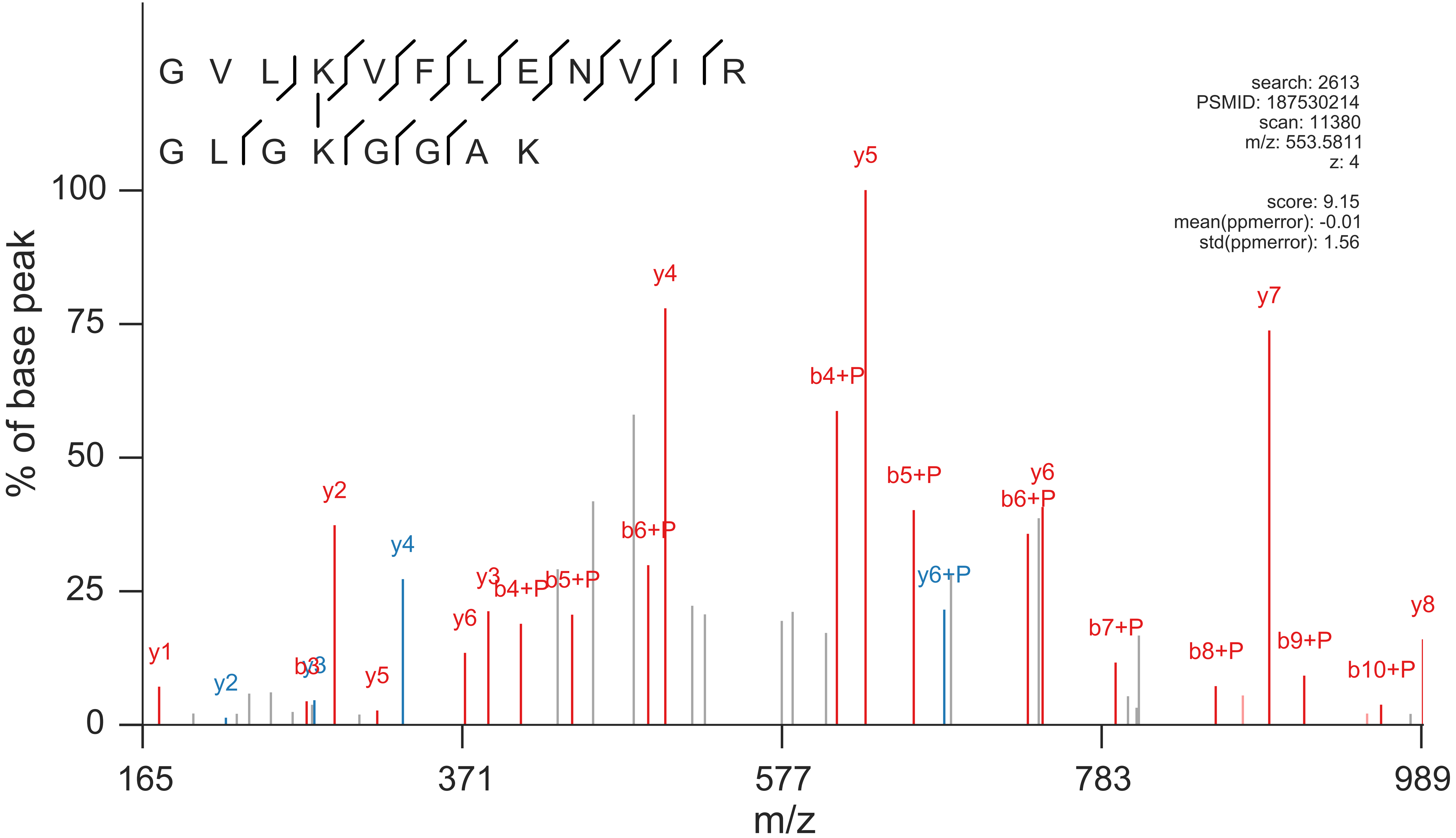

G V L K V F L E N V I R
G L G K G G A K
search: 2613
PSMID: 187530214
scan: 11380
m/z: 553.5811
z: 4
score: 9.15
mean(ppmerror): -0.01
std(ppmerror): 1.56
% of base peak
m/z
100
75
50
25
0
165
371
577
783
989
y1
y2
b3
y3
y2
y5
y4
y6
y3
b4+P
b5+P
b6+P
y4
b4+P
y5
b5+P
y6+P
b6+P
y6
b7+P
b8+P
y7
b9+P
b10+P
y8

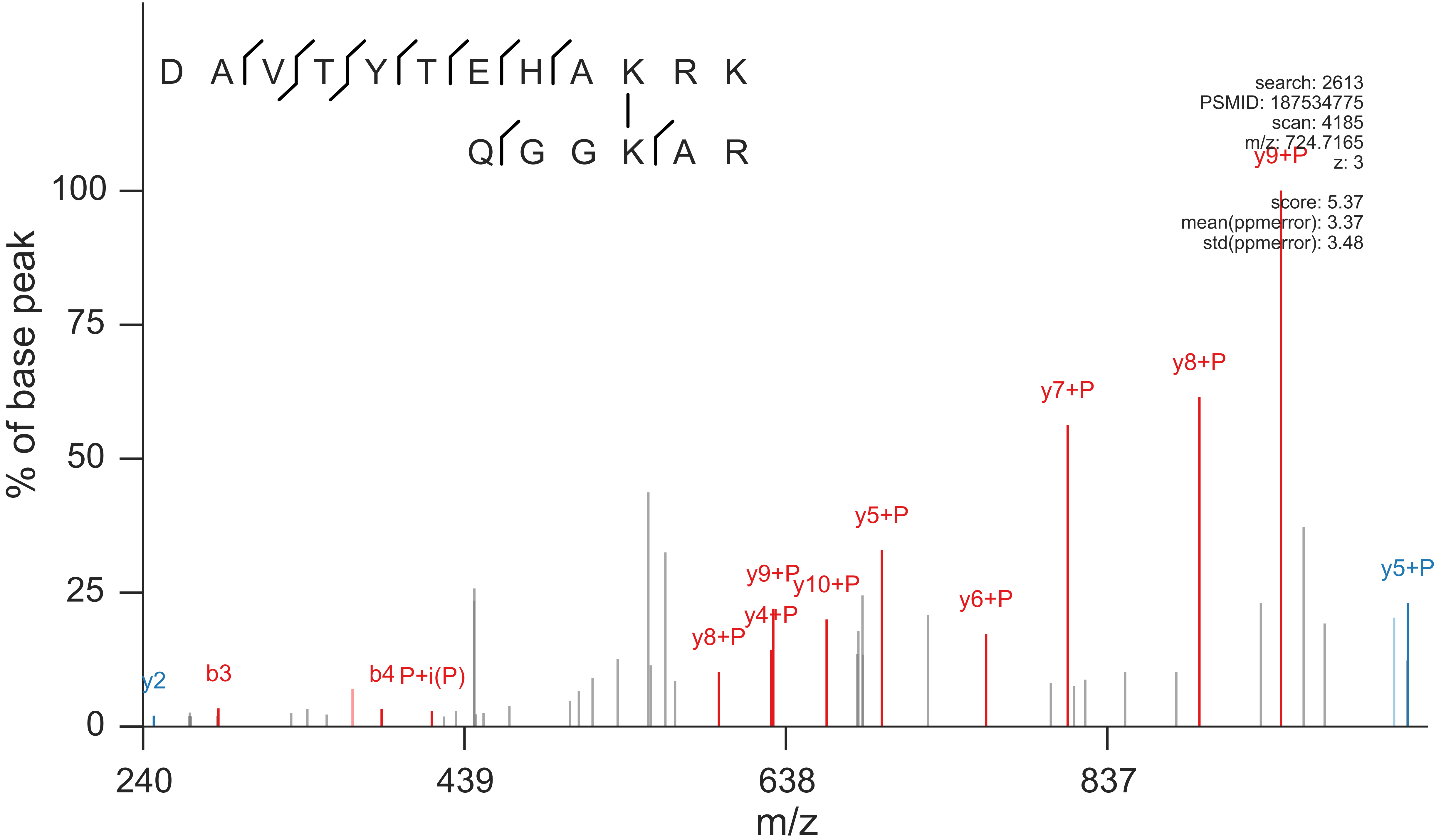

D A V T Y T E H A K R K
Q G G K A R
search: 2613
PSMID: 187534775
scan: 4185
m/z: 724.7165
z: 3
score: 5.37
mean(ppmerror): 3.37
std(ppmerror): 3.48
% of base peak
m/z
0
25
50
75
100
240
439
638
837
y2
b3
b4
P+i(P)
y8+P
y4+P
y9+P
y10+P
y5+P
y6+P
y7+P
y8+P
y9+P
y5+P

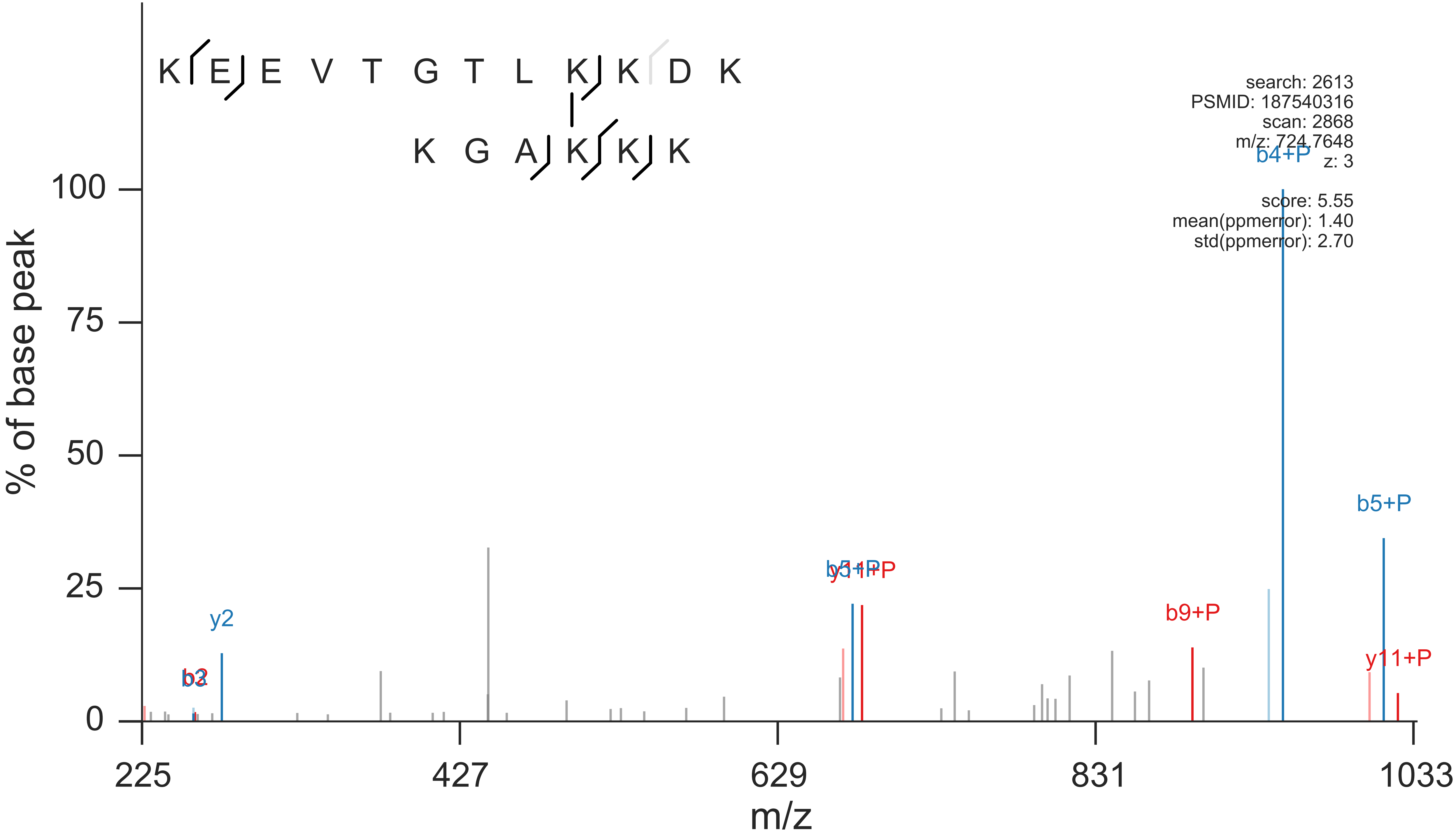

K E E V T G T L K K D K
K G A K K K
search: 2613
PSMID: 187540316
scan: 2868
m/z: 724.7648
z: 3
score: 5.55
mean(ppmerror): 1.40
std(ppmerror): 2.70
% of base peak
100
75
50
25
0
225
427
629
831
1033
m/z
b4+P
b5+P
b9+P
y11+P
y2
b2

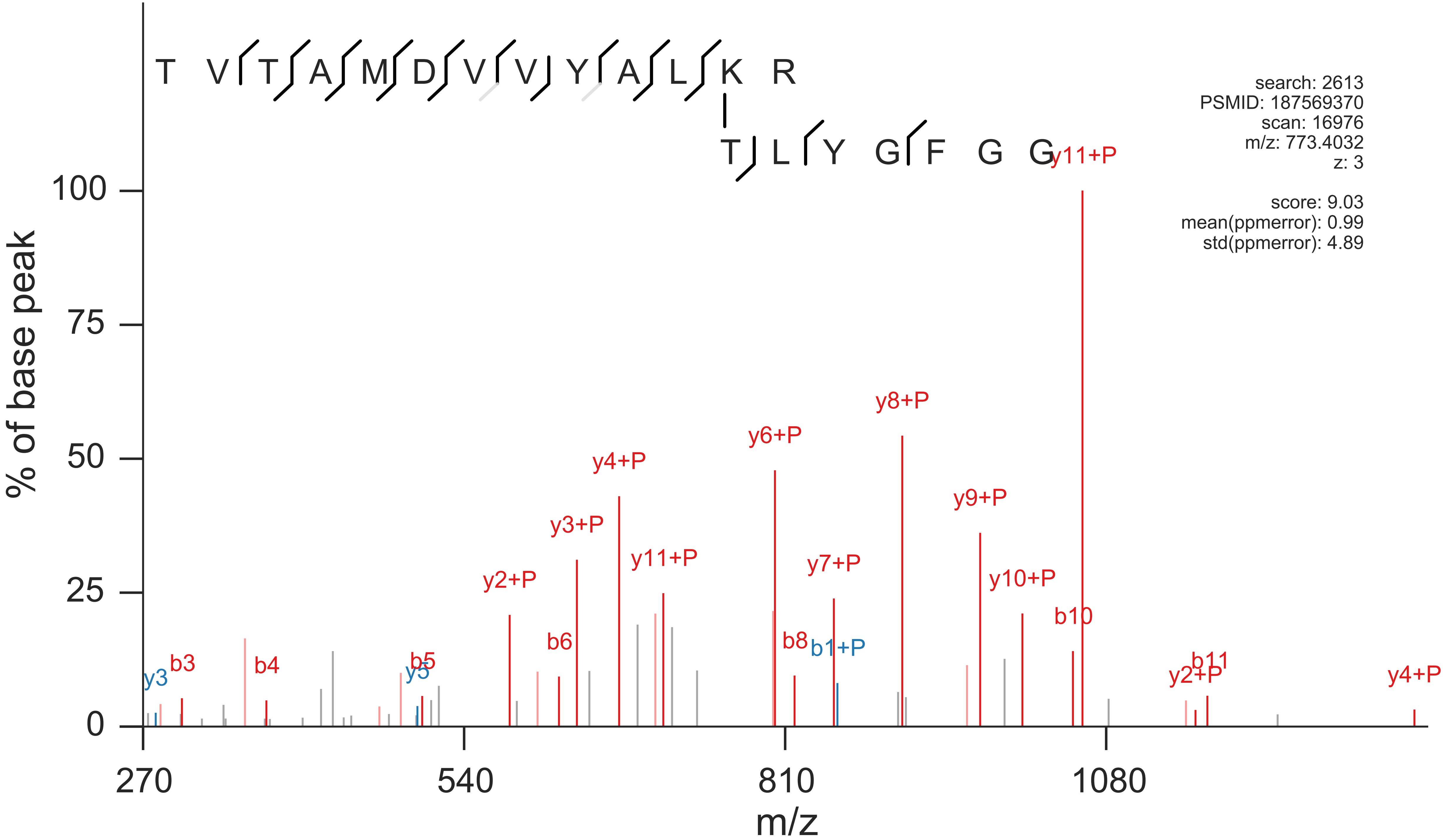

T V T A M D V V Y A L K R
T L Y G F G G
search: 2613
PSMID: 187569370
scan: 16976
m/z: 773.4032
z: 3
score: 9.03
mean(ppmerror): 0.99
std(ppmerror): 4.89
% of base peak
m/z
0
25
50
75
100
270
540
810
1080
y3
b3
b4
y5
b5
y2+P
b6
y3+P
y4+P
y11+P
y6+P
b8
b1+P
y7+P
y8+P
y9+P
y10+P
b10
y11+P
y2+P
b11
y4+P

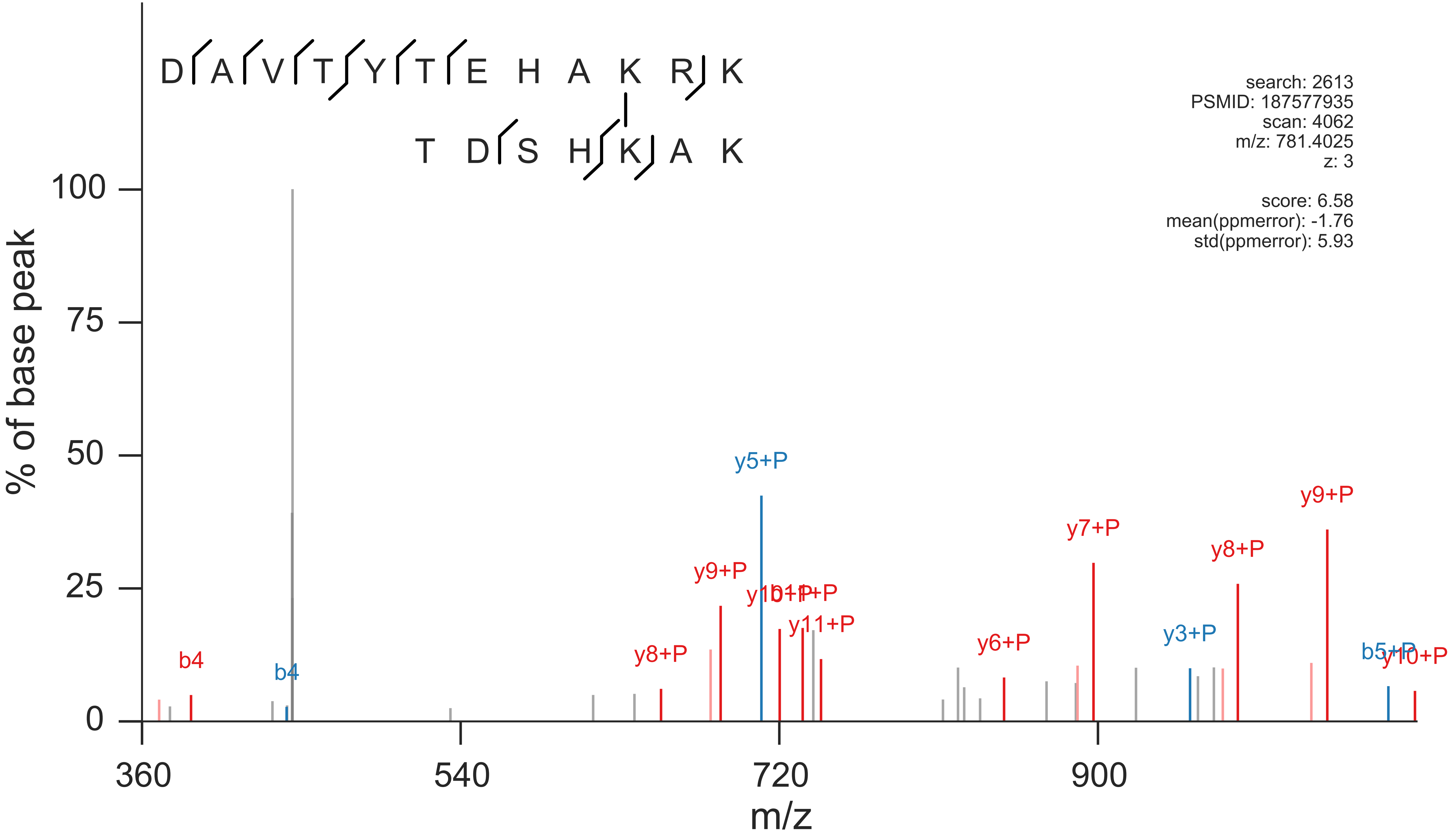

D A V T Y T E H A K R K
T D S H K A K
search: 2613
PSMID: 187577935
scan: 4062
m/z: 781.4025
z: 3
score: 6.58
mean(ppmerror): -1.76
std(ppmerror): 5.93
% of base peak
100
75
50
25
0
360
540
720
900
m/z
b4
b4
y8+P
y9+P
y5+P
y10+P
b11+P
y11+P
y6+P
y7+P
y3+P
y8+P
y9+P
b5+P
y10+P

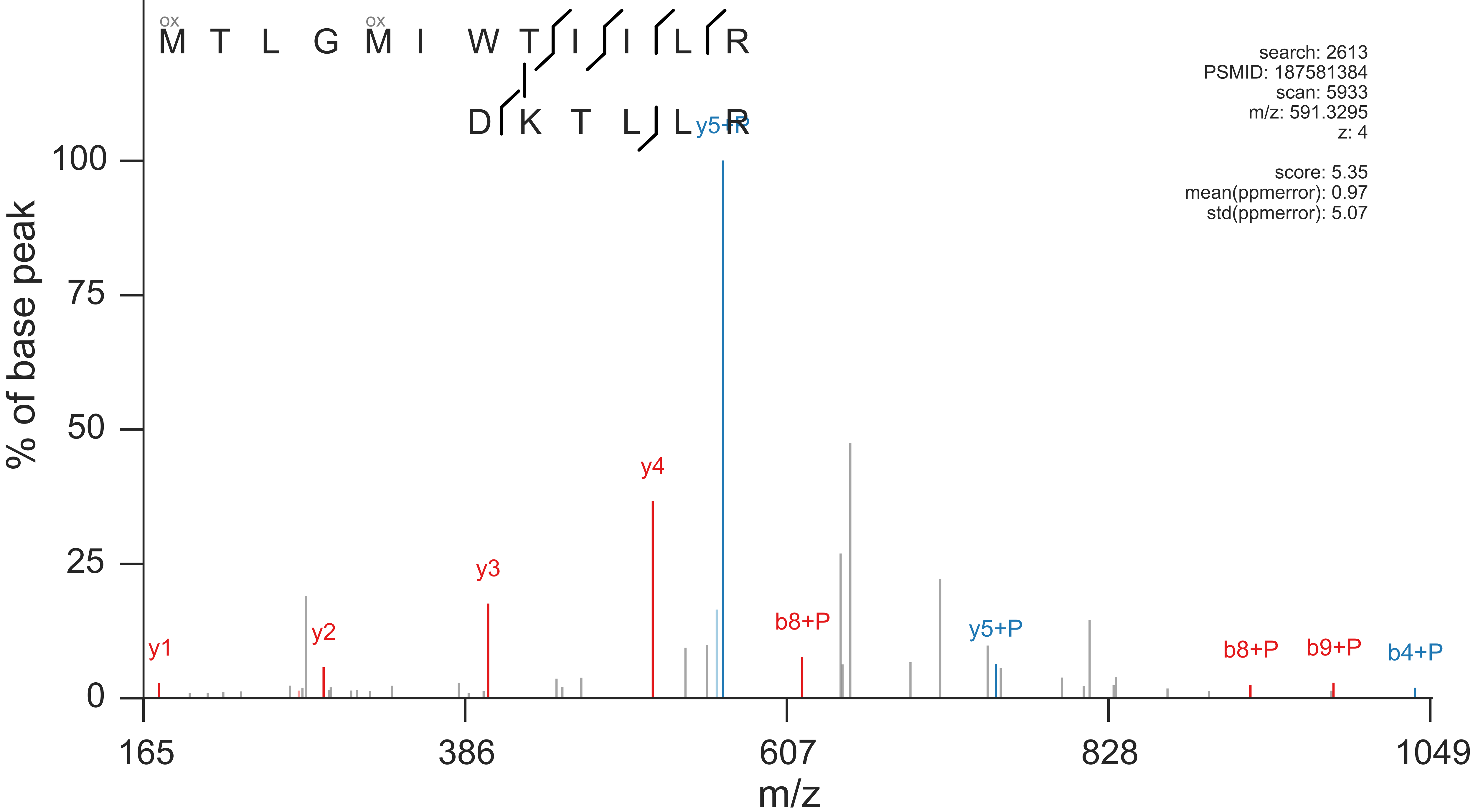

ox ox
M T L G M I W T I I L R
D K T L L R
search: 2613
PSMID: 187581384
scan: 5933
m/z: 591.3295
z: 4
score: 5.35
mean(ppmerror): 0.97
std(ppmerror): 5.07
% of base peak
100
75
50
25
0
y5+P
y4
y3
y2
y1
b8+P
y5+P
b8+P
b9+P
b4+P
165
386
607
828
1049
m/z

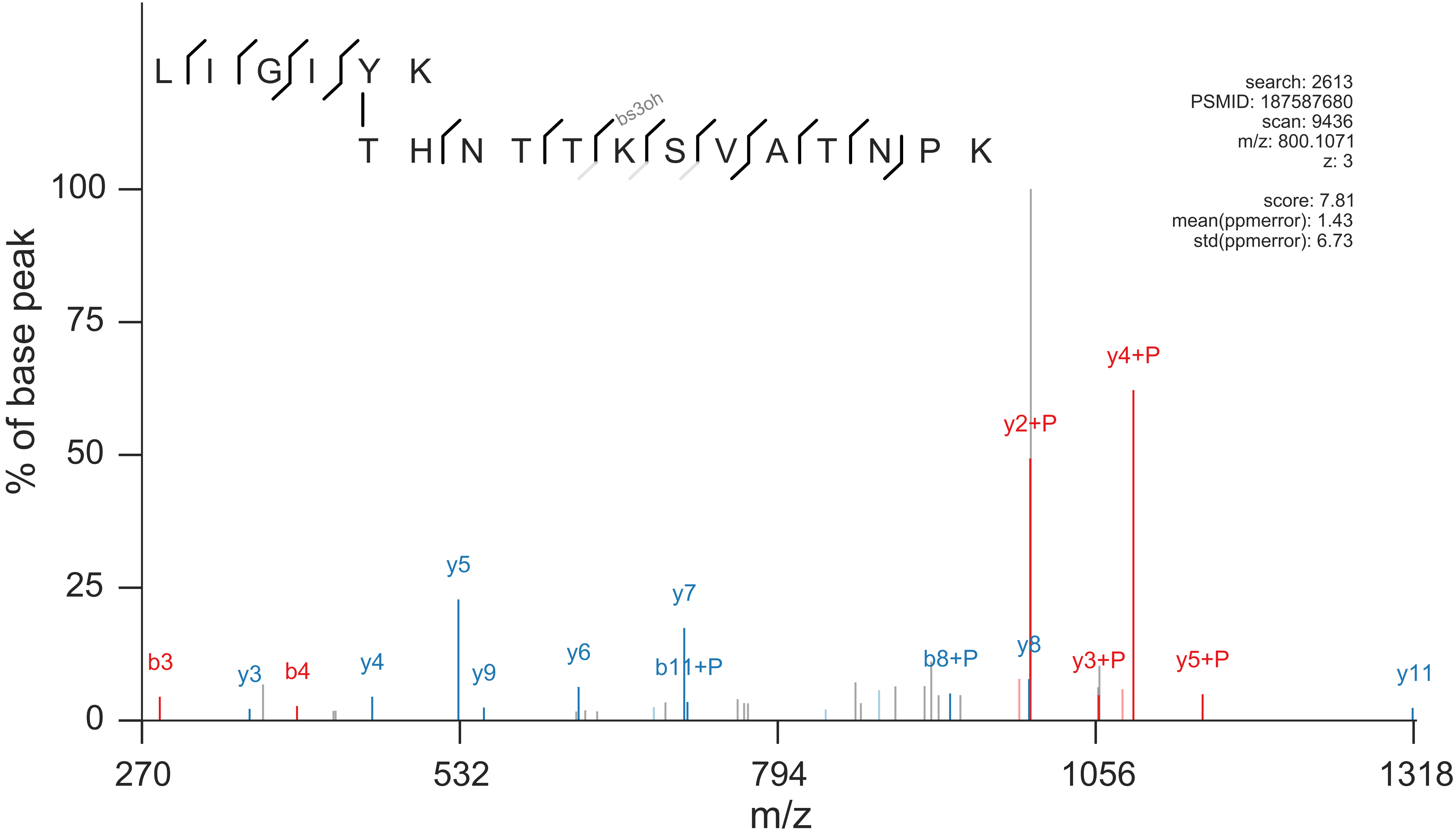

L I G I Y K
T H N T T K S V A T N P K
bs3oh
search: 2613
PSMID: 187587680
scan: 9436
m/z: 800.1071
z: 3
score: 7.81
mean(ppmerror): 1.43
std(ppmerror): 6.73
% of base peak
100
75
50
25
0
b3
y3
b4
y4
y5
y9
y6
y7
b11+P
b8+P
y8
y2+P
y4+P
y3+P
y5+P
y11
270
532
794
1056
1318
m/z

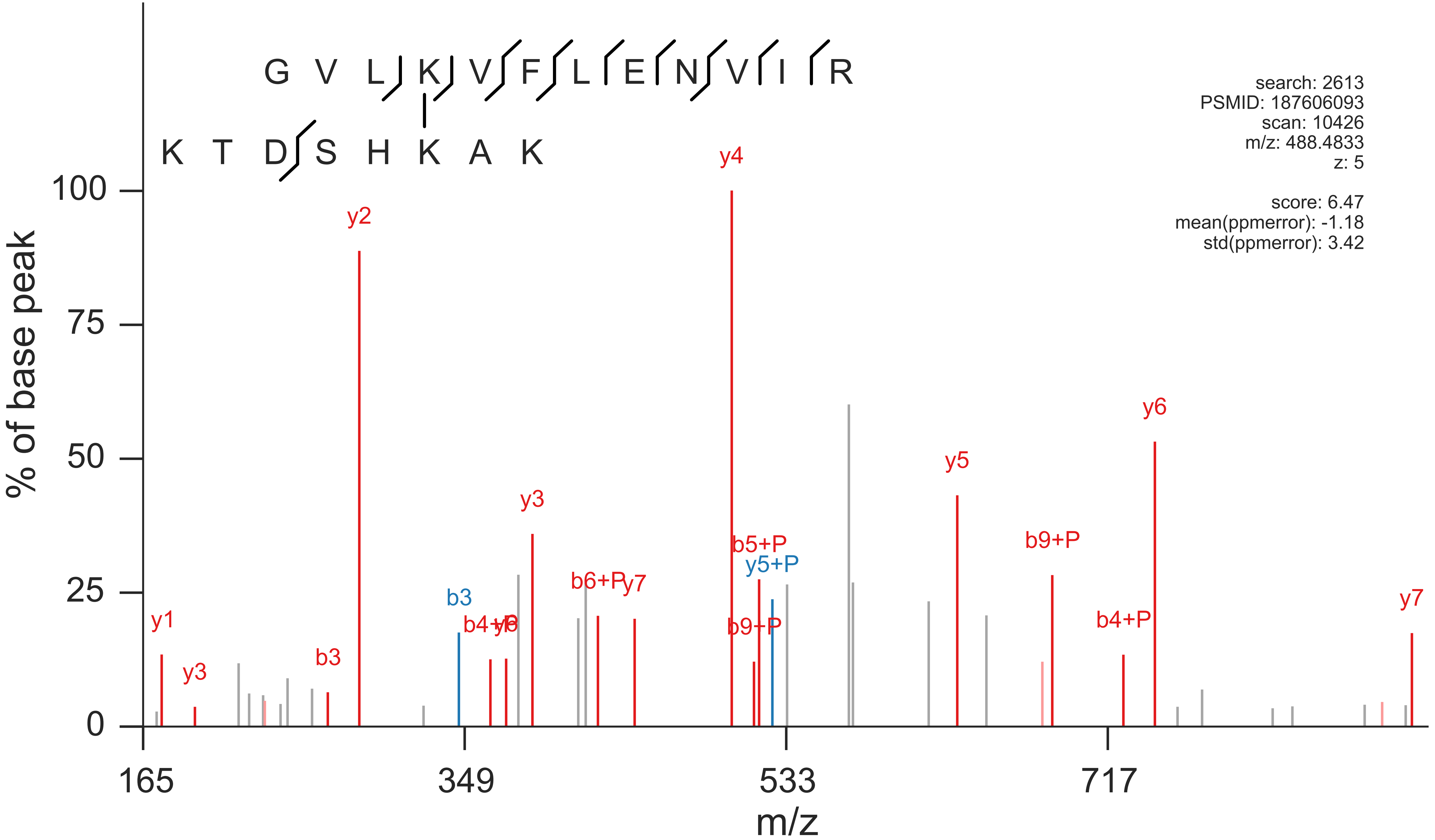

G V L K V F L E N V I R
K T D S H K A K
search: 2613
PSMID: 187606093
scan: 10426
m/z: 488.4833
z: 5
score: 6.47
mean(ppmerror): -1.18
std(ppmerror): 3.42
% of base peak
m/z
0
25
50
75
100
165
349
533
717
y1
y3
b3
y2
b3
b4+P
y3
b6+P
y7
y4
b5+P
y5+P
b9+P
y5
b9+P
b4+P
y6
y7

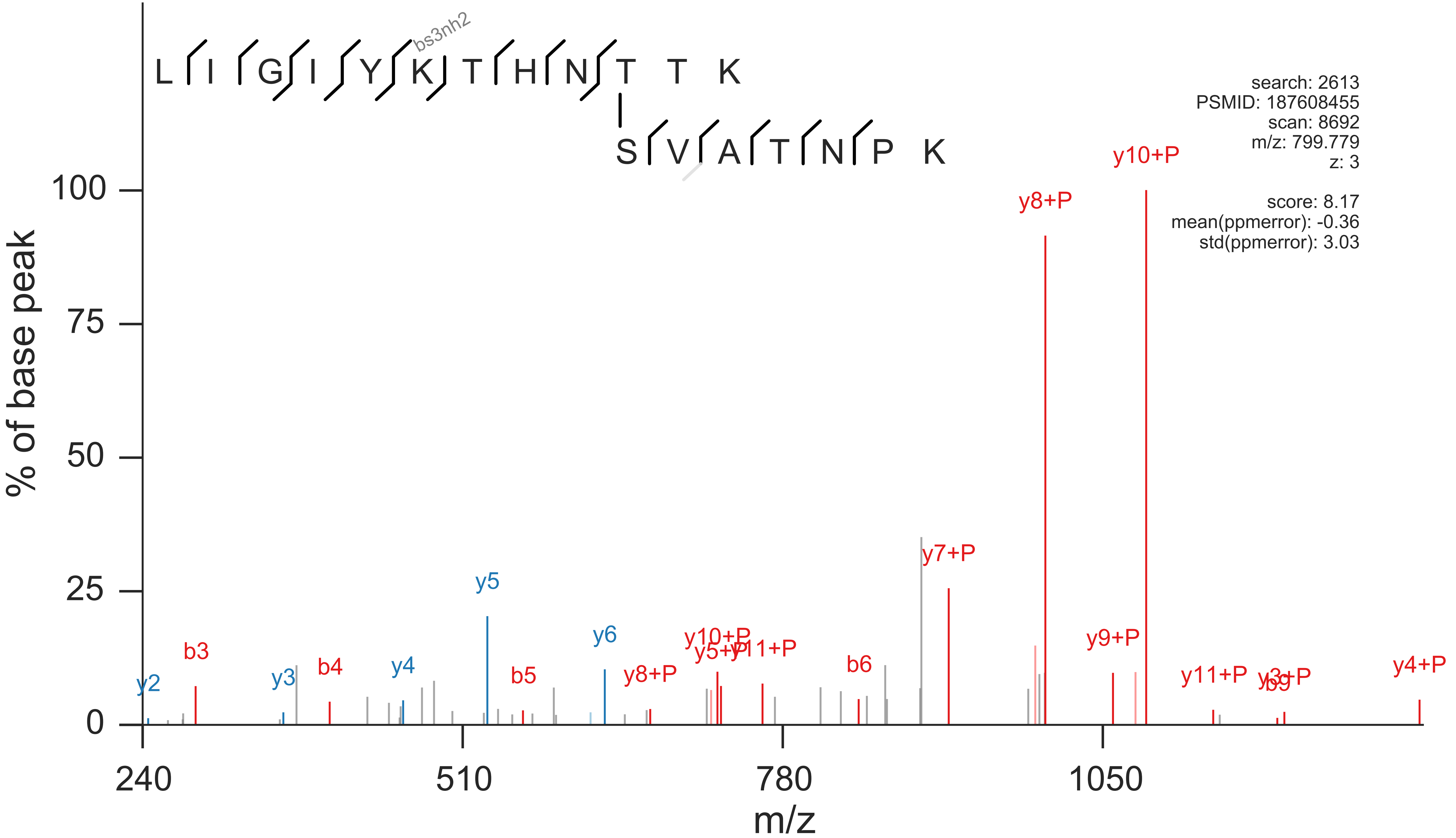

L I G I Y K T H N T T K
S V A T N P K
bs3nh2
search: 2613
PSMID: 187608455
scan: 8692
m/z: 799.779
z: 3
score: 8.17
mean(ppmerror): -0.36
std(ppmerror): 3.03
% of base peak
m/z
y2 y3 y4 y5 y6 b3 b4 b5 b6 y7+P y8+P y9+P y10+P y11+P y4+P y5+P y3+P b9

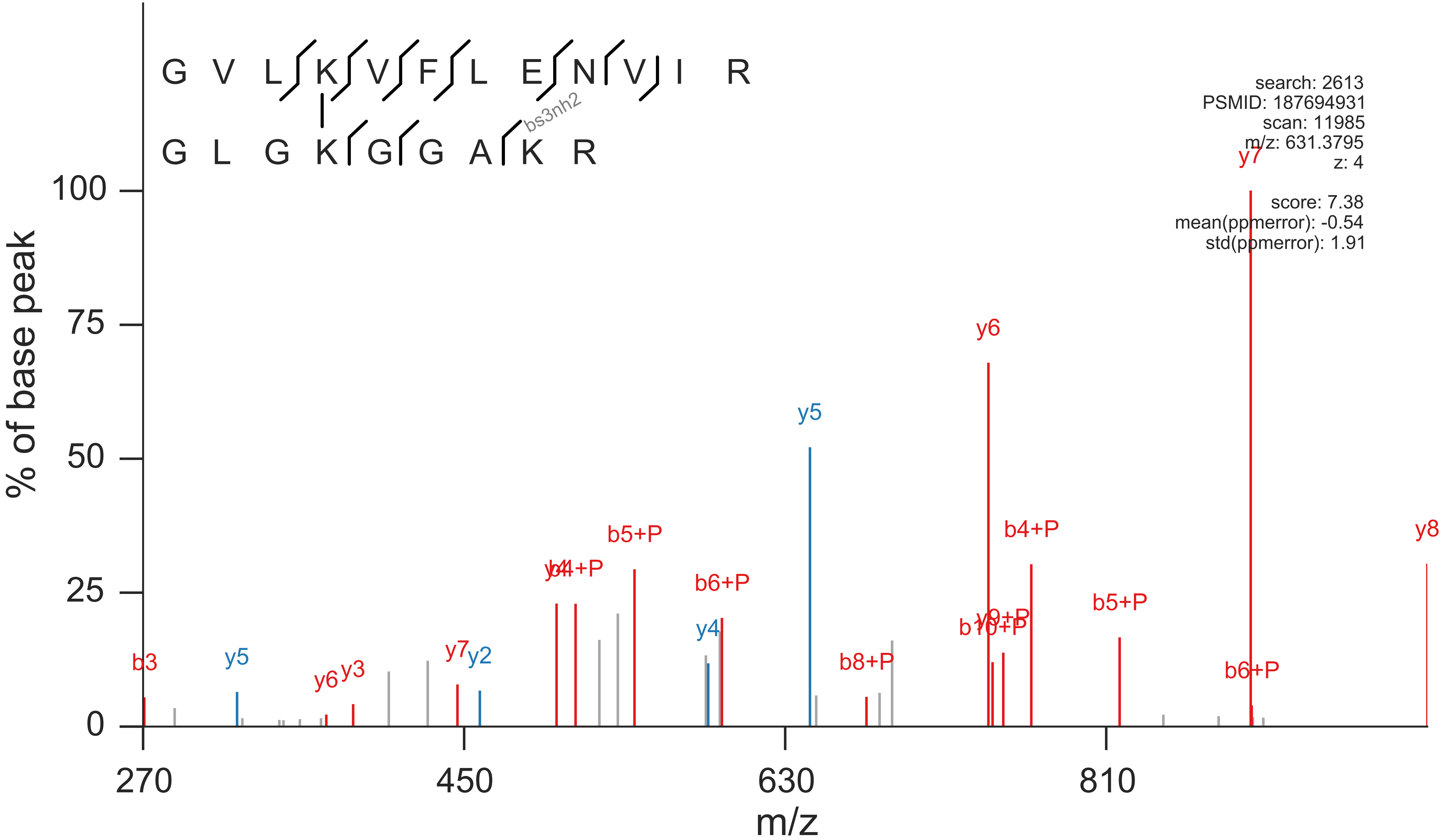

G V L K V F L E N V I R
G L G K G G A K R
bs3nh2
search: 2613
PSMID: 187694931
scan: 11985
m/z: 631.3795
z: 4
score: 7.38
mean(ppmerror): -0.54
std(ppmerror): 1.91
% of base peak
m/z
0
25
50
75
100
270
450
630
810
b3
y5
y6
y3
y7
y2
y4
b4+P
b5+P
b6+P
y4
y5
b8+P
y6
b10+P
y9+P
b4+P
b5+P
y7
b6+P
y8

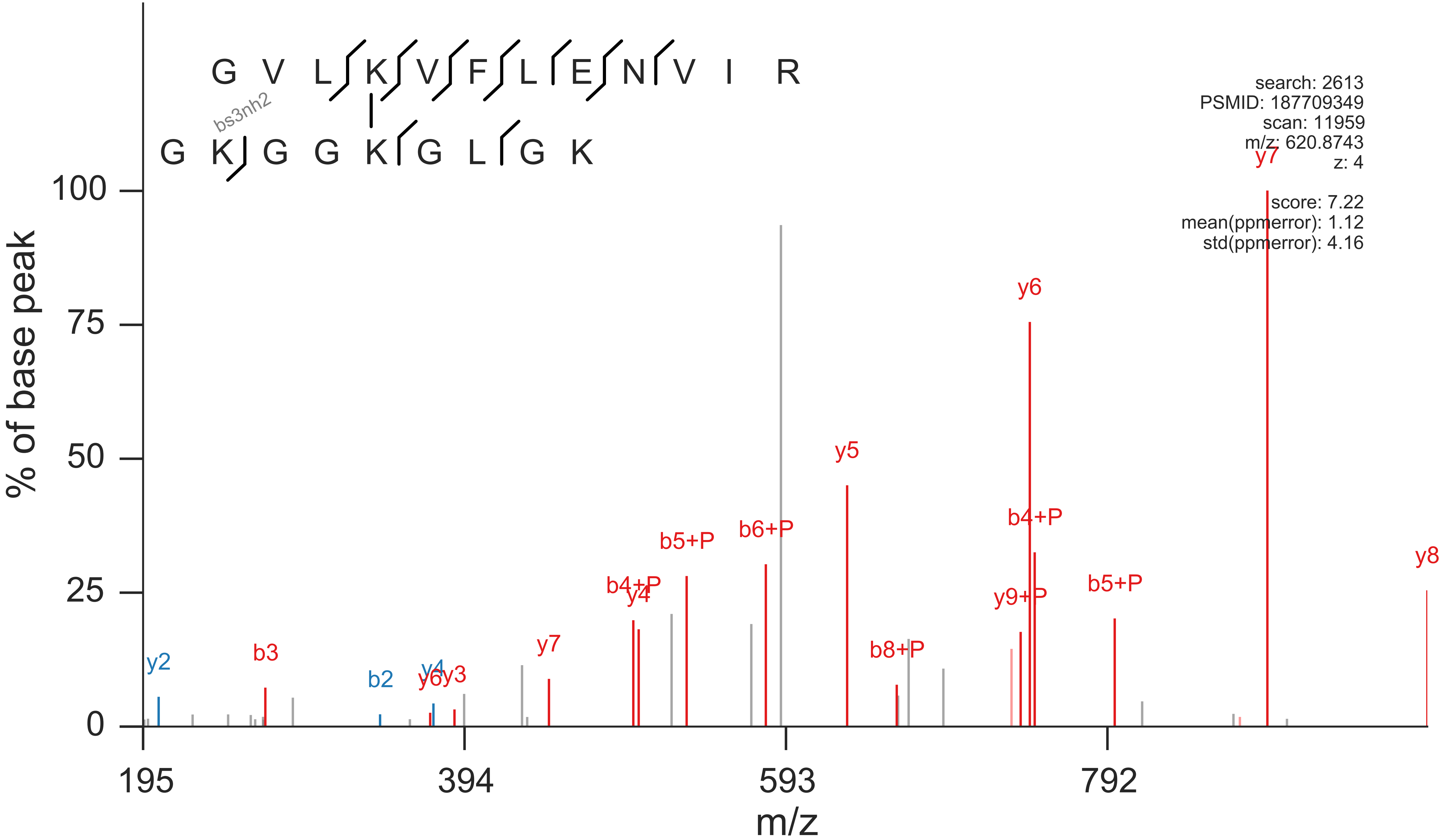

G V L K V F L E N V I R
bs3nh2
G K G G K G L G K
search: 2613
PSMID: 187709349
scan: 11959
m/z: 620.8743
z: 4
score: 7.22
mean(ppmerror): 1.12
std(ppmerror): 4.16
% of base peak
100
75
50
25
0
195
394
593
792
m/z
y2
b3
b2
y4
y6
y3
y7
b4+P
y4
b5+P
b6+P
y5
b8+P
y9+P
b4+P
y6
b5+P
y7
y8

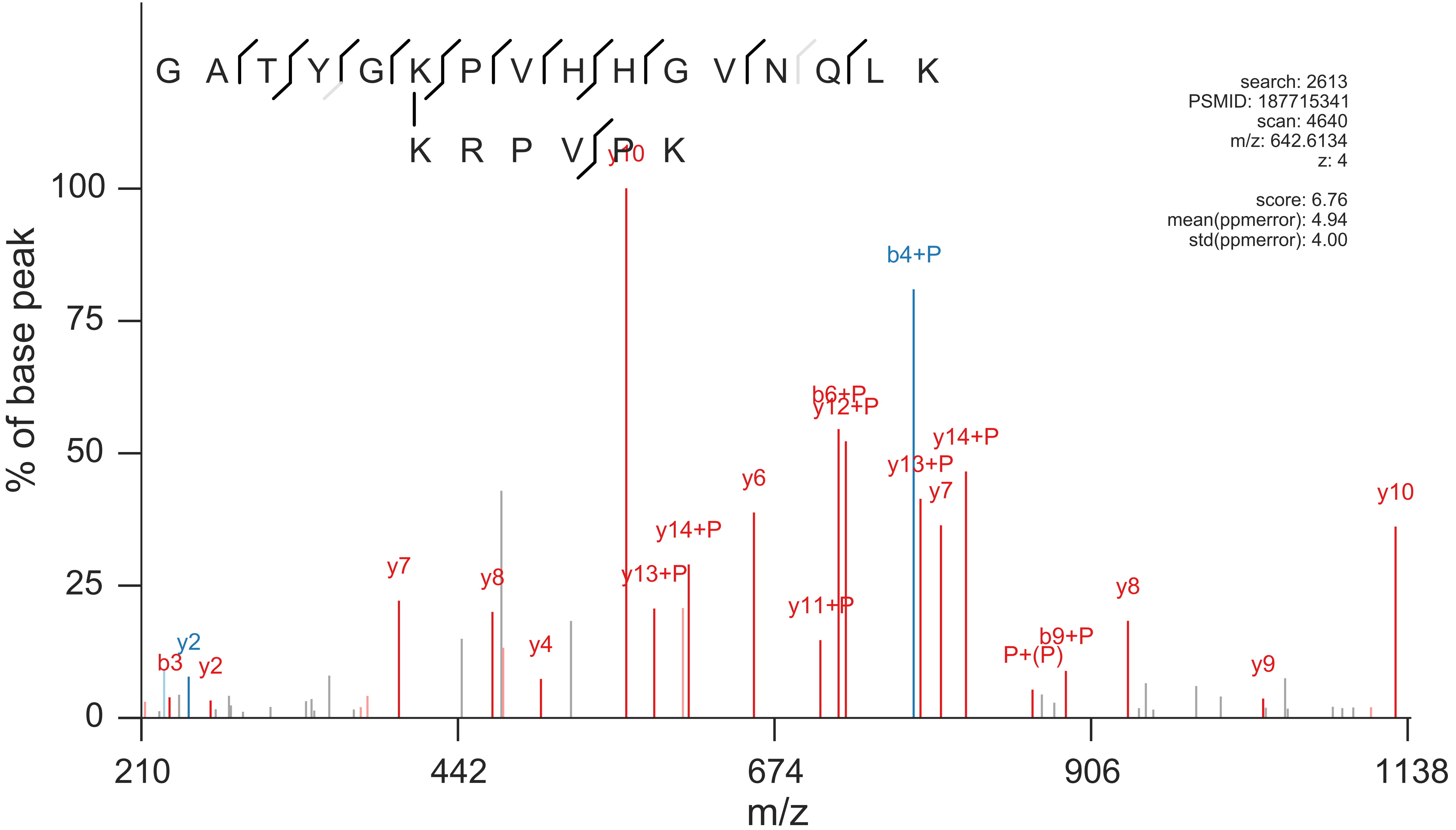

G A T Y G K P V H H G V N Q L K
K R P V P K
search: 2613
PSMID: 187715341
scan: 4640
m/z: 642.6134
z: 4
score: 6.76
mean(ppmerror): 4.94
std(ppmerror): 4.00
% of base peak
m/z
100
75
50
25
0
210
442
674
906
1138
y10
b4+P
b6+P
y12+P
y14+P
y13+P
y6
y7
y10
y14+P
y13+P
y7
y8
y11+P
y8
b9+P
P+(P)
y4
y2
b3
y2
y9

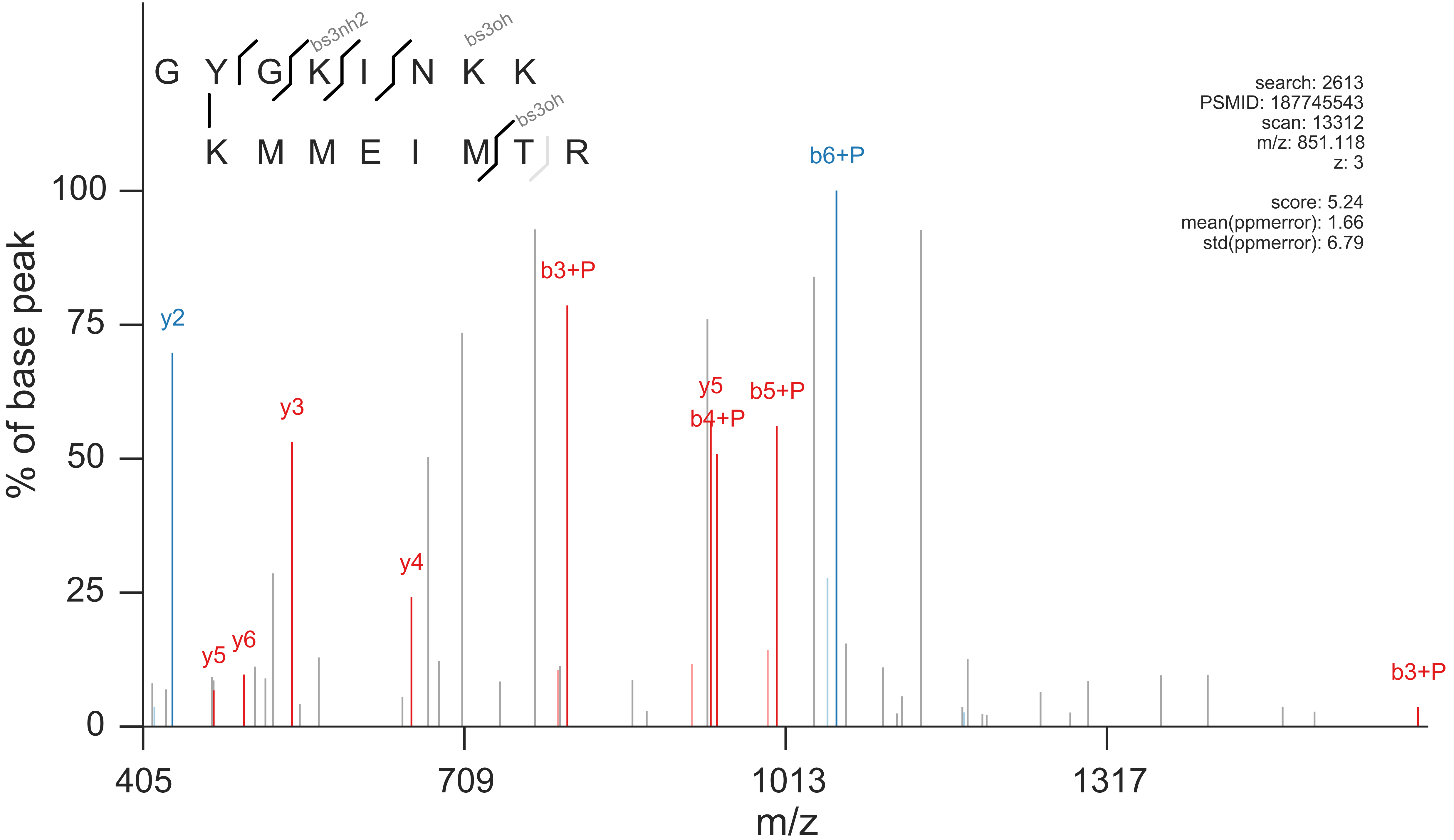

G Y G K I N K K
K M M E I M T R
bs3nh2
bs3oh
bs3oh
search: 2613
PSMID: 187745543
scan: 13312
m/z: 851.118
z: 3
score: 5.24
mean(ppmerror): 1.66
std(ppmerror): 6.79
b6+P
b3+P
y2
y3
y5
b4+P
b5+P
y4
y5
y6
b3+P
% of base peak
100
75
50
25
0
405
709
1013
1317
m/z

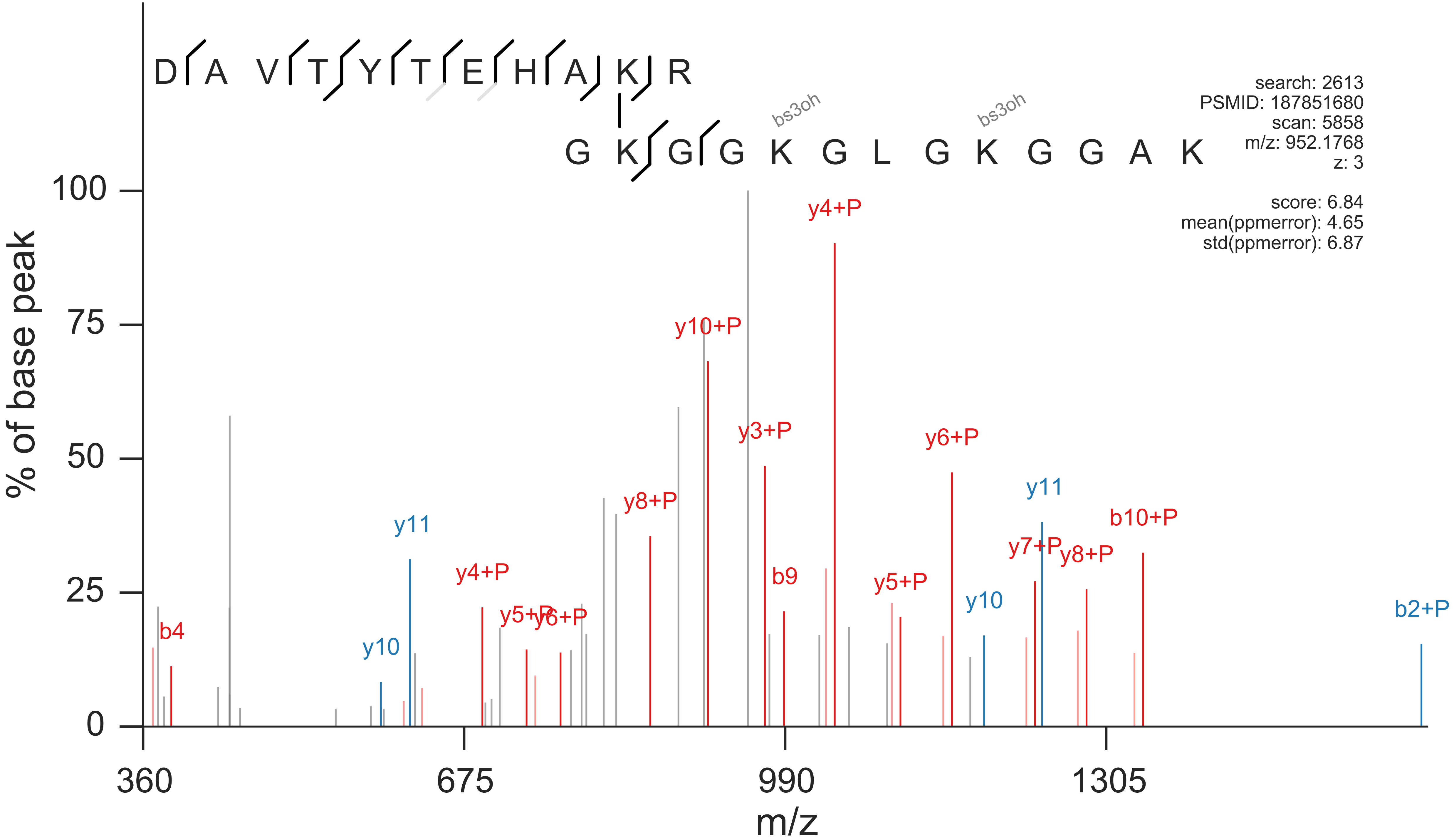

D A V T Y T E H A K R
G K G G K G L G K G G A K
bs3oh
bs3oh
search: 2613
PSMID: 187851680
scan: 5858
m/z: 952.1768
z: 3
score: 6.84
mean(ppmerror): 4.65
std(ppmerror): 6.87
% of base peak
m/z
0
25
50
75
100
360
675
990
1305
b4
y10
y11
y4+P
y5+P
y6+P
y8+P
y10+P
y3+P
b9
y4+P
y5+P
y6+P
y10
y11
y7+P
y8+P
b10+P
b2+P

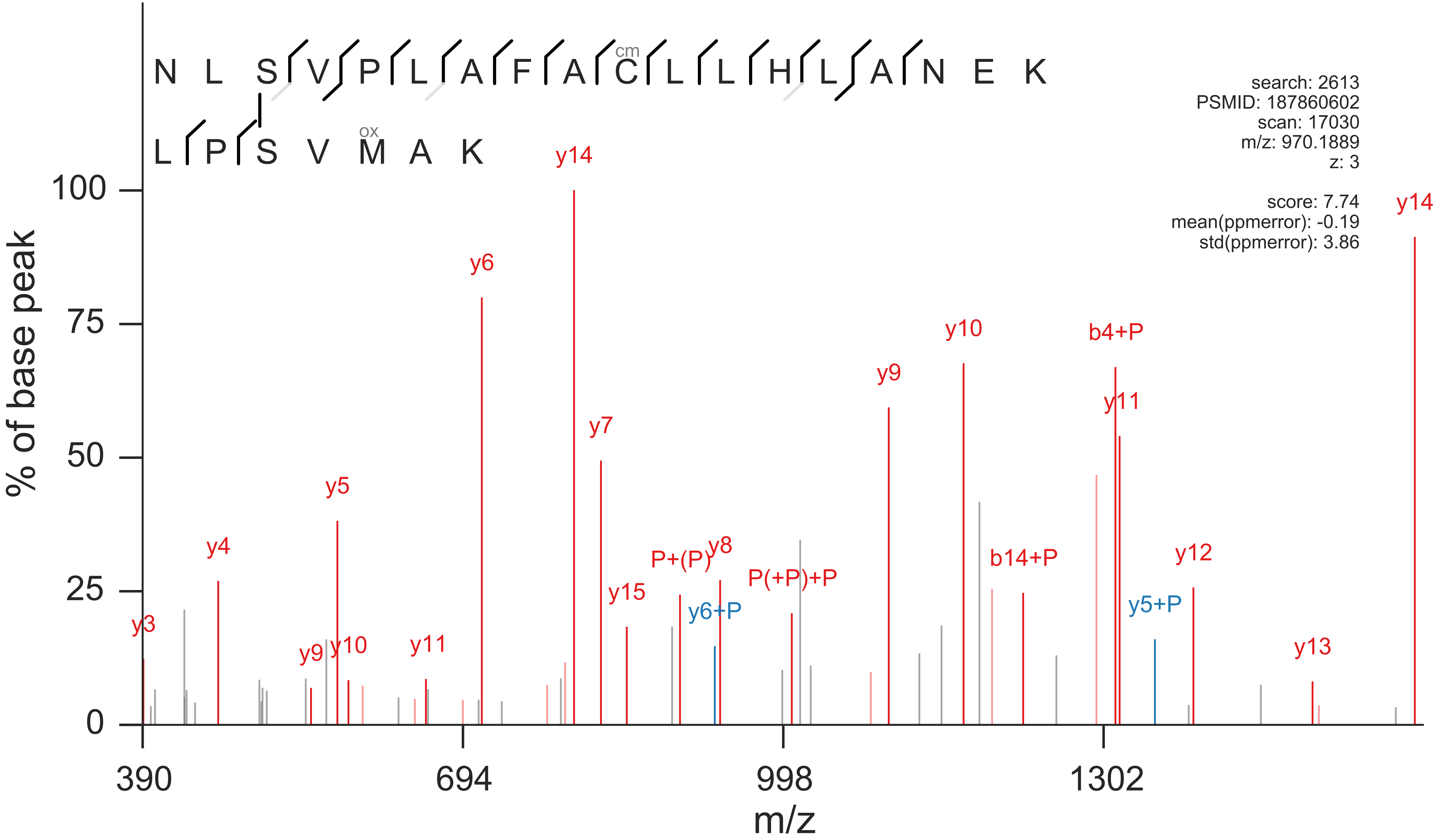

N L S V P L A F A C L L H L A N E K
cm
L P S V M A K
ox
search: 2613
PSMID: 187860602
scan: 17030
m/z: 970.1889
z: 3
score: 7.74
mean(ppmerror): -0.19
std(ppmerror): 3.86
% of base peak
m/z
0
25
50
75
100
390
694
998
1302
y3
y4
y9
y5
y10
y11
y6
y14
y7
y15
P+(P)
y8
y6+P
P(+P)+P
y9
y10
b14+P
b4+P
y11
y5+P
y12
y13
y14

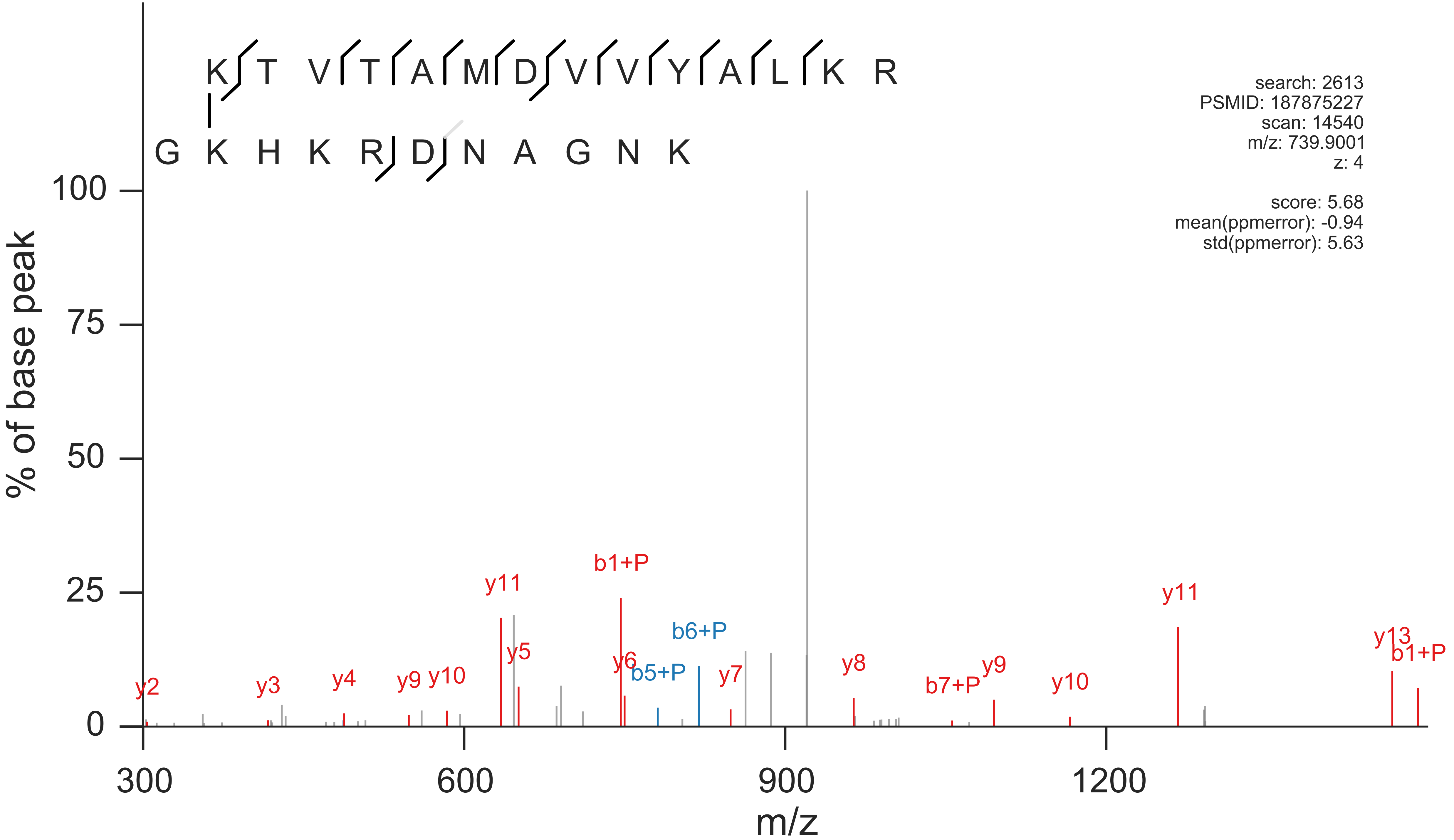

K T V T A M D V V Y A L K R
G K H K R D N A G N K
search: 2613
PSMID: 187875227
scan: 14540
m/z: 739.9001
z: 4
score: 5.68
mean(ppmerror): -0.94
std(ppmerror): 5.63
% of base peak
100
75
50
25
0
300
600
900
1200
m/z
y2
y3
y4
y9
y10
y11
y5
b1+P
y6
b5+P
b6+P
y7
y8
b7+P
y9
y10
y11
y13
b1+P

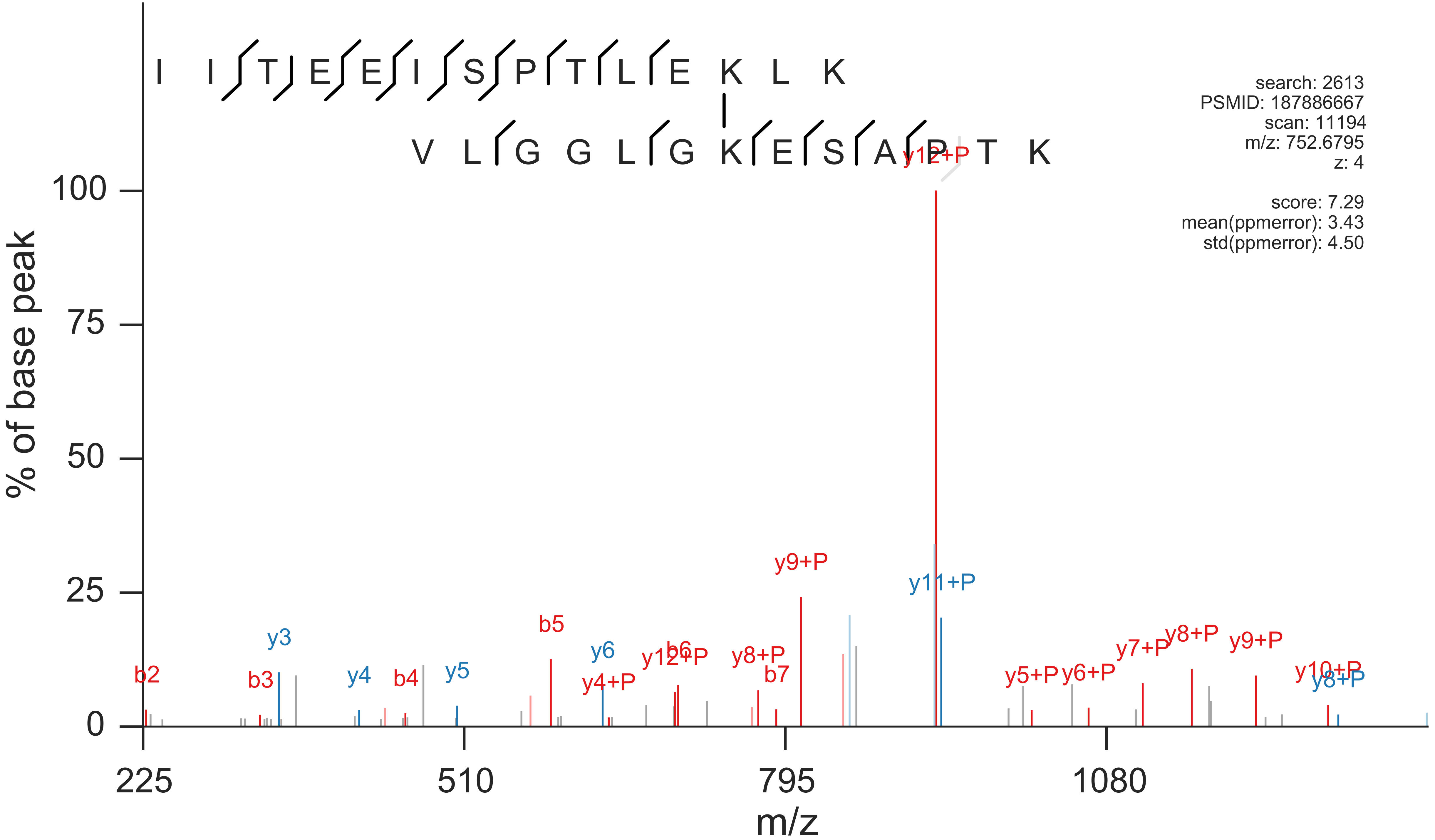
I I T E E I S P T L E K L K
V L G G L G K E S A P T K
search: 2613
PSMID: 187886667
scan: 11194
m/z: 752.6795
z: 4
score: 7.29
mean(ppmerror): 3.43
std(ppmerror): 4.50
% of base peak
m/z
0
25
50
75
100
225
510
795
1080
y12+P
y11+P
y9+P
b5
y3
y6
b6
y12+P
y8+P
b7
b2
b3
y4
b4
y5
y4+P
y5+P
y6+P
y7+P
y8+P
y9+P
y10+P
y8+P

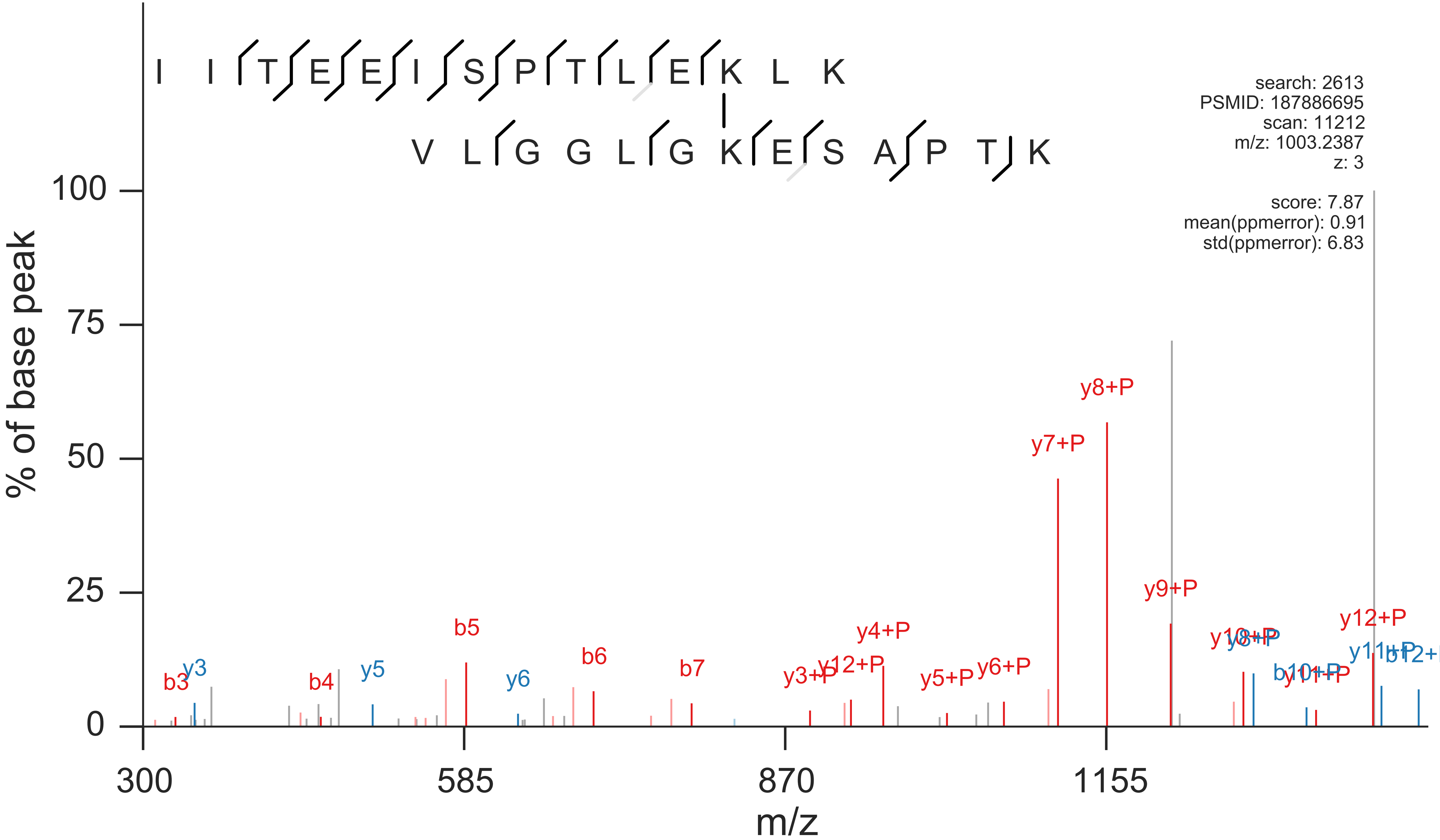
I I T E E I S P T L E K L K
V L G G L G K E S A P T K
search: 2613
PSMID: 187886695
scan: 11212
m/z: 1003.2387
z: 3
score: 7.87
mean(ppmerror): 0.91
std(ppmerror): 6.83
% of base peak
m/z
y8+P
y7+P
y9+P
y12+P
b5
b6
b7
y4+P
y12+P
y3+P
y5+P
y6+P
b3
y3
b4
y5
y6

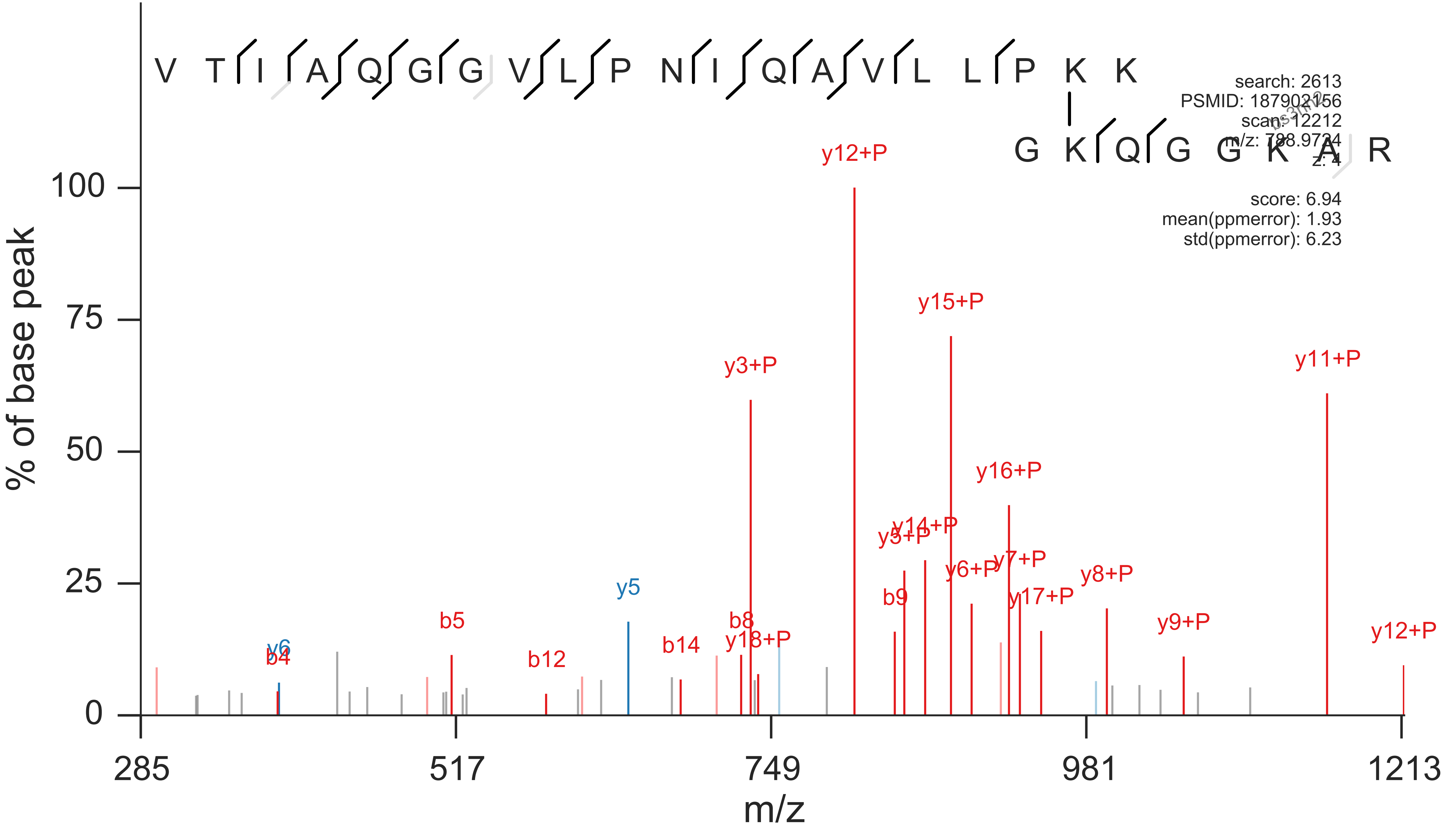

V T I A Q G G V L P N I Q A V L L P K K
G K Q G G K A R
search: 2613
PSMID: 1879021[illegible]6
scan: 12212
m/z: 7[illegible]8.97[illegible]4
z: 4
score: 6.94
mean(ppmerror): 1.93
std(ppmerror): 6.23
% of base peak
m/z
100
75
50
25
0
285
517
749
981
1213
y12+P
y15+P
y3+P
y11+P
y16+P
y14+P
y6+P
y7+P
y8+P
y17+P
y9+P
y12+P
y5
b5
b12
b14
b8
y18+P
b9
y6
b4

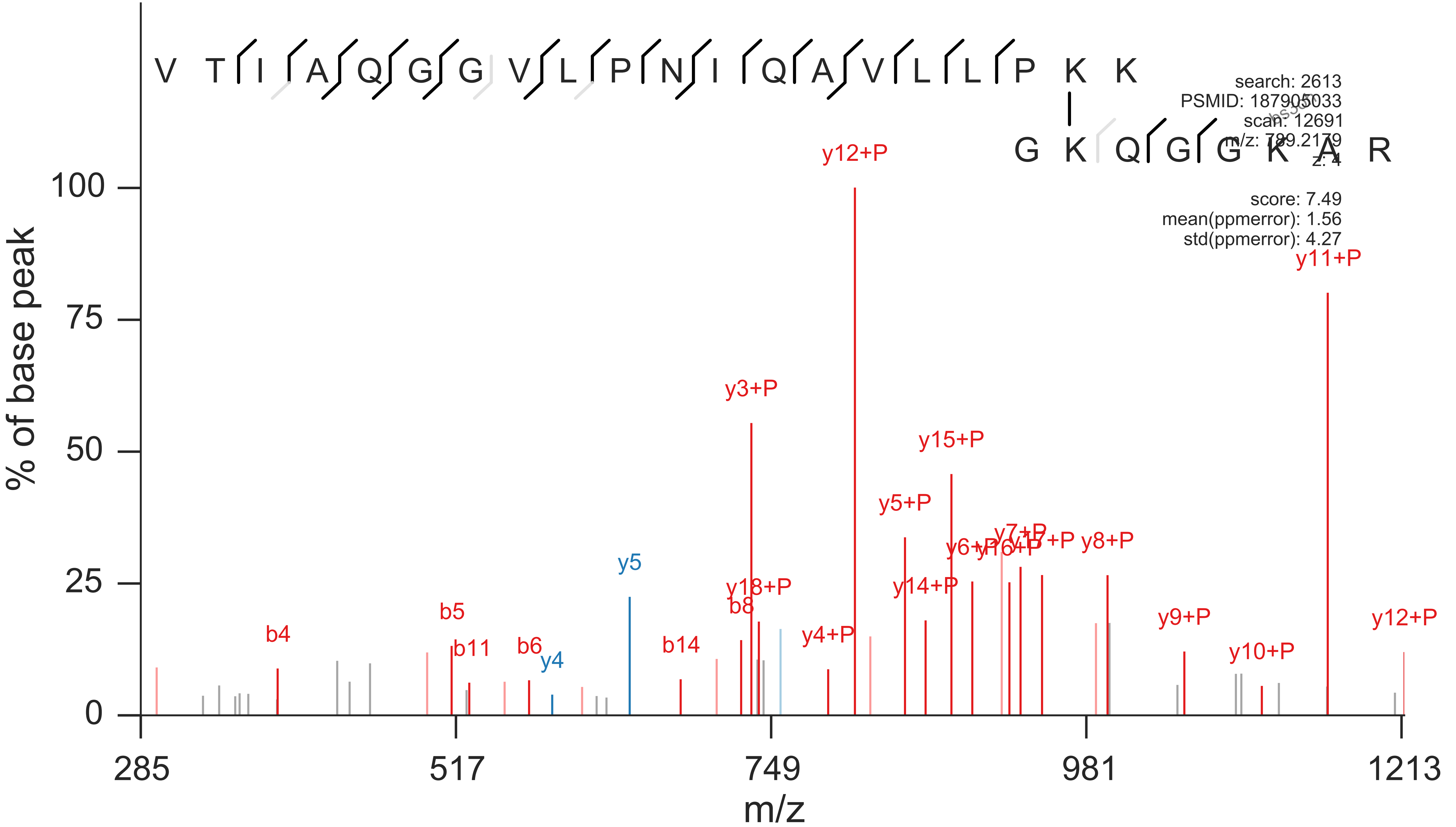
V T I A Q G G V L P N I Q A V L L P K K
G K Q G G K A R
search: 2613
PSMID: 187905033
scan: 12691
m/z: 789.2179
z: 4
score: 7.49
mean(ppmerror): 1.56
std(ppmerror): 4.27
% of base peak
m/z
y12+P
y11+P
y3+P
y15+P
y5+P
y7+P
y8+P
y14+P
y18+P
b8
y5
b4
b5
b11
b6
y4
b14
y4+P
y9+P
y10+P
0
25
50
75
100
285
517
749
981
1213

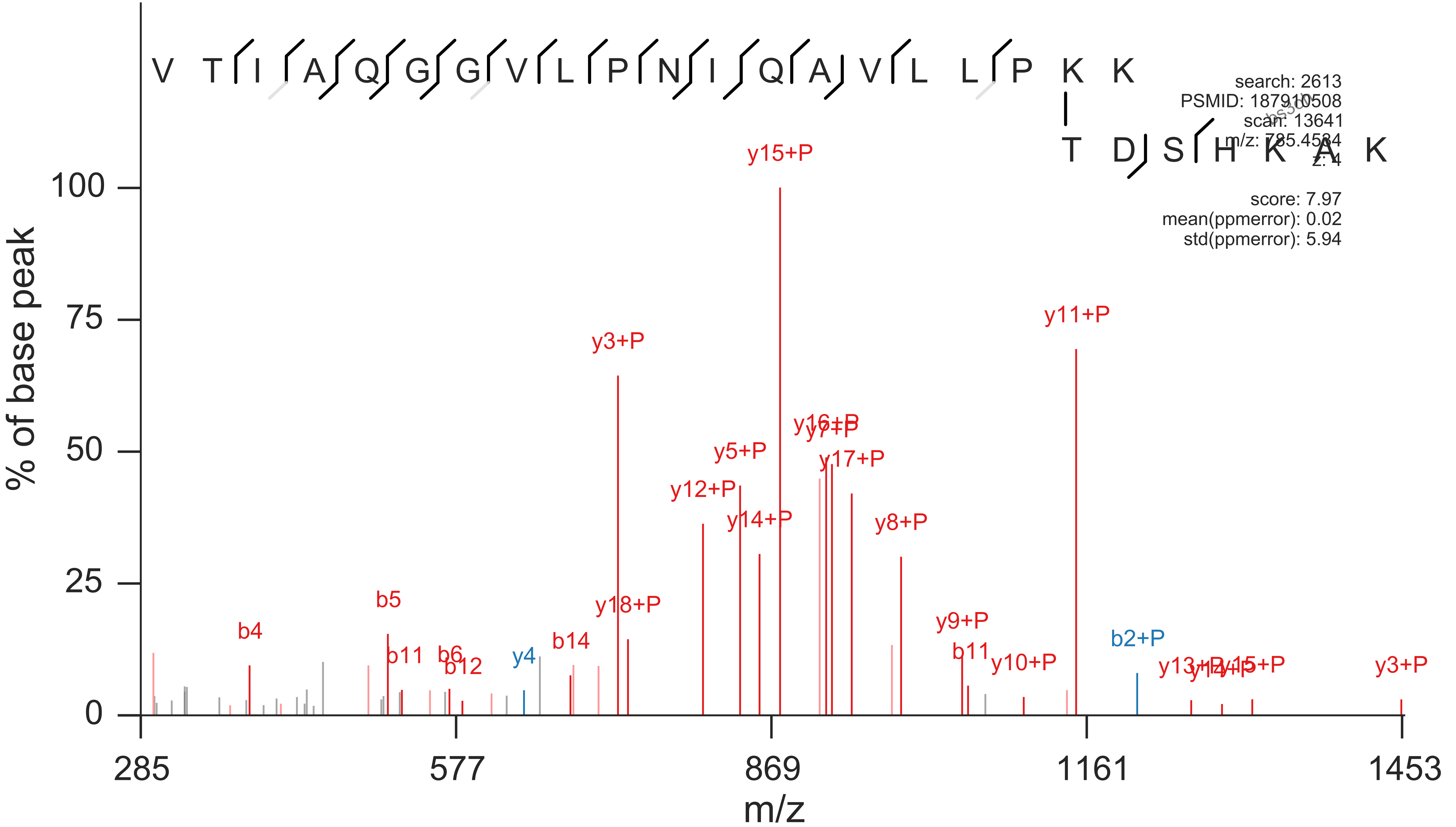
V T I A Q G G V L P N I Q A V L L P K K
T D S H K A K
search: 2613
PSMID: 1879[illegible]0508
scan: 13641
m/z: 785.45[illegible]4
z: 4
score: 7.97
mean(ppmerror): 0.02
std(ppmerror): 5.94
% of base peak
m/z
0
25
50
75
100
285
577
869
1161
1453
y15+P
y11+P
y3+P
y16+P
y7+P
y5+P
y17+P
y12+P
y14+P
y8+P
y18+P
y9+P
b11
y10+P
b2+P
y13+P
y14+P
y15+P
y3+P
b4
b5
b11
b6
b12
y4
b14

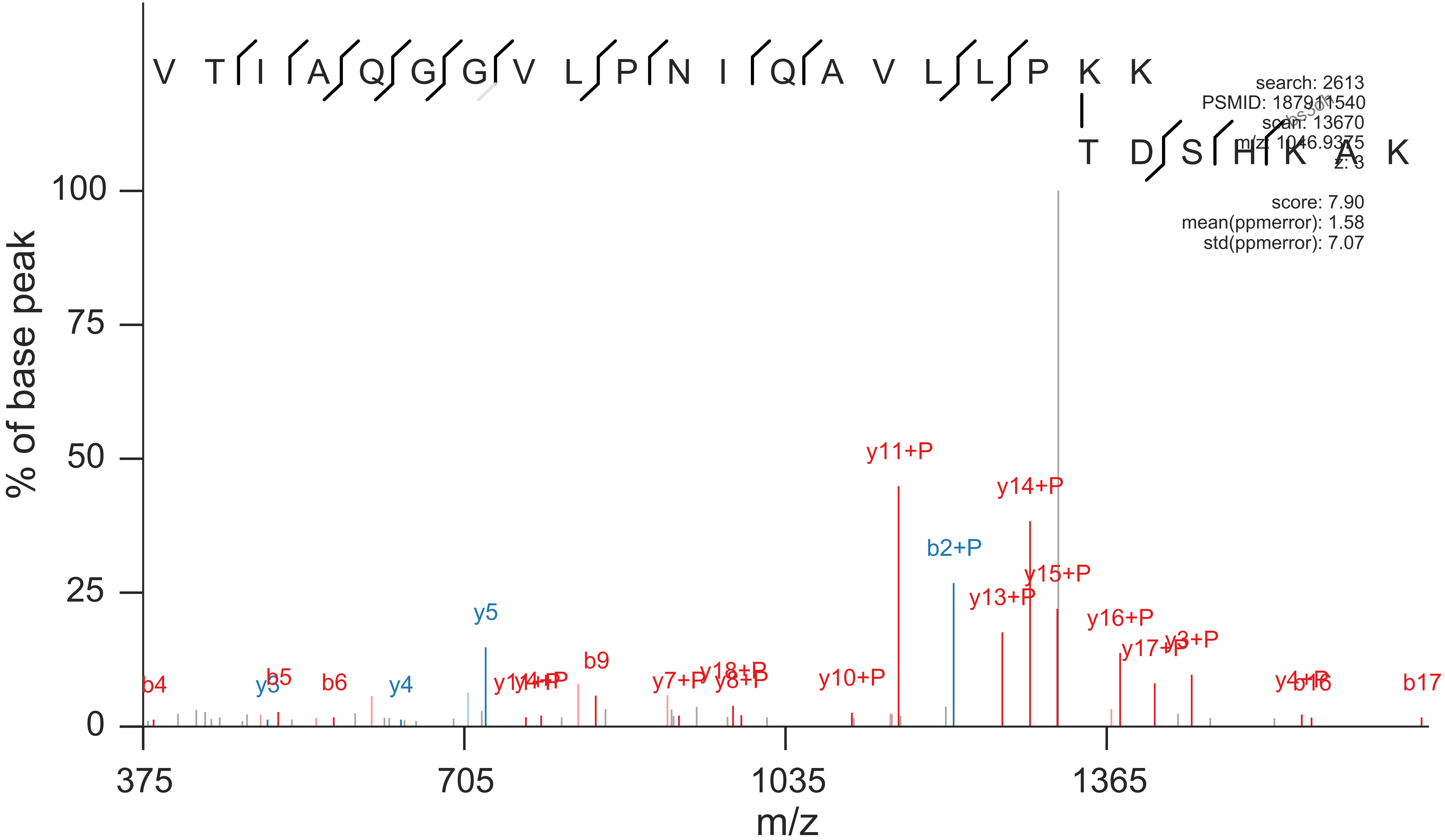

V T I A Q G G V L P N I Q A V L L P K K
T D S H K A K
search: 2613
PSMID: 187911540
scan: 13670
m/z: 1046.9375
z: 3
score: 7.90
mean(ppmerror): 1.58
std(ppmerror): 7.07
% of base peak
100
75
50
25
0
m/z
375
705
1035
1365
y11+P
y14+P
b2+P
y15+P
y13+P
y5
y16+P
y17+P
y3+P
b9
b4
y3
b5
b6
y4
y7+P
y18+P
y8+P
y10+P
b16
b17

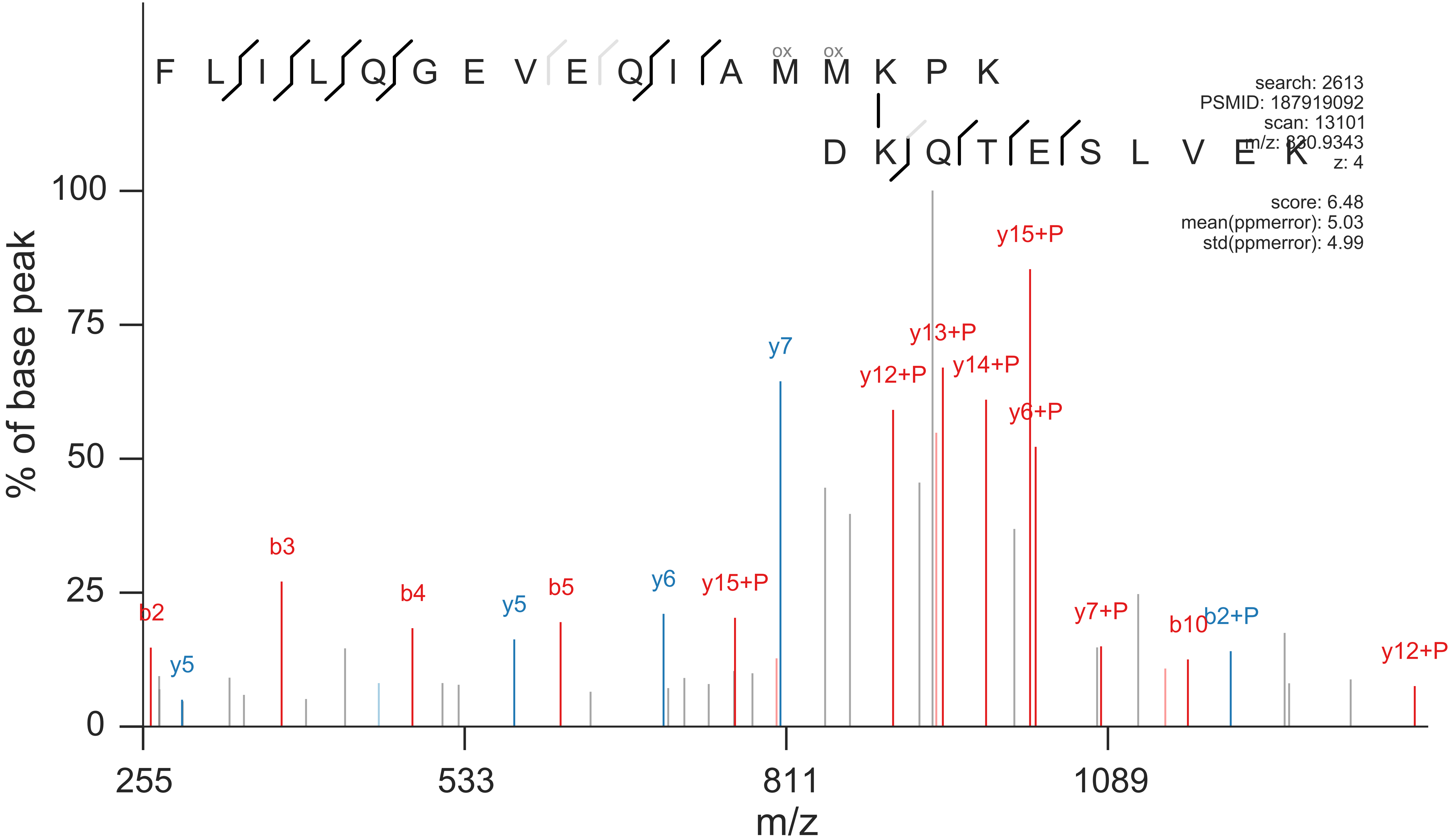

F L I L Q G E V E Q I A M M K P K
ox ox
D K Q T E S L V E K
search: 2613
PSMID: 187919092
scan: 13101
m/z: 830.9343
z: 4
score: 6.48
mean(ppmerror): 5.03
std(ppmerror): 4.99
% of base peak
100
75
50
25
0
255
533
811
1089
m/z
b2
y5
b3
b4
y5
b5
y6
y15+P
y7
y12+P
y13+P
y14+P
y15+P
y6+P
y7+P
b10
b2+P
y12+P

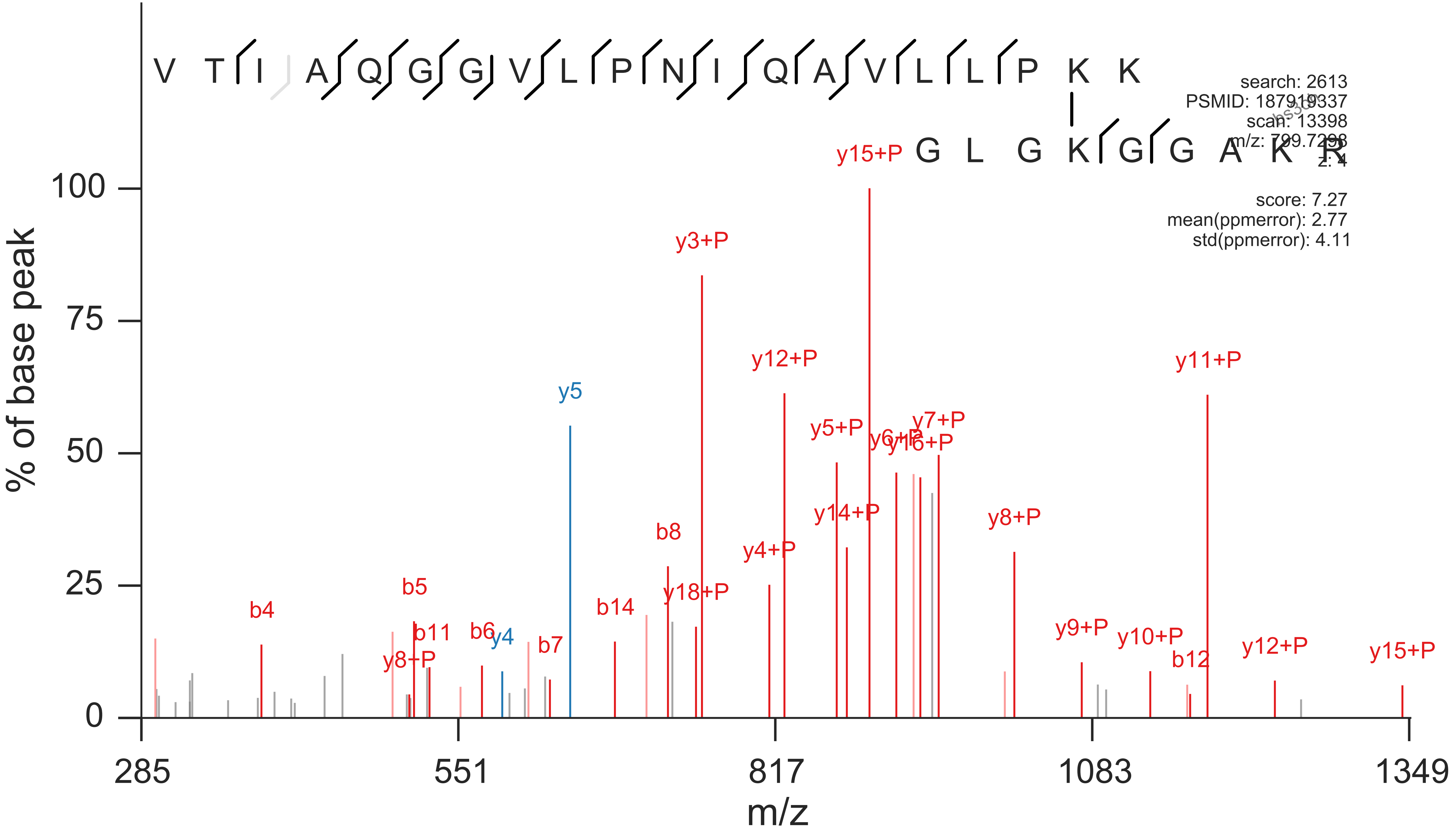
V T I A Q G G V L P N I Q A V L L P K K
G L G K G G A K R
search: 2613
PSMID: 1879193337
scan: 13398
m/z: 799.7298
z: 4
score: 7.27
mean(ppmerror): 2.77
std(ppmerror): 4.11
% of base peak
m/z
100
75
50
25
0
285
551
817
1083
1349
y15+P
y3+P
y12+P
y11+P
y5
y5+P
y6+P
y16+P
y7+P
y14+P
y8+P
b8
y4+P
b5
b4
y18+P
b14
b11
b6
y4
b7
y8+P
y9+P
y10+P
b12
y12+P
y15+P

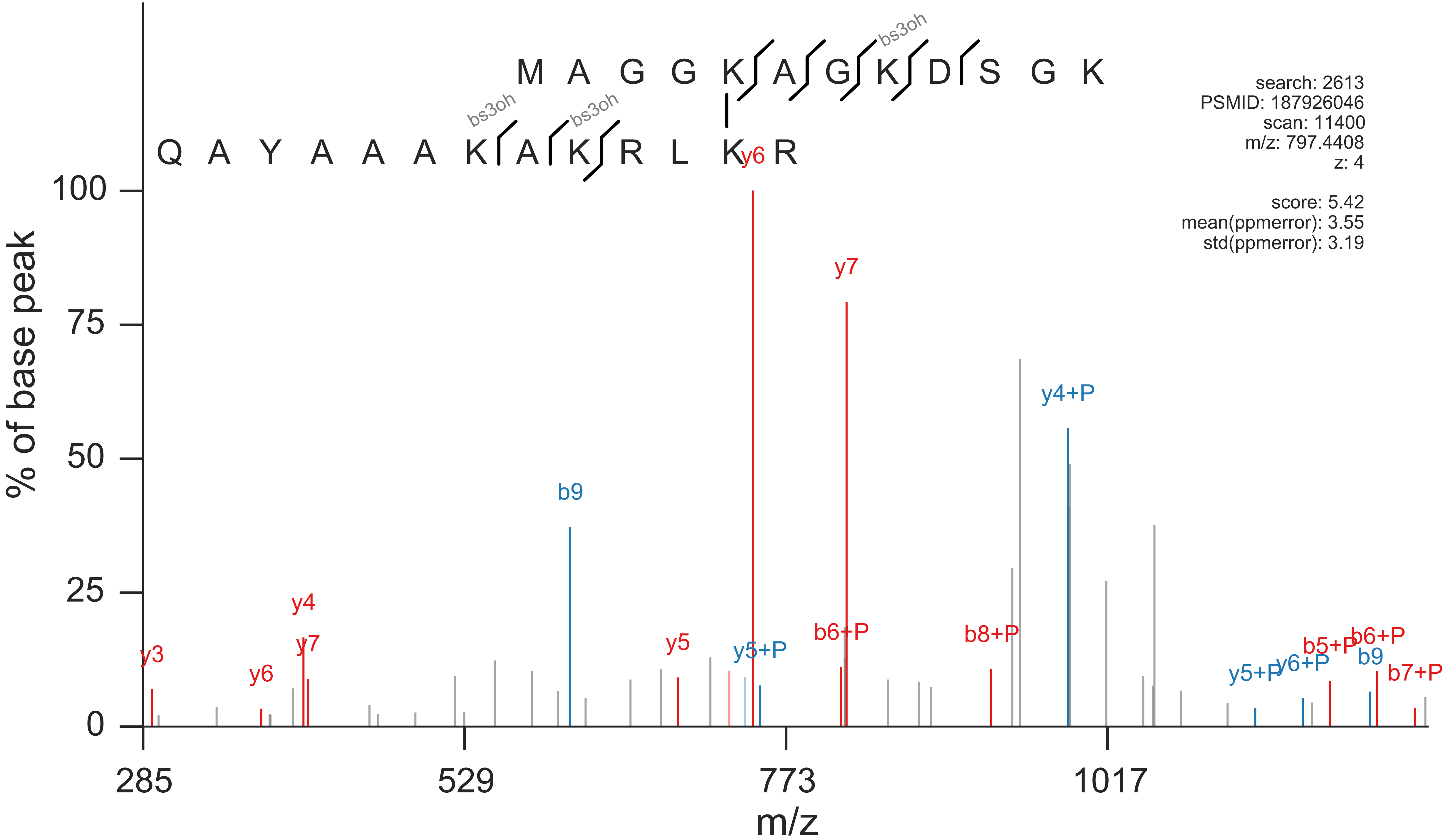
M A G G K A G K D S G K
bs3oh
Q A Y A A A K A K R L K R
bs3oh
bs3oh
search: 2613
PSMID: 187926046
scan: 11400
m/z: 797.4408
z: 4
score: 5.42
mean(ppmerror): 3.55
std(ppmerror): 3.19
% of base peak
100
75
50
25
0
285
529
773
1017
m/z
y6
y7
y4+P
b9
y4
y7
y3
y6
y5
y5+P
b6+P
b8+P
y5+P
y6+P
b5+P
b6+P
b9
b7+P

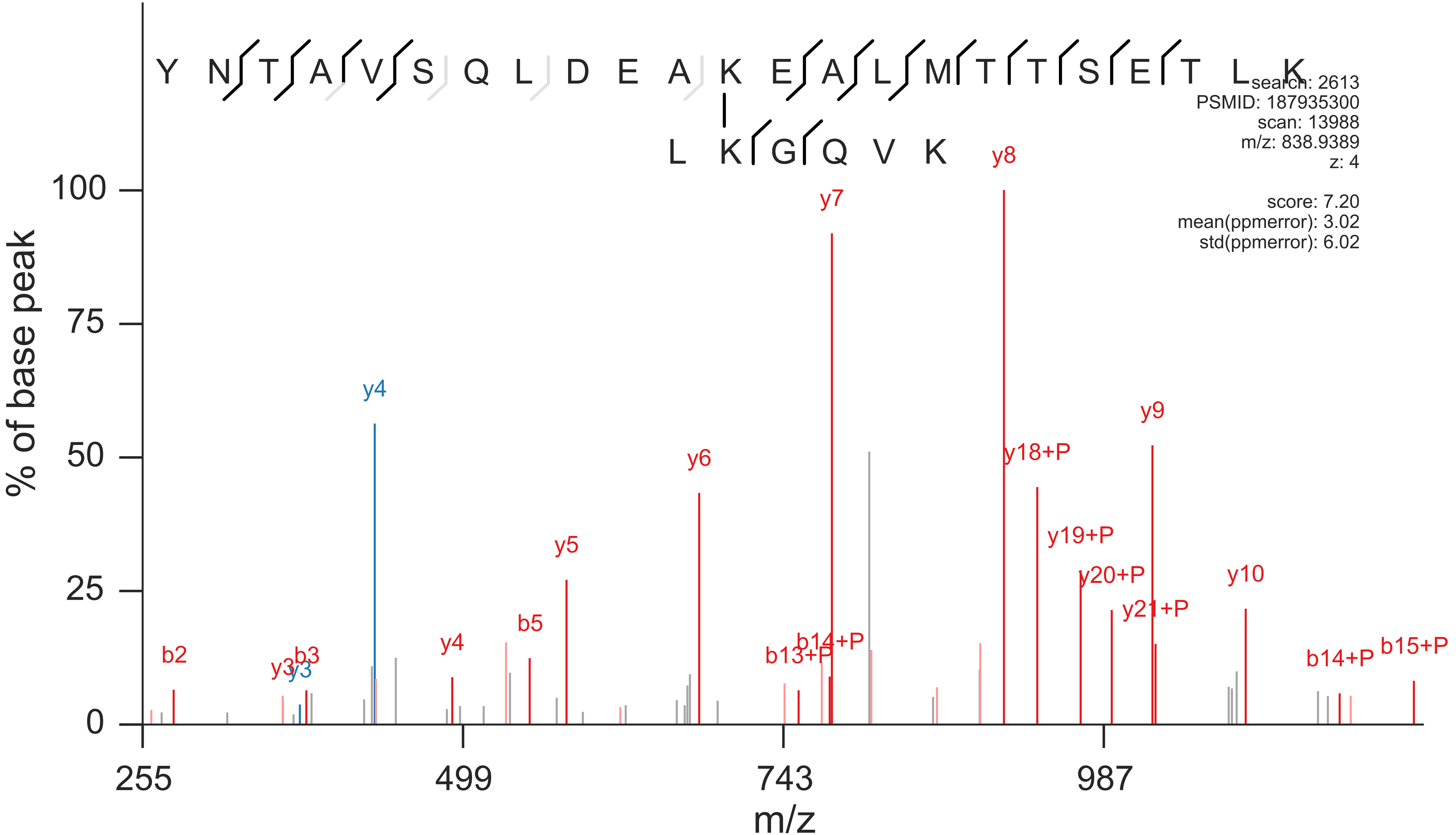

Y N T A V S Q L D E A K E A L M T T S E T L K
L K G Q V K
search: 2613
PSMID: 187935300
scan: 13988
m/z: 838.9389
z: 4
score: 7.20
mean(ppmerror): 3.02
std(ppmerror): 6.02
% of base peak
m/z
0
25
50
75
100
255
499
743
987
b2
y3
y3
b3
y4
y4
b5
y5
y6
b13+P
b14+P
y7
y8
y18+P
y19+P
y20+P
y21+P
y9
y10
b14+P
b15+P

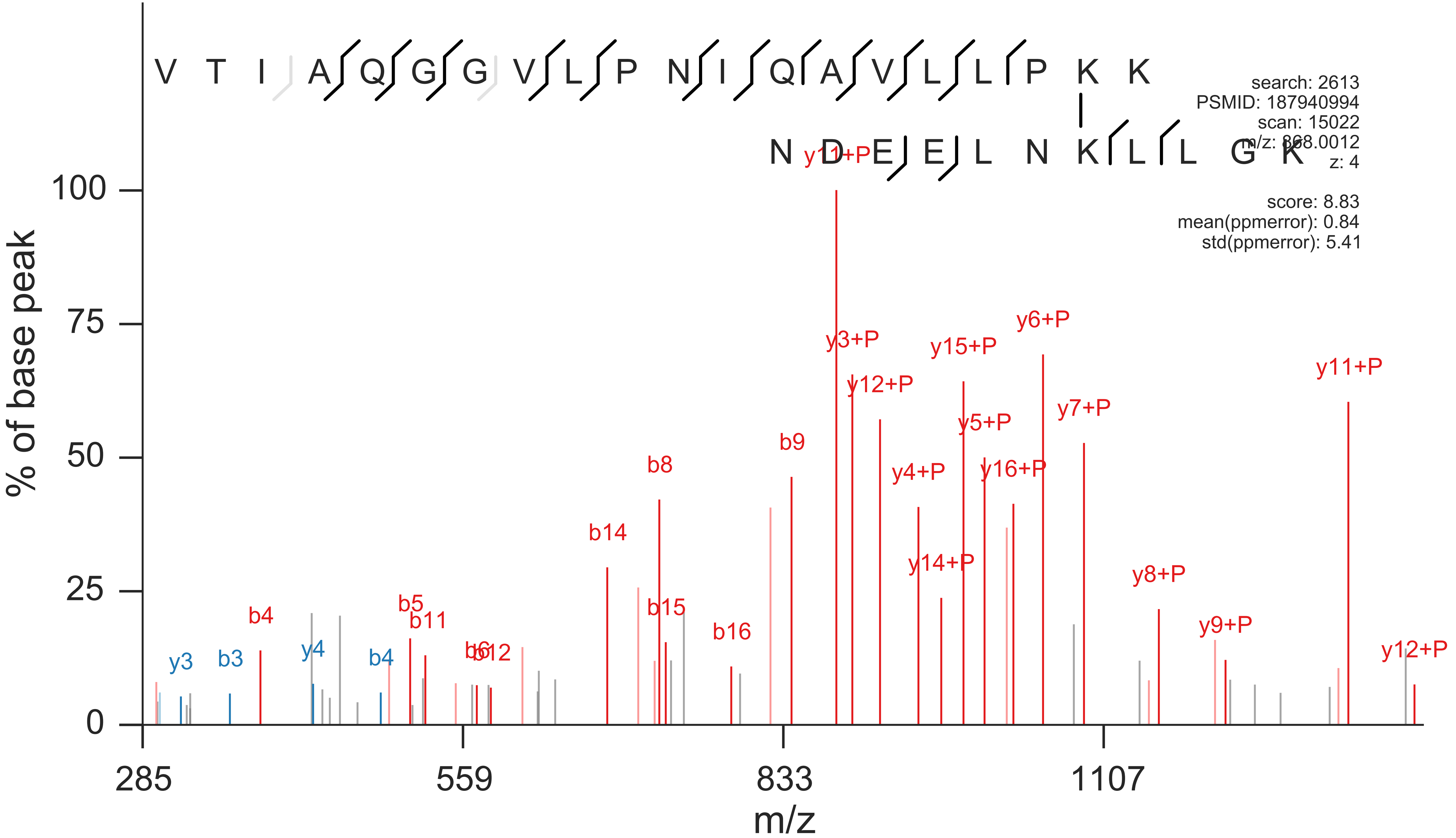
V T I A Q G G V L P N I Q A V L L P K K
N D E E L N K L L G K
search: 2613
PSMID: 187940994
scan: 15022
m/z: 868.0012
z: 4
score: 8.83
mean(ppmerror): 0.84
std(ppmerror): 5.41
% of base peak
m/z
y3 b3 b4 y4 b4 b5 b11 b6 b12 b14 b8 b15 b16 b9 y11+P y3+P y12+P y4+P y14+P y15+P y5+P y16+P y6+P y7+P y8+P y9+P y11+P y12+P
0
25
50
75
100
285
559
833
1107

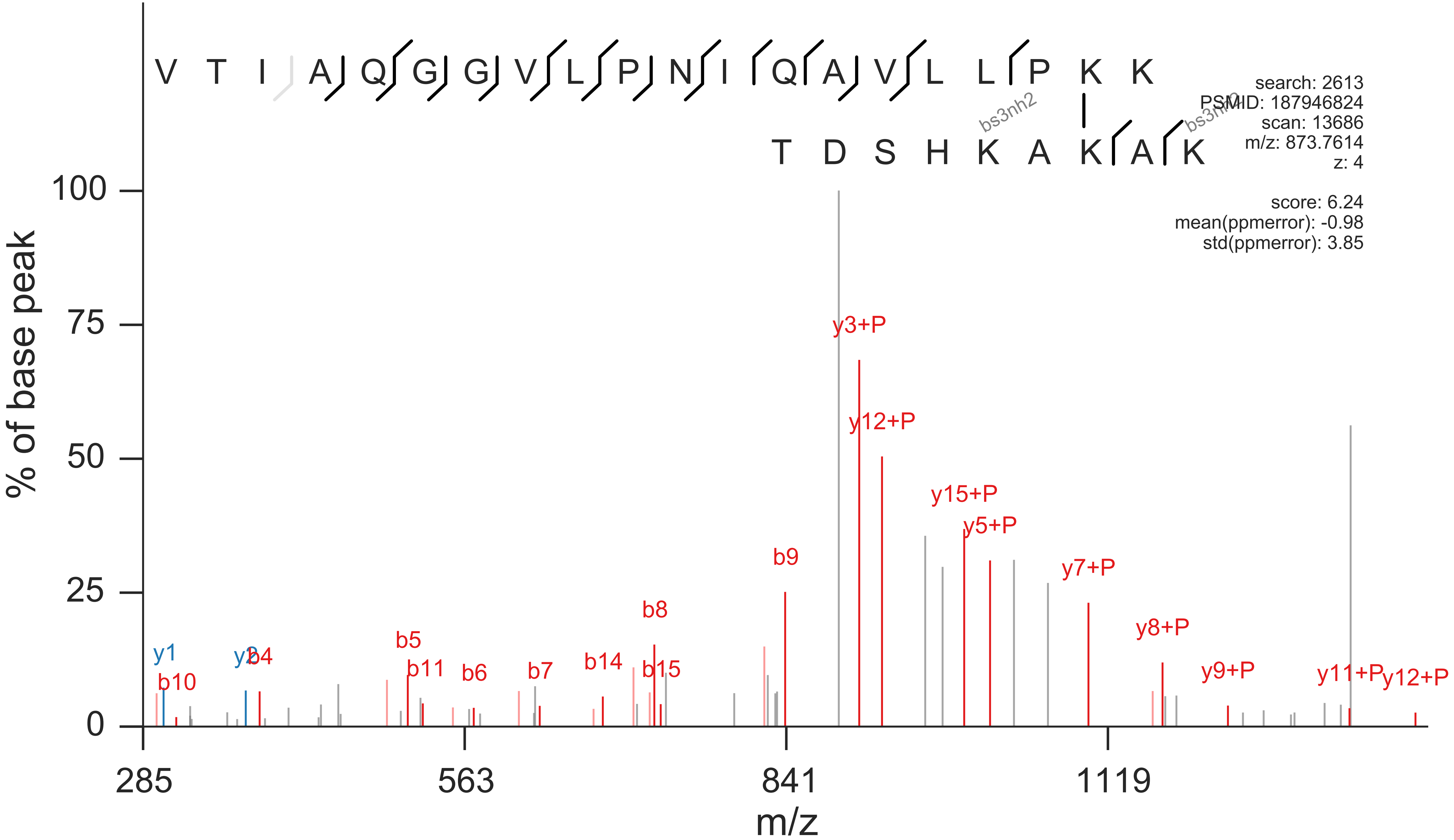

V T I A Q G G V L P N I Q A V L L P K K
T D S H K A K A K
bs3nh2
bs3nh2
search: 2613
PSMID: 187946824
scan: 13686
m/z: 873.7614
z: 4
score: 6.24
mean(ppmerror): -0.98
std(ppmerror): 3.85
% of base peak
m/z
y3+P
y12+P
y15+P
y5+P
y7+P
y8+P
y9+P
y11+P
y12+P
b9
b8
b5
b11
b6
b7
b14
b15
b4
b10
y1
y2

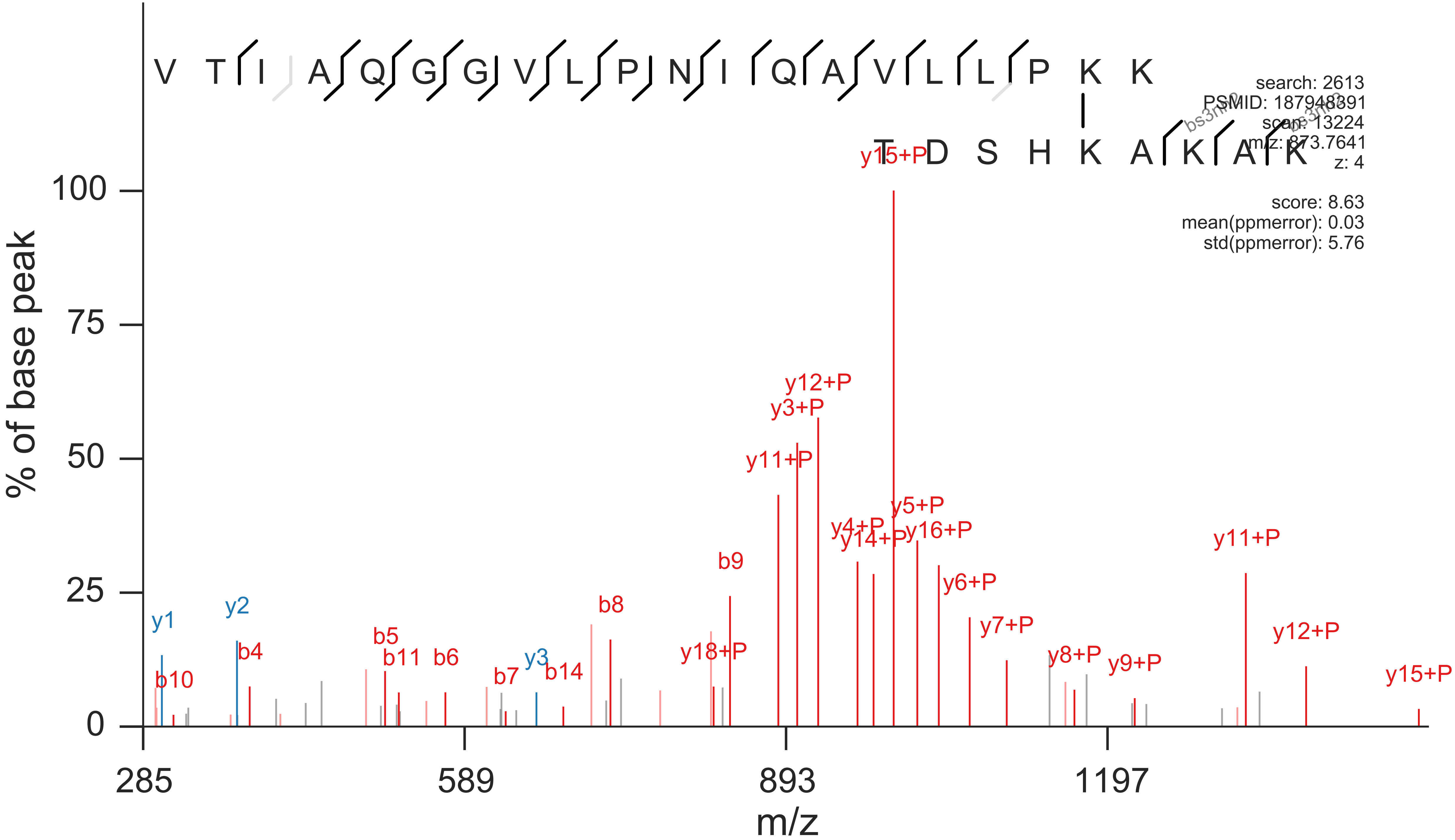

V T I A Q G G V L P N I Q A V L L P K K
T D S H K A K A K
search: 2613
PSMID: 187948391
scan: 13224
m/z: 873.7641
z: 4
score: 8.63
mean(ppmerror): 0.03
std(ppmerror): 5.76
% of base peak
m/z
0
25
50
75
100
285
589
893
1197
y1
y2
y3
b4
b5
b6
b7
b8
b9
b10
b11
b14
y18+P
y11+P
y3+P
y12+P
y4+P
y14+P
y15+P
y5+P
y16+P
y6+P
y7+P
y8+P
y9+P
y11+P
y12+P
y15+P

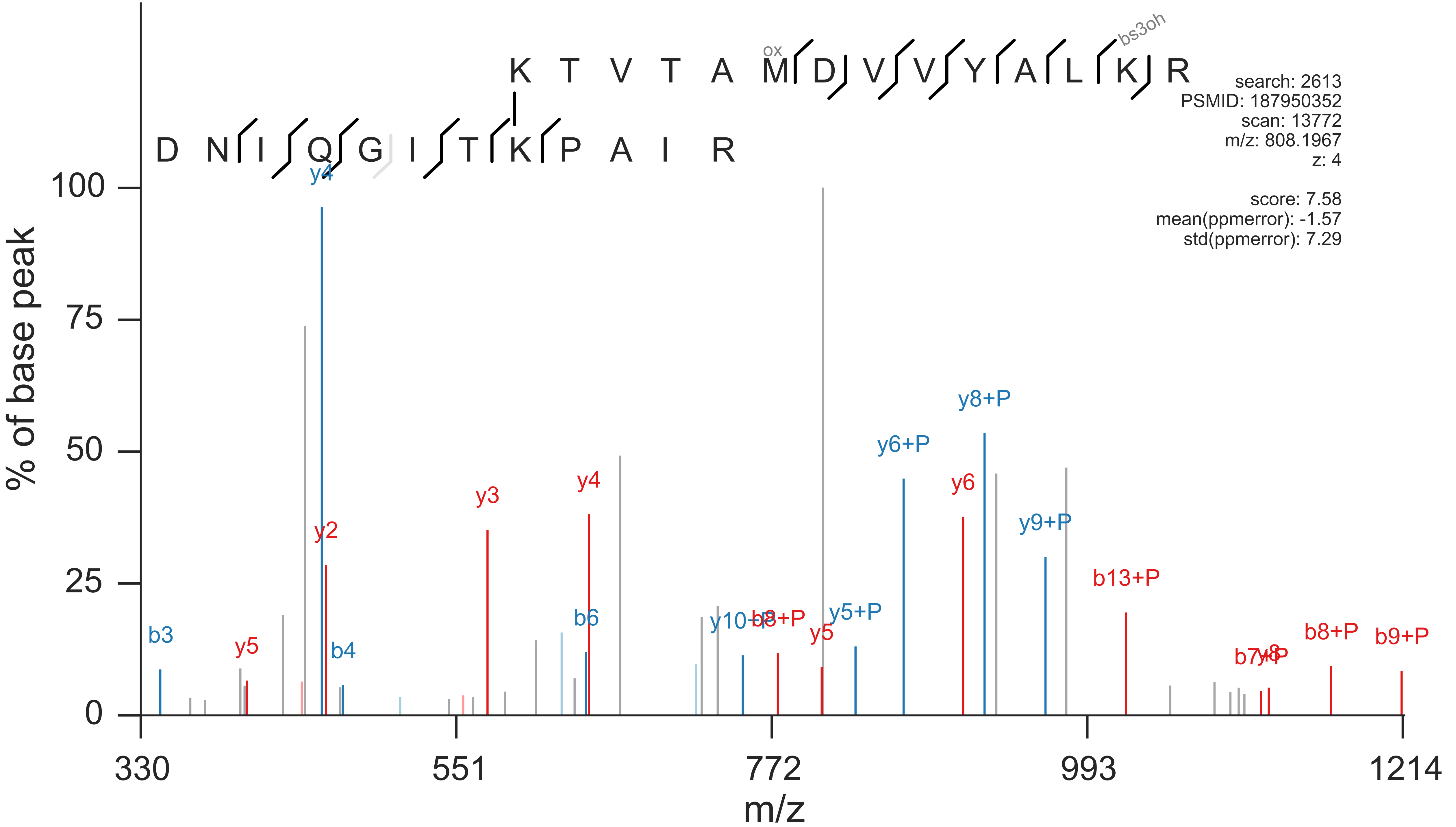

K T V T A M D V V Y A L K R
ox
bs3oh
D N I Q G I T K P A I R
search: 2613
PSMID: 187950352
scan: 13772
m/z: 808.1967
z: 4
score: 7.58
mean(ppmerror): -1.57
std(ppmerror): 7.29
% of base peak
100
75
50
25
0
330
551
772
993
1214
m/z
b3
y5
y4
y2
b4
y3
y4
b6
y10+P
b8+P
y5
y5+P
y6+P
y6
y8+P
y9+P
b13+P
b7+P
y8
b8+P
b9+P

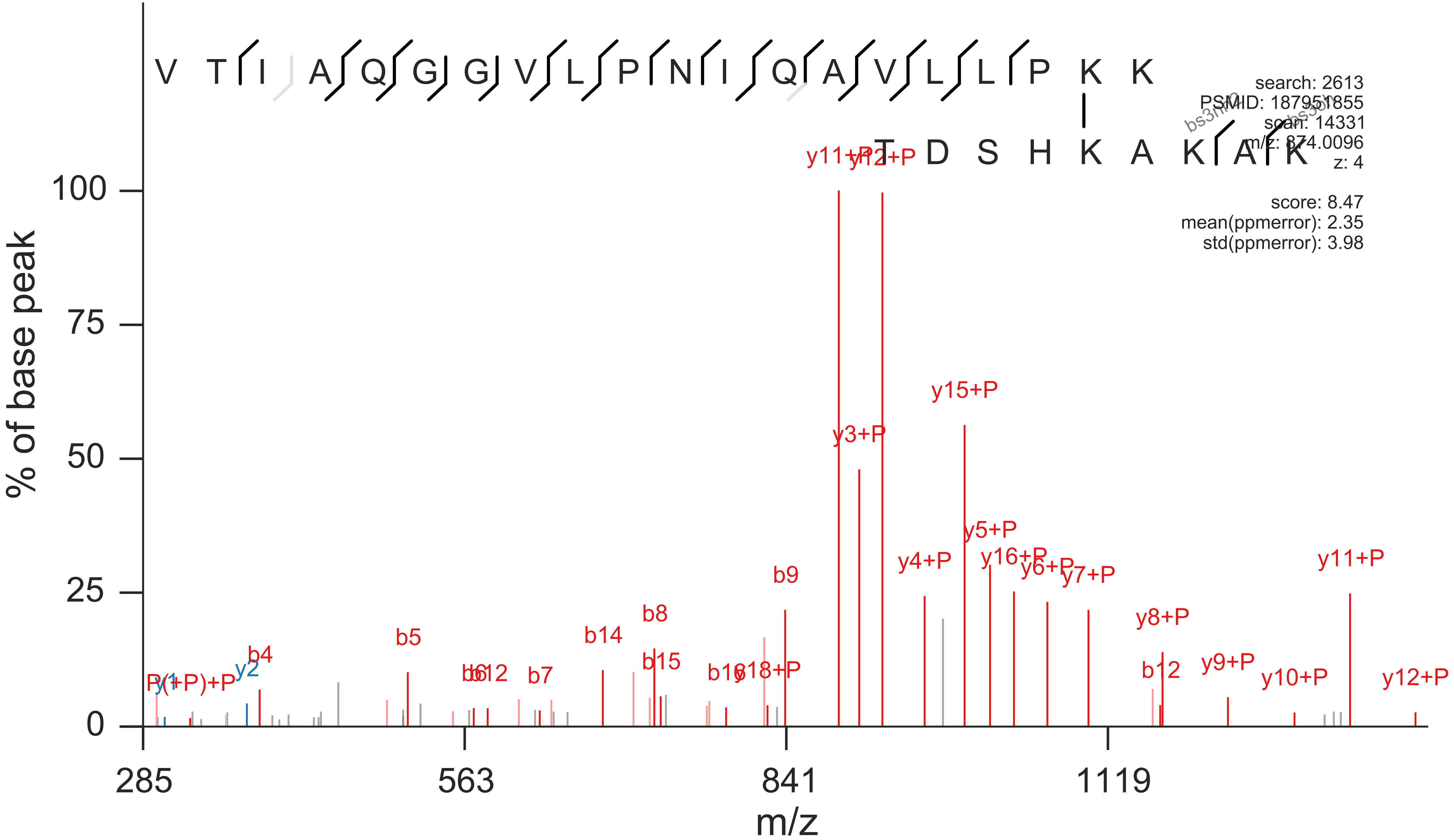

V T I A Q G G V L P N I Q A V L L P K K
T D S H K A K A K
search: 2613
PSMID: 187951855
scan: 14331
m/z: 874.0096
z: 4
score: 8.47
mean(ppmerror): 2.35
std(ppmerror): 3.98
y11+P
y12+P
y15+P
y3+P
y5+P
y4+P
y16+P
y6+P
y7+P
y11+P
b9
b8
b14
b15
b5
b4
y2
b7
b12
y8+P
y9+P
y10+P
y12+P
% of base peak
100
75
50
25
0
285
563
841
1119
m/z

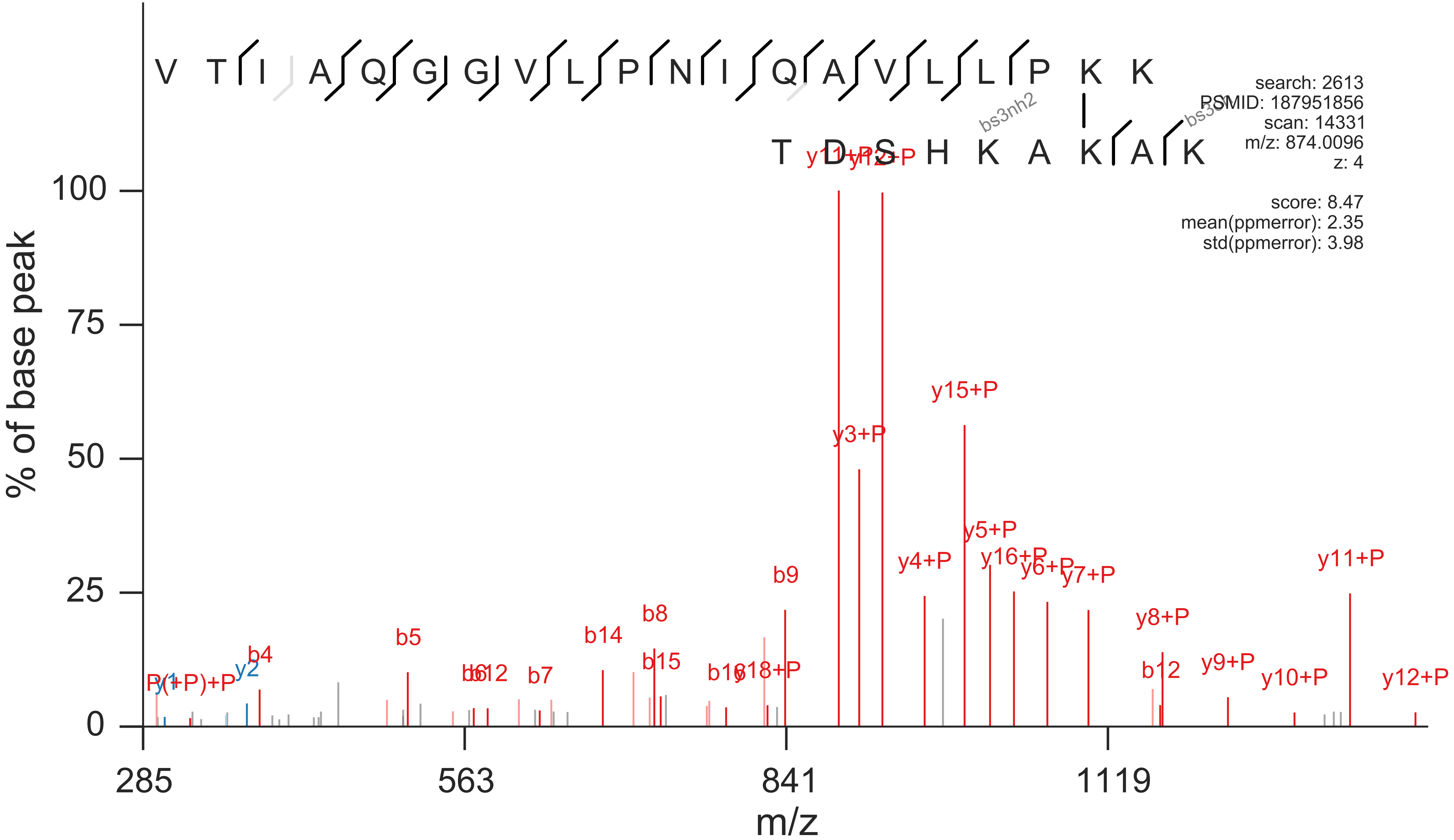

V T I A Q G G V L P N I Q A V L L P K K
T D S H K A K A K
bs3nh2
bs3oh
search: 2613
PSMID: 187951856
scan: 14331
m/z: 874.0096
z: 4
score: 8.47
mean(ppmerror): 2.35
std(ppmerror): 3.98
% of base peak
m/z
0
25
50
75
100
285
563
841
1119
y3+P
y4+P
y5+P
y6+P
y7+P
y8+P
y9+P
y10+P
y11+P
y12+P
y15+P
y16+P
y1
y2
b4
b5
b7
b8
b9
b12
b14
b15

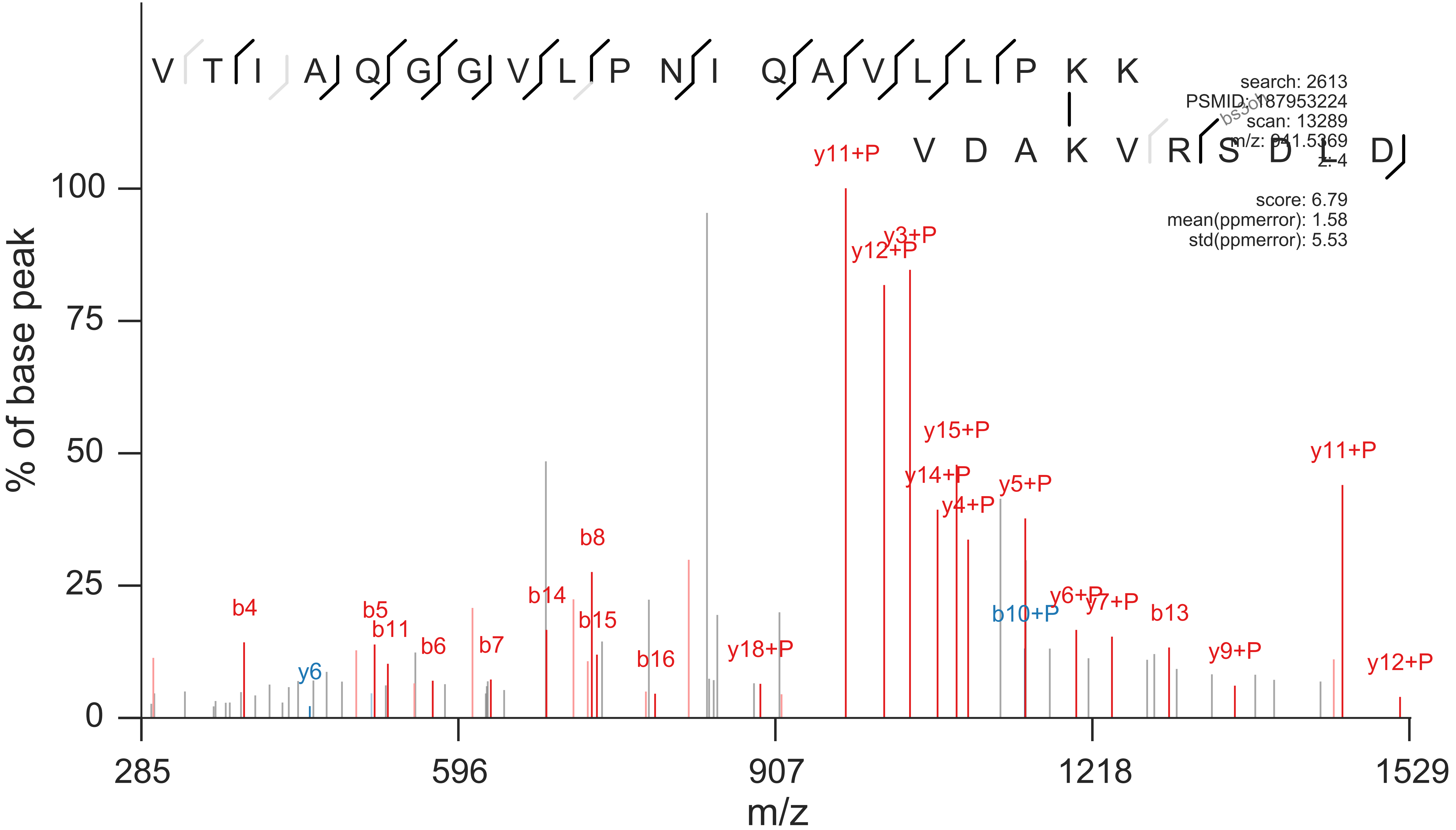

V T I A Q G G V L P N I Q A V L L P K K
V D A K V R S D L D
search: 2613
PSMID: 187953224
scan: 13289
m/z: 941.5369
z: 4
score: 6.79
mean(ppmerror): 1.58
std(ppmerror): 5.53
% of base peak
m/z
b4
y6
b5
b11
b6
b7
b14
b8
b15
b16
y18+P
y11+P
y12+P
y3+P
y15+P
y14+P
y4+P
y5+P
b10+P
y6+P
y7+P
b13
y9+P
y11+P
y12+P

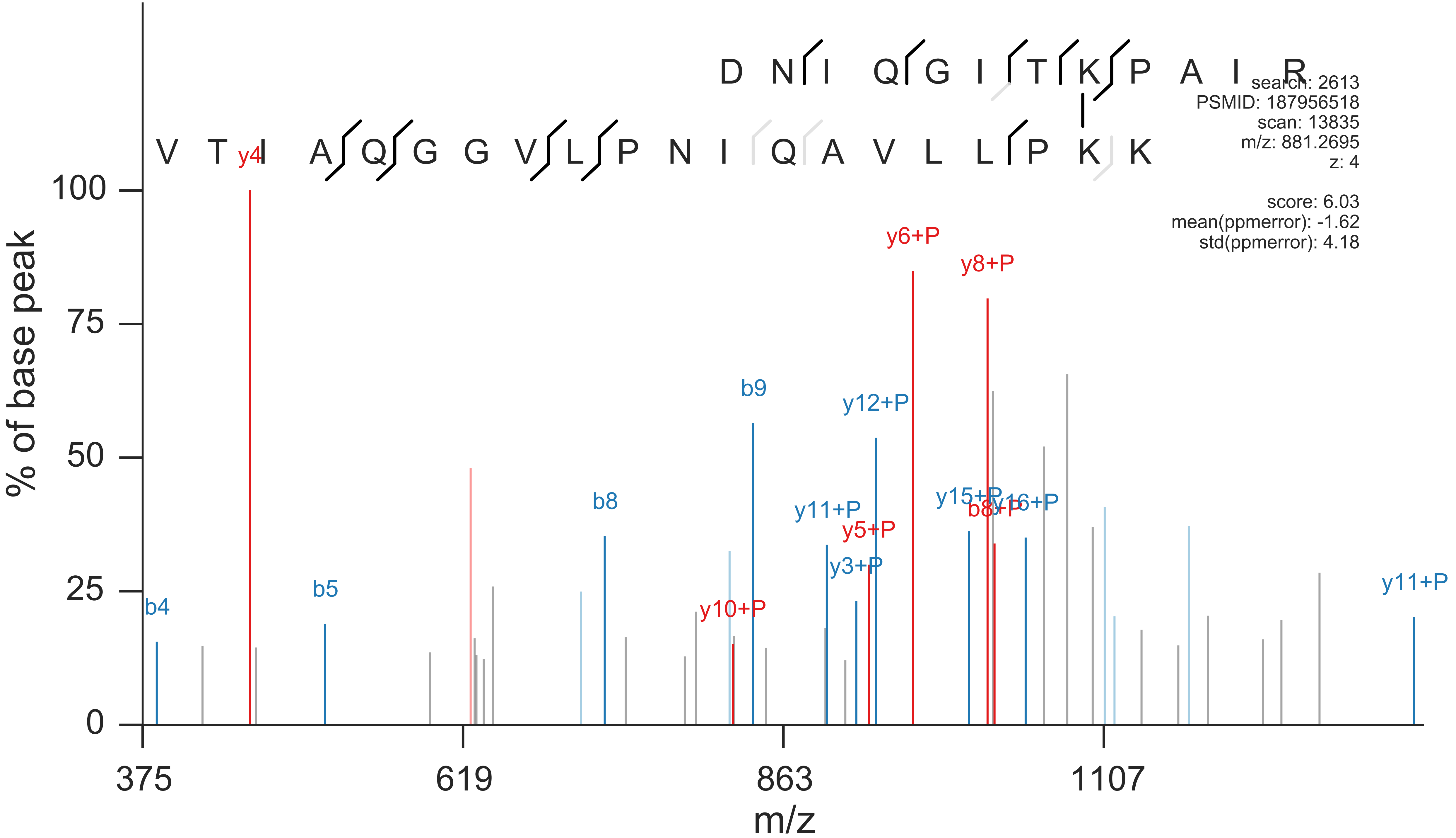

D N I Q G I T K P A I R
V T I A Q G G V L P N I Q A V L L P K K
search: 2613
PSMID: 187956518
scan: 13835
m/z: 881.2695
z: 4
score: 6.03
mean(ppmerror): -1.62
std(ppmerror): 4.18
% of base peak
m/z
100
75
50
25
0
375
619
863
1107
y4
y6+P
y8+P
b9
y12+P
b8
y11+P
y15+P
y16+P
b8+P
y5+P
y3+P
b5
b4
y10+P
y11+P

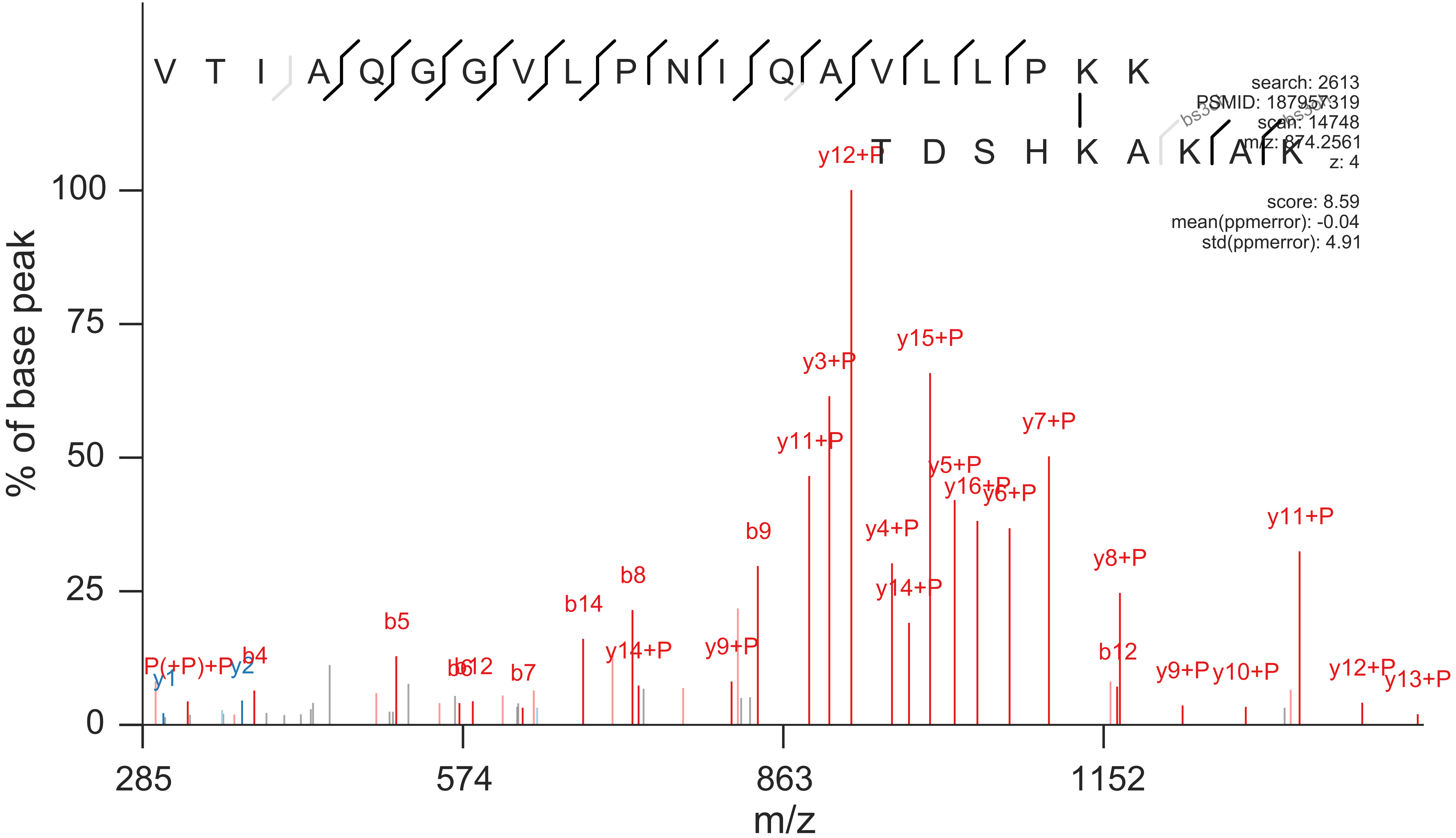

V T I A Q G G V L P N I Q A V L L P K K
T D S H K A K A K
search: 2613
PSMID: 187957319
scan: 14748
m/z: 874.2561
z: 4
score: 8.59
mean(ppmerror): -0.04
std(ppmerror): 4.91
% of base peak
m/z

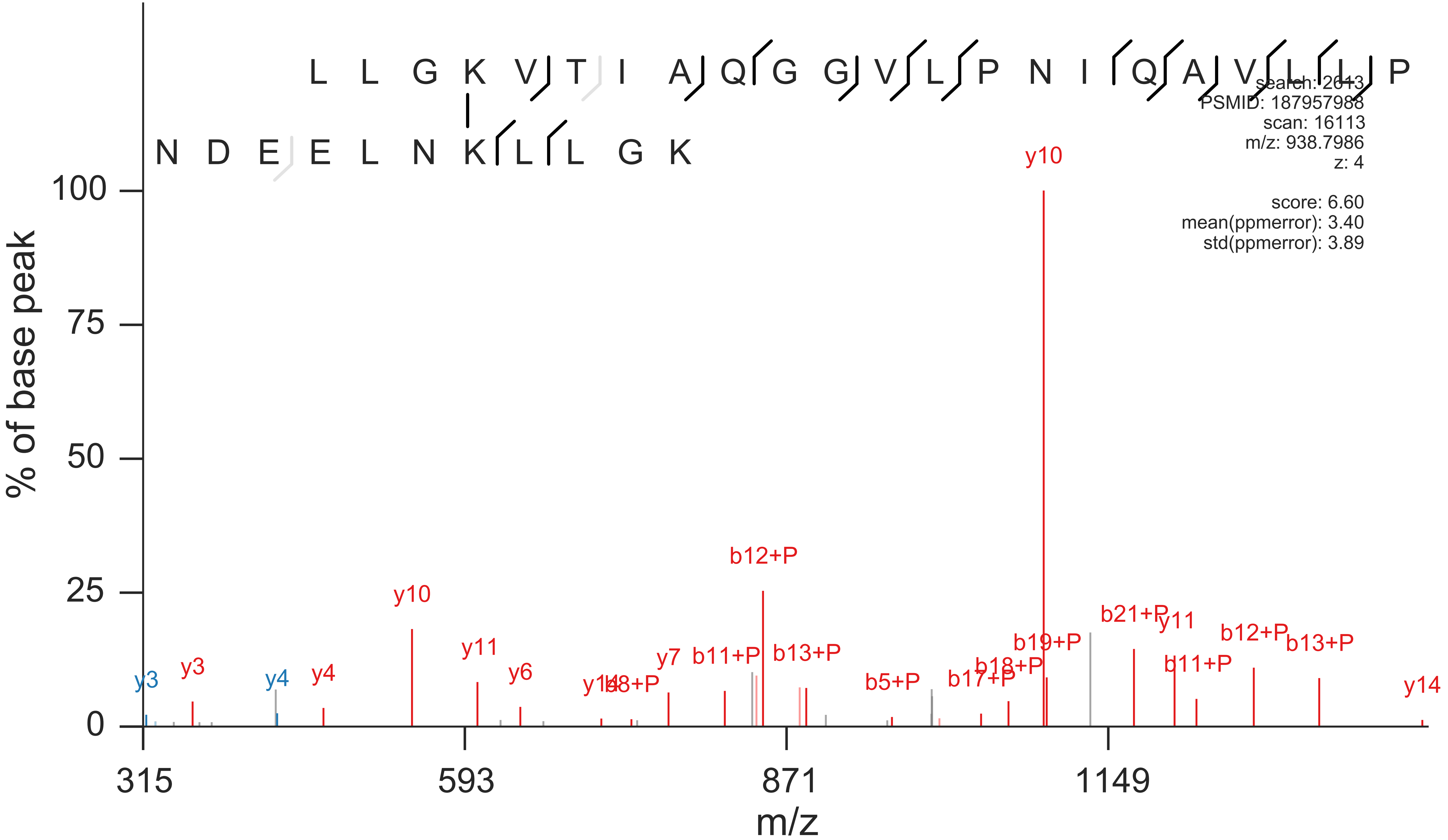

L L G K V T I A Q G G V L P N I Q A V L L P
N D E E L N K L L G K
search: 2613
PSMID: 187957988
scan: 16113
m/z: 938.7986
z: 4
score: 6.60
mean(ppmerror): 3.40
std(ppmerror): 3.89
% of base peak
m/z

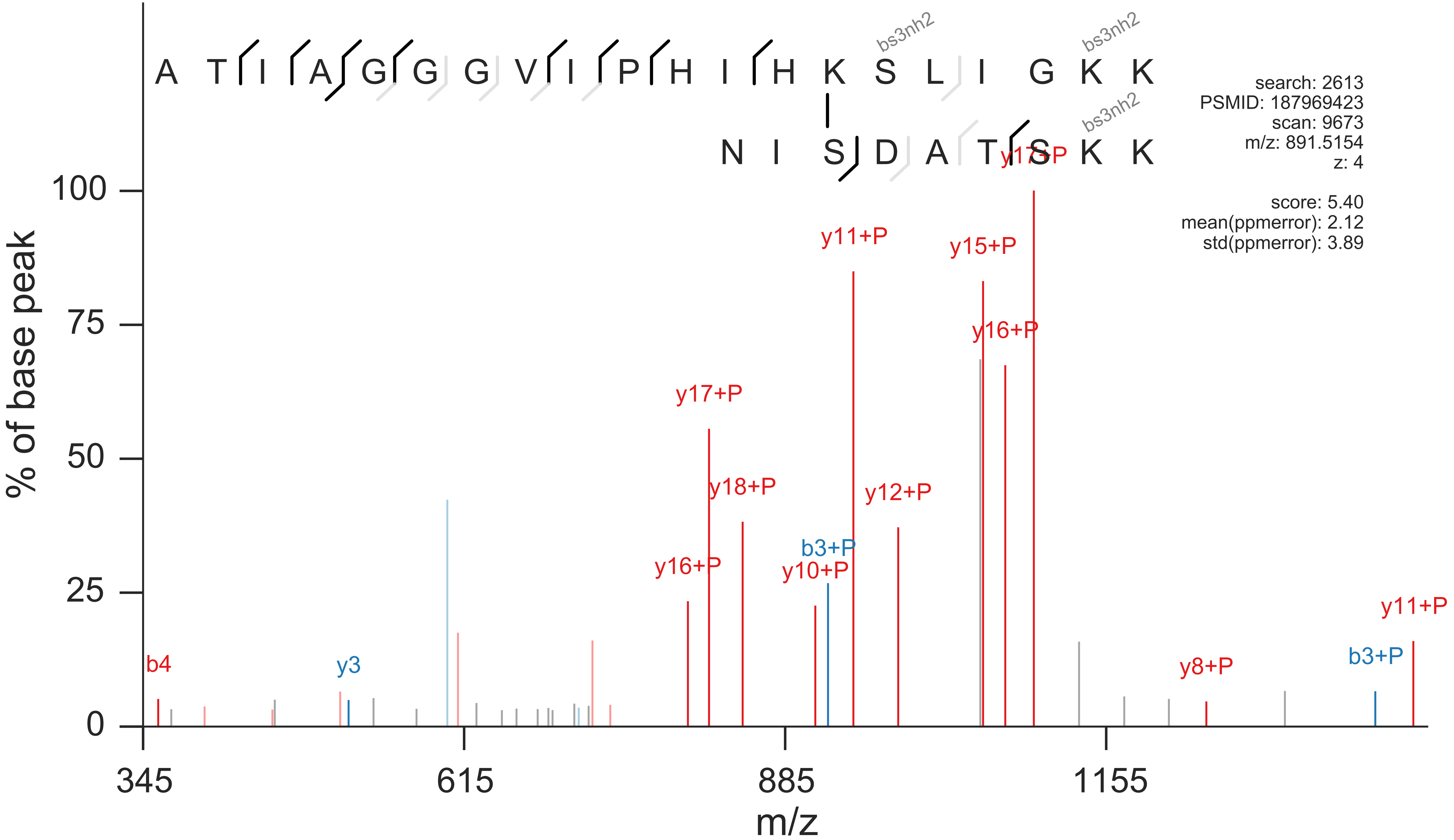

A T I A G G G V I P H I H K S L I G K K
N I S D A T S K K
bs3nh2
bs3nh2
bs3nh2
search: 2613
PSMID: 187969423
scan: 9673
m/z: 891.5154
z: 4
score: 5.40
mean(ppmerror): 2.12
std(ppmerror): 3.89
% of base peak
m/z
100
75
50
25
0
345
615
885
1155
b4
y3
y16+P
y17+P
y18+P
b3+P
y10+P
y11+P
y12+P
y15+P
y16+P
y17+P
y8+P
b3+P
y11+P

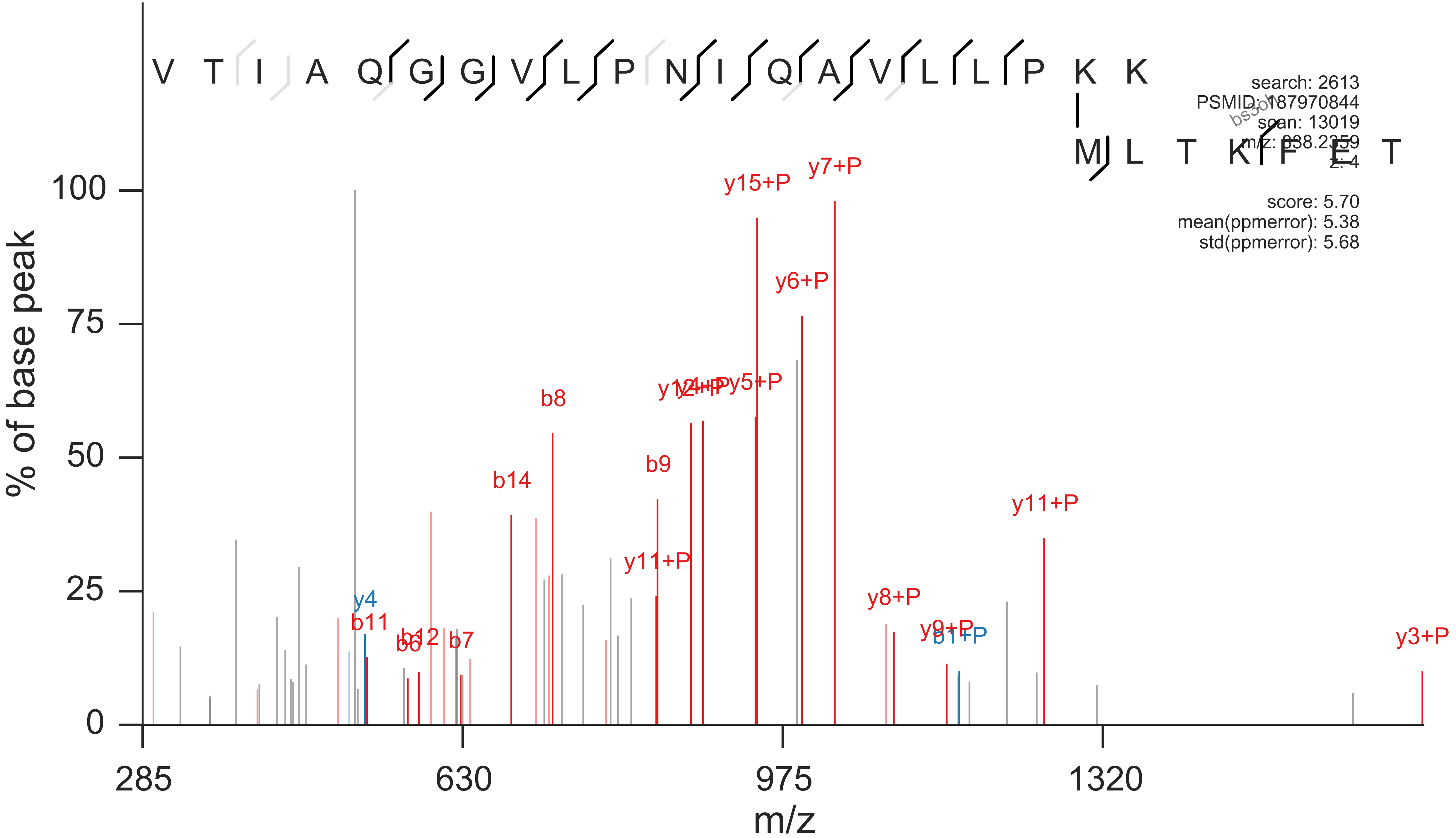
V T I A Q G G V L P N I Q A V L L P K K
M L T K F E T
search: 2613
PSMID: 187970844
scan: 13019
m/z: 838.2359
z: 4
score: 5.70
mean(ppmerror): 5.38
std(ppmerror): 5.68
% of base peak
m/z
0
25
50
75
100
285
630
975
1320
y15+P
y7+P
y6+P
y5+P
b8
b9
b14
y11+P
y4
b11
b6
b12
b7
y8+P
y9+P
b1+P
y3+P

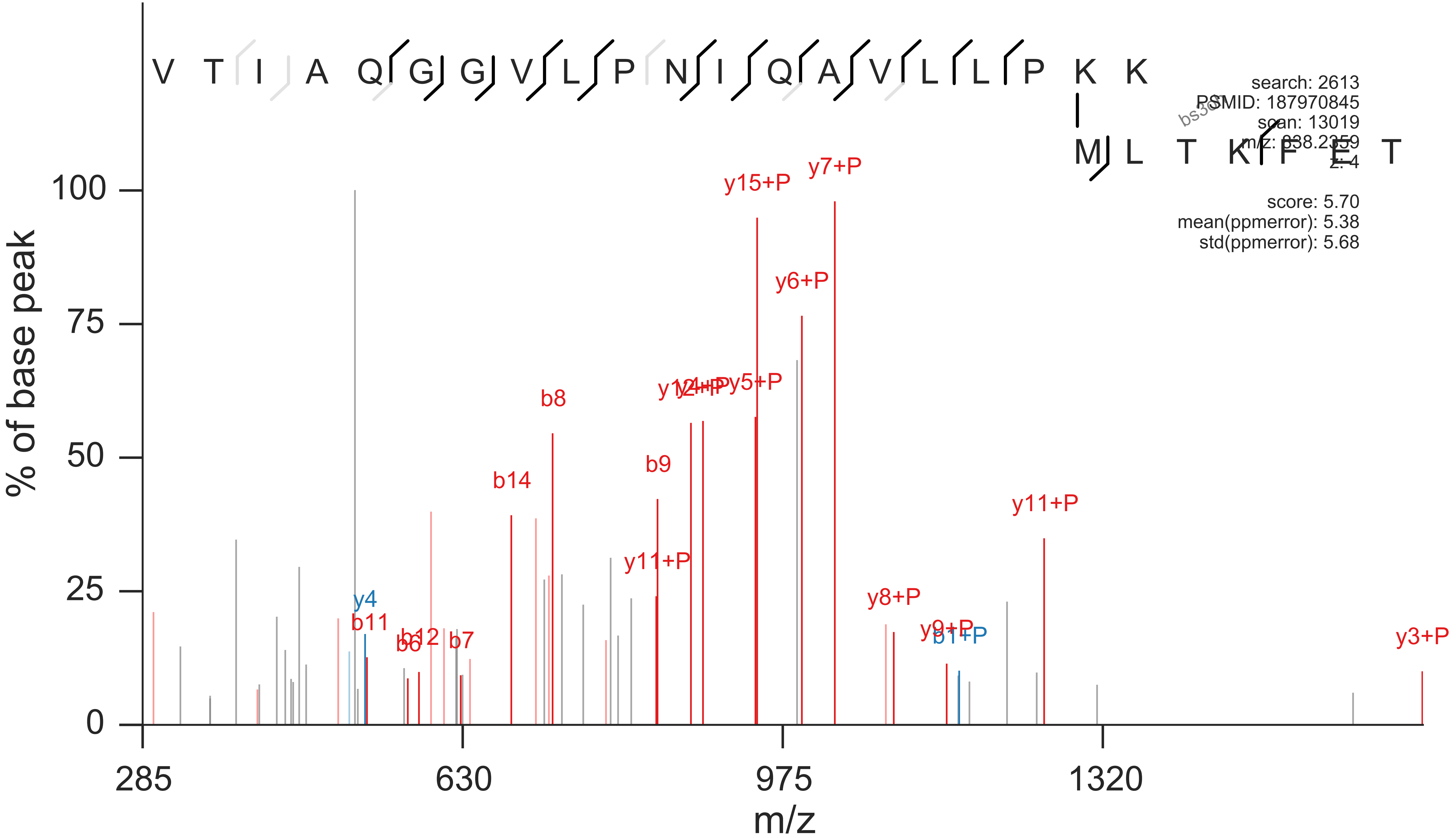

V T I A Q G G V L P N I Q A V L L P K K
M L T K F E T
search: 2613
PSMID: 187970845
scan: 13019
m/z: 838.2359
z: 4
score: 5.70
mean(ppmerror): 5.38
std(ppmerror): 5.68
% of base peak
m/z
y15+P
y7+P
y6+P
y5+P
b8
b9
b14
y11+P
y8+P
y9+P
b1+P
y3+P
y4
b11
b12
b6
b7
0
25
50
75
100
285
630
975
1320

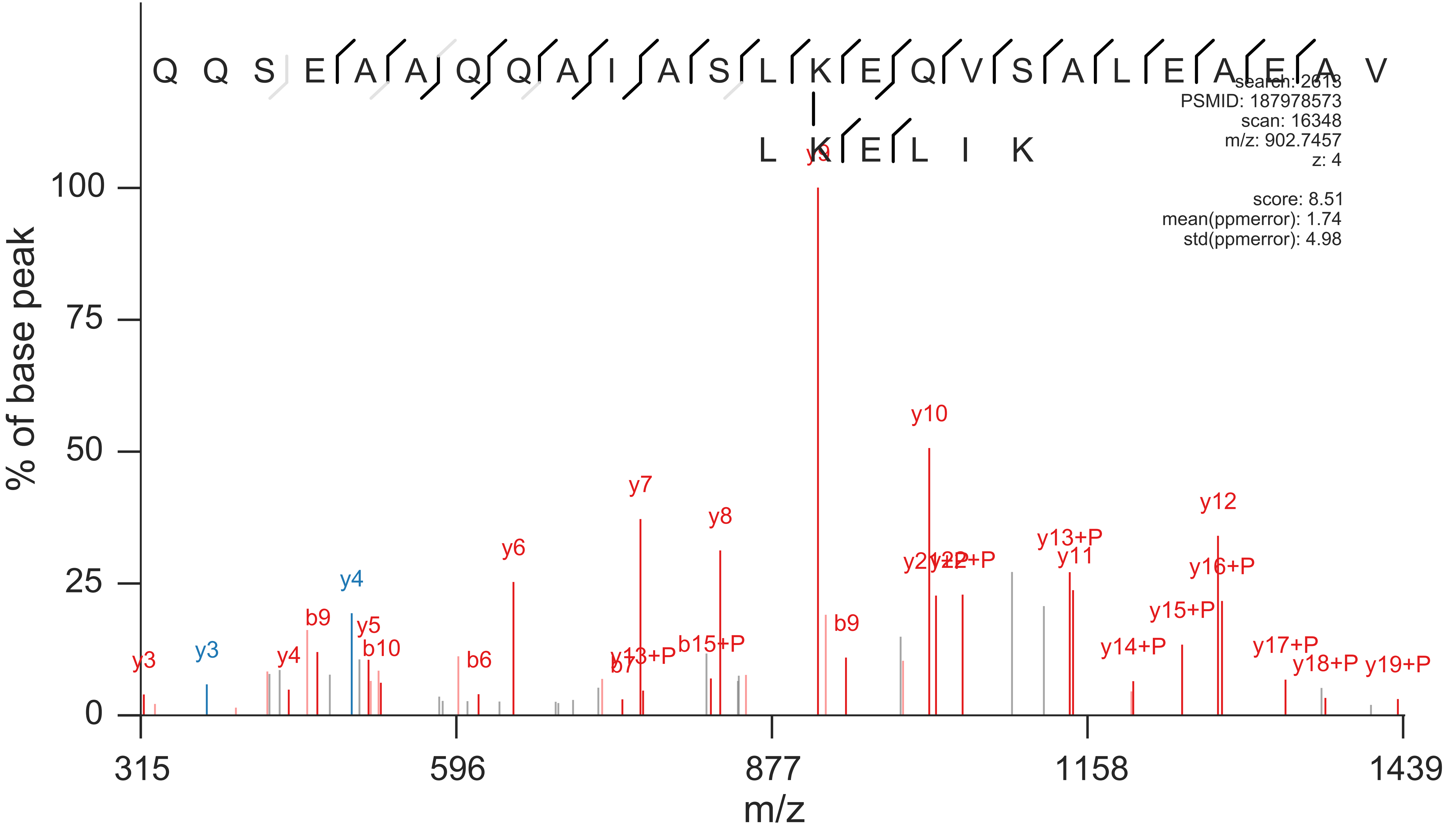

Q Q S E A A Q Q A I A S L K E Q V S A L E A E A V
L K E L I K
search: 2613
PSMID: 187978573
scan: 16348
m/z: 902.7457
z: 4
score: 8.51
mean(ppmerror): 1.74
std(ppmerror): 4.98
% of base peak
m/z
y3 y3 y4 b9 y4 y5 b10 b6 y6 b7 y13+P y7 b15+P y8 y9 b9 y10 y11 y13+P y12 y16+P y15+P y14+P y17+P y18+P y19+P
0
25
50
75
100
315
596
877
1158
1439

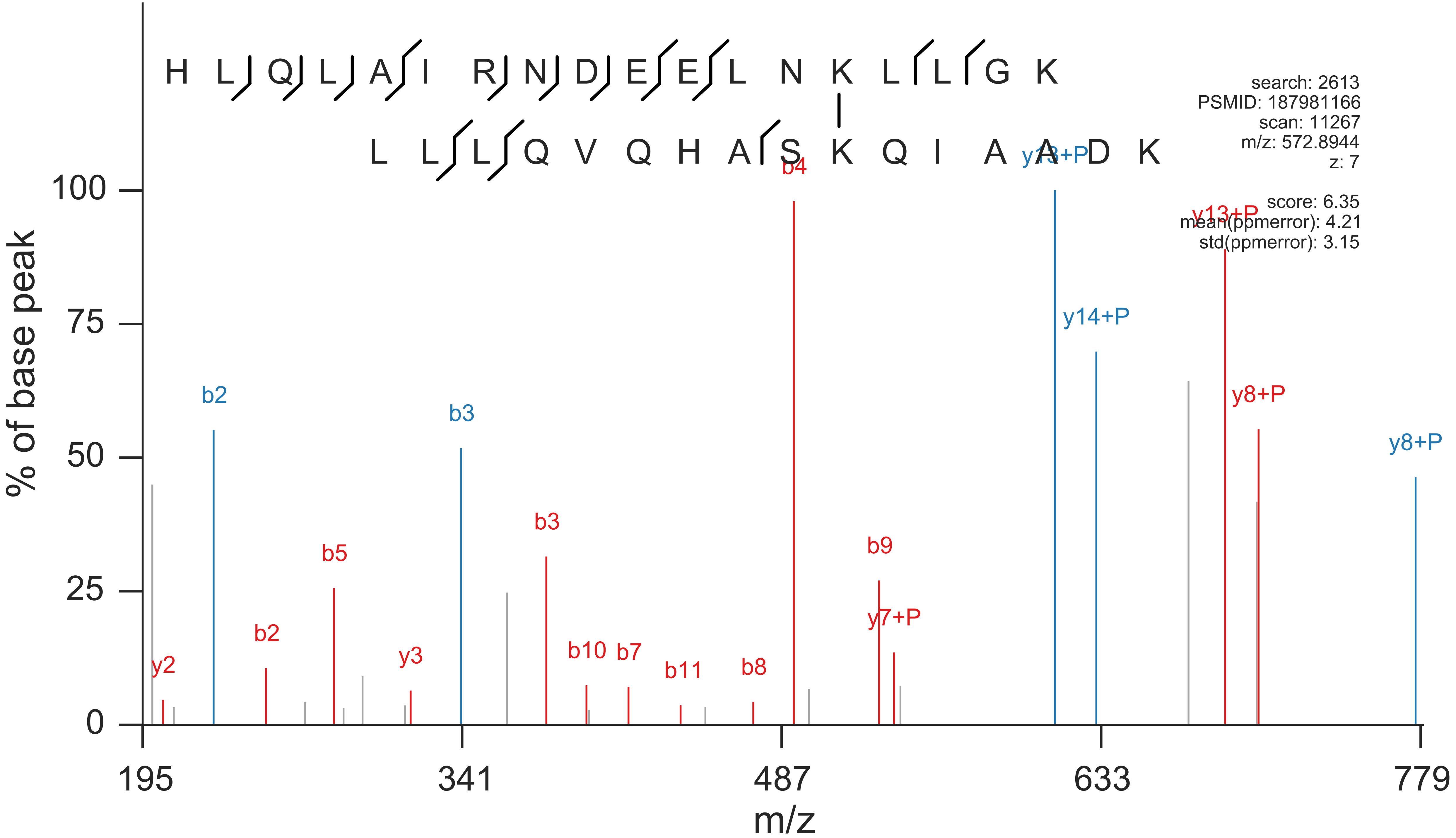

H L Q L A I R N D E E L N K L L G K
L L L Q V Q H A S K Q I A A D K
search: 2613
PSMID: 187981166
scan: 11267
m/z: 572.8944
z: 7
score: 6.35
mean(ppmerror): 4.21
std(ppmerror): 3.15
% of base peak
m/z
100
75
50
25
0
195
341
487
633
779
b2
b3
b4
b5
b3
b2
y2
y3
b10
b7
b11
b8
b9
y7+P
y13+P
y14+P
y13+P
y8+P
y8+P

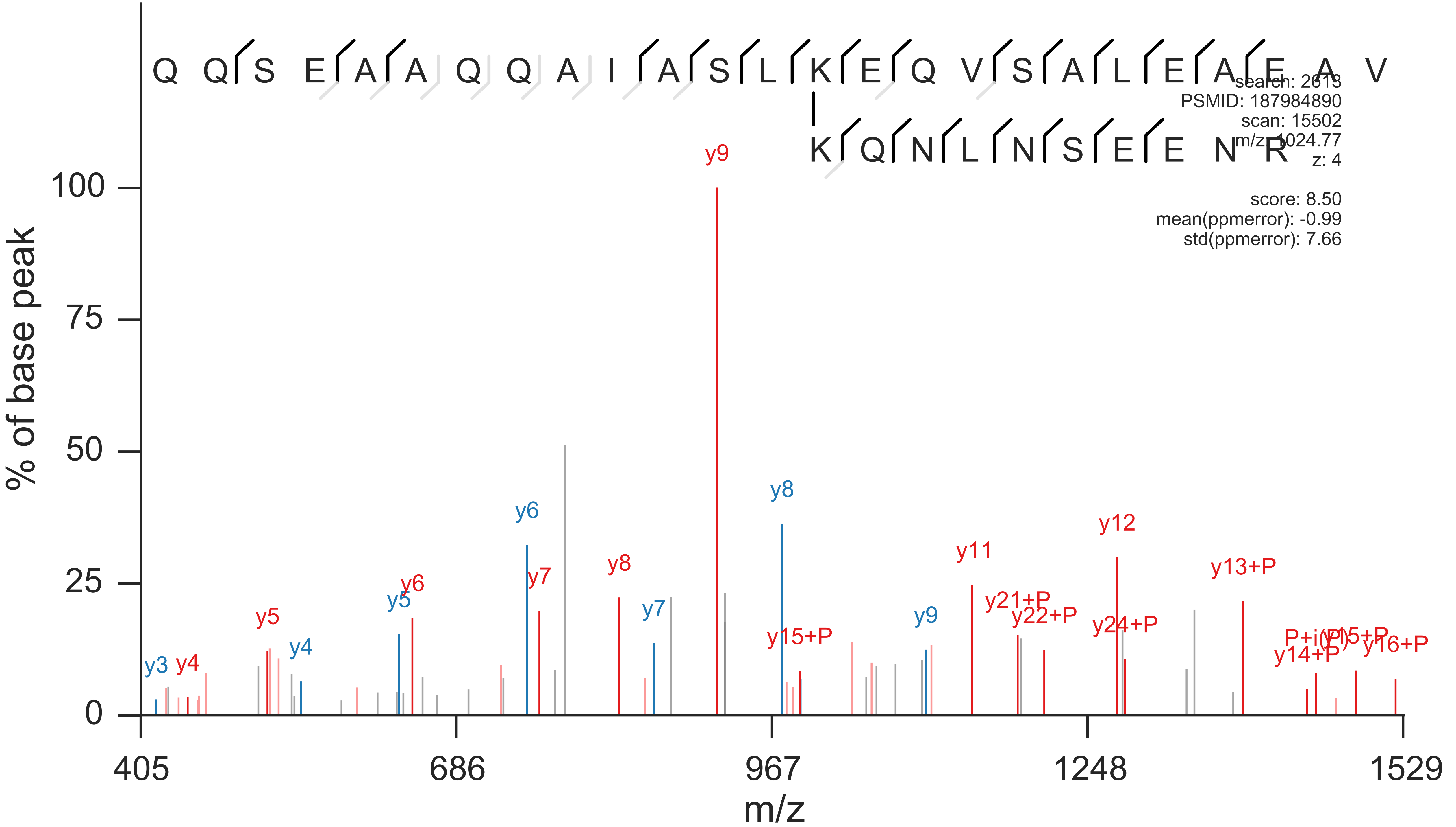

Q Q S E A A Q Q A I A S L K E Q V S A L E A E A V
K Q N L N S E E N R
search: 2613
PSMID: 187984890
scan: 15502
m/z: 1024.77
z: 4
score: 8.50
mean(ppmerror): -0.99
std(ppmerror): 7.66
% of base peak
m/z
405
686
967
1248
1529
0
25
50
75
100
y9
y8
y6
y12
y11
y13+P
y21+P
y22+P
y24+P
y15+P
P+i(P)5+P
y14+P
y16+P
y3
y4
y5
y7

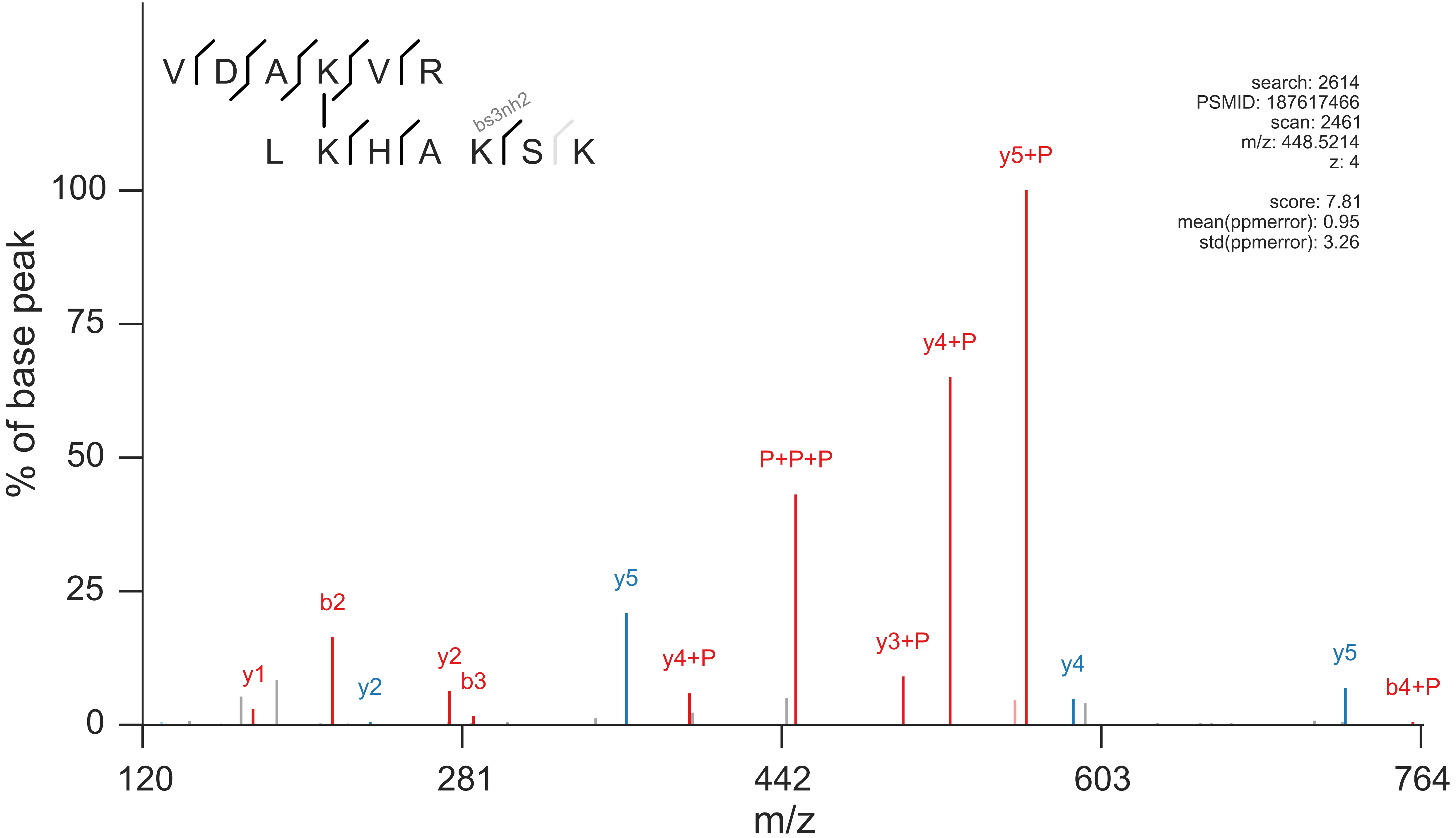
V D A K V R
L K H A K S K
bs3nh2
search: 2614
PSMID: 187617466
scan: 2461
m/z: 448.5214
z: 4
score: 7.81
mean(ppmerror): 0.95
std(ppmerror): 3.26
% of base peak
0
25
50
75
100
m/z
120
281
442
603
764
y1
b2
y2
y2
b3
y5
y4+P
P+P+P
y3+P
y4+P
y5+P
y4
y5
b4+P

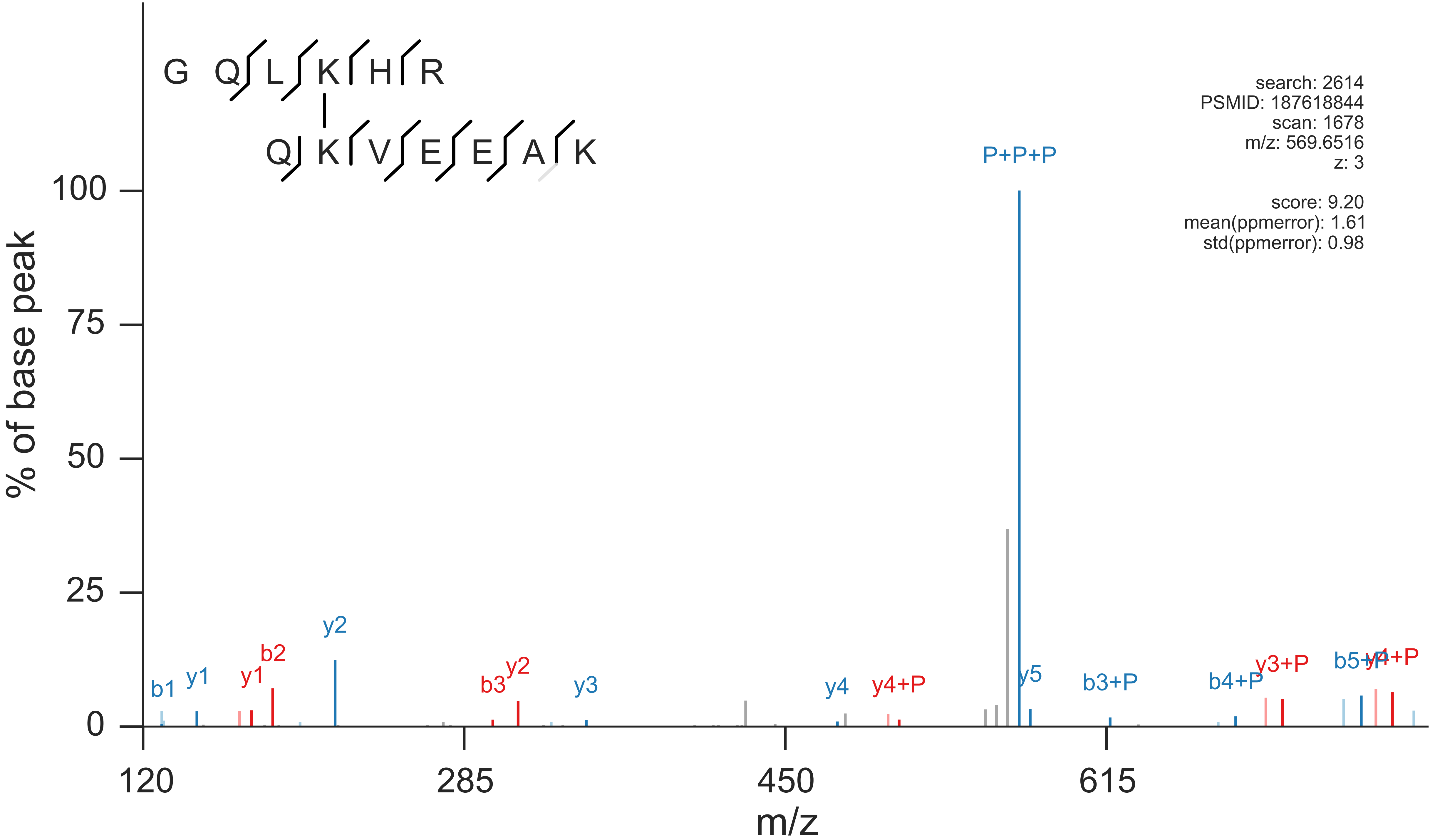

G Q L K H R
Q K V E E A K
search: 2614
PSMID: 187618844
scan: 1678
m/z: 569.6516
z: 3
score: 9.20
mean(ppmerror): 1.61
std(ppmerror): 0.98
P+P+P
% of base peak
100
75
50
25
0
120
285
450
615
m/z
b1
y1
y1
b2
y2
b3
y2
y3
y4
y4+P
y5
b3+P
b4+P
y3+P
b5+P
y4+P

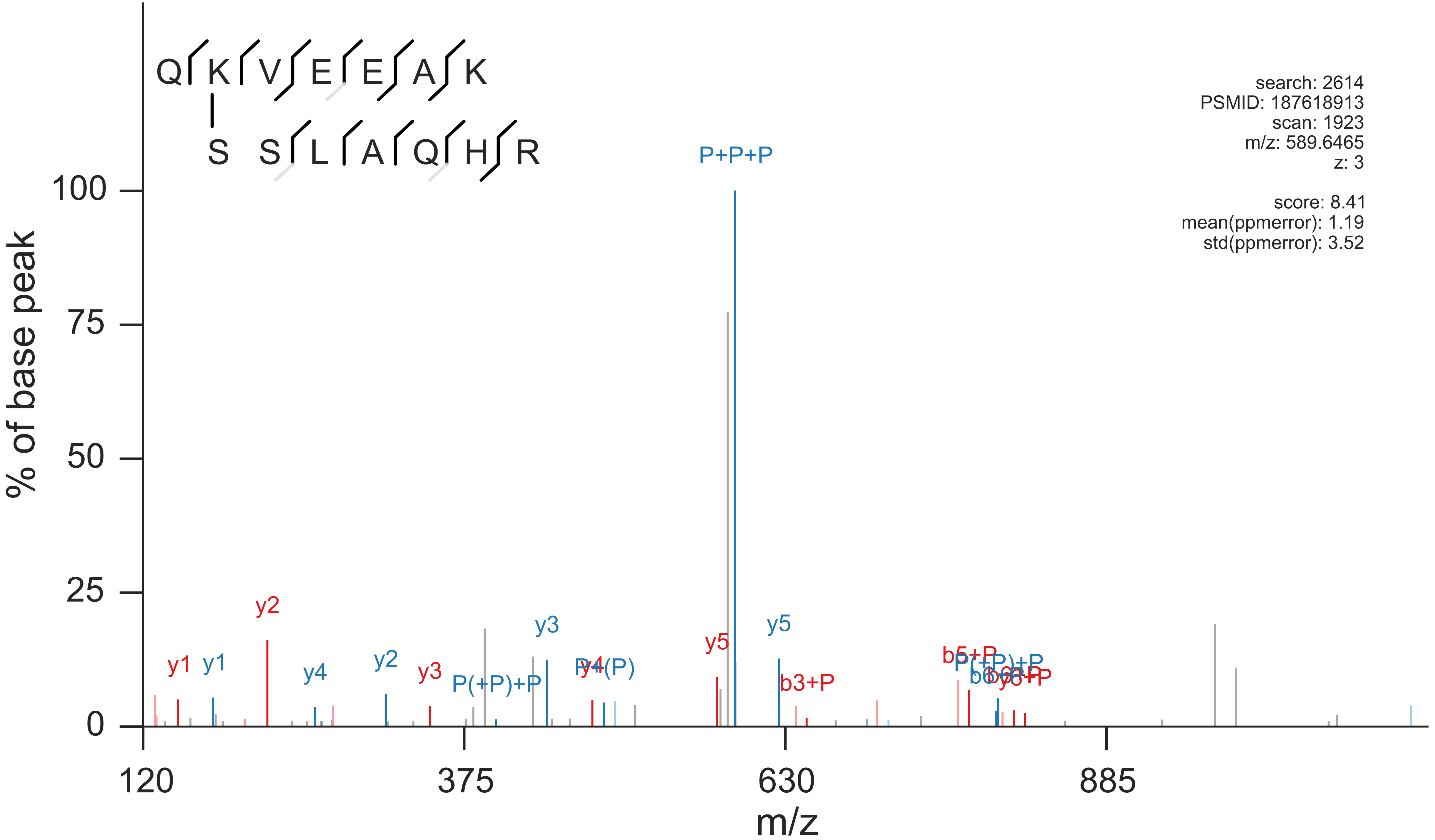

Q K V E E A K
S S L A Q H R
search: 2614
PSMID: 187618913
scan: 1923
m/z: 589.6465
z: 3
score: 8.41
mean(ppmerror): 1.19
std(ppmerror): 3.52
P+P+P
y1
y1
y2
y4
y2
y3
P(+P)+P
y3
P+(P)
y4
y5
y5
b3+P
b5+P
P(+P)+P
% of base peak
m/z
0
25
50
75
100
120
375
630
885

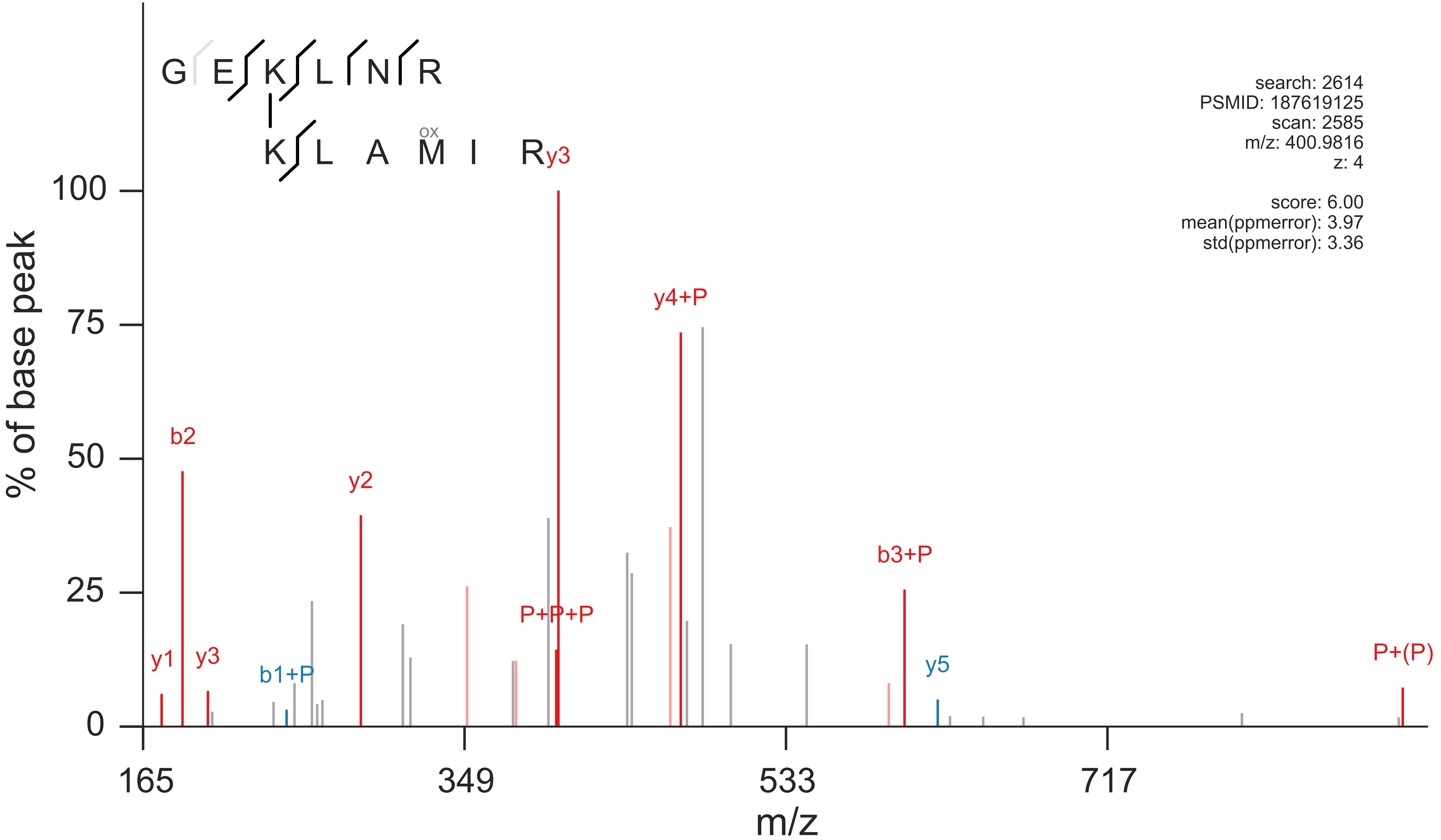

G E K L N R
K L A M I R
ox
search: 2614
PSMID: 187619125
scan: 2585
m/z: 400.9816
z: 4
score: 6.00
mean(ppmerror): 3.97
std(ppmerror): 3.36
% of base peak
m/z
0
25
50
75
100
165
349
533
717
y1
b2
y3
b1+P
y2
P+P+P
y3
y4+P
b3+P
y5
P+(P)

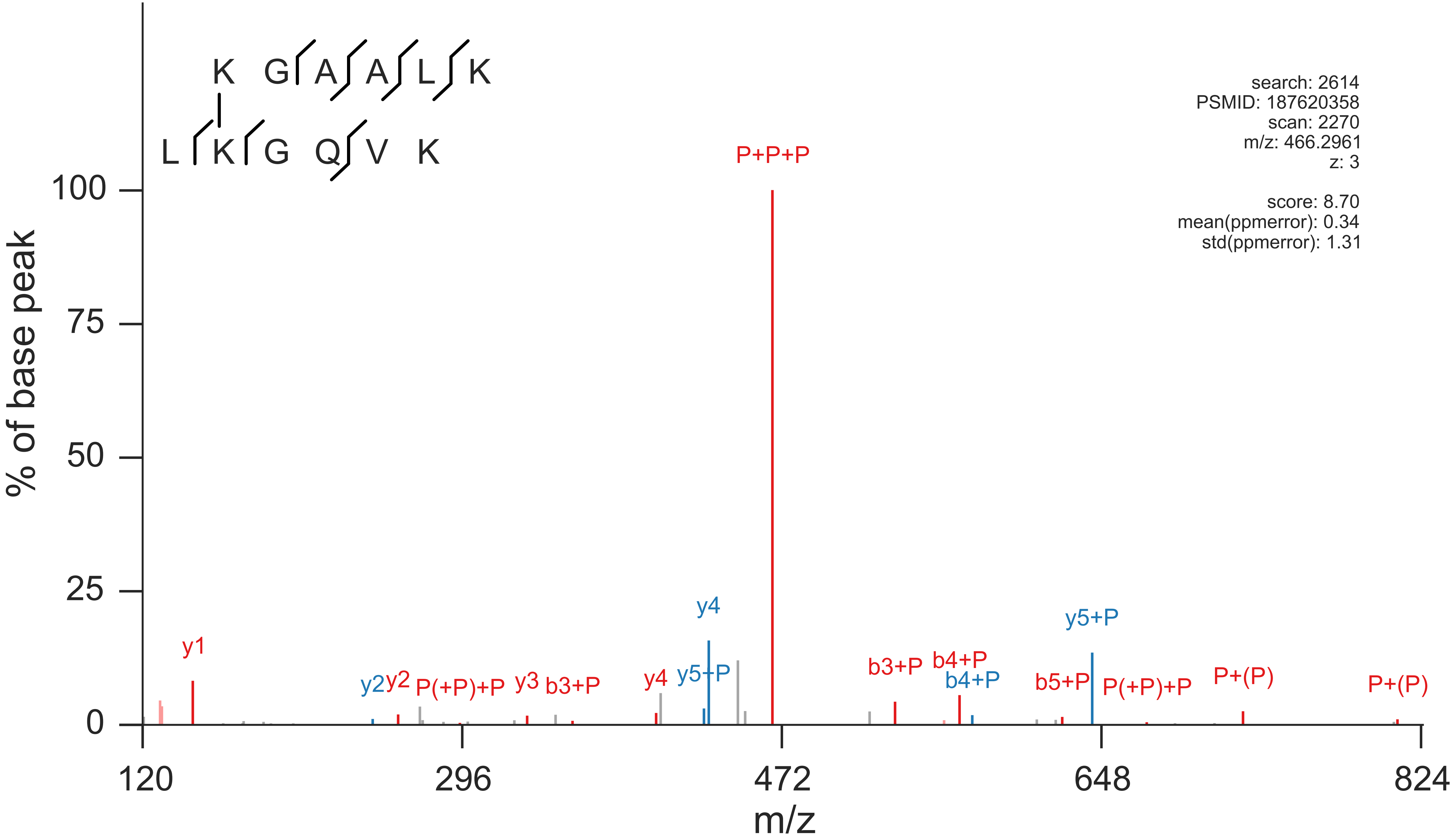

K G A A L K
L K G Q V K
search: 2614
PSMID: 187620358
scan: 2270
m/z: 466.2961
z: 3
score: 8.70
mean(ppmerror): 0.34
std(ppmerror): 1.31
% of base peak
m/z
P+P+P
y1
y2
y2
P(+P)+P
y3
b3+P
y4
y4
y5+P
b3+P
b4+P
b4+P
y5+P
b5+P
P(+P)+P
P+(P)
P+(P)
0
25
50
75
100
120
296
472
648
824

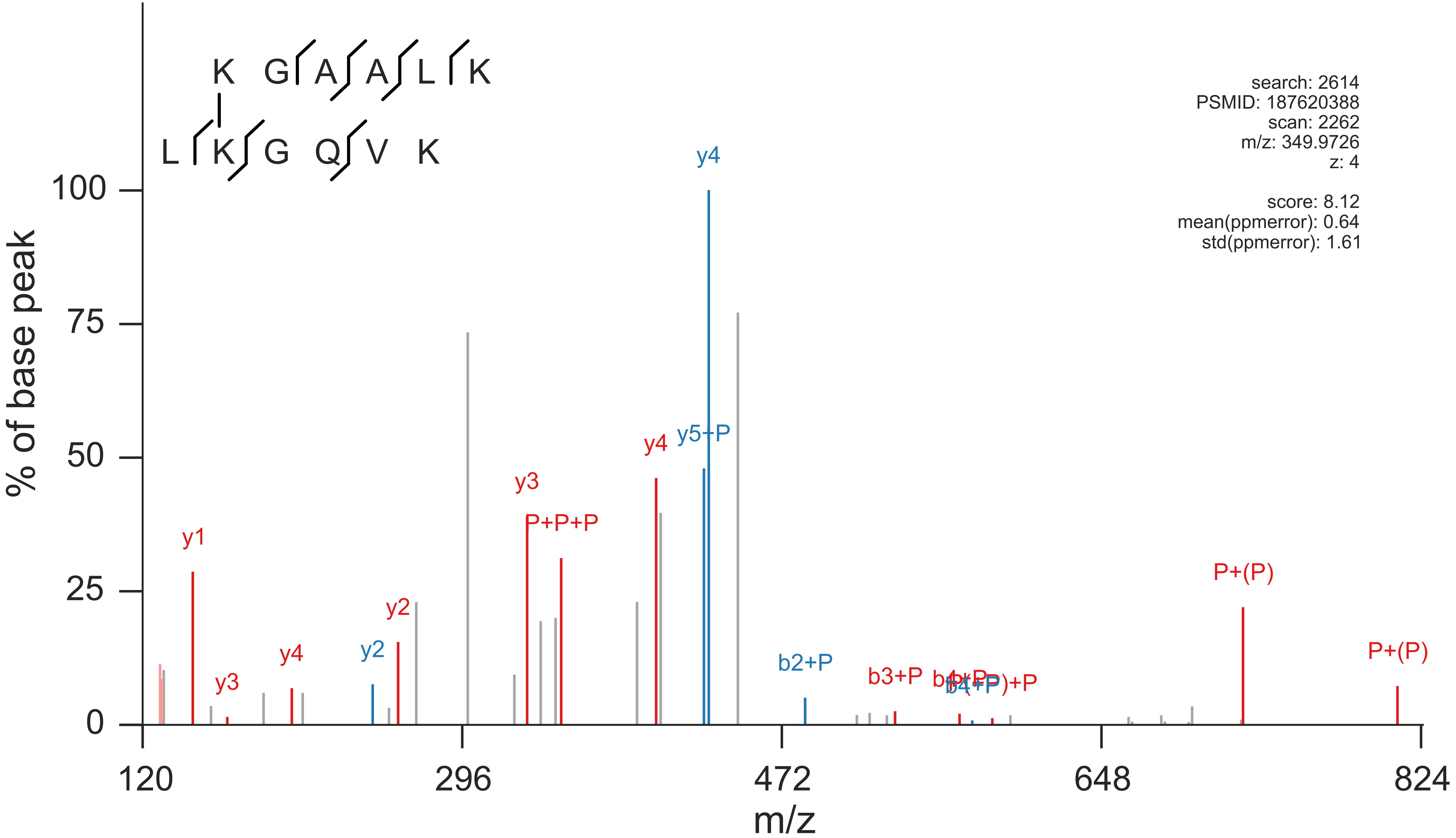

K G A A L K
L K G Q V K
search: 2614
PSMID: 187620388
scan: 2262
m/z: 349.9726
z: 4
score: 8.12
mean(ppmerror): 0.64
std(ppmerror): 1.61
% of base peak
100
75
50
25
0
m/z
120
296
472
648
824
y1
y3
y4
y2
y2
y3
P+P+P
y4
y5+P
y4
b2+P
b3+P
b4+P
P+(P)
P+(P)

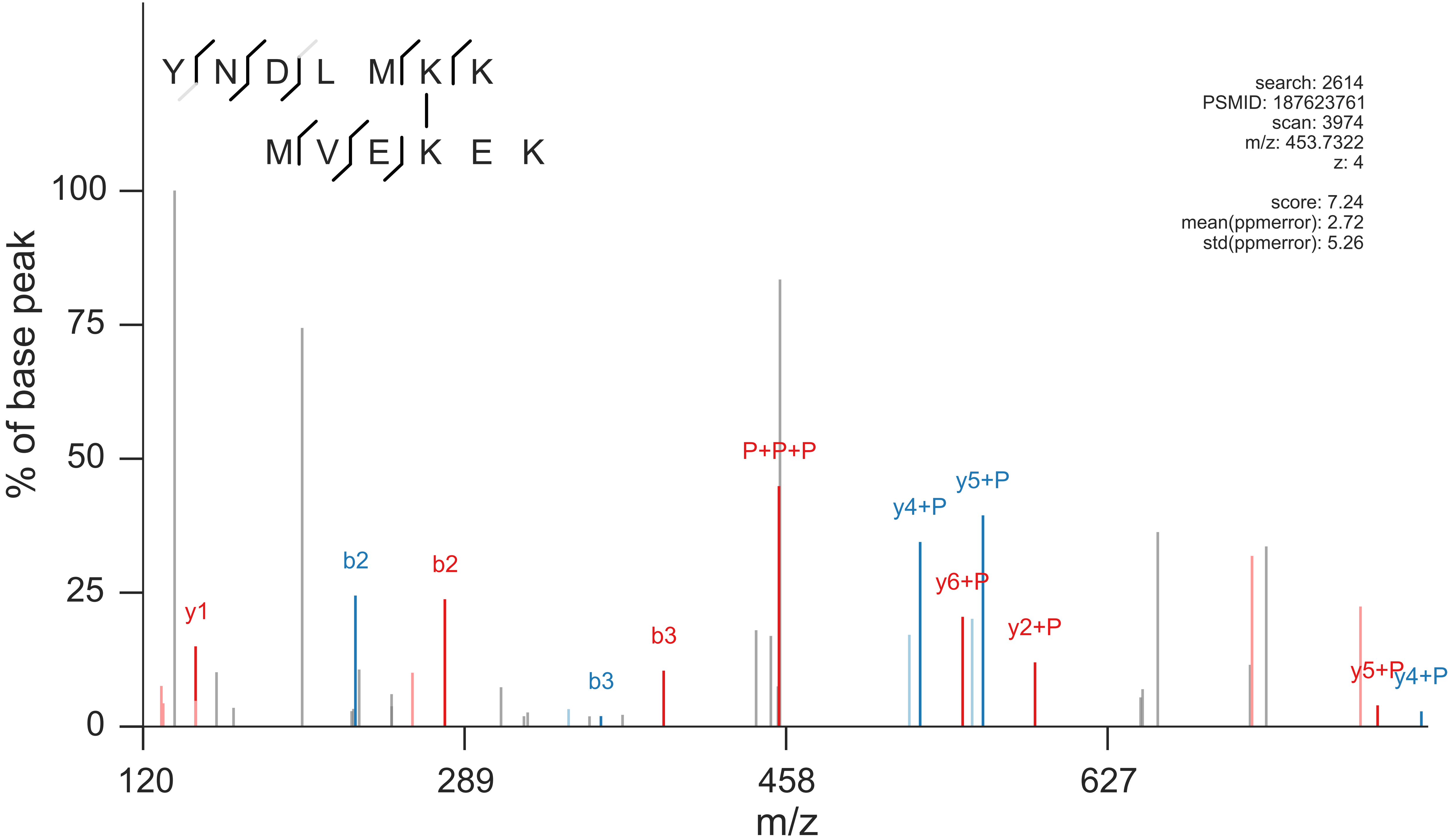

Y N D L M K K
M V E K E K
search: 2614
PSMID: 187623761
scan: 3974
m/z: 453.7322
z: 4
score: 7.24
mean(ppmerror): 2.72
std(ppmerror): 5.26
% of base peak
100
75
50
25
0
120
289
458
627
m/z
y1
b2
b2
b3
b3
P+P+P
y4+P
y5+P
y6+P
y2+P
y5+P
y4+P

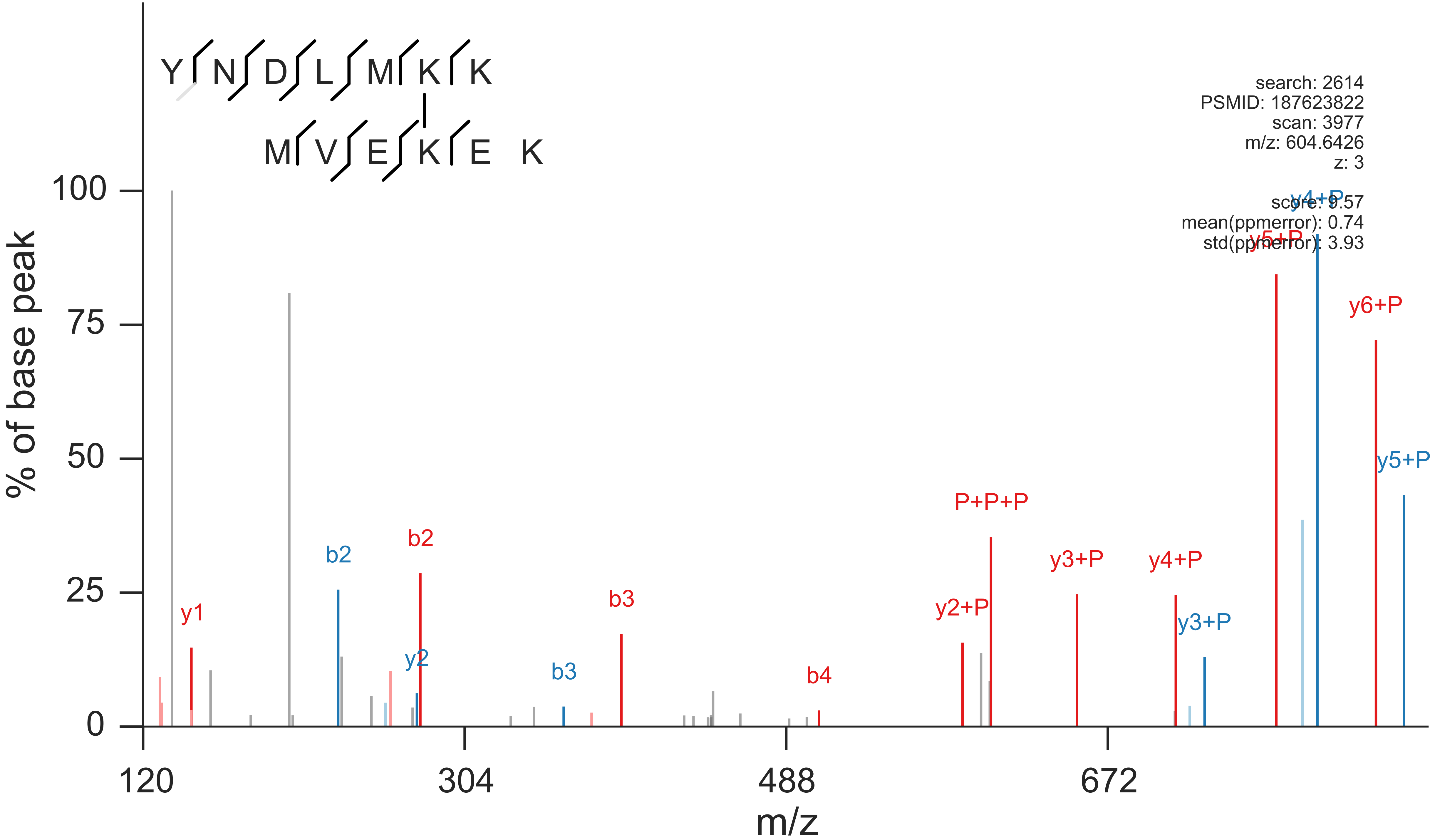
Y N D L M K K
M V E K E K
search: 2614
PSMID: 187623822
scan: 3977
m/z: 604.6426
z: 3
score: 9.57
mean(ppmerror): 0.74
std(ppmerror): 3.93
% of base peak
m/z
100
75
50
25
0
120
304
488
672
y1
b2
b2
y2
b3
b3
b4
y2+P
P+P+P
y3+P
y4+P
y3+P
y5+P
y4+P
y6+P
y5+P

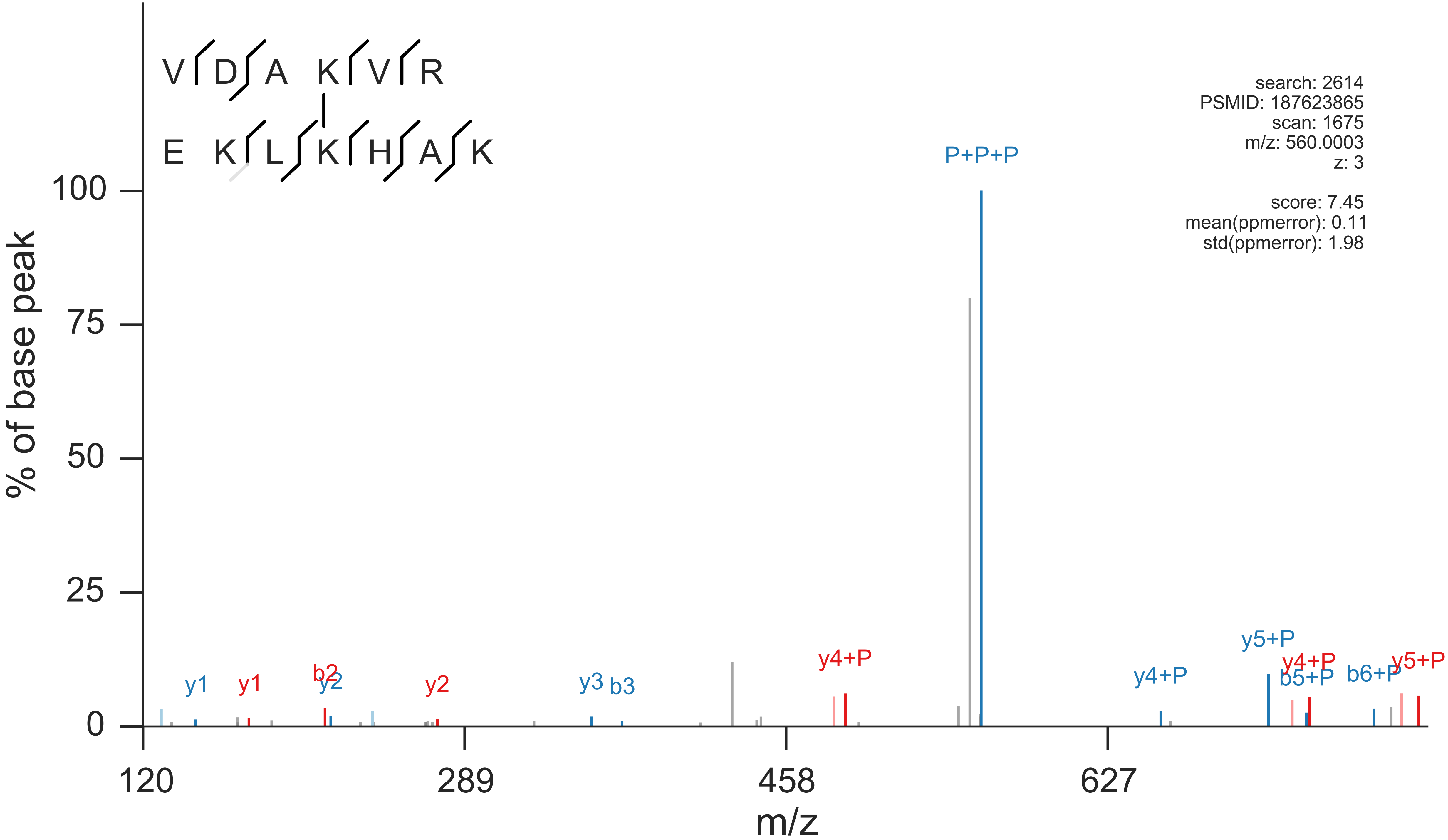

V D A K V R
E K L K H A K
search: 2614
PSMID: 187623865
scan: 1675
m/z: 560.0003
z: 3
score: 7.45
mean(ppmerror): 0.11
std(ppmerror): 1.98
P+P+P
% of base peak
100
75
50
25
0
y1
y1
b2
y2
y2
y3
b3
y4+P
y4+P
y5+P
y4+P
b5+P
b6+P
y5+P
120
289
458
627
m/z

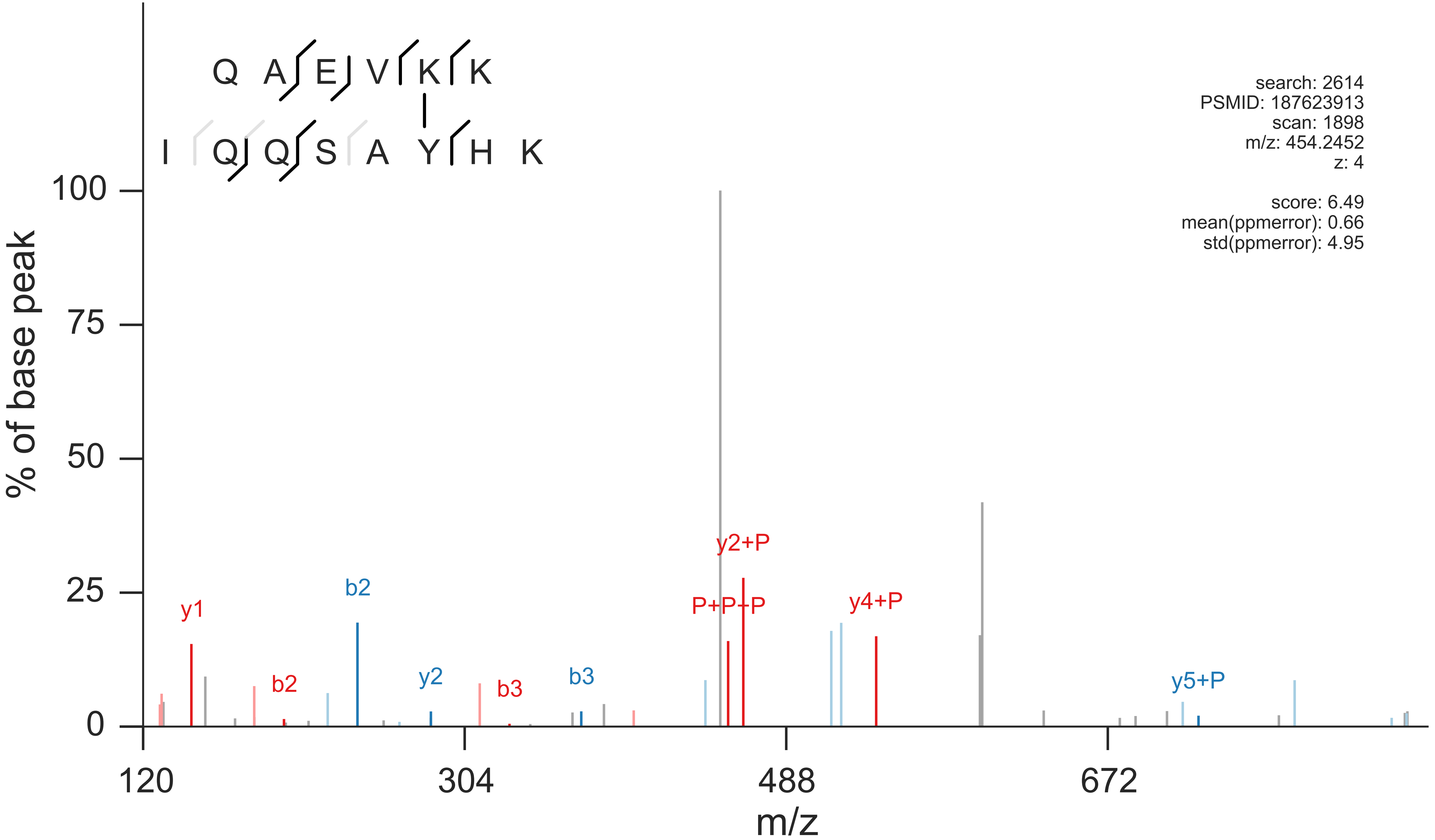
Q A E V K K
I Q Q S A Y H K
search: 2614
PSMID: 187623913
scan: 1898
m/z: 454.2452
z: 4
score: 6.49
mean(ppmerror): 0.66
std(ppmerror): 4.95
% of base peak
100
75
50
25
0
y1
b2
b2
y2
b3
b3
P+P+P
y2+P
y4+P
y5+P
120
304
488
672
m/z

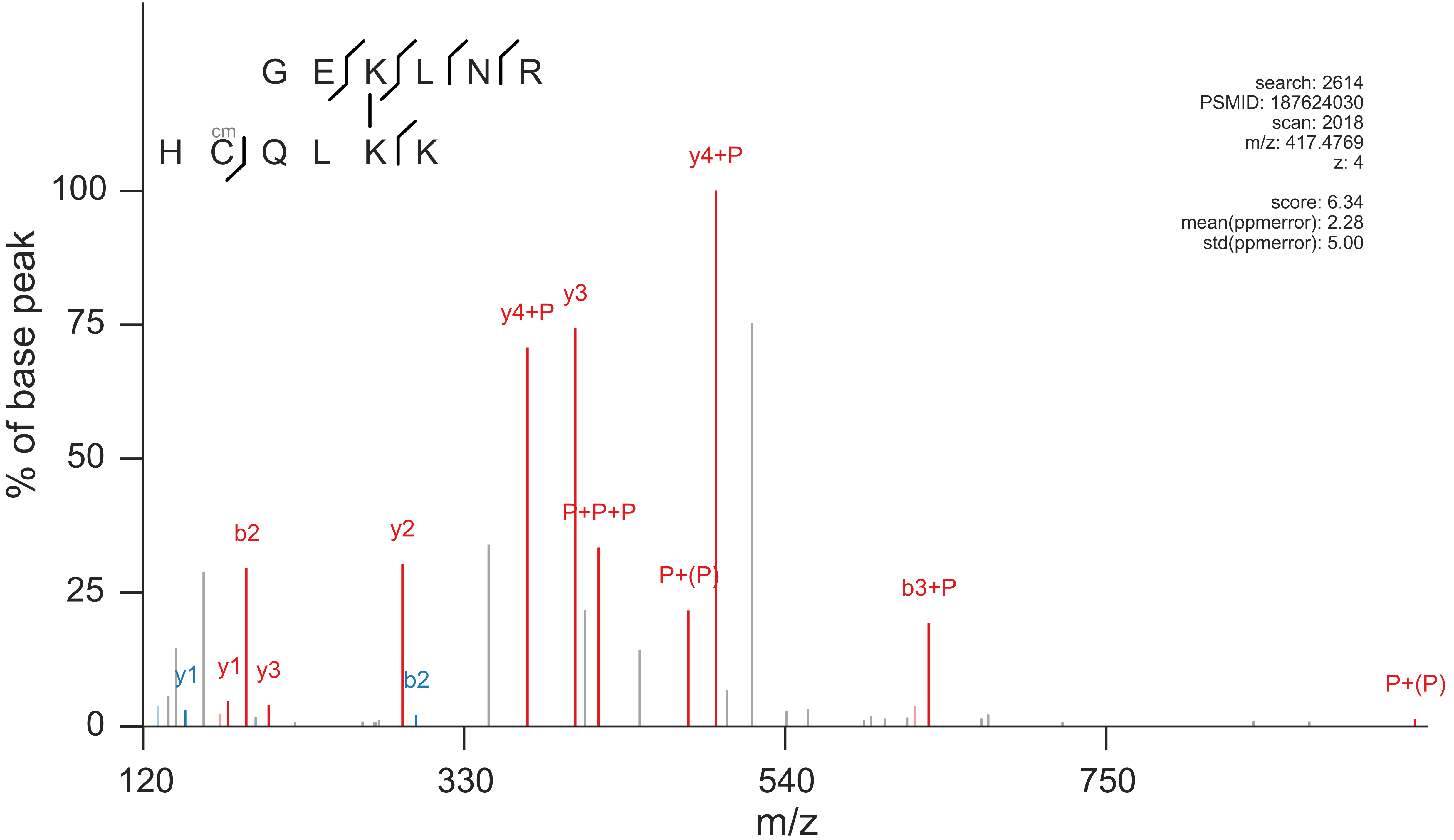

G E K L N R
H C Q L K K
cm
search: 2614
PSMID: 187624030
scan: 2018
m/z: 417.4769
z: 4
score: 6.34
mean(ppmerror): 2.28
std(ppmerror): 5.00
% of base peak
m/z
100
75
50
25
0
120
330
540
750
y4+P
y3
y4+P
P+P+P
P+(P)
b3+P
b2
y2
y1
y1
y3
b2
P+(P)

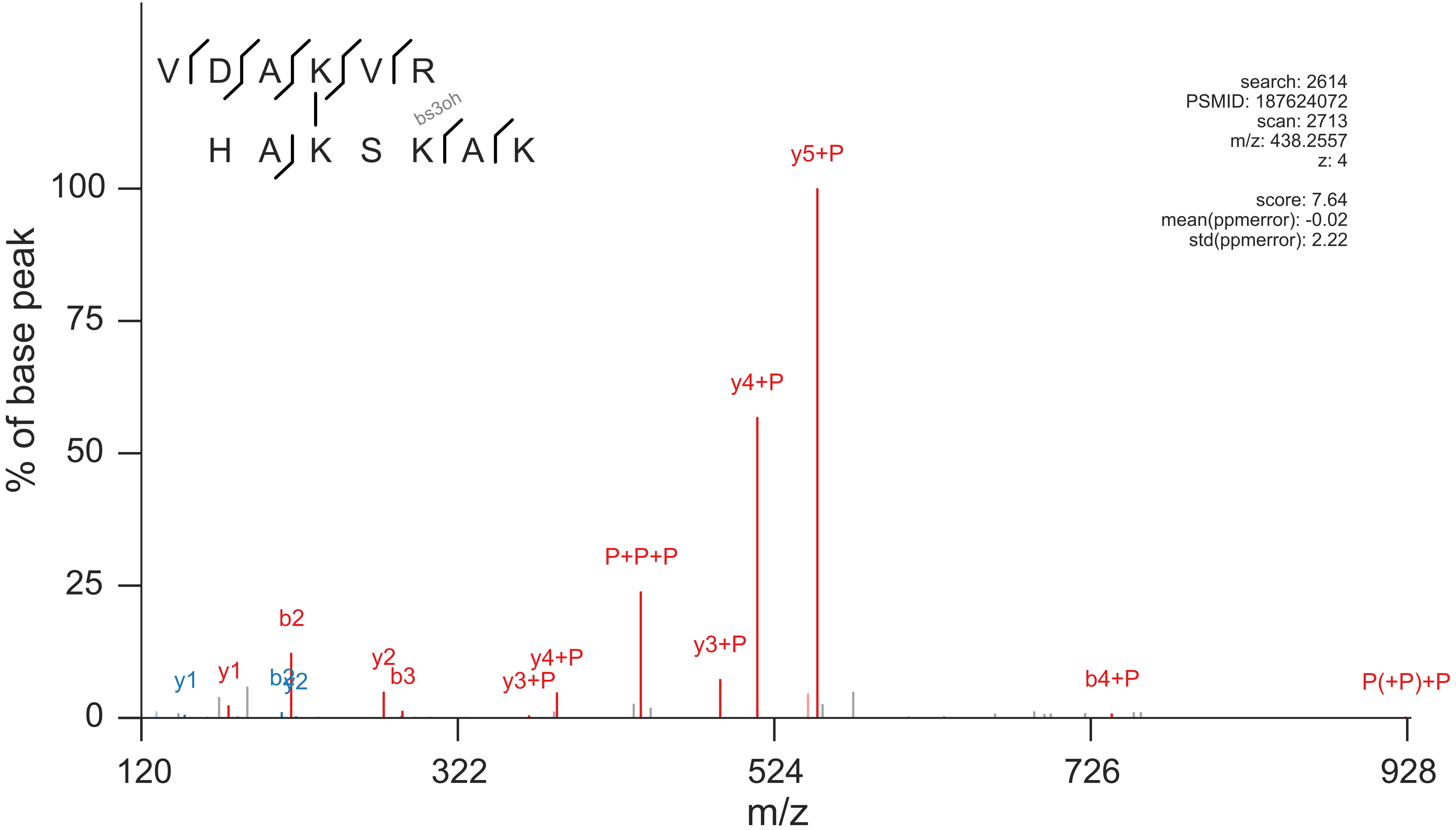

V D A K V R
H A K S K A K
bs3oh
search: 2614
PSMID: 187624072
scan: 2713
m/z: 438.2557
z: 4
score: 7.64
mean(ppmerror): -0.02
std(ppmerror): 2.22
% of base peak
m/z
y5+P
y4+P
P+P+P
y3+P
y4+P
y3+P
b2
y2
b3
y1
y1
b2
y2
b4+P
P(+P)+P

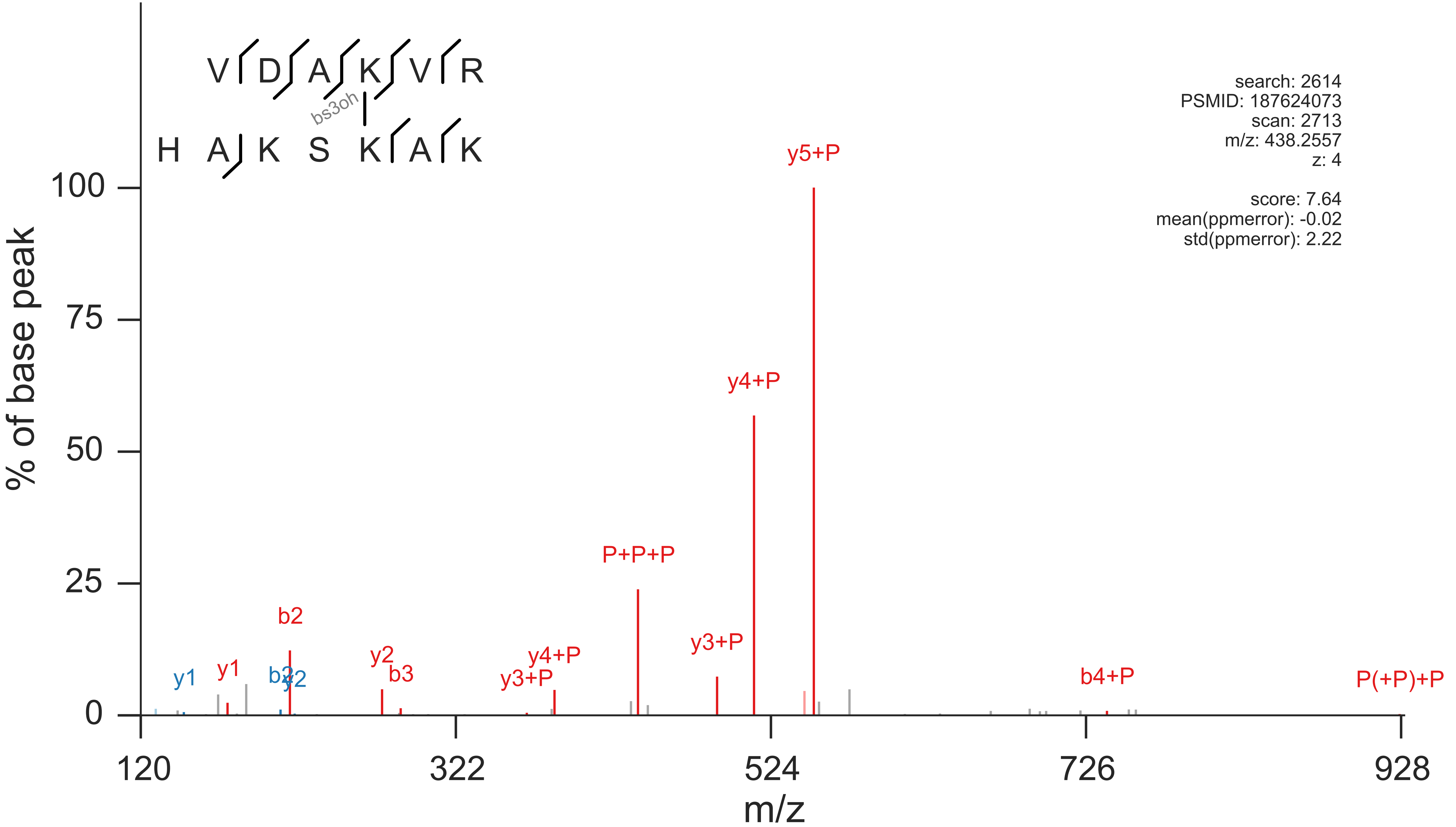

V D A K V R
bs3oh
H A K S K A K
search: 2614
PSMID: 187624073
scan: 2713
m/z: 438.2557
z: 4
score: 7.64
mean(ppmerror): -0.02
std(ppmerror): 2.22
% of base peak
100
75
50
25
0
y5+P
y4+P
P+P+P
y3+P
y4+P
y3+P
b2
y2
b3
y1
y1
b2
y2
b4+P
P(+P)+P
120
322
524
726
928
m/z

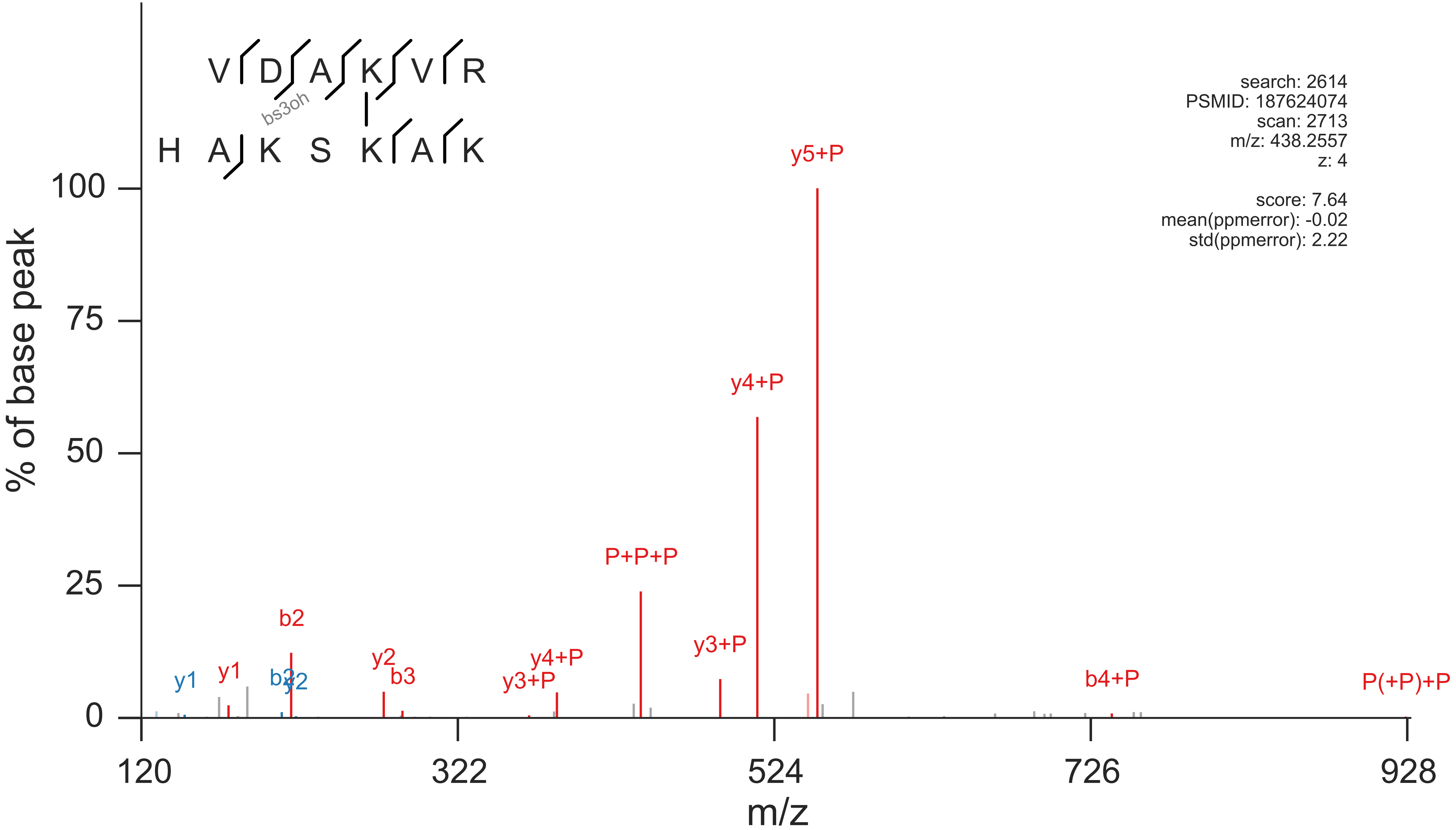

V D A K V R
bs3oh
H A K S K A K
search: 2614
PSMID: 187624074
scan: 2713
m/z: 438.2557
z: 4
score: 7.64
mean(ppmerror): -0.02
std(ppmerror): 2.22
% of base peak
0
25
50
75
100
m/z
120
322
524
726
928
y5+P
y4+P
P+P+P
y3+P
b2
y2
b3
y4+P
y3+P
y1
y1
b2
y2
b4+P
P(+P)+P

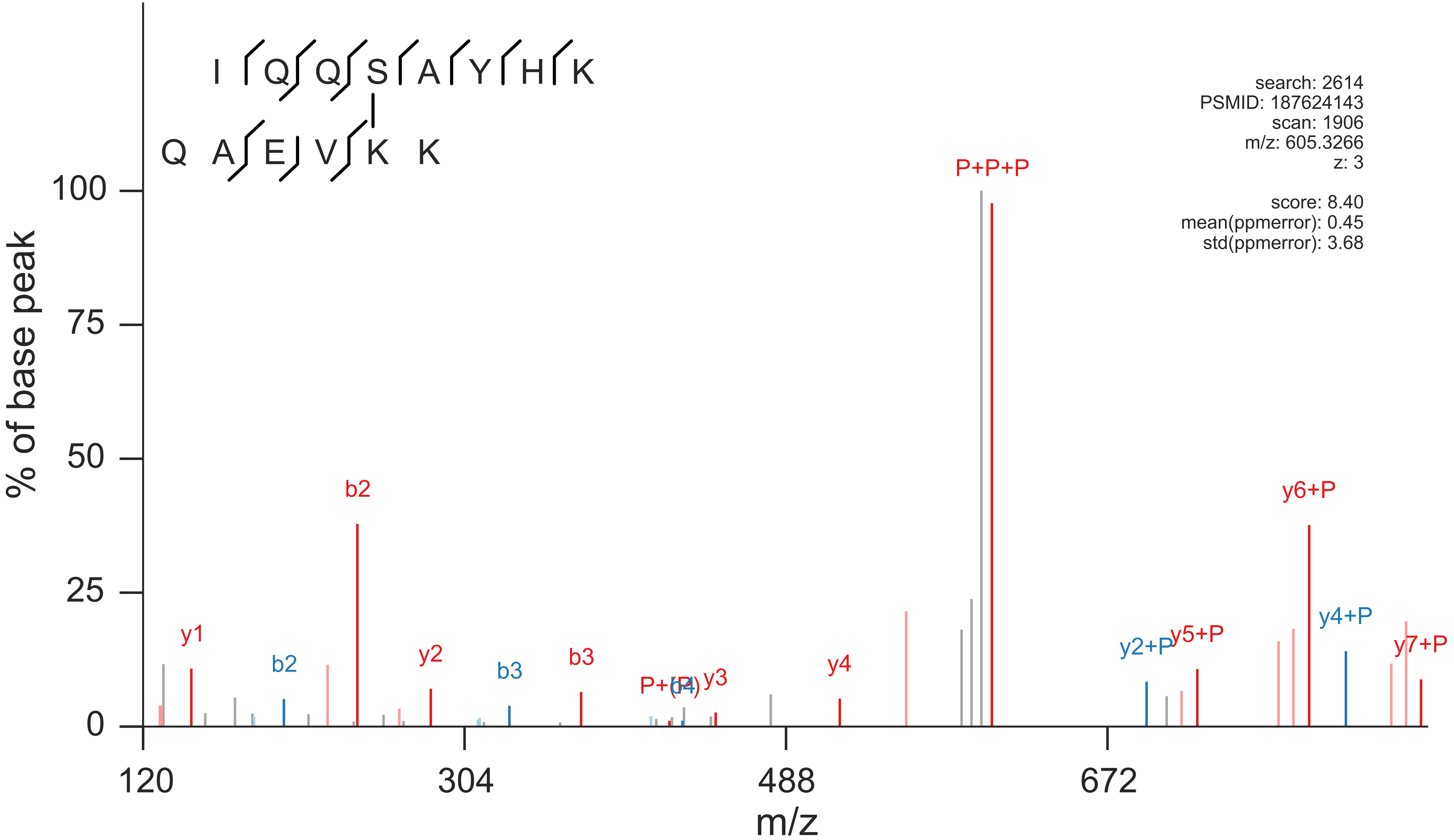
I Q Q S A Y H K
Q A E V K K
search: 2614
PSMID: 187624143
scan: 1906
m/z: 605.3266
z: 3
score: 8.40
mean(ppmerror): 0.45
std(ppmerror): 3.68
P+P+P
b2
y6+P
y1
b2
y2
b3
b3
P+(P)
b4
y3
y4
y2+P
y5+P
y4+P
y7+P
% of base peak
0
25
50
75
100
120
304
488
672
m/z

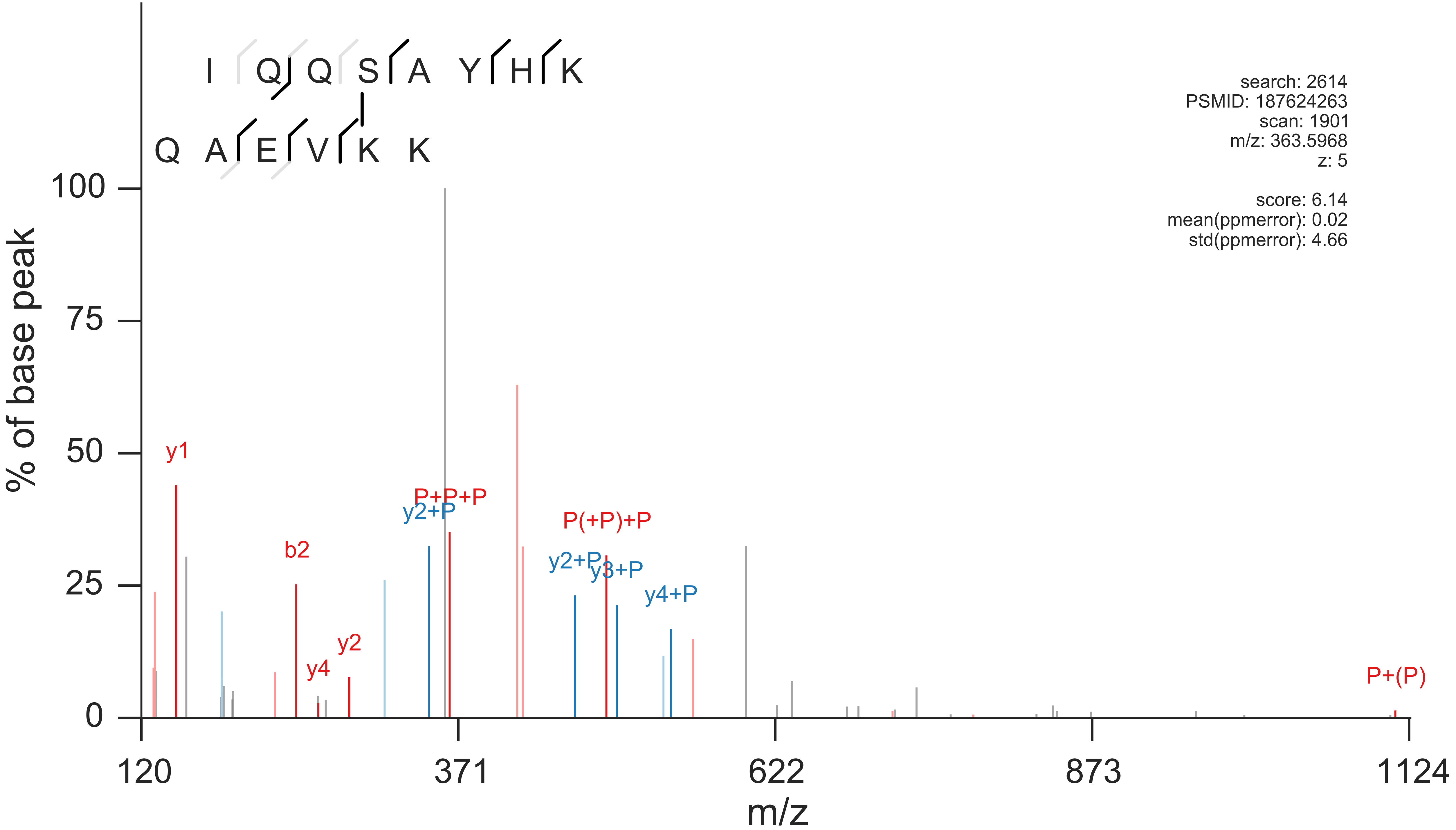

I Q Q S A Y H K
Q A E V K K
search: 2614
PSMID: 187624263
scan: 1901
m/z: 363.5968
z: 5
score: 6.14
mean(ppmerror): 0.02
std(ppmerror): 4.66
% of base peak
100
75
50
25
0
y1
b2
y4
y2
y2+P
P+P+P
P(+P)+P
y2+P
y3+P
y4+P
P+(P)
120
371
622
873
1124
m/z

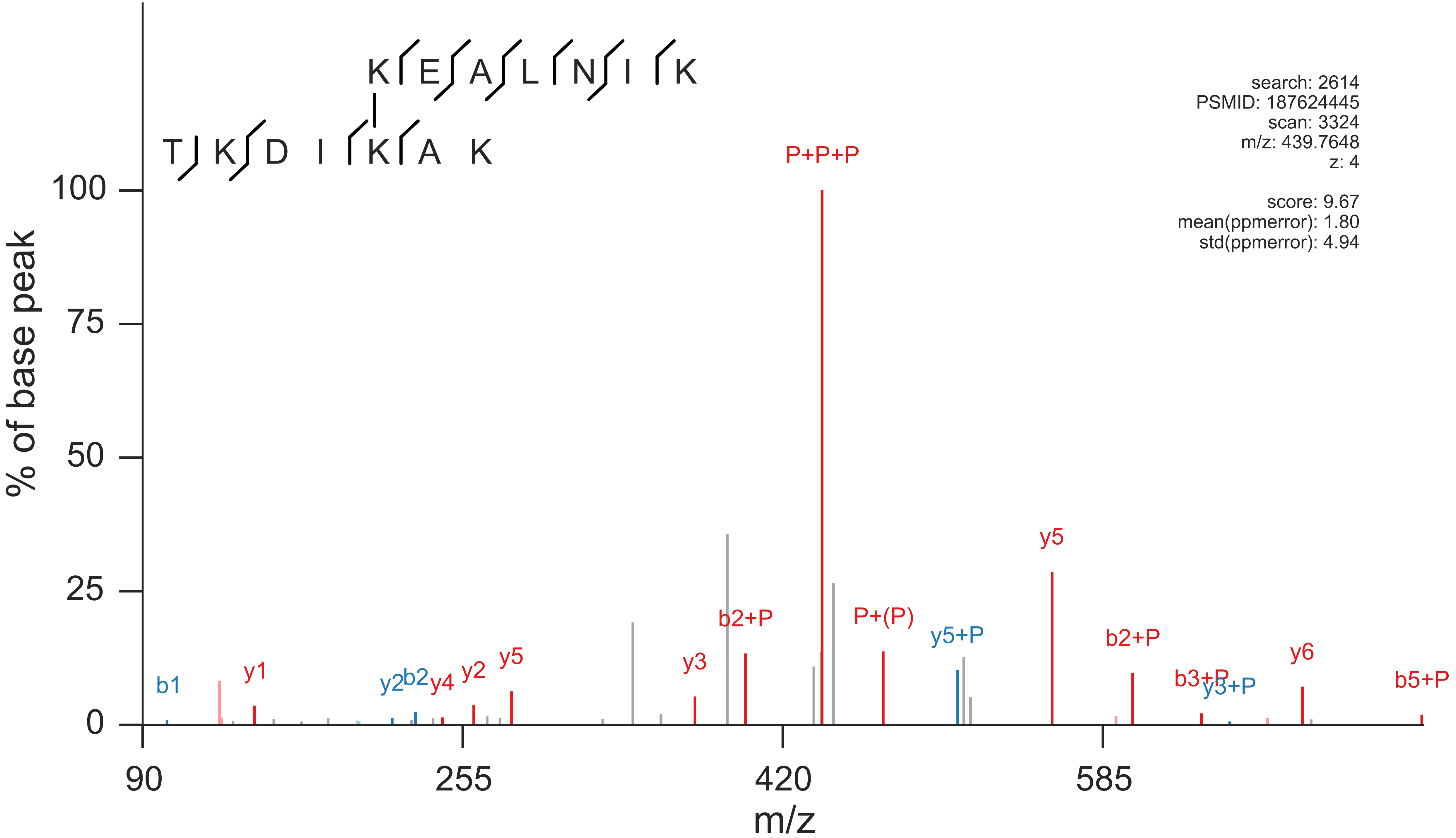
K E A L N I K
T K D I K A K
search: 2614
PSMID: 187624445
scan: 3324
m/z: 439.7648
z: 4
score: 9.67
mean(ppmerror): 1.80
std(ppmerror): 4.94
% of base peak
100
75
50
25
0
90
255
420
585
m/z
P+P+P
b1
y1
y2
b2
y4
y2
y5
y3
b2+P
P+(P)
y5+P
y5
b2+P
b3+P
y3+P
y6
b5+P

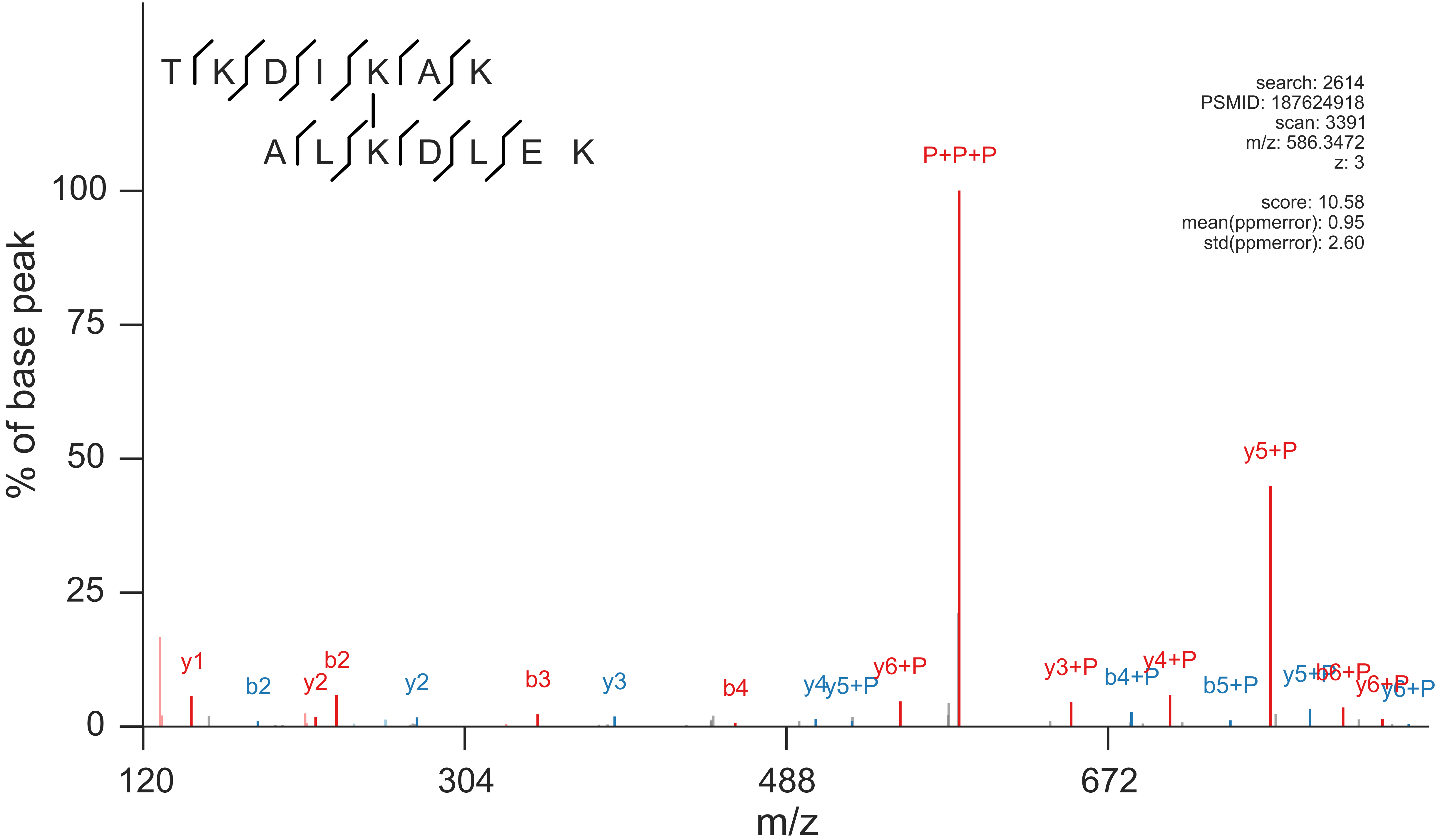
T K D I K A K
A L K D L E K
search: 2614
PSMID: 187624918
scan: 3391
m/z: 586.3472
z: 3
score: 10.58
mean(ppmerror): 0.95
std(ppmerror): 2.60
P+P+P
y5+P
y1
b2
y2
b2
y2
b3
y3
b4
y4
y5+P
y6+P
y3+P
b4+P
y4+P
b5+P
y5+P
b6+P
y6+P
y6+P
% of base peak
100
75
50
25
0
120
304
488
672
m/z

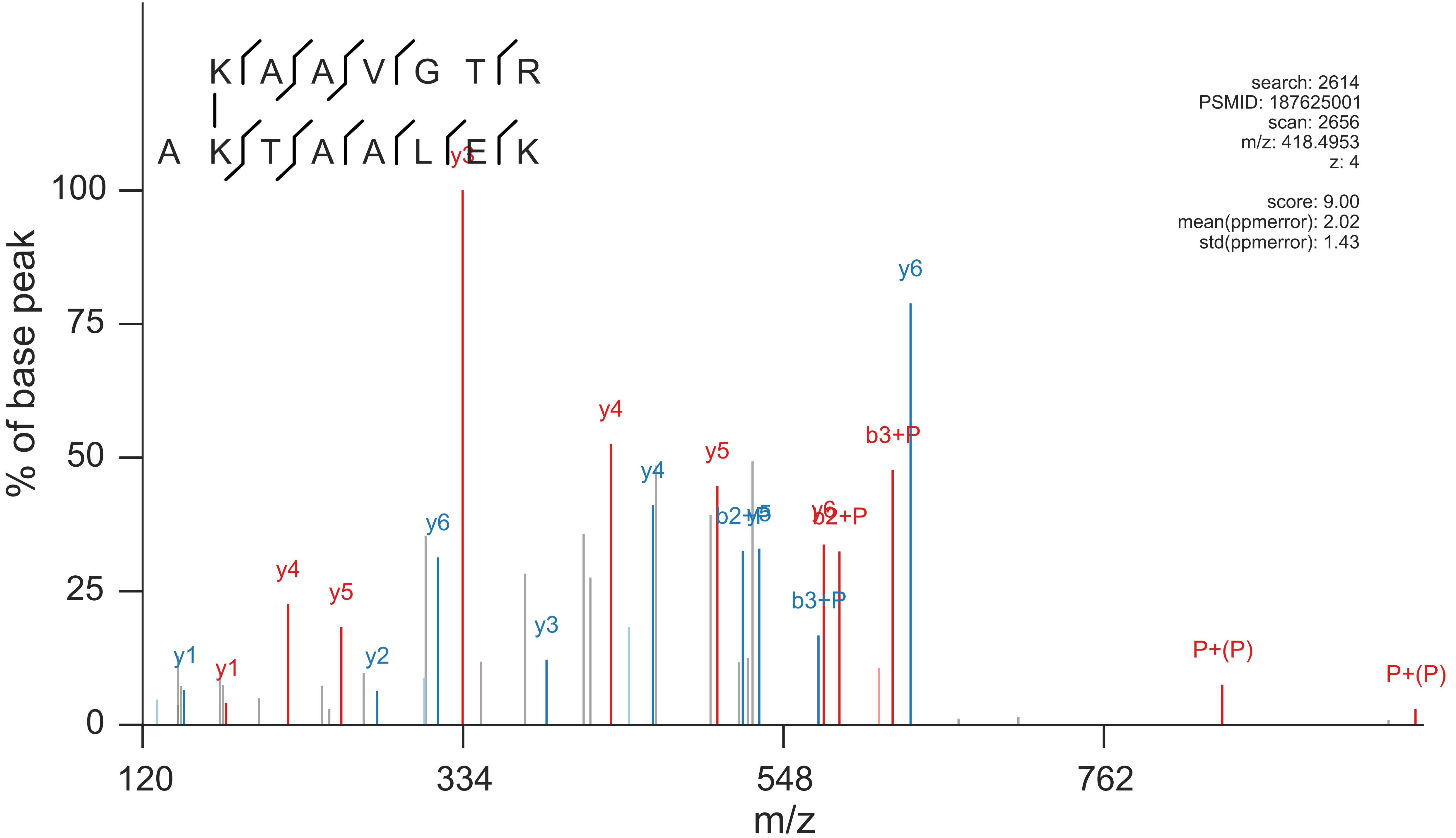

K A A V G T R
A K T A A L E K
search: 2614
PSMID: 187625001
scan: 2656
m/z: 418.4953
z: 4
score: 9.00
mean(ppmerror): 2.02
std(ppmerror): 1.43
% of base peak
m/z
100
75
50
25
0
120
334
548
762
y1
y1
y4
y5
y2
y6
y3
y3
y4
y4
y5
b2+P
y5
y6
b2+P
b3+P
b3+P
y6
P+(P)
P+(P)

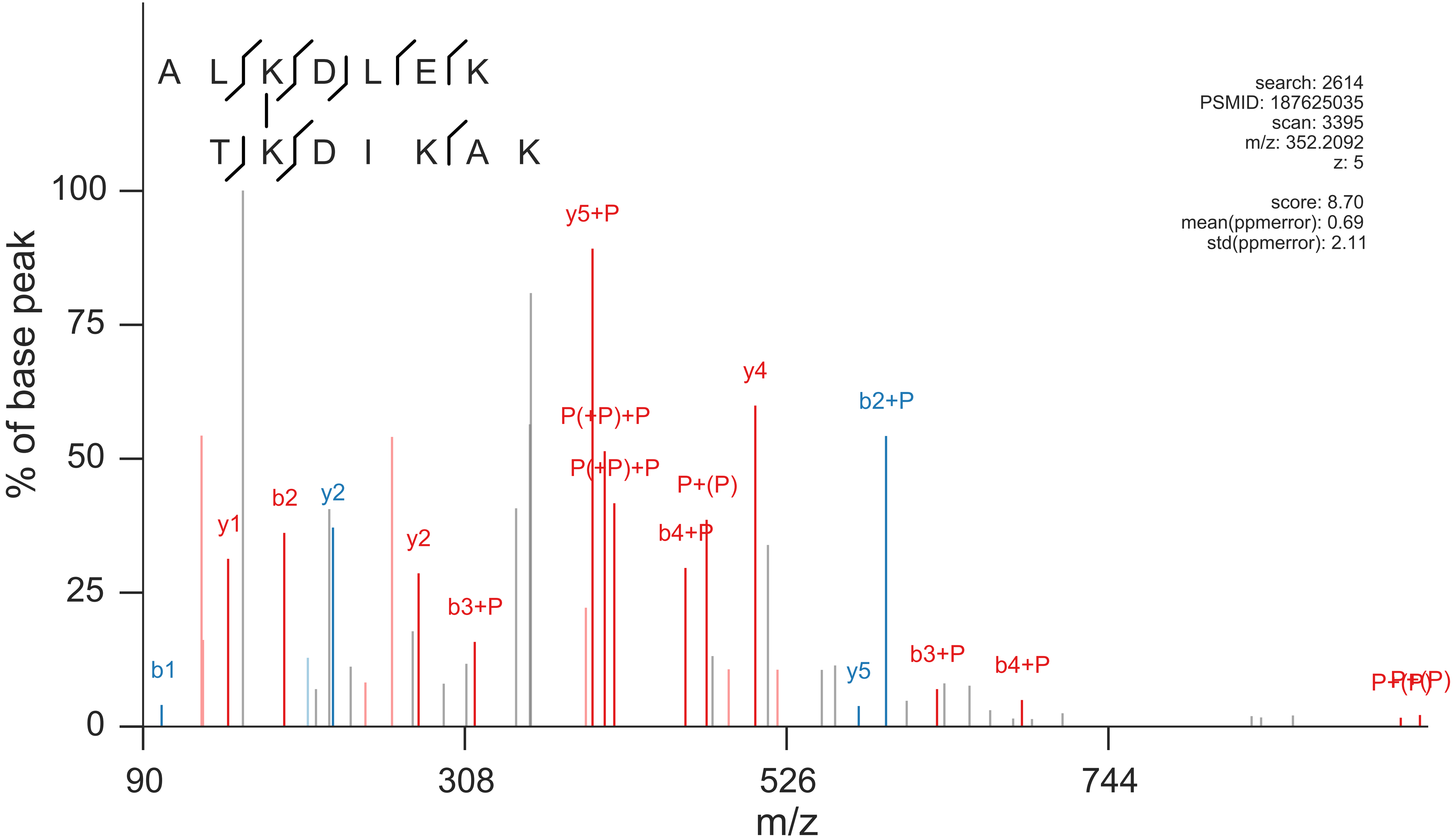

A L K D L E K
T K D I K A K
search: 2614
PSMID: 187625035
scan: 3395
m/z: 352.2092
z: 5
score: 8.70
mean(ppmerror): 0.69
std(ppmerror): 2.11
% of base peak
0
25
50
75
100
m/z
90
308
526
744
b1
y1
b2
y2
y2
b3+P
y5+P
P(+P)+P
P(+P)+P
b4+P
P+(P)
y4
b2+P
y5
b3+P
b4+P
P+P(P)

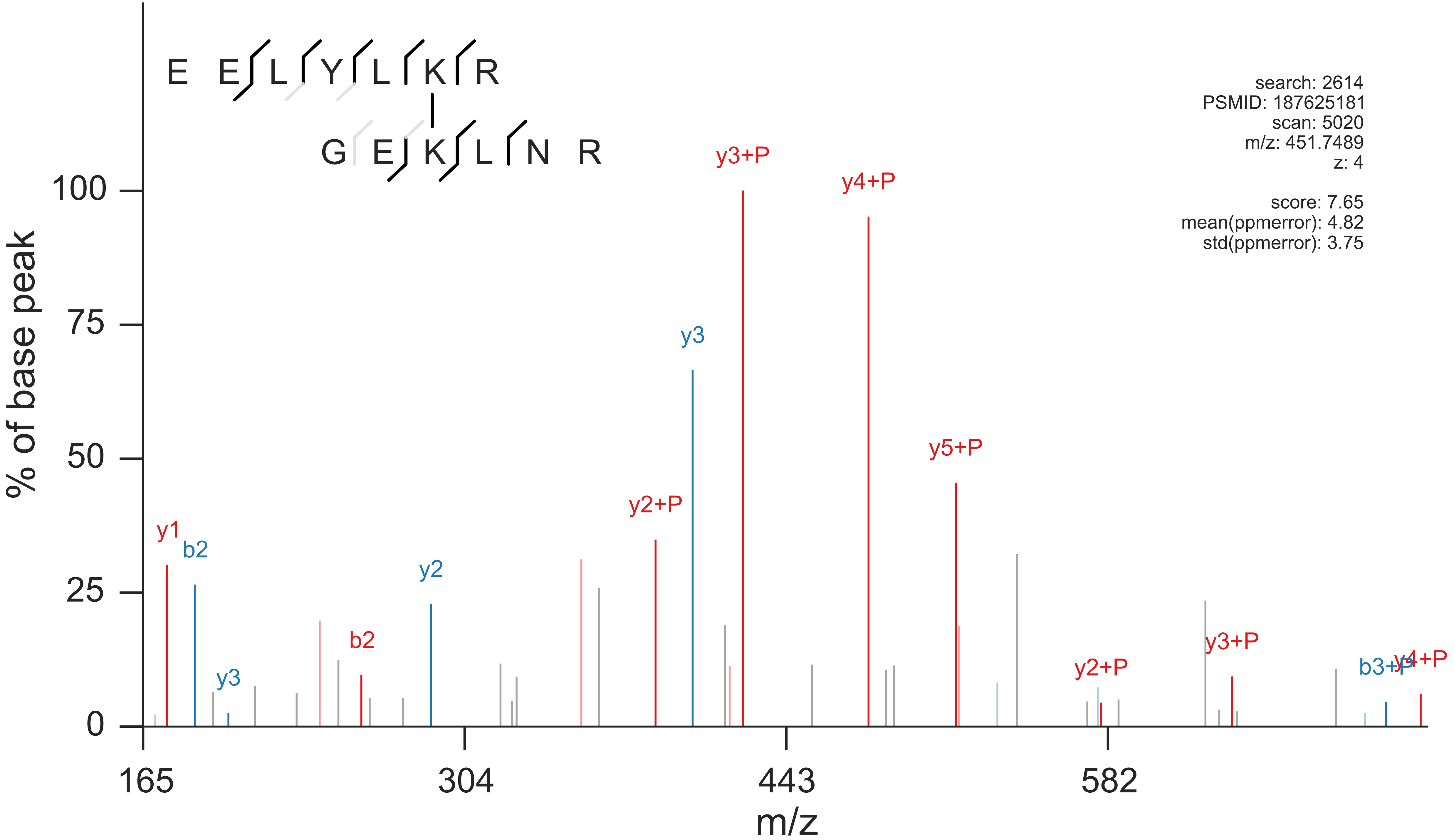

E E L Y L K R
G E K L N R
search: 2614
PSMID: 187625181
scan: 5020
m/z: 451.7489
z: 4
score: 7.65
mean(ppmerror): 4.82
std(ppmerror): 3.75
% of base peak
m/z
0
25
50
75
100
165
304
443
582
y1
b2
y3
b2
y2
y2+P
y3
y3+P
y4+P
y5+P
y2+P
y3+P
b3+P
y4+P

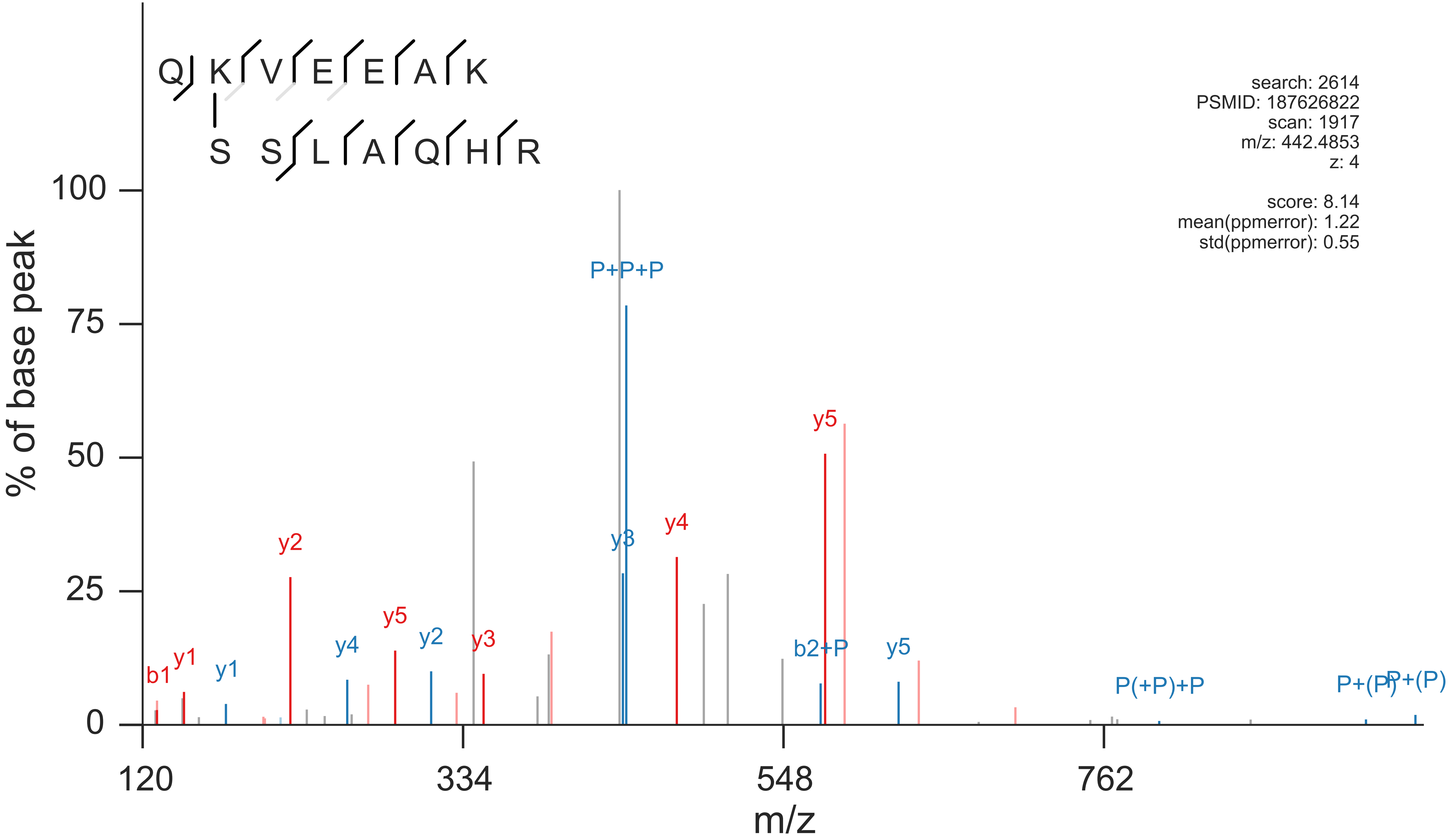

Q K V E E A K
S S L A Q H R
search: 2614
PSMID: 187626822
scan: 1917
m/z: 442.4853
z: 4
score: 8.14
mean(ppmerror): 1.22
std(ppmerror): 0.55
% of base peak
m/z
100
75
50
25
0
120
334
548
762
P+P+P
y5
y4
y3
y2
y5
y2
y3
y4
b1
y1
y1
b2+P
y5
P(+P)+P
P+(P)
P+(P)

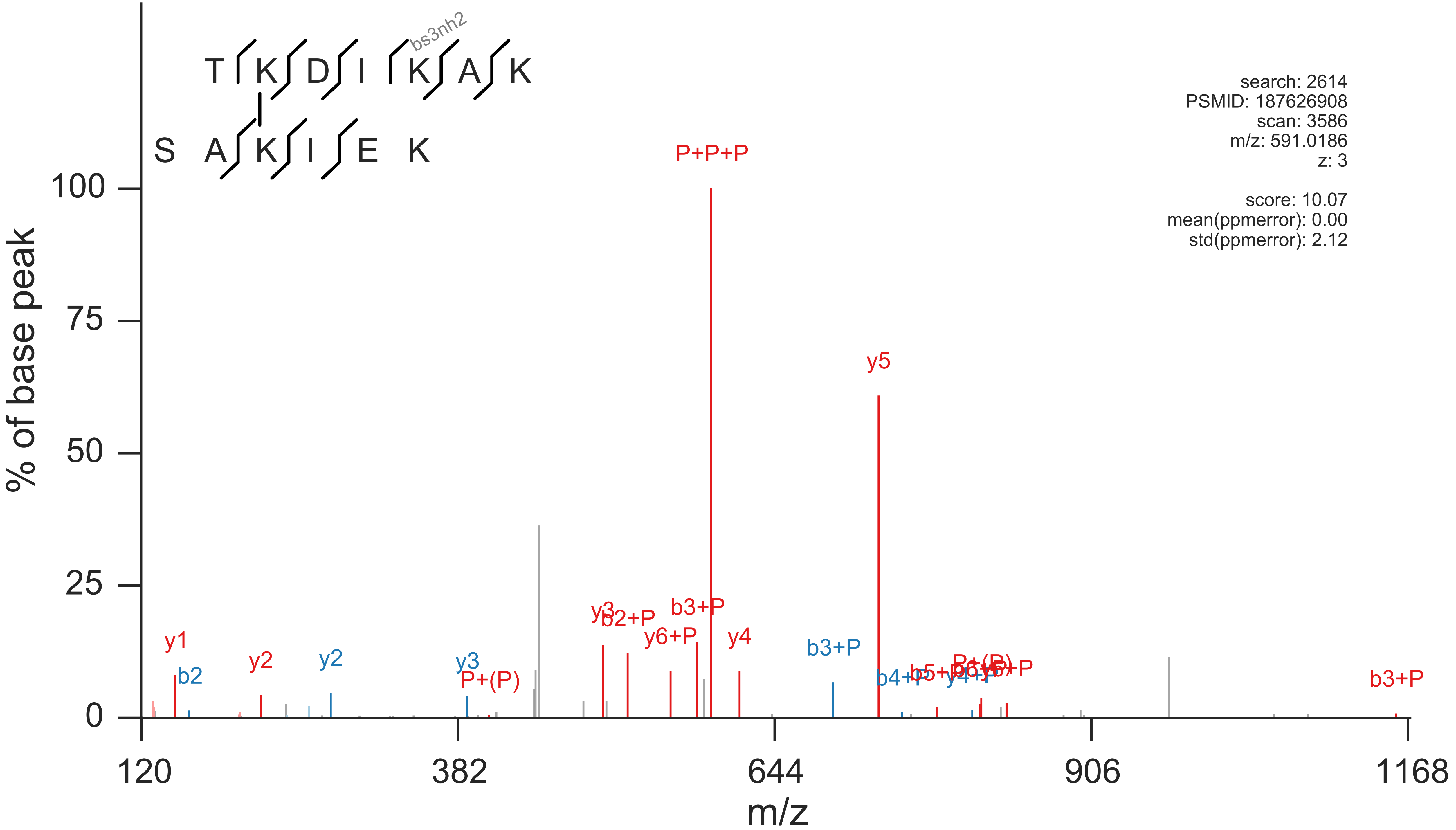

T K D I K A K
bs3nh2
S A K I E K
search: 2614
PSMID: 187626908
scan: 3586
m/z: 591.0186
z: 3
score: 10.07
mean(ppmerror): 0.00
std(ppmerror): 2.12
% of base peak
m/z
0
25
50
75
100
120
382
644
906
1168
P+P+P
y5
y1
b2
y2
y2
y3
P+(P)
y3
b2+P
b3+P
y6+P
y4
b3+P
b3+P

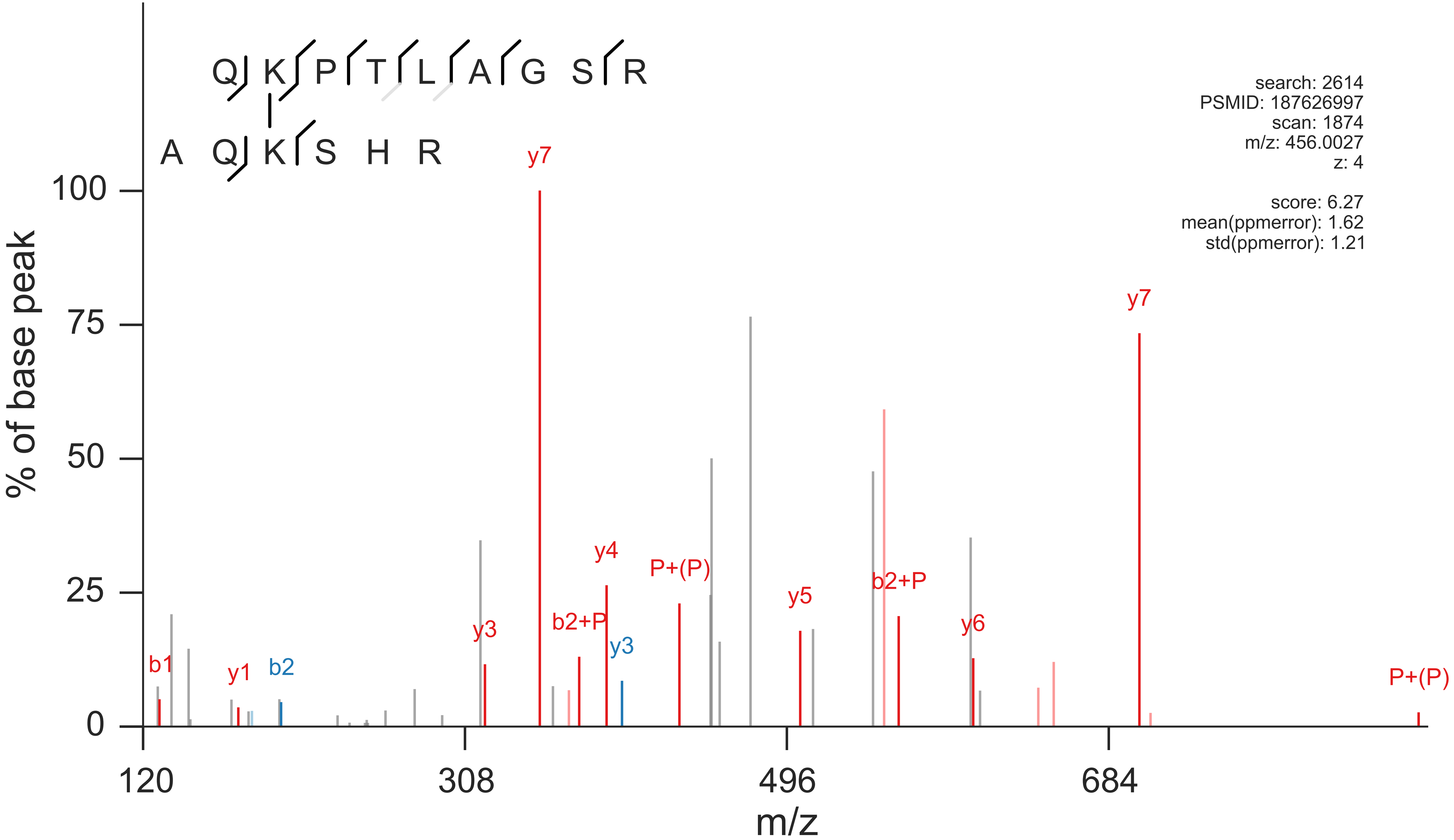

Q K P T L A G S R
A Q K S H R
search: 2614
PSMID: 187626997
scan: 1874
m/z: 456.0027
z: 4
score: 6.27
mean(ppmerror): 1.62
std(ppmerror): 1.21
% of base peak
100
75
50
25
0
120
308
496
684
m/z
y7
y7
y4
P+(P)
y5
b2+P
y6
y3
b2+P
y3
b1
y1
b2
P+(P)

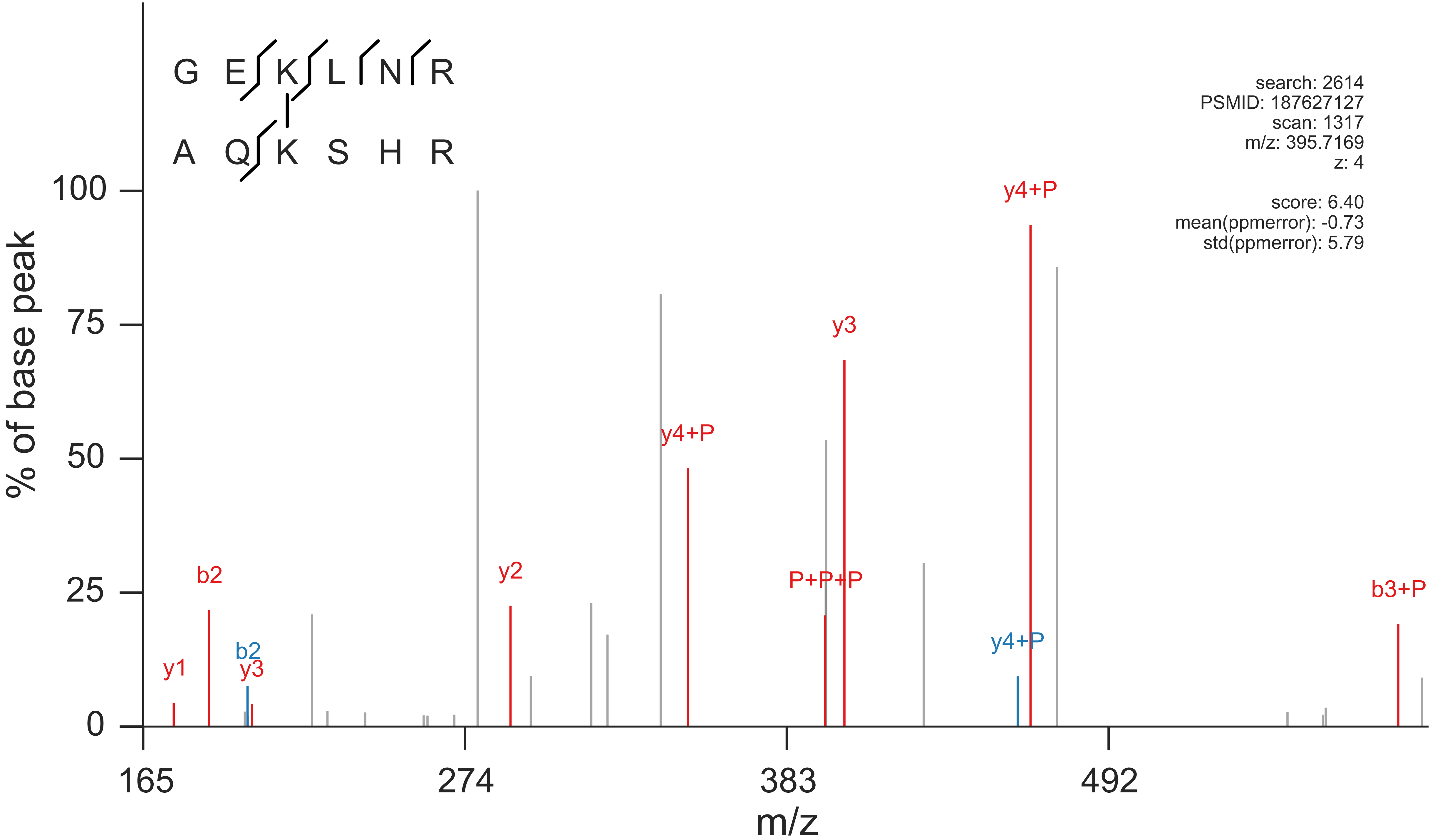

G E K L N R
A Q K S H R
search: 2614
PSMID: 187627127
scan: 1317
m/z: 395.7169
z: 4
score: 6.40
mean(ppmerror): -0.73
std(ppmerror): 5.79
% of base peak
100
75
50
25
0
y1
b2
b2
y3
y2
y4+P
P+P+P
y3
y4+P
y4+P
b3+P
165
274
383
492
m/z

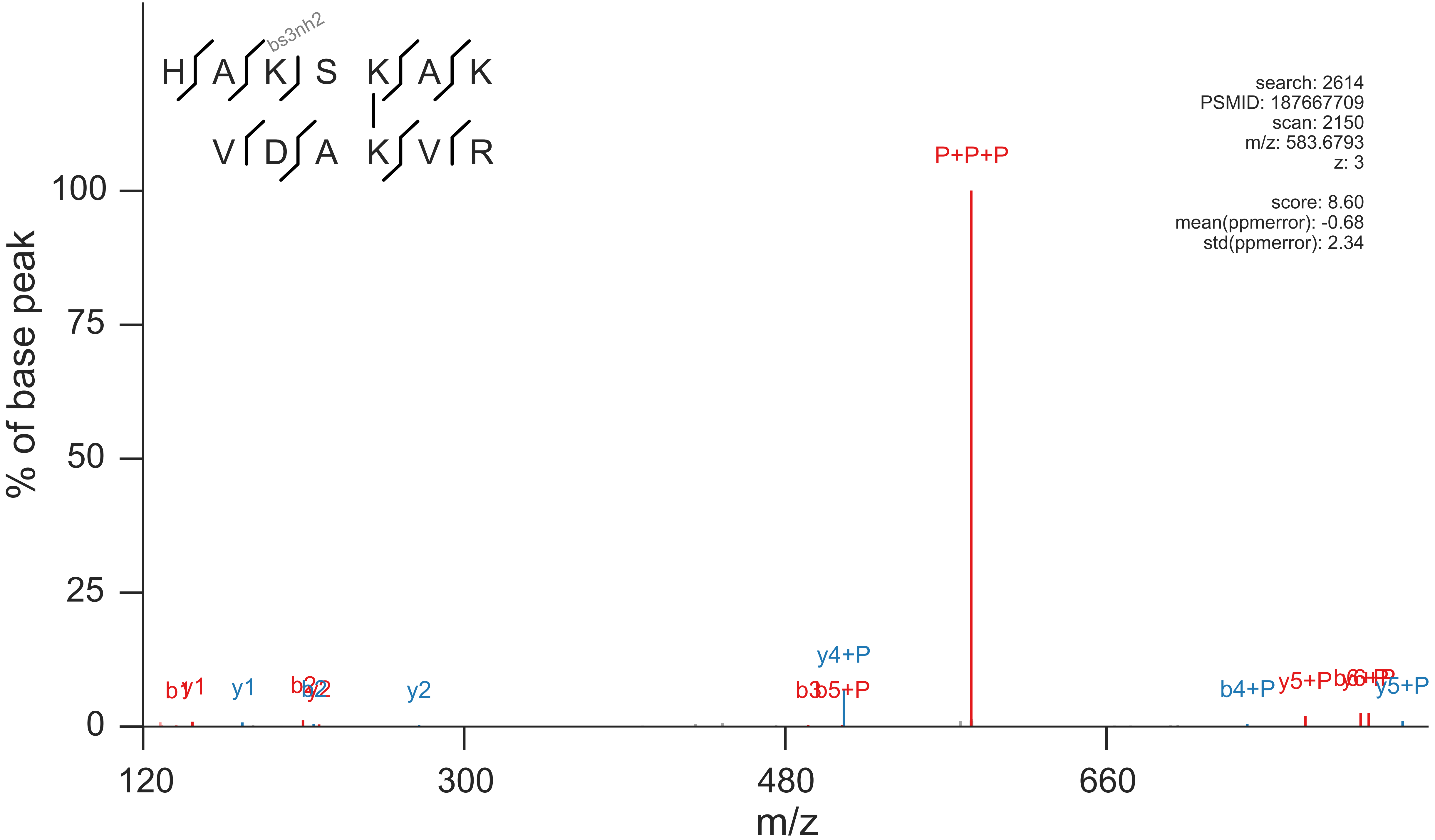

H A K S K A K
V D A K V R
bs3nh2
search: 2614
PSMID: 187667709
scan: 2150
m/z: 583.6793
z: 3
score: 8.60
mean(ppmerror): -0.68
std(ppmerror): 2.34
P+P+P
y4+P
b3
b5+P
b4+P
y5+P
b6+P
y6+P
b1
y1
b2
y2
% of base peak
100
75
50
25
0
120
300
480
660
m/z

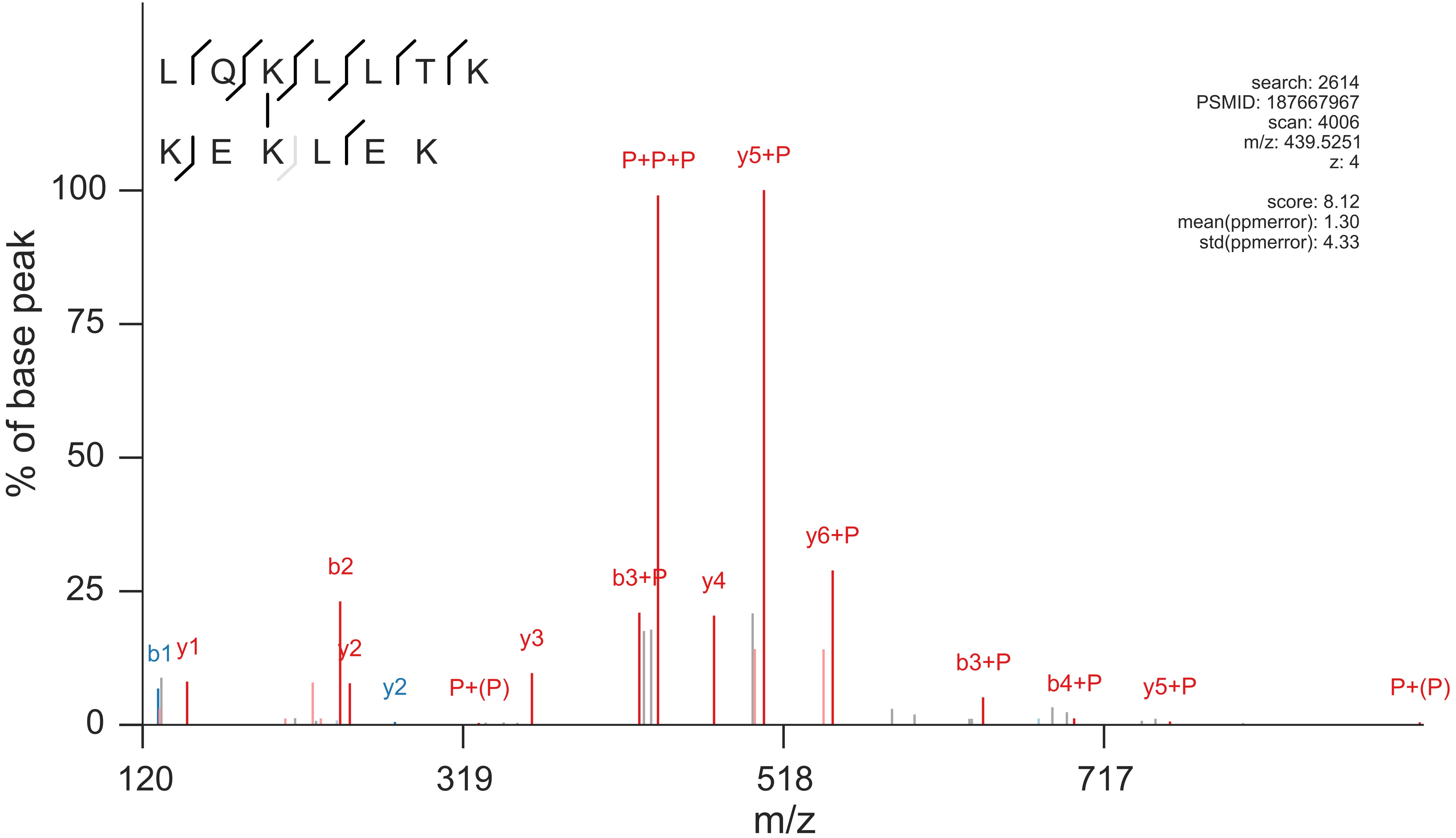

L Q K L L T K
K E K L E K
search: 2614
PSMID: 187667967
scan: 4006
m/z: 439.5251
z: 4
score: 8.12
mean(ppmerror): 1.30
std(ppmerror): 4.33
% of base peak
100
75
50
25
0
120
319
518
717
m/z
b1
y1
b2
y2
y2
P+(P)
y3
b3+P
P+P+P
y4
y5+P
y6+P
b3+P
b4+P
y5+P
P+(P)

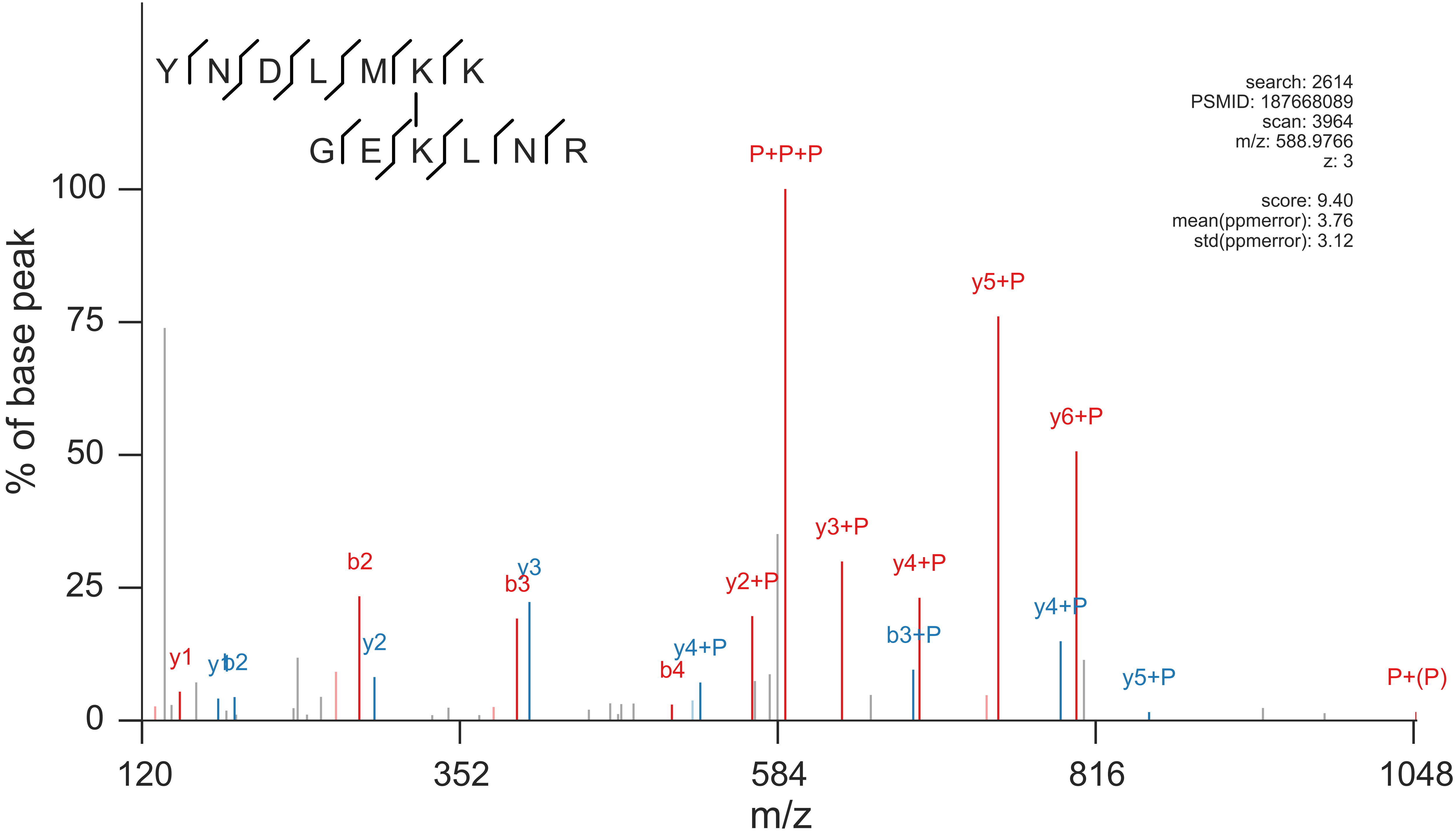

Y N D L M K K
G E K L N R
search: 2614
PSMID: 187668089
scan: 3964
m/z: 588.9766
z: 3
score: 9.40
mean(ppmerror): 3.76
std(ppmerror): 3.12
P+P+P
y5+P
y6+P
y3+P
y4+P
y2+P
b2
y3
b3
y2
y1
y1
b2
y4+P
b4
b3+P
y4+P
y5+P
P+(P)
% of base peak
100
75
50
25
0
120
352
584
816
1048
m/z

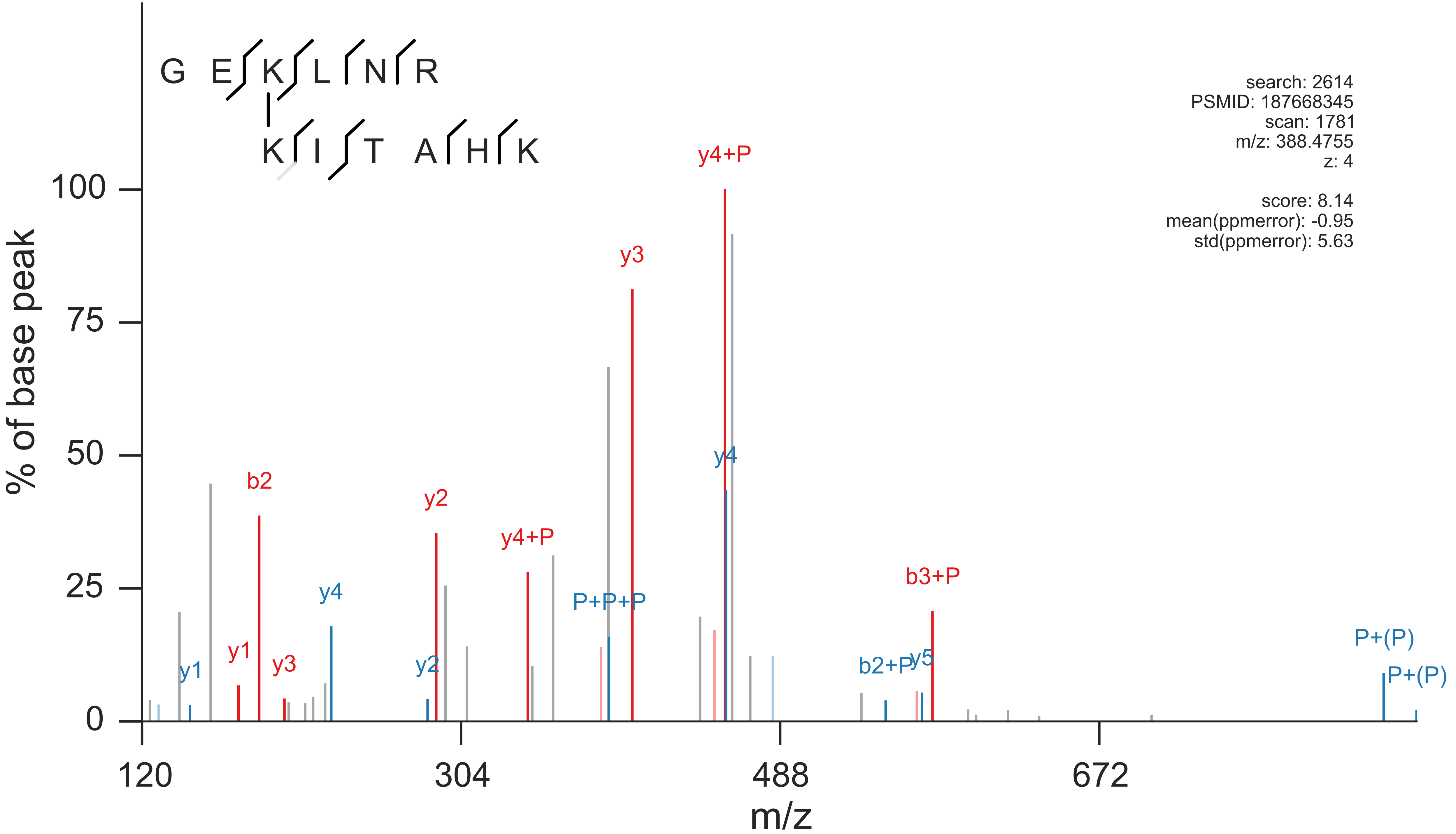

G E K L N R
K I T A H K
search: 2614
PSMID: 187668345
scan: 1781
m/z: 388.4755
z: 4
score: 8.14
mean(ppmerror): -0.95
std(ppmerror): 5.63
% of base peak
100
75
50
25
0
120
304
488
672
m/z
y4+P
y3
y4
b2
y2
y4+P
b3+P
y4
P+P+P
y1
y1
y3
y2
b2+P
y5
P+(P)
P+(P)

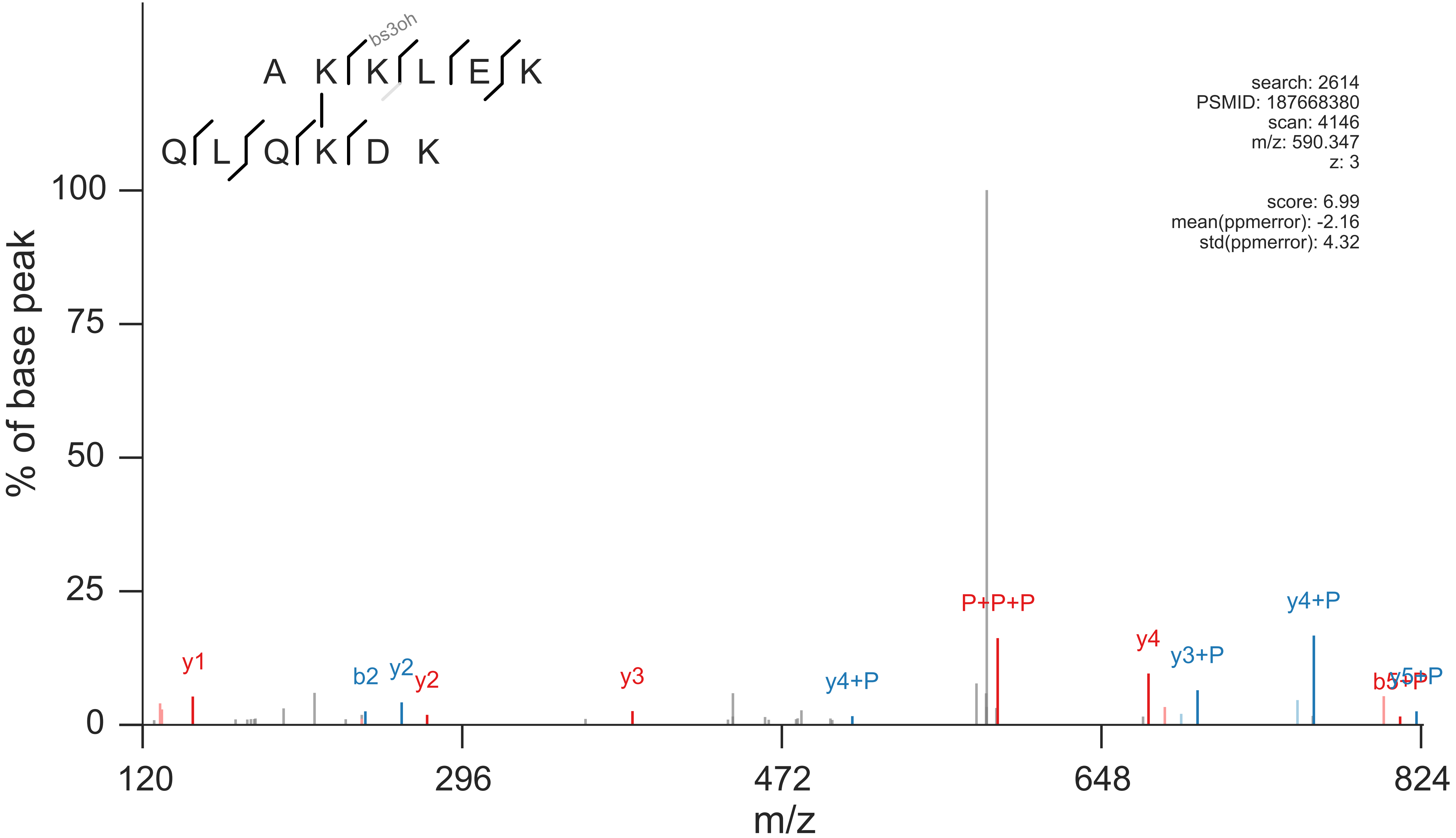

bs3oh
A K K L E K
Q L Q K D K
search: 2614
PSMID: 187668380
scan: 4146
m/z: 590.347
z: 3
score: 6.99
mean(ppmerror): -2.16
std(ppmerror): 4.32
% of base peak
100
75
50
25
0
y1
b2
y2
y2
y3
y4+P
P+P+P
y4
y3+P
y4+P
b5+P
y5+P
120
296
472
648
824
m/z

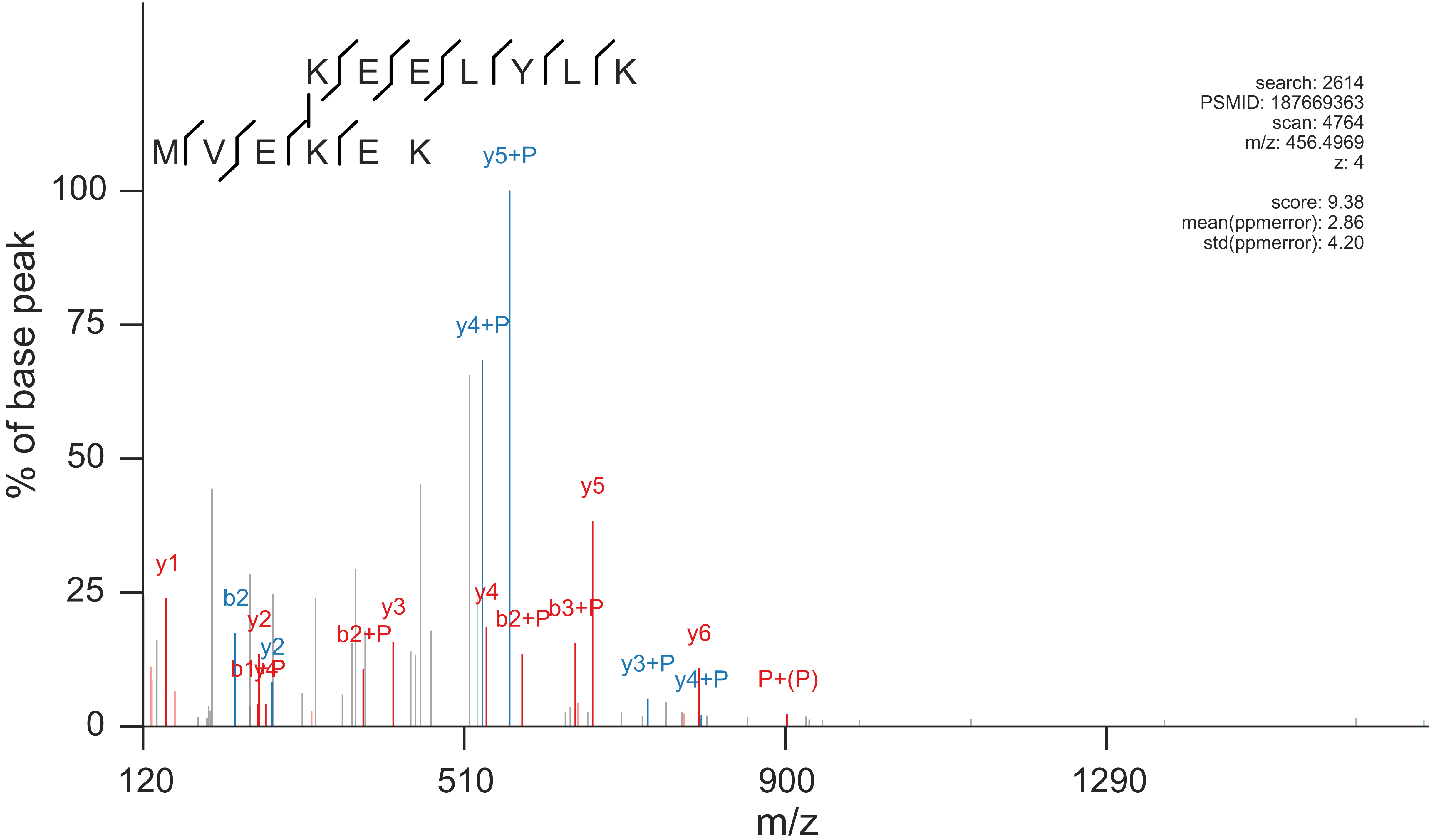

K E E L Y L K
M V E K E K
search: 2614
PSMID: 187669363
scan: 4764
m/z: 456.4969
z: 4
score: 9.38
mean(ppmerror): 2.86
std(ppmerror): 4.20
% of base peak
100
75
50
25
0
120
510
900
1290
m/z
y5+P
y4+P
y5
y1
b2
y2
y2
b1
y4
y3
b2+P
y4
b2+P
b3+P
y6
y3+P
y4+P
P+(P)

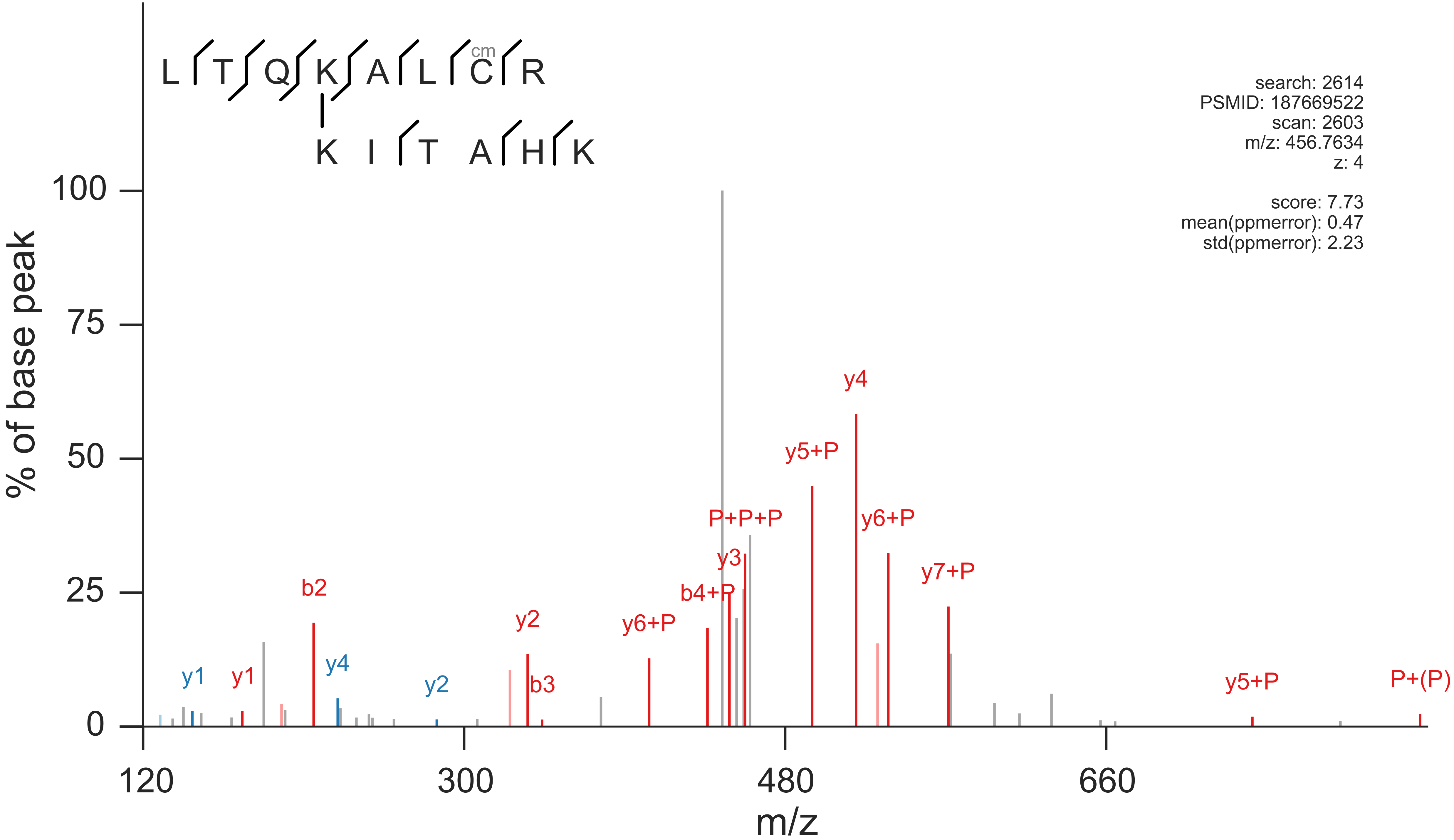

L T Q K A L C R
cm
K I T A H K
search: 2614
PSMID: 187669522
scan: 2603
m/z: 456.7634
z: 4
score: 7.73
mean(ppmerror): 0.47
std(ppmerror): 2.23
% of base peak
100
75
50
25
0
120
300
480
660
m/z
y1
y1
b2
y4
y2
y2
b3
y6+P
b4+P
y3
P+P+P
y5+P
y4
y6+P
y7+P
y5+P
P+(P)

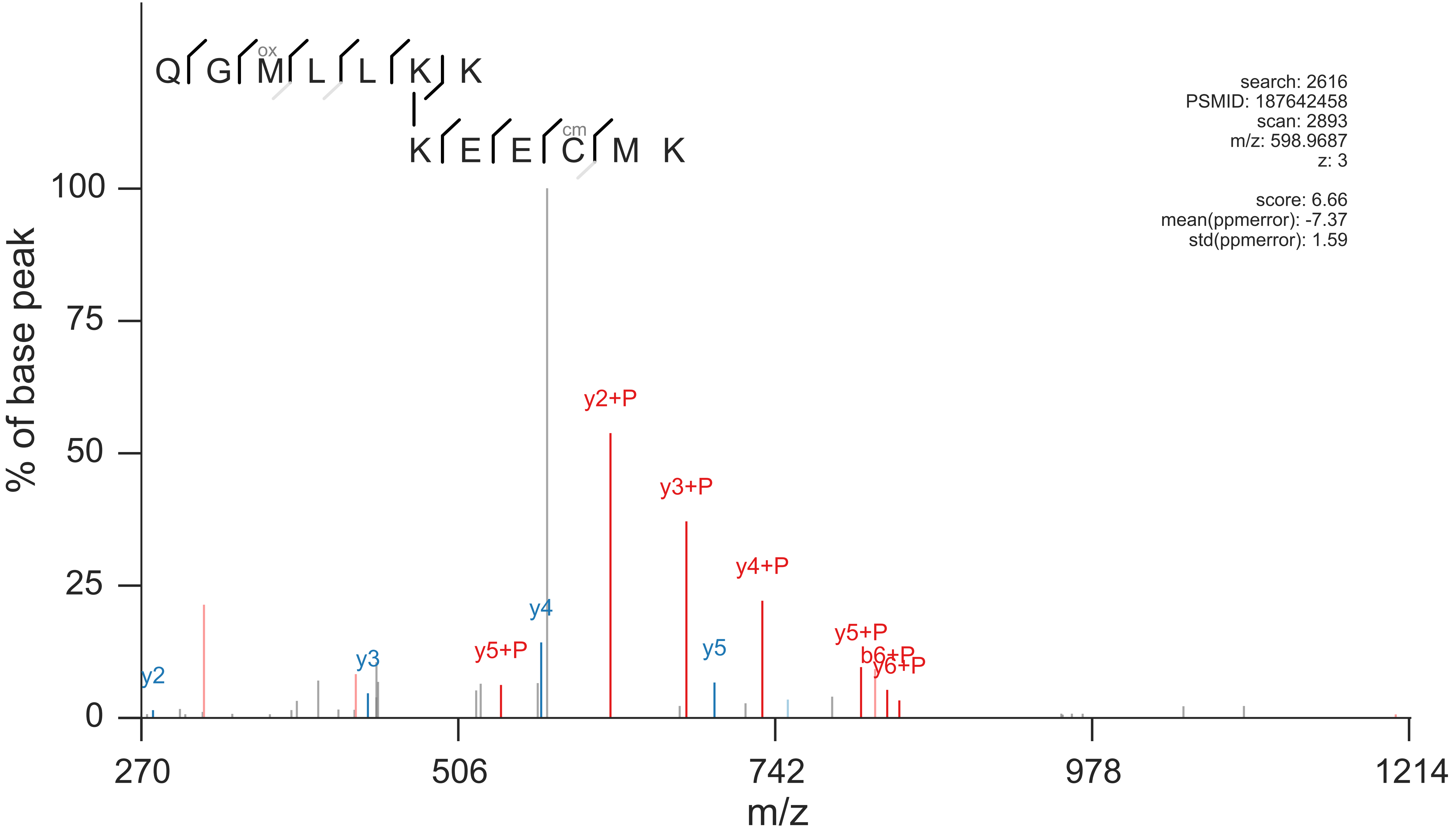

Q G M L L K K
ox
K E E C M K
cm
search: 2616
PSMID: 187642458
scan: 2893
m/z: 598.9687
z: 3
score: 6.66
mean(ppmerror): -7.37
std(ppmerror): 1.59
% of base peak
100
75
50
25
0
y2+P
y3+P
y4+P
y5+P
b6+P
y6+P
y5+P
y4
y3
y2
y5
270
506
742
978
1214
m/z

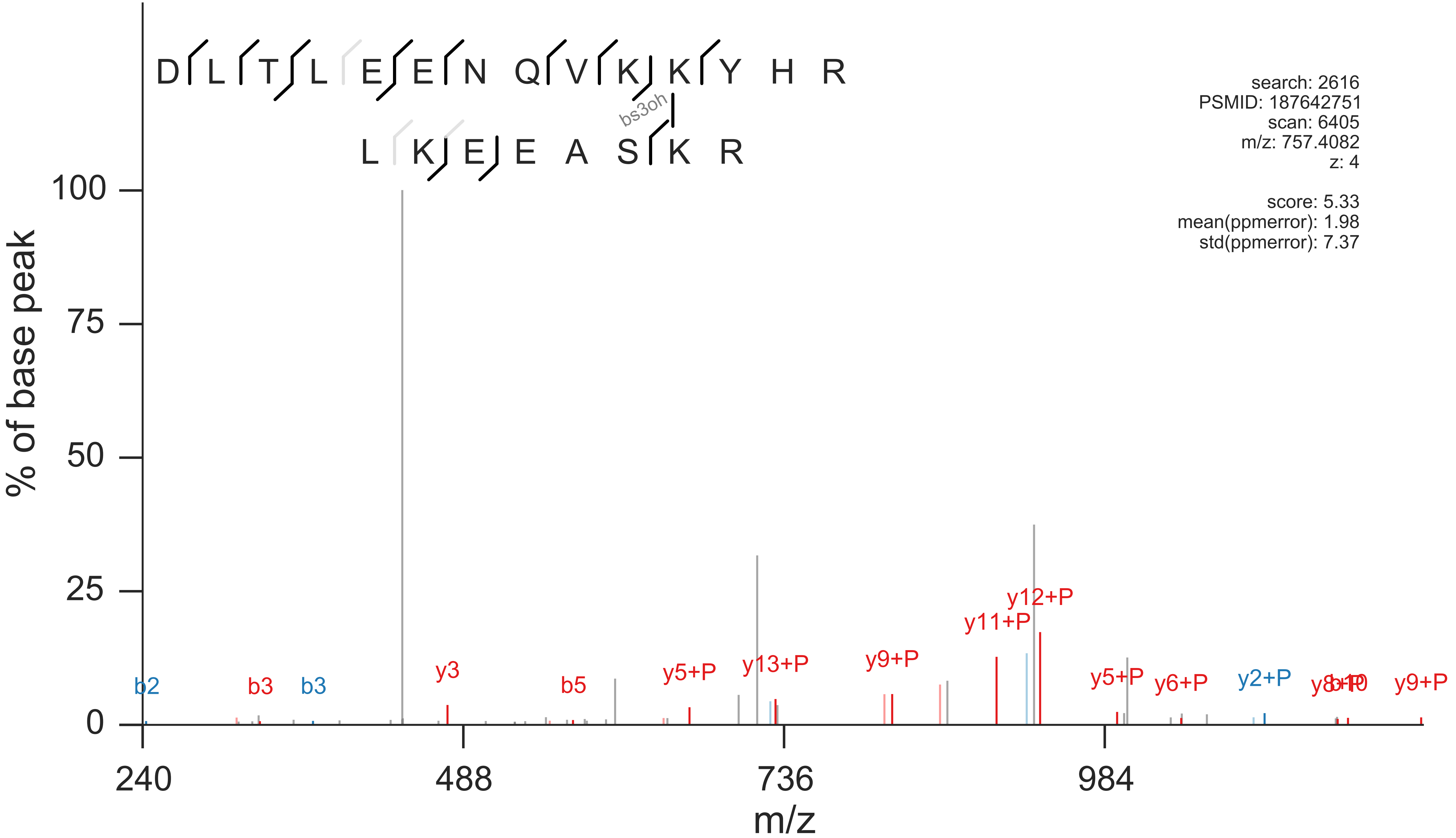

D L T L E E N Q V K K Y H R
bs3oh
L K E E A S K R
search: 2616
PSMID: 187642751
scan: 6405
m/z: 757.4082
z: 4
score: 5.33
mean(ppmerror): 1.98
std(ppmerror): 7.37
% of base peak
100
75
50
25
0
240
488
736
984
m/z
b2
b3
b3
y3
b5
y5+P
y13+P
y9+P
y11+P
y12+P
y5+P
y6+P
y2+P
y9+P

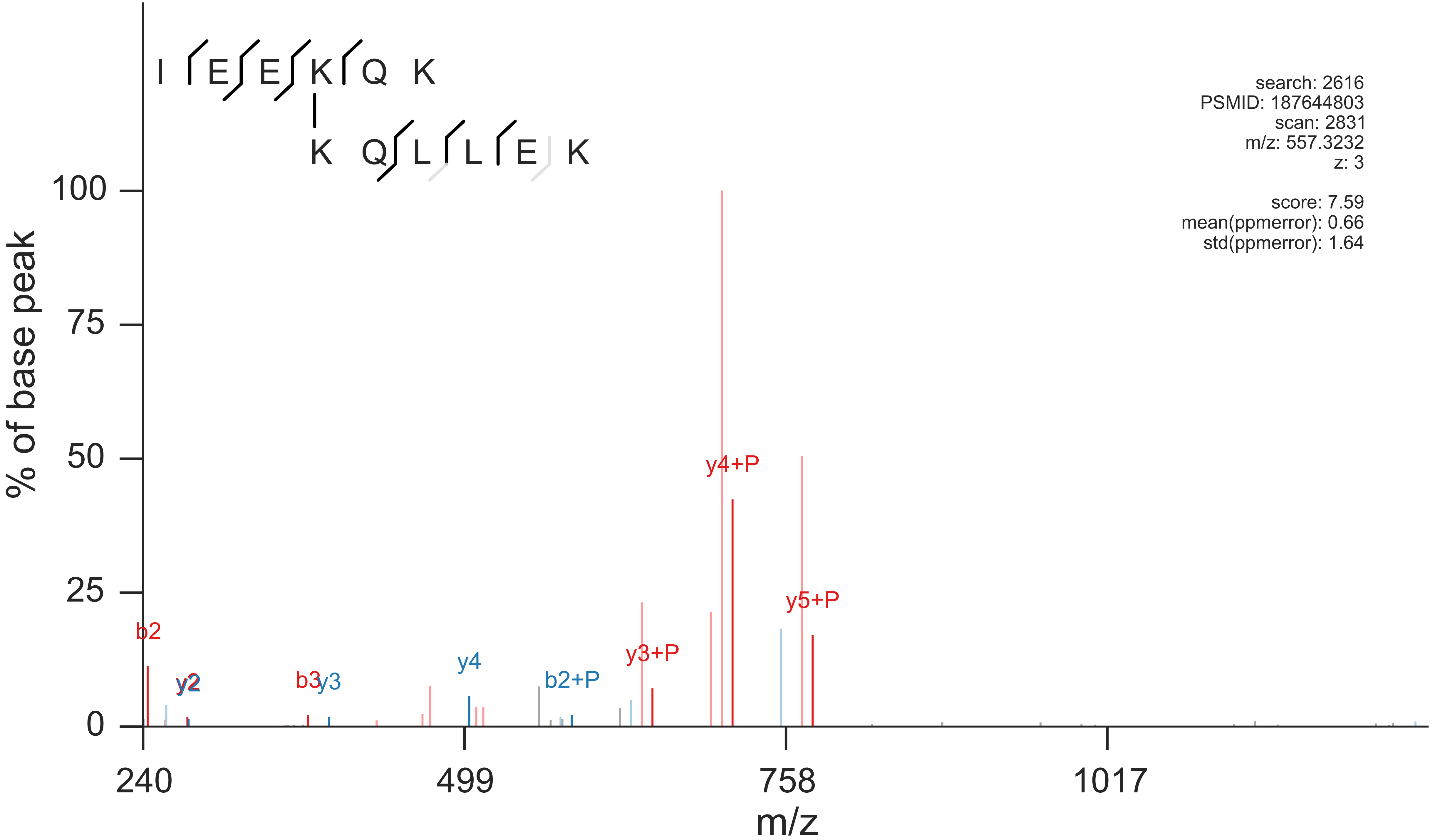
I E E K Q K
K Q L L E K
search: 2616
PSMID: 187644803
scan: 2831
m/z: 557.3232
z: 3
score: 7.59
mean(ppmerror): 0.66
std(ppmerror): 1.64
% of base peak
100
75
50
25
0
b2
y2
b3
y3
y4
b2+P
y3+P
y4+P
y5+P
240
499
758
1017
m/z

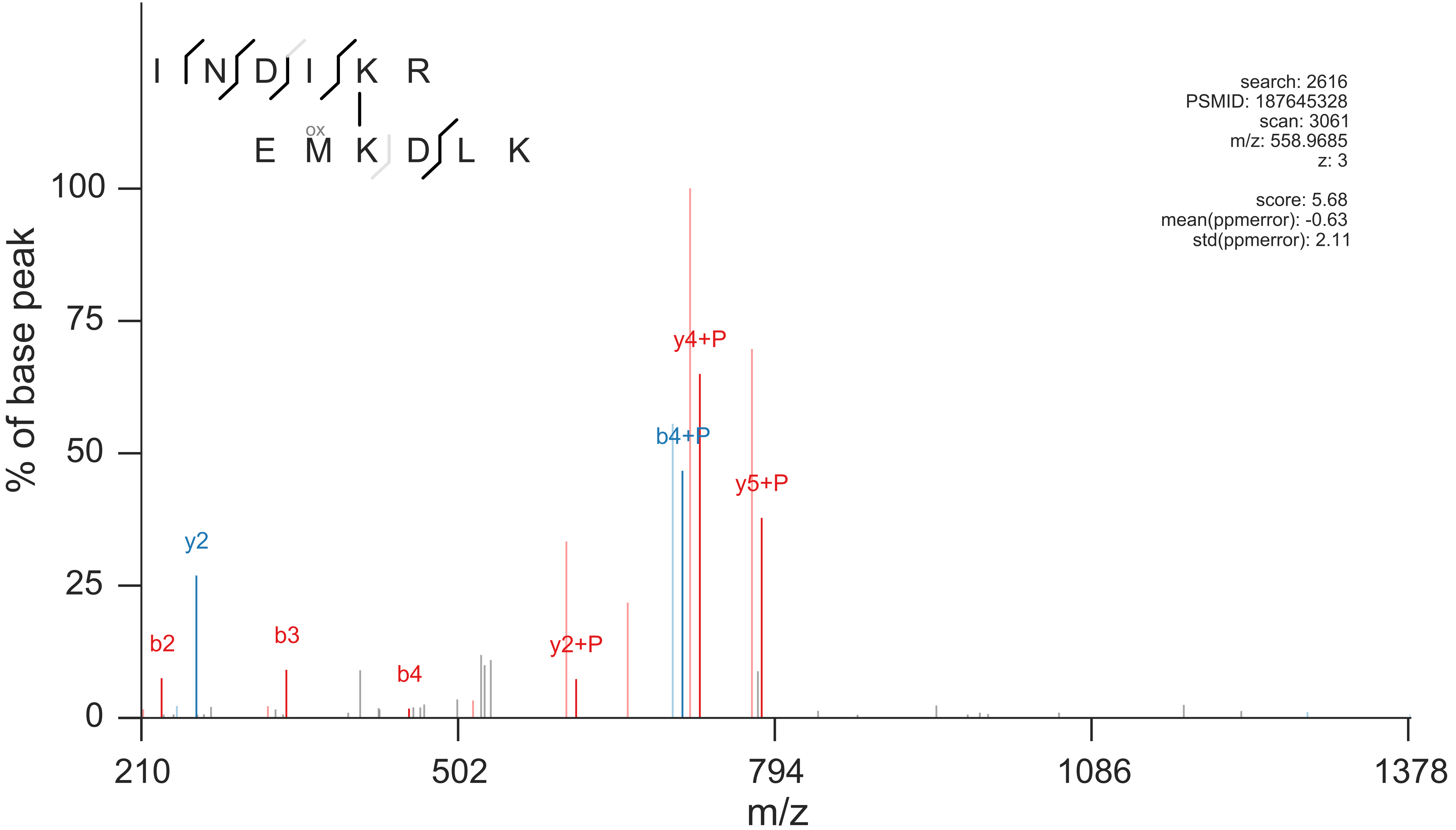

I N D I K R
E M K D L K
ox
search: 2616
PSMID: 187645328
scan: 3061
m/z: 558.9685
z: 3
score: 5.68
mean(ppmerror): -0.63
std(ppmerror): 2.11
% of base peak
100
75
50
25
0
y2
b2
b3
b4
y2+P
b4+P
y4+P
y5+P
210
502
794
1086
1378
m/z

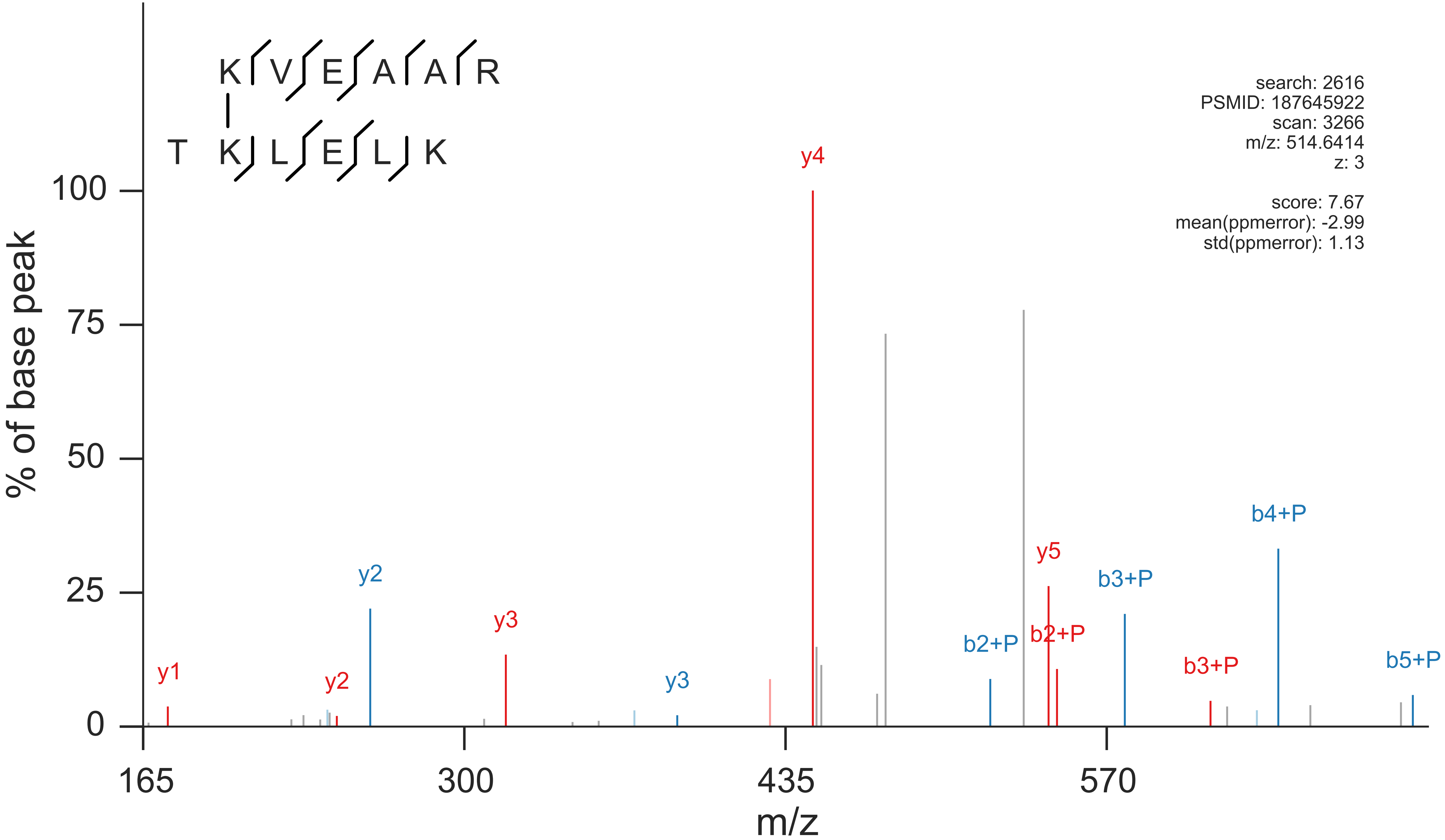

K V E A A R
T K L E L K
search: 2616
PSMID: 187645922
scan: 3266
m/z: 514.6414
z: 3
score: 7.67
mean(ppmerror): -2.99
std(ppmerror): 1.13
% of base peak
100
75
50
25
0
y4
y5
b4+P
b3+P
y2
y3
b2+P
b2+P
b3+P
b5+P
y1
y2
y3
165
300
435
570
m/z

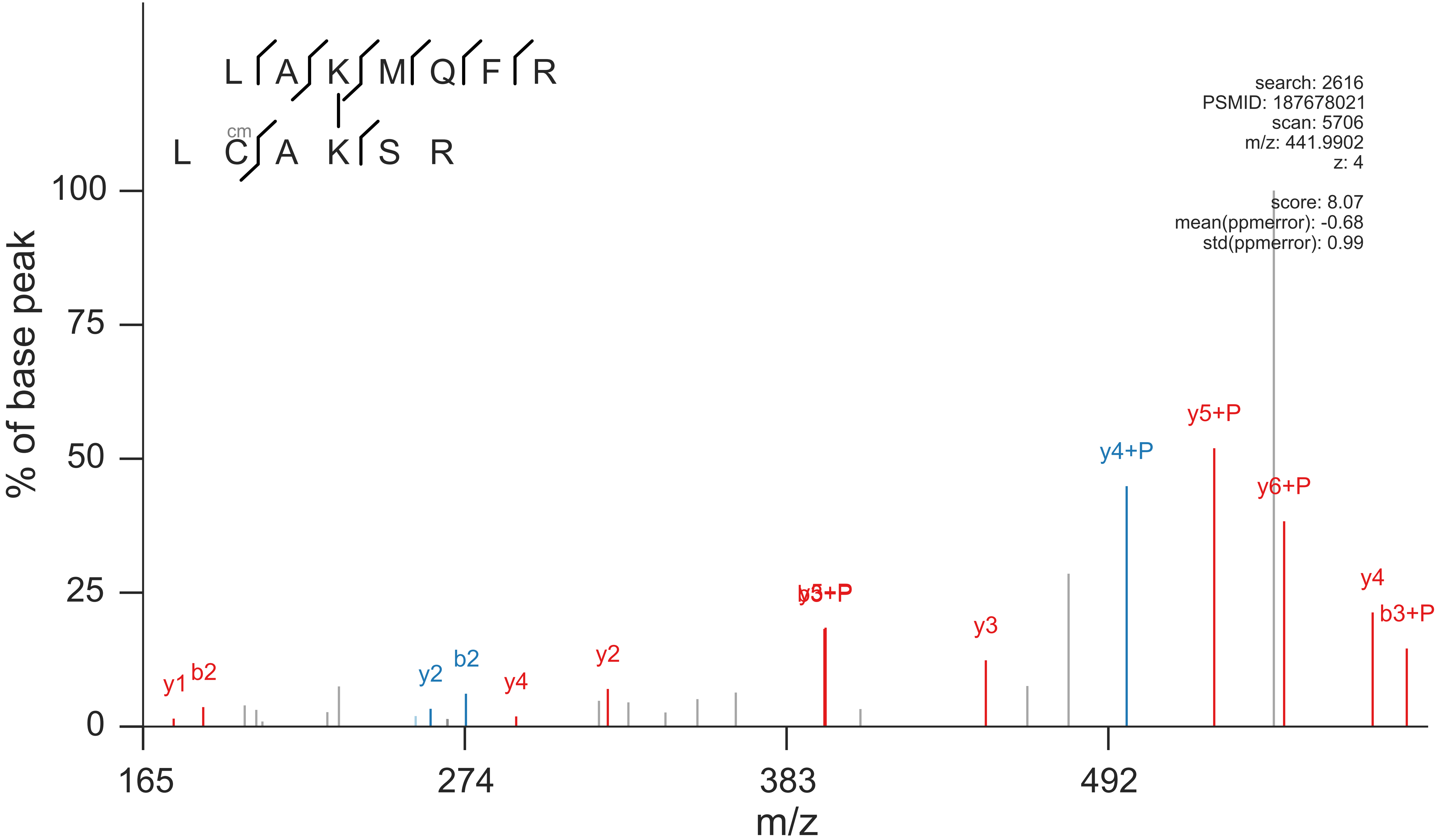

L A K M Q F R
L C A K S R
cm
search: 2616
PSMID: 187678021
scan: 5706
m/z: 441.9902
z: 4
score: 8.07
mean(ppmerror): -0.68
std(ppmerror): 0.99
% of base peak
100
75
50
25
0
y1
b2
y2
b2
y4
y2
y5+P
y3
y4+P
y5+P
y6+P
y4
b3+P
165
274
383
492
m/z

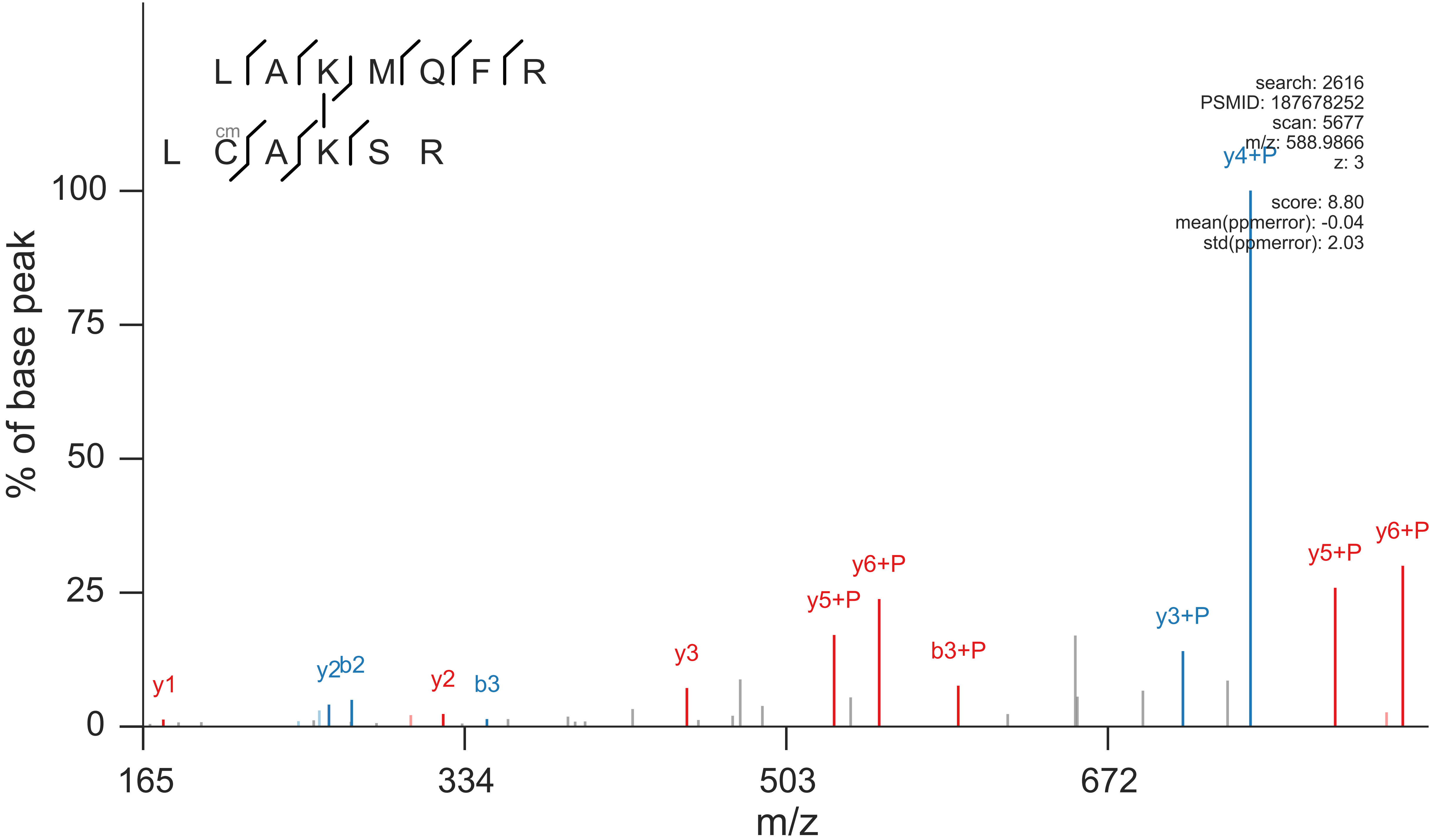

L A K M Q F R
L cm C A K S R
search: 2616
PSMID: 187678252
scan: 5677
m/z: 588.9866
z: 3
score: 8.80
mean(ppmerror): -0.04
std(ppmerror): 2.03
% of base peak
100
75
50
25
0
165
334
503
672
m/z
y1
y2
b2
y2
b3
y3
y5+P
y6+P
b3+P
y3+P
y4+P
y5+P
y6+P

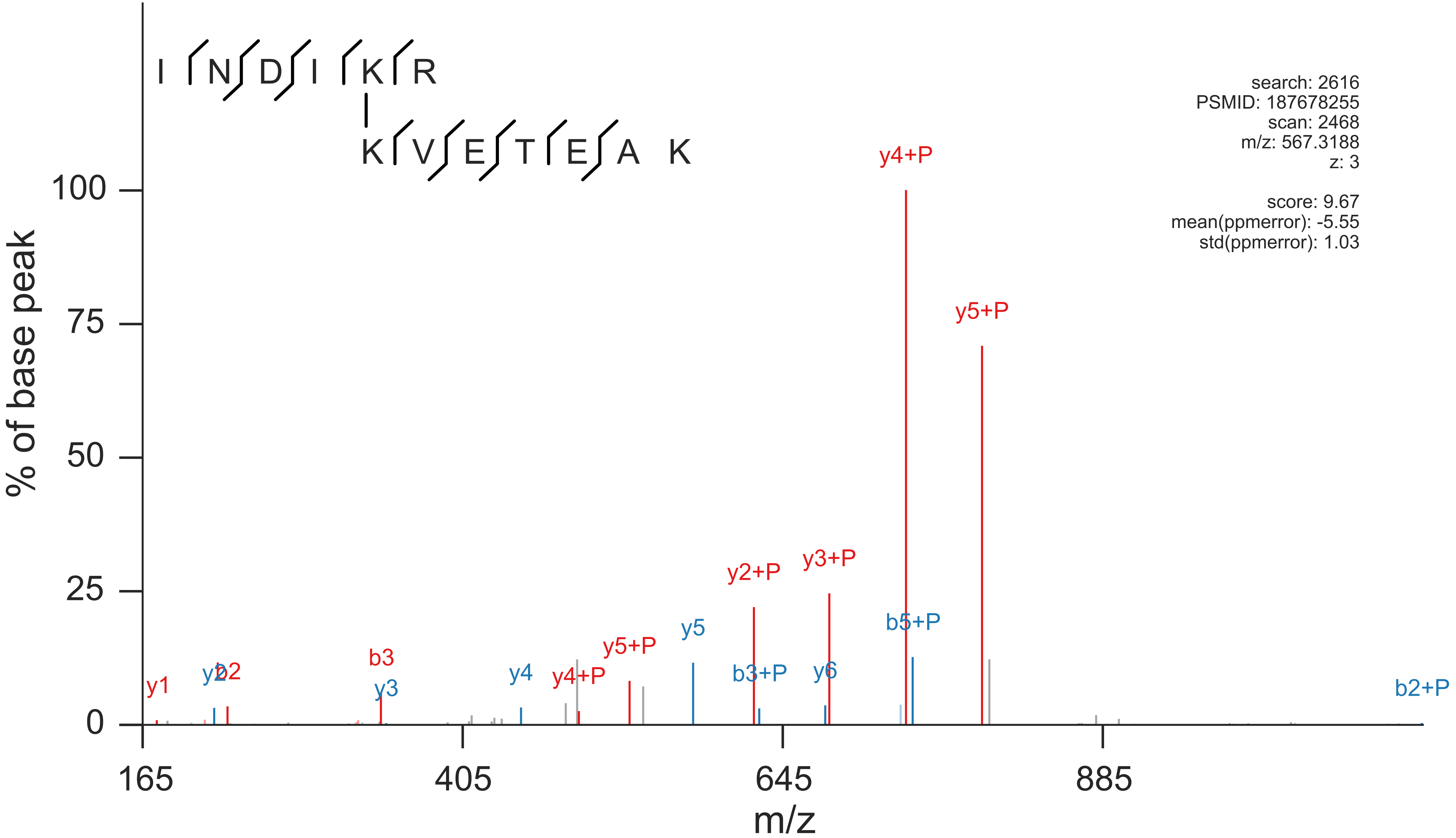

I N D I K R
K V E T E A K
search: 2616
PSMID: 187678255
scan: 2468
m/z: 567.3188
z: 3
score: 9.67
mean(ppmerror): -5.55
std(ppmerror): 1.03
% of base peak
100
75
50
25
0
165
405
645
885
m/z
y4+P
y5+P
y2+P
y3+P
b5+P
y5
y5+P
b3
y1
y2
b2
y3
y4
y4+P
b3+P
y6
b2+P

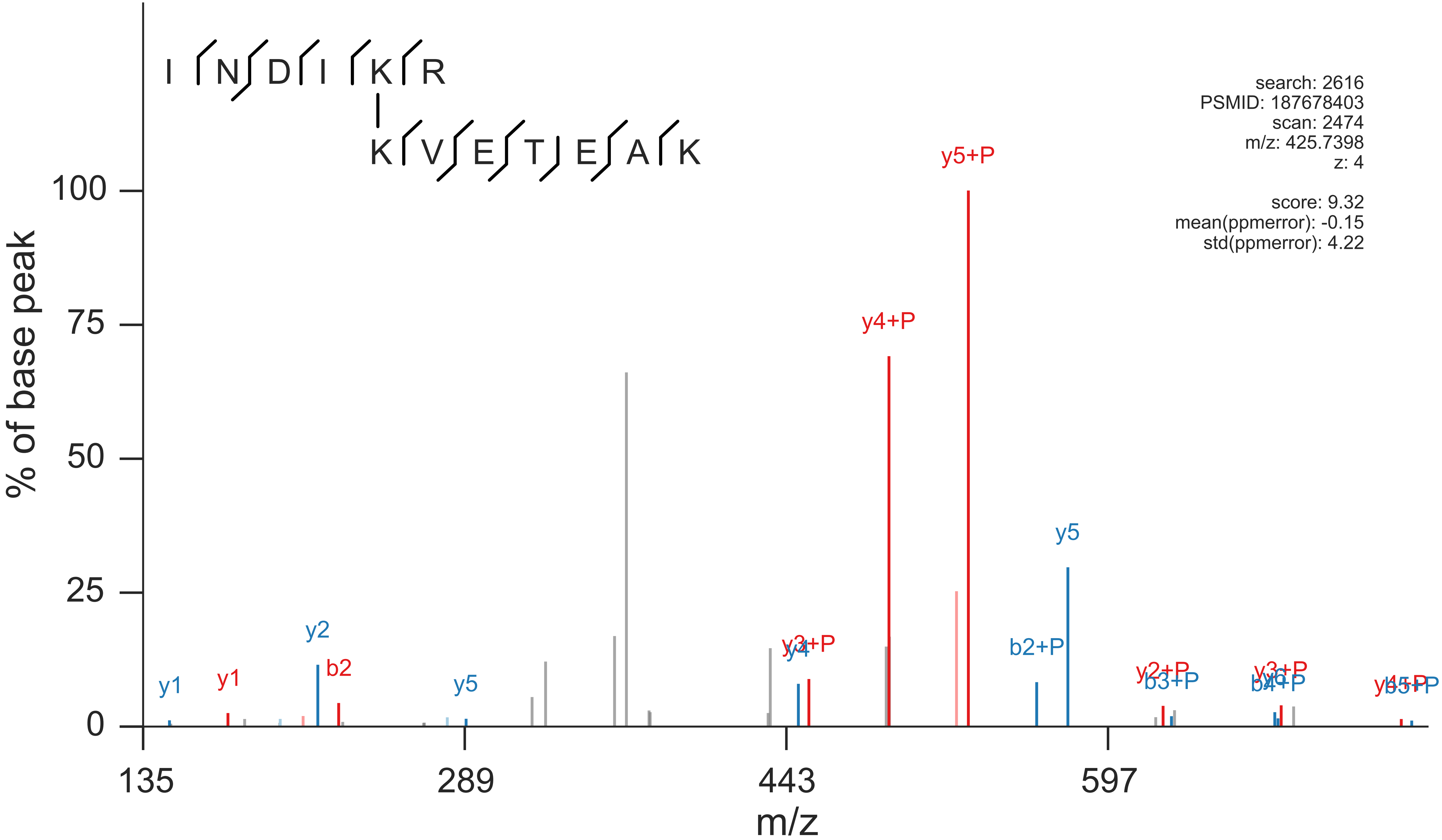

I N D I K R
K V E T E A K
search: 2616
PSMID: 187678403
scan: 2474
m/z: 425.7398
z: 4
score: 9.32
mean(ppmerror): -0.15
std(ppmerror): 4.22
% of base peak
m/z
100
75
50
25
0
135
289
443
597
y5+P
y4+P
y5
y2
b2
y1
y1
y5
y3+P
y4
b2+P
y2+P
b3+P
y3+P
b4+P
y4+P
b5+P

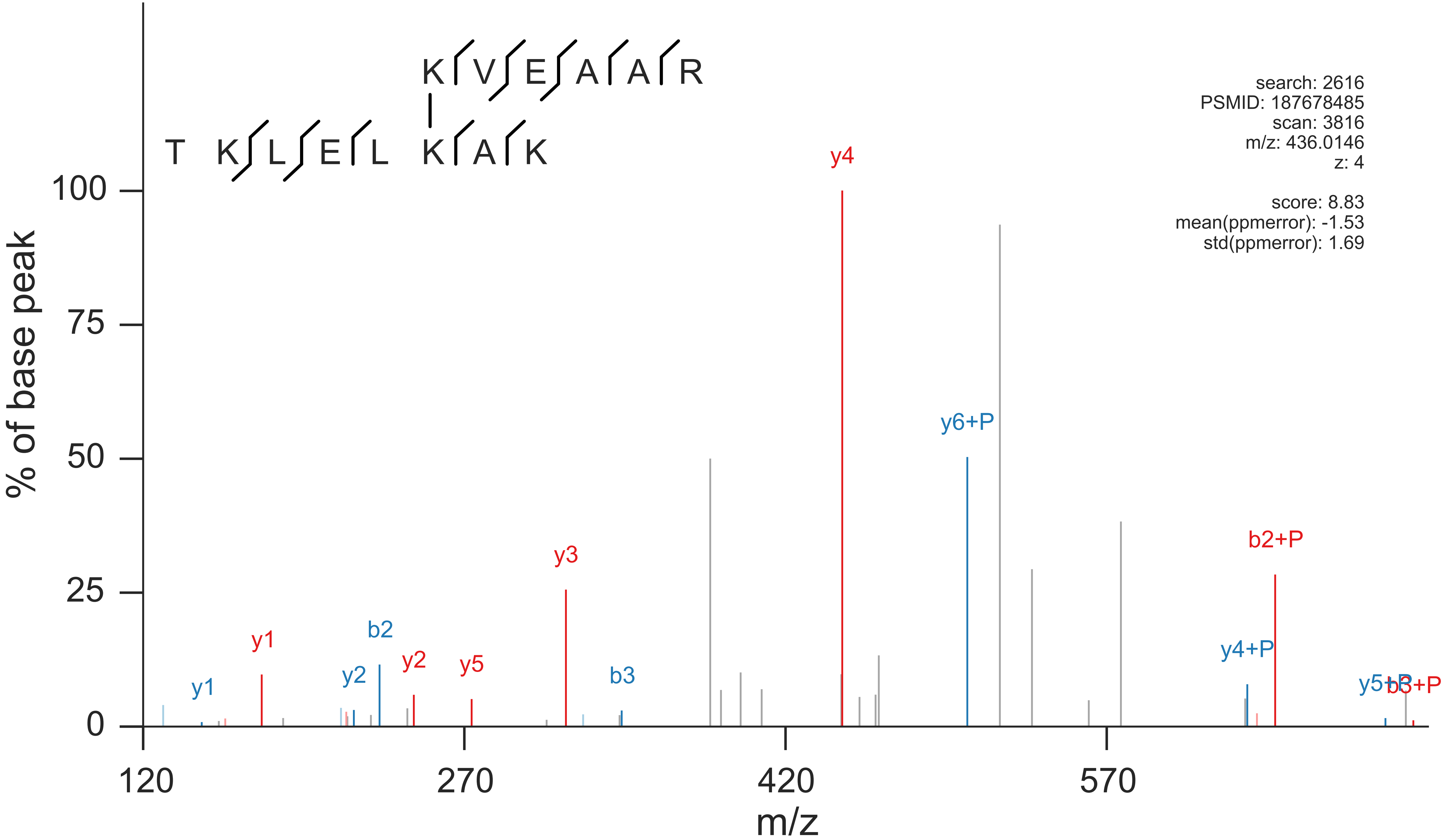

K V E A A R
T K L E L K A K
search: 2616
PSMID: 187678485
scan: 3816
m/z: 436.0146
z: 4
score: 8.83
mean(ppmerror): -1.53
std(ppmerror): 1.69
% of base peak
100
75
50
25
0
120
270
420
570
m/z
y4
y6+P
b2+P
y3
y4+P
y1
b2
y2
y5
b3
y1
y2
y5+P
b3+P

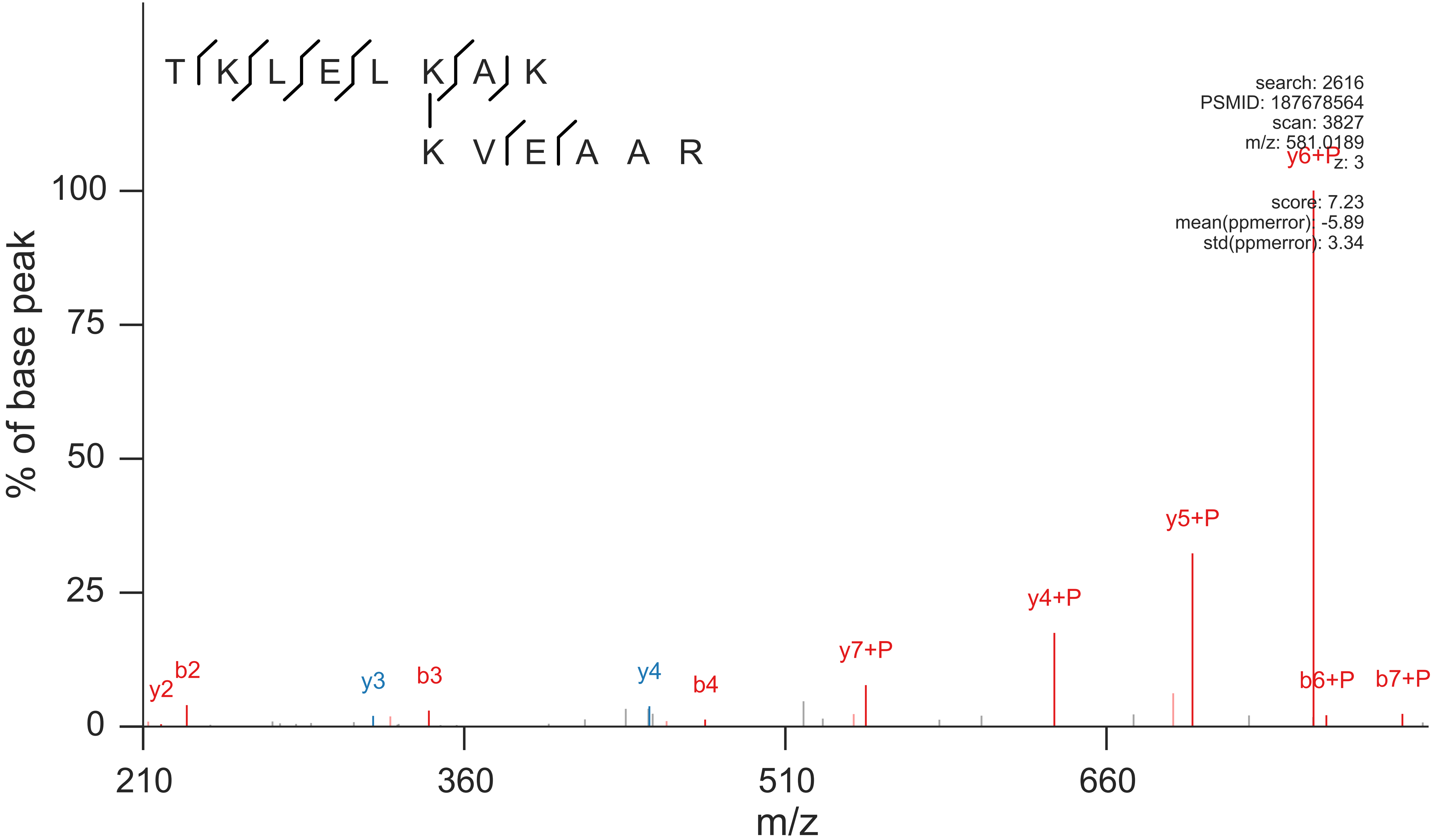

T K L E L K A K
K V E A A R
search: 2616
PSMID: 187678564
scan: 3827
m/z: 581.0189
z: 3
score: 7.23
mean(ppmerror): -5.89
std(ppmerror): 3.34
% of base peak
100
75
50
25
0
y2
b2
y3
b3
y4
b4
y7+P
y4+P
y5+P
y6+P
b6+P
b7+P
210
360
510
660
m/z

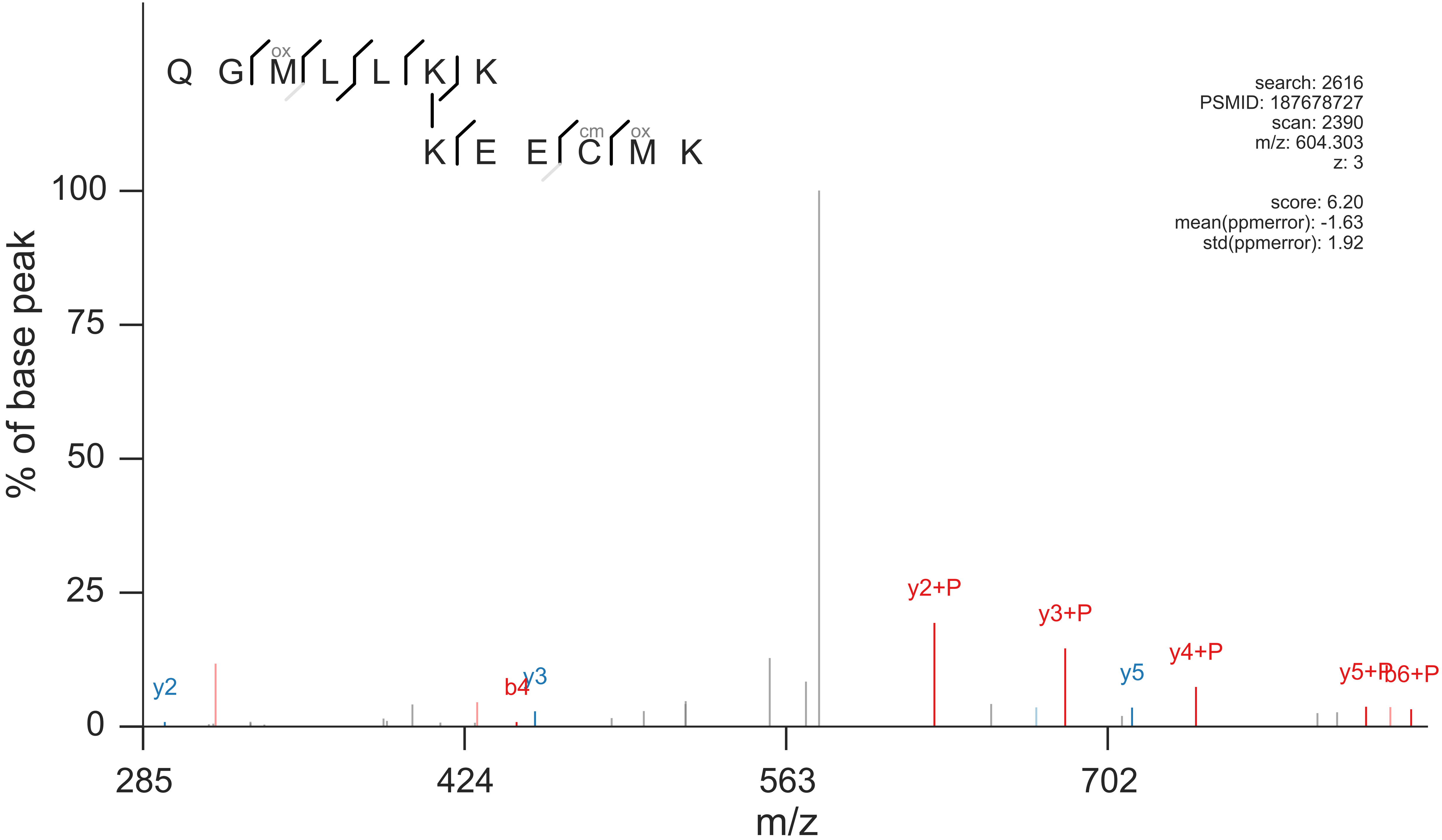

Q G M L L K K
ox
K E E C M K
cm
ox
search: 2616
PSMID: 187678727
scan: 2390
m/z: 604.303
z: 3
score: 6.20
mean(ppmerror): -1.63
std(ppmerror): 1.92
% of base peak
100
75
50
25
0
285
424
563
702
m/z
y2
b4
y3
y2+P
y3+P
y5
y4+P
y5+P
b6+P

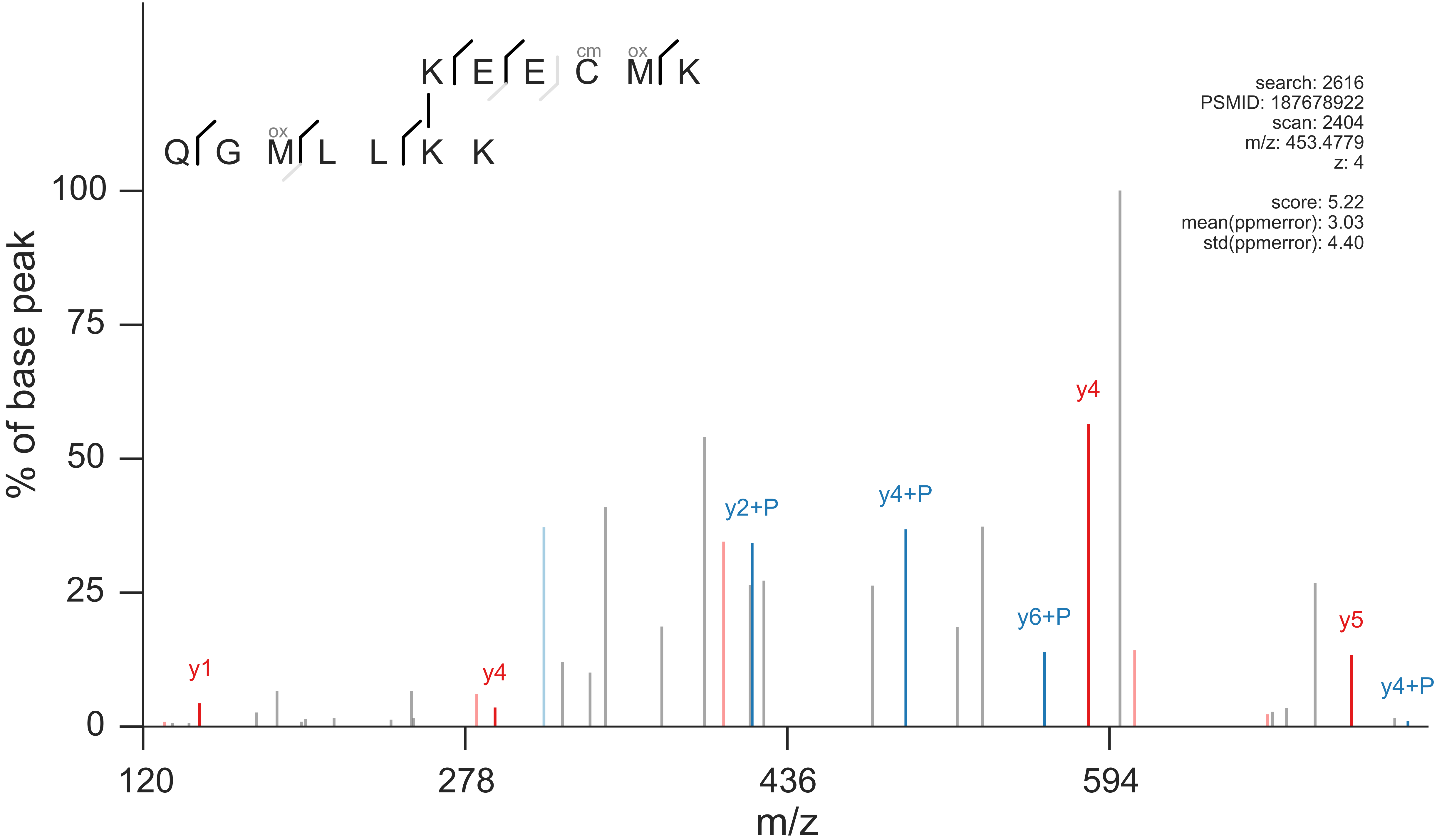

K E E C M K
cm
ox
Q G M L L K K
ox
search: 2616
PSMID: 187678922
scan: 2404
m/z: 453.4779
z: 4
score: 5.22
mean(ppmerror): 3.03
std(ppmerror): 4.40
% of base peak
100
75
50
25
0
y1
y4
y2+P
y4+P
y6+P
y4
y5
y4+P
120
278
436
594
m/z

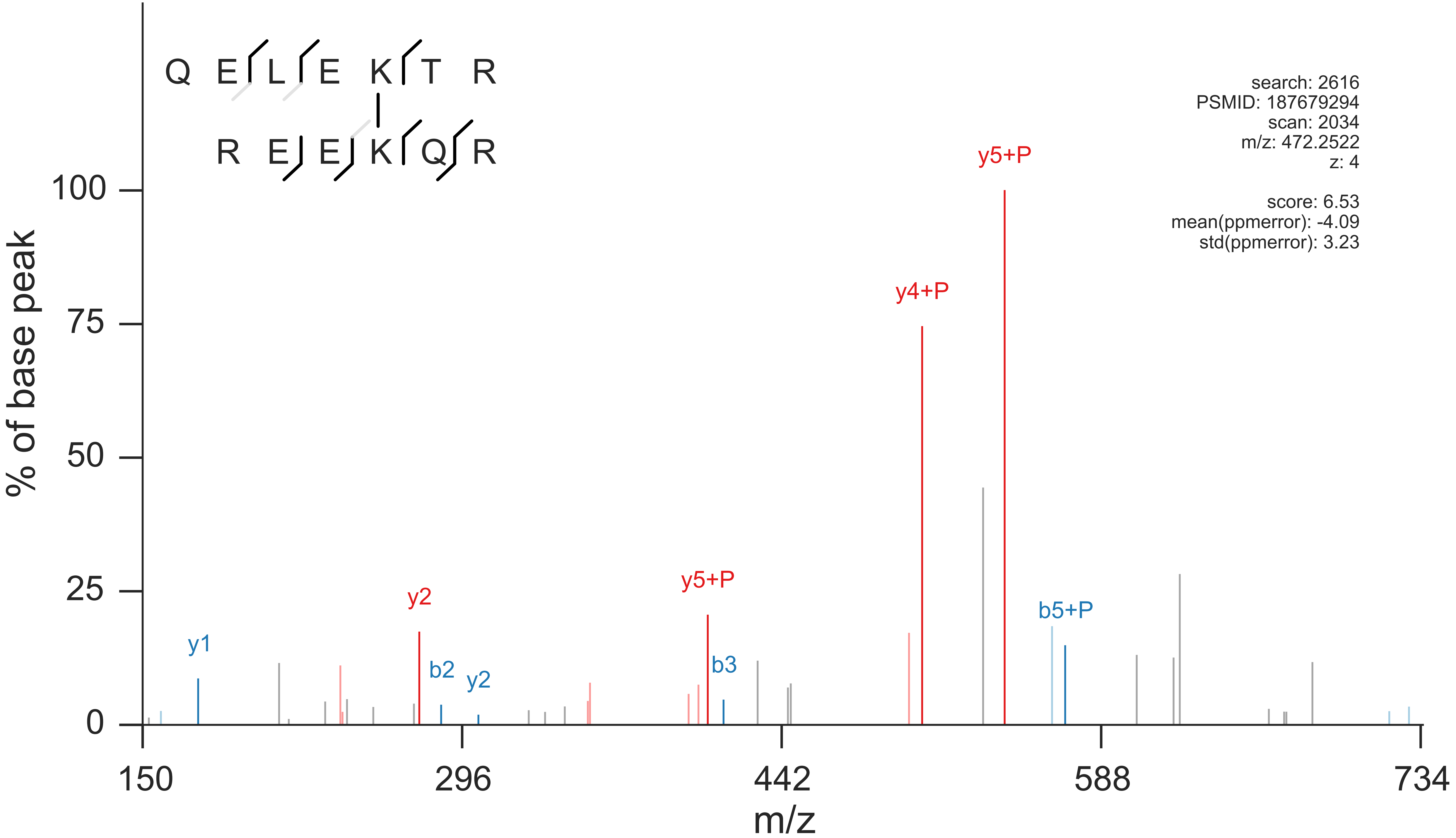

Q E L E K T R
R E E K Q R
search: 2616
PSMID: 187679294
scan: 2034
m/z: 472.2522
z: 4
score: 6.53
mean(ppmerror): -4.09
std(ppmerror): 3.23
% of base peak
m/z
0
25
50
75
100
150
296
442
588
734
y1
y2
b2
y2
y5+P
b3
y4+P
y5+P
b5+P

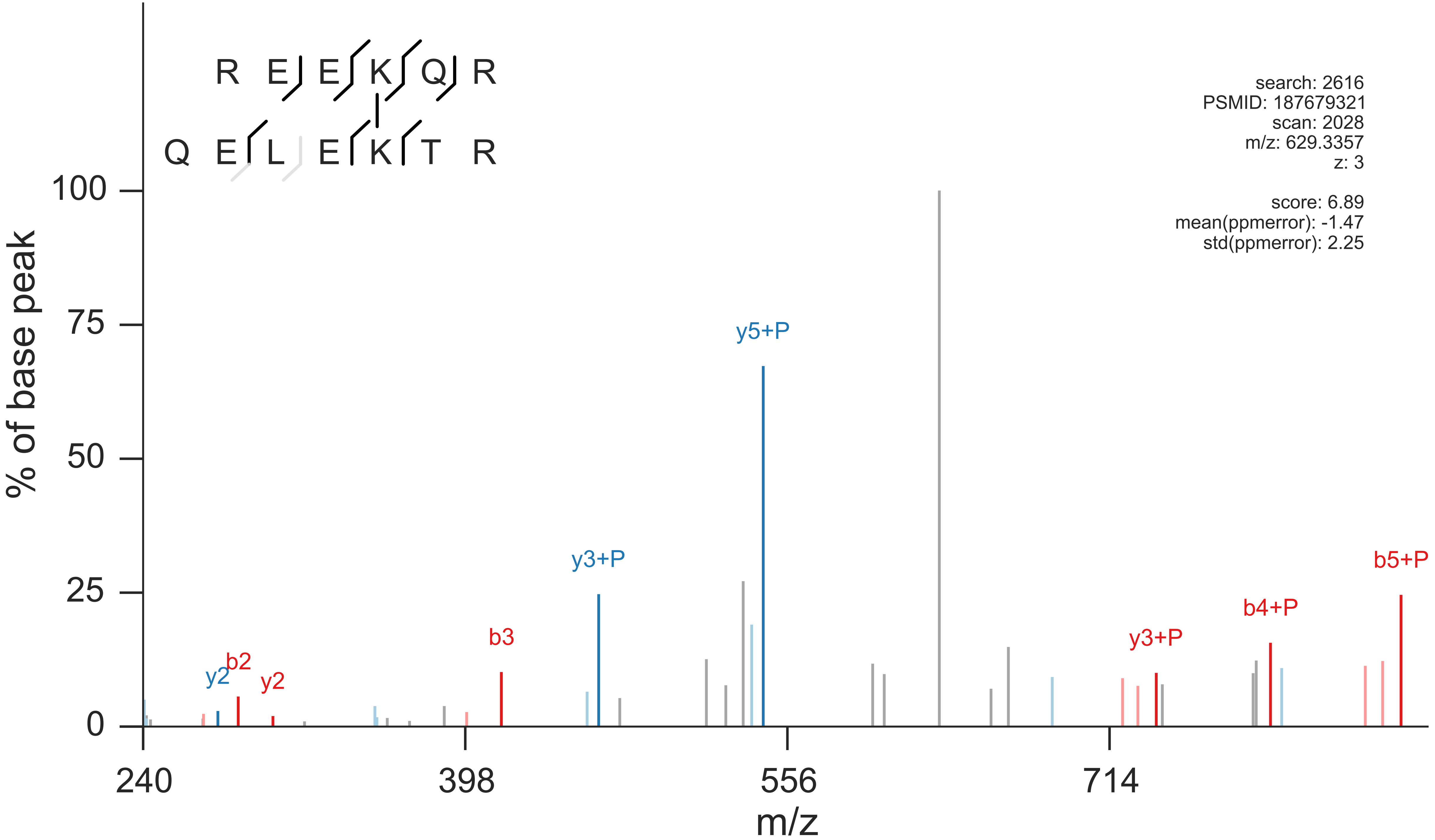

R E E K Q R
Q E L E K T R
search: 2616
PSMID: 187679321
scan: 2028
m/z: 629.3357
z: 3
score: 6.89
mean(ppmerror): -1.47
std(ppmerror): 2.25
% of base peak
100
75
50
25
0
240
398
556
714
m/z
y5+P
y3+P
b5+P
b4+P
y3+P
b3
b2
y2
y2

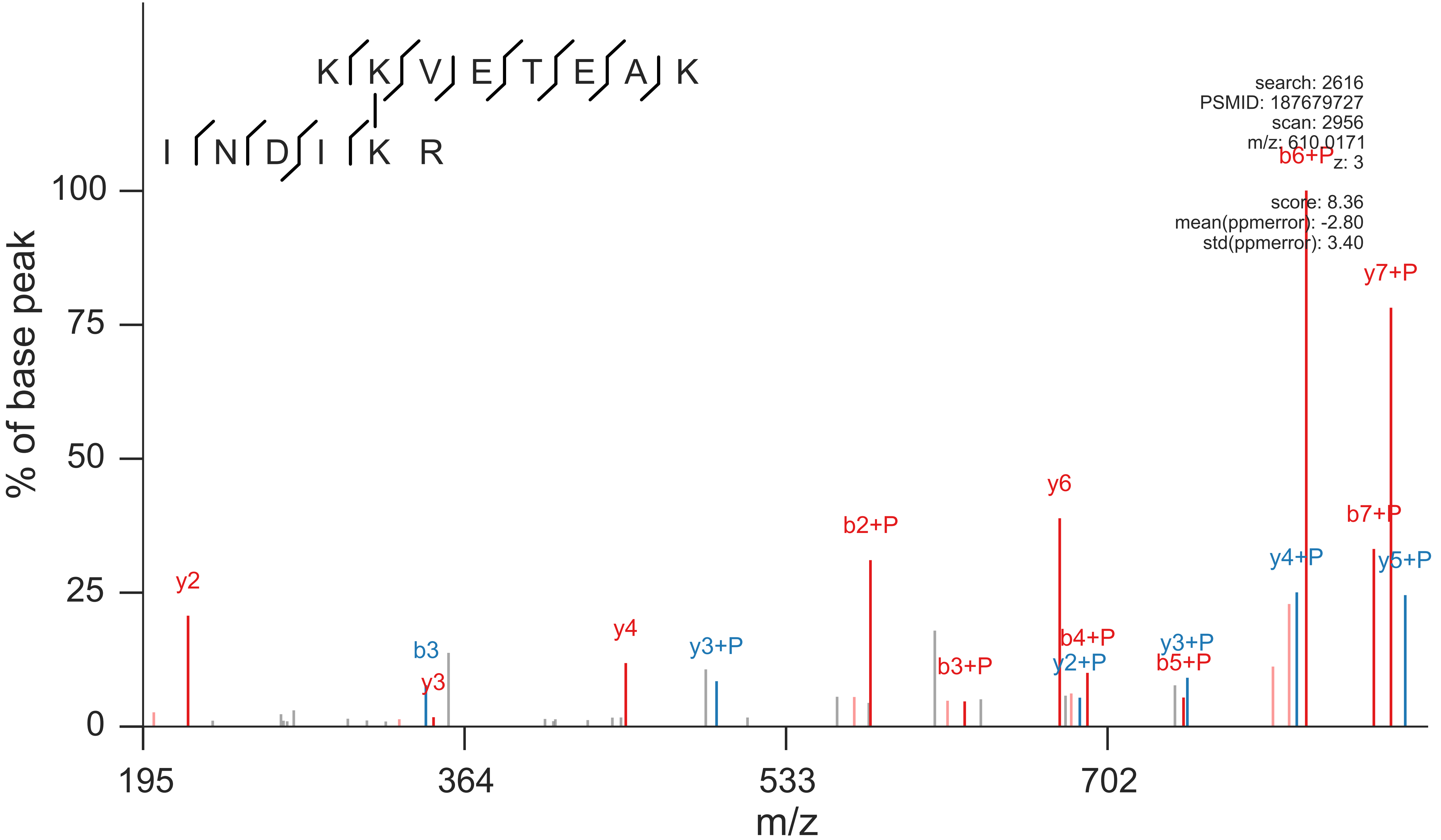
K K V E T E A K
I N D I K R
search: 2616
PSMID: 187679727
scan: 2956
m/z: 610.0171
z: 3
score: 8.36
mean(ppmerror): -2.80
std(ppmerror): 3.40
% of base peak
100
75
50
25
0
195
364
533
702
m/z
y2
b3
y3
y4
y3+P
b2+P
b3+P
y6
b4+P
y2+P
y3+P
b5+P
y4+P
b6+P
b7+P
y7+P
y5+P

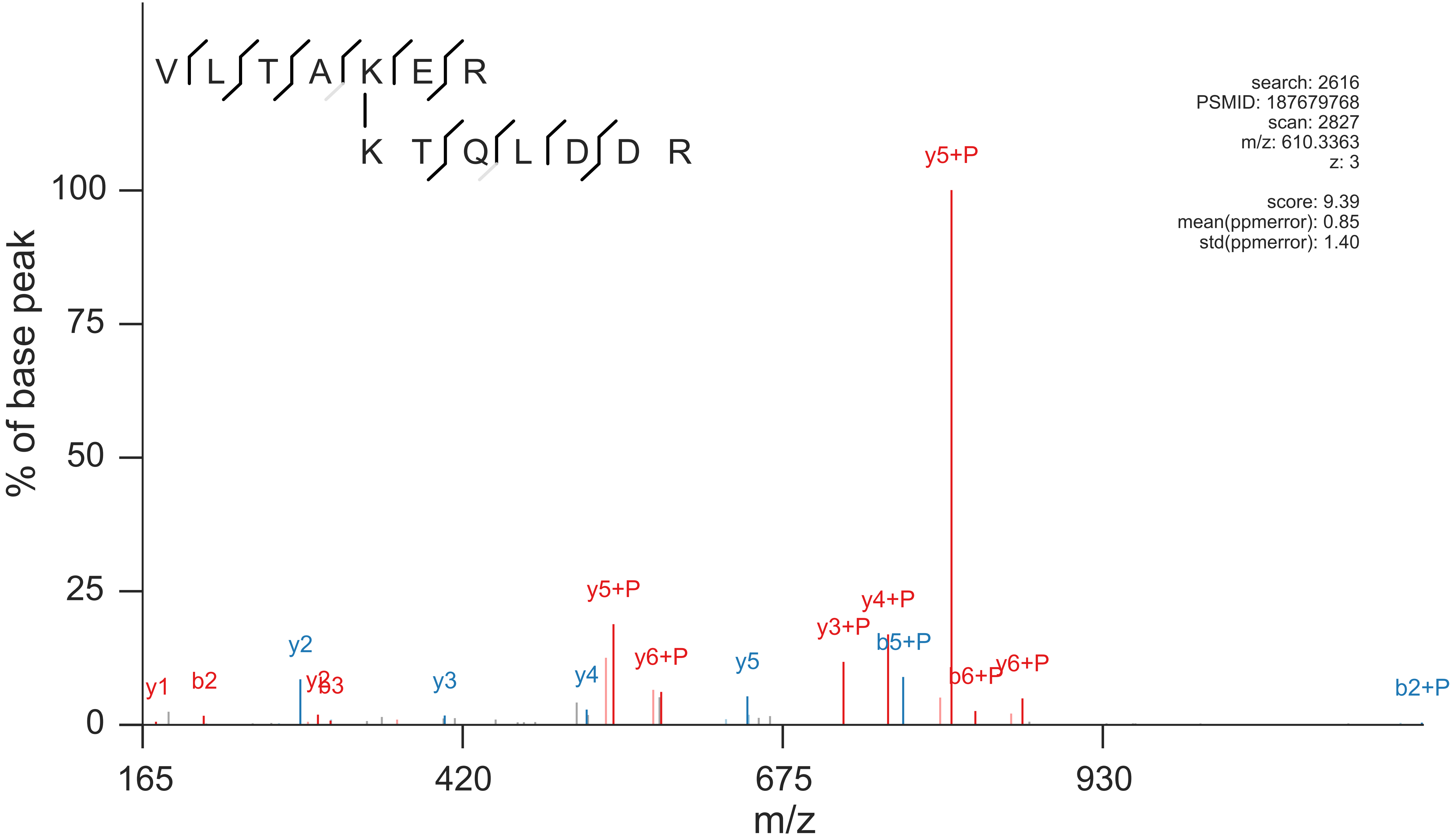

V L T A K E R
K T Q L D D R
search: 2616
PSMID: 187679768
scan: 2827
m/z: 610.3363
z: 3
score: 9.39
mean(ppmerror): 0.85
std(ppmerror): 1.40
% of base peak
m/z
100
75
50
25
0
165
420
675
930
y5+P
y1
b2
y2
y2
b3
y3
y4
y6+P
y5
y3+P
y4+P
b5+P
b6+P
y6+P
b2+P

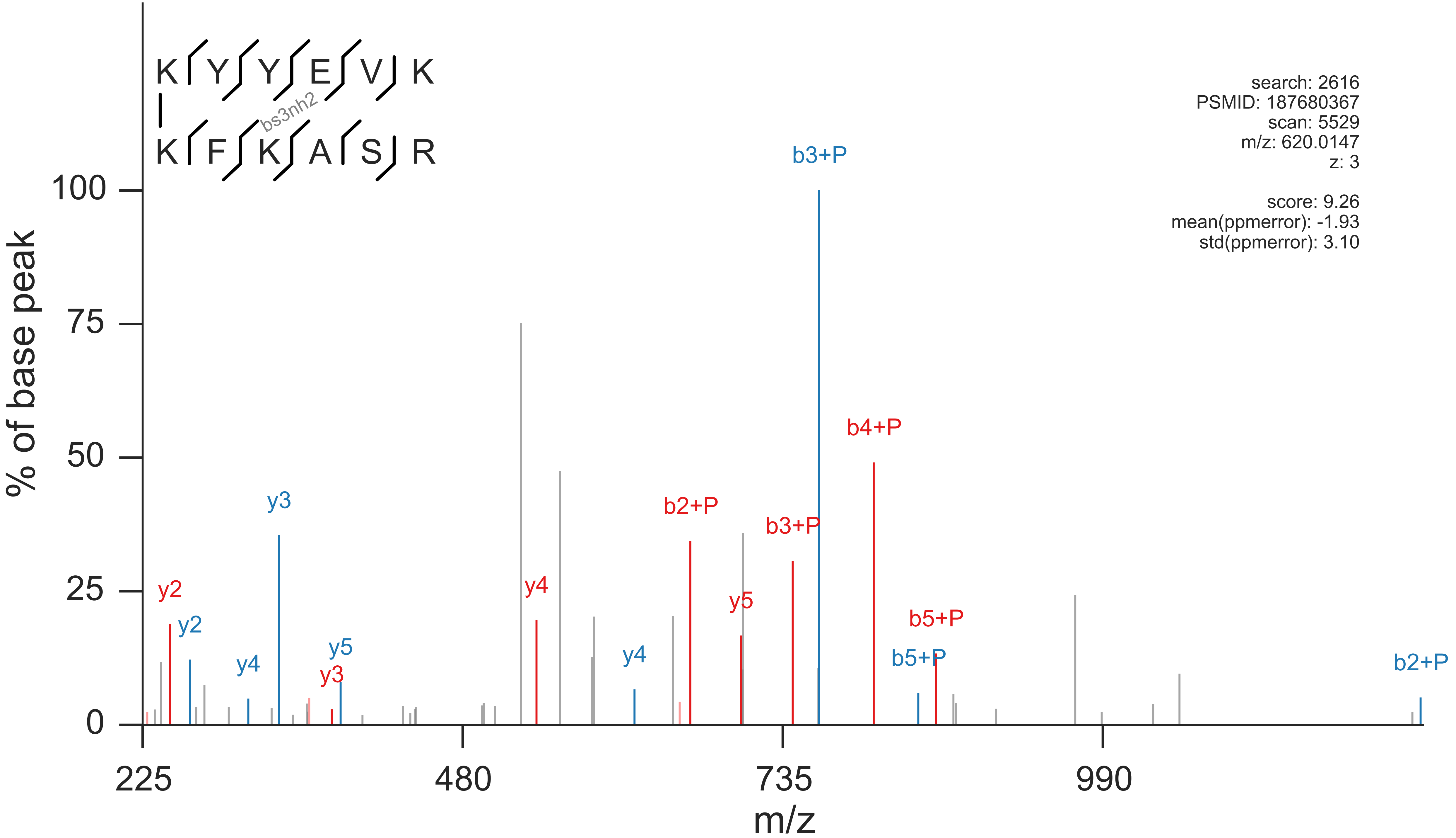

K Y Y E V K
K F K A S R
bs3nh2
search: 2616
PSMID: 187680367
scan: 5529
m/z: 620.0147
z: 3
score: 9.26
mean(ppmerror): -1.93
std(ppmerror): 3.10
% of base peak
m/z
0
25
50
75
100
225
480
735
990
b3+P
b4+P
y3
b2+P
b3+P
y2
y2
y4
y4
y5
y4
y5
y3
b5+P
b5+P
b2+P

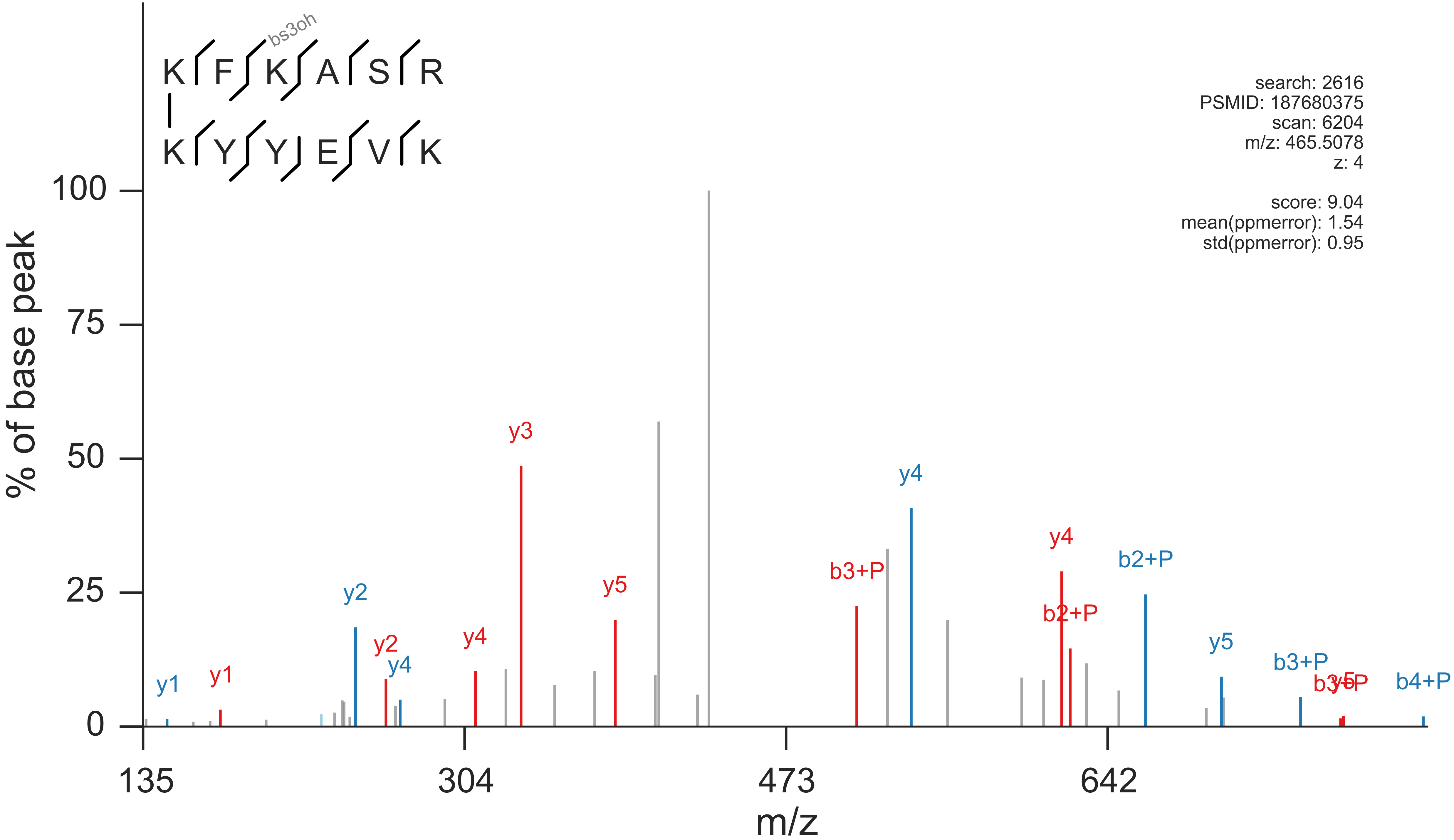

bs3oh
K F K A S R
K Y Y E V K
search: 2616
PSMID: 187680375
scan: 6204
m/z: 465.5078
z: 4
score: 9.04
mean(ppmerror): 1.54
std(ppmerror): 0.95
% of base peak
100
75
50
25
0
y1
y1
y2
y2
y4
y4
y3
y5
b3+P
y4
y4
b2+P
b2+P
y5
b3+P
b3+P
y5
b4+P
135
304
473
642
m/z

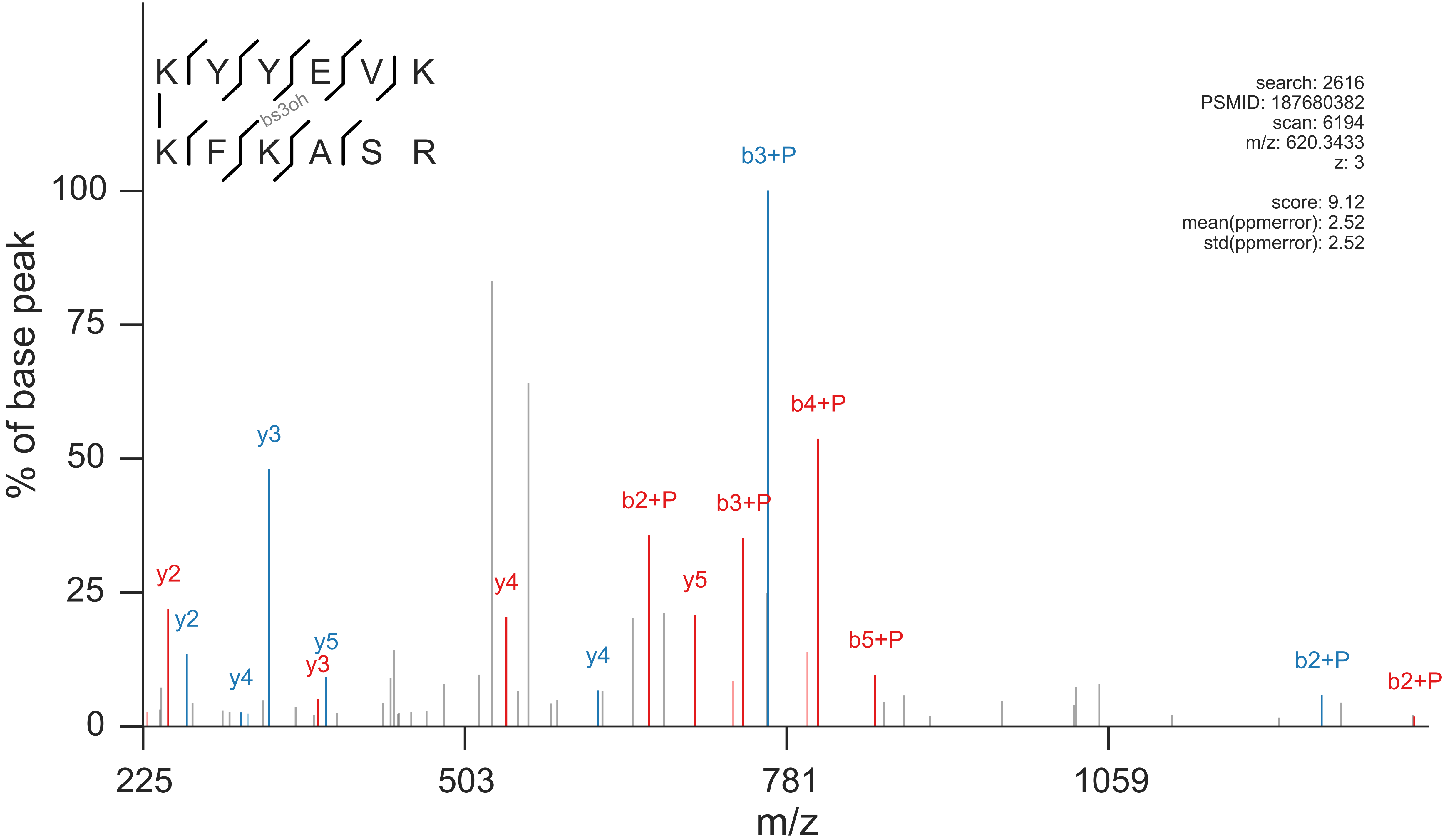

K Y Y E V K
K F K A S R
bs3oh
search: 2616
PSMID: 187680382
scan: 6194
m/z: 620.3433
z: 3
score: 9.12
mean(ppmerror): 2.52
std(ppmerror): 2.52
% of base peak
m/z
b3+P
b4+P
y3
b2+P
b3+P
y2
y4
y5
y2
y5
y3
y4
y4
b5+P
b2+P
b2+P
100
75
50
25
0
225
503
781
1059

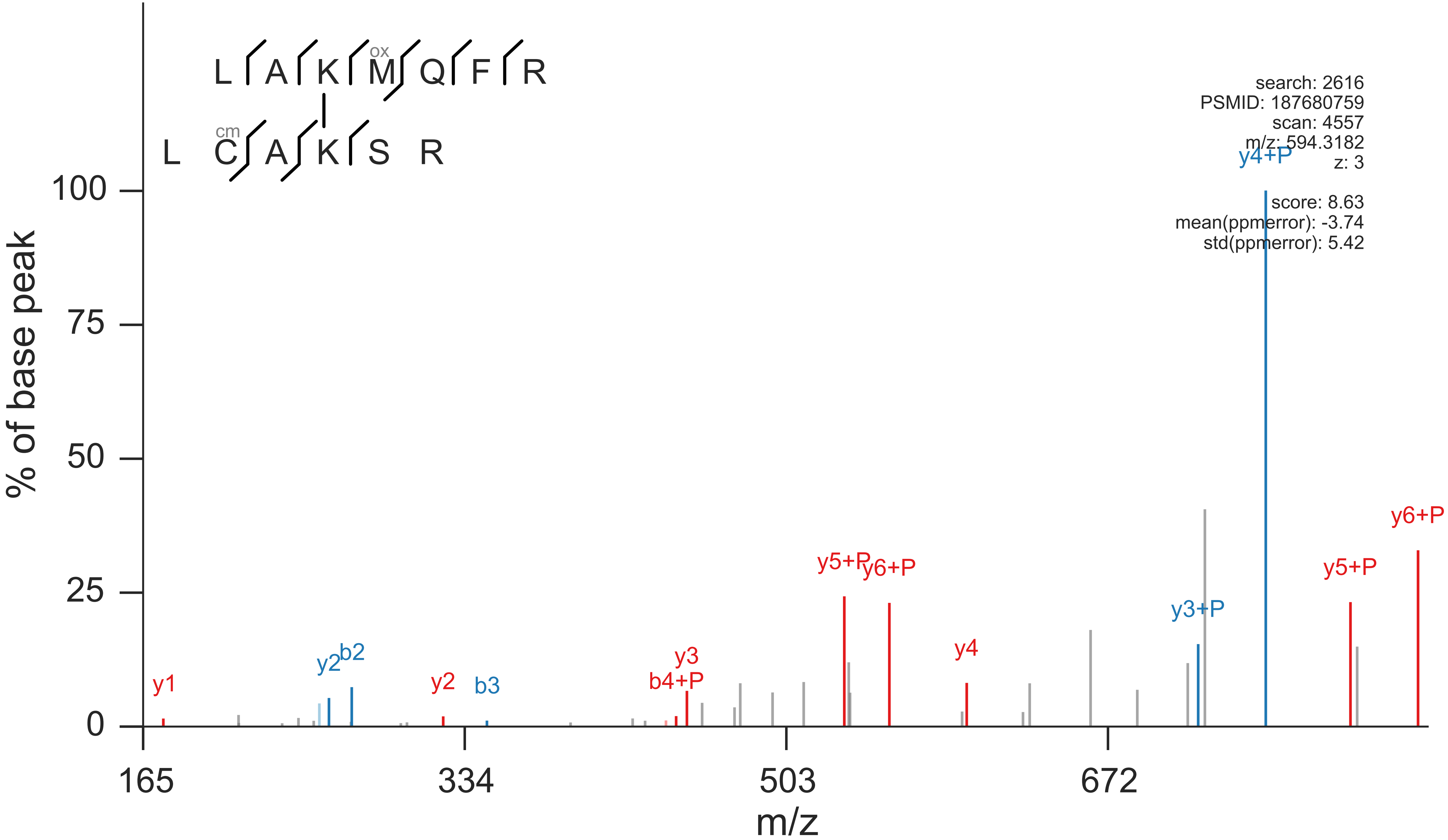

L A K M Q F R
ox
L C A K S R
cm
search: 2616
PSMID: 187680759
scan: 4557
m/z: 594.3182
z: 3
score: 8.63
mean(ppmerror): -3.74
std(ppmerror): 5.42
% of base peak
100
75
50
25
0
m/z
165
334
503
672
y1
y2
b2
y2
b3
y3
b4+P
y5+P
y6+P
y4
y3+P
y4+P
y5+P
y6+P

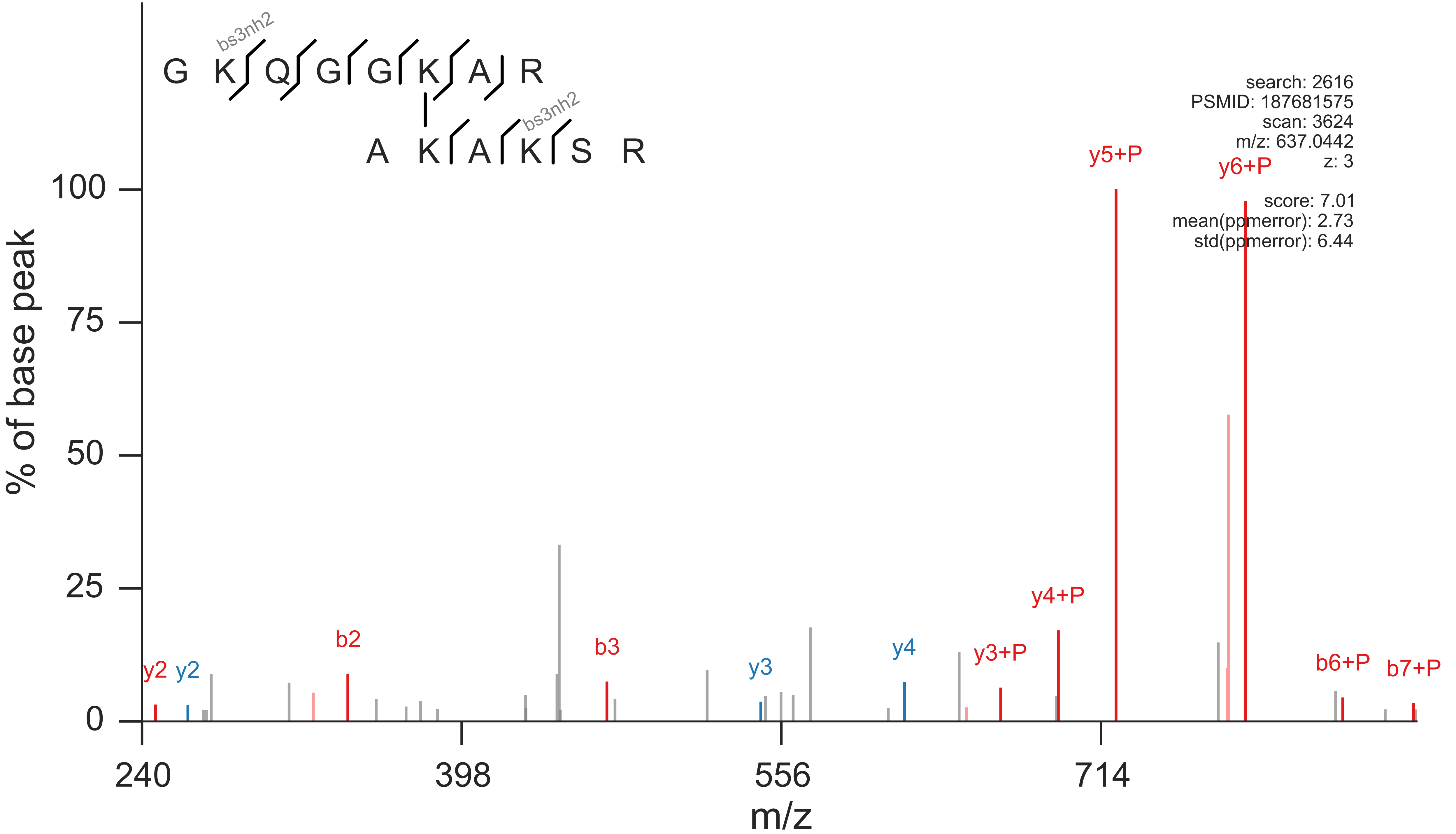
G K Q G G K A R
A K A K S R
bs3nh2
bs3nh2
search: 2616
PSMID: 187681575
scan: 3624
m/z: 637.0442
z: 3
score: 7.01
mean(ppmerror): 2.73
std(ppmerror): 6.44
% of base peak
100
75
50
25
0
y2
y2
b2
b3
y3
y4
y3+P
y4+P
y5+P
y6+P
b6+P
b7+P
240
398
556
714
m/z

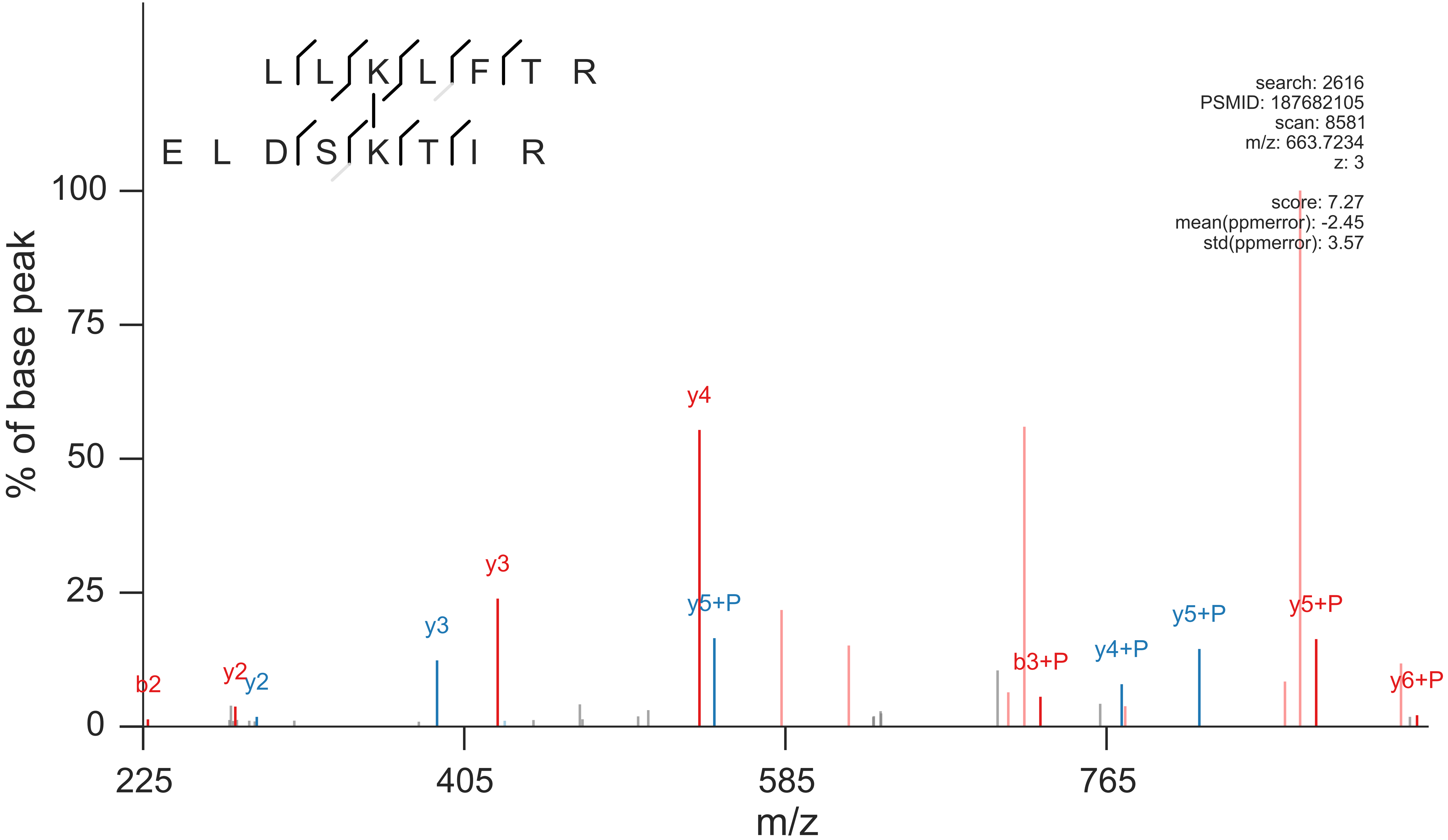

L L K L F T R
E L D S K T I R
search: 2616
PSMID: 187682105
scan: 8581
m/z: 663.7234
z: 3
score: 7.27
mean(ppmerror): -2.45
std(ppmerror): 3.57
% of base peak
100
75
50
25
0
225
405
585
765
m/z
b2
y2
y2
y3
y3
y4
y5+P
b3+P
y4+P
y5+P
y5+P
y6+P

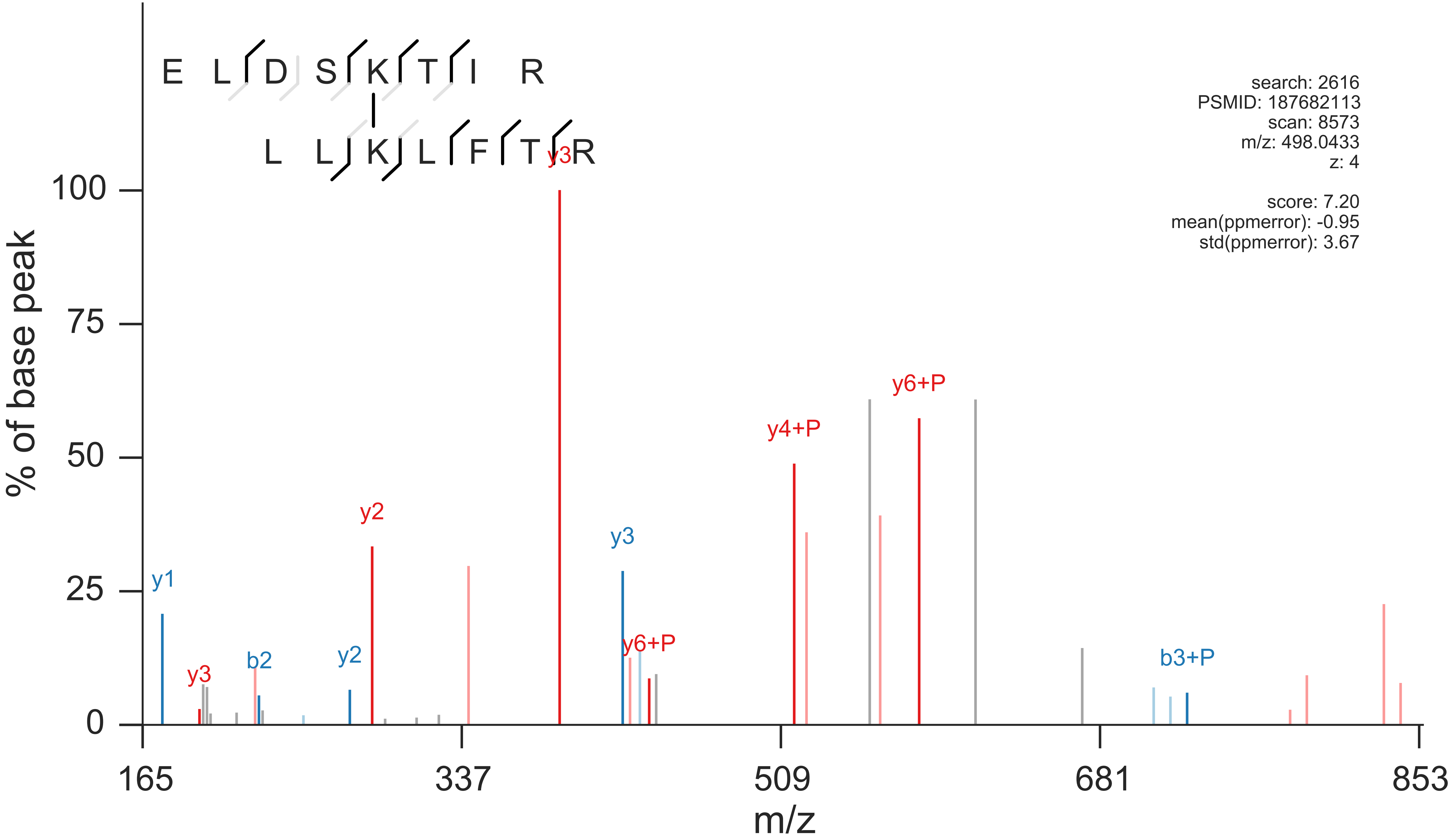

E L D S K T I R
L L K L F T R
search: 2616
PSMID: 187682113
scan: 8573
m/z: 498.0433
z: 4
score: 7.20
mean(ppmerror): -0.95
std(ppmerror): 3.67
% of base peak
100
75
50
25
0
165
337
509
681
853
m/z
y1
y3
b2
y2
y2
y3
y3
y6+P
y4+P
y6+P
b3+P

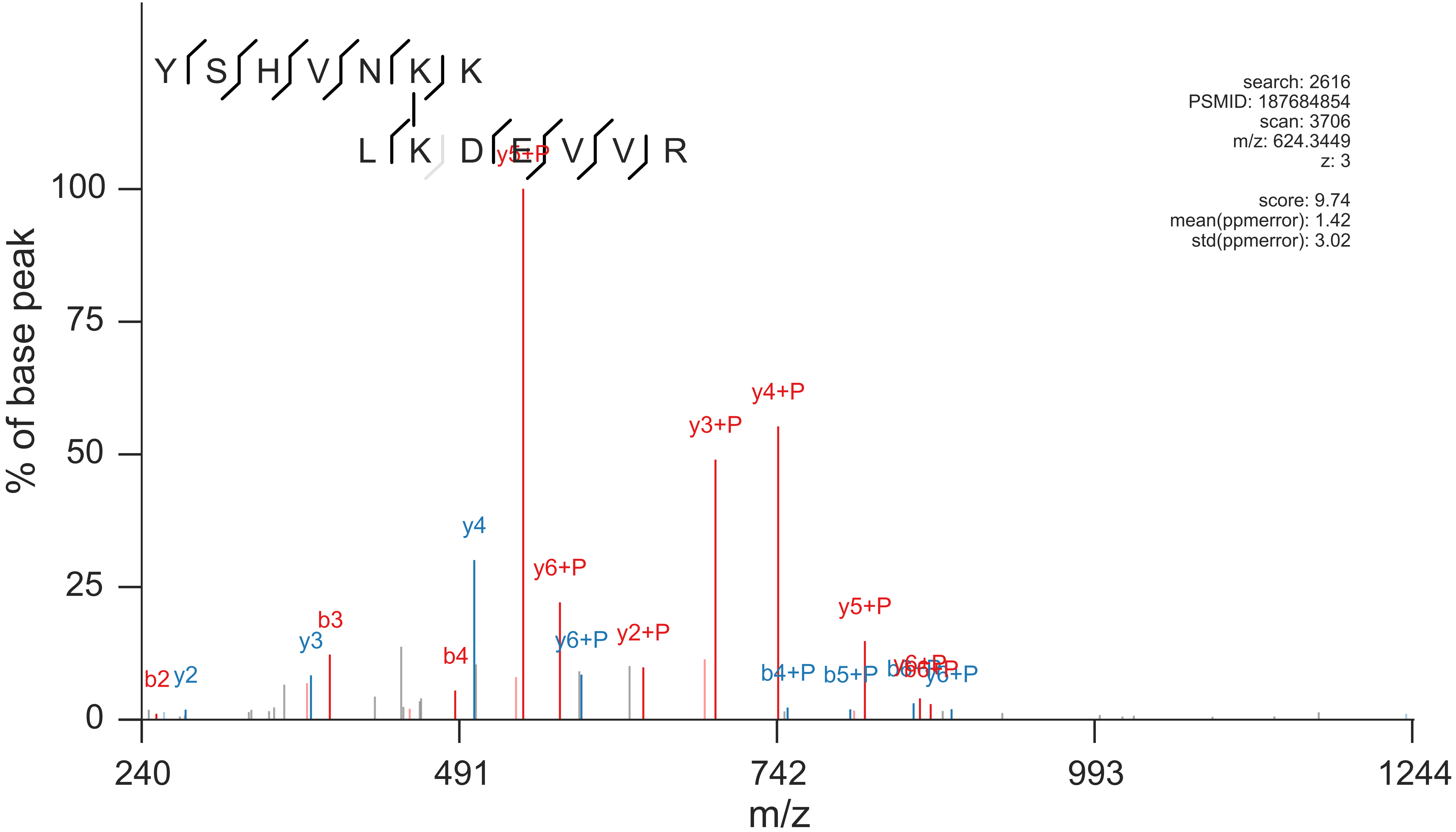

Y S H V N K K
L K D E V V R
search: 2616
PSMID: 187684854
scan: 3706
m/z: 624.3449
z: 3
score: 9.74
mean(ppmerror): 1.42
std(ppmerror): 3.02
% of base peak
100
75
50
25
0
240
491
742
993
1244
m/z
b2
y2
y3
b3
b4
y4
y5+P
y6+P
y6+P
y2+P
y3+P
y4+P
b4+P
b5+P
y5+P
y6+P

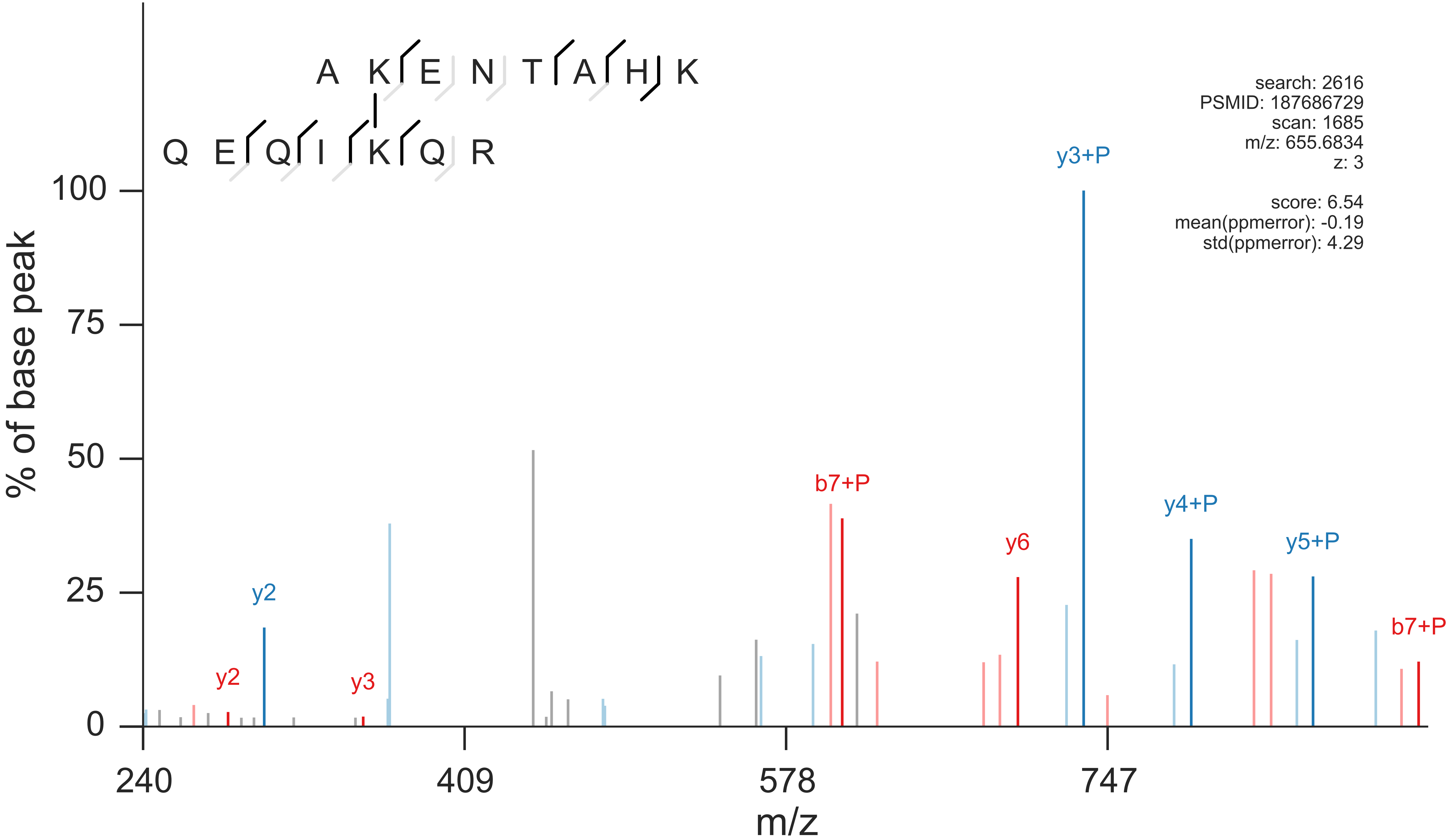

A K E N T A H K
Q E Q I K Q R
search: 2616
PSMID: 187686729
scan: 1685
m/z: 655.6834
z: 3
score: 6.54
mean(ppmerror): -0.19
std(ppmerror): 4.29
% of base peak
100
75
50
25
0
y2
y2
y3
b7+P
y6
y3+P
y4+P
y5+P
b7+P
240
409
578
747
m/z

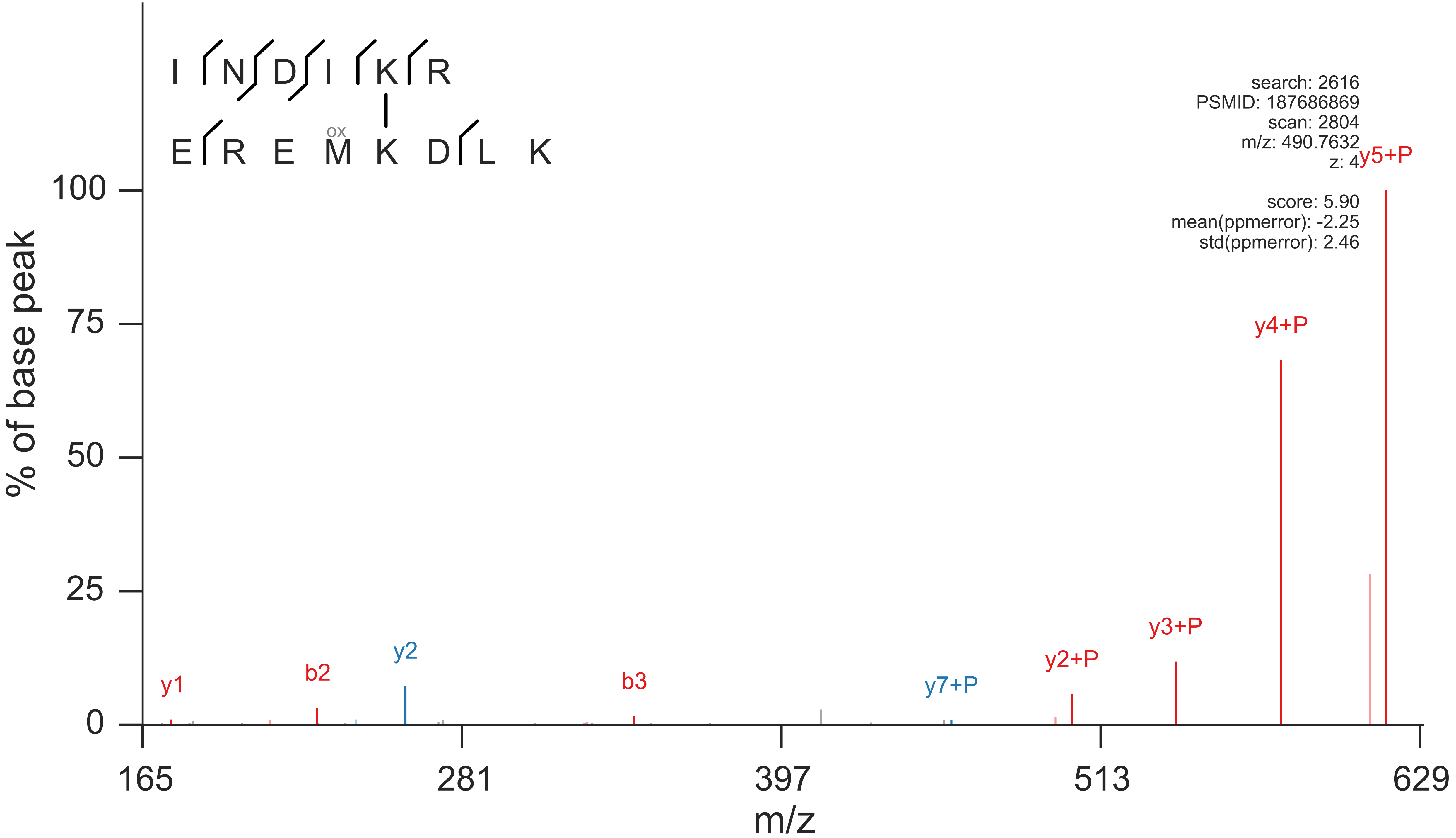

I N D I K R
E R E M K D L K
ox
search: 2616
PSMID: 187686869
scan: 2804
m/z: 490.7632
z: 4
score: 5.90
mean(ppmerror): -2.25
std(ppmerror): 2.46
% of base peak
m/z
y1
b2
y2
b3
y7+P
y2+P
y3+P
y4+P
y5+P
0
25
50
75
100
165
281
397
513
629

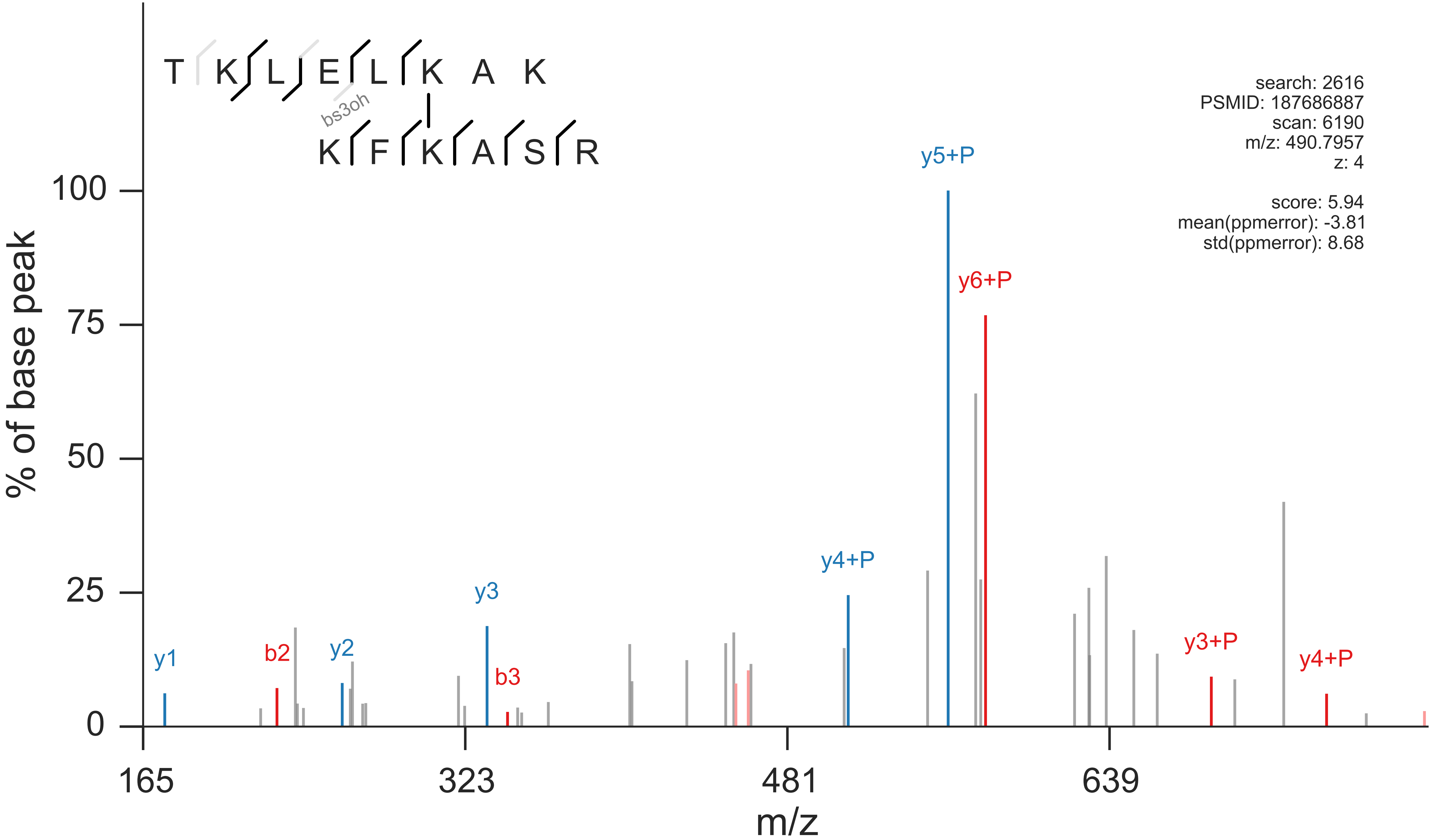

T K L E L K A K
bs3oh
K F K A S R
search: 2616
PSMID: 187686887
scan: 6190
m/z: 490.7957
z: 4
score: 5.94
mean(ppmerror): -3.81
std(ppmerror): 8.68
% of base peak
100
75
50
25
0
y1
b2
y2
y3
b3
y4+P
y5+P
y6+P
y3+P
y4+P
165
323
481
639
m/z

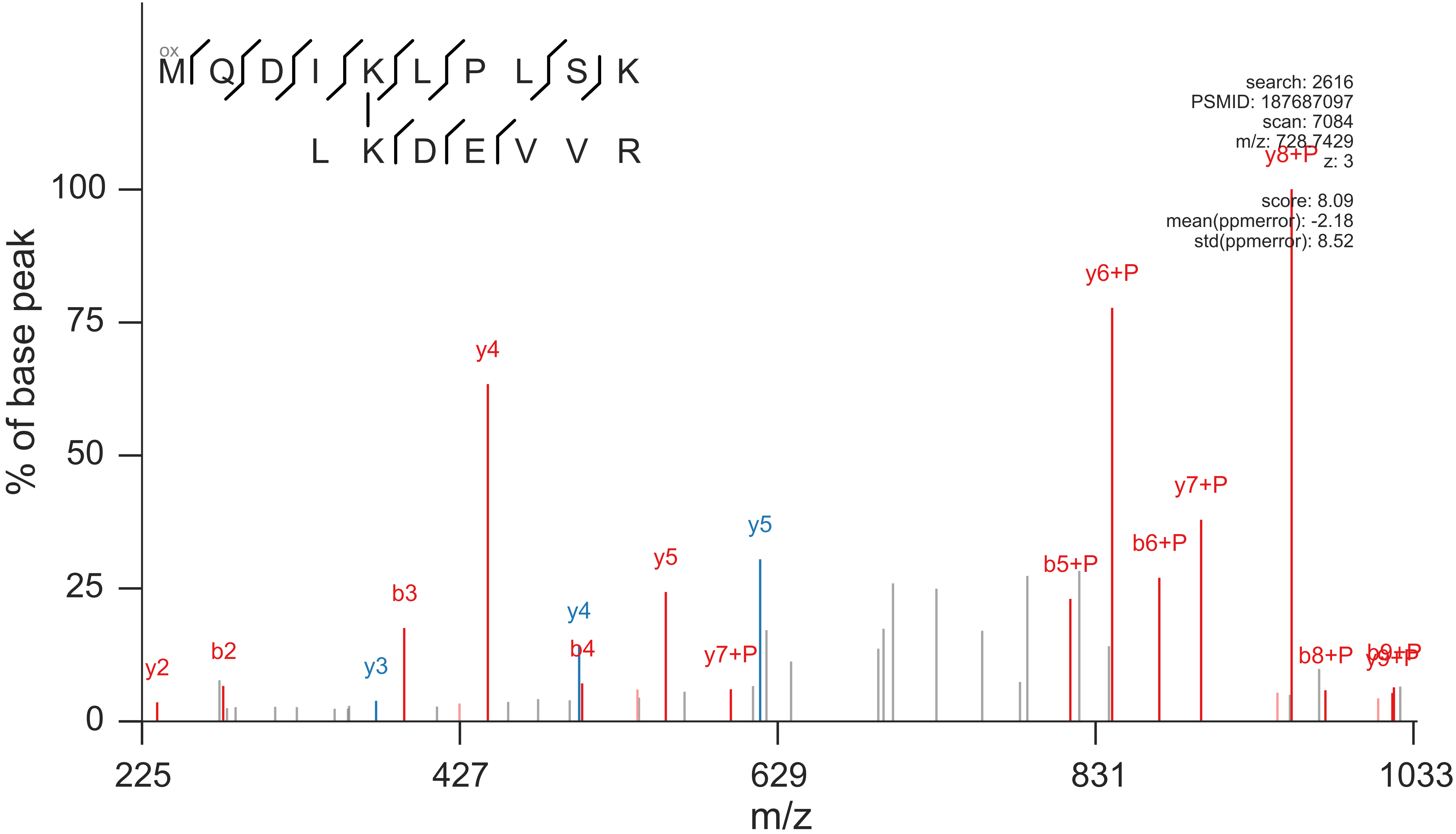

ox
M Q D I K L P L S K
L K D E V V R
search: 2616
PSMID: 187687097
scan: 7084
m/z: 728.7429
z: 3
score: 8.09
mean(ppmerror): -2.18
std(ppmerror): 8.52
% of base peak
m/z
0
25
50
75
100
225
427
629
831
1033
y2
b2
y3
b3
y4
y4
b4
y5
y7+P
y5
b5+P
y6+P
b6+P
y7+P
y8+P
b8+P

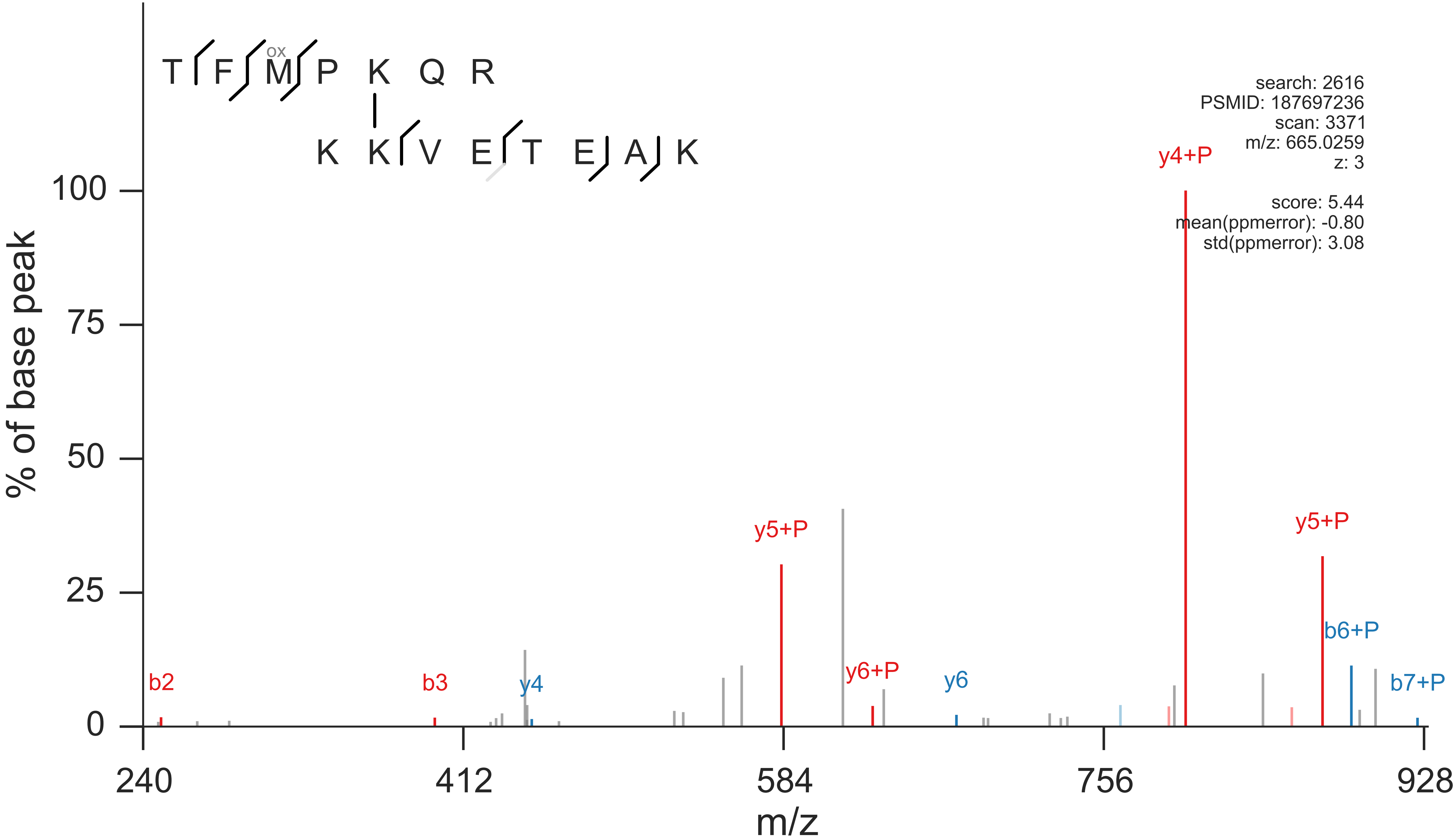

T F M P K Q R
ox
K K V E T E A K
search: 2616
PSMID: 187697236
scan: 3371
m/z: 665.0259
z: 3
score: 5.44
mean(ppmerror): -0.80
std(ppmerror): 3.08
y4+P
y5+P
y5+P
b6+P
b2
b3
y4
y6+P
y6
b7+P
100
75
50
25
0
% of base peak
240
412
584
756
928
m/z

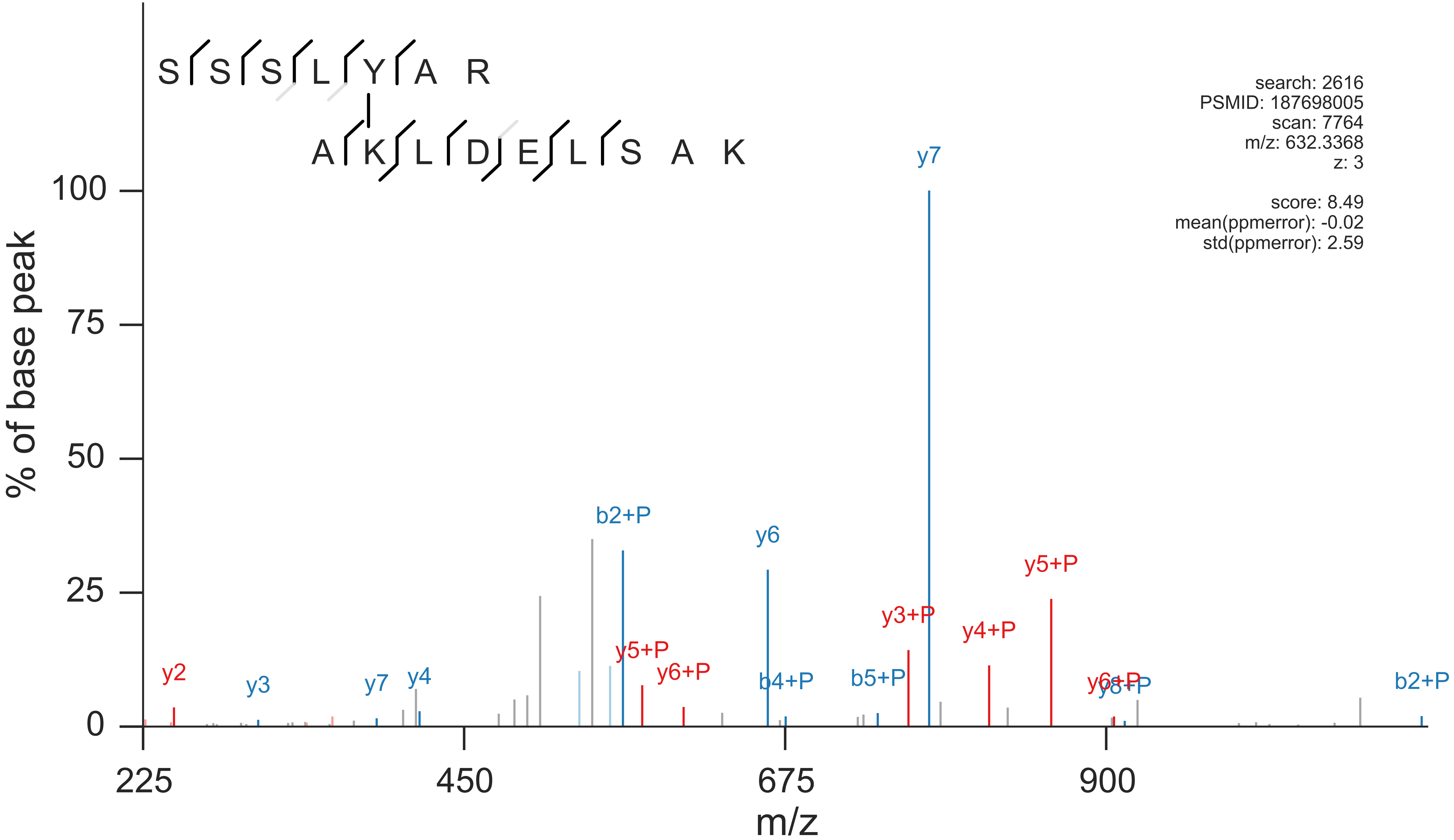

S S S L Y A R
A K L D E L S A K
search: 2616
PSMID: 187698005
scan: 7764
m/z: 632.3368
z: 3
score: 8.49
mean(ppmerror): -0.02
std(ppmerror): 2.59
% of base peak
m/z
100
75
50
25
0
225
450
675
900
y7
b2+P
y6
y5+P
y3+P
y4+P
y5+P
y6+P
y2
y3
y7
y4
b4+P
b5+P
y6+P
y8+P
b2+P

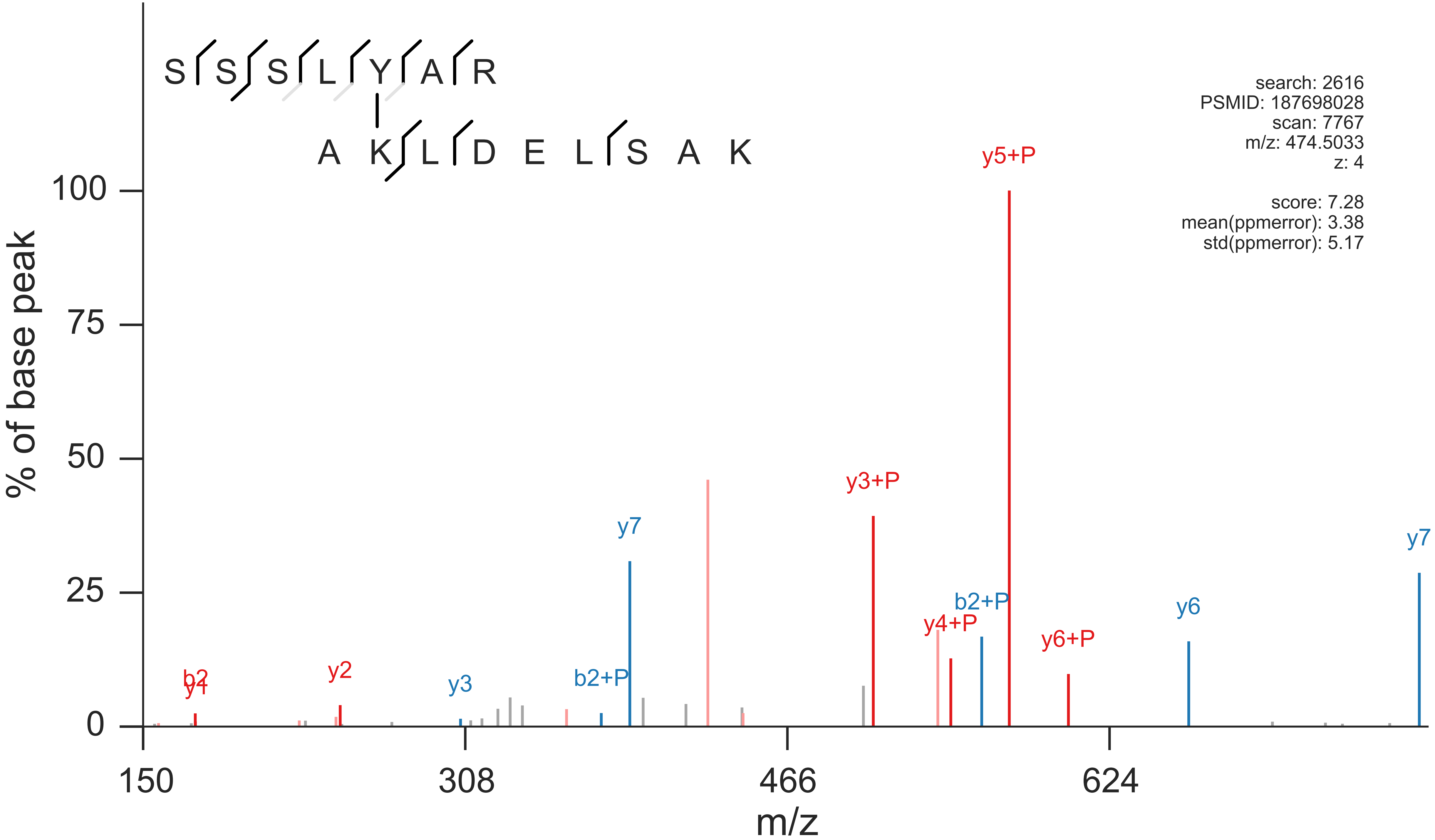

S S S L Y A R
A K L D E L S A K
search: 2616
PSMID: 187698028
scan: 7767
m/z: 474.5033
z: 4
score: 7.28
mean(ppmerror): 3.38
std(ppmerror): 5.17
% of base peak
100
75
50
25
0
150
308
466
624
m/z
b2
y1
y2
y3
b2+P
y7
y3+P
y4+P
b2+P
y5+P
y6+P
y6
y7

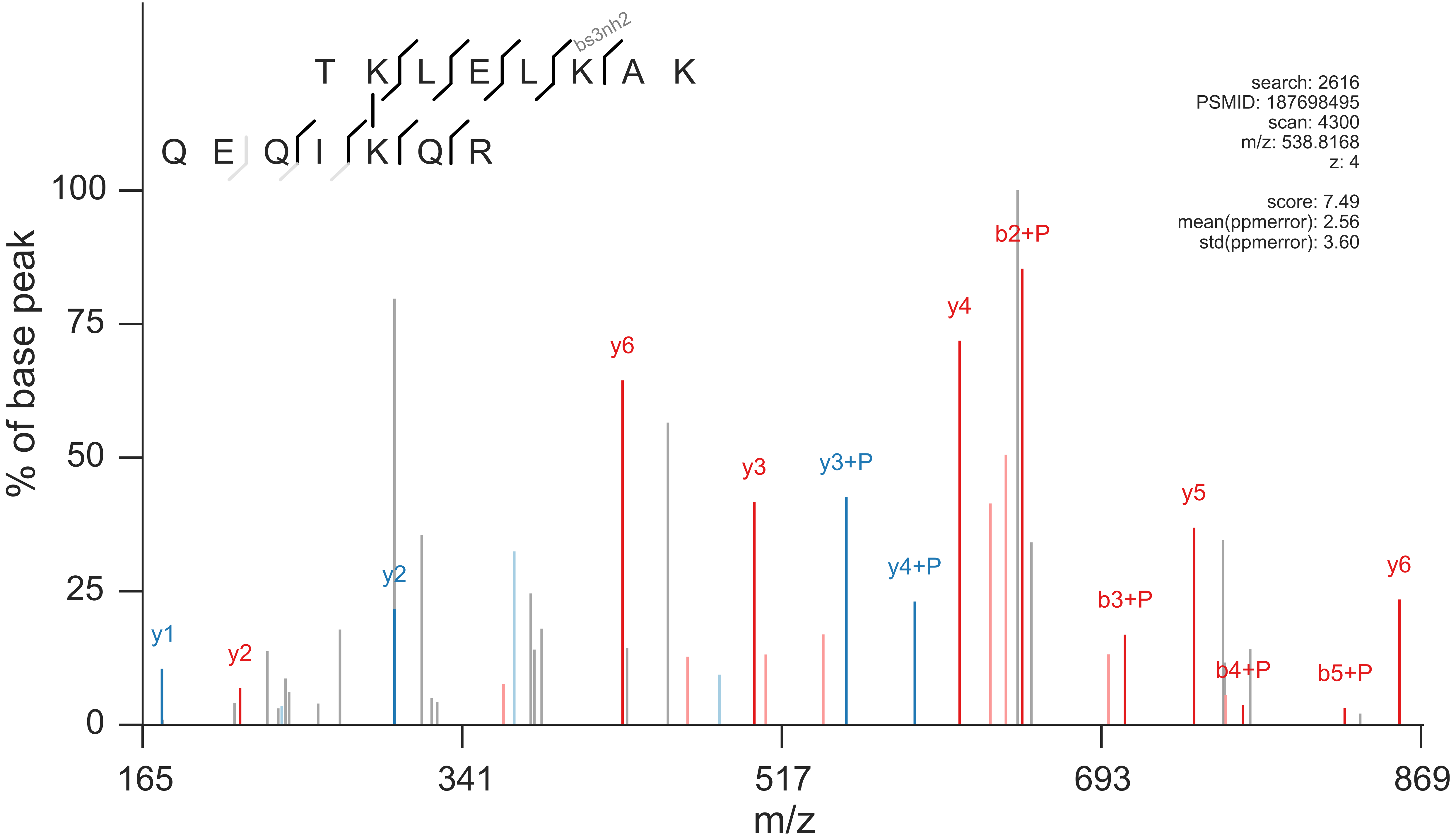

T K L E L K A K
bs3nh2
Q E Q I K Q R
search: 2616
PSMID: 187698495
scan: 4300
m/z: 538.8168
z: 4
score: 7.49
mean(ppmerror): 2.56
std(ppmerror): 3.60
% of base peak
100
75
50
25
0
y1
y2
y2
y6
y3
y3+P
y4+P
y4
b2+P
b3+P
y5
b4+P
b5+P
y6
165
341
517
693
869
m/z

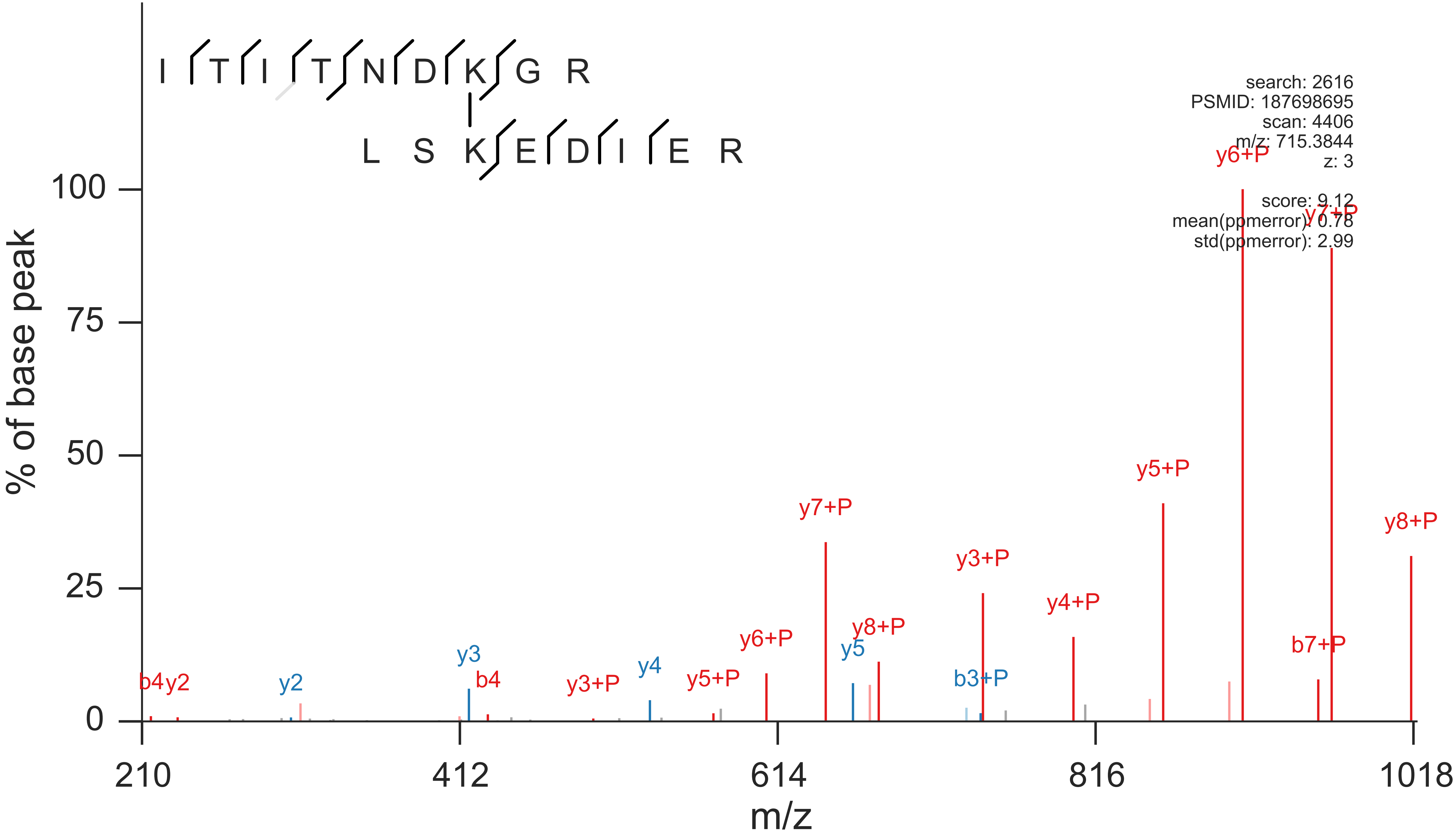

I T I T N D K G R
L S K E D I E R
search: 2616
PSMID: 187698695
scan: 4406
m/z: 715.3844
z: 3
score: 9.12
mean(ppmerror): 0.78
std(ppmerror): 2.99
% of base peak
100
75
50
25
0
210
412
614
816
1018
m/z
b4y2
y2
y3
b4
y3+P
y4
y5+P
y6+P
y7+P
y5
y8+P
b3+P
y3+P
y4+P
y5+P
y6+P
y7+P
b7+P
y8+P

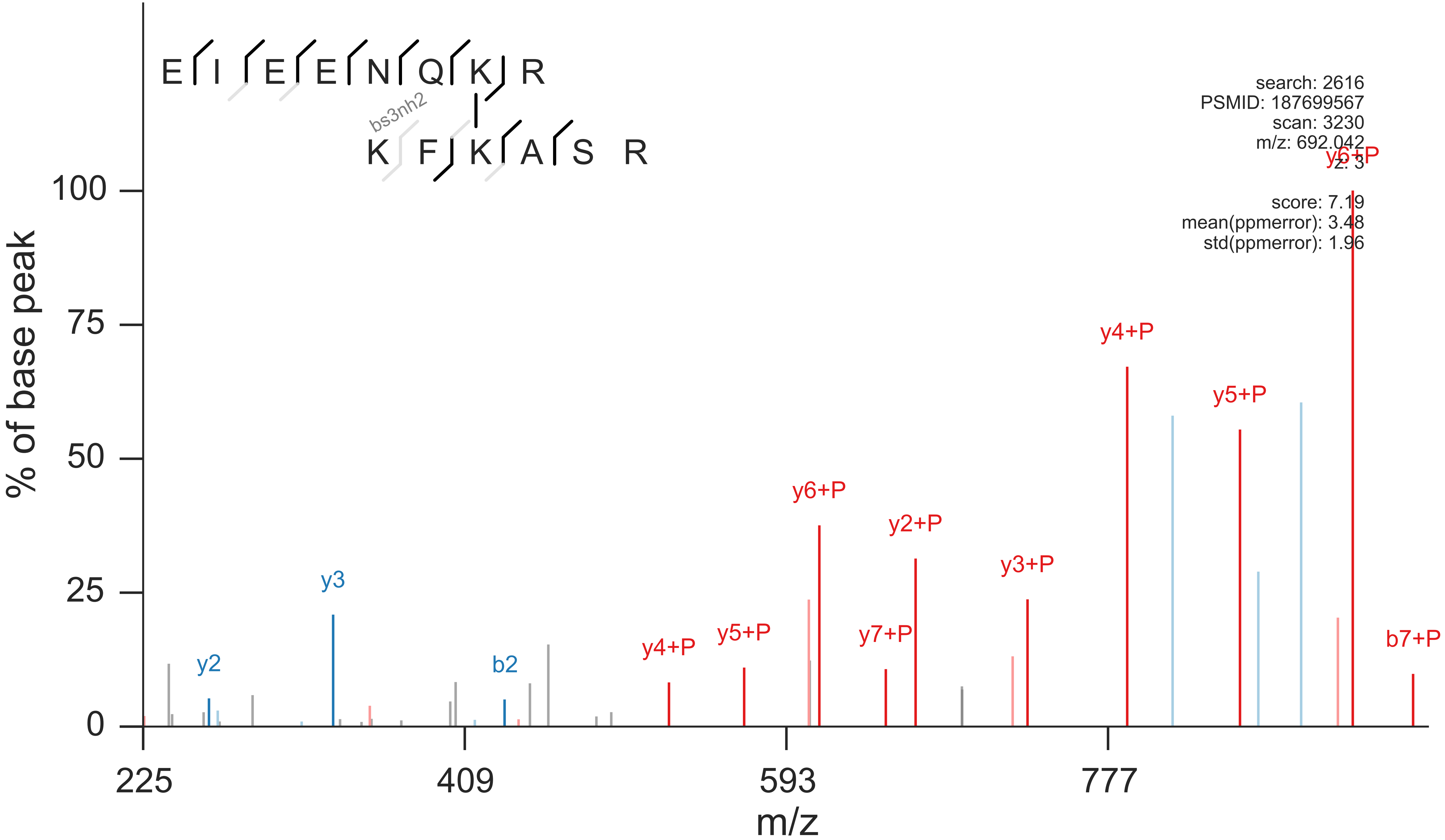

E I E E N Q K R
bs3nh2
K F K A S R
search: 2616
PSMID: 187699567
scan: 3230
m/z: 692.042
z: 3
score: 7.19
mean(ppmerror): 3.48
std(ppmerror): 1.96
% of base peak
100
75
50
25
0
m/z
225
409
593
777
y2
y3
b2
y4+P
y5+P
y6+P
y7+P
y2+P
y3+P
y4+P
y5+P
y6+P
b7+P

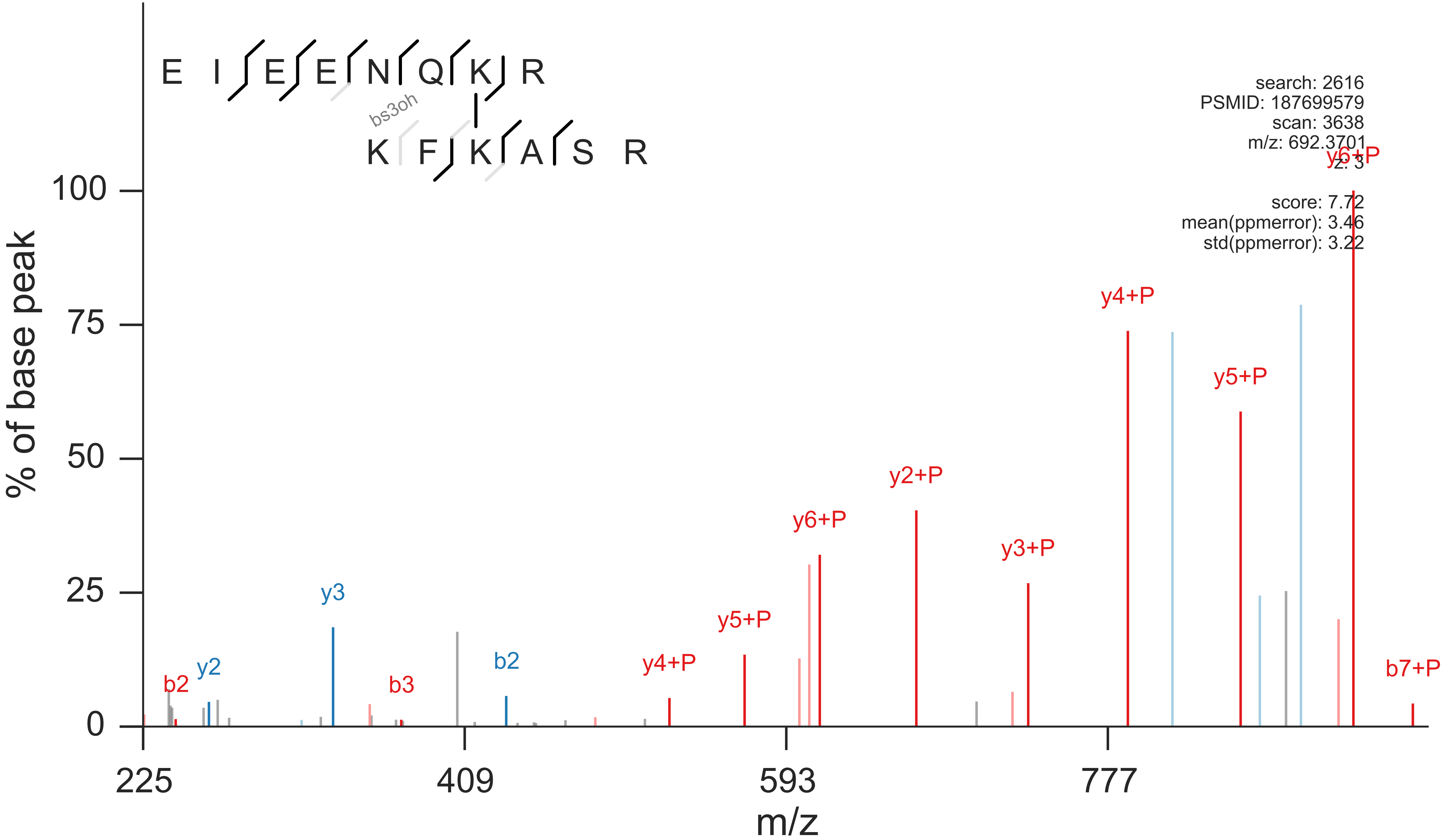

E I E E N Q K R
bs3oh
K F K A S R
search: 2616
PSMID: 187699579
scan: 3638
m/z: 692.3701
z: 3
score: 7.72
mean(ppmerror): 3.46
std(ppmerror): 3.22
% of base peak
100
75
50
25
0
225
409
593
777
m/z
b2
y2
y3
b3
b2
y4+P
y5+P
y6+P
y2+P
y3+P
y4+P
y5+P
y6+P
b7+P

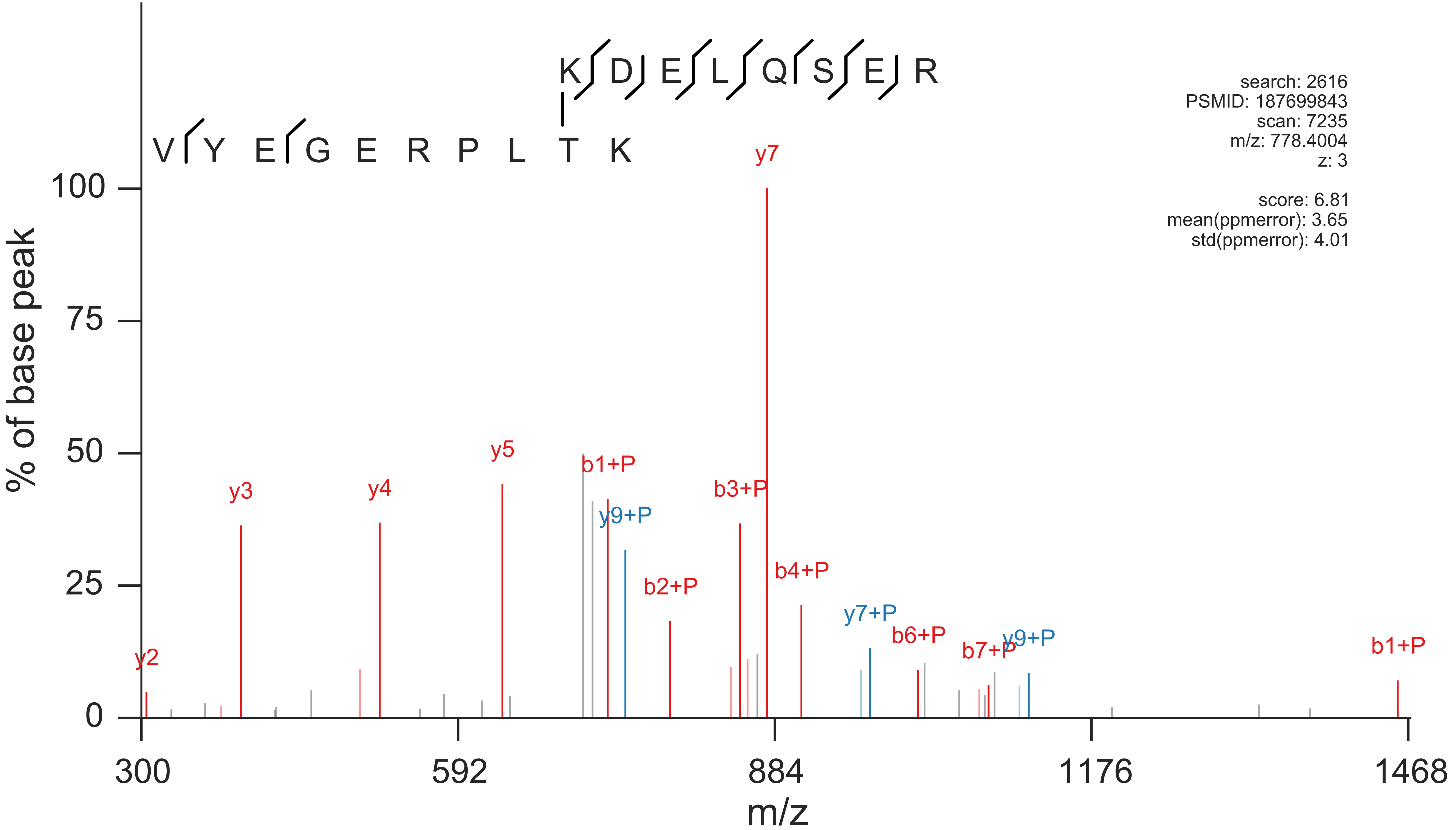
K D E L Q S E R
V Y E G E R P L T K
search: 2616
PSMID: 187699843
scan: 7235
m/z: 778.4004
z: 3
score: 6.81
mean(ppmerror): 3.65
std(ppmerror): 4.01
% of base peak
100
75
50
25
0
y2
y3
y4
y5
b1+P
y9+P
b2+P
b3+P
y7
b4+P
y7+P
b6+P
b7+P
y9+P
b1+P
300
592
884
1176
1468
m/z

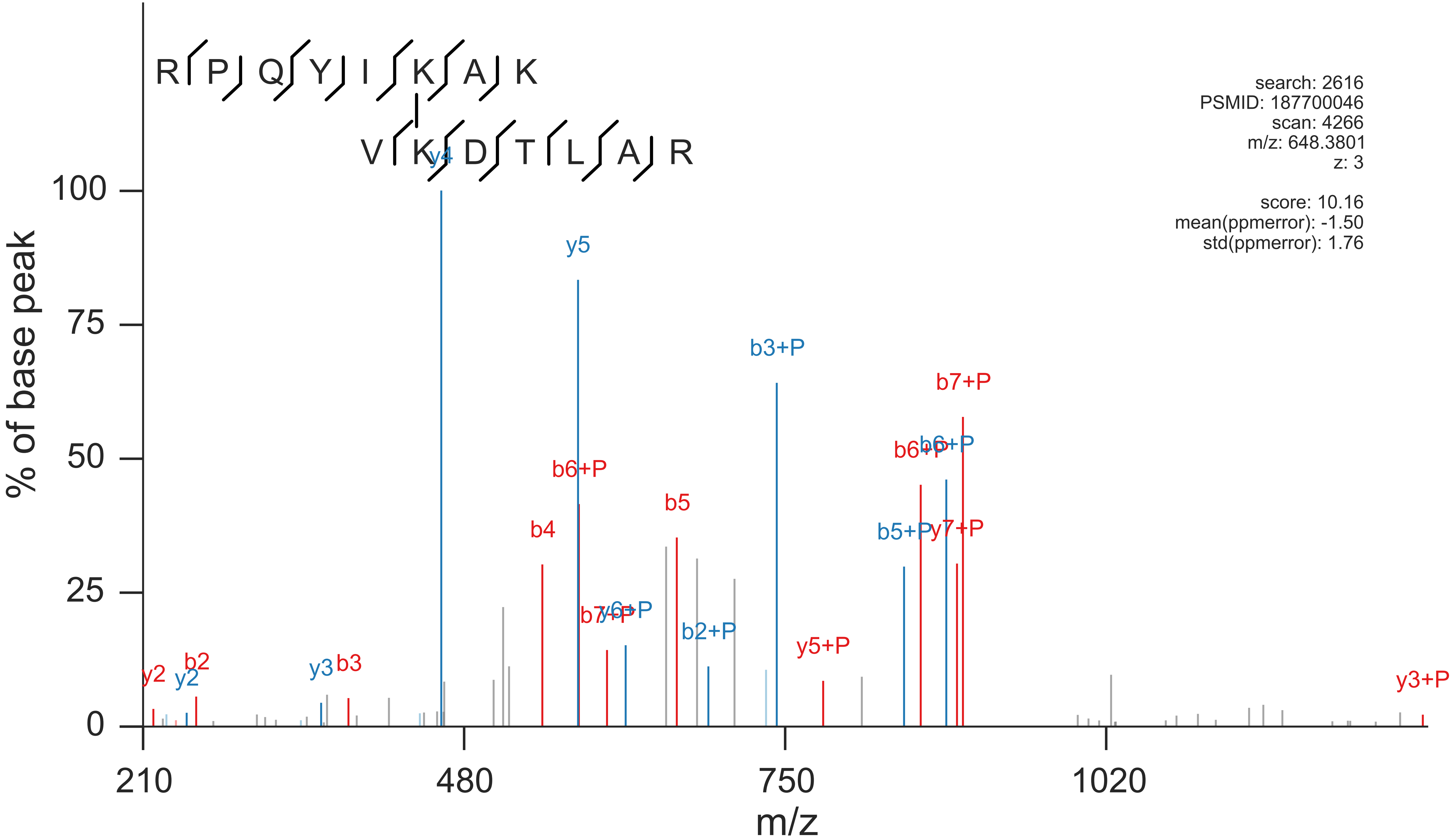

R P Q Y I K A K
V K D T L A R
search: 2616
PSMID: 187700046
scan: 4266
m/z: 648.3801
z: 3
score: 10.16
mean(ppmerror): -1.50
std(ppmerror): 1.76
% of base peak
m/z
y4
y5
b3+P
b7+P
b6+P
b6+P
b5
b4
b5+P
y7+P
b7+P
y6+P
b2+P
y5+P
y2
b2
y2
y3
b3
y3+P
100
75
50
25
0
210
480
750
1020

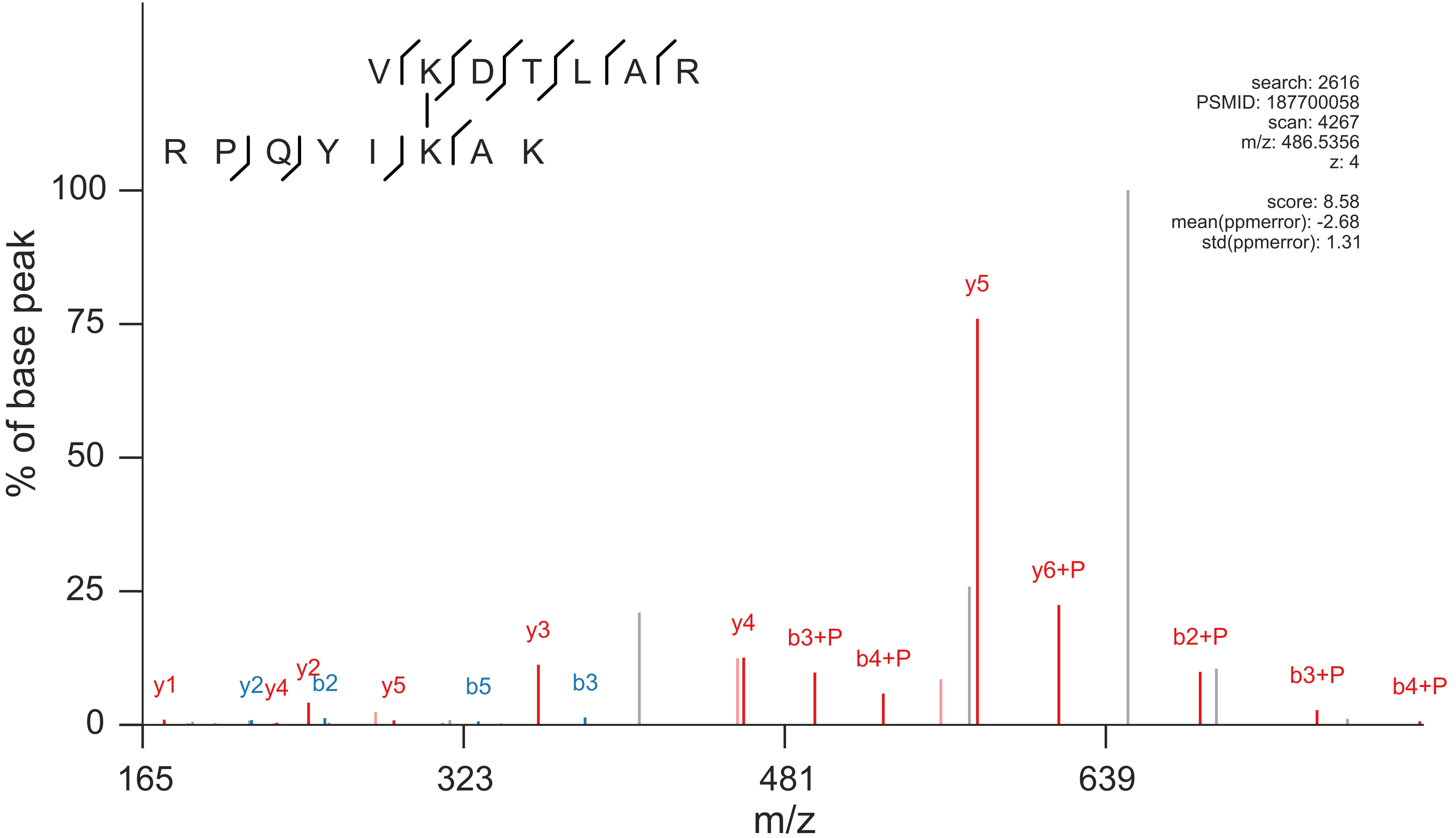

V K D T L A R
R P Q Y I K A K
search: 2616
PSMID: 187700058
scan: 4267
m/z: 486.5356
z: 4
score: 8.58
mean(ppmerror): -2.68
std(ppmerror): 1.31
% of base peak
m/z
0
25
50
75
100
165
323
481
639
y1
y2
y4
y2
b2
y5
b5
y3
b3
y4
b3+P
b4+P
y5
y6+P
b2+P
b3+P
b4+P

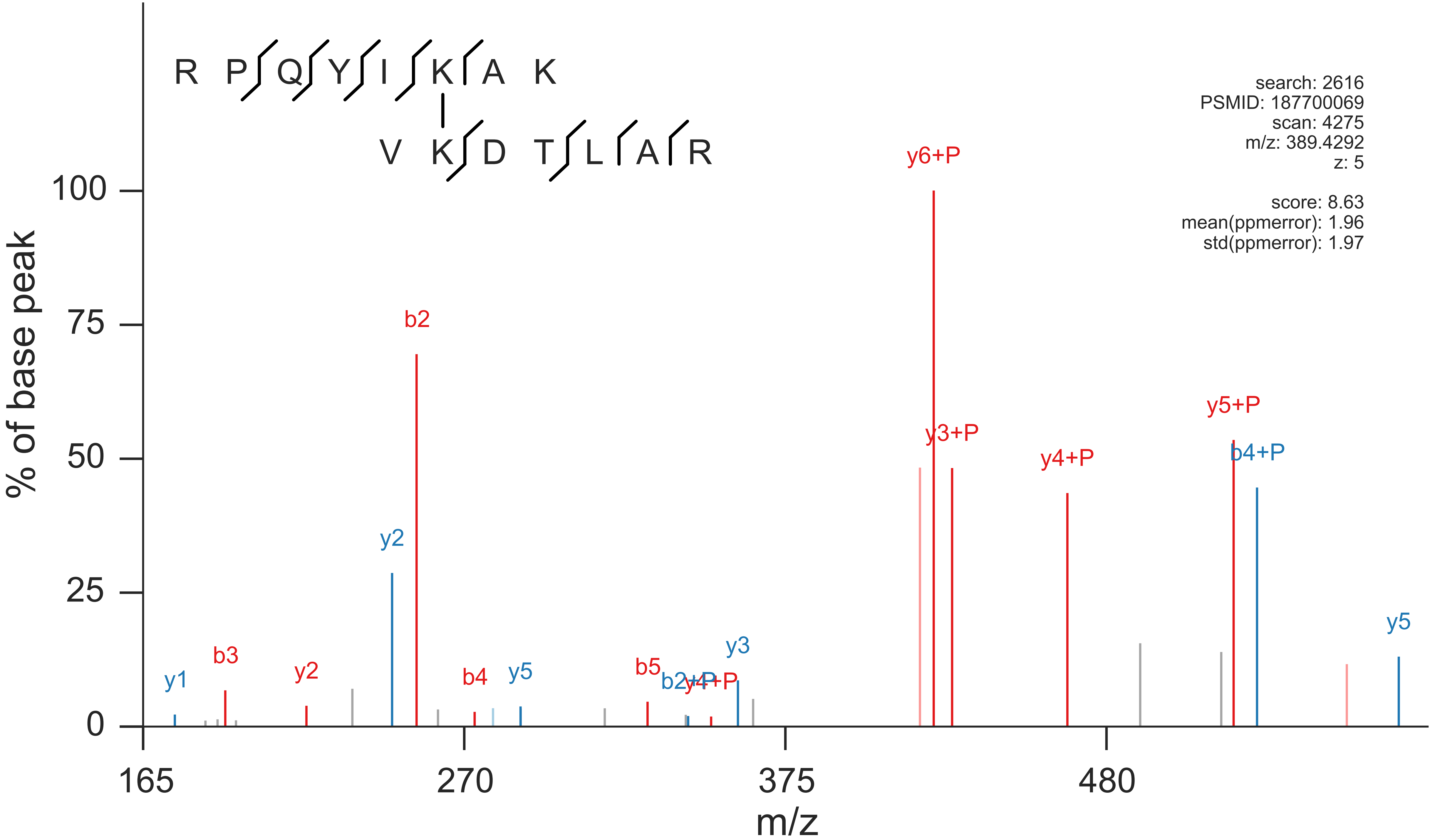

R P Q Y I K A K
V K D T L A R
search: 2616
PSMID: 187700069
scan: 4275
m/z: 389.4292
z: 5
score: 8.63
mean(ppmerror): 1.96
std(ppmerror): 1.97
% of base peak
m/z
0
25
50
75
100
165
270
375
480
y1
b3
y2
y2
b2
b4
y5
b5
b2+P
y4+P
y3
y6+P
y3+P
y4+P
y5+P
b4+P
y5

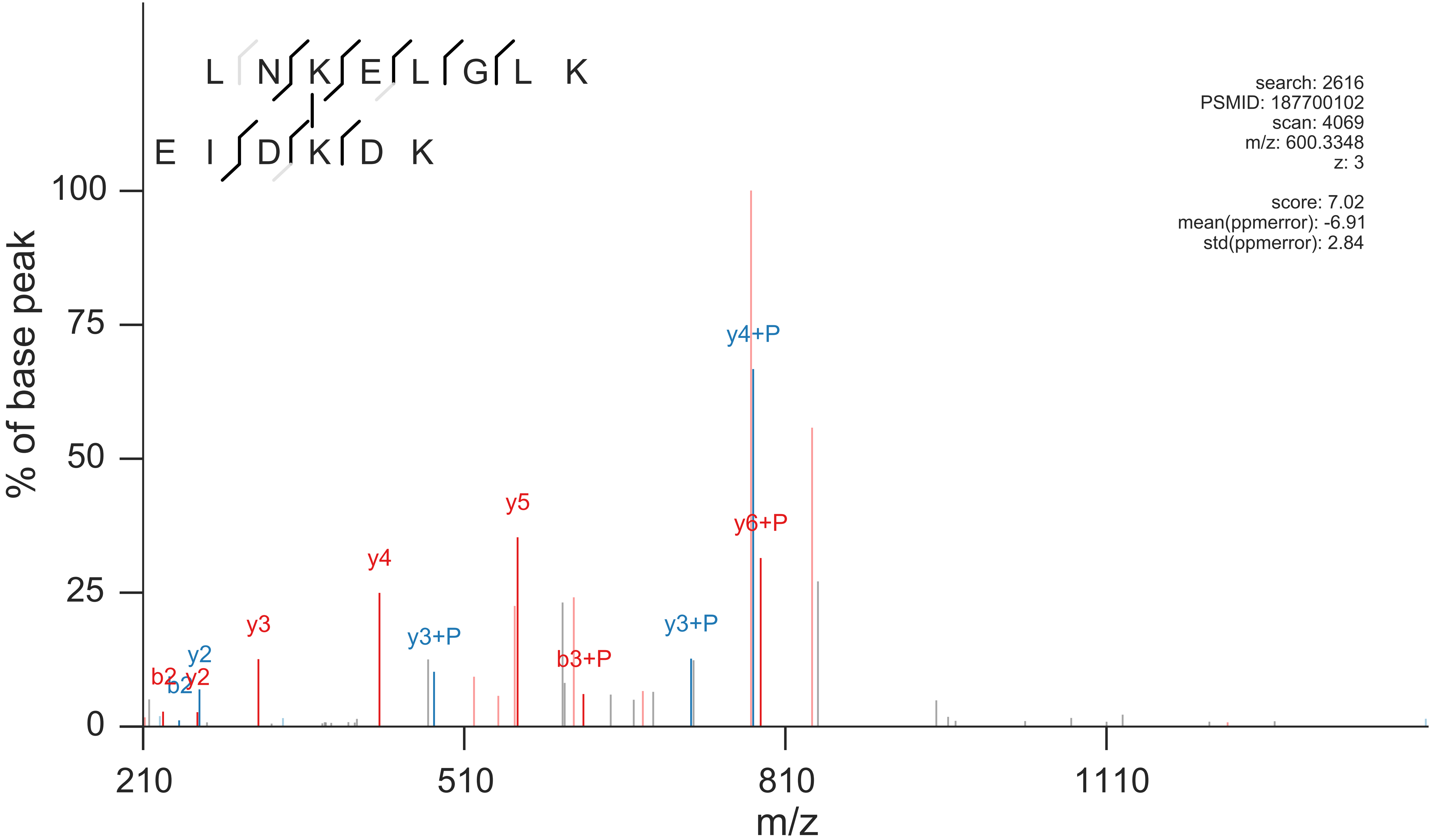

L N K E L G L K
E I D K D K
search: 2616
PSMID: 187700102
scan: 4069
m/z: 600.3348
z: 3
score: 7.02
mean(ppmerror): -6.91
std(ppmerror): 2.84
% of base peak
100
75
50
25
0
y4+P
y5
y6+P
y4
y3
y3+P
y3+P
b3+P
y2
b2
b2
y2
210
510
810
1110
m/z

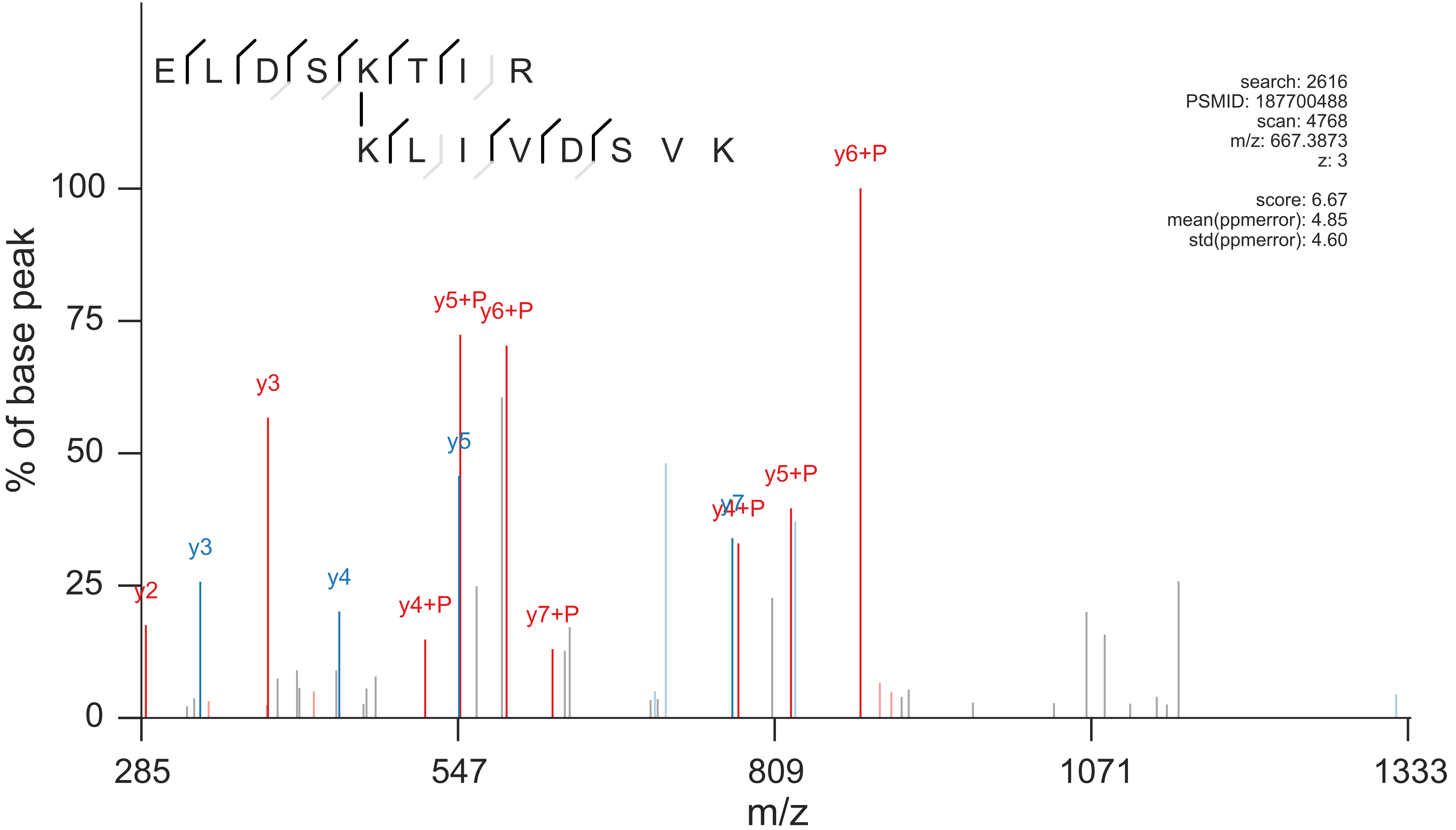

E L D S K T I R
K L I V D S V K
search: 2616
PSMID: 187700488
scan: 4768
m/z: 667.3873
z: 3
score: 6.67
mean(ppmerror): 4.85
std(ppmerror): 4.60
y6+P
y5+P
y6+P
y3
y5
y5+P
y7
y4+P
y3
y4
y4+P
y7+P
y2
% of base peak
100
75
50
25
0
285
547
809
1071
1333
m/z

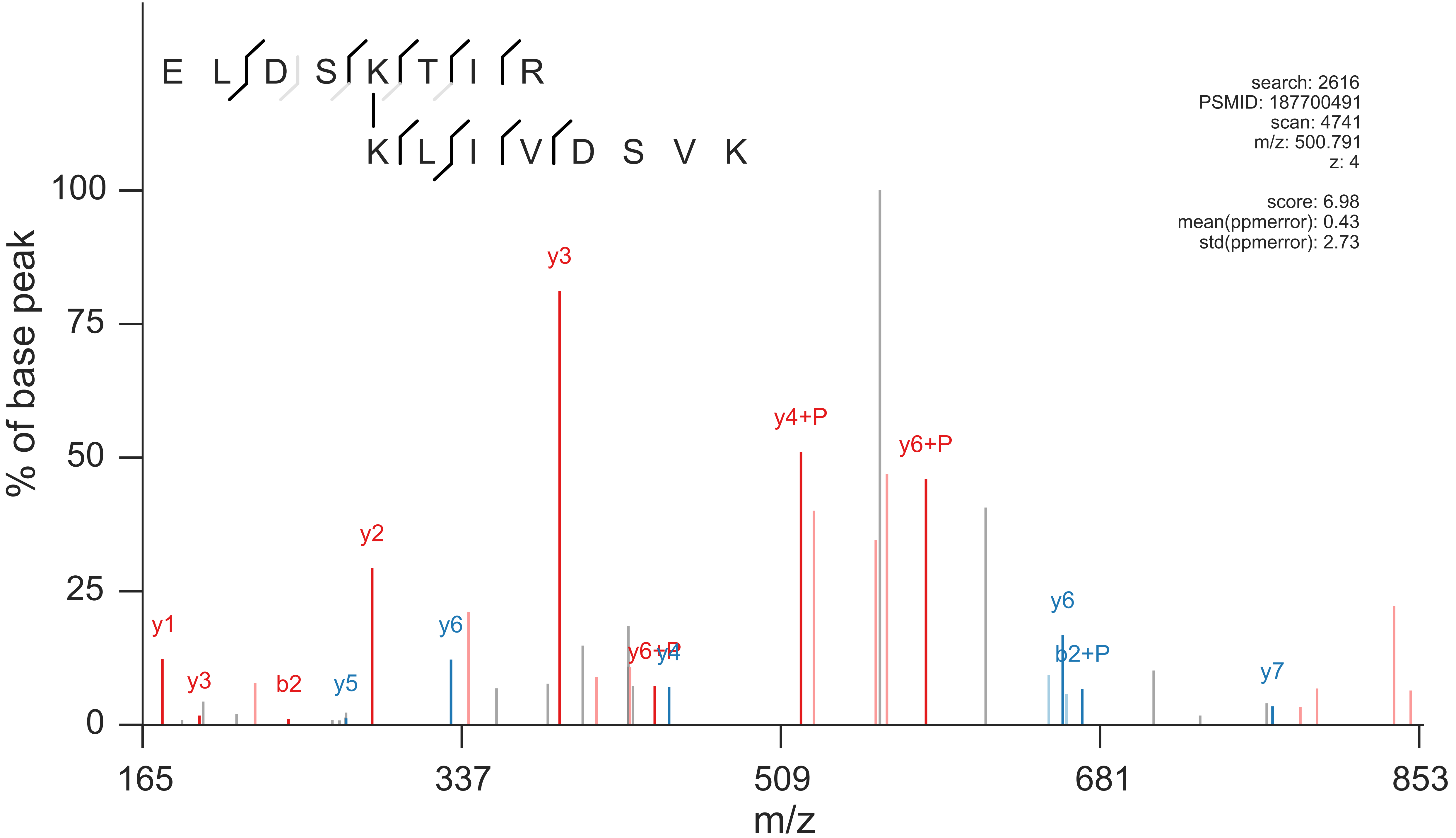

E L D S K T I R
K L I V D S V K
search: 2616
PSMID: 187700491
scan: 4741
m/z: 500.791
z: 4
score: 6.98
mean(ppmerror): 0.43
std(ppmerror): 2.73
% of base peak
100
75
50
25
0
y3
y4+P
y6+P
y2
y1
y3
b2
y5
y6
y6+P
y4
y6
b2+P
y7
165
337
509
681
853
m/z

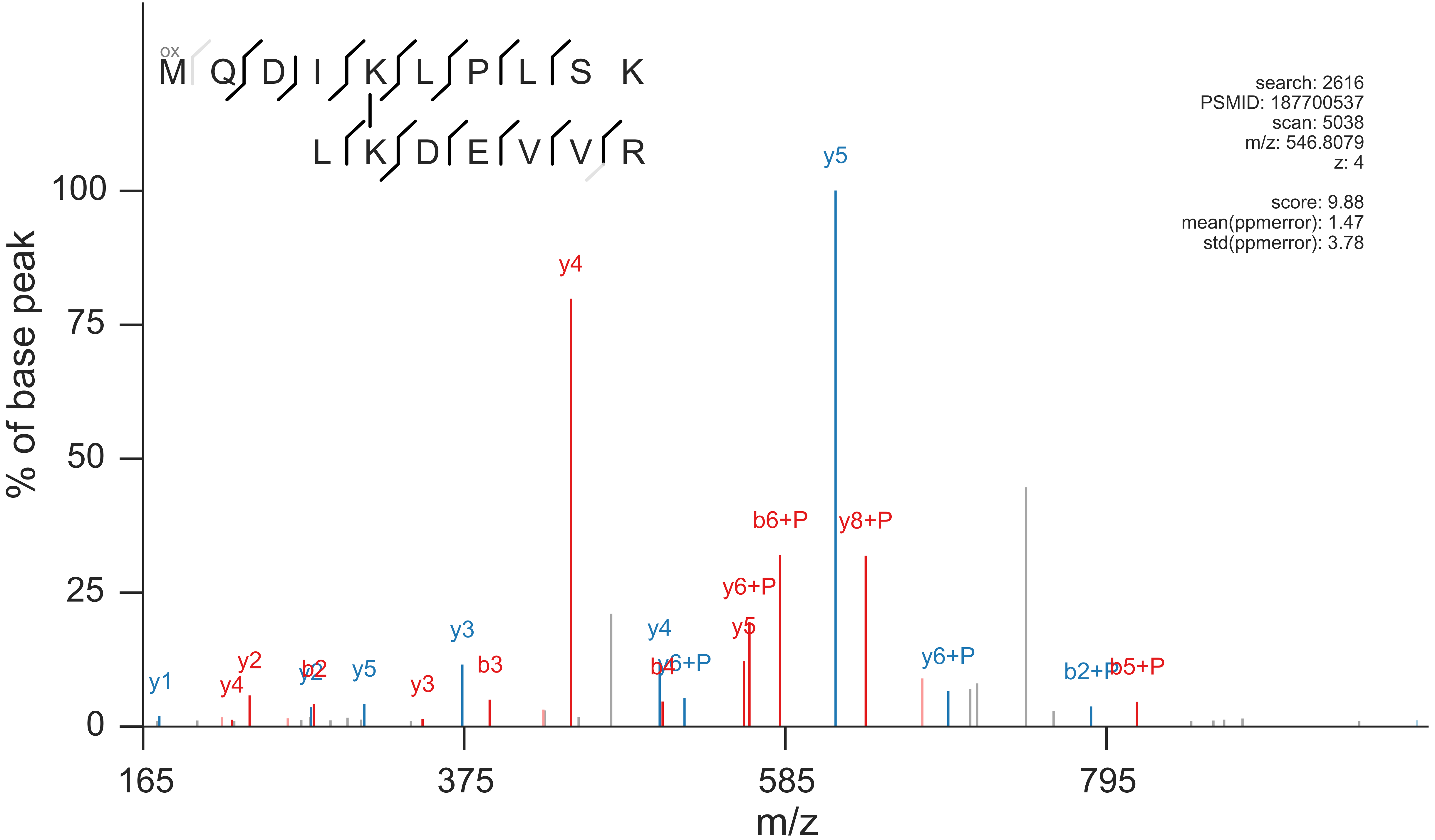
M Q D I K L P L S K
L K D E V V R
search: 2616
PSMID: 187700537
scan: 5038
m/z: 546.8079
z: 4
score: 9.88
mean(ppmerror): 1.47
std(ppmerror): 3.78
% of base peak
m/z
y1
y4
y2
y2
b2
y5
y3
y3
b3
y4
y4
b4
y6+P
y5
y6+P
b6+P
y5
y8+P
y6+P
b2+P
b5+P

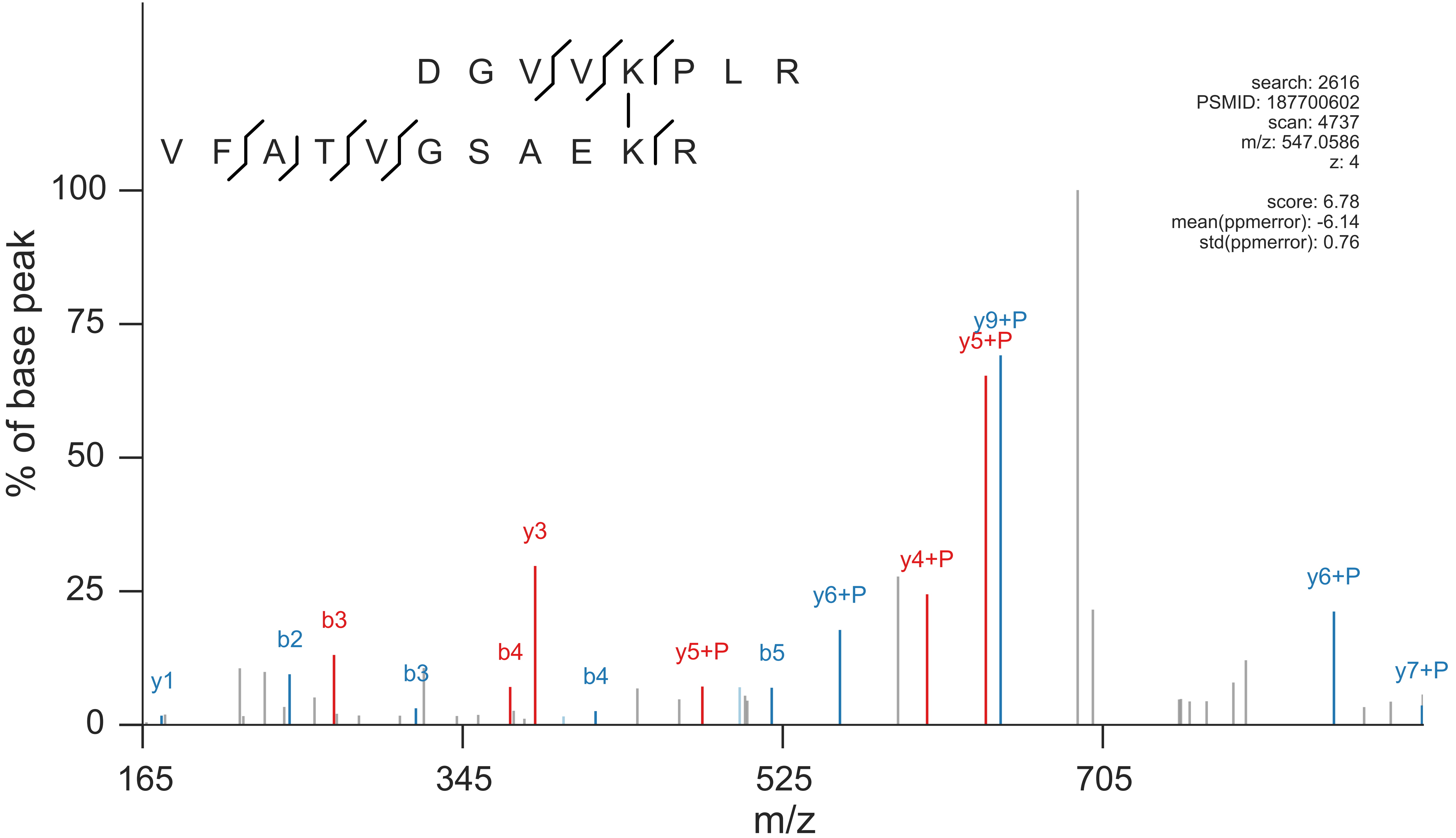

D G V V K P L R
V F A T V G S A E K R
search: 2616
PSMID: 187700602
scan: 4737
m/z: 547.0586
z: 4
score: 6.78
mean(ppmerror): -6.14
std(ppmerror): 0.76
% of base peak
100
75
50
25
0
y9+P
y5+P
y3
y4+P
y6+P
y6+P
y7+P
b2
b3
y1
b3
b4
b4
y5+P
b5
165
345
525
705
m/z

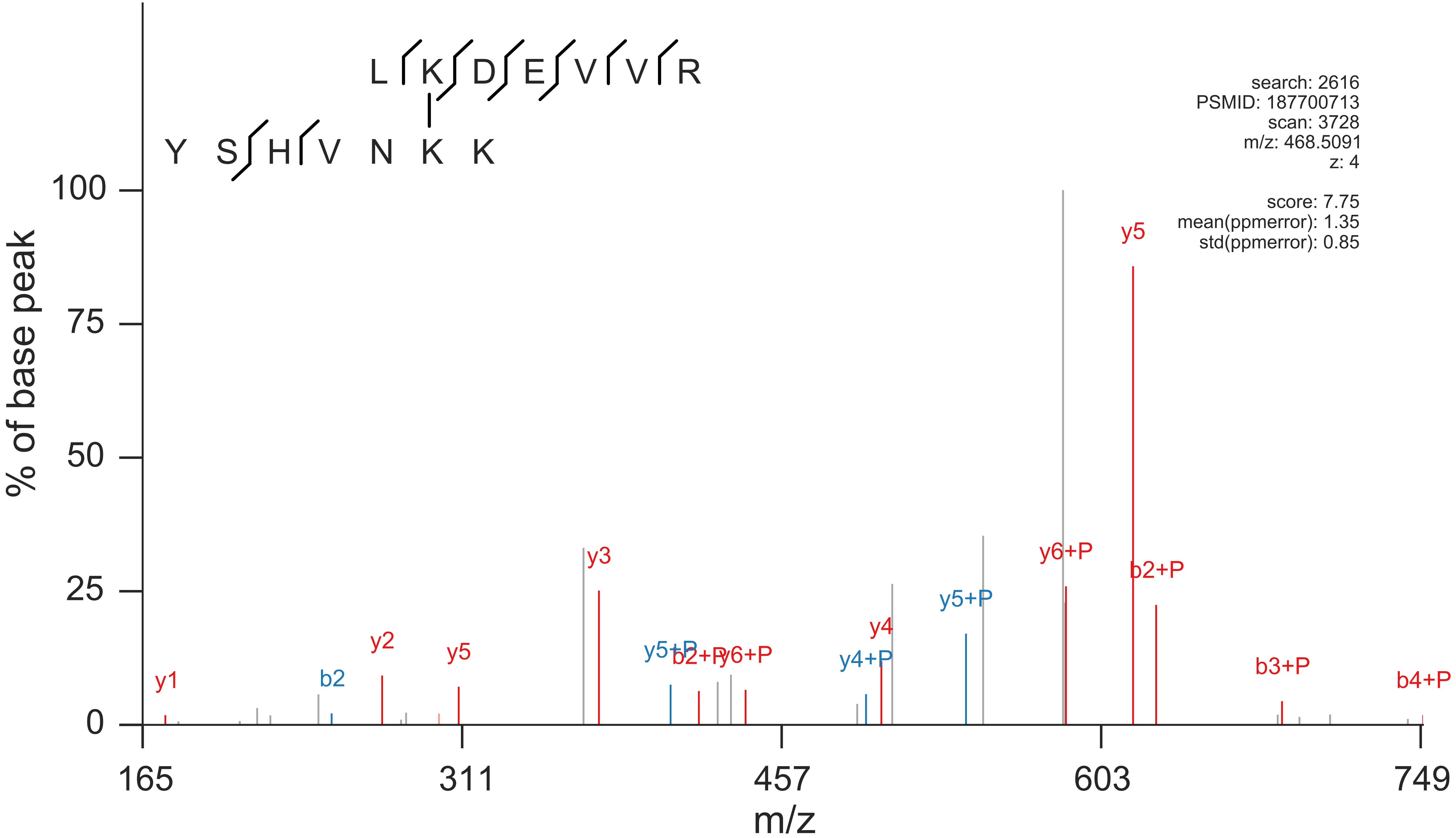

L K D E V V R
Y S H V N K K
search: 2616
PSMID: 187700713
scan: 3728
m/z: 468.5091
z: 4
score: 7.75
mean(ppmerror): 1.35
std(ppmerror): 0.85
% of base peak
100
75
50
25
0
y1
b2
y2
y5
y3
y5+P
b2+P
y6+P
y4+P
y4
y5+P
y6+P
y5
b2+P
b3+P
b4+P
165
311
457
603
749
m/z

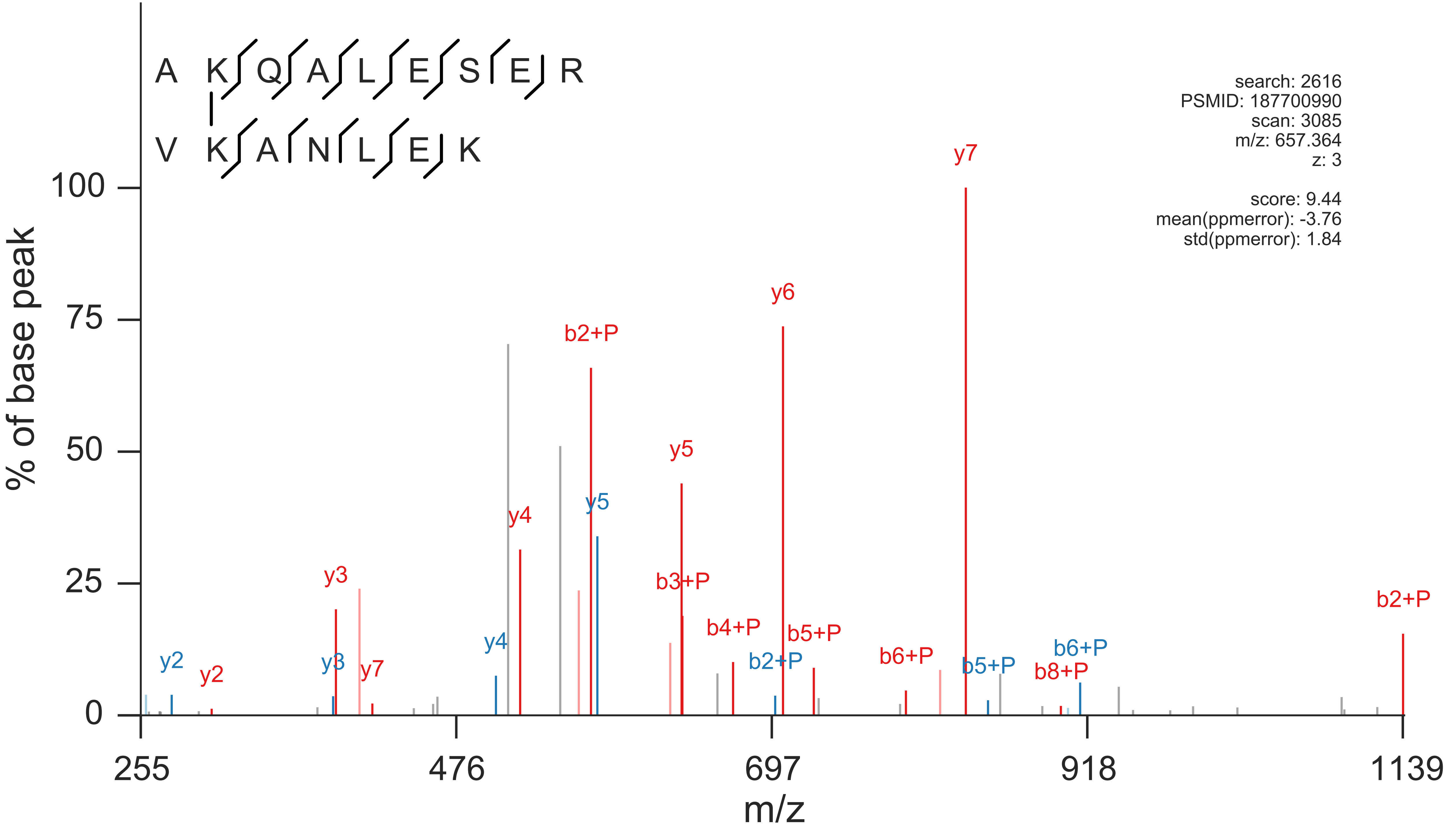

A K Q A L E S E R
V K A N L E K
search: 2616
PSMID: 187700990
scan: 3085
m/z: 657.364
z: 3
score: 9.44
mean(ppmerror): -3.76
std(ppmerror): 1.84
% of base peak
m/z
0
25
50
75
100
255
476
697
918
1139
y2
y2
y3
y3
y7
y4
y4
b2+P
y5
y5
b3+P
b4+P
b2+P
y6
b5+P
b6+P
y7
b5+P
b8+P
b6+P
b2+P

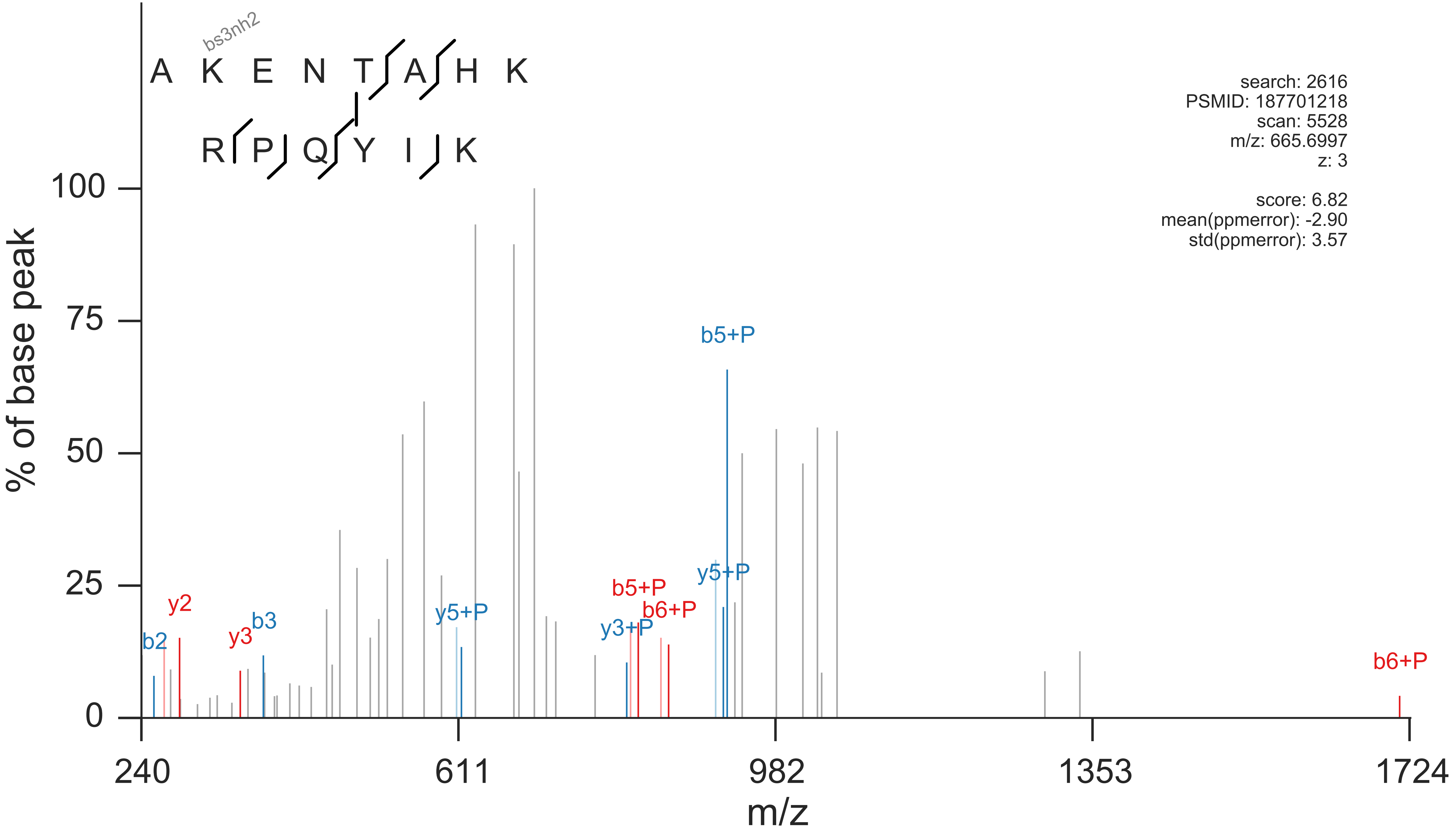
bs3nh2
A K E N T A H K
R P Q Y I K
search: 2616
PSMID: 187701218
scan: 5528
m/z: 665.6997
z: 3
score: 6.82
mean(ppmerror): -2.90
std(ppmerror): 3.57
% of base peak
100
75
50
25
0
b5+P
y5+P
b5+P
b6+P
y5+P
y3+P
y2
b3
y3
b2
b6+P
240
611
982
1353
1724
m/z

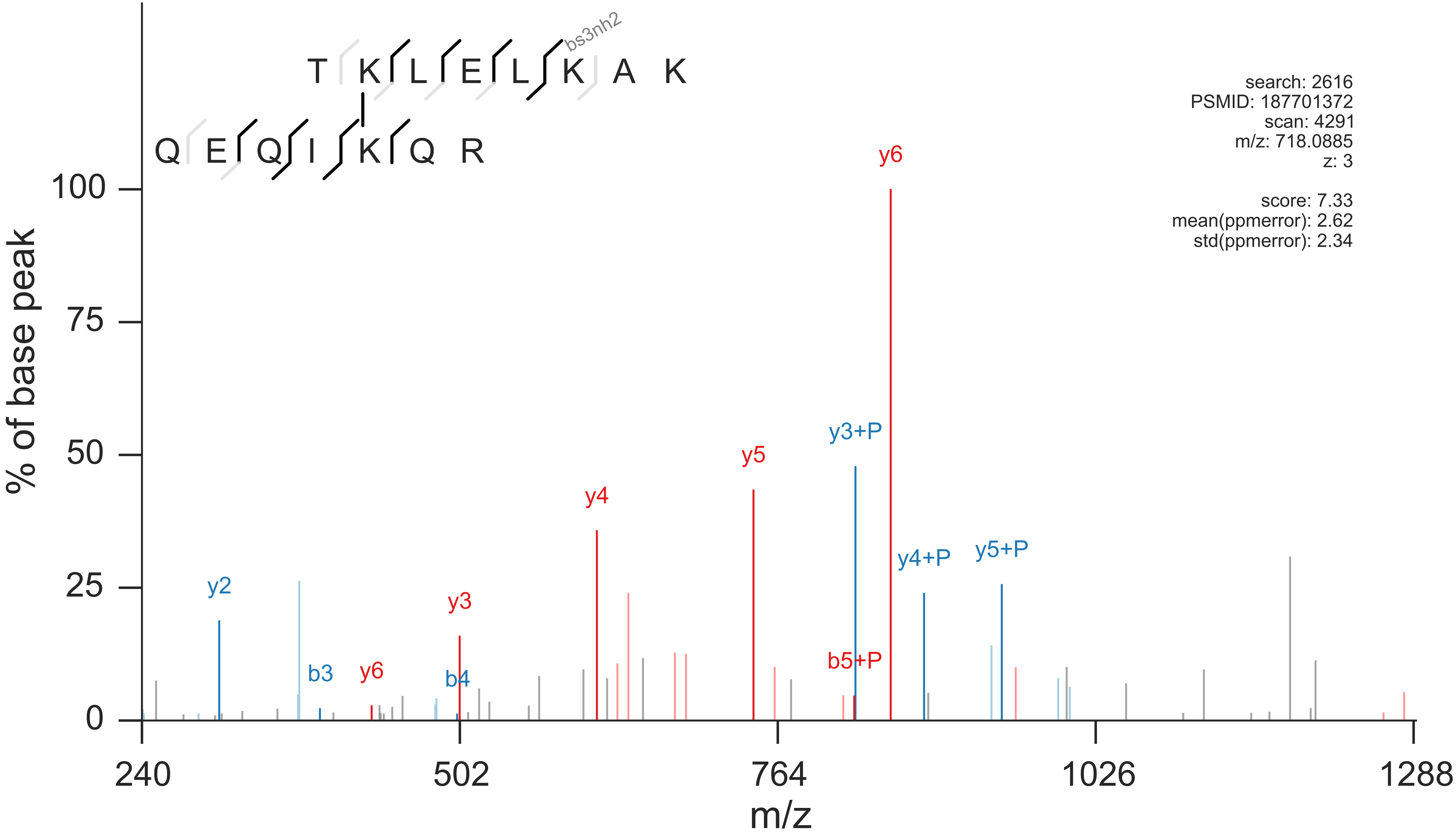

T K L E L K A K
bs3nh2
Q E Q I K Q R
search: 2616
PSMID: 187701372
scan: 4291
m/z: 718.0885
z: 3
score: 7.33
mean(ppmerror): 2.62
std(ppmerror): 2.34
% of base peak
m/z
y6
y3+P
y5
y4
y4+P
y5+P
y2
y3
b3
y6
b4
b5+P
0
25
50
75
100
240
502
764
1026
1288

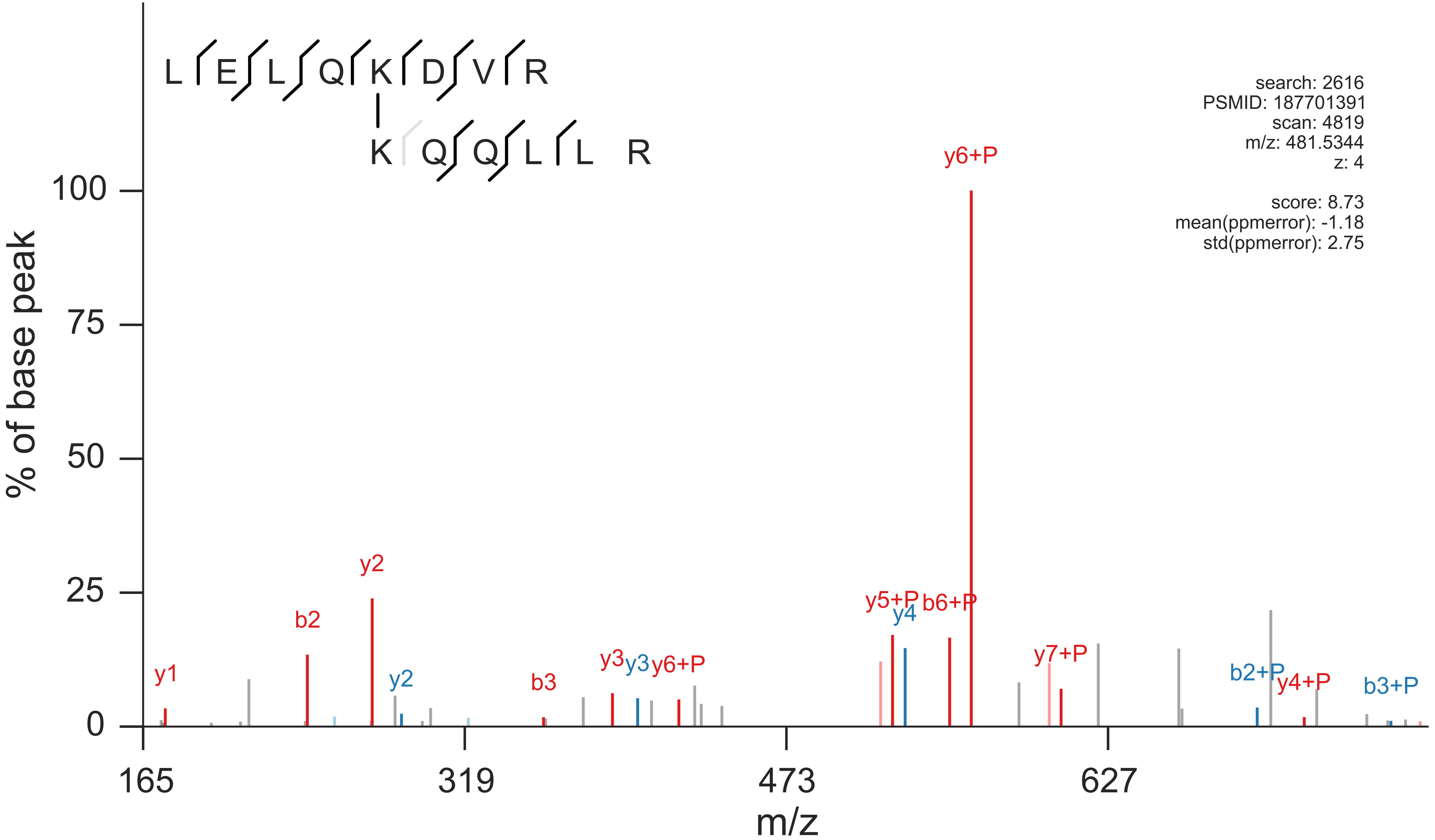

L E L Q K D V R
K Q Q L L R
search: 2616
PSMID: 187701391
scan: 4819
m/z: 481.5344
z: 4
score: 8.73
mean(ppmerror): -1.18
std(ppmerror): 2.75
y6+P
y1
b2
y2
y2
b3
y3
y3
y6+P
y5+P
y4
b6+P
y7+P
b2+P
y4+P
b3+P
% of base peak
100
75
50
25
0
165
319
473
627
m/z

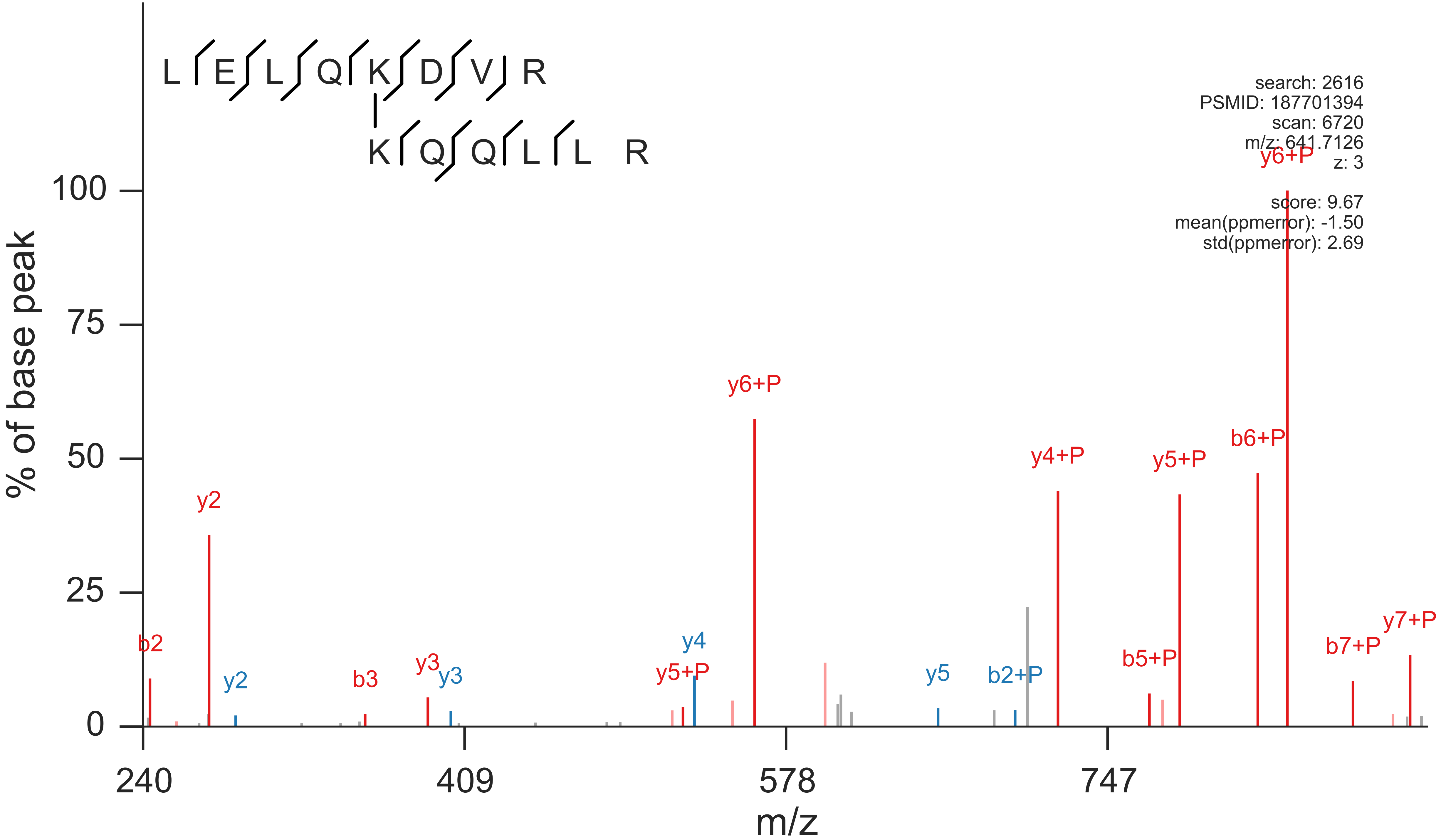

L E L Q K D V R
K Q Q L L R
search: 2616
PSMID: 187701394
scan: 6720
m/z: 641.7126
z: 3
score: 9.67
mean(ppmerror): -1.50
std(ppmerror): 2.69
% of base peak
m/z
100
75
50
25
0
240
409
578
747
b2
y2
y2
b3
y3
y3
y5+P
y4
y6+P
y5
b2+P
y4+P
b5+P
y5+P
b6+P
y6+P
b7+P
y7+P

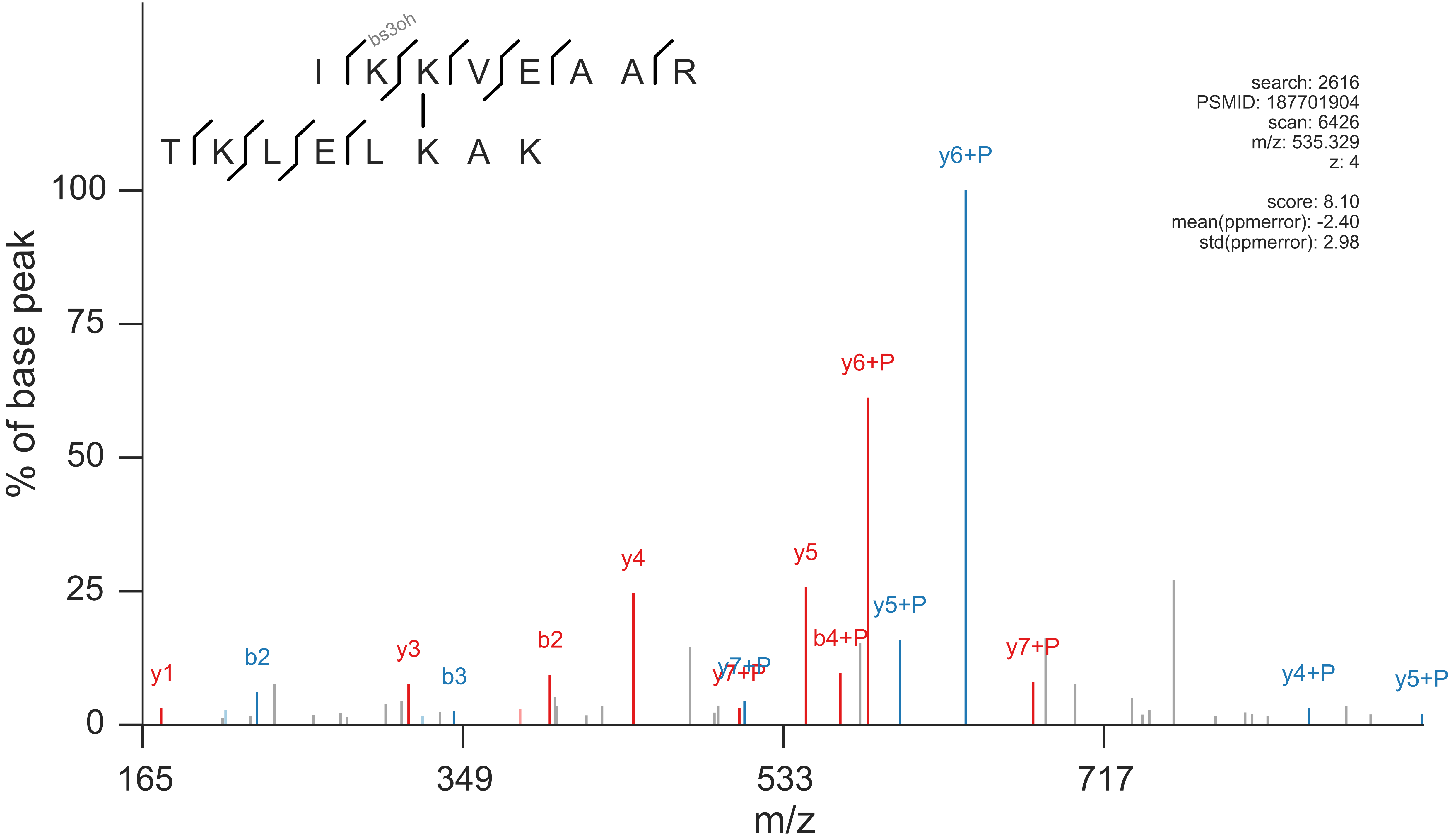

bs3oh
I K K V E A A R
T K L E L K A K
search: 2616
PSMID: 187701904
scan: 6426
m/z: 535.329
z: 4
score: 8.10
mean(ppmerror): -2.40
std(ppmerror): 2.98
% of base peak
m/z
0
25
50
75
100
165
349
533
717
y1
b2
y3
b3
b2
y4
y7+P
y7+P
y5
b4+P
y6+P
y5+P
y6+P
y7+P
y4+P
y5+P

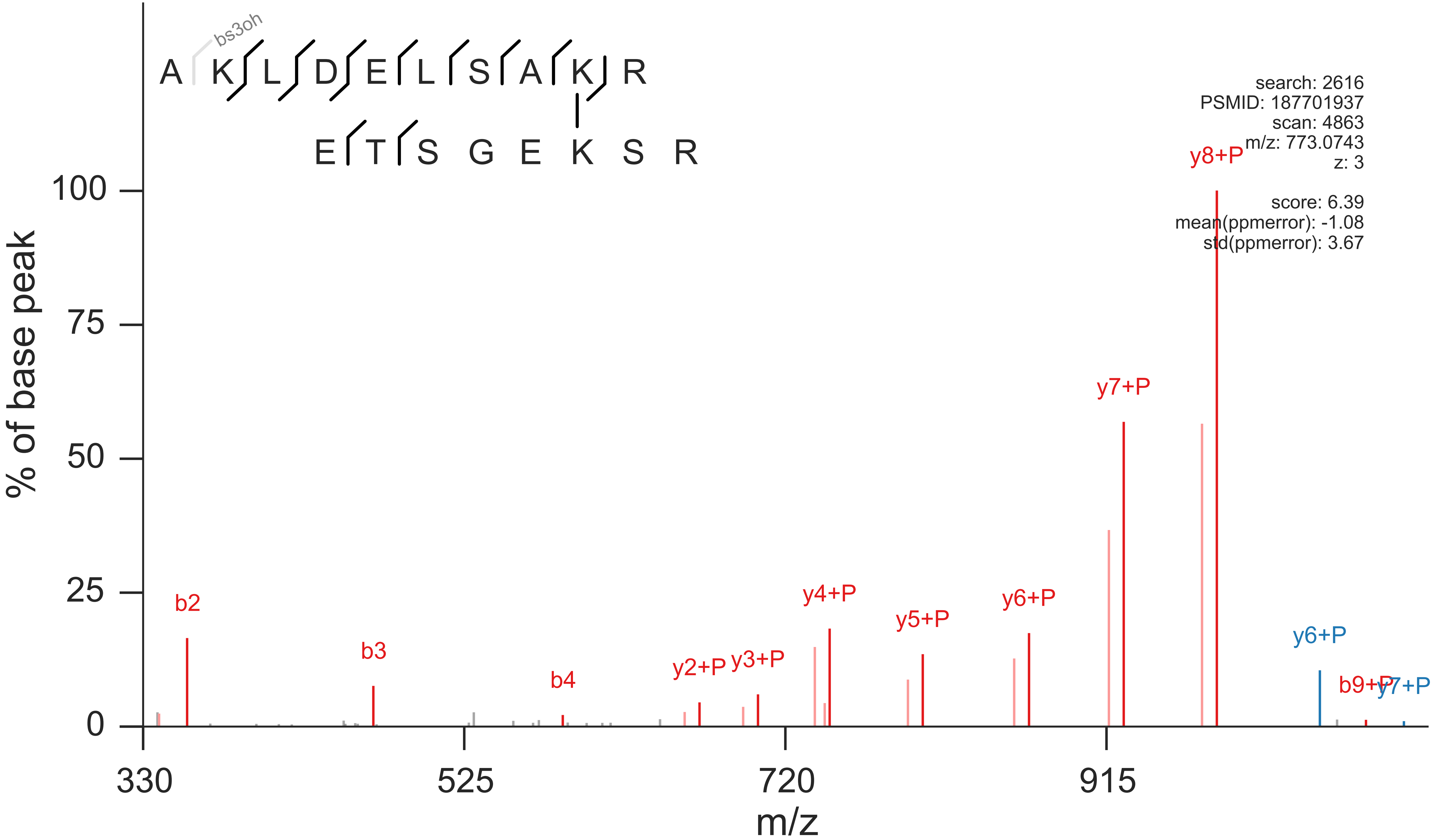

bs3oh
A K L D E L S A K R
E T S G E K S R
search: 2616
PSMID: 187701937
scan: 4863
m/z: 773.0743
z: 3
score: 6.39
mean(ppmerror): -1.08
std(ppmerror): 3.67
% of base peak
100
75
50
25
0
b2
b3
b4
y2+P
y3+P
y4+P
y5+P
y6+P
y7+P
y8+P
y6+P
b9+P
y7+P
330
525
720
915
m/z

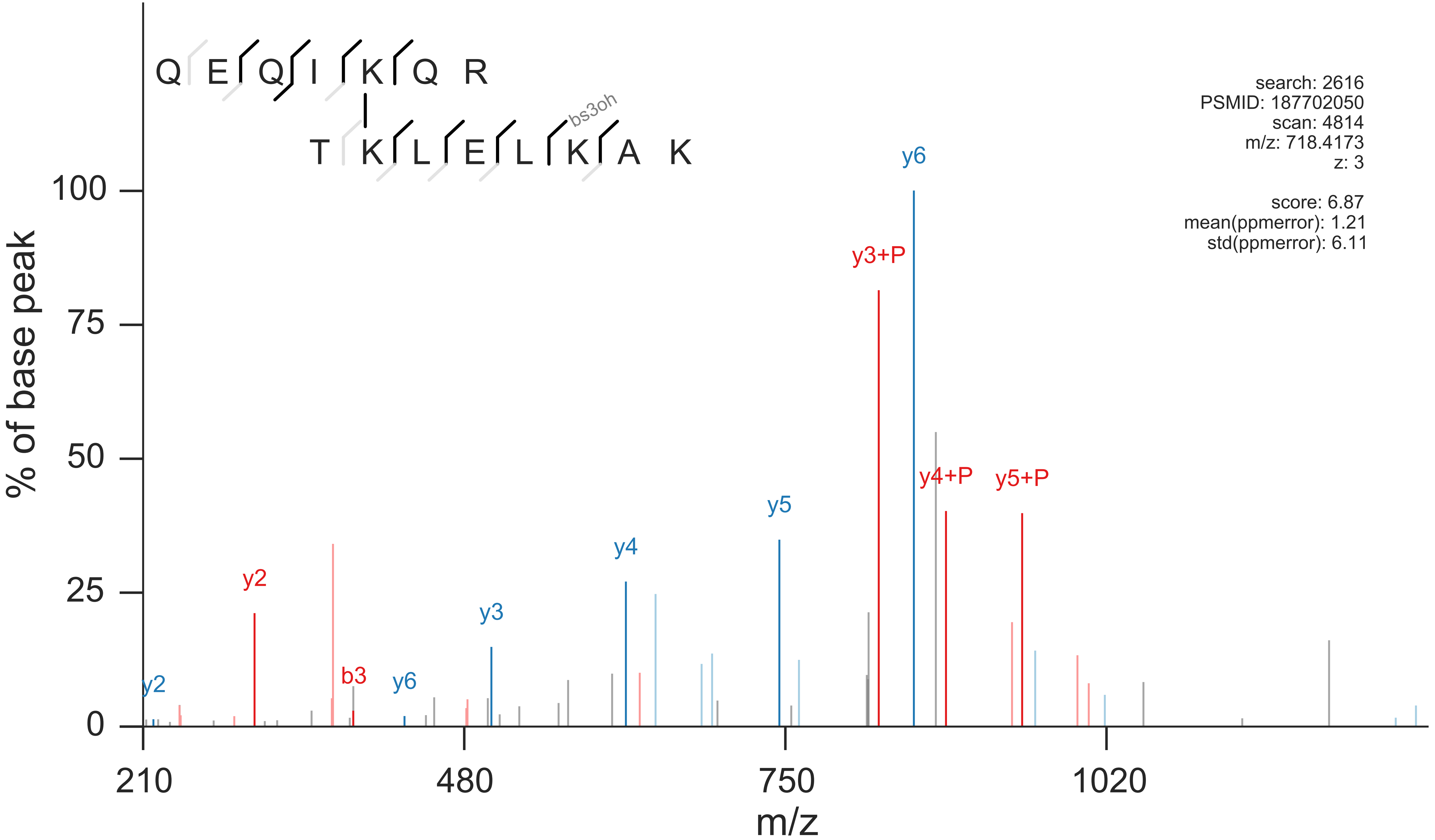

Q E Q I K Q R
T K L E L K A K
bs3oh
search: 2616
PSMID: 187702050
scan: 4814
m/z: 718.4173
z: 3
score: 6.87
mean(ppmerror): 1.21
std(ppmerror): 6.11
% of base peak
m/z
0
25
50
75
100
210
480
750
1020
y2
y2
b3
y6
y3
y4
y5
y3+P
y6
y4+P
y5+P

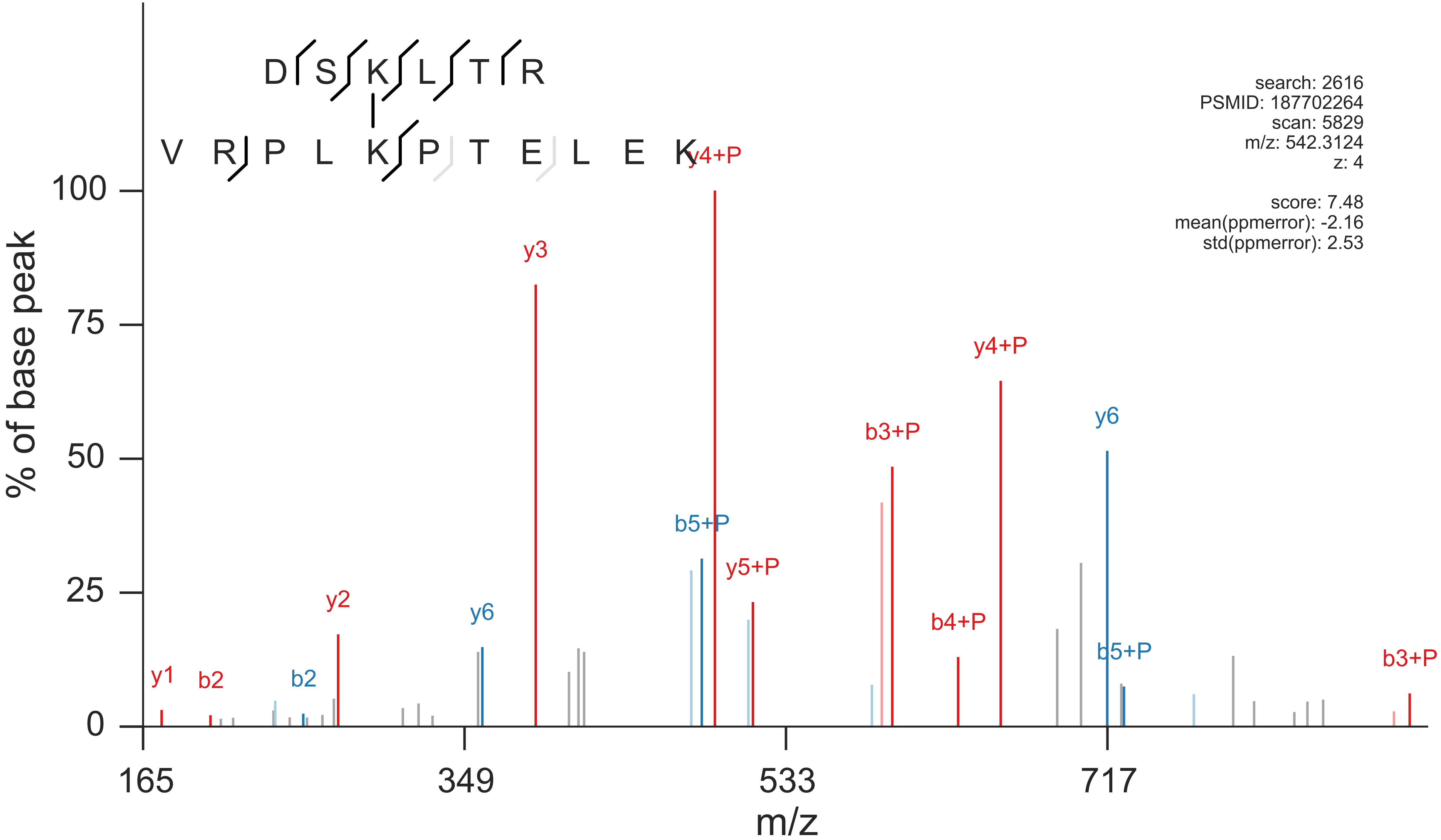

D S K L T R
V R P L K P T E L E K
search: 2616
PSMID: 187702264
scan: 5829
m/z: 542.3124
z: 4
score: 7.48
mean(ppmerror): -2.16
std(ppmerror): 2.53
% of base peak
m/z
0
25
50
75
100
165
349
533
717
y1
b2
b2
y2
y6
y3
b5+P
y4+P
y5+P
b3+P
b4+P
y4+P
y6
b5+P
b3+P

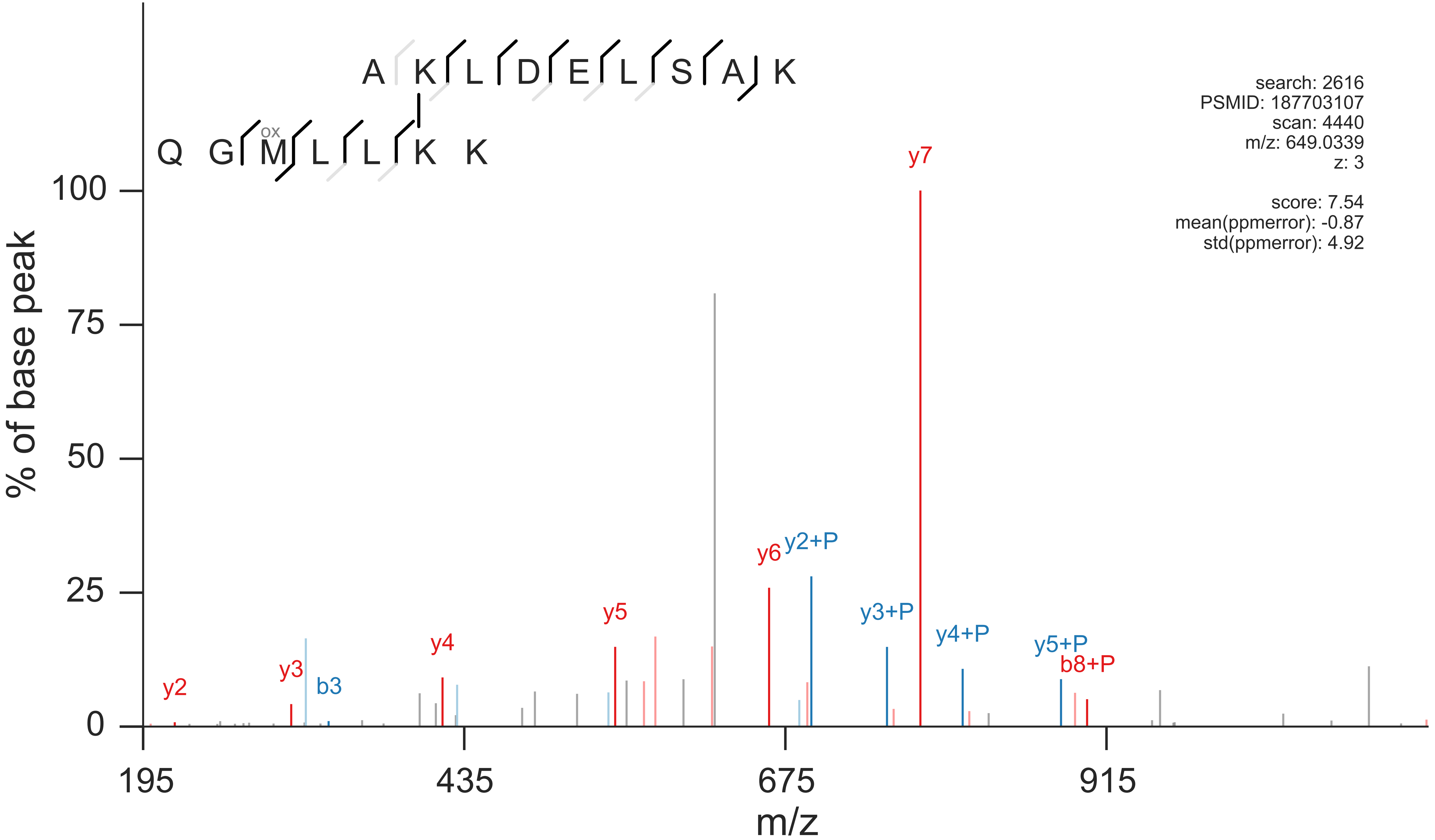

A K L D E L S A K
Q G M L L K K
ox
search: 2616
PSMID: 187703107
scan: 4440
m/z: 649.0339
z: 3
score: 7.54
mean(ppmerror): -0.87
std(ppmerror): 4.92
% of base peak
100
75
50
25
0
y7
y6
y2+P
y5
y3+P
y4+P
y5+P
b8+P
y4
y3
b3
y2
195
435
675
915
m/z

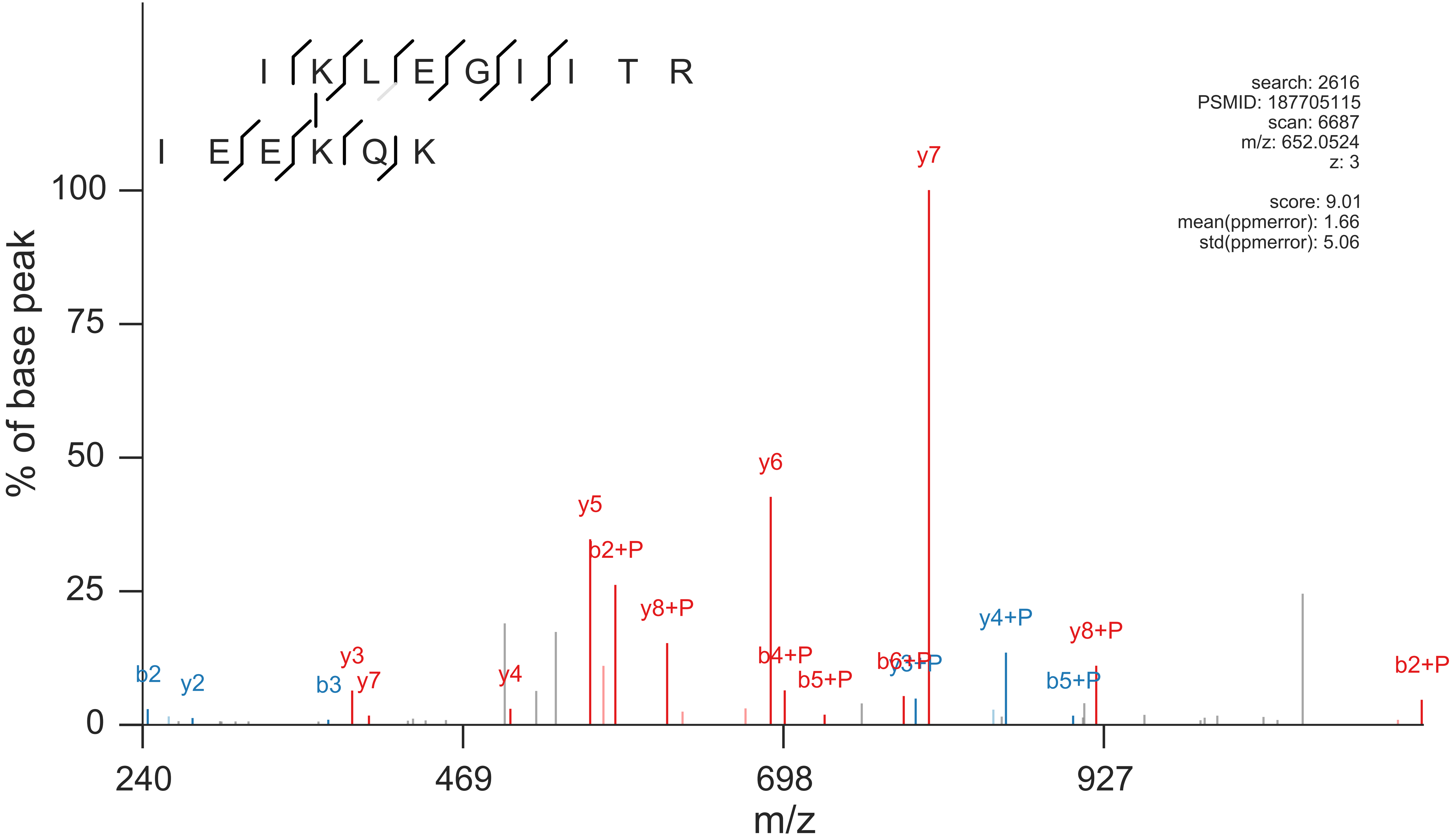
I K L E G I I T R
I E E K Q K
search: 2616
PSMID: 187705115
scan: 6687
m/z: 652.0524
z: 3
score: 9.01
mean(ppmerror): 1.66
std(ppmerror): 5.06
% of base peak
m/z
0
25
50
75
100
240
469
698
927
y7
y6
y5
b2+P
y8+P
y3
y7
y4
b2
y2
b3
b4+P
b5+P
b6+P
y3+P
y4+P
y8+P
b5+P
b2+P

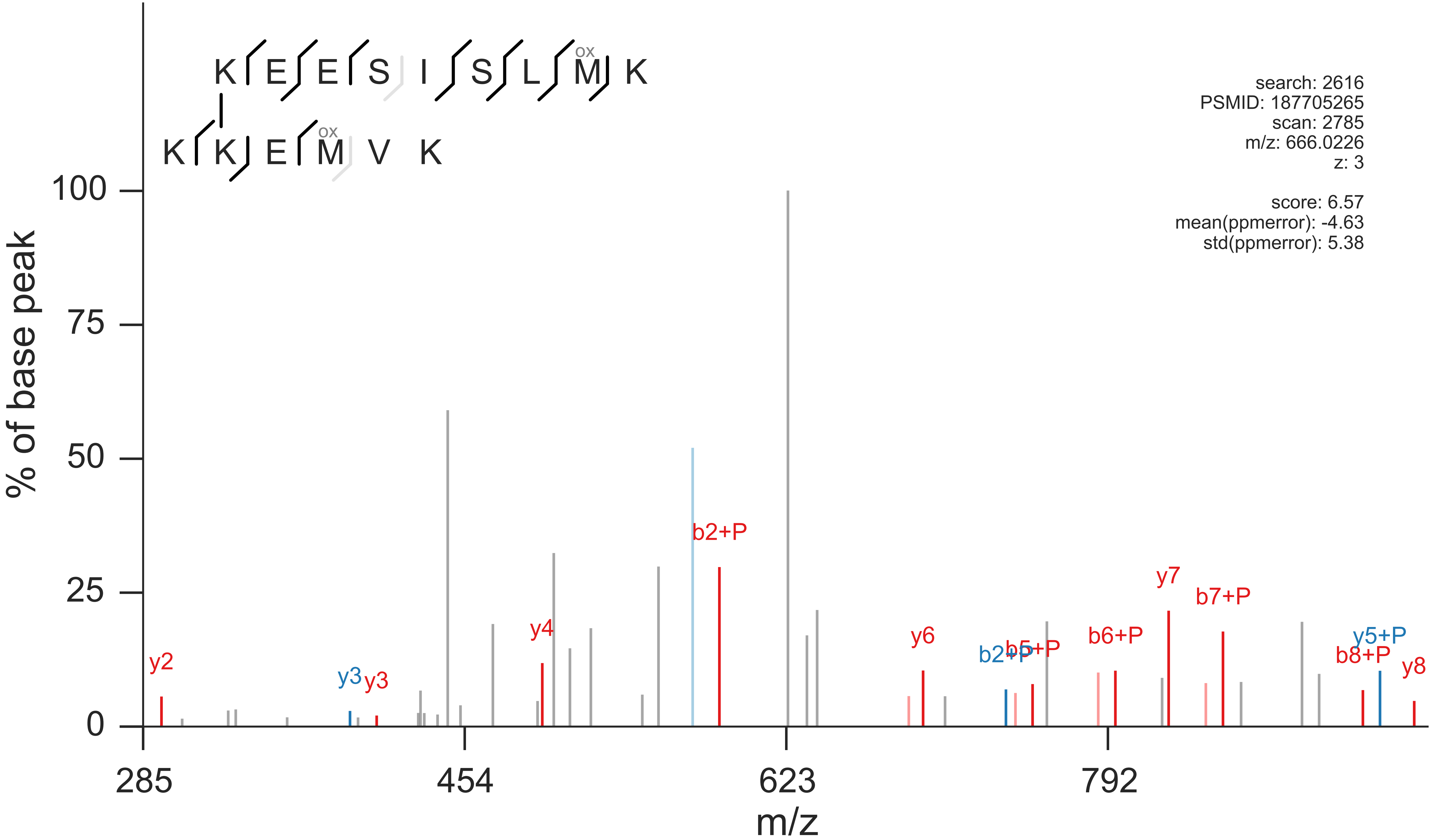
K E E S I S L M K
ox
K K E M V K
ox
search: 2616
PSMID: 187705265
scan: 2785
m/z: 666.0226
z: 3
score: 6.57
mean(ppmerror): -4.63
std(ppmerror): 5.38
% of base peak
100
75
50
25
0
285
454
623
792
m/z
y2
y3
y3
y4
b2+P
y6
b2+P
b5+P
b6+P
y7
b7+P
y5+P
b8+P
y8

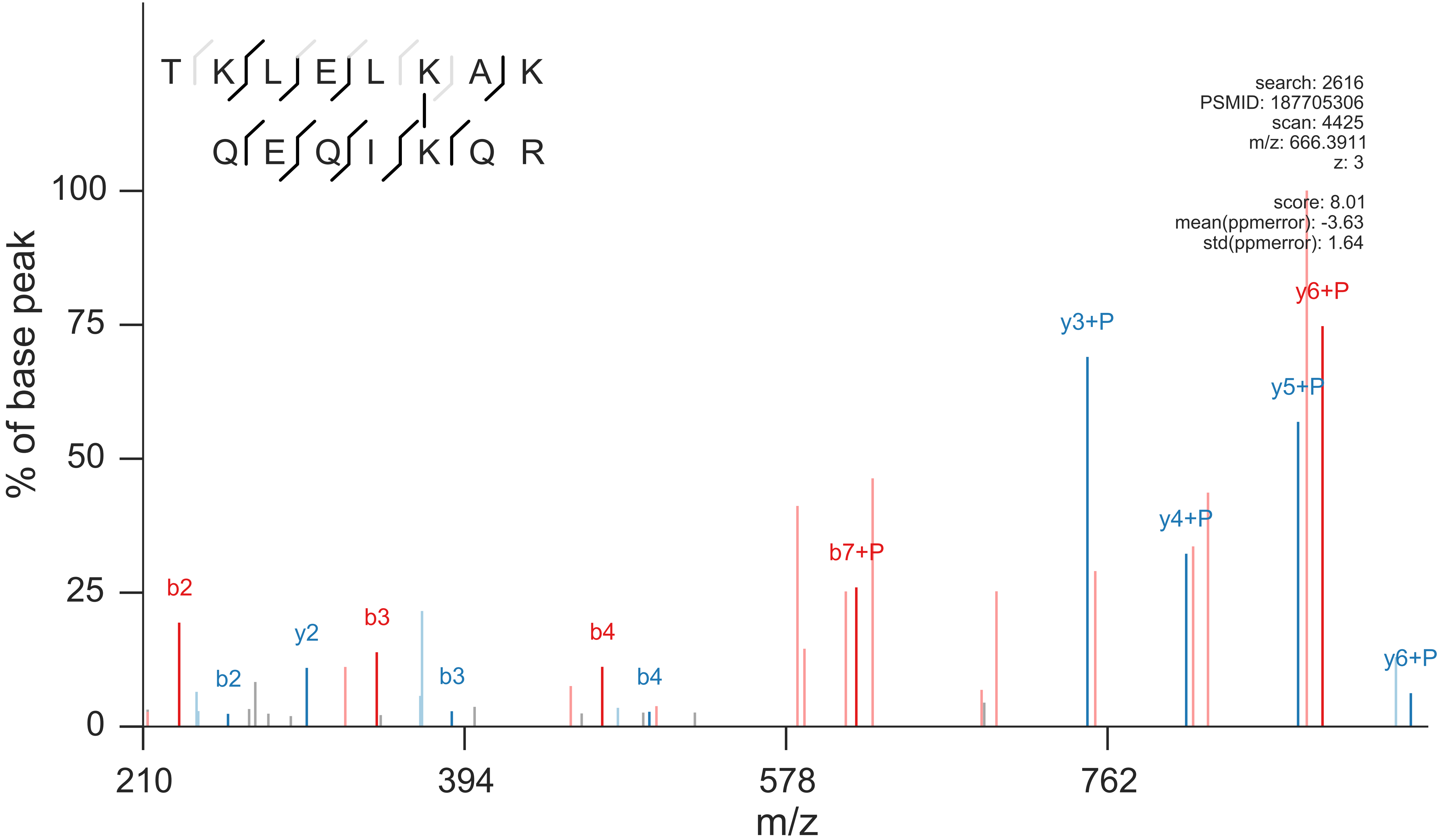

T K L E L K A K
Q E Q I K Q R
search: 2616
PSMID: 187705306
scan: 4425
m/z: 666.3911
z: 3
score: 8.01
mean(ppmerror): -3.63
std(ppmerror): 1.64
% of base peak
100
75
50
25
0
210
394
578
762
m/z
b2
b2
y2
b3
b3
b4
b4
b7+P
y3+P
y4+P
y5+P
y6+P
y6+P

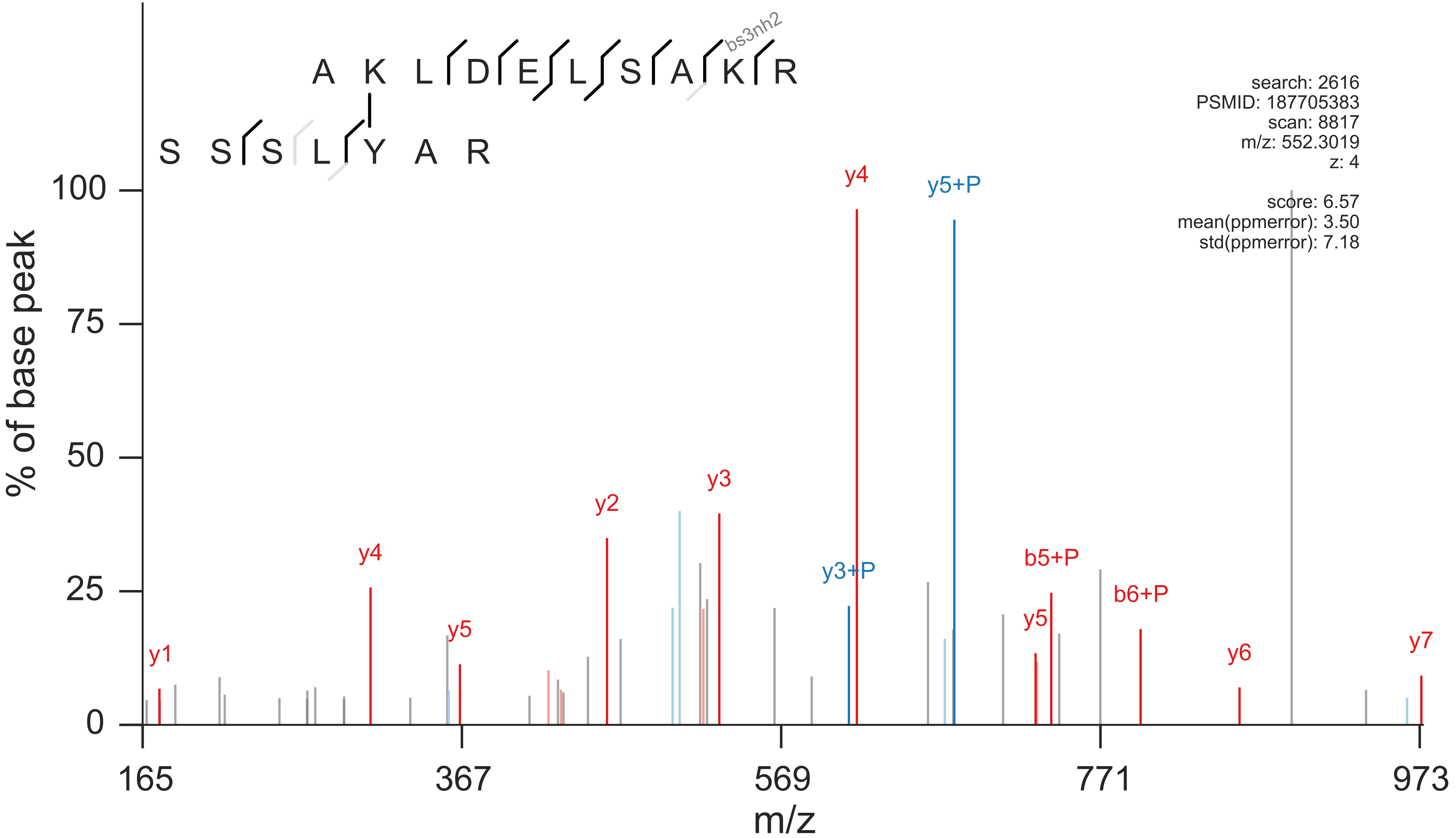

A K L D E L S A K R
bs3nh2
S S S L Y A R
search: 2616
PSMID: 187705383
scan: 8817
m/z: 552.3019
z: 4
score: 6.57
mean(ppmerror): 3.50
std(ppmerror): 7.18
% of base peak
100
75
50
25
0
y1
y4
y5
y2
y3
y4
y3+P
y5+P
b5+P
y5
b6+P
y6
y7
165
367
569
771
973
m/z

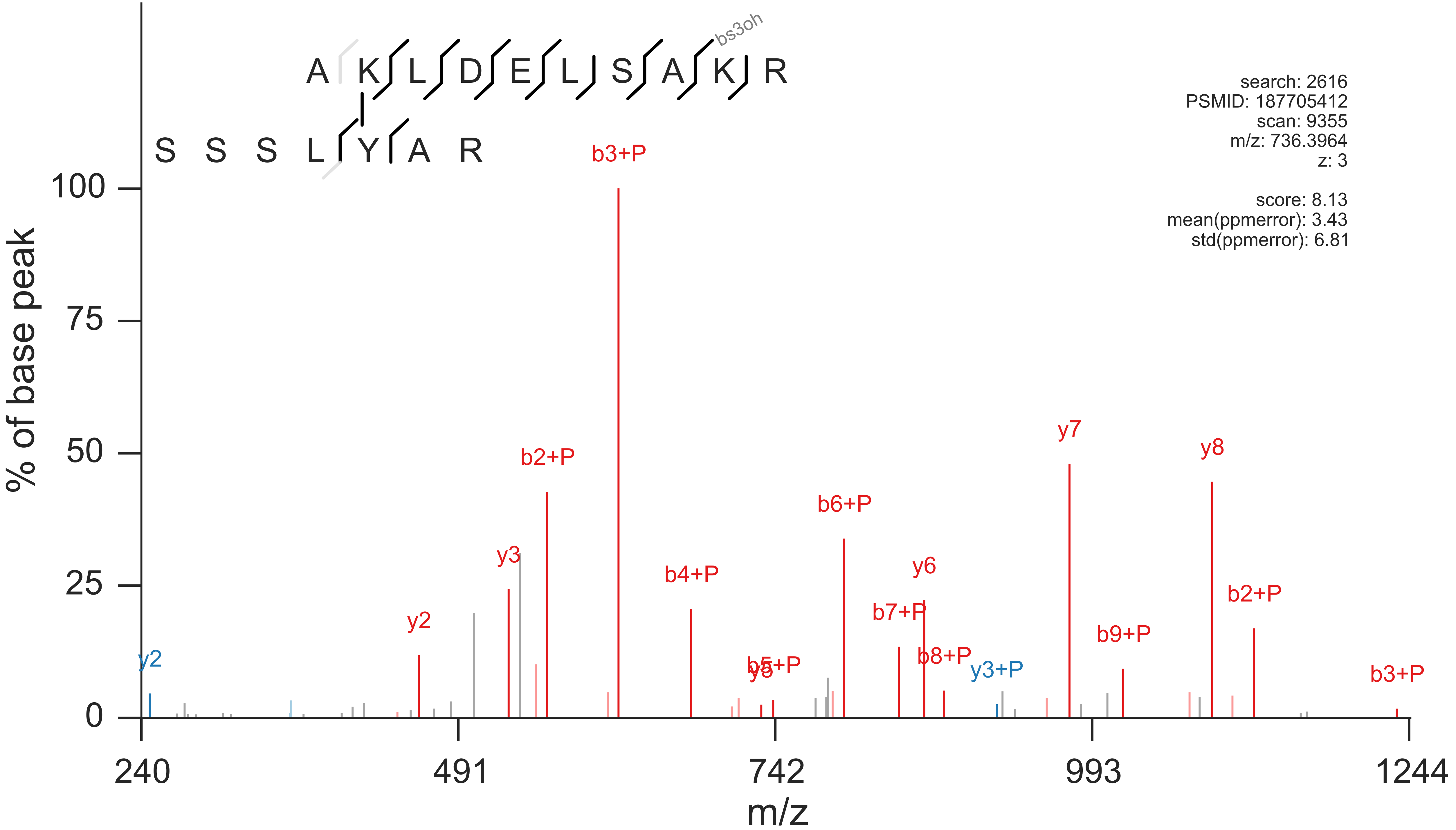

A K L D E L S A K R
bs3oh
S S S L Y A R
search: 2616
PSMID: 187705412
scan: 9355
m/z: 736.3964
z: 3
score: 8.13
mean(ppmerror): 3.43
std(ppmerror): 6.81
% of base peak
m/z
100
75
50
25
0
240
491
742
993
1244
y2
y2
y3
b2+P
b3+P
b4+P
b5+P
y5
b6+P
b7+P
y6
b8+P
y3+P
y7
b9+P
y8
b2+P
b3+P

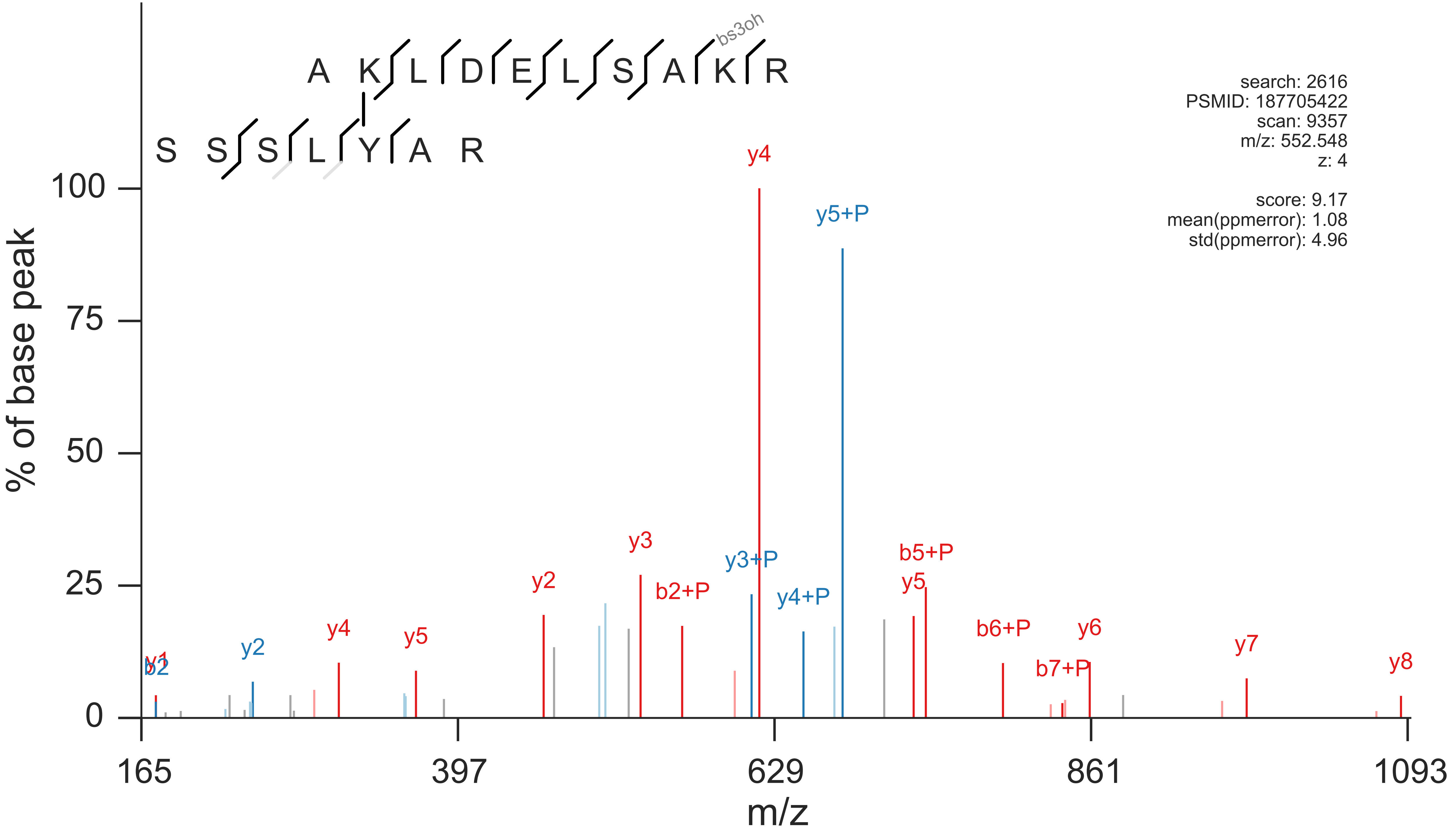

A K L D E L S A K R
bs3oh
S S S L Y A R
search: 2616
PSMID: 187705422
scan: 9357
m/z: 552.548
z: 4
score: 9.17
mean(ppmerror): 1.08
std(ppmerror): 4.96
% of base peak
100
75
50
25
0
y4
y5+P
y3
y3+P
b5+P
y2
b2+P
y5
y4+P
b6+P
y6
y4
y5
y2
y1
b2
b7+P
y7
y8
165
397
629
861
1093
m/z

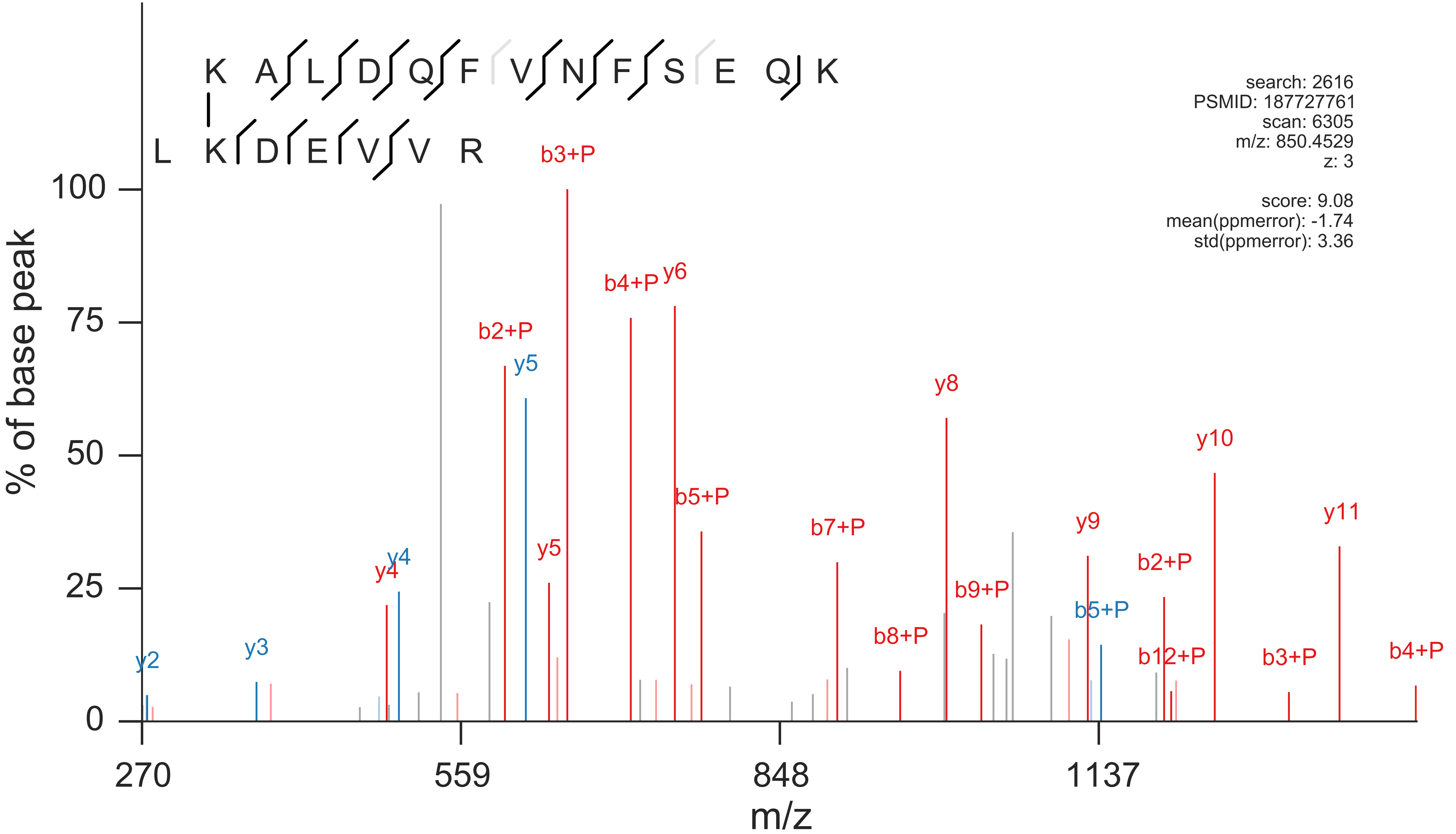
K A L D Q F V N F S E Q K
L K D E V V R
search: 2616
PSMID: 187727761
scan: 6305
m/z: 850.4529
z: 3
score: 9.08
mean(ppmerror): -1.74
std(ppmerror): 3.36
% of base peak
m/z
0
25
50
75
100
270
559
848
1137
y2
y3
y4
y4
b2+P
y5
y5
b3+P
b4+P
y6
b5+P
b7+P
b8+P
y8
b9+P
y9
b5+P
b2+P
b12+P
y10
b3+P
y11
b4+P

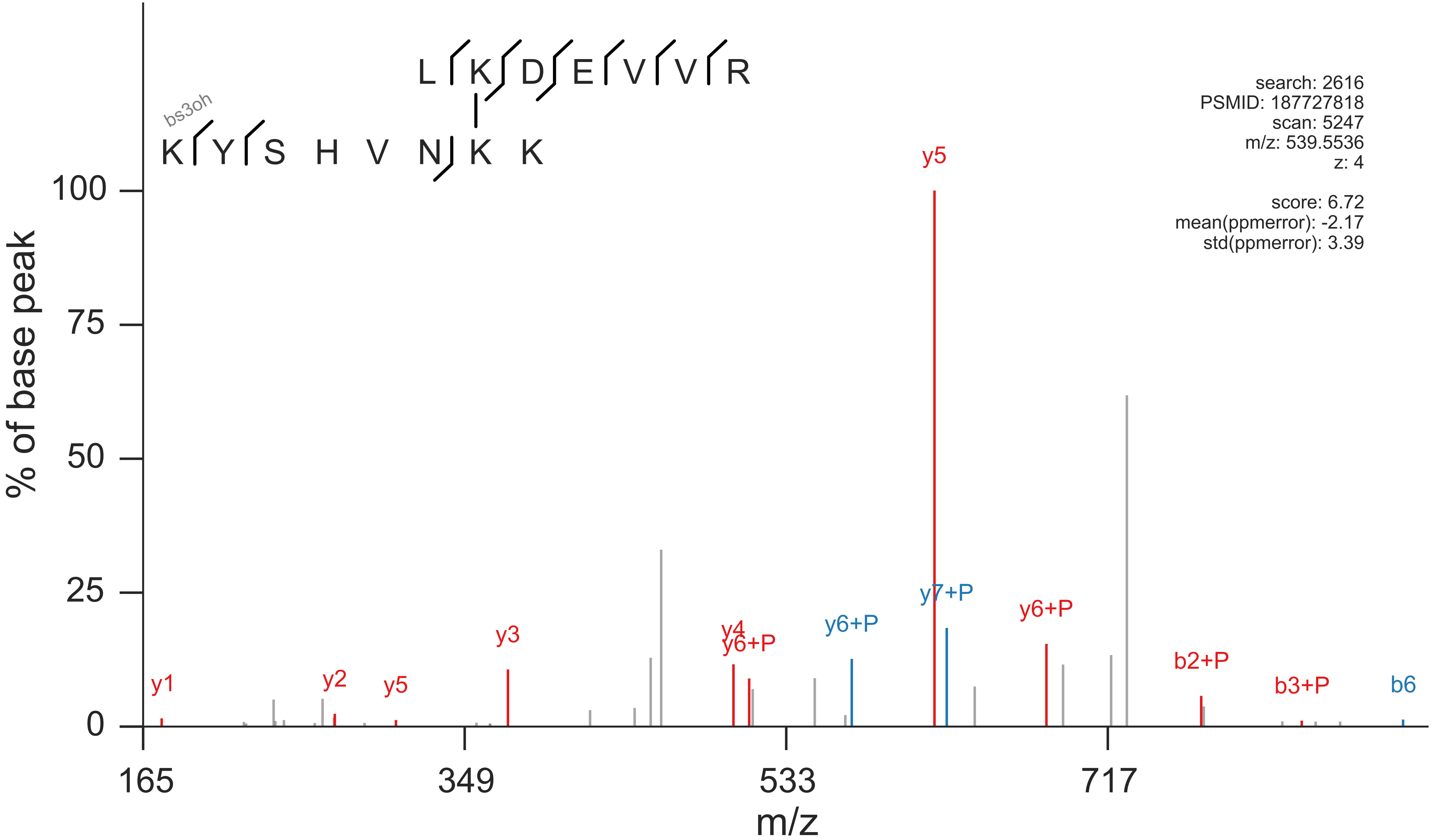

L K D E V V R
bs3oh
K Y S H V N K K
search: 2616
PSMID: 187727818
scan: 5247
m/z: 539.5536
z: 4
score: 6.72
mean(ppmerror): -2.17
std(ppmerror): 3.39
% of base peak
100
75
50
25
0
y1
y2
y5
y3
y4
y6+P
y6+P
y7+P
y5
y6+P
b2+P
b3+P
b6
165
349
533
717
m/z

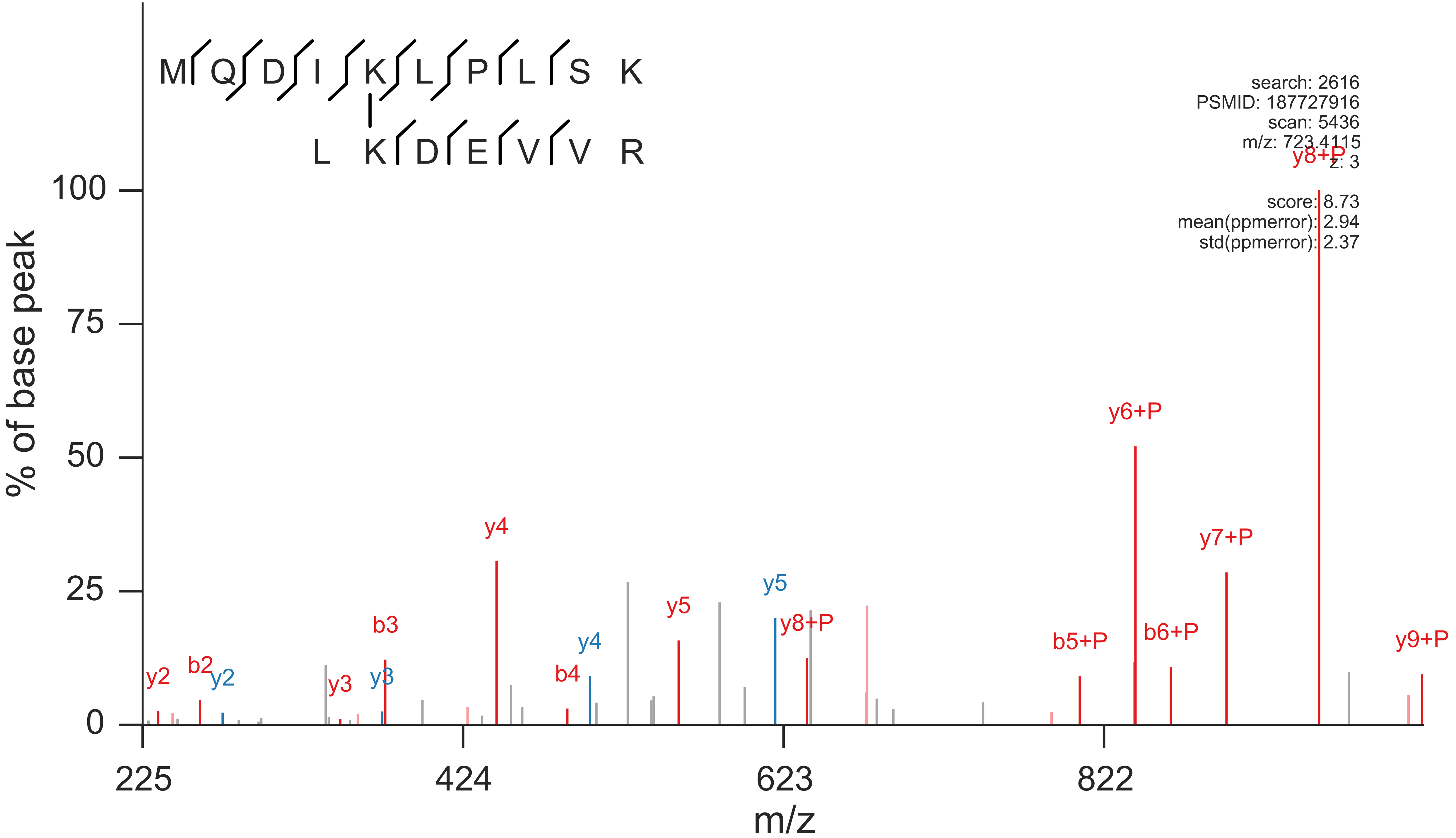
M Q D I K L P L S K
L K D E V V R
search: 2616
PSMID: 187727916
scan: 5436
m/z: 723.4115
z: 3
score: 8.73
mean(ppmerror): 2.94
std(ppmerror): 2.37
% of base peak
m/z
100
75
50
25
0
225
424
623
822
y2
b2
y2
y3
y3
b3
y4
b4
y4
y5
y5
y8+P
b5+P
y6+P
b6+P
y7+P
y8+P
y9+P

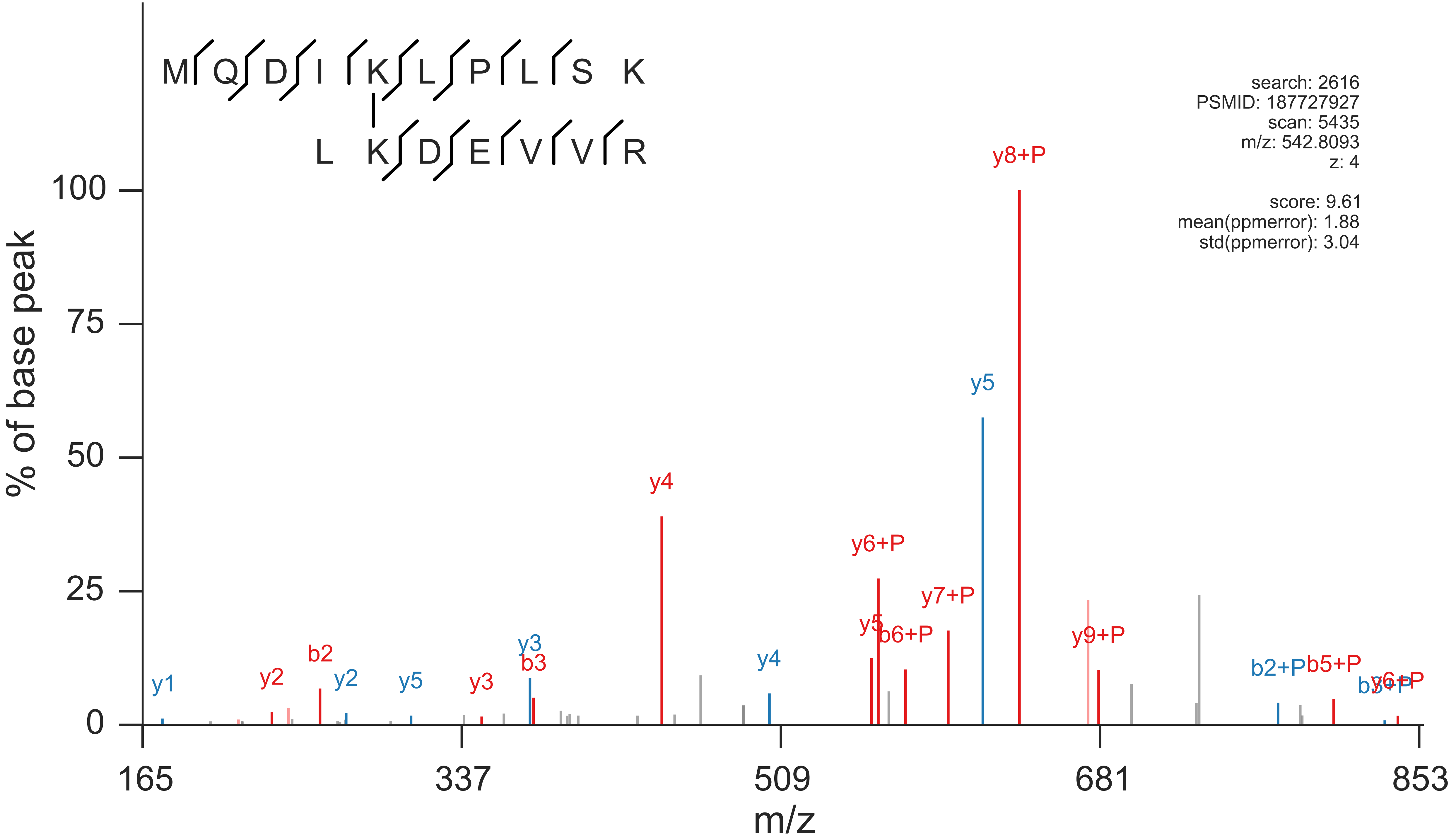

M Q D I K L P L S K
L K D E V V R
search: 2616
PSMID: 187727927
scan: 5435
m/z: 542.8093
z: 4
score: 9.61
mean(ppmerror): 1.88
std(ppmerror): 3.04
% of base peak
m/z
100
75
50
25
0
165
337
509
681
853
y8+P
y5
y4
y6+P
y7+P
y5
b6+P
y9+P
y3
b3
y4
b2
y1
y2
y2
y5
y3
b2+P
b5+P
y6+P

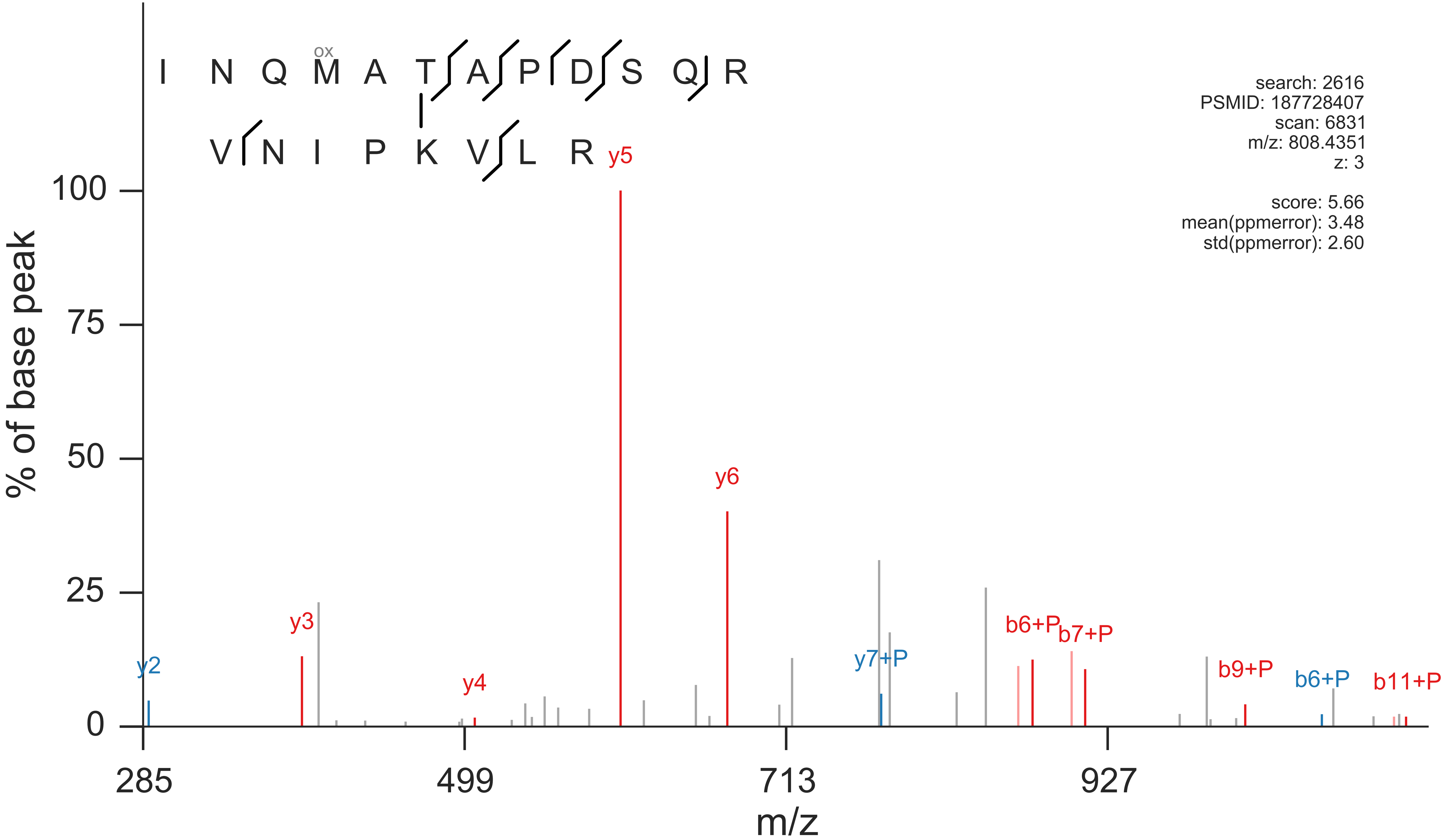
I N Q M A T A P D S Q R
ox
V N I P K V L R
search: 2616
PSMID: 187728407
scan: 6831
m/z: 808.4351
z: 3
score: 5.66
mean(ppmerror): 3.48
std(ppmerror): 2.60
% of base peak
100
75
50
25
0
y5
y6
y3
y2
y4
y7+P
b6+P
b7+P
b9+P
b6+P
b11+P
285
499
713
927
m/z

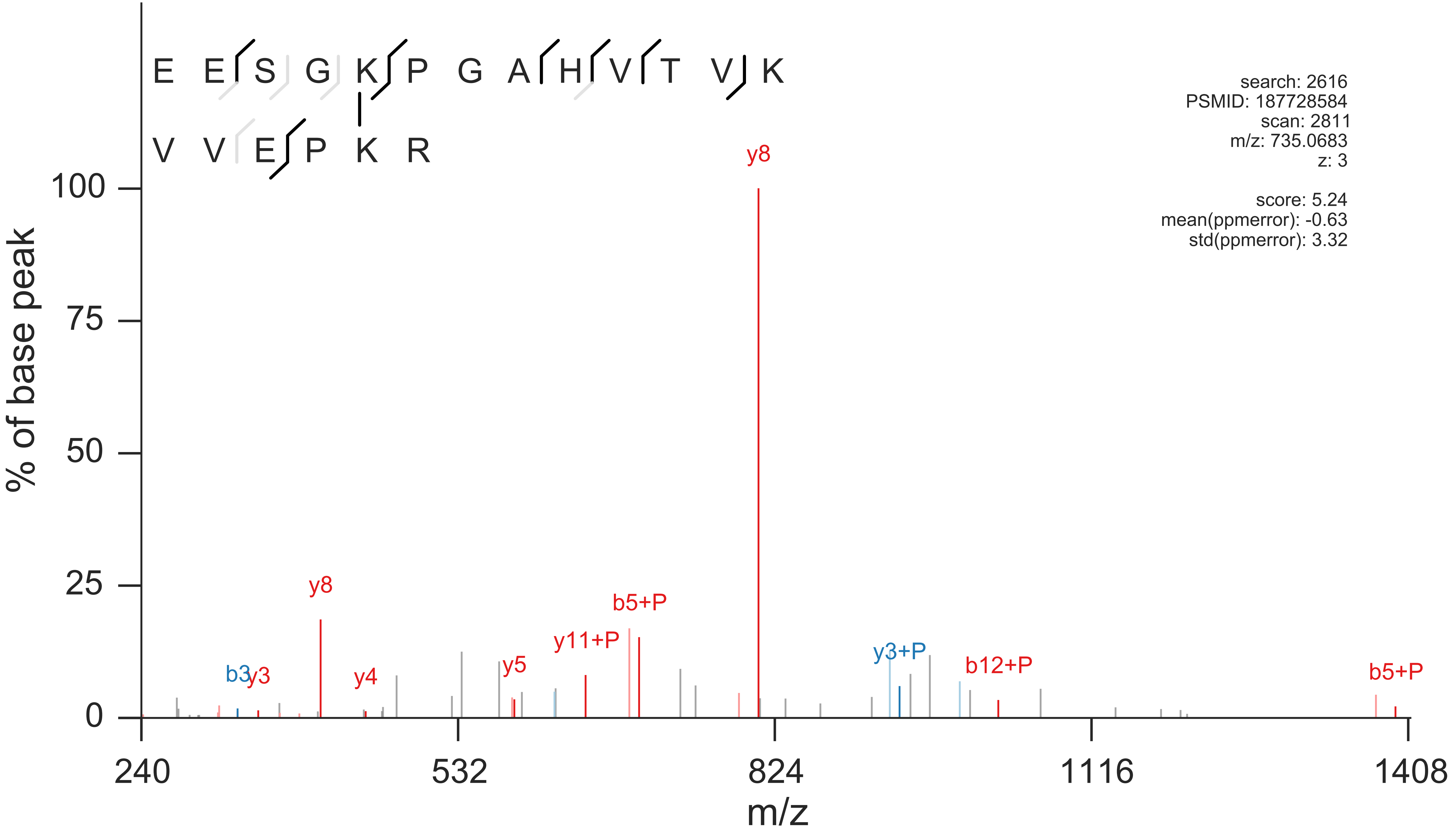

E E S G K P G A H V T V K
V V E P K R
search: 2616
PSMID: 187728584
scan: 2811
m/z: 735.0683
z: 3
score: 5.24
mean(ppmerror): -0.63
std(ppmerror): 3.32
y8
y8
b5+P
y11+P
y5
b3
y3
y4
y3+P
b12+P
b5+P
% of base peak
100
75
50
25
0
240
532
824
1116
1408
m/z

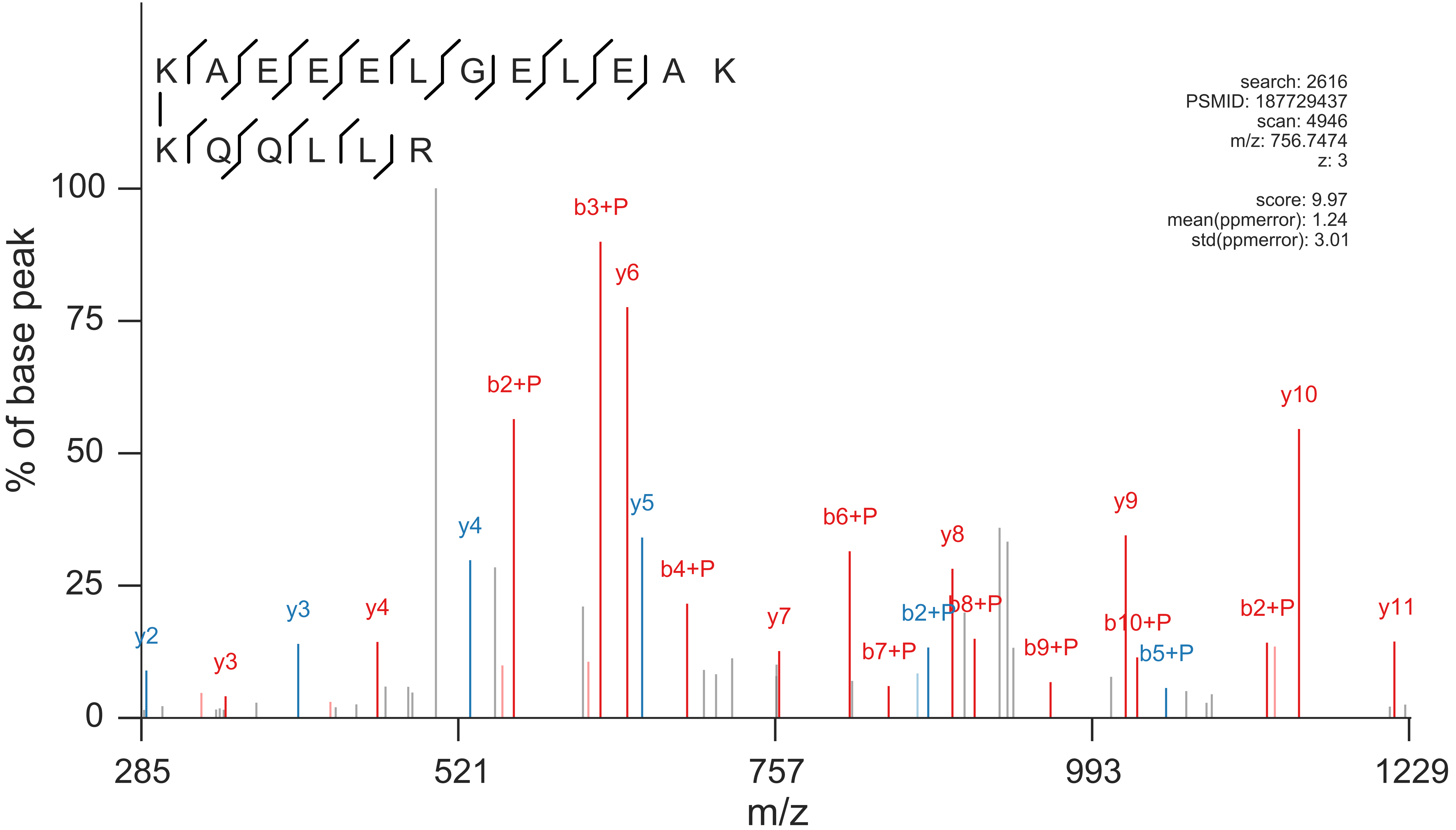

K A E E E L G E L E A K
K Q Q L L R
search: 2616
PSMID: 187729437
scan: 4946
m/z: 756.7474
z: 3
score: 9.97
mean(ppmerror): 1.24
std(ppmerror): 3.01
% of base peak
m/z
0
25
50
75
100
285
521
757
993
1229
y2
y3
y3
y4
y4
b2+P
b3+P
y6
y5
b4+P
y7
b6+P
b7+P
b2+P
y8
b8+P
b9+P
y9
b10+P
b5+P
b2+P
y10
y11

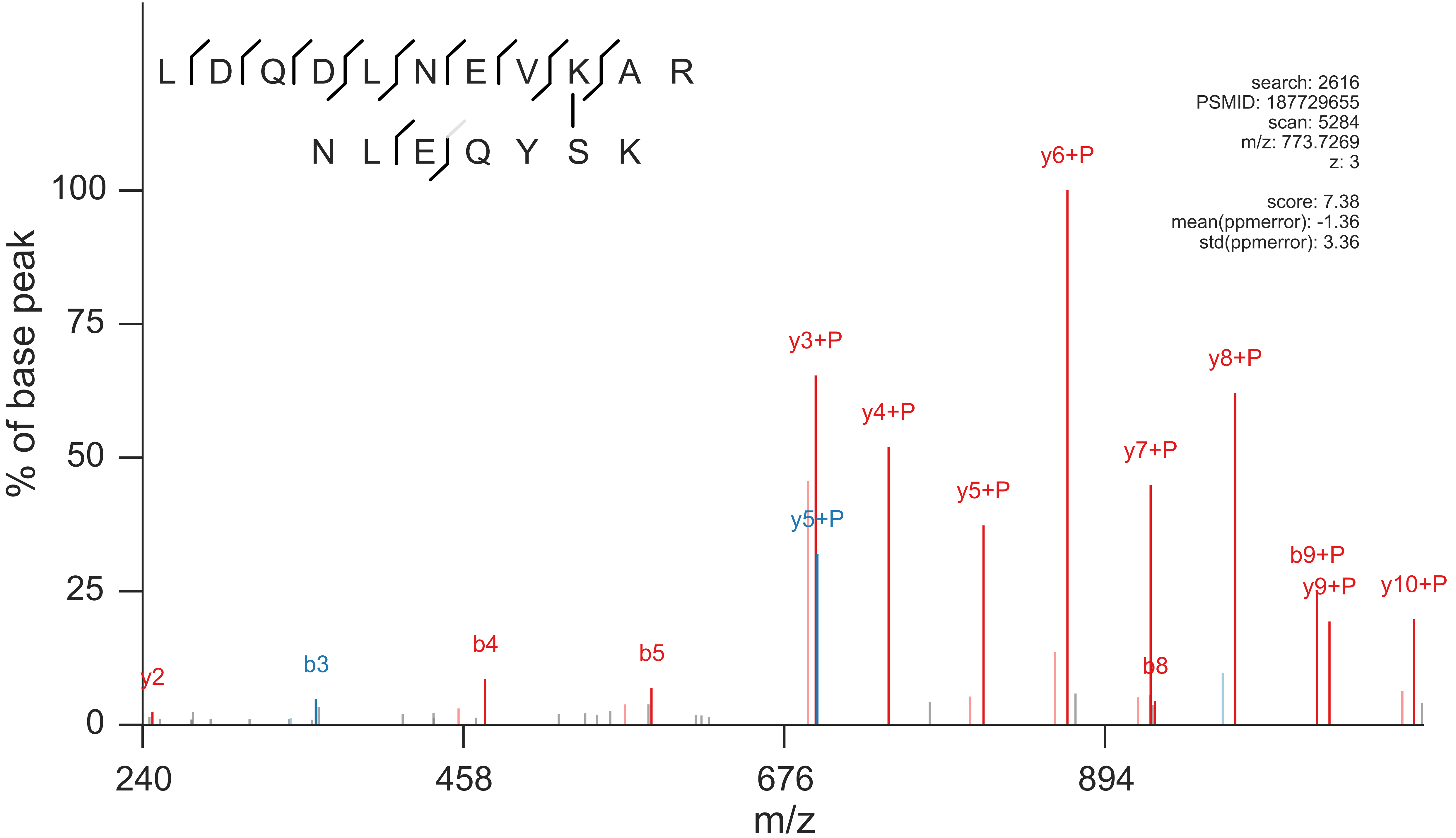

L D Q D L N E V K A R
N L E Q Y S K
search: 2616
PSMID: 187729655
scan: 5284
m/z: 773.7269
z: 3
score: 7.38
mean(ppmerror): -1.36
std(ppmerror): 3.36
% of base peak
100
75
50
25
0
240
458
676
894
m/z
y2
b3
b4
b5
y3+P
y5+P
y4+P
y5+P
y6+P
y7+P
y8+P
b8
b9+P
y9+P
y10+P

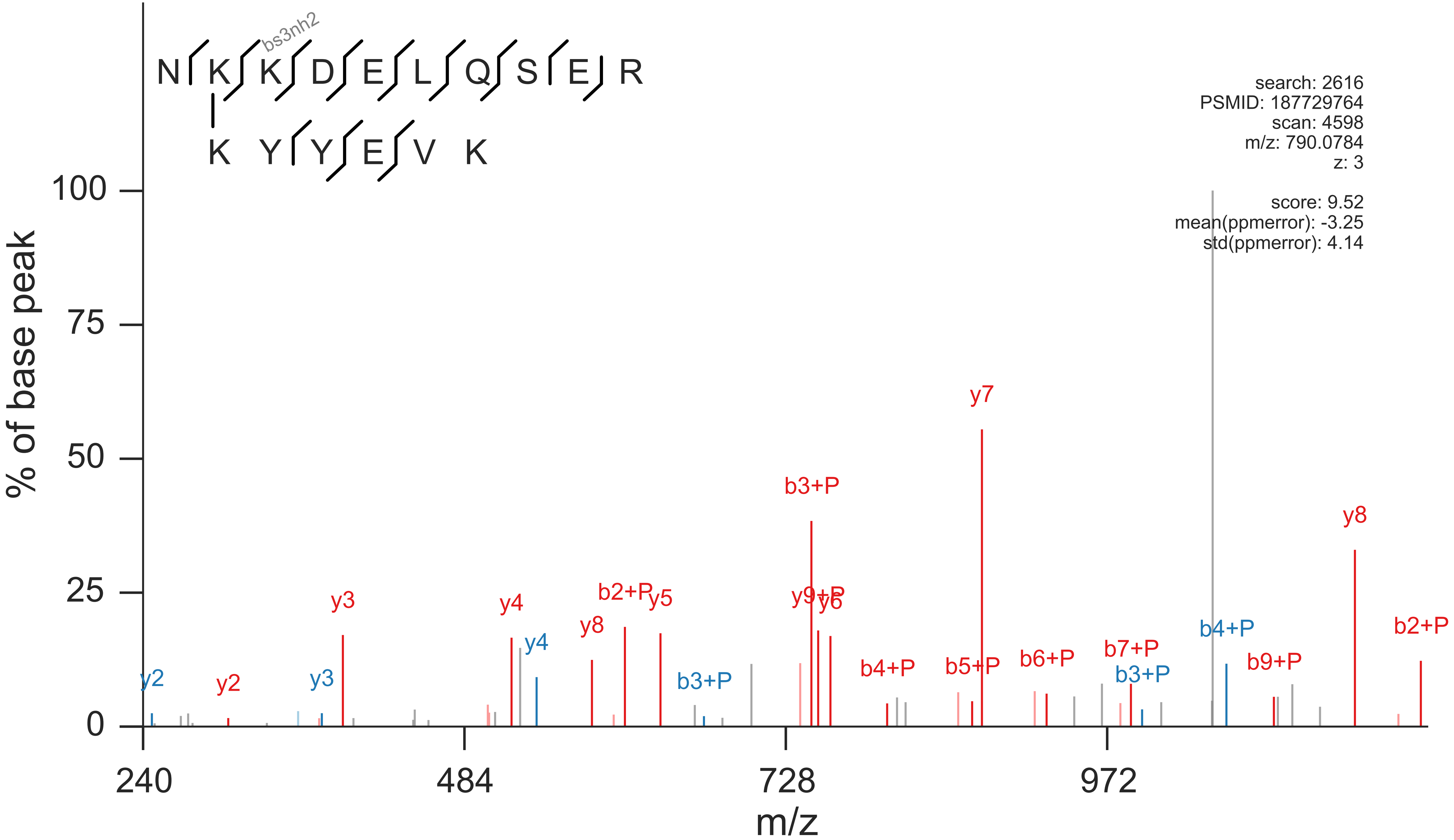

bs3nh2
N K K D E L Q S E R
K Y Y E V K
search: 2616
PSMID: 187729764
scan: 4598
m/z: 790.0784
z: 3
score: 9.52
mean(ppmerror): -3.25
std(ppmerror): 4.14
% of base peak
100
75
50
25
0
240
484
728
972
m/z
y2
y2
y3
y3
y4
y4
y8
b2+P
y5
b3+P
y9+P
y6
b3+P
b4+P
b5+P
y7
b6+P
b7+P
b3+P
b4+P
b9+P
y8
b2+P

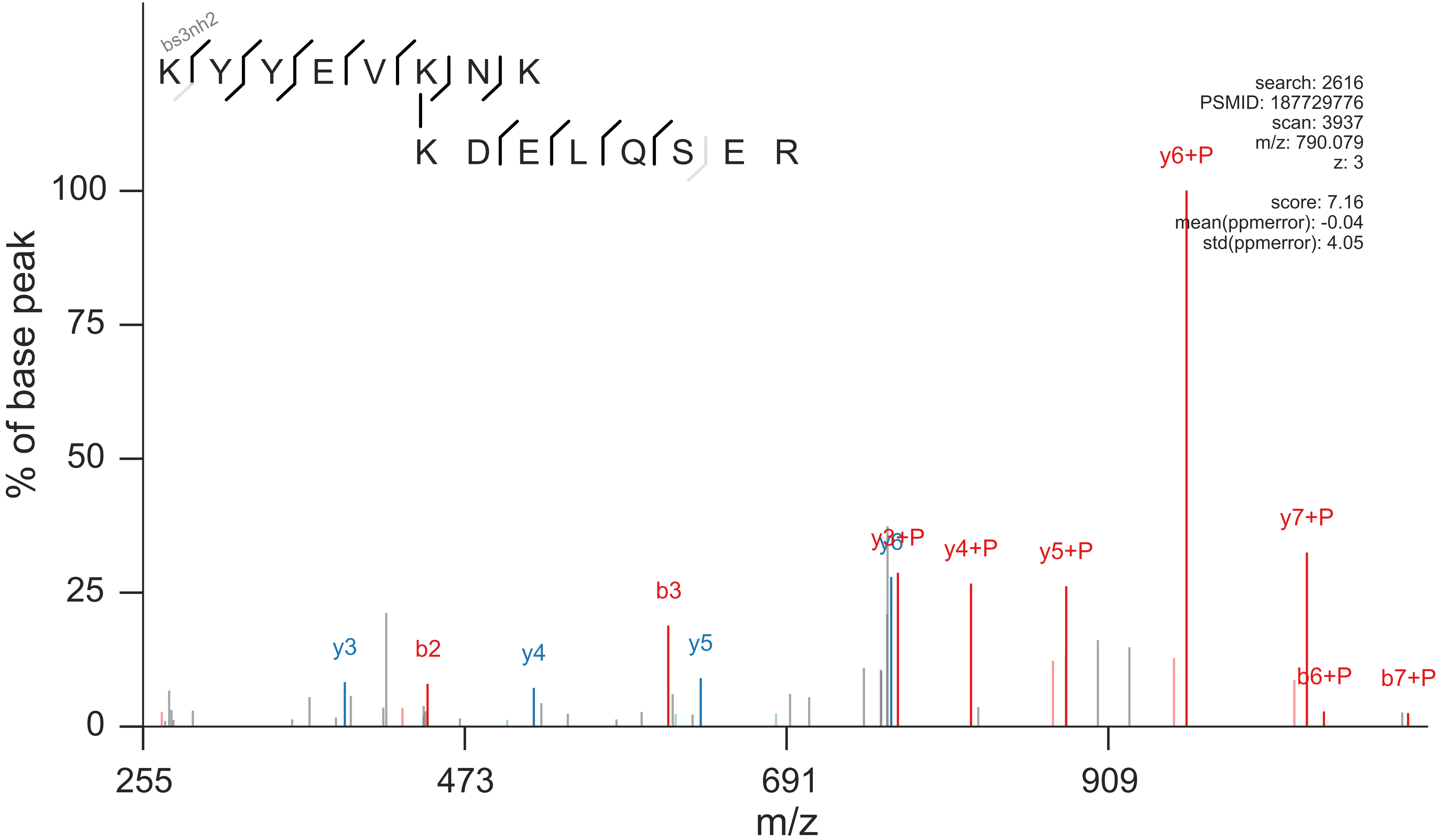
bs3nh2
K Y Y E V K N K
K D E L Q S E R
search: 2616
PSMID: 187729776
scan: 3937
m/z: 790.079
z: 3
score: 7.16
mean(ppmerror): -0.04
std(ppmerror): 4.05
% of base peak
m/z
100
75
50
25
0
255
473
691
909
y3
b2
y4
b3
y5
y6
y3+P
y4+P
y5+P
y6+P
y7+P
b6+P
b7+P

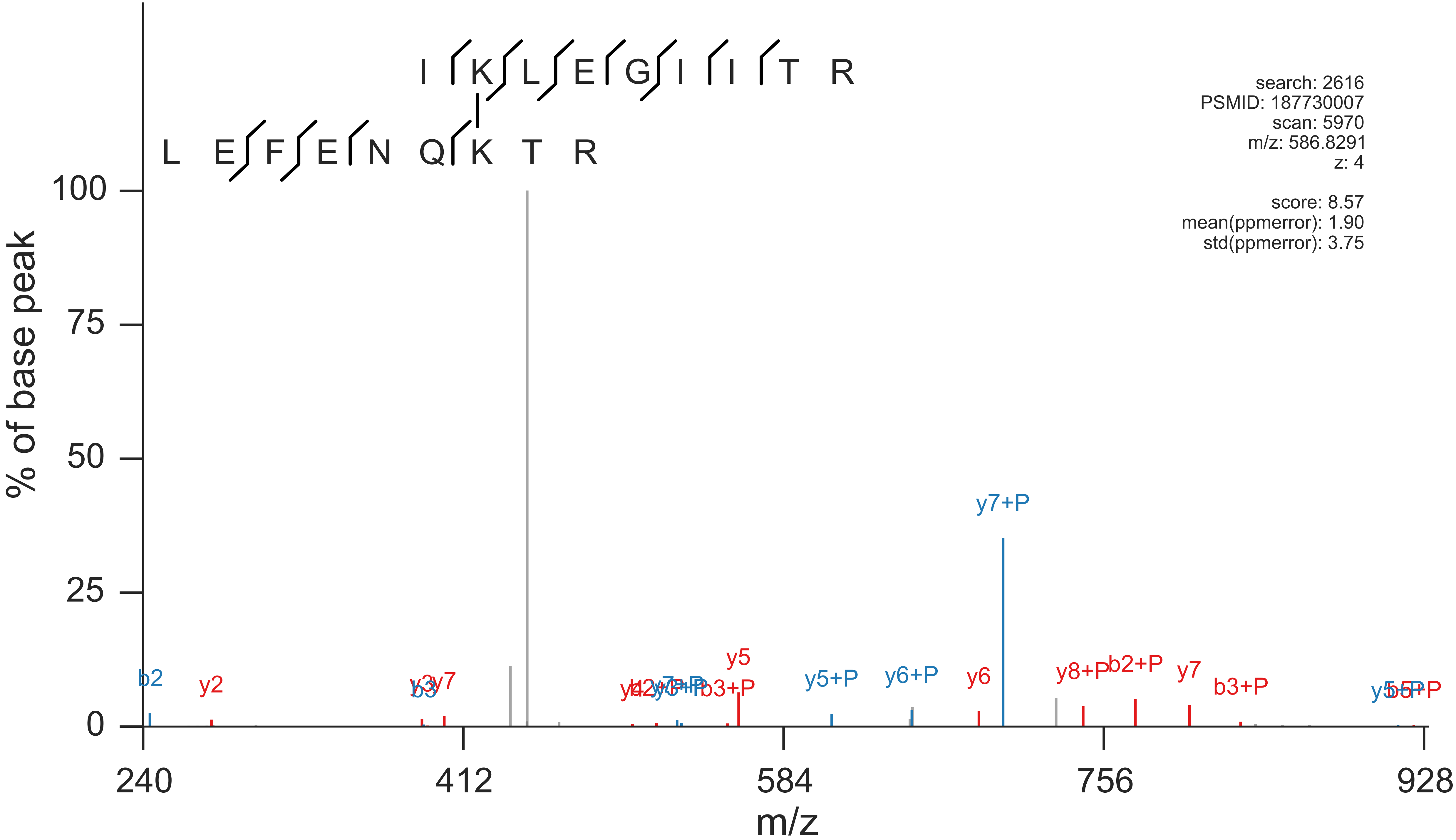

I K L E G I I T R
L E F E N Q K T R
search: 2616
PSMID: 187730007
scan: 5970
m/z: 586.8291
z: 4
score: 8.57
mean(ppmerror): 1.90
std(ppmerror): 3.75
% of base peak
m/z
0
25
50
75
100
240
412
584
756
928
b2
y2
b3
y3
y7
y5
b3+P
y5+P
y6+P
y6
y7+P
y8+P
b2+P
y7
b3+P

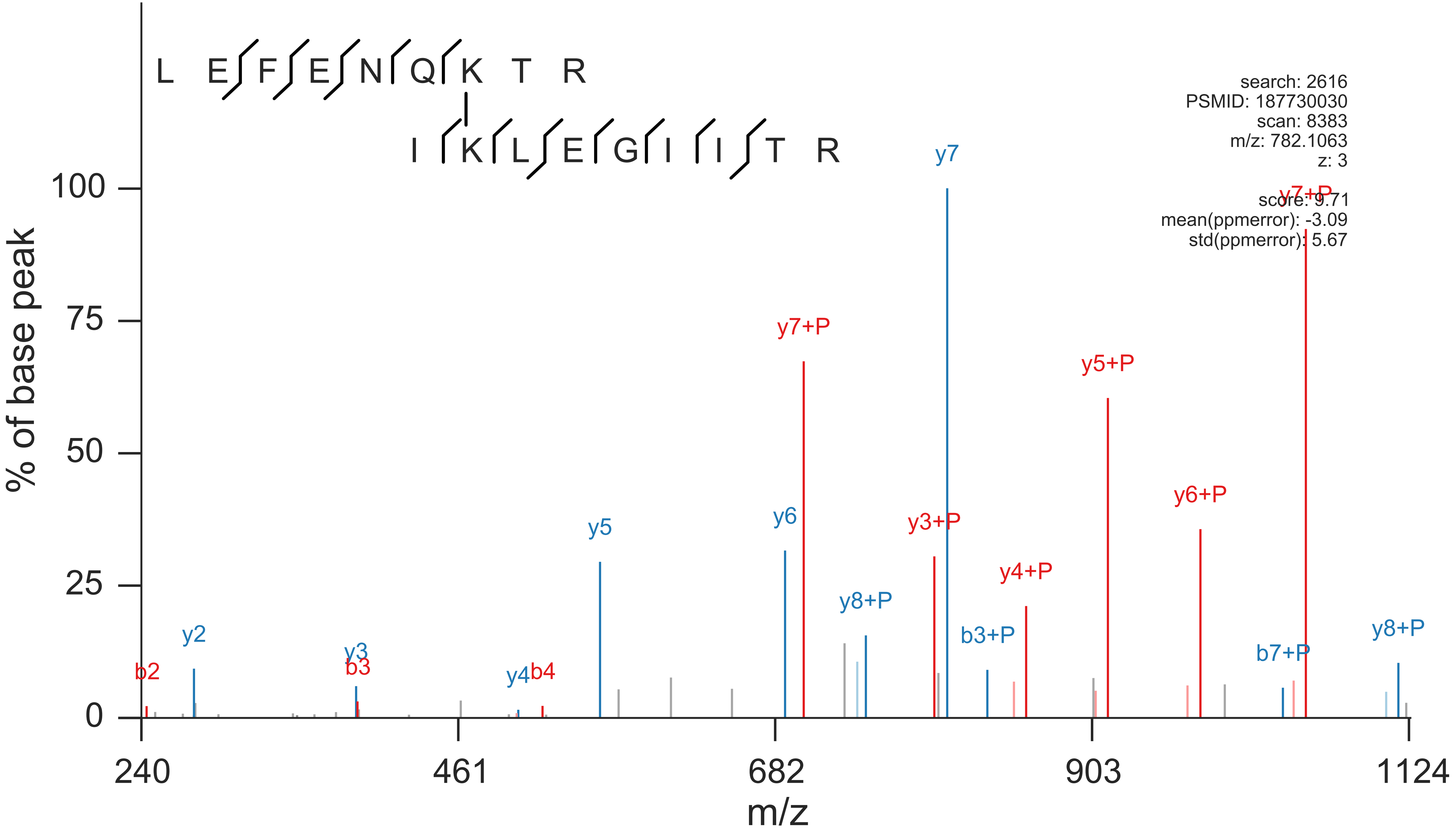

L E F E N Q K T R
I K L E G I I T R
search: 2616
PSMID: 187730030
scan: 8383
m/z: 782.1063
z: 3
score: 9.71
mean(ppmerror): -3.09
std(ppmerror): 5.67
% of base peak
m/z
0
25
50
75
100
240
461
682
903
1124
b2
y2
y3
b3
y4
b4
y5
y6
y7+P
y8+P
y3+P
y7
b3+P
y4+P
y5+P
y6+P
b7+P
y7+P
y8+P

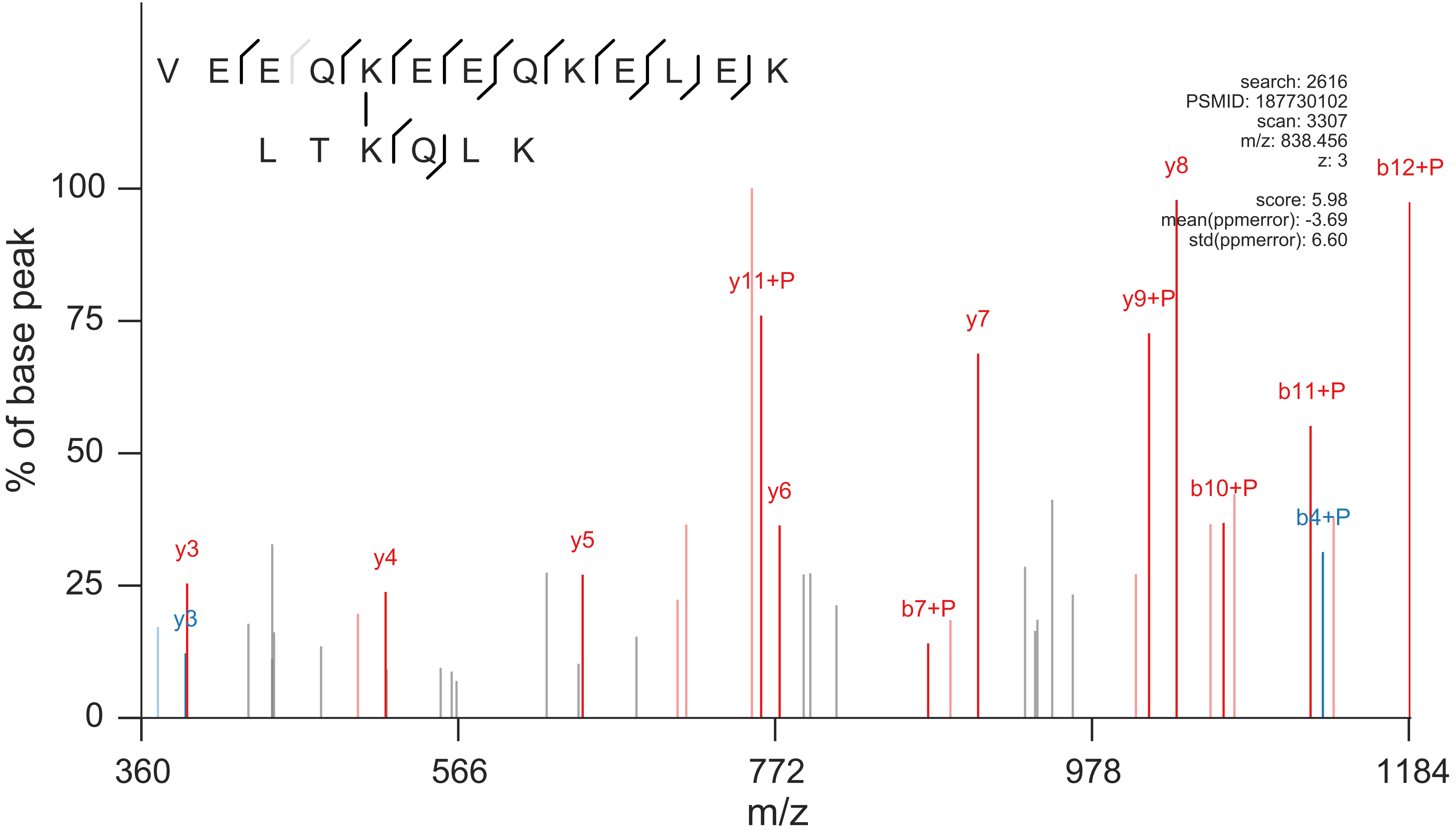

V E E Q K E E Q K E L E K
L T K Q L K
search: 2616
PSMID: 187730102
scan: 3307
m/z: 838.456
z: 3
score: 5.98
mean(ppmerror): -3.69
std(ppmerror): 6.60
% of base peak
m/z
0
25
50
75
100
360
566
772
978
1184
y3
y3
y4
y5
y11+P
y6
b7+P
y7
y9+P
y8
b10+P
b11+P
b4+P
b12+P

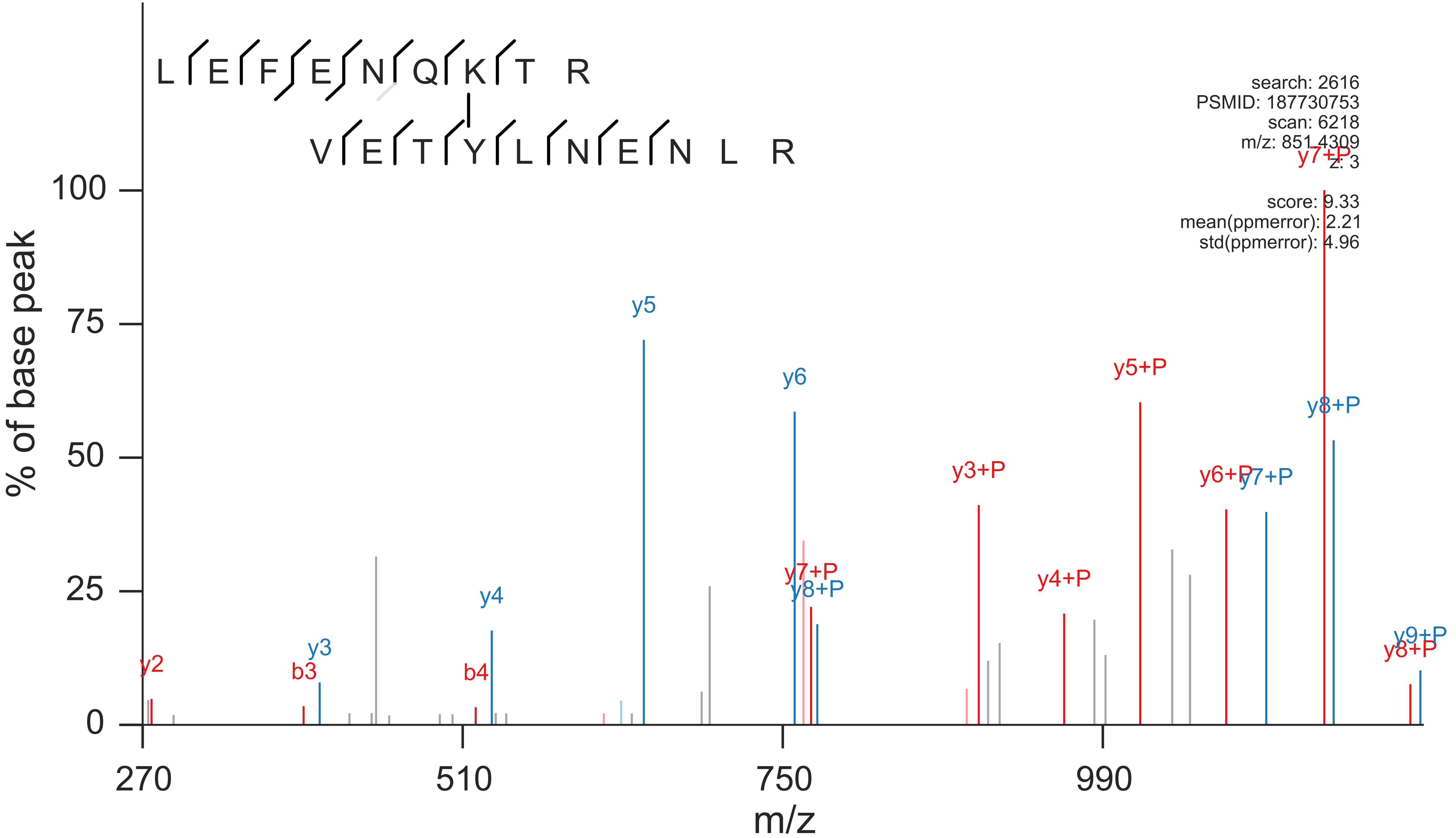

L E F E N Q K T R
V E T Y L N E N L R
search: 2616
PSMID: 187730753
scan: 6218
m/z: 851.4309
z: 3
score: 9.33
mean(ppmerror): 2.21
std(ppmerror): 4.96
% of base peak
m/z
y2
b3
y3
b4
y4
y5
y6
y7+P
y8+P
y3+P
y4+P
y5+P
y6+P
y7+P
y8+P
y7+P
y9+P
y8+P

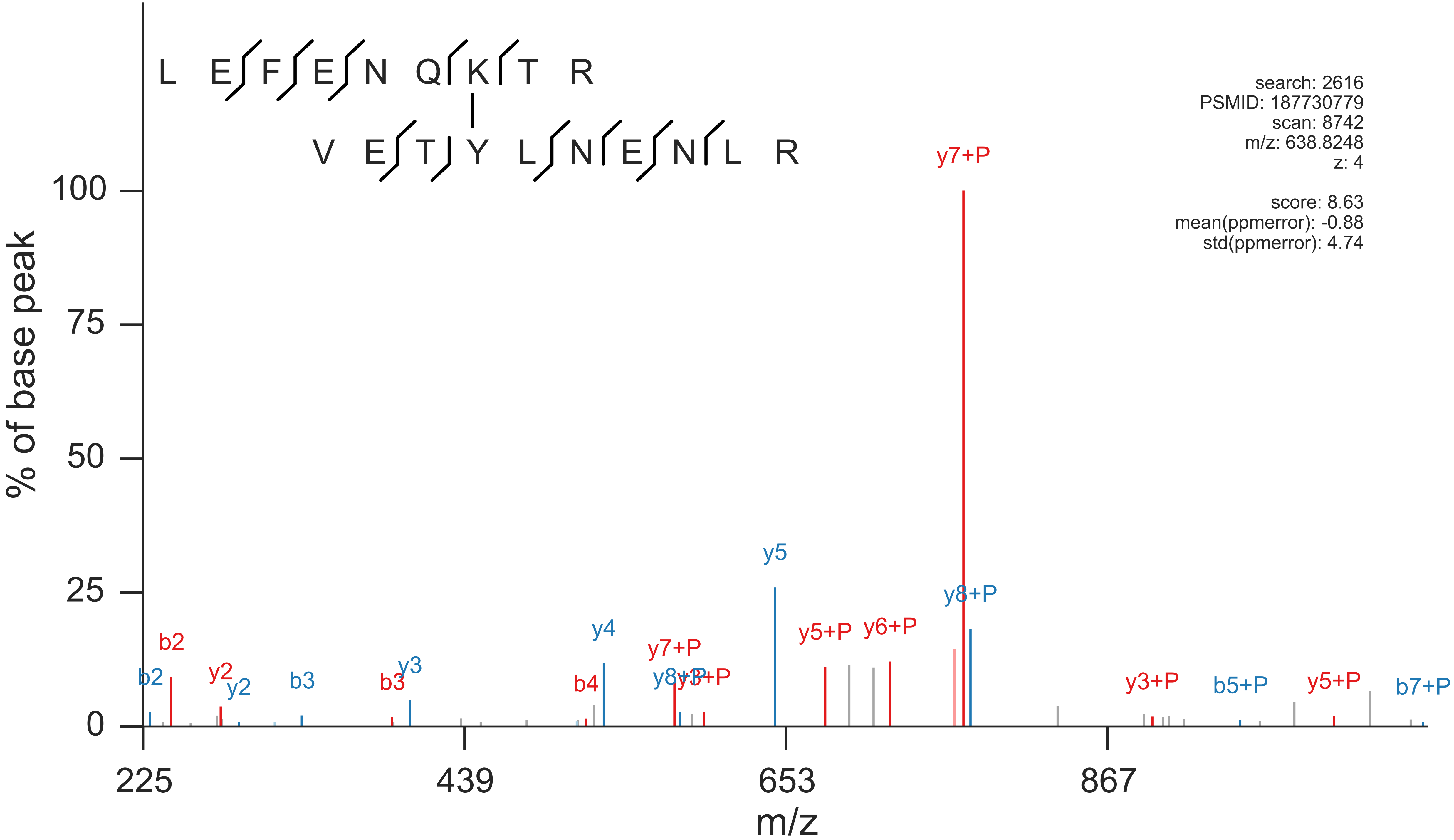

L E F E N Q K T R
V E T Y L N E N L R
search: 2616
PSMID: 187730779
scan: 8742
m/z: 638.8248
z: 4
score: 8.63
mean(ppmerror): -0.88
std(ppmerror): 4.74
% of base peak
m/z
0
25
50
75
100
225
439
653
867
y7+P
y5
y8+P
y4
y7+P
y5+P
y6+P
b2
b2
y2
y2
b3
b3
y3
b4
y8+P
y3+P
y3+P
b5+P
y5+P
b7+P

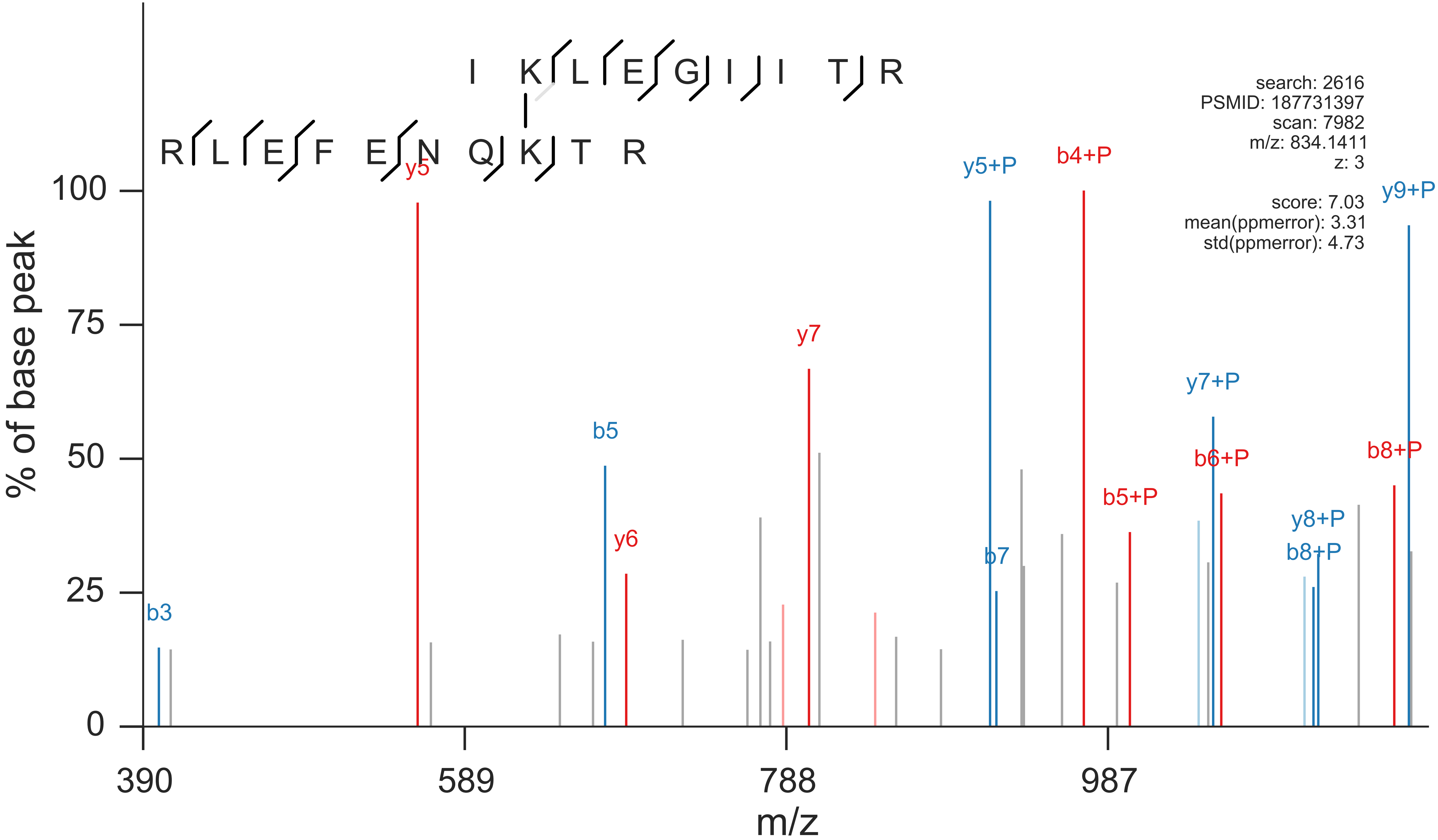

I K L E G I I T R
R L E F E N Q K T R
search: 2616
PSMID: 187731397
scan: 7982
m/z: 834.1411
z: 3
score: 7.03
mean(ppmerror): 3.31
std(ppmerror): 4.73
% of base peak
m/z
390
589
788
987
0
25
50
75
100
b3
y5
b5
y6
y7
b7
y5+P
b4+P
b5+P
y7+P
b6+P
y8+P
b8+P
b8+P
y9+P

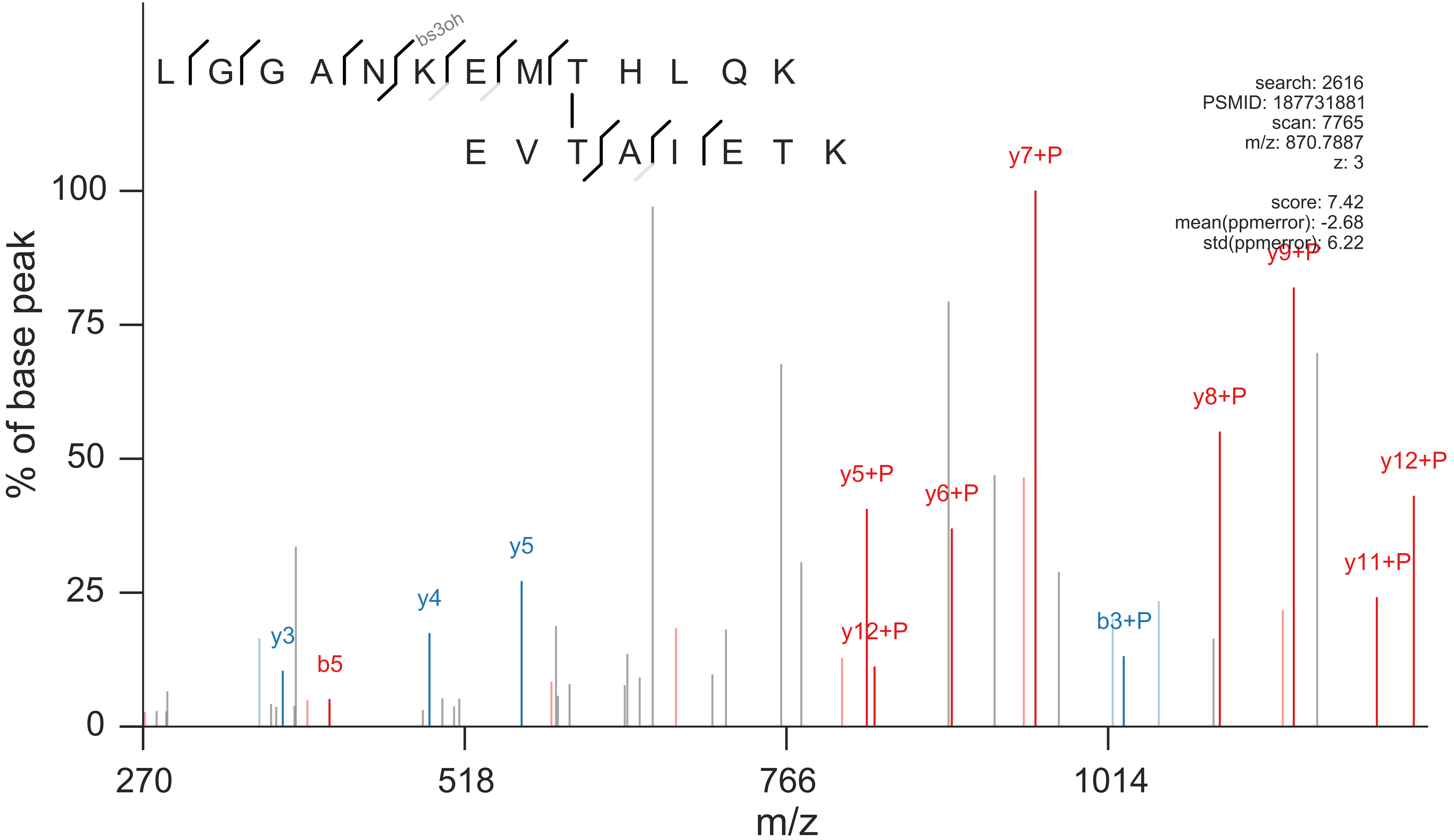

L G G A N K E M T H L Q K
bs3oh
E V T A I E T K
search: 2616
PSMID: 187731881
scan: 7765
m/z: 870.7887
z: 3
score: 7.42
mean(ppmerror): -2.68
std(ppmerror): 6.22
% of base peak
m/z
y3
b5
y4
y5
y5+P
y12+P
y6+P
y7+P
b3+P
y8+P
y9+P
y11+P
y12+P

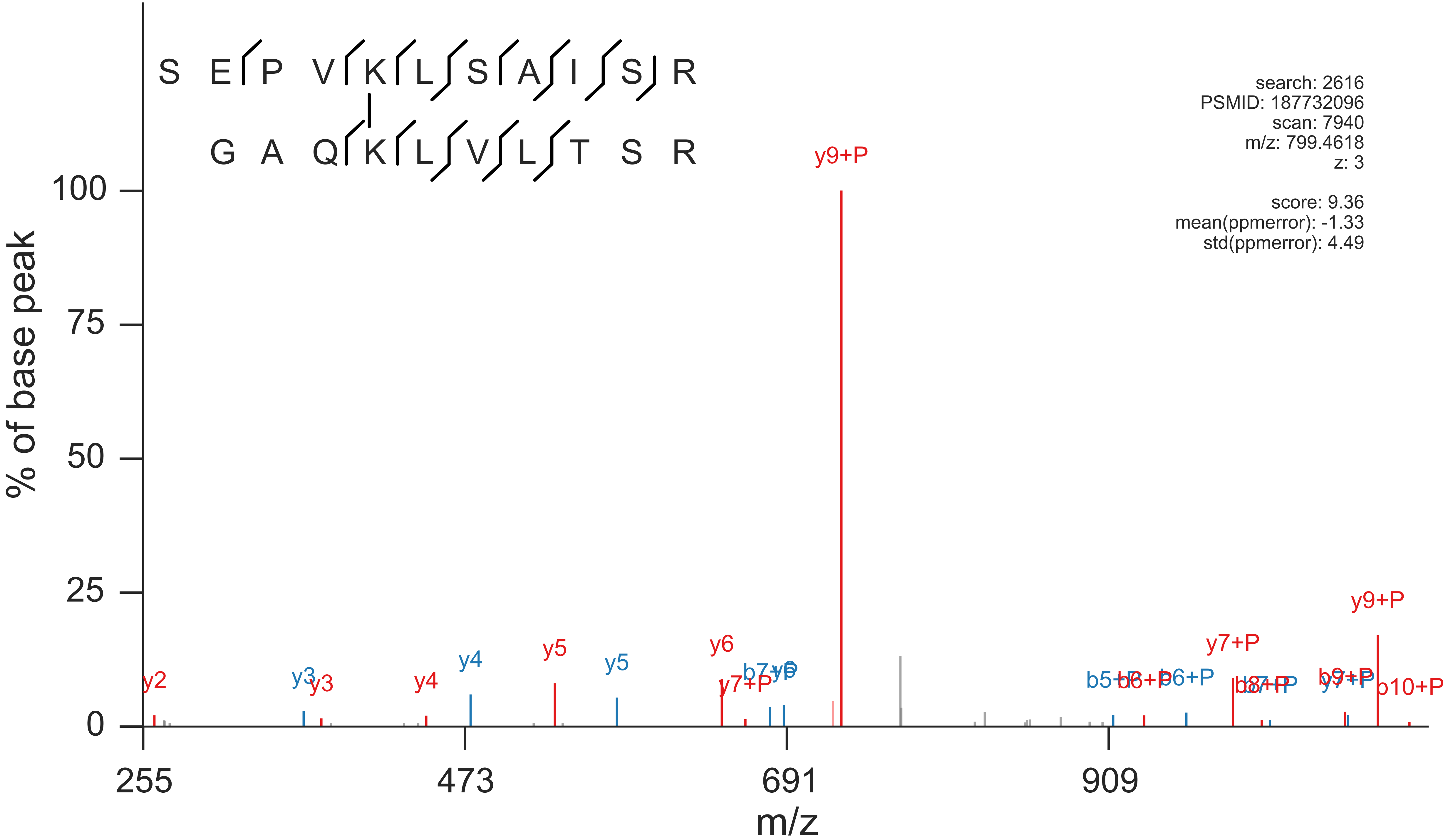
S E P V K L S A I S R
G A Q K L V L T S R
search: 2616
PSMID: 187732096
scan: 7940
m/z: 799.4618
z: 3
score: 9.36
mean(ppmerror): -1.33
std(ppmerror): 4.49
% of base peak
100
75
50
25
0
255
473
691
909
m/z
y9+P
y2
y3
y3
y4
y4
y5
y5
y6
y7+P
y7+P
b10+P

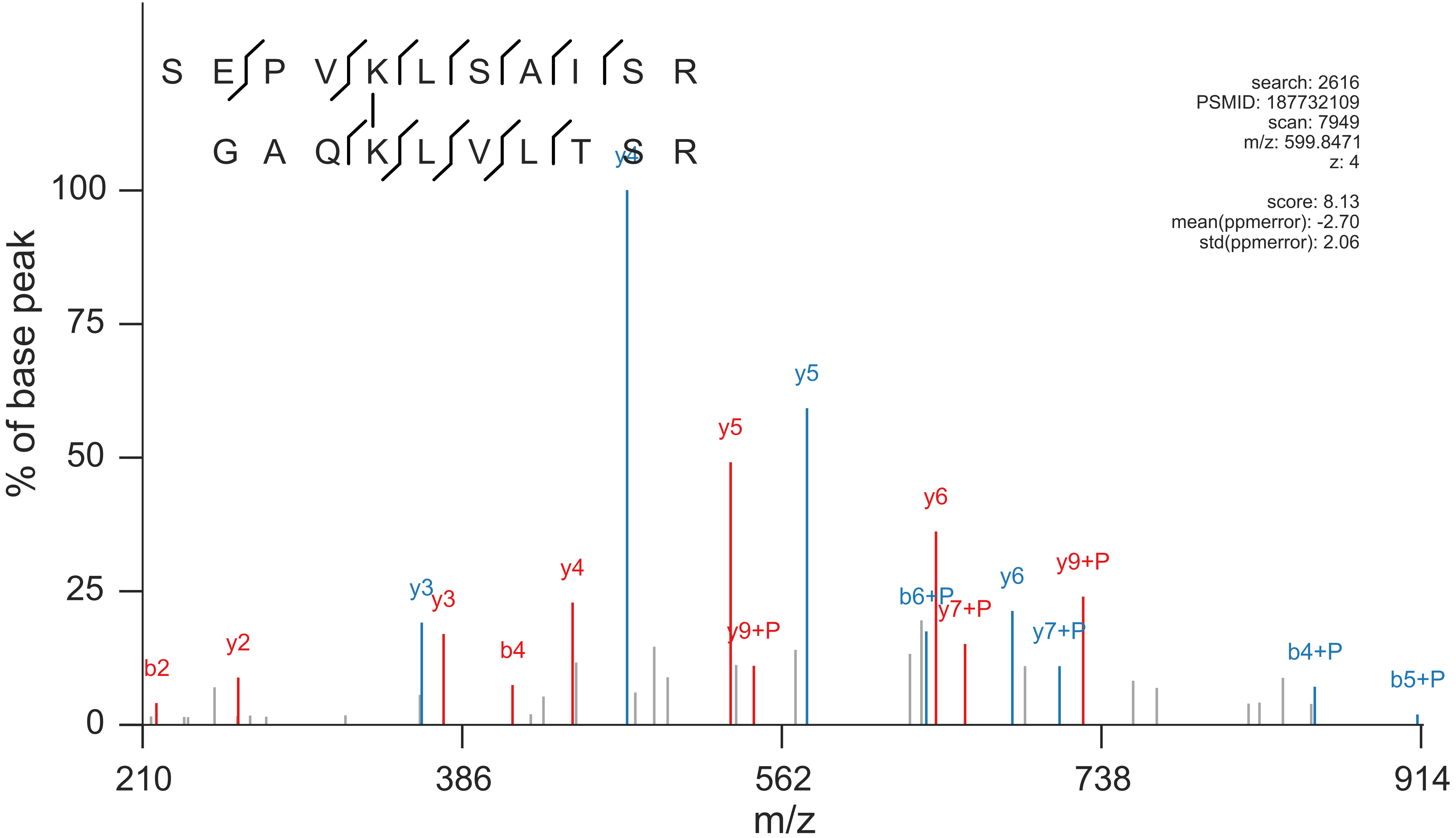

S E P V K L S A I S R
G A Q K L V L T S R
search: 2616
PSMID: 187732109
scan: 7949
m/z: 599.8471
z: 4
score: 8.13
mean(ppmerror): -2.70
std(ppmerror): 2.06
% of base peak
m/z
0
25
50
75
100
210
386
562
738
914
b2
y2
y3
y3
b4
y4
y4
y5
y9+P
y5
b6+P
y6
y7+P
y6
y7+P
y9+P
b4+P
b5+P

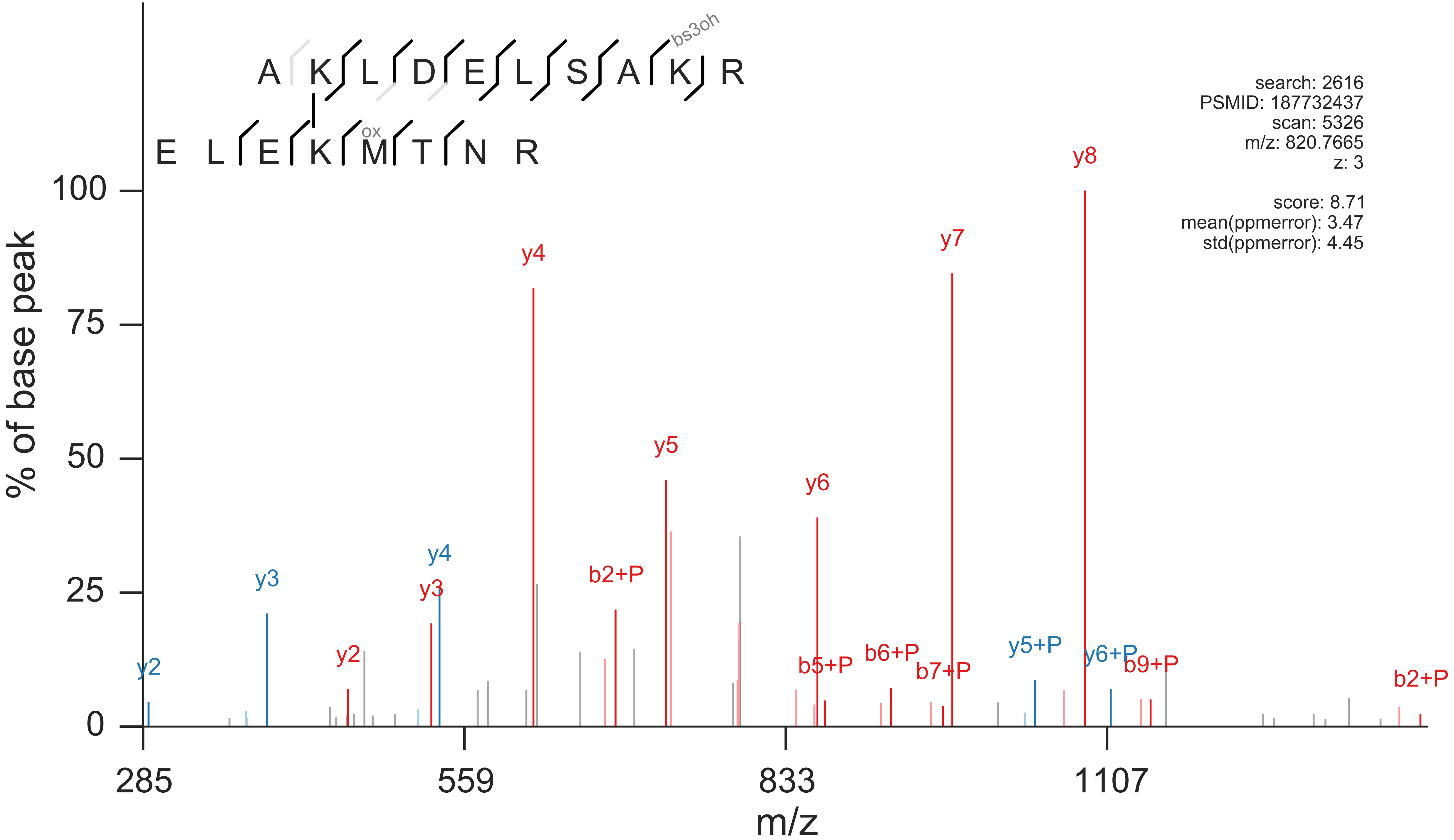

A K L D E L S A K R
bs3oh
E L E K M T N R
ox
search: 2616
PSMID: 187732437
scan: 5326
m/z: 820.7665
z: 3
score: 8.71
mean(ppmerror): 3.47
std(ppmerror): 4.45
% of base peak
m/z
0
25
50
75
100
285
559
833
1107
y2
y3
y4
y5
y6
y7
y8
b2+P
b5+P
b6+P
b7+P
b9+P
y5+P
y6+P

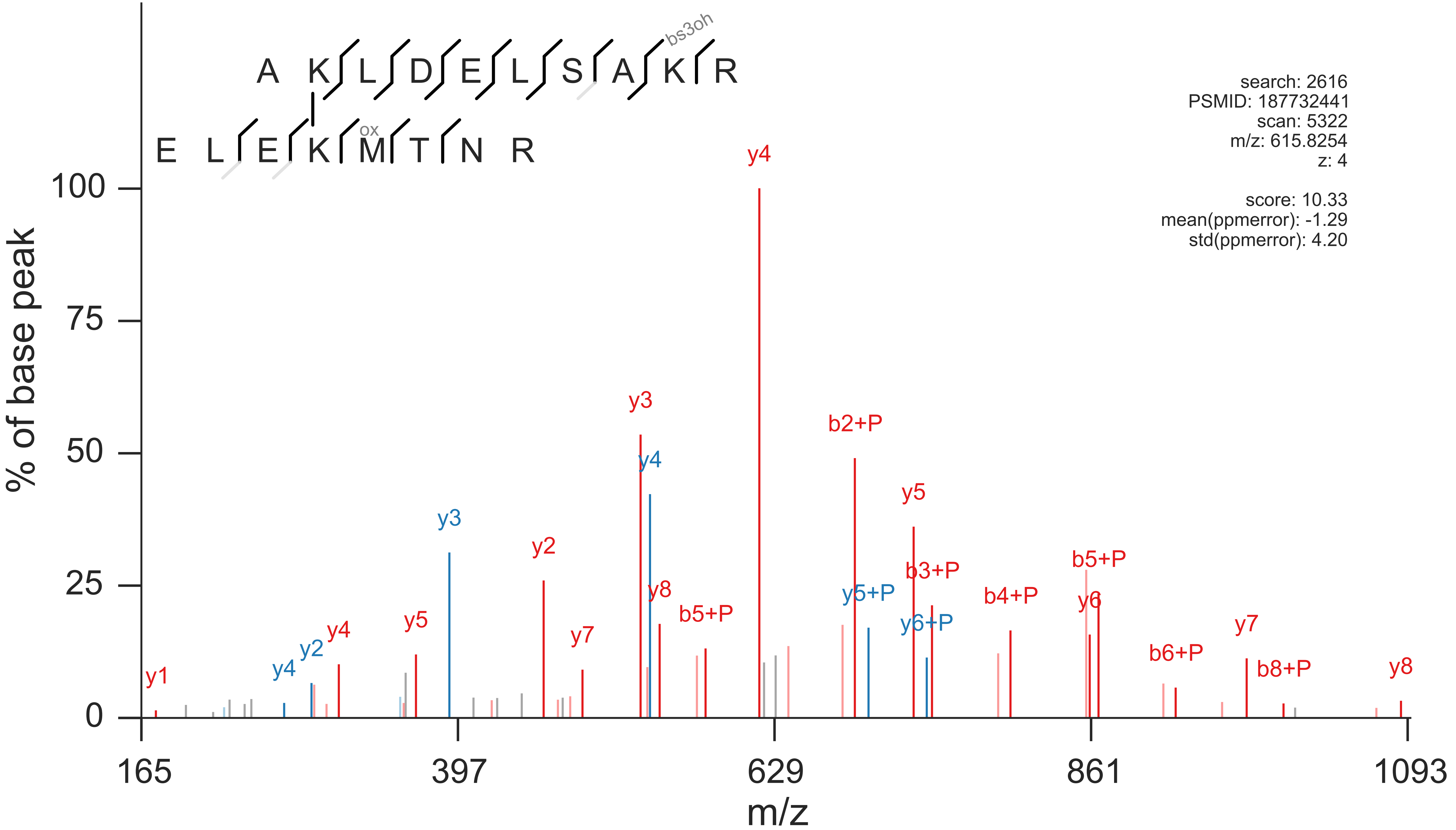

A K L D E L S A K R
bs3oh
E L E K M T N R
ox
search: 2616
PSMID: 187732441
scan: 5322
m/z: 615.8254
z: 4
score: 10.33
mean(ppmerror): -1.29
std(ppmerror): 4.20
% of base peak
100
75
50
25
0
m/z
165
397
629
861
1093
y1
y4
y2
y4
y5
y3
y2
y7
y3
y4
y8
b5+P
y4
b2+P
y5+P
y5
b3+P
y6+P
b4+P
b5+P
y6
b6+P
y7
b8+P
y8

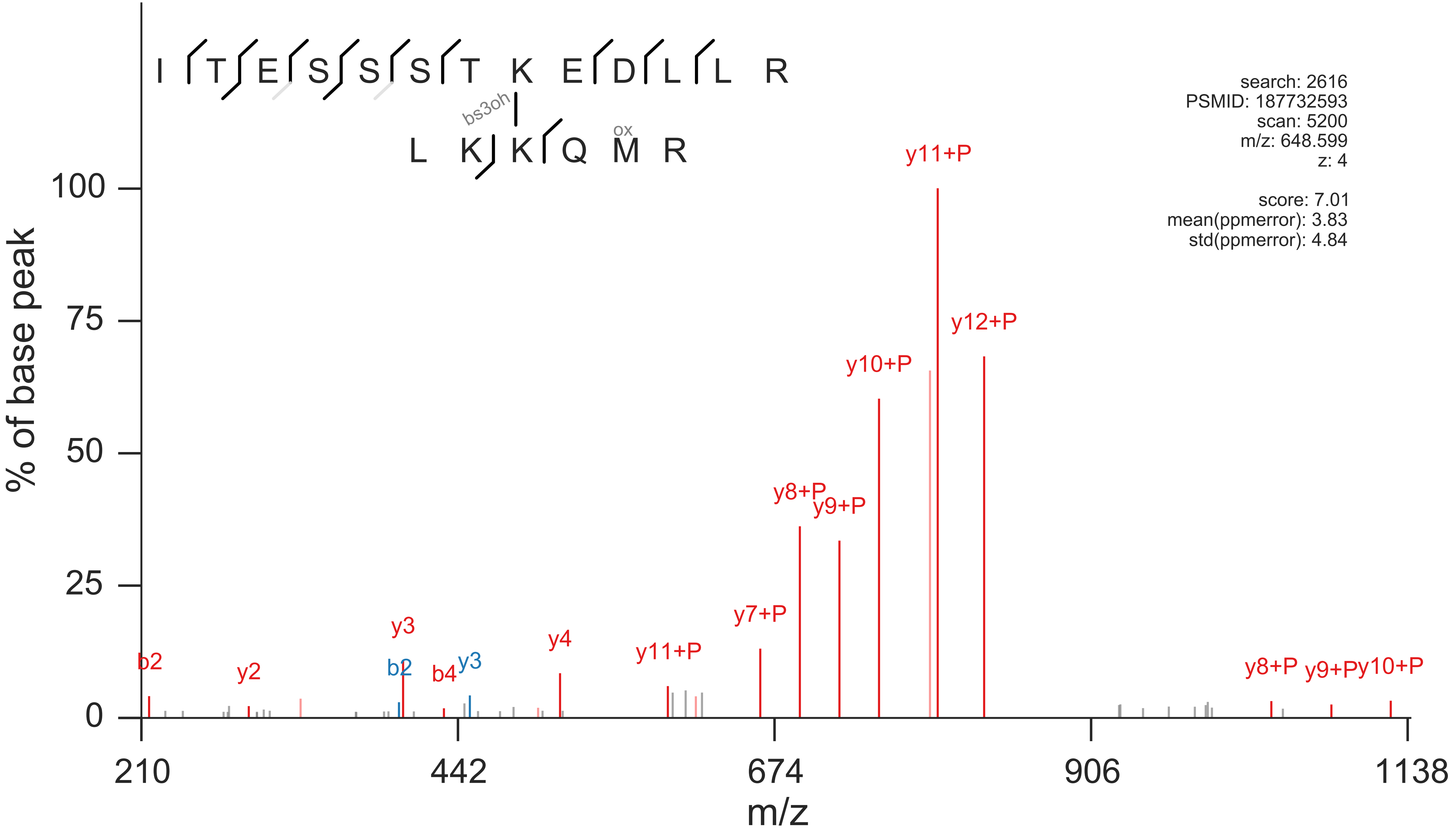
I T E S S S T K E D L L R
bs3oh
L K K Q M R
ox
search: 2616
PSMID: 187732593
scan: 5200
m/z: 648.599
z: 4
score: 7.01
mean(ppmerror): 3.83
std(ppmerror): 4.84
% of base peak
m/z
y11+P
y12+P
y10+P
y8+P
y9+P
y7+P
y11+P
y4
y3
b2
y2
b2
b4
y3
y8+P
y9+P
y10+P
0
25
50
75
100
210
442
674
906
1138

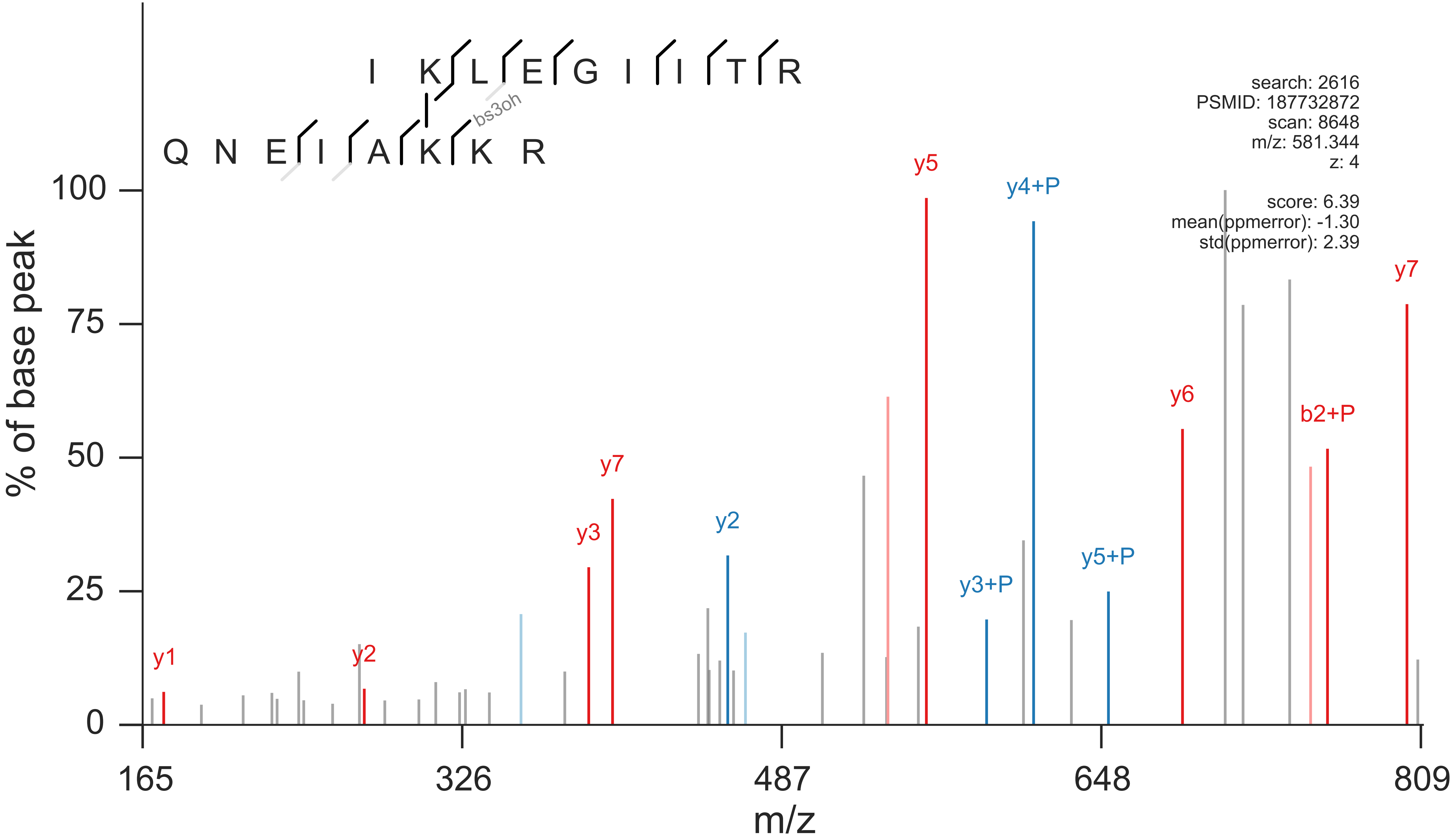
I K L E G I I T R
Q N E I A K K R
bs3oh
search: 2616
PSMID: 187732872
scan: 8648
m/z: 581.344
z: 4
score: 6.39
mean(ppmerror): -1.30
std(ppmerror): 2.39
y1
y2
y3
y7
y2
y5
y3+P
y4+P
y5+P
y6
b2+P
y7
% of base peak
0
25
50
75
100
165
326
487
648
809
m/z

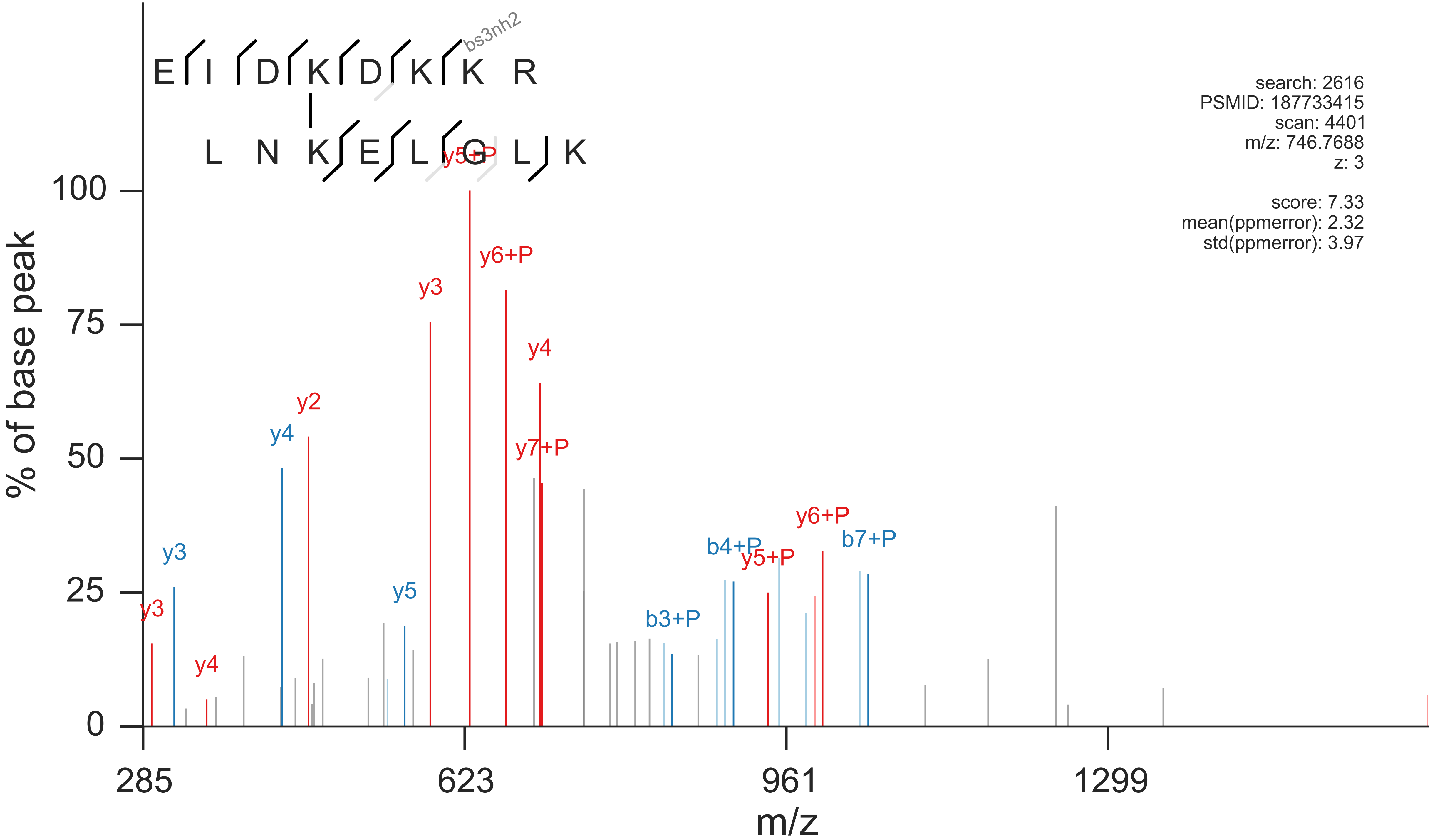

E I D K D K K R
bs3nh2
L N K E L G L K
search: 2616
PSMID: 187733415
scan: 4401
m/z: 746.7688
z: 3
score: 7.33
mean(ppmerror): 2.32
std(ppmerror): 3.97
% of base peak
m/z
0
25
50
75
100
285
623
961
1299
y3
y3
y4
y4
y2
y5
y3
y5+P
y6+P
y4
y7+P
b3+P
b4+P
y5+P
y6+P
b7+P

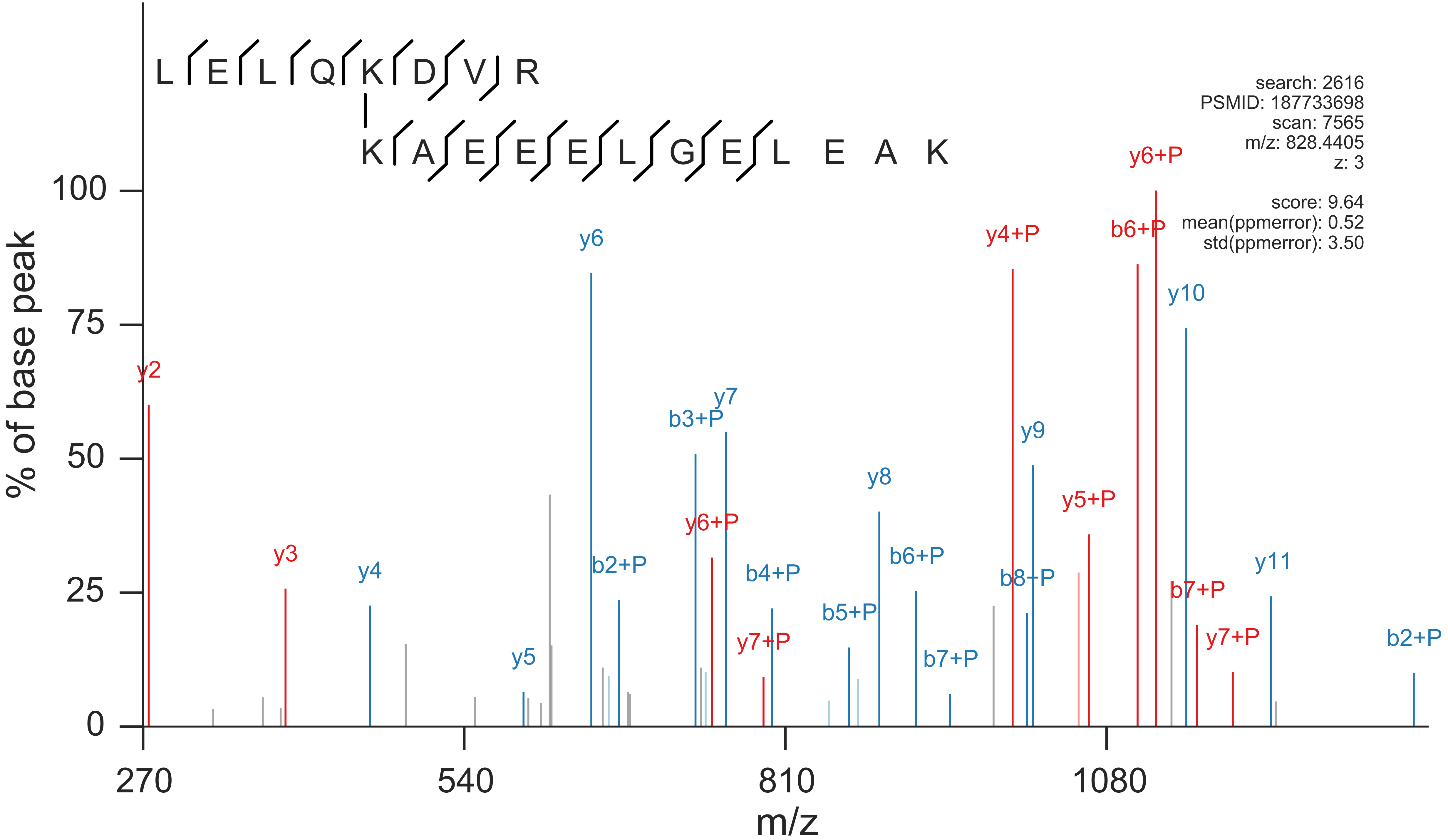

L E L Q K D V R
K A E E E L G E L E A K
search: 2616
PSMID: 187733698
scan: 7565
m/z: 828.4405
z: 3
score: 9.64
mean(ppmerror): 0.52
std(ppmerror): 3.50
% of base peak
m/z
0
25
50
75
100
270
540
810
1080
y2
y3
y4
y5
y6
b2+P
b3+P
y7
y6+P
y7+P
b4+P
b5+P
y8
b6+P
b7+P
y4+P
y9
b8+P
y5+P
b6+P
y6+P
y10
b7+P
y7+P
y11
b2+P

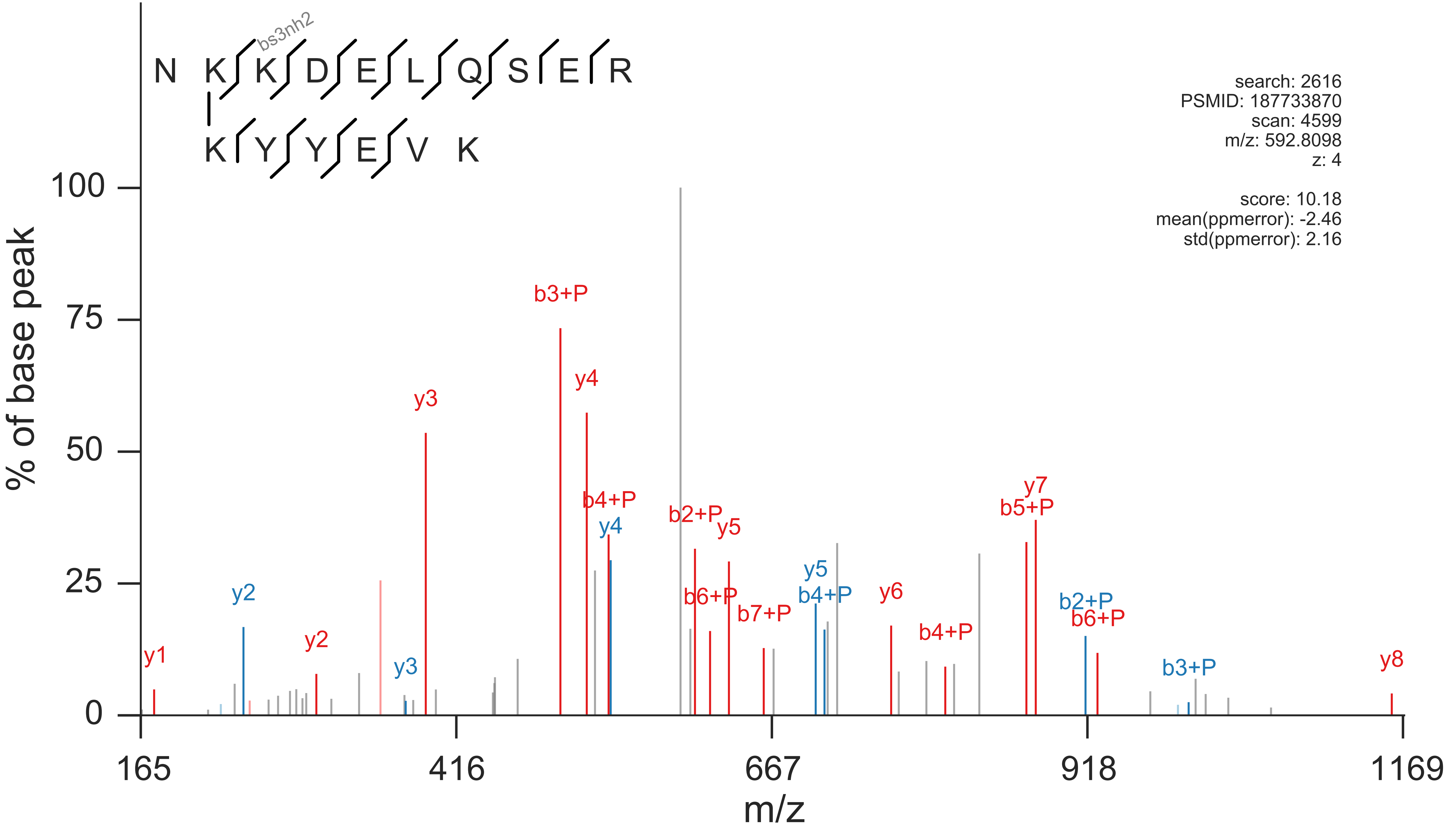
N K K D E L Q S E R
K Y Y E V K
bs3nh2
search: 2616
PSMID: 187733870
scan: 4599
m/z: 592.8098
z: 4
score: 10.18
mean(ppmerror): -2.46
std(ppmerror): 2.16
% of base peak
m/z
0
25
50
75
100
165
416
667
918
1169
y1
y2
y2
y3
y3
b3+P
y4
b4+P
y4
b2+P
y5
b6+P
b7+P
y5
b4+P
y6
b4+P
y7
b5+P
b2+P
b6+P
b3+P
y8

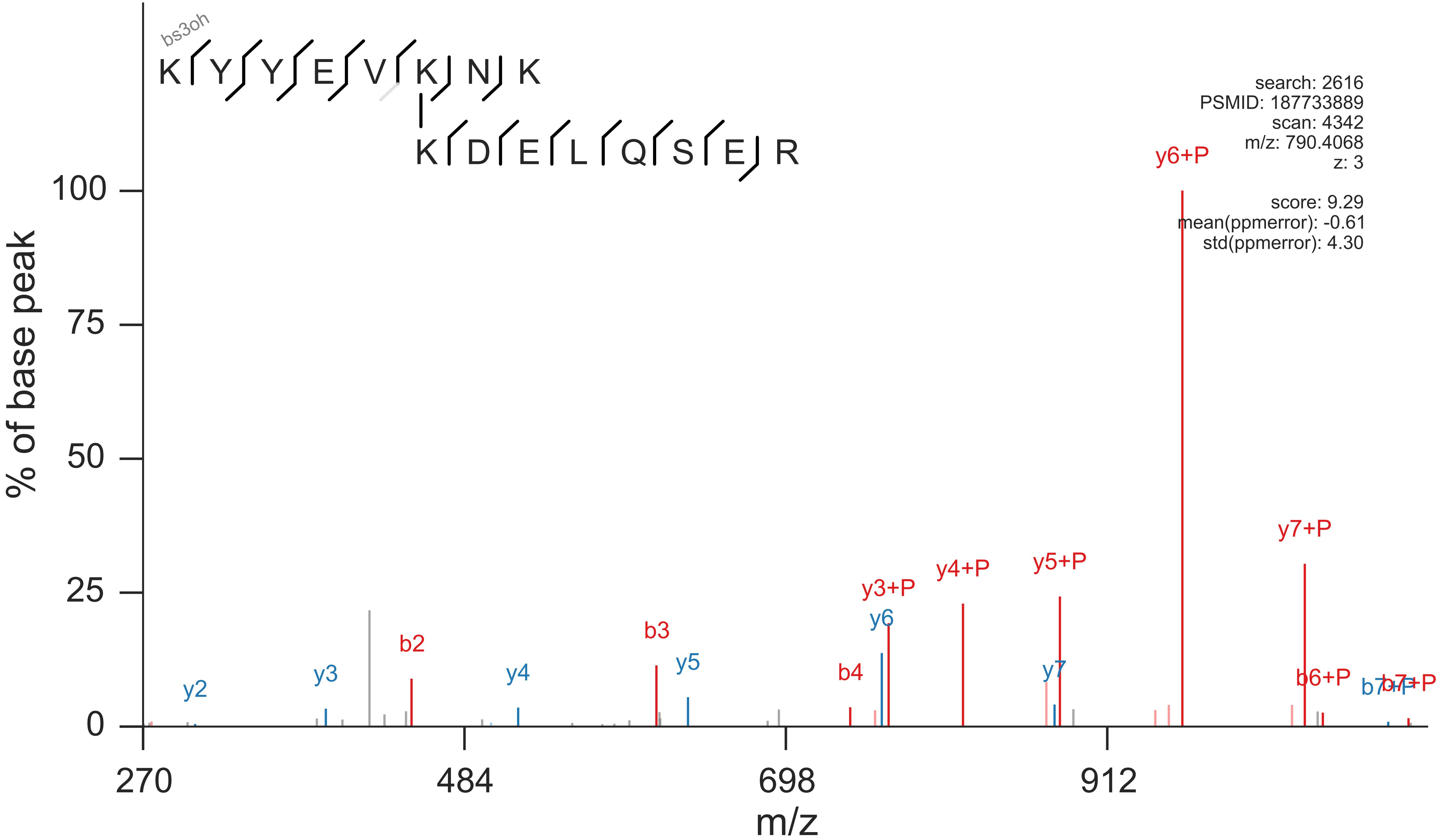
bs3oh
K Y Y E V K N K
K D E L Q S E R
search: 2616
PSMID: 187733889
scan: 4342
m/z: 790.4068
z: 3
score: 9.29
mean(ppmerror): -0.61
std(ppmerror): 4.30
% of base peak
100
75
50
25
0
270
484
698
912
m/z
y6+P
y7+P
y5+P
y4+P
y3+P
y6
b3
b2
y5
y4
y3
y2
b4
y7
b6+P
b7+P

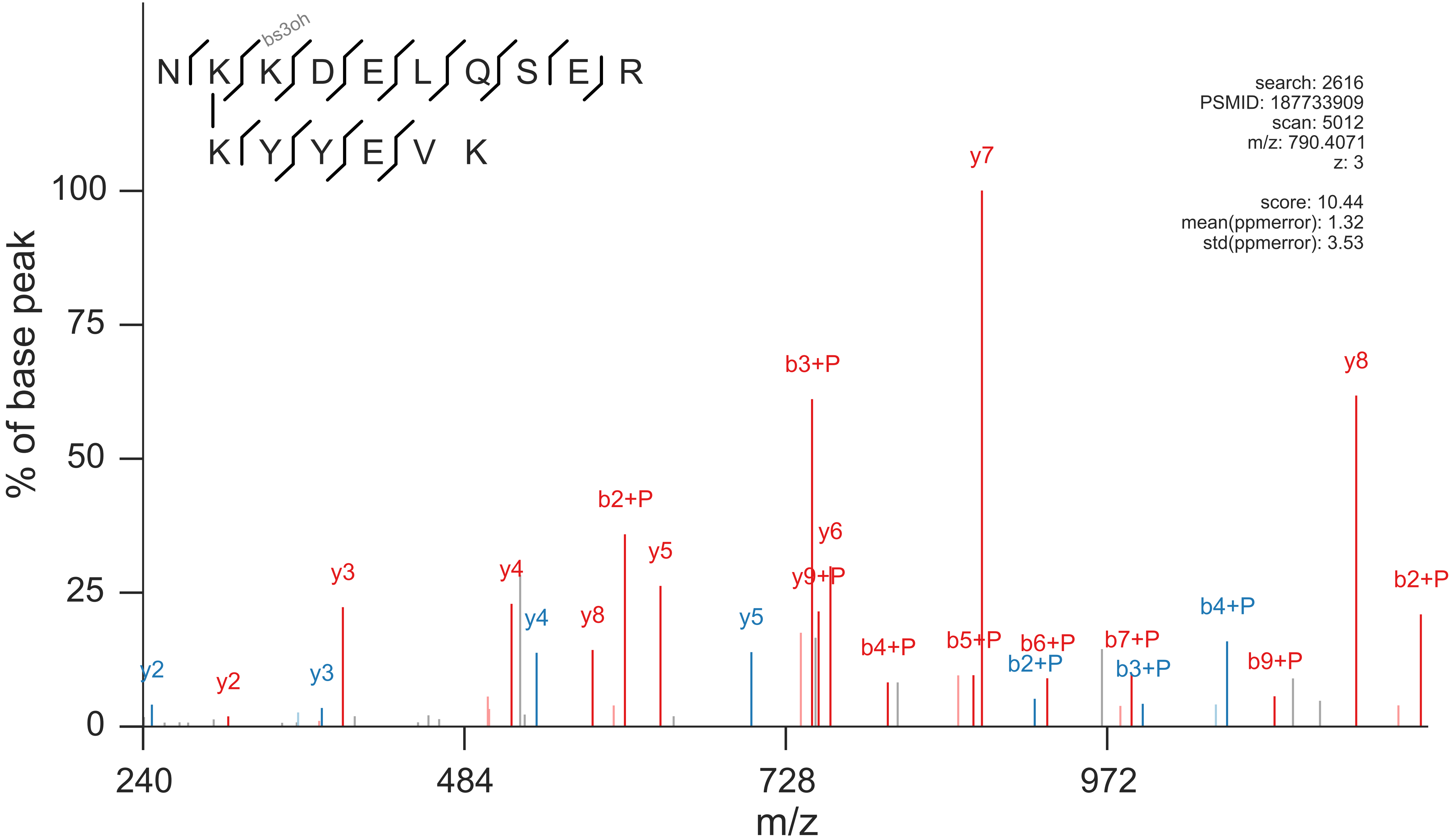

N K K D E L Q S E R
bs3oh
K Y Y E V K
search: 2616
PSMID: 187733909
scan: 5012
m/z: 790.4071
z: 3
score: 10.44
mean(ppmerror): 1.32
std(ppmerror): 3.53
% of base peak
m/z
y7
b3+P
y8
b2+P
y6
y5
y9+P
y3
y4
y4
y8
y5
b2+P
b4+P
b5+P
b6+P
b2+P
b7+P
b3+P
b9+P
y2
y2
y3
0
25
50
75
100
240
484
728
972

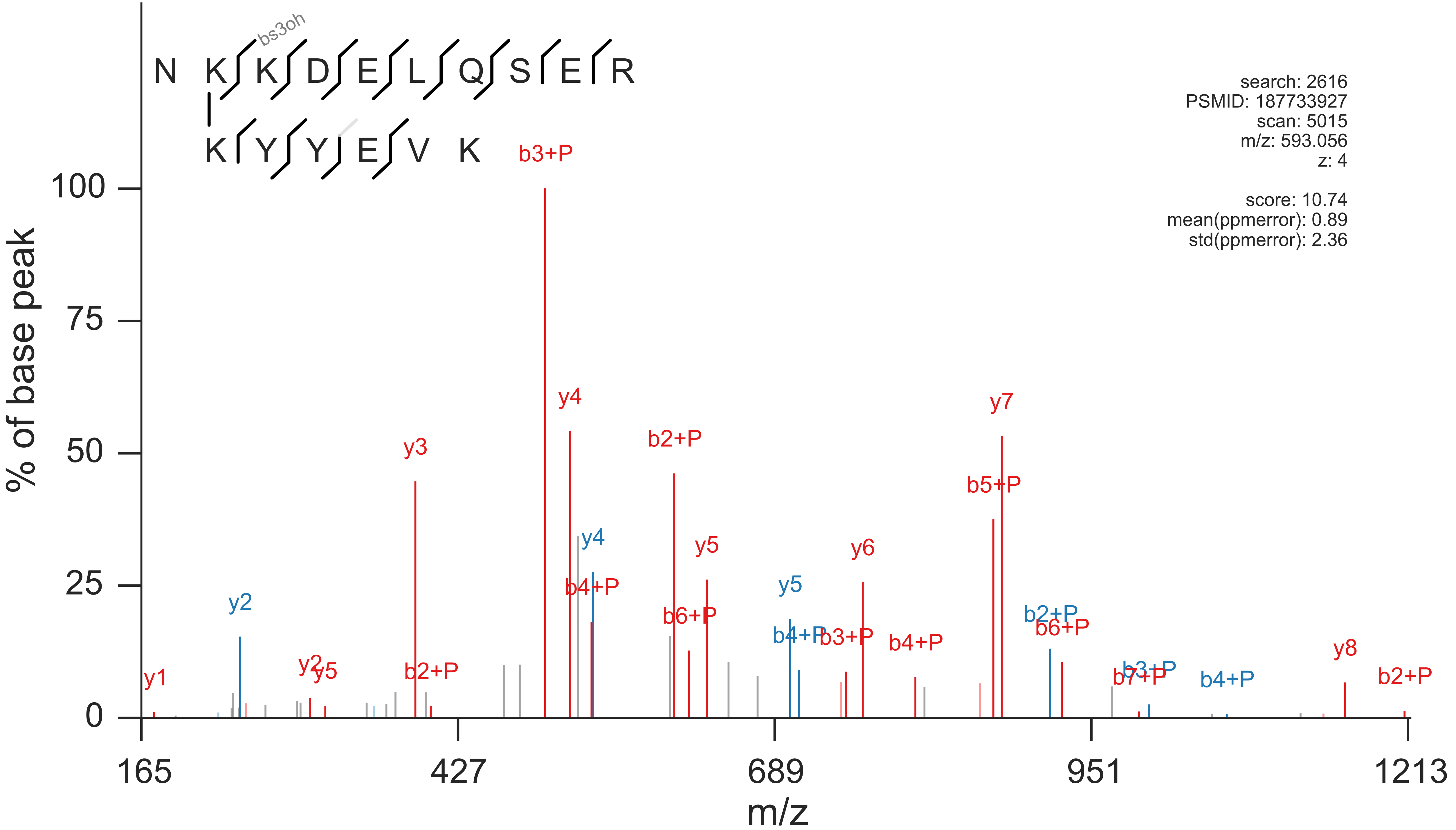

N K K D E L Q S E R
K Y Y E V K
bs3oh
search: 2616
PSMID: 187733927
scan: 5015
m/z: 593.056
z: 4
score: 10.74
mean(ppmerror): 0.89
std(ppmerror): 2.36
% of base peak
m/z
0
25
50
75
100
165
427
689
951
1213
b3+P
y4
y7
b2+P
y3
b5+P
y4
y5
y6
b4+P
y5
y2
b6+P
b2+P
b6+P
b4+P
b3+P
b4+P
y8
y1
y2
y5
b2+P
b3+P
b7+P
b4+P
b2+P

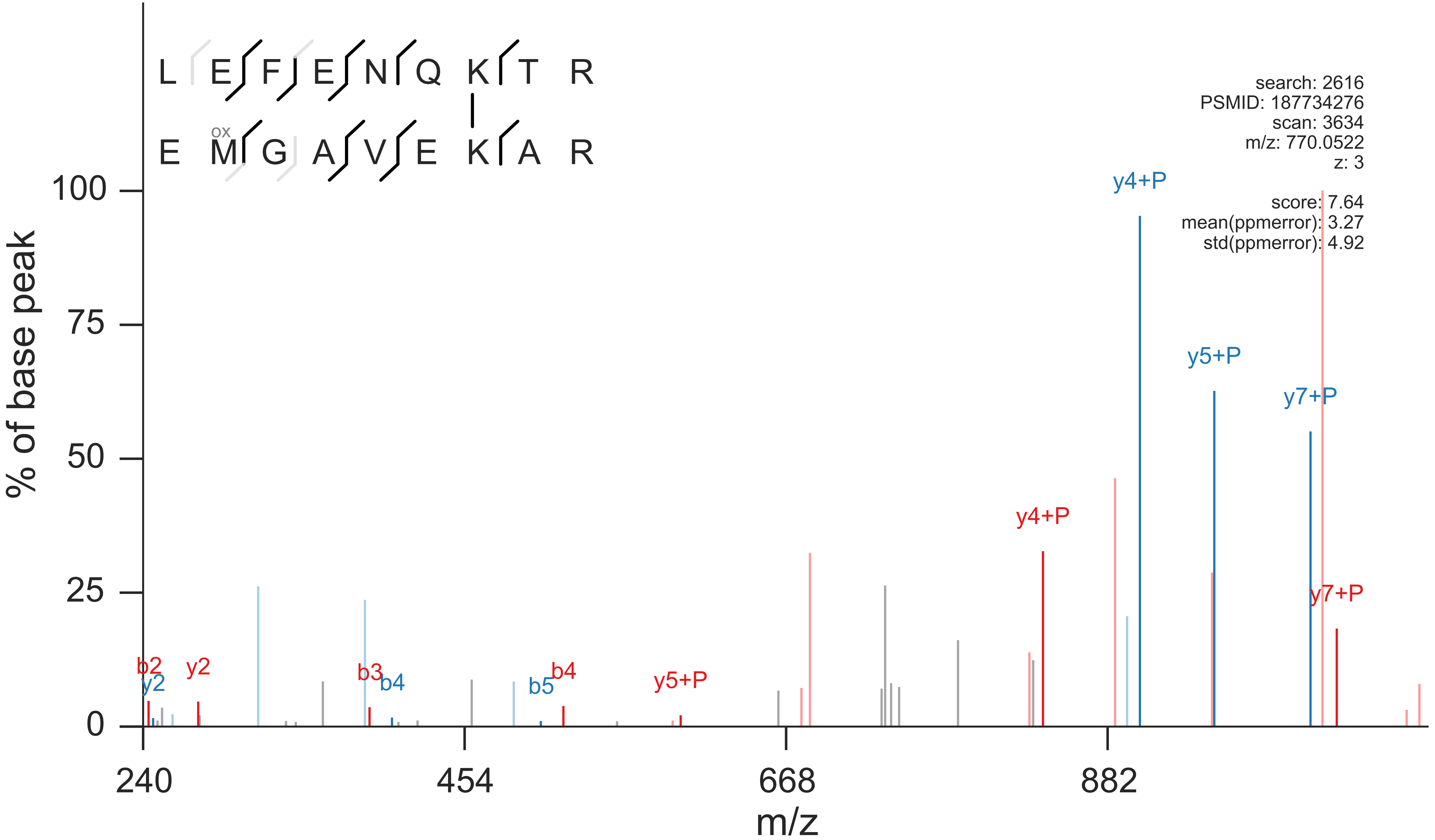

L E F E N Q K T R
E M G A V E K A R
ox
search: 2616
PSMID: 187734276
scan: 3634
m/z: 770.0522
z: 3
score: 7.64
mean(ppmerror): 3.27
std(ppmerror): 4.92
% of base peak
m/z
y4+P
y5+P
y7+P
y4+P
y7+P
b2
y2
y2
b3
b4
b5
b4
y5+P
100
75
50
25
0
240
454
668
882

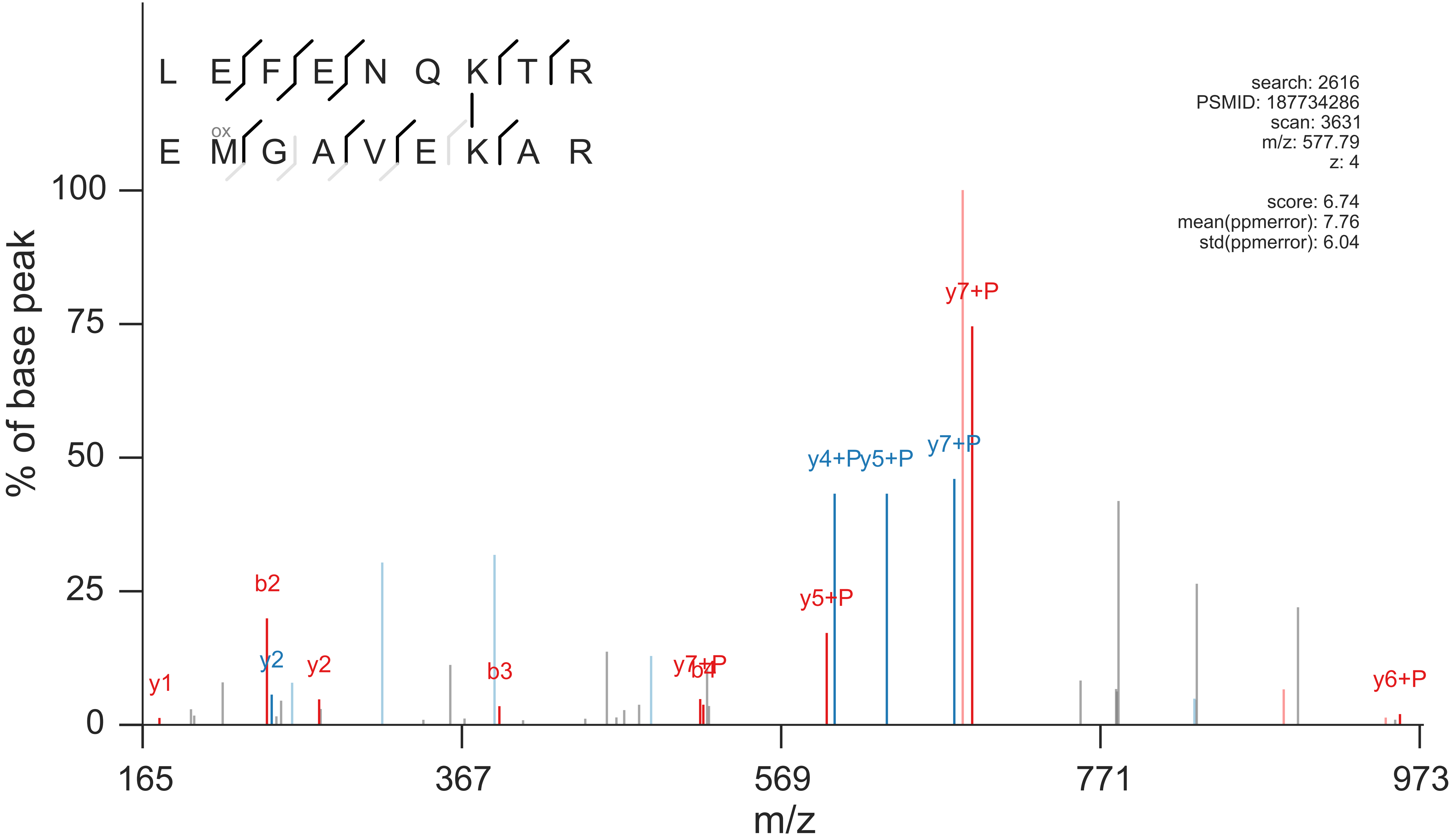
L E F E N Q K T R
E ox M G A V E K A R
search: 2616
PSMID: 187734286
scan: 3631
m/z: 577.79
z: 4
score: 6.74
mean(ppmerror): 7.76
std(ppmerror): 6.04
% of base peak
m/z
0
25
50
75
100
165
367
569
771
973
y1
b2
y2
y2
b3
y7+P
b4
y5+P
y4+P
y5+P
y7+P
y7+P
y6+P

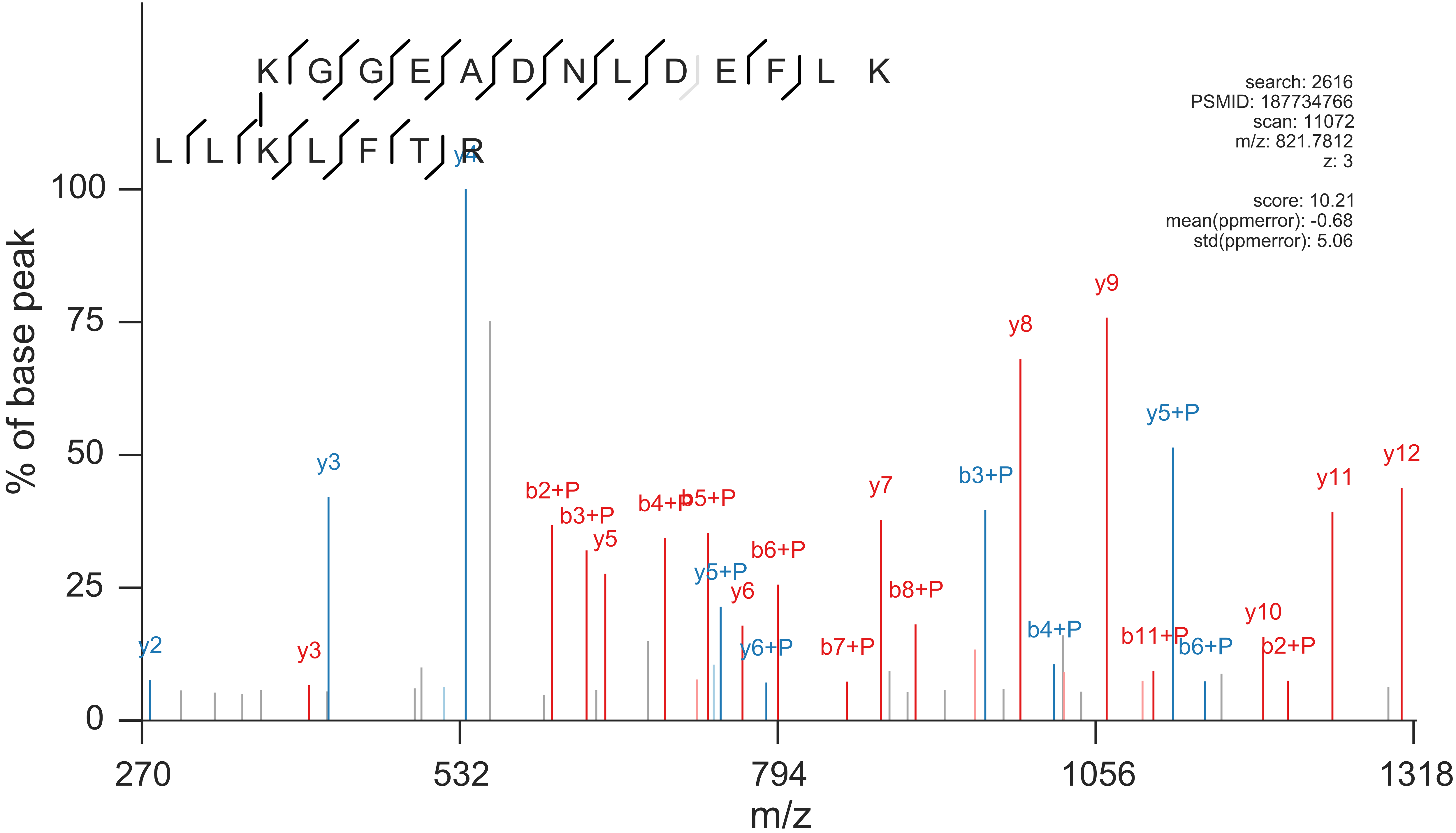
K G G E A D N L D E F L K
L L K L F T R
search: 2616
PSMID: 187734766
scan: 11072
m/z: 821.7812
z: 3
score: 10.21
mean(ppmerror): -0.68
std(ppmerror): 5.06
% of base peak
m/z
y2
y3
y4
b2+P
b3+P
y5
b4+P
b5+P
y5+P
y6
b6+P
y6+P
b7+P
y7
b8+P
b3+P
y8
b4+P
y9
b11+P
y5+P
b6+P
y10
b2+P
y11
y12

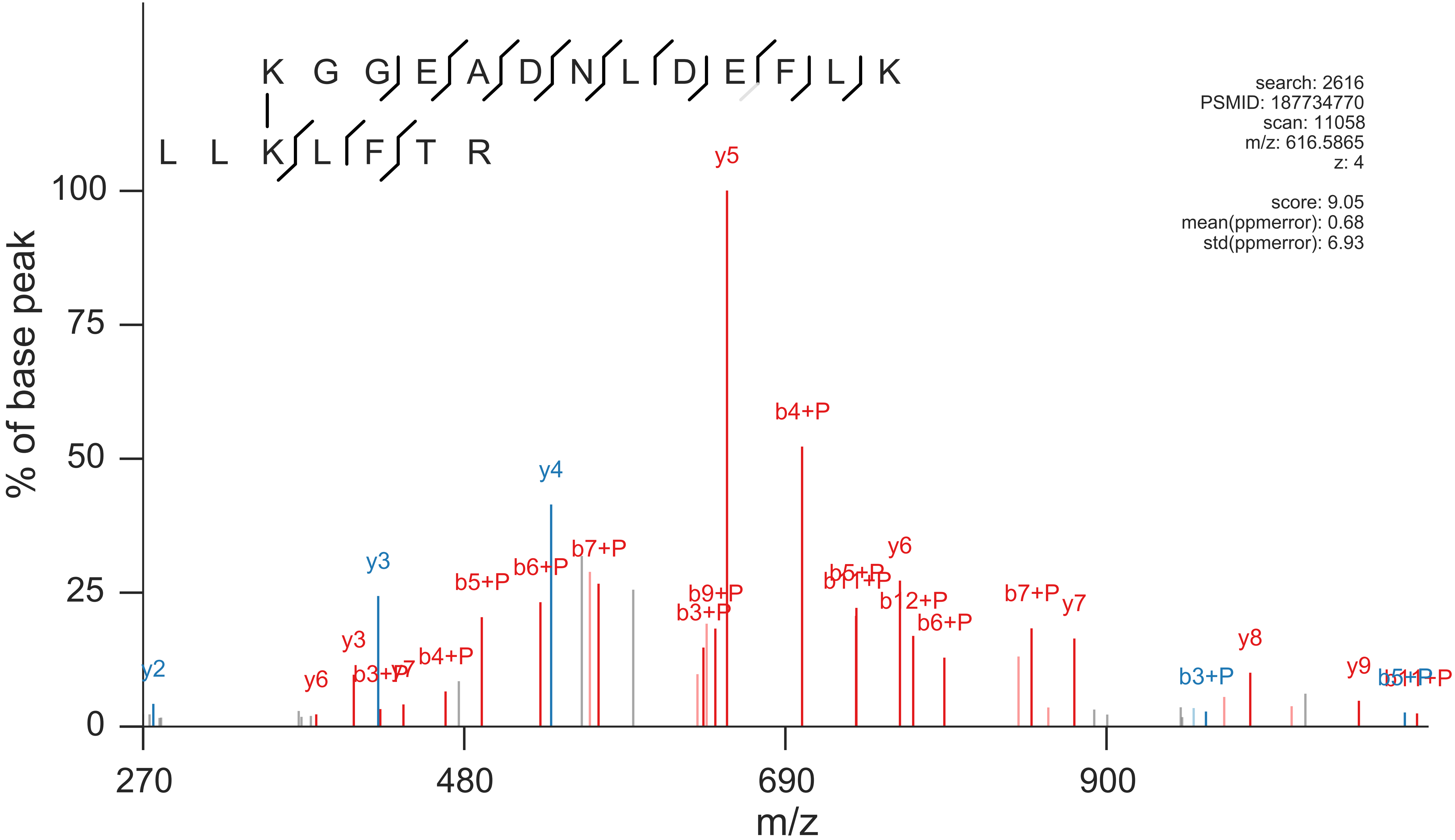

K G G E A D N L D E F L K
L L K L F T R
search: 2616
PSMID: 187734770
scan: 11058
m/z: 616.5865
z: 4
score: 9.05
mean(ppmerror): 0.68
std(ppmerror): 6.93
% of base peak
m/z
0
25
50
75
100
270
480
690
900
y2
y6
y3
y3
b3+P
y7
b4+P
b5+P
b6+P
y4
b7+P
b3+P
b9+P
y5
b4+P
b5+P
b11+P
y6
b12+P
b6+P
b7+P
y7
b3+P
y8
y9
b5+P
b11+P

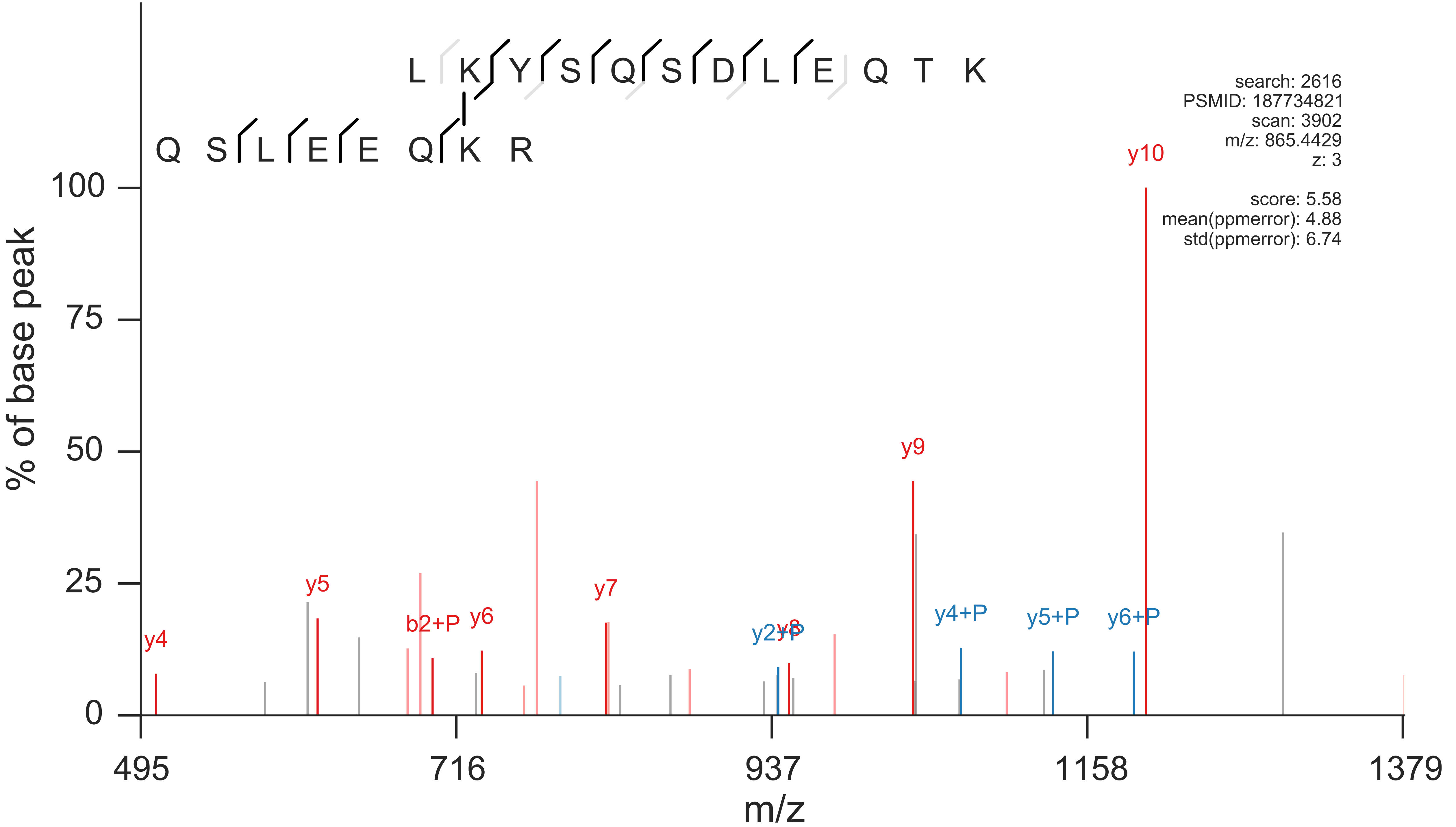
L K Y S Q S D L E Q T K
Q S L E E Q K R
search: 2616
PSMID: 187734821
scan: 3902
m/z: 865.4429
z: 3
score: 5.58
mean(ppmerror): 4.88
std(ppmerror): 6.74
% of base peak
m/z
y4
y5
b2+P
y6
y7
y2+P
y8
y9
y4+P
y5+P
y6+P
y10
0
25
50
75
100
495
716
937
1158
1379

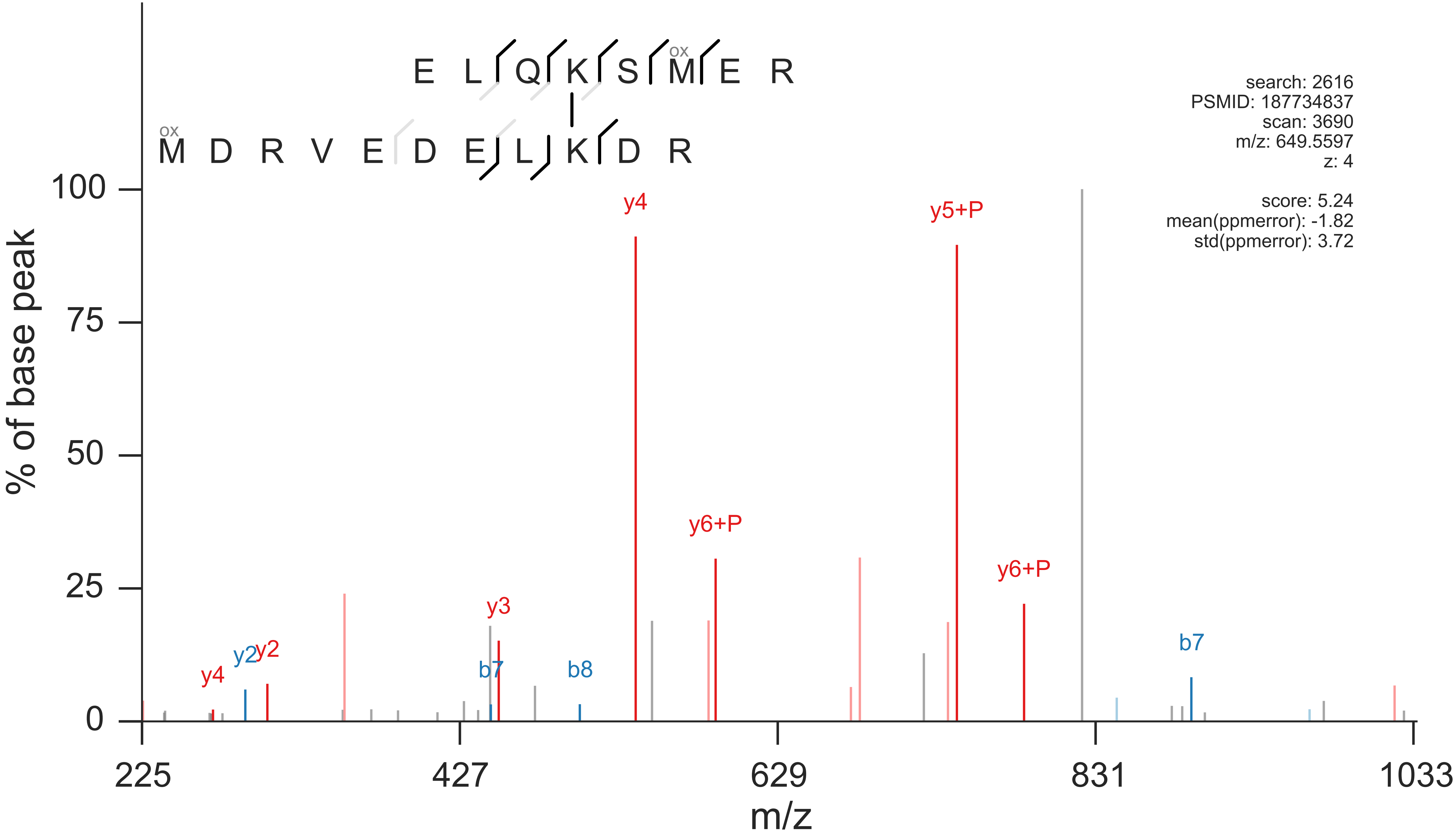

E L Q K S M E R
ox
M D R V E D E L K D R
ox
search: 2616
PSMID: 187734837
scan: 3690
m/z: 649.5597
z: 4
score: 5.24
mean(ppmerror): -1.82
std(ppmerror): 3.72
% of base peak
100
75
50
25
0
y4
y5+P
y6+P
y6+P
y3
y4
y2
y2
b7
b8
b7
225
427
629
831
1033
m/z

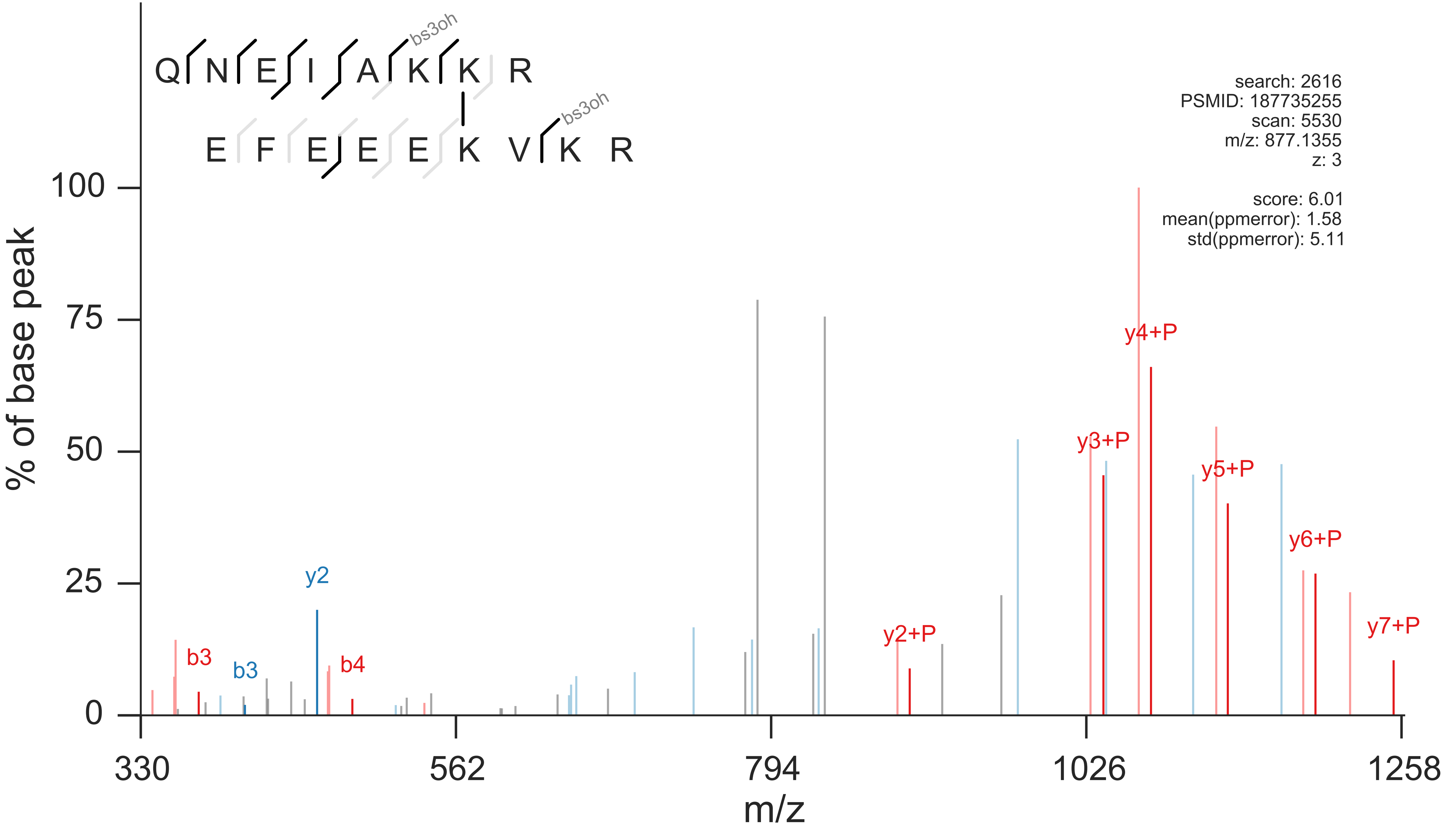

Q N E I A K K R
E F E E E K V K R
bs3oh
bs3oh
search: 2616
PSMID: 187735255
scan: 5530
m/z: 877.1355
z: 3
score: 6.01
mean(ppmerror): 1.58
std(ppmerror): 5.11
% of base peak
m/z
b3
b3
y2
b4
y2+P
y3+P
y4+P
y5+P
y6+P
y7+P

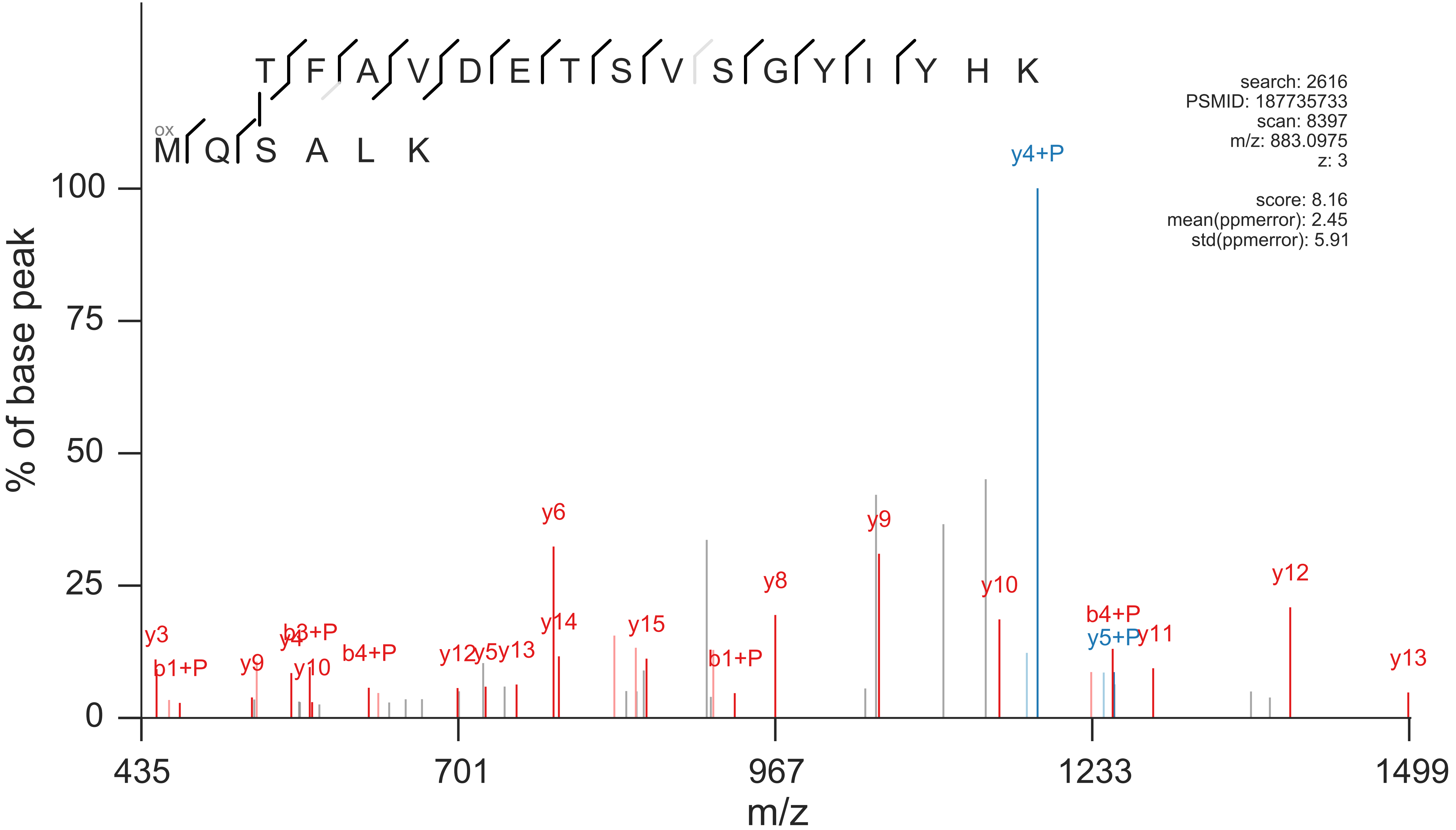

T F A V D E T S V S G Y I Y H K
ox
M Q S A L K
search: 2616
PSMID: 187735733
scan: 8397
m/z: 883.0975
z: 3
score: 8.16
mean(ppmerror): 2.45
std(ppmerror): 5.91
% of base peak
m/z
y4+P
y6
y9
y8
y10
y12
y14
y15
b4+P
y5+P
y11
y13
y3
b1+P
y9
y4
b3+P
y10
b4+P
y12
y5
y13
b1+P

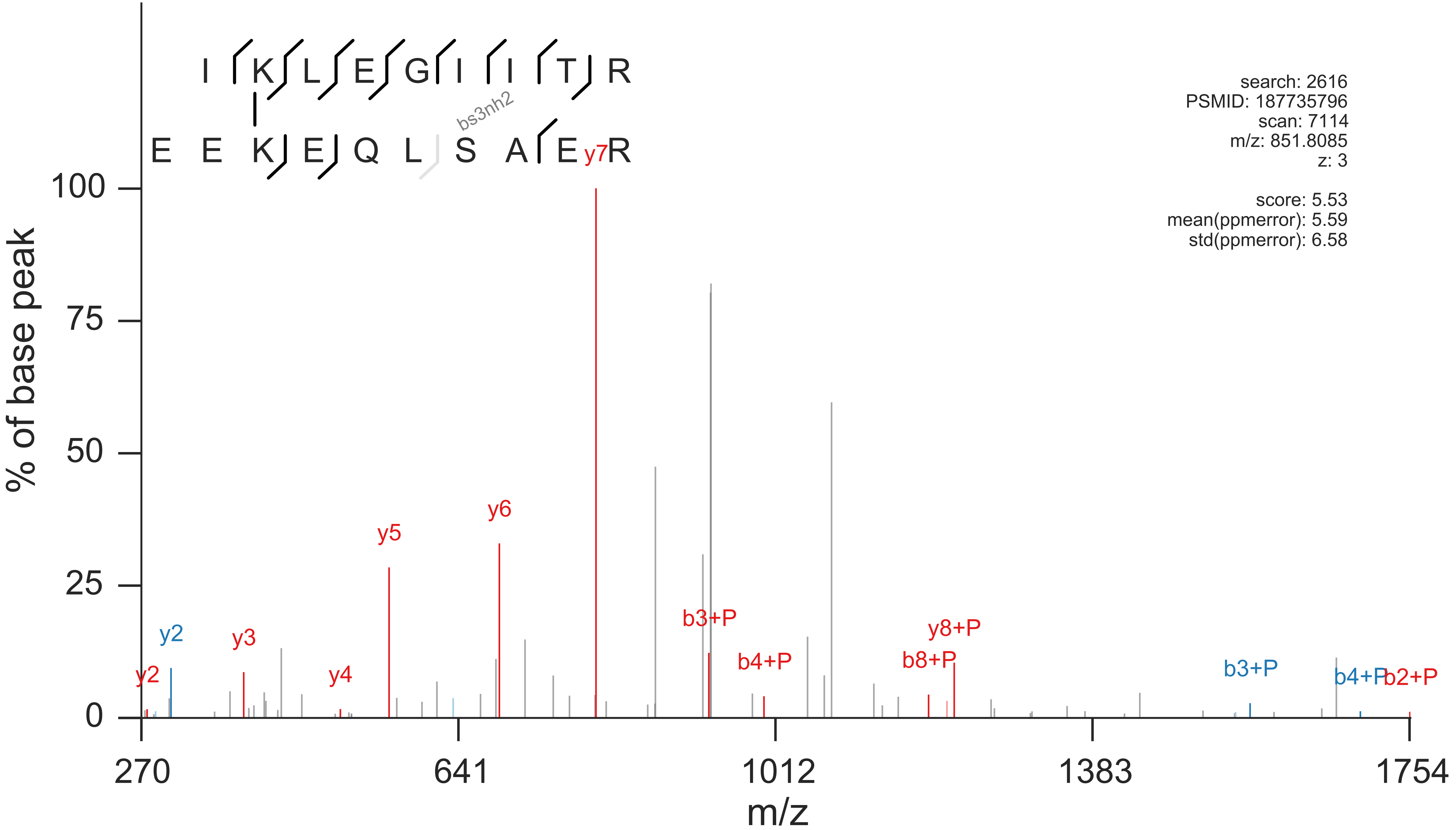

I K L E G I I T R
E E K E Q L S A E R
bs3nh2
search: 2616
PSMID: 187735796
scan: 7114
m/z: 851.8085
z: 3
score: 5.53
mean(ppmerror): 5.59
std(ppmerror): 6.58
% of base peak
m/z
100
75
50
25
0
270
641
1012
1383
1754
y2
y2
y3
y4
y5
y6
y7
b3+P
b4+P
b8+P
y8+P
b3+P
b4+P
b2+P

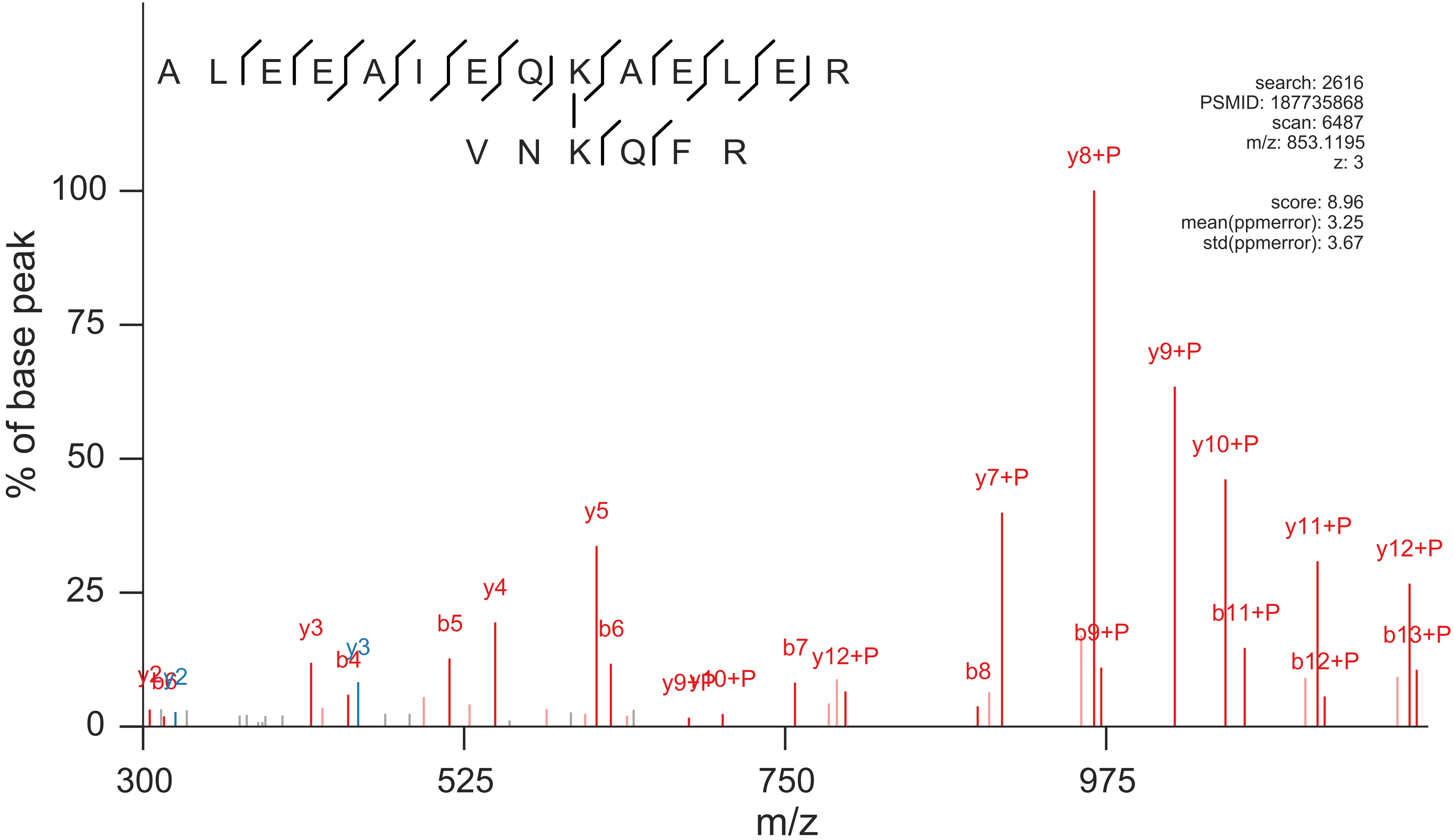

A L E E A I E Q K A E L E R
V N K Q F R
search: 2616
PSMID: 187735868
scan: 6487
m/z: 853.1195
z: 3
score: 8.96
mean(ppmerror): 3.25
std(ppmerror): 3.67
% of base peak
m/z
y8+P
y9+P
y10+P
y7+P
y5
y11+P
y12+P
y4
b11+P
b5
b6
y3
b9+P
b13+P
b7
y12+P
b12+P
b8
y9+P
y10+P

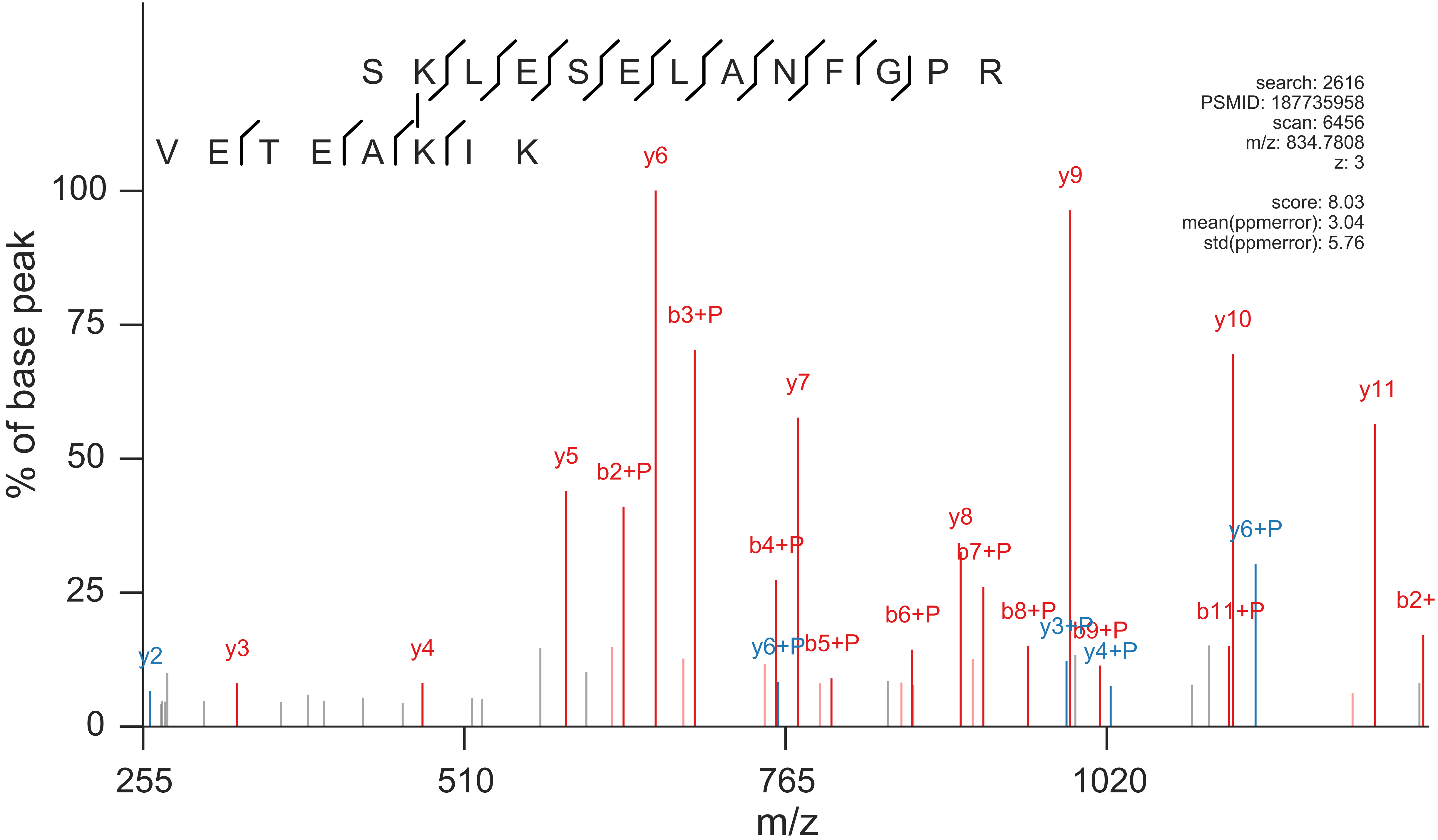

S K L E S E L A N F G P R
V E T E A K I K
search: 2616
PSMID: 187735958
scan: 6456
m/z: 834.7808
z: 3
score: 8.03
mean(ppmerror): 3.04
std(ppmerror): 5.76
% of base peak
m/z
y2
y3
y4
y5
b2+P
y6
b3+P
y6+P
b4+P
y7
b5+P
b6+P
y8
b7+P
b8+P
y3+P
b9+P
y4+P
y9
b11+P
y10
y6+P
y11
b2+P

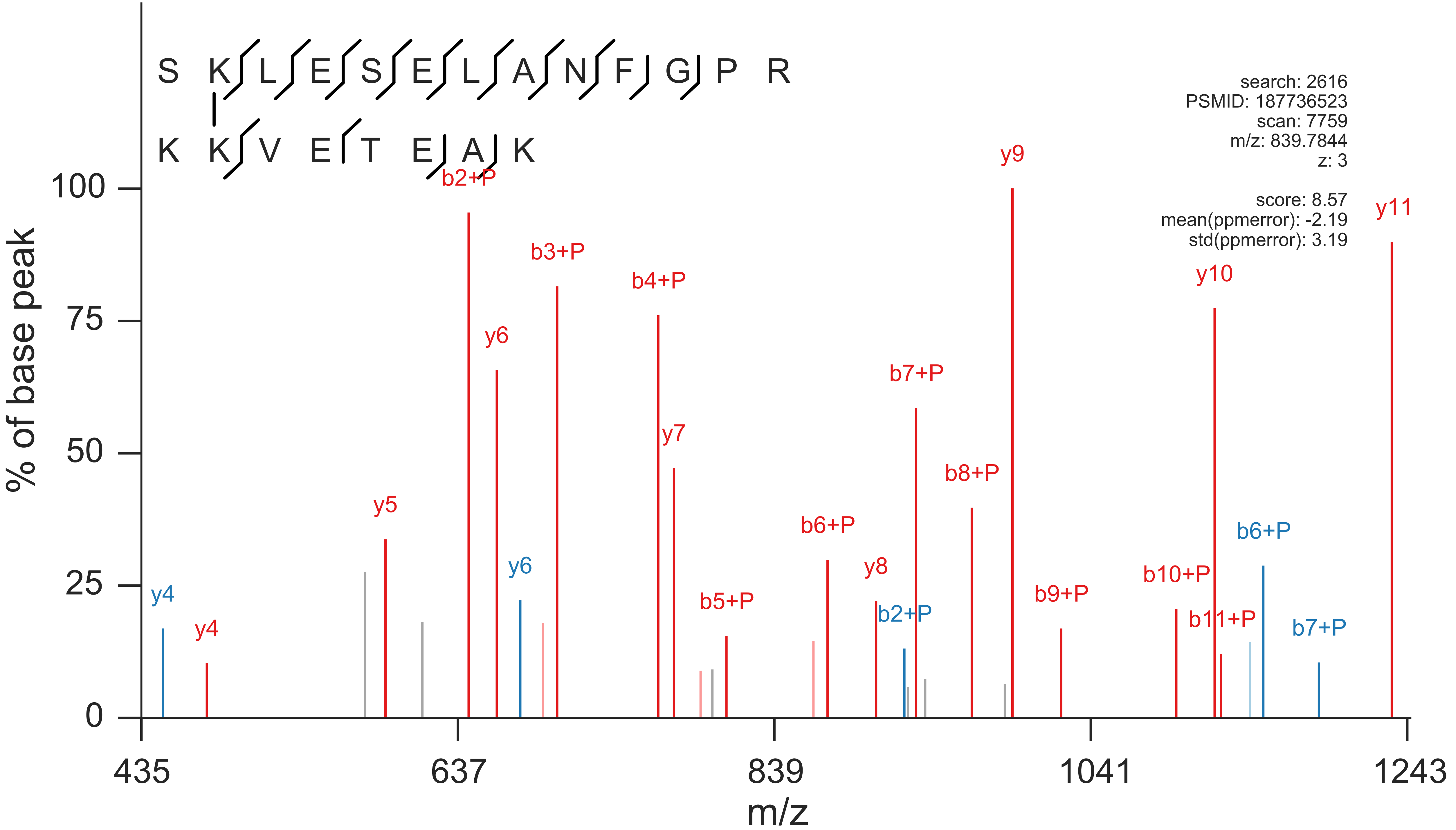

S K L E S E L A N F G P R
K K V E T E A K
search: 2616
PSMID: 187736523
scan: 7759
m/z: 839.7844
z: 3
score: 8.57
mean(ppmerror): -2.19
std(ppmerror): 3.19
% of base peak
m/z
0
25
50
75
100
435
637
839
1041
1243
y4
y4
y5
b2+P
y6
y6
b3+P
b4+P
y7
b5+P
b6+P
y8
b2+P
b7+P
b8+P
y9
b9+P
b10+P
y10
b11+P
b6+P
b7+P
y11

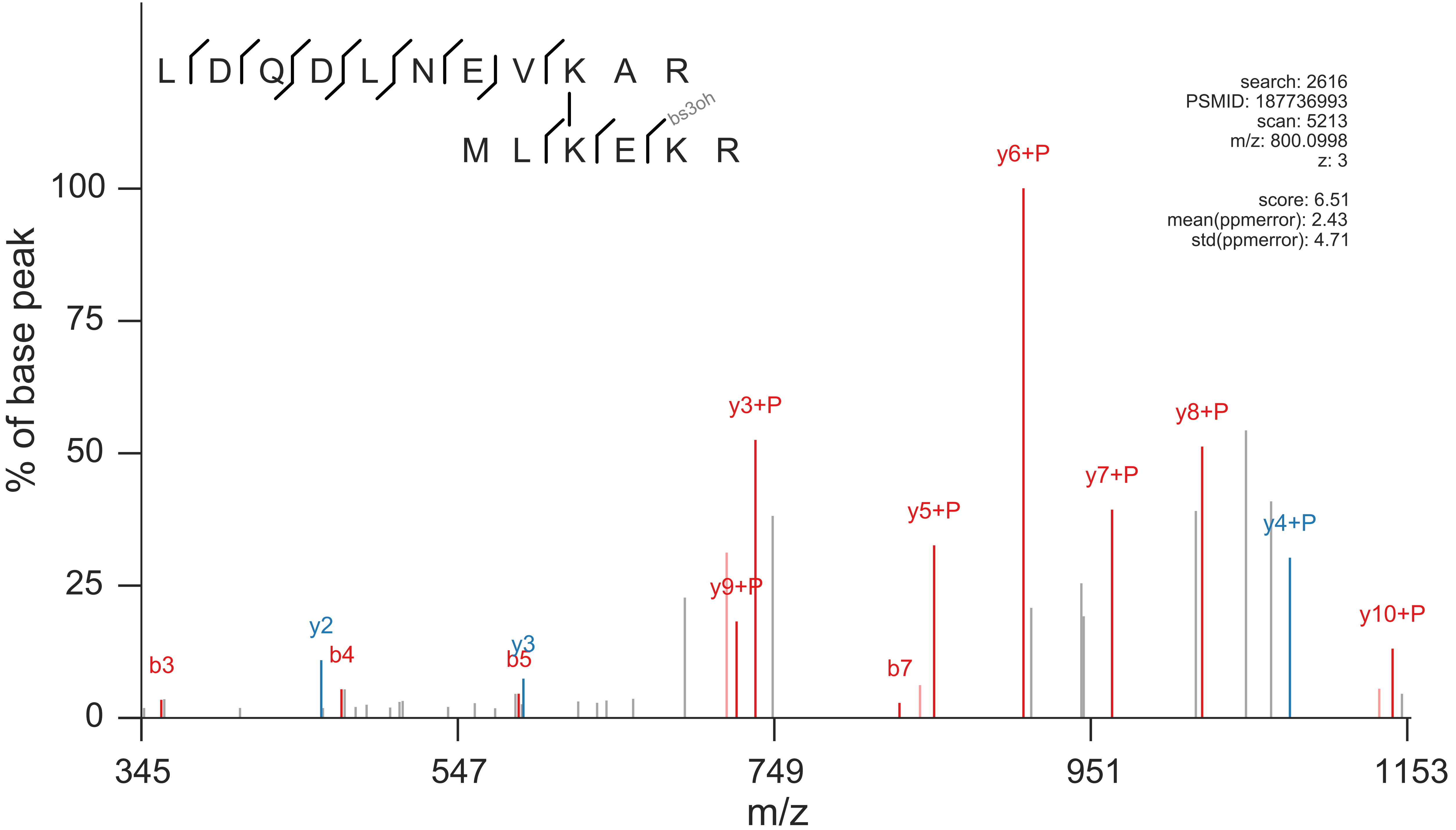
L D Q D L N E V K A R
M L K E K R
bs3oh
search: 2616
PSMID: 187736993
scan: 5213
m/z: 800.0998
z: 3
score: 6.51
mean(ppmerror): 2.43
std(ppmerror): 4.71
% of base peak
100
75
50
25
0
y6+P
y3+P
y8+P
y7+P
y5+P
y4+P
y9+P
y10+P
y2
b4
y3
b5
b3
b7
345
547
749
951
1153
m/z

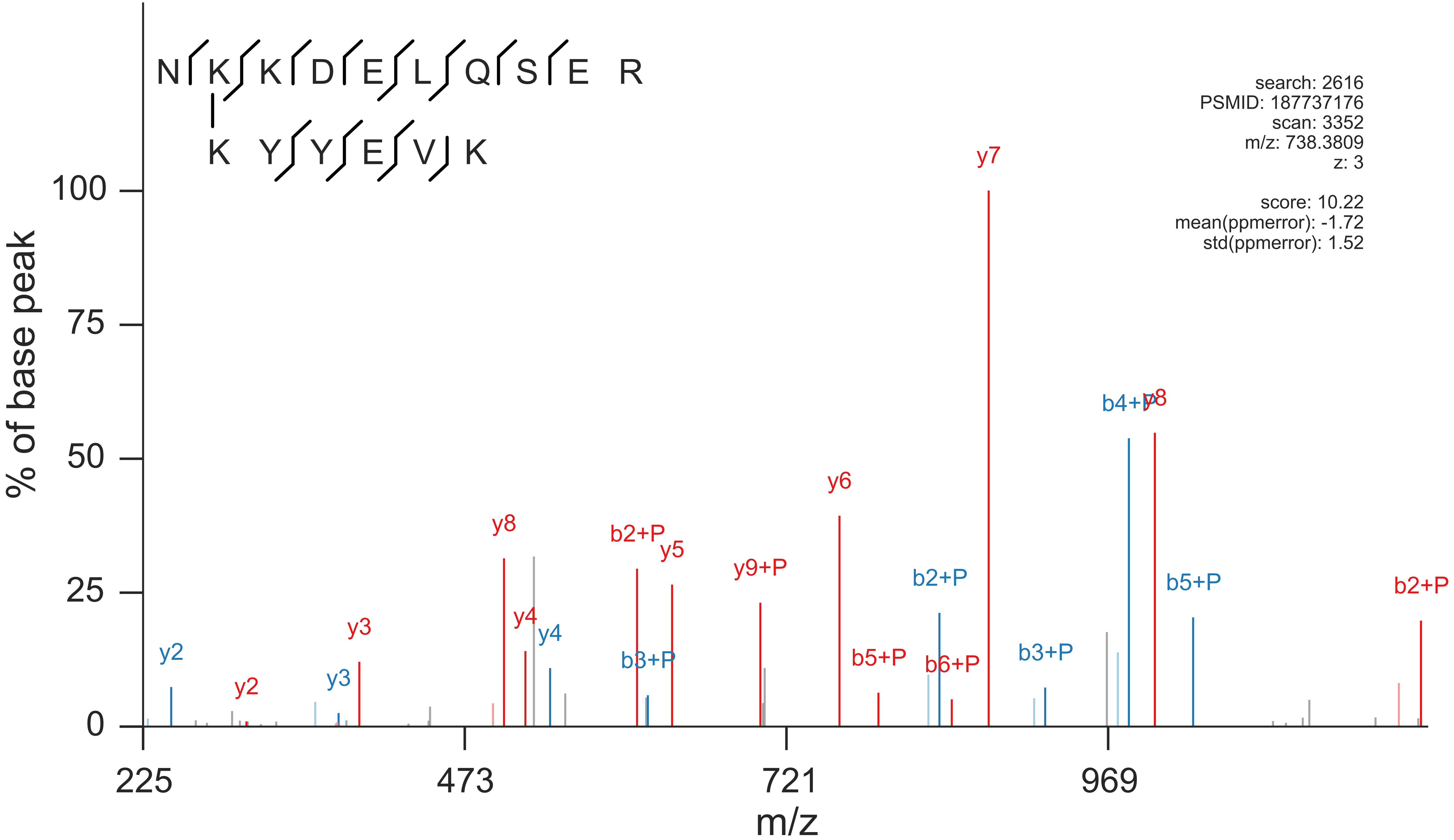

N K K D E L Q S E R
K Y Y E V K
search: 2616
PSMID: 187737176
scan: 3352
m/z: 738.3809
z: 3
score: 10.22
mean(ppmerror): -1.72
std(ppmerror): 1.52
% of base peak
m/z
0
25
50
75
100
225
473
721
969
y2
y2
y3
y3
y8
y4
y4
b2+P
y5
b3+P
y9+P
y6
b5+P
b2+P
b6+P
y7
b3+P
b4+P
y8
b5+P
b2+P

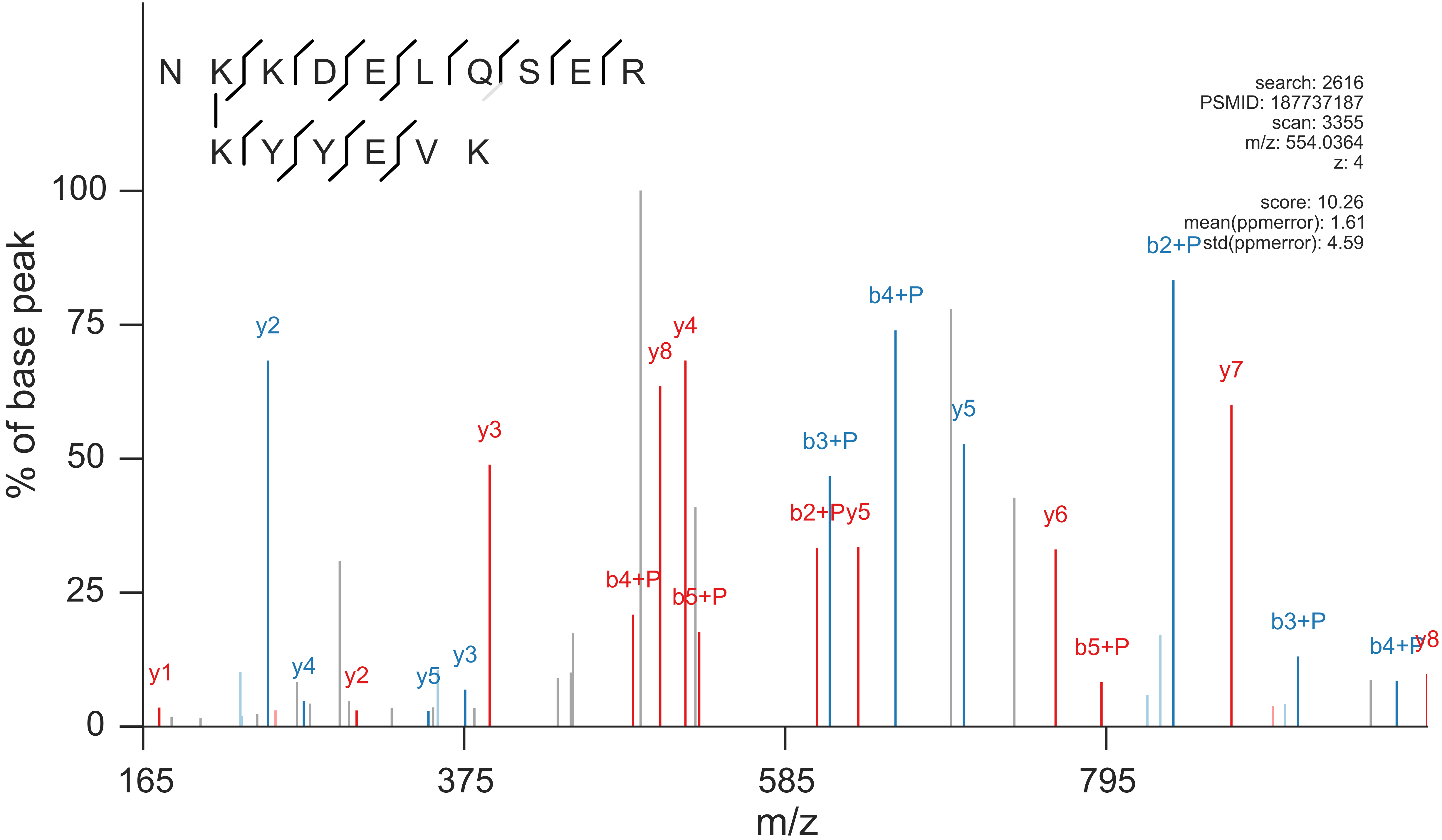

N K K D E L Q S E R
K Y Y E V K
search: 2616
PSMID: 187737187
scan: 3355
m/z: 554.0364
z: 4
score: 10.26
mean(ppmerror): 1.61
std(ppmerror): 4.59
% of base peak
m/z
0
25
50
75
100
165
375
585
795
y1
y2
y4
y2
y5
y3
y3
b4+P
y8
y4
b5+P
b2+P
y5
b3+P
b4+P
y5
y6
b5+P
b2+P
y7
b3+P
b4+P
y8

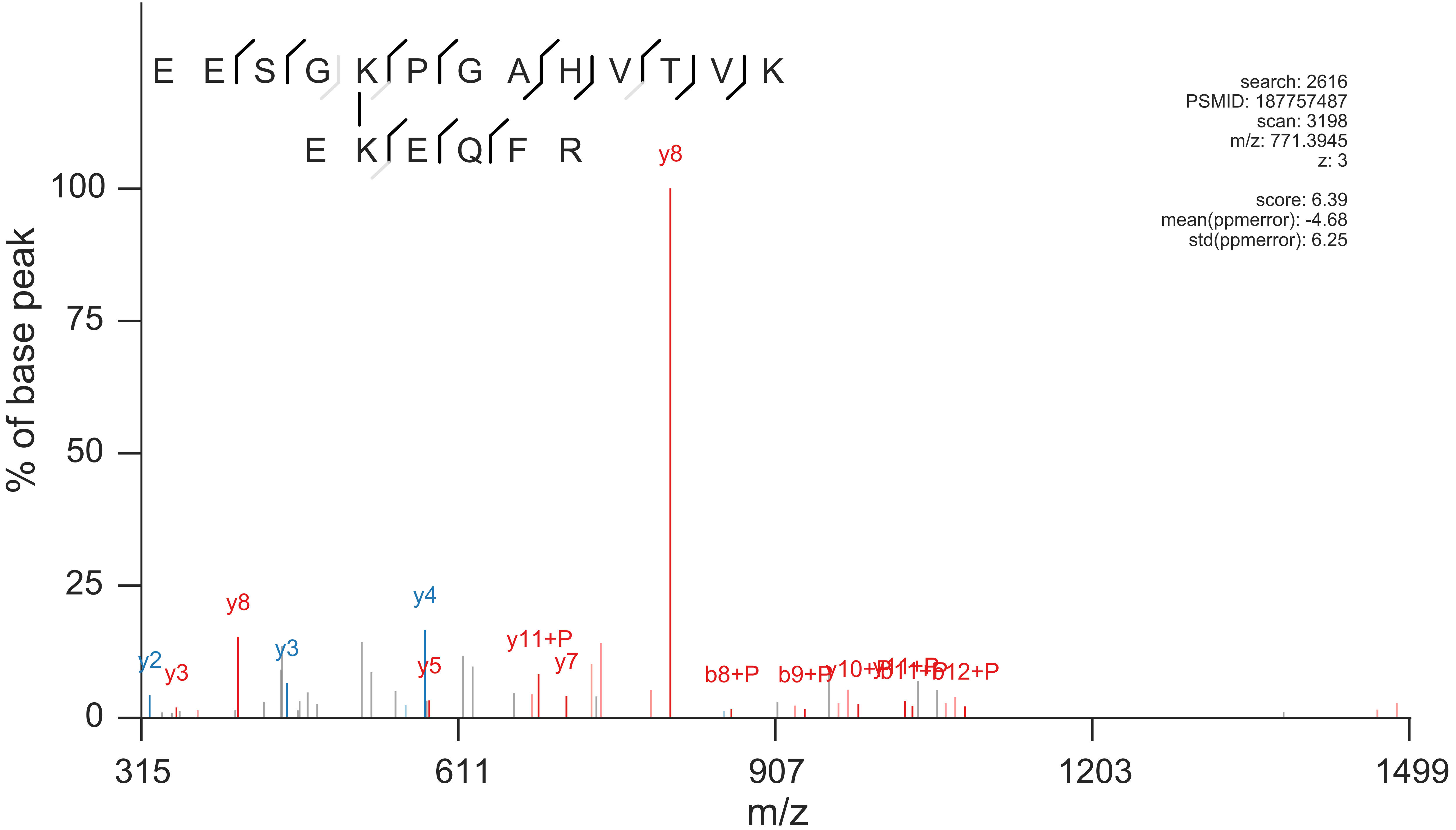
E E S G K P G A H V T V K
E K E Q F R
search: 2616
PSMID: 187757487
scan: 3198
m/z: 771.3945
z: 3
score: 6.39
mean(ppmerror): -4.68
std(ppmerror): 6.25
% of base peak
m/z
y2
y3
y8
y3
y4
y5
y11+P
y7
y8
b8+P
b9+P
b12+P

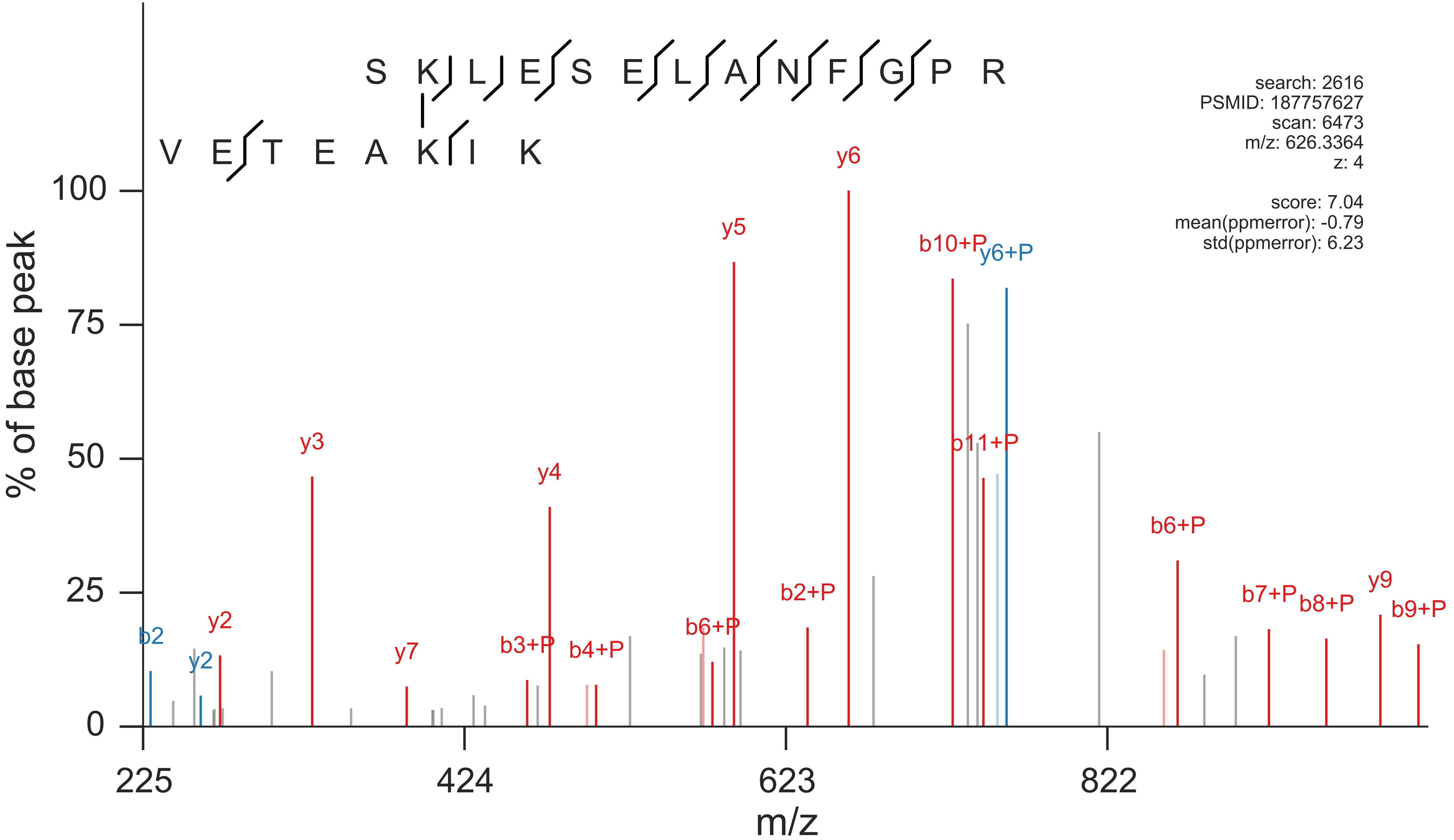

S K L E S E L A N F G P R
V E T E A K I K
search: 2616
PSMID: 187757627
scan: 6473
m/z: 626.3364
z: 4
score: 7.04
mean(ppmerror): -0.79
std(ppmerror): 6.23
% of base peak
m/z
100
75
50
25
0
225
424
623
822
y6
y5
b10+P
y6+P
y3
b11+P
y4
b6+P
b2+P
b7+P
b8+P
y9
b9+P
b2
y2
y2
y7
b3+P
b4+P
b6+P

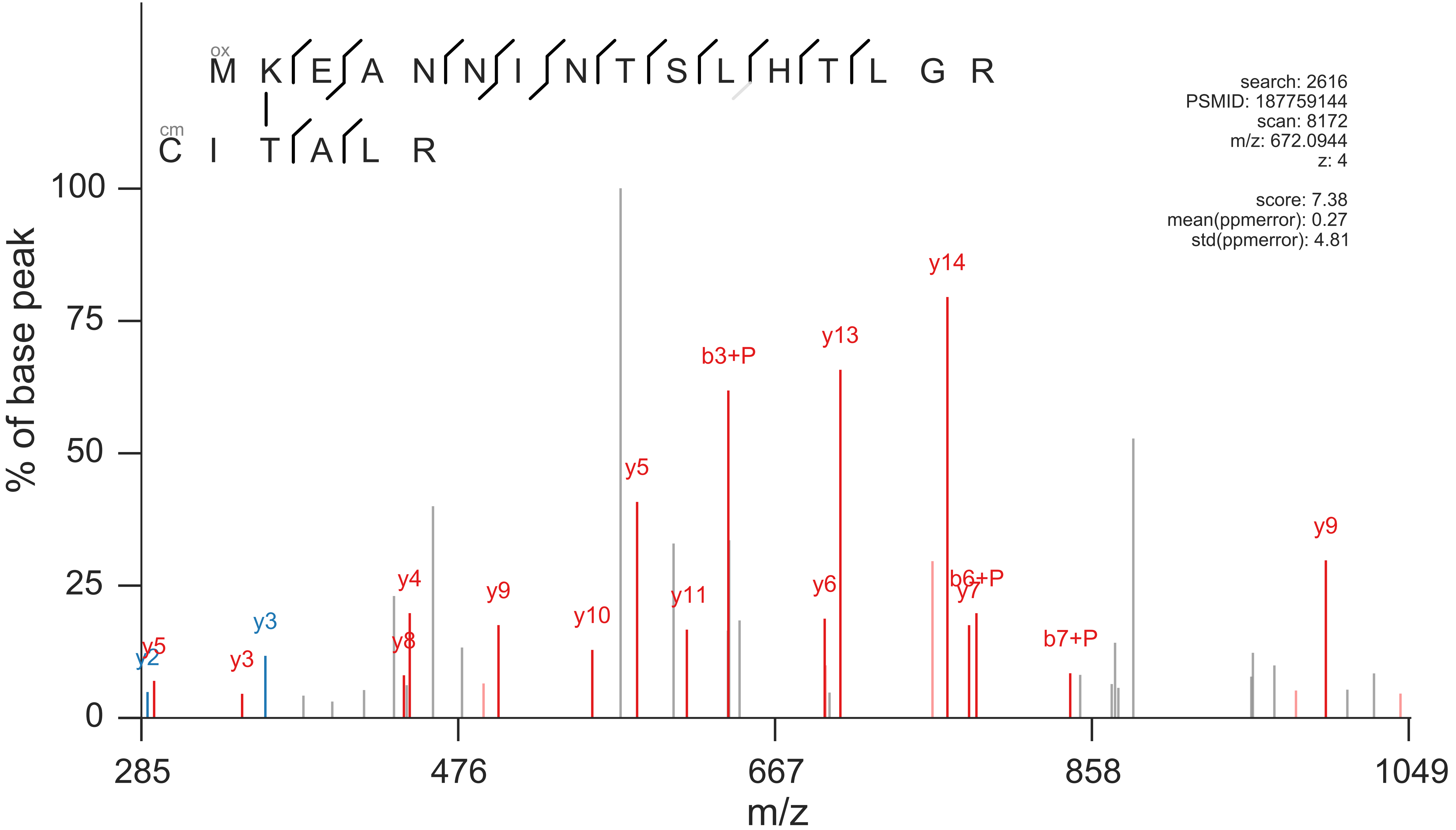

ox
M K E A N N I N T S L H T L G R
cm
C I T A L R
search: 2616
PSMID: 187759144
scan: 8172
m/z: 672.0944
z: 4
score: 7.38
mean(ppmerror): 0.27
std(ppmerror): 4.81
% of base peak
m/z
100
75
50
25
0
285
476
667
858
1049
y2
y5
y3
y3
y4
y8
y9
y10
y5
y11
b3+P
y6
y13
y14
b6+P
y7
b7+P
y9

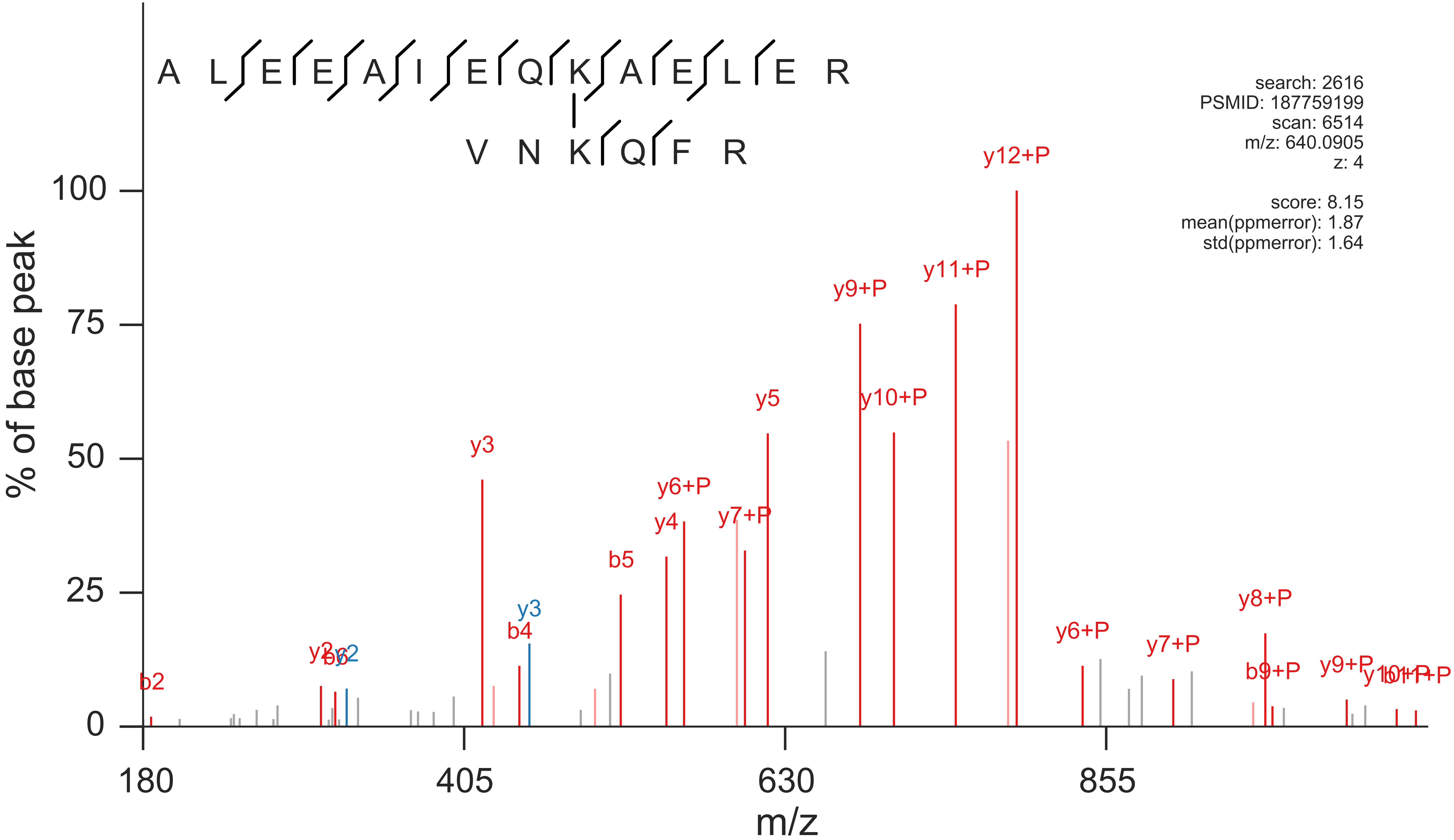

A L E E A I E Q K A E L E R
V N K Q F R
search: 2616
PSMID: 187759199
scan: 6514
m/z: 640.0905
z: 4
score: 8.15
mean(ppmerror): 1.87
std(ppmerror): 1.64
% of base peak
m/z
y12+P
y11+P
y9+P
y10+P
y5
y3
y6+P
y4
y7+P
b5
y3
b4
b2
y6+P
y7+P
y8+P
b9+P
y9+P
0
25
50
75
100
180
405
630
855

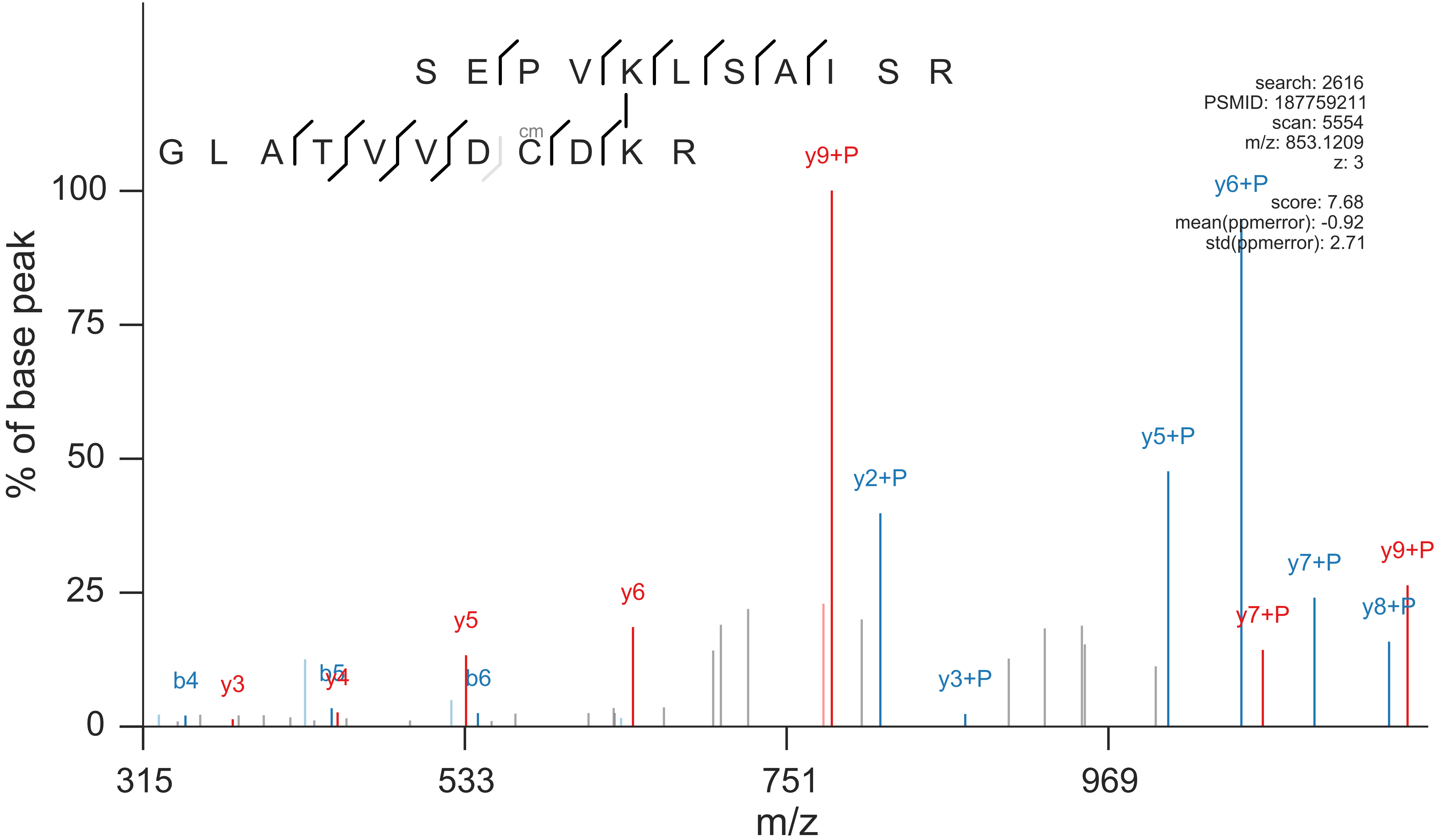

S E P V K L S A I S R
G L A T V V D C D K R
cm
search: 2616
PSMID: 187759211
scan: 5554
m/z: 853.1209
z: 3
score: 7.68
mean(ppmerror): -0.92
std(ppmerror): 2.71
% of base peak
m/z
0
25
50
75
100
315
533
751
969
y9+P
y6+P
y5+P
y2+P
y7+P
y9+P
y8+P
y7+P
y6
y5
y3+P
b4
y3
b5
y4
b6

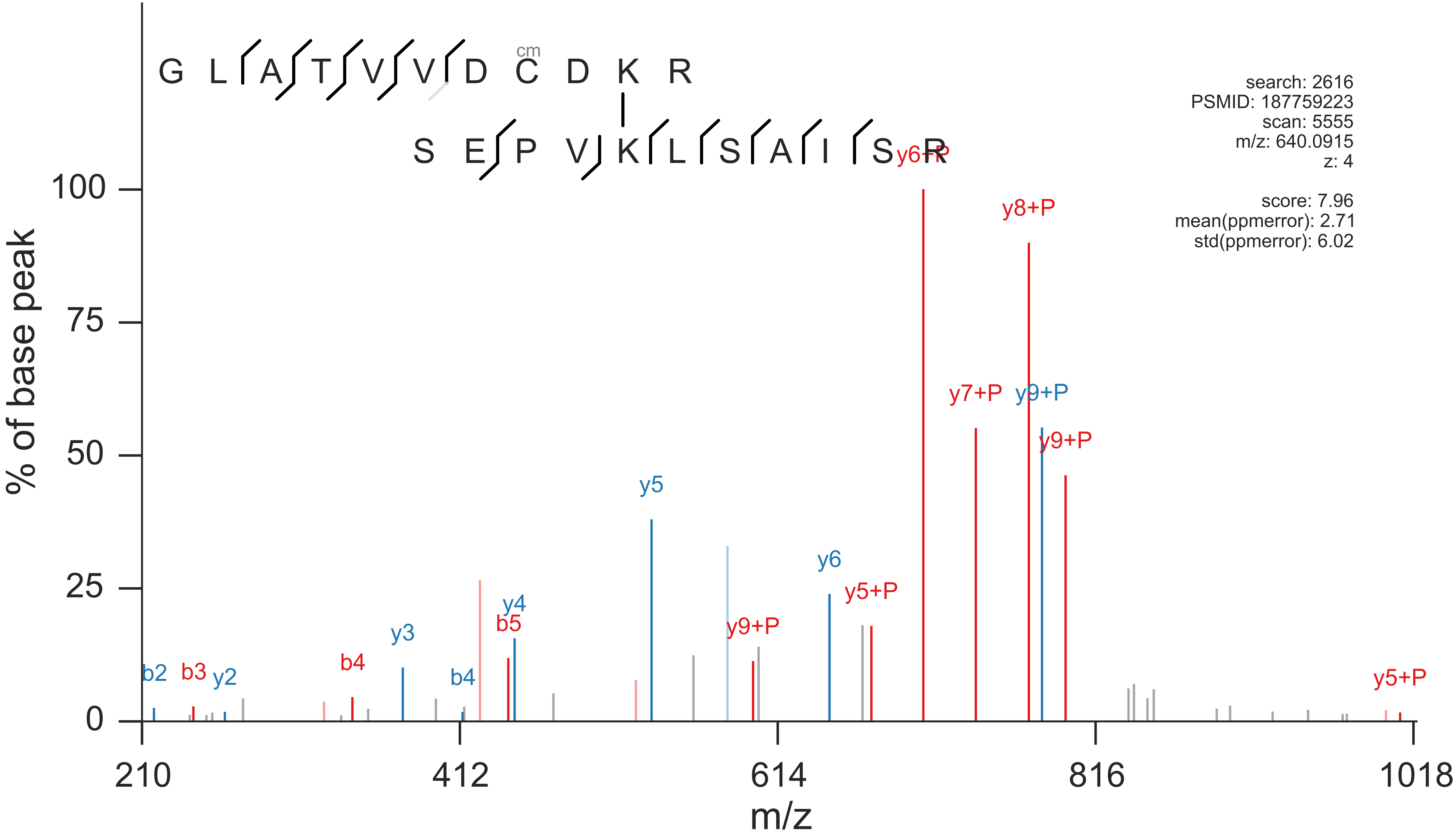

G L A T V V D C D K R
cm
S E P V K L S A I S R
search: 2616
PSMID: 187759223
scan: 5555
m/z: 640.0915
z: 4
score: 7.96
mean(ppmerror): 2.71
std(ppmerror): 6.02
% of base peak
m/z
0
25
50
75
100
210
412
614
816
1018
b2
b3
y2
b4
y3
b4
y4
b5
y5
y9+P
y6
y5+P
y6+P
y7+P
y8+P
y9+P
y9+P
y5+P

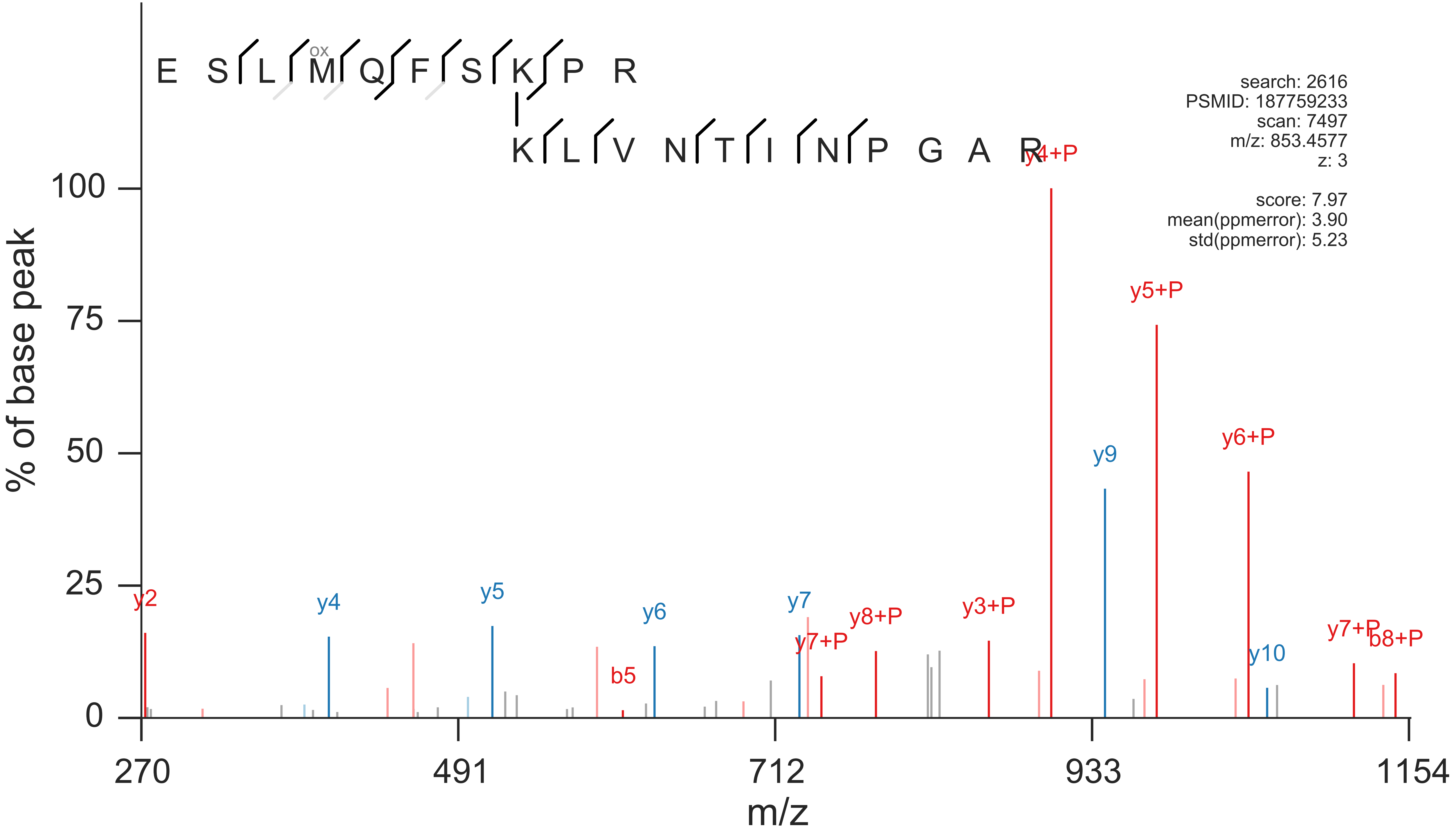

E S L M Q F S K P R
ox
K L V N T I N P G A R
search: 2616
PSMID: 187759233
scan: 7497
m/z: 853.4577
z: 3
score: 7.97
mean(ppmerror): 3.90
std(ppmerror): 5.23
% of base peak
100
75
50
25
0
y4+P
y5+P
y6+P
y9
y2
y4
y5
y6
y7
y7+P
y8+P
y3+P
b5
y10
y7+P
b8+P
270
491
712
933
1154
m/z

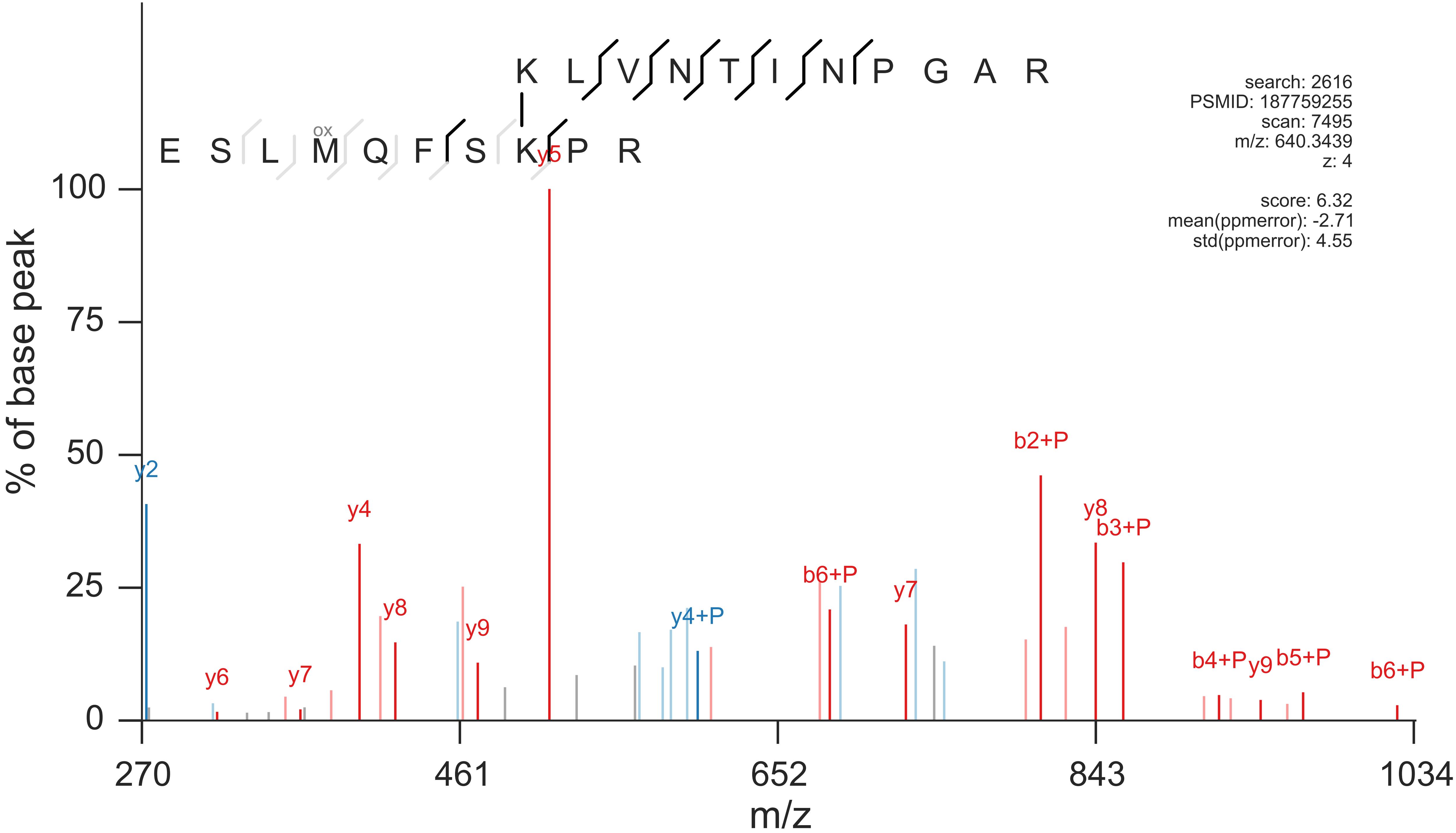

K L V N T I N P G A R
E S L M Q F S K P R
ox
search: 2616
PSMID: 187759255
scan: 7495
m/z: 640.3439
z: 4
score: 6.32
mean(ppmerror): -2.71
std(ppmerror): 4.55
% of base peak
m/z
0
25
50
75
100
270
461
652
843
1034
y2
y6
y7
y4
y8
y9
y5
y4+P
b6+P
y7
b2+P
y8
b3+P
b4+P
y9
b5+P
b6+P

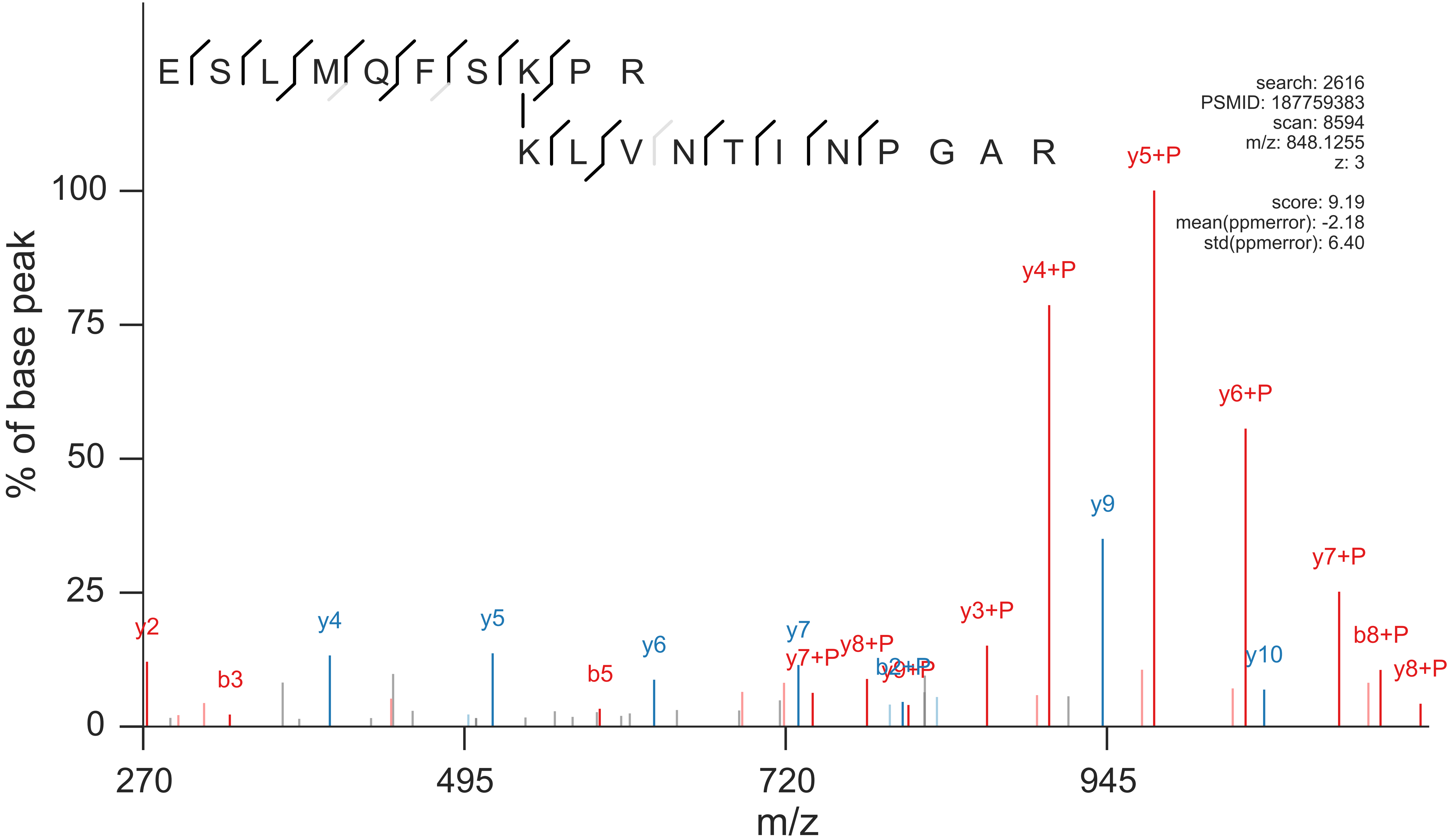

E S L M Q F S K P R
K L V N T I N P G A R
search: 2616
PSMID: 187759383
scan: 8594
m/z: 848.1255
z: 3
score: 9.19
mean(ppmerror): -2.18
std(ppmerror): 6.40
% of base peak
m/z
0
25
50
75
100
270
495
720
945
y2
b3
y4
y5
b5
y6
y7
y7+P
y8+P
y3+P
y4+P
y9
y5+P
y6+P
y10
y7+P
b8+P
y8+P

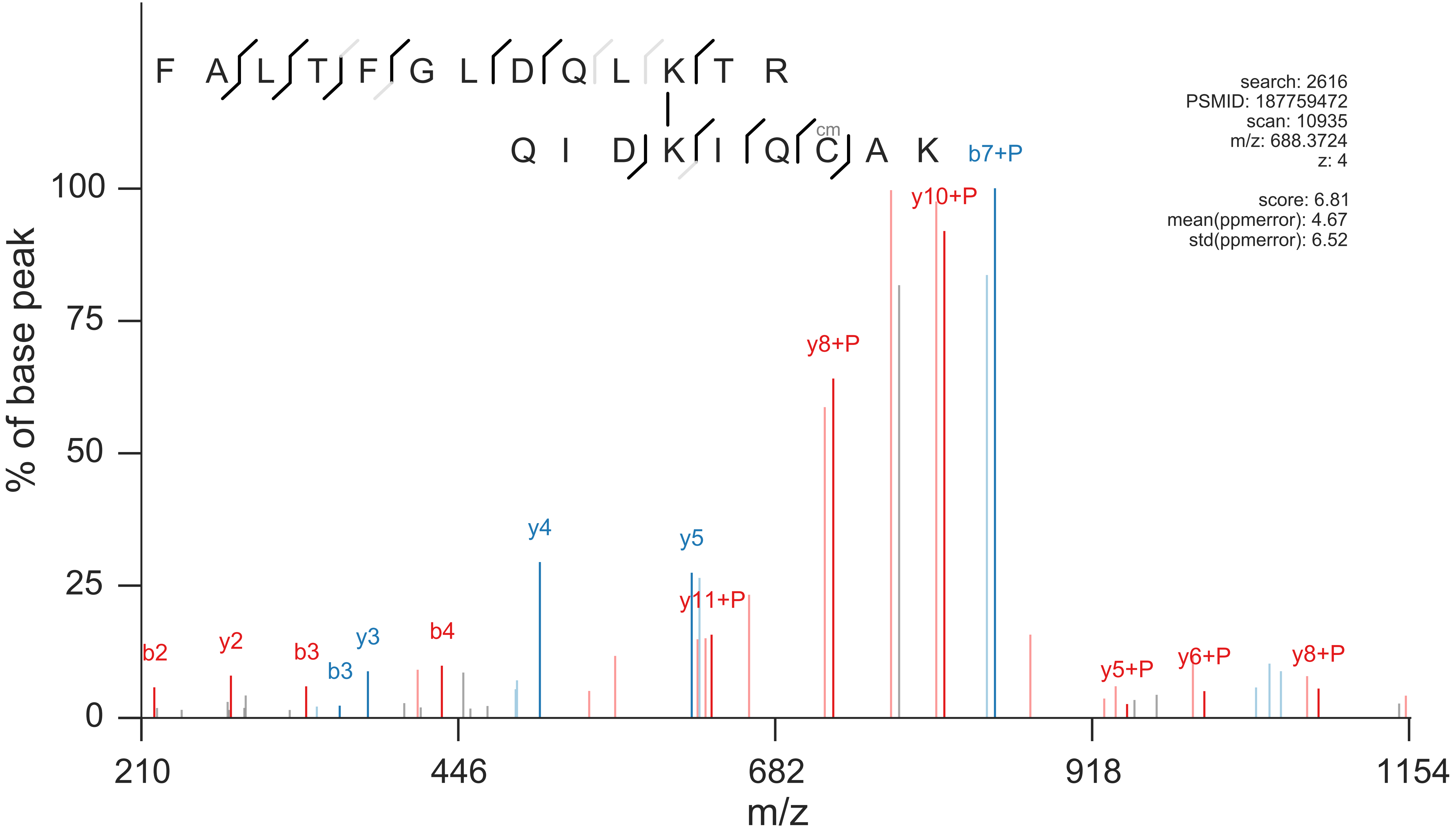

F A L T F G L D Q L K T R
Q I D K I Q C A K
cm
search: 2616
PSMID: 187759472
scan: 10935
m/z: 688.3724
z: 4
score: 6.81
mean(ppmerror): 4.67
std(ppmerror): 6.52
% of base peak
m/z
0
25
50
75
100
210
446
682
918
1154
b2
y2
b3
b3
y3
b4
y4
y5
y11+P
y8+P
y10+P
b7+P
y5+P
y6+P
y8+P

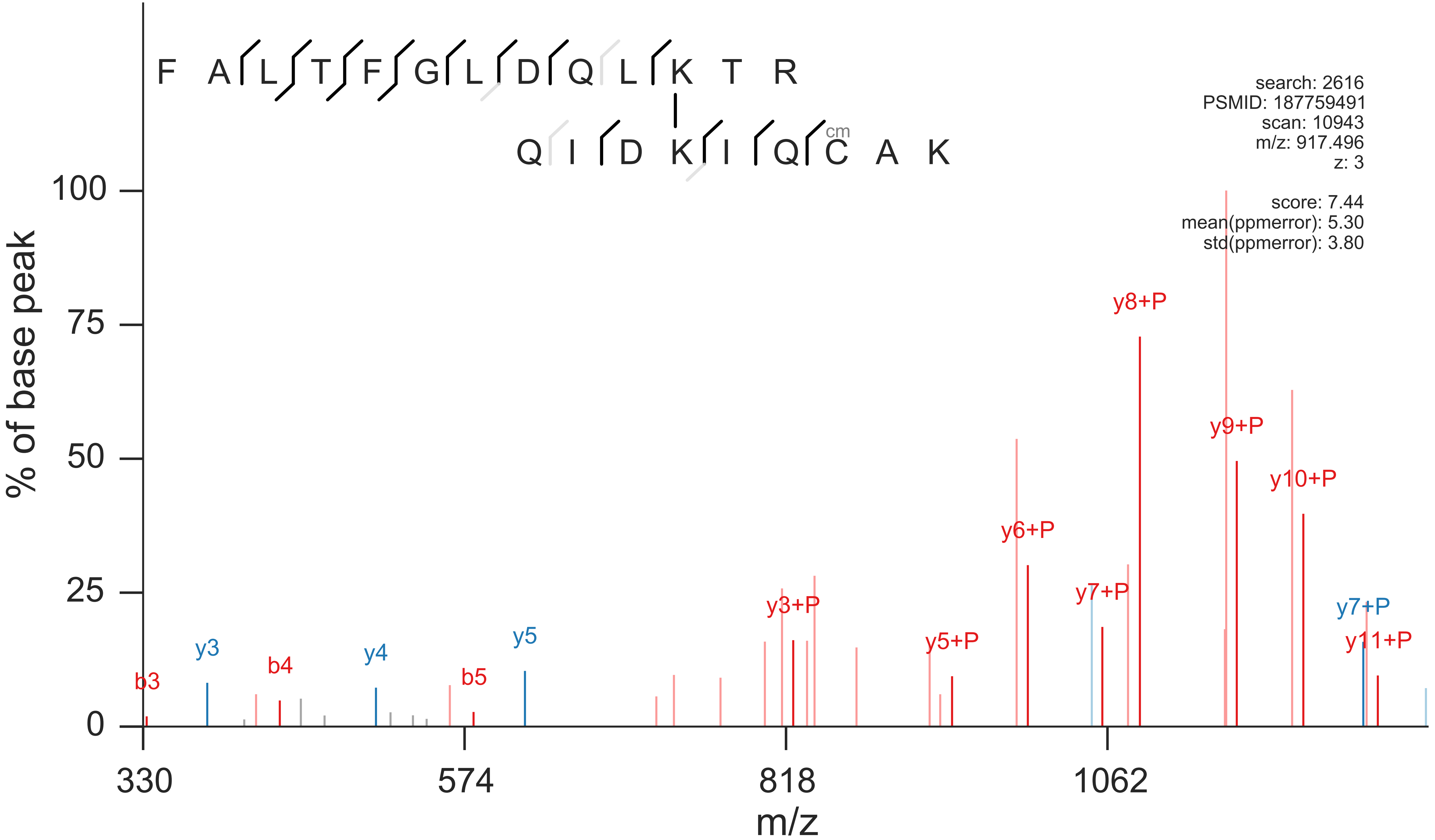

F A L T F G L D Q L K T R
Q I D K I Q C A K
cm
search: 2616
PSMID: 187759491
scan: 10943
m/z: 917.496
z: 3
score: 7.44
mean(ppmerror): 5.30
std(ppmerror): 3.80
% of base peak
100
75
50
25
0
y8+P
y9+P
y10+P
y6+P
y7+P
y3+P
y5+P
y7+P
y11+P
y3
y4
y5
b3
b4
b5
330
574
818
1062
m/z

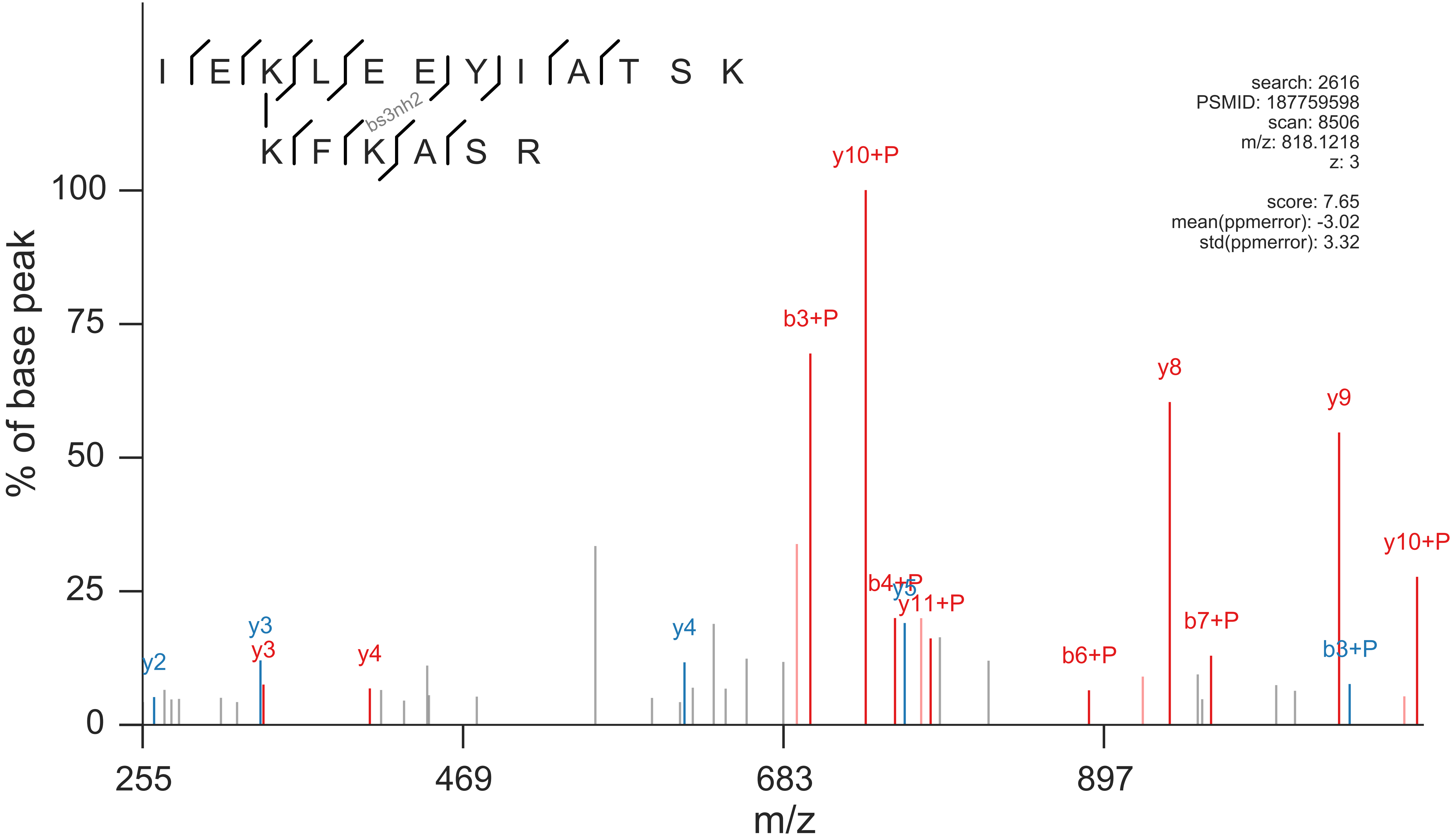

I E K L E E Y I A T S K
K F K A S R
bs3nh2
search: 2616
PSMID: 187759598
scan: 8506
m/z: 818.1218
z: 3
score: 7.65
mean(ppmerror): -3.02
std(ppmerror): 3.32
y10+P
b3+P
y8
y9
y10+P
b4+P
y5
y11+P
b7+P
b6+P
b3+P
y2
y3
y3
y4
y4
% of base peak
100
75
50
25
0
255
469
683
897
m/z

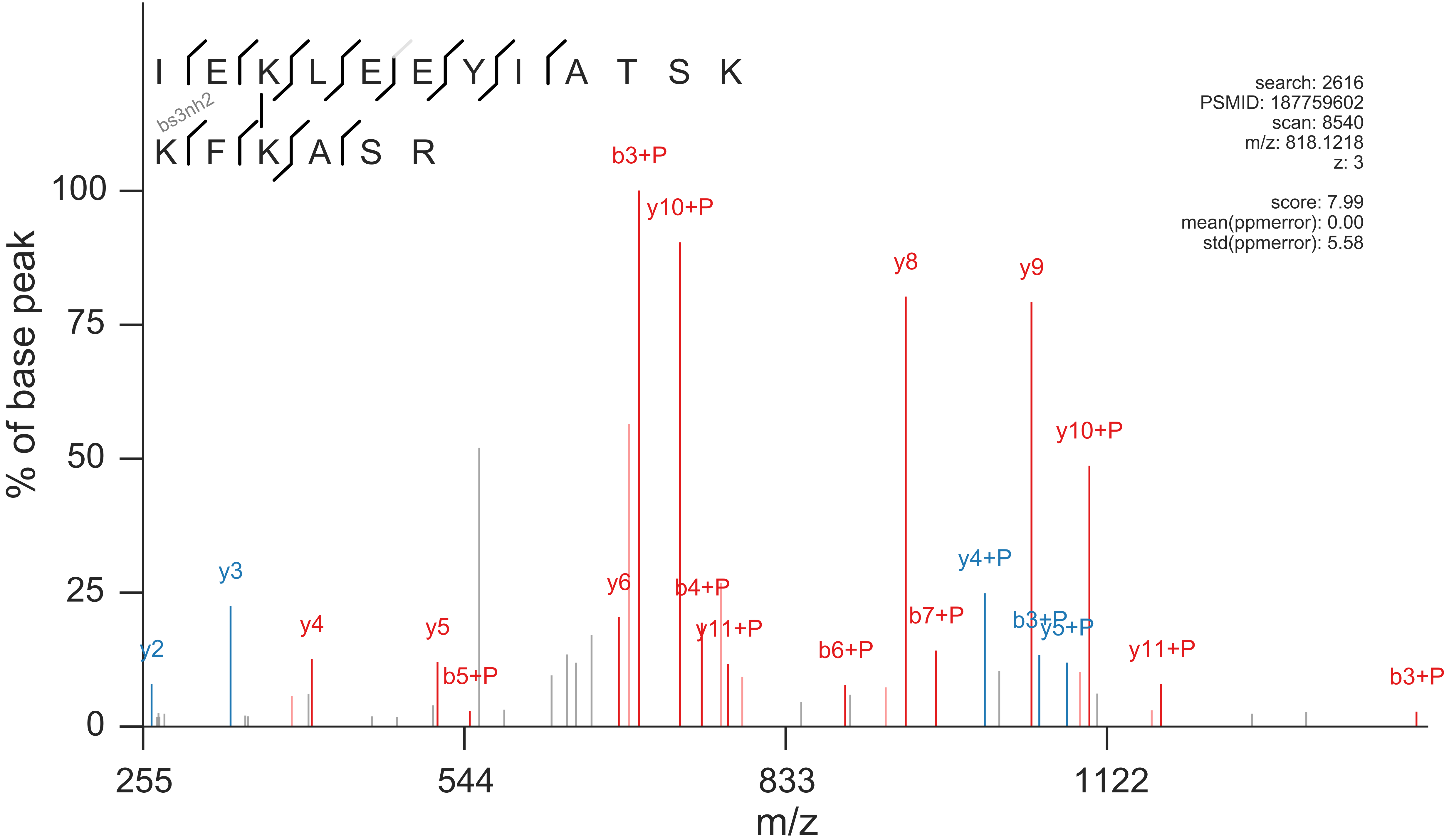

I E K L E E Y I A T S K
bs3nh2
K F K A S R
search: 2616
PSMID: 187759602
scan: 8540
m/z: 818.1218
z: 3
score: 7.99
mean(ppmerror): 0.00
std(ppmerror): 5.58
% of base peak
m/z
100
75
50
25
0
255
544
833
1122
b3+P
y10+P
y8
y9
y10+P
y4+P
y3
y6
b4+P
b7+P
b3+P
y5+P
y2
y4
y5
y11+P
b6+P
y11+P
b5+P
b3+P

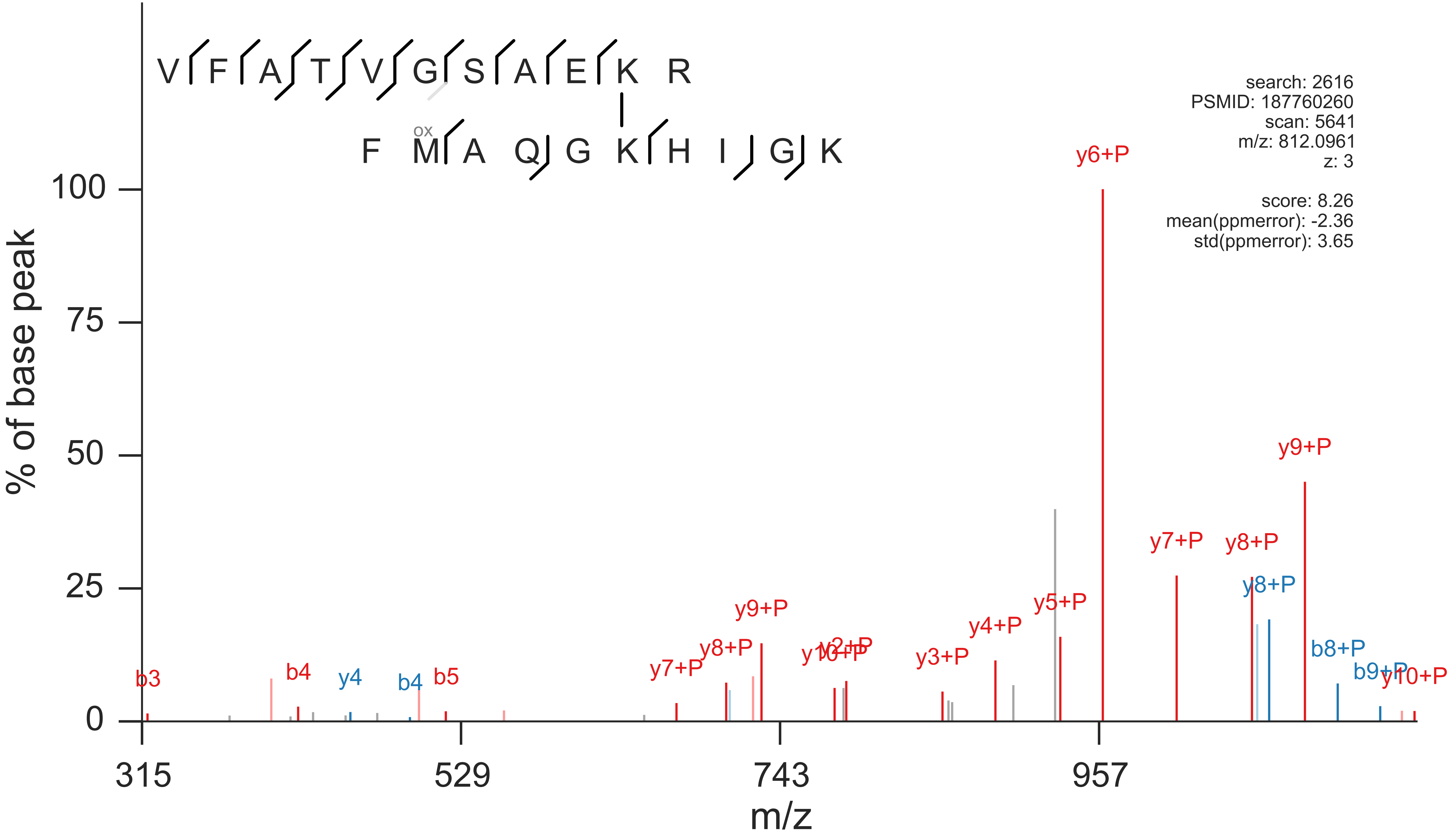
V F A T V G S A E K R
F M A Q G K H I G K
ox
search: 2616
PSMID: 187760260
scan: 5641
m/z: 812.0961
z: 3
score: 8.26
mean(ppmerror): -2.36
std(ppmerror): 3.65
% of base peak
100
75
50
25
0
315
529
743
957
m/z
y6+P
y9+P
y7+P
y8+P
y8+P
y5+P
y4+P
y9+P
y8+P
y3+P
y7+P
b8+P
b9+P
y10+P
b3
b4
y4
b4
b5

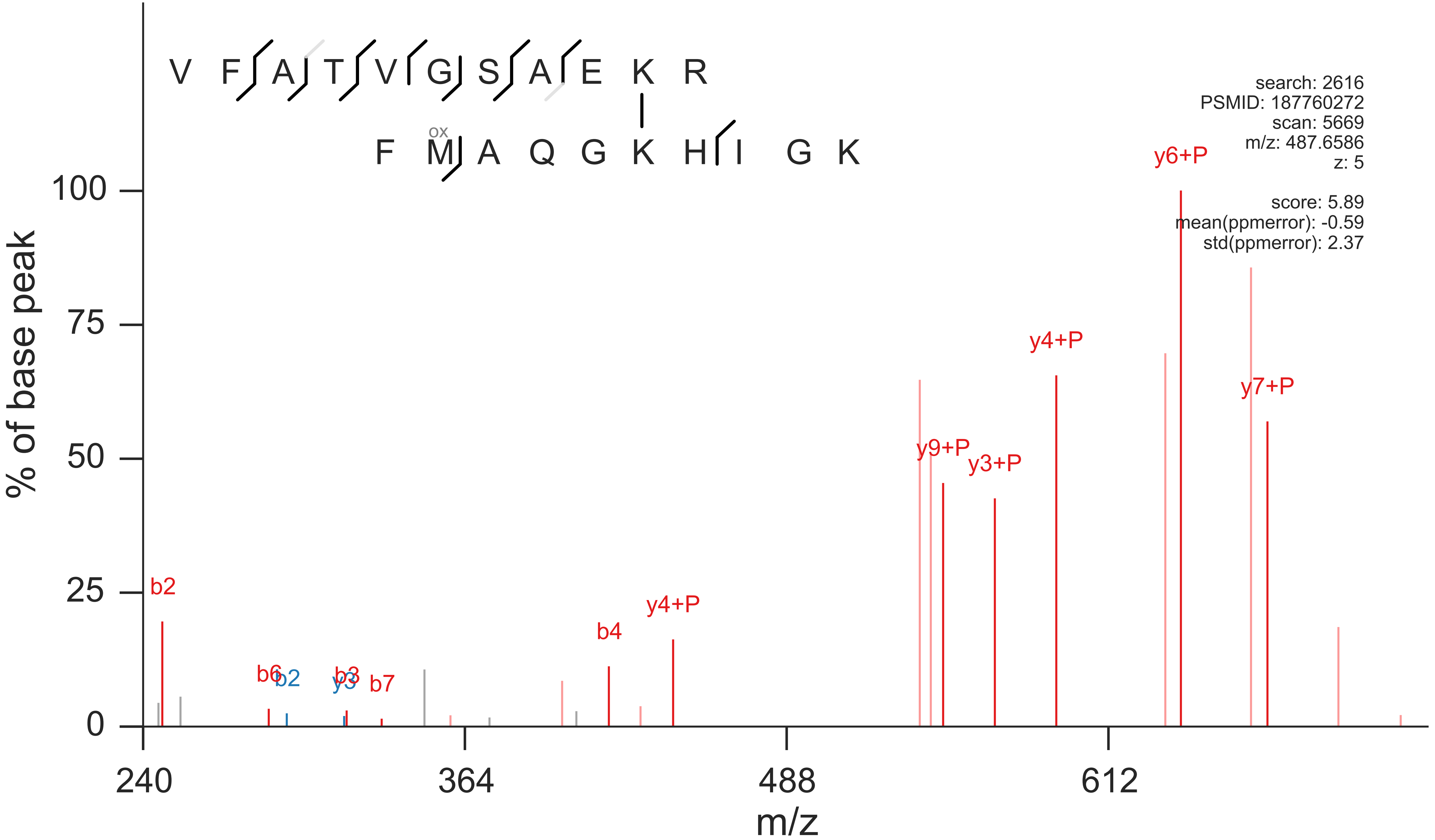

V F A T V G S A E K R
F M A Q G K H I G K
ox
search: 2616
PSMID: 187760272
scan: 5669
m/z: 487.6586
z: 5
score: 5.89
mean(ppmerror): -0.59
std(ppmerror): 2.37
% of base peak
m/z
0
25
50
75
100
240
364
488
612
b2
b6
b2
y3
b3
b7
b4
y4+P
y9+P
y3+P
y4+P
y6+P
y7+P

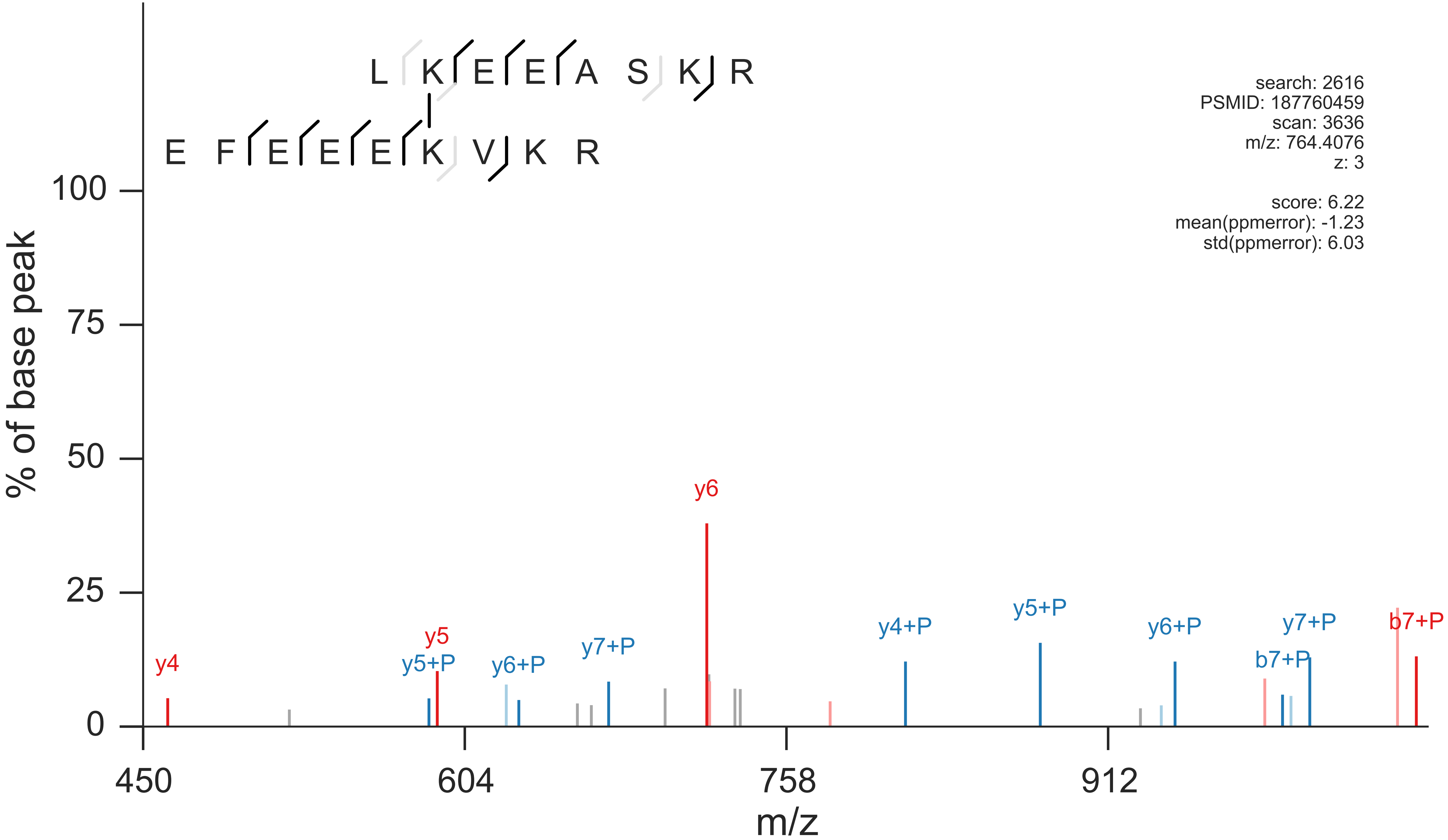

L K E E A S K R
E F E E E K V K R
search: 2616
PSMID: 187760459
scan: 3636
m/z: 764.4076
z: 3
score: 6.22
mean(ppmerror): -1.23
std(ppmerror): 6.03
% of base peak
100
75
50
25
0
y4
y5
y5+P
y6+P
y7+P
y6
y4+P
y5+P
y6+P
b7+P
y7+P
b7+P
450
604
758
912
m/z

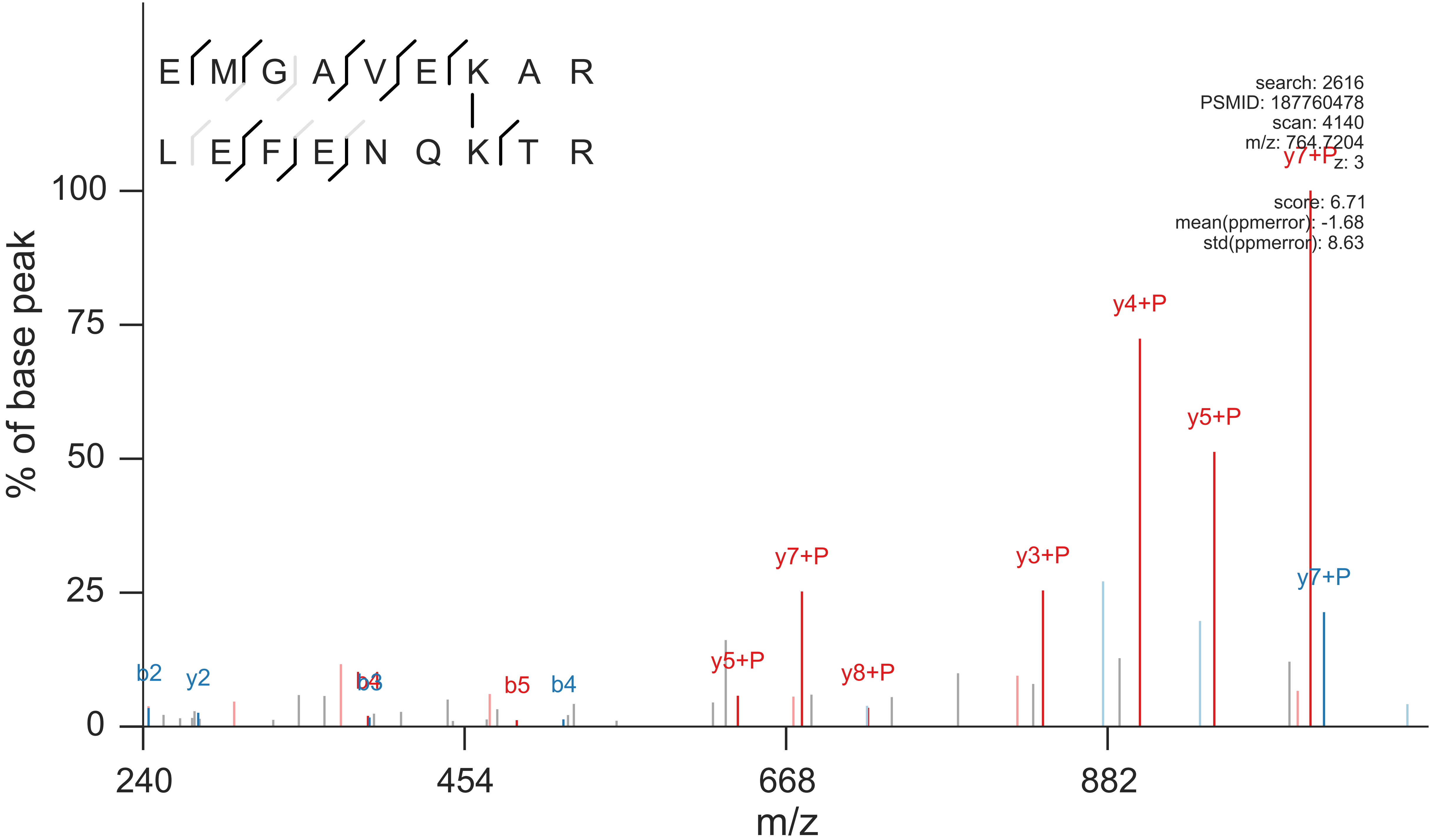

E M G A V E K A R
L E F E N Q K T R
search: 2616
PSMID: 187760478
scan: 4140
m/z: 764.7204
z: 3
score: 6.71
mean(ppmerror): -1.68
std(ppmerror): 8.63
% of base peak
m/z
y7+P
y4+P
y5+P
y7+P
y3+P
y7+P
y5+P
y8+P
b2
y2
b4
b3
b5
b4
100
75
50
25
0
240
454
668
882

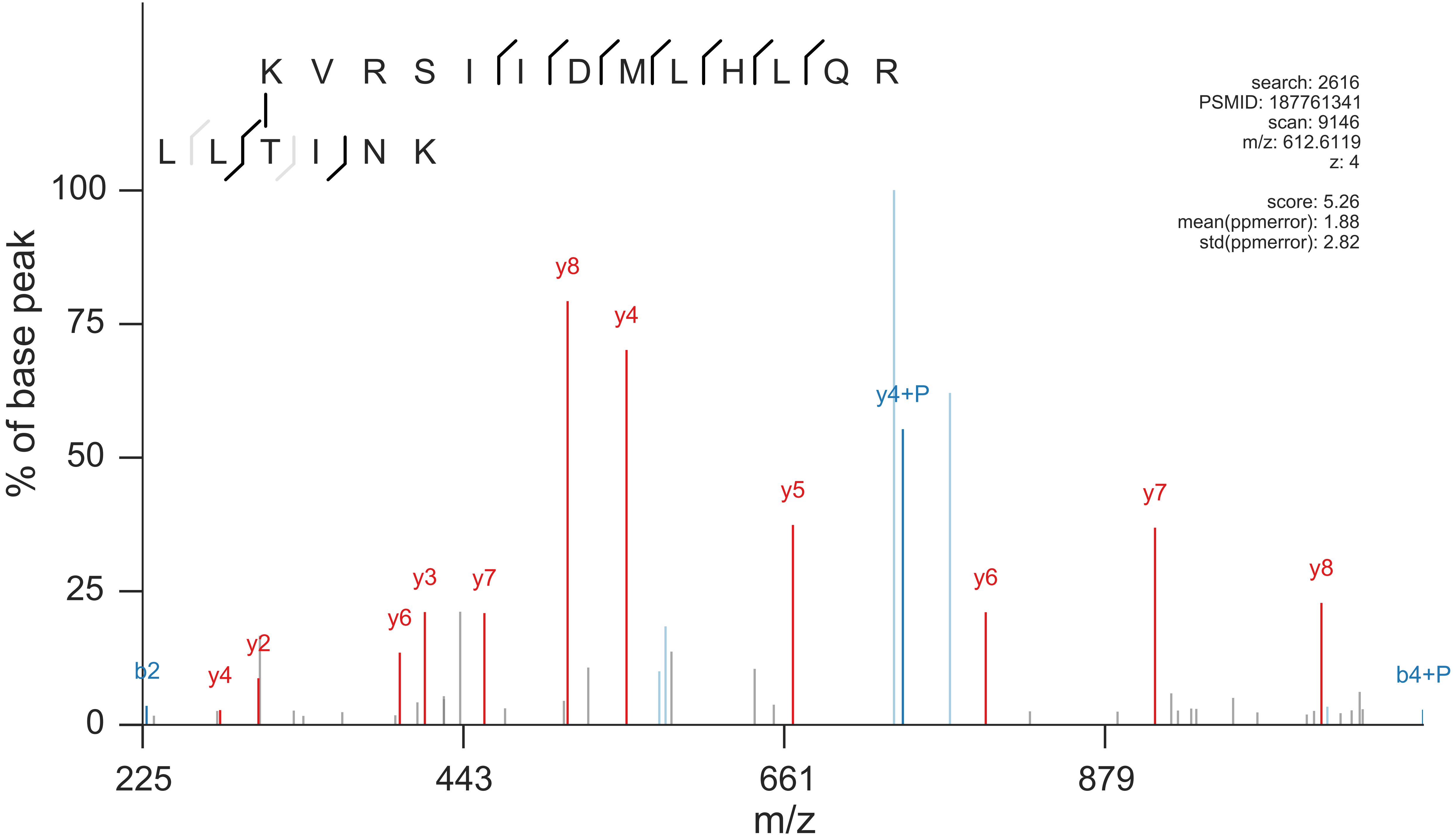
K V R S I I D M L H L Q R
L L T I N K
search: 2616
PSMID: 187761341
scan: 9146
m/z: 612.6119
z: 4
score: 5.26
mean(ppmerror): 1.88
std(ppmerror): 2.82
% of base peak
m/z
b2
y4
y2
y6
y3
y7
y8
y4
y5
y4+P
y6
y7
y8
b4+P
225
443
661
879
0
25
50
75
100

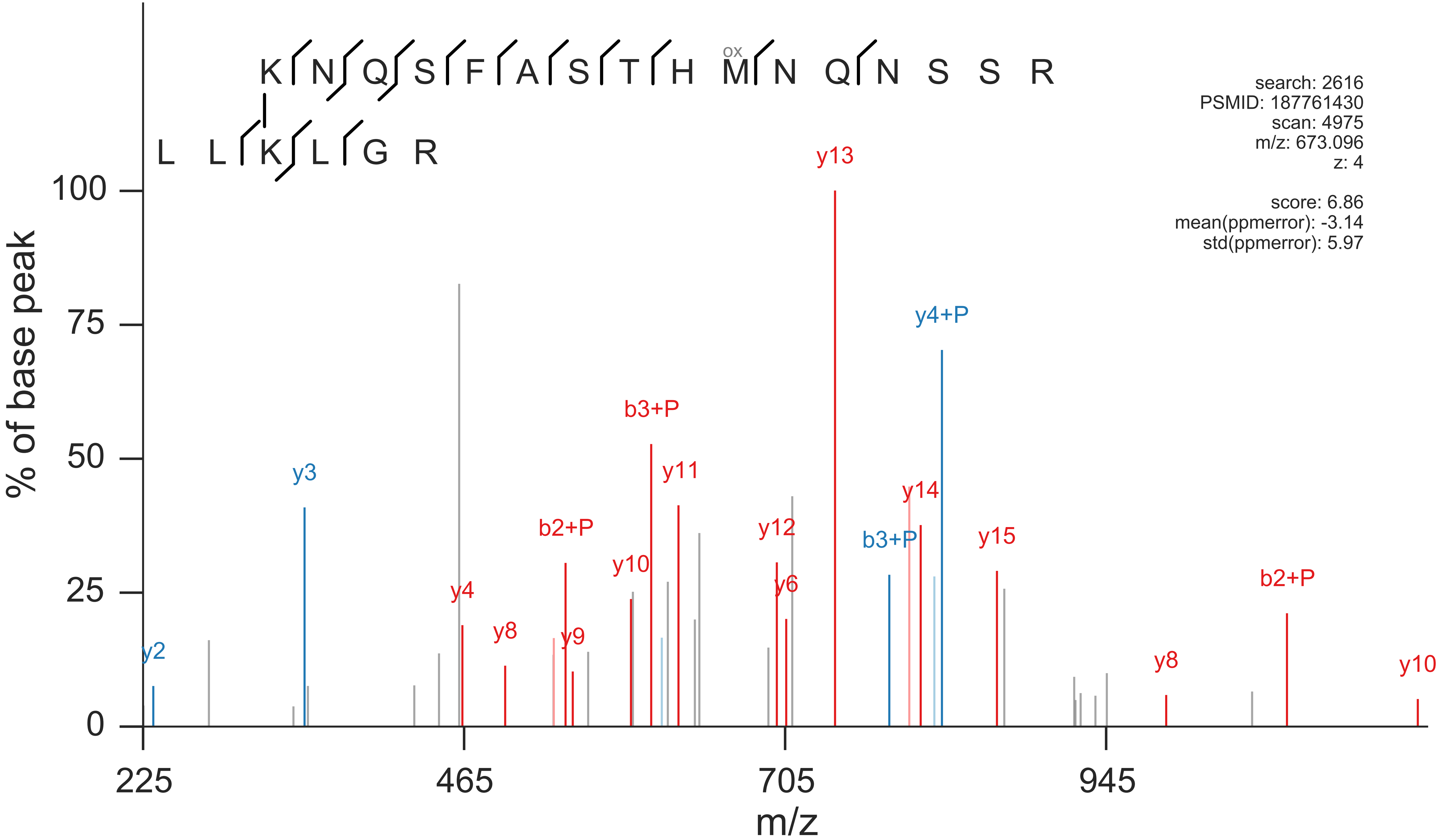

K N Q S F A S T H M N Q N S S R
ox
L L K L G R
search: 2616
PSMID: 187761430
scan: 4975
m/z: 673.096
z: 4
score: 6.86
mean(ppmerror): -3.14
std(ppmerror): 5.97
% of base peak
m/z
y2
y3
y4
y8
b2+P
y9
y10
b3+P
y11
y12
y6
y13
b3+P
y14
y4+P
y15
y8
b2+P
y10
0
25
50
75
100
225
465
705
945

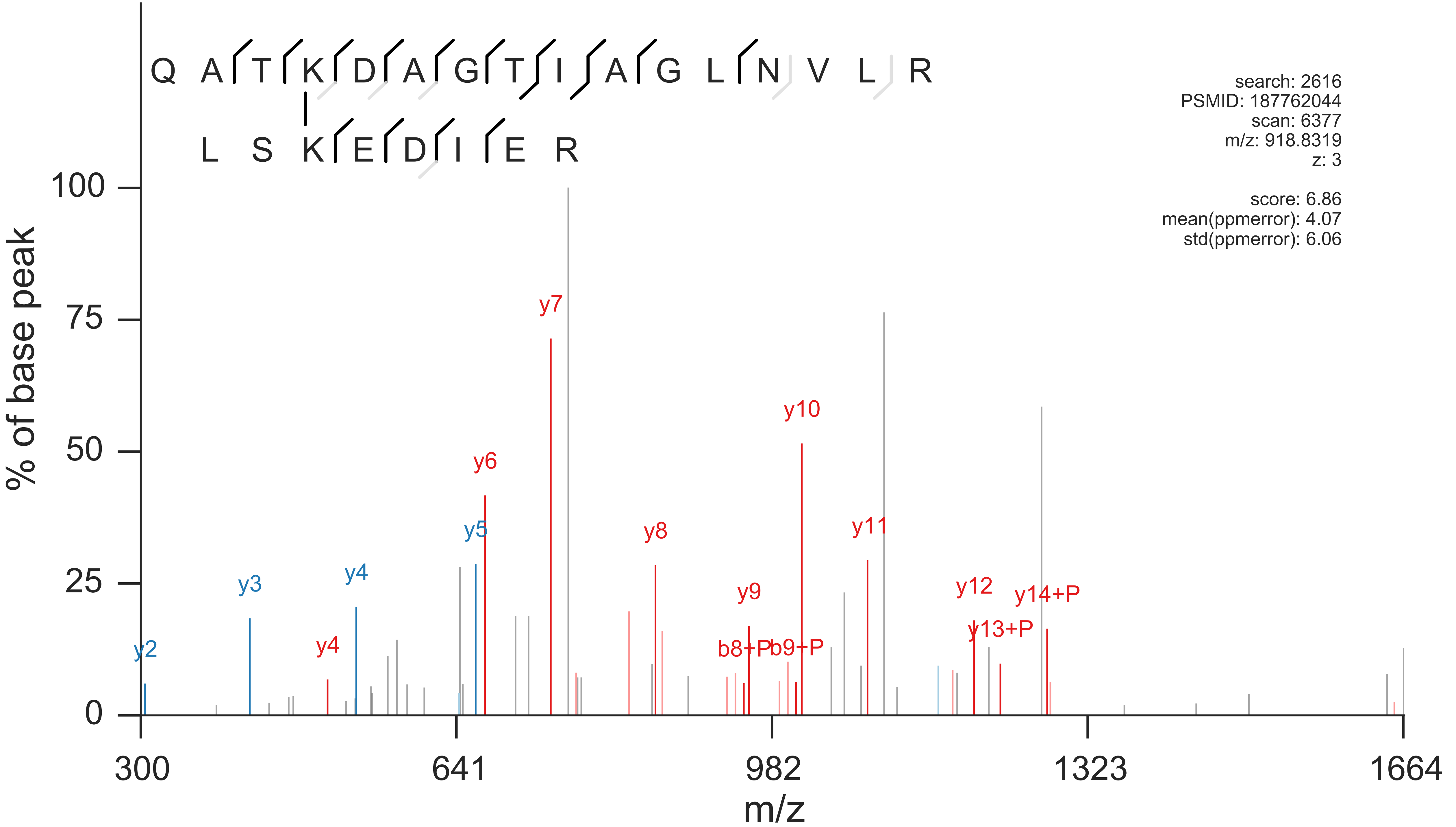
Q A T K D A G T I A G L N V L R
L S K E D I E R
search: 2616
PSMID: 187762044
scan: 6377
m/z: 918.8319
z: 3
score: 6.86
mean(ppmerror): 4.07
std(ppmerror): 6.06
% of base peak
m/z
y2
y3
y4
y5
y4
y6
y7
y8
y9
b8+P
b9+P
y10
y11
y12
y13+P
y14+P

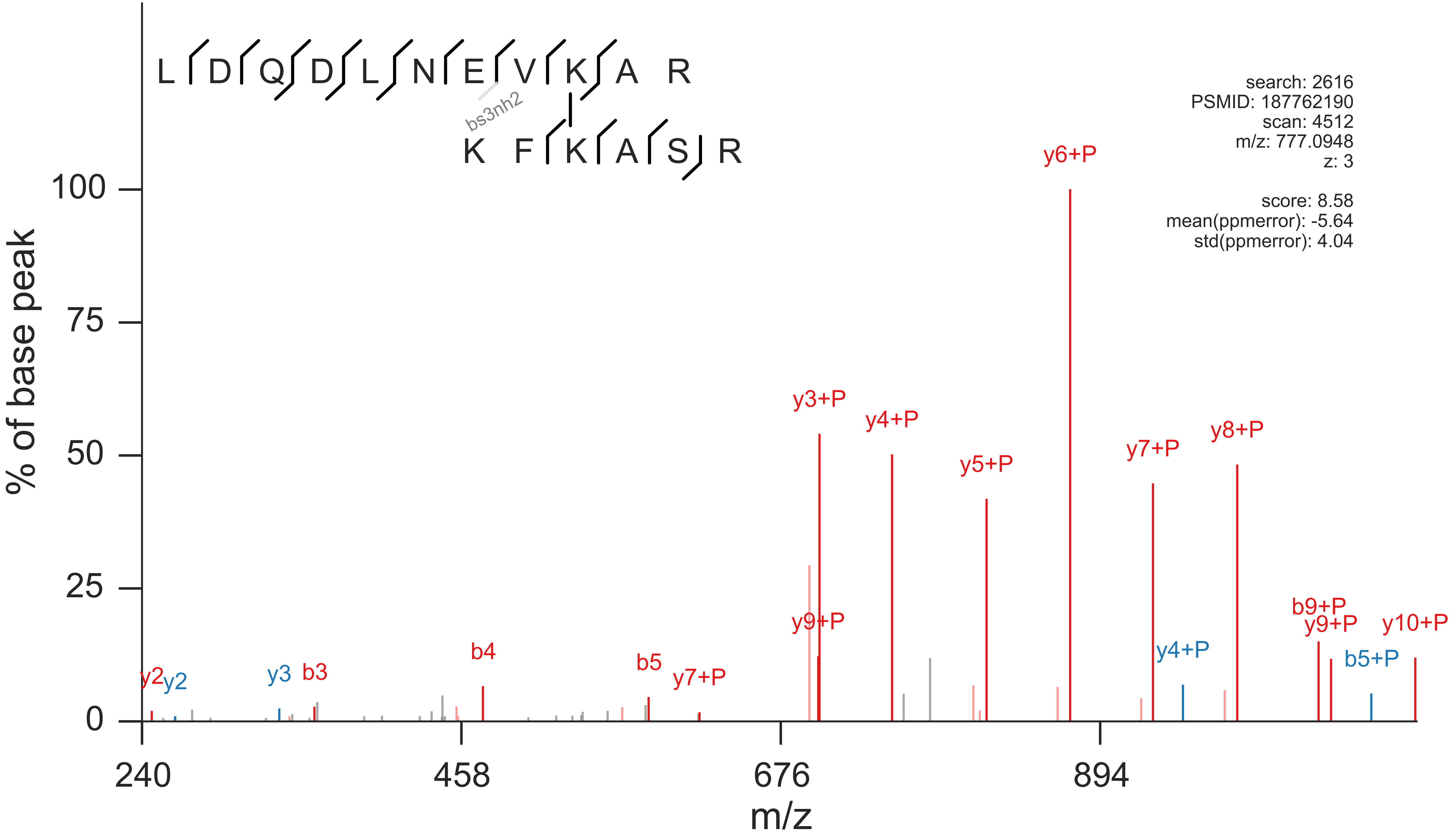

L D Q D L N E V K A R
bs3nh2
K F K A S R
search: 2616
PSMID: 187762190
scan: 4512
m/z: 777.0948
z: 3
score: 8.58
mean(ppmerror): -5.64
std(ppmerror): 4.04
% of base peak
100
75
50
25
0
240
458
676
894
m/z
y6+P
y3+P
y4+P
y5+P
y7+P
y8+P
y9+P
b9+P
y9+P
y10+P
y4+P
b5+P
y2
y2
y3
b3
b4
b5
y7+P

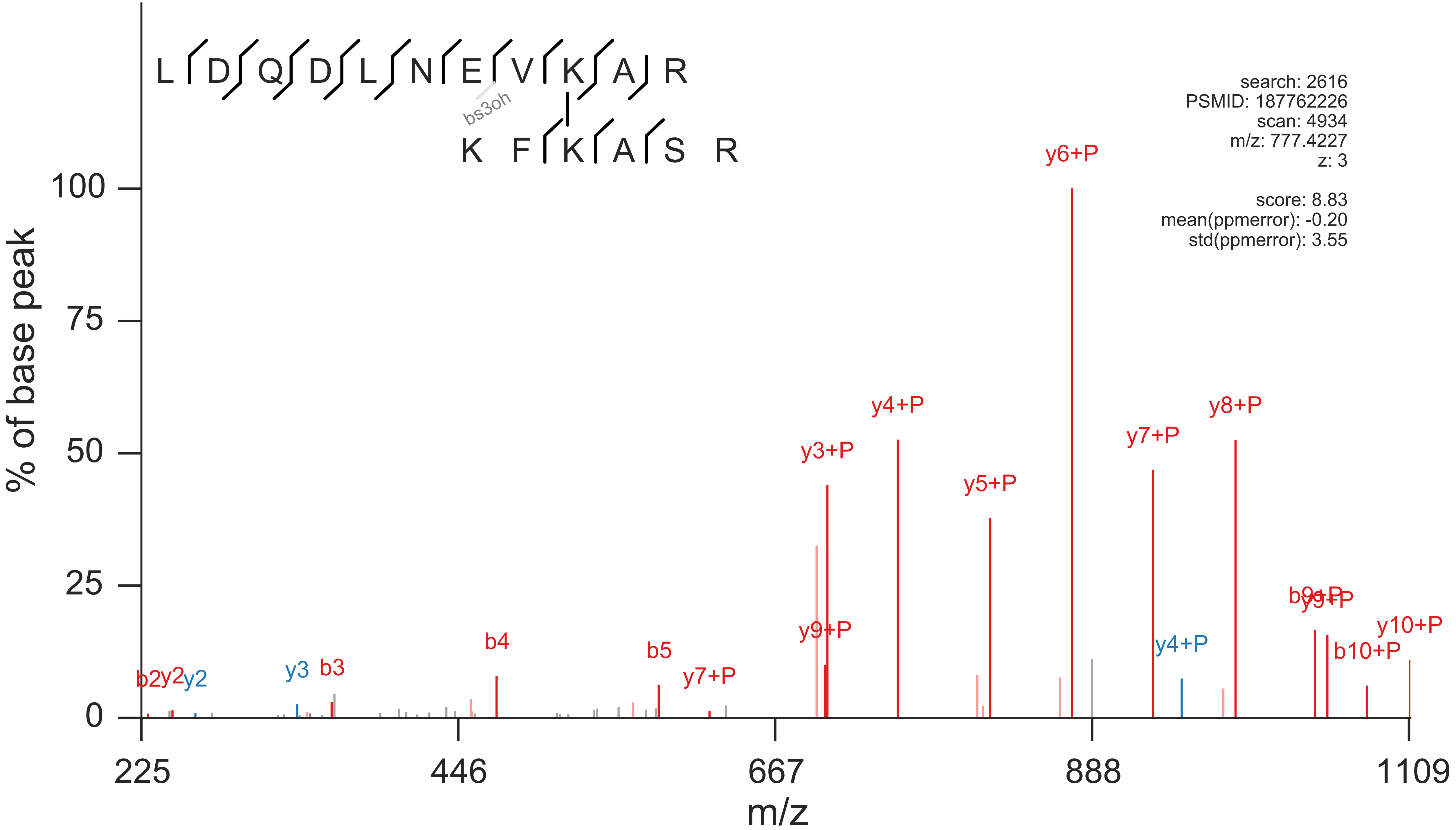
L D Q D L N E V K A R
bs3oh
K F K A S R
search: 2616
PSMID: 187762226
scan: 4934
m/z: 777.4227
z: 3
score: 8.83
mean(ppmerror): -0.20
std(ppmerror): 3.55
% of base peak
100
75
50
25
0
225
446
667
888
1109
m/z
y6+P
y4+P
y8+P
y7+P
y3+P
y5+P
y10+P
b10+P
y9+P
y4+P
b4
b5
y7+P
y3
b3
b2
y2
y2

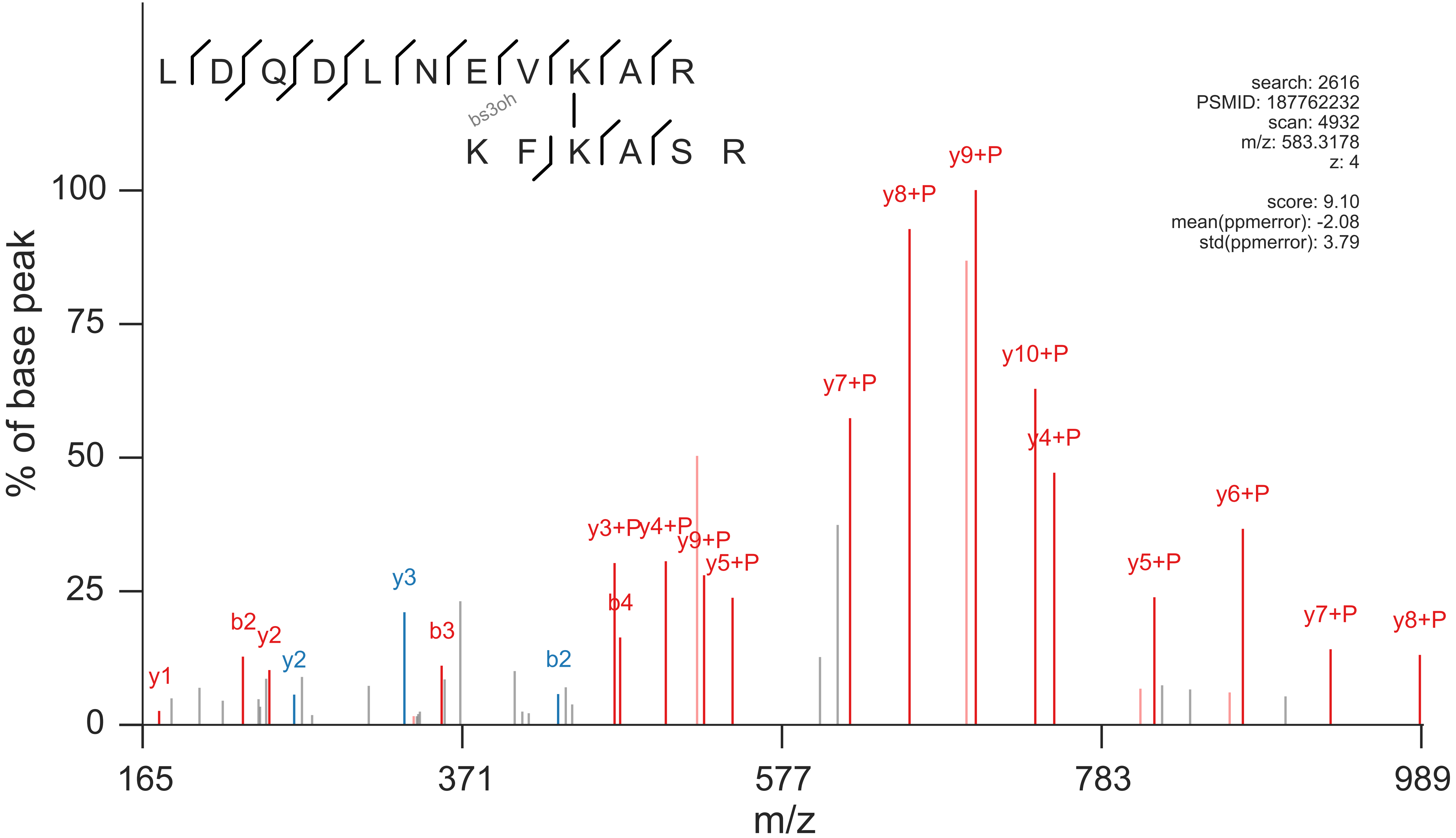

L D Q D L N E V K A R
bs3oh
K F K A S R
search: 2616
PSMID: 187762232
scan: 4932
m/z: 583.3178
z: 4
score: 9.10
mean(ppmerror): -2.08
std(ppmerror): 3.79
% of base peak
100
75
50
25
0
m/z
165
371
577
783
989
y1
b2
y2
y2
y3
b3
b2
y3+P
y4+P
y9+P
b4
y5+P
y7+P
y8+P
y9+P
y10+P
y4+P
y5+P
y6+P
y7+P
y8+P

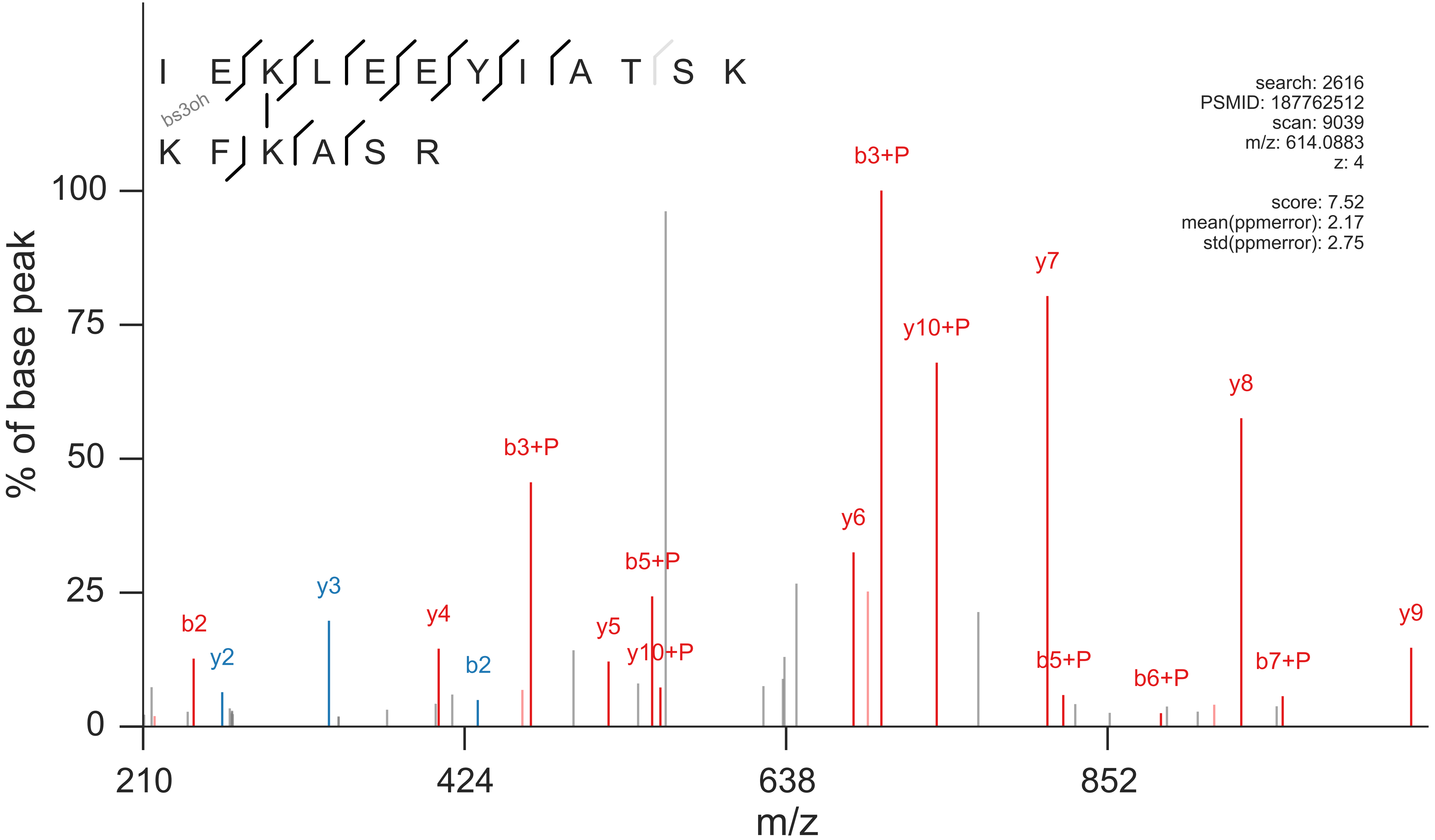

I E K L E E Y I A T S K
bs3oh
K F K A S R
search: 2616
PSMID: 187762512
scan: 9039
m/z: 614.0883
z: 4
score: 7.52
mean(ppmerror): 2.17
std(ppmerror): 2.75
% of base peak
100
75
50
25
0
m/z
210
424
638
852
b2
y2
y3
y4
b2
b3+P
y5
b5+P
y10+P
y6
b3+P
y10+P
y7
b5+P
b6+P
y8
b7+P
y9

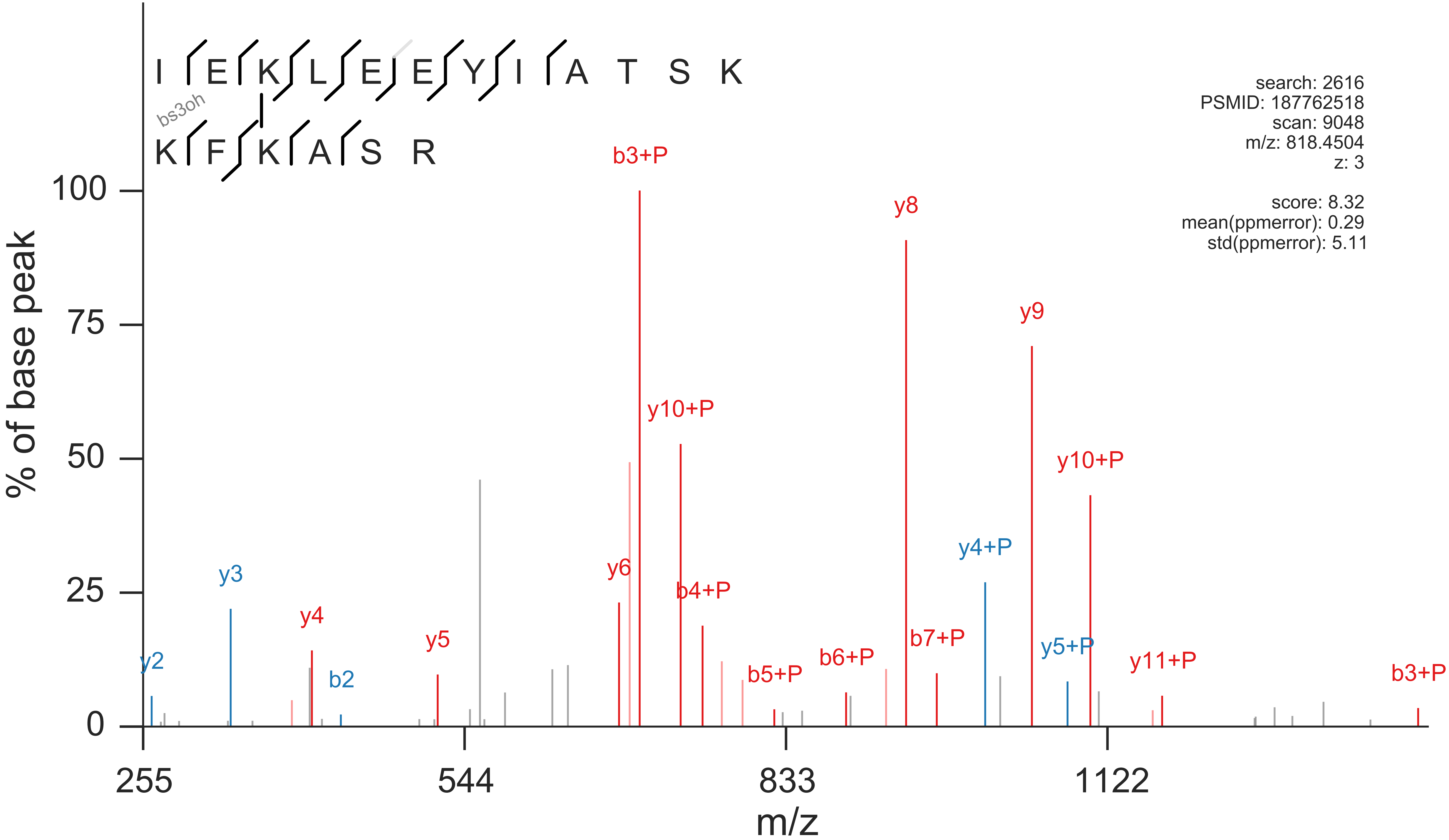
I E K L E E Y I A T S K
bs3oh
K F K A S R
search: 2616
PSMID: 187762518
scan: 9048
m/z: 818.4504
z: 3
score: 8.32
mean(ppmerror): 0.29
std(ppmerror): 5.11
% of base peak
m/z
0
25
50
75
100
255
544
833
1122
b3+P
y8
y9
y10+P
y10+P
y4+P
y6
b4+P
y3
y4
y5
y2
b2
b5+P
b6+P
b7+P
y5+P
y11+P
b3+P

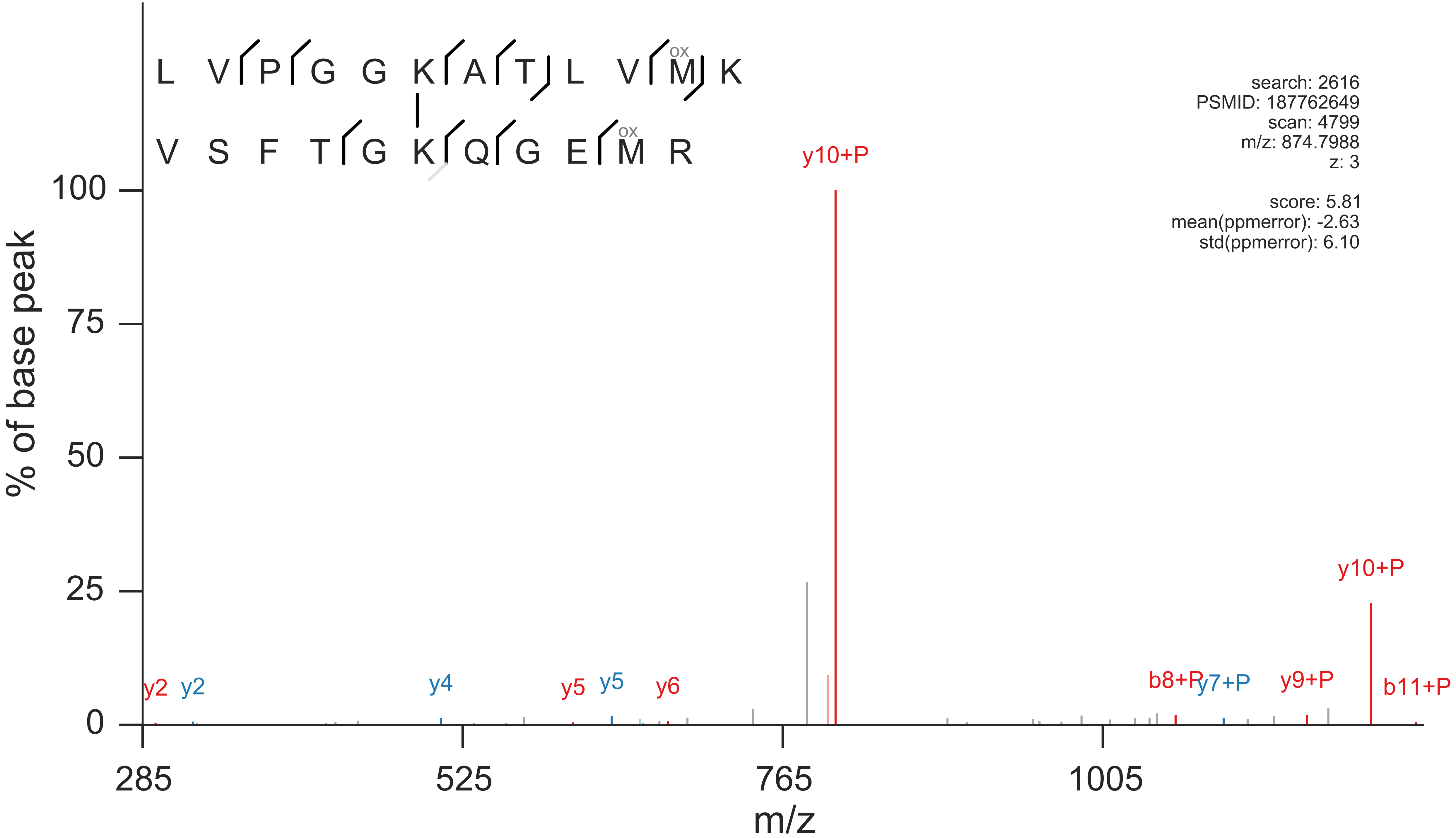
L V P G G K A T L V M K
ox
V S F T G K Q G E M R
ox
search: 2616
PSMID: 187762649
scan: 4799
m/z: 874.7988
z: 3
score: 5.81
mean(ppmerror): -2.63
std(ppmerror): 6.10
y10+P
y2
y2
y4
y5
y5
y6
b8+P
y7+P
y9+P
y10+P
b11+P
% of base peak
0
25
50
75
100
285
525
765
1005
m/z

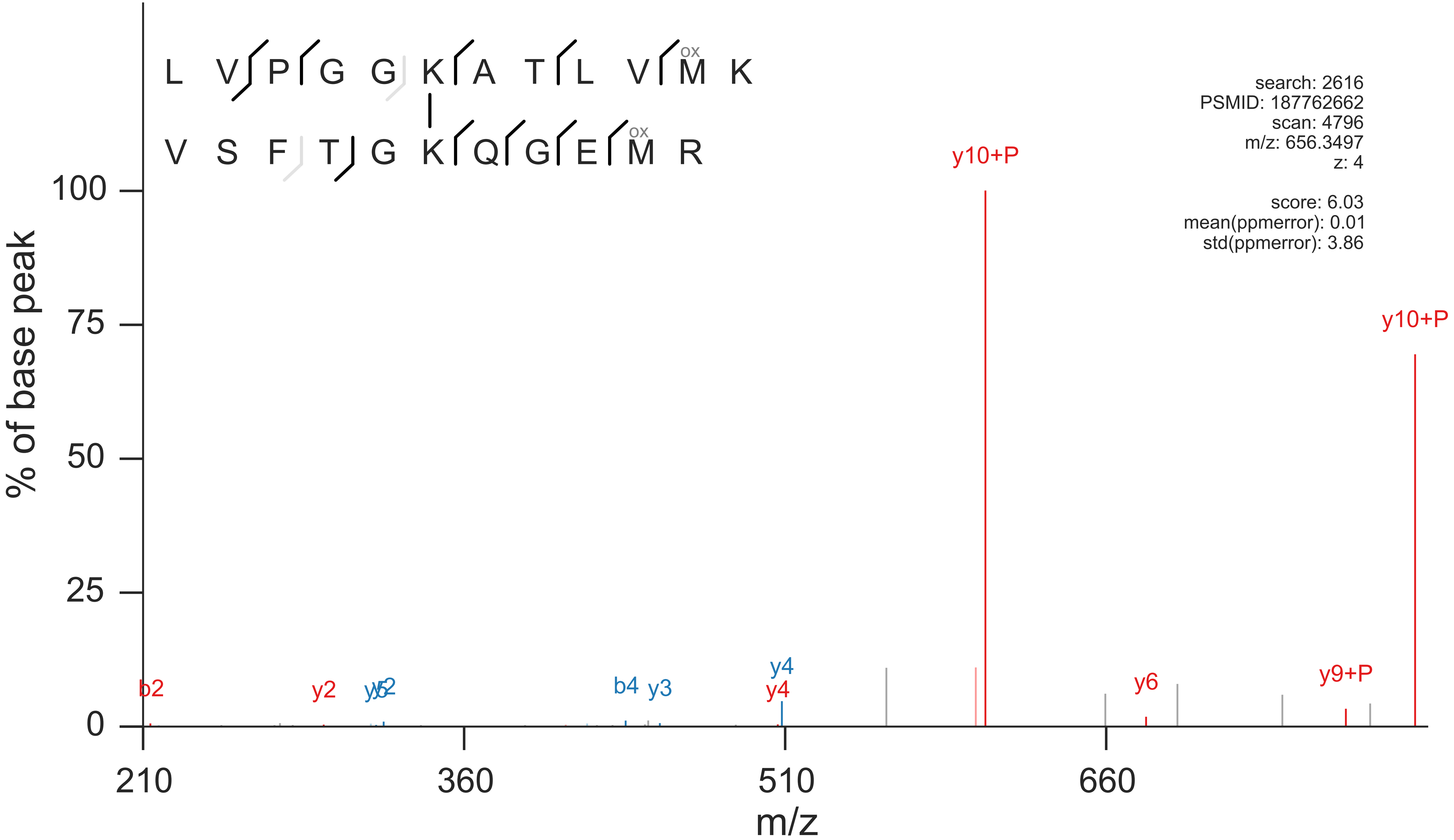

L V P G G K A T L V M K
V S F T G K Q G E M R
ox
search: 2616
PSMID: 187762662
scan: 4796
m/z: 656.3497
z: 4
score: 6.03
mean(ppmerror): 0.01
std(ppmerror): 3.86
y10+P
y10+P
y9+P
y6
y4
y4
b4
y3
y2
b2
% of base peak
100
75
50
25
0
210
360
510
660
m/z

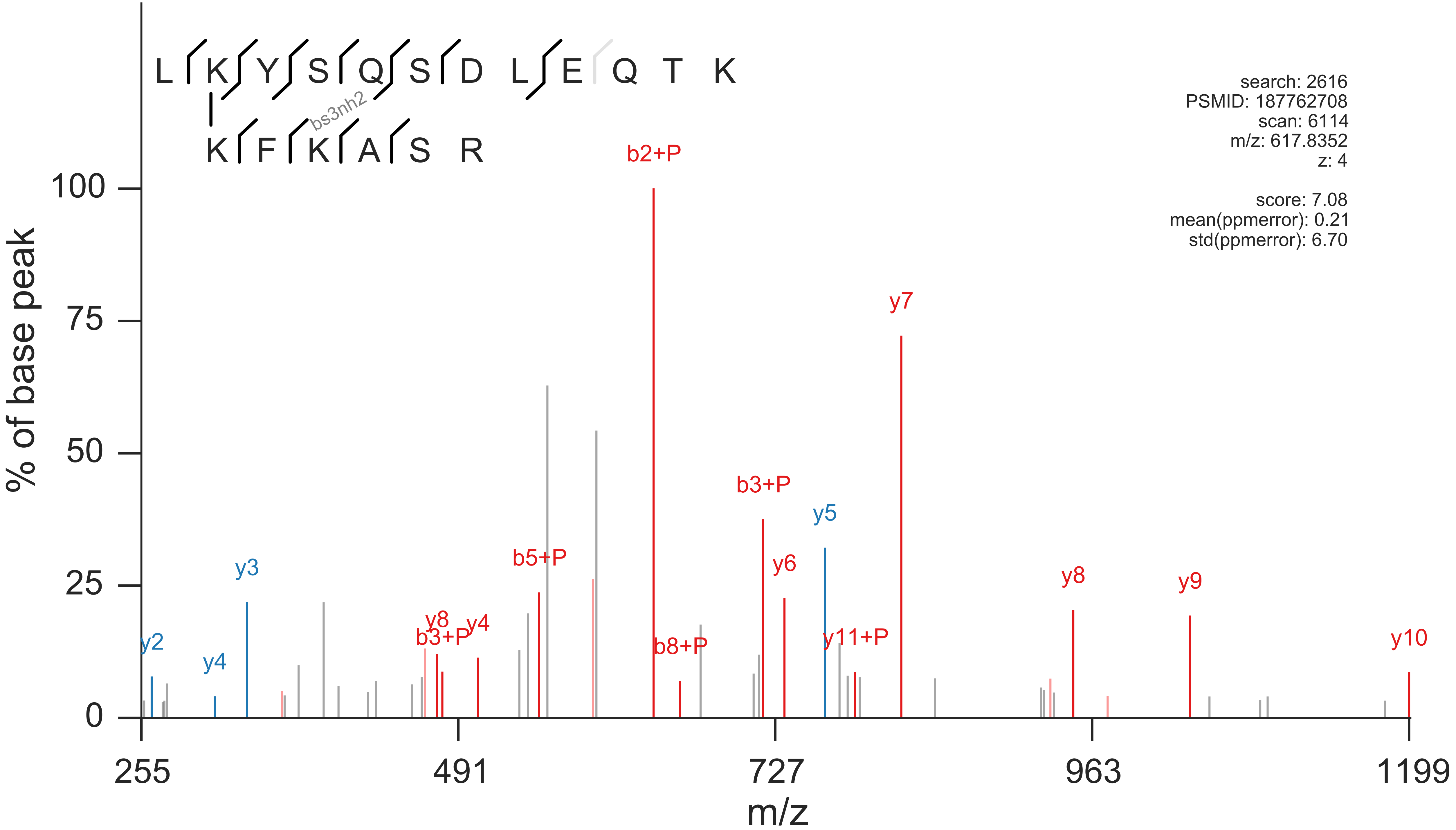

L K Y S Q S D L E Q T K
bs3nh2
K F K A S R
search: 2616
PSMID: 187762708
scan: 6114
m/z: 617.8352
z: 4
score: 7.08
mean(ppmerror): 0.21
std(ppmerror): 6.70
% of base peak
m/z
0
25
50
75
100
255
491
727
963
1199
b2+P
y7
b3+P
y5
y6
b5+P
y3
y2
y4
y8
b3+P
y4
b8+P
y11+P
y8
y9
y10

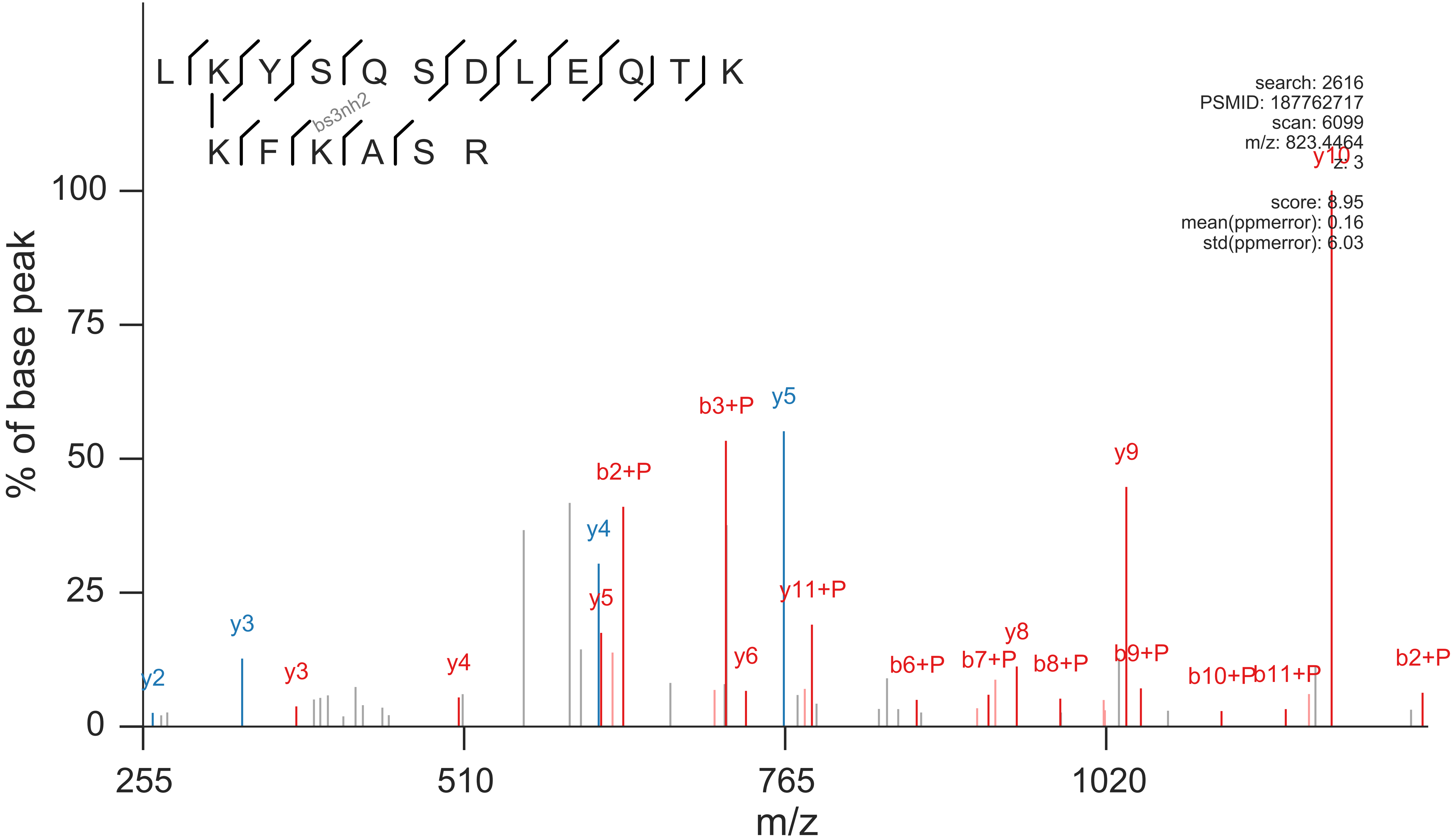

L K Y S Q S D L E Q T K
K F K A S R
bs3nh2
search: 2616
PSMID: 187762717
scan: 6099
m/z: 823.4464
z: 3
score: 8.95
mean(ppmerror): 0.16
std(ppmerror): 6.03
% of base peak
m/z
y2
y3
y3
y4
y4
y5
b2+P
b3+P
y5
y6
y11+P
b6+P
b7+P
y8
b8+P
y9
b9+P
b10+P
b11+P
y10
b2+P

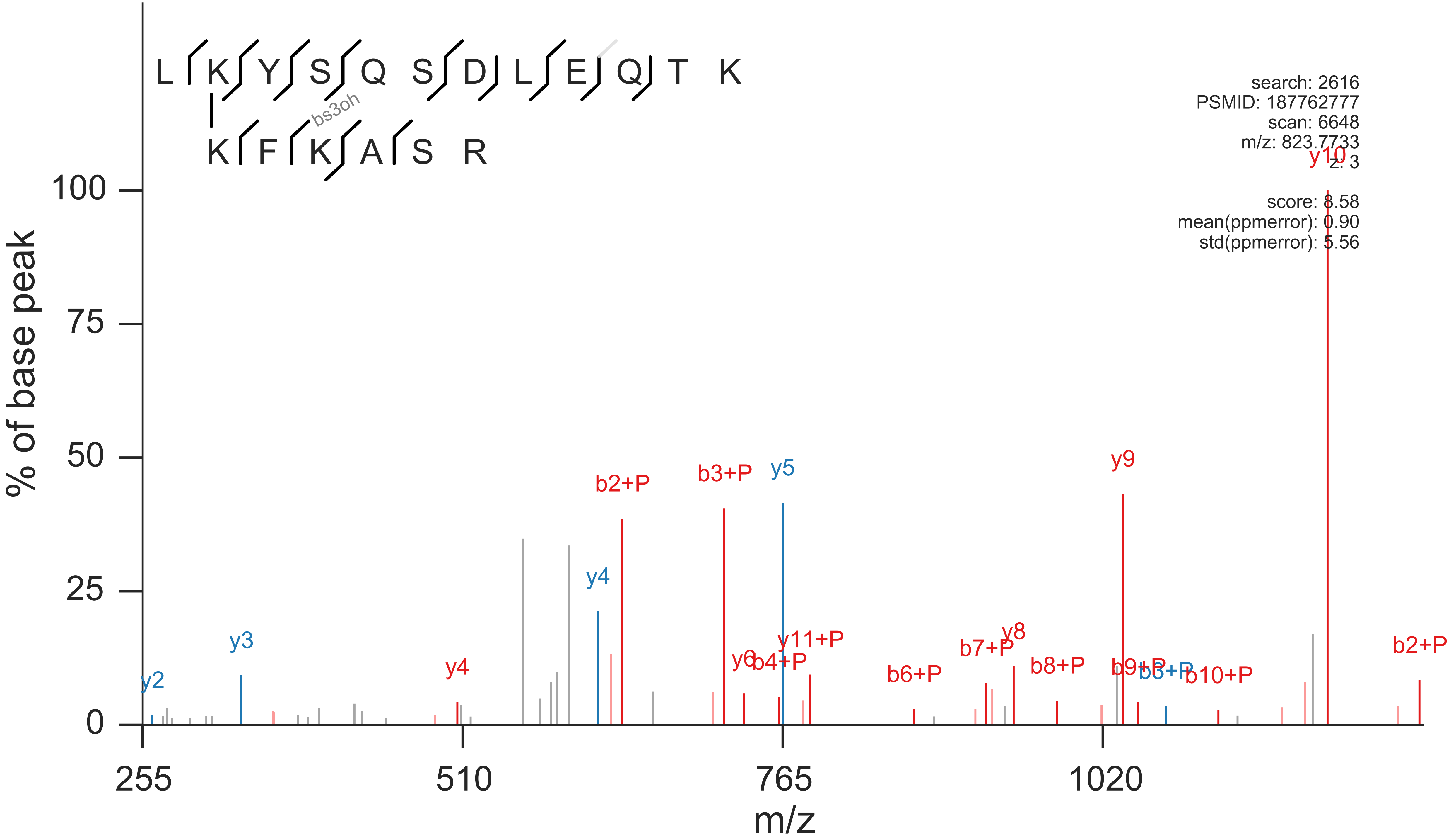

L K Y S Q S D L E Q T K
K F K A S R
bs3oh
search: 2616
PSMID: 187762777
scan: 6648
m/z: 823.7733
z: 3
score: 8.58
mean(ppmerror): 0.90
std(ppmerror): 5.56
% of base peak
m/z
100
75
50
25
0
255
510
765
1020
y2
y3
y4
y4
b2+P
b3+P
y5
y6
b4+P
y11+P
b6+P
b7+P
y8
b8+P
y9
b9+P
b6+P
b10+P
y10
b2+P

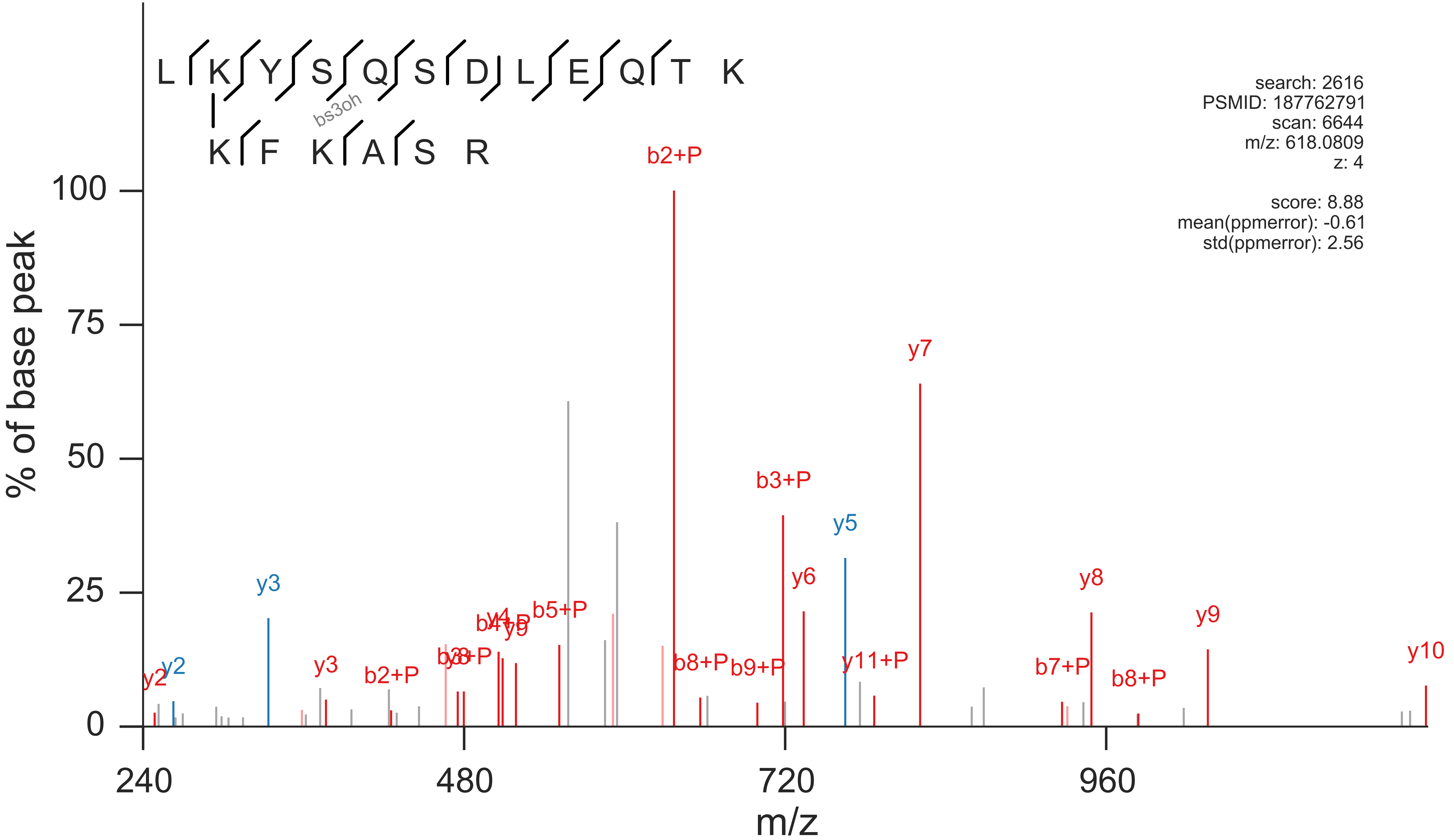

L K Y S Q S D L E Q T K
K F K A S R
bs3oh
search: 2616
PSMID: 187762791
scan: 6644
m/z: 618.0809
z: 4
score: 8.88
mean(ppmerror): -0.61
std(ppmerror): 2.56
% of base peak
m/z
b2+P
y7
b3+P
y5
y6
y8
y9
y10
y3
y2
b5+P
b8+P
b9+P
y11+P
b7+P
b2+P

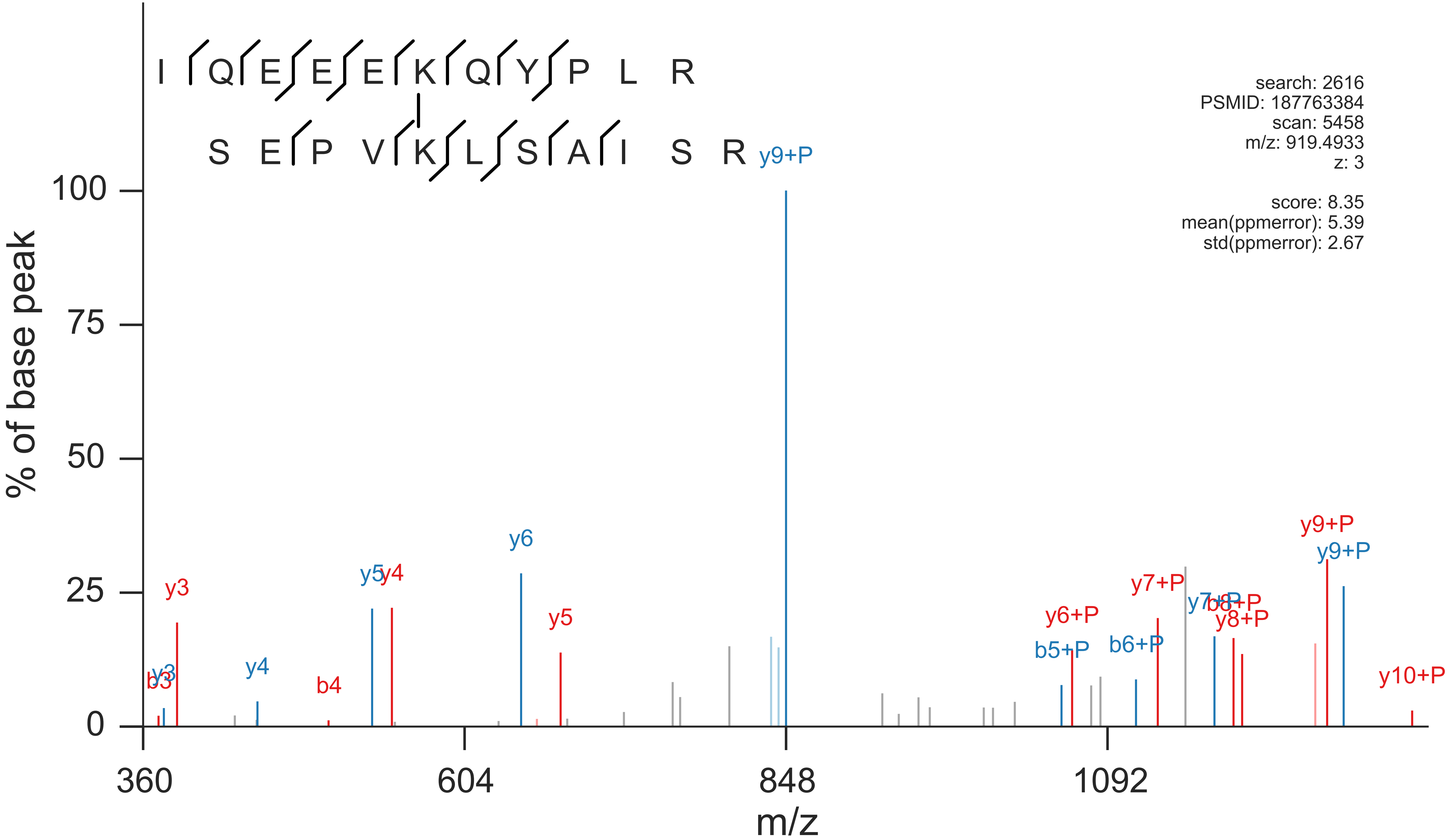

I Q E E E K Q Y P L R
S E P V K L S A I S R
search: 2616
PSMID: 187763384
scan: 5458
m/z: 919.4933
z: 3
score: 8.35
mean(ppmerror): 5.39
std(ppmerror): 2.67
% of base peak
100
75
50
25
0
360
604
848
1092
m/z
y9+P
y3
b3
y3
y4
b4
y5
y4
y6
y5
b5+P
y6+P
b6+P
y7+P
y7+P
b8+P
y8+P
y9+P
y9+P
y10+P

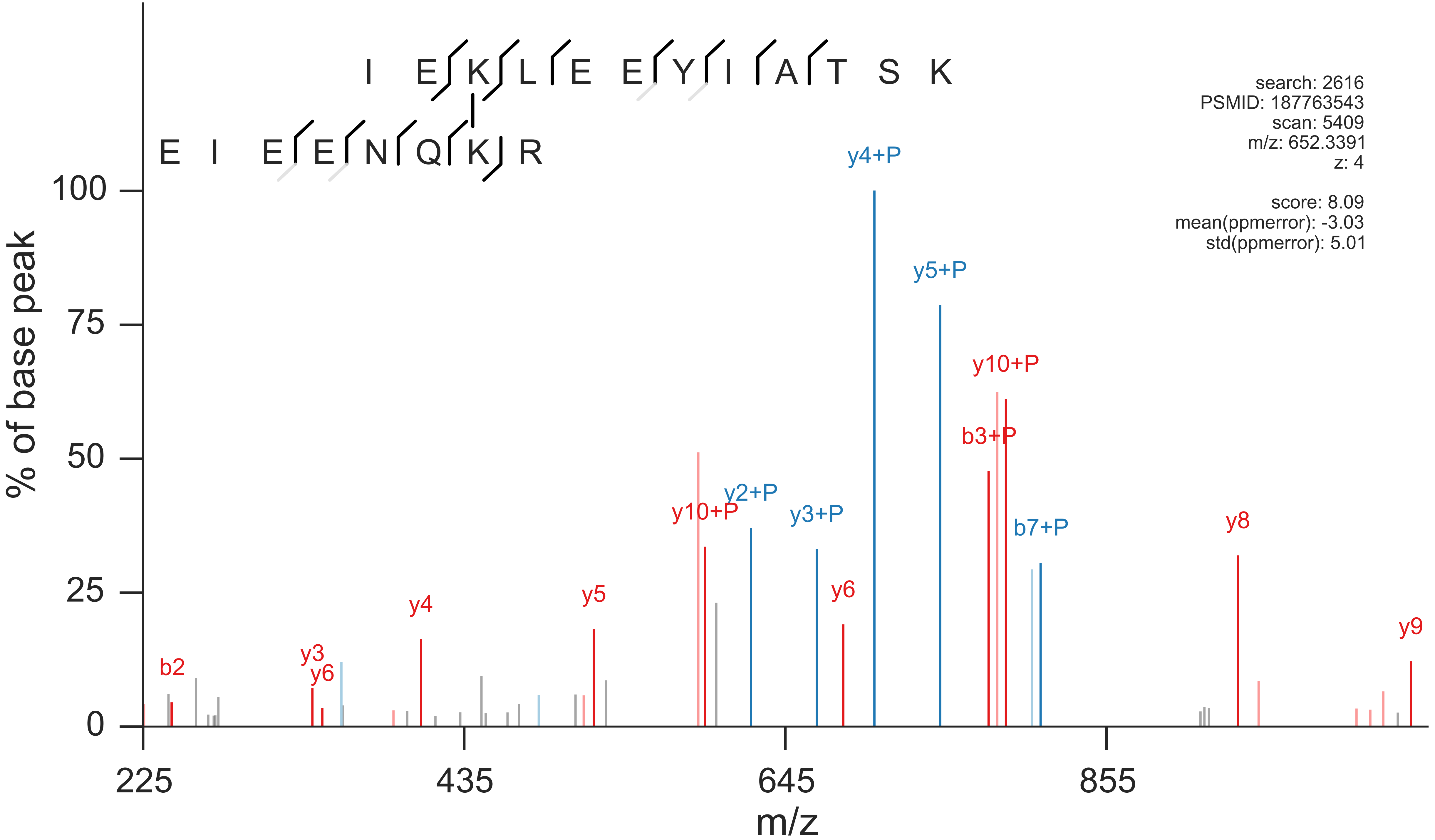

I E K L E E Y I A T S K
E I E E N Q K R
search: 2616
PSMID: 187763543
scan: 5409
m/z: 652.3391
z: 4
score: 8.09
mean(ppmerror): -3.03
std(ppmerror): 5.01
% of base peak
m/z
100
75
50
25
0
225
435
645
855
y4+P
y5+P
y10+P
b3+P
y2+P
y10+P
y3+P
b7+P
y8
y6
y5
y4
y3
y6
b2
y9

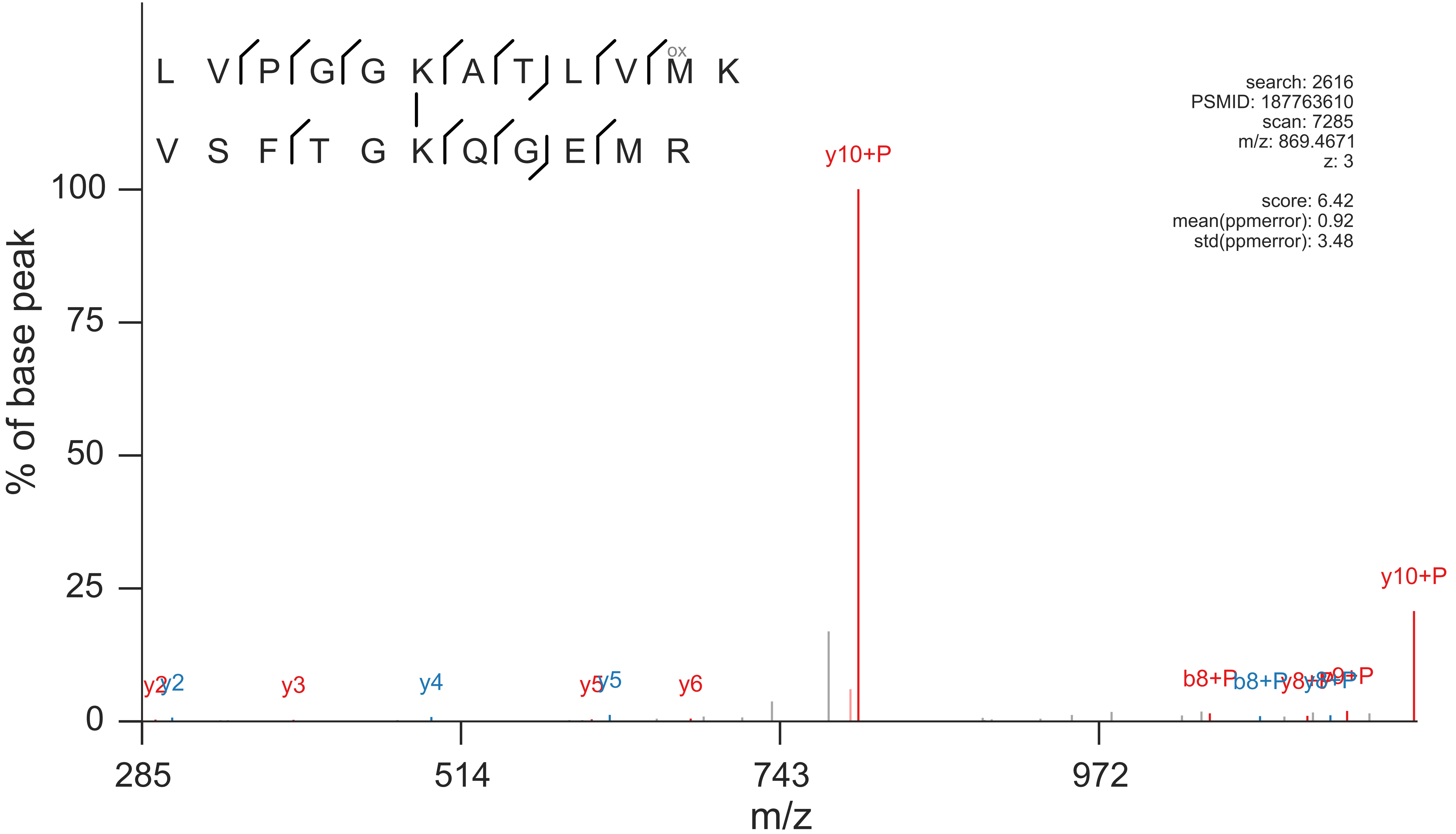

L V P G G K A T L V M K
ox
V S F T G K Q G E M R
search: 2616
PSMID: 187763610
scan: 7285
m/z: 869.4671
z: 3
score: 6.42
mean(ppmerror): 0.92
std(ppmerror): 3.48
y10+P
y10+P
y2
y2
y3
y4
y5
y5
y6
b8+P
b8+P
% of base peak
100
75
50
25
0
285
514
743
972
m/z

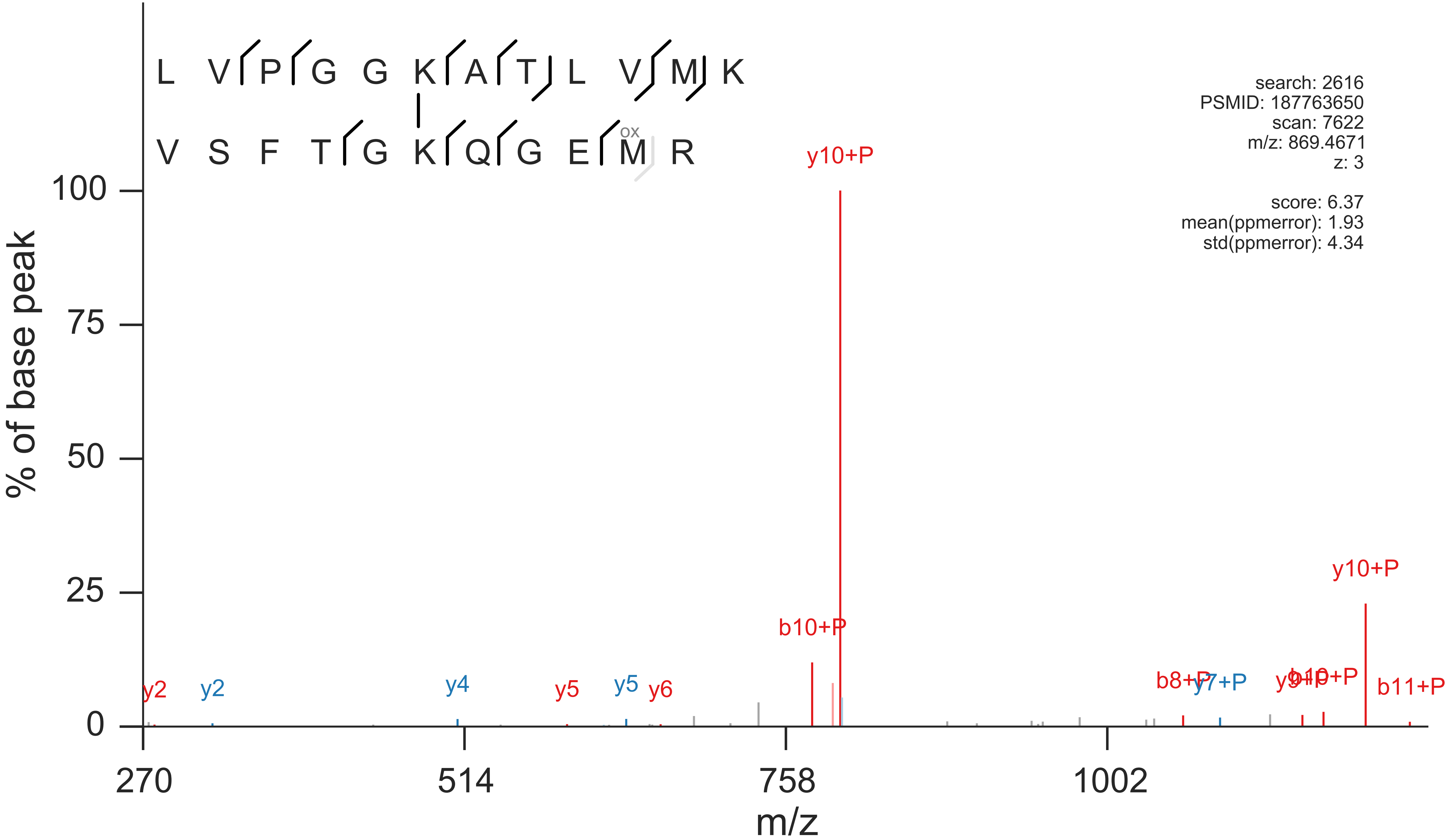

L V P G G K A T L V M K
V S F T G K Q G E M R
ox
search: 2616
PSMID: 187763650
scan: 7622
m/z: 869.4671
z: 3
score: 6.37
mean(ppmerror): 1.93
std(ppmerror): 4.34
% of base peak
100
75
50
25
0
m/z
270
514
758
1002
y10+P
b10+P
y2
y2
y4
y5
y5
y6
b8+P
y7+P
y9+P
b10+P
b11+P
y10+P

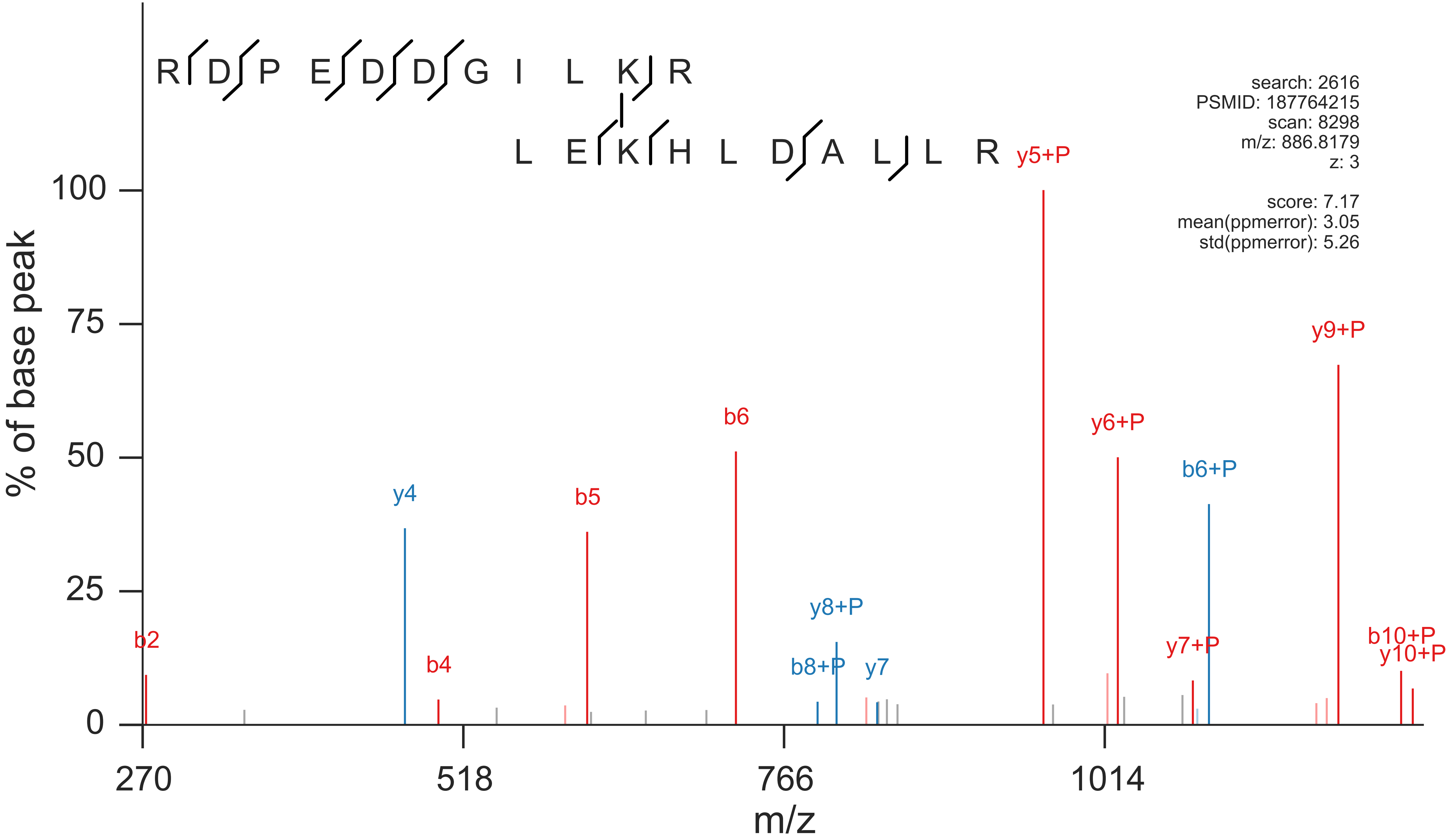

R D P E D D G I L K R
L E K H L D A L L R
search: 2616
PSMID: 187764215
scan: 8298
m/z: 886.8179
z: 3
score: 7.17
mean(ppmerror): 3.05
std(ppmerror): 5.26
% of base peak
m/z
100
75
50
25
0
270
518
766
1014
b2
y4
b4
b5
b6
y8+P
b8+P
y7
y5+P
y6+P
y7+P
b6+P
y9+P
b10+P
y10+P

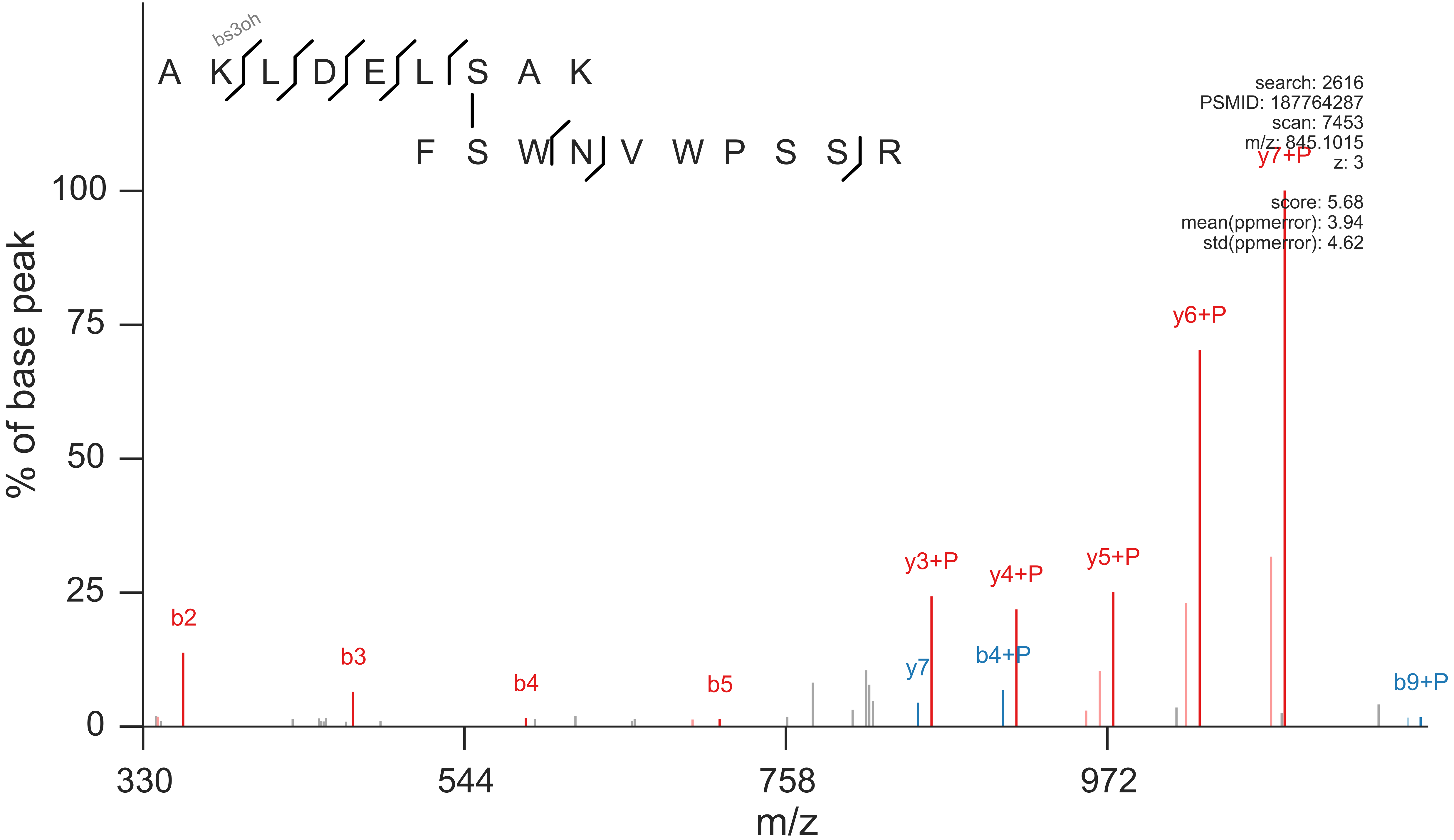
bs3oh
A K L D E L S A K
F S W N V W P S S R
search: 2616
PSMID: 187764287
scan: 7453
m/z: 845.1015
z: 3
score: 5.68
mean(ppmerror): 3.94
std(ppmerror): 4.62
% of base peak
100
75
50
25
0
b2
b3
b4
b5
y7
y3+P
b4+P
y4+P
y5+P
y6+P
y7+P
b9+P
330
544
758
972
m/z

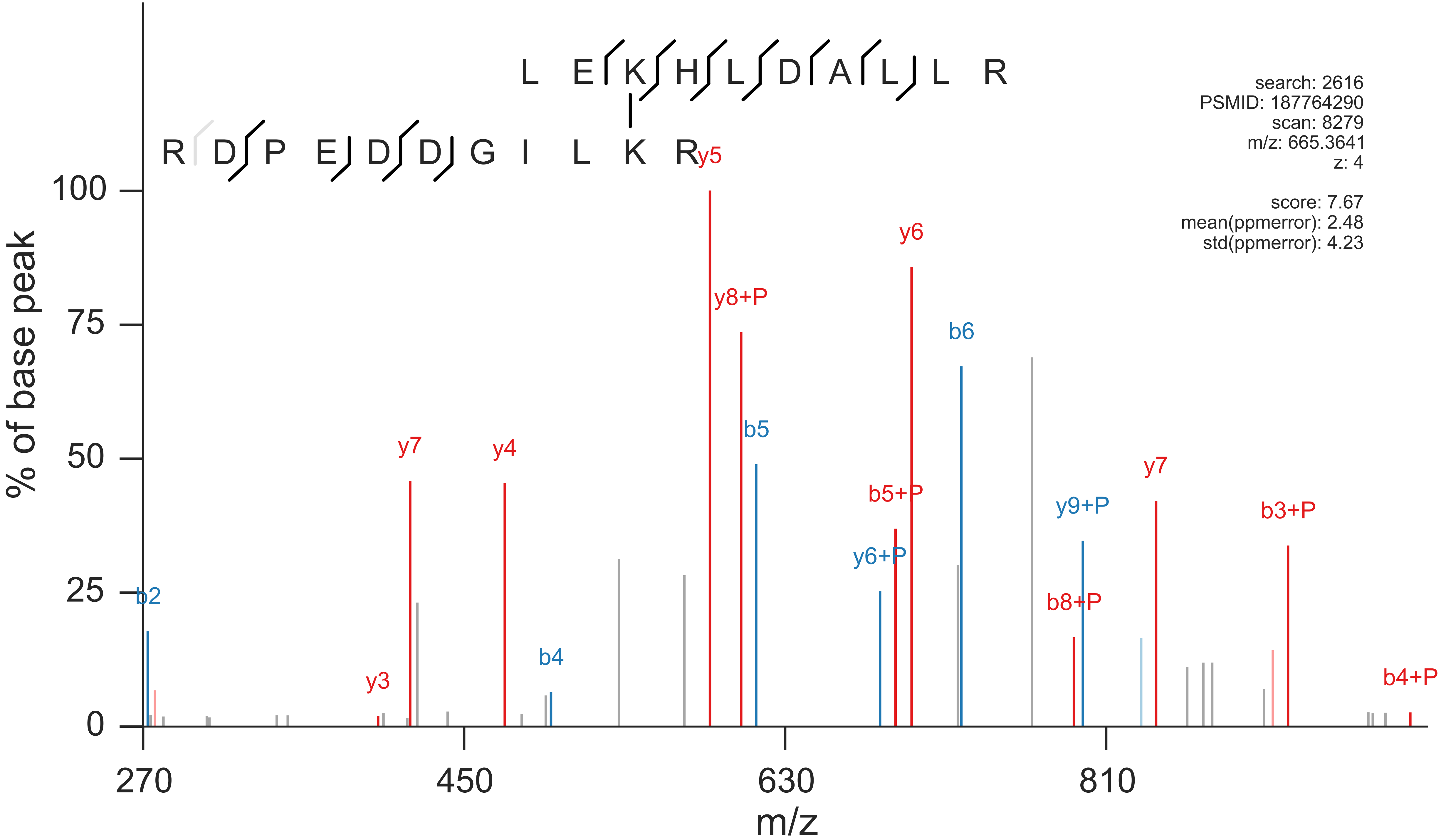
L E K H L D A L L R
R D P E D D G I L K R
search: 2616
PSMID: 187764290
scan: 8279
m/z: 665.3641
z: 4
score: 7.67
mean(ppmerror): 2.48
std(ppmerror): 4.23
% of base peak
m/z
0
25
50
75
100
270
450
630
810
y5
y6
y8+P
b6
b5
y7
y4
b5+P
y7
y9+P
b3+P
y6+P
b2
b8+P
b4
y3
b4+P

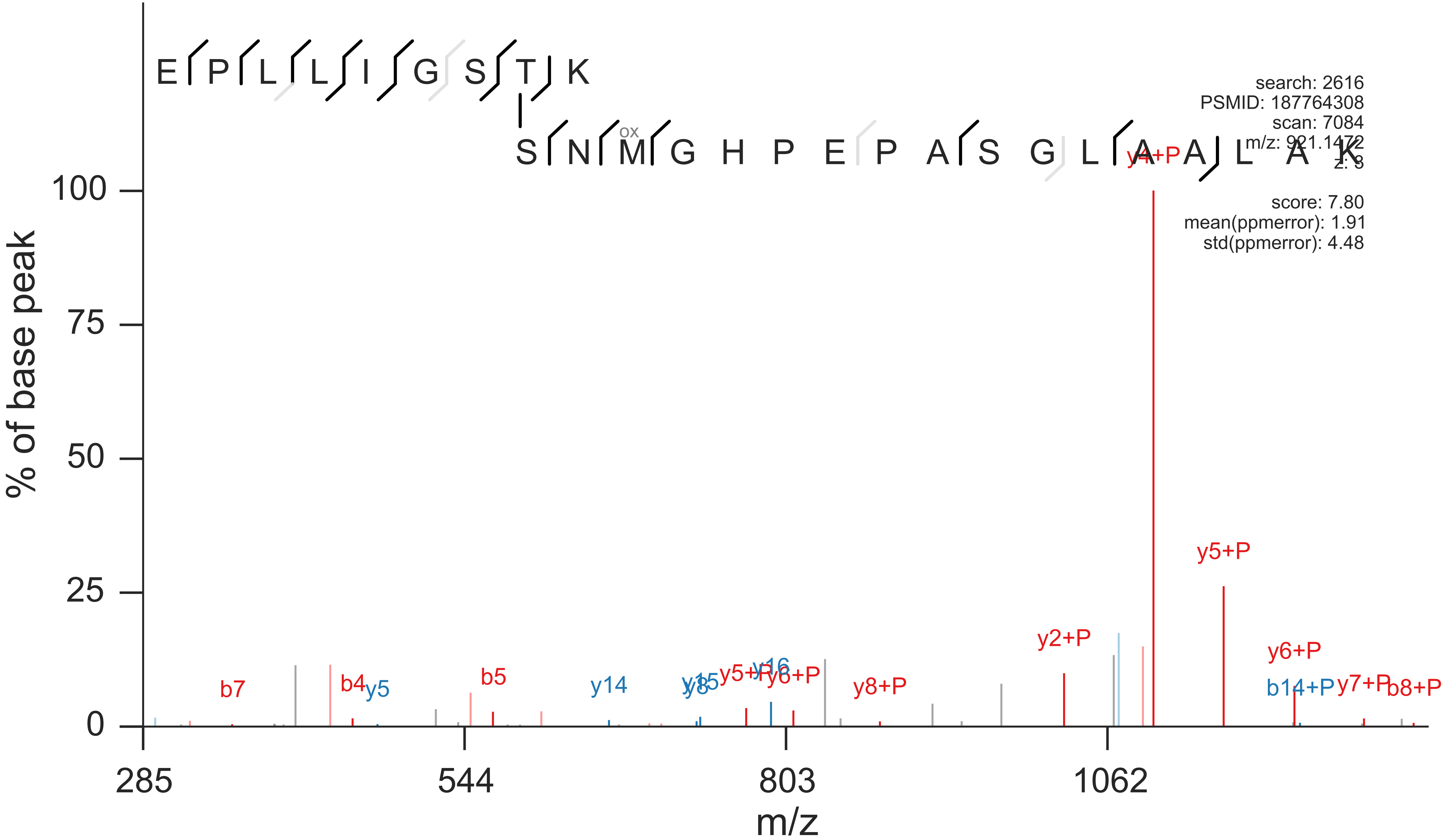

E P L L I G S T K
S N M G H P E P A S G L A A L A K
ox
search: 2616
PSMID: 187764308
scan: 7084
m/z: 921.1472
z: 3
score: 7.80
mean(ppmerror): 1.91
std(ppmerror): 4.48
% of base peak
m/z
100
75
50
25
0
285
544
803
1062
y4+P
y5+P
y2+P
y6+P
b14+P
y7+P
b8+P
y8+P
y6+P
y5+P
y16
y15
y8
y14
b5
b4
y5
b7

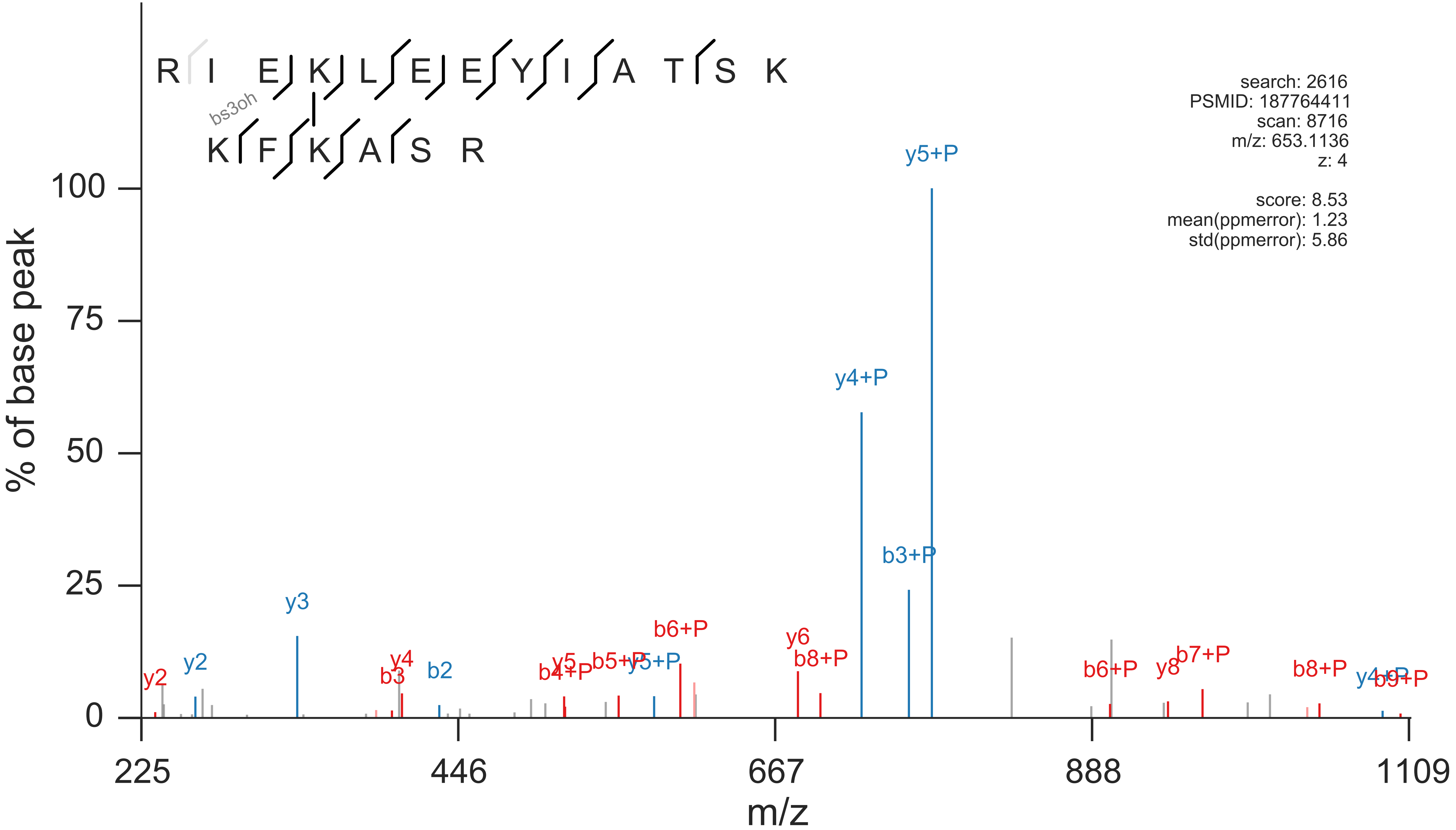
R I E K L E E Y I A T S K
bs3oh
K F K A S R
search: 2616
PSMID: 187764411
scan: 8716
m/z: 653.1136
z: 4
score: 8.53
mean(ppmerror): 1.23
std(ppmerror): 5.86
% of base peak
m/z
0
25
50
75
100
225
446
667
888
1109
y5+P
y4+P
b3+P
y3
y2
y2
y4
b3
b2
y5
b4+P
b5+P
y5+P
b6+P
y6
b8+P
b6+P
y8
b7+P
b8+P
y4+P
b9+P

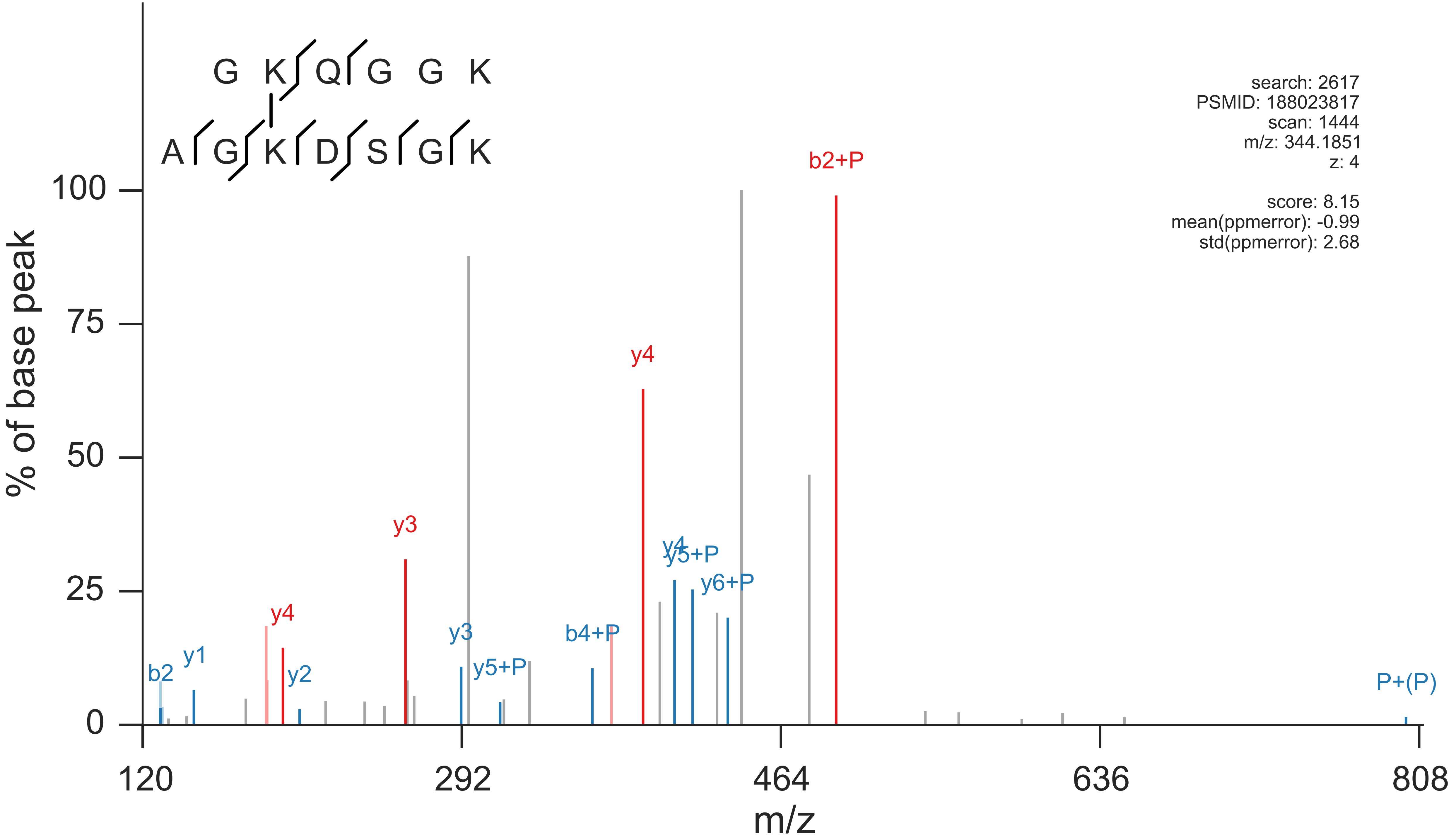
G K Q G G K
A G K D S G K
search: 2617
PSMID: 188023817
scan: 1444
m/z: 344.1851
z: 4
score: 8.15
mean(ppmerror): -0.99
std(ppmerror): 2.68
% of base peak
m/z
b2+P
y4
y3
y4
y5+P
y6+P
b4+P
y3
y5+P
y1
b2
y2
y4
P+(P)
0
25
50
75
100
120
292
464
636
808

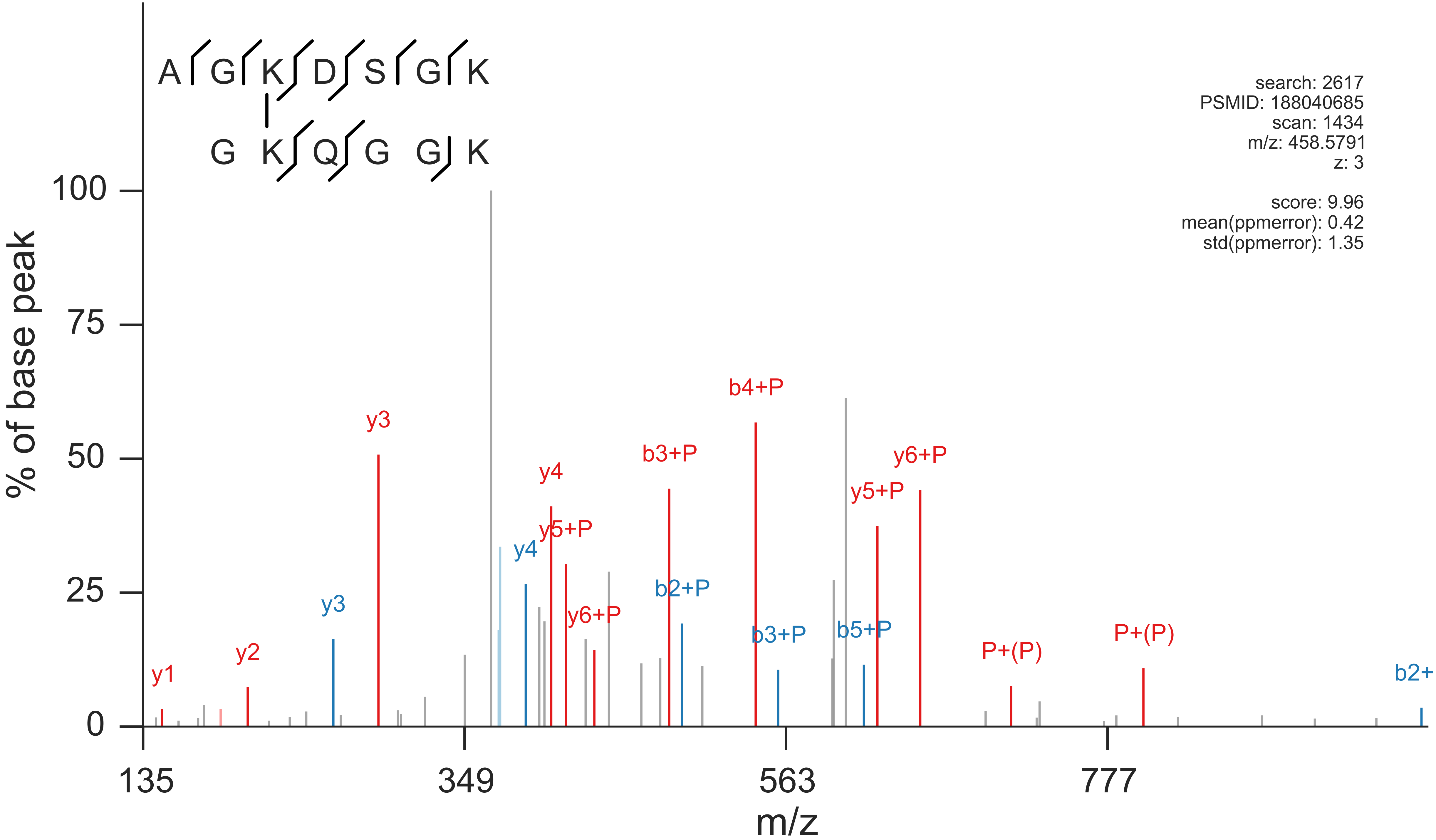

A G K D S G K
G K Q G G K
search: 2617
PSMID: 188040685
scan: 1434
m/z: 458.5791
z: 3
score: 9.96
mean(ppmerror): 0.42
std(ppmerror): 1.35
% of base peak
100
75
50
25
0
y1
y2
y3
y3
y4
y4
y5+P
y6+P
b3+P
b2+P
b4+P
b3+P
b5+P
y5+P
y6+P
P+(P)
P+(P)
b2+P
135
349
563
777
m/z

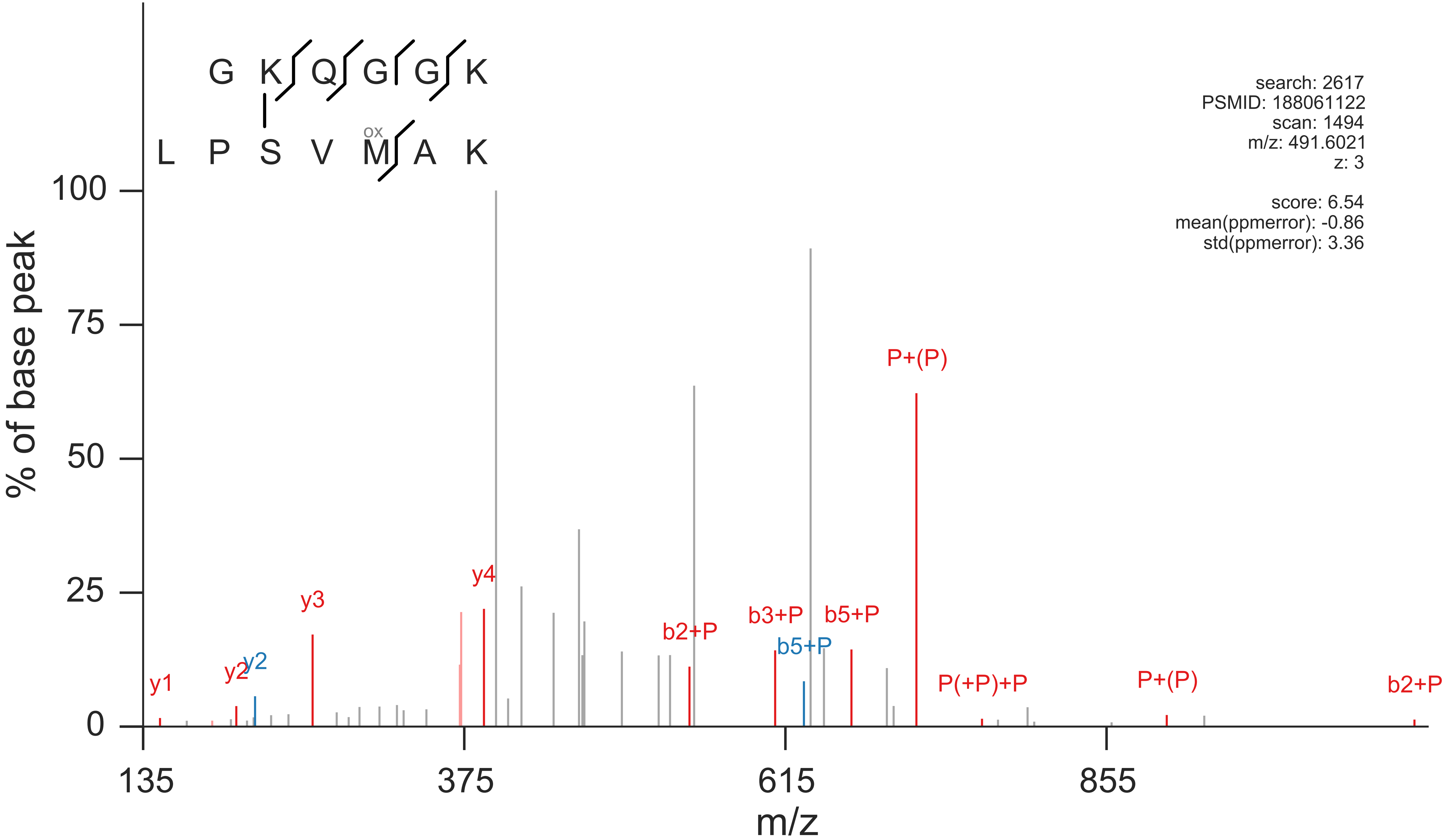

G K Q G G K
L P S V M A K
ox
search: 2617
PSMID: 188061122
scan: 1494
m/z: 491.6021
z: 3
score: 6.54
mean(ppmerror): -0.86
std(ppmerror): 3.36
% of base peak
100
75
50
25
0
135
375
615
855
m/z
y1
y2
y2
y3
y4
b2+P
b3+P
b5+P
b5+P
P+(P)
P(+P)+P
P+(P)
b2+P

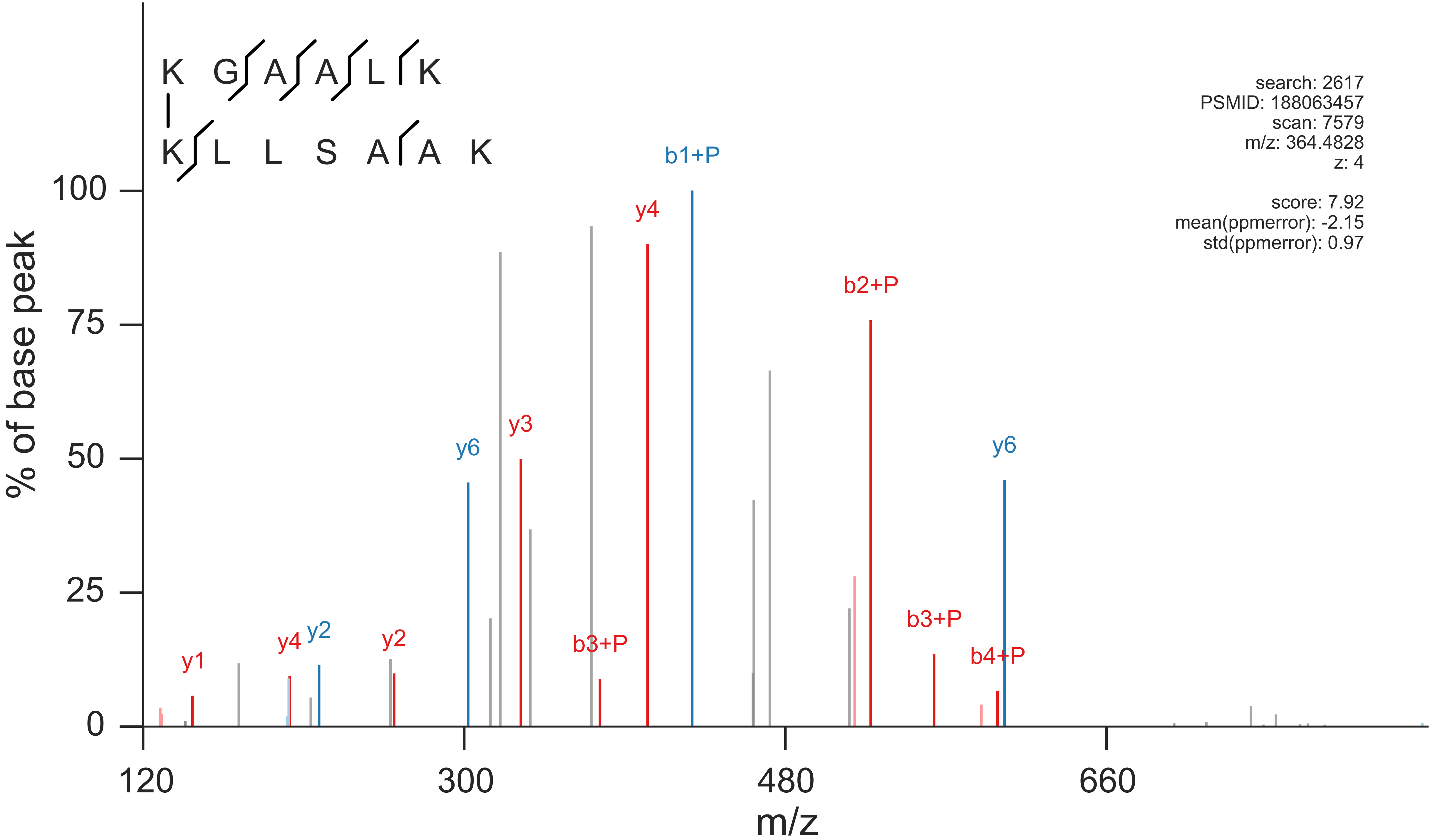
K G A A L K
K L L S A A K
search: 2617
PSMID: 188063457
scan: 7579
m/z: 364.4828
z: 4
score: 7.92
mean(ppmerror): -2.15
std(ppmerror): 0.97
% of base peak
m/z
y1
y4
y2
y2
y6
y3
b3+P
y4
b1+P
b2+P
b3+P
b4+P
y6

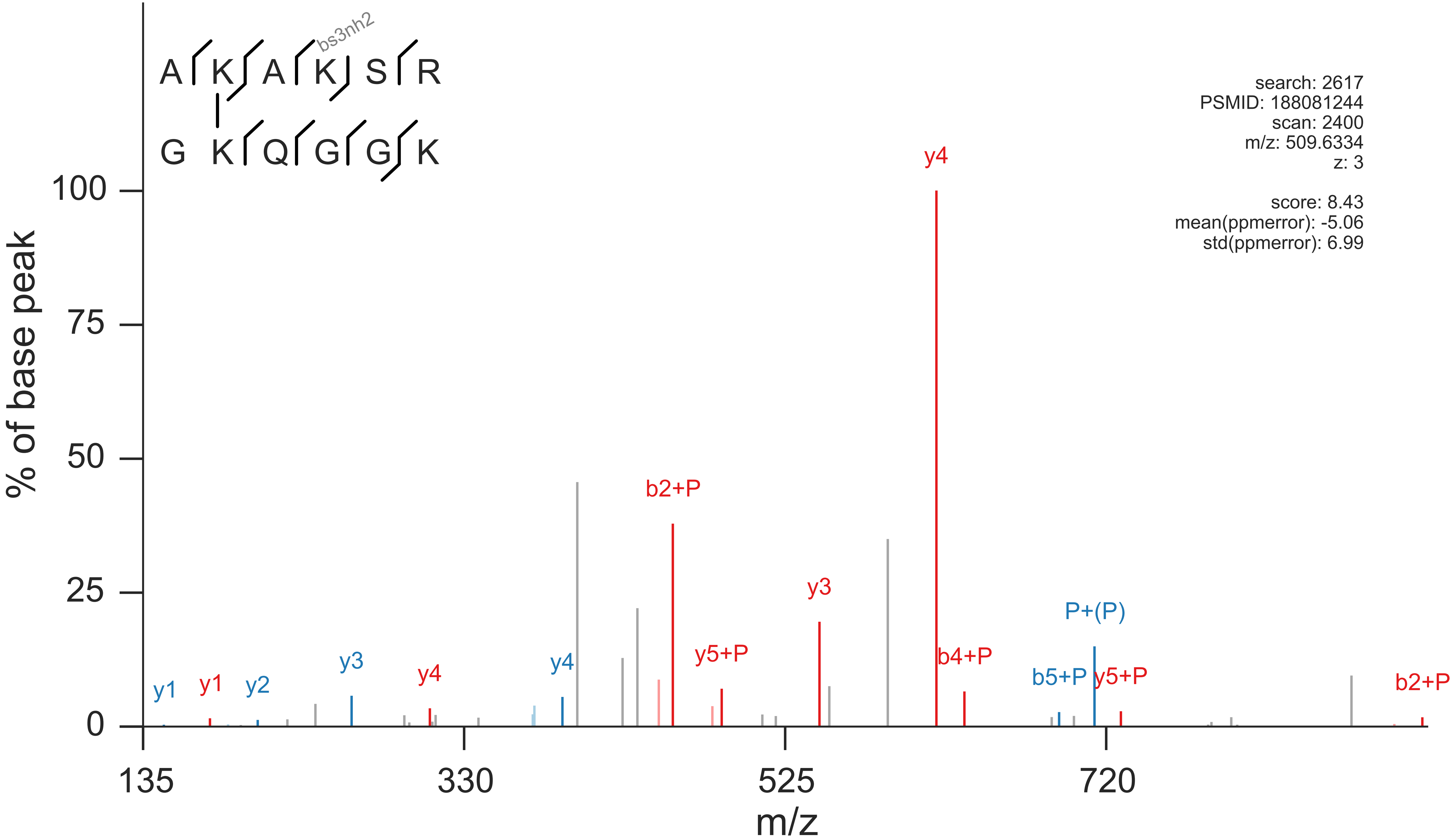

A K A K S R
G K Q G G K
bs3nh2
search: 2617
PSMID: 188081244
scan: 2400
m/z: 509.6334
z: 3
score: 8.43
mean(ppmerror): -5.06
std(ppmerror): 6.99
% of base peak
m/z
0
25
50
75
100
135
330
525
720
y1
y1
y2
y3
y4
y4
b2+P
y5+P
y3
y4
b4+P
b5+P
P+(P)
y5+P
b2+P

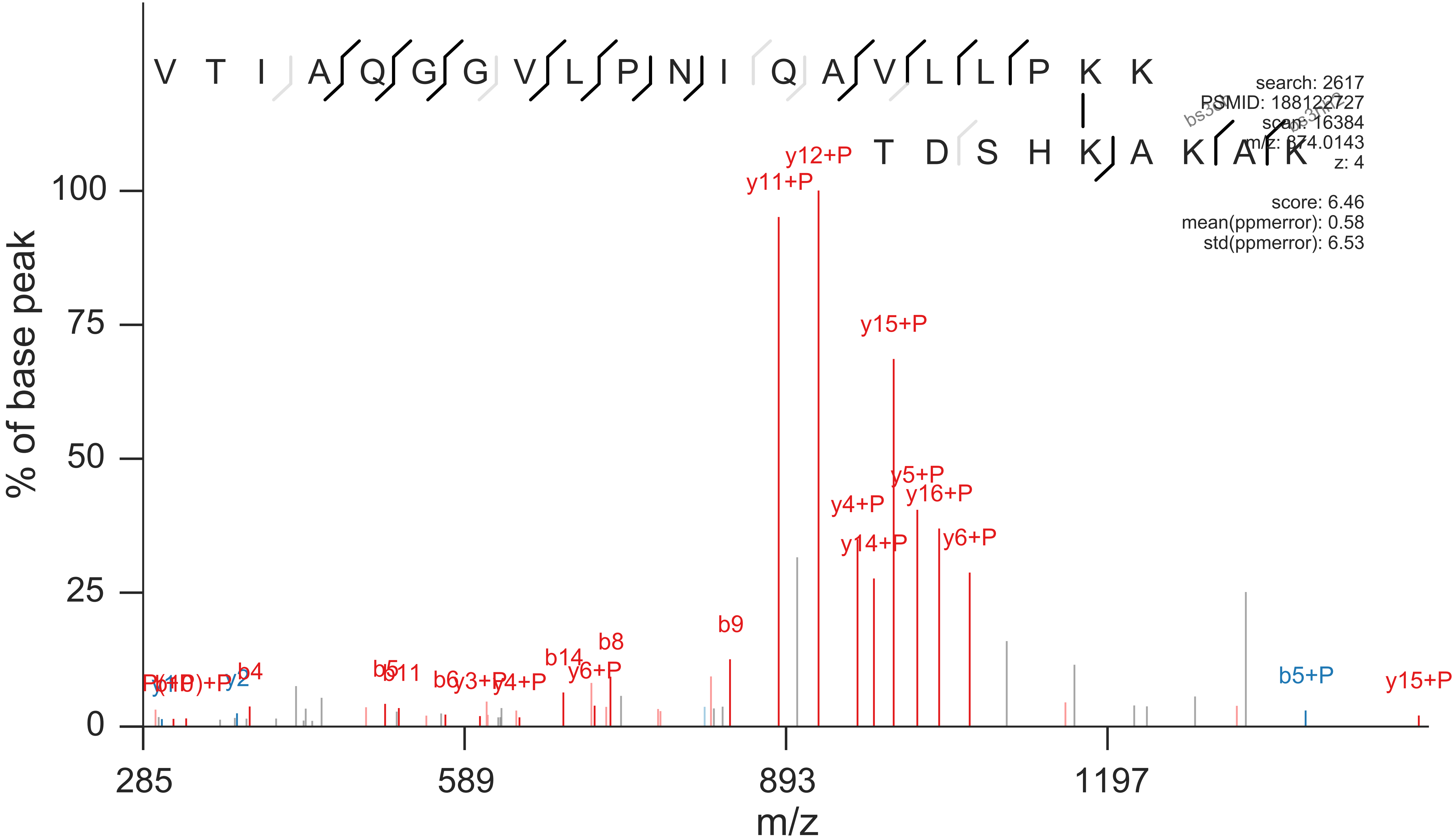

V T I A Q G G V L P N I Q A V L L P K K
T D S H K A K A K
search: 2617
PSMID: 188122727
scan: 16384
m/z: 874.0143
z: 4
score: 6.46
mean(ppmerror): 0.58
std(ppmerror): 6.53
y12+P
y11+P
y15+P
y5+P
y16+P
y4+P
y14+P
y6+P
b9
b8
b14
y6+P
b5
b11
b6
y3+P
y4+P
b4
y2
b5+P
y15+P
% of base peak
100
75
50
25
0
285
589
893
1197
m/z

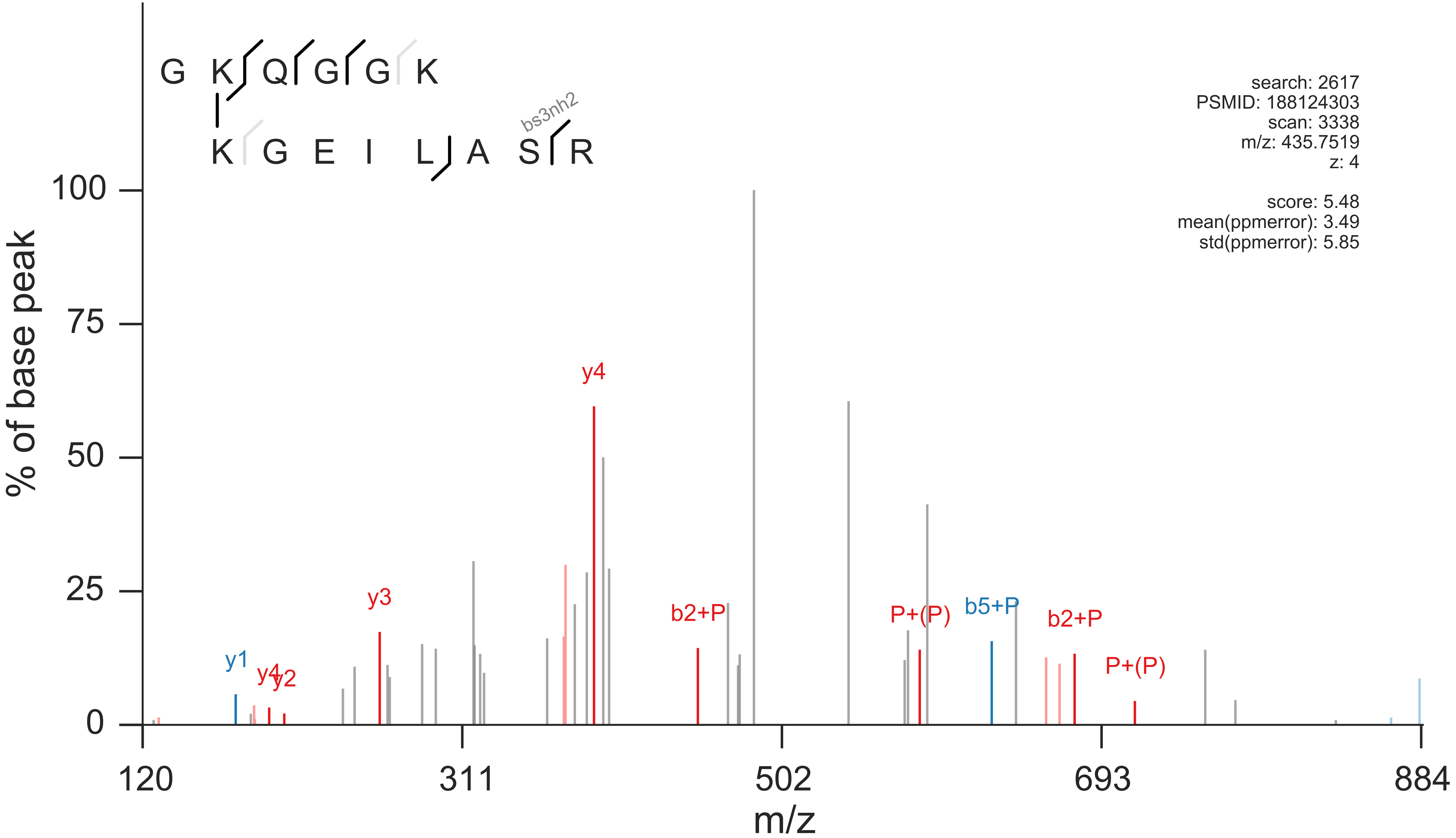

G K Q G G K
K G E I L A S R
bs3nh2
search: 2617
PSMID: 188124303
scan: 3338
m/z: 435.7519
z: 4
score: 5.48
mean(ppmerror): 3.49
std(ppmerror): 5.85
% of base peak
100
75
50
25
0
y1
y4
y2
y3
y4
b2+P
P+(P)
b5+P
b2+P
P+(P)
120
311
502
693
884
m/z

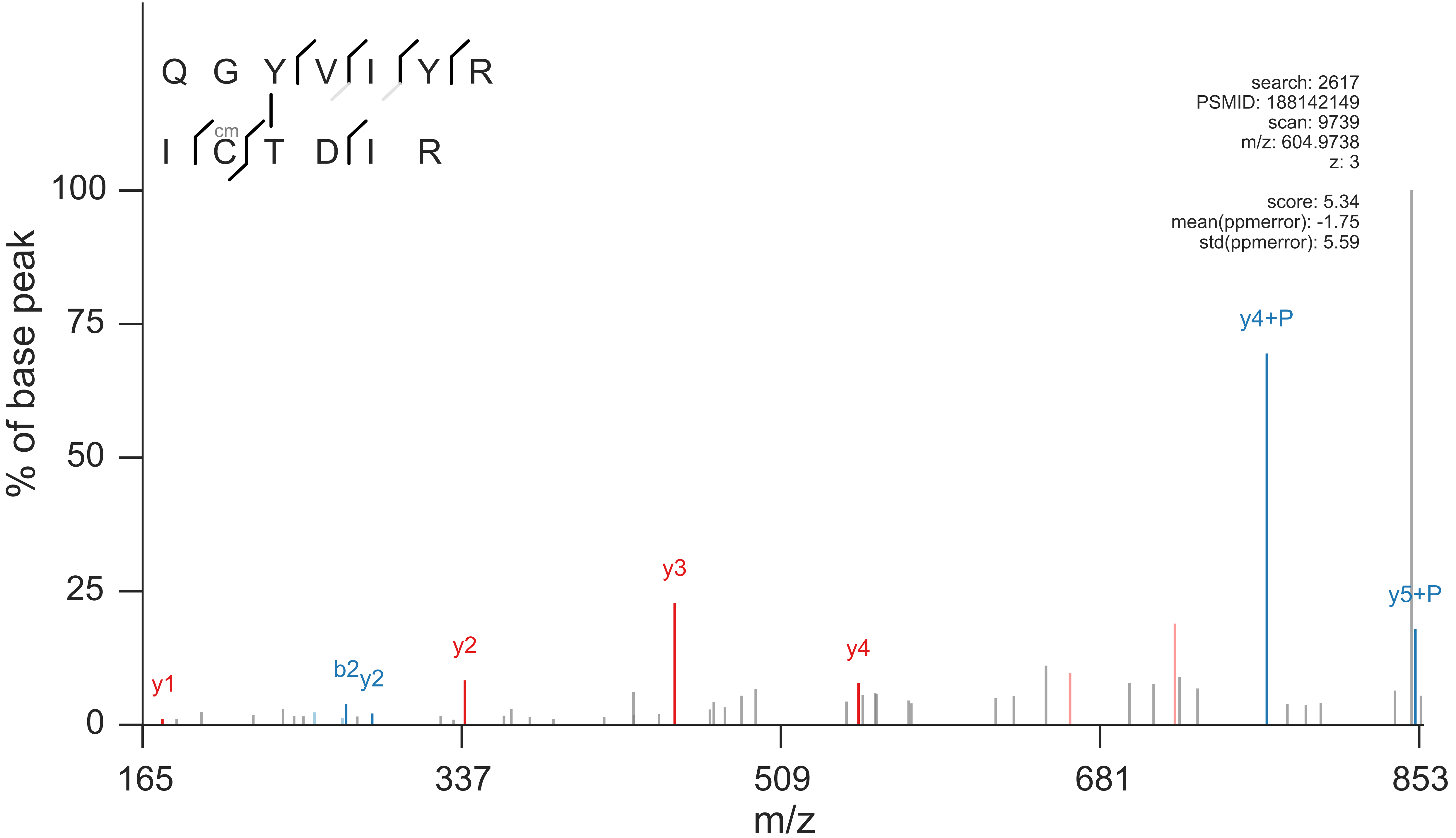

Q G Y V I Y R
I cm C T D I R
search: 2617
PSMID: 188142149
scan: 9739
m/z: 604.9738
z: 3
score: 5.34
mean(ppmerror): -1.75
std(ppmerror): 5.59
% of base peak
100
75
50
25
0
y1
b2
y2
y2
y3
y4
y4+P
y5+P
165
337
509
681
853
m/z

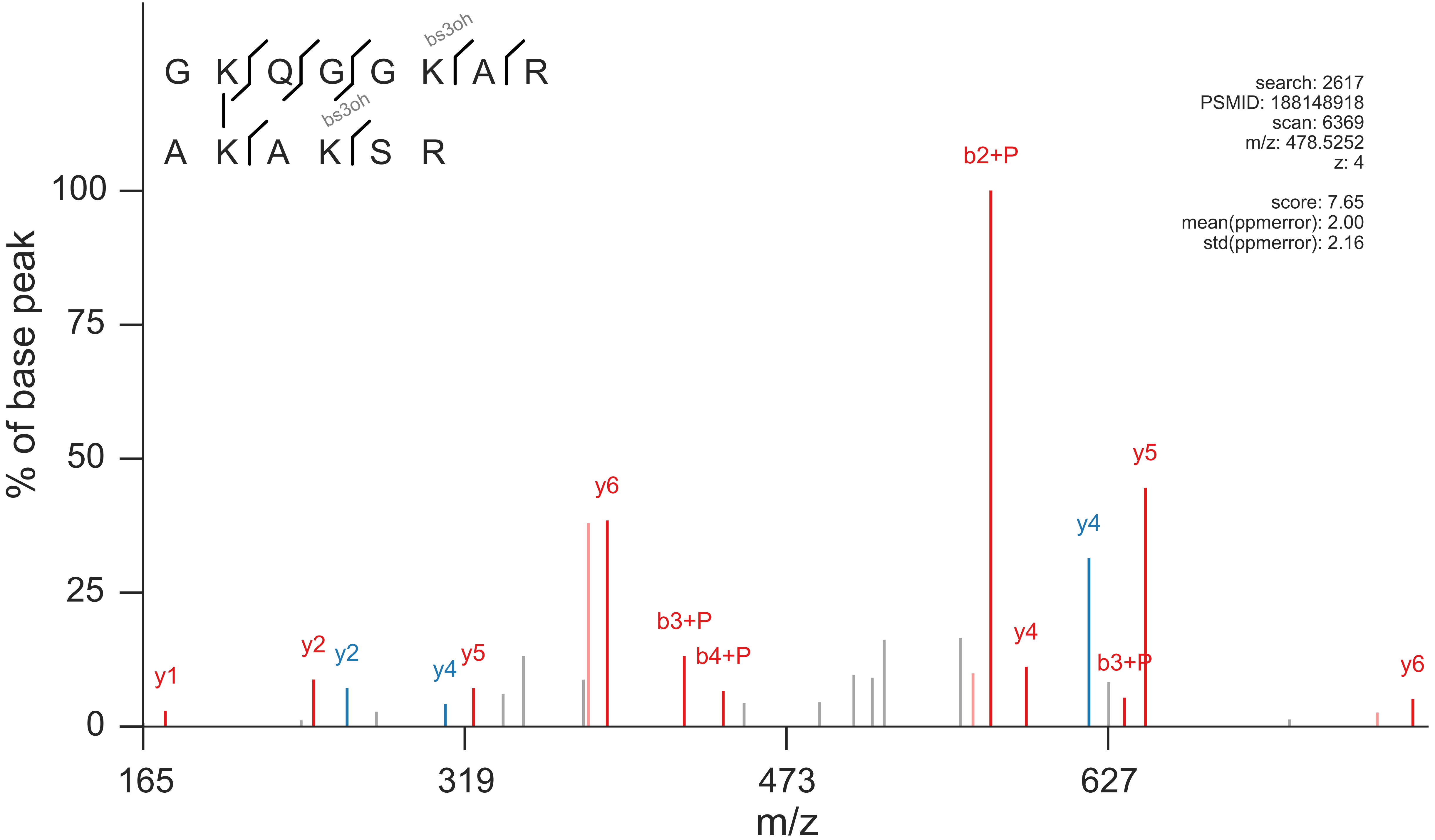

G K Q G G K A R
bs3oh
A K A K S R
bs3oh
search: 2617
PSMID: 188148918
scan: 6369
m/z: 478.5252
z: 4
score: 7.65
mean(ppmerror): 2.00
std(ppmerror): 2.16
% of base peak
100
75
50
25
0
165
319
473
627
m/z
b2+P
y6
y5
y4
b3+P
b4+P
y4
b3+P
y6
y1
y2
y2
y4
y5

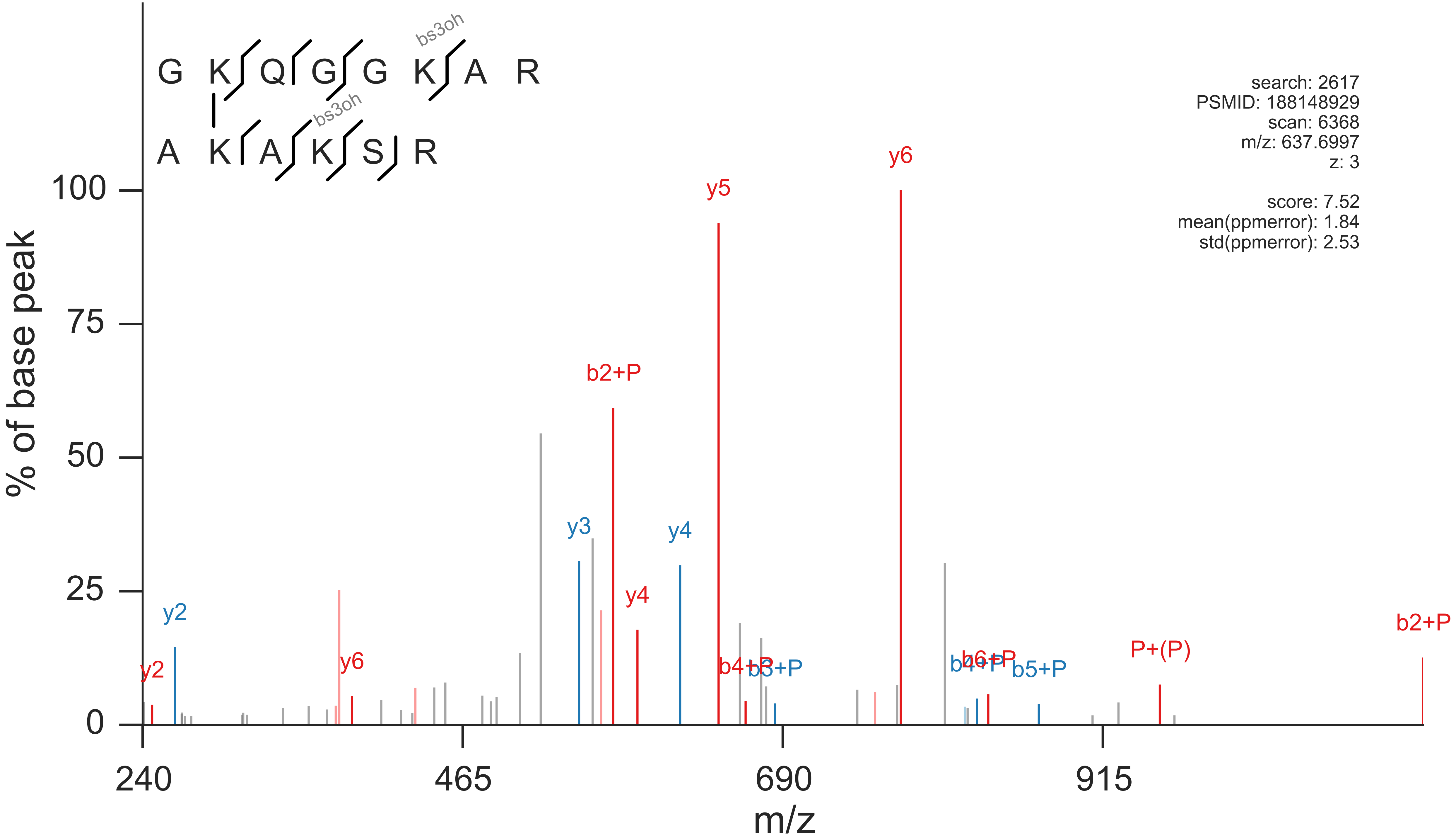

G K Q G G K A R
A K A K S R
bs3oh
bs3oh
search: 2617
PSMID: 188148929
scan: 6368
m/z: 637.6997
z: 3
score: 7.52
mean(ppmerror): 1.84
std(ppmerror): 2.53
% of base peak
m/z
100
75
50
25
0
240
465
690
915
y6
y5
b2+P
y3
y4
y4
y2
y2
y6
b4+P
b3+P
b4+P
b6+P
b5+P
P+(P)
b2+P

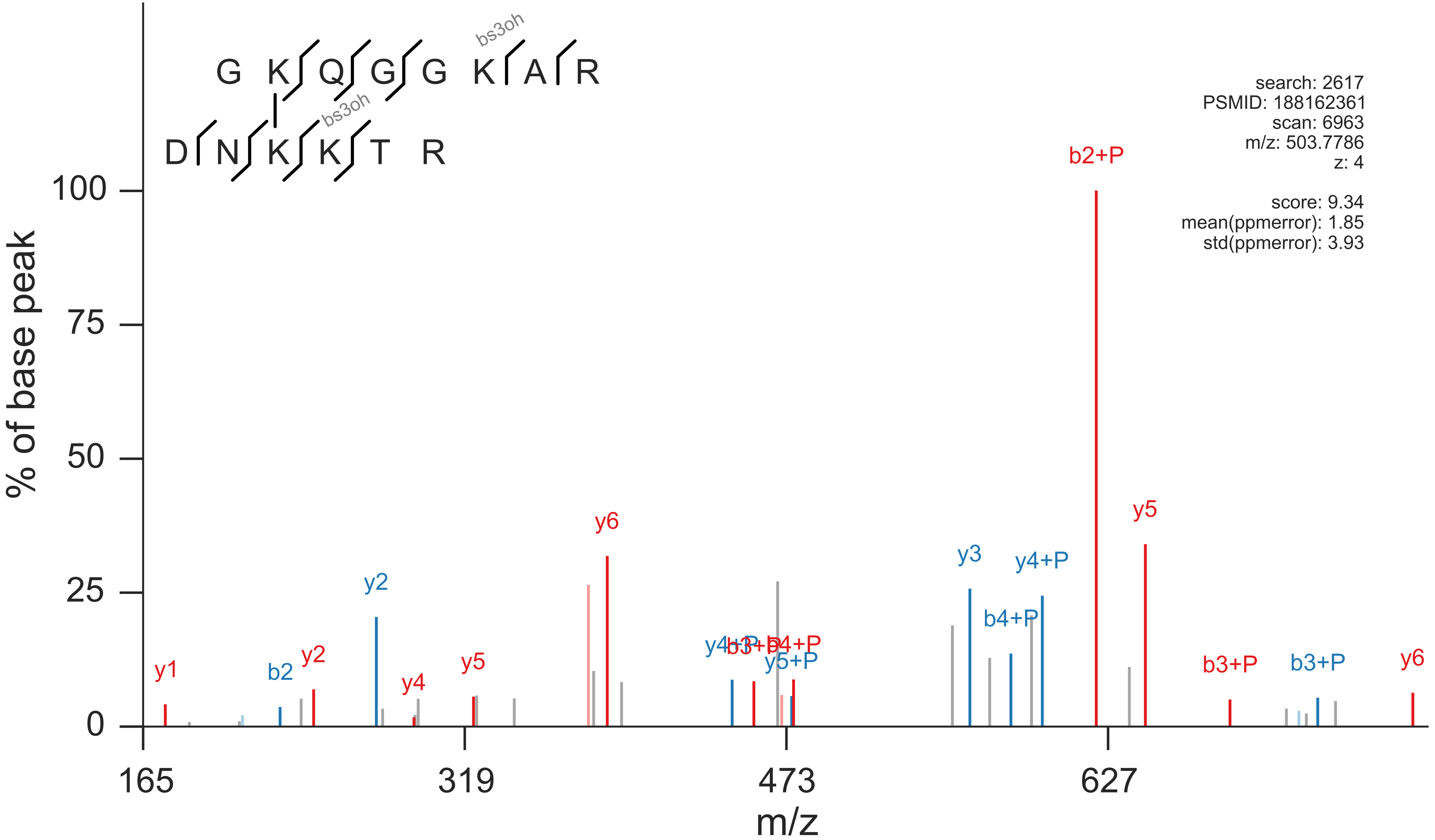

G K Q G G K A R
bs3oh
D N K K T R
bs3oh
search: 2617
PSMID: 188162361
scan: 6963
m/z: 503.7786
z: 4
score: 9.34
mean(ppmerror): 1.85
std(ppmerror): 3.93
% of base peak
100
75
50
25
0
165
319
473
627
m/z
b2+P
y5
y6
y3
y4+P
b4+P
y2
y1
b2
y2
y4
y5
b3+P
b3+P
y6
y5+P

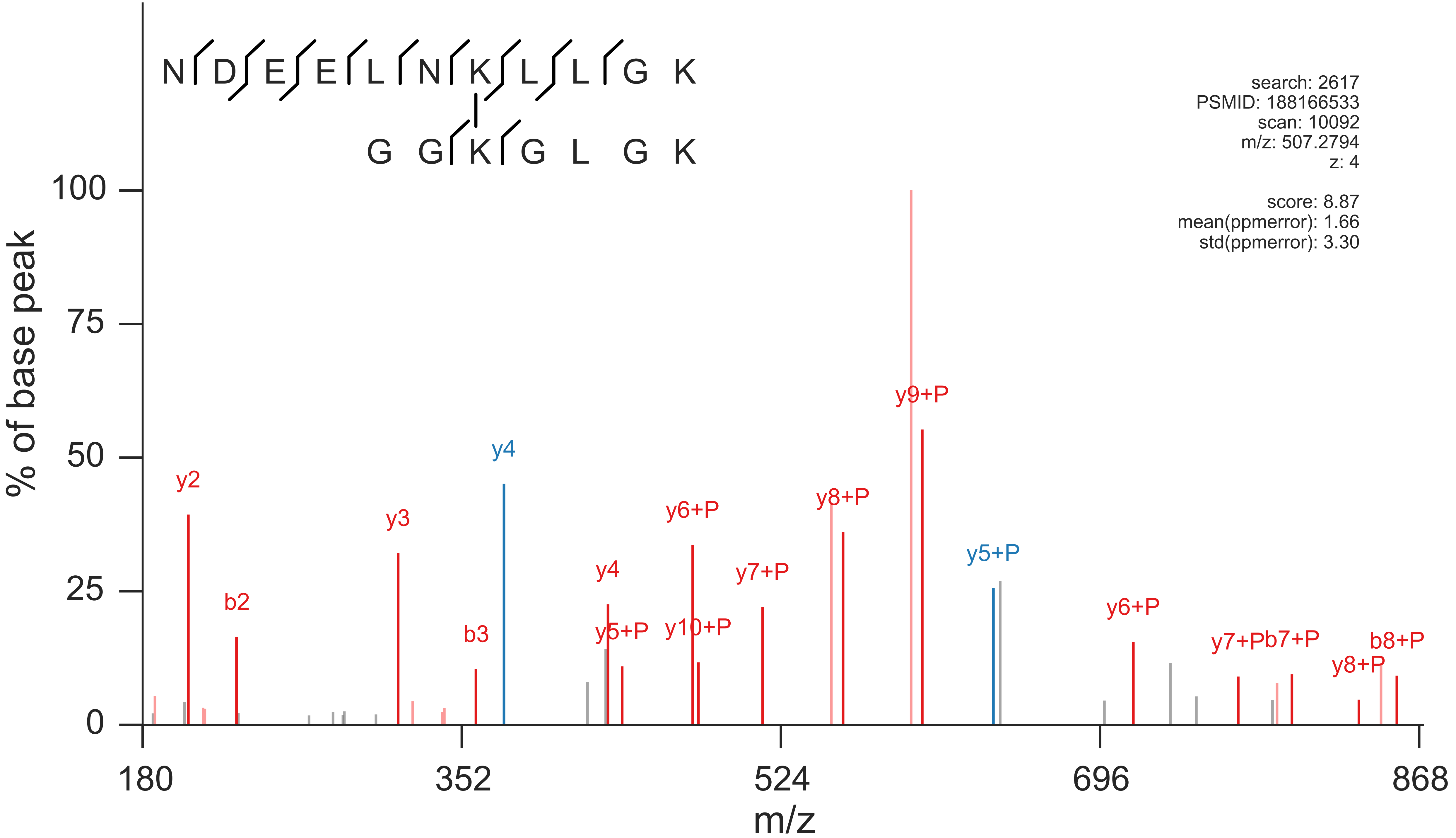

N D E E L N K L L G K
G G K G L G K
search: 2617
PSMID: 188166533
scan: 10092
m/z: 507.2794
z: 4
score: 8.87
mean(ppmerror): 1.66
std(ppmerror): 3.30
% of base peak
m/z
y2
b2
y3
b3
y4
y4
y5+P
y6+P
y10+P
y7+P
y8+P
y9+P
y5+P
y6+P
y7+P
b7+P
y8+P
b8+P

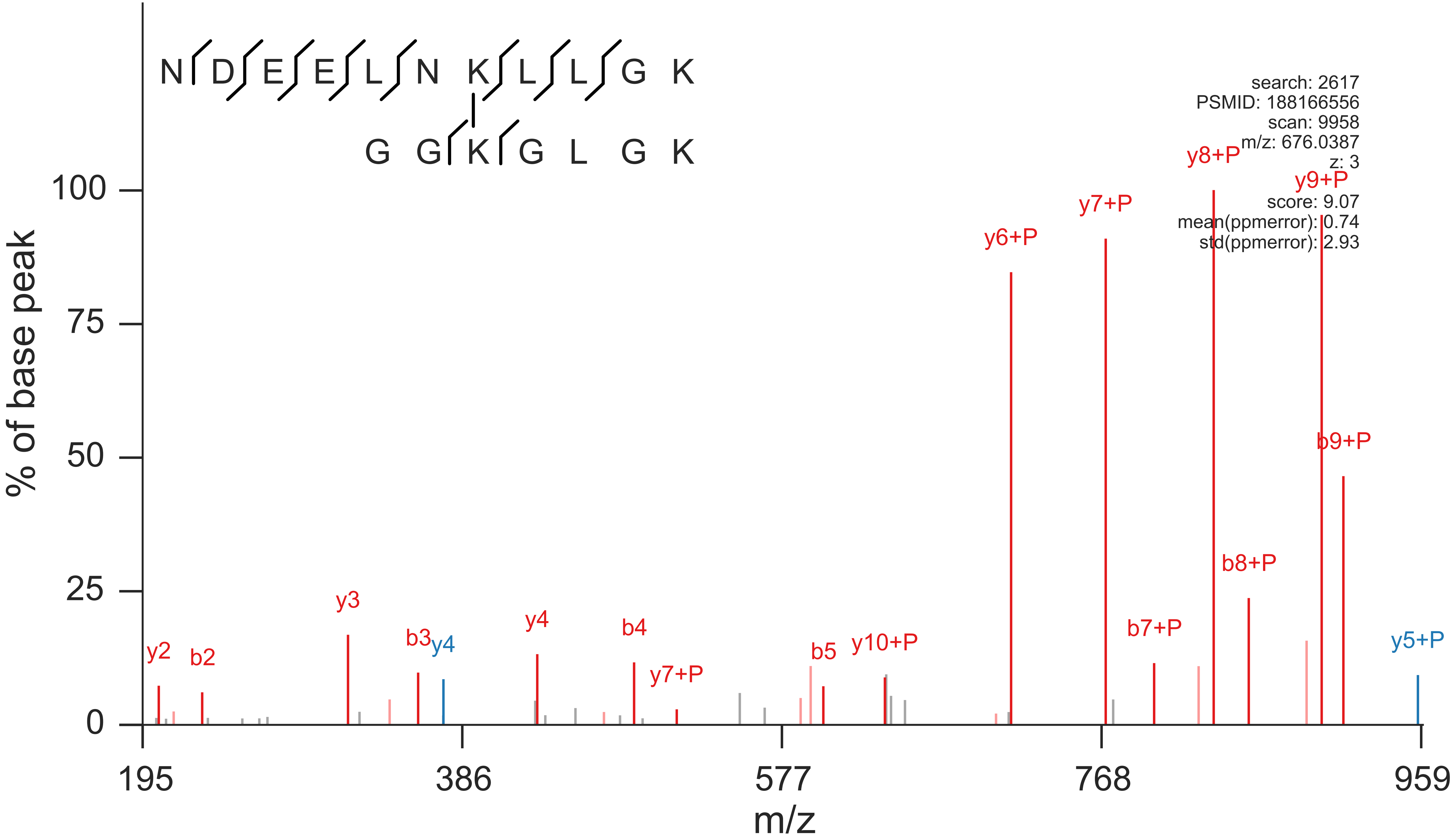

N D E E L N K L L G K
G G K G L G K
search: 2617
PSMID: 188166556
scan: 9958
m/z: 676.0387
z: 3
score: 9.07
mean(ppmerror): 0.74
std(ppmerror): 2.93
% of base peak
m/z
y2
b2
y3
b3
y4
y4
b4
y7+P
b5
y10+P
y6+P
y7+P
b7+P
y8+P
b8+P
y9+P
b9+P
y5+P

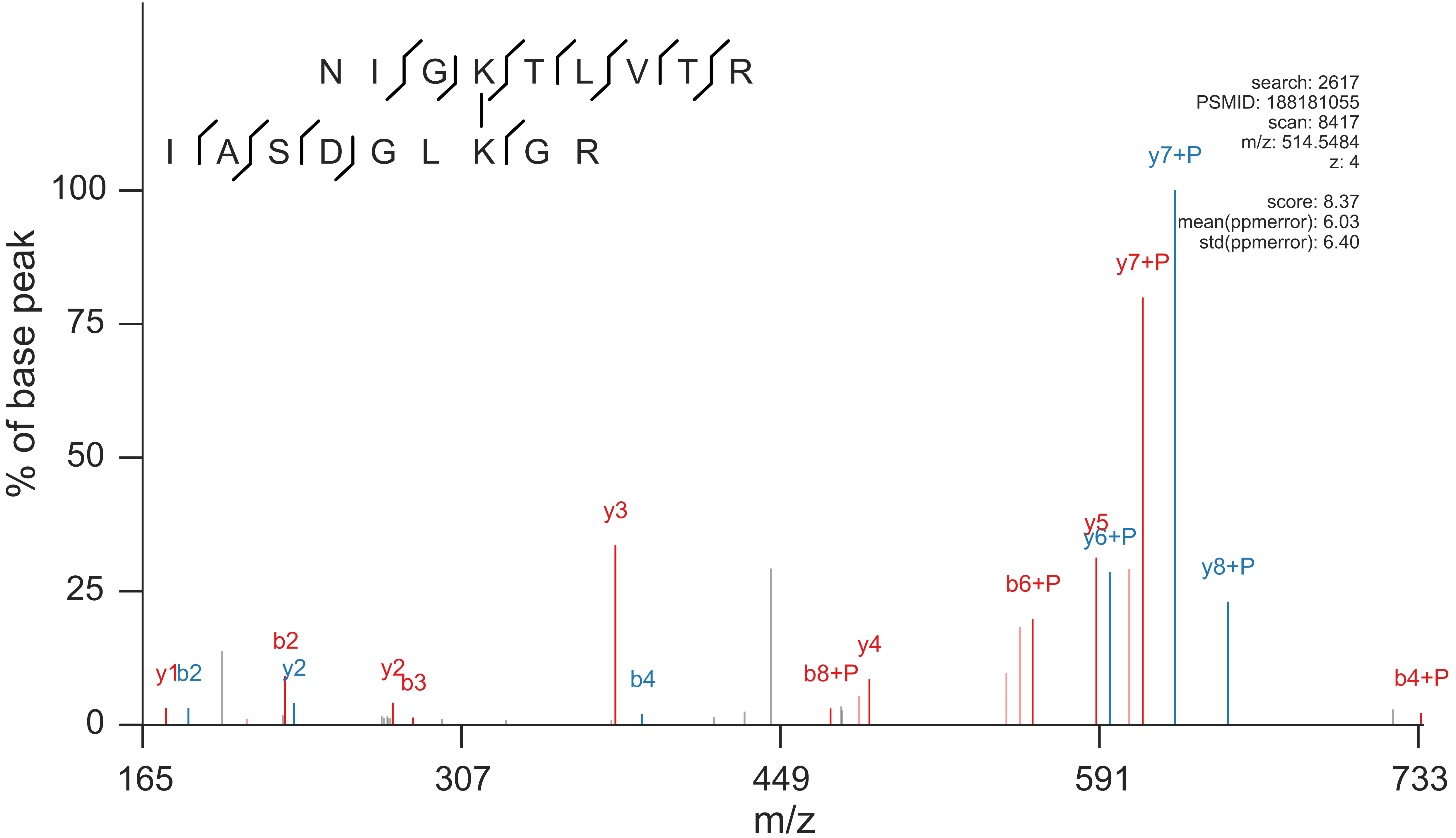

N I G K T L V T R
I A S D G L K G R
search: 2617
PSMID: 188181055
scan: 8417
m/z: 514.5484
z: 4
score: 8.37
mean(ppmerror): 6.03
std(ppmerror): 6.40
% of base peak
100
75
50
25
0
y7+P
y7+P
y3
y5
y6+P
y8+P
b6+P
y4
b8+P
b4+P
b4
y1
b2
b2
y2
y2
b3
165
307
449
591
733
m/z

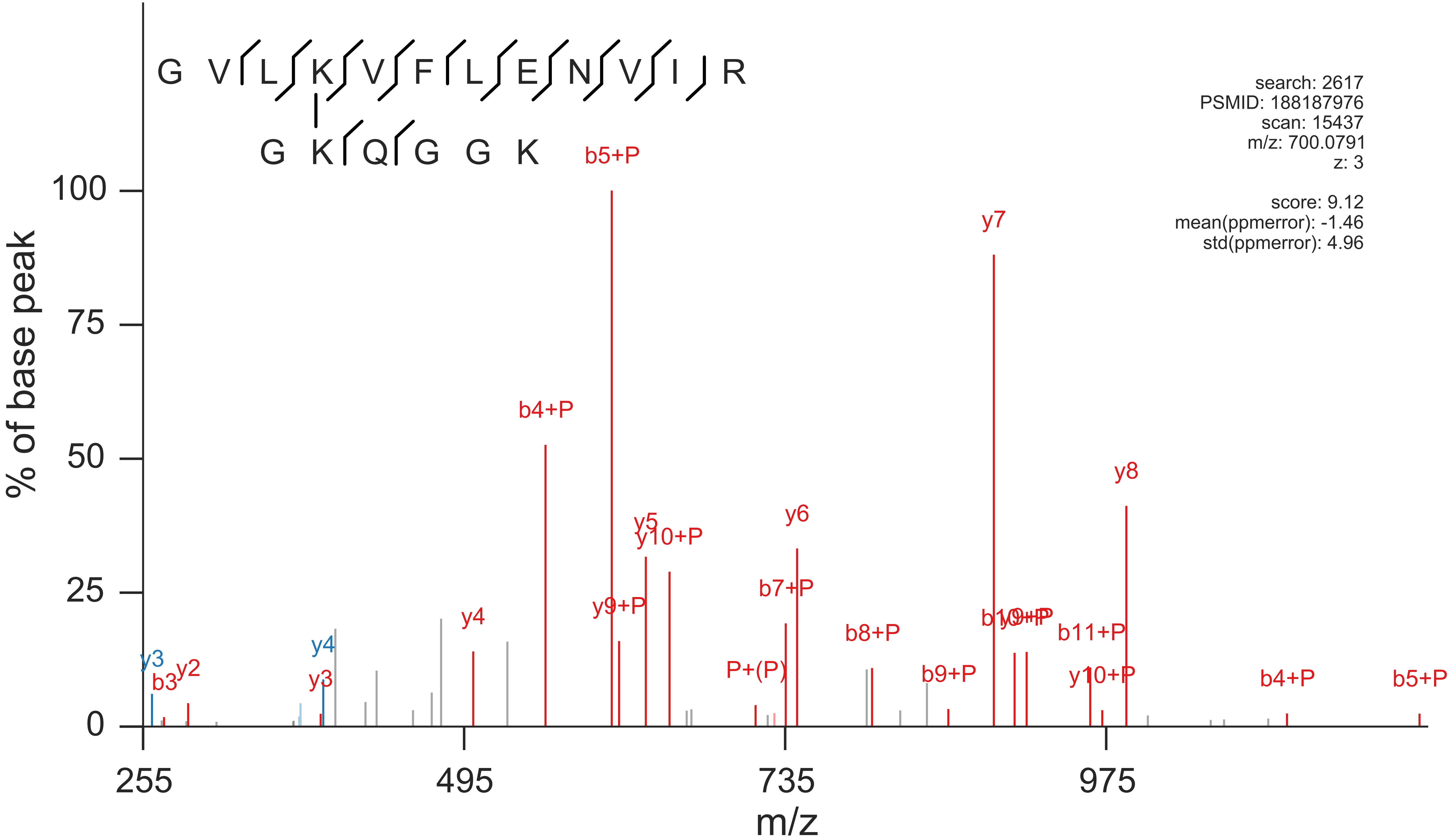

G V L K V F L E N V I R
G K Q G G K
search: 2617
PSMID: 188187976
scan: 15437
m/z: 700.0791
z: 3
score: 9.12
mean(ppmerror): -1.46
std(ppmerror): 4.96
% of base peak
m/z
0
25
50
75
100
255
495
735
975
b5+P
y7
b4+P
y8
y5
y10+P
y6
b7+P
y9+P
y4
b8+P
b11+P
P+(P)
b9+P
y10+P
b4+P
b5+P
y3
b3
y2
y4
y3

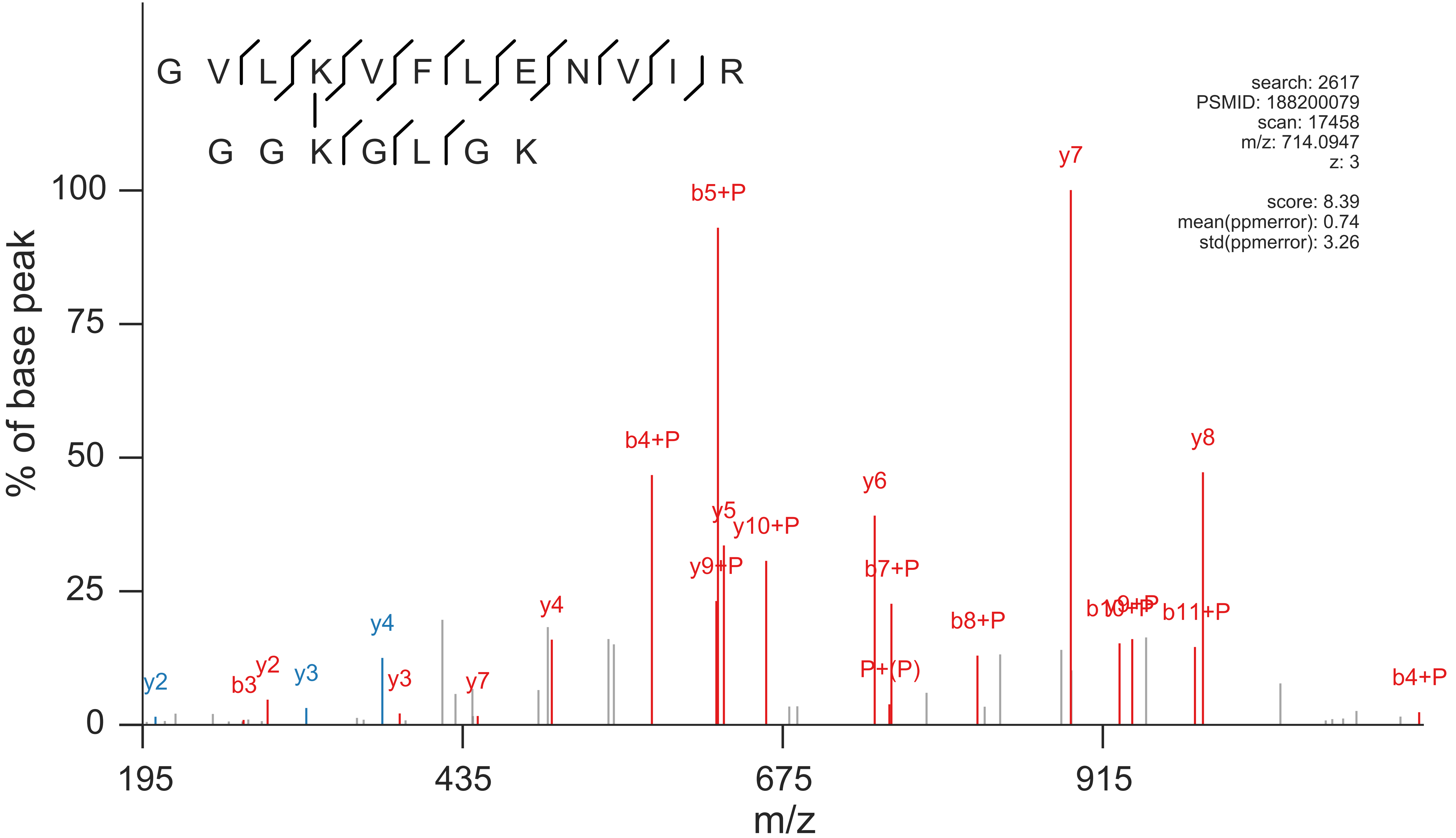

G V L K V F L E N V I R
G G K G L G K
search: 2617
PSMID: 188200079
scan: 17458
m/z: 714.0947
z: 3
score: 8.39
mean(ppmerror): 0.74
std(ppmerror): 3.26
% of base peak
m/z
100
75
50
25
0
195
435
675
915
y7
b5+P
b4+P
y8
y6
y5
y10+P
y9+P
b7+P
b8+P
b11+P
P+(P)
y4
y2
b3
y2
y3
y4
y3
y7
b4+P

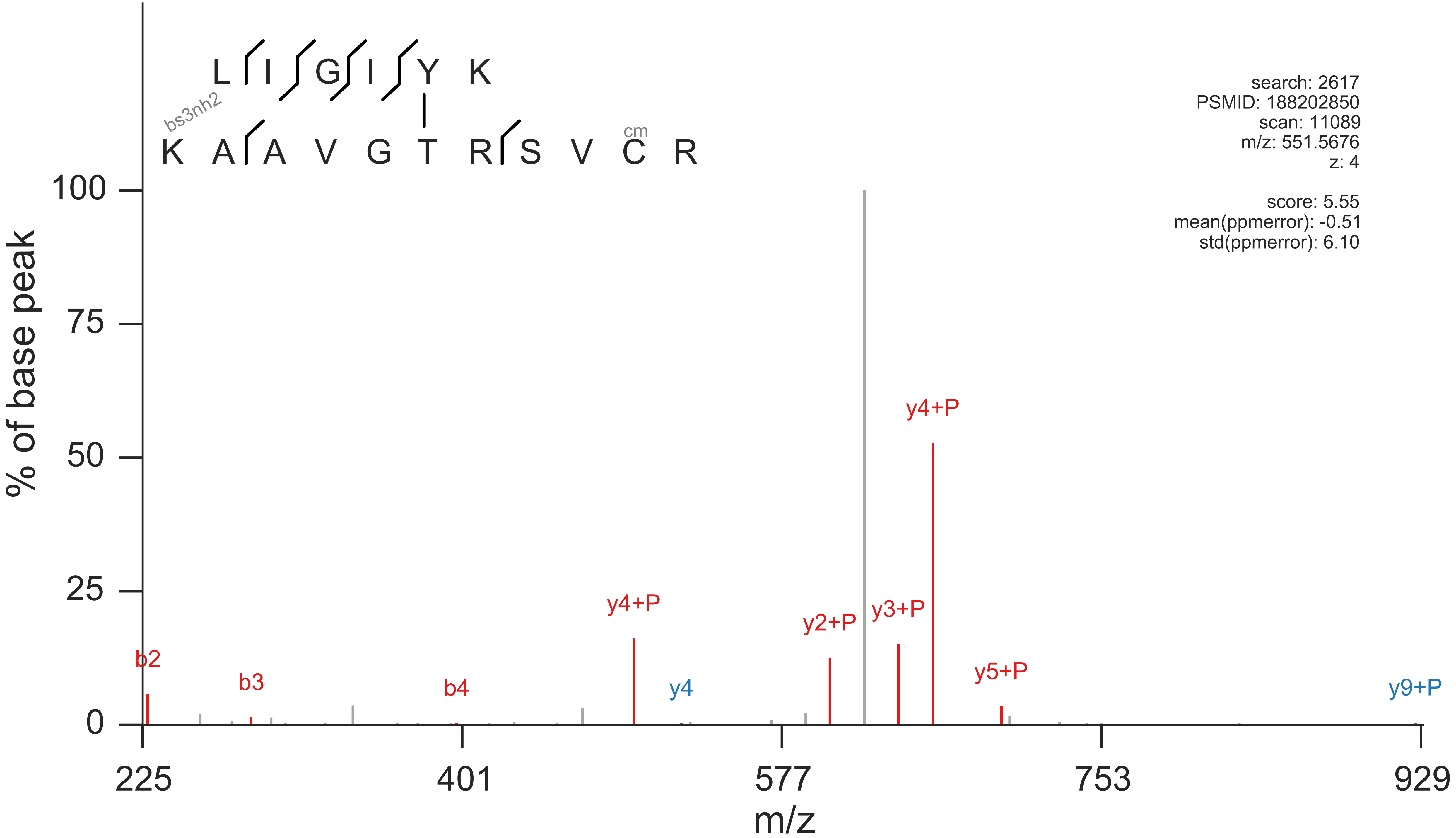
L I G I Y K
bs3nh2
K A A V G T R S V C R
cm
search: 2617
PSMID: 188202850
scan: 11089
m/z: 551.5676
z: 4
score: 5.55
mean(ppmerror): -0.51
std(ppmerror): 6.10
% of base peak
100
75
50
25
0
225
401
577
753
929
m/z
b2
b3
b4
y4+P
y4
y2+P
y3+P
y4+P
y5+P
y9+P

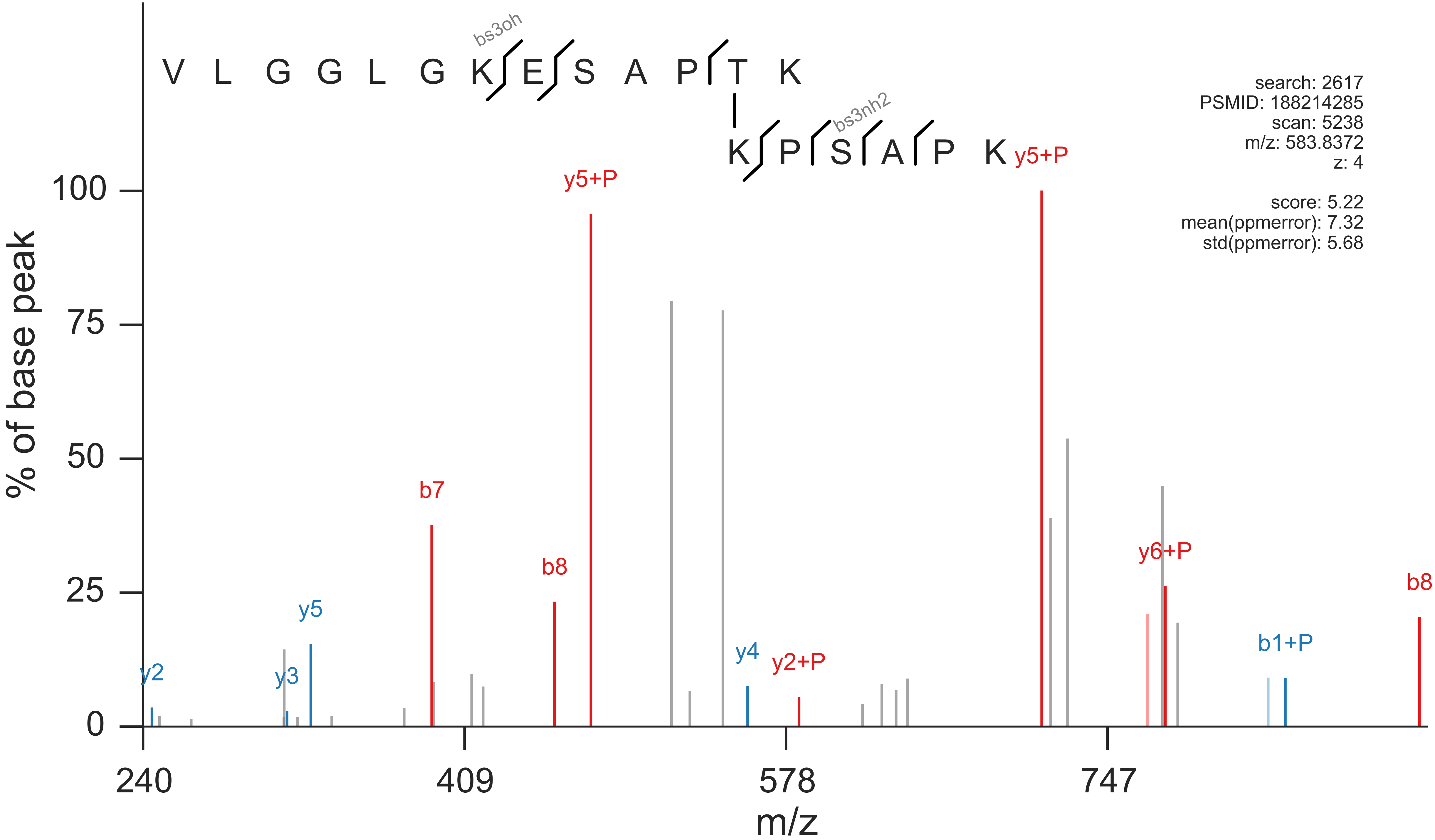

V L G G L G K E S A P T K
bs3oh
K P S A P K
bs3nh2
search: 2617
PSMID: 188214285
scan: 5238
m/z: 583.8372
z: 4
score: 5.22
mean(ppmerror): 7.32
std(ppmerror): 5.68
% of base peak
100
75
50
25
0
m/z
240
409
578
747
y5+P
y5+P
b7
b8
y5
y2
y3
y4
y2+P
y6+P
b1+P
b8

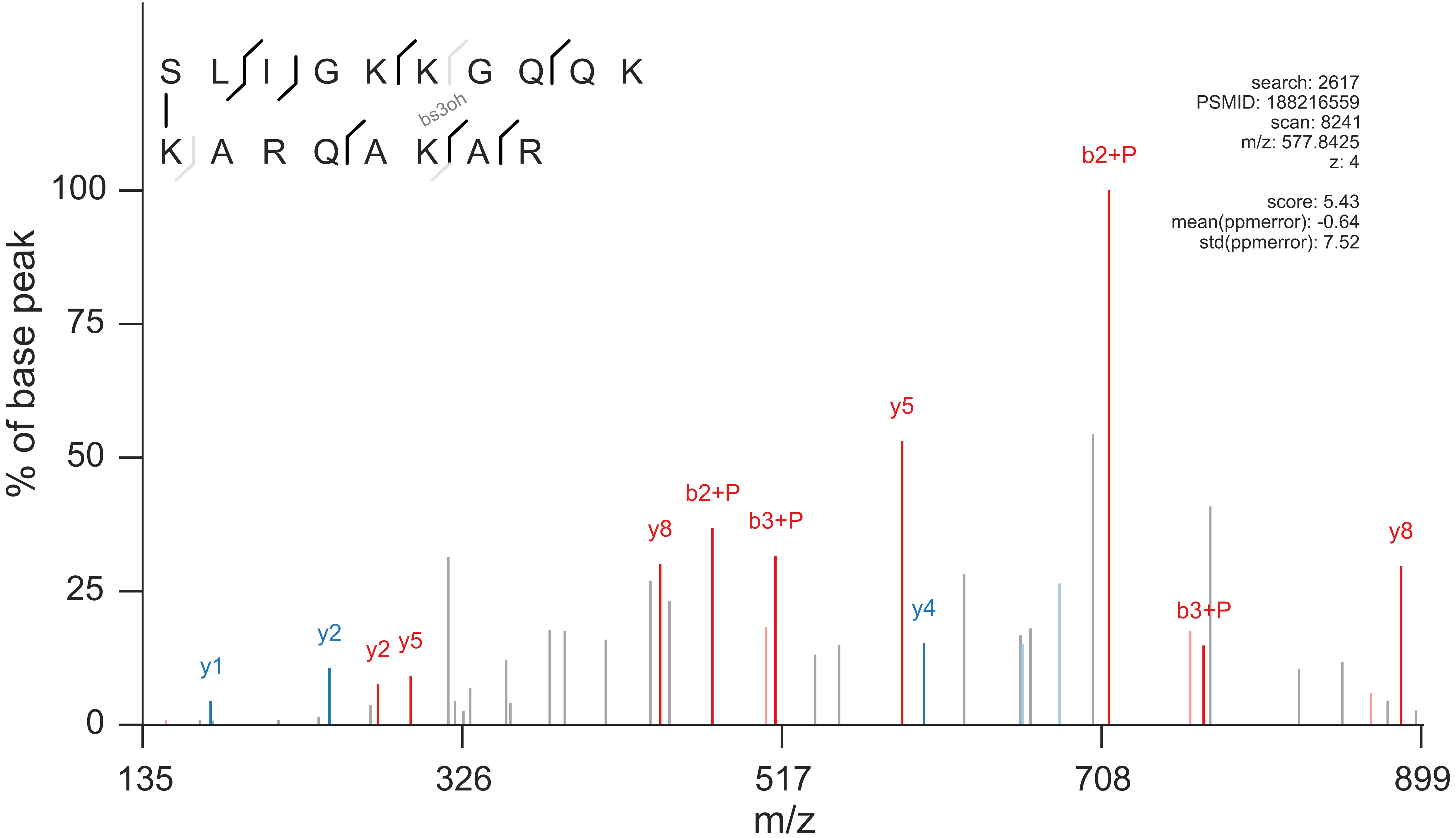
S L I G K K G Q Q K
K A R Q A K A R
bs3oh
search: 2617
PSMID: 188216559
scan: 8241
m/z: 577.8425
z: 4
score: 5.43
mean(ppmerror): -0.64
std(ppmerror): 7.52
% of base peak
m/z
0
25
50
75
100
135
326
517
708
899
y1
y2
y2
y5
y8
b2+P
b3+P
y5
y4
b2+P
b3+P
y8

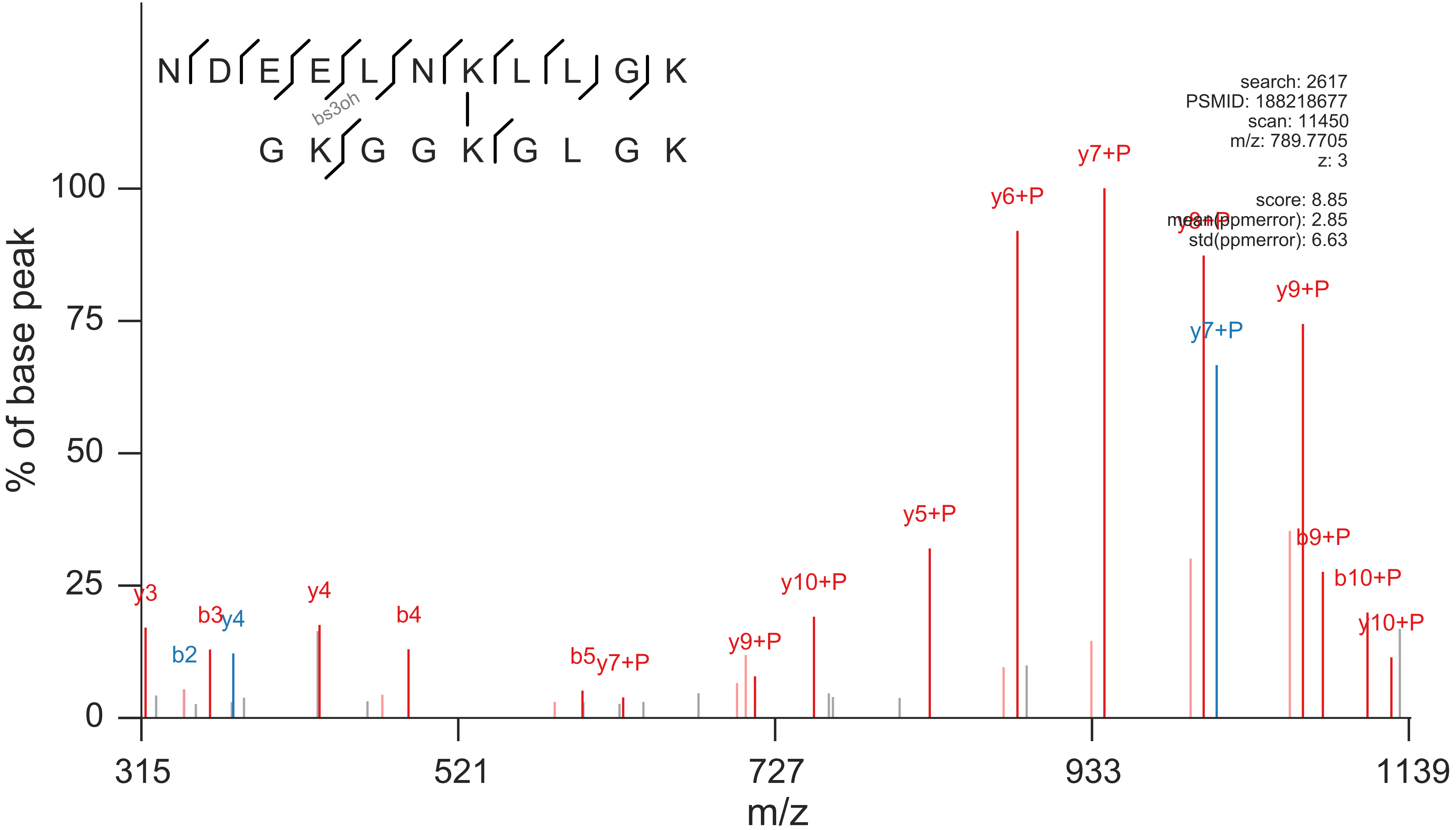

N D E E L N K L L G K
bs3oh
G K G G K G L G K
search: 2617
PSMID: 188218677
scan: 11450
m/z: 789.7705
z: 3
score: 8.85
mean(ppmerror): 2.85
std(ppmerror): 6.63
% of base peak
m/z
0
25
50
75
100
315
521
727
933
1139
y3
b2
b3
y4
y4
b4
b5
y7+P
y9+P
y10+P
y5+P
y6+P
y7+P
y8+P
y7+P
y9+P
b9+P
b10+P
y10+P

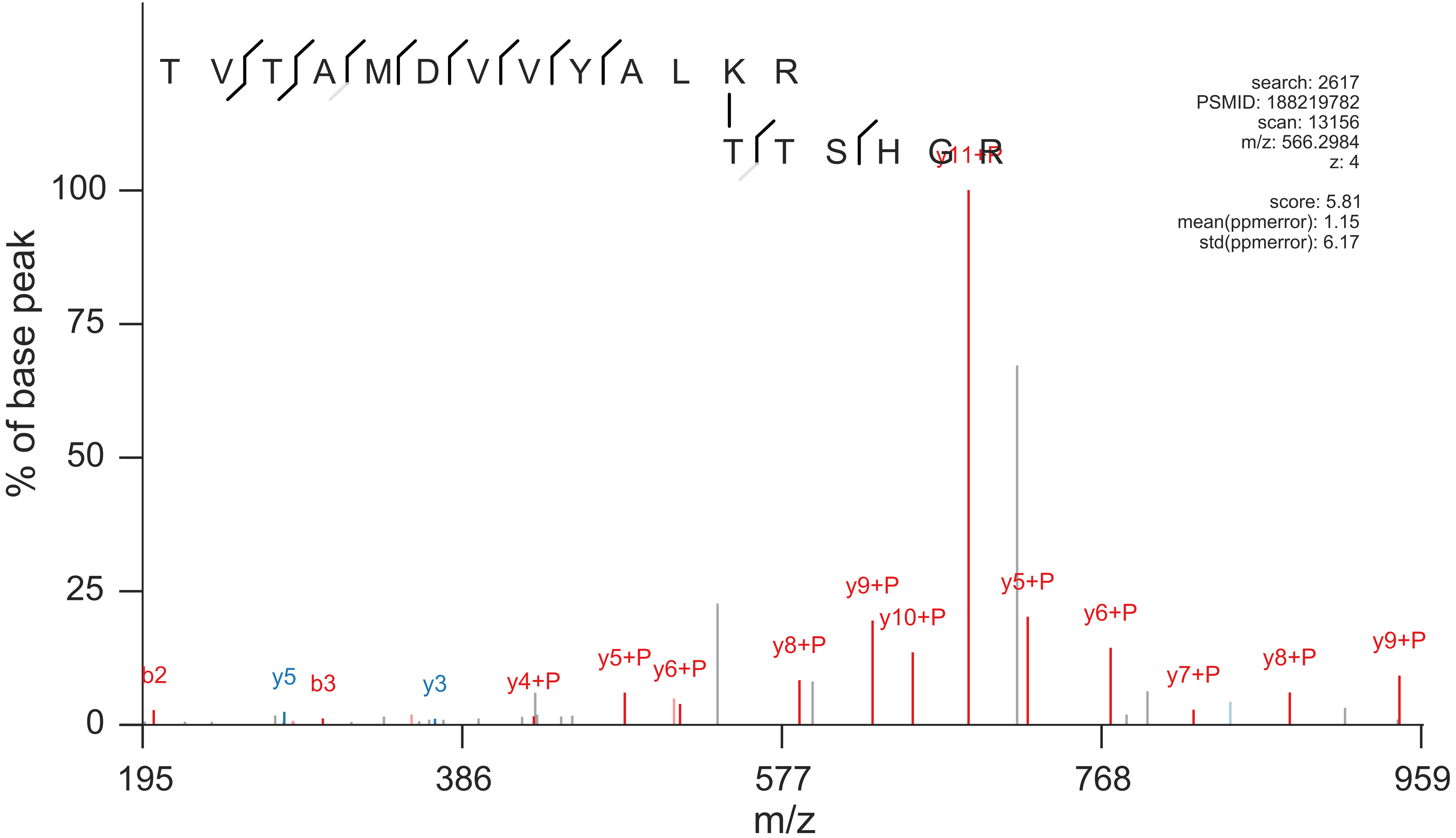

T V T A M D V V Y A L K R
T T S H G R
search: 2617
PSMID: 188219782
scan: 13156
m/z: 566.2984
z: 4
score: 5.81
mean(ppmerror): 1.15
std(ppmerror): 6.17
% of base peak
m/z
0
25
50
75
100
195
386
577
768
959
b2
y5
b3
y3
y4+P
y5+P
y6+P
y8+P
y9+P
y10+P
y11+P
y5+P
y6+P
y7+P
y8+P
y9+P

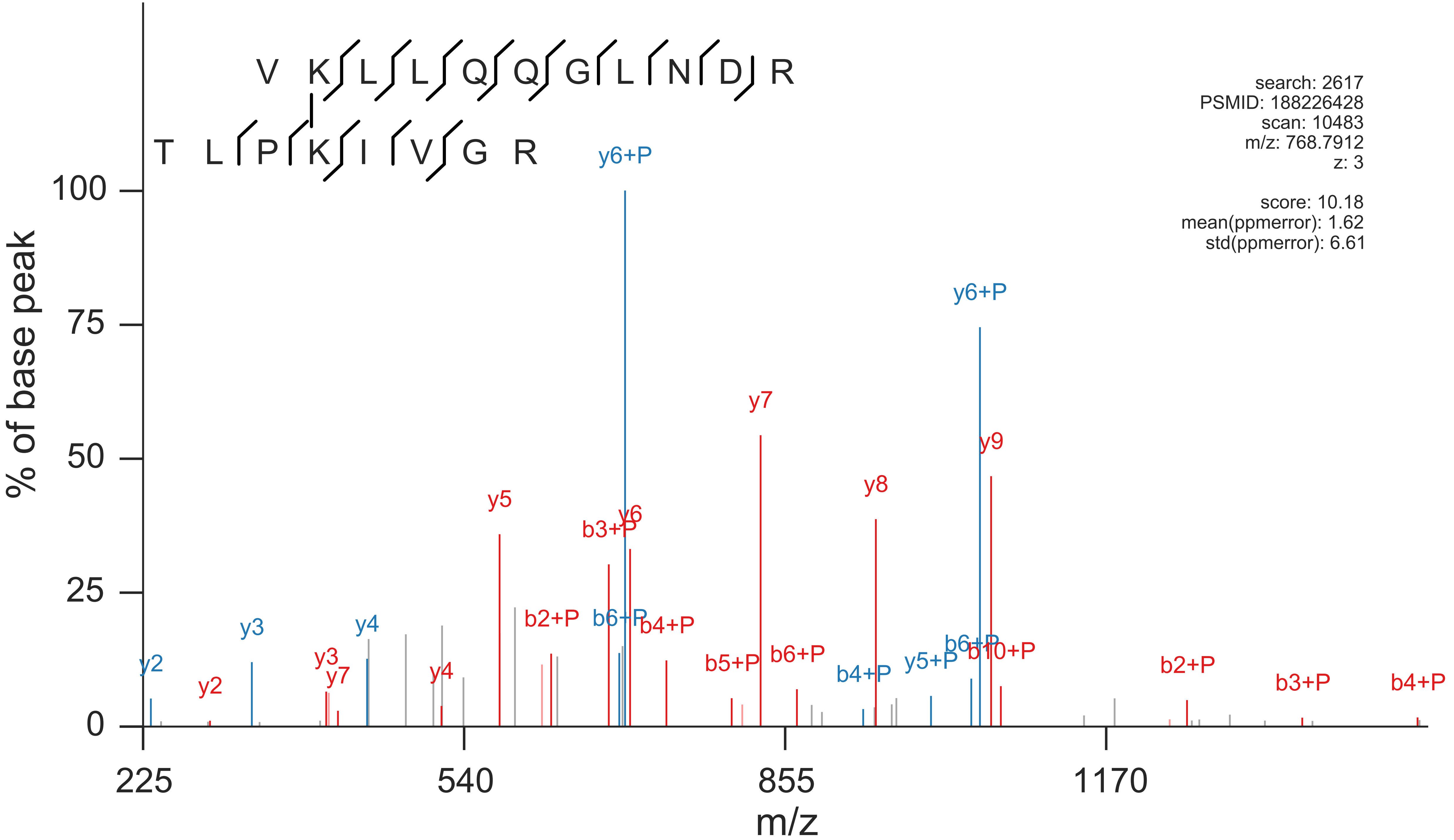
V K L L Q Q G L N D R
T L P K I V G R
search: 2617
PSMID: 188226428
scan: 10483
m/z: 768.7912
z: 3
score: 10.18
mean(ppmerror): 1.62
std(ppmerror): 6.61
% of base peak
m/z
100
75
50
25
0
225
540
855
1170
y6+P
y6+P
y7
y9
y8
y5
y6
b3+P
b2+P
b6+P
b4+P
y3
y4
y2
y2
y3
y7
y4
b5+P
b6+P
b4+P
y5+P
b6+P
b10+P
b2+P
b3+P
b4+P

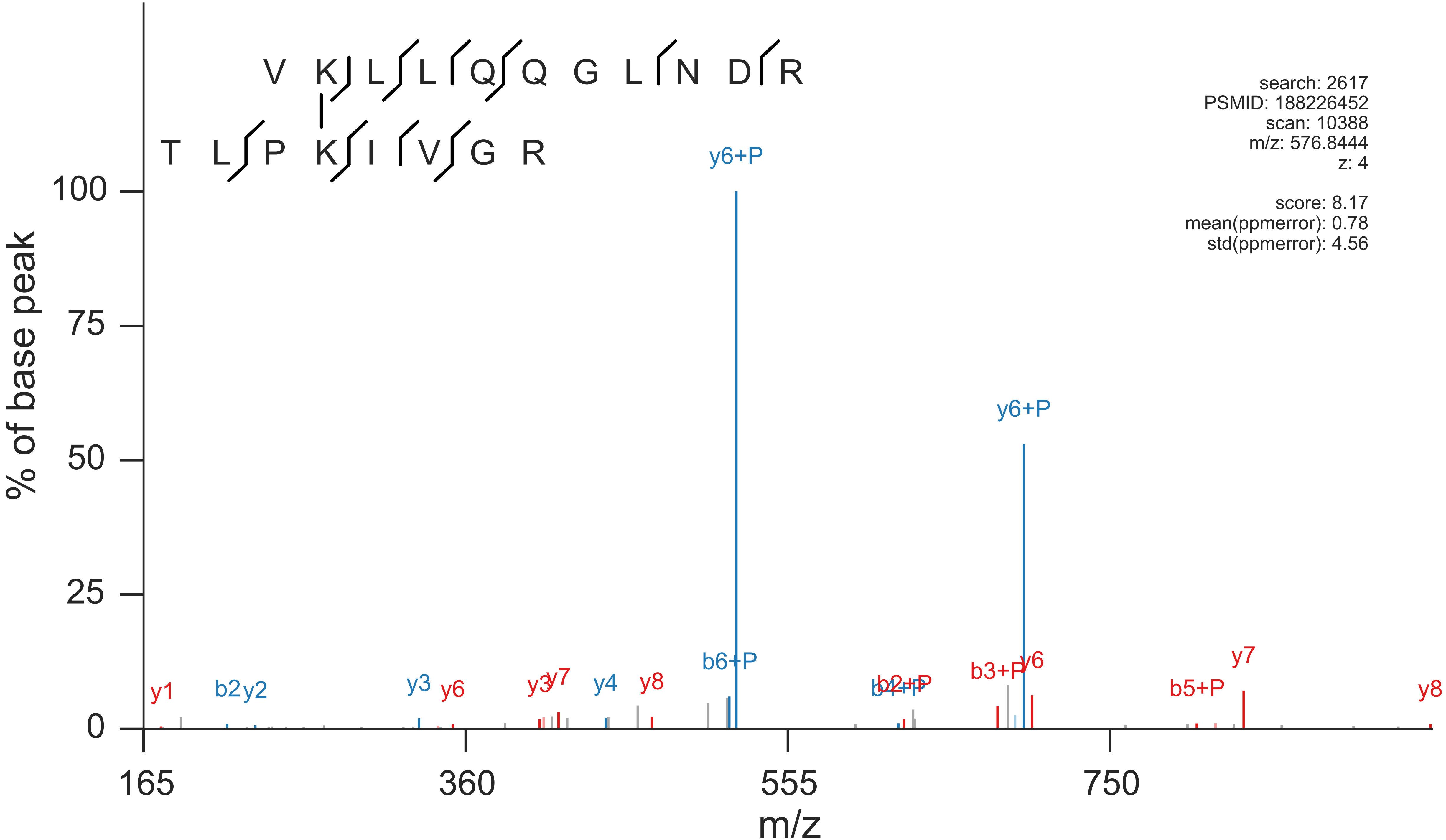
V K L L Q Q G L N D R
T L P K I V G R
search: 2617
PSMID: 188226452
scan: 10388
m/z: 576.8444
z: 4
score: 8.17
mean(ppmerror): 0.78
std(ppmerror): 4.56
% of base peak
m/z
y6+P
y6+P
b6+P
y1
b2
y2
y3
y6
y3
y7
y4
y8
b4+P
b2+P
b3+P
y6
b5+P
y7
y8
0
25
50
75
100
165
360
555
750

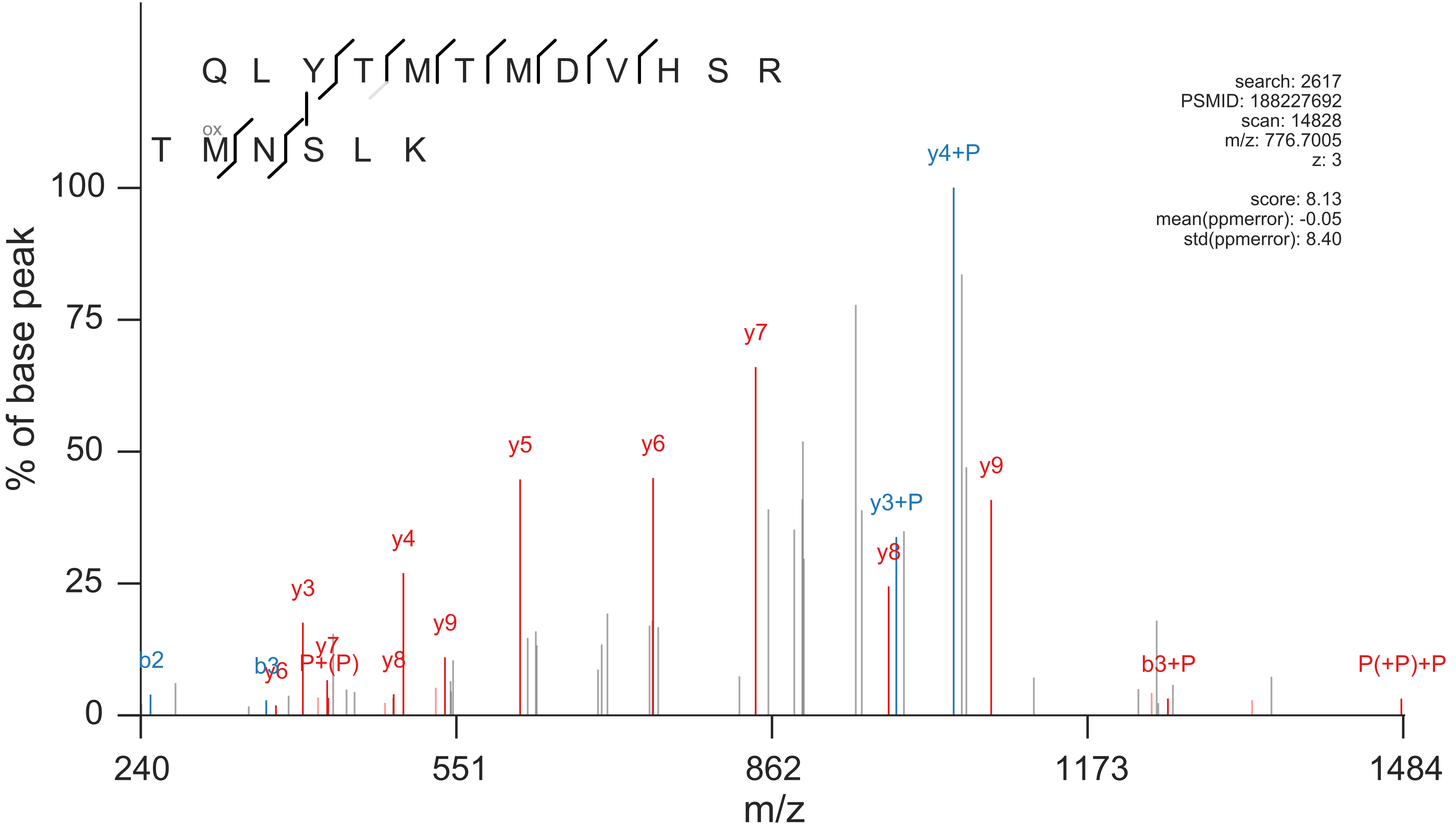

Q L Y T M T M D V H S R
T ox M N S L K
search: 2617
PSMID: 188227692
scan: 14828
m/z: 776.7005
z: 3
score: 8.13
mean(ppmerror): -0.05
std(ppmerror): 8.40
% of base peak
m/z
100
75
50
25
0
240
551
862
1173
1484
b2
b3
y6
y3
y7
P+(P)
y8
y4
y9
y5
y6
y7
y3+P
y8
y4+P
y9
b3+P
P(+P)+P

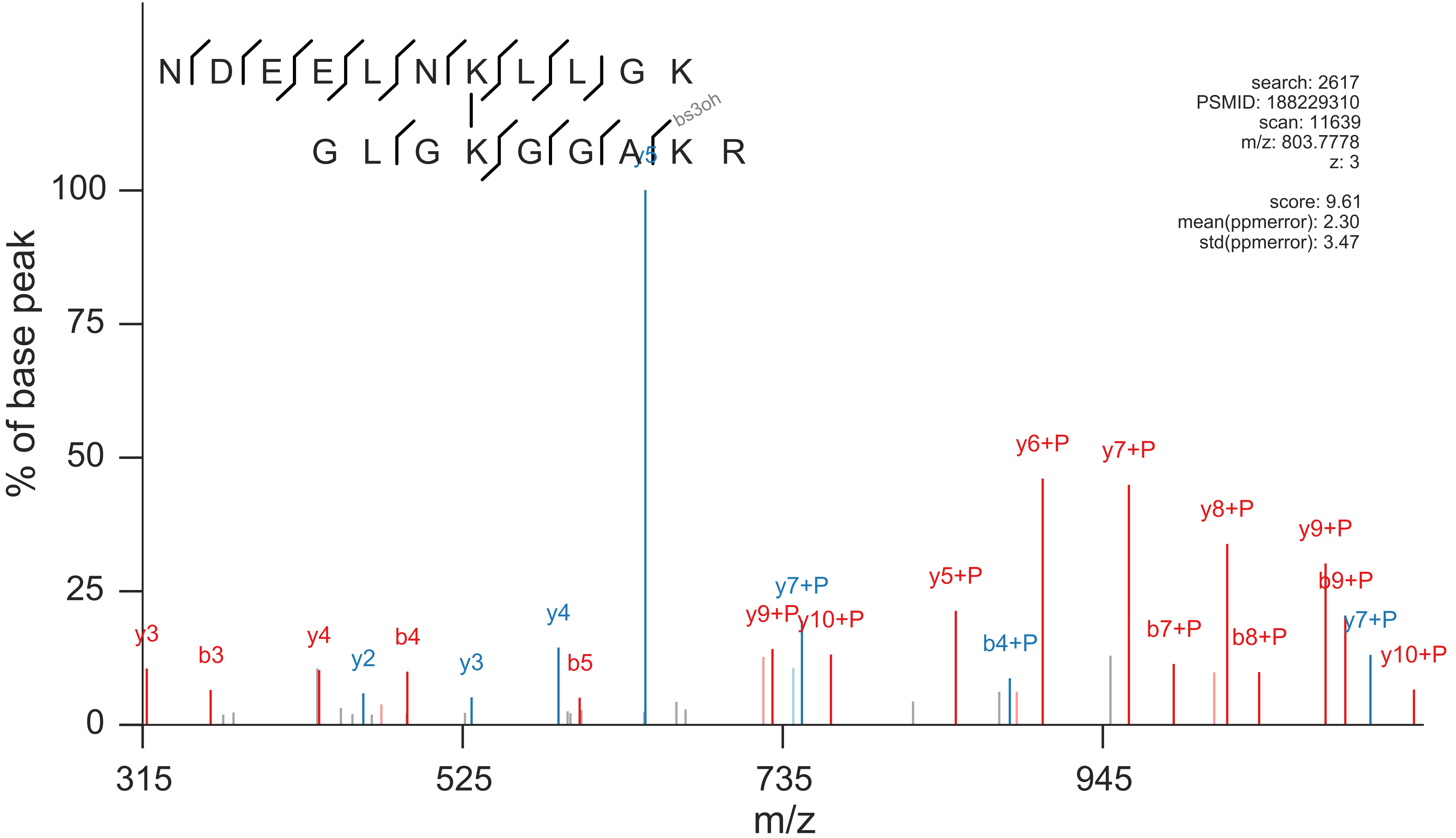

N D E E L N K L L G K
G L G K G G A K R
bs3oh
search: 2617
PSMID: 188229310
scan: 11639
m/z: 803.7778
z: 3
score: 9.61
mean(ppmerror): 2.30
std(ppmerror): 3.47
% of base peak
100
75
50
25
0
315
525
735
945
m/z
y3
b3
y4
y2
b4
y3
y4
b5
y5
y9+P
y7+P
y10+P
y5+P
b4+P
y6+P
y7+P
b7+P
y8+P
b8+P
y9+P
b9+P
y7+P
y10+P

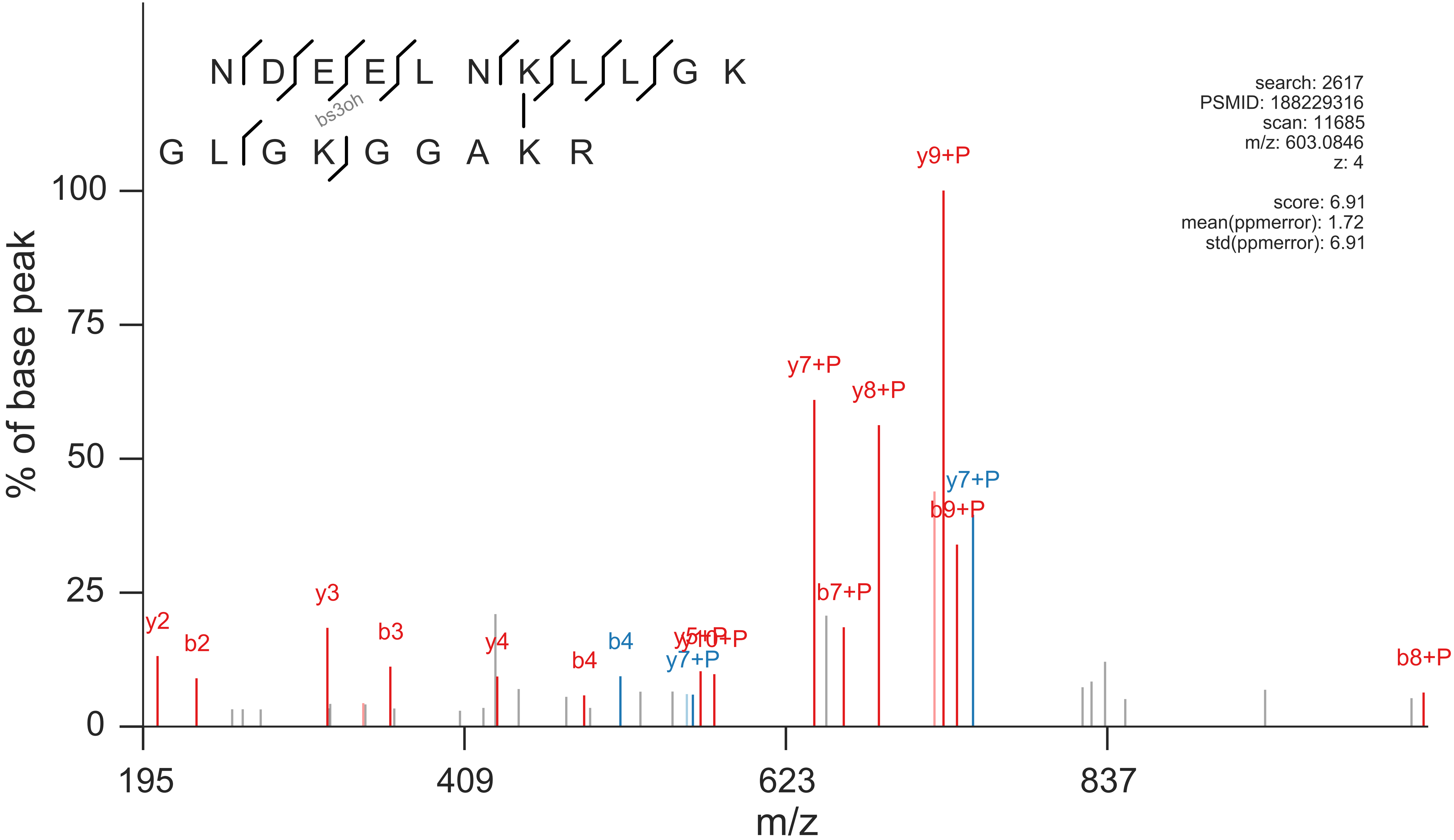

N D E E L N K L L G K
G L G K G G A K R
bs3oh
search: 2617
PSMID: 188229316
scan: 11685
m/z: 603.0846
z: 4
score: 6.91
mean(ppmerror): 1.72
std(ppmerror): 6.91
y9+P
y7+P
y8+P
y7+P
b9+P
b7+P
y2
b2
y3
b3
y4
b4
b4
y5+P
y10+P
y7+P
b8+P
% of base peak
100
75
50
25
0
195
409
623
837
m/z

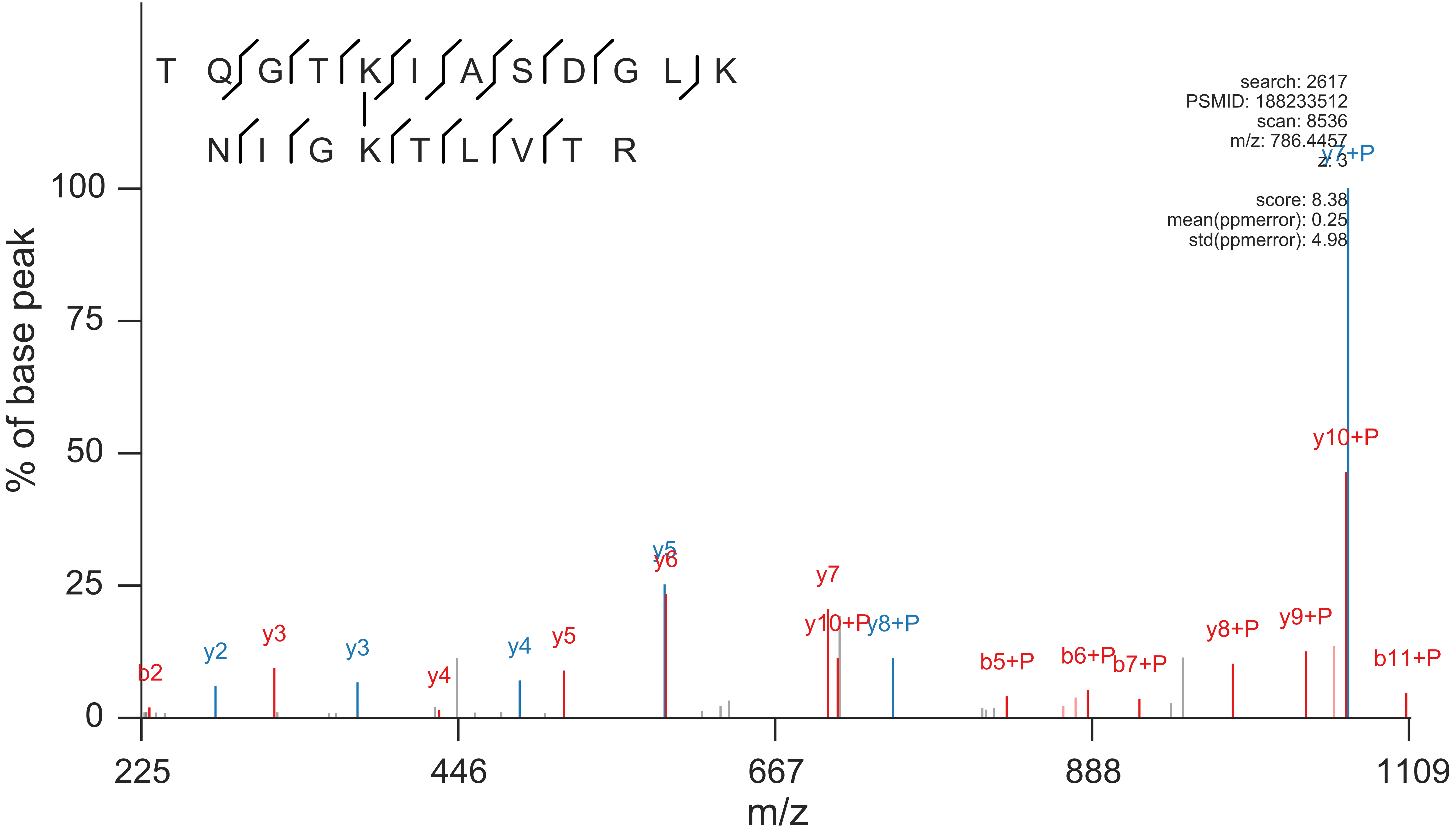
T Q G T K I A S D G L K
N I G K T L V T R
search: 2617
PSMID: 188233512
scan: 8536
m/z: 786.4457
z: 3
score: 8.38
mean(ppmerror): 0.25
std(ppmerror): 4.98
y7+P
y10+P
y5
y6
y7
y10+P
y8+P
b2
y2
y3
y3
y4
y4
y5
b5+P
b6+P
b7+P
y8+P
y9+P
b11+P
% of base peak
100
75
50
25
0
225
446
667
888
1109
m/z

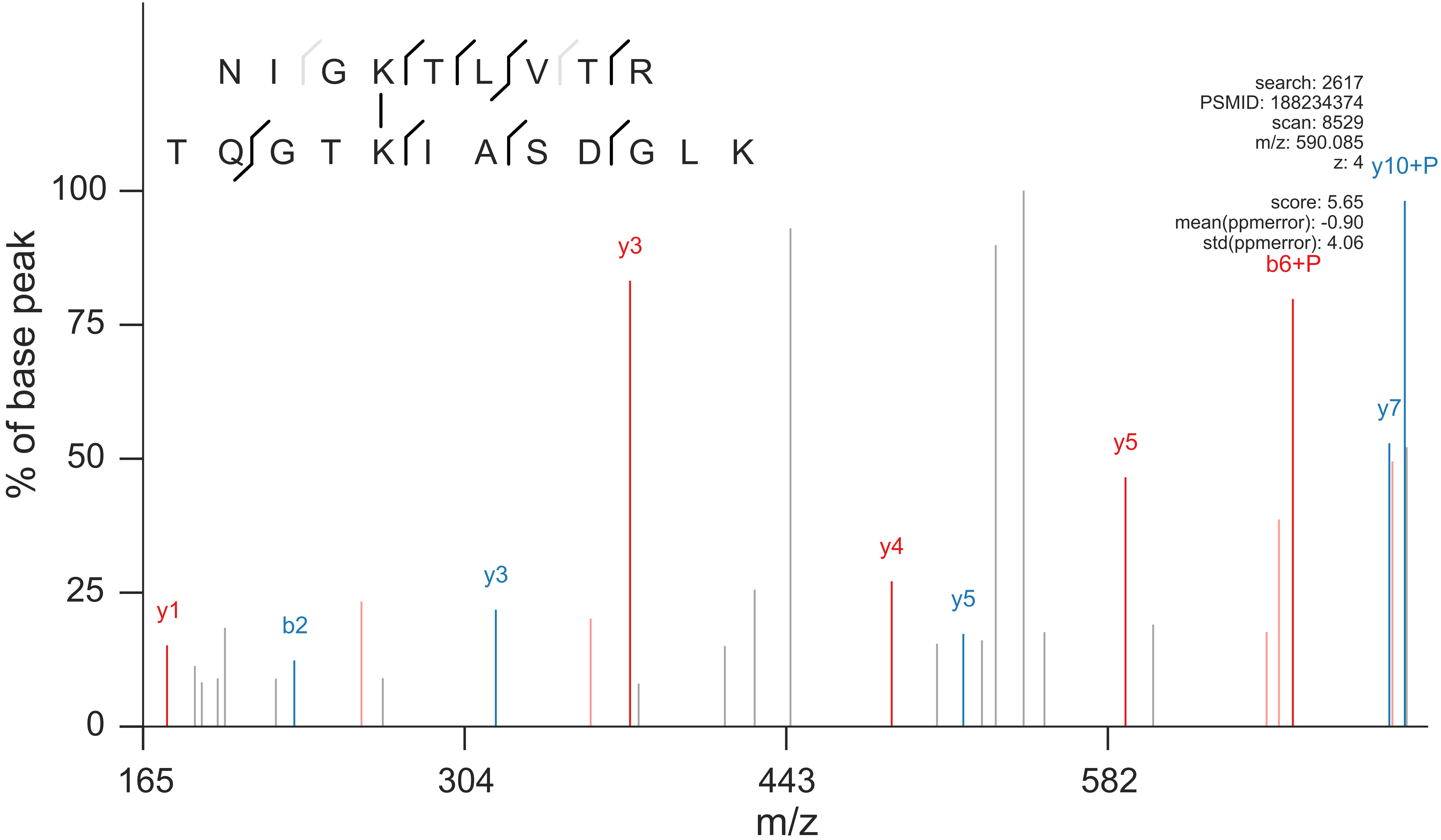

N I G K T L V T R
T Q G T K I A S D G L K
search: 2617
PSMID: 188234374
scan: 8529
m/z: 590.085
z: 4
score: 5.65
mean(ppmerror): -0.90
std(ppmerror): 4.06
y10+P
b6+P
y3
y1
b2
y3
y4
y5
y5
y7
% of base peak
100
75
50
25
0
165
304
443
582
m/z

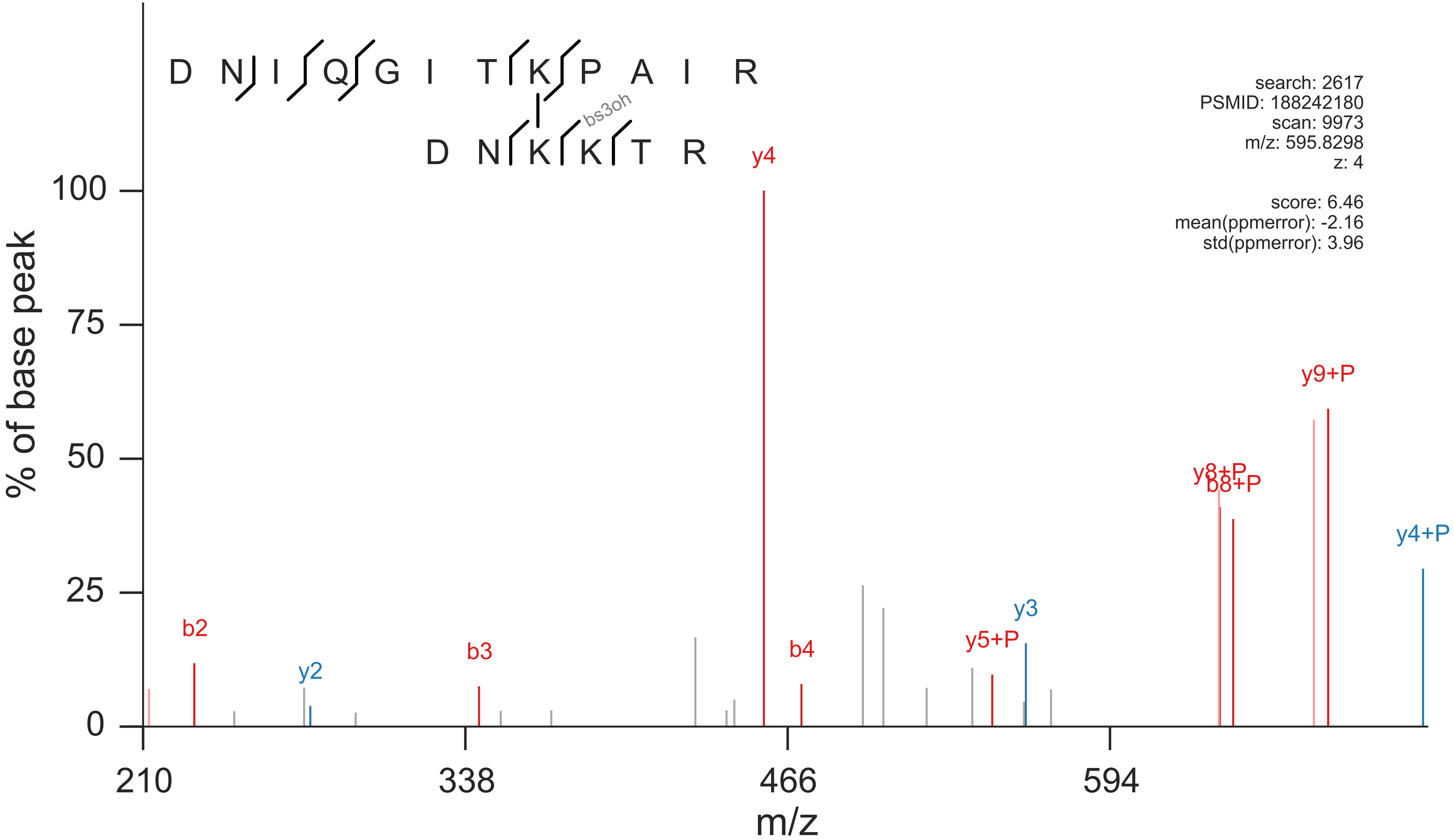

D N I Q G I T K P A I R
bs3oh
D N K K T R
search: 2617
PSMID: 188242180
scan: 9973
m/z: 595.8298
z: 4
score: 6.46
mean(ppmerror): -2.16
std(ppmerror): 3.96
% of base peak
100
75
50
25
0
210
338
466
594
m/z
y4
y9+P
y8+P
b8+P
y4+P
y3
y5+P
b4
b3
b2
y2

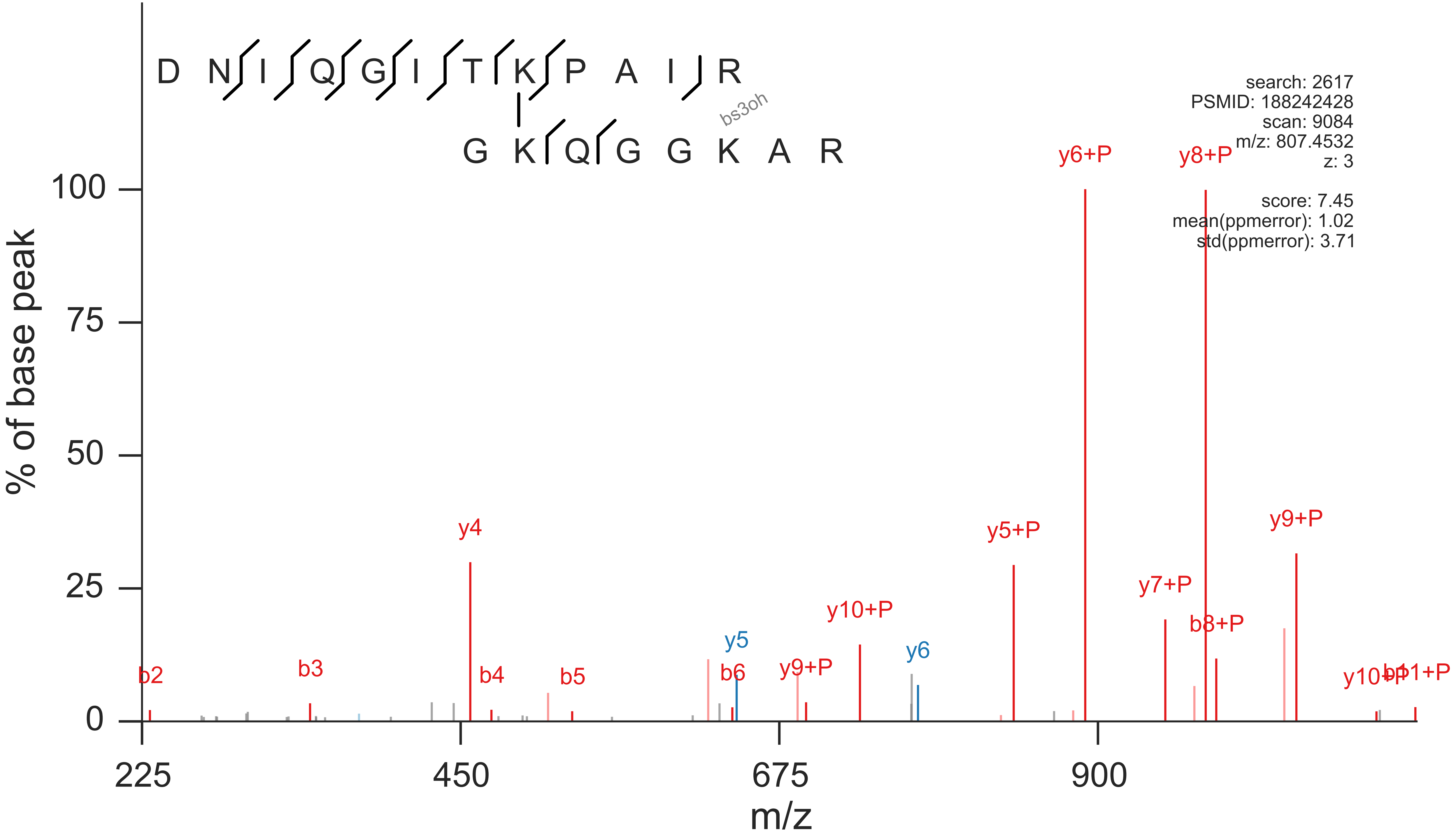

D N I Q G I T K P A I R
G K Q G G K A R
bs3oh
search: 2617
PSMID: 188242428
scan: 9084
m/z: 807.4532
z: 3
score: 7.45
mean(ppmerror): 1.02
std(ppmerror): 3.71
% of base peak
m/z
0
25
50
75
100
225
450
675
900
b2
b3
y4
b4
b5
b6
y5
y9+P
y10+P
y6
y5+P
y6+P
y7+P
y8+P
b8+P
y9+P
y10+P
b11+P

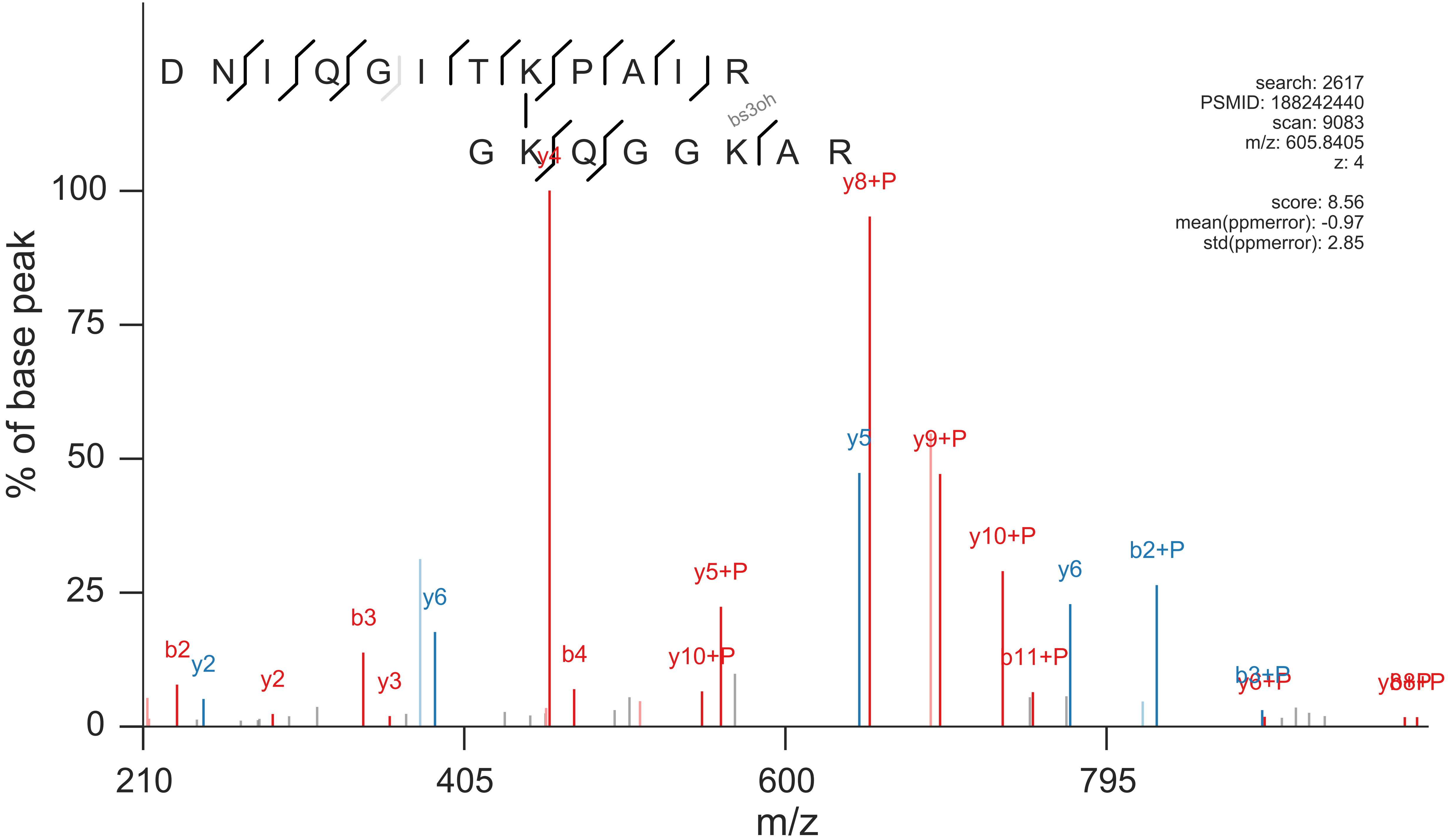

D N I Q G I T K P A I R
G K Q G G K A R
bs3oh
search: 2617
PSMID: 188242440
scan: 9083
m/z: 605.8405
z: 4
score: 8.56
mean(ppmerror): -0.97
std(ppmerror): 2.85
% of base peak
m/z
0
25
50
75
100
210
405
600
795
y4
y8+P
y5
y9+P
y10+P
b2+P
y6
y5+P
b11+P
y10+P
b4
b3
y6
y3
y2
b2
y2
b3+P
y6+P

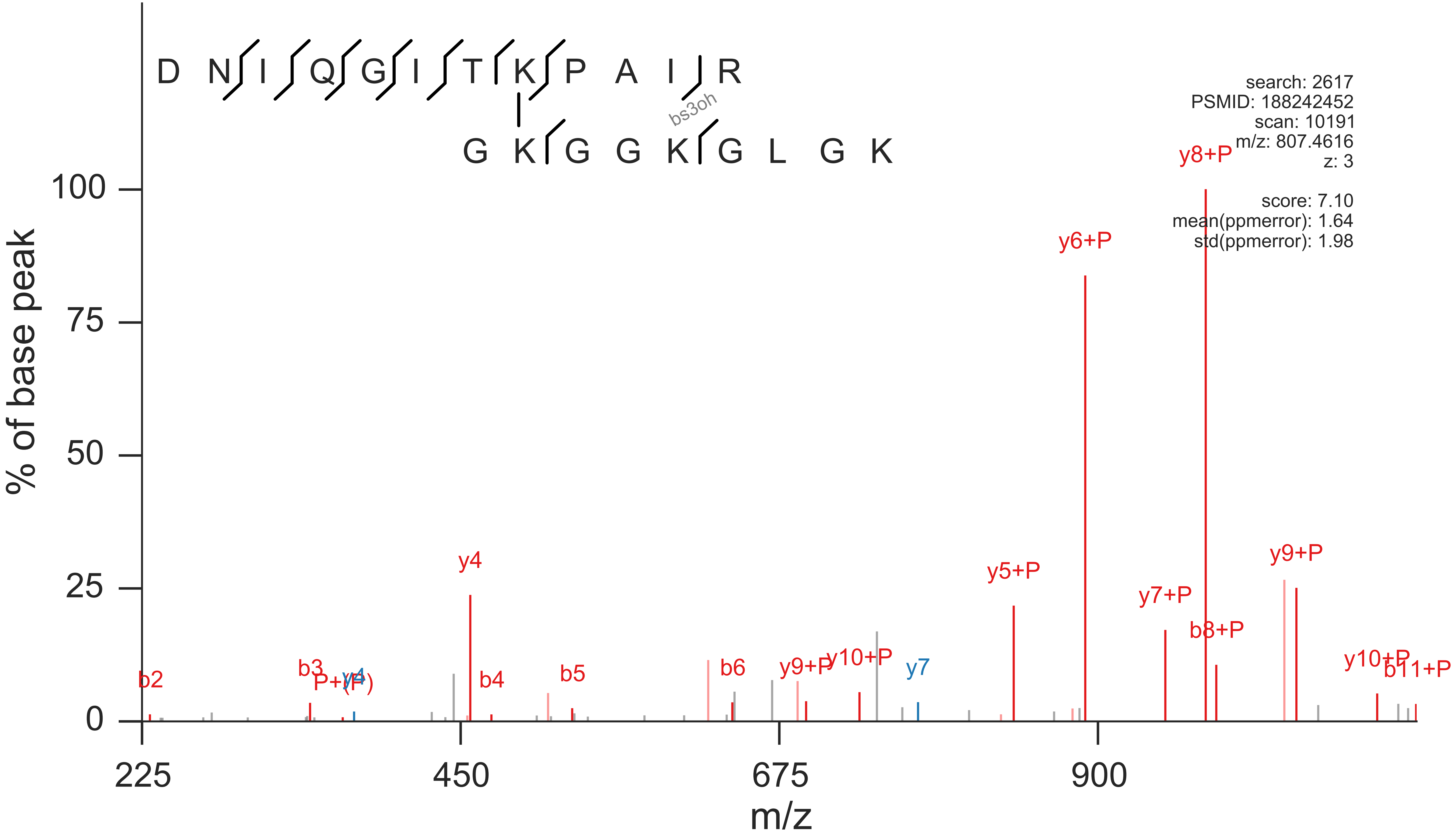

D N I Q G I T K P A I R
bs3oh
G K G G K G L G K
search: 2617
PSMID: 188242452
scan: 10191
m/z: 807.4616
z: 3
score: 7.10
mean(ppmerror): 1.64
std(ppmerror): 1.98
% of base peak
m/z
100
75
50
25
0
225
450
675
900
b2
b3
b4
b5
b6
y4
y7
y4
y5+P
y6+P
y7+P
y8+P
b8+P
y9+P
y10+P
b11+P

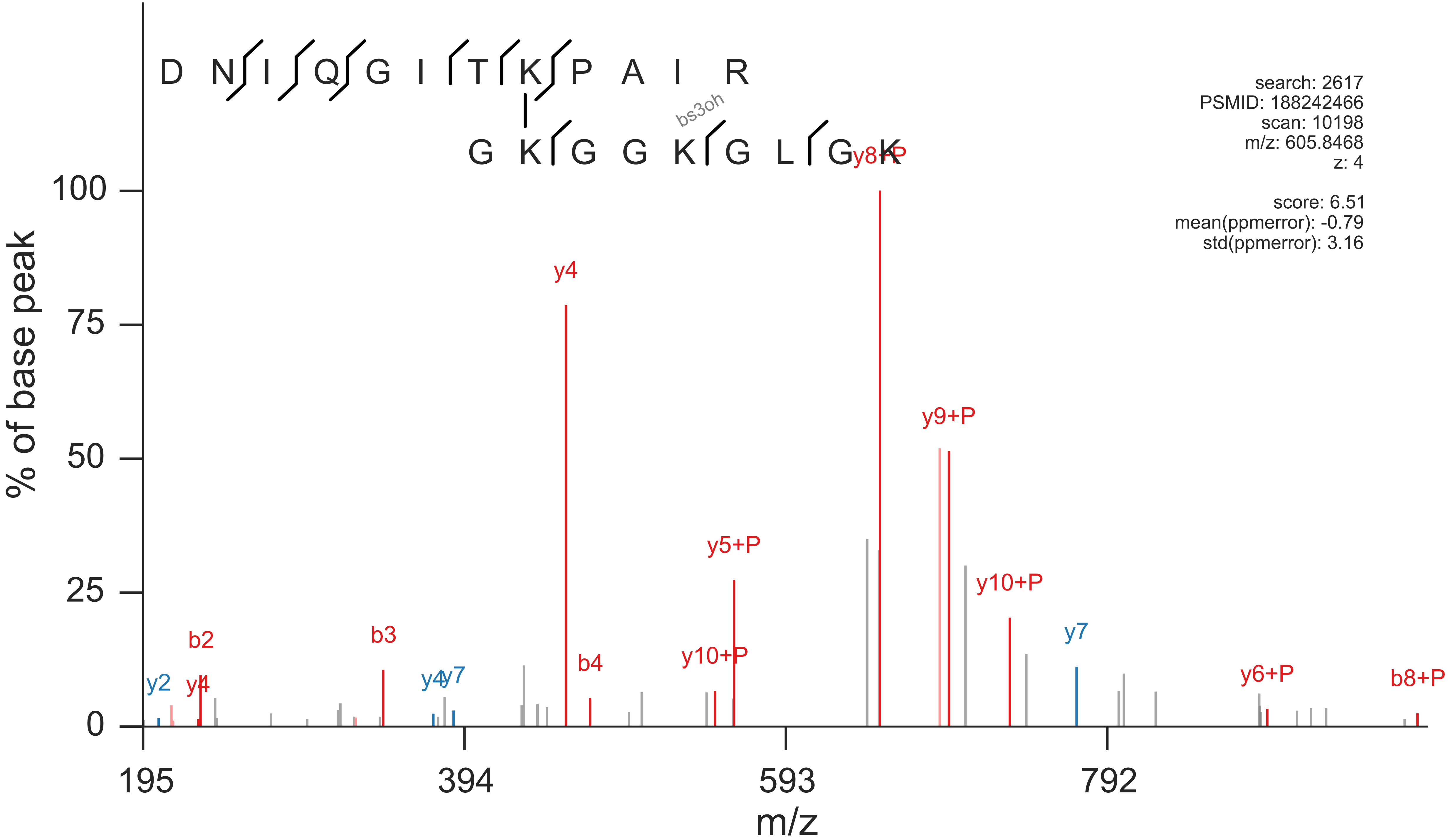

D N I Q G I T K P A I R
bs3oh
G K G G K G L G K
search: 2617
PSMID: 188242466
scan: 10198
m/z: 605.8468
z: 4
score: 6.51
mean(ppmerror): -0.79
std(ppmerror): 3.16
% of base peak
m/z
y8+P
y4
y9+P
y5+P
y10+P
y7
b2
b3
b4
y2
y4
y4
y7
y10+P
y6+P
b8+P

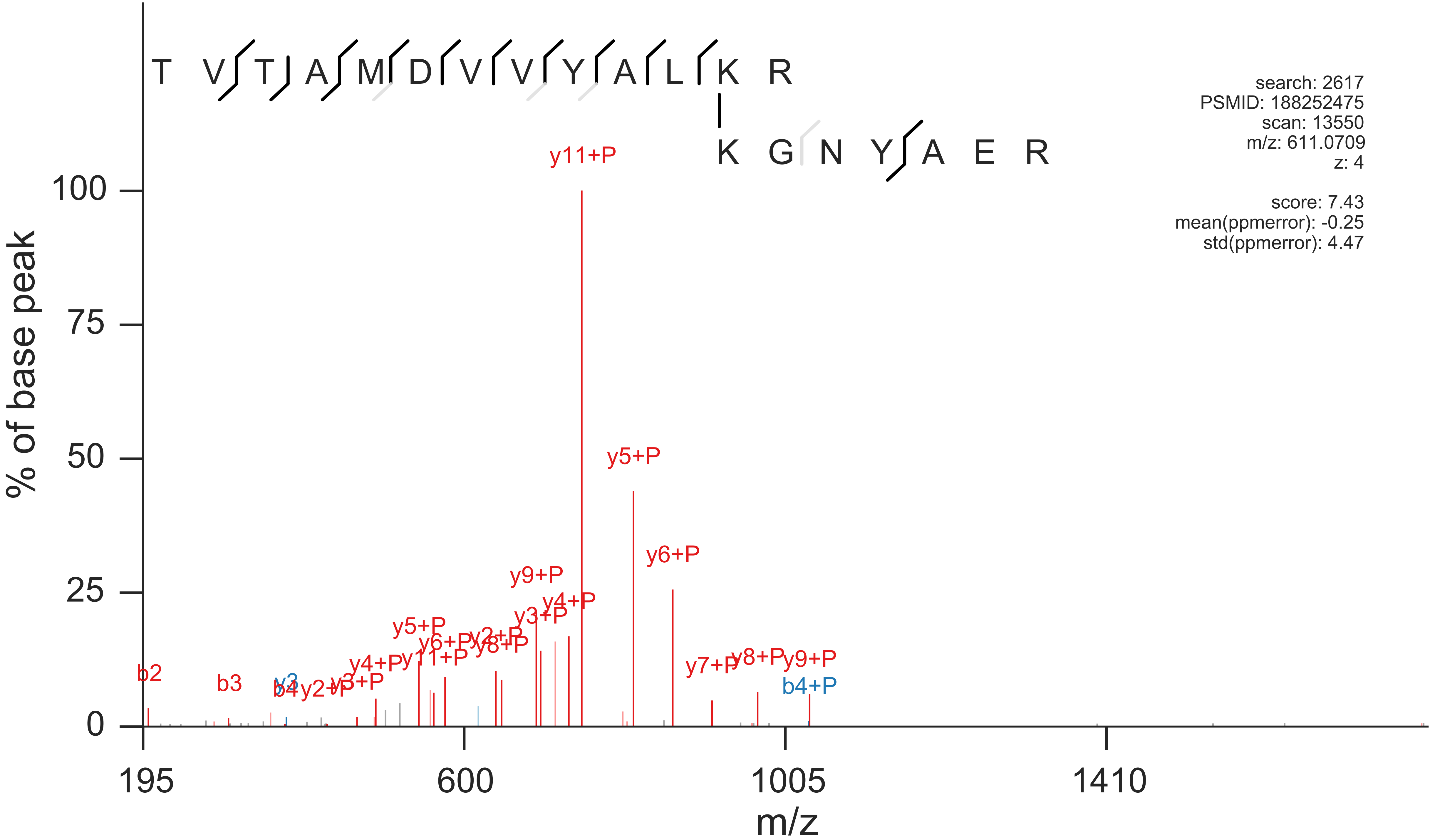
T V T A M D V V Y A L K R
K G N Y A E R
search: 2617
PSMID: 188252475
scan: 13550
m/z: 611.0709
z: 4
score: 7.43
mean(ppmerror): -0.25
std(ppmerror): 4.47
% of base peak
m/z
100
75
50
25
0
195
600
1005
1410
y11+P
y5+P
y6+P
y9+P
y4+P
y3+P
y5+P
y6+P
y2+P
y8+P
y4+P
y11+P
y3+P
y2+P
b2
b3
y2
b4
y7+P
y8+P
y9+P
b4+P

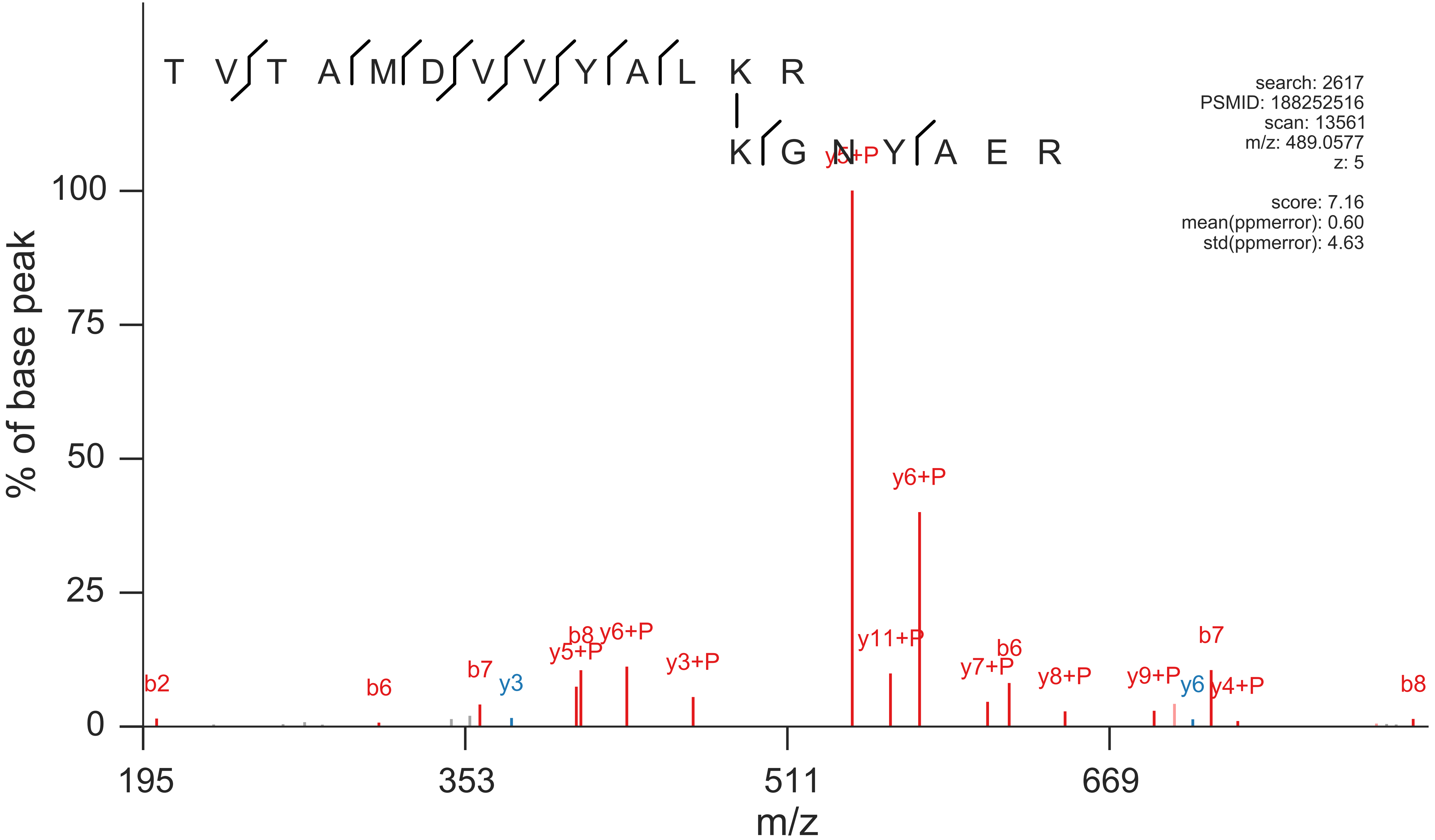
T V T A M D V V Y A L K R
K G N Y A E R
search: 2617
PSMID: 188252516
scan: 13561
m/z: 489.0577
z: 5
score: 7.16
mean(ppmerror): 0.60
std(ppmerror): 4.63
% of base peak
100
75
50
25
0
195
353
511
669
m/z
y5+P
y6+P
b2
b6
b7
y3
y5+P
b8
y6+P
y3+P
y11+P
y7+P
b6
y8+P
y9+P
y6
b7
y4+P
b8

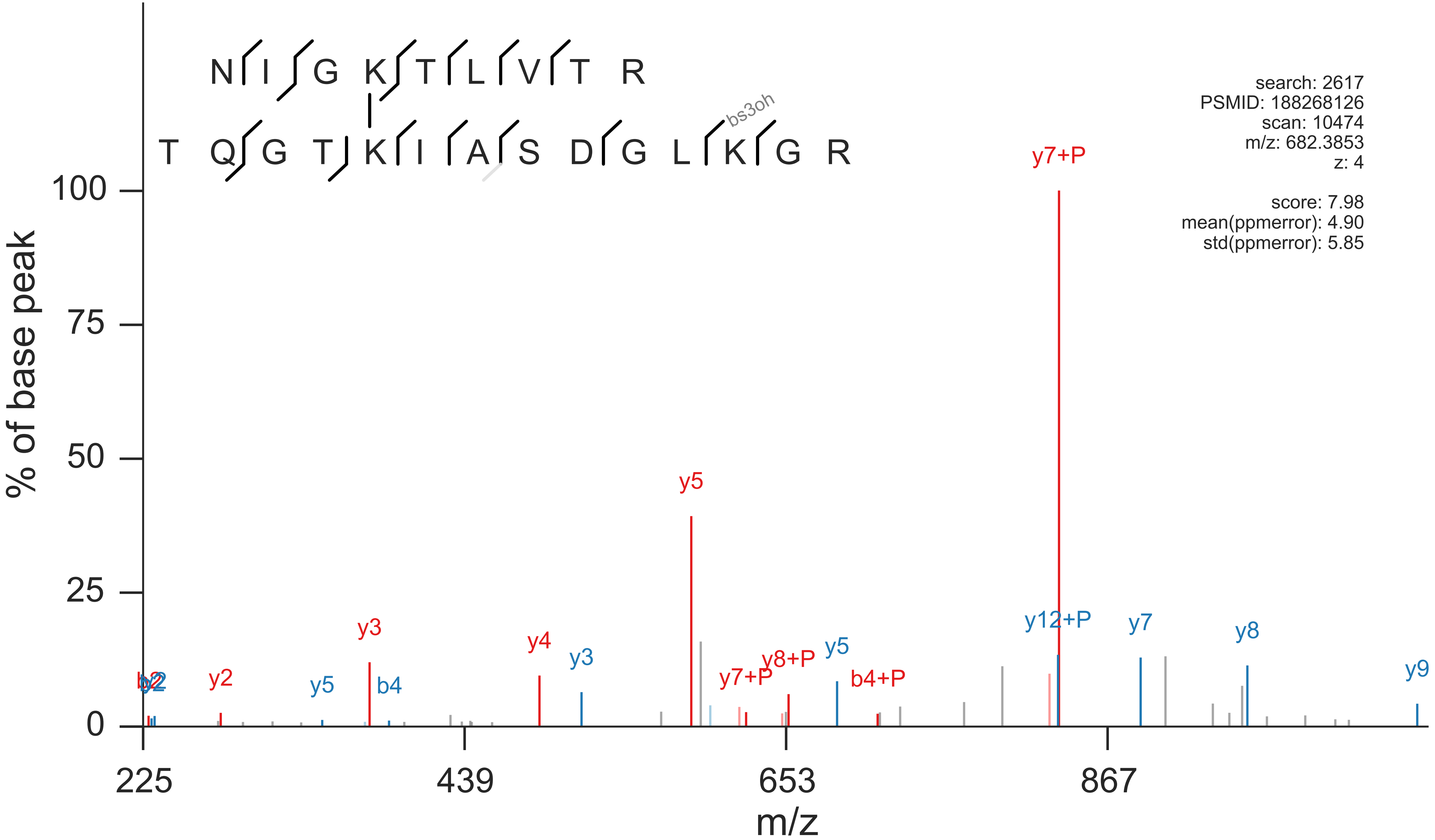

N I G K T L V T R
T Q G T K I A S D G L K G R
bs3oh
search: 2617
PSMID: 188268126
scan: 10474
m/z: 682.3853
z: 4
score: 7.98
mean(ppmerror): 4.90
std(ppmerror): 5.85
% of base peak
100
75
50
25
0
225
439
653
867
m/z
y7+P
y5
y3
y4
y2
b2
y2
y5
b4
y3
y7+P
y8+P
y5
b4+P
y12+P
y7
y8
y9

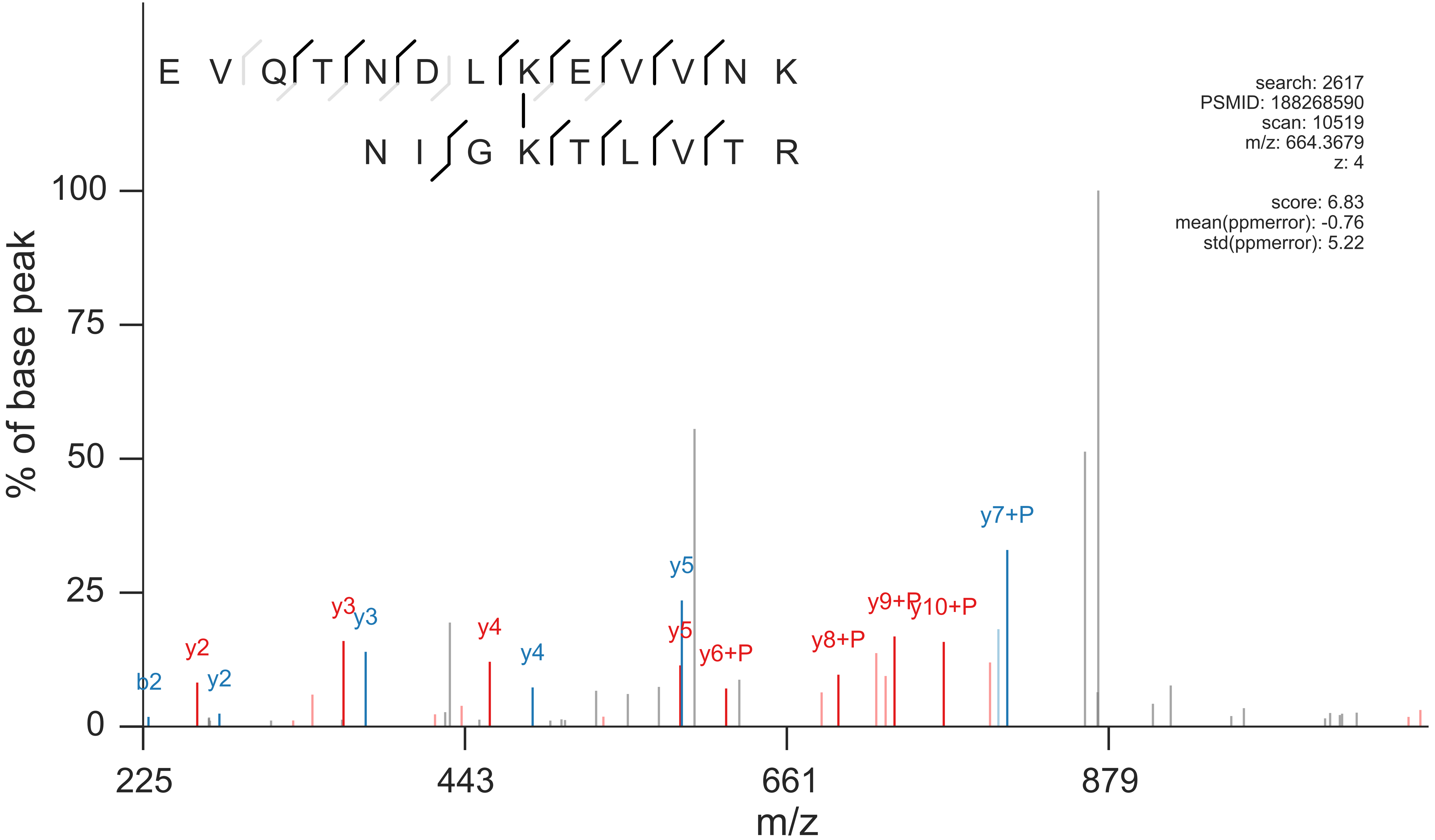
E V Q T N D L K E V V N K
N I G K T L V T R
search: 2617
PSMID: 188268590
scan: 10519
m/z: 664.3679
z: 4
score: 6.83
mean(ppmerror): -0.76
std(ppmerror): 5.22
% of base peak
m/z
0
25
50
75
100
225
443
661
879
b2
y2
y2
y3
y3
y4
y4
y5
y5
y6+P
y8+P
y9+P
y10+P
y7+P

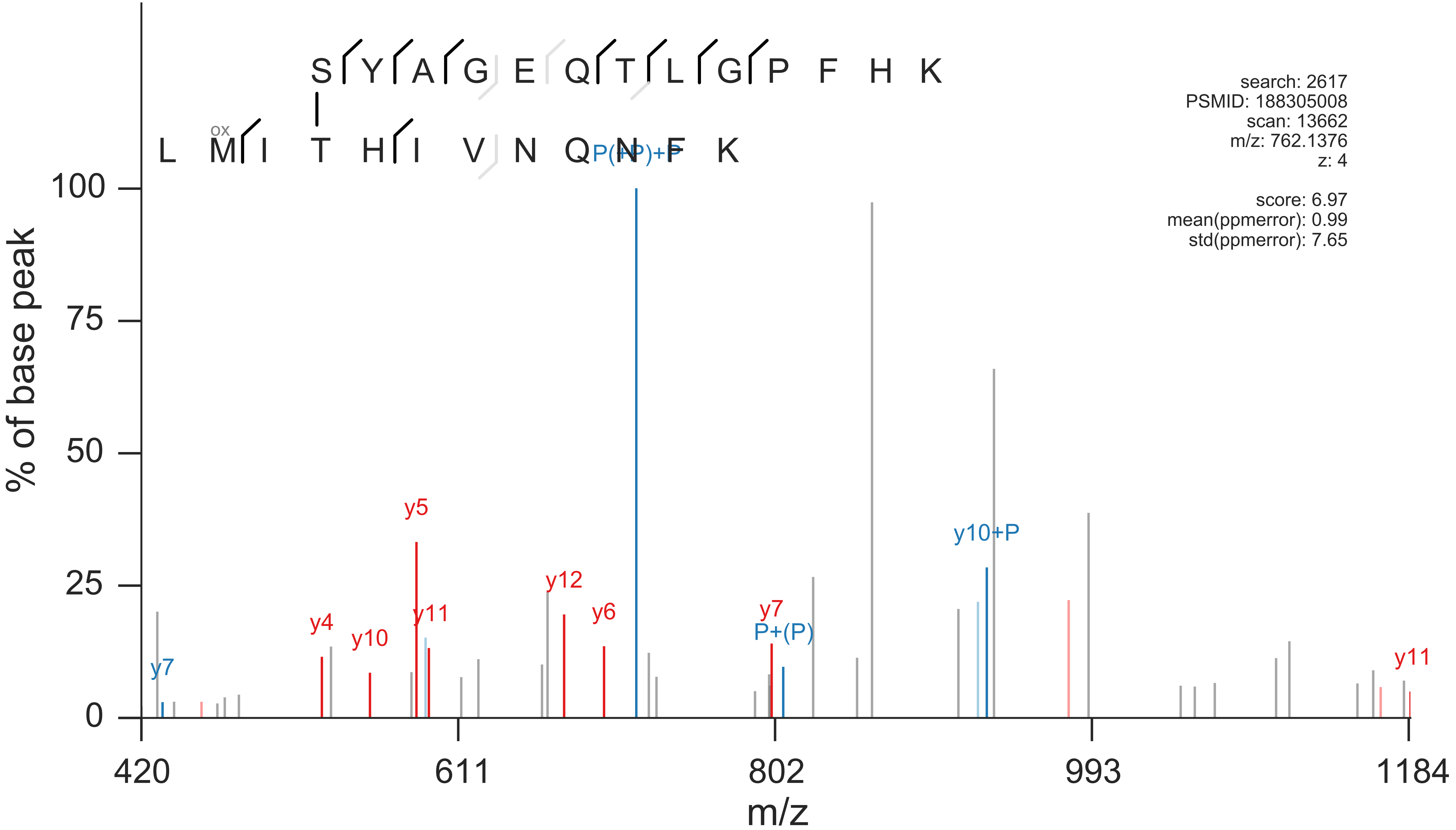

S Y A G E Q T L G P F H K
L M I T H I V N Q N F K
ox
P(+P)+P
search: 2617
PSMID: 188305008
scan: 13662
m/z: 762.1376
z: 4
score: 6.97
mean(ppmerror): 0.99
std(ppmerror): 7.65
% of base peak
100
75
50
25
0
y5
y10+P
y12
y4
y11
y6
y7
y10
P+(P)
y7
y11
420
611
802
993
1184
m/z

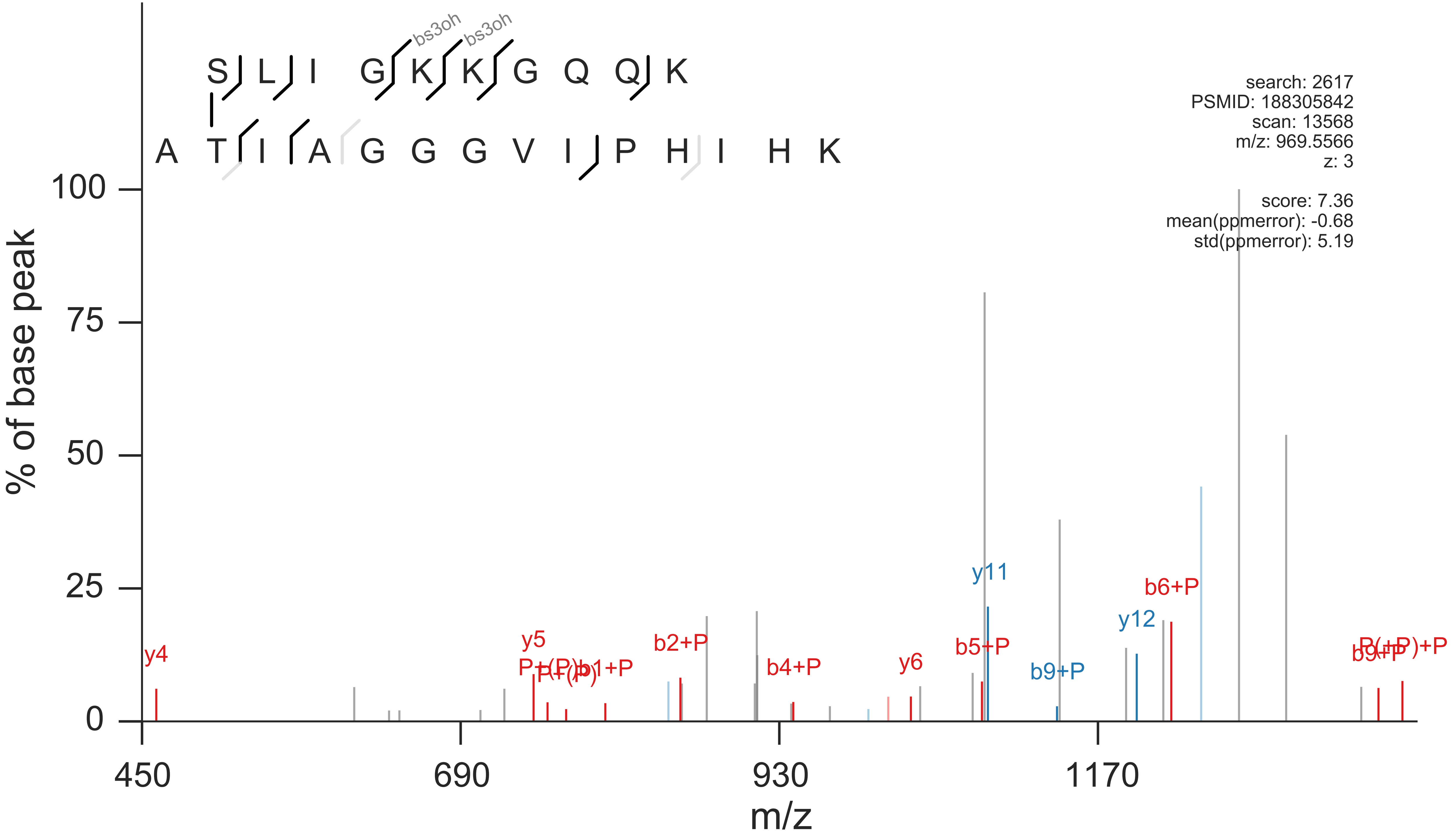

S L I G K K G Q Q K
A T I A G G G V I P H I H K
bs3oh
bs3oh
search: 2617
PSMID: 188305842
scan: 13568
m/z: 969.5566
z: 3
score: 7.36
mean(ppmerror): -0.68
std(ppmerror): 5.19
% of base peak
m/z
y4
y5
b2+P
b4+P
y6
b5+P
y11
b9+P
y12
b6+P

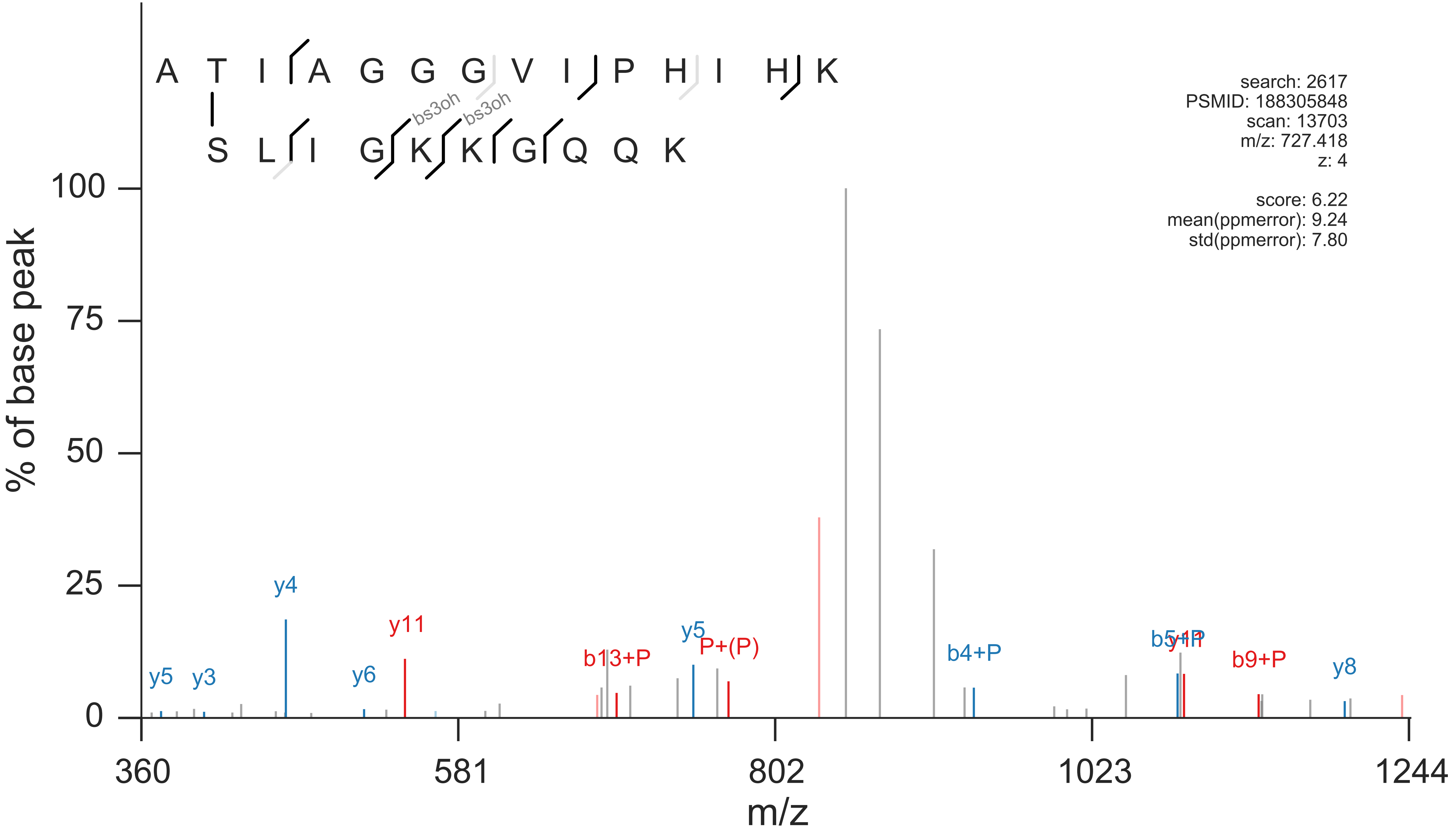

A T I A G G G V I P H I H K
S L I G K K G Q Q K
bs3oh
bs3oh
search: 2617
PSMID: 188305848
scan: 13703
m/z: 727.418
z: 4
score: 6.22
mean(ppmerror): 9.24
std(ppmerror): 7.80
% of base peak
m/z
y5
y3
y4
y6
y11
b13+P
y5
P+(P)
b4+P
b9+P
y8

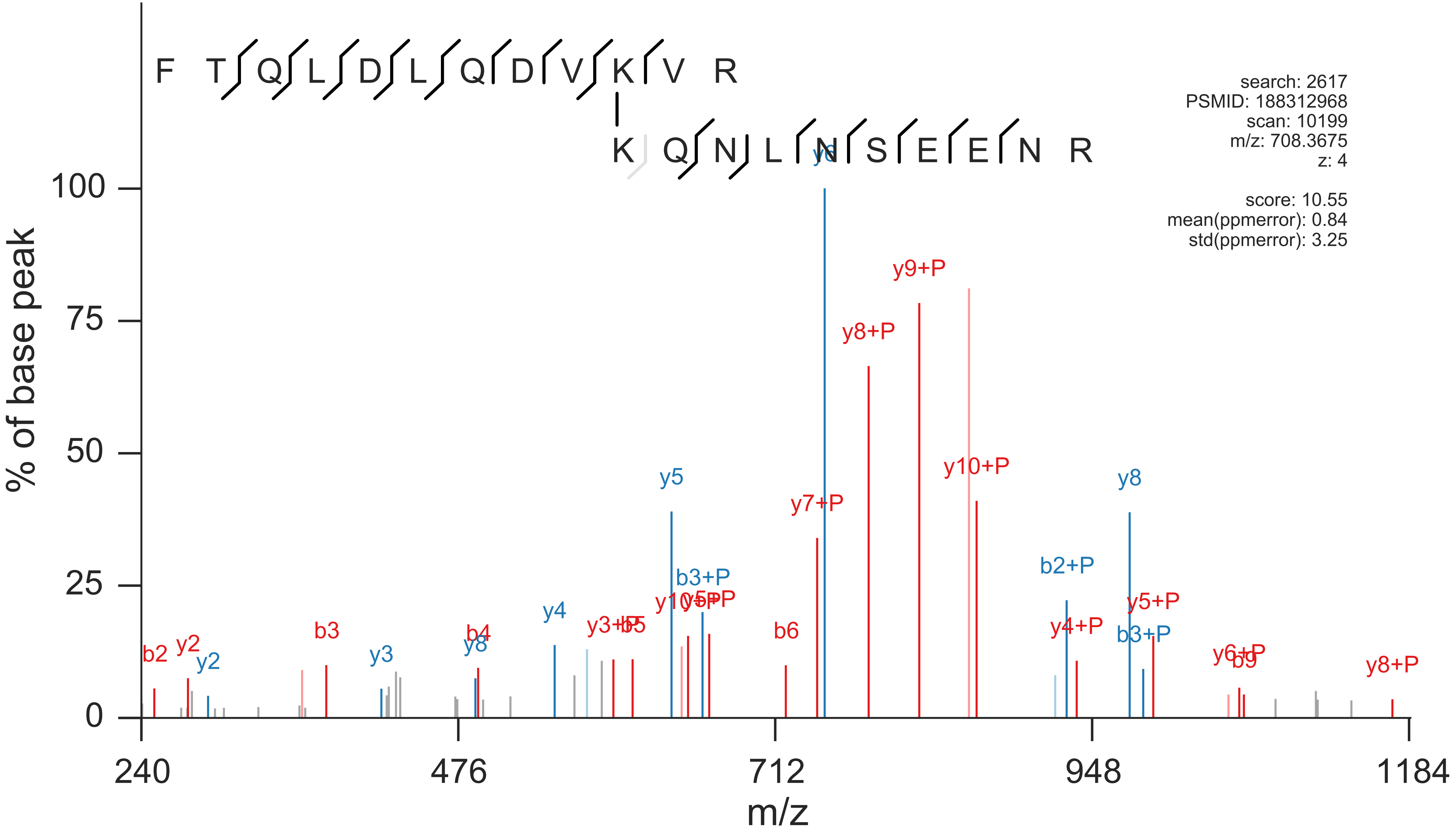
F T Q L D L Q D V K V R
K Q N L N S E E N R
search: 2617
PSMID: 188312968
scan: 10199
m/z: 708.3675
z: 4
score: 10.55
mean(ppmerror): 0.84
std(ppmerror): 3.25
% of base peak
m/z
0
25
50
75
100
240
476
712
948
1184
y6
y9+P
y8+P
y10+P
y7+P
y5
y8
b2+P
b3+P
y5+P
y4+P
b3+P
y4
y3+P
b5
b6
b3
b4
y8
y3
b2
y2
y2
y6+P
b9
y8+P

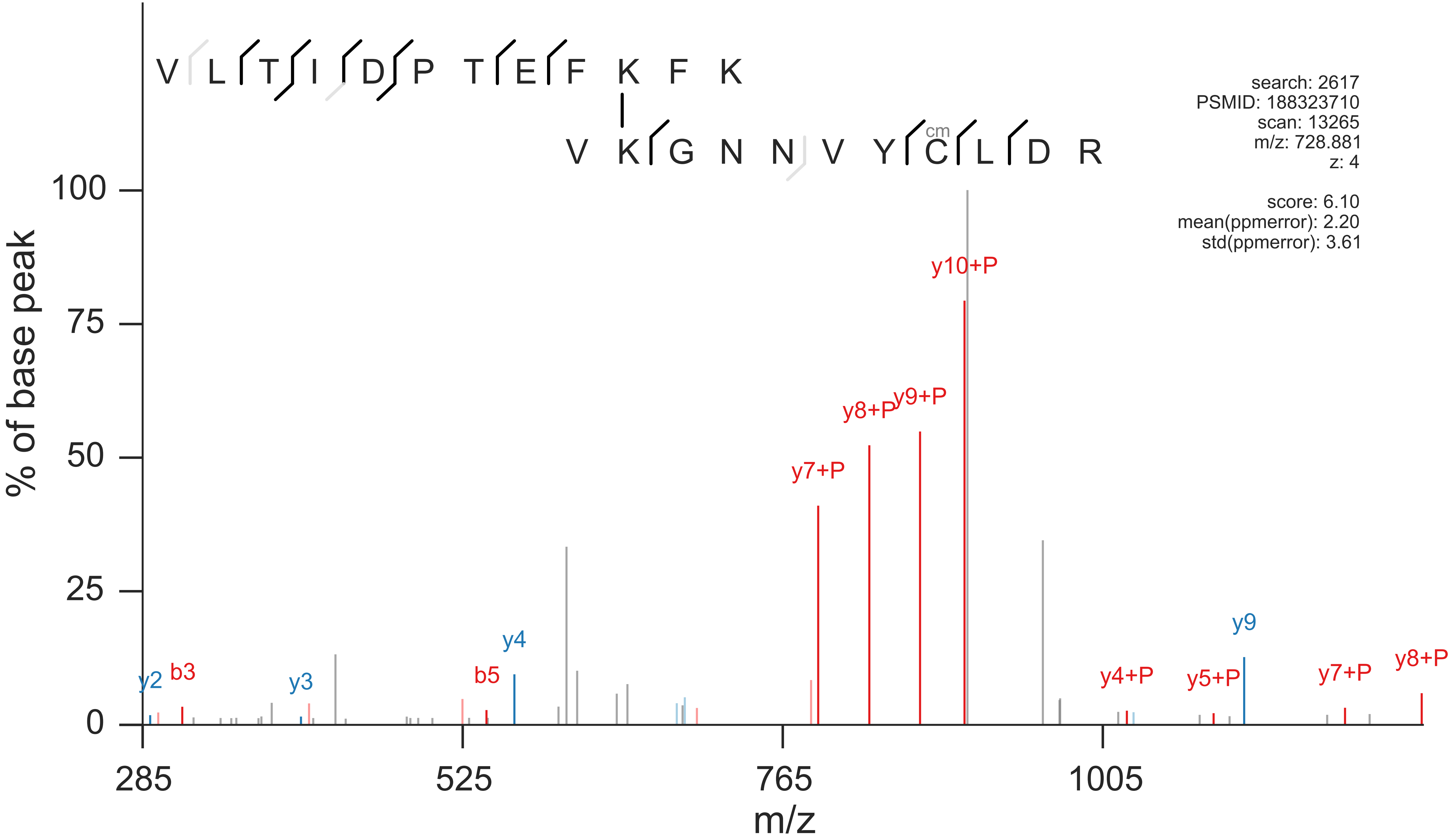

V L T I D P T E F K F K
V K G N N V Y C L D R
cm
search: 2617
PSMID: 188323710
scan: 13265
m/z: 728.881
z: 4
score: 6.10
mean(ppmerror): 2.20
std(ppmerror): 3.61
% of base peak
100
75
50
25
0
285
525
765
1005
m/z
y10+P
y8+P
y9+P
y7+P
y2
b3
y3
b5
y4
y4+P
y5+P
y9
y7+P
y8+P

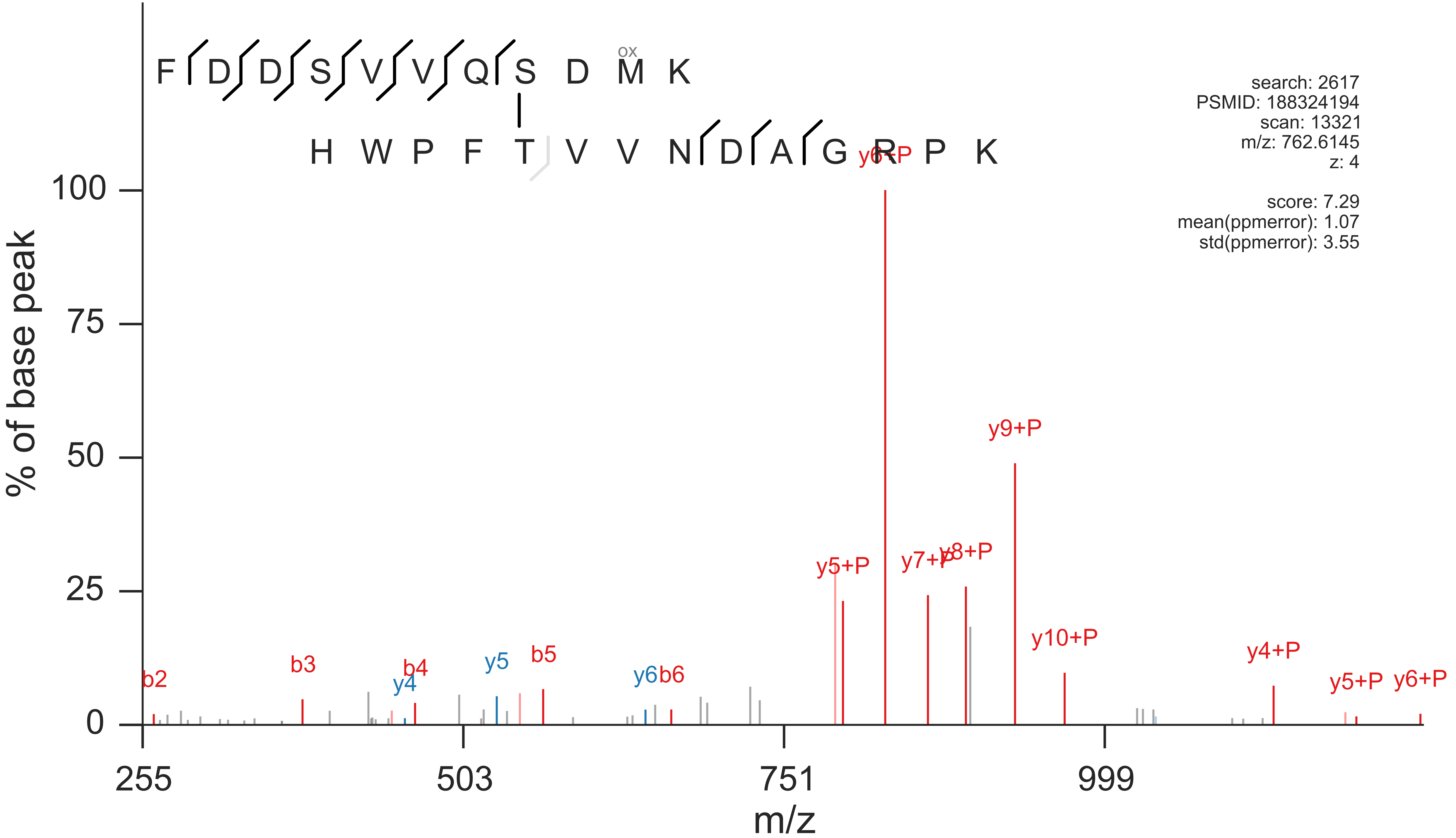

F D D S V V Q S D M K
ox
H W P F T V V N D A G R P K
search: 2617
PSMID: 188324194
scan: 13321
m/z: 762.6145
z: 4
score: 7.29
mean(ppmerror): 1.07
std(ppmerror): 3.55
% of base peak
m/z
100
75
50
25
0
255
503
751
999
b2
b3
b4
y4
y5
b5
y6
b6
y5+P
y6+P
y7+P
y8+P
y9+P
y10+P
y4+P
y5+P
y6+P

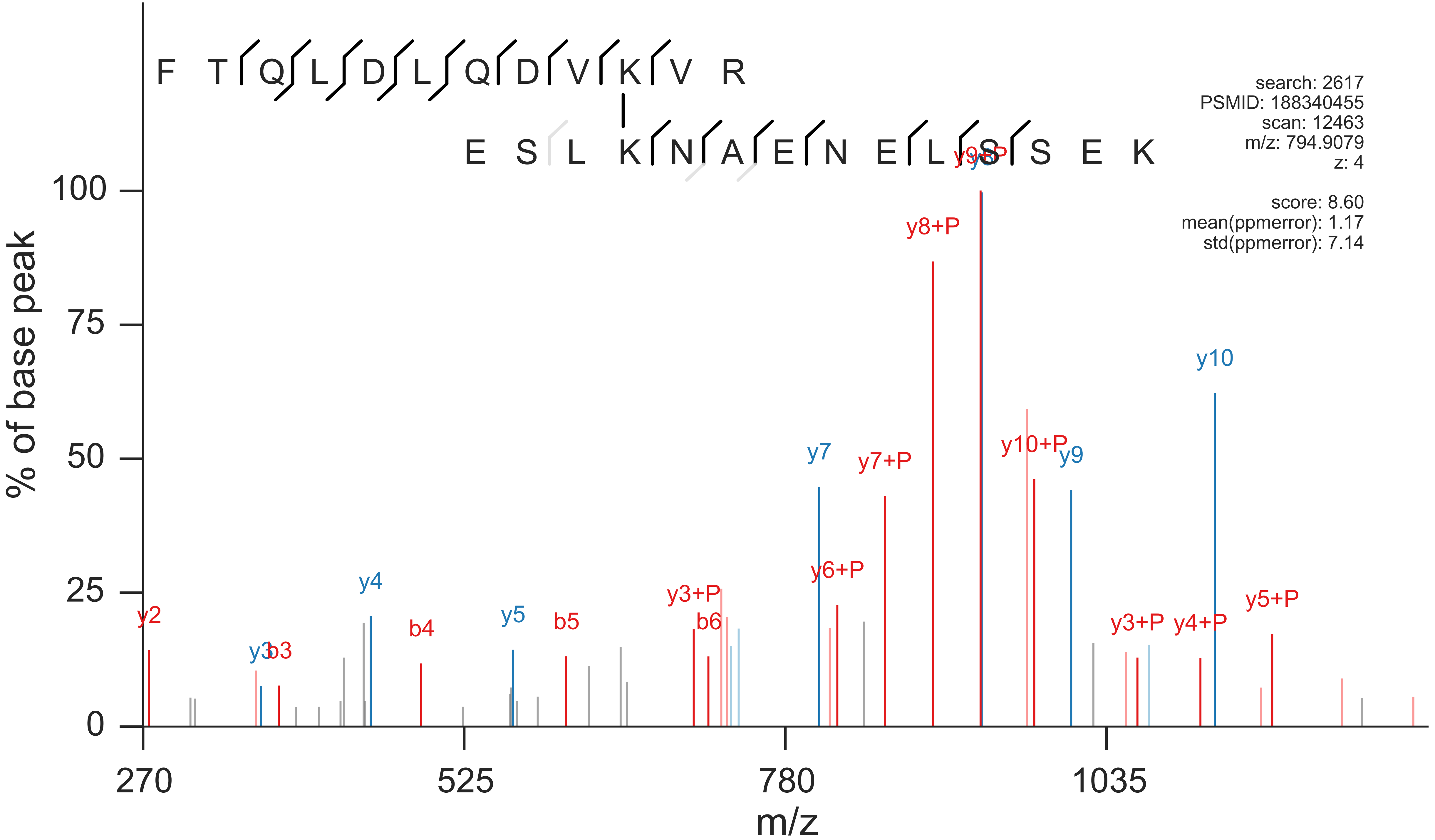

F T Q L D L Q D V K V R
E S L K N A E N E L S S E K
search: 2617
PSMID: 188340455
scan: 12463
m/z: 794.9079
z: 4
score: 8.60
mean(ppmerror): 1.17
std(ppmerror): 7.14
% of base peak
m/z
100
75
50
25
0
270
525
780
1035
y2
y3
b3
y4
b4
y5
b5
y3+P
b6
y7
y6+P
y7+P
y8+P
y9+P
y8
y10+P
y9
y10
y3+P
y4+P
y5+P

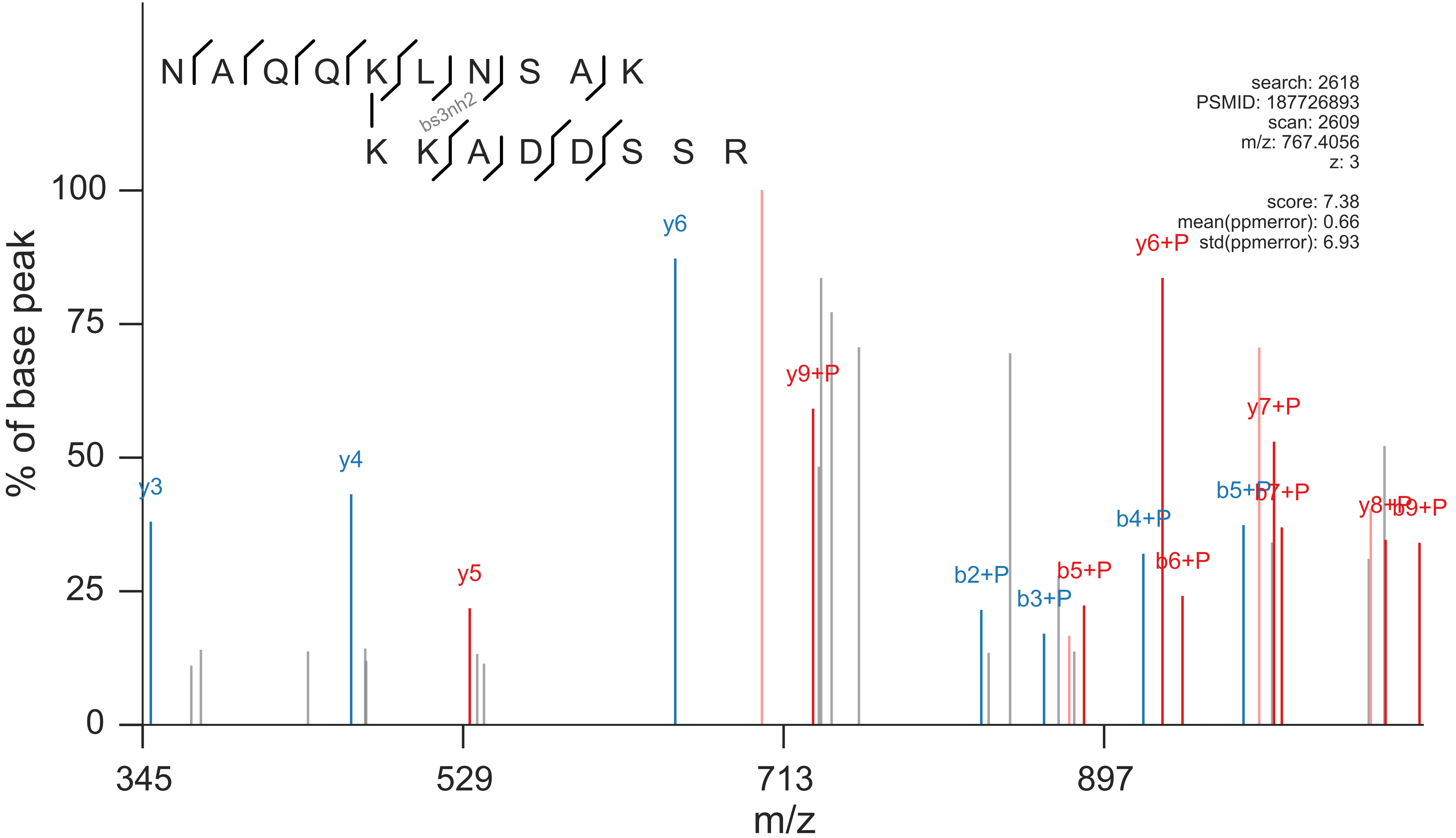

N A Q Q K L N S A K
bs3nh2
K K A D D S S R
search: 2618
PSMID: 187726893
scan: 2609
m/z: 767.4056
z: 3
score: 7.38
mean(ppmerror): 0.66
std(ppmerror): 6.93
% of base peak
m/z
y3
y4
y5
y6
y9+P
y6+P
y7+P
b2+P
b3+P
b4+P
b5+P
b6+P
b7+P
b5+P
y8+P
b9+P

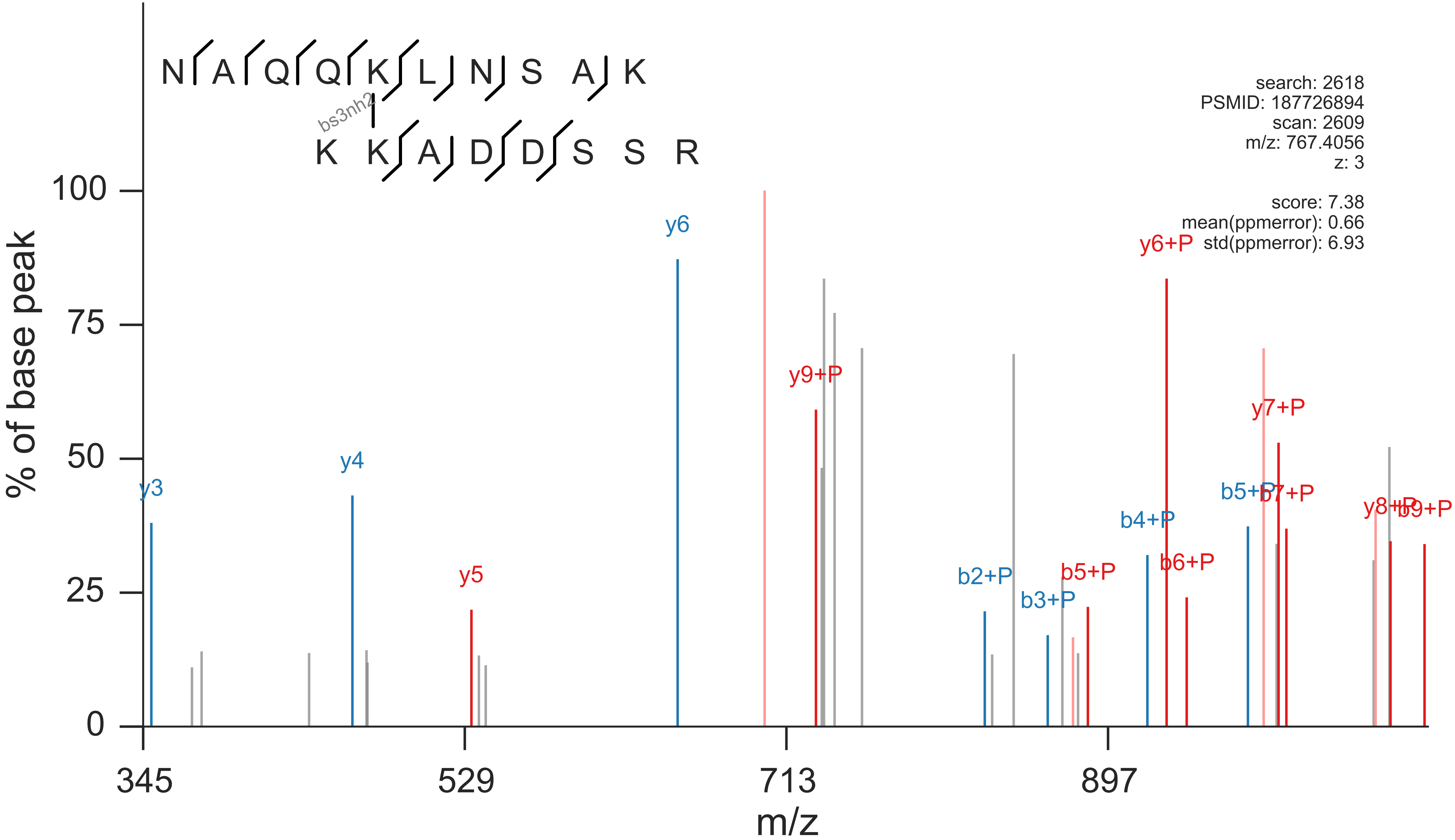

N A Q Q K L N S A K
bs3nh2
K K A D D S S R
search: 2618
PSMID: 187726894
scan: 2609
m/z: 767.4056
z: 3
score: 7.38
mean(ppmerror): 0.66
std(ppmerror): 6.93
% of base peak
m/z
100
75
50
25
0
345
529
713
897
y3
y4
y5
y6
y9+P
y6+P
y7+P
b2+P
b3+P
b4+P
b5+P
b6+P
b7+P
y8+P
b9+P

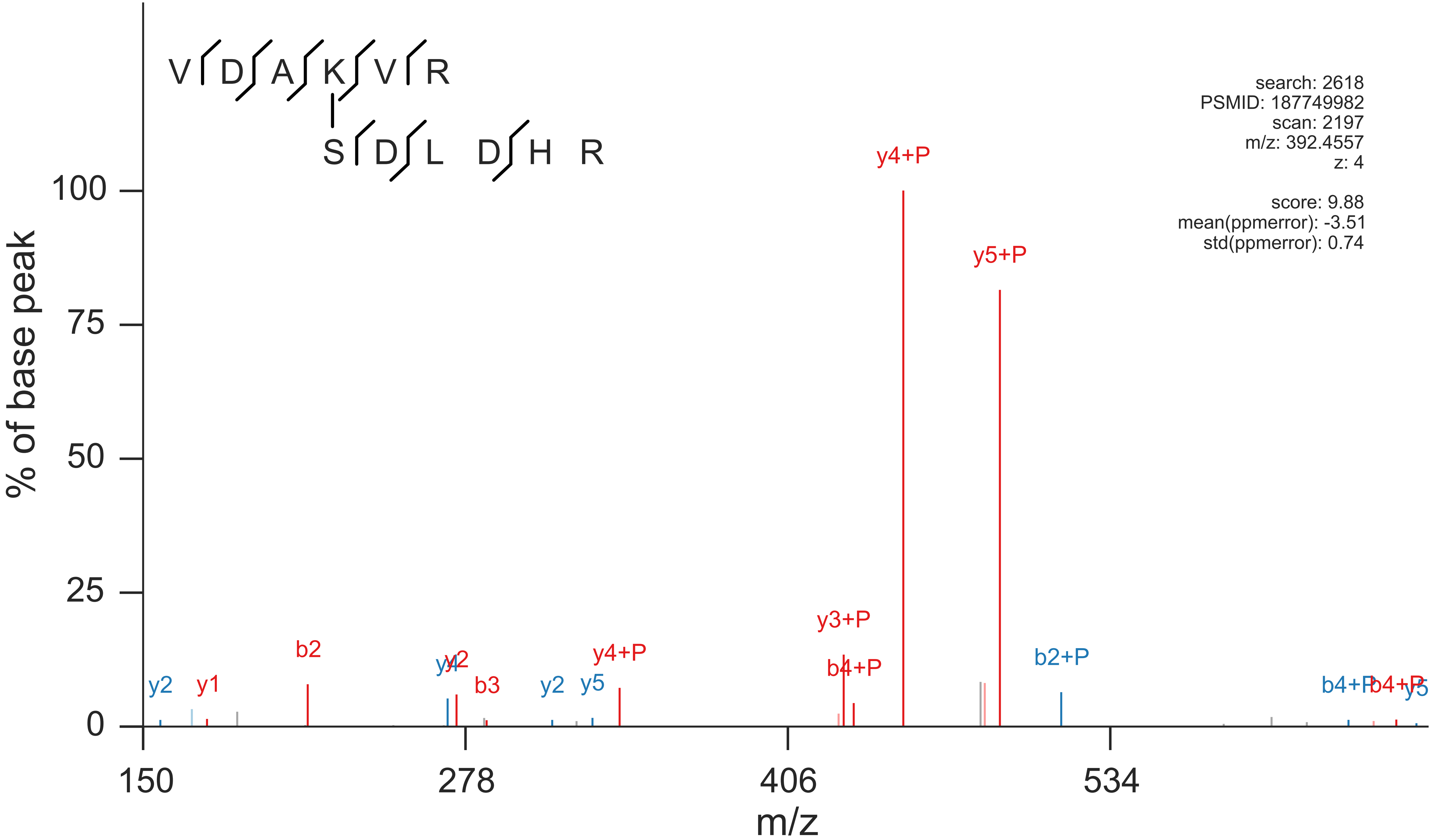

V D A K V R
S D L D H R
search: 2618
PSMID: 187749982
scan: 2197
m/z: 392.4557
z: 4
score: 9.88
mean(ppmerror): -3.51
std(ppmerror): 0.74
% of base peak
m/z
0
25
50
75
100
150
278
406
534
y4+P
y5+P
y3+P
b4+P
b2+P
y2
y1
b2
y1
y2
b3
y2
y5
y4+P
b4+P
b4+P
y5

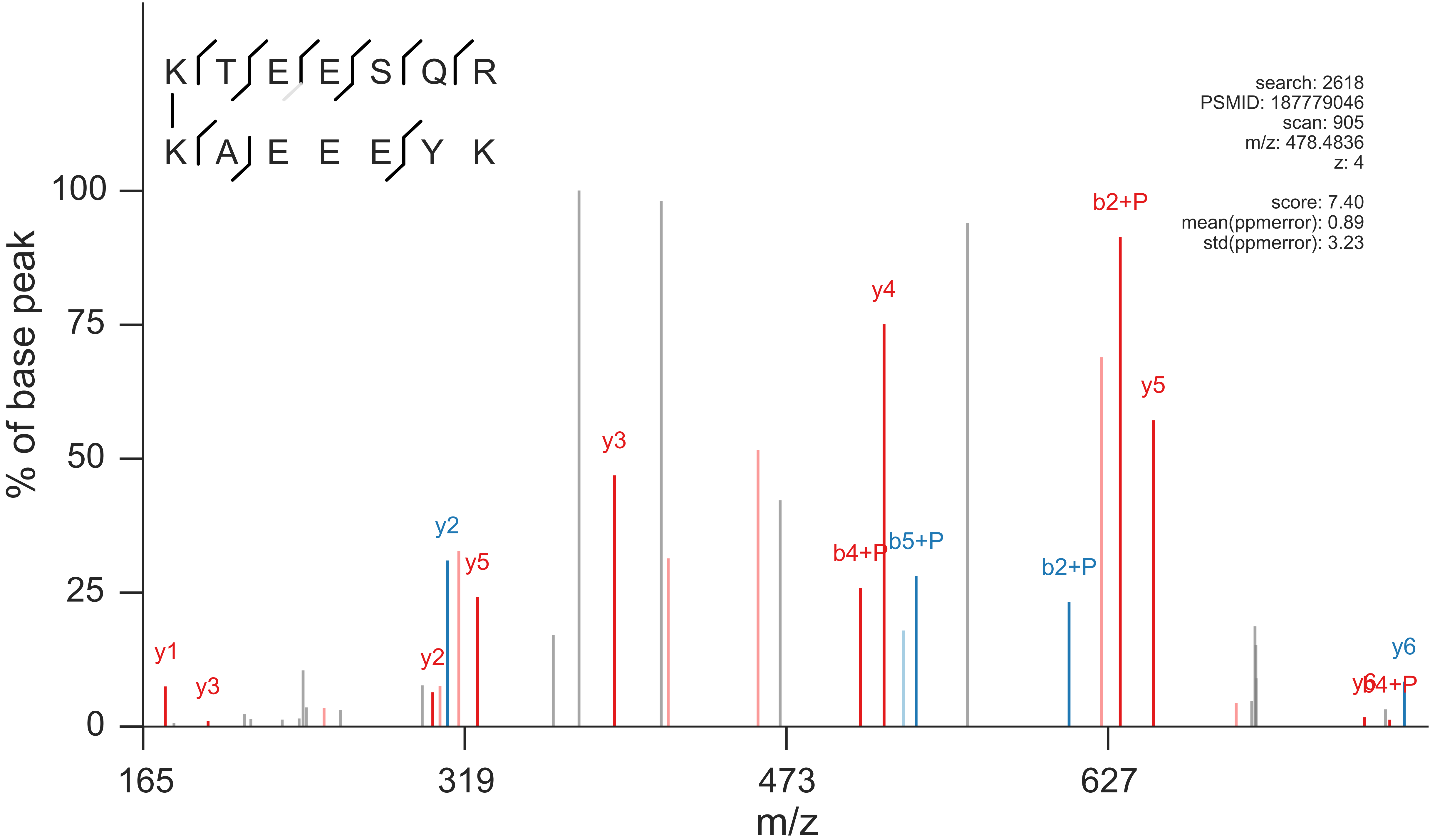

K T E E S Q R
K A E E E Y K
search: 2618
PSMID: 187779046
scan: 905
m/z: 478.4836
z: 4
score: 7.40
mean(ppmerror): 0.89
std(ppmerror): 3.23
% of base peak
m/z
100
75
50
25
0
165
319
473
627
y1
y3
y2
y2
y5
y3
b4+P
y4
b5+P
b2+P
b2+P
y5
y6
b4+P

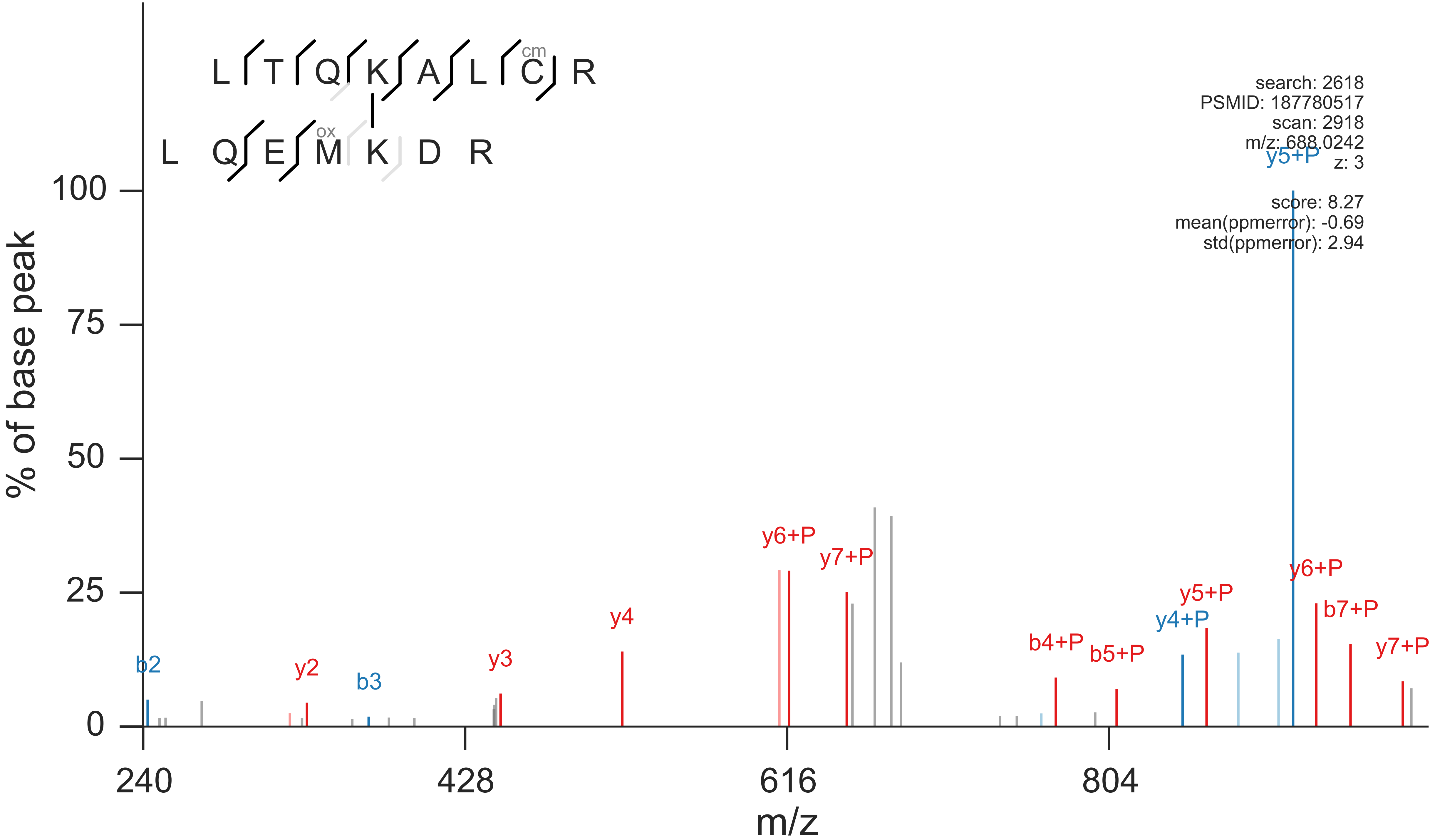

L T Q K A L C R
cm
L Q E M K D R
ox
search: 2618
PSMID: 187780517
scan: 2918
m/z: 688.0242
z: 3
score: 8.27
mean(ppmerror): -0.69
std(ppmerror): 2.94
y5+P
% of base peak
100
75
50
25
0
b2
y2
b3
y3
y4
y6+P
y7+P
b4+P
b5+P
y4+P
y5+P
y6+P
b7+P
y7+P
240
428
616
804
m/z

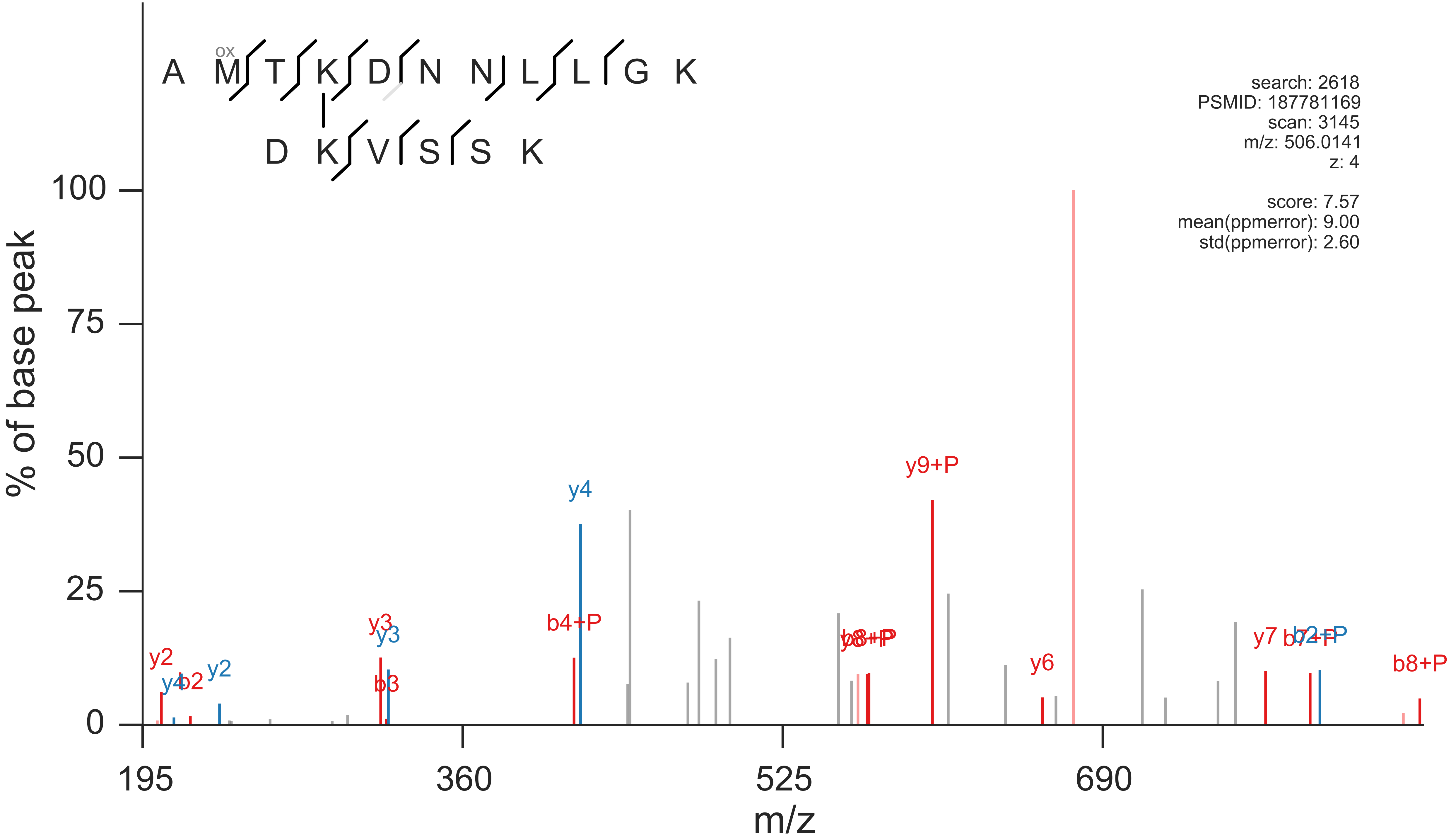

A M T K D N N L L G K
ox
D K V S S K
search: 2618
PSMID: 187781169
scan: 3145
m/z: 506.0141
z: 4
score: 7.57
mean(ppmerror): 9.00
std(ppmerror): 2.60
% of base peak
100
75
50
25
0
195
360
525
690
m/z
y2
y4
b2
y2
y3
y3
b3
b4+P
y4
y9+P
y6
y7
b8+P

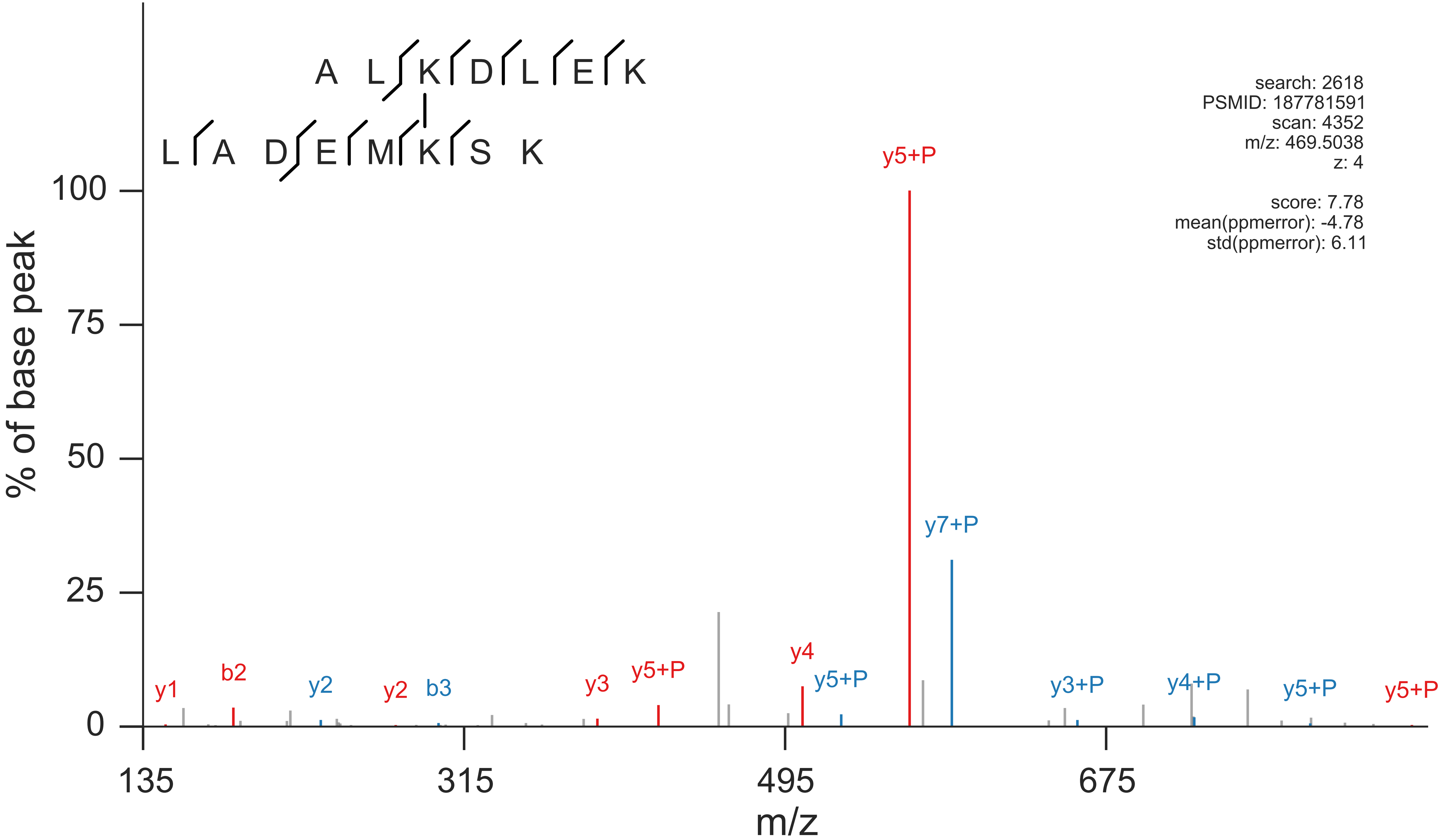

A L K D L E K
L A D E M K S K
search: 2618
PSMID: 187781591
scan: 4352
m/z: 469.5038
z: 4
score: 7.78
mean(ppmerror): -4.78
std(ppmerror): 6.11
y5+P
y7+P
y1
b2
y2
y2
b3
y3
y5+P
y4
y5+P
y3+P
y4+P
y5+P
y5+P
% of base peak
100
75
50
25
0
135
315
495
675
m/z

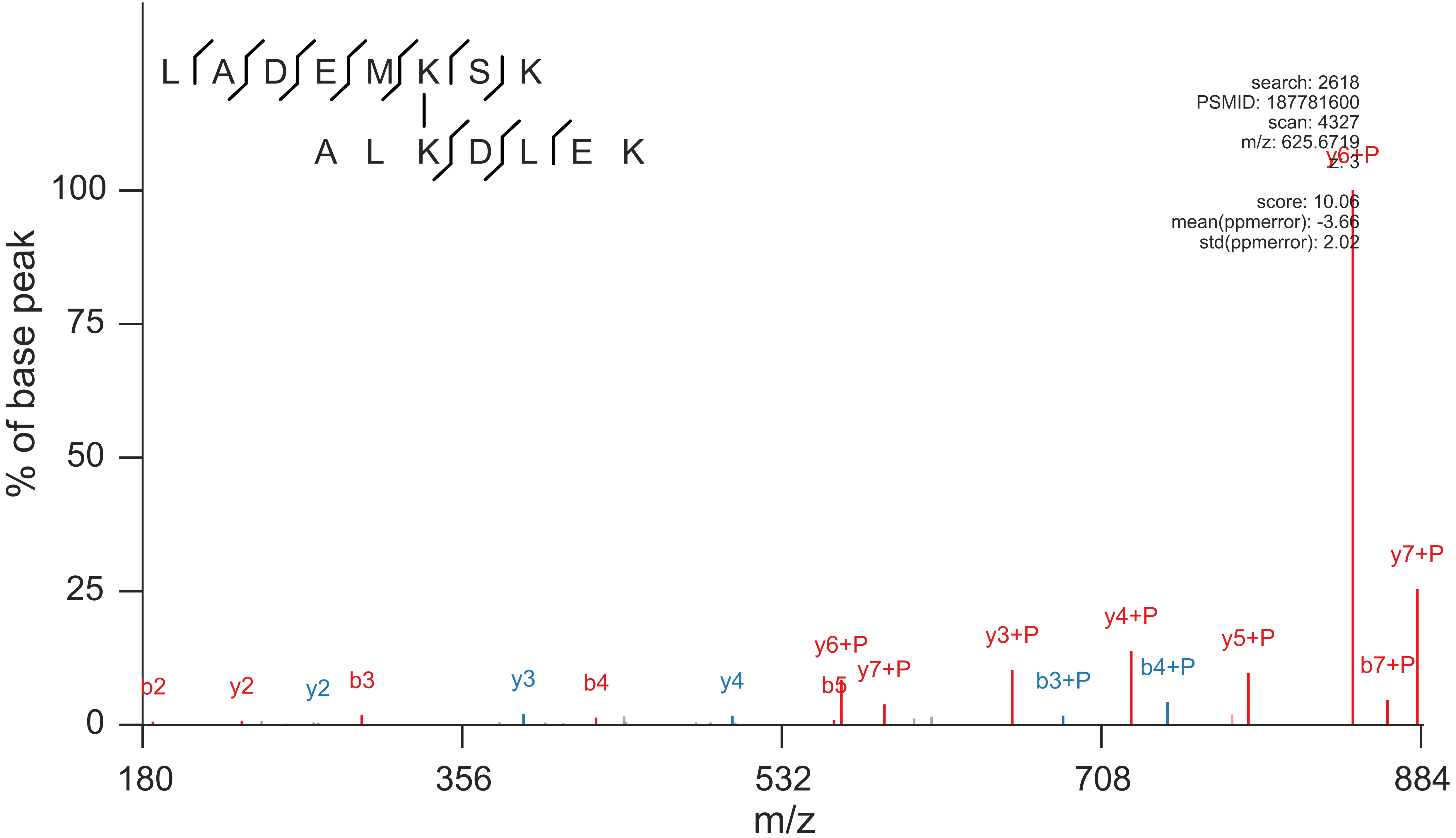

L A D E M K S K
A L K D L E K
search: 2618
PSMID: 187781600
scan: 4327
m/z: 625.6719
z: 3
score: 10.06
mean(ppmerror): -3.66
std(ppmerror): 2.02
% of base peak
m/z
100
75
50
25
0
180
356
532
708
884
b2
y2
y2
b3
y3
b4
y4
b5
y6+P
y7+P
y3+P
b3+P
y4+P
b4+P
y5+P
y6+P
b7+P
y7+P

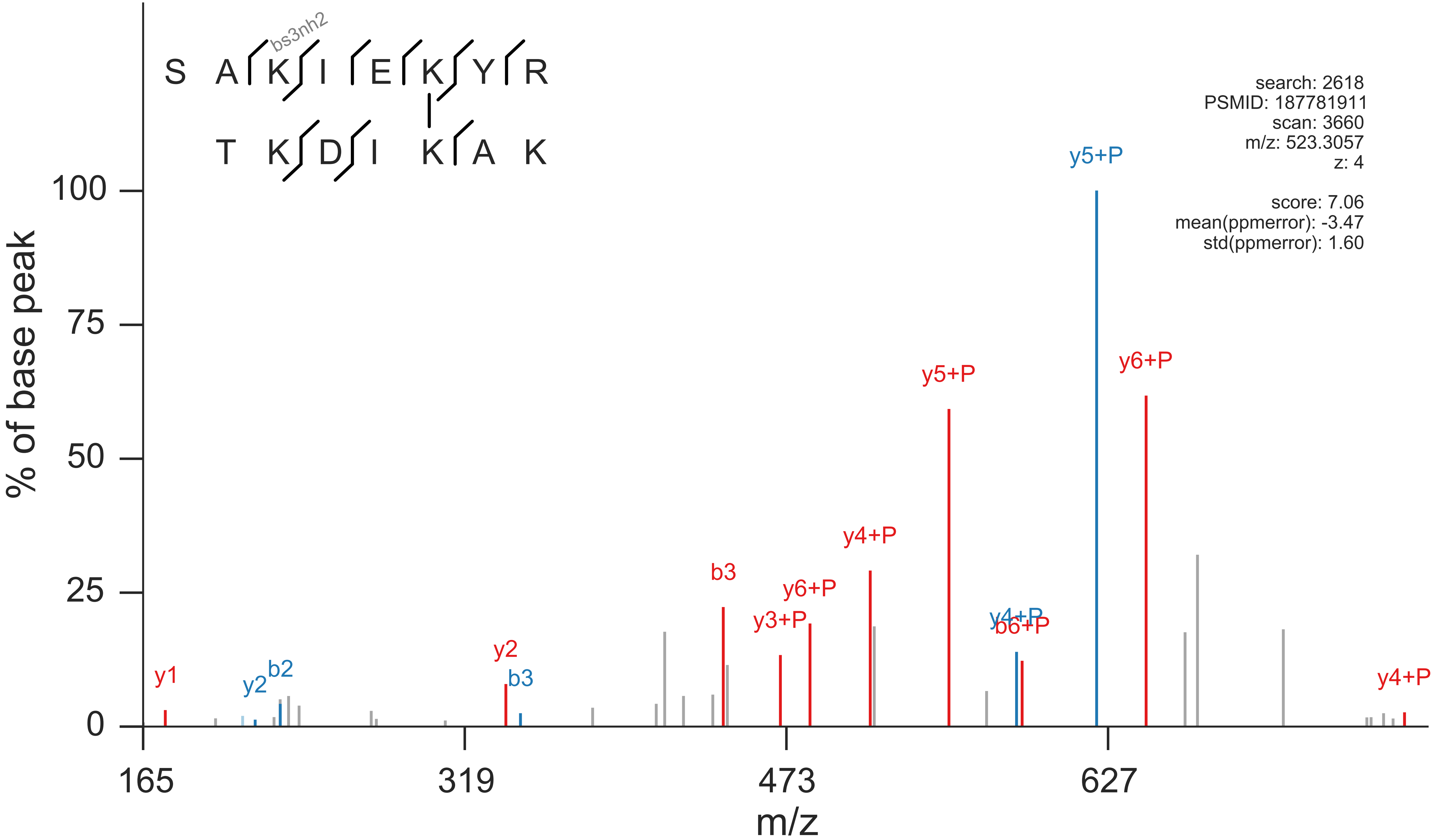

S A K I E K Y R
T K D I K A K
bs3nh2
search: 2618
PSMID: 187781911
scan: 3660
m/z: 523.3057
z: 4
score: 7.06
mean(ppmerror): -3.47
std(ppmerror): 1.60
% of base peak
0
25
50
75
100
165
319
473
627
m/z
y1
y2
b2
y2
b3
b3
y3+P
y6+P
y4+P
y5+P
y4+P
b6+P
y5+P
y6+P
y4+P

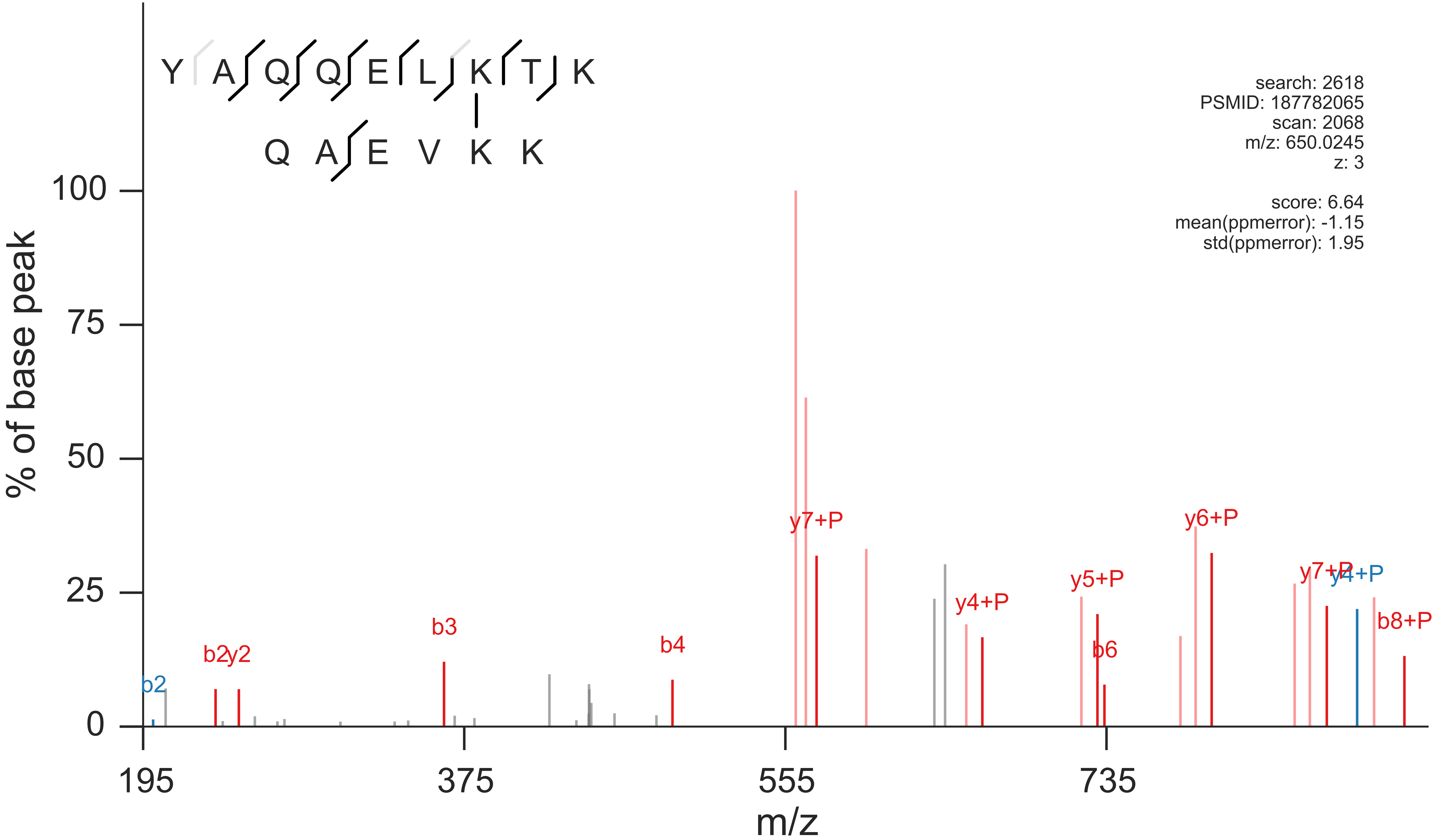

Y A Q Q E L K T K
Q A E V K K
search: 2618
PSMID: 187782065
scan: 2068
m/z: 650.0245
z: 3
score: 6.64
mean(ppmerror): -1.15
std(ppmerror): 1.95
% of base peak
100
75
50
25
0
b2
b2
y2
b3
b4
y7+P
y4+P
y5+P
b6
y6+P
y7+P
y4+P
b8+P
195
375
555
735
m/z

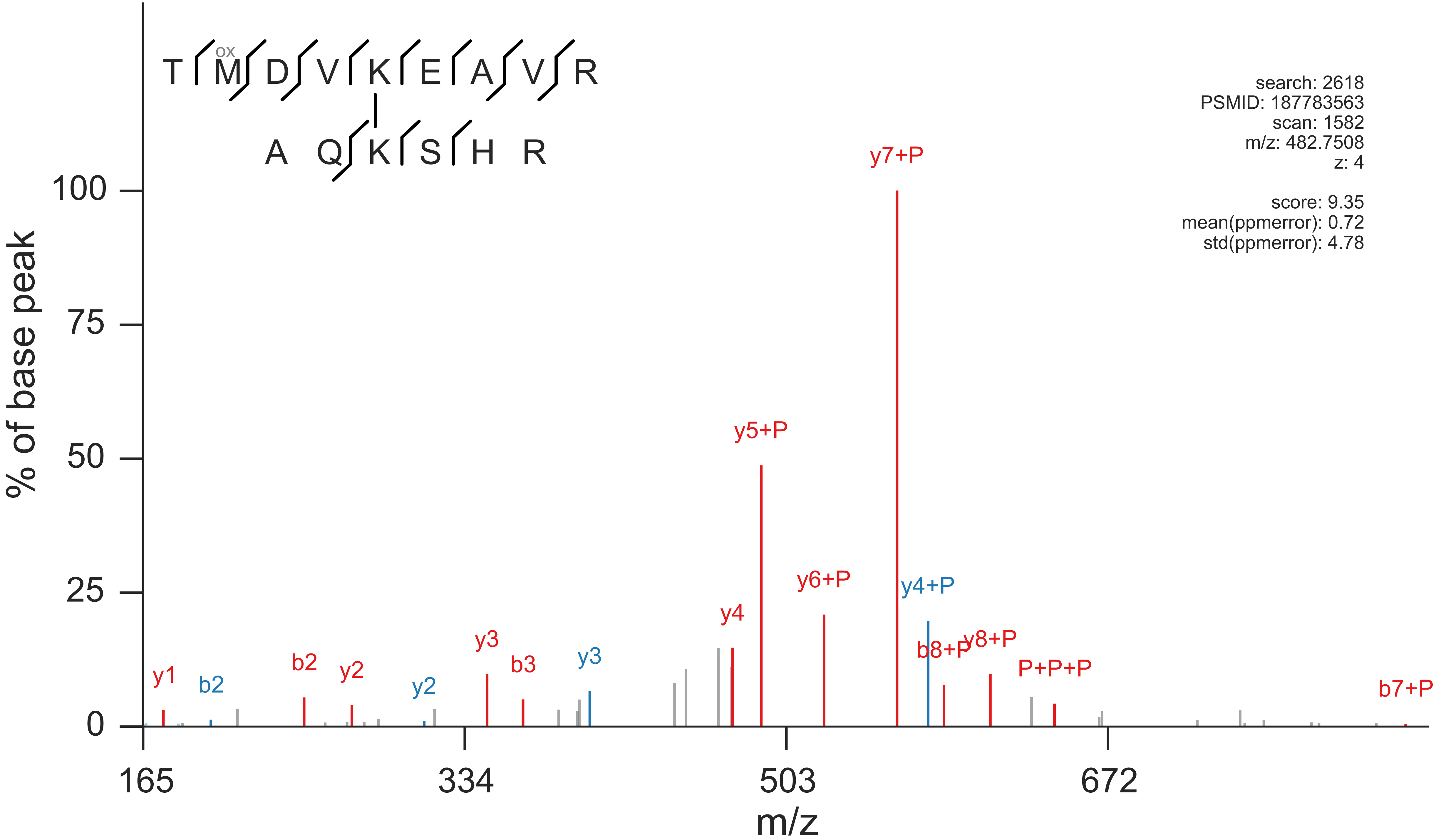

T M D V K E A V R
ox
A Q K S H R
search: 2618
PSMID: 187783563
scan: 1582
m/z: 482.7508
z: 4
score: 9.35
mean(ppmerror): 0.72
std(ppmerror): 4.78
% of base peak
100
75
50
25
0
m/z
165
334
503
672
y1
b2
b2
y2
y2
y3
b3
y3
y4
y5+P
y6+P
y7+P
y4+P
b8+P
y8+P
P+P+P
b7+P

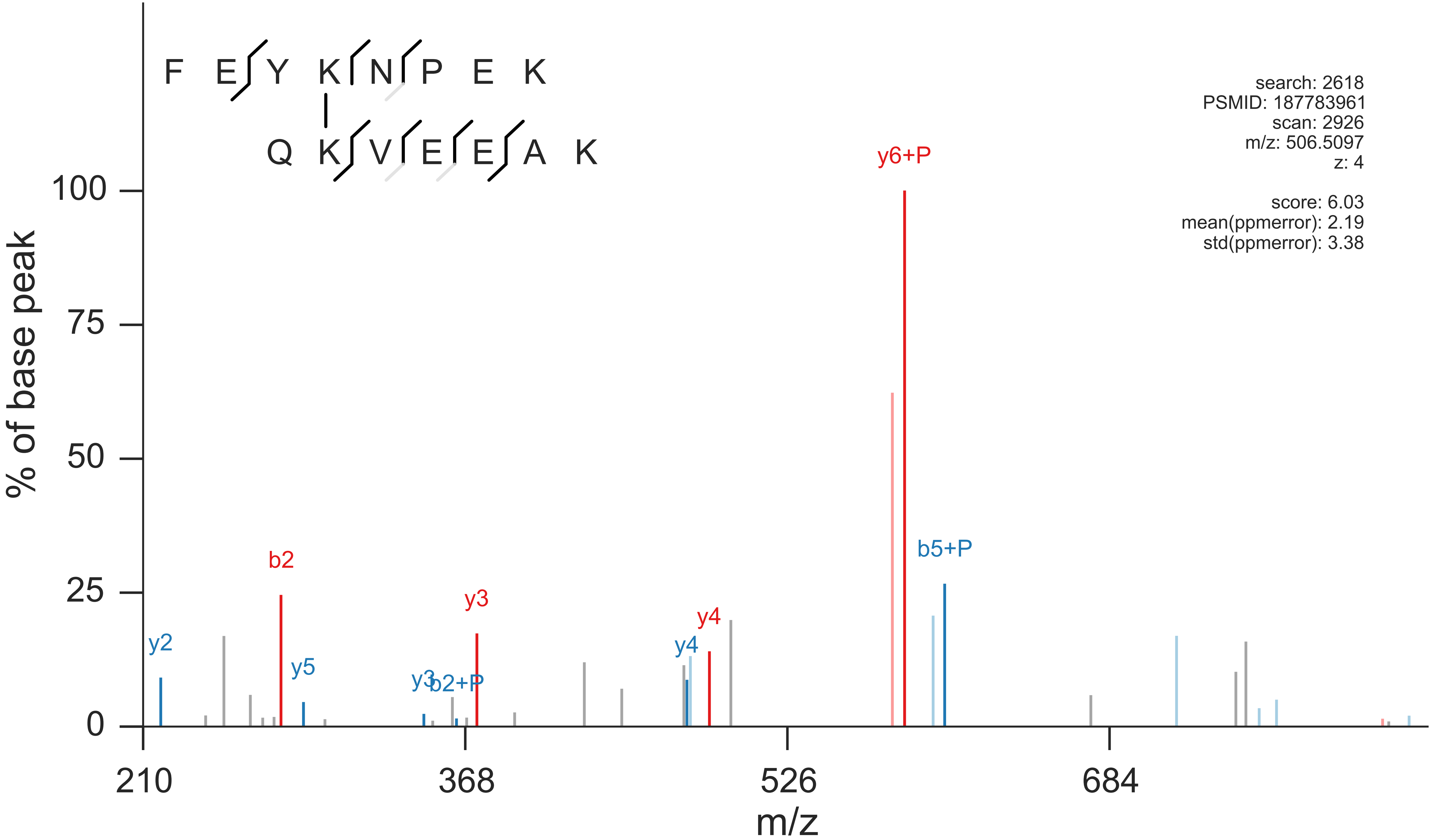

F E Y K N P E K
Q K V E E A K
search: 2618
PSMID: 187783961
scan: 2926
m/z: 506.5097
z: 4
score: 6.03
mean(ppmerror): 2.19
std(ppmerror): 3.38
y6+P
b5+P
b2
y3
y4
y2
y5
y4
y3
b2+P
100
75
50
25
0
% of base peak
210
368
526
684
m/z

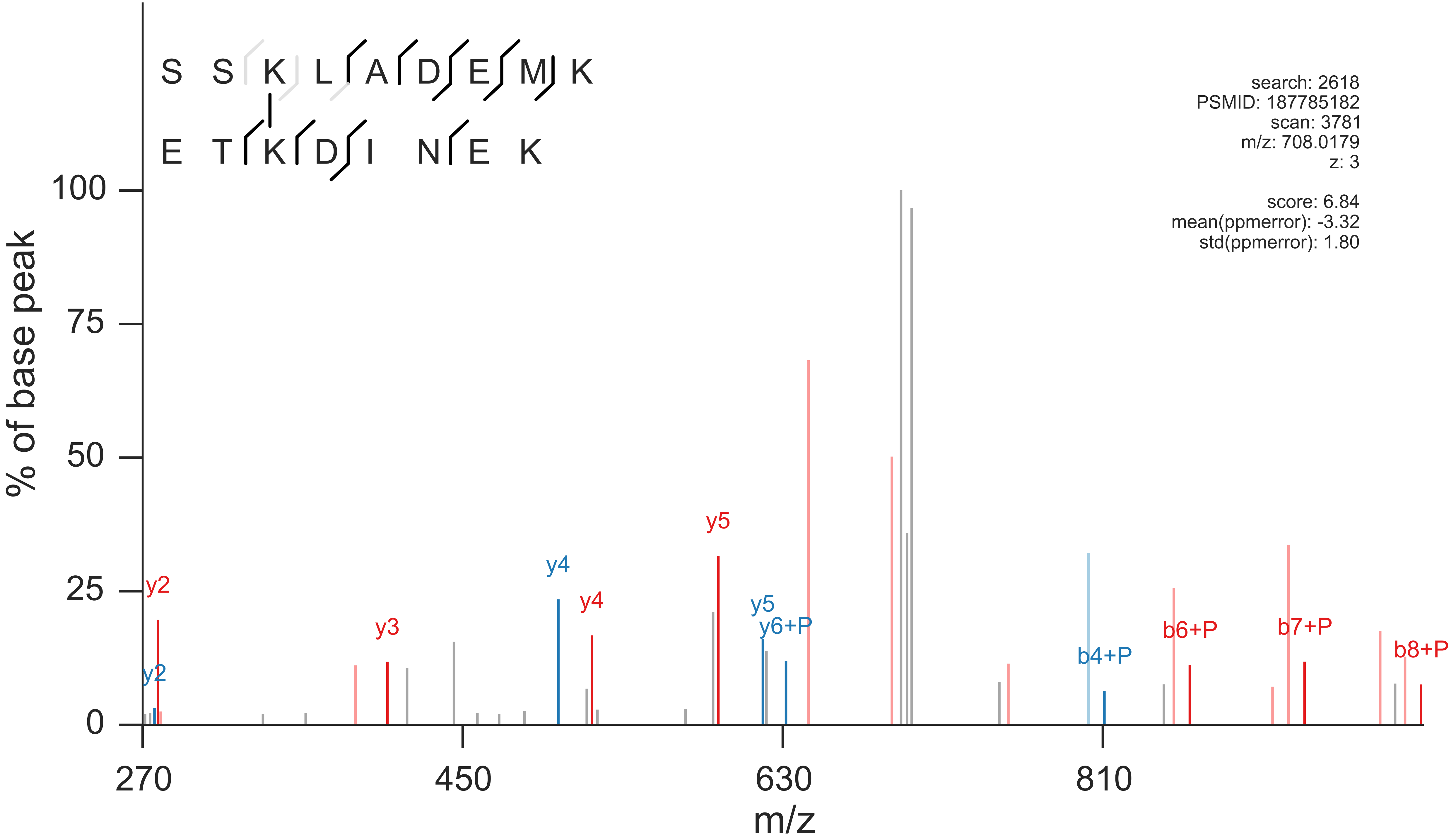
S S K L A D E M K
E T K D I N E K
search: 2618
PSMID: 187785182
scan: 3781
m/z: 708.0179
z: 3
score: 6.84
mean(ppmerror): -3.32
std(ppmerror): 1.80
% of base peak
m/z
y2
y2
y3
y4
y4
y5
y5
y6+P
b4+P
b6+P
b7+P
b8+P
0
25
50
75
100
270
450
630
810

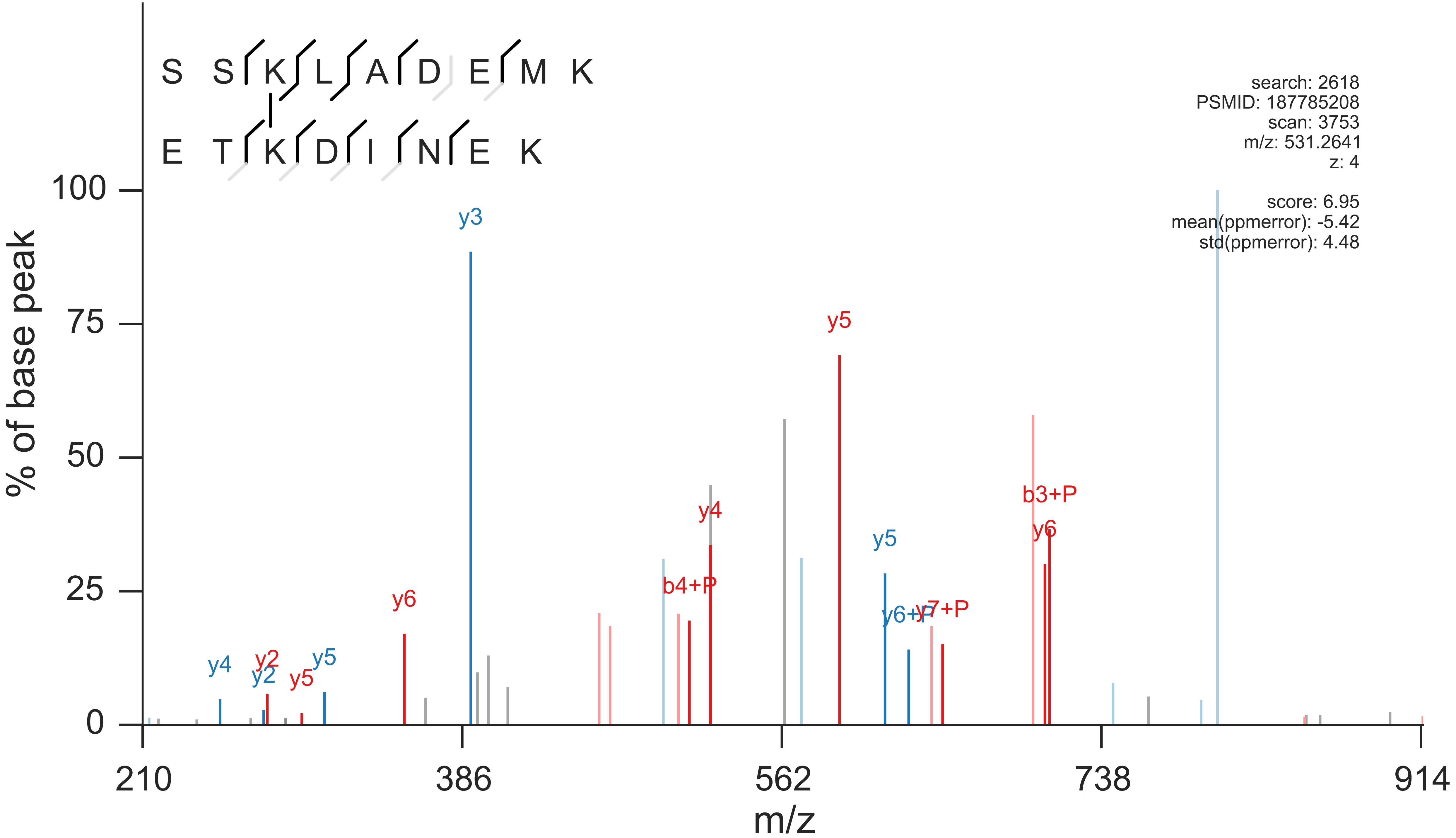

S S K L A D E M K
E T K D I N E K
search: 2618
PSMID: 187785208
scan: 3753
m/z: 531.2641
z: 4
score: 6.95
mean(ppmerror): -5.42
std(ppmerror): 4.48
% of base peak
0
25
50
75
100
210
386
562
738
914
m/z
y3
y5
y4
b3+P
y6
y5
b4+P
y6+P
y7+P
y6
y4
y2
y2
y5
y5

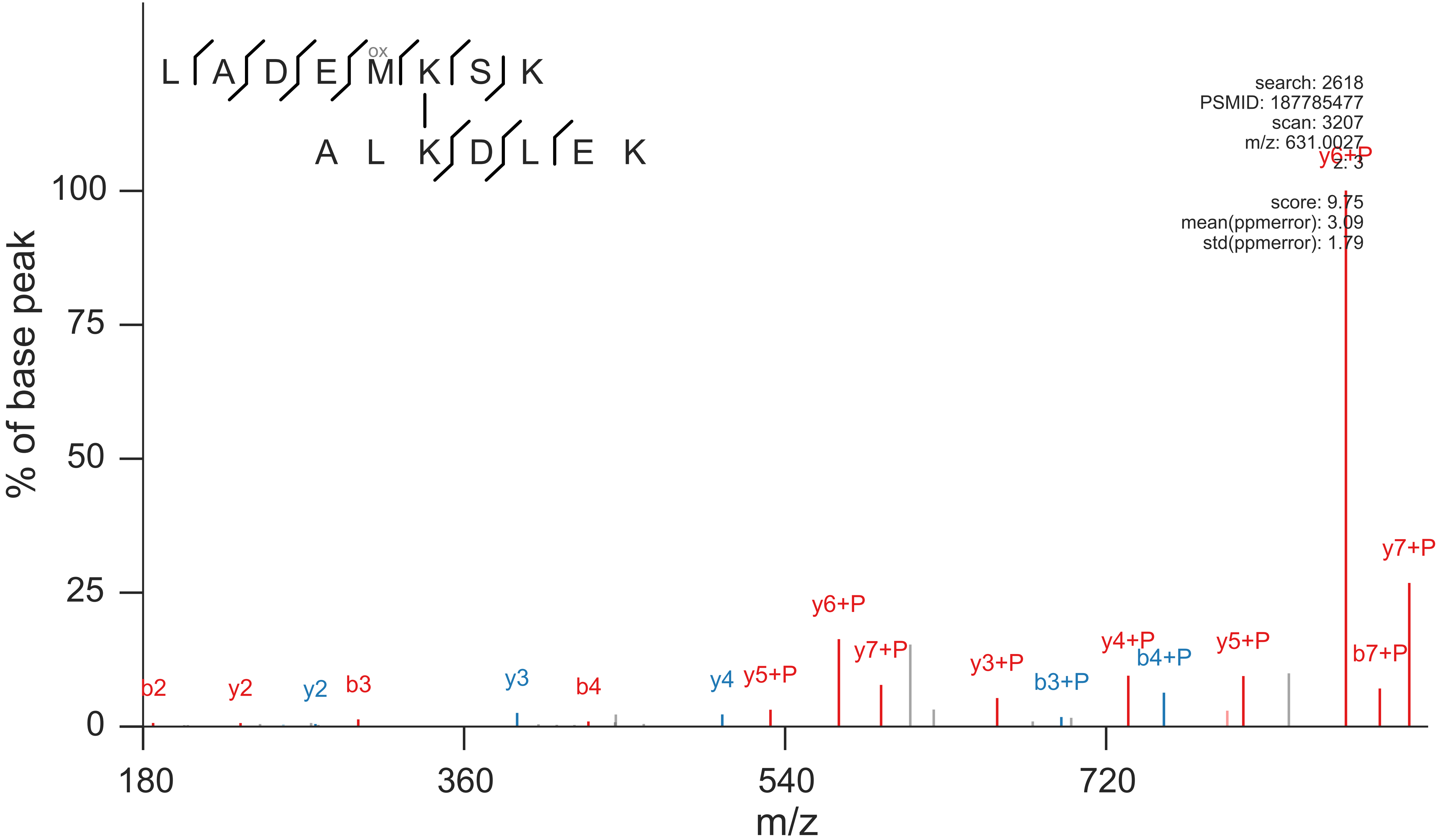

L A D E M K S K
ox
A L K D L E K
search: 2618
PSMID: 187785477
scan: 3207
m/z: 631.0027
z: 3
score: 9.75
mean(ppmerror): 3.09
std(ppmerror): 1.79
% of base peak
100
75
50
25
0
180
360
540
720
m/z
b2
y2
y2
b3
y3
b4
y4
y5+P
y6+P
y7+P
y3+P
b3+P
y4+P
b4+P
y5+P
y6+P
b7+P
y7+P

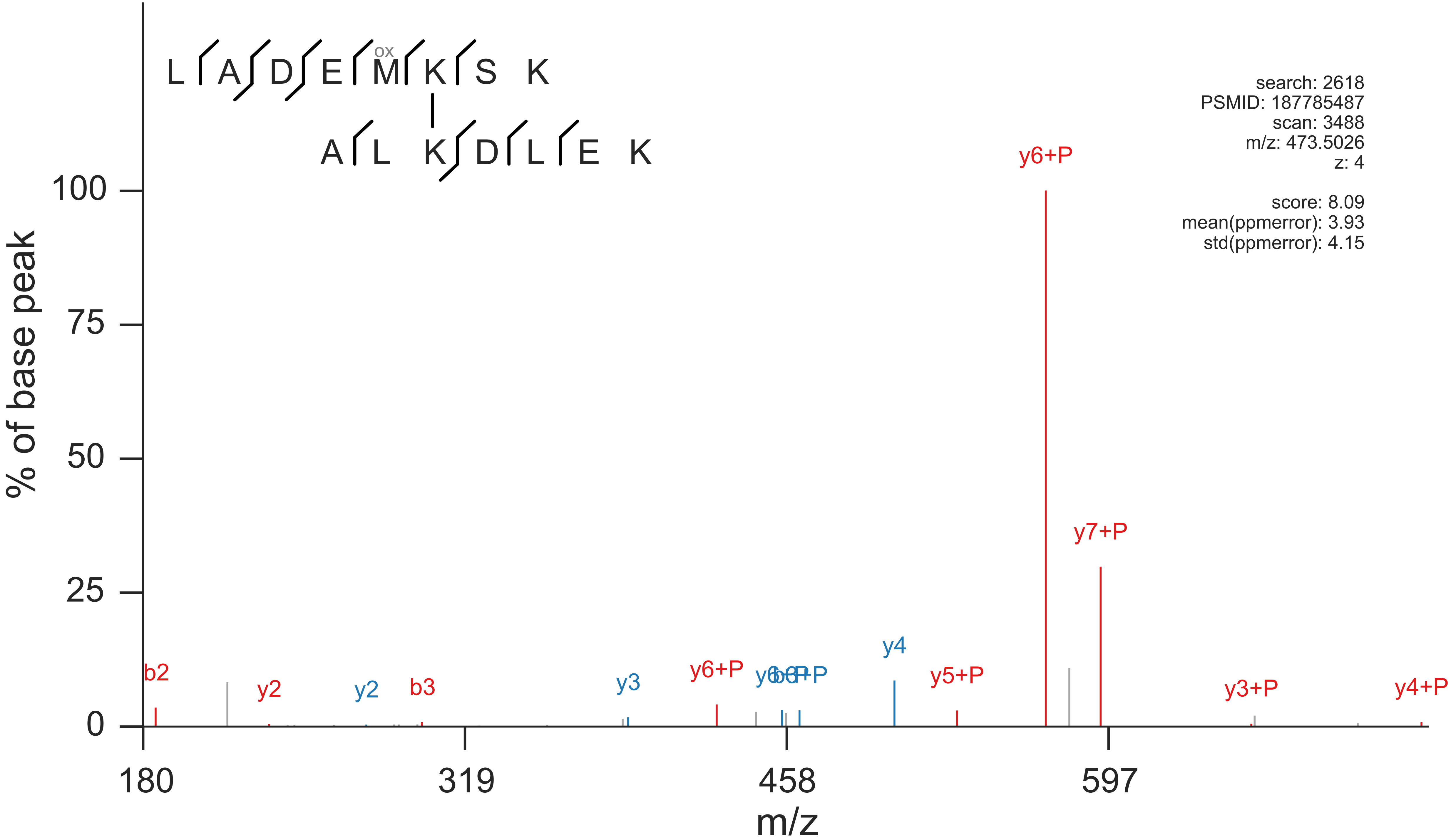

L A D E M K S K
ox
A L K D L E K
search: 2618
PSMID: 187785487
scan: 3488
m/z: 473.5026
z: 4
score: 8.09
mean(ppmerror): 3.93
std(ppmerror): 4.15
% of base peak
100
75
50
25
0
180
319
458
597
m/z
y6+P
y7+P
b2
y2
y2
b3
y3
y6+P
y4
y5+P
y3+P
y4+P

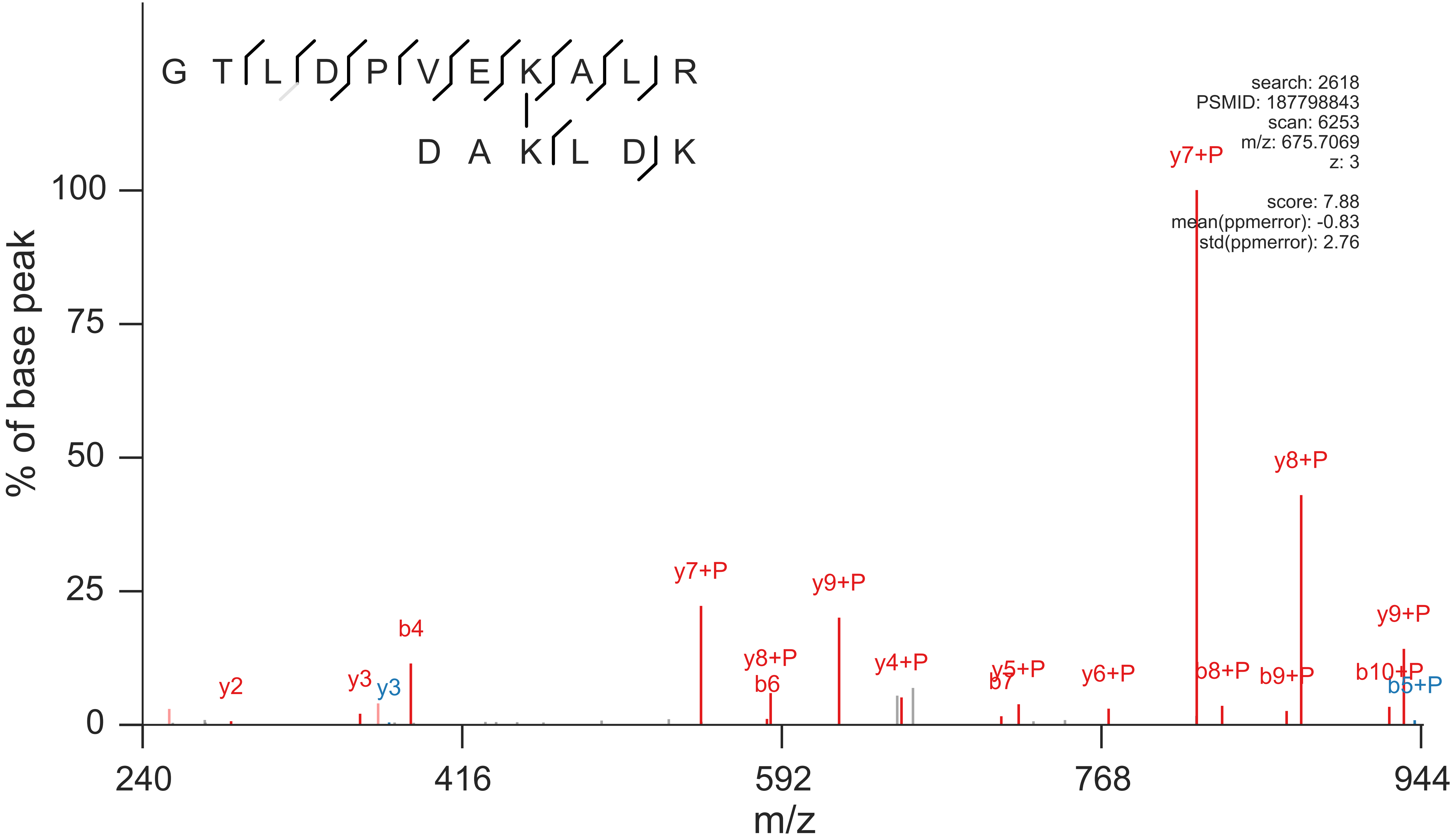

G T L D P V E K A L R
D A K L D K
search: 2618
PSMID: 187798843
scan: 6253
m/z: 675.7069
z: 3
score: 7.88
mean(ppmerror): -0.83
std(ppmerror): 2.76
% of base peak
100
75
50
25
0
y7+P
y8+P
y7+P
y9+P
b4
y8+P
b6
y4+P
y5+P
b7
y6+P
b8+P
b9+P
b10+P
y9+P
b5+P
y2
y3
y3
240
416
592
768
944
m/z

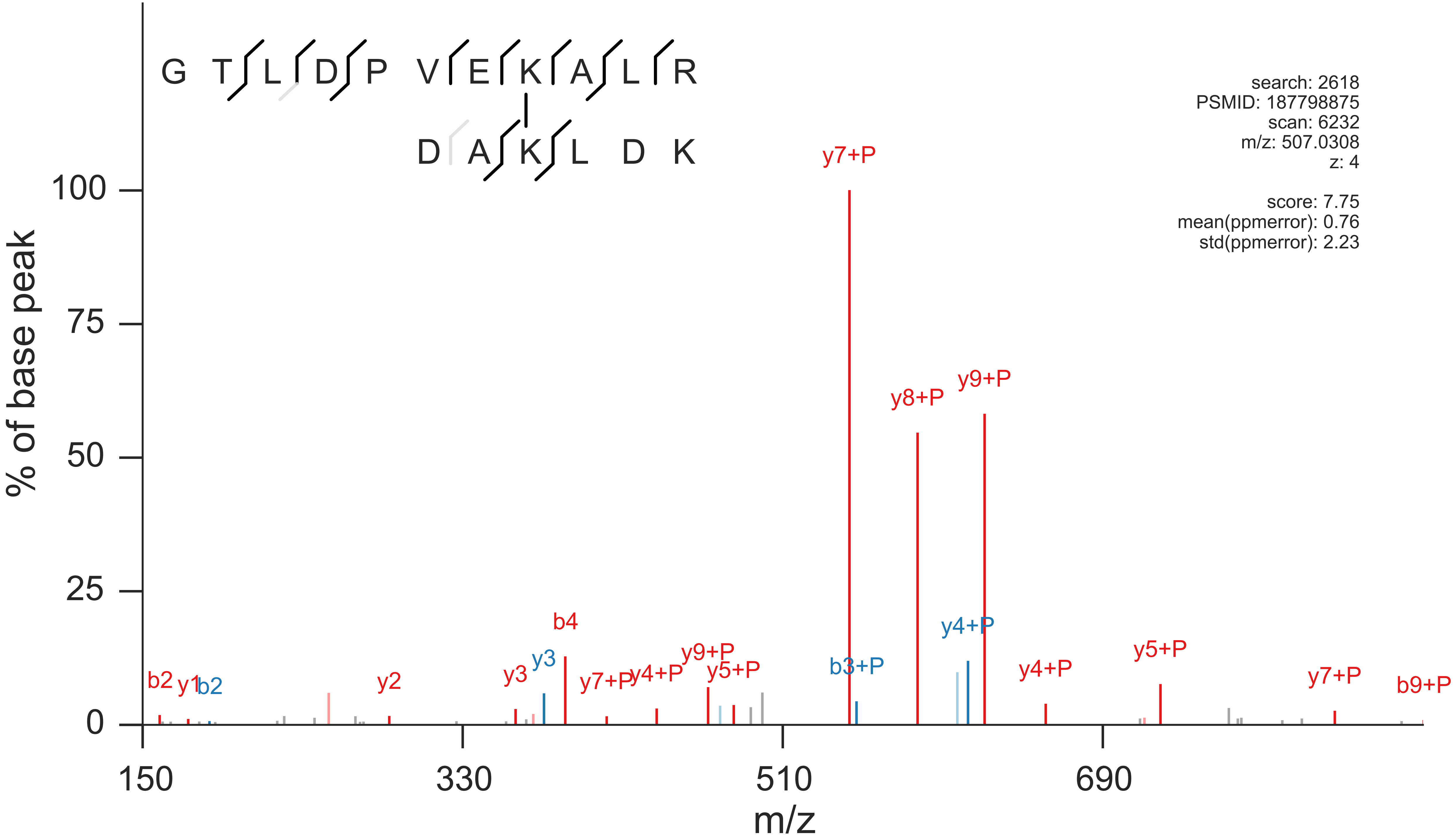

G T L D P V E K A L R
D A K L D K
search: 2618
PSMID: 187798875
scan: 6232
m/z: 507.0308
z: 4
score: 7.75
mean(ppmerror): 0.76
std(ppmerror): 2.23
% of base peak
100
75
50
25
0
150
330
510
690
m/z
y7+P
y8+P
y9+P
y4+P
b4
y3
y9+P
b3+P
y5+P
b2
y1
b2
y2
y3
y7+P
y4+P
y5+P
y4+P
y7+P
b9+P

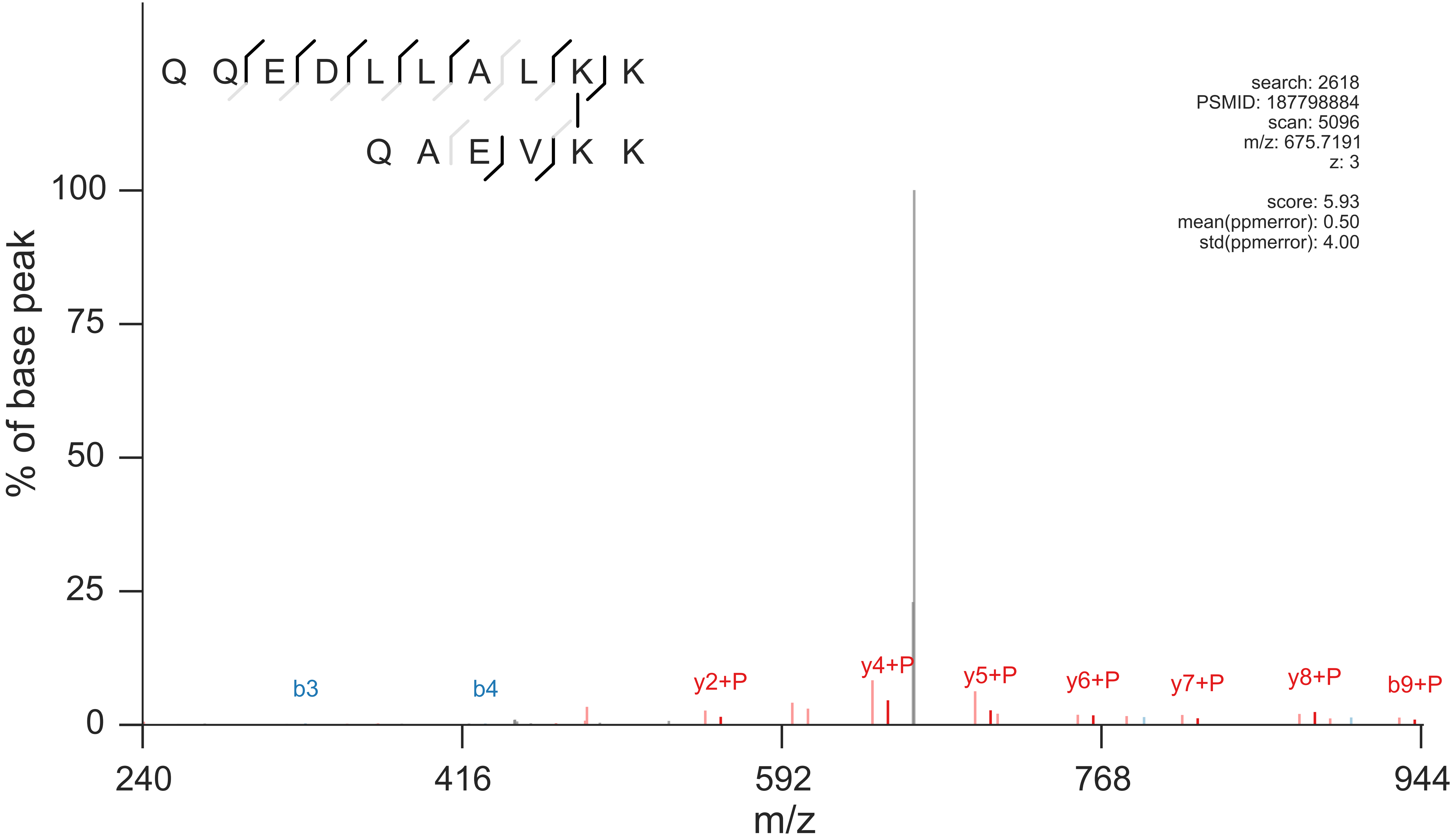

Q Q E D L L A L K K
Q A E V K K
search: 2618
PSMID: 187798884
scan: 5096
m/z: 675.7191
z: 3
score: 5.93
mean(ppmerror): 0.50
std(ppmerror): 4.00
% of base peak
100
75
50
25
0
b3
b4
y2+P
y4+P
y5+P
y6+P
y7+P
y8+P
b9+P
240
416
592
768
944
m/z

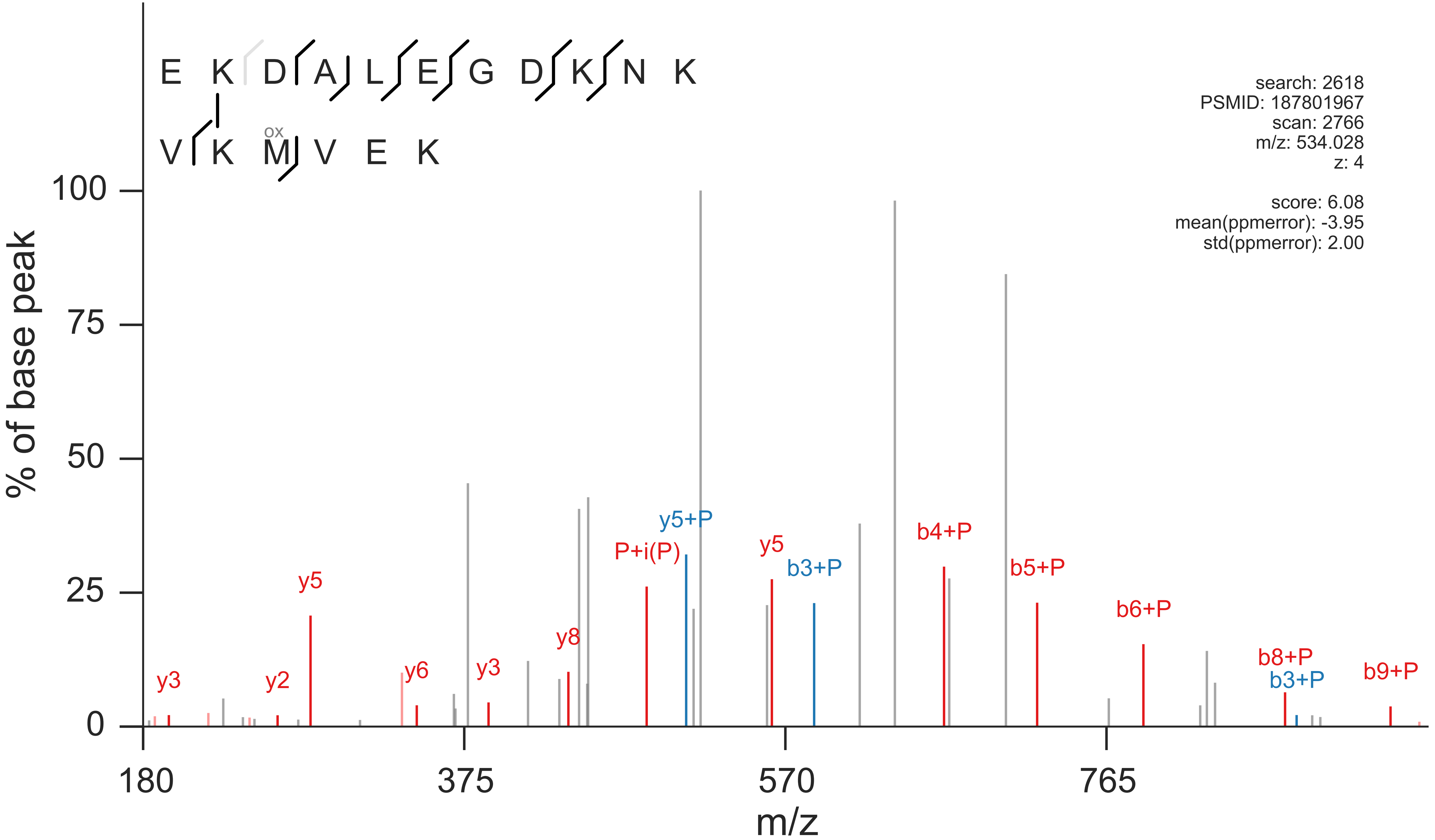

E K D A L E G D K N K
V K M V E K
ox
search: 2618
PSMID: 187801967
scan: 2766
m/z: 534.028
z: 4
score: 6.08
mean(ppmerror): -3.95
std(ppmerror): 2.00
% of base peak
m/z
0
25
50
75
100
180
375
570
765
y3
y2
y5
y6
y3
y8
P+i(P)
y5+P
y5
b3+P
b4+P
b5+P
b6+P
b8+P
b3+P
b9+P

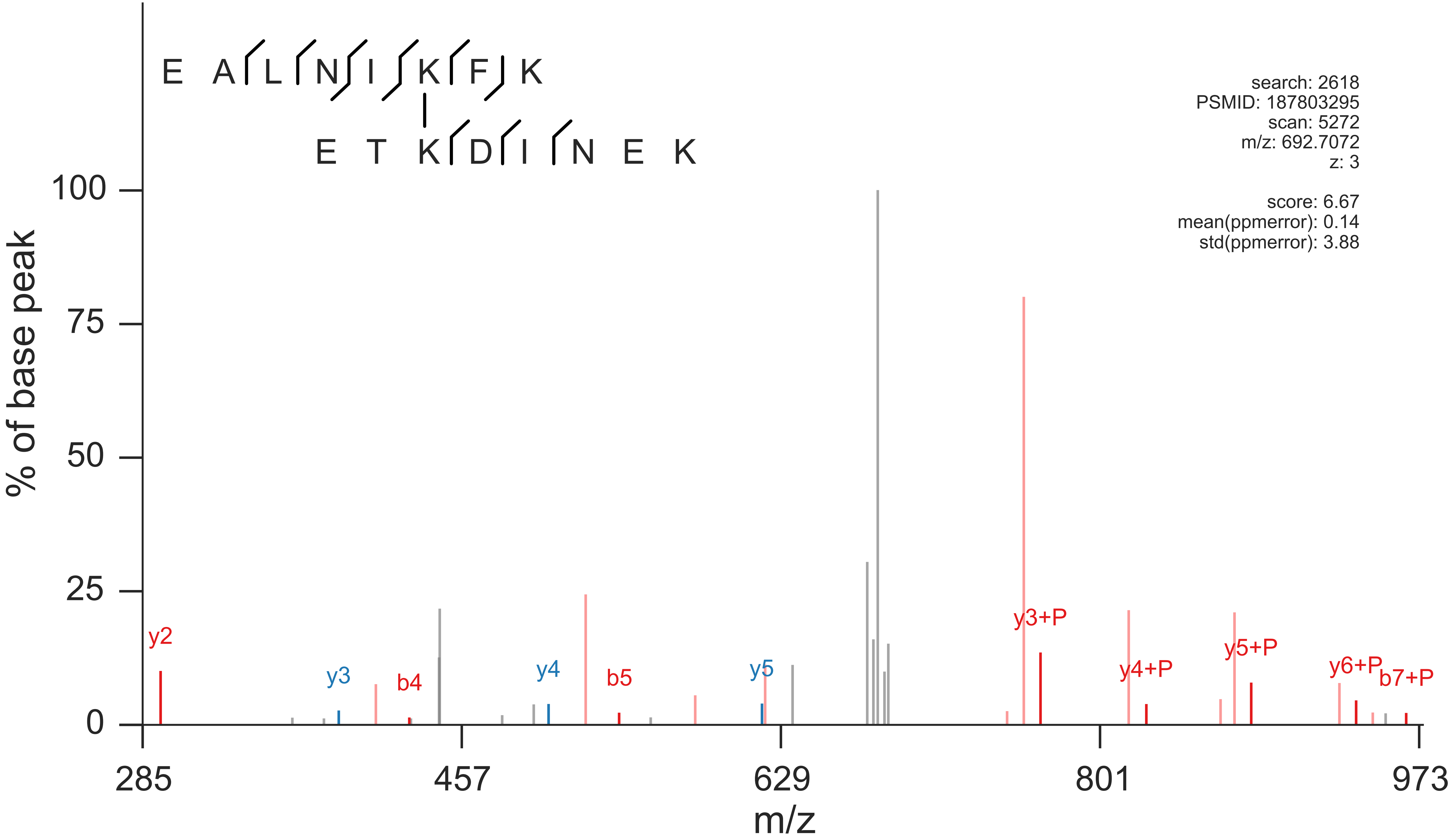

E A L N I K F K
E T K D I N E K
search: 2618
PSMID: 187803295
scan: 5272
m/z: 692.7072
z: 3
score: 6.67
mean(ppmerror): 0.14
std(ppmerror): 3.88
% of base peak
100
75
50
25
0
y2
y3
b4
y4
b5
y5
y3+P
y4+P
y5+P
y6+P
b7+P
285
457
629
801
973
m/z

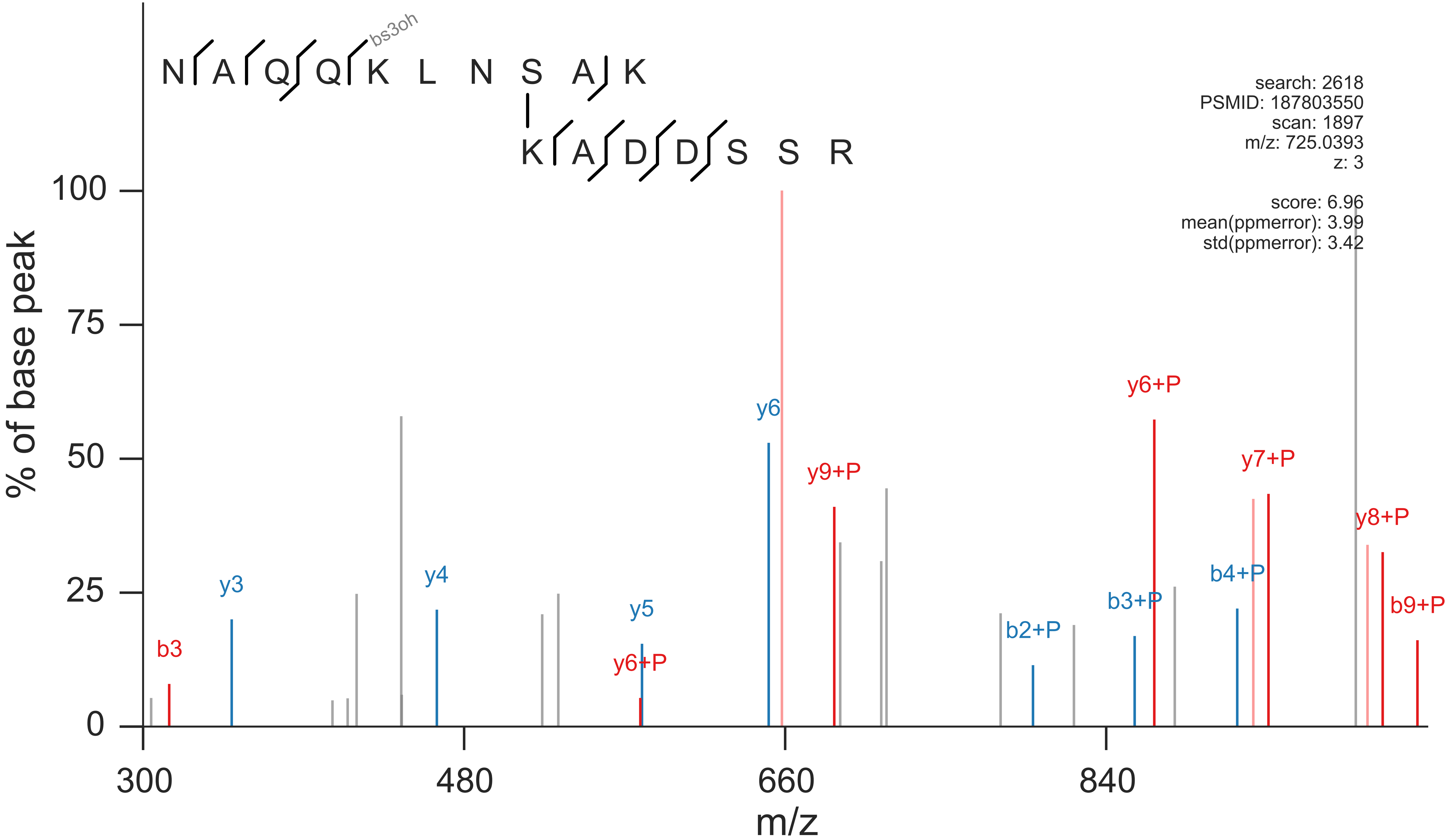

bs3oh
N A Q Q K L N S A K
K A D D S S R
search: 2618
PSMID: 187803550
scan: 1897
m/z: 725.0393
z: 3
score: 6.96
mean(ppmerror): 3.99
std(ppmerror): 3.42
% of base peak
100
75
50
25
0
b3
y3
y4
y5
y6+P
y6
y9+P
b2+P
b3+P
y6+P
b4+P
y7+P
y8+P
b9+P
300
480
660
840
m/z

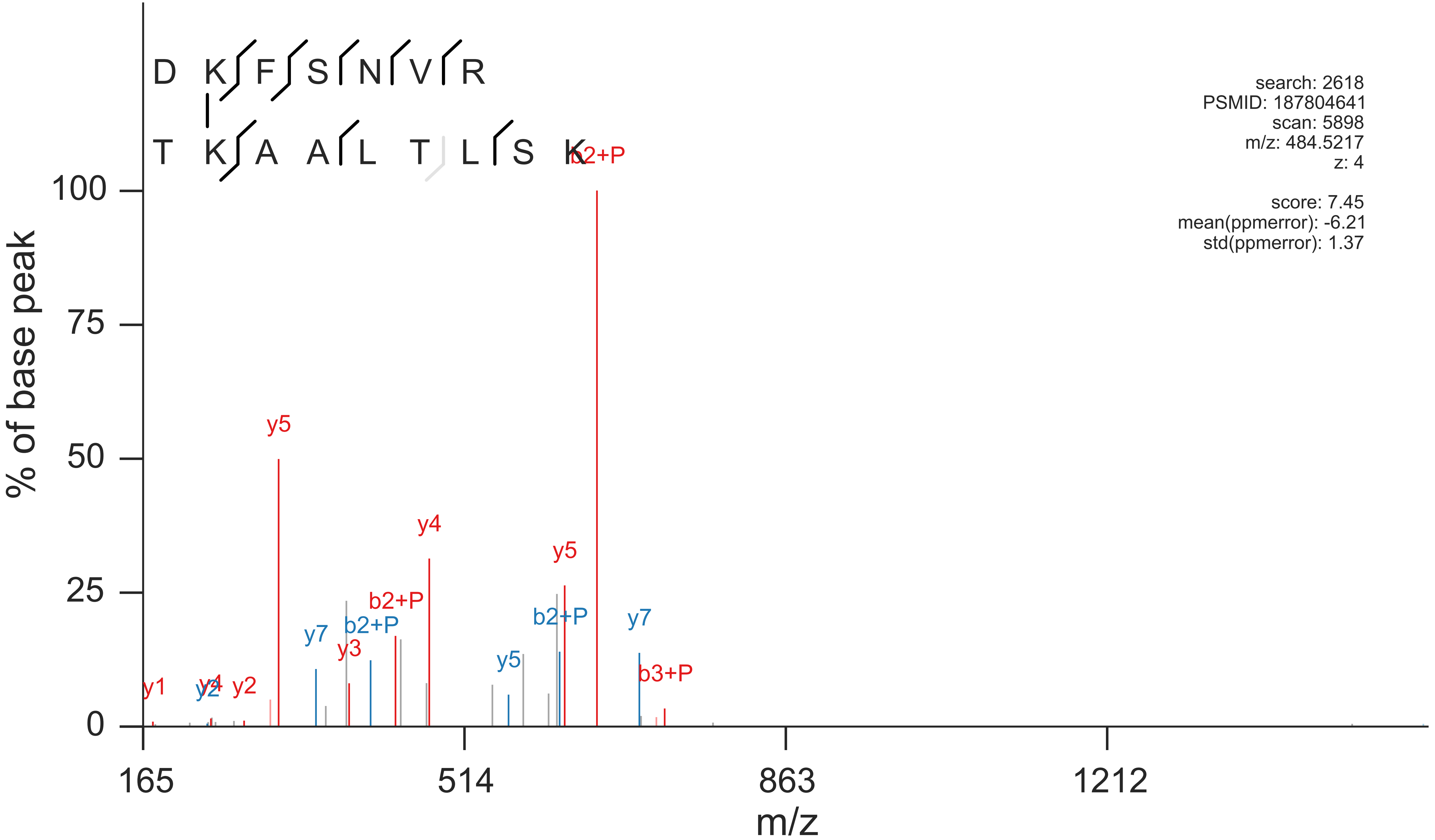

D K F S N V R
T K A A L T L S K
search: 2618
PSMID: 187804641
scan: 5898
m/z: 484.5217
z: 4
score: 7.45
mean(ppmerror): -6.21
std(ppmerror): 1.37
% of base peak
m/z
100
75
50
25
0
165
514
863
1212
b2+P
y5
y4
y5
b2+P
y7
b2+P
y3
b2+P
y7
y5
b3+P
y1
y4
y2
y2

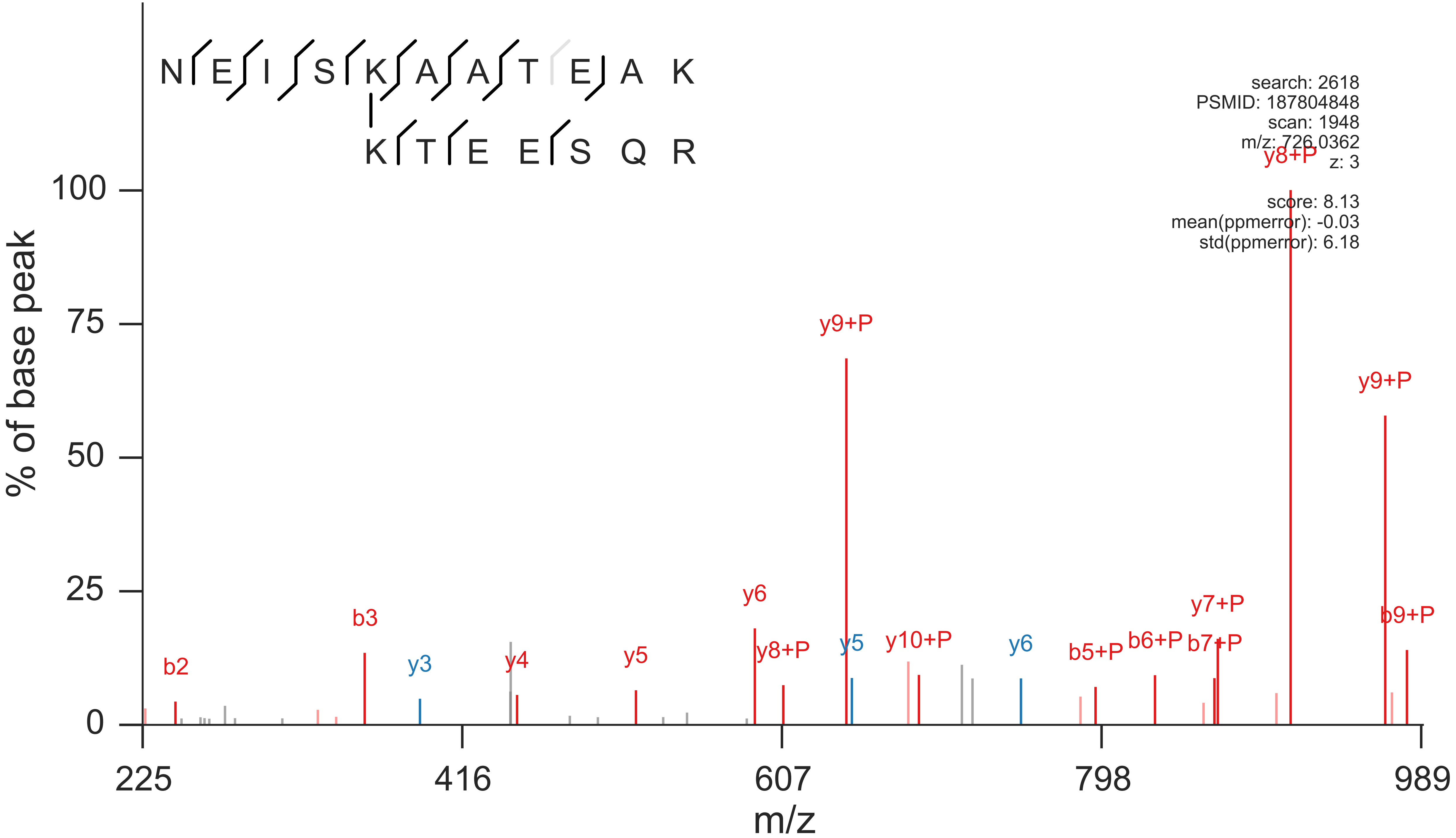

N E I S K A A T E A K
K T E E S Q R
search: 2618
PSMID: 187804848
scan: 1948
m/z: 726.0362
z: 3
score: 8.13
mean(ppmerror): -0.03
std(ppmerror): 6.18
% of base peak
m/z
0
25
50
75
100
225
416
607
798
989
b2
b3
y3
y4
y5
y6
y8+P
y9+P
y5
y10+P
y6
b5+P
b6+P
b7+P
y7+P
y8+P
y9+P
b9+P

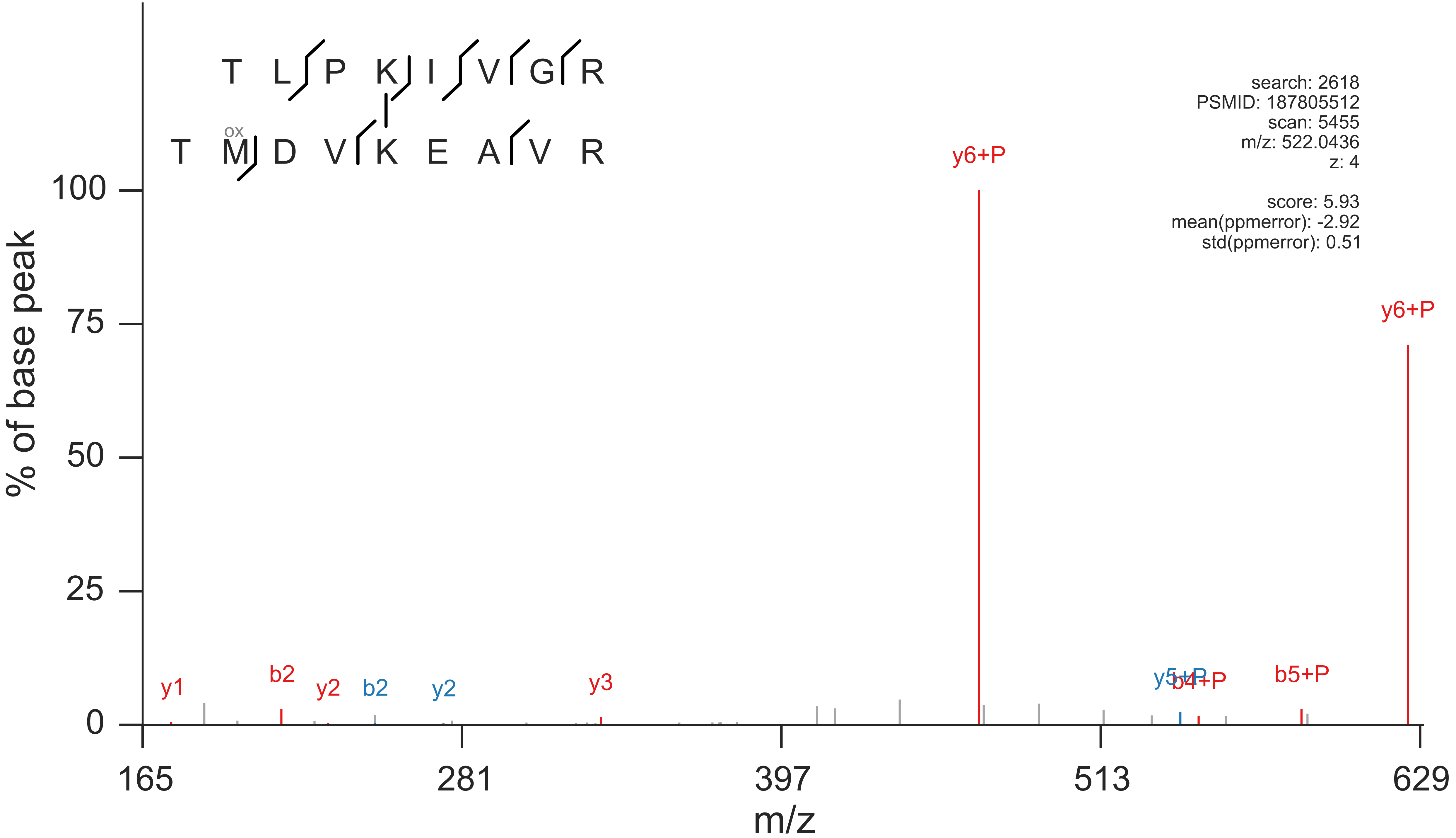

T L P K I V G R
T M D V K E A V R
ox
search: 2618
PSMID: 187805512
scan: 5455
m/z: 522.0436
z: 4
score: 5.93
mean(ppmerror): -2.92
std(ppmerror): 0.51
% of base peak
m/z
y6+P
y6+P
y1
b2
y2
b2
y2
y3
y5+P
b4+P
b5+P
0
25
50
75
100
165
281
397
513
629

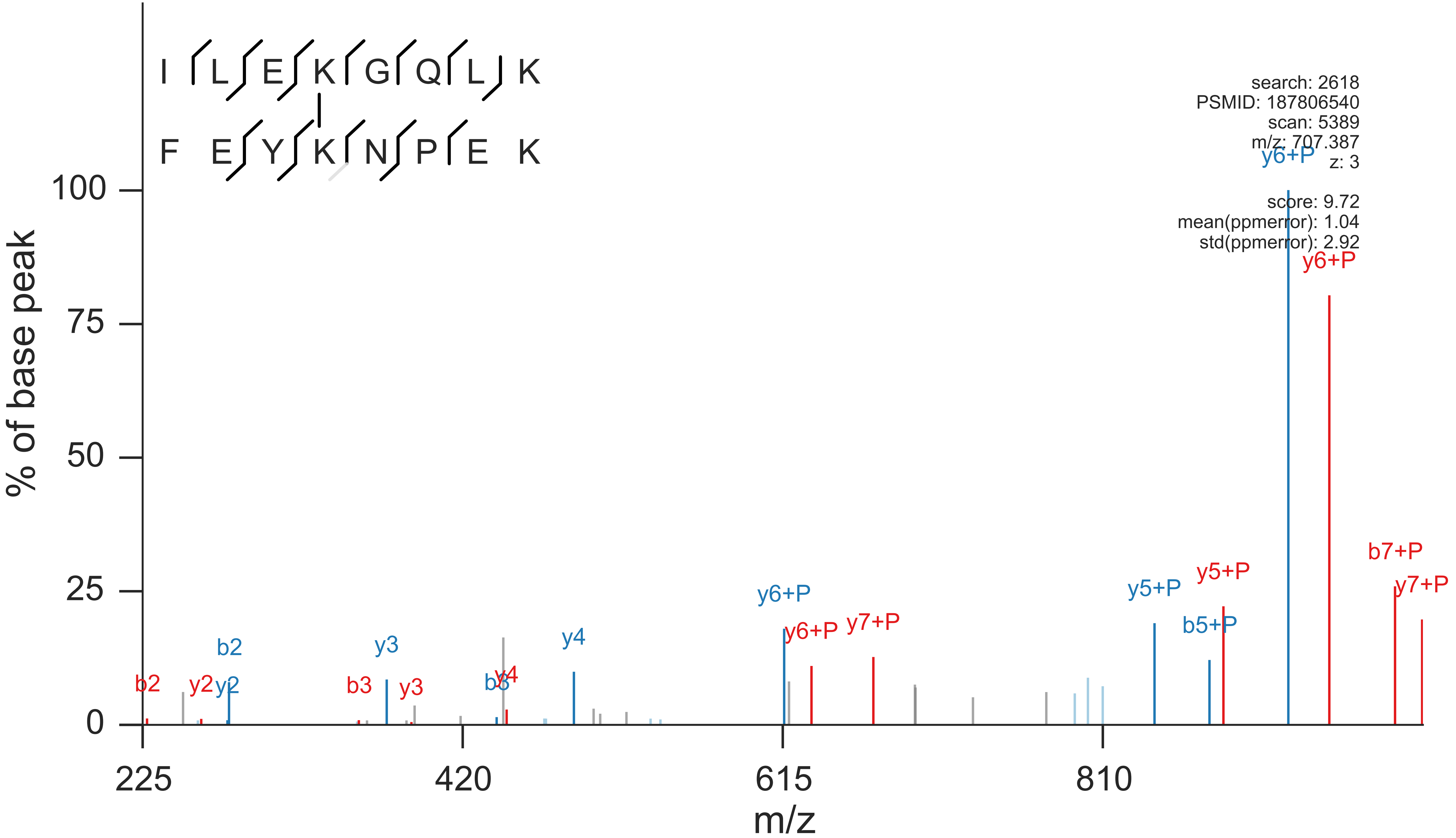

I L E K G Q L K
F E Y K N P E K
search: 2618
PSMID: 187806540
scan: 5389
m/z: 707.387
z: 3
score: 9.72
mean(ppmerror): 1.04
std(ppmerror): 2.92
% of base peak
100
75
50
25
0
225
420
615
810
m/z
y6+P
y6+P
b7+P
y7+P
y5+P
y5+P
b5+P
y6+P
y6+P
y7+P
b2
y3
y4
b2
y2
y2
b3
y3
b3
y4

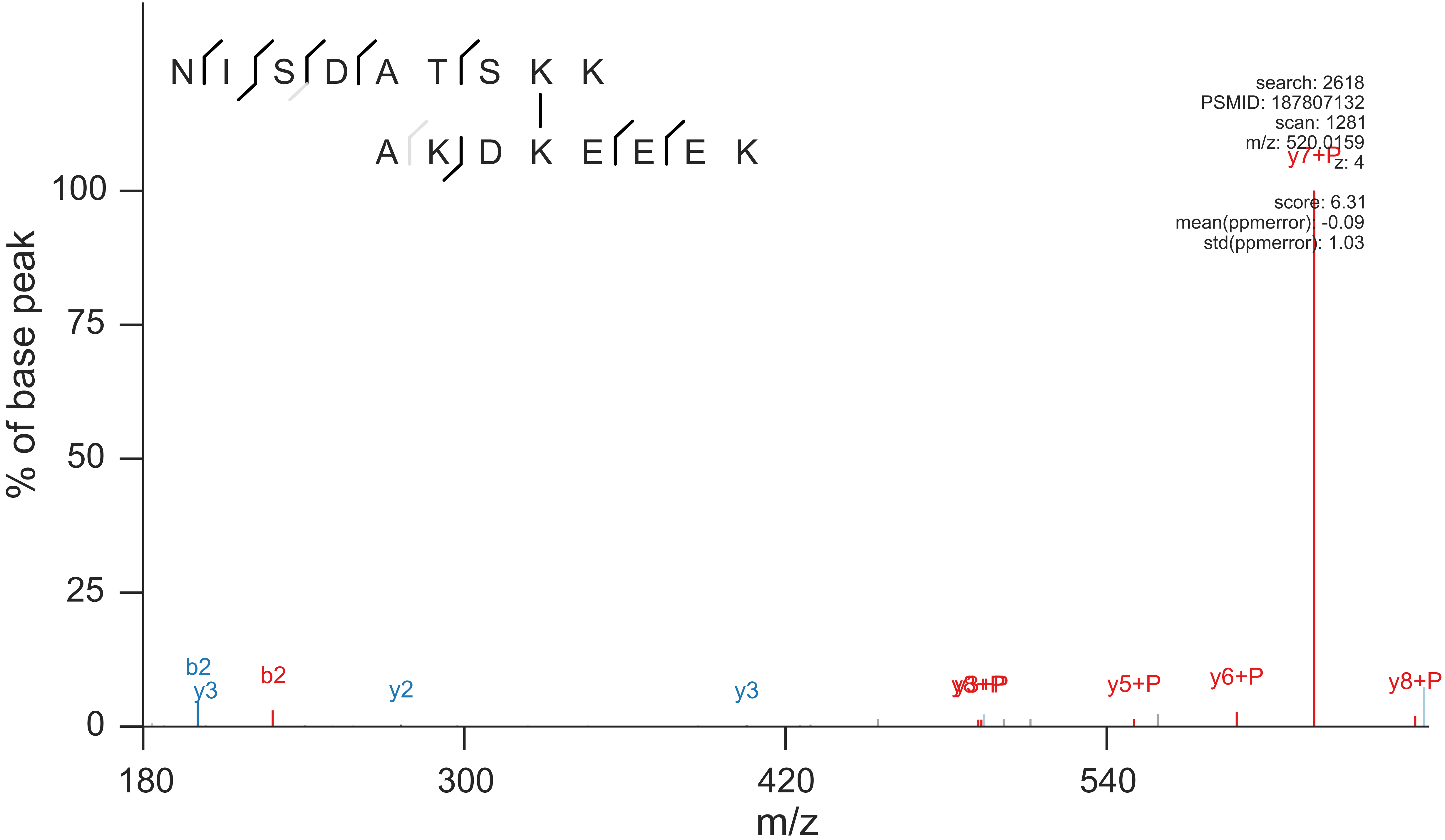

N I S D A T S K K
A K D K E E E K
search: 2618
PSMID: 187807132
scan: 1281
m/z: 520.0159
z: 4
score: 6.31
mean(ppmerror): -0.09
std(ppmerror): 1.03
y7+P
b2
y3
b2
y2
y3
y3+P
y5+P
y6+P
y8+P
% of base peak
100
75
50
25
0
180
300
420
540
m/z

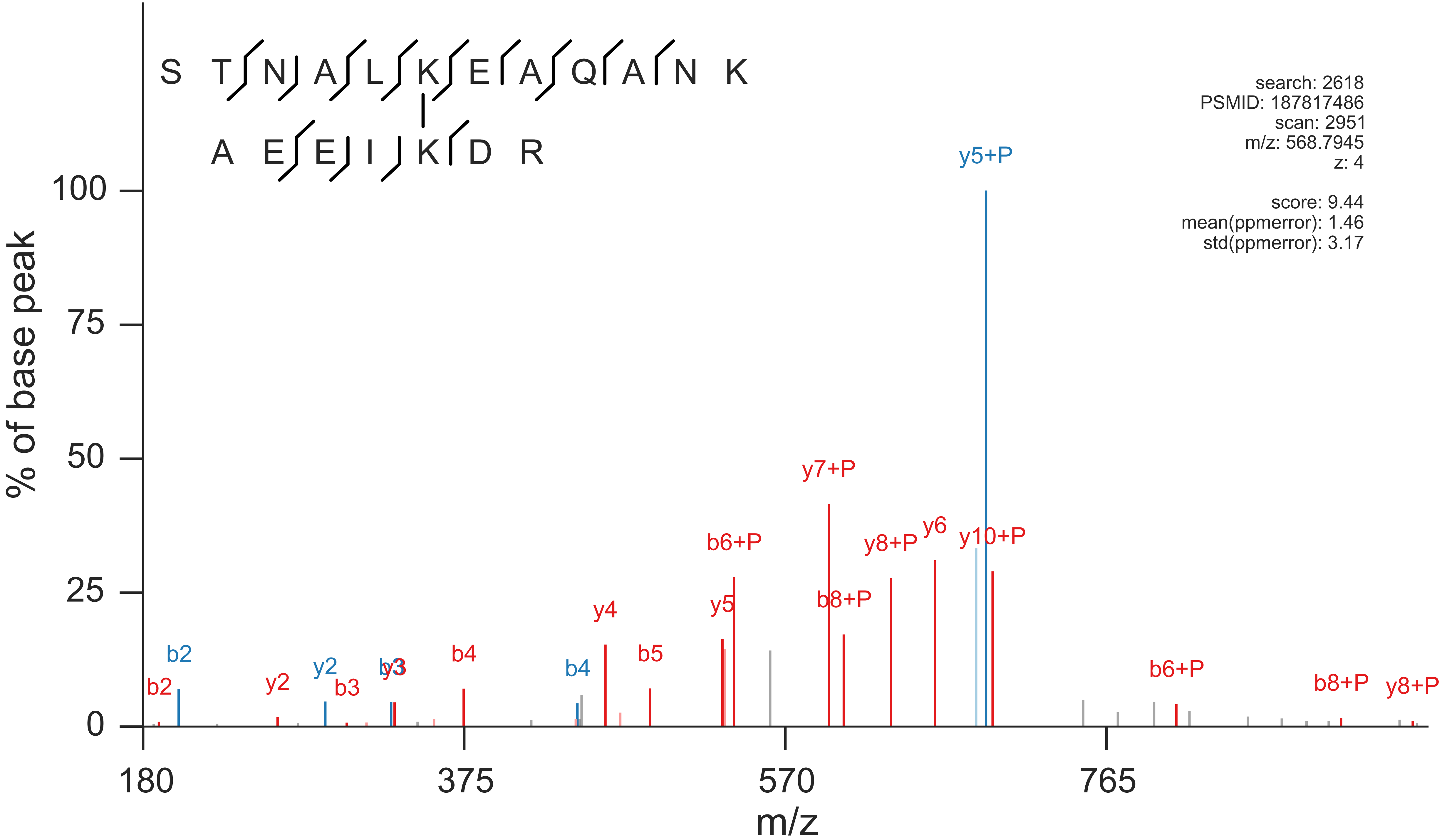

S T N A L K E A Q A N K
A E E I K D R
search: 2618
PSMID: 187817486
scan: 2951
m/z: 568.7945
z: 4
score: 9.44
mean(ppmerror): 1.46
std(ppmerror): 3.17
% of base peak
100
75
50
25
0
180
375
570
765
m/z
y5+P
y7+P
b6+P
y8+P
y6
y10+P
y4
y5
b8+P
b2
y2
b3
y3
b4
b5
b6+P
b8+P
y8+P

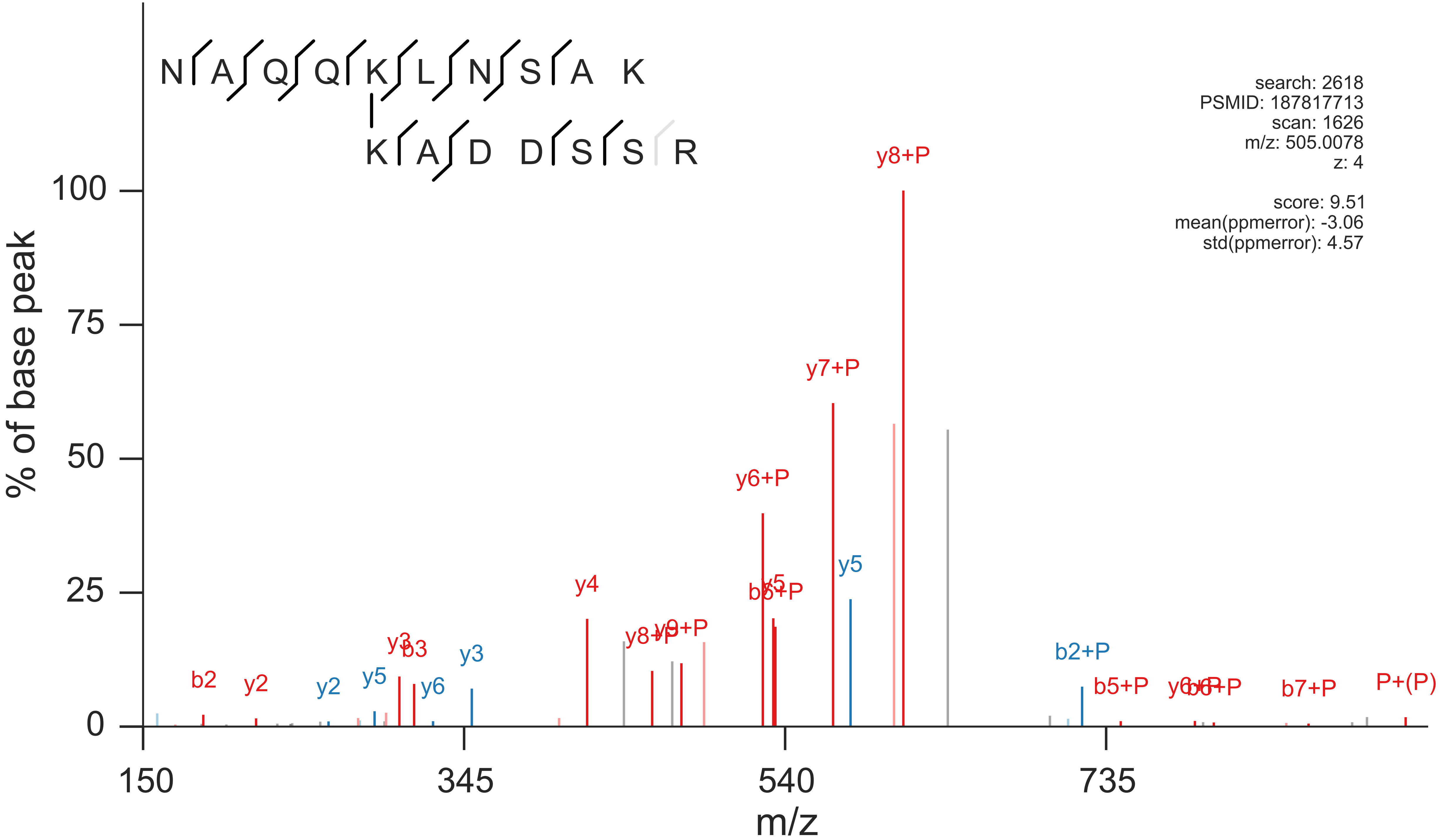

N A Q Q K L N S A K
K A D D S S R
search: 2618
PSMID: 187817713
scan: 1626
m/z: 505.0078
z: 4
score: 9.51
mean(ppmerror): -3.06
std(ppmerror): 4.57
% of base peak
m/z
y8+P
y7+P
y6+P
y5
y4
b2
y2
y3
b3
b2+P
b5+P
b7+P
P+(P)

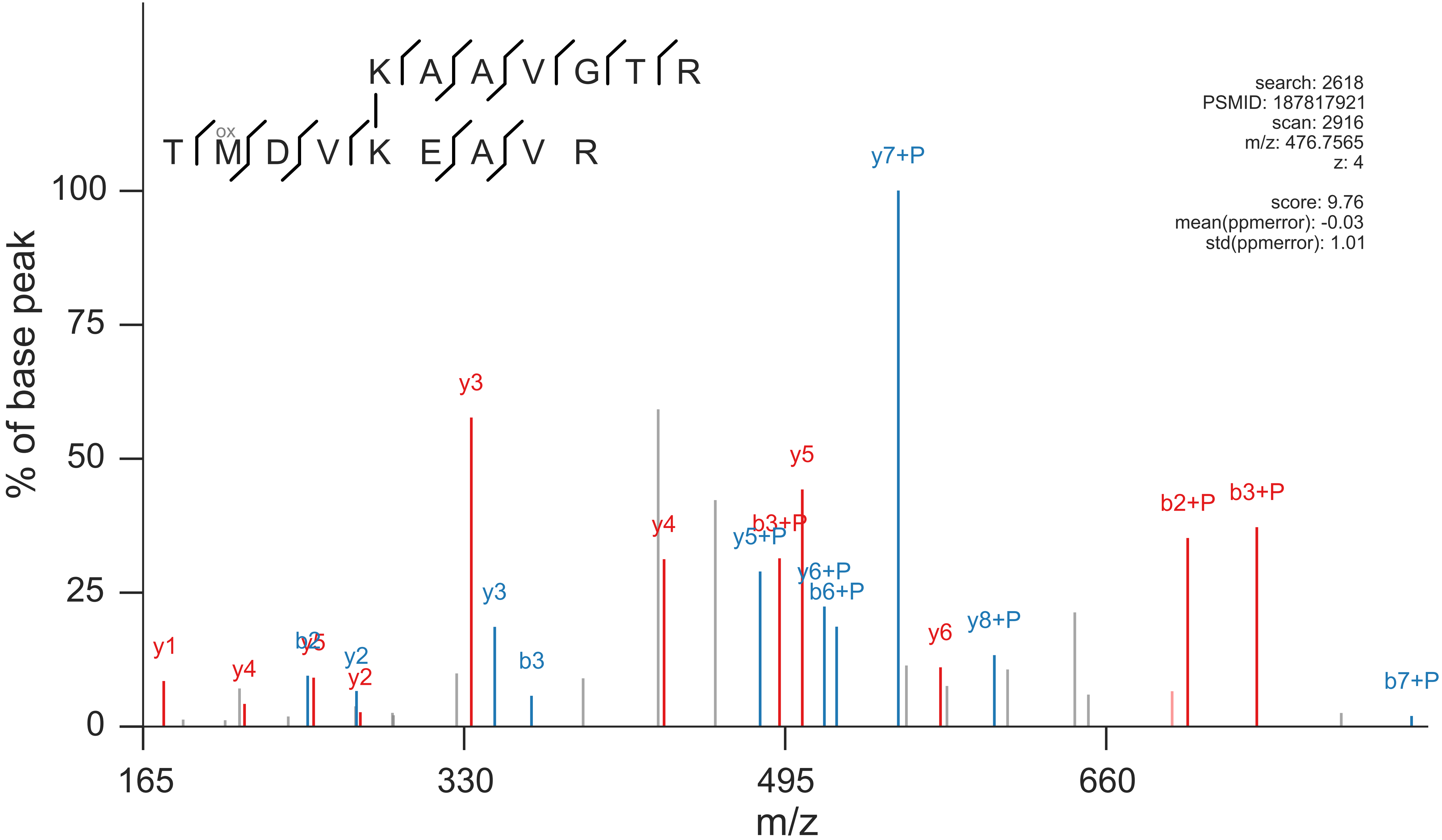
K A A V G T R
T M D V K E A V R
ox
search: 2618
PSMID: 187817921
scan: 2916
m/z: 476.7565
z: 4
score: 9.76
mean(ppmerror): -0.03
std(ppmerror): 1.01
% of base peak
100
75
50
25
0
165
330
495
660
m/z
y1
y4
b2
y5
y2
y2
y3
y3
b3
y4
y5+P
b3+P
y5
y6+P
b6+P
y7+P
y6
y8+P
b2+P
b3+P
b7+P

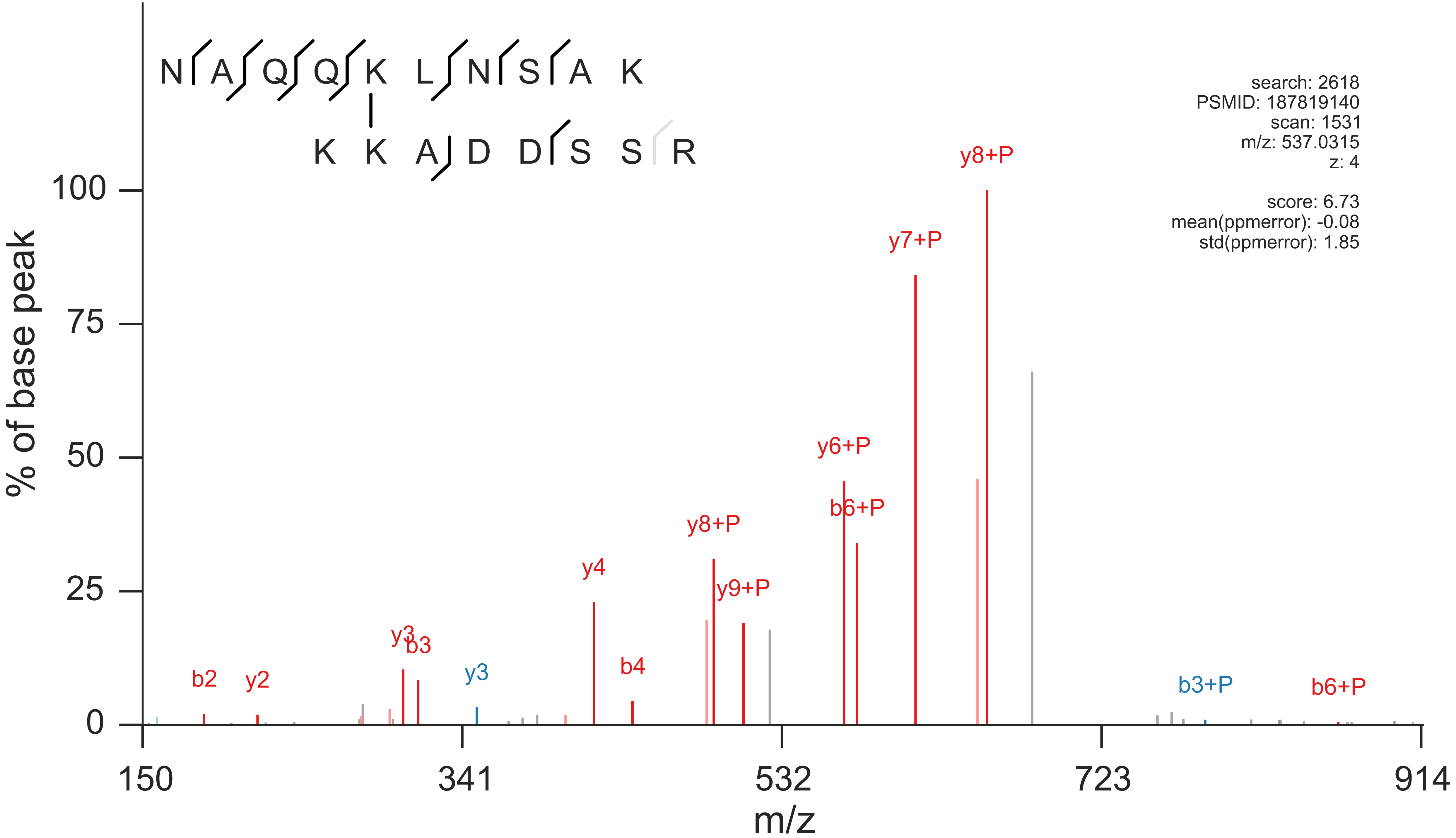

N A Q Q K L N S A K
K K A D D S S R
search: 2618
PSMID: 187819140
scan: 1531
m/z: 537.0315
z: 4
score: 6.73
mean(ppmerror): -0.08
std(ppmerror): 1.85
% of base peak
m/z
0
25
50
75
100
150
341
532
723
914
b2
y2
y3
b3
y3
y4
b4
y8+P
y9+P
y6+P
b6+P
y7+P
y8+P
b3+P
b6+P

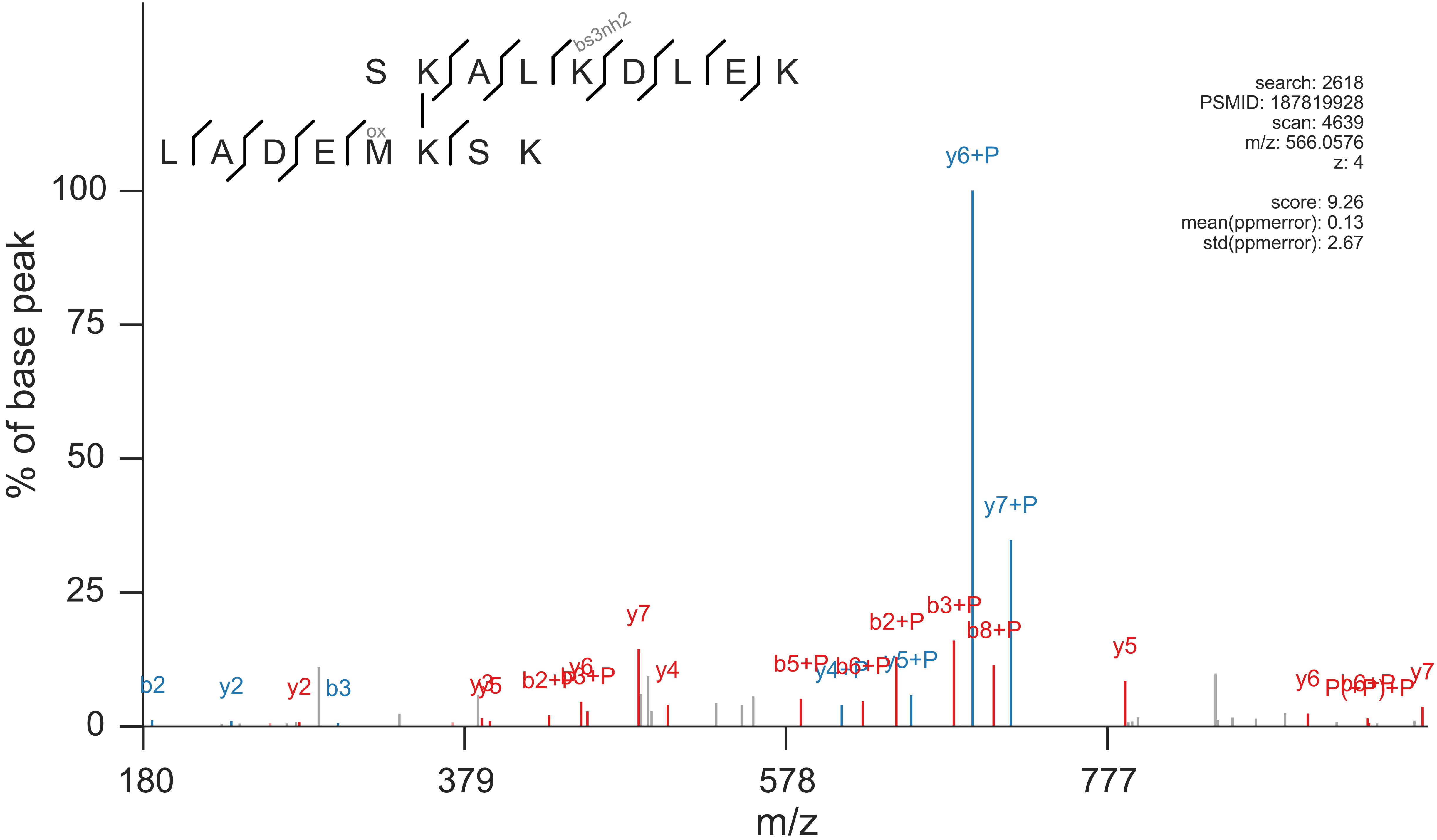

S K A L K D L E K
bs3nh2
L A D E M K S K
ox
search: 2618
PSMID: 187819928
scan: 4639
m/z: 566.0576
z: 4
score: 9.26
mean(ppmerror): 0.13
std(ppmerror): 2.67
% of base peak
m/z
0
25
50
75
100
180
379
578
777
y6+P
y7+P
b3+P
b2+P
b8+P
y7
y5
y4
b5+P
y5+P
b2
y2
y2
b3
y6
y7

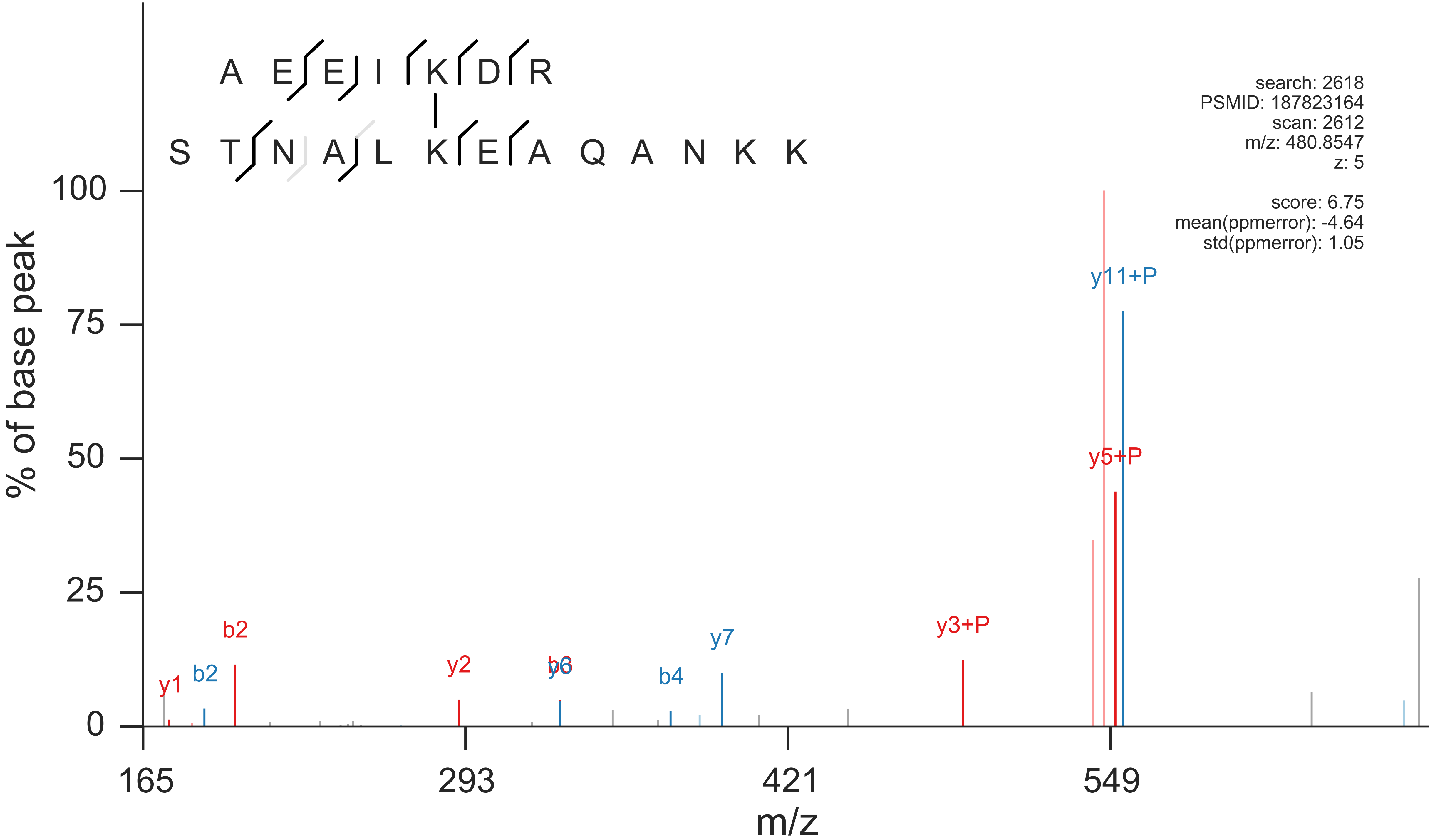

A E E I K D R
S T N A L K E A Q A N K K
search: 2618
PSMID: 187823164
scan: 2612
m/z: 480.8547
z: 5
score: 6.75
mean(ppmerror): -4.64
std(ppmerror): 1.05
% of base peak
100
75
50
25
0
y11+P
y5+P
y3+P
y7
b4
y2
b2
b2
y1
165
293
421
549
m/z

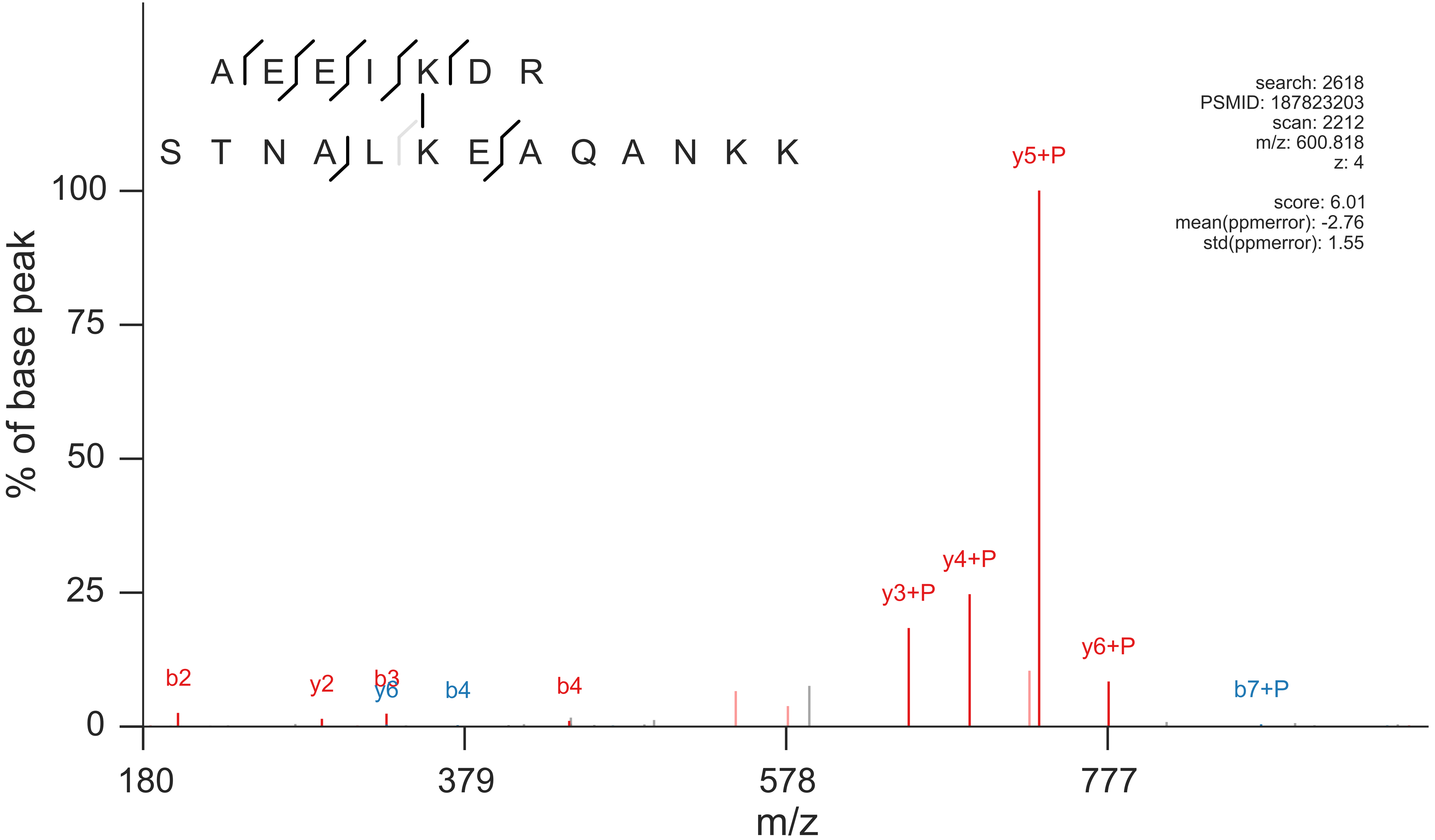

A E E I K D R
S T N A L K E A Q A N K K
search: 2618
PSMID: 187823203
scan: 2212
m/z: 600.818
z: 4
score: 6.01
mean(ppmerror): -2.76
std(ppmerror): 1.55
% of base peak
100
75
50
25
0
y5+P
y4+P
y3+P
y6+P
b2
y2
b3
y6
b4
b4
b7+P
180
379
578
777
m/z

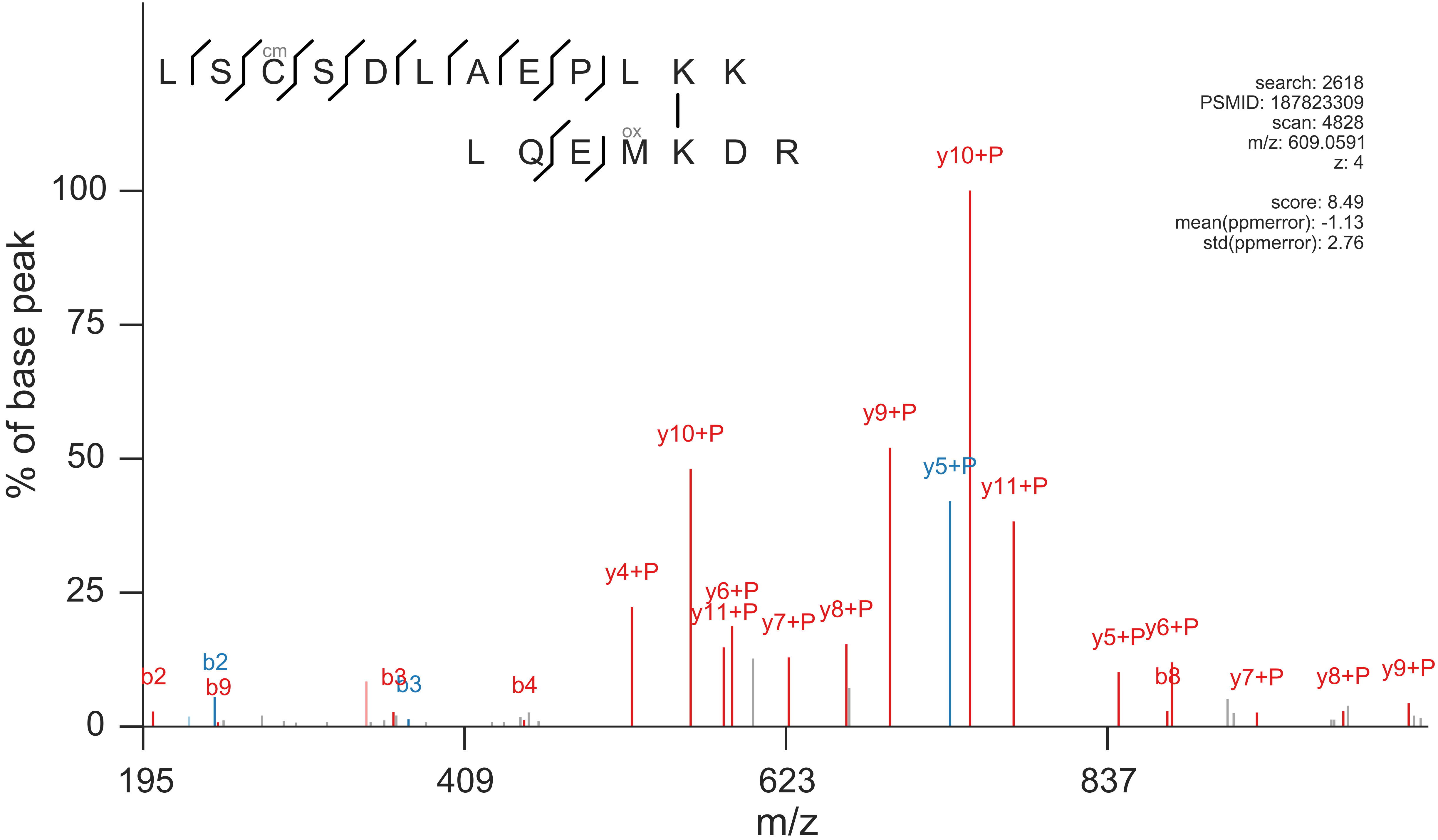

L S C S D L A E P L K K
cm
L Q E M K D R
ox
search: 2618
PSMID: 187823309
scan: 4828
m/z: 609.0591
z: 4
score: 8.49
mean(ppmerror): -1.13
std(ppmerror): 2.76
% of base peak
m/z
0
25
50
75
100
195
409
623
837
b2
b9
b3
b4
y4+P
y10+P
y6+P
y11+P
y7+P
y8+P
y9+P
y5+P
y10+P
y11+P
y5+P
y6+P
b8
y7+P
y8+P
y9+P

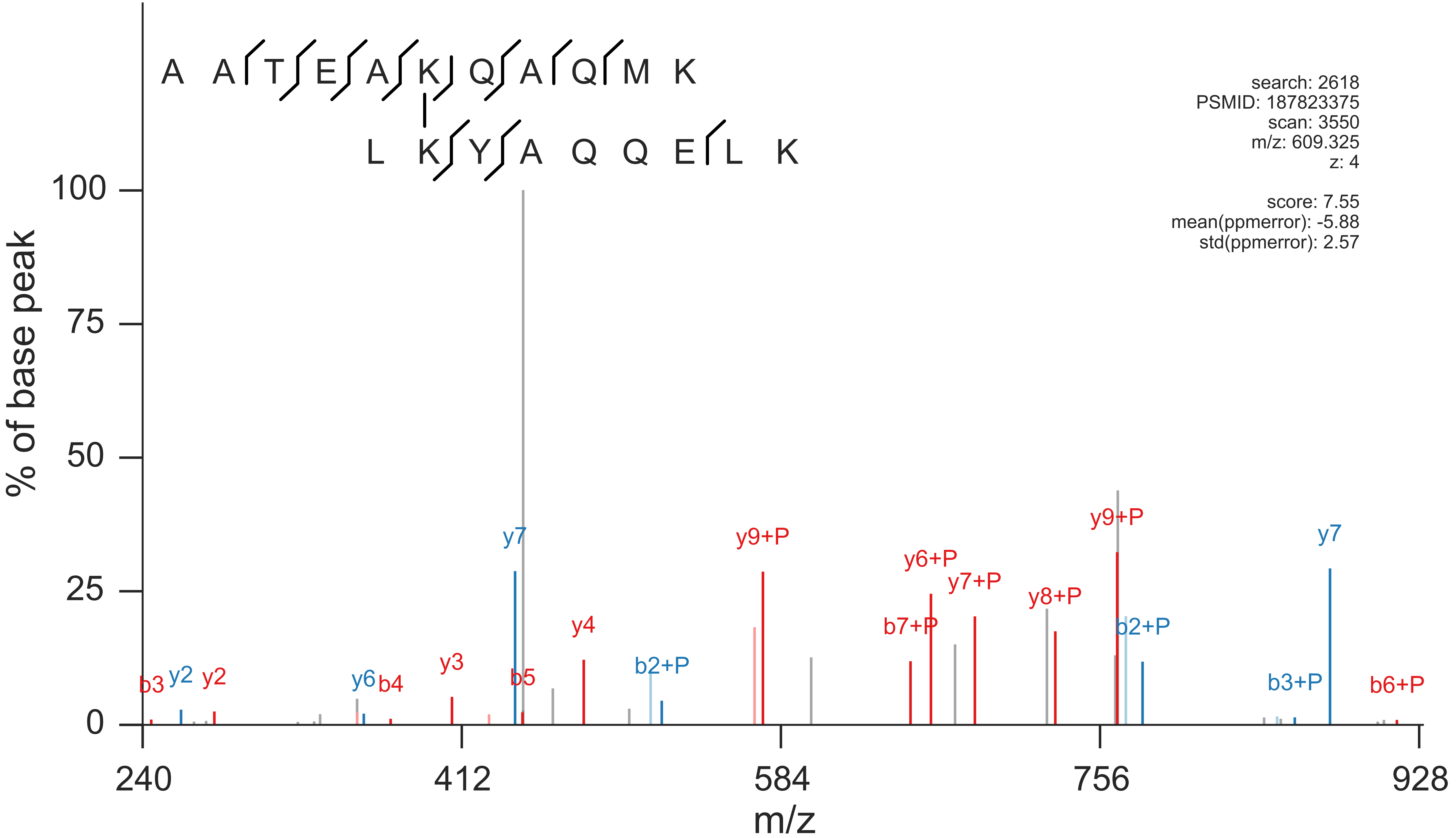

A A T E A K Q A Q M K
L K Y A Q Q E L K
search: 2618
PSMID: 187823375
scan: 3550
m/z: 609.325
z: 4
score: 7.55
mean(ppmerror): -5.88
std(ppmerror): 2.57
% of base peak
m/z
b3
y2
y2
y6
b4
y3
y7
b5
y4
b2+P
y9+P
b7+P
y6+P
y7+P
y8+P
y9+P
b2+P
b3+P
y7
b6+P

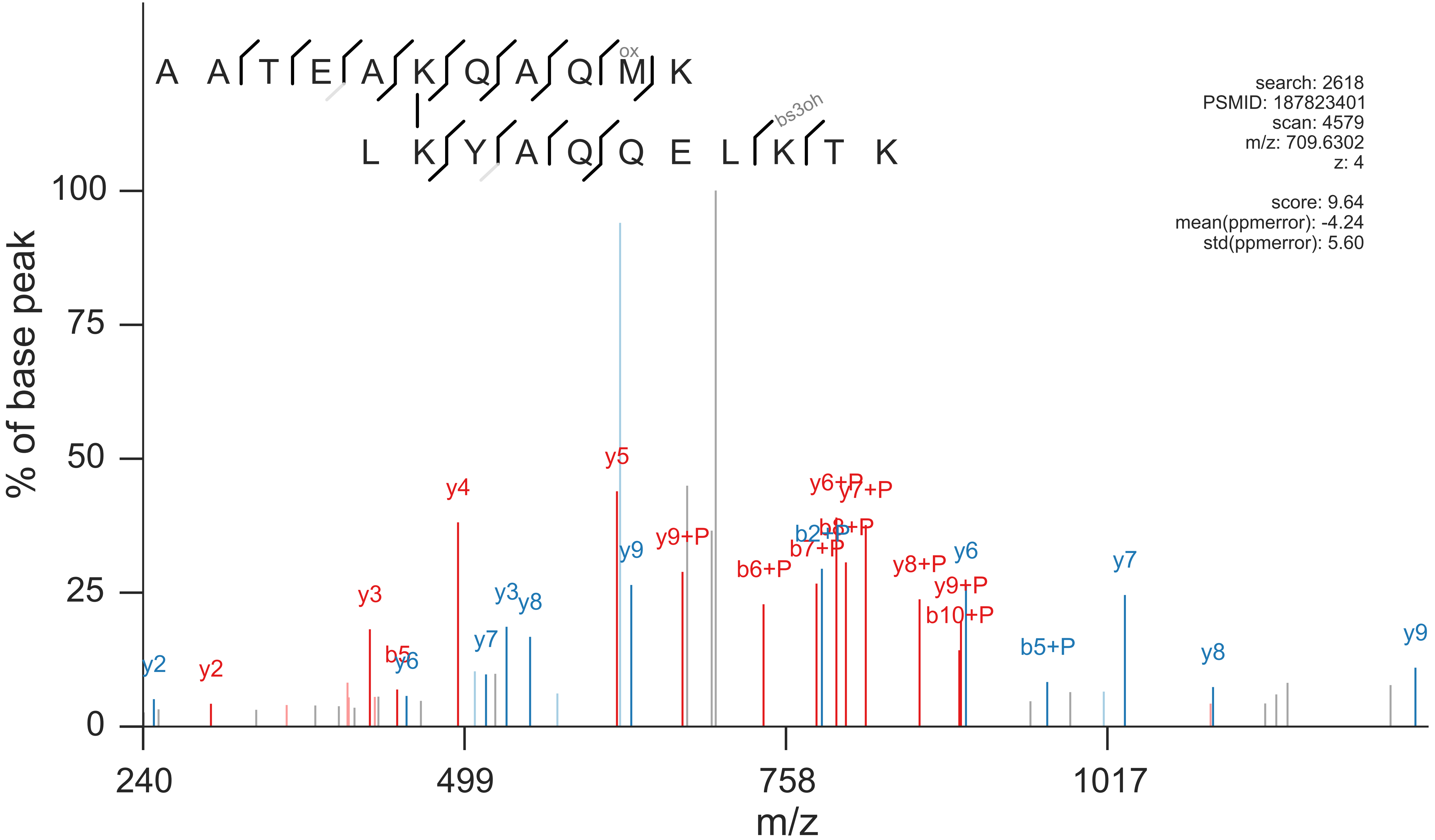
A A T E A K Q A Q M K
ox
L K Y A Q Q E L K T K
bs3oh
search: 2618
PSMID: 187823401
scan: 4579
m/z: 709.6302
z: 4
score: 9.64
mean(ppmerror): -4.24
std(ppmerror): 5.60
% of base peak
m/z
0
25
50
75
100
240
499
758
1017

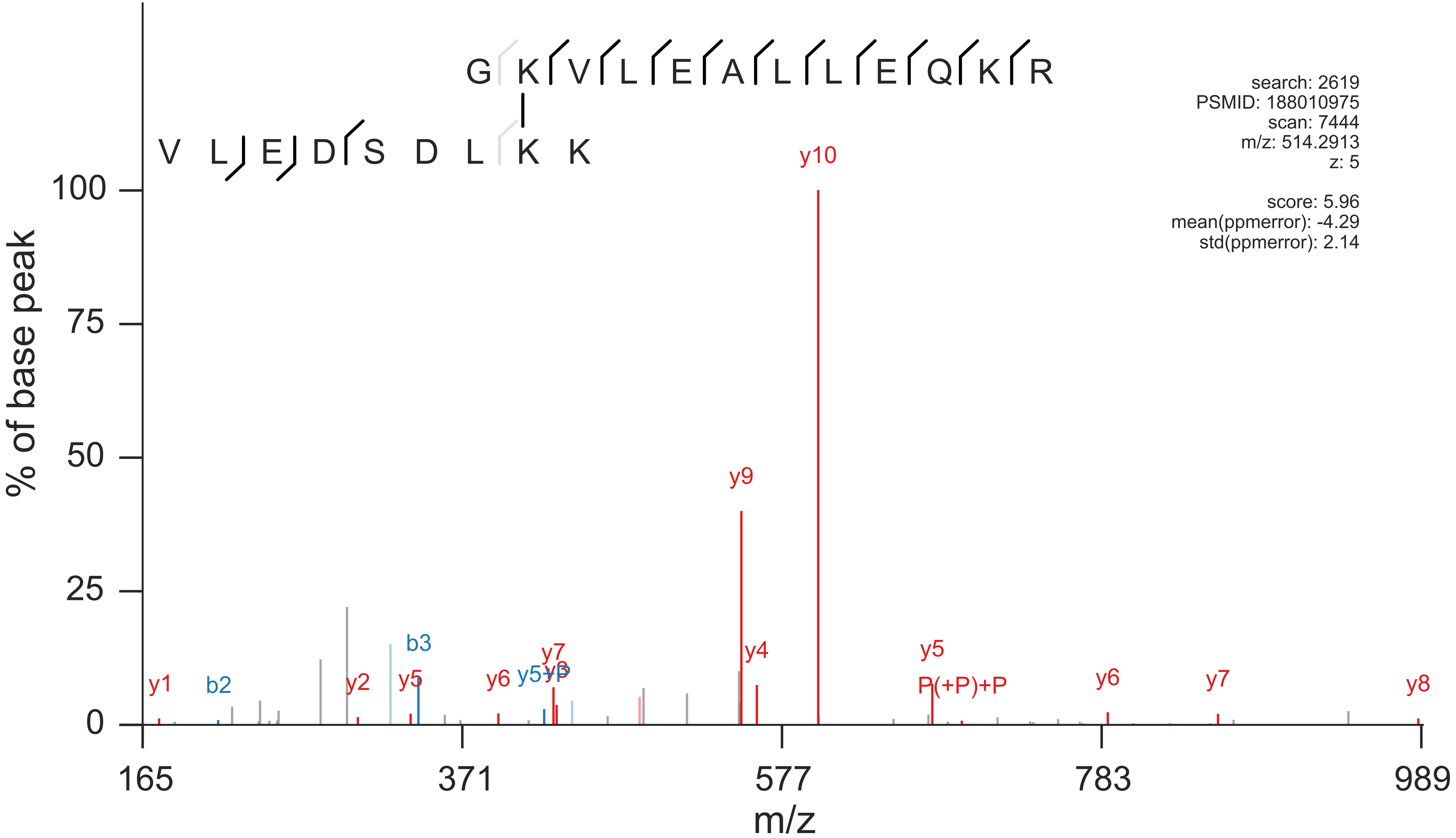

G K V L E A L L E Q K R
V L E D S D L K K
search: 2619
PSMID: 188010975
scan: 7444
m/z: 514.2913
z: 5
score: 5.96
mean(ppmerror): -4.29
std(ppmerror): 2.14
% of base peak
m/z
y10
y9
y4
y5
P(+P)+P
y6
y7
y8
y1
b2
y2
y5
b3
y6
y5+P
y7

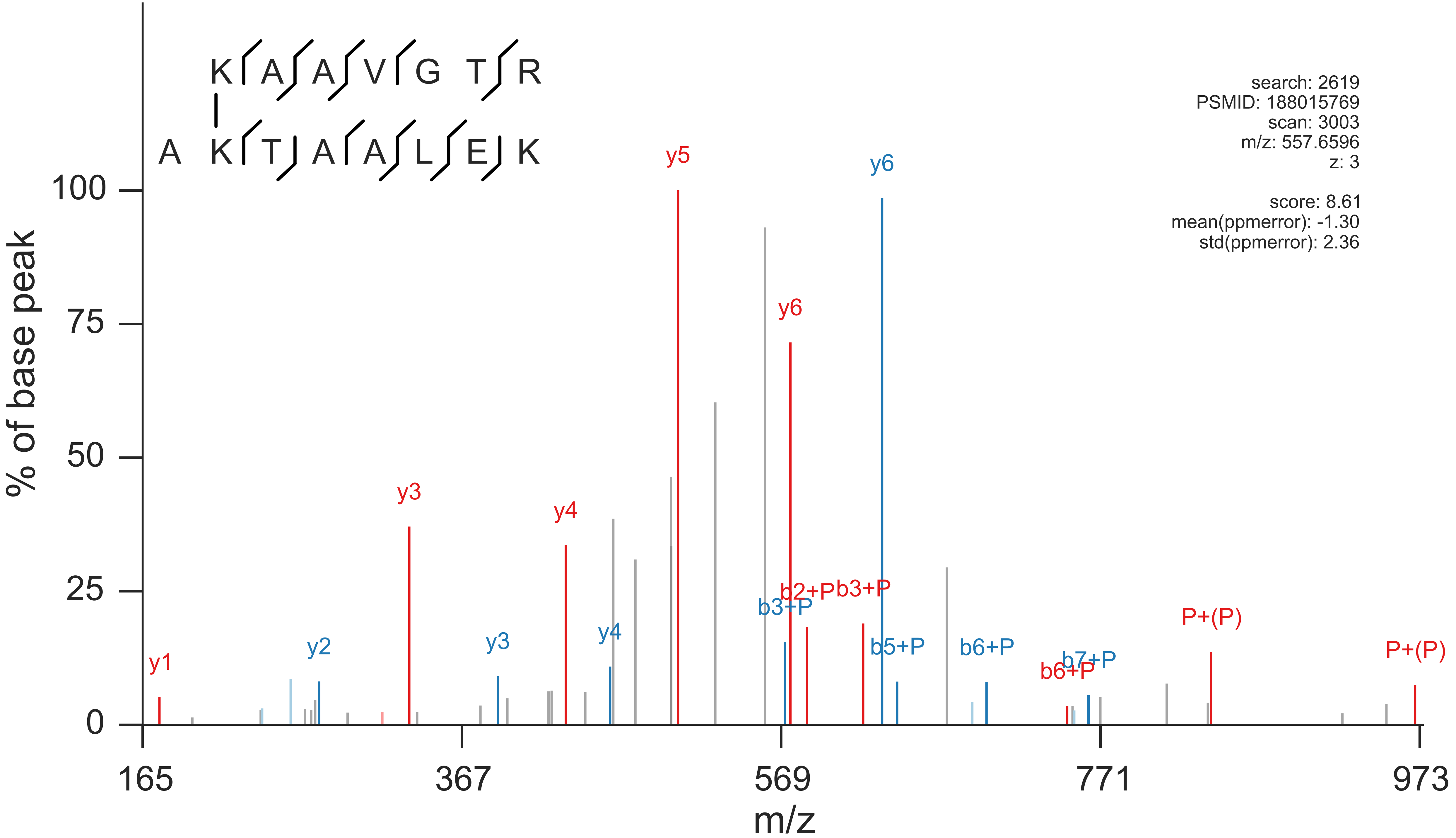

K A A V G T R
A K T A A L E K
search: 2619
PSMID: 188015769
scan: 3003
m/z: 557.6596
z: 3
score: 8.61
mean(ppmerror): -1.30
std(ppmerror): 2.36
% of base peak
m/z
100
75
50
25
0
165
367
569
771
973
y1
y2
y3
y3
y4
y4
y5
y6
y6
b3+P
b2+P
b3+P
b5+P
b6+P
b6+P
b7+P
P+(P)
P+(P)

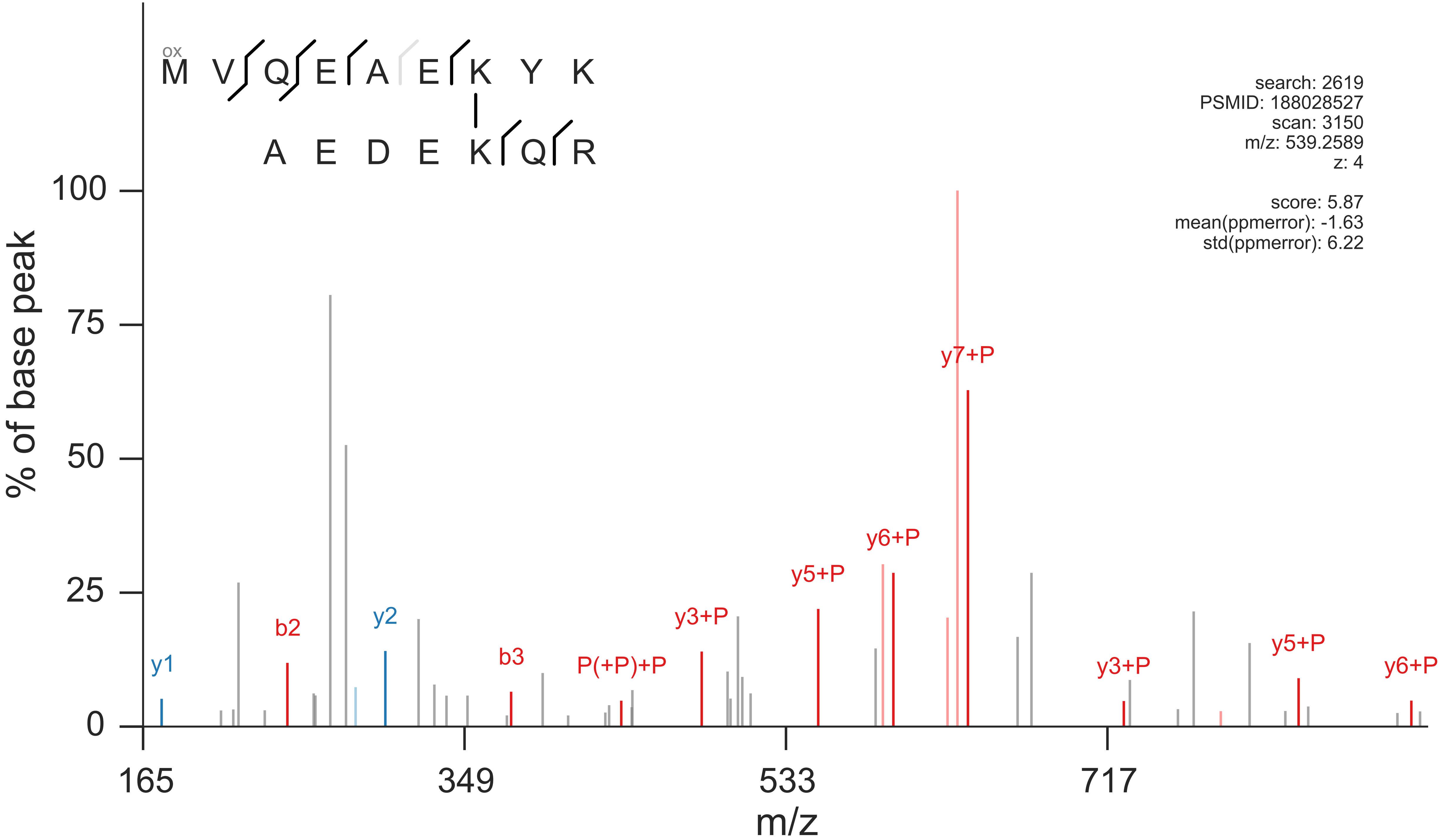

ox
M V Q E A E K Y K
A E D E K Q R
search: 2619
PSMID: 188028527
scan: 3150
m/z: 539.2589
z: 4
score: 5.87
mean(ppmerror): -1.63
std(ppmerror): 6.22
% of base peak
100
75
50
25
0
y1
b2
y2
b3
P(+P)+P
y3+P
y5+P
y6+P
y7+P
y3+P
y5+P
y6+P
165
349
533
717
m/z

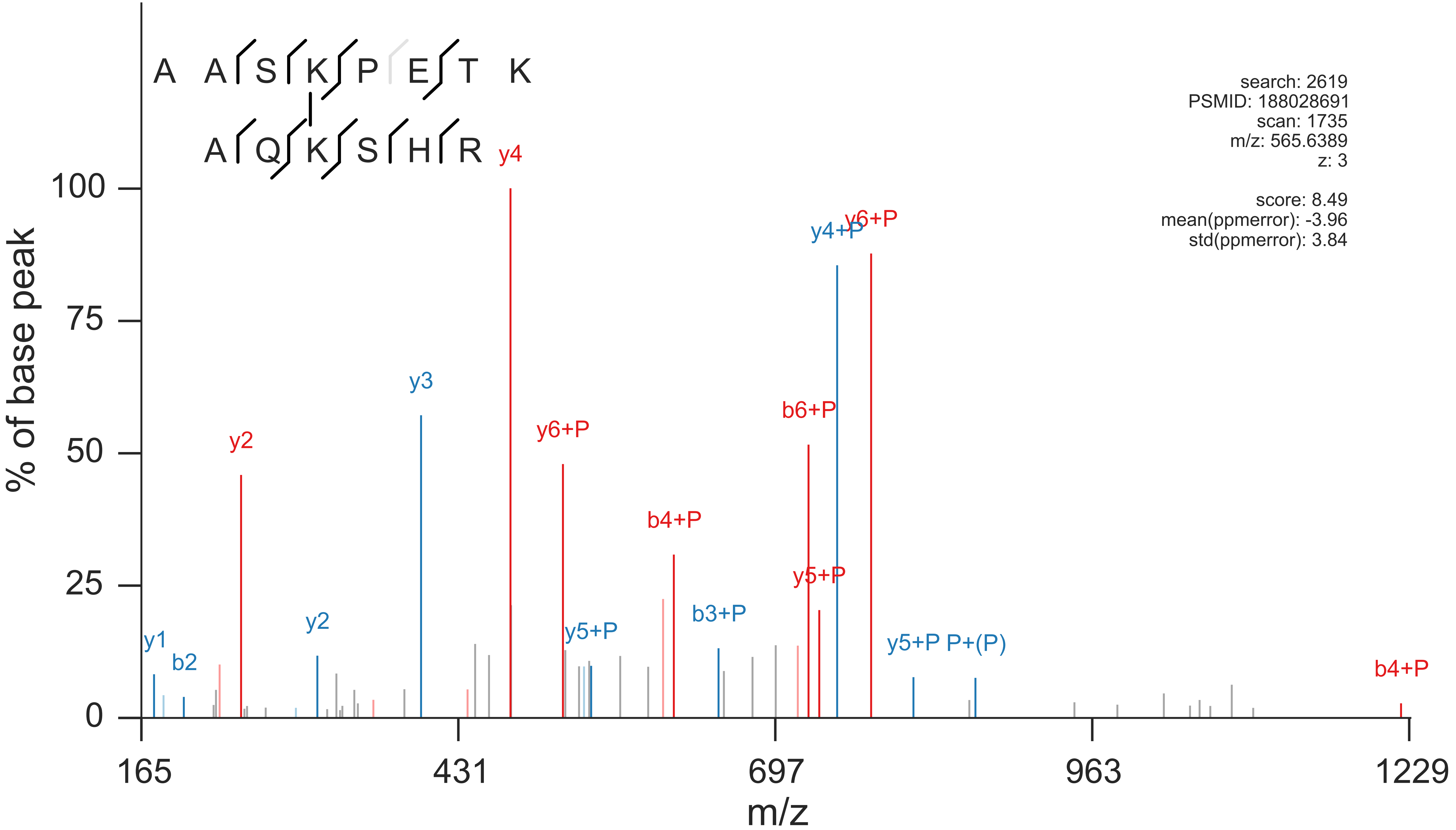
A A S K P E T K
A Q K S H R
search: 2619
PSMID: 188028691
scan: 1735
m/z: 565.6389
z: 3
score: 8.49
mean(ppmerror): -3.96
std(ppmerror): 3.84
% of base peak
m/z
100
75
50
25
0
165
431
697
963
1229
y1
b2
y2
y2
y3
y4
y6+P
y5+P
b4+P
b3+P
b6+P
y5+P
y4+P
y6+P
y5+P P+(P)
b4+P

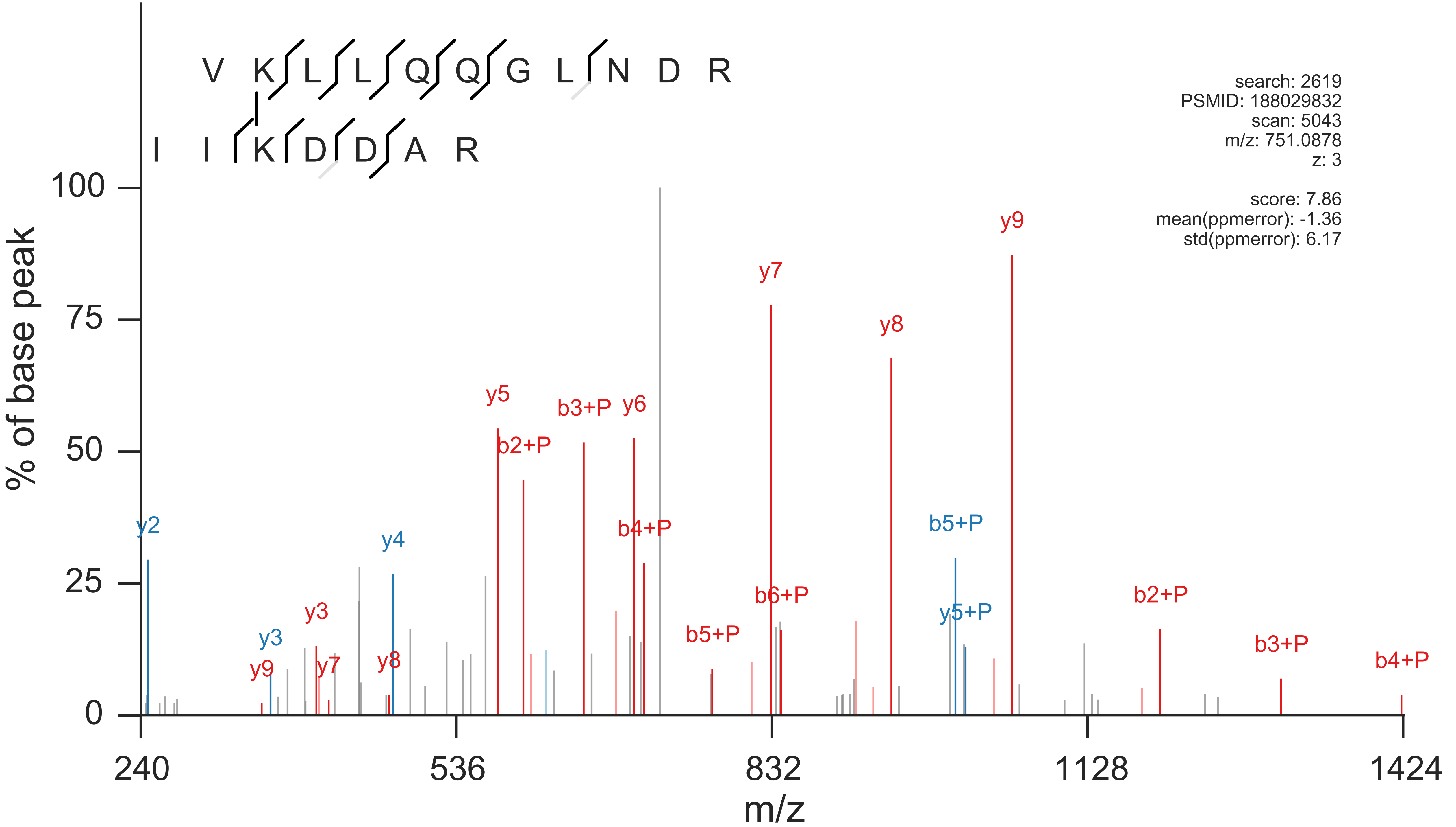

V K L L Q Q G L N D R
I I K D D A R
search: 2619
PSMID: 188029832
scan: 5043
m/z: 751.0878
z: 3
score: 7.86
mean(ppmerror): -1.36
std(ppmerror): 6.17
% of base peak
m/z
0
25
50
75
100
240
536
832
1128
1424
y2
y3
y4
y9
y3
y7
y8
y5
b2+P
b3+P
y6
b4+P
b5+P
b6+P
y7
y8
y9
b5+P
y5+P
b2+P
b3+P
b4+P

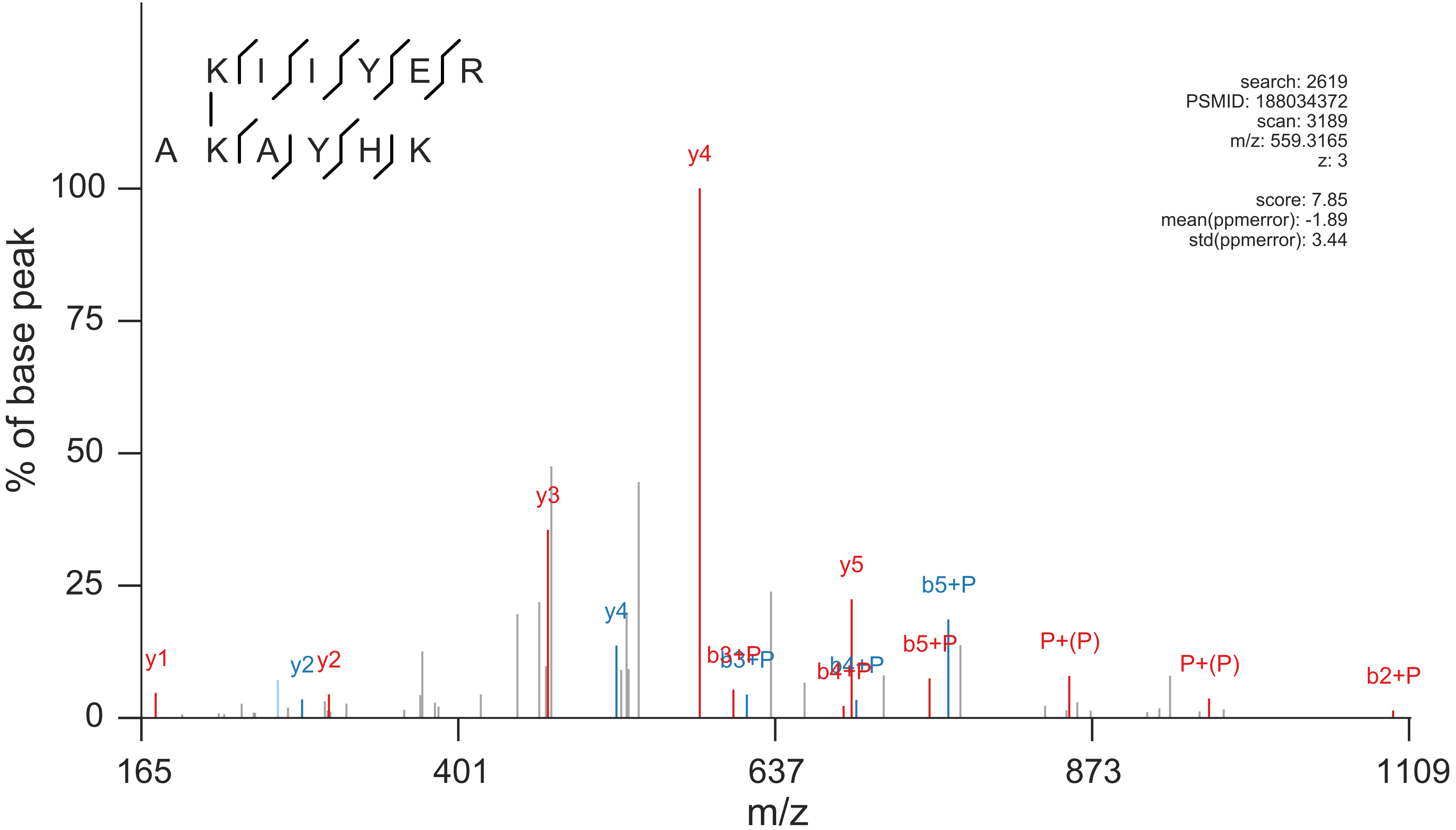
K I I Y E R
A K A Y H K
search: 2619
PSMID: 188034372
scan: 3189
m/z: 559.3165
z: 3
score: 7.85
mean(ppmerror): -1.89
std(ppmerror): 3.44
% of base peak
0
25
50
75
100
165
401
637
873
1109
m/z
y1
y2
y2
y3
y4
y4
b3+P
b3+P
b4+P
b4+P
y5
b5+P
b5+P
P+(P)
P+(P)
b2+P

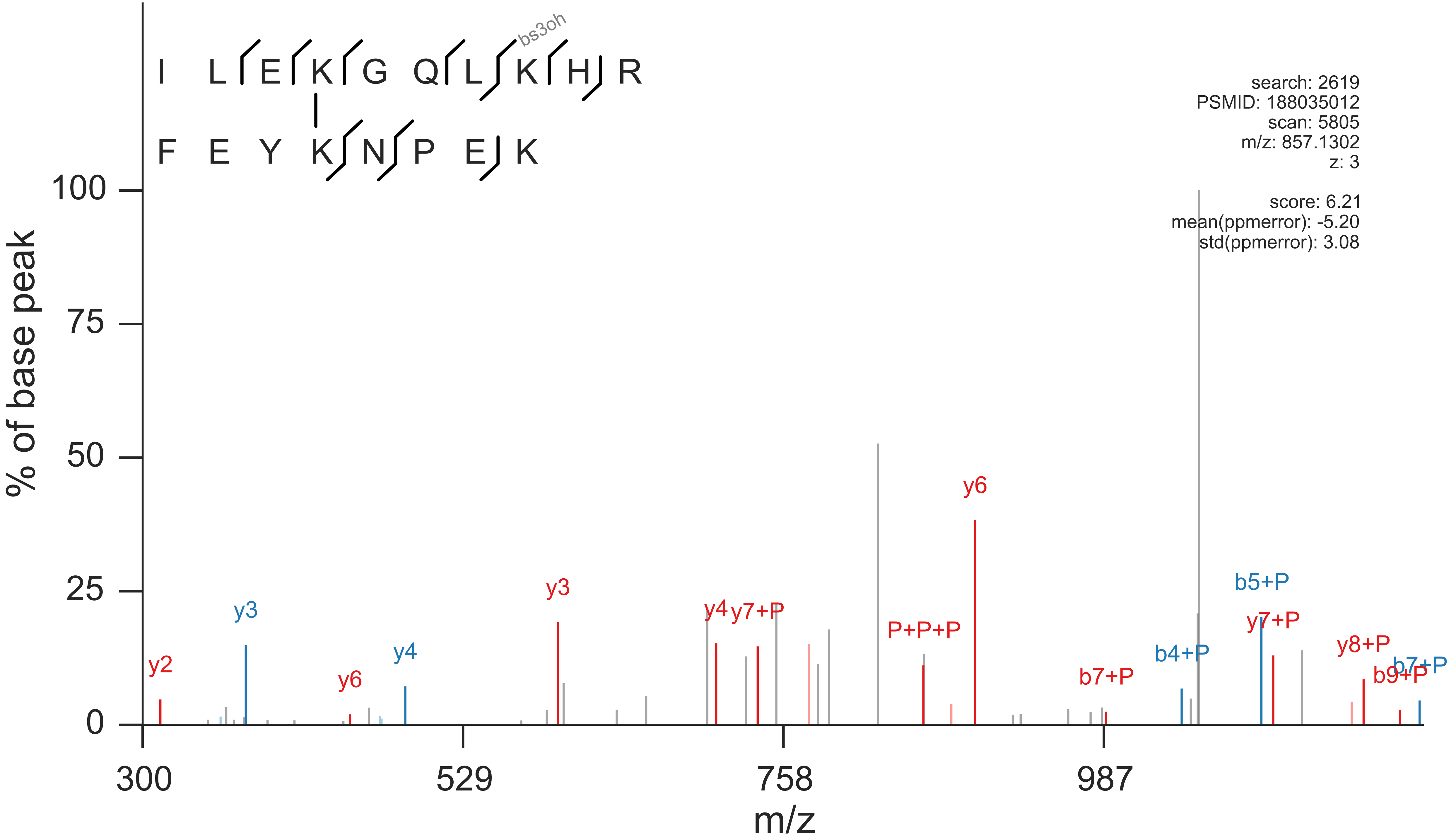

bs3oh
I L E K G Q L K H R
F E Y K N P E K
search: 2619
PSMID: 188035012
scan: 5805
m/z: 857.1302
z: 3
score: 6.21
mean(ppmerror): -5.20
std(ppmerror): 3.08
% of base peak
100
75
50
25
0
y2
y3
y6
y4
y3
y4
y7+P
P+P+P
y6
b7+P
b4+P
b5+P
y7+P
y8+P
b9+P
b7+P
300
529
758
987
m/z

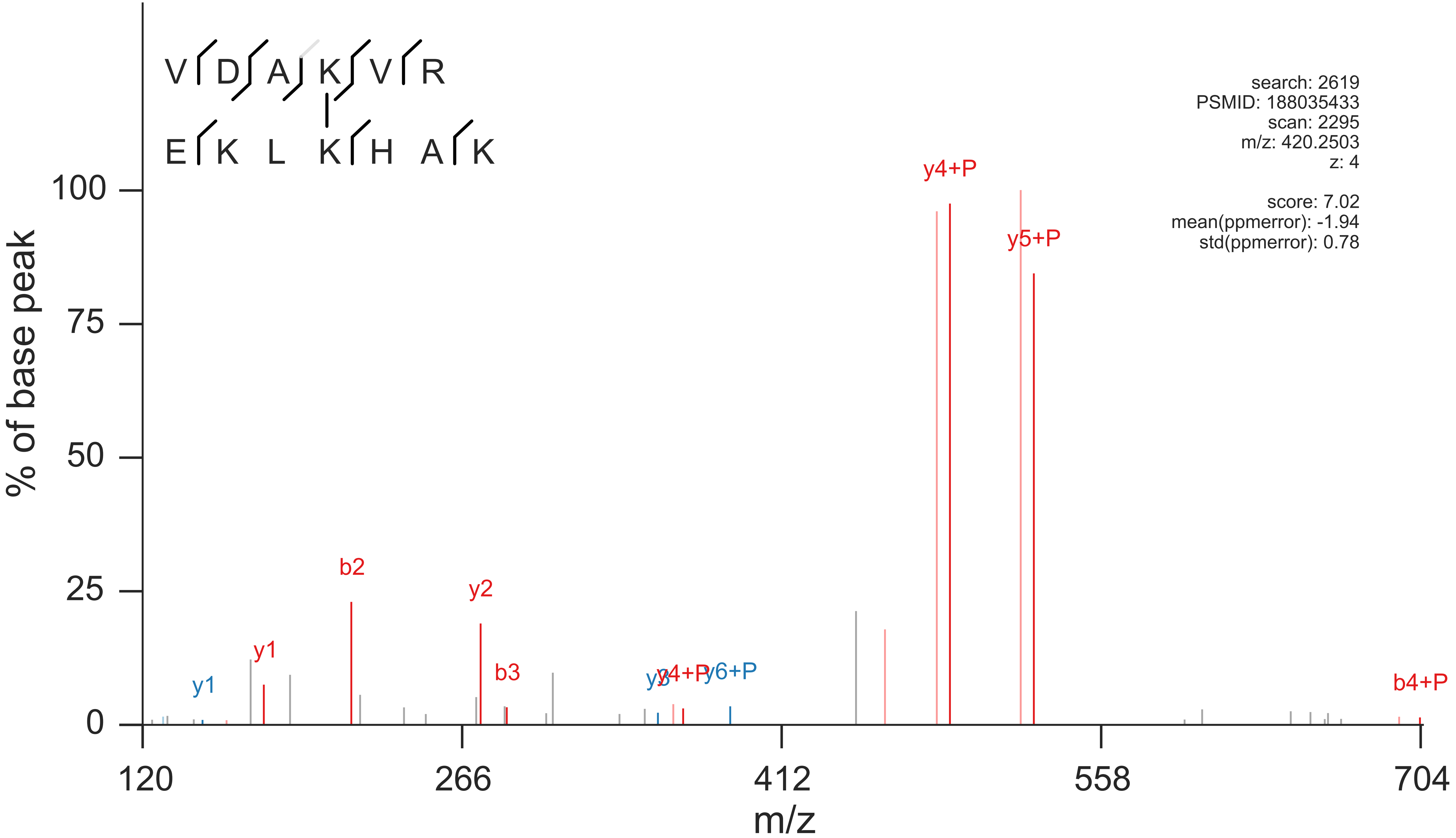
V D A K V R
E K L K H A K
search: 2619
PSMID: 188035433
scan: 2295
m/z: 420.2503
z: 4
score: 7.02
mean(ppmerror): -1.94
std(ppmerror): 0.78
y4+P
y5+P
b2
y2
y1
y1
b3
y3
y4+P
y6+P
b4+P
% of base peak
100
75
50
25
0
120
266
412
558
704
m/z

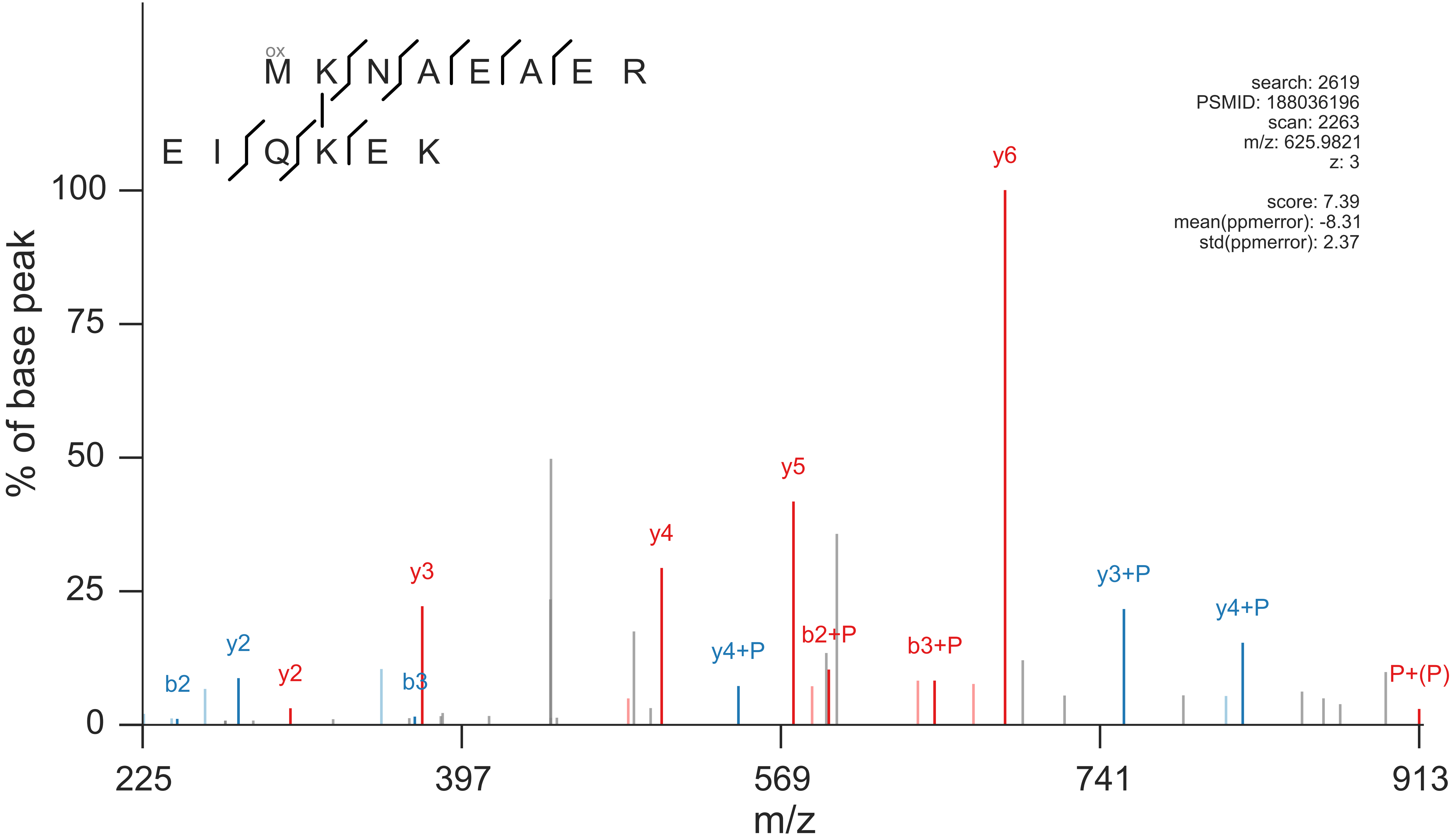

ox
M K N A E A E R
E I Q K E K
search: 2619
PSMID: 188036196
scan: 2263
m/z: 625.9821
z: 3
score: 7.39
mean(ppmerror): -8.31
std(ppmerror): 2.37
% of base peak
100
75
50
25
0
225
397
569
741
913
m/z
b2
y2
y2
b3
y3
y4
y4+P
y5
b2+P
b3+P
y6
y3+P
y4+P
P+(P)

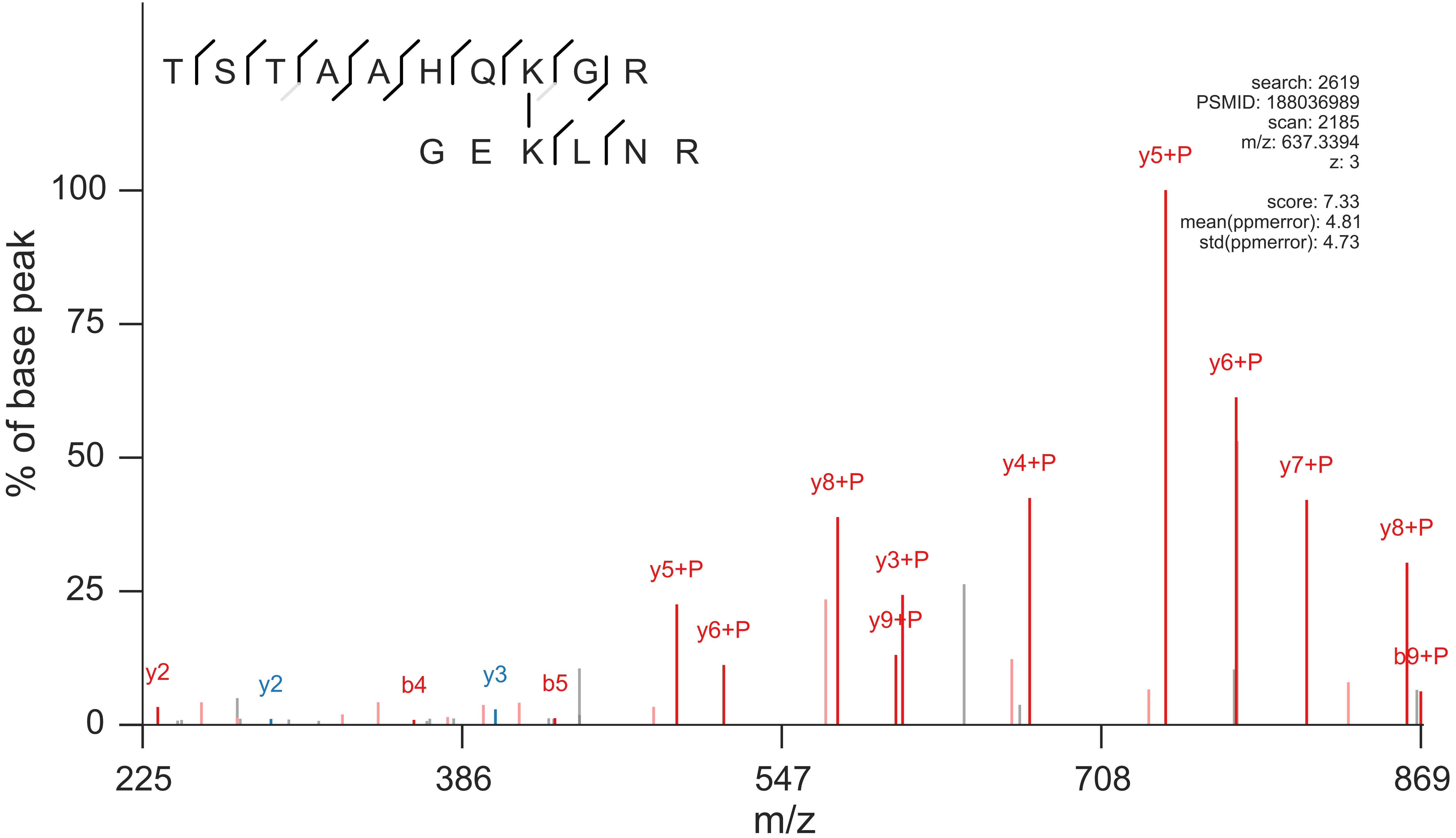

T S T A A H Q K G R
G E K L N R
search: 2619
PSMID: 188036989
scan: 2185
m/z: 637.3394
z: 3
score: 7.33
mean(ppmerror): 4.81
std(ppmerror): 4.73
% of base peak
m/z
100
75
50
25
0
225
386
547
708
869
y2
y2
b4
y3
b5
y5+P
y6+P
y8+P
y3+P
y9+P
y4+P
y5+P
y6+P
y7+P
y8+P
b9+P

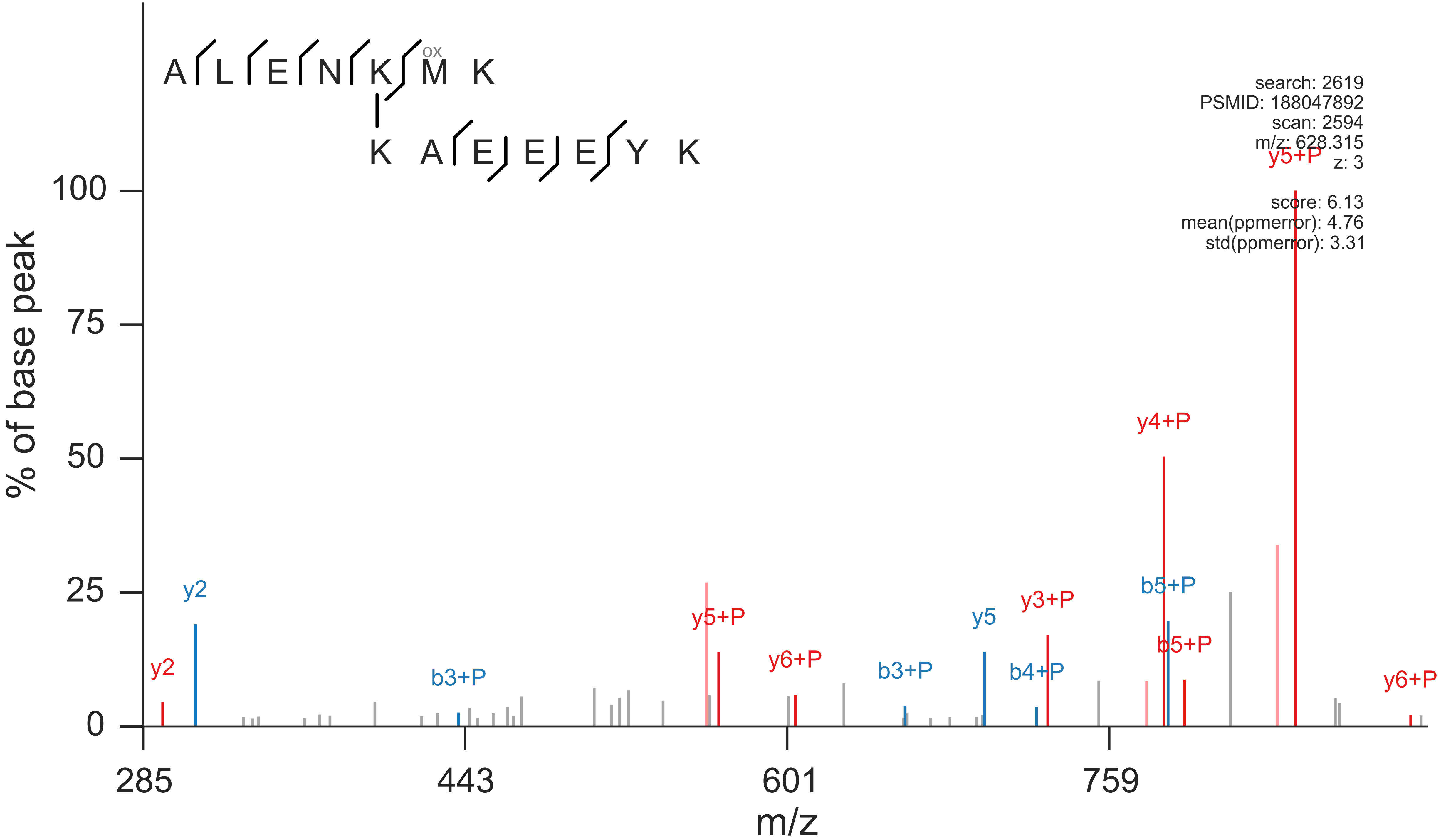

A L E N K M K
ox
K A E E E Y K
search: 2619
PSMID: 188047892
scan: 2594
m/z: 628.315
z: 3
score: 6.13
mean(ppmerror): 4.76
std(ppmerror): 3.31
% of base peak
m/z
0
25
50
75
100
285
443
601
759
y2
y2
b3+P
y5+P
y6+P
b3+P
y5
b4+P
y3+P
b5+P
y4+P
b5+P
y5+P
y6+P

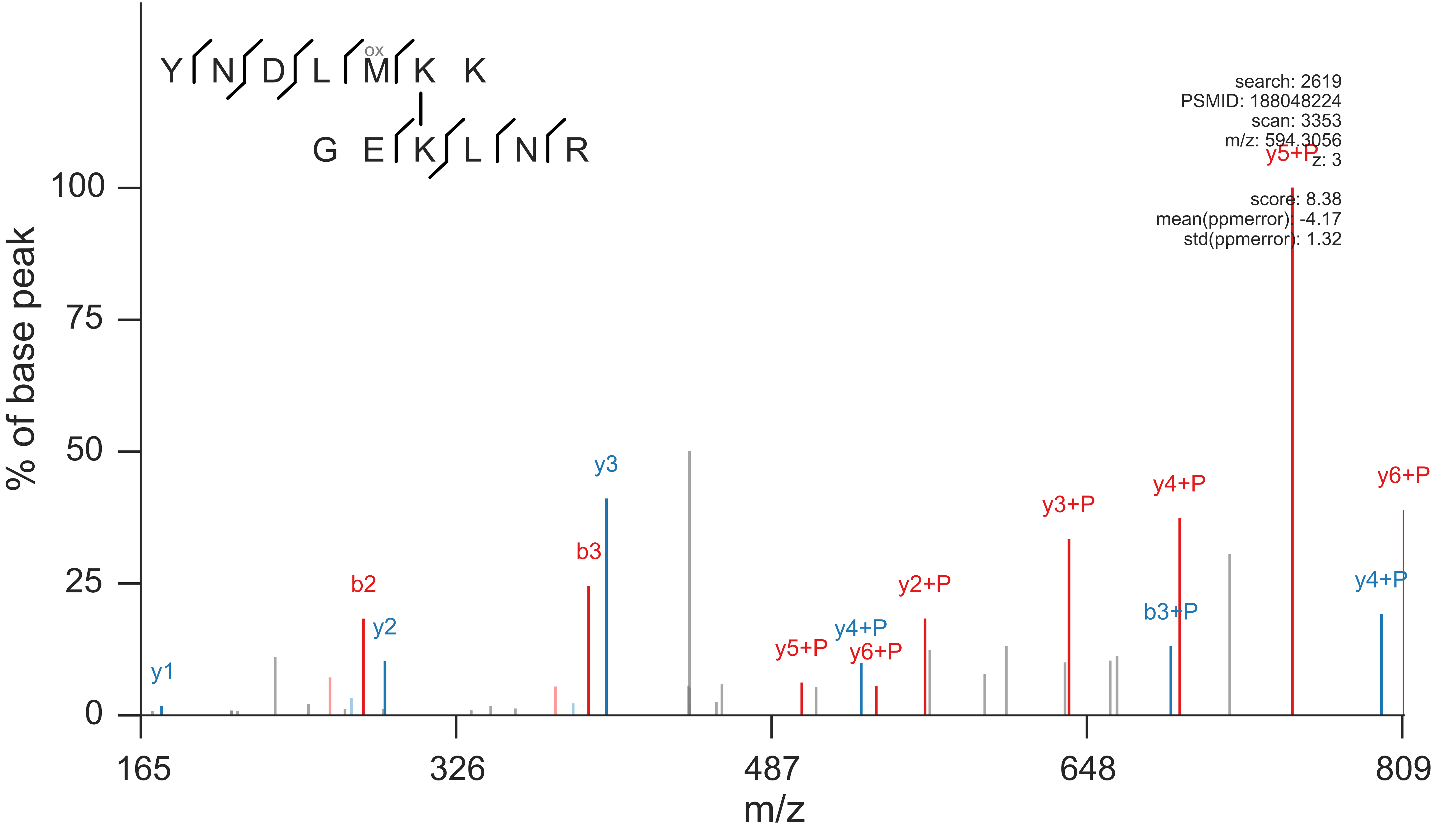
Y N D L M K K
ox
G E K L N R
search: 2619
PSMID: 188048224
scan: 3353
m/z: 594.3056
z: 3
score: 8.38
mean(ppmerror): -4.17
std(ppmerror): 1.32
% of base peak
100
75
50
25
0
165
326
487
648
809
m/z
y1
b2
y2
b3
y3
y5+P
y4+P
y6+P
y2+P
y3+P
y4+P
b3+P
y5+P
y4+P
y6+P

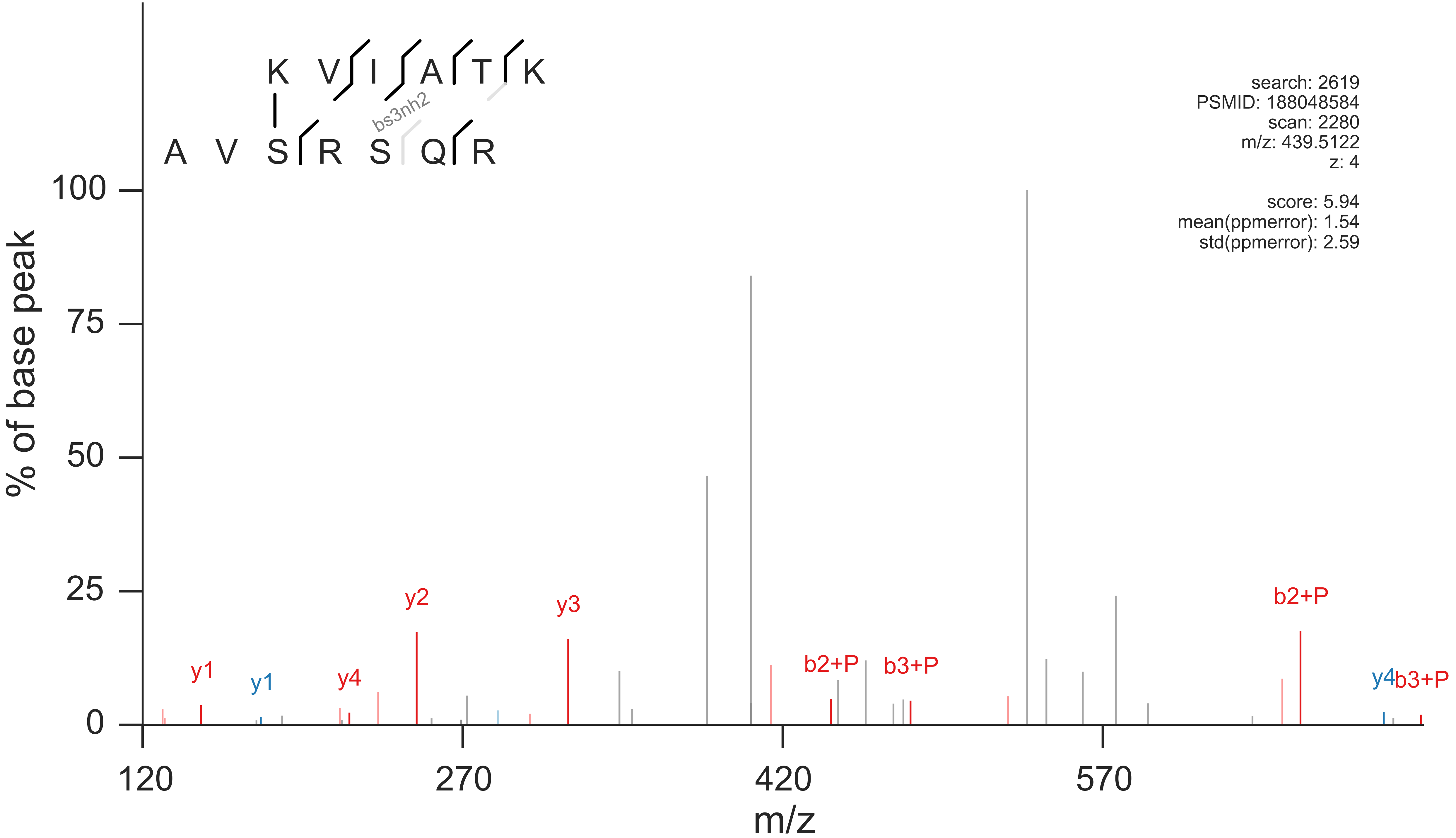

K V I A T K
A V S R S Q R
bs3nh2
search: 2619
PSMID: 188048584
scan: 2280
m/z: 439.5122
z: 4
score: 5.94
mean(ppmerror): 1.54
std(ppmerror): 2.59
% of base peak
100
75
50
25
0
y1
y1
y4
y2
y3
b2+P
b3+P
b2+P
y4
b3+P
120
270
420
570
m/z

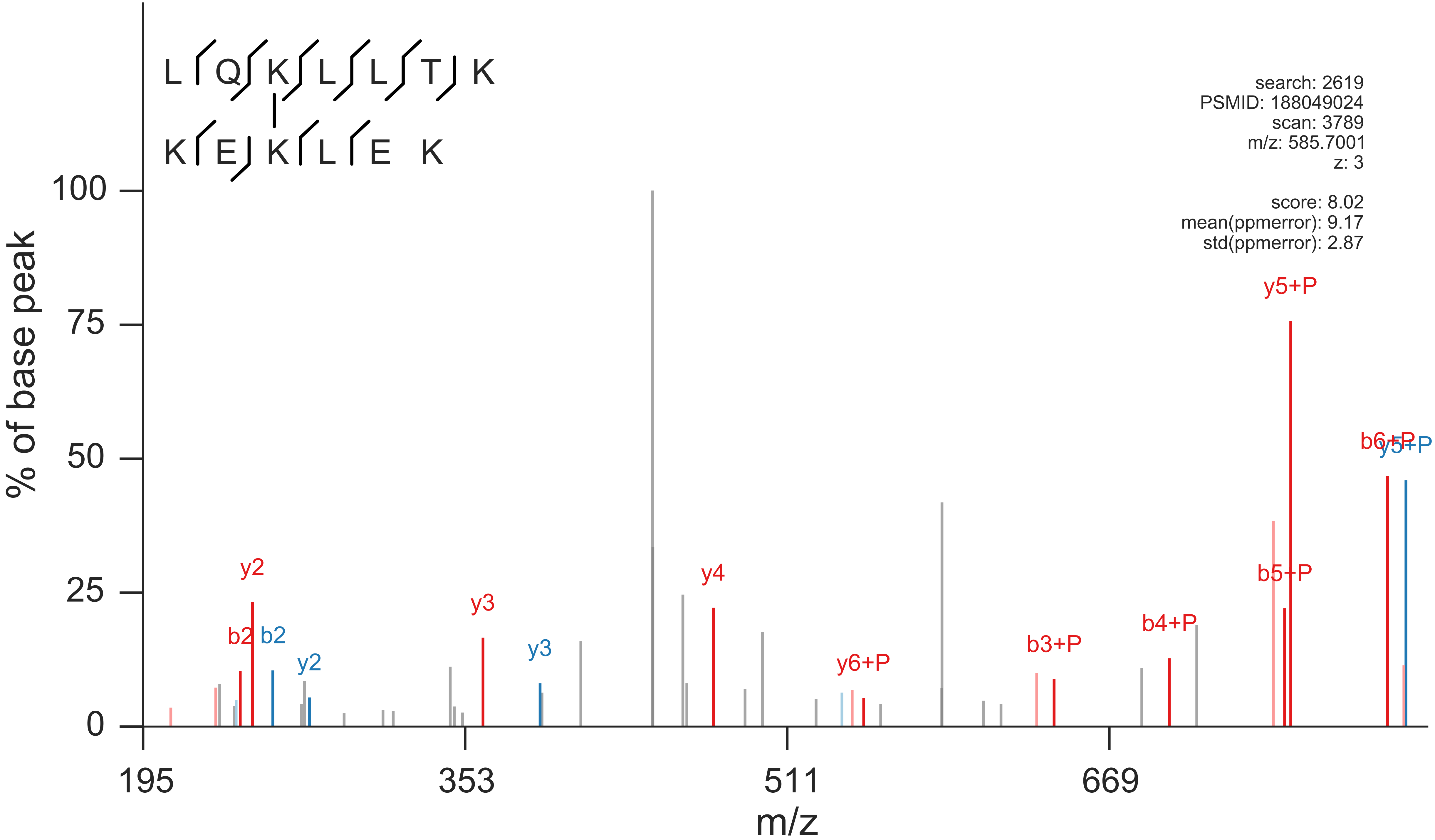
L Q K L L T K
K E K L E K
search: 2619
PSMID: 188049024
scan: 3789
m/z: 585.7001
z: 3
score: 8.02
mean(ppmerror): 9.17
std(ppmerror): 2.87
% of base peak
m/z
100
75
50
25
0
195
353
511
669
y2
b2
b2
y2
y3
y3
y4
y6+P
b3+P
b4+P
b5+P
y5+P
b6+P
y5+P

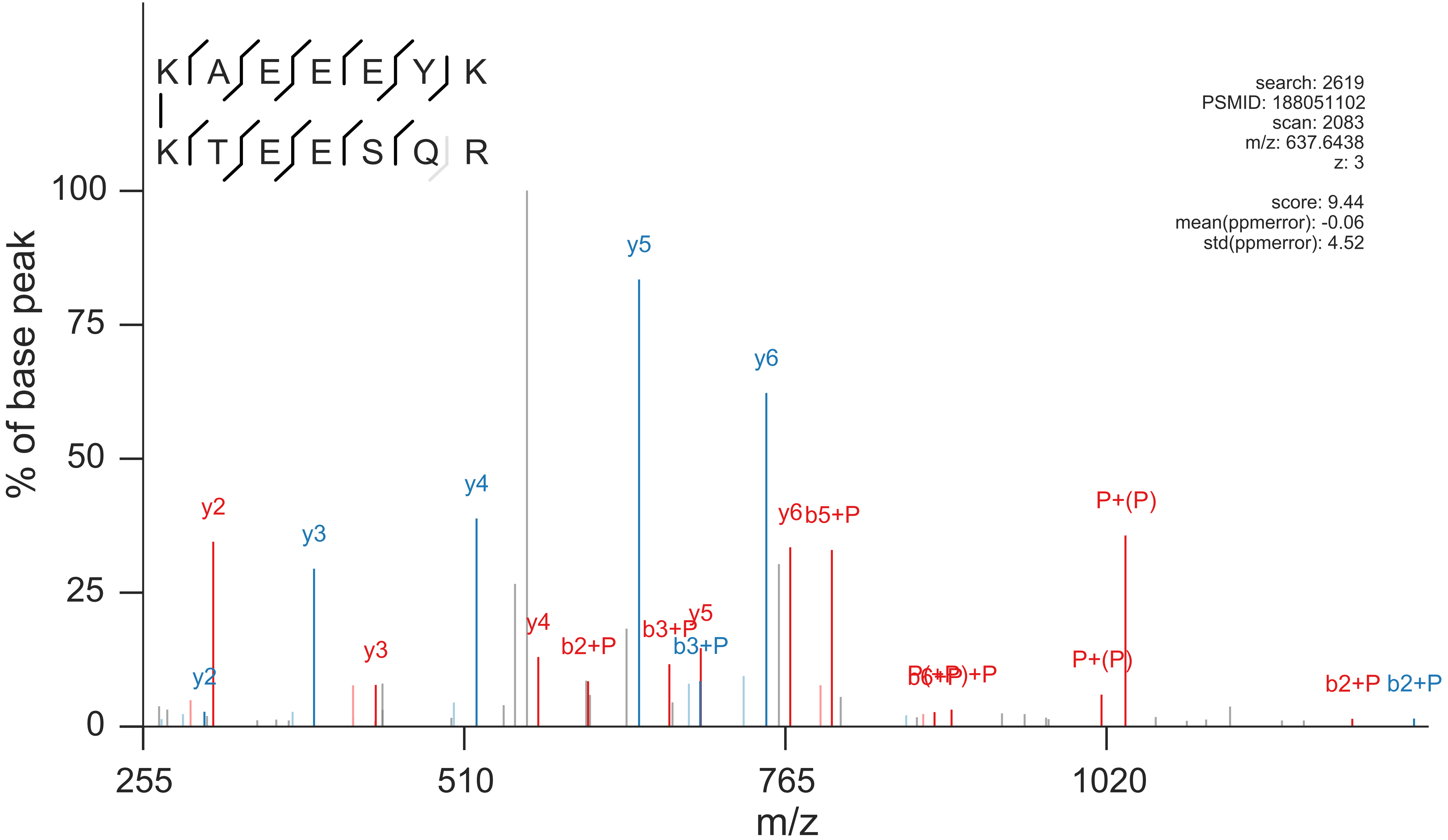

K A E E E Y K
K T E E S Q R
search: 2619
PSMID: 188051102
scan: 2083
m/z: 637.6438
z: 3
score: 9.44
mean(ppmerror): -0.06
std(ppmerror): 4.52
% of base peak
m/z
0
25
50
75
100
255
510
765
1020
y2
y3
y4
y5
y6
b3+P
b2+P
b5+P
P+(P)

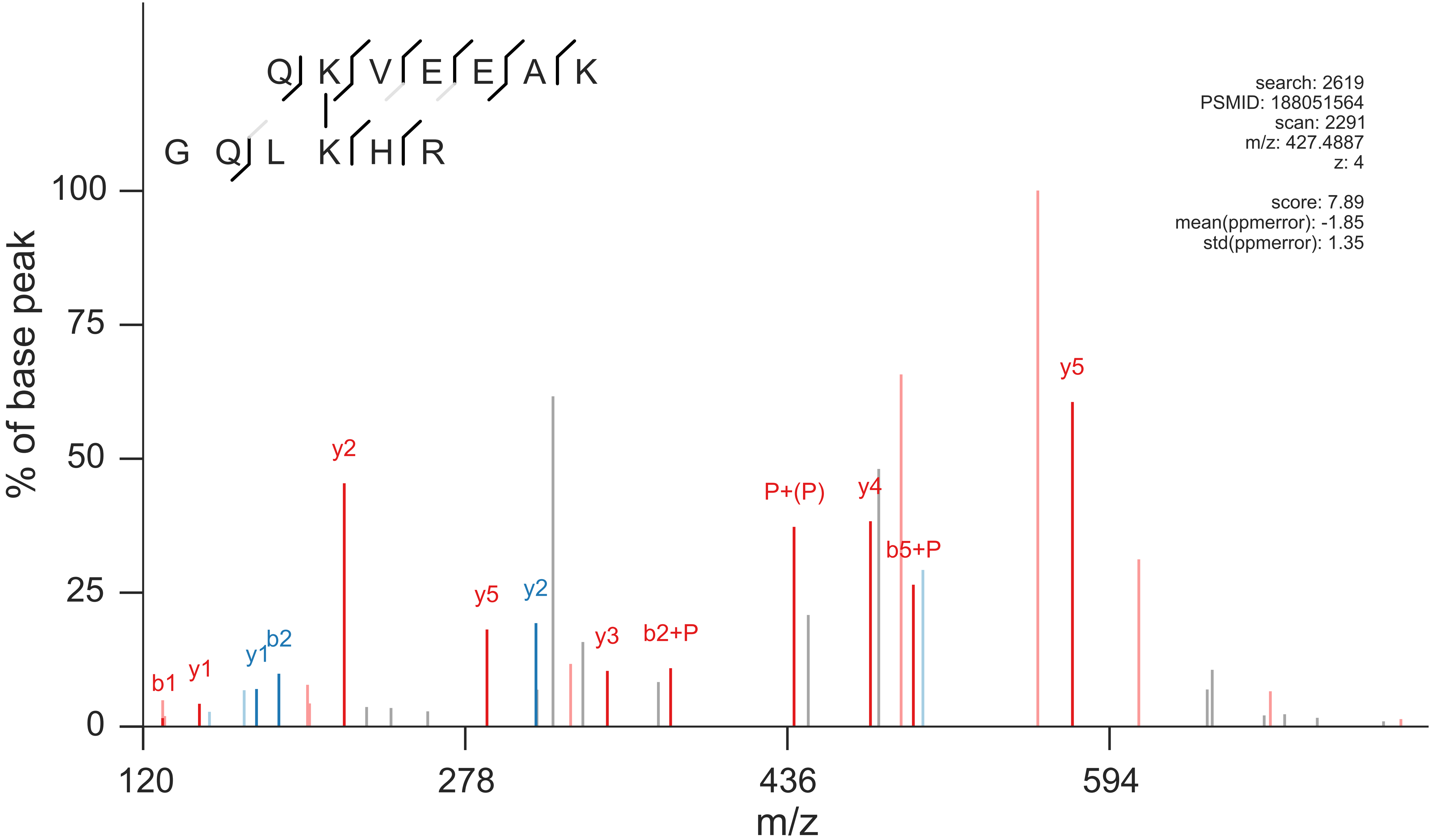

QKVEEAK
GQLKHR
search: 2619
PSMID: 188051564
scan: 2291
m/z: 427.4887
z: 4
score: 7.89
mean(ppmerror): -1.85
std(ppmerror): 1.35
% of base peak
m/z
b1
y1
y1
b2
y2
y5
y2
y3
b2+P
P+(P)
y4
b5+P
y5

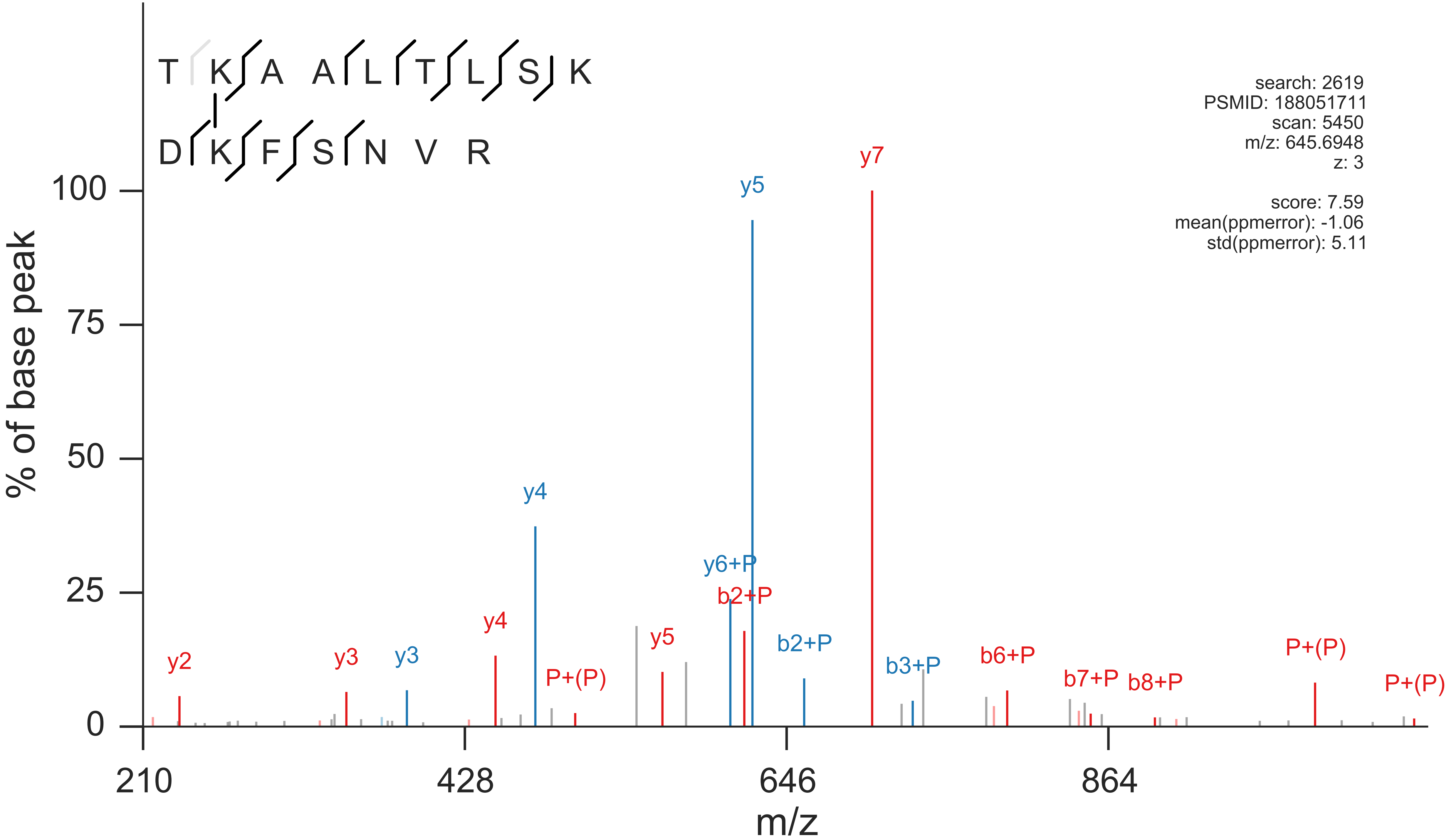

T K A A L T L S K
D K F S N V R
search: 2619
PSMID: 188051711
scan: 5450
m/z: 645.6948
z: 3
score: 7.59
mean(ppmerror): -1.06
std(ppmerror): 5.11
% of base peak
m/z
0
25
50
75
100
210
428
646
864
y2
y3
y3
y4
y4
P+(P)
y5
y6+P
b2+P
y5
b2+P
y7
b3+P
b6+P
b7+P
b8+P
P+(P)
P+(P)

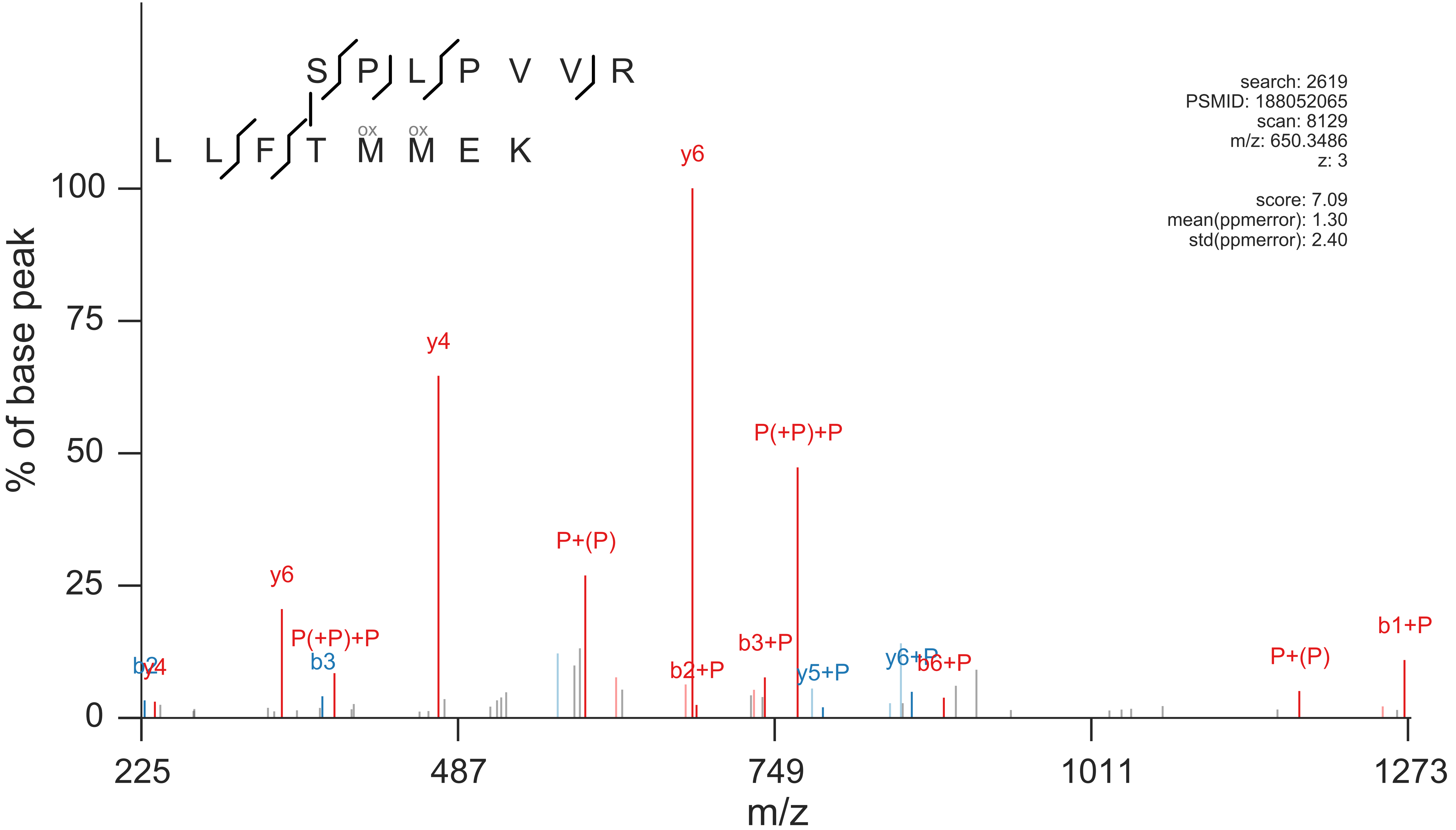

S P L P V V R
L L F T M M E K
ox
ox
search: 2619
PSMID: 188052065
scan: 8129
m/z: 650.3486
z: 3
score: 7.09
mean(ppmerror): 1.30
std(ppmerror): 2.40
% of base peak
m/z
0
25
50
75
100
225
487
749
1011
1273
y6
y4
P(+P)+P
P+(P)
y6
P(+P)+P
b3
b2
y4
b2+P
b3+P
y5+P
y6+P
b6+P
P+(P)
b1+P

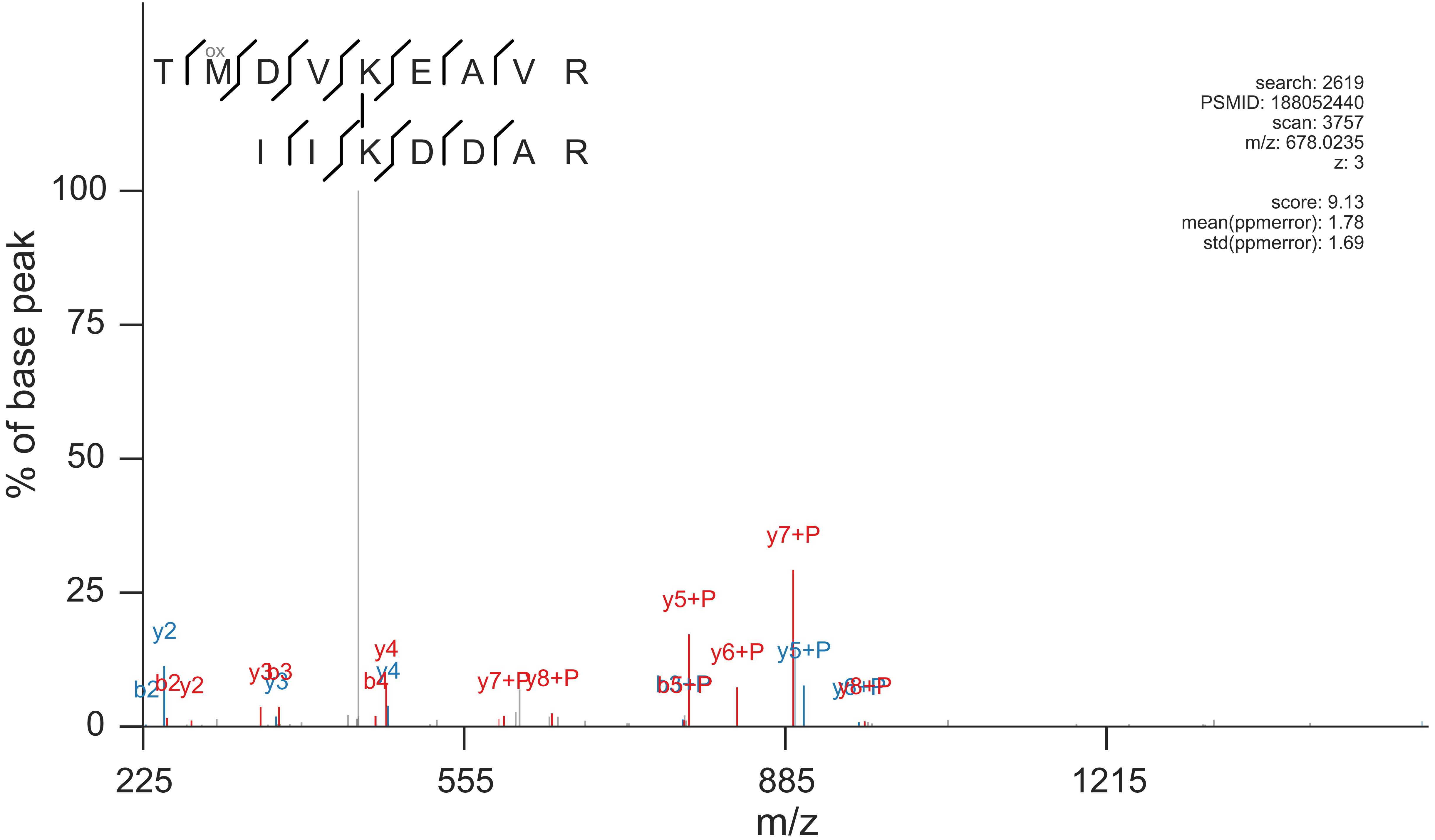

T M D V K E A V R
ox
I I K D D A R
search: 2619
PSMID: 188052440
scan: 3757
m/z: 678.0235
z: 3
score: 9.13
mean(ppmerror): 1.78
std(ppmerror): 1.69
% of base peak
100
75
50
25
0
225
555
885
1215
m/z
y2
b2
b2
y2
y3
b3
y3
y4
b4
y4
y7+P
y8+P
b5+P
y5+P
y6+P
y7+P
y5+P

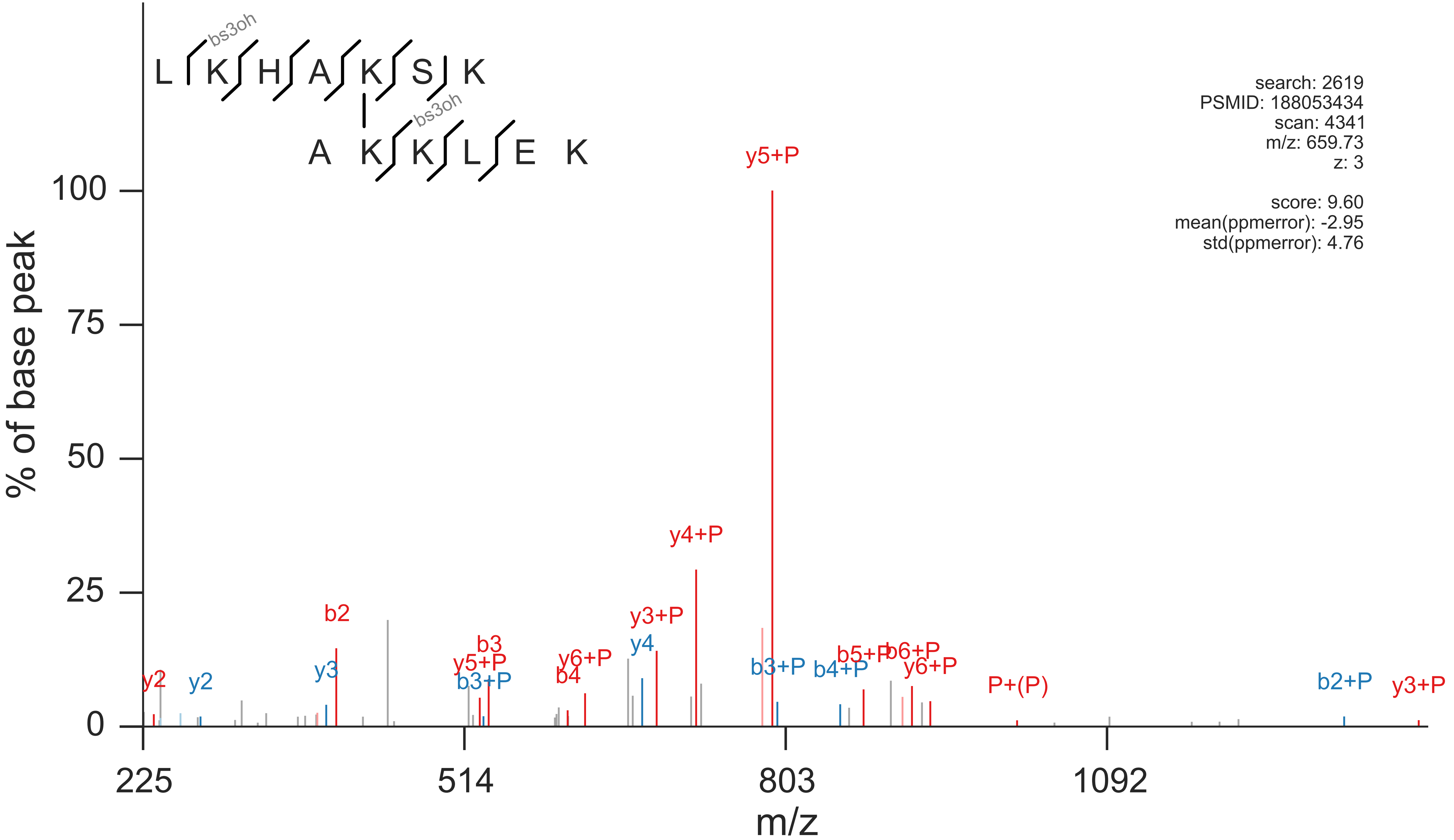

L K H A K S K
bs3oh
A K K L E K
bs3oh
search: 2619
PSMID: 188053434
scan: 4341
m/z: 659.73
z: 3
score: 9.60
mean(ppmerror): -2.95
std(ppmerror): 4.76
% of base peak
m/z
0
25
50
75
100
225
514
803
1092
y5+P
y4+P
y3+P
y4
b2
b3
y5+P
b3+P
y6+P
b4
y3
y2
y2
b3+P
b4+P
b5+P
b6+P
y6+P
P+(P)
b2+P
y3+P

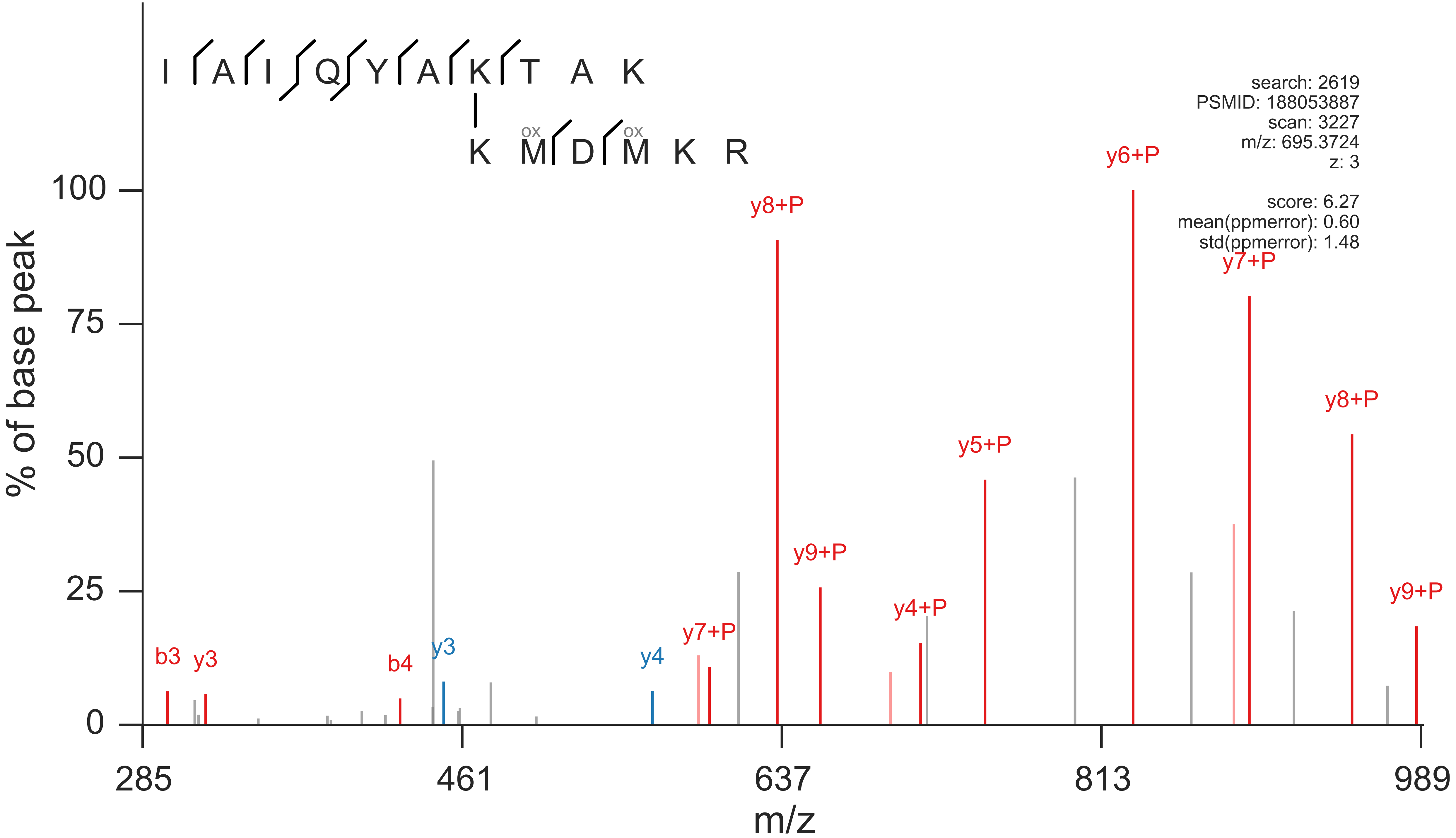
I A I Q Y A K T A K
K M D M K R
ox
ox
search: 2619
PSMID: 188053887
scan: 3227
m/z: 695.3724
z: 3
score: 6.27
mean(ppmerror): 0.60
std(ppmerror): 1.48
% of base peak
100
75
50
25
0
285
461
637
813
989
m/z
b3
y3
b4
y3
y4
y7+P
y8+P
y9+P
y4+P
y5+P
y6+P
y7+P
y8+P
y9+P

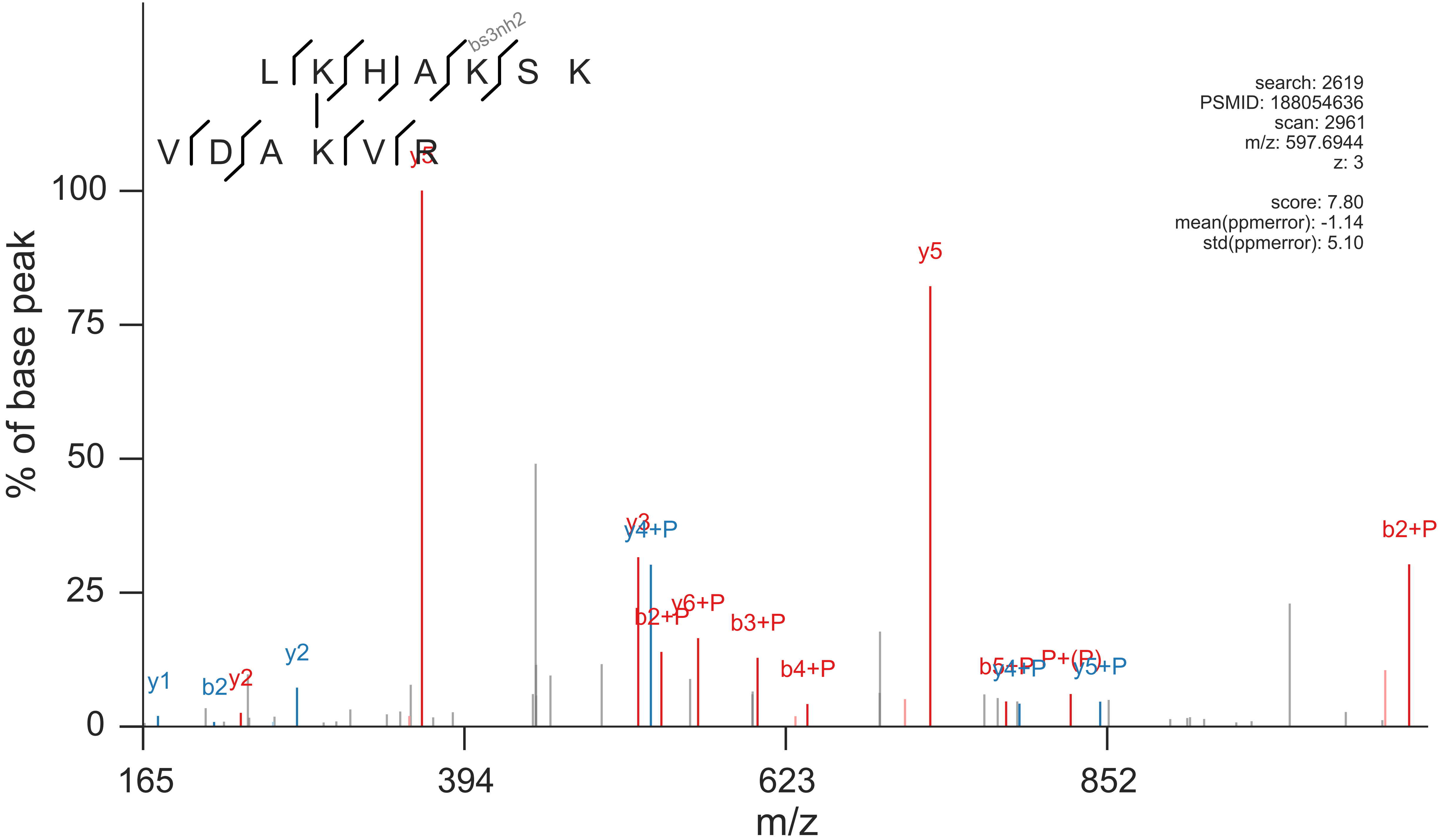

bs3nh2
L K H A K S K
V D A K V R
search: 2619
PSMID: 188054636
scan: 2961
m/z: 597.6944
z: 3
score: 7.80
mean(ppmerror): -1.14
std(ppmerror): 5.10
% of base peak
m/z
100
75
50
25
0
165
394
623
852
y5
y5
y3
y4+P
y6+P
b2+P
b3+P
b4+P
b2+P
y5+P
y1
b2
y2
y2

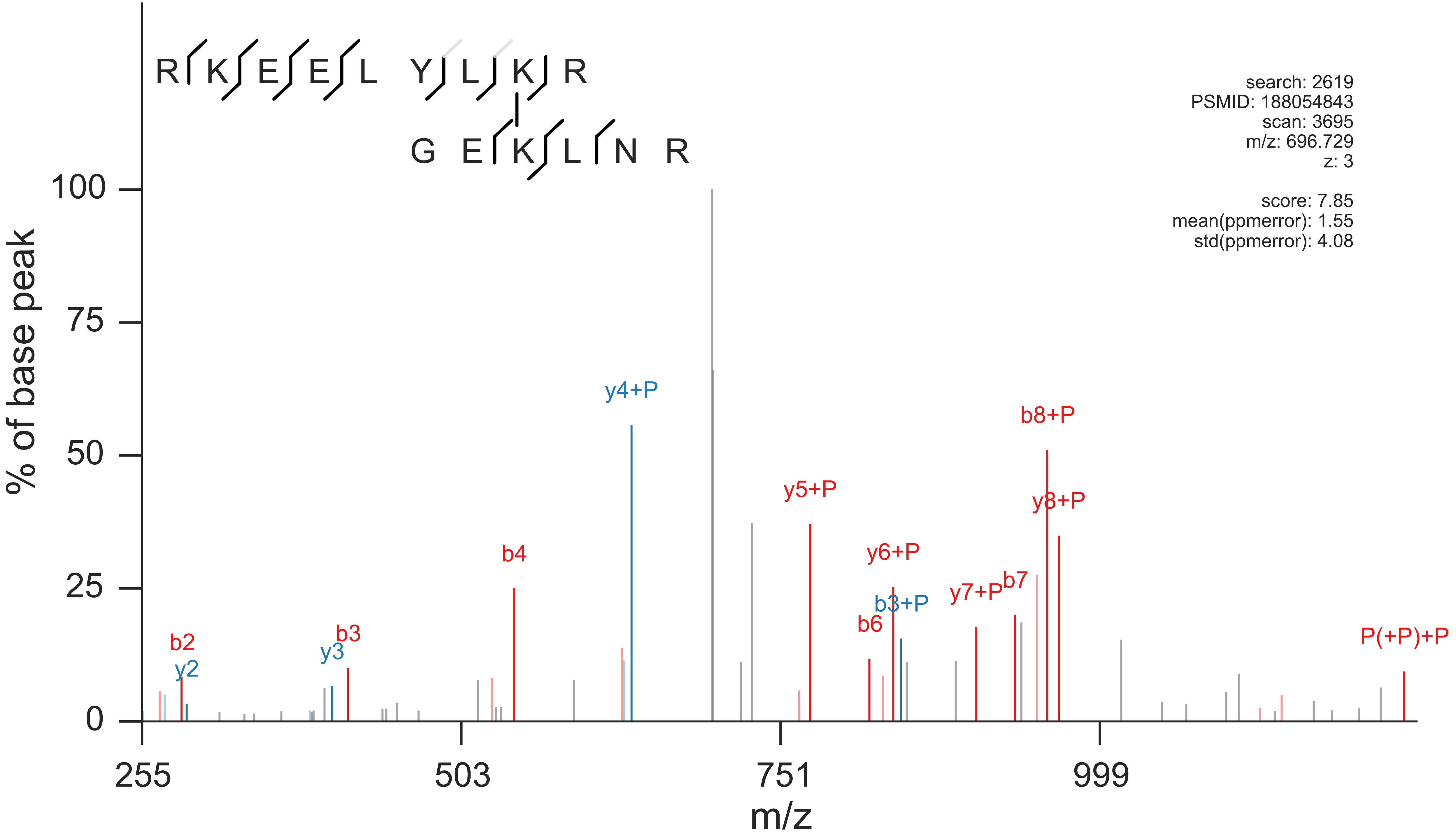
R K E E L Y L K R
G E K L N R
search: 2619
PSMID: 188054843
scan: 3695
m/z: 696.729
z: 3
score: 7.85
mean(ppmerror): 1.55
std(ppmerror): 4.08
% of base peak
m/z
y4+P
b8+P
y5+P
y8+P
b4
y6+P
y7+P
b7
b3+P
b6
b2
y2
y3
b3
P(+P)+P
0
25
50
75
100
255
503
751
999

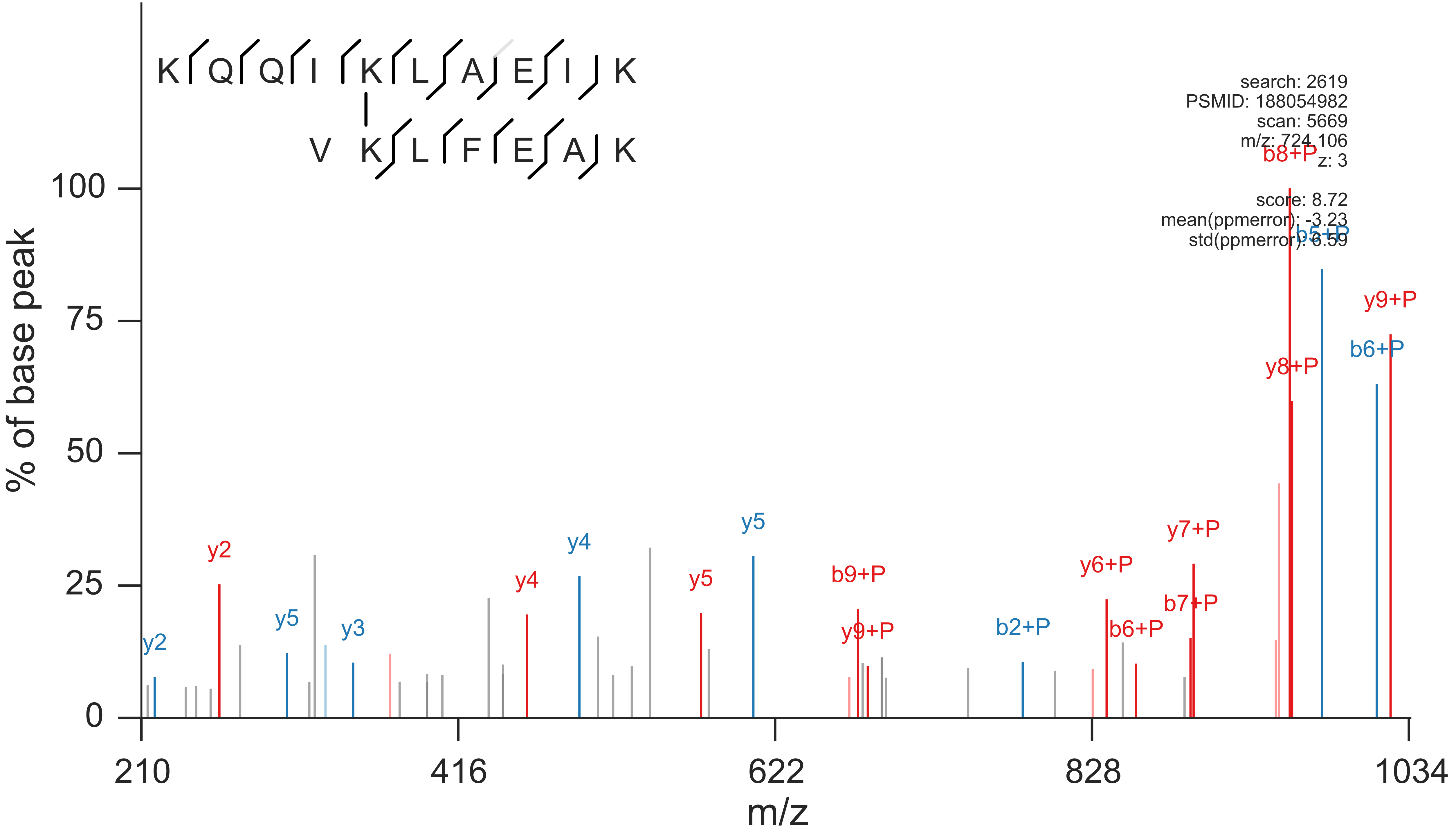

K Q Q I K L A E I K
V K L F E A K
search: 2619
PSMID: 188054982
scan: 5669
m/z: 724.106
z: 3
score: 8.72
mean(ppmerror): -3.23
std(ppmerror): 6.59
% of base peak
m/z
0
25
50
75
100
210
416
622
828
1034
y2
y2
y5
y3
y4
y4
y5
y5
b9+P
y9+P
b2+P
y6+P
b6+P
y7+P
b7+P
b8+P
y8+P
b5+P
b6+P
y9+P

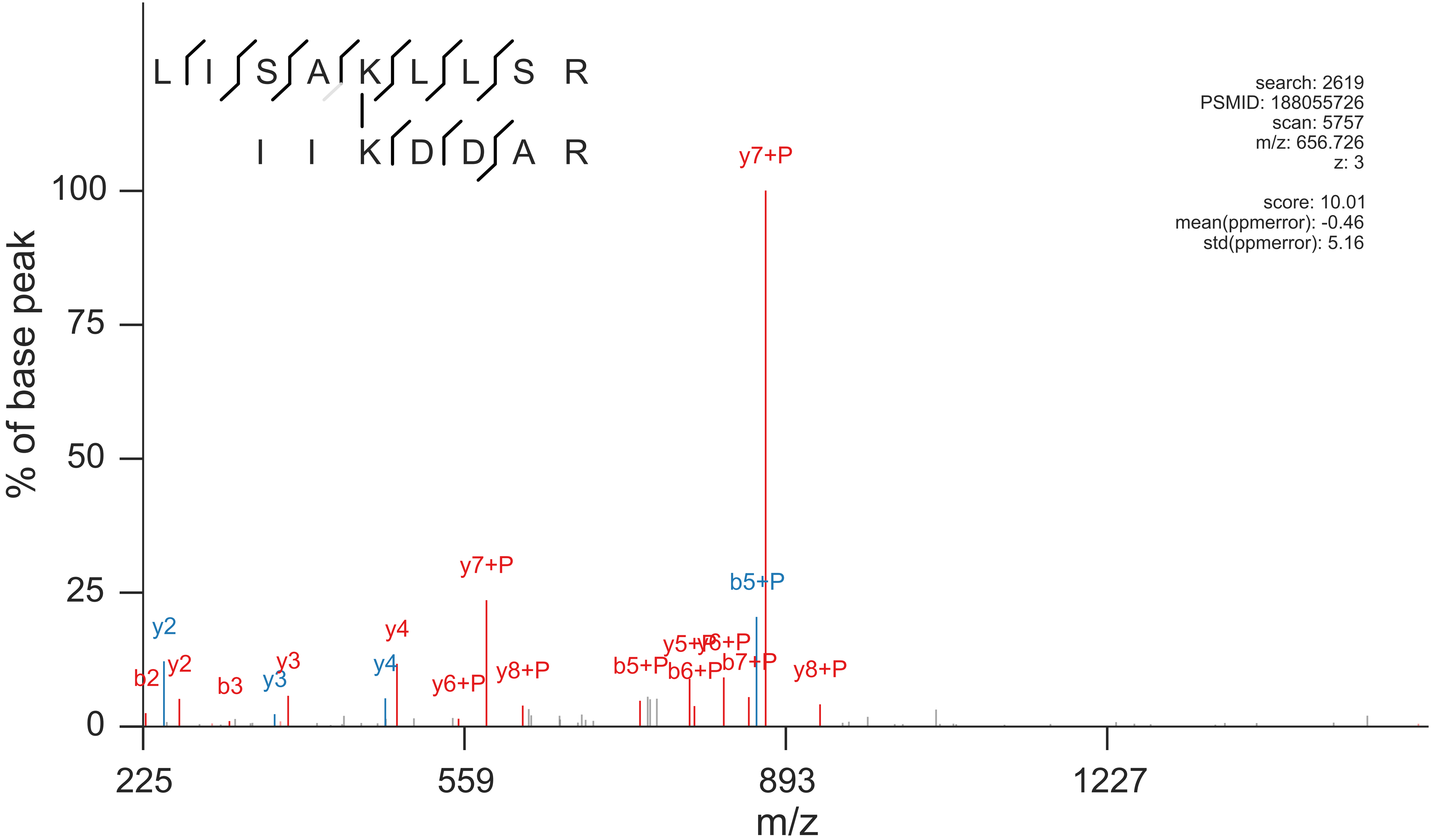

L I S A K L L S R
I I K D D A R
search: 2619
PSMID: 188055726
scan: 5757
m/z: 656.726
z: 3
score: 10.01
mean(ppmerror): -0.46
std(ppmerror): 5.16
% of base peak
m/z
y7+P
y2
b2
y3
b3
y4
y6+P
y8+P
b5+P
b6+P
b7+P

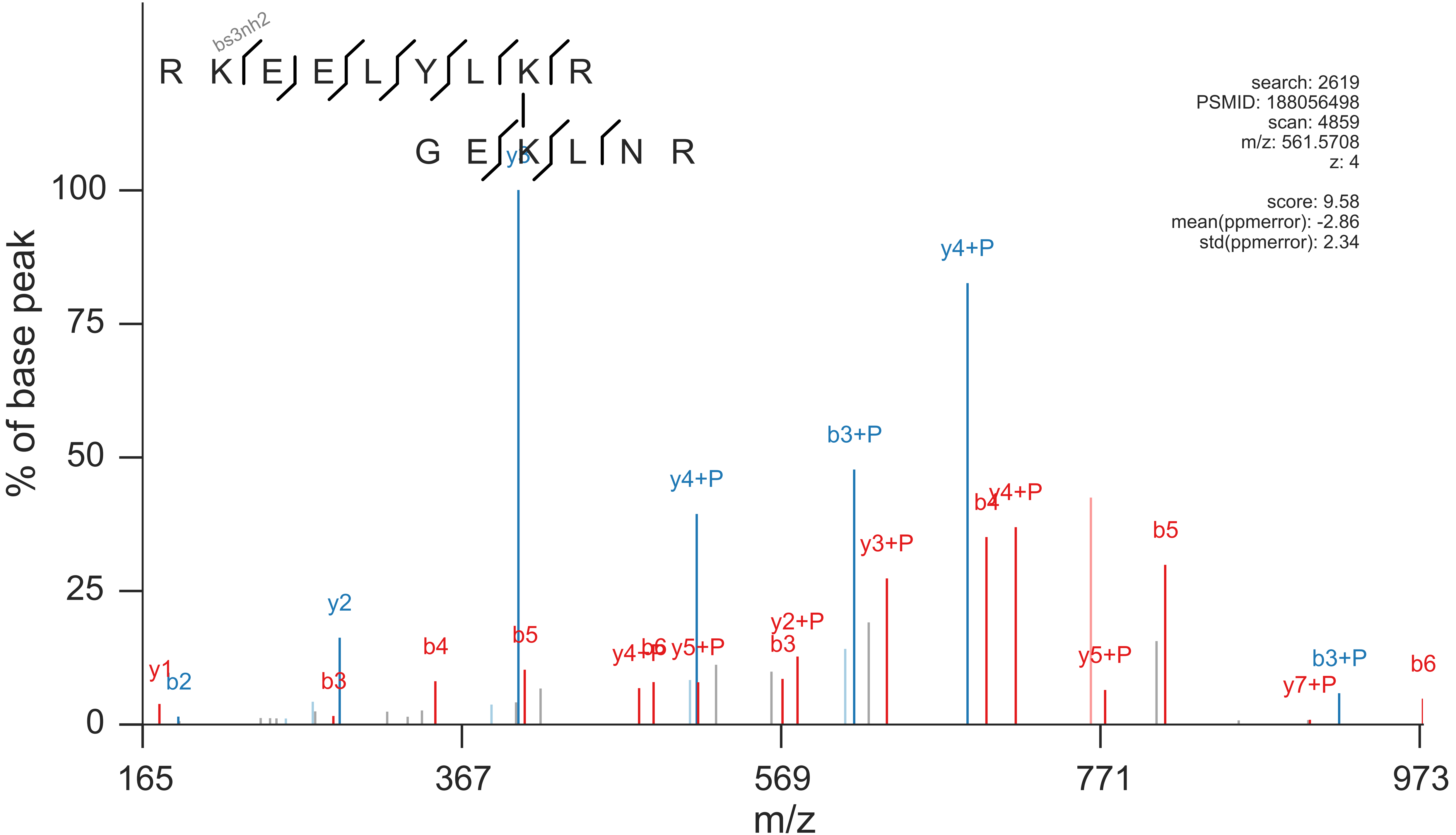

bs3nh2
R K E E L Y L K R
G E K L N R
search: 2619
PSMID: 188056498
scan: 4859
m/z: 561.5708
z: 4
score: 9.58
mean(ppmerror): -2.86
std(ppmerror): 2.34
% of base peak
m/z

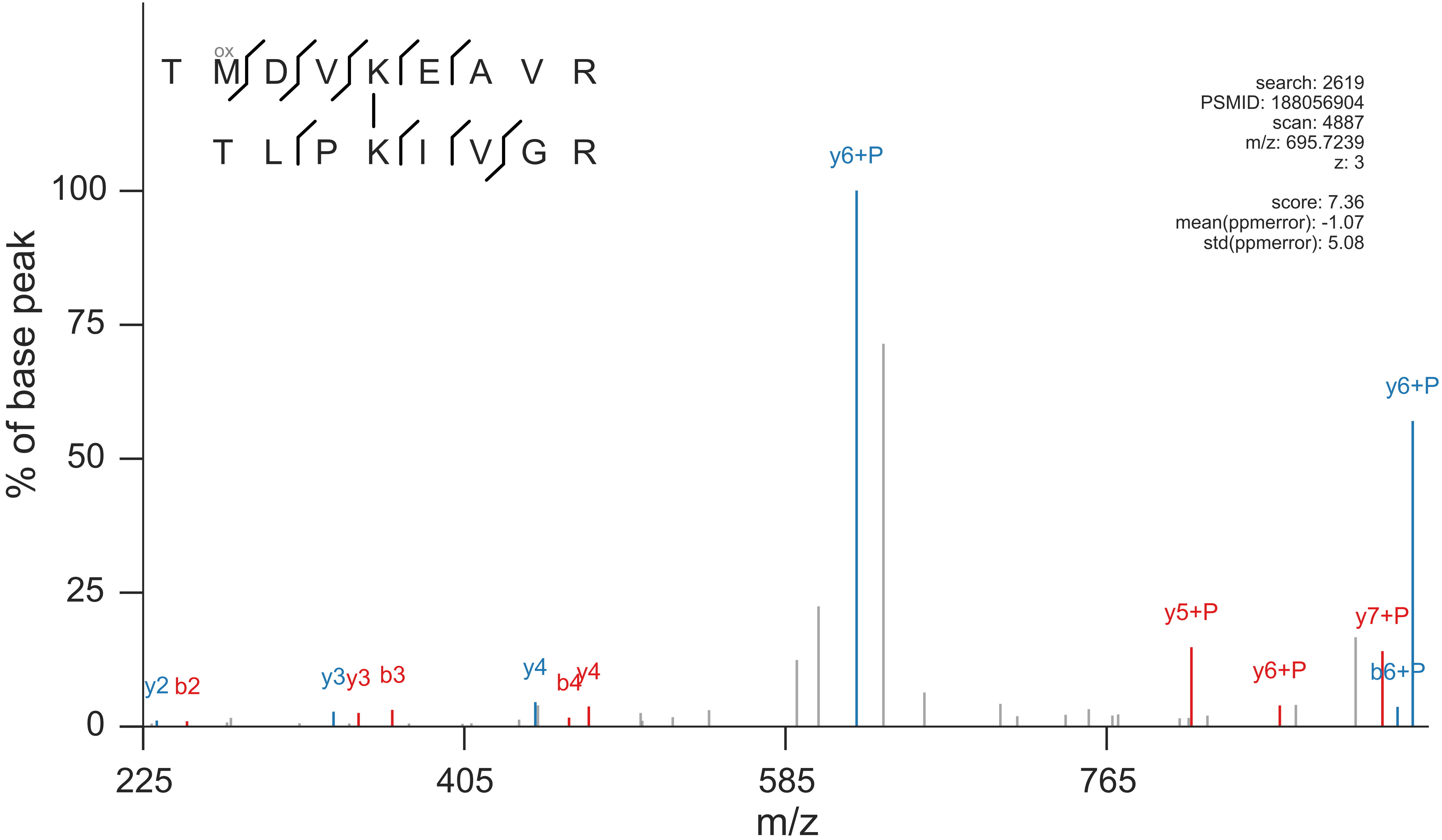

T M D V K E A V R
ox
T L P K I V G R
search: 2619
PSMID: 188056904
scan: 4887
m/z: 695.7239
z: 3
score: 7.36
mean(ppmerror): -1.07
std(ppmerror): 5.08
y6+P
y6+P
y5+P
y7+P
y6+P
b6+P
y2
b2
y3
y3
b3
y4
b4
y4
% of base peak
100
75
50
25
0
225
405
585
765
m/z

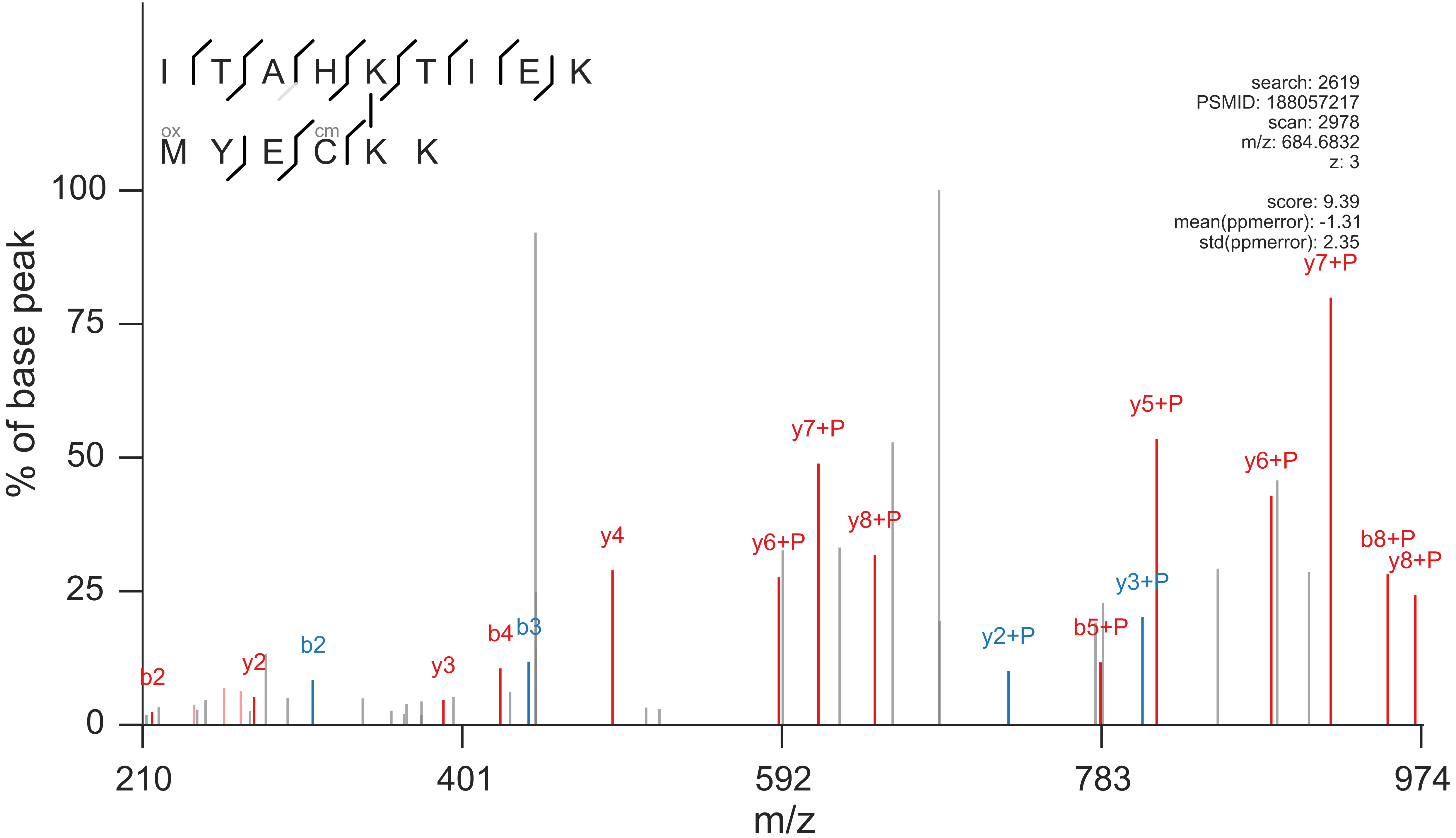

I T A H K T I E K
ox
M Y E C K K
cm
search: 2619
PSMID: 188057217
scan: 2978
m/z: 684.6832
z: 3
score: 9.39
mean(ppmerror): -1.31
std(ppmerror): 2.35
% of base peak
100
75
50
25
0
210
401
592
783
974
m/z
b2
y2
b2
y3
b4
b3
y4
y6+P
y7+P
y8+P
y2+P
b5+P
y3+P
y5+P
y6+P
y7+P
b8+P
y8+P

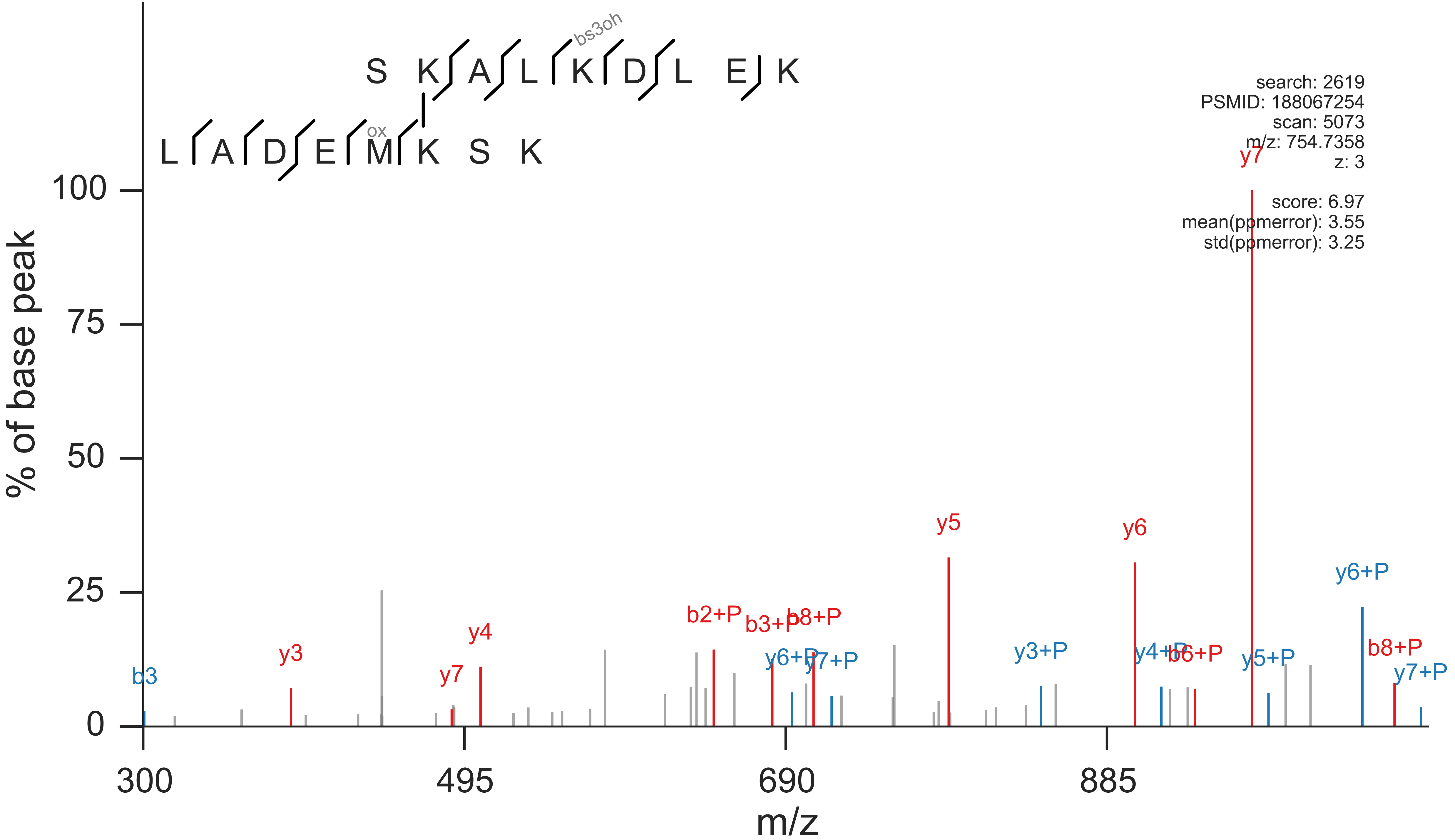
S K A L K D L E K
bs3oh
L A D E M K S K
ox
search: 2619
PSMID: 188067254
scan: 5073
m/z: 754.7358
z: 3
score: 6.97
mean(ppmerror): 3.55
std(ppmerror): 3.25
% of base peak
m/z
0
25
50
75
100
300
495
690
885
b3
y3
y7
y4
b2+P
b3+P
b8+P
y6+P
y7+P
y5
y3+P
y6
y4+P
b6+P
y7
y5+P
y6+P
b8+P
y7+P

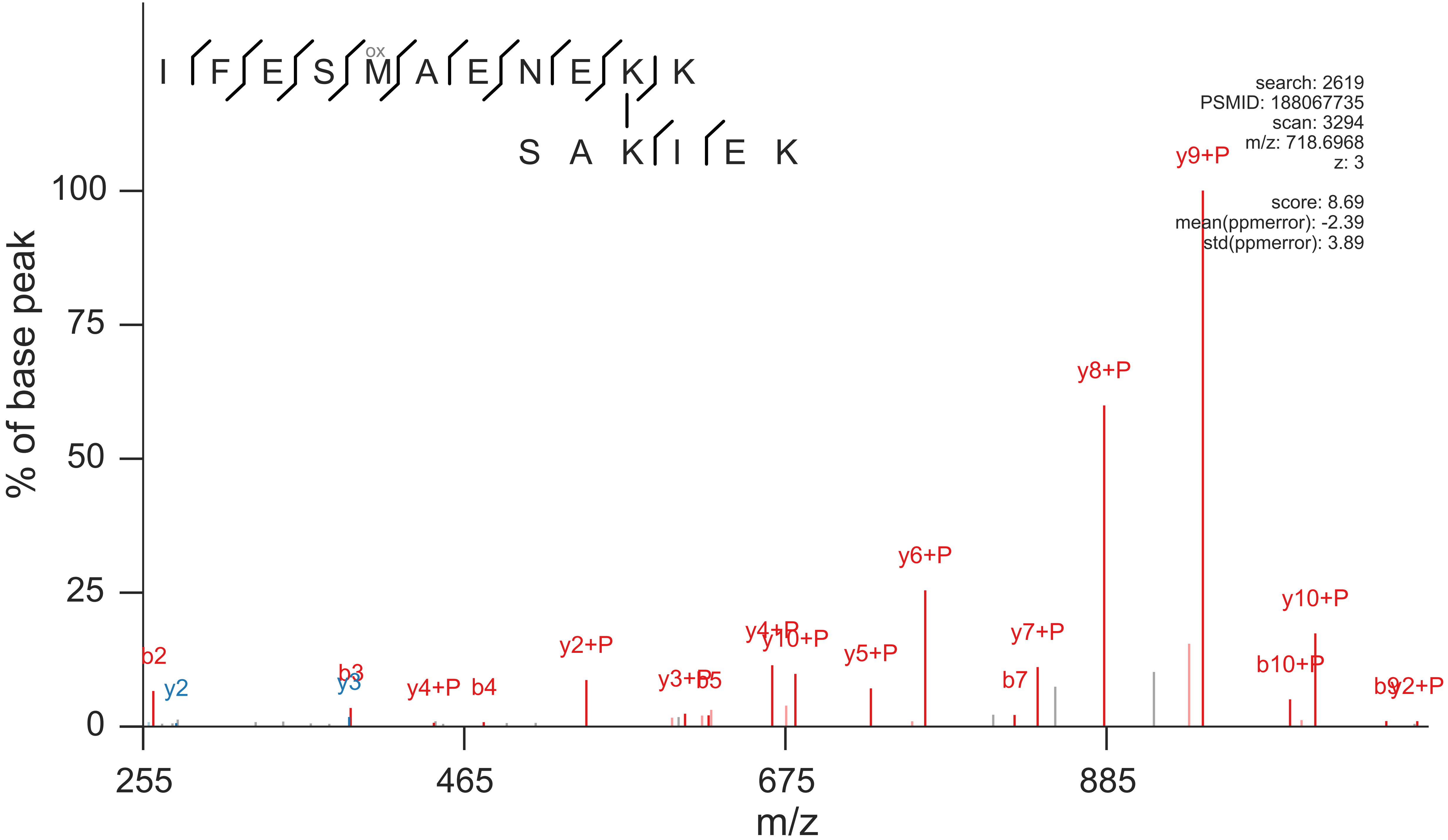

I F E S M A E N E K K
ox
S A K I E K
search: 2619
PSMID: 188067735
scan: 3294
m/z: 718.6968
z: 3
score: 8.69
mean(ppmerror): -2.39
std(ppmerror): 3.89
% of base peak
m/z
0
25
50
75
100
255
465
675
885
b2
y2
b3
y3
y4+P
b4
y2+P
y3+P
b5
y4+P
y10+P
y5+P
y6+P
b7
y7+P
y8+P
y9+P
b10+P
y10+P
b9
y2+P

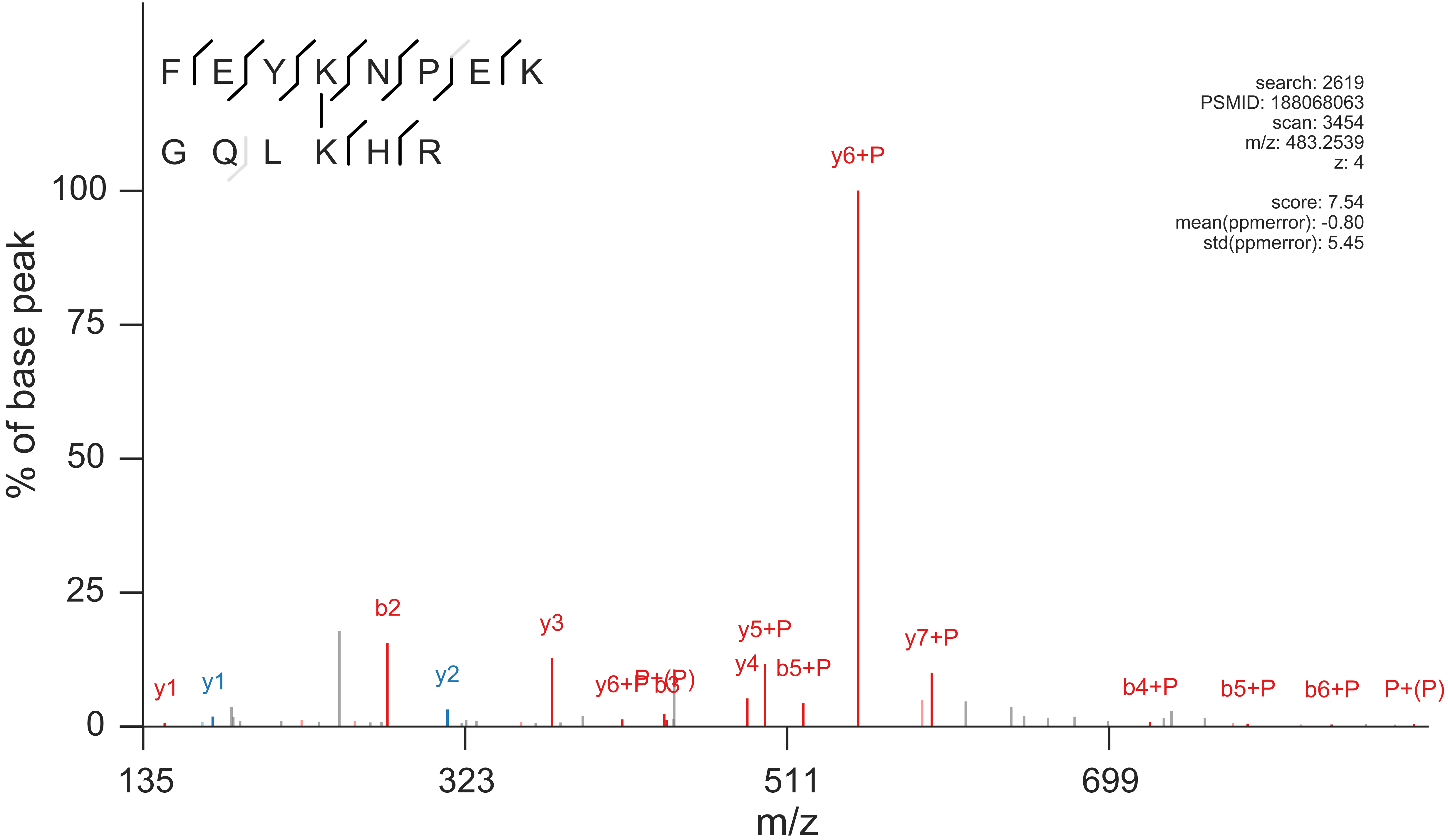

F E Y K N P E K
G Q L K H R
search: 2619
PSMID: 188068063
scan: 3454
m/z: 483.2539
z: 4
score: 7.54
mean(ppmerror): -0.80
std(ppmerror): 5.45
% of base peak
m/z
100
75
50
25
0
135
323
511
699
y1
y1
b2
y2
y3
y6+P
P+(P)
b3
y4
y5+P
b5+P
y6+P
y7+P
b4+P
b5+P
b6+P
P+(P)

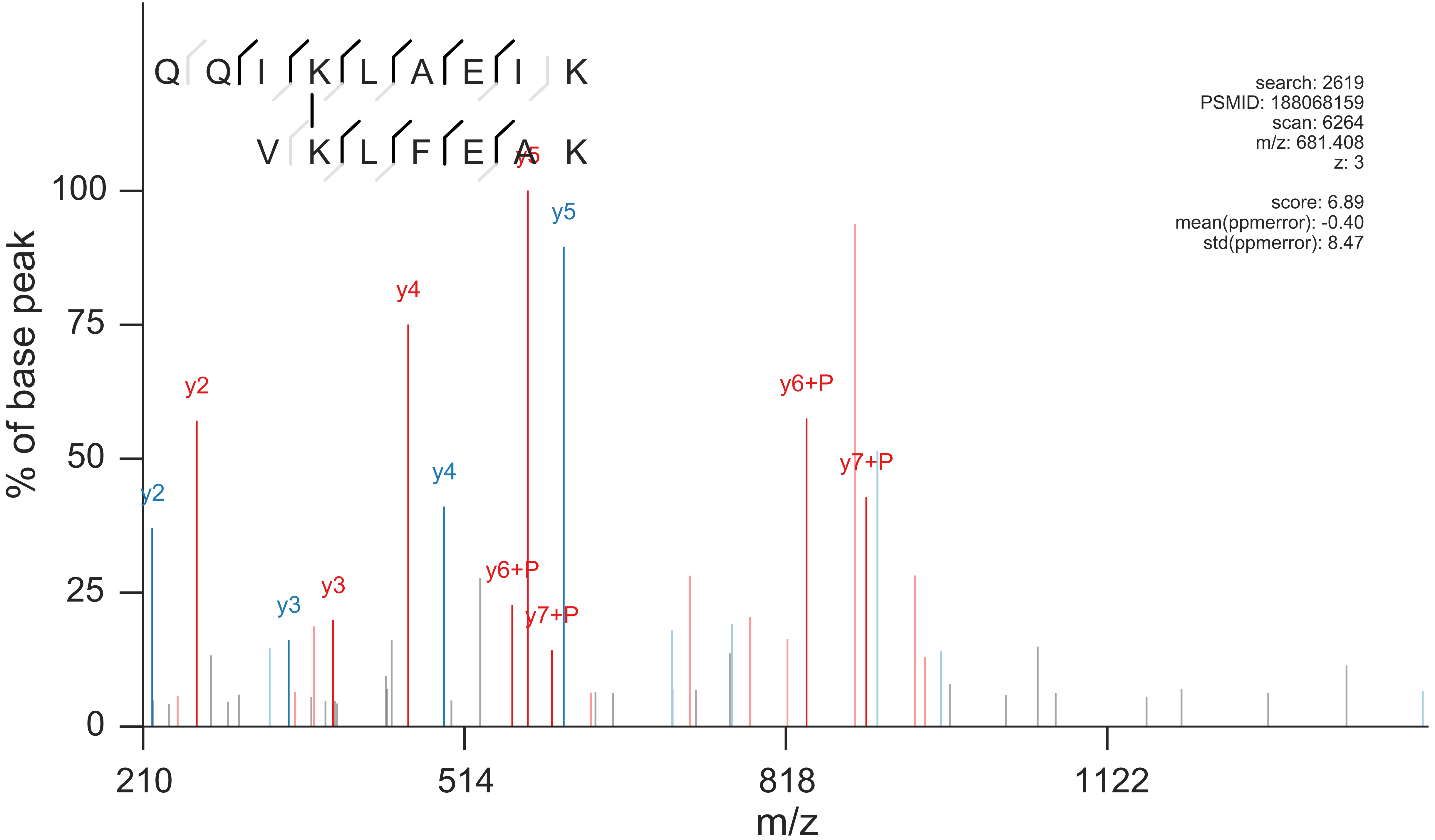

Q Q I K L A E I K
V K L F E A K
search: 2619
PSMID: 188068159
scan: 6264
m/z: 681.408
z: 3
score: 6.89
mean(ppmerror): -0.40
std(ppmerror): 8.47
% of base peak
m/z
0
25
50
75
100
210
514
818
1122
y2
y3
y4
y5
y6+P
y7+P

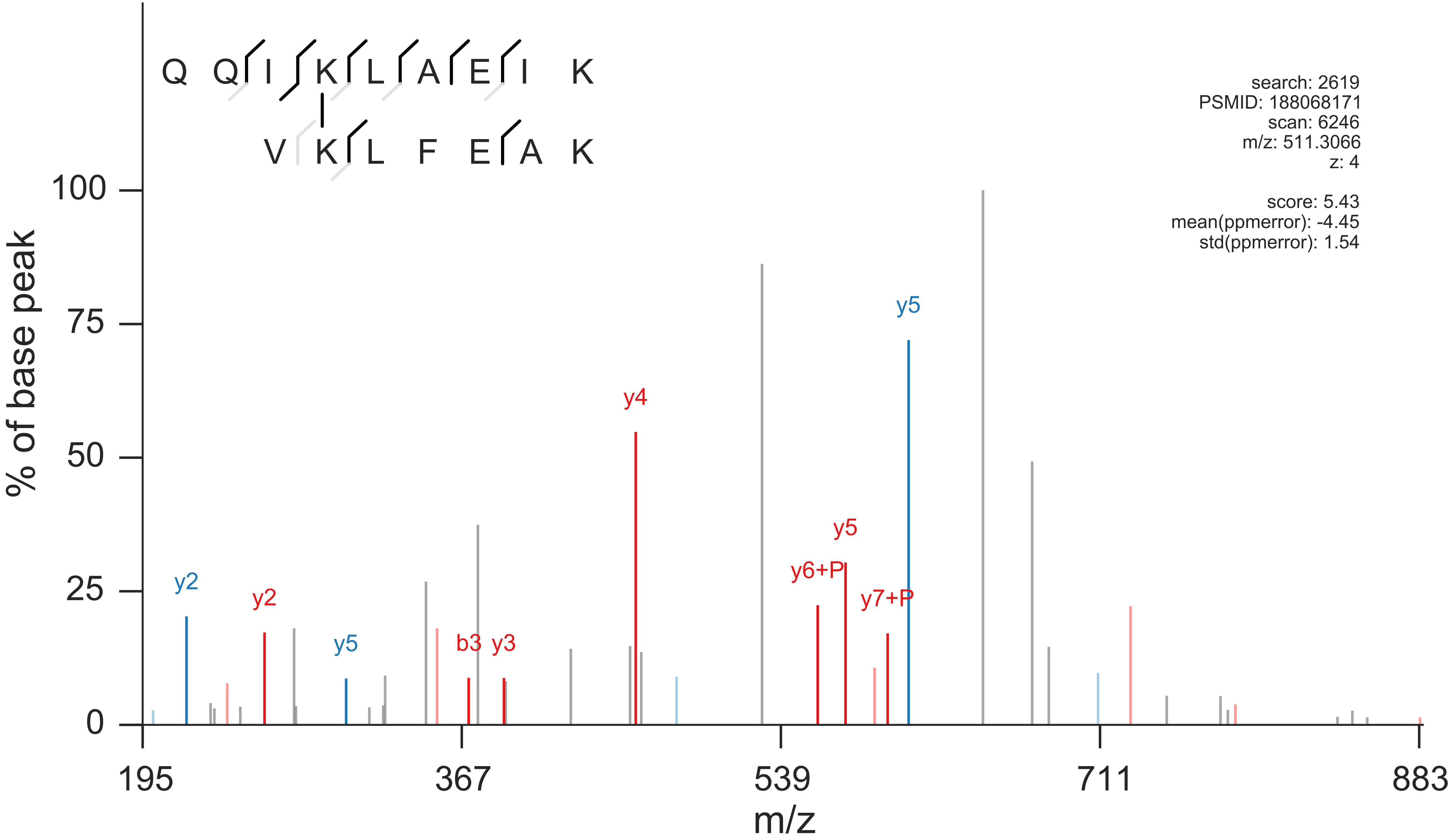

Q Q I K L A E I K
V K L F E A K
search: 2619
PSMID: 188068171
scan: 6246
m/z: 511.3066
z: 4
score: 5.43
mean(ppmerror): -4.45
std(ppmerror): 1.54
% of base peak
100
75
50
25
0
y2
y2
y5
b3
y3
y4
y5
y6+P
y7+P
y5
195
367
539
711
883
m/z

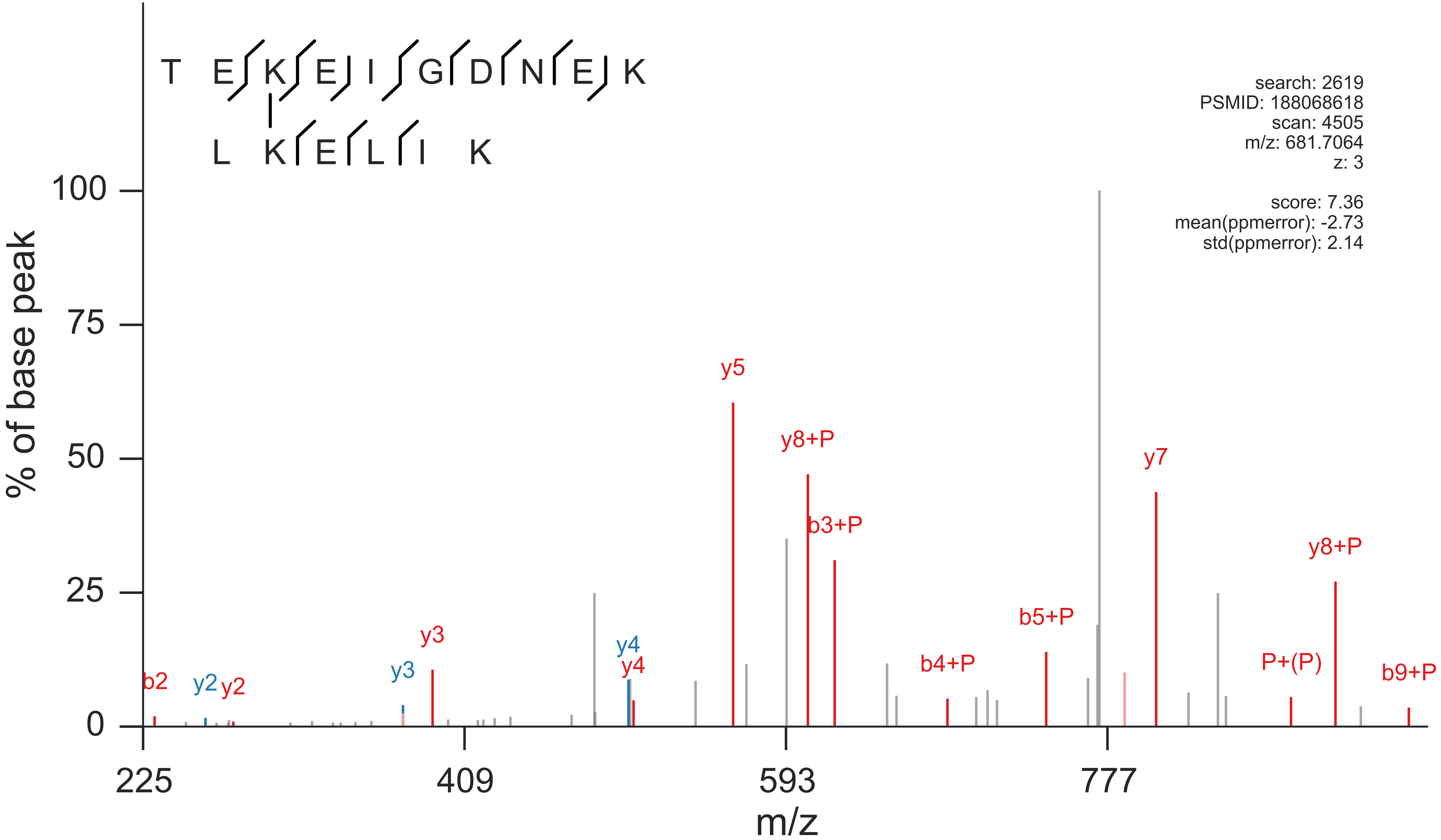

T E K E I G D N E K
L K E L I K
search: 2619
PSMID: 188068618
scan: 4505
m/z: 681.7064
z: 3
score: 7.36
mean(ppmerror): -2.73
std(ppmerror): 2.14
% of base peak
100
75
50
25
0
b2
y2
y2
y3
y3
y4
y4
y5
y8+P
b3+P
b4+P
b5+P
y7
P+(P)
y8+P
b9+P
225
409
593
777
m/z

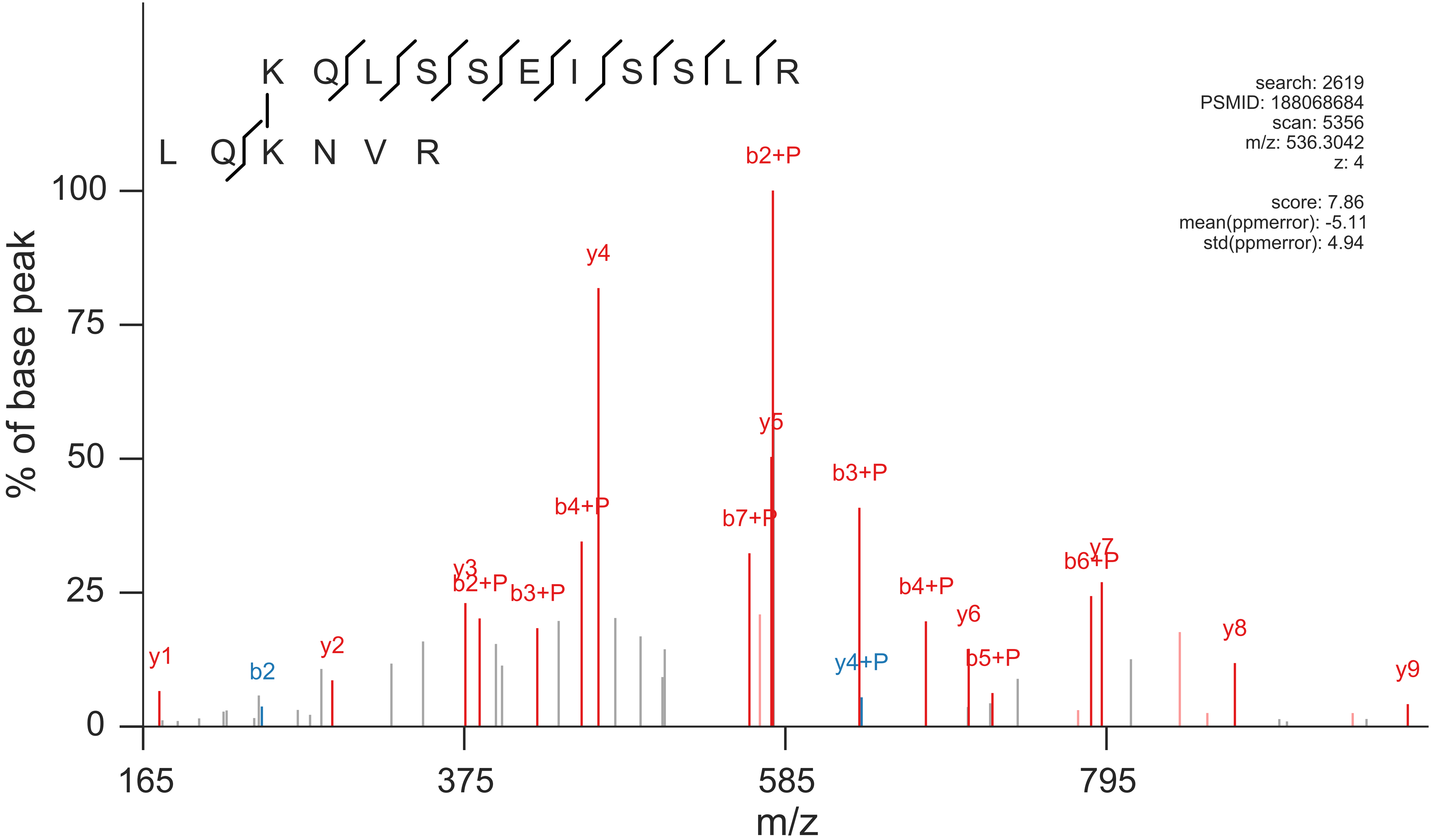

K Q L S S E I S S L R
L Q K N V R
search: 2619
PSMID: 188068684
scan: 5356
m/z: 536.3042
z: 4
score: 7.86
mean(ppmerror): -5.11
std(ppmerror): 4.94
% of base peak
m/z
y1
b2
y2
y3
b2+P
b3+P
b4+P
y4
b7+P
y5
b2+P
b3+P
y4+P
b4+P
y6
b5+P
b6+P
y7
y8
y9

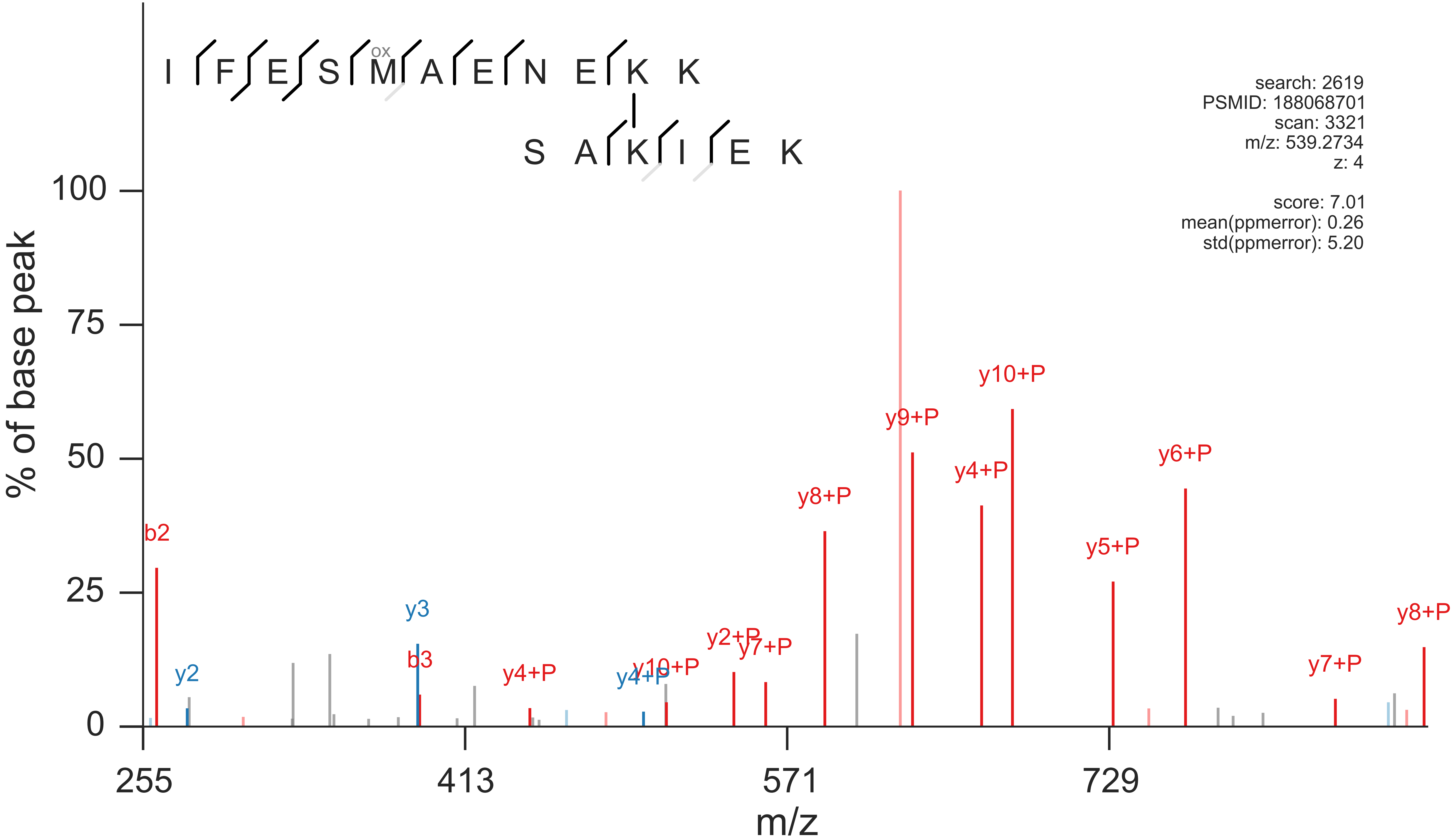

I F E S M A E N E K K
ox
S A K I E K
search: 2619
PSMID: 188068701
scan: 3321
m/z: 539.2734
z: 4
score: 7.01
mean(ppmerror): 0.26
std(ppmerror): 5.20
% of base peak
m/z
100
75
50
25
0
255
413
571
729
b2
y2
y3
b3
y4+P
y4+P
y10+P
y2+P
y7+P
y8+P
y9+P
y4+P
y10+P
y5+P
y6+P
y7+P
y8+P

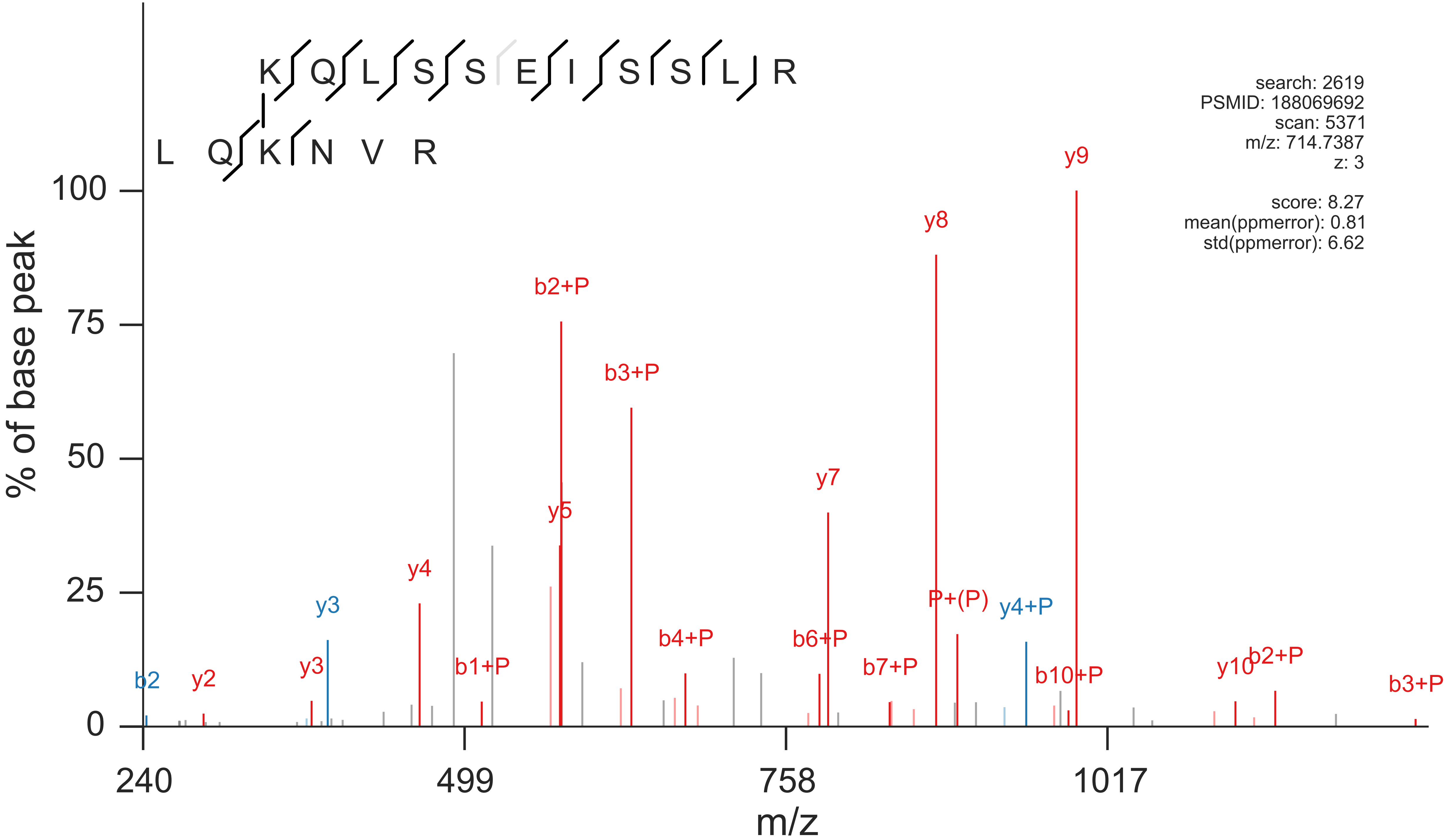
K Q L S S E I S S L R
L Q K N V R
search: 2619
PSMID: 188069692
scan: 5371
m/z: 714.7387
z: 3
score: 8.27
mean(ppmerror): 0.81
std(ppmerror): 6.62
% of base peak
m/z
0
25
50
75
100
240
499
758
1017
y9
y8
b2+P
b3+P
y7
y5
y4
y3
P+(P)
y4+P
b4+P
b6+P
b7+P
b10+P
y10
b2+P
b3+P
b1+P
y3
y2
b2

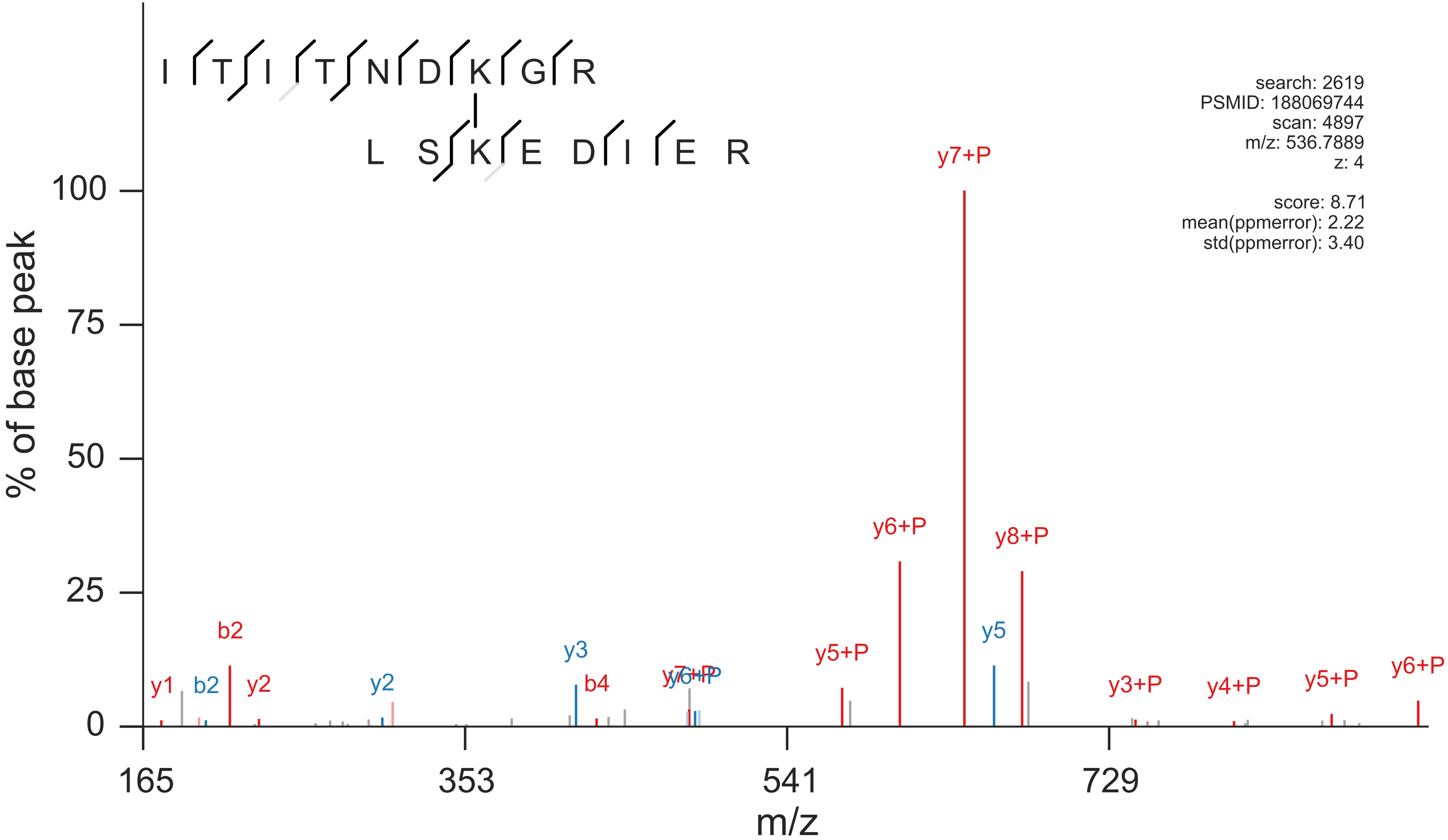
I T I T N D K G R
L S K E D I E R
search: 2619
PSMID: 188069744
scan: 4897
m/z: 536.7889
z: 4
score: 8.71
mean(ppmerror): 2.22
std(ppmerror): 3.40
% of base peak
100
75
50
25
0
165
353
541
729
m/z
y7+P
y6+P
y8+P
b2
y3
y5
y5+P
y1
b2
y2
y2
b4
y3+P
y4+P
y5+P
y6+P

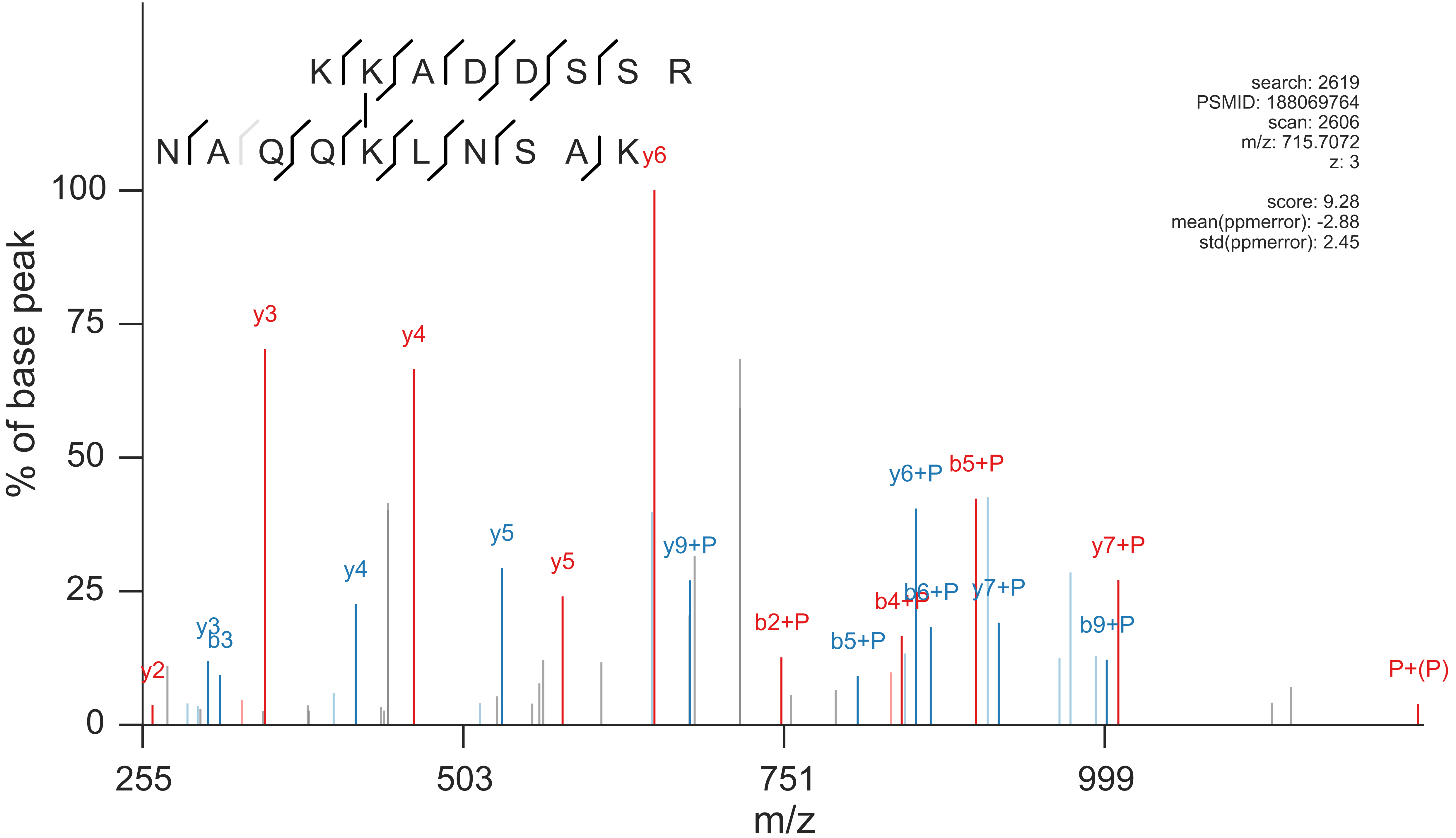

K K A D D S S R
N A Q Q K L N S A K
search: 2619
PSMID: 188069764
scan: 2606
m/z: 715.7072
z: 3
score: 9.28
mean(ppmerror): -2.88
std(ppmerror): 2.45
% of base peak
m/z
0
25
50
75
100
255
503
751
999
y2
y3
b3
y3
y4
y4
y5
y5
y6
y9+P
b2+P
b5+P
b4+P
b6+P
y6+P
b5+P
y7+P
b9+P
y7+P
P+(P)

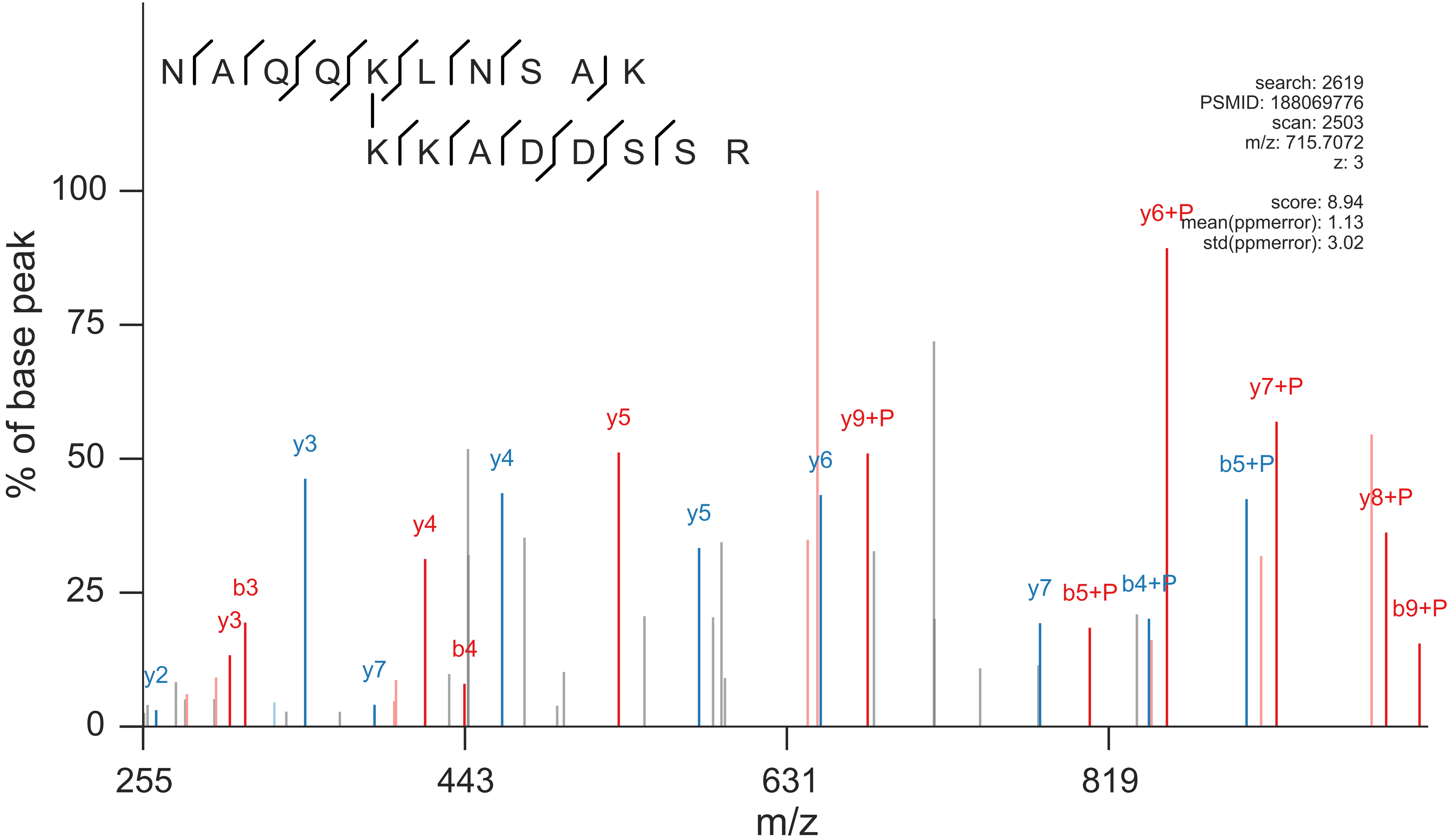

N A Q Q K L N S A K
K K A D D S S R
search: 2619
PSMID: 188069776
scan: 2503
m/z: 715.7072
z: 3
score: 8.94
mean(ppmerror): 1.13
std(ppmerror): 3.02
% of base peak
100
75
50
25
0
255
443
631
819
m/z
y2
y3
b3
y3
y7
y4
b4
y4
y5
y5
y6
y9+P
y6+P
y7
b5+P
b4+P
b5+P
y7+P
y8+P
b9+P

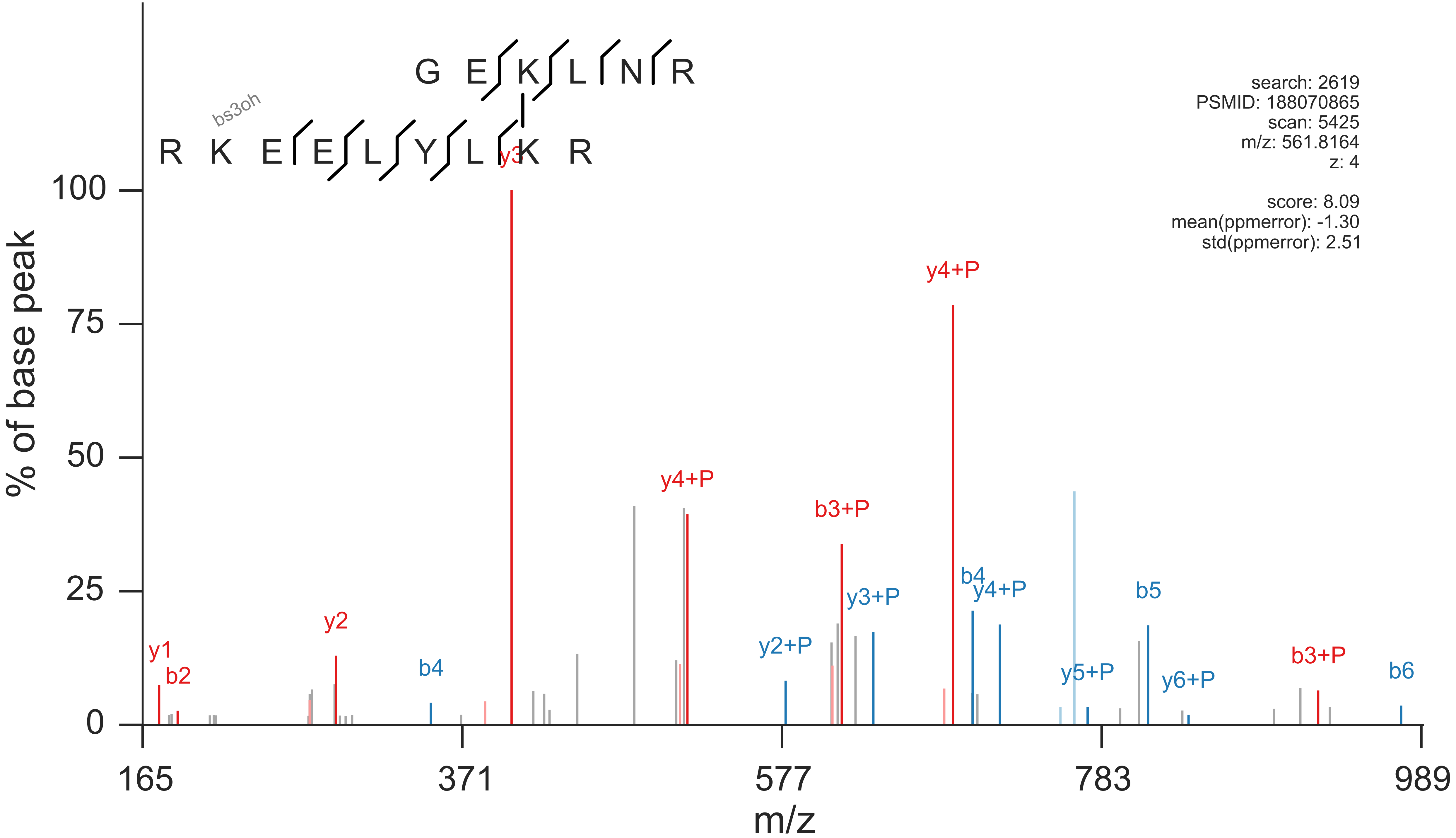

G E K L N R
R K E E L Y L K R
bs3oh
search: 2619
PSMID: 188070865
scan: 5425
m/z: 561.8164
z: 4
score: 8.09
mean(ppmerror): -1.30
std(ppmerror): 2.51
% of base peak
m/z
0
25
50
75
100
165
371
577
783
989
y1
b2
y2
b4
y3
y4+P
b3+P
y2+P
y3+P
y4+P
b4
y4+P
y5+P
b5
y6+P
b3+P
b6

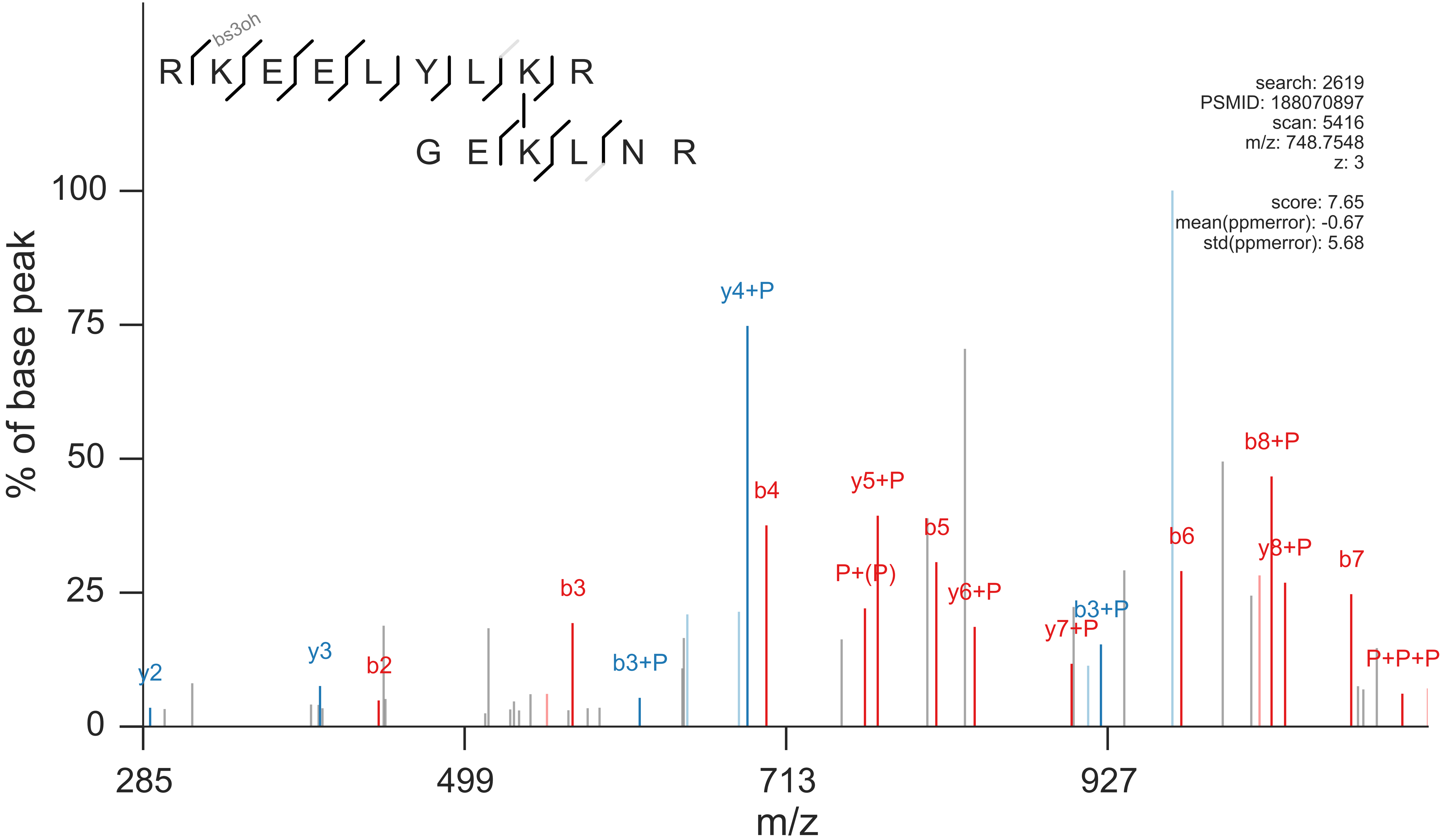

R K E E L Y L K R
bs3oh
G E K L N R
search: 2619
PSMID: 188070897
scan: 5416
m/z: 748.7548
z: 3
score: 7.65
mean(ppmerror): -0.67
std(ppmerror): 5.68
% of base peak
m/z
y2
y3
b2
b3
b3+P
y4+P
b4
P+(P)
y5+P
b5
y6+P
y7+P
b3+P
b6
b8+P
y8+P
b7
P+P+P
0
25
50
75
100
285
499
713
927

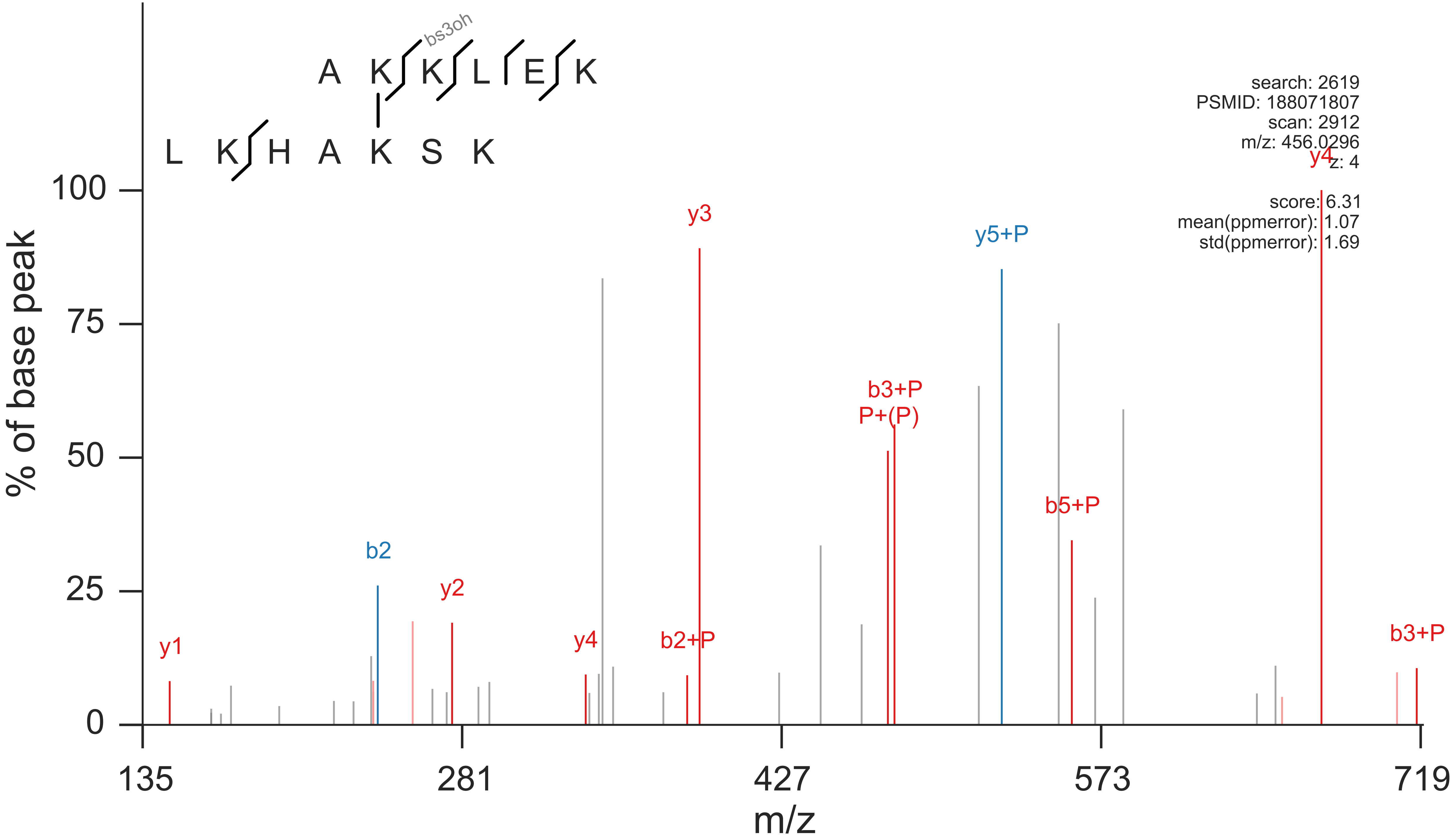

bs3oh
A K K L E K
L K H A K S K
search: 2619
PSMID: 188071807
scan: 2912
m/z: 456.0296
z: 4
score: 6.31
mean(ppmerror): 1.07
std(ppmerror): 1.69
y1
b2
y2
y4
b2+P
y3
b3+P
P+(P)
y5+P
b5+P
y4
b3+P
% of base peak
0
25
50
75
100
135
281
427
573
719
m/z

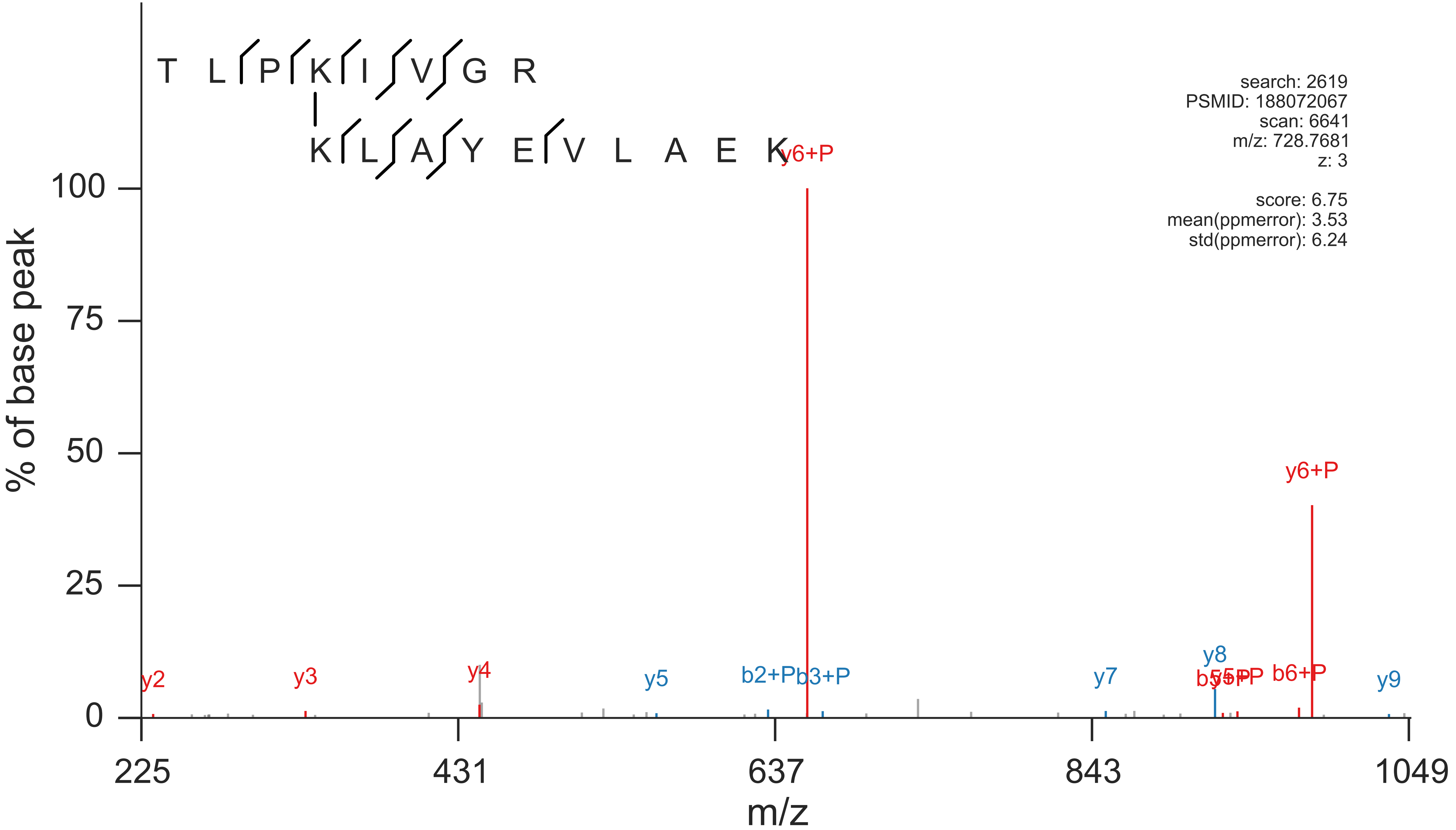

T L P K I V G R
K L A Y E V L A E K
search: 2619
PSMID: 188072067
scan: 6641
m/z: 728.7681
z: 3
score: 6.75
mean(ppmerror): 3.53
std(ppmerror): 6.24
% of base peak
m/z
100
75
50
25
0
225
431
637
843
1049
y6+P
y2
y3
y4
y5
b2+P
b3+P
y7
y8
b6+P
y9

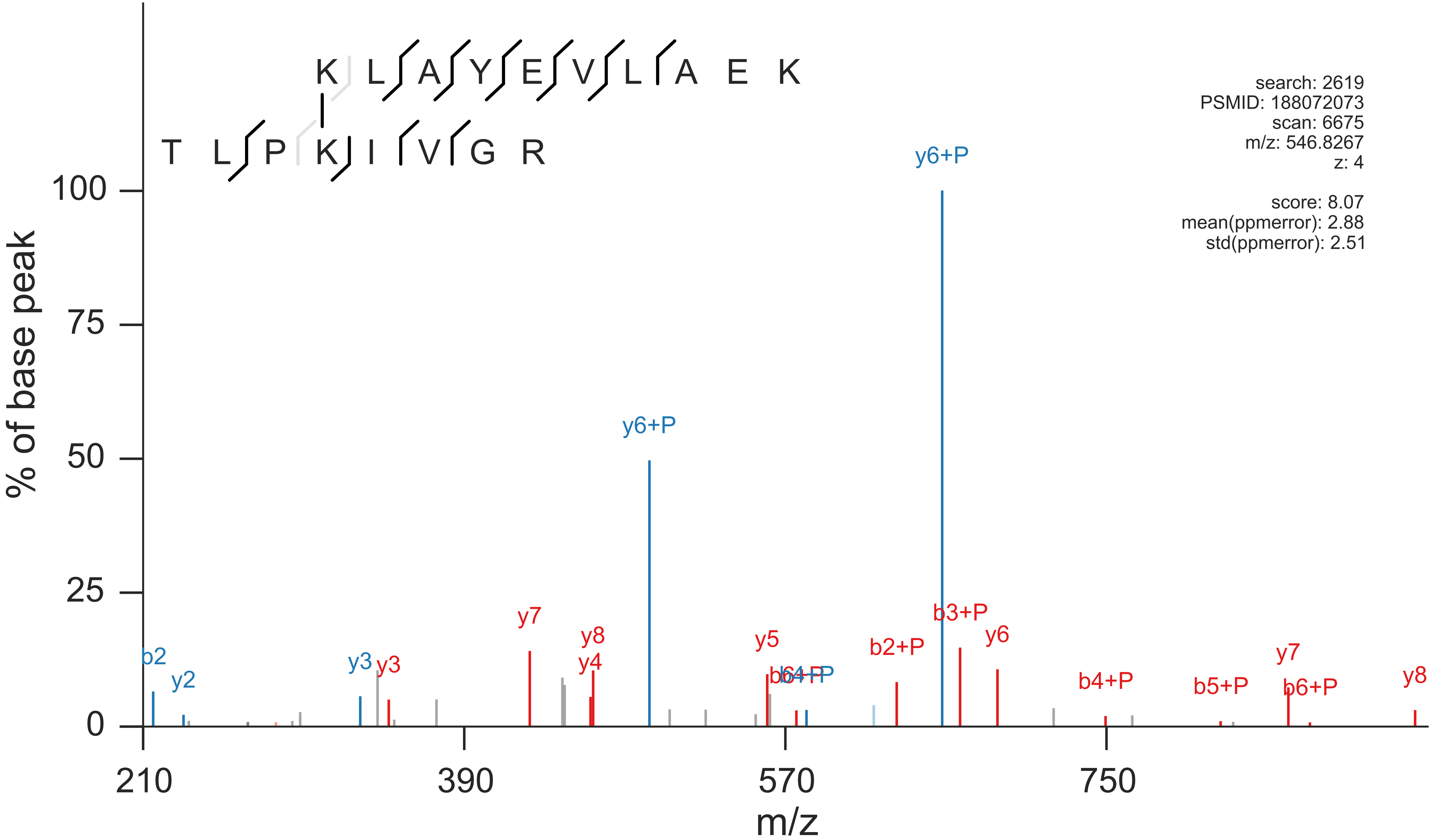

K L A Y E V L A E K
T L P K I V G R
search: 2619
PSMID: 188072073
scan: 6675
m/z: 546.8267
z: 4
score: 8.07
mean(ppmerror): 2.88
std(ppmerror): 2.51
% of base peak
m/z
0
25
50
75
100
210
390
570
750
y6+P
y6+P
b2
y2
y3
y3
y7
y8
y4
y5
b4+P
b3+P
b2+P
y6
b4+P
b5+P
y7
b6+P
y8

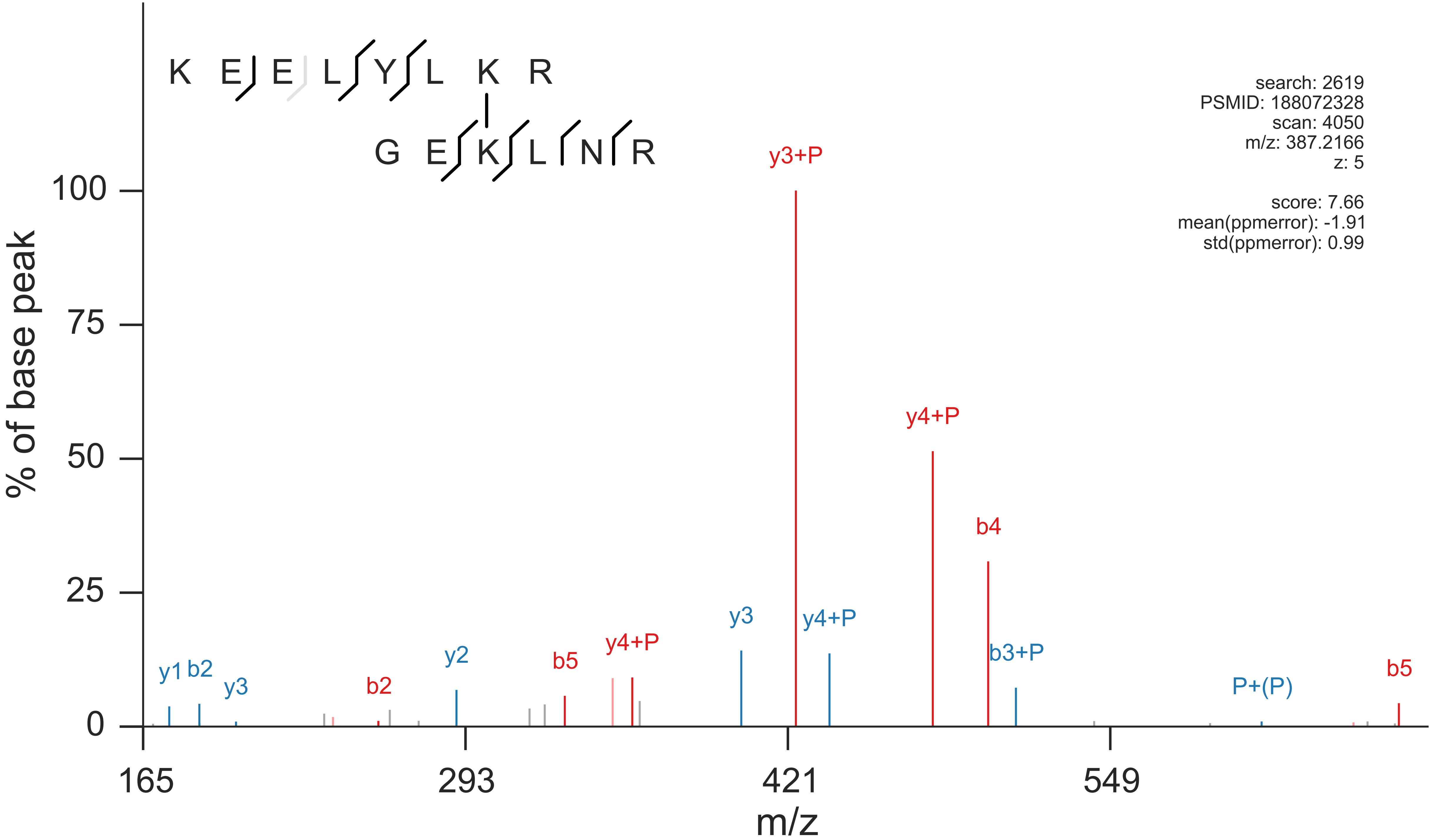

K E E L Y L K R
G E K L N R
search: 2619
PSMID: 188072328
scan: 4050
m/z: 387.2166
z: 5
score: 7.66
mean(ppmerror): -1.91
std(ppmerror): 0.99
% of base peak
m/z
y3+P
y4+P
b4
y1
b2
y3
b2
y2
b5
y4+P
y3
y4+P
b3+P
P+(P)
b5
0
25
50
75
100
165
293
421
549

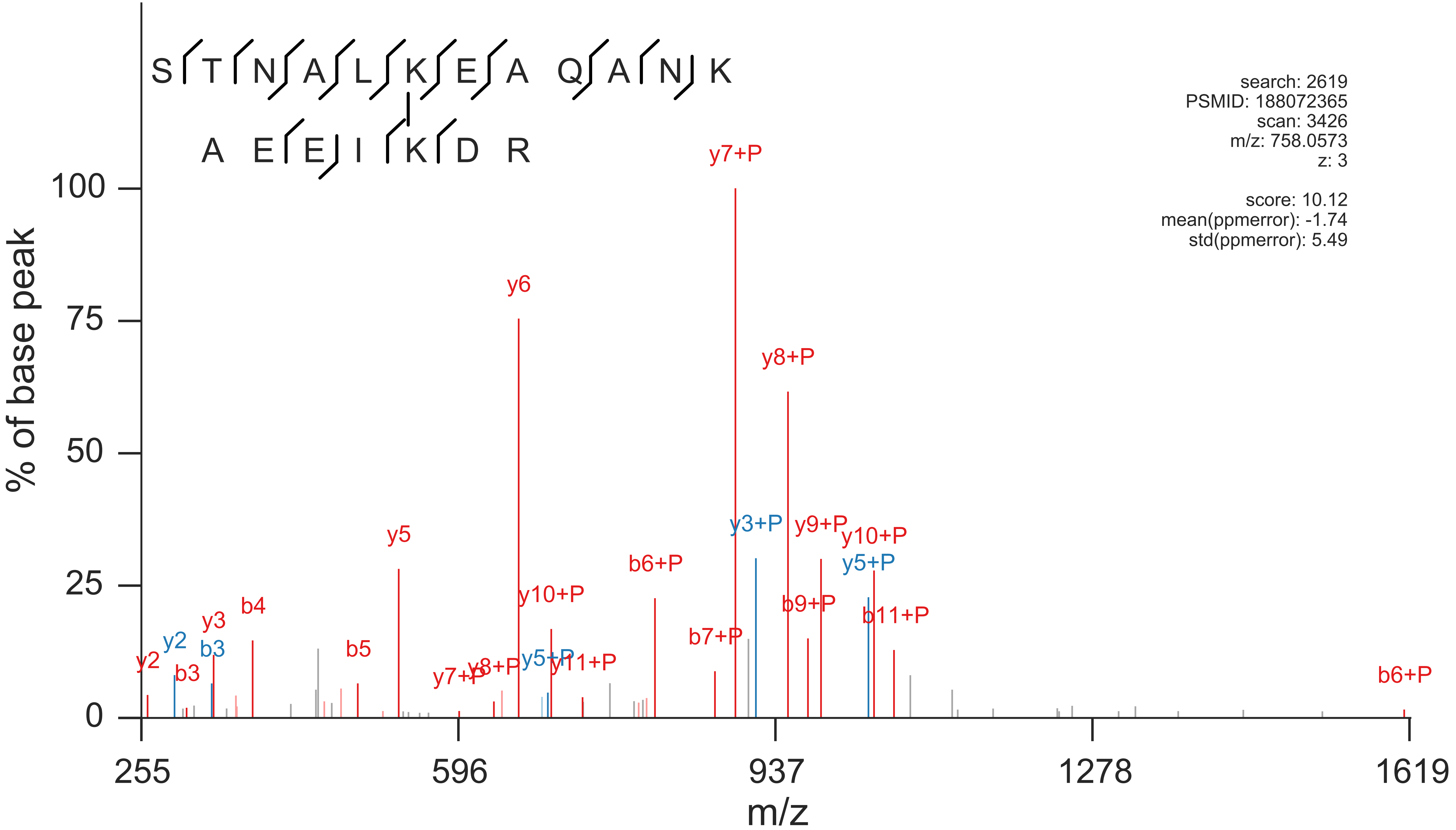

S T N A L K E A Q A N K
A E E I K D R
search: 2619
PSMID: 188072365
scan: 3426
m/z: 758.0573
z: 3
score: 10.12
mean(ppmerror): -1.74
std(ppmerror): 5.49
% of base peak
m/z
100
75
50
25
0
255
596
937
1278
1619
y7+P
y6
y8+P
y5
y3+P
y9+P
y10+P
y5+P
b6+P
y10+P
b9+P
b11+P
y2
y3
b4
b3
b5
b7+P
y2
b3
y7+P
y8+P
y5+P
y11+P
b6+P

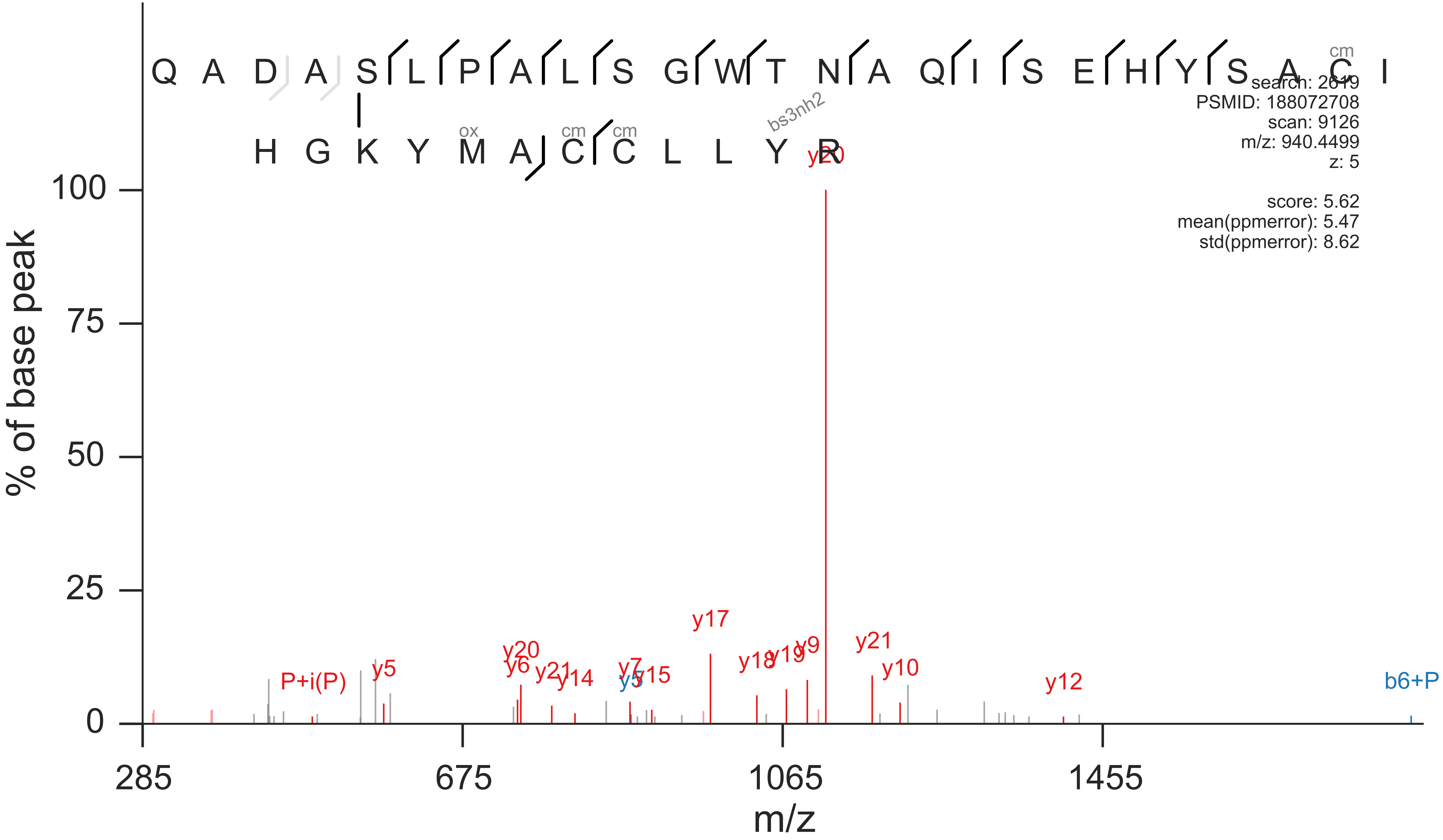

Q A D A S L P A L S G W T N A Q I S E H Y S A C I
H G K Y M A C C L L Y R
ox
cm
cm
cm
bs3nh2
search: 2619
PSMID: 188072708
scan: 9126
m/z: 940.4499
z: 5
score: 5.62
mean(ppmerror): 5.47
std(ppmerror): 8.62
% of base peak
m/z
y20
y17
y21
y10
y12
y5
y6
y14
y7
y15
y18
y19
y9
P+i(P)
b6+P

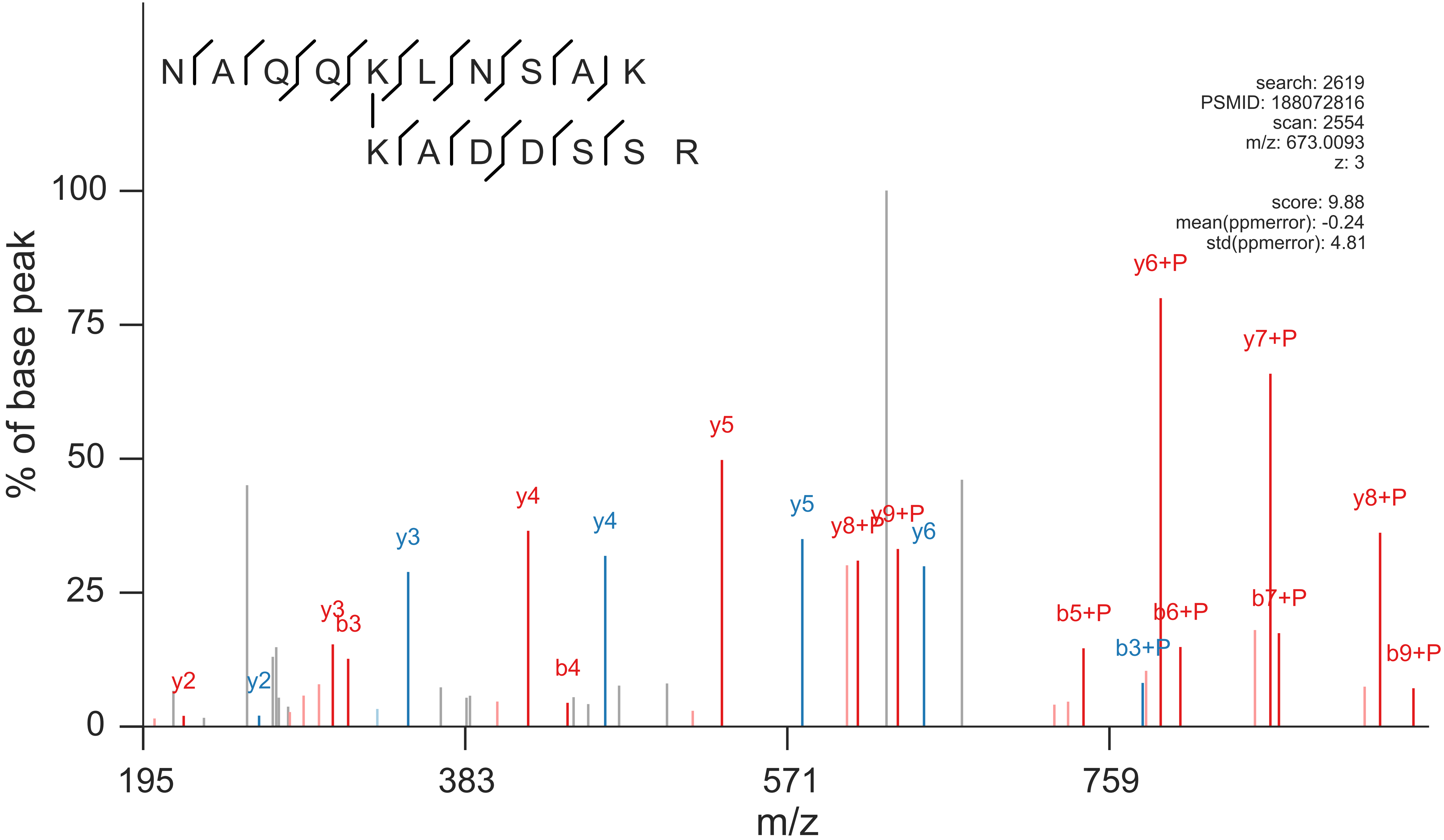
N A Q Q K L N S A K
K A D D S S R
search: 2619
PSMID: 188072816
scan: 2554
m/z: 673.0093
z: 3
score: 9.88
mean(ppmerror): -0.24
std(ppmerror): 4.81
% of base peak
100
75
50
25
0
195
383
571
759
m/z
y2
y2
y3
b3
y3
y4
b4
y4
y5
y5
y8+P
y9+P
y6
b5+P
b3+P
y6+P
b6+P
y7+P
b7+P
y8+P
b9+P

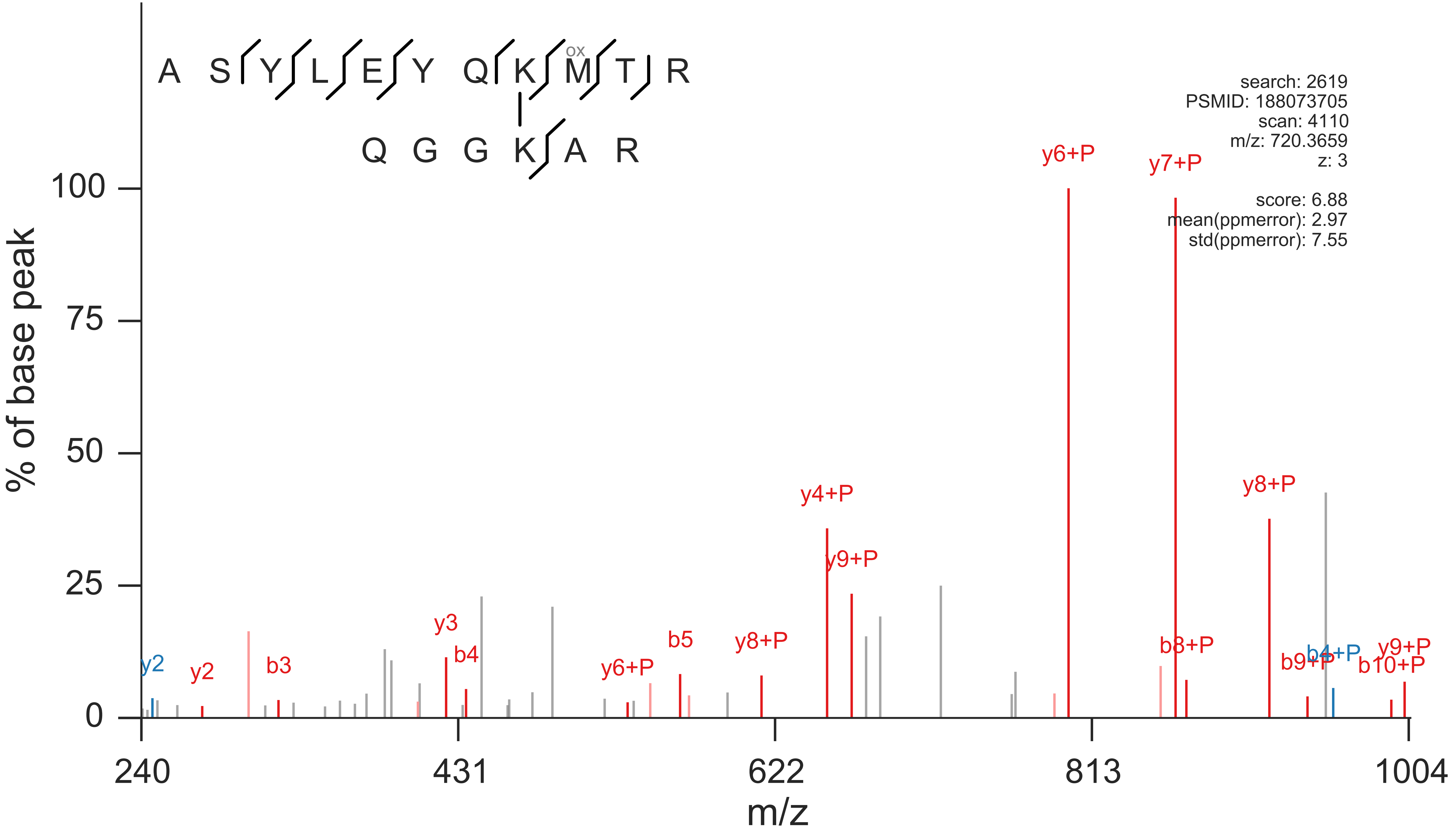
A S Y L E Y Q K M T R
ox
Q G G K A R
search: 2619
PSMID: 188073705
scan: 4110
m/z: 720.3659
z: 3
score: 6.88
mean(ppmerror): 2.97
std(ppmerror): 7.55
% of base peak
m/z
0
25
50
75
100
240
431
622
813
1004
y2
y2
b3
y3
b4
y6+P
b5
y8+P
y4+P
y9+P
y6+P
y7+P
b8+P
y8+P
b9+P
b4+P
y9+P
b10+P

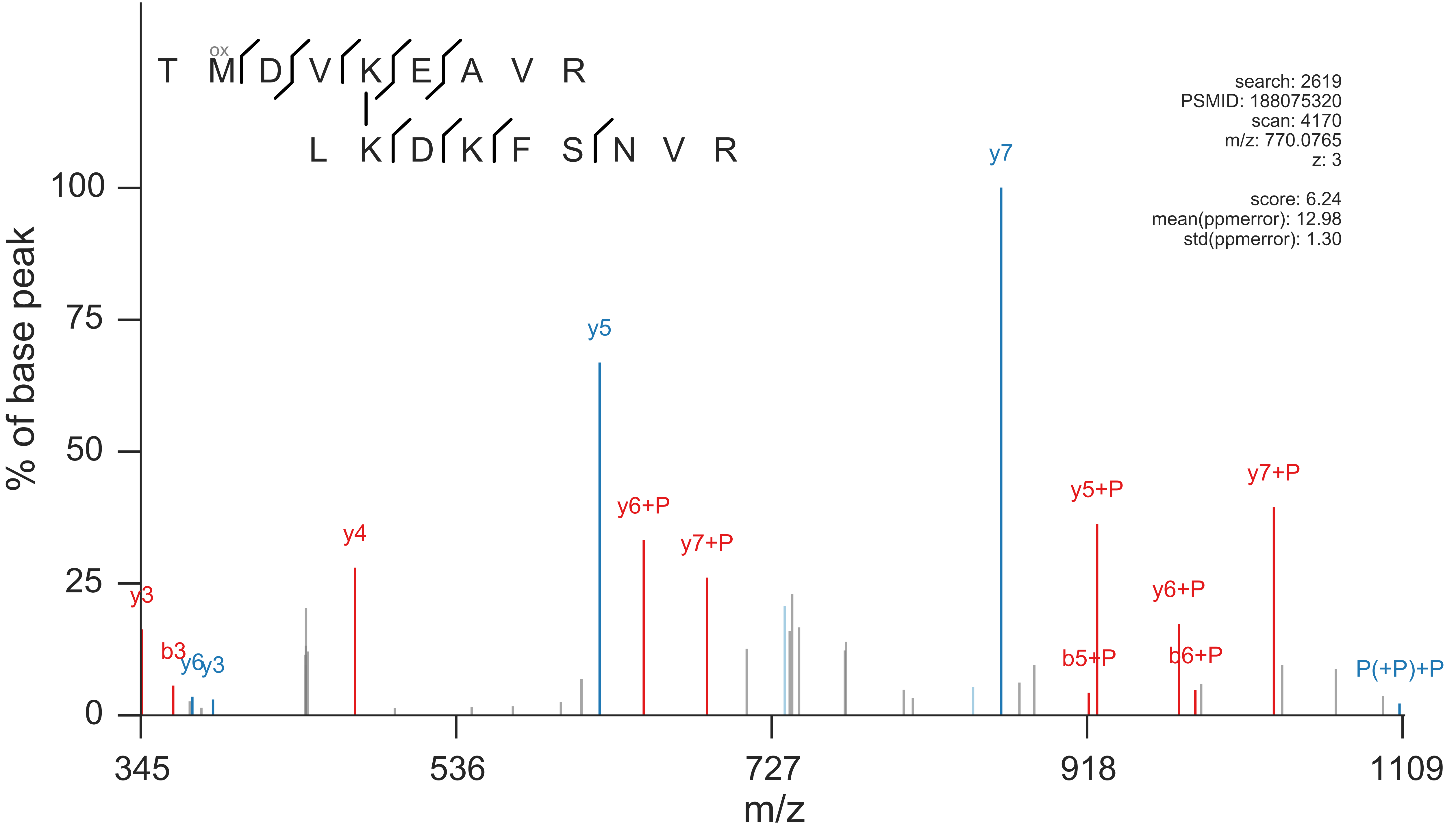

T M D V K E A V R
ox
L K D K F S N V R
search: 2619
PSMID: 188075320
scan: 4170
m/z: 770.0765
z: 3
score: 6.24
mean(ppmerror): 12.98
std(ppmerror): 1.30
% of base peak
0
25
50
75
100
m/z
345
536
727
918
1109
y3
b3
y6
y3
y4
y5
y6+P
y7+P
y7
b5+P
y5+P
y6+P
b6+P
y7+P
P(+P)+P

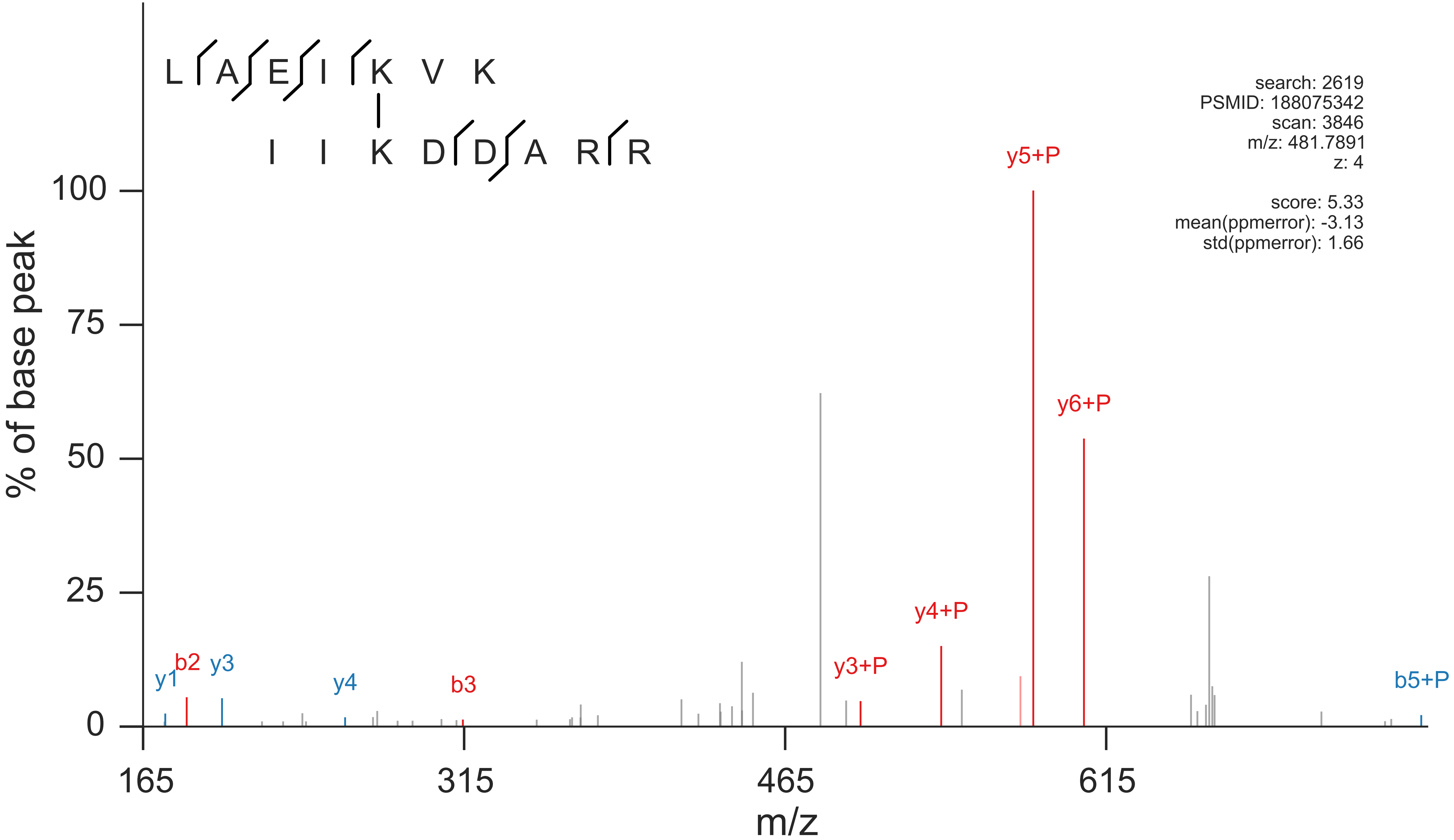

L A E I K V K
I I K D D A R R
search: 2619
PSMID: 188075342
scan: 3846
m/z: 481.7891
z: 4
score: 5.33
mean(ppmerror): -3.13
std(ppmerror): 1.66
y5+P
y6+P
y4+P
y3+P
y1
b2
y3
y4
b3
b5+P
100
75
50
25
0
% of base peak
165
315
465
615
m/z

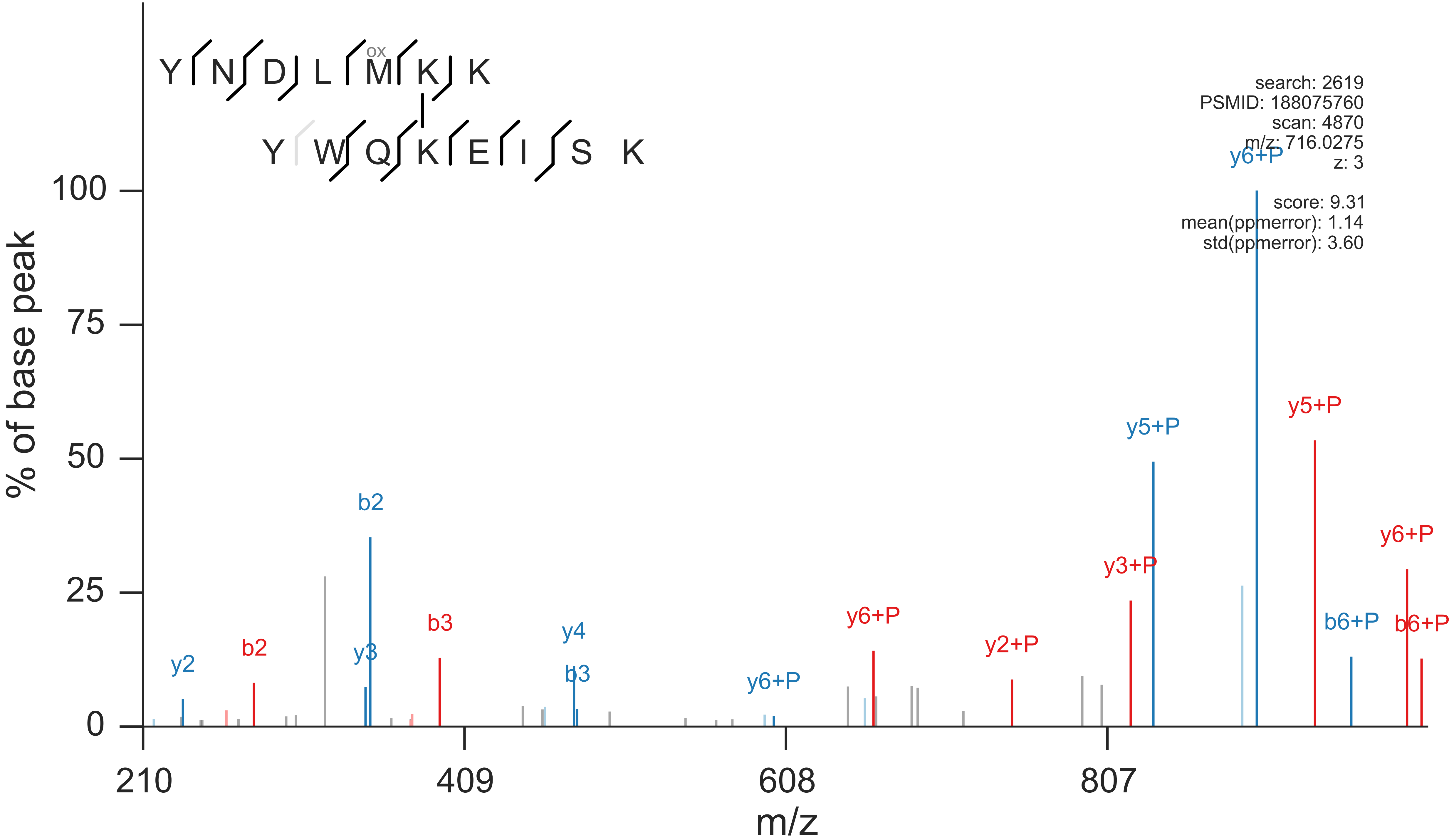

Y N D L M K K
ox
Y W Q K E I S K
search: 2619
PSMID: 188075760
scan: 4870
m/z: 716.0275
z: 3
score: 9.31
mean(ppmerror): 1.14
std(ppmerror): 3.60
% of base peak
100
75
50
25
0
210
409
608
807
m/z
y2
b2
b2
y3
b3
y4
b3
y6+P
y6+P
y2+P
y3+P
y5+P
y6+P
y5+P
b6+P
y6+P
b6+P

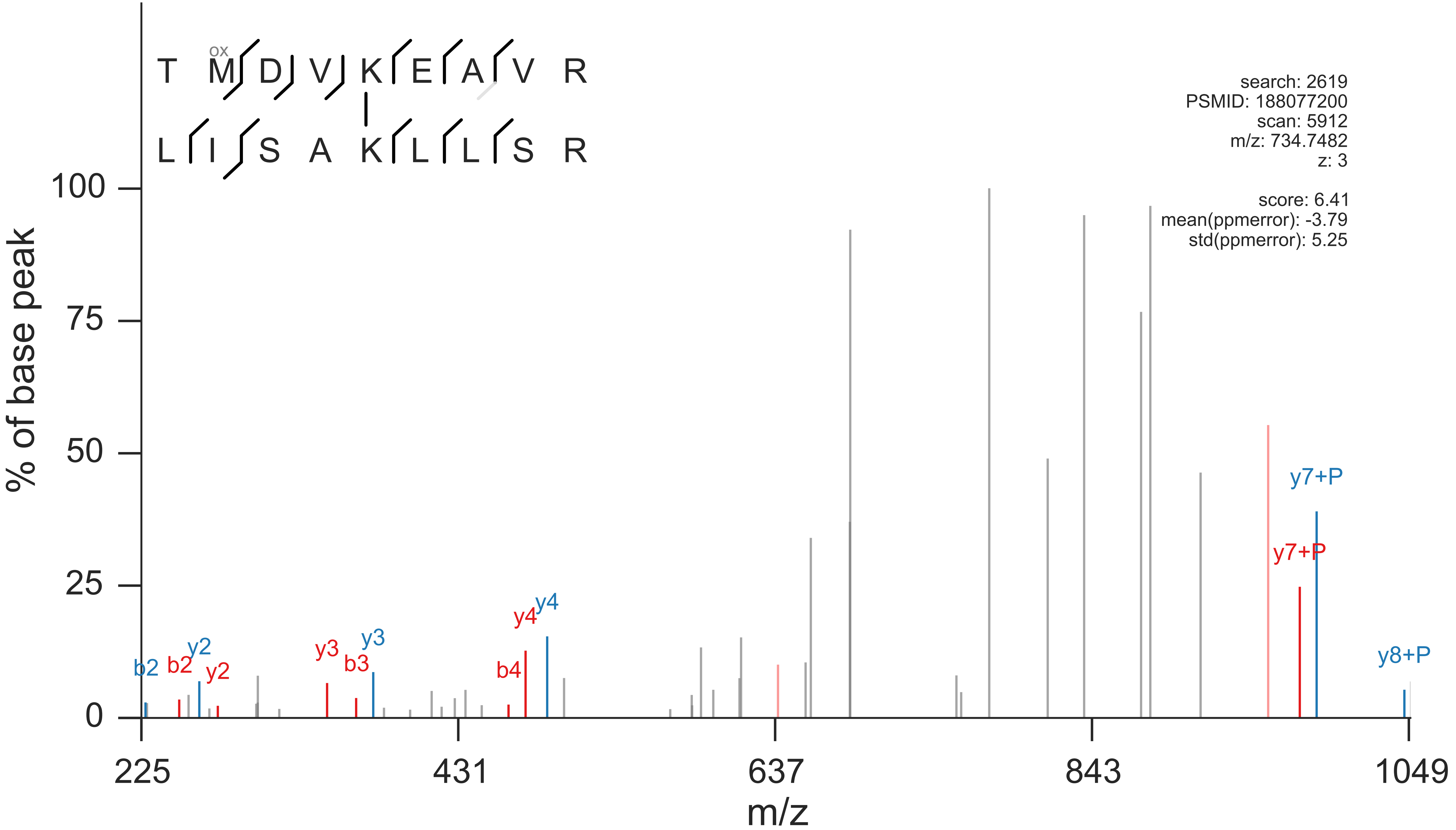

T M D V K E A V R
ox
L I S A K L L S R
search: 2619
PSMID: 188077200
scan: 5912
m/z: 734.7482
z: 3
score: 6.41
mean(ppmerror): -3.79
std(ppmerror): 5.25
% of base peak
100
75
50
25
0
b2
b2
y2
y2
y3
b3
y3
b4
y4
y4
y7+P
y7+P
y8+P
225
431
637
843
1049
m/z

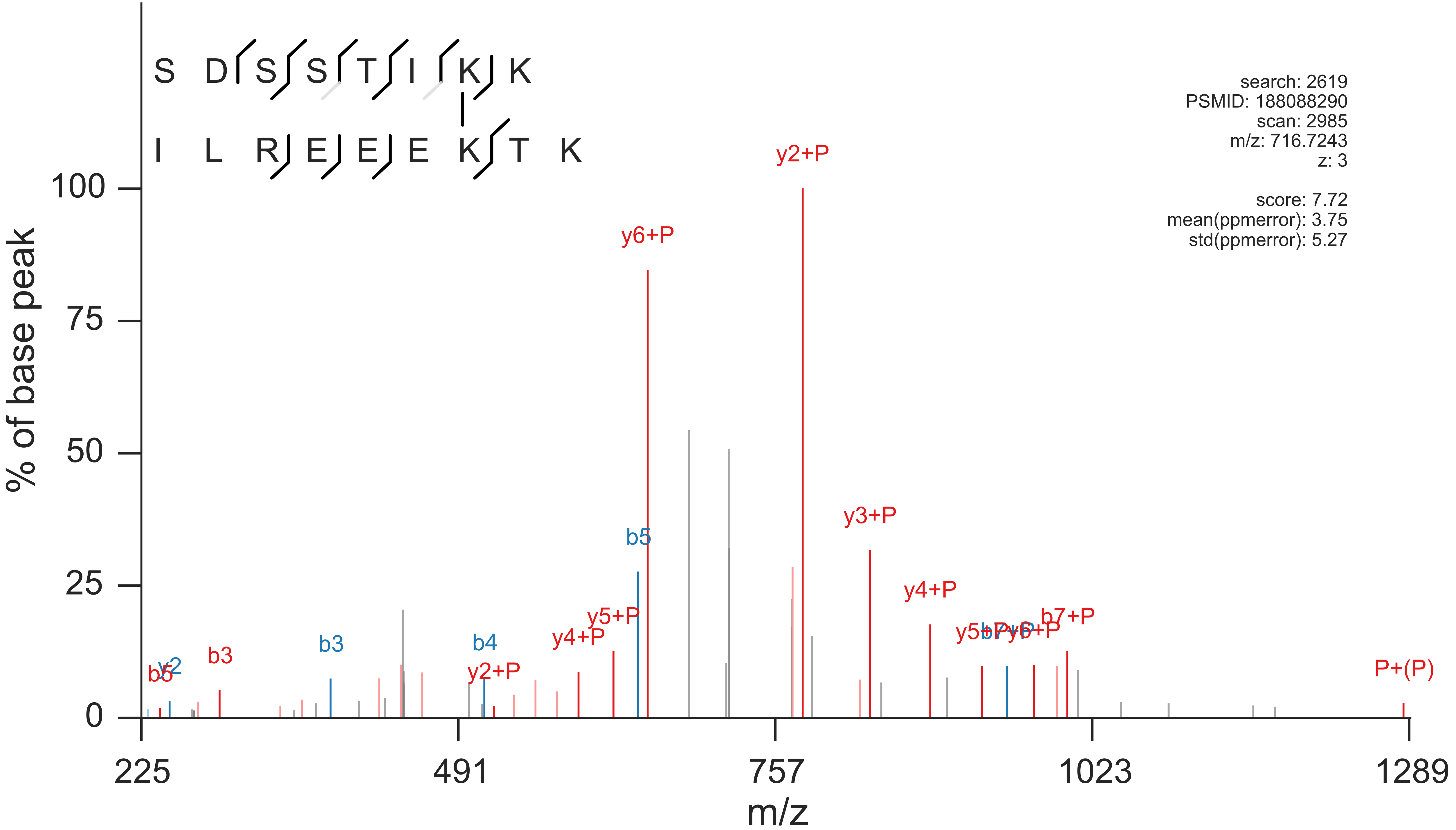

S D S S T I K K
I L R E E E K T K
search: 2619
PSMID: 188088290
scan: 2985
m/z: 716.7243
z: 3
score: 7.72
mean(ppmerror): 3.75
std(ppmerror): 5.27
% of base peak
m/z
y2+P
y6+P
y3+P
y4+P
y5+P
y4+P
b5
b3
b4
b3
y2
b5
y2+P
b7+P
P+(P)

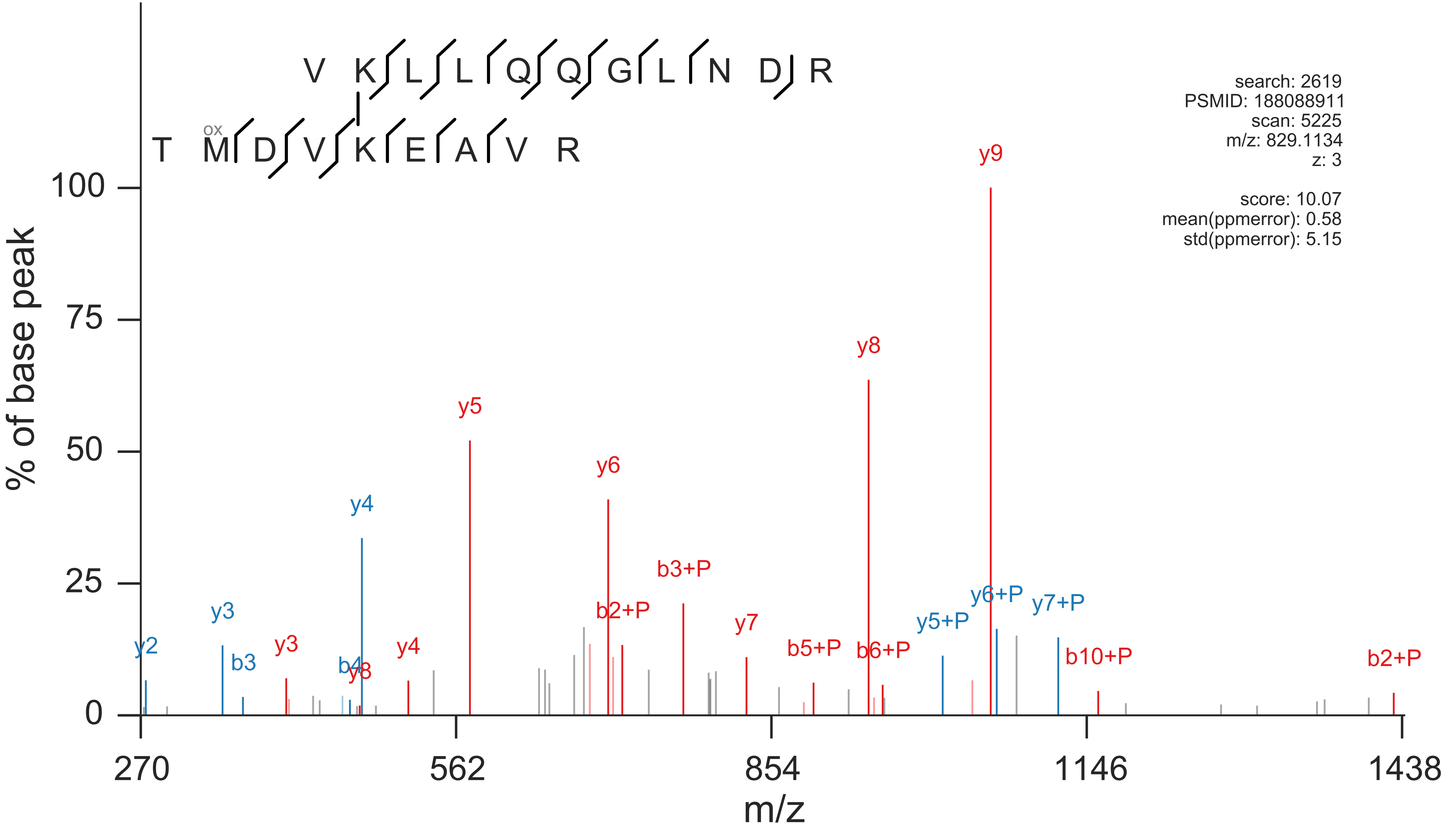

V K L L Q Q G L N D R
T M D V K E A V R
ox
search: 2619
PSMID: 188088911
scan: 5225
m/z: 829.1134
z: 3
score: 10.07
mean(ppmerror): 0.58
std(ppmerror): 5.15
% of base peak
m/z
0
25
50
75
100
270
562
854
1146
1438
y9
y8
y5
y6
y4
y3
y2
b3
y3
b4
y8
y4
b2+P
b3+P
y7
b5+P
b6+P
y5+P
y6+P
y7+P
b10+P
b2+P

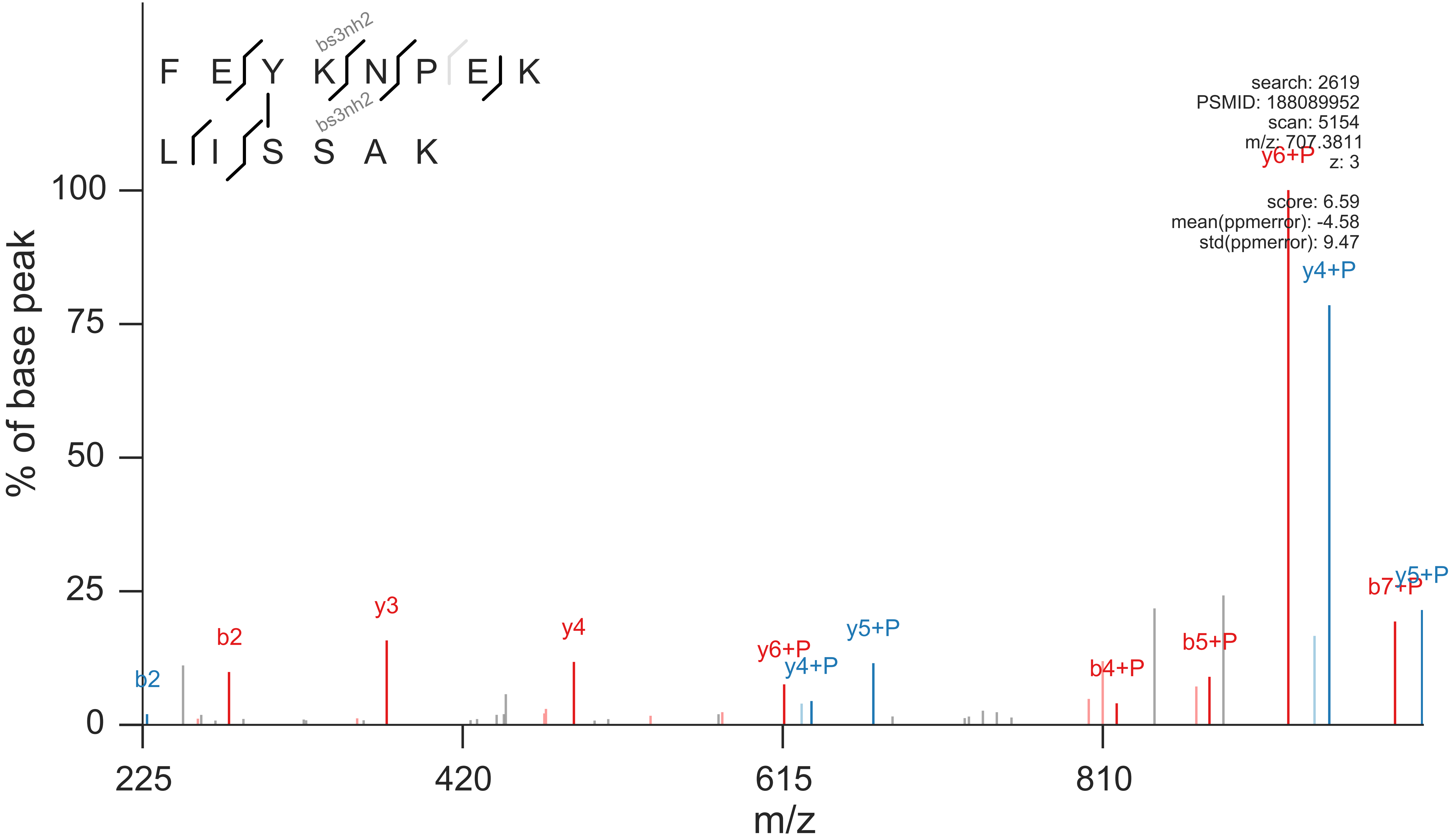

F E Y K N P E K
bs3nh2
bs3nh2
L I S S A K
search: 2619
PSMID: 188089952
scan: 5154
m/z: 707.3811
z: 3
score: 6.59
mean(ppmerror): -4.58
std(ppmerror): 9.47
% of base peak
m/z
0
25
50
75
100
225
420
615
810
b2
b2
y3
y4
y6+P
y4+P
y5+P
b4+P
b5+P
y6+P
y4+P
b7+P
y5+P

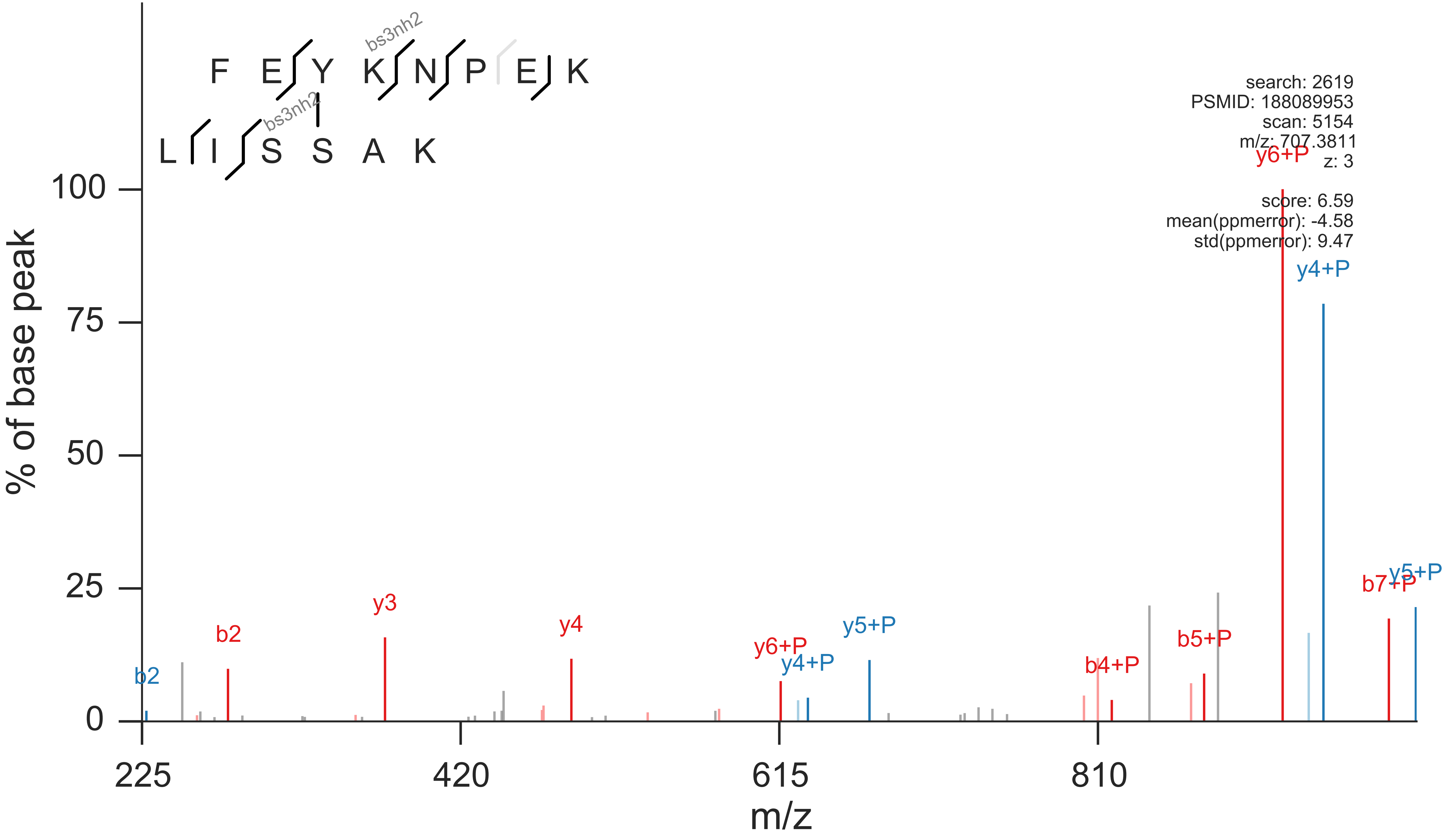

F E Y K N P E K
L I S S A K
bs3nh2
bs3nh2
search: 2619
PSMID: 188089953
scan: 5154
m/z: 707.3811
z: 3
score: 6.59
mean(ppmerror): -4.58
std(ppmerror): 9.47
% of base peak
m/z
0
25
50
75
100
225
420
615
810
b2
b2
y3
y4
y6+P
y4+P
y5+P
b4+P
b5+P
y6+P
y4+P
b7+P
y5+P

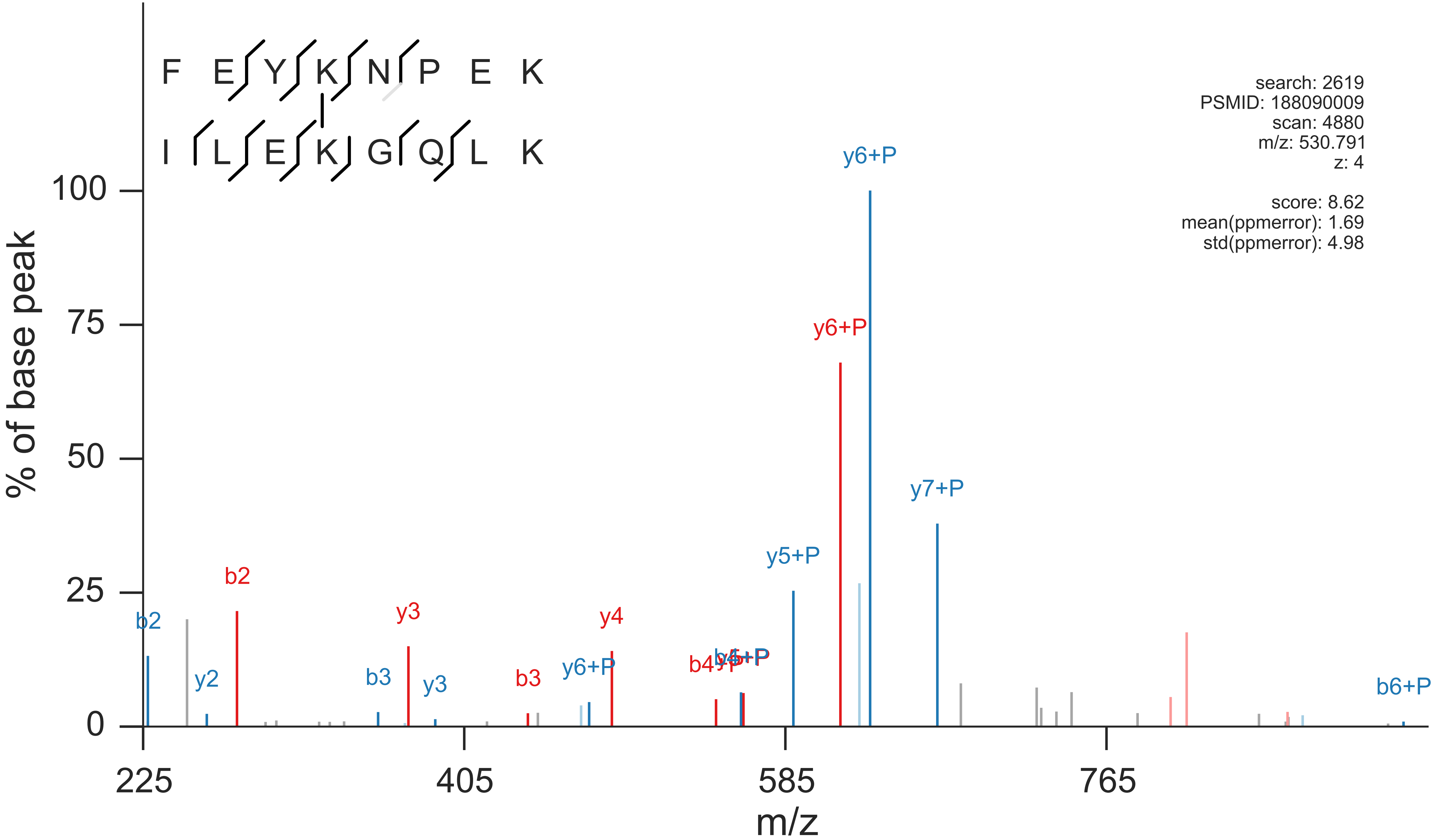
F E Y K N P E K
I L E K G Q L K
search: 2619
PSMID: 188090009
scan: 4880
m/z: 530.791
z: 4
score: 8.62
mean(ppmerror): 1.69
std(ppmerror): 4.98
% of base peak
m/z
0
25
50
75
100
225
405
585
765
b2
y2
b2
y3
b3
y3
b3
y6+P
y4
b4+P
b4+P
y5+P
y6+P
y6+P
y7+P
b6+P

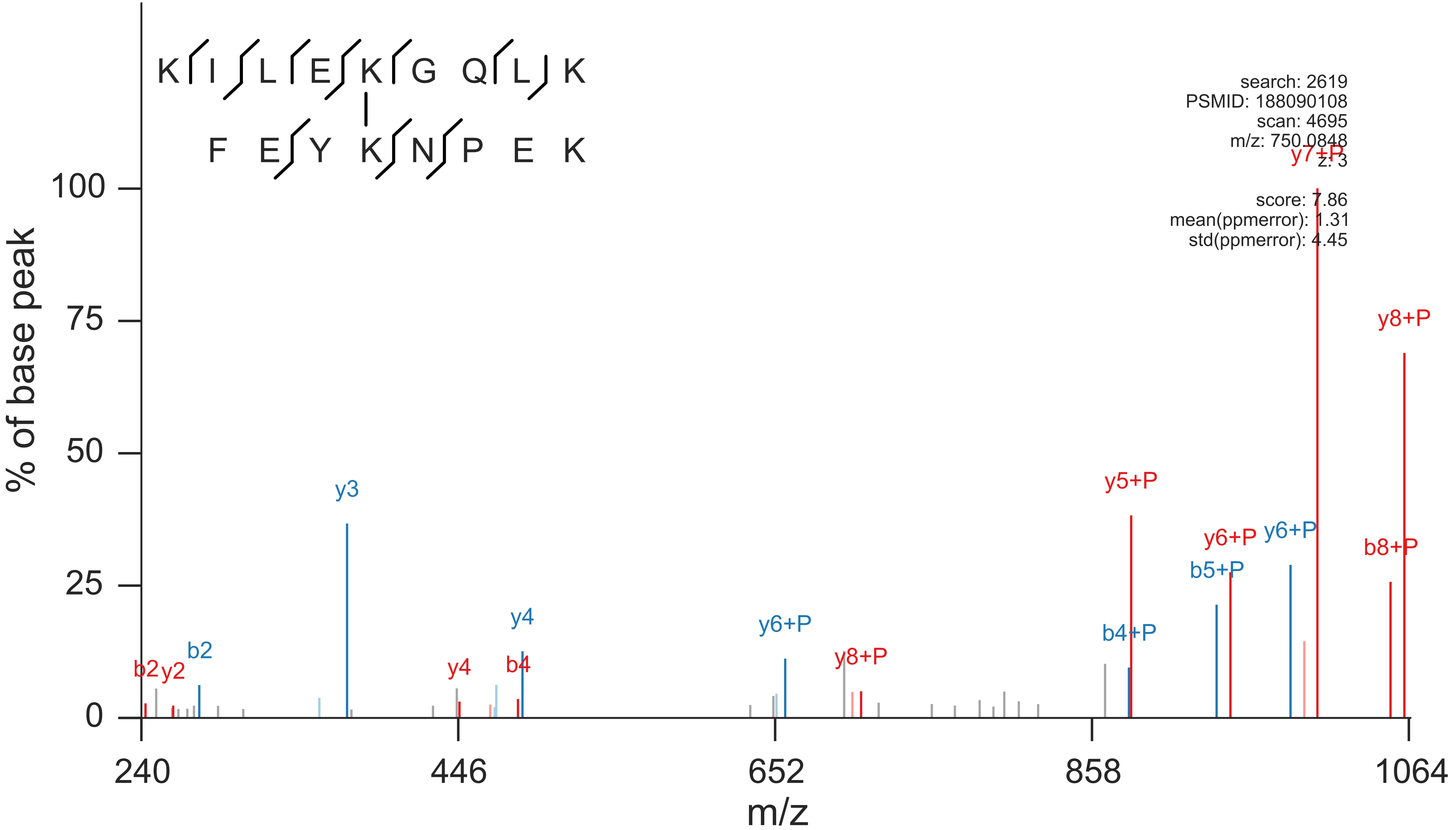

K I L E K G Q L K
F E Y K N P E K
search: 2619
PSMID: 188090108
scan: 4695
m/z: 750.0848
z: 3
score: 7.86
mean(ppmerror): 1.31
std(ppmerror): 4.45
% of base peak
m/z
100
75
50
25
0
240
446
652
858
1064
b2
y2
b2
y3
y4
b4
y4
y6+P
y8+P
b4+P
y5+P
b5+P
y6+P
y6+P
y7+P
b8+P
y8+P

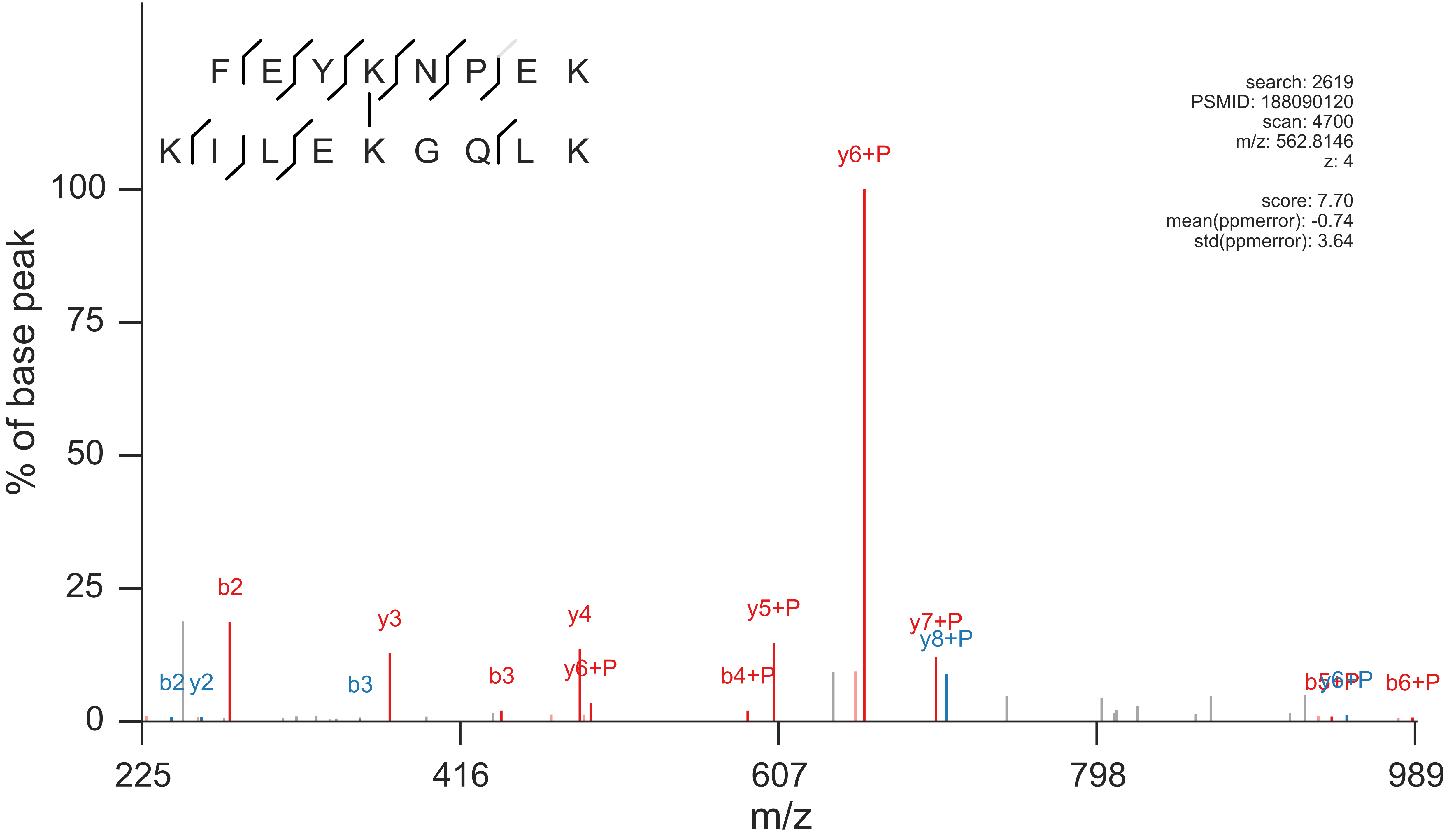

F E Y K N P E K
K I L E K G Q L K
search: 2619
PSMID: 188090120
scan: 4700
m/z: 562.8146
z: 4
score: 7.70
mean(ppmerror): -0.74
std(ppmerror): 3.64
% of base peak
100
75
50
25
0
y6+P
b2
y3
y4
y5+P
y7+P
y8+P
b2
y2
b3
b3
y6+P
b4+P
b6+P
225
416
607
798
989
m/z

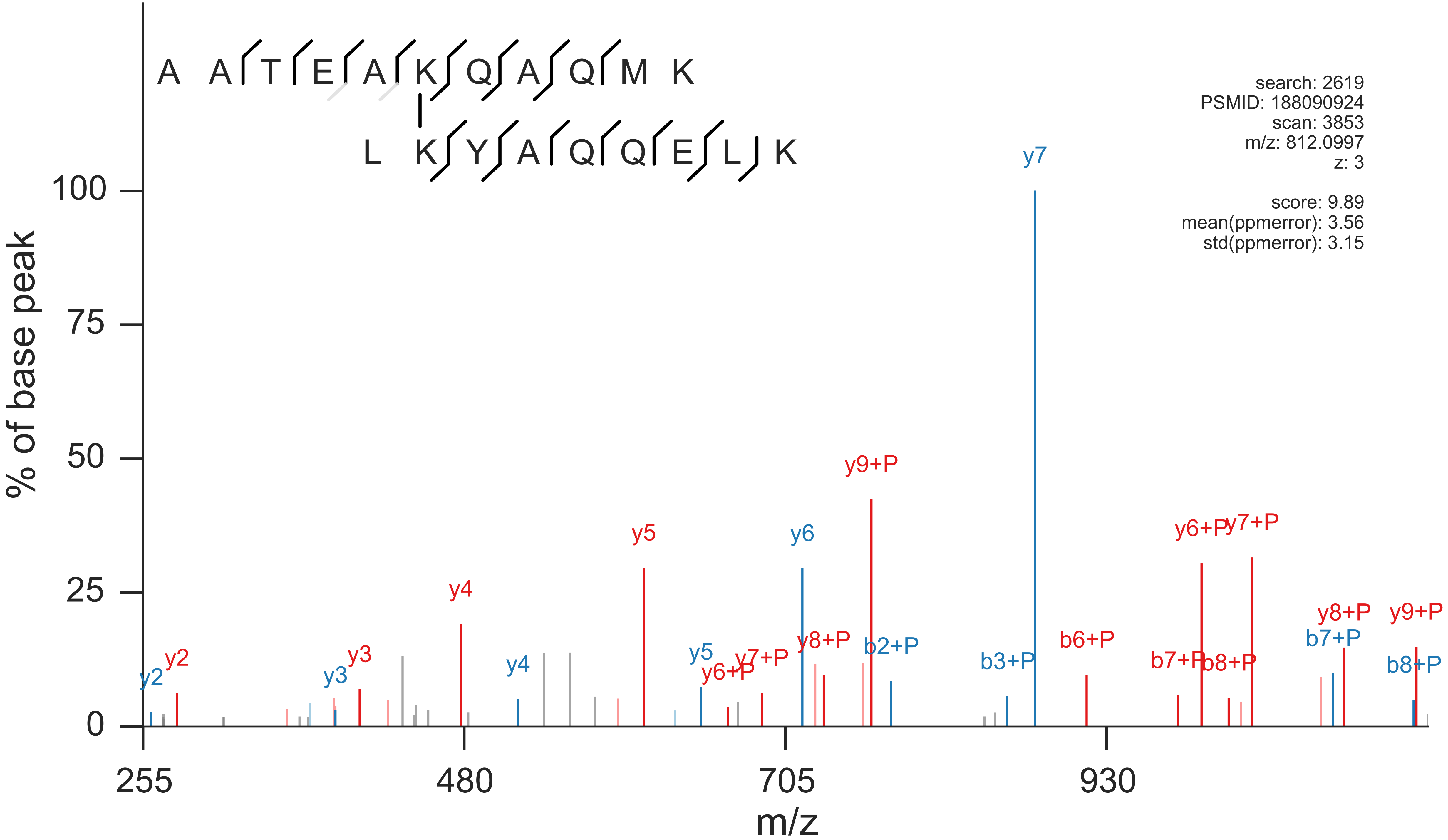
A A T E A K Q A Q M K
L K Y A Q Q E L K
search: 2619
PSMID: 188090924
scan: 3853
m/z: 812.0997
z: 3
score: 9.89
mean(ppmerror): 3.56
std(ppmerror): 3.15
% of base peak
m/z
y2
y2
y3
y3
y4
y4
y5
y5
y6
y6+P
y7+P
y8+P
y9+P
b2+P
b3+P
y7
b6+P
b7+P
b8+P
y6+P
y7+P
y8+P
b7+P
y9+P
b8+P
100
75
50
25
0
255
480
705
930

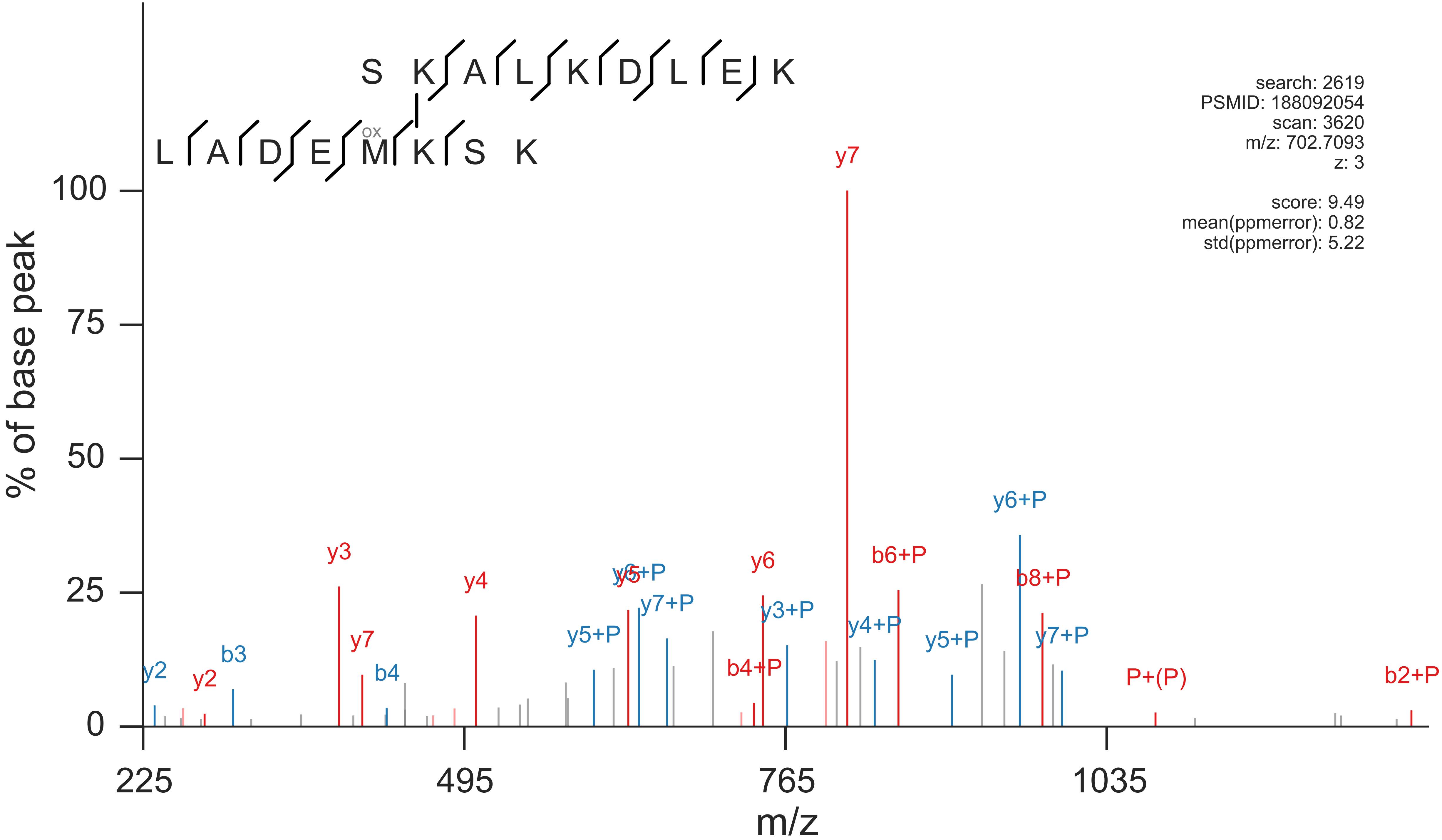
S K A L K D L E K
L A D E M K S K
ox
search: 2619
PSMID: 188092054
scan: 3620
m/z: 702.7093
z: 3
score: 9.49
mean(ppmerror): 0.82
std(ppmerror): 5.22
% of base peak
100
75
50
25
0
225
495
765
1035
m/z
y7
y6+P
b6+P
b8+P
y6
y5
y5+P
y7+P
y3+P
y4+P
y5+P
y7+P
y3
y4
y7
b4+P
y2
y2
b3
b4
P+(P)
b2+P

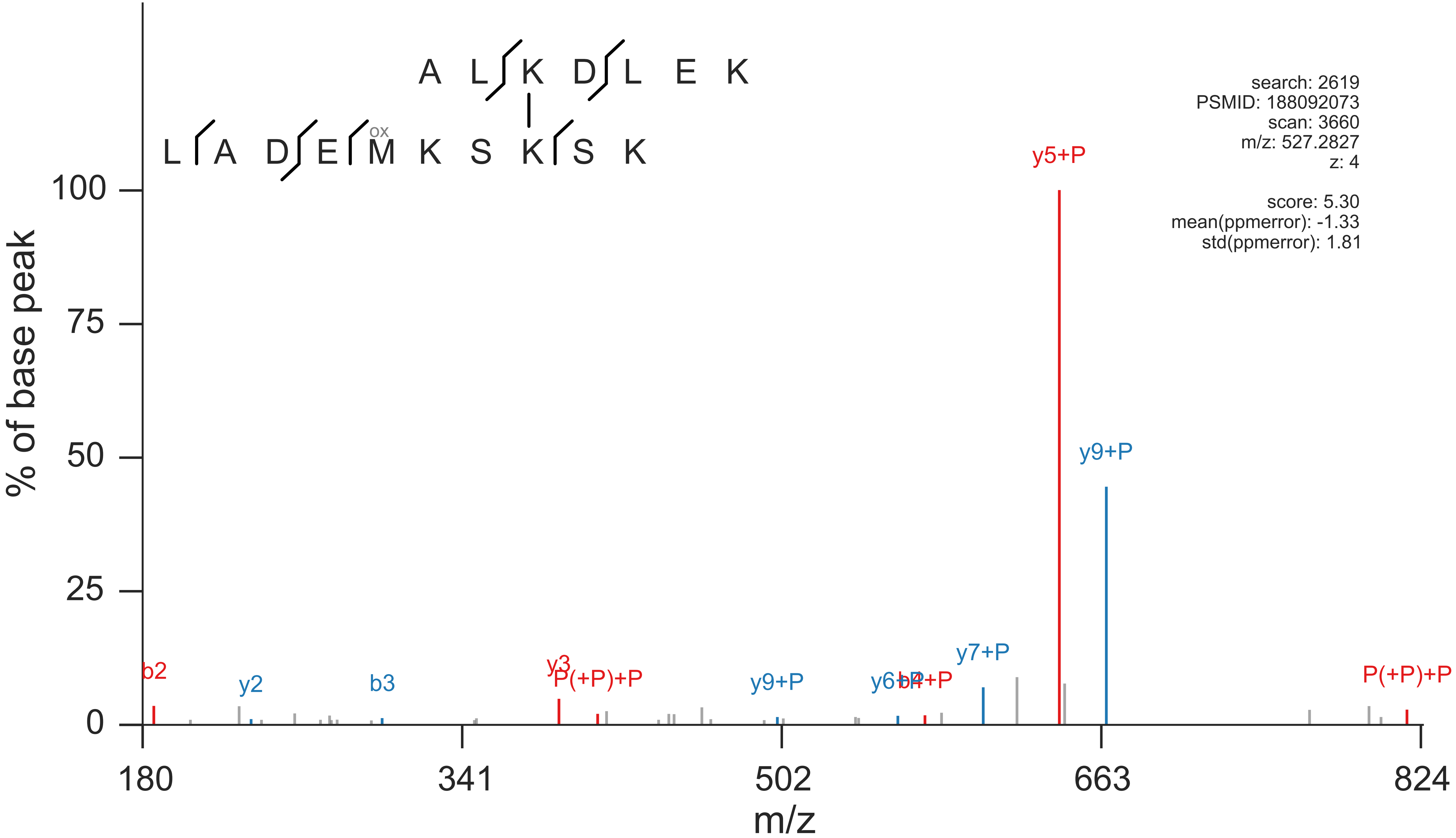

A L K D L E K
L A D E M K S K S K
ox
search: 2619
PSMID: 188092073
scan: 3660
m/z: 527.2827
z: 4
score: 5.30
mean(ppmerror): -1.33
std(ppmerror): 1.81
y5+P
y9+P
b2
y2
b3
y3
P(+P)+P
y9+P
y6+P
b4+P
y7+P
P(+P)+P
% of base peak
100
75
50
25
0
180
341
502
663
824
m/z

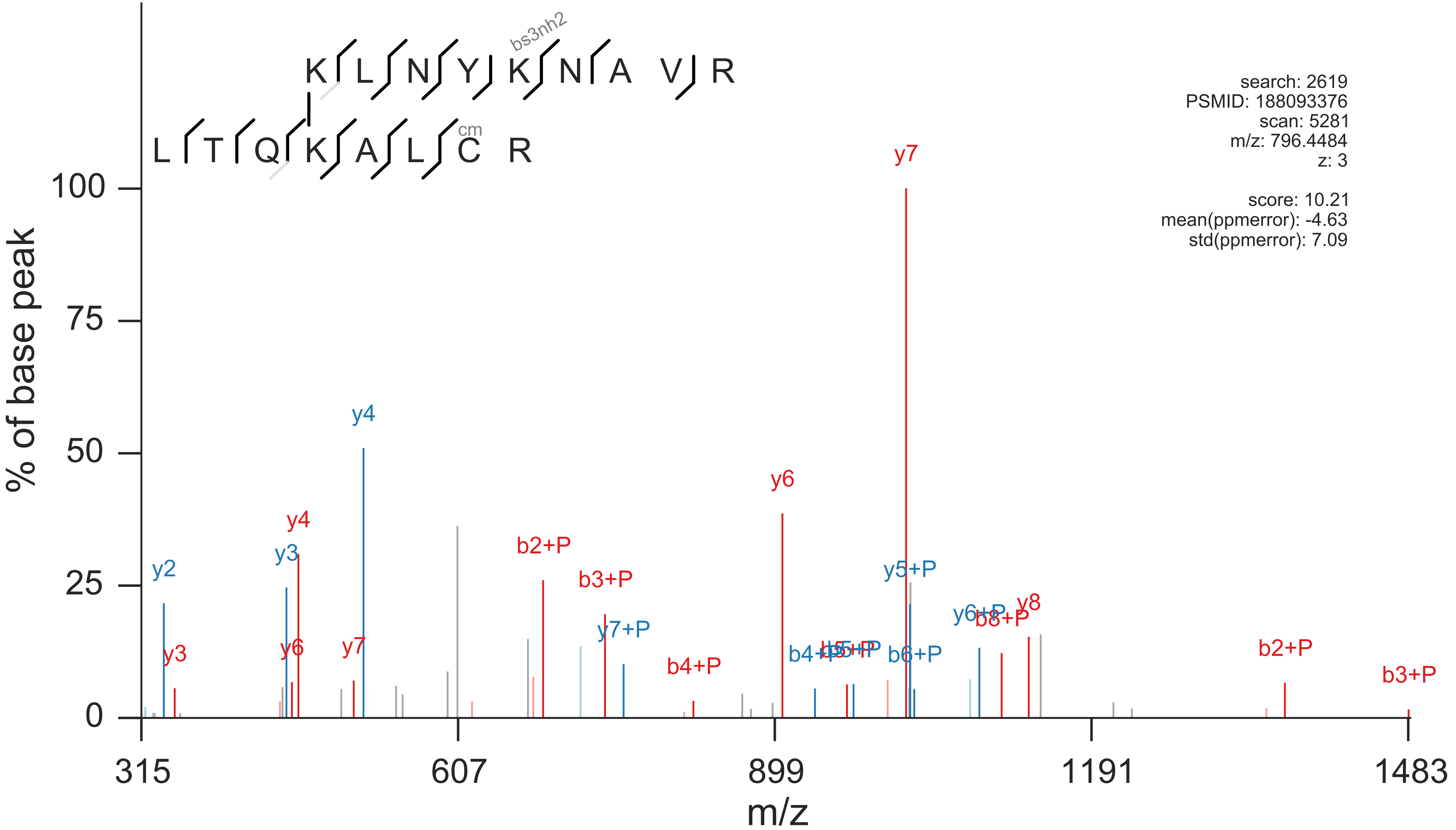

K L N Y K N A V R
bs3nh2
L T Q K A L C R
cm
search: 2619
PSMID: 188093376
scan: 5281
m/z: 796.4484
z: 3
score: 10.21
mean(ppmerror): -4.63
std(ppmerror): 7.09
% of base peak
100
75
50
25
0
315
607
899
1191
1483
m/z
y7
y4
y6
y4
y3
y2
b2+P
b3+P
y5+P
y8
y6+P
b8+P
y7+P
y3
y6
y7
b4+P
b4+P
b6+P
b2+P
b3+P

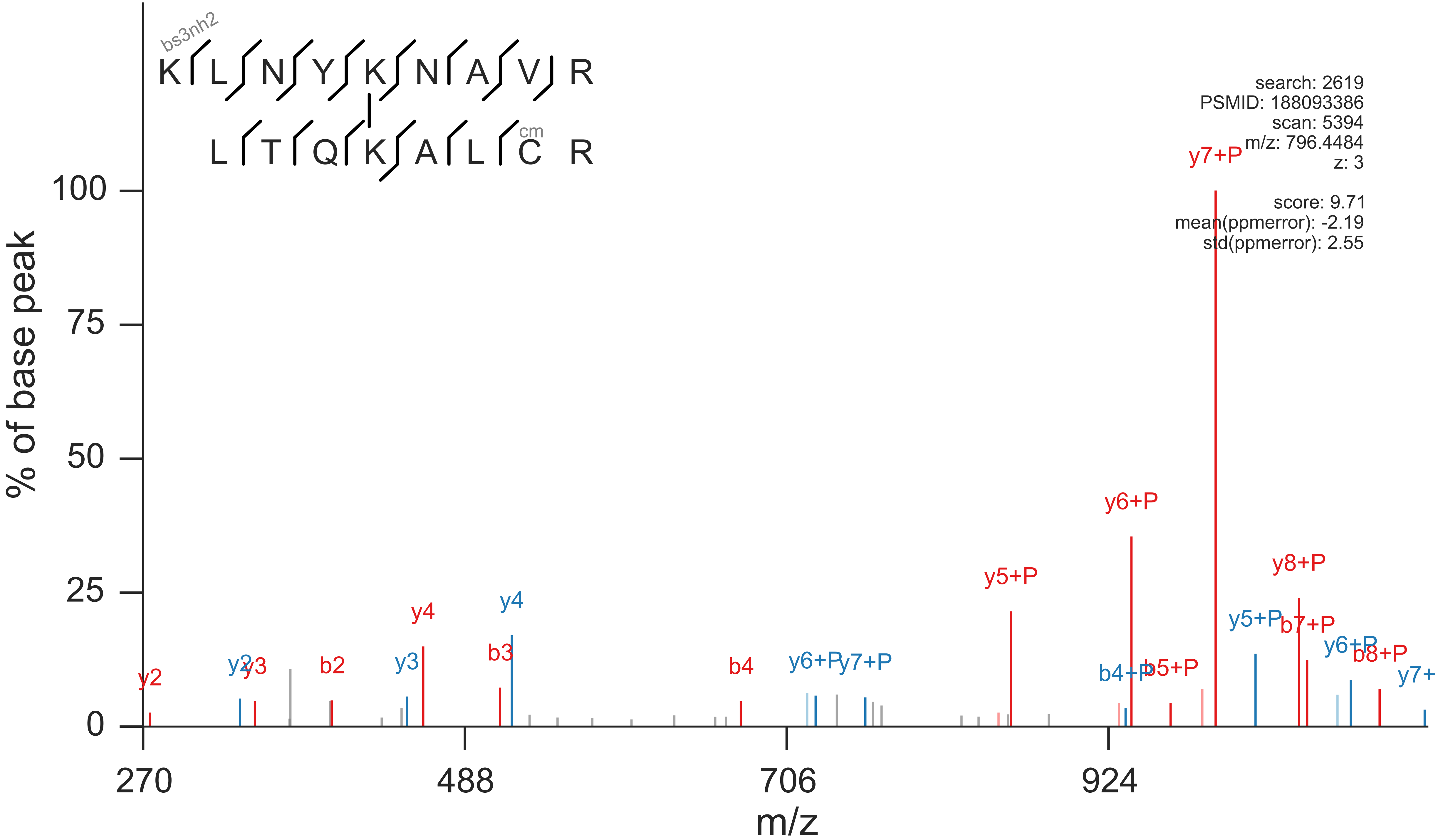

bs3nh2
K L N Y K N A V R
L T Q K A L C R
cm
search: 2619
PSMID: 188093386
scan: 5394
m/z: 796.4484
z: 3
score: 9.71
mean(ppmerror): -2.19
std(ppmerror): 2.55
% of base peak
m/z
y7+P
y6+P
y8+P
y5+P
b7+P
b5+P
b8+P
b4+P
y4
b3
b4
b2
y3
y2

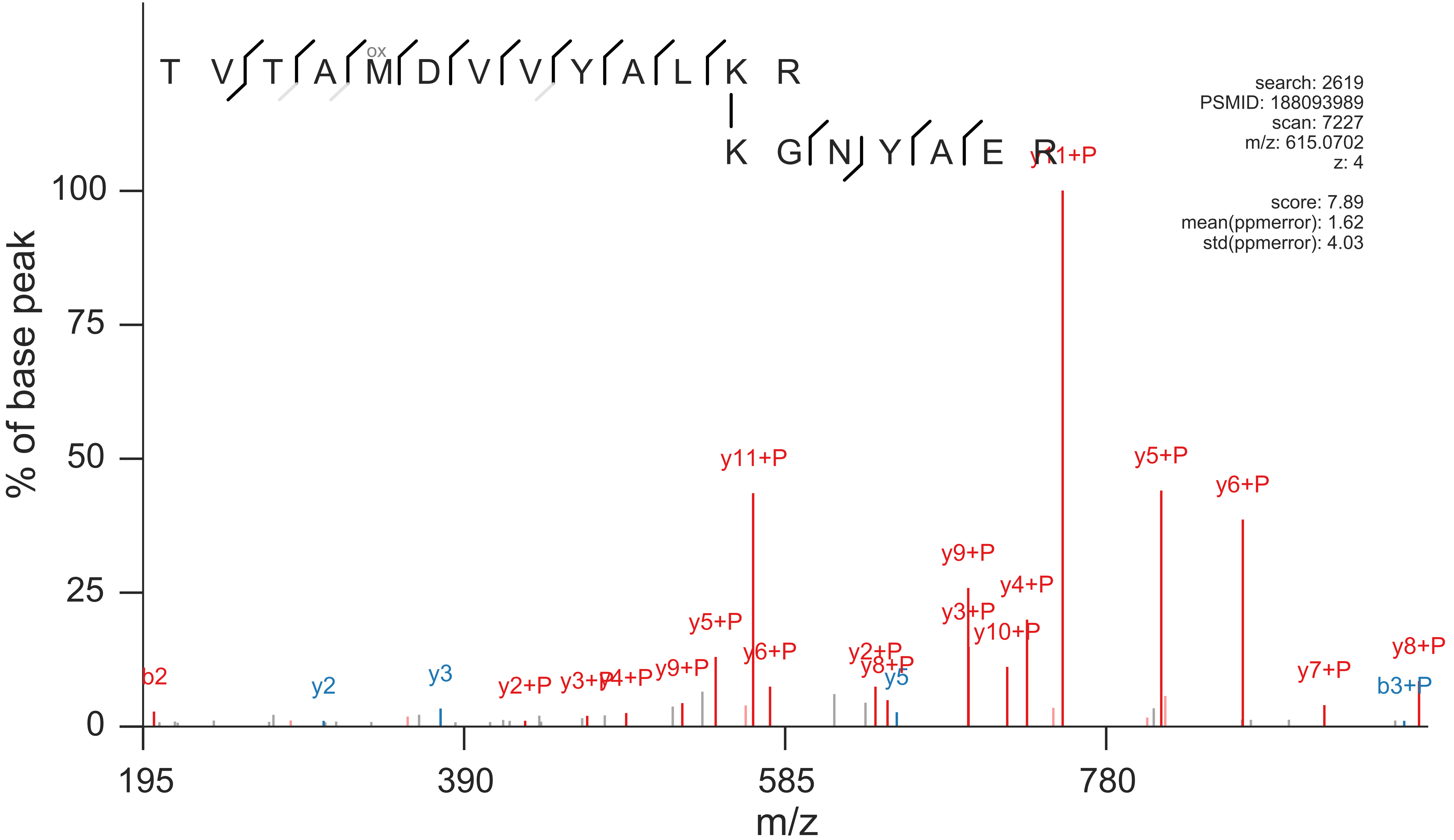

T V T A M D V V Y A L K R
ox
K G N Y A E R
search: 2619
PSMID: 188093989
scan: 7227
m/z: 615.0702
z: 4
score: 7.89
mean(ppmerror): 1.62
std(ppmerror): 4.03
% of base peak
m/z
0
25
50
75
100
195
390
585
780
b2
y2
y3
y2+P
y3+P
y4+P
y9+P
y5+P
y11+P
y6+P
y2+P
y8+P
y5
y9+P
y3+P
y10+P
y4+P
y11+P
y5+P
y6+P
y7+P
b3+P
y8+P

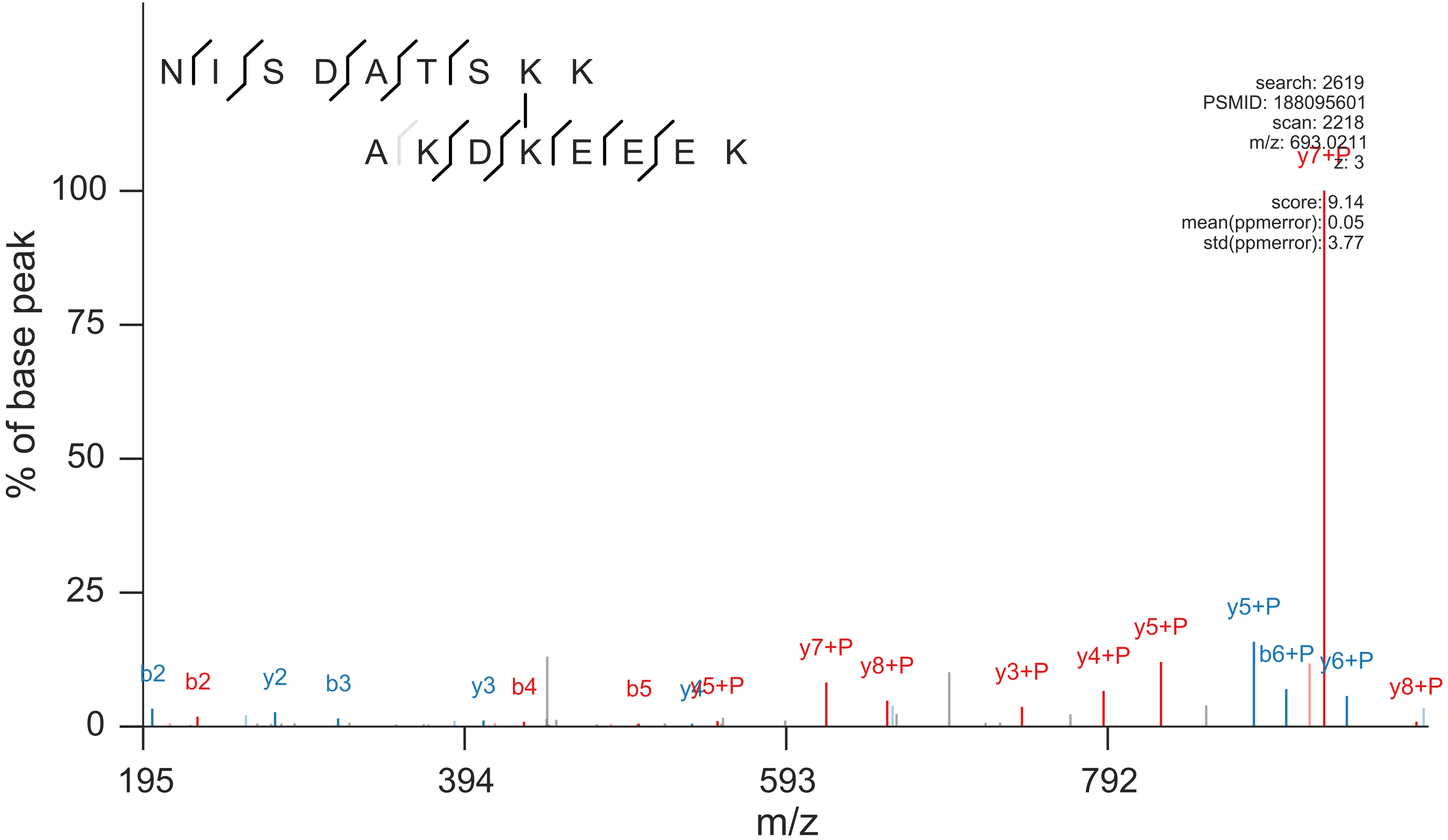

N I S D A T S K K
A K D K E E E K
search: 2619
PSMID: 188095601
scan: 2218
m/z: 693.0211
z: 3
score: 9.14
mean(ppmerror): 0.05
std(ppmerror): 3.77
% of base peak
m/z
0
25
50
75
100
195
394
593
792
b2
b2
y2
b3
y3
b4
b5
y4
y5+P
y7+P
y8+P
y3+P
y4+P
y5+P
y5+P
b6+P
y7+P
y6+P
y8+P

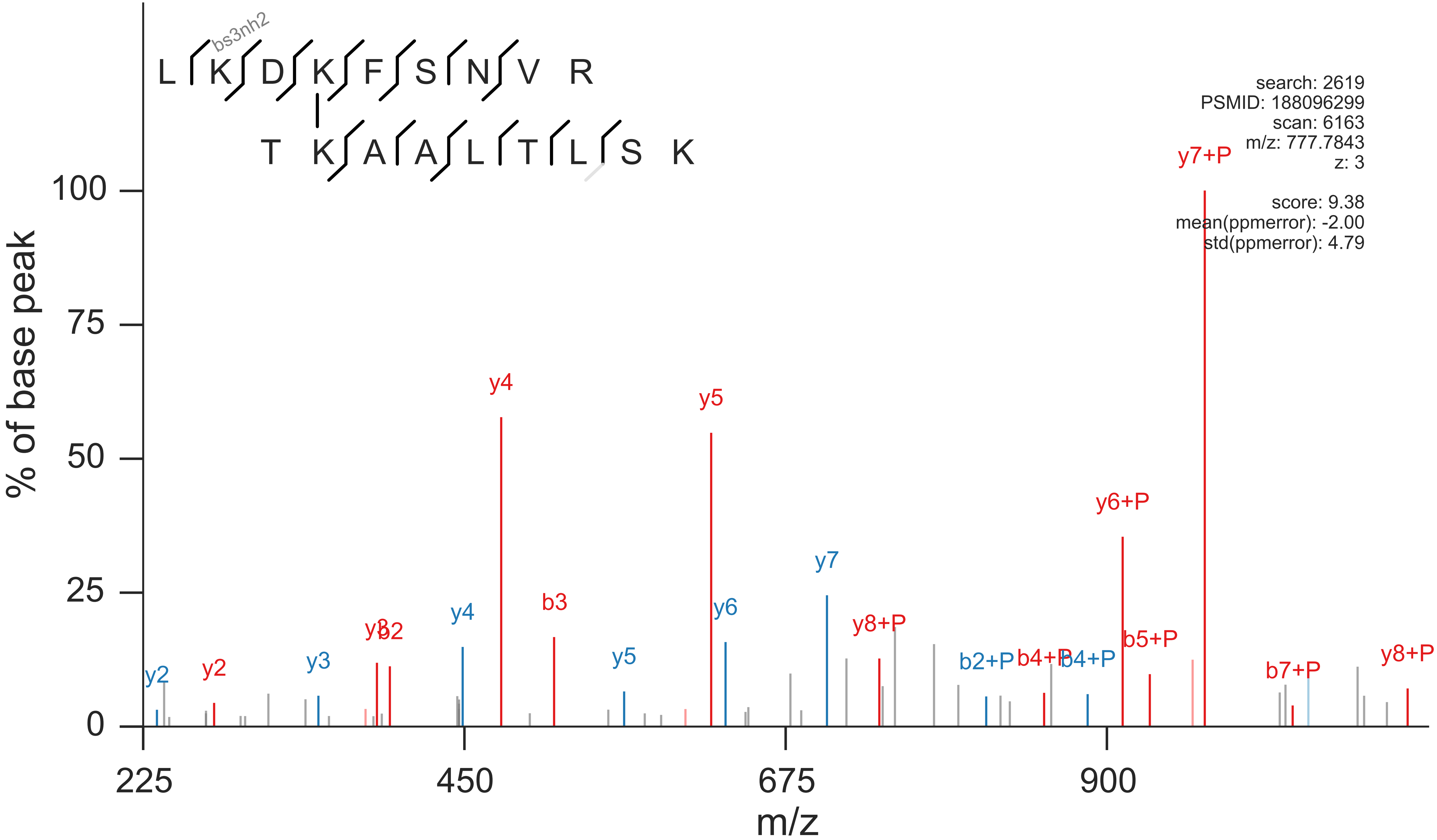
bs3nh2
L K D K F S N V R
T K A A L T L S K
search: 2619
PSMID: 188096299
scan: 6163
m/z: 777.7843
z: 3
score: 9.38
mean(ppmerror): -2.00
std(ppmerror): 4.79
% of base peak
m/z
100
75
50
25
0
225
450
675
900
y7+P
y4
y5
y6+P
y7
y6
y8+P
b3
y4
y3
b2
y3
y2
y2
y5
b2+P
b4+P
b4+P
b5+P
b7+P
y8+P

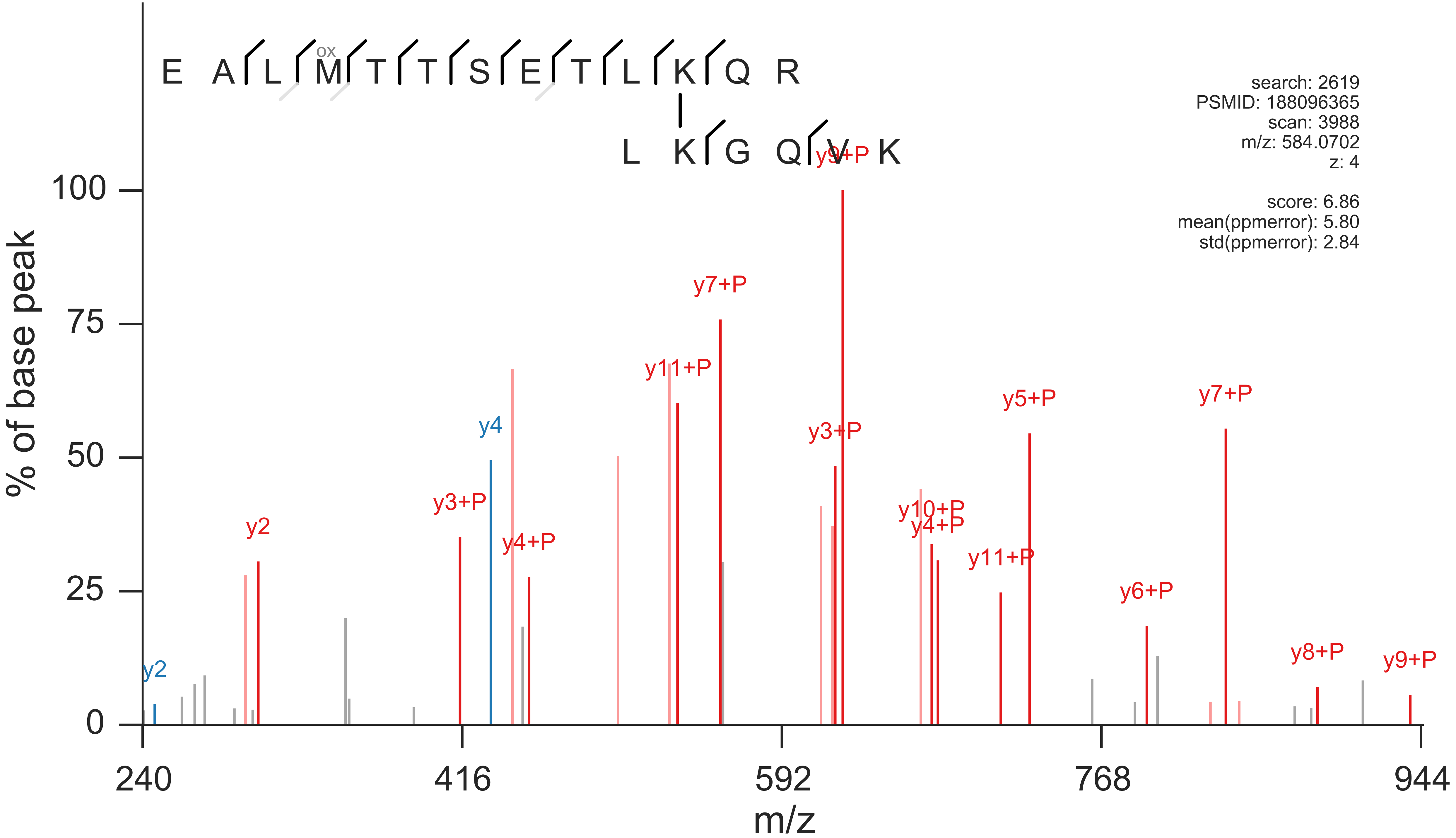

E A L M T T S E T L K Q R
ox
L K G Q V K
search: 2619
PSMID: 188096365
scan: 3988
m/z: 584.0702
z: 4
score: 6.86
mean(ppmerror): 5.80
std(ppmerror): 2.84
% of base peak
m/z
0
25
50
75
100
240
416
592
768
944
y2
y2
y3+P
y4
y4+P
y11+P
y7+P
y3+P
y9+P
y10+P
y4+P
y11+P
y5+P
y6+P
y7+P
y8+P
y9+P

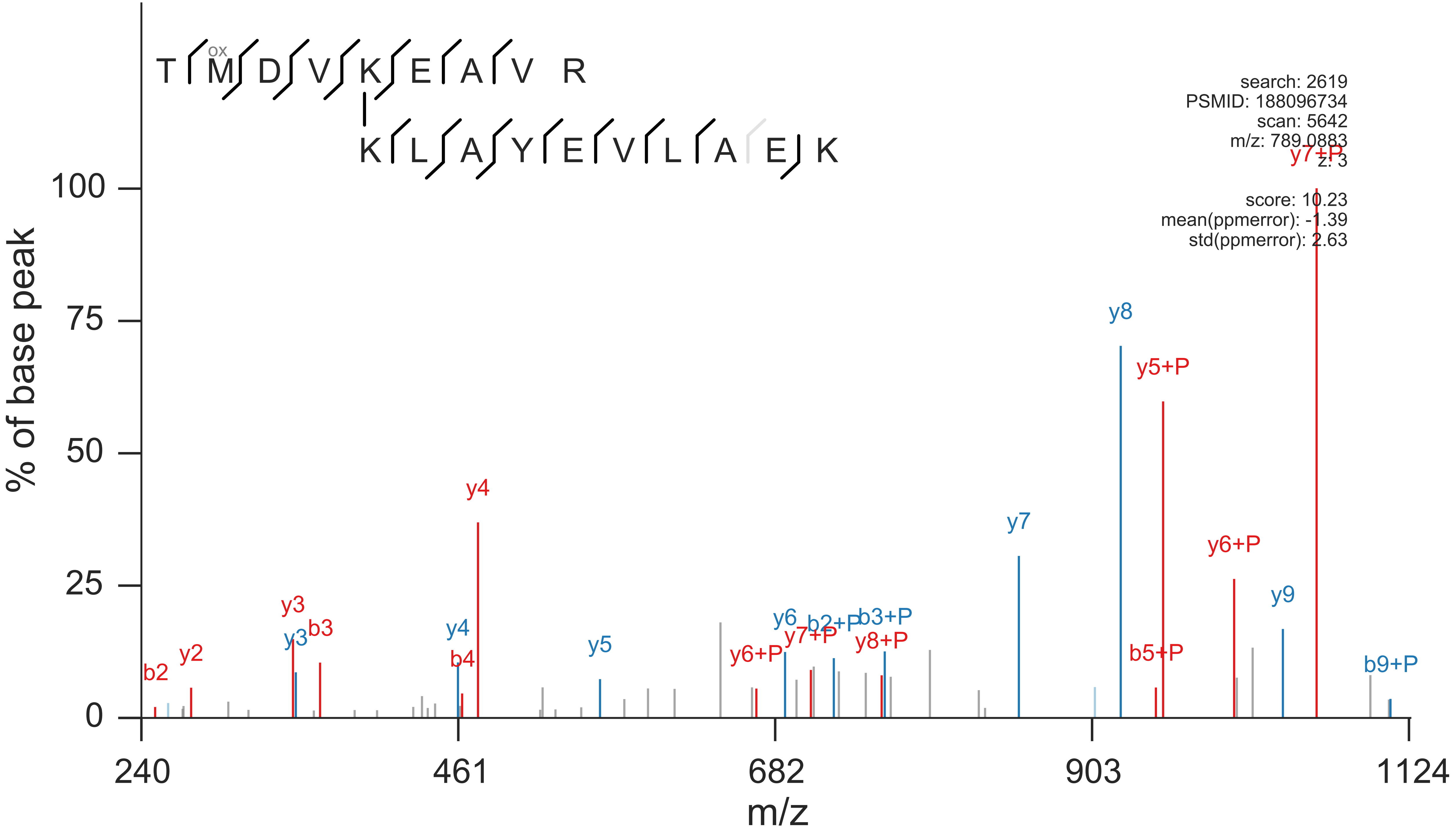

T M D V K E A V R
ox
K L A Y E V L A E K
search: 2619
PSMID: 188096734
scan: 5642
m/z: 789.0883
z: 3
score: 10.23
mean(ppmerror): -1.39
std(ppmerror): 2.63
% of base peak
m/z
100
75
50
25
0
240
461
682
903
1124
b2
y2
y3
y3
b3
y4
y4
b4
y5
y6+P
y6
b2+P
y7+P
b3+P
y8+P
y7
y8
y5+P
b5+P
y6+P
y9
y7+P
b9+P

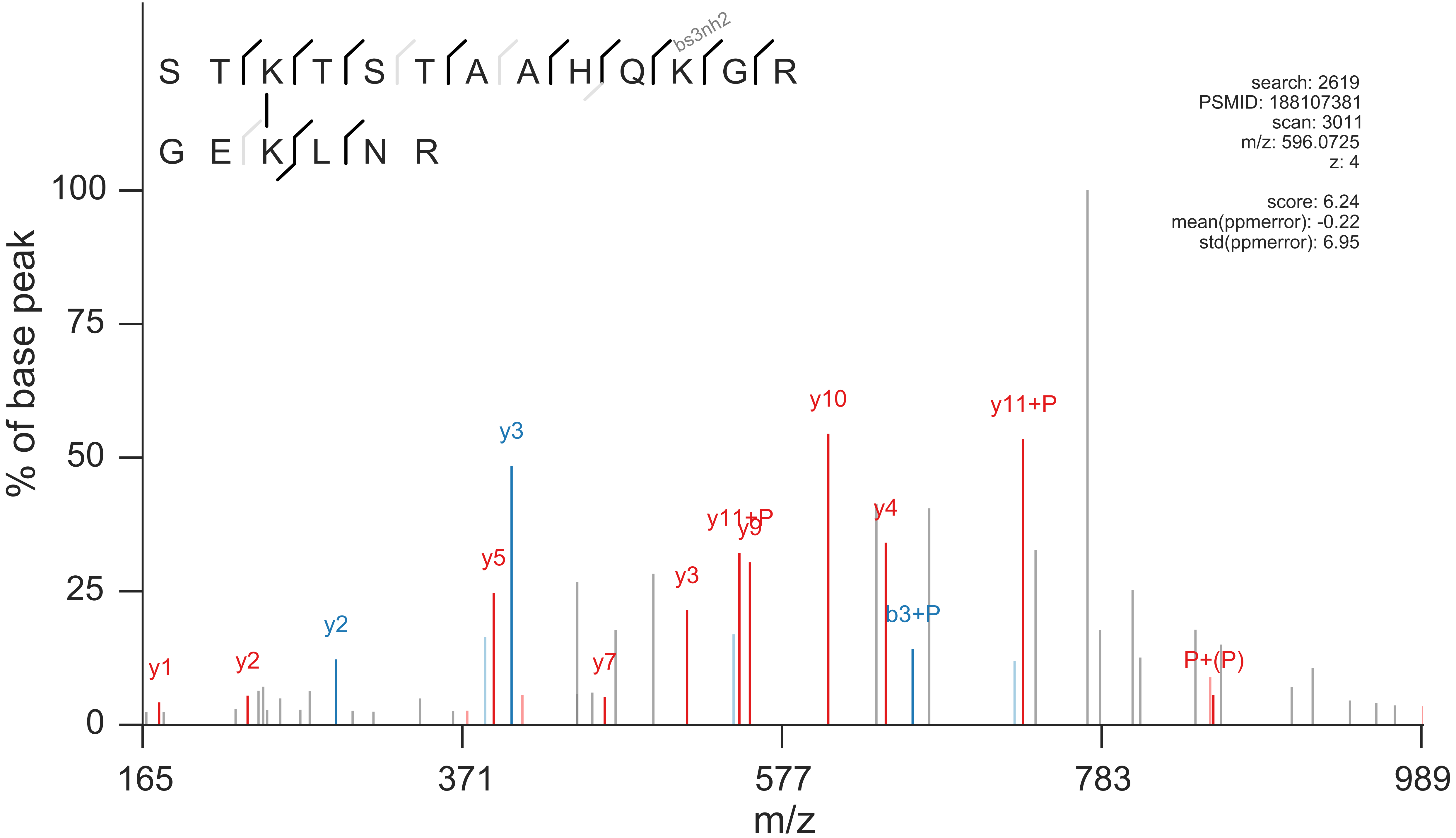

S T K T S T A A H Q K G R
G E K L N R
bs3nh2
search: 2619
PSMID: 188107381
scan: 3011
m/z: 596.0725
z: 4
score: 6.24
mean(ppmerror): -0.22
std(ppmerror): 6.95
% of base peak
100
75
50
25
0
y1
y2
y2
y5
y3
y7
y3
y11+P
y9
y10
y4
b3+P
y11+P
P+(P)
165
371
577
783
989
m/z

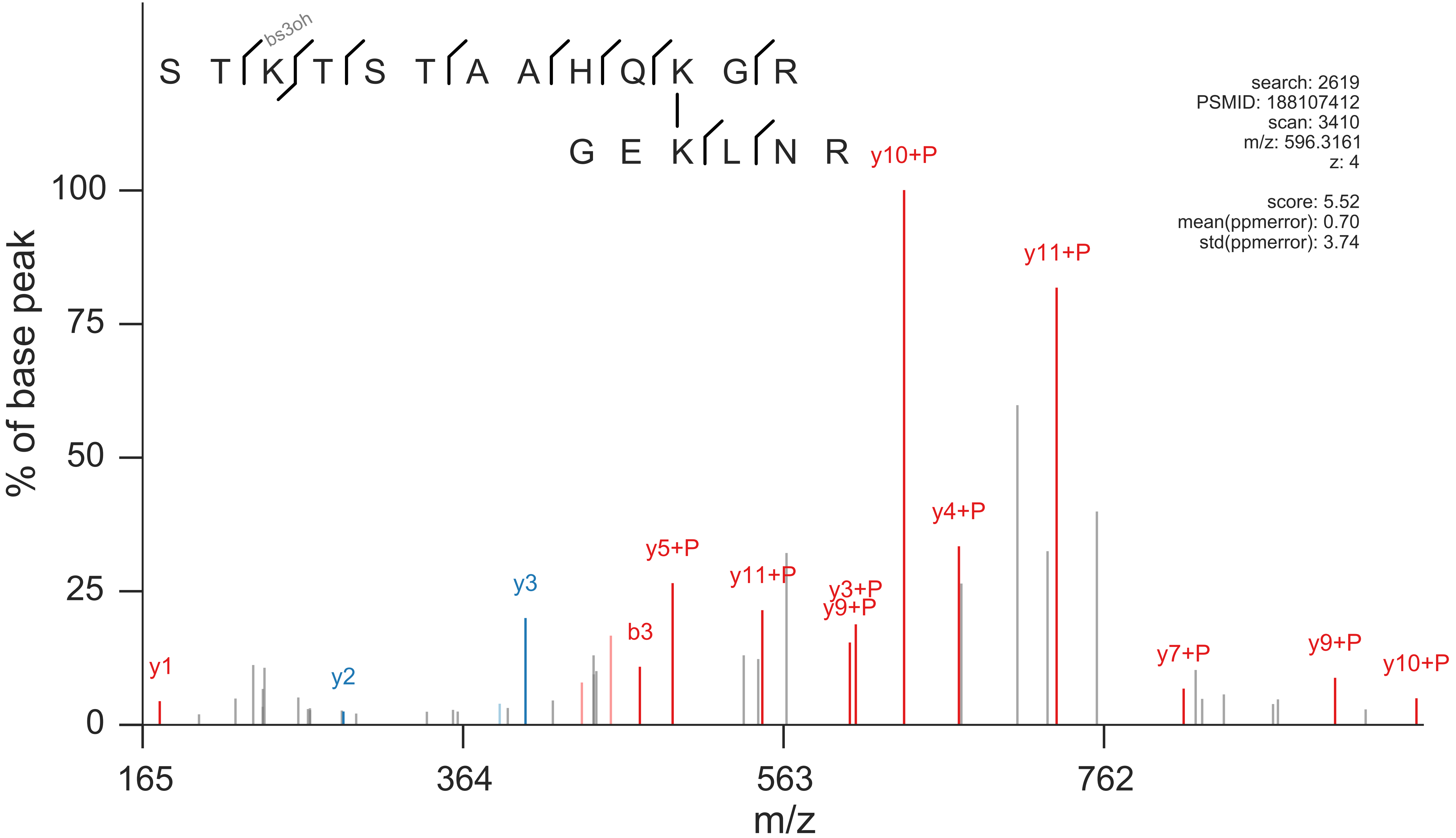

bs3oh
S T K T S T A A H Q K G R
G E K L N R
search: 2619
PSMID: 188107412
scan: 3410
m/z: 596.3161
z: 4
score: 5.52
mean(ppmerror): 0.70
std(ppmerror): 3.74
% of base peak
m/z
y1
y2
y3
b3
y5+P
y11+P
y3+P
y9+P
y10+P
y4+P
y11+P
y7+P
y9+P
y10+P

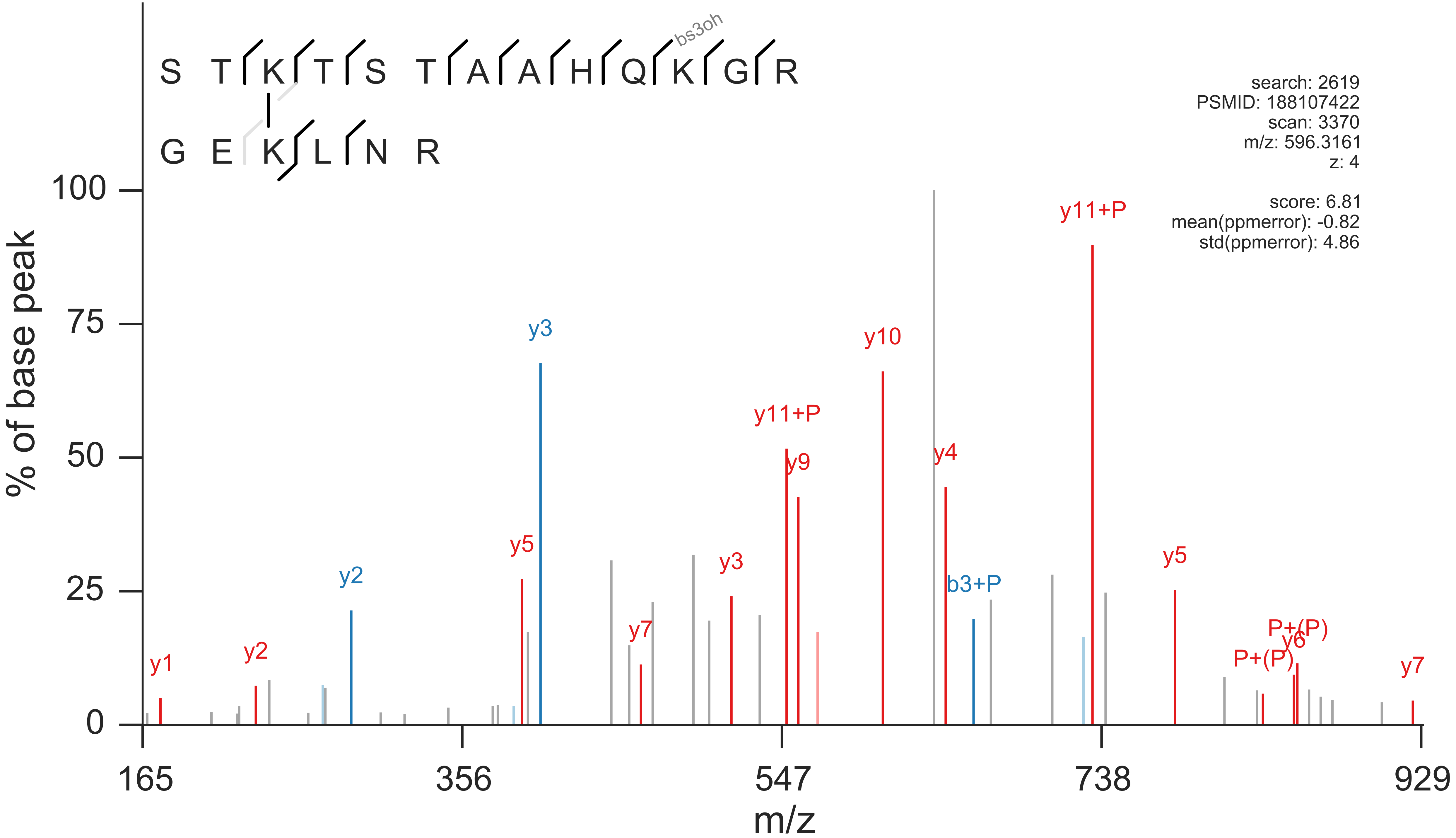

S T K T S T A A H Q K G R
bs3oh
G E K L N R
search: 2619
PSMID: 188107422
scan: 3370
m/z: 596.3161
z: 4
score: 6.81
mean(ppmerror): -0.82
std(ppmerror): 4.86
% of base peak
100
75
50
25
0
y1
y2
y2
y5
y3
y7
y3
y11+P
y9
y10
y4
b3+P
y11+P
y5
P+(P)
y6
P+(P)
y7
165
356
547
738
929
m/z

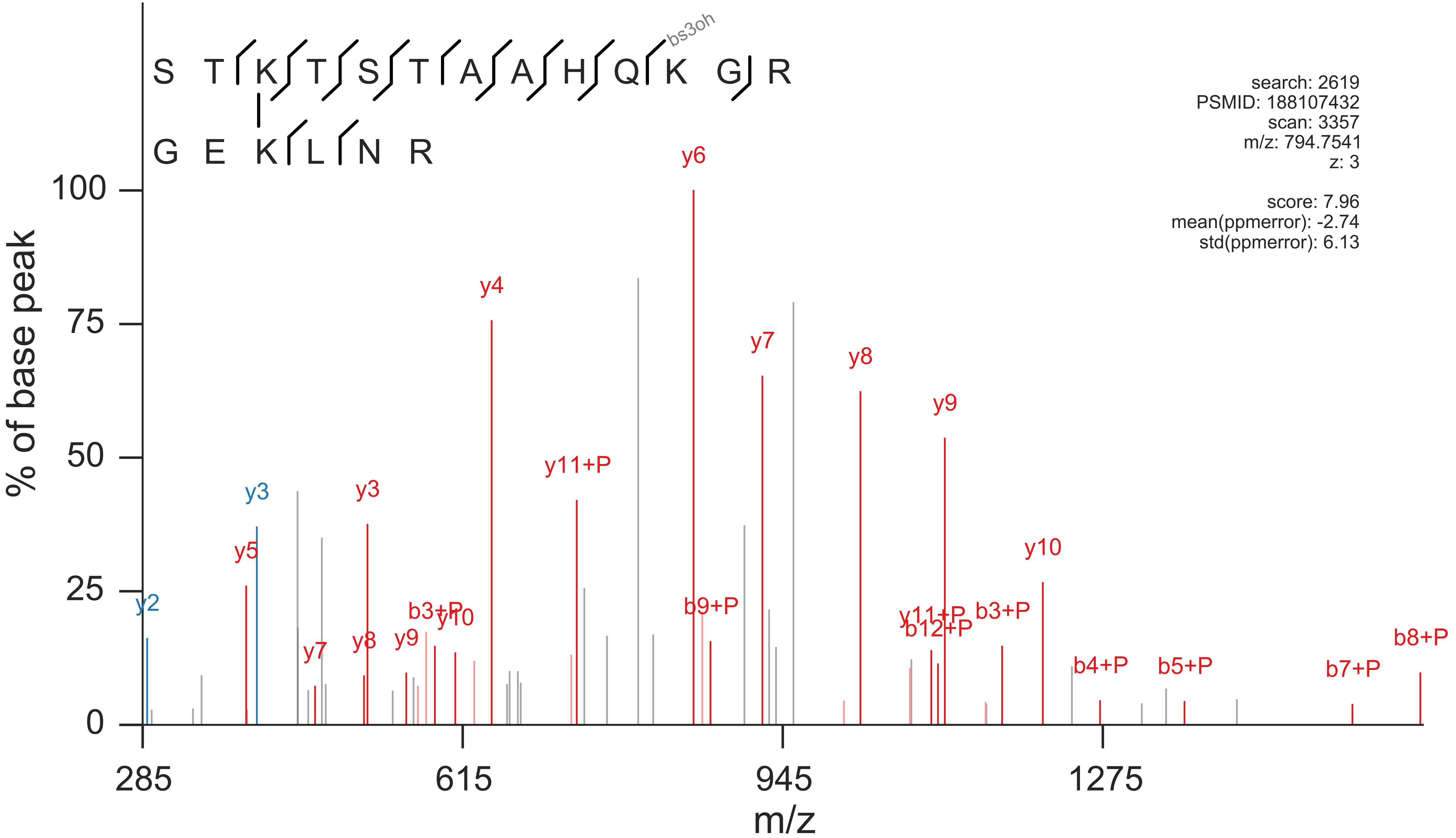

S T K T S T A A H Q K G R
bs3oh
G E K L N R
search: 2619
PSMID: 188107432
scan: 3357
m/z: 794.7541
z: 3
score: 7.96
mean(ppmerror): -2.74
std(ppmerror): 6.13
% of base peak
m/z

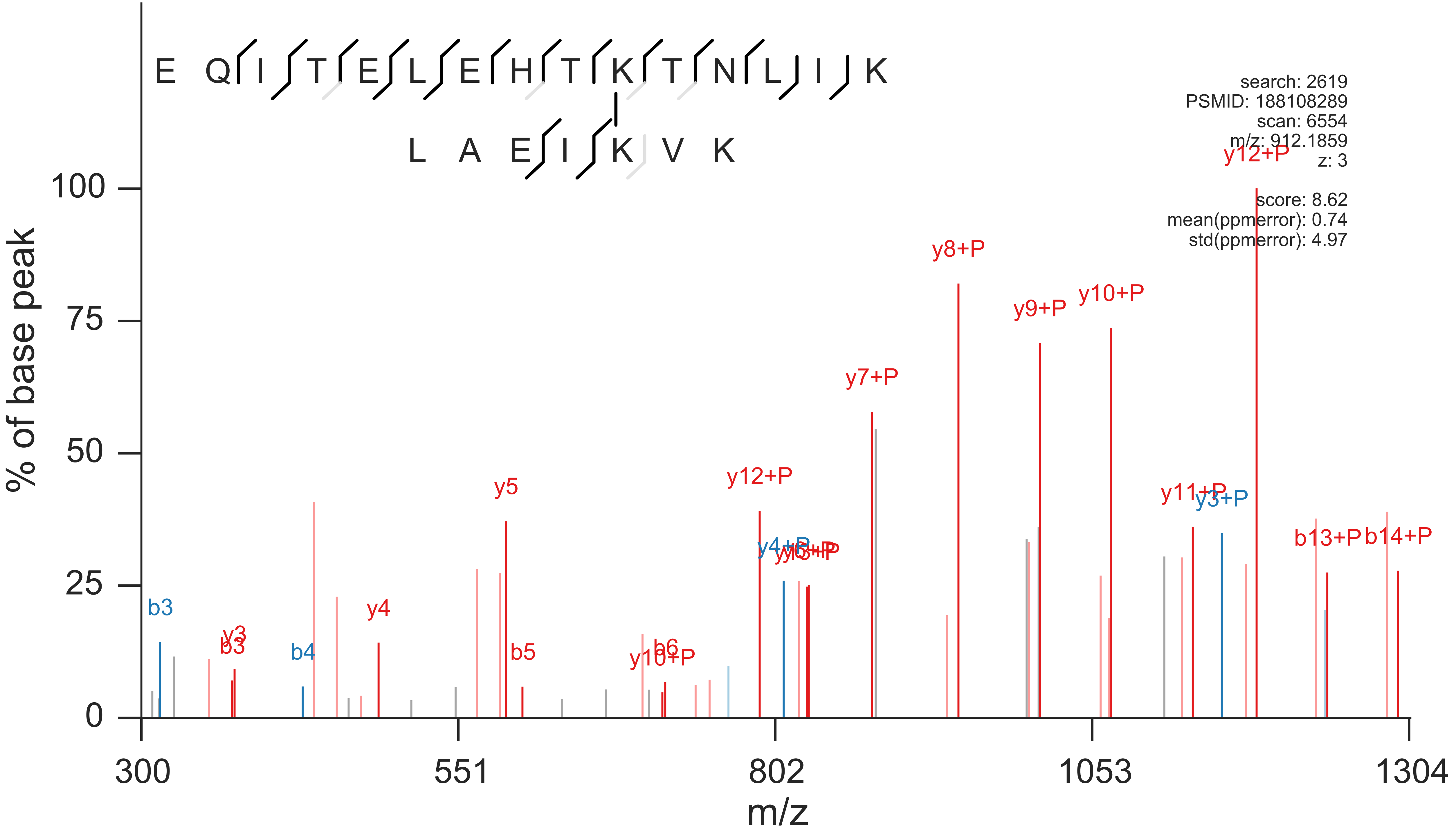

E Q I T E L E H T K T N L I K
L A E I K V K
search: 2619
PSMID: 188108289
scan: 6554
m/z: 912.1859
z: 3
score: 8.62
mean(ppmerror): 0.74
std(ppmerror): 4.97
% of base peak
m/z
y12+P
y8+P
y9+P
y10+P
y7+P
y12+P
y5
y11+P
y3+P
b13+P
b14+P
y4+P
b3
y3
b3
b4
y4
b5
b6
y10+P

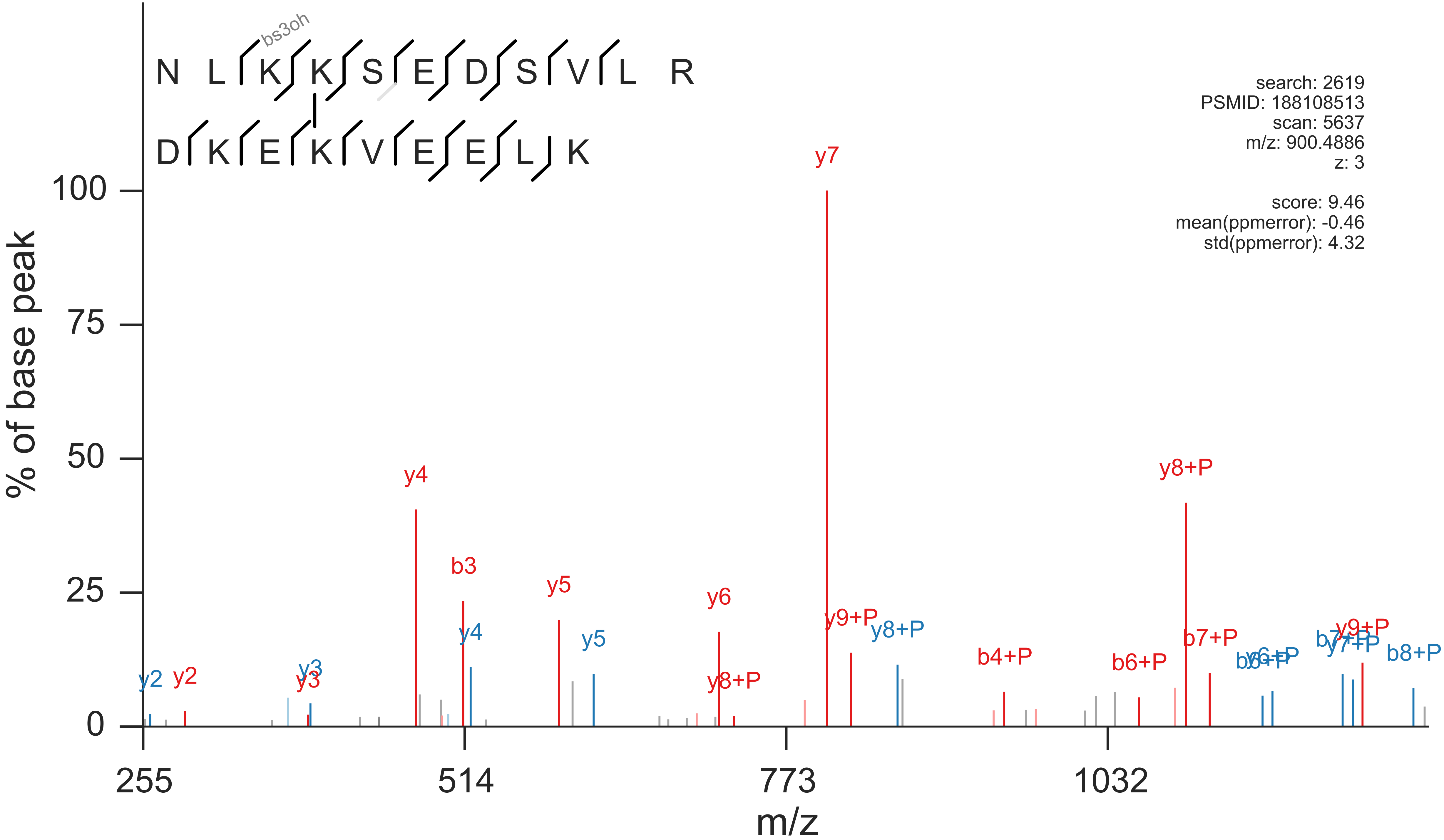

N L K K S E D S V L R
bs3oh
D K E K V E E L K
search: 2619
PSMID: 188108513
scan: 5637
m/z: 900.4886
z: 3
score: 9.46
mean(ppmerror): -0.46
std(ppmerror): 4.32
% of base peak
m/z
0
25
50
75
100
255
514
773
1032
y7
y4
y8+P
b3
y5
y6
y9+P
y8+P
y4
y5
y3
y3
y2
y2
y8+P
b4+P
b6+P
b7+P
y9+P
b8+P

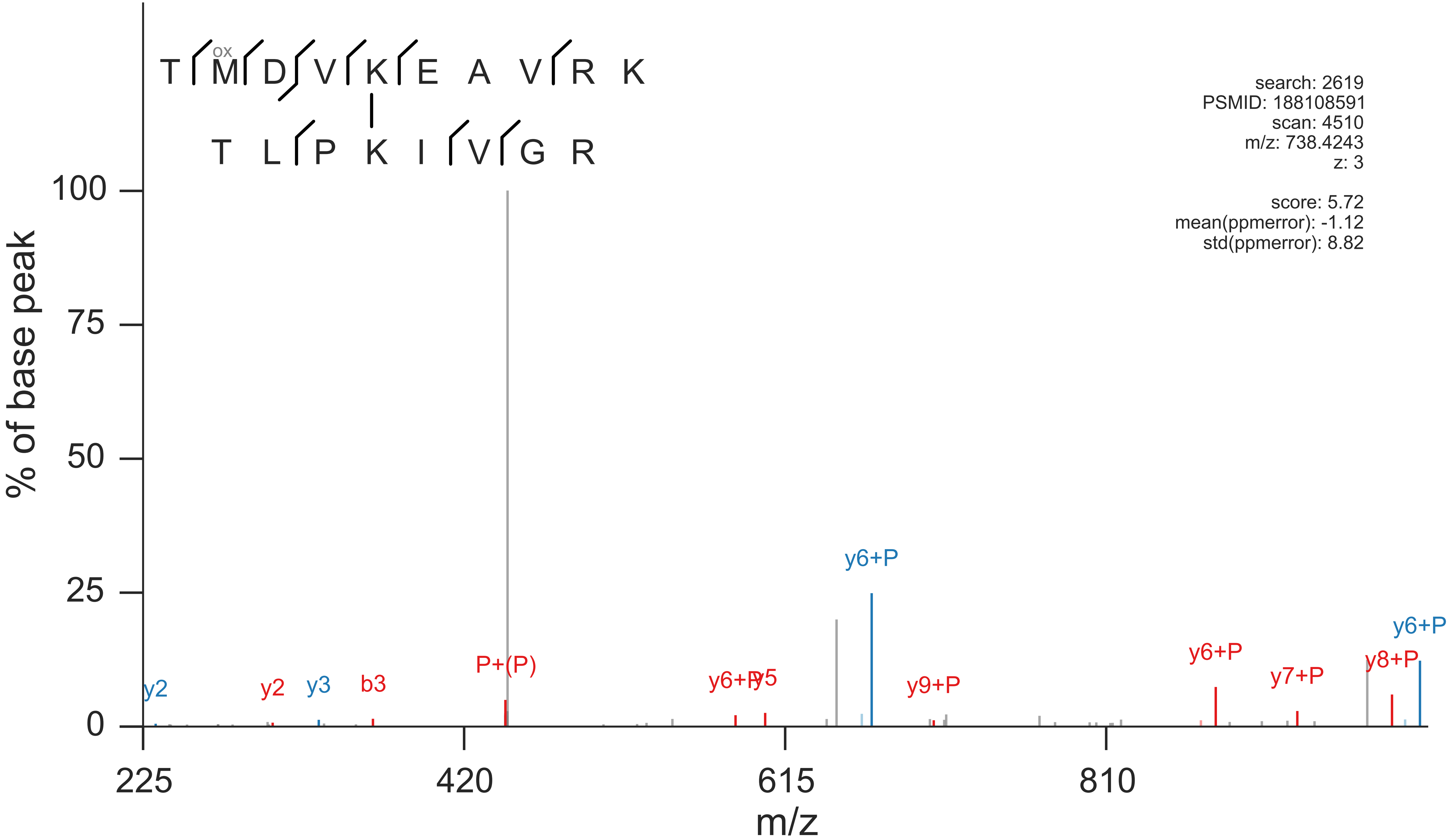

T M D V K E A V R K
ox
T L P K I V G R
search: 2619
PSMID: 188108591
scan: 4510
m/z: 738.4243
z: 3
score: 5.72
mean(ppmerror): -1.12
std(ppmerror): 8.82
% of base peak
100
75
50
25
0
y2
y2
y3
b3
P+(P)
y6+P
y5
y6+P
y9+P
y6+P
y7+P
y8+P
y6+P
225
420
615
810
m/z

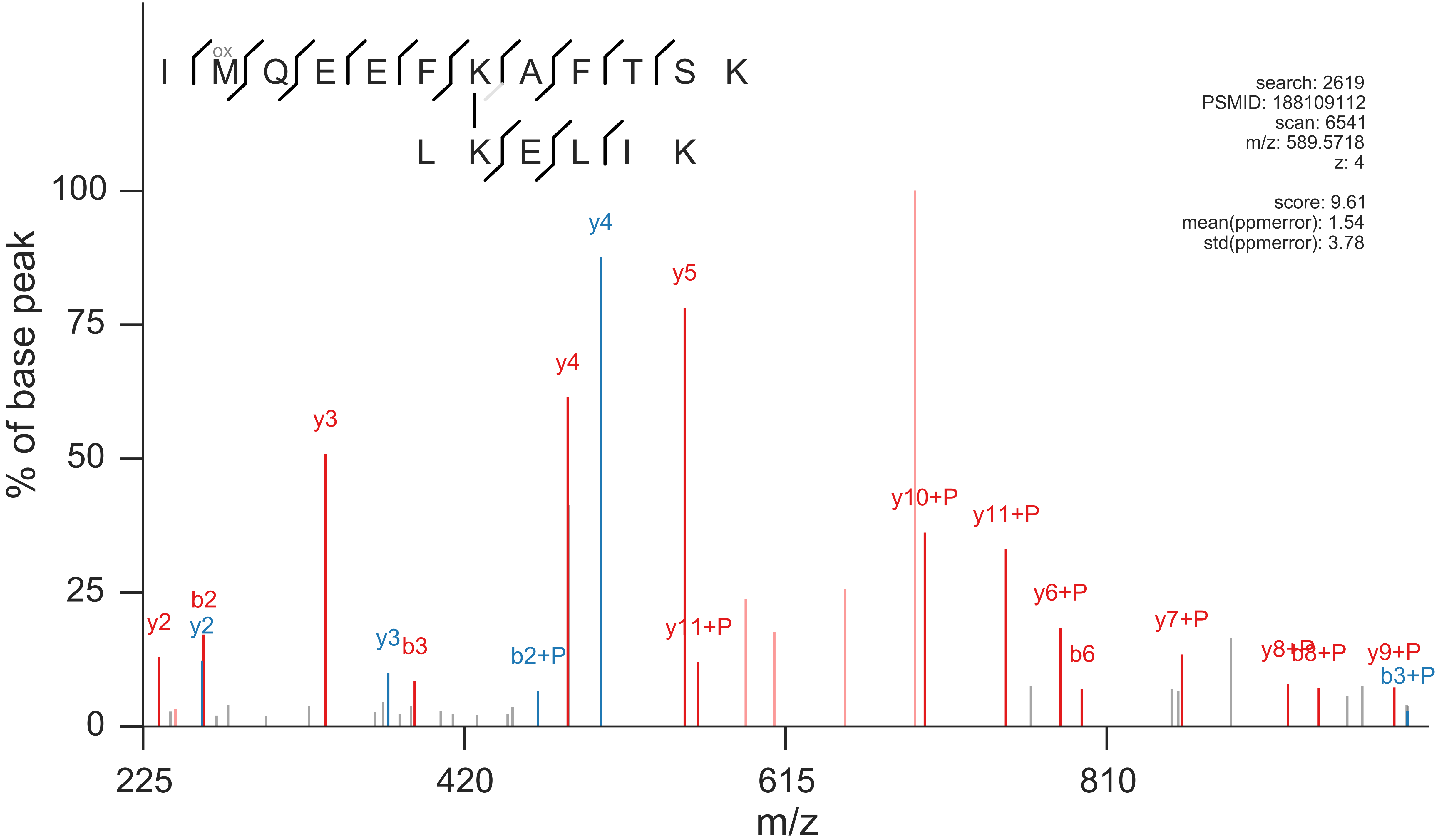
I M Q E E F K A F T S K
ox
L K E L I K
search: 2619
PSMID: 188109112
scan: 6541
m/z: 589.5718
z: 4
score: 9.61
mean(ppmerror): 1.54
std(ppmerror): 3.78
% of base peak
m/z
0
25
50
75
100
225
420
615
810
y2
b2
y2
y3
y3
b3
b2+P
y4
y4
y5
y11+P
y10+P
y11+P
y6+P
b6
y7+P
y8+P
b8+P
y9+P
b3+P

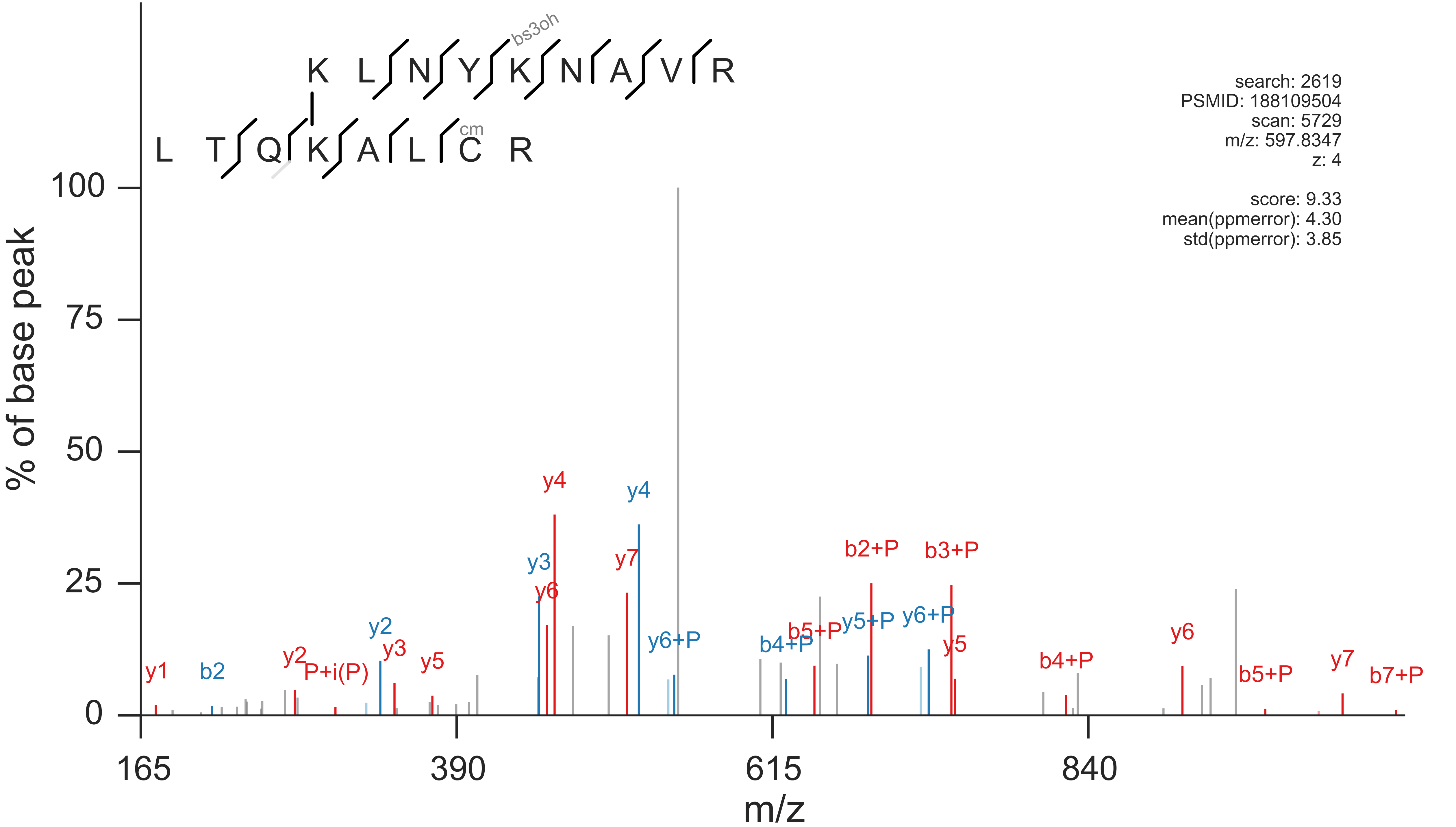
K L N Y K N A V R
L T Q K A L C R
bs3oh
cm
search: 2619
PSMID: 188109504
scan: 5729
m/z: 597.8347
z: 4
score: 9.33
mean(ppmerror): 4.30
std(ppmerror): 3.85
% of base peak
m/z
y1
b2
y2
P+i(P)
y2
y3
y5
y3
y6
y4
y7
y4
y6+P
b4+P
b5+P
y5+P
y6+P
b2+P
b3+P
y5
b4+P
y6
b5+P
y7
b7+P

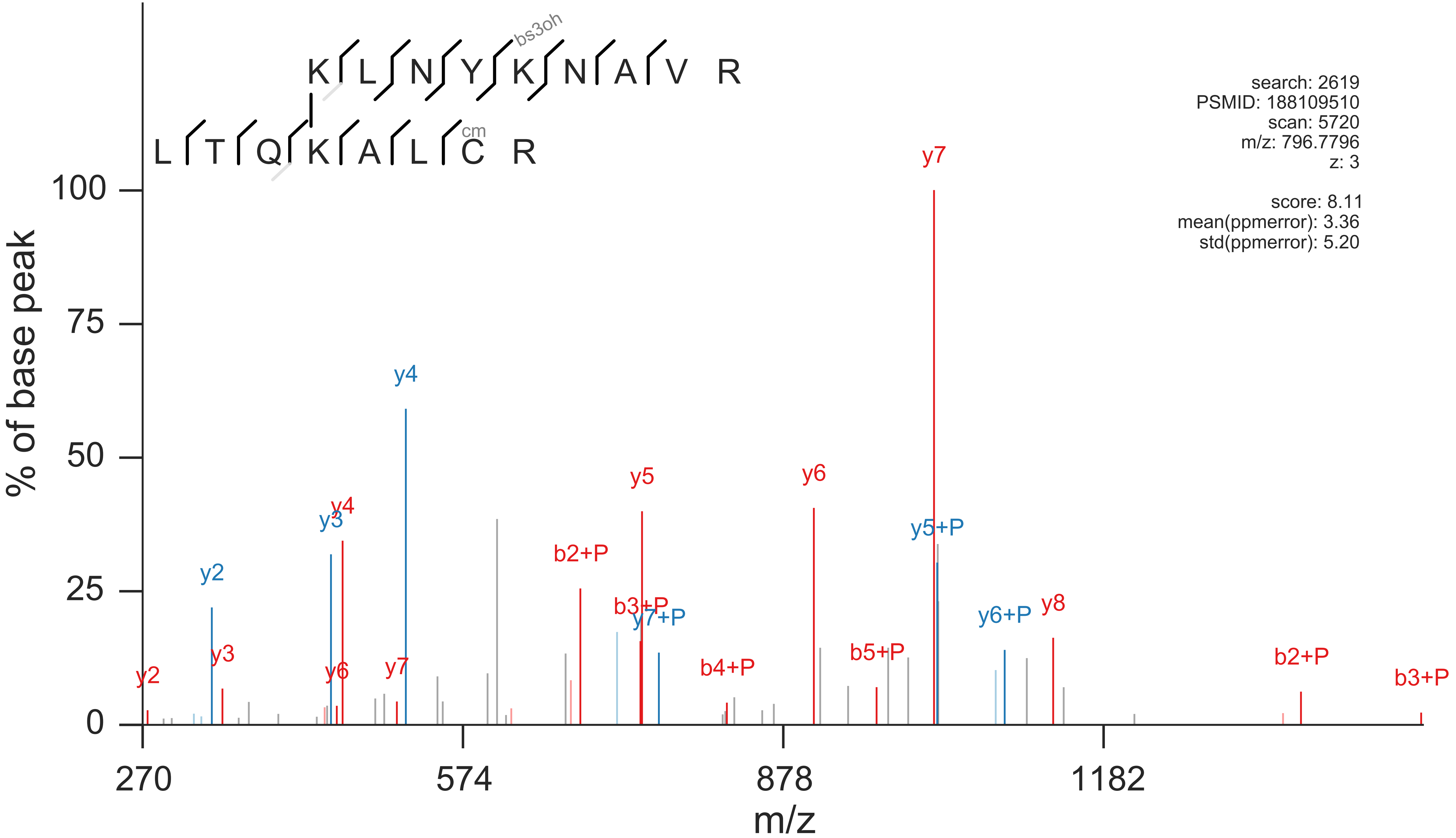

K L N Y K N A V R
bs3oh
L T Q K A L C R
cm
search: 2619
PSMID: 188109510
scan: 5720
m/z: 796.7796
z: 3
score: 8.11
mean(ppmerror): 3.36
std(ppmerror): 5.20
% of base peak
m/z
y7
y4
y5
y6
y4
y3
y5+P
b2+P
y2
b3+P
y7+P
y6+P
y8
y3
b4+P
b5+P
b2+P
y2
y6
y7
b3+P

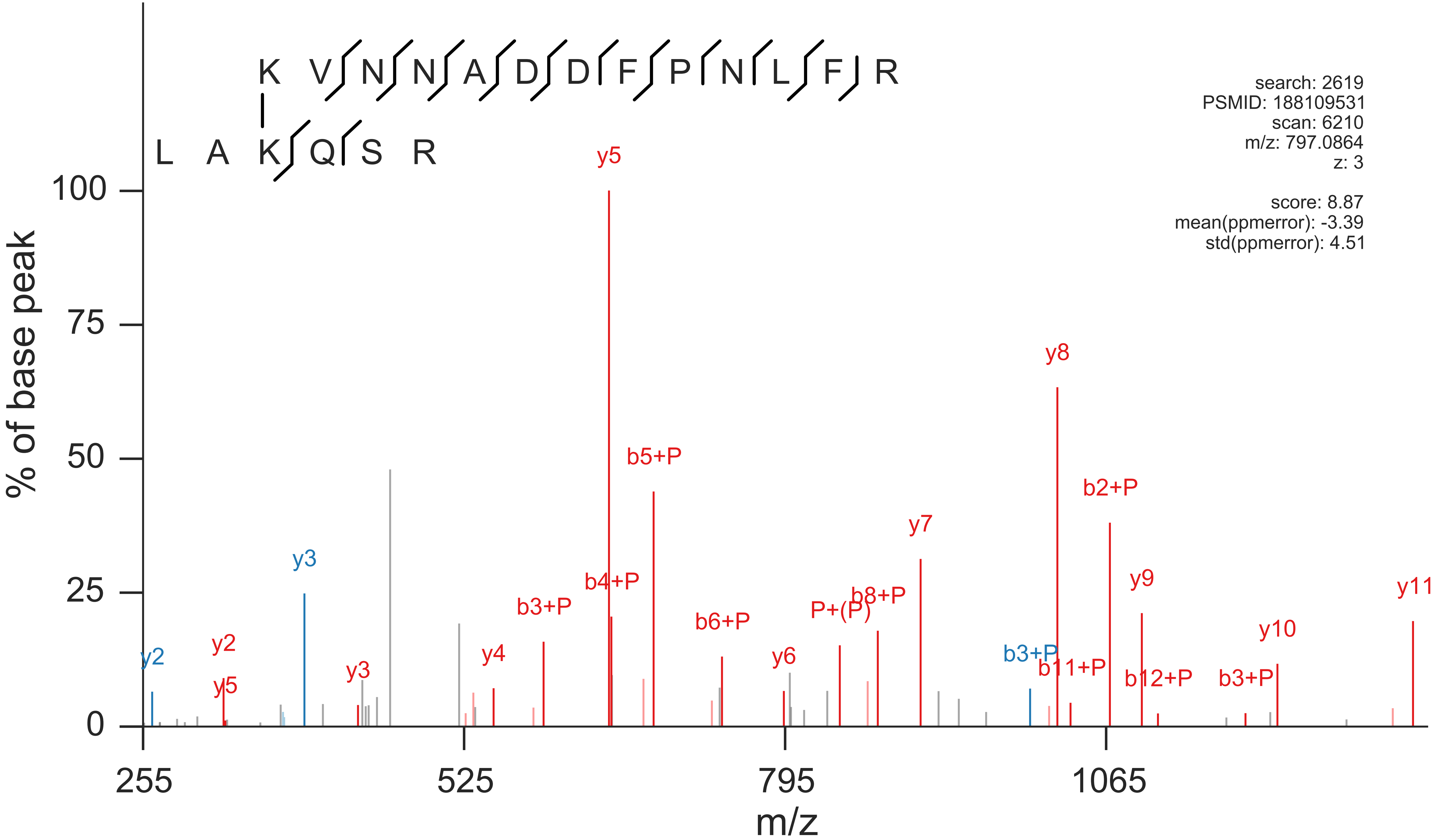

K V N N A D D F P N L F R
L A K Q S R
search: 2619
PSMID: 188109531
scan: 6210
m/z: 797.0864
z: 3
score: 8.87
mean(ppmerror): -3.39
std(ppmerror): 4.51
% of base peak
m/z
y2
y2
y5
y3
y3
y4
b3+P
b4+P
y5
b5+P
b6+P
y6
P+(P)
b8+P
y7
b3+P
y8
b11+P
b2+P
y9
b12+P
b3+P
y10
y11

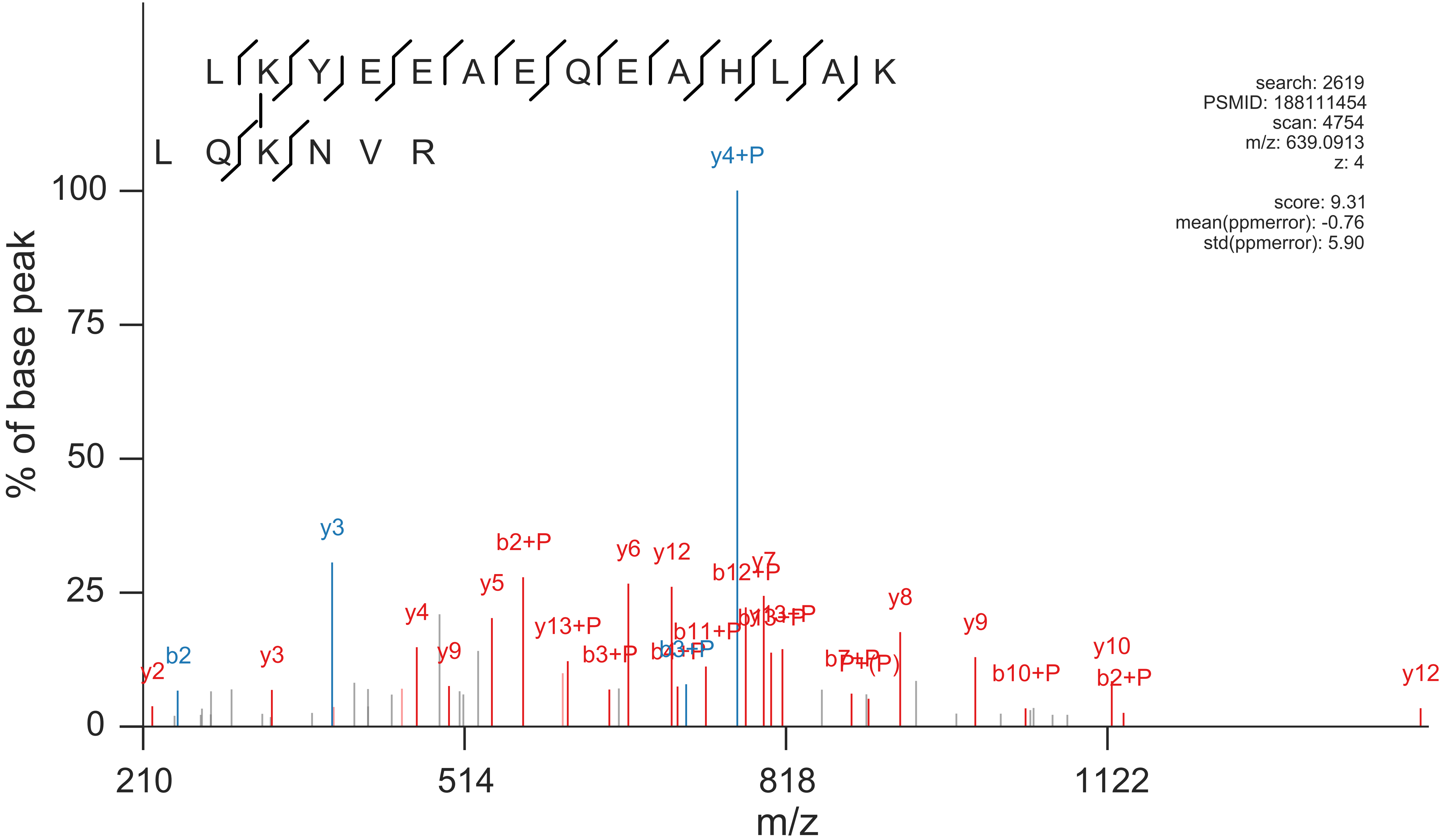

L K Y E E A E Q E A H L A K
L Q K N V R
search: 2619
PSMID: 188111454
scan: 4754
m/z: 639.0913
z: 4
score: 9.31
mean(ppmerror): -0.76
std(ppmerror): 5.90
% of base peak
m/z
0
25
50
75
100
210
514
818
1122
y4+P
y3
b2+P
y6
y12
y7
b12+P
y5
y4
y13+P
y8
y9
b11+P
y10
b10+P
b2+P
y12
y2
b2
y3
b3+P

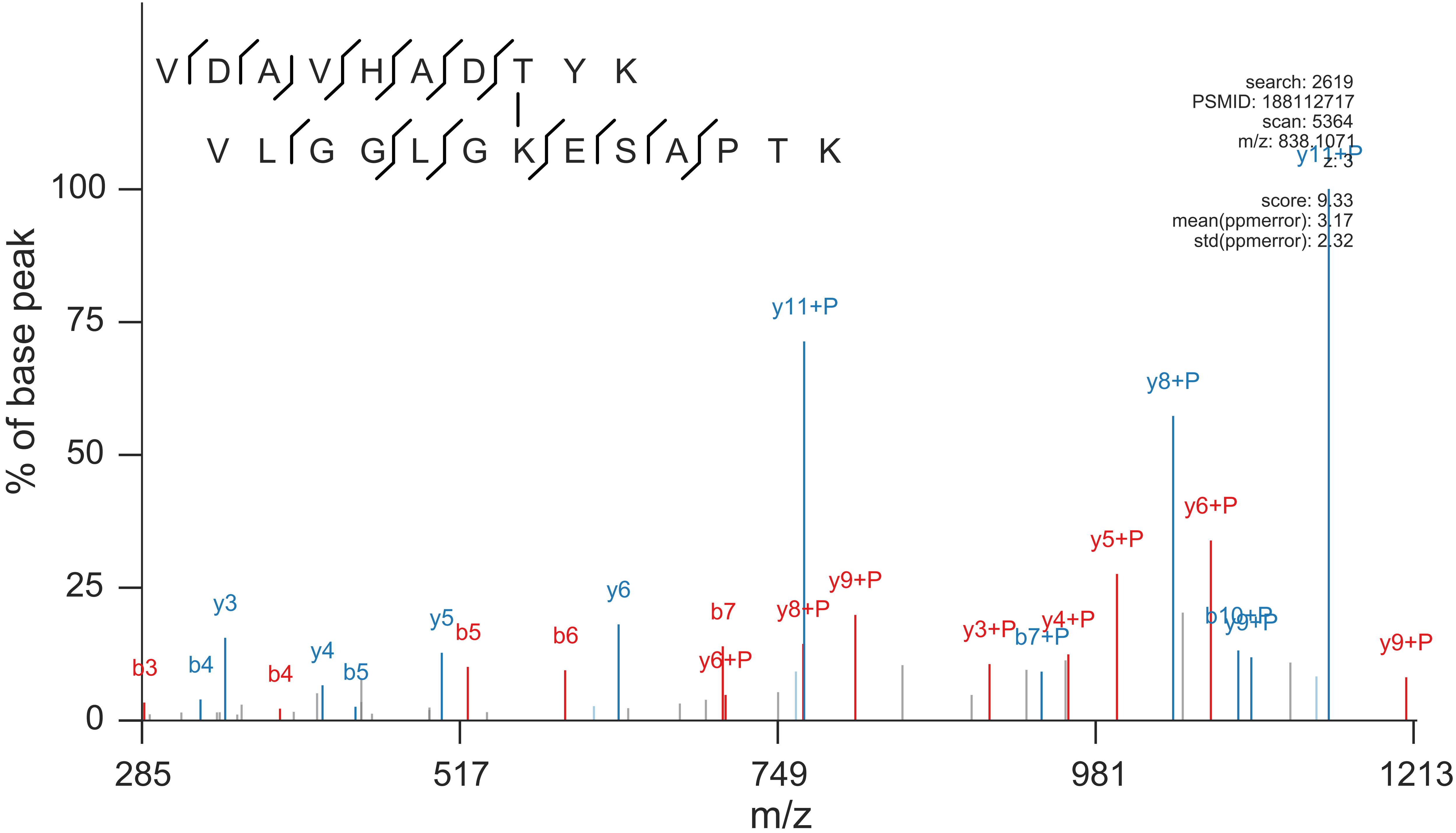

V D A V H A D T Y K
V L G G L G K E S A P T K
search: 2619
PSMID: 188112717
scan: 5364
m/z: 838.1071
z: 3
score: 9.33
mean(ppmerror): 3.17
std(ppmerror): 2.32
% of base peak
m/z
0
25
50
75
100
285
517
749
981
1213
b3
b4
y3
b4
y4
b5
y5
b5
b6
y6
b7
y6+P
y8+P
y11+P
y9+P
y3+P
b7+P
y4+P
y5+P
y8+P
y6+P
b10+P
y9+P
y11+P
y9+P

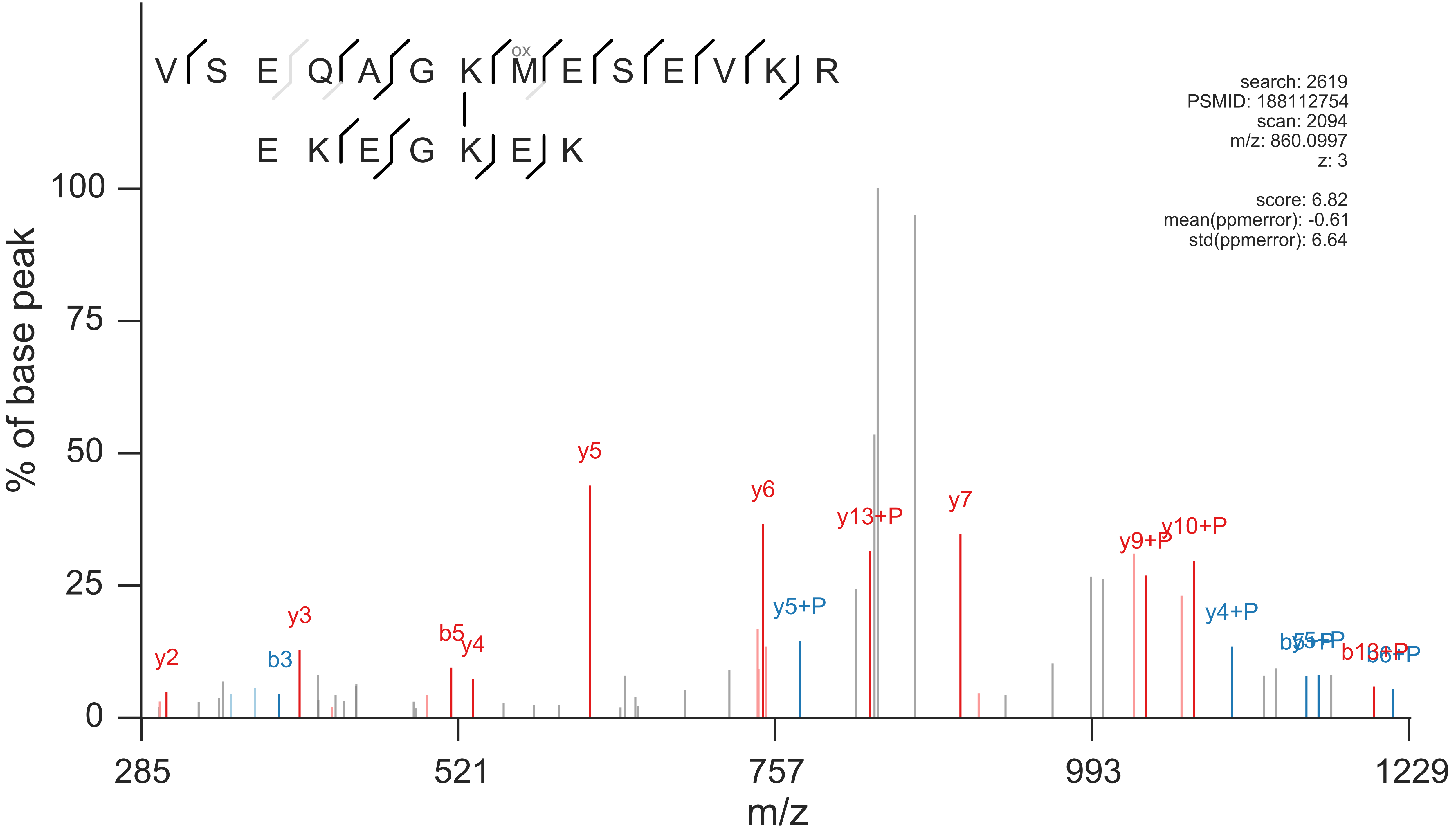
V S E Q A G K M E S E V K R
ox
E K E G K E K
search: 2619
PSMID: 188112754
scan: 2094
m/z: 860.0997
z: 3
score: 6.82
mean(ppmerror): -0.61
std(ppmerror): 6.64
% of base peak
100
75
50
25
0
285
521
757
993
1229
m/z
y2
b3
y3
b5
y4
y5
y6
y5+P
y13+P
y7
y9+P
y10+P
y4+P
b13+P

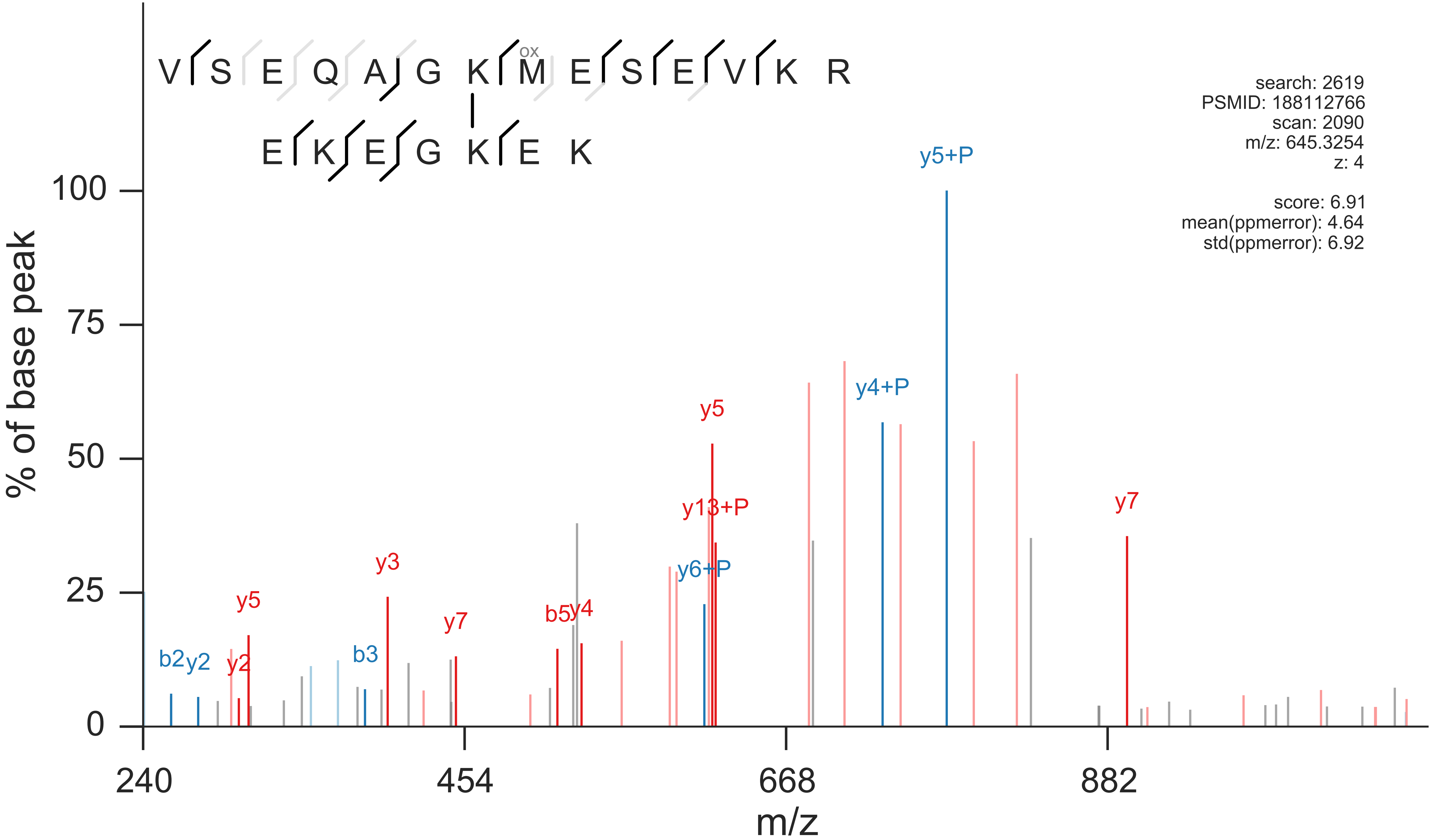
V S E Q A G K M E S E V K R
ox
E K E G K E K
search: 2619
PSMID: 188112766
scan: 2090
m/z: 645.3254
z: 4
score: 6.91
mean(ppmerror): 4.64
std(ppmerror): 6.92
% of base peak
100
75
50
25
0
240
454
668
882
m/z
y5+P
y4+P
y5
y13+P
y6+P
y7
y3
y5
y7
b5
y4
b2
y2
y2
b3

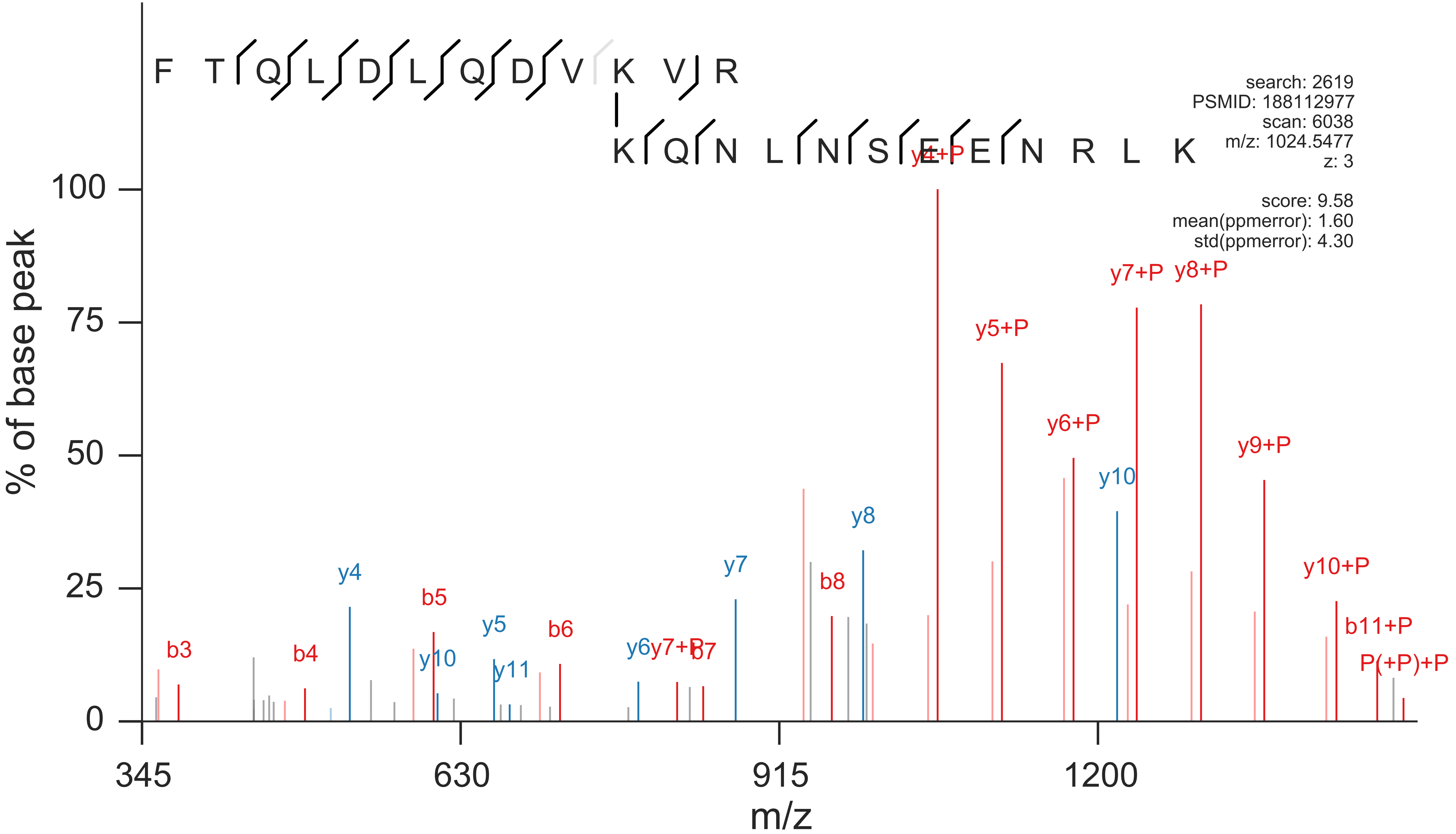

F T Q L D L Q D V K V R
K Q N L N S E E N R L K
search: 2619
PSMID: 188112977
scan: 6038
m/z: 1024.5477
z: 3
score: 9.58
mean(ppmerror): 1.60
std(ppmerror): 4.30
% of base peak
m/z
0
25
50
75
100
345
630
915
1200
y4+P
y5+P
y6+P
y7+P
y8+P
y9+P
y10+P
b11+P
P(+P)+P
y10
y8
y7
b8
y6
y7+P
b7
b6
y5
y11
b5
y4
b4
b3

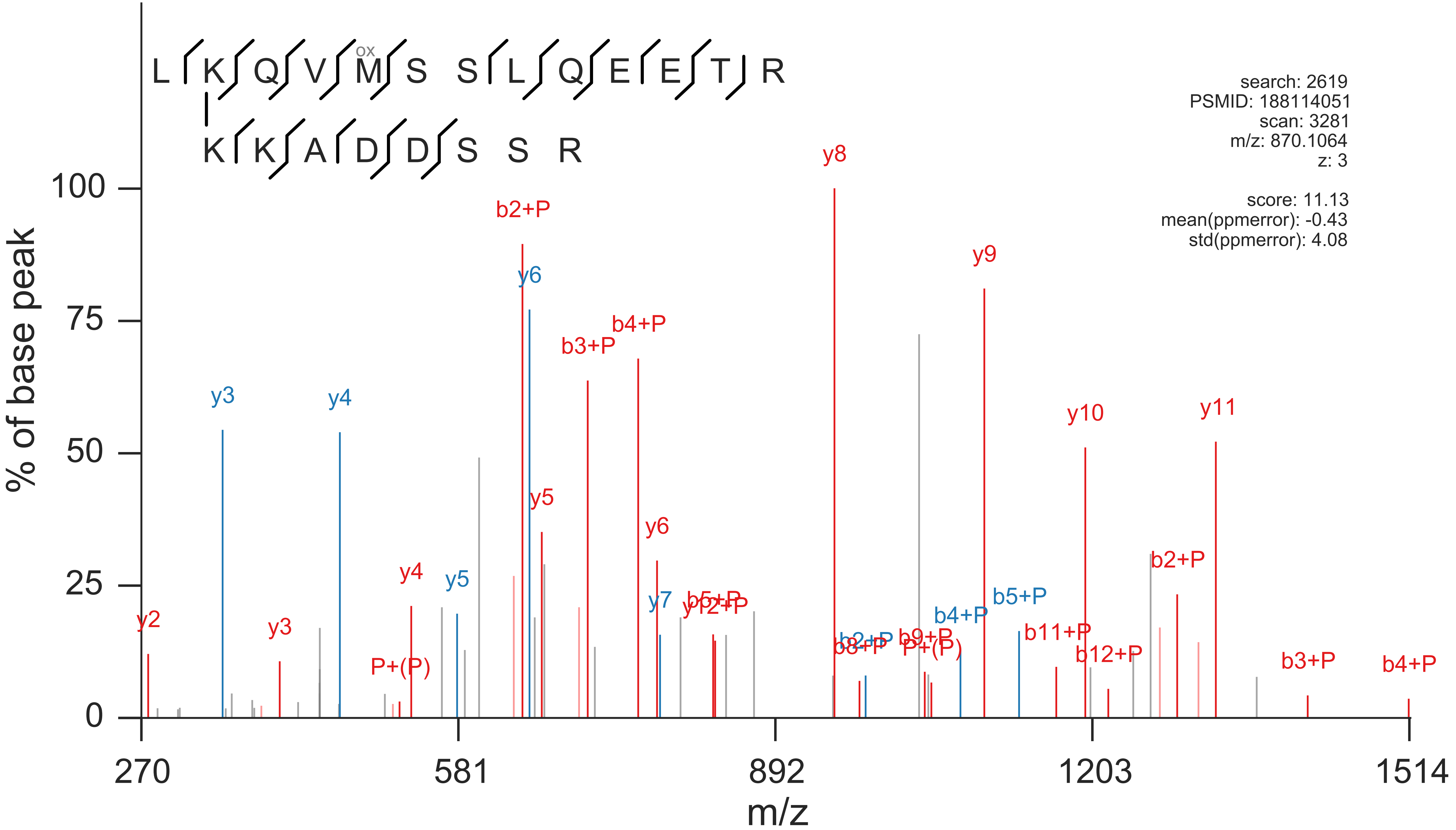

L K Q V M S S L Q E E T R
ox
K K A D D S S R
search: 2619
PSMID: 188114051
scan: 3281
m/z: 870.1064
z: 3
score: 11.13
mean(ppmerror): -0.43
std(ppmerror): 4.08
% of base peak
100
75
50
25
0
270
581
892
1203
1514
m/z
y2
y3
y3
y4
y4
P+(P)
y5
b2+P
y6
y5
b3+P
b4+P
y6
y7
y8
b2+P
b8+P
b9+P
P+(P)
b4+P
y9
b5+P
b11+P
y10
b12+P
b2+P
y11
b3+P
b4+P

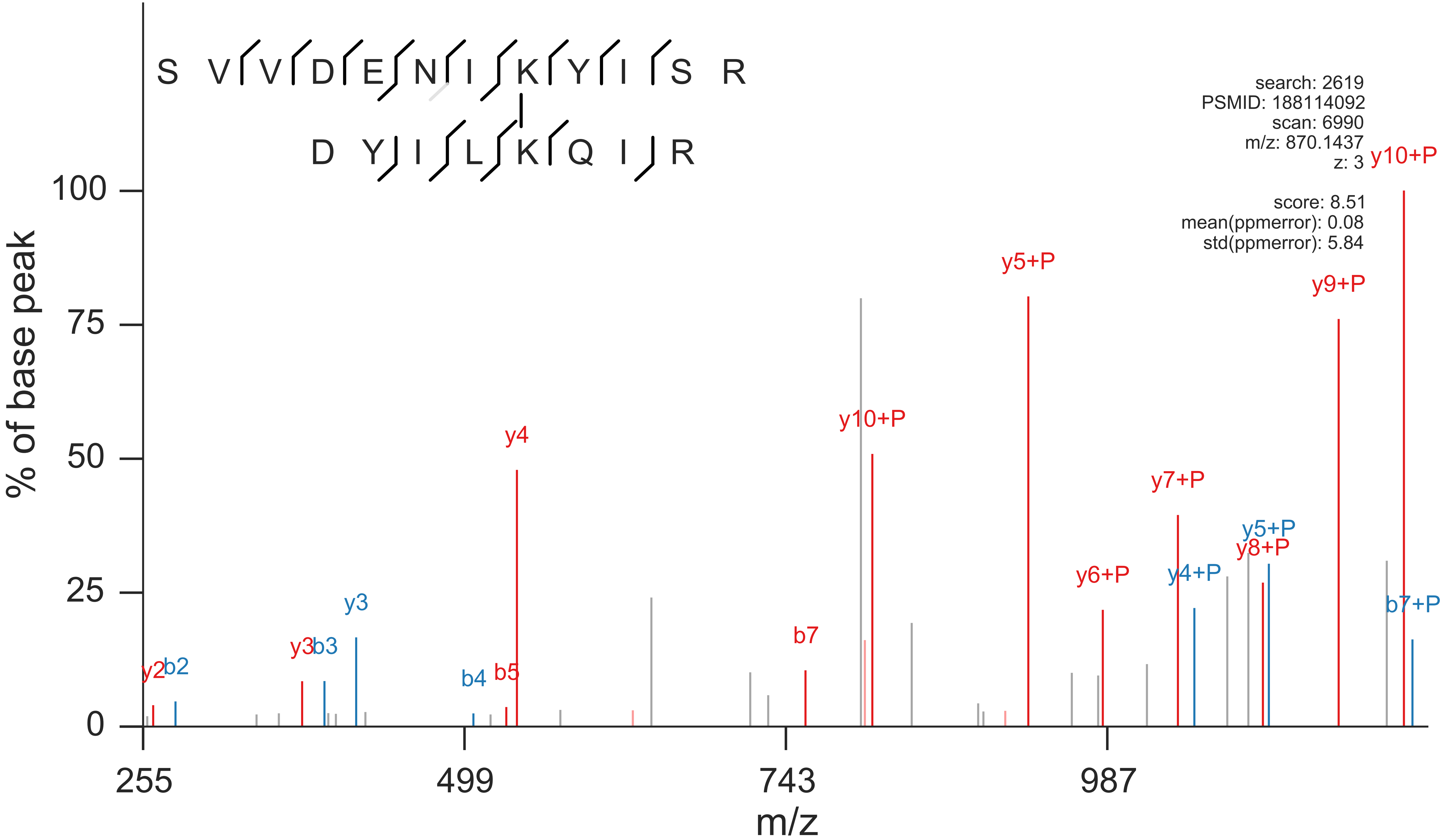
S V V D E N I K Y I S R
D Y I L K Q I R
search: 2619
PSMID: 188114092
scan: 6990
m/z: 870.1437
z: 3
score: 8.51
mean(ppmerror): 0.08
std(ppmerror): 5.84
% of base peak
m/z
y2 b2 y3 b3 y3 b4 b5 y4 b7 y10+P y5+P y6+P y7+P y4+P y5+P y8+P y9+P y10+P b7+P
0
25
50
75
100
255
499
743
987

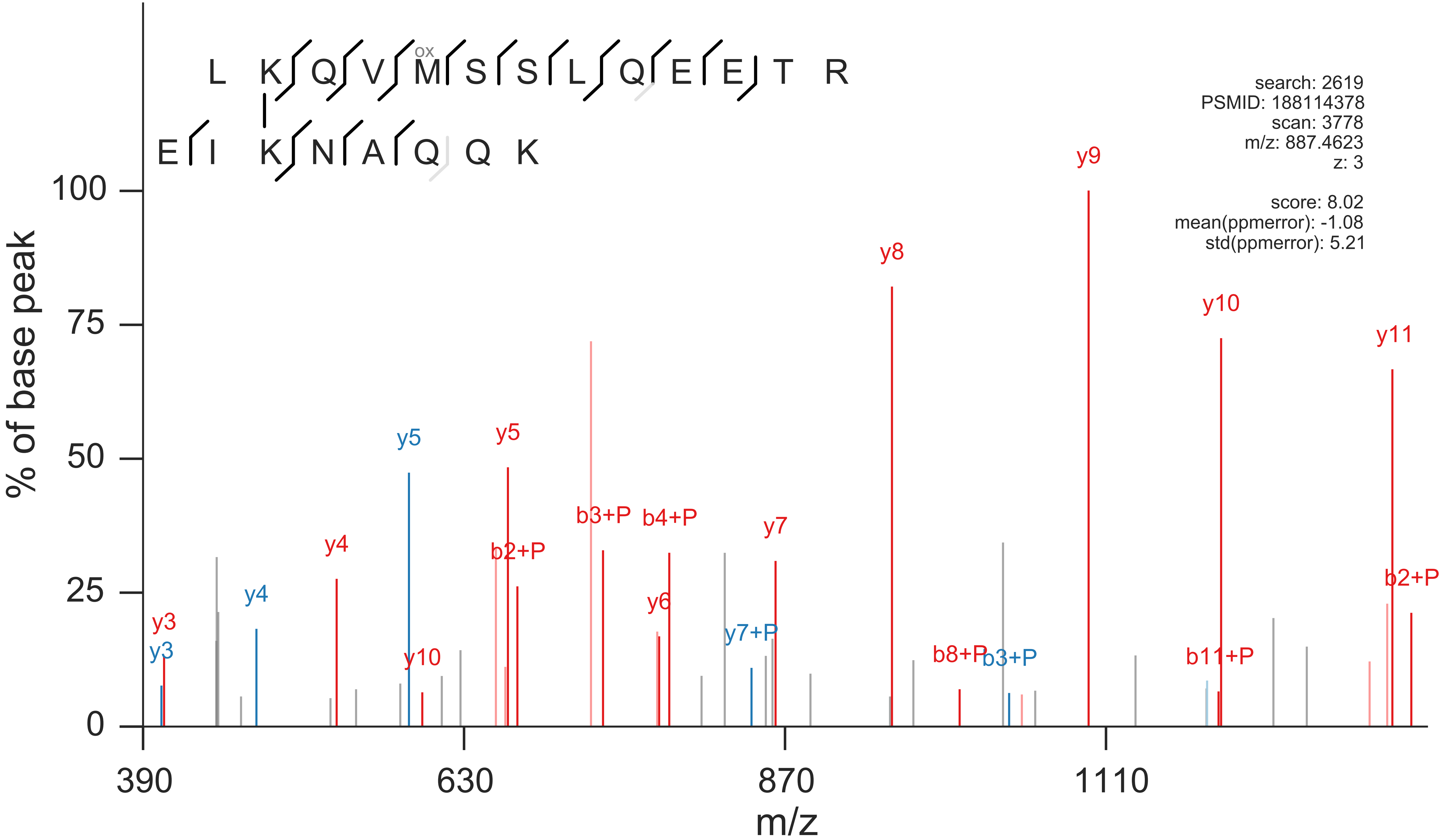

L K Q V M S S L Q E E T R
ox
E I K N A Q Q K
search: 2619
PSMID: 188114378
scan: 3778
m/z: 887.4623
z: 3
score: 8.02
mean(ppmerror): -1.08
std(ppmerror): 5.21
% of base peak
m/z
y9
y8
y10
y11
y5
y5
b3+P
b4+P
y7
y4
b2+P
y4
y6
b2+P
y3
y3
y10
y7+P
b8+P
b3+P
b11+P
100
75
50
25
0
390
630
870
1110

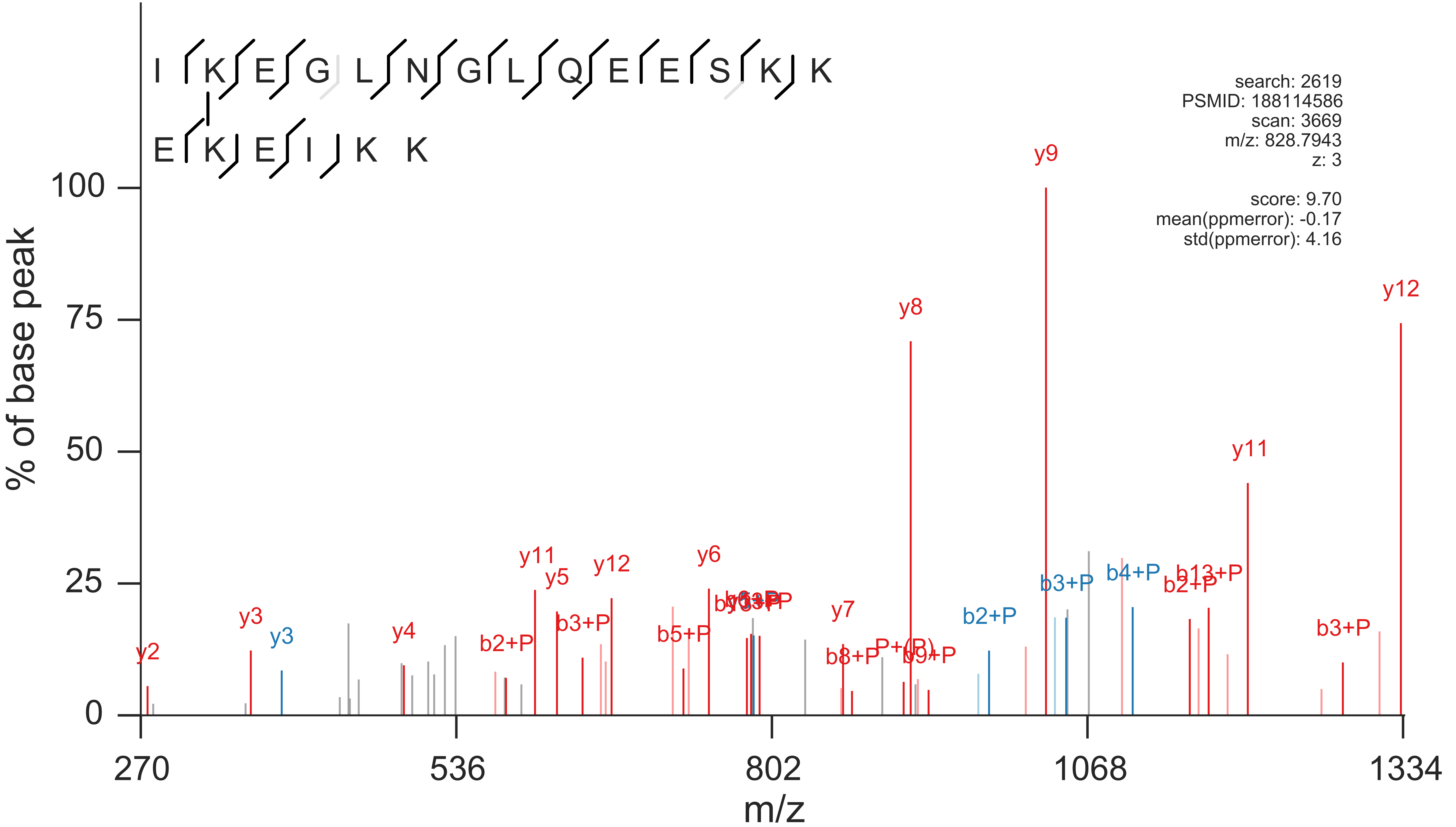

I K E G L N G L Q E E S K K
E K E I K K
search: 2619
PSMID: 188114586
scan: 3669
m/z: 828.7943
z: 3
score: 9.70
mean(ppmerror): -0.17
std(ppmerror): 4.16
% of base peak
m/z
0
25
50
75
100
270
536
802
1068
1334
y2
y3
y3
y4
b2+P
y11
y5
b3+P
y12
b5+P
y6
y7
b8+P
y8
b9+P
y9
b2+P
b3+P
b4+P
b2+P
b13+P
y11
b3+P
y12

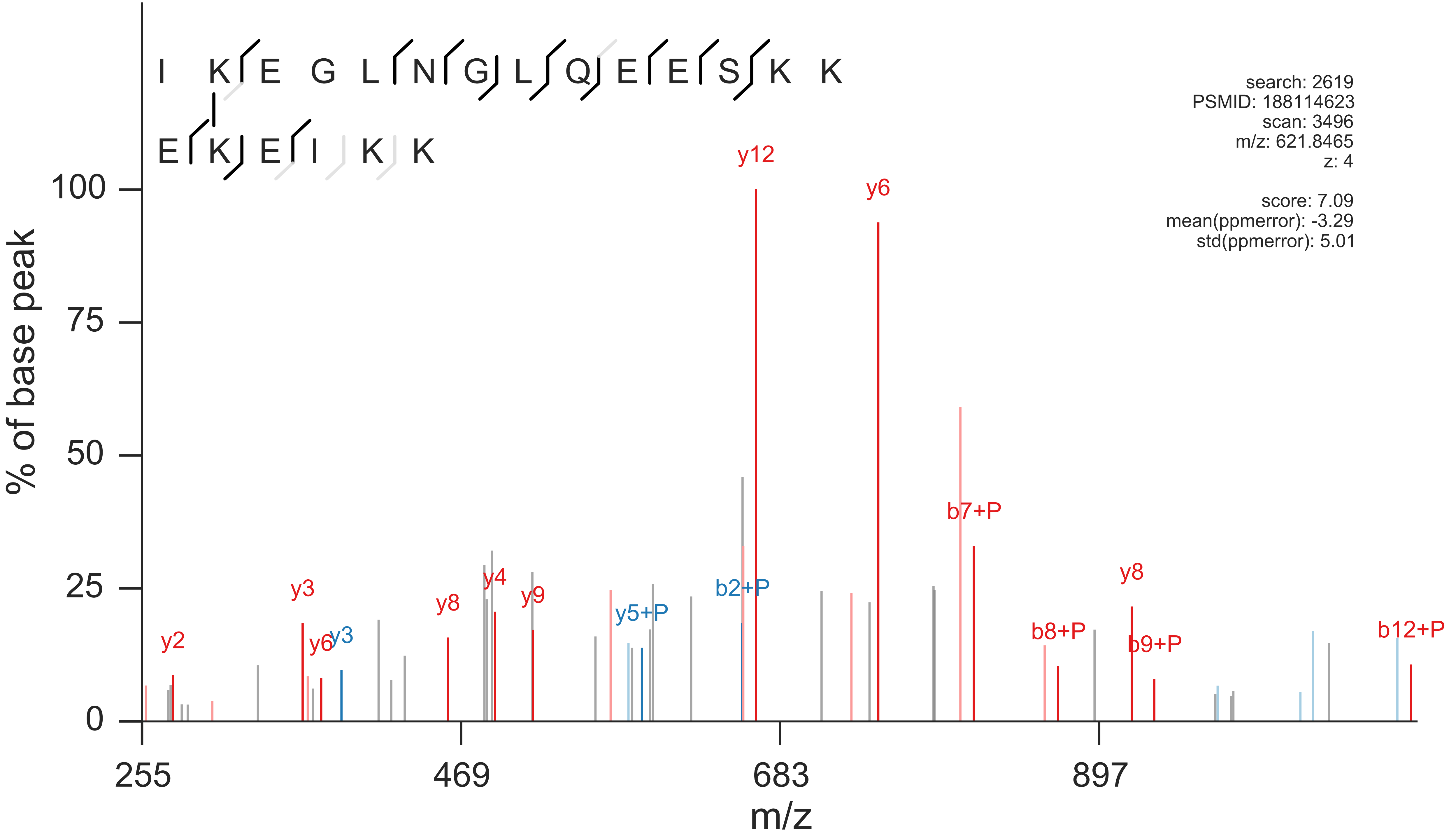

I K E G L N G L Q E E S K K
E K E I K K
search: 2619
PSMID: 188114623
scan: 3496
m/z: 621.8465
z: 4
score: 7.09
mean(ppmerror): -3.29
std(ppmerror): 5.01
% of base peak
100
75
50
25
0
y12
y6
b7+P
y8
y4
y9
y8
y3
y2
y6
y3
y5+P
b2+P
b8+P
b9+P
b12+P
255
469
683
897
m/z

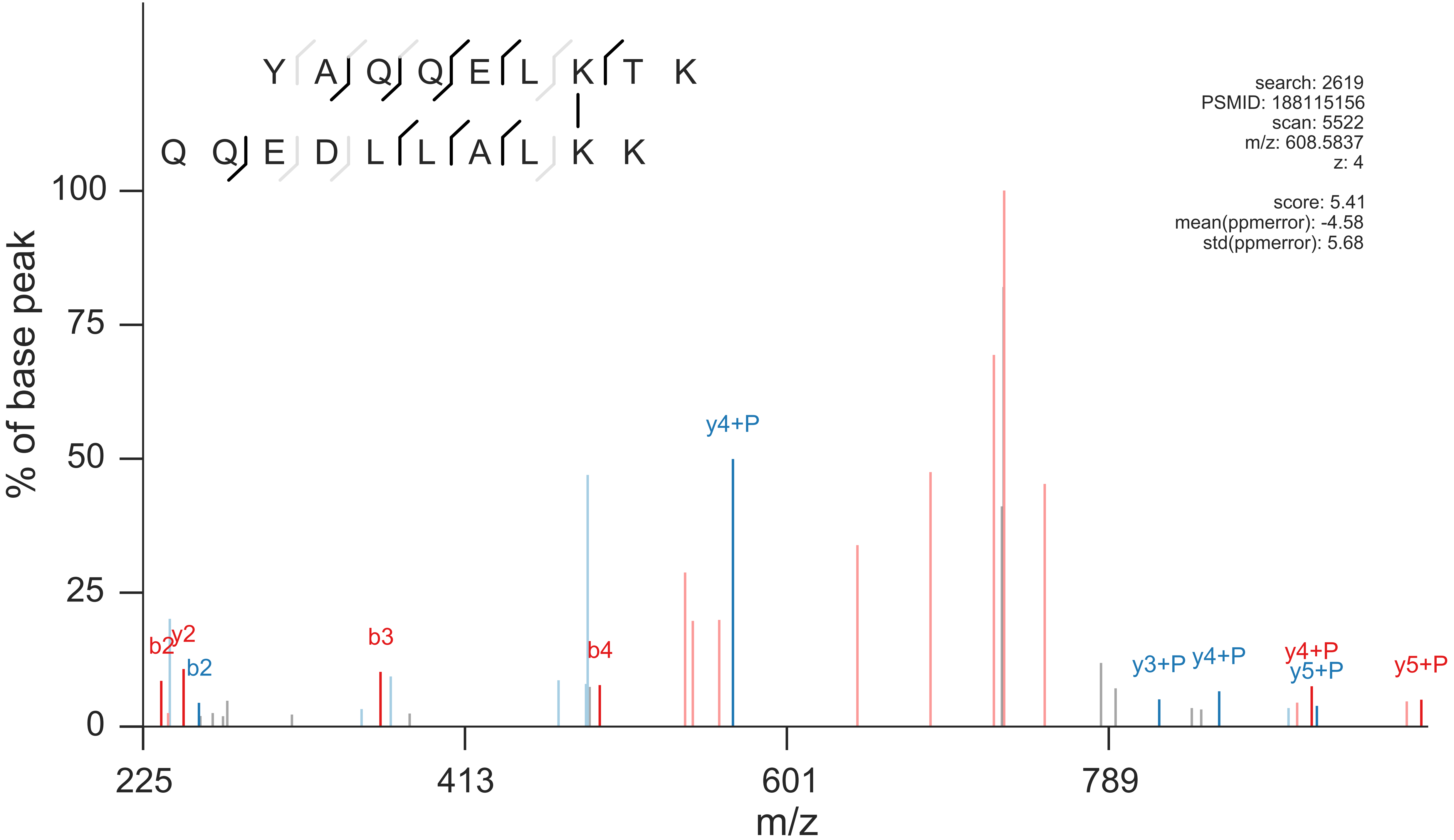

Y A Q Q E L K T K
Q Q E D L L A L K K
search: 2619
PSMID: 188115156
scan: 5522
m/z: 608.5837
z: 4
score: 5.41
mean(ppmerror): -4.58
std(ppmerror): 5.68
% of base peak
100
75
50
25
0
225
413
601
789
m/z
b2
y2
b2
b3
b4
y4+P
y3+P
y4+P
y4+P
y5+P
y5+P

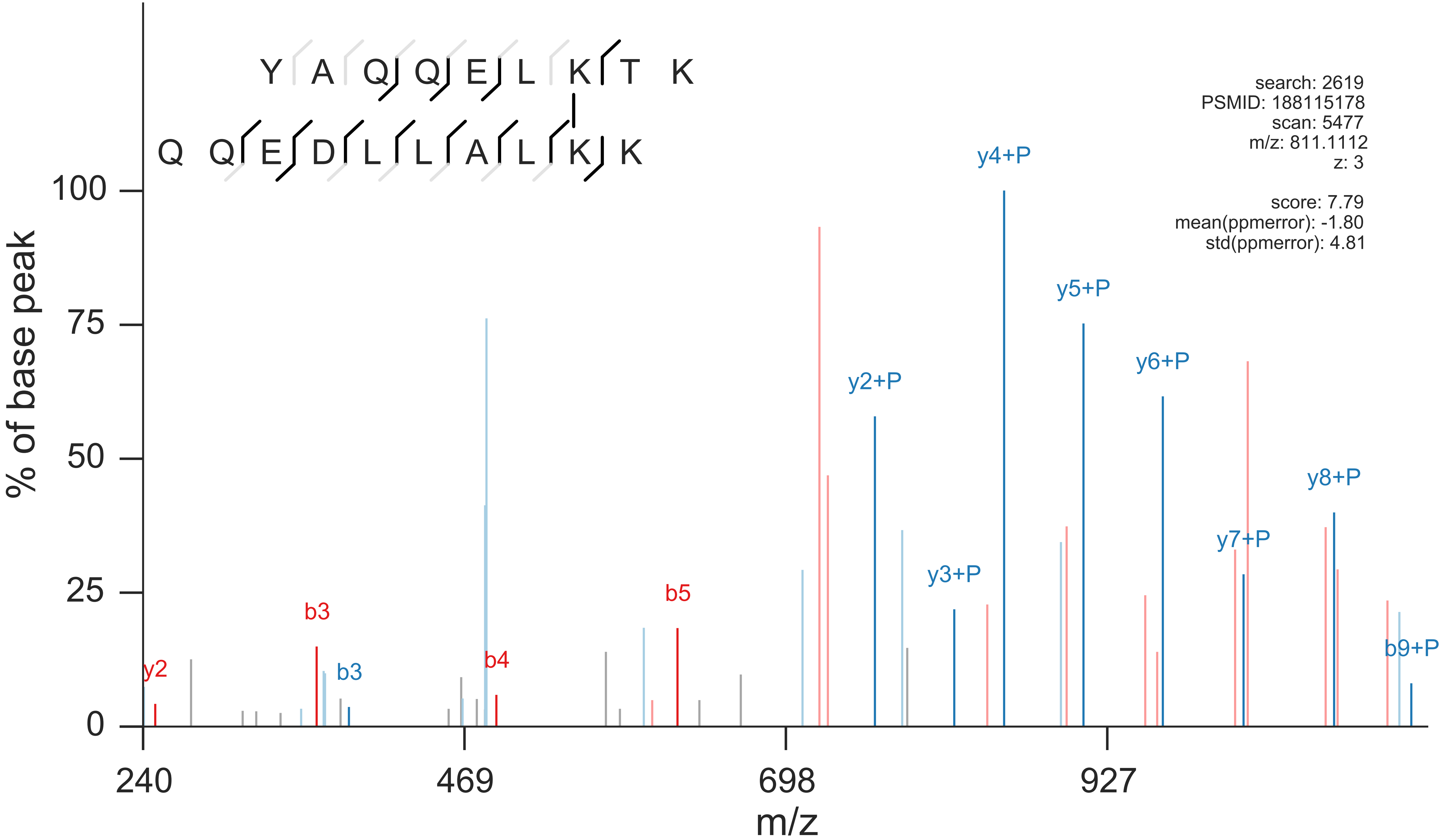

Y A Q Q E L K T K
Q Q E D L L A L K K
search: 2619
PSMID: 188115178
scan: 5477
m/z: 811.1112
z: 3
score: 7.79
mean(ppmerror): -1.80
std(ppmerror): 4.81
% of base peak
m/z
y2
b3
b3
b4
b5
y2+P
y3+P
y4+P
y5+P
y6+P
y7+P
y8+P
b9+P
0
25
50
75
100
240
469
698
927

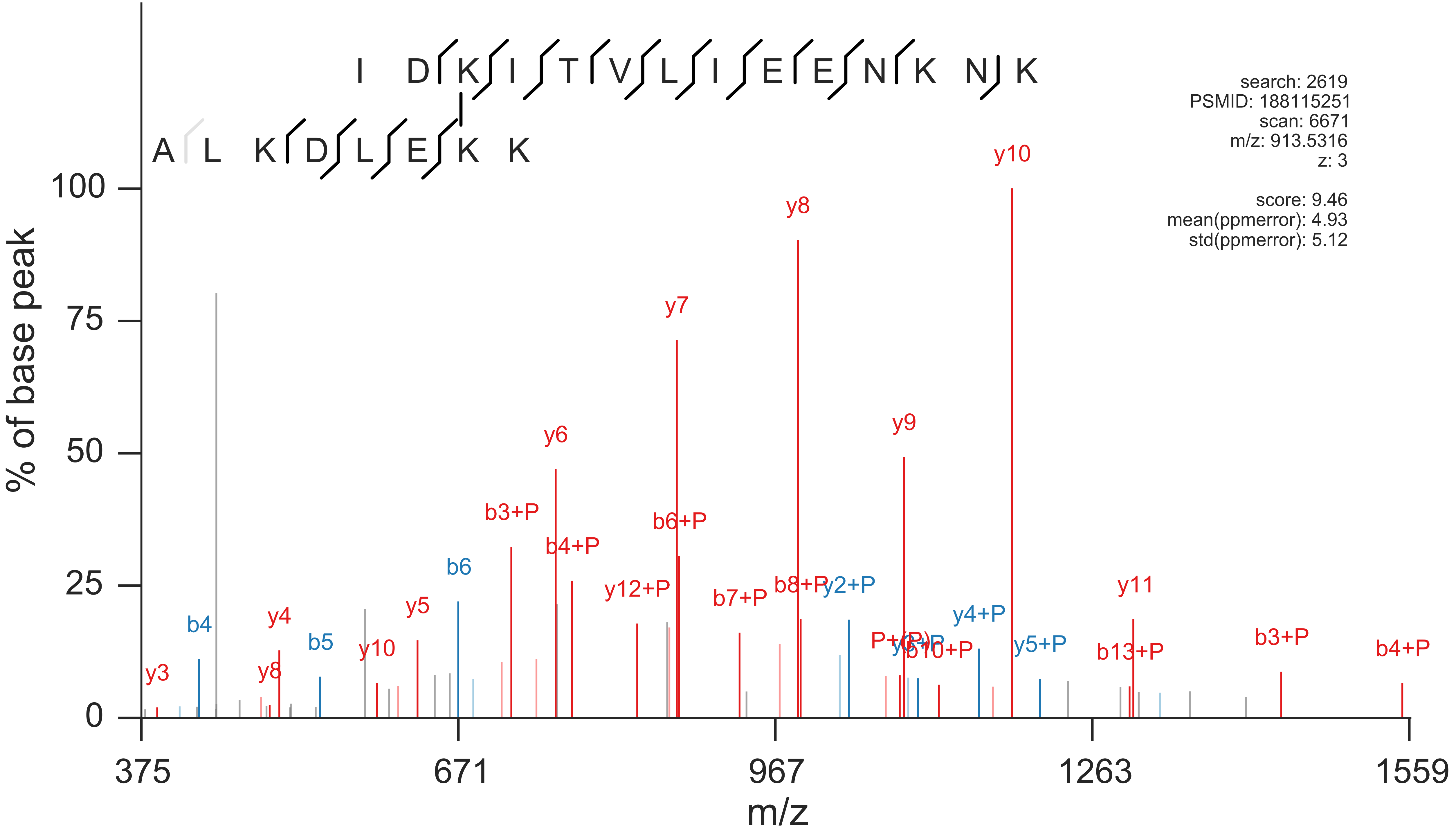

I D K I T V L I E E N K N K
A L K D L E K K
search: 2619
PSMID: 188115251
scan: 6671
m/z: 913.5316
z: 3
score: 9.46
mean(ppmerror): 4.93
std(ppmerror): 5.12
% of base peak
m/z
y10
y8
y7
y9
y6
b3+P
b6+P
b4+P
b6
y12+P
b7+P
b8+P
y2+P
y11
y4+P
y5+P
b13+P
b3+P
b4+P
b10+P
y5
y4
b4
b5
y10
y3
y8

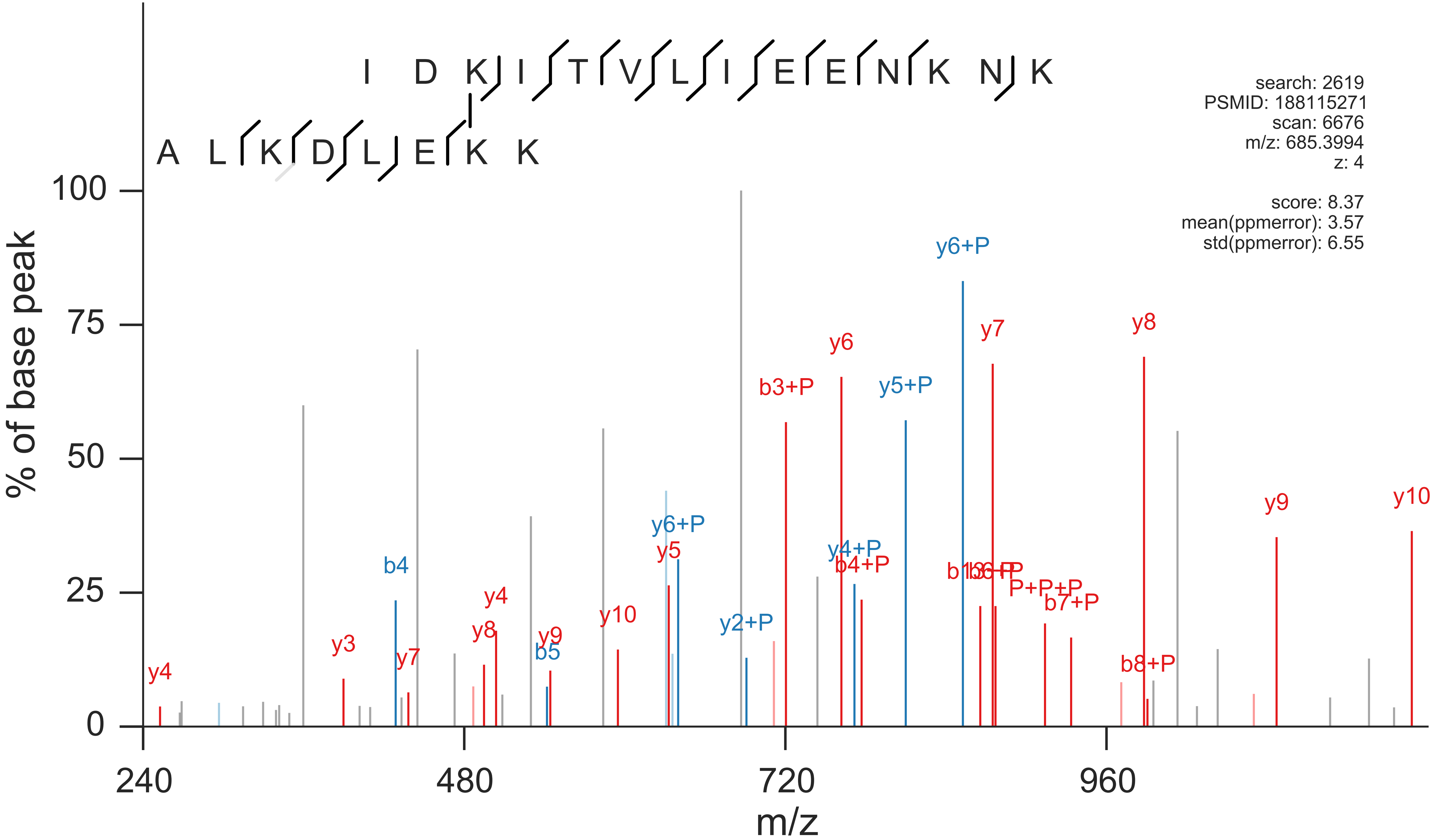
I D K I T V L I E E N K N K
A L K D L E K K
search: 2619
PSMID: 188115271
scan: 6676
m/z: 685.3994
z: 4
score: 8.37
mean(ppmerror): 3.57
std(ppmerror): 6.55
% of base peak
m/z
0
25
50
75
100
240
480
720
960
y4
y3
y7
b4
y8
y4
y9
b5
y10
y6+P
y5
y2+P
b3+P
y6
y4+P
b4+P
y5+P
y6+P
y7
y8
P+P+P
b7+P
b8+P
y9
y10

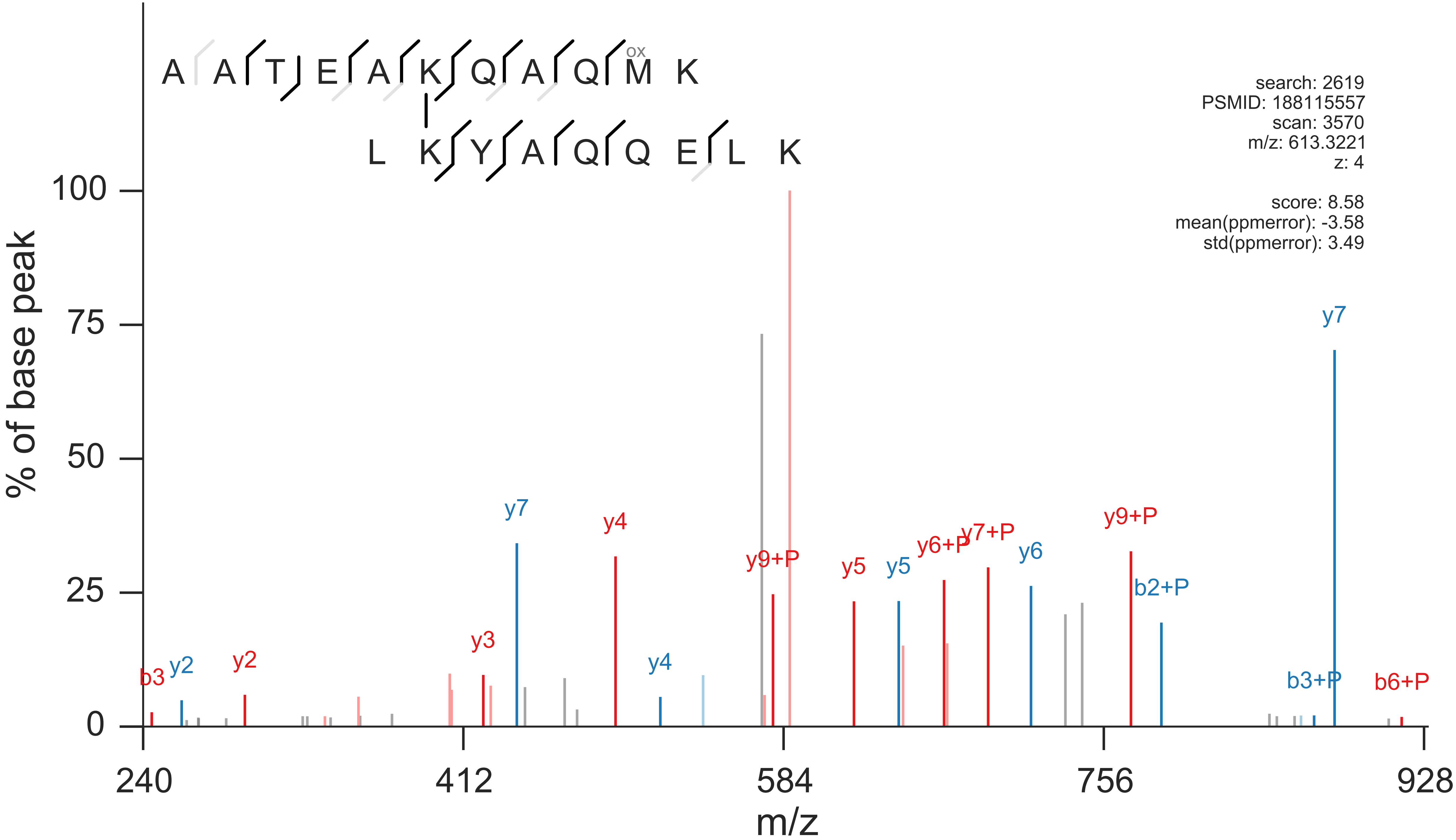

A A T E A K Q A Q M K
ox
L K Y A Q Q E L K
search: 2619
PSMID: 188115557
scan: 3570
m/z: 613.3221
z: 4
score: 8.58
mean(ppmerror): -3.58
std(ppmerror): 3.49
% of base peak
100
75
50
25
0
240
412
584
756
928
m/z
b3
y2
y2
y3
y7
y4
y4
y9+P
y5
y5
y6+P
y7+P
y6
y9+P
b2+P
y7
b3+P
b6+P

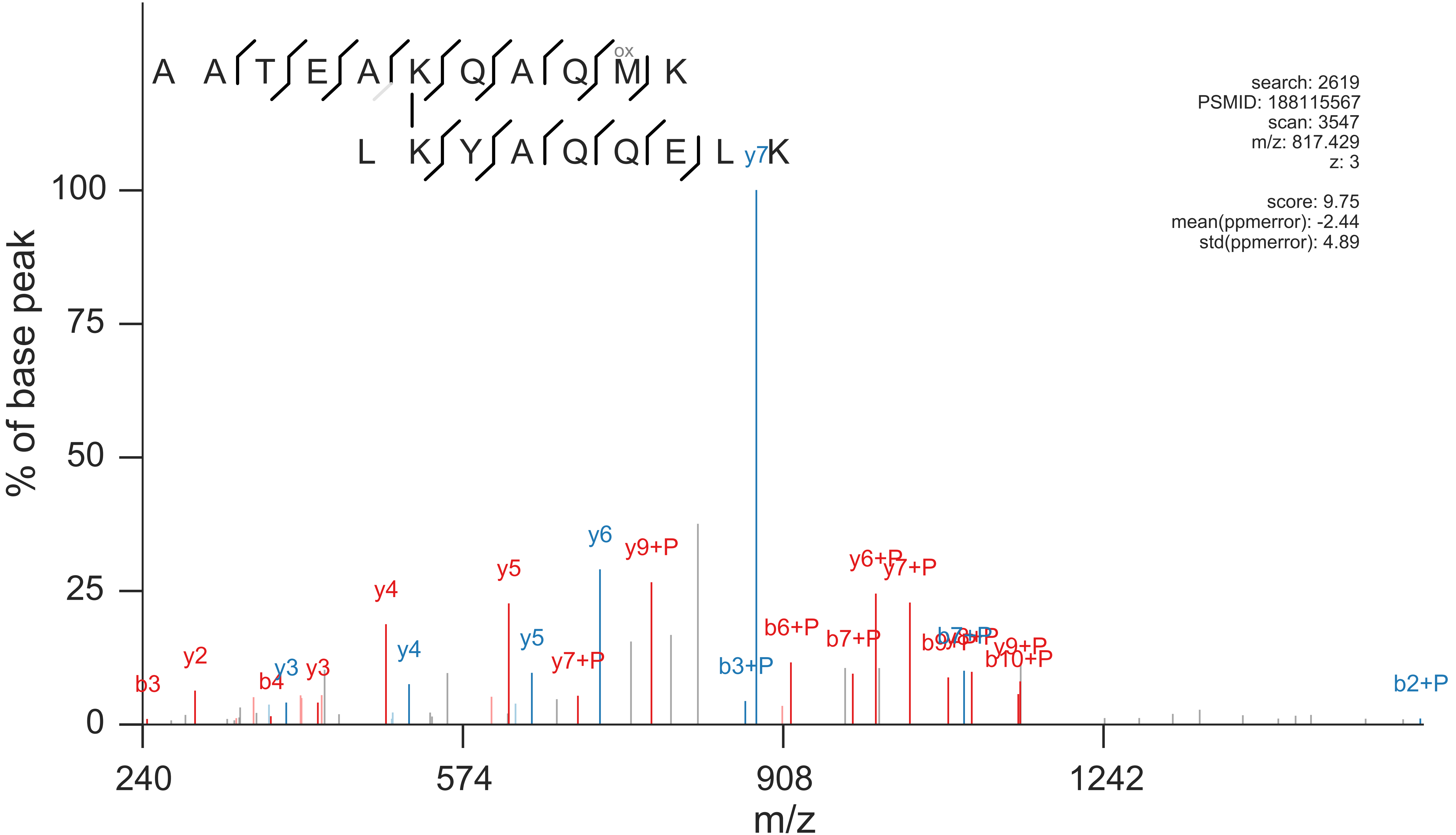

A A T E A K Q A Q M K
ox
L K Y A Q Q E L K
search: 2619
PSMID: 188115567
scan: 3547
m/z: 817.429
z: 3
score: 9.75
mean(ppmerror): -2.44
std(ppmerror): 4.89
% of base peak
m/z
0
25
50
75
100
240
574
908
1242
b3
y2
b4
y3
y3
y4
y4
y5
y5
y6
y7+P
y9+P
y7
b3+P
b6+P
b7+P
y6+P
y7+P
y9+P
b10+P
b2+P

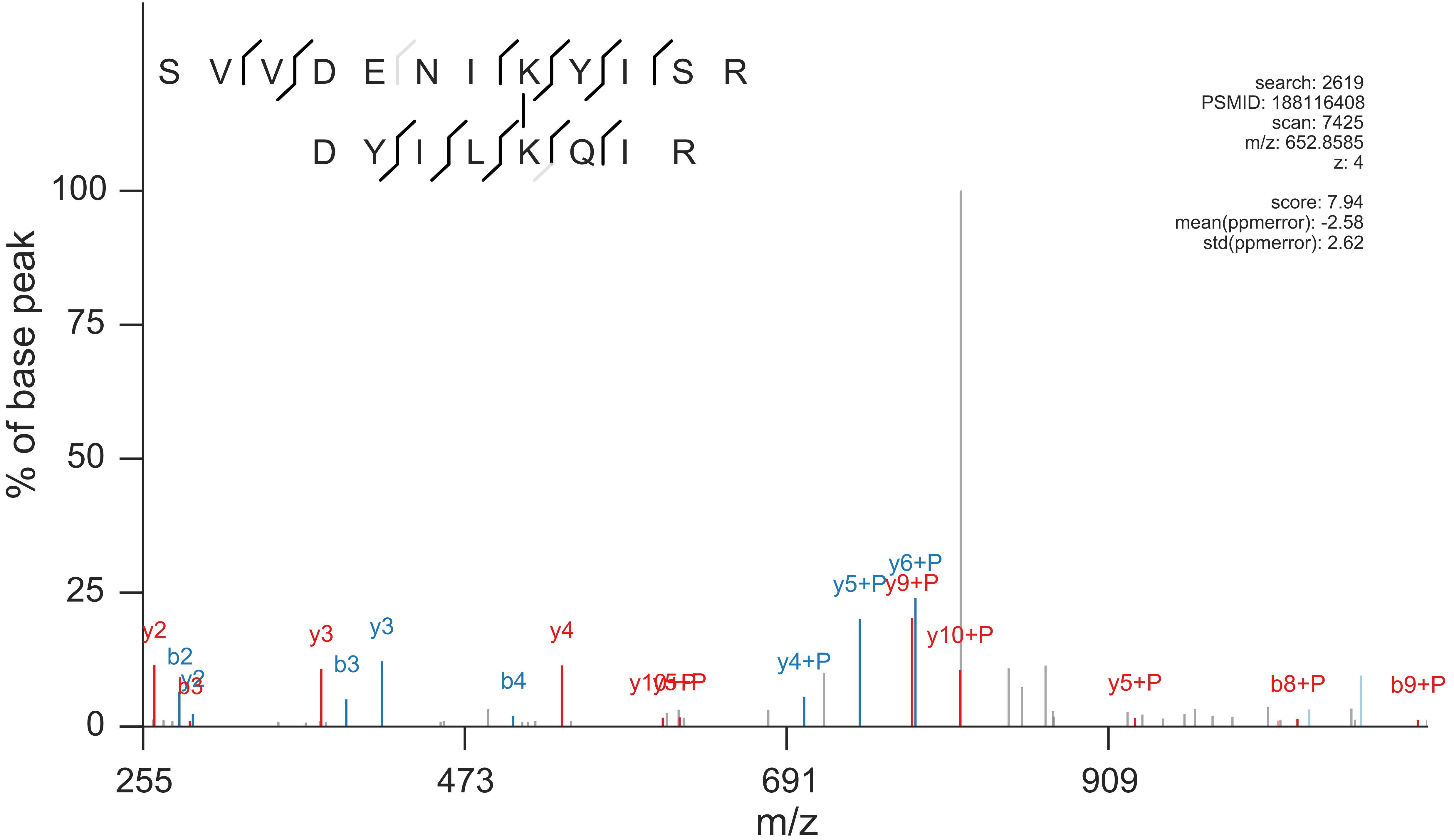
S V V D E N I K Y I S R
D Y I L K Q I R
search: 2619
PSMID: 188116408
scan: 7425
m/z: 652.8585
z: 4
score: 7.94
mean(ppmerror): -2.58
std(ppmerror): 2.62
% of base peak
0
25
50
75
100
255
473
691
909
m/z
y2
b2
y2
b3
y3
b3
y3
b4
y4
y5+P
y10+P
y4+P
y5+P
y6+P
y9+P
y10+P
y5+P
b8+P
b9+P

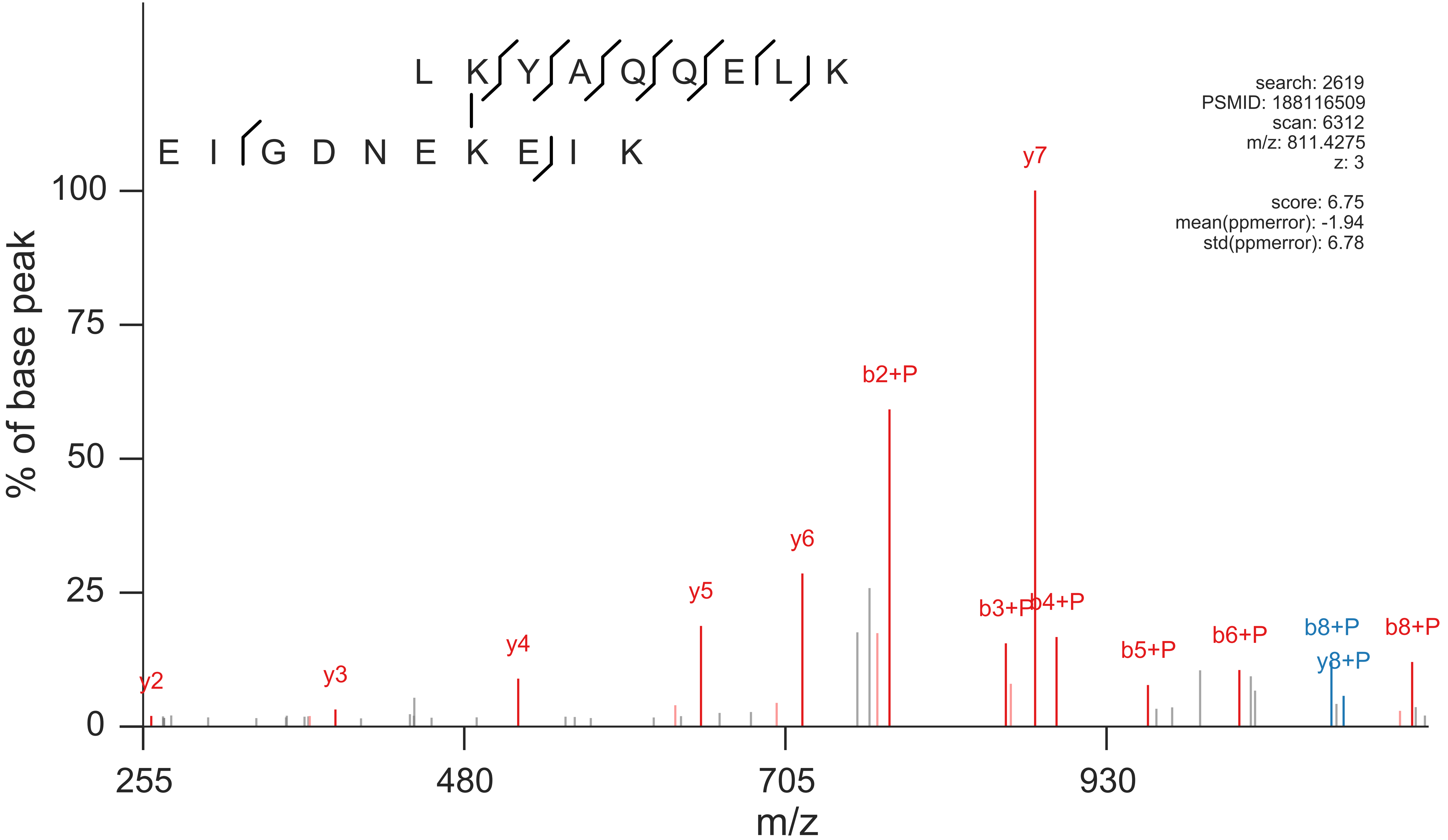

L K Y A Q Q E L K
E I G D N E K E I K
search: 2619
PSMID: 188116509
scan: 6312
m/z: 811.4275
z: 3
score: 6.75
mean(ppmerror): -1.94
std(ppmerror): 6.78
% of base peak
100
75
50
25
0
y7
b2+P
y6
y5
y4
y3
y2
b3+P
b4+P
b5+P
b6+P
b8+P
y8+P
b8+P
255
480
705
930
m/z

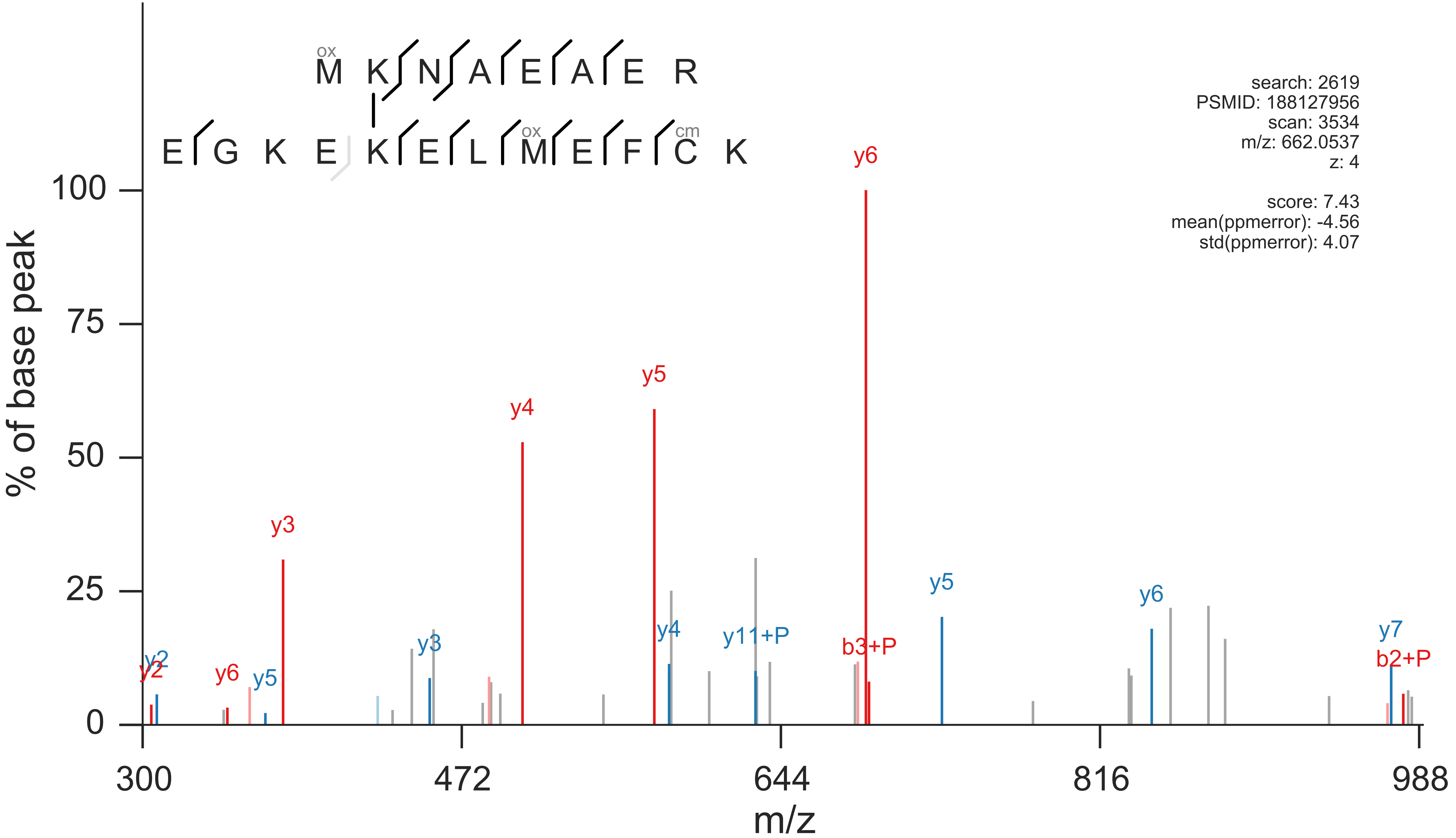

ox
M K N A E A E R
E G K E K E L M E F C K
ox
cm
search: 2619
PSMID: 188127956
scan: 3534
m/z: 662.0537
z: 4
score: 7.43
mean(ppmerror): -4.56
std(ppmerror): 4.07
% of base peak
0
25
50
75
100
m/z
300
472
644
816
988
y2
y2
y6
y5
y3
y3
y4
y5
y4
y11+P
y6
b3+P
y5
y6
y7
b2+P

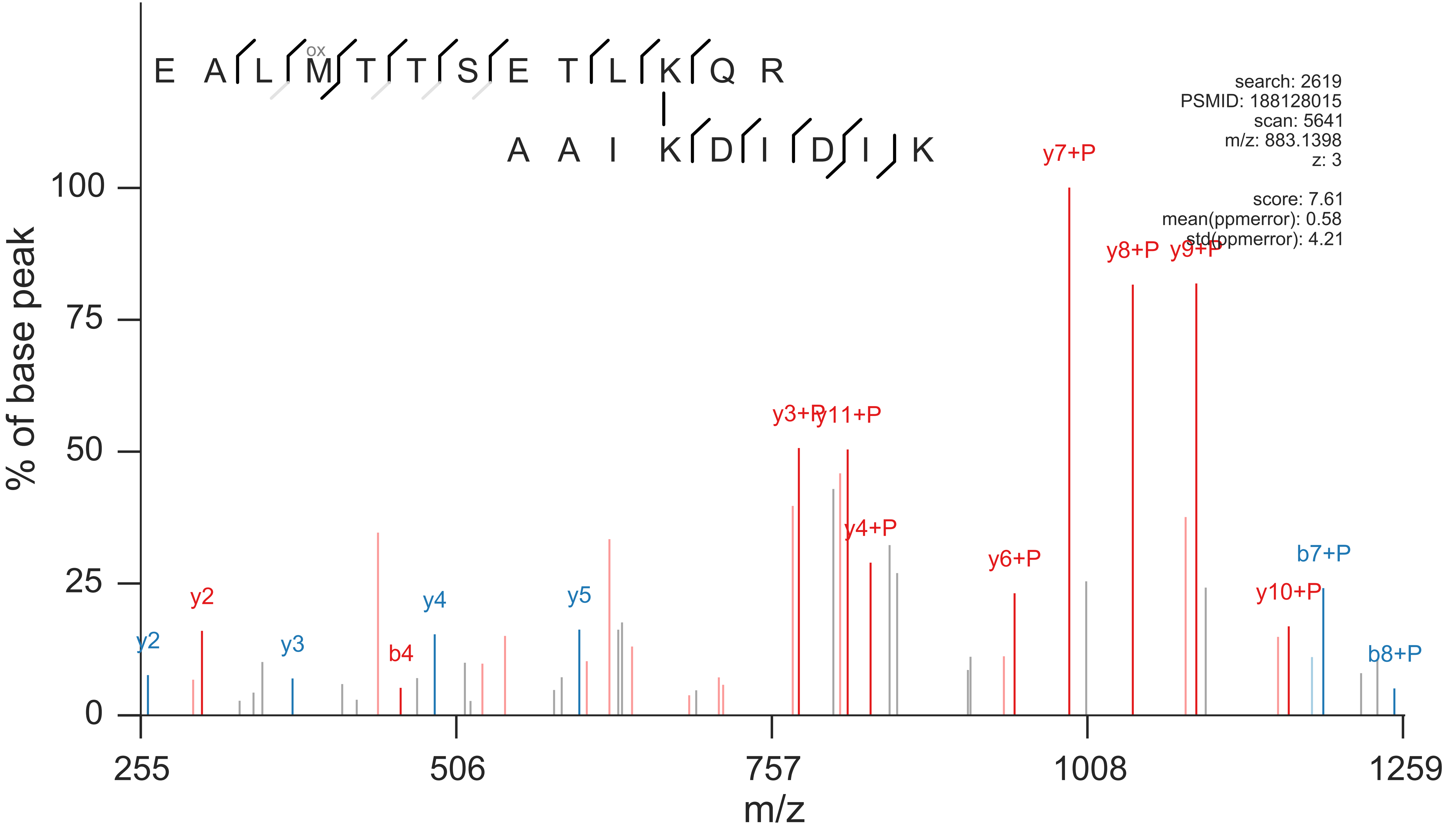

E A L M T T S E T L K Q R
ox
A A I K D I D I K
search: 2619
PSMID: 188128015
scan: 5641
m/z: 883.1398
z: 3
score: 7.61
mean(ppmerror): 0.58
std(ppmerror): 4.21
% of base peak
m/z
y2
y2
y3
b4
y4
y5
y3+P
y11+P
y4+P
y6+P
y7+P
y8+P
y9+P
y10+P
b7+P
b8+P
0
25
50
75
100
255
506
757
1008
1259

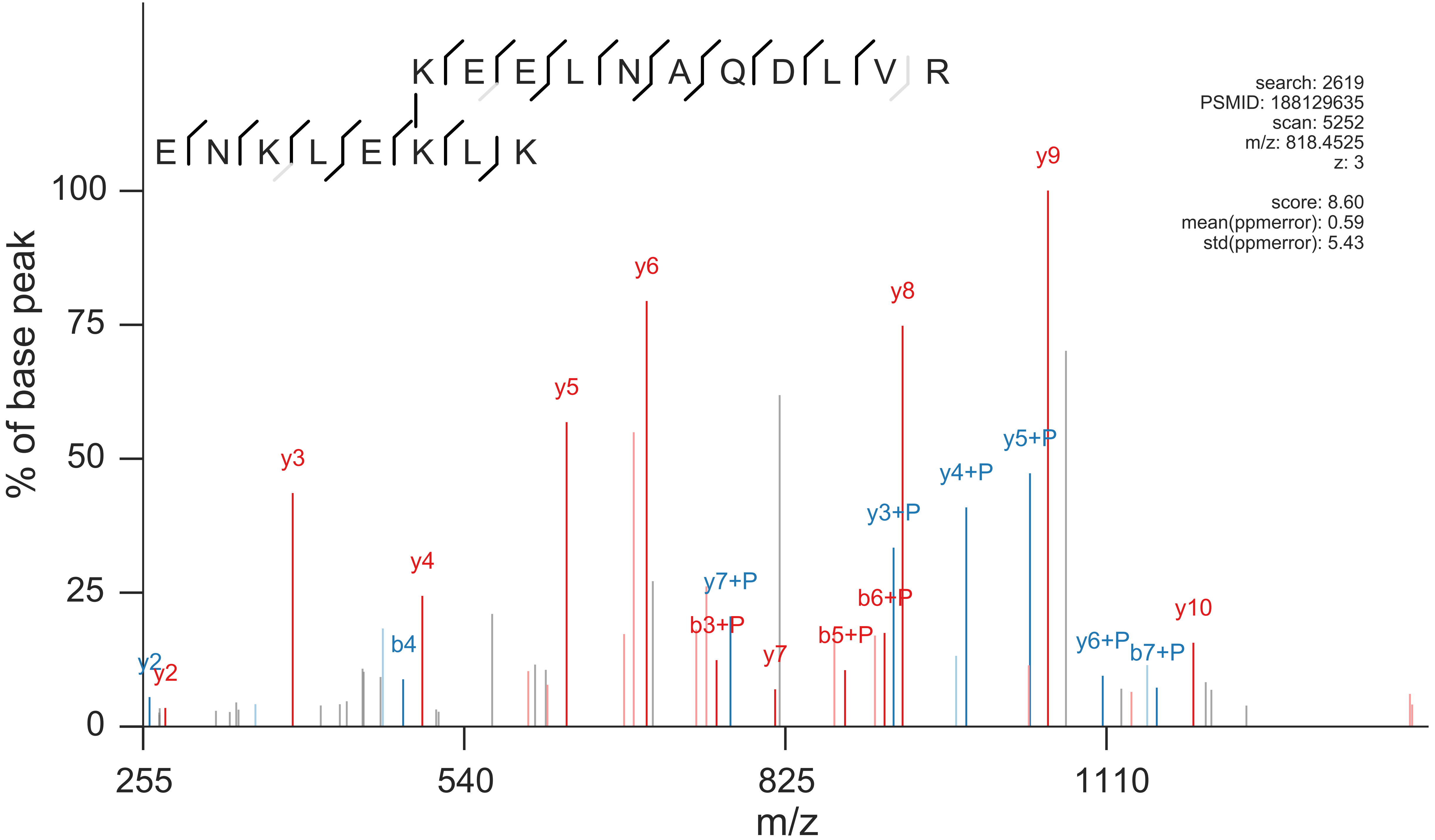

K E E L N A Q D L V R
E N K L E K L K
search: 2619
PSMID: 188129635
scan: 5252
m/z: 818.4525
z: 3
score: 8.60
mean(ppmerror): 0.59
std(ppmerror): 5.43
% of base peak
m/z
0
25
50
75
100
255
540
825
1110
y2
y2
y3
b4
y4
y5
y6
y7+P
b3+P
y7
b5+P
b6+P
y3+P
y8
y4+P
y5+P
y9
y6+P
b7+P
y10

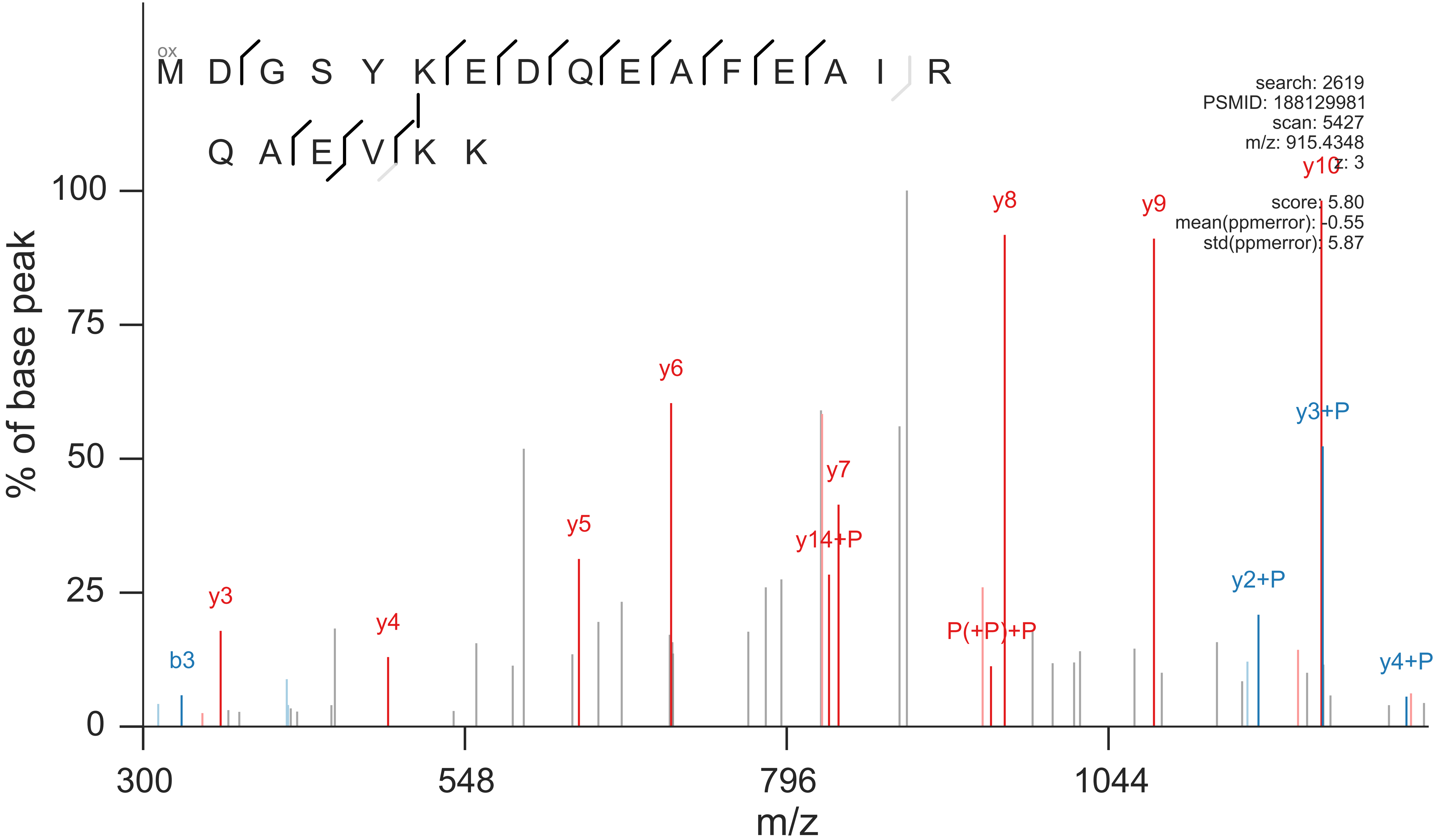

ox
M D G S Y K E D Q E A F E A I R
Q A E V K K
search: 2619
PSMID: 188129981
scan: 5427
m/z: 915.4348
z: 3
score: 5.80
mean(ppmerror): -0.55
std(ppmerror): 5.87
b3
y3
y4
y5
y6
y7
y14+P
y8
y9
y10
P(+P)+P
y2+P
y3+P
y4+P
% of base peak
0
25
50
75
100
300
548
796
1044
m/z

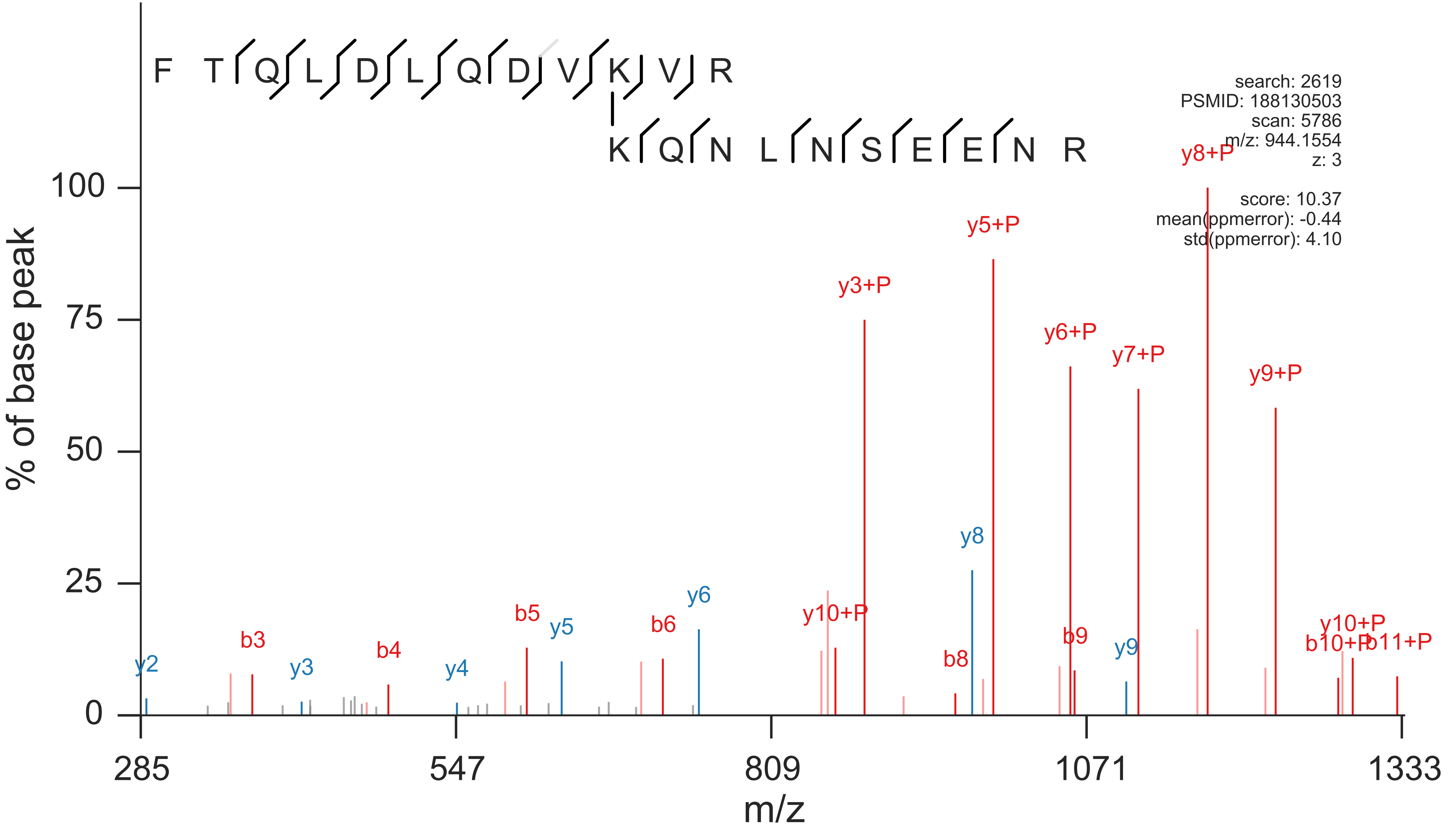
F T Q L D L Q D V K V R
K Q N L N S E E N R
search: 2619
PSMID: 188130503
scan: 5786
m/z: 944.1554
z: 3
score: 10.37
mean(ppmerror): -0.44
std(ppmerror): 4.10
% of base peak
m/z
0
25
50
75
100
285
547
809
1071
1333
y2
b3
y3
b4
y4
b5
y5
b6
y6
y10+P
y3+P
b8
y8
y5+P
b9
y6+P
y9
y7+P
y8+P
y9+P
b10+P
y10+P
b11+P
