# Supplementary material for: A Study into the Collision-induced Dissociation (CID) Behavior of Cross-Linked Peptides
Source: Mol Cell Proteomics. 2015 Dec 30;15(3):1094–104. doi: 10.1074/mcp.M115.049296 (PMC4813691; doi:10.1074/mcp.M115.049296)
Supplement: Supplemental Data [file 10.1074_M115.049296_mcp.M115.049296-1.pdf]

Supplementary Material: A study into the CID behavior of cross-linked peptides

Sven H. Giese[1, 2], Lutz Fischer[1], and Juri Rappsilber*[1, 2]

[1]Department of Bioanalytics, Institute of Biotechnology, Technische Universität Berlin, 13355 Berlin, Germany
[2]Wellcome Trust Centre for Cell Biology, School of Biological Sciences, University of Edinburgh, Edinburgh EH9 3BF, United Kingdom

1 List of Supplementary Material

S1: Output from XiFDR containing tab-delimited information about each PSM

S2: Annotated spectra for all cross-link identifications from S1

S3: Distribution of residues involved in cross-link bonds

S4: Factors influencing the fragmentation similarity

S5: Sensitivity and specificity for transforming the highest intense ions

S6: Single spectra that are used in the manuscript (Fig. 3A)

S7: Linear spectrum corresponding to the alpha peptide in the manuscript (Fig. 4D)

S8: Cross-linker fragmentation observations

S9: Comparison of Figure 1 results based on 910 PSMs wtih results based on 8,301 PSMs

S10: Comparison of Figure 2 results based on 910 PSMs wtih results based on 8,301 PSMs

S11: Comparison of Figure 3 results based on 910 PSMs wtih results based on 8,301 PSMs

S12: Comparison of Figure 4 results based on 910 PSMs wtih results based on 8,301 PSMs

S1: XiFDR output

Available online as separate Excel file.

S2: Annotated spectra

Available online as separate PDF. Peptides at the top are alpha peptides (in red); peptides at the bottom are beta peptides (in blue). Annotation: Loss ions (water, ammonia) are not annotated but the peaks are highlighted with light red/blue colors. Cross-linked fragments are annotated as +P. Precursor ions as P. P+i(P) denotes ions that have a modified lysine rest on one end of the cross-link and on the other the complete second peptide. P(+P) denotes the individual peptide fragments without the cross-linker mass and P+(P) refers to the individual peptides with the cross-linker attached. Mean(ppmerror) and std(ppmerror) refer to the measurement error on the fragment peak matching.

*juri.rappsilber@tu-berlin.de

S3: Cross-linked residue distribution

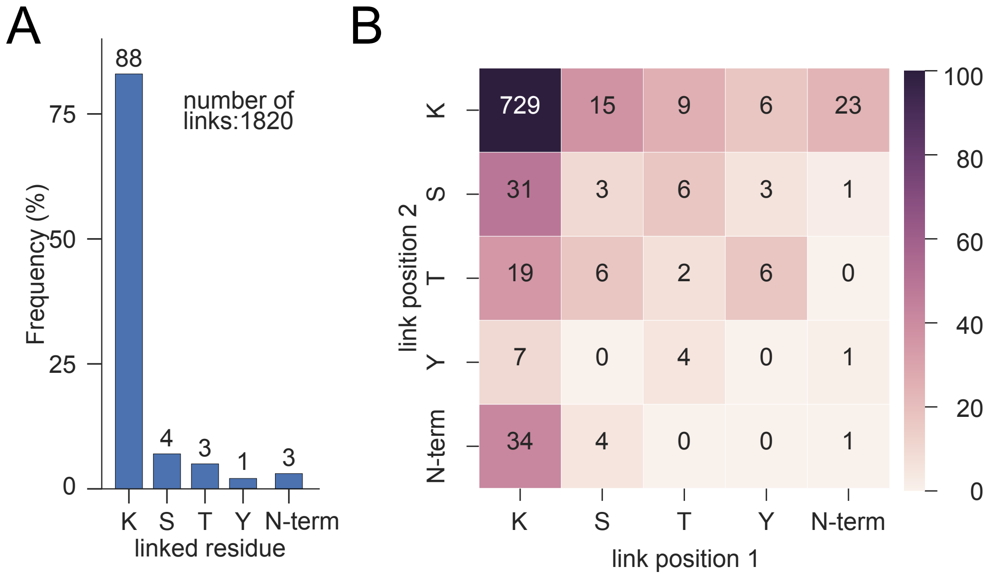

Supplementary Figure 3: Cross-linked residue distribution. Frequency distribution of all observed cross-linked residues from 910 PSMs (**A**). Heatmap showing all observed linkage combinations based on BS3 cross-linking. Annotations in the cells refer to the absolute counts of an observed linkage pair. Cross-links involving serine, threonine or tyrosine in at least one linkage site account for roughly 14% of the cross-links (**B**).

S4: Cross-link factors that influence fragmentation

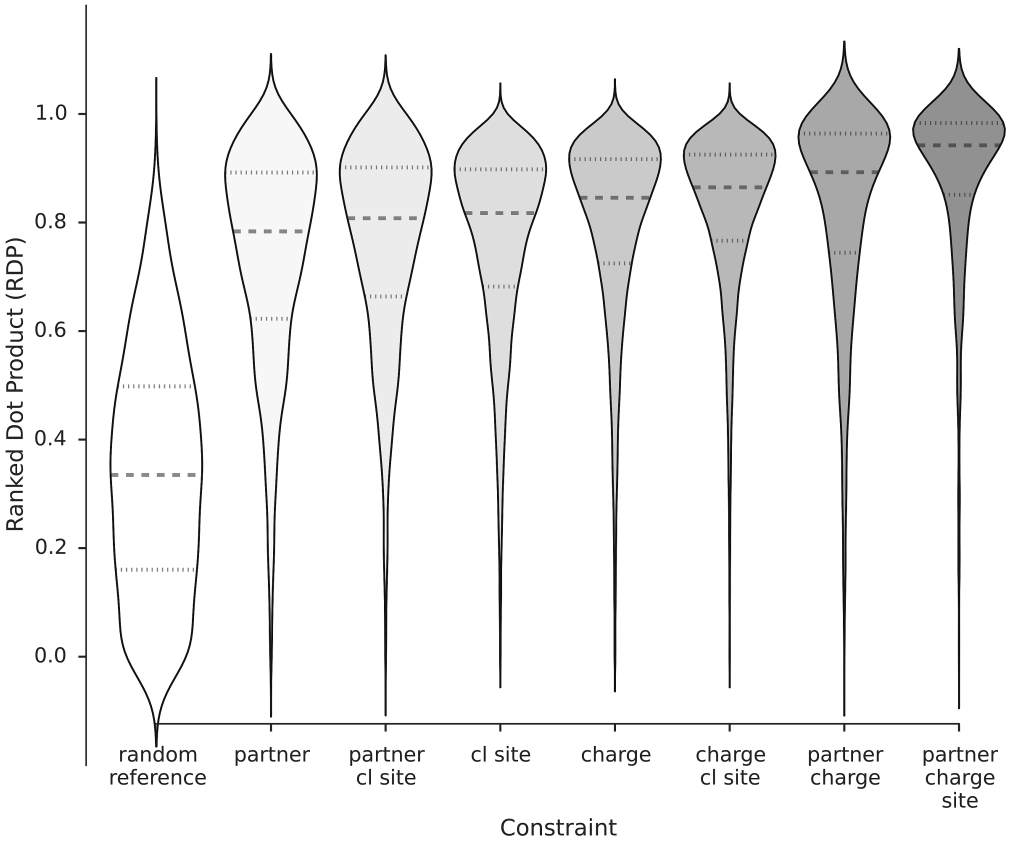

Supplementary Figure 4: Influence on the cross-linking context on the spectral similarity. The x-axis describes the similarity class constraints for the comparison. For example, in the 'partner' class the cross-linked peptides that were compared against each other had to be identified with the same partner. Multiple constraints are simply combinations of individual classes. A reference distribution is derived by randomly comparing spectra of cross-linked peptides. The data was derived from 8,301 high-confidence identifications by XiFDR with a 5% false discovery rate (FDR). In addition, each peptide in a cross-link was required to be six amino acids or longer. Note that this Figure includes data from unpublished acquisitions. Abbreviations: partner - partner peptide in a cross-link, cl site - cross-linking site position. Data were derived from additional unpublished data from our in-house database.

S5: Fragment linearization among the top ten ions

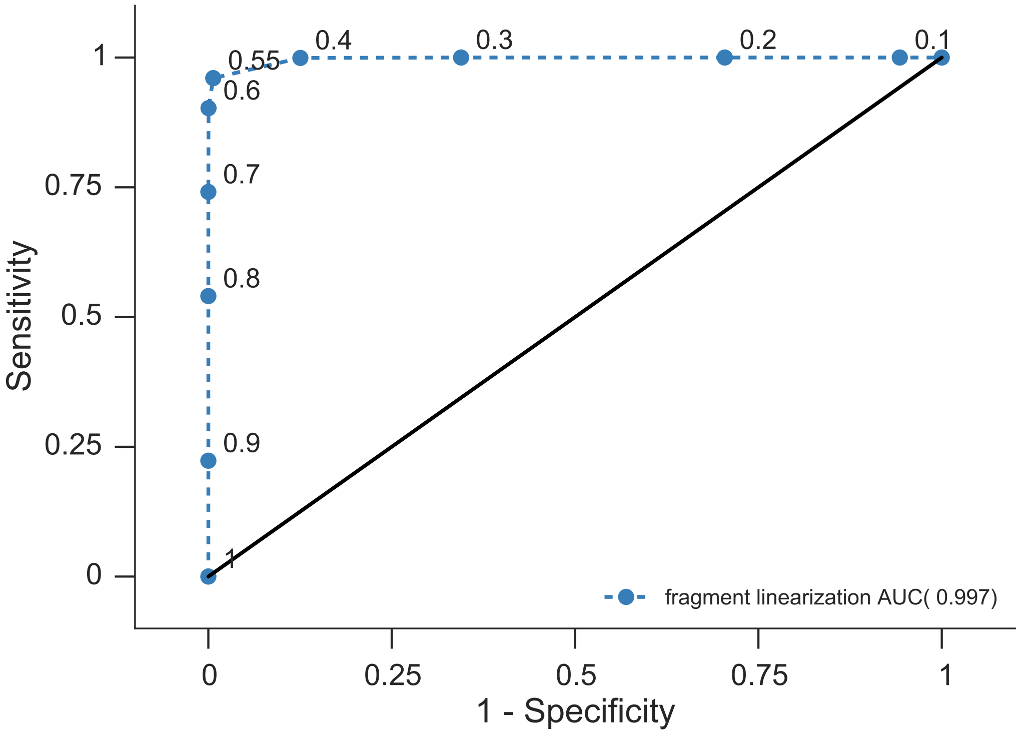

Supplementary Figure 5: ROC curve evaluating the linearization of the ten highest intense (and identified) ions. Sensitivity and specificity are defined as in the manuscript taking only fragments from the alpha peptide into account. Annotations in the plot refer to the relative mass cut-off that is used to decide whether or not to linearize a fragment.

S6: Individual spectra

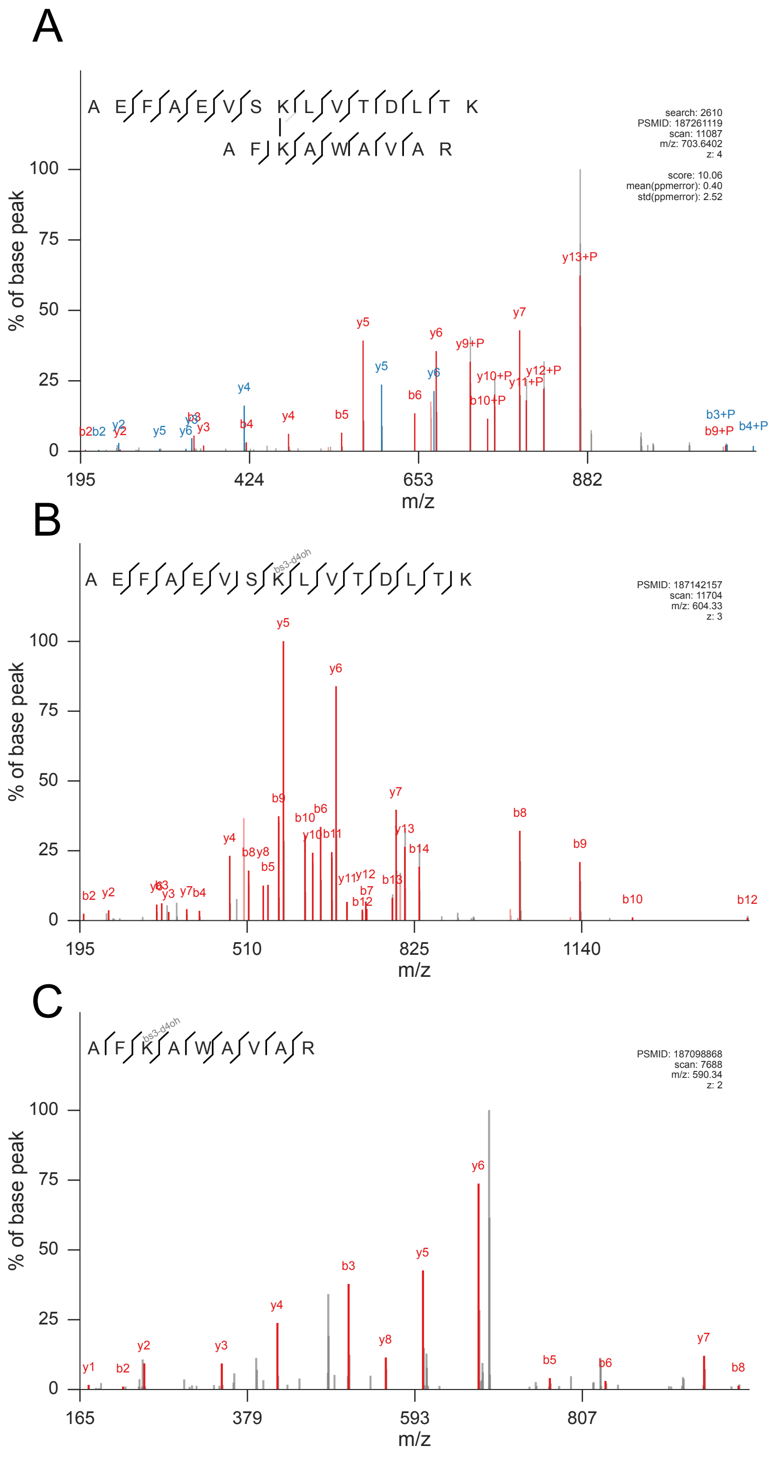

Supplementary Figure 6: Peak annotation for the individual spectra used in the overlay spectrum shown in the manuscript (Fig 3A). The cross-linked PSM **(A)**, with the alpha peptide in red (upper peptide sequence) and the beta peptide in blue (lower peptide sequence), the alpha peptide **(B)** and the beta peptide **(C)** are shown. Additional information such as precursor mass (m/z), precursor charge (z), scan number, and PSMID (unique identifier) are annotated.

S7: Linear spectrum

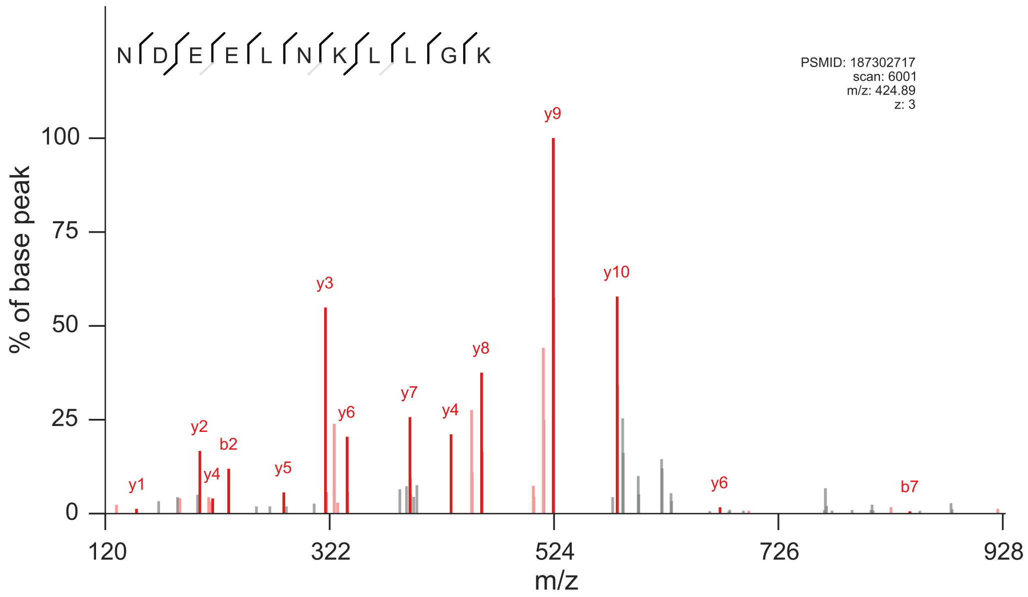

Supplementary Figure 7: Linear spectrum corresponding to the alpha peptide shown in the manuscript (Fig. 4D). Additional information such as precursor mass (m/z), precursor charge (z), scan number, and PSMID (unique identifier) are annotated.

S8: Cross-linker fragmentation

count type / ion type	immonium type (P+i(P))	peptide + cross-linker loss (P(+P))	peptide loss (P+(P))
per cross-link	3%	16%	7%
per peptide	2%	10%	4%

Supplementary Table 1: Cross-linker fragmentation frequencies. P+i(P)) type ions have a modified lysine rest on one end of the cross-link and on the other the complete second peptide. P(+P) refers to the individual peptide fragments without the cross-linker mass and P+(P) refers to the individual peptides with the cross-linker attached.

S9: Figure 1 comparison to a larger sample

Section S9-S12 compare the results form the manuscript with another larger sample from our local database including unpublished data.

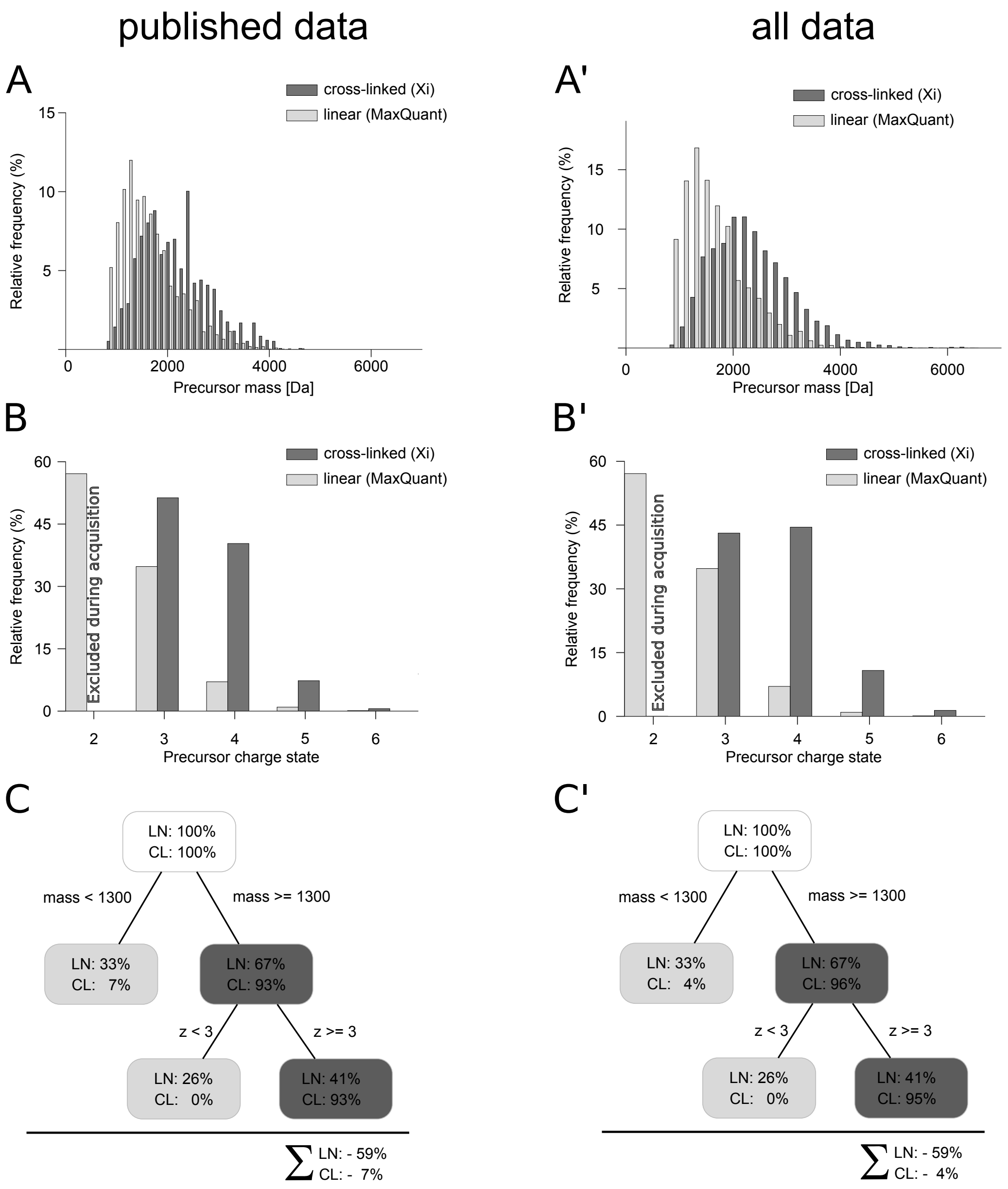

Supplementary Figure 9: Influence of different sample sizes on the results of Figure 1. The panel names refer to the same names in the manuscript. The left column panels (A-C) are the same plots as in the manuscript (published samples). The right column panels (A'-C') are one-to-one copies of the plots from the manuscript but with a larger sample size.

S10: Figure 2 comparison to a larger sample

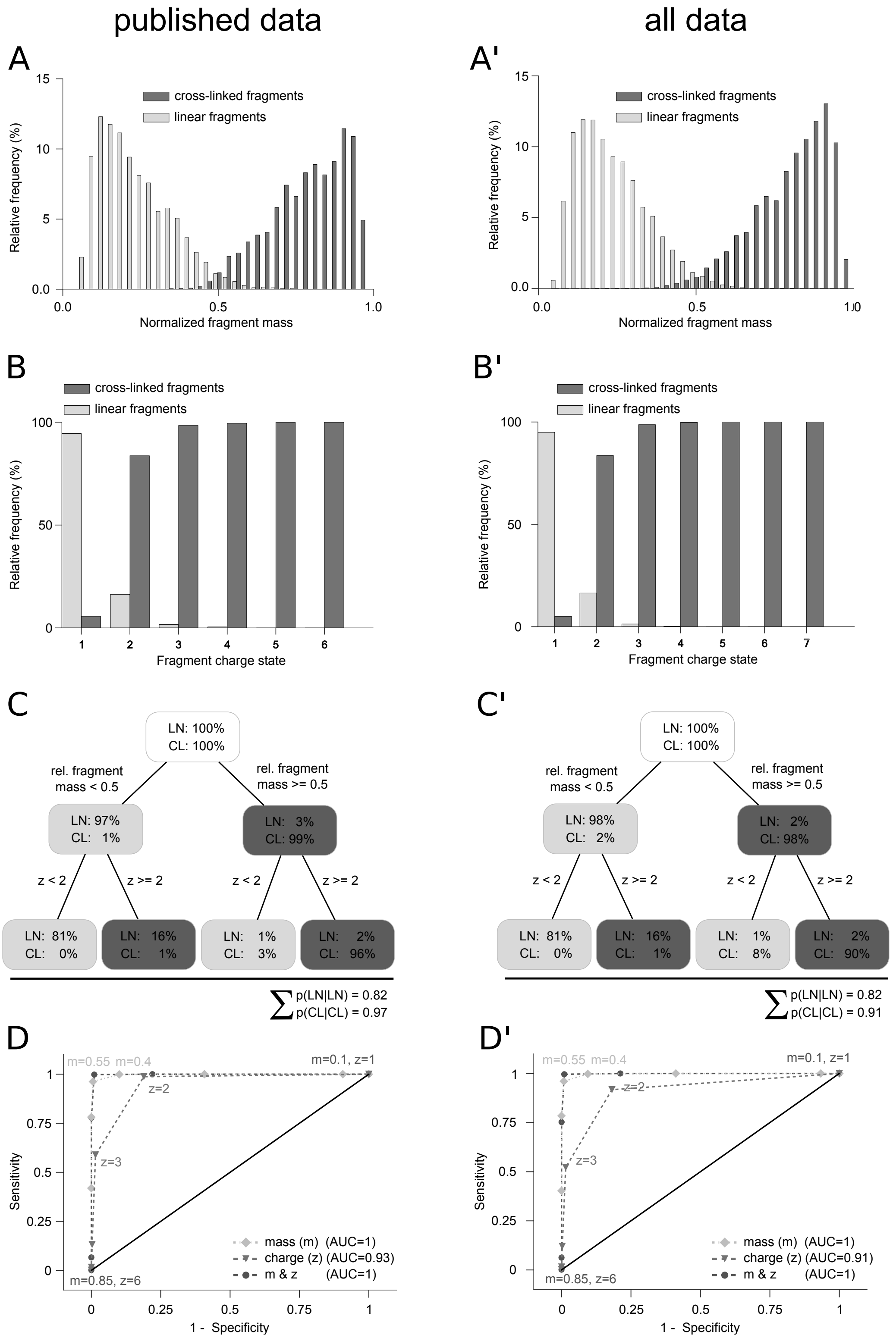

Supplementary Figure 10: Influence of different sample sizes on the results of Figure 2. The panel names refer to the same names in the manuscript. The left column panels (A-D) are the same plots as in the manuscript (published samples). The right column panels (A'-D') are one-to-one copies of the plots from the manuscript but with a larger sample size (all samples).

S11: Figure 3 comparison to a larger sample

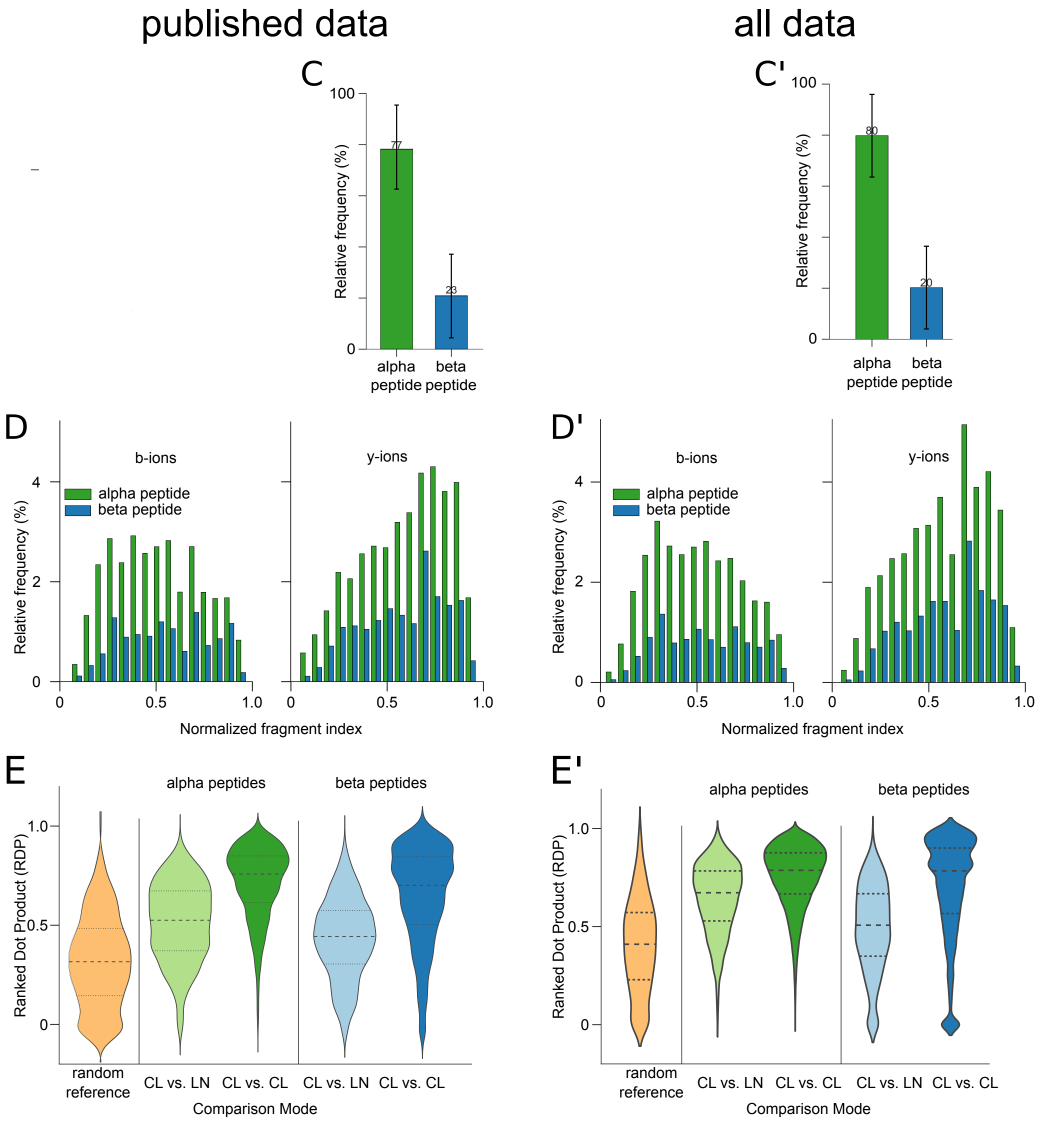

Supplementary Figure 11: Influence of different sample sizes on the results of Figure 3. The panel names refer to the same names in the manuscript. The left column panels (C-E) are the same plots as in the manuscript (published samples). The right column panels (C'-E') are one-to-one copies of the plots from the manuscript but with a larger sample size (all samples).

S12: Figure 4 comparison to a larger sample

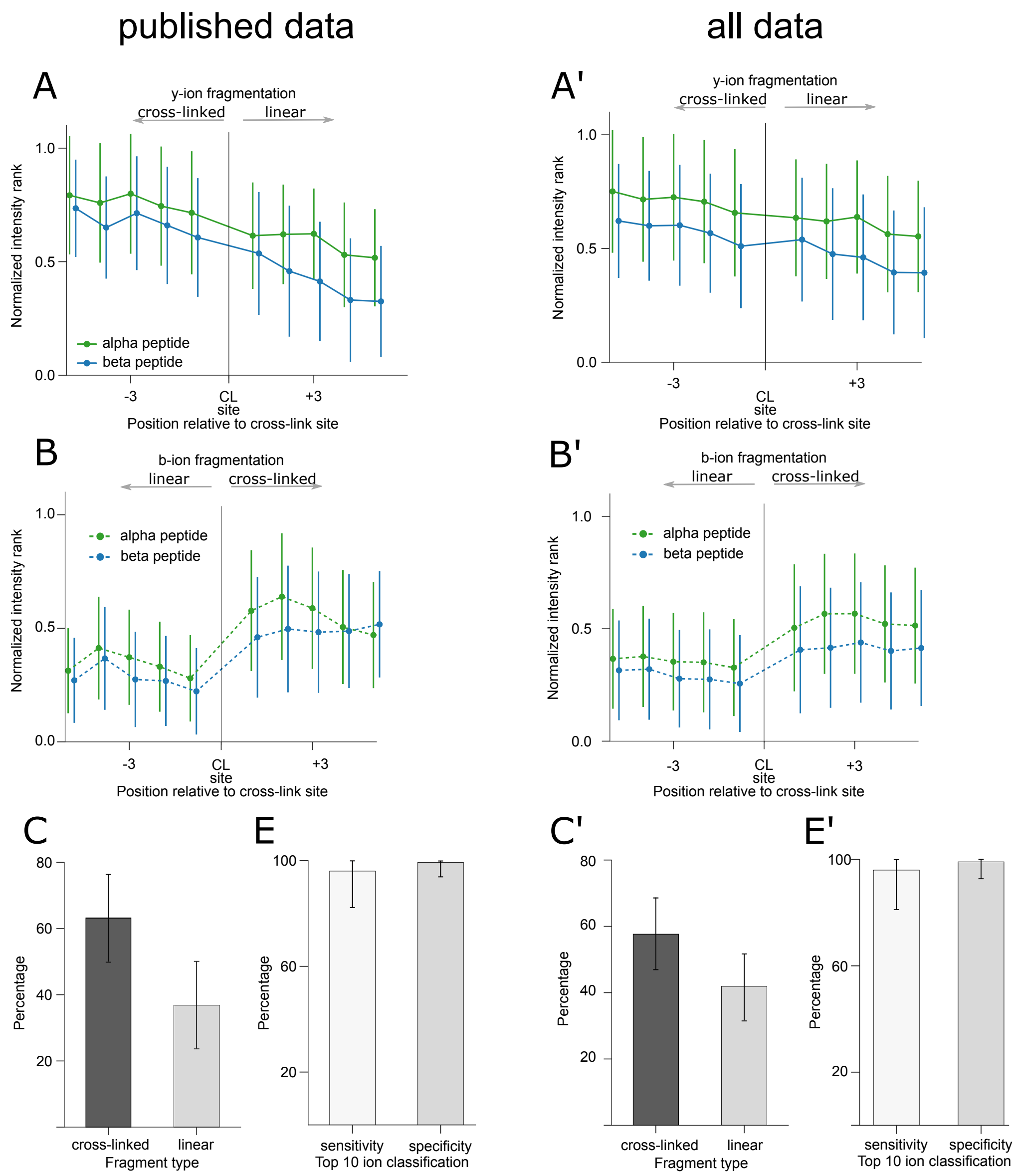

Supplementary Figure 12: Influence of different sample sizes on the results of Figure 4. The panel names refer to the same names in the manuscript. The left column panels (A, B, C, E) are the same plots as in the manuscript (published samples). The right column panels (A', B', C', E') are one-to-one copies of the plots from the manuscript but with a larger sample size (all samples).
